# METSP: A Maximum-Entropy Classifier Based Text Mining Tool for Transporter-Substrate Identification with Semistructured Text

**DOI:** 10.1155/2015/254838

**Published:** 2015-10-01

**Authors:** Min Zhao, Yanming Chen, Dacheng Qu, Hong Qu

**Affiliations:** ^1^School of Engineering, Faculty of Science, Health, Education and Engineering, University of the Sunshine Coast, Maroochydore DC, QLD 4558, Australia; ^2^School of Computer Science & Technology, Beijing Institute of Technology, Beijing 100081, China; ^3^Center for Bioinformatics, State Key Laboratory of Protein and Plant Gene Research, College of Life Sciences, Peking University, Beijing 100871, China

## Abstract

The substrates of a transporter are not only useful for inferring function of the transporter, but also important to discover compound-compound interaction and to reconstruct metabolic pathway. Though plenty of data has been accumulated with the developing of new technologies such as *in vitro* transporter assays, the search for substrates of transporters is far from complete. In this article, we introduce METSP, a maximum-entropy classifier devoted to retrieve transporter-substrate pairs (TSPs) from semistructured text. Based on the high quality annotation from UniProt, METSP achieves high precision and recall in cross-validation experiments. When METSP is applied to 182,829 human transporter annotation sentences in UniProt, it identifies 3942 sentences with transporter and compound information. Finally, 1547 confidential human TSPs are identified for further manual curation, among which 58.37% pairs with novel substrates not annotated in public transporter databases. METSP is the first efficient tool to extract TSPs from semistructured annotation text in UniProt. This tool can help to determine the precise substrates and drugs of transporters, thus facilitating drug-target prediction, metabolic network reconstruction, and literature classification.

## 1. Introduction

Metabolic network analysis and reconstruction have become increasingly prevalent with diverse sources from functional genomics experiments. Plenty of bioinformatic tools were developed to generate high quality metabolic models on metabolic enzyme and pathway annotation for different organisms [1]. However, transporters, as a large group of proteins to exchange metabolite, drug, toxin, and environmental signal between cells [2, 3], are often ignored in metabolic analysis and reconstruction [4, 5]. One possible challenge is the inherently difficult integration of enzyme metabolic system with transporting system. Our previous study built connections between all metabolic enzymes and transporters in human via their shared substrates [6]. Although it is far from complete, it provides a practical solution to link transporter and metabolic enzyme in genome scale. To get more comprehensive metabolic reconstruction with both metabolic enzyme and transporter, more accurate information on substrates of transporters is needed.

Although many transporter databases were developed to store and classify all reported transporters such as TCDB [7] and TransportDB [2], most of them focus on collection of transporters. TCDB contains comprehensive transporter families according to their transporter classification system. And TransportDB includes more comprehensive annotations for transporters from 365 organisms. The important substrate information for transporters is not systematically collected and classified. To get accurate relations between transporters and their substrates, we manually curated transporter-substrate information from UniProt function annotation (TSDB: http://TSdb.cbi.pku.edu.cn/) [6]. Though manual curation gains reliable substrate data for transporters, it is also too time consuming to keep abreast of the growth of transporter-substrates information from published literature and UniProt annotation.

In postgenomic era, biomedical data and literature are growing in an exponential way. To date, the PubMed, the most comprehensive biomedical literature repository, includes over 21 million abstracts [8]. And as the most popular protein database, UniProt [9] records over 1.5 million proteins with various annotations currently. Given the explosion of free text based electronically available publications, increasing strategies in text mining and information extraction were applied to extract biomedical knowledge. Many high efficient tools were developed for recognition of named entity such as protein and gene names in free text, identification of subcellular localization of proteins, extraction of interaction of proteins, and association of genes according to functional concepts such as gene ontology and MeSH terms [10–16].

Here we constructed a standalone tool METSP, a maximum-entropy text mining classifier, to extract TSPs from semistructured text in UniProt protein annotation. As most comprehensive and confidential protein databases, UniProt provides us with more reliable substrate data. In addition, its semistructured text for protein annotation makes information extraction more reliable than those from free text. We believe that it will be useful to help the metabolic network reconstruction [17–19] and disease network analyses [20, 21] by incorporating the transporter-substrate information.

## 2. Results

**Figure 1:**
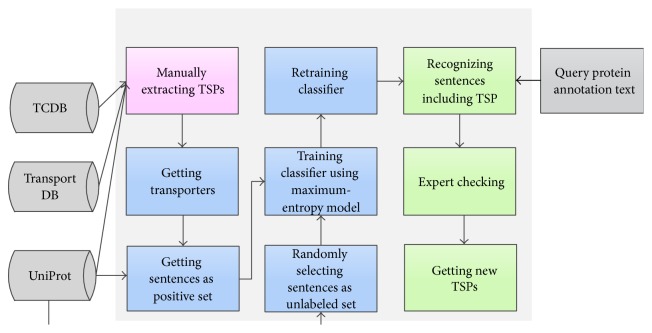
Workflow of design and function of METSP. Step I (highlighted in pink): explicit TSPs were manually collected from UniProt, TCDB, and TransportDB databases. Step II (in blue): the UniProt annotation text of proteins in explicit TSPs and in randomly selecting protein set was processed to get positive and unlabeled sentence training sets. The maximum-entropy model was used to train and retain the classifier. Step III (in green): the classifier was used to recognize TSPs from query protein annotation text. The new TSPs were obtained by further experts checking.

The main goal of METSP is to identify and extract sentences with transporter-substrate information from UniProt entries. Thus, METSP focuses on two tasks: The first task is to extract semistructured annotation sentences of transporters in UniProt and then map transporter and compound names in sentences into standardized protein identifiers in UniProt and compound identifiers in KEGG LIGAND database [22]. The second addresses the judgement of transporter-substrate relationship accurately. For example, when we aim to figure out what substrates are transported by SLC12A8 (Solute carrier family 12 member 8, UniProt entry with AC as A0AV02), first the sentences containing transporter and compound information are extracted by METSP. METSP then extracts two compound names potassium and chloride from functional annotation sentences of SLC12A8 (A0AV02) and maps potassium and chloride to compound identifiers C00238 and C00698, respectively. Next, METSP classifier assigns the correct substrates for the transporter based on the two extracted compounds. In this example, potassium (C00238) curated by human expert is the real substrate of SLC12A8 (A0AV02).  Figure 1 shows a typical workflow for METSP.

### 2.1. Collecting Reliable TSPs from Public Databases

To obtain a comprehensive and reliable TSP dataset, we first retrieved all the known transporter and substrate information from UniProt and the other two popular transporter databases TCDB and TransportDB. Then we mapped substrate names to compound IDs in KEGG LIGAND database and manually checked all the transporter names and their corresponding substrate names and compound IDs one by one. Finally, we compiled 6955 reliable TSPs from all the above three databases (Additional file 1, in Supplementary Material available online at http://dx.doi.org/10.1155/2015/254838, the 6955 TSPs were manually collected from UniProt, TransportDB, and TCDB). As shown in Table 1, the number of extracted transporters, substrates, and TSPs in different data sources is different. Though TransportDB has the largest TSPs, the collected substrates are less than those from UniProt.

### 2.2. Constructing Training Set for TSP Prediction

The training sentences expressing transporting relationship of transporter and substrate are included in protein semistructured annotation text of UniProt. To obtain a reliable training dataset, we first retrieved all text of transporter entries from UniProt based on the accession numbers of transporters in our curated reliable TSPs (Additional file 1). As only annotations from protein name (DE), function annotation (CC), and gene ontology (DR) fields were informative to extract substrate information, we deleted annotations in other fields for training classifier to reduce the size of preprocessing data and to drop negative influence generated by irrelevant fields. Previous study indicated that better performance can be achieved by using sentences as input instead of sentence pairs [23]. Therefore, we split functional annotations into sentences and regarded them as elements of training set, as well as the text related with protein name and gene ontology fields. If a sentence belonging to annotations of a transporter contained one or more substrates which were transported by this transporter and had corresponding compound IDs in KEGG LIGAND database, we considered that this sentence was a positive instance. We collected 13,212 positive instances from the annotations of 5,042 transporters (Additional file 2, the training set includes 41,332 instances in the format of “label + accession number + field flag + a sentence”).

It was expensive to collect negative dataset manually, so we collected unlabeled instances as negative data and combined positive instances to make up training set. There were 525,997 reviewed protein accession numbers in UniProt (checked on May 4, 2011), from which 5,042 protein accession numbers were chosen randomly. Then 28,120 sentences were extracted as unlabeled instances from the annotations of selected proteins which are not overlapping with any proteins in our training set (Additional file 2). Previous study indicated that training set consisting of positive and completely randomly chosen instances could get similar classification with training set consisting of positive and negative instances [24]. Therefore, we used positive and unlabeled datasets to train classifier. However, the performance of the classifier must be influenced by positive instances that existed in unlabeled set. For excluding positive sentences that might exist in unlabeled set, a method of iterating relabelled training set was adopted to make unlabeled instances as similar to negative instances as possible.

In this study, all marked compound names must have compound IDs in KEGG LIGAND database, with a hypothesis that a compound name appearing in sentences could be mapped to an identifier in KEGG LIGAND database and be unique. Besides, compound names in UniProt should be general and in KEGG LIGAND database should be comprehensive, for example, UniProt names compound sucrose “sucrose,” and the other names it “cane sugar,” “saccharose,” and “1-alpha-D-Glucopyranosyl-2-beta-D-fructofuranoside.” For these reasons, we fast tagged compound names in sentences using Trie data structure [25].

### 2.3. The Tenfold Cross-Validation Strategy to Evaluate the Prediction Results

To assess how the predicted results are robust to any independent data set, we conducted a tenfold cross-validation. The collected training set (13,212 positive instances from the annotations of 5,042 transporters) was randomly partitioned into 10 equal sized subsets. Of the 10 subsets, a single subset was used as the validation data to test the output from our statistical model. The remaining 9 sets were used as training data for each run. This cross-validation process was repeated 10 times. As each subset was used exactly once as the validation data, we harvested 10 results from calculation, which were combined by average to generate the final rotation estimation. Our 10-fold cross-validation performed better than that repeated random subsampling because all the collected 13,212 positive instances were used for training and validation. In addition, each observation was used for validation exactly once. For each cross-validation process, there were 1,320 positive instances used as testing set roughly. We calculated the true positive by counting how many prediction results from testing set were the exact same as their original labels (positive or negative, as shown in Additional file 2).

### 2.4. The Comparison of Maximum-Entropy Classifier and Naïve Bayes (NB) Model

We obtained features from sentences with the idea of bag of words [26] that ignored the positions of words in the sentences. In addition, words with the same stem usually have a similar meaning; for example, transporter, transporting, and transported have the same stem “transport.” So we used Porter algorithm [27] to combine all the words with the same stem into a single term to obtain general features. Stop words were removed from sentences by the filter in Mallet Toolkit [28]. The maximum-entropy (ME) model and Naïve Bayes (NB) model in Mallet toolkit software package [28] were applied to construct the classifiers. Given a known probability distribution of a fact dataset, ME model that is consistent with the distribution of this dataset is constructed with even probability distributions of unknown facts [29–31]. NB model assumes that the occurrence of a given word in a sentence is independent of all other words in the sentence [32].

**Figure 2:**
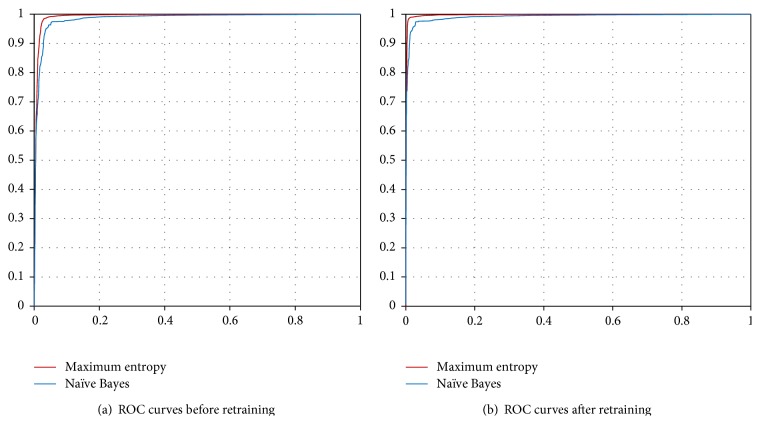
The performance comparison of ME and NB classifiers. ROC curves of maximum-entropy classifier and Naïve Bayes classifier on the original (a) and relabelled datasets (b).

Since there were positive instances in unlabeled dataset, the method of iteration of tagging false negative instances in unlabeled dataset was adopted to reduce their negative effect on the classifier. In each iteration, we rescued the sentences that expressed real transporting relationship in unlabeled dataset, added them into positive set to construct new training dataset, and then obtained the classification results from tenfold cross-validation experiments on the new training sets. The precision and recall [30] of our classifiers were improved step by step until they met steady status after the fourth iteration (Table 2). As unlabeled dataset is close to negative dataset after 4 iterations, the final training set from the last iteration was used to build our classifier to extract TSPs from protein annotation in UniProt. The performance of two classifiers was shown in Figure 2: When original dataset was applied, the accuracy scores of two classifiers were 0.9749 (ME) and 0.9568 (NB), respectively. When retraining dataset was applied, the accuracy scores of two classifiers were 0.9891 (ME) and 0.9698 (NB), respectively. The performance of ME classifier was better than that of NB classifier; thus we adopt ME classifier as the main component of METSP.

### 2.5. The Precomputed Results for All the Human Proteins in UniProt Identified Thousands of Novel Transporter-Substrate Relationships

It is promising to extract specific transporter and substrate information from the wealth of biomedical knowledge in free text due to the increasing number of stored literature in databases such as PubMed and UniProt. To evaluate the results from METSP, we applied our tool on the 23,204 human reviewed protein annotations in UniProt (23 August 2011), which contained 182,829 sentences as input of classifier; 3942 TSPs were extracted (Additional file 3, the 3942 human TSPs were extracted by METSP).

**Figure 3:**
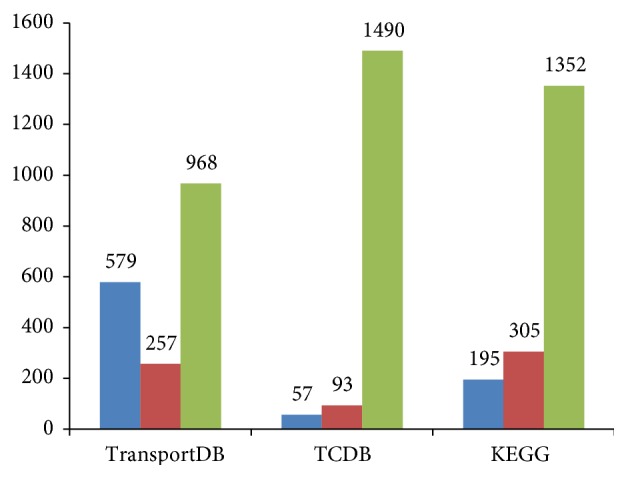
Comparison of TSP data. Comparing human TSPs extracted by METSP with that in three existing transporter-substrate databases (TCDB, TransportDB, and KEGG database). Blue bars represent the number of TSPs extracted by METSP and in the three databases; red bars represent the number of TSPs that were not extracted by METSP but in the three databases; green bars represent the number of TSPs extracted by METSP but not in the three databases.

To identify novel transporter-substrate relationships, we compared our predicted human TSPs with the human TSP data in TCDB, TransportDB, and KEGG (Figure 3). The total percentage of new TSPs is 58.37% against total TSPs in all three other databases. The amount of our TSPs extracted by METSP was more comprehensive than those in other transporter databases. First, it could be explained by the inaccuracy substrate description in other related databases. Many substrate names in these databases are too general such as “organic anion” in TransportDB. As the terms such as “organic anion” are too general and common, it is difficult to map these terms to compound IDs in KEGG LIGAND database. In total, there are 40 human transporters for “organic anion,” 22 for “organic cation,” 17 for “monocarboxylate,” and 45 for “amino acid” in TransportDB. However, the “organic anion” was specified to triiodothyronine (C02465) and sulfobromophthalein (C11363) in our result. On the other hand, the content of transporters in these databases is not comprehensive as these databases are constructed for specific purpose. For instance, TCDB only includes representative transporters; thus it does not collect some transporters with similar functions in different organisms [33]. The UniProt entry P41130 (maltose-binding periplasmic protein) from photorhabdus luminescens is not included in TCDB because it is similar to transporter P0AEX9 (maltose-binding periplasmic protein) from* E. coli*. In summary, our approach utilizes not only accurate substrate information but also more comprehensive transporter data in UniProt. Thus our tool is able to harvest more TSPs from UniProt compared to other transporter databases.

The comprehensive TSPs extracted by METSP are not only able to facilitate access to transporter and substrate researches, but also useful to link transporters with metabolic pathways in KEGG PATHWAY database. For instance, a novel TSP (Q4U2R8 and C00954) summarized by METSP has never been recorded in any existent transporter database such as TCDB and TransportDB. TCDB does not include the entry Q4U2R8. In addition, TransportDB and KEGG databases only annotate substrate with general words as “organic anion” for entry Q4U2R8 (named “NP_004781” in TransportDB, “hsa: 9356” in KEGG), which is useless to assign a compound ID in KEGG LIGAND database for “organic anion” term. However the function annotations of Q4U2R8 in UniProt contain the precise transporting substrate as “indoleacetate” belonging to “organic anion.” Based on its extracted substrate name in the protein annotation, Q4U2R8 could be easier to be associated with four KEGG pathways including “ko00380,” “map01070,” “ko01100,” and “ko04075.” This link of transporter Q4U2R8 to the four pathways may give more accurate clues for this transporter on its substrate flux balance. In total, there are 136 compound IDs in our curated human TSPs, which are not included in other databases and in which 75 are annotated to 88 metabolic pathways. Therefore, our METSP is powerful to discover potential novel linkage between transporters and metabolic pathways.

## 3. Programming

METSP is implemented by using JAVA language and consists of data downloadable module, preprocessing module, ME classifier module, compound name mapping module, and assisting manual validation module. The three main features of METSP are

1. extracting accurate TSPs using the UniProt accession numbers as input;
2. extracting accurate TSPs from local semistructured text, which is similar to the format of text in UniProt, with transporter and substrate information;
3. a command line-based running for user to process big data without minimum deployment.

## 4. Usage

METSP was packed for downloading at http://tsdb.cbi.pku.edu.cn/metsp.cgi. By unzipping the packed tar file, user can find the executable jar file in the extracted directory. User can configure the running environment for better performance. The runnable classifier is included in the folder “classifier.” The folder “compoundNameData” contains the file “compoundName.txt” to construct Tire tree. User can add more compound names to this compound name file for better compound name match. The known TSPs collected from public databases are included in folder “result,” which are useful to evaluate the performance.

METSP provides a few parameters for user implementing the running setting. The threshold of classifier can be set by the parameter “*t*” that is in positive correlation with the precision of METSP and is in negative correlation with the recall of METSP. It can balance the weight of scale and validity of TSPs. The default value of “*t*” is 0.5. User can obtain better precision of the classifier by setting a threshold of parameter *t*. The parameter “*n*” is used to set whether to remove known TSPs from prediction result; the default value is not. The parameter “*f*” is used to obtain input data with the full records in UniProt format. The parameter “*d*” denotes a list of UniProt accession numbers as input. METSP can retrieve semistructured annotation sentences from UniProt entries automatically. The default input is from parameter “*f*” if user does not set parameter “*d*.” The parameter “*c*” is used to set whether to export the scores of instances in output; the default is yes. The parameter “*e*” is used to set whether to extract TSP data in output; the default is yes. An example of input file “input.txt” in UniProt format is also stored in METSP. The default output is a PDF file containing all TSPs with prediction scores from the classifier. The example of the command to run the example file is as follows:

“java -jar METSP.jar -t 0.5 -f input.txt result.pdf”.

## 5. Discussion

In cellular metabolism, transporters are a class of molecules to control metabolite homeostasis and drug delivery. For transporter studies, it is crucial to identify their substrates precisely. In this study, we present a tool METSP focusing on the extraction of transporter and substrate knowledge. The resulting knowledge will be easy to convert into the formatted compound name from KEGG LIGAND database. The multitude of possible applications of METSP makes it a complementary approach to more comprehensive metabolic reconnection of metabolic enzymes and transporters. It should be noted that the organism-specific substrates of transporters are still scarce. With the rapidly expanding transporter and substrate data, the ability to predict transporter and substrate information based on the data from phylogenetic neighbours may be of great help. We believe that the combination of our METSP and sequence alignment tools such as BLAST can achieve a more comprehensive transporter and substrate reconstructions for many uncurated metabolic networks.

Our TSPs were mainly collected from only TCDB, TransportDB, and UniProt. As many reported TSPs in the literature still lack annotation, in the future, we will focus on transplant of our METSP to an abstract-based text mining system. To achieve this, it is necessary to gain reliable protein literature mapping relations. Starting from the mapped protein literature relations, more accurate and comprehensive TSPs will be collected based on our improved METSP system. In general, the next version of METSP will be focused on the extraction substrate information from free text to support growing free full text literature.

## 6. Conclusion

We present METSP, the first text mining tool to extract transporter and substrate information from the semistructured text in UniProt annotation. Using maximum-entropy model, METSP achieves high precision and recall in cross-validation experiments for identification of TSPs. We believe that METSP can be widely applied to help elucidate the relationship between transporter and its substrates including clinical drugs. This tool could have profound implications for the further tool development of the semistructured text mining by focusing on other high quality UniProt annotations such as disease and tissue specificity. The METSP is flexible and freely available at http://tsdb.cbi.pku.edu.cn/metsp.cgi.

## Supplementary Material

In Additional file 1, the 6955 TSPs were manually collected from UniProt, TransportDB, and TCDB. In Additional file 2, the training set includes 41,332 instances in the format of “label + accession number + field flag + a sentence.” In Additional file 3, the 3942 human TSPs were extracted by METSP.

## Figures and Tables

**Table 1:** Summary of reliable TSPs from UniProt, TCDB, and TransportDB.

	UniProt	TCDB	TransportDB	Sum from formula (*∗*)
TSPs	35586	2641	86726	6955
Transporters	25056	1501	57070	5042
Substrates	528	229	351	275

Note: (*∗*): *R* = {*r* | *r* ∈ *R* ^UniProt^∩*R* ^TransportDB^ ∪ *R* ^TCDB^}, where *R* ^UniProt^, *R* ^TCDB^, and *R* ^TransportDB^ refer to all TSPs collected from UniProt, TCDB, and TransportDB, respectively.

**Table 2:** The precision, recall of ME classifier, and the number of “false” negative instances that were actually positive instances in four iterations.

	Iteration 1	Iteration 2	Iteration 3	Iteration 4
Precision	94.93%	98.17%	98.50%	98.54%
Recall	97.52%	97.95%	98.00%	98.02%
FP ratio^*∗*^	546/688	70/250	24/205	16/201

Note: ^*∗*^FP ratio represents the number of “false” negative instances that were actually positive instances.
