# Supplementary material for: METSP: A Maximum-Entropy Classifier Based Text Mining Tool for Transporter-Substrate Identification with Semistructured Text
Source: Biomed Res Int. 2015 Oct 1;2015:254838. doi: 10.1155/2015/254838 (PMC4606149; doi:10.1155/2015/254838)
Supplement: Supplementary file 1 — In Additional file 1, the 6955 TSPs were manually collected from UniProt, TransportDB, and TCDB. In Additional file 2, the training set includes 41,332 instances in the format of “label + accession number + field flag + a sentence.” In Additional file 3, the 3942 human TSPs were extracted by METSP. [file 254838.f1.zip › 254838.f1/254838-1.pdf]

Additional file 1 – The 6955 TSPs were manually collected from UniProt, TransportDB and TCDB.

CompoundID_KEGG	UniProt_AC
C00001	O14520
C00001	O22588
C00001	O28846
C00001	O43315
C00001	O54794
C00001	O68874
C00001	O82316
C00001	O82598
C00001	O94778
C00001	P0CD90
C00001	P0CD91
C00001	P25818
C00001	P26587
C00001	P29972
C00001	P30301
C00001	P30302
C00001	P41181
C00001	P43286
C00001	P43287
C00001	P51180
C00001	P55064
C00001	P55087
C00001	P55088
C00001	P56402
C00001	P60925
C00001	P61837
C00001	P73809
C00001	P93004
C00001	Q02013
C00001	Q06611
C00001	Q08733
C00001	Q0DK16
C00001	Q0IWF3
C00001	Q0JPT5
C00001	Q10M80
C00001	Q13520
C00001	Q39196
C00001	Q40746
C00001	Q41951
C00001	Q41963
C00001	Q41975
C00001	Q5VR89
C00001	Q5Z6F0
C00001	Q5Z9E2
C00001	Q651D5

C00001	Q67WJ8
C00001	Q6EU94
C00001	Q6K215
C00001	Q7EYH7
C00001	Q7MIV9
C00001	Q7N5C1
C00001	Q7NNP3
C00001	Q7W917
C00001	Q7WKG2
C00001	Q7XKI5
C00001	Q7XLR1
C00001	Q7XSQ9
C00001	Q7XU31
C00001	Q7XUA6
C00001	Q7Y1E6
C00001	Q84S07
C00001	Q87MQ5
C00001	Q89EG9
C00001	Q8C4A0
C00001	Q8DB17
C00001	Q8EHC1
C00001	Q8FY85
C00001	Q8GRT8
C00001	Q8H5N9
C00001	Q8LAA6
C00001	Q8LAI1
C00001	Q8LFP7
C00001	Q8R2N1
C00001	Q8SRK2
C00001	Q8VZW1
C00001	Q8W036
C00001	Q8W037
C00001	Q92482
C00001	Q92NM3
C00001	Q92ZW9
C00001	Q96PS8
C00001	Q9ASI1
C00001	Q9C6T0
C00001	Q9FF53
C00001	Q9FIZ9
C00001	Q9FK43
C00001	Q9FWV6
C00001	Q9HWZ3
C00001	Q9L772
C00001	Q9LWR0
C00001	Q9LWR2
C00001	Q9M1K3
C00001	Q9M8W5

C00001	Q9SAI4
C00001	Q9STX9
C00001	Q9SV31
C00001	Q9SV84
C00001	Q9SXF8
C00001	Q9V5Z7
C00001	Q9WTY4
C00001	Q9ZV07
C00001	Q9ZVX8
C00002	B0RZB7
C00002	O05962
C00002	O84068
C00002	O97470
C00002	P02723
C00002	P04710
C00002	P05141
C00002	P12235
C00002	P12236
C00002	P18238
C00002	P18239
C00002	P19568
C00002	P31167
C00002	P40941
C00002	P48962
C00002	P51881
C00002	P92935
C00002	Q09188
C00002	Q39002
C00002	Q3V132
C00002	Q8SRA2
C00002	Q8SUF9
C00002	Q8SUG0
C00002	Q8SUG7
C00002	Q92GI5
C00002	Q92HP9
C00002	Q92HV4
C00002	Q92JI6
C00002	Q9H0C2
C00002	Q9PJP6
C00002	Q9PKX5
C00002	Q9S6V3
C00002	Q9Z7U0
C00002	Q9Z8J2
C00002	Q9ZD47
C00002	Q9ZD67
C00002	Q9ZDF2
C00008	B0RZB7
C00008	O05962

C00008	O84068
C00008	O97470
C00008	P02723
C00008	P04710
C00008	P05141
C00008	P12235
C00008	P12236
C00008	P18238
C00008	P18239
C00008	P19568
C00008	P31167
C00008	P40941
C00008	P48962
C00008	P51881
C00008	P92935
C00008	Q09188
C00008	Q39002
C00008	Q3V132
C00008	Q8SRA2
C00008	Q8SUF9
C00008	Q8SUG0
C00008	Q8SUG7
C00008	Q92GI5
C00008	Q92HP9
C00008	Q92HV4
C00008	Q92JI6
C00008	Q9H0C2
C00008	Q9PJP6
C00008	Q9PKX5
C00008	Q9S6V3
C00008	Q9Z7U0
C00008	Q9Z8J2
C00008	Q9ZD47
C00008	Q9ZD67
C00008	Q9ZDF2
C00009	A0R4C0
C00009	O00476
C00009	O00624
C00009	O05870
C00009	O06411
C00009	O27764
C00009	O28912
C00009	O30499
C00009	O34436
C00009	O34734
C00009	O48639
C00009	O51236
C00009	O53832

C00009	O67154
C00009	O82390
C00009	O86345
C00009	O95436
C00009	P02932
C00009	P05695
C00009	P07654
C00009	P08194
C00009	P09836
C00009	P0A2V8
C00009	P0A5Y2
C00009	P0A5Y3
C00009	P0A626
C00009	P0A627
C00009	P0A628
C00009	P0A629
C00009	P0A630
C00009	P0A631
C00009	P0AAH0
C00009	P0AAH2
C00009	P0AAH3
C00009	P0AFJ9
C00009	P0AG82
C00009	P0AG83
C00009	P0AGC0
C00009	P0AGC1
C00009	P0AGC2
C00009	P0AGH8
C00009	P0AGH9
C00009	P0AGI0
C00009	P0C2M5
C00009	P0CAT6
C00009	P12234
C00009	P12681
C00009	P15710
C00009	P15712
C00009	P16677
C00009	P16682
C00009	P16683
C00009	P25297
C00009	P25360
C00009	P38361
C00009	P40035
C00009	P45190
C00009	P45191
C00009	P45192
C00009	P46339
C00009	P46340

C00009	P46341
C00009	P46342
C00009	P47650
C00009	P47651
C00009	P58655
C00009	P59950
C00009	P63361
C00009	P63362
C00009	P63363
C00009	P63364
C00009	P63365
C00009	P63366
C00009	P63367
C00009	P63368
C00009	P63369
C00009	P63370
C00009	P63371
C00009	P63372
C00009	P63373
C00009	P63375
C00009	P63378
C00009	P69879
C00009	P69881
C00009	P73788
C00009	P75185
C00009	P75186
C00009	P95302
C00009	Q00325
C00009	Q01395
C00009	Q06495
C00009	Q06496
C00009	Q08357
C00009	Q084V3
C00009	Q0A9K1
C00009	Q0ASG3
C00009	Q0BTP1
C00009	Q0C0L5
C00009	Q0IAC3
C00009	Q0IZQ3
C00009	Q0SNU4
C00009	Q0VL18
C00009	Q11NG0
C00009	Q14916
C00009	Q180A5
C00009	Q1LLB5
C00009	Q1MIN0
C00009	Q1MLW4
C00009	Q2FTF8

C00009	Q2KCV5
C00009	Q2LTG0
C00009	Q2QWW7
C00009	Q2RWU0
C00009	Q2SS06
C00009	Q30PC0
C00009	Q31I88
C00009	Q38954
C00009	Q3A6U0
C00009	Q3AAA4
C00009	Q3AJS9
C00009	Q3ATA4
C00009	Q3AXX4
C00009	Q3B3H7
C00009	Q3ILC5
C00009	Q3IM36
C00009	Q3IS07
C00009	Q3J8J2
C00009	Q3JDJ6
C00009	Q3SVB5
C00009	Q3ZA58
C00009	Q3ZWN4
C00009	Q43650
C00009	Q47A37
C00009	Q47Y12
C00009	Q494P0
C00009	Q4FLF6
C00009	Q4K3K9
C00009	Q50046
C00009	Q50097
C00009	Q50098
C00009	Q50173
C00009	Q51546
C00009	Q53P54
C00009	Q54BF6
C00009	Q55195
C00009	Q55196
C00009	Q57AC2
C00009	Q58418
C00009	Q58419
C00009	Q58420
C00009	Q58421
C00009	Q5FM17
C00009	Q5FM18
C00009	Q5FT15
C00009	Q5HAV5
C00009	Q5HG31
C00009	Q5HPF2

C00009	Q5HPF5
C00009	Q5JEP9
C00009	Q5KX47
C00009	Q5LS19
C00009	Q5LZU2
C00009	Q5LZU3
C00009	Q5N1G5
C00009	Q5NCM1
C00009	Q5NNN6
C00009	Q5P1F3
C00009	Q5QVB0
C00009	Q5SZA1
C00009	Q5V0G3
C00009	Q5V225
C00009	Q5Z293
C00009	Q609Z8
C00009	Q61609
C00009	Q61983
C00009	Q62L74
C00009	Q63V79
C00009	Q64SM5
C00009	Q652N5
C00009	Q65HB9
C00009	Q65HC0
C00009	Q662E6
C00009	Q663R5
C00009	Q668E1
C00009	Q67RE7
C00009	Q67RG2
C00009	Q69T94
C00009	Q6AAX3
C00009	Q6ADG4
C00009	Q6AM16
C00009	Q6CYN3
C00009	Q6D2D5
C00009	Q6F1N1
C00009	Q6FCW7
C00009	Q6G0L7
C00009	Q6G4T6
C00009	Q6GQS1
C00009	Q6HDP8
C00009	Q6KIS8
C00009	Q6LSC4
C00009	Q6LU82
C00009	Q6LY93
C00009	Q6MMH0
C00009	Q6MTC1
C00009	Q6N0I5

C00009	Q6NUK1
C00009	Q71WT2
C00009	Q71WT3
C00009	Q72D46
C00009	Q72GX5
C00009	Q743D1
C00009	Q74E68
C00009	Q74KF8
C00009	Q74KF9
C00009	Q7A5Q2
C00009	Q7M9G3
C00009	Q7MFE8
C00009	Q7MNI7
C00009	Q7N9U4
C00009	Q7NC40
C00009	Q7NNG3
C00009	Q7NPP4
C00009	Q7U0Z9
C00009	Q7U172
C00009	Q7U6R4
C00009	Q7UP21
C00009	Q7V1X3
C00009	Q7V7P0
C00009	Q7VCZ3
C00009	Q7VZ66
C00009	Q7W8Q6
C00009	Q7X7V2
C00009	Q7XDZ7
C00009	Q7XRH8
C00009	Q818I7
C00009	Q81LW6
C00009	Q82G23
C00009	Q82VR4
C00009	Q834B3
C00009	Q834B4
C00009	Q83GE8
C00009	Q83HT1
C00009	Q87C88
C00009	Q87C89
C00009	Q87C90
C00009	Q87C91
C00009	Q87G59
C00009	Q87S48
C00009	Q87U31
C00009	Q880A6
C00009	Q88C57
C00009	Q88JJ0
C00009	Q88YK7

C00009	Q88YK8
C00009	Q895Y0
C00009	Q89A24
C00009	Q89VF2
C00009	Q8A853
C00009	Q8BMD8
C00009	Q8CP98
C00009	Q8CPA1
C00009	Q8CVC9
C00009	Q8DGZ3
C00009	Q8DU23
C00009	Q8DU24
C00009	Q8DZV4
C00009	Q8E310
C00009	Q8E5K2
C00009	Q8E9I8
C00009	Q8EG82
C00009	Q8ELT4
C00009	Q8EUJ1
C00009	Q8FMN9
C00009	Q8G7F4
C00009	Q8GSD9
C00009	Q8GYF4
C00009	Q8H074
C00009	Q8H6G7
C00009	Q8H6G8
C00009	Q8H6G9
C00009	Q8H6H0
C00009	Q8H6H2
C00009	Q8H6H4
C00009	Q8K903
C00009	Q8KDZ5
C00009	Q8N130
C00009	Q8NMK1
C00009	Q8PM59
C00009	Q8PVF6
C00009	Q8R9I2
C00009	Q8RCU0
C00009	Q8TSA8
C00009	Q8TUR7
C00009	Q8U242
C00009	Q8UI76
C00009	Q8VEM8
C00009	Q8VYM2
C00009	Q8WUM9
C00009	Q8XMP8
C00009	Q8XZ72
C00009	Q8Y4E9

C00009	Q8YNJ3
C00009	Q8YYE2
C00009	Q8YYE3
C00009	Q8Z9T1
C00009	Q8ZCX5
C00009	Q8ZX91
C00009	Q927Z7
C00009	Q92SA1
C00009	Q94DB8
C00009	Q96243
C00009	Q96303
C00009	Q97IE0
C00009	Q97Q31
C00009	Q97Q34
C00009	Q97ZT9
C00009	Q98FL2
C00009	Q98FL3
C00009	Q98FL4
C00009	Q98FL5
C00009	Q9BV35
C00009	Q9CBE5
C00009	Q9CEW7
C00009	Q9CEW8
C00009	Q9CNJ4
C00009	Q9CNJ5
C00009	Q9CNJ6
C00009	Q9CNJ7
C00009	Q9DBP0
C00009	Q9HML8
C00009	Q9HS13
C00009	Q9K8L5
C00009	Q9KN92
C00009	Q9KU04
C00009	Q9KZW2
C00009	Q9PBK0
C00009	Q9PBK1
C00009	Q9PBK2
C00009	Q9PBK3
C00009	Q9PHQ1
C00009	Q9PQU3
C00009	Q9S735
C00009	Q9SDI4
C00009	Q9SYQ1
C00009	Q9UZU7
C00009	Q9V7S5
C00009	Q9X0Y8
C00009	Q9Y2C5
C00009	Q9YG51

C00009	Q9Z7N9
C00009	Q9ZWT3
C00010	O00400
C00011	Q02094
C00011	Q8I0L6
C00011	Q9NIV0
C00012	P08183
C00012	P0A2J3
C00012	P0A2J4
C00012	P0A2J5
C00012	P0A2J6
C00012	P0AAH6
C00012	P0AAH7
C00012	P0AAI0
C00012	P0AE16
C00012	P0AGH4
C00012	P0AGH7
C00012	P36371
C00012	P36634
C00012	P36636
C00012	P36638
C00012	P43505
C00012	P45285
C00012	P45286
C00012	P45287
C00012	P45288
C00012	P45289
C00012	Q03518
C00012	Q03519
C00012	Q0WML0
C00012	Q51006
C00012	Q51073
C00012	Q8IY34
C00012	Q8N697
C00012	Q8RY46
C00012	Q9JJ59
C00012	Q9LFB8
C00012	Q9M390
C00012	Q9NP78
C00012	Q9P380
C00013	P32977
C00016	P40464
C00019	P38921
C00019	Q08986
C00019	Q5U680
C00019	Q92JG1
C00019	Q9ZE70
C00022	P41036

C00022	P53985
C00023	A3ZKG8
C00023	O31567
C00023	O31568
C00023	O34348
C00023	O34510
C00023	O34832
C00023	O34933
C00023	O81850
C00023	O87656
C00023	P05825
C00023	P06971
C00023	P0A0Y4
C00023	P11461
C00023	P13036
C00023	P16869
C00023	P17315
C00023	P35755
C00023	P42512
C00023	P44513
C00023	P71338
C00023	P72827
C00023	P73182
C00023	P74884
C00023	P75780
C00023	Q0I2Z4
C00023	Q2K8C8
C00023	Q38856
C00023	Q4KC87
C00023	Q4W575
C00023	Q55835
C00023	Q578K3
C00023	Q5FA19
C00023	Q5HGV1
C00023	Q5N0R0
C00023	Q65S66
C00023	Q668Q3
C00023	Q6D2F6
C00023	Q6D734
C00023	Q6H3Z3
C00023	Q6H3Z6
C00023	Q6LKD4
C00023	Q7AH43
C00023	Q7N8B9
C00023	Q82VI7
C00023	Q8DLH9
C00023	Q8FVV5
C00023	Q8LE59

C00023	Q8R0Z5
C00023	Q8RGC8
C00023	Q8U6M1
C00023	Q8YCG3
C00023	Q8ZBM3
C00023	Q8ZCM2
C00023	Q8ZRP7
C00023	Q92UV5
C00023	Q92WJ0
C00023	Q98G43
C00023	Q9CM80
C00023	Q9HFE1
C00023	Q9HXB2
C00023	Q9KLQ5
C00024	O00400
C00024	Q99J27
C00025	O00341
C00025	O30417
C00025	O35544
C00025	O75746
C00025	P0AAG4
C00025	P0AER7
C00025	P0AER9
C00025	P43003
C00025	P43004
C00025	P43006
C00025	P45240
C00025	P48242
C00025	P48243
C00025	P48244
C00025	P48245
C00025	P48664
C00025	P51906
C00025	P56564
C00025	P58229
C00025	Q10901
C00025	Q3TXX4
C00025	Q8BFU8
C00025	Q8BH59
C00025	Q8BLE7
C00025	Q8JZR4
C00025	Q8NDX2
C00025	Q8RQL4
C00025	Q8RQL5
C00025	Q8RQL6
C00025	Q8RQL7
C00025	Q91WN3
C00025	Q9CG19

C00025	Q9D6M3
C00025	Q9DB41
C00025	Q9H1K4
C00025	Q9H936
C00025	Q9P2U7
C00025	Q9P2U8
C00025	Q9QXX4
C00025	Q9UPY5
C00025	Q9WTR6
C00026	Q02978
C00026	Q54PY7
C00026	Q9CR62
C00031	A0PJK1
C00031	O07563
C00031	O25788
C00031	O74969
C00031	P0A283
C00031	P0A284
C00031	P0C105
C00031	P10870
C00031	P11166
C00031	P11168
C00031	P11169
C00031	P13181
C00031	P13866
C00031	P14142
C00031	P14246
C00031	P14672
C00031	P15729
C00031	P17809
C00031	P22732
C00031	P23586
C00031	P31639
C00031	P32037
C00031	P37439
C00031	P39816
C00031	P39924
C00031	P42833
C00031	P45338
C00031	P45618
C00031	P47315
C00031	P53403
C00031	P54862
C00031	P60857
C00031	P60944
C00031	P69785
C00031	P69788
C00031	P75569

C00031	P78831
C00031	Q05BQ3
C00031	Q12300
C00031	Q2M3M2
C00031	Q44840
C00031	Q5HD13
C00031	Q5HFZ9
C00031	Q5HJI3
C00031	Q5HL73
C00031	Q5HLZ2
C00031	Q5HPB5
C00031	Q5SWY8
C00031	Q5TF39
C00031	Q6PXP3
C00031	Q7A3G4
C00031	Q7A807
C00031	Q7CCJ4
C00031	Q814U8
C00031	Q816V0
C00031	Q81JY0
C00031	Q81KM7
C00031	Q89AG6
C00031	Q89B05
C00031	Q8BFW9
C00031	Q8C3K6
C00031	Q8CNF2
C00031	Q8CP79
C00031	Q8CZD5
C00031	Q8IRI6
C00031	Q8K0E3
C00031	Q8K9J0
C00031	Q8KA51
C00031	Q8TDB8
C00031	Q8VCV9
C00031	Q8VDT1
C00031	Q8VHD6
C00031	Q8WWX8
C00031	Q92253
C00031	Q923I7
C00031	Q94AZ2
C00031	Q9BYW1
C00031	Q9JIF3
C00031	Q9KCQ4
C00031	Q9NRM0
C00031	Q9NY64
C00031	Q9NY91
C00031	Q9P3U6
C00031	Q9UGQ3

C00032	P0ABM0
C00032	P0ABM3
C00032	P0ABM4
C00032	P30962
C00032	P30963
C00032	P30964
C00032	P31499
C00032	P45032
C00032	P45033
C00032	P45034
C00032	P45356
C00032	P61377
C00032	P61378
C00032	Q089M3
C00032	Q0A808
C00032	Q0AKQ2
C00032	Q0BV49
C00032	Q0BWF7
C00032	Q0I2G0
C00032	Q0VR80
C00032	Q1LFZ8
C00032	Q1LKR4
C00032	Q1MAL7
C00032	Q2K396
C00032	Q2NSG3
C00032	Q2RYF0
C00032	Q3JCI7
C00032	Q3SVV2
C00032	Q478L3
C00032	Q487I2
C00032	Q4KG27
C00032	Q56991
C00032	Q56992
C00032	Q56993
C00032	Q57FS7
C00032	Q5FQN4
C00032	Q5LR15
C00032	Q5NQX0
C00032	Q5P3L0
C00032	Q5PBP5
C00032	Q5QZP7
C00032	Q5ZX76
C00032	Q60AB3
C00032	Q65V02
C00032	Q668T8
C00032	Q6D606
C00032	Q6G0V9
C00032	Q6G529

C00032	Q6LTL7
C00032	Q6NDA6
C00032	Q6PEM8
C00032	Q73HX8
C00032	Q7MIR0
C00032	Q7VN12
C00032	Q7W736
C00032	Q7WIP8
C00032	Q82WC8
C00032	Q83KD5
C00032	Q83QV3
C00032	Q87BH8
C00032	Q87MK8
C00032	Q87Z03
C00032	Q88EX5
C00032	Q8EK40
C00032	Q8G358
C00032	Q8PK53
C00032	Q8UC12
C00032	Q8XE58
C00032	Q8ZD58
C00032	Q8ZKZ9
C00032	Q92G95
C00032	Q98EA4
C00032	Q9A298
C00032	Q9C8T1
C00032	Q9CPN2
C00032	Q9I3N5
C00032	Q9I3N6
C00032	Q9I3N7
C00032	Q9KQE3
C00032	Q9PAP0
C00032	Q9ZCF9
C00034	A0KG66
C00034	A1IQK4
C00034	A1IQK5
C00034	A6TC38
C00034	B5XVU3
C00034	O34610
C00034	O34946
C00034	O34966
C00034	O69443
C00034	P0A4G2
C00034	P0A771
C00034	P13586
C00034	P38778
C00034	P38925
C00034	P65544

C00034	P65545
C00034	P96593
C00034	P98194
C00034	Q03D26
C00034	Q03DK0
C00034	Q03TH5
C00034	Q0BW85
C00034	Q10177
C00034	Q12067
C00034	Q492G8
C00034	Q50103
C00034	Q55280
C00034	Q55281
C00034	Q55282
C00034	Q553K4
C00034	Q5FA63
C00034	Q5HGX9
C00034	Q5HQ64
C00034	Q668N2
C00034	Q71ZP6
C00034	Q80XR2
C00034	Q87EJ9
C00034	Q89K67
C00034	Q8CPM6
C00034	Q8GH68
C00034	Q8PKX1
C00034	Q8R7S2
C00034	Q8UEM1
C00034	Q8XSF6
C00034	Q8Y651
C00034	Q8Y652
C00034	Q8Y653
C00034	Q8Y773
C00034	Q8Z4X5
C00034	Q8ZCK2
C00034	Q8ZSB0
C00034	Q92BT1
C00034	Q97TN5
C00034	Q98I99
C00034	Q99385
C00034	Q99UZ7
C00034	Q9CFZ5
C00034	Q9KD29
C00034	Q9KD30
C00034	Q9KFG3
C00034	Q9KIJ3
C00034	Q9PEL3
C00034	Q9RPF2

C00034	Q9RPF3
C00034	Q9RPF4
C00034	Q9RTP8
C00036	P32089
C00036	P32332
C00036	Q55BF4
C00036	Q63965
C00036	Q9UTN1
C00037	O35633
C00037	P0AAE1
C00037	P28571
C00037	P48067
C00037	Q9H598
C00037	Q9Y345
C00038	A0KPH6
C00038	A1WXT0
C00038	A4SE48
C00038	O04089
C00038	O05703
C00038	O13918
C00038	O14863
C00038	O23039
C00038	O32219
C00038	O33704
C00038	O64738
C00038	O81123
C00038	O81850
C00038	O82643
C00038	O85143
C00038	O94639
C00038	P0A2U6
C00038	P0A4G4
C00038	P0A9X1
C00038	P0A9X3
C00038	P13511
C00038	P13512
C00038	P20021
C00038	P20107
C00038	P32798
C00038	P32804
C00038	P37617
C00038	P39172
C00038	P39832
C00038	P42360
C00038	P42361
C00038	P42364
C00038	P44526
C00038	P44691

C00038	P44692
C00038	P97441
C00038	Q02DK9
C00038	Q0A9E2
C00038	Q0I4A9
C00038	Q0VTB6
C00038	Q12436
C00038	Q13433
C00038	Q15043
C00038	Q1MEG2
C00038	Q2GFZ6
C00038	Q2GJA5
C00038	Q2K6Q4
C00038	Q2NTI7
C00038	Q31125
C00038	Q31I51
C00038	Q38856
C00038	Q3UVU3
C00038	Q3YSK9
C00038	Q4KKK4
C00038	Q504Y0
C00038	Q576K0
C00038	Q576K1
C00038	Q5GRS1
C00038	Q5HBR8
C00038	Q5LUR8
C00038	Q5PB72
C00038	Q60738
C00038	Q62720
C00038	Q62941
C00038	Q65UG3
C00038	Q66D85
C00038	Q6D4A8
C00038	Q6D7E5
C00038	Q6DBM8
C00038	Q6FFL0
C00038	Q6LTB1
C00038	Q6NXT4
C00038	Q6P5F6
C00038	Q6P5W5
C00038	Q6PML9
C00038	Q6XR72
C00038	Q6ZMH5
C00038	Q73GK9
C00038	Q75N73
C00038	Q78IQ7
C00038	Q7MMN0
C00038	Q7N545

C00038	Q7VLS9
C00038	Q83KR7
C00038	Q83SA2
C00038	Q87RE5
C00038	Q87UN0
C00038	Q88CP1
C00038	Q88RL1
C00038	Q88RT8
C00038	Q89AJ0
C00038	Q89AJ1
C00038	Q8BGG0
C00038	Q8BJM5
C00038	Q8BWY7
C00038	Q8BZH0
C00038	Q8C145
C00038	Q8DFQ4
C00038	Q8FUU5
C00038	Q8FUU6
C00038	Q8IWU4
C00038	Q8K9M5
C00038	Q8K9M6
C00038	Q8K9M7
C00038	Q8LE59
C00038	Q8N1S5
C00038	Q8NEW0
C00038	Q8R4H9
C00038	Q8S3W4
C00038	Q8TAD4
C00038	Q8TJZ4
C00038	Q8UF79
C00038	Q8W245
C00038	Q8W246
C00038	Q8X400
C00038	Q8YDJ7
C00038	Q8YDJ8
C00038	Q8Z5W6
C00038	Q8Z784
C00038	Q8Z8B6
C00038	Q8ZEU2
C00038	Q8ZGY6
C00038	Q8ZNV7
C00038	Q8ZS90
C00038	Q91W10
C00038	Q92504
C00038	Q926D8
C00038	Q92G36
C00038	Q92P76
C00038	Q92T56

C00038	Q94EG9
C00038	Q96H72
C00038	Q99726
C00038	Q99K24
C00038	Q9A0L9
C00038	Q9BRI3
C00038	Q9BRY0
C00038	Q9C0K1
C00038	Q9CP24
C00038	Q9D856
C00038	Q9FIS2
C00038	Q9HT73
C00038	Q9I147
C00038	Q9JKN1
C00038	Q9KQB8
C00038	Q9LTH9
C00038	Q9LXS1
C00038	Q9M271
C00038	Q9NP94
C00038	Q9NUM3
C00038	Q9NY26
C00038	Q9RNI8
C00038	Q9RNI9
C00038	Q9RPX0
C00038	Q9SLG3
C00038	Q9ULF5
C00038	Q9XDA6
C00038	Q9Y6M5
C00038	Q9ZCC4
C00042	P0AE75
C00042	P33303
C00042	Q83JJ9
C00042	Q8XBK4
C00043	O74750
C00043	Q869W7
C00043	Q8CIA5
C00043	Q8R1T4
C00043	Q969S0
C00043	Q9Y2D2
C00050	P37974
C00050	P49279
C00050	P49281
C00050	Q03218
C00050	Q08970
C00050	Q869V1
C00050	Q9C6B2
C00050	Q9FN18
C00050	Q9S9N8

C00050	Q9SAH8
C00050	Q9SN36
C00050	Q9SNV9
C00051	A0ECD9
C00051	A8I268
C00051	O15440
C00051	O88269
C00051	O95255
C00051	P14772
C00051	P32386
C00051	P39109
C00051	Q10185
C00051	Q6J0P5
C00051	Q7DM58
C00051	Q7GB25
C00051	Q92887
C00051	Q96J66
C00051	Q9C8G9
C00051	Q9LK64
C00052	P78381
C00052	P78383
C00052	P87041
C00052	P97858
C00058	P0AC23
C00058	P0AC25
C00058	P35839
C00058	P43756
C00058	Q51330
C00059	O04289
C00059	O04722
C00059	O06984
C00059	P02906
C00059	P08690
C00059	P0A4W2
C00059	P0A4W3
C00059	P0AB93
C00059	P0AEB0
C00059	P0AFR3
C00059	P0AG78
C00059	P0AG79
C00059	P14788
C00059	P16676
C00059	P16700
C00059	P16701
C00059	P23622
C00059	P32332
C00059	P38359
C00059	P40860

C00059	P41031
C00059	P41032
C00059	P50443
C00059	P58735
C00059	P63353
C00059	P63354
C00059	P74547
C00059	P74548
C00059	P92946
C00059	Q01895
C00059	Q01903
C00059	Q07782
C00059	Q12325
C00059	Q55027
C00059	Q5YZY9
C00059	Q609Q1
C00059	Q62273
C00059	Q62K82
C00059	Q63TY1
C00059	Q65T42
C00059	Q668K6
C00059	Q6D201
C00059	Q6F9A8
C00059	Q6NBT1
C00059	Q73XU8
C00059	Q7N6Z2
C00059	Q7NIW1
C00059	Q7VZE5
C00059	Q7W9U5
C00059	Q7WGW1
C00059	Q80ZD3
C00059	Q81GU1
C00059	Q82WT5
C00059	Q86WA9
C00059	Q87DT9
C00059	Q88AS5
C00059	Q88CL2
C00059	Q89UD2
C00059	Q8D0W8
C00059	Q8E8K8
C00059	Q8F6Z1
C00059	Q8GYH8
C00059	Q8PNN4
C00059	Q8R2Z3
C00059	Q8TE54
C00059	Q8UA73
C00059	Q8UH62
C00059	Q8XZP8

C00059	Q8Z0H0
C00059	Q92VJ2
C00059	Q92XW1
C00059	Q94225
C00059	Q94LW6
C00059	Q98K23
C00059	Q9A7X1
C00059	Q9BZW2
C00059	Q9FEP7
C00059	Q9FY46
C00059	Q9H2B4
C00059	Q9I6L0
C00059	Q9JZW0
C00059	Q9K876
C00059	Q9KUI0
C00059	Q9LW86
C00059	Q9MAX3
C00059	Q9PDN2
C00059	Q9SAY1
C00059	Q9SV13
C00059	Q9SXS2
C00059	Q9UKG4
C00060	O15374
C00060	O15375
C00060	O15403
C00060	O15427
C00060	O35308
C00060	O70324
C00060	O70451
C00060	O95907
C00060	P36021
C00060	P53985
C00060	P53986
C00060	P57787
C00060	Q5NC32
C00060	Q6ZSM3
C00060	Q7RTX9
C00060	Q7RTY0
C00060	Q7RTY1
C00060	Q7TM99
C00060	Q8BGC3
C00060	Q8CE94
C00060	Q8K1C7
C00060	Q8NCK7
C00060	Q8TF71
C00065	A6TAU9
C00065	A6TEC8
C00065	A7I2B9

C00065	B5XTX0
C00065	B5XVZ7
C00065	P0AAD7
C00065	P0AAD9
C00065	P44615
C00065	Q02LI9
C00065	Q07ZL7
C00065	Q0I455
C00065	Q0WJ82
C00065	Q5F5G9
C00065	Q5M1F4
C00065	Q83Q28
C00065	Q83QD0
C00065	Q8ZLW1
C00070	B1L487
C00070	O15431
C00070	O15432
C00070	O30085
C00070	O32220
C00070	O59666
C00070	O67203
C00070	O67432
C00070	O94722
C00070	P05425
C00070	P07893
C00070	P31845
C00070	P32113
C00070	P35670
C00070	P38360
C00070	P38865
C00070	P38995
C00070	P40866
C00070	P40867
C00070	P40868
C00070	P40869
C00070	P40870
C00070	P49573
C00070	P55989
C00070	P58341
C00070	P58342
C00070	Q04656
C00070	Q06686
C00070	Q39065
C00070	Q3L890
C00070	Q3MNJ6
C00070	Q5HCZ3
C00070	Q5HKB0
C00070	Q5HL56

C00070	Q72N56
C00070	Q7A3E6
C00070	Q7Y051
C00070	Q8CN02
C00070	Q8CQF7
C00070	Q8K211
C00070	Q8VPE6
C00070	Q8XD24
C00070	Q8Z8S4
C00070	Q8ZCA7
C00070	Q8ZR95
C00070	Q8ZRG7
C00070	Q8ZUJ0
C00070	Q978Z8
C00070	Q97UU7
C00070	Q97VH4
C00070	Q9CPU9
C00070	Q9HYL1
C00070	Q9KPZ7
C00070	Q9P7F9
C00070	Q9S7J8
C00070	Q9SH30
C00070	Q9SZC9
C00070	Q9USV7
C00070	Q9YBZ6
C00072	Q9EPR4
C00072	Q9UGH3
C00072	Q9UHI7
C00072	Q9WTW7
C00072	Q9Z2J0
C00073	O26096
C00073	P38734
C00073	P44785
C00073	P50276
C00073	P63356
C00073	Q02DK6
C00073	Q02ME3
C00073	Q032A0
C00073	Q03A07
C00073	Q03P57
C00073	Q03Z27
C00073	Q043Y8
C00073	Q04B25
C00073	Q04DA7
C00073	Q04F14
C00073	Q0AU85
C00073	Q0I5E9
C00073	Q0KDG3

C00073	Q18C09
C00073	Q1LQF6
C00073	Q1M8E0
C00073	Q2K284
C00073	Q2NRN5
C00073	Q2RWA3
C00073	Q3A9G5
C00073	Q4KBU0
C00073	Q4KK46
C00073	Q4KKK8
C00073	Q579H8
C00073	Q5FKL2
C00073	Q5HHK4
C00073	Q5HIL5
C00073	Q5HQQ9
C00073	Q5HRU5
C00073	Q5KVK2
C00073	Q5L5Z1
C00073	Q5M1F6
C00073	Q5NFU5
C00073	Q5YRD1
C00073	Q5ZUG5
C00073	Q62B84
C00073	Q62M41
C00073	Q63NI4
C00073	Q63S19
C00073	Q65M34
C00073	Q65VG9
C00073	Q667L9
C00073	Q66CQ3
C00073	Q67SV5
C00073	Q6A6X6
C00073	Q6AE21
C00073	Q6D1C4
C00073	Q6D3Q6
C00073	Q6D5H7
C00073	Q6F9P2
C00073	Q6FAN3
C00073	Q6G2E2
C00073	Q6HBS0
C00073	Q6HP89
C00073	Q6LN52
C00073	Q6ME20
C00073	Q6N798
C00073	Q6N9W0
C00073	Q6NJ07
C00073	Q724C0
C00073	Q74IV9

C00073	Q7A6M2
C00073	Q7A7E3
C00073	Q7CHF8
C00073	Q7M816
C00073	Q7MN25
C00073	Q7N8M2
C00073	Q7VI92
C00073	Q7VM95
C00073	Q7VV72
C00073	Q7W4E1
C00073	Q7WFU9
C00073	Q815Y7
C00073	Q81IN8
C00073	Q81IZ6
C00073	Q81VM2
C00073	Q81XL3
C00073	Q81ZF5
C00073	Q823C4
C00073	Q827Y0
C00073	Q832Y6
C00073	Q83MC5
C00073	Q87AL9
C00073	Q87RS1
C00073	Q87UN4
C00073	Q87UV4
C00073	Q88RB3
C00073	Q88RL5
C00073	Q88UV2
C00073	Q88WA5
C00073	Q895C4
C00073	Q89LP2
C00073	Q8CQS7
C00073	Q8CTB2
C00073	Q8DFC3
C00073	Q8DY54
C00073	Q8E3S0
C00073	Q8ELA5
C00073	Q8ELQ6
C00073	Q8ENU2
C00073	Q8EPK1
C00073	Q8FRX8
C00073	Q8G5P8
C00073	Q8NSN2
C00073	Q8PGE8
C00073	Q8RFN2
C00073	Q8U7G2
C00073	Q8Y4L8
C00073	Q8YA75

C00073	Q8Z8R5
C00073	Q8Z990
C00073	Q8ZH38
C00073	Q92EZ6
C00073	Q93DA2
C00073	Q97KD5
C00073	Q9A502
C00073	Q9HT70
C00073	Q9I1C8
C00073	Q9K789
C00073	Q9L1C3
C00073	Q9Z8Q8
C00074	P12681
C00076	A6URW9
C00076	A9BJX0
C00076	O14983
C00076	O22218
C00076	O23087
C00076	O45215
C00076	O55017
C00076	O55143
C00076	O59768
C00076	O59868
C00076	O60721
C00076	O60840
C00076	O64806
C00076	O66938
C00076	O75185
C00076	O81108
C00076	O94759
C00076	O95180
C00076	P11881
C00076	P13586
C00076	P16615
C00076	P20020
C00076	P21817
C00076	P22189
C00076	P22700
C00076	P23634
C00076	P34315
C00076	P34322
C00076	P38929
C00076	P41821
C00076	P48995
C00076	P50077
C00076	P54678
C00076	P57103
C00076	P70227

C00076	P70414
C00076	P91645
C00076	P92939
C00076	P97445
C00076	P98194
C00076	Q00975
C00076	Q01668
C00076	Q01814
C00076	Q13698
C00076	Q14571
C00076	Q14573
C00076	Q14643
C00076	Q15413
C00076	Q15878
C00076	Q16720
C00076	Q19QZ8
C00076	Q19QZ9
C00076	Q19R02
C00076	Q19R03
C00076	Q19R08
C00076	Q19R11
C00076	Q19R12
C00076	Q19R13
C00076	Q19R17
C00076	Q24270
C00076	Q2TAC4
C00076	Q37145
C00076	Q39253
C00076	Q39254
C00076	Q3KR50
C00076	Q3SWS6
C00076	Q4LE63
C00076	Q5KQN0
C00076	Q5KTQ9
C00076	Q5M0A4
C00076	Q5QM84
C00076	Q5TKG3
C00076	Q61056
C00076	Q64518
C00076	Q6J4K2
C00076	Q6K1C4
C00076	Q71RS6
C00076	Q769E5
C00076	Q80XR2
C00076	Q8BWC0
C00076	Q8BWG9
C00076	Q8C261
C00076	Q8CGQ8

C00076	Q8L783
C00076	Q8NFF2
C00076	Q8R429
C00076	Q925Q3
C00076	Q92736
C00076	Q93Z81
C00076	Q945S5
C00076	Q94KI8
C00076	Q96D31
C00076	Q97JK5
C00076	Q99246
C00076	Q99385
C00076	Q9EQJ0
C00076	Q9H1D0
C00076	Q9HC58
C00076	Q9LF79
C00076	Q9LFZ8
C00076	Q9LIK7
C00076	Q9LJI2
C00076	Q9LU41
C00076	Q9LY77
C00076	Q9M2L4
C00076	Q9N694
C00076	Q9NQA5
C00076	Q9QX29
C00076	Q9R0K7
C00076	Q9R244
C00076	Q9SY55
C00076	Q9SYG9
C00076	Q9SZR1
C00076	Q9U6A0
C00076	Q9UBN4
C00076	Q9UI40
C00076	Q9UL62
C00076	Q9UPR5
C00076	Q9XES1
C00076	Q9XTG4
C00076	Q9Z329
C00078	P0AAD3
C00078	P38085
C00078	P38967
C00078	P44614
C00078	Q8XB33
C00080	A0L3E7
C00080	D3DN75
C00080	D3DSQ5
C00080	O02713
C00080	O07837

C00080	O07838
C00080	O22920
C00080	O27598
C00080	O27599
C00080	O27600
C00080	O27601
C00080	O30417
C00080	O30510
C00080	O30513
C00080	O31137
C00080	O31199
C00080	O32715
C00080	O32859
C00080	O35956
C00080	O50471
C00080	O52557
C00080	O52717
C00080	O52718
C00080	O60931
C00080	O66163
C00080	O68441
C00080	O76082
C00080	O76729
C00080	O82855
C00080	O87880
C00080	O88750
C00080	P00830
C00080	P00854
C00080	P00856
C00080	P02945
C00080	P02982
C00080	P02983
C00080	P04817
C00080	P04997
C00080	P05316
C00080	P05626
C00080	P07038
C00080	P07251
C00080	P07775
C00080	P08194
C00080	P09192
C00080	P09193
C00080	P09457
C00080	P09836
C00080	P0A0J7
C00080	P0A0J9
C00080	P0A6E6
C00080	P0AA78

C00080	P0AA82
C00080	P0AAD2
C00080	P0AAD4
C00080	P0AAD6
C00080	P0AAD8
C00080	P0AAE0
C00080	P0AB98
C00080	P0ABA0
C00080	P0ABA4
C00080	P0ABA6
C00080	P0ABB0
C00080	P0ABB4
C00080	P0ABC9
C00080	P0ABP3
C00080	P0AC26
C00080	P0AE16
C00080	P0AEJ0
C00080	P0AFA7
C00080	P0AFC3
C00080	P0AFC7
C00080	P0AFD1
C00080	P0AFD4
C00080	P0AFD6
C00080	P0AFE0
C00080	P0AFE4
C00080	P0AFE8
C00080	P0AFF0
C00080	P0AFF2
C00080	P0AFF4
C00080	P0AG34
C00080	P0AG38
C00080	P0AG90
C00080	P0AGM7
C00080	P0C0L7
C00080	P0C105
C00080	P10861
C00080	P11004
C00080	P11005
C00080	P11551
C00080	P12012
C00080	P12681
C00080	P13511
C00080	P13738
C00080	P14285
C00080	P15365
C00080	P15380
C00080	P15685
C00080	P15686

C00080	P15701
C00080	P15993
C00080	P16033
C00080	P16482
C00080	P17064
C00080	P17551
C00080	P17583
C00080	P18581
C00080	P19072
C00080	P19145
C00080	P20648
C00080	P21306
C00080	P21345
C00080	P22732
C00080	P23173
C00080	P23910
C00080	P24942
C00080	P24943
C00080	P25185
C00080	P25297
C00080	P25360
C00080	P25376
C00080	P25527
C00080	P25714
C00080	P26235
C00080	P27589
C00080	P27611
C00080	P28873
C00080	P30145
C00080	P30902
C00080	P31122
C00080	P31224
C00080	P31414
C00080	P31442
C00080	P31553
C00080	P31675
C00080	P31979
C00080	P32369
C00080	P32487
C00080	P33026
C00080	P33231
C00080	P33599
C00080	P33602
C00080	P33607
C00080	P34579
C00080	P34711
C00080	P36035
C00080	P37460

C00080	P37594
C00080	P37735
C00080	P38077
C00080	P38084
C00080	P38085
C00080	P38124
C00080	P38196
C00080	P38206
C00080	P38680
C00080	P38724
C00080	P38778
C00080	P38971
C00080	P39344
C00080	P39843
C00080	P39886
C00080	P39952
C00080	P39980
C00080	P40812
C00080	P41036
C00080	P41438
C00080	P42086
C00080	P42237
C00080	P42308
C00080	P42670
C00080	P43505
C00080	P43548
C00080	P45562
C00080	P46032
C00080	P46104
C00080	P46133
C00080	P46349
C00080	P48777
C00080	P48813
C00080	P50276
C00080	P50487
C00080	P50537
C00080	P51164
C00080	P51574
C00080	P52002
C00080	P53048
C00080	P53388
C00080	P53985
C00080	P54104
C00080	P54219
C00080	P54417
C00080	P55069
C00080	P60678
C00080	P60682

C00080	P60686
C00080	P60690
C00080	P60694
C00080	P60698
C00080	P61829
C00080	P68699
C00080	P69926
C00080	P70786
C00080	P74072
C00080	P74898
C00080	P74899
C00080	P74900
C00080	P74901
C00080	P74902
C00080	P74903
C00080	P76350
C00080	P76460
C00080	P77589
C00080	P92961
C00080	P94131
C00080	P94633
C00080	P95827
C00080	P96517
C00080	P96796
C00080	P96797
C00080	P97054
C00080	P97055
C00080	Q00538
C00080	Q01440
C00080	Q01818
C00080	Q01857
C00080	Q02563
C00080	Q04733
C00080	Q04895
C00080	Q05085
C00080	Q05181
C00080	Q05998
C00080	Q07307
C00080	Q07394
C00080	Q07396
C00080	Q07492
C00080	Q07824
C00080	Q08299
C00080	Q08986
C00080	Q12165
C00080	Q12349
C00080	Q25272
C00080	Q39608

C00080	Q41515
C00080	Q42400
C00080	Q43390
C00080	Q44659
C00080	Q44660
C00080	Q45082
C00080	Q45153
C00080	Q46M63
C00080	Q47142
C00080	Q48797
C00080	Q50248
C00080	Q50249
C00080	Q50250
C00080	Q50392
C00080	Q51006
C00080	Q51073
C00080	Q51955
C00080	Q52000
C00080	Q52715
C00080	Q52716
C00080	Q52978
C00080	Q52980
C00080	Q52981
C00080	Q52982
C00080	Q52983
C00080	Q53787
C00080	Q53903
C00080	Q54806
C00080	Q55027
C00080	Q56EB3
C00080	Q57038
C00080	Q59574
C00080	Q59575
C00080	Q59576
C00080	Q59577
C00080	Q59578
C00080	Q59579
C00080	Q59580
C00080	Q60183
C00080	Q60184
C00080	Q60185
C00080	Q60186
C00080	Q60187
C00080	Q60188
C00080	Q60189
C00080	Q60961
C00080	Q61168
C00080	Q63089

C00080	Q63965
C00080	Q6FIH7
C00080	Q79VC7
C00080	Q79VE0
C00080	Q8LBP4
C00080	Q8R4H8
C00080	Q93YH1
C00080	Q96241
C00080	Q96247
C00080	Q96T83
C00080	Q99271
C00080	Q99385
C00080	Q9C6B8
C00080	Q9H015
C00080	Q9LU77
C00080	Q9R1U7
C00080	Q9RPV1
C00080	Q9S3J9
C00080	Q9S3K0
C00080	Q9UAC2
C00080	Q9WTM1
C00080	Q9X9D2
C00080	Q9Y860
C00080	Q9Z3Q3
C00080	Q9ZHC9
C00080	Q9ZNG6
C00082	P0AAD5
C00082	P38085
C00082	P44727
C00082	P44747
C00082	Q04671
C00082	Q62052
C00086	O05123
C00086	O94469
C00086	P33413
C00086	Q13336
C00086	Q15849
C00086	Q8VHL0
C00086	Q9URY6
C00088	O82811
C00088	P0AC26
C00088	P0AC27
C00088	P10903
C00088	P37758
C00088	P38043
C00088	P38044
C00088	P38045
C00088	P38046

C00088	Q166T6
C00088	Q93PW1
C00089	A8F6Z4
C00089	B0K2P2
C00089	B0K2P3
C00089	B1L0U0
C00089	B1L0U1
C00089	O14091
C00089	O31427
C00089	O31428
C00089	O80605
C00089	P08470
C00089	P12655
C00089	P15400
C00089	P18767
C00089	P30000
C00089	P40739
C00089	Q0ILJ3
C00089	Q39231
C00089	Q39232
C00089	Q67YF8
C00089	Q6A329
C00089	Q6KYW8
C00089	Q6KYW9
C00089	Q8NMD6
C00089	Q9C8X2
C00089	Q9CJZ2
C00089	Q9FE59
C00089	Q9FG00
C00089	Q9X2W0
C00089	Q9Z3R5
C00089	Q9Z3R6
C00089	Q9Z3R7
C00089	Q9ZVK6
C00092	O43826
C00092	P09836
C00092	P39580
C00093	P57057
C00093	P96335
C00093	Q0K998
C00093	Q1LLP5
C00093	Q1M589
C00093	Q1M8R6
C00093	Q2K1C8
C00093	Q2K4V4
C00093	Q2K6L3
C00093	Q578E9
C00093	Q5LX21

C00093	Q62GB4
C00093	Q63Q62
C00093	Q66FU4
C00093	Q6CZ34
C00093	Q6G194
C00093	Q6G5J0
C00093	Q6NDQ0
C00093	Q74R28
C00093	Q7MLB8
C00093	Q7VYN2
C00093	Q7W6G5
C00093	Q7WID6
C00093	Q89WG0
C00093	Q8FW07
C00093	Q8UBB7
C00093	Q8UII7
C00093	Q8X6U5
C00093	Q8XZX8
C00093	Q8YCB1
C00093	Q8Z245
C00093	Q8ZLF4
C00093	Q92WD6
C00093	Q98G42
C00093	Q9KRT4
C00098	O01840
C00098	O04514
C00098	O31307
C00098	O31309
C00098	O31310
C00098	O31315
C00098	O50927
C00098	O51307
C00098	O51308
C00098	O51310
C00098	O54584
C00098	O82485
C00098	P04285
C00098	P06202
C00098	P08005
C00098	P08006
C00098	P08007
C00098	P0A2U8
C00098	P0A2V4
C00098	P0A2V5
C00098	P0A2V6
C00098	P0A4G0
C00098	P0A4M7
C00098	P0A4M9

C00098	P0A4N7
C00098	P0A4N8
C00098	P0A4N9
C00098	P0A4P0
C00098	P0AFH4
C00098	P0AFH5
C00098	P0AFH7
C00098	P18766
C00098	P18791
C00098	P40897
C00098	P45051
C00098	P45052
C00098	P45053
C00098	P45054
C00098	P46059
C00098	P47323
C00098	P47324
C00098	P47325
C00098	P47326
C00098	P50980
C00098	P71370
C00098	P72479
C00098	P75551
C00098	P75552
C00098	P75553
C00098	P75554
C00098	P75796
C00098	P75797
C00098	P75798
C00098	P75799
C00098	P77348
C00098	Q02VA9
C00098	Q06593
C00098	Q07733
C00098	Q16348
C00098	Q17758
C00098	Q21219
C00098	Q2FZR3
C00098	Q2FZR4
C00098	Q2FZR5
C00098	Q2FZR6
C00098	Q2FZR7
C00098	Q5HG38
C00098	Q5HG39
C00098	Q5HG40
C00098	Q5HG41
C00098	Q7A5Q6
C00098	Q7A5Q7

C00098	Q7A5Q8
C00098	Q7A5Q9
C00098	Q8BPX9
C00098	Q8CBB2
C00098	Q8Z6Q5
C00098	Q91W98
C00098	Q92NE8
C00098	Q92NE9
C00098	Q92NF0
C00098	Q92NF1
C00098	Q9CEK0
C00098	Q9FG72
C00098	Q9FJD1
C00098	Q9FJD2
C00098	Q9FME8
C00098	Q9T095
C00098	Q9WXN4
C00098	Q9WXN5
C00098	Q9WXN6
C00098	Q9WXN7
C00098	Q9WXN8
C00098	Q9WXS6
C00098	Q9WXS7
C00098	Q9WXS8
C00098	Q9WXS9
C00098	Q9WXT0
C00098	Q9X0F3
C00098	Q9X0F4
C00098	Q9X0F5
C00098	Q9X0F6
C00098	Q9X0F7
C00098	Q9X0U6
C00098	Q9X0U7
C00098	Q9X0U8
C00098	Q9X0U9
C00098	Q9X0V0
C00098	Q9X268
C00098	Q9X269
C00098	Q9X270
C00098	Q9X271
C00098	Q9X272
C00099	P46349
C00101	P41438
C00106	P05316
C00106	P0AGM7
C00106	P0AGM8
C00106	P45117
C00106	Q10279

C00106	Q9CPL9
C00107	P0AAG1
C00107	P0AAG2
C00107	P0AEG0
C00107	P0AEG3
C00107	P0AFU1
C00107	P26902
C00107	P26903
C00107	P26904
C00107	P26905
C00107	P26906
C00107	P33913
C00107	P33915
C00107	P33916
C00107	P42061
C00107	P42062
C00107	P42063
C00107	P42064
C00107	P42065
C00107	P45094
C00107	P45095
C00107	P45096
C00107	P51000
C00107	Q5V9R8
C00107	Q5V9R9
C00107	Q5V9S0
C00107	Q5V9S1
C00107	Q5V9S2
C00107	Q64K09
C00107	Q7CQ74
C00107	Q8ZNJ8
C00107	Q8ZNJ9
C00107	Q8ZNK0
C00107	Q9CIK8
C00107	Q9CIK9
C00107	Q9CIL0
C00107	Q9CIL1
C00107	Q9CIL2
C00107	Q9F9T4
C00107	Q9F9T5
C00107	Q9F9T6
C00107	Q9F9T7
C00114	O32243
C00114	O34742
C00114	O34878
C00114	O34992
C00114	P0ABC9
C00114	P0ABD0

C00114	P0AFM2
C00114	P14175
C00114	P14176
C00114	P19807
C00114	P28570
C00114	P39775
C00114	Q45460
C00114	Q45461
C00114	Q45462
C00114	Q87WH3
C00114	Q87WH4
C00114	Q87WH5
C00114	Q8BGY9
C00114	Q8BY89
C00114	Q8IWA5
C00114	Q8Q040
C00114	Q8Q041
C00114	Q8Q042
C00114	Q8Q043
C00114	Q8WWI5
C00114	Q9GZV3
C00114	Q9HTH6
C00114	Q9HTI6
C00114	Q9HTI7
C00114	Q9HTI8
C00116	A3QNP2
C00116	P08194
C00116	P0A3Q7
C00116	P0AER2
C00116	P0AG80
C00116	P10905
C00116	P10906
C00116	P10907
C00116	P19255
C00116	P23900
C00116	P31140
C00116	P33941
C00116	P33951
C00116	P44826
C00116	P47279
C00116	P52280
C00116	P53154
C00116	P75071
C00116	Q08929
C00116	Q26C21
C00116	Q45592
C00116	Q45593
C00116	Q48475

C00116	Q48476
C00116	Q51340
C00116	Q51389
C00116	Q54121
C00116	Q55035
C00116	Q55471
C00116	Q55472
C00116	Q55473
C00116	Q6E7E8
C00116	Q6E7E9
C00116	Q88P38
C00116	Q8K9M9
C00116	Q8KN17
C00116	Q8KN18
C00116	Q8KN19
C00116	Q93GF4
C00116	Q9CE02
C00116	Q9L0Q1
C00116	Q9X1E3
C00120	O70247
C00121	P0A2C5
C00121	P0A2C6
C00121	P44737
C00122	P33303
C00133	P0AAE1
C00134	A0LUE6
C00134	A3CMQ7
C00134	O51587
C00134	O83658
C00134	P0A2C7
C00134	P0A2C8
C00134	P0A2J7
C00134	P0A2J8
C00134	P0AAF1
C00134	P0AFK4
C00134	P0AFK5
C00134	P0AFK6
C00134	P0AFK8
C00134	P0AFK9
C00134	P0AFL0
C00134	P0AFL1
C00134	P31133
C00134	P31134
C00134	P31135
C00134	P40790
C00134	P44731
C00134	P44768
C00134	P45168

C00134	P45169
C00134	P45170
C00134	P45171
C00134	P47288
C00134	P47289
C00134	P47290
C00134	P69874
C00134	P69876
C00134	P69877
C00134	P75057
C00134	P75058
C00134	P75059
C00134	Q02R79
C00134	Q02Z10
C00134	Q03AH0
C00134	Q03JH1
C00134	Q03PF2
C00134	Q03ZQ0
C00134	Q042G7
C00134	Q04BG2
C00134	Q0AGF4
C00134	Q0I3Y9
C00134	Q0RAT5
C00134	Q0SML1
C00134	Q18AM3
C00134	Q2NK31
C00134	Q2SSS4
C00134	Q4K681
C00134	Q5FL41
C00134	Q5HGY5
C00134	Q5HQ70
C00134	Q5L222
C00134	Q5LT05
C00134	Q5LYN4
C00134	Q5ZWE4
C00134	Q60AI1
C00134	Q64SQ6
C00134	Q65UE1
C00134	Q660M8
C00134	Q6D4E2
C00134	Q6F0V4
C00134	Q6HLQ9
C00134	Q6KIP2
C00134	Q6LR20
C00134	Q6MCV4
C00134	Q6MU19
C00134	Q6YPR6
C00134	Q722B1

C00134	Q72FW5
C00134	Q74K65
C00134	Q7A679
C00134	Q7MKU3
C00134	Q7NB11
C00134	Q7VNG4
C00134	Q81GC1
C00134	Q81TH8
C00134	Q82TL6
C00134	Q830W6
C00134	Q83RR7
C00134	Q87PH3
C00134	Q88ZJ6
C00134	Q8A883
C00134	Q8CPN0
C00134	Q8D9J4
C00134	Q8DUF7
C00134	Q8DZJ0
C00134	Q8E554
C00134	Q8ELR4
C00134	Q8EUR3
C00134	Q8RI39
C00134	Q8XIZ5
C00134	Q8Y8T6
C00134	Q8YM92
C00134	Q8Z7H7
C00134	Q92DL6
C00134	Q97KS6
C00134	Q98HF7
C00134	Q98QE1
C00134	Q9CGD4
C00134	Q9CP06
C00134	Q9HY19
C00134	Q9I6T2
C00134	Q9KS33
C00134	Q9PR37
C00134	Q9X196
C00137	P30605
C00137	P30606
C00137	P53794
C00137	P87110
C00137	Q01440
C00137	Q3UHK1
C00137	Q8VM88
C00137	Q96QE2
C00147	Q84MA8
C00147	Q9SRK7
C00148	P0AFM3

C00148	P17327
C00148	P17328
C00149	P50537
C00149	Q02978
C00149	Q54PY7
C00149	Q8LG88
C00149	Q9CR62
C00150	O32209
C00150	P0A5Y1
C00150	P0A625
C00150	P0AF02
C00150	P45321
C00150	P45322
C00150	P45323
C00150	P59738
C00150	Q4KB64
C00150	Q5P4W2
C00150	Q608V9
C00150	Q65SW3
C00150	Q66D71
C00150	Q6D7D0
C00150	Q6LHL2
C00150	Q6N0P7
C00150	Q7ME63
C00150	Q7N6R3
C00150	Q7VKP7
C00150	Q7VUJ5
C00150	Q7W1F4
C00150	Q7WP62
C00150	Q87HN4
C00150	Q881C1
C00150	Q89EW7
C00150	Q89TQ9
C00150	Q8D740
C00150	Q8EAN3
C00150	Q8FXI7
C00150	Q8X4V7
C00150	Q8YE15
C00150	Q8Z8A4
C00150	Q8ZGX6
C00150	Q8ZQR6
C00150	Q92LU2
C00150	Q98KI3
C00150	Q9CMS0
C00150	Q9I2N4
C00150	Q9KLL9
C00152	P0A4W1
C00152	P40812

C00152	Q7VEQ4
C00152	Q9X7P0
C00156	Q43975
C00156	Q51955
C00156	Q9I6Q3
C00158	O35055
C00158	P0A2F8
C00158	P0A2F9
C00158	P0A2G3
C00158	P0A2G4
C00158	P0AE74
C00158	P0AE75
C00158	P16482
C00158	P21608
C00158	P31602
C00158	P38152
C00158	P42308
C00158	P55069
C00158	P94363
C00158	Q13183
C00158	Q48769
C00158	Q67BT3
C00158	Q86YT5
C00158	Q9VVT2
C00159	O07563
C00159	P26379
C00159	P26380
C00159	P26381
C00159	P26382
C00159	P69797
C00159	P69799
C00159	P69800
C00159	P69801
C00159	P69803
C00159	P69804
C00159	P69805
C00159	P69807
C00159	Q01441
C00162	P43505
C00162	P44051
C00162	Q51006
C00162	Q51073
C00175	A3CRB8
C00175	A3CRB9
C00175	A6TDB4
C00175	B5XUV2
C00175	O25901
C00175	O26234

C00175	O27739
C00175	O29529
C00175	O54189
C00175	O85143
C00175	P0A2R8
C00175	P0A2R9
C00175	P0ABI6
C00175	P0ABI7
C00175	P0C0E8
C00175	P13511
C00175	P13512
C00175	P28795
C00175	P32798
C00175	P37617
C00175	P37972
C00175	P44998
C00175	P47425
C00175	P47426
C00175	P96454
C00175	Q032H3
C00175	Q032H4
C00175	Q035B2
C00175	Q035B3
C00175	Q03EE4
C00175	Q03I82
C00175	Q03I83
C00175	Q03PY5
C00175	Q03PY6
C00175	Q03ZL5
C00175	Q03ZL6
C00175	Q045Z7
C00175	Q045Z8
C00175	Q04BY6
C00175	Q04BY7
C00175	Q04EY4
C00175	Q04EY5
C00175	Q04FM1
C00175	Q05594
C00175	Q05595
C00175	Q05596
C00175	Q05598
C00175	Q07418
C00175	Q0AUL1
C00175	Q18CI9
C00175	Q18CJ0
C00175	Q2FNX9
C00175	Q2FRT7
C00175	Q2LY16

C00175	Q2NIT5
C00175	Q2NQF9
C00175	Q2SRI1
C00175	Q2SRI2
C00175	Q3ABN1
C00175	Q491Z9
C00175	Q4A5A4
C00175	Q4A5A5
C00175	Q50293
C00175	Q50294
C00175	Q58488
C00175	Q58490
C00175	Q5FM62
C00175	Q5FM63
C00175	Q5HDY6
C00175	Q5HDY7
C00175	Q5HM27
C00175	Q5HM28
C00175	Q5KG96
C00175	Q5L3Q9
C00175	Q5L3R0
C00175	Q5LXJ3
C00175	Q5LXJ4
C00175	Q65P76
C00175	Q65P77
C00175	Q65VT3
C00175	Q66FY7
C00175	Q67JX3
C00175	Q67JX4
C00175	Q6CZH4
C00175	Q6F1W4
C00175	Q6F1W5
C00175	Q6HPM9
C00175	Q6HPN0
C00175	Q6KHL1
C00175	Q6KHL2
C00175	Q6LX68
C00175	Q6LX70
C00175	Q6MSQ1
C00175	Q6MSQ2
C00175	Q6YR39
C00175	Q71WH7
C00175	Q71WH8
C00175	Q74L61
C00175	Q74L62
C00175	Q7A470
C00175	Q7A471
C00175	Q7M9C0

C00175	Q7MYP0
C00175	Q7NAQ6
C00175	Q7NAQ7
C00175	Q7VFJ7
C00175	Q7VN59
C00175	Q7VRM7
C00175	Q81J15
C00175	Q81J16
C00175	Q81VQ1
C00175	Q81VQ2
C00175	Q839D4
C00175	Q839D5
C00175	Q88XV1
C00175	Q88XV2
C00175	Q890R2
C00175	Q890R3
C00175	Q8CRI6
C00175	Q8CRI7
C00175	Q8DRR9
C00175	Q8DRS0
C00175	Q8DWR3
C00175	Q8DWR4
C00175	Q8E2L2
C00175	Q8E2L3
C00175	Q8ETV6
C00175	Q8ETV7
C00175	Q8EUF1
C00175	Q8R7Y4
C00175	Q8R7Y5
C00175	Q8RHL0
C00175	Q8TI16
C00175	Q8XHV2
C00175	Q8XHV3
C00175	Q8XNY9
C00175	Q8Y454
C00175	Q8Y455
C00175	Q8YQ90
C00175	Q8Z5N3
C00175	Q8Z5N5
C00175	Q8ZAG5
C00175	Q927N8
C00175	Q927N9
C00175	Q97EK8
C00175	Q97EK9
C00175	Q98QH4
C00175	Q98QH5
C00175	Q99XI2
C00175	Q9CLC4

C00175	Q9HPH5
C00175	Q9KGD6
C00175	Q9PPI1
C00175	Q9PPV1
C00175	Q9PPV2
C00175	Q9WZ31
C00175	Q9Z9J3
C00180	O30513
C00180	P07775
C00181	P0AGI6
C00181	P0AGI7
C00181	P45045
C00181	P45046
C00181	P45047
C00181	Q5KYS1
C00181	Q6DB03
C00181	Q6LUY1
C00181	Q7CFR2
C00181	Q83J33
C00181	Q880Z2
C00181	Q8XDM1
C00181	Q8XPK6
C00185	O05506
C00185	P08722
C00185	P24241
C00186	P65254
C00186	Q8Z2E3
C00186	Q8ZL63
C00186	Q9K5Z9
C00187	Q91V24
C00188	O31199
C00188	P0AAD8
C00188	P0AG38
C00188	P0AGE4
C00189	Q53148
C00190	Q8CIA5
C00191	C9RT46
C00191	O82390
C00191	O85018
C00191	P08716
C00191	P0AA78
C00191	P18770
C00191	P23886
C00191	P29018
C00191	P77307
C00191	Q07YH1
C00191	Q09LY6
C00191	Q09LY7

C00191	Q12235
C00191	Q54203
C00191	Q54204
C00191	Q5W7C1
C00191	Q5Z8H2
C00191	Q88RG3
C00191	Q8BFU8
C00191	Q9BYT1
C00191	Q9I2M0
C00191	Q9I2M1
C00191	Q9I2M2
C00191	Q9Y2C5
C00191	Q9Z4Q1
C00191	Q9Z4Q2
C00201	Q9X9D2
C00208	A3MWP1
C00208	A3MWP2
C00208	A5A639
C00208	A8F6Z4
C00208	B0K2P2
C00208	B0K2P3
C00208	B1L0U0
C00208	B1L0U1
C00208	O31427
C00208	O31428
C00208	O32748
C00208	O51923
C00208	O51924
C00208	O51925
C00208	O54396
C00208	P02916
C00208	P0ABM0
C00208	P0ABM7
C00208	P0AEX9
C00208	P0AEY0
C00208	P0CD99
C00208	P0CE00
C00208	P15685
C00208	P18767
C00208	P19566
C00208	P19576
C00208	P19642
C00208	P23212
C00208	P24584
C00208	P24586
C00208	P26467
C00208	P26468
C00208	P30963

C00208	P30964
C00208	P31675
C00208	P33116
C00208	P37608
C00208	P38156
C00208	P39115
C00208	P42332
C00208	P42333
C00208	P42334
C00208	P43109
C00208	P43110
C00208	P58300
C00208	P59213
C00208	P68183
C00208	P68185
C00208	P68186
C00208	P68187
C00208	P68188
C00208	P73329
C00208	P74256
C00208	P74757
C00208	P93280
C00208	P97046
C00208	Q03203
C00208	Q07638
C00208	Q07639
C00208	Q57403
C00208	Q5TLL2
C00208	Q65DD1
C00208	Q65DD3
C00208	Q65QT6
C00208	Q664X5
C00208	Q6KYW8
C00208	Q6KYW9
C00208	Q74RF8
C00208	Q74RF9
C00208	Q7A937
C00208	Q7MFC1
C00208	Q7MFC2
C00208	Q7MFC4
C00208	Q7N983
C00208	Q7N984
C00208	Q7N986
C00208	Q7UBD0
C00208	Q83P81
C00208	Q83XH0
C00208	Q83XH1
C00208	Q87GB5

C00208	Q87GB7
C00208	Q87GB8
C00208	Q8L122
C00208	Q8L124
C00208	Q8L125
C00208	Q8L126
C00208	Q8R6Q4
C00208	Q8R6Q5
C00208	Q8R6Q6
C00208	Q8RJU8
C00208	Q8RJU9
C00208	Q8RJV0
C00208	Q8TZP9
C00208	Q8TZQ0
C00208	Q8TZQ3
C00208	Q8XE58
C00208	Q8Z1U0
C00208	Q8Z1U2
C00208	Q8Z1U3
C00208	Q8ZAS8
C00208	Q93KB7
C00208	Q93KB8
C00208	Q93KB9
C00208	Q93KC0
C00208	Q97UF2
C00208	Q97UF3
C00208	Q97UF4
C00208	Q97UF5
C00208	Q97UY8
C00208	Q97UY9
C00208	Q97UZ0
C00208	Q97UZ1
C00208	Q97ZC0
C00208	Q97ZC1
C00208	Q97ZC2
C00208	Q97ZC3
C00208	Q9AI63
C00208	Q9AI66
C00208	Q9AI67
C00208	Q9AI68
C00208	Q9C8T1
C00208	Q9KL04
C00208	Q9KL06
C00208	Q9KL07
C00208	Q9KWT5
C00208	Q9KWT6
C00208	Q9KWT7
C00208	Q9KWT8

C00208	Q9KWT9
C00208	Q9LF50
C00208	Q9R9Q4
C00208	Q9R9Q5
C00208	Q9R9Q6
C00208	Q9R9Q7
C00208	Q9V297
C00208	Q9X2W0
C00208	Q9Z3R5
C00208	Q9Z3R6
C00208	Q9Z3R7
C00209	Q51330
C00215	P21420
C00230	Q51955
C00238	A0KJR8
C00238	A0KL61
C00238	A0LJ91
C00238	A0LP92
C00238	A0R395
C00238	A1KHG8
C00238	A1KHH0
C00238	A2BDX4
C00238	A2RUL8
C00238	A3KLL5
C00238	A4D0V9
C00238	A4IVV7
C00238	A4SGR1
C00238	A5EHA3
C00238	A5EHG1
C00238	A5EHG2
C00238	A5EQ10
C00238	A5ESW9
C00238	A5VRD1
C00238	A6H8H5
C00238	A6T4I3
C00238	A6T6D7
C00238	A6T6D8
C00238	A6T6D9
C00238	A6TEY9
C00238	A6TG51
C00238	A6U6M1
C00238	A6UM20
C00238	A6UMS8
C00238	A8HY21
C00238	A8MYU2
C00238	A9C190
C00238	A9LNM6
C00238	B2R8T4

C00238	B2RDS2
C00238	B5TJL4
C00238	B5TJL8
C00238	B5XN76
C00238	B5XZE8
C00238	B5XZE9
C00238	B5XZF0
C00238	B5XZK9
C00238	B5Y1Z8
C00238	B9JA25
C00238	B9JKQ7
C00238	B9JKQ9
C00238	B9K2I1
C00238	B9K2I3
C00238	D3DSH5
C00238	D3DTA5
C00238	D3DTQ2
C00238	D3DV90
C00238	D3DVE4
C00238	D3DX04
C00238	O00180
C00238	O08581
C00238	O14649
C00238	O15554
C00238	O18367
C00238	O22397
C00238	O22881
C00238	O27564
C00238	O31658
C00238	O32081
C00238	O32327
C00238	O32328
C00238	O35111
C00238	O35173
C00238	O35174
C00238	O43525
C00238	O43526
C00238	O49423
C00238	O54982
C00238	O60721
C00238	O60741
C00238	O60928
C00238	O64769
C00238	O80739
C00238	O88335
C00238	O88454
C00238	O88703
C00238	O88704

C00238	O88705
C00238	O88932
C00238	O89109
C00238	O95069
C00238	O95259
C00238	O95279
C00238	P03959
C00238	P03960
C00238	P03961
C00238	P05023
C00238	P05026
C00238	P07038
C00238	P0A008
C00238	P0A057
C00238	P0A333
C00238	P0AFZ8
C00238	P0AFZ9
C00238	P12685
C00238	P13637
C00238	P14094
C00238	P14231
C00238	P14415
C00238	P15388
C00238	P16388
C00238	P16389
C00238	P16390
C00238	P17658
C00238	P17970
C00238	P17971
C00238	P17972
C00238	P20648
C00238	P22001
C00238	P22459
C00238	P22460
C00238	P28584
C00238	P34410
C00238	P35561
C00238	P36937
C00238	P40310
C00238	P44843
C00238	P44933
C00238	P47946
C00238	P48048
C00238	P48050
C00238	P48051
C00238	P48543
C00238	P48544
C00238	P48547

C00238	P48549
C00238	P50992
C00238	P50993
C00238	P51164
C00238	P51787
C00238	P54707
C00238	P54709
C00238	P54710
C00238	P55011
C00238	P55012
C00238	P55014
C00238	P56696
C00238	P57683
C00238	P57686
C00238	P57688
C00238	P57698
C00238	P57700
C00238	P57789
C00238	P58390
C00238	P59053
C00238	P59111
C00238	P59219
C00238	P60678
C00238	P60682
C00238	P60686
C00238	P60690
C00238	P60694
C00238	P60698
C00238	P63141
C00238	P63250
C00238	P63252
C00238	P63681
C00238	P63682
C00238	P63684
C00238	P65209
C00238	P65210
C00238	P65211
C00238	P65212
C00238	P65214
C00238	P73867
C00238	P78508
C00238	P86046
C00238	P92960
C00238	P94606
C00238	P97370
C00238	P97438
C00238	P97794
C00238	Q02280

C00238	Q02I77
C00238	Q02KC0
C00238	Q035P5
C00238	Q03719
C00238	Q03721
C00238	Q03S07
C00238	Q03XG1
C00238	Q04646
C00238	Q046Q7
C00238	Q04CF3
C00238	Q08460
C00238	Q09470
C00238	Q09573
C00238	Q0AIM8
C00238	Q0BPL2
C00238	Q0BVS2
C00238	Q0D9S3
C00238	Q0JKV1
C00238	Q0KBR7
C00238	Q0RF16
C00238	Q10065
C00238	Q11PK1
C00238	Q12809
C00238	Q13621
C00238	Q13733
C00238	Q14003
C00238	Q14500
C00238	Q14721
C00238	Q14CL3
C00238	Q14D71
C00238	Q15842
C00238	Q16322
C00238	Q17ST2
C00238	Q18120
C00238	Q186E8
C00238	Q19907
C00238	Q1LP09
C00238	Q1M651
C00238	Q1MEX2
C00238	Q1MKT5
C00238	Q22271
C00238	Q23435
C00238	Q24046
C00238	Q24048
C00238	Q2FQC0
C00238	Q2K2I9
C00238	Q2K711
C00238	Q2KBW4

C00238	Q2NQ98
C00238	Q2NW73
C00238	Q2RV87
C00238	Q2RV89
C00238	Q2YDA1
C00238	Q38849
C00238	Q38898
C00238	Q38998
C00238	Q39128
C00238	Q3APU9
C00238	Q3B1C1
C00238	Q3KNS8
C00238	Q3LS21
C00238	Q3ST91
C00238	Q43390
C00238	Q44659
C00238	Q44660
C00238	Q47A46
C00238	Q47GU1
C00238	Q47H38
C00238	Q47H39
C00238	Q47H40
C00238	Q47IT9
C00238	Q4KHA4
C00238	Q4ZFY1
C00238	Q52978
C00238	Q52980
C00238	Q52981
C00238	Q52982
C00238	Q52983
C00238	Q53SA1
C00238	Q53SU2
C00238	Q53Y30
C00238	Q547S7
C00238	Q57603
C00238	Q57CC4
C00238	Q58I19
C00238	Q5FLF5
C00238	Q5FMK7
C00238	Q5FQL0
C00238	Q5HEC4
C00238	Q5HK63
C00238	Q5HK64
C00238	Q5HK65
C00238	Q5JK32
C00238	Q5JMH0
C00238	Q5KUV3
C00238	Q5NN77

C00238	Q5P3E3
C00238	Q5YZH9
C00238	Q5Z6K9
C00238	Q5ZC87
C00238	Q5ZSY2
C00238	Q5ZTN5
C00238	Q5ZW98
C00238	Q605R1
C00238	Q605R3
C00238	Q60603
C00238	Q60A92
C00238	Q61743
C00238	Q61923
C00238	Q62IJ6
C00238	Q62IJ8
C00238	Q62JX8
C00238	Q63US0
C00238	Q63VS1
C00238	Q63VS3
C00238	Q64YU7
C00238	Q64YU9
C00238	Q652J4
C00238	Q653B6
C00238	Q664Q5
C00238	Q667S4
C00238	Q667S5
C00238	Q66GH5
C00238	Q67UC7
C00238	Q67VS5
C00238	Q69L87
C00238	Q69RI8
C00238	Q6A5S0
C00238	Q6ABJ2
C00238	Q6B014
C00238	Q6CZU5
C00238	Q6D7I3
C00238	Q6D7I5
C00238	Q6DB92
C00238	Q6F793
C00238	Q6H4L9
C00238	Q6H4M2
C00238	Q6H4R6
C00238	Q6HN77
C00238	Q6HN78
C00238	Q6HN79
C00238	Q6J4K2
C00238	Q6MLL0
C00238	Q6N5F2

C00238	Q6N5G6
C00238	Q6N5H0
C00238	Q6N5H2
C00238	Q6N893
C00238	Q6PIE5
C00238	Q6PIU1
C00238	Q6UVM3
C00238	Q6VV64
C00238	Q6VVA6
C00238	Q6YSA9
C00238	Q6YWQ4
C00238	Q6ZPR4
C00238	Q71RS6
C00238	Q71W89
C00238	Q71W90
C00238	Q71W91
C00238	Q725T6
C00238	Q741T7
C00238	Q747C1
C00238	Q74AA5
C00238	Q74AA8
C00238	Q74AB0
C00238	Q74AK4
C00238	Q74LN2
C00238	Q74LN3
C00238	Q75G84
C00238	Q795M8
C00238	Q7A4G4
C00238	Q7N6W5
C00238	Q7N6W6
C00238	Q7N6W7
C00238	Q7TSH7
C00238	Q7VTK0
C00238	Q7VVZ9
C00238	Q7W536
C00238	Q7W7H9
C00238	Q7WCL8
C00238	Q7WKW8
C00238	Q7XIV8
C00238	Q7XLC6
C00238	Q7XPL3
C00238	Q7Z418
C00238	Q80XM3
C00238	Q81HP9
C00238	Q81HQ0
C00238	Q81HQ1
C00238	Q81UW5
C00238	Q81UW7

C00238	Q82PI3
C00238	Q82PI5
C00238	Q837G9
C00238	Q83PJ2
C00238	Q83PY0
C00238	Q83S83
C00238	Q83SQ3
C00238	Q84MS3
C00238	Q84MS4
C00238	Q84TI7
C00238	Q84YJ9
C00238	Q86WJ5
C00238	Q86WJ6
C00238	Q86Y85
C00238	Q87D01
C00238	Q87Y19
C00238	Q883V4
C00238	Q883V6
C00238	Q88FD7
C00238	Q88FD8
C00238	Q88NK7
C00238	Q88SV0
C00238	Q88Z42
C00238	Q89FC2
C00238	Q89FC4
C00238	Q89L53
C00238	Q89NN5
C00238	Q89NN6
C00238	Q8A519
C00238	Q8A521
C00238	Q8BQZ8
C00238	Q8BZN2
C00238	Q8C261
C00238	Q8CFS6
C00238	Q8CGQ8
C00238	Q8DHB0
C00238	Q8DZL1
C00238	Q8E575
C00238	Q8F1M1
C00238	Q8F1M2
C00238	Q8FZT8
C00238	Q8G6U3
C00238	Q8G7Q3
C00238	Q8GXE6
C00238	Q8H3P9
C00238	Q8JZN3
C00238	Q8K3F6
C00238	Q8KAT2

C00238	Q8KU73
C00238	Q8LBL1
C00238	Q8LPL8
C00238	Q8N538
C00238	Q8NCM2
C00238	Q8NFF2
C00238	Q8NSG3
C00238	Q8PHV1
C00238	Q8PPC8
C00238	Q8PPC9
C00238	Q8R1C0
C00238	Q8R1P5
C00238	Q8R8I5
C00238	Q8R8I6
C00238	Q8R8I7
C00238	Q8TAE7
C00238	Q8TDN1
C00238	Q8TDN2
C00238	Q8TL61
C00238	Q8U9D8
C00238	Q8U9D9
C00238	Q8U9E0
C00238	Q8UHH1
C00238	Q8UJL0
C00238	Q8VDN2
C00238	Q8VI23
C00238	Q8VXB1
C00238	Q8VXB5
C00238	Q8W4I4
C00238	Q8X878
C00238	Q8X9F8
C00238	Q8X9F9
C00238	Q8X9G0
C00238	Q8XA20
C00238	Q8XAW9
C00238	Q8XR16
C00238	Q8XU10
C00238	Q8XU11
C00238	Q8XU12
C00238	Q8XYY5
C00238	Q8Y3Z6
C00238	Q8Y3Z7
C00238	Q8Y3Z8
C00238	Q8YI23
C00238	Q8YPE8
C00238	Q8YPE9
C00238	Q8YPF1
C00238	Q8YSD5

C00238	Q8YSD7
C00238	Q8Z1Y7
C00238	Q8Z2R2
C00238	Q8Z8E4
C00238	Q8Z8E5
C00238	Q8Z8E6
C00238	Q8Z9K0
C00238	Q8ZD96
C00238	Q8ZD97
C00238	Q8ZD98
C00238	Q8ZJC4
C00238	Q8ZJT0
C00238	Q8ZKW1
C00238	Q8ZLL3
C00238	Q8ZQW1
C00238	Q8ZQW2
C00238	Q8ZQW3
C00238	Q8ZRW2
C00238	Q91V14
C00238	Q925Q3
C00238	Q927F9
C00238	Q927G0
C00238	Q927G1
C00238	Q92806
C00238	Q92952
C00238	Q92953
C00238	Q92RN0
C00238	Q92XJ0
C00238	Q92Y93
C00238	Q93235
C00238	Q942X8
C00238	Q94526
C00238	Q94A76
C00238	Q96KK3
C00238	Q96L42
C00238	Q96PR1
C00238	Q96RP8
C00238	Q96T54
C00238	Q96T55
C00238	Q97BF5
C00238	Q97BF6
C00238	Q97BF7
C00238	Q98GX5
C00238	Q98GX6
C00238	Q98GX7
C00238	Q98KL7
C00238	Q98KL8
C00238	Q99271

C00238	Q99712
C00238	Q99Z39
C00238	Q9A7X6
C00238	Q9A7X7
C00238	Q9A7X8
C00238	Q9ABT9
C00238	Q9BQ31
C00238	Q9CHU4
C00238	Q9CHU5
C00238	Q9EQR3
C00238	Q9FE38
C00238	Q9FL25
C00238	Q9FWX6
C00238	Q9FY75
C00238	Q9H252
C00238	Q9H2S1
C00238	Q9H2X9
C00238	Q9H3M0
C00238	Q9H427
C00238	Q9HB14
C00238	Q9HB15
C00238	Q9HC58
C00238	Q9I540
C00238	Q9JIS8
C00238	Q9JK45
C00238	Q9JK97
C00238	Q9JM63
C00238	Q9KM59
C00238	Q9L6L2
C00238	Q9LD18
C00238	Q9M7J9
C00238	Q9M7K4
C00238	Q9M8S6
C00238	Q9NPC2
C00238	Q9NPI9
C00238	Q9NR82
C00238	Q9NS40
C00238	Q9NSA2
C00238	Q9NYG8
C00238	Q9NZV8
C00238	Q9P1Z3
C00238	Q9P7I1
C00238	Q9PC78
C00238	Q9RZN6
C00238	Q9RZN7
C00238	Q9RZP0
C00238	Q9S6Z8
C00238	Q9SA05

C00238	Q9SCX5
C00238	Q9SVV6
C00238	Q9U6A0
C00238	Q9UGI6
C00238	Q9UHW9
C00238	Q9UI40
C00238	Q9UIX4
C00238	Q9UJ96
C00238	Q9UK17
C00238	Q9UL51
C00238	Q9ULD8
C00238	Q9ULS6
C00238	Q9UNX9
C00238	Q9UP95
C00238	Q9UQ05
C00238	Q9WVL3
C00238	Q9X8Z8
C00238	Q9X8Z9
C00238	Q9X900
C00238	Q9XFR0
C00238	Q9Y257
C00238	Q9Y2U2
C00238	Q9Y3Q4
C00238	Q9Y666
C00238	Q9Z0V1
C00238	Q9Z0V2
C00238	Q9Z2T1
C00238	Q9Z3Q3
C00238	Q9ZNG6
C00241	O05123
C00242	Q84MA8
C00242	Q9SRK7
C00243	A6MER5
C00243	A6MER6
C00243	A6MER7
C00243	O06980
C00243	O06981
C00243	O34697
C00243	O34741
C00243	O50295
C00243	O50296
C00243	O52853
C00243	P02920
C00243	P07921
C00243	P0A0D4
C00243	P0A0D6
C00243	P11162
C00243	P23936

C00243	P26426
C00243	P29822
C00243	P29823
C00243	P29824
C00243	P31675
C00243	P33026
C00243	P42423
C00243	P42424
C00243	P42953
C00243	P42954
C00243	P50976
C00243	Q01937
C00243	Q09IH8
C00243	Q09IH9
C00243	Q09II0
C00243	Q45404
C00243	Q45405
C00243	Q48597
C00243	Q48598
C00243	Q48599
C00243	Q5HE14
C00243	Q5HE15
C00243	Q5HM39
C00243	Q5HM40
C00243	Q75V13
C00243	Q75V14
C00243	Q75V15
C00243	Q7A2B0
C00243	Q8CNF6
C00243	Q8CNF7
C00243	Q8DQ76
C00243	Q8DQ77
C00243	Q8VUH1
C00243	Q8VUH2
C00243	Q8YT39
C00243	Q99S77
C00243	Q9F443
C00243	Q9F444
C00243	Q9S3K0
C00244	P22152
C00244	P38043
C00244	P38044
C00244	P38045
C00244	P38046
C00244	P42432
C00244	P73265
C00244	P73450
C00244	P73451

C00244	P73452
C00244	Q05085
C00244	Q39608
C00244	Q43390
C00244	Q44292
C00244	Q55108
C00244	Q55463
C00244	Q5HLK7
C00244	Q5MZ53
C00244	Q5MZ54
C00244	Q5MZ55
C00244	Q7A3U8
C00244	Q8CN76
C00244	Q9R6U5
C00244	Q9SWR8
C00245	B2RNU7
C00245	O35316
C00245	P31641
C00245	Q02SA6
C00245	Q1M7R4
C00245	Q2K164
C00245	Q47537
C00245	Q47538
C00245	Q47539
C00245	Q4FMG5
C00245	Q4KK16
C00245	Q5LVM5
C00245	Q62AW4
C00245	Q63JZ3
C00245	Q664P8
C00245	Q83MA0
C00245	Q87UH7
C00245	Q88RA1
C00245	Q8X5I6
C00245	Q8ZJD0
C00245	Q92UX0
C00245	Q98DW6
C00245	Q9HX79
C00253	O88750
C00259	P0AAF5
C00259	P0AE27
C00259	Q62GY9
C00259	Q63QQ7
C00259	Q65WJ1
C00259	Q6D4W8
C00259	Q83KP2
C00259	Q87FK7
C00259	Q882I8

C00259	Q8XVS2
C00259	Q9KEE7
C00259	Q9KEE9
C00259	Q9KEF0
C00288	A0L3E7
C00288	A2AJN7
C00288	O88343
C00288	P02730
C00288	P39660
C00288	P76460
C00288	Q2Y0W8
C00288	Q55027
C00288	Q55106
C00288	Q55107
C00288	Q55108
C00288	Q55460
C00288	Q55461
C00288	Q55462
C00288	Q55463
C00288	Q5DTL9
C00288	Q5MZ53
C00288	Q5MZ54
C00288	Q5MZ55
C00288	Q5MZ56
C00288	Q6U841
C00288	Q8JZR6
C00288	Q8NBS3
C00288	Q9BY07
C00288	Q9R1N3
C00288	Q9Y6M7
C00288	Q9Y6R1
C00291	P0AFA9
C00291	P0AFB0
C00291	P23516
C00291	P28795
C00291	P33590
C00291	P33591
C00291	P33593
C00291	P33594
C00291	P37617
C00291	P37972
C00291	P39642
C00291	Q07418
C00291	Q45247
C00291	Q48262
C00291	Q5KG96
C00291	Q83J77
C00291	Q83J78

C00291	Q88HL0
C00291	Q88HL1
C00291	Q8FVM9
C00291	Q8FVN0
C00291	Q8X4L6
C00291	Q8X5U1
C00291	Q8YCN7
C00291	Q8YCN8
C00293	A0PJK1
C00293	A0QZX3
C00293	A0ZXK6
C00293	A1Z264
C00293	A3M0N3
C00293	B1PM37
C00293	O05176
C00293	O05177
C00293	O07563
C00293	O23492
C00293	O25788
C00293	O52733
C00293	O74969
C00293	P0A283
C00293	P0A284
C00293	P0AAG8
C00293	P0AEE5
C00293	P0C105
C00293	P10870
C00293	P11166
C00293	P11168
C00293	P11169
C00293	P13181
C00293	P13866
C00293	P14142
C00293	P14246
C00293	P14672
C00293	P15729
C00293	P17809
C00293	P21906
C00293	P22732
C00293	P23200
C00293	P23586
C00293	P25548
C00293	P26379
C00293	P26380
C00293	P26381
C00293	P26382
C00293	P26430
C00293	P28288

C00293	P31636
C00293	P31639
C00293	P32037
C00293	P32568
C00293	P33302
C00293	P33897
C00293	P34230
C00293	P37439
C00293	P39816
C00293	P39924
C00293	P40550
C00293	P41820
C00293	P42833
C00293	P43071
C00293	P45338
C00293	P45618
C00293	P47315
C00293	P51533
C00293	P53048
C00293	P53403
C00293	P54862
C00293	P60857
C00293	P60944
C00293	P69783
C00293	P69785
C00293	P69786
C00293	P69788
C00293	P69797
C00293	P69801
C00293	P69805
C00293	P75569
C00293	P78577
C00293	P78595
C00293	P78831
C00293	P96169
C00293	Q01441
C00293	Q02785
C00293	Q05BQ3
C00293	Q06222
C00293	Q07647
C00293	Q12300
C00293	Q2M3M2
C00293	Q2MDH1
C00293	Q39228
C00293	Q44840
C00293	Q48WG5
C00293	Q50614
C00293	Q51485

C00293	Q53922
C00293	Q57071
C00293	Q5ERC7
C00293	Q5HD13
C00293	Q5HFZ9
C00293	Q5HJI3
C00293	Q5HL73
C00293	Q5HLZ2
C00293	Q5HPB5
C00293	Q5SWY8
C00293	Q5TF39
C00293	Q64L87
C00293	Q6PXP3
C00293	Q7A3G4
C00293	Q7A807
C00293	Q7CCJ4
C00293	Q814U8
C00293	Q816V0
C00293	Q81JY0
C00293	Q81KM7
C00293	Q89AG6
C00293	Q89B05
C00293	Q8BFW9
C00293	Q8C3K6
C00293	Q8CNF2
C00293	Q8CP79
C00293	Q8CZD5
C00293	Q8DS05
C00293	Q8G3X1
C00293	Q8IRI6
C00293	Q8K0E3
C00293	Q8K9J0
C00293	Q8KA51
C00293	Q8L6Z8
C00293	Q8LBP4
C00293	Q8TDB8
C00293	Q8VCV9
C00293	Q8VDT1
C00293	Q8VHD6
C00293	Q8VZR6
C00293	Q8WWX8
C00293	Q8X0Z3
C00293	Q8ZQ35
C00293	Q92253
C00293	Q923I7
C00293	Q94AZ2
C00293	Q94FB9
C00293	Q9AGA7

C00293	Q9BYW1
C00293	Q9C757
C00293	Q9FMX3
C00293	Q9JIF3
C00293	Q9KCQ4
C00293	Q9LNV3
C00293	Q9M9E1
C00293	Q9NRM0
C00293	Q9NY64
C00293	Q9NY91
C00293	Q9P3U6
C00293	Q9UGQ3
C00293	Q9XIE2
C00296	A1Z265
C00296	B1PLM1
C00296	P11636
C00296	Q5A8J5
C00296	Q926Q9
C00296	Q9NRM0
C00299	P05316
C00299	P38196
C00300	P48029
C00300	Q8VBW1
C00302	O00341
C00302	O30417
C00302	O35544
C00302	O75746
C00302	P0AAG3
C00302	P0AAG4
C00302	P0AER3
C00302	P0AER5
C00302	P0AER7
C00302	P0AER8
C00302	P0AER9
C00302	P21345
C00302	P24942
C00302	P24943
C00302	P31596
C00302	P37902
C00302	P43003
C00302	P43004
C00302	P43006
C00302	P45240
C00302	P48242
C00302	P48243
C00302	P48244
C00302	P48245
C00302	P48664

C00302	P51906
C00302	P53388
C00302	P56564
C00302	P58229
C00302	Q10901
C00302	Q3TXX4
C00302	Q52663
C00302	Q52664
C00302	Q52665
C00302	Q52666
C00302	Q8BFU8
C00302	Q8BH59
C00302	Q8BLE7
C00302	Q8NDX2
C00302	Q8RQL4
C00302	Q8RQL5
C00302	Q8RQL6
C00302	Q8RQL7
C00302	Q91WN3
C00302	Q9CG19
C00302	Q9D6M3
C00302	Q9DB41
C00302	Q9H1K4
C00302	Q9H936
C00302	Q9P2U7
C00302	Q9P2U8
C00302	Q9QXX4
C00302	Q9UPY5
C00302	Q9WTR6
C00303	P0AAE0
C00303	P0AEQ3
C00303	P0AEQ6
C00303	P0AEQ8
C00303	P0AEQ9
C00303	P10346
C00303	P25376
C00303	P28572
C00303	P30144
C00303	P48813
C00303	Q52663
C00303	Q52664
C00303	Q52665
C00303	Q52666
C00303	Q92J96
C00303	Q9A1H0
C00303	Q9A1H1
C00304	B7ZGM0
C00304	O82855

C00304	P31675
C00304	Q2G140
C00304	Q3V050
C00304	Q5I0E9
C00304	Q86VL8
C00304	Q8K0H1
C00304	Q945F0
C00304	Q96FL8
C00304	Q9I3Y3
C00304	Q9JV27
C00305	A3BV82
C00305	O25901
C00305	O34442
C00305	P0A2R8
C00305	P0A2R9
C00305	P0ABI6
C00305	P0ABI7
C00305	P28795
C00305	P36640
C00305	P40948
C00305	P44998
C00305	P55069
C00305	P87149
C00305	Q01926
C00305	Q02783
C00305	Q058N4
C00305	Q07418
C00305	Q0D2K0
C00305	Q0JBZ6
C00305	Q10D38
C00305	Q10S25
C00305	Q1PE39
C00305	Q2NQF9
C00305	Q304A0
C00305	Q45121
C00305	Q491Z9
C00305	Q58439
C00305	Q5KG96
C00305	Q5NCE8
C00305	Q65VT3
C00305	Q66FY7
C00305	Q67UQ7
C00305	Q6CZH4
C00305	Q6NVV3
C00305	Q7M9C0
C00305	Q7MYP0
C00305	Q7RTP0
C00305	Q7SFQ9

C00305	Q7VFJ7
C00305	Q7VN59
C00305	Q7VRM7
C00305	Q7XQQ1
C00305	Q830V1
C00305	Q8BHK1
C00305	Q8BJA2
C00305	Q8BMW7
C00305	Q8BYR8
C00305	Q8BZF2
C00305	Q8H1G7
C00305	Q8IVJ1
C00305	Q8L4S2
C00305	Q8N8Q9
C00305	Q8S1N1
C00305	Q8ZAG5
C00305	Q923J1
C00305	Q93ZD7
C00305	Q96JW4
C00305	Q96QT4
C00305	Q9AUK4
C00305	Q9BX84
C00305	Q9CLC4
C00305	Q9FLG2
C00305	Q9HD23
C00305	Q9JJC8
C00305	Q9LJN2
C00305	Q9LXD4
C00305	Q9PPI1
C00305	Q9S9N4
C00305	Q9SAH0
C00305	Q9WZ31
C00305	Q9ZPR4
C00315	A0LUE6
C00315	A3CMQ7
C00315	O51587
C00315	O83658
C00315	P0A2C7
C00315	P0A2C8
C00315	P0A2J7
C00315	P0A2J8
C00315	P0AFK4
C00315	P0AFK5
C00315	P0AFK6
C00315	P0AFK8
C00315	P0AFK9
C00315	P0AFL0
C00315	P40790

C00315	P44731
C00315	P45168
C00315	P45169
C00315	P45170
C00315	P45171
C00315	P47288
C00315	P47289
C00315	P47290
C00315	P69874
C00315	P69876
C00315	P69877
C00315	P75057
C00315	P75058
C00315	P75059
C00315	Q02R79
C00315	Q02Z10
C00315	Q03AH0
C00315	Q03JH1
C00315	Q03PF2
C00315	Q03ZQ0
C00315	Q042G7
C00315	Q04BG2
C00315	Q0AGF4
C00315	Q0I3Y9
C00315	Q0RAT5
C00315	Q0SML1
C00315	Q18AM3
C00315	Q2NK31
C00315	Q2SSS4
C00315	Q4K681
C00315	Q5FL41
C00315	Q5HGY5
C00315	Q5HQ70
C00315	Q5L222
C00315	Q5LT05
C00315	Q5LYN4
C00315	Q5ZWE4
C00315	Q60AI1
C00315	Q64SQ6
C00315	Q65UE1
C00315	Q660M8
C00315	Q6D4E2
C00315	Q6F0V4
C00315	Q6HLQ9
C00315	Q6KIP2
C00315	Q6LR20
C00315	Q6MCV4
C00315	Q6MU19

C00315	Q6YPR6
C00315	Q722B1
C00315	Q72FW5
C00315	Q74K65
C00315	Q7A679
C00315	Q7MKU3
C00315	Q7NB11
C00315	Q7VNG4
C00315	Q81GC1
C00315	Q81TH8
C00315	Q82TL6
C00315	Q830W6
C00315	Q83RR7
C00315	Q87PH3
C00315	Q88ZJ6
C00315	Q8A883
C00315	Q8CPN0
C00315	Q8D9J4
C00315	Q8DUF7
C00315	Q8DZJ0
C00315	Q8E554
C00315	Q8ELR4
C00315	Q8EUR3
C00315	Q8RI39
C00315	Q8XIZ5
C00315	Q8Y8T6
C00315	Q8YM92
C00315	Q8Z7H7
C00315	Q92DL6
C00315	Q97KS6
C00315	Q97Q42
C00315	Q98HF7
C00315	Q98QE1
C00315	Q99ZS8
C00315	Q9CGD4
C00315	Q9CP06
C00315	Q9HY19
C00315	Q9I6T2
C00315	Q9KS33
C00315	Q9PR37
C00315	Q9X196
C00316	P0C2U2
C00316	Q8X651
C00318	P0AFM2
C00318	P14175
C00318	P14176
C00320	P0AEB0
C00320	P0AG78

C00320	P16676
C00320	P16700
C00320	P16701
C00320	P32332
C00320	Q7N6Z2
C00320	Q8EBC3
C00320	Q8XBJ8
C00334	O35633
C00334	P30531
C00334	P31648
C00334	P31649
C00334	P32837
C00334	P48065
C00334	P48066
C00334	Q9H598
C00334	Q9NSD5
C00334	Q9VR07
C00338	Q15311
C00338	Q48475
C00338	Q48476
C00342	P0A742
C00366	Q07307
C00366	Q96S37
C00370	O15118
C00370	O35956
C00370	P61916
C00370	Q12200
C00370	Q60961
C00370	Q99PE8
C00370	Q9H221
C00370	Q9H222
C00376	P78363
C00377	O35956
C00377	P33302
C00377	P41820
C00377	P43505
C00377	P51533
C00377	Q51006
C00377	Q51073
C00377	Q9M9E1
C00378	O60779
C00378	P31548
C00378	P31549
C00378	P31550
C00378	P44984
C00378	P44985
C00378	P44986
C00378	Q05998

C00378	Q08579
C00378	Q0I354
C00378	Q0WJE4
C00378	Q2NVW9
C00378	Q57BC2
C00378	Q57BC3
C00378	Q57BC4
C00378	Q65SC9
C00378	Q6D0F3
C00378	Q7MPC5
C00378	Q7N8V0
C00378	Q7VP69
C00378	Q83MG3
C00378	Q87SV4
C00378	Q8FYU9
C00378	Q8FYV0
C00378	Q8FYV1
C00378	Q8UBY6
C00378	Q8XA06
C00378	Q8YJ02
C00378	Q8YJ03
C00378	Q8YJ04
C00378	Q8Z9I6
C00378	Q8ZRV2
C00378	Q92L31
C00378	Q98FA5
C00378	Q99PL8
C00378	Q9CNP9
C00378	Q9KP42
C00380	P0AA82
C00380	P17064
C00380	P40039
C00380	Q12119
C00380	Q8X691
C00382	O05176
C00382	O05177
C00382	P25548
C00382	P32568
C00382	P33302
C00382	P40550
C00382	P41820
C00382	P43071
C00382	P51533
C00382	P78577
C00382	P78595
C00382	Q02785
C00382	Q8X0Z3
C00382	Q9M9E1

C00382	Q9XIE2
C00383	O06931
C00383	O06932
C00383	O32715
C00383	P32332
C00383	P56949
C00385	P42086
C00385	Q07307
C00392	O30831
C00392	O30832
C00392	O30833
C00392	O53899
C00392	O65989
C00392	P00550
C00392	P0A0D8
C00392	P0ADC3
C00392	P27547
C00392	P32010
C00392	P32011
C00392	P46903
C00392	P46904
C00392	P54933
C00392	P69825
C00392	P69827
C00392	P75145
C00392	P75146
C00392	P75957
C00392	P75958
C00392	Q02420
C00392	Q2J9P4
C00392	Q2J9P5
C00392	Q4VWC6
C00392	Q4VWC7
C00392	Q4VWC8
C00392	Q4VWC9
C00392	Q4VWD0
C00392	Q53716
C00392	Q53717
C00392	Q5HE46
C00392	Q5HE48
C00392	Q70J75
C00392	Q70J76
C00392	Q7A4B3
C00392	Q7D911
C00392	Q89A36
C00392	Q8K911
C00392	Q9A1K4
C00392	Q9A1K5

C00392	Q9CIP5
C00392	Q9CIP6
C00392	Q9CLY8
C00392	Q9K678
C00392	Q9K680
C00392	Q9KJ75
C00392	Q9KKQ7
C00395	P0AE16
C00413	B7ZGM0
C00413	O82855
C00413	P31675
C00413	Q2G140
C00413	Q3V050
C00413	Q5I0E9
C00413	Q86VL8
C00413	Q8K0H1
C00413	Q945F0
C00413	Q96FL8
C00413	Q9I3Y3
C00413	Q9JV27
C00420	P18895
C00420	P32015
C00420	P32016
C00420	P43109
C00420	P43110
C00455	P52059
C00473	O70491
C00474	O52717
C00486	A8I268
C00486	O88750
C00486	Q7GB25
C00486	Q92887
C00486	Q9C8G9
C00487	O43772
C00487	O76082
C00487	P31553
C00487	P97521
C00487	Q12289
C00487	Q86VW1
C00487	Q8BL03
C00487	Q8N8R3
C00487	Q93XM7
C00487	Q9H015
C00487	Q9Z2Z6
C00492	A0L4K8
C00492	A0L4L0
C00492	A4YGY2
C00492	A8M8Z1

C00492	A8TDW7
C00492	A8TDX0
C00492	A9QDR6
C00492	A9QDR7
C00492	A9QDR8
C00492	O06989
C00492	O06990
C00492	O06991
C00492	O07009
C00492	O07011
C00492	O07330
C00492	O30489
C00492	O31710
C00492	O31711
C00492	O31712
C00492	O32261
C00492	P0A3G5
C00492	P16552
C00492	P36497
C00492	P43466
C00492	P50332
C00492	P50333
C00492	P68579
C00492	P75830
C00492	P75831
C00492	P94360
C00492	P96483
C00492	Q00564
C00492	Q00749
C00492	Q00750
C00492	Q00751
C00492	Q00752
C00492	Q03727
C00492	Q2SDB0
C00492	Q2SDB1
C00492	Q52899
C00492	Q52900
C00492	Q72H66
C00492	Q72H67
C00492	Q72H68
C00492	Q72KX2
C00492	Q72KX3
C00492	Q72KX4
C00492	Q72L52
C00492	Q73MJ2
C00492	Q73MJ3
C00492	Q73MJ4
C00492	Q7A3X3

C00492	Q7A7X2
C00492	Q7AUQ4
C00492	Q7WWQ6
C00492	Q7WWQ8
C00492	Q7WWQ9
C00492	Q7WWR0
C00492	Q8DNB9
C00492	Q8DNC0
C00492	Q8DT25
C00492	Q8DT26
C00492	Q8DT27
C00492	Q8DT28
C00492	Q8RKC0
C00492	Q8RKC1
C00492	Q8TM30
C00492	Q8TM31
C00492	Q93LN0
C00492	Q93LN1
C00492	Q973J4
C00492	Q9L026
C00492	Q9L027
C00492	Q9S5Y1
C00492	Q9X0S9
C00492	Q9X0T0
C00492	Q9X0T1
C00492	Q9X103
C00492	Q9X2F5
C00492	Q9X9R5
C00492	Q9X9R6
C00492	Q9X9R7
C00493	P76350
C00499	P15365
C00504	O45166
C00504	O60779
C00504	O76729
C00504	P41438
C00504	P41440
C00504	Q07492
C00504	Q17766
C00504	Q25272
C00504	Q54QN2
C00504	Q6PEM8
C00504	Q86I81
C00504	Q8BMG8
C00504	Q96NT5
C00504	Q9H2D1
C00504	Q9UAC2
C00507	B5XZ47

C00507	P27135
C00507	P39952
C00507	Q65Q27
C00507	Q66FF0
C00507	Q6DA13
C00507	Q83PD8
C00507	Q8X3T0
C00507	Q8Z2V6
C00507	Q8ZIZ8
C00528	P0AE16
C00547	P23975
C00572	Q96QT4
C00638	P28288
C00638	P34230
C00698	A6T4V9
C00698	A8K0U7
C00698	A8K7I4
C00698	A8MPY1
C00698	B5Y1L4
C00698	C9JAR3
C00698	O00168
C00698	O00299
C00698	O00591
C00698	O14764
C00698	O15247
C00698	O18276
C00698	O35454
C00698	O43511
C00698	O70496
C00698	O95833
C00698	P02730
C00698	P04919
C00698	P13569
C00698	P13808
C00698	P14867
C00698	P16102
C00698	P18505
C00698	P18507
C00698	P22723
C00698	P22933
C00698	P24046
C00698	P26048
C00698	P26049
C00698	P26361
C00698	P28472
C00698	P28476
C00698	P31641
C00698	P31644

C00698	P34903
C00698	P35523
C00698	P37020
C00698	P40879
C00698	P47869
C00698	P47870
C00698	P48169
C00698	P50571
C00698	P51788
C00698	P51790
C00698	P51791
C00698	P51793
C00698	P51795
C00698	P51798
C00698	P51800
C00698	P51801
C00698	P55011
C00698	P55014
C00698	P55017
C00698	P55018
C00698	P56475
C00698	P58244
C00698	P59585
C00698	P59638
C00698	P59639
C00698	P62812
C00698	P63080
C00698	P63137
C00698	P67730
C00698	P78334
C00698	P92941
C00698	P92942
C00698	P92943
C00698	Q08832
C00698	Q09573
C00698	Q13621
C00698	Q14802
C00698	Q14CN2
C00698	Q16445
C00698	Q17328
C00698	Q1ZXJ0
C00698	Q24352
C00698	Q43390
C00698	Q54AX6
C00698	Q54C67
C00698	Q54LQ4
C00698	Q64347
C00698	Q6PCC3

C00698	Q6PCD2
C00698	Q75JF3
C00698	Q7MDF0
C00698	Q86AZ6
C00698	Q87GZ9
C00698	Q8BG22
C00698	Q8BHB9
C00698	Q8BHJ7
C00698	Q8BXK9
C00698	Q8GX93
C00698	Q8N1C3
C00698	Q8QZW7
C00698	Q8RXR2
C00698	Q8VI23
C00698	Q8X794
C00698	Q8Z4X8
C00698	Q8Z6Y0
C00698	Q8Z9B3
C00698	Q8ZBM0
C00698	Q8ZPK5
C00698	Q8ZRP8
C00698	Q91V14
C00698	Q93IM6
C00698	Q96282
C00698	Q96NY7
C00698	Q99928
C00698	Q9BXP2
C00698	Q9D6F4
C00698	Q9D7P7
C00698	Q9D7Z6
C00698	Q9H2X9
C00698	Q9JIS8
C00698	Q9JLF1
C00698	Q9KM62
C00698	Q9NZA1
C00698	Q9QYB1
C00698	Q9R0A1
C00698	Q9R155
C00698	Q9UHW9
C00698	Q9UN88
C00698	Q9UP95
C00698	Q9UQC9
C00698	Q9WUB7
C00698	Q9WVC8
C00698	Q9WVD4
C00698	Q9WVL3
C00698	Q9Y666
C00698	Q9Y696

C00698	Q9Z1Q5
C00701	Q60961
C00706	O02713
C00706	O35956
C00706	O76082
C00706	Q5RLM2
C00706	Q63089
C00706	Q80UJ1
C00706	Q86VW1
C00706	Q8CFZ5
C00706	Q8IVM8
C00706	Q96S37
C00706	Q9CAT6
C00706	Q9H015
C00706	Q9NSA0
C00706	Q9R0W2
C00706	Q9Y226
C00708	Q8N695
C00708	Q92911
C00711	P50537
C00711	Q02978
C00711	Q48797
C00711	Q53787
C00711	Q54PY7
C00711	Q8LG88
C00711	Q9CR62
C00716	A6TAU9
C00716	A6TEC8
C00716	A7I2B9
C00716	B5XTX0
C00716	B5XVZ7
C00716	P0AAD6
C00716	P0AAD7
C00716	P0AAD9
C00716	P0AAE0
C00716	P0AGE4
C00716	P44615
C00716	Q02LI9
C00716	Q07ZL7
C00716	Q0I455
C00716	Q0WJ82
C00716	Q5F5G9
C00716	Q5M1F4
C00716	Q83Q28
C00716	Q83QD0
C00716	Q8ZLW1
C00719	O32243
C00719	O34742

C00719	O34878
C00719	O34992
C00719	O76082
C00719	P0AFM2
C00719	P0C0L7
C00719	P0C0L8
C00719	P14175
C00719	P14176
C00719	P40862
C00719	P43007
C00719	P46920
C00719	P46921
C00719	P46922
C00719	P48065
C00719	P54417
C00719	Q5HRH0
C00719	Q79VE0
C00719	Q7A771
C00719	Q87WH3
C00719	Q87WH4
C00719	Q87WH5
C00719	Q8Q040
C00719	Q8Q041
C00719	Q8Q042
C00719	Q8Q043
C00719	Q9HTH6
C00719	Q9HTI6
C00719	Q9HTI7
C00719	Q9HTI8
C00725	O70247
C00735	O15118
C00735	P61916
C00735	Q12200
C00738	O04249
C00738	O82390
C00738	P0AA78
C00738	P0AGC0
C00738	P0AGC1
C00738	P0AGC2
C00738	P15686
C00738	P32466
C00738	P32467
C00738	P38695
C00738	P39003
C00738	P39004
C00738	P39924
C00738	P40885
C00738	P40886

C00738	P42833
C00738	P43581
C00738	P47185
C00738	P53631
C00738	P54854
C00738	Q01441
C00738	Q07YH1
C00738	Q12235
C00738	Q8BFU8
C00738	Q8GW61
C00738	Q8L7R8
C00738	Q93Y91
C00738	Q94AZ2
C00738	Q9BYT1
C00738	Q9FMX3
C00738	Q9LNV3
C00738	Q9SBA7
C00738	Q9SX48
C00738	Q9Y2C5
C00738	Q9Z7N9
C00740	P0AAE1
C00767	O13880
C00767	O43000
C00767	P25621
C00767	P42237
C00767	P53241
C00767	P53322
C00767	Q62634
C00767	Q7YQJ7
C00767	Q8FDB7
C00767	Q9JI12
C00767	Q9NRA2
C00768	A1VZQ3
C00768	A3ZI83
C00768	O30506
C00768	O31199
C00768	O34315
C00768	O34406
C00768	O34852
C00768	O34900
C00768	O34931
C00768	O50181
C00768	O50182
C00768	O50183
C00768	P02910
C00768	P02915
C00768	P06775
C00768	P09551

C00768	P0A2I7
C00768	P0A2I8
C00768	P0A2I9
C00768	P0A2J0
C00768	P0AEM9
C00768	P0AEU2
C00768	P0AEU5
C00768	P0AEU6
C00768	P0AFT2
C00768	P0AG34
C00768	P32487
C00768	P37774
C00768	P38090
C00768	P38971
C00768	P39456
C00768	P42087
C00768	P42199
C00768	P42200
C00768	P43548
C00768	P46032
C00768	P46349
C00768	P53388
C00768	P54535
C00768	P54536
C00768	P54537
C00768	P73544
C00768	P73721
C00768	Q03770
C00768	Q08986
C00768	Q0P9X8
C00768	Q12372
C00768	Q43390
C00768	Q88NY2
C00768	Q88NY3
C00768	Q88NY4
C00768	Q88NY5
C00768	Q8IY34
C00768	Q8J266
C00768	Q8N697
C00768	Q8NKC4
C00768	Q8YPM7
C00768	Q8YPM8
C00768	Q8YPM9
C00768	Q8YSA2
C00768	Q91W98
C00768	Q9HZS1
C00770	P39344
C00780	P31645

C00780	P51905
C00780	Q5EE02
C00794	P59784
C00801	O00337
C00801	O32115
C00801	O43868
C00801	P0A927
C00801	P0AFF2
C00801	P0AFF4
C00801	P26430
C00801	P36051
C00801	P39141
C00801	Q14542
C00801	Q60961
C00801	Q62674
C00801	Q62773
C00801	Q99808
C00801	Q99P65
C00801	Q9JIM1
C00801	Q9UA35
C00806	O50649
C00806	P0AAD2
C00806	P0AAD3
C00806	P23173
C00806	P38085
C00806	P38967
C00806	P44614
C00806	Q8XB33
C00855	P31728
C00855	P44785
C00855	P63356
C00855	Q0I5E9
C00855	Q2NRN5
C00855	Q65VG9
C00855	Q667L9
C00855	Q6D1C4
C00855	Q6LN52
C00855	Q7MN25
C00855	Q7N8M2
C00855	Q7VM95
C00855	Q83MC5
C00855	Q87RS1
C00855	Q8DFC3
C00855	Q8X800
C00855	Q8X8V9
C00855	Q8Z990
C00855	Q8Z992
C00855	Q8ZH38

C00855	Q8ZH39
C00855	Q8ZH40
C00864	P25621
C00864	P44963
C00865	O35600
C00865	P02730
C00865	P08183
C00865	P40527
C00865	Q12675
C00865	Q91V24
C00898	P39414
C00898	P70786
C00918	O94528
C00918	P0A4K4
C00918	P31141
C00918	P32482
C00918	P39843
C00918	P40474
C00918	P53389
C00918	P53943
C00918	Q45RG2
C00918	Q56RY7
C00918	Q5JAK9
C00918	Q5SR56
C00918	Q5WX21
C00930	P22340
C00934	P0AGC0
C01019	A1S297
C01019	A1S5F4
C01019	P11551
C01019	P44776
C01019	Q07XD1
C01021	P15993
C01021	P59737
C01021	Q3U9N9
C01021	Q8X968
C01074	Q9KIQ2
C01074	Q9KIQ3
C01074	Q9KIQ4
C01083	A3MWP1
C01083	A3MWP2
C01083	A8F6Z4
C01083	B0K2P2
C01083	B0K2P3
C01083	B1L0U0
C01083	B1L0U1
C01083	O31427
C01083	O31428

C01083	O32748
C01083	O51923
C01083	O51924
C01083	O51925
C01083	P0ABM0
C01083	P0ABM7
C01083	P18767
C01083	P30963
C01083	P30964
C01083	P36672
C01083	P93280
C01083	P97046
C01083	Q07638
C01083	Q07639
C01083	Q6KYW8
C01083	Q6KYW9
C01083	Q8XE58
C01083	Q9C8T1
C01083	Q9X2W0
C01083	Q9Z3R5
C01083	Q9Z3R6
C01083	Q9Z3R7
C01319	Q54464
C01319	Q6UP69
C01319	Q6UP70
C01328	P10861
C01328	P12234
C01330	A0KG33
C01330	A0KL02
C01330	A0KL08
C01330	A0LLE6
C01330	A0M0N2
C01330	A0PJK1
C01330	A0QW18
C01330	A1R4H0
C01330	A2RUN4
C01330	A3KLL5
C01330	A4IVT5
C01330	A5VNX4
C01330	A5X2V1
C01330	A6Q0Z0
C01330	A6T4F6
C01330	A6TAW7
C01330	A6TEC8
C01330	A6TFD1
C01330	A6TGZ7
C01330	A6TJ58
C01330	A6UH53

C01330	A7HZF9
C01330	A7I1I1
C01330	A7I2B9
C01330	A8EUK5
C01330	A8EVL5
C01330	A8I126
C01330	A8IDZ2
C01330	B1PS44
C01330	B2R812
C01330	B2RNU7
C01330	B4DX48
C01330	B5XN31
C01330	B5XQ77
C01330	B5XTX0
C01330	B5XXT7
C01330	B9K2V4
C01330	C9JBG3
C01330	D3DPL4
C01330	D3DTQ2
C01330	D3DVE4
C01330	O00337
C01330	O00341
C01330	O00476
C01330	O00624
C01330	O01635
C01330	O02228
C01330	O02713
C01330	O06923
C01330	O06924
C01330	O06925
C01330	O06926
C01330	O06927
C01330	O06930
C01330	O06931
C01330	O06932
C01330	O16452
C01330	O26076
C01330	O27598
C01330	O27599
C01330	O27600
C01330	O27601
C01330	O32212
C01330	O35055
C01330	O35316
C01330	O35544
C01330	O35874
C01330	O35956
C01330	O43868

C01330	O50649
C01330	O60721
C01330	O60779
C01330	O61365
C01330	O66163
C01330	O70247
C01330	O76082
C01330	O76689
C01330	O82390
C01330	O85187
C01330	O88343
C01330	O88575
C01330	O88576
C01330	O88703
C01330	O88704
C01330	O88705
C01330	O95436
C01330	P05023
C01330	P05026
C01330	P07117
C01330	P09131
C01330	P0A2F8
C01330	P0A2F9
C01330	P0AE74
C01330	P0AER8
C01330	P0AER9
C01330	P0AFA7
C01330	P0AFA8
C01330	P0AFC3
C01330	P0AFC7
C01330	P0AFD1
C01330	P0AFD4
C01330	P0AFD6
C01330	P0AFE0
C01330	P0AFE4
C01330	P0AFE8
C01330	P0AFF0
C01330	P0AFU3
C01330	P0AGE4
C01330	P0AGE5
C01330	P0C0L7
C01330	P10502
C01330	P13587
C01330	P13637
C01330	P13738
C01330	P13866
C01330	P14094
C01330	P14231

C01330	P14415
C01330	P16256
C01330	P19072
C01330	P19634
C01330	P20672
C01330	P21129
C01330	P21608
C01330	P21903
C01330	P21904
C01330	P21905
C01330	P23975
C01330	P24585
C01330	P24942
C01330	P24943
C01330	P25185
C01330	P26235
C01330	P26430
C01330	P26435
C01330	P27611
C01330	P28570
C01330	P28571
C01330	P28572
C01330	P28573
C01330	P29706
C01330	P29707
C01330	P29708
C01330	P29709
C01330	P29710
C01330	P30144
C01330	P30145
C01330	P30531
C01330	P31596
C01330	P31602
C01330	P31636
C01330	P31639
C01330	P31641
C01330	P31645
C01330	P31648
C01330	P31649
C01330	P31661
C01330	P31979
C01330	P32739
C01330	P33599
C01330	P33602
C01330	P33607
C01330	P34315
C01330	P34322
C01330	P34644

C01330	P35449
C01330	P35498
C01330	P35499
C01330	P36606
C01330	P37088
C01330	P39141
C01330	P41438
C01330	P43003
C01330	P43004
C01330	P43005
C01330	P43006
C01330	P43007
C01330	P43436
C01330	P43437
C01330	P43439
C01330	P43455
C01330	P43456
C01330	P43457
C01330	P44581
C01330	P44706
C01330	P44963
C01330	P45174
C01330	P45240
C01330	P45246
C01330	P46903
C01330	P46904
C01330	P48029
C01330	P48065
C01330	P48066
C01330	P48067
C01330	P48664
C01330	P48764
C01330	P50334
C01330	P50992
C01330	P50993
C01330	P51164
C01330	P51168
C01330	P51170
C01330	P51172
C01330	P51905
C01330	P51906
C01330	P51912
C01330	P53794
C01330	P54104
C01330	P54709
C01330	P54710
C01330	P55011
C01330	P55012

C01330	P55014
C01330	P55017
C01330	P55018
C01330	P56564
C01330	P57103
C01330	P58734
C01330	P60675
C01330	P60677
C01330	P60678
C01330	P60681
C01330	P60682
C01330	P60685
C01330	P60686
C01330	P60689
C01330	P60690
C01330	P60693
C01330	P60694
C01330	P60697
C01330	P60698
C01330	P70172
C01330	P70414
C01330	P78348
C01330	P80183
C01330	P80184
C01330	P80185
C01330	P80186
C01330	P80187
C01330	P96169
C01330	P97370
C01330	Q01118
C01330	Q01896
C01330	Q01959
C01330	Q02563
C01330	Q02KW3
C01330	Q02LI9
C01330	Q02UV9
C01330	Q03030
C01330	Q03031
C01330	Q03032
C01330	Q03614
C01330	Q03MC5
C01330	Q04121
C01330	Q04646
C01330	Q06495
C01330	Q06496
C01330	Q06700
C01330	Q07782
C01330	Q07YY4

C01330	Q07ZL7
C01330	Q083H3
C01330	Q09274
C01330	Q0AM65
C01330	Q0BSV4
C01330	Q0BW71
C01330	Q0I3A2
C01330	Q0I455
C01330	Q0KEX1
C01330	Q0P7X3
C01330	Q0P7X4
C01330	Q0RG46
C01330	Q0RHS2
C01330	Q0RPD5
C01330	Q0VQ74
C01330	Q0WJ82
C01330	Q11TX2
C01330	Q12691
C01330	Q12908
C01330	Q13183
C01330	Q13621
C01330	Q13733
C01330	Q14916
C01330	Q14940
C01330	Q14973
C01330	Q15758
C01330	Q16515
C01330	Q1EHB4
C01330	Q1LFB1
C01330	Q1LRW2
C01330	Q1MDR5
C01330	Q21974
C01330	Q22851
C01330	Q24046
C01330	Q24048
C01330	Q25011
C01330	Q2FMC1
C01330	Q2K5Z8
C01330	Q2KCN1
C01330	Q2M3M2
C01330	Q2NVY9
C01330	Q2RY62
C01330	Q2Y0W8
C01330	Q30PM3
C01330	Q39510
C01330	Q3A1R2
C01330	Q3A4S1
C01330	Q3KNW5

C01330	Q3SNN8
C01330	Q3UEZ8
C01330	Q3YL57
C01330	Q3ZCW7
C01330	Q41515
C01330	Q43390
C01330	Q44659
C01330	Q44660
C01330	Q47BQ7
C01330	Q47I87
C01330	Q485F0
C01330	Q48769
C01330	Q49B93
C01330	Q4FLX6
C01330	Q4G0N8
C01330	Q4K9I6
C01330	Q4KBY6
C01330	Q4KH68
C01330	Q4VAM4
C01330	Q4ZJI4
C01330	Q50773
C01330	Q50774
C01330	Q52978
C01330	Q52980
C01330	Q52981
C01330	Q52982
C01330	Q52983
C01330	Q552S0
C01330	Q56582
C01330	Q56584
C01330	Q56586
C01330	Q56587
C01330	Q56589
C01330	Q56724
C01330	Q56725
C01330	Q56EB3
C01330	Q56XP4
C01330	Q57079
C01330	Q57095
C01330	Q57111
C01330	Q57286
C01330	Q57490
C01330	Q58I19
C01330	Q59574
C01330	Q59575
C01330	Q59576
C01330	Q59577
C01330	Q59578

C01330	Q59579
C01330	Q59580
C01330	Q59584
C01330	Q5DTL9
C01330	Q5EE02
C01330	Q5F5G9
C01330	Q5FNE6
C01330	Q5HEK4
C01330	Q5HEM0
C01330	Q5HHD3
C01330	Q5HHD4
C01330	Q5HHD5
C01330	Q5HHD6
C01330	Q5HHD7
C01330	Q5HHD8
C01330	Q5HHD9
C01330	Q5HN32
C01330	Q5HQL0
C01330	Q5HQL1
C01330	Q5HQL2
C01330	Q5HQL3
C01330	Q5HQL4
C01330	Q5HQL5
C01330	Q5HQL6
C01330	Q5M1F4
C01330	Q5NCM1
C01330	Q5NE91
C01330	Q5NRB1
C01330	Q5PT53
C01330	Q5PT54
C01330	Q5PT55
C01330	Q5SWY8
C01330	Q5SZA1
C01330	Q5U4D8
C01330	Q5Z2Z5
C01330	Q61165
C01330	Q61180
C01330	Q61327
C01330	Q61983
C01330	Q62674
C01330	Q62773
C01330	Q63089
C01330	Q64T76
C01330	Q65M31
C01330	Q65RK2
C01330	Q65UC1
C01330	Q65VH3
C01330	Q665P2

C01330	Q66AQ9
C01330	Q66ES8
C01330	Q66FN0
C01330	Q67BT3
C01330	Q68KI4
C01330	Q695T7
C01330	Q6AB77
C01330	Q6AI14
C01330	Q6AQH5
C01330	Q6AS08
C01330	Q6D0B9
C01330	Q6D4M9
C01330	Q6D913
C01330	Q6D9G4
C01330	Q6FB65
C01330	Q6G0H9
C01330	Q6G5K1
C01330	Q6J4K2
C01330	Q6LG82
C01330	Q6LNY8
C01330	Q6LUL9
C01330	Q6NXK8
C01330	Q6PGE7
C01330	Q6PIE5
C01330	Q6QIY3
C01330	Q6U841
C01330	Q6X1Y6
C01330	Q71RS6
C01330	Q74AP9
C01330	Q74U12
C01330	Q7A4P8
C01330	Q7A4Q7
C01330	Q7CG77
C01330	Q7M922
C01330	Q7MGA3
C01330	Q7MLJ3
C01330	Q7N0A2
C01330	Q7N3Z4
C01330	Q7N8X6
C01330	Q7NA72
C01330	Q7TNS7
C01330	Q7UTY3
C01330	Q7VFN0
C01330	Q7VFN1
C01330	Q7VKY3
C01330	Q7VLY3
C01330	Q7VN39
C01330	Q7W1Y0

C01330	Q7WQV8
C01330	Q7XPF8
C01330	Q82EL6
C01330	Q82ZN5
C01330	Q83N58
C01330	Q83P94
C01330	Q83Q34
C01330	Q83RQ5
C01330	Q83SR3
C01330	Q84WG1
C01330	Q86UD5
C01330	Q86VS0
C01330	Q86YT5
C01330	Q87FP8
C01330	Q87N04
C01330	Q87UW8
C01330	Q87WL3
C01330	Q88FQ6
C01330	Q88K49
C01330	Q88NS2
C01330	Q8BG16
C01330	Q8BGY9
C01330	Q8BJI1
C01330	Q8BLV3
C01330	Q8BUE1
C01330	Q8BYF6
C01330	Q8BZ00
C01330	Q8C0X2
C01330	Q8C261
C01330	Q8C3K6
C01330	Q8CGQ8
C01330	Q8CNP2
C01330	Q8CPU8
C01330	Q8CPU9
C01330	Q8CPV0
C01330	Q8CPV1
C01330	Q8CPV2
C01330	Q8CPV3
C01330	Q8CPV4
C01330	Q8D666
C01330	Q8D8Y2
C01330	Q8DAG9
C01330	Q8DY58
C01330	Q8E3S4
C01330	Q8ECL5
C01330	Q8ED79
C01330	Q8EH93
C01330	Q8G2C7

C01330	Q8G5R2
C01330	Q8IVB4
C01330	Q8JZR4
C01330	Q8JZR6
C01330	Q8K0E3
C01330	Q8N130
C01330	Q8N695
C01330	Q8NBS3
C01330	Q8NFF2
C01330	Q8R4D1
C01330	Q8R4H8
C01330	Q8RWU6
C01330	Q8S396
C01330	Q8S397
C01330	Q8VBW1
C01330	Q8VDB9
C01330	Q8VDN2
C01330	Q8VDT1
C01330	Q8VM88
C01330	Q8WWX8
C01330	Q8X5T7
C01330	Q8XA63
C01330	Q8Y2K5
C01330	Q8YFI5
C01330	Q8Z1R2
C01330	Q8Z3L4
C01330	Q8Z684
C01330	Q8Z9N5
C01330	Q8ZA28
C01330	Q8ZJ73
C01330	Q8ZKF8
C01330	Q8ZLX1
C01330	Q8ZP14
C01330	Q8ZRZ3
C01330	Q91Y63
C01330	Q923I7
C01330	Q92581
C01330	Q925H0
C01330	Q925Q3
C01330	Q92911
C01330	Q92Y37
C01330	Q93235
C01330	Q93YH1
C01330	Q96EP9
C01330	Q96FT7
C01330	Q96N87
C01330	Q96T83
C01330	Q98C38

C01330	Q99250
C01330	Q99271
C01330	Q99884
C01330	Q9A1E0
C01330	Q9AAZ2
C01330	Q9BY07
C01330	Q9BZW2
C01330	Q9C007
C01330	Q9CKD8
C01330	Q9CMD9
C01330	Q9CPJ1
C01330	Q9CXB2
C01330	Q9D687
C01330	Q9DBP0
C01330	Q9EPR4
C01330	Q9ER60
C01330	Q9ES88
C01330	Q9GZN6
C01330	Q9GZV3
C01330	Q9H015
C01330	Q9H1V8
C01330	Q9H2J7
C01330	Q9H9F5
C01330	Q9HC58
C01330	Q9I273
C01330	Q9I2S5
C01330	Q9JJV9
C01330	Q9JMA9
C01330	Q9JXB8
C01330	Q9KNC9
C01330	Q9KQU7
C01330	Q9LJI2
C01330	Q9LKW9
C01330	Q9NP91
C01330	Q9NRA2
C01330	Q9NSD5
C01330	Q9NY37
C01330	Q9NY91
C01330	Q9P1Z3
C01330	Q9R0Y1
C01330	Q9R1N3
C01330	Q9S1X8
C01330	Q9S2C8
C01330	Q9SYG9
C01330	Q9U6A0
C01330	Q9UA35
C01330	Q9UBY0
C01330	Q9UGH3

C01330	Q9UHC3
C01330	Q9UHI7
C01330	Q9UI33
C01330	Q9UI40
C01330	Q9UKG4
C01330	Q9UN76
C01330	Q9UPR5
C01330	Q9UQD0
C01330	Q9V7S5
C01330	Q9VR07
C01330	Q9VVT2
C01330	Q9W4C5
C01330	Q9WTU3
C01330	Q9WTW7
C01330	Q9WU38
C01330	Q9WU39
C01330	Q9X929
C01330	Q9Y289
C01330	Q9Y2C5
C01330	Q9Y2E8
C01330	Q9Y345
C01330	Q9Y5Y9
C01330	Q9Y666
C01330	Q9Y6M7
C01330	Q9Y6R1
C01330	Q9Z2J0
C01330	Q9Z3Q3
C01330	Q9ZAA6
C01330	Q9ZAA7
C01330	Q9ZAA8
C01330	Q9ZNG6
C01394	A4HWI7
C01394	A8WEV1
C01394	O05176
C01394	O05177
C01394	O34718
C01394	O74849
C01394	O74969
C01394	O97467
C01394	P0AE24
C01394	P0AGF4
C01394	P0AGI4
C01394	P0AGI6
C01394	P0AGI7
C01394	P10090
C01394	P11166
C01394	P11168
C01394	P15729

C01394	P25548
C01394	P32467
C01394	P32568
C01394	P33302
C01394	P37387
C01394	P37388
C01394	P39003
C01394	P39932
C01394	P40550
C01394	P41820
C01394	P42417
C01394	P43071
C01394	P45045
C01394	P45046
C01394	P49374
C01394	P51533
C01394	P78577
C01394	P78595
C01394	Q01440
C01394	Q02785
C01394	Q0I348
C01394	Q11180
C01394	Q400D8
C01394	Q576H3
C01394	Q5KYS1
C01394	Q663Y5
C01394	Q6DB03
C01394	Q6LUY1
C01394	Q6PXP3
C01394	Q7BEC4
C01394	Q7CFR2
C01394	Q83J33
C01394	Q880Z2
C01394	Q8FUR8
C01394	Q8NJ22
C01394	Q8RXN0
C01394	Q8VZ80
C01394	Q8X0Z3
C01394	Q8XDM1
C01394	Q8XPK6
C01394	Q8YDN0
C01394	Q92339
C01394	Q96QE2
C01394	Q9C8K2
C01394	Q9H221
C01394	Q9H222
C01394	Q9M9E1
C01394	Q9UNQ0

C01394	Q9XIE2
C01401	P0AAE0
C01401	P30144
C01401	P30145
C01413	A0ECD9
C01413	O05469
C01413	O32219
C01413	P13511
C01413	P13512
C01413	P20021
C01413	P20107
C01413	P37617
C01413	P58414
C01413	Q88CP1
C01413	Q88RT8
C01413	Q8TJZ4
C01413	Q8ZS90
C01413	Q92T56
C01413	Q9I147
C01417	O05347
C01417	O07334
C01417	P17583
C01417	Q7BW13
C01420	O60931
C01420	P0AEM9
C01420	P0AFT2
C01420	P37774
C01420	Q9UPY5
C01420	Q9WTR6
C01432	P33231
C01432	P36035
C01432	P53985
C01432	P65254
C01432	P71067
C01432	Q48769
C01432	Q8Z2E3
C01432	Q8ZL63
C01432	Q9K5Z9
C01451	P08722
C01451	P24241
C01452	P37080
C01452	P37081
C01452	P37082
C01452	P37083
C01455	O65943
C01485	P46032
C01485	Q05085
C01485	Q43390

C01496	P17127
C01496	P44714
C01496	P44715
C01496	P47308
C01496	P69812
C01496	P75039
C01496	Q8Z591
C01536	P0AAD4
C01536	P0AAD5
C01536	P38085
C01536	P38967
C01536	P44727
C01536	P44747
C01536	Q04671
C01536	Q62052
C01551	Q04895
C01551	Q93Z75
C01551	Q9ZPR7
C01558	A8I268
C01558	O30510
C01558	O35956
C01558	O95342
C01558	P09131
C01558	P21129
C01558	P26435
C01558	P32369
C01558	P43505
C01558	P70172
C01558	Q12908
C01558	Q14973
C01558	Q22NS1
C01558	Q3KNW5
C01558	Q3UEZ8
C01558	Q51006
C01558	Q51073
C01558	Q5PT53
C01558	Q5PT54
C01558	Q5PT55
C01558	Q7GB25
C01558	Q92887
C01558	Q96EP9
C01558	Q9C8G9
C01558	Q9CXB2
C01582	O05176
C01582	O05177
C01582	O25788
C01582	O34718
C01582	O74849

C01582	O74969
C01582	O97467
C01582	P0AAG8
C01582	P0AAG9
C01582	P0AE24
C01582	P0AEE5
C01582	P0AEE7
C01582	P0AEP1
C01582	P0C105
C01582	P10870
C01582	P11166
C01582	P11168
C01582	P13181
C01582	P13866
C01582	P15685
C01582	P15686
C01582	P22732
C01582	P23200
C01582	P25548
C01582	P28288
C01582	P32568
C01582	P33302
C01582	P33897
C01582	P34230
C01582	P40550
C01582	P41820
C01582	P42417
C01582	P43071
C01582	P44883
C01582	P44884
C01582	P51533
C01582	P53048
C01582	P78577
C01582	P78595
C01582	P95908
C01582	P96169
C01582	Q01440
C01582	Q01441
C01582	Q02785
C01582	Q06222
C01582	Q07647
C01582	Q12300
C01582	Q50614
C01582	Q65UW1
C01582	Q66C83
C01582	Q7CHQ3
C01582	Q896Y2
C01582	Q8REE1

C01582	Q8X0Z3
C01582	Q8ZQ35
C01582	Q92339
C01582	Q94FB9
C01582	Q96QE2
C01582	Q9CM08
C01582	Q9M9E1
C01582	Q9XIE2
C01602	A1VZQ3
C01602	A3ZI83
C01602	O30506
C01602	O32204
C01602	O34315
C01602	O34406
C01602	O34852
C01602	O34900
C01602	O34931
C01602	O50181
C01602	O50182
C01602	O50183
C01602	P02910
C01602	P02915
C01602	P09551
C01602	P0A2I7
C01602	P0A2I9
C01602	P0AAE7
C01602	P0AAF1
C01602	P0AEM9
C01602	P0AFT2
C01602	P18275
C01602	P37774
C01602	P39456
C01602	P42199
C01602	P42200
C01602	P54535
C01602	P54536
C01602	P54537
C01602	P73544
C01602	P73721
C01602	Q0P9X8
C01602	Q46170
C01602	Q88NY2
C01602	Q88NY3
C01602	Q88NY4
C01602	Q88NY5
C01602	Q8YPM7
C01602	Q8YPM8
C01602	Q8YPM9

C01602	Q8YSA2
C01602	Q9BXI2
C01602	Q9WVD5
C01602	Q9Y619
C01606	Q05181
C01609	P0A2J3
C01609	P0A2J5
C01609	P36634
C01609	P36636
C01609	P36638
C01610	P42670
C01610	Q79VC7
C01661	P32010
C01661	P32011
C01672	P0AAE8
C01672	P0AAF0
C01682	P35113
C01682	P35116
C01682	P35118
C01682	P35120
C01697	P0A436
C01697	P0A437
C01697	P37188
C01697	P69814
C01697	P69828
C01697	P69831
C01697	P69832
C01708	Q51104
C01733	O26096
C01733	P38734
C01733	P44785
C01733	P50276
C01733	P63356
C01733	Q02DK6
C01733	Q02ME3
C01733	Q032A0
C01733	Q03A07
C01733	Q03P57
C01733	Q03Z27
C01733	Q043Y8
C01733	Q04B25
C01733	Q04DA7
C01733	Q04F14
C01733	Q0AU85
C01733	Q0I5E9
C01733	Q0KDG3
C01733	Q18C09
C01733	Q1LQF6

C01733	Q1M8E0
C01733	Q2K284
C01733	Q2NRN5
C01733	Q2RWA3
C01733	Q3A9G5
C01733	Q4KBU0
C01733	Q4KK46
C01733	Q4KKK8
C01733	Q579H8
C01733	Q5FKL2
C01733	Q5HHK4
C01733	Q5HIL5
C01733	Q5HQQ9
C01733	Q5HRU5
C01733	Q5KVK2
C01733	Q5L5Z1
C01733	Q5M1F6
C01733	Q5NFU5
C01733	Q5YRD1
C01733	Q5ZUG5
C01733	Q62B84
C01733	Q62M41
C01733	Q63NI4
C01733	Q63S19
C01733	Q65M34
C01733	Q65VG9
C01733	Q667L9
C01733	Q66CQ3
C01733	Q67SV5
C01733	Q6A6X6
C01733	Q6AE21
C01733	Q6D1C4
C01733	Q6D3Q6
C01733	Q6D5H7
C01733	Q6F9P2
C01733	Q6FAN3
C01733	Q6G2E2
C01733	Q6HBS0
C01733	Q6HP89
C01733	Q6LN52
C01733	Q6ME20
C01733	Q6N798
C01733	Q6N9W0
C01733	Q6NJ07
C01733	Q724C0
C01733	Q74IV9
C01733	Q7A6M2
C01733	Q7A7E3

C01733	Q7CHF8
C01733	Q7M816
C01733	Q7MN25
C01733	Q7N8M2
C01733	Q7VI92
C01733	Q7VM95
C01733	Q7VV72
C01733	Q7W4E1
C01733	Q7WFU9
C01733	Q815Y7
C01733	Q81IN8
C01733	Q81IZ6
C01733	Q81VM2
C01733	Q81XL3
C01733	Q81ZF5
C01733	Q823C4
C01733	Q827Y0
C01733	Q832Y6
C01733	Q83MC5
C01733	Q87AL9
C01733	Q87RS1
C01733	Q87UN4
C01733	Q87UV4
C01733	Q88RB3
C01733	Q88RL5
C01733	Q88UV2
C01733	Q88WA5
C01733	Q895C4
C01733	Q89LP2
C01733	Q8CQS7
C01733	Q8CTB2
C01733	Q8DFC3
C01733	Q8DY54
C01733	Q8E3S0
C01733	Q8ELA5
C01733	Q8ELQ6
C01733	Q8ENU2
C01733	Q8EPK1
C01733	Q8FRX8
C01733	Q8G5P8
C01733	Q8NSN2
C01733	Q8PGE8
C01733	Q8RFN2
C01733	Q8U7G2
C01733	Q8Y4L8
C01733	Q8YA75
C01733	Q8Z8R5
C01733	Q8Z990

C01733	Q8ZH38
C01733	Q92EZ6
C01733	Q93DA2
C01733	Q97KD5
C01733	Q9A502
C01733	Q9HT70
C01733	Q9I1C8
C01733	Q9K789
C01733	Q9L1C3
C01733	Q9Z8Q8
C01762	P45562
C01768	P18895
C01780	O15118
C01780	P61916
C01780	Q12200
C01834	O66646
C01834	P45247
C01834	P57031
C01834	P57066
C01834	P61481
C01834	P61482
C01834	P74997
C01834	Q080S4
C01834	Q0A8P9
C01834	Q0APW8
C01834	Q0BSM2
C01834	Q0C1C3
C01834	Q0I3C2
C01834	Q0VQQ0
C01834	Q1LPJ9
C01834	Q1MIJ4
C01834	Q2GE91
C01834	Q2GHT4
C01834	Q2K9R2
C01834	Q2LVM2
C01834	Q2NU23
C01834	Q31GF5
C01834	Q3A558
C01834	Q3ARY3
C01834	Q3ATY5
C01834	Q3B276
C01834	Q3B2U2
C01834	Q3IL62
C01834	Q3J7S3
C01834	Q3SRG8
C01834	Q44613
C01834	Q47C66
C01834	Q47YG8

C01834	Q492R2
C01834	Q4KFA2
C01834	Q57DS9
C01834	Q5F8K2
C01834	Q5FUV5
C01834	Q5HC57
C01834	Q5LUD0
C01834	Q5NHP2
C01834	Q5NZT6
C01834	Q5PBX2
C01834	Q5QU46
C01834	Q5ZT78
C01834	Q604C1
C01834	Q62J04
C01834	Q63SP4
C01834	Q64Z80
C01834	Q65TB7
C01834	Q669P3
C01834	Q6AMR9
C01834	Q6D664
C01834	Q6FZX3
C01834	Q6G3A6
C01834	Q6MD10
C01834	Q6MMY0
C01834	Q6N5P8
C01834	Q6N999
C01834	Q729H7
C01834	Q73L25
C01834	Q7MJ01
C01834	Q7N3A6
C01834	Q7UPK3
C01834	Q7UX73
C01834	Q7VMV4
C01834	Q7VR29
C01834	Q7VZ31
C01834	Q7W8T0
C01834	Q7WK40
C01834	Q82VL9
C01834	Q83CV2
C01834	Q83RS0
C01834	Q87EF4
C01834	Q87R20
C01834	Q884I3
C01834	Q88KY4
C01834	Q89KN0
C01834	Q8A1M1
C01834	Q8EEV5
C01834	Q8F6L8

C01834	Q8PKT0
C01834	Q8RFV0
C01834	Q8X8E3
C01834	Q8Y0C6
C01834	Q8YGM0
C01834	Q8ZFR4
C01834	Q9A6Z7
C01834	Q9A9P4
C01834	Q9EYM2
C01834	Q9HZL7
C01907	P32010
C01907	P32011
C01907	Q01818
C01907	Q48658
C01912	P95827
C01912	Q4VWC6
C01912	Q4VWC7
C01912	Q4VWC8
C01912	Q4VWC9
C01912	Q4VWD0
C01935	Q65QT6
C01935	Q664X5
C01935	Q7MFC4
C01935	Q7N986
C01935	Q7UBD0
C01935	Q8Z1U0
C01935	Q8ZAS8
C01937	P28873
C01937	P41438
C01937	P70502
C01946	P95827
C01946	Q53716
C01946	Q53717
C01996	O17444
C01996	O76082
C01996	P34711
C01996	P97521
C01996	Q08986
C01996	Q16572
C02028	A0KL02
C02028	A4QAL6
C02028	O07837
C02028	O07838
C02028	O35055
C02028	P0ABN5
C02028	P0ABN9
C02028	P0ABP3
C02028	P0AGE5

C02028	P20672
C02028	P22292
C02028	P32739
C02028	P33303
C02028	P37735
C02028	P39414
C02028	P45246
C02028	P50334
C02028	P50537
C02028	Q01857
C02028	Q02UV9
C02028	Q03MC5
C02028	Q0WJ82
C02028	Q13183
C02028	Q21339
C02028	Q2FFH9
C02028	Q2FMC1
C02028	Q41364
C02028	Q485F0
C02028	Q4K9I6
C02028	Q52000
C02028	Q5HEK4
C02028	Q65NC0
C02028	Q65UC1
C02028	Q665P2
C02028	Q6AS08
C02028	Q6D9G4
C02028	Q6FB65
C02028	Q6LG82
C02028	Q7A4P8
C02028	Q7MGA3
C02028	Q7N0A2
C02028	Q7VLY3
C02028	Q82ZN5
C02028	Q83Q34
C02028	Q87FP8
C02028	Q88K49
C02028	Q8DY58
C02028	Q8E3S4
C02028	Q8ECL5
C02028	Q8Z3L4
C02028	Q8ZLX1
C02028	Q91Y63
C02028	Q9A1E0
C02028	Q9CMD9
C02028	Q9ES88
C02028	Q9I273
C02028	Q9JXB8

C02028	Q9KNC9
C02028	Q9QZD8
C02028	Q9UBX3
C02028	Q9VVT2
C02057	P0A187
C02057	P24207
C02057	P39137
C02057	P76037
C02057	Q46065
C02057	Q47689
C02336	P17127
C02336	P20966
C02336	P22732
C02336	P26379
C02336	P26380
C02336	P26381
C02336	P26382
C02336	P32670
C02336	P32672
C02336	P32676
C02336	P44714
C02336	P44715
C02336	P47308
C02336	P69797
C02336	P69801
C02336	P69805
C02336	P69808
C02336	P69811
C02336	P69812
C02336	P69816
C02336	P75039
C02336	P77439
C02336	P77579
C02336	Q01441
C02336	Q8Z591
C02385	A1VZQ3
C02385	A3ZI83
C02385	B5XUC1
C02385	O30506
C02385	O32204
C02385	O34315
C02385	O34406
C02385	O34852
C02385	O34900
C02385	O34931
C02385	O50181
C02385	O50182
C02385	O50183

C02385	O84379
C02385	O84385
C02385	O88750
C02385	P02910
C02385	P02915
C02385	P04817
C02385	P09551
C02385	P0A2I7
C02385	P0A2I9
C02385	P0AAE7
C02385	P0AAF6
C02385	P0AAF8
C02385	P0AAF9
C02385	P0AE30
C02385	P0AE32
C02385	P0AE33
C02385	P0AE34
C02385	P0AE36
C02385	P0AEM9
C02385	P0AFT2
C02385	P18275
C02385	P30859
C02385	P30860
C02385	P37774
C02385	P39456
C02385	P42199
C02385	P42200
C02385	P45089
C02385	P45090
C02385	P45091
C02385	P45092
C02385	P54535
C02385	P54536
C02385	P54537
C02385	P73544
C02385	P73721
C02385	Q0P9X8
C02385	Q46170
C02385	Q5L5E6
C02385	Q822F2
C02385	Q88NY2
C02385	Q88NY3
C02385	Q88NY4
C02385	Q88NY5
C02385	Q8YPM7
C02385	Q8YPM8
C02385	Q8YPM9
C02385	Q8YSA2

C02385	Q8ZGS9
C02385	Q9PK20
C02385	Q9Z6M8
C02480	P94131
C02538	O15118
C02538	P61916
C02926	P0AG34
C02926	P0AG36
C02926	P0AG37
C02926	Q8Z3B4
C03033	A8I268
C03033	P0CE45
C03033	Q7GB25
C03033	Q92887
C03033	Q9C8G9
C03474	O52557
C03474	P13090
C03474	P23054
C03474	P32369
C03474	P37594
C03474	P39886
C03474	P42670
C03474	P76269
C03474	Q00538
C03474	Q9KIH3
C03474	Q9L1B4
C03479	P41438
C03557	Q8Z8W7
C03557	Q8Z8W8
C03557	Q8Z8W9
C03557	Q8Z8X0
C03664	Q46M63
C03758	A2RUN4
C03758	Q01959
C03758	Q02563
C03758	Q61327
C03758	Q79VE0
C04137	P0A2V2
C04137	P0A4F8
C04137	P0A4N5
C04137	P35113
C04137	P35114
C04137	P35116
C04137	P35118
C04137	P35120
C05005	O50295
C05005	O50296
C05005	Q7A2B0

C05005	Q8YT39
C05079	O51918
C05079	O51919
C05079	O69070
C05079	P0AEJ0
C05079	P25594
C05079	P36554
C05079	Q5PU79
C05079	Q7A5M0
C05079	Q939A4
C05079	Q93AT6
C05079	Q9HS33
C05079	Q9RQ29
C05113	P06129
C05122	O88397
C05122	Q00910
C05122	Q9JHI3
C05227	Q95YI5
C05402	A0L4K8
C05402	A0L4L0
C05402	A4YGY2
C05402	A7ZUZ0
C05402	A8M8Z1
C05402	A8TDW7
C05402	A8TDX0
C05402	A9QDR6
C05402	A9QDR7
C05402	A9QDR8
C05402	O06989
C05402	O06990
C05402	O06991
C05402	O07009
C05402	O07011
C05402	O07330
C05402	O30489
C05402	O31710
C05402	O31711
C05402	O31712
C05402	O32261
C05402	P0A3G5
C05402	P30878
C05402	P36497
C05402	P50332
C05402	P50333
C05402	P68579
C05402	P75830
C05402	P75831
C05402	P94360

C05402	P96483
C05402	P96517
C05402	Q00564
C05402	Q00749
C05402	Q00750
C05402	Q00751
C05402	Q00752
C05402	Q03727
C05402	Q2SDB0
C05402	Q2SDB1
C05402	Q52899
C05402	Q52900
C05402	Q72H66
C05402	Q72H67
C05402	Q72H68
C05402	Q72KX2
C05402	Q72KX3
C05402	Q72KX4
C05402	Q72L52
C05402	Q73MJ2
C05402	Q73MJ3
C05402	Q73MJ4
C05402	Q7A3X3
C05402	Q7A7X2
C05402	Q7AUQ4
C05402	Q7WWQ6
C05402	Q7WWQ8
C05402	Q7WWQ9
C05402	Q7WWR0
C05402	Q8DNB9
C05402	Q8DNC0
C05402	Q8DT25
C05402	Q8DT26
C05402	Q8DT27
C05402	Q8DT28
C05402	Q8RKC0
C05402	Q8RKC1
C05402	Q8TM30
C05402	Q8TM31
C05402	Q93LN0
C05402	Q93LN1
C05402	Q973J4
C05402	Q9L026
C05402	Q9L027
C05402	Q9S3J9
C05402	Q9S5Y1
C05402	Q9X0S9
C05402	Q9X0T0

C05402	Q9X0T1
C05402	Q9X103
C05402	Q9X2F5
C05402	Q9X9R5
C05402	Q9X9R6
C05402	Q9X9R7
C05629	P44629
C05776	A6TAH6
C05776	P06609
C05776	P06611
C05776	P63351
C05776	P63352
C05776	Q669Z9
C05776	Q66A01
C05776	Q66EE7
C05776	Q6D1Z3
C05776	Q6D654
C05776	Q6D656
C05776	Q6LQ77
C05776	Q6LUR6
C05776	Q7C1M3
C05776	Q7C1M5
C05776	Q7MLE6
C05776	Q7MLE7
C05776	Q7MNT2
C05776	Q7N3Q3
C05776	Q7N3Q4
C05776	Q7N842
C05776	Q83MD7
C05776	Q87Q38
C05776	Q87Q39
C05776	Q87SE7
C05776	Q8D928
C05776	Q8X4L7
C05776	Q8X5W0
C05776	Q8X8Z0
C05776	Q8Z6I5
C05776	Q8Z9B2
C05776	Q8ZBM3
C05776	Q8ZDX4
C05776	Q8ZDX6
C05776	Q8ZPS8
C05776	Q8ZRP7
C05776	Q9KSL1
C05776	Q9KSL2
C05821	Q08299
C06186	P08722
C06186	P24241

C06228	P06971
C06228	P38731
C06230	Q08299
C06231	O32243
C06231	O34742
C06231	O34878
C06231	O34992
C06232	O30142
C06232	O30143
C06232	O30144
C06232	P09833
C06232	P0AF01
C06232	P37329
C06232	Q0BQ80
C06232	Q0VQ44
C06232	Q1MA70
C06232	Q2K2X0
C06232	Q2RWI9
C06232	Q47CB7
C06232	Q579Z3
C06232	Q88G95
C06232	Q8U4K3
C06232	Q8U4K4
C06232	Q8U4K5
C06232	Q8UCD5
C06232	Q8ZGX6
C06232	Q9V2C0
C06232	Q9V2C1
C06232	Q9V2C4
C06268	O32243
C06268	O34742
C06268	O34878
C06268	O34992
C06268	P0AFM2
C06268	P14175
C06268	P14176
C06268	P31661
C06268	Q87WH3
C06268	Q87WH4
C06268	Q87WH5
C06268	Q8Q040
C06268	Q8Q041
C06268	Q8Q042
C06268	Q8Q043
C06268	Q9HTH6
C06268	Q9HTI6
C06268	Q9HTI7
C06268	Q9HTI8

C06313	O76729
C06313	Q07492
C06313	Q25272
C06313	Q9UAC2
C06413	P0A0J7
C06570	B2YGG2
C06570	O31137
C06570	P02982
C06570	P02983
C06570	P38125
C06570	P38227
C06570	P70187
C06570	Q14728
C06570	Q2FZP8
C06570	Q5SR56
C06570	Q79VC7
C06570	Q8CIA9
C06570	Q96MC6
C06570	Q9KMQ3
C06574	P0AE16
C06685	P0ADL1
C06685	P25744
C06685	P28873
C06685	P31462
C06685	P32071
C06685	P69367
C06685	Q05940
C06685	Q16572
C06685	Q70WR7
C06685	Q797E3
C06685	Q7VW14
C06685	Q8NRB5
C06687	B7ZGM0
C06687	O82855
C06687	Q2G140
C06687	Q3V050
C06687	Q5I0E9
C06687	Q86VL8
C06687	Q8K0H1
C06687	Q945F0
C06687	Q96FL8
C06687	Q9I3Y3
C06687	Q9JV27
C06691	Q53902
C06691	Q53903
C06696	P37617
C06697	O43681
C06697	P08690

C06697	P0AB93
C06697	P0AB94
C06697	P0AB95
C06697	P30329
C06697	P46838
C06697	P63620
C06697	Q06598
C06697	Q54GU0
C06697	Q5HF02
C06697	Q5HRI3
C06697	Q8CQF4
C06701	A0QQ68
C06701	A0QQ70
C06701	A0QQ71
C06701	P16677
C06701	P16682
C06701	P16683
C06701	Q02QE8
C06701	Q02QM1
C06701	Q032D0
C06701	Q035E0
C06701	Q04CG8
C06701	Q0ASQ1
C06701	Q0BZD8
C06701	Q1LJ08
C06701	Q1LQB5
C06701	Q1MMZ3
C06701	Q2KDV1
C06701	Q2SR40
C06701	Q31DV4
C06701	Q3IM24
C06701	Q3ISC1
C06701	Q47F10
C06701	Q5HJM6
C06701	Q5HKQ8
C06701	Q5LVC2
C06701	Q5UW69
C06701	Q5V6B8
C06701	Q5YTW4
C06701	Q62H59
C06701	Q63R24
C06701	Q66D26
C06701	Q6F0P3
C06701	Q6HFB5
C06701	Q6MUF4
C06701	Q6NA00
C06701	Q72AQ6
C06701	Q73P71

C06701	Q74LQ3
C06701	Q7A848
C06701	Q7M8M4
C06701	Q7W148
C06701	Q7WNT8
C06701	Q81A96
C06701	Q81Y10
C06701	Q83P97
C06701	Q882S0
C06701	Q889G0
C06701	Q88PM5
C06701	Q88YN5
C06701	Q89C51
C06701	Q8CMU4
C06701	Q8CUY0
C06701	Q8NQH4
C06701	Q8REG7
C06701	Q8UIW7
C06701	Q8XDV7
C06701	Q8YK28
C06701	Q8YUI9
C06701	Q8YUV1
C06701	Q8ZGU5
C06701	Q92V71
C06701	Q98GF5
C06701	Q9AB70
C06701	Q9HNI8
C06701	Q9HYL7
C06701	Q9HYT0
C06701	Q9K8N1
C06701	Q9KFL0
C06701	Q9KFN9
C06702	P0AFK4
C06702	P0AFK6
C06702	P0AFK9
C06702	P0AFM2
C06702	P14175
C06702	P14176
C06702	P69874
C06702	Q07824
C06702	Q87WH3
C06702	Q87WH4
C06702	Q87WH5
C06702	Q8Q040
C06702	Q8Q041
C06702	Q8Q042
C06702	Q8Q043
C06702	Q9HTH6

C06702	Q9HTI6
C06702	Q9HTI7
C06702	Q9HTI8
C06703	O32243
C06703	O34742
C06703	O34878
C06703	O34992
C06703	P0AFM2
C06703	P14175
C06703	P14176
C06703	P39775
C06703	P46920
C06703	P46921
C06703	P46922
C06703	Q45153
C06703	Q45460
C06703	Q45461
C06703	Q45462
C06703	Q87WH3
C06703	Q87WH4
C06703	Q87WH5
C06703	Q8Q040
C06703	Q8Q041
C06703	Q8Q042
C06703	Q8Q043
C06703	Q9HTH6
C06703	Q9HTI6
C06703	Q9HTI7
C06703	Q9HTI8
C06703	Q9RQ05
C06703	Q9RQ06
C06705	P24584
C06705	P24586
C06707	P42953
C06707	P42954
C06707	Q5HR97
C06707	Q65E84
C06707	Q720Z5
C06707	Q7A713
C06707	Q81K31
C06707	Q831L8
C06707	Q88ZH4
C06707	Q8CTM4
C06707	Q8Y843
C06707	Q92CV8
C06707	Q9CH26
C06708	Q5RLM2
C06708	Q63089

C06708	Q80UJ1
C06708	Q86VW1
C06708	Q8CFZ5
C06708	Q8IVM8
C06708	Q96S37
C06708	Q9CAT6
C06708	Q9NSA0
C06708	Q9R0W2
C06708	Q9Y226
C06710	Q9ZHC9
C06767	O68877
C06767	P31499
C06767	Q02FW7
C06767	Q1LC89
C06767	Q1MCZ1
C06767	Q2K551
C06767	Q2NSR0
C06767	Q31J97
C06767	Q3ICT8
C06767	Q3SQ65
C06767	Q4K5Z7
C06767	Q56991
C06767	Q56992
C06767	Q56993
C06767	Q5QXD0
C06767	Q5YVL8
C06767	Q62A98
C06767	Q63NR0
C06767	Q66FK0
C06767	Q6D645
C06767	Q6G098
C06767	Q6G475
C06767	Q6LQC0
C06767	Q6N7Y6
C06767	Q7MFA1
C06767	Q7N3S7
C06767	Q7NN36
C06767	Q7W025
C06767	Q7W359
C06767	Q7WEH6
C06767	Q87J32
C06767	Q88DY1
C06767	Q8EB59
C06767	Q8UCM5
C06767	Q8X5N2
C06767	Q92N13
C06767	Q93SH7
C06767	Q98L75

C06767	Q9KL34
C06767	Q9RKQ4
C06812	P46104
C06812	P95827
C06812	Q8Y9K8
C06821	P0A0J7
C06821	P0AEY8
C06821	P23910
C06821	P31122
C06821	P33532
C06821	P34711
C06821	P38124
C06821	P54219
C06821	Q01818
C06821	Q07824
C06821	Q9S3J9
C06956	P46720
C06956	P46721
C06956	Q6ZQN7
C06956	Q8UVG4
C06956	Q9EPZ7
C06956	Q9NPD5
C07597	P39980
C08215	O88397
C08215	Q9JHI3
C08353	A6VKS8
C08353	A6VKS9
C08353	A6VKT0
C08353	A8H4W5
C08353	A8H4W6
C08353	A8H4W7
C08353	O80725
C08353	O83340
C08353	O83341
C08353	O83342
C08353	O83343
C08353	O94911
C08353	O95342
C08353	P02925
C08353	P04983
C08353	P08183
C08353	P0AGI1
C08353	P0AGI3
C08353	P13568
C08353	P21439
C08353	P25714
C08353	P29724
C08353	P34358

C08353	P41233
C08353	P44735
C08353	P44736
C08353	P60949
C08353	P78363
C08353	Q02XM9
C08353	Q03CA4
C08353	Q0HIQ5
C08353	Q0HIQ6
C08353	Q0HIQ7
C08353	Q0HIQ8
C08353	Q0WML0
C08353	Q1M360
C08353	Q1M4Q7
C08353	Q1M4Q8
C08353	Q1M4Q9
C08353	Q1M5X4
C08353	Q1MHS1
C08353	Q2K0S7
C08353	Q2K204
C08353	Q2K9A3
C08353	Q5HJA6
C08353	Q5KUX3
C08353	Q62K91
C08353	Q63P06
C08353	Q65E55
C08353	Q6DB87
C08353	Q6HNE7
C08353	Q6LH11
C08353	Q7CG00
C08353	Q7MEV1
C08353	Q7NA79
C08353	Q7UU57
C08353	Q81HW8
C08353	Q81V36
C08353	Q825P1
C08353	Q82CM5
C08353	Q82ZT8
C08353	Q87H79
C08353	Q88J90
C08353	Q891M1
C08353	Q8DU36
C08353	Q8DU37
C08353	Q8DU38
C08353	Q8E281
C08353	Q8E7N9
C08353	Q8ENB3
C08353	Q8NR12

C08353	Q8RD43
C08353	Q8T5Z7
C08353	Q8U949
C08353	Q8UA86
C08353	Q8X5Q4
C08353	Q8XAW7
C08353	Q8XJX3
C08353	Q8Z2R4
C08353	Q8ZKV9
C08353	Q90Z35
C08353	Q92UI2
C08353	Q92W60
C08353	Q92WK1
C08353	Q92WK2
C08353	Q92WK4
C08353	Q987E7
C08353	Q98K15
C08353	Q99758
C08353	Q9BZC7
C08353	Q9CL63
C08353	Q9CP98
C08353	Q9K6J9
C08353	Q9KN37
C08353	Q9LJX0
C08353	Q9RDI1
C08353	Q9WXX0
C08353	Q9X051
C08353	Q9ZR72
C10896	P28873
C11161	A5H8A5
C11161	B7ZGM0
C11161	D1QCY9
C11161	O32182
C11161	O35018
C11161	O82855
C11161	P39843
C11161	P71678
C11161	Q01818
C11161	Q2G140
C11161	Q3V050
C11161	Q48658
C11161	Q50392
C11161	Q5I0E9
C11161	Q86VL8
C11161	Q8K0H1
C11161	Q945F0
C11161	Q96FL8
C11161	Q9I3Y3

C11161	Q9JV27
C11161	Q9ZGB6
C11227	P38206
C11259	P28246
C11261	O52557
C11261	P13090
C11261	P23054
C11261	P32369
C11261	P37594
C11261	P39886
C11261	P42670
C11261	P76269
C11261	Q00538
C11261	Q9KIH3
C11261	Q9L1B4
C11476	P0AAF5
C11476	P0AE27
C11476	Q62GY9
C11476	Q63QQ7
C11476	Q65WJ1
C11476	Q6D4W8
C11476	Q83KP2
C11476	Q87FK7
C11476	Q882I8
C11476	Q8XVS2
C11476	Q9KEE7
C11476	Q9KEE9
C11476	Q9KEF0
C11477	O04036
C11477	O25797
C11477	O95528
C11477	P31436
C11477	P31675
C11477	P33026
C11477	P33027
C11477	P39932
C11477	P44535
C11477	P58529
C11477	P58530
C11477	P58531
C11477	Q02SS7
C11477	Q39228
C11477	Q4F7G0
C11477	Q65S74
C11477	Q6CZ44
C11477	Q7UCG7
C11477	Q888L8
C11477	Q88K63

C11477	Q8BIV7
C11477	Q8LPQ8
C11477	Q8VZ80
C11477	Q8VZT3
C11477	Q921R7
C11477	Q94CI6
C11477	Q94CI7
C11477	Q94KE0
C11477	Q96290
C11477	Q9D321
C11477	Q9HWR7
C11477	Q9LT15
C11477	Q9S3K0
C11477	Q9SCW7
C11477	Q9SD00
C11477	Q9SFG0
C11477	Q9Y2W3
C12044	O52557
C12044	Q54806
C12268	P33951
C14701	Q07824
C14818	O25396
C14818	P28795
C14818	P40988
C14818	Q07418
C14818	Q38856
C14818	Q5KG96
C14818	Q5ZS62
C14818	Q7A3F2
C14818	Q83PW3
C14818	Q8F332
C14818	Q8XED4
C14819	O54368
C14819	O54369
C14819	O54370
C14819	O54371
C14819	O54372
C14819	P0A4G4
C14819	P21408
C14819	P21409
C14819	P21410
C14819	P33650
C14819	P38993
C14819	P40088
C14819	P44531
C14819	Q56952
C14819	Q56953
C14819	Q56954

C14819	Q56955
C14819	Q57341
C14819	Q9RNI8
C14819	Q9RNI9
C14819	Q9Z4R9
C15025	O32243
C15025	O34742
C15025	O34878
C15025	O34992
C15025	P0AFM2
C15025	P14175
C15025	P14176
C15025	Q87WH3
C15025	Q87WH4
C15025	Q87WH5
C15025	Q8Q040
C15025	Q8Q041
C15025	Q8Q042
C15025	Q8Q043
C15025	Q9HTH6
C15025	Q9HTI6
C15025	Q9HTI7
C15025	Q9HTI8
C15587	P17064
C15587	P48777
C15587	P50487
C15587	Q12119
C15587	Q84MA8
C15587	Q9SRK7
C15643	O15118
C15643	P61916
C15643	Q12200
C15773	Q79VC7
C16433	O35544
C16433	O75746
C16433	P0AAG3
C16433	P0AAG4
C16433	P0ABN5
C16433	P0AER3
C16433	P0AER5
C16433	P0AER7
C16433	P21345
C16433	P24942
C16433	P24943
C16433	P37902
C16433	P43004
C16433	P43006
C16433	P48664

C16433	P51906
C16433	Q52663
C16433	Q52664
C16433	Q52665
C16433	Q52666
C16433	Q8BH59
C16433	Q91WN3
C16433	Q9QXX4
C16434	P04816
C16434	P0A9S7
C16434	P0AD96
C16434	P0AEX7
C16434	P19072
C16434	P22729
C16434	P22731
C16434	P25185
C16434	P38084
C16434	P41815
C16434	P54104
C16435	P07117
C16435	P0AFM2
C16435	P0C0L7
C16435	P0C0L8
C16435	P10502
C16435	P14175
C16435	P14176
C16435	P15380
C16435	P28573
C16435	P37460
C16435	P40862
C16435	P45174
C16435	P92961
C16435	Q5HEM0
C16435	Q5HIA2
C16435	Q5HN32
C16435	Q5HRH0
C16435	Q6PGE7
C16435	Q79VE0
C16435	Q7A4Q7
C16435	Q7A771
C16435	Q87WH3
C16435	Q87WH4
C16435	Q87WH5
C16435	Q8CNP2
C16435	Q8Q040
C16435	Q8Q041
C16435	Q8Q042
C16435	Q8Q043

C16435	Q99884
C16435	Q9HTH6
C16435	Q9HTI6
C16435	Q9HTI7
C16435	Q9HTI8
C16436	P04816
C16436	P0A9S7
C16436	P0AD96
C16436	P0AEX7
C16436	P19072
C16436	P22729
C16436	P22731
C16436	P25185
C16436	P38084
C16436	P38085
C16436	P41815
C16436	P54104
C16438	P25376
C16438	P40812
C16438	P53388
C16438	Q52663
C16438	Q52664
C16438	Q52665
C16438	Q52666
C16439	P04816
C16439	P0A9S7
C16439	P0AD96
C16439	P0AD98
C16439	P0AEX7
C16439	P17215
C16439	P19072
C16439	P22729
C16439	P22731
C16439	P25185
C16439	P38084
C16439	P54104
C16439	Q03770
C16440	A1VZQ3
C16440	A3ZI83
C16440	A6TDS8
C16440	O30506
C16440	O34315
C16440	O34406
C16440	O34852
C16440	O34900
C16440	O34931
C16440	O50181
C16440	O50182

C16440	Q50183
C16440	P02910
C16440	P02915
C16440	P09551
C16440	P0A2I7
C16440	P0A2I9
C16440	P0AAE8
C16440	P0AAF0
C16440	P0AEM9
C16440	P0AFT2
C16440	P25737
C16440	P32487
C16440	P35865
C16440	P37774
C16440	P39456
C16440	P42199
C16440	P42200
C16440	P54535
C16440	P54536
C16440	P54537
C16440	P73544
C16440	P73721
C16440	P94633
C16440	Q0P9X8
C16440	Q666Q4
C16440	Q6D090
C16440	Q6NHP1
C16440	Q7N183
C16440	Q7UBP8
C16440	Q88NY2
C16440	Q88NY3
C16440	Q88NY4
C16440	Q88NY5
C16440	Q8RQM4
C16440	Q8XD10
C16440	Q8YPM7
C16440	Q8YPM8
C16440	Q8YPM9
C16440	Q8YSA2
C16440	Q8Z3W2
C16440	Q8ZHH6
C16440	Q8ZM68
C16692	Q44381
C16692	Q44382
C16692	Q44383
C16692	Q44384
