# Supplementary material for: METSP: A Maximum-Entropy Classifier Based Text Mining Tool for Transporter-Substrate Identification with Semistructured Text
Source: Biomed Res Int. 2015 Oct 1;2015:254838. doi: 10.1155/2015/254838 (PMC4606149; doi:10.1155/2015/254838)
Supplement: Supplementary file 1 — In Additional file 1, the 6955 TSPs were manually collected from UniProt, TransportDB, and TCDB. In Additional file 2, the training set includes 41,332 instances in the format of “label + accession number + field flag + a sentence.” In Additional file 3, the 3942 human TSPs were extracted by METSP. [file 254838.f1.zip › 254838.f1/254838-2.pdf]

Additional file 2 – Training set includes 41,332 instances in the format of "label + accession number + field flag + a sentence".

Positive_or_negative	UniProt_AC	Annotatation_field	Annotation_detail
P	A0KG33	CC	Na+/H+ antiporter that extrudes sodium in exchange for external protons (By similarity).
P	A0KG33	DE	Na+/H+ antiporter nhaA;
P	A0KG33	DE	Sodium/proton antiporter nhaA;
P	A0KG66	CC	H+-stimulated, highly selective, manganese uptake system (By similarity).
P	A0KG66	DE	Probable manganese transport protein mntH;
P	A0KJR8	CC	Transport of potassium into the cell (By similarity).
P	A0KJR8	DE	Probable potassium transport system protein kup 1;
P	A0KJR8	DR	potassium ion transmembrane transporter activity
P	A0KL02	CC	Involved in the import of serine and threonine into the cell, with the concomitant import of sodium (symport system) (By similarity).
P	A0KL02	DE	Na+/serine-threonine symporter;
P	A0KL02	DR	sodium:dicarboxylate symporter activity
P	A0KL02	DR	sodium:dicarboxylate symporter activity
P	A0KL08	CC	Na+/H+ antiporter that extrudes sodium in exchange for external protons (By similarity).
P	A0KL08	DE	Na+/H+ antiporter nhaB;
P	A0KL08	DE	Sodium/proton antiporter nhaB;
P	A0KL08	DR	sodium:hydrogen antiporter activity
P	A0KL61	CC	Transport of potassium into the cell (By similarity).
P	A0KL61	DE	Probable potassium transport system protein kup 2;
P	A0KL61	DR	potassium ion transmembrane transporter activity
P	A0KPH6	CC	Part of the ABC transporter complex ZnuABC involved in zinc import
P	A0KPH6	DE	Zinc import ATP-binding protein ZnuC;
P	A0KPH6	DR	zinc transporting ATPase activity
P	A0LJ91	CC	Transport of potassium into the cell (By similarity).
P	A0LJ91	DE	Probable potassium transport system protein kup 1;
P	A0LJ91	DR	potassium ion transmembrane transporter activity
P	A0LLE6	CC	Na+/H+ antiporter that extrudes sodium in exchange for external protons (By similarity).
P	A0LLE6	DE	Na+/H+ antiporter nhaA;
P	A0LLE6	DE	Sodium/proton antiporter nhaA;
P	A0LP92	CC	Transport of potassium into the cell (By similarity).
P	A0LP92	DE	Probable potassium transport system protein kup 2;
P	A0LP92	DR	potassium ion transmembrane transporter activity
P	A0LUE6	CC	Part of the ABC transporter complex PotABCD involved in spermidine/putrescine import
P	A0LUE6	CC	Part of the ABC transporter complex PotABCD involved in spermidine/putrescine import
P	A0LUE6	DE	Spermidine/putrescine import ATP-binding protein PotA;
P	A0LUE6	DE	Spermidine/putrescine import ATP-binding protein PotA;
P	A0M0N2	CC	Na+/H+ antiporter that extrudes sodium in exchange for external protons (By similarity).
P	A0M0N2	DE	Na+/H+ antiporter nhaA;
P	A0M0N2	DE	Sodium/proton antiporter nhaA;
P	A0PJK1	DE	Na+/glucose cotransporter 5;
P	A0PJK1	DE	Na+/glucose cotransporter 5;
P	A0PJK1	DE	Sodium/glucose cotransporter 5;
P	A0PJK1	DE	Sodium/glucose cotransporter 5;
P	A0QQ68	DR	phosphonate transmembrane transporter activity
P	A0QQ70	DR	phosphonate transmembrane-transporting ATPase activity
P	A0QQ71	DR	phosphonate transmembrane transporter activity
P	A0QW18	CC	Na+/H+ antiporter that extrudes sodium in exchange for external protons (By similarity).
P	A0QW18	DE	Na+/H+ antiporter nhaA;
P	A0QW18	DE	Sodium/proton antiporter nhaA;
P	A0R395	CC	One of the components of the high-affinity ATP-driven potassium transport (or KDP) system, which catalyzes the hydrolysis of ATP coupled with the exchange of hydrogen and potassium ions (By similarity).
P	A0R395	DE	Potassium-binding and translocating subunit A;
P	A0R395	DE	Potassium-translocating ATPase A chain;
P	A0R395	DE	Potassium-transporting ATPase A chain;
P	A0R395	DR	potassium-transporting ATPase activity

P	A0R4C0	CC	Part of the ABC transporter complex PstSACB involved in phosphate import
P	A0R4C0	DE	ABC phosphate transporter;
P	A0R4C0	DE	Phosphate import ATP-binding protein PstB;
P	A0R4C0	DE	Phosphate-transporting ATPase;
P	A0R4C0	DR	inorganic phosphate transmembrane transporter activity
P	A0R4C0	DR	phosphate transmembrane-transporting ATPase activity
P	A1KHG8	CC	One of the components of the high-affinity ATP-driven potassium transport (or KDP) system, which catalyzes the hydrolysis of ATP coupled with the exchange of hydrogen and potassium ions (By similarity).
P	A1KHG8	DE	Potassium-binding and translocating subunit A;
P	A1KHG8	DE	Potassium-translocating ATPase A chain;
P	A1KHG8	DE	Potassium-transporting ATPase A chain;
P	A1KHG8	DR	potassium-transporting ATPase activity
P	A1KHH0	CC	One of the components of the high-affinity ATP-driven potassium transport (or KDP) system, which catalyzes the hydrolysis of ATP coupled with the exchange of hydrogen and potassium ions
P	A1KHH0	DE	Potassium-binding and translocating subunit C;
P	A1KHH0	DE	Potassium-translocating ATPase C chain;
P	A1KHH0	DE	Potassium-transporting ATPase C chain;
P	A1KHH0	DR	potassium-transporting ATPase activity
P	A1R4H0	CC	Na+/H+ antiporter that extrudes sodium in exchange for external protons (By similarity).
P	A1R4H0	DE	Na+/H+ antiporter nhaA;
P	A1R4H0	DE	Sodium/proton antiporter nhaA;
P	A1WXT0	CC	Part of the ABC transporter complex ZnuABC involved in zinc import
P	A1WXT0	DE	Zinc import ATP-binding protein ZnuC;
P	A1WXT0	DR	zinc transporting ATPase activity
P	A2AJN7	DE	Bicarbonate transporter-related protein 1;
P	A2BDX4	CC	Probable potassium channel subunit
P	A2BDX4	DE	Potassium voltage-gated channel subfamily G member 1;
P	A2BDX4	DE	Voltage-gated potassium channel subunit Kv6.1;
P	A2BDX4	DR	voltage-gated potassium channel activity
P	A2RUL8	DE	Potassium voltage-gated channel, delayed-rectifier, subfamily S, member 1, isoform CRA_b;
P	A2RUL8	DE	cDNA FLJ75995, highly similar to Homo sapiens potassium voltage-gated channel, delayed-rectifier, subfamily S, member 1 (KCNS1), mRNA;
P	A2RUL8	DR	voltage-gated potassium channel activity
P	A3BV82	CC	Putative magnesium transporter (By similarity).
P	A3BV82	DE	Putative magnesium transporter MRS2-G;
P	A3CMQ7	CC	Part of the ABC transporter complex PotABCD involved in spermidine/putrescine import
P	A3CMQ7	CC	Part of the ABC transporter complex PotABCD involved in spermidine/putrescine import
P	A3CMQ7	DE	Spermidine/putrescine import ATP-binding protein PotA;
P	A3CMQ7	DE	Spermidine/putrescine import ATP-binding protein PotA;
P	A3KLL5	CC	This is the non-catalytic component of the active enzyme, which catalyzes the hydrolysis of ATP coupled with the exchange of Na+ and K+ ions across the plasma membrane (By similarity).
P	A3KLL5	CC	This is the non-catalytic component of the active enzyme, which catalyzes the hydrolysis of ATP coupled with the exchange of Na+ and K+ ions across the plasma membrane (By similarity).
P	A3KLL5	DE	ATPase, Na+/K+ transporting, beta 1 polypeptide, isoform CRA_a;
P	A3KLL5	DE	ATPase, Na+/K+ transporting, beta 1 polypeptide, isoform CRA_a;
P	A3KLL5	DE	cDNA FLJ76773, highly similar to Homo sapiens ATPase, Na+/K+ transporting, beta 1 polypeptide (ATP1B1), transcript variant 1, mRNA;
P	A3KLL5	DE	cDNA FLJ76773, highly similar to Homo sapiens ATPase, Na+/K+ transporting, beta 1 polypeptide (ATP1B1), transcript variant 1, mRNA;
P	A3M0N3	DE	Glucose transporter/sensor;
P	A4D0V9	DE	Potassium voltage-gated channel, Shal-related subfamily, member 2;
P	A4IVT5	CC	Na+/H+ antiporter that extrudes sodium in exchange for external protons (By similarity).
P	A4IVT5	DE	Na+/H+ antiporter nhaA;
P	A4IVT5	DE	Sodium/proton antiporter nhaA;
P	A4IVV7	CC	One of the components of the high-affinity ATP-driven potassium transport (or KDP) system, which catalyzes the hydrolysis of ATP coupled with the exchange of hydrogen and potassium ions (By similarity).

P	A4IVV7	DE	Potassium-binding and translocating subunit A;
P	A4IVV7	DE	Potassium-translocating ATPase A chain;
P	A4IVV7	DE	Potassium-transporting ATPase A chain;
P	A4IVV7	DR	potassium-transporting ATPase activity
P	A4SE48	CC	Mediates zinc uptake
P	A4SE48	DE	Zinc transporter ZupT;
P	A4SGR1	CC	Transport of potassium into the cell (By similarity).
P	A4SGR1	DE	Probable potassium transport system protein kup;
P	A4SGR1	DR	potassium ion transmembrane transporter activity
P	A5EHA3	CC	Transport of potassium into the cell (By similarity).
P	A5EHA3	DE	Probable potassium transport system protein kup 1;
P	A5EHA3	DR	potassium ion transmembrane transporter activity
P	A5EHG1	CC	Transport of potassium into the cell (By similarity).
P	A5EHG1	DE	Probable potassium transport system protein kup 2;
P	A5EHG1	DR	potassium ion transmembrane transporter activity
P	A5EHG2	CC	Transport of potassium into the cell (By similarity).
P	A5EHG2	DE	Probable potassium transport system protein kup 3;
P	A5EHG2	DR	potassium ion transmembrane transporter activity
P	A5EQ10	CC	One of the components of the high-affinity ATP-driven potassium transport (or KDP) system, which catalyzes the hydrolysis of ATP coupled with the exchange of hydrogen and potassium ions
P	A5EQ10	DE	Potassium-binding and translocating subunit C;
P	A5EQ10	DE	Potassium-translocating ATPase C chain;
P	A5EQ10	DE	Potassium-transporting ATPase C chain;
P	A5EQ10	DR	potassium-transporting ATPase activity
P	A5ESW9	CC	Transport of potassium into the cell (By similarity).
P	A5ESW9	DE	Probable potassium transport system protein kup 4;
P	A5ESW9	DR	potassium ion transmembrane transporter activity
P	A5VNX4	CC	Na+/H+ antiporter that extrudes sodium in exchange for external protons (By similarity).
P	A5VNX4	DE	Na+/H+ antiporter nhaA;
P	A5VNX4	DE	Sodium/proton antiporter nhaA;
P	A5VRD1	CC	Transport of potassium into the cell (By similarity).
P	A5VRD1	DE	Probable potassium transport system protein kup;
P	A5VRD1	DR	potassium ion transmembrane transporter activity
P	A5X2V1	DE	Amiloride-sensitive sodium channel gamma-subunit;
P	A5X2V1	DE	Nasal epithelial sodium channel gamma subunit;
P	A5X2V1	DE	Sodium channel, nonvoltage-gated 1, gamma, isoform CRA_a;
P	A6H8H5	CC	Channels open or close in response to the voltage difference across the membrane, letting potassium ions pass in accordance with their electrochemical gradient (By similarity).
P	A6H8H5	CC	Mediates the voltage-dependent potassium ion permeability of excitable membranes
P	A6H8H5	DE	Potassium voltage-gated channel subfamily B member 2;
P	A6H8H5	DE	Voltage-gated potassium channel subunit Kv2.2;
P	A6H8H5	DR	voltage-gated potassium channel activity
P	A6Q0Z0	CC	Na+/H+ antiporter that extrudes sodium in exchange for external protons (By similarity).
P	A6Q0Z0	DE	Na+/H+ antiporter nhaA;
P	A6Q0Z0	DE	Sodium/proton antiporter nhaA;
P	A6T4F6	CC	Na+/H+ antiporter that extrudes sodium in exchange for external protons (By similarity).
P	A6T4F6	DE	Na+/H+ antiporter nhaA 1;
P	A6T4F6	DE	Sodium/proton antiporter nhaA 1;
P	A6T4I3	CC	Transport system that facilitates potassium-efflux, possibly by potassium-proton antiport (By similarity).
P	A6T4I3	DE	Glutathione-regulated potassium-efflux system protein kefC;
P	A6T4I3	DE	K+/H+ antiporter;
P	A6T4V9	CC	Functions as antiport system and exchanges two chloride ions for 1 proton
P	A6T4V9	CC	Proton-coupled chloride transporter
P	A6T4V9	DR	voltage-gated chloride channel activity
P	A6T6D7	CC	One of the components of the high-affinity ATP-driven potassium transport (or KDP) system, which catalyzes the hydrolysis of ATP coupled with the exchange of hydrogen and potassium ions
P	A6T6D7	DE	Potassium-binding and translocating subunit C;
P	A6T6D7	DE	Potassium-translocating ATPase C chain;

P	A6T6D7	DE	Potassium-transporting ATPase C chain;
P	A6T6D7	DR	potassium-transporting ATPase activity
P	A6T6D8	CC	One of the components of the high-affinity ATP-driven potassium transport (or KDP) system, which catalyzes the hydrolysis of ATP coupled with the exchange of hydrogen and potassium ions (By similarity).
P	A6T6D8	DE	Potassium-binding and translocating subunit B;
P	A6T6D8	DE	Potassium-translocating ATPase B chain;
P	A6T6D8	DE	Potassium-transporting ATPase B chain;
P	A6T6D8	DR	potassium-transporting ATPase activity
P	A6T6D9	CC	One of the components of the high-affinity ATP-driven potassium transport (or KDP) system, which catalyzes the hydrolysis of ATP coupled with the exchange of hydrogen and potassium ions (By similarity).
P	A6T6D9	DE	Potassium-binding and translocating subunit A;
P	A6T6D9	DE	Potassium-translocating ATPase A chain;
P	A6T6D9	DE	Potassium-transporting ATPase A chain;
P	A6T6D9	DR	potassium-transporting ATPase activity
P	A6TAH6	CC	Part of the ABC transporter complex BtuCDF involved in vitamin B12 import
P	A6TAH6	DE	Vitamin B12 import system permease protein BtuC;
P	A6TAU9	CC	Involved in the import of threonine and serine into the cell, with the concomitant import of a proton (symport system) (By similarity).
P	A6TAU9	DE	Threonine/serine transporter TdcC;
P	A6TAW7	CC	Na+/H+ antiporter that extrudes sodium in exchange for external protons (By similarity).
P	A6TAW7	DE	Na+/H+ antiporter nhaB;
P	A6TAW7	DE	Sodium/proton antiporter nhaB;
P	A6TAW7	DR	sodium:hydrogen antiporter activity
P	A6TC38	CC	H+-stimulated, highly selective, manganese uptake system (By similarity).
P	A6TC38	DE	Probable manganese transport protein mntH;
P	A6TDB4	DR	cobalt ion transmembrane transporter activity
P	A6TDS8	CC	Important to control the intracellular level of arginine and the correct balance between arginine and lysine (By similarity).
P	A6TEC8	CC	Involved in the import of serine and threonine into the cell, with the concomitant import of sodium (symport system) (By similarity).
P	A6TEC8	CC	Involved in the import of serine and threonine into the cell, with the concomitant import of sodium (symport system) (By similarity).
P	A6TEC8	DE	Na+/serine-threonine symporter;
P	A6TEC8	DE	Na+/serine-threonine symporter;
P	A6TEC8	DE	Serine/threonine transporter sstT;
P	A6TEC8	DR	sodium:dicarboxylate symporter activity
P	A6TEY9	CC	Transport system that facilitates potassium-efflux, possibly by potassium-proton antiport (By similarity).
P	A6TEY9	DE	Glutathione-regulated potassium-efflux system protein kefB;
P	A6TEY9	DE	K+/H+ antiporter;
P	A6TEY9	DE	NEM-activatable K+/H+ antiporter;
P	A6TEY9	DR	glutathione-regulated potassium exporter activity
P	A6TFD1	DR	sodium:dicarboxylate symporter activity
P	A6TG51	CC	Responsible for the low-affinity transport of potassium into the cell, with the probable concomitant uptake of protons (symport system) (By similarity).
P	A6TG51	DE	Kup system potassium uptake protein;
P	A6TG51	DE	Low affinity potassium transport system protein kup;
P	A6TG51	DR	potassium ion transmembrane transporter activity
P	A6TJ58	CC	Na+/H+ antiporter that extrudes sodium in exchange for external protons (By similarity).
P	A6TJ58	DE	Na+/H+ antiporter nhaA 2;
P	A6TJ58	DE	Sodium/proton antiporter nhaA 2;
P	A6U6M1	CC	Transport of potassium into the cell (By similarity).
P	A6U6M1	DE	Probable potassium transport system protein kup 1;
P	A6U6M1	DR	potassium ion transmembrane transporter activity
P	A6UH53	DR	sodium:dicarboxylate symporter activity
P	A6UM20	CC	Transport of potassium into the cell (By similarity).
P	A6UM20	DE	Probable potassium transport system protein kup 2;
P	A6UM20	DR	potassium ion transmembrane transporter activity
P	A6UMS8	CC	Transport of potassium into the cell (By similarity).
P	A6UMS8	DE	Probable potassium transport system protein kup 3;
P	A6UMS8	DR	potassium ion transmembrane transporter activity
P	A6VKS8	CC	Part of the ABC transporter complex RbsABCD involved in ribose import
P	A7HZF9	CC	Na+/H+ antiporter that extrudes sodium in exchange for external protons (By similarity).

P	A7HZF9	DE	Na+/H+ antiporter nhaA 1;
P	A7HZF9	DE	Sodium/proton antiporter nhaA 1;
P	A7I1I1	CC	Na+/H+ antiporter that extrudes sodium in exchange for external protons (By similarity)
P	A7I1I1	DE	Na+/H+ antiporter nhaA 2;
P	A7I1I1	DE	Sodium/proton antiporter nhaA 2;
P	A7I2B9	CC	Involved in the import of serine and threonine into the cell, with the concomitant import of sodium (symport system) (By similarity).
P	A7I2B9	CC	Involved in the import of serine and threonine into the cell, with the concomitant import of sodium (symport system) (By similarity).
P	A7I2B9	DE	Na+/serine-threonine symporter;
P	A7I2B9	DE	Na+/serine-threonine symporter;
P	A7I2B9	DE	Serine/threonine transporter sstT;
P	A7I2B9	DR	sodium:dicarboxylate symporter activity
P	A7ZUZ0	DE	Melibiose carrier protein;
P	A8EUK5	CC	Na+/H+ antiporter that extrudes sodium in exchange for external protons (By similarity)
P	A8EUK5	DE	Na+/H+ antiporter nhaA 1;
P	A8EUK5	DE	Sodium/proton antiporter nhaA 1;
P	A8EVL5	CC	Na+/H+ antiporter that extrudes sodium in exchange for external protons (By similarity)
P	A8EVL5	DE	Na+/H+ antiporter nhaA 2;
P	A8EVL5	DE	Sodium/proton antiporter nhaA 2;
P	A8HY21	CC	One of the components of the high-affinity ATP-driven potassium transport (or KDP) system, which catalyzes the hydrolysis of ATP coupled with the exchange of hydrogen and potassium ions
P	A8HY21	DE	Potassium-binding and translocating subunit C;
P	A8HY21	DE	Potassium-translocating ATPase C chain;
P	A8HY21	DE	Potassium-transporting ATPase C chain;
P	A8HY21	DR	potassium-transporting ATPase activity
P	A8I126	CC	Na+/H+ antiporter that extrudes sodium in exchange for external protons (By similarity)
P	A8I126	DE	Na+/H+ antiporter nhaA;
P	A8I126	DE	Sodium/proton antiporter nhaA;
P	A8IDZ2	DR	sodium:dicarboxylate symporter activity
P	A8K7I4	DR	chloride channel activity
P	A8MPY1	CC	GABA, the major inhibitory neurotransmitter in the vertebrate brain, mediates neuronal inhibition by binding to the GABA/benzodiazepine receptor and opening an integral chloride channel (By similarity).
P	A8MPY1	DR	chloride channel activity
P	A8MYU2	CC	Testis-specific potassium channel activated by both intracellular pH and membrane voltage that mediates export of K+
P	A8MYU2	DE	Potassium channel subfamily U member 1;
P	A9BJX0	DE	Calcium-translocating P-type ATPase, PMCA-type;
P	A9BJX0	DR	calcium-transporting ATPase activity
P	A9C190	CC	One of the components of the high-affinity ATP-driven potassium transport (or KDP) system, which catalyzes the hydrolysis of ATP coupled with the exchange of hydrogen and potassium ions (By similarity).
P	A9C190	DE	Potassium-binding and translocating subunit A;
P	A9C190	DE	Potassium-translocating ATPase A chain;
P	A9C190	DE	Potassium-transporting ATPase A chain;
P	A9C190	DR	potassium-transporting ATPase activity
P	A9LNM6	DE	Sodium activated potassium channel;
P	B0RZB7	DE	ATP/ADP translocase;
P	B0RZB7	DE	ATP/ADP translocase;
P	B0RZB7	DR	ATP:ADP antiporter activity
P	B0RZB7	DR	ATP:ADP antiporter activity
P	B1L487	DR	copper-exporting ATPase activity
P	B1PM37	DE	Glucose sensor;
P	B1PS44	DE	Sodium channel nonvoltage-gated 1 delta;
P	B1PS44	DE	cDNA FLJ45431 fis, clone BRHIP3039509, highly similar to Amiloride-sensitive sodium channel subunit delta;
P	B1PS44	DR	ligand-gated sodium channel activity
P	B2R812	DE	Amiloride-sensitive sodium channel subunit beta 1;
P	B2R812	DE	Sodium channel, nonvoltage-gated 1, beta (Liddle syndrome), isoform CRA_b;
P	B2R812	DE	cDNA, FLJ93691, highly similar to Homo sapiens sodium channel, nonvoltage-gated 1, beta (Liddle syndrome) (SCNN1B), mRNA;
P	B2R8T4	DE	K2P10.1 potassium channel isoform 2;
P	B2R8T4	DE	Potassium channel, subfamily K, member 10, isoform CRA_b;

P	B2R8T4	DE	cDNA, FLJ94053, Homo sapiens potassium channel, subfamily K, member 10 (KCNK10),transcript variant 2, mRNA;
P	B2RDS2	DE	K2P6.1 potassium channel;
P	B2RDS2	DE	Potassium channel, subfamily K, member 6;
P	B2RDS2	DE	cDNA, FLJ96744, Homo sapiens potassium channel, subfamily K, member 6 (KCNK6),mRNA;
P	B2RNU7	DR	neurotransmitter:sodium symporter activity
P	B2YGG2	DE	Tetracycline resistance protein;
P	B2YGG2	DR	tetracycline:hydrogen antiporter activity
P	B4DX48	DR	neurotransmitter:sodium symporter activity
P	B5TJL4	DE	K2P10.1 potassium channel isoform 1;
P	B5TJL4	DE	Potassium channel, subfamily K, member 10, isoform CRA_c;
P	B5TJL4	DR	potassium channel activity
P	B5TJL8	DE	K2P13.1 potassium channel;
P	B5TJL8	DE	Potassium channel, subfamily K, member 13;
P	B5TJL8	DR	potassium channel activity
P	B5XN31	DR	sodium:dicarboxylate symporter activity
P	B5XN76	CC	Transport system that facilitates potassium-efflux, possibly by potassium-proton antiport (By similarity).
P	B5XN76	DE	Glutathione-regulated potassium-efflux system protein kefB;
P	B5XN76	DE	K+/H+ antiporter;
P	B5XN76	DE	NEM-activatable K+/H+ antiporter;
P	B5XN76	DR	glutathione-regulated potassium exporter activity
P	B5XQ77	CC	Na+/H+ antiporter that extrudes sodium in exchange for external protons (By similarity).
P	B5XQ77	DE	Na+/H+ antiporter nhaB;
P	B5XQ77	DE	Sodium/proton antiporter nhaB;
P	B5XQ77	DR	sodium:hydrogen antiporter activity
P	B5XTX0	CC	Involved in the import of serine and threonine into the cell, with the concomitant import of sodium (symport system) (By similarity).
P	B5XTX0	CC	Involved in the import of serine and threonine into the cell, with the concomitant import of sodium (symport system) (By similarity).
P	B5XTX0	DE	Na+/serine-threonine symporter;
P	B5XTX0	DE	Na+/serine-threonine symporter;
P	B5XTX0	DE	Serine/threonine transporter sstT;
P	B5XTX0	DR	sodium:dicarboxylate symporter activity
P	B5XUC1	CC	Important to control the intracellular level of arginine and the correct balance between arginine and lysine (By similarity).
P	B5XUC1	CC	Involved in the export of arginine
P	B5XUC1	DE	Arginine exporter protein ArgO;
P	B5XUV2	DR	cobalt ion transmembrane transporter activity
P	B5XVU3	CC	H+-stimulated, highly selective, manganese uptake system (By similarity).
P	B5XVU3	DE	Probable manganese transport protein mntH;
P	B5XVZ7	CC	Involved in the import of threonine and serine into the cell, with the concomitant import of a proton (symport system) (By similarity).
P	B5XVZ7	DE	Threonine/serine transporter TdcC;
P	B5XZ47	CC	Uptake of L-rhamnose across the boundary membrane with the concomitant transport of protons into the cell (symport system) (By similarity).
P	B5XZ47	DE	L-rhamnose-H+ transport protein;
P	B5XZ47	DE	L-rhamnose-proton symporter;
P	B5XZE8	CC	One of the components of the high-affinity ATP-driven potassium transport (or KDP) system, which catalyzes the hydrolysis of ATP coupled with the exchange of hydrogen and potassium ions (By similarity).
P	B5XZE8	DE	Potassium-binding and translocating subunit A;
P	B5XZE8	DE	Potassium-translocating ATPase A chain;
P	B5XZE8	DE	Potassium-transporting ATPase A chain;
P	B5XZE8	DR	potassium-transporting ATPase activity
P	B5XZE9	CC	One of the components of the high-affinity ATP-driven potassium transport (or KDP) system, which catalyzes the hydrolysis of ATP coupled with the exchange of hydrogen and potassium ions (By similarity).
P	B5XZE9	DE	Potassium-binding and translocating subunit B;
P	B5XZE9	DE	Potassium-translocating ATPase B chain;
P	B5XZE9	DE	Potassium-transporting ATPase B chain;
P	B5XZE9	DR	potassium-transporting ATPase activity

P	B5XZF0	CC	One of the components of the high-affinity ATP-driven potassium transport (or KDP) system, which catalyzes the hydrolysis of ATP coupled with the exchange of hydrogen and potassium ions
P	B5XZF0	DE	Potassium-binding and translocating subunit C;
P	B5XZF0	DE	Potassium-translocating ATPase C chain;
P	B5XZF0	DE	Potassium-transporting ATPase C chain;
P	B5XZF0	DR	potassium-transporting ATPase activity
P	B5XZK9	CC	Responsible for the low-affinity transport of potassium into the cell, with the probable concomitant uptake of protons (symport system) (By similarity).
P	B5XZK9	DE	Kup system potassium uptake protein;
P	B5XZK9	DE	Low affinity potassium transport system protein kup;
P	B5XZK9	DR	potassium ion transmembrane transporter activity
P	B5Y1L4	CC	Functions as antiport system and exchanges two chloride ions for 1 proton
P	B5Y1L4	CC	Proton-coupled chloride transporter
P	B5Y1L4	DR	voltage-gated chloride channel activity
P	B5Y1Z8	CC	Transport system that facilitates potassium-efflux, possibly by potassium-proton antiport (By similarity).
P	B5Y1Z8	DE	Glutathione-regulated potassium-efflux system protein kefC;
P	B5Y1Z8	DE	K+/H+ antiporter;
P	B9JA25	CC	Transport of potassium into the cell (By similarity).
P	B9JA25	DE	Probable potassium transport system protein kup;
P	B9JA25	DR	potassium ion transmembrane transporter activity
P	B9JKQ7	CC	One of the components of the high-affinity ATP-driven potassium transport (or KDP) system, which catalyzes the hydrolysis of ATP coupled with the exchange of hydrogen and potassium ions (By similarity).
P	B9JKQ7	DE	Potassium-binding and translocating subunit A;
P	B9JKQ7	DE	Potassium-translocating ATPase A chain;
P	B9JKQ7	DE	Potassium-transporting ATPase A chain;
P	B9JKQ7	DR	potassium-transporting ATPase activity
P	B9JKQ9	CC	One of the components of the high-affinity ATP-driven potassium transport (or KDP) system, which catalyzes the hydrolysis of ATP coupled with the exchange of hydrogen and potassium ions
P	B9JKQ9	DE	Potassium-binding and translocating subunit C;
P	B9JKQ9	DE	Potassium-translocating ATPase C chain;
P	B9JKQ9	DE	Potassium-transporting ATPase C chain;
P	B9JKQ9	DR	potassium-transporting ATPase activity
P	B9K2I1	CC	One of the components of the high-affinity ATP-driven potassium transport (or KDP) system, which catalyzes the hydrolysis of ATP coupled with the exchange of hydrogen and potassium ions
P	B9K2I1	DE	Potassium-binding and translocating subunit C;
P	B9K2I1	DE	Potassium-translocating ATPase C chain;
P	B9K2I1	DE	Potassium-transporting ATPase C chain;
P	B9K2I1	DR	potassium-transporting ATPase activity
P	B9K2I3	CC	One of the components of the high-affinity ATP-driven potassium transport (or KDP) system, which catalyzes the hydrolysis of ATP coupled with the exchange of hydrogen and potassium ions (By similarity).
P	B9K2I3	DE	Potassium-binding and translocating subunit A;
P	B9K2I3	DE	Potassium-translocating ATPase A chain;
P	B9K2I3	DE	Potassium-transporting ATPase A chain;
P	B9K2I3	DR	potassium-transporting ATPase activity
P	B9K2V4	DR	sodium:dicarboxylate symporter activity
P	O00168	CC	Induces a hyperpolarization-activated chloride current when expressed in Xenopus oocytes
P	O00168	DR	chloride channel activity
P	O00180	CC	Weakly inward rectifying potassium channel.
P	O00180	DE	Inward rectifying potassium channel protein TWIK-1;
P	O00180	DE	Potassium channel KCNO1;
P	O00180	DE	Potassium channel subfamily K member 1;
P	O00180	DR	inward rectifier potassium channel activity
P	O00299	CC	Can insert into membranes and form chloride ion channels
P	O00299	DE	Chloride channel ABP;
P	O00299	DE	Chloride intracellular channel protein 1;
P	O00299	DE	Nuclear chloride ion channel 27;
P	O00299	DE	Regulatory nuclear chloride ion channel protein;
P	O00299	DR	voltage-gated chloride channel activity

P	O00337	CC	Exhibits the transport characteristics of the nucleoside transport system cit or N2 subtype (N2/cit) (selective for pyrimidine nucleosides and adenosine)
P	O00337	CC	Sodium-dependent and pyrimidine-selective
P	O00337	DE	Concentrative nucleoside transporter 1;
P	O00337	DE	Na+/nucleoside cotransporter 1;
P	O00337	DE	Na+/nucleoside cotransporter 1;
P	O00337	DE	Sodium-coupled nucleoside transporter 1;
P	O00337	DE	Sodium-coupled nucleoside transporter 1;
P	O00337	DE	Sodium/nucleoside cotransporter 1;
P	O00337	DE	Sodium/nucleoside cotransporter 1;
P	O00341	CC	Transports L-glutamate; the L-glutamate uptake is sodium- and voltage-dependent and chloride-independent
P	O00341	CC	Transports L-glutamate; the L-glutamate uptake is sodium- and voltage-dependent and chloride-independent
P	O00341	DE	Retinal glutamate transporter;
P	O00341	DR	high-affinity glutamate transmembrane transporter activity
P	O00341	DR	sodium:dicarboxylate symporter activity
P	O00400	CC	Probable acetyl-CoA transporter necessary for O- acetylation of gangliosides.
P	O00400	DE	Acetyl-CoA transporter 1;
P	O00400	DR	acetyl-CoA transporter activity
P	O00476	CC	May be involved in actively transporting phosphate into cells via Na+ cotransport (By similarity).
P	O00476	CC	May be involved in actively transporting phosphate into cells via Na+ cotransport (By similarity).
P	O00476	DE	Na+/PI cotransporter 4;
P	O00476	DE	Sodium-dependent phosphate transport protein 4;
P	O00476	DE	Sodium-dependent phosphate transport protein 4;
P	O00476	DE	Sodium/phosphate cotransporter 4;
P	O00476	DE	Sodium/phosphate cotransporter 4;
P	O00476	DR	sodium:phosphate symporter activity
P	O00476	DR	sodium:phosphate symporter activity
P	O00591	CC	GABA, the major inhibitory neurotransmitter in the vertebrate brain, mediates neuronal inhibition by binding to the GABA/benzodiazepine receptor and opening an integral chloride channel
P	O00591	DR	chloride channel activity
P	O00624	CC	May be involved in actively transporting phosphate into cells via Na+ cotransport (By similarity).
P	O00624	CC	May be involved in actively transporting phosphate into cells via Na+ cotransport (By similarity).
P	O00624	DE	Na+/PI cotransporter 3;
P	O00624	DE	Sodium-dependent phosphate transport protein 3;
P	O00624	DE	Sodium-dependent phosphate transport protein 3;
P	O00624	DE	Sodium/phosphate cotransporter 3;
P	O00624	DE	Sodium/phosphate cotransporter 3;
P	O00624	DR	sodium:phosphate symporter activity
P	O00624	DR	sodium:phosphate symporter activity
P	O01635	DR	sodium channel activity
P	O01840	CC	Neuron-specific, H+-coupled oligopeptide transporter
P	O01840	DE	Oligopeptide transporter 3;
P	O02228	CC	Sodium ion and chloride ion dependent.
P	O04036	CC	Sugar transporter (Potential).
P	O04036	DE	Sugar transporter ERD6;
P	O04036	DE	Sugar transporter-like protein 1;
P	O04089	CC	May play a role in the transport of zinc in the plastids
P	O04089	DE	Zinc transporter 4, chloroplastic;
P	O04089	DR	zinc ion transmembrane transporter activity
P	O04249	DE	Hexose transporter 7;
P	O04289	CC	H+/sulfate cotransporter that may play a role in the regulation of sulfate [illegible]
P	O04289	DE	Sulfate transporter 3.2;
P	O04289	DR	secondary active sulfate transmembrane transporter activity
P	O04514	DE	Oligopeptide transporter 2;
P	O04722	CC	Low-affinity H+/sulfate cotransporter that may be involved in root-to-shoot translocation of sulfate
P	O04722	CC	Plays a central role in the regulation of sulfate assimilation.
P	O04722	DE	Sulfate transporter 2.1;

P	O04722	DR	secondary active sulfate transmembrane transporter activity
P	O05703	CC	Part of the ATP-driven transport system AdcABC for zinc
P	O05703	DE	Zinc-binding lipoprotein AdcA;
P	O05870	CC	Part of the ABC transporter complex pstSACB involved in phosphate import (By similarity).
P	O05870	DE	Phosphate-binding protein pstS 2;
P	O05870	DR	inorganic phosphate transmembrane transporter activity
P	O05962	CC	Provides the rickettsial cell with host ATP in exchange for rickettsial ADP
P	O05962	CC	Provides the rickettsial cell with host ATP in exchange for rickettsial ADP
P	O05962	DE	ADP,ATP carrier protein 5;
P	O05962	DE	ADP/ATP translocase 5;
P	O05962	DE	ADP/ATP translocase 5;
P	O05962	DR	ATP:ADP antiporter activity
P	O05962	DR	ATP:ADP antiporter activity
P	O06411	CC	Low-affinity inorganic phosphate transport (By similarity).
P	O06411	DE	Probable low-affinity inorganic phosphate transporter;
P	O06411	DR	inorganic phosphate transmembrane transporter activity
P	O06984	DE	Putative sulfate transporter yvdB;
P	O06984	DR	secondary active sulfate transmembrane transporter activity
P	O07563	CC	Can transport glucose, mannose, 2-deoxyglucose and methyl alpha-glucoside, but not galactose.
P	O07563	CC	Can transport glucose, mannose, 2-deoxyglucose and methyl alpha-glucoside, but not galactose.
P	O07563	DE	Glucose/mannose transporter glcP;
P	O07563	DE	Glucose/mannose transporter glcP;
P	O07563	DE	Glucose/mannose:H+ symporter;
P	O07563	DE	Glucose/mannose:H+ symporter;
P	O08581	CC	Weak inwardly rectifying potassium channel.
P	O08581	DE	Inward rectifying potassium channel protein TWIK-1;
P	O08581	DE	Potassium channel subfamily K member 1;
P	O08581	DR	potassium channel activity
P	O13918	CC	Involved in zinc homeostasis, where it plays a role in its accumulation in the endoplasmic reticulum/nucleus
P	O13918	DE	Zinc homeostasis factor 1;
P	O13918	DR	zinc ion transmembrane transporter activity
P	O14091	CC	Responsible for the transport of maltose and sucrose into the cell, with the concomitant uptake of protons (symport system).
P	O14520	CC	Forms a channel for water and glycerol.
P	O14520	DR	water channel activity
P	O14649	CC	Acts as an outward rectifier when external potassium concentration is low
P	O14649	CC	Rectification direction results from potassium ion concentration on either side of the membrane
P	O14649	CC	When external potassium concentration is high, current is inward.
P	O14649	CC	pH-dependent, voltage-insensitive, background potassium channel protein
P	O14649	DE	Acid-sensitive potassium channel protein TASK-1;
P	O14649	DE	Potassium channel subfamily K member 3;
P	O14649	DE	TWIK-related acid-sensitive K+ channel 1;
P	O14649	DE	Two pore K+ channel KT3.1;
P	O14649	DE	Two pore potassium channel KT3.1;
P	O14764	CC	GABA, the major inhibitory neurotransmitter in the vertebrate brain, mediates neuronal inhibition by binding to the GABA/benzodiazepine receptor and opening an integral chloride channel.
P	O14764	DR	chloride channel activity
P	O14863	CC	Probably involved in zinc transport out of the cytoplasm, maybe by sequestration into an intracellular compartment.
P	O14863	DE	Zinc transporter 4;
P	O14863	DR	zinc ion transmembrane transporter activity
P	O14983	CC	Contributes to calcium sequestration involved in muscular excitation/contraction.
P	O14983	CC	This magnesium-dependent enzyme catalyzes the hydrolysis of ATP coupled with the translocation of calcium from the cytosol to the sarcoplasmic reticulum lumen
P	O14983	DE	Calcium pump 1;
P	O14983	DE	Calcium-transporting ATPase sarcoplasmic reticulum type, fast twitch skeletal muscle isoform;
P	O14983	DE	Endoplasmic reticulum class 1/2 Ca2+ ATPase;
P	O14983	DE	SR Ca2+-ATPase 1;

P	O14983	DE	Sarcoplasmic/endoplasmic reticulum calcium ATPase 1;
P	O14983	DR	calcium-transporting ATPase activity
P	O15118	DR	sterol transporter activity
P	O15247	CC	Can insert into membranes and form chloride ion channels
P	O15247	DE	Chloride intracellular channel protein 2;
P	O15247	DR	voltage-gated chloride channel activity
P	O15374	CC	Proton-linked monocarboxylate transporter
P	O15374	DE	Monocarboxylate transporter 4;
P	O15374	DE	Monocarboxylate transporter 5;
P	O15375	CC	Proton-linked monocarboxylate transporter
P	O15375	DE	Monocarboxylate transporter 5;
P	O15375	DE	Monocarboxylate transporter 6;
P	O15375	DR	secondary active monocarboxylate transmembrane transporter activity
P	O15403	CC	Proton-linked monocarboxylate transporter
P	O15403	DE	Monocarboxylate transporter 6;
P	O15403	DE	Monocarboxylate transporter 7;
P	O15427	CC	Proton-linked monocarboxylate transporter
P	O15427	DE	Monocarboxylate transporter 3;
P	O15427	DE	Monocarboxylate transporter 4;
P	O15427	DR	secondary active monocarboxylate transmembrane transporter activity
P	O15431	CC	High-affinity, saturable copper transporter involved in dietary copper uptake.
P	O15431	DE	Copper transporter 1;
P	O15431	DE	High affinity copper uptake protein 1;
P	O15431	DR	copper ion transmembrane transporter activity
P	O15432	CC	Involved in low-affinity copper uptake (Potential).
P	O15432	DE	Copper transporter 2;
P	O15432	DE	Probable low affinity copper uptake protein 2;
P	O15432	DR	copper ion transmembrane transporter activity
P	O15554	CC	Forms a voltage-independent potassium channel that is activated by intracellular calcium
P	O16452	DE	Na+-H+ exchanger protein 3;
P	O16452	DE	Probable Na+/H+ antiporter nhx-3;
P	O16452	DR	sodium:hydrogen antiporter activity
P	O17444	CC	Involved in acetylcholine transport into synaptic vesicles.
P	O17444	DE	Vesicular acetylcholine transporter;
P	O18276	CC	GABA, an inhibitory neurotransmitter, mediates neuronal inhibition by binding to the GABA receptor and opening an integral chloride channel (By similarity).
P	O18276	DR	chloride channel activity
P	O22218	CC	This magnesium-dependent enzyme catalyzes the hydrolysis of ATP coupled with the translocation of calcium from the cytosol into small vacuoles.
P	O22218	DE	Ca2+-ATPase isoform 4;
P	O22218	DE	Calcium-transporting ATPase 4, plasma membrane-type;
P	O22218	DR	calcium-transporting ATPase activity
P	O22397	CC	High-affinity potassium transporter that could play a major role in the uptake of potassium from the rhizosphere
P	O22397	CC	May act as a low-affinity potassium transporter under high potassium concentrations
P	O22397	DE	Potassium transporter 1;
P	O22397	DR	potassium ion transmembrane transporter activity
P	O22588	CC	Aquaporins facilitate the transport of water and small neutral solutes across cell membranes (By similarity).
P	O22881	CC	Could mediate the potassium-dependent cell expansion in growing tissues.
P	O22881	CC	Low-affinity potassium transporter
P	O22881	DE	Potassium transporter 2;
P	O22881	DR	potassium ion transmembrane transporter activity
P	O22920	CC	May operate as a K+/H+ symporter.
P	O22920	DE	Cation/H+ symporter 13;
P	O22920	DE	Protein CATION/H+ EXCHANGER 13;
P	O23039	CC	Probably mediates zinc uptake from the rhizosphere (By similarity).
P	O23039	DE	Zinc transporter 5;
P	O23039	DR	zinc ion transmembrane transporter activity
P	O23087	CC	This magnesium-dependent enzyme catalyzes the hydrolysis of ATP coupled with the translocation of calcium from the cytosol to an endomembrane compartment
P	O23087	DE	Calcium-transporting ATPase 2, endoplasmic reticulum-type;
P	O23087	DR	calcium-transporting ATPase activity
P	O25788	CC	Intake of glucose and galactose (Potential).
P	O25788	DE	Putative glucose/galactose transporter;

P	O25788	DE	Putative glucose/galactose transporter;
P	O25788	DR	galactose transmembrane transporter activity
P	O25788	DR	glucose transmembrane transporter activity
P	O25797	CC	The physiological role may be the reduction of the intracellular concentration of toxic sugars or sugar metabolites (By similarity).
P	O25797	DE	Probable sugar efflux transporter;
P	O25901	CC	Mediates influx of magnesium ions
P	O25901	CC	Plays a key role in the adaptation to the low magnesium conditions predominant in the gastric environment.
P	O25901	DE	Magnesium transport protein CorA;
P	O25901	DR	cobalt ion transmembrane transporter activity
P	O25901	DR	magnesium ion transmembrane transporter activity
P	O26076	CC	Na+/H+ antiporter that extrudes sodium in exchange for external protons (By similarity)
P	O26076	DE	Na+/H+ antiporter nhaA;
P	O26076	DE	Sodium/proton antiporter nhaA;
P	O26096	CC	Part of the ABC transporter complex MetNIQ involved in methionine import
P	O26096	DE	Methionine import ATP-binding protein MetN;
P	O26234	DR	cobalt ion transmembrane transporter activity
P	O27764	CC	Part of the ABC transporter complex PstSACB involved in phosphate import
P	O27764	DE	ABC phosphate transporter;
P	O27764	DE	Phosphate import ATP-binding protein PstB;
P	O27764	DE	Phosphate-transporting ATPase;
P	O27764	DR	inorganic phosphate transmembrane transporter activity
P	O27764	DR	phosphate transmembrane-transporting ATPase activity
P	O28846	CC	Channel that permits osmotically driven movement of water in both directions (By similarity).
P	O28912	CC	Part of the ABC transporter complex PstSACB involved in phosphate import
P	O28912	DE	ABC phosphate transporter;
P	O28912	DE	Phosphate import ATP-binding protein PstB;
P	O28912	DE	Phosphate-transporting ATPase;
P	O28912	DR	inorganic phosphate transmembrane transporter activity
P	O28912	DR	phosphate transmembrane-transporting ATPase activity
P	O29529	DR	cobalt ion transmembrane transporter activity
P	O30085	CC	Probably involved in copper export.
P	O30085	DE	Probable copper-exporting P-type ATPase B;
P	O30085	DR	copper-exporting ATPase activity
P	O30142	CC	Binds tungstate and molybdate.
P	O30142	CC	Part of the ABC transporter complex wtpABC involved in molybdate/tungstate import
P	O30142	DE	Molybdate/tungstate-binding protein wtpA;
P	O30143	CC	Part of the ABC transporter complex wtpABC involved in molybdate/tungstate import
P	O30143	DE	Molybdate/tungstate transport system permease protein wtpB;
P	O30144	CC	Part of the ABC transporter complex WtpABC involved in molybdate/tungstate import
P	O30144	DE	Molybdate/tungstate import ATP-binding protein WtpC;
P	O30417	CC	Involved in glutamate-dependent acid resistance
P	O30417	CC	May be involved in antiport of glutamate and glutamate-gamma- aminobutyrate (GABA)
P	O30417	DE	Glutamate/gamma-aminobutyrate antiporter;
P	O30499	CC	Low-affinity inorganic phosphate transport (Probable).
P	O30499	DE	Probable low-affinity inorganic phosphate transporter;
P	O30499	DR	inorganic phosphate transmembrane transporter activity
P	O30506	CC	Part of the arginine-inducible binding-protein-dependent transport system for arginine and ornithine
P	O30506	CC	Part of the arginine-inducible binding-protein-dependent transport system for arginine and ornithine
P	O30506	DE	Arginine/ornithine transport ATP-binding protein AotP;
P	O30506	DE	Arginine/ornithine transport ATP-binding protein AotP;
P	O30510	DE	Putative bile salt transporter;
P	O30513	CC	Probable uptake of benzoate.
P	O30513	DE	Benzoate transport protein;
P	O30832	DE	Sorbitol/mannitol transport inner membrane protein;
P	O30833	DE	Sorbitol/mannitol transport inner membrane protein;
P	O31137	DE	Tetracycline-resistance determinant tetV;
P	O31199	DE	Inducible histidine transporter;
P	O31307	DE	Oligopeptide ABC transporter, permease protein (OppB-1);
P	O31307	DE	Oligopeptide permease homolog B;
P	O31309	DE	Oligopeptide ABC transporter, ATP-binding protein (OppD);

P	O31309	DE	Oligopeptide permease homolog D;
P	O31310	DE	Oligopeptide ABC transporter, ATP-binding protein (OppF);
P	O31310	DE	Oligopeptide permease homolog F;
P	O31315	DE	Oligopeptide ABC transporter, periplasmic oligopeptide-binding protein (OppAIV);
P	O31567	CC	Part of the ABC transporter complex yfiYZ/yfhA/yusV involved in import of the iron-hydroxamate siderophores schizokinen, arthrobactin and corprogen
P	O31567	DR	iron ion transmembrane transporter activity
P	O31568	CC	Part of the ABC transporter complex yfiYZ/yfhA/yusV involved in import of the iron-hydroxamate siderophores schizokinen, arthrobactin and corprogen (Probable).
P	O31658	CC	Integral membrane subunit of the KtrCD potassium uptake transporter
P	O31658	CC	The 2 major potassium transporter complexes KtrAB and KtrCD confer resistance to both suddenly imposed and prolonged osmotic stress.
P	O31658	DE	K+-uptake protein ktrD;
P	O31658	DE	Ktr system potassium uptake protein D;
P	O32081	CC	Integral membrane subunit of the KtrAB potassium uptake transporter
P	O32081	CC	The 2 major potassium transporter complexes KtrAB and KtrCD confer resistance to both suddenly imposed and prolonged osmotic stress.
P	O32081	DE	K+-uptake protein ktrB;
P	O32081	DE	Ktr system potassium uptake protein B;
P	O32115	DR	nucleoside:sodium symporter activity
P	O32204	CC	Catalyzes an electroneutral exchange between arginine and ornithine to allow high-efficiency energy conversion in the arginine deiminase pathway (By similarity).
P	O32204	CC	Catalyzes an electroneutral exchange between arginine and ornithine to allow high-efficiency energy conversion in the arginine deiminase pathway (By similarity).
P	O32204	DE	Putative arginine/ornithine antiporter;
P	O32204	DE	Putative arginine/ornithine antiporter;
P	O32209	CC	could be part of the binding-protein-dependent transport system for molybdenum; probably responsible for the translocation of the substrate across the membrane (By similarity).
P	O32209	DE	Putative molybdenum transport system permease protein yvgM;
P	O32212	CC	Transporter involved in the efflux of sodium, potassium, lithium and rubidium.
P	O32212	DE	Sodium, potassium, lithium and rubidium/H+ antiporter;
P	O32219	CC	Couples the hydrolysis of ATP with the transport of cadmium, zinc and cobalt out of the cell
P	O32219	CC	Couples the hydrolysis of ATP with the transport of cadmium, zinc and cobalt out of the cell
P	O32219	DE	Cadmium, zinc and cobalt-transporting ATPase;
P	O32219	DE	Cadmium, zinc and cobalt-transporting ATPase;
P	O32219	DR	cadmium-exporting ATPase activity
P	O32219	DR	zinc-exporting ATPase activity
P	O32220	CC	Involved in copper export.
P	O32220	DE	Copper-exporting P-type ATPase A;
P	O32220	DR	copper-exporting ATPase activity
P	O32243	CC	Member of a high affinity multicomponent binding- protein-dependent transport system for glycine betaine, carnitine, and choline.
P	O32243	CC	Member of a high affinity multicomponent binding- protein-dependent transport system for glycine betaine, carnitine, and choline.
P	O32243	DE	Glycine betaine/carnitine/choline-binding protein;
P	O32243	DE	Glycine betaine/carnitine/choline-binding protein;
P	O32327	CC	One of the components of the high-affinity ATP-driven potassium transport (or KDP) system, which catalyzes the hydrolysis of ATP coupled with the exchange of hydrogen and potassium ions (By similarity).
P	O32327	DE	Potassium-binding and translocating subunit A;
P	O32327	DE	Potassium-translocating ATPase A chain;
P	O32327	DE	Potassium-transporting ATPase A chain;
P	O32327	DR	potassium-transporting ATPase activity
P	O32328	CC	One of the components of the high-affinity ATP-driven potassium transport (or KDP) system, which catalyzes the hydrolysis of ATP coupled with the exchange of hydrogen and potassium ions.
P	O32328	DE	Potassium-binding and translocating subunit B;
P	O32328	DE	Potassium-translocating ATPase B chain;
P	O32328	DE	Potassium-transporting ATPase B chain;
P	O32328	DR	potassium-transporting ATPase activity
P	O33704	DE	Zinc ABC transporter, permease protein;
P	O34348	DR	iron ion transmembrane transporter activity

P	O34436	CC	Low-affinity inorganic phosphate transport (By similarity).
P	O34436	DE	Probable low-affinity inorganic phosphate transporter;
P	O34436	DR	inorganic phosphate transmembrane transporter activity
P	O34442	DE	Magnesium transporter mgtE;
P	O34734	DR	inorganic phosphate transmembrane transporter activity
P	O34742	CC	Involved in a high affinity multicomponent binding- protein-dependent transport system for glycine betaine, carnitine and choline; probably responsible for the translocation of the substrate across the membrane.
P	O34742	CC	Involved in a high affinity multicomponent binding- protein-dependent transport system for glycine betaine, carnitine and choline; probably responsible for the translocation of the substrate across the membrane.
P	O34742	DE	Glycine betaine/carnitine/choline transport system permease protein opuCD;
P	O34742	DE	Glycine betaine/carnitine/choline transport system permease protein opuCD;
P	O34878	CC	Involved in a high affinity multicomponent binding- protein-dependent transport system for glycine betaine, carnitine and choline; probably responsible for the translocation of the substrate across the membrane.
P	O34878	CC	Involved in a high affinity multicomponent binding- protein-dependent transport system for glycine betaine, carnitine and choline; probably responsible for the translocation of the substrate across the membrane.
P	O34878	DE	Glycine betaine/carnitine/choline transport system permease protein opuCB;
P	O34878	DE	Glycine betaine/carnitine/choline transport system permease protein opuCB;
P	O34992	CC	Involved in a high affinity multicomponent binding- protein-dependent transport system for glycine betaine, carnitine and choline; probably responsible for energy coupling to the transport system.
P	O34992	CC	Involved in a high affinity multicomponent binding- protein-dependent transport system for glycine betaine, carnitine and choline; probably responsible for energy coupling to the transport system.
P	O34992	DE	Glycine betaine/carnitine/choline transport ATP-binding protein OpuCA;
P	O34992	DE	Glycine betaine/carnitine/choline transport ATP-binding protein OpuCA;
P	O35055	CC	AT LEAST ONE Na+ SEEMS TO BIND TO THE TRANSPORTER BEFORE THE SUBSTRATE.
P	O35055	CC	IT MAY HAVE A SODIUM:SUBSTRATE STOICHIOMETRY OF 3:1
P	O35055	CC	MAY MEDIATE ELECTROGENIC, SODIUM-DEPENDENT TRANSPORT OF MOST KREBS CYCLE INTERMEDIATES, INCLUDING CITRATE, SUCCINATE, ALPHA-KETOGLUTARATE, AND OXALOACETATE
P	O35055	CC	MAY MEDIATE ELECTROGENIC, SODIUM-DEPENDENT TRANSPORT OF MOST KREBS CYCLE INTERMEDIATES, INCLUDING CITRATE, SUCCINATE, ALPHA-KETOGLUTARATE, AND OXALOACETATE
P	O35055	CC	TRANSPORT OF CITRATE WAS PH SENSITIVE
P	O35055	DE	Sodium-dependent dicarboxylate transporter;
P	O35055	DE	Sodium-dependent dicarboxylate transporter;
P	O35055	DE	Sodium-dicarboxylate cotransporter SDCT1;
P	O35055	DE	Solute carrier family 13 (Sodium-dependent dicarboxylate transporter), member 2, isoform CRA_a;
P	O35111	CC	Acts as an outward rectifier when external potassium concentration is low
P	O35111	CC	Rectification direction results from potassium ion concentration on either side of the membrane
P	O35111	CC	When external potassium concentration is high, current is inward (By similarity).
P	O35111	CC	pH-dependent, voltage-insensitive, background potassium channel protein
P	O35111	DE	Acid-sensitive potassium channel protein TASK-1;
P	O35111	DE	Cardiac two pore background K+ channel;
P	O35111	DE	Potassium channel subfamily K member 3;
P	O35111	DE	TWIK-related acid-sensitive K+ channel 1;
P	O35111	DE	Two pore K+ channel KT3.1;
P	O35111	DE	Two pore potassium channel KT3.1;
P	O35173	CC	Potassium channel subunit
P	O35173	DE	Delayed-rectifier K+ channel alpha subunit 1;
P	O35173	DE	Potassium voltage-gated channel subfamily S member 1;
P	O35173	DE	Voltage-gated potassium channel subunit Kv9.1;
P	O35173	DR	delayed rectifier potassium channel activity
P	O35173	DR	potassium channel regulator activity
P	O35174	CC	Potassium channel subunit
P	O35174	DE	Delayed-rectifier K+ channel alpha subunit 2;
P	O35174	DE	Potassium voltage-gated channel subfamily S member 2;
P	O35174	DE	Voltage-gated potassium channel subunit Kv9.2;

P	O35174	DR	voltage-gated potassium channel activity
P	O35308	CC	Proton-linked monocarboxylate transporter
P	O35308	DE	Monocarboxylate transporter 3;
P	O35308	DE	Proton-coupled monocarboxylate transporter 3;
P	O35308	DR	secondary active monocarboxylate transmembrane transporter activity
P	O35316	CC	Required for the uptake of taurine
P	O35316	CC	Transports both taurine and beta-alanine which requires sodium ions
P	O35316	CC	Transports both taurine and beta-alanine which requires sodium ions
P	O35316	DE	Sodium- and chloride-dependent taurine transporter;
P	O35316	DE	Sodium- and chloride-dependent taurine transporter;
P	O35316	DR	neurotransmitter:sodium symporter activity
P	O35454	CC	Chloride transport protein, initially identified as voltage-gated chloride channel
P	O35454	DE	Chloride channel protein 6;
P	O35454	DE	Chloride transport protein 6;
P	O35454	DR	voltage-gated chloride channel activity
P	O35544	CC	Seems to act as a symport by cotransporting sodium (By similarity).
P	O35544	CC	Transports L-glutamate and also L- and D-aspartate
P	O35544	DE	High-affinity neuronal glutamate transporter;
P	O35544	DE	Sodium-dependent glutamate/aspartate transporter;
P	O35544	DE	Sodium-dependent glutamate/aspartate transporter;
P	O35544	DE	Sodium-dependent glutamate/aspartate transporter;
P	O35544	DR	L-glutamate transmembrane transporter activity
P	O35544	DR	sodium:dicarboxylate symporter activity
P	O35600	DR	phospholipid-translocating ATPase activity
P	O35633	CC	Involved in the uptake of GABA and glycine into the synaptic vesicles.
P	O35633	CC	Involved in the uptake of GABA and glycine into the synaptic vesicles.
P	O35633	DE	GABA and glycine transporter;
P	O35633	DE	GABA and glycine transporter;
P	O35633	DE	Vesicular GABA transporter;
P	O35633	DR	gamma-aminobutyric acid:hydrogen symporter activity
P	O35633	DR	glycine transmembrane transporter activity
P	O35874	CC	Exhibits sodium dependence (By similarity).
P	O35874	DR	sodium:dicarboxylate symporter activity
P	O35956	CC	Mediates the sodium-independent uptake of 2,3-dimercapto-1-propanesulfonic acid (DMPS), 9-(2- phosphonylmethoxyethyl) guanine (PMEG), 9-(2- phosphonylmethoxyethyl) diaminopurine (PMEDAP), ochratoxin (OTA), acyclovir (ACV), 3'-azido-3-'deoxythymidine (AZT), cimetidine (CMD) and edaravone sulfate
P	O35956	CC	Mediates the sodium-independent uptake of p-aminohippurate (PAH), cidofovir, adefovir, 2,4- dichloro-phenoxyacetate (2,4-D), hippurate (HA), indoleacetate (IA), indoxyl sulfate (IS) and 3-carboxy-4-methyl-5-propyl-2- furanpropionate (CMPF)
P	O35956	DR	sodium-independent organic anion transmembrane transporter activity
P	O43315	DR	water channel activity
P	O43511	CC	Sodium-independent transporter of chloride and iodide.
P	O43511	DE	Sodium-independent chloride/iodide transporter;
P	O43511	DR	chloride transmembrane transporter activity
P	O43525	CC	Associates with KCNQ2 or KCNQ5 to form a potassium channel with essentially identical properties to the channel underlying the native M-current, a slowly activating and deactivating potassium conductance which plays a critical role in determining the subthreshold electrical excitability of neurons as well as the responsiveness to synaptic inputs.
P	O43525	DE	Potassium channel subunit alpha KvLQT3;
P	O43525	DE	Potassium voltage-gated channel subfamily KQT member 3;
P	O43525	DE	Voltage-gated potassium channel subunit Kv7.3;
P	O43525	DR	voltage-gated potassium channel activity
P	O43526	CC	Associates with KCNQ3 to form a potassium channel with essentially identical properties to the channel underlying the native M-current, a slowly activating and deactivating potassium conductance which plays a critical role in determining the subthreshold electrical excitability of neurons as well as the responsiveness to synaptic inputs
P	O43526	DE	Neuroblastoma-specific potassium channel subunit alpha KvLQT2;
P	O43526	DE	Potassium voltage-gated channel subfamily KQT member 2;
P	O43526	DE	Voltage-gated potassium channel subunit Kv7.2;
P	O43526	DR	voltage-gated potassium channel activity
P	O43681	DE	Arsenite-stimulated ATPase;
P	O43681	DR	arsenite transmembrane transporter activity
P	O43772	DE	Carnitine/acylcarnitine translocase;

P	O43772	DE	Mitochondrial carnitine/acylcarnitine carrier protein;
P	O43772	DR	acyl carnitine transporter activity
P	O43868	CC	Exhibits the transport characteristics of the nucleoside transport system cif or N1 subtype (N1/cif) (selective for purine nucleosides and uridine)
P	O43868	CC	Sodium-dependent and purine-selective transporter
P	O43868	DE	Concentrative nucleoside transporter 2;
P	O43868	DE	Na+/nucleoside cotransporter 2;
P	O43868	DE	Na+/nucleoside cotransporter 2;
P	O43868	DE	Sodium-coupled nucleoside transporter 2;
P	O43868	DE	Sodium-coupled nucleoside transporter 2;
P	O43868	DE	Sodium/nucleoside cotransporter 2;
P	O43868	DE	Sodium/nucleoside cotransporter 2;
P	O43868	DE	Sodium/purine nucleoside co-transporter;
P	O43868	DR	nucleoside:sodium symporter activity
P	O43868	DR	nucleoside:sodium symporter activity
P	O45166	DE	Folate-like transporter 2;
P	O45166	DR	reduced folate carrier activity
P	O45215	DE	Calcium ATPase;
P	O45215	DR	calcium-transporting ATPase activity
P	O48639	CC	High-affinity transporter for external inorganic phosphate (By similarity).
P	O48639	DE	Probable inorganic phosphate transporter 1-3;
P	O48639	DR	inorganic phosphate transmembrane transporter activity
P	O49423	CC	Putative potassium transporter.
P	O49423	DE	Potassium transporter 9;
P	O49423	DR	potassium ion transmembrane transporter activity
P	O50181	DE	Arginine and ornithine binding protein;
P	O50181	DE	Arginine and ornithine binding protein;
P	O50181	DE	Arginine/ornithine binding protein AotJ;
P	O50181	DE	Arginine/ornithine binding protein AotJ;
P	O50183	DE	Arginine/ornithine transport protein AotM;
P	O50183	DE	Arginine/ornithine transport protein AotM;
P	O50649	DR	neurotransmitter:sodium symporter activity
P	O50927	DE	Oligopeptide ABC transporter, periplasmic oligopeptide-binding protein (OppAV);
P	O51236	CC	Part of the ABC transporter complex PstSACB involved in phosphate import
P	O51236	DE	ABC phosphate transporter;
P	O51236	DE	Phosphate import ATP-binding protein PstB;
P	O51236	DE	Phosphate-transporting ATPase;
P	O51236	DR	inorganic phosphate transmembrane transporter activity
P	O51236	DR	phosphate transmembrane-transporting ATPase activity
P	O51307	DE	Oligopeptide ABC transporter, periplasmic oligopeptide-binding protein (OppA-1);
P	O51308	DE	Oligopeptide ABC transporter, periplasmic oligopeptide-binding protein (OppA-2);
P	O51310	DE	Oligopeptide ABC transporter, permease protein (OppC-1);
P	O51587	CC	Part of the ABC transporter complex PotABCD involved in spermidine/putrescine import
P	O51587	CC	Part of the ABC transporter complex PotABCD involved in spermidine/putrescine import
P	O51587	DE	Spermidine/putrescine import ATP-binding protein PotA;
P	O51587	DE	Spermidine/putrescine import ATP-binding protein PotA;
P	O51923	DE	Trehalose/maltose binding protein;
P	O51923	DE	Trehalose/maltose binding protein;
P	O51925	DE	Trehalose/maltose transport inner membrane protein;
P	O51925	DE	Trehalose/maltose transport inner membrane protein;
P	O52717	DE	Ribitol transporter;
P	O53832	CC	Part of the ABC transporter complex PstSACB involved in phosphate import
P	O53832	DE	ABC phosphate transporter 1;
P	O53832	DE	Phosphate import ATP-binding protein PstB 1;
P	O53832	DE	Phosphate-transporting ATPase 1;
P	O53832	DR	inorganic phosphate transmembrane transporter activity
P	O53832	DR	phosphate transmembrane-transporting ATPase activity
P	O54189	DR	cobalt ion transmembrane transporter activity
P	O54584	DE	Oligopeptide ABC transporter, periplasmic oligopeptide-binding protein (OppA-2);
P	O54584	DE	Oligopeptide permease periplasmic binding protein;
P	O54794	CC	Forms a channel for water and glycerol.
P	O54794	DR	water channel activity
P	O54982	CC	Testis-specific potassium channel activated by both intracellular pH and membrane voltage that mediates export of K+
P	O54982	DE	Potassium channel subfamily U member 1;

P	O54982	DE	pH-sensitive maxi potassium channel;
P	O55017	CC	Calcium channels containing alpha-1B subunit may play a role in directed migration of immature neurons.
P	O55017	CC	N-type calcium channels belong to the 'high-voltage activated' (HVA) group and are blocked by omega-conotoxin-GVIA (omega-CTx-GVIA) and by omega-agatoxin- IIIA (omega-Aga-IIIA)
P	O55017	CC	The isoform alpha-1B gives rise to N-type calcium currents
P	O55017	CC	Voltage-sensitive calcium channels (VSCC) mediate the entry of calcium ions into excitable cells and are also involved in a variety of calcium-dependent processes, including muscle contraction, hormone or neurotransmitter release, gene expression, cell motility, cell division and cell death
P	O55017	DE	Brain calcium channel III;
P	O55017	DE	Calcium channel, L type, alpha-1 polypeptide isoform 5;
P	O55017	DE	Voltage-dependent N-type calcium channel subunit alpha-1B;
P	O55017	DE	Voltage-gated calcium channel subunit alpha Cav2.2;
P	O55017	DR	voltage-gated calcium channel activity
P	O55143	CC	This magnesium-dependent enzyme catalyzes the hydrolysis of ATP coupled with the translocation of calcium from the cytosol to the sarcoplasmic reticulum lumen
P	O55143	DE	Calcium pump 2;
P	O55143	DE	Calcium-transporting ATPase sarcoplasmic reticulum type, slow twitch skeletal muscle isoform;
P	O55143	DE	Endoplasmic reticulum class 1/2 Ca2+ ATPase;
P	O55143	DE	SR Ca2+-ATPase 2;
P	O55143	DE	Sarcoplasmic/endoplasmic reticulum calcium ATPase 2;
P	O55143	DR	calcium-transporting ATPase activity
P	O59666	CC	Probably involved in copper transport and in the regulation of cellular copper level
P	O59666	CC	Retrieves copper from the metallochaperone atx1 and incorporates it into trans-Golgi vesicles (By similarity).
P	O59666	DE	Copper-transporting ATPase ccc2;
P	O59666	DR	copper-exporting ATPase activity
P	O59768	CC	Has a role in promoting intracellular calcium ion sequestration via the exchange of calcium ions for hydrogen ions across the vacuolar membrane
P	O59768	DE	Vacuolar Ca2+/H+ exchanger;
P	O59768	DE	Vacuolar calcium ion transporter;
P	O59868	CC	Regulates cell morphogenesis through control of manganese and calcium homeostasis
P	O59868	CC	Transports calcium and manganese ions into the cell
P	O59868	DE	Calcium-transporting ATPase 1;
P	O59868	DE	Golgi Ca2+-ATPase;
P	O59868	DR	calcium-transporting ATPase activity
P	O60721	CC	Critical component of the visual transduction cascade, controlling the calcium concentration of outer segments during light and darkness
P	O60721	CC	Light causes a rapid lowering of cytosolic free calcium in the outer segment of both retinal rod and cone photoreceptors and the light-induced lowering of calcium is caused by extrusion via this protein which plays a key role in the process of light adaptation
P	O60721	CC	Transports 1 Ca2+ and 1 K+ in exchange for 4 Na+.
P	O60721	CC	Transports 1 Ca2+ and 1 K+ in exchange for 4 Na+.
P	O60721	CC	Transports 1 Ca2+ and 1 K+ in exchange for 4 Na+.
P	O60721	DE	Na+/K+/Ca2+-exchange protein 1;
P	O60721	DE	Na+/K+/Ca2+-exchange protein 1;
P	O60721	DE	Na+/K+/Ca2+-exchange protein 1;
P	O60721	DE	Sodium/potassium/calcium exchanger 1;
P	O60721	DE	Sodium/potassium/calcium exchanger 1;
P	O60721	DE	Sodium/potassium/calcium exchanger 1;
P	O60721	DR	calcium, potassium:sodium antiporter activity
P	O60721	DR	calcium, potassium:sodium antiporter activity
P	O60721	DR	calcium, potassium:sodium antiporter activity
P	O60741	CC	Hyperpolarization-activated ion channel exhibiting weak selectivity for potassium over sodium ions
P	O60741	DE	Potassium/sodium hyperpolarization-activated cyclic nucleotide-gated channel 1;
P	O60741	DR	voltage-gated potassium channel activity
P	O60779	CC	High-affinity transporter for the intake of thiamine.
P	O60779	DE	Thiamine carrier 1;
P	O60779	DE	Thiamine transporter 1;
P	O60779	DR	folic acid transporter activity
P	O60779	DR	reduced folate carrier activity

P	O60779	DR	thiamine uptake transmembrane transporter activity
P	O60840	CC	Long-lasting (L-type) calcium channels belong to the 'high-voltage activated' (HVA) group
P	O60840	CC	The isoform alpha-1F gives rise to L-type calcium currents
P	O60840	CC	Voltage-sensitive calcium channels (VSCC) mediate the entry of calcium ions into excitable cells and are also involved in a variety of calcium-dependent processes, including muscle contraction, hormone or neurotransmitter release, gene expression, cell motility, cell division and cell death
P	O60840	DE	Voltage-dependent L-type calcium channel subunit alpha-1F;
P	O60840	DE	Voltage-gated calcium channel subunit alpha Cav1.4;
P	O60840	DR	voltage-gated calcium channel activity
P	O60928	CC	Inward rectifier potassium channels are characterized by a greater tendency to allow potassium to flow into the cell rather than out of it
P	O60928	CC	Their voltage dependence is regulated by the concentration of extracellular potassium; as external potassium is raised, the voltage range of the channel opening shifts to more positive voltages
P	O60928	DE	Inward rectifier K+ channel Kir7.1;
P	O60928	DE	Inward rectifier potassium channel 13;
P	O60928	DE	Potassium channel, inwardly rectifying subfamily J member 13;
P	O60928	DR	inward rectifier potassium channel activity
P	O60931	CC	Thought to transport cystine out of lysosomes.
P	O61365	CC	Probable role in sodium transport.
P	O61365	DE	Sodium channel protein Nach;
P	O61365	DR	sodium channel activity
P	O64738	CC	May play a role in the transport of zinc in the plastids (By similarity).
P	O64738	DE	Zinc transporter 6, chloroplastic;
P	O64738	DR	zinc ion transmembrane transporter activity
P	O64769	CC	Probable potassium transporter.
P	O64769	DE	Potassium transporter 11;
P	O64769	DR	potassium ion transmembrane transporter activity
P	O64806	CC	This magnesium-dependent enzyme catalyzes the hydrolysis of ATP coupled with the translocation of calcium from the cytosol out of the cell or into organelles (By similarity).
P	O64806	DE	Ca2+-ATPase isoform 7;
P	O64806	DE	Putative calcium-transporting ATPase 7, plasma membrane-type;
P	O64806	DR	calcium-transporting ATPase activity
P	O65989	CC	This system is involved in mannitol transport (By similarity).
P	O65989	DE	Mannitol permease IIC component;
P	O65989	DE	Mannitol-specific phosphotransferase enzyme IIB component;
P	O65989	DE	PTS system mannitol-specific EIIB component;
P	O65989	DE	PTS system mannitol-specific EIIC component;
P	O65989	DE	PTS system mannitol-specific EIICB component;
P	O66646	DE	Lipoprotein-releasing system ATP-binding protein LolD;
P	O66646	DR	lipoprotein transporter activity
P	O67154	CC	Part of the ABC transporter complex PstSACB involved in phosphate import
P	O67154	DE	ABC phosphate transporter;
P	O67154	DE	Phosphate import ATP-binding protein PstB;
P	O67154	DE	Phosphate-transporting ATPase;
P	O67154	DR	inorganic phosphate transmembrane transporter activity
P	O67154	DR	phosphate transmembrane-transporting ATPase activity
P	O67203	DR	copper-exporting ATPase activity
P	O67432	DR	copper-exporting ATPase activity
P	O68874	CC	Channel that permits osmotically driven movement of water in both directions
P	O68874	CC	It mediates rapid entry or exit of water in response to abrupt changes in osmolarity (By similarity).
P	O68877	CC	Part of the ABC transporter complex HmuTUV involved in hemin import
P	O68877	DE	Hemin import ATP-binding protein HmuV;
P	O69443	CC	H+-stimulated, highly selective, manganese uptake system (By similarity).
P	O69443	DE	Probable manganese transport protein mntH;
P	O70247	CC	Transports pantothenate, biotin and lipoate in the presence of sodium.
P	O70247	CC	Transports pantothenate, biotin and lipoate in the presence of sodium.
P	O70247	DE	Na+-dependent multivitamin transporter;
P	O70247	DE	Sodium-dependent multivitamin transporter;
P	O70247	DR	sodium-dependent multivitamin transmembrane transporter activity
P	O70324	DE	Monocarboxylate transporter 8;
P	O70451	CC	Proton-linked monocarboxylate transporter

P	O70451	DE	Monocarboxylate transporter 2;
P	O70451	DR	secondary active monocarboxylate transmembrane transporter activity
P	O70491	CC	Acts by removing retinol from RBP/RBP4 and transports it across the plasma membrane, where it can be metabolized
P	O70491	CC	Increases cellular retinol uptake from the retinol-RBP complex (By similarity).
P	O70491	CC	May act as a high-affinity cell-surface receptor for the complex retinol-retinol binding protein (RBP/RBP4)
P	O70496	CC	Mediates the exchange of chloride ions against protons
P	O70496	DE	Chloride channel protein 7;
P	O70496	DR	voltage-gated chloride channel activity
P	O74750	CC	Sugar transporter that specifically mediates the transport of UDP-N-acetylglucosamine (UDP-GlcNAc) and is required for cell wall chitin synthesis (By similarity)
P	O74750	DE	UDP-N-acetylglucosamine transporter yea4;
P	O74969	CC	High-affinity glucose transporter.
P	O74969	DE	High-affinity glucose transporter ght2;
P	O75185	CC	This magnesium-dependent enzyme catalyzes the hydrolysis of ATP coupled with the transport of calcium (By similarity).
P	O75185	DE	Calcium-transporting ATPase type 2C member 2;
P	O75185	DE	Secretory pathway Ca2+-ATPase 2;
P	O75185	DR	calcium-transporting ATPase activity
P	O75746	CC	Calcium-dependent mitochondrial aspartate and glutamate carrier
P	O75746	DE	Mitochondrial aspartate glutamate carrier 1;
P	O75746	DR	L-glutamate transmembrane transporter activity
P	O76082	CC	Also relative uptake activity ratio of carnitine to TEA is 11.3.
P	O76082	CC	Also transports organic cations such as tetraethylammonium (TEA) without the involvement of sodium
P	O76082	CC	Involved in the active cellular uptake of carnitine
P	O76082	CC	Sodium-ion dependent, high affinity carnitine transporter
P	O76082	CC	Sodium-ion dependent, high affinity carnitine transporter
P	O76082	CC	Transports one sodium ion with one molecule of carnitine
P	O76082	CC	Transports one sodium ion with one molecule of carnitine
P	O76082	DE	High-affinity sodium-dependent carnitine cotransporter;
P	O76082	DE	High-affinity sodium-dependent carnitine cotransporter;
P	O76082	DE	Organic cation/carnitine transporter 2;
P	O76082	DR	carnitine transporter activity
P	O76689	CC	Mediates sodium-dependent uptake of acetylcholine at neuromuscular junctions during periods of increased synaptic activity, may also prevent spillover to adjacent synaptic sites
P	O76689	DE	Sodium-dependent acetylcholine transporter;
P	O76689	DE	Sodium:neurotransmitter symporter family protein 6;
P	O76689	DR	neurotransmitter:sodium symporter activity
P	O76729	DE	Biopterin transporter;
P	O80605	CC	Responsible for the transport of sucrose into the cell, with the concomitant uptake of protons (symport system)
P	O80605	DE	Sucrose permease 3;
P	O80605	DE	Sucrose transport protein SUC3;
P	O80605	DE	Sucrose transporter 2;
P	O80605	DE	Sucrose-proton symporter 3;
P	O80605	DR	sucrose transmembrane transporter activity
P	O80739	CC	Putative potassium transporter.
P	O80739	DE	Putative potassium transporter 12;
P	O80739	DR	potassium ion transmembrane transporter activity
P	O81108	CC	This magnesium-dependent enzyme catalyzes the hydrolysis of ATP coupled with the translocation of calcium from the cytosol into the endoplasmic reticulum
P	O81108	DE	Ca2+-ATPase isoform 2;
P	O81108	DE	Calcium-transporting ATPase 2, plasma membrane-type;
P	O81108	DR	calcium-transporting ATPase activity
P	O81123	CC	Mediates zinc uptake from the rhizosphere
P	O81123	DE	Zinc transporter 1;
P	O81123	DR	zinc ion transmembrane transporter activity
P	O81850	CC	Could also be capable of transporting zinc ions.
P	O81850	CC	High-affinity iron transporter that mediates under iron- deficiency the iron uptake from the rhizosphere across the plasma membrane in the root epidermal layer
P	O81850	DE	Iron-regulated transporter 2;
P	O81850	DR	iron ion transmembrane transporter activity

P	O81850	DR	zinc ion transmembrane transporter activity
P	O82316	CC	Aquaporins facilitate the transport of water and small neutral solutes across cell membranes
P	O82390	CC	Specific for inorganic phosphate transport across the thylakoid membrane in a sodium dependent manner
P	O82390	CC	Specific for inorganic phosphate transport across the thylakoid membrane in a sodium dependent manner
P	O82390	DE	Na+/PI cotransporter 1;
P	O82390	DE	Phosphate transporter PHT4;1;
P	O82390	DE	Sodium-dependent phosphate transport protein 1, chloroplastic;
P	O82390	DE	Sodium-dependent phosphate transport protein 1, chloroplastic;
P	O82390	DE	Sodium/phosphate cotransporter 1;
P	O82390	DE	Sodium/phosphate cotransporter 1;
P	O82390	DR	inorganic phosphate transmembrane transporter activity
P	O82485	DE	Oligopeptide transporter 7;
P	O82485	DR	oligopeptide transporter activity
P	O82598	CC	Potential aquaporin, which may facilitate the transport of water and small neutral solutes across cell membranes (By similarity).
P	O82598	DR	water channel activity
P	O82643	CC	Probably mediates zinc uptake from the rhizosphere (By similarity).
P	O82643	DE	Zinc transporter 9;
P	O82643	DR	zinc ion transmembrane transporter activity
P	O82855	CC	Confers resistance to several drugs, such as norfloxacin, ciprofloxacin, ethidium, kanamycin and streptomycin.
P	O82855	CC	Confers resistance to several drugs, such as norfloxacin, ciprofloxacin, ethidium, kanamycin and streptomycin.
P	O83341	DE	Ribose/galactose ABC transporter, ATP-binding protein (RbsA-2);
P	O83342	DE	Ribose/galactose ABC transporter, permease protein (RbsC-1);
P	O83343	DE	Ribose/galactose ABC transporter, permease protein (RbsC-2);
P	O83658	CC	Part of the ABC transporter complex PotABCD involved in spermidine/putrescine import
P	O83658	CC	Part of the ABC transporter complex PotABCD involved in spermidine/putrescine import
P	O83658	DE	Spermidine/putrescine import ATP-binding protein PotA;
P	O83658	DE	Spermidine/putrescine import ATP-binding protein PotA;
P	O84068	DE	ADP,ATP carrier protein 1;
P	O84068	DE	ADP/ATP translocase 1;
P	O84068	DE	ADP/ATP translocase 1;
P	O84068	DR	ATP:ADP antiporter activity
P	O84068	DR	ATP:ADP antiporter activity
P	O84379	CC	The arginine uptake by the bacterium in the macrophage may be a virulence factor against the host innate immune response (By similarity).
P	O84379	DE	Arginine/agmatine antiporter;
P	O84385	CC	Binds arginine (By similarity)
P	O84385	CC	Probably part of an ABC transporter complex involved in arginine transport
P	O84385	DE	Probable ABC transporter arginine-binding protein ArtJ;
P	O85187	CC	Na+/H+ antiporter that extrudes sodium in exchange for external protons
P	O85187	DE	Na+/H+ antiporter nhaA;
P	O85187	DE	Sodium/proton antiporter nhaA;
P	O86345	CC	Part of the binding-protein-dependent transport system for phosphate; probably responsible for the translocation of the substrate across the membrane (By similarity)
P	O86345	DE	Phosphate transport system permease protein pstA 1;
P	O86345	DR	inorganic phosphate transmembrane transporter activity
P	O87656	DE	Iron(III)-hydroxamate import system permease protein fhuB;
P	O88335	CC	In the kidney, probably plays a major role in potassium homeostasis
P	O88335	CC	Inward rectifier potassium channels are characterized by a greater tendency to allow potassium to flow into the cell rather than out of it
P	O88335	CC	Their voltage dependence is regulated by the concentration of extracellular potassium; as external potassium is raised, the voltage range of the channel opening shifts to more positive voltages
P	O88335	DE	ATP-regulated potassium channel ROM-K;
P	O88335	DE	ATP-sensitive inward rectifier potassium channel 1;
P	O88335	DE	Inward rectifier K+ channel Kir1.1;
P	O88335	DE	Potassium channel, inwardly rectifying subfamily J member 1;
P	O88335	DR	inward rectifier potassium channel activity
P	O88343	CC	Electrogenic sodium/bicarbonate cotransporter with a Na+:HCO3(-) stoichiometry varying from 1:2 to 1:3

P	O88343	CC	Electrogenic sodium/bicarbonate cotransporter with a Na+:HCO3(-) stoichiometry varying from 1:2 to 1:3
P	O88343	CC	May regulate bicarbonate influx/efflux at the basolateral membrane of cells and regulate intracellular pH.
P	O88343	DE	Na+/HCO3(-) cotransporter;
P	O88397	CC	Mediates the Na+-independent transport of organic anions such as taurocholate and thyroid hormones.
P	O88397	DR	thyroid hormone transmembrane transporter activity
P	O88454	CC	Outward rectification is reversed at high external K+ concentrations.
P	O88454	CC	Voltage insensitive, instantaneous, outwardly rectifying potassium channel
P	O88454	DE	Potassium channel subfamily K member 4;
P	O88454	DE	TWIK-related arachidonic acid-stimulated potassium channel protein;
P	O88454	DR	potassium channel activity
P	O88575	DE	Sodium- and chloride-dependent transporter XTRP3B;
P	O88575	DR	neurotransmitter:sodium symporter activity
P	O88576	DE	Sodium- and chloride-dependent transporter XTRP2;
P	O88576	DE	Sodium-dependent neutral amino acid transporter B(0)AT3;
P	O88576	DR	neurotransmitter:sodium symporter activity
P	O88703	CC	Hyperpolarization-activated ion channel exhibiting weak selectivity for potassium over sodium ions
P	O88703	DE	Potassium/sodium hyperpolarization-activated cyclic nucleotide-gated channel 2;
P	O88703	DE	Potassium/sodium hyperpolarization-activated cyclic nucleotide-gated channel 2;
P	O88703	DR	sodium channel activity
P	O88703	DR	voltage-gated potassium channel activity
P	O88704	CC	Hyperpolarization-activated ion channel exhibiting weak selectivity for potassium over sodium ions
P	O88704	DE	Potassium/sodium hyperpolarization-activated cyclic nucleotide-gated channel 1;
P	O88704	DE	Potassium/sodium hyperpolarization-activated cyclic nucleotide-gated channel 1;
P	O88704	DR	sodium channel activity
P	O88704	DR	voltage-gated potassium channel activity
P	O88705	CC	Putative hyperpolarization-activated ion channel exhibiting weak selectivity for potassium over sodium ions.
P	O88705	DE	Potassium/sodium hyperpolarization-activated cyclic nucleotide-gated channel 3;
P	O88705	DE	Potassium/sodium hyperpolarization-activated cyclic nucleotide-gated channel 3;
P	O88705	DR	sodium channel activity
P	O88705	DR	voltage-gated potassium channel activity
P	O88932	CC	Inward rectifier potassium channels are characterized by a greater tendency to allow potassium to flow into the cell rather than out of it
P	O88932	CC	Their voltage dependence is regulated by the concentration of extracellular potassium; as external potassium is raised, the voltage range of the channel opening shifts to more positive voltages
P	O88932	DE	ATP-sensitive inward rectifier potassium channel 15;
P	O88932	DE	Inward rectifier K+ channel Kir4.2;
P	O88932	DE	Potassium channel, inwardly rectifying subfamily J member 15;
P	O89109	CC	Forms a voltage-independent potassium channel that is activated by intracellular calcium
P	O94469	CC	Involved in active transport of urea (By similarity).
P	O94469	DE	Probable urea active transporter 1;
P	O94639	CC	High-affinity zinc transport protein
P	O94639	CC	Regulates intracellular zinc levels.
P	O94639	DE	High-affinity zinc transport protein zrt1;
P	O94639	DE	Zinc-regulated transporter 1;
P	O94639	DR	zinc ion transmembrane transporter activity
P	O94722	CC	Required for high affinity copper (probably reduced Cu I) transport into the cell.
P	O94722	DE	Copper transport protein ctr4;
P	O94722	DE	Copper transporter 4;
P	O94722	DR	copper ion transmembrane transporter activity
P	O94759	CC	Extracellular calcium passes through the channel and acts from the intracellular side as a positive regulator in channel activation
P	O94759	CC	Nonselective, voltage-independent cation channel mediating sodium and calcium ion influx in response to oxidative stress
P	O94759	DR	calcium channel activity
P	O94778	CC	Forms a water-specific channel; mercury-sensitive
P	O94778	DR	water channel activity
P	O95180	CC	T-type calcium channels belong to the "low-voltage activated (LVA)" group and are strongly blocked by nickel and mibefradil

P	O95180	CC	T-type channels serve pacemaking functions in both central neurons and cardiac nodal cells and support calcium signaling in secretory cells and vascular smooth muscle
P	O95180	CC	The isoform alpha-1H gives rise to T-type calcium currents
P	O95180	CC	Voltage-sensitive calcium channels (VSCC) mediate the entry of calcium ions into excitable cells and are also involved in a variety of calcium-dependent processes, including muscle contraction, hormone or neurotransmitter release, gene expression, cell motility, cell division and cell death
P	O95180	DE	Low-voltage-activated calcium channel alpha1 3.2 subunit;
P	O95180	DE	Voltage-dependent T-type calcium channel subunit alpha-1H;
P	O95180	DE	Voltage-gated calcium channel subunit alpha Cav3.2;
P	O95180	DR	low voltage-gated calcium channel activity
P	O95255	CC	Transports glutathione conjugates as leukotriene-c4 (LTC4) and N-ethylmaleimide S-glutathione (NEM-GS).
P	O95259	CC	Pore-forming (alpha) subunit of voltage-gated non- inactivating delayed rectifier potassium channel
P	O95259	DE	Ether-a-go-go potassium channel 1;
P	O95259	DE	Potassium voltage-gated channel subfamily H member 1;
P	O95259	DE	Voltage-gated potassium channel subunit Kv10.1;
P	O95259	DR	delayed rectifier potassium channel activity
P	O95279	CC	Outward rectification is lost at high external K+ concentrations.
P	O95279	CC	pH-dependent, voltage insensitive, outwardly rectifying potassium channel
P	O95279	DE	Acid-sensitive potassium channel protein TASK-2;
P	O95279	DE	Potassium channel subfamily K member 5;
P	O95279	DE	TWIK-related acid-sensitive K+ channel 2;
P	O95279	DR	potassium channel activity
P	O95342	DE	Bile salt export pump;
P	O95342	DR	bile acid-exporting ATPase activity
P	O95342	DR	canalicular bile acid transmembrane transporter activity
P	O95436	CC	It may be the main phosphate transport protein in the intestinal brush border membrane
P	O95436	CC	May be involved in actively transporting phosphate into cells via Na+ cotransport
P	O95436	CC	May be involved in actively transporting phosphate into cells via Na+ cotransport
P	O95436	DE	Na+-dependent phosphate cotransporter 2B;
P	O95436	DE	Na+-dependent phosphate cotransporter 2B;
P	O95436	DE	Na+/Pi cotransporter 2B;
P	O95436	DE	Sodium-dependent phosphate transport protein 2B;
P	O95436	DE	Sodium-dependent phosphate transport protein 2B;
P	O95436	DE	Sodium-phosphate transport protein 2B;
P	O95436	DE	Sodium/phosphate cotransporter 2B;
P	O95436	DE	Sodium/phosphate cotransporter 2B;
P	O95436	DR	sodium-dependent phosphate transmembrane transporter activity
P	O95436	DR	sodium-dependent phosphate transmembrane transporter activity
P	O95436	DR	sodium:phosphate symporter activity
P	O95436	DR	sodium:phosphate symporter activity
P	O95528	DR	sugar:hydrogen symporter activity
P	O95833	CC	Can insert into membranes and form chloride ion channels
P	O95833	DE	Chloride intracellular channel protein 3;
P	O95833	DR	voltage-gated chloride channel activity
P	O95907	CC	Proton-linked monocarboxylate transporter
P	O95907	DE	Monocarboxylate transporter 3;
P	O95907	DR	secondary active monocarboxylate transmembrane transporter activity
P	O97470	CC	Catalyzes the exchange of ADP and ATP across the mitochondrial inner membrane
P	O97470	CC	Catalyzes the exchange of ADP and ATP across the mitochondrial inner membrane
P	O97470	DE	ADP,ATP carrier protein;
P	O97470	DE	ADP/ATP translocase;
P	O97470	DE	ADP/ATP translocase;
P	P00550	CC	This system is involved in mannitol transport.
P	P00550	DE	Mannitol permease IIC component;
P	P00550	DE	Mannitol-specific phosphotransferase enzyme IIA component;
P	P00550	DE	Mannitol-specific phosphotransferase enzyme IIB component;
P	P00550	DE	PTS system mannitol-specific EIIA component;
P	P00550	DE	PTS system mannitol-specific EIIB component;
P	P00550	DE	PTS system mannitol-specific EIIC component;
P	P00550	DE	PTS system mannitol-specific EIICBA component;
P	P02723	CC	Catalyzes the exchange of ADP and ATP across the mitochondrial inner membrane
P	P02723	CC	Catalyzes the exchange of ADP and ATP across the mitochondrial inner membrane

P	P02723	DE	ADP,ATP carrier protein;
P	P02723	DE	ADP/ATP translocase;
P	P02723	DE	ADP/ATP translocase;
P	P02730	DR	chloride transmembrane transporter activity
P	P02906	CC	This protein specifically binds sulfate and is involved in its transmembrane transport
P	P02906	DE	Sulfate-binding protein;
P	P02906	DR	secondary active sulfate transmembrane transporter activity
P	P02906	DR	sulfate transmembrane-transporting ATPase activity
P	P02910	CC	Component of the high-affinity histidine permease, a binding-protein-dependent transport system
P	P02910	DE	Histidine-binding periplasmic protein;
P	P02915	CC	Part of the binding-protein-dependent transport system for histidine
P	P02915	DE	Histidine transport ATP-binding protein HisP;
P	P02916	CC	Part of the binding-protein-dependent transport system for maltose; probably responsible for the translocation of the substrate across the membrane.
P	P02916	DE	Maltose transport system permease protein malF;
P	P02916	DR	maltose-transporting ATPase activity
P	P02920	DE	Lactose permease;
P	P02920	DE	Lactose-proton symport;
P	P02920	DR	lactose:hydrogen symporter activity
P	P02932	CC	Uptake of inorganic phosphate, phosphorylated compounds, and some other negatively charged solutes.
P	P02982	CC	Resistance to tetracycline by an active tetracycline efflux
P	P02982	CC	This protein functions as a metal-tetracycline/H+ antiporter.
P	P02982	DE	Tetracycline resistance protein, class A;
P	P02982	DR	tetracycline:hydrogen antiporter activity
P	P02983	CC	Resistance to tetracycline by an active tetracycline efflux
P	P02983	CC	This protein functions as a metal-tetracycline/H+ antiporter.
P	P02983	DE	Tetracycline efflux protein;
P	P02983	DE	Tetracycline resistance protein;
P	P02983	DR	tetracycline:hydrogen antiporter activity
P	P03959	CC	One of the components of the high-affinity ATP-driven potassium transport (or KDP) system, which catalyzes the hydrolysis of ATP coupled with the exchange of hydrogen and potassium ions.
P	P03959	DE	Potassium-binding and translocating subunit A;
P	P03959	DE	Potassium-translocating ATPase A chain;
P	P03959	DE	Potassium-transporting ATPase A chain;
P	P03959	DR	potassium-transporting ATPase activity
P	P03960	CC	One of the components of the high-affinity ATP-driven potassium transport (or KDP) system, which catalyzes the hydrolysis of ATP coupled with the exchange of hydrogen and potassium ions.
P	P03960	DE	Potassium-binding and translocating subunit B;
P	P03960	DE	Potassium-translocating ATPase B chain;
P	P03960	DE	Potassium-transporting ATPase B chain;
P	P03960	DR	potassium-transporting ATPase activity
P	P03961	CC	One of the components of the high-affinity ATP-driven potassium transport (or KDP) system, which catalyzes the hydrolysis of ATP coupled with the exchange of hydrogen and potassium ions
P	P03961	DE	Potassium-binding and translocating subunit C;
P	P03961	DE	Potassium-translocating ATPase C chain;
P	P03961	DE	Potassium-transporting ATPase C chain;
P	P03961	DR	potassium-transporting ATPase activity
P	P04285	DE	Oligopeptide transport ATP-binding protein OppD;
P	P04710	CC	Catalyzes the exchange of ADP and ATP across the mitochondrial inner membrane
P	P04710	CC	Catalyzes the exchange of ADP and ATP across the mitochondrial inner membrane
P	P04710	DE	ADP,ATP carrier protein 1;
P	P04710	DE	ADP/ATP translocase 1;
P	P04710	DE	ADP/ATP translocase 1;
P	P04710	DR	ATP:ADP antiporter activity
P	P04710	DR	ATP:ADP antiporter activity
P	P04816	CC	This protein is a component of the leucine-specific transport system, which is one of the two periplasmic binding protein-dependent transport systems of the high-affinity transport of the branched-chain amino acids in E.coli.
P	P04816	DE	Leucine-specific-binding protein;
P	P04817	CC	High-affinity permease for arginine.

P	P04817	DE	Arginine permease;
P	P04817	DR	arginine transmembrane transporter activity
P	P04919	DR	chloride transmembrane transporter activity
P	P04983	CC	Part of the ABC transporter complex RbsABCD involved in ribose import
P	P04983	DE	Ribose import ATP-binding protein RbsA;
P	P05023	CC	This action creates the electrochemical gradient of sodium and potassium ions, providing the energy for active transport of various nutrients.
P	P05023	CC	This action creates the electrochemical gradient of sodium and potassium ions, providing the energy for active transport of various nutrients.
P	P05023	CC	This is the catalytic component of the active enzyme, which catalyzes the hydrolysis of ATP coupled with the exchange of sodium and potassium ions across the plasma membrane
P	P05023	CC	This is the catalytic component of the active enzyme, which catalyzes the hydrolysis of ATP coupled with the exchange of sodium and potassium ions across the plasma membrane
P	P05023	DE	Na+/K+ ATPase alpha-1 subunit;
P	P05023	DE	Na+/K+ ATPase alpha-1 subunit;
P	P05023	DE	Sodium pump subunit alpha-1;
P	P05023	DE	Sodium/potassium-transporting ATPase subunit alpha-1;
P	P05023	DE	Sodium/potassium-transporting ATPase subunit alpha-1;
P	P05023	DR	sodium:potassium-exchanging ATPase activity
P	P05023	DR	sodium:potassium-exchanging ATPase activity
P	P05026	CC	The beta subunit regulates, through assembly of alpha/beta heterodimers, the number of sodium pumps transported to the plasma membrane.
P	P05026	CC	This is the non-catalytic component of the active enzyme, which catalyzes the hydrolysis of ATP coupled with the exchange of Na+ and K+ ions across the plasma membrane
P	P05026	CC	This is the non-catalytic component of the active enzyme, which catalyzes the hydrolysis of ATP coupled with the exchange of Na+ and K+ ions across the plasma membrane
P	P05026	DE	Sodium/potassium-dependent ATPase subunit beta-1;
P	P05026	DE	Sodium/potassium-dependent ATPase subunit beta-1;
P	P05026	DE	Sodium/potassium-transporting ATPase subunit beta-1;
P	P05026	DE	Sodium/potassium-transporting ATPase subunit beta-1;
P	P05026	DR	sodium:potassium-exchanging ATPase activity
P	P05026	DR	sodium:potassium-exchanging ATPase activity
P	P05141	CC	Catalyzes the exchange of ADP and ATP across the mitochondrial inner membrane
P	P05141	CC	Catalyzes the exchange of ADP and ATP across the mitochondrial inner membrane
P	P05141	DE	ADP,ATP carrier protein 2;
P	P05141	DE	ADP,ATP carrier protein, fibroblast isoform;
P	P05141	DE	ADP/ATP translocase 2;
P	P05141	DE	ADP/ATP translocase 2;
P	P05316	CC	Transport of uracil.
P	P05316	DE	Uracil permease;
P	P05316	DR	uracil:cation symporter activity
P	P05425	CC	Involved in copper export
P	P05425	DE	Copper-exporting P-type ATPase B;
P	P05425	DR	copper-exporting ATPase activity
P	P05695	CC	Anion specific, the binding site has higher affinity for phosphate than chloride ions
P	P05695	CC	Porin O has a higher affinity for polyphosphates (tripolyphosphate and pyrophosphate) while porin P has a higher affinity for orthophosphate.
P	P05825	CC	This protein is involved in the initial step of iron uptake by binding ferrienterobactin (Fe-ENT), an iron chelatin siderophore that allows E.coli to extract iron from the environment
P	P06202	CC	This protein is a component of the oligopeptide permease, a binding protein-dependent transport system, it binds peptides up to five amino acids long with high affinity
P	P06202	DE	Periplasmic oligopeptide-binding protein;
P	P06609	CC	Part of the ABC transporter complex BtuCDF involved in vitamin B12 import
P	P06609	DE	Vitamin B12 import system permease protein BtuC;
P	P06611	CC	Part of the ABC transporter complex BtuCDF involved in vitamin B12 import
P	P06611	DE	Vitamin B12 import ATP-binding protein BtuD;
P	P06611	DE	Vitamin B12-transporting ATPase;
P	P06775	CC	High-affinity permease for histidine.
P	P06775	DE	Histidine permease;
P	P06971	CC	It interacts with the tonB protein, which is responsible for energy coupling of the ferrichrome-promoted iron transport system
P	P06971	CC	It interacts with the tonB protein, which is responsible for energy coupling of the ferrichrome-promoted iron transport system

P	P06971	CC	This receptor binds the ferrichrome-iron ligand
P	P06971	DE	Ferrichrome-iron receptor;
P	P07038	CC	The proton gradient it generates drives the active transport of nutrients by H+-symport
P	P07117	CC	Catalyzes the sodium-dependent uptake of extracellular L-proline
P	P07117	DE	Proline permease;
P	P07117	DE	Sodium/proline symporter;
P	P07117	DE	Sodium/proline symporter;
P	P07117	DR	proline:sodium symporter activity
P	P07117	DR	proline:sodium symporter activity
P	P07654	CC	Part of the binding-protein-dependent transport system for phosphate; probably responsible for the translocation of the substrate across the membrane.
P	P07654	DE	Phosphate transport system permease protein pstA;
P	P07654	DR	inorganic phosphate transmembrane transporter activity
P	P07775	CC	Probably involved in the transport of benzoate.
P	P07775	DE	Benzoate membrane transport protein;
P	P07893	CC	Involved in copper transport.
P	P07893	DE	Probable copper-transporting ATPase SynA;
P	P07893	DR	copper-exporting ATPase activity
P	P07921	CC	LAC12 mediates the transport of lactose and it would appear that the permease works in part by a proton symport mechanism.
P	P07921	DE	Lactose permease;
P	P08005	DE	Oligopeptide transport system permease protein oppB;
P	P08006	DE	Oligopeptide transport system permease protein oppC;
P	P08007	DE	Oligopeptide transport ATP-binding protein OppF;
P	P08194	DR	glycerol-phosphate:inorganic phosphate antiporter activity
P	P08194	DR	glycerol-phosphate:inorganic phosphate antiporter activity
P	P08470	CC	This system is involved in sucrose transport.
P	P08470	DE	PTS system sucrose-specific EIIB component;
P	P08470	DE	PTS system sucrose-specific EIIBC component;
P	P08470	DE	PTS system sucrose-specific EIIC component;
P	P08470	DE	Sucrose permease IIC component;
P	P08470	DE	Sucrose-specific phosphotransferase enzyme IIB component;
P	P08690	CC	Catalyzes the extrusion of the oxyanions arsenite, antimonite and arsenate
P	P08690	DE	Arsenite-translocating ATPase;
P	P08690	DE	Arsenite-transporting ATPase;
P	P08690	DR	arsenite-transporting ATPase activity
P	P09131	DR	bile acid:sodium symporter activity
P	P09131	DR	bile acid:sodium symporter activity
P	P09551	CC	ArgT and histidine-binding protein J (hisJ) interact with a common membrane-bound receptor, hisP.
P	P09551	CC	This periplasmic binding protein is involved in an arginine transport system
P	P09551	DE	Lysine-arginine-ornithine-binding periplasmic protein;
P	P09833	DR	molybdate transmembrane-transporting ATPase activity
P	P0A008	CC	One of the components of the high-affinity ATP-driven potassium transport (or KDP) system, which catalyzes the hydrolysis of ATP coupled with the exchange of hydrogen and potassium ions (By similarity).
P	P0A008	DE	Potassium-binding and translocating subunit B 1;
P	P0A008	DE	Potassium-translocating ATPase B chain 1;
P	P0A008	DE	Potassium-transporting ATPase B chain 1;
P	P0A008	DR	potassium-transporting ATPase activity
P	P0A057	CC	One of the components of the high-affinity ATP-driven potassium transport (or KDP) system, which catalyzes the hydrolysis of ATP coupled with the exchange of hydrogen and potassium ions (By similarity).
P	P0A057	DE	Potassium-binding and translocating subunit A 2;
P	P0A057	DE	Potassium-translocating ATPase A chain 2;
P	P0A057	DE	Potassium-transporting ATPase A chain 2;
P	P0A057	DR	potassium-transporting ATPase activity
P	P0A0D4	CC	This system is involved in lactose transport (By similarity).
P	P0A0D4	DE	Lactose-specific phosphotransferase enzyme IIA component;
P	P0A0D4	DE	PTS system lactose-specific EIIA component;
P	P0A0D6	CC	This system is involved in lactose transport (By similarity).
P	P0A0D6	DE	Lactose-specific phosphotransferase enzyme IIA component;
P	P0A0D6	DE	PTS system lactose-specific EIIA component;
P	P0A0D8	CC	This system is involved in mannitol transport (By similarity).
P	P0A0D8	DE	Mannitol-specific phosphotransferase enzyme IIA component;

P	P0A0D8	DE	PTS system mannitol-specific EIIA component;
P	P0A0Y4	CC	This protein may be a central component in the iron- acquisition system (By similarity).
P	P0A0Y4	DE	Iron(III) periplasmic-binding protein;
P	P0A0Y4	DE	Major ferric iron-binding protein;
P	P0A0Y4	DE	Major iron-regulated protein;
P	P0A283	CC	This system is involved in glucose transport.
P	P0A283	DE	Glucose-specific phosphotransferase enzyme IIA component;
P	P0A283	DE	PTS system glucose-specific EIIA component;
P	P0A284	CC	This system is involved in glucose transport (By similarity).
P	P0A284	DE	Glucose-specific phosphotransferase enzyme IIA component;
P	P0A284	DE	PTS system glucose-specific EIIA component;
P	P0A2C5	CC	Involved in the high-affinity D-ribose membrane transport system and also serves as the primary chemoreceptor for chemotaxis.
P	P0A2C5	DE	D-ribose-binding periplasmic protein;
P	P0A2C6	CC	Involved in the high-affinity D-ribose membrane transport system and also serves as the primary chemoreceptor for chemotaxis (By similarity).
P	P0A2C6	DE	D-ribose-binding periplasmic protein;
P	P0A2C7	CC	Required for the activity of the bacterial periplasmic transport system of putrescine and spermidine
P	P0A2C7	CC	Required for the activity of the bacterial periplasmic transport system of putrescine and spermidine
P	P0A2C7	DE	Spermidine/putrescine-binding periplasmic protein;
P	P0A2C7	DE	Spermidine/putrescine-binding periplasmic protein;
P	P0A2C8	CC	Required for the activity of the bacterial periplasmic transport system of putrescine and spermidine
P	P0A2C8	CC	Required for the activity of the bacterial periplasmic transport system of putrescine and spermidine
P	P0A2C8	DE	Spermidine/putrescine-binding periplasmic protein;
P	P0A2C8	DE	Spermidine/putrescine-binding periplasmic protein;
P	P0A2F8	CC	Uptake of citrate across the boundary membrane with the concomitant uptake of a sodium ion (symport system) (By similarity).
P	P0A2F8	CC	Uptake of citrate across the boundary membrane with the concomitant uptake of a sodium ion (symport system) (By similarity).
P	P0A2F8	DE	Citrate carrier protein;
P	P0A2F8	DE	Citrate transporter;
P	P0A2F8	DE	Citrate-sodium symporter;
P	P0A2F9	CC	Uptake of citrate across the boundary membrane with the concomitant uptake of a sodium ion (symport system) (By similarity).
P	P0A2F9	CC	Uptake of citrate across the boundary membrane with the concomitant uptake of a sodium ion (symport system) (By similarity).
P	P0A2F9	DE	Citrate carrier protein;
P	P0A2F9	DE	Citrate transporter;
P	P0A2F9	DE	Citrate-sodium symporter;
P	P0A2G3	CC	Uptake of citrate across the boundary membrane with the concomitant transport of protons into the cell (symport system).
P	P0A2G3	DE	Citrate carrier protein;
P	P0A2G3	DE	Citrate transporter;
P	P0A2G3	DE	Citrate utilization determinant;
P	P0A2G3	DE	Citrate utilization protein A;
P	P0A2G3	DE	Citrate-proton symporter;
P	P0A2G4	CC	Uptake of citrate across the boundary membrane with the concomitant transport of protons into the cell (symport system) (By similarity).
P	P0A2G4	DE	Citrate carrier protein;
P	P0A2G4	DE	Citrate transporter;
P	P0A2G4	DE	Citrate utilization determinant;
P	P0A2G4	DE	Citrate utilization protein A;
P	P0A2G4	DE	Citrate-proton symporter;
P	P0A2I7	CC	Part of the binding-protein-dependent transport system for histidine; probably responsible for the translocation of the substrate across the membrane.
P	P0A2I7	DE	Histidine transport system permease protein hisM;
P	P0A2I8	CC	Part of the binding-protein-dependent transport system for histidine; probably responsible for the translocation of the substrate across the membrane (By similarity).
P	P0A2I8	DE	Histidine transport system permease protein hisM;
P	P0A2I9	CC	Part of the binding-protein-dependent transport system for histidine; probably responsible for the translocation of the substrate across the membrane.

P	P0A2I9	DE	Histidine transport system permease protein hisQ;
P	P0A2J0	CC	Part of the binding-protein-dependent transport system for histidine; probably responsible for the translocation of the substrate across the membrane (By similarity).
P	P0A2J0	DE	Histidine transport system permease protein hisQ;
P	P0A2J3	CC	Involved in a peptide intake transport system that plays a role in the resistance to antimicrobial peptides.
P	P0A2J3	DE	Peptide transport system permease protein sapB;
P	P0A2J4	CC	Involved in a peptide intake transport system that plays a role in the resistance to antimicrobial peptides (By similarity).
P	P0A2J4	DE	Peptide transport system permease protein sapB;
P	P0A2J5	CC	Involved in a peptide intake transport system that plays a role in the resistance to antimicrobial peptides.
P	P0A2J5	DE	Peptide transport system permease protein sapC;
P	P0A2J6	CC	Involved in a peptide intake transport system that plays a role in the resistance to antimicrobial peptides (By similarity).
P	P0A2J6	DE	Peptide transport system permease protein sapC;
P	P0A2J8	CC	Required for the activity of the bacterial periplasmic transport system of putrescine and spermidine (By similarity).
P	P0A2J8	CC	Required for the activity of the bacterial periplasmic transport system of putrescine and spermidine (By similarity).
P	P0A2J8	DE	Spermidine/putrescine transport system permease protein PotB;
P	P0A2J8	DE	Spermidine/putrescine transport system permease protein PotB;
P	P0A2R8	CC	Mediates both influx and efflux of magnesium ions
P	P0A2R8	DE	Magnesium transport protein CorA;
P	P0A2R8	DR	cobalt ion transmembrane transporter activity
P	P0A2R8	DR	magnesium ion transmembrane transporter activity
P	P0A2R9	CC	Mediates influx of magnesium ions (By similarity).
P	P0A2R9	DE	Magnesium transport protein CorA;
P	P0A2R9	DR	cobalt ion transmembrane transporter activity
P	P0A2R9	DR	magnesium ion transmembrane transporter activity
P	P0A2U6	CC	Part of the ATP-driven transport system AdcABC for zinc
P	P0A2U6	DE	Zinc transport system ATP-binding protein AdcC;
P	P0A2U8	DE	Oligopeptide transport ATP-binding protein AmiE;
P	P0A2V2	CC	Component of the octopine active transport system probably consisting of four subunits: Q, M, P and T.
P	P0A2V2	DE	Octopine permease ATP-binding protein P;
P	P0A2V4	DE	Oligopeptide transport ATP-binding protein OppF;
P	P0A2V5	DE	Oligopeptide transport ATP-binding protein OppF;
P	P0A2V6	DE	Oligopeptide transport ATP-binding protein OppF;
P	P0A2V8	CC	Part of the ABC transporter complex PstSACB involved in phosphate import
P	P0A2V8	DE	ABC phosphate transporter 3;
P	P0A2V8	DE	Phosphate import ATP-binding protein PstB 3;
P	P0A2V8	DE	Phosphate-transporting ATPase 3;
P	P0A2V8	DR	inorganic phosphate transmembrane transporter activity
P	P0A2V8	DR	phosphate transmembrane-transporting ATPase activity
P	P0A333	DE	Voltage-gated potassium channel;
P	P0A333	DR	voltage-gated potassium channel activity
P	P0A3Q7	CC	Glycerol enters the cell via the glycerol diffusion facilitator protein
P	P0A3Q7	CC	This membrane protein facilitates the movement of glycerol across the cytoplasmic membrane (By similarity).
P	P0A3Q7	DE	Glycerol uptake facilitator protein;
P	P0A436	CC	This system is involved in galactitol transport (By similarity).
P	P0A436	DE	Galactitol-specific phosphotransferase enzyme IIB component;
P	P0A436	DE	PTS system galactitol-specific EIIB component;
P	P0A437	CC	This system is involved in galactitol transport (By similarity).
P	P0A437	DE	Galactitol-specific phosphotransferase enzyme IIB component;
P	P0A437	DE	PTS system galactitol-specific EIIB component;
P	P0A4F8	CC	Component of the octopine active transport system probably consisting of four subunits: Q, M, P and T.
P	P0A4F8	DE	Octopine-binding periplasmic protein;
P	P0A4G0	CC	Part of the binding-protein-dependent transport system for oligopeptides; probably an oligopeptide binding protein.
P	P0A4G0	DE	Oligopeptide-binding protein AliB;
P	P0A4G2	CC	Part of an ATP-driven transport system for manganese
P	P0A4G2	DE	Manganese ABC transporter substrate-binding lipoprotein;

P	P0A4M7	DE	Oligopeptide transport system permease protein AmiC;
P	P0A4M9	DE	Oligopeptide transport system permease protein AmiD;
P	P0A4N5	CC	Component of the octopine active transport system probably consisting of four subunits: Q, M, P and T.
P	P0A4N5	DE	Octopine transport system permease protein occQ;
P	P0A4N7	DE	Oligopeptide transport system permease protein oppB;
P	P0A4N8	DE	Oligopeptide transport system permease protein oppB;
P	P0A4N9	DE	Oligopeptide transport system permease protein oppC;
P	P0A4P0	DE	Oligopeptide transport system permease protein oppC;
P	P0A4W1	DE	L-asparagine permease 2;
P	P0A4W1	DE	L-asparagine transport protein 2;
P	P0A4W2	CC	Part of the ABC transporter complex CysAWTP involved in sulfate/thiosulfate import
P	P0A4W2	DE	Sulfate-transporting ATPase;
P	P0A4W2	DE	Sulfate/thiosulfate import ATP-binding protein CysA;
P	P0A4W2	DR	sulfate transmembrane-transporting ATPase activity
P	P0A4W3	CC	Part of the ABC transporter complex CysAWTP involved in sulfate/thiosulfate import
P	P0A4W3	DE	Sulfate-transporting ATPase;
P	P0A4W3	DE	Sulfate/thiosulfate import ATP-binding protein CysA;
P	P0A4W3	DR	sulfate transmembrane-transporting ATPase activity
P	P0A5Y1	CC	Involved in the transport of molybdenum into the cell
P	P0A5Y2	CC	Part of the ABC transporter complex pstSACB involved in phosphate import (By similarity).
P	P0A5Y2	DE	Phosphate-binding protein pstS 3;
P	P0A5Y2	DR	inorganic phosphate transmembrane transporter activity
P	P0A5Y3	CC	Part of the ABC transporter complex pstSACB involved in phosphate import (By similarity).
P	P0A5Y3	DE	Phosphate-binding protein pstS 3;
P	P0A5Y3	DR	inorganic phosphate transmembrane transporter activity
P	P0A625	CC	Part of the binding-protein-dependent transport system modABCD for molybdenum; probably responsible for the translocation of the substrate across the membrane
P	P0A625	DE	Molybdenum transport system permease protein modB;
P	P0A626	CC	Part of the binding-protein-dependent transport system for phosphate; probably responsible for the translocation of the substrate across the membrane (By similarity).
P	P0A626	DE	Phosphate transport system permease protein pstA 2;
P	P0A626	DR	inorganic phosphate transmembrane transporter activity
P	P0A627	CC	Part of the binding-protein-dependent transport system for phosphate; probably responsible for the translocation of the substrate across the membrane (By similarity).
P	P0A627	DE	Phosphate transport system permease protein pstA 2;
P	P0A627	DR	inorganic phosphate transmembrane transporter activity
P	P0A628	CC	Part of the binding-protein-dependent transport system for phosphate; probably responsible for the translocation of the substrate across the membrane (By similarity).
P	P0A628	DE	Phosphate transport system permease protein pstC 1;
P	P0A628	DR	inorganic phosphate transmembrane transporter activity
P	P0A629	CC	Part of the binding-protein-dependent transport system for phosphate; probably responsible for the translocation of the substrate across the membrane (By similarity).
P	P0A629	DE	Phosphate transport system permease protein pstC 1;
P	P0A629	DR	inorganic phosphate transmembrane transporter activity
P	P0A630	CC	Part of the binding-protein-dependent transport system for phosphate; probably responsible for the translocation of the substrate across the membrane (By similarity).
P	P0A630	DE	Phosphate transport system permease protein pstC 2;
P	P0A630	DR	inorganic phosphate transmembrane transporter activity
P	P0A631	CC	Part of the binding-protein-dependent transport system for phosphate; probably responsible for the translocation of the substrate across the membrane (By similarity).
P	P0A631	DE	Phosphate transport system permease protein pstC 2;
P	P0A631	DR	inorganic phosphate transmembrane transporter activity
P	P0A771	CC	H+-stimulated, highly selective, manganese uptake system (By similarity).
P	P0A771	DE	Manganese transport protein mntH;
P	P0A927	DE	Nucleoside-specific channel-forming protein tsx;
P	P0A927	DR	nucleoside transmembrane transporter activity
P	P0A9S7	CC	Component of the leucine-specific transport system.
P	P0A9X1	CC	Part of the ABC transporter complex ZnuABC involved in zinc import
P	P0A9X1	DE	Zinc import ATP-binding protein ZnuC;
P	P0A9X1	DR	zinc transporting ATPase activity
P	P0A9X3	CC	Part of the ABC transporter complex ZnuABC involved in zinc import
P	P0A9X3	DE	Zinc import ATP-binding protein ZnuC;

P	P0A9X3	DR	zinc transporting ATPase activity
P	P0AA82	CC	Required for cytosine transport into the cell.
P	P0AA82	DE	Cytosine permease;
P	P0AAD3	CC	Involved in transporting tryptophan across the cytoplasmic membrane (By similarity).
P	P0AAD3	DE	Tryptophan permease;
P	P0AAD3	DE	Tryptophan-specific transport protein;
P	P0AAD5	CC	Involved in transporting tyrosine across the cytoplasmic membrane (By similarity).
P	P0AAD5	DE	Tyrosine permease;
P	P0AAD5	DE	Tyrosine-specific transport protein;
P	P0AAD6	CC	Involved in the import of serine into the cell
P	P0AAD6	DE	Serine transporter;
P	P0AAD7	CC	Involved in the import of serine into the cell
P	P0AAD7	DE	Serine transporter;
P	P0AAD8	DE	H+/threonine-serine symporter;
P	P0AAD9	CC	Involved in the import of threonine and serine into the cell, with the concomitant import of a proton (symport system) (By similarity).
P	P0AAD9	DE	Threonine/serine transporter TdcC;
P	P0AAE1	CC	Permease that is involved in the transport across the cytoplasmic membrane of D-alanine, D-serine and glycine (By similarity).
P	P0AAE1	CC	Permease that is involved in the transport across the cytoplasmic membrane of D-alanine, D-serine and glycine (By similarity).
P	P0AAE1	CC	Permease that is involved in the transport across the cytoplasmic membrane of D-alanine, D-serine and glycine (By similarity).
P	P0AAE1	DE	D-serine/D-alanine/glycine transporter;
P	P0AAE1	DE	D-serine/D-alanine/glycine transporter;
P	P0AAE1	DE	D-serine/D-alanine/glycine transporter;
P	P0AAE7	CC	Catalyzes an electroneutral exchange between arginine and ornithine to allow high-efficiency energy conversion in the arginine deiminase pathway (By similarity).
P	P0AAE7	CC	Catalyzes an electroneutral exchange between arginine and ornithine to allow high-efficiency energy conversion in the arginine deiminase pathway (By similarity).
P	P0AAE7	DE	Putative arginine/ornithine antiporter;
P	P0AAE7	DE	Putative arginine/ornithine antiporter;
P	P0AAE8	CC	Probable cadaverine/lysine antiporter or part of it.
P	P0AAE8	CC	Probable cadaverine/lysine antiporter or part of it.
P	P0AAE8	DE	Probable cadaverine/lysine antiporter;
P	P0AAE8	DE	Probable cadaverine/lysine antiporter;
P	P0AAF0	CC	Probable cadaverine/lysine antiporter or part of it (By similarity).
P	P0AAF0	CC	Probable cadaverine/lysine antiporter or part of it (By similarity).
P	P0AAF0	DE	Probable cadaverine/lysine antiporter;
P	P0AAF0	DE	Probable cadaverine/lysine antiporter;
P	P0AAF1	CC	Probable putrescine-ornithine antiporter.
P	P0AAF1	DE	Putrescine transport protein;
P	P0AAF1	DE	Putrescine-ornithine antiporter;
P	P0AAF5	DR	L-arabinose-importing ATPase activity
P	P0AAF6	CC	Part of the ABC transporter complex ArtPIQMJ involved in arginine transport
P	P0AAF6	DE	Arginine transport ATP-binding protein ArtP;
P	P0AAF8	CC	Part of the ABC transporter complex ArtPIQMJ involved in arginine transport
P	P0AAF8	DE	Arginine transport ATP-binding protein ArtP;
P	P0AAF9	CC	Part of the ABC transporter complex ArtPIQMJ involved in arginine transport
P	P0AAF9	DE	Arginine transport ATP-binding protein ArtP;
P	P0AAG1	DE	Dipeptide transport ATP-binding protein DppD;
P	P0AAG2	DE	Dipeptide transport ATP-binding protein DppD;
P	P0AAG3	CC	Part of the binding-protein-dependent transport system for glutamate and aspartate
P	P0AAG3	CC	Part of the binding-protein-dependent transport system for glutamate and aspartate
P	P0AAG3	DE	Glutamate/aspartate transport ATP-binding protein GltL;
P	P0AAG3	DE	Glutamate/aspartate transport ATP-binding protein GltL;
P	P0AAG4	CC	Part of the binding-protein-dependent transport system for glutamate and aspartate
P	P0AAG4	CC	Part of the binding-protein-dependent transport system for glutamate and aspartate
P	P0AAG4	DE	Glutamate/aspartate transport ATP-binding protein GltL;
P	P0AAG4	DE	Glutamate/aspartate transport ATP-binding protein GltL;
P	P0AAG8	CC	Part of the ABC transporter complex MglABC involved in galactose/methyl galactoside import
P	P0AAG8	DE	Galactose/methyl galactoside import ATP-binding protein MglA;
P	P0AAG9	CC	Part of the ABC transporter complex MglABC involved in galactose/methyl galactoside import

P	P0AAG9	DE	Galactose/methyl galactoside import ATP-binding protein MglA;
P	P0AAH0	CC	Part of the ABC transporter complex PstSACB involved in phosphate import
P	P0AAH0	DE	ABC phosphate transporter;
P	P0AAH0	DE	Phosphate import ATP-binding protein PstB;
P	P0AAH0	DE	Phosphate-transporting ATPase;
P	P0AAH0	DR	inorganic phosphate transmembrane transporter activity
P	P0AAH0	DR	phosphate transmembrane-transporting ATPase activity
P	P0AAH2	CC	Part of the ABC transporter complex PstSACB involved in phosphate import
P	P0AAH2	DE	ABC phosphate transporter;
P	P0AAH2	DE	Phosphate import ATP-binding protein PstB;
P	P0AAH2	DE	Phosphate-transporting ATPase;
P	P0AAH2	DR	inorganic phosphate transmembrane transporter activity
P	P0AAH2	DR	phosphate transmembrane-transporting ATPase activity
P	P0AAH3	CC	Part of the ABC transporter complex PstSACB involved in phosphate import
P	P0AAH3	DE	ABC phosphate transporter;
P	P0AAH3	DE	Phosphate import ATP-binding protein PstB;
P	P0AAH3	DE	Phosphate-transporting ATPase;
P	P0AAH3	DR	inorganic phosphate transmembrane transporter activity
P	P0AAH3	DR	phosphate transmembrane-transporting ATPase activity
P	P0AAH6	CC	Involved in a peptide intake transport system that plays a role in the resistance to antimicrobial peptides (By similarity).
P	P0AAH6	DE	Peptide transport system ATP-binding protein SapD;
P	P0AAH6	DR	peptide transporter activity
P	P0AAH7	CC	Involved in a peptide intake transport system that plays a role in the resistance to antimicrobial peptides (By similarity).
P	P0AAH7	DE	Peptide transport system ATP-binding protein SapD;
P	P0AAH7	DR	peptide transporter activity
P	P0AAI0	CC	Involved in a peptide intake transport system that plays a role in the resistance to antimicrobial peptides (By similarity).
P	P0AAI0	DE	Peptide transport system ATP-binding protein SapF;
P	P0AB93	CC	Thought to form the channel of an arsenite pump (By similarity).
P	P0AB93	DR	arsenite secondary active transmembrane transporter activity
P	P0AB94	CC	Thought to form the channel of an arsenite pump (By similarity).
P	P0AB94	DR	arsenite transmembrane transporter activity
P	P0AB95	CC	Thought to form the channel of an arsenite pump (By similarity).
P	P0AB95	DR	arsenite transmembrane transporter activity
P	P0ABC9	CC	High-affinity uptake of choline driven by a proton- motive force.
P	P0ABC9	DE	High-affinity choline transport protein;
P	P0ABD0	CC	High-affinity uptake of choline driven by a proton- motive force (By similarity).
P	P0ABD0	DE	High-affinity choline transport protein;
P	P0ABI6	CC	Mediates influx of magnesium ions (By similarity).
P	P0ABI6	DE	Magnesium transport protein CorA;
P	P0ABI6	DR	cobalt ion transmembrane transporter activity
P	P0ABI6	DR	magnesium ion transmembrane transporter activity
P	P0ABI7	CC	Mediates influx of magnesium ions (By similarity).
P	P0ABI7	DE	Magnesium transport protein CorA;
P	P0ABI7	DR	cobalt ion transmembrane transporter activity
P	P0ABI7	DR	magnesium ion transmembrane transporter activity
P	P0ABM0	CC	Required for the export of heme to the periplasm for the biogenesis of c-type cytochromes (By similarity).
P	P0ABM0	DE	Heme exporter protein B;
P	P0ABM0	DR	heme transporter activity
P	P0ABM3	CC	Required for the export of heme to the periplasm for the biogenesis of c-type cytochromes (By similarity).
P	P0ABM3	DE	Heme exporter protein C;
P	P0ABM3	DR	heme transporter activity
P	P0ABM4	CC	Required for the export of heme to the periplasm for the biogenesis of c-type cytochromes (By similarity).
P	P0ABM4	DE	Heme exporter protein C;
P	P0ABM4	DR	heme transporter activity
P	P0AC23	CC	Involved in the bidirectional transport of formate.
P	P0AC23	DE	Formate channel 1;
P	P0AC23	DE	Probable formate transporter 1;
P	P0AC23	DR	formate transmembrane transporter activity
P	P0AC25	CC	Involved in the bidirectional transport of formate (By similarity).

P	P0AC25	DE	Formate channel 1;
P	P0AC25	DE	Probable formate transporter 1;
P	P0AC26	DE	Probable nitrite transporter;
P	P0AC26	DR	nitrite uptake transmembrane transporter activity
P	P0AC27	DE	Probable nitrite transporter;
P	P0AD96	CC	This protein is a component of the leucine, isoleucine, valine, (threonine) transport system, which is one of the two periplasmic binding protein-dependent transport systems of the high-affinity transport of the branched-chain amino acids
P	P0AD96	CC	This protein is a component of the leucine, isoleucine, valine, (threonine) transport system, which is one of the two periplasmic binding protein-dependent transport systems of the high-affinity transport of the branched-chain amino acids
P	P0AD96	CC	This protein is a component of the leucine, isoleucine, valine, (threonine) transport system, which is one of the two periplasmic binding protein-dependent transport systems of the high-affinity transport of the branched-chain amino acids
P	P0AD98	CC	This protein is a component of the leucine, isoleucine, valine, (threonine) transport system, which is one of the two periplasmic binding protein-dependent transport systems of the high-affinity transport of the branched-chain amino acids (By similarity)
P	P0AE27	CC	Part of the binding-protein-dependent transport system for L-arabinose
P	P0AE27	DE	L-arabinose transport system permease protein AraH;
P	P0AE30	CC	Part of the ABC transporter complex ArtPIQMJ involved in arginine transport
P	P0AE30	DE	Arginine ABC transporter permease protein ArtM;
P	P0AE32	CC	Part of the ABC transporter complex ArtPIQMJ involved in arginine transport
P	P0AE32	DE	Arginine ABC transporter permease protein ArtM;
P	P0AE33	CC	Part of the ABC transporter complex ArtPIQMJ involved in arginine transport
P	P0AE33	DE	Arginine ABC transporter permease protein ArtM;
P	P0AE34	CC	Part of the ABC transporter complex ArtPIQMJ involved in arginine transport
P	P0AE34	DE	Arginine ABC transporter permease protein ArtQ;
P	P0AE36	CC	Part of the ABC transporter complex ArtPIQMJ involved in arginine transport
P	P0AE36	DE	Arginine ABC transporter permease protein ArtQ;
P	P0AE74	CC	Responsible for the uptake of citrate in exchange to the efflux of succinate
P	P0AE74	DE	Citrate carrier;
P	P0AE74	DE	Citrate transporter;
P	P0AE74	DE	Citrate/succinate antiporter;
P	P0AE75	CC	Responsible for the uptake of citrate in exchange to the efflux of succinate
P	P0AE75	CC	Responsible for the uptake of citrate in exchange to the efflux of succinate
P	P0AE75	DE	Citrate carrier;
P	P0AE75	DE	Citrate transporter;
P	P0AE75	DE	Citrate/succinate antiporter;
P	P0AE75	DE	Citrate/succinate antiporter;
P	P0AEB0	CC	Part of the ABC transporter complex CysAWTP (TC 3.A.1.6.1) involved in sulfate/thiosulfate import
P	P0AEB0	CC	Part of the ABC transporter complex CysAWTP (TC 3.A.1.6.1) involved in sulfate/thiosulfate import
P	P0AEB0	DE	Sulfate transport system permease protein CysW;
P	P0AEB0	DR	sulfate transmembrane transporter activity
P	P0AEE5	CC	This protein is involved in the active transport of galactose and glucose
P	P0AEE7	CC	This protein is involved in the active transport of galactose and glucose
P	P0AEG0	DE	Dipeptide transport system permease protein dppB;
P	P0AEG3	DE	Dipeptide transport system permease protein dppC;
P	P0AEM9	CC	Part of a binding-protein-dependent transport system for cystine.
P	P0AEM9	DE	Cystine-binding periplasmic protein;
P	P0AEP1	CC	Uptake of galactose across the boundary membrane with the concomitant transport of protons into the cell (symport system).
P	P0AEP1	DE	Galactose transporter;
P	P0AEP1	DE	Galactose-proton symporter;
P	P0AEP1	DR	galactose:hydrogen symporter activity
P	P0AEQ3	CC	Involved in a glutamine-transport system glnHPQ.
P	P0AEQ3	DE	Glutamine-binding periplasmic protein;
P	P0AEQ6	CC	Part of the binding-protein-dependent transport system for glutamine; probably responsible for the translocation of the substrate across the membrane.
P	P0AEQ6	DE	Glutamine transport system permease protein glnP;
P	P0AEQ8	CC	Part of the binding-protein-dependent transport system for glutamine; probably responsible for the translocation of the substrate across the membrane (By similarity)
P	P0AEQ8	DE	Glutamine transport system permease protein glnP;

P	P0AEQ9	CC	Part of the binding-protein-dependent transport system for glutamine; probably responsible for the translocation of the substrate across the membrane (By similarity)
P	P0AEQ9	DE	Glutamine transport system permease protein glnP;
P	P0AER2	CC	Transporter of glycerol across the cytoplasmic membrane, with limited permeability to water and small uncharged compounds such as polyols (By similarity)
P	P0AER2	DE	Glycerol uptake facilitator protein;
P	P0AER3	CC	Part of the binding-protein-dependent transport system for aspartate/glutamate; probably responsible for the translocation of the substrate across the membrane.
P	P0AER3	CC	Part of the binding-protein-dependent transport system for aspartate/glutamate; probably responsible for the translocation of the substrate across the membrane.
P	P0AER3	DE	Glutamate/aspartate transport system permease protein gltJ;
P	P0AER3	DE	Glutamate/aspartate transport system permease protein gltJ;
P	P0AER5	CC	Part of the binding-protein-dependent transport system for aspartate/glutamate; probably responsible for the translocation of the substrate across the membrane.
P	P0AER5	CC	Part of the binding-protein-dependent transport system for aspartate/glutamate; probably responsible for the translocation of the substrate across the membrane.
P	P0AER5	DE	Glutamate/aspartate transport system permease protein gltK;
P	P0AER5	DE	Glutamate/aspartate transport system permease protein gltK;
P	P0AER7	CC	Part of the binding-protein-dependent transport system for aspartate/glutamate; probably responsible for the translocation of the substrate across the membrane (By similarity)
P	P0AER7	CC	Part of the binding-protein-dependent transport system for aspartate/glutamate; probably responsible for the translocation of the substrate across the membrane (By similarity)
P	P0AER7	DE	Glutamate/aspartate transport system permease protein gltK;
P	P0AER7	DE	Glutamate/aspartate transport system permease protein gltK;
P	P0AER8	CC	Catalyzes the sodium-dependent uptake of extracellular glutamate.
P	P0AER8	CC	Catalyzes the sodium-dependent uptake of extracellular glutamate.
P	P0AER8	DE	Glutamate permease;
P	P0AER8	DE	Sodium/glutamate symport carrier protein;
P	P0AER8	DE	Sodium/glutamate symport carrier protein;
P	P0AER8	DR	glutamate:sodium symporter activity
P	P0AER8	DR	glutamate:sodium symporter activity
P	P0AER9	CC	Catalyzes the sodium-dependent uptake of extracellular glutamate (By similarity).
P	P0AER9	CC	Catalyzes the sodium-dependent uptake of extracellular glutamate (By similarity).
P	P0AER9	DE	Glutamate permease;
P	P0AER9	DE	Sodium/glutamate symport carrier protein;
P	P0AER9	DE	Sodium/glutamate symport carrier protein;
P	P0AER9	DR	glutamate:sodium symporter activity
P	P0AER9	DR	glutamate:sodium symporter activity
P	P0AEU2	CC	Component of the high-affinity histidine permease, a binding-protein-dependent transport system
P	P0AEU2	DE	Histidine-binding periplasmic protein;
P	P0AEU5	CC	Part of the binding-protein-dependent transport system for histidine; probably responsible for the translocation of the substrate across the membrane (By similarity)
P	P0AEU5	DE	Histidine transport system permease protein hisM;
P	P0AEU6	CC	Part of the binding-protein-dependent transport system for histidine; probably responsible for the translocation of the substrate across the membrane (By similarity)
P	P0AEU6	DE	Histidine transport system permease protein hisM;
P	P0AEX9	CC	Involved in the high-affinity maltose membrane transport system malEFGK
P	P0AEX9	DE	Maltose-binding periplasmic protein;
P	P0AEX9	DR	maltose transmembrane transporter activity
P	P0AEY0	CC	Involved in the high-affinity maltose membrane transport system malEFGK
P	P0AEY0	DE	Maltose-binding periplasmic protein;
P	P0AEY0	DR	maltose transmembrane transporter activity
P	P0AF01	DR	molybdate ion transmembrane transporter activity
P	P0AF02	CC	Part of the binding-protein-dependent transport system for molybdenum; probably responsible for the translocation of the substrate across the membrane (By similarity)
P	P0AF02	DE	Molybdenum transport system permease protein modB;
P	P0AFA7	CC	Catalyzes the exchange of 3 H+ per 2 Na+
P	P0AFA7	CC	Catalyzes the exchange of 3 H+ per 2 Na+
P	P0AFA7	CC	Has a high affinity for sodium, but can also transport lithium
P	P0AFA7	CC	Na+/H+ antiporter that extrudes sodium in exchange for external protons
P	P0AFA7	CC	Na+/H+ antiporter that extrudes sodium in exchange for external protons
P	P0AFA7	DE	Na+/H+ antiporter nhaB;
P	P0AFA7	DE	Na+/H+ antiporter nhaB;
P	P0AFA7	DE	Sodium/proton antiporter nhaB;

P	P0AFA7	DR	sodium:hydrogen antiporter activity
P	P0AFA8	CC	Na+/H+ antiporter that extrudes sodium in exchange for external protons (By similarity)
P	P0AFA8	DE	Na+/H+ antiporter nhaB;
P	P0AFA8	DE	Sodium/proton antiporter nhaB;
P	P0AFA8	DR	sodium:hydrogen antiporter activity
P	P0AFA9	CC	Involved in a nickel transport system, probably translocates nickel through the bacterial inner membrane.
P	P0AFA9	DE	Nickel transport system permease protein nikC;
P	P0AFB0	CC	Involved in a nickel transport system, probably translocates nickel through the bacterial inner membrane (By similarity).
P	P0AFB0	DE	Nickel transport system permease protein nikC;
P	P0AFF2	DE	Nucleoside permease nupC;
P	P0AFF2	DE	Nucleoside-transport system protein nupC;
P	P0AFF2	DR	nucleoside:sodium symporter activity
P	P0AFF4	DE	Nucleoside permease nupG;
P	P0AFF4	DE	Nucleoside-transport system protein nupG;
P	P0AFH4	DE	Oligopeptide transport system permease protein oppB;
P	P0AFH5	DE	Oligopeptide transport system permease protein oppB;
P	P0AFH7	DE	Oligopeptide transport system permease protein oppC;
P	P0AFJ9	CC	Low-affinity inorganic phosphate transport
P	P0AFJ9	DE	Low-affinity inorganic phosphate transporter 1;
P	P0AFJ9	DR	inorganic phosphate transmembrane transporter activity
P	P0AFK4	CC	Required for the activity of the bacterial periplasmic transport system of putrescine and spermidine.
P	P0AFK4	CC	Required for the activity of the bacterial periplasmic transport system of putrescine and spermidine.
P	P0AFK4	DE	Spermidine/putrescine transport system permease protein PotB;
P	P0AFK4	DE	Spermidine/putrescine transport system permease protein PotB;
P	P0AFK4	DR	polyamine-transporting ATPase activity
P	P0AFK5	CC	Required for the activity of the bacterial periplasmic transport system of putrescine and spermidine (By similarity).
P	P0AFK5	CC	Required for the activity of the bacterial periplasmic transport system of putrescine and spermidine (By similarity).
P	P0AFK5	DE	Spermidine/putrescine transport system permease protein PotB;
P	P0AFK5	DE	Spermidine/putrescine transport system permease protein PotB;
P	P0AFK6	CC	Required for the activity of the bacterial periplasmic transport system of putrescine and spermidine.
P	P0AFK6	CC	Required for the activity of the bacterial periplasmic transport system of putrescine and spermidine.
P	P0AFK6	DE	Spermidine/putrescine transport system permease protein PotC;
P	P0AFK6	DE	Spermidine/putrescine transport system permease protein PotC;
P	P0AFK6	DR	polyamine-transporting ATPase activity
P	P0AFK8	CC	Required for the activity of the bacterial periplasmic transport system of putrescine and spermidine (By similarity).
P	P0AFK8	CC	Required for the activity of the bacterial periplasmic transport system of putrescine and spermidine (By similarity).
P	P0AFK8	DE	Spermidine/putrescine transport system permease protein PotC;
P	P0AFK8	DE	Spermidine/putrescine transport system permease protein PotC;
P	P0AFK9	CC	Polyamine binding protein.
P	P0AFK9	CC	Required for the activity of the bacterial periplasmic transport system of putrescine and spermidine
P	P0AFK9	CC	Required for the activity of the bacterial periplasmic transport system of putrescine and spermidine
P	P0AFK9	DE	Spermidine/putrescine-binding periplasmic protein;
P	P0AFK9	DE	Spermidine/putrescine-binding periplasmic protein;
P	P0AFK9	DR	polyamine-transporting ATPase activity
P	P0AFL0	CC	Required for the activity of the bacterial periplasmic transport system of putrescine and spermidine
P	P0AFL0	CC	Required for the activity of the bacterial periplasmic transport system of putrescine and spermidine
P	P0AFL0	DE	Spermidine/putrescine-binding periplasmic protein;
P	P0AFL0	DE	Spermidine/putrescine-binding periplasmic protein;
P	P0AFL1	CC	Required for the activity of the bacterial periplasmic transport system of putrescine
P	P0AFL1	DE	Putrescine transport system permease protein PotI;
P	P0AFL1	DR	putrescine-importing ATPase activity

P	P0AFM2	CC	Member of a multicomponent binding-protein-dependent transport system (the proU transporter) which serves as the glycine betaine/L-proline transporter.
P	P0AFM2	DE	Glycine betaine-binding periplasmic protein;
P	P0AFM3	CC	Member of a multicomponent binding-protein-dependent transport system (the proU transporter) which serves as the glycine betaine/L-proline transporter (By similarity).
P	P0AFR3	CC	Possible sulfate transporter (By similarity).
P	P0AFR3	DE	Putative sulfate transporter ychM;
P	P0AFR3	DR	secondary active sulfate transmembrane transporter activity
P	P0AFZ8	CC	Interacts with trk system potassium uptake protein trkA and requires trkE for transport activity (By similarity).
P	P0AFZ8	CC	Low-affinity potassium transport system
P	P0AFZ8	DE	Trk system potassium uptake protein trkH;
P	P0AFZ9	CC	Interacts with trk system potassium uptake protein trkA and requires trkE for transport activity (By similarity).
P	P0AFZ9	CC	Low-affinity potassium transport system
P	P0AFZ9	DE	Trk system potassium uptake protein trkH;
P	P0AG34	CC	Conducts the efflux of homoserine and homoserine lactone.
P	P0AG34	DE	Homoserine/homoserine lactone efflux protein;
P	P0AG36	CC	Conducts the efflux of homoserine and homoserine lactone (By similarity).
P	P0AG36	DE	Homoserine/homoserine lactone efflux protein;
P	P0AG37	CC	Conducts the efflux of homoserine and homoserine lactone (By similarity).
P	P0AG37	DE	Homoserine/homoserine lactone efflux protein;
P	P0AG78	CC	This protein specifically binds sulfate and is involved in its transmembrane transport
P	P0AG78	DE	Sulfate starvation-induced protein 2;
P	P0AG78	DE	Sulfate-binding protein;
P	P0AG78	DR	secondary active sulfate transmembrane transporter activity
P	P0AG78	DR	sulfate transmembrane-transporting ATPase activity
P	P0AG79	CC	This protein specifically binds sulfate and is involved in its transmembrane transport (By similarity).
P	P0AG79	DE	Sulfate starvation-induced protein 2;
P	P0AG79	DE	Sulfate-binding protein;
P	P0AG79	DR	secondary active sulfate transmembrane transporter activity
P	P0AG79	DR	sulfate transmembrane-transporting ATPase activity
P	P0AG82	CC	Part of the ABC transporter complex pstSACB involved in phosphate import.
P	P0AG82	DE	Phosphate-binding protein pstS;
P	P0AG82	DR	inorganic phosphate transmembrane transporter activity
P	P0AG83	CC	Part of the ABC transporter complex pstSACB involved in phosphate import (By similarity).
P	P0AG83	DE	Phosphate-binding protein pstS;
P	P0AG83	DR	inorganic phosphate transmembrane transporter activity
P	P0AGC0	CC	Transport protein for sugar phosphate uptake.
P	P0AGC0	DE	Hexose phosphate transport protein;
P	P0AGC0	DR	hexose-phosphate:inorganic phosphate antiporter activity
P	P0AGC0	DR	hexose-phosphate:inorganic phosphate antiporter activity
P	P0AGC1	DE	Hexose phosphate transport protein;
P	P0AGC2	DE	Hexose phosphate transport protein;
P	P0AGE4	CC	Involved in the import of serine and threonine into the cell, with the concomitant import of sodium (symport system).
P	P0AGE4	CC	Involved in the import of serine and threonine into the cell, with the concomitant import of sodium (symport system).
P	P0AGE4	DE	Na+/serine-threonine symporter;
P	P0AGE4	DE	Na+/serine-threonine symporter;
P	P0AGE4	DE	Serine/threonine transporter sstT;
P	P0AGE4	DR	neutral amino acid:sodium symporter activity
P	P0AGE4	DR	sodium:dicarboxylate symporter activity
P	P0AGE5	CC	Involved in the import of serine and threonine into the cell, with the concomitant import of sodium (symport system) (By similarity).
P	P0AGE5	DE	Na+/serine-threonine symporter;
P	P0AGE5	DR	sodium:dicarboxylate symporter activity
P	P0AGE5	DR	sodium:dicarboxylate symporter activity
P	P0AGH4	CC	Involved in a peptide intake transport system that plays a role in the resistance to antimicrobial peptides (By similarity).
P	P0AGH4	DE	Peptide transport system permease protein sapB;
P	P0AGH7	CC	Involved in a peptide intake transport system that plays a role in the resistance to antimicrobial peptides (By similarity).

P	P0AGH7	DE	Peptide transport system permease protein sapC;
P	P0AGH8	CC	Part of the binding-protein-dependent transport system for phosphate; probably responsible for the translocation of the substrate across the membrane.
P	P0AGH8	DE	Phosphate transport system permease protein pstC;
P	P0AGH8	DR	inorganic phosphate transmembrane transporter activity
P	P0AGH9	CC	Part of the binding-protein-dependent transport system for phosphate; probably responsible for the translocation of the substrate across the membrane (By similarity)
P	P0AGH9	DE	Phosphate transport system permease protein pstC;
P	P0AGH9	DR	inorganic phosphate transmembrane transporter activity
P	P0AGI0	CC	Part of the binding-protein-dependent transport system for phosphate; probably responsible for the translocation of the substrate across the membrane (By similarity)
P	P0AGI0	DE	Phosphate transport system permease protein pstC;
P	P0AGI0	DR	inorganic phosphate transmembrane transporter activity
P	P0AGI1	CC	Part of the binding-protein-dependent transport system for ribose
P	P0AGI1	DE	Ribose transport system permease protein rbsC;
P	P0AGI3	CC	Part of the binding-protein-dependent transport system for ribose
P	P0AGI3	DE	Ribose transport system permease protein rbsC;
P	P0AGI4	DE	Xylose transport system permease protein xylH;
P	P0AGI6	CC	Part of the binding-protein-dependent transport system for D-xylose
P	P0AGI6	DE	Xylose transport system permease protein xylH;
P	P0AGI7	CC	Part of the binding-protein-dependent transport system for D-xylose
P	P0AGI7	DE	Xylose transport system permease protein xylH;
P	P0AGM7	CC	Transport of uracil in the cell.
P	P0AGM7	DE	Uracil permease;
P	P0AGM7	DE	Uracil transporter;
P	P0AGM8	CC	Transport of uracil in the cell (By similarity).
P	P0AGM8	DE	Uracil permease;
P	P0AGM8	DE	Uracil transporter;
P	P0C0L7	CC	Proton symporter that senses osmotic shifts and responds by importing osmolytes such as proline, glycine betaine, stachydrine, pipecolic acid, ectoine and taurine
P	P0C0L7	DE	Proline porter II;
P	P0C0L7	DE	Proline/betaine transporter;
P	P0C0L7	DE	Proline/betaine transporter;
P	P0C0L8	CC	Proton symporter that senses osmotic shifts and responds by importing osmolytes such as proline, glycine betaine, stachydrine, pipecolic acid, ectoine and taurine
P	P0C0L8	DE	Proline porter II;
P	P0C0L8	DE	Proline/betaine transporter;
P	P0C0L8	DE	Proline/betaine transporter;
P	P0C105	CC	Intake of glucose and galactose (Potential).
P	P0C105	DE	Glucose/galactose transporter;
P	P0C105	DE	Glucose/galactose transporter;
P	P0C105	DR	galactose transmembrane transporter activity
P	P0C105	DR	glucose transmembrane transporter activity
P	P0C2M5	CC	Part of the ABC transporter complex pstSACB involved in phosphate import (Probable)
P	P0C2M5	DE	Phosphate-binding protein pstS 2;
P	P0C2U2	DE	Di-/tripeptide transporter;
P	P0CAT6	CC	Part of the ABC transporter complex PstSACB involved in phosphate import
P	P0CAT6	DE	ABC phosphate transporter;
P	P0CAT6	DE	Phosphate import ATP-binding protein PstB;
P	P0CAT6	DE	Phosphate-transporting ATPase;
P	P0CAT6	DR	inorganic phosphate transmembrane transporter activity
P	P0CAT6	DR	phosphate transmembrane-transporting ATPase activity
P	P0CD90	CC	Water channel required to facilitate the transport of water across membranes
P	P0CD91	CC	Water channel required to facilitate the transport of water across membranes
P	P0CD99	CC	High-affinity uptake of maltose and maltotriose
P	P0CD99	DE	Maltose transport protein 2;
P	P0CD99	DR	maltose transmembrane transporter activity
P	P0CE00	CC	High-affinity uptake of maltose and maltotriose
P	P0CE00	DE	Maltose transport protein 3;
P	P0CE45	CC	Responsible for the transport of glucuronide into the cell energized by the proton motive force (probably by symport)
P	P0CE45	DE	Glucuronide carrier protein;
P	P0CE45	DE	Glucuronide permease;
P	P10346	CC	Part of the binding-protein-dependent transport system for glutamine
P	P10346	DE	Glutamine transport ATP-binding protein GlnQ;

P	P10502	CC	Catalyzes the sodium-dependent uptake of extracellular L-proline.
P	P10502	DE	Proline permease;
P	P10502	DE	Sodium/proline symporter;
P	P10502	DE	Sodium/proline symporter;
P	P10502	DR	proline:sodium symporter activity
P	P10502	DR	proline:sodium symporter activity
P	P10870	CC	Can function as a negative regulator of glucose transport
P	P10870	CC	High-affinity glucose transporter
P	P10870	CC	Inhibition of RGT1 by low levels of glucose, and hence induction of HTX2 and HTX4 expression, requires SNF3.
P	P10870	CC	Serves as a sensor that generates an intracellular signal in the presence of low level of glucose
P	P10870	DE	High-affinity glucose transporter SNF3;
P	P10903	CC	Involved in excretion of nitrite produced by the dissimilatory reduction of nitrate.
P	P10903	DE	Nitrite extrusion protein 1;
P	P10903	DE	Nitrite facilitator 1;
P	P10903	DR	nitrite efflux transmembrane transporter activity
P	P10903	DR	nitrite uptake transmembrane transporter activity
P	P11162	CC	This system is involved in lactose transport.
P	P11162	DE	Lactose permease IIC component;
P	P11162	DE	Lactose-specific phosphotransferase enzyme IIB component;
P	P11162	DE	PTS system lactose-specific EIIB component;
P	P11162	DE	PTS system lactose-specific EIIC component;
P	P11162	DE	PTS system lactose-specific EIICB component;
P	P11166	CC	Facilitative glucose transporter
P	P11166	CC	This isoform may be responsible for constitutive or basal glucose uptake
P	P11166	DE	Glucose transporter type 1, erythrocyte/brain;
P	P11166	DE	HepG2 glucose transporter;
P	P11166	DE	Solute carrier family 2, facilitated glucose transporter member 1;
P	P11166	DR	D-glucose transmembrane transporter activity
P	P11168	CC	Facilitative glucose transporter
P	P11168	CC	May also participate with the Na+/glucose cotransporter in the transcellular transport of glucose in the small intestine and kidney.
P	P11168	CC	This isoform likely mediates the bidirectional transfer of glucose across the plasma membrane of hepatocytes and is responsible for uptake of glucose by the beta cells; may comprise part of the glucose-sensing mechanism of the beta cell
P	P11168	DE	Glucose transporter type 2, liver;
P	P11168	DE	Solute carrier family 2, facilitated glucose transporter member 2;
P	P11168	DR	D-glucose transmembrane transporter activity
P	P11169	CC	Facilitative glucose transporter
P	P11169	CC	Probably a neuronal glucose transporter.
P	P11169	DE	Glucose transporter type 3, brain;
P	P11169	DE	Solute carrier family 2, facilitated glucose transporter member 3;
P	P11169	DR	D-glucose transmembrane transporter activity
P	P11461	CC	Anguibactin receptor, essential component of the iron uptake system of V. anguillarum
P	P11551	CC	Can also transport L-galactose and D-arabinose, but at reduced rates compared with L-fucose
P	P11551	CC	Mediates the uptake of L-fucose across the boundary membrane with the concomitant transport of protons into the cell (symport system)
P	P11551	DE	6-deoxy-L-galactose permease;
P	P11551	DE	L-fucose permease;
P	P11551	DE	L-fucose-proton symporter;
P	P11636	DE	Quinate permease;
P	P11636	DE	Quinate transporter;
P	P11881	CC	Intracellular channel that mediates calcium release from the endoplasmic reticulum following stimulation by inositol 1,4,5- trisphosphate.
P	P11881	DR	inositol 1,4,5-trisphosphate-sensitive calcium-release channel activity
P	P12234	CC	Phosphate is cotransported with H+.
P	P12234	CC	Transport of phosphate groups from the cytosol to the mitochondrial matrix
P	P12234	DE	Phosphate carrier protein, mitochondrial;
P	P12234	DE	Phosphate transport protein;
P	P12235	CC	Catalyzes the exchange of ADP and ATP across the mitochondrial inner membrane
P	P12235	CC	Catalyzes the exchange of ADP and ATP across the mitochondrial inner membrane
P	P12235	DE	ADP,ATP carrier protein 1;
P	P12235	DE	ADP,ATP carrier protein, heart/skeletal muscle isoform T1;

P	P12235	DE	ADP/ATP translocase 1;
P	P12235	DE	ADP/ATP translocase 1;
P	P12236	CC	Catalyzes the exchange of ADP and ATP across the mitochondrial inner membrane
P	P12236	CC	Catalyzes the exchange of ADP and ATP across the mitochondrial inner membrane
P	P12236	DE	ADP,ATP carrier protein 3;
P	P12236	DE	ADP,ATP carrier protein, isoform T2;
P	P12236	DE	ADP/ATP translocase 3;
P	P12236	DE	ADP/ATP translocase 3;
P	P12236	DR	ATP:ADP antiporter activity
P	P12236	DR	ATP:ADP antiporter activity
P	P12655	CC	This system is involved in sucrose transport.
P	P12655	DE	PTS system sucrose-specific EIIA component;
P	P12655	DE	PTS system sucrose-specific EIIB component;
P	P12655	DE	PTS system sucrose-specific EIIBCA component;
P	P12655	DE	PTS system sucrose-specific EIIC component;
P	P12655	DE	Sucrose permease IIC component;
P	P12655	DE	Sucrose-specific phosphotransferase enzyme IIA component;
P	P12655	DE	Sucrose-specific phosphotransferase enzyme IIB component;
P	P12685	CC	This protein is required for high-affinity potassium transport.
P	P12685	DE	High-affinity potassium transport protein;
P	P12685	DR	potassium ion transmembrane transporter activity
P	P13036	DE	Iron(III) dicitrate transport protein fecA;
P	P13090	CC	Appears to confer resistance only to aminotriazole.
P	P13090	CC	Putative component of the machinery responsible for pumping aminotriazole (and possibly other toxic compounds) out of the cell
P	P13090	DE	Aminotriazole resistance protein;
P	P13181	CC	GAL2 is a facilitated diffusion transporter required for both the high-affinity galactokinase-dependent and low-affinity galactokinase-independent galactose transport processes.
P	P13181	DE	Galactose permease;
P	P13181	DE	Galactose transporter;
P	P13181	DR	galactose transmembrane transporter activity
P	P13181	DR	glucose transmembrane transporter activity
P	P13511	CC	CzcA and CzcB together would act in zinc efflux nearly as effectively as the complete CZC efflux system (CzcABC).
P	P13511	CC	Has a low cation transport activity for cobalt, it is essential for the expression of cobalt, zinc, and cadmium resistance
P	P13511	CC	Has a low cation transport activity for cobalt, it is essential for the expression of cobalt, zinc, and cadmium resistance
P	P13569	CC	Involved in the transport of chloride ions
P	P13569	DE	cAMP-dependent chloride channel;
P	P13569	DR	ATP-binding and phosphorylation-dependent chloride channel activity
P	P13569	DR	chloride channel regulator activity
P	P13586	CC	This magnesium-dependent enzyme catalyzes the hydrolysis of ATP coupled with the transport of calcium
P	P13586	DE	Calcium-transporting ATPase 1;
P	P13586	DE	Golgi Ca2+-ATPase;
P	P13586	DR	calcium-transporting ATPase activity
P	P13586	DR	manganese-transporting ATPase activity
P	P13587	CC	This magnesium-dependent enzyme catalyzes the hydrolysis of ATP coupled with the transport of the sodium or lithium ions to allow salt tolerance
P	P13587	DE	Sodium transport ATPase 1;
P	P13587	DR	sodium-exporting ATPase activity, phosphorylative mechanism
P	P13637	CC	This action creates the electrochemical gradient of sodium and potassium ions, providing the energy for active transport of various nutrients.
P	P13637	CC	This action creates the electrochemical gradient of sodium and potassium ions, providing the energy for active transport of various nutrients.
P	P13637	CC	This is the catalytic component of the active enzyme, which catalyzes the hydrolysis of ATP coupled with the exchange of sodium and potassium ions across the plasma membrane
P	P13637	CC	This is the catalytic component of the active enzyme, which catalyzes the hydrolysis of ATP coupled with the exchange of sodium and potassium ions across the plasma membrane
P	P13637	DE	Na+/K+ ATPase alpha(III) subunit;
P	P13637	DE	Na+/K+ ATPase alpha(III) subunit;

P	P13637	DE	Na+/K+ ATPase alpha-3 subunit;
P	P13637	DE	Na+/K+ ATPase alpha-3 subunit;
P	P13637	DE	Sodium pump subunit alpha-3;
P	P13637	DE	Sodium/potassium-transporting ATPase subunit alpha-3;
P	P13637	DE	Sodium/potassium-transporting ATPase subunit alpha-3;
P	P13637	DR	sodium:potassium-exchanging ATPase activity
P	P13637	DR	sodium:potassium-exchanging ATPase activity
P	P13738	CC	Can mediate sodium uptake when a transmembrane pH gradient is applied
P	P13738	CC	Catalyzes the exchange of 2 H+ per Na+
P	P13738	CC	Catalyzes the exchange of 2 H+ per Na+
P	P13738	CC	Na+/H+ antiporter that extrudes sodium in exchange for external protons
P	P13738	CC	Na+/H+ antiporter that extrudes sodium in exchange for external protons
P	P13738	DE	Na+/H+ antiporter nhaA;
P	P13738	DE	Na+/H+ antiporter nhaA;
P	P13738	DE	Sodium/proton antiporter nhaA;
P	P13808	DR	chloride transmembrane transporter activity
P	P13866	CC	Actively transports glucose into cells by Na+ cotransport with a Na+ to glucose coupling ratio of 2:1
P	P13866	CC	Actively transports glucose into cells by Na+ cotransport with a Na+ to glucose coupling ratio of 2:1
P	P13866	CC	Efficient substrate transport in mammalian kidney is provided by the concerted action of a low affinity high capacity and a high affinity low capacity Na+/glucose cotransporter arranged in series along kidney proximal tubules.
P	P13866	CC	Efficient substrate transport in mammalian kidney is provided by the concerted action of a low affinity high capacity and a high affinity low capacity Na+/glucose cotransporter arranged in series along kidney proximal tubules.
P	P13866	DE	High affinity sodium-glucose cotransporter;
P	P13866	DE	Na+/glucose cotransporter 1;
P	P13866	DE	Na+/glucose cotransporter 1;
P	P13866	DE	Sodium/glucose cotransporter 1;
P	P13866	DE	Sodium/glucose cotransporter 1;
P	P13866	DR	glucose:sodium symporter activity
P	P13866	DR	glucose:sodium symporter activity
P	P14094	CC	The beta subunit regulates, through assembly of alpha/beta heterodimers, the number of sodium pumps transported to the plasma membrane.
P	P14094	CC	This is the non-catalytic component of the active enzyme, which catalyzes the hydrolysis of ATP coupled with the exchange of Na+ and K+ ions across the plasma membrane
P	P14094	CC	This is the non-catalytic component of the active enzyme, which catalyzes the hydrolysis of ATP coupled with the exchange of Na+ and K+ ions across the plasma membrane
P	P14094	DE	Sodium/potassium-dependent ATPase subunit beta-1;
P	P14094	DE	Sodium/potassium-dependent ATPase subunit beta-1;
P	P14094	DE	Sodium/potassium-transporting ATPase subunit beta-1;
P	P14094	DE	Sodium/potassium-transporting ATPase subunit beta-1;
P	P14142	CC	Insulin-regulated facilitative glucose transporter.
P	P14142	DE	Glucose transporter type 4, insulin-responsive;
P	P14142	DE	Solute carrier family 2, facilitated glucose transporter member 4;
P	P14142	DR	insulin-responsive hydrogen:glucose symporter activity
P	P14175	CC	Involved in a multicomponent binding-protein-dependent transport system for glycine betaine/L-proline.
P	P14175	DE	Glycine betaine/L-proline transport ATP-binding protein ProV;
P	P14176	CC	Involved in a multicomponent binding-protein-dependent transport system for glycine betaine/L-proline.
P	P14176	DE	Glycine betaine/L-proline transport system permease protein proW;
P	P14231	CC	This is the non-catalytic component of the active enzyme, which catalyzes the hydrolysis of ATP coupled with the exchange of Na+ and K+ ions across the plasma membrane
P	P14231	CC	This is the non-catalytic component of the active enzyme, which catalyzes the hydrolysis of ATP coupled with the exchange of Na+ and K+ ions across the plasma membrane
P	P14231	DE	Sodium/potassium-dependent ATPase subunit beta-2;
P	P14231	DE	Sodium/potassium-dependent ATPase subunit beta-2;
P	P14231	DE	Sodium/potassium-transporting ATPase subunit beta-2;
P	P14231	DE	Sodium/potassium-transporting ATPase subunit beta-2;
P	P14231	DR	sodium:potassium-exchanging ATPase activity
P	P14231	DR	sodium:potassium-exchanging ATPase activity
P	P14246	CC	Facilitative glucose transporter

P	P14246	CC	May also participate with the Na+/glucose cotransporter in the transcellular transport of glucose in the small intestine and kidney.
P	P14246	CC	This isoform likely mediates the bidirectional transfer of glucose across the plasma membrane of hepatocytes and is responsible for uptake of glucose by the beta cells; may comprise part of the glucose-sensing mechanism of the beta cell
P	P14246	DE	Glucose transporter type 2, liver;
P	P14246	DE	Solute carrier family 2, facilitated glucose transporter member 2;
P	P14246	DR	D-glucose transmembrane transporter activity
P	P14415	CC	This is the non-catalytic component of the active enzyme, which catalyzes the hydrolysis of ATP coupled with the exchange of Na+ and K+ ions across the plasma membrane
P	P14415	CC	This is the non-catalytic component of the active enzyme, which catalyzes the hydrolysis of ATP coupled with the exchange of Na+ and K+ ions across the plasma membrane
P	P14415	DE	Sodium/potassium-dependent ATPase subunit beta-2;
P	P14415	DE	Sodium/potassium-dependent ATPase subunit beta-2;
P	P14415	DE	Sodium/potassium-transporting ATPase subunit beta-2;
P	P14415	DE	Sodium/potassium-transporting ATPase subunit beta-2;
P	P14415	DR	sodium:potassium-exchanging ATPase activity
P	P14415	DR	sodium:potassium-exchanging ATPase activity
P	P14672	CC	Insulin-regulated facilitative glucose transporter.
P	P14672	DE	Glucose transporter type 4, insulin-responsive;
P	P14672	DE	Solute carrier family 2, facilitated glucose transporter member 4;
P	P14672	DR	D-glucose transmembrane transporter activity
P	P14772	CC	Cooperates for the ATP-dependent vacuolar transport of bilirubin and glutathione conjugates.
P	P14788	CC	Part of the ABC transporter complex CysAWTP involved in sulfate/thiosulfate import
P	P14788	DE	Sulfate-transporting ATPase;
P	P14788	DE	Sulfate/thiosulfate import ATP-binding protein CysA;
P	P14788	DR	sulfate transmembrane-transporting ATPase activity
P	P14867	CC	GABA, the major inhibitory neurotransmitter in the vertebrate brain, mediates neuronal inhibition by binding to the GABA/benzodiazepine receptor and opening an integral chloride channel.
P	P14867	DR	chloride channel activity
P	P15365	CC	Component of the allantoate transport system.
P	P15365	DE	Allantoate permease;
P	P15365	DR	allantoate transmembrane transporter activity
P	P15380	CC	May be responsible for proline recognition and probably also for proline translocation across the plasma membrane
P	P15380	CC	Required for high-affinity proline transport
P	P15380	DE	Proline-specific permease;
P	P15388	CC	Assuming opened or closed conformations in response to the voltage difference across the membrane, the protein forms a potassium-selective channel through which potassium ions may pass in accordance with their electrochemical gradient.
P	P15388	CC	Mediates the voltage-dependent potassium ion permeability of excitable membranes.
P	P15388	DE	Potassium voltage-gated channel subfamily C member 1;
P	P15388	DE	Voltage-gated potassium channel subunit Kv3.1;
P	P15388	DE	Voltage-gated potassium channel subunit Kv4;
P	P15388	DR	voltage-gated potassium channel activity
P	P15685	CC	Transporter for maltose.
P	P15685	DE	Maltose permease MAL61;
P	P15685	DE	Maltose transport protein MAL61;
P	P15686	DE	H+/hexose cotransporter 1;
P	P15686	DE	H+/hexose cotransporter 1;
P	P15710	CC	These include this high-affinity phosphate permease, which is part of a functional entity called ptsII
P	P15710	DE	Phosphate-repressible phosphate permease;
P	P15710	DR	inorganic phosphate transmembrane transporter activity
P	P15712	CC	Part of the ABC transporter complex pstSACB involved in phosphate import (By similarity).
P	P15712	DE	Phosphate-binding protein pstS 1;
P	P15712	DR	inorganic phosphate transmembrane transporter activity
P	P15729	DE	Glucose transport protein;
P	P15993	DE	Aromatic amino acid transport protein AroP;
P	P15993	DE	General aromatic amino acid permease;
P	P16102	CC	Halorhodopsin is a light-driven chloride pump.
P	P16256	CC	Catalyzes the sodium-dependent uptake of extracellular pantothenate.

P	P16256	DE	Sodium/pantothenate symporter;
P	P16256	DR	sodium ion transmembrane transporter activity
P	P16388	CC	Assuming opened or closed conformations in response to the voltage difference across the membrane, the protein forms a potassium-selective channel through which potassium ions may pass in accordance with their electrochemical gradient
P	P16388	CC	Mediates the voltage-dependent potassium ion permeability of excitable membranes
P	P16388	DE	Potassium voltage-gated channel subfamily A member 1;
P	P16388	DE	Voltage-gated potassium channel subunit Kv1.1;
P	P16389	CC	Assuming opened or closed conformations in response to the voltage difference across the membrane, the protein forms a potassium-selective channel through which potassium ions may pass in accordance with their electrochemical gradient
P	P16389	CC	Mediates the voltage-dependent potassium ion permeability of excitable membranes
P	P16389	DE	Potassium voltage-gated channel subfamily A member 2;
P	P16389	DE	Voltage-gated K+ channel HuKIV;
P	P16389	DE	Voltage-gated potassium channel HBK5;
P	P16389	DE	Voltage-gated potassium channel subunit Kv1.2;
P	P16389	DR	delayed rectifier potassium channel activity
P	P16390	CC	Assuming opened or closed conformations in response to the voltage difference across the membrane, the protein forms a potassium-selective channel through which potassium ions may pass in accordance with their electrochemical gradient
P	P16390	CC	Mediates the voltage-dependent potassium ion permeability of excitable membranes
P	P16390	DE	Potassium voltage-gated channel subfamily A member 3;
P	P16390	DE	Voltage-gated potassium channel subunit Kv1.3;
P	P16482	CC	Uptake of citrate across the boundary membrane with the concomitant transport of protons into the cell (symport system).
P	P16482	DE	Citrate carrier protein;
P	P16482	DE	Citrate transporter;
P	P16482	DE	Citrate-proton symporter;
P	P16552	CC	Responsible for transport of raffinose into the cell, with the concomitant import of a proton (symport system).
P	P16552	DE	Raffinose permease;
P	P16615	CC	This magnesium-dependent enzyme catalyzes the hydrolysis of ATP coupled with the translocation of calcium from the cytosol to the sarcoplasmic reticulum lumen
P	P16615	DE	Calcium pump 2;
P	P16615	DE	Calcium-transporting ATPase sarcoplasmic reticulum type, slow twitch skeletal muscle isoform;
P	P16615	DE	Endoplasmic reticulum class 1/2 Ca2+ ATPase;
P	P16615	DE	SR Ca2+-ATPase 2;
P	P16615	DE	Sarcoplasmic/endoplasmic reticulum calcium ATPase 2;
P	P16615	DR	calcium-transporting ATPase activity
P	P16676	CC	Part of the ABC transporter complex CysAWTP involved in sulfate/thiosulfate import
P	P16676	CC	Part of the ABC transporter complex CysAWTP involved in sulfate/thiosulfate import
P	P16676	DE	Sulfate-transporting ATPase;
P	P16676	DE	Sulfate/thiosulfate import ATP-binding protein CysA;
P	P16676	DE	Sulfate/thiosulfate import ATP-binding protein CysA;
P	P16676	DR	sulfate transmembrane-transporting ATPase activity
P	P16677	CC	Part of the ABC transporter complex PhnCDE involved in phosphonates, phosphate esters, phosphite and phosphate import
P	P16677	DR	phosphonate transmembrane-transporting ATPase activity
P	P16700	CC	Part of the ABC transporter complex CysAWTP (TC 3.A.1.6.1) involved in sulfate/thiosulfate import
P	P16700	CC	Part of the ABC transporter complex CysAWTP (TC 3.A.1.6.1) involved in sulfate/thiosulfate import
P	P16700	CC	This protein specifically binds thiosulfate and is involved in its transmembrane transport
P	P16700	DE	Thiosulfate-binding protein;
P	P16700	DR	secondary active sulfate transmembrane transporter activity
P	P16700	DR	sulfate transmembrane-transporting ATPase activity
P	P16701	CC	Part of the ABC transporter complex CysAWTP (TC 3.A.1.6.1) involved in sulfate/thiosulfate import
P	P16701	CC	Part of the ABC transporter complex CysAWTP (TC 3.A.1.6.1) involved in sulfate/thiosulfate import
P	P16701	DE	Sulfate transport system permease protein CysT;
P	P16701	DR	sulfate transmembrane transporter activity
P	P17064	CC	This permease has a broad specificity towards purines, and also transport cytosine and 5-methylcytosine but neither uracil nor thymine.

P	P17064	DE	Cytosine/purine transport protein FCY2;
P	P17064	DE	Cytosine/purine transport protein FCY2;
P	P17064	DE	Purine-cytosine permease FCY2;
P	P17127	CC	This system is involved in fructose transport (By similarity).
P	P17127	DE	Fructose-specific phosphotransferase enzyme IIA component;
P	P17127	DE	PTS system fructose-specific EIIA component;
P	P17215	CC	This protein is a component of the leucine, isoleucine, valine, threonine transport system, which is one of the two periplasmic binding protein-dependent transport systems of the high-affinity transport of the branched-chain amino acids (By similarity).
P	P17315	CC	Postulated to participate in iron transport
P	P17327	CC	Involved in a multicomponent binding-protein-dependent transport system for glycine betaine/L-proline.
P	P17327	DE	Glycine betaine/L-proline transport system permease protein proW;
P	P17328	CC	Involved in a multicomponent binding-protein-dependent transport system for glycine betaine/L-proline.
P	P17328	DE	Glycine betaine/L-proline transport ATP-binding protein ProV;
P	P17583	CC	This protein is part of an active transport system that transports exogenous cyanate into E.coli cells.
P	P17583	DE	Cyanate transport protein CynX;
P	P17658	CC	Assuming opened or closed conformations in response to the voltage difference across the membrane, the protein forms a potassium-selective channel through which potassium ions may pass in accordance with their electrochemical gradient
P	P17658	CC	Mediates the voltage-dependent potassium ion permeability of excitable membranes
P	P17658	DE	Potassium voltage-gated channel subfamily A member 6;
P	P17658	DE	Voltage-gated potassium channel HBK2;
P	P17658	DE	Voltage-gated potassium channel subunit Kv1.6;
P	P17658	DR	voltage-gated potassium channel activity
P	P17809	CC	Facilitative glucose transporter
P	P17809	CC	This isoform may be responsible for constitutive or basal glucose uptake
P	P17809	DE	Glucose transporter type 1, erythrocyte/brain;
P	P17809	DE	Solute carrier family 2, facilitated glucose transporter member 1;
P	P17809	DR	D-glucose transmembrane transporter activity
P	P17970	CC	Assuming opened or closed conformations in response to the voltage difference across the membrane, the protein forms a potassium-selective channel through which potassium ions may pass in accordance with their electrochemical gradient
P	P17970	CC	Mediates the voltage-dependent potassium ion permeability of excitable membranes
P	P17970	DE	Potassium voltage-gated channel protein Shab;
P	P17970	DR	voltage-gated potassium channel activity
P	P17971	CC	Assuming opened or closed conformations in response to the voltage difference across the membrane, the protein forms a potassium-selective channel through which potassium ions may pass in accordance with their electrochemical gradient
P	P17971	CC	Mediates the voltage-dependent potassium ion permeability of excitable membranes
P	P17971	DE	Potassium voltage-gated channel protein Shal;
P	P17971	DR	A-type (transient outward) potassium channel activity
P	P17972	CC	Assuming opened or closed conformations in response to the voltage difference across the membrane, the protein forms a potassium-selective channel through which potassium ions may pass in accordance with their electrochemical gradient
P	P17972	CC	Mediates the voltage-dependent potassium ion permeability of excitable membranes
P	P17972	DE	Potassium voltage-gated channel protein Shaw;
P	P17972	DR	voltage-gated potassium channel activity
P	P18238	CC	Catalyzes the exchange of ADP and ATP across the mitochondrial inner membrane
P	P18238	CC	Catalyzes the exchange of ADP and ATP across the mitochondrial inner membrane
P	P18238	DE	ADP,ATP carrier protein 3;
P	P18238	DE	ADP/ATP translocase 3;
P	P18238	DE	ADP/ATP translocase 3;
P	P18239	CC	Catalyzes the exchange of ADP and ATP across the mitochondrial inner membrane
P	P18239	CC	Catalyzes the exchange of ADP and ATP across the mitochondrial inner membrane
P	P18239	DE	ADP,ATP carrier protein 2;
P	P18239	DE	ADP/ATP translocase 2;
P	P18239	DE	ADP/ATP translocase 2;
P	P18239	DR	ATP:ADP antiporter activity
P	P18239	DR	ATP:ADP antiporter activity
P	P18275	CC	Catalyzes an electroneutral exchange between arginine and ornithine to allow high-efficiency energy conversion in the arginine deiminase pathway.

P	P18275	CC	Catalyzes an electroneutral exchange between arginine and ornithine to allow high-efficiency energy conversion in the arginine deiminase pathway.
P	P18275	DE	Arginine/ornithine antiporter;
P	P18275	DE	Arginine/ornithine antiporter;
P	P18505	CC	GABA, the major inhibitory neurotransmitter in the vertebrate brain, mediates neuronal inhibition by binding to the GABA/benzodiazepine receptor and opening an integral chloride channel.
P	P18505	DR	chloride channel activity
P	P18507	CC	GABA, the major inhibitory neurotransmitter in the vertebrate brain, mediates neuronal inhibition by binding to the GABA/benzodiazepine receptor and opening an integral chloride channel.
P	P18507	DR	chloride channel activity
P	P18766	DE	Oligopeptide transport ATP-binding protein AmiF;
P	P18791	CC	Part of the binding-protein-dependent transport system for oligopeptides; probably an oligopeptide binding protein.
P	P18791	DE	Oligopeptide-binding protein AmiA;
P	P18895	DE	Alginate production protein AlgE;
P	P19072	CC	BraB is specific for isoleucine, leucine and valine
P	P19072	CC	BraB is specific for isoleucine, leucine and valine
P	P19072	CC	BraB is specific for isoleucine, leucine and valine
P	P19072	CC	The LIV-II transport system is coupled to sodium and lithium ions.
P	P19255	CC	Glycerol enters the cell via the glycerol diffusion facilitator protein
P	P19255	CC	This membrane protein facilitates the movement of glycerol across the cytoplasmic membrane (By similarity).
P	P19255	DE	Probable glycerol uptake facilitator protein;
P	P19566	CC	Part of the ABC transporter complex MalEFGK involved in maltose/maltodextrin import
P	P19566	DE	Maltose/maltodextrin import ATP-binding protein MalK;
P	P19566	DR	maltose-transporting ATPase activity
P	P19568	CC	Provides the rickettsial cell with host ATP in exchange for rickettsial ADP
P	P19568	CC	Provides the rickettsial cell with host ATP in exchange for rickettsial ADP
P	P19568	DE	ADP,ATP carrier protein 1;
P	P19568	DE	ADP/ATP translocase 1;
P	P19568	DE	ADP/ATP translocase 1;
P	P19568	DR	ATP:ADP antiporter activity
P	P19568	DR	ATP:ADP antiporter activity
P	P19576	CC	Involved in the high-affinity maltose membrane transport system
P	P19576	DE	Maltose-binding periplasmic protein;
P	P19576	DR	maltose transmembrane transporter activity
P	P19634	CC	Major proton extruding system driven by the inward sodium ion chemical gradient
P	P19634	DE	Na+/H+ antiporter, amiloride-sensitive;
P	P19634	DE	Na+/H+ exchanger 1;
P	P19634	DE	Sodium/hydrogen exchanger 1;
P	P19634	DR	sodium:hydrogen antiporter activity
P	P19642	CC	This system is involved in maltose and glucose transport.
P	P19642	DE	Maltose and glucose permease IIC component;
P	P19642	DE	Maltose- and glucose-specific phosphotransferase enzyme IIB component;
P	P19642	DE	PTS system maltose- and glucose-specific EIIB component;
P	P19642	DE	PTS system maltose- and glucose-specific EIIC component;
P	P19642	DE	PTS system maltose- and glucose-specific EIICB component;
P	P19807	CC	Sole choline transporter in yeast.
P	P19807	DE	Choline transport protein;
P	P19807	DR	choline transmembrane transporter activity
P	P20020	CC	This magnesium-dependent enzyme catalyzes the hydrolysis of ATP coupled with the transport of calcium out of the cell.
P	P20020	DE	Plasma membrane calcium ATPase isoform 1;
P	P20020	DE	Plasma membrane calcium pump isoform 1;
P	P20020	DE	Plasma membrane calcium-transporting ATPase 1;
P	P20020	DR	calcium-transporting ATPase activity
P	P20021	CC	This electroneutral antiporter ejects one cadmium ion while accumulating two protons by an energy-dependent efflux mechanism.
P	P20021	DE	Cadmium efflux ATPase;
P	P20021	DE	Probable cadmium-transporting ATPase;
P	P20021	DR	cadmium-exporting ATPase activity
P	P20107	CC	Probably responsible for the uptake of zinc and cadmium ions.
P	P20107	CC	Probably responsible for the uptake of zinc and cadmium ions.

P	P20107	DE	Zinc/cadmium resistance protein;
P	P20107	DE	Zinc/cadmium resistance protein;
P	P20107	DR	zinc ion transmembrane transporter activity
P	P20648	CC	Catalyzes the hydrolysis of ATP coupled with the exchange of H+ and K+ ions across the plasma membrane
P	P20648	CC	Catalyzes the hydrolysis of ATP coupled with the exchange of H+ and K+ ions across the plasma membrane
P	P20648	DE	Gastric H+/K+ ATPase subunit alpha;
P	P20648	DE	Gastric H+/K+ ATPase subunit alpha;
P	P20648	DE	Potassium-transporting ATPase alpha chain 1;
P	P20648	DR	hydrogen:potassium-exchanging ATPase activity
P	P20672	DR	sodium:dicarboxylate symporter activity
P	P20672	DR	sodium:dicarboxylate symporter activity
P	P21129	DR	bile acid:sodium symporter activity
P	P21129	DR	bile acid:sodium symporter activity
P	P21345	CC	This carrier protein is part of the Na+-independent, binding-protein-independent glutamate-aspartate transport system.
P	P21345	DE	Glutamate-aspartate carrier protein;
P	P21345	DE	Proton glutamate symport protein;
P	P21608	CC	Uptake of citrate across the boundary membrane with the concomitant uptake of a sodium ion (symport system).
P	P21608	CC	Uptake of citrate across the boundary membrane with the concomitant uptake of a sodium ion (symport system).
P	P21608	DE	Citrate carrier protein;
P	P21608	DE	Citrate transporter;
P	P21608	DE	Citrate-sodium symporter;
P	P21817	CC	Contraction of skeletal muscle is triggered by release of calcium ions from SR following depolarization of T-tubules.
P	P21817	DE	Skeletal muscle calcium release channel;
P	P21817	DR	ryanodine-sensitive calcium-release channel activity
P	P21903	DE	ATP synthase subunit a, sodium ion specific;
P	P21904	CC	F(1)F(0) ATP synthase produces ATP from ADP in the presence of a proton or sodium gradient
P	P21904	DE	ATP synthase subunit b, sodium ion specific;
P	P21905	CC	During catalysis, ATP synthesis in the catalytic domain of F(1) is coupled via a rotary mechanism of the central stalk subunits to sodium translocation.
P	P21905	CC	F(1)F(0) ATP synthase produces ATP from ADP in the presence of a proton or sodium gradient
P	P21905	CC	F-type ATPases consist of two structural domains, F(1) containing the extramembraneous catalytic core and F(0) containing the membrane sodium channel, linked together by a central stalk and a peripheral stalk
P	P21905	DE	ATP synthase subunit c, sodium ion specific;
P	P21906	DE	Glucose facilitated diffusion protein;
P	P22001	CC	Assuming opened or closed conformations in response to the voltage difference across the membrane, the protein forms a potassium-selective channel through which potassium ions may pass in accordance with their electrochemical gradient
P	P22001	CC	Mediates the voltage-dependent potassium ion permeability of excitable membranes
P	P22001	DE	Potassium voltage-gated channel subfamily A member 3;
P	P22001	DE	Voltage-gated K+ channel HuKIII;
P	P22001	DE	Voltage-gated potassium channel subunit Kv1.3;
P	P22001	DR	delayed rectifier potassium channel activity
P	P22152	CC	Permease for nitrate uptake.
P	P22152	DE	Nitrate permease;
P	P22152	DE	Nitrate transporter;
P	P22189	CC	This magnesium-dependent enzyme catalyzes the hydrolysis of ATP coupled with the transport of the calcium.
P	P22189	DE	Calcium-transporting ATPase 3;
P	P22189	DR	calcium-transporting ATPase activity
P	P22459	CC	Assuming opened or closed conformations in response to the voltage difference across the membrane, the protein forms a potassium-selective channel through which potassium ions may pass in accordance with their electrochemical gradient
P	P22459	CC	Mediates the voltage-dependent potassium ion permeability of excitable membranes
P	P22459	DE	Potassium voltage-gated channel subfamily A member 4;
P	P22459	DE	Voltage-gated K+ channel HuKII;
P	P22459	DE	Voltage-gated potassium channel HBK4;

P	P22459	DE	Voltage-gated potassium channel HK1;
P	P22459	DE	Voltage-gated potassium channel subunit Kv1.4;
P	P22459	DR	voltage-gated potassium channel activity
P	P22460	CC	Assuming opened or closed conformations in response to the voltage difference across the membrane, the protein forms a potassium-selective channel through which potassium ions may pass in accordance with their electrochemical gradient
P	P22460	CC	Mediates the voltage-dependent potassium ion permeability of excitable membranes
P	P22460	DE	Potassium voltage-gated channel subfamily A member 5;
P	P22460	DE	Voltage-gated potassium channel HK2;
P	P22460	DE	Voltage-gated potassium channel subunit Kv1.5;
P	P22460	DR	delayed rectifier potassium channel activity
P	P22700	CC	This magnesium-dependent enzyme catalyzes the hydrolysis of ATP coupled with the transport of calcium.
P	P22700	DE	Calcium pump;
P	P22700	DE	Calcium-transporting ATPase sarcoplasmic/endoplasmic reticulum type;
P	P22700	DR	calcium-transporting ATPase activity
P	P22723	CC	GABA, the major inhibitory neurotransmitter in the vertebrate brain, mediates neuronal inhibition by binding to the GABA/benzodiazepine receptor and opening an integral chloride channel.
P	P22723	DR	chloride channel activity
P	P22731	CC	Component of the leucine-specific transport system.
P	P22732	DE	Glucose transporter type 5, small intestine;
P	P22732	DE	Solute carrier family 2, facilitated glucose transporter member 5;
P	P22732	DR	glucose transmembrane transporter activity
P	P22933	CC	GABA, the major inhibitory neurotransmitter in the vertebrate brain, mediates neuronal inhibition by binding to the GABA/benzodiazepine receptor and opening an integral chloride channel.
P	P22933	DR	chloride channel activity
P	P23516	CC	High-affinity nickel transporter responsible for nickel uptake
P	P23516	DE	High-affinity nickel transport protein;
P	P23516	DR	nickel ion transmembrane transporter activity
P	P23586	CC	Can transport glucose, 3-O- methylglucose, fructose, xylose, mannose, galactose, fucose, 2- deoxyglucose and arabinose
P	P23586	DE	Glucose transporter;
P	P23622	CC	Uptake of sulfate into the cell.
P	P23622	DE	Sulfate permease 2;
P	P23622	DE	Sulfate permease II;
P	P23622	DR	secondary active sulfate transmembrane transporter activity
P	P23634	CC	This magnesium-dependent enzyme catalyzes the hydrolysis of ATP coupled with the transport of calcium out of the cell.
P	P23634	DE	Plasma membrane calcium ATPase isoform 4;
P	P23634	DE	Plasma membrane calcium pump isoform 4;
P	P23634	DE	Plasma membrane calcium-transporting ATPase 4;
P	P23634	DR	calcium-transporting ATPase activity
P	P23900	CC	Channel protein for glycerol
P	P23900	CC	Has a role in both glycerol influx and efflux
P	P23900	CC	Plays a role in osmoregulation: under osmotic stress the channel is apparently closed to allow accumulation of glycerol in the cell under hyperosmotic conditions
P	P23900	DE	Glycerol uptake/efflux facilitator protein;
P	P23900	DR	glycerol transmembrane transporter activity
P	P23936	DE	Lactose permease;
P	P23936	DE	Lactose transport protein;
P	P23936	DE	Lactose-proton symporter;
P	P23975	CC	Terminates the action of noradrenaline by its high affinity sodium-dependent reuptake into presynaptic terminals.
P	P23975	CC	Terminates the action of noradrenaline by its high affinity sodium-dependent reuptake into presynaptic terminals.
P	P23975	DE	Norepinephrine transporter;
P	P23975	DE	Sodium-dependent noradrenaline transporter;
P	P23975	DE	Sodium-dependent noradrenaline transporter;
P	P23975	DR	norepinephrine:sodium symporter activity
P	P23975	DR	norepinephrine:sodium symporter activity
P	P24046	CC	GABA, the major inhibitory neurotransmitter in the vertebrate brain, mediates neuronal inhibition by binding to the GABA/benzodiazepine receptor and opening an integral chloride channel

P	P24046	DR	chloride channel activity
P	P24207	CC	Permease that is involved in the transport across the cytoplasmic membrane of phenylalanine.
P	P24207	DE	Phenylalanine-specific permease;
P	P24241	CC	This system is involved in arbutin, cellobiose, and salicin transport.
P	P24241	CC	This system is involved in arbutin, cellobiose, and salicin transport.
P	P24241	CC	This system is involved in arbutin, cellobiose, and salicin transport.
P	P24241	DE	Arbutin, cellobiose, and salicin permease IIC component;
P	P24241	DE	Arbutin, cellobiose, and salicin permease IIC component;
P	P24241	DE	Arbutin, cellobiose, and salicin permease IIC component;
P	P24241	DE	Arbutin-, cellobiose-, and salicin-specific phosphotransferase enzyme IIB component;
P	P24241	DE	Arbutin-, cellobiose-, and salicin-specific phosphotransferase enzyme IIB component;
P	P24241	DE	PTS system arbutin-, cellobiose-, and salicin-specific EIIB component;
P	P24241	DE	PTS system arbutin-, cellobiose-, and salicin-specific EIIB component;
P	P24241	DE	PTS system arbutin-, cellobiose-, and salicin-specific EIIBC component;
P	P24241	DE	PTS system arbutin-, cellobiose-, and salicin-specific EIIBC component;
P	P24241	DE	PTS system arbutin-, cellobiose-, and salicin-specific EIIC component;
P	P24241	DE	PTS system arbutin-, cellobiose-, and salicin-specific EIIC component;
P	P24585	CC	Probable sodium channel subunit
P	P24585	DR	sodium channel activity
P	P24942	CC	Acts as a symport by cotransporting sodium.
P	P24942	CC	Essential for terminating the postsynaptic action of glutamate by rapidly removing released glutamate from the synaptic cleft
P	P24942	DE	Glial glutamate transporter;
P	P24942	DE	Sodium-dependent glutamate/aspartate transporter 1;
P	P24942	DE	Sodium-dependent glutamate/aspartate transporter 1;
P	P24942	DE	Sodium-dependent glutamate/aspartate transporter 1;
P	P24942	DR	high-affinity glutamate transmembrane transporter activity
P	P24942	DR	sodium:dicarboxylate symporter activity
P	P24943	CC	This carrier protein is part of the Na+-dependent, binding-protein-independent glutamate-aspartate transport system.
P	P24943	CC	This carrier protein is part of the Na+-dependent, binding-protein-independent glutamate-aspartate transport system.
P	P24943	DE	Glutamate-aspartate carrier protein;
P	P24943	DE	Proton/sodium-glutamate symport protein;
P	P24943	DR	sodium:dicarboxylate symporter activity
P	P25185	CC	BraZ is specific for isoleucine and valine
P	P25185	CC	BraZ is specific for isoleucine and valine
P	P25185	CC	The LIV-III transport system may be H+-coupled.
P	P25297	CC	High-affinity transporter for external inorganic phosphate
P	P25297	DE	Inorganic phosphate transporter PHO84;
P	P25297	DR	inorganic phosphate transmembrane transporter activity
P	P25360	CC	Involved in the uptake of inorganic phosphate.
P	P25360	DE	Inorganic phosphate transporter PHO87;
P	P25360	DR	inorganic phosphate transmembrane transporter activity
P	P25376	CC	Broad substrate range permease which transports asparagine and glutamine with intermediate specificity
P	P25376	CC	Broad substrate range permease which transports asparagine and glutamine with intermediate specificity
P	P25376	DE	Asparagine/glutamine permease;
P	P25376	DE	Asparagine/glutamine permease;
P	P25621	CC	Transports pantothenate into the cell
P	P25621	DE	Pantothenate transporter FEN2;
P	P25621	DR	pantothenate transmembrane transporter activity
P	P25737	CC	Permease that is involved in the transport across the cytoplasmic membrane of lysine.
P	P25737	DE	Lysine-specific permease;
P	P25818	CC	Water channel required to facilitate the transport of water, diffusion of amino acids and/or peptides from the vacuolar compartment to the cytoplasm
P	P25818	DR	water channel activity
P	P26048	CC	GABA, the major inhibitory neurotransmitter in the vertebrate brain, mediates neuronal inhibition by binding to the GABA/benzodiazepine receptor and opening an integral chloride channel.
P	P26048	DR	chloride channel activity

P	P26049	CC	GABA, the major inhibitory neurotransmitter in the vertebrate brain, mediates neuronal inhibition by binding to the GABA/benzodiazepine receptor and opening an integral chloride channel.
P	P26049	DR	chloride channel activity
P	P26235	CC	It can mediate sodium uptake when a transmembrane pH gradient is applied.
P	P26235	CC	This is a Na+/H+ antiporter
P	P26235	CC	This is a Na+/H+ antiporter
P	P26235	DE	Na+/H+ antiporter;
P	P26235	DE	Na+/H+ antiporter;
P	P26361	CC	Involved in the transport of chloride ions
P	P26361	DE	cAMP-dependent chloride channel;
P	P26426	CC	This system is involved in lactose transport.
P	P26426	DE	Lactose-specific phosphotransferase enzyme IIA component;
P	P26426	DE	PTS system lactose-specific EIIA component;
P	P26430	CC	Actively transports uridine into cells by Na+ cotransport
P	P26430	DE	Na+/glucose cotransporter 2;
P	P26430	DE	Na+/glucose cotransporter 2;
P	P26430	DE	Sodium/nucleoside cotransporter;
P	P26430	DE	Sodium/nucleoside cotransporter;
P	P26435	CC	It is strictly dependent on the extracellular presence of sodium.
P	P26435	CC	The hepatic sodium/bile acid uptake system exhibits broad substrate specificity and transports various non-bile acid organic compounds as well
P	P26435	CC	The hepatic sodium/bile acid uptake system exhibits broad substrate specificity and transports various non-bile acid organic compounds as well
P	P26435	DE	Na+/bile acid cotransporter;
P	P26435	DE	Na+/bile acid cotransporter;
P	P26435	DE	Na+/taurocholate transport protein;
P	P26435	DE	Sodium/bile acid cotransporter;
P	P26435	DE	Sodium/bile acid cotransporter;
P	P26435	DE	Sodium/taurocholate cotransporting polypeptide;
P	P26435	DR	bile acid:sodium symporter activity
P	P26435	DR	bile acid:sodium symporter activity
P	P26467	CC	Part of the binding-protein-dependent transport system for maltose; probably responsible for the translocation of the substrate across the membrane.
P	P26467	DE	Maltose transport system permease protein malF;
P	P26468	CC	Part of the binding-protein-dependent transport system for maltose; probably responsible for the translocation of the substrate across the membrane.
P	P26468	DE	Maltose transport system permease protein malG;
P	P26587	CC	Water channel required to facilitate the transport of water from the vacuolar compartment to the cytoplasm.
P	P26903	DE	Dipeptide transport system permease protein dppB;
P	P26904	DE	Dipeptide transport system permease protein dppC;
P	P26905	DE	Dipeptide transport ATP-binding protein DppD;
P	P26906	DE	Dipeptide-binding protein dppE;
P	P27135	CC	Uptake of L-rhamnose across the boundary membrane with the concomitant transport of protons into the cell (symport system).
P	P27135	DE	L-rhamnose-H+ transport protein;
P	P27135	DE	L-rhamnose-proton symporter;
P	P27547	CC	This system is involved in mannitol transport (By similarity).
P	P27547	DE	Mannitol-specific phosphotransferase enzyme IIA component;
P	P27547	DE	PTS system mannitol-specific EIIA component;
P	P27611	CC	Involved in pH homeostasis and sodium extrusion.
P	P27611	CC	Na+/H+ antiporter
P	P27611	CC	Na+/H+ antiporter
P	P27611	DE	Na+/H+ antiporter nhaC;
P	P27611	DE	Na+/H+ antiporter nhaC;
P	P27611	DE	Sodium/hydrogen antiporter;
P	P27611	DE	Sodium/proton antiporter;
P	P27611	DR	sodium:hydrogen antiporter activity
P	P28246	CC	Involved in sulfonamide (sulfathiazole) and bicyclomycin resistance
P	P28246	DE	Bicyclomycin resistance protein;
P	P28472	CC	GABA, the major inhibitory neurotransmitter in the vertebrate brain, mediates neuronal inhibition by binding to the GABA/benzodiazepine receptor and opening an integral chloride channel.
P	P28472	DR	chloride channel activity

P	P28476	CC	GABA, the major inhibitory neurotransmitter in the vertebrate brain, mediates neuronal inhibition by binding to the GABA/benzodiazepine receptor and opening an integral chloride channel
P	P28476	DR	chloride channel activity
P	P28570	DE	Sodium- and chloride-dependent creatine transporter 1;
P	P28570	DR	choline transmembrane transporter activity
P	P28570	DR	creatine:sodium symporter activity
P	P28570	DR	neurotransmitter:sodium symporter activity
P	P28571	CC	May play a role in regulation of glycine levels in NMDA receptor-mediated neurotransmission.
P	P28571	CC	Terminates the action of glycine by its high affinity sodium-dependent reuptake into presynaptic terminals
P	P28571	CC	Terminates the action of glycine by its high affinity sodium-dependent reuptake into presynaptic terminals
P	P28571	DE	Sodium- and chloride-dependent glycine transporter 1;
P	P28571	DE	Sodium- and chloride-dependent glycine transporter 1;
P	P28571	DR	glycine transmembrane transporter activity
P	P28571	DR	neurotransmitter:sodium symporter activity
P	P28571	DR	sodium:amino acid symporter activity
P	P28572	CC	Terminates the action of glycine by its high affinity sodium-dependent reuptake into presynaptic terminals
P	P28572	DE	Sodium- and chloride-dependent glycine transporter 1;
P	P28572	DR	neurotransmitter:sodium symporter activity
P	P28572	DR	sodium:amino acid symporter activity
P	P28573	CC	Terminates the action of proline by its high affinity sodium-dependent reuptake into presynaptic terminals.
P	P28573	CC	Terminates the action of proline by its high affinity sodium-dependent reuptake into presynaptic terminals.
P	P28573	DE	Sodium-dependent proline transporter;
P	P28573	DE	Sodium-dependent proline transporter;
P	P28573	DR	neurotransmitter:sodium symporter activity
P	P28584	CC	This protein is required for low-affinity potassium transport.
P	P28584	DE	Low-affinity potassium transport protein;
P	P28584	DR	potassium ion transmembrane transporter activity
P	P28873	CC	Confers resistance to benomyl and methotrexate.
P	P28873	CC	Confers resistance to benomyl and methotrexate.
P	P28873	DE	Benomyl/methotrexate resistance protein;
P	P28873	DE	Benomyl/methotrexate resistance protein;
P	P29706	CC	Produces ATP from ADP in the presence of a sodium ion gradient across the membrane
P	P29706	DE	ATP synthase subunit alpha, sodium ion specific;
P	P29706	DE	Na+-translocating ATPase subunit alpha;
P	P29707	CC	Produces ATP from ADP in the presence of a sodium ion gradient across the membrane
P	P29707	DE	ATP synthase subunit beta, sodium ion specific;
P	P29707	DE	Na+-translocating ATPase subunit beta;
P	P29708	CC	F(1)F(0) ATP synthase produces ATP from ADP in the presence of a proton or sodium gradient
P	P29708	DE	ATP synthase subunit delta, sodium ion specific;
P	P29709	CC	Produces ATP from ADP in the presence of a sodium gradient across the membrane
P	P29709	DE	ATP synthase epsilon chain, sodium ion specific;
P	P29709	DE	F-ATPase epsilon subunit, sodium ion specific;
P	P29709	DE	Na+-translocating ATPase subunit epsilon;
P	P29710	DE	ATP synthase gamma chain, sodium ion specific;
P	P29710	DE	F-ATPase gamma subunit, sodium ion specific;
P	P29822	CC	Part of the binding-protein-dependent transport system for lactose.
P	P29822	DE	Lactose-binding protein;
P	P29823	CC	Part of the binding-protein-dependent transport system for lactose
P	P29823	DE	Lactose transport system permease protein lacF;
P	P29824	CC	Part of the binding-protein-dependent transport system for lactose
P	P29824	DE	Lactose transport system permease protein lacG;
P	P29972	CC	Forms a water-specific channel that provides the plasma membranes of red cells and kidney proximal tubules with high permeability to water, thereby permitting water to move in the direction of an osmotic gradient.
P	P29972	DE	Urine water channel;
P	P29972	DE	Water channel protein for red blood cells and kidney proximal tubule;
P	P29972	DR	water channel activity

P	P30000	CC	Has an essential role in sucrose metabolism.
P	P30000	DE	Sucrose permease;
P	P30000	DE	Sucrose transport protein;
P	P30144	CC	Catalyzes the sodium-dependent uptake of extracellular D-alanine and glycine.
P	P30144	DE	Na+-linked D-alanine glycine permease;
P	P30144	DR	sodium:amino acid symporter activity
P	P30145	CC	Protein responsible for alanine transport
P	P30145	CC	Uses hydrogen and sodium cations as coupling cations for cotransport.
P	P30145	DE	Sodium/proton-dependent alanine carrier protein;
P	P30145	DE	Sodium/proton-dependent alanine carrier protein;
P	P30145	DR	sodium:amino acid symporter activity
P	P30301	CC	Water channel
P	P30302	CC	Water channel required to facilitate the transport of water across cell membrane; mercury-insensitive.
P	P30302	DE	Water stress-induced tonoplast intrinsic protein;
P	P30302	DR	water channel activity
P	P30329	CC	Thought to form the channel of an arsenite pump.
P	P30329	DR	arsenite transmembrane transporter activity
P	P30531	CC	Terminates the action of GABA by its high affinity sodium-dependent reuptake into presynaptic terminals.
P	P30531	CC	Terminates the action of GABA by its high affinity sodium-dependent reuptake into presynaptic terminals.
P	P30531	DE	Sodium- and chloride-dependent GABA transporter 1;
P	P30531	DE	Sodium- and chloride-dependent GABA transporter 1;
P	P30531	DR	gamma-aminobutyric acid:sodium symporter activity
P	P30531	DR	gamma-aminobutyric acid:sodium symporter activity
P	P30531	DR	neurotransmitter:sodium symporter activity
P	P30605	CC	Major transporter for myo-inositol.
P	P30605	DE	Myo-inositol transporter 1;
P	P30605	DR	myo-inositol transmembrane transporter activity
P	P30606	CC	Minor transporter for myo-inositol.
P	P30606	DE	Myo-inositol transporter 2;
P	P30606	DR	myo-inositol transmembrane transporter activity
P	P30859	CC	Part of the ABC transporter complex ArtPIQMJ involved in arginine transport.
P	P30859	DE	Putative ABC transporter arginine-binding protein 2;
P	P30860	CC	Part of the ABC transporter complex ArtPIQMJ involved in arginine transport
P	P30860	DE	ABC transporter arginine-binding protein 1;
P	P30878	CC	Responsible for melibiose transport
P	P30878	DE	Melibiose carrier protein;
P	P30878	DE	Melibiose permease;
P	P30878	DE	Melibiose transporter;
P	P30878	DE	Na+ (Li+)/melibiose symporter;
P	P30962	CC	Required for the export of heme to the periplasm for the biogenesis of c-type cytochromes.
P	P30962	DE	Heme exporter protein C;
P	P30962	DR	heme transporter activity
P	P30963	CC	Part of the ABC transporter complex CcmAB involved in the biogenesis of c-type cytochromes; once thought to export heme, this seems not to be the case, but its exact role is uncertain
P	P30963	DE	Heme exporter protein A;
P	P30963	DR	heme-transporting ATPase activity
P	P30964	CC	Required for the export of heme to the periplasm for the biogenesis of c-type cytochromes.
P	P30964	DE	Heme exporter protein B;
P	P30964	DR	heme transporter activity
P	P31133	CC	Required for the activity of the bacterial periplasmic transport system of putrescine
P	P31133	DE	Putrescine-binding periplasmic protein;
P	P31133	DR	putrescine-importing ATPase activity
P	P31134	CC	Part of the binding-protein-dependent transport system for putrescine
P	P31134	DE	Putrescine transport ATP-binding protein PotG;
P	P31134	DR	putrescine-importing ATPase activity
P	P31135	CC	Required for the activity of the bacterial periplasmic transport system of putrescine
P	P31135	DE	Putrescine transport system permease protein PotH;
P	P31135	DR	putrescine-importing ATPase activity

P	P31140	CC	Transporter of glycerol across the cytoplasmic membrane, with limited permeability to water and small uncharged compounds such as polyols (By similarity)
P	P31140	DE	Glycerol uptake facilitator protein;
P	P31141	DE	Chloramphenicol resistance protein;
P	P31167	CC	Catalyzes the exchange of ADP and ATP across the mitochondrial inner membrane
P	P31167	CC	Catalyzes the exchange of ADP and ATP across the mitochondrial inner membrane
P	P31167	DE	ADP,ATP carrier protein 1, mitochondrial;
P	P31167	DE	ADP/ATP translocase 1;
P	P31167	DE	ADP/ATP translocase 1;
P	P31167	DR	ATP:ADP antiporter activity
P	P31167	DR	ATP:ADP antiporter activity
P	P31414	CC	Contributes to the transtonoplast (from cytosol to vacuole lumen) H+-electrochemical potential difference
P	P31414	CC	It establishes a proton gradient of similar and often greater magnitude than the H+-ATPase on the same membrane
P	P31414	DE	H+-PPase 1;
P	P31436	CC	The physiological role may be the detoxification of non-metabolizable sugar analogs
P	P31436	DE	Sugar efflux transporter C;
P	P31436	DR	sugar efflux transmembrane transporter activity
P	P31499	CC	This protein is involved in the initial step of iron uptake by binding hemin, an iron chelatin siderophore that allows the bacteria to extract iron from the environment
P	P31499	DE	Hemin receptor;
P	P31548	CC	Part of the ABC transporter complex ThiBPQ involved in thiamine import
P	P31548	DE	Thiamine import ATP-binding protein ThiQ;
P	P31549	CC	Part of the ABC transporter complex thiBPQ involved in thiamine import
P	P31549	DE	Thiamine transport system permease protein thiP;
P	P31550	CC	Part of the ABC transporter complex thiBPQ involved in thiamine import.
P	P31550	DE	Thiamine-binding periplasmic protein;
P	P31553	DR	carnitine transporter activity
P	P31596	CC	Acts as a symport by cotransporting sodium.
P	P31596	CC	Essential for terminating the postsynaptic action of glutamate by rapidly removing released glutamate from the synaptic cleft
P	P31596	DE	Sodium-dependent glutamate/aspartate transporter 2;
P	P31596	DE	Sodium-dependent glutamate/aspartate transporter 2;
P	P31596	DR	high-affinity glutamate transmembrane transporter activity
P	P31596	DR	sodium:dicarboxylate symporter activity
P	P31602	CC	Uptake of citrate across the boundary membrane with the concomitant uptake of a sodium ion (symport system).
P	P31602	CC	Uptake of citrate across the boundary membrane with the concomitant uptake of a sodium ion (symport system).
P	P31602	DE	Citrate carrier protein;
P	P31602	DE	Citrate transporter;
P	P31602	DE	Citrate-sodium symporter;
P	P31636	CC	Has a Na+ to glucose coupling ratio of 1:1.
P	P31636	CC	Has a Na+ to glucose coupling ratio of 1:1.
P	P31636	CC	Sodium-dependent glucose transporter
P	P31636	CC	Sodium-dependent glucose transporter
P	P31636	DE	Low affinity sodium-glucose cotransporter;
P	P31636	DE	Na+/glucose cotransporter 3;
P	P31636	DE	Na+/glucose cotransporter 3;
P	P31636	DE	Sodium/glucose cotransporter 3;
P	P31636	DE	Sodium/glucose cotransporter 3;
P	P31639	CC	Has a Na+ to glucose coupling ratio of 1:1.
P	P31639	CC	Has a Na+ to glucose coupling ratio of 1:1.
P	P31639	CC	Sodium-dependent glucose transporter
P	P31639	CC	Sodium-dependent glucose transporter
P	P31639	DE	Low affinity sodium-glucose cotransporter;
P	P31639	DE	Na+/glucose cotransporter 2;
P	P31639	DE	Na+/glucose cotransporter 2;
P	P31639	DE	Sodium/glucose cotransporter 2;
P	P31639	DE	Sodium/glucose cotransporter 2;
P	P31639	DR	low-affinity glucose:sodium symporter activity
P	P31639	DR	low-affinity glucose:sodium symporter activity
P	P31641	CC	Chloride ions are necessary for optimal uptake.
P	P31641	CC	Required for the uptake of taurine

P	P31641	CC	Transports both taurine and beta-alanine which requires sodium ions
P	P31641	CC	Transports both taurine and beta-alanine which requires sodium ions
P	P31641	DE	Sodium- and chloride-dependent taurine transporter;
P	P31641	DE	Sodium- and chloride-dependent taurine transporter;
P	P31641	DE	Sodium- and chloride-dependent taurine transporter;
P	P31641	DR	neurotransmitter:sodium symporter activity
P	P31641	DR	taurine:sodium symporter activity
P	P31641	DR	taurine:sodium symporter activity
P	P31644	CC	GABA, the major inhibitory neurotransmitter in the vertebrate brain, mediates neuronal inhibition by binding to the GABA/benzodiazepine receptor and opening an integral chloride channel.
P	P31644	DR	chloride channel activity
P	P31645	CC	Plays a key role in mediating regulation of the availability of serotonin to other receptors of serotonergic systems
P	P31645	CC	Serotonin transporter whose primary function in the central nervous system involves the regulation of serotonergic signaling via transport of serotonin molecules from the synaptic cleft back into the pre-synaptic terminal for re-utilization
P	P31645	CC	Terminates the action of serotonin and recycles it in a sodium-dependent manner.
P	P31645	CC	Terminates the action of serotonin and recycles it in a sodium-dependent manner.
P	P31645	DE	Sodium-dependent serotonin transporter;
P	P31645	DE	Sodium-dependent serotonin transporter;
P	P31645	DR	serotonin transmembrane transporter activity
P	P31645	DR	serotonin:sodium symporter activity
P	P31645	DR	serotonin:sodium symporter activity
P	P31648	CC	Terminates the action of GABA by its high affinity sodium-dependent reuptake into presynaptic terminals.
P	P31648	CC	Terminates the action of GABA by its high affinity sodium-dependent reuptake into presynaptic terminals.
P	P31648	DE	Sodium- and chloride-dependent GABA transporter 1;
P	P31648	DE	Sodium- and chloride-dependent GABA transporter 1;
P	P31648	DR	neurotransmitter:sodium symporter activity
P	P31649	CC	Terminates the action of GABA by its high affinity sodium-dependent reuptake into presynaptic terminals
P	P31649	CC	Terminates the action of GABA by its high affinity sodium-dependent reuptake into presynaptic terminals
P	P31649	DE	Sodium- and chloride-dependent GABA transporter 2;
P	P31649	DE	Sodium- and chloride-dependent GABA transporter 2;
P	P31649	DE	Sodium- and chloride-dependent GABA transporter 3;
P	P31649	DE	Sodium- and chloride-dependent GABA transporter 3;
P	P31649	DR	neurotransmitter:sodium symporter activity
P	P31661	DE	Sodium- and chloride-dependent creatine transporter 1;
P	P31661	DR	creatine:sodium symporter activity
P	P31661	DR	neurotransmitter:sodium symporter activity
P	P31675	CC	Can transport IPTG, lactose and glucose
P	P31675	CC	The physiological role may be the detoxification of non-metabolizable sugar analogs
P	P31675	DE	Sugar efflux transporter A;
P	P31675	DR	sugar efflux transmembrane transporter activity
P	P31728	CC	This protein is a component of a D-methionine permease, a binding protein-dependent, ATP-driven transport system (By similarity).
P	P31728	DE	Probable D-methionine-binding lipoprotein metQ;
P	P32010	CC	Part of the ABC transporter complex DrrAB involved in daunorubicin and doxorubicin resistance
P	P32010	DE	Daunorubicin/doxorubicin resistance ATP-binding protein DrrA;
P	P32010	DE	Daunorubicin/doxorubicin resistance ATP-binding protein DrrA;
P	P32011	CC	Part of the ABC transporter complex drrAB involved in daunorubicin and doxorubicin resistance
P	P32011	DE	Daunorubicin/doxorubicin resistance ABC transporter permease protein drrB;
P	P32011	DE	Daunorubicin/doxorubicin resistance ABC transporter permease protein drrB;
P	P32015	CC	May form an ATP-driven capsule polysaccharide export apparatus, in association with the CtrB and CtrD proteins.
P	P32015	DE	Capsule polysaccharide export inner-membrane protein CtrC;
P	P32016	CC	Putative ATP-binding protein, and an energy-coupling component of capsule polysaccharide export apparatus.
P	P32016	DE	Capsule polysaccharide export ATP-binding protein CtrD;
P	P32037	CC	Facilitative glucose transporter

P	P32037	CC	Probably a neuronal glucose transporter.
P	P32037	DE	Glucose transporter type 3, brain;
P	P32037	DE	Solute carrier family 2, facilitated glucose transporter member 3;
P	P32037	DR	D-glucose transmembrane transporter activity
P	P32071	CC	Confers resistance to cycloheximide.
P	P32071	DE	Cycloheximide resistance protein;
P	P32113	CC	Probably involved in copper import under copper limiting conditions.
P	P32113	DE	Probable copper-importing P-type ATPase A;
P	P32113	DR	copper-exporting ATPase activity
P	P32332	CC	Transports oxaloacetate and sulfate.
P	P32332	DE	Mitochondrial oxaloacetate transport protein;
P	P32332	DR	oxaloacetate secondary active transmembrane transporter activity
P	P32332	DR	secondary active sulfate transmembrane transporter activity
P	P32369	DE	Bile acid transporter;
P	P32467	CC	Can also transport xylose.
P	P32482	DE	Chloramphenicol resistance protein;
P	P32487	CC	High-affinity permease for lysine.
P	P32487	DE	Lysine-specific permease;
P	P32739	CC	High-affinity sodium-dicarboxylate cotransporter that accepts a range of tricarboxylic acid-cycle intermediates with 4-5 carbon atoms
P	P32739	DE	Na+/dicarboxylate cotransporter 2;
P	P32739	DE	Na+/dicarboxylate cotransporter 2;
P	P32739	DE	Sodium-dependent high-affinity dicarboxylate transporter 2;
P	P32739	DE	Sodium-dependent high-affinity dicarboxylate transporter 2;
P	P32798	DR	cobalt ion transmembrane transporter activity
P	P32798	DR	zinc ion transmembrane transporter activity
P	P32804	CC	High-affinity zinc transport protein.
P	P32804	DE	High-affinity zinc transport protein ZRT1;
P	P32804	DE	Zinc-regulated transporter 1;
P	P32804	DR	high affinity zinc uptake transmembrane transporter activity
P	P32837	CC	Required for high-affinity, high-specificity GABA transport
P	P32837	DE	GABA-specific permease;
P	P32837	DE	GABA-specific transport protein;
P	P32837	DR	gamma-aminobutyric acid:hydrogen symporter activity
P	P32977	CC	Porin O has a higher affinity for polyphosphates (tripolyphosphate and pyrophosphate) while porin P has a higher affinity for orthophosphate.
P	P33026	CC	Can transport lactose and glucose.
P	P33026	CC	The physiological role may be the detoxification of non-metabolizable sugar analogs
P	P33026	DE	Sugar efflux transporter B;
P	P33026	DR	sugar efflux transmembrane transporter activity
P	P33027	CC	The physiological role may be the detoxification of non-metabolizable sugar analogs
P	P33027	DE	Sugar efflux transporter B;
P	P33027	DR	sugar efflux transmembrane transporter activity
P	P33231	DR	lactate transmembrane transporter activity
P	P33303	CC	Transports cytoplasmic succinate, derived from isocitrate by the action of isocitrate lyase in the cytosol, into the mitochondrial matrix in exchange for fumarate
P	P33303	CC	Transports cytoplasmic succinate, derived from isocitrate by the action of isocitrate lyase in the cytosol, into the mitochondrial matrix in exchange for fumarate
P	P33303	DE	Succinate/fumarate mitochondrial transporter;
P	P33303	DE	Succinate/fumarate mitochondrial transporter;
P	P33303	DR	succinate:fumarate antiporter activity
P	P33303	DR	succinate:fumarate antiporter activity
P	P33413	CC	Required for active transport of urea.
P	P33413	DE	Urea active transporter;
P	P33413	DR	urea transmembrane transporter activity
P	P33532	CC	Also imports, but does not export, amiloride and so confers sensitivity.
P	P33532	DE	Amiloride transporter car1;
P	P33532	DE	Changed amiloride resistance protein 1;
P	P33590	CC	Involved in a nickel transport system, probably represents the nickel binder.
P	P33590	DE	Nickel-binding periplasmic protein;
P	P33591	CC	Involved in a nickel transport system, probably translocates nickel through the bacterial inner membrane.
P	P33591	DE	Nickel transport system permease protein nikB;
P	P33593	CC	Part of the ABC transporter complex NikABCDE involved in nickel import
P	P33593	DE	Nickel import ATP-binding protein NikD;

P	P33593	DR	nickel-transporting ATPase activity
P	P33594	CC	Part of the ABC transporter complex NikABCDE involved in nickel import
P	P33594	DE	Nickel import ATP-binding protein NikE;
P	P33594	DR	nickel-transporting ATPase activity
P	P33951	CC	ATP-driven efflux pump necessary for the secretion of syringomycin
P	P33951	CC	May specifically bind syringomycin and translocate it to the periplasmic space
P	P34230	DE	Peroxisomal long-chain fatty acid import protein 1;
P	P34315	DE	Na+/Ca2+-exchange protein 6;
P	P34315	DE	Na+/Ca2+-exchange protein 6;
P	P34315	DE	Putative sodium/calcium exchanger 6;
P	P34315	DE	Putative sodium/calcium exchanger 6;
P	P34322	DE	Na+/Ca2+-exchange protein 7;
P	P34322	DE	Na+/Ca2+-exchange protein 7;
P	P34322	DE	Putative sodium/calcium exchanger 7;
P	P34322	DE	Putative sodium/calcium exchanger 7;
P	P34410	DE	TWiK family of potassium channels protein 7;
P	P34410	DR	potassium channel activity
P	P34711	CC	Involved in acetylcholine transport into synaptic vesicles.
P	P34711	DE	Vesicular acetylcholine transporter unc-17;
P	P34711	DR	acetylcholine transmembrane transporter activity
P	P34903	CC	GABA, the major inhibitory neurotransmitter in the vertebrate brain, mediates neuronal inhibition by binding to the GABA/benzodiazepine receptor and opening an integral chloride channel.
P	P34903	DR	chloride channel activity
P	P35113	CC	Component of the nopaline active transport system probably consisting of four subunits: Q, M, P and T
P	P35113	CC	This system is also capable of transporting octopine provided that catabolic functions are induced with nopaline.
P	P35113	CC	This system is also capable of transporting octopine provided that catabolic functions are induced with nopaline.
P	P35113	DE	Nopaline transport system permease protein nocM;
P	P35114	CC	Component of the octopine active transport system probably consisting of four subunits: Q, M, P and T.
P	P35114	DE	Octopine transport system permease protein occM;
P	P35116	CC	Component of the nopaline active transport system probably consisting of four subunits: Q, M, P and T
P	P35116	CC	This system is also capable of transporting octopine provided that catabolic functions are induced with nopaline.
P	P35116	CC	This system is also capable of transporting octopine provided that catabolic functions are induced with nopaline.
P	P35116	DE	Nopaline permease ATP-binding protein P;
P	P35118	CC	Component of the nopaline active transport system probably consisting of four subunits: Q, M, P and T
P	P35118	CC	This system is also capable of transporting octopine provided that catabolic functions are induced with nopaline.
P	P35118	CC	This system is also capable of transporting octopine provided that catabolic functions are induced with nopaline.
P	P35118	DE	Nopaline transport system permease protein nocQ;
P	P35120	CC	Component of the nopaline active transport system probably consisting of four subunits: Q, M, P and T
P	P35120	CC	This system is also capable of transporting octopine provided that catabolic functions are induced with nopaline.
P	P35120	CC	This system is also capable of transporting octopine provided that catabolic functions are induced with nopaline.
P	P35120	DE	Nopaline-binding periplasmic protein;
P	P35449	DE	Na+-H+ exchanger protein 9;
P	P35449	DE	Probable Na+/H+ antiporter nhx-9;
P	P35449	DR	sodium:hydrogen antiporter activity
P	P35498	CC	Assuming opened or closed conformations in response to the voltage difference across the membrane, the protein forms a sodium-selective channel through which Na+ ions may pass in accordance with their electrochemical gradient.
P	P35498	CC	Mediates the voltage-dependent sodium ion permeability of excitable membranes
P	P35498	DE	Sodium channel protein brain I subunit alpha;
P	P35498	DE	Sodium channel protein type 1 subunit alpha;
P	P35498	DE	Sodium channel protein type I subunit alpha;

P	P35498	DE	Voltage-gated sodium channel subunit alpha Nav1.1;
P	P35498	DR	voltage-gated sodium channel activity
P	P35499	CC	Assuming opened or closed conformations in response to the voltage difference across the membrane, the protein forms a sodium-selective channel through which Na+ ions may pass in accordance with their electrochemical gradient
P	P35499	CC	This protein mediates the voltage-dependent sodium ion permeability of excitable membranes
P	P35499	CC	This sodium channel may be present in both denervated and innervated skeletal muscle
P	P35499	DE	Sodium channel protein skeletal muscle subunit alpha;
P	P35499	DE	Sodium channel protein type 4 subunit alpha;
P	P35499	DE	Sodium channel protein type IV subunit alpha;
P	P35499	DE	Voltage-gated sodium channel subunit alpha Nav1.4;
P	P35499	DR	voltage-gated sodium channel activity
P	P35523	CC	Chloride channels have several functions including the regulation of cell volume; membrane potential stabilization, signal transduction and transepithelial transport
P	P35523	CC	Voltage-gated chloride channel
P	P35523	DE	Chloride channel protein 1;
P	P35523	DE	Chloride channel protein, skeletal muscle;
P	P35523	DR	voltage-gated chloride channel activity
P	P35561	CC	Inward rectifier potassium channels are characterized by a greater tendency to allow potassium to flow into the cell rather than out of it
P	P35561	CC	Their voltage dependence is regulated by the concentration of extracellular potassium; as external potassium is raised, the voltage range of the channel opening shifts to more positive voltages
P	P35561	DE	Inward rectifier K+ channel Kir2.1;
P	P35561	DE	Inward rectifier potassium channel 2;
P	P35561	DE	Potassium channel, inwardly rectifying subfamily J member 2;
P	P35561	DR	inward rectifier potassium channel activity
P	P35670	CC	Involved in the export of copper out of the cells, such as the efflux of hepatic copper into the bile.
P	P35670	DE	Copper pump 2;
P	P35670	DE	Copper-transporting ATPase 2;
P	P35670	DR	copper-exporting ATPase activity
P	P35755	DE	Iron-regulated 40 kDa protein;
P	P35755	DE	Iron-utilization periplasmic protein;
P	P35755	DE	Major ferric iron-binding protein;
P	P35839	DE	Probable formate transporter;
P	P35865	CC	Permease that is involved in the transport across the membrane of lysine.
P	P36021	DE	Monocarboxylate transporter 7;
P	P36021	DE	Monocarboxylate transporter 8;
P	P36035	CC	Essential to lactate transport.
P	P36035	DR	lactate transmembrane transporter activity
P	P36371	CC	Also acts as a molecular scaffold for the final stage of MHC class I folding, namely the binding of peptide
P	P36371	DE	Antigen peptide transporter 2;
P	P36371	DR	peptide transporter activity
P	P36606	CC	Sodium export from cell, takes up external protons in exchange for internal sodium ions
P	P36606	DE	Na+/H+ antiporter;
P	P36606	DR	sodium:hydrogen antiporter activity
P	P36634	CC	Involved in a peptide intake transport system that plays a role in the resistance to antimicrobial peptides.
P	P36634	DE	Peptide transport periplasmic protein sapA;
P	P36636	CC	Involved in a peptide intake transport system that plays a role in the resistance to antimicrobial peptides.
P	P36636	DE	Peptide transport system ATP-binding protein SapD;
P	P36636	DR	peptide transporter activity
P	P36638	CC	Involved in a peptide intake transport system that plays a role in the resistance to antimicrobial peptides.
P	P36638	DE	Peptide transport system ATP-binding protein SapF;
P	P36640	CC	Mediates magnesium influx to the cytosol.
P	P36640	DE	Magnesium-transporting ATPase, P-type 1;
P	P36640	DE	Mg2+ transport ATPase, P-type 1;
P	P36640	DR	magnesium-importing ATPase activity
P	P36672	CC	This system is involved in trehalose transport.
P	P36672	DE	PTS system trehalose-specific EIIB component;

P	P36672	DE	PTS system trehalose-specific EIIBC component;
P	P36672	DE	PTS system trehalose-specific EIIC component;
P	P36672	DE	Trehalose permease IIC component;
P	P36672	DE	Trehalose-specific phosphotransferase enzyme IIB component;
P	P36672	DR	trehalose transmembrane transporter activity
P	P37020	CC	Potential voltage-gated chloride channel.
P	P37020	DE	Voltage-gated chloride channel;
P	P37020	DR	voltage-gated chloride channel activity
P	P37080	CC	This system is involved in sorbose transport.
P	P37080	DE	PTS system sorbose-specific EIIA component;
P	P37080	DE	Sorbose-specific phosphotransferase enzyme IIA component;
P	P37081	CC	This system is involved in sorbose transport.
P	P37081	DE	PTS system sorbose-specific EIIB component;
P	P37081	DE	Sorbose-specific phosphotransferase enzyme IIB component;
P	P37082	CC	This system is involved in sorbose transport.
P	P37082	DE	PTS system sorbose-specific EIIC component;
P	P37082	DE	Sorbose permease IIC component;
P	P37083	CC	This system is involved in sorbose transport.
P	P37083	DE	PTS system sorbose-specific EIID component;
P	P37083	DE	Sorbose permease IID component;
P	P37088	CC	Controls the reabsorption of sodium in kidney, colon, lung and sweat glands
P	P37088	CC	Sodium permeable non-voltage-sensitive ion channel inhibited by the diuretic amiloride
P	P37088	DE	Amiloride-sensitive sodium channel subunit alpha;
P	P37088	DE	Epithelial Na+ channel subunit alpha;
P	P37088	DE	Nonvoltage-gated sodium channel 1 subunit alpha;
P	P37188	CC	This system is involved in galactitol transport.
P	P37188	DE	Galactitol-specific phosphotransferase enzyme IIB component;
P	P37188	DE	PTS system galactitol-specific EIIB component;
P	P37329	CC	Binds molybdate with high specificity and affinity.
P	P37329	DE	Molybdate-binding periplasmic protein;
P	P37329	DR	molybdate transmembrane-transporting ATPase activity
P	P37387	CC	Binds with high affinity to xylose.
P	P37388	CC	Part of the ABC transporter complex XylFGH involved in xylose import
P	P37388	CC	The XylFGH system can also transport ribose in absence of xylose.
P	P37388	DE	Xylose import ATP-binding protein XylG;
P	P37439	CC	This system is involved in glucose transport (By similarity).
P	P37439	DE	Glucose permease IIC component;
P	P37439	DE	Glucose-specific phosphotransferase enzyme IIB component;
P	P37439	DE	PTS system glucose-specific EIIB component;
P	P37439	DE	PTS system glucose-specific EIIC component;
P	P37439	DE	PTS system glucose-specific EIICB component;
P	P37439	DR	glucose transmembrane transporter activity
P	P37460	CC	Permease that is involved in the transport across the cytoplasmic membrane of proline
P	P37460	DE	Proline-specific permease ProY;
P	P37617	CC	Involved in export of lead, cadmium, zinc and mercury.
P	P37617	CC	Involved in export of lead, cadmium, zinc and mercury.
P	P37617	CC	Involved in export of lead, cadmium, zinc and mercury.
P	P37617	DE	Lead, cadmium, zinc and mercury-transporting ATPase;
P	P37617	DE	Lead, cadmium, zinc and mercury-transporting ATPase;
P	P37617	DE	Lead, cadmium, zinc and mercury-transporting ATPase;
P	P37617	DR	cadmium-exporting ATPase activity
P	P37617	DR	zinc-exporting ATPase activity
P	P37758	CC	Involved in excretion of nitrite produced by the dissimilatory reduction of nitrate.
P	P37758	DE	Nitrite extrusion protein 2;
P	P37758	DE	Nitrite facilitator 2;
P	P37758	DR	nitrite efflux transmembrane transporter activity
P	P37902	CC	Binds to both aspartate and glutamate.
P	P37902	CC	Part of the binding-protein-dependent transport system for glutamate and aspartate
P	P37902	CC	Part of the binding-protein-dependent transport system for glutamate and aspartate
P	P37902	DE	Glutamate/aspartate periplasmic-binding protein;
P	P37902	DE	Glutamate/aspartate periplasmic-binding protein;
P	P37972	DE	Nickel and cobalt resistance protein CnrA;
P	P38043	CC	Essential component of the nitrate-transporting system
P	P38043	DE	Nitrate transport protein NrtA;

P	P38044	CC	Probably part of a high-affinity binding-protein- dependent transport system for nitrate; probably responsible for the translocation of the substrate across the membrane (By similarity).
P	P38044	DE	Nitrate transport permease protein nrtB;
P	P38044	DR	nitrate transmembrane transporter activity
P	P38045	CC	Probably part of a high-affinity binding-protein- dependent transport system for nitrate
P	P38045	DE	Nitrate transport ATP-binding protein NrtC;
P	P38045	DR	nitrate transmembrane transporter activity
P	P38046	CC	Probably part of a high-affinity binding-protein- dependent transport system for nitrate
P	P38046	DE	Nitrate transport ATP-binding protein NrtD;
P	P38046	DR	nitrate transmembrane transporter activity
P	P38084	CC	Permease for leucine, valine and isoleucine
P	P38084	CC	Permease for leucine, valine and isoleucine
P	P38084	CC	Permease for leucine, valine and isoleucine
P	P38085	CC	High-affinity transport of valine and tyrosine
P	P38085	CC	High-affinity transport of valine and tyrosine
P	P38085	CC	Low- affinity transport of tryptophan
P	P38085	DE	Tyrosine and tryptophan amino acid transporter 1;
P	P38085	DE	Tyrosine and tryptophan amino acid transporter 1;
P	P38085	DE	Valine/tyrosine/tryptophan amino-acid permease 1;
P	P38085	DE	Valine/tyrosine/tryptophan amino-acid permease 1;
P	P38085	DE	Valine/tyrosine/tryptophan amino-acid permease 1;
P	P38085	DR	low-affinity tryptophan transmembrane transporter activity
P	P38152	CC	Transport of citrate across inner mitochondrial membrane.
P	P38152	DE	Citrate transport protein;
P	P38156	CC	High-affinity uptake of maltose and maltotriose
P	P38156	DE	Maltose permease MAL31;
P	P38156	DE	Maltose transport protein MAL31;
P	P38196	CC	High-affinity transport of uridine.
P	P38196	DE	Uridine permease;
P	P38196	DR	uridine transmembrane transporter activity
P	P38359	CC	High affinity uptake of sulfate into the cell.
P	P38359	DE	High-affinity sulfate transporter 1;
P	P38359	DE	Sulfate permease 1;
P	P38359	DR	secondary active sulfate transmembrane transporter activity
P	P38360	CC	Probably involved in copper transport and in the regulation of cellular copper level
P	P38360	DE	Probable copper-transporting ATPase;
P	P38360	DR	copper-exporting ATPase activity
P	P38361	DE	Phosphate permease PHO89;
P	P38361	DR	inorganic phosphate transmembrane transporter activity
P	P38361	DR	sodium:inorganic phosphate symporter activity
P	P38731	CC	Involved in the transport of siderophore ferrichrome and so has a role in iron homeostasis.
P	P38731	DE	Ferrichrome permease;
P	P38734	CC	Very low affinity permease for methionine.
P	P38734	DE	Low-affinity methionine permease;
P	P38734	DR	L-methionine transmembrane transporter activity
P	P38778	CC	High-affinity manganese transporter involved in mobilizing manganese from vesicular stores iin conditions of low manganese ion concentrations.
P	P38778	DE	Manganese transporter SMF2;
P	P38865	CC	Provides bioavailable copper via mobilization of vacuolar copper stores and export to the cytoplasm.
P	P38865	DE	Copper transport protein CTR2;
P	P38865	DE	Copper transporter 2;
P	P38865	DR	copper uptake transmembrane transporter activity
P	P38921	DR	S-adenosylmethionine transmembrane transporter activity
P	P38925	CC	High-affinity manganese transporter involved in manganese uptake from the extracellular environment
P	P38925	DE	Manganese transporter SMF1;
P	P38929	CC	This magnesium-dependent enzyme catalyzes the hydrolysis of ATP coupled with the transport of calcium
P	P38929	CC	Transports the calcium to the vacuole and participates in the control of the cytosolic free calcium.
P	P38929	DE	Calcium-transporting ATPase 2;
P	P38929	DE	Vacuolar Ca2+-ATPase;

P	P38929	DR	calcium-transporting ATPase activity
P	P38967	CC	Required for high-affinity tryptophan transport
P	P38967	DE	Tryptophan amino acid transporter;
P	P38967	DE	Tryptophan permease;
P	P38967	DR	high-affinity tryptophan transmembrane transporter activity
P	P38995	CC	Probably involved in copper transport and in the regulation of cellular copper level
P	P38995	CC	Retrieves copper from the metallochaperone ATX1 and incorporates it into trans-Golgi vesicles.
P	P38995	DE	Copper-transporting ATPase;
P	P38995	DR	copper-exporting ATPase activity
P	P39003	DE	High-affinity hexose transporter HXT6;
P	P39004	DE	High-affinity hexose transporter HXT6;
P	P39109	CC	Cooperates for the ATP-dependent vacuolar transport of bilirubin and glutathione conjugates.
P	P39109	DR	glutathione S-conjugate-exporting ATPase activity
P	P39141	DR	nucleoside:sodium symporter activity
P	P39141	DR	nucleoside:sodium symporter activity
P	P39172	CC	Involved in the high-affinity zinc uptake transport system.
P	P39172	DE	High-affinity zinc uptake system protein znuA;
P	P39344	CC	Transports L-idonate, D-gluconate and 5-keto-D- gluconate, from the periplasm across the inner membrane.
P	P39344	DE	Gnt-II system L-idonate transporter;
P	P39344	DR	L-idonate transmembrane transporter activity
P	P39414	CC	Catalyzes the uptake of tartrate in exchange for intracellular succinate
P	P39414	DE	Tartrate carrier;
P	P39414	DE	Tartrate transporter;
P	P39660	CC	Binds bicarbonate with high affinity.
P	P39660	CC	Part of the ABC transporter complex CmpABCD involved in bicarbonate transport
P	P39660	DE	Bicarbonate-binding protein CmpA;
P	P39775	CC	Involved in a high affinity multicomponent binding- protein-dependent transport system for choline; probably responsible for the translocation of the substrate across the membrane.
P	P39775	DE	Choline transport system permease protein opuBD;
P	P39816	DR	glucose transmembrane transporter activity
P	P39832	CC	Involved in the high-affinity zinc uptake transport system.
P	P39832	DE	High-affinity zinc uptake system membrane protein znuB;
P	P39843	CC	Causes the efflux of a variety of toxic substances, including such structurally diverse compounds as ethidium bromide, rhodamine and acridine dyes, tetraphenylphosphonium, puromycin, chloramphenicol, doxorubicin, and fluoroquinolone antibiotics
P	P39924	CC	Probable glucose transporter.
P	P39924	DE	Hexose transporter HXT13;
P	P39924	DR	glucose transmembrane transporter activity
P	P39932	DE	Sugar transporter STL1;
P	P39980	CC	Involved in the transport of siderophore ferrioxamine B and so has a role in iron homeostasis.
P	P39980	DE	Ferrioxamine B permease;
P	P40035	CC	Transport of phosphate groups from the cytosol to the mitochondrial matrix.
P	P40035	DE	Mitochondrial phosphate carrier protein 2;
P	P40035	DE	Phosphate transport protein 2;
P	P40035	DR	inorganic phosphate transmembrane transporter activity
P	P40039	DE	Cytosine/purine transport protein FCY21;
P	P40310	CC	Outwardly rectifying potassium channel.
P	P40310	DE	Outward-rectifier potassium channel TOK1;
P	P40310	DE	Two-domain outward rectifier K+ channel YORK;
P	P40310	DR	potassium channel activity
P	P40527	DE	Probable phospholipid-transporting ATPase NEO1;
P	P40527	DR	phospholipid-translocating ATPase activity
P	P40790	CC	Part of the ABC transporter complex PotABCD involved in spermidine/putrescine import
P	P40790	CC	Part of the ABC transporter complex PotABCD involved in spermidine/putrescine import
P	P40790	DE	Spermidine/putrescine import ATP-binding protein PotA;
P	P40790	DE	Spermidine/putrescine import ATP-binding protein PotA;
P	P40812	DE	L-asparagine permease;
P	P40812	DE	L-asparagine transport protein;
P	P40860	CC	Part of the ABC transporter complex CysAWTP involved in sulfate/thiosulfate import
P	P40860	DE	Sulfate-transporting ATPase;

P	P40860	DE	Sulfate/thiosulfate import ATP-binding protein CysA;
P	P40860	DR	sulfate transmembrane-transporting ATPase activity
P	P40862	CC	Proton symporter that senses osmotic shifts and responds by importing osmolytes such as proline, glycine betaine, stachydrine, pipecolic acid, ectoine and taurine
P	P40862	DE	Proline porter II;
P	P40862	DE	Proline/betaine transporter;
P	P40862	DE	Proline/betaine transporter;
P	P40879	CC	Chloride/bicarbonate exchanger
P	P40879	DE	Chloride anion exchanger;
P	P40885	DE	Hexose transporter HXT9;
P	P40886	DE	Hexose transporter HXT8;
P	P40897	DE	Oligopeptide transporter 1;
P	P40897	DR	proton-dependent oligopeptide secondary active transmembrane transporter activity
P	P40941	CC	Catalyzes the exchange of ADP and ATP across the mitochondrial inner membrane
P	P40941	CC	Catalyzes the exchange of ADP and ATP across the mitochondrial inner membrane
P	P40941	DE	ADP,ATP carrier protein 2, mitochondrial;
P	P40941	DE	ADP/ATP translocase 2;
P	P40941	DE	ADP/ATP translocase 2;
P	P40941	DR	ATP:ADP antiporter activity
P	P40941	DR	ATP:ADP antiporter activity
P	P40948	CC	Mediates influx of magnesium ions (By similarity).
P	P40948	DE	Magnesium transport protein CorA;
P	P40988	DE	Low-affinity Fe(II) transport protein;
P	P41031	CC	Part of the ABC transporter complex CysAWTP (TC 3.A.1.6.1) involved in sulfate/thiosulfate import
P	P41031	DR	secondary active sulfate transmembrane transporter activity
P	P41031	DR	sulfate transmembrane-transporting ATPase activity
P	P41032	CC	Part of the ABC transporter complex CysAWTP (TC 3.A.1.6.1) involved in sulfate/thiosulfate import
P	P41032	DE	Sulfate transport system permease protein CysT;
P	P41032	DR	sulfate transmembrane transporter activity
P	P41181	CC	Forms a water-specific channel that provides the plasma membranes of renal collecting duct with high permeability to water, thereby permitting water to move in the direction of an osmotic gradient.
P	P41181	DE	ADH water channel;
P	P41181	DE	Collecting duct water channel protein;
P	P41181	DE	Water channel protein for renal collecting duct;
P	P41181	DR	water channel activity
P	P41438	CC	Transporter for the intake of folate, reduced folates and methotrexate.
P	P41438	CC	Transporter for the intake of folate, reduced folates and methotrexate.
P	P41438	DE	Folate transporter 1;
P	P41438	DE	Intestinal folate carrier 1;
P	P41438	DE	Reduced folate carrier 1;
P	P41438	DR	reduced folate carrier activity
P	P41440	CC	Transporter for the intake of folate
P	P41440	CC	Uptake of folate in human placental choriocarcinoma cells occurs by a novel mechanism called potocytosis which functionally couples three components, namely the folate receptor, the folate transporter, and a V-type H+-pump.
P	P41440	DE	Folate transporter 1;
P	P41440	DE	Intestinal folate carrier 1;
P	P41440	DE	Placental folate transporter;
P	P41440	DE	Reduced folate carrier protein;
P	P41440	DR	folic acid transporter activity
P	P41440	DR	reduced folate carrier activity
P	P41815	CC	Involved in transport of isoleucine, leucine, valine, cysteine, methionine, phenylalanine, tyrosine and tryptophan.
P	P41815	CC	Involved in transport of isoleucine, leucine, valine, cysteine, methionine, phenylalanine, tyrosine and tryptophan.
P	P41815	DE	Valine amino-acid permease;
P	P41821	CC	Calcium-permeable, cation-selective stretch-activated channel (SAC)
P	P41821	CC	Functions together with CCH1 to ensure that adequate levels of Ca2+ are supplied to PMR1 to sustain secretion and growth
P	P41821	CC	Required for calcium influx and for vitality of MATa cells in a late, pheromone-induced event of the mating process requiring calcium induced signaling
P	P41821	CC	Required for growth in low- calcium environments

P	P41821	DR	calcium channel activity
P	P42086	CC	Transport of xanthine in the cell.
P	P42086	DE	Xanthine permease;
P	P42087	DE	Putative histidine permease;
P	P42237	DE	Probable glucarate transporter;
P	P42308	CC	Probably cotransports citrate and at least three or four protons
P	P42308	CC	The citrate uptake is inhibited by the presence of magnesium ions.
P	P42308	CC	Transports the free citrate anion
P	P42308	DE	Citrate transporter;
P	P42308	DR	citrate transmembrane transporter activity
P	P42432	DE	Nitrate transporter;
P	P42670	CC	In addition, it could be implicated in secreting N-acetylpuromycin, the last intermediate of the puromycin biosynthesis pathway, to the environment.
P	P42670	CC	May be involved in active puromycin efflux energized by a proton-dependent electrochemical gradient
P	P42670	DE	Puromycin resistance protein pur8;
P	P42833	CC	Probable glucose transporter.
P	P42833	DE	Hexose transporter HXT14;
P	P42953	DE	Teichoic acid translocation permease protein tagG;
P	P42954	DE	Teichoic acid-transporting ATPase;
P	P43003	CC	Acts as a symport by cotransporting sodium.
P	P43003	CC	Essential for terminating the postsynaptic action of glutamate by rapidly removing released glutamate from the synaptic cleft
P	P43003	CC	Transports L-glutamate and also L- and D-aspartate
P	P43003	DE	Sodium-dependent glutamate/aspartate transporter 1;
P	P43003	DE	Sodium-dependent glutamate/aspartate transporter 1;
P	P43003	DR	high-affinity glutamate transmembrane transporter activity
P	P43003	DR	sodium:dicarboxylate symporter activity
P	P43004	CC	Acts as a symport by cotransporting sodium.
P	P43004	CC	Essential for terminating the postsynaptic action of glutamate by rapidly removing released glutamate from the synaptic cleft
P	P43004	CC	Transports L-glutamate and also L- and D-aspartate
P	P43004	DE	Glutamate/aspartate transporter II;
P	P43004	DE	Glutamate/aspartate transporter II;
P	P43004	DE	Sodium-dependent glutamate/aspartate transporter 2;
P	P43004	DE	Sodium-dependent glutamate/aspartate transporter 2;
P	P43004	DE	Sodium-dependent glutamate/aspartate transporter 2;
P	P43004	DR	high-affinity glutamate transmembrane transporter activity
P	P43004	DR	sodium:dicarboxylate symporter activity
P	P43005	CC	Acts as a symport by cotransporting sodium
P	P43005	DE	Sodium-dependent glutamate/aspartate transporter 3;
P	P43005	DR	sodium:dicarboxylate symporter activity
P	P43006	CC	Acts as a symport by cotransporting sodium.
P	P43006	CC	Essential for terminating the postsynaptic action of glutamate by rapidly removing released glutamate from the synaptic cleft
P	P43006	CC	Transports L-glutamate and also L- and D-aspartate
P	P43006	DE	Sodium-dependent glutamate/aspartate transporter 2;
P	P43006	DE	Sodium-dependent glutamate/aspartate transporter 2;
P	P43006	DE	Sodium-dependent glutamate/aspartate transporter 2;
P	P43006	DR	glutamate:sodium symporter activity
P	P43006	DR	glutamate:sodium symporter activity
P	P43006	DR	sodium:dicarboxylate symporter activity
P	P43007	CC	Exhibits sodium dependence.
P	P43007	DR	sodium:dicarboxylate symporter activity
P	P43109	CC	May form an ATP-driven Vi polysaccharide export apparatus, in association with the vexA, vexC and vexD proteins.
P	P43109	DE	Vi polysaccharide export inner-membrane protein vexB;
P	P43110	CC	May form an ATP-driven Vi polysaccharide export apparatus, in association with the VexA, VexB and VexD proteins.
P	P43110	DE	Vi polysaccharide export ATP-binding protein VexC;
P	P43286	CC	Probably involved in root water uptake
P	P43286	CC	Water channel required to facilitate the transport of water across cell membrane
P	P43286	DR	water channel activity
P	P43287	CC	Plays an predominant role in root water uptake process in conditions of reduced transpiration, and in osmotic fluid transport

P	P43287	CC	Water channel required to facilitate the transport of water across cell membrane
P	P43287	DR	water channel activity
P	P43436	DE	Na+-translocating ATPase subunit E;
P	P43436	DE	V-type sodium ATPase subunit E;
P	P43436	DE	V-type sodium pump subunit E;
P	P43437	DE	Na+-translocating ATPase subunit F;
P	P43437	DE	V-type sodium ATPase subunit F;
P	P43437	DE	V-type sodium pump subunit F;
P	P43439	DE	Na+-translocating ATPase subunit I;
P	P43439	DE	V-type sodium ATPase subunit I;
P	P43439	DE	V-type sodium pump subunit I;
P	P43455	DE	Na+-translocating ATPase subunit G;
P	P43455	DE	V-type sodium ATPase subunit G;
P	P43455	DE	V-type sodium pump subunit G;
P	P43456	DE	Na+-translocating ATPase subunit C;
P	P43456	DE	V-type sodium ATPase subunit C;
P	P43456	DE	V-type sodium pump subunit C;
P	P43457	DE	Na+-translocating ATPase subunit K;
P	P43457	DE	Sodium ATPase proteolipid component;
P	P43457	DE	V-type sodium ATPase subunit K;
P	P43466	DE	Raffinose carrier protein;
P	P43466	DE	Raffinose permease;
P	P43581	DE	Hexose transporter HXT10;
P	P43756	CC	Involved in the bidirectional transport of formate (By similarity).
P	P43756	DE	Formate channel;
P	P43756	DE	Probable formate transporter;
P	P44051	DR	short-chain fatty acid transporter activity
P	P44526	CC	Involved in the high-affinity zinc uptake transport system (By similarity).
P	P44526	DE	High-affinity zinc uptake system protein znuA;
P	P44535	CC	The physiological role may be the reduction of the intracellular concentration of toxic sugars or sugar metabolites (By similarity).
P	P44535	DE	Probable sugar efflux transporter;
P	P44581	CC	Na+/H+ antiporter that extrudes sodium in exchange for external protons (By [illegible]
P	P44581	DE	Na+/H+ antiporter nhaA;
P	P44581	DE	Sodium/proton antiporter nhaA;
P	P44614	CC	Involved in transporting tryptophan across the cytoplasmic membrane (By [illegible]
P	P44614	DE	Tryptophan permease;
P	P44614	DE	Tryptophan-specific transport protein;
P	P44615	CC	Involved in the import of serine into the cell (By similarity).
P	P44615	DE	Serine transporter;
P	P44629	DE	Probable 3-phenylpropionic acid transporter;
P	P44691	CC	Involved in the high-affinity zinc uptake transport system (By similarity).
P	P44691	DE	High-affinity zinc uptake system membrane protein znuB;
P	P44692	CC	Part of the ABC transporter complex ZnuABC involved in zinc import
P	P44692	DE	Zinc import ATP-binding protein ZnuC;
P	P44692	DR	zinc transporting ATPase activity
P	P44706	CC	Na+/H+ antiporter that extrudes sodium in exchange for external protons (By [illegible]
P	P44706	DE	Na+/H+ antiporter nhaB;
P	P44706	DE	Sodium/proton antiporter nhaB;
P	P44706	DR	sodium:hydrogen antiporter activity
P	P44714	CC	This system is involved in fructose transport.
P	P44714	DE	Fructose permease IIC component;
P	P44714	DE	Fructose-specific phosphotransferase enzyme IIB component;
P	P44714	DE	PTS system fructose-specific EIIB component;
P	P44714	DE	PTS system fructose-specific EIIBC component;
P	P44714	DE	PTS system fructose-specific EIIC component;
P	P44715	CC	This system is involved in fructose transport (By similarity).
P	P44715	DE	Fructose PTS diphosphoryl transfer protein;
P	P44715	DE	Fructose-specific phosphotransferase enzyme IIA component;
P	P44715	DE	PTS system fructose-specific EIIA component;
P	P44727	CC	Involved in transporting tyrosine across the cytoplasmic membrane (By [illegible]
P	P44727	DE	Tyrosine permease 1;
P	P44727	DE	Tyrosine-specific transport protein 1;
P	P44731	CC	Required for the activity of the bacterial periplasmic transport system of putrescine and spermidine

P	P44731	CC	Required for the activity of the bacterial periplasmic transport system of putrescine and spermidine
P	P44731	DE	Spermidine/putrescine-binding periplasmic protein 2;
P	P44731	DE	Spermidine/putrescine-binding periplasmic protein 2;
P	P44735	CC	Part of the ABC transporter complex RbsABCD involved in ribose import
P	P44735	DE	Ribose import ATP-binding protein RbsA;
P	P44736	CC	Part of the binding-protein-dependent transport system for ribose
P	P44736	DE	Ribose transport system permease protein rbsC;
P	P44737	CC	Involved in the high-affinity D-ribose membrane transport system and also serves as the primary chemoreceptor for chemotaxis (By similarity).
P	P44737	DE	D-ribose-binding periplasmic protein;
P	P44747	CC	Involved in transporting tyrosine across the cytoplasmic membrane (By similarity)
P	P44747	DE	Tyrosine permease 2;
P	P44747	DE	Tyrosine-specific transport protein 2;
P	P44768	CC	Probable putrescine-ornithine antiporter (By similarity).
P	P44768	DE	Putrescine transport protein;
P	P44768	DE	Putrescine-ornithine antiporter;
P	P44776	CC	Transport of L-fucose into the cell.
P	P44776	DE	L-fucose permease;
P	P44785	CC	Part of the ABC transporter complex MetNIQ involved in methionine import
P	P44785	DE	Methionine import ATP-binding protein MetN;
P	P44785	DR	D-methionine transmembrane transporter activity
P	P44826	CC	Glycerol enters the cell via the glycerol diffusion facilitator protein
P	P44826	CC	This membrane protein facilitates the movement of glycerol across the cytoplasmic membrane (By similarity).
P	P44826	DE	Glycerol uptake facilitator protein;
P	P44843	CC	Interacts with trk system potassium uptake protein trkA and requires trkE for transport activity (By similarity).
P	P44843	CC	Low-affinity potassium transport system
P	P44843	DE	Trk system potassium uptake protein trkH;
P	P44883	CC	This protein is involved in the active transport of galactose and glucose (By similarity)
P	P44884	CC	Part of the ABC transporter complex MglABC involved in galactose/methyl galactoside import
P	P44884	DE	Galactose/methyl galactoside import ATP-binding protein MglA;
P	P44933	CC	Transport system that facilitate potassium-efflux, possibly by potassium-proton antiport (By similarity).
P	P44933	DE	Glutathione-regulated potassium-efflux system protein;
P	P44933	DE	K+/H+ antiporter;
P	P44963	CC	Catalyzes the sodium-dependent uptake of extracellular pantothenate (By similarity)
P	P44963	CC	Catalyzes the sodium-dependent uptake of extracellular pantothenate (By similarity)
P	P44963	DE	Pantothenate permease;
P	P44963	DE	Sodium/pantothenate symporter;
P	P44963	DE	Sodium/pantothenate symporter;
P	P44963	DR	pantothenate transmembrane transporter activity
P	P44963	DR	sodium ion transmembrane transporter activity
P	P44984	CC	Part of the ABC transporter complex thiBPQ involved in thiamine import (By similarity)
P	P44984	DE	Thiamine-binding periplasmic protein;
P	P44985	CC	Part of the ABC transporter complex thiBPQ involved in thiamine import
P	P44985	DE	Thiamine transport system permease protein thiP;
P	P44986	CC	Part of the ABC transporter complex ThiBPQ involved in thiamine import
P	P44986	DE	Thiamine import ATP-binding protein ThiQ;
P	P44998	CC	Mediates influx of magnesium ions (By similarity).
P	P44998	DE	Magnesium transport protein CorA;
P	P44998	DR	cobalt ion transmembrane transporter activity
P	P44998	DR	magnesium ion transmembrane transporter activity
P	P45032	CC	Part of the ABC transporter complex CcmAB involved in the biogenesis of c-type cytochromes; once thought to export heme, this seems not to be the case, but its exact role is uncertain
P	P45032	DE	Heme exporter protein A;
P	P45032	DR	heme-transporting ATPase activity
P	P45033	CC	Required for the export of heme to the periplasm for the biogenesis of c-type cytochromes (By similarity).
P	P45033	DE	Heme exporter protein B;
P	P45033	DR	heme transporter activity

P	P45034	CC	Required for the export of heme to the periplasm for the biogenesis of c-type cytochromes (By similarity).
P	P45034	DE	Heme exporter protein C;
P	P45034	DR	heme transporter activity
P	P45045	CC	Part of the binding-protein-dependent transport system for D-xylose
P	P45045	DE	Xylose transport system permease protein xylH;
P	P45046	CC	Part of the ABC transporter complex XylFGH involved in xylose import
P	P45046	DE	Xylose import ATP-binding protein XylG;
P	P45046	DR	D-xylose-importing ATPase activity
P	P45047	CC	Involved in the high-affinity D-xylose membrane transport system
P	P45047	DE	D-xylose-binding periplasmic protein;
P	P45051	DE	Oligopeptide transport ATP-binding protein OppF;
P	P45052	DE	Oligopeptide transport ATP-binding protein OppD;
P	P45053	DE	Oligopeptide transport system permease protein oppC;
P	P45054	DE	Oligopeptide transport system permease protein oppB;
P	P45089	CC	Part of the ABC transporter complex ArtPIQM involved in arginine transport
P	P45089	DE	Arginine ABC transporter permease protein ArtM;
P	P45090	CC	Part of the ABC transporter complex ArtPIQM involved in arginine transport
P	P45090	DE	Arginine ABC transporter permease protein ArtQ;
P	P45091	CC	Part of the ABC transporter complex ArtPIQM involved in arginine transport (By similarity).
P	P45091	DE	ABC transporter arginine-binding protein;
P	P45092	CC	Part of the ABC transporter complex ArtPIQM involved in arginine transport
P	P45092	DE	Arginine transport ATP-binding protein ArtP;
P	P45094	DE	Dipeptide transport ATP-binding protein DppF;
P	P45095	DE	Dipeptide transport ATP-binding protein DppD;
P	P45096	DE	Dipeptide transport system permease protein dppB;
P	P45117	CC	Transport of uracil in the cell (By similarity).
P	P45117	DE	Probable uracil permease;
P	P45117	DE	Uracil transporter;
P	P45168	CC	Required for the activity of the bacterial periplasmic transport system of putrescine and spermidine
P	P45168	CC	Required for the activity of the bacterial periplasmic transport system of putrescine and spermidine
P	P45168	DE	Spermidine/putrescine-binding periplasmic protein 1;
P	P45168	DE	Spermidine/putrescine-binding periplasmic protein 1;
P	P45169	CC	Required for the activity of the bacterial periplasmic transport system of putrescine and spermidine (By similarity).
P	P45169	CC	Required for the activity of the bacterial periplasmic transport system of putrescine and spermidine (By similarity).
P	P45169	DE	Spermidine/putrescine transport system permease protein PotC;
P	P45169	DE	Spermidine/putrescine transport system permease protein PotC;
P	P45170	CC	Required for the activity of the bacterial periplasmic transport system of putrescine and spermidine (By similarity).
P	P45170	CC	Required for the activity of the bacterial periplasmic transport system of putrescine and spermidine (By similarity).
P	P45170	DE	Spermidine/putrescine transport system permease protein PotB;
P	P45170	DE	Spermidine/putrescine transport system permease protein PotB;
P	P45171	CC	Part of the ABC transporter complex PotABCD involved in spermidine/putrescine import
P	P45171	CC	Part of the ABC transporter complex PotABCD involved in spermidine/putrescine import
P	P45171	DE	Spermidine/putrescine import ATP-binding protein PotA;
P	P45171	DE	Spermidine/putrescine import ATP-binding protein PotA;
P	P45174	CC	Catalyzes the sodium-dependent uptake of extracellular L-proline (By similarity).
P	P45174	DE	Proline permease;
P	P45174	DE	Sodium/proline symporter;
P	P45174	DE	Sodium/proline symporter;
P	P45174	DR	proline:sodium symporter activity
P	P45174	DR	proline:sodium symporter activity
P	P45190	CC	Part of the binding-protein-dependent transport system for phosphate; probably responsible for the translocation of the substrate across the membrane (By similarity).
P	P45190	DE	Phosphate transport system permease protein pstA;
P	P45190	DR	inorganic phosphate transmembrane transporter activity
P	P45191	CC	Part of the binding-protein-dependent transport system for phosphate; probably responsible for the translocation of the substrate across the membrane (By similarity).
P	P45191	DE	Phosphate transport system permease protein pstC;

P	P45191	DR	inorganic phosphate transmembrane transporter activity
P	P45192	CC	Part of the ABC transporter complex pstSACB involved in phosphate import (By similarity).
P	P45192	DE	Phosphate-binding protein pstS;
P	P45192	DR	inorganic phosphate transmembrane transporter activity
P	P45240	CC	Catalyzes the sodium-dependent uptake of extracellular glutamate (By similarity).
P	P45240	CC	Catalyzes the sodium-dependent uptake of extracellular glutamate (By similarity).
P	P45240	DE	Glutamate permease;
P	P45240	DE	Sodium/glutamate symport carrier protein;
P	P45240	DE	Sodium/glutamate symport carrier protein;
P	P45240	DR	glutamate:sodium symporter activity
P	P45240	DR	glutamate:sodium symporter activity
P	P45246	CC	Involved in the import of serine and threonine into the cell, with the concomitant import of sodium (symport system) (By similarity).
P	P45246	DE	Na+/serine-threonine symporter;
P	P45246	DR	sodium:dicarboxylate symporter activity
P	P45246	DR	sodium:dicarboxylate symporter activity
P	P45247	DE	Lipoprotein-releasing system ATP-binding protein LolD;
P	P45247	DR	lipoprotein transporter activity
P	P45285	CC	Involved in a peptide intake transport system that plays a role in the resistance to antimicrobial peptides (By similarity).
P	P45285	DE	Peptide transport periplasmic protein sapA;
P	P45286	CC	Involved in a peptide intake transport system that plays a role in the resistance to antimicrobial peptides (By similarity).
P	P45286	DE	Peptide transport system permease protein sapB;
P	P45287	CC	Involved in a peptide intake transport system that plays a role in the resistance to antimicrobial peptides (By similarity).
P	P45287	DE	Peptide transport system permease protein sapC;
P	P45288	CC	Involved in a peptide intake transport system that plays a role in the resistance to antimicrobial peptides (By similarity).
P	P45288	DE	Peptide transport system ATP-binding protein SapD;
P	P45288	DR	peptide transporter activity
P	P45289	CC	Involved in a peptide intake transport system that plays a role in the resistance to antimicrobial peptides (By similarity).
P	P45289	DE	Peptide transport system ATP-binding protein SapF;
P	P45321	CC	Part of the ABC transporter complex ModABC involved in molybdenum import
P	P45321	DE	Molybdenum import ATP-binding protein ModC;
P	P45321	DR	molybdenum ion transmembrane transporter activity
P	P45322	CC	Part of the binding-protein-dependent transport system for molybdenum; probably responsible for the translocation of the substrate across the membrane (By similarity).
P	P45322	DE	Molybdenum transport system permease protein modB;
P	P45323	CC	Involved in the transport of molybdenum into the cell (By similarity).
P	P45338	CC	This system is involved in glucose transport (By similarity).
P	P45338	DE	Glucose-specific phosphotransferase enzyme IIA component;
P	P45338	DE	PTS system glucose-specific EIIA component;
P	P45356	DE	Heme/hemopexin transporter protein huxB;
P	P45562	DE	Xanthosine permease;
P	P45562	DE	Xanthosine transporter;
P	P45618	CC	This system is involved in glucose transport.
P	P45618	DE	Glucose-specific phosphotransferase enzyme IIA component;
P	P45618	DE	PTS system glucose-specific EIIA component;
P	P46032	CC	Can also transport histidine.
P	P46032	DE	Histidine-transporting protein;
P	P46059	DE	Oligopeptide transporter, small intestine isoform;
P	P46104	CC	May mediate the efflux of lincomycin.
P	P46104	DE	Lincomycin resistance protein;
P	P46339	DR	inorganic phosphate transmembrane transporter activity
P	P46340	DR	inorganic phosphate transmembrane transporter activity
P	P46341	CC	Part of the ABC transporter complex PstSACB involved in phosphate import
P	P46341	DE	ABC phosphate transporter 2;
P	P46341	DE	Phosphate import ATP-binding protein PstB 2;
P	P46341	DE	Phosphate-transporting ATPase 2;
P	P46341	DR	inorganic phosphate transmembrane transporter activity
P	P46341	DR	phosphate transmembrane-transporting ATPase activity
P	P46342	CC	Part of the ABC transporter complex PstSACB involved in phosphate import

P	P46342	DE	ABC phosphate transporter 1;
P	P46342	DE	Phosphate import ATP-binding protein PstB 1;
P	P46342	DE	Phosphate-transporting ATPase 1;
P	P46342	DR	inorganic phosphate transmembrane transporter activity
P	P46342	DR	phosphate transmembrane-transporting ATPase activity
P	P46838	DR	arsenite transmembrane transporter activity
P	P46903	CC	Involved in ATP-dependent electrogenic sodium extrusion.
P	P46903	DE	Na+ ABC transporter;
P	P46904	CC	Involved in ATP-dependent electrogenic sodium extrusion.
P	P46920	CC	Involved in a multicomponent binding-protein-dependent transport system for glycine betaine
P	P46920	DE	Glycine betaine transport ATP-binding protein OpuAA;
P	P46921	CC	Involved in a multicomponent binding-protein-dependent transport system for glycine betaine; probably responsible for the translocation of the substrate across the membrane
P	P46921	DE	Glycine betaine transport system permease protein opuAB;
P	P46922	CC	Involved in a multicomponent binding-protein-dependent transport system for glycine betaine.
P	P46922	DE	Glycine betaine-binding protein;
P	P47185	DE	Hexose transporter HXT16;
P	P47279	CC	Glycerol enters the cell via the glycerol diffusion facilitator protein
P	P47279	CC	This membrane protein facilitates the movement of glycerol across the cytoplasmic membrane (By similarity).
P	P47279	DE	Probable glycerol uptake facilitator protein;
P	P47288	CC	Part of the ABC transporter complex PotABCD involved in spermidine/putrescine import
P	P47288	CC	Part of the ABC transporter complex PotABCD involved in spermidine/putrescine import
P	P47288	DE	Spermidine/putrescine import ATP-binding protein PotA;
P	P47288	DE	Spermidine/putrescine import ATP-binding protein PotA;
P	P47289	CC	Required for the activity of the bacterial transport system of putrescine and spermidine (By similarity).
P	P47289	CC	Required for the activity of the bacterial transport system of putrescine and spermidine (By similarity).
P	P47289	DE	Spermidine/putrescine transport system permease protein PotB homolog;
P	P47289	DE	Spermidine/putrescine transport system permease protein PotB homolog;
P	P47290	CC	Required for the activity of the bacterial transport system of putrescine and spermidine (By similarity).
P	P47290	CC	Required for the activity of the bacterial transport system of putrescine and spermidine (By similarity).
P	P47290	DE	Spermidine/putrescine transport system permease protein PotC homolog;
P	P47290	DE	Spermidine/putrescine transport system permease protein PotC homolog;
P	P47308	CC	This system is involved in fructose transport.
P	P47308	DE	Fructose permease IIC component;
P	P47308	DE	Fructose-specific phosphotransferase enzyme IIA component;
P	P47308	DE	Fructose-specific phosphotransferase enzyme IIB component;
P	P47308	DE	PTS system fructose-specific EIIA component;
P	P47308	DE	PTS system fructose-specific EIIABC component;
P	P47308	DE	PTS system fructose-specific EIIB component;
P	P47308	DE	PTS system fructose-specific EIIC component;
P	P47315	CC	This system is involved in glucose transport.
P	P47315	DE	Glucose permease IIC component;
P	P47315	DE	Glucose-specific phosphotransferase enzyme IIA component;
P	P47315	DE	Glucose-specific phosphotransferase enzyme IIB component;
P	P47315	DE	PTS system glucose-specific EIIA component;
P	P47315	DE	PTS system glucose-specific EIIB component;
P	P47315	DE	PTS system glucose-specific EIIC component;
P	P47315	DE	PTS system glucose-specific EIICBA component;
P	P47323	DE	Oligopeptide transport system permease protein oppB;
P	P47324	DE	Oligopeptide transport system permease protein oppC;
P	P47325	DE	Oligopeptide transport ATP-binding protein OppD;
P	P47326	DE	Oligopeptide transport ATP-binding protein OppF;
P	P47650	CC	Part of the ABC transporter complex PstSACB involved in phosphate import
P	P47650	DE	ABC phosphate transporter;
P	P47650	DE	Phosphate import ATP-binding protein PstB;
P	P47650	DE	Phosphate-transporting ATPase;
P	P47650	DR	inorganic phosphate transmembrane transporter activity
P	P47650	DR	phosphate transmembrane-transporting ATPase activity

P	P47651	CC	Could be part of a binding-protein-dependent transport system for phosphate; probably responsible for the translocation of the substrate across the membrane (By similarity)
P	P47651	DE	Phosphate transport system permease protein pstA homolog;
P	P47651	DR	inorganic phosphate transmembrane transporter activity
P	P47869	CC	GABA, the major inhibitory neurotransmitter in the vertebrate brain, mediates neuronal inhibition by binding to the GABA/benzodiazepine receptor and opening an integral chloride channel.
P	P47869	DR	chloride channel activity
P	P47870	CC	GABA, the major inhibitory neurotransmitter in the vertebrate brain, mediates neuronal inhibition by binding to the GABA/benzodiazepine receptor and opening an integral chloride channel.
P	P47870	DR	chloride channel activity
P	P47946	DE	Potassium transport protein 1;
P	P47946	DR	potassium ion transmembrane transporter activity
P	P48029	CC	Required for the uptake of creatine in muscles and brain.
P	P48029	DE	Creatine transporter 1;
P	P48029	DE	Sodium- and chloride-dependent creatine transporter 1;
P	P48029	DE	Sodium- and chloride-dependent creatine transporter 1;
P	P48029	DR	creatine:sodium symporter activity
P	P48029	DR	creatine:sodium symporter activity
P	P48029	DR	neurotransmitter:sodium symporter activity
P	P48048	CC	In the kidney, probably plays a major role in potassium homeostasis
P	P48048	CC	Inward rectifier potassium channels are characterized by a greater tendency to allow potassium to flow into the cell rather than out of it
P	P48048	CC	Their voltage dependence is regulated by the concentration of extracellular potassium; as external potassium is raised, the voltage range of the channel opening shifts to more positive voltages
P	P48048	DE	ATP-regulated potassium channel ROM-K;
P	P48048	DE	ATP-sensitive inward rectifier potassium channel 1;
P	P48048	DE	Inward rectifier K+ channel Kir1.1;
P	P48048	DE	Potassium channel, inwardly rectifying subfamily J member 1;
P	P48048	DR	inward rectifier potassium channel activity
P	P48050	CC	Inward rectifier potassium channels are characterized by a greater tendency to allow potassium to flow into the cell rather than out of it
P	P48050	CC	Their voltage dependence is regulated by the concentration of extracellular potassium; as external potassium is raised, the voltage range of the channel opening shifts to more positive voltages
P	P48050	DE	Inward rectifier K+ channel Kir2.3;
P	P48050	DE	Inward rectifier potassium channel 4;
P	P48050	DE	Potassium channel, inwardly rectifying subfamily J member 4;
P	P48050	DR	inward rectifier potassium channel activity
P	P48051	CC	Inward rectifier potassium channels are characterized by a greater tendency to allow potassium to flow into the cell rather than out of it
P	P48051	CC	Their voltage dependence is regulated by the concentration of extracellular potassium; as external potassium is raised, the voltage range of the channel opening shifts to more positive voltages
P	P48051	CC	This potassium channel may be involved in the regulation of insulin secretion by glucose and/or neurotransmitters acting through G-protein-coupled receptors
P	P48051	DE	G protein-activated inward rectifier potassium channel 2;
P	P48051	DE	Inward rectifier K+ channel Kir3.2;
P	P48051	DE	Potassium channel, inwardly rectifying subfamily J member 6;
P	P48051	DR	G-protein activated inward rectifier potassium channel activity
P	P48065	CC	May have a role in regulation of GABAergic transmission in the brain through the reuptake of GABA into presynaptic terminals, as well as in osmotic regulation
P	P48065	CC	Transports betaine and GABA
P	P48065	CC	Transports betaine and GABA
P	P48065	DE	Na+/Cl(-) betaine/GABA transporter;
P	P48065	DE	Na+/Cl(-) betaine/GABA transporter;
P	P48065	DE	Na+/Cl(-) betaine/GABA transporter;
P	P48065	DE	Sodium- and chloride-dependent betaine transporter;
P	P48065	DE	Sodium- and chloride-dependent betaine transporter;
P	P48065	DR	gamma-aminobutyric acid:sodium symporter activity
P	P48065	DR	gamma-aminobutyric acid:sodium symporter activity
P	P48065	DR	neurotransmitter:sodium symporter activity

P	P48066	CC	Terminates the action of GABA by its high affinity sodium-dependent reuptake into presynaptic terminals.
P	P48066	CC	Terminates the action of GABA by its high affinity sodium-dependent reuptake into presynaptic terminals.
P	P48066	DE	Sodium- and chloride-dependent GABA transporter 3;
P	P48066	DE	Sodium- and chloride-dependent GABA transporter 3;
P	P48066	DR	gamma-aminobutyric acid:sodium symporter activity
P	P48066	DR	gamma-aminobutyric acid:sodium symporter activity
P	P48066	DR	neurotransmitter:sodium symporter activity
P	P48067	CC	May play a role in regulation of glycine levels in NMDA receptor-mediated neurotransmission.
P	P48067	CC	Terminates the action of glycine by its high affinity sodium-dependent reuptake into presynaptic terminals
P	P48067	CC	Terminates the action of glycine by its high affinity sodium-dependent reuptake into presynaptic terminals
P	P48067	DE	Sodium- and chloride-dependent glycine transporter 1;
P	P48067	DE	Sodium- and chloride-dependent glycine transporter 1;
P	P48067	DR	glycine:sodium symporter activity
P	P48067	DR	glycine:sodium symporter activity
P	P48067	DR	neurotransmitter:sodium symporter activity
P	P48169	CC	GABA, the major inhibitory neurotransmitter in the vertebrate brain, mediates neuronal inhibition by binding to the GABA/benzodiazepine receptor and opening an integral chloride channel.
P	P48169	DR	chloride channel activity
P	P48242	CC	Part of the binding-protein-dependent transport system for glutamate.
P	P48243	CC	Part of the binding-protein-dependent transport system for glutamate
P	P48243	DE	Glutamate transport ATP-binding protein GluA;
P	P48244	CC	Part of the binding-protein-dependent transport system for glutamate; probably responsible for the translocation of the substrate across the membrane.
P	P48244	DE	Glutamate transport system permease protein gluC;
P	P48245	CC	Part of the binding-protein-dependent transport system for glutamate; probably responsible for the translocation of the substrate across the membrane.
P	P48245	DE	Glutamate transport system permease protein gluD;
P	P48543	CC	Inward rectifier potassium channels are characterized by a greater tendency to allow potassium to flow into the cell rather than out of it
P	P48543	CC	Their voltage dependence is regulated by the concentration of extracellular potassium; as external potassium is raised, the voltage range of the channel opening shifts to more positive voltages
P	P48543	DE	G protein-activated inward rectifier potassium channel 3;
P	P48543	DE	Inward rectifier K+ channel Kir3.3;
P	P48543	DE	Potassium channel, inwardly rectifying subfamily J member 9;
P	P48543	DR	G-protein activated inward rectifier potassium channel activity
P	P48544	CC	Inward rectifier potassium channels are characterized by a greater tendency to allow potassium to flow into the cell rather than out of it
P	P48544	CC	Their voltage dependence is regulated by the concentration of extracellular potassium; as external potassium is raised, the voltage range of the channel opening shifts to more positive voltages
P	P48544	CC	This potassium channel is controlled by G proteins
P	P48544	DE	G protein-activated inward rectifier potassium channel 4;
P	P48544	DE	Inward rectifier K+ channel Kir3.4;
P	P48544	DE	Potassium channel, inwardly rectifying subfamily J member 5;
P	P48544	DR	G-protein activated inward rectifier potassium channel activity
P	P48547	CC	Assuming opened or closed conformations in response to the voltage difference across the membrane, the protein forms a potassium-selective channel through which potassium ions may pass in accordance with their electrochemical gradient
P	P48547	CC	Mediates the voltage-dependent potassium ion permeability of excitable membranes
P	P48547	DE	Potassium voltage-gated channel subfamily C member 1;
P	P48547	DE	Voltage-gated potassium channel subunit Kv3.1;
P	P48547	DE	Voltage-gated potassium channel subunit Kv4;
P	P48547	DR	voltage-gated potassium channel activity
P	P48549	CC	Inward rectifier potassium channels are characterized by a greater tendency to allow potassium to flow into the cell rather than out of it
P	P48549	CC	Their voltage dependence is regulated by the concentration of extracellular potassium; as external potassium is raised, the voltage range of the channel opening shifts to more positive voltages

P	P48549	CC	This potassium channel is controlled by G proteins
P	P48549	DE	G protein-activated inward rectifier potassium channel 1;
P	P48549	DE	Inward rectifier K+ channel Kir3.1;
P	P48549	DE	Potassium channel, inwardly rectifying subfamily J member 3;
P	P48549	DR	G-protein activated inward rectifier potassium channel activity
P	P48664	CC	Seems to act as a symport by cotransporting sodium.
P	P48664	CC	Transports L-glutamate and also L- and D-aspartate
P	P48664	DE	Sodium-dependent glutamate/aspartate transporter;
P	P48664	DE	Sodium-dependent glutamate/aspartate transporter;
P	P48664	DE	Sodium-dependent glutamate/aspartate transporter;
P	P48664	DR	high-affinity glutamate transmembrane transporter activity
P	P48664	DR	sodium:dicarboxylate symporter activity
P	P48764	CC	Major proton extruding system driven by the inward sodium ion chemical gradient
P	P48764	DE	Na+/H+ exchanger 3;
P	P48764	DE	Sodium/hydrogen exchanger 3;
P	P48764	DR	sodium:hydrogen antiporter activity
P	P48777	DE	Purine permease;
P	P48813	CC	High affinity transport of glutamine
P	P48813	DE	High-affinity glutamine permease;
P	P48962	CC	Catalyzes the exchange of ADP and ATP across the mitochondrial inner membrane
P	P48962	CC	Catalyzes the exchange of ADP and ATP across the mitochondrial inner membrane
P	P48962	DE	ADP,ATP carrier protein 1;
P	P48962	DE	ADP,ATP carrier protein, heart/skeletal muscle isoform T1;
P	P48962	DE	ADP/ATP translocase 1;
P	P48962	DE	ADP/ATP translocase 1;
P	P48995	CC	Seems to be also activated by intracellular calcium store depletion.
P	P48995	CC	Thought to form a receptor-activated non-selective calcium permeant cation channel
P	P48995	DR	store-operated calcium channel activity
P	P49279	CC	Divalent transition metal (iron and manganese) transporter involved in iron metabolism and host resistance to certain pathogens
P	P49279	DR	metal ion:hydrogen antiporter activity
P	P49281	CC	Important in metal transport, in particular iron
P	P49573	CC	Required for high affinity copper (probably reduced Cu I) transport into the cell.
P	P49573	DE	Copper transport protein CTR1;
P	P49573	DE	Copper transporter 1;
P	P49573	DR	copper uptake transmembrane transporter activity
P	P50077	CC	Functions together with MID1 to ensure that adequate levels of Ca2+ are supplied to PMR1 to sustain secretion and growth
P	P50077	CC	Voltage-gated, high-affinity calcium channel involved in calcium influx in response to some environmental stresses as well as exposure to mating pheromones
P	P50077	DE	Calcium-channel protein CCH1;
P	P50077	DR	calcium channel activity
P	P50276	CC	High affinity permease for methionine.
P	P50276	DE	High-affinity methionine permease;
P	P50276	DR	L-methionine secondary active transmembrane transporter activity
P	P50334	DR	sodium:dicarboxylate symporter activity
P	P50334	DR	sodium:dicarboxylate symporter activity
P	P50443	CC	Sulfate transporter
P	P50443	DE	Sulfate transporter;
P	P50443	DR	secondary active sulfate transmembrane transporter activity
P	P50487	DE	Putative purine permease CPE0397;
P	P50537	CC	Permease for malate and other C4 dicarboxylic acids.
P	P50537	DE	Malate permease;
P	P50537	DE	Malic acid transport protein;
P	P50537	DR	malate transmembrane transporter activity
P	P50571	CC	GABA, the major inhibitory neurotransmitter in the vertebrate brain, mediates neuronal inhibition by binding to the GABA/benzodiazepine receptor and opening an integral chloride channel.
P	P50571	DR	chloride channel activity
P	P50976	CC	This system is involved in lactose transport.
P	P50976	DE	Lactose permease IIC component;
P	P50976	DE	Lactose-specific phosphotransferase enzyme IIB component;
P	P50976	DE	PTS system lactose-specific EIIB component;
P	P50976	DE	PTS system lactose-specific EIIC component;
P	P50976	DE	PTS system lactose-specific EIICB component;

P	P50980	DE	Oligopeptide transport ATP-binding protein OppD;
P	P50992	DE	Gastric H+/K+ ATPase subunit beta;
P	P50992	DE	Potassium-transporting ATPase subunit beta;
P	P50992	DR	sodium:potassium-exchanging ATPase activity
P	P50992	DR	sodium:potassium-exchanging ATPase activity
P	P50993	CC	This action creates the electrochemical gradient of sodium and potassium, providing the energy for active transport of various nutrients.
P	P50993	CC	This action creates the electrochemical gradient of sodium and potassium, providing the energy for active transport of various nutrients.
P	P50993	CC	This is the catalytic component of the active enzyme, which catalyzes the hydrolysis of ATP coupled with the exchange of sodium and potassium ions across the plasma membrane
P	P50993	CC	This is the catalytic component of the active enzyme, which catalyzes the hydrolysis of ATP coupled with the exchange of sodium and potassium ions across the plasma membrane
P	P50993	DE	Na+/K+ ATPase alpha-2 subunit;
P	P50993	DE	Na+/K+ ATPase alpha-2 subunit;
P	P50993	DE	Sodium pump subunit alpha-2;
P	P50993	DE	Sodium/potassium-transporting ATPase subunit alpha-2;
P	P50993	DE	Sodium/potassium-transporting ATPase subunit alpha-2;
P	P50993	DR	sodium:potassium-exchanging ATPase activity
P	P50993	DR	sodium:potassium-exchanging ATPase activity
P	P51000	DE	Dipeptide transport system permease protein dppC;
P	P51164	DE	Gastric H+/K+ ATPase subunit beta;
P	P51164	DE	Gastric H+/K+ ATPase subunit beta;
P	P51164	DE	Potassium-transporting ATPase subunit beta;
P	P51164	DR	hydrogen:potassium-exchanging ATPase activity
P	P51164	DR	sodium:potassium-exchanging ATPase activity
P	P51164	DR	sodium:potassium-exchanging ATPase activity
P	P51168	CC	Controls the reabsorption of sodium in kidney, colon, lung and sweat glands
P	P51168	CC	Sodium permeable non-voltage-sensitive ion channel inhibited by the diuretic amiloride
P	P51168	DE	Amiloride-sensitive sodium channel subunit beta;
P	P51168	DE	Epithelial Na+ channel subunit beta;
P	P51168	DE	Nonvoltage-gated sodium channel 1 subunit beta;
P	P51168	DR	ligand-gated sodium channel activity
P	P51172	CC	Controls the reabsorption of sodium in kidney, colon, lung and sweat glands
P	P51172	CC	Sodium permeable non-voltage-sensitive ion channel inhibited by the diuretic amiloride
P	P51172	DE	Amiloride-sensitive sodium channel subunit delta;
P	P51172	DE	Epithelial Na+ channel subunit delta;
P	P51172	DE	Nonvoltage-gated sodium channel 1 subunit delta;
P	P51172	DR	ligand-gated sodium channel activity
P	P51180	CC	Water channel
P	P51180	DR	water channel activity
P	P51574	DE	Intestinal H+/peptide cotransporter;
P	P51787	CC	Associates with KCNE1 (MinK) to form the I(Ks) cardiac potassium current
P	P51787	CC	Elicits a rapidly activating, potassium-selective outward current
P	P51787	CC	May associate also with KCNE3 (MiRP2) to form the potassium channel that is important for cyclic AMP-stimulated intestinal secretion of chloride ions, which is reduced in cystic fibrosis and pathologically stimulated in cholera and other forms of secretory diarrhea.
P	P51787	DE	IKs producing slow voltage-gated potassium channel subunit alpha KvLQT1;
P	P51787	DE	Potassium voltage-gated channel subfamily KQT member 1;
P	P51787	DE	Voltage-gated potassium channel subunit Kv7.1;
P	P51787	DR	delayed rectifier potassium channel activity
P	P51788	CC	Chloride channels have several functions including the regulation of cell volume; membrane potential stabilization, signal transduction and transepithelial transport
P	P51788	CC	Voltage-gated chloride channel
P	P51788	DE	Chloride channel protein 2;
P	P51788	DR	voltage-gated chloride channel activity
P	P51790	CC	Mediates the exchange of chloride ions against protons
P	P51790	DE	Chloride channel protein 3;
P	P51790	DE	Chloride transporter ClC-3;
P	P51790	DR	voltage-gated chloride channel activity
P	P51791	CC	Mediates the exchange of chloride ions against protons
P	P51791	DE	Chloride channel protein 3;

P	P51791	DE	Chloride transporter ClC-3;
P	P51791	DR	voltage-gated chloride channel activity
P	P51793	CC	Functions as antiport system and exchanges chloride ions against protons.
P	P51793	CC	Proton-coupled chloride transporter
P	P51793	DE	Chloride channel protein 4;
P	P51793	DE	Chloride transporter ClC-4;
P	P51793	DR	voltage-gated chloride channel activity
P	P51795	CC	Functions as antiport system and exchanges chloride ions against protons
P	P51795	CC	Proton-coupled chloride transporter
P	P51795	DE	Chloride channel protein 5;
P	P51795	DE	Chloride transporter ClC-5;
P	P51798	CC	Mediates the exchange of chloride ions against protons
P	P51798	DE	Chloride channel protein 7;
P	P51798	DR	voltage-gated chloride channel activity
P	P51800	CC	Chloride channels have several functions including the regulation of cell volume; membrane potential stabilization, signal transduction and transepithelial transport
P	P51800	CC	Voltage-gated chloride channel
P	P51800	DE	Chloride channel Ka;
P	P51800	DE	Chloride channel protein ClC-Ka;
P	P51800	DR	voltage-gated chloride channel activity
P	P51801	CC	Chloride channels have several functions including the regulation of cell volume; membrane potential stabilization, signal transduction and transepithelial transport
P	P51801	CC	Voltage-gated chloride channel
P	P51801	DE	Chloride channel Kb;
P	P51801	DE	Chloride channel protein ClC-Kb;
P	P51801	DR	voltage-gated chloride channel activity
P	P51881	CC	Catalyzes the exchange of ADP and ATP across the mitochondrial inner membrane
P	P51881	CC	Catalyzes the exchange of ADP and ATP across the mitochondrial inner membrane
P	P51881	DE	ADP,ATP carrier protein 2;
P	P51881	DE	ADP/ATP translocase 2;
P	P51881	DE	ADP/ATP translocase 2;
P	P51905	CC	Terminates the action of serotonin by its high affinity sodium-dependent reuptake into presynaptic terminals.
P	P51905	CC	Terminates the action of serotonin by its high affinity sodium-dependent reuptake into presynaptic terminals.
P	P51905	DE	Cocaine-sensitive serotonin transporter;
P	P51905	DE	Sodium-dependent serotonin transporter;
P	P51905	DE	Sodium-dependent serotonin transporter;
P	P51905	DR	neurotransmitter:sodium symporter activity
P	P51905	DR	serotonin transmembrane transporter activity
P	P51906	CC	Acts as a symport by cotransporting sodium
P	P51906	CC	Essential for terminating the postsynaptic action of glutamate by rapidly removing released glutamate from the synaptic cleft
P	P51906	CC	Transports L-glutamate and also L- and D-aspartate
P	P51906	DE	Sodium-dependent glutamate/aspartate transporter 3;
P	P51906	DE	Sodium-dependent glutamate/aspartate transporter 3;
P	P51906	DE	Sodium-dependent glutamate/aspartate transporter 3;
P	P51906	DR	glutamate:sodium symporter activity
P	P51906	DR	glutamate:sodium symporter activity
P	P51906	DR	sodium:dicarboxylate symporter activity
P	P51912	DE	ASC-like Na+-dependent neutral amino acid transporter ASCT2;
P	P51912	DE	Sodium-dependent neutral amino acid transporter type 2;
P	P51912	DR	sodium:dicarboxylate symporter activity
P	P52280	CC	Glycerol enters the cell via the glycerol diffusion facilitator protein
P	P52280	CC	This membrane protein facilitates the movement of glycerol across the cytoplasmic membrane (By similarity).
P	P52280	DE	Probable glycerol uptake facilitator protein;
P	P53048	CC	High-affinity uptake of alpha-glucosides such as maltose, turanose, isomaltose, alpha-methylglucoside, maltotriose, palatinose, trehalose, melezitose and glucose
P	P53154	CC	Involved in active uptake of glycerol driven by electrogenic proton symport.
P	P53154	DE	Glycerol uptake protein 1;
P	P53388	CC	Can transport glutamate, aspartate, glutamine, asparagine, serine, alanine and glycine
P	P53388	CC	Can transport glutamate, aspartate, glutamine, asparagine, serine, alanine and glycine
P	P53403	CC	Facilitative glucose transporter.
P	P53403	DE	Glucose transporter type 3;

P	P53403	DE	Glucose transporter-like protein;
P	P53631	DE	Hexose transporter HXT17;
P	P53794	CC	Prevents intracellular accumulation of high concentrations of myo-inositol (an osmolyte) that result in impairment of cellular function.
P	P53794	DE	Na+/myo-inositol cotransporter;
P	P53794	DE	Na+/myo-inositol cotransporter;
P	P53794	DE	Sodium/myo-inositol cotransporter;
P	P53794	DE	Sodium/myo-inositol cotransporter;
P	P53794	DR	myo-inositol:sodium symporter activity
P	P53794	DR	myo-inositol:sodium symporter activity
P	P53985	CC	Catalyzes the rapid transport across the plasma membrane of many monocarboxylates such as lactate, pyruvate, branched-chain oxo acids derived from leucine, valine and isoleucine, and the ketone bodies acetoacetate, beta-hydroxybutyrate and acetate
P	P53985	CC	Proton-linked monocarboxylate transporter
P	P53985	DE	Monocarboxylate transporter 1;
P	P53985	DR	secondary active monocarboxylate transmembrane transporter activity
P	P53986	CC	Proton-linked monocarboxylate transporter
P	P53986	DE	Monocarboxylate transporter 1;
P	P53986	DR	secondary active monocarboxylate transmembrane transporter activity
P	P54104	CC	Component of the transport system for branched-chain amino acids (leucine, isoleucine and valine) Which is coupled to a proton motive force.
P	P54104	CC	Component of the transport system for branched-chain amino acids (leucine, isoleucine and valine) Which is coupled to a proton motive force.
P	P54417	CC	High-affinity uptake of glycine betaine.
P	P54417	DE	Glycine betaine transporter OpuD;
P	P54535	CC	Part of a binding-protein-dependent transport system for arginine.
P	P54535	DE	Arginine-binding extracellular protein ArtP;
P	P54536	CC	Part of a binding-protein-dependent transport system for arginine
P	P54536	DE	Arginine transport system permease protein ArtQ;
P	P54537	CC	Part of a binding-protein-dependent transport system for arginine
P	P54537	DE	Arginine transport ATP-binding protein ArtM;
P	P54678	CC	Calcium ATPase involved in Ca2+ homeostasis as a component of the contractile vacuole complex.
P	P54678	DE	Calcium-transporting ATPase PAT1;
P	P54678	DR	calcium-transporting ATPase activity
P	P54707	CC	Catalyzes the hydrolysis of ATP coupled with the exchange of H+ and K+ ions across the plasma membrane
P	P54707	CC	Responsible for potassium absorption in various tissues.
P	P54707	DE	Non-gastric H+/K+ ATPase subunit alpha;
P	P54707	DE	Potassium-transporting ATPase alpha chain 2;
P	P54707	DR	hydrogen:potassium-exchanging ATPase activity
P	P54709	CC	This is the non-catalytic component of the active enzyme, which catalyzes the hydrolysis of ATP coupled with the exchange of Na+ and K+ ions across the plasma membrane
P	P54709	CC	This is the non-catalytic component of the active enzyme, which catalyzes the hydrolysis of ATP coupled with the exchange of Na+ and K+ ions across the plasma membrane
P	P54709	DE	Sodium/potassium-dependent ATPase subunit beta-3;
P	P54709	DE	Sodium/potassium-dependent ATPase subunit beta-3;
P	P54709	DE	Sodium/potassium-transporting ATPase subunit beta-3;
P	P54709	DE	Sodium/potassium-transporting ATPase subunit beta-3;
P	P54709	DR	sodium:potassium-exchanging ATPase activity
P	P54709	DR	sodium:potassium-exchanging ATPase activity
P	P54710	CC	May be involved in forming the receptor site for cardiac glycoside binding or may modulate the transport function of the sodium ATPase.
P	P54710	DE	Na+/K+ ATPase subunit gamma;
P	P54710	DE	Na+/K+ ATPase subunit gamma;
P	P54710	DE	Sodium pump gamma chain;
P	P54710	DE	Sodium/potassium-transporting ATPase subunit gamma;
P	P54710	DE	Sodium/potassium-transporting ATPase subunit gamma;
P	P54710	DR	sodium:potassium-exchanging ATPase activity
P	P54710	DR	sodium:potassium-exchanging ATPase activity
P	P54854	DE	Hexose transporter HXT15;
P	P54862	CC	Probable glucose transporter.
P	P54862	DE	Low-affinity glucose transporter LGT3;
P	P54862	DR	glucose transmembrane transporter activity
P	P55011	CC	Mediates sodium and chloride reabsorption

P	P55011	CC	Mediates sodium and chloride reabsorption
P	P55011	DE	Bumetanide-sensitive sodium-(potassium)-chloride cotransporter 1;
P	P55011	DR	sodium:potassium:chloride symporter activity
P	P55011	DR	sodium:potassium:chloride symporter activity
P	P55011	DR	sodium:potassium:chloride symporter activity
P	P55012	CC	Mediates sodium and chloride reabsorption
P	P55012	DE	Bumetanide-sensitive sodium-(potassium)-chloride cotransporter 1;
P	P55014	CC	Mediates sodium and chloride reabsorption
P	P55014	CC	Mediates sodium and chloride reabsorption
P	P55014	DE	Bumetanide-sensitive sodium-(potassium)-chloride cotransporter 2;
P	P55017	CC	Mediates sodium and chloride reabsorption.
P	P55017	CC	Mediates sodium and chloride reabsorption.
P	P55017	DE	Thiazide-sensitive sodium-chloride cotransporter;
P	P55017	DR	sodium:chloride symporter activity
P	P55017	DR	sodium:chloride symporter activity
P	P55018	CC	Mediates sodium and chloride reabsorption.
P	P55018	CC	Mediates sodium and chloride reabsorption.
P	P55018	DE	Thiazide-sensitive sodium-chloride cotransporter;
P	P55018	DR	sodium:chloride symporter activity
P	P55018	DR	sodium:chloride symporter activity
P	P55064	CC	Forms a water-specific channel
P	P55064	DR	water channel activity
P	P55069	CC	Can also transport citrate in complex with Ni(2+), Mn(2+), Co(2+), and Zn(2+).
P	P55069	CC	Cotransports at least two protons per Mg2+-citrate complex
P	P55069	CC	Proton motive force-driven secondary transporter that mediates the transport of citrate complexed to Mg2+
P	P55069	CC	Proton motive force-driven secondary transporter that mediates the transport of citrate complexed to Mg2+
P	P55069	DE	Mg2+/citrate complex secondary transporter;
P	P55069	DE	Mg2+/citrate complex secondary transporter;
P	P55069	DR	citrate transmembrane transporter activity
P	P55087	CC	Forms a water-specific channel
P	P55087	CC	Osmoreceptor which regulates body water balance and mediates water flow within the central nervous system.
P	P55087	DE	Mercurial-insensitive water channel;
P	P55087	DR	water channel activity
P	P55088	CC	Forms a water-specific channel
P	P55088	CC	Osmoreceptor which regulates body water balance and mediates water flow within the central nervous system.
P	P55088	DE	Mercurial-insensitive water channel;
P	P55088	DR	water channel activity
P	P55989	CC	Probably involved in copper export.
P	P55989	DE	Copper-transporting ATPase;
P	P55989	DR	copper-exporting ATPase activity
P	P56402	CC	Forms a water-specific channel that provides the plasma membranes of renal collecting duct with high permeability to water, thereby permitting water to move in the direction of an osmotic gradient (By similarity).
P	P56402	DE	ADH water channel;
P	P56402	DE	Collecting duct water channel protein;
P	P56402	DE	Water channel protein for renal collecting duct;
P	P56402	DR	water channel activity
P	P56475	CC	GABA, the major inhibitory neurotransmitter in the vertebrate brain, mediates neuronal inhibition by binding to the GABA/benzodiazepine receptor and opening an integral chloride channel
P	P56475	DR	chloride channel activity
P	P56564	CC	Acts as a symport by cotransporting sodium.
P	P56564	CC	Essential for terminating the postsynaptic action of glutamate by rapidly removing released glutamate from the synaptic cleft
P	P56564	CC	Transports L-glutamate and also L- and D-aspartate
P	P56564	DE	Glial high affinity glutamate transporter;
P	P56564	DE	High-affinity neuronal glutamate transporter;
P	P56564	DE	Sodium-dependent glutamate/aspartate transporter 1;
P	P56564	DE	Sodium-dependent glutamate/aspartate transporter 1;
P	P56564	DR	high-affinity glutamate transmembrane transporter activity
P	P56564	DR	sodium:dicarboxylate symporter activity

P	P56696	CC	May underlie a potassium current involved in regulating the excitability of sensory cells of the cochlea
P	P56696	DE	Potassium channel subunit alpha KvLQT4;
P	P56696	DE	Potassium voltage-gated channel subfamily KQT member 4;
P	P56696	DE	Voltage-gated potassium channel subunit Kv7.4;
P	P56949	DE	Putative malonate transporter;
P	P57031	DE	Lipoprotein-releasing system ATP-binding protein LolD;
P	P57031	DR	lipoprotein transporter activity
P	P57057	DE	Glycerol-3-phosphate permease;
P	P57057	DE	Glycerol-3-phosphate transporter;
P	P57066	DE	Lipoprotein-releasing system ATP-binding protein LolD;
P	P57066	DR	lipoprotein transporter activity
P	P57103	CC	Ca2+ is extruded from the cell during relaxation so as to prevent overloading of intracellular stores (By similarity).
P	P57103	CC	Rapidly transports Ca2+ during excitation-contraction coupling
P	P57103	DE	Na+/Ca2+-exchange protein 3;
P	P57103	DE	Na+/Ca2+-exchange protein 3;
P	P57103	DE	Sodium/calcium exchanger 3;
P	P57103	DE	Sodium/calcium exchanger 3;
P	P57103	DR	calcium:sodium antiporter activity
P	P57103	DR	calcium:sodium antiporter activity
P	P57683	CC	One of the components of the high-affinity ATP-driven potassium transport (or KDP) system, which catalyzes the hydrolysis of ATP coupled with the exchange of hydrogen and potassium ions (By similarity).
P	P57683	DE	Potassium-binding and translocating subunit A;
P	P57683	DE	Potassium-translocating ATPase A chain;
P	P57683	DE	Potassium-transporting ATPase A chain;
P	P57683	DR	potassium-transporting ATPase activity
P	P57686	CC	One of the components of the high-affinity ATP-driven potassium transport (or KDP) system, which catalyzes the hydrolysis of ATP coupled with the exchange of hydrogen and potassium ions
P	P57686	DE	Potassium-binding and translocating subunit C;
P	P57686	DE	Potassium-translocating ATPase C chain;
P	P57686	DE	Potassium-transporting ATPase C chain;
P	P57686	DR	potassium-transporting ATPase activity
P	P57688	CC	One of the components of the high-affinity ATP-driven potassium transport (or KDP) system, which catalyzes the hydrolysis of ATP coupled with the exchange of hydrogen and potassium ions
P	P57688	DE	Potassium-binding and translocating subunit C;
P	P57688	DE	Potassium-translocating ATPase C chain;
P	P57688	DE	Potassium-transporting ATPase C chain;
P	P57688	DR	potassium-transporting ATPase activity
P	P57698	CC	One of the components of the high-affinity ATP-driven potassium transport (or KDP) system, which catalyzes the hydrolysis of ATP coupled with the exchange of hydrogen and potassium ions (By similarity).
P	P57698	DE	Potassium-binding and translocating subunit B;
P	P57698	DE	Potassium-translocating ATPase B chain;
P	P57698	DE	Potassium-transporting ATPase B chain;
P	P57698	DR	potassium-transporting ATPase activity
P	P57700	CC	One of the components of the high-affinity ATP-driven potassium transport (or KDP) system, which catalyzes the hydrolysis of ATP coupled with the exchange of hydrogen and potassium ions (By similarity).
P	P57700	DE	Potassium-binding and translocating subunit B;
P	P57700	DE	Potassium-translocating ATPase B chain;
P	P57700	DE	Potassium-transporting ATPase B chain;
P	P57700	DR	potassium-transporting ATPase activity
P	P57787	CC	Proton-linked monocarboxylate transporter
P	P57787	DE	Monocarboxylate transporter 4;
P	P57787	DR	secondary active monocarboxylate transmembrane transporter activity
P	P57789	CC	Outward rectifying potassium channel
P	P57789	CC	Produces rapidly activating and non-inactivating outward rectifier K+ currents
P	P57789	DE	Outward rectifying potassium channel protein TREK-2;
P	P57789	DE	Potassium channel subfamily K member 10;
P	P57789	DE	TREK-2 K+ channel subunit;
P	P57789	DR	potassium channel activity

P	P58229	CC	Imports glutamate inside the cell while simultaneously exporting to the periplasm the GABA produced by gadA and gadB
P	P58229	CC	Involved in glutamate-dependent acid resistance
P	P58229	DE	Probable glutamate/gamma-aminobutyrate antiporter;
P	P58244	CC	Functions as antiport system and exchanges two chloride ions for 1 proton
P	P58244	CC	Proton-coupled chloride transporter
P	P58244	DR	voltage-gated chloride channel activity
P	P58300	CC	Binds maltotriose much more tightly than maltose.
P	P58300	DR	maltose transmembrane transporter activity
P	P58341	CC	Involved in copper transport (By similarity).
P	P58341	DE	Copper-transporting ATPase 1;
P	P58341	DR	copper-exporting ATPase activity
P	P58342	CC	Involved in copper transport (By similarity).
P	P58342	DE	Copper-transporting ATPase 2;
P	P58342	DR	copper-exporting ATPase activity
P	P58390	CC	Forms a voltage-independent potassium channel activated by intracellular calcium
P	P58414	CC	This electroneutral antiporter ejects one cadmium ion while accumulating two protons by an energy-dependent efflux mechanism (By similarity).
P	P58414	DE	Cadmium efflux ATPase;
P	P58414	DE	Probable cadmium-transporting ATPase;
P	P58414	DR	cadmium-exporting ATPase activity
P	P58529	CC	The physiological role may be the reduction of the intracellular concentration of toxic sugars or sugar metabolites (By similarity).
P	P58529	DE	Probable sugar efflux transporter;
P	P58530	CC	The physiological role may be the reduction of the intracellular concentration of toxic sugars or sugar metabolites (By similarity).
P	P58530	DE	Probable sugar efflux transporter;
P	P58531	CC	The physiological role may be the reduction of the intracellular concentration of toxic sugars or sugar metabolites (By similarity).
P	P58531	DE	Probable sugar efflux transporter;
P	P58655	CC	Part of a binding-protein-dependent transport system for phosphate; probably responsible for the translocation of the substrate across the membrane (By similarity)
P	P58655	DE	Phosphate transport system permease protein pstA;
P	P58655	DR	inorganic phosphate transmembrane transporter activity
P	P58734	DR	sodium:dicarboxylate symporter activity
P	P58735	CC	High affinity uptake of sulfate
P	P58735	DE	Sulfate anion transporter 1;
P	P58735	DR	secondary active sulfate transmembrane transporter activity
P	P59053	CC	Potassium channel subunit
P	P59053	DE	Potassium voltage-gated channel subfamily G member 3;
P	P59053	DE	Voltage-gated potassium channel subunit Kv10.1;
P	P59053	DE	Voltage-gated potassium channel subunit Kv6.3;
P	P59111	CC	Pore-forming (alpha) subunit of voltage-gated potassium channel
P	P59111	DE	Ether-a-go-go-like potassium channel 3;
P	P59111	DE	Potassium voltage-gated channel subfamily H member 8;
P	P59111	DE	Voltage-gated potassium channel subunit Kv12.1;
P	P59213	CC	Binding protein for maltose and maltodextrin; involved in its cellular uptake.
P	P59213	DE	Maltose/maltodextrin-binding protein;
P	P59213	DR	maltose transmembrane transporter activity
P	P59219	CC	One of the components of the high-affinity ATP-driven potassium transport (or KDP) system, which catalyzes the hydrolysis of ATP coupled with the exchange of hydrogen and potassium ions (By similarity).
P	P59219	DE	Potassium-binding and translocating subunit B;
P	P59219	DE	Potassium-translocating ATPase B chain;
P	P59219	DE	Potassium-transporting ATPase B chain;
P	P59219	DR	potassium-transporting ATPase activity
P	P59585	DR	voltage-gated chloride channel activity
P	P59638	DE	Voltage-gated ClC-type chloride channel ClcB;
P	P59638	DR	voltage-gated chloride channel activity
P	P59639	CC	Functions as antiport system and exchanges two chloride ions for 1 proton
P	P59639	CC	Proton-coupled chloride transporter
P	P59639	DR	voltage-gated chloride channel activity
P	P59737	DE	Aromatic amino acid transport protein AroP;
P	P59737	DE	General aromatic amino acid permease;
P	P59738	CC	Part of the ABC transporter complex ModABC involved in molybdenum import

P	P59738	DE	Molybdenum import ATP-binding protein ModC;
P	P59738	DR	molybdenum ion transmembrane transporter activity
P	P59784	CC	This system is involved in glucitol/sorbitol transport (By similarity).
P	P59784	DE	Glucitol/sorbitol-specific phosphotransferase enzyme IIA component;
P	P59784	DE	PTS system glucitol/sorbitol-specific EIIA component;
P	P59950	CC	Low-affinity inorganic phosphate transport (By similarity).
P	P59950	DE	Probable low-affinity inorganic phosphate transporter;
P	P59950	DR	inorganic phosphate transmembrane transporter activity
P	P60675	CC	Mnh complex is a Na+/H+ antiporter involved in Na+ excretion (By similarity).
P	P60675	DE	Na+/H+ antiporter subunit A1;
P	P60677	CC	Mnh complex is a Na+/H+ antiporter involved in Na+ excretion (By similarity).
P	P60677	DE	Na+/H+ antiporter subunit B1;
P	P60678	CC	Does not transport K+, Ca2+ or Mg2+.
P	P60678	CC	Mnh complex is a Na+Li+/H+ antiporter involved in Na+ and/or Li+ excretion
P	P60678	CC	Mnh complex is a Na+Li+/H+ antiporter involved in Na+ and/or Li+ excretion
P	P60678	CC	Na+/H+ antiport consumes a transmembrane electrical potential, and is thus inferred to be electrogenic
P	P60678	CC	Na+/H+ antiport consumes a transmembrane electrical potential, and is thus inferred to be electrogenic
P	P60678	DE	Na+/H+ antiporter subunit B1;
P	P60678	DE	Na+/H+ antiporter subunit B1;
P	P60681	CC	Mnh complex is a Na+/H+ antiporter involved in Na+ excretion (By similarity).
P	P60681	DE	Na+/H+ antiporter subunit C1;
P	P60682	CC	Does not transport K+, Ca2+ or Mg2+.
P	P60682	CC	Mnh complex is a Na+Li+/H+ antiporter involved in Na+ and/or Li+ excretion
P	P60682	CC	Mnh complex is a Na+Li+/H+ antiporter involved in Na+ and/or Li+ excretion
P	P60682	CC	Na+/H+ antiport consumes a transmembrane electrical potential, and is thus inferred to be electrogenic
P	P60682	CC	Na+/H+ antiport consumes a transmembrane electrical potential, and is thus inferred to be electrogenic
P	P60682	DE	Na+/H+ antiporter subunit C1;
P	P60682	DE	Na+/H+ antiporter subunit C1;
P	P60685	CC	Mnh complex is a Na+/H+ antiporter involved in Na+ excretion (By similarity).
P	P60685	DE	Na+/H+ antiporter subunit D1;
P	P60686	CC	Does not transport K+, Ca2+ or Mg2+.
P	P60686	CC	Mnh complex is a Na+Li+/H+ antiporter involved in Na+ and/or Li+ excretion
P	P60686	CC	Mnh complex is a Na+Li+/H+ antiporter involved in Na+ and/or Li+ excretion
P	P60686	CC	Na+/H+ antiport consumes a transmembrane electrical potential, and is thus inferred to be electrogenic
P	P60686	CC	Na+/H+ antiport consumes a transmembrane electrical potential, and is thus inferred to be electrogenic
P	P60686	DE	Na+/H+ antiporter subunit D1;
P	P60686	DE	Na+/H+ antiporter subunit D1;
P	P60686	DR	potassium:hydrogen antiporter activity
P	P60689	CC	Mnh complex is a Na+/H+ antiporter involved in Na+ excretion (By similarity).
P	P60689	DE	Na+/H+ antiporter subunit E1;
P	P60690	CC	Does not transport K+, Ca2+ or Mg2+.
P	P60690	CC	Mnh complex is a Na+Li+/H+ antiporter involved in Na+ and/or Li+ excretion
P	P60690	CC	Mnh complex is a Na+Li+/H+ antiporter involved in Na+ and/or Li+ excretion
P	P60690	CC	Na+/H+ antiport consumes a transmembrane electrical potential, and is thus inferred to be electrogenic
P	P60690	CC	Na+/H+ antiport consumes a transmembrane electrical potential, and is thus inferred to be electrogenic
P	P60690	DE	Na+/H+ antiporter subunit E1;
P	P60690	DE	Na+/H+ antiporter subunit E1;
P	P60693	CC	Mnh complex is a Na+/H+ antiporter involved in Na+ excretion (By similarity).
P	P60693	DE	Na+/H+ antiporter subunit F1;
P	P60694	CC	Does not transport K+, Ca2+ or Mg2+.
P	P60694	CC	Mnh complex is a Na+Li+/H+ antiporter involved in Na+ and/or Li+ excretion
P	P60694	CC	Mnh complex is a Na+Li+/H+ antiporter involved in Na+ and/or Li+ excretion
P	P60694	CC	Na+/H+ antiport consumes a transmembrane electrical potential, and is thus inferred to be electrogenic
P	P60694	CC	Na+/H+ antiport consumes a transmembrane electrical potential, and is thus inferred to be electrogenic
P	P60694	DE	Na+/H+ antiporter subunit F1;

P	P60694	DE	Na+/H+ antiporter subunit F1;
P	P60697	CC	Mnh complex is a Na+/H+ antiporter involved in Na+ excretion (By similarity).
P	P60697	DE	Na+/H+ antiporter subunit G1;
P	P60698	CC	Does not transport K+, Ca2+ or Mg2+.
P	P60698	CC	Mnh complex is a Na+Li+/H+ antiporter involved in Na+ and/or Li+ excretion
P	P60698	CC	Mnh complex is a Na+Li+/H+ antiporter involved in Na+ and/or Li+ excretion
P	P60698	CC	Na+/H+ antiport consumes a transmembrane electrical potential, and is thus inferred to be electrogenic
P	P60698	CC	Na+/H+ antiport consumes a transmembrane electrical potential, and is thus inferred to be electrogenic
P	P60698	DE	Na+/H+ antiporter subunit G1;
P	P60698	DE	Na+/H+ antiporter subunit G1;
P	P60857	CC	This system is involved in glucose transport (By similarity).
P	P60857	DE	Glucose-specific phosphotransferase enzyme IIA component;
P	P60857	DE	PTS system glucose-specific EIIA component;
P	P60925	CC	Channel that permits osmotically driven movement of water in both directions
P	P60925	CC	It mediates rapid entry or exit of water in response to abrupt changes in osmolarity (By similarity).
P	P60944	CC	Involved in the uptake of glucose (By similarity).
P	P60944	DE	Probable glucose uptake protein glcU;
P	P60949	CC	Could be involved in the uptake of ribose (By similarity).
P	P60949	DE	Putative ribose uptake protein rbsU;
P	P61377	CC	Part of the ABC transporter complex CcmAB involved in the biogenesis of c-type cytochromes; once thought to export heme, this seems not to be the case, but its exact role is uncertain
P	P61377	DE	Heme exporter protein A;
P	P61377	DR	heme-transporting ATPase activity
P	P61378	CC	Part of the ABC transporter complex CcmAB involved in the biogenesis of c-type cytochromes; once thought to export heme, this seems not to be the case, but its exact role is uncertain
P	P61378	DE	Heme exporter protein A 2;
P	P61378	DR	heme-transporting ATPase activity
P	P61481	DE	Lipoprotein-releasing system ATP-binding protein LolD;
P	P61481	DR	lipoprotein transporter activity
P	P61482	DE	Lipoprotein-releasing system ATP-binding protein LolD;
P	P61482	DR	lipoprotein transporter activity
P	P61837	CC	Water channel required to facilitate the transport of water across cell membrane
P	P61837	DR	water channel activity
P	P62812	CC	GABA, the major inhibitory neurotransmitter in the vertebrate brain, mediates neuronal inhibition by binding to the GABA/benzodiazepine receptor and opening an integral chloride channel.
P	P62812	DR	chloride channel activity
P	P63080	CC	GABA, the major inhibitory neurotransmitter in the vertebrate brain, mediates neuronal inhibition by binding to the GABA/benzodiazepine receptor and opening an integral chloride channel (By similarity).
P	P63080	DR	chloride channel activity
P	P63137	CC	GABA, the major inhibitory neurotransmitter in the vertebrate brain, mediates neuronal inhibition by binding to the GABA/benzodiazepine receptor and opening an integral chloride channel.
P	P63137	DR	chloride channel activity
P	P63141	CC	Assuming opened or closed conformations in response to the voltage difference across the membrane, the protein forms a potassium-selective channel through which potassium ions may pass in accordance with their electrochemical gradient
P	P63141	CC	Mediates the voltage-dependent potassium ion permeability of excitable membranes
P	P63141	DE	Potassium voltage-gated channel subfamily A member 2;
P	P63141	DE	Voltage-gated potassium channel subunit Kv1.2;
P	P63250	CC	Inward rectifier potassium channels are characterized by a greater tendency to allow potassium to flow into the cell rather than out of it
P	P63250	CC	Their voltage dependence is regulated by the concentration of extracellular potassium; as external potassium is raised, the voltage range of the channel opening shifts to more positive voltages
P	P63250	CC	This potassium channel is controlled by G proteins
P	P63250	DE	G protein-activated inward rectifier potassium channel 1;
P	P63250	DE	Inward rectifier K+ channel Kir3.1;
P	P63250	DE	Potassium channel, inwardly rectifying subfamily J member 3;

P	P63250	DR	G-protein activated inward rectifier potassium channel activity
P	P63252	CC	Inward rectifier potassium channels are characterized by a greater tendency to allow potassium to flow into the cell rather than out of it
P	P63252	CC	Their voltage dependence is regulated by the concentration of extracellular potassium; as external potassium is raised, the voltage range of the channel opening shifts to more positive voltages
P	P63252	DE	Cardiac inward rectifier potassium channel;
P	P63252	DE	Inward rectifier K+ channel Kir2.1;
P	P63252	DE	Inward rectifier potassium channel 2;
P	P63252	DE	Potassium channel, inwardly rectifying subfamily J member 2;
P	P63252	DR	inward rectifier potassium channel activity
P	P63351	CC	Part of the ABC transporter complex BtuCDF involved in vitamin B12 import
P	P63351	DE	Vitamin B12 import ATP-binding protein BtuD;
P	P63351	DE	Vitamin B12-transporting ATPase;
P	P63352	CC	Part of the ABC transporter complex BtuCDF involved in vitamin B12 import
P	P63352	DE	Vitamin B12 import ATP-binding protein BtuD;
P	P63352	DE	Vitamin B12-transporting ATPase;
P	P63353	CC	Part of the ABC transporter complex CysAWTP involved in sulfate/thiosulfate import
P	P63353	DE	Sulfate-transporting ATPase;
P	P63353	DE	Sulfate/thiosulfate import ATP-binding protein CysA;
P	P63353	DR	sulfate transmembrane-transporting ATPase activity
P	P63354	CC	Part of the ABC transporter complex CysAWTP involved in sulfate/thiosulfate import
P	P63354	DE	Sulfate-transporting ATPase;
P	P63354	DE	Sulfate/thiosulfate import ATP-binding protein CysA;
P	P63354	DR	sulfate transmembrane-transporting ATPase activity
P	P63356	CC	Part of the ABC transporter complex MetNIQ involved in methionine import
P	P63356	DE	Methionine import ATP-binding protein MetN;
P	P63356	DR	D-methionine transmembrane transporter activity
P	P63361	CC	Part of the ABC transporter complex PstSACB involved in phosphate import
P	P63361	DE	ABC phosphate transporter;
P	P63361	DE	Phosphate import ATP-binding protein PstB;
P	P63361	DE	Phosphate-transporting ATPase;
P	P63361	DR	inorganic phosphate transmembrane transporter activity
P	P63361	DR	phosphate transmembrane-transporting ATPase activity
P	P63362	CC	Part of the ABC transporter complex PstSACB involved in phosphate import
P	P63362	DE	ABC phosphate transporter;
P	P63362	DE	Phosphate import ATP-binding protein PstB;
P	P63362	DE	Phosphate-transporting ATPase;
P	P63362	DR	inorganic phosphate transmembrane transporter activity
P	P63362	DR	phosphate transmembrane-transporting ATPase activity
P	P63363	CC	Part of the ABC transporter complex PstSACB involved in phosphate import
P	P63363	DE	ABC phosphate transporter 1;
P	P63363	DE	Phosphate import ATP-binding protein PstB 1;
P	P63363	DE	Phosphate-transporting ATPase 1;
P	P63363	DR	inorganic phosphate transmembrane transporter activity
P	P63363	DR	phosphate transmembrane-transporting ATPase activity
P	P63364	CC	Part of the ABC transporter complex PstSACB involved in phosphate import
P	P63364	DE	ABC phosphate transporter 1;
P	P63364	DE	Phosphate import ATP-binding protein PstB 1;
P	P63364	DE	Phosphate-transporting ATPase 1;
P	P63364	DR	inorganic phosphate transmembrane transporter activity
P	P63364	DR	phosphate transmembrane-transporting ATPase activity
P	P63365	CC	Part of the ABC transporter complex PstSACB involved in phosphate import
P	P63365	DE	ABC phosphate transporter;
P	P63365	DE	Phosphate import ATP-binding protein PstB;
P	P63365	DE	Phosphate-transporting ATPase;
P	P63365	DR	inorganic phosphate transmembrane transporter activity
P	P63365	DR	phosphate transmembrane-transporting ATPase activity
P	P63366	CC	Part of the ABC transporter complex PstSACB involved in phosphate import
P	P63366	DE	ABC phosphate transporter;
P	P63366	DE	Phosphate import ATP-binding protein PstB;
P	P63366	DE	Phosphate-transporting ATPase;
P	P63366	DR	inorganic phosphate transmembrane transporter activity
P	P63366	DR	phosphate transmembrane-transporting ATPase activity
P	P63367	CC	Part of the ABC transporter complex PstSACB involved in phosphate import

P	P63367	DE	ABC phosphate transporter 1;
P	P63367	DE	Phosphate import ATP-binding protein PstB 1;
P	P63367	DE	Phosphate-transporting ATPase 1;
P	P63367	DR	inorganic phosphate transmembrane transporter activity
P	P63367	DR	phosphate transmembrane-transporting ATPase activity
P	P63368	CC	Part of the ABC transporter complex PstSACB involved in phosphate import
P	P63368	DE	ABC phosphate transporter 1;
P	P63368	DE	Phosphate import ATP-binding protein PstB 1;
P	P63368	DE	Phosphate-transporting ATPase 1;
P	P63368	DR	inorganic phosphate transmembrane transporter activity
P	P63368	DR	phosphate transmembrane-transporting ATPase activity
P	P63369	CC	Part of the ABC transporter complex PstSACB involved in phosphate import
P	P63369	DE	ABC phosphate transporter 2;
P	P63369	DE	Phosphate import ATP-binding protein PstB 2;
P	P63369	DE	Phosphate-transporting ATPase 2;
P	P63369	DR	inorganic phosphate transmembrane transporter activity
P	P63369	DR	phosphate transmembrane-transporting ATPase activity
P	P63370	CC	Part of the ABC transporter complex PstSACB involved in phosphate import
P	P63370	DE	ABC phosphate transporter 2;
P	P63370	DE	Phosphate import ATP-binding protein PstB 2;
P	P63370	DE	Phosphate-transporting ATPase 2;
P	P63370	DR	inorganic phosphate transmembrane transporter activity
P	P63370	DR	phosphate transmembrane-transporting ATPase activity
P	P63371	CC	Part of the ABC transporter complex PstSACB involved in phosphate import
P	P63371	DE	ABC phosphate transporter 3;
P	P63371	DE	Phosphate import ATP-binding protein PstB 3;
P	P63371	DE	Phosphate-transporting ATPase 3;
P	P63371	DR	inorganic phosphate transmembrane transporter activity
P	P63371	DR	phosphate transmembrane-transporting ATPase activity
P	P63372	CC	Part of the ABC transporter complex PstSACB involved in phosphate import
P	P63372	DE	ABC phosphate transporter 3;
P	P63372	DE	Phosphate import ATP-binding protein PstB 3;
P	P63372	DE	Phosphate-transporting ATPase 3;
P	P63372	DR	inorganic phosphate transmembrane transporter activity
P	P63372	DR	phosphate transmembrane-transporting ATPase activity
P	P63373	CC	Part of the ABC transporter complex PstSACB involved in phosphate import
P	P63373	DE	ABC phosphate transporter 1;
P	P63373	DE	Phosphate import ATP-binding protein PstB 1;
P	P63373	DE	Phosphate-transporting ATPase 1;
P	P63373	DR	inorganic phosphate transmembrane transporter activity
P	P63373	DR	phosphate transmembrane-transporting ATPase activity
P	P63375	CC	Part of the ABC transporter complex PstSACB involved in phosphate import
P	P63375	DE	ABC phosphate transporter 1;
P	P63375	DE	Phosphate import ATP-binding protein PstB 1;
P	P63375	DE	Phosphate-transporting ATPase 1;
P	P63375	DR	inorganic phosphate transmembrane transporter activity
P	P63375	DR	phosphate transmembrane-transporting ATPase activity
P	P63378	CC	Part of the ABC transporter complex PstSACB involved in phosphate import
P	P63378	DE	ABC phosphate transporter 2;
P	P63378	DE	Phosphate import ATP-binding protein PstB 2;
P	P63378	DE	Phosphate-transporting ATPase 2;
P	P63378	DR	inorganic phosphate transmembrane transporter activity
P	P63378	DR	phosphate transmembrane-transporting ATPase activity
P	P63620	CC	Thought to form the channel of an arsenite pump.
P	P63620	DR	arsenite transmembrane transporter activity
P	P63681	CC	One of the components of the high-affinity ATP-driven potassium transport (or KDP) system, which catalyzes the hydrolysis of ATP coupled with the exchange of hydrogen and potassium ions (By similarity).
P	P63681	DE	Potassium-binding and translocating subunit B;
P	P63681	DE	Potassium-translocating ATPase B chain;
P	P63681	DE	Potassium-transporting ATPase B chain;
P	P63681	DR	potassium-transporting ATPase activity
P	P63682	CC	One of the components of the high-affinity ATP-driven potassium transport (or KDP) system, which catalyzes the hydrolysis of ATP coupled with the exchange of hydrogen and potassium ions (By similarity).

P	P63682	DE	Potassium-binding and translocating subunit B;
P	P63682	DE	Potassium-translocating ATPase B chain;
P	P63682	DE	Potassium-transporting ATPase B chain;
P	P63682	DR	potassium-transporting ATPase activity
P	P63684	CC	One of the components of the high-affinity ATP-driven potassium transport (or KDP) system, which catalyzes the hydrolysis of ATP coupled with the exchange of hydrogen and potassium ions (By similarity).
P	P63684	DE	Potassium-binding and translocating subunit B 2;
P	P63684	DE	Potassium-translocating ATPase B chain 2;
P	P63684	DE	Potassium-transporting ATPase B chain 2;
P	P63684	DR	potassium-transporting ATPase activity
P	P65209	CC	One of the components of the high-affinity ATP-driven potassium transport (or KDP) system, which catalyzes the hydrolysis of ATP coupled with the exchange of hydrogen and potassium ions (By similarity).
P	P65209	DE	Potassium-binding and translocating subunit A;
P	P65209	DE	Potassium-translocating ATPase A chain;
P	P65209	DE	Potassium-transporting ATPase A chain;
P	P65209	DR	potassium-transporting ATPase activity
P	P65210	CC	One of the components of the high-affinity ATP-driven potassium transport (or KDP) system, which catalyzes the hydrolysis of ATP coupled with the exchange of hydrogen and potassium ions (By similarity).
P	P65210	DE	Potassium-binding and translocating subunit A;
P	P65210	DE	Potassium-translocating ATPase A chain;
P	P65210	DE	Potassium-transporting ATPase A chain;
P	P65210	DR	potassium-transporting ATPase activity
P	P65211	CC	One of the components of the high-affinity ATP-driven potassium transport (or KDP) system, which catalyzes the hydrolysis of ATP coupled with the exchange of hydrogen and potassium ions
P	P65211	DE	Potassium-binding and translocating subunit C;
P	P65211	DE	Potassium-translocating ATPase C chain;
P	P65211	DE	Potassium-transporting ATPase C chain;
P	P65211	DR	potassium-transporting ATPase activity
P	P65212	CC	One of the components of the high-affinity ATP-driven potassium transport (or KDP) system, which catalyzes the hydrolysis of ATP coupled with the exchange of hydrogen and potassium ions
P	P65212	DE	Potassium-binding and translocating subunit C;
P	P65212	DE	Potassium-translocating ATPase C chain;
P	P65212	DE	Potassium-transporting ATPase C chain;
P	P65212	DR	potassium-transporting ATPase activity
P	P65214	CC	One of the components of the high-affinity ATP-driven potassium transport (or KDP) system, which catalyzes the hydrolysis of ATP coupled with the exchange of hydrogen and potassium ions
P	P65214	DE	Potassium-binding and translocating subunit C 1;
P	P65214	DE	Potassium-translocating ATPase C chain 1;
P	P65214	DE	Potassium-transporting ATPase C chain 1;
P	P65214	DR	potassium-transporting ATPase activity
P	P65254	CC	Transports L-lactate across the membrane
P	P65254	DE	L-lactate permease;
P	P65254	DR	lactate transmembrane transporter activity
P	P65544	CC	H+-stimulated, highly selective, manganese uptake system (By similarity).
P	P65544	DE	Probable manganese transport protein mntH;
P	P65545	CC	H+-stimulated, highly selective, manganese uptake system (By similarity).
P	P65545	DE	Probable manganese transport protein mntH;
P	P67730	DR	voltage-gated chloride channel activity
P	P68183	CC	Part of the binding-protein-dependent transport system for maltose; probably responsible for the translocation of the substrate across the membrane.
P	P68183	DE	Maltose transport system permease protein malG;
P	P68183	DR	maltose-transporting ATPase activity
P	P68185	CC	Part of the binding-protein-dependent transport system for maltose; probably responsible for the translocation of the substrate across the membrane.
P	P68185	DE	Maltose transport system permease protein malG;
P	P68186	CC	Part of the binding-protein-dependent transport system for maltose; probably responsible for the translocation of the substrate across the membrane.
P	P68186	DE	Maltose transport system permease protein malG;
P	P68187	CC	Part of the ABC transporter complex MalEFGK involved in maltose/maltodextrin import

P	P68187	DE	Maltose/maltodextrin import ATP-binding protein MalK;
P	P68187	DR	maltose-transporting ATPase activity
P	P68188	CC	Part of the ABC transporter complex MalEFGK involved in maltose/maltodextrin import
P	P68188	DE	Maltose/maltodextrin import ATP-binding protein MalK;
P	P68188	DR	maltose-transporting ATPase activity
P	P69783	CC	This system is involved in glucose transport.
P	P69783	DE	Glucose-specific phosphotransferase enzyme IIA component;
P	P69783	DE	PTS system glucose-specific EIIA component;
P	P69785	CC	This system is involved in glucose transport (By similarity).
P	P69785	DE	Glucose-specific phosphotransferase enzyme IIA component;
P	P69785	DE	PTS system glucose-specific EIIA component;
P	P69786	CC	This system is involved in glucose transport
P	P69786	DE	Glucose permease IIC component;
P	P69786	DE	Glucose-specific phosphotransferase enzyme IIB component;
P	P69786	DE	PTS system glucose-specific EIIB component;
P	P69786	DE	PTS system glucose-specific EIIC component;
P	P69786	DE	PTS system glucose-specific EIICB component;
P	P69786	DR	glucose transmembrane transporter activity
P	P69788	CC	This system is involved in glucose transport
P	P69788	DE	Glucose permease IIC component;
P	P69788	DE	Glucose-specific phosphotransferase enzyme IIB component;
P	P69788	DE	PTS system glucose-specific EIIB component;
P	P69788	DE	PTS system glucose-specific EIIC component;
P	P69788	DE	PTS system glucose-specific EIICB component;
P	P69788	DR	glucose transmembrane transporter activity
P	P69797	CC	This system is involved in mannose transport.
P	P69797	DE	Mannose-specific phosphotransferase enzyme IIA component;
P	P69797	DE	Mannose-specific phosphotransferase enzyme IIB component;
P	P69797	DE	PTS system mannose-specific EIIA component;
P	P69797	DE	PTS system mannose-specific EIIAB component;
P	P69797	DE	PTS system mannose-specific EIIB component;
P	P69799	CC	This system is involved in mannose transport (By similarity).
P	P69799	DE	Mannose-specific phosphotransferase enzyme IIA component;
P	P69799	DE	Mannose-specific phosphotransferase enzyme IIB component;
P	P69799	DE	PTS system mannose-specific EIIA component;
P	P69799	DE	PTS system mannose-specific EIIAB component;
P	P69799	DE	PTS system mannose-specific EIIB component;
P	P69800	CC	This system is involved in mannose transport (By similarity).
P	P69800	DE	Mannose-specific phosphotransferase enzyme IIA component;
P	P69800	DE	Mannose-specific phosphotransferase enzyme IIB component;
P	P69800	DE	PTS system mannose-specific EIIA component;
P	P69800	DE	PTS system mannose-specific EIIAB component;
P	P69800	DE	PTS system mannose-specific EIIB component;
P	P69801	CC	This system is involved in mannose transport.
P	P69801	DE	Mannose permease IIC component;
P	P69801	DE	PTS system mannose-specific EIIC component;
P	P69803	CC	This system is involved in mannose transport (By similarity).
P	P69803	DE	Mannose permease IIC component;
P	P69803	DE	PTS system mannose-specific EIIC component;
P	P69804	CC	This system is involved in mannose transport (By similarity).
P	P69804	DE	Mannose permease IIC component;
P	P69804	DE	PTS system mannose-specific EIIC component;
P	P69805	CC	This system is involved in mannose transport.
P	P69805	DE	Mannose permease IID component;
P	P69805	DE	PTS system mannose-specific EIID component;
P	P69807	CC	This system is involved in mannose transport (By similarity).
P	P69807	DE	Mannose permease IID component;
P	P69807	DE	PTS system mannose-specific EIID component;
P	P69812	CC	This system is involved in fructose transport (By similarity).
P	P69812	DE	Fructose-specific phosphotransferase enzyme IIA component;
P	P69812	DE	PTS system fructose-specific EIIA component;
P	P69814	CC	This system is involved in galactitol transport (By similarity).
P	P69814	DE	Galactitol-specific phosphotransferase enzyme IIA component;
P	P69814	DE	PTS system galactitol-specific EIIA component;
P	P69825	CC	This system is involved in mannitol transport (By similarity).

P	P69825	DE	Mannitol-specific cryptic phosphotransferase enzyme IIA component;
P	P69825	DE	PTS system mannitol-specific EIIA component;
P	P69827	CC	This system is involved in mannitol transport (By similarity).
P	P69827	DE	Mannitol permease IIC component;
P	P69827	DE	Mannitol-specific phosphotransferase enzyme IIB component;
P	P69827	DE	PTS system mannitol-specific EIIB component;
P	P69827	DE	PTS system mannitol-specific EIIC component;
P	P69827	DE	PTS system mannitol-specific cryptic EIICB component;
P	P69828	CC	This system is involved in galactitol transport.
P	P69828	DE	Galactitol-specific phosphotransferase enzyme IIA component;
P	P69828	DE	PTS system galactitol-specific EIIA component;
P	P69831	CC	This system is involved in galactitol transport.
P	P69831	DE	Galactitol permease IIC component;
P	P69831	DE	PTS system galactitol-specific EIIC component;
P	P69832	CC	This system is involved in galactitol transport (By similarity).
P	P69832	DE	Galactitol permease IIC component;
P	P69832	DE	PTS system galactitol-specific EIIC component;
P	P69874	CC	Part of the ABC transporter complex PotABCD involved in spermidine/putrescine import
P	P69874	CC	Part of the ABC transporter complex PotABCD involved in spermidine/putrescine import
P	P69874	DE	Spermidine/putrescine import ATP-binding protein PotA;
P	P69874	DE	Spermidine/putrescine import ATP-binding protein PotA;
P	P69874	DR	polyamine-transporting ATPase activity
P	P69876	CC	Part of the ABC transporter complex PotABCD involved in spermidine/putrescine import
P	P69876	CC	Part of the ABC transporter complex PotABCD involved in spermidine/putrescine import
P	P69876	DE	Spermidine/putrescine import ATP-binding protein PotA;
P	P69876	DE	Spermidine/putrescine import ATP-binding protein PotA;
P	P69877	CC	Part of the ABC transporter complex PotABCD involved in spermidine/putrescine import
P	P69877	CC	Part of the ABC transporter complex PotABCD involved in spermidine/putrescine import
P	P69877	DE	Spermidine/putrescine import ATP-binding protein PotA;
P	P69877	DE	Spermidine/putrescine import ATP-binding protein PotA;
P	P69879	CC	Part of the ABC transporter complex PstSACB involved in phosphate import
P	P69879	DE	ABC phosphate transporter;
P	P69879	DE	Phosphate import ATP-binding protein PstB;
P	P69879	DE	Phosphate-transporting ATPase;
P	P69879	DR	inorganic phosphate transmembrane transporter activity
P	P69879	DR	phosphate transmembrane-transporting ATPase activity
P	P69881	CC	Part of the ABC transporter complex PstSACB involved in phosphate import
P	P69881	DE	ABC phosphate transporter;
P	P69881	DE	Phosphate import ATP-binding protein PstB;
P	P69881	DE	Phosphate-transporting ATPase;
P	P69881	DR	inorganic phosphate transmembrane transporter activity
P	P69881	DR	phosphate transmembrane-transporting ATPase activity
P	P70172	CC	Plays a critical role in the sodium-dependent reabsorption of bile acids from the lumen of the small intestine
P	P70172	DE	Apical sodium-dependent bile acid transporter;
P	P70172	DE	Apical sodium-dependent bile acid transporter;
P	P70172	DE	Ileal Na+/bile acid cotransporter;
P	P70172	DE	Ileal Na+/bile acid cotransporter;
P	P70172	DE	Ileal sodium-dependent bile acid transporter;
P	P70172	DE	Ileal sodium-dependent bile acid transporter;
P	P70172	DE	Ileal sodium/bile acid cotransporter;
P	P70172	DE	Ileal sodium/bile acid cotransporter;
P	P70172	DE	Na+-dependent ileal bile acid transporter;
P	P70172	DE	Na+-dependent ileal bile acid transporter;
P	P70172	DE	Sodium/taurocholate cotransporting polypeptide, ileal;
P	P70187	DR	tetracycline:hydrogen antiporter activity
P	P70227	CC	Receptor for inositol 1,4,5-trisphosphate, a second messenger that mediates the release of intracellular calcium.
P	P70227	DR	inositol 1,4,5-trisphosphate-sensitive calcium-release channel activity
P	P70414	CC	Ca2+ is extruded from the cell during relaxation so as to prevent overloading of intracellular stores.
P	P70414	CC	Rapidly transports Ca2+ during excitation-contraction coupling
P	P70414	DE	Na+/Ca2+-exchange protein 1;
P	P70414	DE	Na+/Ca2+-exchange protein 1;
P	P70414	DE	Sodium/calcium exchanger 1;

P	P70414	DE	Sodium/calcium exchanger 1;
P	P70414	DR	calcium:sodium antiporter activity
P	P70414	DR	calcium:sodium antiporter activity
P	P70786	CC	Component of the tartrate utilization system and may allow entry of tartrate and tartrate dehydrogenase.
P	P70786	DE	Putative tartrate transporter;
P	P71067	DR	lactate transmembrane transporter activity
P	P71338	DE	Iron(III)-transport system permease protein fbpB 2;
P	P71370	CC	This protein is a component of the oligopeptide permease, a binding protein-dependent transport system, it binds peptides up to five amino acids long with high affinity (By similarity).
P	P71370	DE	Periplasmic oligopeptide-binding protein;
P	P71678	CC	Confers resistance to ethidium bromide
P	P72479	DE	Oligopeptide transport ATP-binding protein OppF;
P	P72827	CC	Plays an important role in protecting the acceptor side of photosystem II (PSII) against oxidative damage, especially under iron-limiting growth conditions (Probable).
P	P72827	DE	Iron uptake protein A1;
P	P73182	DE	Ferrous iron transport protein B homolog;
P	P73182	DR	ferrous iron transmembrane transporter activity
P	P73265	CC	Probably part of a high-affinity binding-protein- dependent transport system for nitrate
P	P73265	DE	Putative nitrate transport ATP-binding protein NrtD;
P	P73450	CC	Probably part of a high-affinity binding-protein- dependent transport system for nitrate
P	P73450	DE	Nitrate transport ATP-binding protein NrtC;
P	P73450	DR	nitrate transmembrane transporter activity
P	P73451	CC	Probably part of a high-affinity binding-protein- dependent transport system for nitrate; probably responsible for the translocation of the substrate across the membrane (By similarity).
P	P73451	DE	Nitrate transport permease protein nrtB;
P	P73451	DR	nitrate transmembrane transporter activity
P	P73452	CC	Essential component of the nitrate-transporting system
P	P73452	DE	Nitrate transport protein NrtA;
P	P73788	CC	Part of the ABC transporter complex PstSACB involved in phosphate import
P	P73788	DE	ABC phosphate transporter 3;
P	P73788	DE	Phosphate import ATP-binding protein PstB 3;
P	P73788	DE	Phosphate-transporting ATPase 3;
P	P73788	DR	inorganic phosphate transmembrane transporter activity
P	P73788	DR	phosphate transmembrane-transporting ATPase activity
P	P73809	CC	Channel that permits osmotically driven movement of water in both directions
P	P73809	CC	It mediates rapid entry or exit of water in response to abrupt changes in osmolarity (By similarity).
P	P73867	CC	One of the components of the high-affinity ATP-driven potassium transport (or KDP) system, which catalyzes the hydrolysis of ATP coupled with the exchange of hydrogen and potassium ions (By similarity).
P	P73867	DE	Potassium-binding and translocating subunit B;
P	P73867	DE	Potassium-translocating ATPase B chain;
P	P73867	DE	Potassium-transporting ATPase B chain;
P	P73867	DR	potassium-transporting ATPase activity
P	P74072	DE	H+/Ca2+ exchanger;
P	P74547	CC	Part of the ABC transporter complex CysAWTP (TC 3.A.1.6.1) involved in sulfate/thiosulfate import
P	P74547	DE	Sulfate transport system permease protein CysW;
P	P74547	DR	sulfate transmembrane transporter activity
P	P74548	CC	Part of the ABC transporter complex CysAWTP involved in sulfate/thiosulfate import
P	P74548	DE	Sulfate-transporting ATPase;
P	P74548	DE	Sulfate/thiosulfate import ATP-binding protein CysA;
P	P74548	DR	sulfate transmembrane-transporting ATPase activity
P	P74884	DE	Ferrous iron transport protein B;
P	P74884	DR	ferrous iron transmembrane transporter activity
P	P74997	DE	Lipoprotein-releasing system ATP-binding protein LolD;
P	P74997	DR	lipoprotein transporter activity
P	P75039	CC	This system is involved in fructose transport (By similarity).
P	P75039	DE	Fructose permease IIC component;
P	P75039	DE	Fructose-specific phosphotransferase enzyme IIA component;
P	P75039	DE	Fructose-specific phosphotransferase enzyme IIB component;
P	P75039	DE	PTS system fructose-specific EIIA component;

P	P75039	DE	PTS system fructose-specific EIIABC component;
P	P75039	DE	PTS system fructose-specific EIIB component;
P	P75039	DE	PTS system fructose-specific EIIC component;
P	P75057	CC	Required for the activity of the bacterial transport system of putrescine and spermidine (By similarity).
P	P75057	CC	Required for the activity of the bacterial transport system of putrescine and spermidine (By similarity).
P	P75057	DE	Spermidine/putrescine transport system permease protein PotC homolog;
P	P75057	DE	Spermidine/putrescine transport system permease protein PotC homolog;
P	P75058	CC	Required for the activity of the bacterial transport system of putrescine and spermidine (By similarity).
P	P75058	CC	Required for the activity of the bacterial transport system of putrescine and spermidine (By similarity).
P	P75058	DE	Spermidine/putrescine transport system permease protein PotB homolog;
P	P75058	DE	Spermidine/putrescine transport system permease protein PotB homolog;
P	P75059	CC	Part of the ABC transporter complex PotABCD involved in spermidine/putrescine import
P	P75059	CC	Part of the ABC transporter complex PotABCD involved in spermidine/putrescine import
P	P75059	DE	Spermidine/putrescine import ATP-binding protein PotA;
P	P75059	DE	Spermidine/putrescine import ATP-binding protein PotA;
P	P75071	CC	Glycerol enters the cell via the glycerol diffusion facilitator protein
P	P75071	CC	This membrane protein facilitates the movement of glycerol across the cytoplasmic membrane (By similarity).
P	P75071	DE	Probable glycerol uptake facilitator protein;
P	P75145	CC	This system is involved in mannitol transport (By similarity).
P	P75145	DE	Mannitol-specific phosphotransferase enzyme IIA component;
P	P75145	DE	PTS system mannitol-specific EIIA component;
P	P75146	CC	This system is involved in mannitol transport (By similarity).
P	P75146	DE	Mannitol permease IIC component;
P	P75146	DE	Mannitol-specific phosphotransferase enzyme IIB component;
P	P75146	DE	PTS system mannitol-specific EIIB component;
P	P75146	DE	PTS system mannitol-specific EIIC component;
P	P75146	DE	PTS system mannitol-specific EIICB component;
P	P75185	CC	Could be part of a binding-protein-dependent transport system for phosphate; probably responsible for the translocation of the substrate across the membrane (By similarity)
P	P75185	DE	Phosphate transport system permease protein pstA homolog;
P	P75185	DR	inorganic phosphate transmembrane transporter activity
P	P75186	CC	Part of the ABC transporter complex PstSACB involved in phosphate import
P	P75186	DE	ABC phosphate transporter;
P	P75186	DE	Phosphate import ATP-binding protein PstB;
P	P75186	DE	Phosphate-transporting ATPase;
P	P75186	DR	inorganic phosphate transmembrane transporter activity
P	P75186	DR	phosphate transmembrane-transporting ATPase activity
P	P75551	DE	Oligopeptide transport ATP-binding protein OppF;
P	P75552	DE	Oligopeptide transport ATP-binding protein OppD;
P	P75553	DE	Oligopeptide transport system permease protein oppC;
P	P75554	DE	Oligopeptide transport system permease protein oppB;
P	P75569	CC	This system is involved in glucose transport (By similarity).
P	P75569	DE	Glucose permease IIC component;
P	P75569	DE	Glucose-specific phosphotransferase enzyme IIA component;
P	P75569	DE	Glucose-specific phosphotransferase enzyme IIB component;
P	P75569	DE	PTS system glucose-specific EIIA component;
P	P75569	DE	PTS system glucose-specific EIIB component;
P	P75569	DE	PTS system glucose-specific EIIC component;
P	P75569	DE	PTS system glucose-specific EIICBA component;
P	P75780	CC	Involved in the active transport across the outer membrane of iron complexed with catecholate siderophores such as dihydroxybenzoylserine and dihydroxybenzoate
P	P75780	DE	Ferric iron uptake protein;
P	P76350	DE	Shikimate transporter;
P	P77348	CC	The transport is effected by the oligopeptide permease system.
P	P78334	CC	GABA, the major inhibitory neurotransmitter in the vertebrate brain, mediates neuronal inhibition by binding to the GABA/benzodiazepine receptor and opening an integral chloride channel.
P	P78334	DR	chloride channel activity
P	P78348	CC	Cation channel with high affinity for sodium, which is gated by extracellular protons and inhibited by the diuretic amiloride

P	P78348	DE	Brain sodium channel 2;
P	P78348	DR	ligand-gated sodium channel activity
P	P78363	CC	Once transported to the cytoplasmic surface, ATR is reduced to vitamin A by trans- retinol dehydrogenase (tRDH) and then transferred to the retinal pigment epithelium (RPE) where it is converted to 11-cis-retinal
P	P78363	DE	Retinal-specific ATP-binding cassette transporter;
P	P78381	DE	UDP-galactose translocator;
P	P78381	DE	UDP-galactose transporter;
P	P78381	DR	UDP-galactose transmembrane transporter activity
P	P78383	DE	UDP-galactose transporter-related protein 1;
P	P78383	DR	UDP-galactose transmembrane transporter activity
P	P78508	CC	Inward rectifier potassium channels are characterized by a greater tendency to allow potassium to flow into the cell rather than out of it
P	P78508	CC	May be responsible for potassium buffering action of glial cells in the brain
P	P78508	CC	Their voltage dependence is regulated by the concentration of extracellular potassium; as external potassium is raised, the voltage range of the channel opening shifts to more positive voltages
P	P78508	DE	ATP-dependent inwardly rectifying potassium channel Kir4.1;
P	P78508	DE	ATP-sensitive inward rectifier potassium channel 10;
P	P78508	DE	Inward rectifier K+ channel Kir1.2;
P	P78508	DE	Potassium channel, inwardly rectifying subfamily J member 10;
P	P78508	DR	ATP-activated inward rectifier potassium channel activity
P	P78831	CC	High-affinity glucose transporter.
P	P78831	DE	High-affinity glucose transporter ght5;
P	P80183	CC	This is an energy-conserving, sodium-ion translocating step.
P	P80184	CC	This is an energy-conserving, sodium-ion translocating step.
P	P80185	CC	This is an energy-conserving, sodium-ion translocating step.
P	P80186	CC	This is an energy-conserving, sodium-ion translocating step.
P	P80187	CC	This is an energy-conserving, sodium-ion translocating step
P	P86046	CC	Inward rectifier potassium channels are characterized by a greater tendency to allow potassium to flow into the cell rather than out of it
P	P86046	CC	Their voltage dependence is regulated by the concentration of extracellular potassium; as external potassium is raised, the voltage range of the channel opening shifts to more positive voltages
P	P86046	DE	Inward rectifier K+ channel Kir7.1;
P	P86046	DE	Inward rectifier potassium channel 13;
P	P86046	DE	Potassium channel, inwardly rectifying subfamily J member 13;
P	P86046	DR	inward rectifier potassium channel activity
P	P87041	CC	Essential for the transport of UDP-galactose into the lumen of Golgi apparatus.
P	P87041	DE	UDP-galactose transporter;
P	P87041	DR	UDP-galactose transmembrane transporter activity
P	P87110	CC	Transporter for myo-inositol.
P	P87110	DE	Myo-inositol transporter 2;
P	P87110	DR	myo-inositol transmembrane transporter activity
P	P87149	CC	Mitochondrial inner membrane magnesium transporter required for mitochondrial magnesium homeostasis
P	P87149	DE	Mitochondrial inner membrane magnesium transporter mrs2;
P	P87149	DR	magnesium ion transmembrane transporter activity
P	P91645	CC	Voltage-sensitive calcium channels (VSCC) mediate the entry of calcium ions into excitable cells and are also involved in a variety of calcium-dependent processes, including muscle contraction, neurotransmitter release, gene expression, cell motility, cell division and cell death (By similarity)
P	P91645	DE	Voltage-dependent calcium channel type A subunit alpha-1;
P	P91645	DR	voltage-gated calcium channel activity
P	P92935	DE	ADP,ATP carrier protein 2, chloroplastic;
P	P92935	DE	ADP/ATP translocase 2;
P	P92935	DE	ADP/ATP translocase 2;
P	P92935	DR	ATP:ADP antiporter activity
P	P92935	DR	ATP:ADP antiporter activity
P	P92939	CC	This magnesium-dependent enzyme catalyzes the hydrolysis of ATP coupled with the translocation of calcium from the cytosol to the endoplasmic reticulum lumen
P	P92939	DE	Calcium-transporting ATPase 1, endoplasmic reticulum-type;
P	P92939	DR	calcium-transporting ATPase activity
P	P92941	CC	Voltage-gated chloride channel that could play a role in the regulation of nitrate content
P	P92941	DE	Chloride channel protein CLC-a;

P	P92941	DR	voltage-gated chloride channel activity
P	P92942	CC	Voltage-gated chloride channel.
P	P92942	DE	Chloride channel protein CLC-b;
P	P92942	DR	voltage-gated chloride channel activity
P	P92943	CC	Voltage-gated chloride channel.
P	P92943	DE	Chloride channel protein CLC-d;
P	P92943	DR	voltage-gated chloride channel activity
P	P92946	CC	Low-affinity H+/sulfate cotransporter that may be involved in the distribution of sulfate from vascular bundles to the palisade cells of the leaves
P	P92946	CC	Plays a central role in the regulation of sulfate assimilation.
P	P92946	DE	Sulfate transporter 2.2;
P	P92946	DR	secondary active sulfate transmembrane transporter activity
P	P92960	CC	Could mediate potassium uptake from the soil solution by plant roots in association with AKT1.
P	P92960	CC	Probable modulatory (alpha) subunit of inward-rectifying potassium channels
P	P92960	DE	Potassium channel KAT3;
P	P92960	DE	Potassium channel TKC;
P	P92960	DR	voltage-gated potassium channel activity
P	P92961	DE	Proline transporter 1;
P	P93004	CC	Water channel required to facilitate the transport of water across cell membrane
P	P93004	DR	water channel activity
P	P94131	DE	Cis,cis-muconate transport protein;
P	P94363	CC	Proton motive force-driven secondary transporter that catalyzes the uptake of both citrate and malate
P	P94363	DE	Citrate/malate transporter;
P	P94363	DE	Citrate/malate-proton symporter;
P	P94606	CC	One of the components of the high-affinity ATP-driven potassium transport (or KDP) system, which catalyzes the hydrolysis of ATP coupled with the exchange of hydrogen and potassium ions
P	P94606	DE	Potassium-binding and translocating subunit C;
P	P94606	DE	Potassium-translocating ATPase C chain;
P	P94606	DE	Potassium-transporting ATPase C chain;
P	P94606	DR	potassium-transporting ATPase activity
P	P94633	DE	Lysine exporter protein;
P	P95302	CC	Part of the ABC transporter complex PstSACB involved in phosphate import
P	P95302	DE	ABC phosphate transporter 2;
P	P95302	DE	Phosphate import ATP-binding protein PstB 2;
P	P95302	DE	Phosphate-transporting ATPase 2;
P	P95302	DR	inorganic phosphate transmembrane transporter activity
P	P95302	DR	phosphate transmembrane-transporting ATPase activity
P	P96169	CC	Actively transports glucose into cells by Na+ cotransport (By similarity).
P	P96169	CC	Actively transports glucose into cells by Na+ cotransport (By similarity).
P	P96169	DE	Na+/glucose symporter;
P	P96169	DE	Na+/glucose symporter;
P	P96169	DE	Sodium/glucose cotransporter;
P	P96169	DE	Sodium/glucose cotransporter;
P	P96335	CC	Responsible for glycerol-3-phosphate uptake (By similarity).
P	P96335	DE	Glycerol-3-phosphate transporter;
P	P96335	DR	glycerol-3-phosphate transmembrane transporter activity
P	P96593	CC	H+-stimulated, highly selective, manganese uptake system
P	P96593	CC	May be the predominant transporter of manganese during logarithmic phase growth
P	P96593	DE	Manganese transport protein mntH;
P	P97370	CC	This is the non-catalytic component of the active enzyme, which catalyzes the hydrolysis of ATP coupled with the exchange of Na+ and K+ ions across the plasma membrane
P	P97370	CC	This is the non-catalytic component of the active enzyme, which catalyzes the hydrolysis of ATP coupled with the exchange of Na+ and K+ ions across the plasma membrane
P	P97370	DE	Sodium/potassium-dependent ATPase subunit beta-3;
P	P97370	DE	Sodium/potassium-dependent ATPase subunit beta-3;
P	P97370	DE	Sodium/potassium-transporting ATPase subunit beta-3;
P	P97370	DE	Sodium/potassium-transporting ATPase subunit beta-3;
P	P97370	DR	sodium:potassium-exchanging ATPase activity
P	P97370	DR	sodium:potassium-exchanging ATPase activity
P	P97438	CC	Outward rectifying potassium channel.
P	P97438	DE	Outward rectifying potassium channel protein TREK-1;
P	P97438	DE	Potassium channel subfamily K member 2;

P	P97438	DE	TREK-1 K+ channel subunit;
P	P97438	DE	Two pore potassium channel TPKC1;
P	P97438	DR	voltage-gated potassium channel activity
P	P97441	CC	Involved in accumulation of zinc in synaptic vesicles.
P	P97441	DE	Zinc transporter 3;
P	P97441	DR	zinc ion transmembrane transporter activity
P	P97445	CC	P/Q-type calcium channels belong to the 'high-voltage activated' (HVA) group and are blocked by the funnel toxin (Ftx) and by the omega-agatoxin- IVA (omega-Aga-IVA)
P	P97445	CC	The isoform alpha-1A gives rise to P and/or Q-type calcium currents
P	P97445	CC	Voltage-sensitive calcium channels (VSCC) mediate the entry of calcium ions into excitable cells and are also involved in a variety of calcium-dependent processes, including muscle contraction, hormone or neurotransmitter release, gene expression, cell motility, cell division and cell death
P	P97445	DE	Brain calcium channel I;
P	P97445	DE	Calcium channel, L type, alpha-1 polypeptide isoform 4;
P	P97445	DE	Voltage-dependent P/Q-type calcium channel subunit alpha-1A;
P	P97445	DE	Voltage-gated calcium channel subunit alpha Cav2.1;
P	P97445	DR	high voltage-gated calcium channel activity
P	P97521	DE	Carnitine/acylcarnitine translocase;
P	P97521	DE	Mitochondrial carnitine/acylcarnitine carrier protein;
P	P97794	CC	Inward rectifier potassium channels are characterized by a greater tendency to allow potassium to flow into the cell rather than out of it
P	P97794	CC	Their voltage dependence is regulated by the concentration of extracellular potassium; as external potassium is raised, the voltage range of the channel opening shifts to more positive voltages
P	P97794	CC	This potassium channel is controlled by G proteins
P	P97794	DE	ATP-sensitive inward rectifier potassium channel 8;
P	P97794	DE	Inward rectifier K+ channel Kir6.1;
P	P97794	DE	Potassium channel, inwardly rectifying subfamily J member 8;
P	P97858	DE	UDP-galactose translocator 2;
P	P97858	DE	UDP-galactose transporter-related protein 1;
P	P98194	CC	This magnesium-dependent enzyme catalyzes the hydrolysis of ATP coupled with the transport of the calcium.
P	P98194	DE	ATP-dependent Ca2+ pump PMR1;
P	P98194	DE	Calcium-transporting ATPase type 2C member 1;
P	P98194	DR	calcium-transporting ATPase activity
P	P98194	DR	manganese-transporting ATPase activity
P	Q00325	CC	Phosphate is cotransported with H+.
P	Q00325	CC	Transport of phosphate groups from the cytosol to the mitochondrial matrix
P	Q00325	DE	Phosphate carrier protein, mitochondrial;
P	Q00325	DE	Phosphate transport protein;
P	Q00325	DR	phosphate carrier activity
P	Q00749	CC	Involved in a binding protein-dependent transport system responsible for the uptake of melibiose, raffinose and isomaltotriose.
P	Q00749	CC	Involved in a binding protein-dependent transport system responsible for the uptake of melibiose, raffinose and isomaltotriose.
P	Q00750	CC	Involved in a binding protein-dependent transport system responsible for the uptake of melibiose, raffinose and isomaltotriose.
P	Q00750	CC	Involved in a binding protein-dependent transport system responsible for the uptake of melibiose, raffinose and isomaltotriose.
P	Q00751	CC	Involved in a binding protein-dependent transport system responsible for the uptake of melibiose, raffinose and isomaltotriose.
P	Q00751	CC	Involved in a binding protein-dependent transport system responsible for the uptake of melibiose, raffinose and isomaltotriose.
P	Q00752	CC	Involved in a binding protein-dependent transport system responsible for the uptake of melibiose, raffinose and isomaltotriose
P	Q00752	CC	Involved in a binding protein-dependent transport system responsible for the uptake of melibiose, raffinose and isomaltotriose
P	Q00975	CC	Calcium channels containing alpha-1B subunit may play a role in directed migration of immature neurons.
P	Q00975	CC	N-type calcium channels belong to the 'high-voltage activated' (HVA) group and are blocked by omega-conotoxin-GVIA (omega-CTx-GVIA) and by omega-agatoxin- IIIA (omega-Aga-IIIA)
P	Q00975	CC	The isoform alpha-1B gives rise to N-type calcium currents

P	Q00975	CC	Voltage-sensitive calcium channels (VSCC) mediate the entry of calcium ions into excitable cells and are also involved in a variety of calcium-dependent processes, including muscle contraction, hormone or neurotransmitter release, gene expression, cell motility, cell division and cell death
P	Q00975	DE	Brain calcium channel III;
P	Q00975	DE	Calcium channel, L type, alpha-1 polypeptide isoform 5;
P	Q00975	DE	Voltage-dependent N-type calcium channel subunit alpha-1B;
P	Q00975	DE	Voltage-gated calcium channel subunit alpha Cav2.2;
P	Q00975	DR	voltage-gated calcium channel activity
P	Q01118	CC	Assuming opened or closed conformations in response to the voltage difference across the membrane, the protein forms a sodium-selective channel through which Na+ ions may pass in accordance with their electrochemical gradient.
P	Q01118	CC	Mediates the voltage-dependent sodium ion permeability of excitable membranes
P	Q01118	DE	Putative voltage-gated sodium channel subunit alpha Nax;
P	Q01118	DE	Sodium channel protein cardiac and skeletal muscle subunit alpha;
P	Q01118	DE	Sodium channel protein type 7 subunit alpha;
P	Q01118	DE	Sodium channel protein type VII subunit alpha;
P	Q01118	DR	voltage-gated sodium channel activity
P	Q01395	DE	Repressible high-affinity phosphate permease;
P	Q01395	DR	inorganic phosphate transmembrane transporter activity
P	Q01668	CC	Long-lasting (L-type) calcium channels belong to the 'high-voltage activated' (HVA) group
P	Q01668	CC	The isoform alpha-1D gives rise to L-type calcium currents
P	Q01668	CC	Voltage-sensitive calcium channels (VSCC) mediate the entry of calcium ions into excitable cells and are also involved in a variety of calcium-dependent processes, including muscle contraction, hormone or neurotransmitter release, gene expression, cell motility, cell division and cell death
P	Q01668	DE	Calcium channel, L type, alpha-1 polypeptide, isoform 2;
P	Q01668	DE	Voltage-dependent L-type calcium channel subunit alpha-1D;
P	Q01668	DE	Voltage-gated calcium channel subunit alpha Cav1.3;
P	Q01668	DR	voltage-gated calcium channel activity
P	Q01814	CC	This magnesium-dependent enzyme catalyzes the hydrolysis of ATP coupled with the transport of calcium out of the cell.
P	Q01814	DE	Plasma membrane calcium ATPase isoform 2;
P	Q01814	DE	Plasma membrane calcium pump isoform 2;
P	Q01814	DE	Plasma membrane calcium-transporting ATPase 2;
P	Q01814	DR	calcium-transporting ATPase activity
P	Q01857	DR	sodium:dicarboxylate symporter activity
P	Q01895	CC	Part of the ABC transporter complex CysAWTP (TC 3.A.1.6.1) involved in sulfate/thiosulfate import
P	Q01895	DE	Sulfate transport system permease protein CysT;
P	Q01895	DR	sulfate transmembrane transporter activity
P	Q01896	CC	This magnesium-dependent enzyme catalyzes the hydrolysis of ATP coupled with the transport of sodium or lithiums ions to allow salt tolerance.
P	Q01896	DE	Sodium transport ATPase 2;
P	Q01896	DR	sodium-exporting ATPase activity, phosphorylative mechanism
P	Q01903	CC	This protein specifically binds sulfate and is involved in its transmembrane transport
P	Q01903	DE	Sulfate-binding protein;
P	Q01903	DR	secondary active sulfate transmembrane transporter activity
P	Q01903	DR	sulfate transmembrane-transporting ATPase activity
P	Q01926	CC	Magnesium transporter that mediates the influx of magnesium into the mitochondrial matrix
P	Q01926	DE	Magnesium transporter MRS2, mitochondrial;
P	Q01926	DR	magnesium ion transmembrane transporter activity
P	Q01937	CC	Part of the binding-protein-dependent transport system for lactose
P	Q01937	DE	Lactose transport ATP-binding protein LacK;
P	Q01959	CC	Terminates the action of dopamine by its high affinity sodium-dependent reuptake into presynaptic terminals.
P	Q01959	CC	Terminates the action of dopamine by its high affinity sodium-dependent reuptake into presynaptic terminals.
P	Q01959	DE	Sodium-dependent dopamine transporter;
P	Q01959	DE	Sodium-dependent dopamine transporter;
P	Q02013	CC	Forms a water-specific channel that provides the plasma membranes of red cells and kidney proximal tubules with high permeability to water, thereby permitting water to move in the direction of an osmotic gradient.

P	Q02013	DE	Water channel protein for red blood cells and kidney proximal tubule;
P	Q02013	DR	water channel activity
P	Q02280	CC	Mediates the potassium permeability of membranes; potassium current is regulated by CaMKII and CASK
P	Q02280	CC	Structural component of a potassium channel
P	Q02280	DE	Potassium voltage-gated channel protein eag;
P	Q02280	DR	voltage-gated potassium channel activity
P	Q02420	CC	This system is involved in mannitol transport (By similarity).
P	Q02420	DE	Mannitol-specific phosphotransferase enzyme IIA component;
P	Q02420	DE	PTS system mannitol-specific EIIA component;
P	Q02783	CC	Mitochondrial inner membrane magnesium transporter required for mitochondrial magnesium homeostasis
P	Q02783	DE	Mitochondrial inner membrane magnesium transporter LPE10;
P	Q02783	DR	magnesium ion transmembrane transporter activity
P	Q02978	CC	Catalyzes the transport of 2-oxoglutarate across the inner mitochondrial membrane in an electroneutral exchange for malate or other dicarboxylic acids, and plays an important role in several metabolic processes, including the malate-aspartate shuttle, the oxoglutarate/isocitrate shuttle, in gluconeogenesis from lactate, and in nitrogen metabolism.
P	Q02978	CC	Catalyzes the transport of 2-oxoglutarate across the inner mitochondrial membrane in an electroneutral exchange for malate or other dicarboxylic acids, and plays an important role in several metabolic processes, including the malate-aspartate shuttle, the oxoglutarate/isocitrate shuttle, in gluconeogenesis from lactate, and in nitrogen metabolism.
P	Q02978	DE	Mitochondrial 2-oxoglutarate/malate carrier protein;
P	Q02978	DE	Mitochondrial 2-oxoglutarate/malate carrier protein;
P	Q02978	DR	oxoglutarate:malate antiporter activity
P	Q02DK6	CC	Part of the ABC transporter complex MetNIQ involved in methionine import
P	Q02DK6	DE	Methionine import ATP-binding protein MetN 2;
P	Q02DK9	CC	Part of the ABC transporter complex ZnuABC involved in zinc import
P	Q02DK9	DE	Zinc import ATP-binding protein ZnuC;
P	Q02DK9	DR	zinc transporting ATPase activity
P	Q02FW7	CC	Part of the ABC transporter complex HmuTUV involved in hemin import
P	Q02FW7	DE	Hemin import ATP-binding protein HmuV;
P	Q02I77	CC	Transport of potassium into the cell (By similarity).
P	Q02I77	DE	Probable potassium transport system protein kup;
P	Q02I77	DR	potassium ion transmembrane transporter activity
P	Q02KC0	CC	One of the components of the high-affinity ATP-driven potassium transport (or KDP) system, which catalyzes the hydrolysis of ATP coupled with the exchange of hydrogen and potassium ions
P	Q02KC0	DE	Potassium-binding and translocating subunit C;
P	Q02KC0	DE	Potassium-translocating ATPase C chain;
P	Q02KC0	DE	Potassium-transporting ATPase C chain;
P	Q02KC0	DR	potassium-transporting ATPase activity
P	Q02KW3	CC	Na+/H+ antiporter that extrudes sodium in exchange for external protons (By similarity).
P	Q02KW3	DE	Na+/H+ antiporter nhaB;
P	Q02KW3	DE	Sodium/proton antiporter nhaB;
P	Q02KW3	DR	sodium:hydrogen antiporter activity
P	Q02LI9	CC	Involved in the import of serine and threonine into the cell, with the concomitant import of sodium (symport system) (By similarity).
P	Q02LI9	CC	Involved in the import of serine and threonine into the cell, with the concomitant import of sodium (symport system) (By similarity).
P	Q02LI9	DE	Na+/serine-threonine symporter;
P	Q02LI9	DE	Na+/serine-threonine symporter;
P	Q02LI9	DE	Serine/threonine transporter sstT;
P	Q02LI9	DR	sodium:dicarboxylate symporter activity
P	Q02ME3	CC	Part of the ABC transporter complex MetNIQ involved in methionine import
P	Q02ME3	DE	Methionine import ATP-binding protein MetN 1;
P	Q02QE8	DR	phosphonate transmembrane-transporting ATPase activity
P	Q02QM1	DR	phosphonate transmembrane-transporting ATPase activity
P	Q02R79	CC	Part of the ABC transporter complex PotABCD involved in spermidine/putrescine import
P	Q02R79	CC	Part of the ABC transporter complex PotABCD involved in spermidine/putrescine import
P	Q02R79	DE	Spermidine/putrescine import ATP-binding protein PotA;
P	Q02R79	DE	Spermidine/putrescine import ATP-binding protein PotA;
P	Q02SA6	CC	Part of the ABC transporter complex TauABC involved in taurine import

P	Q02SA6	DE	Taurine import ATP-binding protein TauB;
P	Q02SA6	DR	taurine-transporting ATPase activity
P	Q02SS7	CC	The physiological role may be the reduction of the intracellular concentration of toxic sugars or sugar metabolites (By similarity).
P	Q02SS7	DE	Probable sugar efflux transporter;
P	Q02UV9	DR	sodium:dicarboxylate symporter activity
P	Q02UV9	DR	sodium:dicarboxylate symporter activity
P	Q02VA9	CC	This protein is a component of the oligopeptide permease, a binding protein-dependent transport system, it binds peptides up to five amino acids long with high affinity
P	Q02VA9	DE	Oligopeptide-binding protein oppA;
P	Q02XM9	CC	Part of the ABC transporter complex RbsABCD involved in ribose import
P	Q02XM9	DE	Ribose import ATP-binding protein RbsA;
P	Q02Z10	CC	Part of the ABC transporter complex PotABCD involved in spermidine/putrescine import
P	Q02Z10	CC	Part of the ABC transporter complex PotABCD involved in spermidine/putrescine import
P	Q02Z10	DE	Spermidine/putrescine import ATP-binding protein PotA;
P	Q02Z10	DE	Spermidine/putrescine import ATP-binding protein PotA;
P	Q03030	CC	Lyase and sodium transporter.
P	Q03031	CC	Lyase and sodium transporter.
P	Q03032	CC	Lyase and sodium transporter.
P	Q03032	DR	sodium ion transmembrane transporter activity
P	Q03218	CC	Mitochondrial metal transporter involved in mitochondrial iron accumulation.
P	Q03218	DE	Mitochondrial metal transporter 1;
P	Q032A0	CC	Part of the ABC transporter complex MetNIQ involved in methionine import
P	Q032A0	DE	Methionine import ATP-binding protein MetN;
P	Q032D0	DR	phosphonate transmembrane-transporting ATPase activity
P	Q03518	CC	Also acts as a molecular scaffold for the final stage of MHC class I folding, namely the binding of peptide
P	Q03518	CC	Expression of TAP1 is down-regulated by human Epstein-Barr virus vIL-10 protein, thereby affecting the transport of peptides into the endoplasmic reticulum and subsequent peptide loading by MHC class I molecules.
P	Q03518	CC	Inhibited by human cytomegalovirus US6 glycoprotein, which binds to the lumenal side of the TAP complex and inhibits peptide translocation by specifically blocking ATP-binding to TAP1 and prevents the conformational rearrangement of TAP induced by peptide binding
P	Q03518	CC	Inhibited by the covalent attachment of herpes simplex virus ICP47 protein, which blocks the peptide-binding site of TAP
P	Q03518	DE	Antigen peptide transporter 1;
P	Q03518	DE	Peptide supply factor 1;
P	Q03518	DE	Peptide transporter PSF1;
P	Q03518	DE	Peptide transporter TAP1;
P	Q03518	DE	Peptide transporter involved in antigen processing 1;
P	Q03519	CC	Also acts as a molecular scaffold for the final stage of MHC class I folding, namely the binding of peptide
P	Q03519	CC	Inhibited by human cytomegalovirus US6 glycoprotein, which binds to the lumenal side of the TAP complex and inhibits peptide translocation by specifically blocking ATP-binding to TAP1 and prevents the conformational rearrangement of TAP induced by peptide binding
P	Q03519	CC	Inhibited by the covalent attachment of herpes simplex virus ICP47 protein, which blocks the peptide-binding site of TAP
P	Q03519	DE	Antigen peptide transporter 2;
P	Q03519	DE	Peptide supply factor 2;
P	Q03519	DE	Peptide transporter PSF2;
P	Q03519	DE	Peptide transporter TAP2;
P	Q03519	DE	Peptide transporter involved in antigen processing 2;
P	Q03519	DR	peptide antigen-transporting ATPase activity
P	Q035E0	DR	phosphonate transmembrane-transporting ATPase activity
P	Q035P5	CC	Transport of potassium into the cell (By similarity).
P	Q035P5	DE	Probable potassium transport system protein kup;
P	Q035P5	DR	potassium ion transmembrane transporter activity
P	Q03614	CC	Terminates the action of dopamine by its high affinity sodium-dependent reuptake into presynaptic terminals.
P	Q03614	DE	Sodium-dependent dopamine transporter;
P	Q03614	DR	dopamine:sodium symporter activity
P	Q03719	CC	Pore-forming (alpha) subunit of voltage-gated rapidly inactivating A-type potassium channels

P	Q03719	DE	Potassium voltage-gated channel subfamily D member 1;
P	Q03719	DE	Voltage-gated potassium channel subunit Kv4.1;
P	Q03719	DR	voltage-gated potassium channel activity
P	Q03721	CC	Assuming opened or closed conformations in response to the voltage difference across the membrane, the protein forms a potassium-selective channel through which potassium ions may pass in accordance with their electrochemical gradient
P	Q03721	CC	This protein mediates the voltage-dependent potassium ion permeability of excitable membranes
P	Q03721	DE	Potassium voltage-gated channel subfamily C member 4;
P	Q03721	DE	Voltage-gated potassium channel subunit Kv3.4;
P	Q03721	DR	voltage-gated potassium channel activity
P	Q03A07	CC	Part of the ABC transporter complex MetNIQ involved in methionine import
P	Q03A07	DE	Methionine import ATP-binding protein MetN;
P	Q03AH0	CC	Part of the ABC transporter complex PotABCD involved in spermidine/putrescine import
P	Q03AH0	CC	Part of the ABC transporter complex PotABCD involved in spermidine/putrescine import
P	Q03AH0	DE	Spermidine/putrescine import ATP-binding protein PotA;
P	Q03AH0	DE	Spermidine/putrescine import ATP-binding protein PotA;
P	Q03CA4	CC	Part of the ABC transporter complex RbsABCD involved in ribose import
P	Q03CA4	DE	Ribose import ATP-binding protein RbsA;
P	Q03D26	CC	H+-stimulated, highly selective, manganese uptake system (By similarity).
P	Q03D26	DE	Probable manganese transport protein mntH;
P	Q03DK0	CC	H+-stimulated, highly selective, manganese uptake system (By similarity).
P	Q03DK0	DE	Probable manganese transport protein mntH;
P	Q03JH1	CC	Part of the ABC transporter complex PotABCD involved in spermidine/putrescine import
P	Q03JH1	CC	Part of the ABC transporter complex PotABCD involved in spermidine/putrescine import
P	Q03JH1	DE	Spermidine/putrescine import ATP-binding protein PotA;
P	Q03JH1	DE	Spermidine/putrescine import ATP-binding protein PotA;
P	Q03MC5	CC	Involved in the import of serine and threonine into the cell, with the concomitant import of sodium (symport system) (By similarity).
P	Q03MC5	DE	Na+/serine-threonine symporter;
P	Q03MC5	DR	sodium:dicarboxylate symporter activity
P	Q03MC5	DR	sodium:dicarboxylate symporter activity
P	Q03P57	CC	Part of the ABC transporter complex MetNIQ involved in methionine import
P	Q03P57	DE	Methionine import ATP-binding protein MetN;
P	Q03PF2	CC	Part of the ABC transporter complex PotABCD involved in spermidine/putrescine import
P	Q03PF2	CC	Part of the ABC transporter complex PotABCD involved in spermidine/putrescine import
P	Q03PF2	DE	Spermidine/putrescine import ATP-binding protein PotA;
P	Q03PF2	DE	Spermidine/putrescine import ATP-binding protein PotA;
P	Q03S07	CC	Transport of potassium into the cell (By similarity).
P	Q03S07	DE	Probable potassium transport system protein kup;
P	Q03S07	DR	potassium ion transmembrane transporter activity
P	Q03TH5	CC	H+-stimulated, highly selective, manganese uptake system (By similarity).
P	Q03TH5	DE	Probable manganese transport protein mntH;
P	Q03XG1	CC	Transport of potassium into the cell (By similarity).
P	Q03XG1	DE	Probable potassium transport system protein kup;
P	Q03XG1	DR	potassium ion transmembrane transporter activity
P	Q03Z27	CC	Part of the ABC transporter complex MetNIQ involved in methionine import
P	Q03Z27	DE	Methionine import ATP-binding protein MetN;
P	Q03ZQ0	CC	Part of the ABC transporter complex PotABCD involved in spermidine/putrescine import
P	Q03ZQ0	CC	Part of the ABC transporter complex PotABCD involved in spermidine/putrescine import
P	Q03ZQ0	DE	Spermidine/putrescine import ATP-binding protein PotA;
P	Q03ZQ0	DE	Spermidine/putrescine import ATP-binding protein PotA;
P	Q04121	CC	Endosomal/prevacuolar electroneutral Na+/H+ exchanger which mediates intracellular sequestration of Na+ cations, regulates vacuolar pH and contributes to osmotolerance following sudden exposure to hyperosmotic media
P	Q04121	DE	Endosomal/prevacuolar Na+/H+ exchanger;
P	Q04121	DE	Endosomal/prevacuolar sodium/hydrogen exchanger;
P	Q04121	DR	sodium:hydrogen antiporter activity
P	Q042G7	CC	Part of the ABC transporter complex PotABCD involved in spermidine/putrescine import
P	Q042G7	CC	Part of the ABC transporter complex PotABCD involved in spermidine/putrescine import
P	Q042G7	DE	Spermidine/putrescine import ATP-binding protein PotA;
P	Q042G7	DE	Spermidine/putrescine import ATP-binding protein PotA;
P	Q043Y8	CC	Part of the ABC transporter complex MetNIQ involved in methionine import
P	Q043Y8	DE	Methionine import ATP-binding protein MetN;

P	Q04646	CC	May be involved in forming the receptor site for cardiac glycoside binding or may modulate the transport function of the sodium ATPase.
P	Q04646	DE	Na+/K+ ATPase subunit gamma;
P	Q04646	DE	Na+/K+ ATPase subunit gamma;
P	Q04646	DE	Sodium pump gamma chain;
P	Q04646	DE	Sodium/potassium-transporting ATPase subunit gamma;
P	Q04646	DE	Sodium/potassium-transporting ATPase subunit gamma;
P	Q04656	CC	May supply copper to copper-requiring proteins within the secretory pathway, when localized in the trans-Golgi network
P	Q04656	CC	Under conditions of elevated extracellular copper, it relocalized to the plasma membrane where it functions in the efflux of copper from cells.
P	Q04656	DE	Copper pump 1;
P	Q04656	DE	Copper-transporting ATPase 1;
P	Q04656	DR	copper-exporting ATPase activity
P	Q04656	DR	superoxide dismutase copper chaperone activity
P	Q04671	CC	Could be involved in the transport of tyrosine, the precursor to melanin synthesis, within the melanocyte
P	Q04671	DR	L-tyrosine transmembrane transporter activity
P	Q046Q7	CC	Transport of potassium into the cell (By similarity).
P	Q046Q7	DE	Probable potassium transport system protein kup;
P	Q046Q7	DR	potassium ion transmembrane transporter activity
P	Q04895	CC	Transport of allantoin.
P	Q04895	DE	Allantoin permease;
P	Q04895	DE	Allantoin transport protein;
P	Q04895	DR	allantoin uptake transmembrane transporter activity
P	Q04B25	CC	Part of the ABC transporter complex MetNIQ involved in methionine import
P	Q04B25	DE	Methionine import ATP-binding protein MetN;
P	Q04BG2	CC	Part of the ABC transporter complex PotABCD involved in spermidine/putrescine import
P	Q04BG2	CC	Part of the ABC transporter complex PotABCD involved in spermidine/putrescine import
P	Q04BG2	DE	Spermidine/putrescine import ATP-binding protein PotA;
P	Q04BG2	DE	Spermidine/putrescine import ATP-binding protein PotA;
P	Q04CF3	CC	Transport of potassium into the cell (By similarity).
P	Q04CF3	DE	Probable potassium transport system protein kup;
P	Q04CF3	DR	potassium ion transmembrane transporter activity
P	Q04CG8	DR	phosphonate transmembrane-transporting ATPase activity
P	Q04DA7	CC	Part of the ABC transporter complex MetNIQ involved in methionine import
P	Q04DA7	DE	Methionine import ATP-binding protein MetN 2;
P	Q04F14	CC	Part of the ABC transporter complex MetNIQ involved in methionine import
P	Q04F14	DE	Methionine import ATP-binding protein MetN 1;
P	Q05085	CC	Displays an auxin transport facilitation inhibited by high nitrate concentration
P	Q05085	CC	Dual affinity nitrate transporter
P	Q05085	CC	Involved in proton- dependent nitrate uptake and in the regulation of the nitrate transporter NRT2.1
P	Q05085	CC	Required to prevent auxin accumulation in preemerged lateral root primordia and young lateral roots when external nitrate concentration is low or null
P	Q05085	DE	Nitrate transporter 1.1;
P	Q05085	DE	Nitrate/chlorate transporter;
P	Q05085	DE	Nitrate/chlorate transporter;
P	Q05085	DR	nitrate transmembrane transporter activity
P	Q05181	DE	Phthalate transporter;
P	Q05595	DR	cobalt ion transmembrane transporter activity
P	Q05598	DR	cobalt ion transmembrane transporter activity
P	Q058N4	CC	High-affinity magnesium transporter that mediates the influx of magnesium in chloroplast
P	Q058N4	DE	Magnesium Transporter 10;
P	Q058N4	DE	Magnesium transporter MRS2-11, chloroplastic;
P	Q058N4	DR	magnesium ion transmembrane transporter activity
P	Q05998	CC	Responsible for intake of thiamine.
P	Q05998	DE	Thiamine transporter;
P	Q05998	DR	thiamine transmembrane transporter activity
P	Q05BQ3	DR	glucose transmembrane transporter activity
P	Q06222	CC	Facilitative glucose transporter.
P	Q06222	DE	Glucose transporter 2A;
P	Q06495	CC	May be involved in actively transporting phosphate into cells via Na+ cotransport in the renal brush border membrane

P	Q06495	CC	May be involved in actively transporting phosphate into cells via Na+ cotransport in the renal brush border membrane
P	Q06495	DE	Na+-dependent phosphate cotransporter 2A;
P	Q06495	DE	Na+-dependent phosphate cotransporter 2A;
P	Q06495	DE	Na+/Pi cotransporter 2A;
P	Q06495	DE	Sodium-dependent phosphate transport protein 2A;
P	Q06495	DE	Sodium-dependent phosphate transport protein 2A;
P	Q06495	DE	Sodium-phosphate transport protein 2A;
P	Q06495	DE	Sodium/phosphate cotransporter 2A;
P	Q06495	DE	Sodium/phosphate cotransporter 2A;
P	Q06495	DR	sodium-dependent phosphate transmembrane transporter activity
P	Q06495	DR	sodium-dependent phosphate transmembrane transporter activity
P	Q06496	CC	May be involved in actively transporting phosphate into cells via Na+ cotransport in the renal brush border membrane
P	Q06496	CC	May be involved in actively transporting phosphate into cells via Na+ cotransport in the renal brush border membrane
P	Q06496	DE	Na+-dependent phosphate cotransporter 2A;
P	Q06496	DE	Na+-dependent phosphate cotransporter 2A;
P	Q06496	DE	Na+/Pi cotransporter 2A;
P	Q06496	DE	Sodium-dependent phosphate transport protein 2A;
P	Q06496	DE	Sodium-dependent phosphate transport protein 2A;
P	Q06496	DE	Sodium-phosphate transport protein 2A;
P	Q06496	DE	Sodium/phosphate cotransporter 2A;
P	Q06496	DE	Sodium/phosphate cotransporter 2A;
P	Q06496	DR	sodium-dependent phosphate transmembrane transporter activity
P	Q06496	DR	sodium-dependent phosphate transmembrane transporter activity
P	Q06593	DE	Oligopeptide transporter 2;
P	Q06593	DR	oligopeptide transporter activity
P	Q06598	DR	arsenite transmembrane transporter activity
P	Q06611	CC	Essential for the water permeability of the plasma membrane and for the morphology of the root system
P	Q06611	CC	Water channel required to facilitate the transport of water across cell membrane
P	Q06611	DR	water channel activity
P	Q06686	CC	Required for high affinity copper (probably reduced Cu I) transport into the cell.
P	Q06686	DE	Copper transport protein CTR3;
P	Q06686	DE	Copper transporter 3;
P	Q06686	DR	copper uptake transmembrane transporter activity
P	Q06700	CC	Decarboxylase subunit of the primary sodium pump glutaconyl-CoA decarboxylase (GCD).
P	Q07307	CC	Uric acid-xanthine transporter.
P	Q07307	DE	Uric acid-xanthine permease;
P	Q07307	DR	urate transmembrane transporter activity
P	Q07647	CC	Facilitative glucose transporter
P	Q07647	CC	Probably a neuronal glucose transporter.
P	Q07647	DE	Glucose transporter type 3, brain;
P	Q07647	DE	Solute carrier family 2, facilitated glucose transporter member 3;
P	Q07733	DE	Oligopeptide transport ATP-binding protein OppD;
P	Q07782	CC	Sodium/sulfate cotransporter that mediates sulfate reabsorption in the kidney.
P	Q07782	CC	Sodium/sulfate cotransporter that mediates sulfate reabsorption in the kidney.
P	Q07782	DE	Na+/sulfate cotransporter;
P	Q07782	DE	Na+/sulfate cotransporter;
P	Q07782	DE	Renal sodium/sulfate cotransporter;
P	Q07782	DE	Renal sodium/sulfate cotransporter;
P	Q07782	DR	sodium:sulfate symporter activity
P	Q07782	DR	sodium:sulfate symporter activity
P	Q07824	CC	Catalyzes polyamine uptake at alkaline pH and excretion at acidic pH
P	Q07824	CC	Cell membrane polyamine/proton antiporter, involved in the detoxification of excess polyamines in the cytoplasm
P	Q07824	CC	Recognizes spermidine, spermine and putrescine, the polyamine analogs methylglyoxal bis(guanylhydrazone) (MGBG) and paraquat, the antimalarial drug quinidine, and cycloheximide
P	Q07824	CC	Recognizes spermidine, spermine and putrescine, the polyamine analogs methylglyoxal bis(guanylhydrazone) (MGBG) and paraquat, the antimalarial drug quinidine, and cycloheximide
P	Q07824	DE	Polyamine transporter 1;

P	Q07YY4	CC	Na+/H+ antiporter that extrudes sodium in exchange for external protons (By similarity)
P	Q07YY4	DE	Na+/H+ antiporter nhaA;
P	Q07YY4	DE	Sodium/proton antiporter nhaA;
P	Q07ZL7	CC	Involved in the import of serine and threonine into the cell, with the concomitant import of sodium (symport system) (By similarity).
P	Q07ZL7	CC	Involved in the import of serine and threonine into the cell, with the concomitant import of sodium (symport system) (By similarity).
P	Q07ZL7	DE	Na+/serine-threonine symporter;
P	Q07ZL7	DE	Na+/serine-threonine symporter;
P	Q07ZL7	DE	Serine/threonine transporter sstT;
P	Q07ZL7	DR	sodium:dicarboxylate symporter activity
P	Q080S4	DE	Lipoprotein-releasing system ATP-binding protein LolD;
P	Q080S4	DR	lipoprotein transporter activity
P	Q08299	CC	Involved in the transport of siderophore enterobactin and so has a role in iron homeostasis.
P	Q08299	DE	Enterobactin permease;
P	Q08357	CC	In vitro, sodium-dependent phosphate uptake is not siginificantly affected by acidic and alkaline conditions, however sodium-independent phosphate uptake occurs at acidic conditions
P	Q08357	CC	Sodium-phosphate symporter which seems to play a fundamental housekeeping role in phosphate transport by absorbing phosphate from interstitial fluid for normal cellular functions such as cellular metabolism, signal transduction, and nucleic acid and lipid synthesis
P	Q08357	DE	Phosphate transporter 2;
P	Q08357	DE	Sodium-dependent phosphate transporter 2;
P	Q08357	DR	inorganic phosphate transmembrane transporter activity
P	Q08357	DR	sodium-dependent phosphate transmembrane transporter activity
P	Q08357	DR	sodium:phosphate symporter activity
P	Q083H3	CC	Na+/H+ antiporter that extrudes sodium in exchange for external protons (By similarity)
P	Q083H3	DE	Na+/H+ antiporter nhaB;
P	Q083H3	DE	Sodium/proton antiporter nhaB;
P	Q083H3	DR	sodium:hydrogen antiporter activity
P	Q08460	CC	Potassium channel activated by both membrane depolarization or increase in cytosolic Ca2+ that mediates export of K+
P	Q08460	DR	voltage-gated potassium channel activity
P	Q084V3	CC	Part of the ABC transporter complex PstSACB involved in phosphate import
P	Q084V3	DE	ABC phosphate transporter;
P	Q084V3	DE	Phosphate import ATP-binding protein PstB;
P	Q084V3	DE	Phosphate-transporting ATPase;
P	Q084V3	DR	inorganic phosphate transmembrane transporter activity
P	Q084V3	DR	phosphate transmembrane-transporting ATPase activity
P	Q08579	CC	It is possible that the primary function is the uptake of closely related compounds and that thiamine transport is a secondary activity of these proteins.
P	Q08579	CC	Low affinity thiamine transporter responsible for intake of thiamine
P	Q08579	DE	Thiamine transporter THI72;
P	Q08733	CC	Water channel required to facilitate the transport of water across cell membrane
P	Q08733	DR	water channel activity
P	Q08832	CC	GABA, an inhibitory neurotransmitter, mediates neuronal inhibition by binding to the GABA receptor and opening an integral chloride channel
P	Q08832	DR	chloride channel activity
P	Q08929	CC	Involved in active uptake of glycerol driven by electrogenic proton symport.
P	Q08929	DE	Glycerol uptake protein 2;
P	Q08970	CC	Mitochondrial metal transporter involved in mitochondrial iron accumulation.
P	Q08970	DE	Mitochondrial metal transporter 2;
P	Q08986	CC	High-affinity S-adenosylmethionine permease, required for utilization of S-adenosylmethionine as a sulfur source.
P	Q08986	DE	S-adenosylmethionine metabolism protein 3;
P	Q08986	DE	S-adenosylmethionine permease SAM3;
P	Q08986	DR	S-adenosylmethionine transmembrane transporter activity
P	Q089M3	CC	Part of the ABC transporter complex CcmAB involved in the biogenesis of c-type cytochromes; once thought to export heme, this seems not to be the case, but its exact role is uncertain
P	Q089M3	DE	Heme exporter protein A;
P	Q089M3	DR	heme-transporting ATPase activity
P	Q09188	CC	Catalyzes the exchange of ADP and ATP across the mitochondrial inner membrane

P	Q09188	CC	Catalyzes the exchange of ADP and ATP across the mitochondrial inner membrane
P	Q09188	DE	ADP,ATP carrier protein;
P	Q09188	DE	ADP/ATP translocase;
P	Q09188	DE	ADP/ATP translocase;
P	Q09188	DR	ATP:ADP antiporter activity
P	Q09188	DR	ATP:ADP antiporter activity
P	Q09274	DR	sodium channel activity
P	Q09470	CC	Assuming opened or closed conformations in response to the voltage difference across the membrane, the protein forms a potassium-selective channel through which potassium ions may pass in accordance with their electrochemical gradient
P	Q09470	CC	Mediates the voltage-dependent potassium ion permeability of excitable membranes
P	Q09470	DE	Potassium voltage-gated channel subfamily A member 1;
P	Q09470	DE	Voltage-gated K+ channel HuKI;
P	Q09470	DE	Voltage-gated potassium channel HBK1;
P	Q09470	DE	Voltage-gated potassium channel subunit Kv1.1;
P	Q09470	DR	delayed rectifier potassium channel activity
P	Q09470	DR	potassium ion transmembrane transporter activity
P	Q09573	DE	Sodium/chloride cotransporter 3;
P	Q0A808	CC	Part of the ABC transporter complex CcmAB involved in the biogenesis of c-type cytochromes; once thought to export heme, this seems not to be the case, but its exact role is uncertain
P	Q0A808	DE	Heme exporter protein A;
P	Q0A808	DR	heme-transporting ATPase activity
P	Q0A8P9	DE	Lipoprotein-releasing system ATP-binding protein LolD;
P	Q0A8P9	DR	lipoprotein transporter activity
P	Q0A9E2	CC	Part of the ABC transporter complex ZnuABC involved in zinc import
P	Q0A9E2	DE	Zinc import ATP-binding protein ZnuC;
P	Q0A9E2	DR	zinc transporting ATPase activity
P	Q0A9K1	CC	Part of the ABC transporter complex PstSACB involved in phosphate import
P	Q0A9K1	DE	ABC phosphate transporter;
P	Q0A9K1	DE	Phosphate import ATP-binding protein PstB;
P	Q0A9K1	DE	Phosphate-transporting ATPase;
P	Q0A9K1	DR	inorganic phosphate transmembrane transporter activity
P	Q0A9K1	DR	phosphate transmembrane-transporting ATPase activity
P	Q0AGF4	CC	Part of the ABC transporter complex PotABCD involved in spermidine/putrescine import
P	Q0AGF4	CC	Part of the ABC transporter complex PotABCD involved in spermidine/putrescine import
P	Q0AGF4	DE	Spermidine/putrescine import ATP-binding protein PotA;
P	Q0AGF4	DE	Spermidine/putrescine import ATP-binding protein PotA;
P	Q0AIM8	CC	Transport of potassium into the cell (By similarity).
P	Q0AIM8	DE	Probable potassium transport system protein kup;
P	Q0AIM8	DR	potassium ion transmembrane transporter activity
P	Q0AKQ2	CC	Part of the ABC transporter complex CcmAB involved in the biogenesis of c-type cytochromes; once thought to export heme, this seems not to be the case, but its exact role is uncertain
P	Q0AKQ2	DE	Heme exporter protein A;
P	Q0AKQ2	DR	heme-transporting ATPase activity
P	Q0AM65	CC	Na+/H+ antiporter that extrudes sodium in exchange for external protons (By similarity)
P	Q0AM65	DE	Na+/H+ antiporter nhaA;
P	Q0AM65	DE	Sodium/proton antiporter nhaA;
P	Q0APW8	DE	Lipoprotein-releasing system ATP-binding protein LolD;
P	Q0APW8	DR	lipoprotein transporter activity
P	Q0ASG3	CC	Part of the ABC transporter complex PstSACB involved in phosphate import
P	Q0ASG3	DE	ABC phosphate transporter;
P	Q0ASG3	DE	Phosphate import ATP-binding protein PstB;
P	Q0ASG3	DE	Phosphate-transporting ATPase;
P	Q0ASG3	DR	inorganic phosphate transmembrane transporter activity
P	Q0ASG3	DR	phosphate transmembrane-transporting ATPase activity
P	Q0ASQ1	DR	phosphonate transmembrane-transporting ATPase activity
P	Q0AU85	CC	Part of the ABC transporter complex MetNIQ involved in methionine import
P	Q0AU85	DE	Methionine import ATP-binding protein MetN;
P	Q0BPL2	CC	Transport of potassium into the cell (By similarity).
P	Q0BPL2	DE	Probable potassium transport system protein kup;
P	Q0BPL2	DR	potassium ion transmembrane transporter activity
P	Q0BQ80	DR	molybdate transmembrane-transporting ATPase activity
P	Q0BSM2	DE	Lipoprotein-releasing system ATP-binding protein LolD;

P	Q0BSM2	DR	lipoprotein transporter activity
P	Q0BSV4	CC	Na+/H+ antiporter that extrudes sodium in exchange for external protons (By similarity)
P	Q0BSV4	DE	Na+/H+ antiporter nhaA;
P	Q0BSV4	DE	Sodium/proton antiporter nhaA;
P	Q0BTP1	CC	Part of the ABC transporter complex PstSACB involved in phosphate import
P	Q0BTP1	DE	ABC phosphate transporter;
P	Q0BTP1	DE	Phosphate import ATP-binding protein PstB;
P	Q0BTP1	DE	Phosphate-transporting ATPase;
P	Q0BTP1	DR	inorganic phosphate transmembrane transporter activity
P	Q0BTP1	DR	phosphate transmembrane-transporting ATPase activity
P	Q0BV49	CC	Part of the ABC transporter complex CcmAB involved in the biogenesis of c-type cytochromes; once thought to export heme, this seems not to be the case, but its exact role is uncertain
P	Q0BV49	DE	Heme exporter protein A;
P	Q0BV49	DR	heme-transporting ATPase activity
P	Q0BVS2	CC	One of the components of the high-affinity ATP-driven potassium transport (or KDP) system, which catalyzes the hydrolysis of ATP coupled with the exchange of hydrogen and potassium ions (By similarity).
P	Q0BVS2	DE	Potassium-binding and translocating subunit A;
P	Q0BVS2	DE	Potassium-translocating ATPase A chain;
P	Q0BVS2	DE	Potassium-transporting ATPase A chain;
P	Q0BVS2	DR	potassium-transporting ATPase activity
P	Q0BW71	DR	sodium:dicarboxylate symporter activity
P	Q0BW85	CC	H+-stimulated, highly selective, manganese uptake system (By similarity).
P	Q0BW85	DE	Probable manganese transport protein mntH;
P	Q0BWF7	CC	Part of the ABC transporter complex CcmAB involved in the biogenesis of c-type cytochromes; once thought to export heme, this seems not to be the case, but its exact role is uncertain
P	Q0BWF7	DE	Heme exporter protein A;
P	Q0BWF7	DR	heme-transporting ATPase activity
P	Q0BZD8	DR	phosphonate transmembrane-transporting ATPase activity
P	Q0C0L5	CC	Part of the ABC transporter complex PstSACB involved in phosphate import
P	Q0C0L5	DE	ABC phosphate transporter;
P	Q0C0L5	DE	Phosphate import ATP-binding protein PstB;
P	Q0C0L5	DE	Phosphate-transporting ATPase;
P	Q0C0L5	DR	inorganic phosphate transmembrane transporter activity
P	Q0C0L5	DR	phosphate transmembrane-transporting ATPase activity
P	Q0C1C3	DE	Lipoprotein-releasing system ATP-binding protein LolD;
P	Q0C1C3	DR	lipoprotein transporter activity
P	Q0D2K0	CC	Can also transport other divalent cations such as Ba(2+), Mn(2+), Sr(2+) and Co(2+) but to a much less extent than Mg2+ (By similarity)
P	Q0D2K0	DE	Magnesium transporter NIPA4;
P	Q0D9S3	CC	Seems to act as an high-affinity sodium transporter, which mediates increased sodium uptake in roots under potassium deficiency and contributes to sodium accumulation and salt toxicity
P	Q0D9S3	CC	Seems to be involved in regulation of K+/Na+ homeostasis
P	Q0D9S3	DR	potassium ion transmembrane transporter activity
P	Q0DK16	CC	Aquaporins facilitate the transport of water and small neutral solutes across cell membranes (By similarity).
P	Q0I2G0	CC	Part of the ABC transporter complex CcmAB involved in the biogenesis of c-type cytochromes; once thought to export heme, this seems not to be the case, but its exact role is uncertain
P	Q0I2G0	DE	Heme exporter protein A;
P	Q0I2G0	DR	heme-transporting ATPase activity
P	Q0I348	CC	Part of the ABC transporter complex XylFGH involved in xylose import
P	Q0I348	DE	Xylose import ATP-binding protein XylG;
P	Q0I354	CC	Part of the ABC transporter complex ThiBPQ involved in thiamine import
P	Q0I354	DE	Thiamine import ATP-binding protein ThiQ;
P	Q0I3A2	CC	Na+/H+ antiporter that extrudes sodium in exchange for external protons (By similarity)
P	Q0I3A2	DE	Na+/H+ antiporter nhaA;
P	Q0I3A2	DE	Sodium/proton antiporter nhaA;
P	Q0I3C2	DE	Lipoprotein-releasing system ATP-binding protein LolD;
P	Q0I3C2	DR	lipoprotein transporter activity
P	Q0I3Y9	CC	Part of the ABC transporter complex PotABCD involved in spermidine/putrescine import
P	Q0I3Y9	CC	Part of the ABC transporter complex PotABCD involved in spermidine/putrescine import

P	Q0I3Y9	DE	Spermidine/putrescine import ATP-binding protein PotA;
P	Q0I3Y9	DE	Spermidine/putrescine import ATP-binding protein PotA;
P	Q0I455	CC	Involved in the import of serine and threonine into the cell, with the concomitant import of sodium (symport system) (By similarity).
P	Q0I455	CC	Involved in the import of serine and threonine into the cell, with the concomitant import of sodium (symport system) (By similarity).
P	Q0I455	DE	Na+/serine-threonine symporter;
P	Q0I455	DE	Na+/serine-threonine symporter;
P	Q0I455	DE	Serine/threonine transporter sstT;
P	Q0I455	DR	sodium:dicarboxylate symporter activity
P	Q0I4A9	CC	Part of the ABC transporter complex ZnuABC involved in zinc import
P	Q0I4A9	DE	Zinc import ATP-binding protein ZnuC;
P	Q0I4A9	DR	zinc transporting ATPase activity
P	Q0I5E9	CC	Part of the ABC transporter complex MetNIQ involved in methionine import
P	Q0I5E9	DE	Methionine import ATP-binding protein MetN;
P	Q0I5E9	DR	D-methionine transmembrane transporter activity
P	Q0IAC3	CC	Part of the ABC transporter complex PstSACB involved in phosphate import
P	Q0IAC3	DE	ABC phosphate transporter;
P	Q0IAC3	DE	Phosphate import ATP-binding protein PstB;
P	Q0IAC3	DE	Phosphate-transporting ATPase;
P	Q0IAC3	DR	inorganic phosphate transmembrane transporter activity
P	Q0IAC3	DR	phosphate transmembrane-transporting ATPase activity
P	Q0ILJ3	CC	Responsible for the transport of sucrose into the cell, with the concomitant uptake of protons (symport system)
P	Q0ILJ3	DE	Sucrose permease 2;
P	Q0ILJ3	DE	Sucrose transport protein SUT2;
P	Q0ILJ3	DE	Sucrose transporter 2;
P	Q0ILJ3	DE	Sucrose-proton symporter 2;
P	Q0ILJ3	DR	sucrose transmembrane transporter activity
P	Q0IWF3	CC	Aquaporins facilitate the transport of water and small neutral solutes across cell membranes (By similarity).
P	Q0IZQ3	DE	Phosphate transporter 4;5;
P	Q0JBZ6	CC	Magnesium transporter that may mediate the influx of magnesium (By similarity).
P	Q0JBZ6	DE	Magnesium transporter MRS2-C;
P	Q0JKV1	CC	Highly selective inward-rectifying potassium channel that mediates potassium uptake by plant roots
P	Q0JKV1	CC	May be a major salt-sensitive potassium channel in roots.
P	Q0JKV1	DE	Potassium channel AKT1;
P	Q0JKV1	DR	voltage-gated potassium channel activity
P	Q0JPT5	CC	Aquaporins facilitate the transport of water and small neutral solutes across cell membranes (By similarity).
P	Q0K998	DR	glycerol-3-phosphate-transporting ATPase activity
P	Q0KBR7	CC	Transport of potassium into the cell (By similarity).
P	Q0KBR7	DE	Probable potassium transport system protein kup 1;
P	Q0KBR7	DR	potassium ion transmembrane transporter activity
P	Q0KDG3	CC	Part of the ABC transporter complex MetNIQ involved in methionine import
P	Q0KDG3	DE	Methionine import ATP-binding protein MetN;
P	Q0KEX1	DR	sodium:dicarboxylate symporter activity
P	Q0P7X3	CC	Na+/H+ antiporter that extrudes sodium in exchange for external protons (By similarity)
P	Q0P7X3	DE	Na+/H+ antiporter nhaA 2;
P	Q0P7X3	DE	Sodium/proton antiporter nhaA 2;
P	Q0P7X4	CC	Na+/H+ antiporter that extrudes sodium in exchange for external protons (By similarity)
P	Q0P7X4	DE	Na+/H+ antiporter nhaA 1;
P	Q0P7X4	DE	Sodium/proton antiporter nhaA 1;
P	Q0RAT5	CC	Part of the ABC transporter complex PotABCD involved in spermidine/putrescine import
P	Q0RAT5	CC	Part of the ABC transporter complex PotABCD involved in spermidine/putrescine import
P	Q0RAT5	DE	Spermidine/putrescine import ATP-binding protein PotA;
P	Q0RAT5	DE	Spermidine/putrescine import ATP-binding protein PotA;
P	Q0RF16	CC	One of the components of the high-affinity ATP-driven potassium transport (or KDP) system, which catalyzes the hydrolysis of ATP coupled with the exchange of hydrogen and potassium ions (By similarity).
P	Q0RF16	DE	Potassium-binding and translocating subunit A;
P	Q0RF16	DE	Potassium-translocating ATPase A chain;
P	Q0RF16	DE	Potassium-transporting ATPase A chain;
P	Q0RF16	DR	potassium-transporting ATPase activity

P	Q0RG46	CC	Na+/H+ antiporter that extrudes sodium in exchange for external protons (By similarity)
P	Q0RG46	DE	Na+/H+ antiporter nhaA 3;
P	Q0RG46	DE	Sodium/proton antiporter nhaA 3;
P	Q0RHS2	CC	Na+/H+ antiporter that extrudes sodium in exchange for external protons (By similarity)
P	Q0RHS2	DE	Na+/H+ antiporter nhaA 2;
P	Q0RHS2	DE	Sodium/proton antiporter nhaA 2;
P	Q0RPD5	CC	Na+/H+ antiporter that extrudes sodium in exchange for external protons (By similarity)
P	Q0RPD5	DE	Na+/H+ antiporter nhaA 1;
P	Q0RPD5	DE	Sodium/proton antiporter nhaA 1;
P	Q0SML1	CC	Part of the ABC transporter complex PotABCD involved in spermidine/putrescine import
P	Q0SML1	CC	Part of the ABC transporter complex PotABCD involved in spermidine/putrescine import
P	Q0SML1	DE	Spermidine/putrescine import ATP-binding protein PotA;
P	Q0SML1	DE	Spermidine/putrescine import ATP-binding protein PotA;
P	Q0SNU4	CC	Part of the ABC transporter complex PstSACB involved in phosphate import
P	Q0SNU4	DE	ABC phosphate transporter;
P	Q0SNU4	DE	Phosphate import ATP-binding protein PstB;
P	Q0SNU4	DE	Phosphate-transporting ATPase;
P	Q0SNU4	DR	inorganic phosphate transmembrane transporter activity
P	Q0SNU4	DR	phosphate transmembrane-transporting ATPase activity
P	Q0VL18	CC	Part of the ABC transporter complex PstSACB involved in phosphate import
P	Q0VL18	DE	ABC phosphate transporter;
P	Q0VL18	DE	Phosphate import ATP-binding protein PstB;
P	Q0VL18	DE	Phosphate-transporting ATPase;
P	Q0VL18	DR	inorganic phosphate transmembrane transporter activity
P	Q0VL18	DR	phosphate transmembrane-transporting ATPase activity
P	Q0VQ44	DR	molybdate transmembrane-transporting ATPase activity
P	Q0VQ74	CC	Na+/H+ antiporter that extrudes sodium in exchange for external protons (By similarity)
P	Q0VQ74	DE	Na+/H+ antiporter nhaB;
P	Q0VQ74	DE	Sodium/proton antiporter nhaB;
P	Q0VQ74	DR	sodium:hydrogen antiporter activity
P	Q0VQQ0	DE	Lipoprotein-releasing system ATP-binding protein LolD;
P	Q0VQQ0	DR	lipoprotein transporter activity
P	Q0VR80	CC	Part of the ABC transporter complex CcmAB involved in the biogenesis of c-type cytochromes; once thought to export heme, this seems not to be the case, but its exact role is uncertain
P	Q0VR80	DE	Heme exporter protein A;
P	Q0VR80	DR	heme-transporting ATPase activity
P	Q0VTB6	CC	Part of the ABC transporter complex ZnuABC involved in zinc import
P	Q0VTB6	DE	Zinc import ATP-binding protein ZnuC;
P	Q0VTB6	DR	zinc transporting ATPase activity
P	Q0WJ82	CC	Involved in the import of serine and threonine into the cell, with the concomitant import of sodium (symport system) (By similarity).
P	Q0WJ82	CC	Involved in the import of serine and threonine into the cell, with the concomitant import of sodium (symport system) (By similarity).
P	Q0WJ82	DE	Na+/serine-threonine symporter;
P	Q0WJ82	DE	Na+/serine-threonine symporter;
P	Q0WJ82	DE	Serine/threonine transporter sstT;
P	Q0WJ82	DR	sodium:dicarboxylate symporter activity
P	Q0WJ82	DR	sodium:dicarboxylate symporter activity
P	Q0WJE4	CC	Part of the ABC transporter complex ThiBPQ involved in thiamine import
P	Q0WJE4	DE	Thiamine import ATP-binding protein ThiQ;
P	Q0WML0	DE	Antigen peptide transporter-like 2;
P	Q10065	DE	Potassium transport protein 2;
P	Q10065	DR	potassium ion transmembrane transporter activity
P	Q10177	CC	Regulates cell morphogenesis through control of manganese homeostasis.
P	Q10177	CC	Transports manganese ions into the cell
P	Q10177	DE	Manganese transporter pdt1;
P	Q10177	DR	manganese ion transmembrane transporter activity
P	Q10185	CC	Involved in vacuolar sequestration of glutathione S- conjugates
P	Q10185	DE	ATP-energized glutathione S-conjugate pump abc2;
P	Q10185	DE	Glutathione S-conjugate-transporting ATPase abc2;
P	Q10185	DR	glutathione S-conjugate-exporting ATPase activity
P	Q10279	CC	Transport of uracil.
P	Q10279	DE	Uracil permease;

P	Q10901	CC	Essential for terminating the postsynaptic action of glutamate by rapidly removing released glutamate from the synaptic cleft
P	Q10901	CC	Transports L-glutamate and also L- and D-aspartate
P	Q10901	DE	Sodium-dependent glutamate/ aspartate transporter;
P	Q10901	DR	L-glutamate transmembrane transporter activity
P	Q10D38	CC	Magnesium transporter that may mediate the influx of magnesium (By similarity)
P	Q10D38	DE	Magnesium transporter MRS2-I;
P	Q10M80	CC	Aquaporins facilitate the transport of water and small neutral solutes across cell membranes (By similarity).
P	Q10S25	CC	Putative magnesium transporter (By similarity).
P	Q10S25	DE	Putative magnesium transporter MRS2-H;
P	Q11NG0	CC	Part of the ABC transporter complex PstSACB involved in phosphate import
P	Q11NG0	DE	ABC phosphate transporter;
P	Q11NG0	DE	Phosphate import ATP-binding protein PstB;
P	Q11NG0	DE	Phosphate-transporting ATPase;
P	Q11NG0	DR	inorganic phosphate transmembrane transporter activity
P	Q11NG0	DR	phosphate transmembrane-transporting ATPase activity
P	Q11PK1	CC	Transport of potassium into the cell (By similarity).
P	Q11PK1	DE	Probable potassium transport system protein kup;
P	Q11PK1	DR	potassium ion transmembrane transporter activity
P	Q11TX2	CC	Na+/H+ antiporter that extrudes sodium in exchange for external protons (By similarity)
P	Q11TX2	DE	Na+/H+ antiporter nhaA;
P	Q11TX2	DE	Sodium/proton antiporter nhaA;
P	Q12067	CC	Functions in the homeostasis of manganese ions.
P	Q12067	DR	manganese ion transmembrane transporter activity
P	Q12119	CC	Probable purine-cytosine permease (By similarity).
P	Q12119	DE	Cytosine/purine transport protein FCY22;
P	Q12119	DE	Cytosine/purine transport protein FCY22;
P	Q12119	DE	Purine-cytosine permease FCY22;
P	Q12289	CC	Transports carnitine, acetylcarnitine, propionylcarnitine and to a much lower extent medium- and long- chain acylcarnitines.
P	Q12289	DE	Mitochondrial carnitine carrier;
P	Q12289	DR	carnitine:acyl carnitine antiporter activity
P	Q12300	CC	Can function as a sensor that generates an intracellular signal in the presence of high level of glucose
P	Q12300	CC	Low-affinity glucose transporter
P	Q12300	CC	Required for maximal expression of the high glucose-induced HXT1 protein.
P	Q12300	DE	High-affinity glucose transporter RGT2;
P	Q12325	CC	High affinity uptake of sulfate into the cell.
P	Q12325	DE	High-affinity sulfate transporter 2;
P	Q12325	DE	Sulfate permease 2;
P	Q12325	DR	secondary active sulfate transmembrane transporter activity
P	Q12436	CC	Active in zinc- replete cells and is time-, temperature- and concentration-dependent and prefers zinc over other metals as its substrate.
P	Q12436	CC	Low-affinity zinc transport protein
P	Q12436	DE	Low-affinity zinc transport protein ZRT2;
P	Q12436	DE	Zinc-regulated transporter 2;
P	Q12436	DR	low-affinity zinc ion transmembrane transporter activity
P	Q12675	DE	Probable phospholipid-transporting ATPase DNF2;
P	Q12675	DR	phospholipid-translocating ATPase activity
P	Q12691	CC	This magnesium-dependent enzyme catalyzes the hydrolysis of ATP coupled with the transport of sodium or lithium ions to allow salt tolerance (By similarity)
P	Q12691	DE	Sodium transport ATPase 5;
P	Q12691	DR	sodium-exporting ATPase activity, phosphorylative mechanism
P	Q12809	CC	Mediates the rapidly activating component of the delayed rectifying potassium current in heart (IKr)
P	Q12809	CC	Pore-forming (alpha) subunit of voltage-gated inwardly rectifying potassium channel
P	Q12809	DE	Ether-a-go-go-related gene potassium channel 1;
P	Q12809	DE	Potassium voltage-gated channel subfamily H member 2;
P	Q12809	DE	Voltage-gated potassium channel subunit Kv11.1;
P	Q12809	DR	delayed rectifier potassium channel activity
P	Q12908	CC	Plays a critical role in the sodium-dependent reabsorption of bile acids from the lumen of the small intestine
P	Q12908	DE	Apical sodium-dependent bile acid transporter;
P	Q12908	DE	Apical sodium-dependent bile acid transporter;

P	Q12908	DE	Ileal Na+/bile acid cotransporter;
P	Q12908	DE	Ileal Na+/bile acid cotransporter;
P	Q12908	DE	Ileal sodium-dependent bile acid transporter;
P	Q12908	DE	Ileal sodium-dependent bile acid transporter;
P	Q12908	DE	Ileal sodium/bile acid cotransporter;
P	Q12908	DE	Ileal sodium/bile acid cotransporter;
P	Q12908	DE	Na+-dependent ileal bile acid transporter;
P	Q12908	DE	Na+-dependent ileal bile acid transporter;
P	Q12908	DE	Sodium/taurocholate cotransporting polypeptide, ileal;
P	Q12908	DR	bile acid:sodium symporter activity
P	Q12908	DR	bile acid:sodium symporter activity
P	Q13183	CC	Cotransport of sodium ions and dicarboxylates such as succinate and citrate.
P	Q13183	CC	Cotransport of sodium ions and dicarboxylates such as succinate and citrate.
P	Q13183	DE	Na+/dicarboxylate cotransporter 1;
P	Q13183	DE	Na+/dicarboxylate cotransporter 1;
P	Q13183	DE	Renal sodium/dicarboxylate cotransporter;
P	Q13183	DE	Renal sodium/dicarboxylate cotransporter;
P	Q13183	DR	low affinity sodium:dicarboxylate symporter activity
P	Q13183	DR	low affinity sodium:dicarboxylate symporter activity
P	Q13336	CC	Mediates urea transport in erythrocytes.
P	Q13336	CC	Specialized low-affinity urea transporter
P	Q13336	DE	Urea transporter 1;
P	Q13336	DE	Urea transporter, erythrocyte;
P	Q13336	DR	urea transmembrane transporter activity
P	Q13433	DE	Zinc transporter ZIP6;
P	Q13433	DR	zinc ion transmembrane transporter activity
P	Q13520	CC	Forms a water-specific channel that participates in distinct physiological functions such as glomerular filtration, tubular endocytosis and acid-base metabolism (By similarity)
P	Q13520	DR	water channel activity
P	Q13621	CC	Mediates sodium and chloride reabsorption
P	Q13621	CC	Mediates sodium and chloride reabsorption
P	Q13621	DE	Bumetanide-sensitive sodium-(potassium)-chloride cotransporter 2;
P	Q13621	DR	sodium:potassium:chloride symporter activity
P	Q13621	DR	sodium:potassium:chloride symporter activity
P	Q13621	DR	sodium:potassium:chloride symporter activity
P	Q13698	CC	Calcium channels containing the alpha-1S subunit play an important role in excitation-contraction coupling in skeletal muscle.
P	Q13698	CC	Long-lasting (L-type) calcium channels belong to the 'high-voltage activated' (HVA) group
P	Q13698	CC	The isoform alpha-1S gives rise to L-type calcium currents
P	Q13698	CC	Voltage-sensitive calcium channels (VSCC) mediate the entry of calcium ions into excitable cells and are also involved in a variety of calcium-dependent processes, including muscle contraction, hormone or neurotransmitter release, gene expression, cell motility, cell division and cell death
P	Q13698	DE	Calcium channel, L type, alpha-1 polypeptide, isoform 3, skeletal muscle;
P	Q13698	DE	Voltage-dependent L-type calcium channel subunit alpha-1S;
P	Q13698	DE	Voltage-gated calcium channel subunit alpha Cav1.1;
P	Q13698	DR	high voltage-gated calcium channel activity
P	Q13733	CC	This action creates the electrochemical gradient of sodium and potassium ions, providing the energy for active transport of various nutrients
P	Q13733	CC	This action creates the electrochemical gradient of sodium and potassium ions, providing the energy for active transport of various nutrients
P	Q13733	CC	This is the catalytic component of the active enzyme, which catalyzes the hydrolysis of ATP coupled with the exchange of sodium and potassium ions across the plasma membrane
P	Q13733	CC	This is the catalytic component of the active enzyme, which catalyzes the hydrolysis of ATP coupled with the exchange of sodium and potassium ions across the plasma membrane
P	Q13733	DE	Na+/K+ ATPase alpha-4 subunit;
P	Q13733	DE	Na+/K+ ATPase alpha-4 subunit;
P	Q13733	DE	Sodium pump subunit alpha-4;
P	Q13733	DE	Sodium/potassium-transporting ATPase subunit alpha-4;
P	Q13733	DE	Sodium/potassium-transporting ATPase subunit alpha-4;
P	Q13733	DR	sodium:potassium-exchanging ATPase activity
P	Q13733	DR	sodium:potassium-exchanging ATPase activity

P	Q14003	CC	Assuming opened or closed conformations in response to the voltage difference across the membrane, the protein forms a potassium-selective channel through which potassium ions may pass in accordance with their electrochemical gradient
P	Q14003	CC	This protein mediates the voltage-dependent potassium ion permeability of excitable membranes
P	Q14003	DE	Potassium voltage-gated channel subfamily C member 3;
P	Q14003	DE	Voltage-gated potassium channel subunit Kv3.3;
P	Q14003	DR	voltage-gated potassium channel activity
P	Q14500	CC	Inward rectifier potassium channels are characterized by a greater tendency to allow potassium to flow into the cell rather than out of it
P	Q14500	CC	Their voltage dependence is regulated by the concentration of extracellular potassium; as external potassium is raised, the voltage range of the channel opening shifts to more positive voltages
P	Q14500	DE	ATP-sensitive inward rectifier potassium channel 12;
P	Q14500	DE	Inward rectifier K+ channel Kir2.2;
P	Q14500	DE	Inward rectifier K+ channel Kir2.2v;
P	Q14500	DE	Potassium channel, inwardly rectifying subfamily J member 12;
P	Q14500	DR	inward rectifier potassium channel activity
P	Q14500	DR	potassium channel regulator activity
P	Q14542	DE	Equilibrative NBMPR-insensitive nucleoside transporter;
P	Q14542	DE	Equilibrative nitrobenzylmercaptopurine riboside-insensitive nucleoside transporter;
P	Q14542	DE	Equilibrative nucleoside transporter 2;
P	Q14542	DE	Nucleoside transporter, ei-type;
P	Q14542	DR	nucleoside transmembrane transporter activity
P	Q14571	CC	Receptor for inositol 1,4,5-trisphosphate, a second messenger that mediates the release of intracellular calcium.
P	Q14571	DR	calcium ion transmembrane transporter activity
P	Q14571	DR	inositol 1,4,5-trisphosphate-sensitive calcium-release channel activity
P	Q14573	CC	Receptor for inositol 1,4,5-trisphosphate, a second messenger that mediates the release of intracellular calcium.
P	Q14573	DR	inositol 1,4,5-trisphosphate-sensitive calcium-release channel activity
P	Q14643	CC	Intracellular channel that mediates calcium release from the endoplasmic reticulum following stimulation by inositol 1,4,5- trisphosphate.
P	Q14643	DR	calcium ion transmembrane transporter activity
P	Q14643	DR	inositol 1,4,5-trisphosphate-sensitive calcium-release channel activity
P	Q14721	CC	Channels open or close in response to the voltage difference across the membrane, letting potassium ions pass in accordance with their electrochemical gradient
P	Q14721	CC	Mediates the voltage-dependent potassium ion permeability of excitable membranes
P	Q14721	DE	Delayed rectifier potassium channel 1;
P	Q14721	DE	Potassium voltage-gated channel subfamily B member 1;
P	Q14721	DE	Voltage-gated potassium channel subunit Kv2.1;
P	Q14721	DR	voltage-gated potassium channel activity
P	Q14728	DE	Tetracycline transporter-like protein;
P	Q14728	DR	tetracycline transporter activity
P	Q14802	CC	Induces a hyperpolarization-activated chloride current when expressed in Xenopus oocytes
P	Q14802	DE	Chloride conductance inducer protein Mat-8;
P	Q14802	DR	chloride channel activity
P	Q14916	CC	Important for the resorption of phosphate by the kidney
P	Q14916	CC	May be involved in actively transporting phosphate into cells via Na+ cotransport in the renal brush border membrane.
P	Q14916	CC	May be involved in actively transporting phosphate into cells via Na+ cotransport in the renal brush border membrane.
P	Q14916	DE	Na+/PI cotransporter 1;
P	Q14916	DE	Renal Na+-dependent phosphate cotransporter 1;
P	Q14916	DE	Renal Na+-dependent phosphate cotransporter 1;
P	Q14916	DE	Renal sodium-dependent phosphate transport protein 1;
P	Q14916	DE	Renal sodium-dependent phosphate transport protein 1;
P	Q14916	DE	Renal sodium-phosphate transport protein 1;
P	Q14916	DE	Sodium-dependent phosphate transport protein 1;
P	Q14916	DE	Sodium-dependent phosphate transport protein 1;
P	Q14916	DE	Sodium/phosphate cotransporter 1;
P	Q14916	DE	Sodium/phosphate cotransporter 1;
P	Q14916	DR	sodium-dependent phosphate transmembrane transporter activity
P	Q14916	DR	sodium-dependent phosphate transmembrane transporter activity

P	Q14940	CC	Major proton extruding system driven by the inward sodium ion chemical gradient
P	Q14940	DE	Na+/H+ exchanger 5;
P	Q14940	DE	Sodium/hydrogen exchanger 5;
P	Q14940	DR	sodium:hydrogen antiporter activity
P	Q14973	CC	It is strictly dependent on the extracellular presence of sodium.
P	Q14973	CC	The hepatic sodium/bile acid uptake system exhibits broad substrate specificity and transports various non-bile acid organic compounds as well
P	Q14973	CC	The hepatic sodium/bile acid uptake system exhibits broad substrate specificity and transports various non-bile acid organic compounds as well
P	Q14973	DE	Na+/bile acid cotransporter;
P	Q14973	DE	Na+/bile acid cotransporter;
P	Q14973	DE	Na+/taurocholate transport protein;
P	Q14973	DE	Sodium/bile acid cotransporter;
P	Q14973	DE	Sodium/bile acid cotransporter;
P	Q14973	DE	Sodium/taurocholate cotransporting polypeptide;
P	Q14973	DR	bile acid:sodium symporter activity
P	Q14973	DR	bile acid:sodium symporter activity
P	Q14CL3	DE	Potassium voltage-gated channel, subfamily H (Eag-related), member 1, isoform CRA_a;
P	Q14CL3	DE	Potassium voltage-gated channel, subfamily H (Eag-related), member 1;
P	Q14CL3	DR	voltage-gated potassium channel activity
P	Q14CN2	DR	chloride channel activity
P	Q14D71	DE	Potassium voltage-gated channel, Shal-related subfamily, member 3, isoform CRA_b;
P	Q14D71	DE	Potassium voltage-gated channel, Shal-related subfamily, member 3;
P	Q15043	DE	LIV-1 subfamily of ZIP zinc transporter 4;
P	Q15043	DE	Zinc transporter ZIP14;
P	Q15043	DR	zinc ion transmembrane transporter activity
P	Q15413	CC	Contraction of skeletal muscle is triggered by release of calcium ions from SR following depolarization of T-tubules (By similarity).
P	Q15413	DR	ryanodine-sensitive calcium-release channel activity
P	Q15758	CC	Has a broad substrate specificity, a preference for zwitterionic amino acids, and a sodium-dependence
P	Q15758	DE	Sodium-dependent neutral amino acid transporter type 2;
P	Q15758	DR	sodium:dicarboxylate symporter activity
P	Q15842	CC	Inward rectifier potassium channels are characterized by a greater tendency to allow potassium to flow into the cell rather than out of it
P	Q15842	CC	Their voltage dependence is regulated by the concentration of extracellular potassium; as external potassium is raised, the voltage range of the channel opening shifts to more positive voltages
P	Q15842	CC	This potassium channel is controlled by G proteins
P	Q15842	DE	ATP-sensitive inward rectifier potassium channel 8;
P	Q15842	DE	Inward rectifier K+ channel Kir6.1;
P	Q15842	DE	Potassium channel, inwardly rectifying subfamily J member 8;
P	Q15849	CC	Mediates rapid transepithelial urea transport across the inner medullary collecting duct and plays a major role in the urinary concentrating mechanism.
P	Q15849	CC	Specialized low-affinity vasopressin-regulated urea transporter
P	Q15849	DE	Urea transporter 2;
P	Q15849	DE	Urea transporter, kidney;
P	Q15849	DR	urea transmembrane transporter activity
P	Q15878	CC	Calcium channels containing alpha-1E subunit could be involved in the modulation of firing patterns of neurons which is important for information processing
P	Q15878	CC	R-type calcium channels belong to the 'high-voltage activated' (HVA) group and are blocked by nickel, and partially by omega-agatoxin-IIIA (omega-Aga-IIIA)
P	Q15878	CC	The isoform alpha-1E gives rise to R-type calcium currents
P	Q15878	CC	Voltage-sensitive calcium channels (VSCC) mediate the entry of calcium ions into excitable cells and are also involved in a variety of calcium-dependent processes, including muscle contraction, hormone or neurotransmitter release, gene expression, cell motility, cell division and cell death
P	Q15878	DE	Brain calcium channel II;
P	Q15878	DE	Calcium channel, L type, alpha-1 polypeptide, isoform 6;
P	Q15878	DE	Voltage-dependent R-type calcium channel subunit alpha-1E;
P	Q15878	DE	Voltage-gated calcium channel subunit alpha Cav2.3;
P	Q15878	DR	voltage-gated calcium channel activity
P	Q16322	CC	Assuming opened or closed conformations in response to the voltage difference across the membrane, the protein forms a potassium-selective channel through which potassium ions may pass in accordance with their electrochemical gradient

P	Q16322	CC	Mediates voltage-dependent potassium ion permeability of excitable membranes
P	Q16322	DE	Potassium voltage-gated channel subfamily A member 10;
P	Q16322	DE	Voltage-gated potassium channel subunit Kv1.8;
P	Q16322	DR	voltage-gated potassium channel activity
P	Q16348	DE	Oligopeptide transporter, kidney isoform;
P	Q16445	CC	GABA, the major inhibitory neurotransmitter in the vertebrate brain, mediates neuronal inhibition by binding to the GABA/benzodiazepine receptor and opening an integral chloride channel.
P	Q16445	DR	chloride channel activity
P	Q16515	CC	Cation channel with high affinity for sodium, which is gated by extracellular protons and inhibited by the diuretic amiloride
P	Q16515	DE	Amiloride-sensitive brain sodium channel;
P	Q16515	DE	Brain sodium channel 1;
P	Q16515	DR	ligand-gated sodium channel activity
P	Q16572	CC	Involved in acetylcholine transport into synaptic vesicles.
P	Q16572	DE	Vesicular acetylcholine transporter;
P	Q16572	DR	acetylcholine transmembrane transporter activity
P	Q166T6	DE	Nitrite extrusion protein;
P	Q16720	CC	This magnesium-dependent enzyme catalyzes the hydrolysis of ATP coupled with the transport of calcium out of the cell.
P	Q16720	DE	Plasma membrane calcium ATPase isoform 3;
P	Q16720	DE	Plasma membrane calcium pump isoform 3;
P	Q16720	DE	Plasma membrane calcium-transporting ATPase 3;
P	Q16720	DR	calcium-transporting ATPase activity
P	Q17328	CC	Glutamate gated chloride channel that is dual gated by glutamate and avermectin.
P	Q17328	DE	Avermectin-sensitive glutamate-gated chloride channel subunit;
P	Q17328	DE	Glutamate-gated chloride channel subunit beta;
P	Q17328	DR	chloride channel activity
P	Q17758	DE	Oligopeptide transporter 1;
P	Q17758	DR	oligopeptide transporter activity
P	Q17766	CC	Folate transporter.
P	Q17766	DE	Folate transporter 1;
P	Q17766	DR	reduced folate carrier activity
P	Q17ST2	CC	Assuming opened or closed conformations in response to the voltage difference across the membrane, the protein forms a potassium-selective channel through which potassium ions may pass in accordance with their electrochemical gradient
P	Q17ST2	CC	Mediates the voltage-dependent potassium ion permeability of excitable membranes
P	Q17ST2	DE	Potassium voltage-gated channel subfamily A member 7;
P	Q17ST2	DE	Voltage-gated potassium channel subunit Kv1.7;
P	Q17ST2	DR	voltage-gated potassium channel activity
P	Q180A5	CC	Part of the ABC transporter complex PstSACB involved in phosphate import
P	Q180A5	DE	ABC phosphate transporter;
P	Q180A5	DE	Phosphate import ATP-binding protein PstB;
P	Q180A5	DE	Phosphate-transporting ATPase;
P	Q180A5	DR	inorganic phosphate transmembrane transporter activity
P	Q180A5	DR	phosphate transmembrane-transporting ATPase activity
P	Q18120	CC	Outwardly rectifying potassium channel protein; activity is sharply augmented by increase in temperature.
P	Q18120	DE	TWiK family of potassium channels protein 18;
P	Q18120	DR	outward rectifier potassium channel activity
P	Q186E8	CC	One of the components of the high-affinity ATP-driven potassium transport (or KDP) system, which catalyzes the hydrolysis of ATP coupled with the exchange of hydrogen and potassium ions
P	Q186E8	DE	Potassium-binding and translocating subunit C;
P	Q186E8	DE	Potassium-translocating ATPase C chain;
P	Q186E8	DE	Potassium-transporting ATPase C chain;
P	Q186E8	DR	potassium-transporting ATPase activity
P	Q18AM3	CC	Part of the ABC transporter complex PotABCD involved in spermidine/putrescine import
P	Q18AM3	CC	Part of the ABC transporter complex PotABCD involved in spermidine/putrescine import
P	Q18AM3	DE	Spermidine/putrescine import ATP-binding protein PotA;
P	Q18AM3	DE	Spermidine/putrescine import ATP-binding protein PotA;
P	Q18C09	CC	Part of the ABC transporter complex MetNIQ involved in methionine import
P	Q18C09	DE	Methionine import ATP-binding protein MetN;
P	Q19907	DE	TWiK family of potassium channels protein 12;
P	Q19907	DR	potassium channel activity

P	Q19QZ8	DE	T calcium channel alpha 1G subunit variant 221;
P	Q19QZ8	DR	voltage-gated calcium channel activity
P	Q19QZ9	DE	T calcium channel alpha 1G subunit variant 217;
P	Q19QZ9	DR	voltage-gated calcium channel activity
P	Q19R02	DE	T calcium channel alpha 1G subunit variant 189;
P	Q19R02	DR	voltage-gated calcium channel activity
P	Q19R03	DE	T calcium channel alpha 1G subunit variant 185;
P	Q19R03	DR	voltage-gated calcium channel activity
P	Q19R08	DE	T calcium channel alpha 1G subunit variant 153;
P	Q19R08	DR	voltage-gated calcium channel activity
P	Q19R11	DE	T calcium channel alpha 1G subunit variant 93;
P	Q19R11	DR	voltage-gated calcium channel activity
P	Q19R12	DE	T calcium channel alpha 1G subunit variant 89;
P	Q19R12	DR	voltage-gated calcium channel activity
P	Q19R13	DE	T calcium channel alpha 1G subunit variant 88;
P	Q19R13	DR	voltage-gated calcium channel activity
P	Q19R17	DE	T calcium channel alpha 1G subunit variant 25;
P	Q19R17	DR	voltage-gated calcium channel activity
P	Q1EHB4	CC	Acts as an electroneutral and low-affinity sodium (Na+)-dependent sodium-coupled solute transporter
P	Q1EHB4	CC	Mediates electroneutral uptake of lactate, with a stoichiometry of 2 Na+ for each lactate (By similarity).
P	Q1EHB4	DE	Electroneutral sodium monocarboxylate cotransporter;
P	Q1EHB4	DE	Low-affinity sodium-lactate cotransporter;
P	Q1EHB4	DE	Sodium-coupled monocarboxylate transporter 2;
P	Q1LC89	CC	Part of the ABC transporter complex HmuTUV involved in hemin import
P	Q1LC89	DE	Hemin import ATP-binding protein HmuV;
P	Q1LFB1	DR	sodium:dicarboxylate symporter activity
P	Q1LFZ8	CC	Part of the ABC transporter complex CcmAB involved in the biogenesis of c-type cytochromes; once thought to export heme, this seems not to be the case, but its exact role is uncertain
P	Q1LFZ8	DE	Heme exporter protein A 2;
P	Q1LFZ8	DR	heme-transporting ATPase activity
P	Q1LJ08	DR	phosphonate transmembrane-transporting ATPase activity
P	Q1LKR4	CC	Part of the ABC transporter complex CcmAB involved in the biogenesis of c-type cytochromes; once thought to export heme, this seems not to be the case, but its exact role is uncertain
P	Q1LKR4	DE	Heme exporter protein A 1;
P	Q1LKR4	DR	heme-transporting ATPase activity
P	Q1LLB5	CC	Part of the ABC transporter complex PstSACB involved in phosphate import
P	Q1LLB5	DE	ABC phosphate transporter;
P	Q1LLB5	DE	Phosphate import ATP-binding protein PstB;
P	Q1LLB5	DE	Phosphate-transporting ATPase;
P	Q1LLB5	DR	inorganic phosphate transmembrane transporter activity
P	Q1LLB5	DR	phosphate transmembrane-transporting ATPase activity
P	Q1LLP5	DR	glycerol-3-phosphate-transporting ATPase activity
P	Q1LP09	CC	Transport of potassium into the cell (By similarity).
P	Q1LP09	DE	Probable potassium transport system protein kup;
P	Q1LP09	DR	potassium ion transmembrane transporter activity
P	Q1LPJ9	DE	Lipoprotein-releasing system ATP-binding protein LolD;
P	Q1LPJ9	DR	lipoprotein transporter activity
P	Q1LQB5	DR	phosphonate transmembrane-transporting ATPase activity
P	Q1LQF6	CC	Part of the ABC transporter complex MetNIQ involved in methionine import
P	Q1LQF6	DE	Methionine import ATP-binding protein MetN;
P	Q1LRW2	DR	sodium:dicarboxylate symporter activity
P	Q1M360	CC	Part of the ABC transporter complex RbsABCD involved in ribose import
P	Q1M360	DE	Ribose import ATP-binding protein RbsA 3;
P	Q1M589	DR	glycerol-3-phosphate-transporting ATPase activity
P	Q1M5X4	CC	Part of the ABC transporter complex RbsABCD involved in ribose import
P	Q1M5X4	DE	Ribose import ATP-binding protein RbsA 2;
P	Q1M651	CC	Transport of potassium into the cell (By similarity).
P	Q1M651	DE	Probable potassium transport system protein kup 3;
P	Q1M651	DR	potassium ion transmembrane transporter activity
P	Q1M7R4	CC	Part of the ABC transporter complex TauABC involved in taurine import
P	Q1M7R4	DE	Taurine import ATP-binding protein TauB;

P	Q1M7R4	DR	taurine-transporting ATPase activity
P	Q1M8E0	CC	Part of the ABC transporter complex MetNIQ involved in methionine import
P	Q1M8E0	DE	Methionine import ATP-binding protein MetN;
P	Q1M8R6	DR	glycerol-3-phosphate-transporting ATPase activity
P	Q1MA70	DR	molybdate transmembrane-transporting ATPase activity
P	Q1MAL7	CC	Part of the ABC transporter complex CcmAB involved in the biogenesis of c-type cytochromes; once thought to export heme, this seems not to be the case, but its exact role is uncertain
P	Q1MAL7	DE	Heme exporter protein A;
P	Q1MAL7	DR	heme-transporting ATPase activity
P	Q1MCZ1	CC	Part of the ABC transporter complex HmuTUV involved in hemin import
P	Q1MCZ1	DE	Hemin import ATP-binding protein HmuV;
P	Q1MDR5	DR	sodium:dicarboxylate symporter activity
P	Q1MEG2	CC	Part of the ABC transporter complex ZnuABC involved in zinc import
P	Q1MEG2	DE	Zinc import ATP-binding protein ZnuC;
P	Q1MEG2	DR	zinc transporting ATPase activity
P	Q1MEX2	CC	Transport of potassium into the cell (By similarity).
P	Q1MEX2	DE	Probable potassium transport system protein kup 2;
P	Q1MEX2	DR	potassium ion transmembrane transporter activity
P	Q1MHS1	CC	Part of the ABC transporter complex RbsABCD involved in ribose import
P	Q1MHS1	DE	Ribose import ATP-binding protein RbsA 1;
P	Q1MIJ4	DE	Lipoprotein-releasing system ATP-binding protein LolD;
P	Q1MIJ4	DR	lipoprotein transporter activity
P	Q1MIN0	CC	Part of the ABC transporter complex PstSACB involved in phosphate import
P	Q1MIN0	DE	ABC phosphate transporter 2;
P	Q1MIN0	DE	Phosphate import ATP-binding protein PstB 2;
P	Q1MIN0	DE	Phosphate-transporting ATPase 2;
P	Q1MIN0	DR	inorganic phosphate transmembrane transporter activity
P	Q1MIN0	DR	phosphate transmembrane-transporting ATPase activity
P	Q1MKT5	CC	Transport of potassium into the cell (By similarity).
P	Q1MKT5	DE	Probable potassium transport system protein kup 1;
P	Q1MKT5	DR	potassium ion transmembrane transporter activity
P	Q1MLW4	CC	Part of the ABC transporter complex PstSACB involved in phosphate import
P	Q1MLW4	DE	ABC phosphate transporter 1;
P	Q1MLW4	DE	Phosphate import ATP-binding protein PstB 1;
P	Q1MLW4	DE	Phosphate-transporting ATPase 1;
P	Q1MLW4	DR	inorganic phosphate transmembrane transporter activity
P	Q1MLW4	DR	phosphate transmembrane-transporting ATPase activity
P	Q1MMZ3	DR	phosphonate transmembrane-transporting ATPase activity
P	Q1PE39	CC	Magnesium transporter that mediates the influx/efflux of magnesium in a concentration-dependent manner
P	Q1PE39	DE	Magnesium Transporter 5;
P	Q1PE39	DE	Magnesium transporter MRS2-6, mitochondrial;
P	Q1PE39	DR	magnesium ion transmembrane transporter activity
P	Q1ZXJ0	CC	Chloride channels may have several functions including the regulation of cell volume, membrane potential stabilization and signal transduction (By similarity)
P	Q1ZXJ0	CC	Voltage-gated chloride channel
P	Q1ZXJ0	DE	Chloride channel protein D;
P	Q1ZXJ0	DR	voltage-gated chloride channel activity
P	Q21219	DE	Oligopeptide transporter 1;
P	Q21339	DE	Na+/dicarboxylate cotransporter 3;
P	Q21339	DE	Sodium-dependent high-affinity dicarboxylate transporter 3;
P	Q21339	DR	high affinity sodium:dicarboxylate symporter activity
P	Q21974	DR	sodium channel activity
P	Q22271	CC	Has a role in mobility, possibly in the transport of potassium in muscles.
P	Q22271	DR	potassium channel activity
P	Q22851	DR	sodium channel activity
P	Q23435	CC	Potassium channel protein that may be component of regulatory network that controls ray development and function.
P	Q23435	DE	TWiK family of potassium channels protein 9;
P	Q23435	DR	potassium channel activity
P	Q24046	CC	The beta subunit regulates, through assembly of alpha/beta heterodimers, the number of sodium pumps transported to the plasma membrane.
P	Q24046	CC	This is the non-catalytic component of the active enzyme, which catalyzes the hydrolysis of ATP coupled with the exchange of Na+ and K+ ions across the plasma membrane

P	Q24046	CC	This is the non-catalytic component of the active enzyme, which catalyzes the hydrolysis of ATP coupled with the exchange of Na+ and K+ ions across the plasma membrane
P	Q24046	DE	Sodium/potassium-dependent ATPase subunit beta-1;
P	Q24046	DE	Sodium/potassium-dependent ATPase subunit beta-1;
P	Q24046	DE	Sodium/potassium-transporting ATPase subunit beta-1;
P	Q24046	DE	Sodium/potassium-transporting ATPase subunit beta-1;
P	Q24046	DR	sodium:potassium-exchanging ATPase activity
P	Q24046	DR	sodium:potassium-exchanging ATPase activity
P	Q24048	CC	The beta subunit regulates, through assembly of alpha/beta heterodimers, the number of sodium pumps transported to the plasma membrane.
P	Q24048	CC	This is the non-catalytic component of the active enzyme, which catalyzes the hydrolysis of ATP coupled with the exchange of Na+ and K+ ions across the plasma membrane
P	Q24048	CC	This is the non-catalytic component of the active enzyme, which catalyzes the hydrolysis of ATP coupled with the exchange of Na+ and K+ ions across the plasma membrane
P	Q24048	DE	Sodium/potassium-dependent ATPase subunit beta-2;
P	Q24048	DE	Sodium/potassium-dependent ATPase subunit beta-2;
P	Q24048	DE	Sodium/potassium-transporting ATPase subunit beta-2;
P	Q24048	DE	Sodium/potassium-transporting ATPase subunit beta-2;
P	Q24048	DR	sodium:potassium-exchanging ATPase activity
P	Q24048	DR	sodium:potassium-exchanging ATPase activity
P	Q24270	CC	Voltage-sensitive calcium channels (VSCC) mediate the entry of calcium ions into excitable cells and are also involved in a variety of calcium-dependent processes, including muscle contraction, hormone or neurotransmitter release, gene expression, cell motility, cell division and cell death
P	Q24270	DE	Voltage-dependent calcium channel type D subunit alpha-1;
P	Q24270	DR	voltage-gated calcium channel activity
P	Q24352	CC	GABA, an inhibitory neurotransmitter, mediates neuronal inhibition by binding to the GABA receptor and opening an integral chloride channel
P	Q24352	DR	chloride channel activity
P	Q25011	DE	FMRFamide-activated amiloride-sensitive sodium channel;
P	Q25011	DR	sodium channel activity
P	Q2FFH9	DE	Na+/dicarboxylate symporter;
P	Q2FFH9	DE	Sodium-dependent dicarboxylate transporter sdcS;
P	Q2FQC0	CC	Transport of potassium into the cell (By similarity).
P	Q2FQC0	DE	Probable potassium transport system protein kup;
P	Q2FQC0	DR	potassium ion transmembrane transporter activity
P	Q2FTF8	CC	Part of the ABC transporter complex PstSACB involved in phosphate import
P	Q2FTF8	DE	ABC phosphate transporter;
P	Q2FTF8	DE	Phosphate import ATP-binding protein PstB;
P	Q2FTF8	DE	Phosphate-transporting ATPase;
P	Q2FTF8	DR	inorganic phosphate transmembrane transporter activity
P	Q2FTF8	DR	phosphate transmembrane-transporting ATPase activity
P	Q2FZR3	DE	Oligopeptide ABC transporter, substrate-binding protein, putative;
P	Q2FZR4	DE	Oligopeptide ABC transporter, ATP-binding protein, putative;
P	Q2G140	CC	Multidrug resistance efflux protein involved in transporting several clinically relevant monovalent and divalent biocides and the fluoroquinolone antimicrobial agents norfloxacin and ciprofloxacin
P	Q2GE91	DE	Lipoprotein-releasing system ATP-binding protein LolD;
P	Q2GE91	DR	lipoprotein transporter activity
P	Q2GFZ6	CC	Part of the ABC transporter complex ZnuABC involved in zinc import
P	Q2GFZ6	DE	Zinc import ATP-binding protein ZnuC;
P	Q2GFZ6	DR	zinc transporting ATPase activity
P	Q2GHT4	DE	Lipoprotein-releasing system ATP-binding protein LolD;
P	Q2GHT4	DR	lipoprotein transporter activity
P	Q2GJA5	CC	Part of the ABC transporter complex ZnuABC involved in zinc import
P	Q2GJA5	DE	Zinc import ATP-binding protein ZnuC;
P	Q2GJA5	DR	zinc transporting ATPase activity
P	Q2K0S7	CC	Part of the ABC transporter complex RbsABCD involved in ribose import
P	Q2K0S7	DE	Ribose import ATP-binding protein RbsA 3;
P	Q2K164	CC	Part of the ABC transporter complex TauABC involved in taurine import
P	Q2K164	DE	Taurine import ATP-binding protein TauB;
P	Q2K164	DR	taurine-transporting ATPase activity
P	Q2K1C8	DR	glycerol-3-phosphate-transporting ATPase activity
P	Q2K204	CC	Part of the ABC transporter complex RbsABCD involved in ribose import
P	Q2K204	DE	Ribose import ATP-binding protein RbsA 2;

P	Q2K284	CC	Part of the ABC transporter complex MetNIQ involved in methionine import
P	Q2K284	DE	Methionine import ATP-binding protein MetN;
P	Q2K2I9	CC	Transport of potassium into the cell (By similarity).
P	Q2K2I9	DE	Probable potassium transport system protein kup 3;
P	Q2K2I9	DR	potassium ion transmembrane transporter activity
P	Q2K2X0	DR	molybdate transmembrane-transporting ATPase activity
P	Q2K396	CC	Part of the ABC transporter complex CcmAB involved in the biogenesis of c-type cytochromes; once thought to export heme, this seems not to be the case, but its exact role is uncertain
P	Q2K396	DE	Heme exporter protein A;
P	Q2K396	DR	heme-transporting ATPase activity
P	Q2K4V4	DR	glycerol-3-phosphate-transporting ATPase activity
P	Q2K551	CC	Part of the ABC transporter complex HmuTUV involved in hemin import
P	Q2K551	DE	Hemin import ATP-binding protein HmuV;
P	Q2K5Z8	DR	sodium:dicarboxylate symporter activity
P	Q2K6L3	DR	glycerol-3-phosphate-transporting ATPase activity
P	Q2K6Q4	CC	Part of the ABC transporter complex ZnuABC involved in zinc import
P	Q2K6Q4	DE	Zinc import ATP-binding protein ZnuC;
P	Q2K6Q4	DR	zinc transporting ATPase activity
P	Q2K711	CC	Transport of potassium into the cell (By similarity).
P	Q2K711	DE	Probable potassium transport system protein kup 2;
P	Q2K711	DR	potassium ion transmembrane transporter activity
P	Q2K9A3	CC	Part of the ABC transporter complex RbsABCD involved in ribose import
P	Q2K9A3	DE	Ribose import ATP-binding protein RbsA 1;
P	Q2K9R2	DE	Lipoprotein-releasing system ATP-binding protein LolD;
P	Q2K9R2	DR	lipoprotein transporter activity
P	Q2KBW4	CC	Transport of potassium into the cell (By similarity).
P	Q2KBW4	DE	Probable potassium transport system protein kup 1;
P	Q2KBW4	DR	potassium ion transmembrane transporter activity
P	Q2KCN1	CC	Na+/H+ antiporter that extrudes sodium in exchange for external protons (By similarity).
P	Q2KCN1	DE	Na+/H+ antiporter nhaA;
P	Q2KCN1	DE	Sodium/proton antiporter nhaA;
P	Q2KCV5	CC	Part of the ABC transporter complex PstSACB involved in phosphate import
P	Q2KCV5	DE	ABC phosphate transporter;
P	Q2KCV5	DE	Phosphate import ATP-binding protein PstB;
P	Q2KCV5	DE	Phosphate-transporting ATPase;
P	Q2KCV5	DR	inorganic phosphate transmembrane transporter activity
P	Q2KCV5	DR	phosphate transmembrane-transporting ATPase activity
P	Q2KDV1	DR	phosphonate transmembrane-transporting ATPase activity
P	Q2LTG0	CC	Part of the ABC transporter complex PstSACB involved in phosphate import
P	Q2LTG0	DE	ABC phosphate transporter;
P	Q2LTG0	DE	Phosphate import ATP-binding protein PstB;
P	Q2LTG0	DE	Phosphate-transporting ATPase;
P	Q2LTG0	DR	inorganic phosphate transmembrane transporter activity
P	Q2LTG0	DR	phosphate transmembrane-transporting ATPase activity
P	Q2LVM2	DE	Lipoprotein-releasing system ATP-binding protein LolD;
P	Q2LVM2	DR	lipoprotein transporter activity
P	Q2M3M2	CC	Involved in sodium-dependent transport of D-mannose, D- glucose and D-fructose
P	Q2M3M2	CC	Involved in sodium-dependent transport of D-mannose, D- glucose and D-fructose
P	Q2M3M2	DE	Na+/glucose cotransporter 4;
P	Q2M3M2	DE	Na+/glucose cotransporter 4;
P	Q2M3M2	DE	Sodium/glucose cotransporter 4;
P	Q2M3M2	DE	Sodium/glucose cotransporter 4;
P	Q2M3M2	DR	low-affinity glucose:sodium symporter activity
P	Q2M3M2	DR	low-affinity glucose:sodium symporter activity
P	Q2MDH1	DE	Glucose/xylose facilitator 1;
P	Q2NK31	CC	Part of the ABC transporter complex PotABCD involved in spermidine/putrescine import
P	Q2NK31	CC	Part of the ABC transporter complex PotABCD involved in spermidine/putrescine import
P	Q2NK31	DE	Spermidine/putrescine import ATP-binding protein PotA;
P	Q2NK31	DE	Spermidine/putrescine import ATP-binding protein PotA;
P	Q2NQ98	CC	Responsible for the low-affinity transport of potassium into the cell, with the probable concomitant uptake of protons (symport system) (By similarity).
P	Q2NQ98	DE	Kup system potassium uptake protein;
P	Q2NQ98	DE	Low affinity potassium transport system protein kup;
P	Q2NQ98	DR	potassium ion transmembrane transporter activity

P	Q2NQF9	CC	Mediates influx of magnesium ions (By similarity).
P	Q2NQF9	DE	Magnesium transport protein CorA;
P	Q2NQF9	DR	cobalt ion transmembrane transporter activity
P	Q2NQF9	DR	magnesium ion transmembrane transporter activity
P	Q2NRN5	CC	Part of the ABC transporter complex MetNIQ involved in methionine import
P	Q2NRN5	DE	Methionine import ATP-binding protein MetN;
P	Q2NRN5	DR	D-methionine transmembrane transporter activity
P	Q2NSG3	CC	Part of the ABC transporter complex CcmAB involved in the biogenesis of c-type cytochromes; once thought to export heme, this seems not to be the case, but its exact role is uncertain
P	Q2NSG3	DE	Heme exporter protein A;
P	Q2NSG3	DR	heme-transporting ATPase activity
P	Q2NSR0	CC	Part of the ABC transporter complex HmuTUV involved in hemin import
P	Q2NSR0	DE	Hemin import ATP-binding protein HmuV;
P	Q2NTI7	CC	Part of the ABC transporter complex ZnuABC involved in zinc import
P	Q2NTI7	DE	Zinc import ATP-binding protein ZnuC;
P	Q2NTI7	DR	zinc transporting ATPase activity
P	Q2NU23	DE	Lipoprotein-releasing system ATP-binding protein LolD;
P	Q2NU23	DR	lipoprotein transporter activity
P	Q2NVW9	CC	Part of the ABC transporter complex ThiBPQ involved in thiamine import
P	Q2NVW9	DE	Thiamine import ATP-binding protein ThiQ;
P	Q2NVY9	CC	Na+/H+ antiporter that extrudes sodium in exchange for external protons (By similarity).
P	Q2NVY9	DE	Na+/H+ antiporter nhaA;
P	Q2NVY9	DE	Sodium/proton antiporter nhaA;
P	Q2NW73	CC	One of the components of the high-affinity ATP-driven potassium transport (or KDP) system, which catalyzes the hydrolysis of ATP coupled with the exchange of hydrogen and potassium ions
P	Q2NW73	DE	Potassium-binding and translocating subunit C;
P	Q2NW73	DE	Potassium-translocating ATPase C chain;
P	Q2NW73	DE	Potassium-transporting ATPase C chain;
P	Q2NW73	DR	potassium-transporting ATPase activity
P	Q2QWW7	DE	Phosphate transporter 4;7;
P	Q2RV87	CC	One of the components of the high-affinity ATP-driven potassium transport (or KDP) system, which catalyzes the hydrolysis of ATP coupled with the exchange of hydrogen and potassium ions
P	Q2RV87	DE	Potassium-binding and translocating subunit C;
P	Q2RV87	DE	Potassium-translocating ATPase C chain;
P	Q2RV87	DE	Potassium-transporting ATPase C chain;
P	Q2RV87	DR	potassium-transporting ATPase activity
P	Q2RV89	CC	One of the components of the high-affinity ATP-driven potassium transport (or KDP) system, which catalyzes the hydrolysis of ATP coupled with the exchange of hydrogen and potassium ions (By similarity).
P	Q2RV89	DE	Potassium-binding and translocating subunit A;
P	Q2RV89	DE	Potassium-translocating ATPase A chain;
P	Q2RV89	DE	Potassium-transporting ATPase A chain;
P	Q2RV89	DR	potassium-transporting ATPase activity
P	Q2RWA3	CC	Part of the ABC transporter complex MetNIQ involved in methionine import
P	Q2RWA3	DE	Methionine import ATP-binding protein MetN;
P	Q2RWI9	DR	molybdate transmembrane-transporting ATPase activity
P	Q2RWU0	CC	Part of the ABC transporter complex PstSACB involved in phosphate import
P	Q2RWU0	DE	ABC phosphate transporter;
P	Q2RWU0	DE	Phosphate import ATP-binding protein PstB;
P	Q2RWU0	DE	Phosphate-transporting ATPase;
P	Q2RWU0	DR	inorganic phosphate transmembrane transporter activity
P	Q2RWU0	DR	phosphate transmembrane-transporting ATPase activity
P	Q2RY62	CC	Na+/H+ antiporter that extrudes sodium in exchange for external protons (By similarity).
P	Q2RY62	DE	Na+/H+ antiporter nhaA;
P	Q2RY62	DE	Sodium/proton antiporter nhaA;
P	Q2RYF0	CC	Part of the ABC transporter complex CcmAB involved in the biogenesis of c-type cytochromes; once thought to export heme, this seems not to be the case, but its exact role is uncertain
P	Q2RYF0	DE	Heme exporter protein A;
P	Q2RYF0	DR	heme-transporting ATPase activity
P	Q2SR40	DR	phosphonate transmembrane-transporting ATPase activity
P	Q2SS06	CC	Part of the ABC transporter complex PstSACB involved in phosphate import

P	Q2SS06	DE	ABC phosphate transporter;
P	Q2SS06	DE	Phosphate import ATP-binding protein PstB;
P	Q2SS06	DE	Phosphate-transporting ATPase;
P	Q2SS06	DR	inorganic phosphate transmembrane transporter activity
P	Q2SS06	DR	phosphate transmembrane-transporting ATPase activity
P	Q2SSS4	CC	Part of the ABC transporter complex PotABCD involved in spermidine/putrescine import
P	Q2SSS4	CC	Part of the ABC transporter complex PotABCD involved in spermidine/putrescine import
P	Q2SSS4	DE	Spermidine/putrescine import ATP-binding protein PotA;
P	Q2SSS4	DE	Spermidine/putrescine import ATP-binding protein PotA;
P	Q2TAC4	DE	Calcium channel, voltage-dependent, T type, alpha 1G subunit;
P	Q2TAC4	DE	T calcium channel alpha 1G subunit variant 57;
P	Q2TAC4	DR	voltage-gated calcium channel activity
P	Q2Y0W8	CC	Mediates electroneutral sodium- and carbonate-dependent choride-HCO3(-) exchange with a Na+:HCO3(-) stoichiometry of 2:1
P	Q2Y0W8	DE	Electroneutral Na+-driven Cl-HCO3 exchanger;
P	Q2YDA1	DR	potassium channel activity
P	Q304A0	CC	Low-affinity magnesium transporter that mediates the influx of magnesium.
P	Q304A0	DE	Magnesium Transporter 7;
P	Q304A0	DE	Magnesium transporter MRS2-7;
P	Q30PC0	CC	Part of the ABC transporter complex PstSACB involved in phosphate import
P	Q30PC0	DE	ABC phosphate transporter;
P	Q30PC0	DE	Phosphate import ATP-binding protein PstB;
P	Q30PC0	DE	Phosphate-transporting ATPase;
P	Q30PC0	DR	inorganic phosphate transmembrane transporter activity
P	Q30PC0	DR	phosphate transmembrane-transporting ATPase activity
P	Q30PM3	CC	Na+/H+ antiporter that extrudes sodium in exchange for external protons (By similarity)
P	Q30PM3	DE	Na+/H+ antiporter nhaA;
P	Q30PM3	DE	Sodium/proton antiporter nhaA;
P	Q31125	DE	Zinc transporter SLC39A7;
P	Q31DV4	DR	phosphonate transmembrane-transporting ATPase activity
P	Q31GF5	DE	Lipoprotein-releasing system ATP-binding protein LolD;
P	Q31GF5	DR	lipoprotein transporter activity
P	Q31I51	CC	Part of the ABC transporter complex ZnuABC involved in zinc import
P	Q31I51	DE	Zinc import ATP-binding protein ZnuC;
P	Q31I51	DR	zinc transporting ATPase activity
P	Q31I88	CC	Part of the ABC transporter complex PstSACB involved in phosphate import
P	Q31I88	DE	ABC phosphate transporter;
P	Q31I88	DE	Phosphate import ATP-binding protein PstB;
P	Q31I88	DE	Phosphate-transporting ATPase;
P	Q31I88	DR	inorganic phosphate transmembrane transporter activity
P	Q31I88	DR	phosphate transmembrane-transporting ATPase activity
P	Q31J97	CC	Part of the ABC transporter complex HmuTUV involved in hemin import
P	Q31J97	DE	Hemin import ATP-binding protein HmuV;
P	Q37145	CC	This magnesium-dependent enzyme catalyzes the hydrolysis of ATP coupled with the translocation of calcium from the cytosol out of the cell or into organelles
P	Q37145	DE	Ca2+-ATPase isoform 1;
P	Q37145	DE	Calcium-transporting ATPase 1, chloroplastic;
P	Q37145	DR	calcium channel activity
P	Q37145	DR	calcium-transporting ATPase activity
P	Q38849	CC	Highly selective inward-rectifying potassium channel
P	Q38849	CC	This voltage-dependent channel could mediate long-term potassium influx into guard cells leading to stomatal opening
P	Q38849	DE	Potassium channel KAT2;
P	Q38849	DR	voltage-gated potassium channel activity
P	Q38856	CC	Acts as the principal regulator of iron homeostasis in planta
P	Q38856	CC	Also mediates the heavy metals uptake under iron-deficiency by its ability to transport cobalt, cadmium, manganese and/or zinc ions.
P	Q38856	CC	Also mediates the heavy metals uptake under iron-deficiency by its ability to transport cobalt, cadmium, manganese and/or zinc ions.
P	Q38856	CC	High-affinity iron transporter that plays a key role in the uptake of iron from the rhizosphere across the plasma membrane in the root epidermal layer
P	Q38856	DE	Fe(II) transport protein 1;
P	Q38856	DE	Iron-regulated transporter 1;
P	Q38856	DR	iron ion transmembrane transporter activity
P	Q38856	DR	zinc ion transmembrane transporter activity

P	Q38898	CC	Dephosphorylation by PP2CA not only leads to the inhibition of potassium currents but also to an increase of the voltage- dependence of the channel.
P	Q38898	CC	Highly selective and weak inward-rectifying potassium channel
P	Q38898	CC	Plays a role in both loading and unloading potassium into/from the phloem sap
P	Q38898	DE	Potassium channel AKT2/3;
P	Q38898	DR	voltage-gated potassium channel activity
P	Q38954	CC	Involved in inorganic phosphate (orthophosphate, Pi) uptake in green parts of plants in Pi-sufficient conditions
P	Q38954	DE	Inorganic phosphate transporter 2-1, chloroplastic;
P	Q38954	DR	inorganic phosphate transmembrane transporter activity
P	Q38954	DR	low affinity phosphate transmembrane transporter activity
P	Q38998	CC	Highly selective inward-rectifying potassium channel that mediate potassium uptake by plant roots in response to low K+ conditions, by a calcium-, CBL-, and CIPK-dependent pathway
P	Q38998	DE	Potassium channel AKT1;
P	Q38998	DR	inward rectifier potassium channel activity
P	Q39002	DE	ADP,ATP carrier protein 1, chloroplastic;
P	Q39002	DE	ADP/ATP translocase 1;
P	Q39002	DE	ADP/ATP translocase 1;
P	Q39002	DR	ATP:ADP antiporter activity
P	Q39002	DR	ATP:ADP antiporter activity
P	Q39065	CC	Copper transporter involved in copper acquisition and transport in leaves
P	Q39065	CC	Required for copper homeostasis and normal plant growth and development.
P	Q39065	DE	Copper transporter 1;
P	Q39065	DR	copper ion transmembrane transporter activity
P	Q39128	CC	Highly selective inward-rectifying potassium channel
P	Q39128	CC	This voltage-gated channel could mediate long-term potassium influx into guard cells leading to stomatal opening
P	Q39128	DE	Potassium channel KAT1;
P	Q39128	DR	voltage-gated potassium channel activity
P	Q39196	CC	Aquaporins facilitate the transport of water and small neutral solutes across cell membranes (By similarity).
P	Q39228	CC	Can transport glucose, methylglucose, galactose, xylose and mannose, but not fructose
P	Q39228	CC	Mediates an active uptake of hexoses, probably by sugar/hydrogen symport
P	Q39228	DE	Sugar transport protein 4;
P	Q39231	CC	Required for apoplastic phloem sucrose loading in source tissues (e.g leaves) in order to transport it to sink tissues (e.g
P	Q39231	CC	Responsible for the transport of sucrose into the cell, with the concomitant uptake of protons (symport system)
P	Q39231	DE	Sucrose permease 2;
P	Q39231	DE	Sucrose transport protein SUC2;
P	Q39231	DE	Sucrose transporter 1;
P	Q39231	DE	Sucrose-proton symporter 2;
P	Q39231	DR	sucrose:hydrogen symporter activity
P	Q39232	CC	Required for normal pollen germination and anthocyanin accumulation induced by sucrose.
P	Q39232	CC	Responsible for the transport of sucrose into the cell, with the concomitant uptake of protons (symport system)
P	Q39232	DE	Sucrose permease 1;
P	Q39232	DE	Sucrose transport protein SUC1;
P	Q39232	DE	Sucrose-proton symporter 1;
P	Q39232	DR	sucrose:hydrogen symporter activity
P	Q39253	CC	Translocates Ca2+ and other metal ions into vacuoles using the proton gradient formed by H+-ATPase and H+-pyrophosphatase
P	Q39253	DE	Ca2+/H+ antiporter CAX1;
P	Q39253	DE	Ca2+/H+ exchanger 1;
P	Q39253	DR	calcium:hydrogen antiporter activity
P	Q39254	CC	Translocates Ca2+ and other metal ions into vacuoles using the proton gradient formed by H+-ATPase and H+-pyrophosphatase.
P	Q39254	DE	Ca2+/H+ antiporter CAX2;
P	Q39254	DE	Ca2+/H+ exchanger 2;
P	Q39254	DR	calcium:hydrogen antiporter activity
P	Q39608	DE	Nitrate transporter component;
P	Q3A1R2	CC	Na+/H+ antiporter that extrudes sodium in exchange for external protons (By similarity)
P	Q3A1R2	DE	Na+/H+ antiporter nhaA 2;

P	Q3A1R2	DE	Sodium/proton antiporter nhaA 2;
P	Q3A4S1	CC	Na+/H+ antiporter that extrudes sodium in exchange for external protons (By similarity).
P	Q3A4S1	DE	Na+/H+ antiporter nhaA 1;
P	Q3A4S1	DE	Sodium/proton antiporter nhaA 1;
P	Q3A558	DE	Lipoprotein-releasing system ATP-binding protein LolD;
P	Q3A558	DR	lipoprotein transporter activity
P	Q3A6U0	CC	Part of the ABC transporter complex PstSACB involved in phosphate import
P	Q3A6U0	DE	ABC phosphate transporter;
P	Q3A6U0	DE	Phosphate import ATP-binding protein PstB;
P	Q3A6U0	DE	Phosphate-transporting ATPase;
P	Q3A6U0	DR	inorganic phosphate transmembrane transporter activity
P	Q3A6U0	DR	phosphate transmembrane-transporting ATPase activity
P	Q3A9G5	CC	Part of the ABC transporter complex MetNIQ involved in methionine import
P	Q3A9G5	DE	Methionine import ATP-binding protein MetN;
P	Q3AAA4	CC	Part of the ABC transporter complex PstSACB involved in phosphate import
P	Q3AAA4	DE	ABC phosphate transporter;
P	Q3AAA4	DE	Phosphate import ATP-binding protein PstB;
P	Q3AAA4	DE	Phosphate-transporting ATPase;
P	Q3AAA4	DR	inorganic phosphate transmembrane transporter activity
P	Q3AAA4	DR	phosphate transmembrane-transporting ATPase activity
P	Q3AJS9	CC	Part of the ABC transporter complex PstSACB involved in phosphate import
P	Q3AJS9	DE	ABC phosphate transporter;
P	Q3AJS9	DE	Phosphate import ATP-binding protein PstB;
P	Q3AJS9	DE	Phosphate-transporting ATPase;
P	Q3AJS9	DR	inorganic phosphate transmembrane transporter activity
P	Q3AJS9	DR	phosphate transmembrane-transporting ATPase activity
P	Q3APU9	CC	Transport of potassium into the cell (By similarity).
P	Q3APU9	DE	Probable potassium transport system protein kup;
P	Q3APU9	DR	potassium ion transmembrane transporter activity
P	Q3ARY3	DE	Lipoprotein-releasing system ATP-binding protein LolD 2;
P	Q3ARY3	DR	lipoprotein transporter activity
P	Q3ATA4	CC	Part of the ABC transporter complex PstSACB involved in phosphate import
P	Q3ATA4	DE	ABC phosphate transporter;
P	Q3ATA4	DE	Phosphate import ATP-binding protein PstB;
P	Q3ATA4	DE	Phosphate-transporting ATPase;
P	Q3ATA4	DR	inorganic phosphate transmembrane transporter activity
P	Q3ATA4	DR	phosphate transmembrane-transporting ATPase activity
P	Q3ATY5	DE	Lipoprotein-releasing system ATP-binding protein LolD 1;
P	Q3ATY5	DR	lipoprotein transporter activity
P	Q3AXX4	CC	Part of the ABC transporter complex PstSACB involved in phosphate import
P	Q3AXX4	DE	ABC phosphate transporter;
P	Q3AXX4	DE	Phosphate import ATP-binding protein PstB;
P	Q3AXX4	DE	Phosphate-transporting ATPase;
P	Q3AXX4	DR	inorganic phosphate transmembrane transporter activity
P	Q3AXX4	DR	phosphate transmembrane-transporting ATPase activity
P	Q3B1C1	CC	Transport of potassium into the cell (By similarity).
P	Q3B1C1	DE	Probable potassium transport system protein kup;
P	Q3B1C1	DR	potassium ion transmembrane transporter activity
P	Q3B276	DE	Lipoprotein-releasing system ATP-binding protein LolD 2;
P	Q3B276	DR	lipoprotein transporter activity
P	Q3B2U2	DE	Lipoprotein-releasing system ATP-binding protein LolD 1;
P	Q3B2U2	DR	lipoprotein transporter activity
P	Q3B3H7	CC	Part of the ABC transporter complex PstSACB involved in phosphate import
P	Q3B3H7	DE	ABC phosphate transporter;
P	Q3B3H7	DE	Phosphate import ATP-binding protein PstB;
P	Q3B3H7	DE	Phosphate-transporting ATPase;
P	Q3B3H7	DR	inorganic phosphate transmembrane transporter activity
P	Q3B3H7	DR	phosphate transmembrane-transporting ATPase activity
P	Q3ICT8	CC	Part of the ABC transporter complex HmuTUV involved in hemin import
P	Q3ICT8	DE	Hemin import ATP-binding protein HmuV;
P	Q3IL62	DE	Lipoprotein-releasing system ATP-binding protein LolD;
P	Q3IL62	DR	lipoprotein transporter activity
P	Q3ILC5	CC	Part of the ABC transporter complex PstSACB involved in phosphate import
P	Q3ILC5	DE	ABC phosphate transporter;
P	Q3ILC5	DE	Phosphate import ATP-binding protein PstB;

P	Q3ILC5	DE	Phosphate-transporting ATPase;
P	Q3ILC5	DR	inorganic phosphate transmembrane transporter activity
P	Q3ILC5	DR	phosphate transmembrane-transporting ATPase activity
P	Q3IM24	DR	phosphonate transmembrane-transporting ATPase activity
P	Q3IM36	CC	Part of the ABC transporter complex PstSACB involved in phosphate import
P	Q3IM36	DE	ABC phosphate transporter 3;
P	Q3IM36	DE	Phosphate import ATP-binding protein PstB 3;
P	Q3IM36	DE	Phosphate-transporting ATPase 3;
P	Q3IM36	DR	inorganic phosphate transmembrane transporter activity
P	Q3IM36	DR	phosphate transmembrane-transporting ATPase activity
P	Q3IS07	CC	Part of the ABC transporter complex PstSACB involved in phosphate import
P	Q3IS07	DE	ABC phosphate transporter 1;
P	Q3IS07	DE	Phosphate import ATP-binding protein PstB 1;
P	Q3IS07	DE	Phosphate-transporting ATPase 1;
P	Q3IS07	DR	inorganic phosphate transmembrane transporter activity
P	Q3IS07	DR	phosphate transmembrane-transporting ATPase activity
P	Q3ISC1	DR	phosphonate transmembrane-transporting ATPase activity
P	Q3J7S3	DE	Lipoprotein-releasing system ATP-binding protein LolD;
P	Q3J7S3	DR	lipoprotein transporter activity
P	Q3J8J2	CC	Part of the ABC transporter complex PstSACB involved in phosphate import
P	Q3J8J2	DE	ABC phosphate transporter 2;
P	Q3J8J2	DE	Phosphate import ATP-binding protein PstB 2;
P	Q3J8J2	DE	Phosphate-transporting ATPase 2;
P	Q3J8J2	DR	inorganic phosphate transmembrane transporter activity
P	Q3J8J2	DR	phosphate transmembrane-transporting ATPase activity
P	Q3JCI7	CC	Part of the ABC transporter complex CcmAB involved in the biogenesis of c-type cytochromes; once thought to export heme, this seems not to be the case, but its exact role is uncertain
P	Q3JCI7	DE	Heme exporter protein A;
P	Q3JCI7	DR	heme-transporting ATPase activity
P	Q3JDJ6	CC	Part of the ABC transporter complex PstSACB involved in phosphate import
P	Q3JDJ6	DE	ABC phosphate transporter 1;
P	Q3JDJ6	DE	Phosphate import ATP-binding protein PstB 1;
P	Q3JDJ6	DE	Phosphate-transporting ATPase 1;
P	Q3JDJ6	DR	phosphate transmembrane-transporting ATPase activity
P	Q3KNS8	DE	Potassium voltage-gated channel, Shaw-related subfamily, member 1;
P	Q3KNS8	DR	voltage-gated potassium channel activity
P	Q3KNW5	CC	Transports sulfoconjugated steroid hormones, as well as taurolithocholic acid-3-sulfate and sulfoconjugated pyrenes in a sodium-dependent manner.
P	Q3KNW5	DE	Sodium-dependent organic anion transporter;
P	Q3KNW5	DR	bile acid:sodium symporter activity
P	Q3KNW5	DR	bile acid:sodium symporter activity
P	Q3KR50	DR	calcium channel activity
P	Q3LS21	CC	pH-dependent, voltage-insensitive, background potassium channel protein (By similarity)
P	Q3LS21	DE	Acid-sensitive potassium channel protein TASK-3;
P	Q3LS21	DE	Potassium channel subfamily K member 9;
P	Q3LS21	DE	TWIK-related acid-sensitive K+ channel 3;
P	Q3LS21	DR	potassium channel activity
P	Q3MNJ6	DR	copper-exporting ATPase activity
P	Q3SNN8	CC	Na+/H+ antiporter that extrudes sodium in exchange for external protons (By similarity)
P	Q3SNN8	DE	Na+/H+ antiporter nhaA;
P	Q3SNN8	DE	Sodium/proton antiporter nhaA;
P	Q3SQ65	CC	Part of the ABC transporter complex HmuTUV involved in hemin import
P	Q3SQ65	DE	Hemin import ATP-binding protein HmuV;
P	Q3SRG8	DE	Lipoprotein-releasing system ATP-binding protein LolD;
P	Q3SRG8	DR	lipoprotein transporter activity
P	Q3ST91	CC	Transport of potassium into the cell (By similarity).
P	Q3ST91	DE	Probable potassium transport system protein kup;
P	Q3ST91	DR	potassium ion transmembrane transporter activity
P	Q3SVB5	CC	Part of the ABC transporter complex PstSACB involved in phosphate import
P	Q3SVB5	DE	ABC phosphate transporter;
P	Q3SVB5	DE	Phosphate import ATP-binding protein PstB;
P	Q3SVB5	DE	Phosphate-transporting ATPase;
P	Q3SVB5	DR	inorganic phosphate transmembrane transporter activity
P	Q3SVB5	DR	phosphate transmembrane-transporting ATPase activity

P	Q3SVV2	CC	Part of the ABC transporter complex CcmAB involved in the biogenesis of c-type cytochromes; once thought to export heme, this seems not to be the case, but its exact role is uncertain
P	Q3SVV2	DE	Heme exporter protein A;
P	Q3SVV2	DR	heme-transporting ATPase activity
P	Q3SWS6	DR	calcium channel activity
P	Q3TXX4	CC	Mediates the uptake of glutamate into synaptic vesicles at presynaptic nerve terminals of excitatory neural cells
P	Q3TXX4	DE	Vesicular glutamate transporter 1;
P	Q3U9N9	DE	Aromatic amino acid transporter 1;
P	Q3UEZ8	DE	Na+/bile acid cotransporter 4;
P	Q3UEZ8	DE	Na+/bile acid cotransporter 4;
P	Q3UEZ8	DE	Sodium/bile acid cotransporter 4;
P	Q3UEZ8	DE	Sodium/bile acid cotransporter 4;
P	Q3UEZ8	DR	bile acid:sodium symporter activity
P	Q3UEZ8	DR	bile acid:sodium symporter activity
P	Q3UHK1	DE	Proton myo-inositol cotransporter;
P	Q3UVU3	CC	May be involved in zinc transport out of the cell, being a zinc-efflux transporter (By similarity).
P	Q3UVU3	DE	Zinc transporter 10;
P	Q3V132	CC	Catalyzes the exchange of ADP and ATP across the mitochondrial inner membrane
P	Q3V132	CC	Catalyzes the exchange of ADP and ATP across the mitochondrial inner membrane
P	Q3V132	DE	ADP,ATP carrier protein 4;
P	Q3V132	DE	ADP/ATP translocase 4;
P	Q3V132	DE	ADP/ATP translocase 4;
P	Q3YL57	DE	Na+/H+ exchanger 8;
P	Q3YL57	DE	Sodium/hydrogen exchanger 8;
P	Q3YSK9	CC	Part of the ABC transporter complex ZnuABC involved in zinc import
P	Q3YSK9	DE	Zinc import ATP-binding protein ZnuC;
P	Q3YSK9	DR	zinc transporting ATPase activity
P	Q3ZA58	CC	Part of the ABC transporter complex PstSACB involved in phosphate import
P	Q3ZA58	DE	ABC phosphate transporter;
P	Q3ZA58	DE	Phosphate import ATP-binding protein PstB;
P	Q3ZA58	DE	Phosphate-transporting ATPase;
P	Q3ZA58	DR	inorganic phosphate transmembrane transporter activity
P	Q3ZA58	DR	phosphate transmembrane-transporting ATPase activity
P	Q3ZCW7	DE	Sodium/hydrogen exchanger;
P	Q3ZCW7	DR	sodium:hydrogen antiporter activity
P	Q3ZWN4	CC	Part of the ABC transporter complex PstSACB involved in phosphate import
P	Q3ZWN4	DE	ABC phosphate transporter;
P	Q3ZWN4	DE	Phosphate import ATP-binding protein PstB;
P	Q3ZWN4	DE	Phosphate-transporting ATPase;
P	Q3ZWN4	DR	inorganic phosphate transmembrane transporter activity
P	Q3ZWN4	DR	phosphate transmembrane-transporting ATPase activity
P	Q40746	CC	Aquaporins facilitate the transport of water and small neutral solutes across cell membranes (By similarity).
P	Q41951	CC	Aquaporin required to facilitate the transport of water from the vacuolar compartment to the cytoplasm
P	Q41951	DR	water channel activity
P	Q41963	CC	Water channel required to facilitate the transport of water across cell membrane
P	Q41975	CC	Aquaporins facilitate the transport of water and small neutral solutes across cell membranes (By similarity).
P	Q43650	DE	Inorganic phosphate transporter 1;
P	Q43650	DR	inorganic phosphate transmembrane transporter activity
P	Q43975	DE	4-hydroxybenzoate transporter;
P	Q44292	CC	Essential component of the nitrate-transporting system
P	Q44292	DE	Nitrate transport protein NrtA;
P	Q44613	DE	Lipoprotein-releasing system ATP-binding protein LolD;
P	Q44613	DR	lipoprotein transporter activity
P	Q44840	CC	This system is involved in glucose transport (By similarity).
P	Q44840	DE	Glucose-specific phosphotransferase enzyme IIA component;
P	Q44840	DE	PTS system glucose-specific EIIA component;
P	Q45153	DE	Quaternary amine transporter;
P	Q45247	CC	Involved in nickel incorporation/metabolism into the hydrogenase apoprotein.
P	Q45247	DE	Hydrogenase nickel incorporation protein hupN;

P	Q45247	DR	nickel ion transmembrane transporter activity
P	Q45460	CC	Involved in a high affinity multicomponent binding- protein-dependent transport system for choline
P	Q45460	DE	Choline transport ATP-binding protein OpuBA;
P	Q45461	CC	Involved in a high affinity multicomponent binding- protein-dependent transport system for choline; probably responsible for the translocation of the substrate across the membrane.
P	Q45461	DE	Choline transport system permease protein opuBB;
P	Q45462	CC	Member of a high affinity multicomponent binding- protein-dependent transport system for choline.
P	Q45462	DE	Choline-binding protein;
P	Q46170	CC	Catalyzes an electroneutral exchange between arginine and ornithine to allow high-efficiency energy conversion in the arginine deiminase pathway (By similarity).
P	Q46170	CC	Catalyzes an electroneutral exchange between arginine and ornithine to allow high-efficiency energy conversion in the arginine deiminase pathway (By similarity).
P	Q46170	DE	Arginine/ornithine antiporter;
P	Q46170	DE	Arginine/ornithine antiporter;
P	Q46M63	DE	2,4-D transport protein;
P	Q47537	CC	Part of a binding-protein-dependent transport system for taurine.
P	Q47537	DE	Taurine-binding periplasmic protein;
P	Q47538	CC	Part of the ABC transporter complex TauABC involved in taurine import
P	Q47538	DE	Taurine import ATP-binding protein TauB;
P	Q47538	DR	taurine-transporting ATPase activity
P	Q47539	CC	Part of a binding-protein-dependent transport system for taurine
P	Q47539	DE	Taurine transport system permease protein tauC;
P	Q478L3	CC	Part of the ABC transporter complex CcmAB involved in the biogenesis of c-type cytochromes; once thought to export heme, this seems not to be the case, but its exact role is uncertain
P	Q478L3	DE	Heme exporter protein A;
P	Q478L3	DR	heme-transporting ATPase activity
P	Q47A37	CC	Part of the ABC transporter complex PstSACB involved in phosphate import
P	Q47A37	DE	ABC phosphate transporter;
P	Q47A37	DE	Phosphate import ATP-binding protein PstB;
P	Q47A37	DE	Phosphate-transporting ATPase;
P	Q47A37	DR	inorganic phosphate transmembrane transporter activity
P	Q47A37	DR	phosphate transmembrane-transporting ATPase activity
P	Q47A46	CC	Transport of potassium into the cell (By similarity).
P	Q47A46	DE	Probable potassium transport system protein kup 3;
P	Q47A46	DR	potassium ion transmembrane transporter activity
P	Q47BQ7	DR	sodium:dicarboxylate symporter activity
P	Q47C66	DE	Lipoprotein-releasing system ATP-binding protein LolD;
P	Q47C66	DR	lipoprotein transporter activity
P	Q47CB7	DR	molybdate transmembrane-transporting ATPase activity
P	Q47F10	DR	phosphonate transmembrane-transporting ATPase activity
P	Q47GU1	CC	Transport of potassium into the cell (By similarity).
P	Q47GU1	DE	Probable potassium transport system protein kup 2;
P	Q47GU1	DR	potassium ion transmembrane transporter activity
P	Q47H38	CC	One of the components of the high-affinity ATP-driven potassium transport (or KDP) system, which catalyzes the hydrolysis of ATP coupled with the exchange of hydrogen and potassium ions (By similarity).
P	Q47H38	DE	Potassium-binding and translocating subunit A;
P	Q47H38	DE	Potassium-translocating ATPase A chain;
P	Q47H38	DE	Potassium-transporting ATPase A chain;
P	Q47H38	DR	potassium-transporting ATPase activity
P	Q47H39	CC	One of the components of the high-affinity ATP-driven potassium transport (or KDP) system, which catalyzes the hydrolysis of ATP coupled with the exchange of hydrogen and potassium ions (By similarity).
P	Q47H39	DE	Potassium-binding and translocating subunit B;
P	Q47H39	DE	Potassium-translocating ATPase B chain;
P	Q47H39	DE	Potassium-transporting ATPase B chain;
P	Q47H39	DR	potassium-transporting ATPase activity
P	Q47H40	CC	One of the components of the high-affinity ATP-driven potassium transport (or KDP) system, which catalyzes the hydrolysis of ATP coupled with the exchange of hydrogen and potassium ions
P	Q47H40	DE	Potassium-binding and translocating subunit C;

P	Q47H40	DE	Potassium-translocating ATPase C chain;
P	Q47H40	DE	Potassium-transporting ATPase C chain;
P	Q47H40	DR	potassium-transporting ATPase activity
P	Q47I87	CC	Na+/H+ antiporter that extrudes sodium in exchange for external protons (By similarity)
P	Q47I87	DE	Na+/H+ antiporter nhaA;
P	Q47I87	DE	Sodium/proton antiporter nhaA;
P	Q47IT9	CC	Transport of potassium into the cell (By similarity).
P	Q47IT9	DE	Probable potassium transport system protein kup 1;
P	Q47IT9	DR	potassium ion transmembrane transporter activity
P	Q47Y12	CC	Part of the ABC transporter complex PstSACB involved in phosphate import
P	Q47Y12	DE	ABC phosphate transporter;
P	Q47Y12	DE	Phosphate import ATP-binding protein PstB;
P	Q47Y12	DE	Phosphate-transporting ATPase;
P	Q47Y12	DR	inorganic phosphate transmembrane transporter activity
P	Q47Y12	DR	phosphate transmembrane-transporting ATPase activity
P	Q47YG8	DE	Lipoprotein-releasing system ATP-binding protein LolD;
P	Q47YG8	DR	lipoprotein transporter activity
P	Q48262	CC	High-affinity nickel intake protein
P	Q48262	CC	Imports nickel ions in an energy-dependent fashion
P	Q48262	DE	High-affinity nickel-transport protein nixA;
P	Q48262	DR	nickel ion transmembrane transporter activity
P	Q485F0	CC	Involved in the import of serine and threonine into the cell, with the concomitant import of sodium (symport system) (By similarity).
P	Q485F0	DE	Na+/serine-threonine symporter;
P	Q485F0	DR	sodium:dicarboxylate symporter activity
P	Q485F0	DR	sodium:dicarboxylate symporter activity
P	Q48769	DE	Citrate permease;
P	Q487I2	CC	Part of the ABC transporter complex CcmAB involved in the biogenesis of c-type cytochromes; once thought to export heme, this seems not to be the case, but its exact role is uncertain
P	Q487I2	DE	Heme exporter protein A;
P	Q487I2	DR	heme-transporting ATPase activity
P	Q48WG5	DE	PTS system, glucose-specific IIABC component;
P	Q491Z9	CC	Mediates influx of magnesium ions (By similarity).
P	Q491Z9	DE	Magnesium transport protein CorA;
P	Q491Z9	DR	cobalt ion transmembrane transporter activity
P	Q491Z9	DR	magnesium ion transmembrane transporter activity
P	Q492G8	CC	H+-stimulated, highly selective, manganese uptake system (By similarity).
P	Q492G8	DE	Probable manganese transport protein mntH;
P	Q492R2	DE	Lipoprotein-releasing system ATP-binding protein LolD;
P	Q492R2	DR	lipoprotein transporter activity
P	Q494P0	CC	High-affinity transporter for external inorganic phosphate (By similarity).
P	Q494P0	DE	Probable inorganic phosphate transporter 1-7;
P	Q494P0	DR	inorganic phosphate transmembrane transporter activity
P	Q49B93	CC	Acts as an electroneutral and low-affinity sodium (Na+)-dependent sodium-coupled solute transporter
P	Q49B93	CC	Mediates electroneutral uptake of lactate, with a stoichiometry of 2 Na+ for each lactate
P	Q49B93	DE	Electroneutral sodium monocarboxylate cotransporter;
P	Q49B93	DE	Low-affinity sodium-lactate cotransporter;
P	Q49B93	DE	Sodium-coupled monocarboxylate transporter 2;
P	Q4F7G0	CC	Sugar transporter (Potential).
P	Q4F7G0	DE	Sugar transporter ERD6-like 2;
P	Q4F7G0	DE	Sugar transporter-like protein 3;
P	Q4FLF6	CC	Part of the ABC transporter complex PstSACB involved in phosphate import
P	Q4FLF6	DE	ABC phosphate transporter;
P	Q4FLF6	DE	Phosphate import ATP-binding protein PstB;
P	Q4FLF6	DE	Phosphate-transporting ATPase;
P	Q4FLF6	DR	inorganic phosphate transmembrane transporter activity
P	Q4FLF6	DR	phosphate transmembrane-transporting ATPase activity
P	Q4FLX6	CC	Na+/H+ antiporter that extrudes sodium in exchange for external protons (By similarity)
P	Q4FLX6	DE	Na+/H+ antiporter nhaA;
P	Q4FLX6	DE	Sodium/proton antiporter nhaA;
P	Q4FMG5	CC	Part of the ABC transporter complex TauABC involved in taurine import
P	Q4FMG5	DE	Taurine import ATP-binding protein TauB;
P	Q4FMG5	DR	taurine-transporting ATPase activity

P	Q4G0N8	CC	Sperm-specific sodium/hydrogen exchanger involved in intracellular pH regulation of spermatozoa
P	Q4G0N8	DE	Na+/H+ exchanger 10;
P	Q4G0N8	DE	Sodium/hydrogen exchanger 10;
P	Q4G0N8	DE	Sperm-specific Na+/H+ exchanger;
P	Q4K3K9	CC	Part of the ABC transporter complex PstSACB involved in phosphate import
P	Q4K3K9	DE	ABC phosphate transporter;
P	Q4K3K9	DE	Phosphate import ATP-binding protein PstB;
P	Q4K3K9	DE	Phosphate-transporting ATPase;
P	Q4K3K9	DR	inorganic phosphate transmembrane transporter activity
P	Q4K3K9	DR	phosphate transmembrane-transporting ATPase activity
P	Q4K5Z7	CC	Part of the ABC transporter complex HmuTUV involved in hemin import
P	Q4K5Z7	DE	Hemin import ATP-binding protein HmuV;
P	Q4K681	CC	Part of the ABC transporter complex PotABCD involved in spermidine/putrescine import
P	Q4K681	CC	Part of the ABC transporter complex PotABCD involved in spermidine/putrescine import
P	Q4K681	DE	Spermidine/putrescine import ATP-binding protein PotA;
P	Q4K681	DE	Spermidine/putrescine import ATP-binding protein PotA;
P	Q4K9I6	CC	Involved in the import of serine and threonine into the cell, with the concomitant import of sodium (symport system) (By similarity).
P	Q4K9I6	DE	Na+/serine-threonine symporter;
P	Q4K9I6	DR	sodium:dicarboxylate symporter activity
P	Q4K9I6	DR	sodium:dicarboxylate symporter activity
P	Q4KB64	CC	Part of the ABC transporter complex ModABC involved in molybdenum import
P	Q4KB64	DE	Molybdenum import ATP-binding protein ModC;
P	Q4KB64	DR	molybdenum ion transmembrane transporter activity
P	Q4KBU0	CC	Part of the ABC transporter complex MetNIQ involved in methionine import
P	Q4KBU0	DE	Methionine import ATP-binding protein MetN 3;
P	Q4KBY6	CC	Na+/H+ antiporter that extrudes sodium in exchange for external protons (By similarity)
P	Q4KBY6	DE	Na+/H+ antiporter nhaB;
P	Q4KBY6	DE	Sodium/proton antiporter nhaB;
P	Q4KBY6	DR	sodium:hydrogen antiporter activity
P	Q4KFA2	DE	Lipoprotein-releasing system ATP-binding protein LolD;
P	Q4KFA2	DR	lipoprotein transporter activity
P	Q4KG27	CC	Part of the ABC transporter complex CcmAB involved in the biogenesis of c-type cytochromes; once thought to export heme, this seems not to be the case, but its exact role is uncertain
P	Q4KG27	DE	Heme exporter protein A;
P	Q4KG27	DR	heme-transporting ATPase activity
P	Q4KH68	CC	Na+/H+ antiporter that extrudes sodium in exchange for external protons (By similarity)
P	Q4KH68	DE	Na+/H+ antiporter nhaA;
P	Q4KH68	DE	Sodium/proton antiporter nhaA;
P	Q4KHA4	CC	Transport of potassium into the cell (By similarity).
P	Q4KHA4	DE	Probable potassium transport system protein kup;
P	Q4KHA4	DR	potassium ion transmembrane transporter activity
P	Q4KK16	CC	Part of the ABC transporter complex TauABC involved in taurine import
P	Q4KK16	DE	Taurine import ATP-binding protein TauB;
P	Q4KK16	DR	taurine-transporting ATPase activity
P	Q4KK46	CC	Part of the ABC transporter complex MetNIQ involved in methionine import
P	Q4KK46	DE	Methionine import ATP-binding protein MetN 2;
P	Q4KKK4	CC	Part of the ABC transporter complex ZnuABC involved in zinc import
P	Q4KKK4	DE	Zinc import ATP-binding protein ZnuC;
P	Q4KKK4	DR	zinc transporting ATPase activity
P	Q4KKK8	CC	Part of the ABC transporter complex MetNIQ involved in methionine import
P	Q4KKK8	DE	Methionine import ATP-binding protein MetN 1;
P	Q4LE63	DR	calcium-transporting ATPase activity
P	Q4VAM4	DR	neurotransmitter:sodium symporter activity
P	Q4ZFY1	DE	cDNA, FLJ95252, Homo sapiens potassium voltage-gated channel, delayed-rectifier,subfamily S, member 3 (KCNS3), mRNA;
P	Q4ZFY1	DR	voltage-gated potassium channel activity
P	Q4ZJI4	DE	Na+/H+ exchanger-like domain-containing protein 1;
P	Q4ZJI4	DE	Sodium/hydrogen exchanger-like domain-containing protein 1;
P	Q50046	CC	Part of the ABC transporter complex PstSACB involved in phosphate import
P	Q50046	DE	ABC phosphate transporter;
P	Q50046	DE	Phosphate import ATP-binding protein PstB;
P	Q50046	DE	Phosphate-transporting ATPase;

P	Q50046	DR	inorganic phosphate transmembrane transporter activity
P	Q50046	DR	phosphate transmembrane-transporting ATPase activity
P	Q50097	CC	Part of the binding-protein-dependent transport system for phosphate; probably responsible for the translocation of the substrate across the membrane (By similarity)
P	Q50097	DE	Phosphate transport system permease protein pstA;
P	Q50097	DR	inorganic phosphate transmembrane transporter activity
P	Q50098	CC	Part of a binding-protein-dependent transport system for phosphate; probably responsible for the translocation of the substrate across the membrane (By similarity)
P	Q50098	DE	Phosphate transport system permease protein pstC;
P	Q50098	DR	inorganic phosphate transmembrane transporter activity
P	Q50103	CC	H+-stimulated, highly selective, manganese uptake system (By similarity).
P	Q50103	DE	Probable manganese transport protein mntH;
P	Q50173	CC	Low-affinity inorganic phosphate transport (By similarity).
P	Q50173	DE	Probable low-affinity inorganic phosphate transporter;
P	Q50173	DR	inorganic phosphate transmembrane transporter activity
P	Q50773	CC	This is an energy-conserving, sodium-ion translocating step.
P	Q50774	CC	This is an energy-conserving, sodium-ion translocating step.
P	Q51104	DE	Hemoglobin receptor;
P	Q51330	CC	Anion transporter that carries out the exchange of divalent oxalate with monovalent formate, the product of oxalate decarboxylation, at the plasma membrane, and in doing so catalyzes the vectorial portion of a proton-motive metabolic cycle that drives ATP synthesis.
P	Q51330	CC	Anion transporter that carries out the exchange of divalent oxalate with monovalent formate, the product of oxalate decarboxylation, at the plasma membrane, and in doing so catalyzes the vectorial portion of a proton-motive metabolic cycle that drives ATP synthesis.
P	Q51330	DE	Oxalate:formate antiport protein;
P	Q51330	DE	Oxalate:formate antiport protein;
P	Q51330	DE	Oxalate:formate antiporter;
P	Q51330	DE	Oxalate:formate antiporter;
P	Q51330	DE	Oxalate:formate exchange protein;
P	Q51330	DE	Oxalate:formate exchange protein;
P	Q51389	CC	Glycerol enters the cell via the glycerol diffusion facilitator protein
P	Q51389	CC	This membrane protein facilitates the movement of glycerol across the cytoplasmic membrane.
P	Q51389	DE	Glycerol diffusion facilitator;
P	Q51389	DE	Glycerol uptake facilitator protein;
P	Q51485	CC	Facilitates glucose diffusion across the outer membrane.
P	Q51485	CC	Involved in the transport of glucose, mannitol, fructose and glycerol (sugars able to support the growth of P.aeruginosa)
P	Q51485	DE	Glucose porin;
P	Q51546	CC	Part of the ABC transporter complex PstSACB involved in phosphate import
P	Q51546	DE	ABC phosphate transporter;
P	Q51546	DE	Phosphate import ATP-binding protein PstB;
P	Q51546	DE	Phosphate-transporting ATPase;
P	Q51546	DR	inorganic phosphate transmembrane transporter activity
P	Q51546	DR	phosphate transmembrane-transporting ATPase activity
P	Q51955	CC	Transporter for 4-hydroxybenzoate
P	Q51955	DE	4-hydroxybenzoate transporter;
P	Q52000	DE	Dicarboxylic acid transport protein;
P	Q52663	CC	Part of a binding-protein-dependent transport system for glutamate, glutamine, aspartate and asparagine.
P	Q52663	CC	Part of a binding-protein-dependent transport system for glutamate, glutamine, aspartate and asparagine.
P	Q52663	CC	Part of a binding-protein-dependent transport system for glutamate, glutamine, aspartate and asparagine.
P	Q52663	DE	Glutamate/glutamine/aspartate/asparagine-binding protein BztA;
P	Q52663	DE	Glutamate/glutamine/aspartate/asparagine-binding protein BztA;
P	Q52663	DE	Glutamate/glutamine/aspartate/asparagine-binding protein BztA;
P	Q52663	DE	Glutamate/glutamine/aspartate/asparagine-binding protein BztA;
P	Q52664	CC	Part of a binding-protein-dependent transport system for glutamate, glutamine, aspartate and asparagine
P	Q52664	CC	Part of a binding-protein-dependent transport system for glutamate, glutamine, aspartate and asparagine

P	Q52664	CC	Part of a binding-protein-dependent transport system for glutamate, glutamine, aspartate and asparagine
P	Q52664	DE	Glutamate/glutamine/aspartate/asparagine transport system permease protein BztB;
P	Q52664	DE	Glutamate/glutamine/aspartate/asparagine transport system permease protein BztB;
P	Q52664	DE	Glutamate/glutamine/aspartate/asparagine transport system permease protein BztB;
P	Q52664	DE	Glutamate/glutamine/aspartate/asparagine transport system permease protein BztB;
P	Q52665	CC	Part of a binding-protein-dependent transport system for glutamate, glutamine, aspartate and asparagine
P	Q52665	CC	Part of a binding-protein-dependent transport system for glutamate, glutamine, aspartate and asparagine
P	Q52665	CC	Part of a binding-protein-dependent transport system for glutamate, glutamine, aspartate and asparagine
P	Q52665	DE	Glutamate/glutamine/aspartate/asparagine transport system permease protein BztC;
P	Q52665	DE	Glutamate/glutamine/aspartate/asparagine transport system permease protein BztC;
P	Q52665	DE	Glutamate/glutamine/aspartate/asparagine transport system permease protein BztC;
P	Q52665	DE	Glutamate/glutamine/aspartate/asparagine transport system permease protein BztC;
P	Q52666	CC	Part of a binding-protein-dependent transport system for glutamate, glutamine, aspartate, asparagine
P	Q52666	CC	Part of a binding-protein-dependent transport system for glutamate, glutamine, aspartate, asparagine
P	Q52666	CC	Part of a binding-protein-dependent transport system for glutamate, glutamine, aspartate, asparagine
P	Q52666	CC	Part of a binding-protein-dependent transport system for glutamate, glutamine, aspartate, asparagine
P	Q52666	DE	Glutamate/glutamine/aspartate/asparagine transport ATP-binding protein BztD;
P	Q52666	DE	Glutamate/glutamine/aspartate/asparagine transport ATP-binding protein BztD;
P	Q52666	DE	Glutamate/glutamine/aspartate/asparagine transport ATP-binding protein BztD;
P	Q52666	DE	Glutamate/glutamine/aspartate/asparagine transport ATP-binding protein BztD;
P	Q52978	CC	Part of a K+ efflux system which is required for the adaptation of R.meliloti to alkaline pH as well as for the infection process during symbiotic nodule development
P	Q52978	DE	Probable K+/H+ antiporter subunit A/B;
P	Q52978	DE	Probable K+/H+ antiporter subunit A/B;
P	Q52978	DE	pH adaptation potassium efflux system protein A/B;
P	Q52980	CC	Part of a K+ efflux system which is required for the adaptation of R.meliloti to alkaline pH as well as for the infection process during symbiotic nodule development
P	Q52980	DE	Probable K+/H+ antiporter subunit C;
P	Q52980	DE	Probable K+/H+ antiporter subunit C;
P	Q52980	DE	pH adaptation potassium efflux system protein C;
P	Q52981	CC	Part of a K+ efflux system which is required for the adaptation of R.meliloti to alkaline pH as well as for the infection process during symbiotic nodule development
P	Q52981	DE	Probable K+/H+ antiporter subunit D;
P	Q52981	DE	Probable K+/H+ antiporter subunit D;
P	Q52981	DE	pH adaptation potassium efflux system protein D;
P	Q52981	DR	potassium:hydrogen antiporter activity
P	Q52982	CC	Part of a K+ efflux system which is required for the adaptation of R.meliloti to alkaline pH as well as for the infection process during symbiotic nodule development
P	Q52982	DE	Probable K+/H+ antiporter subunit E;
P	Q52982	DE	Probable K+/H+ antiporter subunit E;
P	Q52982	DE	pH adaptation potassium efflux system protein E;
P	Q52983	CC	Part of a K+ efflux system which is required for the adaptation of R.meliloti to alkaline pH as well as for the infection process during symbiotic nodule development
P	Q52983	DE	Probable K+/H+ antiporter subunit F;
P	Q52983	DE	Probable K+/H+ antiporter subunit F;
P	Q52983	DE	pH adaptation potassium efflux system protein F;
P	Q53148	DE	Ethanolamine permease;
P	Q53922	CC	Can also transport glucose, but not galactose, fructose, mannose, cellobiose, sucrose, maltose, lactose, melibiose and trehalose, as well as N-acetylglucosamine
P	Q53922	DR	glucose transmembrane transporter activity
P	Q53P54	DE	Phosphate transporter 4;6;
P	Q53SA1	DE	Inwardly-rectifying potassium channel Kir7.1;
P	Q53SA1	DE	Potassium inwardly-rectifying channel, subfamily J, member 13;
P	Q53SA1	DE	cDNA, FLJ94677, highly similar to Homo sapiens potassium inwardly-rectifying channel, subfamily J, member 13 (KCNJ13), mRNA;
P	Q53SA1	DR	inward rectifier potassium channel activity
P	Q53Y30	DE	Potassium voltage-gated channel, KQT-like subfamily, member 2, isoform CRA_e;

P	Q53Y30	DE	Potassium voltage-gated channel, KQT-like subfamily, member 2;
P	Q53Y30	DR	voltage-gated potassium channel activity
P	Q547S7	DE	Potassium voltage-gated channel, subfamily G, member 4, isoform CRA_b;
P	Q547S7	DE	Voltage-gated potassium channel subunit Kv6.3;
P	Q547S7	DR	voltage-gated potassium channel activity
P	Q54AX6	CC	Chloride channels may have several functions including the regulation of cell volume, membrane potential stabilization and signal transduction (By similarity).
P	Q54AX6	CC	Voltage-gated chloride channel
P	Q54AX6	DE	Chloride channel protein A;
P	Q54AX6	DR	voltage-gated chloride channel activity
P	Q54BF6	CC	Phosphate is cotransported with H+ (By similarity).
P	Q54BF6	CC	Transports phosphate groups from the cytosol to the mitochondrial matrix
P	Q54C67	CC	Chloride channels may have several functions including the regulation of cell volume, membrane potential stabilization and signal transduction (By similarity).
P	Q54C67	CC	Voltage-gated chloride channel
P	Q54C67	DE	Chloride channel protein F;
P	Q54C67	DR	voltage-gated chloride channel activity
P	Q54GU0	DR	arsenite transmembrane transporter activity
P	Q54LQ4	CC	Chloride channels may have several functions including the regulation of cell volume, membrane potential stabilization and signal transduction (By similarity).
P	Q54LQ4	CC	Voltage-gated chloride channel
P	Q54LQ4	DE	Chloride channel protein E;
P	Q54LQ4	DR	voltage-gated chloride channel activity
P	Q54PY7	CC	Catalyzes the transport of 2-oxoglutarate across the inner mitochondrial membrane in an electroneutral exchange for malate or other dicarboxylic acids, and plays an important role in several metabolic processes, including the malate-aspartate shuttle, the oxoglutarate/isocitrate shuttle, in gluconeogenesis from lactate, and in nitrogen metabolism (By similarity).
P	Q54PY7	CC	Catalyzes the transport of 2-oxoglutarate across the inner mitochondrial membrane in an electroneutral exchange for malate or other dicarboxylic acids, and plays an important role in several metabolic processes, including the malate-aspartate shuttle, the oxoglutarate/isocitrate shuttle, in gluconeogenesis from lactate, and in nitrogen metabolism (By similarity).
P	Q54PY7	DE	Probable mitochondrial 2-oxoglutarate/malate carrier protein;
P	Q54PY7	DE	Probable mitochondrial 2-oxoglutarate/malate carrier protein;
P	Q54QN2	CC	Transports folate across the inner membranes of mitochondria (By similarity).
P	Q55106	CC	Part of the ABC transporter complex CmpABCD involved in bicarbonate transport
P	Q55106	DE	Bicarbonate transport system permease protein CmpB;
P	Q55107	CC	Part of the ABC transporter complex CmpABCD involved in bicarbonate transport
P	Q55107	DE	Bicarbonate transport ATP-binding protein CmpC;
P	Q55108	CC	Part of the ABC transporter complex CmpABCD involved in bicarbonate transport
P	Q55108	DE	Bicarbonate transport ATP-binding protein CmpD;
P	Q55108	DR	nitrate transmembrane transporter activity
P	Q55195	CC	Part of the ABC transporter complex PstSACB involved in phosphate import
P	Q55195	DE	ABC phosphate transporter 2;
P	Q55195	DE	Phosphate import ATP-binding protein PstB 2;
P	Q55195	DE	Phosphate-transporting ATPase 2;
P	Q55195	DR	inorganic phosphate transmembrane transporter activity
P	Q55195	DR	phosphate transmembrane-transporting ATPase activity
P	Q55196	CC	Part of the ABC transporter complex PstSACB involved in phosphate import
P	Q55196	DE	ABC phosphate transporter 1;
P	Q55196	DE	Phosphate import ATP-binding protein PstB 1;
P	Q55196	DE	Phosphate-transporting ATPase 1;
P	Q55196	DR	inorganic phosphate transmembrane transporter activity
P	Q55196	DR	phosphate transmembrane-transporting ATPase activity
P	Q55281	CC	Part of an ATP-driven transport system for manganese.
P	Q55281	DE	Manganese transport system ATP-binding protein MntA;
P	Q55282	CC	Part of an ATP-driven transport system for manganese.
P	Q55282	DE	Manganese transport system membrane protein mntB;
P	Q552S0	DE	Sodium/hydrogen exchanger 1;
P	Q552S0	DR	sodium:hydrogen antiporter activity
P	Q553K4	CC	Divalent transition metal (iron and manganese) transporter (By similarity).
P	Q55460	CC	Binds bicarbonate with high affinity (By similarity).
P	Q55460	CC	Part of the ABC transporter complex CmpABCD involved in bicarbonate transport
P	Q55460	DE	Bicarbonate-binding protein CmpA;

P	Q55461	CC	Part of the ABC transporter complex CmpABCD involved in bicarbonate transport
P	Q55461	DE	Bicarbonate transport system permease protein CmpB;
P	Q55462	CC	Part of the ABC transporter complex CmpABCD involved in bicarbonate transport
P	Q55462	DE	Bicarbonate transport ATP-binding protein CmpC;
P	Q55463	CC	Part of the ABC transporter complex CmpABCD involved in bicarbonate transport
P	Q55463	DE	Bicarbonate transport ATP-binding protein CmpD;
P	Q55463	DR	nitrate transmembrane transporter activity
P	Q55835	CC	Probably part of a periplasmic ABC transporter complex futA1A2BC (TC 3.A.1.10.2) involved in Fe(3+) ion import (ferric iron)
P	Q55835	DE	Iron deficiency-induced protein A;
P	Q55835	DE	Iron uptake protein A2;
P	Q55BF4	CC	Transports oxaloacetate and sulfate (By similarity).
P	Q56582	CC	NQR complex catalyzes the reduction of ubiquinone-1 to ubiquinol by two successive reactions, coupled with the transport of Na+ ions from the cytoplasm to the periplasm
P	Q56582	DE	Na+-translocating NADH-quinone reductase subunit C;
P	Q56582	DE	Na+-translocating NADH-quinone reductase subunit gamma;
P	Q56584	CC	NQR complex catalyzes the reduction of ubiquinone-1 to ubiquinol by two successive reactions, coupled with the transport of Na+ ions from the cytoplasm to the periplasm
P	Q56584	DE	Na+-translocating NADH-quinone reductase subunit F;
P	Q56584	DE	Na+-translocating NADH-quinone reductase subunit beta;
P	Q56586	CC	NQR complex catalyzes the reduction of ubiquinone-1 to ubiquinol by two successive reactions, coupled with the transport of Na+ ions from the cytoplasm to the periplasm
P	Q56586	DE	Na+-translocating NADH-quinone reductase subunit A;
P	Q56586	DE	Na+-translocating NADH-quinone reductase subunit alpha;
P	Q56587	CC	NQR complex catalyzes the reduction of ubiquinone-1 to ubiquinol by two successive reactions, coupled with the transport of Na+ ions from the cytoplasm to the periplasm
P	Q56587	DE	Na+-translocating NADH-quinone reductase subunit B;
P	Q56589	CC	NQR complex catalyzes the reduction of ubiquinone-1 to ubiquinol by two successive reactions, coupled with the transport of Na+ ions from the cytoplasm to the periplasm
P	Q56589	DE	Na+-translocating NADH-quinone reductase subunit E;
P	Q56724	DR	sodium ion transmembrane transporter activity
P	Q56725	CC	Na+/H+ antiporter that extrudes sodium in exchange for external protons
P	Q56725	DE	Na+/H+ antiporter nhaA;
P	Q56725	DE	Sodium/proton antiporter nhaA;
P	Q56991	CC	Part of the binding-protein-dependent transport system for hemin.
P	Q56991	DE	Hemin-binding periplasmic protein hmuT;
P	Q56992	CC	Part of the binding-protein-dependent transport system for hemin; probably responsible for the translocation of the substrate across the membrane.
P	Q56992	DE	Hemin transport system permease protein hmuU;
P	Q56993	CC	Part of the ABC transporter complex HmuTUV involved in hemin import
P	Q56993	DE	Hemin import ATP-binding protein HmuV;
P	Q56XP4	CC	Involved in vacuolar ion compartmentalization necessary for cell volume regulation and cytoplasmic Na+ detoxification.
P	Q56XP4	DE	Na+/H+ exchanger 2;
P	Q56XP4	DE	Sodium/hydrogen exchanger 2;
P	Q56XP4	DR	sodium:hydrogen antiporter activity
P	Q57071	CC	This system is involved in glucose transport
P	Q57071	DE	Glucose permease IIC component;
P	Q57071	DE	Glucose-specific phosphotransferase enzyme IIA component;
P	Q57071	DE	Glucose-specific phosphotransferase enzyme IIB component;
P	Q57071	DE	PTS system glucose-specific EIIA component;
P	Q57071	DE	PTS system glucose-specific EIIB component;
P	Q57071	DE	PTS system glucose-specific EIIC component;
P	Q57071	DE	PTS system glucose-specific EIICBA component;
P	Q57071	DR	glucose transmembrane transporter activity
P	Q57095	CC	NQR complex catalyzes the reduction of ubiquinone-1 to ubiquinol by two successive reactions, coupled with the transport of Na+ ions from the cytoplasm to the periplasm
P	Q57095	DE	Na+-translocating NADH-quinone reductase subunit D;
P	Q57603	CC	Voltage-gated potassium-selective channel opened by hyperpolarization.
P	Q57603	DE	Hyperpolarization-activated voltage-gated potassium channel;
P	Q57603	DR	voltage-gated potassium channel activity
P	Q576H3	CC	Part of the ABC transporter complex XylFGH involved in xylose import
P	Q576H3	DE	Xylose import ATP-binding protein XylG;
P	Q576K0	CC	Part of the ABC transporter complex ZnuABC involved in zinc import
P	Q576K0	DE	Zinc import ATP-binding protein ZnuC;

P	Q576K0	DR	zinc transporting ATPase activity
P	Q576K1	CC	Involved in the high-affinity zinc uptake transport system
P	Q576K1	DE	High-affinity zinc uptake system protein znuA;
P	Q578E9	DR	glycerol-3-phosphate-transporting ATPase activity
P	Q579H8	CC	Part of the ABC transporter complex MetNIQ involved in methionine import
P	Q579H8	DE	Methionine import ATP-binding protein MetN;
P	Q579Z3	DR	molybdate transmembrane-transporting ATPase activity
P	Q57AC2	CC	Part of the ABC transporter complex PstSACB involved in phosphate import
P	Q57AC2	DE	ABC phosphate transporter;
P	Q57AC2	DE	Phosphate import ATP-binding protein PstB;
P	Q57AC2	DE	Phosphate-transporting ATPase;
P	Q57AC2	DR	inorganic phosphate transmembrane transporter activity
P	Q57AC2	DR	phosphate transmembrane-transporting ATPase activity
P	Q57BC2	CC	Part of the ABC transporter complex ThiBPQ involved in thiamine import
P	Q57BC2	DE	Thiamine import ATP-binding protein ThiQ;
P	Q57BC3	CC	Part of the ABC transporter complex thiBPQ involved in thiamine import
P	Q57BC3	DE	Thiamine transport system permease protein thiP;
P	Q57BC4	CC	Part of the ABC transporter complex thiBPQ involved in thiamine import (By similarity)
P	Q57BC4	DE	Thiamine-binding periplasmic protein;
P	Q57CC4	CC	Transport of potassium into the cell (By similarity).
P	Q57CC4	DE	Probable potassium transport system protein kup;
P	Q57CC4	DR	potassium ion transmembrane transporter activity
P	Q57DS9	DE	Lipoprotein-releasing system ATP-binding protein LolD;
P	Q57DS9	DR	lipoprotein transporter activity
P	Q57FS7	CC	Part of the ABC transporter complex CcmAB involved in the biogenesis of c-type cytochromes; once thought to export heme, this seems not to be the case, but its exact role is uncertain
P	Q57FS7	DE	Heme exporter protein A;
P	Q57FS7	DR	heme-transporting ATPase activity
P	Q58418	CC	Part of the ABC transporter complex PstSACB involved in phosphate import
P	Q58418	DE	ABC phosphate transporter;
P	Q58418	DE	Phosphate import ATP-binding protein PstB;
P	Q58418	DE	Phosphate-transporting ATPase;
P	Q58418	DR	inorganic phosphate transmembrane transporter activity
P	Q58418	DR	phosphate transmembrane-transporting ATPase activity
P	Q58419	CC	Part of the binding-protein-dependent transport system for phosphate; probably responsible for the translocation of the substrate across the membrane (By similarity)
P	Q58419	DE	Probable phosphate transport system permease protein pstA;
P	Q58419	DR	inorganic phosphate transmembrane transporter activity
P	Q58420	CC	Part of the binding-protein-dependent transport system for phosphate; probably responsible for the translocation of the substrate across the membrane (By similarity)
P	Q58420	DE	Probable phosphate transport system permease protein pstC;
P	Q58420	DR	inorganic phosphate transmembrane transporter activity
P	Q58421	CC	Part of the ABC transporter complex pstSACB involved in phosphate import (By similarity).
P	Q58421	DE	Phosphate-binding protein pstS;
P	Q58421	DR	inorganic phosphate transmembrane transporter activity
P	Q58439	CC	Mediates influx of magnesium ions.
P	Q58439	DE	Magnesium transport protein CorA;
P	Q58439	DR	magnesium ion transmembrane transporter activity
P	Q58490	DR	cobalt ion transmembrane transporter activity
P	Q58I19	DE	Na+/K+ transporting ATPase beta 2 polypeptide;
P	Q58I19	DE	Na+/K+ transporting ATPase beta 2 polypeptide;
P	Q58I19	DR	sodium:potassium-exchanging ATPase activity
P	Q58I19	DR	sodium:potassium-exchanging ATPase activity
P	Q59584	CC	This is an energy-conserving, sodium-ion translocating step.
P	Q5DTL9	CC	Electrogenic sodium/bicarbonate cotransporter in exchange for intracellular chloride
P	Q5DTL9	CC	Electrogenic sodium/bicarbonate cotransporter in exchange for intracellular chloride
P	Q5DTL9	DE	Sodium-driven chloride bicarbonate exchanger;
P	Q5EE02	DR	serotonin:sodium symporter activity
P	Q5EE02	DR	serotonin:sodium symporter activity
P	Q5ERC7	DE	Glucose transporter 9b;
P	Q5ERC7	DR	glucose transmembrane transporter activity
P	Q5F5G9	CC	Involved in the import of serine and threonine into the cell, with the concomitant import of sodium (symport system) (By similarity).

P	Q5F5G9	CC	Involved in the import of serine and threonine into the cell, with the concomitant import of sodium (symport system) (By similarity).
P	Q5F5G9	DE	Na+/serine-threonine symporter;
P	Q5F5G9	DE	Na+/serine-threonine symporter;
P	Q5F5G9	DE	Serine/threonine transporter sstT;
P	Q5F5G9	DR	sodium:dicarboxylate symporter activity
P	Q5F8K2	DE	Lipoprotein-releasing system ATP-binding protein LolD;
P	Q5F8K2	DR	lipoprotein transporter activity
P	Q5FKL2	CC	Part of the ABC transporter complex MetNIQ involved in methionine import
P	Q5FKL2	DE	Methionine import ATP-binding protein MetN;
P	Q5FL41	CC	Part of the ABC transporter complex PotABCD involved in spermidine/putrescine import
P	Q5FL41	CC	Part of the ABC transporter complex PotABCD involved in spermidine/putrescine import
P	Q5FL41	DE	Spermidine/putrescine import ATP-binding protein PotA;
P	Q5FL41	DE	Spermidine/putrescine import ATP-binding protein PotA;
P	Q5FLF5	CC	Transport of potassium into the cell (By similarity).
P	Q5FLF5	DE	Probable potassium transport system protein kup 2;
P	Q5FLF5	DR	potassium ion transmembrane transporter activity
P	Q5FM17	CC	Part of the ABC transporter complex PstSACB involved in phosphate import
P	Q5FM17	DE	ABC phosphate transporter 2;
P	Q5FM17	DE	Phosphate import ATP-binding protein PstB 2;
P	Q5FM17	DE	Phosphate-transporting ATPase 2;
P	Q5FM17	DR	inorganic phosphate transmembrane transporter activity
P	Q5FM17	DR	phosphate transmembrane-transporting ATPase activity
P	Q5FM18	CC	Part of the ABC transporter complex PstSACB involved in phosphate import
P	Q5FM18	DE	ABC phosphate transporter 1;
P	Q5FM18	DE	Phosphate import ATP-binding protein PstB 1;
P	Q5FM18	DE	Phosphate-transporting ATPase 1;
P	Q5FM18	DR	inorganic phosphate transmembrane transporter activity
P	Q5FM18	DR	phosphate transmembrane-transporting ATPase activity
P	Q5FMK7	CC	Transport of potassium into the cell (By similarity).
P	Q5FMK7	DE	Probable potassium transport system protein kup 1;
P	Q5FMK7	DR	potassium ion transmembrane transporter activity
P	Q5FNE6	CC	Na+/H+ antiporter that extrudes sodium in exchange for external protons (By similarity).
P	Q5FNE6	DE	Na+/H+ antiporter nhaA;
P	Q5FNE6	DE	Sodium/proton antiporter nhaA;
P	Q5FQL0	CC	Transport of potassium into the cell (By similarity).
P	Q5FQL0	DE	Probable potassium transport system protein kup;
P	Q5FQL0	DR	potassium ion transmembrane transporter activity
P	Q5FQN4	CC	Part of the ABC transporter complex CcmAB involved in the biogenesis of c-type cytochromes; once thought to export heme, this seems not to be the case, but its exact role is uncertain
P	Q5FQN4	DE	Heme exporter protein A;
P	Q5FQN4	DR	heme-transporting ATPase activity
P	Q5FT15	CC	Part of the ABC transporter complex PstSACB involved in phosphate import
P	Q5FT15	DE	ABC phosphate transporter;
P	Q5FT15	DE	Phosphate import ATP-binding protein PstB;
P	Q5FT15	DE	Phosphate-transporting ATPase;
P	Q5FT15	DR	inorganic phosphate transmembrane transporter activity
P	Q5FT15	DR	phosphate transmembrane-transporting ATPase activity
P	Q5FUV5	DE	Lipoprotein-releasing system ATP-binding protein LolD;
P	Q5FUV5	DR	lipoprotein transporter activity
P	Q5GRS1	CC	Part of the ABC transporter complex ZnuABC involved in zinc import
P	Q5GRS1	DE	Zinc import ATP-binding protein ZnuC;
P	Q5GRS1	DR	zinc transporting ATPase activity
P	Q5HAV5	CC	Part of the ABC transporter complex PstSACB involved in phosphate import
P	Q5HAV5	DE	ABC phosphate transporter;
P	Q5HAV5	DE	Phosphate import ATP-binding protein PstB;
P	Q5HAV5	DE	Phosphate-transporting ATPase;
P	Q5HAV5	DR	inorganic phosphate transmembrane transporter activity
P	Q5HAV5	DR	phosphate transmembrane-transporting ATPase activity
P	Q5HBR8	CC	Part of the ABC transporter complex ZnuABC involved in zinc import
P	Q5HBR8	DE	Zinc import ATP-binding protein ZnuC;
P	Q5HBR8	DR	zinc transporting ATPase activity
P	Q5HC57	DE	Lipoprotein-releasing system ATP-binding protein LolD;
P	Q5HC57	DR	lipoprotein transporter activity

P	Q5HCZ3	CC	Involved in copper export (By similarity).
P	Q5HCZ3	DE	Copper-exporting P-type ATPase A;
P	Q5HCZ3	DR	copper-exporting ATPase activity
P	Q5HD13	DR	glucose transmembrane transporter activity
P	Q5HE14	CC	This system is involved in lactose transport (By similarity).
P	Q5HE14	DE	Lactose-specific phosphotransferase enzyme IIA component;
P	Q5HE14	DE	PTS system lactose-specific EIIA component;
P	Q5HE15	CC	This system is involved in lactose transport.
P	Q5HE15	DE	Lactose permease IIC component;
P	Q5HE15	DE	Lactose-specific phosphotransferase enzyme IIB component;
P	Q5HE15	DE	PTS system lactose-specific EIIB component;
P	Q5HE15	DE	PTS system lactose-specific EIIC component;
P	Q5HE15	DE	PTS system lactose-specific EIICB component;
P	Q5HE46	CC	This system is involved in mannitol transport (By similarity).
P	Q5HE46	DE	Mannitol-specific phosphotransferase enzyme IIA component;
P	Q5HE46	DE	PTS system mannitol-specific EIIA component;
P	Q5HE48	CC	This system is involved in mannitol transport.
P	Q5HE48	DE	Mannitol permease IIC component;
P	Q5HE48	DE	Mannitol-specific phosphotransferase enzyme IIB component;
P	Q5HE48	DE	PTS system mannitol-specific EIIB component;
P	Q5HE48	DE	PTS system mannitol-specific EIIC component;
P	Q5HE48	DE	PTS system mannitol-specific EIICB component;
P	Q5HEC4	CC	One of the components of the high-affinity ATP-driven potassium transport (or KDP) system, which catalyzes the hydrolysis of ATP coupled with the exchange of hydrogen and potassium ions (By similarity).
P	Q5HEC4	DE	Potassium-binding and translocating subunit B;
P	Q5HEC4	DE	Potassium-translocating ATPase B chain;
P	Q5HEC4	DE	Potassium-transporting ATPase B chain;
P	Q5HEC4	DR	potassium-transporting ATPase activity
P	Q5HEK4	CC	Mediates the transport of the dicarboxylates fumarate, malate, and succinate across the cytoplasmic membrane via a Na+- electrochemical gradient (By similarity).
P	Q5HEK4	DE	Na+/dicarboxylate symporter;
P	Q5HEK4	DE	Na+/dicarboxylate symporter;
P	Q5HEK4	DE	Sodium-dependent dicarboxylate transporter sdcS;
P	Q5HEK4	DE	Sodium-dependent dicarboxylate transporter sdcS;
P	Q5HEM0	CC	Catalyzes the sodium-dependent uptake of extracellular L-proline (By similarity)
P	Q5HEM0	CC	Since most S.aureus strains are L- proline auxotrophs, this transporter may aid the bacterial persistence during an infection of tissues with low proline concentrations (By similarity).
P	Q5HEM0	DE	Proline permease;
P	Q5HEM0	DE	Sodium/proline symporter;
P	Q5HEM0	DE	Sodium/proline symporter;
P	Q5HEM0	DR	proline:sodium symporter activity
P	Q5HEM0	DR	proline:sodium symporter activity
P	Q5HF02	CC	Thought to form the channel of an arsenite pump.
P	Q5HF02	DR	arsenite transmembrane transporter activity
P	Q5HFZ9	CC	This system is involved in glucose transport (By similarity).
P	Q5HFZ9	DE	Glucose-specific phosphotransferase enzyme IIA component;
P	Q5HFZ9	DE	PTS system glucose-specific EIIA component;
P	Q5HG31	CC	Part of the ABC transporter complex pstSACB involved in phosphate import (By similarity).
P	Q5HG31	DE	Phosphate-binding protein pstS;
P	Q5HG38	CC	Part of a transport system that may be involved in oligopeptide uptake
P	Q5HG38	DE	Putative oligopeptide transport system permease protein oppB2;
P	Q5HG39	CC	Part of a transport system that may be involved in oligopeptide uptake
P	Q5HG39	DE	Putative oligopeptide transport system permease protein oppC2;
P	Q5HG40	CC	Part of a transport system that may be involved in oligopeptide uptake
P	Q5HG40	DE	Putative oligopeptide transport ATP-binding protein oppD2;
P	Q5HG41	CC	Part of a transport system that may be involved in oligopeptide uptake
P	Q5HG41	DE	Putative oligopeptide transport ATP-binding protein oppF2;
P	Q5HGV1	DE	Iron-regulated surface determinant protein E;
P	Q5HGV1	DE	Staphylococcal iron-regulated protein F;
P	Q5HGV1	DR	iron ion transmembrane transporter activity
P	Q5HGX9	CC	H+-stimulated, highly selective, manganese uptake system (By similarity).
P	Q5HGX9	DE	Probable manganese transport protein mntH;

P	Q5HGY5	CC	Part of the ABC transporter complex PotABCD involved in spermidine/putrescine import
P	Q5HGY5	CC	Part of the ABC transporter complex PotABCD involved in spermidine/putrescine import
P	Q5HGY5	DE	Spermidine/putrescine import ATP-binding protein PotA;
P	Q5HGY5	DE	Spermidine/putrescine import ATP-binding protein PotA;
P	Q5HHD3	CC	Mnh complex is a Na+/H+ antiporter involved in Na+ excretion (By similarity).
P	Q5HHD3	DE	Na+/H+ antiporter subunit A1;
P	Q5HHD4	CC	Mnh complex is a Na+/H+ antiporter involved in Na+ excretion (By similarity).
P	Q5HHD4	DE	Na+/H+ antiporter subunit B1;
P	Q5HHD5	CC	Mnh complex is a Na+/H+ antiporter involved in Na+ excretion (By similarity).
P	Q5HHD5	DE	Na+/H+ antiporter subunit C1;
P	Q5HHD6	CC	Mnh complex is a Na+/H+ antiporter involved in Na+ excretion (By similarity).
P	Q5HHD6	DE	Na+/H+ antiporter subunit D1;
P	Q5HHD7	CC	Mnh complex is a Na+/H+ antiporter involved in Na+ excretion (By similarity).
P	Q5HHD7	DE	Na+/H+ antiporter subunit E1;
P	Q5HHD8	CC	Mnh complex is a Na+/H+ antiporter involved in Na+ excretion (By similarity).
P	Q5HHD8	DE	Na+/H+ antiporter subunit F1;
P	Q5HHD9	CC	Mnh complex is a Na+/H+ antiporter involved in Na+ excretion (By similarity).
P	Q5HHD9	DE	Na+/H+ antiporter subunit G1;
P	Q5HHK4	CC	Part of the ABC transporter complex MetNIQ involved in methionine import
P	Q5HHK4	DE	Methionine import ATP-binding protein MetN 2;
P	Q5HIA2	DE	Putative proline/betaine transporter;
P	Q5HIL5	CC	Part of the ABC transporter complex MetNIQ involved in methionine import
P	Q5HIL5	DE	Methionine import ATP-binding protein MetN 1;
P	Q5HJA6	CC	Could be involved in the uptake of ribose (By similarity).
P	Q5HJA6	DE	Putative ribose uptake protein rbsU;
P	Q5HJI3	CC	This system is involved in glucose transport (By similarity).
P	Q5HJI3	DE	Glucose permease IIC component;
P	Q5HJI3	DE	Glucose-specific phosphotransferase enzyme IIA component;
P	Q5HJI3	DE	Glucose-specific phosphotransferase enzyme IIB component;
P	Q5HJI3	DE	PTS system glucose-specific EIIA component;
P	Q5HJI3	DE	PTS system glucose-specific EIIB component;
P	Q5HJI3	DE	PTS system glucose-specific EIIC component;
P	Q5HJI3	DE	PTS system glucose-specific EIICBA component;
P	Q5HJI3	DR	glucose transmembrane transporter activity
P	Q5HJM6	DR	phosphonate transmembrane-transporting ATPase activity
P	Q5HK63	CC	One of the components of the high-affinity ATP-driven potassium transport (or KDP) system, which catalyzes the hydrolysis of ATP coupled with the exchange of hydrogen and potassium ions (By similarity).
P	Q5HK63	DE	Potassium-binding and translocating subunit A;
P	Q5HK63	DE	Potassium-translocating ATPase A chain;
P	Q5HK63	DE	Potassium-transporting ATPase A chain;
P	Q5HK63	DR	potassium-transporting ATPase activity
P	Q5HK64	CC	One of the components of the high-affinity ATP-driven potassium transport (or KDP) system, which catalyzes the hydrolysis of ATP coupled with the exchange of hydrogen and potassium ions (By similarity).
P	Q5HK64	DE	Potassium-binding and translocating subunit B;
P	Q5HK64	DE	Potassium-translocating ATPase B chain;
P	Q5HK64	DE	Potassium-transporting ATPase B chain;
P	Q5HK64	DR	potassium-transporting ATPase activity
P	Q5HK65	CC	One of the components of the high-affinity ATP-driven potassium transport (or KDP) system, which catalyzes the hydrolysis of ATP coupled with the exchange of hydrogen and potassium ions
P	Q5HK65	DE	Potassium-binding and translocating subunit C;
P	Q5HK65	DE	Potassium-translocating ATPase C chain;
P	Q5HK65	DE	Potassium-transporting ATPase C chain;
P	Q5HK65	DR	potassium-transporting ATPase activity
P	Q5HKB0	CC	Involved in copper transport (By similarity).
P	Q5HKB0	DE	Probable copper-transporting P-type ATPase B;
P	Q5HKB0	DR	copper-exporting ATPase activity
P	Q5HKQ8	DR	phosphonate transmembrane-transporting ATPase activity
P	Q5HL56	CC	Involved in copper export (By similarity).
P	Q5HL56	DE	Copper-exporting P-type ATPase A;
P	Q5HL56	DR	copper-exporting ATPase activity
P	Q5HL73	CC	This system is involved in glucose transport (By similarity).
P	Q5HL73	DE	Glucose permease IIC component;

P	Q5HL73	DE	Glucose-specific phosphotransferase enzyme IIA component;
P	Q5HL73	DE	Glucose-specific phosphotransferase enzyme IIB component;
P	Q5HL73	DE	PTS system glucose-specific EIIA component;
P	Q5HL73	DE	PTS system glucose-specific EIIB component;
P	Q5HL73	DE	PTS system glucose-specific EIIC component;
P	Q5HL73	DE	PTS system glucose-specific EIICBA component;
P	Q5HL73	DR	glucose transmembrane transporter activity
P	Q5HLK7	CC	Also possibly involved in excretion of nitrite produced by the dissimilatory reduction of nitrate (By similarity).
P	Q5HLK7	CC	Probably required for nitrate uptake under anoxic conditions
P	Q5HLK7	DE	Probable nitrate transporter narT;
P	Q5HLZ2	CC	Involved in the uptake of glucose (By similarity).
P	Q5HLZ2	DE	Probable glucose uptake protein glcU;
P	Q5HM39	CC	This system is involved in lactose transport (By similarity).
P	Q5HM39	DE	Lactose-specific phosphotransferase enzyme IIA component;
P	Q5HM39	DE	PTS system lactose-specific EIIA component;
P	Q5HM40	CC	This system is involved in lactose transport.
P	Q5HM40	DE	Lactose permease IIC component;
P	Q5HM40	DE	Lactose-specific phosphotransferase enzyme IIB component;
P	Q5HM40	DE	PTS system lactose-specific EIIB component;
P	Q5HM40	DE	PTS system lactose-specific EIIC component;
P	Q5HM40	DE	PTS system lactose-specific EIICB component;
P	Q5HN32	CC	Catalyzes the sodium-dependent uptake of extracellular L-proline (By similarity).
P	Q5HN32	DE	Proline permease;
P	Q5HN32	DE	Sodium/proline symporter;
P	Q5HN32	DE	Sodium/proline symporter;
P	Q5HN32	DR	proline:sodium symporter activity
P	Q5HN32	DR	proline:sodium symporter activity
P	Q5HPB5	CC	This system is involved in glucose transport (By similarity).
P	Q5HPB5	DE	Glucose-specific phosphotransferase enzyme IIA component;
P	Q5HPB5	DE	PTS system glucose-specific EIIA component;
P	Q5HPF2	CC	Part of the ABC transporter complex pstSACB involved in phosphate import (By similarity).
P	Q5HPF2	DE	Phosphate-binding protein pstS;
P	Q5HPF5	CC	Part of the ABC transporter complex PstSACB involved in phosphate import
P	Q5HPF5	DE	ABC phosphate transporter;
P	Q5HPF5	DE	Phosphate import ATP-binding protein PstB;
P	Q5HPF5	DE	Phosphate-transporting ATPase;
P	Q5HPF5	DR	inorganic phosphate transmembrane transporter activity
P	Q5HPF5	DR	phosphate transmembrane-transporting ATPase activity
P	Q5HQ64	CC	H+-stimulated, highly selective, manganese uptake system (By similarity).
P	Q5HQ64	DE	Probable manganese transport protein mntH;
P	Q5HQ70	CC	Part of the ABC transporter complex PotABCD involved in spermidine/putrescine import
P	Q5HQ70	CC	Part of the ABC transporter complex PotABCD involved in spermidine/putrescine import
P	Q5HQ70	DE	Spermidine/putrescine import ATP-binding protein PotA;
P	Q5HQ70	DE	Spermidine/putrescine import ATP-binding protein PotA;
P	Q5HQL0	CC	Mnh complex is a Na+/H+ antiporter involved in Na+ excretion (By similarity).
P	Q5HQL0	DE	Na+/H+ antiporter subunit A1;
P	Q5HQL1	CC	Mnh complex is a Na+/H+ antiporter involved in Na+ excretion (By similarity).
P	Q5HQL1	DE	Na+/H+ antiporter subunit B1;
P	Q5HQL2	CC	Mnh complex is a Na+/H+ antiporter involved in Na+ excretion (By similarity).
P	Q5HQL2	DE	Na+/H+ antiporter subunit C1;
P	Q5HQL3	CC	Mnh complex is a Na+/H+ antiporter involved in Na+ excretion (By similarity).
P	Q5HQL3	DE	Na+/H+ antiporter subunit D1;
P	Q5HQL4	CC	Mnh complex is a Na+/H+ antiporter involved in Na+ excretion (By similarity).
P	Q5HQL4	DE	Na+/H+ antiporter subunit E1;
P	Q5HQL5	CC	Mnh complex is a Na+/H+ antiporter involved in Na+ excretion (By similarity).
P	Q5HQL5	DE	Na+/H+ antiporter subunit F1;
P	Q5HQL6	CC	Mnh complex is a Na+/H+ antiporter involved in Na+ excretion (By similarity).
P	Q5HQL6	DE	Na+/H+ antiporter subunit G1;
P	Q5HQQ9	CC	Part of the ABC transporter complex MetNIQ involved in methionine import
P	Q5HQQ9	DE	Methionine import ATP-binding protein MetN 2;
P	Q5HRH0	CC	May be a proton symporter invoved in the uptake of osmolytes such as proline and glycine betaine (By similarity).
P	Q5HRH0	DE	Putative proline/betaine transporter;

P	Q5HRH0	DE	Putative proline/betaine transporter;
P	Q5HRI3	CC	Thought to form the channel of an arsenite pump.
P	Q5HRI3	DR	arsenite transmembrane transporter activity
P	Q5HRU5	CC	Part of the ABC transporter complex MetNIQ involved in methionine import
P	Q5HRU5	DE	Methionine import ATP-binding protein MetN 1;
P	Q5JEP9	CC	Part of the ABC transporter complex PstSACB involved in phosphate import
P	Q5JEP9	DE	ABC phosphate transporter;
P	Q5JEP9	DE	Phosphate import ATP-binding protein PstB;
P	Q5JEP9	DE	Phosphate-transporting ATPase;
P	Q5JEP9	DR	inorganic phosphate transmembrane transporter activity
P	Q5JEP9	DR	phosphate transmembrane-transporting ATPase activity
P	Q5JK32	CC	High-affinity potassium transporter (By similarity).
P	Q5JK32	DE	Potassium transporter 5;
P	Q5JK32	DR	potassium ion transmembrane transporter activity
P	Q5JMH0	CC	High-affinity potassium transporter (By similarity).
P	Q5JMH0	DE	Potassium transporter 6;
P	Q5JMH0	DR	potassium ion transmembrane transporter activity
P	Q5KQN0	CC	Translocates Ca2+ and other metal ions into vacuoles using the proton gradient formed by H+-ATPase and H+-pyrophosphatase.
P	Q5KQN0	DE	Ca2+/H+ exchanger 2;
P	Q5KTQ9	CC	Translocates Ca2+ and other metal ions into vacuoles using the proton gradient formed by H+-ATPase and H+-pyrophosphatase.
P	Q5KTQ9	DE	Ca2+/H+ exchanger 1c;
P	Q5KUV3	CC	One of the components of the high-affinity ATP-driven potassium transport (or KDP) system, which catalyzes the hydrolysis of ATP coupled with the exchange of hydrogen and potassium ions (By similarity).
P	Q5KUV3	DE	Potassium-binding and translocating subunit A;
P	Q5KUV3	DE	Potassium-translocating ATPase A chain;
P	Q5KUV3	DE	Potassium-transporting ATPase A chain;
P	Q5KUV3	DR	potassium-transporting ATPase activity
P	Q5KUX3	CC	Part of the ABC transporter complex RbsABCD involved in ribose import
P	Q5KUX3	DE	Ribose import ATP-binding protein RbsA;
P	Q5KVK2	CC	Part of the ABC transporter complex MetNIQ involved in methionine import
P	Q5KVK2	DE	Methionine import ATP-binding protein MetN;
P	Q5KX47	CC	Part of the ABC transporter complex PstSACB involved in phosphate import
P	Q5KX47	DE	ABC phosphate transporter;
P	Q5KX47	DE	Phosphate import ATP-binding protein PstB;
P	Q5KX47	DE	Phosphate-transporting ATPase;
P	Q5KX47	DR	inorganic phosphate transmembrane transporter activity
P	Q5KX47	DR	phosphate transmembrane-transporting ATPase activity
P	Q5KYS1	CC	Part of the ABC transporter complex XylFGH involved in xylose import
P	Q5KYS1	DE	Xylose import ATP-binding protein XylG;
P	Q5KYS1	DR	D-xylose-importing ATPase activity
P	Q5L222	CC	Part of the ABC transporter complex PotABCD involved in spermidine/putrescine import
P	Q5L222	CC	Part of the ABC transporter complex PotABCD involved in spermidine/putrescine import
P	Q5L222	DE	Spermidine/putrescine import ATP-binding protein PotA;
P	Q5L222	DE	Spermidine/putrescine import ATP-binding protein PotA;
P	Q5L5E6	CC	The arginine uptake by the bacterium in the macrophage may be a virulence factor against the host innate immune response (By similarity).
P	Q5L5E6	DE	Arginine/agmatine antiporter;
P	Q5L5Z1	CC	Part of the ABC transporter complex MetNIQ involved in methionine import
P	Q5L5Z1	DE	Methionine import ATP-binding protein MetN;
P	Q5LR15	CC	Part of the ABC transporter complex CcmAB involved in the biogenesis of c-type cytochromes; once thought to export heme, this seems not to be the case, but its exact role is uncertain
P	Q5LR15	DE	Heme exporter protein A;
P	Q5LR15	DR	heme-transporting ATPase activity
P	Q5LS19	CC	Part of the ABC transporter complex PstSACB involved in phosphate import
P	Q5LS19	DE	ABC phosphate transporter;
P	Q5LS19	DE	Phosphate import ATP-binding protein PstB;
P	Q5LS19	DE	Phosphate-transporting ATPase;
P	Q5LS19	DR	inorganic phosphate transmembrane transporter activity
P	Q5LS19	DR	phosphate transmembrane-transporting ATPase activity
P	Q5LT05	CC	Part of the ABC transporter complex PotABCD involved in spermidine/putrescine import
P	Q5LT05	CC	Part of the ABC transporter complex PotABCD involved in spermidine/putrescine import

P	Q5LT05	DE	Spermidine/putrescine import ATP-binding protein PotA;
P	Q5LT05	DE	Spermidine/putrescine import ATP-binding protein PotA;
P	Q5LUD0	DE	Lipoprotein-releasing system ATP-binding protein LolD;
P	Q5LUD0	DR	lipoprotein transporter activity
P	Q5LUR8	CC	Part of the ABC transporter complex ZnuABC involved in zinc import
P	Q5LUR8	DE	Zinc import ATP-binding protein ZnuC;
P	Q5LUR8	DR	zinc transporting ATPase activity
P	Q5LVC2	DR	phosphonate transmembrane-transporting ATPase activity
P	Q5LVM5	CC	Part of the ABC transporter complex TauABC involved in taurine import
P	Q5LVM5	DE	Taurine import ATP-binding protein TauB;
P	Q5LVM5	DR	taurine-transporting ATPase activity
P	Q5LX21	DR	glycerol-3-phosphate-transporting ATPase activity
P	Q5LYN4	CC	Part of the ABC transporter complex PotABCD involved in spermidine/putrescine import
P	Q5LYN4	CC	Part of the ABC transporter complex PotABCD involved in spermidine/putrescine import
P	Q5LYN4	DE	Spermidine/putrescine import ATP-binding protein PotA;
P	Q5LYN4	DE	Spermidine/putrescine import ATP-binding protein PotA;
P	Q5LZU2	CC	Part of the ABC transporter complex PstSACB involved in phosphate import
P	Q5LZU2	DE	ABC phosphate transporter 2;
P	Q5LZU2	DE	Phosphate import ATP-binding protein PstB 2;
P	Q5LZU2	DE	Phosphate-transporting ATPase 2;
P	Q5LZU2	DR	inorganic phosphate transmembrane transporter activity
P	Q5LZU2	DR	phosphate transmembrane-transporting ATPase activity
P	Q5LZU3	CC	Part of the ABC transporter complex PstSACB involved in phosphate import
P	Q5LZU3	DE	ABC phosphate transporter 1;
P	Q5LZU3	DE	Phosphate import ATP-binding protein PstB 1;
P	Q5LZU3	DE	Phosphate-transporting ATPase 1;
P	Q5LZU3	DR	inorganic phosphate transmembrane transporter activity
P	Q5LZU3	DR	phosphate transmembrane-transporting ATPase activity
P	Q5M0A4	DE	Calcium transporter P-type ATPase;
P	Q5M0A4	DR	calcium-transporting ATPase activity
P	Q5M1F4	CC	Involved in the import of serine and threonine into the cell, with the concomitant import of sodium (symport system) (By similarity).
P	Q5M1F4	CC	Involved in the import of serine and threonine into the cell, with the concomitant import of sodium (symport system) (By similarity).
P	Q5M1F4	DE	Na+/serine-threonine symporter;
P	Q5M1F4	DE	Na+/serine-threonine symporter;
P	Q5M1F4	DE	Serine/threonine transporter sstT;
P	Q5M1F4	DR	sodium:dicarboxylate symporter activity
P	Q5M1F6	CC	Part of the ABC transporter complex MetNIQ involved in methionine import
P	Q5M1F6	DE	Methionine import ATP-binding protein MetN;
P	Q5MZ53	CC	Part of the ABC transporter complex CmpABCD involved in bicarbonate transport
P	Q5MZ53	DE	Bicarbonate transport ATP-binding protein CmpD;
P	Q5MZ53	DR	nitrate transmembrane transporter activity
P	Q5MZ54	CC	Part of the ABC transporter complex CmpABCD involved in bicarbonate transport
P	Q5MZ54	DE	Bicarbonate transport ATP-binding protein CmpC;
P	Q5MZ54	DR	nitrate transmembrane transporter activity
P	Q5MZ55	CC	Part of the ABC transporter complex CmpABCD involved in bicarbonate transport
P	Q5MZ55	DE	Bicarbonate transport system permease protein CmpB;
P	Q5MZ55	DR	nitrate transmembrane transporter activity
P	Q5MZ56	CC	Binds bicarbonate with high affinity (By similarity).
P	Q5MZ56	CC	Part of the ABC transporter complex CmpABCD involved in bicarbonate transport
P	Q5MZ56	DE	Bicarbonate-binding protein CmpA;
P	Q5N0R0	CC	Plays an important role in protecting the acceptor side of photosystem II (PSII) against oxidative damage, especially under iron-limiting growth conditions (By similarity).
P	Q5N0R0	DE	Iron deficiency-induced protein A;
P	Q5N1G5	CC	Part of the ABC transporter complex PstSACB involved in phosphate import
P	Q5N1G5	DE	ABC phosphate transporter;
P	Q5N1G5	DE	Phosphate import ATP-binding protein PstB;
P	Q5N1G5	DE	Phosphate-transporting ATPase;
P	Q5N1G5	DR	inorganic phosphate transmembrane transporter activity
P	Q5N1G5	DR	phosphate transmembrane-transporting ATPase activity
P	Q5NC32	CC	Proton-linked monocarboxylate transporter
P	Q5NC32	DE	Monocarboxylate transporter 11;
P	Q5NCE8	CC	Magnesium transporter that may mediate the influx of magnesium into the mitochondrial matrix (By similarity).

P	Q5NCE8	DE	Magnesium transporter MRS2 homolog, mitochondrial;
P	Q5NCM1	CC	May be involved in actively transporting phosphate into cells via Na+ cotransport (By similarity).
P	Q5NCM1	CC	May be involved in actively transporting phosphate into cells via Na+ cotransport (By similarity).
P	Q5NCM1	DE	Putative small intestine sodium-dependent phosphate transport protein;
P	Q5NCM1	DE	Putative small intestine sodium-dependent phosphate transport protein;
P	Q5NE91	CC	Na+/H+ antiporter that extrudes sodium in exchange for external protons (By similarity)
P	Q5NE91	DE	Na+/H+ antiporter nhaA;
P	Q5NE91	DE	Sodium/proton antiporter nhaA;
P	Q5NFU5	CC	Part of the ABC transporter complex MetNIQ involved in methionine import
P	Q5NFU5	DE	Methionine import ATP-binding protein MetN;
P	Q5NHP2	DE	Lipoprotein-releasing system ATP-binding protein LolD;
P	Q5NHP2	DR	lipoprotein transporter activity
P	Q5NN77	CC	Transport of potassium into the cell (By similarity).
P	Q5NN77	DE	Probable potassium transport system protein kup;
P	Q5NN77	DR	potassium ion transmembrane transporter activity
P	Q5NNN6	CC	Part of the ABC transporter complex PstSACB involved in phosphate import
P	Q5NNN6	DE	ABC phosphate transporter;
P	Q5NNN6	DE	Phosphate import ATP-binding protein PstB;
P	Q5NNN6	DE	Phosphate-transporting ATPase;
P	Q5NNN6	DR	inorganic phosphate transmembrane transporter activity
P	Q5NNN6	DR	phosphate transmembrane-transporting ATPase activity
P	Q5NQX0	CC	Part of the ABC transporter complex CcmAB involved in the biogenesis of c-type cytochromes; once thought to export heme, this seems not to be the case, but its exact role is uncertain
P	Q5NQX0	DE	Heme exporter protein A;
P	Q5NQX0	DR	heme-transporting ATPase activity
P	Q5NRB1	CC	Na+/H+ antiporter that extrudes sodium in exchange for external protons (By similarity)
P	Q5NRB1	DE	Na+/H+ antiporter nhaA;
P	Q5NRB1	DE	Sodium/proton antiporter nhaA;
P	Q5NZT6	DE	Lipoprotein-releasing system ATP-binding protein LolD;
P	Q5NZT6	DR	lipoprotein transporter activity
P	Q5P1F3	CC	Part of the ABC transporter complex PstSACB involved in phosphate import
P	Q5P1F3	DE	ABC phosphate transporter;
P	Q5P1F3	DE	Phosphate import ATP-binding protein PstB;
P	Q5P1F3	DE	Phosphate-transporting ATPase;
P	Q5P1F3	DR	inorganic phosphate transmembrane transporter activity
P	Q5P1F3	DR	phosphate transmembrane-transporting ATPase activity
P	Q5P3E3	CC	Transport of potassium into the cell (By similarity).
P	Q5P3E3	DE	Probable potassium transport system protein kup;
P	Q5P3E3	DR	potassium ion transmembrane transporter activity
P	Q5P3L0	CC	Part of the ABC transporter complex CcmAB involved in the biogenesis of c-type cytochromes; once thought to export heme, this seems not to be the case, but its exact role is uncertain
P	Q5P3L0	DE	Heme exporter protein A;
P	Q5P3L0	DR	heme-transporting ATPase activity
P	Q5P4W2	CC	Part of the ABC transporter complex ModABC involved in molybdenum import
P	Q5P4W2	DE	Molybdenum import ATP-binding protein ModC;
P	Q5P4W2	DR	molybdenum ion transmembrane transporter activity
P	Q5PB72	CC	Part of the ABC transporter complex ZnuABC involved in zinc import
P	Q5PB72	DE	Zinc import ATP-binding protein ZnuC;
P	Q5PB72	DR	zinc transporting ATPase activity
P	Q5PBP5	CC	Part of the ABC transporter complex CcmAB involved in the biogenesis of c-type cytochromes; once thought to export heme, this seems not to be the case, but its exact role is uncertain
P	Q5PBP5	DE	Heme exporter protein A;
P	Q5PBP5	DR	heme-transporting ATPase activity
P	Q5PBX2	DE	Lipoprotein-releasing system ATP-binding protein LolD;
P	Q5PBX2	DR	lipoprotein transporter activity
P	Q5PT53	DE	Na+/bile acid cotransporter 7;
P	Q5PT53	DE	Na+/bile acid cotransporter 7;
P	Q5PT53	DE	Sodium/bile acid cotransporter 7;
P	Q5PT53	DE	Sodium/bile acid cotransporter 7;
P	Q5PT53	DR	bile acid:sodium symporter activity

P	Q5PT53	DR	bile acid:sodium symporter activity
P	Q5PT54	DE	Na+/bile acid cotransporter 5;
P	Q5PT54	DE	Na+/bile acid cotransporter 5;
P	Q5PT54	DE	Sodium/bile acid cotransporter 5;
P	Q5PT54	DE	Sodium/bile acid cotransporter 5;
P	Q5PT54	DR	bile acid:sodium symporter activity
P	Q5PT54	DR	bile acid:sodium symporter activity
P	Q5PT55	DE	Na+/bile acid cotransporter 5;
P	Q5PT55	DE	Na+/bile acid cotransporter 5;
P	Q5PT55	DE	Sodium/bile acid cotransporter 5;
P	Q5PT55	DE	Sodium/bile acid cotransporter 5;
P	Q5PT55	DR	bile acid:sodium symporter activity
P	Q5PT55	DR	bile acid:sodium symporter activity
P	Q5QM84	CC	Acts as the major ROS-responsive Ca2+ channel and is the possible target of Al-dependent inhibition
P	Q5QM84	CC	May be involved in the regulation of cytosolic Ca2+ and in growth and development
P	Q5QM84	CC	May function as one of the major voltage-gated Ca2+ channel (VDCC) across the plasma membrane
P	Q5QM84	DE	Two pore calcium channel protein 1;
P	Q5QM84	DE	Voltage-dependent calcium channel protein TPC1;
P	Q5QM84	DR	calcium channel activity
P	Q5QU46	DE	Lipoprotein-releasing system ATP-binding protein LolD;
P	Q5QU46	DR	lipoprotein transporter activity
P	Q5QVB0	CC	Part of the ABC transporter complex PstSACB involved in phosphate import
P	Q5QVB0	DE	ABC phosphate transporter;
P	Q5QVB0	DE	Phosphate import ATP-binding protein PstB;
P	Q5QVB0	DE	Phosphate-transporting ATPase;
P	Q5QVB0	DR	inorganic phosphate transmembrane transporter activity
P	Q5QVB0	DR	phosphate transmembrane-transporting ATPase activity
P	Q5QXD0	CC	Part of the ABC transporter complex HmuTUV involved in hemin import
P	Q5QXD0	DE	Hemin import ATP-binding protein HmuV;
P	Q5QZP7	CC	Part of the ABC transporter complex CcmAB involved in the biogenesis of c-type cytochromes; once thought to export heme, this seems not to be the case, but its exact role is uncertain
P	Q5QZP7	DE	Heme exporter protein A;
P	Q5QZP7	DR	heme-transporting ATPase activity
P	Q5SR56	DR	tetracycline:hydrogen antiporter activity
P	Q5SWY8	DE	Na+/glucose cotransporter 5;
P	Q5SWY8	DE	Na+/glucose cotransporter 5;
P	Q5SWY8	DE	Sodium/glucose cotransporter 5;
P	Q5SWY8	DE	Sodium/glucose cotransporter 5;
P	Q5SZA1	CC	Important for the resorption of phosphate by the kidney
P	Q5SZA1	CC	May be involved in actively transporting phosphate into cells via Na+ cotransport in the renal brush border membrane (By similarity).
P	Q5SZA1	CC	May be involved in actively transporting phosphate into cells via Na+ cotransport in the renal brush border membrane (By similarity).
P	Q5SZA1	DE	Na+/PI cotransporter 3;
P	Q5SZA1	DE	Sodium-dependent phosphate transport protein 3;
P	Q5SZA1	DE	Sodium-dependent phosphate transport protein 3;
P	Q5SZA1	DE	Sodium/phosphate cotransporter 3;
P	Q5SZA1	DE	Sodium/phosphate cotransporter 3;
P	Q5TF39	CC	May function as a sodium-dependent glucose transporter (By similarity).
P	Q5TF39	DE	Sodium-dependent glucose transporter 1;
P	Q5TKG3	CC	Translocates Ca2+ and other metal ions into vacuoles using the proton gradient formed by H+-ATPase and H+-pyrophosphatase.
P	Q5TKG3	DE	Ca2+/H+ exchanger 1b;
P	Q5U4D8	CC	Transports pantothenate, biotin and lipoate in the presence of sodium.
P	Q5U4D8	DE	Na+-dependent multivitamin transporter;
P	Q5U4D8	DE	Sodium-dependent multivitamin transporter;
P	Q5U680	DE	Mitochondrial S-adenosylmethionine transporter;
P	Q5U680	DE	S-adenosylmethionine mitochondrial carrier protein;
P	Q5U680	DR	S-adenosylmethionine transmembrane transporter activity
P	Q5UW69	DR	phosphonate transmembrane-transporting ATPase activity
P	Q5V0G3	CC	Part of the ABC transporter complex PstSACB involved in phosphate import
P	Q5V0G3	DE	ABC phosphate transporter 2;

P	Q5V0G3	DE	Phosphate import ATP-binding protein PstB 2;
P	Q5V0G3	DE	Phosphate-transporting ATPase 2;
P	Q5V0G3	DR	inorganic phosphate transmembrane transporter activity
P	Q5V0G3	DR	phosphate transmembrane-transporting ATPase activity
P	Q5V225	CC	Part of the ABC transporter complex PstSACB involved in phosphate import
P	Q5V225	DE	ABC phosphate transporter 1;
P	Q5V225	DE	Phosphate import ATP-binding protein PstB 1;
P	Q5V225	DE	Phosphate-transporting ATPase 1;
P	Q5V225	DR	inorganic phosphate transmembrane transporter activity
P	Q5V225	DR	phosphate transmembrane-transporting ATPase activity
P	Q5V6B8	DR	phosphonate transmembrane-transporting ATPase activity
P	Q5VR89	CC	Aquaporins facilitate the transport of water and small neutral solutes across cell membranes (By similarity).
P	Q5YRD1	CC	Part of the ABC transporter complex MetNIQ involved in methionine import
P	Q5YRD1	DE	Methionine import ATP-binding protein MetN;
P	Q5YTW4	DR	phosphonate transmembrane-transporting ATPase activity
P	Q5YVL8	CC	Part of the ABC transporter complex HmuTUV involved in hemin import
P	Q5YVL8	DE	Hemin import ATP-binding protein HmuV;
P	Q5YZH9	CC	One of the components of the high-affinity ATP-driven potassium transport (or KDP) system, which catalyzes the hydrolysis of ATP coupled with the exchange of hydrogen and potassium ions (By similarity).
P	Q5YZH9	DE	Potassium-binding and translocating subunit A;
P	Q5YZH9	DE	Potassium-translocating ATPase A chain;
P	Q5YZH9	DE	Potassium-transporting ATPase A chain;
P	Q5YZH9	DR	potassium-transporting ATPase activity
P	Q5YZY9	CC	Part of the ABC transporter complex CysAWTP involved in sulfate/thiosulfate import
P	Q5YZY9	DE	Sulfate-transporting ATPase;
P	Q5YZY9	DE	Sulfate/thiosulfate import ATP-binding protein CysA;
P	Q5YZY9	DR	sulfate transmembrane-transporting ATPase activity
P	Q5Z293	CC	Part of the ABC transporter complex PstSACB involved in phosphate import
P	Q5Z293	DE	ABC phosphate transporter;
P	Q5Z293	DE	Phosphate import ATP-binding protein PstB;
P	Q5Z293	DE	Phosphate-transporting ATPase;
P	Q5Z293	DR	inorganic phosphate transmembrane transporter activity
P	Q5Z293	DR	phosphate transmembrane-transporting ATPase activity
P	Q5Z2Z5	CC	Na+/H+ antiporter that extrudes sodium in exchange for external protons (By similarity).
P	Q5Z2Z5	DE	Na+/H+ antiporter nhaA;
P	Q5Z2Z5	DE	Sodium/proton antiporter nhaA;
P	Q5Z6F0	CC	Aquaporins facilitate the transport of water and small neutral solutes across cell membranes
P	Q5Z6K9	CC	High-affinity potassium transporter (By similarity).
P	Q5Z6K9	DE	Potassium transporter 24;
P	Q5Z6K9	DR	potassium ion transmembrane transporter activity
P	Q5Z9E2	CC	Aquaporins facilitate the transport of water and small neutral solutes across cell membranes (By similarity).
P	Q5ZC87	CC	High-affinity potassium transporter (By similarity).
P	Q5ZC87	DE	Probable potassium transporter 3;
P	Q5ZC87	DR	potassium ion transmembrane transporter activity
P	Q5ZSY2	CC	Transport of potassium into the cell (By similarity).
P	Q5ZSY2	DE	Probable potassium transport system protein kup 3;
P	Q5ZSY2	DR	potassium ion transmembrane transporter activity
P	Q5ZT78	DE	Lipoprotein-releasing system ATP-binding protein LolD;
P	Q5ZT78	DR	lipoprotein transporter activity
P	Q5ZTN5	CC	Transport of potassium into the cell (By similarity).
P	Q5ZTN5	DE	Probable potassium transport system protein kup 2;
P	Q5ZTN5	DR	potassium ion transmembrane transporter activity
P	Q5ZUG5	CC	Part of the ABC transporter complex MetNIQ involved in methionine import
P	Q5ZUG5	DE	Methionine import ATP-binding protein MetN;
P	Q5ZW98	CC	Transport of potassium into the cell (By similarity).
P	Q5ZW98	DE	Probable potassium transport system protein kup 1;
P	Q5ZW98	DR	potassium ion transmembrane transporter activity
P	Q5ZWE4	CC	Part of the ABC transporter complex PotABCD involved in spermidine/putrescine import
P	Q5ZWE4	CC	Part of the ABC transporter complex PotABCD involved in spermidine/putrescine import
P	Q5ZWE4	DE	Spermidine/putrescine import ATP-binding protein PotA;
P	Q5ZWE4	DE	Spermidine/putrescine import ATP-binding protein PotA;

P	Q5ZX76	CC	Part of the ABC transporter complex CcmAB involved in the biogenesis of c-type cytochromes; once thought to export heme, this seems not to be the case, but its exact role is uncertain
P	Q5ZX76	DE	Heme exporter protein A;
P	Q5ZX76	DR	heme-transporting ATPase activity
P	Q604C1	DE	Lipoprotein-releasing system ATP-binding protein LolD;
P	Q604C1	DR	lipoprotein transporter activity
P	Q605R1	CC	One of the components of the high-affinity ATP-driven potassium transport (or KDP) system, which catalyzes the hydrolysis of ATP coupled with the exchange of hydrogen and potassium ions (By similarity).
P	Q605R1	DE	Potassium-binding and translocating subunit A;
P	Q605R1	DE	Potassium-translocating ATPase A chain;
P	Q605R1	DE	Potassium-transporting ATPase A chain;
P	Q605R1	DR	potassium-transporting ATPase activity
P	Q605R3	CC	One of the components of the high-affinity ATP-driven potassium transport (or KDP) system, which catalyzes the hydrolysis of ATP coupled with the exchange of hydrogen and potassium ions
P	Q605R3	DE	Potassium-binding and translocating subunit C;
P	Q605R3	DE	Potassium-translocating ATPase C chain;
P	Q605R3	DE	Potassium-transporting ATPase C chain;
P	Q605R3	DR	potassium-transporting ATPase activity
P	Q60603	CC	Pore-forming (alpha) subunit of voltage-gated non- inactivating delayed rectifier potassium channel
P	Q60603	DE	Ether-a-go-go potassium channel 1;
P	Q60603	DE	Potassium voltage-gated channel subfamily H member 1;
P	Q60603	DE	Voltage-gated potassium channel subunit Kv10.1;
P	Q60603	DR	voltage-gated potassium channel activity
P	Q60738	CC	Lethality of knockout early in gestation suggests a role of the protein in fetal zinc acquisition and retention.
P	Q60738	CC	May be involved in zinc transport out of the cell
P	Q60738	DE	Zinc transporter 1;
P	Q608V9	CC	Part of the ABC transporter complex ModABC involved in molybdenum import
P	Q608V9	DE	Molybdenum import ATP-binding protein ModC;
P	Q608V9	DR	molybdenum ion transmembrane transporter activity
P	Q60961	CC	May function in the transport of nucleosides and/or nucleoside derivatives between the cytosol and the lumen of an intracellular membrane-bound compartment
P	Q609Q1	CC	Part of the ABC transporter complex CysAWTP involved in sulfate/thiosulfate import
P	Q609Q1	DE	Sulfate-transporting ATPase;
P	Q609Q1	DE	Sulfate/thiosulfate import ATP-binding protein CysA;
P	Q609Q1	DR	sulfate transmembrane-transporting ATPase activity
P	Q609Z8	CC	Part of the ABC transporter complex PstSACB involved in phosphate import
P	Q609Z8	DE	ABC phosphate transporter;
P	Q609Z8	DE	Phosphate import ATP-binding protein PstB;
P	Q609Z8	DE	Phosphate-transporting ATPase;
P	Q609Z8	DR	inorganic phosphate transmembrane transporter activity
P	Q609Z8	DR	phosphate transmembrane-transporting ATPase activity
P	Q60A92	CC	Transport of potassium into the cell (By similarity).
P	Q60A92	DE	Probable potassium transport system protein kup;
P	Q60A92	DR	potassium ion transmembrane transporter activity
P	Q60AB3	CC	Part of the ABC transporter complex CcmAB involved in the biogenesis of c-type cytochromes; once thought to export heme, this seems not to be the case, but its exact role is uncertain
P	Q60AB3	DE	Heme exporter protein A;
P	Q60AB3	DR	heme-transporting ATPase activity
P	Q60AI1	CC	Part of the ABC transporter complex PotABCD involved in spermidine/putrescine import
P	Q60AI1	CC	Part of the ABC transporter complex PotABCD involved in spermidine/putrescine import
P	Q60AI1	DE	Spermidine/putrescine import ATP-binding protein PotA;
P	Q60AI1	DE	Spermidine/putrescine import ATP-binding protein PotA;
P	Q61056	CC	Seems to be also activated by intracellular calcium store depletion.
P	Q61056	CC	Thought to form a receptor-activated non-selective calcium permeant cation channel
P	Q61056	DR	store-operated calcium channel activity
P	Q61165	CC	Major proton extruding system driven by the inward sodium ion chemical gradient
P	Q61165	DE	Na+/H+ exchanger 1;
P	Q61165	DE	Sodium/hydrogen exchanger 1;
P	Q61165	DR	sodium:hydrogen antiporter activity

P	Q61180	CC	Controls the reabsorption of sodium in kidney, colon, lung and sweat glands
P	Q61180	CC	Sodium permeable non-voltage-sensitive ion channel inhibited by the diuretic amiloride
P	Q61180	DE	Amiloride-sensitive sodium channel subunit alpha;
P	Q61180	DE	Epithelial Na+ channel subunit alpha;
P	Q61180	DE	Nonvoltage-gated sodium channel 1 subunit alpha;
P	Q61180	DR	ligand-gated sodium channel activity
P	Q61327	CC	Terminates the action of dopamine by its high affinity sodium-dependent reuptake into presynaptic terminals (By similarity).
P	Q61327	CC	Terminates the action of dopamine by its high affinity sodium-dependent reuptake into presynaptic terminals (By similarity).
P	Q61327	DE	Sodium-dependent dopamine transporter;
P	Q61327	DE	Sodium-dependent dopamine transporter;
P	Q61609	CC	Sodium-phosphate symporter which plays a fundamental housekeeping role in phosphate transport, such as absorbing phosphate from interstitial fluid for normal cellular functions such as cellular metabolism, signal transduction, and nucleic acid and lipid synthesis
P	Q61609	DE	Phosphate transporter 1;
P	Q61609	DE	Sodium-dependent phosphate transporter 1;
P	Q61609	DR	inorganic phosphate transmembrane transporter activity
P	Q61743	CC	Inward rectifier potassium channels are characterized by a greater tendency to allow potassium to flow into the cell rather than out of it
P	Q61743	CC	Their voltage dependence is regulated by the concentration of extracellular potassium; as external potassium is raised, the voltage range of the channel opening shifts to more positive voltages
P	Q61743	DE	ATP-sensitive inward rectifier potassium channel 11;
P	Q61743	DE	Inward rectifier K+ channel Kir6.2;
P	Q61743	DE	Potassium channel, inwardly rectifying subfamily J member 11;
P	Q61743	DR	ATP-activated inward rectifier potassium channel activity
P	Q61923	CC	Assuming opened or closed conformations in response to the voltage difference across the membrane, the protein forms a potassium-selective channel through which potassium ions may pass in accordance with their electrochemical gradient
P	Q61923	CC	Mediates the voltage-dependent potassium ion permeability of excitable membranes
P	Q61923	DE	Potassium voltage-gated channel subfamily A member 6;
P	Q61923	DE	Voltage-gated potassium channel subunit Kv1.6;
P	Q61923	DR	voltage-gated potassium channel activity
P	Q61983	CC	Important for the resorption of phosphate by the kidney
P	Q61983	CC	May be involved in actively transporting phosphate into cells via Na+ cotransport in the renal brush border membrane.
P	Q61983	CC	May be involved in actively transporting phosphate into cells via Na+ cotransport in the renal brush border membrane.
P	Q61983	DE	Na+/PI cotransporter 1;
P	Q61983	DE	Renal Na+-dependent phosphate cotransporter 1;
P	Q61983	DE	Renal Na+-dependent phosphate cotransporter 1;
P	Q61983	DE	Renal sodium-dependent phosphate transport protein 1;
P	Q61983	DE	Renal sodium-dependent phosphate transport protein 1;
P	Q61983	DE	Renal sodium-phosphate transport protein 1;
P	Q61983	DE	Sodium-dependent phosphate transport protein 1;
P	Q61983	DE	Sodium-dependent phosphate transport protein 1;
P	Q61983	DE	Sodium/phosphate cotransporter 1;
P	Q61983	DE	Sodium/phosphate cotransporter 1;
P	Q61983	DR	phosphate transmembrane transporter activity
P	Q62052	CC	Could be involved in the transport of tyrosine, the precursor to melanin synthesis, within the melanocyte
P	Q62273	CC	Sulfate transporter
P	Q62273	DE	Sulfate transporter;
P	Q62273	DR	secondary active sulfate transmembrane transporter activity
P	Q62674	CC	Exhibits the transport characteristics of the nucleoside transport system cit or N2 subtype (N2/cit) (selective for pyrimidine nucleosides and adenosine)
P	Q62674	CC	Sodium-dependent and pyrimidine-selective
P	Q62674	DE	Concentrative nucleoside transporter 1;
P	Q62674	DE	Na+/nucleoside cotransporter 1;
P	Q62674	DE	Na+/nucleoside cotransporter 1;
P	Q62674	DE	Sodium-coupled nucleoside transporter 1;
P	Q62674	DE	Sodium-coupled nucleoside transporter 1;
P	Q62674	DE	Sodium/nucleoside cotransporter 1;
P	Q62674	DE	Sodium/nucleoside cotransporter 1;

P	Q62674	DR	pyrimidine- and adenine-specific:sodium symporter activity
P	Q62720	CC	May be involved in zinc transport out of the cell.
P	Q62720	DE	Zinc transporter 1;
P	Q62720	DR	zinc ion transmembrane transporter activity
P	Q62773	CC	Exhibits the transport characteristics of the nucleoside transport system cif or N1 subtype (N1/cif) (selective for purine nucleosides and uridine)
P	Q62773	CC	Sodium-dependent and purine-selective
P	Q62773	DE	Concentrative nucleoside transporter 2;
P	Q62773	DE	Na+/nucleoside cotransporter 2;
P	Q62773	DE	Na+/nucleoside cotransporter 2;
P	Q62773	DE	Sodium-coupled nucleoside transporter 2;
P	Q62773	DE	Sodium-coupled nucleoside transporter 2;
P	Q62773	DE	Sodium/nucleoside cotransporter 2;
P	Q62773	DE	Sodium/nucleoside cotransporter 2;
P	Q62773	DE	Sodium/purine nucleoside cotransporter;
P	Q62773	DR	nucleoside:sodium symporter activity
P	Q62773	DR	nucleoside:sodium symporter activity
P	Q62941	CC	Involved in accumulation of zinc in endosomal/lysosomal vesicles.
P	Q62941	DE	Zinc transporter 2;
P	Q62941	DR	zinc ion transmembrane transporter activity
P	Q62A98	CC	Part of the ABC transporter complex HmuTUV involved in hemin import
P	Q62A98	DE	Hemin import ATP-binding protein HmuV;
P	Q62AW4	CC	Part of the ABC transporter complex TauABC involved in taurine import
P	Q62AW4	DE	Taurine import ATP-binding protein TauB;
P	Q62AW4	DR	taurine-transporting ATPase activity
P	Q62B84	CC	Part of the ABC transporter complex MetNIQ involved in methionine import
P	Q62B84	DE	Methionine import ATP-binding protein MetN 2;
P	Q62GB4	DR	glycerol-3-phosphate-transporting ATPase activity
P	Q62GY9	DR	L-arabinose-importing ATPase activity
P	Q62H59	DR	phosphonate transmembrane-transporting ATPase activity
P	Q62IJ6	CC	One of the components of the high-affinity ATP-driven potassium transport (or KDP) system, which catalyzes the hydrolysis of ATP coupled with the exchange of hydrogen and potassium ions (By similarity).
P	Q62IJ6	DE	Potassium-binding and translocating subunit A;
P	Q62IJ6	DE	Potassium-translocating ATPase A chain;
P	Q62IJ6	DE	Potassium-transporting ATPase A chain;
P	Q62IJ6	DR	potassium-transporting ATPase activity
P	Q62IJ8	CC	One of the components of the high-affinity ATP-driven potassium transport (or KDP) system, which catalyzes the hydrolysis of ATP coupled with the exchange of hydrogen and potassium ions
P	Q62IJ8	DE	Potassium-binding and translocating subunit C;
P	Q62IJ8	DE	Potassium-translocating ATPase C chain;
P	Q62IJ8	DE	Potassium-transporting ATPase C chain;
P	Q62IJ8	DR	potassium-transporting ATPase activity
P	Q62J04	DE	Lipoprotein-releasing system ATP-binding protein LolD;
P	Q62J04	DR	lipoprotein transporter activity
P	Q62JX8	CC	Transport of potassium into the cell (By similarity).
P	Q62JX8	DE	Probable potassium transport system protein kup;
P	Q62JX8	DR	potassium ion transmembrane transporter activity
P	Q62K82	CC	Part of the ABC transporter complex CysAWTP involved in sulfate/thiosulfate import
P	Q62K82	DE	Sulfate-transporting ATPase;
P	Q62K82	DE	Sulfate/thiosulfate import ATP-binding protein CysA;
P	Q62K82	DR	sulfate transmembrane-transporting ATPase activity
P	Q62K91	CC	Part of the ABC transporter complex RbsABCD involved in ribose import
P	Q62K91	DE	Ribose import ATP-binding protein RbsA;
P	Q62L74	CC	Part of the ABC transporter complex PstSACB involved in phosphate import
P	Q62L74	DE	ABC phosphate transporter;
P	Q62L74	DE	Phosphate import ATP-binding protein PstB;
P	Q62L74	DE	Phosphate-transporting ATPase;
P	Q62L74	DR	inorganic phosphate transmembrane transporter activity
P	Q62L74	DR	phosphate transmembrane-transporting ATPase activity
P	Q62M41	CC	Part of the ABC transporter complex MetNIQ involved in methionine import
P	Q62M41	DE	Methionine import ATP-binding protein MetN 1;
P	Q63JZ3	CC	Part of the ABC transporter complex TauABC involved in taurine import
P	Q63JZ3	DE	Taurine import ATP-binding protein TauB;

P	Q63JZ3	DR	taurine-transporting ATPase activity
P	Q63NI4	CC	Part of the ABC transporter complex MetNIQ involved in methionine import
P	Q63NI4	DE	Methionine import ATP-binding protein MetN 2;
P	Q63NR0	CC	Part of the ABC transporter complex HmuTUV involved in hemin import
P	Q63NR0	DE	Hemin import ATP-binding protein HmuV;
P	Q63P06	CC	Part of the ABC transporter complex RbsABCD involved in ribose import
P	Q63P06	DE	Ribose import ATP-binding protein RbsA;
P	Q63Q62	DR	glycerol-3-phosphate-transporting ATPase activity
P	Q63QQ7	DR	L-arabinose-importing ATPase activity
P	Q63R24	DR	phosphonate transmembrane-transporting ATPase activity
P	Q63S19	CC	Part of the ABC transporter complex MetNIQ involved in methionine import
P	Q63S19	DE	Methionine import ATP-binding protein MetN 1;
P	Q63SP4	DE	Lipoprotein-releasing system ATP-binding protein LolD;
P	Q63SP4	DR	lipoprotein transporter activity
P	Q63TY1	CC	Part of the ABC transporter complex CysAWTP involved in sulfate/thiosulfate import
P	Q63TY1	DE	Sulfate-transporting ATPase;
P	Q63TY1	DE	Sulfate/thiosulfate import ATP-binding protein CysA;
P	Q63TY1	DR	sulfate transmembrane-transporting ATPase activity
P	Q63US0	CC	Transport of potassium into the cell (By similarity).
P	Q63US0	DE	Probable potassium transport system protein kup;
P	Q63US0	DR	potassium ion transmembrane transporter activity
P	Q63V79	CC	Part of the ABC transporter complex PstSACB involved in phosphate import
P	Q63V79	DE	ABC phosphate transporter;
P	Q63V79	DE	Phosphate import ATP-binding protein PstB;
P	Q63V79	DE	Phosphate-transporting ATPase;
P	Q63V79	DR	inorganic phosphate transmembrane transporter activity
P	Q63V79	DR	phosphate transmembrane-transporting ATPase activity
P	Q63VS1	CC	One of the components of the high-affinity ATP-driven potassium transport (or KDP) system, which catalyzes the hydrolysis of ATP coupled with the exchange of hydrogen and potassium ions
P	Q63VS1	DE	Potassium-binding and translocating subunit C;
P	Q63VS1	DE	Potassium-translocating ATPase C chain;
P	Q63VS1	DE	Potassium-transporting ATPase C chain;
P	Q63VS1	DR	potassium-transporting ATPase activity
P	Q63VS3	CC	One of the components of the high-affinity ATP-driven potassium transport (or KDP) system, which catalyzes the hydrolysis of ATP coupled with the exchange of hydrogen and potassium ions (By similarity).
P	Q63VS3	DE	Potassium-binding and translocating subunit A;
P	Q63VS3	DE	Potassium-translocating ATPase A chain;
P	Q63VS3	DE	Potassium-transporting ATPase A chain;
P	Q63VS3	DR	potassium-transporting ATPase activity
P	Q64347	CC	Chloride channels have several functions including the regulation of cell volume; membrane potential stabilization, signal transduction and transepithelial transport
P	Q64347	CC	Voltage-gated chloride channel
P	Q64347	DE	Chloride channel protein 1;
P	Q64347	DE	Chloride channel protein, skeletal muscle;
P	Q64347	DR	voltage-gated chloride channel activity
P	Q64518	CC	Contributes to calcium sequestration involved in muscular excitation/contraction.
P	Q64518	CC	This magnesium-dependent enzyme catalyzes the hydrolysis of ATP coupled with the transport of the calcium
P	Q64518	CC	Transports calcium ions from the cytosol into the sarcoplasmic/endoplasmic reticulum lumen
P	Q64518	DE	Calcium pump 3;
P	Q64518	DE	SR Ca2+-ATPase 3;
P	Q64518	DE	Sarcoplasmic/endoplasmic reticulum calcium ATPase 3;
P	Q64518	DR	calcium-transporting ATPase activity
P	Q64SM5	CC	Part of the ABC transporter complex PstSACB involved in phosphate import
P	Q64SM5	DE	ABC phosphate transporter;
P	Q64SM5	DE	Phosphate import ATP-binding protein PstB;
P	Q64SM5	DE	Phosphate-transporting ATPase;
P	Q64SM5	DR	inorganic phosphate transmembrane transporter activity
P	Q64SM5	DR	phosphate transmembrane-transporting ATPase activity
P	Q64SQ6	CC	Part of the ABC transporter complex PotABCD involved in spermidine/putrescine import
P	Q64SQ6	CC	Part of the ABC transporter complex PotABCD involved in spermidine/putrescine import
P	Q64SQ6	DE	Spermidine/putrescine import ATP-binding protein PotA;

P	Q64SQ6	DE	Spermidine/putrescine import ATP-binding protein PotA;
P	Q64T76	CC	Na+/H+ antiporter that extrudes sodium in exchange for external protons (By similarity).
P	Q64T76	DE	Na+/H+ antiporter nhaA;
P	Q64T76	DE	Sodium/proton antiporter nhaA;
P	Q64YU7	CC	One of the components of the high-affinity ATP-driven potassium transport (or KDP) system, which catalyzes the hydrolysis of ATP coupled with the exchange of hydrogen and potassium ions
P	Q64YU7	DE	Potassium-binding and translocating subunit C;
P	Q64YU7	DE	Potassium-translocating ATPase C chain;
P	Q64YU7	DE	Potassium-transporting ATPase C chain;
P	Q64YU7	DR	potassium-transporting ATPase activity
P	Q64YU9	CC	One of the components of the high-affinity ATP-driven potassium transport (or KDP) system, which catalyzes the hydrolysis of ATP coupled with the exchange of hydrogen and potassium ions (By similarity).
P	Q64YU9	DE	Potassium-binding and translocating subunit A;
P	Q64YU9	DE	Potassium-translocating ATPase A chain;
P	Q64YU9	DE	Potassium-transporting ATPase A chain;
P	Q64YU9	DR	potassium-transporting ATPase activity
P	Q64Z80	DE	Lipoprotein-releasing system ATP-binding protein LolD;
P	Q64Z80	DR	lipoprotein transporter activity
P	Q651D5	CC	Aquaporins facilitate the transport of water and small neutral solutes across cell membranes (By similarity).
P	Q652J4	CC	High-affinity potassium transporter (By similarity).
P	Q652J4	DE	Probable potassium transporter 13;
P	Q652J4	DR	potassium ion transmembrane transporter activity
P	Q652N5	DE	Phosphate transporter 4;4;
P	Q653B6	CC	High-affinity potassium transporter (By similarity).
P	Q653B6	DE	Potassium transporter 18;
P	Q653B6	DR	potassium ion transmembrane transporter activity
P	Q65E55	CC	Part of the ABC transporter complex RbsABCD involved in ribose import
P	Q65E55	DE	Ribose import ATP-binding protein RbsA;
P	Q65HB9	CC	Part of the ABC transporter complex PstSACB involved in phosphate import
P	Q65HB9	DE	ABC phosphate transporter 2;
P	Q65HB9	DE	Phosphate import ATP-binding protein PstB 2;
P	Q65HB9	DE	Phosphate-transporting ATPase 2;
P	Q65HB9	DR	inorganic phosphate transmembrane transporter activity
P	Q65HB9	DR	phosphate transmembrane-transporting ATPase activity
P	Q65HC0	CC	Part of the ABC transporter complex PstSACB involved in phosphate import
P	Q65HC0	DE	ABC phosphate transporter 1;
P	Q65HC0	DE	Phosphate import ATP-binding protein PstB 1;
P	Q65HC0	DE	Phosphate-transporting ATPase 1;
P	Q65HC0	DR	inorganic phosphate transmembrane transporter activity
P	Q65HC0	DR	phosphate transmembrane-transporting ATPase activity
P	Q65M31	DR	sodium:dicarboxylate symporter activity
P	Q65M34	CC	Part of the ABC transporter complex MetNIQ involved in methionine import
P	Q65M34	DE	Methionine import ATP-binding protein MetN 1;
P	Q65Q27	CC	Uptake of L-rhamnose across the boundary membrane with the concomitant transport of protons into the cell (symport system) (By similarity).
P	Q65Q27	DE	L-rhamnose-H+ transport protein;
P	Q65Q27	DE	L-rhamnose-proton symporter;
P	Q65QT6	CC	Part of the ABC transporter complex MalEFGK involved in maltose/maltodextrin import
P	Q65QT6	CC	Part of the ABC transporter complex MalEFGK involved in maltose/maltodextrin import
P	Q65QT6	DE	Maltose/maltodextrin import ATP-binding protein MalK;
P	Q65QT6	DE	Maltose/maltodextrin import ATP-binding protein MalK;
P	Q65QT6	DR	maltose-transporting ATPase activity
P	Q65RK2	CC	Na+/H+ antiporter that extrudes sodium in exchange for external protons (By similarity).
P	Q65RK2	DE	Na+/H+ antiporter nhaA;
P	Q65RK2	DE	Sodium/proton antiporter nhaA;
P	Q65S74	CC	The physiological role may be the reduction of the intracellular concentration of toxic sugars or sugar metabolites (By similarity).
P	Q65S74	DE	Probable sugar efflux transporter;
P	Q65SC9	CC	Part of the ABC transporter complex ThiBPQ involved in thiamine import
P	Q65SC9	DE	Thiamine import ATP-binding protein ThiQ;
P	Q65SW3	CC	Part of the ABC transporter complex ModABC involved in molybdenum import
P	Q65SW3	DE	Molybdenum import ATP-binding protein ModC;

P	Q65SW3	DR	molybdenum ion transmembrane transporter activity
P	Q65T42	CC	Part of the ABC transporter complex CysAWTP involved in sulfate/thiosulfate import
P	Q65T42	DE	Sulfate-transporting ATPase;
P	Q65T42	DE	Sulfate/thiosulfate import ATP-binding protein CysA;
P	Q65T42	DR	sulfate transmembrane-transporting ATPase activity
P	Q65TB7	DE	Lipoprotein-releasing system ATP-binding protein LolD;
P	Q65TB7	DR	lipoprotein transporter activity
P	Q65UC1	CC	Involved in the import of serine and threonine into the cell, with the concomitant import of sodium (symport system) (By similarity).
P	Q65UC1	DE	Na+/serine-threonine symporter;
P	Q65UC1	DR	sodium:dicarboxylate symporter activity
P	Q65UC1	DR	sodium:dicarboxylate symporter activity
P	Q65UE1	CC	Part of the ABC transporter complex PotABCD involved in spermidine/putrescine import
P	Q65UE1	CC	Part of the ABC transporter complex PotABCD involved in spermidine/putrescine import
P	Q65UE1	DE	Spermidine/putrescine import ATP-binding protein PotA;
P	Q65UE1	DE	Spermidine/putrescine import ATP-binding protein PotA;
P	Q65UG3	CC	Part of the ABC transporter complex ZnuABC involved in zinc import
P	Q65UG3	DE	Zinc import ATP-binding protein ZnuC;
P	Q65UG3	DR	zinc transporting ATPase activity
P	Q65UW1	CC	Part of the ABC transporter complex MglABC involved in galactose/methyl galactoside import
P	Q65UW1	DE	Galactose/methyl galactoside import ATP-binding protein MglA;
P	Q65V02	CC	Part of the ABC transporter complex CcmAB involved in the biogenesis of c-type cytochromes; once thought to export heme, this seems not to be the case, but its exact role is uncertain
P	Q65V02	DE	Heme exporter protein A;
P	Q65V02	DR	heme-transporting ATPase activity
P	Q65VG9	CC	Part of the ABC transporter complex MetNIQ involved in methionine import
P	Q65VG9	DE	Methionine import ATP-binding protein MetN;
P	Q65VG9	DR	D-methionine transmembrane transporter activity
P	Q65VH3	CC	Na+/H+ antiporter that extrudes sodium in exchange for external protons (By similarity).
P	Q65VH3	DE	Na+/H+ antiporter nhaB;
P	Q65VH3	DE	Sodium/proton antiporter nhaB;
P	Q65VH3	DR	sodium:hydrogen antiporter activity
P	Q65VT3	CC	Mediates influx of magnesium ions (By similarity).
P	Q65VT3	DE	Magnesium transport protein CorA;
P	Q65VT3	DR	cobalt ion transmembrane transporter activity
P	Q65VT3	DR	magnesium ion transmembrane transporter activity
P	Q65WJ1	DR	L-arabinose-importing ATPase activity
P	Q660M8	CC	Part of the ABC transporter complex PotABCD involved in spermidine/putrescine import
P	Q660M8	CC	Part of the ABC transporter complex PotABCD involved in spermidine/putrescine import
P	Q660M8	DE	Spermidine/putrescine import ATP-binding protein PotA;
P	Q660M8	DE	Spermidine/putrescine import ATP-binding protein PotA;
P	Q662E6	CC	Part of the ABC transporter complex PstSACB involved in phosphate import
P	Q662E6	DE	ABC phosphate transporter;
P	Q662E6	DE	Phosphate import ATP-binding protein PstB;
P	Q662E6	DE	Phosphate-transporting ATPase;
P	Q662E6	DR	inorganic phosphate transmembrane transporter activity
P	Q662E6	DR	phosphate transmembrane-transporting ATPase activity
P	Q663R5	CC	Part of the ABC transporter complex PstSACB involved in phosphate import
P	Q663R5	DE	ABC phosphate transporter 2;
P	Q663R5	DE	Phosphate import ATP-binding protein PstB 2;
P	Q663R5	DE	Phosphate-transporting ATPase 2;
P	Q663R5	DR	inorganic phosphate transmembrane transporter activity
P	Q663R5	DR	phosphate transmembrane-transporting ATPase activity
P	Q663Y5	CC	Part of the ABC transporter complex XylFGH involved in xylose import
P	Q663Y5	DE	Xylose import ATP-binding protein XylG;
P	Q664P8	CC	Part of the ABC transporter complex TauABC involved in taurine import
P	Q664P8	DE	Taurine import ATP-binding protein TauB;
P	Q664P8	DR	taurine-transporting ATPase activity
P	Q664Q5	CC	Transport system that facilitates potassium-efflux, possibly by potassium-proton antiport (By similarity).
P	Q664Q5	DE	Glutathione-regulated potassium-efflux system protein kefB;
P	Q664Q5	DE	K+/H+ antiporter;
P	Q664Q5	DE	NEM-activable K+/H+ antiporter;

P	Q664Q5	DR	glutathione-regulated potassium exporter activity
P	Q664X5	CC	Part of the ABC transporter complex MalEFGK involved in maltose/maltodextrin import
P	Q664X5	CC	Part of the ABC transporter complex MalEFGK involved in maltose/maltodextrin import
P	Q664X5	DE	Maltose/maltodextrin import ATP-binding protein MalK;
P	Q664X5	DE	Maltose/maltodextrin import ATP-binding protein MalK;
P	Q664X5	DR	maltose-transporting ATPase activity
P	Q665P2	CC	Involved in the import of serine and threonine into the cell, with the concomitant import of sodium (symport system) (By similarity).
P	Q665P2	DE	Na+/serine-threonine symporter;
P	Q665P2	DR	sodium:dicarboxylate symporter activity
P	Q665P2	DR	sodium:dicarboxylate symporter activity
P	Q666Q4	CC	Important to control the intracellular level of arginine and the correct balance between arginine and lysine (By similarity).
P	Q667L9	CC	Part of the ABC transporter complex MetNIQ involved in methionine import
P	Q667L9	DE	Methionine import ATP-binding protein MetN 2;
P	Q667L9	DR	D-methionine transmembrane transporter activity
P	Q667S4	CC	One of the components of the high-affinity ATP-driven potassium transport (or KDP) system, which catalyzes the hydrolysis of ATP coupled with the exchange of hydrogen and potassium ions (By similarity).
P	Q667S4	DE	Potassium-binding and translocating subunit B;
P	Q667S4	DE	Potassium-translocating ATPase B chain;
P	Q667S4	DE	Potassium-transporting ATPase B chain;
P	Q667S4	DR	potassium-transporting ATPase activity
P	Q667S5	CC	One of the components of the high-affinity ATP-driven potassium transport (or KDP) system, which catalyzes the hydrolysis of ATP coupled with the exchange of hydrogen and potassium ions (By similarity).
P	Q667S5	DE	Potassium-binding and translocating subunit A;
P	Q667S5	DE	Potassium-translocating ATPase A chain;
P	Q667S5	DE	Potassium-transporting ATPase A chain;
P	Q667S5	DR	potassium-transporting ATPase activity
P	Q668E1	CC	Part of the ABC transporter complex PstSACB involved in phosphate import
P	Q668E1	DE	ABC phosphate transporter 1;
P	Q668E1	DE	Phosphate import ATP-binding protein PstB 1;
P	Q668E1	DE	Phosphate-transporting ATPase 1;
P	Q668E1	DR	inorganic phosphate transmembrane transporter activity
P	Q668E1	DR	phosphate transmembrane-transporting ATPase activity
P	Q668K6	CC	Part of the ABC transporter complex CysAWTP involved in sulfate/thiosulfate import
P	Q668K6	DE	Sulfate-transporting ATPase;
P	Q668K6	DE	Sulfate/thiosulfate import ATP-binding protein CysA;
P	Q668K6	DR	sulfate transmembrane-transporting ATPase activity
P	Q668N2	CC	H+-stimulated, highly selective, manganese uptake system (By similarity).
P	Q668N2	DE	Probable manganese transport protein mntH;
P	Q668T8	CC	Part of the ABC transporter complex CcmAB involved in the biogenesis of c-type cytochromes; once thought to export heme, this seems not to be the case, but its exact role is uncertain
P	Q668T8	DE	Heme exporter protein A;
P	Q668T8	DR	heme-transporting ATPase activity
P	Q669P3	DE	Lipoprotein-releasing system ATP-binding protein LolD;
P	Q669P3	DR	lipoprotein transporter activity
P	Q669Z9	CC	Part of the ABC transporter complex BtuCDF involved in vitamin B12 import
P	Q669Z9	DE	Vitamin B12 import system permease protein BtuC;
P	Q66A01	CC	Part of the ABC transporter complex BtuCDF involved in vitamin B12 import
P	Q66A01	DE	Vitamin B12 import ATP-binding protein BtuD;
P	Q66A01	DE	Vitamin B12-transporting ATPase;
P	Q66AQ9	CC	Na+/H+ antiporter that extrudes sodium in exchange for external protons (By similarity)
P	Q66AQ9	DE	Na+/H+ antiporter nhaB;
P	Q66AQ9	DE	Sodium/proton antiporter nhaB;
P	Q66AQ9	DR	sodium:hydrogen antiporter activity
P	Q66C83	CC	Part of the ABC transporter complex MglABC involved in galactose/methyl galactoside import
P	Q66C83	DE	Galactose/methyl galactoside import ATP-binding protein MglA;
P	Q66CQ3	CC	Part of the ABC transporter complex MetNIQ involved in methionine import
P	Q66CQ3	DE	Methionine import ATP-binding protein MetN 1;
P	Q66D26	DR	phosphonate transmembrane-transporting ATPase activity
P	Q66D71	CC	Part of the ABC transporter complex ModABC involved in molybdenum import

P	Q66D71	DE	Molybdenum import ATP-binding protein ModC;
P	Q66D71	DR	molybdenum ion transmembrane transporter activity
P	Q66D85	CC	Involved in zinc efflux across the cytoplasmic membrane, thus reducing zinc accumulation in the cytoplasm and rendering bacteria more resistant to zinc
P	Q66D85	CC	It may contribute to zinc homeostasis at low concentrations of zinc (By similarity)
P	Q66D85	DE	Zinc transporter zitB;
P	Q66EE7	CC	Binds vitamin B12 and delivers it to the periplasmic surface of BtuC (By similarity)
P	Q66EE7	CC	Part of the ABC transporter complex BtuCDF involved in vitamin B12 import
P	Q66ES8	CC	Na+/H+ antiporter that extrudes sodium in exchange for external protons (By similarity)
P	Q66ES8	DE	Na+/H+ antiporter nhaA;
P	Q66ES8	DE	Sodium/proton antiporter nhaA;
P	Q66FF0	CC	Uptake of L-rhamnose across the boundary membrane with the concomitant transport of protons into the cell (symport system) (By similarity).
P	Q66FF0	DE	L-rhamnose-H+ transport protein;
P	Q66FF0	DE	L-rhamnose-proton symporter;
P	Q66FK0	CC	Part of the ABC transporter complex HmuTUV involved in hemin import
P	Q66FK0	DE	Hemin import ATP-binding protein HmuV;
P	Q66FU4	DR	glycerol-3-phosphate-transporting ATPase activity
P	Q66FY7	CC	Mediates influx of magnesium ions (By similarity).
P	Q66FY7	DE	Magnesium transport protein CorA;
P	Q66FY7	DR	cobalt ion transmembrane transporter activity
P	Q66FY7	DR	magnesium ion transmembrane transporter activity
P	Q66GH5	CC	Responsible for the low-affinity transport of potassium into the cell, with the probable concomitant uptake of protons (symport system) (By similarity).
P	Q66GH5	DE	Kup system potassium uptake protein;
P	Q66GH5	DE	Low affinity potassium transport system protein kup;
P	Q66GH5	DR	potassium ion transmembrane transporter activity
P	Q67BT3	CC	High-affinity sodium/citrate cotransporter that mediates citrate entry into cells
P	Q67BT3	CC	High-affinity sodium/citrate cotransporter that mediates citrate entry into cells
P	Q67BT3	CC	May facilitate the utilization of circulating citrate for the generation of metabolic energy and for the synthesis of fatty acids and cholesterol (By similarity).
P	Q67BT3	CC	The transport process is electrogenic; it is the trivalent form of citrate rather than the divalent form that is recognized as a substrate
P	Q67BT3	DE	Na+/citrate cotransporter;
P	Q67BT3	DE	Na+/citrate cotransporter;
P	Q67BT3	DE	Sodium-coupled citrate transporter;
P	Q67BT3	DE	Sodium-coupled citrate transporter;
P	Q67BT3	DE	Sodium-dependent citrate transporter;
P	Q67BT3	DE	Sodium-dependent citrate transporter;
P	Q67BT3	DR	citrate transmembrane transporter activity
P	Q67RE7	CC	Part of the ABC transporter complex PstSACB involved in phosphate import
P	Q67RE7	DE	ABC phosphate transporter 2;
P	Q67RE7	DE	Phosphate import ATP-binding protein PstB 2;
P	Q67RE7	DE	Phosphate-transporting ATPase 2;
P	Q67RE7	DR	inorganic phosphate transmembrane transporter activity
P	Q67RE7	DR	phosphate transmembrane-transporting ATPase activity
P	Q67RG2	CC	Part of the ABC transporter complex PstSACB involved in phosphate import
P	Q67RG2	DE	ABC phosphate transporter 1;
P	Q67RG2	DE	Phosphate import ATP-binding protein PstB 1;
P	Q67RG2	DE	Phosphate-transporting ATPase 1;
P	Q67RG2	DR	inorganic phosphate transmembrane transporter activity
P	Q67RG2	DR	phosphate transmembrane-transporting ATPase activity
P	Q67SV5	CC	Part of the ABC transporter complex MetNIQ involved in methionine import
P	Q67SV5	DE	Methionine import ATP-binding protein MetN;
P	Q67UC7	CC	High-affinity potassium transporter (By similarity).
P	Q67UC7	DE	Probable potassium transporter 17;
P	Q67UC7	DR	potassium ion transmembrane transporter activity
P	Q67UQ7	CC	Magnesium transporter that may mediate the influx of magnesium (By similarity)
P	Q67UQ7	DE	Magnesium transporter MRS2-B;
P	Q67VS5	CC	High-affinity potassium transporter.
P	Q67VS5	DE	Potassium transporter 10;
P	Q67VS5	DR	potassium ion transmembrane transporter activity
P	Q67WJ8	CC	Aquaporins facilitate the transport of water and small neutral solutes across cell membranes (By similarity).
P	Q67YF8	CC	Does not seem to transport sucrose.

P	Q67YF8	DE	Sucrose permease 7;
P	Q67YF8	DE	Sucrose transport protein SUC7;
P	Q67YF8	DE	Sucrose-proton symporter 7;
P	Q67YF8	DR	sucrose transmembrane transporter activity
P	Q68KI4	CC	Involved in vacuolar ion compartmentalization necessary for cell volume regulation and cytoplasmic Na+ detoxification
P	Q68KI4	DE	Na+/H+ exchanger 1;
P	Q68KI4	DE	Sodium/hydrogen exchanger 1;
P	Q68KI4	DR	sodium:hydrogen antiporter activity
P	Q695T7	CC	Uptake of leucine is sodium- dependent
P	Q695T7	DE	Sodium-dependent neutral amino acid transporter B(0)AT1;
P	Q695T7	DR	neurotransmitter:sodium symporter activity
P	Q69L87	CC	High-affinity potassium transporter (By similarity).
P	Q69L87	DE	Potassium transporter 22;
P	Q69L87	DR	potassium ion transmembrane transporter activity
P	Q69RI8	CC	High-affinity potassium transporter (By similarity).
P	Q69RI8	DE	Probable potassium transporter 14;
P	Q69RI8	DR	potassium ion transmembrane transporter activity
P	Q69T94	CC	High-affinity transporter for external inorganic phosphate (By similarity).
P	Q69T94	DE	Probable inorganic phosphate transporter 1-10;
P	Q6A329	CC	Does not seem to transport sucrose.
P	Q6A329	DE	Putative sucrose transport protein SUC6;
P	Q6A329	DE	Sucrose permease 6;
P	Q6A329	DE	Sucrose-proton symporter 6;
P	Q6A329	DR	sucrose transmembrane transporter activity
P	Q6A5S0	CC	Transport of potassium into the cell (By similarity).
P	Q6A5S0	DE	Probable potassium transport system protein kup;
P	Q6A5S0	DR	potassium ion transmembrane transporter activity
P	Q6A6X6	CC	Part of the ABC transporter complex MetNIQ involved in methionine import
P	Q6A6X6	DE	Methionine import ATP-binding protein MetN;
P	Q6AAX3	CC	Part of the ABC transporter complex PstSACB involved in phosphate import
P	Q6AAX3	DE	ABC phosphate transporter;
P	Q6AAX3	DE	Phosphate import ATP-binding protein PstB;
P	Q6AAX3	DE	Phosphate-transporting ATPase;
P	Q6AAX3	DR	inorganic phosphate transmembrane transporter activity
P	Q6AAX3	DR	phosphate transmembrane-transporting ATPase activity
P	Q6AB77	CC	Na+/H+ antiporter that extrudes sodium in exchange for external protons (By similarity)
P	Q6AB77	DE	Na+/H+ antiporter nhaA;
P	Q6AB77	DE	Sodium/proton antiporter nhaA;
P	Q6ABJ2	CC	One of the components of the high-affinity ATP-driven potassium transport (or KDP) system, which catalyzes the hydrolysis of ATP coupled with the exchange of hydrogen and potassium ions (By similarity).
P	Q6ABJ2	DE	Potassium-binding and translocating subunit A;
P	Q6ABJ2	DE	Potassium-translocating ATPase A chain;
P	Q6ABJ2	DE	Potassium-transporting ATPase A chain;
P	Q6ABJ2	DR	potassium-transporting ATPase activity
P	Q6ADG4	CC	Part of the ABC transporter complex PstSACB involved in phosphate import
P	Q6ADG4	DE	ABC phosphate transporter;
P	Q6ADG4	DE	Phosphate import ATP-binding protein PstB;
P	Q6ADG4	DE	Phosphate-transporting ATPase;
P	Q6ADG4	DR	inorganic phosphate transmembrane transporter activity
P	Q6ADG4	DR	phosphate transmembrane-transporting ATPase activity
P	Q6AE21	CC	Part of the ABC transporter complex MetNIQ involved in methionine import
P	Q6AE21	DE	Methionine import ATP-binding protein MetN;
P	Q6AI14	CC	In apical cell membrane may be involved in mediating sodium absorption
P	Q6AI14	CC	Major proton extruding system driven by the inward sodium ion chemical gradient
P	Q6AI14	DE	Na+/H+ exchanger 4;
P	Q6AI14	DE	Sodium/hydrogen exchanger 4;
P	Q6AI14	DR	sodium:hydrogen antiporter activity
P	Q6AM16	CC	Part of the ABC transporter complex PstSACB involved in phosphate import
P	Q6AM16	DE	ABC phosphate transporter;
P	Q6AM16	DE	Phosphate import ATP-binding protein PstB;
P	Q6AM16	DE	Phosphate-transporting ATPase;
P	Q6AM16	DR	inorganic phosphate transmembrane transporter activity
P	Q6AM16	DR	phosphate transmembrane-transporting ATPase activity

P	Q6AMR9	DE	Lipoprotein-releasing system ATP-binding protein LolD;
P	Q6AMR9	DR	lipoprotein transporter activity
P	Q6AQH5	CC	Na+/H+ antiporter that extrudes sodium in exchange for external protons (By similarity)
P	Q6AQH5	DE	Na+/H+ antiporter nhaA;
P	Q6AQH5	DE	Sodium/proton antiporter nhaA;
P	Q6AS08	CC	Involved in the import of serine and threonine into the cell, with the concomitant import of sodium (symport system) (By similarity).
P	Q6AS08	DE	Na+/serine-threonine symporter;
P	Q6AS08	DR	sodium:dicarboxylate symporter activity
P	Q6AS08	DR	sodium:dicarboxylate symporter activity
P	Q6B014	DE	K2P10.1 potassium channel isoform 3;
P	Q6B014	DE	Potassium channel, subfamily K, member 10, isoform 3;
P	Q6B014	DE	Potassium channel, subfamily K, member 10, isoform CRA_a;
P	Q6B014	DR	potassium channel activity
P	Q6CYN3	CC	Part of the ABC transporter complex PstSACB involved in phosphate import
P	Q6CYN3	DE	ABC phosphate transporter 2;
P	Q6CYN3	DE	Phosphate import ATP-binding protein PstB 2;
P	Q6CYN3	DE	Phosphate-transporting ATPase 2;
P	Q6CYN3	DR	inorganic phosphate transmembrane transporter activity
P	Q6CYN3	DR	phosphate transmembrane-transporting ATPase activity
P	Q6CZ34	DR	glycerol-3-phosphate-transporting ATPase activity
P	Q6CZ44	CC	The physiological role may be the reduction of the intracellular concentration of toxic sugars or sugar metabolites (By similarity).
P	Q6CZ44	DE	Probable sugar efflux transporter;
P	Q6CZH4	CC	Mediates influx of magnesium ions (By similarity).
P	Q6CZH4	DE	Magnesium transport protein CorA;
P	Q6CZH4	DR	cobalt ion transmembrane transporter activity
P	Q6CZH4	DR	magnesium ion transmembrane transporter activity
P	Q6CZU5	CC	Transport system that facilitates potassium-efflux, possibly by potassium-proton antiport (By similarity).
P	Q6CZU5	DE	Glutathione-regulated potassium-efflux system protein kefB;
P	Q6CZU5	DE	K+/H+ antiporter;
P	Q6CZU5	DE	NEM-activable K+/H+ antiporter;
P	Q6CZU5	DR	glutathione-regulated potassium exporter activity
P	Q6D090	CC	Important to control the intracellular level of arginine and the correct balance between arginine and lysine (By similarity).
P	Q6D0B9	CC	Na+/H+ antiporter that extrudes sodium in exchange for external protons (By similarity)
P	Q6D0B9	DE	Na+/H+ antiporter nhaA;
P	Q6D0B9	DE	Sodium/proton antiporter nhaA;
P	Q6D0F3	CC	Part of the ABC transporter complex ThiBPQ involved in thiamine import
P	Q6D0F3	DE	Thiamine import ATP-binding protein ThiQ;
P	Q6D1C4	CC	Part of the ABC transporter complex MetNIQ involved in methionine import
P	Q6D1C4	DE	Methionine import ATP-binding protein MetN 3;
P	Q6D1C4	DR	D-methionine transmembrane transporter activity
P	Q6D1Z3	CC	Binds vitamin B12 and delivers it to the periplasmic surface of BtuC (By similarity)
P	Q6D1Z3	CC	Part of the ABC transporter complex BtuCDF involved in vitamin B12 import
P	Q6D201	CC	Part of the ABC transporter complex CysAWTP involved in sulfate/thiosulfate import
P	Q6D201	DE	Sulfate-transporting ATPase;
P	Q6D201	DE	Sulfate/thiosulfate import ATP-binding protein CysA;
P	Q6D201	DR	sulfate transmembrane-transporting ATPase activity
P	Q6D2D5	CC	Part of the ABC transporter complex PstSACB involved in phosphate import
P	Q6D2D5	DE	ABC phosphate transporter 1;
P	Q6D2D5	DE	Phosphate import ATP-binding protein PstB 1;
P	Q6D2D5	DE	Phosphate-transporting ATPase 1;
P	Q6D2D5	DR	inorganic phosphate transmembrane transporter activity
P	Q6D2D5	DR	phosphate transmembrane-transporting ATPase activity
P	Q6D3Q6	CC	Part of the ABC transporter complex MetNIQ involved in methionine import
P	Q6D3Q6	DE	Methionine import ATP-binding protein MetN 2;
P	Q6D4A8	CC	Part of the ABC transporter complex ZnuABC involved in zinc import
P	Q6D4A8	DE	Zinc import ATP-binding protein ZnuC;
P	Q6D4A8	DR	zinc transporting ATPase activity
P	Q6D4E2	CC	Part of the ABC transporter complex PotABCD involved in spermidine/putrescine import
P	Q6D4E2	CC	Part of the ABC transporter complex PotABCD involved in spermidine/putrescine import
P	Q6D4E2	DE	Spermidine/putrescine import ATP-binding protein PotA;
P	Q6D4E2	DE	Spermidine/putrescine import ATP-binding protein PotA;

P	Q6D4M9	CC	Na+/H+ antiporter that extrudes sodium in exchange for external protons (By similarity)
P	Q6D4M9	DE	Na+/H+ antiporter nhaB;
P	Q6D4M9	DE	Sodium/proton antiporter nhaB;
P	Q6D4M9	DR	sodium:hydrogen antiporter activity
P	Q6D4W8	DR	L-arabinose-importing ATPase activity
P	Q6D5H7	CC	Part of the ABC transporter complex MetNIQ involved in methionine import
P	Q6D5H7	DE	Methionine import ATP-binding protein MetN 1;
P	Q6D606	CC	Part of the ABC transporter complex CcmAB involved in the biogenesis of c-type cytochromes; once thought to export heme, this seems not to be the case, but its exact role is uncertain
P	Q6D606	DE	Heme exporter protein A;
P	Q6D606	DR	heme-transporting ATPase activity
P	Q6D645	CC	Part of the ABC transporter complex HmuTUV involved in hemin import
P	Q6D645	DE	Hemin import ATP-binding protein HmuV;
P	Q6D654	CC	Part of the ABC transporter complex BtuCDF involved in vitamin B12 import
P	Q6D654	DE	Vitamin B12 import ATP-binding protein BtuD;
P	Q6D654	DE	Vitamin B12-transporting ATPase;
P	Q6D656	CC	Part of the ABC transporter complex BtuCDF involved in vitamin B12 import
P	Q6D656	DE	Vitamin B12 import system permease protein BtuC;
P	Q6D664	DE	Lipoprotein-releasing system ATP-binding protein LolD;
P	Q6D664	DR	lipoprotein transporter activity
P	Q6D7D0	CC	Part of the ABC transporter complex ModABC involved in molybdenum import
P	Q6D7D0	DE	Molybdenum import ATP-binding protein ModC;
P	Q6D7D0	DR	molybdenum ion transmembrane transporter activity
P	Q6D7E5	CC	Involved in zinc efflux across the cytoplasmic membrane, thus reducing zinc accumulation in the cytoplasm and rendering bacteria more resistant to zinc
P	Q6D7E5	CC	It may contribute to zinc homeostasis at low concentrations of zinc (By similarity)
P	Q6D7E5	DE	Zinc transporter zitB;
P	Q6D7I3	CC	One of the components of the high-affinity ATP-driven potassium transport (or KDP) system, which catalyzes the hydrolysis of ATP coupled with the exchange of hydrogen and potassium ions (By similarity).
P	Q6D7I3	DE	Potassium-binding and translocating subunit A;
P	Q6D7I3	DE	Potassium-translocating ATPase A chain;
P	Q6D7I3	DE	Potassium-transporting ATPase A chain;
P	Q6D7I3	DR	potassium-transporting ATPase activity
P	Q6D7I5	CC	One of the components of the high-affinity ATP-driven potassium transport (or KDP) system, which catalyzes the hydrolysis of ATP coupled with the exchange of hydrogen and potassium ions
P	Q6D7I5	DE	Potassium-binding and translocating subunit C;
P	Q6D7I5	DE	Potassium-translocating ATPase C chain;
P	Q6D7I5	DE	Potassium-transporting ATPase C chain;
P	Q6D7I5	DR	potassium-transporting ATPase activity
P	Q6D9G4	CC	Involved in the import of serine and threonine into the cell, with the concomitant import of sodium (symport system) (By similarity).
P	Q6D9G4	DE	Na+/serine-threonine symporter;
P	Q6D9G4	DR	sodium:dicarboxylate symporter activity
P	Q6D9G4	DR	sodium:dicarboxylate symporter activity
P	Q6DA13	CC	Uptake of L-rhamnose across the boundary membrane with the concomitant transport of protons into the cell (symport system) (By similarity).
P	Q6DA13	DE	L-rhamnose-H+ transport protein;
P	Q6DA13	DE	L-rhamnose-proton symporter;
P	Q6DB03	CC	Part of the ABC transporter complex XylFGH involved in xylose import
P	Q6DB03	DE	Xylose import ATP-binding protein XylG;
P	Q6DB03	DR	D-xylose-importing ATPase activity
P	Q6DB87	CC	Part of the ABC transporter complex RbsABCD involved in ribose import
P	Q6DB87	DE	Ribose import ATP-binding protein RbsA;
P	Q6DB92	CC	Responsible for the low-affinity transport of potassium into the cell, with the probable concomitant uptake of protons (symport system) (By similarity).
P	Q6DB92	DE	Kup system potassium uptake protein;
P	Q6DB92	DE	Low affinity potassium transport system protein kup;
P	Q6DB92	DR	potassium ion transmembrane transporter activity
P	Q6DBM8	CC	Involved in sequestration of excess zinc in the cytoplasm into vacuoles to maintain zinc homeostasis (By similarity).
P	Q6EU94	CC	May function as water channel to facilitate the transport of water across cell membrane
P	Q6EU94	DE	Water channel protein RWC1;

P	Q6F0P3	DR	phosphonate transmembrane-transporting ATPase activity
P	Q6F0V4	CC	Part of the ABC transporter complex PotABCD involved in spermidine/putrescine import
P	Q6F0V4	CC	Part of the ABC transporter complex PotABCD involved in spermidine/putrescine import
P	Q6F0V4	DE	Spermidine/putrescine import ATP-binding protein PotA;
P	Q6F0V4	DE	Spermidine/putrescine import ATP-binding protein PotA;
P	Q6F1N1	CC	Part of the ABC transporter complex PstSACB involved in phosphate import
P	Q6F1N1	DE	ABC phosphate transporter;
P	Q6F1N1	DE	Phosphate import ATP-binding protein PstB;
P	Q6F1N1	DE	Phosphate-transporting ATPase;
P	Q6F1N1	DR	inorganic phosphate transmembrane transporter activity
P	Q6F1N1	DR	phosphate transmembrane-transporting ATPase activity
P	Q6F793	CC	Transport of potassium into the cell (By similarity).
P	Q6F793	DE	Probable potassium transport system protein kup;
P	Q6F793	DR	potassium ion transmembrane transporter activity
P	Q6F9A8	CC	Part of the ABC transporter complex CysAWTP involved in sulfate/thiosulfate import
P	Q6F9A8	DE	Sulfate-transporting ATPase;
P	Q6F9A8	DE	Sulfate/thiosulfate import ATP-binding protein CysA;
P	Q6F9A8	DR	sulfate transmembrane-transporting ATPase activity
P	Q6F9P2	CC	Part of the ABC transporter complex MetNIQ involved in methionine import
P	Q6F9P2	DE	Methionine import ATP-binding protein MetN 2;
P	Q6FAN3	CC	Part of the ABC transporter complex MetNIQ involved in methionine import
P	Q6FAN3	DE	Methionine import ATP-binding protein MetN 1;
P	Q6FB65	CC	Involved in the import of serine and threonine into the cell, with the concomitant import of sodium (symport system) (By similarity).
P	Q6FB65	DE	Na+/serine-threonine symporter;
P	Q6FB65	DR	sodium:dicarboxylate symporter activity
P	Q6FB65	DR	sodium:dicarboxylate symporter activity
P	Q6FCW7	CC	Part of the ABC transporter complex PstSACB involved in phosphate import
P	Q6FCW7	DE	ABC phosphate transporter;
P	Q6FCW7	DE	Phosphate import ATP-binding protein PstB;
P	Q6FCW7	DE	Phosphate-transporting ATPase;
P	Q6FCW7	DR	inorganic phosphate transmembrane transporter activity
P	Q6FCW7	DR	phosphate transmembrane-transporting ATPase activity
P	Q6FFL0	CC	Part of the ABC transporter complex ZnuABC involved in zinc import
P	Q6FFL0	DE	Zinc import ATP-binding protein ZnuC;
P	Q6FFL0	DR	zinc transporting ATPase activity
P	Q6FIH7	DE	ATP synthase, H+ transporting, mitochondrial F0 complex, subunit C1 (Subunit 9), isoform CRA_a;
P	Q6FIH7	DE	cDNA, FLJ92113, Homo sapiens ATP synthase, H+ transporting, mitochondrial F0complex, subunit c (subunit 9), isoform 1 (ATP5G1), mRNA;
P	Q6FZX3	DE	Lipoprotein-releasing system ATP-binding protein LolD;
P	Q6FZX3	DR	lipoprotein transporter activity
P	Q6G098	CC	Part of the ABC transporter complex HmuTUV involved in hemin import
P	Q6G098	DE	Hemin import ATP-binding protein HmuV;
P	Q6G0H9	CC	Na+/H+ antiporter that extrudes sodium in exchange for external protons (By similarity)
P	Q6G0H9	DE	Na+/H+ antiporter nhaA;
P	Q6G0H9	DE	Sodium/proton antiporter nhaA;
P	Q6G0L7	CC	Part of the ABC transporter complex PstSACB involved in phosphate import
P	Q6G0L7	DE	ABC phosphate transporter;
P	Q6G0L7	DE	Phosphate import ATP-binding protein PstB;
P	Q6G0L7	DE	Phosphate-transporting ATPase;
P	Q6G0L7	DR	inorganic phosphate transmembrane transporter activity
P	Q6G0L7	DR	phosphate transmembrane-transporting ATPase activity
P	Q6G0V9	CC	Part of the ABC transporter complex CcmAB involved in the biogenesis of c-type cytochromes; once thought to export heme, this seems not to be the case, but its exact role is uncertain
P	Q6G0V9	DE	Heme exporter protein A;
P	Q6G0V9	DR	heme-transporting ATPase activity
P	Q6G194	DR	glycerol-3-phosphate-transporting ATPase activity
P	Q6G2E2	CC	Part of the ABC transporter complex MetNIQ involved in methionine import
P	Q6G2E2	DE	Methionine import ATP-binding protein MetN;
P	Q6G3A6	DE	Lipoprotein-releasing system ATP-binding protein LolD;
P	Q6G3A6	DR	lipoprotein transporter activity
P	Q6G475	CC	Part of the ABC transporter complex HmuTUV involved in hemin import
P	Q6G475	DE	Hemin import ATP-binding protein HmuV;

P	Q6G4T6	CC	Part of the ABC transporter complex PstSACB involved in phosphate import
P	Q6G4T6	DE	ABC phosphate transporter;
P	Q6G4T6	DE	Phosphate import ATP-binding protein PstB;
P	Q6G4T6	DE	Phosphate-transporting ATPase;
P	Q6G4T6	DR	inorganic phosphate transmembrane transporter activity
P	Q6G4T6	DR	phosphate transmembrane-transporting ATPase activity
P	Q6G529	CC	Part of the ABC transporter complex CcmAB involved in the biogenesis of c-type cytochromes; once thought to export heme, this seems not to be the case, but its exact role is uncertain
P	Q6G529	DE	Heme exporter protein A;
P	Q6G529	DR	heme-transporting ATPase activity
P	Q6G5J0	DR	glycerol-3-phosphate-transporting ATPase activity
P	Q6G5K1	CC	Na+/H+ antiporter that extrudes sodium in exchange for external protons (By similarity)
P	Q6G5K1	DE	Na+/H+ antiporter nhaA;
P	Q6G5K1	DE	Sodium/proton antiporter nhaA;
P	Q6GQS1	CC	May act as a ATP-Mg/Pi exchanger that mediates the transport of Mg-ATP in exchange for phosphate, catalyzing the net uptake or efflux of adenine nucleotides into or from the mitochondria (By similarity).
P	Q6H3Z3	CC	Involved in Fe(3+) uptake from the rhizosphere and phloem transport of iron
P	Q6H3Z3	CC	Plays a important role in iron homeostasis during the early stages of growth
P	Q6H3Z3	DE	Iron-phytosiderophore transporter YSL15;
P	Q6H3Z6	CC	Involved in the phloem transport of iron and manganese and their translocation into the grain
P	Q6H3Z6	CC	Transports iron- and manganese-nicotianamine chelates, but not iron-phytosiderophore
P	Q6H4L9	CC	High-affinity potassium transporter (By similarity).
P	Q6H4L9	DE	Potassium transporter 20;
P	Q6H4L9	DR	potassium ion transmembrane transporter activity
P	Q6H4M2	CC	High-affinity potassium transporter (By similarity).
P	Q6H4M2	DE	Potassium transporter 19;
P	Q6H4M2	DR	potassium ion transmembrane transporter activity
P	Q6H4R6	CC	High-affinity potassium transporter (By similarity).
P	Q6H4R6	DE	Potassium transporter 23;
P	Q6H4R6	DR	potassium ion transmembrane transporter activity
P	Q6HBS0	CC	Part of the ABC transporter complex MetNIQ involved in methionine import
P	Q6HBS0	DE	Methionine import ATP-binding protein MetN 2;
P	Q6HDP8	CC	Part of the ABC transporter complex PstSACB involved in phosphate import
P	Q6HDP8	DE	ABC phosphate transporter;
P	Q6HDP8	DE	Phosphate import ATP-binding protein PstB;
P	Q6HDP8	DE	Phosphate-transporting ATPase;
P	Q6HDP8	DR	inorganic phosphate transmembrane transporter activity
P	Q6HDP8	DR	phosphate transmembrane-transporting ATPase activity
P	Q6HFB5	DR	phosphonate transmembrane-transporting ATPase activity
P	Q6HLQ9	CC	Part of the ABC transporter complex PotABCD involved in spermidine/putrescine import
P	Q6HLQ9	CC	Part of the ABC transporter complex PotABCD involved in spermidine/putrescine import
P	Q6HLQ9	DE	Spermidine/putrescine import ATP-binding protein PotA;
P	Q6HLQ9	DE	Spermidine/putrescine import ATP-binding protein PotA;
P	Q6HN77	CC	One of the components of the high-affinity ATP-driven potassium transport (or KDP) system, which catalyzes the hydrolysis of ATP coupled with the exchange of hydrogen and potassium ions
P	Q6HN77	DE	Potassium-binding and translocating subunit C;
P	Q6HN77	DE	Potassium-translocating ATPase C chain;
P	Q6HN77	DE	Potassium-transporting ATPase C chain;
P	Q6HN77	DR	potassium-transporting ATPase activity
P	Q6HN78	CC	One of the components of the high-affinity ATP-driven potassium transport (or KDP) system, which catalyzes the hydrolysis of ATP coupled with the exchange of hydrogen and potassium ions (By similarity).
P	Q6HN78	DE	Potassium-binding and translocating subunit B;
P	Q6HN78	DE	Potassium-translocating ATPase B chain;
P	Q6HN78	DE	Potassium-transporting ATPase B chain;
P	Q6HN78	DR	potassium-transporting ATPase activity
P	Q6HN79	CC	One of the components of the high-affinity ATP-driven potassium transport (or KDP) system, which catalyzes the hydrolysis of ATP coupled with the exchange of hydrogen and potassium ions (By similarity).
P	Q6HN79	DE	Potassium-binding and translocating subunit A;
P	Q6HN79	DE	Potassium-translocating ATPase A chain;

P	Q6HN79	DE	Potassium-transporting ATPase A chain;
P	Q6HN79	DR	potassium-transporting ATPase activity
P	Q6HNE7	CC	Part of the ABC transporter complex RbsABCD involved in ribose import
P	Q6HNE7	DE	Ribose import ATP-binding protein RbsA;
P	Q6HP89	CC	Part of the ABC transporter complex MetNIQ involved in methionine import
P	Q6HP89	DE	Methionine import ATP-binding protein MetN 1;
P	Q6J4K2	CC	In contrast to other members of the family its function is independent of K+.
P	Q6J4K2	CC	Transports Ca2+ in exchange for either Li+ or Na+, explaining how Li+ catalyzes Ca2+ exchange
P	Q6J4K2	CC	Transports Ca2+ in exchange for either Li+ or Na+, explaining how Li+ catalyzes Ca2+ exchange
P	Q6J4K2	DE	Na+/K+/Ca2+-exchange protein 6;
P	Q6J4K2	DE	Na+/K+/Ca2+-exchange protein 6;
P	Q6J4K2	DE	Na+/K+/Ca2+-exchange protein 6;
P	Q6J4K2	DE	Sodium/potassium/calcium exchanger 6;
P	Q6J4K2	DE	Sodium/potassium/calcium exchanger 6;
P	Q6J4K2	DE	Sodium/potassium/calcium exchanger 6;
P	Q6J4K2	DR	calcium:cation antiporter activity
P	Q6K1C4	CC	Translocates Ca2+ and other metal ions into vacuoles using the proton gradient formed by H+-ATPase and H+-pyrophosphatase.
P	Q6K1C4	DE	Ca2+/H+ exchanger 3;
P	Q6K215	CC	Aquaporins facilitate the transport of water and small neutral solutes across cell membranes (By similarity).
P	Q6KIP2	CC	Part of the ABC transporter complex PotABCD involved in spermidine/putrescine import
P	Q6KIP2	CC	Part of the ABC transporter complex PotABCD involved in spermidine/putrescine import
P	Q6KIP2	DE	Spermidine/putrescine import ATP-binding protein PotA;
P	Q6KIP2	DE	Spermidine/putrescine import ATP-binding protein PotA;
P	Q6KIS8	CC	Part of the ABC transporter complex PstSACB involved in phosphate import
P	Q6KIS8	DE	ABC phosphate transporter;
P	Q6KIS8	DE	Phosphate import ATP-binding protein PstB;
P	Q6KIS8	DE	Phosphate-transporting ATPase;
P	Q6KIS8	DR	inorganic phosphate transmembrane transporter activity
P	Q6KIS8	DR	phosphate transmembrane-transporting ATPase activity
P	Q6LG82	CC	Involved in the import of serine and threonine into the cell, with the concomitant import of sodium (symport system) (By similarity).
P	Q6LG82	DE	Na+/serine-threonine symporter;
P	Q6LG82	DR	sodium:dicarboxylate symporter activity
P	Q6LG82	DR	sodium:dicarboxylate symporter activity
P	Q6LH11	CC	Part of the ABC transporter complex RbsABCD involved in ribose import
P	Q6LH11	DE	Ribose import ATP-binding protein RbsA;
P	Q6LHL2	CC	Part of the ABC transporter complex ModABC involved in molybdenum import
P	Q6LHL2	DE	Molybdenum import ATP-binding protein ModC;
P	Q6LHL2	DR	molybdenum ion transmembrane transporter activity
P	Q6LN52	CC	Part of the ABC transporter complex MetNIQ involved in methionine import
P	Q6LN52	DE	Methionine import ATP-binding protein MetN;
P	Q6LN52	DR	D-methionine transmembrane transporter activity
P	Q6LNY8	CC	Na+/H+ antiporter that extrudes sodium in exchange for external protons (By similarity).
P	Q6LNY8	DE	Na+/H+ antiporter nhaB;
P	Q6LNY8	DE	Sodium/proton antiporter nhaB;
P	Q6LNY8	DR	sodium:hydrogen antiporter activity
P	Q6LQ77	CC	Part of the ABC transporter complex BtuCDF involved in vitamin B12 import
P	Q6LQ77	DE	Vitamin B12 import ATP-binding protein BtuD;
P	Q6LQ77	DE	Vitamin B12-transporting ATPase;
P	Q6LQC0	CC	Part of the ABC transporter complex HmuTUV involved in hemin import
P	Q6LQC0	DE	Hemin import ATP-binding protein HmuV;
P	Q6LR20	CC	Part of the ABC transporter complex PotABCD involved in spermidine/putrescine import
P	Q6LR20	CC	Part of the ABC transporter complex PotABCD involved in spermidine/putrescine import
P	Q6LR20	DE	Spermidine/putrescine import ATP-binding protein PotA;
P	Q6LR20	DE	Spermidine/putrescine import ATP-binding protein PotA;
P	Q6LSC4	CC	Part of the ABC transporter complex PstSACB involved in phosphate import
P	Q6LSC4	DE	ABC phosphate transporter 2;
P	Q6LSC4	DE	Phosphate import ATP-binding protein PstB 2;
P	Q6LSC4	DE	Phosphate-transporting ATPase 2;
P	Q6LSC4	DR	inorganic phosphate transmembrane transporter activity
P	Q6LSC4	DR	phosphate transmembrane-transporting ATPase activity

P	Q6LTB1	CC	Part of the ABC transporter complex ZnuABC involved in zinc import
P	Q6LTB1	DE	Zinc import ATP-binding protein ZnuC;
P	Q6LTB1	DR	zinc transporting ATPase activity
P	Q6LTL7	CC	Part of the ABC transporter complex CcmAB involved in the biogenesis of c-type cytochromes; once thought to export heme, this seems not to be the case, but its exact role is uncertain
P	Q6LTL7	DE	Heme exporter protein A;
P	Q6LTL7	DR	heme-transporting ATPase activity
P	Q6LU82	CC	Part of the ABC transporter complex PstSACB involved in phosphate import
P	Q6LU82	DE	ABC phosphate transporter 1;
P	Q6LU82	DE	Phosphate import ATP-binding protein PstB 1;
P	Q6LU82	DE	Phosphate-transporting ATPase 1;
P	Q6LU82	DR	inorganic phosphate transmembrane transporter activity
P	Q6LU82	DR	phosphate transmembrane-transporting ATPase activity
P	Q6LUL9	CC	Na+/H+ antiporter that extrudes sodium in exchange for external protons (By similarity)
P	Q6LUL9	DE	Na+/H+ antiporter nhaA;
P	Q6LUL9	DE	Sodium/proton antiporter nhaA;
P	Q6LUR6	CC	Binds vitamin B12 and delivers it to the periplasmic surface of BtuC (By similarity)
P	Q6LUR6	CC	Part of the ABC transporter complex BtuCDF involved in vitamin B12 import
P	Q6LUY1	CC	Part of the ABC transporter complex XylFGH involved in xylose import
P	Q6LUY1	DE	Xylose import ATP-binding protein XylG;
P	Q6LUY1	DR	D-xylose-importing ATPase activity
P	Q6LX70	DR	cobalt ion transmembrane transporter activity
P	Q6LY93	CC	Part of the ABC transporter complex PstSACB involved in phosphate import
P	Q6LY93	DE	ABC phosphate transporter;
P	Q6LY93	DE	Phosphate import ATP-binding protein PstB;
P	Q6LY93	DE	Phosphate-transporting ATPase;
P	Q6LY93	DR	inorganic phosphate transmembrane transporter activity
P	Q6LY93	DR	phosphate transmembrane-transporting ATPase activity
P	Q6MCV4	CC	Part of the ABC transporter complex PotABCD involved in spermidine/putrescine import
P	Q6MCV4	CC	Part of the ABC transporter complex PotABCD involved in spermidine/putrescine import
P	Q6MCV4	DE	Spermidine/putrescine import ATP-binding protein PotA;
P	Q6MCV4	DE	Spermidine/putrescine import ATP-binding protein PotA;
P	Q6MD10	DE	Lipoprotein-releasing system ATP-binding protein LolD;
P	Q6MD10	DR	lipoprotein transporter activity
P	Q6ME20	CC	Part of the ABC transporter complex MetNIQ involved in methionine import
P	Q6ME20	DE	Methionine import ATP-binding protein MetN;
P	Q6MLL0	CC	Transport of potassium into the cell (By similarity).
P	Q6MLL0	DE	Probable potassium transport system protein kup;
P	Q6MLL0	DR	potassium ion transmembrane transporter activity
P	Q6MMH0	CC	Part of the ABC transporter complex PstSACB involved in phosphate import
P	Q6MMH0	DE	ABC phosphate transporter;
P	Q6MMH0	DE	Phosphate import ATP-binding protein PstB;
P	Q6MMH0	DE	Phosphate-transporting ATPase;
P	Q6MMH0	DR	inorganic phosphate transmembrane transporter activity
P	Q6MMH0	DR	phosphate transmembrane-transporting ATPase activity
P	Q6MMY0	DE	Lipoprotein-releasing system ATP-binding protein LolD;
P	Q6MMY0	DR	lipoprotein transporter activity
P	Q6MTC1	CC	Part of the ABC transporter complex PstSACB involved in phosphate import
P	Q6MTC1	DE	ABC phosphate transporter;
P	Q6MTC1	DE	Phosphate import ATP-binding protein PstB;
P	Q6MTC1	DE	Phosphate-transporting ATPase;
P	Q6MTC1	DR	inorganic phosphate transmembrane transporter activity
P	Q6MTC1	DR	phosphate transmembrane-transporting ATPase activity
P	Q6MU19	CC	Part of the ABC transporter complex PotABCD involved in spermidine/putrescine import
P	Q6MU19	CC	Part of the ABC transporter complex PotABCD involved in spermidine/putrescine import
P	Q6MU19	DE	Spermidine/putrescine import ATP-binding protein PotA;
P	Q6MU19	DE	Spermidine/putrescine import ATP-binding protein PotA;
P	Q6MUF4	DR	phosphonate transmembrane-transporting ATPase activity
P	Q6N0I5	CC	Part of the ABC transporter complex PstSACB involved in phosphate import
P	Q6N0I5	DE	ABC phosphate transporter;
P	Q6N0I5	DE	Phosphate import ATP-binding protein PstB;
P	Q6N0I5	DE	Phosphate-transporting ATPase;
P	Q6N0I5	DR	inorganic phosphate transmembrane transporter activity
P	Q6N0I5	DR	phosphate transmembrane-transporting ATPase activity

P	Q6N0P7	CC	Part of the ABC transporter complex ModABC involved in molybdenum import
P	Q6N0P7	DE	Molybdenum import ATP-binding protein ModC;
P	Q6N0P7	DR	molybdenum ion transmembrane transporter activity
P	Q6N5F2	CC	Transport of potassium into the cell (By similarity).
P	Q6N5F2	DE	Probable potassium transport system protein kup 1;
P	Q6N5F2	DR	potassium ion transmembrane transporter activity
P	Q6N5G6	CC	Transport of potassium into the cell (By similarity).
P	Q6N5G6	DE	Probable potassium transport system protein kup 2;
P	Q6N5G6	DR	potassium ion transmembrane transporter activity
P	Q6N5H0	CC	One of the components of the high-affinity ATP-driven potassium transport (or KDP) system, which catalyzes the hydrolysis of ATP coupled with the exchange of hydrogen and potassium ions (By similarity).
P	Q6N5H0	DE	Potassium-binding and translocating subunit A;
P	Q6N5H0	DE	Potassium-translocating ATPase A chain;
P	Q6N5H0	DE	Potassium-transporting ATPase A chain;
P	Q6N5H0	DR	potassium-transporting ATPase activity
P	Q6N5H2	CC	One of the components of the high-affinity ATP-driven potassium transport (or KDP) system, which catalyzes the hydrolysis of ATP coupled with the exchange of hydrogen and potassium ions
P	Q6N5H2	DE	Potassium-binding and translocating subunit C;
P	Q6N5H2	DE	Potassium-translocating ATPase C chain;
P	Q6N5H2	DE	Potassium-transporting ATPase C chain;
P	Q6N5H2	DR	potassium-transporting ATPase activity
P	Q6N5P8	DE	Lipoprotein-releasing system ATP-binding protein LolD 2;
P	Q6N5P8	DR	lipoprotein transporter activity
P	Q6N798	CC	Part of the ABC transporter complex MetNIQ involved in methionine import
P	Q6N798	DE	Methionine import ATP-binding protein MetN 2;
P	Q6N7Y6	CC	Part of the ABC transporter complex HmuTUV involved in hemin import
P	Q6N7Y6	DE	Hemin import ATP-binding protein HmuV;
P	Q6N893	CC	Transport of potassium into the cell (By similarity).
P	Q6N893	DE	Probable potassium transport system protein kup 3;
P	Q6N893	DR	potassium ion transmembrane transporter activity
P	Q6N999	DE	Lipoprotein-releasing system ATP-binding protein LolD 1;
P	Q6N999	DR	lipoprotein transporter activity
P	Q6N9W0	CC	Part of the ABC transporter complex MetNIQ involved in methionine import
P	Q6N9W0	DE	Methionine import ATP-binding protein MetN 1;
P	Q6NA00	DR	phosphonate transmembrane-transporting ATPase activity
P	Q6NBT1	CC	Part of the ABC transporter complex CysAWTP involved in sulfate/thiosulfate import
P	Q6NBT1	DE	Sulfate-transporting ATPase;
P	Q6NBT1	DE	Sulfate/thiosulfate import ATP-binding protein CysA;
P	Q6NBT1	DR	sulfate transmembrane-transporting ATPase activity
P	Q6NDA6	CC	Part of the ABC transporter complex CcmAB involved in the biogenesis of c-type cytochromes; once thought to export heme, this seems not to be the case, but its exact role is uncertain
P	Q6NDA6	DE	Heme exporter protein A;
P	Q6NDA6	DR	heme-transporting ATPase activity
P	Q6NDQ0	DR	glycerol-3-phosphate-transporting ATPase activity
P	Q6NHP1	DE	Lysine exporter protein;
P	Q6NJ07	CC	Part of the ABC transporter complex MetNIQ involved in methionine import
P	Q6NJ07	DE	Methionine import ATP-binding protein MetN;
P	Q6NUK1	CC	May act as a ATP-Mg/Pi exchanger that mediates the transport of Mg-ATP in exchange for phosphate, catalyzing the net uptake or efflux of adenine nucleotides into or from the mitochondria.
P	Q6NVV3	CC	Can also transport other divalent cations such as Fe(2+), Sr(2+), Ba(2+), Mn(2+), Cu(2+) and Co(2+) but to a much less extent than Mg2+ (By similarity).
P	Q6NVV3	DE	Magnesium transporter NIPA3;
P	Q6NXK8	CC	Cation channel with high affinity for sodium, which is gated by extracellular protons and inhibited by the diuretic amiloride
P	Q6NXK8	DE	Brain sodium channel 2;
P	Q6NXK8	DR	ligand-gated sodium channel activity
P	Q6NXT4	CC	Zinc-efflux transporter which allocates the cytoplasmic zinc to the trans-Golgi network (TGN) as well as the vesicular compartment (By similarity).
P	Q6NXT4	DE	Zinc transporter 6;
P	Q6NXT4	DR	zinc ion transmembrane transporter activity
P	Q6P5F6	DE	Zinc transporter ZIP10;

P	Q6P5W5	CC	Plays an important role in cellular zinc homeostasis as a zinc transporter
P	Q6P5W5	CC	Regulated in response to zinc availability (By similarity).
P	Q6P5W5	DE	Zinc transporter ZIP4;
P	Q6P5W5	DR	zinc ion transmembrane transporter activity
P	Q6PEM8	CC	Dietary heme iron is an important nutritional source of iron
P	Q6PEM8	CC	Has been shown to act both as an intestinal proton- coupled high-affinity folate transporter and as an intestinal heme transporter which mediates heme uptake from the gut lumen into duodenal epithelial cells
P	Q6PEM8	CC	Has been shown to act both as an intestinal proton- coupled high-affinity folate transporter and as an intestinal heme transporter which mediates heme uptake from the gut lumen into duodenal epithelial cells
P	Q6PEM8	CC	Shows a higher affinity for folate than heme.
P	Q6PEM8	CC	Shows a higher affinity for folate than heme.
P	Q6PEM8	CC	The iron is then released from heme and may be transported into the bloodstream
P	Q6PEM8	DE	Heme carrier protein 1;
P	Q6PEM8	DE	Proton-coupled folate transporter;
P	Q6PEM8	DR	folic acid transporter activity
P	Q6PEM8	DR	heme transporter activity
P	Q6PGE7	CC	Terminates the action of proline by its high affinity sodium-dependent reuptake into presynaptic terminals (By similarity).
P	Q6PGE7	CC	Terminates the action of proline by its high affinity sodium-dependent reuptake into presynaptic terminals (By similarity).
P	Q6PGE7	DE	Sodium-dependent proline transporter;
P	Q6PGE7	DE	Sodium-dependent proline transporter;
P	Q6PGE7	DR	neurotransmitter:sodium symporter activity
P	Q6PIE5	CC	This action creates the electrochemical gradient of sodium and potassium ions, providing the energy for active transport of various nutrients (By similarity).
P	Q6PIE5	CC	This action creates the electrochemical gradient of sodium and potassium ions, providing the energy for active transport of various nutrients (By similarity).
P	Q6PIE5	CC	This is the catalytic component of the active enzyme, which catalyzes the hydrolysis of ATP coupled with the exchange of sodium and potassium ions across the plasma membrane
P	Q6PIE5	CC	This is the catalytic component of the active enzyme, which catalyzes the hydrolysis of ATP coupled with the exchange of sodium and potassium ions across the plasma membrane
P	Q6PIE5	DE	Na+/K+ ATPase alpha(+) subunit;
P	Q6PIE5	DE	Na+/K+ ATPase alpha(+) subunit;
P	Q6PIE5	DE	Na+/K+ ATPase alpha-2 subunit;
P	Q6PIE5	DE	Na+/K+ ATPase alpha-2 subunit;
P	Q6PIE5	DE	Sodium pump subunit alpha-2;
P	Q6PIE5	DE	Sodium/potassium-transporting ATPase subunit alpha-2;
P	Q6PIE5	DE	Sodium/potassium-transporting ATPase subunit alpha-2;
P	Q6PIE5	DR	sodium:potassium-exchanging ATPase activity
P	Q6PIE5	DR	sodium:potassium-exchanging ATPase activity
P	Q6PIU1	CC	Potassium channel subunit that does not form functional channels by itself
P	Q6PIU1	DE	Neuronal potassium channel alpha subunit HNKA;
P	Q6PIU1	DE	Potassium voltage-gated channel subfamily V member 1;
P	Q6PIU1	DE	Voltage-gated potassium channel subunit Kv8.1;
P	Q6PIU1	DR	potassium channel regulator activity
P	Q6PIU1	DR	voltage-gated potassium channel activity
P	Q6PML9	DE	Zinc transporter 9;
P	Q6PXP3	CC	High-affinity transporter for glucose and fructose Does not transport galactose, 2-deoxy-d-glucose and xylose.
P	Q6PXP3	DE	Glucose transporter type 7;
P	Q6PXP3	DE	Solute carrier family 2, facilitated glucose transporter member 7;
P	Q6QIY3	CC	Assuming opened or closed conformations in response to the voltage difference across the membrane, the protein forms a sodium-selective channel through which sodium ions may pass in accordance with their electrochemical gradient
P	Q6QIY3	CC	It is a tetrodotoxin-resistant sodium channel isoform
P	Q6QIY3	CC	This protein mediates the voltage-dependent sodium ion permeability of excitable membranes
P	Q6QIY3	DE	Peripheral nerve sodium channel 3;
P	Q6QIY3	DE	Sensory neuron sodium channel;
P	Q6QIY3	DE	Sodium channel protein type 10 subunit alpha;
P	Q6QIY3	DE	Sodium channel protein type X subunit alpha;

P	Q6QIY3	DE	Voltage-gated sodium channel subunit alpha Nav1.8;
P	Q6QIY3	DR	voltage-gated sodium channel activity
P	Q6U841	CC	Electrogenic sodium/bicarbonate cotransporter in exchange for intracellular chloride
P	Q6U841	CC	Electrogenic sodium/bicarbonate cotransporter in exchange for intracellular chloride
P	Q6U841	DE	Sodium-driven chloride bicarbonate exchanger;
P	Q6UP69	DE	Mercury ion transport protein;
P	Q6UP69	DR	mercury ion transmembrane transporter activity
P	Q6UP70	DE	Periplasmic mercury ion binding protein;
P	Q6UP70	DR	mercury ion transmembrane transporter activity
P	Q6UVM3	CC	Outward rectifying potassium channel
P	Q6UVM3	CC	Produces rapidly activating outward rectifier K+ currents
P	Q6UVM3	DE	Potassium channel subfamily T member 2;
P	Q6UVM3	DE	Sequence like an intermediate conductance potassium channel subunit;
P	Q6UVM3	DE	Sodium and chloride-activated ATP-sensitive potassium channel Slo2.1;
P	Q6VV64	CC	May function as background potassium channel that sets the resting membrane potential
P	Q6VV64	CC	Outward rectifying potassium channel
P	Q6VV64	CC	Produces rapidly activating outward rectifier K+ currents
P	Q6VV64	DE	Potassium channel subfamily K member 18;
P	Q6VV64	DE	Two-pore-domain potassium channel TRESK;
P	Q6VVA6	CC	High-affinity potassium transporter
P	Q6VVA6	DE	Potassium transporter 1;
P	Q6VVA6	DR	potassium ion transmembrane transporter activity
P	Q6X1Y6	CC	Cation channel with high affinity for sodium, which is gated by extracellular protons and inhibited by the diuretic amiloride
P	Q6XR72	CC	May be involved in zinc transport out of the cell, being a zinc-efflux transporter (By similarity).
P	Q6XR72	DE	Zinc transporter 10;
P	Q6YPR6	CC	Part of the ABC transporter complex PotABCD involved in spermidine/putrescine import
P	Q6YPR6	CC	Part of the ABC transporter complex PotABCD involved in spermidine/putrescine import
P	Q6YPR6	DE	Spermidine/putrescine import ATP-binding protein PotA;
P	Q6YPR6	DE	Spermidine/putrescine import ATP-binding protein PotA;
P	Q6YSA9	CC	High-affinity potassium transporter (By similarity).
P	Q6YSA9	DE	Probable potassium transporter 4;
P	Q6YSA9	DR	potassium ion transmembrane transporter activity
P	Q6YWQ4	CC	High-affinity potassium transporter (By similarity).
P	Q6YWQ4	DE	Potassium transporter 25;
P	Q6YWQ4	DR	potassium ion transmembrane transporter activity
P	Q6ZMH5	CC	May play a role in polarized cells by carrying out serosal-to-mucosal zinc transport
P	Q6ZMH5	CC	Seems to play a central role in controlling organismal zinc status (By similarity).
P	Q6ZMH5	DE	Zinc transporter ZIP5;
P	Q6ZPR4	CC	Outwardly rectifying potassium channel subunit that may co-assemble with other Slo-type channel subunits
P	Q6ZPR4	DE	Potassium channel subfamily T member 1;
P	Q6ZQN7	CC	May be involved in the first step of the transport pathway of digoxin and various compounds into the urine in the kidney
P	Q6ZSM3	CC	Proton-linked monocarboxylate transporter
P	Q6ZSM3	DE	Monocarboxylate transporter 12;
P	Q71RS6	CC	Probably transports 1 Ca2+ and 1 K+ in exchange for 4 Na+.
P	Q71RS6	CC	Probably transports 1 Ca2+ and 1 K+ in exchange for 4 Na+.
P	Q71RS6	CC	Probably transports 1 Ca2+ and 1 K+ in exchange for 4 Na+.
P	Q71RS6	DE	Na+/K+/Ca2+-exchange protein 5;
P	Q71RS6	DE	Na+/K+/Ca2+-exchange protein 5;
P	Q71RS6	DE	Na+/K+/Ca2+-exchange protein 5;
P	Q71RS6	DE	Sodium/potassium/calcium exchanger 5;
P	Q71RS6	DE	Sodium/potassium/calcium exchanger 5;
P	Q71RS6	DE	Sodium/potassium/calcium exchanger 5;
P	Q71RS6	DR	calcium, potassium:sodium antiporter activity
P	Q71RS6	DR	calcium, potassium:sodium antiporter activity
P	Q71RS6	DR	calcium, potassium:sodium antiporter activity
P	Q71W89	CC	One of the components of the high-affinity ATP-driven potassium transport (or KDP) system, which catalyzes the hydrolysis of ATP coupled with the exchange of hydrogen and potassium ions (By similarity).
P	Q71W89	DE	Potassium-binding and translocating subunit A;
P	Q71W89	DE	Potassium-translocating ATPase A chain;
P	Q71W89	DE	Potassium-transporting ATPase A chain;

P	Q71W89	DR	potassium-transporting ATPase activity
P	Q71W90	CC	One of the components of the high-affinity ATP-driven potassium transport (or KDP) system, which catalyzes the hydrolysis of ATP coupled with the exchange of hydrogen and potassium ions (By similarity).
P	Q71W90	DE	Potassium-binding and translocating subunit B;
P	Q71W90	DE	Potassium-translocating ATPase B chain;
P	Q71W90	DE	Potassium-transporting ATPase B chain;
P	Q71W90	DR	potassium-transporting ATPase activity
P	Q71W91	CC	One of the components of the high-affinity ATP-driven potassium transport (or KDP) system, which catalyzes the hydrolysis of ATP coupled with the exchange of hydrogen and potassium ions
P	Q71W91	DE	Potassium-binding and translocating subunit C;
P	Q71W91	DE	Potassium-translocating ATPase C chain;
P	Q71W91	DE	Potassium-transporting ATPase C chain;
P	Q71W91	DR	potassium-transporting ATPase activity
P	Q71WH7	DR	cobalt ion transmembrane transporter activity
P	Q71WH8	DR	cobalt ion transmembrane transporter activity
P	Q71WT2	CC	Part of the ABC transporter complex PstSACB involved in phosphate import
P	Q71WT2	DE	ABC phosphate transporter 2;
P	Q71WT2	DE	Phosphate import ATP-binding protein PstB 2;
P	Q71WT2	DE	Phosphate-transporting ATPase 2;
P	Q71WT2	DR	inorganic phosphate transmembrane transporter activity
P	Q71WT2	DR	phosphate transmembrane-transporting ATPase activity
P	Q71WT3	CC	Part of the ABC transporter complex PstSACB involved in phosphate import
P	Q71WT3	DE	ABC phosphate transporter 1;
P	Q71WT3	DE	Phosphate import ATP-binding protein PstB 1;
P	Q71WT3	DE	Phosphate-transporting ATPase 1;
P	Q71WT3	DR	inorganic phosphate transmembrane transporter activity
P	Q71WT3	DR	phosphate transmembrane-transporting ATPase activity
P	Q71ZP6	CC	H+-stimulated, highly selective, manganese uptake system (By similarity).
P	Q71ZP6	DE	Probable manganese transport protein mntH;
P	Q722B1	CC	Part of the ABC transporter complex PotABCD involved in spermidine/putrescine import
P	Q722B1	CC	Part of the ABC transporter complex PotABCD involved in spermidine/putrescine import
P	Q722B1	DE	Spermidine/putrescine import ATP-binding protein PotA;
P	Q722B1	DE	Spermidine/putrescine import ATP-binding protein PotA;
P	Q724C0	CC	Part of the ABC transporter complex MetNIQ involved in methionine import
P	Q724C0	DE	Methionine import ATP-binding protein MetN 1;
P	Q725T6	CC	One of the components of the high-affinity ATP-driven potassium transport (or KDP) system, which catalyzes the hydrolysis of ATP coupled with the exchange of hydrogen and potassium ions (By similarity).
P	Q725T6	DE	Potassium-binding and translocating subunit A;
P	Q725T6	DE	Potassium-translocating ATPase A chain;
P	Q725T6	DE	Potassium-transporting ATPase A chain;
P	Q725T6	DR	potassium-transporting ATPase activity
P	Q729H7	DE	Lipoprotein-releasing system ATP-binding protein LolD;
P	Q729H7	DR	lipoprotein transporter activity
P	Q72AQ6	DR	phosphonate transmembrane-transporting ATPase activity
P	Q72D46	CC	Part of the ABC transporter complex PstSACB involved in phosphate import
P	Q72D46	DE	ABC phosphate transporter;
P	Q72D46	DE	Phosphate import ATP-binding protein PstB;
P	Q72D46	DE	Phosphate-transporting ATPase;
P	Q72D46	DR	inorganic phosphate transmembrane transporter activity
P	Q72D46	DR	phosphate transmembrane-transporting ATPase activity
P	Q72FW5	CC	Part of the ABC transporter complex PotABCD involved in spermidine/putrescine import
P	Q72FW5	CC	Part of the ABC transporter complex PotABCD involved in spermidine/putrescine import
P	Q72FW5	DE	Spermidine/putrescine import ATP-binding protein PotA;
P	Q72FW5	DE	Spermidine/putrescine import ATP-binding protein PotA;
P	Q72GX5	CC	Part of the ABC transporter complex PstSACB involved in phosphate import
P	Q72GX5	DE	ABC phosphate transporter;
P	Q72GX5	DE	Phosphate import ATP-binding protein PstB;
P	Q72GX5	DE	Phosphate-transporting ATPase;
P	Q72GX5	DR	inorganic phosphate transmembrane transporter activity
P	Q72GX5	DR	phosphate transmembrane-transporting ATPase activity
P	Q72N56	DR	copper-exporting ATPase activity
P	Q73GK9	CC	Part of the ABC transporter complex ZnuABC involved in zinc import

P	Q73GK9	DE	Zinc import ATP-binding protein ZnuC;
P	Q73GK9	DR	zinc transporting ATPase activity
P	Q73HX8	CC	Part of the ABC transporter complex CcmAB involved in the biogenesis of c-type cytochromes; once thought to export heme, this seems not to be the case, but its exact role is uncertain
P	Q73HX8	DE	Heme exporter protein A;
P	Q73HX8	DR	heme-transporting ATPase activity
P	Q73L25	DE	Lipoprotein-releasing system ATP-binding protein LolD;
P	Q73L25	DR	lipoprotein transporter activity
P	Q73P71	DR	phosphonate transmembrane-transporting ATPase activity
P	Q73XU8	CC	Part of the ABC transporter complex CysAWTP involved in sulfate/thiosulfate import
P	Q73XU8	DE	Sulfate-transporting ATPase;
P	Q73XU8	DE	Sulfate/thiosulfate import ATP-binding protein CysA;
P	Q73XU8	DR	sulfate transmembrane-transporting ATPase activity
P	Q741T7	CC	One of the components of the high-affinity ATP-driven potassium transport (or KDP) system, which catalyzes the hydrolysis of ATP coupled with the exchange of hydrogen and potassium ions (By similarity).
P	Q741T7	DE	Potassium-binding and translocating subunit A;
P	Q741T7	DE	Potassium-translocating ATPase A chain;
P	Q741T7	DE	Potassium-transporting ATPase A chain;
P	Q741T7	DR	potassium-transporting ATPase activity
P	Q743D1	CC	Part of the ABC transporter complex PstSACB involved in phosphate import
P	Q743D1	DE	ABC phosphate transporter;
P	Q743D1	DE	Phosphate import ATP-binding protein PstB;
P	Q743D1	DE	Phosphate-transporting ATPase;
P	Q743D1	DR	inorganic phosphate transmembrane transporter activity
P	Q743D1	DR	phosphate transmembrane-transporting ATPase activity
P	Q747C1	CC	Transport of potassium into the cell (By similarity).
P	Q747C1	DE	Probable potassium transport system protein kup 3;
P	Q747C1	DR	potassium ion transmembrane transporter activity
P	Q74AA5	CC	Transport of potassium into the cell (By similarity).
P	Q74AA5	DE	Probable potassium transport system protein kup 2;
P	Q74AA5	DR	potassium ion transmembrane transporter activity
P	Q74AA8	CC	One of the components of the high-affinity ATP-driven potassium transport (or KDP) system, which catalyzes the hydrolysis of ATP coupled with the exchange of hydrogen and potassium ions
P	Q74AA8	DE	Potassium-binding and translocating subunit C;
P	Q74AA8	DE	Potassium-translocating ATPase C chain;
P	Q74AA8	DE	Potassium-transporting ATPase C chain;
P	Q74AA8	DR	potassium-transporting ATPase activity
P	Q74AB0	CC	One of the components of the high-affinity ATP-driven potassium transport (or KDP) system, which catalyzes the hydrolysis of ATP coupled with the exchange of hydrogen and potassium ions (By similarity).
P	Q74AB0	DE	Potassium-binding and translocating subunit A;
P	Q74AB0	DE	Potassium-translocating ATPase A chain;
P	Q74AB0	DE	Potassium-transporting ATPase A chain;
P	Q74AB0	DR	potassium-transporting ATPase activity
P	Q74AK4	CC	Transport of potassium into the cell (By similarity).
P	Q74AK4	DE	Probable potassium transport system protein kup 1;
P	Q74AK4	DR	potassium ion transmembrane transporter activity
P	Q74AP9	CC	Na+/H+ antiporter that extrudes sodium in exchange for external protons (By similarity)
P	Q74AP9	DE	Na+/H+ antiporter nhaA;
P	Q74AP9	DE	Sodium/proton antiporter nhaA;
P	Q74E68	CC	Part of the ABC transporter complex PstSACB involved in phosphate import
P	Q74E68	DE	ABC phosphate transporter;
P	Q74E68	DE	Phosphate import ATP-binding protein PstB;
P	Q74E68	DE	Phosphate-transporting ATPase;
P	Q74E68	DR	inorganic phosphate transmembrane transporter activity
P	Q74E68	DR	phosphate transmembrane-transporting ATPase activity
P	Q74IV9	CC	Part of the ABC transporter complex MetNIQ involved in methionine import
P	Q74IV9	DE	Methionine import ATP-binding protein MetN;
P	Q74K65	CC	Part of the ABC transporter complex PotABCD involved in spermidine/putrescine import
P	Q74K65	CC	Part of the ABC transporter complex PotABCD involved in spermidine/putrescine import
P	Q74K65	DE	Spermidine/putrescine import ATP-binding protein PotA;
P	Q74K65	DE	Spermidine/putrescine import ATP-binding protein PotA;

P	Q74KF8	CC	Part of the ABC transporter complex PstSACB involved in phosphate import
P	Q74KF8	DE	ABC phosphate transporter 2;
P	Q74KF8	DE	Phosphate import ATP-binding protein PstB 2;
P	Q74KF8	DE	Phosphate-transporting ATPase 2;
P	Q74KF8	DR	inorganic phosphate transmembrane transporter activity
P	Q74KF8	DR	phosphate transmembrane-transporting ATPase activity
P	Q74KF9	CC	Part of the ABC transporter complex PstSACB involved in phosphate import
P	Q74KF9	DE	ABC phosphate transporter 1;
P	Q74KF9	DE	Phosphate import ATP-binding protein PstB 1;
P	Q74KF9	DE	Phosphate-transporting ATPase 1;
P	Q74KF9	DR	inorganic phosphate transmembrane transporter activity
P	Q74KF9	DR	phosphate transmembrane-transporting ATPase activity
P	Q74LN2	CC	Transport of potassium into the cell (By similarity).
P	Q74LN2	DE	Probable potassium transport system protein kup 2;
P	Q74LN2	DR	potassium ion transmembrane transporter activity
P	Q74LN3	CC	Transport of potassium into the cell (By similarity).
P	Q74LN3	DE	Probable potassium transport system protein kup 1;
P	Q74LN3	DR	potassium ion transmembrane transporter activity
P	Q74LQ3	DR	phosphonate transmembrane-transporting ATPase activity
P	Q74R28	DR	glycerol-3-phosphate-transporting ATPase activity
P	Q74RF8	DE	Maltose transport system permease protein malG;
P	Q74RF9	DE	Maltose transport system permease protein malF;
P	Q74U12	CC	Na+/H+ antiporter that extrudes sodium in exchange for external protons (By similarity).
P	Q74U12	DE	Na+/H+ antiporter nhaB;
P	Q74U12	DE	Sodium/proton antiporter nhaB;
P	Q74U12	DR	sodium:hydrogen antiporter activity
P	Q75G84	CC	High-affinity potassium transporter (By similarity).
P	Q75G84	DE	Potassium transporter 21;
P	Q75G84	DR	potassium ion transmembrane transporter activity
P	Q75JF3	CC	Chloride channels may have several functions including the regulation of cell volume, membrane potential stabilization and signal transduction (By similarity).
P	Q75JF3	CC	Voltage-gated chloride channel
P	Q75JF3	DE	Chloride channel protein C;
P	Q75JF3	DR	voltage-gated chloride channel activity
P	Q75N73	DE	Zinc transporter ZIP14;
P	Q75N73	DR	zinc ion transmembrane transporter activity
P	Q769E5	CC	Translocates Ca2+ and other metal ions into vacuoles using the proton gradient formed by H+-ATPase and H+-pyrophosphatase.
P	Q769E5	DE	Ca2+/H+ exchanger 1a;
P	Q78IQ7	CC	Plays an important role in cellular zinc homeostasis as a zinc transporter
P	Q78IQ7	CC	Regulated in response to zinc availability.
P	Q78IQ7	DE	Zinc transporter ZIP4;
P	Q78IQ7	DR	zinc ion transmembrane transporter activity
P	Q795M8	DE	Putative potassium channel protein yugO;
P	Q795M8	DR	voltage-gated potassium channel activity
P	Q79VE0	DE	Choline-glycine betaine transporter and EctP protein;
P	Q79VE0	DE	Ectoine/proline/glycine betaine carrier EctP;
P	Q79VE0	DE	Ectoine/proline/glycine betaine carrier EctP;
P	Q7A3E6	CC	Involved in copper export (By similarity).
P	Q7A3E6	DE	Copper-exporting P-type ATPase A;
P	Q7A3E6	DR	copper-exporting ATPase activity
P	Q7A3G4	DR	glucose transmembrane transporter activity
P	Q7A3U8	CC	Also possibly involved in excretion of nitrite produced by the dissimilatory reduction of nitrate (By similarity).
P	Q7A3U8	CC	Probably required for nitrate uptake under anoxic conditions
P	Q7A3U8	DE	Probable nitrate transporter narT;
P	Q7A4B3	CC	This system is involved in mannitol transport.
P	Q7A4B3	DE	Mannitol permease IIC component;
P	Q7A4B3	DE	Mannitol-specific phosphotransferase enzyme IIB component;
P	Q7A4B3	DE	PTS system mannitol-specific EIIB component;
P	Q7A4B3	DE	PTS system mannitol-specific EIIC component;
P	Q7A4B3	DE	PTS system mannitol-specific EIICB component;
P	Q7A4G4	CC	One of the components of the high-affinity ATP-driven potassium transport (or KDP) system, which catalyzes the hydrolysis of ATP coupled with the exchange of hydrogen and potassium ions (By similarity).

P	Q7A4G4	DE	Potassium-binding and translocating subunit A 1;
P	Q7A4G4	DE	Potassium-translocating ATPase A chain 1;
P	Q7A4G4	DE	Potassium-transporting ATPase A chain 1;
P	Q7A4G4	DR	potassium-transporting ATPase activity
P	Q7A4P8	CC	Mediates the transport of the dicarboxylates fumarate, malate, and succinate across the cytoplasmic membrane via a Na+- electrochemical gradient (By similarity)
P	Q7A4P8	DE	Na+/dicarboxylate symporter;
P	Q7A4P8	DE	Na+/dicarboxylate symporter;
P	Q7A4P8	DE	Sodium-dependent dicarboxylate transporter sdcS;
P	Q7A4P8	DE	Sodium-dependent dicarboxylate transporter sdcS;
P	Q7A4Q7	CC	Catalyzes the sodium-dependent uptake of extracellular L-proline (By similarity)
P	Q7A4Q7	CC	Since most S.aureus strains are L- proline auxotrophs, this transporter may aid the bacterial persistence during an infection of tissues with low proline concentrations (By similarity).
P	Q7A4Q7	DE	Proline permease;
P	Q7A4Q7	DE	Sodium/proline symporter;
P	Q7A4Q7	DE	Sodium/proline symporter;
P	Q7A4Q7	DR	proline:sodium symporter activity
P	Q7A4Q7	DR	proline:sodium symporter activity
P	Q7A5Q2	CC	Part of the ABC transporter complex pstSACB involved in phosphate import (By similarity).
P	Q7A5Q2	DE	Phosphate-binding protein pstS;
P	Q7A5Q6	CC	Part of a transport system that may be involved in oligopeptide uptake
P	Q7A5Q6	DE	Putative oligopeptide transport system permease protein oppB2;
P	Q7A5Q7	CC	Part of a transport system that may be involved in oligopeptide uptake
P	Q7A5Q7	DE	Putative oligopeptide transport system permease protein oppC2;
P	Q7A5Q8	CC	Part of a transport system that may be involved in oligopeptide uptake
P	Q7A5Q8	DE	Putative oligopeptide transport ATP-binding protein oppD2;
P	Q7A5Q9	CC	Part of a transport system that may be involved in oligopeptide uptake
P	Q7A5Q9	DE	Putative oligopeptide transport ATP-binding protein oppF2;
P	Q7A679	CC	Part of the ABC transporter complex PotABCD involved in spermidine/putrescine import
P	Q7A679	CC	Part of the ABC transporter complex PotABCD involved in spermidine/putrescine import
P	Q7A679	DE	Spermidine/putrescine import ATP-binding protein PotA;
P	Q7A679	DE	Spermidine/putrescine import ATP-binding protein PotA;
P	Q7A6M2	CC	Part of the ABC transporter complex MetNIQ involved in methionine import
P	Q7A6M2	DE	Methionine import ATP-binding protein MetN 2;
P	Q7A771	CC	May be a proton symporter invoved in the uptake of osmolytes such as proline and glycine betaine (By similarity).
P	Q7A771	DE	Putative proline/betaine transporter;
P	Q7A771	DE	Putative proline/betaine transporter;
P	Q7A7E3	CC	Part of the ABC transporter complex MetNIQ involved in methionine import
P	Q7A7E3	DE	Methionine import ATP-binding protein MetN 1;
P	Q7A807	CC	This system is involved in glucose transport (By similarity).
P	Q7A807	DE	Glucose permease IIC component;
P	Q7A807	DE	Glucose-specific phosphotransferase enzyme IIA component;
P	Q7A807	DE	Glucose-specific phosphotransferase enzyme IIB component;
P	Q7A807	DE	PTS system glucose-specific EIIA component;
P	Q7A807	DE	PTS system glucose-specific EIIB component;
P	Q7A807	DE	PTS system glucose-specific EIIC component;
P	Q7A807	DE	PTS system glucose-specific EIICBA component;
P	Q7A807	DR	glucose transmembrane transporter activity
P	Q7A848	DR	phosphonate transmembrane-transporting ATPase activity
P	Q7A937	DE	Maltose transport system permease protein malF;
P	Q7C1M3	CC	Part of the ABC transporter complex BtuCDF involved in vitamin B12 import
P	Q7C1M3	DE	Vitamin B12 import ATP-binding protein BtuD;
P	Q7C1M3	DE	Vitamin B12-transporting ATPase;
P	Q7C1M5	CC	Part of the ABC transporter complex BtuCDF involved in vitamin B12 import
P	Q7C1M5	DE	Vitamin B12 import system permease protein BtuC;
P	Q7CCJ4	CC	This system is involved in glucose transport (By similarity).
P	Q7CCJ4	DE	Glucose permease IIC component;
P	Q7CCJ4	DE	Glucose-specific phosphotransferase enzyme IIA component;
P	Q7CCJ4	DE	Glucose-specific phosphotransferase enzyme IIB component;
P	Q7CCJ4	DE	PTS system glucose-specific EIIA component;
P	Q7CCJ4	DE	PTS system glucose-specific EIIB component;
P	Q7CCJ4	DE	PTS system glucose-specific EIIC component;

P	Q7CCJ4	DE	PTS system glucose-specific EIICBA component;
P	Q7CCJ4	DR	glucose transmembrane transporter activity
P	Q7CFR2	CC	Part of the ABC transporter complex XylFGH involved in xylose import
P	Q7CFR2	DE	Xylose import ATP-binding protein XylG;
P	Q7CFR2	DR	D-xylose-importing ATPase activity
P	Q7CG00	CC	Part of the ABC transporter complex RbsABCD involved in ribose import
P	Q7CG00	DE	Ribose import ATP-binding protein RbsA;
P	Q7CG77	CC	Na+/H+ antiporter that extrudes sodium in exchange for external protons (By similarity)
P	Q7CG77	DE	Na+/H+ antiporter nhaA;
P	Q7CG77	DE	Sodium/proton antiporter nhaA;
P	Q7CHF8	CC	Part of the ABC transporter complex MetNIQ involved in methionine import
P	Q7CHF8	DE	Methionine import ATP-binding protein MetN 2;
P	Q7CHQ3	CC	Part of the ABC transporter complex MglABC involved in galactose/methyl galactoside import
P	Q7CHQ3	DE	Galactose/methyl galactoside import ATP-binding protein MglA;
P	Q7CQ74	DE	Putative ABC-type dipeptide/oligopeptide/nickel transport systems, permease component;
P	Q7DM58	DE	ATP-energized glutathione S-conjugate pump 4;
P	Q7DM58	DE	Glutathione S-conjugate-transporting ATPase 4;
P	Q7EYH7	CC	Aquaporins facilitate the transport of water and small neutral solutes across cell membranes (By similarity).
P	Q7GB25	CC	Pump for glutathione S-conjugates
P	Q7GB25	DE	ATP-energized glutathione S-conjugate pump 5;
P	Q7GB25	DE	Glutathione S-conjugate-transporting ATPase 5;
P	Q7M816	CC	Part of the ABC transporter complex MetNIQ involved in methionine import
P	Q7M816	DE	Methionine import ATP-binding protein MetN;
P	Q7M8M4	DR	phosphonate transmembrane-transporting ATPase activity
P	Q7M922	CC	Na+/H+ antiporter that extrudes sodium in exchange for external protons (By similarity)
P	Q7M922	DE	Na+/H+ antiporter nhaA;
P	Q7M922	DE	Sodium/proton antiporter nhaA;
P	Q7M9C0	CC	Mediates influx of magnesium ions (By similarity).
P	Q7M9C0	DE	Magnesium transport protein CorA;
P	Q7M9C0	DR	cobalt ion transmembrane transporter activity
P	Q7M9C0	DR	magnesium ion transmembrane transporter activity
P	Q7M9G3	CC	Part of the ABC transporter complex PstSACB involved in phosphate import
P	Q7M9G3	DE	ABC phosphate transporter;
P	Q7M9G3	DE	Phosphate import ATP-binding protein PstB;
P	Q7M9G3	DE	Phosphate-transporting ATPase;
P	Q7M9G3	DR	inorganic phosphate transmembrane transporter activity
P	Q7M9G3	DR	phosphate transmembrane-transporting ATPase activity
P	Q7MDF0	CC	Functions as antiport system and exchanges two chloride ions for 1 proton
P	Q7MDF0	CC	Proton-coupled chloride transporter
P	Q7MDF0	DR	voltage-gated chloride channel activity
P	Q7ME63	CC	Part of the ABC transporter complex ModABC involved in molybdenum import
P	Q7ME63	DE	Molybdenum import ATP-binding protein ModC;
P	Q7ME63	DR	molybdenum ion transmembrane transporter activity
P	Q7MEV1	CC	Part of the ABC transporter complex RbsABCD involved in ribose import
P	Q7MEV1	DE	Ribose import ATP-binding protein RbsA;
P	Q7MFA1	CC	Part of the ABC transporter complex HmuTUV involved in hemin import
P	Q7MFA1	DE	Hemin import ATP-binding protein HmuV;
P	Q7MFC1	DE	Maltose transport system permease protein malG;
P	Q7MFC2	DE	Maltose transport system permease protein malF;
P	Q7MFC4	CC	Part of the ABC transporter complex MalEFGK involved in maltose/maltodextrin import
P	Q7MFC4	CC	Part of the ABC transporter complex MalEFGK involved in maltose/maltodextrin import
P	Q7MFC4	DE	Maltose/maltodextrin import ATP-binding protein MalK;
P	Q7MFC4	DE	Maltose/maltodextrin import ATP-binding protein MalK;
P	Q7MFC4	DR	maltose-transporting ATPase activity
P	Q7MFE8	CC	Part of the ABC transporter complex PstSACB involved in phosphate import
P	Q7MFE8	DE	ABC phosphate transporter 2;
P	Q7MFE8	DE	Phosphate import ATP-binding protein PstB 2;
P	Q7MFE8	DE	Phosphate-transporting ATPase 2;
P	Q7MFE8	DR	inorganic phosphate transmembrane transporter activity
P	Q7MFE8	DR	phosphate transmembrane-transporting ATPase activity
P	Q7MGA3	CC	Involved in the import of serine and threonine into the cell, with the concomitant import of sodium (symport system) (By similarity).
P	Q7MGA3	DE	Na+/serine-threonine symporter;

P	Q7MGA3	DR	sodium:dicarboxylate symporter activity
P	Q7MGA3	DR	sodium:dicarboxylate symporter activity
P	Q7MIR0	CC	Part of the ABC transporter complex CcmAB involved in the biogenesis of c-type cytochromes; once thought to export heme, this seems not to be the case, but its exact role is uncertain
P	Q7MIR0	DE	Heme exporter protein A;
P	Q7MIR0	DR	heme-transporting ATPase activity
P	Q7MIV9	CC	Channel that permits osmotically driven movement of water in both directions
P	Q7MIV9	CC	It mediates rapid entry or exit of water in response to abrupt changes in osmolarity (By similarity).
P	Q7MJ01	DE	Lipoprotein-releasing system ATP-binding protein LolD;
P	Q7MJ01	DR	lipoprotein transporter activity
P	Q7MKU3	CC	Part of the ABC transporter complex PotABCD involved in spermidine/putrescine import
P	Q7MKU3	CC	Part of the ABC transporter complex PotABCD involved in spermidine/putrescine import
P	Q7MKU3	DE	Spermidine/putrescine import ATP-binding protein PotA;
P	Q7MKU3	DE	Spermidine/putrescine import ATP-binding protein PotA;
P	Q7MLB8	DR	glycerol-3-phosphate-transporting ATPase activity
P	Q7MLE6	CC	Part of the ABC transporter complex BtuCDF involved in vitamin B12 import
P	Q7MLE6	DE	Vitamin B12 import ATP-binding protein BtuD;
P	Q7MLE6	DE	Vitamin B12-transporting ATPase;
P	Q7MLE7	CC	Part of the ABC transporter complex BtuCDF involved in vitamin B12 import
P	Q7MLE7	DE	Vitamin B12 import system permease protein BtuC;
P	Q7MLJ3	CC	Na+/H+ antiporter that extrudes sodium in exchange for external protons (By similarity).
P	Q7MLJ3	DE	Na+/H+ antiporter nhaA;
P	Q7MLJ3	DE	Sodium/proton antiporter nhaA;
P	Q7MMN0	CC	Part of the ABC transporter complex ZnuABC involved in zinc import
P	Q7MMN0	DE	Zinc import ATP-binding protein ZnuC;
P	Q7MMN0	DR	zinc transporting ATPase activity
P	Q7MN25	CC	Part of the ABC transporter complex MetNIQ involved in methionine import
P	Q7MN25	DE	Methionine import ATP-binding protein MetN;
P	Q7MN25	DR	D-methionine transmembrane transporter activity
P	Q7MNI7	CC	Part of the ABC transporter complex PstSACB involved in phosphate import
P	Q7MNI7	DE	ABC phosphate transporter 1;
P	Q7MNI7	DE	Phosphate import ATP-binding protein PstB 1;
P	Q7MNI7	DE	Phosphate-transporting ATPase 1;
P	Q7MNI7	DR	inorganic phosphate transmembrane transporter activity
P	Q7MNI7	DR	phosphate transmembrane-transporting ATPase activity
P	Q7MNT2	CC	Binds vitamin B12 and delivers it to the periplasmic surface of BtuC (By similarity).
P	Q7MNT2	CC	Part of the ABC transporter complex BtuCDF involved in vitamin B12 import
P	Q7MPC5	CC	Part of the ABC transporter complex ThiBPQ involved in thiamine import
P	Q7MPC5	DE	Thiamine import ATP-binding protein ThiQ;
P	Q7MYP0	CC	Mediates influx of magnesium ions (By similarity).
P	Q7MYP0	DE	Magnesium transport protein CorA;
P	Q7MYP0	DR	cobalt ion transmembrane transporter activity
P	Q7MYP0	DR	magnesium ion transmembrane transporter activity
P	Q7N0A2	CC	Involved in the import of serine and threonine into the cell, with the concomitant import of sodium (symport system) (By similarity).
P	Q7N0A2	DE	Na+/serine-threonine symporter;
P	Q7N0A2	DR	sodium:dicarboxylate symporter activity
P	Q7N0A2	DR	sodium:dicarboxylate symporter activity
P	Q7N183	CC	Important to control the intracellular level of arginine and the correct balance between arginine and lysine (By similarity).
P	Q7N3A6	DE	Lipoprotein-releasing system ATP-binding protein LolD;
P	Q7N3A6	DR	lipoprotein transporter activity
P	Q7N3Q3	CC	Part of the ABC transporter complex BtuCDF involved in vitamin B12 import
P	Q7N3Q3	DE	Vitamin B12 import system permease protein BtuC;
P	Q7N3Q4	CC	Part of the ABC transporter complex BtuCDF involved in vitamin B12 import
P	Q7N3Q4	DE	Vitamin B12 import ATP-binding protein BtuD;
P	Q7N3Q4	DE	Vitamin B12-transporting ATPase;
P	Q7N3S7	CC	Part of the ABC transporter complex HmuTUV involved in hemin import
P	Q7N3S7	DE	Hemin import ATP-binding protein HmuV;
P	Q7N3Z4	CC	Na+/H+ antiporter that extrudes sodium in exchange for external protons (By similarity).
P	Q7N3Z4	DE	Na+/H+ antiporter nhaB;
P	Q7N3Z4	DE	Sodium/proton antiporter nhaB;
P	Q7N3Z4	DR	sodium:hydrogen antiporter activity

P	Q7N545	CC	Part of the ABC transporter complex ZnuABC involved in zinc import
P	Q7N545	DE	Zinc import ATP-binding protein ZnuC;
P	Q7N545	DR	zinc transporting ATPase activity
P	Q7N5C1	CC	Channel that permits osmotically driven movement of water in both directions
P	Q7N5C1	CC	It mediates rapid entry or exit of water in response to abrupt changes in osmolarity (By similarity).
P	Q7N6R3	CC	Part of the ABC transporter complex ModABC involved in molybdenum import
P	Q7N6R3	DE	Molybdenum import ATP-binding protein ModC;
P	Q7N6R3	DR	molybdenum ion transmembrane transporter activity
P	Q7N6W5	CC	One of the components of the high-affinity ATP-driven potassium transport (or KDP) system, which catalyzes the hydrolysis of ATP coupled with the exchange of hydrogen and potassium ions (By similarity).
P	Q7N6W5	DE	Potassium-binding and translocating subunit A;
P	Q7N6W5	DE	Potassium-translocating ATPase A chain;
P	Q7N6W5	DE	Potassium-transporting ATPase A chain;
P	Q7N6W5	DR	potassium-transporting ATPase activity
P	Q7N6W6	CC	One of the components of the high-affinity ATP-driven potassium transport (or KDP) system, which catalyzes the hydrolysis of ATP coupled with the exchange of hydrogen and potassium ions (By similarity).
P	Q7N6W6	DE	Potassium-binding and translocating subunit B;
P	Q7N6W6	DE	Potassium-translocating ATPase B chain;
P	Q7N6W6	DE	Potassium-transporting ATPase B chain;
P	Q7N6W6	DR	potassium-transporting ATPase activity
P	Q7N6W7	CC	One of the components of the high-affinity ATP-driven potassium transport (or KDP) system, which catalyzes the hydrolysis of ATP coupled with the exchange of hydrogen and potassium ions
P	Q7N6W7	DE	Potassium-binding and translocating subunit C;
P	Q7N6W7	DE	Potassium-translocating ATPase C chain;
P	Q7N6W7	DE	Potassium-transporting ATPase C chain;
P	Q7N6W7	DR	potassium-transporting ATPase activity
P	Q7N6Z2	CC	Part of the ABC transporter complex CysAWTP involved in sulfate/thiosulfate import
P	Q7N6Z2	CC	Part of the ABC transporter complex CysAWTP involved in sulfate/thiosulfate import
P	Q7N6Z2	DE	Sulfate-transporting ATPase;
P	Q7N6Z2	DE	Sulfate/thiosulfate import ATP-binding protein CysA;
P	Q7N6Z2	DE	Sulfate/thiosulfate import ATP-binding protein CysA;
P	Q7N6Z2	DR	sulfate transmembrane-transporting ATPase activity
P	Q7N842	CC	Binds vitamin B12 and delivers it to the periplasmic surface of BtuC (By similarity)
P	Q7N842	CC	Part of the ABC transporter complex BtuCDF involved in vitamin B12 import
P	Q7N8M2	CC	Part of the ABC transporter complex MetNIQ involved in methionine import
P	Q7N8M2	DE	Methionine import ATP-binding protein MetN;
P	Q7N8M2	DR	D-methionine transmembrane transporter activity
P	Q7N8V0	CC	Part of the ABC transporter complex ThiBPQ involved in thiamine import
P	Q7N8V0	DE	Thiamine import ATP-binding protein ThiQ;
P	Q7N8X6	CC	Na+/H+ antiporter that extrudes sodium in exchange for external protons (By similarity)
P	Q7N8X6	DE	Na+/H+ antiporter nhaA;
P	Q7N8X6	DE	Sodium/proton antiporter nhaA;
P	Q7N983	DE	Maltose transport system permease protein malG;
P	Q7N984	DE	Maltose transport system permease protein malF;
P	Q7N986	CC	Part of the ABC transporter complex MalEFGK involved in maltose/maltodextrin import
P	Q7N986	CC	Part of the ABC transporter complex MalEFGK involved in maltose/maltodextrin import
P	Q7N986	DE	Maltose/maltodextrin import ATP-binding protein MalK;
P	Q7N986	DE	Maltose/maltodextrin import ATP-binding protein MalK;
P	Q7N986	DR	maltose-transporting ATPase activity
P	Q7N9U4	CC	Part of the ABC transporter complex PstSACB involved in phosphate import
P	Q7N9U4	DE	ABC phosphate transporter;
P	Q7N9U4	DE	Phosphate import ATP-binding protein PstB;
P	Q7N9U4	DE	Phosphate-transporting ATPase;
P	Q7N9U4	DR	inorganic phosphate transmembrane transporter activity
P	Q7N9U4	DR	phosphate transmembrane-transporting ATPase activity
P	Q7NA79	CC	Part of the ABC transporter complex RbsABCD involved in ribose import
P	Q7NA79	DE	Ribose import ATP-binding protein RbsA;
P	Q7NB11	CC	Part of the ABC transporter complex PotABCD involved in spermidine/putrescine import
P	Q7NB11	CC	Part of the ABC transporter complex PotABCD involved in spermidine/putrescine import
P	Q7NB11	DE	Spermidine/putrescine import ATP-binding protein PotA;
P	Q7NB11	DE	Spermidine/putrescine import ATP-binding protein PotA;

P	Q7NC40	CC	Part of the ABC transporter complex PstSACB involved in phosphate import
P	Q7NC40	DE	ABC phosphate transporter;
P	Q7NC40	DE	Phosphate import ATP-binding protein PstB;
P	Q7NC40	DE	Phosphate-transporting ATPase;
P	Q7NC40	DR	inorganic phosphate transmembrane transporter activity
P	Q7NC40	DR	phosphate transmembrane-transporting ATPase activity
P	Q7NIW1	CC	Part of the ABC transporter complex CysAWTP involved in sulfate/thiosulfate import
P	Q7NIW1	DE	Sulfate-transporting ATPase;
P	Q7NIW1	DE	Sulfate/thiosulfate import ATP-binding protein CysA;
P	Q7NIW1	DR	sulfate transmembrane-transporting ATPase activity
P	Q7NN36	CC	Part of the ABC transporter complex HmuTUV involved in hemin import
P	Q7NN36	DE	Hemin import ATP-binding protein HmuV;
P	Q7NNG3	CC	Part of the ABC transporter complex PstSACB involved in phosphate import
P	Q7NNG3	DE	ABC phosphate transporter 2;
P	Q7NNG3	DE	Phosphate import ATP-binding protein PstB 2;
P	Q7NNG3	DE	Phosphate-transporting ATPase 2;
P	Q7NNG3	DR	inorganic phosphate transmembrane transporter activity
P	Q7NNG3	DR	phosphate transmembrane-transporting ATPase activity
P	Q7NNP3	CC	Channel that permits osmotically driven movement of water in both directions
P	Q7NNP3	CC	It mediates rapid entry or exit of water in response to abrupt changes in osmolarity (By similarity).
P	Q7NPP4	CC	Part of the ABC transporter complex PstSACB involved in phosphate import
P	Q7NPP4	DE	ABC phosphate transporter 1;
P	Q7NPP4	DE	Phosphate import ATP-binding protein PstB 1;
P	Q7NPP4	DE	Phosphate-transporting ATPase 1;
P	Q7NPP4	DR	inorganic phosphate transmembrane transporter activity
P	Q7NPP4	DR	phosphate transmembrane-transporting ATPase activity
P	Q7RTP0	CC	Can also transport other divalent cations such as Fe(2+), Sr(2+), Ba(2+), Mn(2+) and Co(2+) but to a much less extent than Mg2+ (By similarity).
P	Q7RTP0	DE	Magnesium transporter NIPA1;
P	Q7RTX9	CC	Proton-linked monocarboxylate transporter
P	Q7RTX9	DE	Monocarboxylate transporter 14;
P	Q7RTY0	CC	Proton-linked monocarboxylate transporter
P	Q7RTY0	DE	Monocarboxylate transporter 13;
P	Q7RTY1	CC	Proton-linked monocarboxylate transporter
P	Q7RTY1	DE	Monocarboxylate transporter 9;
P	Q7SFQ9	CC	Mitochondrial inner membrane magnesium transporter required for mitochondrial magnesium homeostasis
P	Q7SFQ9	DE	Mitochondrial inner membrane magnesium transporter mrs2;
P	Q7SFQ9	DR	magnesium ion transmembrane transporter activity
P	Q7TM99	CC	Proton-linked monocarboxylate transporter
P	Q7TM99	DE	Monocarboxylate transporter 9;
P	Q7TNS7	CC	Probable cation channel with high affinity for sodium.
P	Q7TNS7	DR	sodium channel activity
P	Q7TSH7	CC	Putative voltage-gated potassium channel (By similarity).
P	Q7TSH7	DE	Potassium voltage-gated channel subfamily F member 1;
P	Q7TSH7	DE	Voltage-gated potassium channel subunit Kv5.1;
P	Q7TSH7	DR	voltage-gated potassium channel activity
P	Q7U0Z9	CC	Part of the ABC transporter complex PstSACB involved in phosphate import
P	Q7U0Z9	DE	ABC phosphate transporter 2;
P	Q7U0Z9	DE	Phosphate import ATP-binding protein PstB 2;
P	Q7U0Z9	DE	Phosphate-transporting ATPase 2;
P	Q7U0Z9	DR	inorganic phosphate transmembrane transporter activity
P	Q7U0Z9	DR	phosphate transmembrane-transporting ATPase activity
P	Q7U172	CC	Part of the ABC transporter complex PstSACB involved in phosphate import
P	Q7U172	DE	ABC phosphate transporter 1;
P	Q7U172	DE	Phosphate import ATP-binding protein PstB 1;
P	Q7U172	DE	Phosphate-transporting ATPase 1;
P	Q7U172	DR	inorganic phosphate transmembrane transporter activity
P	Q7U172	DR	phosphate transmembrane-transporting ATPase activity
P	Q7U6R4	CC	Part of the ABC transporter complex PstSACB involved in phosphate import
P	Q7U6R4	DE	ABC phosphate transporter;
P	Q7U6R4	DE	Phosphate import ATP-binding protein PstB;
P	Q7U6R4	DE	Phosphate-transporting ATPase;
P	Q7U6R4	DR	inorganic phosphate transmembrane transporter activity

P	Q7U6R4	DR	phosphate transmembrane-transporting ATPase activity
P	Q7UBD0	CC	Part of the ABC transporter complex MalEFGK involved in maltose/maltodextrin import
P	Q7UBD0	CC	Part of the ABC transporter complex MalEFGK involved in maltose/maltodextrin import
P	Q7UBD0	DE	Maltose/maltodextrin import ATP-binding protein MalK;
P	Q7UBD0	DE	Maltose/maltodextrin import ATP-binding protein MalK;
P	Q7UBD0	DR	maltose-transporting ATPase activity
P	Q7UBP8	CC	Important to control the intracellular level of arginine and the correct balance between arginine and lysine (By similarity).
P	Q7UCG7	CC	The physiological role may be the reduction of the intracellular concentration of toxic sugars or sugar metabolites (By similarity).
P	Q7UCG7	DE	Probable sugar efflux transporter;
P	Q7UP21	CC	Part of the ABC transporter complex PstSACB involved in phosphate import
P	Q7UP21	DE	ABC phosphate transporter;
P	Q7UP21	DE	Phosphate import ATP-binding protein PstB;
P	Q7UP21	DE	Phosphate-transporting ATPase;
P	Q7UP21	DR	inorganic phosphate transmembrane transporter activity
P	Q7UP21	DR	phosphate transmembrane-transporting ATPase activity
P	Q7UPK3	DE	Lipoprotein-releasing system ATP-binding protein LolD 2;
P	Q7UPK3	DR	lipoprotein transporter activity
P	Q7UTY3	CC	Na+/H+ antiporter that extrudes sodium in exchange for external protons (By similarity).
P	Q7UTY3	DE	Na+/H+ antiporter nhaA;
P	Q7UTY3	DE	Sodium/proton antiporter nhaA;
P	Q7UU57	CC	Part of the ABC transporter complex RbsABCD involved in ribose import
P	Q7UU57	DE	Ribose import ATP-binding protein RbsA;
P	Q7UX73	DE	Lipoprotein-releasing system ATP-binding protein LolD 1;
P	Q7UX73	DR	lipoprotein transporter activity
P	Q7V1X3	CC	Part of the ABC transporter complex PstSACB involved in phosphate import
P	Q7V1X3	DE	ABC phosphate transporter;
P	Q7V1X3	DE	Phosphate import ATP-binding protein PstB;
P	Q7V1X3	DE	Phosphate-transporting ATPase;
P	Q7V1X3	DR	inorganic phosphate transmembrane transporter activity
P	Q7V1X3	DR	phosphate transmembrane-transporting ATPase activity
P	Q7V7P0	CC	Part of the ABC transporter complex PstSACB involved in phosphate import
P	Q7V7P0	DE	ABC phosphate transporter;
P	Q7V7P0	DE	Phosphate import ATP-binding protein PstB;
P	Q7V7P0	DE	Phosphate-transporting ATPase;
P	Q7V7P0	DR	inorganic phosphate transmembrane transporter activity
P	Q7V7P0	DR	phosphate transmembrane-transporting ATPase activity
P	Q7VCZ3	CC	Part of the ABC transporter complex PstSACB involved in phosphate import
P	Q7VCZ3	DE	ABC phosphate transporter;
P	Q7VCZ3	DE	Phosphate import ATP-binding protein PstB;
P	Q7VCZ3	DE	Phosphate-transporting ATPase;
P	Q7VCZ3	DR	inorganic phosphate transmembrane transporter activity
P	Q7VCZ3	DR	phosphate transmembrane-transporting ATPase activity
P	Q7VEQ4	DE	L-asparagine permease 1;
P	Q7VEQ4	DE	L-asparagine transport protein 1;
P	Q7VFJ7	CC	Mediates influx of magnesium ions (By similarity).
P	Q7VFJ7	DE	Magnesium transport protein CorA;
P	Q7VFJ7	DR	cobalt ion transmembrane transporter activity
P	Q7VFJ7	DR	magnesium ion transmembrane transporter activity
P	Q7VFN0	CC	Na+/H+ antiporter that extrudes sodium in exchange for external protons (By similarity).
P	Q7VFN0	DE	Na+/H+ antiporter nhaA 2;
P	Q7VFN0	DE	Sodium/proton antiporter nhaA 2;
P	Q7VFN1	CC	Na+/H+ antiporter that extrudes sodium in exchange for external protons (By similarity).
P	Q7VFN1	DE	Na+/H+ antiporter nhaA 1;
P	Q7VFN1	DE	Sodium/proton antiporter nhaA 1;
P	Q7VI92	CC	Part of the ABC transporter complex MetNIQ involved in methionine import
P	Q7VI92	DE	Methionine import ATP-binding protein MetN;
P	Q7VKP7	CC	Part of the ABC transporter complex ModABC involved in molybdenum import
P	Q7VKP7	DE	Molybdenum import ATP-binding protein ModC;
P	Q7VKP7	DR	molybdenum ion transmembrane transporter activity
P	Q7VKY3	CC	Na+/H+ antiporter that extrudes sodium in exchange for external protons (By similarity).
P	Q7VKY3	DE	Na+/H+ antiporter nhaB;
P	Q7VKY3	DE	Sodium/proton antiporter nhaB;
P	Q7VKY3	DR	sodium:hydrogen antiporter activity

P	Q7VLS9	CC	Part of the ABC transporter complex ZnuABC involved in zinc import
P	Q7VLS9	DE	Zinc import ATP-binding protein ZnuC;
P	Q7VLS9	DR	zinc transporting ATPase activity
P	Q7VLY3	CC	Involved in the import of serine and threonine into the cell, with the concomitant import of sodium (symport system) (By similarity).
P	Q7VLY3	DE	Na+/serine-threonine symporter;
P	Q7VLY3	DR	sodium:dicarboxylate symporter activity
P	Q7VLY3	DR	sodium:dicarboxylate symporter activity
P	Q7VM95	CC	Part of the ABC transporter complex MetNIQ involved in methionine import
P	Q7VM95	DE	Methionine import ATP-binding protein MetN;
P	Q7VM95	DR	D-methionine transmembrane transporter activity
P	Q7VMV4	DE	Lipoprotein-releasing system ATP-binding protein LolD;
P	Q7VMV4	DR	lipoprotein transporter activity
P	Q7VN12	CC	Part of the ABC transporter complex CcmAB involved in the biogenesis of c-type cytochromes; once thought to export heme, this seems not to be the case, but its exact role is uncertain
P	Q7VN12	DE	Heme exporter protein A;
P	Q7VN12	DR	heme-transporting ATPase activity
P	Q7VN39	CC	Na+/H+ antiporter that extrudes sodium in exchange for external protons (By similarity)
P	Q7VN39	DE	Na+/H+ antiporter nhaA;
P	Q7VN39	DE	Sodium/proton antiporter nhaA;
P	Q7VN59	CC	Mediates influx of magnesium ions (By similarity).
P	Q7VN59	DE	Magnesium transport protein CorA;
P	Q7VN59	DR	cobalt ion transmembrane transporter activity
P	Q7VN59	DR	magnesium ion transmembrane transporter activity
P	Q7VNG4	CC	Part of the ABC transporter complex PotABCD involved in spermidine/putrescine import
P	Q7VNG4	CC	Part of the ABC transporter complex PotABCD involved in spermidine/putrescine import
P	Q7VNG4	DE	Spermidine/putrescine import ATP-binding protein PotA;
P	Q7VNG4	DE	Spermidine/putrescine import ATP-binding protein PotA;
P	Q7VP69	CC	Part of the ABC transporter complex ThiBPQ involved in thiamine import
P	Q7VP69	DE	Thiamine import ATP-binding protein ThiQ;
P	Q7VR29	DE	Lipoprotein-releasing system ATP-binding protein LolD;
P	Q7VR29	DR	lipoprotein transporter activity
P	Q7VRM7	CC	Mediates influx of magnesium ions (By similarity).
P	Q7VRM7	DE	Magnesium transport protein CorA;
P	Q7VRM7	DR	cobalt ion transmembrane transporter activity
P	Q7VRM7	DR	magnesium ion transmembrane transporter activity
P	Q7VTK0	CC	Transport of potassium into the cell (By similarity).
P	Q7VTK0	DE	Probable potassium transport system protein kup;
P	Q7VTK0	DR	potassium ion transmembrane transporter activity
P	Q7VUJ5	CC	Part of the ABC transporter complex ModABC involved in molybdenum import
P	Q7VUJ5	DE	Molybdenum import ATP-binding protein ModC;
P	Q7VUJ5	DR	molybdenum ion transmembrane transporter activity
P	Q7VV72	CC	Part of the ABC transporter complex MetNIQ involved in methionine import
P	Q7VV72	DE	Methionine import ATP-binding protein MetN;
P	Q7VVZ9	CC	One of the components of the high-affinity ATP-driven potassium transport (or KDP) system, which catalyzes the hydrolysis of ATP coupled with the exchange of hydrogen and potassium ions (By similarity).
P	Q7VVZ9	DE	Potassium-binding and translocating subunit A;
P	Q7VVZ9	DE	Potassium-translocating ATPase A chain;
P	Q7VVZ9	DE	Potassium-transporting ATPase A chain;
P	Q7VVZ9	DR	potassium-transporting ATPase activity
P	Q7VYN2	DR	glycerol-3-phosphate-transporting ATPase activity
P	Q7VZ31	DE	Lipoprotein-releasing system ATP-binding protein LolD;
P	Q7VZ31	DR	lipoprotein transporter activity
P	Q7VZ66	CC	Part of the ABC transporter complex PstSACB involved in phosphate import
P	Q7VZ66	DE	ABC phosphate transporter;
P	Q7VZ66	DE	Phosphate import ATP-binding protein PstB;
P	Q7VZ66	DE	Phosphate-transporting ATPase;
P	Q7VZ66	DR	inorganic phosphate transmembrane transporter activity
P	Q7VZ66	DR	phosphate transmembrane-transporting ATPase activity
P	Q7VZE5	CC	Part of the ABC transporter complex CysAWTP involved in sulfate/thiosulfate import
P	Q7VZE5	DE	Sulfate-transporting ATPase;
P	Q7VZE5	DE	Sulfate/thiosulfate import ATP-binding protein CysA;
P	Q7VZE5	DR	sulfate transmembrane-transporting ATPase activity

P	Q7W025	CC	Part of the ABC transporter complex HmuTUV involved in hemin import
P	Q7W025	DE	Hemin import ATP-binding protein HmuV;
P	Q7W148	DR	phosphonate transmembrane-transporting ATPase activity
P	Q7W1F4	CC	Part of the ABC transporter complex ModABC involved in molybdenum import
P	Q7W1F4	DE	Molybdenum import ATP-binding protein ModC;
P	Q7W1F4	DR	molybdenum ion transmembrane transporter activity
P	Q7W1Y0	CC	Na+/H+ antiporter that extrudes sodium in exchange for external protons (By similarity)
P	Q7W1Y0	DE	Na+/H+ antiporter nhaA;
P	Q7W1Y0	DE	Sodium/proton antiporter nhaA;
P	Q7W359	CC	Part of the ABC transporter complex HmuTUV involved in hemin import
P	Q7W359	DE	Hemin import ATP-binding protein HmuV;
P	Q7W4E1	CC	Part of the ABC transporter complex MetNIQ involved in methionine import
P	Q7W4E1	DE	Methionine import ATP-binding protein MetN;
P	Q7W536	CC	One of the components of the high-affinity ATP-driven potassium transport (or KDP) system, which catalyzes the hydrolysis of ATP coupled with the exchange of hydrogen and potassium ions (By similarity).
P	Q7W536	DE	Potassium-binding and translocating subunit A;
P	Q7W536	DE	Potassium-translocating ATPase A chain;
P	Q7W536	DE	Potassium-transporting ATPase A chain;
P	Q7W536	DR	potassium-transporting ATPase activity
P	Q7W6G5	DR	glycerol-3-phosphate-transporting ATPase activity
P	Q7W736	CC	Part of the ABC transporter complex CcmAB involved in the biogenesis of c-type cytochromes; once thought to export heme, this seems not to be the case, but its exact role is uncertain
P	Q7W736	DE	Heme exporter protein A;
P	Q7W736	DR	heme-transporting ATPase activity
P	Q7W7H9	CC	Transport of potassium into the cell (By similarity).
P	Q7W7H9	DE	Probable potassium transport system protein kup;
P	Q7W7H9	DR	potassium ion transmembrane transporter activity
P	Q7W8Q6	CC	Part of the ABC transporter complex PstSACB involved in phosphate import
P	Q7W8Q6	DE	ABC phosphate transporter;
P	Q7W8Q6	DE	Phosphate import ATP-binding protein PstB;
P	Q7W8Q6	DE	Phosphate-transporting ATPase;
P	Q7W8Q6	DR	inorganic phosphate transmembrane transporter activity
P	Q7W8Q6	DR	phosphate transmembrane-transporting ATPase activity
P	Q7W8T0	DE	Lipoprotein-releasing system ATP-binding protein LolD;
P	Q7W8T0	DR	lipoprotein transporter activity
P	Q7W917	CC	Channel that permits osmotically driven movement of water in both directions
P	Q7W917	CC	It mediates rapid entry or exit of water in response to abrupt changes in osmolarity (By similarity).
P	Q7W9U5	CC	Part of the ABC transporter complex CysAWTP involved in sulfate/thiosulfate import
P	Q7W9U5	DE	Sulfate-transporting ATPase;
P	Q7W9U5	DE	Sulfate/thiosulfate import ATP-binding protein CysA;
P	Q7W9U5	DR	sulfate transmembrane-transporting ATPase activity
P	Q7WCL8	CC	One of the components of the high-affinity ATP-driven potassium transport (or KDP) system, which catalyzes the hydrolysis of ATP coupled with the exchange of hydrogen and potassium ions (By similarity).
P	Q7WCL8	DE	Potassium-binding and translocating subunit A;
P	Q7WCL8	DE	Potassium-translocating ATPase A chain;
P	Q7WCL8	DE	Potassium-transporting ATPase A chain;
P	Q7WCL8	DR	potassium-transporting ATPase activity
P	Q7WEH6	CC	Part of the ABC transporter complex HmuTUV involved in hemin import
P	Q7WEH6	DE	Hemin import ATP-binding protein HmuV;
P	Q7WFU9	CC	Part of the ABC transporter complex MetNIQ involved in methionine import
P	Q7WFU9	DE	Methionine import ATP-binding protein MetN;
P	Q7WGW1	CC	Part of the ABC transporter complex CysAWTP involved in sulfate/thiosulfate import
P	Q7WGW1	DE	Sulfate-transporting ATPase;
P	Q7WGW1	DE	Sulfate/thiosulfate import ATP-binding protein CysA;
P	Q7WGW1	DR	sulfate transmembrane-transporting ATPase activity
P	Q7WID6	DR	glycerol-3-phosphate-transporting ATPase activity
P	Q7WIP8	CC	Part of the ABC transporter complex CcmAB involved in the biogenesis of c-type cytochromes; once thought to export heme, this seems not to be the case, but its exact role is uncertain
P	Q7WIP8	DE	Heme exporter protein A;
P	Q7WIP8	DR	heme-transporting ATPase activity

P	Q7WK40	DE	Lipoprotein-releasing system ATP-binding protein LolD;
P	Q7WK40	DR	lipoprotein transporter activity
P	Q7WKG2	CC	Channel that permits osmotically driven movement of water in both directions
P	Q7WKG2	CC	It mediates rapid entry or exit of water in response to abrupt changes in osmolarity (By similarity).
P	Q7WKW8	CC	Transport of potassium into the cell (By similarity).
P	Q7WKW8	DE	Probable potassium transport system protein kup;
P	Q7WKW8	DR	potassium ion transmembrane transporter activity
P	Q7WNT8	DR	phosphonate transmembrane-transporting ATPase activity
P	Q7WP62	CC	Part of the ABC transporter complex ModABC involved in molybdenum import
P	Q7WP62	DE	Molybdenum import ATP-binding protein ModC;
P	Q7WP62	DR	molybdenum ion transmembrane transporter activity
P	Q7WQV8	CC	Na+/H+ antiporter that extrudes sodium in exchange for external protons (By similarity).
P	Q7WQV8	DE	Na+/H+ antiporter nhaA;
P	Q7WQV8	DE	Sodium/proton antiporter nhaA;
P	Q7X7V2	CC	High-affinity transporter for external inorganic phosphate (By similarity).
P	Q7X7V2	DE	Probable inorganic phosphate transporter 1-5;
P	Q7X7V2	DR	inorganic phosphate transmembrane transporter activity
P	Q7XDZ7	CC	High-affinity transporter for external inorganic phosphate (By similarity).
P	Q7XDZ7	DE	Probable inorganic phosphate transporter 1-3;
P	Q7XDZ7	DR	inorganic phosphate transmembrane transporter activity
P	Q7XIV8	CC	High-affinity potassium transporter (By similarity).
P	Q7XIV8	DE	Probable potassium transporter 9;
P	Q7XIV8	DR	potassium ion transmembrane transporter activity
P	Q7XKI5	CC	Aquaporins facilitate the transport of water and small neutral solutes across cell membranes
P	Q7XLC6	CC	High-affinity potassium transporter (By similarity).
P	Q7XLC6	DE	Probable potassium transporter 11;
P	Q7XLC6	DR	potassium ion transmembrane transporter activity
P	Q7XLR1	CC	Aquaporins facilitate the transport of water and small neutral solutes across cell membranes (By similarity).
P	Q7XPF8	CC	May be involved in regulation of K+/Na+ homeostasis
P	Q7XPF8	CC	Seems to act as a low-affinity sodium transporter.
P	Q7XPL3	CC	High-affinity potassium transporter (By similarity).
P	Q7XPL3	DE	Probable potassium transporter 15;
P	Q7XPL3	DR	potassium ion transmembrane transporter activity
P	Q7XQQ1	CC	Putative magnesium transporter (By similarity).
P	Q7XQQ1	DE	Putative magnesium transporter MRS2-D;
P	Q7XRH8	CC	High-affinity transporter for external inorganic phosphate (By similarity).
P	Q7XRH8	DE	Putative inorganic phosphate transporter 1-13;
P	Q7XSQ9	CC	Aquaporins facilitate the transport of water and small neutral solutes across cell membranes (By similarity).
P	Q7XU31	CC	Aquaporins facilitate the transport of water and small neutral solutes across cell membranes
P	Q7XUA6	CC	Aquaporins facilitate the transport of water and small neutral solutes across cell membranes (By similarity).
P	Q7Y051	DR	copper-exporting ATPase activity
P	Q7Y1E6	CC	Aquaporins facilitate the transport of water and small neutral solutes across cell membranes (By similarity).
P	Q7Z418	CC	May function as background potassium channel that sets the resting membrane potential
P	Q7Z418	CC	Outward rectifying potassium channel
P	Q7Z418	CC	Produces rapidly activating outward rectifier K+ currents
P	Q7Z418	DE	Potassium channel subfamily K member 18;
P	Q7Z418	DE	TWIK-related individual potassium channel;
P	Q7Z418	DE	TWIK-related spinal cord potassium channel;
P	Q80XM3	CC	Potassium channel subunit
P	Q80XM3	DE	Potassium voltage-gated channel subfamily G member 4;
P	Q80XM3	DE	Voltage-gated potassium channel subunit Kv6.4;
P	Q80XM3	DR	voltage-gated potassium channel activity
P	Q80XR2	CC	This magnesium-dependent enzyme catalyzes the hydrolysis of ATP coupled with the transport of the calcium (By similarity).
P	Q80XR2	DE	ATP-dependent Ca2+ pump PMR1;
P	Q80XR2	DE	Calcium-transporting ATPase type 2C member 1;
P	Q80XR2	DR	calcium-transporting ATPase activity
P	Q80XR2	DR	manganese-transporting ATPase activity

P	Q80ZD3	CC	Exhibits sodium-independent sulfate anion transporter activity that may cooperate with SLC26A2 to mediate DIDS-sensitive sulfate uptake into high endothelial venules endothelial cells (HEVEC) (By similarity).
P	Q80ZD3	DE	Sodium-independent sulfate anion transporter;
P	Q80ZD3	DR	secondary active sulfate transmembrane transporter activity
P	Q814U8	CC	This system is involved in glucose transport (By similarity).
P	Q814U8	DE	Glucose-specific phosphotransferase enzyme IIA component;
P	Q814U8	DE	PTS system glucose-specific EIIA component;
P	Q815Y7	CC	Part of the ABC transporter complex MetNIQ involved in methionine import
P	Q815Y7	DE	Methionine import ATP-binding protein MetN 3;
P	Q816V0	CC	Involved in the uptake of glucose (By similarity).
P	Q816V0	DE	Probable glucose uptake protein glcU;
P	Q818I7	CC	Part of the ABC transporter complex PstSACB involved in phosphate import
P	Q818I7	DE	ABC phosphate transporter;
P	Q818I7	DE	Phosphate import ATP-binding protein PstB;
P	Q818I7	DE	Phosphate-transporting ATPase;
P	Q818I7	DR	inorganic phosphate transmembrane transporter activity
P	Q818I7	DR	phosphate transmembrane-transporting ATPase activity
P	Q81A96	DR	phosphonate transmembrane-transporting ATPase activity
P	Q81GC1	CC	Part of the ABC transporter complex PotABCD involved in spermidine/putrescine import
P	Q81GC1	CC	Part of the ABC transporter complex PotABCD involved in spermidine/putrescine import
P	Q81GC1	DE	Spermidine/putrescine import ATP-binding protein PotA;
P	Q81GC1	DE	Spermidine/putrescine import ATP-binding protein PotA;
P	Q81GU1	CC	Part of the ABC transporter complex CysAWTP involved in sulfate/thiosulfate import
P	Q81GU1	DE	Sulfate-transporting ATPase;
P	Q81GU1	DE	Sulfate/thiosulfate import ATP-binding protein CysA;
P	Q81GU1	DR	sulfate transmembrane-transporting ATPase activity
P	Q81HP9	CC	One of the components of the high-affinity ATP-driven potassium transport (or KDP) system, which catalyzes the hydrolysis of ATP coupled with the exchange of hydrogen and potassium ions
P	Q81HP9	DE	Potassium-binding and translocating subunit C;
P	Q81HP9	DE	Potassium-translocating ATPase C chain;
P	Q81HP9	DE	Potassium-transporting ATPase C chain;
P	Q81HP9	DR	potassium-transporting ATPase activity
P	Q81HQ0	CC	One of the components of the high-affinity ATP-driven potassium transport (or KDP) system, which catalyzes the hydrolysis of ATP coupled with the exchange of hydrogen and potassium ions (By similarity).
P	Q81HQ0	DE	Potassium-binding and translocating subunit B;
P	Q81HQ0	DE	Potassium-translocating ATPase B chain;
P	Q81HQ0	DE	Potassium-transporting ATPase B chain;
P	Q81HQ0	DR	potassium-transporting ATPase activity
P	Q81HQ1	CC	One of the components of the high-affinity ATP-driven potassium transport (or KDP) system, which catalyzes the hydrolysis of ATP coupled with the exchange of hydrogen and potassium ions (By similarity).
P	Q81HQ1	DE	Potassium-binding and translocating subunit A;
P	Q81HQ1	DE	Potassium-translocating ATPase A chain;
P	Q81HQ1	DE	Potassium-transporting ATPase A chain;
P	Q81HQ1	DR	potassium-transporting ATPase activity
P	Q81HW8	CC	Part of the ABC transporter complex RbsABCD involved in ribose import
P	Q81HW8	DE	Ribose import ATP-binding protein RbsA;
P	Q81IN8	CC	Part of the ABC transporter complex MetNIQ involved in methionine import
P	Q81IN8	DE	Methionine import ATP-binding protein MetN 2;
P	Q81IZ6	CC	Part of the ABC transporter complex MetNIQ involved in methionine import
P	Q81IZ6	DE	Methionine import ATP-binding protein MetN 1;
P	Q81JY0	CC	This system is involved in glucose transport (By similarity).
P	Q81JY0	DE	Glucose-specific phosphotransferase enzyme IIA component;
P	Q81JY0	DE	PTS system glucose-specific EIIA component;
P	Q81KM7	CC	Involved in the uptake of glucose (By similarity).
P	Q81KM7	DE	Probable glucose uptake protein glcU;
P	Q81LW6	CC	Part of the ABC transporter complex PstSACB involved in phosphate import
P	Q81LW6	DE	ABC phosphate transporter;
P	Q81LW6	DE	Phosphate import ATP-binding protein PstB;
P	Q81LW6	DE	Phosphate-transporting ATPase;
P	Q81LW6	DR	inorganic phosphate transmembrane transporter activity
P	Q81LW6	DR	phosphate transmembrane-transporting ATPase activity

P	Q81TH8	CC	Part of the ABC transporter complex PotABCD involved in spermidine/putrescine import
P	Q81TH8	CC	Part of the ABC transporter complex PotABCD involved in spermidine/putrescine import
P	Q81TH8	DE	Spermidine/putrescine import ATP-binding protein PotA;
P	Q81TH8	DE	Spermidine/putrescine import ATP-binding protein PotA;
P	Q81UW5	CC	One of the components of the high-affinity ATP-driven potassium transport (or KDP) system, which catalyzes the hydrolysis of ATP coupled with the exchange of hydrogen and potassium ions
P	Q81UW5	DE	Potassium-binding and translocating subunit C;
P	Q81UW5	DE	Potassium-translocating ATPase C chain;
P	Q81UW5	DE	Potassium-transporting ATPase C chain;
P	Q81UW5	DR	potassium-transporting ATPase activity
P	Q81UW7	CC	One of the components of the high-affinity ATP-driven potassium transport (or KDP) system, which catalyzes the hydrolysis of ATP coupled with the exchange of hydrogen and potassium ions (By similarity).
P	Q81UW7	DE	Potassium-binding and translocating subunit A;
P	Q81UW7	DE	Potassium-translocating ATPase A chain;
P	Q81UW7	DE	Potassium-transporting ATPase A chain;
P	Q81UW7	DR	potassium-transporting ATPase activity
P	Q81V36	CC	Part of the ABC transporter complex RbsABCD involved in ribose import
P	Q81V36	DE	Ribose import ATP-binding protein RbsA;
P	Q81VM2	CC	Part of the ABC transporter complex MetNIQ involved in methionine import
P	Q81VM2	DE	Methionine import ATP-binding protein MetN 1;
P	Q81XL3	CC	Part of the ABC transporter complex MetNIQ involved in methionine import
P	Q81XL3	DE	Methionine import ATP-binding protein MetN 3;
P	Q81Y10	DR	phosphonate transmembrane-transporting ATPase activity
P	Q81ZF5	CC	Part of the ABC transporter complex MetNIQ involved in methionine import
P	Q81ZF5	DE	Methionine import ATP-binding protein MetN 2;
P	Q822F2	CC	The arginine uptake by the bacterium in the macrophage may be a virulence factor against the host innate immune response (By similarity).
P	Q822F2	DE	Arginine/agmatine antiporter;
P	Q823C4	CC	Part of the ABC transporter complex MetNIQ involved in methionine import
P	Q823C4	DE	Methionine import ATP-binding protein MetN;
P	Q825P1	CC	Part of the ABC transporter complex RbsABCD involved in ribose import
P	Q825P1	DE	Ribose import ATP-binding protein RbsA 2;
P	Q827Y0	CC	Part of the ABC transporter complex MetNIQ involved in methionine import
P	Q827Y0	DE	Methionine import ATP-binding protein MetN;
P	Q82CM5	CC	Part of the ABC transporter complex RbsABCD involved in ribose import
P	Q82CM5	DE	Ribose import ATP-binding protein RbsA 1;
P	Q82EL6	CC	Na+/H+ antiporter that extrudes sodium in exchange for external protons (By similarity).
P	Q82EL6	DE	Na+/H+ antiporter nhaA;
P	Q82EL6	DE	Sodium/proton antiporter nhaA;
P	Q82G23	CC	Part of the ABC transporter complex PstSACB involved in phosphate import
P	Q82G23	DE	ABC phosphate transporter;
P	Q82G23	DE	Phosphate import ATP-binding protein PstB;
P	Q82G23	DE	Phosphate-transporting ATPase;
P	Q82G23	DR	inorganic phosphate transmembrane transporter activity
P	Q82G23	DR	phosphate transmembrane-transporting ATPase activity
P	Q82PI3	CC	One of the components of the high-affinity ATP-driven potassium transport (or KDP) system, which catalyzes the hydrolysis of ATP coupled with the exchange of hydrogen and potassium ions (By similarity).
P	Q82PI3	DE	Potassium-binding and translocating subunit A;
P	Q82PI3	DE	Potassium-translocating ATPase A chain;
P	Q82PI3	DE	Potassium-transporting ATPase A chain;
P	Q82PI3	DR	potassium-transporting ATPase activity
P	Q82PI5	CC	One of the components of the high-affinity ATP-driven potassium transport (or KDP) system, which catalyzes the hydrolysis of ATP coupled with the exchange of hydrogen and potassium ions
P	Q82PI5	DE	Potassium-binding and translocating subunit C;
P	Q82PI5	DE	Potassium-translocating ATPase C chain;
P	Q82PI5	DE	Potassium-transporting ATPase C chain;
P	Q82PI5	DR	potassium-transporting ATPase activity
P	Q82TL6	CC	Part of the ABC transporter complex PotABCD involved in spermidine/putrescine import
P	Q82TL6	CC	Part of the ABC transporter complex PotABCD involved in spermidine/putrescine import
P	Q82TL6	DE	Spermidine/putrescine import ATP-binding protein PotA;
P	Q82TL6	DE	Spermidine/putrescine import ATP-binding protein PotA;

P	Q82VL9	DE	Lipoprotein-releasing system ATP-binding protein LolD;
P	Q82VL9	DR	lipoprotein transporter activity
P	Q82VR4	CC	Part of the ABC transporter complex PstSACB involved in phosphate import
P	Q82VR4	DE	ABC phosphate transporter;
P	Q82VR4	DE	Phosphate import ATP-binding protein PstB;
P	Q82VR4	DE	Phosphate-transporting ATPase;
P	Q82VR4	DR	inorganic phosphate transmembrane transporter activity
P	Q82VR4	DR	phosphate transmembrane-transporting ATPase activity
P	Q82WC8	CC	Part of the ABC transporter complex CcmAB involved in the biogenesis of c-type cytochromes; once thought to export heme, this seems not to be the case, but its exact role is uncertain
P	Q82WC8	DE	Heme exporter protein A;
P	Q82WC8	DR	heme-transporting ATPase activity
P	Q82WT5	CC	Part of the ABC transporter complex CysAWTP involved in sulfate/thiosulfate import
P	Q82WT5	DE	Sulfate-transporting ATPase;
P	Q82WT5	DE	Sulfate/thiosulfate import ATP-binding protein CysA;
P	Q82WT5	DR	sulfate transmembrane-transporting ATPase activity
P	Q82ZN5	CC	Involved in the import of serine and threonine into the cell, with the concomitant import of sodium (symport system) (By similarity).
P	Q82ZN5	DE	Na+/serine-threonine symporter;
P	Q82ZN5	DR	sodium:dicarboxylate symporter activity
P	Q82ZN5	DR	sodium:dicarboxylate symporter activity
P	Q82ZT8	CC	Could be involved in the uptake of ribose (By similarity).
P	Q82ZT8	DE	Putative ribose uptake protein rbsU;
P	Q830V1	DE	Magnesium transporter mgtE;
P	Q830W6	CC	Part of the ABC transporter complex PotABCD involved in spermidine/putrescine import
P	Q830W6	CC	Part of the ABC transporter complex PotABCD involved in spermidine/putrescine import
P	Q830W6	DE	Spermidine/putrescine import ATP-binding protein PotA;
P	Q830W6	DE	Spermidine/putrescine import ATP-binding protein PotA;
P	Q832Y6	CC	Part of the ABC transporter complex MetNIQ involved in methionine import
P	Q832Y6	DE	Methionine import ATP-binding protein MetN 1;
P	Q834B3	CC	Part of the ABC transporter complex PstSACB involved in phosphate import
P	Q834B3	DE	ABC phosphate transporter 2;
P	Q834B3	DE	Phosphate import ATP-binding protein PstB 2;
P	Q834B3	DE	Phosphate-transporting ATPase 2;
P	Q834B3	DR	inorganic phosphate transmembrane transporter activity
P	Q834B3	DR	phosphate transmembrane-transporting ATPase activity
P	Q834B4	CC	Part of the ABC transporter complex PstSACB involved in phosphate import
P	Q834B4	DE	ABC phosphate transporter 1;
P	Q834B4	DE	Phosphate import ATP-binding protein PstB 1;
P	Q834B4	DE	Phosphate-transporting ATPase 1;
P	Q834B4	DR	inorganic phosphate transmembrane transporter activity
P	Q834B4	DR	phosphate transmembrane-transporting ATPase activity
P	Q837G9	CC	Transport of potassium into the cell (By similarity).
P	Q837G9	DE	Probable potassium transport system protein kup;
P	Q837G9	DR	potassium ion transmembrane transporter activity
P	Q83CV2	DE	Lipoprotein-releasing system ATP-binding protein LolD;
P	Q83CV2	DR	lipoprotein transporter activity
P	Q83GE8	CC	Part of the ABC transporter complex PstSACB involved in phosphate import
P	Q83GE8	DE	ABC phosphate transporter;
P	Q83GE8	DE	Phosphate import ATP-binding protein PstB;
P	Q83GE8	DE	Phosphate-transporting ATPase;
P	Q83GE8	DR	phosphate transmembrane-transporting ATPase activity
P	Q83HT1	CC	Part of the ABC transporter complex PstSACB involved in phosphate import
P	Q83HT1	DE	ABC phosphate transporter;
P	Q83HT1	DE	Phosphate import ATP-binding protein PstB;
P	Q83HT1	DE	Phosphate-transporting ATPase;
P	Q83HT1	DR	phosphate transmembrane-transporting ATPase activity
P	Q83J33	CC	Part of the ABC transporter complex XylFGH involved in xylose import
P	Q83J33	DE	Xylose import ATP-binding protein XylG;
P	Q83J33	DR	D-xylose-importing ATPase activity
P	Q83J77	CC	Part of the ABC transporter complex NikABCDE involved in nickel import
P	Q83J77	DE	Nickel import ATP-binding protein NikE;
P	Q83J77	DR	nickel-transporting ATPase activity
P	Q83J78	CC	Part of the ABC transporter complex NikABCDE involved in nickel import

P	Q83J78	DE	Nickel import ATP-binding protein NikD;
P	Q83J78	DR	nickel-transporting ATPase activity
P	Q83JJ9	CC	Catalyzes the uptake of tartrate in exchange for intracellular succinate
P	Q83JJ9	DE	L-tartrate/succinate antiporter;
P	Q83KD5	CC	Part of the ABC transporter complex CcmAB involved in the biogenesis of c-type cytochromes; once thought to export heme, this seems not to be the case, but its exact role is uncertain
P	Q83KD5	DE	Heme exporter protein A;
P	Q83KD5	DR	heme-transporting ATPase activity
P	Q83KP2	DR	L-arabinose-importing ATPase activity
P	Q83KR7	CC	Part of the ABC transporter complex ZnuABC involved in zinc import
P	Q83KR7	DE	Zinc import ATP-binding protein ZnuC;
P	Q83KR7	DR	zinc transporting ATPase activity
P	Q83MA0	CC	Part of the ABC transporter complex TauABC involved in taurine import
P	Q83MA0	DE	Taurine import ATP-binding protein TauB;
P	Q83MA0	DR	taurine-transporting ATPase activity
P	Q83MC5	CC	Part of the ABC transporter complex MetNIQ involved in methionine import
P	Q83MC5	DE	Methionine import ATP-binding protein MetN;
P	Q83MC5	DR	D-methionine transmembrane transporter activity
P	Q83MD7	CC	Binds vitamin B12 and delivers it to the periplasmic surface of BtuC (By similarity)
P	Q83MD7	CC	Part of the ABC transporter complex BtuCDF involved in vitamin B12 import
P	Q83MG3	CC	Part of the ABC transporter complex ThiBPQ involved in thiamine import
P	Q83MG3	DE	Thiamine import ATP-binding protein ThiQ;
P	Q83N58	CC	Na+/H+ antiporter that extrudes sodium in exchange for external protons (By similarity)
P	Q83N58	DE	Na+/H+ antiporter nhaA;
P	Q83N58	DE	Sodium/proton antiporter nhaA;
P	Q83P81	CC	Part of the binding-protein-dependent transport system for maltose; probably responsible for the translocation of the substrate across the membrane (By similarity)
P	Q83P81	DE	Maltose transport system permease protein malF;
P	Q83P97	DR	phosphonate transmembrane-transporting ATPase activity
P	Q83PD8	CC	Uptake of L-rhamnose across the boundary membrane with the concomitant transport of protons into the cell (symport system) (By similarity).
P	Q83PD8	DE	L-rhamnose-H+ transport protein;
P	Q83PD8	DE	L-rhamnose-proton symporter;
P	Q83PJ2	CC	Responsible for the low-affinity transport of potassium into the cell, with the probable concomitant uptake of protons (symport system) (By similarity).
P	Q83PJ2	DE	Kup system potassium uptake protein;
P	Q83PJ2	DE	Low affinity potassium transport system protein kup;
P	Q83PJ2	DR	potassium ion transmembrane transporter activity
P	Q83PY0	CC	Transport system that facilitates potassium-efflux, possibly by potassium-proton antiport (By similarity).
P	Q83PY0	DE	K+/H+ antiporter;
P	Q83PY0	DE	NEM-activable K+/H+ antiporter;
P	Q83PY0	DE	Putative glutathione-regulated potassium-efflux system protein kefB;
P	Q83PY0	DR	glutathione-regulated potassium exporter activity
P	Q83Q28	CC	Involved in the import of threonine and serine into the cell, with the concomitant import of a proton (symport system) (By similarity).
P	Q83Q28	DE	Threonine/serine transporter TdcC;
P	Q83Q34	CC	Involved in the import of serine and threonine into the cell, with the concomitant import of sodium (symport system) (By similarity).
P	Q83Q34	DE	Na+/serine-threonine symporter;
P	Q83Q34	DR	sodium:dicarboxylate symporter activity
P	Q83Q34	DR	sodium:dicarboxylate symporter activity
P	Q83QD0	CC	Involved in the import of serine into the cell
P	Q83QD0	DE	Serine transporter;
P	Q83QV3	CC	Required for the export of heme to the periplasm for the biogenesis of c-type cytochromes (By similarity).
P	Q83QV3	DE	Heme exporter protein B;
P	Q83QV3	DR	heme transporter activity
P	Q83RQ5	CC	Na+/H+ antiporter that extrudes sodium in exchange for external protons (By similarity)
P	Q83RQ5	DE	Na+/H+ antiporter nhaB;
P	Q83RQ5	DE	Sodium/proton antiporter nhaB;
P	Q83RQ5	DR	sodium:hydrogen antiporter activity
P	Q83RR7	CC	Required for the activity of the bacterial periplasmic transport system of putrescine and spermidine (By similarity).

P	Q83RR7	CC	Required for the activity of the bacterial periplasmic transport system of putrescine and spermidine (By similarity).
P	Q83RR7	DE	Spermidine/putrescine transport system permease protein PotC;
P	Q83RR7	DE	Spermidine/putrescine transport system permease protein PotC;
P	Q83RS0	DE	Lipoprotein-releasing system ATP-binding protein LolD;
P	Q83RS0	DR	lipoprotein transporter activity
P	Q83S83	CC	One of the components of the high-affinity ATP-driven potassium transport (or KDP) system, which catalyzes the hydrolysis of ATP coupled with the exchange of hydrogen and potassium ions (By similarity).
P	Q83S83	DE	Potassium-binding and translocating subunit A;
P	Q83S83	DE	Potassium-translocating ATPase A chain;
P	Q83S83	DE	Potassium-transporting ATPase A chain;
P	Q83S83	DR	potassium-transporting ATPase activity
P	Q83SA2	CC	Involved in zinc efflux across the cytoplasmic membrane, thus reducing zinc accumulation in the cytoplasm and rendering bacteria more resistant to zinc [illegible]
P	Q83SA2	CC	It may contribute to zinc homeostasis at low concentrations of zinc (By [illegible]
P	Q83SA2	DE	Zinc transporter zitB;
P	Q83SQ3	CC	Transport system that facilitates potassium-efflux, possibly by potassium-proton antiport (By similarity).
P	Q83SQ3	DE	Glutathione-regulated potassium-efflux system protein kefC;
P	Q83SQ3	DE	K+/H+ antiporter;
P	Q83SR3	CC	Na+/H+ antiporter that extrudes sodium in exchange for external protons (By [illegible]
P	Q83SR3	DE	Na+/H+ antiporter nhaA;
P	Q83SR3	DE	Sodium/proton antiporter nhaA;
P	Q84MA8	CC	Transports natural purines (adenine and guanine) as well as purine analogs
P	Q84MA8	DE	Adenine/guanine permease AZG2;
P	Q84MA8	DE	Adenine/guanine permease AZG2;
P	Q84MA8	DR	purine base transmembrane transporter activity
P	Q84MS3	CC	High-affinity potassium transporter (By similarity).
P	Q84MS3	DE	Probable potassium transporter 16;
P	Q84MS3	DR	potassium ion transmembrane transporter activity
P	Q84MS4	CC	High-affinity potassium transporter (By similarity).
P	Q84MS4	DE	Potassium transporter 27;
P	Q84MS4	DR	potassium ion transmembrane transporter activity
P	Q84S07	CC	Aquaporins facilitate the transport of water and small neutral solutes across cell membranes (By similarity).
P	Q84TI7	CC	Does not transport K+ but regulates K+ nutrient status via its ability to facilitate Na+ homeostasis
P	Q84TI7	DR	potassium ion transmembrane transporter activity
P	Q84WG1	DE	Na+/H+ exchanger 3;
P	Q84WG1	DE	Sodium/hydrogen exchanger 3;
P	Q84WG1	DR	sodium:hydrogen antiporter activity
P	Q84YJ9	CC	High-affinity potassium transporter (By similarity).
P	Q84YJ9	DE	Potassium transporter 26;
P	Q84YJ9	DR	potassium ion transmembrane transporter activity
P	Q869V1	CC	May rather act as a symporter of protons and metal cations in an ATP-dependent process
P	Q869V1	DE	Metal transporter nramp1 homolog;
P	Q869W7	CC	Sugar transporter that specifically mediates the transport of UDP-N-acetylglucosamine (UDP-GlcNAc) from cytosol into Golgi (By similarity).
P	Q869W7	DE	UDP-N-acetylglucosamine transporter slc35b4;
P	Q86AZ6	CC	Chloride channels may have several functions including the regulation of cell volume, membrane potential stabilization and signal transduction (By similarity).
P	Q86AZ6	CC	Voltage-gated chloride channel
P	Q86AZ6	DE	Chloride channel protein B;
P	Q86AZ6	DR	voltage-gated chloride channel activity
P	Q86I81	CC	Transports folate across the inner membranes of mitochondria (By similarity).
P	Q86I81	DE	Mitochondrial folate transporter A;
P	Q86UD5	CC	Electroneutral exchange of protons for Na+ and Li+ across the inner mitochondrial membrane
P	Q86UD5	DE	Mitochondrial Na+/H+ exchanger NHA2;
P	Q86UD5	DE	Mitochondrial sodium/hydrogen exchanger NHA2;
P	Q86UD5	DE	Na+/H+ exchanger-like domain-containing protein 2;
P	Q86UD5	DE	Sodium/hydrogen exchanger-like domain-containing protein 2;
P	Q86VS0	DE	Sodium/hydrogen exchanger;

P	Q86VS0	DR	sodium:hydrogen antiporter activity
P	Q86VW1	CC	High affinity carnitine transporter; the uptake is partially sodium-ion dependent
P	Q86VW1	DE	Carnitine transporter 2;
P	Q86VW1	DE	Organic cation/carnitine transporter 6;
P	Q86VW1	DR	carnitine transporter activity
P	Q86WA9	CC	Exhibits sodium-independent sulfate anion transporter activity that may cooperate with SLC26A2 to mediate DIDS-sensitive sulfate uptake into high endothelial venules endothelial cells (HEVEC).
P	Q86WA9	DE	Sodium-independent sulfate anion transporter;
P	Q86WA9	DR	secondary active sulfate transmembrane transporter activity
P	Q86WJ5	DE	Hyperpolarization activated cyclic nucleotide-gated potassium channel;
P	Q86WJ5	DR	voltage-gated potassium channel activity
P	Q86WJ6	DE	Hyperpolarization activated cyclic nucleotide-gated potassium channel;
P	Q86WJ6	DR	voltage-gated potassium channel activity
P	Q86Y85	DE	Potassium voltage-gated channel, subfamily G, member 1;
P	Q86Y85	DR	voltage-gated potassium channel activity
P	Q86YT5	CC	High-affinity sodium/citrate cotransporter that mediates citrate entry into cells
P	Q86YT5	CC	High-affinity sodium/citrate cotransporter that mediates citrate entry into cells
P	Q86YT5	CC	May facilitate the utilization of circulating citrate for the generation of metabolic energy and for the synthesis of fatty acids and cholesterol.
P	Q86YT5	CC	The transport process is electrogenic; it is the trivalent form of citrate rather than the divalent form that is recognized as a substrate
P	Q86YT5	DE	Na+/citrate cotransporter;
P	Q86YT5	DE	Na+/citrate cotransporter;
P	Q86YT5	DE	Sodium-coupled citrate transporter;
P	Q86YT5	DE	Sodium-coupled citrate transporter;
P	Q86YT5	DE	Sodium-dependent citrate transporter;
P	Q86YT5	DE	Sodium-dependent citrate transporter;
P	Q86YT5	DR	citrate transmembrane transporter activity
P	Q87AL9	CC	Part of the ABC transporter complex MetNIQ involved in methionine import
P	Q87AL9	DE	Methionine import ATP-binding protein MetN;
P	Q87BH8	CC	Part of the ABC transporter complex CcmAB involved in the biogenesis of c-type cytochromes; once thought to export heme, this seems not to be the case, but its exact role is uncertain
P	Q87BH8	DE	Heme exporter protein A;
P	Q87BH8	DR	heme-transporting ATPase activity
P	Q87C88	CC	Part of the ABC transporter complex PstSACB involved in phosphate import
P	Q87C88	DE	ABC phosphate transporter;
P	Q87C88	DE	Phosphate import ATP-binding protein PstB;
P	Q87C88	DE	Phosphate-transporting ATPase;
P	Q87C88	DR	inorganic phosphate transmembrane transporter activity
P	Q87C88	DR	phosphate transmembrane-transporting ATPase activity
P	Q87C89	CC	Part of a binding-protein-dependent transport system for phosphate; probably responsible for the translocation of the substrate across the membrane (By similarity)
P	Q87C89	DE	Phosphate transport system permease protein pstA;
P	Q87C89	DR	inorganic phosphate transmembrane transporter activity
P	Q87C90	CC	Part of a binding-protein-dependent transport system for phosphate; probably responsible for the translocation of the substrate across the membrane (By similarity)
P	Q87C90	DE	Phosphate transport system permease protein pstC;
P	Q87C90	DR	inorganic phosphate transmembrane transporter activity
P	Q87C91	CC	Part of the ABC transporter complex pstSACB involved in phosphate import (By similarity).
P	Q87C91	DE	Phosphate-binding protein pstS;
P	Q87C91	DR	inorganic phosphate transmembrane transporter activity
P	Q87D01	CC	Transport of potassium into the cell (By similarity).
P	Q87D01	DE	Probable potassium transport system protein kup;
P	Q87D01	DR	potassium ion transmembrane transporter activity
P	Q87DT9	CC	Part of the ABC transporter complex CysAWTP involved in sulfate/thiosulfate import
P	Q87DT9	DE	Sulfate-transporting ATPase;
P	Q87DT9	DE	Sulfate/thiosulfate import ATP-binding protein CysA;
P	Q87DT9	DR	sulfate transmembrane-transporting ATPase activity
P	Q87EF4	DE	Lipoprotein-releasing system ATP-binding protein LolD;
P	Q87EF4	DR	lipoprotein transporter activity
P	Q87EJ9	CC	H+-stimulated, highly selective, manganese uptake system (By similarity).
P	Q87EJ9	DE	Probable manganese transport protein mntH;

P	Q87FK7	DR	L-arabinose-importing ATPase activity
P	Q87FP8	CC	Involved in the import of serine and threonine into the cell, with the concomitant import of sodium (symport system) (By similarity).
P	Q87FP8	DE	Na+/serine-threonine symporter;
P	Q87FP8	DR	sodium:dicarboxylate symporter activity
P	Q87FP8	DR	sodium:dicarboxylate symporter activity
P	Q87G59	CC	Part of the ABC transporter complex PstSACB involved in phosphate import
P	Q87G59	DE	ABC phosphate transporter 2;
P	Q87G59	DE	Phosphate import ATP-binding protein PstB 2;
P	Q87G59	DE	Phosphate-transporting ATPase 2;
P	Q87G59	DR	inorganic phosphate transmembrane transporter activity
P	Q87G59	DR	phosphate transmembrane-transporting ATPase activity
P	Q87GB5	CC	Part of the ABC transporter complex MalEFGK involved in maltose/maltodextrin import
P	Q87GB5	DE	Maltose/maltodextrin import ATP-binding protein MalK;
P	Q87GB5	DR	maltose-transporting ATPase activity
P	Q87GB7	DE	Maltose transport system permease protein malF;
P	Q87GB8	DE	Maltose transport system permease protein malG;
P	Q87GZ9	CC	Functions as antiport system and exchanges two chloride ions for 1 proton
P	Q87GZ9	CC	Proton-coupled chloride transporter
P	Q87GZ9	DR	voltage-gated chloride channel activity
P	Q87H79	CC	Part of the ABC transporter complex RbsABCD involved in ribose import
P	Q87H79	DE	Ribose import ATP-binding protein RbsA;
P	Q87HN4	CC	Part of the ABC transporter complex ModABC involved in molybdenum import
P	Q87HN4	DE	Molybdenum import ATP-binding protein ModC;
P	Q87HN4	DR	molybdenum ion transmembrane transporter activity
P	Q87J32	CC	Part of the ABC transporter complex HmuTUV involved in hemin import
P	Q87J32	DE	Hemin import ATP-binding protein HmuV;
P	Q87MK8	CC	Part of the ABC transporter complex CcmAB involved in the biogenesis of c-type cytochromes; once thought to export heme, this seems not to be the case, but its exact role is uncertain
P	Q87MK8	DE	Heme exporter protein A;
P	Q87MK8	DR	heme-transporting ATPase activity
P	Q87MQ5	CC	Channel that permits osmotically driven movement of water in both directions
P	Q87MQ5	CC	It mediates rapid entry or exit of water in response to abrupt changes in osmolarity (By similarity).
P	Q87N04	CC	Na+/H+ antiporter that extrudes sodium in exchange for external protons.
P	Q87N04	DE	Na+/H+ antiporter nhaB;
P	Q87N04	DE	Sodium/proton antiporter nhaB;
P	Q87N04	DR	sodium:hydrogen antiporter activity
P	Q87PH3	CC	Part of the ABC transporter complex PotABCD involved in spermidine/putrescine import
P	Q87PH3	CC	Part of the ABC transporter complex PotABCD involved in spermidine/putrescine import
P	Q87PH3	DE	Spermidine/putrescine import ATP-binding protein PotA;
P	Q87PH3	DE	Spermidine/putrescine import ATP-binding protein PotA;
P	Q87Q38	CC	Part of the ABC transporter complex BtuCDF involved in vitamin B12 import
P	Q87Q38	DE	Vitamin B12 import ATP-binding protein BtuD;
P	Q87Q38	DE	Vitamin B12-transporting ATPase;
P	Q87Q39	CC	Part of the ABC transporter complex BtuCDF involved in vitamin B12 import
P	Q87Q39	DE	Vitamin B12 import system permease protein BtuC;
P	Q87R20	DE	Lipoprotein-releasing system ATP-binding protein LolD;
P	Q87R20	DR	lipoprotein transporter activity
P	Q87RE5	CC	Part of the ABC transporter complex ZnuABC involved in zinc import
P	Q87RE5	DE	Zinc import ATP-binding protein ZnuC;
P	Q87RE5	DR	zinc transporting ATPase activity
P	Q87RS1	CC	Part of the ABC transporter complex MetNIQ involved in methionine import
P	Q87RS1	DE	Methionine import ATP-binding protein MetN;
P	Q87RS1	DR	D-methionine transmembrane transporter activity
P	Q87S48	CC	Part of the ABC transporter complex PstSACB involved in phosphate import
P	Q87S48	DE	ABC phosphate transporter 1;
P	Q87S48	DE	Phosphate import ATP-binding protein PstB 1;
P	Q87S48	DE	Phosphate-transporting ATPase 1;
P	Q87S48	DR	inorganic phosphate transmembrane transporter activity
P	Q87S48	DR	phosphate transmembrane-transporting ATPase activity
P	Q87SE7	CC	Binds vitamin B12 and delivers it to the periplasmic surface of BtuC (By similarity)
P	Q87SE7	CC	Part of the ABC transporter complex BtuCDF involved in vitamin B12 import
P	Q87SV4	CC	Part of the ABC transporter complex ThiBPQ involved in thiamine import

P	Q87SV4	DE	Thiamine import ATP-binding protein ThiQ;
P	Q87U31	CC	Part of the ABC transporter complex PstSACB involved in phosphate import
P	Q87U31	DE	ABC phosphate transporter 2;
P	Q87U31	DE	Phosphate import ATP-binding protein PstB 2;
P	Q87U31	DE	Phosphate-transporting ATPase 2;
P	Q87U31	DR	inorganic phosphate transmembrane transporter activity
P	Q87U31	DR	phosphate transmembrane-transporting ATPase activity
P	Q87UH7	CC	Part of the ABC transporter complex TauABC involved in taurine import
P	Q87UH7	DE	Taurine import ATP-binding protein TauB;
P	Q87UH7	DR	taurine-transporting ATPase activity
P	Q87UN0	CC	Part of the ABC transporter complex ZnuABC involved in zinc import
P	Q87UN0	DE	Zinc import ATP-binding protein ZnuC;
P	Q87UN0	DR	zinc transporting ATPase activity
P	Q87UN4	CC	Part of the ABC transporter complex MetNIQ involved in methionine import
P	Q87UN4	DE	Methionine import ATP-binding protein MetN 2;
P	Q87UV4	CC	Part of the ABC transporter complex MetNIQ involved in methionine import
P	Q87UV4	DE	Methionine import ATP-binding protein MetN 1;
P	Q87UW8	CC	Na+/H+ antiporter that extrudes sodium in exchange for external protons (By similarity)
P	Q87UW8	DE	Na+/H+ antiporter nhaA 2;
P	Q87UW8	DE	Sodium/proton antiporter nhaA 2;
P	Q87WH3	DE	Glycine betaine/choline OpuC ABC transporter, periplasmic substrate-binding protein;
P	Q87WH3	DE	Glycine betaine/choline OpuC ABC transporter, periplasmic substrate-binding protein;
P	Q87WH4	DE	Glycine betaine/choline OpuC ABC transporter, permease protein;
P	Q87WH4	DE	Glycine betaine/choline OpuC ABC transporter, permease protein;
P	Q87WH5	DE	Glycine betaine/choline OpuC ABC transporter, ATP-binding protein;
P	Q87WH5	DE	Glycine betaine/choline OpuC ABC transporter, ATP-binding protein;
P	Q87WL3	CC	Na+/H+ antiporter that extrudes sodium in exchange for external protons (By similarity)
P	Q87WL3	DE	Na+/H+ antiporter nhaA 1;
P	Q87WL3	DE	Sodium/proton antiporter nhaA 1;
P	Q87Y19	CC	Transport of potassium into the cell (By similarity).
P	Q87Y19	DE	Probable potassium transport system protein kup;
P	Q87Y19	DR	potassium ion transmembrane transporter activity
P	Q87Z03	CC	Part of the ABC transporter complex CcmAB involved in the biogenesis of c-type cytochromes; once thought to export heme, this seems not to be the case, but its exact role is uncertain
P	Q87Z03	DE	Heme exporter protein A;
P	Q87Z03	DR	heme-transporting ATPase activity
P	Q880A6	CC	Part of the ABC transporter complex PstSACB involved in phosphate import
P	Q880A6	DE	ABC phosphate transporter 1;
P	Q880A6	DE	Phosphate import ATP-binding protein PstB 1;
P	Q880A6	DE	Phosphate-transporting ATPase 1;
P	Q880A6	DR	inorganic phosphate transmembrane transporter activity
P	Q880A6	DR	phosphate transmembrane-transporting ATPase activity
P	Q880Z2	CC	Part of the ABC transporter complex XylFGH involved in xylose import
P	Q880Z2	DE	Xylose import ATP-binding protein XylG;
P	Q880Z2	DR	D-xylose-importing ATPase activity
P	Q881C1	CC	Part of the ABC transporter complex ModABC involved in molybdenum import
P	Q881C1	DE	Molybdenum import ATP-binding protein ModC;
P	Q881C1	DR	molybdenum ion transmembrane transporter activity
P	Q882I8	DR	L-arabinose-importing ATPase activity
P	Q882S0	DR	phosphonate transmembrane-transporting ATPase activity
P	Q883V4	CC	One of the components of the high-affinity ATP-driven potassium transport (or KDP) system, which catalyzes the hydrolysis of ATP coupled with the exchange of hydrogen and potassium ions
P	Q883V4	DE	Potassium-binding and translocating subunit C;
P	Q883V4	DE	Potassium-translocating ATPase C chain;
P	Q883V4	DE	Potassium-transporting ATPase C chain;
P	Q883V4	DR	potassium-transporting ATPase activity
P	Q883V6	CC	One of the components of the high-affinity ATP-driven potassium transport (or KDP) system, which catalyzes the hydrolysis of ATP coupled with the exchange of hydrogen and potassium ions (By similarity).
P	Q883V6	DE	Potassium-binding and translocating subunit A;
P	Q883V6	DE	Potassium-translocating ATPase A chain;
P	Q883V6	DE	Potassium-transporting ATPase A chain;
P	Q883V6	DR	potassium-transporting ATPase activity

P	Q884I3	DE	Lipoprotein-releasing system ATP-binding protein LolD;
P	Q884I3	DR	lipoprotein transporter activity
P	Q888L8	CC	The physiological role may be the reduction of the intracellular concentration of toxic sugars or sugar metabolites (By similarity).
P	Q888L8	DE	Probable sugar efflux transporter;
P	Q889G0	DR	phosphonate transmembrane-transporting ATPase activity
P	Q88AS5	CC	Part of the ABC transporter complex CysAWTP involved in sulfate/thiosulfate import
P	Q88AS5	DE	Sulfate-transporting ATPase;
P	Q88AS5	DE	Sulfate/thiosulfate import ATP-binding protein CysA;
P	Q88AS5	DR	sulfate transmembrane-transporting ATPase activity
P	Q88C57	CC	Part of the ABC transporter complex PstSACB involved in phosphate import
P	Q88C57	DE	ABC phosphate transporter 2;
P	Q88C57	DE	Phosphate import ATP-binding protein PstB 2;
P	Q88C57	DE	Phosphate-transporting ATPase 2;
P	Q88C57	DR	inorganic phosphate transmembrane transporter activity
P	Q88C57	DR	phosphate transmembrane-transporting ATPase activity
P	Q88CL2	CC	Part of the ABC transporter complex CysAWTP involved in sulfate/thiosulfate import
P	Q88CL2	DE	Sulfate-transporting ATPase;
P	Q88CL2	DE	Sulfate/thiosulfate import ATP-binding protein CysA;
P	Q88CL2	DR	sulfate transmembrane-transporting ATPase activity
P	Q88CP1	DE	Cadmium translocating P-type ATPase;
P	Q88DY1	CC	Part of the ABC transporter complex HmuTUV involved in hemin import
P	Q88DY1	DE	Hemin import ATP-binding protein HmuV;
P	Q88EX5	CC	Part of the ABC transporter complex CcmAB involved in the biogenesis of c-type cytochromes; once thought to export heme, this seems not to be the case, but its exact role is uncertain
P	Q88EX5	DE	Heme exporter protein A;
P	Q88EX5	DR	heme-transporting ATPase activity
P	Q88FD7	CC	One of the components of the high-affinity ATP-driven potassium transport (or KDP) system, which catalyzes the hydrolysis of ATP coupled with the exchange of hydrogen and potassium ions (By similarity).
P	Q88FD7	DE	Potassium-binding and translocating subunit A;
P	Q88FD7	DE	Potassium-translocating ATPase A chain;
P	Q88FD7	DE	Potassium-transporting ATPase A chain;
P	Q88FD7	DR	potassium-transporting ATPase activity
P	Q88FD8	CC	One of the components of the high-affinity ATP-driven potassium transport (or KDP) system, which catalyzes the hydrolysis of ATP coupled with the exchange of hydrogen and potassium ions
P	Q88FD8	DE	Potassium-binding and translocating subunit C;
P	Q88FD8	DE	Potassium-translocating ATPase C chain;
P	Q88FD8	DE	Potassium-transporting ATPase C chain;
P	Q88FD8	DR	potassium-transporting ATPase activity
P	Q88FQ6	CC	Na+/H+ antiporter that extrudes sodium in exchange for external protons (By similarity)
P	Q88FQ6	DE	Na+/H+ antiporter nhaB;
P	Q88FQ6	DE	Sodium/proton antiporter nhaB;
P	Q88FQ6	DR	sodium:hydrogen antiporter activity
P	Q88G95	DR	molybdate transmembrane-transporting ATPase activity
P	Q88HL0	CC	Part of the ABC transporter complex NikABCDE involved in nickel import
P	Q88HL0	DE	Nickel import ATP-binding protein NikE;
P	Q88HL0	DR	nickel-transporting ATPase activity
P	Q88HL1	CC	Part of the ABC transporter complex NikABCDE involved in nickel import
P	Q88HL1	DE	Nickel import ATP-binding protein NikD;
P	Q88HL1	DR	nickel-transporting ATPase activity
P	Q88J90	CC	Part of the ABC transporter complex RbsABCD involved in ribose import
P	Q88J90	DE	Ribose import ATP-binding protein RbsA;
P	Q88JJ0	CC	Part of the ABC transporter complex PstSACB involved in phosphate import
P	Q88JJ0	DE	ABC phosphate transporter 1;
P	Q88JJ0	DE	Phosphate import ATP-binding protein PstB 1;
P	Q88JJ0	DE	Phosphate-transporting ATPase 1;
P	Q88JJ0	DR	inorganic phosphate transmembrane transporter activity
P	Q88JJ0	DR	phosphate transmembrane-transporting ATPase activity
P	Q88K49	CC	Involved in the import of serine and threonine into the cell, with the concomitant import of sodium (symport system) (By similarity).
P	Q88K49	DE	Na+/serine-threonine symporter;
P	Q88K49	DR	sodium:dicarboxylate symporter activity

P	Q88K49	DR	sodium:dicarboxylate symporter activity
P	Q88K63	CC	The physiological role may be the reduction of the intracellular concentration of toxic sugars or sugar metabolites (By similarity).
P	Q88K63	DE	Probable sugar efflux transporter;
P	Q88KY4	DE	Lipoprotein-releasing system ATP-binding protein LolD;
P	Q88KY4	DR	lipoprotein transporter activity
P	Q88NK7	CC	Transport of potassium into the cell (By similarity).
P	Q88NK7	DE	Probable potassium transport system protein kup;
P	Q88NK7	DR	potassium ion transmembrane transporter activity
P	Q88NS2	CC	Na+/H+ antiporter that extrudes sodium in exchange for external protons (By similarity).
P	Q88NS2	DE	Na+/H+ antiporter nhaA 1;
P	Q88NS2	DE	Sodium/proton antiporter nhaA 1;
P	Q88PM5	DR	phosphonate transmembrane-transporting ATPase activity
P	Q88RA1	CC	Part of the ABC transporter complex TauABC involved in taurine import
P	Q88RA1	DE	Taurine import ATP-binding protein TauB;
P	Q88RA1	DR	taurine-transporting ATPase activity
P	Q88RB3	CC	Part of the ABC transporter complex MetNIQ involved in methionine import
P	Q88RB3	DE	Methionine import ATP-binding protein MetN 2;
P	Q88RL1	CC	Part of the ABC transporter complex ZnuABC involved in zinc import
P	Q88RL1	DE	Zinc import ATP-binding protein ZnuC;
P	Q88RL1	DR	zinc transporting ATPase activity
P	Q88RL5	CC	Part of the ABC transporter complex MetNIQ involved in methionine import
P	Q88RL5	DE	Methionine import ATP-binding protein MetN 1;
P	Q88RT8	DE	Cadmium translocating P-type ATPase;
P	Q88SV0	CC	Transport of potassium into the cell (By similarity).
P	Q88SV0	DE	Probable potassium transport system protein kup 2;
P	Q88SV0	DR	potassium ion transmembrane transporter activity
P	Q88UV2	CC	Part of the ABC transporter complex MetNIQ involved in methionine import
P	Q88UV2	DE	Methionine import ATP-binding protein MetN 2;
P	Q88WA5	CC	Part of the ABC transporter complex MetNIQ involved in methionine import
P	Q88WA5	DE	Methionine import ATP-binding protein MetN 1;
P	Q88YK7	CC	Part of the ABC transporter complex PstSACB involved in phosphate import
P	Q88YK7	DE	ABC phosphate transporter 2;
P	Q88YK7	DE	Phosphate import ATP-binding protein PstB 2;
P	Q88YK7	DE	Phosphate-transporting ATPase 2;
P	Q88YK7	DR	inorganic phosphate transmembrane transporter activity
P	Q88YK7	DR	phosphate transmembrane-transporting ATPase activity
P	Q88YK8	CC	Part of the ABC transporter complex PstSACB involved in phosphate import
P	Q88YK8	DE	ABC phosphate transporter 1;
P	Q88YK8	DE	Phosphate import ATP-binding protein PstB 1;
P	Q88YK8	DE	Phosphate-transporting ATPase 1;
P	Q88YK8	DR	inorganic phosphate transmembrane transporter activity
P	Q88YK8	DR	phosphate transmembrane-transporting ATPase activity
P	Q88YN5	DR	phosphonate transmembrane-transporting ATPase activity
P	Q88Z42	CC	Transport of potassium into the cell (By similarity).
P	Q88Z42	DE	Probable potassium transport system protein kup 1;
P	Q88Z42	DR	potassium ion transmembrane transporter activity
P	Q88ZJ6	CC	Part of the ABC transporter complex PotABCD involved in spermidine/putrescine import
P	Q88ZJ6	CC	Part of the ABC transporter complex PotABCD involved in spermidine/putrescine import
P	Q88ZJ6	DE	Spermidine/putrescine import ATP-binding protein PotA;
P	Q88ZJ6	DE	Spermidine/putrescine import ATP-binding protein PotA;
P	Q891M1	CC	Part of the ABC transporter complex RbsABCD involved in ribose import
P	Q891M1	DE	Ribose import ATP-binding protein RbsA;
P	Q895C4	CC	Part of the ABC transporter complex MetNIQ involved in methionine import
P	Q895C4	DE	Methionine import ATP-binding protein MetN;
P	Q895Y0	CC	Part of the ABC transporter complex PstSACB involved in phosphate import
P	Q895Y0	DE	ABC phosphate transporter;
P	Q895Y0	DE	Phosphate import ATP-binding protein PstB;
P	Q895Y0	DE	Phosphate-transporting ATPase;
P	Q895Y0	DR	inorganic phosphate transmembrane transporter activity
P	Q895Y0	DR	phosphate transmembrane-transporting ATPase activity
P	Q896Y2	CC	Part of the ABC transporter complex MglABC involved in galactose/methyl galactoside import
P	Q896Y2	DE	Galactose/methyl galactoside import ATP-binding protein MglA;
P	Q89A24	CC	Low-affinity inorganic phosphate transport (By similarity).

P	Q89A24	DE	Low-affinity inorganic phosphate transporter;
P	Q89A24	DR	inorganic phosphate transmembrane transporter activity
P	Q89A36	CC	This system is involved in mannitol transport (By similarity).
P	Q89A36	DE	Mannitol permease IIC component;
P	Q89A36	DE	Mannitol-specific phosphotransferase enzyme IIA component;
P	Q89A36	DE	Mannitol-specific phosphotransferase enzyme IIB component;
P	Q89A36	DE	PTS system mannitol-specific EIIA component;
P	Q89A36	DE	PTS system mannitol-specific EIIB component;
P	Q89A36	DE	PTS system mannitol-specific EIIC component;
P	Q89A36	DE	PTS system mannitol-specific EIICBA component;
P	Q89AG6	CC	This system is involved in glucose transport (By similarity).
P	Q89AG6	DE	Glucose permease IIC component;
P	Q89AG6	DE	Glucose-specific phosphotransferase enzyme IIB component;
P	Q89AG6	DE	PTS system glucose-specific EIIB component;
P	Q89AG6	DE	PTS system glucose-specific EIIC component;
P	Q89AG6	DE	PTS system glucose-specific EIICB component;
P	Q89AG6	DR	glucose transmembrane transporter activity
P	Q89AJ0	CC	Part of the ABC transporter complex ZnuABC involved in zinc import
P	Q89AJ0	DE	Zinc import ATP-binding protein ZnuC;
P	Q89AJ0	DR	zinc transporting ATPase activity
P	Q89AJ1	CC	Involved in the high-affinity zinc uptake transport system (By similarity).
P	Q89AJ1	DE	High-affinity zinc uptake system membrane protein znuB;
P	Q89B05	CC	This system is involved in glucose transport (By similarity).
P	Q89B05	DE	Glucose-specific phosphotransferase enzyme IIA component;
P	Q89B05	DE	PTS system glucose-specific EIIA component;
P	Q89C51	DR	phosphonate transmembrane-transporting ATPase activity
P	Q89EG9	CC	Channel that permits osmotically driven movement of water in both directions
P	Q89EG9	CC	It mediates rapid entry or exit of water in response to abrupt changes in osmolarity (By similarity).
P	Q89EW7	CC	Part of the ABC transporter complex ModABC involved in molybdenum import
P	Q89EW7	DE	Molybdenum import ATP-binding protein ModC 2;
P	Q89EW7	DR	molybdenum ion transmembrane transporter activity
P	Q89FC2	CC	One of the components of the high-affinity ATP-driven potassium transport (or KDP) system, which catalyzes the hydrolysis of ATP coupled with the exchange of hydrogen and potassium ions (By similarity).
P	Q89FC2	DE	Potassium-binding and translocating subunit A;
P	Q89FC2	DE	Potassium-translocating ATPase A chain;
P	Q89FC2	DE	Potassium-transporting ATPase A chain;
P	Q89FC2	DR	potassium-transporting ATPase activity
P	Q89FC4	CC	One of the components of the high-affinity ATP-driven potassium transport (or KDP) system, which catalyzes the hydrolysis of ATP coupled with the exchange of hydrogen and potassium ions
P	Q89FC4	DE	Potassium-binding and translocating subunit C;
P	Q89FC4	DE	Potassium-translocating ATPase C chain;
P	Q89FC4	DE	Potassium-transporting ATPase C chain;
P	Q89FC4	DR	potassium-transporting ATPase activity
P	Q89K67	CC	H+-stimulated, highly selective, manganese uptake system (By similarity).
P	Q89K67	DE	Probable manganese transport protein mntH;
P	Q89KN0	DE	Lipoprotein-releasing system ATP-binding protein LolD;
P	Q89KN0	DR	lipoprotein transporter activity
P	Q89L53	CC	Transport of potassium into the cell (By similarity).
P	Q89L53	DE	Probable potassium transport system protein kup 3;
P	Q89L53	DR	potassium ion transmembrane transporter activity
P	Q89LP2	CC	Part of the ABC transporter complex MetNIQ involved in methionine import
P	Q89LP2	DE	Methionine import ATP-binding protein MetN;
P	Q89NN5	CC	Transport of potassium into the cell (By similarity).
P	Q89NN5	DE	Probable potassium transport system protein kup 2;
P	Q89NN5	DR	potassium ion transmembrane transporter activity
P	Q89NN6	CC	Transport of potassium into the cell (By similarity).
P	Q89NN6	DE	Probable potassium transport system protein kup 1;
P	Q89NN6	DR	potassium ion transmembrane transporter activity
P	Q89TQ9	CC	Part of the ABC transporter complex ModABC involved in molybdenum import
P	Q89TQ9	DE	Molybdenum import ATP-binding protein ModC 1;
P	Q89TQ9	DR	molybdenum ion transmembrane transporter activity
P	Q89UD2	CC	Part of the ABC transporter complex CysAWTP involved in sulfate/thiosulfate [illegible]

P	Q89UD2	DE	Sulfate-transporting ATPase;
P	Q89UD2	DE	Sulfate/thiosulfate import ATP-binding protein CysA;
P	Q89UD2	DR	sulfate transmembrane-transporting ATPase activity
P	Q89VF2	CC	Part of the ABC transporter complex PstSACB involved in phosphate import
P	Q89VF2	DE	ABC phosphate transporter;
P	Q89VF2	DE	Phosphate import ATP-binding protein PstB;
P	Q89VF2	DE	Phosphate-transporting ATPase;
P	Q89VF2	DR	inorganic phosphate transmembrane transporter activity
P	Q89VF2	DR	phosphate transmembrane-transporting ATPase activity
P	Q89WG0	DR	glycerol-3-phosphate-transporting ATPase activity
P	Q8A1M1	DE	Lipoprotein-releasing system ATP-binding protein LolD;
P	Q8A1M1	DR	lipoprotein transporter activity
P	Q8A519	CC	One of the components of the high-affinity ATP-driven potassium transport (or KDP) system, which catalyzes the hydrolysis of ATP coupled with the exchange of hydrogen and potassium ions (By similarity).
P	Q8A519	DE	Potassium-binding and translocating subunit A;
P	Q8A519	DE	Potassium-translocating ATPase A chain;
P	Q8A519	DE	Potassium-transporting ATPase A chain;
P	Q8A519	DR	potassium-transporting ATPase activity
P	Q8A521	CC	One of the components of the high-affinity ATP-driven potassium transport (or KDP) system, which catalyzes the hydrolysis of ATP coupled with the exchange of hydrogen and potassium ions
P	Q8A521	DE	Potassium-binding and translocating subunit C;
P	Q8A521	DE	Potassium-translocating ATPase C chain;
P	Q8A521	DE	Potassium-transporting ATPase C chain;
P	Q8A521	DR	potassium-transporting ATPase activity
P	Q8A853	CC	Part of the ABC transporter complex PstSACB involved in phosphate import
P	Q8A853	DE	ABC phosphate transporter;
P	Q8A853	DE	Phosphate import ATP-binding protein PstB;
P	Q8A853	DE	Phosphate-transporting ATPase;
P	Q8A853	DR	inorganic phosphate transmembrane transporter activity
P	Q8A853	DR	phosphate transmembrane-transporting ATPase activity
P	Q8A883	CC	Part of the ABC transporter complex PotABCD involved in spermidine/putrescine import
P	Q8A883	CC	Part of the ABC transporter complex PotABCD involved in spermidine/putrescine import
P	Q8A883	DE	Spermidine/putrescine import ATP-binding protein PotA;
P	Q8A883	DE	Spermidine/putrescine import ATP-binding protein PotA;
P	Q8BFU8	CC	Mediates the uptake of glutamate into synaptic vesicles at presynaptic nerve terminals of excitatory neural cells
P	Q8BFU8	DE	Vesicular glutamate transporter 3;
P	Q8BFU8	DR	L-glutamate transmembrane transporter activity
P	Q8BFW9	CC	Facilitative glucose transporter (By similarity).
P	Q8BFW9	DE	Glucose transporter type 12;
P	Q8BFW9	DE	Solute carrier family 2, facilitated glucose transporter member 12;
P	Q8BG16	CC	Mediates the saturable, pH-sensitive and electrogenic cotransport of proline and sodium ions with a stoichiometry of 1:1
P	Q8BG16	DE	Sodium- and chloride-dependent neurotransmitter transporter NTT73;
P	Q8BG16	DE	Sodium-dependent neutral amino acid transporter B(0)AT2;
P	Q8BG16	DR	neurotransmitter:sodium symporter activity
P	Q8BG22	CC	Play a role in modulating chloride current across the plasma membrane in a calcium-dependent manner, and cell adhesion
P	Q8BG22	DR	chloride channel activity
P	Q8BGC3	CC	Proton-linked monocarboxylate transporter
P	Q8BGC3	DE	Monocarboxylate transporter 12;
P	Q8BGG0	CC	Facilitates the accumulation of zinc from the cytoplasm into intracellular vesicles, being a zinc-efflux transporter
P	Q8BGG0	CC	May be a major component for providing zinc to insulin maturation and/or storage processes in insulin-secreting pancreatic beta- cells (By similarity).
P	Q8BGG0	DE	Zinc transporter 8;
P	Q8BGY9	CC	Imports choline from the extracellular space to the neuron with high affinity
P	Q8BGY9	CC	Sodium ion and chloride ion dependent.
P	Q8BGY9	DE	Hemicholinium-3-sensitive choline transporter;
P	Q8BGY9	DE	High affinity choline transporter 1;
P	Q8BH59	CC	Calcium-dependent mitochondrial aspartate and glutamate carrier
P	Q8BH59	DE	Mitochondrial aspartate glutamate carrier 1;
P	Q8BH59	DR	L-glutamate transmembrane transporter activity

P	Q8BHB9	CC	May insert into membranes and form chloride ion channels
P	Q8BHB9	CC	May play a critical role in water-secreting cells, possibly through the regulation of chloride ion transport (By similarity).
P	Q8BHB9	DE	Chloride intracellular channel protein 6;
P	Q8BHB9	DR	voltage-gated chloride channel activity
P	Q8BHJ7	CC	GABA, the major inhibitory neurotransmitter in the vertebrate brain, mediates neuronal inhibition by binding to the GABA/benzodiazepine receptor and opening an integral chloride channel (By similarity).
P	Q8BHJ7	DR	chloride channel activity
P	Q8BHK1	CC	Can also transport other divalent cations such as Fe(2+), Sr(2+), Ba(2+), Mn(2+) and Co(2+) but to a much less extent than Mg2+.
P	Q8BHK1	DE	Magnesium transporter NIPA1;
P	Q8BIV7	DE	Proton-associated sugar transporter A;
P	Q8BJA2	CC	Acts as a magnesium transporter that is responsive to magnesium balance (By [illegible]
P	Q8BJI1	DE	Sodium-dependent neurotransmitter transporter NTT4;
P	Q8BJI1	DE	Sodium-dependent neutral amino acid transporter SLC6A17;
P	Q8BJI1	DR	neurotransmitter:sodium symporter activity
P	Q8BJM5	CC	Zinc-efflux transporter which allocates the cytoplasmic zinc to the trans-Golgi network (TGN) as well as the vesicular compartment.
P	Q8BJM5	DE	Zinc transporter 6;
P	Q8BJM5	DR	zinc ion transmembrane transporter activity
P	Q8BL03	DE	Mitochondrial carnitine/acylcarnitine carrier protein CACL;
P	Q8BL03	DR	acyl carnitine transporter activity
P	Q8BLE7	CC	Mediates the uptake of glutamate into synaptic vesicles at presynaptic nerve terminals of excitatory neural cells
P	Q8BLE7	DE	Vesicular glutamate transporter 2;
P	Q8BLE7	DR	L-glutamate transmembrane transporter activity
P	Q8BLV3	CC	Mediates electroneutral exchange of protons for Na+ and K+ across [illegible]
P	Q8BLV3	DE	Na+/H+ exchanger 7;
P	Q8BLV3	DE	Sodium/hydrogen exchanger 7;
P	Q8BLV3	DR	sodium:hydrogen antiporter activity
P	Q8BMD8	CC	May act as a ATP-Mg/Pi exchanger that mediates the transport of Mg-ATP in exchange for phosphate, catalyzing the net uptake or efflux of adenine nucleotides into or from the mitochondria (By similarity).
P	Q8BMG8	CC	Transports folate across the inner membranes of mitochondria (By similarity).
P	Q8BMG8	DE	Mitochondrial folate transporter/carrier;
P	Q8BMW7	CC	Can also transport other divalent cations such as Fe(2+), Sr(2+), Ba(2+), Mn(2+), Cu(2+) and Co(2+) but to a much less extent than Mg2+.
P	Q8BMW7	DE	Magnesium transporter NIPA3;
P	Q8BPX9	CC	Proton oligopeptide cotransporter
P	Q8BQZ8	CC	Potassium channel subunit
P	Q8BQZ8	DE	Potassium voltage-gated channel subfamily S member 3;
P	Q8BQZ8	DE	Voltage-gated potassium channel subunit Kv9.3;
P	Q8BQZ8	DR	voltage-gated potassium channel activity
P	Q8BUE1	CC	In apical cell membrane may be involved in mediating sodium absorption
P	Q8BUE1	CC	Major proton extruding system driven by the inward sodium ion chemical [illegible]
P	Q8BUE1	DE	Na+/H+ exchanger 4;
P	Q8BUE1	DE	Sodium/hydrogen exchanger 4;
P	Q8BUE1	DR	sodium:hydrogen antiporter activity
P	Q8BWC0	CC	[illegible] acid adenine dinucleotide phosphate (NAADP) receptor that may function as one of the major voltage-gated Ca2+ channels (VDCC) across the [illegible]
P	Q8BWC0	DE	Two pore calcium channel protein 2;
P	Q8BWC0	DE	Voltage-dependent calcium channel protein TPC2;
P	Q8BWC0	DR	calcium channel activity
P	Q8BWG9	CC	CRAC channels are the main pathway for Ca2+ influx in T-cells and promote the immune response to pathogens by activating the transcription factor NFAT (By similarity).
P	Q8BWG9	CC	Ca2+ release-activated Ca2+ (CRAC) channel subunit which mediates Ca2+ influx following depletion of intracellular Ca2+ stores and channel activation by the Ca2+ sensor, STIM1
P	Q8BWG9	DE	Calcium release-activated calcium channel protein 1;
P	Q8BWG9	DR	store-operated calcium channel activity
P	Q8BWY7	DE	Zinc transporter ZIP11;
P	Q8BXK9	CC	Can insert into membranes and form poorly selective ion channels that may also transport chloride ions

P	Q8BXK9	DE	Chloride intracellular channel protein 5;
P	Q8BXK9	DR	voltage-gated chloride channel activity
P	Q8BY89	CC	Putative choline transporter (By similarity).
P	Q8BY89	DE	Choline transporter-like protein 2;
P	Q8BYF6	CC	Acts as an electrogenic sodium (Na+) and chloride (Cl-)-dependent sodium-coupled solute transporter, including transport of monocarboxylates (short-chain fatty acids including L-lactate, D-lactate, pyruvate, acetate, propionate, valerate and butyrate), lactate, mocarboxylate drugs (nicotinate, benzoate, salicylate and 5-aminosalicylate) and ketone bodies (beta-D- hydroxybutyrate, acetoacetate and alpha-ketoisocaproate), with a Na+:substrate stoichiometry of between 4:1 and 2:1
P	Q8BYF6	CC	May play a critical role in the entry of L-lactate and ketone bodies into neurons by a process driven by an electrochemical Na+ gradient and hence contribute to the maintenance of the energy status and function of neurons.
P	Q8BYF6	DE	Electrogenic sodium monocarboxylate cotransporter;
P	Q8BYF6	DE	Sodium-coupled monocarboxylate transporter 1;
P	Q8BYR8	CC	Acts as a plasma-membrane magnesium transporter (By similarity).
P	Q8BZ00	CC	May act in electroneutral exchange of protons for Na+ across membranes
P	Q8BZ00	DE	Na+/H+ exchanger 9;
P	Q8BZ00	DE	Sodium/hydrogen exchanger 9;
P	Q8BZ00	DR	sodium:hydrogen antiporter activity
P	Q8BZF2	CC	Can also transport other divalent cations such as Ba(2+), Mn(2+), Sr(2+) and Co(2+) but to a much less extent than Mg2+
P	Q8BZF2	DE	Magnesium transporter NIPA4;
P	Q8BZH0	DE	Zinc transporter ZIP13;
P	Q8BZN2	CC	Potassium channel subunit that does not form functional channels by itself
P	Q8BZN2	DE	Potassium voltage-gated channel subfamily V member 1;
P	Q8BZN2	DE	Voltage-gated potassium channel subunit Kv8.1;
P	Q8BZN2	DR	voltage-gated potassium channel activity
P	Q8C0X2	DE	Na+/H+ exchanger-like domain-containing protein 1;
P	Q8C0X2	DE	Sodium/hydrogen exchanger-like domain-containing protein 1;
P	Q8C145	DE	Zinc transporter ZIP6;
P	Q8C261	CC	Probably transports 1 Ca2+ and 1 K+ in exchange for 4 Na+ (By similarity).
P	Q8C261	CC	Probably transports 1 Ca2+ and 1 K+ in exchange for 4 Na+ (By similarity).
P	Q8C261	CC	Probably transports 1 Ca2+ and 1 K+ in exchange for 4 Na+ (By similarity).
P	Q8C261	DE	Na+/K+/Ca2+-exchange protein 5;
P	Q8C261	DE	Na+/K+/Ca2+-exchange protein 5;
P	Q8C261	DE	Na+/K+/Ca2+-exchange protein 5;
P	Q8C261	DE	Sodium/potassium/calcium exchanger 5;
P	Q8C261	DE	Sodium/potassium/calcium exchanger 5;
P	Q8C261	DE	Sodium/potassium/calcium exchanger 5;
P	Q8C3K6	CC	Actively transports glucose into cells by Na+ cotransport with a Na+ to glucose coupling ratio of 2:1
P	Q8C3K6	CC	Actively transports glucose into cells by Na+ cotransport with a Na+ to glucose coupling ratio of 2:1
P	Q8C3K6	CC	Efficient substrate transport in mammalian kidney is provided by the concerted action of a low affinity high capacity and a high affinity low capacity Na+/glucose cotransporter arranged in series along kidney proximal tubules.
P	Q8C3K6	CC	Efficient substrate transport in mammalian kidney is provided by the concerted action of a low affinity high capacity and a high affinity low capacity Na+/glucose cotransporter arranged in series along kidney proximal tubules.
P	Q8C3K6	DE	High affinity sodium-glucose cotransporter;
P	Q8C3K6	DE	Na+/glucose cotransporter 1;
P	Q8C3K6	DE	Na+/glucose cotransporter 1;
P	Q8C3K6	DE	Sodium/glucose cotransporter 1;
P	Q8C3K6	DE	Sodium/glucose cotransporter 1;
P	Q8C4A0	CC	Forms a water-specific channel that participates in distinct physiological functions such as glomerular filtration, tubular endocytosis and acid-base metabolism (By similarity).
P	Q8C4A0	DR	water channel activity
P	Q8CBB2	CC	Proton oligopeptide cotransporter (Potential).
P	Q8CE94	CC	Proton-linked monocarboxylate transporter
P	Q8CE94	DE	Monocarboxylate transporter 13;
P	Q8CFS6	CC	Potassium channel subunit
P	Q8CFS6	DE	Potassium voltage-gated channel subfamily V member 2;
P	Q8CFS6	DE	Voltage-gated potassium channel subunit Kv8.2;
P	Q8CFS6	DR	voltage-gated potassium channel activity

P	Q8CGQ8	CC	Transports 1 Ca2+ and 1 K+ in exchange for 4 Na+.
P	Q8CGQ8	CC	Transports 1 Ca2+ and 1 K+ in exchange for 4 Na+.
P	Q8CGQ8	CC	Transports 1 Ca2+ and 1 K+ in exchange for 4 Na+.
P	Q8CGQ8	DE	Na+/K+/Ca2+-exchange protein 4;
P	Q8CGQ8	DE	Na+/K+/Ca2+-exchange protein 4;
P	Q8CGQ8	DE	Na+/K+/Ca2+-exchange protein 4;
P	Q8CGQ8	DE	Sodium/potassium/calcium exchanger 4;
P	Q8CGQ8	DE	Sodium/potassium/calcium exchanger 4;
P	Q8CGQ8	DE	Sodium/potassium/calcium exchanger 4;
P	Q8CIA5	CC	Sugar transporter that specifically mediates the transport of UDP-xylose (UDP-Xyl) and UDP-N-acetylglucosamine (UDP-GlcNAc) from cytosol into Golgi (By similarity).
P	Q8CIA5	CC	Sugar transporter that specifically mediates the transport of UDP-xylose (UDP-Xyl) and UDP-N-acetylglucosamine (UDP-GlcNAc) from cytosol into Golgi (By similarity).
P	Q8CIA5	DE	UDP-xylose and UDP-N-acetylglucosamine transporter;
P	Q8CIA5	DE	UDP-xylose and UDP-N-acetylglucosamine transporter;
P	Q8CIA9	DR	tetracycline:hydrogen antiporter activity
P	Q8CMU4	DR	phosphonate transmembrane-transporting ATPase activity
P	Q8CN02	CC	Involved in copper export (By similarity).
P	Q8CN02	DE	Copper-exporting P-type ATPase A;
P	Q8CN02	DR	copper-exporting ATPase activity
P	Q8CN76	CC	Also possibly involved in excretion of nitrite produced by the dissimilatory reduction of nitrate (By similarity).
P	Q8CN76	CC	Probably required for nitrate uptake under anoxic conditions
P	Q8CN76	DE	Probable nitrate transporter narT;
P	Q8CNF2	CC	Involved in the uptake of glucose (By similarity).
P	Q8CNF2	DE	Probable glucose uptake protein glcU;
P	Q8CNF6	CC	This system is involved in lactose transport (By similarity).
P	Q8CNF6	DE	Lactose-specific phosphotransferase enzyme IIA component;
P	Q8CNF6	DE	PTS system lactose-specific EIIA component;
P	Q8CNF7	CC	This system is involved in lactose transport.
P	Q8CNF7	DE	Lactose permease IIC component;
P	Q8CNF7	DE	Lactose-specific phosphotransferase enzyme IIB component;
P	Q8CNF7	DE	PTS system lactose-specific EIIB component;
P	Q8CNF7	DE	PTS system lactose-specific EIIC component;
P	Q8CNF7	DE	PTS system lactose-specific EIICB component;
P	Q8CNP2	CC	Catalyzes the sodium-dependent uptake of extracellular L-proline (By similarity).
P	Q8CNP2	DE	Proline permease;
P	Q8CNP2	DE	Sodium/proline symporter;
P	Q8CNP2	DE	Sodium/proline symporter;
P	Q8CNP2	DR	proline:sodium symporter activity
P	Q8CNP2	DR	proline:sodium symporter activity
P	Q8CP79	CC	This system is involved in glucose transport (By similarity).
P	Q8CP79	DE	Glucose-specific phosphotransferase enzyme IIA component;
P	Q8CP79	DE	PTS system glucose-specific EIIA component;
P	Q8CP98	CC	Part of the ABC transporter complex pstSACB involved in phosphate import (By similarity).
P	Q8CP98	DE	Phosphate-binding protein pstS;
P	Q8CPA1	CC	Part of the ABC transporter complex PstSACB involved in phosphate import
P	Q8CPA1	DE	ABC phosphate transporter;
P	Q8CPA1	DE	Phosphate import ATP-binding protein PstB;
P	Q8CPA1	DE	Phosphate-transporting ATPase;
P	Q8CPA1	DR	inorganic phosphate transmembrane transporter activity
P	Q8CPA1	DR	phosphate transmembrane-transporting ATPase activity
P	Q8CPM6	CC	H+-stimulated, highly selective, manganese uptake system (By similarity).
P	Q8CPM6	DE	Probable manganese transport protein mntH;
P	Q8CPN0	CC	Part of the ABC transporter complex PotABCD involved in spermidine/putrescine import
P	Q8CPN0	CC	Part of the ABC transporter complex PotABCD involved in spermidine/putrescine import
P	Q8CPN0	DE	Spermidine/putrescine import ATP-binding protein PotA;
P	Q8CPN0	DE	Spermidine/putrescine import ATP-binding protein PotA;
P	Q8CPU8	CC	Mnh complex is a Na+/H+ antiporter involved in Na+ excretion (By similarity).
P	Q8CPU8	DE	Na+/H+ antiporter subunit A1;
P	Q8CPU9	CC	Mnh complex is a Na+/H+ antiporter involved in Na+ excretion (By similarity).
P	Q8CPU9	DE	Na+/H+ antiporter subunit B1;
P	Q8CPV0	CC	Mnh complex is a Na+/H+ antiporter involved in Na+ excretion (By similarity).
P	Q8CPV0	DE	Na+/H+ antiporter subunit C1;

P	Q8CPV1	CC	Mnh complex is a Na+/H+ antiporter involved in Na+ excretion (By similarity).
P	Q8CPV1	DE	Na+/H+ antiporter subunit D1;
P	Q8CPV2	CC	Mnh complex is a Na+/H+ antiporter involved in Na+ excretion (By similarity).
P	Q8CPV2	DE	Na+/H+ antiporter subunit E1;
P	Q8CPV3	CC	Mnh complex is a Na+/H+ antiporter involved in Na+ excretion (By similarity).
P	Q8CPV3	DE	Na+/H+ antiporter subunit F1;
P	Q8CPV4	CC	Mnh complex is a Na+/H+ antiporter involved in Na+ excretion (By similarity).
P	Q8CPV4	DE	Na+/H+ antiporter subunit G1;
P	Q8CQF4	CC	Thought to form the channel of an arsenite pump.
P	Q8CQF4	DR	arsenite transmembrane transporter activity
P	Q8CQF7	CC	Involved in copper transport (By similarity).
P	Q8CQF7	DE	Probable copper-transporting P-type ATPase B;
P	Q8CQF7	DR	copper-exporting ATPase activity
P	Q8CQS7	CC	Part of the ABC transporter complex MetNIQ involved in methionine import
P	Q8CQS7	DE	Methionine import ATP-binding protein MetN 2;
P	Q8CTB2	CC	Part of the ABC transporter complex MetNIQ involved in methionine import
P	Q8CTB2	DE	Methionine import ATP-binding protein MetN 1;
P	Q8CUY0	DR	phosphonate transmembrane-transporting ATPase activity
P	Q8CVC9	CC	Part of the ABC transporter complex pstSACB involved in phosphate import (By similarity).
P	Q8CVC9	DE	Phosphate-binding protein pstS 2;
P	Q8CZD5	CC	This system is involved in glucose transport (By similarity).
P	Q8CZD5	DE	Glucose-specific phosphotransferase enzyme IIA component;
P	Q8CZD5	DE	PTS system glucose-specific EIIA component;
P	Q8D0W8	CC	Part of the ABC transporter complex CysAWTP involved in sulfate/thiosulfate import
P	Q8D0W8	DE	Sulfate-transporting ATPase;
P	Q8D0W8	DE	Sulfate/thiosulfate import ATP-binding protein CysA;
P	Q8D0W8	DR	sulfate transmembrane-transporting ATPase activity
P	Q8D666	CC	Na+/H+ antiporter that extrudes sodium in exchange for external protons (By similarity)
P	Q8D666	DE	Na+/H+ antiporter nhaA 2;
P	Q8D666	DE	Sodium/proton antiporter nhaA 2;
P	Q8D740	CC	Part of the ABC transporter complex ModABC involved in molybdenum import
P	Q8D740	DE	Molybdenum import ATP-binding protein ModC;
P	Q8D740	DR	molybdenum ion transmembrane transporter activity
P	Q8D8Y2	CC	Na+/H+ antiporter that extrudes sodium in exchange for external protons (By similarity)
P	Q8D8Y2	DE	Na+/H+ antiporter nhaA 1;
P	Q8D8Y2	DE	Sodium/proton antiporter nhaA 1;
P	Q8D928	CC	Part of the ABC transporter complex BtuCDF involved in vitamin B12 import
P	Q8D928	DE	Vitamin B12 import ATP-binding protein BtuD;
P	Q8D928	DE	Vitamin B12-transporting ATPase;
P	Q8D9J4	CC	Part of the ABC transporter complex PotABCD involved in spermidine/putrescine import
P	Q8D9J4	CC	Part of the ABC transporter complex PotABCD involved in spermidine/putrescine import
P	Q8D9J4	DE	Spermidine/putrescine import ATP-binding protein PotA;
P	Q8D9J4	DE	Spermidine/putrescine import ATP-binding protein PotA;
P	Q8DAG9	CC	Na+/H+ antiporter that extrudes sodium in exchange for external protons (By similarity)
P	Q8DAG9	DE	Na+/H+ antiporter nhaB;
P	Q8DAG9	DE	Sodium/proton antiporter nhaB;
P	Q8DAG9	DR	sodium:hydrogen antiporter activity
P	Q8DB17	CC	Channel that permits osmotically driven movement of water in both directions
P	Q8DB17	CC	It mediates rapid entry or exit of water in response to abrupt changes in osmolarity (By similarity).
P	Q8DFC3	CC	Part of the ABC transporter complex MetNIQ involved in methionine import
P	Q8DFC3	DE	Methionine import ATP-binding protein MetN;
P	Q8DFC3	DR	D-methionine transmembrane transporter activity
P	Q8DFQ4	CC	Part of the ABC transporter complex ZnuABC involved in zinc import
P	Q8DFQ4	DE	Zinc import ATP-binding protein ZnuC;
P	Q8DFQ4	DR	zinc transporting ATPase activity
P	Q8DGZ3	CC	Part of the ABC transporter complex PstSACB involved in phosphate import
P	Q8DGZ3	DE	ABC phosphate transporter;
P	Q8DGZ3	DE	Phosphate import ATP-binding protein PstB;
P	Q8DGZ3	DE	Phosphate-transporting ATPase;
P	Q8DGZ3	DR	inorganic phosphate transmembrane transporter activity
P	Q8DGZ3	DR	phosphate transmembrane-transporting ATPase activity
P	Q8DHB0	CC	Transport of potassium into the cell (By similarity).
P	Q8DHB0	DE	Probable potassium transport system protein kup;

P	Q8DHB0	DR	potassium ion transmembrane transporter activity
P	Q8DLH9	CC	Plays an important role in protecting the acceptor side of photosystem II (PSII) against oxidative damage, especially under iron-limiting growth conditions (By similarity).
P	Q8DLH9	DE	Iron deficiency-induced protein A;
P	Q8DS05	DE	Putative PTS system, glucose-specific IIABC component;
P	Q8DU23	CC	Part of the ABC transporter complex PstSACB involved in phosphate import
P	Q8DU23	DE	ABC phosphate transporter 2;
P	Q8DU23	DE	Phosphate import ATP-binding protein PstB 2;
P	Q8DU23	DE	Phosphate-transporting ATPase 2;
P	Q8DU23	DR	inorganic phosphate transmembrane transporter activity
P	Q8DU23	DR	phosphate transmembrane-transporting ATPase activity
P	Q8DU24	CC	Part of the ABC transporter complex PstSACB involved in phosphate import
P	Q8DU24	DE	ABC phosphate transporter 1;
P	Q8DU24	DE	Phosphate import ATP-binding protein PstB 1;
P	Q8DU24	DE	Phosphate-transporting ATPase 1;
P	Q8DU24	DR	inorganic phosphate transmembrane transporter activity
P	Q8DU24	DR	phosphate transmembrane-transporting ATPase activity
P	Q8DUF7	CC	Part of the ABC transporter complex PotABCD involved in spermidine/putrescine import
P	Q8DUF7	CC	Part of the ABC transporter complex PotABCD involved in spermidine/putrescine import
P	Q8DUF7	DE	Spermidine/putrescine import ATP-binding protein PotA;
P	Q8DUF7	DE	Spermidine/putrescine import ATP-binding protein PotA;
P	Q8DY54	CC	Part of the ABC transporter complex MetNIQ involved in methionine import
P	Q8DY54	DE	Methionine import ATP-binding protein MetN;
P	Q8DY58	CC	Involved in the import of serine and threonine into the cell, with the concomitant import of sodium (symport system) (By similarity).
P	Q8DY58	DE	Na+/serine-threonine symporter;
P	Q8DY58	DR	sodium:dicarboxylate symporter activity
P	Q8DY58	DR	sodium:dicarboxylate symporter activity
P	Q8DZJ0	CC	Part of the ABC transporter complex PotABCD involved in spermidine/putrescine import
P	Q8DZJ0	CC	Part of the ABC transporter complex PotABCD involved in spermidine/putrescine import
P	Q8DZJ0	DE	Spermidine/putrescine import ATP-binding protein PotA;
P	Q8DZJ0	DE	Spermidine/putrescine import ATP-binding protein PotA;
P	Q8DZL1	CC	Transport of potassium into the cell (By similarity).
P	Q8DZL1	DE	Probable potassium transport system protein kup;
P	Q8DZL1	DR	potassium ion transmembrane transporter activity
P	Q8DZV4	CC	Part of the ABC transporter complex pstSACB involved in phosphate import (By similarity).
P	Q8DZV4	DE	Phosphate-binding protein pstS 1;
P	Q8E281	CC	Part of the ABC transporter complex RbsABCD involved in ribose import
P	Q8E281	DE	Ribose import ATP-binding protein RbsA;
P	Q8E310	CC	Part of the ABC transporter complex pstSACB involved in phosphate import (By similarity).
P	Q8E310	DE	Phosphate-binding protein pstS 2;
P	Q8E3S0	CC	Part of the ABC transporter complex MetNIQ involved in methionine import
P	Q8E3S0	DE	Methionine import ATP-binding protein MetN;
P	Q8E3S4	CC	Involved in the import of serine and threonine into the cell, with the concomitant import of sodium (symport system) (By similarity).
P	Q8E3S4	DE	Na+/serine-threonine symporter;
P	Q8E3S4	DR	sodium:dicarboxylate symporter activity
P	Q8E3S4	DR	sodium:dicarboxylate symporter activity
P	Q8E554	CC	Part of the ABC transporter complex PotABCD involved in spermidine/putrescine import
P	Q8E554	CC	Part of the ABC transporter complex PotABCD involved in spermidine/putrescine import
P	Q8E554	DE	Spermidine/putrescine import ATP-binding protein PotA;
P	Q8E554	DE	Spermidine/putrescine import ATP-binding protein PotA;
P	Q8E575	CC	Transport of potassium into the cell (By similarity).
P	Q8E575	DE	Probable potassium transport system protein kup;
P	Q8E575	DR	potassium ion transmembrane transporter activity
P	Q8E5K2	CC	Part of the ABC transporter complex pstSACB involved in phosphate import (By similarity).
P	Q8E5K2	DE	Phosphate-binding protein pstS 1;
P	Q8E7N9	CC	Part of the ABC transporter complex RbsABCD involved in ribose import
P	Q8E7N9	DE	Ribose import ATP-binding protein RbsA;
P	Q8E8K8	CC	Part of the ABC transporter complex CysAWTP involved in sulfate/thiosulfate import
P	Q8E8K8	DE	Sulfate-transporting ATPase 2;
P	Q8E8K8	DE	Sulfate/thiosulfate import ATP-binding protein CysA 2;

P	Q8E8K8	DR	sulfate transmembrane-transporting ATPase activity
P	Q8E9I8	CC	Part of the ABC transporter complex PstSACB involved in phosphate import
P	Q8E9I8	DE	ABC phosphate transporter 2;
P	Q8E9I8	DE	Phosphate import ATP-binding protein PstB 2;
P	Q8E9I8	DE	Phosphate-transporting ATPase 2;
P	Q8E9I8	DR	inorganic phosphate transmembrane transporter activity
P	Q8E9I8	DR	phosphate transmembrane-transporting ATPase activity
P	Q8EAN3	CC	Part of the ABC transporter complex ModABC involved in molybdenum import
P	Q8EAN3	DE	Molybdenum import ATP-binding protein ModC;
P	Q8EAN3	DR	molybdenum ion transmembrane transporter activity
P	Q8EB59	CC	Part of the ABC transporter complex HmuTUV involved in hemin import
P	Q8EB59	DE	Hemin import ATP-binding protein HmuV;
P	Q8EBC3	CC	Part of the ABC transporter complex CysAWTP involved in sulfate/thiosulfate import
P	Q8EBC3	DE	Sulfate/thiosulfate import ATP-binding protein CysA 1;
P	Q8ECL5	CC	Involved in the import of serine and threonine into the cell, with the concomitant import of sodium (symport system) (By similarity).
P	Q8ECL5	DE	Na+/serine-threonine symporter;
P	Q8ECL5	DR	sodium:dicarboxylate symporter activity
P	Q8ECL5	DR	sodium:dicarboxylate symporter activity
P	Q8ED79	CC	Na+/H+ antiporter that extrudes sodium in exchange for external protons (By similarity)
P	Q8ED79	DE	Na+/H+ antiporter nhaB;
P	Q8ED79	DE	Sodium/proton antiporter nhaB;
P	Q8ED79	DR	sodium:hydrogen antiporter activity
P	Q8EEV5	DE	Lipoprotein-releasing system ATP-binding protein LolD;
P	Q8EEV5	DR	lipoprotein transporter activity
P	Q8EG82	CC	Part of the ABC transporter complex PstSACB involved in phosphate import
P	Q8EG82	DE	ABC phosphate transporter 1;
P	Q8EG82	DE	Phosphate import ATP-binding protein PstB 1;
P	Q8EG82	DE	Phosphate-transporting ATPase 1;
P	Q8EG82	DR	inorganic phosphate transmembrane transporter activity
P	Q8EG82	DR	phosphate transmembrane-transporting ATPase activity
P	Q8EH93	CC	Na+/H+ antiporter that extrudes sodium in exchange for external protons (By similarity)
P	Q8EH93	DE	Na+/H+ antiporter nhaA;
P	Q8EH93	DE	Sodium/proton antiporter nhaA;
P	Q8EHC1	CC	Channel that permits osmotically driven movement of water in both directions
P	Q8EHC1	CC	It mediates rapid entry or exit of water in response to abrupt changes in osmolarity (By similarity).
P	Q8EK40	CC	Part of the ABC transporter complex CcmAB involved in the biogenesis of c-type cytochromes; once thought to export heme, this seems not to be the case, but its exact role is uncertain
P	Q8EK40	DE	Heme exporter protein A;
P	Q8EK40	DR	heme-transporting ATPase activity
P	Q8ELA5	CC	Part of the ABC transporter complex MetNIQ involved in methionine import
P	Q8ELA5	DE	Methionine import ATP-binding protein MetN 4;
P	Q8ELQ6	CC	Part of the ABC transporter complex MetNIQ involved in methionine import
P	Q8ELQ6	DE	Methionine import ATP-binding protein MetN 3;
P	Q8ELR4	CC	Part of the ABC transporter complex PotABCD involved in spermidine/putrescine import
P	Q8ELR4	CC	Part of the ABC transporter complex PotABCD involved in spermidine/putrescine import
P	Q8ELR4	DE	Spermidine/putrescine import ATP-binding protein PotA;
P	Q8ELR4	DE	Spermidine/putrescine import ATP-binding protein PotA;
P	Q8ELT4	CC	Part of the ABC transporter complex PstSACB involved in phosphate import
P	Q8ELT4	DE	ABC phosphate transporter;
P	Q8ELT4	DE	Phosphate import ATP-binding protein PstB;
P	Q8ELT4	DE	Phosphate-transporting ATPase;
P	Q8ELT4	DR	inorganic phosphate transmembrane transporter activity
P	Q8ELT4	DR	phosphate transmembrane-transporting ATPase activity
P	Q8ENB3	CC	Part of the ABC transporter complex RbsABCD involved in ribose import
P	Q8ENB3	DE	Ribose import ATP-binding protein RbsA;
P	Q8ENU2	CC	Part of the ABC transporter complex MetNIQ involved in methionine import
P	Q8ENU2	DE	Methionine import ATP-binding protein MetN 2;
P	Q8EPK1	CC	Part of the ABC transporter complex MetNIQ involved in methionine import
P	Q8EPK1	DE	Methionine import ATP-binding protein MetN 1;
P	Q8EUJ1	CC	Part of the ABC transporter complex PstSACB involved in phosphate import
P	Q8EUJ1	DE	ABC phosphate transporter;
P	Q8EUJ1	DE	Phosphate import ATP-binding protein PstB;

P	Q8EUJ1	DE	Phosphate-transporting ATPase;
P	Q8EUJ1	DR	inorganic phosphate transmembrane transporter activity
P	Q8EUJ1	DR	phosphate transmembrane-transporting ATPase activity
P	Q8EUR3	CC	Part of the ABC transporter complex PotABCD involved in spermidine/putrescine import
P	Q8EUR3	CC	Part of the ABC transporter complex PotABCD involved in spermidine/putrescine import
P	Q8EUR3	DE	Spermidine/putrescine import ATP-binding protein PotA;
P	Q8EUR3	DE	Spermidine/putrescine import ATP-binding protein PotA;
P	Q8F1M1	CC	One of the components of the high-affinity ATP-driven potassium transport (or KDP) system, which catalyzes the hydrolysis of ATP coupled with the exchange of hydrogen and potassium ions (By similarity).
P	Q8F1M1	DE	Potassium-binding and translocating subunit A;
P	Q8F1M1	DE	Potassium-translocating ATPase A chain;
P	Q8F1M1	DE	Potassium-transporting ATPase A chain;
P	Q8F1M1	DR	potassium-transporting ATPase activity
P	Q8F1M2	CC	One of the components of the high-affinity ATP-driven potassium transport (or KDP) system, which catalyzes the hydrolysis of ATP coupled with the exchange of hydrogen and potassium ions
P	Q8F1M2	DE	Potassium-binding and translocating subunit C;
P	Q8F1M2	DE	Potassium-translocating ATPase C chain;
P	Q8F1M2	DE	Potassium-transporting ATPase C chain;
P	Q8F1M2	DR	potassium-transporting ATPase activity
P	Q8F6L8	DE	Lipoprotein-releasing system ATP-binding protein LolD;
P	Q8F6L8	DR	lipoprotein transporter activity
P	Q8F6Z1	CC	Part of the ABC transporter complex CysAWTP involved in sulfate/thiosulfate import
P	Q8F6Z1	DE	Sulfate-transporting ATPase;
P	Q8F6Z1	DE	Sulfate/thiosulfate import ATP-binding protein CysA;
P	Q8F6Z1	DR	sulfate transmembrane-transporting ATPase activity
P	Q8FMN9	CC	Part of the ABC transporter complex PstSACB involved in phosphate import
P	Q8FMN9	DE	ABC phosphate transporter;
P	Q8FMN9	DE	Phosphate import ATP-binding protein PstB;
P	Q8FMN9	DE	Phosphate-transporting ATPase;
P	Q8FMN9	DR	inorganic phosphate transmembrane transporter activity
P	Q8FMN9	DR	phosphate transmembrane-transporting ATPase activity
P	Q8FRX8	CC	Part of the ABC transporter complex MetNIQ involved in methionine import
P	Q8FRX8	DE	Methionine import ATP-binding protein MetN;
P	Q8FUR8	CC	Part of the ABC transporter complex XylFGH involved in xylose import
P	Q8FUR8	DE	Xylose import ATP-binding protein XylG;
P	Q8FUU5	CC	Part of the ABC transporter complex ZnuABC involved in zinc import
P	Q8FUU5	DE	Zinc import ATP-binding protein ZnuC;
P	Q8FUU5	DR	zinc transporting ATPase activity
P	Q8FUU6	CC	Involved in the high-affinity zinc uptake transport system
P	Q8FUU6	DE	High-affinity zinc uptake system protein znuA;
P	Q8FVM9	CC	Part of the ABC transporter complex NikABCDE involved in nickel import
P	Q8FVM9	DE	Nickel import ATP-binding protein NikD;
P	Q8FVM9	DR	nickel-transporting ATPase activity
P	Q8FVN0	CC	Part of the ABC transporter complex NikABCDE involved in nickel import
P	Q8FVN0	DE	Nickel import ATP-binding protein NikE;
P	Q8FVN0	DR	nickel-transporting ATPase activity
P	Q8FW07	DR	glycerol-3-phosphate-transporting ATPase activity
P	Q8FXI7	CC	Part of the ABC transporter complex ModABC involved in molybdenum import
P	Q8FXI7	DE	Molybdenum import ATP-binding protein ModC;
P	Q8FXI7	DR	molybdenum ion transmembrane transporter activity
P	Q8FY85	CC	Transport of water across the membrane
P	Q8FYU9	CC	Part of the ABC transporter complex ThiBPQ involved in thiamine import
P	Q8FYU9	DE	Thiamine import ATP-binding protein ThiQ;
P	Q8FYV0	CC	Part of the ABC transporter complex thiBPQ involved in thiamine import
P	Q8FYV0	DE	Thiamine transport system permease protein thiP;
P	Q8FYV1	CC	Part of the ABC transporter complex thiBPQ involved in thiamine import (By similarity).
P	Q8FYV1	DE	Thiamine-binding periplasmic protein;
P	Q8FZT8	CC	Transport of potassium into the cell (By similarity).
P	Q8FZT8	DE	Probable potassium transport system protein kup;
P	Q8FZT8	DR	potassium ion transmembrane transporter activity
P	Q8G2C7	CC	Na+/H+ antiporter that extrudes sodium in exchange for external protons (By similarity).
P	Q8G2C7	DE	Na+/H+ antiporter nhaA;
P	Q8G2C7	DE	Sodium/proton antiporter nhaA;

P	Q8G358	CC	Part of the ABC transporter complex CcmAB involved in the biogenesis of c-type cytochromes; once thought to export heme, this seems not to be the case, but its exact role is uncertain
P	Q8G358	DE	Heme exporter protein A;
P	Q8G358	DR	heme-transporting ATPase activity
P	Q8G5P8	CC	Part of the ABC transporter complex MetNIQ involved in methionine import
P	Q8G5P8	DE	Methionine import ATP-binding protein MetN;
P	Q8G5R2	CC	Na+/H+ antiporter that extrudes sodium in exchange for external protons (By similarity)
P	Q8G5R2	DE	Na+/H+ antiporter nhaA;
P	Q8G5R2	DE	Sodium/proton antiporter nhaA;
P	Q8G6U3	CC	Transport of potassium into the cell (By similarity).
P	Q8G6U3	DE	Probable potassium transport system protein kup 1;
P	Q8G6U3	DR	potassium ion transmembrane transporter activity
P	Q8G7F4	CC	Part of the ABC transporter complex PstSACB involved in phosphate import
P	Q8G7F4	DE	ABC phosphate transporter;
P	Q8G7F4	DE	Phosphate import ATP-binding protein PstB;
P	Q8G7F4	DE	Phosphate-transporting ATPase;
P	Q8G7F4	DR	inorganic phosphate transmembrane transporter activity
P	Q8G7F4	DR	phosphate transmembrane-transporting ATPase activity
P	Q8G7Q3	CC	Transport of potassium into the cell (By similarity).
P	Q8G7Q3	DE	Probable potassium transport system protein kup 2;
P	Q8G7Q3	DR	potassium ion transmembrane transporter activity
P	Q8GH68	CC	H+-stimulated, highly selective, manganese uptake system (By similarity).
P	Q8GH68	DE	Probable manganese transport protein mntH;
P	Q8GRT8	CC	May play a role in root water uptake.
P	Q8GRT8	CC	Water channel required to facilitate the transport of water across cell membrane
P	Q8GSD9	CC	Acts as a H+:phosphate symporter.
P	Q8GSD9	CC	Low-affinity transporter for inorganic phosphate (Pi)
P	Q8GSD9	DE	Inorganic phosphate transporter 1-2;
P	Q8GSD9	DR	inorganic phosphate transmembrane transporter activity
P	Q8GW61	DE	Hexose transporter 14;
P	Q8GX93	CC	Voltage-gated chloride channel.
P	Q8GX93	DE	Chloride channel protein CLC-e;
P	Q8GX93	DR	voltage-gated chloride channel activity
P	Q8GXE6	CC	Highly selective inward-rectifying potassium channel that could mediate potassium uptake in the pollen membrane
P	Q8GXE6	DE	Potassium channel AKT6;
P	Q8GXE6	DE	Potassium channel SPIK;
P	Q8GXE6	DE	Shaker pollen inward rectifier K+ channel;
P	Q8GXE6	DR	voltage-gated potassium channel activity
P	Q8GYF4	CC	High-affinity transporter for external inorganic phosphate (By similarity).
P	Q8GYF4	DE	Probable inorganic phosphate transporter 1-5;
P	Q8GYF4	DR	inorganic phosphate transmembrane transporter activity
P	Q8GYH8	CC	H+/sulfate cotransporter that may play a role in the regulation of sulfate assimilation (By similarity).
P	Q8GYH8	DE	Probable sulfate transporter 4.2;
P	Q8GYH8	DR	secondary active sulfate transmembrane transporter activity
P	Q8H074	CC	High-affinity transporter for external inorganic phosphate (By similarity).
P	Q8H074	DE	Probable inorganic phosphate transporter 1-12;
P	Q8H074	DR	inorganic phosphate transmembrane transporter activity
P	Q8H1G7	CC	Magnesium transporter that may mediate the influx of magnesium (By similarity)
P	Q8H1G7	DE	Magnesium Transporter 8;
P	Q8H1G7	DE	Magnesium transporter MRS2-8;
P	Q8H3P9	CC	High-affinity potassium transporter.
P	Q8H3P9	DE	Potassium transporter 7;
P	Q8H3P9	DR	potassium ion transmembrane transporter activity
P	Q8H5N9	CC	Aquaporins facilitate the transport of water and small neutral solutes across cell membranes (By similarity).
P	Q8H6G7	CC	High-affinity transporter for external inorganic phosphate (By similarity).
P	Q8H6G7	DE	Probable inorganic phosphate transporter 1-9;
P	Q8H6G8	CC	High-affinity transporter for external inorganic phosphate (By similarity).
P	Q8H6G8	DE	Probable inorganic phosphate transporter 1-8;
P	Q8H6G8	DR	inorganic phosphate transmembrane transporter activity
P	Q8H6G9	CC	High-affinity transporter for external inorganic phosphate (By similarity).
P	Q8H6G9	DE	Probable inorganic phosphate transporter 1-7;

P	Q8H6G9	DR	inorganic phosphate transmembrane transporter activity
P	Q8H6H0	CC	High-affinity transporter for external inorganic phosphate (Pi)
P	Q8H6H0	DE	Inorganic phosphate transporter 1-6;
P	Q8H6H0	DR	inorganic phosphate transmembrane transporter activity
P	Q8H6H2	CC	High-affinity transporter for external inorganic phosphate (By similarity).
P	Q8H6H2	DE	Probable inorganic phosphate transporter 1-4;
P	Q8H6H2	DR	inorganic phosphate transmembrane transporter activity
P	Q8H6H4	CC	High-affinity transporter for external inorganic phosphate (By similarity)
P	Q8H6H4	CC	Required for phosphate acquisition in plant.
P	Q8H6H4	DE	Inorganic phosphate transporter 1-1;
P	Q8H6H4	DR	inorganic phosphate transmembrane transporter activity
P	Q8I0L6	CC	May be a carbon dioxide/bicarbonate transporter (By similarity).
P	Q8IRI6	CC	Facilitative glucose transporter.
P	Q8IRI6	DE	Glucose transporter type 1;
P	Q8IVB4	CC	May act in electroneutral exchange of protons for Na+ across membranes
P	Q8IVB4	DE	Na+/H+ exchanger 9;
P	Q8IVB4	DE	Sodium/hydrogen exchanger 9;
P	Q8IVB4	DR	sodium:hydrogen antiporter activity
P	Q8IVJ1	CC	Acts as a magnesium transporter that is responsive to magnesium balance.
P	Q8IVJ1	DR	magnesium ion transmembrane transporter activity
P	Q8IWA5	CC	Putative choline transporter.
P	Q8IWA5	DE	Choline transporter-like protein 2;
P	Q8IWA5	DR	choline transmembrane transporter activity
P	Q8IWU4	CC	Facilitates the accumulation of zinc from the cytoplasm into intracellular vesicles, being a zinc-efflux transporter
P	Q8IWU4	CC	May be a major component for providing zinc to insulin maturation and/or storage processes in insulin-secreting pancreatic beta- cells.
P	Q8IWU4	DE	Zinc transporter 8;
P	Q8IWU4	DR	zinc ion transmembrane transporter activity
P	Q8IY34	CC	Transports free histidine and certain di- and tripeptides (By similarity).
P	Q8IY34	DE	Peptide transporter 3;
P	Q8IY34	DE	Peptide/histidine transporter 2;
P	Q8IY34	DE	Peptide/histidine transporter 2;
P	Q8IY34	DR	peptide:hydrogen symporter activity
P	Q8JZN3	CC	Inward rectifier potassium channels are characterized by a greater tendency to allow potassium to flow into the cell rather than out of it
P	Q8JZN3	CC	Their voltage dependence is regulated by the concentration of extracellular potassium; as external potassium is raised, the voltage range of the channel opening shifts to more positive voltages
P	Q8JZN3	DE	ATP-sensitive inward rectifier potassium channel 14;
P	Q8JZN3	DE	Inward rectifier K+ channel Kir2.4;
P	Q8JZN3	DE	Potassium channel, inwardly rectifying subfamily J member 14;
P	Q8JZN3	DR	inward rectifier potassium channel activity
P	Q8JZR4	CC	Transports L-glutamate; the L-glutamate uptake is sodium- and voltage-dependent and chloride-independent
P	Q8JZR4	CC	Transports L-glutamate; the L-glutamate uptake is sodium- and voltage-dependent and chloride-independent
P	Q8JZR4	DR	sodium:dicarboxylate symporter activity
P	Q8JZR6	CC	Mediates electroneutral sodium- and carbonate-dependent choride-HCO3(-) exchange with a Na+:HCO3(-) stoichiometry of 2:1
P	Q8JZR6	DE	Electroneutral Na+-driven Cl-HCO3 exchanger;
P	Q8K0E3	CC	Exhibits stereospecific cotransport of both D-glucose and D- xylose
P	Q8K0E3	CC	Involved in the sodium-dependent cotransport of myo- inositol (MI) with a Na+:MI stoichiometry of 2:1
P	Q8K0E3	DE	Na+/myo-inositol cotransporter 2;
P	Q8K0E3	DE	Sodium-dependent glucose cotransporter;
P	Q8K0E3	DE	Sodium-dependent glucose cotransporter;
P	Q8K0E3	DE	Sodium/glucose cotransporter KST1;
P	Q8K0E3	DE	Sodium/glucose cotransporter KST1;
P	Q8K0E3	DE	Sodium/myo-inositol cotransporter 2;
P	Q8K1C7	CC	Proton-linked monocarboxylate transporter
P	Q8K1C7	DE	Monocarboxylate transporter 14;
P	Q8K211	CC	High-affinity, saturable copper transporter involved in dietary copper uptake.
P	Q8K211	DE	Copper transporter 1;
P	Q8K211	DE	High affinity copper uptake protein 1;

P	Q8K211	DR	copper ion transmembrane transporter activity
P	Q8K3F6	CC	Associates with KCNQ2 to form a potassium channel with essentially identical properties to the channel underlying the native M-current, a slowly activating and deactivating potassium conductance which plays a critical role in determining the subthreshold electrical excitability of neurons as well as the responsiveness to synaptic inputs (By similarity).
P	Q8K3F6	DE	Potassium channel subunit alpha KvLQT3;
P	Q8K3F6	DE	Potassium voltage-gated channel subfamily KQT member 3;
P	Q8K3F6	DE	Voltage-gated potassium channel subunit Kv7.3;
P	Q8K3F6	DR	voltage-gated potassium channel activity
P	Q8K903	CC	Low-affinity inorganic phosphate transport (By similarity).
P	Q8K903	DE	Low-affinity inorganic phosphate transporter;
P	Q8K903	DR	inorganic phosphate transmembrane transporter activity
P	Q8K911	CC	This system is involved in mannitol transport (By similarity).
P	Q8K911	DE	Mannitol permease IIC component;
P	Q8K911	DE	Mannitol-specific phosphotransferase enzyme IIA component;
P	Q8K911	DE	Mannitol-specific phosphotransferase enzyme IIB component;
P	Q8K911	DE	PTS system mannitol-specific EIIB component;
P	Q8K911	DE	PTS system mannitol-specific EIIC component;
P	Q8K911	DE	PTS system mannitol-specific EIICBA component;
P	Q8K911	DE	PTS system, mannitol-specific EIIA component;
P	Q8K9J0	CC	This system is involved in glucose transport (By similarity).
P	Q8K9J0	DE	Glucose permease IIC component;
P	Q8K9J0	DE	Glucose-specific phosphotransferase enzyme IIB component;
P	Q8K9J0	DE	PTS system glucose-specific EIIB component;
P	Q8K9J0	DE	PTS system glucose-specific EIIC component;
P	Q8K9J0	DE	PTS system glucose-specific EIICB component;
P	Q8K9J0	DR	glucose transmembrane transporter activity
P	Q8K9M5	CC	Involved in the high-affinity zinc uptake transport system (By similarity).
P	Q8K9M5	DE	High-affinity zinc uptake system protein znuA;
P	Q8K9M6	CC	Part of the ABC transporter complex ZnuABC involved in zinc import
P	Q8K9M6	DE	Zinc import ATP-binding protein ZnuC;
P	Q8K9M6	DR	zinc transporting ATPase activity
P	Q8K9M7	CC	Involved in the high-affinity zinc uptake transport system (By similarity).
P	Q8K9M7	DE	High-affinity zinc uptake system membrane protein znuB;
P	Q8K9M9	CC	Glycerol enters the cell via the glycerol diffusion facilitator protein
P	Q8K9M9	CC	This membrane protein facilitates the movement of glycerol across the cytoplasmic membrane (By similarity).
P	Q8K9M9	DE	Glycerol uptake facilitator protein;
P	Q8KA51	CC	This system is involved in glucose transport (By similarity).
P	Q8KA51	DE	Glucose-specific phosphotransferase enzyme IIA component;
P	Q8KA51	DE	PTS system glucose-specific EIIA component;
P	Q8KAT2	CC	Transport of potassium into the cell (By similarity).
P	Q8KAT2	DE	Probable potassium transport system protein kup;
P	Q8KAT2	DR	potassium ion transmembrane transporter activity
P	Q8KDZ5	CC	Part of the ABC transporter complex PstSACB involved in phosphate import
P	Q8KDZ5	DE	ABC phosphate transporter;
P	Q8KDZ5	DE	Phosphate import ATP-binding protein PstB;
P	Q8KDZ5	DE	Phosphate-transporting ATPase;
P	Q8KDZ5	DR	inorganic phosphate transmembrane transporter activity
P	Q8KDZ5	DR	phosphate transmembrane-transporting ATPase activity
P	Q8KU73	CC	One of the components of the high-affinity ATP-driven potassium transport (or KDP) system, which catalyzes the hydrolysis of ATP coupled with the exchange of hydrogen and potassium ions (By similarity).
P	Q8KU73	DE	Potassium-binding and translocating subunit B;
P	Q8KU73	DE	Potassium-translocating ATPase B chain;
P	Q8KU73	DE	Potassium-transporting ATPase B chain;
P	Q8KU73	DR	potassium-transporting ATPase activity
P	Q8L4S2	CC	Magnesium transporter that may mediate the influx of magnesium (By similarity)
P	Q8L4S2	DE	Magnesium transporter MRS2-F;
P	Q8L6Z8	DR	glucose transmembrane transporter activity
P	Q8L783	CC	Translocates Ca2+ and other metal ions into vacuoles using the proton gradient formed by H+-ATPase and H+-pyrophosphatase (By similarity).
P	Q8L783	DE	Ca2+/H+ antiporter CAX5;
P	Q8L783	DE	Ca2+/H+ exchanger 5;

P	Q8L7R8	DE	Hexose transporter 3;
P	Q8LAA6	CC	Aquaporins facilitate the transport of water and small neutral solutes across cell membranes (By similarity).
P	Q8LAI1	CC	Aquaporins facilitate the transport of water and small neutral solutes across cell membranes (By similarity).
P	Q8LBL1	CC	Calcium-activated outward rectifying potassium channel
P	Q8LBL1	DE	Calcium-activated outward-rectifying potassium channel 1;
P	Q8LBL1	DR	potassium channel activity
P	Q8LE59	CC	May play a role in the transport of iron in the plastids (By similarity).
P	Q8LE59	DE	Iron-regulated transporter 3;
P	Q8LE59	DR	iron ion transmembrane transporter activity
P	Q8LE59	DR	zinc ion transmembrane transporter activity
P	Q8LFP7	CC	Water channel probably required to promote glycerol permeability and water transport across cell membranes.
P	Q8LG88	CC	Putative carrier protein indirectly involved in the uptake of malate and fumarate to the vacuole, probably by regulating the energization across the tonoplast
P	Q8LG88	CC	Uptake of malate to vacuoles is inhibited by citrate and by the uncoupler carbonyl- cyanide m-chlorophenylhydrazone, but seems to be not affected by sodium
P	Q8LG88	DE	Vacuolar malate transporter;
P	Q8LG88	DR	malate transmembrane transporter activity
P	Q8LPL8	CC	Probable potassium transporter.
P	Q8LPL8	DE	Potassium transporter 13;
P	Q8LPL8	DR	potassium ion transmembrane transporter activity
P	Q8LPQ8	DE	Sugar transporter MSSP2;
P	Q8N130	CC	May be involved in actively transporting phosphate into cells via Na+ cotransport in the renal brush border membrane
P	Q8N130	CC	May be involved in actively transporting phosphate into cells via Na+ cotransport in the renal brush border membrane
P	Q8N130	DE	Na+-dependent phosphate cotransporter 2C;
P	Q8N130	DE	Na+-dependent phosphate cotransporter 2C;
P	Q8N130	DE	Na+/Pi cotransporter 2C;
P	Q8N130	DE	Sodium-dependent phosphate transport protein 2C;
P	Q8N130	DE	Sodium-dependent phosphate transport protein 2C;
P	Q8N130	DE	Sodium-phosphate transport protein 2C;
P	Q8N130	DE	Sodium/inorganic phosphate cotransporter IIC;
P	Q8N130	DE	Sodium/inorganic phosphate cotransporter IIC;
P	Q8N130	DE	Sodium/phosphate cotransporter 2C;
P	Q8N130	DE	Sodium/phosphate cotransporter 2C;
P	Q8N130	DR	sodium-dependent phosphate transmembrane transporter activity
P	Q8N130	DR	sodium-dependent phosphate transmembrane transporter activity
P	Q8N130	DR	sodium:phosphate symporter activity
P	Q8N130	DR	sodium:phosphate symporter activity
P	Q8N1C3	CC	GABA, the major inhibitory neurotransmitter in the vertebrate brain, mediates neuronal inhibition by binding to the GABA/benzodiazepine receptor and opening an integral chloride channel.
P	Q8N1C3	DR	chloride channel activity
P	Q8N1S5	DE	Zinc transporter ZIP11;
P	Q8N538	DE	Potassium inwardly-rectifying channel, subfamily J, member 16;
P	Q8N538	DR	inward rectifier potassium channel activity
P	Q8N695	CC	Acts as an electrogenic sodium (Na+) and chloride (Cl-)-dependent sodium-coupled solute transporter, including transport of monocarboxylates (short-chain fatty acids including L-lactate, D-lactate, pyruvate, acetate, propionate, valerate and butyrate), lactate, mocarboxylate drugs (nicotinate, benzoate, salicylate and 5-aminosalicylate) and ketone bodies (beta-D- hydroxybutyrate, acetoacetate and alpha-ketoisocaproate), with a Na+:substrate stoichiometry of between 4:1 and 2:1
P	Q8N695	CC	Catalyzes passive carrier mediated diffusion of iodide
P	Q8N695	CC	May play a critical role in the entry of L-lactate and ketone bodies into neurons by a process driven by an electrochemical Na+ gradient and hence contribute to the maintenance of the energy status and function of neurons.
P	Q8N695	CC	Mediates iodide transport from the thyrocyte into the colloid lumen through the apical membrane
P	Q8N695	DE	Apical iodide transporter;
P	Q8N695	DE	Electrogenic sodium monocarboxylate cotransporter;
P	Q8N695	DE	Sodium-coupled monocarboxylate transporter 1;
P	Q8N697	CC	Transports free histidine and certain di- and tripeptides.

P	Q8N697	DE	Peptide transporter 4;
P	Q8N697	DE	Peptide/histidine transporter 1;
P	Q8N697	DE	Peptide/histidine transporter 1;
P	Q8N697	DR	peptide:hydrogen symporter activity
P	Q8N8Q9	CC	Acts as a selective Mg2+ transporter (By similarity).
P	Q8N8Q9	DE	Magnesium transporter NIPA2;
P	Q8N8R3	DE	Mitochondrial carnitine/acylcarnitine carrier protein CACL;
P	Q8NBS3	CC	In the kidney, is essential for urinary concentration, mediates a sodium flux into the thin descending limb of Henle loop to allow countercurrent multiplication by osmotic equilibration (By similarity)
P	Q8NBS3	CC	In the presence of borate functions as an electrogenic Na+ coupled borate cotransporter
P	Q8NBS3	CC	Transporter which plays an important role in sodium- mediated fluid transport in different organs
P	Q8NBS3	DE	Bicarbonate transporter-related protein 1;
P	Q8NBS3	DE	Sodium borate cotransporter 1;
P	Q8NBS3	DR	bicarbonate transmembrane transporter activity
P	Q8NBS3	DR	sodium channel activity
P	Q8NCK7	CC	Proton-linked monocarboxylate transporter
P	Q8NCK7	DE	Monocarboxylate transporter 11;
P	Q8NCM2	CC	Pore-forming (alpha) subunit of voltage-gated potassium channel
P	Q8NCM2	DE	Ether-a-go-go potassium channel 2;
P	Q8NCM2	DE	Potassium voltage-gated channel subfamily H member 5;
P	Q8NCM2	DE	Voltage-gated potassium channel subunit Kv10.2;
P	Q8NCM2	DR	voltage-gated potassium channel activity
P	Q8NDX2	CC	Mediates the uptake of glutamate into synaptic vesicles at presynaptic nerve terminals of excitatory neural cells
P	Q8NDX2	DE	Vesicular glutamate transporter 3;
P	Q8NDX2	DR	L-glutamate transmembrane transporter activity
P	Q8NEW0	CC	Partly regulates cellular zinc homeostasis
P	Q8NEW0	CC	Required with ZNT5 for the activation of zinc- requiring enzymes, alkaline phosphatases (ALPs)
P	Q8NEW0	CC	Seems to facilitate zinc transport from the cytoplasm into the Golgi apparatus
P	Q8NEW0	CC	Transports zinc into the lumens of the Golgi apparatus and the vesicular compartments where ALPs locate, thus, converting apoALPs to holoALPs
P	Q8NEW0	DE	Zinc transporter 7;
P	Q8NFF2	CC	Transports 1 Ca2+ and 1 K+ in exchange for 4 Na+.
P	Q8NFF2	CC	Transports 1 Ca2+ and 1 K+ in exchange for 4 Na+.
P	Q8NFF2	CC	Transports 1 Ca2+ and 1 K+ in exchange for 4 Na+.
P	Q8NFF2	DE	Na+/K+/Ca2+-exchange protein 4;
P	Q8NFF2	DE	Na+/K+/Ca2+-exchange protein 4;
P	Q8NFF2	DE	Na+/K+/Ca2+-exchange protein 4;
P	Q8NFF2	DE	Sodium/potassium/calcium exchanger 4;
P	Q8NFF2	DE	Sodium/potassium/calcium exchanger 4;
P	Q8NFF2	DE	Sodium/potassium/calcium exchanger 4;
P	Q8NFF2	DR	calcium, potassium:sodium antiporter activity
P	Q8NFF2	DR	calcium, potassium:sodium antiporter activity
P	Q8NFF2	DR	calcium, potassium:sodium antiporter activity
P	Q8NMD6	DE	ENZYME II SUCROSE PROTEIN;
P	Q8NMK1	CC	Part of the ABC transporter complex PstSACB involved in phosphate import
P	Q8NMK1	DE	ABC phosphate transporter;
P	Q8NMK1	DE	Phosphate import ATP-binding protein PstB;
P	Q8NMK1	DE	Phosphate-transporting ATPase;
P	Q8NMK1	DR	inorganic phosphate transmembrane transporter activity
P	Q8NMK1	DR	phosphate transmembrane-transporting ATPase activity
P	Q8NQH4	DR	phosphonate transmembrane-transporting ATPase activity
P	Q8NR12	CC	Part of the ABC transporter complex RbsABCD involved in ribose import
P	Q8NR12	DE	Ribose import ATP-binding protein RbsA;
P	Q8NSG3	CC	Transport of potassium into the cell (By similarity).
P	Q8NSG3	DE	Probable potassium transport system protein kup;
P	Q8NSG3	DR	potassium ion transmembrane transporter activity
P	Q8NSN2	CC	Part of the ABC transporter complex MetNIQ involved in methionine import
P	Q8NSN2	DE	Methionine import ATP-binding protein MetN;
P	Q8PGE8	CC	Part of the ABC transporter complex MetNIQ involved in methionine import
P	Q8PGE8	DE	Methionine import ATP-binding protein MetN;
P	Q8PHV1	CC	Transport of potassium into the cell (By similarity).

P	Q8PHV1	DE	Probable potassium transport system protein kup;
P	Q8PHV1	DR	potassium ion transmembrane transporter activity
P	Q8PK53	CC	Part of the ABC transporter complex CcmAB involved in the biogenesis of c-type cytochromes; once thought to export heme, this seems not to be the case, but its exact role is uncertain
P	Q8PK53	DE	Heme exporter protein A;
P	Q8PK53	DR	heme-transporting ATPase activity
P	Q8PKT0	DE	Lipoprotein-releasing system ATP-binding protein LolD;
P	Q8PKT0	DR	lipoprotein transporter activity
P	Q8PKX1	CC	H+-stimulated, highly selective, manganese uptake system (By similarity).
P	Q8PKX1	DE	Probable manganese transport protein mntH;
P	Q8PM59	CC	Part of the ABC transporter complex PstSACB involved in phosphate import
P	Q8PM59	DE	ABC phosphate transporter;
P	Q8PM59	DE	Phosphate import ATP-binding protein PstB;
P	Q8PM59	DE	Phosphate-transporting ATPase;
P	Q8PM59	DR	inorganic phosphate transmembrane transporter activity
P	Q8PM59	DR	phosphate transmembrane-transporting ATPase activity
P	Q8PNN4	CC	Part of the ABC transporter complex CysAWTP involved in sulfate/thiosulfate import
P	Q8PNN4	DE	Sulfate-transporting ATPase;
P	Q8PNN4	DE	Sulfate/thiosulfate import ATP-binding protein CysA;
P	Q8PNN4	DR	sulfate transmembrane-transporting ATPase activity
P	Q8PPC8	CC	One of the components of the high-affinity ATP-driven potassium transport (or KDP) system, which catalyzes the hydrolysis of ATP coupled with the exchange of hydrogen and potassium ions
P	Q8PPC8	DE	Potassium-binding and translocating subunit C;
P	Q8PPC8	DE	Potassium-translocating ATPase C chain;
P	Q8PPC8	DE	Potassium-transporting ATPase C chain;
P	Q8PPC8	DR	potassium-transporting ATPase activity
P	Q8PPC9	CC	One of the components of the high-affinity ATP-driven potassium transport (or KDP) system, which catalyzes the hydrolysis of ATP coupled with the exchange of hydrogen and potassium ions (By similarity).
P	Q8PPC9	DE	Potassium-binding and translocating subunit B;
P	Q8PPC9	DE	Potassium-translocating ATPase B chain;
P	Q8PPC9	DE	Potassium-transporting ATPase B chain;
P	Q8PPC9	DR	potassium-transporting ATPase activity
P	Q8PVF6	CC	Part of the ABC transporter complex PstSACB involved in phosphate import
P	Q8PVF6	DE	ABC phosphate transporter;
P	Q8PVF6	DE	Phosphate import ATP-binding protein PstB;
P	Q8PVF6	DE	Phosphate-transporting ATPase;
P	Q8PVF6	DR	inorganic phosphate transmembrane transporter activity
P	Q8PVF6	DR	phosphate transmembrane-transporting ATPase activity
P	Q8Q040	DE	Glycine betaine-binding protein;
P	Q8Q041	DE	Glycine betaine transporter, ATP-binding protein;
P	Q8Q042	DE	Glycine betaine transport system, permease protein;
P	Q8Q043	DE	Glycine betaine transport system, permease protein;
P	Q8QZW7	CC	GABA, the major inhibitory neurotransmitter in the vertebrate brain, mediates neuronal inhibition by binding to the GABA/benzodiazepine receptor and opening an integral chloride channel
P	Q8QZW7	DR	chloride channel activity
P	Q8R0Z5	CC	Mitochondrial iron transporter that mediates iron uptake
P	Q8R0Z5	CC	The iron delivered into the mitochondria, presumably as Fe(2+), is then probably delivered to ferrochelatase to catalyze Fe(2+) incorporation into protoprophyrin IX to make heme (Probable).
P	Q8R0Z5	DE	Mitochondrial iron transporter 2;
P	Q8R1C0	CC	Assuming opened or closed conformations in response to the voltage difference across the membrane, the protein forms a potassium-selective channel through which potassium ions may pass in accordance with their electrochemical gradient (By similarity).
P	Q8R1C0	CC	This protein mediates the voltage-dependent potassium ion permeability of excitable membranes
P	Q8R1C0	DE	Potassium voltage-gated channel subfamily C member 4;
P	Q8R1C0	DE	Voltage-gated potassium channel subunit Kv3.4;
P	Q8R1C0	DR	voltage-gated potassium channel activity
P	Q8R1P5	CC	Potassium channel displaying weak inward rectification in symmetrical K+ solution (By similarity).
P	Q8R1P5	DE	Potassium channel subfamily K member 13;

P	Q8R1P5	DE	Tandem pore domain halothane-inhibited potassium channel 1;
P	Q8R1P5	DR	potassium channel activity
P	Q8R1T4	DE	UDP-N-acetylglucosamine transporter;
P	Q8R2N1	CC	It may play an important role in gastrointestinal tract water transport and in glycerol metabolism.
P	Q8R2N1	CC	Provides kidney medullary collecting duct with high permeability to water, thereby permitting water to move in the direction of an osmotic gradient
P	Q8R2N1	CC	Water channel required to promote glycerol permeability and water transport across cell membranes
P	Q8R2N1	DR	water channel activity
P	Q8R2Z3	CC	Acts as a sodium-independent DIDS-sensitive anion exchanger mediating bicarbonate, chloride, sulfate and oxalate transport
P	Q8R429	CC	Contributes to calcium sequestration involved in muscular excitation/contraction (By similarity).
P	Q8R429	CC	This magnesium-dependent enzyme catalyzes the hydrolysis of ATP coupled with the translocation of calcium from the cytosol to the sarcoplasmic reticulum lumen
P	Q8R429	DE	Calcium pump 1;
P	Q8R429	DE	Calcium-transporting ATPase sarcoplasmic reticulum type, fast twitch skeletal muscle isoform;
P	Q8R429	DE	Endoplasmic reticulum class 1/2 Ca2+ ATPase;
P	Q8R429	DE	SR Ca2+-ATPase 1;
P	Q8R429	DE	Sarcoplasmic/endoplasmic reticulum calcium ATPase 1;
P	Q8R429	DR	calcium-transporting ATPase activity
P	Q8R4D1	CC	Major proton extruding system driven by the inward sodium ion chemical gradient
P	Q8R4D1	DE	Na+/H+ exchanger 8;
P	Q8R4D1	DE	Sodium/hydrogen exchanger 8;
P	Q8R4D1	DR	sodium:hydrogen antiporter activity
P	Q8R4H8	DE	Sodium/hydrogen exchanger;
P	Q8R4H8	DR	sodium:hydrogen antiporter activity
P	Q8R4H9	CC	May be a transporter of zinc into beta cells in order to form insulin crystals
P	Q8R4H9	CC	Partly regulates cellular zinc homeostasis
P	Q8R4H9	CC	Required with ZNT7 for the activation of zinc-requiring enzymes, alkaline phosphatases (ALPs)
P	Q8R4H9	CC	Transports zinc into the lumens of the Golgi apparatus and vesicular compartments where ALPs locate, thus, converting apoALPs to holoALPs
P	Q8R4H9	DE	Zinc transporter 5;
P	Q8R7S2	CC	H+-stimulated, highly selective, manganese uptake system (By similarity).
P	Q8R7S2	DE	Probable manganese transport protein mntH;
P	Q8R8I5	CC	One of the components of the high-affinity ATP-driven potassium transport (or KDP) system, which catalyzes the hydrolysis of ATP coupled with the exchange of hydrogen and potassium ions (By similarity).
P	Q8R8I5	DE	Potassium-binding and translocating subunit A;
P	Q8R8I5	DE	Potassium-translocating ATPase A chain;
P	Q8R8I5	DE	Potassium-transporting ATPase A chain;
P	Q8R8I5	DR	potassium-transporting ATPase activity
P	Q8R8I6	CC	One of the components of the high-affinity ATP-driven potassium transport (or KDP) system, which catalyzes the hydrolysis of ATP coupled with the exchange of hydrogen and potassium ions (By similarity).
P	Q8R8I6	DE	Potassium-binding and translocating subunit B;
P	Q8R8I6	DE	Potassium-translocating ATPase B chain;
P	Q8R8I6	DE	Potassium-transporting ATPase B chain;
P	Q8R8I6	DR	potassium-transporting ATPase activity
P	Q8R8I7	CC	One of the components of the high-affinity ATP-driven potassium transport (or KDP) system, which catalyzes the hydrolysis of ATP coupled with the exchange of hydrogen and potassium ions
P	Q8R8I7	DE	Potassium-binding and translocating subunit C;
P	Q8R8I7	DE	Potassium-translocating ATPase C chain;
P	Q8R8I7	DE	Potassium-transporting ATPase C chain;
P	Q8R8I7	DR	potassium-transporting ATPase activity
P	Q8R9I2	CC	Part of the ABC transporter complex PstSACB involved in phosphate import
P	Q8R9I2	DE	ABC phosphate transporter 2;
P	Q8R9I2	DE	Phosphate import ATP-binding protein PstB 2;
P	Q8R9I2	DE	Phosphate-transporting ATPase 2;
P	Q8R9I2	DR	inorganic phosphate transmembrane transporter activity
P	Q8R9I2	DR	phosphate transmembrane-transporting ATPase activity

P	Q8RCU0	CC	Part of the ABC transporter complex PstSACB involved in phosphate import
P	Q8RCU0	DE	ABC phosphate transporter 1;
P	Q8RCU0	DE	Phosphate import ATP-binding protein PstB 1;
P	Q8RCU0	DE	Phosphate-transporting ATPase 1;
P	Q8RCU0	DR	phosphate transmembrane-transporting ATPase activity
P	Q8RD43	CC	Part of the ABC transporter complex RbsABCD involved in ribose import
P	Q8RD43	DE	Ribose import ATP-binding protein RbsA;
P	Q8REE1	CC	Part of the ABC transporter complex MglABC involved in galactose/methyl galactoside import
P	Q8REE1	DE	Galactose/methyl galactoside import ATP-binding protein MglA;
P	Q8REG7	DR	phosphonate transmembrane-transporting ATPase activity
P	Q8RFN2	CC	Part of the ABC transporter complex MetNIQ involved in methionine import
P	Q8RFN2	DE	Methionine import ATP-binding protein MetN;
P	Q8RFV0	DE	Lipoprotein-releasing system ATP-binding protein LolD;
P	Q8RFV0	DR	lipoprotein transporter activity
P	Q8RI39	CC	Part of the ABC transporter complex PotABCD involved in spermidine/putrescine import
P	Q8RI39	CC	Part of the ABC transporter complex PotABCD involved in spermidine/putrescine import
P	Q8RI39	DE	Spermidine/putrescine import ATP-binding protein PotA;
P	Q8RI39	DE	Spermidine/putrescine import ATP-binding protein PotA;
P	Q8RQL4	CC	Part of the binding-protein-dependent transport system for glutamate; probably responsible for the translocation of the substrate across the membrane (By similarity)
P	Q8RQL4	DE	Glutamate transport system permease protein gluD;
P	Q8RQL5	CC	Part of the binding-protein-dependent transport system for glutamate; probably responsible for the translocation of the substrate across the membrane (By similarity)
P	Q8RQL5	DE	Glutamate transport system permease protein gluC;
P	Q8RQL6	CC	Part of the binding-protein-dependent transport system for glutamate (By similarity)
P	Q8RQL7	CC	Part of the binding-protein-dependent transport system for glutamate
P	Q8RQL7	DE	Glutamate transport ATP-binding protein GluA;
P	Q8RQM4	DE	Lysine exporter protein;
P	Q8RWU6	DE	Na+/H+ exchanger 6;
P	Q8RWU6	DE	Sodium/hydrogen exchanger 6;
P	Q8RWU6	DR	sodium:hydrogen antiporter activity
P	Q8RXR2	CC	Voltage-gated chloride channel.
P	Q8RXR2	DE	Chloride channel protein CLC-f;
P	Q8RXR2	DR	voltage-gated chloride channel activity
P	Q8RY46	DE	Antigen peptide transporter-like 1;
P	Q8S1N1	CC	Magnesium transporter that may mediate the influx of magnesium (By similarity)
P	Q8S1N1	DE	Magnesium transporter MRS2-E;
P	Q8S396	CC	Involved in vacuolar ion compartmentalization necessary for cell volume regulation and cytoplasmic Na+ detoxification.
P	Q8S396	DE	Na+/H+ exchanger 5;
P	Q8S396	DE	Sodium/hydrogen exchanger 5;
P	Q8S396	DR	sodium:hydrogen antiporter activity
P	Q8S397	DE	Na+/H+ exchanger 4;
P	Q8S397	DE	Sodium/hydrogen exchanger 4;
P	Q8S397	DR	sodium:hydrogen antiporter activity
P	Q8S3W4	CC	Probably mediates zinc uptake from the rhizosphere (By similarity).
P	Q8S3W4	DE	Probable zinc transporter 8;
P	Q8S3W4	DR	zinc ion transmembrane transporter activity
P	Q8SRA2	CC	ATP transporter involved in the uptake of ATP from the host cell cytoplasm
P	Q8SRA2	CC	Provides the microsporidian cell with host ATP in exchange for ADP
P	Q8SRA2	CC	Provides the microsporidian cell with host ATP in exchange for ADP
P	Q8SRA2	DE	ADP,ATP carrier protein 1;
P	Q8SRA2	DE	ADP/ATP translocase 1;
P	Q8SRA2	DE	ADP/ATP translocase 1;
P	Q8SRK2	CC	Water channel required to facilitate the transport of water across membranes
P	Q8SUF9	CC	ATP transporter involved in the uptake of ATP from the host cell cytoplasm
P	Q8SUF9	CC	Provides the microsporidian cell with host ATP in exchange for ADP
P	Q8SUF9	CC	Provides the microsporidian cell with host ATP in exchange for ADP
P	Q8SUF9	DE	ADP,ATP carrier protein 2;
P	Q8SUF9	DE	ADP/ATP translocase 2;
P	Q8SUF9	DE	ADP/ATP translocase 2;
P	Q8SUG0	CC	ATP transporter involved in the uptake of ATP from the parasite cell cytoplasm into the mitosome matrix
P	Q8SUG0	DE	ADP,ATP carrier protein 3;

P	Q8SUG0	DE	ADP/ATP translocase 3;
P	Q8SUG0	DE	ADP/ATP translocase 3;
P	Q8SUG7	CC	ATP transporter involved in the uptake of ATP from the host cell cytoplasm
P	Q8SUG7	CC	Provides the microsporidian cell with host ATP in exchange for ADP
P	Q8SUG7	CC	Provides the microsporidian cell with host ATP in exchange for ADP
P	Q8SUG7	DE	ADP,ATP carrier protein 4;
P	Q8SUG7	DE	ADP/ATP translocase 4;
P	Q8SUG7	DE	ADP/ATP translocase 4;
P	Q8TAD4	CC	May be a transporter of zinc into beta cells in order to form insulin crystals
P	Q8TAD4	CC	Partly regulates cellular zinc homeostasis
P	Q8TAD4	CC	Required with ZNT7 for the activation of zinc-requiring enzymes, alkaline phosphatases (ALPs)
P	Q8TAD4	CC	Transports zinc into the lumens of the Golgi apparatus and vesicular compartments where ALPs locate, thus, converting apoALPs to holoALPs
P	Q8TAD4	DE	Zinc transporter 5;
P	Q8TAD4	DR	zinc ion transmembrane transporter activity
P	Q8TAE7	CC	Potassium channel subunit
P	Q8TAE7	DE	Potassium voltage-gated channel subfamily G member 3;
P	Q8TAE7	DE	Voltage-gated potassium channel subunit Kv10.1;
P	Q8TAE7	DE	Voltage-gated potassium channel subunit Kv6.3;
P	Q8TDB8	CC	Facilitative glucose transporter (By similarity)
P	Q8TDB8	DE	Glucose transporter type 14;
P	Q8TDB8	DE	Solute carrier family 2, facilitated glucose transporter member 14;
P	Q8TDB8	DR	glucose transmembrane transporter activity
P	Q8TDN1	CC	Potassium channel subunit
P	Q8TDN1	DE	Potassium voltage-gated channel subfamily G member 4;
P	Q8TDN1	DE	Voltage-gated potassium channel subunit Kv6.4;
P	Q8TDN1	DR	voltage-gated potassium channel activity
P	Q8TDN2	CC	Potassium channel subunit
P	Q8TDN2	DE	Potassium voltage-gated channel subfamily V member 2;
P	Q8TDN2	DE	Voltage-gated potassium channel subunit Kv8.2;
P	Q8TDN2	DR	voltage-gated potassium channel activity
P	Q8TE54	CC	Acts as a sodium-independent DIDS-sensitive anion exchanger mediating bicarbonate, chloride, sulfate and oxalate transport
P	Q8TE54	DR	sulfate transmembrane transporter activity
P	Q8TF71	DE	Monocarboxylate transporter 10;
P	Q8TJZ4	DE	Cadmium efflux ATPase;
P	Q8TL61	CC	Transport of potassium into the cell (By similarity).
P	Q8TL61	DE	Probable potassium transport system protein kup;
P	Q8TL61	DR	potassium ion transmembrane transporter activity
P	Q8TSA8	CC	Part of the ABC transporter complex PstSACB involved in phosphate import
P	Q8TSA8	DE	ABC phosphate transporter;
P	Q8TSA8	DE	Phosphate import ATP-binding protein PstB;
P	Q8TSA8	DE	Phosphate-transporting ATPase;
P	Q8TSA8	DR	inorganic phosphate transmembrane transporter activity
P	Q8TSA8	DR	phosphate transmembrane-transporting ATPase activity
P	Q8TUR7	CC	Part of the ABC transporter complex PstSACB involved in phosphate import
P	Q8TUR7	DE	ABC phosphate transporter;
P	Q8TUR7	DE	Phosphate import ATP-binding protein PstB;
P	Q8TUR7	DE	Phosphate-transporting ATPase;
P	Q8TUR7	DR	phosphate transmembrane-transporting ATPase activity
P	Q8U242	CC	Part of the ABC transporter complex PstSACB involved in phosphate import
P	Q8U242	DE	ABC phosphate transporter;
P	Q8U242	DE	Phosphate import ATP-binding protein PstB;
P	Q8U242	DE	Phosphate-transporting ATPase;
P	Q8U242	DR	inorganic phosphate transmembrane transporter activity
P	Q8U242	DR	phosphate transmembrane-transporting ATPase activity
P	Q8U4K3	CC	Part of the ABC transporter complex WtpABC involved in molybdate/tungstate import
P	Q8U4K3	DE	Molybdate/tungstate import ATP-binding protein WtpC;
P	Q8U4K3	DR	molybdate transmembrane-transporting ATPase activity
P	Q8U4K4	CC	Part of the ABC transporter complex wtpABC involved in molybdate/tungstate import
P	Q8U4K4	DE	Molybdate/tungstate transport system permease protein wtpB;
P	Q8U4K5	CC	Binds tungstate and molybdate, with a preference for tungstate.
P	Q8U4K5	CC	Part of the ABC transporter complex wtpABC involved in molybdate/tungstate import
P	Q8U4K5	DE	Molybdate/tungstate-binding protein wtpA;

P	Q8U7G2	CC	Part of the ABC transporter complex MetNIQ involved in methionine import
P	Q8U7G2	DE	Methionine import ATP-binding protein MetN;
P	Q8U949	CC	Part of the ABC transporter complex RbsABCD involved in ribose import
P	Q8U949	DE	Ribose import ATP-binding protein RbsA 2;
P	Q8U9D8	CC	One of the components of the high-affinity ATP-driven potassium transport (or KDP) system, which catalyzes the hydrolysis of ATP coupled with the exchange of hydrogen and potassium ions (By similarity).
P	Q8U9D8	DE	Potassium-binding and translocating subunit A;
P	Q8U9D8	DE	Potassium-translocating ATPase A chain;
P	Q8U9D8	DE	Potassium-transporting ATPase A chain;
P	Q8U9D8	DR	potassium-transporting ATPase activity
P	Q8U9D9	CC	One of the components of the high-affinity ATP-driven potassium transport (or KDP) system, which catalyzes the hydrolysis of ATP coupled with the exchange of hydrogen and potassium ions (By similarity).
P	Q8U9D9	DE	Potassium-binding and translocating subunit B;
P	Q8U9D9	DE	Potassium-translocating ATPase B chain;
P	Q8U9D9	DE	Potassium-transporting ATPase B chain;
P	Q8U9D9	DR	potassium-transporting ATPase activity
P	Q8U9E0	CC	One of the components of the high-affinity ATP-driven potassium transport (or KDP) system, which catalyzes the hydrolysis of ATP coupled with the exchange of hydrogen and potassium ions
P	Q8U9E0	DE	Potassium-binding and translocating subunit C;
P	Q8U9E0	DE	Potassium-translocating ATPase C chain;
P	Q8U9E0	DE	Potassium-transporting ATPase C chain;
P	Q8U9E0	DR	potassium-transporting ATPase activity
P	Q8UA73	CC	Part of the ABC transporter complex CysAWTP involved in sulfate/thiosulfate import
P	Q8UA73	DE	Sulfate-transporting ATPase 2;
P	Q8UA73	DE	Sulfate/thiosulfate import ATP-binding protein CysA 2;
P	Q8UA73	DR	sulfate transmembrane-transporting ATPase activity
P	Q8UA86	CC	Part of the ABC transporter complex RbsABCD involved in ribose import
P	Q8UA86	DE	Ribose import ATP-binding protein RbsA 1;
P	Q8UBB7	DR	glycerol-3-phosphate-transporting ATPase activity
P	Q8UBY6	CC	Part of the ABC transporter complex ThiBPQ involved in thiamine import
P	Q8UBY6	DE	Thiamine import ATP-binding protein ThiQ;
P	Q8UC12	CC	Part of the ABC transporter complex CcmAB involved in the biogenesis of c-type cytochromes; once thought to export heme, this seems not to be the case, but its exact role is uncertain
P	Q8UC12	DE	Heme exporter protein A;
P	Q8UC12	DR	heme-transporting ATPase activity
P	Q8UCD5	DR	molybdate transmembrane-transporting ATPase activity
P	Q8UCM5	CC	Part of the ABC transporter complex HmuTUV involved in hemin import
P	Q8UCM5	DE	Hemin import ATP-binding protein HmuV;
P	Q8UEM1	CC	H+-stimulated, highly selective, manganese uptake system (By similarity).
P	Q8UEM1	DE	Probable manganese transport protein mntH;
P	Q8UF79	CC	Part of the ABC transporter complex ZnuABC involved in zinc import
P	Q8UF79	DE	Zinc import ATP-binding protein ZnuC;
P	Q8UF79	DR	zinc transporting ATPase activity
P	Q8UH62	CC	Part of the ABC transporter complex CysAWTP involved in sulfate/thiosulfate import
P	Q8UH62	DE	Sulfate-transporting ATPase 1;
P	Q8UH62	DE	Sulfate/thiosulfate import ATP-binding protein CysA 1;
P	Q8UH62	DR	sulfate transmembrane-transporting ATPase activity
P	Q8UHH1	CC	Transport of potassium into the cell (By similarity).
P	Q8UHH1	DE	Probable potassium transport system protein kup 1;
P	Q8UHH1	DR	potassium ion transmembrane transporter activity
P	Q8UI76	CC	Part of the ABC transporter complex PstSACB involved in phosphate import
P	Q8UI76	DE	ABC phosphate transporter;
P	Q8UI76	DE	Phosphate import ATP-binding protein PstB;
P	Q8UI76	DE	Phosphate-transporting ATPase;
P	Q8UI76	DR	inorganic phosphate transmembrane transporter activity
P	Q8UI76	DR	phosphate transmembrane-transporting ATPase activity
P	Q8UII7	DR	glycerol-3-phosphate-transporting ATPase activity
P	Q8UIW7	DR	phosphonate transmembrane-transporting ATPase activity
P	Q8UJL0	CC	Transport of potassium into the cell (By similarity).
P	Q8UJL0	DE	Probable potassium transport system protein kup 2;
P	Q8UJL0	DR	potassium ion transmembrane transporter activity

P	Q8VBW1	CC	Plays an important role in supplying creatine to the brain via the blood-brain barrier
P	Q8VBW1	CC	Required for the uptake of creatine
P	Q8VBW1	DE	Creatine transporter 1;
P	Q8VBW1	DE	Sodium- and chloride-dependent creatine transporter 1;
P	Q8VBW1	DE	Sodium- and chloride-dependent creatine transporter 1;
P	Q8VBW1	DR	neurotransmitter:sodium symporter activity
P	Q8VCV9	CC	May function as a sodium-dependent glucose transporter (By similarity).
P	Q8VCV9	DE	Sodium-dependent glucose transporter 1A;
P	Q8VDB9	DE	Sodium- and chloride-dependent transporter XTRP3A;
P	Q8VDB9	DR	neurotransmitter:sodium symporter activity
P	Q8VDN2	CC	This action creates the electrochemical gradient of sodium and potassium ions, providing the energy for active transport of various nutrients (By similarity)
P	Q8VDN2	CC	This action creates the electrochemical gradient of sodium and potassium ions, providing the energy for active transport of various nutrients (By similarity)
P	Q8VDN2	CC	This is the catalytic component of the active enzyme, which catalyzes the hydrolysis of ATP coupled with the exchange of sodium and potassium ions across the plasma membrane
P	Q8VDN2	CC	This is the catalytic component of the active enzyme, which catalyzes the hydrolysis of ATP coupled with the exchange of sodium and potassium ions across the plasma membrane
P	Q8VDN2	DE	Na+/K+ ATPase alpha-1 subunit;
P	Q8VDN2	DE	Na+/K+ ATPase alpha-1 subunit;
P	Q8VDN2	DE	Sodium pump subunit alpha-1;
P	Q8VDN2	DE	Sodium/potassium-transporting ATPase subunit alpha-1;
P	Q8VDN2	DE	Sodium/potassium-transporting ATPase subunit alpha-1;
P	Q8VDN2	DR	sodium:potassium-exchanging ATPase activity
P	Q8VDN2	DR	sodium:potassium-exchanging ATPase activity
P	Q8VDT1	CC	Involved in sodium-dependent transport of D-mannose, D- glucose and D-fructose (By similarity).
P	Q8VDT1	CC	Involved in sodium-dependent transport of D-mannose, D- glucose and D-fructose (By similarity).
P	Q8VDT1	DE	Na+/glucose cotransporter 4;
P	Q8VDT1	DE	Na+/glucose cotransporter 4;
P	Q8VDT1	DE	Sodium/glucose cotransporter 4;
P	Q8VDT1	DE	Sodium/glucose cotransporter 4;
P	Q8VEM8	CC	Phosphate is cotransported with H+ (By similarity).
P	Q8VEM8	CC	Transport of phosphate groups from the cytosol to mitochondrial matrix
P	Q8VEM8	DE	Phosphate carrier protein, mitochondrial;
P	Q8VEM8	DE	Phosphate transport protein;
P	Q8VHD6	CC	Facilitative glucose transporter (By similarity).
P	Q8VHD6	DE	Glucose transporter type 10;
P	Q8VHD6	DE	Solute carrier family 2, facilitated glucose transporter member 10;
P	Q8VHL0	CC	Mediates urea transport in erythrocytes (By similarity).
P	Q8VHL0	CC	Specialized low-affinity urea transporter
P	Q8VHL0	DE	Urea transporter 1;
P	Q8VHL0	DE	Urea transporter B;
P	Q8VHL0	DE	Urea transporter, erythrocyte;
P	Q8VHL0	DR	urea transmembrane transporter activity
P	Q8VI23	CC	Cation/chloride cotransporter that may play a role in the control of keratinocyte proliferation (By similarity).
P	Q8VPE6	DR	copper-exporting ATPase activity
P	Q8VXB1	CC	High-affinity potassium transporter (By similarity).
P	Q8VXB1	DE	Putative potassium transporter 12;
P	Q8VXB1	DR	potassium ion transmembrane transporter activity
P	Q8VXB5	CC	High-affinity potassium transporter (By similarity).
P	Q8VXB5	DE	Putative potassium transporter 8;
P	Q8VXB5	DR	potassium ion transmembrane transporter activity
P	Q8VYM2	CC	Acts as a H+:phosphate symporter in both low- and high-Pi conditions
P	Q8VYM2	CC	High-affinity transporter for external inorganic phosphate
P	Q8VYM2	DE	Inorganic phosphate transporter 1-1;
P	Q8VYM2	DR	inorganic phosphate transmembrane transporter activity
P	Q8VZ80	CC	Can transport the cyclic polyol myo- inositol and different hexoses, pentoses (including ribose), tetroses and sugar alcohols.
P	Q8VZ80	CC	Plasma membrane broad-spectrum sugar-proton symporter
P	Q8VZ80	DE	Sugar-proton symporter PLT5;

P	Q8VZ80	DR	sugar:hydrogen symporter activity
P	Q8VZT3	CC	Sugar transporter (Potential).
P	Q8VZT3	DE	Sugar transporter ERD6-like 12;
P	Q8VZT3	DE	Sugar transporter-like protein 5;
P	Q8VZW1	CC	Water channel probably required to promote glycerol permeability and water transport across cell membranes.
P	Q8VZW1	DR	water channel activity
P	Q8W036	CC	Aquaporins facilitate the transport of water and small neutral solutes across cell membranes (By similarity).
P	Q8W037	CC	Low water transport activity in yeast cells.
P	Q8W245	CC	Probably mediates zinc uptake from the rhizosphere (By similarity).
P	Q8W245	DE	Probable zinc transporter 10;
P	Q8W245	DR	zinc ion transmembrane transporter activity
P	Q8W246	CC	Probably mediates zinc uptake from the rhizosphere (By similarity).
P	Q8W246	DE	Zinc transporter 7;
P	Q8W246	DR	zinc ion transmembrane transporter activity
P	Q8W4I4	CC	Probable potassium transporter.
P	Q8W4I4	DE	Potassium transporter 6;
P	Q8W4I4	DR	potassium ion transmembrane transporter activity
P	Q8WUM9	CC	Sodium-phosphate symporter which plays a fundamental housekeeping role in phosphate transport, such as absorbing phosphate from interstitial fluid for normal cellular functions such as cellular metabolism, signal transduction, and nucleic acid and lipid synthesis
P	Q8WUM9	DE	Phosphate transporter 1;
P	Q8WUM9	DE	Sodium-dependent phosphate transporter 1;
P	Q8WUM9	DR	inorganic phosphate transmembrane transporter activity
P	Q8WUM9	DR	sodium-dependent phosphate transmembrane transporter activity
P	Q8WWI5	CC	Choline transporter
P	Q8WWI5	DE	Choline transporter-like protein 1;
P	Q8WWI5	DR	choline transmembrane transporter activity
P	Q8WWX8	CC	Exhibits stereospecific cotransport of both D-glucose and D- xylose
P	Q8WWX8	CC	Involved in the sodium-dependent cotransport of myo- inositol (MI) with a Na+:MI stoichiometry of 2:1
P	Q8WWX8	DE	Na+/myo-inositol cotransporter 2;
P	Q8WWX8	DE	Sodium-dependent glucose cotransporter;
P	Q8WWX8	DE	Sodium-dependent glucose cotransporter;
P	Q8WWX8	DE	Sodium/glucose cotransporter KST1;
P	Q8WWX8	DE	Sodium/glucose cotransporter KST1;
P	Q8WWX8	DE	Sodium/myo-inositol cotransporter 2;
P	Q8X3T0	CC	Uptake of L-rhamnose across the boundary membrane with the concomitant transport of protons into the cell (symport system) (By similarity).
P	Q8X3T0	DE	L-rhamnose-H+ transport protein;
P	Q8X3T0	DE	L-rhamnose-proton symporter;
P	Q8X400	CC	Involved in zinc efflux across the cytoplasmic membrane, thus reducing zinc accumulation in the cytoplasm and rendering bacteria more resistant to zinc
P	Q8X400	CC	It may contribute to zinc homeostasis at low concentrations of zinc (By similarity)
P	Q8X400	DE	Zinc transporter zitB;
P	Q8X4L6	CC	Part of the ABC transporter complex NikABCDE involved in nickel import
P	Q8X4L6	DE	Nickel import ATP-binding protein NikE;
P	Q8X4L6	DR	nickel-transporting ATPase activity
P	Q8X4L7	CC	Part of the ABC transporter complex BtuCDF involved in vitamin B12 import
P	Q8X4L7	DE	Vitamin B12 import system permease protein BtuC;
P	Q8X4V7	CC	Part of the ABC transporter complex ModABC involved in molybdenum import
P	Q8X4V7	DE	Molybdenum import ATP-binding protein ModC;
P	Q8X4V7	DR	molybdenum ion transmembrane transporter activity
P	Q8X5I6	CC	Part of the ABC transporter complex TauABC involved in taurine import
P	Q8X5I6	DE	Taurine import ATP-binding protein TauB;
P	Q8X5I6	DR	taurine-transporting ATPase activity
P	Q8X5N2	CC	Part of the ABC transporter complex HmuTUV involved in hemin import
P	Q8X5N2	DE	Hemin import ATP-binding protein HmuV;
P	Q8X5Q4	CC	Part of the ABC transporter complex RbsABCD involved in ribose import
P	Q8X5Q4	DE	Ribose import ATP-binding protein RbsA 2;
P	Q8X5U1	CC	Part of the ABC transporter complex NikABCDE involved in nickel import
P	Q8X5U1	DE	Nickel import ATP-binding protein NikD;
P	Q8X5U1	DR	nickel-transporting ATPase activity
P	Q8X5W0	CC	Part of the ABC transporter complex BtuCDF involved in vitamin B12 import

P	Q8X5W0	DE	Vitamin B12 import ATP-binding protein BtuD;
P	Q8X5W0	DE	Vitamin B12-transporting ATPase;
P	Q8X691	CC	Required for cytosine transport into the cell.
P	Q8X691	DE	Cytosine permease;
P	Q8X6U5	DR	glycerol-3-phosphate-transporting ATPase activity
P	Q8X794	DE	Voltage-gated ClC-type chloride channel ClcB;
P	Q8X794	DR	voltage-gated chloride channel activity
P	Q8X800	CC	Part of the binding-protein-dependent transport system for D-methionine and the toxic methionine analog alpha-methyl- methionine
P	Q8X800	DE	D-methionine transport system permease protein metI;
P	Q8X878	CC	Transport system that facilitates potassium-efflux, possibly by potassium-proton antiport (By similarity).
P	Q8X878	DE	Glutathione-regulated potassium-efflux system protein kefB;
P	Q8X878	DE	K+/H+ antiporter;
P	Q8X878	DE	NEM-activable K+/H+ antiporter;
P	Q8X878	DR	glutathione-regulated potassium exporter activity
P	Q8X8E3	DE	Lipoprotein-releasing system ATP-binding protein LolD;
P	Q8X8E3	DR	lipoprotein transporter activity
P	Q8X8V9	CC	This protein is a component of a D-methionine permease, a binding protein-dependent, ATP-driven transport system (By similarity).
P	Q8X8V9	DE	D-methionine-binding lipoprotein metQ;
P	Q8X8Z0	CC	Binds vitamin B12 and delivers it to the periplasmic surface of BtuC (By similarity)
P	Q8X8Z0	CC	Part of the ABC transporter complex BtuCDF involved in vitamin B12 import
P	Q8X968	DE	Aromatic amino acid transport protein AroP;
P	Q8X968	DE	General aromatic amino acid permease;
P	Q8X9F8	CC	One of the components of the high-affinity ATP-driven potassium transport (or KDP) system, which catalyzes the hydrolysis of ATP coupled with the exchange of hydrogen and potassium ions (By similarity).
P	Q8X9F8	DE	Potassium-binding and translocating subunit A;
P	Q8X9F8	DE	Potassium-translocating ATPase A chain;
P	Q8X9F8	DE	Potassium-transporting ATPase A chain;
P	Q8X9F8	DR	potassium-transporting ATPase activity
P	Q8X9F9	CC	One of the components of the high-affinity ATP-driven potassium transport (or KDP) system, which catalyzes the hydrolysis of ATP coupled with the exchange of hydrogen and potassium ions (By similarity).
P	Q8X9F9	DE	Potassium-binding and translocating subunit B;
P	Q8X9F9	DE	Potassium-translocating ATPase B chain;
P	Q8X9F9	DE	Potassium-transporting ATPase B chain;
P	Q8X9F9	DR	potassium-transporting ATPase activity
P	Q8X9G0	CC	One of the components of the high-affinity ATP-driven potassium transport (or KDP) system, which catalyzes the hydrolysis of ATP coupled with the exchange of hydrogen and potassium ions
P	Q8X9G0	DE	Potassium-binding and translocating subunit C;
P	Q8X9G0	DE	Potassium-translocating ATPase C chain;
P	Q8X9G0	DE	Potassium-transporting ATPase C chain;
P	Q8X9G0	DR	potassium-transporting ATPase activity
P	Q8XA06	CC	Part of the ABC transporter complex ThiBPQ involved in thiamine import
P	Q8XA06	DE	Thiamine import ATP-binding protein ThiQ;
P	Q8XA20	CC	Transport system that facilitates potassium-efflux, possibly by potassium-proton antiport (By similarity).
P	Q8XA20	DE	Glutathione-regulated potassium-efflux system protein kefC;
P	Q8XA20	DE	K+/H+ antiporter;
P	Q8XA63	CC	Na+/H+ antiporter that extrudes sodium in exchange for external protons (By similarity)
P	Q8XA63	DE	Na+/H+ antiporter nhaA;
P	Q8XA63	DE	Sodium/proton antiporter nhaA;
P	Q8XAW7	CC	Part of the ABC transporter complex RbsABCD involved in ribose import
P	Q8XAW7	DE	Ribose import ATP-binding protein RbsA 1;
P	Q8XAW9	CC	Responsible for the low-affinity transport of potassium into the cell, with the probable concomitant uptake of protons (symport system) (By similarity).
P	Q8XAW9	DE	Kup system potassium uptake protein;
P	Q8XAW9	DE	Low affinity potassium transport system protein kup;
P	Q8XAW9	DR	potassium ion transmembrane transporter activity
P	Q8XB33	CC	Involved in tryptophan transport across the cytoplasmic membrane
P	Q8XB33	CC	Plays a role in transporting tryptophan which is to be used catabolically (By similarity)
P	Q8XB33	DE	Low affinity tryptophan permease;

P	Q8XBJ8	CC	Part of the ABC transporter complex CysAWTP involved in sulfate/thiosulfate import
P	Q8XBJ8	DE	Sulfate/thiosulfate import ATP-binding protein CysA;
P	Q8XBK4	CC	Catalyzes the uptake of tartrate in exchange for intracellular succinate
P	Q8XBK4	DE	L-tartrate/succinate antiporter;
P	Q8XD10	CC	Important to control the intracellular level of arginine and the correct balance between arginine and lysine (By similarity).
P	Q8XD24	CC	Involved in copper export (By similarity).
P	Q8XD24	DE	Copper-exporting P-type ATPase A;
P	Q8XD24	DR	copper-exporting ATPase activity
P	Q8XDM1	CC	Part of the ABC transporter complex XylFGH involved in xylose import
P	Q8XDM1	DE	Xylose import ATP-binding protein XylG;
P	Q8XDM1	DR	D-xylose-importing ATPase activity
P	Q8XDV7	DR	phosphonate transmembrane-transporting ATPase activity
P	Q8XE58	CC	Part of the ABC transporter complex CcmAB involved in the biogenesis of c-type cytochromes; once thought to export heme, this seems not to be the case, but its exact role is uncertain
P	Q8XE58	DE	Heme exporter protein A;
P	Q8XE58	DR	heme-transporting ATPase activity
P	Q8XIZ5	CC	Part of the ABC transporter complex PotABCD involved in spermidine/putrescine import
P	Q8XIZ5	CC	Part of the ABC transporter complex PotABCD involved in spermidine/putrescine import
P	Q8XIZ5	DE	Spermidine/putrescine import ATP-binding protein PotA;
P	Q8XIZ5	DE	Spermidine/putrescine import ATP-binding protein PotA;
P	Q8XJX3	CC	Part of the ABC transporter complex RbsABCD involved in ribose import
P	Q8XJX3	DE	Ribose import ATP-binding protein RbsA;
P	Q8XMP8	CC	Part of the ABC transporter complex PstSACB involved in phosphate import
P	Q8XMP8	DE	ABC phosphate transporter;
P	Q8XMP8	DE	Phosphate import ATP-binding protein PstB;
P	Q8XMP8	DE	Phosphate-transporting ATPase;
P	Q8XMP8	DR	inorganic phosphate transmembrane transporter activity
P	Q8XMP8	DR	phosphate transmembrane-transporting ATPase activity
P	Q8XNY9	DR	cobalt ion transmembrane transporter activity
P	Q8XPK6	CC	Part of the ABC transporter complex XylFGH involved in xylose import
P	Q8XPK6	DE	Xylose import ATP-binding protein XylG;
P	Q8XPK6	DR	D-xylose-importing ATPase activity
P	Q8XR16	CC	Transport of potassium into the cell (By similarity).
P	Q8XR16	DE	Probable potassium transport system protein kup 1;
P	Q8XR16	DR	potassium ion transmembrane transporter activity
P	Q8XSF6	CC	H+-stimulated, highly selective, manganese uptake system (By similarity).
P	Q8XSF6	DE	Probable manganese transport protein mntH;
P	Q8XU10	CC	One of the components of the high-affinity ATP-driven potassium transport (or KDP) system, which catalyzes the hydrolysis of ATP coupled with the exchange of hydrogen and potassium ions
P	Q8XU10	DE	Potassium-binding and translocating subunit C;
P	Q8XU10	DE	Potassium-translocating ATPase C chain;
P	Q8XU10	DE	Potassium-transporting ATPase C chain;
P	Q8XU10	DR	potassium-transporting ATPase activity
P	Q8XU11	CC	One of the components of the high-affinity ATP-driven potassium transport (or KDP) system, which catalyzes the hydrolysis of ATP coupled with the exchange of hydrogen and potassium ions (By similarity).
P	Q8XU11	DE	Potassium-binding and translocating subunit B;
P	Q8XU11	DE	Potassium-translocating ATPase B chain;
P	Q8XU11	DE	Potassium-transporting ATPase B chain;
P	Q8XU11	DR	potassium-transporting ATPase activity
P	Q8XU12	CC	One of the components of the high-affinity ATP-driven potassium transport (or KDP) system, which catalyzes the hydrolysis of ATP coupled with the exchange of hydrogen and potassium ions (By similarity).
P	Q8XU12	DE	Potassium-binding and translocating subunit A;
P	Q8XU12	DE	Potassium-translocating ATPase A chain;
P	Q8XU12	DE	Potassium-transporting ATPase A chain;
P	Q8XU12	DR	potassium-transporting ATPase activity
P	Q8XVS2	DR	L-arabinose-importing ATPase activity
P	Q8XYY5	CC	Transport of potassium into the cell (By similarity).
P	Q8XYY5	DE	Probable potassium transport system protein kup 2;
P	Q8XYY5	DR	potassium ion transmembrane transporter activity
P	Q8XZ72	CC	Part of the ABC transporter complex PstSACB involved in phosphate import

P	Q8XZ72	DE	ABC phosphate transporter;
P	Q8XZ72	DE	Phosphate import ATP-binding protein PstB;
P	Q8XZ72	DE	Phosphate-transporting ATPase;
P	Q8XZ72	DR	inorganic phosphate transmembrane transporter activity
P	Q8XZ72	DR	phosphate transmembrane-transporting ATPase activity
P	Q8XZP8	CC	Part of the ABC transporter complex CysAWTP involved in sulfate/thiosulfate import
P	Q8XZP8	DE	Sulfate-transporting ATPase;
P	Q8XZP8	DE	Sulfate/thiosulfate import ATP-binding protein CysA;
P	Q8XZP8	DR	sulfate transmembrane-transporting ATPase activity
P	Q8XZX8	DR	glycerol-3-phosphate-transporting ATPase activity
P	Q8Y0C6	DE	Lipoprotein-releasing system ATP-binding protein LolD;
P	Q8Y0C6	DR	lipoprotein transporter activity
P	Q8Y2K5	DR	sodium:dicarboxylate symporter activity
P	Q8Y3Z6	CC	One of the components of the high-affinity ATP-driven potassium transport (or KDP) system, which catalyzes the hydrolysis of ATP coupled with the exchange of hydrogen and potassium ions (By similarity).
P	Q8Y3Z6	DE	Potassium-binding and translocating subunit A;
P	Q8Y3Z6	DE	Potassium-translocating ATPase A chain;
P	Q8Y3Z6	DE	Potassium-transporting ATPase A chain;
P	Q8Y3Z6	DR	potassium-transporting ATPase activity
P	Q8Y3Z7	CC	One of the components of the high-affinity ATP-driven potassium transport (or KDP) system, which catalyzes the hydrolysis of ATP coupled with the exchange of hydrogen and potassium ions (By similarity).
P	Q8Y3Z7	DE	Potassium-binding and translocating subunit B;
P	Q8Y3Z7	DE	Potassium-translocating ATPase B chain;
P	Q8Y3Z7	DE	Potassium-transporting ATPase B chain;
P	Q8Y3Z7	DR	potassium-transporting ATPase activity
P	Q8Y3Z8	CC	One of the components of the high-affinity ATP-driven potassium transport (or KDP) system, which catalyzes the hydrolysis of ATP coupled with the exchange of hydrogen and potassium ions
P	Q8Y3Z8	DE	Potassium-binding and translocating subunit C;
P	Q8Y3Z8	DE	Potassium-translocating ATPase C chain;
P	Q8Y3Z8	DE	Potassium-transporting ATPase C chain;
P	Q8Y3Z8	DR	potassium-transporting ATPase activity
P	Q8Y4E9	CC	Part of the ABC transporter complex PstSACB involved in phosphate import
P	Q8Y4E9	DE	ABC phosphate transporter 2;
P	Q8Y4E9	DE	Phosphate import ATP-binding protein PstB 2;
P	Q8Y4E9	DE	Phosphate-transporting ATPase 2;
P	Q8Y4E9	DR	inorganic phosphate transmembrane transporter activity
P	Q8Y4E9	DR	phosphate transmembrane-transporting ATPase activity
P	Q8Y4L8	CC	Part of the ABC transporter complex MetNIQ involved in methionine import
P	Q8Y4L8	DE	Methionine import ATP-binding protein MetN 2;
P	Q8Y651	CC	This protein is probably a component of a manganese permease, a binding protein-dependent, ATP-driven transport system
P	Q8Y651	DE	Manganese transport system ATP-binding protein MntB;
P	Q8Y652	CC	This protein is probably a component of a manganese permease, a binding protein-dependent, ATP-driven transport system (By similarity).
P	Q8Y652	DE	Manganese transport system membrane protein mntC;
P	Q8Y653	CC	This protein is probably a component of a manganese permease, a binding protein-dependent, ATP-driven transport system (By similarity).
P	Q8Y653	DE	Manganese-binding lipoprotein mntA;
P	Q8Y773	CC	H+-stimulated, highly selective, manganese uptake system (By similarity).
P	Q8Y773	DE	Probable manganese transport protein mntH;
P	Q8Y8T6	CC	Part of the ABC transporter complex PotABCD involved in spermidine/putrescine import
P	Q8Y8T6	CC	Part of the ABC transporter complex PotABCD involved in spermidine/putrescine import
P	Q8Y8T6	DE	Spermidine/putrescine import ATP-binding protein PotA;
P	Q8Y8T6	DE	Spermidine/putrescine import ATP-binding protein PotA;
P	Q8Y9K8	CC	May mediate the efflux of lincomycin (By similarity).
P	Q8Y9K8	DE	Lincomycin resistance protein lmrB;
P	Q8YA75	CC	Part of the ABC transporter complex MetNIQ involved in methionine import
P	Q8YA75	DE	Methionine import ATP-binding protein MetN 1;
P	Q8YCB1	DR	glycerol-3-phosphate-transporting ATPase activity
P	Q8YCN7	CC	Part of the ABC transporter complex NikABCDE involved in nickel import
P	Q8YCN7	DE	Nickel import ATP-binding protein NikE;
P	Q8YCN7	DR	nickel-transporting ATPase activity

P	Q8YCN8	CC	Part of the ABC transporter complex NikABCDE involved in nickel import
P	Q8YCN8	DE	Nickel import ATP-binding protein NikD;
P	Q8YCN8	DR	nickel-transporting ATPase activity
P	Q8YDJ7	CC	Involved in the high-affinity zinc uptake transport system
P	Q8YDJ7	DE	High-affinity zinc uptake system protein znuA;
P	Q8YDJ8	CC	Part of the ABC transporter complex ZnuABC involved in zinc import
P	Q8YDJ8	DE	Zinc import ATP-binding protein ZnuC;
P	Q8YDJ8	DR	zinc transporting ATPase activity
P	Q8YDN0	CC	Part of the ABC transporter complex XylFGH involved in xylose import
P	Q8YDN0	DE	Xylose import ATP-binding protein XylG;
P	Q8YE15	CC	Part of the ABC transporter complex ModABC involved in molybdenum import
P	Q8YE15	DE	Molybdenum import ATP-binding protein ModC;
P	Q8YE15	DR	molybdenum ion transmembrane transporter activity
P	Q8YFI5	CC	Na+/H+ antiporter that extrudes sodium in exchange for external protons (By similarity)
P	Q8YFI5	DE	Na+/H+ antiporter nhaA;
P	Q8YFI5	DE	Sodium/proton antiporter nhaA;
P	Q8YGM0	DE	Lipoprotein-releasing system ATP-binding protein LolD;
P	Q8YGM0	DR	lipoprotein transporter activity
P	Q8YI23	CC	Transport of potassium into the cell (By similarity).
P	Q8YI23	DE	Probable potassium transport system protein kup;
P	Q8YI23	DR	potassium ion transmembrane transporter activity
P	Q8YJ02	CC	Part of the ABC transporter complex thiBPQ involved in thiamine import (By similarity)
P	Q8YJ02	DE	Thiamine-binding periplasmic protein;
P	Q8YJ03	CC	Part of the ABC transporter complex thiBPQ involved in thiamine import
P	Q8YJ03	DE	Thiamine transport system permease protein thiP;
P	Q8YJ04	CC	Part of the ABC transporter complex ThiBPQ involved in thiamine import
P	Q8YJ04	DE	Thiamine import ATP-binding protein ThiQ;
P	Q8YK28	DR	phosphonate transmembrane-transporting ATPase activity
P	Q8YM92	CC	Part of the ABC transporter complex PotABCD involved in spermidine/putrescine import
P	Q8YM92	CC	Part of the ABC transporter complex PotABCD involved in spermidine/putrescine import
P	Q8YM92	DE	Spermidine/putrescine import ATP-binding protein PotA;
P	Q8YM92	DE	Spermidine/putrescine import ATP-binding protein PotA;
P	Q8YNJ3	CC	Part of the ABC transporter complex PstSACB involved in phosphate import
P	Q8YNJ3	DE	ABC phosphate transporter 3;
P	Q8YNJ3	DE	Phosphate import ATP-binding protein PstB 3;
P	Q8YNJ3	DE	Phosphate-transporting ATPase 3;
P	Q8YNJ3	DR	inorganic phosphate transmembrane transporter activity
P	Q8YNJ3	DR	phosphate transmembrane-transporting ATPase activity
P	Q8YPE8	CC	One of the components of the high-affinity ATP-driven potassium transport (or KDP) system, which catalyzes the hydrolysis of ATP coupled with the exchange of hydrogen and potassium ions (By similarity).
P	Q8YPE8	DE	Potassium-binding and translocating subunit A;
P	Q8YPE8	DE	Potassium-translocating ATPase A chain;
P	Q8YPE8	DE	Potassium-transporting ATPase A chain;
P	Q8YPE8	DR	potassium-transporting ATPase activity
P	Q8YPE9	CC	One of the components of the high-affinity ATP-driven potassium transport (or KDP) system, which catalyzes the hydrolysis of ATP coupled with the exchange of hydrogen and potassium ions (By similarity).
P	Q8YPE9	DE	Potassium-binding and translocating subunit B 1;
P	Q8YPE9	DE	Potassium-translocating ATPase B chain 1;
P	Q8YPE9	DE	Potassium-transporting ATPase B chain 1;
P	Q8YPE9	DR	potassium-transporting ATPase activity
P	Q8YPF1	CC	One of the components of the high-affinity ATP-driven potassium transport (or KDP) system, which catalyzes the hydrolysis of ATP coupled with the exchange of hydrogen and potassium ions
P	Q8YPF1	DE	Potassium-binding and translocating subunit C 2;
P	Q8YPF1	DE	Potassium-translocating ATPase C chain 2;
P	Q8YPF1	DE	Potassium-transporting ATPase C chain 2;
P	Q8YPF1	DR	potassium-transporting ATPase activity
P	Q8YQ90	DR	cobalt ion transmembrane transporter activity
P	Q8YSD5	CC	One of the components of the high-affinity ATP-driven potassium transport (or KDP) system, which catalyzes the hydrolysis of ATP coupled with the exchange of hydrogen and potassium ions (By similarity).
P	Q8YSD5	DE	Potassium-binding and translocating subunit B 2;
P	Q8YSD5	DE	Potassium-translocating ATPase B chain 2;

P	Q8YSD5	DE	Potassium-transporting ATPase B chain 2;
P	Q8YSD5	DR	potassium-transporting ATPase activity
P	Q8YSD7	CC	One of the components of the high-affinity ATP-driven potassium transport (or KDP) system, which catalyzes the hydrolysis of ATP coupled with the exchange of hydrogen and potassium ions
P	Q8YSD7	DE	Potassium-binding and translocating subunit C 1;
P	Q8YSD7	DE	Potassium-translocating ATPase C chain 1;
P	Q8YSD7	DE	Potassium-transporting ATPase C chain 1;
P	Q8YSD7	DR	potassium-transporting ATPase activity
P	Q8YUI9	DR	phosphonate transmembrane-transporting ATPase activity
P	Q8YUV1	DR	phosphonate transmembrane-transporting ATPase activity
P	Q8YYE2	CC	Part of the ABC transporter complex PstSACB involved in phosphate import
P	Q8YYE2	DE	ABC phosphate transporter 2;
P	Q8YYE2	DE	Phosphate import ATP-binding protein PstB 2;
P	Q8YYE2	DE	Phosphate-transporting ATPase 2;
P	Q8YYE2	DR	inorganic phosphate transmembrane transporter activity
P	Q8YYE2	DR	phosphate transmembrane-transporting ATPase activity
P	Q8YYE3	CC	Part of the ABC transporter complex PstSACB involved in phosphate import
P	Q8YYE3	DE	ABC phosphate transporter 1;
P	Q8YYE3	DE	Phosphate import ATP-binding protein PstB 1;
P	Q8YYE3	DE	Phosphate-transporting ATPase 1;
P	Q8YYE3	DR	phosphate transmembrane-transporting ATPase activity
P	Q8Z0H0	CC	Part of the ABC transporter complex CysAWTP involved in sulfate/thiosulfate import
P	Q8Z0H0	DE	Sulfate-transporting ATPase;
P	Q8Z0H0	DE	Sulfate/thiosulfate import ATP-binding protein CysA;
P	Q8Z0H0	DR	sulfate transmembrane-transporting ATPase activity
P	Q8Z1U0	CC	Part of the ABC transporter complex MalEFGK involved in maltose/maltodextrin import
P	Q8Z1U0	CC	Part of the ABC transporter complex MalEFGK involved in maltose/maltodextrin import
P	Q8Z1U0	DE	Maltose/maltodextrin import ATP-binding protein MalK;
P	Q8Z1U0	DE	Maltose/maltodextrin import ATP-binding protein MalK;
P	Q8Z1U0	DR	maltose-transporting ATPase activity
P	Q8Z1U2	DE	Maltose transport system permease protein malF;
P	Q8Z1U3	DE	Maltose transport system permease protein malG;
P	Q8Z1Y7	CC	Transport system that facilitates potassium-efflux, possibly by potassium-proton antiport (By similarity).
P	Q8Z1Y7	DE	Glutathione-regulated potassium-efflux system protein kefB;
P	Q8Z1Y7	DE	K+/H+ antiporter;
P	Q8Z1Y7	DE	NEM-activable K+/H+ antiporter;
P	Q8Z1Y7	DR	glutathione-regulated potassium exporter activity
P	Q8Z245	DR	glycerol-3-phosphate-transporting ATPase activity
P	Q8Z2E3	CC	Transports L-lactate across the membrane
P	Q8Z2E3	DE	L-lactate permease;
P	Q8Z2E3	DR	lactate transmembrane transporter activity
P	Q8Z2R2	CC	Responsible for the low-affinity transport of potassium into the cell, with the probable concomitant uptake of protons (symport system) (By similarity).
P	Q8Z2R2	DE	Kup system potassium uptake protein;
P	Q8Z2R2	DE	Low affinity potassium transport system protein kup;
P	Q8Z2R2	DR	potassium ion transmembrane transporter activity
P	Q8Z2R4	CC	Part of the ABC transporter complex RbsABCD involved in ribose import
P	Q8Z2R4	DE	Ribose import ATP-binding protein RbsA;
P	Q8Z2V6	CC	Uptake of L-rhamnose across the boundary membrane with the concomitant transport of protons into the cell (symport system) (By similarity).
P	Q8Z2V6	DE	L-rhamnose-H+ transport protein;
P	Q8Z2V6	DE	L-rhamnose-proton symporter;
P	Q8Z3B4	CC	Conducts the efflux of homoserine and homoserine lactone (By similarity).
P	Q8Z3B4	DE	Homoserine/homoserine lactone efflux protein;
P	Q8Z3L4	CC	Involved in the import of serine and threonine into the cell, with the concomitant import of sodium (symport system) (By similarity).
P	Q8Z3L4	DE	Na+/serine-threonine symporter;
P	Q8Z3L4	DR	sodium:dicarboxylate symporter activity
P	Q8Z3L4	DR	sodium:dicarboxylate symporter activity
P	Q8Z3W2	CC	Important to control the intracellular level of arginine and the correct balance between arginine and lysine (By similarity).
P	Q8Z4X5	CC	H+-stimulated, highly selective, manganese uptake system (By similarity).
P	Q8Z4X5	DE	Manganese transport protein mntH;

P	Q8Z4X8	DR	voltage-gated chloride channel activity
P	Q8Z591	CC	This system is involved in fructose transport (By similarity).
P	Q8Z591	DE	Fructose-specific phosphotransferase enzyme IIA component;
P	Q8Z591	DE	PTS system fructose-specific EIIA component;
P	Q8Z5N3	DR	cobalt ion transmembrane transporter activity
P	Q8Z5W6	CC	Part of the ABC transporter complex ZnuABC involved in zinc import
P	Q8Z5W6	DE	Zinc import ATP-binding protein ZnuC;
P	Q8Z5W6	DR	zinc transporting ATPase activity
P	Q8Z684	CC	Na+/H+ antiporter that extrudes sodium in exchange for external protons (By similarity).
P	Q8Z684	DE	Na+/H+ antiporter nhaB;
P	Q8Z684	DE	Sodium/proton antiporter nhaB;
P	Q8Z684	DR	sodium:hydrogen antiporter activity
P	Q8Z6I5	CC	Part of the ABC transporter complex BtuCDF involved in vitamin B12 import
P	Q8Z6I5	DE	Vitamin B12 import system permease protein BtuC;
P	Q8Z6Y0	DE	Voltage-gated ClC-type chloride channel ClcB;
P	Q8Z6Y0	DR	voltage-gated chloride channel activity
P	Q8Z784	CC	Mediates efflux of zinc ions (By similarity).
P	Q8Z784	DE	Zinc transport protein ZntB;
P	Q8Z7H7	CC	Part of the ABC transporter complex PotABCD involved in spermidine/putrescine import
P	Q8Z7H7	CC	Part of the ABC transporter complex PotABCD involved in spermidine/putrescine import
P	Q8Z7H7	DE	Spermidine/putrescine import ATP-binding protein PotA;
P	Q8Z7H7	DE	Spermidine/putrescine import ATP-binding protein PotA;
P	Q8Z8A4	CC	Part of the ABC transporter complex ModABC involved in molybdenum import
P	Q8Z8A4	DE	Molybdenum import ATP-binding protein ModC;
P	Q8Z8A4	DR	molybdenum ion transmembrane transporter activity
P	Q8Z8B6	CC	Involved in zinc efflux across the cytoplasmic membrane, thus reducing zinc accumulation in the cytoplasm and rendering bacteria more resistant to zinc
P	Q8Z8B6	CC	It may contribute to zinc homeostasis at low concentrations of zinc (By similarity).
P	Q8Z8B6	DE	Zinc transporter zitB;
P	Q8Z8E4	CC	One of the components of the high-affinity ATP-driven potassium transport (or KDP) system, which catalyzes the hydrolysis of ATP coupled with the exchange of hydrogen and potassium ions (By similarity).
P	Q8Z8E4	DE	Potassium-binding and translocating subunit A;
P	Q8Z8E4	DE	Potassium-translocating ATPase A chain;
P	Q8Z8E4	DE	Potassium-transporting ATPase A chain;
P	Q8Z8E4	DR	potassium-transporting ATPase activity
P	Q8Z8E5	CC	One of the components of the high-affinity ATP-driven potassium transport (or KDP) system, which catalyzes the hydrolysis of ATP coupled with the exchange of hydrogen and potassium ions (By similarity).
P	Q8Z8E5	DE	Potassium-binding and translocating subunit B;
P	Q8Z8E5	DE	Potassium-translocating ATPase B chain;
P	Q8Z8E5	DE	Putative potassium-transporting ATPase B chain;
P	Q8Z8E5	DR	potassium-transporting ATPase activity
P	Q8Z8E6	CC	One of the components of the high-affinity ATP-driven potassium transport (or KDP) system, which catalyzes the hydrolysis of ATP coupled with the exchange of hydrogen and potassium ions
P	Q8Z8E6	DE	Potassium-binding and translocating subunit C;
P	Q8Z8E6	DE	Potassium-translocating ATPase C chain;
P	Q8Z8E6	DE	Potassium-transporting ATPase C chain;
P	Q8Z8E6	DR	potassium-transporting ATPase activity
P	Q8Z8R5	CC	Part of the ABC transporter complex MetNIQ involved in methionine import
P	Q8Z8R5	DE	Methionine import ATP-binding protein MetN 2;
P	Q8Z8S4	CC	Involved in copper export (By similarity).
P	Q8Z8S4	DE	Copper-exporting P-type ATPase A;
P	Q8Z8S4	DR	copper-exporting ATPase activity
P	Q8Z8W7	CC	Probably part of the phnSTUV complex (TC 3.A.1.11.5) involved in 2-aminoethylphosphonate import (By similarity).
P	Q8Z8W7	DE	Putative 2-aminoethylphosphonate-binding periplasmic protein;
P	Q8Z8W8	CC	Probably part of the PhnSTUV complex (TC 3.A.1.11.5) involved in 2-aminoethylphosphonate import
P	Q8Z8W8	DE	Putative 2-aminoethylphosphonate import ATP-binding protein PhnT;
P	Q8Z8W9	CC	Probably part of the phnSTUV complex (TC 3.A.1.11.5) involved in 2-aminoethylphosphonate import
P	Q8Z8W9	DE	Putative 2-aminoethylphosphonate transport system permease protein phnU;

P	Q8Z8X0	CC	Probably part of the phnSTUV complex (TC 3.A.1.11.5) involved in 2-aminoethylphosphonate import
P	Q8Z8X0	DE	Putative 2-aminoethylphosphonate transport system permease protein phnV;
P	Q8Z990	CC	Part of the ABC transporter complex MetNIQ involved in methionine import
P	Q8Z990	DE	Methionine import ATP-binding protein MetN 1;
P	Q8Z990	DR	D-methionine transmembrane transporter activity
P	Q8Z992	CC	This protein is a component of a D-methionine permease, a binding protein-dependent, ATP-driven transport system (By similarity).
P	Q8Z992	DE	D-methionine-binding lipoprotein metQ;
P	Q8Z9B2	CC	Binds vitamin B12 and delivers it to the periplasmic surface of BtuC (By similarity)
P	Q8Z9B2	CC	Part of the ABC transporter complex BtuCDF involved in vitamin B12 import
P	Q8Z9B3	CC	Functions as antiport system and exchanges two chloride ions for 1 proton
P	Q8Z9B3	CC	Proton-coupled chloride transporter
P	Q8Z9B3	DR	voltage-gated chloride channel activity
P	Q8Z9I6	CC	Part of the ABC transporter complex ThiBPQ involved in thiamine import
P	Q8Z9I6	DE	Thiamine import ATP-binding protein ThiQ;
P	Q8Z9K0	CC	Transport system that facilitates potassium-efflux, possibly by potassium-proton antiport (By similarity).
P	Q8Z9K0	DE	Glutathione-regulated potassium-efflux system protein kefC;
P	Q8Z9K0	DE	K+/H+ antiporter;
P	Q8Z9N5	CC	Na+/H+ antiporter that extrudes sodium in exchange for external protons (By similarity)
P	Q8Z9N5	DE	Na+/H+ antiporter nhaA;
P	Q8Z9N5	DE	Sodium/proton antiporter nhaA;
P	Q8Z9T1	CC	Part of the ABC transporter complex PstSACB involved in phosphate import
P	Q8Z9T1	DE	ABC phosphate transporter 2;
P	Q8Z9T1	DE	Phosphate import ATP-binding protein PstB 2;
P	Q8Z9T1	DE	Phosphate-transporting ATPase 2;
P	Q8Z9T1	DR	inorganic phosphate transmembrane transporter activity
P	Q8Z9T1	DR	phosphate transmembrane-transporting ATPase activity
P	Q8ZA28	DR	sodium:dicarboxylate symporter activity
P	Q8ZAG5	CC	Mediates influx of magnesium ions (By similarity).
P	Q8ZAG5	DE	Magnesium transport protein CorA;
P	Q8ZAG5	DR	cobalt ion transmembrane transporter activity
P	Q8ZAG5	DR	magnesium ion transmembrane transporter activity
P	Q8ZAS8	CC	Part of the ABC transporter complex MalEFGK involved in maltose/maltodextrin import
P	Q8ZAS8	CC	Part of the ABC transporter complex MalEFGK involved in maltose/maltodextrin import
P	Q8ZAS8	DE	Maltose/maltodextrin import ATP-binding protein MalK;
P	Q8ZAS8	DE	Maltose/maltodextrin import ATP-binding protein MalK;
P	Q8ZAS8	DR	maltose-transporting ATPase activity
P	Q8ZBM0	CC	Functions as antiport system and exchanges two chloride ions for 1 proton
P	Q8ZBM0	CC	Proton-coupled chloride transporter
P	Q8ZBM0	DR	voltage-gated chloride channel activity
P	Q8ZBM3	CC	Binds vitamin B12 and delivers it to the periplasmic surface of BtuC (By similarity)
P	Q8ZBM3	CC	Part of the ABC transporter complex BtuCDF involved in vitamin B12 import
P	Q8ZBM3	DR	iron ion transmembrane transporter activity
P	Q8ZCA7	CC	Involved in copper export (By similarity).
P	Q8ZCA7	DE	Copper-exporting P-type ATPase A;
P	Q8ZCA7	DR	copper-exporting ATPase activity
P	Q8ZCK2	CC	H+-stimulated, highly selective, manganese uptake system (By similarity).
P	Q8ZCK2	DE	Probable manganese transport protein mntH;
P	Q8ZCX5	CC	Part of the ABC transporter complex PstSACB involved in phosphate import
P	Q8ZCX5	DE	ABC phosphate transporter 1;
P	Q8ZCX5	DE	Phosphate import ATP-binding protein PstB 1;
P	Q8ZCX5	DE	Phosphate-specific transport component 1;
P	Q8ZCX5	DE	Phosphate-transporting ATPase 1;
P	Q8ZCX5	DR	inorganic phosphate transmembrane transporter activity
P	Q8ZCX5	DR	phosphate transmembrane-transporting ATPase activity
P	Q8ZD58	CC	Part of the ABC transporter complex CcmAB involved in the biogenesis of c-type cytochromes; once thought to export heme, this seems not to be the case, but its exact role is uncertain
P	Q8ZD58	DE	Heme exporter protein A;
P	Q8ZD58	DR	heme-transporting ATPase activity
P	Q8ZD96	CC	One of the components of the high-affinity ATP-driven potassium transport (or KDP) system, which catalyzes the hydrolysis of ATP coupled with the exchange of hydrogen and potassium ions (By similarity).

P	Q8ZD96	DE	Potassium-binding and translocating subunit A;
P	Q8ZD96	DE	Potassium-translocating ATPase A chain;
P	Q8ZD96	DE	Potassium-transporting ATPase A chain;
P	Q8ZD96	DR	potassium-transporting ATPase activity
P	Q8ZD97	CC	One of the components of the high-affinity ATP-driven potassium transport (or KDP) system, which catalyzes the hydrolysis of ATP coupled with the exchange of hydrogen and potassium ions (By similarity).
P	Q8ZD97	DE	Potassium-binding and translocating subunit B;
P	Q8ZD97	DE	Potassium-translocating ATPase B chain;
P	Q8ZD97	DE	Potassium-transporting ATPase B chain;
P	Q8ZD97	DR	potassium-transporting ATPase activity
P	Q8ZD98	CC	One of the components of the high-affinity ATP-driven potassium transport (or KDP) system, which catalyzes the hydrolysis of ATP coupled with the exchange of hydrogen and potassium ions
P	Q8ZD98	DE	Potassium-binding and translocating subunit C;
P	Q8ZD98	DE	Potassium-translocating ATPase C chain;
P	Q8ZD98	DE	Potassium-transporting ATPase C chain;
P	Q8ZD98	DR	potassium-transporting ATPase activity
P	Q8ZDX4	CC	Part of the ABC transporter complex BtuCDF involved in vitamin B12 import
P	Q8ZDX4	DE	Vitamin B12 import system permease protein BtuC;
P	Q8ZDX6	CC	Part of the ABC transporter complex BtuCDF involved in vitamin B12 import
P	Q8ZDX6	DE	Vitamin B12 import ATP-binding protein BtuD;
P	Q8ZDX6	DE	Vitamin B12-transporting ATPase;
P	Q8ZEU2	CC	Involved in the high-affinity zinc uptake transport system (By similarity).
P	Q8ZEU2	DE	High-affinity zinc uptake system protein znuA;
P	Q8ZFR4	DE	Lipoprotein-releasing system ATP-binding protein LolD;
P	Q8ZFR4	DR	lipoprotein transporter activity
P	Q8ZGS9	CC	Exchanges extracellular arginine for its intracellular decarboxylation product agmatine (Agm) thereby expelling intracellular protons (By similarity).
P	Q8ZGS9	DE	Arginine/agmatine antiporter;
P	Q8ZGU5	DR	phosphonate transmembrane-transporting ATPase activity
P	Q8ZGX6	CC	Part of the ABC transporter complex ModABC involved in molybdenum import
P	Q8ZGX6	DE	Molybdenum import ATP-binding protein ModC;
P	Q8ZGX6	DR	molybdate transmembrane-transporting ATPase activity
P	Q8ZGX6	DR	molybdenum ion transmembrane transporter activity
P	Q8ZGY6	CC	Involved in zinc efflux across the cytoplasmic membrane, thus reducing zinc accumulation in the cytoplasm and rendering bacteria more resistant to zinc
P	Q8ZGY6	CC	It may contribute to zinc homeostasis at low concentrations of zinc (By similarity)
P	Q8ZGY6	DE	Zinc transporter zitB;
P	Q8ZH38	CC	Part of the ABC transporter complex MetNIQ involved in methionine import
P	Q8ZH38	DE	Methionine import ATP-binding protein MetN 1;
P	Q8ZH38	DR	D-methionine transmembrane transporter activity
P	Q8ZH39	CC	Part of the binding-protein-dependent transport system for D-methionine and the toxic methionine analog alpha-methyl- methionine
P	Q8ZH39	DE	D-methionine transport system permease protein metI;
P	Q8ZH40	CC	This protein is a component of a D-methionine permease, a binding protein-dependent, ATP-driven transport system (By similarity).
P	Q8ZH40	DE	D-methionine-binding lipoprotein metQ;
P	Q8ZHH6	CC	Important to control the intracellular level of arginine and the correct balance between arginine and lysine (By similarity).
P	Q8ZIZ8	CC	Uptake of L-rhamnose across the boundary membrane with the concomitant transport of protons into the cell (symport system) (By similarity).
P	Q8ZIZ8	DE	L-rhamnose-H+ transport protein;
P	Q8ZIZ8	DE	L-rhamnose-proton symporter;
P	Q8ZJC4	CC	Transport system that facilitates potassium-efflux, possibly by potassium-proton antiport (By similarity).
P	Q8ZJC4	DE	Glutathione-regulated potassium-efflux system protein kefB;
P	Q8ZJC4	DE	K+/H+ antiporter;
P	Q8ZJC4	DE	NEM-activable K+/H+ antiporter;
P	Q8ZJC4	DR	glutathione-regulated potassium exporter activity
P	Q8ZJD0	CC	Part of the ABC transporter complex TauABC involved in taurine import
P	Q8ZJD0	DE	Taurine import ATP-binding protein TauB;
P	Q8ZJD0	DR	taurine-transporting ATPase activity
P	Q8ZJT0	CC	Responsible for the low-affinity transport of potassium into the cell, with the probable concomitant uptake of protons (symport system) (By similarity).

P	Q8ZJT0	DE	Kup system potassium uptake protein;
P	Q8ZJT0	DE	Low affinity potassium transport system protein kup;
P	Q8ZJT0	DR	potassium ion transmembrane transporter activity
P	Q8ZKV9	CC	Part of the ABC transporter complex RbsABCD involved in ribose import
P	Q8ZKV9	DE	Ribose import ATP-binding protein RbsA;
P	Q8ZKW1	CC	Responsible for the low-affinity transport of potassium into the cell, with the probable concomitant uptake of protons (symport system) (By similarity).
P	Q8ZKW1	DE	Kup system potassium uptake protein;
P	Q8ZKW1	DE	Low affinity potassium transport system protein kup;
P	Q8ZKW1	DR	potassium ion transmembrane transporter activity
P	Q8ZKZ9	CC	Part of the ABC transporter complex CcmAB involved in the biogenesis of c-type cytochromes; once thought to export heme, this seems not to be the case, but its exact role is uncertain
P	Q8ZKZ9	DE	Heme exporter protein A 1;
P	Q8ZKZ9	DR	heme-transporting ATPase activity
P	Q8ZL63	CC	Transports L-lactate across the membrane
P	Q8ZL63	DE	L-lactate permease;
P	Q8ZL63	DR	lactate transmembrane transporter activity
P	Q8ZLF4	DR	glycerol-3-phosphate-transporting ATPase activity
P	Q8ZLL3	CC	Transport system that facilitates potassium-efflux, possibly by potassium-proton antiport (By similarity).
P	Q8ZLL3	DE	Glutathione-regulated potassium-efflux system protein kefB;
P	Q8ZLL3	DE	K+/H+ antiporter;
P	Q8ZLL3	DE	NEM-activable K+/H+ antiporter;
P	Q8ZLL3	DR	glutathione-regulated potassium exporter activity
P	Q8ZLW1	CC	Involved in the import of threonine and serine into the cell, with the concomitant import of a proton (symport system) (By similarity).
P	Q8ZLW1	DE	Threonine/serine transporter TdcC;
P	Q8ZLX1	CC	Involved in the import of serine and threonine into the cell, with the concomitant import of sodium (symport system) (By similarity).
P	Q8ZLX1	DE	Na+/serine-threonine symporter;
P	Q8ZLX1	DR	sodium:dicarboxylate symporter activity
P	Q8ZLX1	DR	sodium:dicarboxylate symporter activity
P	Q8ZM68	CC	Important to control the intracellular level of arginine and the correct balance between arginine and lysine (By similarity).
P	Q8ZNJ9	DE	Putative ABC-type dipeptide/oligopeptide/nickel transport systems, permease component;
P	Q8ZNV7	CC	Part of the ABC transporter complex ZnuABC involved in zinc import
P	Q8ZNV7	DE	Zinc import ATP-binding protein ZnuC;
P	Q8ZNV7	DR	zinc transporting ATPase activity
P	Q8ZP14	CC	Na+/H+ antiporter that extrudes sodium in exchange for external protons (By similarity)
P	Q8ZP14	DE	Na+/H+ antiporter nhaB;
P	Q8ZP14	DE	Sodium/proton antiporter nhaB;
P	Q8ZP14	DR	sodium:hydrogen antiporter activity
P	Q8ZPK5	DE	Voltage-gated ClC-type chloride channel ClcB;
P	Q8ZPK5	DR	voltage-gated chloride channel activity
P	Q8ZPS8	CC	Part of the ABC transporter complex BtuCDF involved in vitamin B12 import
P	Q8ZPS8	DE	Vitamin B12 import system permease protein BtuC;
P	Q8ZQ35	DE	Putative sodium/glucose cotransporter;
P	Q8ZQR6	CC	Part of the ABC transporter complex ModABC involved in molybdenum import
P	Q8ZQR6	DE	Molybdenum import ATP-binding protein ModC;
P	Q8ZQR6	DR	molybdenum ion transmembrane transporter activity
P	Q8ZQW1	CC	One of the components of the high-affinity ATP-driven potassium transport (or KDP) system, which catalyzes the hydrolysis of ATP coupled with the exchange of hydrogen and potassium ions (By similarity).
P	Q8ZQW1	DE	Potassium-binding and translocating subunit A;
P	Q8ZQW1	DE	Potassium-translocating ATPase A chain;
P	Q8ZQW1	DE	Potassium-transporting ATPase A chain;
P	Q8ZQW1	DR	potassium-transporting ATPase activity
P	Q8ZQW2	CC	One of the components of the high-affinity ATP-driven potassium transport (or KDP) system, which catalyzes the hydrolysis of ATP coupled with the exchange of hydrogen and potassium ions (By similarity).
P	Q8ZQW2	DE	Potassium-binding and translocating subunit B;
P	Q8ZQW2	DE	Potassium-translocating ATPase B chain;
P	Q8ZQW2	DE	Potassium-transporting ATPase B chain;
P	Q8ZQW2	DR	potassium-transporting ATPase activity

P	Q8ZQW3	CC	One of the components of the high-affinity ATP-driven potassium transport (or KDP) system, which catalyzes the hydrolysis of ATP coupled with the exchange of hydrogen and potassium ions
P	Q8ZQW3	DE	Potassium-binding and translocating subunit C;
P	Q8ZQW3	DE	Potassium-translocating ATPase C chain;
P	Q8ZQW3	DE	Potassium-transporting ATPase C chain;
P	Q8ZQW3	DR	potassium-transporting ATPase activity
P	Q8ZR95	CC	Essential for copper tolerance under both aerobic and anaerobic conditions.
P	Q8ZR95	CC	Involved in copper export (By similarity)
P	Q8ZR95	DE	Copper-exporting P-type ATPase A;
P	Q8ZR95	DR	copper-exporting ATPase activity
P	Q8ZRG7	DR	copper-exporting ATPase activity
P	Q8ZRP7	CC	Binds vitamin B12 and delivers it to the periplasmic surface of BtuC (By similarity)
P	Q8ZRP7	CC	Part of the ABC transporter complex BtuCDF involved in vitamin B12 import
P	Q8ZRP7	DR	iron ion transmembrane transporter activity
P	Q8ZRP8	CC	Functions as antiport system and exchanges two chloride ions for 1 proton
P	Q8ZRP8	CC	Proton-coupled chloride transporter
P	Q8ZRP8	DR	voltage-gated chloride channel activity
P	Q8ZRV2	CC	Part of the ABC transporter complex ThiBPQ involved in thiamine import
P	Q8ZRV2	DE	Thiamine import ATP-binding protein ThiQ;
P	Q8ZRW2	CC	Transport system that facilitates potassium-efflux, possibly by potassium-proton antiport (By similarity).
P	Q8ZRW2	DE	Glutathione-regulated potassium-efflux system protein kefC;
P	Q8ZRW2	DE	K+/H+ antiporter;
P	Q8ZRZ3	CC	Na+/H+ antiporter that extrudes sodium in exchange for external protons (By similarity)
P	Q8ZRZ3	DE	Na+/H+ antiporter nhaA;
P	Q8ZRZ3	DE	Sodium/proton antiporter nhaA;
P	Q8ZSB0	CC	H+-stimulated, highly selective, manganese uptake system (By similarity).
P	Q8ZSB0	DE	Probable manganese transport protein mntH;
P	Q8ZUJ0	DR	copper-exporting ATPase activity
P	Q8ZX91	CC	Part of the ABC transporter complex PstSACB involved in phosphate import
P	Q8ZX91	DE	ABC phosphate transporter;
P	Q8ZX91	DE	Phosphate import ATP-binding protein PstB;
P	Q8ZX91	DE	Phosphate-transporting ATPase;
P	Q8ZX91	DR	phosphate transmembrane-transporting ATPase activity
P	Q91V14	CC	Mediates electroneutral potassium-chloride cotransport in mature neurons
P	Q91V14	DE	Electroneutral potassium-chloride cotransporter 2;
P	Q91V14	DR	potassium:chloride symporter activity
P	Q91V14	DR	potassium:chloride symporter activity
P	Q91V24	CC	Binds APOA1 and may function in apolipoprotein-mediated phospholipid efflux from cells
P	Q91V24	CC	May also mediate cholesterol efflux
P	Q91V24	DR	phospholipid transporter activity
P	Q91W10	DE	Zinc transporter ZIP8;
P	Q91W98	CC	Proton oligopeptide cotransporter
P	Q91W98	CC	Transports free histidine and certain di- and tripeptides (By similarity).
P	Q91W98	DE	Peptide/histidine transporter 1;
P	Q91WN3	DE	Sodium-independent aspartate/glutamate transporter 1;
P	Q91WN3	DE	Sodium-independent aspartate/glutamate transporter 1;
P	Q91Y63	CC	High-affinity sodium-dicarboxylate cotransporter that accepts a range of substrates with 4-5 carbon atoms
P	Q91Y63	CC	The stoichiometry is probably 3 Na+ for 1 divalent succinate.
P	Q91Y63	DE	Na+/dicarboxylate cotransporter 3;
P	Q91Y63	DE	Na+/dicarboxylate cotransporter 3;
P	Q91Y63	DE	Sodium-dependent high-affinity dicarboxylate transporter 2;
P	Q91Y63	DE	Sodium-dependent high-affinity dicarboxylate transporter 2;
P	Q91Y63	DR	high affinity sodium:dicarboxylate symporter activity
P	Q91Y63	DR	high affinity sodium:dicarboxylate symporter activity
P	Q921R7	DR	sugar:hydrogen symporter activity
P	Q92253	CC	Probable glucose transporter
P	Q92253	DE	Probable glucose transporter rco-3;
P	Q923I7	CC	Efficient substrate transport in mammalian kidney is provided by the concerted action of a low affinity high capacity and a high affinity low capacity Na+/glucose cotransporter arranged in series along kidney proximal tubules (By similarity)

P	Q923I7	CC	Efficient substrate transport in mammalian kidney is provided by the concerted action of a low affinity high capacity and a high affinity low capacity Na+/glucose cotransporter arranged in series along kidney proximal tubules (By similarity).
P	Q923I7	DE	Low affinity sodium-glucose cotransporter;
P	Q923I7	DE	Na+/glucose cotransporter 2;
P	Q923I7	DE	Na+/glucose cotransporter 2;
P	Q923I7	DE	Sodium/glucose cotransporter 2;
P	Q923I7	DE	Sodium/glucose cotransporter 2;
P	Q923J1	CC	Divalent cation channel permeable to calcium and magnesium
P	Q923J1	CC	Has a central role in magnesium ion homeostasis and in the regulation of anoxic neuronal cell death
P	Q92482	CC	It may play an important role in gastrointestinal tract water transport and in glycerol metabolism (By similarity).
P	Q92482	CC	Provides kidney medullary collecting duct with high permeability to water, thereby permitting water to move in the direction of an osmotic gradient
P	Q92482	CC	Water channel required to promote glycerol permeability and water transport across cell membranes
P	Q92482	DR	water channel activity
P	Q92504	DE	Zinc transporter SLC39A7;
P	Q92504	DR	zinc ion transmembrane transporter activity
P	Q92581	CC	Electroneutral exchange of protons for Na+ and K+ across the early and recycling endosome membranes
P	Q92581	DE	Na+/H+ exchanger 6;
P	Q92581	DE	Sodium/hydrogen exchanger 6;
P	Q92581	DR	sodium:hydrogen antiporter activity
P	Q925H0	CC	Cation channel with high affinity for sodium, which is gated by extracellular protons and inhibited by the diuretic amiloride
P	Q925H0	DE	Amiloride-sensitive brain sodium channel;
P	Q925H0	DE	Brain sodium channel 1;
P	Q925H0	DR	ligand-gated sodium channel activity
P	Q925Q3	CC	In contrast to other members of the family its function is independent of K+ (By similarity).
P	Q925Q3	CC	Transports Ca2+ in exchange for either Li+ or Na+, explaining how Li+ catalyzes Ca2+ exchange
P	Q925Q3	CC	Transports Ca2+ in exchange for either Li+ or Na+, explaining how Li+ catalyzes Ca2+ exchange
P	Q925Q3	DE	Na+/K+/Ca2+-exchange protein 6;
P	Q925Q3	DE	Na+/K+/Ca2+-exchange protein 6;
P	Q925Q3	DE	Na+/K+/Ca2+-exchange protein 6;
P	Q925Q3	DE	Sodium/potassium/calcium exchanger 6;
P	Q925Q3	DE	Sodium/potassium/calcium exchanger 6;
P	Q925Q3	DE	Sodium/potassium/calcium exchanger 6;
P	Q926D8	CC	Involved in a zinc uptake transport system (Probable).
P	Q926D8	DE	Zinc uptake system ATP-binding protein ZurA;
P	Q92736	CC	Contraction of cardiac muscle is triggered by release of calcium ions from SR following depolarization of T- tubules (By similarity).
P	Q92736	DR	ryanodine-sensitive calcium-release channel activity
P	Q927F9	CC	One of the components of the high-affinity ATP-driven potassium transport (or KDP) system, which catalyzes the hydrolysis of ATP coupled with the exchange of hydrogen and potassium ions (By similarity).
P	Q927F9	DE	Potassium-binding and translocating subunit A;
P	Q927F9	DE	Potassium-translocating ATPase A chain;
P	Q927F9	DE	Potassium-transporting ATPase A chain;
P	Q927F9	DR	potassium-transporting ATPase activity
P	Q927G0	CC	One of the components of the high-affinity ATP-driven potassium transport (or KDP) system, which catalyzes the hydrolysis of ATP coupled with the exchange of hydrogen and potassium ions (By similarity).
P	Q927G0	DE	Potassium-binding and translocating subunit B 1;
P	Q927G0	DE	Potassium-translocating ATPase B chain 1;
P	Q927G0	DE	Potassium-transporting ATPase B chain 1;
P	Q927G0	DR	potassium-transporting ATPase activity
P	Q927G1	CC	One of the components of the high-affinity ATP-driven potassium transport (or KDP) system, which catalyzes the hydrolysis of ATP coupled with the exchange of hydrogen and potassium ions
P	Q927G1	DE	Potassium-binding and translocating subunit C;

P	Q927G1	DE	Potassium-translocating ATPase C chain;
P	Q927G1	DE	Potassium-transporting ATPase C chain;
P	Q927G1	DR	potassium-transporting ATPase activity
P	Q927Z7	CC	Part of the ABC transporter complex PstSACB involved in phosphate import
P	Q927Z7	DE	ABC phosphate transporter 2;
P	Q927Z7	DE	Phosphate import ATP-binding protein PstB 2;
P	Q927Z7	DE	Phosphate-transporting ATPase 2;
P	Q927Z7	DR	inorganic phosphate transmembrane transporter activity
P	Q927Z7	DR	phosphate transmembrane-transporting ATPase activity
P	Q92806	CC	Inward rectifier potassium channels are characterized by a greater tendency to allow potassium to flow into the cell rather than out of it
P	Q92806	CC	Their voltage dependence is regulated by the concentration of extracellular potassium; as external potassium is raised, the voltage range of the channel opening shifts to more positive voltages
P	Q92806	DE	G protein-activated inward rectifier potassium channel 3;
P	Q92806	DE	Inward rectifier K+ channel Kir3.3;
P	Q92806	DE	Potassium channel, inwardly rectifying subfamily J member 9;
P	Q92806	DR	G-protein activated inward rectifier potassium channel activity
P	Q92911	CC	Mediates iodide uptake in the thyroid gland.
P	Q92911	DE	Na+/I(-) cotransporter;
P	Q92911	DE	Na+/I(-) symporter;
P	Q92911	DE	Sodium-iodide symporter;
P	Q92911	DE	Sodium/iodide cotransporter;
P	Q92911	DE	Sodium/iodide cotransporter;
P	Q92911	DR	iodide transmembrane transporter activity
P	Q92911	DR	sodium:iodide symporter activity
P	Q92911	DR	sodium:iodide symporter activity
P	Q92952	CC	Forms a voltage-independent potassium channel activated by intracellular calcium
P	Q92953	CC	Channels open or close in response to the voltage difference across the membrane, letting potassium ions pass in accordance with their electrochemical gradient
P	Q92953	CC	Mediates the voltage-dependent potassium ion permeability of excitable membranes
P	Q92953	DE	Potassium voltage-gated channel subfamily B member 2;
P	Q92953	DE	Voltage-gated potassium channel subunit Kv2.2;
P	Q92953	DR	delayed rectifier potassium channel activity
P	Q92BT1	CC	H+-stimulated, highly selective, manganese uptake system (By similarity).
P	Q92BT1	DE	Probable manganese transport protein mntH;
P	Q92DL6	CC	Part of the ABC transporter complex PotABCD involved in spermidine/putrescine import
P	Q92DL6	CC	Part of the ABC transporter complex PotABCD involved in spermidine/putrescine import
P	Q92DL6	DE	Spermidine/putrescine import ATP-binding protein PotA;
P	Q92DL6	DE	Spermidine/putrescine import ATP-binding protein PotA;
P	Q92EZ6	CC	Part of the ABC transporter complex MetNIQ involved in methionine import
P	Q92EZ6	DE	Methionine import ATP-binding protein MetN 1;
P	Q92G36	CC	Part of the ABC transporter complex ZnuABC involved in zinc import
P	Q92G36	DE	Zinc import ATP-binding protein ZnuC;
P	Q92G36	DR	zinc transporting ATPase activity
P	Q92G95	CC	Part of the ABC transporter complex CcmAB involved in the biogenesis of c-type cytochromes; once thought to export heme, this seems not to be the case, but its exact role is uncertain
P	Q92G95	DE	Heme exporter protein A;
P	Q92G95	DR	heme-transporting ATPase activity
P	Q92GI5	CC	Provides the rickettsial cell with host ATP in exchange for rickettsial ADP
P	Q92GI5	CC	Provides the rickettsial cell with host ATP in exchange for rickettsial ADP
P	Q92GI5	DE	ADP,ATP carrier protein 5;
P	Q92GI5	DE	ADP/ATP translocase 5;
P	Q92GI5	DE	ADP/ATP translocase 5;
P	Q92GI5	DR	ATP:ADP antiporter activity
P	Q92GI5	DR	ATP:ADP antiporter activity
P	Q92HP9	CC	Provides the rickettsial cell with host ATP in exchange for rickettsial ADP
P	Q92HP9	CC	Provides the rickettsial cell with host ATP in exchange for rickettsial ADP
P	Q92HP9	DE	ADP,ATP carrier protein 3;
P	Q92HP9	DE	ADP/ATP translocase 3;
P	Q92HP9	DE	ADP/ATP translocase 3;
P	Q92HP9	DR	ATP:ADP antiporter activity
P	Q92HP9	DR	ATP:ADP antiporter activity
P	Q92HV4	CC	Provides the rickettsial cell with host ATP in exchange for rickettsial ADP

P	Q92HV4	CC	Provides the rickettsial cell with host ATP in exchange for rickettsial ADP
P	Q92HV4	DE	ADP,ATP carrier protein 4;
P	Q92HV4	DE	ADP/ATP translocase 4;
P	Q92HV4	DE	ADP/ATP translocase 4;
P	Q92HV4	DR	ATP:ADP antiporter activity
P	Q92HV4	DR	ATP:ADP antiporter activity
P	Q92J96	CC	Part of the binding-protein-dependent transport system for glutamine; probably responsible for the translocation of the substrate across the membrane (By similarity)
P	Q92J96	DE	Putative glutamine transport system permease protein glnP;
P	Q92JG1	CC	Transports S-adenosylmethionine (By similarity).
P	Q92JG1	DE	S-adenosylmethionine uptake transporter;
P	Q92JI6	CC	Provides the rickettsial cell with host ATP in exchange for rickettsial ADP
P	Q92JI6	CC	Provides the rickettsial cell with host ATP in exchange for rickettsial ADP
P	Q92JI6	DE	ADP,ATP carrier protein 1;
P	Q92JI6	DE	ADP/ATP translocase 1;
P	Q92JI6	DE	ADP/ATP translocase 1;
P	Q92JI6	DR	ATP:ADP antiporter activity
P	Q92JI6	DR	ATP:ADP antiporter activity
P	Q92L31	CC	Part of the ABC transporter complex ThiBPQ involved in thiamine import
P	Q92L31	DE	Thiamine import ATP-binding protein ThiQ;
P	Q92LU2	CC	Part of the ABC transporter complex ModABC involved in molybdenum import
P	Q92LU2	DE	Molybdenum import ATP-binding protein ModC;
P	Q92LU2	DR	molybdenum ion transmembrane transporter activity
P	Q92N13	CC	Part of the ABC transporter complex HmuTUV involved in hemin import
P	Q92N13	DE	Hemin import ATP-binding protein HmuV;
P	Q92NM3	CC	Channel that permits osmotically driven movement of water in both directions
P	Q92NM3	CC	It mediates rapid entry or exit of water in response to abrupt changes in osmolarity (By similarity).
P	Q92P76	CC	Part of the ABC transporter complex ZnuABC involved in zinc import
P	Q92P76	DE	Zinc import ATP-binding protein ZnuC;
P	Q92P76	DR	zinc transporting ATPase activity
P	Q92RN0	CC	Transport of potassium into the cell (By similarity).
P	Q92RN0	DE	Probable potassium transport system protein kup 1;
P	Q92RN0	DR	potassium ion transmembrane transporter activity
P	Q92SA1	CC	Part of the ABC transporter complex PstSACB involved in phosphate import
P	Q92SA1	DE	ABC phosphate transporter;
P	Q92SA1	DE	Phosphate import ATP-binding protein PstB;
P	Q92SA1	DE	Phosphate-transporting ATPase;
P	Q92SA1	DR	inorganic phosphate transmembrane transporter activity
P	Q92SA1	DR	phosphate transmembrane-transporting ATPase activity
P	Q92UI2	CC	Part of the ABC transporter complex RbsABCD involved in ribose import
P	Q92UI2	DE	Ribose import ATP-binding protein RbsA 3;
P	Q92UX0	CC	Part of the ABC transporter complex TauABC involved in taurine import
P	Q92UX0	DE	Taurine import ATP-binding protein TauB;
P	Q92UX0	DR	taurine-transporting ATPase activity
P	Q92V71	DR	phosphonate transmembrane-transporting ATPase activity
P	Q92VJ2	CC	Part of the ABC transporter complex CysAWTP involved in sulfate/thiosulfate import
P	Q92VJ2	DE	Sulfate-transporting ATPase 2;
P	Q92VJ2	DE	Sulfate/thiosulfate import ATP-binding protein CysA 2;
P	Q92VJ2	DR	sulfate transmembrane-transporting ATPase activity
P	Q92W60	CC	Part of the ABC transporter complex RbsABCD involved in ribose import
P	Q92W60	DE	Ribose import ATP-binding protein RbsA 2;
P	Q92WD6	DR	glycerol-3-phosphate-transporting ATPase activity
P	Q92XJ0	CC	One of the components of the high-affinity ATP-driven potassium transport (or KDP) system, which catalyzes the hydrolysis of ATP coupled with the exchange of hydrogen and potassium ions (By similarity).
P	Q92XJ0	DE	Potassium-binding and translocating subunit B;
P	Q92XJ0	DE	Potassium-translocating ATPase B chain;
P	Q92XJ0	DE	Potassium-transporting ATPase B chain;
P	Q92XJ0	DR	potassium-transporting ATPase activity
P	Q92XW1	CC	Part of the ABC transporter complex CysAWTP involved in sulfate/thiosulfate import
P	Q92XW1	DE	Sulfate-transporting ATPase 1;
P	Q92XW1	DE	Sulfate/thiosulfate import ATP-binding protein CysA 1;
P	Q92XW1	DR	sulfate transmembrane-transporting ATPase activity
P	Q92Y37	CC	Na+/H+ antiporter that extrudes sodium in exchange for external protons (By similarity)

P	Q92Y37	DE	Na+/H+ antiporter nhaA;
P	Q92Y37	DE	Sodium/proton antiporter nhaA;
P	Q92Y93	CC	Transport of potassium into the cell (By similarity).
P	Q92Y93	DE	Probable potassium transport system protein kup 2;
P	Q92Y93	DR	potassium ion transmembrane transporter activity
P	Q92ZW9	CC	Channel that permits osmotically driven movement of water in both directions
P	Q92ZW9	CC	It mediates rapid entry or exit of water in response to abrupt changes in osmolarity (By similarity).
P	Q93235	CC	Non-catalytic component of the active enzyme, which catalyzes the hydrolysis of ATP coupled with the exchange of Na+ and K+ ions across the plasma membrane
P	Q93235	CC	Non-catalytic component of the active enzyme, which catalyzes the hydrolysis of ATP coupled with the exchange of Na+ and K+ ions across the plasma membrane
P	Q93235	CC	The beta subunit regulates, through assembly of alpha/beta heterodimers, the number of sodium pumps transported to the plasma membrane (By similarity)
P	Q93235	DE	Sodium/potassium-dependent ATPase subunit beta-1;
P	Q93235	DE	Sodium/potassium-dependent ATPase subunit beta-1;
P	Q93235	DE	Sodium/potassium-transporting ATPase subunit beta-1;
P	Q93235	DE	Sodium/potassium-transporting ATPase subunit beta-1;
P	Q93235	DR	sodium:potassium-exchanging ATPase activity
P	Q93235	DR	sodium:potassium-exchanging ATPase activity
P	Q93DA2	CC	Part of the ABC transporter complex MetNIQ involved in methionine import
P	Q93DA2	DE	Methionine import ATP-binding protein MetN;
P	Q93IM6	DR	voltage-gated chloride channel activity
P	Q93SH7	CC	Part of the ABC transporter complex HmuTUV involved in hemin import
P	Q93SH7	DE	Hemin import ATP-binding protein HmuV;
P	Q93XM7	CC	May transport carnitine or acylcarnitine from the cytosol to the mitochondrial matrix as an alternative or a complement to the succinate-producing glyoxylate cycle
P	Q93XM7	DE	Carnitine/acylcarnitine translocase-like protein;
P	Q93XM7	DE	Mitochondrial carnitine/acylcarnitine carrier-like protein;
P	Q93Y91	DE	Hexose transporter 5;
P	Q93YH1	DE	Sodium/hydrogen exchanger;
P	Q93YH1	DR	sodium:hydrogen antiporter activity
P	Q93Z75	DR	allantoin uptake transmembrane transporter activity
P	Q93Z81	CC	Translocates Ca2+ and other metal ions into vacuoles using the proton gradient formed by H+-ATPase and H+-pyrophosphatase (By similarity)
P	Q93Z81	DE	Ca2+/H+ antiporter CAX3;
P	Q93Z81	DE	Ca2+/H+ exchanger 3;
P	Q93Z81	DR	calcium:hydrogen antiporter activity
P	Q93ZD7	CC	Magnesium transporter that may mediate the influx of magnesium (By similarity)
P	Q93ZD7	DE	Magnesium Transporter 6;
P	Q93ZD7	DE	Magnesium transporter MRS2-4;
P	Q93ZD7	DR	magnesium ion transmembrane transporter activity
P	Q94225	CC	Possible sulfate transporter.
P	Q94225	DE	Sulfate permease family protein 3;
P	Q94225	DR	secondary active sulfate transmembrane transporter activity
P	Q942X8	CC	High-affinity potassium transporter (By similarity).
P	Q942X8	DE	Probable potassium transporter 2;
P	Q942X8	DR	potassium ion transmembrane transporter activity
P	Q94526	CC	Acts as an outwardly rectifying channel but as external potassium levels increase, this is reversed.
P	Q94526	CC	Background potassium channel
P	Q94526	CC	Rectification is dependent on external potassium concentration
P	Q94526	DE	Open rectifier potassium channel protein 1;
P	Q94526	DE	Two pore domain potassium channel Ork1;
P	Q94526	DR	potassium channel activity
P	Q945S5	CC	Translocates Ca2+ and other metal ions into vacuoles using the proton gradient formed by H+-ATPase and H+-pyrophosphatase
P	Q945S5	DE	Ca2+/H+ antiporter CAX4;
P	Q945S5	DE	Ca2+/H+ exchanger 4;
P	Q945S5	DR	calcium:hydrogen antiporter activity
P	Q94A76	CC	Conductance of the channel is modulated in a potassium-dependent fashion
P	Q94A76	CC	Major selective outward-rectifying potassium channel of the guard cell membrane
P	Q94A76	DE	Guard cell outward rectifying K+ channel;
P	Q94A76	DE	Potassium channel GORK;
P	Q94A76	DR	outward rectifier potassium channel activity

P	Q94AZ2	DE	Hexose transporter 13;
P	Q94AZ2	DR	high-affinity hydrogen:glucose symporter activity
P	Q94CI6	CC	Sugar transporter (Potential).
P	Q94CI6	DE	Sugar transporter ERD6-like 18;
P	Q94CI6	DE	Sugar-porter family protein 2;
P	Q94CI7	CC	Sugar transporter (Potential).
P	Q94CI7	DE	Sugar transporter ERD6-like 17;
P	Q94CI7	DE	Sugar-porter family protein 1;
P	Q94DB8	CC	Symbiosis-specific regulated inorganic phosphate (Pi) transporter
P	Q94DB8	DE	Inorganic phosphate transporter 1-11;
P	Q94EG9	CC	Probably mediates zinc uptake from the rhizosphere (By similarity).
P	Q94EG9	DE	Zinc transporter 11;
P	Q94KE0	CC	Sugar transporter (Potential).
P	Q94KE0	DE	Sugar transporter ERD6-like 3;
P	Q94KE0	DE	Sugar transporter-like protein 2;
P	Q94KI8	CC	Acts as the major ROS-responsive Ca2+ channel and is the possible target of Al-dependent inhibition
P	Q94KI8	CC	Functions as a voltage-gated inward-rectifying Ca2+ channel (VDCC) across the vacuole membrane
P	Q94KI8	DE	Calcium channel protein 1;
P	Q94KI8	DE	Two pore calcium channel protein 1;
P	Q94KI8	DE	Voltage-dependent calcium channel protein TPC1;
P	Q94KI8	DR	voltage-gated calcium channel activity
P	Q94LW6	CC	H+/sulfate cotransporter that may play a role in the regulation of sulfate assimilation (By similarity).
P	Q94LW6	DE	Probable sulfate transporter 3.5;
P	Q94LW6	DR	secondary active sulfate transmembrane transporter activity
P	Q95YI5	DE	UDP-sugar transporter UST74c;
P	Q96243	CC	High-affinity transporter for external inorganic phosphate (By similarity).
P	Q96243	DE	Probable inorganic phosphate transporter 1-2;
P	Q96243	DR	inorganic phosphate transmembrane transporter activity
P	Q96282	CC	Voltage-gated chloride channel.
P	Q96282	DE	Chloride channel protein CLC-c;
P	Q96282	DR	voltage-gated chloride channel activity
P	Q96290	DE	Sugar transporter MSSP1;
P	Q96290	DE	Sugar transporter MT1;
P	Q96303	CC	Acts as a H+:phosphate symporter in both low- and high-Pi conditions
P	Q96303	CC	High-affinity transporter for external inorganic phosphate
P	Q96303	DE	Inorganic phosphate transporter 1-4;
P	Q96303	DR	inorganic phosphate transmembrane transporter activity
P	Q969S0	CC	Sugar transporter that specifically mediates the transport of UDP-xylose (UDP-Xyl) and UDP-N-acetylglucosamine (UDP-GlcNAc) from cytosol into Golgi.
P	Q969S0	DE	UDP-xylose and UDP-N-acetylglucosamine transporter;
P	Q969S0	DR	UDP-N-acetylglucosamine transmembrane transporter activity
P	Q96D31	CC	CRAC channels are the main pathway for Ca2+ influx in T-cells and promote the immune response to pathogens by activating the transcription factor NFAT.
P	Q96D31	CC	Ca2+ release-activated Ca2+ (CRAC) channel subunit which mediates Ca2+ influx following depletion of intracellular Ca2+ stores and channel activation by the Ca2+ sensor, STIM1
P	Q96D31	DE	Calcium release-activated calcium channel protein 1;
P	Q96D31	DR	store-operated calcium channel activity
P	Q96EP9	DE	Na+/bile acid cotransporter 4;
P	Q96EP9	DE	Na+/bile acid cotransporter 4;
P	Q96EP9	DE	Sodium/bile acid cotransporter 4;
P	Q96EP9	DE	Sodium/bile acid cotransporter 4;
P	Q96EP9	DR	bile acid:sodium symporter activity
P	Q96EP9	DR	bile acid:sodium symporter activity
P	Q96FT7	CC	Probable cation channel with high affinity for sodium
P	Q96FT7	DR	sodium channel activity
P	Q96FT7	DR	sodium ion transmembrane transporter activity
P	Q96H72	DE	LIV-1 subfamily of ZIP zinc transporter 9;
P	Q96H72	DE	Zinc transporter ZIP13;

P	Q96J66	CC	Stimulates the ATP-dependent uptake of a range of physiological and synthetic lipophilic anions, including the glutathione S-conjugates leukotriene C4 and dinitrophenyl S-glutathione, steroid sulfates such as dehydroepiandrosterone 3-sulfate (DHEAS) and estrone 3-sulfate, glucuronides such as estradiol 17-beta-D-glucuronide (E(2)17betaG), the monoanionic bile acids glycocholate and taurocholate, and methotrexate
P	Q96JW4	CC	Acts as a plasma-membrane magnesium transporter.
P	Q96JW4	DR	magnesium ion transmembrane transporter activity
P	Q96KK3	CC	Potassium channel subunit
P	Q96KK3	DE	Delayed-rectifier K+ channel alpha subunit 1;
P	Q96KK3	DE	Potassium voltage-gated channel subfamily S member 1;
P	Q96KK3	DE	Voltage-gated potassium channel subunit Kv9.1;
P	Q96KK3	DR	delayed rectifier potassium channel activity
P	Q96KK3	DR	potassium channel regulator activity
P	Q96L42	CC	Pore-forming (alpha) subunit of voltage-gated potassium channel
P	Q96L42	DE	Ether-a-go-go-like potassium channel 3;
P	Q96L42	DE	Potassium voltage-gated channel subfamily H member 8;
P	Q96L42	DE	Voltage-gated potassium channel subunit Kv12.1;
P	Q96MC6	DE	Putative tetracycline transporter-like protein;
P	Q96MC6	DR	tetracycline:hydrogen antiporter activity
P	Q96N87	DE	Sodium- and chloride-dependent transporter XTRP2;
P	Q96N87	DE	Sodium-dependent neutral amino acid transporter B(0)AT3;
P	Q96N87	DR	neurotransmitter:sodium symporter activity
P	Q96NT5	CC	Has been shown to act both as an intestinal proton- coupled high-affinity folate transporter and as an intestinal heme transporter which mediates heme uptake from the gut lumen into duodenal epithelial cells
P	Q96NT5	CC	Shows a higher affinity for folate than heme.
P	Q96NT5	DE	Proton-coupled folate transporter;
P	Q96NT5	DR	folic acid transporter activity
P	Q96NY7	CC	May insert into membranes and form chloride ion channels
P	Q96NY7	CC	May play a critical role in water-secreting cells, possibly through the regulation of chloride ion transport (By similarity).
P	Q96NY7	DE	Chloride intracellular channel protein 6;
P	Q96NY7	DR	voltage-gated chloride channel activity
P	Q96PR1	CC	Assuming opened or closed conformations in response to the voltage difference across the membrane, the protein forms a potassium-selective channel through which potassium ions may pass in accordance with their electrochemical gradient
P	Q96PR1	CC	Mediates the voltage-dependent potassium ion permeability of excitable membranes
P	Q96PR1	DE	Potassium voltage-gated channel subfamily C member 2;
P	Q96PR1	DE	Voltage-gated potassium channel Kv3.2;
P	Q96PR1	DR	voltage-gated potassium channel activity
P	Q96PS8	CC	May contribute to water transport in the upper portion of small intestine
P	Q96PS8	CC	Water channel required to promote glycerol permeability and water transport across cell membranes
P	Q96QE2	DE	Proton myo-inositol cotransporter;
P	Q96QE2	DR	myo-inositol:hydrogen symporter activity
P	Q96QT4	CC	Divalent cation channel permeable to calcium and magnesium
P	Q96QT4	CC	Divalent cation channel permeable to calcium and magnesium
P	Q96QT4	CC	Has a central role in magnesium ion homeostasis and in the regulation of anoxic neuronal cell death
P	Q96QT4	CC	May be involved in a fundamental process that adjusts plasma membrane divalent cation fluxes according to the metabolic state of the cell
P	Q96RP8	CC	Assuming opened or closed conformations in response to the voltage difference across the membrane, the protein forms a potassium-selective channel through which potassium ions may pass in accordance with their electrochemical gradient (By similarity)
P	Q96RP8	CC	Mediates the voltage-dependent potassium ion permeability of excitable membranes
P	Q96RP8	DE	Potassium voltage-gated channel subfamily A member 7;
P	Q96RP8	DE	Voltage-gated potassium channel subunit Kv1.7;
P	Q96RP8	DR	voltage-gated potassium channel activity
P	Q96S37	CC	Mediates saturable urate uptake by facilitating the exchange of urate against organic anions.
P	Q96S37	CC	Regulates blood urate levels
P	Q96S37	CC	Required for efficient urate re-absorption in the kidney
P	Q96S37	DE	Urate anion exchanger 1;
P	Q96S37	DR	urate transmembrane transporter activity
P	Q96T54	CC	Outward rectifying potassium channel

P	Q96T54	CC	Produces rapidly activating and non-inactivating outward rectifier K+ currents.
P	Q96T54	DE	2P domain potassium channel Talk-2;
P	Q96T54	DE	Acid-sensitive potassium channel protein TASK-4;
P	Q96T54	DE	Potassium channel subfamily K member 17;
P	Q96T54	DE	TWIK-related acid-sensitive K+ channel 4;
P	Q96T54	DE	TWIK-related alkaline pH-activated K+ channel 2;
P	Q96T54	DR	potassium channel activity
P	Q96T55	CC	Outward rectifying potassium channel
P	Q96T55	CC	Produces rapidly activating and non-inactivating outward rectifier K+ currents.
P	Q96T55	DE	2P domain potassium channel Talk-1;
P	Q96T55	DE	Potassium channel subfamily K member 16;
P	Q96T55	DE	TWIK-related alkaline pH-activated K+ channel 1;
P	Q96T55	DR	potassium channel activity
P	Q96T83	CC	Mediates electroneutral exchange of protons for Na+ and K+ across endomembranes
P	Q96T83	DE	Na+/H+ exchanger 7;
P	Q96T83	DE	Na+/H+ exchanger 7;
P	Q96T83	DE	Sodium/hydrogen exchanger 7;
P	Q96T83	DR	sodium:hydrogen antiporter activity
P	Q978Z8	DR	copper-exporting ATPase activity
P	Q97BF5	CC	One of the components of the high-affinity ATP-driven potassium transport (or KDP) system, which catalyzes the hydrolysis of ATP coupled with the exchange of hydrogen and potassium ions
P	Q97BF5	DE	Potassium-binding and translocating subunit C;
P	Q97BF5	DE	Potassium-translocating ATPase C chain;
P	Q97BF5	DE	Potassium-transporting ATPase C chain;
P	Q97BF5	DR	potassium-transporting ATPase activity
P	Q97BF6	CC	One of the components of the high-affinity ATP-driven potassium transport (or KDP) system, which catalyzes the hydrolysis of ATP coupled with the exchange of hydrogen and potassium ions (By similarity).
P	Q97BF6	DE	Potassium-binding and translocating subunit B;
P	Q97BF6	DE	Potassium-translocating ATPase B chain;
P	Q97BF6	DE	Potassium-transporting ATPase B chain;
P	Q97BF6	DR	potassium-transporting ATPase activity
P	Q97BF7	CC	One of the components of the high-affinity ATP-driven potassium transport (or KDP) system, which catalyzes the hydrolysis of ATP coupled with the exchange of hydrogen and potassium ions (By similarity).
P	Q97BF7	DE	Potassium-binding and translocating subunit A;
P	Q97BF7	DE	Potassium-translocating ATPase A chain;
P	Q97BF7	DE	Potassium-transporting ATPase A chain;
P	Q97BF7	DR	potassium-transporting ATPase activity
P	Q97IE0	CC	Part of the ABC transporter complex PstSACB involved in phosphate import
P	Q97IE0	DE	ABC phosphate transporter;
P	Q97IE0	DE	Phosphate import ATP-binding protein PstB;
P	Q97IE0	DE	Phosphate-transporting ATPase;
P	Q97IE0	DR	inorganic phosphate transmembrane transporter activity
P	Q97IE0	DR	phosphate transmembrane-transporting ATPase activity
P	Q97KD5	CC	Part of the ABC transporter complex MetNIQ involved in methionine import
P	Q97KD5	DE	Methionine import ATP-binding protein MetN;
P	Q97KS6	CC	Part of the ABC transporter complex PotABCD involved in spermidine/putrescine import
P	Q97KS6	CC	Part of the ABC transporter complex PotABCD involved in spermidine/putrescine import
P	Q97KS6	DE	Spermidine/putrescine import ATP-binding protein PotA;
P	Q97KS6	DE	Spermidine/putrescine import ATP-binding protein PotA;
P	Q97Q31	CC	Part of the ABC transporter complex pstSACB involved in phosphate import (By similarity).
P	Q97Q31	DE	Phosphate-binding protein pstS 1;
P	Q97Q34	CC	Part of the ABC transporter complex PstSACB involved in phosphate import
P	Q97Q34	DE	ABC phosphate transporter 2;
P	Q97Q34	DE	Phosphate import ATP-binding protein PstB 2;
P	Q97Q34	DE	Phosphate-transporting ATPase 2;
P	Q97Q34	DR	inorganic phosphate transmembrane transporter activity
P	Q97Q34	DR	phosphate transmembrane-transporting ATPase activity
P	Q97Q42	CC	Part of the ABC transporter complex PotABCD involved in spermidine/putrescine import
P	Q97Q42	DE	Spermidine/putrescine import ATP-binding protein PotA;
P	Q97TN5	CC	H+-stimulated, highly selective, manganese uptake system (By similarity).
P	Q97TN5	DE	Probable manganese transport protein mntH;

P	Q97UU7	DE	Copper-transporting ATPase;
P	Q97UU7	DR	copper-exporting ATPase activity
P	Q97VH4	DR	copper-exporting ATPase activity
P	Q97ZC2	DE	Maltose transport inner membrane protein;
P	Q97ZT9	CC	Part of the ABC transporter complex PstSACB involved in phosphate import
P	Q97ZT9	DE	ABC phosphate transporter;
P	Q97ZT9	DE	Phosphate import ATP-binding protein PstB;
P	Q97ZT9	DE	Phosphate-transporting ATPase;
P	Q97ZT9	DR	phosphate transmembrane-transporting ATPase activity
P	Q987E7	CC	Part of the ABC transporter complex RbsABCD involved in ribose import
P	Q987E7	DE	Ribose import ATP-binding protein RbsA 2;
P	Q98C38	CC	Na+/H+ antiporter that extrudes sodium in exchange for external protons (By similarity).
P	Q98C38	DE	Na+/H+ antiporter nhaA;
P	Q98C38	DE	Sodium/proton antiporter nhaA;
P	Q98DW6	CC	Part of the ABC transporter complex TauABC involved in taurine import
P	Q98DW6	DE	Taurine import ATP-binding protein TauB;
P	Q98DW6	DR	taurine-transporting ATPase activity
P	Q98EA4	CC	Part of the ABC transporter complex CcmAB involved in the biogenesis of c-type cytochromes; once thought to export heme, this seems not to be the case, but its exact role is uncertain
P	Q98EA4	DE	Heme exporter protein A;
P	Q98EA4	DR	heme-transporting ATPase activity
P	Q98FA5	CC	Part of the ABC transporter complex ThiBPQ involved in thiamine import
P	Q98FA5	DE	Thiamine import ATP-binding protein ThiQ;
P	Q98FL2	CC	Part of the ABC transporter complex pstSACB involved in phosphate import (By similarity).
P	Q98FL2	DE	Phosphate-binding protein pstS;
P	Q98FL2	DR	inorganic phosphate transmembrane transporter activity
P	Q98FL3	CC	Part of a binding-protein-dependent transport system for phosphate; probably responsible for the translocation of the substrate across the membrane (By similarity).
P	Q98FL3	DE	Phosphate transport system permease protein pstC;
P	Q98FL3	DR	inorganic phosphate transmembrane transporter activity
P	Q98FL4	CC	Part of a binding-protein-dependent transport system for phosphate; probably responsible for the translocation of the substrate across the membrane (By similarity).
P	Q98FL4	DE	Phosphate transport system permease protein pstA;
P	Q98FL4	DR	inorganic phosphate transmembrane transporter activity
P	Q98FL5	CC	Part of the ABC transporter complex PstSACB involved in phosphate import
P	Q98FL5	DE	ABC phosphate transporter;
P	Q98FL5	DE	Phosphate import ATP-binding protein PstB;
P	Q98FL5	DE	Phosphate-transporting ATPase;
P	Q98FL5	DR	inorganic phosphate transmembrane transporter activity
P	Q98FL5	DR	phosphate transmembrane-transporting ATPase activity
P	Q98G42	DR	glycerol-3-phosphate-transporting ATPase activity
P	Q98GF5	DR	phosphonate transmembrane-transporting ATPase activity
P	Q98GX5	CC	One of the components of the high-affinity ATP-driven potassium transport (or KDP) system, which catalyzes the hydrolysis of ATP coupled with the exchange of hydrogen and potassium ions (By similarity).
P	Q98GX5	DE	Potassium-binding and translocating subunit A;
P	Q98GX5	DE	Potassium-translocating ATPase A chain;
P	Q98GX5	DE	Potassium-transporting ATPase A chain;
P	Q98GX5	DR	potassium-transporting ATPase activity
P	Q98GX6	CC	One of the components of the high-affinity ATP-driven potassium transport (or KDP) system, which catalyzes the hydrolysis of ATP coupled with the exchange of hydrogen and potassium ions (By similarity).
P	Q98GX6	DE	Potassium-binding and translocating subunit B;
P	Q98GX6	DE	Potassium-translocating ATPase B chain;
P	Q98GX6	DE	Potassium-transporting ATPase B chain;
P	Q98GX6	DR	potassium-transporting ATPase activity
P	Q98GX7	CC	One of the components of the high-affinity ATP-driven potassium transport (or KDP) system, which catalyzes the hydrolysis of ATP coupled with the exchange of hydrogen and potassium ions
P	Q98GX7	DE	Potassium-binding and translocating subunit C;
P	Q98GX7	DE	Potassium-translocating ATPase C chain;
P	Q98GX7	DE	Potassium-transporting ATPase C chain;
P	Q98GX7	DR	potassium-transporting ATPase activity

P	Q98HF7	CC	Part of the ABC transporter complex PotABCD involved in spermidine/putrescine import
P	Q98HF7	CC	Part of the ABC transporter complex PotABCD involved in spermidine/putrescine import
P	Q98HF7	DE	Spermidine/putrescine import ATP-binding protein PotA;
P	Q98HF7	DE	Spermidine/putrescine import ATP-binding protein PotA;
P	Q98I99	CC	H+-stimulated, highly selective, manganese uptake system (By similarity).
P	Q98I99	DE	Probable manganese transport protein mntH;
P	Q98K15	CC	Part of the ABC transporter complex RbsABCD involved in ribose import
P	Q98K15	DE	Ribose import ATP-binding protein RbsA 1;
P	Q98K23	CC	Part of the ABC transporter complex CysAWTP involved in sulfate/thiosulfate import
P	Q98K23	DE	Sulfate-transporting ATPase;
P	Q98K23	DE	Sulfate/thiosulfate import ATP-binding protein CysA;
P	Q98K23	DR	sulfate transmembrane-transporting ATPase activity
P	Q98KI3	CC	Part of the ABC transporter complex ModABC involved in molybdenum import
P	Q98KI3	DE	Molybdenum import ATP-binding protein ModC;
P	Q98KI3	DR	molybdenum ion transmembrane transporter activity
P	Q98KL7	CC	Transport of potassium into the cell (By similarity).
P	Q98KL7	DE	Probable potassium transport system protein kup 2;
P	Q98KL7	DR	potassium ion transmembrane transporter activity
P	Q98KL8	CC	Transport of potassium into the cell (By similarity).
P	Q98KL8	DE	Probable potassium transport system protein kup 1;
P	Q98KL8	DR	potassium ion transmembrane transporter activity
P	Q98L75	CC	Part of the ABC transporter complex HmuTUV involved in hemin import
P	Q98L75	DE	Hemin import ATP-binding protein HmuV;
P	Q98QE1	CC	Part of the ABC transporter complex PotABCD involved in spermidine/putrescine import
P	Q98QE1	CC	Part of the ABC transporter complex PotABCD involved in spermidine/putrescine import
P	Q98QE1	DE	Spermidine/putrescine import ATP-binding protein PotA;
P	Q98QE1	DE	Spermidine/putrescine import ATP-binding protein PotA;
P	Q99246	CC	Long-lasting (L-type) calcium channels belong to the 'high-voltage activated' (HVA) group
P	Q99246	CC	The isoform alpha-1D gives rise to L-type calcium currents
P	Q99246	CC	Voltage-sensitive calcium channels (VSCC) mediate the entry of calcium ions into excitable cells and are also involved in a variety of calcium-dependent processes, including muscle contraction, hormone or neurotransmitter release, gene expression, cell motility, cell division and cell death
P	Q99246	DE	Calcium channel, L type, alpha-1 polypeptide isoform 2;
P	Q99246	DE	Voltage-dependent L-type calcium channel subunit alpha-1D;
P	Q99246	DE	Voltage-gated calcium channel subunit alpha Cav1.3;
P	Q99246	DR	high voltage-gated calcium channel activity
P	Q99250	CC	Assuming opened or closed conformations in response to the voltage difference across the membrane, the protein forms a sodium-selective channel through which Na+ ions may pass in accordance with their electrochemical gradient.
P	Q99250	CC	Mediates the voltage-dependent sodium ion permeability of excitable membranes
P	Q99250	DE	Sodium channel protein brain II subunit alpha;
P	Q99250	DE	Sodium channel protein type 2 subunit alpha;
P	Q99250	DE	Sodium channel protein type II subunit alpha;
P	Q99250	DE	Voltage-gated sodium channel subunit alpha Nav1.2;
P	Q99250	DR	voltage-gated sodium channel activity
P	Q99271	CC	Sodium export from cell, takes up external protons in exchange for internal sodium ions (By similarity).
P	Q99271	DE	Na+/H+ antiporter;
P	Q99271	DE	Na+/H+ antiporter;
P	Q99271	DR	sodium:hydrogen antiporter activity
P	Q99385	CC	Has a role in promoting intracellular calcium ion sequestration via the exchange of calcium ions for hydrogen ions across the vacuolar membrane
P	Q99385	CC	Involved also in manganese ion homeostasis via its uptake into the vacuole.
P	Q99385	DE	High copy number undoes manganese protein 1;
P	Q99385	DE	Manganese resistance 1 protein;
P	Q99385	DE	Vacuolar Ca2+/H+ exchanger;
P	Q99385	DE	Vacuolar Ca2+/H+ exchanger;
P	Q99385	DE	Vacuolar calcium ion transporter;
P	Q99385	DR	calcium:hydrogen antiporter activity
P	Q99712	CC	Inward rectifier potassium channels are characterized by a greater tendency to allow potassium to flow into the cell rather than out of it

P	Q99712	CC	Their voltage dependence is regulated by the concentration of extracellular potassium; as external potassium is raised, the voltage range of the channel opening shifts to more positive voltages
P	Q99712	DE	ATP-sensitive inward rectifier potassium channel 15;
P	Q99712	DE	Inward rectifier K+ channel Kir1.3;
P	Q99712	DE	Inward rectifier K+ channel Kir4.2;
P	Q99712	DE	Potassium channel, inwardly rectifying subfamily J member 15;
P	Q99712	DR	inward rectifier potassium channel activity
P	Q99726	CC	Involved in accumulation of zinc in synaptic vesicles (By similarity).
P	Q99726	DE	Zinc transporter 3;
P	Q99726	DR	zinc transporting ATPase activity
P	Q99808	DE	Equilibrative NBMPR-sensitive nucleoside transporter;
P	Q99808	DE	Equilibrative nitrobenzylmercaptopurine riboside-sensitive nucleoside transporter;
P	Q99808	DE	Equilibrative nucleoside transporter 1;
P	Q99808	DE	Nucleoside transporter, es-type;
P	Q99808	DR	nucleoside transmembrane transporter activity
P	Q99884	CC	Terminates the action of proline by its high affinity sodium-dependent reuptake into presynaptic terminals.
P	Q99884	CC	Terminates the action of proline by its high affinity sodium-dependent reuptake into presynaptic terminals.
P	Q99884	DE	Sodium-dependent proline transporter;
P	Q99884	DE	Sodium-dependent proline transporter;
P	Q99884	DR	neurotransmitter:sodium symporter activity
P	Q99884	DR	proline:sodium symporter activity
P	Q99884	DR	proline:sodium symporter activity
P	Q99928	CC	GABA, the major inhibitory neurotransmitter in the vertebrate brain, mediates neuronal inhibition by binding to the GABA/benzodiazepine receptor and opening an integral chloride channel.
P	Q99928	DR	chloride channel activity
P	Q99J27	CC	Probable acetyl-CoA transporter necessary for O- acetylation of gangliosides.
P	Q99J27	DE	Acetyl-CoA transporter 1;
P	Q99K24	DE	Zinc transporter ZIP3;
P	Q99K24	DR	zinc ion transmembrane transporter activity
P	Q99P65	DE	Equilibrative nucleoside transporter 3;
P	Q99PE8	CC	Transporter that appears to play an indispensable role in the selective transport of the dietary cholesterol in and out of the enterocytes and in the selective sterol excretion by the liver into bile.
P	Q99PL8	CC	Mediates high affinity thiamine uptake, probably via a proton anti-port mechanism
P	Q99PL8	DE	Thiamine transporter 2;
P	Q99PL8	DR	thiamine uptake transmembrane transporter activity
P	Q99S77	CC	This system is involved in lactose transport.
P	Q99S77	DE	Lactose permease IIC component;
P	Q99S77	DE	Lactose-specific phosphotransferase enzyme IIB component;
P	Q99S77	DE	PTS system lactose-specific EIIB component;
P	Q99S77	DE	PTS system lactose-specific EIIC component;
P	Q99S77	DE	PTS system lactose-specific EIICB component;
P	Q99UZ7	CC	H+-stimulated, highly selective, manganese uptake system (By similarity).
P	Q99UZ7	DE	Probable manganese transport protein mntH;
P	Q99Z39	CC	Transport of potassium into the cell (By similarity).
P	Q99Z39	DE	Probable potassium transport system protein kup;
P	Q99Z39	DR	potassium ion transmembrane transporter activity
P	Q99ZS8	CC	Part of the ABC transporter complex PotABCD involved in spermidine/putrescine import
P	Q99ZS8	DE	Spermidine/putrescine import ATP-binding protein PotA;
P	Q9A0L9	CC	Part of the ATP-driven transport system AdcABC for zinc.
P	Q9A0L9	DE	Zinc-binding protein AdcA;
P	Q9A1E0	CC	Involved in the import of serine and threonine into the cell, with the concomitant import of sodium (symport system) (By similarity).
P	Q9A1E0	DE	Na+/serine-threonine symporter;
P	Q9A1E0	DR	sodium:dicarboxylate symporter activity
P	Q9A1E0	DR	sodium:dicarboxylate symporter activity
P	Q9A1H0	DE	Putative glutamine-binding periplasmic protein;
P	Q9A298	CC	Part of the ABC transporter complex CcmAB involved in the biogenesis of c-type cytochromes; once thought to export heme, this seems not to be the case, but its exact role is uncertain
P	Q9A298	DE	Heme exporter protein A;

P	Q9A298	DR	heme-transporting ATPase activity
P	Q9A502	CC	Part of the ABC transporter complex MetNIQ involved in methionine import
P	Q9A502	DE	Methionine import ATP-binding protein MetN;
P	Q9A6Z7	DE	Lipoprotein-releasing system ATP-binding protein LolD 2;
P	Q9A6Z7	DR	lipoprotein transporter activity
P	Q9A7X1	CC	Part of the ABC transporter complex CysAWTP involved in sulfate/thiosulfate import
P	Q9A7X1	DE	Sulfate-transporting ATPase;
P	Q9A7X1	DE	Sulfate/thiosulfate import ATP-binding protein CysA;
P	Q9A7X1	DR	sulfate transmembrane-transporting ATPase activity
P	Q9A7X6	CC	One of the components of the high-affinity ATP-driven potassium transport (or KDP) system, which catalyzes the hydrolysis of ATP coupled with the exchange of hydrogen and potassium ions
P	Q9A7X6	DE	Potassium-binding and translocating subunit C;
P	Q9A7X6	DE	Potassium-translocating ATPase C chain;
P	Q9A7X6	DE	Potassium-transporting ATPase C chain;
P	Q9A7X6	DR	potassium-transporting ATPase activity
P	Q9A7X7	CC	One of the components of the high-affinity ATP-driven potassium transport (or KDP) system, which catalyzes the hydrolysis of ATP coupled with the exchange of hydrogen and potassium ions (By similarity).
P	Q9A7X7	DE	Potassium-binding and translocating subunit B;
P	Q9A7X7	DE	Potassium-translocating ATPase B chain;
P	Q9A7X7	DE	Potassium-transporting ATPase B chain;
P	Q9A7X7	DR	potassium-transporting ATPase activity
P	Q9A7X8	CC	One of the components of the high-affinity ATP-driven potassium transport (or KDP) system, which catalyzes the hydrolysis of ATP coupled with the exchange of hydrogen and potassium ions (By similarity).
P	Q9A7X8	DE	Potassium-binding and translocating subunit A;
P	Q9A7X8	DE	Potassium-translocating ATPase A chain;
P	Q9A7X8	DE	Potassium-transporting ATPase A chain;
P	Q9A7X8	DR	potassium-transporting ATPase activity
P	Q9A9P4	DE	Lipoprotein-releasing system ATP-binding protein LolD 1;
P	Q9A9P4	DR	lipoprotein transporter activity
P	Q9AAZ2	CC	Na+/H+ antiporter that extrudes sodium in exchange for external protons (By similarity).
P	Q9AAZ2	DE	Na+/H+ antiporter nhaA;
P	Q9AAZ2	DE	Sodium/proton antiporter nhaA;
P	Q9AB70	DR	phosphonate transmembrane-transporting ATPase activity
P	Q9ABT9	CC	Transport of potassium into the cell (By similarity).
P	Q9ABT9	DE	Probable potassium transport system protein kup;
P	Q9ABT9	DR	potassium ion transmembrane transporter activity
P	Q9ASI1	CC	Aquaporins facilitate the transport of water and small neutral solutes across cell membranes (By similarity).
P	Q9AUK4	CC	Magnesium transporter that may mediate the influx of magnesium in chloroplast (By similarity).
P	Q9AUK4	DE	Magnesium transporter MRS2-A, chloroplastic;
P	Q9BQ31	CC	Potassium channel subunit
P	Q9BQ31	DE	Delayed-rectifier K+ channel alpha subunit 3;
P	Q9BQ31	DE	Potassium voltage-gated channel subfamily S member 3;
P	Q9BQ31	DE	Voltage-gated potassium channel subunit Kv9.3;
P	Q9BQ31	DR	delayed rectifier potassium channel activity
P	Q9BQ31	DR	potassium channel regulator activity
P	Q9BRI3	DE	Zinc transporter 2;
P	Q9BRY0	DE	Zinc transporter ZIP3;
P	Q9BRY0	DR	zinc ion transmembrane transporter activity
P	Q9BV35	CC	May act as a ATP-Mg/Pi exchanger that mediates the transport of Mg-ATP in exchange for phosphate, catalyzing the net uptake or efflux of adenine nucleotides into or from the mitochondria.
P	Q9BX84	CC	Crucial for magnesium homeostasis
P	Q9BX84	CC	Has an important role in epithelial magnesium transport and in the active magnesium absorption in the gut and kidney
P	Q9BXI2	CC	Ornithine transport across inner mitochondrial membrane, from the cytoplasm to the matrix.
P	Q9BXI2	DE	Mitochondrial ornithine transporter 2;
P	Q9BXP2	DR	cation:chloride symporter activity
P	Q9BY07	CC	May play a role in mediating Na+:HCO3(-) cotransport in hepatocytes and intrahepatic cholangiocytes

P	Q9BY07	CC	Mediates sodium- and bicarbonate-dependent electrogenic sodium bicarbonate cotransport, with a Na+:HCO3(-) stoichiometry of 2:1
P	Q9BY07	CC	Mediates sodium- and bicarbonate-dependent electrogenic sodium bicarbonate cotransport, with a Na+:HCO3(-) stoichiometry of 2:1
P	Q9BY07	DR	sodium:bicarbonate symporter activity
P	Q9BY07	DR	sodium:bicarbonate symporter activity
P	Q9BYW1	CC	Facilitative glucose transporter (By similarity).
P	Q9BYW1	DE	Glucose transporter type 10;
P	Q9BYW1	DE	Glucose transporter type 11;
P	Q9BYW1	DE	Solute carrier family 2, facilitated glucose transporter member 11;
P	Q9BZW2	CC	Sodium/sulfate cotransporter that mediates sulfate reabsorption in the kidney.
P	Q9BZW2	CC	Sodium/sulfate cotransporter that mediates sulfate reabsorption in the kidney.
P	Q9BZW2	DE	Na+/sulfate cotransporter;
P	Q9BZW2	DE	Na+/sulfate cotransporter;
P	Q9BZW2	DE	Renal sodium/sulfate cotransporter;
P	Q9BZW2	DE	Renal sodium/sulfate cotransporter;
P	Q9BZW2	DR	sodium:sulfate symporter activity
P	Q9BZW2	DR	sodium:sulfate symporter activity
P	Q9C007	DE	Voltage-gated sodium channel alpha subunit splice variant SCN3A-s;
P	Q9C007	DR	voltage-gated sodium channel activity
P	Q9C0K1	DE	LIV-1 subfamily of ZIP zinc transporter 6;
P	Q9C0K1	DE	Zinc transporter ZIP8;
P	Q9C0K1	DR	zinc ion transmembrane transporter activity
P	Q9C6B2	DE	Metal transporter Nramp2;
P	Q9C6T0	CC	Aquaporins facilitate the transport of water and small neutral solutes across cell membranes (By similarity).
P	Q9C8G9	CC	Pump for glutathione S-conjugates
P	Q9C8G9	DE	ATP-energized glutathione S-conjugate pump 1;
P	Q9C8G9	DE	Glutathione S-conjugate-transporting ATPase 1;
P	Q9C8T1	CC	Part of the ABC transporter complex CcmAB involved in the biogenesis of c-type cytochromes; once thought to export heme, this seems not to be the case, but its exact role is uncertain
P	Q9C8T1	DR	heme-transporting ATPase activity
P	Q9C8X2	CC	Responsible for the transport of sucrose into the cell, with the concomitant uptake of protons (symport system)
P	Q9C8X2	DE	Sucrose permease 5;
P	Q9C8X2	DE	Sucrose transport protein SUC5;
P	Q9C8X2	DE	Sucrose-proton symporter 5;
P	Q9C8X2	DR	sucrose transmembrane transporter activity
P	Q9CBE5	CC	Part of the ABC transporter complex pstSACB involved in phosphate import (By similarity).
P	Q9CBE5	DE	Phosphate-binding protein pstS 3;
P	Q9CBE5	DR	inorganic phosphate transmembrane transporter activity
P	Q9CE02	CC	Mixed channel protein that transports both water and glycerol (By similarity).
P	Q9CE02	DE	Glycerol facilitator-aquaporin gla;
P	Q9CEK0	CC	This protein is a component of the oligopeptide permease, a binding protein-dependent transport system, it binds peptides up to five amino acids long with high affinity
P	Q9CEK0	DE	Oligopeptide-binding protein oppA;
P	Q9CEW7	CC	Part of the ABC transporter complex PstSACB involved in phosphate import
P	Q9CEW7	DE	ABC phosphate transporter 2;
P	Q9CEW7	DE	Phosphate import ATP-binding protein PstB 2;
P	Q9CEW7	DE	Phosphate-transporting ATPase 2;
P	Q9CEW7	DR	inorganic phosphate transmembrane transporter activity
P	Q9CEW7	DR	phosphate transmembrane-transporting ATPase activity
P	Q9CEW8	CC	Part of the ABC transporter complex PstSACB involved in phosphate import
P	Q9CEW8	DE	ABC phosphate transporter 1;
P	Q9CEW8	DE	Phosphate import ATP-binding protein PstB 1;
P	Q9CEW8	DE	Phosphate-transporting ATPase 1;
P	Q9CEW8	DR	inorganic phosphate transmembrane transporter activity
P	Q9CEW8	DR	phosphate transmembrane-transporting ATPase activity
P	Q9CFZ5	CC	Part of an ATP-driven transport system for a metal; probably for manganese.
P	Q9CG19	CC	Involved in glutamate-dependent acid resistance
P	Q9CG19	CC	May be involved in antiport of glutamate and glutamate-gamma- aminobutyrate (GABA)
P	Q9CG19	DE	Glutamate/gamma-aminobutyrate antiporter;
P	Q9CGD4	CC	Part of the ABC transporter complex PotABCD involved in spermidine/putrescine import

P	Q9CGD4	CC	Part of the ABC transporter complex PotABCD involved in spermidine/putrescine import
P	Q9CGD4	DE	Spermidine/putrescine import ATP-binding protein PotA;
P	Q9CGD4	DE	Spermidine/putrescine import ATP-binding protein PotA;
P	Q9CHU4	CC	Transport of potassium into the cell (By similarity).
P	Q9CHU4	DE	Probable potassium transport system protein kup 2;
P	Q9CHU4	DR	potassium ion transmembrane transporter activity
P	Q9CHU5	CC	Transport of potassium into the cell (By similarity).
P	Q9CHU5	DE	Probable potassium transport system protein kup 1;
P	Q9CHU5	DR	potassium ion transmembrane transporter activity
P	Q9CJZ2	CC	This system is involved in sucrose transport (By similarity).
P	Q9CJZ2	DE	PTS system sucrose-specific EIIB component;
P	Q9CJZ2	DE	PTS system sucrose-specific EIIBC component;
P	Q9CJZ2	DE	PTS system sucrose-specific EIIC component;
P	Q9CJZ2	DE	Sucrose permease IIC component;
P	Q9CJZ2	DE	Sucrose-specific phosphotransferase enzyme IIB component;
P	Q9CKD8	CC	Na+/H+ antiporter that extrudes sodium in exchange for external protons (By similarity).
P	Q9CKD8	DE	Na+/H+ antiporter nhaA;
P	Q9CKD8	DE	Sodium/proton antiporter nhaA;
P	Q9CL63	CC	Part of the ABC transporter complex RbsABCD involved in ribose import
P	Q9CL63	DE	Ribose import ATP-binding protein RbsA 2;
P	Q9CLC4	CC	Mediates influx of magnesium ions (By similarity).
P	Q9CLC4	DE	Magnesium transport protein CorA;
P	Q9CLC4	DR	cobalt ion transmembrane transporter activity
P	Q9CLC4	DR	magnesium ion transmembrane transporter activity
P	Q9CLY8	CC	This system is involved in mannitol transport (By similarity).
P	Q9CLY8	DE	Mannitol permease IIC component;
P	Q9CLY8	DE	Mannitol-specific phosphotransferase enzyme IIA component;
P	Q9CLY8	DE	Mannitol-specific phosphotransferase enzyme IIB component;
P	Q9CLY8	DE	PTS system mannitol-specific EIIA component;
P	Q9CLY8	DE	PTS system mannitol-specific EIIB component;
P	Q9CLY8	DE	PTS system mannitol-specific EIIC component;
P	Q9CLY8	DE	PTS system mannitol-specific EIICBA component;
P	Q9CM08	CC	Part of the ABC transporter complex MglABC involved in galactose/methyl galactoside import
P	Q9CM08	DE	Galactose/methyl galactoside import ATP-binding protein MglA;
P	Q9CMD9	CC	Involved in the import of serine and threonine into the cell, with the concomitant import of sodium (symport system) (By similarity).
P	Q9CMD9	DE	Na+/serine-threonine symporter;
P	Q9CMD9	DR	sodium:dicarboxylate symporter activity
P	Q9CMD9	DR	sodium:dicarboxylate symporter activity
P	Q9CMS0	CC	Part of the ABC transporter complex ModABC involved in molybdenum import
P	Q9CMS0	DE	Molybdenum import ATP-binding protein ModC;
P	Q9CMS0	DR	molybdenum ion transmembrane transporter activity
P	Q9CNJ4	CC	Part of the ABC transporter complex pstSACB involved in phosphate import (By similarity).
P	Q9CNJ4	DE	Phosphate-binding protein pstS;
P	Q9CNJ4	DR	inorganic phosphate transmembrane transporter activity
P	Q9CNJ5	CC	Part of the binding-protein-dependent transport system for phosphate; probably responsible for the translocation of the substrate across the membrane (By similarity).
P	Q9CNJ5	DE	Phosphate transport system permease protein pstC;
P	Q9CNJ5	DR	inorganic phosphate transmembrane transporter activity
P	Q9CNJ6	CC	Part of a binding-protein-dependent transport system for phosphate; probably responsible for the translocation of the substrate across the membrane (By similarity).
P	Q9CNJ6	DE	Phosphate transport system permease protein pstA;
P	Q9CNJ6	DR	inorganic phosphate transmembrane transporter activity
P	Q9CNJ7	CC	Part of the ABC transporter complex PstSACB involved in phosphate import
P	Q9CNJ7	DE	ABC phosphate transporter;
P	Q9CNJ7	DE	Phosphate import ATP-binding protein PstB;
P	Q9CNJ7	DE	Phosphate-transporting ATPase;
P	Q9CNJ7	DR	inorganic phosphate transmembrane transporter activity
P	Q9CNJ7	DR	phosphate transmembrane-transporting ATPase activity
P	Q9CNP9	CC	Part of the ABC transporter complex ThiBPQ involved in thiamine import
P	Q9CNP9	DE	Thiamine import ATP-binding protein ThiQ;
P	Q9CP06	CC	Part of the ABC transporter complex PotABCD involved in spermidine/putrescine import
P	Q9CP06	CC	Part of the ABC transporter complex PotABCD involved in spermidine/putrescine import

P	Q9CP06	DE	Spermidine/putrescine import ATP-binding protein PotA;
P	Q9CP06	DE	Spermidine/putrescine import ATP-binding protein PotA;
P	Q9CP24	CC	Part of the ABC transporter complex ZnuABC involved in zinc import
P	Q9CP24	DE	Zinc import ATP-binding protein ZnuC;
P	Q9CP24	DR	zinc transporting ATPase activity
P	Q9CP98	CC	Part of the ABC transporter complex RbsABCD involved in ribose import
P	Q9CP98	DE	Ribose import ATP-binding protein RbsA 1;
P	Q9CPJ1	CC	Na+/H+ antiporter that extrudes sodium in exchange for external protons (By similarity)
P	Q9CPJ1	DE	Na+/H+ antiporter nhaB;
P	Q9CPJ1	DE	Sodium/proton antiporter nhaB;
P	Q9CPJ1	DR	sodium:hydrogen antiporter activity
P	Q9CPL9	CC	Transport of uracil in the cell (By similarity).
P	Q9CPL9	DE	Probable uracil permease;
P	Q9CPL9	DE	Uracil transporter;
P	Q9CPN2	CC	Part of the ABC transporter complex CcmAB involved in the biogenesis of c-type cytochromes; once thought to export heme, this seems not to be the case, but its exact role is uncertain
P	Q9CPN2	DE	Heme exporter protein A;
P	Q9CPN2	DR	heme-transporting ATPase activity
P	Q9CPU9	CC	Involved in low-affinity copper uptake (Potential).
P	Q9CPU9	DE	Copper transporter 2;
P	Q9CPU9	DE	Probable low affinity copper uptake protein 2;
P	Q9CPU9	DR	copper ion transmembrane transporter activity
P	Q9CR62	CC	Catalyzes the transport of 2-oxoglutarate across the inner mitochondrial membrane in an electroneutral exchange for malate or other dicarboxylic acids, and plays an important role in several metabolic processes, including the malate-aspartate shuttle, the oxoglutarate/isocitrate shuttle, in gluconeogenesis from lactate, and in nitrogen metabolism (By similarity).
P	Q9CR62	CC	Catalyzes the transport of 2-oxoglutarate across the inner mitochondrial membrane in an electroneutral exchange for malate or other dicarboxylic acids, and plays an important role in several metabolic processes, including the malate-aspartate shuttle, the oxoglutarate/isocitrate shuttle, in gluconeogenesis from lactate, and in nitrogen metabolism (By similarity).
P	Q9CR62	DE	Mitochondrial 2-oxoglutarate/malate carrier protein;
P	Q9CR62	DE	Mitochondrial 2-oxoglutarate/malate carrier protein;
P	Q9CXB2	CC	Transports sulfoconjugated steroid hormones, as well as taurolithocholic acid-3-sulfate and sulfoconjugated pyrenes in a sodium-dependent manner (By similarity)
P	Q9CXB2	DE	Sodium-dependent organic anion transporter;
P	Q9CXB2	DR	bile acid:sodium symporter activity
P	Q9CXB2	DR	bile acid:sodium symporter activity
P	Q9D321	DR	sugar:hydrogen symporter activity
P	Q9D687	CC	Uptake of leucine is sodium- dependent
P	Q9D687	DE	Sodium-dependent neutral amino acid transporter B(0)AT1;
P	Q9D687	DR	neurotransmitter:sodium symporter activity
P	Q9D6F4	CC	GABA, the major inhibitory neurotransmitter in the vertebrate brain, mediates neuronal inhibition by binding to the GABA/benzodiazepine receptor and opening an integral chloride channel.
P	Q9D6F4	DR	chloride channel activity
P	Q9D6M3	CC	Glutamate is cotransported with H+ (By similarity).
P	Q9D6M3	CC	Involved in the transport of glutamate across the inner mitochondrial membrane
P	Q9D6M3	DE	Glutamate/H+ symporter 1;
P	Q9D6M3	DE	Mitochondrial glutamate carrier 1;
P	Q9D6M3	DR	L-glutamate transmembrane transporter activity
P	Q9D7P7	CC	Can insert into membranes and form chloride ion channels
P	Q9D7P7	DE	Chloride intracellular channel protein 3;
P	Q9D7P7	DR	voltage-gated chloride channel activity
P	Q9D856	CC	May play a role in polarized cells by carrying out serosal-to-mucosal zinc transport
P	Q9D856	CC	Seems to play a central role in controlling organismal zinc status.
P	Q9D856	DE	Zinc transporter ZIP5;
P	Q9D856	DR	zinc ion transmembrane transporter activity
P	Q9DB41	CC	Glutamate is cotransported with H+ (By similarity).
P	Q9DB41	CC	Involved in the transport of glutamate across the inner mitochondrial membrane
P	Q9DB41	DE	Glutamate/H+ symporter 2;
P	Q9DB41	DE	Mitochondrial glutamate carrier 2;
P	Q9DBP0	CC	It may be the main phosphate transport protein in the intestinal brush border membrane

P	Q9DBP0	CC	May be involved in actively transporting phosphate into cells via Na+ cotransport
P	Q9DBP0	CC	May be involved in actively transporting phosphate into cells via Na+ cotransport
P	Q9DBP0	DE	Na+-dependent phosphate cotransporter 2B;
P	Q9DBP0	DE	Na+-dependent phosphate cotransporter 2B;
P	Q9DBP0	DE	Na+/Pi cotransporter 2B;
P	Q9DBP0	DE	Sodium-dependent phosphate transport protein 2B;
P	Q9DBP0	DE	Sodium-dependent phosphate transport protein 2B;
P	Q9DBP0	DE	Sodium-phosphate transport protein 2B;
P	Q9DBP0	DE	Sodium/phosphate cotransporter 2B;
P	Q9DBP0	DE	Sodium/phosphate cotransporter 2B;
P	Q9DBP0	DR	sodium-dependent phosphate transmembrane transporter activity
P	Q9DBP0	DR	sodium-dependent phosphate transmembrane transporter activity
P	Q9EPR4	CC	Mediates electrogenic uptake of vitamin C, with a stoichiometry of 2 Na+ for each ascorbate (By similarity).
P	Q9EPR4	CC	Mediates electrogenic uptake of vitamin C, with a stoichiometry of 2 Na+ for each ascorbate (By similarity).
P	Q9EPR4	CC	Sodium/ascorbate cotransporter
P	Q9EPR4	CC	Sodium/ascorbate cotransporter
P	Q9EPR4	DE	Na+/L-ascorbic acid transporter 2;
P	Q9EPR4	DE	Na+/L-ascorbic acid transporter 2;
P	Q9EPR4	DE	Sodium-dependent vitamin C transporter 2;
P	Q9EPR4	DE	Sodium-dependent vitamin C transporter 2;
P	Q9EPR4	DR	L-ascorbate:sodium symporter activity
P	Q9EPR4	DR	L-ascorbate:sodium symporter activity
P	Q9EQJ0	CC	Nicotinic acid adenine dinucleotide phosphate (NAADP) receptor that may function as one of the major voltage-gated Ca2+ channels (VDCC) across the lysosomal and endosomal membrane (By similarity).
P	Q9EQJ0	DE	Two pore calcium channel protein 1;
P	Q9EQJ0	DE	Voltage-dependent calcium channel protein TPC1;
P	Q9EQJ0	DR	calcium channel activity
P	Q9EQR3	CC	Forms a voltage-independent potassium channel activated by intracellular calcium
P	Q9ER60	CC	Assuming opened or closed conformations in response to the voltage difference across the membrane, the protein forms a sodium-selective channel through which Na+ ions may pass in accordance with their electrochemical gradient
P	Q9ER60	CC	This protein mediates the voltage-dependent sodium ion permeability of excitable membranes
P	Q9ER60	CC	This sodium channel may be present in both denervated and innervated skeletal muscle
P	Q9ER60	DE	Sodium channel protein skeletal muscle subunit alpha;
P	Q9ER60	DE	Sodium channel protein type 4 subunit alpha;
P	Q9ER60	DE	Sodium channel protein type IV subunit alpha;
P	Q9ER60	DE	Voltage-gated sodium channel subunit alpha Nav1.4;
P	Q9ER60	DR	voltage-gated sodium channel activity
P	Q9ES88	CC	Cotransport of sodium ions and dicarboxylates such as succinate and citrate.
P	Q9ES88	DE	Na+/dicarboxylate cotransporter 1;
P	Q9ES88	DE	Na+/dicarboxylate cotransporter 1;
P	Q9ES88	DE	Renal sodium/dicarboxylate cotransporter;
P	Q9ES88	DE	Renal sodium/dicarboxylate cotransporter;
P	Q9EYM2	DE	Lipoprotein-releasing system ATP-binding protein LolD;
P	Q9EYM2	DR	lipoprotein transporter activity
P	Q9FE38	CC	High-affinity potassium transporter required for tip growth of root hairs.
P	Q9FE38	DE	Potassium transporter 3;
P	Q9FE38	DR	potassium ion transmembrane transporter activity
P	Q9FE59	CC	Responsible for the transport of sucrose into the cell, with the concomitant uptake of protons (symport system)
P	Q9FE59	DE	Sucrose permease 4;
P	Q9FE59	DE	Sucrose transport protein SUC4;
P	Q9FE59	DE	Sucrose transporter 4;
P	Q9FE59	DE	Sucrose-proton symporter 4;
P	Q9FE59	DR	sucrose transmembrane transporter activity
P	Q9FEP7	CC	High-affinity H+/sulfate cotransporter that mediates the loading of sulfate into the sieve tube
P	Q9FEP7	CC	Plays a central role in the regulation of sulfate assimilation.
P	Q9FEP7	DE	Sulfate transporter 1.3;
P	Q9FEP7	DR	secondary active sulfate transmembrane transporter activity

P	Q9FF53	CC	Aquaporins facilitate the transport of water and small neutral solutes across cell membranes (By similarity).
P	Q9FG00	CC	High-affinity sucrose transporter
P	Q9FG00	CC	Responsible for the transport of sucrose into the cell, with the concomitant uptake of protons (symport system)
P	Q9FG00	DE	Sucrose permease 9;
P	Q9FG00	DE	Sucrose transport protein SUC9;
P	Q9FG00	DE	Sucrose-proton symporter 9;
P	Q9FG00	DR	sucrose transmembrane transporter activity
P	Q9FG72	DE	Oligopeptide transporter 1;
P	Q9FG72	DR	oligopeptide transporter activity
P	Q9FIS2	CC	Probably mediates zinc uptake from the rhizosphere (By similarity).
P	Q9FIS2	DE	Probable zinc transporter 12;
P	Q9FIS2	DR	zinc ion transmembrane transporter activity
P	Q9FIZ9	CC	Potential aquaporin, which may facilitate the transport of water and small neutral solutes across cell membranes (By similarity).
P	Q9FJD1	DE	Oligopeptide transporter 8;
P	Q9FJD2	DE	Oligopeptide transporter 9;
P	Q9FK43	CC	Water channel required to facilitate the transport of water across cell membrane.
P	Q9FK43	DR	water channel activity
P	Q9FL25	CC	Calcium-activated outward rectifying potassium channel
P	Q9FL25	DE	Probable calcium-activated outward-rectifying potassium channel 2;
P	Q9FL25	DR	potassium channel activity
P	Q9FLG2	CC	Low-affinity magnesium transporter that mediates the influx of magnesium
P	Q9FLG2	DE	Magnesium Transporter 9;
P	Q9FLG2	DE	Magnesium transporter MRS2-2;
P	Q9FLG2	DR	magnesium ion transmembrane transporter activity
P	Q9FME8	DE	Oligopeptide transporter 4;
P	Q9FME8	DR	oligopeptide transporter activity
P	Q9FMX3	CC	Can transport glucose, galactose, mannose, xylose and 3-O-methylglucose, but not fructose and ribose.
P	Q9FMX3	DE	Hexose transporter 11;
P	Q9FN18	CC	Regulates metal accumulation under Fe starvation
P	Q9FN18	CC	Vacuolar metal transporter involved in intracellular metal homeostasis
P	Q9FN18	DE	Metal transporter Nramp4;
P	Q9FWV6	CC	Aquaporins facilitate the transport of water and small neutral solutes across cell membranes
P	Q9FWX6	CC	Outward rectifying potassium channel
P	Q9FWX6	DE	Outward-rectifying potassium channel 4;
P	Q9FWX6	DE	Two-pore potassium channel 4;
P	Q9FWX6	DR	potassium channel activity
P	Q9FY46	CC	H+/sulfate cotransporter that may play a role in the regulation of sulfate assimilation
P	Q9FY46	DE	Sulfate transporter 4.1, chloroplastic;
P	Q9FY46	DR	secondary active sulfate transmembrane transporter activity
P	Q9FY75	CC	Probable potassium transporter.
P	Q9FY75	DE	Potassium transporter 7;
P	Q9FY75	DR	potassium ion transmembrane transporter activity
P	Q9GZN6	DE	Orphan sodium- and chloride-dependent neurotransmitter transporter NTT5;
P	Q9GZN6	DR	neurotransmitter:sodium symporter activity
P	Q9GZV3	CC	Choline uptake is the rate-limiting step in acetylcholine synthesis
P	Q9GZV3	CC	Imports choline from the extracellular space to the neuron with high affinity
P	Q9GZV3	CC	Sodium ion- and chloride ion- dependent.
P	Q9GZV3	DE	Hemicholinium-3-sensitive choline transporter;
P	Q9GZV3	DE	High affinity choline transporter 1;
P	Q9GZV3	DR	choline:sodium symporter activity
P	Q9GZV3	DR	choline:sodium symporter activity
P	Q9H015	CC	Also transports organic cations such as tetraethylammonium (TEA) without the involvement of sodium
P	Q9H015	CC	Probably transports one sodium ion with one molecule of carnitine
P	Q9H015	CC	Probably transports one sodium ion with one molecule of carnitine
P	Q9H015	CC	Relative uptake activity ratio of carnitine to TEA is 1.78
P	Q9H015	CC	Sodium-ion dependent, low affinity carnitine transporter
P	Q9H015	CC	Sodium-ion dependent, low affinity carnitine transporter
P	Q9H015	DE	Organic cation/carnitine transporter 1;
P	Q9H015	DR	carnitine transporter activity

P	Q9H0C2	CC	Catalyzes the exchange of ADP and ATP across the mitochondrial inner membrane
P	Q9H0C2	CC	Catalyzes the exchange of ADP and ATP across the mitochondrial inner membrane
P	Q9H0C2	DE	ADP,ATP carrier protein 4;
P	Q9H0C2	DE	ADP/ATP translocase 4;
P	Q9H0C2	DE	ADP/ATP translocase 4;
P	Q9H1D0	CC	Calcium selective cation channel probably involved in Ca2+ uptake in various tissues, including Ca2+ reabsorption in intestine
P	Q9H1D0	CC	Inactivation includes both, a rapid Ca2+-dependent and a slower Ca2+-calmodulin- dependent mechanism, the latter may be regulated by phosphorylation
P	Q9H1D0	CC	The channel is activated by low internal calcium level, probably including intracellular calcium store depletion, and the current exhibits an inward rectification
P	Q9H1D0	DE	Calcium transport protein 1;
P	Q9H1D0	DE	Epithelial calcium channel 2;
P	Q9H1D0	DR	calcium channel activity
P	Q9H1K4	CC	Glutamate is cotransported with H+.
P	Q9H1K4	CC	Involved in the transport of glutamate across the inner mitochondrial membrane
P	Q9H1K4	DE	Glutamate/H+ symporter 2;
P	Q9H1K4	DE	Mitochondrial glutamate carrier 2;
P	Q9H1V8	DE	Sodium-dependent neurotransmitter transporter NTT4;
P	Q9H1V8	DE	Sodium-dependent neutral amino acid transporter SLC6A17;
P	Q9H1V8	DR	neurotransmitter:sodium symporter activity
P	Q9H221	CC	Transporter that appears to play an indispensable role in the selective transport of the dietary cholesterol in and out of the enterocytes and in the selective sterol excretion by the liver into bile.
P	Q9H222	CC	Transporter that appears to play an indispensable role in the selective transport of the dietary cholesterol in and out of the enterocytes and in the selective sterol excretion by the liver into bile.
P	Q9H252	CC	Pore-forming (alpha) subunit of voltage-gated potassium channel
P	Q9H252	DE	Ether-a-go-go-related gene potassium channel 2;
P	Q9H252	DE	Potassium voltage-gated channel subfamily H member 6;
P	Q9H252	DE	Voltage-gated potassium channel subunit Kv11.2;
P	Q9H2B4	CC	High affinity uptake of sulfate
P	Q9H2B4	DE	Sulfate anion transporter 1;
P	Q9H2B4	DR	secondary active sulfate transmembrane transporter activity
P	Q9H2D1	CC	Transports folate across the inner membranes of mitochondria.
P	Q9H2D1	DE	Mitochondrial folate transporter/carrier;
P	Q9H2D1	DR	folic acid transporter activity
P	Q9H2J7	CC	Mediates the saturable, pH-sensitive and electrogenic cotransport of proline and sodium ions with a stoichiometry of 1:1
P	Q9H2J7	DE	Sodium- and chloride-dependent neurotransmitter transporter NTT73;
P	Q9H2J7	DE	Sodium-coupled branched-chain amino-acid transporter 1;
P	Q9H2J7	DE	Sodium-dependent neutral amino acid transporter B(0)AT2;
P	Q9H2J7	DR	neurotransmitter:sodium symporter activity
P	Q9H2S1	CC	Forms a voltage-independent potassium channel activated by intracellular calcium
P	Q9H2X9	CC	Mediates electroneutral potassium-chloride cotransport in mature neurons
P	Q9H2X9	DE	Electroneutral potassium-chloride cotransporter 2;
P	Q9H2X9	DR	potassium:chloride symporter activity
P	Q9H2X9	DR	potassium:chloride symporter activity
P	Q9H3M0	CC	Putative voltage-gated potassium channel.
P	Q9H3M0	DE	Potassium voltage-gated channel subfamily F member 1;
P	Q9H3M0	DE	Voltage-gated potassium channel subunit Kv5.1;
P	Q9H3M0	DR	voltage-gated potassium channel activity
P	Q9H427	CC	Probable potassium channel subunit
P	Q9H427	DE	Acid-sensitive potassium channel protein TASK-5;
P	Q9H427	DE	Potassium channel subfamily K member 15;
P	Q9H427	DE	TWIK-related acid-sensitive K+ channel 5;
P	Q9H427	DE	Two pore K+ channel KT3.3;
P	Q9H427	DE	Two pore potassium channel KT3.3;
P	Q9H427	DR	potassium channel activity
P	Q9H598	CC	Involved in the uptake of GABA and glycine into the synaptic vesicles.
P	Q9H598	CC	Involved in the uptake of GABA and glycine into the synaptic vesicles.
P	Q9H598	DE	GABA and glycine transporter;
P	Q9H598	DE	GABA and glycine transporter;
P	Q9H598	DE	Vesicular GABA transporter;
P	Q9H936	CC	Glutamate is cotransported with H+.

P	Q9H936	CC	Involved in the transport of glutamate across the inner mitochondrial membrane
P	Q9H936	DE	Glutamate/H+ symporter 1;
P	Q9H936	DE	Mitochondrial glutamate carrier 1;
P	Q9H936	DR	L-glutamate transmembrane transporter activity
P	Q9H9F5	DR	neurotransmitter:sodium symporter activity
P	Q9HB14	CC	Potassium channel displaying weak inward rectification in symmetrical K+ solution (By similarity).
P	Q9HB14	DE	Potassium channel subfamily K member 13;
P	Q9HB14	DE	Tandem pore domain halothane-inhibited potassium channel 1;
P	Q9HB14	DR	potassium channel activity
P	Q9HB15	CC	Probable potassium channel subunit
P	Q9HB15	DE	Potassium channel subfamily K member 12;
P	Q9HB15	DE	Tandem pore domain halothane-inhibited potassium channel 2;
P	Q9HB15	DR	potassium channel activity
P	Q9HC58	CC	Transports 1 Ca2+ and 1 K+ in exchange for 4 Na+ (By similarity).
P	Q9HC58	CC	Transports 1 Ca2+ and 1 K+ in exchange for 4 Na+ (By similarity).
P	Q9HC58	CC	Transports 1 Ca2+ and 1 K+ in exchange for 4 Na+ (By similarity).
P	Q9HC58	DE	Na+/K+/Ca2+-exchange protein 3;
P	Q9HC58	DE	Na+/K+/Ca2+-exchange protein 3;
P	Q9HC58	DE	Na+/K+/Ca2+-exchange protein 3;
P	Q9HC58	DE	Sodium/potassium/calcium exchanger 3;
P	Q9HC58	DE	Sodium/potassium/calcium exchanger 3;
P	Q9HC58	DE	Sodium/potassium/calcium exchanger 3;
P	Q9HC58	DR	calcium, potassium:sodium antiporter activity
P	Q9HC58	DR	calcium, potassium:sodium antiporter activity
P	Q9HC58	DR	calcium, potassium:sodium antiporter activity
P	Q9HD23	CC	Magnesium transporter that may mediate the influx of magnesium into the mitochondrial matrix.
P	Q9HD23	DE	Magnesium transporter MRS2 homolog, mitochondrial;
P	Q9HFE1	DE	Low-affinity iron/zinc ion transport protein fet4;
P	Q9HML8	CC	Part of the ABC transporter complex PstSACB involved in phosphate import
P	Q9HML8	DE	ABC phosphate transporter 2;
P	Q9HML8	DE	Phosphate import ATP-binding protein PstB 2;
P	Q9HML8	DE	Phosphate-transporting ATPase 2;
P	Q9HML8	DR	inorganic phosphate transmembrane transporter activity
P	Q9HML8	DR	phosphate transmembrane-transporting ATPase activity
P	Q9HNI8	DR	phosphonate transmembrane-transporting ATPase activity
P	Q9HPH5	DR	cobalt ion transmembrane transporter activity
P	Q9HS13	CC	Part of the ABC transporter complex PstSACB involved in phosphate import
P	Q9HS13	DE	ABC phosphate transporter 1;
P	Q9HS13	DE	Phosphate import ATP-binding protein PstB 1;
P	Q9HS13	DE	Phosphate-transporting ATPase 1;
P	Q9HS13	DR	inorganic phosphate transmembrane transporter activity
P	Q9HS13	DR	phosphate transmembrane-transporting ATPase activity
P	Q9HT70	CC	Part of the ABC transporter complex MetNIQ involved in methionine import
P	Q9HT70	DE	Methionine import ATP-binding protein MetN 2;
P	Q9HT73	CC	Part of the ABC transporter complex ZnuABC involved in zinc import
P	Q9HT73	DE	Zinc import ATP-binding protein ZnuC;
P	Q9HT73	DR	zinc transporting ATPase activity
P	Q9HWR7	CC	The physiological role may be the reduction of the intracellular concentration of toxic sugars or sugar metabolites (By similarity).
P	Q9HWR7	DE	Probable sugar efflux transporter;
P	Q9HWZ3	CC	Channel that permits osmotically driven movement of water in both directions
P	Q9HWZ3	CC	It mediates rapid entry or exit of water in response to abrupt changes in osmolarity (By similarity).
P	Q9HX79	CC	Part of the ABC transporter complex TauABC involved in taurine import
P	Q9HX79	DE	Taurine import ATP-binding protein TauB;
P	Q9HX79	DR	taurine-transporting ATPase activity
P	Q9HY19	CC	Part of the ABC transporter complex PotABCD involved in spermidine/putrescine import
P	Q9HY19	CC	Part of the ABC transporter complex PotABCD involved in spermidine/putrescine import
P	Q9HY19	DE	Spermidine/putrescine import ATP-binding protein PotA 2;
P	Q9HY19	DE	Spermidine/putrescine import ATP-binding protein PotA 2;
P	Q9HYL1	CC	Involved in copper processing and transport; in the assembly of the copper chromophores of nitrous oxide reductase.
P	Q9HYL1	DE	Copper-binding periplasmic protein;

P	Q9HYL7	DR	phosphonate transmembrane-transporting ATPase activity
P	Q9HYT0	DR	phosphonate transmembrane-transporting ATPase activity
P	Q9HZL7	DE	Lipoprotein-releasing system ATP-binding protein LolD;
P	Q9HZL7	DR	lipoprotein transporter activity
P	Q9HZS1	CC	Part of the binding-protein-dependent transport system for histidine
P	Q9HZS1	DE	Histidine transport ATP-binding protein HisP;
P	Q9I1C8	CC	Part of the ABC transporter complex MetNIQ involved in methionine import
P	Q9I1C8	DE	Methionine import ATP-binding protein MetN 1;
P	Q9I273	CC	Involved in the import of serine and threonine into the cell, with the concomitant import of sodium (symport system) (By similarity).
P	Q9I273	DE	Na+/serine-threonine symporter;
P	Q9I273	DR	sodium:dicarboxylate symporter activity
P	Q9I273	DR	sodium:dicarboxylate symporter activity
P	Q9I2N4	CC	Part of the ABC transporter complex ModABC involved in molybdenum import
P	Q9I2N4	DE	Molybdenum import ATP-binding protein ModC;
P	Q9I2N4	DR	molybdenum ion transmembrane transporter activity
P	Q9I2S5	CC	Na+/H+ antiporter that extrudes sodium in exchange for external protons
P	Q9I2S5	DE	Na+/H+ antiporter nhaB;
P	Q9I2S5	DE	Sodium/proton antiporter nhaB;
P	Q9I2S5	DR	sodium:hydrogen antiporter activity
P	Q9I3N5	CC	Required for the export of heme to the periplasm for the biogenesis of c-type cytochromes (By similarity).
P	Q9I3N5	DE	Heme exporter protein C;
P	Q9I3N5	DR	heme transporter activity
P	Q9I3N6	CC	Required for the export of heme to the periplasm for the biogenesis of c-type cytochromes (By similarity).
P	Q9I3N6	DE	Heme exporter protein B;
P	Q9I3N6	DR	heme transporter activity
P	Q9I3N7	CC	Part of the ABC transporter complex CcmAB involved in the biogenesis of c-type cytochromes; once thought to export heme, this seems not to be the case, but its exact role is uncertain
P	Q9I3N7	DE	Heme exporter protein A;
P	Q9I3N7	DR	heme-transporting ATPase activity
P	Q9I3Y3	CC	Confers resistance to benzalkonium chloride, fluoroquinolones, ethidium bromide, acriflavine and tetraphenylphosphonium chloride.
P	Q9I540	CC	Transport of potassium into the cell (By similarity).
P	Q9I540	DE	Probable potassium transport system protein kup;
P	Q9I540	DR	potassium ion transmembrane transporter activity
P	Q9I6L0	CC	Part of the ABC transporter complex CysAWTP involved in sulfate/thiosulfate import
P	Q9I6L0	DE	Sulfate-transporting ATPase;
P	Q9I6L0	DE	Sulfate/thiosulfate import ATP-binding protein CysA;
P	Q9I6L0	DR	sulfate transmembrane-transporting ATPase activity
P	Q9I6Q3	CC	Transporter for 4-hydroxybenzoate
P	Q9I6Q3	DE	4-hydroxybenzoate transporter;
P	Q9I6T2	CC	Part of the ABC transporter complex PotABCD involved in spermidine/putrescine import
P	Q9I6T2	CC	Part of the ABC transporter complex PotABCD involved in spermidine/putrescine import
P	Q9I6T2	DE	Spermidine/putrescine import ATP-binding protein PotA 1;
P	Q9I6T2	DE	Spermidine/putrescine import ATP-binding protein PotA 1;
P	Q9JIF3	CC	Binds cytochalasin B in a glucose-inhibitable manner
P	Q9JIF3	CC	Insulin-regulated facilitative glucose transporter
P	Q9JIF3	DE	Glucose transporter type 8;
P	Q9JIF3	DE	Glucose transporter type X1;
P	Q9JIF3	DE	Solute carrier family 2, facilitated glucose transporter member 8;
P	Q9JIF3	DR	glucose transmembrane transporter activity
P	Q9JIM1	DE	Equilibrative NBMPR-sensitive nucleoside transporter;
P	Q9JIM1	DE	Equilibrative nitrobenzylmercaptopurine riboside-sensitive nucleoside transporter;
P	Q9JIM1	DE	Equilibrative nucleoside transporter 1;
P	Q9JIM1	DE	Nucleoside transporter, es-type;
P	Q9JIM1	DR	nucleoside transmembrane transporter activity
P	Q9JIS8	CC	Mediates electroneutral potassium-chloride cotransport when activated by cell swelling
P	Q9JIS8	DE	Electroneutral potassium-chloride cotransporter 1;
P	Q9JIS8	DR	cation:chloride symporter activity
P	Q9JJC8	CC	Acts as a selective Mg2+ transporter.
P	Q9JJC8	DE	Magnesium transporter NIPA2;

P	Q9JJV9	CC	Assuming opened or closed conformations in response to the voltage difference across the membrane, the protein forms a sodium-selective channel through which Na+ ions may pass in accordance with their electrochemical gradient
P	Q9JJV9	CC	It is a tetrodotoxin-resistant Na+ channel isoform.
P	Q9JJV9	CC	This protein mediates the voltage-dependent sodium ion permeability of excitable membranes
P	Q9JJV9	DE	Sodium channel protein cardiac muscle subunit alpha;
P	Q9JJV9	DE	Sodium channel protein type 5 subunit alpha;
P	Q9JJV9	DE	Sodium channel protein type V subunit alpha;
P	Q9JJV9	DE	Voltage-gated sodium channel subunit alpha Nav1.5;
P	Q9JJV9	DR	voltage-gated sodium channel activity
P	Q9JK45	CC	Associates with KCNQ3 to form a potassium channel which contributes to M-type current, a slowly activating and deactivating potassium conductance which plays a critical role in determining the subthreshold electrical excitability of neurons
P	Q9JK45	CC	May contribute, with other potassium channels, to the molecular diversity of an heterogeneous population of M-channels, varying in kinetic and pharmacological properties, which underlie this physiologically important current (By similarity).
P	Q9JK45	DE	Potassium channel subunit alpha KvLQT5;
P	Q9JK45	DE	Potassium voltage-gated channel subfamily KQT member 5;
P	Q9JK45	DE	Voltage-gated potassium channel subunit Kv7.5;
P	Q9JK45	DR	voltage-gated potassium channel activity
P	Q9JK97	CC	May underlie a potassium current involved in regulating the excitability of sensory cells of the cochlea.
P	Q9JK97	DE	Potassium channel subunit alpha KvLQT4;
P	Q9JK97	DE	Potassium voltage-gated channel subfamily KQT member 4;
P	Q9JK97	DE	Voltage-gated potassium channel subunit Kv7.4;
P	Q9JK97	DR	voltage-gated potassium channel activity
P	Q9JKN1	CC	Partly regulates cellular zinc homeostasis
P	Q9JKN1	CC	Required with ZNT5 for the activation of zinc- requiring enzymes, alkaline phosphatases (ALPs)
P	Q9JKN1	CC	Seems to facilitate zinc transport from the cytoplasm into the Golgi apparatus
P	Q9JKN1	CC	Transports zinc into the lumens of the Golgi apparatus and the vesicular compartments where ALPs locate, thus, converting apoALPs to holoALPs
P	Q9JKN1	DE	Zinc transporter 7;
P	Q9JKN1	DR	zinc ion transmembrane transporter activity
P	Q9JLF1	CC	GABA, the major inhibitory neurotransmitter in the vertebrate brain, mediates neuronal inhibition by binding to the GABA/benzodiazepine receptor and opening an integral chloride channel.
P	Q9JLF1	DR	chloride channel activity
P	Q9JM63	CC	Inward rectifier potassium channels are characterized by a greater tendency to allow potassium to flow into the cell rather than out of it
P	Q9JM63	CC	May be responsible for potassium buffering action of glial cells in the brain
P	Q9JM63	CC	Their voltage dependence is regulated by the concentration of extracellular potassium; as external potassium is raised, the voltage range of the channel opening shifts to more positive voltages
P	Q9JM63	DE	ATP-sensitive inward rectifier potassium channel 10;
P	Q9JM63	DE	Inward rectifier K+ channel Kir4.1;
P	Q9JM63	DE	Potassium channel, inwardly rectifying subfamily J member 10;
P	Q9JM63	DR	inward rectifier potassium channel activity
P	Q9JMA9	CC	Mediates the uptake of a broad range of neutral and cationic amino acids (with the exception of proline) in a Na+/Cl(-)-dependent manner.
P	Q9JMA9	DE	Sodium- and chloride-dependent neutral and basic amino acid transporter B(0+);
P	Q9JMA9	DR	neurotransmitter:sodium symporter activity
P	Q9JXB8	CC	Involved in the import of serine and threonine into the cell, with the concomitant import of sodium (symport system) (By similarity).
P	Q9JXB8	DE	Na+/serine-threonine symporter;
P	Q9JXB8	DR	sodium:dicarboxylate symporter activity
P	Q9JXB8	DR	sodium:dicarboxylate symporter activity
P	Q9JZW0	CC	Part of the ABC transporter complex CysAWTP involved in sulfate/thiosulfate import
P	Q9JZW0	DE	Sulfate-transporting ATPase;
P	Q9JZW0	DE	Sulfate/thiosulfate import ATP-binding protein CysA;
P	Q9JZW0	DR	sulfate transmembrane-transporting ATPase activity
P	Q9K5Z9	CC	May play a role in L-lactate transport.
P	Q9K5Z9	DE	L-lactate permease;
P	Q9K5Z9	DR	lactate transmembrane transporter activity

P	Q9K678	CC	This system is involved in mannitol transport (By similarity).
P	Q9K678	DE	Mannitol permease IIC component;
P	Q9K678	DE	Mannitol-specific phosphotransferase enzyme IIB component;
P	Q9K678	DE	PTS system mannitol-specific EIIB component;
P	Q9K678	DE	PTS system mannitol-specific EIIC component;
P	Q9K678	DE	PTS system mannitol-specific EIICB component;
P	Q9K680	CC	This system is involved in mannitol transport (By similarity).
P	Q9K680	DE	Mannitol-specific phosphotransferase enzyme IIA component;
P	Q9K680	DE	PTS system mannitol-specific EIIA component;
P	Q9K6J9	CC	Part of the ABC transporter complex RbsABCD involved in ribose import
P	Q9K6J9	DE	Ribose import ATP-binding protein RbsA;
P	Q9K789	CC	Part of the ABC transporter complex MetNIQ involved in methionine import
P	Q9K789	DE	Methionine import ATP-binding protein MetN;
P	Q9K876	CC	Part of the ABC transporter complex CysAWTP involved in sulfate/thiosulfate import
P	Q9K876	DE	Sulfate-transporting ATPase;
P	Q9K876	DE	Sulfate/thiosulfate import ATP-binding protein CysA;
P	Q9K876	DR	sulfate transmembrane-transporting ATPase activity
P	Q9K8L5	CC	Part of the ABC transporter complex PstSACB involved in phosphate import
P	Q9K8L5	DE	ABC phosphate transporter;
P	Q9K8L5	DE	Phosphate import ATP-binding protein PstB;
P	Q9K8L5	DE	Phosphate-transporting ATPase;
P	Q9K8L5	DR	inorganic phosphate transmembrane transporter activity
P	Q9K8L5	DR	phosphate transmembrane-transporting ATPase activity
P	Q9K8N1	DR	phosphonate transmembrane-transporting ATPase activity
P	Q9KCQ4	CC	This system is involved in glucose transport (By similarity).
P	Q9KCQ4	DE	Glucose-specific phosphotransferase enzyme IIA component;
P	Q9KCQ4	DE	PTS system glucose-specific EIIA component;
P	Q9KD29	CC	This protein is probably a component of a manganese permease, a binding protein-dependent, ATP-driven transport system (By similarity).
P	Q9KD29	DE	Manganese transport system membrane protein mntC;
P	Q9KD30	CC	This protein is probably a component of a manganese permease, a binding protein-dependent, ATP-driven transport system
P	Q9KD30	DE	Manganese transport system ATP-binding protein MntB;
P	Q9KEE7	CC	Part of the binding-protein-dependent transport system for L-arabinose (By similarity)
P	Q9KEE9	CC	Part of the binding-protein-dependent transport system for L-arabinose
P	Q9KEE9	DE	L-arabinose transport system permease protein AraQ;
P	Q9KEF0	CC	Part of the binding-protein-dependent transport system for L-arabinose
P	Q9KEF0	DE	L-arabinose transport system permease protein AraP;
P	Q9KFG3	CC	This protein is probably a component of a manganese permease, a binding protein-dependent, ATP-driven transport system (By similarity).
P	Q9KFG3	DE	Manganese-binding lipoprotein mntA;
P	Q9KFL0	DR	phosphonate transmembrane-transporting ATPase activity
P	Q9KFN9	DR	phosphonate transmembrane-transporting ATPase activity
P	Q9KIJ3	CC	Part of an ATP-driven transport system for a metal; probably for manganese
P	Q9KJ75	CC	This system is involved in mannitol transport (By similarity).
P	Q9KJ75	DE	Mannitol permease IIC component;
P	Q9KJ75	DE	Mannitol-specific phosphotransferase enzyme IIB component;
P	Q9KJ75	DE	PTS system mannitol-specific EIIB component;
P	Q9KJ75	DE	PTS system mannitol-specific EIIC component;
P	Q9KJ75	DE	PTS system mannitol-specific EIICB component;
P	Q9KKQ7	CC	This system is involved in mannitol transport (By similarity).
P	Q9KKQ7	DE	Mannitol permease IIC component;
P	Q9KKQ7	DE	Mannitol-specific phosphotransferase enzyme IIA component;
P	Q9KKQ7	DE	Mannitol-specific phosphotransferase enzyme IIB component;
P	Q9KKQ7	DE	PTS system mannitol-specific EIIA component;
P	Q9KKQ7	DE	PTS system mannitol-specific EIIB component;
P	Q9KKQ7	DE	PTS system mannitol-specific EIIC component;
P	Q9KKQ7	DE	PTS system mannitol-specific EIICBA component;
P	Q9KL04	CC	Part of the ABC transporter complex MalEFGK involved in maltose/maltodextrin import
P	Q9KL04	DE	Maltose/maltodextrin import ATP-binding protein MalK;
P	Q9KL04	DR	maltose-transporting ATPase activity
P	Q9KL06	DE	Maltose transport system permease protein malF;
P	Q9KL07	DE	Maltose transport system permease protein malG;
P	Q9KL34	CC	Part of the ABC transporter complex HmuTUV involved in hemin import
P	Q9KL34	DE	Hemin import ATP-binding protein HmuV;

P	Q9KLL9	CC	Part of the ABC transporter complex ModABC involved in molybdenum import
P	Q9KLL9	DE	Molybdenum import ATP-binding protein ModC;
P	Q9KLL9	DR	molybdenum ion transmembrane transporter activity
P	Q9KM59	CC	Transport of potassium into the cell (By similarity).
P	Q9KM59	DE	Probable potassium transport system protein kup;
P	Q9KM59	DR	potassium ion transmembrane transporter activity
P	Q9KM62	CC	Functions as antiport system and exchanges two chloride ions for 1 proton
P	Q9KM62	CC	Proton-coupled chloride transporter
P	Q9KM62	DR	voltage-gated chloride channel activity
P	Q9KN37	CC	Part of the ABC transporter complex RbsABCD involved in ribose import
P	Q9KN37	DE	Ribose import ATP-binding protein RbsA;
P	Q9KN92	CC	Part of the ABC transporter complex PstSACB involved in phosphate import
P	Q9KN92	DE	ABC phosphate transporter 2;
P	Q9KN92	DE	Phosphate import ATP-binding protein PstB 2;
P	Q9KN92	DE	Phosphate-transporting ATPase 2;
P	Q9KN92	DR	inorganic phosphate transmembrane transporter activity
P	Q9KN92	DR	phosphate transmembrane-transporting ATPase activity
P	Q9KNC9	CC	Involved in the import of serine and threonine into the cell, with the concomitant import of sodium (symport system) (By similarity).
P	Q9KNC9	DE	Na+/serine-threonine symporter;
P	Q9KNC9	DR	sodium:dicarboxylate symporter activity
P	Q9KNC9	DR	sodium:dicarboxylate symporter activity
P	Q9KP42	CC	Part of the ABC transporter complex ThiBPQ involved in thiamine import
P	Q9KP42	DE	Thiamine import ATP-binding protein ThiQ;
P	Q9KPZ7	CC	Involved in copper export (By similarity).
P	Q9KPZ7	DE	Copper-exporting P-type ATPase A;
P	Q9KPZ7	DR	copper-exporting ATPase activity
P	Q9KQB8	CC	Part of the ABC transporter complex ZnuABC involved in zinc import
P	Q9KQB8	DE	Zinc import ATP-binding protein ZnuC;
P	Q9KQB8	DR	zinc transporting ATPase activity
P	Q9KQE3	CC	Part of the ABC transporter complex CcmAB involved in the biogenesis of c-type cytochromes; once thought to export heme, this seems not to be the case, but its exact role is uncertain
P	Q9KQE3	DE	Heme exporter protein A;
P	Q9KQE3	DR	heme-transporting ATPase activity
P	Q9KQU7	CC	Na+/H+ antiporter that extrudes sodium in exchange for external protons.
P	Q9KQU7	DE	Na+/H+ antiporter nhaB;
P	Q9KQU7	DE	Sodium/proton antiporter nhaB;
P	Q9KQU7	DR	sodium:hydrogen antiporter activity
P	Q9KRT4	DR	glycerol-3-phosphate-transporting ATPase activity
P	Q9KS33	CC	Part of the ABC transporter complex PotABCD involved in spermidine/putrescine import
P	Q9KS33	CC	Part of the ABC transporter complex PotABCD involved in spermidine/putrescine import
P	Q9KS33	DE	Spermidine/putrescine import ATP-binding protein PotA;
P	Q9KS33	DE	Spermidine/putrescine import ATP-binding protein PotA;
P	Q9KSL1	CC	Part of the ABC transporter complex BtuCDF involved in vitamin B12 import
P	Q9KSL1	DE	Vitamin B12 import ATP-binding protein BtuD;
P	Q9KSL1	DE	Vitamin B12-transporting ATPase;
P	Q9KSL2	CC	Part of the ABC transporter complex BtuCDF involved in vitamin B12 import
P	Q9KSL2	DE	Vitamin B12 import system permease protein BtuC;
P	Q9KU04	CC	Part of the ABC transporter complex PstSACB involved in phosphate import
P	Q9KU04	DE	ABC phosphate transporter 1;
P	Q9KU04	DE	Phosphate import ATP-binding protein PstB 1;
P	Q9KU04	DE	Phosphate-transporting ATPase 1;
P	Q9KU04	DR	inorganic phosphate transmembrane transporter activity
P	Q9KU04	DR	phosphate transmembrane-transporting ATPase activity
P	Q9KUI0	CC	Part of the ABC transporter complex CysAWTP involved in sulfate/thiosulfate import
P	Q9KUI0	DE	Sulfate-transporting ATPase;
P	Q9KUI0	DE	Sulfate/thiosulfate import ATP-binding protein CysA;
P	Q9KUI0	DR	sulfate transmembrane-transporting ATPase activity
P	Q9KZW2	CC	Part of the ABC transporter complex PstSACB involved in phosphate import
P	Q9KZW2	DE	ABC phosphate transporter;
P	Q9KZW2	DE	Phosphate import ATP-binding protein PstB;
P	Q9KZW2	DE	Phosphate-transporting ATPase;
P	Q9KZW2	DR	inorganic phosphate transmembrane transporter activity
P	Q9KZW2	DR	phosphate transmembrane-transporting ATPase activity

P	Q9L1C3	CC	Part of the ABC transporter complex MetNIQ involved in methionine import
P	Q9L1C3	DE	Methionine import ATP-binding protein MetN;
P	Q9L6L2	CC	Interacts with trk system potassium uptake protein trkA and requires trkE for transport activity (By similarity).
P	Q9L6L2	CC	Low-affinity potassium transport system
P	Q9L6L2	DE	Trk system potassium uptake protein trkH;
P	Q9L772	CC	Transport of water across the membrane
P	Q9LD18	CC	High-affinity potassium transporter.
P	Q9LD18	DE	Potassium transporter 4;
P	Q9LD18	DR	potassium ion transmembrane transporter activity
P	Q9LF50	CC	Essential for the conversion of starch to sucrose in leaves at night, probably via the export of maltose from the chloroplast
P	Q9LF50	CC	Probable maltose transporter
P	Q9LF50	DE	Maltose excess protein 1, chloroplastic;
P	Q9LF50	DR	maltose transmembrane transporter activity
P	Q9LF79	CC	This magnesium-dependent enzyme catalyzes the hydrolysis of ATP coupled with the translocation of calcium from the cytosol out of the cell.
P	Q9LF79	DE	Ca2+-ATPase isoform 8;
P	Q9LF79	DE	Calcium-transporting ATPase 8, plasma membrane-type;
P	Q9LF79	DR	calcium-transporting ATPase activity
P	Q9LFB8	CC	Peptide transporter
P	Q9LFB8	DE	Peptide transporter PTR5;
P	Q9LFZ8	CC	Translocates Ca2+ and other metal ions into vacuoles using the proton gradient formed by H+-ATPase and H+-pyrophosphatase (By similarity).
P	Q9LFZ8	DE	Ca2+/H+ antiporter CAX6;
P	Q9LFZ8	DE	Ca2+/H+ exchanger 6;
P	Q9LIK7	CC	This magnesium-dependent enzyme catalyzes the hydrolysis of ATP coupled with the translocation of calcium from the cytosol out of the cell or into organelles (By similarity).
P	Q9LIK7	DE	Ca2+-ATPase isoform 13;
P	Q9LIK7	DE	Putative calcium-transporting ATPase 13, plasma membrane-type;
P	Q9LIK7	DR	calcium-transporting ATPase activity
P	Q9LJI2	DE	Cation/calcium exchanger 3;
P	Q9LJI2	DE	Protein CATION CALCIUM EXCHANGER 3;
P	Q9LJI2	DR	sodium ion transmembrane transporter activity
P	Q9LJN2	CC	Magnesium transporter that may mediate the influx of magnesium (By similarity).
P	Q9LJN2	DE	Magnesium Transporter 4;
P	Q9LJN2	DE	Magnesium transporter MRS2-3;
P	Q9LJN2	DR	magnesium ion transmembrane transporter activity
P	Q9LK64	CC	Mediates the transport of glutathione conjugates such as chlorodinitrobenzene-GS (DNB-GS), and of chlorophyll catabolites such as Bn-NCC-1
P	Q9LK64	CC	Pump for glutathione S-conjugates
P	Q9LK64	DE	ATP-energized glutathione S-conjugate pump 3;
P	Q9LK64	DE	Glutathione S-conjugate-transporting ATPase 3;
P	Q9LK64	DR	glutathione S-conjugate-exporting ATPase activity
P	Q9LKW9	CC	Involved in Na+ and K+ homeostasis
P	Q9LKW9	CC	Regulates Na+ content of the xylem sap.
P	Q9LKW9	CC	Required for cytoplasmic Na+ and Li+ detoxification by secreting them from the cytoplasm to the extracellular space
P	Q9LKW9	DE	Na+/H+ exchanger 7;
P	Q9LKW9	DE	Sodium/hydrogen exchanger 7;
P	Q9LNV3	CC	Can transport glucose, 3-O-methylglucose, xylose, mannose, fructose and galactose
P	Q9LNV3	DE	Hexose transporter 2;
P	Q9LT15	CC	Mediates an active uptake of hexoses, probably by sugar/hydrogen symport (By similarity).
P	Q9LT15	DE	Sugar transport protein 10;
P	Q9LTH9	CC	Mediates zinc uptake
P	Q9LTH9	DE	Zinc transporter 2;
P	Q9LU41	CC	This magnesium-dependent enzyme catalyzes the hydrolysis of ATP coupled with the translocation of calcium from the cytosol out of the cell or into organelles (By similarity).
P	Q9LU41	DE	Ca2+-ATPase isoform 9;
P	Q9LU41	DE	Calcium-transporting ATPase 9, plasma membrane-type;
P	Q9LU41	DR	calcium-transporting ATPase activity
P	Q9LW86	CC	H+/sulfate cotransporter that may play a role in the regulation of sulfate assimilation (By similarity).
P	Q9LW86	DE	Probable sulfate transporter 3.4;

P	Q9LW86	DR	secondary active sulfate transmembrane transporter activity
P	Q9LWR0	CC	Aquaporins facilitate the transport of water and small neutral solutes across cell membranes
P	Q9LWR2	CC	Aquaporins facilitate the transport of water and small neutral solutes across cell membranes
P	Q9LXD4	CC	Putative magnesium transporter.
P	Q9LXD4	DE	Putative magnesium transporter MRS2-9;
P	Q9LXS1	CC	Involved in sequestration of excess zinc in the cytoplasm into vacuoles to maintain zinc homeostasis.
P	Q9LY77	CC	This magnesium-dependent enzyme catalyzes the hydrolysis of ATP coupled with the translocation of calcium from the cytosol out of the cell or into organelles (By similarity)
P	Q9LY77	DE	Ca2+-ATPase isoform 12;
P	Q9LY77	DE	Calcium-transporting ATPase 12, plasma membrane-type;
P	Q9LY77	DR	calcium-transporting ATPase activity
P	Q9M1K3	CC	Water channel required to facilitate the transport of water across cell membrane
P	Q9M271	CC	Involved in sequestration of excess zinc in the cytoplasm into vacuoles to maintain zinc homeostasis (By similarity).
P	Q9M2L4	CC	This magnesium-dependent enzyme catalyzes the hydrolysis of ATP coupled with the translocation of calcium from the cytosol out of the cell or into organelles (By similarity)
P	Q9M2L4	DE	Ca2+-ATPase isoform 11;
P	Q9M2L4	DE	Putative calcium-transporting ATPase 11, plasma membrane-type;
P	Q9M2L4	DR	calcium-transporting ATPase activity
P	Q9M390	CC	Peptide transporter
P	Q9M390	DE	Peptide transporter PTR1;
P	Q9M7J9	CC	Probable potassium transporter.
P	Q9M7J9	DE	Potassium transporter 8;
P	Q9M7J9	DR	potassium ion transmembrane transporter activity
P	Q9M7K4	CC	High-affinity potassium transporter
P	Q9M7K4	DE	Potassium transporter 5;
P	Q9M7K4	DR	potassium:sodium symporter activity
P	Q9M8S6	CC	Highly selective outward-rectifying potassium channel
P	Q9M8S6	CC	Involved in potassium release into the xylem sap toward the shoots
P	Q9M8S6	DE	Potassium channel SKOR;
P	Q9M8S6	DE	Stelar K+ outward rectifying channel;
P	Q9M8S6	DR	voltage-gated potassium channel activity
P	Q9M8W5	CC	Water channel required to facilitate the transport of water across cell membrane.
P	Q9M8W5	DR	water channel activity
P	Q9MAX3	CC	High-affinity H+/sulfate cotransporter that mediates the uptake of the environmental sulfate by plant roots
P	Q9MAX3	CC	Plays a central role in the regulation of sulfate assimilation.
P	Q9MAX3	DE	Sulfate transporter 1.2;
P	Q9MAX3	DR	secondary active sulfate transmembrane transporter activity
P	Q9N694	DE	Ca2+-ATPase;
P	Q9N694	DR	calcium-transporting ATPase activity
P	Q9NIV0	CC	May be a carbon dioxide/bicarbonate transporter (By similarity).
P	Q9NP91	DE	Sodium- and chloride-dependent transporter XTRP3;
P	Q9NP91	DE	Sodium/imino-acid transporter 1;
P	Q9NP91	DR	neurotransmitter:sodium symporter activity
P	Q9NP94	CC	Mediates zinc uptake
P	Q9NP94	CC	Zinc uptake may be mediated by a Zn(2+)-HCO(3)(-) symport mechanism and can function in the presence of albumin
P	Q9NP94	DE	Zinc transporter ZIP2;
P	Q9NP94	DR	zinc ion transmembrane transporter activity
P	Q9NPC2	CC	pH-dependent, voltage-insensitive, background potassium channel protein.
P	Q9NPC2	DE	Acid-sensitive potassium channel protein TASK-3;
P	Q9NPC2	DE	Potassium channel subfamily K member 9;
P	Q9NPC2	DE	TWIK-related acid-sensitive K+ channel 3;
P	Q9NPC2	DE	Two pore K+ channel KT3.2;
P	Q9NPC2	DE	Two pore potassium channel KT3.2;
P	Q9NPC2	DR	potassium channel activity
P	Q9NPI9	CC	Inward rectifier potassium channels are characterized by a greater tendency to allow potassium to flow into the cell rather than out of it
P	Q9NPI9	CC	Their voltage dependence is regulated by the concentration of extracellular potassium; as external potassium is raised, the voltage range of the channel opening shifts to more positive voltages

P	Q9NPI9	DE	Inward rectifier K+ channel Kir5.1;
P	Q9NPI9	DE	Inward rectifier potassium channel 16;
P	Q9NPI9	DE	Potassium channel, inwardly rectifying subfamily J member 16;
P	Q9NPI9	DR	inward rectifier potassium channel activity
P	Q9NQA5	CC	A Ca2+- dependent feedback regulation includes fast channel inactivation and slow current decay
P	Q9NQA5	CC	Constitutively active calcium selective cation channel thought to be involved in Ca2+ reabsorption in kidney and intestine
P	Q9NQA5	CC	The channel is activated by low internal calcium level and the current exhibits an inward rectification
P	Q9NQA5	DE	Calcium transport protein 2;
P	Q9NQA5	DE	Epithelial calcium channel 1;
P	Q9NQA5	DR	calcium channel activity
P	Q9NR82	CC	Associates with KCNQ3 to form a potassium channel which contributes to M-type current, a slowly activating and deactivating potassium conductance which plays a critical role in determining the subthreshold electrical excitability of neurons
P	Q9NR82	CC	May contribute, with other potassium channels, to the molecular diversity of an heterogeneous population of M-channels, varying in kinetic and pharmacological properties, which underlie this physiologically important current
P	Q9NR82	DE	Potassium channel subunit alpha KvLQT5;
P	Q9NR82	DE	Potassium voltage-gated channel subfamily KQT member 5;
P	Q9NR82	DE	Voltage-gated potassium channel subunit Kv7.5;
P	Q9NR82	DR	inward rectifier potassium channel activity
P	Q9NRA2	DE	Sodium/sialic acid cotransporter;
P	Q9NRM0	CC	Also transports glucose at low rate.
P	Q9NRM0	DE	Glucose transporter type 9;
P	Q9NRM0	DE	Solute carrier family 2, facilitated glucose transporter member 9;
P	Q9NS40	CC	Pore-forming (alpha) subunit of voltage-gated potassium channel
P	Q9NS40	DE	Ether-a-go-go-related gene potassium channel 3;
P	Q9NS40	DE	Potassium voltage-gated channel subfamily H member 7;
P	Q9NS40	DE	Voltage-gated potassium channel subunit Kv11.3;
P	Q9NSA2	CC	Pore-forming (alpha) subunit of voltage-gated rapidly inactivating A-type potassium channels
P	Q9NSA2	DE	Potassium voltage-gated channel subfamily D member 1;
P	Q9NSA2	DE	Voltage-gated potassium channel subunit Kv4.1;
P	Q9NSA2	DR	voltage-gated potassium channel activity
P	Q9NSD5	CC	Terminates the action of GABA by its high affinity sodium-dependent reuptake into presynaptic terminals.
P	Q9NSD5	CC	Terminates the action of GABA by its high affinity sodium-dependent reuptake into presynaptic terminals.
P	Q9NSD5	DE	Sodium- and chloride-dependent GABA transporter 2;
P	Q9NSD5	DE	Sodium- and chloride-dependent GABA transporter 2;
P	Q9NSD5	DR	gamma-aminobutyric acid:sodium symporter activity
P	Q9NSD5	DR	gamma-aminobutyric acid:sodium symporter activity
P	Q9NSD5	DR	neurotransmitter:sodium symporter activity
P	Q9NUM3	DE	Zinc transporter ZIP9;
P	Q9NY26	CC	May function as a major endogenous zinc uptake transporter in many cells of the body
P	Q9NY26	CC	Mediates zinc uptake
P	Q9NY26	CC	Responsible for the rapid uptake and accumulation of physiologically effective zinc in prostate cells.
P	Q9NY26	DE	Zinc transporter ZIP1;
P	Q9NY26	DE	Zinc-iron-regulated transporter-like;
P	Q9NY26	DR	zinc ion transmembrane transporter activity
P	Q9NY37	CC	The activated channel exhibits selectivity for sodium, and is inhibited by amiloride
P	Q9NY37	DE	Human intestine Na+ channel;
P	Q9NY64	CC	Binds cytochalasin B in a glucose-inhibitable manner
P	Q9NY64	CC	Insulin-regulated facilitative glucose transporter
P	Q9NY64	DE	Glucose transporter type 8;
P	Q9NY64	DE	Glucose transporter type X1;
P	Q9NY64	DE	Solute carrier family 2, facilitated glucose transporter member 8;
P	Q9NY64	DR	D-glucose transmembrane transporter activity
P	Q9NY91	CC	Sodium-dependent glucose transporter (By similarity).
P	Q9NY91	CC	Sodium-dependent glucose transporter (By similarity).
P	Q9NY91	DE	Low affinity sodium-glucose cotransporter;
P	Q9NY91	DE	Na+/glucose cotransporter 3;

P	Q9NY91	DE	Na+/glucose cotransporter 3;
P	Q9NY91	DE	Sodium/glucose cotransporter 3;
P	Q9NY91	DE	Sodium/glucose cotransporter 3;
P	Q9NYG8	CC	Outward rectification is reversed at high external K+ concentrations (By similarity)
P	Q9NYG8	CC	Voltage insensitive, instantaneous, outwardly rectifying potassium channel
P	Q9NYG8	DE	Potassium channel subfamily K member 4;
P	Q9NYG8	DE	TWIK-related arachidonic acid-stimulated potassium channel protein;
P	Q9NYG8	DE	Two pore K+ channel KT4.1;
P	Q9NYG8	DE	Two pore potassium channel KT4.1;
P	Q9NYG8	DR	potassium channel activity
P	Q9NZA1	CC	Can insert into membranes and form poorly selective ion channels that may also transport chloride ions
P	Q9NZA1	DE	Chloride intracellular channel protein 5;
P	Q9NZA1	DR	voltage-gated chloride channel activity
P	Q9NZV8	CC	Pore-forming (alpha) subunit of voltage-gated rapidly inactivating A-type potassium channels
P	Q9NZV8	DE	Potassium voltage-gated channel subfamily D member 2;
P	Q9NZV8	DE	Voltage-gated potassium channel subunit Kv4.2;
P	Q9P1Z3	CC	Putative hyperpolarization-activated ion channel exhibiting weak selectivity for potassium over sodium ions (By similarity).
P	Q9P1Z3	DE	Potassium/sodium hyperpolarization-activated cyclic nucleotide-gated channel 3;
P	Q9P1Z3	DE	Potassium/sodium hyperpolarization-activated cyclic nucleotide-gated channel 3;
P	Q9P1Z3	DR	sodium channel activity
P	Q9P1Z3	DR	voltage-gated potassium channel activity
P	Q9P2U7	CC	Mediates the uptake of glutamate into synaptic vesicles at presynaptic nerve terminals of excitatory neural cells
P	Q9P2U7	DE	Vesicular glutamate transporter 1;
P	Q9P2U7	DR	L-glutamate transmembrane transporter activity
P	Q9P2U8	CC	Mediates the uptake of glutamate into synaptic vesicles at presynaptic nerve terminals of excitatory neural cells
P	Q9P2U8	DE	Vesicular glutamate transporter 2;
P	Q9P2U8	DR	L-glutamate transmembrane transporter activity
P	Q9P380	DE	Peptide permease ptr2;
P	Q9P380	DE	Probable peptide transporter ptr2;
P	Q9P3U6	CC	High-affinity glucose transporter.
P	Q9P3U6	DE	High-affinity glucose transporter ght1;
P	Q9P7F9	CC	Required for high affinity copper (probably reduced Cu I) transport into the cell.
P	Q9P7F9	DE	Copper transport protein ctr5;
P	Q9P7F9	DE	Copper transporter 5;
P	Q9P7F9	DR	copper ion transmembrane transporter activity
P	Q9P7I1	CC	Potassium-proton antiport (By similarity).
P	Q9P7I1	DE	K+/H+ antiporter 1;
P	Q9PAP0	CC	Part of the ABC transporter complex CcmAB involved in the biogenesis of c-type cytochromes; once thought to export heme, this seems not to be the case, but its exact role is uncertain
P	Q9PAP0	DE	Heme exporter protein A;
P	Q9PAP0	DR	heme-transporting ATPase activity
P	Q9PBK0	CC	Part of the ABC transporter complex PstSACB involved in phosphate import
P	Q9PBK0	DE	ABC phosphate transporter;
P	Q9PBK0	DE	Phosphate import ATP-binding protein PstB;
P	Q9PBK0	DE	Phosphate-transporting ATPase;
P	Q9PBK0	DR	inorganic phosphate transmembrane transporter activity
P	Q9PBK0	DR	phosphate transmembrane-transporting ATPase activity
P	Q9PBK1	CC	Part of a binding-protein-dependent transport system for phosphate; probably responsible for the translocation of the substrate across the membrane (By similarity)
P	Q9PBK1	DE	Phosphate transport system permease protein pstA;
P	Q9PBK1	DR	inorganic phosphate transmembrane transporter activity
P	Q9PBK2	CC	Part of a binding-protein-dependent transport system for phosphate; probably responsible for the translocation of the substrate across the membrane (By similarity)
P	Q9PBK2	DE	Phosphate transport system permease protein pstC;
P	Q9PBK2	DR	inorganic phosphate transmembrane transporter activity
P	Q9PBK3	CC	Part of the ABC transporter complex pstSACB involved in phosphate import (By similarity).
P	Q9PBK3	DE	Phosphate-binding protein pstS;
P	Q9PBK3	DR	inorganic phosphate transmembrane transporter activity

P	Q9PC78	CC	Transport of potassium into the cell (By similarity).
P	Q9PC78	DE	Probable potassium transport system protein kup;
P	Q9PC78	DR	potassium ion transmembrane transporter activity
P	Q9PDN2	CC	Part of the ABC transporter complex CysAWTP involved in sulfate/thiosulfate import
P	Q9PDN2	DE	Sulfate-transporting ATPase;
P	Q9PDN2	DE	Sulfate/thiosulfate import ATP-binding protein CysA;
P	Q9PDN2	DR	sulfate transmembrane-transporting ATPase activity
P	Q9PEL3	CC	H+-stimulated, highly selective, manganese uptake system (By similarity).
P	Q9PEL3	DE	Probable manganese transport protein mntH;
P	Q9PHQ1	CC	Part of the ABC transporter complex PstSACB involved in phosphate import
P	Q9PHQ1	DE	ABC phosphate transporter;
P	Q9PHQ1	DE	Phosphate import ATP-binding protein PstB;
P	Q9PHQ1	DE	Phosphate-transporting ATPase;
P	Q9PHQ1	DR	inorganic phosphate transmembrane transporter activity
P	Q9PHQ1	DR	phosphate transmembrane-transporting ATPase activity
P	Q9PJP6	DE	ADP,ATP carrier protein 2;
P	Q9PJP6	DE	ADP/ATP translocase 2;
P	Q9PJP6	DE	ADP/ATP translocase 2;
P	Q9PJP6	DR	ATP:ADP antiporter activity
P	Q9PJP6	DR	ATP:ADP antiporter activity
P	Q9PK20	CC	The arginine uptake by the bacterium in the macrophage may be a virulence factor against the host innate immune response (By similarity).
P	Q9PK20	DE	Arginine/agmatine antiporter;
P	Q9PKX5	DE	ADP,ATP carrier protein 1;
P	Q9PKX5	DE	ADP/ATP translocase 1;
P	Q9PKX5	DE	ADP/ATP translocase 1;
P	Q9PKX5	DR	ATP:ADP antiporter activity
P	Q9PKX5	DR	ATP:ADP antiporter activity
P	Q9PPI1	CC	Mediates influx of magnesium ions (By similarity).
P	Q9PPI1	DE	Magnesium transport protein CorA;
P	Q9PPI1	DR	cobalt ion transmembrane transporter activity
P	Q9PPI1	DR	magnesium ion transmembrane transporter activity
P	Q9PQU3	CC	Part of the ABC transporter complex PstSACB involved in phosphate import
P	Q9PQU3	DE	ABC phosphate transporter;
P	Q9PQU3	DE	Phosphate import ATP-binding protein PstB;
P	Q9PQU3	DE	Phosphate-transporting ATPase;
P	Q9PQU3	DR	inorganic phosphate transmembrane transporter activity
P	Q9PQU3	DR	phosphate transmembrane-transporting ATPase activity
P	Q9PR37	CC	Part of the ABC transporter complex PotABCD involved in spermidine/putrescine import
P	Q9PR37	CC	Part of the ABC transporter complex PotABCD involved in spermidine/putrescine import
P	Q9PR37	DE	Spermidine/putrescine import ATP-binding protein PotA;
P	Q9PR37	DE	Spermidine/putrescine import ATP-binding protein PotA;
P	Q9QX29	CC	Has also been shown to be calcium-selective
P	Q9QX29	CC	May also be activated by intracellular calcium store depletion.
P	Q9QX29	CC	Thought to form a receptor-activated non-selective calcium permeant cation channel
P	Q9QX29	DE	Capacitative calcium entry channel 2;
P	Q9QX29	DR	calcium channel activity
P	Q9QXX4	CC	Calcium-dependent mitochondrial aspartate and glutamate carrier
P	Q9QXX4	DE	Mitochondrial aspartate glutamate carrier 2;
P	Q9QXX4	DR	L-glutamate transmembrane transporter activity
P	Q9QYB1	CC	Can insert into membranes and form poorly selective ion channels that may also transport chloride ions
P	Q9QYB1	DE	Chloride intracellular channel protein 4;
P	Q9QYB1	DR	voltage-gated chloride channel activity
P	Q9QZD8	DE	Mitochondrial dicarboxylate carrier;
P	Q9R0A1	CC	Chloride channels have several functions including the regulation of cell volume; membrane potential stabilization, signal transduction and transepithelial transport
P	Q9R0A1	CC	Voltage-gated chloride channel
P	Q9R0A1	DE	Chloride channel protein 2;
P	Q9R0A1	DR	voltage-gated chloride channel activity
P	Q9R0K7	CC	This magnesium-dependent enzyme catalyzes the hydrolysis of ATP coupled with the transport of calcium out of the cell
P	Q9R0K7	DE	Plasma membrane calcium ATPase isoform 2;
P	Q9R0K7	DE	Plasma membrane calcium pump isoform 2;
P	Q9R0K7	DE	Plasma membrane calcium-transporting ATPase 2;

P	Q9R0K7	DR	calcium-dependent ATPase activity
P	Q9R0K7	DR	calcium-transporting ATPase activity
P	Q9R0Y1	CC	The activated channel exhibits selectivity for sodium and lithium, and is inhibited by amiloride (By similarity).
P	Q9R0Y1	DE	Brain-liver-intestine amiloride-sensitive Na+ channel;
P	Q9R155	CC	Sodium-independent transporter of chloride and iodide.
P	Q9R155	DE	Sodium-independent chloride/iodide transporter;
P	Q9R1N3	CC	Electroneutral sodium- and bicarbonate-dependent cotransporter with a Na+:HCO3(-) 1:1 stoichiometry
P	Q9R1N3	CC	Electroneutral sodium- and bicarbonate-dependent cotransporter with a Na+:HCO3(-) 1:1 stoichiometry
P	Q9R1N3	CC	May also have an associated sodium channel activity.
P	Q9R1N3	CC	Regulates intracellular pH and may play a role in bicarbonate salvage in secretory epithelia
P	Q9R244	CC	May also be activated by intracellular calcium store depletion.
P	Q9R244	CC	Thought to form a receptor-activated non-selective calcium permeant cation [illegible]
P	Q9R244	DR	calcium channel activity
P	Q9R244	DR	store-operated calcium channel activity
P	Q9R6U5	DE	Nitrate transporter;
P	Q9RDI1	CC	Part of the ABC transporter complex RbsABCD involved in ribose import
P	Q9RDI1	DE	Ribose import ATP-binding protein RbsA 2;
P	Q9RKQ4	CC	Part of the ABC transporter complex HmuTUV involved in hemin import
P	Q9RKQ4	DE	Hemin import ATP-binding protein HmuV;
P	Q9RPF2	CC	H+-stimulated, highly selective, manganese uptake system (By similarity).
P	Q9RPF2	DE	Manganese transport protein mntH2;
P	Q9RPF3	CC	H+-stimulated, highly selective, manganese uptake system (By similarity).
P	Q9RPF3	DE	Manganese transport protein mntH1;
P	Q9RPF4	CC	H+-stimulated, highly selective, manganese uptake system (By similarity).
P	Q9RPF4	DE	Manganese transport protein mntH;
P	Q9RPX0	CC	Involved in the high-affinity zinc uptake transport system.
P	Q9RPX0	DE	High-affinity zinc uptake system protein znuA;
P	Q9RTP8	CC	H+-stimulated, highly selective, manganese uptake system (By similarity).
P	Q9RTP8	DE	Probable manganese transport protein mntH;
P	Q9RZN6	CC	One of the components of the high-affinity ATP-driven potassium transport (or KDP) system, which catalyzes the hydrolysis of ATP coupled with the exchange of hydrogen and potassium ions
P	Q9RZN6	DE	Potassium-binding and translocating subunit C;
P	Q9RZN6	DE	Potassium-translocating ATPase C chain;
P	Q9RZN6	DE	Potassium-transporting ATPase C chain;
P	Q9RZN6	DR	potassium-transporting ATPase activity
P	Q9RZN7	CC	One of the components of the high-affinity ATP-driven potassium transport (or KDP) system, which catalyzes the hydrolysis of ATP coupled with the exchange of hydrogen and potassium ions (By similarity).
P	Q9RZN7	DE	Potassium-binding and translocating subunit A;
P	Q9RZN7	DE	Potassium-translocating ATPase A chain;
P	Q9RZN7	DE	Potassium-transporting ATPase A chain;
P	Q9RZN7	DR	potassium-transporting ATPase activity
P	Q9RZP0	CC	One of the components of the high-affinity ATP-driven potassium transport (or KDP) system, which catalyzes the hydrolysis of ATP coupled with the exchange of hydrogen and potassium ions (By similarity).
P	Q9RZP0	DE	Potassium-binding and translocating subunit B;
P	Q9RZP0	DE	Potassium-translocating ATPase B chain;
P	Q9RZP0	DE	Potassium-transporting ATPase B chain;
P	Q9RZP0	DR	potassium-transporting ATPase activity
P	Q9S1X8	CC	Na+/H+ antiporter that extrudes sodium in exchange for external protons (By [illegible]
P	Q9S1X8	DE	Na+/H+ antiporter nhaA 1/4;
P	Q9S1X8	DE	Sodium/proton antiporter nhaA 1;
P	Q9S2C8	CC	Na+/H+ antiporter that extrudes sodium in exchange for external protons (By [illegible]
P	Q9S2C8	DE	Na+/H+ antiporter nhaA 2;
P	Q9S2C8	DE	Sodium/proton antiporter nhaA 2;
P	Q9S3K0	CC	The physiological role may be the reduction of the intracellular concentration of toxic sugars or sugar metabolites
P	Q9S3K0	CC	Transports IPTG, lactose and arabinose.
P	Q9S3K0	DE	Sugar efflux transporter A;
P	Q9S3K0	DR	sugar efflux transmembrane transporter activity

P	Q9S6V3	DR	ATP:ADP antiporter activity
P	Q9S6V3	DR	ATP:ADP antiporter activity
P	Q9S6Z8	CC	Outward rectifying potassium channel
P	Q9S6Z8	DE	Probable calcium-activated outward-rectifying potassium channel 5, chloroplastic;
P	Q9S6Z8	DR	potassium channel activity
P	Q9S735	CC	High-affinity transporter for external inorganic phosphate (By similarity).
P	Q9S735	DE	Probable inorganic phosphate transporter 1-9;
P	Q9S7J8	CC	Acts by delivering copper to create functional hormone receptors.
P	Q9S7J8	CC	Essential for ethylene signaling, which requires copper
P	Q9S7J8	CC	Involved in copper import into the cell
P	Q9S7J8	DE	Copper-transporting ATPase RAN1;
P	Q9S7J8	DR	copper-exporting ATPase activity
P	Q9S9N4	CC	Magnesium transporter that may mediate the influx of magnesium.
P	Q9S9N4	DE	Magnesium Transporter 2;
P	Q9S9N4	DE	Magnesium transporter MRS2-1;
P	Q9S9N4	DR	magnesium ion transmembrane transporter activity
P	Q9S9N8	DE	Metal transporter Nramp6;
P	Q9SA05	CC	Putative potassium transporter.
P	Q9SA05	DE	Potassium transporter 10;
P	Q9SA05	DR	potassium ion transmembrane transporter activity
P	Q9SAH0	CC	High-affinity magnesium transporter that mediates the influx of magnesium
P	Q9SAH0	DE	Magnesium Transporter 1;
P	Q9SAH0	DE	Magnesium transporter MRS2-10;
P	Q9SAH0	DR	magnesium ion transmembrane transporter activity
P	Q9SAH8	DE	Metal transporter Nramp1;
P	Q9SAI4	CC	Very low water transport activity.
P	Q9SAY1	CC	High-affinity H+/sulfate cotransporter that mediates the uptake of the environmental sulfate by plant roots under low- sulfur conditions
P	Q9SAY1	CC	Plays a central role in the regulation of sulfate assimilation.
P	Q9SAY1	DE	High-affinity sulfate transporter 1;
P	Q9SAY1	DE	Sulfate transporter 1.1;
P	Q9SAY1	DR	secondary active sulfate transmembrane transporter activity
P	Q9SBA7	DE	Hexose transporter 8;
P	Q9SCW7	CC	Sugar transporter (Potential).
P	Q9SCW7	DE	Sugar transporter ERD6-like 1;
P	Q9SCW7	DE	Sugar transporter-like protein 4;
P	Q9SCX5	CC	Probable potassium channel
P	Q9SCX5	DE	Probable potassium channel AKT5;
P	Q9SCX5	DR	voltage-gated potassium channel activity
P	Q9SD00	DE	Sugar transporter MSSP3;
P	Q9SDI4	DE	Phosphate transporter 4;1;
P	Q9SFG0	CC	Mediates an active uptake of hexoses, probably by sugar/hydrogen symport
P	Q9SFG0	DE	Sugar transport protein 6;
P	Q9SH30	CC	Involved in copper import into the cell.
P	Q9SH30	DE	Putative copper-transporting ATPase 3;
P	Q9SH30	DR	copper-exporting ATPase activity
P	Q9SLG3	CC	Mediates zinc uptake from the rhizosphere
P	Q9SLG3	DE	Zinc transporter 3;
P	Q9SLG3	DR	zinc ion transmembrane transporter activity
P	Q9SN36	DE	Metal transporter Nramp5;
P	Q9SNV9	CC	Regulates metal accumulation under Fe starvation
P	Q9SNV9	CC	Vacuolar metal transporter involved in intracellular metal homeostasis
P	Q9SNV9	DE	Metal transporter Nramp3;
P	Q9SRK7	CC	Transports natural purines (adenine and guanine) as well as purine analogs
P	Q9SRK7	DE	Adenine/guanine permease AZG1;
P	Q9SRK7	DE	Adenine/guanine permease AZG1;
P	Q9SRK7	DR	purine base transmembrane transporter activity
P	Q9STX9	CC	Potential aquaporin, which may facilitate the transport of water and small neutral solutes across cell membranes (By similarity).
P	Q9STX9	DR	water channel activity
P	Q9SV13	CC	H+/sulfate cotransporter that may play a role in the regulation of sulfate assimilation
P	Q9SV13	DE	Sulfate transporter 3.1;
P	Q9SV13	DR	secondary active sulfate transmembrane transporter activity
P	Q9SV31	CC	Aquaporins facilitate the transport of water and small neutral solutes across cell membranes (By similarity).

P	Q9SV84	CC	Low water transport activity
P	Q9SV84	DR	water channel activity
P	Q9SVV6	CC	Calcium-activated outward rectifying potassium channel
P	Q9SVV6	DE	Probable calcium-activated outward-rectifying potassium channel 6;
P	Q9SVV6	DR	potassium channel activity
P	Q9SWR8	DE	Nitrate transporter;
P	Q9SX48	DE	Hexose transporter 9;
P	Q9SXF8	CC	Increases the capacity for root water uptake under water deficit
P	Q9SXF8	CC	Water channel required to facilitate the transport of water across cell membrane
P	Q9SXF8	DE	Water channel protein RWC3;
P	Q9SXS2	CC	H+/sulfate cotransporter that may play a role in the regulation of sulfate assimilation (By similarity).
P	Q9SXS2	DE	Probable sulfate transporter 3.3;
P	Q9SXS2	DR	secondary active sulfate transmembrane transporter activity
P	Q9SY55	CC	Involved in calcium-enhanced root growth, in tolerance to toxic levels of manganese and in secretory processes
P	Q9SY55	CC	This magnesium-dependent enzyme catalyzes the hydrolysis of ATP coupled with the translocation of calcium from the cytosol to an endomembrane compartment
P	Q9SY55	DE	Calcium-transporting ATPase 3, endoplasmic reticulum-type;
P	Q9SY55	DR	calcium-transporting ATPase activity
P	Q9SYG9	DE	Cation/calcium exchanger 4;
P	Q9SYG9	DE	Protein CATION CALCIUM EXCHANGER 4;
P	Q9SYQ1	CC	High-affinity transporter for external inorganic phosphate (By similarity).
P	Q9SYQ1	DE	Probable inorganic phosphate transporter 1-8;
P	Q9SZC9	CC	Involved in copper transport (Potential).
P	Q9SZC9	DE	Putative copper-transporting ATPase PAA1;
P	Q9SZC9	DR	copper-exporting ATPase activity
P	Q9SZR1	CC	This magnesium-dependent enzyme catalyzes the hydrolysis of ATP coupled with the translocation of calcium from the cytosol into the endoplasmic reticulum (By similarity).
P	Q9SZR1	DE	Ca2+-ATPase isoform 10;
P	Q9SZR1	DE	Calcium-transporting ATPase 10, plasma membrane-type;
P	Q9SZR1	DR	calcium-transporting ATPase activity
P	Q9T095	DE	Oligopeptide transporter 6;
P	Q9T095	DR	oligopeptide transporter activity
P	Q9U6A0	CC	May function in the removal and maintenance of calcium homeostasis during signaling in the adult and in signaling events during embryogenesis and patterning of imaginal disks
P	Q9U6A0	CC	Transports one Ca2+ and 1 K+ in exchange for 4 Na+.
P	Q9U6A0	CC	Transports one Ca2+ and 1 K+ in exchange for 4 Na+.
P	Q9U6A0	CC	Transports one Ca2+ and 1 K+ in exchange for 4 Na+.
P	Q9U6A0	DE	Na+/K+/Ca2+-exchange protein Nckx30C;
P	Q9U6A0	DE	Na+/K+/Ca2+-exchange protein Nckx30C;
P	Q9U6A0	DE	Na+/K+/Ca2+-exchange protein Nckx30C;
P	Q9U6A0	DE	Sodium/potassium/calcium exchanger Nckx30C;
P	Q9U6A0	DE	Sodium/potassium/calcium exchanger Nckx30C;
P	Q9U6A0	DE	Sodium/potassium/calcium exchanger Nckx30C;
P	Q9U6A0	DR	calcium, potassium:sodium antiporter activity
P	Q9U6A0	DR	calcium, potassium:sodium antiporter activity
P	Q9U6A0	DR	calcium, potassium:sodium antiporter activity
P	Q9UA35	CC	Employs a 2:1 sodium/nucleoside ratio
P	Q9UA35	CC	Employs a 2:1 sodium/nucleoside ratio
P	Q9UA35	CC	Exhibits the transport characteristics of the nucleoside transport system cib or N3 subtype (N3/cib) (with marked transport of both thymidine and inosine)
P	Q9UA35	CC	Sodium-dependent, pyrimidine- and purine-selective
P	Q9UA35	DE	Concentrative Na+-nucleoside cotransporter 3;
P	Q9UA35	DR	nucleoside:sodium symporter activity
P	Q9UA35	DR	nucleoside:sodium symporter activity
P	Q9UAC2	DE	Folate/methotrexate transporter FT1;
P	Q9UBN4	CC	Acts as a cell-cell contact-dependent endothelial calcium entry channel
P	Q9UBN4	CC	Form a receptor-activated non-selective calcium permeant cation channel
P	Q9UBN4	CC	May also be activated by intracellular calcium store depletion.
P	Q9UBN4	DR	store-operated calcium channel activity
P	Q9UBX3	DE	Mitochondrial dicarboxylate carrier;
P	Q9UBY0	CC	Major proton extruding system driven by the inward sodium ion chemical gradient
P	Q9UBY0	CC	Seems to play an important role in colonic sodium absorption.
P	Q9UBY0	DE	Na+/H+ exchanger 2;

P	Q9UBY0	DE	Sodium/hydrogen exchanger 2;
P	Q9UBY0	DR	sodium:hydrogen antiporter activity
P	Q9UGH3	CC	Mediates electrogenic uptake of vitamin C, with a stoichiometry of 2 Na+ for each ascorbate.
P	Q9UGH3	CC	Mediates electrogenic uptake of vitamin C, with a stoichiometry of 2 Na+ for each ascorbate.
P	Q9UGH3	CC	Sodium/ascorbate cotransporter
P	Q9UGH3	CC	Sodium/ascorbate cotransporter
P	Q9UGH3	DE	Na+/L-ascorbic acid transporter 2;
P	Q9UGH3	DE	Na+/L-ascorbic acid transporter 2;
P	Q9UGH3	DE	Sodium-dependent vitamin C transporter 2;
P	Q9UGH3	DE	Sodium-dependent vitamin C transporter 2;
P	Q9UGH3	DR	sodium-dependent L-ascorbate transmembrane transporter activity
P	Q9UGH3	DR	sodium-dependent L-ascorbate transmembrane transporter activity
P	Q9UGH3	DR	sodium-dependent multivitamin transmembrane transporter activity
P	Q9UGI6	CC	Forms a voltage-independent potassium channel activated by intracellular calcium
P	Q9UGQ3	CC	Facilitative glucose transporter; binds cytochalasin B with low affinity.
P	Q9UGQ3	DE	Glucose transporter type 6;
P	Q9UGQ3	DE	Glucose transporter type 9;
P	Q9UGQ3	DE	Solute carrier family 2, facilitated glucose transporter member 6;
P	Q9UGQ3	DR	D-glucose transmembrane transporter activity
P	Q9UHC3	CC	Cation channel with high affinity for sodium, which is gated by extracellular protons and inhibited by the diuretic amiloride
P	Q9UHC3	DE	Testis sodium channel 1;
P	Q9UHC3	DR	ligand-gated sodium channel activity
P	Q9UHI7	CC	Mediates electrogenic uptake of vitamin C, with a stoichiometry of 2 Na+ for each ascorbate.
P	Q9UHI7	CC	Mediates electrogenic uptake of vitamin C, with a stoichiometry of 2 Na+ for each ascorbate.
P	Q9UHI7	CC	Sodium/ascorbate cotransporter
P	Q9UHI7	CC	Sodium/ascorbate cotransporter
P	Q9UHI7	DE	Na+/L-ascorbic acid transporter 1;
P	Q9UHI7	DE	Na+/L-ascorbic acid transporter 1;
P	Q9UHI7	DE	Sodium-dependent vitamin C transporter 1;
P	Q9UHI7	DE	Sodium-dependent vitamin C transporter 1;
P	Q9UHI7	DR	L-ascorbate:sodium symporter activity
P	Q9UHI7	DR	L-ascorbate:sodium symporter activity
P	Q9UHI7	DR	sodium-dependent L-ascorbate transmembrane transporter activity
P	Q9UHI7	DR	sodium-dependent L-ascorbate transmembrane transporter activity
P	Q9UHW9	CC	Mediates electroneutral potassium-chloride cotransport
P	Q9UHW9	DE	Electroneutral potassium-chloride cotransporter 3;
P	Q9UHW9	DR	potassium:chloride symporter activity
P	Q9UHW9	DR	potassium:chloride symporter activity
P	Q9UI33	CC	Assuming opened or closed conformations in response to the voltage difference across the membrane, the protein forms a sodium-selective channel through which sodium ions may pass in accordance with their electrochemical gradient
P	Q9UI33	CC	It is a tetrodotoxin-resistant sodium channel isoform
P	Q9UI33	CC	This protein mediates the voltage-dependent sodium ion permeability of excitable membranes
P	Q9UI33	DE	Peripheral nerve sodium channel 5;
P	Q9UI33	DE	Sensory neuron sodium channel 2;
P	Q9UI33	DE	Sodium channel protein type 11 subunit alpha;
P	Q9UI33	DE	Sodium channel protein type XI subunit alpha;
P	Q9UI33	DE	Voltage-gated sodium channel subunit alpha Nav1.9;
P	Q9UI33	DR	voltage-gated sodium channel activity
P	Q9UI40	CC	Critical component of the visual transduction cascade, controlling the calcium concentration of outer segments during light and darkness
P	Q9UI40	CC	Light causes a rapid lowering of cytosolic free calcium in the outer segment of both retinal rod and cone photoreceptors and the light-induced lowering of calcium is caused by extrusion via this protein which plays a key role in the process of light adaptation
P	Q9UI40	CC	Transports 1 Ca2+ and 1 K+ in exchange for 4 Na+.
P	Q9UI40	CC	Transports 1 Ca2+ and 1 K+ in exchange for 4 Na+.
P	Q9UI40	CC	Transports 1 Ca2+ and 1 K+ in exchange for 4 Na+.
P	Q9UI40	DE	Na+/K+/Ca2+-exchange protein 2;
P	Q9UI40	DE	Na+/K+/Ca2+-exchange protein 2;

P	Q9UI40	DE	Na+/K+/Ca2+-exchange protein 2;
P	Q9UI40	DE	Sodium/potassium/calcium exchanger 2;
P	Q9UI40	DE	Sodium/potassium/calcium exchanger 2;
P	Q9UI40	DE	Sodium/potassium/calcium exchanger 2;
P	Q9UI40	DR	calcium, potassium:sodium antiporter activity
P	Q9UI40	DR	calcium, potassium:sodium antiporter activity
P	Q9UI40	DR	calcium, potassium:sodium antiporter activity
P	Q9UIX4	CC	Probable potassium channel subunit
P	Q9UIX4	DE	Potassium voltage-gated channel subfamily G member 1;
P	Q9UIX4	DE	Voltage-gated potassium channel subunit Kv6.1;
P	Q9UIX4	DR	voltage-gated potassium channel activity
P	Q9UJ96	CC	Potassium channel subunit
P	Q9UJ96	DE	Cardiac potassium channel subunit;
P	Q9UJ96	DE	Potassium voltage-gated channel subfamily G member 2;
P	Q9UJ96	DE	Voltage-gated potassium channel subunit Kv6.2;
P	Q9UJ96	DR	delayed rectifier potassium channel activity
P	Q9UK17	CC	Pore-forming (alpha) subunit of voltage-gated rapidly inactivating A-type potassium channels
P	Q9UK17	DE	Potassium voltage-gated channel subfamily D member 3;
P	Q9UK17	DE	Voltage-gated potassium channel subunit Kv4.3;
P	Q9UK17	DR	A-type (transient outward) potassium channel activity
P	Q9UKG4	CC	Sodium/sulfate cotransporter that mediates sulfate reabsorption in the high endothelial venules (HEV).
P	Q9UKG4	CC	Sodium/sulfate cotransporter that mediates sulfate reabsorption in the high endothelial venules (HEV).
P	Q9UKG4	DE	Na+/sulfate cotransporter SUT-1;
P	Q9UKG4	DE	Na+/sulfate cotransporter SUT-1;
P	Q9UKG4	DR	sodium:sulfate symporter activity
P	Q9UKG4	DR	sodium:sulfate symporter activity
P	Q9UL51	CC	Hyperpolarization-activated ion channel exhibiting weak selectivity for potassium over sodium ions
P	Q9UL51	DE	Potassium/sodium hyperpolarization-activated cyclic nucleotide-gated channel 2;
P	Q9UL51	DR	voltage-gated potassium channel activity
P	Q9UL62	CC	Has also been shown to be calcium-selective (By similarity)
P	Q9UL62	CC	May also be activated by intracellular calcium store depletion.
P	Q9UL62	CC	Thought to form a receptor-activated non-selective calcium permeant cation channel
P	Q9UL62	DR	store-operated calcium channel activity
P	Q9ULD8	CC	Pore-forming (alpha) subunit of voltage-gated potassium channel
P	Q9ULD8	DE	Ether-a-go-go-like potassium channel 2;
P	Q9ULD8	DE	Potassium voltage-gated channel subfamily H member 3;
P	Q9ULD8	DE	Voltage-gated potassium channel subunit Kv12.2;
P	Q9ULD8	DR	voltage-gated potassium channel activity
P	Q9ULF5	DE	Zinc transporter ZIP10;
P	Q9ULS6	CC	Potassium channel subunit
P	Q9ULS6	DE	Delayed-rectifier K+ channel alpha subunit 2;
P	Q9ULS6	DE	Potassium voltage-gated channel subfamily S member 2;
P	Q9ULS6	DE	Voltage-gated potassium channel subunit Kv9.2;
P	Q9ULS6	DR	voltage-gated potassium channel activity
P	Q9UN76	CC	Mediates the uptake of a broad range of neutral and cationic amino acids (with the exception of proline) in a Na+/Cl(-)-dependent manner.
P	Q9UN76	DE	Sodium- and chloride-dependent neutral and basic amino acid transporter B(0+);
P	Q9UN76	DR	neurotransmitter:sodium symporter activity
P	Q9UN88	CC	GABA, the major inhibitory neurotransmitter in the vertebrate brain, mediates neuronal inhibition by binding to the GABA/benzodiazepine receptor and opening an integral chloride channel.
P	Q9UN88	DR	chloride channel activity
P	Q9UNX9	CC	Inward rectifier potassium channels are characterized by a greater tendency to allow potassium to flow into the cell rather than out of it
P	Q9UNX9	CC	Their voltage dependence is regulated by the concentration of extracellular potassium; as external potassium is raised, the voltage range of the channel opening shifts to more positive voltages
P	Q9UNX9	DE	ATP-sensitive inward rectifier potassium channel 14;
P	Q9UNX9	DE	Inward rectifier K+ channel Kir2.4;
P	Q9UNX9	DE	Potassium channel, inwardly rectifying subfamily J member 14;
P	Q9UNX9	DR	inward rectifier potassium channel activity

P	Q9UP95	CC	Mediates electroneutral potassium-chloride cotransport when activated by cell swelling
P	Q9UP95	DE	Electroneutral potassium-chloride cotransporter 1;
P	Q9UP95	DR	potassium:chloride symporter activity
P	Q9UP95	DR	potassium:chloride symporter activity
P	Q9UPR5	CC	Ca2+ is extruded from the cell during relaxation so as to prevent overloading of intracellular stores (By similarity).
P	Q9UPR5	CC	Rapidly transports Ca2+ during excitation-contraction coupling
P	Q9UPR5	DE	Na+/Ca2+-exchange protein 2;
P	Q9UPR5	DE	Na+/Ca2+-exchange protein 2;
P	Q9UPR5	DE	Sodium/calcium exchanger 2;
P	Q9UPR5	DE	Sodium/calcium exchanger 2;
P	Q9UPR5	DR	calcium:sodium antiporter activity
P	Q9UPR5	DR	calcium:sodium antiporter activity
P	Q9UPY5	CC	Sodium-independent, high-affinity exchange of anionic amino acids with high specificity for anionic form of cystine and glutamate.
P	Q9UPY5	CC	Sodium-independent, high-affinity exchange of anionic amino acids with high specificity for anionic form of cystine and glutamate.
P	Q9UPY5	DE	Cystine/glutamate transporter;
P	Q9UPY5	DE	Cystine/glutamate transporter;
P	Q9UPY5	DR	cystine:glutamate antiporter activity
P	Q9UPY5	DR	cystine:glutamate antiporter activity
P	Q9UQ05	CC	Pore-forming (alpha) subunit of voltage-gated potassium channel
P	Q9UQ05	DE	Ether-a-go-go-like potassium channel 1;
P	Q9UQ05	DE	Potassium voltage-gated channel subfamily H member 4;
P	Q9UQ05	DE	Voltage-gated potassium channel subunit Kv12.3;
P	Q9UQ05	DR	voltage-gated potassium channel activity
P	Q9UQC9	CC	Play a role in modulating chloride current across the plasma membrane in a calcium-dependent manner, and cell adhesion
P	Q9UQC9	DR	chloride channel activity
P	Q9UQD0	CC	Assuming opened or closed conformations in response to the voltage difference across the membrane, the protein forms a sodium-selective channel through which Na+ ions may pass in accordance with their electrochemical gradient
P	Q9UQD0	CC	Mediates the voltage-dependent sodium ion permeability of excitable membranes
P	Q9UQD0	DE	Sodium channel protein type 8 subunit alpha;
P	Q9UQD0	DE	Sodium channel protein type VIII subunit alpha;
P	Q9UQD0	DE	Voltage-gated sodium channel subunit alpha Nav1.6;
P	Q9UQD0	DR	voltage-gated sodium channel activity
P	Q9URY6	CC	Involved in active transport of urea (By similarity).
P	Q9URY6	DE	Probable urea active transporter 3;
P	Q9USV7	CC	Mobilizes stored copper from the vacuole to the cytoplasm under conditions of copper limitation.
P	Q9USV7	DE	Copper transport protein ctr6;
P	Q9USV7	DE	Copper transporter 6;
P	Q9USV7	DR	copper ion transmembrane transporter activity
P	Q9UTN1	CC	Transports oxaloacetate and sulfate (By similarity).
P	Q9UTN1	DE	Mitochondrial oxaloacetate transport protein;
P	Q9UZU7	CC	Part of the ABC transporter complex PstSACB involved in phosphate import
P	Q9UZU7	DE	ABC phosphate transporter;
P	Q9UZU7	DE	Phosphate import ATP-binding protein PstB;
P	Q9UZU7	DE	Phosphate-transporting ATPase;
P	Q9UZU7	DR	inorganic phosphate transmembrane transporter activity
P	Q9UZU7	DR	phosphate transmembrane-transporting ATPase activity
P	Q9V297	DR	maltose transmembrane transporter activity
P	Q9V2C0	CC	Part of the ABC transporter complex WtpABC involved in molybdate/tungstate import
P	Q9V2C0	DE	Molybdate/tungstate import ATP-binding protein WtpC;
P	Q9V2C0	DR	molybdate transmembrane-transporting ATPase activity
P	Q9V2C1	CC	Part of the ABC transporter complex wtpABC involved in molybdate/tungstate import
P	Q9V2C1	DE	Molybdate/tungstate transport system permease protein wtpB;
P	Q9V2C4	CC	Binds tungstate and molybdate (By similarity).
P	Q9V2C4	CC	Part of the ABC transporter complex wtpABC involved in molybdate/tungstate import
P	Q9V2C4	DE	Molybdate/tungstate-binding protein wtpA;
P	Q9V5Z7	CC	Forms a water-specific channel (By similarity).
P	Q9V5Z7	DR	water channel activity
P	Q9V7S5	CC	May be an inorganic phosphate cotransporter.
P	Q9V7S5	DE	Putative inorganic phosphate cotransporter;

P	Q9V7S5	DR	inorganic phosphate transmembrane transporter activity
P	Q9VR07	CC	Ine negatively regulates neuronal sodium channels
P	Q9VR07	DE	Sodium- and chloride-dependent GABA transporter ine;
P	Q9VR07	DE	Sodium- and chloride-dependent GABA transporter ine;
P	Q9VR07	DR	neurotransmitter:sodium symporter activity
P	Q9VVT2	CC	Affinity for substrates is citrate > succinate > pyruvate
P	Q9VVT2	CC	Cation-independent electroneutral transporter (not associated with membrane depolarization) of a variety of tricarboxylic and dicarboxylic acid-cycle intermediates
P	Q9VVT2	CC	Transport mechanism that is not coupled to Na+, K+, or Cl(-)
P	Q9VVT2	DR	citrate transmembrane transporter activity
P	Q9W4C5	CC	Unusual broad substrate spectrum amino acid:sodium cotransporter that promotes absorption of the D isomers of essential amino acids
P	Q9W4C5	DE	Sodium-dependent nutrient amino acid transporter 1;
P	Q9W4C5	DR	neurotransmitter:sodium symporter activity
P	Q9W4C5	DR	sodium:amino acid symporter activity
P	Q9WTR6	CC	Sodium-independent, high-affinity exchange of anionic amino acids with high specificity for anionic form of cystine and glutamate.
P	Q9WTR6	CC	Sodium-independent, high-affinity exchange of anionic amino acids with high specificity for anionic form of cystine and glutamate.
P	Q9WTR6	DE	Cystine/glutamate transporter;
P	Q9WTR6	DE	Cystine/glutamate transporter;
P	Q9WTU3	CC	Assuming opened or closed conformations in response to the voltage difference across the membrane, the protein forms a sodium-selective channel through which Na+ ions may pass in accordance with their electrochemical gradient
P	Q9WTU3	CC	Mediates the voltage-dependent sodium ion permeability of excitable membranes
P	Q9WTU3	DE	Sodium channel protein type 8 subunit alpha;
P	Q9WTU3	DE	Sodium channel protein type VIII subunit alpha;
P	Q9WTU3	DE	Voltage-gated sodium channel subunit alpha Nav1.6;
P	Q9WTU3	DR	voltage-gated sodium channel activity
P	Q9WTW7	CC	Mediates electrogenic uptake of vitamin C, with a stoichiometry of 2 Na+ for each ascorbate.
P	Q9WTW7	CC	Mediates electrogenic uptake of vitamin C, with a stoichiometry of 2 Na+ for each ascorbate.
P	Q9WTW7	CC	Sodium/ascorbate cotransporter
P	Q9WTW7	CC	Sodium/ascorbate cotransporter
P	Q9WTW7	DE	Na+/L-ascorbic acid transporter 1;
P	Q9WTW7	DE	Na+/L-ascorbic acid transporter 1;
P	Q9WTW7	DE	Sodium-dependent vitamin C transporter 1;
P	Q9WTW7	DE	Sodium-dependent vitamin C transporter 1;
P	Q9WTW7	DR	L-ascorbate:sodium symporter activity
P	Q9WTW7	DR	L-ascorbate:sodium symporter activity
P	Q9WTW7	DR	L-ascorbic acid transporter activity
P	Q9WTY4	CC	Forms a water-specific channel
P	Q9WTY4	DR	water channel activity
P	Q9WU38	CC	Controls the reabsorption of sodium in kidney, colon, lung and sweat glands
P	Q9WU38	CC	Sodium permeable non-voltage-sensitive ion channel inhibited by the diuretic amiloride
P	Q9WU38	DE	Amiloride-sensitive sodium channel subunit beta;
P	Q9WU38	DE	Epithelial Na+ channel subunit beta;
P	Q9WU38	DE	Nonvoltage-gated sodium channel 1 subunit beta;
P	Q9WU38	DR	ligand-gated sodium channel activity
P	Q9WU39	CC	Controls the reabsorption of sodium in kidney, colon, lung and sweat glands
P	Q9WU39	CC	Sodium permeable non-voltage-sensitive ion channel inhibited by the diuretic amiloride
P	Q9WU39	DE	Amiloride-sensitive sodium channel subunit gamma;
P	Q9WU39	DE	Epithelial Na+ channel subunit gamma;
P	Q9WU39	DE	Nonvoltage-gated sodium channel 1 subunit gamma;
P	Q9WU39	DR	ligand-gated sodium channel activity
P	Q9WUB7	CC	Chloride channels have several functions including the regulation of cell volume; membrane potential stabilization, signal transduction and transepithelial transport
P	Q9WUB7	CC	May be the basolateral chloride channel mediating net chloride absorption in CTAL cells
P	Q9WUB7	CC	Voltage-gated chloride channel
P	Q9WUB7	DE	Chloride channel Ka;
P	Q9WUB7	DE	Chloride channel protein ClC-Ka;
P	Q9WUB7	DR	voltage-gated chloride channel activity
P	Q9WVC8	CC	Chloride/bicarbonate exchanger
P	Q9WVC8	DE	Chloride anion exchanger;

P	Q9WVD4	CC	Functions as antiport system and exchanges chloride ions against protons
P	Q9WVD4	CC	Proton-coupled chloride transporter
P	Q9WVD4	DE	Chloride channel protein 5;
P	Q9WVD4	DE	Chloride transporter ClC-5;
P	Q9WVD5	CC	Ornithine transport across inner mitochondrial membrane, from the cytoplasm to the matrix.
P	Q9WVD5	DE	Mitochondrial ornithine transporter 1;
P	Q9WVL3	CC	May mediate K+ uptake into Deiters' cells in the cochlea and contribute to K+ recycling in the inner ear
P	Q9WVL3	CC	Mediates electroneutral potassium-chloride cotransport when activated by cell swelling (By similarity)
P	Q9WVL3	DE	Electroneutral potassium-chloride cotransporter 4;
P	Q9WVL3	DR	cation:chloride symporter activity
P	Q9WXN4	DE	Oligopeptide ABC transporter, ATP-binding protein;
P	Q9WXN5	DE	Oligopeptide ABC transporter, ATP-binding protein;
P	Q9WXN6	DE	Oligopeptide ABC transporter, permease protein;
P	Q9WXN7	DE	Oligopeptide ABC transporter, permease protein;
P	Q9WXN8	DE	Oligopeptide ABC transporter, periplasmic oligopeptide-binding protein;
P	Q9WXS6	DE	Oligopeptide ABC transporter, periplasmic oligopeptide-binding protein;
P	Q9WXS7	DE	Oligopeptide ABC transporter, permease protein;
P	Q9WXS8	DE	Oligopeptide ABC transporter, permease protein;
P	Q9WXS9	DE	Oligopeptide ABC transporter, ATP-binding protein;
P	Q9WXT0	DE	Oligopeptide ABC transporter, ATP-binding protein;
P	Q9WXX0	CC	Part of the ABC transporter complex RbsABCD involved in ribose import
P	Q9WXX0	DE	Ribose import ATP-binding protein RbsA 1;
P	Q9WZ31	CC	Mediates influx of magnesium ions (By similarity).
P	Q9WZ31	DE	Magnesium transport protein CorA;
P	Q9WZ31	DR	cobalt ion transmembrane transporter activity
P	Q9WZ31	DR	magnesium ion transmembrane transporter activity
P	Q9X051	CC	Part of the ABC transporter complex RbsABCD involved in ribose import
P	Q9X051	DE	Ribose import ATP-binding protein RbsA 2;
P	Q9X0F3	DE	Oligopeptide ABC transporter, ATP-binding protein;
P	Q9X0F4	DE	Oligopeptide ABC transporter, ATP-binding protein;
P	Q9X0F5	DE	Oligopeptide ABC transporter, permease protein;
P	Q9X0F6	DE	Oligopeptide ABC transporter, permease protein;
P	Q9X0F7	DE	Oligopeptide ABC transporter, periplasmic oligopeptide-binding protein;
P	Q9X0U6	DE	Oligopeptide ABC transporter, ATP-binding protein;
P	Q9X0U7	DE	Oligopeptide ABC transporter, ATP-binding protein;
P	Q9X0U8	DE	Oligopeptide ABC transporter, permease protein;
P	Q9X0U9	DE	Oligopeptide ABC transporter, permease protein;
P	Q9X0V0	DE	Oligopeptide ABC transporter, periplasmic oligopeptide-binding protein;
P	Q9X0Y8	CC	Part of the ABC transporter complex PstSACB involved in phosphate import
P	Q9X0Y8	DE	ABC phosphate transporter;
P	Q9X0Y8	DE	Phosphate import ATP-binding protein PstB;
P	Q9X0Y8	DE	Phosphate-transporting ATPase;
P	Q9X0Y8	DR	inorganic phosphate transmembrane transporter activity
P	Q9X0Y8	DR	phosphate transmembrane-transporting ATPase activity
P	Q9X196	CC	Part of the ABC transporter complex PotABCD involved in spermidine/putrescine import
P	Q9X196	CC	Part of the ABC transporter complex PotABCD involved in spermidine/putrescine import
P	Q9X196	DE	Spermidine/putrescine import ATP-binding protein PotA;
P	Q9X196	DE	Spermidine/putrescine import ATP-binding protein PotA;
P	Q9X1E3	CC	Glycerol enters the cell via the glycerol diffusion facilitator protein
P	Q9X1E3	CC	This membrane protein facilitates the movement of glycerol across the cytoplasmic membrane (By similarity).
P	Q9X1E3	DE	Probable glycerol uptake facilitator protein;
P	Q9X268	DE	Oligopeptide ABC transporter, periplasmic oligopeptide-binding protein;
P	Q9X269	DE	Oligopeptide ABC transporter, permease protein;
P	Q9X270	DE	Oligopeptide ABC transporter, permease protein;
P	Q9X271	DE	Oligopeptide ABC transporter, ATP-binding protein;
P	Q9X272	DE	Oligopeptide ABC transporter, ATP-binding protein;
P	Q9X7P0	DE	L-asparagine permease;
P	Q9X7P0	DE	L-asparagine transport protein;
P	Q9X8Z8	CC	One of the components of the high-affinity ATP-driven potassium transport (or KDP) system, which catalyzes the hydrolysis of ATP coupled with the exchange of hydrogen and potassium ions

P	Q9X8Z8	DE	Potassium-binding and translocating subunit C;
P	Q9X8Z8	DE	Potassium-translocating ATPase C chain;
P	Q9X8Z8	DE	Potassium-transporting ATPase C chain;
P	Q9X8Z8	DR	potassium-transporting ATPase activity
P	Q9X8Z9	CC	One of the components of the high-affinity ATP-driven potassium transport (or KDP) system, which catalyzes the hydrolysis of ATP coupled with the exchange of hydrogen and potassium ions (By similarity).
P	Q9X8Z9	DE	Potassium-binding and translocating subunit B;
P	Q9X8Z9	DE	Potassium-translocating ATPase B chain;
P	Q9X8Z9	DE	Potassium-transporting ATPase B chain;
P	Q9X8Z9	DR	potassium-transporting ATPase activity
P	Q9X900	CC	One of the components of the high-affinity ATP-driven potassium transport (or KDP) system, which catalyzes the hydrolysis of ATP coupled with the exchange of hydrogen and potassium ions (By similarity).
P	Q9X900	DE	Potassium-binding and translocating subunit A;
P	Q9X900	DE	Potassium-translocating ATPase A chain;
P	Q9X900	DE	Potassium-transporting ATPase A chain;
P	Q9X900	DR	potassium-transporting ATPase activity
P	Q9X929	CC	Na+/H+ antiporter that extrudes sodium in exchange for external protons (By similarity)
P	Q9X929	DE	Na+/H+ antiporter nhaA 3;
P	Q9X929	DE	Sodium/proton antiporter nhaA 3;
P	Q9X9D2	DE	Nucleoside triphosphate transport protein 2;
P	Q9XDA6	CC	Involved in a zinc uptake transport system (Probable).
P	Q9XDA6	DE	Zinc uptake system ATP-binding protein ZurA;
P	Q9XES1	CC	This magnesium-dependent enzyme catalyzes the hydrolysis of ATP coupled with the translocation of calcium from the cytosol to an endomembrane compartment
P	Q9XES1	DE	Calcium-transporting ATPase 4, endoplasmic reticulum-type;
P	Q9XES1	DR	calcium-transporting ATPase activity
P	Q9XFR0	DE	Probable calcium-activated outward-rectifying potassium channel 3;
P	Q9XFR0	DR	potassium channel activity
P	Q9XTG4	DR	calcium-transporting ATPase activity
P	Q9Y257	CC	Exhibits outward rectification in a physiological K+ gradient and mild inward rectification in symmetrical K+ conditions.
P	Q9Y257	DE	Inward rectifying potassium channel protein TWIK-2;
P	Q9Y257	DE	Potassium channel subfamily K member 6;
P	Q9Y257	DR	inward rectifier potassium channel activity
P	Q9Y289	CC	Transports pantothenate, biotin and lipoate in the presence of sodium.
P	Q9Y289	DE	Na+-dependent multivitamin transporter;
P	Q9Y289	DE	Sodium-dependent multivitamin transporter;
P	Q9Y289	DR	sodium-dependent multivitamin transmembrane transporter activity
P	Q9Y2C5	CC	May be involved in actively transporting phosphate into cells via Na+ cotransport (By similarity).
P	Q9Y2C5	CC	May be involved in actively transporting phosphate into cells via Na+ cotransport (By similarity).
P	Q9Y2C5	DE	Putative small intestine sodium-dependent phosphate transport protein;
P	Q9Y2C5	DE	Putative small intestine sodium-dependent phosphate transport protein;
P	Q9Y2C5	DR	sodium:phosphate symporter activity
P	Q9Y2C5	DR	sodium:phosphate symporter activity
P	Q9Y2D2	DE	UDP-N-acetylglucosamine transporter;
P	Q9Y2D2	DR	UDP-N-acetylglucosamine transmembrane transporter activity
P	Q9Y2E8	CC	Major proton extruding system driven by the inward sodium ion chemical gradient
P	Q9Y2E8	DE	Na+/H+ exchanger 8;
P	Q9Y2E8	DE	Sodium/hydrogen exchanger 8;
P	Q9Y2E8	DR	sodium:hydrogen antiporter activity
P	Q9Y2U2	CC	Probable potassium channel subunit
P	Q9Y2U2	DE	Potassium channel subfamily K member 7;
P	Q9Y2U2	DR	potassium channel activity
P	Q9Y2W3	DE	Proton-associated sugar transporter A;
P	Q9Y345	CC	Terminates the action of glycine by its high affinity sodium-dependent reuptake into presynaptic terminals
P	Q9Y345	CC	Terminates the action of glycine by its high affinity sodium-dependent reuptake into presynaptic terminals
P	Q9Y345	DE	Sodium- and chloride-dependent glycine transporter 2;
P	Q9Y345	DE	Sodium- and chloride-dependent glycine transporter 2;
P	Q9Y345	DR	glycine:sodium symporter activity

P	Q9Y345	DR	glycine:sodium symporter activity
P	Q9Y345	DR	neurotransmitter:sodium symporter activity
P	Q9Y3Q4	CC	Hyperpolarization-activated ion channel with very slow activation and inactivation exhibiting weak selectivity for potassium over sodium ions
P	Q9Y3Q4	DE	Potassium/sodium hyperpolarization-activated cyclic nucleotide-gated channel 4;
P	Q9Y3Q4	DR	voltage-gated potassium channel activity
P	Q9Y5Y9	CC	Assuming opened or closed conformations in response to the voltage difference across the membrane, the protein forms a sodium-selective channel through which sodium ions may pass in accordance with their electrochemical gradient
P	Q9Y5Y9	CC	It is a tetrodotoxin-resistant sodium channel isoform
P	Q9Y5Y9	CC	This protein mediates the voltage-dependent sodium ion permeability of excitable membranes
P	Q9Y5Y9	DE	Peripheral nerve sodium channel 3;
P	Q9Y5Y9	DE	Sodium channel protein type 10 subunit alpha;
P	Q9Y5Y9	DE	Sodium channel protein type X subunit alpha;
P	Q9Y5Y9	DE	Voltage-gated sodium channel subunit alpha Nav1.8;
P	Q9Y619	CC	Ornithine transport across inner mitochondrial membrane, from the cytoplasm to the matrix.
P	Q9Y619	DE	Mitochondrial ornithine transporter 1;
P	Q9Y666	CC	May mediate K+ uptake into Deiters' cells in the cochlea and contribute to K+ recycling in the inner ear
P	Q9Y666	CC	Mediates electroneutral potassium-chloride cotransport when activated by cell swelling
P	Q9Y666	DE	Electroneutral potassium-chloride cotransporter 4;
P	Q9Y666	DR	potassium:chloride symporter activity
P	Q9Y666	DR	potassium:chloride symporter activity
P	Q9Y696	CC	Can insert into membranes and form poorly selective ion channels that may also transport chloride ions
P	Q9Y696	DE	Chloride intracellular channel protein 4;
P	Q9Y696	DE	Intracellular chloride ion channel protein p64H1;
P	Q9Y696	DR	voltage-gated chloride channel activity
P	Q9Y6M5	CC	May be involved in zinc transport out of the cell.
P	Q9Y6M5	DE	Zinc transporter 1;
P	Q9Y6M5	DR	zinc ion transmembrane transporter activity
P	Q9Y6M7	CC	Electroneutral sodium- and bicarbonate-dependent cotransporter with a Na+:HCO3(-) 1:1 stoichiometry
P	Q9Y6M7	CC	Electroneutral sodium- and bicarbonate-dependent cotransporter with a Na+:HCO3(-) 1:1 stoichiometry
P	Q9Y6M7	CC	May also have an associated sodium channel activity.
P	Q9Y6M7	CC	Regulates intracellular pH and may play a role in bicarbonate salvage in secretory epithelia
P	Q9Y6M7	DE	Bicarbonate transporter;
P	Q9Y6M7	DR	sodium:bicarbonate symporter activity
P	Q9Y6M7	DR	sodium:bicarbonate symporter activity
P	Q9Y6R1	CC	Electrogenic sodium/bicarbonate cotransporter with a Na+:HCO3(-) stoichiometry varying from 1:2 to 1:3
P	Q9Y6R1	CC	Electrogenic sodium/bicarbonate cotransporter with a Na+:HCO3(-) stoichiometry varying from 1:2 to 1:3
P	Q9Y6R1	CC	May regulate bicarbonate influx/efflux at the basolateral membrane of cells and regulate intracellular pH.
P	Q9Y6R1	DE	Na+/HCO3(-) cotransporter;
P	Q9Y6R1	DR	sodium:bicarbonate symporter activity
P	Q9Y6R1	DR	sodium:bicarbonate symporter activity
P	Q9YBZ6	DR	copper-exporting ATPase activity
P	Q9YG51	CC	Part of the ABC transporter complex PstSACB involved in phosphate import
P	Q9YG51	DE	ABC phosphate transporter;
P	Q9YG51	DE	Phosphate import ATP-binding protein PstB;
P	Q9YG51	DE	Phosphate-transporting ATPase;
P	Q9YG51	DR	phosphate transmembrane-transporting ATPase activity
P	Q9Z0V1	CC	Pore-forming (alpha) subunit of voltage-gated rapidly inactivating A-type potassium channels
P	Q9Z0V1	DE	Potassium voltage-gated channel subfamily D member 3;
P	Q9Z0V1	DE	Voltage-gated potassium channel subunit Kv4.3;
P	Q9Z0V2	CC	Pore-forming (alpha) subunit of voltage-gated rapidly inactivating A-type potassium channels
P	Q9Z0V2	DE	Potassium voltage-gated channel subfamily D member 2;

P	Q9Z0V2	DE	Voltage-gated potassium channel subunit Kv4.2;
P	Q9Z1Q5	CC	Can insert into membranes and form chloride ion channels
P	Q9Z1Q5	DE	Chloride intracellular channel protein 1;
P	Q9Z1Q5	DE	Nuclear chloride ion channel 27;
P	Q9Z1Q5	DR	voltage-gated chloride channel activity
P	Q9Z2J0	CC	Mediates electrogenic uptake of vitamin C, with a stoichiometry of 2 Na+ for each ascorbate (By similarity).
P	Q9Z2J0	CC	Mediates electrogenic uptake of vitamin C, with a stoichiometry of 2 Na+ for each ascorbate (By similarity).
P	Q9Z2J0	CC	Sodium/ascorbate cotransporter
P	Q9Z2J0	CC	Sodium/ascorbate cotransporter
P	Q9Z2J0	DE	Na+/L-ascorbic acid transporter 1;
P	Q9Z2J0	DE	Na+/L-ascorbic acid transporter 1;
P	Q9Z2J0	DE	Sodium-dependent vitamin C transporter 1;
P	Q9Z2J0	DE	Sodium-dependent vitamin C transporter 1;
P	Q9Z2J0	DR	L-ascorbic acid transporter activity
P	Q9Z2T1	CC	Probable potassium channel subunit
P	Q9Z2T1	DE	Double-pore K+ channel 3;
P	Q9Z2T1	DE	Neuromuscular two p domain potassium channel;
P	Q9Z2T1	DE	Potassium channel subfamily K member 7;
P	Q9Z2T1	DE	Putative potassium channel DP3;
P	Q9Z2T1	DR	potassium channel activity
P	Q9Z2Z6	DE	Carnitine/acylcarnitine translocase;
P	Q9Z2Z6	DE	Mitochondrial carnitine/acylcarnitine carrier protein;
P	Q9Z329	CC	Isoform 3 has neither inositol 1,4,5-trisphosphate binding activity nor calcium releasing activity.
P	Q9Z329	CC	Receptor for inositol 1,4,5-trisphosphate, a second messenger that mediates the release of intracellular calcium
P	Q9Z329	DR	inositol 1,4,5-trisphosphate-sensitive calcium-release channel activity
P	Q9Z3Q3	CC	Part of a K+ efflux system which is required for the adaptation of R.meliloti to alkaline pH as well as for the infection process during symbiotic nodule development
P	Q9Z3Q3	DE	Probable K+/H+ antiporter subunit G;
P	Q9Z3Q3	DE	Probable K+/H+ antiporter subunit G;
P	Q9Z3Q3	DE	pH adaptation potassium efflux system protein G;
P	Q9Z3R5	CC	Part of the binding-protein-dependent transport system for alpha-glucosides such as sucrose, maltose and trehalose.
P	Q9Z3R5	CC	Part of the binding-protein-dependent transport system for alpha-glucosides such as sucrose, maltose and trehalose.
P	Q9Z3R6	CC	Part of the binding-protein-dependent transport system for alpha-glucosides such as sucrose, maltose and trehalose
P	Q9Z3R6	CC	Part of the binding-protein-dependent transport system for alpha-glucosides such as sucrose, maltose and trehalose
P	Q9Z3R7	CC	Part of the binding-protein-dependent transport system for alpha-glucosides such as sucrose, maltose and trehalose
P	Q9Z3R7	CC	Part of the binding-protein-dependent transport system for alpha-glucosides such as sucrose, maltose and trehalose
P	Q9Z6M8	CC	The arginine uptake by the bacterium in the macrophage may be a virulence factor against the host innate immune response.
P	Q9Z6M8	DE	Arginine/agmatine antiporter;
P	Q9Z7N9	DE	Probable hexose phosphate transport protein;
P	Q9Z7U0	DE	ADP,ATP carrier protein 2;
P	Q9Z7U0	DE	ADP/ATP translocase 2;
P	Q9Z7U0	DE	ADP/ATP translocase 2;
P	Q9Z7U0	DR	ATP:ADP antiporter activity
P	Q9Z7U0	DR	ATP:ADP antiporter activity
P	Q9Z8J2	DE	ADP,ATP carrier protein 1;
P	Q9Z8J2	DE	ADP/ATP translocase 1;
P	Q9Z8J2	DE	ADP/ATP translocase 1;
P	Q9Z8J2	DR	ATP:ADP antiporter activity
P	Q9Z8J2	DR	ATP:ADP antiporter activity
P	Q9Z8Q8	CC	Part of the ABC transporter complex MetNIQ involved in methionine import
P	Q9Z8Q8	DE	Methionine import ATP-binding protein MetN;
P	Q9ZAA6	CC	Tunnel subunit of the primary sodium pump glutaconyl-CoA decarboxylase (GCD)
P	Q9ZAA7	CC	Biotin carrier subunit of the primary sodium pump glutaconyl-CoA decarboxylase (GCD)
P	Q9ZAA8	CC	Makes part of the primary sodium pump glutaconyl-CoA decarboxylase (GCD)

P	Q9ZAA8	DR	sodium ion transmembrane transporter activity
P	Q9ZCC4	CC	Part of the ABC transporter complex ZnuABC involved in zinc import
P	Q9ZCC4	DE	Zinc import ATP-binding protein ZnuC;
P	Q9ZCC4	DR	zinc transporting ATPase activity
P	Q9ZCF9	CC	Part of the ABC transporter complex CcmAB involved in the biogenesis of c-type cytochromes; once thought to export heme, this seems not to be the case, but its exact role is uncertain
P	Q9ZCF9	DE	Heme exporter protein A;
P	Q9ZCF9	DR	heme-transporting ATPase activity
P	Q9ZD47	CC	Provides the rickettsial cell with host ATP in exchange for rickettsial ADP
P	Q9ZD47	CC	Provides the rickettsial cell with host ATP in exchange for rickettsial ADP
P	Q9ZD47	DE	ADP,ATP carrier protein 4;
P	Q9ZD47	DE	ADP/ATP translocase 4;
P	Q9ZD47	DE	ADP/ATP translocase 4;
P	Q9ZD47	DR	ATP:ADP antiporter activity
P	Q9ZD47	DR	ATP:ADP antiporter activity
P	Q9ZD67	CC	Provides the rickettsial cell with host ATP in exchange for rickettsial ADP
P	Q9ZD67	CC	Provides the rickettsial cell with host ATP in exchange for rickettsial ADP
P	Q9ZD67	DE	ADP,ATP carrier protein 3;
P	Q9ZD67	DE	ADP/ATP translocase 3;
P	Q9ZD67	DE	ADP/ATP translocase 3;
P	Q9ZD67	DR	ATP:ADP antiporter activity
P	Q9ZD67	DR	ATP:ADP antiporter activity
P	Q9ZDF2	CC	Provides the rickettsial cell with host ATP in exchange for rickettsial ADP
P	Q9ZDF2	CC	Provides the rickettsial cell with host ATP in exchange for rickettsial ADP
P	Q9ZDF2	DE	ADP,ATP carrier protein 2;
P	Q9ZDF2	DE	ADP/ATP translocase 2;
P	Q9ZDF2	DE	ADP/ATP translocase 2;
P	Q9ZDF2	DR	ATP:ADP antiporter activity
P	Q9ZDF2	DR	ATP:ADP antiporter activity
P	Q9ZE70	CC	Transports S-adenosylmethionine.
P	Q9ZE70	DE	S-adenosylmethionine uptake transporter;
P	Q9ZHC9	CC	Component of the sil cation-efflux system that confers resistance to silver
P	Q9ZNG6	CC	Does not transport K+, Ca2+ or Mg2+.
P	Q9ZNG6	CC	Mnh complex is a Na+ Li+/H+ antiporter involved in Na+ and/or Li+ excretion
P	Q9ZNG6	CC	Mnh complex is a Na+ Li+/H+ antiporter involved in Na+ and/or Li+ excretion
P	Q9ZNG6	CC	Na+/H+ antiport consumes a transmembrane electrical potential, and is thus inferred to be electrogenic
P	Q9ZNG6	CC	Na+/H+ antiport consumes a transmembrane electrical potential, and is thus inferred to be electrogenic
P	Q9ZNG6	DE	Na+/H+ antiporter subunit A1;
P	Q9ZNG6	DE	Na+/H+ antiporter subunit A1;
P	Q9ZPR4	CC	Magnesium transporter that may mediate the influx of magnesium (By similarity)
P	Q9ZPR4	DE	Magnesium Transporter 3;
P	Q9ZPR4	DE	Magnesium transporter MRS2-5;
P	Q9ZPR4	DR	magnesium ion transmembrane transporter activity
P	Q9ZPR7	CC	Transports a wide spectrum of oxo derivatives of heterocyclic nitrogen compounds, including allantoin, uric acid and xanthine, but not adenine
P	Q9ZPR7	DR	allantoin uptake transmembrane transporter activity
P	Q9ZV07	CC	Aquaporins facilitate the transport of water and small neutral solutes across cell membranes (By similarity).
P	Q9ZVK6	CC	Responsible for the transport of sucrose into the cell, with the concomitant uptake of protons (symport system)
P	Q9ZVK6	DE	Sucrose permease 8;
P	Q9ZVK6	DE	Sucrose transport protein SUC8;
P	Q9ZVK6	DE	Sucrose-proton symporter 8;
P	Q9ZVK6	DR	sucrose transmembrane transporter activity
P	Q9ZVX8	CC	Aquaporins facilitate the transport of water and small neutral solutes across cell membranes (By similarity).
P	Q9ZWT3	CC	High-affinity transporter for external inorganic phosphate (By similarity).
P	Q9ZWT3	DE	Probable inorganic phosphate transporter 1-6;
P	Q9ZWT3	DR	inorganic phosphate transmembrane transporter activity
N	A0A322	CC	F(1)F(0) ATP synthase produces ATP from ADP in the presence of a proton or sodium gradient

N	A0A322	CC	During catalysis, ATP synthesis in the catalytic domain of F(1) is coupled via a rotary mechanism of the central stalk subunits to proton translocation (By similarity).
N	A0A322	CC	F-type ATPases consist of two structural domains, F(1) containing the extramembraneous catalytic core and F(0) containing the membrane proton channel, linked together by a central stalk and a peripheral stalk
N	A0A322	DE	ATP synthase F(0) sector subunit c;
N	A0A322	DE	ATP synthase subunit c, chloroplastic;
N	A0A322	DE	ATPase subunit III;
N	A0A322	DE	F-ATPase subunit c;
N	A0A322	DE	F-type ATPase subunit c;
N	A0A322	DE	Lipid-binding protein;
N	A0A322	DR	ATP hydrolysis coupled proton transport
N	A0A322	DR	ATP synthesis coupled proton transport
N	A0A322	DR	hydrogen ion transmembrane transporter activity
N	A0A322	DR	lipid binding
N	A0AH37	DE	Pantothenate kinase;
N	A0AH37	DE	Pantothenic acid kinase;
N	A0AH37	DR	coenzyme A biosynthetic process
N	A0AH37	DR	pantothenate kinase activity
N	A0AII5	CC	Catalyzes the 1,3-allylic rearrangement of the homoallylic substrate isopentenyl (IPP) to its allylic isomer, dimethylallyl diphosphate (DMAPP) (By similarity).
N	A0AII5	DE	IPP isomerase;
N	A0AII5	DE	Isopentenyl pyrophosphate isomerase;
N	A0AII5	DE	Isopentenyl-diphosphate delta-isomerase;
N	A0AII5	DR	isopentenyl-diphosphate delta-isomerase activity
N	A0AII5	DR	isoprenoid biosynthetic process
N	A0AII5	DR	oxidoreductase activity
N	A0AK41	CC	Major role in the synthesis of nucleoside triphosphates other than ATP
N	A0AK41	CC	The ATP gamma phosphate is transferred to the NDP beta phosphate via a ping-pong mechanism, using a phosphorylated active-site intermediate.
N	A0AK41	DE	NDP kinase;
N	A0AK41	DE	Nucleoside diphosphate kinase;
N	A0AK41	DE	Nucleoside-2-P kinase;
N	A0AK41	DR	CTP biosynthetic process
N	A0AK41	DR	GTP biosynthetic process
N	A0AK41	DR	UTP biosynthetic process
N	A0AK41	DR	metal ion binding
N	A0AK41	DR	nucleoside diphosphate kinase activity
N	A0AMG5	DE	50S ribosomal protein L34;
N	A0AMG5	DR	structural constituent of ribosome
N	A0JWY6	CC	Component of the proteasome core, a large protease complex with broad specificity involved in protein degradation (By similarity).
N	A0JWY6	DE	20S proteasome beta subunit;
N	A0JWY6	DE	Proteasome core protein PrcB;
N	A0JWY6	DE	Proteasome subunit beta;
N	A0JWY6	DR	proteasomal protein catabolic process
N	A0JWY6	DR	threonine-type endopeptidase activity
N	A0JWY8	CC	Catalyzes the covalent attachment of the prokaryotic ubiquitin-like protein modifier Pup to the proteasomal substrate proteins, thereby targeting them for proteasomal degradation.
N	A0JWY8	CC	The ligation reaction involves the side-chain carboxylate of the C-terminal glutamate of Pup and the side-chain amino group of a substrate lysine (By similarity).
N	A0JWY8	CC	This tagging system is termed pupylation
N	A0JWY8	DE	Proteasome accessory factor A;
N	A0JWY8	DE	Pup--protein ligase;
N	A0JWY8	DE	Pup-conjugating enzyme;
N	A0JWY8	DR	acid-amino acid ligase activity
N	A0JWY8	DR	modification-dependent protein catabolic process
N	A0JWY8	DR	proteasomal protein catabolic process
N	A0K487	CC	Cell wall formation (By similarity).
N	A0K487	DE	UDP-N-acetylmuramate--L-alanine ligase;
N	A0K487	DE	UDP-N-acetylmuramoyl-L-alanine synthetase;
N	A0K487	DR	UDP-N-acetylmuramate-L-alanine ligase activity
N	A0K487	DR	cell division
N	A0K487	DR	cellular cell wall organization
N	A0K487	DR	peptidoglycan biosynthetic process

N	A0K487	DR	regulation of cell shape
N	A0K6Q0	CC	Part of an ABC transporter complex involved in carbohydrate import
N	A0K6Q0	CC	Could be involved in ribose, galactose and/or methyl galactoside import
N	A0K6Q0	CC	Responsible for energy coupling to the transport system (By similarity).
N	A0K6Q0	DE	Putative ribose/galactose/methyl galactoside import ATP-binding protein 1;
N	A0K6Q0	DR	monosaccharide-transporting ATPase activity
N	A0K8B3	CC	Catalyzes the synthesis of GMP from XMP (By similarity).
N	A0K8B3	DE	GMP synthase [glutamine-hydrolyzing];
N	A0K8B3	DE	GMP synthetase;
N	A0K8B3	DE	Glutamine amidotransferase;
N	A0K8B3	DR	GMP biosynthetic process
N	A0K8B3	DR	GMP synthase (glutamine-hydrolyzing) activity
N	A0K8B3	DR	glutamine metabolic process
N	A0KGJ2	DE	AICAR transformylase;
N	A0KGJ2	DE	Bifunctional purine biosynthesis protein purH;
N	A0KGJ2	DE	IMP cyclohydrolase;
N	A0KGJ2	DE	IMP synthase;
N	A0KGJ2	DE	Inosinicase;
N	A0KGJ2	DE	Phosphoribosylaminoimidazolecarboxamide formyltransferase;
N	A0KGJ2	DR	IMP biosynthetic process
N	A0KGJ2	DR	IMP cyclohydrolase activity
N	A0KGJ2	DR	phosphoribosylaminoimidazolecarboxamide formyltransferase activity
N	A0KR79	CC	One of the proteins required for the normal export of preproteins out of the cell cytoplasm
N	A0KR79	CC	It also specifically binds to its receptor secA (By similarity).
N	A0KR79	CC	It is a molecular chaperone that binds to a subset of precursor proteins, maintaining them in a translocation-competent state
N	A0KR79	DE	Protein-export protein secB;
N	A0KR79	DR	protein tetramerization
N	A0KR79	DR	protein transport
N	A0KR79	DR	transmembrane transport
N	A0KR79	DR	unfolded protein binding
N	A0KSQ0	DE	Thiopurine S-methyltransferase;
N	A0KSQ0	DE	Thiopurine methyltransferase;
N	A0KSQ0	DR	thiopurine S-methyltransferase activity
N	A0KTX7	CC	The natural substrate for this enzyme may be peptidyl- tRNAs which drop off the ribosome during protein synthesis (By similarity).
N	A0KTX7	DE	Peptidyl-tRNA hydrolase;
N	A0KTX7	DR	aminoacyl-tRNA hydrolase activity
N	A0KWB1	DE	1-(5-phosphoribosyl)-5-[(5-phosphoribosylamino)methylideneamino] imidazole-4-carboxamide isomerase;
N	A0KWB1	DE	Phosphoribosylformimino-5-aminoimidazole carboxamide ribotide isomerase;
N	A0KWB1	DR	1-(5-phosphoribosyl)-5-[(5-phosphoribosylamino)methylideneamino]imidazole-4-carboxamide isomerase activity
N	A0KWB1	DR	histidine biosynthetic process
N	A0L4K6	CC	Nucleotidase that shows phosphatase activity on nucleoside 5'-monophosphates (By similarity).
N	A0L4K6	DE	5'-nucleotidase surE;
N	A0L4K6	DE	Nucleoside 5'-monophosphate phosphohydrolase;
N	A0L4K6	DR	5'-nucleotidase activity
N	A0L4K6	DR	metal ion binding
N	A0L4K6	DR	nucleotide binding
N	A0L6F7	DE	Lysine--tRNA ligase;
N	A0L6F7	DE	Lysyl-tRNA synthetase;
N	A0L6F7	DR	lysine-tRNA ligase activity
N	A0L6F7	DR	lysyl-tRNA aminoacylation
N	A0L6F7	DR	metal ion binding
N	A0L6F7	DR	nucleic acid binding
N	A0LQZ7	CC	Converts 4-diphosphocytidyl-2-C-methyl-D-erythritol 2- phosphate into 2-C-methyl-D-erythritol 2,4-cyclodiphosphate (MECDP) and CMP (By similarity).
N	A0LQZ7	DE	2-C-methyl-D-erythritol 2,4-cyclodiphosphate synthase;
N	A0LQZ7	DE	MECDP-synthase;
N	A0LQZ7	DR	2-C-methyl-D-erythritol 2,4-cyclodiphosphate synthase activity
N	A0LQZ7	DR	metal ion binding
N	A0LQZ7	DR	terpenoid biosynthetic process

N	A0LRK0	DE	UPF0234 protein Acel_0286;
N	A0LRM3	CC	One of the primary rRNA binding proteins
N	A0LRM3	CC	It has been suggested to have peptidyltransferase activity; this is somewhat controversial
N	A0LRM3	CC	Makes several contacts with the 16S rRNA in the 70S ribosome (By similarity).
N	A0LRM3	CC	Required for association of the 30S and 50S subunits to form the 70S ribosome, for tRNA binding and peptide bond formation
N	A0LRM3	DE	50S ribosomal protein L2;
N	A0LRM3	DR	structural constituent of ribosome
N	A0LRM3	DR	transferase activity
N	A0M2N6	CC	Exhibits a very high intrinsic GTPase hydrolysis rate
N	A0M2N6	CC	Involved in the addition of a carboxymethylaminomethyl (cmnm) group at the wobble position (U34) of certain tRNAs, forming tRNA- cmnm(5)s(2)U34 (By similarity).
N	A0M2N6	DE	tRNA modification GTPase mnmE;
N	A0M2N6	DR	GTPase activity
N	A0M2N6	DR	metal ion binding
N	A0M2N6	DR	small GTPase mediated signal transduction
N	A0M2N6	DR	tRNA modification
N	A0PLC7	CC	Catalyzes the transfer of acetyl from acetyl-CoA to desacetylmycothiol (Cys-GlcN-Ins) to form mycothiol (By similarity).
N	A0PLC7	DE	MSH acetyltransferase;
N	A0PLC7	DE	Mycothiol acetyltransferase;
N	A0PLC7	DE	Mycothiol synthase;
N	A0PLC7	DR	N-acetyltransferase activity
N	A0PRV0	CC	Exhibits S-adenosyl-L-methionine-dependent methyltransferase activity (By similarity).
N	A0PRV0	DE	Putative S-adenosyl-L-methionine-dependent methyltransferase MUL_2766;
N	A0PRV0	DR	methyltransferase activity
N	A0PT76	CC	Represses a number of genes involved in the response to DNA damage (SOS response), including recA and lexA
N	A0PT76	CC	In the presence of single-stranded DNA, recA interacts with lexA causing an autocatalytic cleavage which disrupts the DNA-binding part of lexA, leading to derepression of the SOS regulon and eventually DNA repair (By similarity).
N	A0PT76	DE	LexA repressor;
N	A0PT76	DR	DNA replication
N	A0PT76	DR	serine-type endopeptidase activity
N	A0PX72	DE	50S ribosomal protein L34;
N	A0PX72	DR	structural constituent of ribosome
N	A0Q0Y3	DE	30S ribosomal protein S16;
N	A0Q0Y3	DR	structural constituent of ribosome
N	A0Q2A0	CC	Condenses 4-methyl-5-(beta-hydroxyethyl)thiazole monophosphate (THZ-P) and 2-methyl-4-amino-5-hydroxymethyl pyrimidine pyrophosphate (HMP-PP) to form thiamine monophosphate (TMP) (By similarity).
N	A0Q2A0	DE	TMP pyrophosphorylase;
N	A0Q2A0	DE	Thiamine-phosphate pyrophosphorylase;
N	A0Q2A0	DE	Thiamine-phosphate synthase;
N	A0Q2A0	DR	metal ion binding
N	A0Q2A0	DR	thiamine biosynthetic process
N	A0Q2A0	DR	thiamine-phosphate diphosphorylase activity
N	A0Q6R7	CC	Necessary for normal cell division and for the maintenance of normal septation (By similarity).
N	A0Q6R7	DE	Probable GTP-binding protein EngB;
N	A0Q6R7	DR	barrier septum formation
N	A0Q753	CC	NAD-binding protein involved in the addition of a carboxymethylaminomethyl (cmnm) group at the wobble position (U34) of certain tRNAs, forming tRNA-cmnm(5)s(2)U34 (By similarity).
N	A0Q753	DE	Glucose-inhibited division protein A;
N	A0Q753	DE	tRNA uridine 5-carboxymethylaminomethyl modification enzyme MnmG;
N	A0Q753	DR	flavin adenine dinucleotide binding
N	A0Q753	DR	tRNA wobble uridine modification
N	A0Q8F6	CC	Catalyzes the decarboxylation of four acetate groups of uroporphyrinogen-III to yield coproporphyrinogen-III (By similarity).
N	A0Q8F6	DE	Uroporphyrinogen decarboxylase;
N	A0Q8F6	DR	porphyrin biosynthetic process
N	A0Q8F6	DR	uroporphyrinogen decarboxylase activity
N	A0QJI1	DE	O-phosphoserine phosphohydrolase;
N	A0QJI1	DE	Phosphoserine phosphatase;

N	A0QJI1	DR	L-serine biosynthetic process
N	A0QJI1	DR	amino acid binding
N	A0QJI1	DR	metal ion binding
N	A0QJI1	DR	phosphoserine phosphatase activity
N	A0QL54	DE	50S ribosomal protein L10;
N	A0QL54	DR	ribosome biogenesis
N	A0QL54	DR	structural constituent of ribosome
N	A0QU58	CC	Peptide chain release factor 2 directs the termination of translation in response to the peptide chain termination codons UGA and UAA (By similarity).
N	A0QU58	DE	Peptide chain release factor 2;
N	A0QU58	DR	translation release factor activity, codon specific
N	A0R9A0	CC	Catalyzes the attachment of proline to tRNA(Pro) in a two-step reaction: proline is first activated by ATP to form Pro- AMP and then transferred to the acceptor end of tRNA(Pro) (By similarity).
N	A0R9A0	DE	Proline--tRNA ligase 2;
N	A0R9A0	DE	Prolyl-tRNA synthetase 2;
N	A0R9A0	DR	proline-tRNA ligase activity
N	A0R9A0	DR	prolyl-tRNA aminoacylation
N	A0RA13	CC	One of the components of the high-affinity ATP-driven potassium transport (or KDP) system, which catalyzes the hydrolysis of ATP coupled with the exchange of hydrogen and potassium ions (By similarity).
N	A0RA13	DE	ATP phosphohydrolase [potassium-transporting] B chain;
N	A0RA13	DE	Potassium-binding and translocating subunit B;
N	A0RA13	DE	Potassium-translocating ATPase B chain;
N	A0RA13	DE	Potassium-transporting ATPase B chain;
N	A0RA13	DR	ATP biosynthetic process
N	A0RA13	DR	magnesium ion binding
N	A0RA13	DR	potassium-transporting ATPase activity
N	A0RQD0	CC	Involved in mRNA degradation
N	A0RQD0	CC	Hydrolyzes single-stranded polyribonucleotides processively in the 3'- to 5'-direction (By similarity).
N	A0RQD0	DE	Polynucleotide phosphorylase;
N	A0RQD0	DE	Polyribonucleotide nucleotidyltransferase;
N	A0RQD0	DR	3'-5'-exoribonuclease activity
N	A0RQD0	DR	RNA processing
N	A0RQD0	DR	mRNA catabolic process
N	A0RQD0	DR	polyribonucleotide nucleotidyltransferase activity
N	A0RQJ3	CC	This protein promotes the GTP-dependent binding of aminoacyl-tRNA to the A-site of ribosomes during protein biosynthesis.
N	A0RQJ3	DE	Elongation factor Tu;
N	A0RQJ3	DR	GTPase activity
N	A0RQJ3	DR	translation elongation factor activity
N	A0RQU7	CC	Associates with the EF-Tu.GDP complex and induces the exchange of GDP to GTP
N	A0RQU7	CC	It remains bound to the aminoacyl-tRNA.EF- Tu.GTP complex up to the GTP hydrolysis stage on the ribosome.
N	A0RQU7	DE	Elongation factor Ts;
N	A0RQU7	DR	translation elongation factor activity
N	A0RY02	CC	One of the primary rRNA binding proteins, it binds directly to 16S rRNA where it nucleates assembly of the body of the 30S subunit (By similarity).
N	A0RY02	DE	30S ribosomal protein S4P;
N	A0RY02	DR	structural constituent of ribosome
N	A1A8P2	CC	Catalyzes the formation of 2-(5''-triphosphoribosyl)-3'- dephosphocoenzyme-A, the precursor of the prosthetic group of the holo-acyl carrier protein (gamma chain) of citrate lyase, from ATP and dephospho-CoA (By similarity).
N	A1A8P2	DE	2-(5''-triphosphoribosyl)-3'-dephospho-CoA synthase;
N	A1A8P2	DE	2-(5''-triphosphoribosyl)-3'-dephosphocoenzyme-A synthase;
N	A1A8P2	DR	phosphorylation
N	A1A8P2	DR	triphosphoribosyl-dephospho-CoA synthase activity
N	A1A9L2	CC	Catalyzes the desulfonation of aliphatic sulfonates (By similarity).
N	A1A9L2	DE	Alkanesulfonate monooxygenase;
N	A1A9L2	DE	FMNH2-dependent aliphatic sulfonate monooxygenase;
N	A1A9L2	DR	alkanesulfonate monooxygenase activity
N	A1A9L2	DR	oxidation-reduction process
N	A1AGY4	CC	sn-glycerol-3-phosphate and glycerophosphoryl diester- binding protein interacts with the binding protein-dependent transport system ugpACE (By similarity).

N	A1AGY4	DE	sn-glycerol-3-phosphate-binding periplasmic protein ugpB;
N	A1AGY4	DR	transporter activity
N	A1AHK5	DE	Adenine aminase;
N	A1AHK5	DE	Adenine deaminase;
N	A1AHK5	DR	adenine catabolic process
N	A1AHK5	DR	adenine deaminase activity
N	A1AIE7	CC	Catalyzes the formation of 5-methyl-uridine at position 54 (M-5-U54) in all tRNAs (By similarity).
N	A1AIE7	DE	tRNA (uracil-5-)-methyltransferase;
N	A1AIE7	DE	tRNA(M-5-U54)-methyltransferase;
N	A1AIE7	DR	tRNA (uracil-5-)-methyltransferase activity
N	A1AW36	CC	Catalyzes the isomerization between 2-isopropylmalate and 3-isopropylmalate, via the formation of 2-isopropylmaleate.
N	A1AW36	DE	3-isopropylmalate dehydratase large subunit;
N	A1AW36	DE	Alpha-IPM isomerase;
N	A1AW36	DE	Isopropylmalate isomerase;
N	A1AW36	DR	3-isopropylmalate dehydratase activity
N	A1AW36	DR	4 iron, 4 sulfur cluster binding
N	A1AW36	DR	leucine biosynthetic process
N	A1AW36	DR	metal ion binding
N	A1AWI6	DE	UPF0250 protein Rmag_0541;
N	A1AX52	DE	Argininosuccinate lyase;
N	A1AX52	DE	Arginosuccinase;
N	A1AX52	DR	arginine biosynthetic process via ornithine
N	A1AX52	DR	argininosuccinate lyase activity
N	A1B036	CC	One of the primary rRNA binding proteins, it binds specifically to the 5'-end of 16S ribosomal RNA (By similarity).
N	A1B036	DE	30S ribosomal protein S17;
N	A1B036	DR	structural constituent of ribosome
N	A1BD21	DE	50S ribosomal protein L10;
N	A1BD21	DR	ribosome biogenesis
N	A1BD21	DR	structural constituent of ribosome
N	A1BHN8	DE	50S ribosomal protein L27;
N	A1BHN8	DR	structural constituent of ribosome
N	A1BI66	DE	UPF0235 protein Cpha266_2081;
N	A1BJ29	CC	This protein binds specifically to 23S rRNA; its binding is stimulated by other ribosomal proteins, e.g., L4, L17, and L20
N	A1BJ29	CC	It is important during the early stages of 50S assembly
N	A1BJ29	CC	It makes multiple contacts with different domains of the 23S rRNA in the assembled 50S subunit and ribosome (By similarity).
N	A1BJ29	DE	50S ribosomal protein L22;
N	A1BJ29	DR	structural constituent of ribosome
N	A1BJ36	CC	This protein promotes the GTP-dependent binding of aminoacyl-tRNA to the A-site of ribosomes during protein biosynthesis.
N	A1BJ36	DE	Elongation factor Tu;
N	A1BJ36	DR	GTPase activity
N	A1BJ36	DR	translation elongation factor activity
N	A1BJW3	CC	F(1)F(0) ATP synthase produces ATP from ADP in the presence of a proton or sodium gradient
N	A1BJW3	CC	During catalysis, ATP synthesis in the catalytic domain of F(1) is coupled via a rotary mechanism of the central stalk subunits to proton translocation (By similarity).
N	A1BJW3	CC	F-type ATPases consist of two structural domains, F(1) containing the extramembraneous catalytic core and F(0) containing the membrane proton channel, linked together by a central stalk and a peripheral stalk
N	A1BJW3	DE	ATP synthase F(1) sector subunit delta;
N	A1BJW3	DE	ATP synthase subunit delta;
N	A1BJW3	DE	F-ATPase subunit delta;
N	A1BJW3	DE	F-type ATPase subunit delta;
N	A1BJW3	DR	ATP synthesis coupled proton transport
N	A1BJW3	DR	hydrogen ion transporting ATP synthase activity, rotational mechanism
N	A1BJY3	CC	Catalyzes the addition of meso-diaminopimelic acid to the nucleotide precursor UDP-N-acetylmuramoyl-L-alanyl-D-glutamate (UMAG) in the biosynthesis of bacterial cell-wall peptidoglycan (By similarity).
N	A1BJY3	DE	Meso-A2pm-adding enzyme;
N	A1BJY3	DE	Meso-diaminopimelate-adding enzyme;

N	A1BJY3	DE	UDP-MurNAc-L-Ala-D-Glu:meso-diaminopimelate ligase;
N	A1BJY3	DE	UDP-MurNAc-tripeptide synthetase;
N	A1BJY3	DE	UDP-N-acetylmuramoyl-L-alanyl-D-glutamate--2,6-diaminopimelate ligase;
N	A1BJY3	DE	UDP-N-acetylmuramyl-tripeptide synthetase;
N	A1BJY3	DR	UDP-N-acetylmuramoylalanyl-D-glutamate-2,6-diaminopimelate ligase activity
N	A1BJY3	DR	cell division
N	A1BJY3	DR	cellular cell wall organization
N	A1BJY3	DR	peptidoglycan biosynthetic process
N	A1BJY3	DR	regulation of cell shape
N	A1C8U0	CC	Endo-1,4-mannanase, a crucial enzyme for depolymerization of seed galactomannans and wood galactoglucomannans (By similarity).
N	A1C8U0	DE	Endo-beta-1,4-mannanase F;
N	A1C8U0	DE	Mannan endo-1,4-beta-mannosidase F;
N	A1C8U0	DR	carbohydrate metabolic process
N	A1C8U0	DR	cation binding
N	A1C8U0	DR	cellulose binding
N	A1C8U0	DR	mannan endo-1,4-beta-mannosidase activity
N	A1CCU0	CC	Endo-1,4-beta-xylanase involved in the hydrolysis of xylan, a major structural heterogeneous polysaccharide found in plant biomass representing the second most abundant polysaccharide in the biosphere, after cellulose (By similarity).
N	A1CCU0	DE	1,4-beta-D-xylan xylanohydrolase A;
N	A1CCU0	DE	Probable endo-1,4-beta-xylanase A;
N	A1CCU0	DE	Xylanase A;
N	A1CCU0	DR	endo-1,4-beta-xylanase activity
N	A1CCU0	DR	xylan catabolic process
N	A1CF18	CC	Positively regulates the activity of the minus-end directed microtubule motor protein dynein
N	A1CF18	CC	May enhances dynein- mediated microtubule sliding by targeting dynein to the microtubule plus end
N	A1CF18	CC	Recruits additional proteins to the dynein complex at SPBs (By similarity).
N	A1CF18	CC	Required for localization of dynein to the mitotic spindle poles
N	A1CF18	CC	Required for nuclear migration during vegetative growth as well as development
N	A1CF18	CC	Required for retrograde early endosome (EE) transport from the hyphal tip
N	A1CF18	DE	Lissencephaly-1 homolog 2;
N	A1CF18	DE	Nuclear distribution protein nudF 2;
N	A1CF18	DR	cell division
N	A1CYR9	CC	Polynucleotide 5'-kinase involved in rRNA processing (By similarity).
N	A1CYR9	DE	Polynucleotide 5'-hydroxyl-kinase grc3;
N	A1CYR9	DR	polynucleotide 5'-hydroxyl-kinase activity
N	A1CYR9	DR	rRNA processing
N	A1DZY4	CC	Regulator of rDNA transcription (By similarity).
N	A1DZY4	DE	Ras-like protein family member 11A-like;
N	A1DZY4	DR	GTPase activity
N	A1DZY4	DR	positive regulation of transcription from RNA polymerase I promoter
N	A1DZY4	DR	small GTPase mediated signal transduction
N	A1E295	CC	Thiol protease which is believed to participate in intracellular degradation and turnover of proteins
N	A1E295	CC	Has also been implicated in tumor invasion and metastasis (By similarity).
N	A1E295	DE	Cathepsin B heavy chain;
N	A1E295	DE	Cathepsin B light chain;
N	A1E295	DE	Cathepsin B;
N	A1E295	DR	cysteine-type endopeptidase activity
N	A1E295	DR	regulation of catalytic activity
N	A1E9L2	DE	30S ribosomal protein S18, chloroplastic;
N	A1E9L2	DR	structural constituent of ribosome
N	A1E9U6	DE	30S ribosomal protein S18, chloroplastic;
N	A1E9U6	DR	structural constituent of ribosome
N	A1EA12	CC	NDH shuttles electrons from NAD(P)H:plastoquinone, via FMN and iron-sulfur (Fe-S) centers, to quinones in the photosynthetic chain and possibly in a chloroplast respiratory chain
N	A1EA12	CC	Couples the redox reaction to proton translocation, and thus conserves the redox energy in a proton gradient (By similarity).
N	A1EA12	CC	The immediate electron acceptor for the enzyme in this species is believed to be plastoquinone
N	A1EA12	DE	NAD(P)H dehydrogenase subunit K;

N	A1EA12	DE	NAD(P)H-quinone oxidoreductase subunit K, chloroplastic;
N	A1EA12	DE	NADH-plastoquinone oxidoreductase subunit K;
N	A1EA12	DR	4 iron, 4 sulfur cluster binding
N	A1EA12	DR	NADH dehydrogenase (ubiquinone) activity
N	A1EA12	DR	metal ion binding
N	A1EA12	DR	oxidation-reduction process
N	A1EA12	DR	quinone binding
N	A1IGU4	CC	May act as a guanine nucleotide exchange factor (GEF) (By similarity).
N	A1IGU4	DE	Rho guanine nucleotide exchange factor 37;
N	A1IGU4	DR	Rho guanyl-nucleotide exchange factor activity
N	A1IGU4	DR	regulation of Rho protein signal transduction
N	A1JIQ8	CC	3'-to-5' exoribonuclease specific for small oligoribonucleotides (By similarity).
N	A1JIQ8	DE	Oligoribonuclease;
N	A1JIQ8	DR	exonuclease activity
N	A1JIQ8	DR	nucleic acid binding
N	A1JJD0	CC	Transaldolase is important for the balance of metabolites in the pentose-phosphate pathway (By similarity).
N	A1JJD0	DE	Transaldolase;
N	A1JJD0	DR	pentose-phosphate shunt
N	A1JJD0	DR	sedoheptulose-7-phosphate:D-glyceraldehyde-3-phosphate glyceronetransferase activity
N	A1JKD8	DE	Urea amidohydrolase subunit beta;
N	A1JKD8	DE	Urease subunit beta;
N	A1JKD8	DR	nickel ion binding
N	A1JKD8	DR	nitrogen compound metabolic process
N	A1JKD8	DR	urease activity
N	A1JKS2	CC	Converts 2C-methyl-D-erythritol 2,4-cyclodiphosphate (ME-2,4cPP) into 1-hydroxy-2-methyl-2-(E)-butenyl 4-diphosphate (By similarity).
N	A1JKS2	DE	1-hydroxy-2-methyl-2-(E)-butenyl 4-diphosphate synthase;
N	A1JKS2	DE	4-hydroxy-3-methylbut-2-en-1-yl diphosphate synthase;
N	A1JKS2	DR	4 iron, 4 sulfur cluster binding
N	A1JKS2	DR	4-hydroxy-3-methylbut-2-en-1-yl diphosphate synthase activity
N	A1JKS2	DR	iron ion binding
N	A1JKS2	DR	oxidation-reduction process
N	A1JKS2	DR	terpenoid biosynthetic process
N	A1JRU3	CC	Synthesis of 3-octaprenyl-4-hydroxybenzoate (By similarity).
N	A1JRU3	DE	4-HB polyprenyltransferase;
N	A1JRU3	DE	4-hydroxybenzoate octaprenyltransferase;
N	A1JRU3	DR	prenyltransferase activity
N	A1JRU3	DR	ubiquinone biosynthetic process
N	A1JT62	CC	Catalyzes the NADPH-dependent reduction of glyoxylate and hydroxypyruvate into glycolate and glycerate, respectively (By similarity).
N	A1JT62	DE	Glyoxylate/hydroxypyruvate reductase B;
N	A1JT62	DR	glyoxylate reductase (NADP) activity
N	A1JT62	DR	hydroxypyruvate reductase activity
N	A1JT62	DR	oxidation-reduction process
N	A1K1B4	CC	Plays an important role in the initiation and regulation of chromosomal replication
N	A1K1B4	CC	Binds to the origin of replication; it binds specifically double-stranded DNA at a 9 bp consensus (dnaA box): 5'-TTATC[CA]A[CA]A-3'
N	A1K1B4	CC	DnaA binds to ATP and to acidic phospholipids (By similarity).
N	A1K1B4	DE	Chromosomal replication initiator protein DnaA;
N	A1K1B4	DR	DNA replication origin binding
N	A1K1B4	DR	DNA-dependent DNA replication initiation
N	A1K1B4	DR	nucleoside-triphosphatase activity
N	A1K1B4	DR	regulation of DNA replication
N	A1K6Y5	CC	Catalyzes the conversion of acetaldehyde to acetyl-CoA, using NAD(+) and coenzyme A
N	A1K6Y5	CC	Is the final enzyme in the meta- cleavage pathway for the degradation of aromatic compounds (By similarity).
N	A1K6Y5	DE	Acetaldehyde dehydrogenase 2/3;
N	A1K6Y5	DE	Acetaldehyde dehydrogenase [acetylating] 2/3;
N	A1K6Y5	DR	acetaldehyde dehydrogenase (acetylating) activity
N	A1K6Y5	DR	aromatic compound catabolic process
N	A1K6Y5	DR	cellular amino acid metabolic process
N	A1K6Y5	DR	oxidation-reduction process

N	A1KFN9	CC	Catalyzes the rearrangement of 1-deoxy-D-xylulose 5- phosphate (DXP) to produce the thiazole phosphate moiety of thiamine
N	A1KFN9	CC	In vitro, sulfur can be provided by H(2)S (By similarity).
N	A1KFN9	CC	Sulfur is provided by the thiocarboxylate moiety of the carrier protein ThiS
N	A1KFN9	DE	Thiazole synthase;
N	A1KFN9	DR	lyase activity
N	A1KFN9	DR	thiamine biosynthetic process
N	A1KKQ5	CC	Presumably involved in the processing and regular turnover of intracellular [illegible]
N	A1KKQ5	CC	Catalyzes the removal of unsubstituted N-terminal amino acids from various peptides (By similarity).
N	A1KKQ5	DE	Leucine aminopeptidase;
N	A1KKQ5	DE	Leucyl aminopeptidase;
N	A1KKQ5	DE	Probable cytosol aminopeptidase;
N	A1KKQ5	DR	aminopeptidase activity
N	A1KKQ5	DR	manganese ion binding
N	A1KKQ5	DR	metalloexopeptidase activity
N	A1KMN3	CC	Associates with the EF-Tu.GDP complex and induces the exchange of GDP to [illegible]
N	A1KMN3	CC	It remains bound to the aminoacyl-tRNA.EF- Tu.GTP complex up to the GTP hydrolysis stage on the ribosome.
N	A1KMN3	DE	Elongation factor Ts;
N	A1KMN3	DR	translation elongation factor activity
N	A1KNZ9	CC	Structural subunit of pili, which are thin, flexible, coiled-coil, aggregative fibers
N	A1KNZ9	CC	Mediates adhesion to the extracellular matrix, an event that would facilitate direct interaction with the host epithelium during infection in the lung or other tissues (By similarity).
N	A1KNZ9	DE	Pili structural subunit;
N	A1KNZ9	DR	cell adhesion
N	A1KPD0	CC	This protein is one of the early assembly proteins of the 50S ribosomal subunit, although it is not seen to bind rRNA by itself
N	A1KPD0	CC	It is important during the early stages of 50S assembly (By similarity).
N	A1KPD0	DE	50S ribosomal protein L13;
N	A1KPD0	DR	structural constituent of ribosome
N	A1KS98	CC	Catalyzes the formation of S-adenosylmethionine from methionine and ATP
N	A1KS98	CC	The overall synthetic reaction is composed of two sequential steps, AdoMet formation and the subsequent tripolyphosphate hydrolysis which occurs prior to release of AdoMet from the enzyme (By similarity).
N	A1KS98	DE	AdoMet synthase;
N	A1KS98	DE	Methionine adenosyltransferase;
N	A1KS98	DE	S-adenosylmethionine synthase;
N	A1KS98	DR	metal ion binding
N	A1KS98	DR	methionine adenosyltransferase activity
N	A1KS98	DR	one-carbon metabolic process
N	A1KST1	CC	Phosphorylation of dTMP to form dTDP in both de novo and salvage pathways of dTTP synthesis (By similarity).
N	A1KST1	DE	Thymidylate kinase;
N	A1KST1	DE	dTMP kinase;
N	A1KST1	DR	dTDP biosynthetic process
N	A1KST1	DR	thymidylate kinase activity
N	A1KT74	CC	Catalyzes the decarboxylation of orotidine 5'- monophosphate (OMP) to uridine 5'-monophosphate (UMP) (By similarity).
N	A1KT74	DE	OMP decarboxylase;
N	A1KT74	DE	Orotidine 5'-phosphate decarboxylase;
N	A1KT74	DR	'de novo' UMP biosynthetic process
N	A1KT74	DR	'de novo' pyrimidine base biosynthetic process
N	A1KT74	DR	orotidine-5'-phosphate decarboxylase activity
N	A1KU16	CC	Modifies, by uridylylation or deuridylylation the PII (glnB) regulatory protein (By similarity).
N	A1KU16	DE	PII uridylyl-transferase;
N	A1KU16	DE	Uridylyl-removing enzyme;
N	A1KU16	DE	[Protein-PII] uridylyltransferase;
N	A1KU16	DR	[protein-PII] uridylyltransferase activity
N	A1KU16	DR	nitrogen compound metabolic process
N	A1KU52	CC	The ruvA-ruvB complex in the presence of ATP renatures cruciform structure in supercoiled DNA with palindromic sequence, indicating that it may promote strand exchange reactions in homologous recombination

N	A1KU52	CC	RuvAB is an helicase that mediates the Holliday junction migration by localized denaturation and reannealing (By similarity).
N	A1KU52	DE	Holliday junction ATP-dependent DNA helicase ruvB;
N	A1KU52	DR	DNA recombination
N	A1KU52	DR	four-way junction helicase activity
N	A1KW12	CC	Produces ATP from ADP in the presence of a proton gradient across the membrane
N	A1KW12	CC	The gamma chain is believed to be important in regulating ATPase activity and the flow of protons through the CF(0) complex (By similarity).
N	A1KW12	DE	ATP synthase F1 sector gamma subunit;
N	A1KW12	DE	ATP synthase gamma chain;
N	A1KW12	DE	F-ATPase gamma subunit;
N	A1KW12	DR	ATP synthesis coupled proton transport
N	A1KW12	DR	hydrogen ion transporting ATP synthase activity, rotational mechanism
N	A1KW12	DR	proton-transporting ATPase activity, rotational mechanism
N	A1L1N5	CC	Transcription factor, probably binds to the inverted palindrome 5'-GTTAATNATTAAC-3' (By similarity)
N	A1L1N5	CC	Required for induction of rhombomere r5/r6 gene expression in the hindbrain.
N	A1L1N5	DE	HNF-1-beta-A;
N	A1L1N5	DE	Hepatocyte nuclear factor 1-beta-2;
N	A1L1N5	DE	Hepatocyte nuclear factor 1-beta-A;
N	A1L1N5	DE	Transcription factor 2;
N	A1L1N5	DE	Variant hepatic nuclear factor 1;
N	A1L1N5	DR	anterior/posterior pattern formation
N	A1L1N5	DR	digestive tract morphogenesis
N	A1L1N5	DR	embryonic digestive tract development
N	A1L1N5	DR	endoderm formation
N	A1L1N5	DR	hindbrain morphogenesis
N	A1L1N5	DR	liver development
N	A1L1N5	DR	otic placode formation
N	A1L1N5	DR	otolith morphogenesis
N	A1L1N5	DR	pancreas development
N	A1L1N5	DR	regulation of transcription, DNA-dependent
N	A1L1N5	DR	rhombomere 5 morphogenesis
N	A1L1N5	DR	rhombomere 6 morphogenesis
N	A1L1N5	DR	semicircular canal morphogenesis
N	A1L1N5	DR	sequence-specific DNA binding
N	A1L1N5	DR	sequence-specific DNA binding transcription factor activity
N	A1L1N5	DR	transcription activator activity
N	A1L1N5	DR	tube formation
N	A1L253	DE	Protein FAM149B1;
N	A1L3K1	CC	Catalyzes the covalent attachment of ubiquitin to other proteins
N	A1L3K1	CC	Acts as an essential factor of the anaphase promoting complex/cyclosome (APC/C), a cell cycle-regulated ubiquitin ligase that controls progression through mitosis
N	A1L3K1	CC	Acts by specifically elongating 'Lys-11'-linked polyubiquitin chains initiated by the E2 enzyme ube2c/ubch10 on APC/C substrates, enhancing the degradation of APC/C substrates by the proteasome and promoting mitotic exit (By similarity).
N	A1L3K1	DE	Ubiquitin carrier protein S-A;
N	A1L3K1	DE	Ubiquitin-conjugating enzyme E2 S-A;
N	A1L3K1	DE	Ubiquitin-protein ligase S-A;
N	A1L3K1	DR	activation of anaphase-promoting complex activity
N	A1L3K1	DR	anaphase-promoting complex-dependent proteasomal ubiquitin-dependent protein catabolic process
N	A1L3K1	DR	cell division
N	A1L3K1	DR	exit from mitosis
N	A1L3K1	DR	free ubiquitin chain polymerization
N	A1L3K1	DR	post-translational protein modification
N	A1L3K1	DR	protein K11-linked ubiquitination
N	A1L3K1	DR	ubiquitin-protein ligase activity
N	A1L3X0	CC	Condensing enzyme that catalyzes the synthesis of saturated and polyunsaturated very long chain fatty acids
N	A1L3X0	CC	Highest activity toward C18 acyl-CoAs, especially C18:3(n-3) acyl-CoAs.
N	A1L3X0	DE	3-keto acyl-CoA synthase ELOVL7;
N	A1L3X0	DE	Elongation of very long chain fatty acids protein 7;
N	A1L3X0	DR	fatty acid elongase activity
N	A1L3X0	DR	fatty acid elongation, polyunsaturated fatty acid

N	A1L3X0	DR	long-chain fatty-acyl-CoA biosynthetic process
N	A1L3X0	DR	protein binding
N	A1L3X0	DR	triglyceride biosynthetic process
N	A1L3X0	DR	very long-chain fatty acid biosynthetic process
N	A1QZD0	DE	30S ribosomal protein S9;
N	A1QZD0	DR	structural constituent of ribosome
N	A1R6H1	DE	Phosphoglycerate kinase;
N	A1R6H1	DR	phosphoglycerate kinase activity
N	A1RET8	CC	Phosphorolytic exoribonuclease that removes nucleotide residues following the -CCA terminus of tRNA and adds nucleotides to the ends of RNA molecules by using nucleoside diphosphates as substrates (By similarity).
N	A1RET8	DE	Ribonuclease PH;
N	A1RET8	DE	tRNA nucleotidyltransferase;
N	A1RET8	DR	3'-5'-exoribonuclease activity
N	A1RET8	DR	tRNA nucleotidyltransferase activity
N	A1RET8	DR	tRNA processing
N	A1RET8	DR	tRNA-specific ribonuclease activity
N	A1RFM0	CC	Responsible for the addition of glutamate residues to the C-terminus of ribosomal protein S6 (By similarity).
N	A1RFM0	DE	Ribosomal protein S6 modification protein 1;
N	A1RFM0	DR	ligase activity
N	A1RFM0	DR	metal ion binding
N	A1RFM0	DR	protein modification process
N	A1RIP7	CC	Catalyzes a salvage reaction resulting in the formation of AMP, that is energically less costly than de novo synthesis (By similarity).
N	A1RIP7	DE	Adenine phosphoribosyltransferase;
N	A1RIP7	DR	adenine phosphoribosyltransferase activity
N	A1RIP7	DR	adenine salvage
N	A1RIP7	DR	purine ribonucleoside salvage
N	A1RMT4	CC	Catalyzes two reactions: the first one is the production of beta-formyl glycinamide ribonucleotide (GAR) from formate, ATP and beta GAR; the second, a side reaction, is the production of acetyl phosphate and ADP from acetate and ATP (By similarity).
N	A1RMT4	DE	5'-phosphoribosylglycinamide transformylase 2;
N	A1RMT4	DE	Formate-dependent GAR transformylase;
N	A1RMT4	DE	GAR transformylase 2;
N	A1RMT4	DE	Phosphoribosylglycinamide formyltransferase 2;
N	A1RMT4	DR	hydroxymethyl-, formyl- and related transferase activity
N	A1RMT4	DR	ligase activity
N	A1RMT4	DR	magnesium ion binding
N	A1RMT4	DR	purine ribonucleotide biosynthetic process
N	A1RMV7	DE	50S ribosomal protein L27;
N	A1RMV7	DR	structural constituent of ribosome
N	A1RNM8	CC	Displays ATPase and GTPase activities (By similarity).
N	A1RNM8	DE	UPF0042 nucleotide-binding protein Sputw3181_3461;
N	A1RQQ2	DE	FGAM synthase II;
N	A1RQQ2	DE	Phosphoribosylformylglycinamidine synthase 2;
N	A1RQQ2	DE	Phosphoribosylformylglycinamidine synthase II;
N	A1RQQ2	DR	'de novo' IMP biosynthetic process
N	A1RQQ2	DR	phosphoribosylformylglycinamidine synthase activity
N	A1RU37	CC	With S4 and S12 plays an important role in translational accuracy (By similarity).
N	A1RU37	DE	30S ribosomal protein S5P;
N	A1RU37	DR	structural constituent of ribosome
N	A1RWQ7	CC	One of the primary rRNA binding proteins
N	A1RWQ7	CC	It has been suggested to have peptidyltransferase activity; this is somewhat controversial
N	A1RWQ7	CC	Makes several contacts with the 16S rRNA in the 70S ribosome (By similarity).
N	A1RWQ7	CC	Required for association of the 30S and 50S subunits to form the 70S ribosome, for tRNA binding and peptide bond formation
N	A1RWQ7	DE	50S ribosomal protein L2P;
N	A1RWQ7	DR	structural constituent of ribosome
N	A1RWX6	DE	50S ribosomal protein L40e;
N	A1S227	CC	One of the primary rRNA binding proteins, it binds specifically to the 5'-end of 16S ribosomal RNA (By similarity).
N	A1S227	DE	30S ribosomal protein S17;
N	A1S227	DR	structural constituent of ribosome
N	A1S467	CC	Involved in mRNA degradation

N	A1S467	CC	Hydrolyzes single-stranded polyribonucleotides processively in the 3'- to 5'-direction (By similarity).
N	A1S467	DE	Polynucleotide phosphorylase;
N	A1S467	DE	Polyribonucleotide nucleotidyltransferase;
N	A1S467	DR	3'-5'-exoribonuclease activity
N	A1S467	DR	RNA processing
N	A1S467	DR	mRNA catabolic process
N	A1S467	DR	polyribonucleotide nucleotidyltransferase activity
N	A1S474	CC	Catalyzes a reversible aldol reaction between acetaldehyde and D-glyceraldehyde 3-phosphate to generate 2-deoxy- D-ribose 5-phosphate (By similarity).
N	A1S474	DE	2-deoxy-D-ribose 5-phosphate aldolase;
N	A1S474	DE	Deoxyriboaldolase;
N	A1S474	DE	Deoxyribose-phosphate aldolase;
N	A1S474	DE	Phosphodeoxyriboaldolase;
N	A1S474	DR	deoxyribonucleotide catabolic process
N	A1S474	DR	deoxyribose-phosphate aldolase activity
N	A1S579	DE	Acylphosphatase;
N	A1S579	DE	Acylphosphate phosphohydrolase;
N	A1S579	DR	acylphosphatase activity
N	A1S859	CC	GTPase that plays an essential role in the late steps of ribosome biogenesis (By similarity).
N	A1S859	DE	GTP-binding protein EngA;
N	A1S859	DR	nucleoside-triphosphatase activity
N	A1S859	DR	ribosome biogenesis
N	A1SAS5	DE	Dihydroxy-acid dehydratase;
N	A1SAS5	DR	4 iron, 4 sulfur cluster binding
N	A1SAS5	DR	branched chain family amino acid biosynthetic process
N	A1SAS5	DR	dihydroxy-acid dehydratase activity
N	A1SAS5	DR	metal ion binding
N	A1SBV0	CC	Specifically methylates the N7 position of guanosine in position 527 of 16S rRNA (By similarity).
N	A1SBV0	DE	16S rRNA 7-methylguanosine methyltransferase;
N	A1SBV0	DE	16S rRNA m7G methyltransferase;
N	A1SBV0	DE	Ribosomal RNA small subunit methyltransferase G;
N	A1SBV0	DR	rRNA methyltransferase activity
N	A1SHI6	CC	F(1)F(0) ATP synthase produces ATP from ADP in the presence of a proton or sodium gradient
N	A1SHI6	CC	During catalysis, ATP synthesis in the catalytic domain of F(1) is coupled via a rotary mechanism of the central stalk subunits to proton translocation (By similarity).
N	A1SHI6	CC	F-type ATPases consist of two structural domains, F(1) containing the extramembraneous catalytic core and F(0) containing the membrane proton channel, linked together by a central stalk and a peripheral stalk
N	A1SHI6	DE	ATP synthase F(0) sector subunit c;
N	A1SHI6	DE	ATP synthase subunit c;
N	A1SHI6	DE	F-ATPase subunit c;
N	A1SHI6	DE	F-type ATPase subunit c;
N	A1SHI6	DE	Lipid-binding protein;
N	A1SHI6	DR	ATP hydrolysis coupled proton transport
N	A1SHI6	DR	ATP synthesis coupled proton transport
N	A1SHI6	DR	hydrogen ion transmembrane transporter activity
N	A1SHI6	DR	lipid binding
N	A1SL79	CC	Cell wall formation (By similarity).
N	A1SL79	DE	UDP-N-acetylmuramate--L-alanine ligase;
N	A1SL79	DE	UDP-N-acetylmuramoyl-L-alanine synthetase;
N	A1SL79	DR	UDP-N-acetylmuramate-L-alanine ligase activity
N	A1SL79	DR	cell division
N	A1SL79	DR	cellular cell wall organization
N	A1SL79	DR	peptidoglycan biosynthetic process
N	A1SL79	DR	regulation of cell shape
N	A1SNC7	CC	Negatively regulates transcription of bacterial ribonucleotide reductase nrd genes and operons by binding to NrdR- boxes (By similarity).
N	A1SNC7	DE	Transcriptional repressor NrdR;
N	A1SNC7	DR	transcription repressor activity
N	A1SNC7	DR	zinc ion binding
N	A1SR48	DE	Mannitol-1-phosphate 5-dehydrogenase;

N	A1SR48	DR	coenzyme binding
N	A1SR48	DR	mannitol-1-phosphate 5-dehydrogenase activity
N	A1SR48	DR	oxidation-reduction process
N	A1SRU1	CC	Catalyzes the interconversion of beta-pyran and beta- furan forms of D-ribose (By similarity).
N	A1SRU1	DE	D-ribose pyranase;
N	A1SRU1	DR	carbohydrate metabolic process
N	A1SRU1	DR	carbohydrate transport
N	A1SRU1	DR	intramolecular lyase activity
N	A1T1A5	CC	Exhibits S-adenosyl-L-methionine-dependent methyltransferase activity (By similarity).
N	A1T1A5	DE	Putative S-adenosyl-L-methionine-dependent methyltransferase Mvan_0104;
N	A1T1A5	DR	methyltransferase activity
N	A1T5Z3	DE	3-phosphoshikimate 1-carboxyvinyltransferase;
N	A1T5Z3	DE	5-enolpyruvylshikimate-3-phosphate synthase;
N	A1T5Z3	DE	EPSP synthase;
N	A1T5Z3	DR	3-phosphoshikimate 1-carboxyvinyltransferase activity
N	A1T5Z3	DR	aromatic amino acid family biosynthetic process
N	A1T8F3	DE	5-enolpyruvylshikimate-3-phosphate phospholyase;
N	A1T8F3	DE	Chorismate synthase;
N	A1T8F3	DR	aromatic amino acid family biosynthetic process
N	A1T8F3	DR	chorismate synthase activity
N	A1TJA3	CC	ATP-dependent carboxylate-amine ligase (By similarity).
N	A1TJA3	DE	Carboxylate-amine ligase Aave_0434;
N	A1TJA3	DR	glutamate-cysteine ligase activity
N	A1TJA3	DR	glutathione biosynthetic process
N	A1TLL7	CC	NDH-1 shuttles electrons from NADH, via FMN and iron- sulfur (Fe-S) centers, to quinones in the respiratory chain
N	A1TLL7	CC	Couples the redox reaction to proton translocation (for every two electrons transferred, four hydrogen ions are translocated across the cytoplasmic membrane), and thus conserves the redox energy in a proton gradient (By similarity).
N	A1TLL7	DE	NADH dehydrogenase I subunit B;
N	A1TLL7	DE	NADH-quinone oxidoreductase subunit B;
N	A1TLL7	DE	NDH-1 subunit B;
N	A1TLL7	DR	4 iron, 4 sulfur cluster binding
N	A1TLL7	DR	NADH dehydrogenase (ubiquinone) activity
N	A1TLL7	DR	metal ion binding
N	A1TLL7	DR	oxidation-reduction process
N	A1TLL7	DR	quinone binding
N	A1TPW5	DE	D-serine deaminase;
N	A1TPW5	DE	Probable D-serine dehydratase;
N	A1TPW5	DR	D-amino acid metabolic process
N	A1TPW5	DR	D-serine ammonia-lyase activity
N	A1TPW5	DR	pyridoxal phosphate binding
N	A1TQ96	CC	Catalyzes the reversible transfer of the terminal phosphate group between ATP and AMP
N	A1TQ96	CC	This small ubiquitous enzyme involved in the energy metabolism and nucleotide synthesis, is essential for maintenance and cell growth (By similarity).
N	A1TQ96	DE	ATP-AMP transphosphorylase;
N	A1TQ96	DE	Adenylate kinase;
N	A1TQ96	DR	adenylate kinase activity
N	A1TQ96	DR	nucleotide biosynthetic process
N	A1TRE4	DE	Histidine ammonia-lyase;
N	A1TRE4	DR	biosynthetic process
N	A1TRE4	DR	histidine ammonia-lyase activity
N	A1TRE4	DR	histidine catabolic process
N	A1TT41	CC	Hydrolyzes non-standard nucleotides such as XTP and dITP/ITP
N	A1TT41	CC	Might exclude non-standard purines from DNA precursor pool, preventing thus incorporation into DNA and avoiding chromosomal lesions (By similarity).
N	A1TT41	DE	Nucleoside triphosphate phosphohydrolase;
N	A1TT41	DE	Nucleoside-triphosphatase;
N	A1TT41	DR	nucleoside-triphosphatase activity
N	A1TWI0	CC	Catalyzes a salvage reaction resulting in the formation of AMP, that is energically less costly than de novo synthesis (By similarity).
N	A1TWI0	DE	Adenine phosphoribosyltransferase;
N	A1TWI0	DR	adenine phosphoribosyltransferase activity

N	A1TWI0	DR	adenine salvage
N	A1TWI0	DR	purine ribonucleoside salvage
N	A1TZ73	DE	AIR synthase;
N	A1TZ73	DE	Phosphoribosyl-aminoimidazole synthetase;
N	A1TZ73	DE	Phosphoribosylformylglycinamidine cyclo-ligase;
N	A1TZ73	DR	'de novo' IMP biosynthetic process
N	A1TZ73	DR	phosphoribosylformylglycinamidine cyclo-ligase activity
N	A1TZ94	CC	Could accelerate the degradation of some genes transcripts potentially through selective RNA binding (By similarity).
N	A1TZ94	DE	Carbon storage regulator homolog;
N	A1TZ94	DR	mRNA catabolic process
N	A1TZ94	DR	regulation of carbohydrate metabolic process
N	A1UA84	CC	Plays an important role in the de novo pathway of purine nucleotide biosynthesis
N	A1UA84	CC	Catalyzes the first committed step in the biosynthesis of AMP from IMP (By similarity)
N	A1UA84	DE	Adenylosuccinate synthetase;
N	A1UA84	DE	IMP--aspartate ligase;
N	A1UA84	DR	adenylosuccinate synthase activity
N	A1UA84	DR	magnesium ion binding
N	A1UA84	DR	purine nucleotide biosynthetic process
N	A1UC06	CC	Catalyzes the conversion of glucosamine-6-phosphate to glucosamine-1-phosphate (By similarity).
N	A1UC06	DE	Phosphoglucosamine mutase;
N	A1UC06	DR	carbohydrate metabolic process
N	A1UC06	DR	magnesium ion binding
N	A1UC06	DR	phosphoglucosamine mutase activity
N	A1UED9	DE	Acylphosphatase;
N	A1UED9	DE	Acylphosphate phosphohydrolase;
N	A1UED9	DR	acylphosphatase activity
N	A1UMI0	CC	Catalyzes the condensation of pantoate with beta-alanine in an ATP-dependent reaction via a pantoyl-adenylate intermediate (By similarity).
N	A1UMI0	DE	Pantoate--beta-alanine ligase;
N	A1UMI0	DE	Pantoate-activating enzyme;
N	A1UMI0	DE	Pantothenate synthetase;
N	A1UMI0	DR	pantoate-beta-alanine ligase activity
N	A1UMI0	DR	pantothenate biosynthetic process
N	A1USQ7	CC	Binds 16S rRNA, required for the assembly of 30S particles and may also be responsible for determining the conformation of the 16S rRNA at the A site (By similarity)
N	A1USQ7	DE	30S ribosomal protein S14;
N	A1USQ7	DR	structural constituent of ribosome
N	A1UUA2	DE	Nicotinate phosphoribosyltransferase;
N	A1UUA2	DR	NAD biosynthetic process
N	A1UUA2	DR	nicotinate nucleotide biosynthetic process
N	A1UUA2	DR	nicotinate phosphoribosyltransferase activity
N	A1UUA2	DR	nicotinate-nucleotide diphosphorylase (carboxylating) activity
N	A1V878	CC	One of the primary rRNA binding proteins, it binds directly to 16S rRNA where it nucleates assembly of the body of the 30S subunit (By similarity).
N	A1V878	DE	30S ribosomal protein S4;
N	A1V878	DR	structural constituent of ribosome
N	A1V905	CC	Probably deamidates glutamine residues to glutamate on methyl-accepting chemotaxis receptors (MCPs), playing an important role in chemotaxis (By similarity)
N	A1V905	DE	Probable chemoreceptor glutamine deamidase CheD;
N	A1V905	DR	protein-glutamine glutaminase activity
N	A1VDQ7	CC	Protease subunit of a proteasome-like degradation complex believed to be a general protein degrading machinery (By similarity).
N	A1VDQ7	DE	ATP-dependent protease subunit HslV;
N	A1VDQ7	DR	metal ion binding
N	A1VDQ7	DR	proteolysis involved in cellular protein catabolic process
N	A1VDQ7	DR	threonine-type endopeptidase activity
N	A1VE87	CC	Synthesizes selenophosphate from selenide and ATP (By similarity).
N	A1VE87	DE	Selenide, water dikinase;
N	A1VE87	DE	Selenium donor protein;
N	A1VE87	DE	Selenophosphate synthase;
N	A1VE87	DR	selenide, water dikinase activity
N	A1VEA9	CC	Binds 23S rRNA and is also seen to make contacts with the A and possibly P site tRNAs (By similarity).

N	A1VEA9	DE	50S ribosomal protein L16;
N	A1VEA9	DR	structural constituent of ribosome
N	A1VEB3	CC	One of the primary rRNA binding proteins
N	A1VEB3	CC	It has been suggested to have peptidyltransferase activity; this is somewhat controversial
N	A1VEB3	CC	Makes several contacts with the 16S rRNA in the 70S ribosome (By similarity).
N	A1VEB3	CC	Required for association of the 30S and 50S subunits to form the 70S ribosome, for tRNA binding and peptide bond formation
N	A1VEB3	DE	50S ribosomal protein L2;
N	A1VEB3	DR	structural constituent of ribosome
N	A1VEB3	DR	transferase activity
N	A1VEP0	CC	Is an aliphatic amidase with a restricted substrate specificity, as it only hydrolyzes formamide (By similarity).
N	A1VEP0	DE	Formamidase;
N	A1VEP0	DE	Formamide amidohydrolase;
N	A1VEP0	DR	formamidase activity
N	A1VEP0	DR	nitrogen compound metabolic process
N	A1VXZ8	CC	Involved in the biosynthesis of lipid A, a phosphorylated glycolipid that anchors the lipopolysaccharide to the outer membrane of the cell (By similarity).
N	A1VXZ8	DE	Acyl-[acyl-carrier-protein]--UDP-N-acetylglucosamine O-acyltransferase;
N	A1VXZ8	DE	UDP-N-acetylglucosamine acyltransferase;
N	A1VXZ8	DR	acyl-[acyl-carrier-protein]-UDP-N-acetylglucosamine O-acyltransferase activity
N	A1VXZ8	DR	lipid A biosynthetic process
N	A1W0I1	CC	Allows the formation of correctly charged Asn-tRNA(Asn) or Gln-tRNA(Gln) through the transamidation of misacylated Asp- tRNA(Asn) or Glu-tRNA(Gln) in organisms which lack either or both of asparaginyl-tRNA or glutaminyl-tRNA synthetases
N	A1W0I1	CC	The reaction takes place in the presence of glutamine and ATP through an activated phospho-Asp-tRNA(Asn) or phospho-Glu-tRNA(Gln) (By similarity).
N	A1W0I1	DE	Asp/Glu-ADT subunit B;
N	A1W0I1	DE	Aspartyl/glutamyl-tRNA(Asn/Gln) amidotransferase subunit B;
N	A1W0I1	DR	carbon-nitrogen ligase activity, with glutamine as amido-N-donor
N	A1W0X9	CC	The ruvA-ruvB complex in the presence of ATP renatures cruciform structure in supercoiled DNA with palindromic sequence, indicating that it may promote strand exchange reactions in homologous recombination
N	A1W0X9	CC	RuvAB is an helicase that mediates the Holliday junction migration by localized denaturation and reannealing (By similarity).
N	A1W0X9	DE	Holliday junction ATP-dependent DNA helicase ruvB;
N	A1W0X9	DR	DNA recombination
N	A1W0X9	DR	four-way junction helicase activity
N	A1W1V8	CC	One of the early assembly proteins it binds 23S rRNA
N	A1W1V8	CC	Forms the main docking site for trigger factor binding to the ribosome (By similarity).
N	A1W1V8	CC	One of the proteins that surrounds the polypeptide exit tunnel on the outside of the ribosome
N	A1W1V8	DE	50S ribosomal protein L23;
N	A1W1V8	DR	nucleotide binding
N	A1W1V8	DR	structural constituent of ribosome
N	A1W2Z7	DE	Anthranilate phosphoribosyltransferase;
N	A1W2Z7	DR	anthranilate phosphoribosyltransferase activity
N	A1W2Z7	DR	tryptophan biosynthetic process
N	A1W333	CC	DNA-dependent RNA polymerase catalyzes the transcription of DNA into RNA using the four ribonucleoside triphosphates as substrates.
N	A1W333	DE	DNA-directed RNA polymerase subunit alpha;
N	A1W333	DE	RNA polymerase subunit alpha;
N	A1W333	DE	RNAP subunit alpha;
N	A1W333	DE	Transcriptase subunit alpha;
N	A1W333	DR	DNA-directed RNA polymerase activity
N	A1W333	DR	protein dimerization activity
N	A1WBI2	CC	Catalyzes the pyruvoyl-dependent decarboxylation of aspartate to produce beta-alanine (By similarity).
N	A1WBI2	DE	Aspartate 1-decarboxylase alpha chain;
N	A1WBI2	DE	Aspartate 1-decarboxylase beta chain;
N	A1WBI2	DE	Aspartate 1-decarboxylase;
N	A1WBI2	DE	Aspartate alpha-decarboxylase;
N	A1WBI2	DR	alanine biosynthetic process
N	A1WBI2	DR	aspartate 1-decarboxylase activity
N	A1WBI2	DR	pantothenate biosynthetic process

N	A1WGK3	CC	Binds as a heterodimer with protein S6 to the central domain of the 16S rRNA, where it helps stabilize the platform of the 30S subunit (By similarity).
N	A1WGK3	DE	30S ribosomal protein S18;
N	A1WGK3	DR	structural constituent of ribosome
N	A1WH89	DE	Acetylglutamate kinase;
N	A1WH89	DE	N-acetyl-L-glutamate 5-phosphotransferase;
N	A1WH89	DR	acetylglutamate kinase activity
N	A1WH89	DR	arginine biosynthetic process
N	A1WH89	DR	glutamate 5-kinase activity
N	A1WH89	DR	proline biosynthetic process
N	A1WX11	CC	Involved in the biosynthesis of lipid A, a phosphorylated glycolipid that anchors the lipopolysaccharide to the outer membrane of the cell (By similarity).
N	A1WX11	DE	Acyl-[acyl-carrier-protein]--UDP-N-acetylglucosamine O-acyltransferase;
N	A1WX11	DE	UDP-N-acetylglucosamine acyltransferase;
N	A1WX11	DR	acyl-[acyl-carrier-protein]-UDP-N-acetylglucosamine O-acyltransferase activity
N	A1WX11	DR	lipid A biosynthetic process
N	A1WXV0	CC	Associates with free 30S ribosomal subunits (but not with 30S subunits that are part of 70S ribosomes or polysomes)
N	A1WXV0	CC	Essential for efficient processing of 16S rRNA
N	A1WXV0	CC	May interact with the 5'-terminal helix region of 16S rRNA (By similarity).
N	A1WXV0	DE	Ribosome-binding factor A;
N	A1WXV0	DR	rRNA processing
N	A1WYT0	CC	Catalyzes the decarboxylation of S-adenosylmethionine to S-adenosylmethioninamine (dcAdoMet), the propylamine donor required for the synthesis of the polyamines spermine and spermidine from the diamine putrescine (By similarity).
N	A1WYT0	DE	S-adenosylmethionine decarboxylase alpha chain;
N	A1WYT0	DE	S-adenosylmethionine decarboxylase beta chain;
N	A1WYT0	DE	S-adenosylmethionine decarboxylase proenzyme;
N	A1WYT0	DR	adenosylmethionine decarboxylase activity
N	A1WYT0	DR	spermidine biosynthetic process
N	A1WZT3	CC	Produces ATP from ADP in the presence of a proton gradient across the membrane
N	A1WZT3	CC	The alpha chain is a regulatory subunit (By similarity).
N	A1WZT3	DE	ATP synthase F1 sector subunit alpha;
N	A1WZT3	DE	ATP synthase subunit alpha;
N	A1WZT3	DE	F-ATPase subunit alpha;
N	A1WZT3	DR	ATP hydrolysis coupled proton transport
N	A1WZT3	DR	ATP synthesis coupled proton transport
N	A1WZT3	DR	hydrogen ion transporting ATP synthase activity, rotational mechanism
N	A1WZT3	DR	proton-transporting ATPase activity, rotational mechanism
N	A1XFV1	CC	One of the components of the core antenna complex of photosystem II
N	A1XFV1	CC	It binds chlorophyll and helps catalyze the primary light-induced photochemical processes of photosystem II (By similarity).
N	A1XFV1	DE	PSII 43 kDa protein;
N	A1XFV1	DE	Photosystem II 44 kDa reaction center protein;
N	A1XFV1	DE	Photosystem II CP43 chlorophyll apoprotein;
N	A1XFV1	DE	Protein CP-43;
N	A1XFV1	DR	chlorophyll binding
N	A1XFV1	DR	electron transporter, transferring electrons within the cyclic electron transport pathway of photosynthesis activity
N	A1XFV1	DR	photosynthetic electron transport in photosystem II
N	A1XFV1	DR	protein-chromophore linkage
N	A1YGR5	CC	Transfer of galactose from UDP-galactose to an acceptor molecule (R).
N	A1YGR5	DE	Galactosyltransferase;
N	A1YGR5	DE	N-acetyllactosaminide alpha-1,3-galactosyltransferase;
N	A1YGR5	DE	UDP-galactose:beta-D-galactosyl-1,4-N-acetyl-D-glucosaminide alpha-1,3-galactosyltransferase;
N	A1YGR5	DR	N-acetyllactosaminide 3-alpha-galactosyltransferase activity
N	A1YGR5	DR	carbohydrate metabolic process
N	A1YGR5	DR	metal ion binding
N	A1ZB42	CC	Component of the Mediator complex, a coactivator involved in the regulated transcription of nearly all RNA polymerase II-dependent genes
N	A1ZB42	CC	Mediator functions as a bridge to convey information from gene-specific regulatory proteins to the basal RNA polymerase II transcription machinery

N	A1ZB42	CC	Mediator is recruited to promoters by direct interactions with regulatory proteins and serves as a scaffold for the assembly of a functional preinitiation complex with RNA polymerase II and the general transcription factors (By similarity).
N	A1ZB42	DE	Mediator complex subunit 9;
N	A1ZB42	DE	Mediator of RNA polymerase II transcription subunit 9;
N	A1ZB42	DR	RNA polymerase II transcription mediator activity
N	A1ZB42	DR	regulation of transcription from RNA polymerase II promoter
N	A1ZB42	DR	transcription from RNA polymerase II promoter
N	A2BGT0	CC	May participate in the maintenance of mitochondrial morphology (By similarity).
N	A2BGT0	DE	Deubiquitinating enzyme 30;
N	A2BGT0	DE	Ub-specific protease 30;
N	A2BGT0	DE	Ubiquitin carboxyl-terminal hydrolase 30;
N	A2BGT0	DE	Ubiquitin thiolesterase 30;
N	A2BGT0	DE	Ubiquitin-specific-processing protease 30;
N	A2BGT0	DR	cysteine-type peptidase activity
N	A2BGT0	DR	ubiquitin thiolesterase activity
N	A2BGT0	DR	ubiquitin-dependent protein catabolic process
N	A2BN64	CC	Binds directly to 23S rRNA
N	A2BN64	CC	Probably involved in E site tRNA release (By similarity).
N	A2BN64	DE	50S ribosomal protein L1P;
N	A2BN64	DR	RNA processing
N	A2BN64	DR	regulation of translation
N	A2BN64	DR	structural constituent of ribosome
N	A2BQY1	DE	Anthranilate phosphoribosyltransferase;
N	A2BQY1	DR	anthranilate phosphoribosyltransferase activity
N	A2BQY1	DR	tryptophan biosynthetic process
N	A2BSP9	CC	Catalyzes the transfer of an acyl group from acyl- phosphate (acyl-PO(4)) to glycerol-3-phosphate (G3P) to form lysophosphatidic acid (LPA)
N	A2BSP9	CC	This enzyme utilizes acyl-phosphate as fatty acyl donor, but not acyl-CoA or acyl-ACP (By similarity).
N	A2BSP9	DE	Acyl-PO4 G3P acyltransferase;
N	A2BSP9	DE	Acyl-phosphate--glycerol-3-phosphate acyltransferase;
N	A2BSP9	DE	G3P acyltransferase;
N	A2BSP9	DE	Glycerol-3-phosphate acyltransferase;
N	A2BSP9	DE	LPA synthase;
N	A2BSP9	DE	Lysophosphatidic acid synthase;
N	A2BSP9	DR	phospholipid biosynthetic process
N	A2BSP9	DR	transferase activity, transferring acyl groups other than amino-acyl groups
N	A2BV61	CC	Catalyzes the transfer of endogenously produced octanoic acid from octanoyl-acyl-carrier-protein onto the lipoyl domains of lipoate-dependent enzymes
N	A2BV61	CC	Lipoyl-ACP can also act as a substrate although octanoyl-ACP is likely to be the physiological substrate (By similarity).
N	A2BV61	DE	Lipoate-protein ligase B;
N	A2BV61	DE	Lipoyl/octanoyl transferase;
N	A2BV61	DE	Octanoyl-[acyl-carrier-protein]-protein N-octanoyltransferase;
N	A2BV61	DE	Octanoyltransferase;
N	A2BV61	DR	lipoate biosynthetic process
N	A2BV61	DR	lipoyl(octanoyl) transferase activity
N	A2BV61	DR	octanoyltransferase activity
N	A2BV61	DR	protein modification process
N	A2BV78	DE	Malate dehydrogenase [quinone];
N	A2BV78	DE	Probable malate:quinone oxidoreductase;
N	A2BV78	DR	malate dehydrogenase (menaquinone) activity
N	A2BV78	DR	malate dehydrogenase (quinone) activity
N	A2BV78	DR	tricarboxylic acid cycle
N	A2BWM0	DE	N-acetyl-gamma-glutamyl-phosphate reductase;
N	A2BWM0	DE	N-acetyl-glutamate semialdehyde dehydrogenase;
N	A2BWM0	DE	NAGSA dehydrogenase;
N	A2BWM0	DR	N-acetyl-gamma-glutamyl-phosphate reductase activity
N	A2BWM0	DR	arginine biosynthetic process
N	A2BWM0	DR	oxidation-reduction process
N	A2BWM0	DR	protein dimerization activity
N	A2C4J7	CC	F(1)F(0) ATP synthase produces ATP from ADP in the presence of a proton or sodium gradient

N	A2C4J7	CC	During catalysis, ATP synthesis in the catalytic domain of F(1) is coupled via a rotary mechanism of the central stalk subunits to proton translocation (By similarity).
N	A2C4J7	CC	F-type ATPases consist of two structural domains, F(1) containing the extramembraneous catalytic core and F(0) containing the membrane proton channel, linked together by a central stalk and a peripheral stalk
N	A2C4J7	DE	ATP synthase F(0) sector subunit b;
N	A2C4J7	DE	ATP synthase subunit b;
N	A2C4J7	DE	ATPase subunit I;
N	A2C4J7	DE	F-ATPase subunit b;
N	A2C4J7	DE	F-type ATPase subunit b;
N	A2C4J7	DR	ATP synthesis coupled proton transport
N	A2C4J7	DR	hydrogen ion transmembrane transporter activity
N	A2C4M0	CC	Specifically methylates the N7 position of a guanosine in 16S rRNA (By similarity).
N	A2C4M0	DE	16S rRNA 7-methylguanosine methyltransferase;
N	A2C4M0	DE	16S rRNA m7G methyltransferase;
N	A2C4M0	DE	Ribosomal RNA small subunit methyltransferase G;
N	A2C4M0	DR	rRNA methyltransferase activity
N	A2CBT5	DE	Photosystem II reaction center protein M;
N	A2CBT5	DR	photosynthesis, light reaction
N	A2CC86	CC	Catalyzes the GTP-dependent ribosomal translocation step during translation elongation.
N	A2CC86	CC	Catalyzes the coordinated movement of the two tRNA molecules, the mRNA and conformational changes in the ribosome (By similarity).
N	A2CC86	CC	During this step, the ribosome changes from the pre-translocational (PRE) to the post- translocational (POST) state as the newly formed A-site-bound peptidyl-tRNA and P-site-bound deacylated tRNA move to the P and E sites, respectively
N	A2CC86	DE	Elongation factor G;
N	A2CC86	DR	GTPase activity
N	A2CC86	DR	translation elongation factor activity
N	A2CI35	CC	May induce both caspase-dependent apoptosis and caspase- independent cell death (By similarity).
N	A2CI35	DE	Dual serine/threonine and tyrosine protein kinase;
N	A2CI35	DE	Dusty protein kinase;
N	A2CI35	DE	Receptor-interacting serine/threonine-protein kinase 5;
N	A2CI35	DR	protein phosphorylation
N	A2CI35	DR	protein serine/threonine kinase activity
N	A2CI35	DR	protein tyrosine kinase activity
N	A2ICR5	CC	Crystallins are the dominant structural components of the vertebrate eye lens (By similarity).
N	A2ICR5	DE	Beta-A4 crystallin;
N	A2ICR5	DE	Beta-crystallin A4;
N	A2ICR5	DR	structural constituent of eye lens
N	A2QUJ2	CC	Catalytic subunit of the slx1-slx4 structure-specific endonuclease that resolves DNA secondary structures generated during DNA repair and recombination
N	A2QUJ2	CC	Has endonuclease activity towards branched DNA substrates, introducing single-strand cuts in duplex DNA close to junctions with ss-DNA (By similarity).
N	A2QUJ2	DE	Structure-specific endonuclease subunit slx1;
N	A2QUJ2	DR	DNA recombination
N	A2QUJ2	DR	endonuclease activity
N	A2QUJ2	DR	metal ion binding
N	A2QWA6	CC	Involved in cytoplasm to vacuole transport (Cvt)
N	A2QWA6	CC	Probably involved in the switching machinery regulating the conversion between the Cvt pathway and autophagy (By similarity).
N	A2QWA6	DE	Autophagy-related protein 13;
N	A2QWA6	DR	protein transport
N	A2RC52	CC	DNA-dependent RNA polymerase catalyzes the transcription of DNA into RNA using the four ribonucleoside triphosphates as substrates.
N	A2RC52	DE	DNA-directed RNA polymerase subunit beta';
N	A2RC52	DE	RNA polymerase subunit beta';
N	A2RC52	DE	RNAP subunit beta';
N	A2RC52	DE	Transcriptase subunit beta';
N	A2RC52	DR	DNA-directed RNA polymerase activity
N	A2RIV7	CC	Zinc phosphodiesterase, which displays some tRNA 3'-processing endonuclease activity.
N	A2RIV7	CC	Probably involved in tRNA maturation, by removing a 3'-trailer from precursor tRNA (By similarity).
N	A2RIV7	DE	Ribonuclease Z;

N	A2RIV7	DE	tRNA 3 endonuclease;
N	A2RIV7	DR	endoribonuclease activity, producing 5'-phosphomonoesters
N	A2RIV7	DR	metal ion binding
N	A2RIV7	DR	tRNA 3'-trailer cleavage
N	A2RKP9	CC	Site-specific tyrosine recombinase, which acts by catalyzing the cutting and rejoining of the recombining DNA molecules
N	A2RKP9	CC	Essential to convert dimers of the bacterial chromosome into monomers to permit their segregation at cell division (By similarity).
N	A2RKP9	DE	Tyrosine recombinase xerS;
N	A2RKP9	DR	DNA integration
N	A2RKP9	DR	DNA recombination
N	A2RKP9	DR	cell division
N	A2RKP9	DR	chromosome segregation
N	A2S3E6	CC	Probable reductase (By similarity).
N	A2S3E6	DE	Putative reductase BMA10229_A0467;
N	A2S3E6	DR	oxidation-reduction process
N	A2S3E6	DR	oxidoreductase activity
N	A2S8D3	DE	50S ribosomal protein L34;
N	A2S8D3	DR	structural constituent of ribosome
N	A2SFW9	DE	Fd-NADP+ reductase;
N	A2SFW9	DE	Ferredoxin--NADP reductase;
N	A2SFW9	DR	ferredoxin-NADP+ reductase activity
N	A2SFW9	DR	oxidation-reduction process
N	A2SJJ7	CC	RuBisCO catalyzes two reactions: the carboxylation of D- ribulose 1,5-bisphosphate, the primary event in carbon dioxide fixation, as well as the oxidative fragmentation of the pentose substrate
N	A2SJJ7	CC	Both reactions occur simultaneously and in competition at the same active site (By similarity).
N	A2SJJ7	DE	Ribulose bisphosphate carboxylase large chain 2;
N	A2SJJ7	DE	RuBisCO large subunit 2;
N	A2SJJ7	DR	magnesium ion binding
N	A2SJJ7	DR	monooxygenase activity
N	A2SJJ7	DR	oxidation-reduction process
N	A2SJJ7	DR	reductive pentose-phosphate cycle
N	A2SJJ7	DR	ribulose-bisphosphate carboxylase activity
N	A2SPM2	CC	With S4 and S12 plays an important role in translational accuracy (By similarity).
N	A2SPM2	DE	30S ribosomal protein S5P;
N	A2SPM2	DR	structural constituent of ribosome
N	A2SQM4	CC	Specifically catalyzes the AdoMet-dependent 2'-O-ribose methylation of cytidine at position 56 in tRNAs (By similarity).
N	A2SQM4	DE	tRNA ribose 2'-O-methyltransferase aTrm56;
N	A2SQM4	DR	tRNA methyltransferase activity
N	A2SQM4	DR	tRNA nucleoside ribose methylation
N	A2SR38	CC	DNA ligase that seals nicks in double-stranded DNA during DNA replication, DNA recombination and DNA repair (By similarity).
N	A2SR38	DE	Polydeoxyribonucleotide synthase [ATP];
N	A2SR38	DR	DNA ligase (ATP) activity
N	A2SR38	DR	DNA recombination
N	A2SR38	DR	DNA replication
N	A2SR38	DR	cell division
N	A2SR38	DR	metal ion binding
N	A2SST4	CC	Produces ATP from ADP in the presence of a proton gradient across the membrane
N	A2SST4	DE	V-type ATP synthase subunit F;
N	A2SST4	DE	V-type ATPase subunit F;
N	A2SST4	DR	ATP biosynthetic process
N	A2SST4	DR	ATP hydrolysis coupled proton transport
N	A2SST4	DR	hydrogen ion transporting ATP synthase activity, rotational mechanism
N	A2SST4	DR	proton-transporting ATPase activity, rotational mechanism
N	A2ST72	DE	UPF0147 protein Mlab_1361;
N	A2SU54	CC	Possesses two activities: a DNA synthesis (polymerase) and an exonucleolytic activity that degrades single stranded DNA in the 3'- to 5'-direction
N	A2SU54	CC	Has a template-primer preference which is characteristic of a replicative DNA polymerase (By similarity).
N	A2SU54	DE	DNA polymerase II large subunit;
N	A2SU54	DR	DNA catabolic process

N	A2SU54	DR	DNA replication
N	A2SU54	DR	DNA-directed DNA polymerase activity
N	A2SU54	DR	exonuclease activity
N	A2T318	CC	F(1)F(0) ATP synthase produces ATP from ADP in the presence of a proton or sodium gradient
N	A2T318	CC	During catalysis, ATP synthesis in the catalytic domain of F(1) is coupled via a rotary mechanism of the central stalk subunits to proton translocation (By similarity)
N	A2T318	CC	F-type ATPases consist of two structural domains, F(1) containing the extramembraneous catalytic core and F(0) containing the membrane proton channel, linked together by a central stalk and a peripheral stalk
N	A2T318	DE	ATP synthase F(0) sector subunit b;
N	A2T318	DE	ATP synthase subunit b, chloroplastic;
N	A2T318	DE	ATPase subunit I;
N	A2T318	DR	ATP synthesis coupled proton transport
N	A2T318	DR	hydrogen ion transmembrane transporter activity
N	A2T381	DE	50S ribosomal protein L32, chloroplastic;
N	A2T381	DR	structural constituent of ribosome
N	A2VDC7	CC	Endoribonuclease which preferentially cleaves ApU and ApG phosphodiester bonds (By similarity).
N	A2VDC7	DE	RNase kappa-A;
N	A2VDC7	DE	Ribonuclease kappa-A;
N	A2VDC7	DR	endoribonuclease activity
N	A2VDC7	DR	rRNA transcription
N	A2XX73	CC	Catalyzes the post-translational methylation of isoprenylated C-terminal cysteine residues (By similarity).
N	A2XX73	DE	Isoprenylcysteine carboxylmethyltransferase;
N	A2XX73	DE	Prenylated protein carboxyl methyltransferase;
N	A2XX73	DE	Prenylcysteine carboxyl methyltransferase;
N	A2XX73	DE	Probable protein-S-isoprenylcysteine O-methyltransferase;
N	A2XX73	DR	protein C-terminal S-isoprenylcysteine carboxyl O-methyltransferase activity
N	A2XYY8	CC	Arginine methyltransferase that can both catalyze the formation of omega-N monomethylarginine (MMA) and asymmetrical dimethylarginine (aDMA) (By similarity)
N	A2XYY8	DE	Probable protein arginine N-methyltransferase 6.1;
N	A2XYY8	DR	protein methyltransferase activity
N	A2YFN7	CC	Magnesium transporter that may mediate the influx of magnesium (By similarity)
N	A2YFN7	DE	Magnesium transporter MRS2-B;
N	A2YFN7	DR	metal ion transmembrane transporter activity
N	A3C6D7	CC	Actins are highly conserved proteins that are involved in various types of cell motility and are ubiquitously expressed in all eukaryotic cells.
N	A3CK64	CC	One of the primary rRNA binding proteins, this protein initially binds near the 5'-end of the 23S rRNA
N	A3CK64	CC	It is important during the early stages of 50S assembly
N	A3CK64	CC	It makes multiple contacts with different domains of the 23S rRNA in the assembled 50S subunit and ribosome (By similarity).
N	A3CK64	DE	50S ribosomal protein L4;
N	A3CK64	DR	structural constituent of ribosome
N	A3CLP4	CC	Catalyzes the attachment of isoleucine to tRNA(Ile)
N	A3CLP4	CC	As IleRS can inadvertently accommodate and process structurally similar amino acids such as valine, to avoid such errors it has two additional distinct tRNA(Ile)-dependent editing activities
N	A3CLP4	CC	One activity is designated as 'pretransfer' editing and involves the hydrolysis of activated Val-AMP
N	A3CLP4	CC	The other activity is designated 'posttransfer' editing and involves deacylation of mischarged Val-tRNA(Ile) (By similarity).
N	A3CLP4	DE	Isoleucine--tRNA ligase;
N	A3CLP4	DE	Isoleucyl-tRNA synthetase;
N	A3CLP4	DR	isoleucine-tRNA ligase activity
N	A3CLP4	DR	isoleucyl-tRNA aminoacylation
N	A3CLP4	DR	metal ion binding
N	A3CM38	CC	May be required for sporulation (By similarity).
N	A3CM38	DE	Putative sporulation transcription regulator whiA;
N	A3CPH6	DE	Galactose-6-phosphate isomerase subunit lacB;
N	A3CPH6	DR	galactose-6-phosphate isomerase activity
N	A3CPH6	DR	lactose catabolic process
N	A3CPV8	CC	Modulates recA activity (By similarity).

N	A3CPV8	DE	Regulatory protein recX;
N	A3CPV8	DR	regulation of DNA repair
N	A3CTR4	CC	Part of the RFC clamp loader complex which loads the PCNA sliding clamp onto DNA (By similarity).
N	A3CTR4	DE	Clamp loader large subunit;
N	A3CTR4	DE	RFC large subunit;
N	A3CTR4	DE	Replication factor C large subunit;
N	A3CTR4	DR	DNA replication
N	A3CTR4	DR	nucleoside-triphosphatase activity
N	A3D289	DE	ADP-glucose pyrophosphorylase;
N	A3D289	DE	ADP-glucose synthase;
N	A3D289	DE	ADPGlc PPase;
N	A3D289	DE	Glucose-1-phosphate adenylyltransferase;
N	A3D289	DR	glucose-1-phosphate adenylyltransferase activity
N	A3D289	DR	glycogen biosynthetic process
N	A3DE29	CC	First step of the lipid cycle reactions in the biosynthesis of the cell wall peptidoglycan (By similarity).
N	A3DE29	DE	Phospho-N-acetylmuramoyl-pentapeptide-transferase;
N	A3DE29	DE	UDP-MurNAc-pentapeptide phosphotransferase;
N	A3DE29	DR	UDP-N-acetylmuramoyl-L-alanyl-D-glutamyl-meso-2,6-diaminopimelyl-D-alanyl-D-alanine:undecaprenyl-phosphate transferase activity
N	A3DE29	DR	cell division
N	A3DE29	DR	cellular cell wall organization
N	A3DE29	DR	peptidoglycan biosynthetic process
N	A3DE29	DR	phospho-N-acetylmuramoyl-pentapeptide-transferase activity
N	A3DE29	DR	regulation of cell shape
N	A3DE59	DE	30S ribosomal protein S2;
N	A3DE59	DR	structural constituent of ribosome
N	A3DIF3	DE	Glutamate-1-semialdehyde 2,1-aminomutase;
N	A3DIF3	DE	Glutamate-1-semialdehyde aminotransferase;
N	A3DIF3	DR	glutamate-1-semialdehyde 2,1-aminomutase activity
N	A3DIF3	DR	porphyrin biosynthetic process
N	A3DIF3	DR	pyridoxal phosphate binding
N	A3DIF3	DR	transaminase activity
N	A3DN28	CC	Catalyzes the NAD(P)H-dependent reduction of dihydroxyacetonephosphate (DHAP or glycerone phosphate) to glycerol-1-phosphate (G1P)
N	A3DN28	CC	The G1P thus generated is used as the glycerophosphate backbone of phospholipids in the cellular membranes of Archaea (By similarity).
N	A3DN28	DE	Enantiomeric glycerophosphate synthase;
N	A3DN28	DE	G1P dehydrogenase;
N	A3DN28	DE	Glycerol-1-phosphate dehydrogenase [NAD(P)+];
N	A3DN28	DE	sn-glycerol-1-phosphate dehydrogenase;
N	A3DN28	DR	glycerol-1-phosphate dehydrogenase [NAD(P)+] activity
N	A3DN28	DR	metal ion binding
N	A3DN28	DR	oxidation-reduction process
N	A3DN28	DR	phospholipid biosynthetic process
N	A3EX94	CC	S1 region attaches the virion to the cell membrane by interacting with cell receptors, initiating the infection (By similarity).
N	A3EX94	DE	Peplomer protein;
N	A3EX94	DE	S glycoprotein;
N	A3EX94	DE	Spike glycoprotein;
N	A3EX94	DE	Spike protein S1;
N	A3EX94	DE	Spike protein S2;
N	A3EX94	DR	cellular membrane fusion
N	A3EX94	DR	viral envelope fusion with host membrane
N	A3EX94	DR	virion attachment, binding of host cell surface receptor
N	A3FPG8	CC	Esterifies acyl-group from acyl-ACP to the sn-1 position of glycerol-3-phosphate, an essential step in glycerolipid biosynthesis
N	A3FPG8	CC	Active against both saturated and unsaturated long- chain fatty acyl-CoAs (By similarity).
N	A3FPG8	DE	1-AGP acyltransferase 6;
N	A3FPG8	DE	1-acylglycerol-3-phosphate O-acyltransferase 6;
N	A3FPG8	DE	Acyl-CoA:glycerol-3-phosphate acyltransferase 4;
N	A3FPG8	DE	Glycerol-3-phosphate acyltransferase 4;
N	A3FPG8	DE	LPAAT-zeta;

N	A3FPG8	DE	Lysophosphatidic acid acyltransferase zeta;
N	A3FPG8	DR	glycerol-3-phosphate O-acyltransferase activity
N	A3LZZ0	CC	Core component of nucleosome which plays a central role in DNA double strand break (DSB) repair
N	A3LZZ0	CC	DNA accessibility is regulated via a complex set of post-translational modifications of histones, also called histone code, and nucleosome remodeling.
N	A3LZZ0	CC	Histones thereby play a central role in transcription regulation, DNA repair, DNA replication and chromosomal stability
N	A3LZZ0	CC	Nucleosomes wrap and compact DNA into chromatin, limiting DNA accessibility to the cellular machineries which require DNA as a template
N	A3LZZ0	DE	Histone H2A.1;
N	A3LZZ0	DR	nucleosome assembly
N	A3M2T7	CC	Catalyzes the phosphorylation of pantothenate (Pan), the first step in CoA biosynthesis (By similarity).
N	A3M2T7	DE	Pantothenic acid kinase;
N	A3M2T7	DE	Type III pantothenate kinase;
N	A3M2T7	DR	coenzyme A biosynthetic process
N	A3M2T7	DR	pantothenate kinase activity
N	A3M2T7	DR	transcription activator activity
N	A3MA34	CC	Riboflavin synthase is a bifunctional enzyme complex catalyzing the formation of riboflavin from 5-amino-6-(1'-D)- ribityl-amino-2,4(1H,3H)-pyrimidinedione and L-3,4-dihydrohy-2- butanone-4-phosphate via 6,7-dimethyl-8-lumazine
N	A3MA34	CC	The beta subunit catalyzes the condensation of 5-amino-6-(1'-D)-ribityl-amino-2,4(1H,3H)-pyrimidinedione with L-3,4-dihydrohy-2-butanone-4- phosphate yielding 6,7-dimethyl-8-lumazine (By similarity).
N	A3MA34	DE	6,7-dimethyl-8-ribityllumazine synthase;
N	A3MA34	DE	DMRL synthase;
N	A3MA34	DE	Lumazine synthase;
N	A3MA34	DE	Riboflavin synthase beta chain;
N	A3MA34	DR	riboflavin biosynthetic process
N	A3MA34	DR	riboflavin synthase activity
N	A3MNG2	DE	DTB synthetase;
N	A3MNG2	DE	Dethiobiotin synthase;
N	A3MNG2	DE	Dethiobiotin synthetase;
N	A3MNG2	DR	biotin biosynthetic process
N	A3MNG2	DR	dethiobiotin synthase activity
N	A3MNG2	DR	magnesium ion binding
N	A3MQB6	CC	Catalyzes the phosphorylation of the position 2 hydroxy group of 4-diphosphocytidyl-2C-methyl-D-erythritol (By similarity).
N	A3MQB6	DE	4-(cytidine-5'-diphospho)-2-C-methyl-D-erythritol kinase;
N	A3MQB6	DE	4-diphosphocytidyl-2-C-methyl-D-erythritol kinase;
N	A3MQB6	DR	4-(cytidine 5'-diphospho)-2-C-methyl-D-erythritol kinase activity
N	A3MQB6	DR	terpenoid biosynthetic process
N	A3MRF1	CC	Catalyzes the reaction of cyanate with bicarbonate to produce ammonia and carbon dioxide (By similarity).
N	A3MRF1	DE	Cyanate hydratase;
N	A3MRF1	DE	Cyanate hydrolase;
N	A3MRF1	DE	Cyanate lyase;
N	A3MRF1	DR	cyanate hydratase activity
N	A3MRF1	DR	cyanate metabolic process
N	A3MRF1	DR	hydro-lyase activity
N	A3MYB2	CC	An essential GTPase which binds GTP, GDP and possibly (p)ppGpp with moderate affinity, with high nucleotide exchange rates and a fairly low GTP hydrolysis rate (By similarity)
N	A3MYB2	CC	It may play a role in control of the cell cycle, stress response, ribosome biogenesis and in those bacteria that undergo differentiation, in morphogenesis control (Potential)
N	A3MYB2	DE	GTP-binding protein obg;
N	A3MYB2	DR	GTPase activity
N	A3MYB2	DR	magnesium ion binding
N	A3MYK2	CC	May play a role in 30S ribosomal subunit biogenesis
N	A3MYK2	CC	Unusual circulary permuted GTPase that catalyzes rapid hydrolysis of GTP with a slow catalytic turnover (By similarity).
N	A3MYK2	DE	Putative ribosome biogenesis GTPase RsgA;
N	A3MYK2	DR	GTPase activity
N	A3MYK2	DR	metal ion binding

N	A3N186	CC	Catalyzes the formation of S-adenosylmethionine from methionine and ATP
N	A3N186	CC	The overall synthetic reaction is composed of two sequential steps, AdoMet formation and the subsequent tripolyphosphate hydrolysis which occurs prior to release of AdoMet from the enzyme (By similarity).
N	A3N186	DE	AdoMet synthase;
N	A3N186	DE	Methionine adenosyltransferase;
N	A3N186	DE	S-adenosylmethionine synthase;
N	A3N186	DR	metal ion binding
N	A3N186	DR	methionine adenosyltransferase activity
N	A3N186	DR	one-carbon metabolic process
N	A3N3T8	DE	50S ribosomal protein L27;
N	A3N3T8	DR	structural constituent of ribosome
N	A3NC12	CC	Oxidative deamination of D-amino acids (By similarity).
N	A3NC12	DE	D-amino acid dehydrogenase small subunit;
N	A3NC12	DR	D-amino-acid dehydrogenase activity
N	A3NC12	DR	oxidation-reduction process
N	A3NCY6	CC	Negatively regulates transcription of bacterial ribonucleotide reductase nrd genes and operons by binding to NrdR- boxes (By similarity).
N	A3NCY6	DE	Transcriptional repressor NrdR;
N	A3NCY6	DR	transcription repressor activity
N	A3NCY6	DR	zinc ion binding
N	A3NPX0	DE	UPF0161 protein BURPS1106A_0106;
N	A3NQ45	CC	Furnishes a means for formation of correctly charged Gln-tRNA(Gln) through the transamidation of misacylated Glu- tRNA(Gln) in organisms which lack glutaminyl-tRNA synthetase
N	A3NQ45	CC	The reaction takes place in the presence of glutamine and ATP through an activated gamma-phospho-Glu-tRNA(Gln) (By similarity).
N	A3NQ45	DE	Glu-ADT subunit A;
N	A3NQ45	DE	Glutamyl-tRNA(Gln) amidotransferase subunit A;
N	A3NQ45	DR	carbon-nitrogen ligase activity, with glutamine as amido-N-donor
N	A3NQS3	CC	Catalyzes the decarboxylative condensation of pimeloyl- CoA and L-alanine to produce 8-amino-7-oxononanoate (AON), coenzyme A, and carbon dioxide (By similarity).
N	A3NQS3	DE	7-KAP synthase;
N	A3NQS3	DE	7-keto-8-amino-pelargonic acid synthase;
N	A3NQS3	DE	8-amino-7-ketopelargonate synthase;
N	A3NQS3	DE	8-amino-7-oxononanoate synthase;
N	A3NQS3	DE	KAPA synthase;
N	A3NQS3	DE	L-alanine--pimeloyl-CoA ligase;
N	A3NQS3	DR	8-amino-7-oxononanoate synthase activity
N	A3NQS3	DR	biotin biosynthetic process
N	A3NQS3	DR	pyridoxal phosphate binding
N	A3NQS3	DR	transferase activity, transferring nitrogenous groups
N	A3NS83	CC	Binds directly to 16S ribosomal RNA (By similarity).
N	A3NS83	DE	30S ribosomal protein S20;
N	A3NS83	DR	structural constituent of ribosome
N	A3NWL8	CC	Involved in the biosynthesis of lipid A, a phosphorylated glycolipid that anchors the lipopolysaccharide to the outer membrane of the cell (By similarity).
N	A3NWL8	DE	Acyl-[acyl-carrier-protein]--UDP-N-acetylglucosamine O-acyltransferase;
N	A3NWL8	DE	UDP-N-acetylglucosamine acyltransferase;
N	A3NWL8	DR	acyl-[acyl-carrier-protein]-UDP-N-acetylglucosamine O-acyltransferase activity
N	A3NWL8	DR	lipid A biosynthetic process
N	A3NY89	CC	Involved in the transcription termination process (By similarity).
N	A3NY89	DE	N utilization substance protein B homolog;
N	A3NY89	DE	Protein nusB;
N	A3NY89	DR	regulation of transcription, DNA-dependent
N	A3NY89	DR	transcription termination factor activity
N	A3P0A1	CC	This is 1 of the proteins that binds and probably mediates the attachment of the 5S RNA into the large ribosomal subunit, where it forms part of the central protuberance
N	A3P0A1	CC	Contacts the P site tRNA; the 5S rRNA and some of its associated proteins might help stabilize positioning of ribosome-bound tRNAs (By similarity).
N	A3P0A1	CC	In the 70S ribosome it contacts protein S13 of the 30S subunit (bridge B1b), connecting the 2 subunits; this bridge is implicated in subunit movement
N	A3P0A1	DE	50S ribosomal protein L5;
N	A3P0A1	DR	structural constituent of ribosome
N	A3PA97	CC	Major role in the synthesis of nucleoside triphosphates other than ATP

N	A3PA97	CC	The ATP gamma phosphate is transferred to the NDP beta phosphate via a ping-pong mechanism, using a phosphorylated active-site intermediate.
N	A3PA97	DE	NDP kinase;
N	A3PA97	DE	Nucleoside diphosphate kinase;
N	A3PA97	DE	Nucleoside-2-P kinase;
N	A3PA97	DR	CTP biosynthetic process
N	A3PA97	DR	GTP biosynthetic process
N	A3PA97	DR	UTP biosynthetic process
N	A3PA97	DR	metal ion binding
N	A3PA97	DR	nucleoside diphosphate kinase activity
N	A3PDM1	CC	Catalyzes the folate-dependent formation of 5-methyl- uridine at position 54 (M-5-U54) in all tRNAs (By similarity).
N	A3PDM1	DE	Folate-dependent tRNA (uracil-5-)-methyltransferase;
N	A3PDM1	DE	Folate-dependent tRNA(M-5-U54)-methyltransferase;
N	A3PDM1	DE	Methylenetetrahydrofolate--tRNA-(uracil-5-)-methyltransferase TrmFO;
N	A3PDM1	DR	flavin adenine dinucleotide binding
N	A3PDM1	DR	methylenetetrahydrofolate-tRNA-(uracil-5-)-methyltransferase (FADH2-oxidizing) activity
N	A3PDM1	DR	tRNA processing
N	A3PGM0	CC	One of the primary rRNA binding proteins, it binds specifically to the 5'-end of 16S ribosomal RNA (By similarity).
N	A3PGM0	DE	30S ribosomal protein S17;
N	A3PGM0	DR	structural constituent of ribosome
N	A3PNB5	DE	Acetylglutamate kinase;
N	A3PNB5	DE	N-acetyl-L-glutamate 5-phosphotransferase;
N	A3PNB5	DR	acetylglutamate kinase activity
N	A3PNB5	DR	arginine biosynthetic process
N	A3PNB5	DR	glutamate 5-kinase activity
N	A3PNB5	DR	proline biosynthetic process
N	A3PQJ2	CC	Catalyzes the NADPH dependent reduction of L-gamma- glutamyl 5-phosphate into L-glutamate 5-semialdehyde and phosphate
N	A3PQJ2	CC	The product spontaneously undergoes cyclization to form 1-pyrroline-5-carboxylate
N	A3PQJ2	DE	GSA dehydrogenase;
N	A3PQJ2	DE	Gamma-glutamyl phosphate reductase;
N	A3PQJ2	DE	Glutamate-5-semialdehyde dehydrogenase;
N	A3PQJ2	DE	Glutamyl-gamma-semialdehyde dehydrogenase;
N	A3PQJ2	DR	glutamate-5-semialdehyde dehydrogenase activity
N	A3PQJ2	DR	oxidation-reduction process
N	A3PQJ2	DR	proline biosynthetic process
N	A3PXU3	DE	Acylphosphatase;
N	A3PXU3	DE	Acylphosphate phosphohydrolase;
N	A3PXU3	DR	acylphosphatase activity
N	A3PXX6	DE	UPF0102 protein Mjls_1965;
N	A3PXX6	DR	nuclease activity
N	A3QC90	CC	Could accelerate the degradation of some genes transcripts potentially through selective RNA binding (By similarity).
N	A3QC90	DE	Carbon storage regulator homolog;
N	A3QC90	DR	mRNA catabolic process
N	A3QC90	DR	regulation of carbohydrate metabolic process
N	A3QCX7	CC	Involved in the import of serine and threonine into the cell, with the concomitant import of sodium (symport system) (By similarity).
N	A3QCX7	DE	Na(+)/serine-threonine symporter;
N	A3QCX7	DE	Serine/threonine transporter sstT;
N	A3QCX7	DR	amino acid transport
N	A3QCX7	DR	sodium:dicarboxylate symporter activity
N	A3QDA2	CC	Specifically methylates the cytosine at position 1407 (m5C1407) of 16S rRNA (By similarity).
N	A3QDA2	DE	16S rRNA m5C1407 methyltransferase;
N	A3QDA2	DE	Ribosomal RNA small subunit methyltransferase F;
N	A3QDA2	DE	rRNA (cytosine-C(5)-)-methyltransferase rsmF;
N	A3QDA2	DR	rRNA methyltransferase activity
N	A3QEC1	DE	CMP kinase;
N	A3QEC1	DE	Cytidine monophosphate kinase;
N	A3QEC1	DE	Cytidylate kinase;
N	A3QEC1	DR	cytidylate kinase activity

N	A3QIQ6	CC	Phosphorolytic exoribonuclease that removes nucleotide residues following the -CCA terminus of tRNA and adds nucleotides to the ends of RNA molecules by using nucleoside diphosphates as substrates (By similarity).
N	A3QIQ6	DE	Ribonuclease PH;
N	A3QIQ6	DE	tRNA nucleotidyltransferase;
N	A3QIQ6	DR	3'-5'-exoribonuclease activity
N	A3QIQ6	DR	tRNA nucleotidyltransferase activity
N	A3QIQ6	DR	tRNA processing
N	A3QIQ6	DR	tRNA-specific ribonuclease activity
N	A3R064	CC	DOK proteins are enzymatically inert adaptor or scaffolding proteins
N	A3R064	CC	Plays a role as negative regulator of the mobilization of calcium ions and of calcium signaling.
N	A3R064	CC	They provide a docking platform for the assembly of multimolecular signaling complexes
N	A3R064	DE	Docking protein 3;
N	A3R064	DE	Downstream of tyrosine kinase 3;
N	A3R064	DR	insulin receptor binding
N	A4D9J5	CC	Component of the Mediator complex, a coactivator involved in the regulated transcription of nearly all RNA polymerase II-dependent genes
N	A4D9J5	CC	Mediator functions as a bridge to convey information from gene-specific regulatory proteins to the basal RNA polymerase II transcription machinery
N	A4D9J5	CC	Mediator is recruited to promoters by direct interactions with regulatory proteins and serves as a scaffold for the assembly of a functional preinitiation complex with RNA polymerase II and the general transcription factors (By similarity).
N	A4D9J5	DE	Mediator complex subunit 16;
N	A4D9J5	DE	Mediator of RNA polymerase II transcription subunit 16;
N	A4F7F9	CC	Facilitates the functional incorporation of the urease nickel metallocenter
N	A4F7F9	CC	This process requires GTP hydrolysis, probably effectuated by ureG (By similarity).
N	A4F7F9	DE	Urease accessory protein ureG 1;
N	A4F7F9	DR	nickel ion binding
N	A4FAB4	DE	Glutaminase;
N	A4FAB4	DR	glutaminase activity
N	A4FAB4	DR	glutamine metabolic process
N	A4FBY0	CC	Protein modifier that is covalently attached to lysine residues of substrate proteins, thereby targeting them for proteasomal degradation
N	A4FBY0	CC	The tagging system is termed pupylation (By similarity).
N	A4FBY0	DE	Bacterial ubiquitin-like modifier 1;
N	A4FBY0	DE	Prokaryotic ubiquitin-like protein Pup 1;
N	A4FBZ2	CC	Required for correct localization of precursor proteins bearing signal peptides with the twin arginine conserved motif S/T-R-R-X-F-L-K
N	A4FBZ2	CC	This sec-independent pathway is termed TAT for twin-arginine translocation system
N	A4FBZ2	CC	This system mainly transports proteins with bound cofactors that require folding prior to export (By similarity).
N	A4FBZ2	DE	Sec-independent protein translocase protein tatA/E homolog;
N	A4FBZ2	DR	protein secretion
N	A4FBZ2	DR	protein transporter activity
N	A4FBZ2	DR	transmembrane transport
N	A4FK76	CC	Catalyzes the 1,3-allylic rearrangement of the homoallylic substrate isopentenyl (IPP) to its highly electrophilic allylic isomer, dimethylallyl diphosphate (DMAPP) (By similarity).
N	A4FK76	DE	IPP isomerase;
N	A4FK76	DE	IPP:DMAPP isomerase;
N	A4FK76	DE	Isopentenyl pyrophosphate isomerase;
N	A4FK76	DE	Isopentenyl-diphosphate Delta-isomerase;
N	A4FK76	DR	hydrolase activity
N	A4FK76	DR	isopentenyl-diphosphate delta-isomerase activity
N	A4FK76	DR	isoprenoid biosynthetic process
N	A4FK76	DR	metal ion binding
N	A4FM36	CC	Required for maturation of 30S ribosomal subunits (By similarity).
N	A4FM36	DE	Ribosome maturation factor rimP;
N	A4FM36	DR	ribosome biogenesis
N	A4FMD4	CC	Responsible for the release of ribosomes from messenger RNA at the termination of protein biosynthesis
N	A4FMD4	CC	May increase the efficiency of translation by recycling ribosomes from one round of translation to another (By similarity).
N	A4FMD4	DE	Ribosome-recycling factor;
N	A4FMD4	DE	Ribosome-releasing factor;

N	A4FWA1	CC	With S4 and S12 plays an important role in translational accuracy (By similarity).
N	A4FWA1	DE	30S ribosomal protein S5P;
N	A4FWA1	DR	structural constituent of ribosome
N	A4G096	DE	50S ribosomal protein L21e;
N	A4G096	DR	structural constituent of ribosome
N	A4G1L0	CC	Catalyzes the transfer of a ribosyl phosphate group from 5-phosphoribose 1-diphosphate to orotate, leading to the formation of orotidine monophosphate (OMP) (By similarity).
N	A4G1L0	DE	Orotate phosphoribosyltransferase;
N	A4G1L0	DR	nucleoside metabolic process
N	A4G1L0	DR	orotate phosphoribosyltransferase activity
N	A4G1L0	DR	pyrimidine nucleotide biosynthetic process
N	A4G4H8	DE	Acetohydroxy-acid isomeroreductase;
N	A4G4H8	DE	Alpha-keto-beta-hydroxylacil reductoisomerase;
N	A4G4H8	DE	Ketol-acid reductoisomerase;
N	A4G4H8	DR	branched chain family amino acid biosynthetic process
N	A4G4H8	DR	coenzyme binding
N	A4G4H8	DR	ketol-acid reductoisomerase activity
N	A4G4H8	DR	oxidation-reduction process
N	A4G778	DE	Lysine--tRNA ligase;
N	A4G778	DE	Lysyl-tRNA synthetase;
N	A4G778	DR	lysine-tRNA ligase activity
N	A4G778	DR	lysyl-tRNA aminoacylation
N	A4G778	DR	metal ion binding
N	A4G778	DR	nucleic acid binding
N	A4G8X1	DE	Aspartate--tRNA ligase;
N	A4G8X1	DE	Aspartyl-tRNA synthetase;
N	A4G8X1	DR	aspartate-tRNA ligase activity
N	A4G8X1	DR	aspartyl-tRNA aminoacylation
N	A4G8X1	DR	nucleic acid binding
N	A4GGA6	CC	DNA-dependent RNA polymerase catalyzes the transcription of DNA into RNA using the four ribonucleoside triphosphates as substrates (By similarity).
N	A4GGA6	DE	DNA-directed RNA polymerase subunit beta';
N	A4GGA6	DE	Plastid-encoded RNA polymerase subunit beta';
N	A4GGA6	DE	RNA polymerase subunit beta';
N	A4GGA6	DR	DNA-directed RNA polymerase activity
N	A4GGB2	CC	Produces ATP from ADP in the presence of a proton gradient across the membrane.
N	A4GGB2	CC	The alpha chain is a regulatory subunit (By similarity).
N	A4GGB2	DE	ATP synthase F1 sector subunit alpha;
N	A4GGB2	DE	ATP synthase subunit alpha, chloroplastic;
N	A4GGB2	DE	F-ATPase subunit alpha;
N	A4GGB2	DR	ATP hydrolysis coupled proton transport
N	A4GGB2	DR	ATP synthesis coupled proton transport
N	A4GGB2	DR	hydrogen ion transporting ATP synthase activity, rotational mechanism
N	A4GGB2	DR	proton-transporting ATPase activity, rotational mechanism
N	A4GGF5	CC	One of the primary rRNA binding proteins, it binds directly to 16S rRNA where it nucleates assembly of the head domain of the 30S subunit (By similarity).
N	A4GGF5	DE	30S ribosomal protein S7, chloroplastic;
N	A4GGF5	DR	structural constituent of ribosome
N	A4H207	CC	Has antimicrobial activity (By similarity).
N	A4H207	DE	Beta-defensin 105A;
N	A4H207	DE	Defensin, beta 105;
N	A4H207	DE	Defensin, beta 105A;
N	A4H207	DR	defense response to bacterium
N	A4HGN7	CC	Catalyzes the dehydration of methylthioribulose-1- phosphate (MTRu-1-P) into 2,3-diketo-5-methylthiopentyl-1- phosphate (DK-MTP-1-P) (By similarity).
N	A4HGN7	DE	MTRu-1-P dehydratase;
N	A4HGN7	DE	Probable methylthioribulose-1-phosphate dehydratase;
N	A4HGN7	DR	L-methionine salvage
N	A4HGN7	DR	metal ion binding
N	A4HGN7	DR	methylthioribulose 1-phosphate dehydratase activity
N	A4IF87	DE	Acyl-CoA:dihydroxyacetonephosphateacyltransferase;
N	A4IF87	DE	Dihydroxyacetone phosphate acyltransferase;
N	A4IF87	DE	Glycerone-phosphate O-acyltransferase;
N	A4IF87	DR	glycerone-phosphate O-acyltransferase activity
N	A4IFB4	DE	BTB/POZ domain-containing protein KCTD7;

N	A4IFB4	DR	voltage-gated potassium channel activity
N	A4IHK6	CC	Sodium-, chloride, pH-independent high affinity transport of large neutral amino acids (By similarity).
N	A4IHK6	DE	L-type amino acid transporter 4;
N	A4IHK6	DE	Large neutral amino acids transporter small subunit 4;
N	A4IHK6	DE	Solute carrier family 43 member 2;
N	A4IHK6	DR	amino acid transport
N	A4IHK6	DR	transmembrane transport
N	A4IQ61	CC	Catalyzes the reversible reaction in which hydroxymethyl group from 5,10-methylenetetrahydrofolate is tranferred onto alpha-ketoisovalerate to form ketopantoate (By similarity).
N	A4IQ61	DE	3-methyl-2-oxobutanoate hydroxymethyltransferase;
N	A4IQ61	DE	Ketopantoate hydroxymethyltransferase;
N	A4IQ61	DR	3-methyl-2-oxobutanoate hydroxymethyltransferase activity
N	A4IQ61	DR	metal ion binding
N	A4IQ61	DR	methyltransferase activity
N	A4IQ61	DR	pantothenate biosynthetic process
N	A4ITI0	CC	NDH-1 shuttles electrons from NADH, via FMN and iron- sulfur (Fe-S) centers, to quinones in the respiratory chain
N	A4ITI0	CC	Couples the redox reaction to proton translocation (for every two electrons transferred, four hydrogen ions are translocated across the cytoplasmic membrane), and thus conserves the redox energy in a proton gradient (By similarity)
N	A4ITI0	CC	The immediate electron acceptor for the enzyme in this species is believed to be a menaquinone
N	A4ITI0	DE	NADH dehydrogenase I subunit K;
N	A4ITI0	DE	NADH-quinone oxidoreductase subunit K;
N	A4ITI0	DE	NDH-1 subunit K;
N	A4ITI0	DR	ATP synthesis coupled electron transport
N	A4ITI0	DR	NADH dehydrogenase (quinone) activity
N	A4ITI0	DR	quinone binding
N	A4IXL1	CC	One of the proteins required for the normal export of preproteins out of the cell cytoplasm
N	A4IXL1	CC	It also specifically binds to its receptor secA (By similarity).
N	A4IXL1	CC	It is a molecular chaperone that binds to a subset of precursor proteins, maintaining them in a translocation-competent state
N	A4IXL1	DE	Protein-export protein secB 1;
N	A4IXL1	DR	protein tetramerization
N	A4IXL1	DR	protein transport
N	A4IXL1	DR	transmembrane transport
N	A4IXL1	DR	unfolded protein binding
N	A4IZR8	CC	This is one of the proteins that binds and probably mediates the attachment of the 5S RNA into the large ribosomal subunit, where it forms part of the central protuberance (By similarity).
N	A4IZR8	DE	50S ribosomal protein L18;
N	A4IZR8	DR	structural constituent of ribosome
N	A4J5U0	DE	Putative competence-damage inducible protein;
N	A4J5U0	DR	Mo-molybdopterin cofactor biosynthetic process
N	A4J9D2	DE	Arginine--tRNA ligase;
N	A4J9D2	DE	Arginyl-tRNA synthetase;
N	A4J9D2	DR	arginine-tRNA ligase activity
N	A4J9D2	DR	arginyl-tRNA aminoacylation
N	A4J9S5	DE	50S ribosomal protein L34;
N	A4J9S5	DR	structural constituent of ribosome
N	A4JA22	CC	NAD-binding protein involved in the addition of a carboxymethylaminomethyl (cmnm) group at the wobble position (U34) of certain tRNAs, forming tRNA-cmnm(5)s(2)U34 (By similarity).
N	A4JA22	DE	Glucose-inhibited division protein A;
N	A4JA22	DE	tRNA uridine 5-carboxymethylaminomethyl modification enzyme MnmG;
N	A4JA22	DR	flavin adenine dinucleotide binding
N	A4JA22	DR	tRNA wobble uridine modification
N	A4JA69	CC	The glycine cleavage system catalyzes the degradation of glycine
N	A4JA69	CC	The P protein binds the alpha-amino group of glycine through its pyridoxal phosphate cofactor; CO(2) is released and the remaining methylamine moiety is then transferred to the lipoamide cofactor of the H protein (By similarity).
N	A4JA69	DE	Glycine cleavage system P-protein;

N	A4JA69	DE	Glycine decarboxylase;
N	A4JA69	DE	Glycine dehydrogenase [decarboxylating];
N	A4JA69	DR	glycine dehydrogenase (decarboxylating) activity
N	A4JA69	DR	glycine metabolic process
N	A4JA69	DR	oxidation-reduction process
N	A4JA69	DR	pyridoxal phosphate binding
N	A4JAN2	CC	DNA-dependent RNA polymerase catalyzes the transcription of DNA into RNA using the four ribonucleoside triphosphates as substrates.
N	A4JAN2	DE	DNA-directed RNA polymerase subunit beta;
N	A4JAN2	DE	RNA polymerase subunit beta;
N	A4JAN2	DE	RNAP subunit beta;
N	A4JAN2	DE	Transcriptase subunit beta;
N	A4JAN2	DR	DNA-directed RNA polymerase activity
N	A4JAN2	DR	ribonucleoside binding
N	A4JAP5	CC	This protein binds specifically to 23S rRNA; its binding is stimulated by other ribosomal proteins, e.g., L4, L17, and L20
N	A4JAP5	CC	It is important during the early stages of 50S assembly
N	A4JAP5	CC	It makes multiple contacts with different domains of the 23S rRNA in the assembled 50S subunit and ribosome (By similarity).
N	A4JAP5	DE	50S ribosomal protein L22;
N	A4JAP5	DR	structural constituent of ribosome
N	A4JB51	CC	Catalyzes the NADPH-dependent reduction of glutamyl- tRNA(Glu) to glutamate 1-semialdehyde (GSA) (By similarity).
N	A4JB51	DE	Glutamyl-tRNA reductase;
N	A4JB51	DR	glutamyl-tRNA reductase activity
N	A4JB51	DR	oxidation-reduction process
N	A4JB51	DR	porphyrin biosynthetic process
N	A4JB51	DR	shikimate 5-dehydrogenase activity
N	A4JBD7	CC	Methylates ribosomal protein L11 (By similarity).
N	A4JBD7	DE	Ribosomal protein L11 methyltransferase;
N	A4JBD7	DR	protein methyltransferase activity
N	A4JCP8	CC	Catalyzes the condensation reaction of fatty acid synthesis by the addition to an acyl acceptor of two carbons from malonyl-ACP
N	A4JCP8	CC	Catalyzes the first condensation reaction which initiates fatty acid synthesis and may therefore play a role in governing the total rate of fatty acid production
N	A4JCP8	CC	Its substrate specificity determines the biosynthesis of branched- chain and/or straight-chain of fatty acids (By similarity).
N	A4JCP8	CC	Possesses both acetoacetyl-ACP synthase and acetyl transacylase activities
N	A4JCP8	DE	3-oxoacyl-[acyl-carrier-protein] synthase 3;
N	A4JCP8	DE	3-oxoacyl-[acyl-carrier-protein] synthase III;
N	A4JCP8	DE	Beta-ketoacyl-ACP synthase III;
N	A4JCP8	DR	3-oxoacyl-[acyl-carrier-protein] synthase activity
N	A4JCP8	DR	fatty acid biosynthetic process
N	A4QC57	CC	Transport of potassium into the cell (By similarity).
N	A4QC57	DE	Probable potassium transport system protein kup;
N	A4QC57	DR	potassium ion transmembrane transporter activity
N	A4QDU2	CC	Reversibly transfers an adenylyl group from ATP to 4'- phosphopantetheine, yielding dephospho-CoA (dPCoA) and pyrophosphate (By similarity).
N	A4QDU2	DE	Dephospho-CoA pyrophosphorylase;
N	A4QDU2	DE	Pantetheine-phosphate adenylyltransferase;
N	A4QDU2	DE	Phosphopantetheine adenylyltransferase;
N	A4QDU2	DR	coenzyme A biosynthetic process
N	A4QDU2	DR	pantetheine-phosphate adenylyltransferase activity
N	A4QGX4	CC	Catalyzes the transfer of acetyl from acetyl-CoA to desacetylmycothiol (Cys-GlcN-Ins) to form mycothiol (By similarity).
N	A4QGX4	DE	MSH acetyltransferase;
N	A4QGX4	DE	Mycothiol acetyltransferase;
N	A4QGX4	DE	Mycothiol synthase;
N	A4QGX4	DR	N-acetyltransferase activity
N	A4QH41	DE	Cysteine--tRNA ligase;
N	A4QH41	DE	Cysteinyl-tRNA synthetase;
N	A4QH41	DR	cysteine-tRNA ligase activity
N	A4QH41	DR	cysteinyl-tRNA aminoacylation
N	A4QH41	DR	metal ion binding
N	A4QJC2	CC	Produces ATP from ADP in the presence of a proton gradient across the [illegible]

N	A4QJC2	CC	The catalytic sites are hosted primarily by the beta subunits (By similarity).
N	A4QJC2	DE	ATP synthase F1 sector subunit beta;
N	A4QJC2	DE	ATP synthase subunit beta, chloroplastic;
N	A4QJC2	DE	F-ATPase subunit beta;
N	A4QJC2	DR	ATP hydrolysis coupled proton transport
N	A4QJC2	DR	ATP synthesis coupled proton transport
N	A4QJC2	DR	hydrogen ion transporting ATP synthase activity, rotational mechanism
N	A4QJC2	DR	hydrogen-exporting ATPase activity, phosphorylative mechanism
N	A4QJC2	DR	proton-transporting ATPase activity, rotational mechanism
N	A4QJM7	DE	Protein psbN;
N	A4QJM7	DR	photosynthesis
N	A4QJQ3	CC	NDH shuttles electrons from NAD(P)H:plastoquinone, via FMN and iron-sulfur (Fe-S) centers, to quinones in the photosynthetic chain and possibly in a chloroplast respiratory chain
N	A4QJQ3	CC	Couples the redox reaction to proton translocation, and thus conserves the redox energy in a proton gradient (By similarity).
N	A4QJQ3	CC	The immediate electron acceptor for the enzyme in this species is believed to be plastoquinone
N	A4QJQ3	DE	NAD(P)H dehydrogenase subunit 6;
N	A4QJQ3	DE	NAD(P)H-quinone oxidoreductase subunit 6, chloroplastic;
N	A4QJQ3	DE	NADH-plastoquinone oxidoreductase subunit 6;
N	A4QJQ3	DR	NADH dehydrogenase (ubiquinone) activity
N	A4QJQ3	DR	oxidation-reduction process
N	A4QJQ3	DR	quinone binding
N	A4QKL5	CC	Binds directly to 23S ribosomal RNA and is necessary for the in vitro assembly process of the 50S ribosomal subunit
N	A4QKL5	CC	It is not involved in the protein synthesizing functions of that subunit (By similarity)
N	A4QKL5	DE	50S ribosomal protein L20, chloroplastic;
N	A4QKL5	DR	structural constituent of ribosome
N	A4QKN3	DE	50S ribosomal protein L2, chloroplastic;
N	A4QKN3	DR	structural constituent of ribosome
N	A4QKN3	DR	transferase activity
N	A4QKQ3	CC	Unknown
N	A4QKQ3	CC	Its presence in a non-photosynthetic plant (Epifagus virginiana) and experiments in tobacco indicate that it may have a function which is probably not related to photosynthesis.
N	A4QKQ3	DE	Putative membrane protein ycf1;
N	A4R9U5	CC	Involved in the biogenesis of the 60S ribosomal subunit (By similarity).
N	A4R9U5	DE	Nucleolar protein 16;
N	A4R9U5	DR	rRNA processing
N	A4RC23	CC	Component of the nascent polypeptide-associated complex (NAC), a dynamic component of the ribosomal exit tunnel, protecting the emerging polypeptides from interaction with other cytoplasmic proteins to ensure appropriate nascent protein targeting (By similarity)
N	A4RC23	CC	EGD1 may act as a transcription factor that exert a negative effect on the expression of several genes that are transcribed by RNA polymerase II (By similarity)
N	A4RC23	CC	The NAC complex also promotes mitochondrial protein import by enhancing productive ribosome interactions with the outer mitochondrial membrane and blocks the inappropriate interaction of ribosomes translating non-secretory nascent polypeptides with translocation sites in the membrane of the endoplasmic reticulum (By similarity)
N	A4RC23	DE	Nascent polypeptide-associated complex subunit beta;
N	A4RC23	DR	protein transport
N	A4RK68	CC	Component of the eukaryotic translation initiation factor 3 (eIF-3) complex, which is involved in protein synthesis and, together with other initiation factors, stimulates binding of mRNA and methionyl-tRNAi to the 40S ribosome (By similarity)
N	A4RK68	DE	Eukaryotic translation initiation factor 3 subunit M;
N	A4RK68	DR	translation initiation factor activity
N	A4SCQ4	CC	With S4 and S5 plays an important role in translational accuracy (By similarity).
N	A4SCQ4	DE	30S ribosomal protein S12;
N	A4SCQ4	DR	structural constituent of ribosome
N	A4SFT2	CC	This protein binds to 23S rRNA in the presence of protein L20 (By similarity).
N	A4SFT2	DE	50S ribosomal protein L21;
N	A4SFT2	DR	structural constituent of ribosome
N	A4SHU9	CC	DNA-dependent RNA polymerase catalyzes the transcription of DNA into RNA using the four ribonucleoside triphosphates as substrates.

N	A4SHU9	DE	DNA-directed RNA polymerase subunit beta;
N	A4SHU9	DE	RNA polymerase subunit beta;
N	A4SHU9	DE	RNAP subunit beta;
N	A4SHU9	DE	Transcriptase subunit beta;
N	A4SHU9	DR	DNA-directed RNA polymerase activity
N	A4SHU9	DR	ribonucleoside binding
N	A4SNB5	CC	Catalyzes the transfer of a methyl group from 5- methyltetrahydrofolate to homocysteine resulting in methionine formation (By similarity).
N	A4SNB5	DE	5-methyltetrahydropteroyltriglutamate--homocysteine methyltransferase;
N	A4SNB5	DE	Cobalamin-independent methionine synthase;
N	A4SNB5	DE	Methionine synthase, vitamin-B12 independent isozyme;
N	A4SNB5	DR	5-methyltetrahydropteroyltriglutamate-homocysteine S-methyltransferase activity
N	A4SNB5	DR	methionine biosynthetic process
N	A4SNB5	DR	zinc ion binding
N	A4ST17	CC	Shows carboxylesterase activity with a preference for short chain fatty acid esters (acyl chain length of up to 6 carbons)
N	A4ST17	CC	Also displays a weak thioesterase activity
N	A4ST17	CC	Can form a complex with CoA, and may be involved in the condensation of CoA and pimelic acid into pimeloyl-CoA, a precursor in biotin biosynthesis (By similarity)
N	A4ST17	DE	Biotin synthesis protein BioH;
N	A4ST17	DE	Carboxylesterase BioH;
N	A4ST17	DR	biotin biosynthetic process
N	A4ST17	DR	methyl indole-3-acetate esterase activity
N	A4ST17	DR	methyl jasmonate esterase activity
N	A4ST17	DR	methyl salicylate esterase activity
N	A4SUV0	CC	This protein binds directly to 23S ribosomal RNA (By similarity).
N	A4SUV0	DE	50S ribosomal protein L11;
N	A4SUV0	DR	structural constituent of ribosome
N	A4SXH5	CC	Involved in cell division; probably involved in intracellular septation (By similarity)
N	A4SXH5	DE	Probable intracellular septation protein;
N	A4SXH5	DR	barrier septum formation
N	A4T2C4	CC	Negative regulator of class I heat shock genes (grpE- dnaK-dnaJ and groELS operons)
N	A4T2C4	CC	Prevents heat-shock induction of these operons (By similarity).
N	A4T2C4	DE	Heat-inducible transcription repressor hrcA;
N	A4T2C4	DR	regulation of transcription, DNA-dependent
N	A4T2C4	DR	response to stress
N	A4T8H8	DE	UPF0286 protein Mflv_2337;
N	A4TIM3	CC	Catalyzes the transfer of 4-deoxy-4-formamido-L- arabinose from UDP to undecaprenyl phosphate
N	A4TIM3	CC	The modified arabinose is attached to lipid A and is required for resistance to polymyxin and cationic antimicrobial peptides (By similarity).
N	A4TIM3	DE	Ara4FN transferase;
N	A4TIM3	DE	Undecaprenyl-phosphate 4-deoxy-4-formamido-L-arabinose transferase;
N	A4TIM3	DE	Undecaprenyl-phosphate Ara4FN transferase;
N	A4TIM3	DR	lipid A biosynthetic process
N	A4TIM3	DR	response to antibiotic
N	A4TIM3	DR	transferase activity, transferring glycosyl groups
N	A4TIQ4	CC	Is the main repressor of the genes involved in the de novo synthesis of purine nucleotides, regulating purB, purC, purEK, purF, purHD, purL, purMN and guaBA expression
N	A4TIQ4	CC	PurR is allosterically activated to bind its cognate DNA by binding the purine corepressors, hypoxanthine or guanine, thereby effecting transcription repression (By similarity).
N	A4TIQ4	DE	HTH-type transcriptional repressor purR;
N	A4TIQ4	DE	Pur regulon repressor;
N	A4TIQ4	DE	Purine nucleotide synthesis repressor;
N	A4TIQ4	DR	purine nucleotide biosynthetic process
N	A4TIQ4	DR	regulation of transcription, DNA-dependent
N	A4TIQ4	DR	sequence-specific DNA binding transcription factor activity
N	A4TJK3	DE	UPF0082 protein YPDSF_1067;
N	A4TMV1	CC	Co-chaperone involved in the maturation of iron-sulfur cluster-containing proteins
N	A4TMV1	CC	Seems to help targeting proteins to be folded toward hscA (By similarity).
N	A4TMV1	DE	Co-chaperone protein hscB;
N	A4TMV1	DR	chaperone binding
N	A4TMV1	DR	heat shock protein binding

N	A4TMV1	DR	protein folding
N	A4TPJ4	CC	Catalyzes the specific phosphorylation of the 3-hydroxyl group of shikimic acid using ATP as a cosubstrate (By similarity).
N	A4TPJ4	DE	Shikimate kinase 2;
N	A4TPJ4	DR	aromatic amino acid family biosynthetic process
N	A4TPJ4	DR	metal ion binding
N	A4TPJ4	DR	shikimate kinase activity
N	A4TRR0	CC	Prevents misfolding and promotes the refolding and proper assembly of unfolded polypeptides generated under stress conditions (By similarity).
N	A4TRR0	DE	60 kDa chaperonin;
N	A4TRR0	DE	GroEL protein;
N	A4TRR0	DE	Protein Cpn60;
N	A4TRR0	DR	protein refolding
N	A4VIR7	CC	Together with moaA, is involved in the conversion of a guanosine derivative (5'-GTP) into molybdopterin precursor Z (By similarity).
N	A4VIR7	DE	Molybdenum cofactor biosynthesis protein C;
N	A4VIR7	DR	Mo-molybdopterin cofactor biosynthetic process
N	A4VPC3	CC	Catalyzes the phosphorylation of the position 2 hydroxy group of 4-diphosphocytidyl-2C-methyl-D-erythritol (By similarity).
N	A4VPC3	DE	4-(cytidine-5'-diphospho)-2-C-methyl-D-erythritol kinase;
N	A4VPC3	DE	4-diphosphocytidyl-2-C-methyl-D-erythritol kinase;
N	A4VPC3	DR	4-(cytidine 5'-diphospho)-2-C-methyl-D-erythritol kinase activity
N	A4VPC3	DR	terpenoid biosynthetic process
N	A4VS81	CC	Exhibits a very high intrinsic GTPase hydrolysis rate
N	A4VS81	CC	Involved in the addition of a carboxymethylaminomethyl (cmnm) group at the wobble position (U34) of certain tRNAs, forming tRNA- cmnm(5)s(2)U34 (By similarity)
N	A4VS81	DE	tRNA modification GTPase mnmE;
N	A4VS81	DR	GTPase activity
N	A4VS81	DR	metal ion binding
N	A4VS81	DR	tRNA modification
N	A4VS85	DE	50S ribosomal protein L34;
N	A4VS85	DR	structural constituent of ribosome
N	A4VUD1	CC	The heterodimer acts as both an ATP-dependent DNA helicase and an ATP-dependent, dual-direction single-stranded exonuclease
N	A4VUD1	CC	Recognizes the chi site generating a DNA molecule suitable for the initiation of homologous recombination
N	A4VUD1	CC	This subunit has 5' -> 3' nuclease activity (By similarity).
N	A4VUD1	DE	ATP-dependent helicase/deoxyribonuclease subunit B;
N	A4VUD1	DE	ATP-dependent helicase/nuclease RexB;
N	A4VUD1	DR	exonuclease activity
N	A4VUD1	DR	hydrolase activity, acting on acid anhydrides
N	A4VZC6	CC	Formation of pseudouridine at positions 38, 39 and 40 in the anticodon stem and loop of transfer RNAs (By similarity).
N	A4VZC6	DE	tRNA pseudouridine synthase A;
N	A4VZC6	DE	tRNA pseudouridylate synthase I;
N	A4VZC6	DE	tRNA-uridine isomerase I;
N	A4VZC6	DR	pseudouridine synthase activity
N	A4VZC6	DR	pseudouridine synthesis
N	A4VZC6	DR	tRNA processing
N	A4W0M6	CC	The heterodimer acts as both an ATP-dependent DNA helicase and an ATP-dependent, dual-direction single-stranded exonuclease
N	A4W0M6	CC	Recognizes the chi site generating a DNA molecule suitable for the initiation of homologous recombination
N	A4W0M6	CC	This subunit has 5' -> 3' nuclease activity (By similarity).
N	A4W0M6	DE	ATP-dependent helicase/deoxyribonuclease subunit B;
N	A4W0M6	DE	ATP-dependent helicase/nuclease RexB;
N	A4W0M6	DR	exonuclease activity
N	A4W0M6	DR	hydrolase activity, acting on acid anhydrides
N	A4W5C7	DE	Homoserine O-succinyltransferase;
N	A4W5C7	DE	Homoserine O-transsuccinylase;
N	A4W5C7	DR	L-methionine biosynthetic process from homoserine via O-succinyl-L-homoserine and cystathionine
N	A4W5C7	DR	homoserine O-succinyltransferase activity
N	A4W6I4	DE	Protein MraZ;

N	A4W6V2	CC	Thiolesterase that catalyzes the hydrolysis of S-D- lactoyl-glutathione to form glutathione and D-lactic acid (By similarity).
N	A4W6V2	DE	Glyoxalase II;
N	A4W6V2	DE	Hydroxyacylglutathione hydrolase;
N	A4W6V2	DR	hydroxyacylglutathione hydrolase activity
N	A4W6V2	DR	zinc ion binding
N	A4WCC2	DE	Multidrug resistance protein MdtC;
N	A4WCC2	DE	Multidrug transporter MdtC;
N	A4WCC2	DR	transporter activity
N	A4WER5	CC	Regulates transcription of many different genes
N	A4WER5	CC	In the absence of autoinducer 2 (AI-2), represses transcription of the lsrACDBFG operon and its own transcription
N	A4WER5	CC	In the presence of AI- 2, lsrR is inactivated by binding phospho-AI-2, leading to the transcription of the lsr genes (By similarity).
N	A4WER5	DE	Transcriptional regulator lsrR;
N	A4WER5	DR	carbohydrate binding
N	A4WER5	DR	transcription regulator activity
N	A4WFD8	CC	Part of a sulfur-relay system required for 2-thiolation of 5-methylaminomethyl-2-thiouridine (mnm(5)s(2)U) at tRNA wobble positions
N	A4WFD8	CC	Accepts sulfur from tusA and transfers it in turn to tusE (By similarity).
N	A4WFD8	DE	Sulfurtransferase tusD;
N	A4WFD8	DE	tRNA 2-thiouridine synthesizing protein D;
N	A4WFD8	DR	sulfurtransferase activity
N	A4WFD8	DR	tRNA processing
N	A4WFL5	CC	Catalyzes the formation of the alpha-1,6-glucosidic linkages in glycogen by scission of a 1,4-alpha-linked oligosaccharide from growing alpha-1,4-glucan chains and the subsequent attachment of the oligosaccharide to the alpha-1,6 position (By similarity).
N	A4WFL5	DE	1,4-alpha-D-glucan:1,4-alpha-D-glucan 6-glucosyl-transferase;
N	A4WFL5	DE	1,4-alpha-glucan-branching enzyme;
N	A4WFL5	DE	Glycogen-branching enzyme;
N	A4WFL5	DR	1,4-alpha-glucan branching enzyme activity
N	A4WFL5	DR	cation binding
N	A4WFL5	DR	glycogen biosynthetic process
N	A4WFL5	DR	hydrolase activity, hydrolyzing O-glycosyl compounds
N	A4WG69	CC	Globally modulates RNA abundance by binding to RNase E (Rne) and regulating its endonucleolytic activity
N	A4WG69	CC	Can modulate Rne action in a substrate-dependent manner by altering the composition of the degradosome
N	A4WG69	CC	Modulates RNA-binding and helicase activities of the degradosome (By similarity).
N	A4WG69	DE	Regulator of ribonuclease activity A;
N	A4WG69	DR	regulation of RNA metabolic process
N	A4WG69	DR	ribonuclease inhibitor activity
N	A4WH03	DE	30S ribosomal protein S17e;
N	A4WH03	DR	structural constituent of ribosome
N	A4WNY9	CC	F(1)F(0) ATP synthase produces ATP from ADP in the presence of a proton or sodium gradient
N	A4WNY9	CC	During catalysis, ATP synthesis in the catalytic domain of F(1) is coupled via a rotary mechanism of the central stalk subunits to proton translocation (By similarity).
N	A4WNY9	CC	F-type ATPases consist of two structural domains, F(1) containing the extramembraneous catalytic core and F(0) containing the membrane proton channel, linked together by a central stalk and a peripheral stalk
N	A4WNY9	DE	ATP synthase F(0) sector subunit b;
N	A4WNY9	DE	ATP synthase subunit b;
N	A4WNY9	DE	ATPase subunit I;
N	A4WNY9	DE	F-ATPase subunit b;
N	A4WNY9	DE	F-type ATPase subunit b;
N	A4WNY9	DR	ATP synthesis coupled proton transport
N	A4WNY9	DR	hydrogen ion transmembrane transporter activity
N	A4WPR4	DE	UPF0102 protein Rsph17025_0472;
N	A4WPR4	DR	nuclease activity
N	A4WRD4	CC	Catalyzes the methylthiolation of an aspartic acid residue of ribosomal protein S12 (By similarity).
N	A4WRD4	DE	Ribosomal protein S12 methylthiotransferase RimO;
N	A4WRD4	DE	Ribosome maturation factor RimO;
N	A4WRD4	DE	S12 MTTase;

N	A4WRD4	DE	S12 methylthiotransferase;
N	A4WRD4	DR	4 iron, 4 sulfur cluster binding
N	A4WRD4	DR	RNA modification
N	A4WRD4	DR	metal ion binding
N	A4WRD4	DR	peptidyl-L-beta-methylthioaspartic acid biosynthetic process from peptidyl-aspartic acid
N	A4WRD4	DR	transferase activity
N	A4X4I2	CC	Endonuclease that specifically degrades the RNA of RNA- DNA hybrids (By similarity).
N	A4X4I2	DE	Ribonuclease HII;
N	A4X4I2	DR	metal ion binding
N	A4X4I2	DR	ribonuclease H activity
N	A4XAW2	CC	Produces ATP from ADP in the presence of a proton gradient across the membrane
N	A4XAW2	CC	The catalytic sites are hosted primarily by the beta subunits (By similarity).
N	A4XAW2	DE	ATP synthase F1 sector subunit beta;
N	A4XAW2	DE	ATP synthase subunit beta;
N	A4XAW2	DE	F-ATPase subunit beta;
N	A4XAW2	DR	ATP hydrolysis coupled proton transport
N	A4XAW2	DR	ATP synthesis coupled proton transport
N	A4XAW2	DR	hydrogen ion transporting ATP synthase activity, rotational mechanism
N	A4XAW2	DR	hydrogen-exporting ATPase activity, phosphorylative mechanism
N	A4XAW2	DR	proton-transporting ATPase activity, rotational mechanism
N	A4XG37	DE	UPF0251 protein Csac_0224;
N	A4XI01	CC	First step of the lipid cycle reactions in the biosynthesis of the cell wall peptidoglycan (By similarity).
N	A4XI01	DE	Phospho-N-acetylmuramoyl-pentapeptide-transferase;
N	A4XI01	DE	UDP-MurNAc-pentapeptide phosphotransferase;
N	A4XI01	DR	UDP-N-acetylmuramoyl-L-alanyl-D-glutamyl-meso-2,6-diaminopimelyl-D-alanyl-D-alanine:undecaprenyl-phosphate transferase activity
N	A4XI01	DR	cell division
N	A4XI01	DR	cellular cell wall organization
N	A4XI01	DR	peptidoglycan biosynthetic process
N	A4XI01	DR	phospho-N-acetylmuramoyl-pentapeptide-transferase activity
N	A4XI01	DR	regulation of cell shape
N	A4XIA5	CC	Binds directly to 16S ribosomal RNA (By similarity).
N	A4XIA5	DE	30S ribosomal protein S20;
N	A4XIA5	DR	structural constituent of ribosome
N	A4XS59	DE	NH(3)-dependent NAD(+) synthetase;
N	A4XS59	DR	NAD biosynthetic process
N	A4XS59	DR	NAD+ synthase (glutamine-hydrolyzing) activity
N	A4XS59	DR	NAD+ synthase activity
N	A4XV44	CC	Catalyzes the transfer of selenium from selenophosphate for conversion of 2-thiouridine to 2-selenouridine at the wobble position in tRNA (By similarity).
N	A4XV44	DE	Selenophosphate-dependent tRNA 2-selenouridine synthase;
N	A4XV44	DE	tRNA 2-selenouridine synthase;
N	A4XV44	DR	selenium binding
N	A4XV44	DR	selenium compound metabolic process
N	A4XV44	DR	transferase activity, transferring selenium-containing groups
N	A4XWT1	DE	UDP-3-O-[3-hydroxymyristoyl] glucosamine N-acyltransferase;
N	A4XWT1	DR	acyltransferase activity
N	A4XWT1	DR	lipid A biosynthetic process
N	A4XYV5	CC	Condenses 4-methyl-5-(beta-hydroxyethyl)thiazole monophosphate (THZ-P) and 2-methyl-4-amino-5-hydroxymethyl pyrimidine pyrophosphate (HMP-PP) to form thiamine monophosphate (TMP) (By similarity).
N	A4XYV5	DE	TMP pyrophosphorylase;
N	A4XYV5	DE	Thiamine-phosphate pyrophosphorylase;
N	A4XYV5	DE	Thiamine-phosphate synthase;
N	A4XYV5	DR	metal ion binding
N	A4XYV5	DR	thiamine biosynthetic process
N	A4XYV5	DR	thiamine-phosphate diphosphorylase activity
N	A4XZ66	CC	One of the primary rRNA binding proteins, it binds directly to 16S rRNA where it nucleates assembly of the body of the 30S subunit (By similarity).
N	A4XZ66	DE	30S ribosomal protein S4;
N	A4XZ66	DR	structural constituent of ribosome
N	A4Y187	CC	Produces ATP from ADP in the presence of a proton gradient across the membrane
N	A4Y187	CC	The catalytic sites are hosted primarily by the beta subunits (By similarity).

N	A4Y187	DE	ATP synthase F1 sector subunit beta;
N	A4Y187	DE	ATP synthase subunit beta;
N	A4Y187	DE	F-ATPase subunit beta;
N	A4Y187	DR	ATP hydrolysis coupled proton transport
N	A4Y187	DR	ATP synthesis coupled proton transport
N	A4Y187	DR	hydrogen ion transporting ATP synthase activity, rotational mechanism
N	A4Y187	DR	hydrogen-exporting ATPase activity, phosphorylative mechanism
N	A4Y187	DR	proton-transporting ATPase activity, rotational mechanism
N	A4Y2P4	CC	Part of the Sec protein translocase complex
N	A4Y2P4	CC	Has a central role in coupling the hydrolysis of ATP to the transfer of proteins into and across the cell membrane, serving both as a receptor for the preprotein-secB complex and as an ATP-driven molecular motor driving the stepwise translocation of polypeptide chains across the membrane (By similarity).
N	A4Y2P4	CC	Interacts with the secYEG preprotein conducting channel
N	A4Y2P4	DE	Protein translocase subunit secA;
N	A4Y2P4	DR	metal ion binding
N	A4Y2P4	DR	protein import
N	A4Y2P4	DR	protein targeting
N	A4Y2P4	DR	transmembrane transport
N	A4YBF5	CC	Site-specific tyrosine recombinase, which acts by catalyzing the cutting and rejoining of the recombining DNA molecules
N	A4YBF5	CC	It also contributes to the segregational stability of plasmids (By similarity).
N	A4YBF5	CC	The xerC-xerD complex is essential to convert dimers of the bacterial chromosome into monomers to permit their segregation at cell division
N	A4YBF5	DE	Tyrosine recombinase xerC;
N	A4YBF5	DR	DNA integration
N	A4YBF5	DR	DNA recombination
N	A4YBF5	DR	cell division
N	A4YBF5	DR	chromosome segregation
N	A4YBX9	CC	Protein S19 forms a complex with S13 that binds strongly to the 16S ribosomal RNA (By similarity).
N	A4YBX9	DE	30S ribosomal protein S19;
N	A4YBX9	DR	structural constituent of ribosome
N	A4YVC7	DE	2-dehydro-3-deoxyphosphooctonate aldolase;
N	A4YVC7	DE	3-deoxy-D-manno-octulosonic acid 8-phosphate synthase;
N	A4YVC7	DE	KDO 8-P synthase;
N	A4YVC7	DE	KDO-8-phosphate synthase;
N	A4YVC7	DE	Phospho-2-dehydro-3-deoxyoctonate aldolase;
N	A4YVC7	DR	3-deoxy-8-phosphooctulonate synthase activity
N	A4YVC7	DR	lipopolysaccharide biosynthetic process
N	A5CES0	CC	NDH-1 shuttles electrons from NADH, via FMN and iron- sulfur (Fe-S) centers, to quinones in the respiratory chain
N	A5CES0	CC	Couples the redox reaction to proton translocation (for every two electrons transferred, four hydrogen ions are translocated across the cytoplasmic membrane), and thus conserves the redox energy in a proton gradient (By similarity).
N	A5CES0	DE	NADH dehydrogenase I subunit B;
N	A5CES0	DE	NADH-quinone oxidoreductase subunit B;
N	A5CES0	DE	NDH-1 subunit B;
N	A5CES0	DR	4 iron, 4 sulfur cluster binding
N	A5CES0	DR	NADH dehydrogenase (ubiquinone) activity
N	A5CES0	DR	metal ion binding
N	A5CES0	DR	oxidation-reduction process
N	A5CES0	DR	quinone binding
N	A5CW34	CC	Tetrapolymerization of the monopyrrole PBG into the hydroxymethylbilane pre-uroporphyrinogen in several discrete steps (By similarity).
N	A5CW34	DE	Hydroxymethylbilane synthase;
N	A5CW34	DE	Porphobilinogen deaminase;
N	A5CW34	DE	Pre-uroporphyrinogen synthase;
N	A5CW34	DR	hydroxymethylbilane synthase activity
N	A5CW34	DR	peptidyl-pyrromethane cofactor linkage
N	A5CW34	DR	porphyrin biosynthetic process
N	A5CXG4	CC	NDH-1 shuttles electrons from NADH, via FMN and iron- sulfur (Fe-S) centers, to quinones in the respiratory chain

N	A5CXG4	CC	Couples the redox reaction to proton translocation (for every two electrons transferred, four hydrogen ions are translocated across the cytoplasmic membrane), and thus conserves the redox energy in a proton gradient
N	A5CXG4	CC	The immediate electron acceptor for the enzyme in this species is believed to be ubiquinone
N	A5CXG4	CC	This subunit may bind ubiquinone (By similarity).
N	A5CXG4	DE	NADH dehydrogenase I subunit H;
N	A5CXG4	DE	NADH-quinone oxidoreductase subunit H;
N	A5CXG4	DE	NDH-1 subunit H;
N	A5CXG4	DR	NADH dehydrogenase (quinone) activity
N	A5CXG4	DR	oxidation-reduction process
N	A5CXG4	DR	quinone binding
N	A5CXR1	CC	Catalyzes the phosphorylation of the position 2 hydroxy group of 4-diphosphocytidyl-2C-methyl-D-erythritol (By similarity).
N	A5CXR1	DE	4-(cytidine-5'-diphospho)-2-C-methyl-D-erythritol kinase;
N	A5CXR1	DE	4-diphosphocytidyl-2-C-methyl-D-erythritol kinase;
N	A5CXR1	DR	4-(cytidine 5'-diphospho)-2-C-methyl-D-erythritol kinase activity
N	A5CXR1	DR	terpenoid biosynthetic process
N	A5D3L5	CC	Tetrapolymerization of the monopyrrole PBG into the hydroxymethylbilane pre-uroporphyrinogen in several discrete steps (By similarity).
N	A5D3L5	DE	Hydroxymethylbilane synthase;
N	A5D3L5	DE	Porphobilinogen deaminase;
N	A5D3L5	DE	Pre-uroporphyrinogen synthase;
N	A5D3L5	DR	hydroxymethylbilane synthase activity
N	A5D3L5	DR	peptidyl-pyrromethane cofactor linkage
N	A5D3L5	DR	porphyrin biosynthetic process
N	A5D3W7	CC	Involved in DNA repair and recF pathway recombination (By similarity).
N	A5D3W7	DE	DNA repair protein recO;
N	A5D3W7	DE	Recombination protein O;
N	A5D3W7	DR	ARF GTPase activator activity
N	A5D3W7	DR	DNA recombination
N	A5D3W7	DR	regulation of ARF GTPase activity
N	A5D3W7	DR	zinc ion binding
N	A5D7C3	CC	Mediates homophilic cell-cell adhesion (By similarity).
N	A5D7C3	DE	Myelin protein zero-like protein 3;
N	A5D7C3	DR	cell adhesion
N	A5D7F6	CC	Acts as a transcriptional coactivator for NOTCH proteins
N	A5D7F6	CC	Has been shown to amplify NOTCH-induced transcription of HES1
N	A5D7F6	CC	Potentiates activation by NOTCH3 and NOTCH4 more efficiently than MAML1 or MAML3 (By similarity).
N	A5D7F6	DE	Mastermind-like protein 2;
N	A5D7F6	DR	Notch signaling pathway
N	A5D9D4	CC	Involved in the regulation of mitochondrial distribution and morphology (By similarity).
N	A5D9D4	DE	Protein misato homolog 1;
N	A5D9D4	DR	mitochondrion distribution
N	A5D9D4	DR	protein polymerization
N	A5DDB7	CC	Negative regulator of transcription elongation (By similarity).
N	A5DDB7	DE	Transcription factor BYE1;
N	A5DDB7	DR	zinc ion binding
N	A5DJX5	CC	Positively regulates the activity of the minus-end directed microtubule motor protein dynein
N	A5DJX5	CC	Plays a central role in positioning the mitotic spindle at the bud neck during cell division
N	A5DJX5	CC	Targets cytoplasmic dynein to microtubule plus ends, thereby promoting dynein-mediated microtubule sliding along the bud cortex and consequently the movement of the mitotic spindle to the bud neck (By similarity).
N	A5DJX5	DE	Lissencephaly-1 homolog;
N	A5DJX5	DE	Nuclear distribution protein PAC1;
N	A5DJX5	DE	nudF homolog;
N	A5DJX5	DR	cell division
N	A5DUA7	CC	Bifunctional enzyme that catalyzes the enolization of 2,3-diketo-5-methylthiopentyl-1-phosphate (DK-MTP-1-P) into the intermediate 2-hydroxy-3-keto-5-methylthiopentenyl-1-phosphate (HK-MTPenyl-1-P), which is then dephosphorylated to form the acireductone 1,2-dihydroxy-3-keto-5-methylthiopentene (DHK-MTPene) (By similarity).
N	A5DUA7	DE	2,3-diketo-5-methylthio-1-phosphopentane phosphatase;
N	A5DUA7	DE	Enolase-phosphatase E1;

N	A5DUA7	DR	L-methionine salvage from methylthioadenosine
N	A5DUA7	DR	acireductone synthase activity
N	A5DUA7	DR	metal ion binding
N	A5E8J0	CC	Negative regulator of class I heat shock genes (grpE- dnaK-dnaJ and groELS operons)
N	A5E8J0	CC	Prevents heat-shock induction of these operons (By similarity).
N	A5E8J0	DE	Heat-inducible transcription repressor hrcA;
N	A5E8J0	DR	regulation of transcription, DNA-dependent
N	A5E8J0	DR	response to stress
N	A5ELN9	DE	50S ribosomal protein L10;
N	A5ELN9	DR	ribosome biogenesis
N	A5ELN9	DR	structural constituent of ribosome
N	A5EV42	DE	Glycine--tRNA ligase alpha subunit;
N	A5EV42	DE	Glycyl-tRNA synthetase alpha subunit;
N	A5EV42	DR	glycine-tRNA ligase activity
N	A5EV42	DR	glycyl-tRNA aminoacylation
N	A5EVB5	DE	High frequency lysogenization protein hflD homolog;
N	A5EVB8	CC	Excises uracil residues from the DNA which can arise as a result of misincorporation of dUMP residues by DNA polymerase or due to deamination of cytosine (By similarity).
N	A5EVB8	DE	Uracil-DNA glycosylase;
N	A5EVB8	DR	base-excision repair
N	A5EVB8	DR	uracil DNA N-glycosylase activity
N	A5EX94	CC	DNA-dependent RNA polymerase catalyzes the transcription of DNA into RNA using the four ribonucleoside triphosphates as substrates.
N	A5EX94	DE	DNA-directed RNA polymerase subunit alpha;
N	A5EX94	DE	RNA polymerase subunit alpha;
N	A5EX94	DE	RNAP subunit alpha;
N	A5EX94	DE	Transcriptase subunit alpha;
N	A5EX94	DR	DNA-directed RNA polymerase activity
N	A5EX94	DR	protein dimerization activity
N	A5EXA5	CC	Involved in rRNA and/or ribosome maturation and assembly
N	A5EXA5	CC	May have metal-dependent hydrolase activity (By similarity).
N	A5EXA5	CC	Required for normal 5' and 3' processing of 16S, 23S and 5S rRNAs
N	A5EXA5	DE	Probable rRNA maturation factor;
N	A5EXA5	DR	metal ion binding
N	A5EXA5	DR	metalloendopeptidase activity
N	A5EXA5	DR	rRNA processing
N	A5F3H1	CC	Transfers and isomerizes the ribose moiety from AdoMet to the 7-aminomethyl group of 7-deazaguanine (preQ1-tRNA) to give epoxyqueuosine (oQ-tRNA) (By similarity).
N	A5F3H1	DE	Queuosine biosynthesis protein queA;
N	A5F3H1	DE	S-adenosylmethionine:tRNA ribosyltransferase-isomerase;
N	A5F3H1	DR	isomerase activity
N	A5F3H1	DR	queuosine biosynthetic process
N	A5F3H1	DR	transferase activity
N	A5F3P3	DE	50S ribosomal protein L10;
N	A5F3P3	DR	ribosome biogenesis
N	A5F3P3	DR	structural constituent of ribosome
N	A5F3R3	CC	Conversion of pppGpp to ppGpp
N	A5F3R3	CC	Guanosine pentaphosphate (pppGpp) is a cytoplasmic signaling molecule which together with ppGpp controls the "stringent response", an adaptive process that allows bacteria to respond to amino acid starvation, resulting in the coordinated regulation of numerous cellular activities (By similarity).
N	A5F3R3	DE	Guanosine pentaphosphate phosphohydrolase;
N	A5F3R3	DE	Guanosine-5'-triphosphate,3'-diphosphate pyrophosphatase;
N	A5F3R3	DE	pppGpp-5'-phosphohydrolase;
N	A5F3R3	DR	guanosine-5'-triphosphate,3'-diphosphate diphosphatase activity
N	A5F3Z4	CC	Catalyzes the interconversion between ADP-D-glycero- beta-D-manno-heptose and ADP-L-glycero-beta-D-manno-heptose via an epimerization at carbon 6 of the heptose (By similarity).
N	A5F3Z4	DE	ADP-L-glycero-D-manno-heptose-6-epimerase;
N	A5F3Z4	DE	ADP-L-glycero-beta-D-manno-heptose-6-epimerase;
N	A5F3Z4	DE	ADP-glyceromanno-heptose 6-epimerase;
N	A5F3Z4	DE	ADP-hep 6-epimerase;
N	A5F3Z4	DR	ADP-glyceromanno-heptose 6-epimerase activity
N	A5F3Z4	DR	carbohydrate metabolic process
N	A5F3Z4	DR	cellular metabolic process

N	A5F475	CC	F(1)F(0) ATP synthase produces ATP from ADP in the presence of a proton or sodium gradient
N	A5F475	CC	During catalysis, ATP synthesis in the catalytic domain of F(1) is coupled via a rotary mechanism of the central stalk subunits to proton translocation (By similarity)
N	A5F475	CC	F-type ATPases consist of two structural domains, F(1) containing the extramembraneous catalytic core and F(0) containing the membrane proton channel, linked together by a central stalk and a peripheral stalk
N	A5F475	DE	ATP synthase F(0) sector subunit b;
N	A5F475	DE	ATP synthase subunit b;
N	A5F475	DE	ATPase subunit I;
N	A5F475	DE	F-ATPase subunit b;
N	A5F475	DE	F-type ATPase subunit b;
N	A5F475	DR	ATP synthesis coupled proton transport
N	A5F475	DR	hydrogen ion transmembrane transporter activity
N	A5F5B4	CC	Cell wall formation
N	A5F5B4	CC	Adds enolpyruvyl to UDP-N- acetylglucosamine (By similarity).
N	A5F5B4	DE	Enoylpyruvate transferase;
N	A5F5B4	DE	UDP-N-acetylglucosamine 1-carboxyvinyltransferase;
N	A5F5B4	DE	UDP-N-acetylglucosamine enolpyruvyl transferase;
N	A5F5B4	DR	UDP-N-acetylgalactosamine biosynthetic process
N	A5F5B4	DR	UDP-N-acetylglucosamine 1-carboxyvinyltransferase activity
N	A5F5B4	DR	cell division
N	A5F5B4	DR	cellular cell wall organization
N	A5F5B4	DR	peptidoglycan biosynthetic process
N	A5F5B4	DR	regulation of cell shape
N	A5F5F3	CC	Folate-binding protein involved in regulating the level of ATP-dnaA and in the modification of some tRNAs
N	A5F5F3	CC	It is probably a key factor in regulatory networks that act via tRNA modification, such as initiation of chromosomal replication (By similarity).
N	A5F5F3	DE	tRNA-modifying protein ygfZ;
N	A5F5F3	DR	aminomethyltransferase activity
N	A5F5F3	DR	folic acid binding
N	A5F5F3	DR	glycine catabolic process
N	A5F5F3	DR	tRNA processing
N	A5FEF4	CC	Interconversion of serine and glycine (By similarity).
N	A5FEF4	DE	Serine hydroxymethyltransferase;
N	A5FEF4	DE	Serine methylase;
N	A5FEF4	DR	L-serine metabolic process
N	A5FEF4	DR	glycine hydroxymethyltransferase activity
N	A5FEF4	DR	glycine metabolic process
N	A5FEF4	DR	one-carbon metabolic process
N	A5FEF4	DR	pyridoxal phosphate binding
N	A5FGL1	CC	Acts as a chaperone (By similarity).
N	A5FGL1	DE	Chaperone protein DnaK;
N	A5FGL1	DE	Heat shock 70 kDa protein;
N	A5FGL1	DE	Heat shock protein 70;
N	A5FGL1	DR	protein folding
N	A5FGL1	DR	response to stress
N	A5FGL1	DR	unfolded protein binding
N	A5FIJ4	CC	DNA-dependent RNA polymerase catalyzes the transcription of DNA into RNA using the four ribonucleoside triphosphates as substrates (By similarity).
N	A5FIJ4	DE	DNA-directed RNA polymerase subunit beta';
N	A5FIJ4	DE	RNA polymerase subunit beta';
N	A5FIJ4	DE	RNAP subunit beta';
N	A5FIJ4	DE	Transcriptase subunit beta';
N	A5FIJ4	DR	DNA-directed RNA polymerase activity
N	A5FIJ9	CC	This protein promotes the GTP-dependent binding of aminoacyl-tRNA to the A-site of ribosomes during protein biosynthesis (By similarity).
N	A5FIJ9	DE	Elongation factor Tu;
N	A5FIJ9	DR	GTPase activity
N	A5FIJ9	DR	translation elongation factor activity
N	A5FQS4	CC	Plays an important role in the de novo pathway of purine nucleotide biosynthesis
N	A5FQS4	CC	Catalyzes the first committed step in the biosynthesis of AMP from IMP (By similarity)
N	A5FQS4	DE	Adenylosuccinate synthetase;
N	A5FQS4	DE	IMP--aspartate ligase;

N	A5FQS4	DR	adenylosuccinate synthase activity
N	A5FQS4	DR	magnesium ion binding
N	A5FQS4	DR	purine nucleotide biosynthetic process
N	A5FRQ3	CC	Produces ATP from ADP in the presence of a proton gradient across the membrane
N	A5FRQ3	CC	The alpha chain is a regulatory subunit (By similarity).
N	A5FRQ3	DE	ATP synthase F1 sector subunit alpha;
N	A5FRQ3	DE	ATP synthase subunit alpha;
N	A5FRQ3	DE	F-ATPase subunit alpha;
N	A5FRQ3	DR	ATP hydrolysis coupled proton transport
N	A5FRQ3	DR	ATP synthesis coupled proton transport
N	A5FRQ3	DR	hydrogen ion transporting ATP synthase activity, rotational mechanism
N	A5FRQ3	DR	proton-transporting ATPase activity, rotational mechanism
N	A5FX21	CC	NDH-1 shuttles electrons from NADH, via FMN and iron- sulfur (Fe-S) centers, to quinones in the respiratory chain
N	A5FX21	CC	Couples the redox reaction to proton translocation (for every two electrons transferred, four hydrogen ions are translocated across the cytoplasmic membrane), and thus conserves the redox energy in a proton gradient (By similarity).
N	A5FX21	CC	The immediate electron acceptor for the enzyme in this species is believed to be ubiquinone
N	A5FX21	DE	NADH dehydrogenase I subunit N 1;
N	A5FX21	DE	NADH-quinone oxidoreductase subunit N 1;
N	A5FX21	DE	NDH-1 subunit N 1;
N	A5FX21	DR	ATP synthesis coupled electron transport
N	A5FX21	DR	NADH dehydrogenase (ubiquinone) activity
N	A5FX21	DR	quinone binding
N	A5FYE1	DE	Imidazoleglycerol-phosphate dehydratase;
N	A5FYE1	DR	histidine biosynthetic process
N	A5FYE1	DR	imidazoleglycerol-phosphate dehydratase activity
N	A5G962	CC	Catalyzes the synthesis of alpha-ribazole-5'-phosphate from nicotinate mononucleotide (NAMN) and 5,6- dimethylbenzimidazole (DMB) (By similarity).
N	A5G962	DE	N(1)-alpha-phosphoribosyltransferase;
N	A5G962	DE	NN:DBI PRT;
N	A5G962	DE	Nicotinate-nucleotide--dimethylbenzimidazole phosphoribosyltransferase;
N	A5G962	DR	cobalamin biosynthetic process
N	A5G962	DR	nicotinate-nucleotide-dimethylbenzimidazole phosphoribosyltransferase activity
N	A5G962	DR	nucleoside biosynthetic process
N	A5GBW0	DE	UPF0234 protein Gura_0717;
N	A5GC19	CC	Plays an important role in the de novo pathway of purine nucleotide biosynthesis
N	A5GC19	CC	Catalyzes the first committed step in the biosynthesis of AMP from IMP (By similarity).
N	A5GC19	DE	Adenylosuccinate synthetase;
N	A5GC19	DE	IMP--aspartate ligase;
N	A5GC19	DR	adenylosuccinate synthase activity
N	A5GC19	DR	magnesium ion binding
N	A5GC19	DR	purine nucleotide biosynthetic process
N	A5GIT9	CC	Binds the lower part of the 30S subunit head
N	A5GIT9	CC	Binds mRNA in the 70S ribosome, positioning it for translation (By similarity).
N	A5GIT9	DE	30S ribosomal protein S3;
N	A5GIT9	DR	structural constituent of ribosome
N	A5GJP1	CC	NDH-1 shuttles electrons from an unknown electron donor, via FMN and iron- sulfur (Fe-S) centers, to quinones in the respiratory and/or the photosynthetic chain
N	A5GJP1	CC	Couples the redox reaction to proton translocation, and thus conserves the redox energy in a proton gradient
N	A5GJP1	CC	Cyanobacterial NDH-1 also plays a role in inorganic carbon- concentration (By similarity).
N	A5GJP1	CC	The immediate electron acceptor for the enzyme in this species is believed to be plastoquinone
N	A5GJP1	DE	NAD(P)H dehydrogenase I subunit L;
N	A5GJP1	DE	NAD(P)H-quinone oxidoreductase subunit L;
N	A5GJP1	DE	NDH-1 subunit L;
N	A5GJP1	DR	oxidation-reduction process
N	A5GJP1	DR	oxidoreductase activity, acting on NADH or NADPH, quinone or similar compound as acceptor
N	A5GJP1	DR	quinone binding

N	A5GL22	CC	Component of the cytochrome b6-f complex, which mediates electron transfer between photosystem II (PSII) and photosystem I (PSI), cyclic electron flow around PSI, and state transitions (By similarity).
N	A5GL22	DE	Cytochrome b6-f complex subunit 8;
N	A5GL22	DE	Cytochrome b6-f complex subunit VIII;
N	A5GL22	DE	Cytochrome b6-f complex subunit petN;
N	A5GL22	DR	cytochrome complex assembly
N	A5GL22	DR	electron transport chain
N	A5GL22	DR	electron transporter, transferring electrons within cytochrome b6/f complex of photosystem II activity
N	A5GL22	DR	photosynthesis
N	A5GQA2	DE	Histidine--tRNA ligase;
N	A5GQA2	DE	Histidyl-tRNA synthetase;
N	A5GQA2	DR	histidine-tRNA ligase activity
N	A5GQA2	DR	histidyl-tRNA aminoacylation
N	A5GR50	CC	Catalyzes a trans-dehydration via an enolate intermediate (By similarity).
N	A5GR50	DE	3-dehydroquinase;
N	A5GR50	DE	3-dehydroquinate dehydratase;
N	A5GR50	DE	Type II DHQase;
N	A5GR50	DR	3-dehydroquinate dehydratase activity
N	A5GR50	DR	aromatic amino acid family biosynthetic process
N	A5I9H9	CC	Oxygenase that introduces the hydroxyl group at carbon five of 2-nonaprenyl-3-methyl-6-methoxy-1,4-benzoquinol resulting in the formation of 2-nonaprenyl-3-methyl-5-hydroxy-6-methoxy-1,4- benzoquinol (By similarity).
N	A5I9H9	DE	2-nonaprenyl-3-methyl-6-methoxy-1,4-benzoquinol hydroxylase;
N	A5I9H9	DE	5-demethoxyubiquinone hydroxylase;
N	A5I9H9	DE	DMQ hydroxylase;
N	A5I9H9	DR	monooxygenase activity
N	A5I9H9	DR	oxidation-reduction process
N	A5I9H9	DR	protein metabolic process
N	A5I9H9	DR	transition metal ion binding
N	A5I9H9	DR	ubiquinone biosynthetic process
N	A5ICZ5	DE	UPF0341 protein LPC_1282;
N	A5IHR9	CC	With S4 and S5 plays an important role in translational accuracy (By similarity).
N	A5IHR9	DE	30S ribosomal protein S12;
N	A5IHR9	DR	structural constituent of ribosome
N	A5IMA0	CC	With S4 and S12 plays an important role in translational accuracy (By similarity).
N	A5IMA0	DE	30S ribosomal protein S5;
N	A5IMA0	DR	structural constituent of ribosome
N	A5ISE3	CC	Responsible for the release of ribosomes from messenger RNA at the termination of protein biosynthesis
N	A5ISE3	CC	May increase the efficiency of translation by recycling ribosomes from one round of translation to another (By similarity).
N	A5ISE3	DE	Ribosome-recycling factor;
N	A5ISE3	DE	Ribosome-releasing factor;
N	A5IU80	DE	NH(3)-dependent NAD(+) synthetase;
N	A5IU80	DR	NAD biosynthetic process
N	A5IU80	DR	NAD+ synthase (glutamine-hydrolyzing) activity
N	A5IU80	DR	NAD+ synthase activity
N	A5IVK3	CC	Transforms pimelate into pimeloyl-CoA (By similarity).
N	A5IVK3	DE	6-carboxyhexanoate--CoA ligase;
N	A5IVK3	DE	Pimeloyl-CoA synthase;
N	A5IVK3	DR	6-carboxyhexanoate-CoA ligase activity
N	A5IVK3	DR	biotin biosynthetic process
N	A5IXT6	CC	Catalyzes the folate-dependent formation of 5-methyl- uridine at position 54 (M-5-U54) in all tRNAs (By similarity).
N	A5IXT6	DE	Folate-dependent tRNA (uracil-5-)-methyltransferase;
N	A5IXT6	DE	Folate-dependent tRNA(M-5-U54)-methyltransferase;
N	A5IXT6	DE	Methylenetetrahydrofolate--tRNA-(uracil-5-)-methyltransferase TrmFO;
N	A5IXT6	DR	flavin adenine dinucleotide binding
N	A5IXT6	DR	methylenetetrahydrofolate-tRNA-(uracil-5-)-methyltransferase (FADH2-oxidizing) activity
N	A5IXT6	DR	tRNA processing
N	A5N451	CC	Exhibits a very high intrinsic GTPase hydrolysis rate

N	A5N451	CC	Involved in the addition of a carboxymethylaminomethyl (cmnm) group at the wobble position (U34) of certain tRNAs, forming tRNA- cmnm(5)s(2)U34 (By similarity).
N	A5N451	DE	tRNA modification GTPase mnmE;
N	A5N451	DR	GTPase activity
N	A5N451	DR	metal ion binding
N	A5N451	DR	tRNA modification
N	A5N4I4	CC	Could be involved in septation (By similarity).
N	A5N4I4	DE	Putative septation protein spoVG;
N	A5N4I4	DR	barrier septum formation
N	A5N4I4	DR	sporulation resulting in formation of a cellular spore
N	A5N4S0	CC	No specific function has so far been attributed to this initiation factor; however, it seems to stimulate more or less all the activities of the other two initiation factors, IF-2 and IF-3.
N	A5N4S0	DE	Translation initiation factor IF-1;
N	A5N4S0	DR	translation initiation factor activity
N	A5N6A9	DE	3-phosphoshikimate 1-carboxyvinyltransferase;
N	A5N6A9	DE	5-enolpyruvylshikimate-3-phosphate synthase;
N	A5N6A9	DE	EPSP synthase;
N	A5N6A9	DR	3-phosphoshikimate 1-carboxyvinyltransferase activity
N	A5N6A9	DR	aromatic amino acid family biosynthetic process
N	A5N7V5	CC	Negatively regulates transcription of bacterial ribonucleotide reductase nrd genes and operons by binding to NrdR- boxes (By similarity).
N	A5N7V5	DE	Transcriptional repressor NrdR;
N	A5N7V5	DR	transcription repressor activity
N	A5N7V5	DR	zinc ion binding
N	A5PJJ5	DE	Leucine-rich repeat-containing protein 14;
N	A5PJU6	DE	Aminoglycoside phosphotransferase domain-containing protein 1;
N	A5PJU6	DR	kinase activity
N	A5PK16	CC	Substrate recognition component of a SCF (SKP1-CUL1-F- box protein) E3 ubiquitin-protein ligase complex which mediates the ubiquitination and subsequent proteasomal degradation of CP110 during G2 phase, thereby acting as an inhibitor of centrosome reduplication (By similarity).
N	A5PK16	DR	SCF-dependent proteasomal ubiquitin-dependent protein catabolic process
N	A5PK16	DR	cell division
N	A5PK16	DR	negative regulation of centrosome duplication
N	A5PK16	DR	protein ubiquitination
N	A5U030	CC	Involved in the conversion of a cis-olefin into a trans- olefin with concomitant introduction of an allylic methyl branch at the proximal position of the precursor to both the methoxy and ketomycolic acids
N	A5U030	CC	It directly affects the cis- to trans ratio and indirectly affects the keto to methoxy ratio (By similarity).
N	A5U030	DE	Mycolic acid methyltransferase MmaA1;
N	A5U030	DE	S-adenosylmethionine-dependent methyltransferase;
N	A5U030	DR	lipid biosynthetic process
N	A5U030	DR	methyltransferase activity
N	A5U2R2	CC	Seems to be involved in the anchoring of the catalytic components of the fumarate reductase complex to the cytoplasmic membrane (By similarity).
N	A5U2R2	DE	Fumarate reductase subunit D;
N	A5U2R2	DR	fumarate metabolic process
N	A5U2V0	CC	Catalyzes the condensation of iminoaspartate with dihydroxyacetone phosphate to form quinolinate (By similarity).
N	A5U2V0	DE	Quinolinate synthase A;
N	A5U2V0	DR	4 iron, 4 sulfur cluster binding
N	A5U2V0	DR	NAD biosynthetic process
N	A5U2V0	DR	metal ion binding
N	A5U2V0	DR	quinolinate synthetase A activity
N	A5U2V0	DR	transferase activity, transferring alkyl or aryl (other than methyl) groups
N	A5U313	DE	Ornithine carbamoyltransferase;
N	A5U313	DR	amino acid binding
N	A5U313	DR	arginine biosynthetic process
N	A5U313	DR	ornithine carbamoyltransferase activity
N	A5U871	CC	Catalyzes the synthesis of GMP from XMP (By similarity).
N	A5U871	DE	GMP synthase [glutamine-hydrolyzing];
N	A5U871	DE	GMP synthetase;
N	A5U871	DE	Glutamine amidotransferase;
N	A5U871	DR	GMP biosynthetic process

N	A5U871	DR	GMP synthase (glutamine-hydrolyzing) activity
N	A5U871	DR	glutamine metabolic process
N	A5UAB7	DE	Histidine--tRNA ligase;
N	A5UAB7	DE	Histidyl-tRNA synthetase;
N	A5UAB7	DR	histidine-tRNA ligase activity
N	A5UAB7	DR	histidyl-tRNA aminoacylation
N	A5UEV0	DE	UPF0181 protein CGSHiGG_01050;
N	A5UEY9	DE	UPF0268 protein CGSHiGG_01275;
N	A5UFF5	CC	Required for insertion of 4Fe-4S clusters for at least ispG (By similarity).
N	A5UFF5	DE	Iron-sulfur cluster insertion protein erpA;
N	A5UFF5	DR	iron-sulfur cluster assembly
N	A5UFF5	DR	iron-sulfur cluster binding
N	A5UFF5	DR	metal ion binding
N	A5UFF5	DR	structural molecule activity
N	A5UHF7	CC	Non-essential, abundant cell division factor that is required for proper Z-ring formation
N	A5UHF7	CC	It is recruited early to the divisome by direct interaction with FtsZ, stimulating Z-ring assembly and thereby promoting cell division earlier in the cell cycle
N	A5UHF7	CC	Its recruitment to the Z-ring requires functional FtsA or ZipA (By similarity).
N	A5UHF7	DE	Cell division protein ZapB;
N	A5UHF7	DR	barrier septum formation
N	A5UHF7	DR	cytokinesis by binary fission
N	A5UHR4	CC	Could be a mediator in iron transactions between iron acquisition and iron-requiring processes, such as synthesis and/or repair of Fe-S clusters in biosynthetic enzymes (By similarity).
N	A5UHR4	DE	Probable Fe(2+)-trafficking protein;
N	A5UHR4	DR	iron ion binding
N	A5UI35	DE	UPF0243 zinc-binding protein CGSHiGG_08005;
N	A5UI35	DR	regulation of transcription, DNA-dependent
N	A5UI35	DR	sequence-specific DNA binding transcription factor activity
N	A5UI35	DR	zinc ion binding
N	A5UIC3	DE	UPF0246 protein CGSHiGG_08495;
N	A5UIY9	CC	Forms oxaloacetate, a four-carbon dicarboxylic acid source for the tricarboxylic acid cycle.
N	A5UIY9	DE	Phosphoenolpyruvate carboxylase;
N	A5UIY9	DR	carbon fixation
N	A5UIY9	DR	phosphoenolpyruvate carboxylase activity
N	A5UIY9	DR	tricarboxylic acid cycle
N	A5UJ22	CC	Plays a critical role in the incorporation of lipoproteins in the outer membrane after they are released by the lolA protein (By similarity).
N	A5UJ22	DE	Outer-membrane lipoprotein lolB;
N	A5UJ22	DR	protein transport
N	A5V5X4	DE	50S ribosomal protein L17;
N	A5V5X4	DR	structural constituent of ribosome
N	A5V876	CC	Involved in the tonB-independent uptake of proteins (By similarity).
N	A5V876	DE	Protein tolB;
N	A5V876	DR	protein import
N	A5VAC0	CC	Responsible for the transport of dicarboxylates such as succinate, fumarate, and malate from the periplasm across the membrane (By similarity).
N	A5VAC0	DE	C4-dicarboxylate transport protein;
N	A5VAC0	DR	carbohydrate transport
N	A5VAC0	DR	sodium:dicarboxylate symporter activity
N	A5VIL5	DE	Glucose-6-phosphate isomerase;
N	A5VIL5	DE	Phosphoglucose isomerase;
N	A5VIL5	DE	Phosphohexose isomerase;
N	A5VIL5	DR	gluconeogenesis
N	A5VIL5	DR	glucose-6-phosphate isomerase activity
N	A5VJA0	CC	Zinc phosphodiesterase, which displays some tRNA 3'-processing endonuclease activity
N	A5VJA0	CC	Probably involved in tRNA maturation, by removing a 3'-trailer from precursor tRNA (By similarity).
N	A5VJA0	DE	Ribonuclease Z;
N	A5VJA0	DE	tRNA 3 endonuclease;
N	A5VJA0	DR	endoribonuclease activity, producing 5'-phosphomonoesters
N	A5VJA0	DR	metal ion binding
N	A5VJA0	DR	tRNA 3'-trailer cleavage
N	A5VKN9	CC	Might take part in the signal recognition particle (SRP) pathway

N	A5VKN9	CC	May be a regulatory protein.
N	A5VKN9	CC	This is inferred from the conservation of its genetic proximity to ftsY/ffh
N	A5VKN9	DE	UPF0122 protein Lreu_1156;
N	A5VKN9	DR	regulation of transcription, DNA-dependent
N	A5VKN9	DR	sequence-specific DNA binding transcription factor activity
N	A5VKN9	DR	sigma factor activity
N	A5VKN9	DR	transcription initiation, DNA-dependent
N	A5VKT4	CC	Catalyzes the attachment of glutamate to tRNA(Glu) in a two-step reaction: glutamate is first activated by ATP to form Glu-AMP and then transferred to the acceptor end of tRNA(Glu) (By similarity).
N	A5VKT4	DE	Glutamate--tRNA ligase;
N	A5VKT4	DE	Glutamyl-tRNA synthetase;
N	A5VKT4	DR	glutamate-tRNA ligase activity
N	A5VKT4	DR	glutamyl-tRNA aminoacylation
N	A5VP26	CC	Binds as a heterodimer with protein S6 to the central domain of the 16S rRNA, where it helps stabilize the platform of the 30S subunit (By similarity).
N	A5VP26	DE	30S ribosomal protein S18;
N	A5VP26	DR	structural constituent of ribosome
N	A5VSG4	DE	30S ribosomal protein S16;
N	A5VSG4	DR	structural constituent of ribosome
N	A5VSU9	DE	UPF0178 protein BOV_1904;
N	A5VSZ7	CC	Component of the acetyl coenzyme A carboxylase (ACC) complex
N	A5VSZ7	CC	First, biotin carboxylase catalyzes the carboxylation of biotin on its carrier protein (BCCP) and then the CO(2) group is transferred by the carboxyltransferase to acetyl-CoA to form malonyl-CoA (By similarity).
N	A5VSZ7	DE	ACCase subunit alpha;
N	A5VSZ7	DE	Acetyl-CoA carboxylase carboxyltransferase subunit alpha;
N	A5VSZ7	DE	Acetyl-coenzyme A carboxylase carboxyl transferase subunit alpha;
N	A5VSZ7	DR	acetyl-CoA carboxylase activity
N	A5VSZ7	DR	fatty acid biosynthetic process
N	A5VT60	CC	Component of the acetyl coenzyme A carboxylase (ACC) complex
N	A5VT60	CC	Biotin carboxylase (BC) catalyzes the carboxylation of biotin on its carrier protein (BCCP) and then the CO(2) group is transferred by the transcarboxylase to acetyl-CoA to form malonyl- CoA (By similarity).
N	A5VT60	DE	ACCase subunit beta;
N	A5VT60	DE	Acetyl-CoA carboxylase carboxyltransferase subunit beta;
N	A5VT60	DE	Acetyl-coenzyme A carboxylase carboxyl transferase subunit beta;
N	A5VT60	DR	acetyl-CoA carboxylase activity
N	A5VT60	DR	fatty acid biosynthetic process
N	A5VZX1	DE	Phosphoribosylaminoimidazole-succinocarboxamide synthase;
N	A5VZX1	DE	SAICAR synthetase;
N	A5VZX1	DR	phosphoribosylaminoimidazolesuccinocarboxamide synthase activity
N	A5VZX1	DR	purine nucleotide biosynthetic process
N	A5W2C0	CC	Catalyzes the hydrolysis of short-chain aliphatic amides to their corresponding organic acids with release of ammonia (By similarity).
N	A5W2C0	DE	Acylamide amidohydrolase;
N	A5W2C0	DE	Aliphatic amidase;
N	A5W2C0	DR	amidase activity
N	A5W2C0	DR	nitrogen compound metabolic process
N	A5W488	DE	UPF0145 protein Pput_2816;
N	A5W838	CC	Involved in the biosynthesis of lipid A, a phosphorylated glycolipid that anchors the lipopolysaccharide to the outer membrane of the cell (By similarity).
N	A5W838	DE	Acyl-[acyl-carrier-protein]--UDP-N-acetylglucosamine O-acyltransferase;
N	A5W838	DE	UDP-N-acetylglucosamine acyltransferase;
N	A5W838	DR	acyl-[acyl-carrier-protein]-UDP-N-acetylglucosamine O-acyltransferase activity
N	A5W838	DR	lipid A biosynthetic process
N	A5W8J7	CC	Catalyzes the decarboxylation of oxaloacetate into pyruvate
N	A5W8J7	CC	Seems to play a role in maintaining cellular concentrations of bicarbonate and pyruvate (By similarity).
N	A5W8J7	DE	Oxaloacetate decarboxylase;
N	A5W8J7	DR	metal ion binding
N	A5W8J7	DR	oxaloacetate decarboxylase activity
N	A5WBA8	CC	F(1)F(0) ATP synthase produces ATP from ADP in the presence of a proton or sodium gradient

N	A5WBA8	CC	During catalysis, ATP synthesis in the catalytic domain of F(1) is coupled via a rotary mechanism of the central stalk subunits to proton translocation (By similarity).
N	A5WBA8	CC	F-type ATPases consist of two structural domains, F(1) containing the extramembraneous catalytic core and F(0) containing the membrane proton channel, linked together by a central stalk and a peripheral stalk
N	A5WBA8	DE	ATP synthase F(0) sector subunit c;
N	A5WBA8	DE	ATP synthase subunit c;
N	A5WBA8	DE	F-ATPase subunit c;
N	A5WBA8	DE	F-type ATPase subunit c;
N	A5WBA8	DE	Lipid-binding protein;
N	A5WBA8	DR	ATP hydrolysis coupled proton transport
N	A5WBA8	DR	ATP synthesis coupled proton transport
N	A5WBA8	DR	hydrogen ion transmembrane transporter activity
N	A5WBA8	DR	lipid binding
N	A5WBB7	CC	Required for the insertion of integral membrane proteins into the membrane
N	A5WBB7	CC	Involved in integration of membrane proteins that insert dependently and independently of the Sec translocase complex (By similarity).
N	A5WBB7	CC	Probably plays an essential role in the integration of proteins of the respiratory chain complexes
N	A5WBB7	DE	Inner membrane protein oxaA;
N	A5WBB7	DR	protein insertion into membrane
N	A5WBG4	CC	Required for disulfide bond formation in some periplasmic proteins
N	A5WBG4	CC	Acts by oxidizing the DsbA protein (By similarity).
N	A5WBG4	DE	Disulfide bond formation protein B;
N	A5WBG4	DE	Disulfide oxidoreductase;
N	A5WBG4	DR	electron transport chain
N	A5WBG4	DR	protein disulfide oxidoreductase activity
N	A5WG42	CC	NDH-1 shuttles electrons from NADH, via FMN and iron- sulfur (Fe-S) centers, to quinones in the respiratory chain
N	A5WG42	CC	Couples the redox reaction to proton translocation (for every two electrons transferred, four hydrogen ions are translocated across the cytoplasmic membrane), and thus conserves the redox energy in a proton gradient
N	A5WG42	CC	The immediate electron acceptor for the enzyme in this species is believed to be ubiquinone
N	A5WG42	CC	This subunit may bind ubiquinone (By similarity).
N	A5WG42	DE	NADH dehydrogenase I subunit H;
N	A5WG42	DE	NADH-quinone oxidoreductase subunit H;
N	A5WG42	DE	NDH-1 subunit H;
N	A5WG42	DR	NADH dehydrogenase (quinone) activity
N	A5WG42	DR	oxidation-reduction process
N	A5WG42	DR	quinone binding
N	A6GZ72	DE	50S ribosomal protein L17;
N	A6GZ72	DR	structural constituent of ribosome
N	A6H098	CC	Catalyzes the oxidation of either pyridoxine 5'-phosphate (PNP) or pyridoxamine 5'-phosphate (PMP) into pyridoxal 5'-phosphate (PLP) (By similarity).
N	A6H098	DE	PNP/PMP oxidase;
N	A6H098	DE	Pyridoxal 5'-phosphate synthase;
N	A6H098	DE	Pyridoxine/pyridoxamine 5'-phosphate oxidase;
N	A6H098	DR	oxidation-reduction process
N	A6H098	DR	pyridoxamine-phosphate oxidase activity
N	A6H098	DR	pyridoxine biosynthetic process
N	A6H0P1	CC	Binds to the 23S rRNA (By similarity).
N	A6H0P1	DE	50S ribosomal protein L9;
N	A6H0P1	DR	structural constituent of ribosome
N	A6H5Y1	DE	M-phase phosphoprotein 9;
N	A6L4L7	CC	Produces ATP from ADP in the presence of a proton gradient across the membrane
N	A6L4L7	CC	The catalytic sites are hosted primarily by the beta subunits (By similarity).
N	A6L4L7	DE	ATP synthase F1 sector subunit beta;
N	A6L4L7	DE	ATP synthase subunit beta;
N	A6L4L7	DE	F-ATPase subunit beta;
N	A6L4L7	DR	ATP hydrolysis coupled proton transport
N	A6L4L7	DR	ATP synthesis coupled proton transport
N	A6L4L7	DR	hydrogen ion transporting ATP synthase activity, rotational mechanism
N	A6L4L7	DR	hydrogen-exporting ATPase activity, phosphorylative mechanism
N	A6L4L7	DR	proton-transporting ATPase activity, rotational mechanism

N	A6L8J2	CC	Catalyzes the oxidation of 5,10- methylenetetrahydrofolate to 5,10- methenyltetrahydrofolate and then the hydrolysis of 5,10- methenyltetrahydrofolate to 10- formyltetrahydrofolate (By similarity).
N	A6L8J2	DE	Bifunctional protein FolD;
N	A6L8J2	DE	Methenyltetrahydrofolate cyclohydrolase;
N	A6L8J2	DE	Methylenetetrahydrofolate dehydrogenase;
N	A6L8J2	DR	folic acid-containing compound biosynthetic process
N	A6L8J2	DR	histidine biosynthetic process
N	A6L8J2	DR	methenyltetrahydrofolate cyclohydrolase activity
N	A6L8J2	DR	methionine biosynthetic process
N	A6L8J2	DR	methylenetetrahydrofolate dehydrogenase (NADP+) activity
N	A6L8J2	DR	one-carbon metabolic process
N	A6L8J2	DR	oxidation-reduction process
N	A6L8J2	DR	purine nucleotide biosynthetic process
N	A6L8T2	CC	Cleaves the N-terminal amino acid of tripeptides (By similarity).
N	A6L8T2	DE	Aminotripeptidase;
N	A6L8T2	DE	Peptidase T;
N	A6L8T2	DE	Tripeptidase;
N	A6L8T2	DE	Tripeptide aminopeptidase;
N	A6L8T2	DR	metallopeptidase activity
N	A6L8T2	DR	peptide metabolic process
N	A6L8T2	DR	tripeptide aminopeptidase activity
N	A6L8T2	DR	zinc ion binding
N	A6LIG7	CC	Catalyzes the attachment of valine to tRNA(Val)
N	A6LIG7	CC	As ValRS can inadvertently accommodate and process structurally similar amino acids such as threonine, to avoid such errors, it has a "posttransfer" editing activity that hydrolyzes mischarged Thr-tRNA(Val) in a tRNA-dependent manner (By similarity)
N	A6LIG7	DE	Valine--tRNA ligase;
N	A6LIG7	DE	Valyl-tRNA synthetase;
N	A6LIG7	DR	valine-tRNA ligase activity
N	A6LIG7	DR	valyl-tRNA aminoacylation
N	A6LPN9	CC	Catalyzes the formation of 4-diphosphocytidyl-2-C- methyl-D-erythritol from CTP and 2-C-methyl-D-erythritol 4- phosphate (MEP) (By similarity).
N	A6LPN9	DE	2-C-methyl-D-erythritol 4-phosphate cytidylyltransferase;
N	A6LPN9	DE	4-diphosphocytidyl-2C-methyl-D-erythritol synthase;
N	A6LPN9	DE	MEP cytidylyltransferase;
N	A6LPN9	DR	2-C-methyl-D-erythritol 4-phosphate cytidylyltransferase activity
N	A6LPN9	DR	isoprenoid biosynthetic process
N	A6LPQ5	CC	DNA-dependent RNA polymerase catalyzes the transcription of DNA into RNA using the four ribonucleoside triphosphates as substrates (By similarity).
N	A6LPQ5	DE	DNA-directed RNA polymerase subunit beta';
N	A6LPQ5	DE	RNA polymerase subunit beta';
N	A6LPQ5	DE	RNAP subunit beta';
N	A6LPQ5	DE	Transcriptase subunit beta';
N	A6LPQ5	DR	DNA-directed RNA polymerase activity
N	A6LPS1	CC	Binds to 23S rRNA
N	A6LPS1	CC	Forms part of two intersubunit bridges in the 70S ribosome (By similarity).
N	A6LPS1	DE	50S ribosomal protein L14;
N	A6LPS1	DR	structural constituent of ribosome
N	A6LQR6	CC	This protein binds to 23S rRNA in the presence of protein L20 (By similarity).
N	A6LQR6	DE	50S ribosomal protein L21;
N	A6LQR6	DR	structural constituent of ribosome
N	A6M273	DE	Deoxycytidine triphosphate deaminase;
N	A6M273	DE	dCTP deaminase;
N	A6M273	DR	dCTP deaminase activity
N	A6M273	DR	dUTP biosynthetic process
N	A6M931	CC	ATP-dependent RNA helicase
N	A6M931	CC	Binds spliced mRNA in sequence-independent manner, 20-24 nucleotides upstream of mRNA exon-exon junctions
N	A6M931	CC	Component of a splicing- dependent multiprotein exon junction complex (EJC) deposited at splice junction on mRNAs
N	A6M931	CC	Constitutes at least part of the platform anchoring other EJC proteins to spliced mRNAs

N	A6M931	CC	Core components of the EJC, that remains bound to spliced mRNAs throughout all stages of mRNA metabolism, functions to mark the position of the exon-exon junction in the mature mRNA and thereby influences downstream processes of gene expression including mRNA splicing, nuclear mRNA export, subcellular mRNA localization, translation efficiency and nonsense-mediated mRNA decay (NMD)
N	A6M931	CC	Involved in translational enhancement of spliced mRNAs after formation of the 80S ribosome complex
N	A6M931	CC	Its RNA-dependent ATPase and RNA-helicase activities are induced by CASC3, but abolished in presence of the MAGOH/RBM8A heterodimer, thereby trapping the ATP- bound EJC core onto spliced mRNA in a stable conformation
N	A6M931	CC	Shows higher affinity for single-stranded RNA in an ATP-bound core EJC complex than after the ATP is hydrolyzed (By similarity).
N	A6M931	CC	The EJC is a dynamic structure consisting of a few core proteins and several more peripheral nuclear and cytoplasmic associated factors that join the complex only transiently either during EJC assembly or during subsequent mRNA metabolism
N	A6M931	CC	The inhibition of ATPase activity by the MAGOH/RBM8A heterodimer increases the RNA-binding affinity of the EJC
N	A6M931	DE	ATP-dependent RNA helicase DDX48;
N	A6M931	DE	ATP-dependent RNA helicase eIF4A-3;
N	A6M931	DE	DEAD box protein 48;
N	A6M931	DE	Eukaryotic initiation factor 4A-III;
N	A6M931	DE	Eukaryotic translation initiation factor 4A isoform 3;
N	A6M931	DE	eIF-4A-III;
N	A6M931	DR	mRNA processing
N	A6M931	DR	mRNA transport
N	A6M931	DR	nuclear-transcribed mRNA catabolic process, nonsense-mediated decay
N	A6M931	DR	rRNA processing
N	A6MMP1	DE	50S ribosomal protein L36, chloroplastic;
N	A6MMP1	DR	structural constituent of ribosome
N	A6MMV5	CC	Seems to be required for the assembly of the photosystem I complex (By similarity).
N	A6MMV5	DE	Photosystem I assembly protein ycf4;
N	A6MMV5	DR	photosynthesis
N	A6NFC5	DE	Transmembrane protein 235;
N	A6NFC5	DR	structural molecule activity
N	A6NHJ4	CC	May be involved in transcriptional regulation (By similarity).
N	A6NHJ4	DE	Zinc finger protein 860;
N	A6NHJ4	DR	regulation of transcription, DNA-dependent
N	A6NHJ4	DR	zinc ion binding
N	A6NHP6	DE	NPIP-like protein LOC729602;
N	A6NIL9	DE	Putative uncharacterized protein encoded by NCRNA00169;
N	A6NNM8	CC	Polyglutamylase which preferentially modifies alpha- tubulin
N	A6NNM8	CC	Involved in the side-chain elongation step of the polyglutamylation reaction rather than in the initiation step (By similarity).
N	A6NNM8	DE	Tubulin polyglutamylase TTLL13;
N	A6NNM8	DE	Tubulin--tyrosine ligase-like protein 13;
N	A6NNM8	DR	protein modification process
N	A6NNM8	DR	tubulin-tyrosine ligase activity
N	A6Q169	CC	Binds directly to 23S ribosomal RNA and is necessary for the in vitro assembly process of the 50S ribosomal subunit
N	A6Q169	CC	It is not involved in the protein synthesizing functions of that subunit (By similarity).
N	A6Q169	DE	50S ribosomal protein L20;
N	A6Q169	DR	structural constituent of ribosome
N	A6Q1P4	CC	NDH-1 shuttles electrons from NADH, via FMN and iron- sulfur (Fe-S) centers, to quinones in the respiratory chain
N	A6Q1P4	CC	Couples the redox reaction to proton translocation (for every two electrons transferred, four hydrogen ions are translocated across the cytoplasmic membrane), and thus conserves the redox energy in a proton gradient (By similarity).
N	A6Q1P4	CC	The immediate electron acceptor for the enzyme in this species is believed to be ubiquinone
N	A6Q1P4	DE	NADH dehydrogenase I subunit B;
N	A6Q1P4	DE	NADH-quinone oxidoreductase subunit B;
N	A6Q1P4	DE	NDH-1 subunit B;
N	A6Q1P4	DR	4 iron, 4 sulfur cluster binding
N	A6Q1P4	DR	NADH dehydrogenase (ubiquinone) activity
N	A6Q1P4	DR	metal ion binding

N	A6Q1P4	DR	oxidation-reduction process
N	A6Q1P4	DR	quinone binding
N	A6Q639	CC	Catalyzes the transfer of a ribosyl phosphate group from 5-phosphoribose 1-diphosphate to orotate, leading to the formation of orotidine monophosphate (OMP) (By similarity).
N	A6Q639	DE	Orotate phosphoribosyltransferase;
N	A6Q639	DR	nucleoside metabolic process
N	A6Q639	DR	orotate phosphoribosyltransferase activity
N	A6Q639	DR	pyrimidine base biosynthetic process
N	A6Q639	DR	pyrimidine nucleotide biosynthetic process
N	A6Q826	CC	Catalyzes the decarboxylation of orotidine 5'- monophosphate (OMP) to uridine 5'-monophosphate (UMP) (By similarity).
N	A6Q826	DE	OMP decarboxylase;
N	A6Q826	DE	Orotidine 5'-phosphate decarboxylase;
N	A6Q826	DR	'de novo' UMP biosynthetic process
N	A6Q826	DR	'de novo' pyrimidine base biosynthetic process
N	A6Q826	DR	orotidine-5'-phosphate decarboxylase activity
N	A6QHN4	CC	Component of the acetyl coenzyme A carboxylase (ACC) complex
N	A6QHN4	CC	First, biotin carboxylase catalyzes the carboxylation of biotin on its carrier protein (BCCP) and then the CO(2) group is transferred by the carboxyltransferase to acetyl-CoA to form malonyl-CoA (By similarity).
N	A6QHN4	DE	ACCase subunit alpha;
N	A6QHN4	DE	Acetyl-CoA carboxylase carboxyltransferase subunit alpha;
N	A6QHN4	DE	Acetyl-coenzyme A carboxylase carboxyl transferase subunit alpha;
N	A6QHN4	DR	acetyl-CoA carboxylase activity
N	A6QHN4	DR	fatty acid biosynthetic process
N	A6QJH4	CC	Metallothiol transferase which confers resistance to fosfomycin by catalyzing the addition of a thiol cofactor to fosfomycin
N	A6QJH4	CC	L-cysteine is probably the physiological thiol donor (By similarity).
N	A6QJH4	DE	Fosfomycin resistance protein;
N	A6QJH4	DE	Metallothiol transferase fosB;
N	A6QJH4	DR	metal ion binding
N	A6QJH4	DR	response to antibiotic
N	A6QJH4	DR	transferase activity, transferring alkyl or aryl (other than methyl) groups
N	A6QLQ8	CC	Endoribonuclease that cleaves single-stranded RNAs at uridylates and releases products that have 2'-3'-cyclic phosphate termini (By similarity).
N	A6QLQ8	DE	Poly(U)-specific endoribonuclease;
N	A6QLQ8	DE	Protein endoU;
N	A6QLQ8	DE	Uridylate-specific endoribonuclease;
N	A6QLQ8	DR	endoribonuclease activity
N	A6QLQ8	DR	immune response
N	A6QLQ8	DR	metal ion binding
N	A6QLQ8	DR	polysaccharide binding
N	A6QLQ8	DR	scavenger receptor activity
N	A6QPA0	CC	Plays an essential role in the maturation of presomitic mesoderm cells by individual attenuation of both FGF and WNT signaling (By similarity).
N	A6QPA0	DE	Protein shisa-2 homolog;
N	A6QPA0	DE	Transmembrane protein 46;
N	A6QPA0	DR	multicellular organismal development
N	A6QQ93	DE	Membrane protein FAM159B;
N	A6QQL9	CC	Signal-recognition-particle assembly has a crucial role in targeting secretory proteins to the rough endoplasmic reticulum membrane
N	A6QQL9	CC	SRP9 together with SRP14 and the Alu portion of the SRP RNA, constitutes the elongation arrest domain of SRP
N	A6QQL9	CC	The complex of SRP9 and SRP14 is required for SRP RNA binding (By similarity).
N	A6QQL9	DE	Signal recognition particle 14 kDa protein;
N	A6QQL9	DR	7S RNA binding
N	A6QQL9	DR	SRP-dependent cotranslational protein targeting to membrane
N	A6QQL9	DR	endoplasmic reticulum signal peptide binding
N	A6QQL9	DR	negative regulation of translational elongation
N	A6QXC1	CC	ATP-dependent RNA helicase required for 60S ribosomal subunit synthesis
N	A6QXC1	CC	Involved in efficient pre-rRNA processing, predominantly at site A3, which is necessary for the normal formation of 25S and 5.8S rRNAs (By similarity).
N	A6QXC1	DE	ATP-dependent RNA helicase DBP3;
N	A6QXC1	DR	ATP-dependent helicase activity
N	A6QXC1	DR	rRNA processing

N	A6SED8	CC	Type-I myosin implicated in the organization of the actin cytoskeleton
N	A6SED8	CC	At the cell cortex, assembles in patch-like structures together with proteins from the actin-polymerizing machinery and promotes actin assembly
N	A6SED8	CC	Functions as actin nucleation-promoting factor (NPF) for the Arp2/3 complex (By similarity).
N	A6SED8	CC	Required for proper actin cytoskeleton polarization
N	A6SED8	DE	Class I unconventional myosin;
N	A6SED8	DE	Type I myosin;
N	A6SED8	DR	actin binding
N	A6SED8	DR	motor activity
N	A6SMQ7	CC	Forms a chaperone-bound H2A.Z-H2B complex that acts as a source for SWR1 complex-dependent H2A to H2A.Z histone replacement in chromatin (By similarity)
N	A6SMQ7	DE	Histone H2A.Z-specific chaperone chz1;
N	A6SUY3	DE	Succinyl-CoA ligase [ADP-forming] subunit beta;
N	A6SUY3	DE	Succinyl-CoA synthetase subunit beta;
N	A6SUY3	DR	metal ion binding
N	A6SUY3	DR	succinate-CoA ligase (ADP-forming) activity
N	A6SUY3	DR	tricarboxylic acid cycle
N	A6T4E3	CC	Catalyzes the ATP-dependent phosphorylation of L- homoserine to L-homoserine phosphate (By similarity).
N	A6T4E3	DE	Homoserine kinase;
N	A6T4E3	DR	homoserine kinase activity
N	A6T4E3	DR	threonine biosynthetic process
N	A6T6L5	CC	Catalyzes the conversion of dethiobiotin (DTB) to biotin by the insertion of a sulfur atom into dethiobiotin via a radical- based mechanism (By similarity).
N	A6T6L5	DE	Biotin synthase;
N	A6T6L5	DR	2 iron, 2 sulfur cluster binding
N	A6T6L5	DR	4 iron, 4 sulfur cluster binding
N	A6T6L5	DR	biotin biosynthetic process
N	A6T6L5	DR	biotin synthase activity
N	A6T6L5	DR	metal ion binding
N	A6T766	CC	Specifically methylates the cytosine at position 1962 (m5C1962) of 23S rRNA (By similarity).
N	A6T766	DE	23S rRNA m5C1962 methyltransferase;
N	A6T766	DE	Ribosomal RNA large subunit methyltransferase I;
N	A6T766	DE	rRNA (cytosine-C(5)-)-methyltransferase RlmI;
N	A6T766	DR	rRNA methyltransferase activity
N	A6TE36	CC	Catalyzes the dephosphorylation of undecaprenyl diphosphate (UPP)
N	A6TE36	CC	Confers resistance to bacitracin (By similarity).
N	A6TE36	DE	Bacitracin resistance protein;
N	A6TE36	DE	Undecaprenyl pyrophosphate phosphatase;
N	A6TE36	DE	Undecaprenyl-diphosphatase;
N	A6TE36	DR	cellular cell wall organization
N	A6TE36	DR	dephosphorylation
N	A6TE36	DR	peptidoglycan biosynthetic process
N	A6TE36	DR	regulation of cell shape
N	A6TE36	DR	response to antibiotic
N	A6TE36	DR	undecaprenyl-diphosphatase activity
N	A6TQJ6	CC	An essential GTPase which binds GTP, GDP and possibly (p)ppGpp with moderate affinity, with high nucleotide exchange rates and a fairly low GTP hydrolysis rate (By similarity)
N	A6TQJ6	CC	It may play a role in control of the cell cycle, stress response, ribosome biogenesis and in those bacteria that undergo differentiation, in morphogenesis control (Potential)
N	A6TQJ6	DE	GTP-binding protein obg;
N	A6TQJ6	DR	GTPase activity
N	A6TQJ6	DR	magnesium ion binding
N	A6TWF2	DE	50S ribosomal protein L17;
N	A6TWF2	DR	structural constituent of ribosome
N	A6TXC8	DE	UPF0133 protein Amet_4780;
N	A6U041	CC	Involved in the biosynthesis of D-alanyl-lipoteichoic acid (LTA)
N	A6U041	CC	Activated D-alanyl-Dcp donates its D-alanyl substituent to membrane-associated LTA (By similarity).
N	A6U041	DE	D-alanine--poly(phosphoribitol) ligase subunit 2;
N	A6U041	DE	D-alanyl carrier protein;
N	A6U041	DR	D-alanine-poly(phosphoribitol) ligase activity

N	A6U041	DR	acyl carrier activity
N	A6U041	DR	cellular cell wall organization
N	A6U041	DR	cofactor binding
N	A6U041	DR	regulation of cell shape
N	A6U041	DR	teichoic acid biosynthetic process
N	A6U0U8	CC	Involved in heme (porphyrin) scavenging
N	A6U0U8	CC	Binds hemoglobin and almost exclusively free-base protoporphyrin IX
N	A6U0U8	CC	Hemin-free isdC (apo-isdC) acquires hemin from hemin-containing isdA (holo-isdA) probably through the activated holo-isdA-apo-isdC complex and due to the higher affinity of apo-isdC for the cofactor
N	A6U0U8	CC	Probably has a role as the central conduit of the isd heme uptake system, i.e., mediates the transfer of the iron-containing nutrient from isdABH to the membrane translocation system isdDEF
N	A6U0U8	CC	The reaction is reversible (By similarity).
N	A6U0U8	DE	Iron-regulated surface determinant protein C;
N	A6U0U8	DE	Staphylococcal iron-regulated protein D;
N	A6U0U8	DR	heme transport
N	A6U0U8	DR	hemoglobin binding
N	A6U0U8	DR	metal ion binding
N	A6U1F6	CC	Binds 16S rRNA, required for the assembly of 30S particles and may also be responsible for determining the conformation of the 16S rRNA at the A site (By similarity)
N	A6U1F6	DE	30S ribosomal protein S14;
N	A6U1F6	DR	structural constituent of ribosome
N	A6U305	CC	Furnishes a means for formation of correctly charged Gln-tRNA(Gln) through the transamidation of misacylated Glu- tRNA(Gln) in organisms which lack glutaminyl-tRNA synthetase
N	A6U305	CC	The reaction takes place in the presence of glutamine and ATP through an activated gamma-phospho-Glu-tRNA(Gln) (By similarity).
N	A6U305	DE	Glu-ADT subunit A;
N	A6U305	DE	Glutamyl-tRNA(Gln) amidotransferase subunit A;
N	A6U305	DR	carbon-nitrogen ligase activity, with glutamine as amido-N-donor
N	A6U3E7	DE	Protein sprT-like;
N	A6U3E7	DR	metal ion binding
N	A6U5I6	CC	Formation of pseudouridine at positions 38, 39 and 40 in the anticodon stem and loop of transfer RNAs (By similarity).
N	A6U5I6	DE	tRNA pseudouridine synthase A;
N	A6U5I6	DE	tRNA pseudouridylate synthase I;
N	A6U5I6	DE	tRNA-uridine isomerase I;
N	A6U5I6	DR	pseudouridine synthase activity
N	A6U5I6	DR	pseudouridine synthesis
N	A6U5I6	DR	tRNA processing
N	A6UB63	CC	Catalyzes the condensation of pantoate with beta-alanine in an ATP-dependent reaction via a pantoyl-adenylate intermediate (By similarity).
N	A6UB63	DE	Pantoate--beta-alanine ligase;
N	A6UB63	DE	Pantoate-activating enzyme;
N	A6UB63	DE	Pantothenate synthetase;
N	A6UB63	DR	pantoate-beta-alanine ligase activity
N	A6UB63	DR	pantothenate biosynthetic process
N	A6UDM7	CC	Transaldolase is important for the balance of metabolites in the pentose-phosphate pathway (By similarity).
N	A6UDM7	DE	Probable transaldolase;
N	A6UDM7	DR	pentose-phosphate shunt
N	A6UDM7	DR	sedoheptulose-7-phosphate:D-glyceraldehyde-3-phosphate glyceronetransferase activity
N	A6UE79	CC	Converts 2C-methyl-D-erythritol 2,4-cyclodiphosphate (ME-2,4cPP) into 1-hydroxy-2-methyl-2-(E)-butenyl 4-diphosphate (By similarity).
N	A6UE79	DE	1-hydroxy-2-methyl-2-(E)-butenyl 4-diphosphate synthase;
N	A6UE79	DE	4-hydroxy-3-methylbut-2-en-1-yl diphosphate synthase;
N	A6UE79	DR	4 iron, 4 sulfur cluster binding
N	A6UE79	DR	4-hydroxy-3-methylbut-2-en-1-yl diphosphate synthase activity
N	A6UE79	DR	iron ion binding
N	A6UE79	DR	oxidation-reduction process
N	A6UE79	DR	terpenoid biosynthetic process
N	A6URL2	DE	Glutamate-1-semialdehyde 2,1-aminomutase;
N	A6URL2	DE	Glutamate-1-semialdehyde aminotransferase;
N	A6URL2	DR	glutamate-1-semialdehyde 2,1-aminomutase activity

N	A6URL2	DR	porphyrin biosynthetic process
N	A6URL2	DR	pyridoxal phosphate binding
N	A6URL2	DR	transaminase activity
N	A6US52	DE	30S ribosomal protein S24e;
N	A6US52	DR	nucleotide binding
N	A6US52	DR	structural constituent of ribosome
N	A6UU96	DE	UPF0264 protein Maeo_0482;
N	A6UU96	DR	catalytic activity
N	A6UVT6	CC	Involved in F420 biosynthesis through the oxidation of lactaldehyde to lactate (By similarity).
N	A6UVT6	DE	Lactaldehyde dehydrogenase;
N	A6UVT6	DR	lactaldehyde dehydrogenase activity
N	A6UVT6	DR	oxidation-reduction process
N	A6V0W4	CC	GTPase that plays an essential role in the late steps of ribosome biogenesis (By similarity).
N	A6V0W4	DE	GTP-binding protein EngA;
N	A6V0W4	DR	nucleoside-triphosphatase activity
N	A6V0W4	DR	ribosome biogenesis
N	A6V1J0	DE	50S ribosomal protein L36 2;
N	A6V1J0	DR	structural constituent of ribosome
N	A6V383	CC	Catalyzes the final step of fatty acid oxidation in which acetyl-CoA is released and the CoA ester of a fatty acid two carbons shorter is formed (By similarity).
N	A6V383	DE	3-ketoacyl-CoA thiolase;
N	A6V383	DE	Acetyl-CoA acyltransferase;
N	A6V383	DE	Beta-ketothiolase;
N	A6V383	DE	Fatty acid oxidation complex subunit beta;
N	A6V383	DR	acetyl-CoA C-acyltransferase activity
N	A6V383	DR	fatty acid metabolic process
N	A6V383	DR	lipid catabolic process
N	A6V3C9	CC	Catalyzes the reversible formation of acyl-phosphate (acyl-PO(4)) from acyl-[acyl-carrier-protein] (acyl-ACP)
N	A6V3C9	CC	This enzyme utilizes acyl-ACP as fatty acyl donor, but not acyl-CoA (By similarity).
N	A6V3C9	DE	Acyl-ACP phosphotransacylase;
N	A6V3C9	DE	Acyl-[acyl-carrier-protein]--phosphate acyltransferase;
N	A6V3C9	DE	Phosphate acyltransferase;
N	A6V3C9	DE	Phosphate-acyl-ACP acyltransferase;
N	A6V3C9	DR	fatty acid biosynthetic process
N	A6V3C9	DR	phospholipid biosynthetic process
N	A6V3C9	DR	transferase activity, transferring acyl groups other than amino-acyl groups
N	A6V720	CC	Involved in protein export
N	A6V720	CC	Acts as a chaperone by maintaining the newly synthesized protein in an open conformation (By similarity).
N	A6V720	DE	Trigger factor;
N	A6V720	DR	cell division
N	A6V720	DR	peptidyl-prolyl cis-trans isomerase activity
N	A6V720	DR	protein folding
N	A6V720	DR	protein transport
N	A6VC67	CC	This is one of the proteins that binds to the 5S RNA in the ribosome where it forms part of the central protuberance (By similarity).
N	A6VC67	DE	50S ribosomal protein L25;
N	A6VC67	DE	General stress protein CTC;
N	A6VC67	DR	5S rRNA binding
N	A6VC67	DR	structural constituent of ribosome
N	A6VQ73	DE	1-(5-phosphoribosyl)-5-[(5-phosphoribosylamino)methylideneamino] imidazole-4-carboxamide isomerase;
N	A6VQ73	DE	Phosphoribosylformimino-5-aminoimidazole carboxamide ribotide isomerase;
N	A6VQ73	DR	1-(5-phosphoribosyl)-5-[(5-phosphoribosylamino)methylideneamino]imidazole-4-carboxamide isomerase activity
N	A6VQ73	DR	histidine biosynthetic process
N	A6W2W4	CC	Catalyzes the synthesis of GMP from XMP (By similarity).
N	A6W2W4	DE	GMP synthase [glutamine-hydrolyzing];
N	A6W2W4	DE	GMP synthetase;
N	A6W2W4	DE	Glutamine amidotransferase;
N	A6W2W4	DR	GMP biosynthetic process
N	A6W2W4	DR	GMP synthase (glutamine-hydrolyzing) activity

N	A6W2W4	DR	glutamine metabolic process
N	A6WP56	CC	Is required not only for elongation of protein synthesis but also for the initiation of all mRNA translation through initiator tRNA(fMet) aminoacylation (By similarity)
N	A6WP56	DE	Methionine--tRNA ligase;
N	A6WP56	DE	Methionyl-tRNA synthetase;
N	A6WP56	DR	metal ion binding
N	A6WP56	DR	methionine-tRNA ligase activity
N	A6WP56	DR	methionyl-tRNA aminoacylation
N	A6WXX0	CC	Produces ATP from ADP in the presence of a proton gradient across the membrane
N	A6WXX0	CC	The gamma chain is believed to be important in regulating ATPase activity and the flow of protons through the CF(0) complex (By similarity).
N	A6WXX0	DE	ATP synthase F1 sector gamma subunit;
N	A6WXX0	DE	ATP synthase gamma chain;
N	A6WXX0	DE	F-ATPase gamma subunit;
N	A6WXX0	DR	ATP synthesis coupled proton transport
N	A6WXX0	DR	hydrogen ion transporting ATP synthase activity, rotational mechanism
N	A6WXX0	DR	proton-transporting ATPase activity, rotational mechanism
N	A6WZD8	CC	Catalyzes a salvage reaction resulting in the formation of AMP, that is energically less costly than de novo synthesis (By similarity).
N	A6WZD8	DE	Adenine phosphoribosyltransferase;
N	A6WZD8	DR	adenine phosphoribosyltransferase activity
N	A6WZD8	DR	adenine salvage
N	A6WZD8	DR	purine ribonucleoside salvage
N	A6X0A5	CC	This protein binds directly to 23S ribosomal RNA (By similarity).
N	A6X0A5	DE	50S ribosomal protein L11;
N	A6X0A5	DR	structural constituent of ribosome
N	A6X1M5	CC	NDH-1 shuttles electrons from NADH, via FMN and iron- sulfur (Fe-S) centers, to quinones in the respiratory chain
N	A6X1M5	CC	Couples the redox reaction to proton translocation (for every two electrons transferred, four hydrogen ions are translocated across the cytoplasmic membrane), and thus conserves the redox energy in a proton gradient (By similarity)
N	A6X1M5	CC	The immediate electron acceptor for the enzyme in this species is believed to be ubiquinone
N	A6X1M5	DE	NADH dehydrogenase I subunit I;
N	A6X1M5	DE	NADH-quinone oxidoreductase subunit I;
N	A6X1M5	DE	NDH-1 subunit I;
N	A6X1M5	DR	4 iron, 4 sulfur cluster binding
N	A6X1M5	DR	NADH dehydrogenase (quinone) activity
N	A6X1M5	DR	electron carrier activity
N	A6X1M5	DR	metal ion binding
N	A6X1M5	DR	oxidation-reduction process
N	A6X1M5	DR	quinone binding
N	A6YG90	DE	Protein psbN;
N	A6YG90	DR	photosynthesis
N	A6ZQX9	CC	Forms a chaperone-bound H2A.Z-H2B complex that acts as a source for SWR1 complex-dependent H2A to H2A.Z histone replacement in chromatin (By similarity)
N	A6ZQX9	DE	Histone H2A.Z-specific chaperone CHZ1;
N	A6ZRZ6	CC	Component of the PAN1 actin cytoskeleton-regulatory complex required for the internalization of endosomes during actin-coupled endocytosis
N	A6ZRZ6	CC	END3 regulates PAN1 function by preventing phosphorylation of PAN1 by PKR1 and is also involved in the correct localization of SLA1 to the cell cortex, in the bipolar budding of diploid cells and the correct distribution of chitin at the cell surface (By similarity)
N	A6ZRZ6	CC	Mediates uptake of external molecules and vacuolar degradation of plasma membrane proteins
N	A6ZRZ6	CC	Plays a role in the proper organization of the cell membrane-associated actin cytoskeleton and promotes its destabilization
N	A6ZRZ6	CC	The complex links the site of endocytosis to the cell membrane-associated actin cytoskeleton
N	A6ZRZ6	DE	Actin cytoskeleton-regulatory complex protein END3;
N	A6ZRZ6	DE	Endocytosis protein 3;
N	A6ZRZ6	DR	actin binding
N	A6ZRZ6	DR	calcium ion binding

N	A6ZWL1	CC	Component of the nascent polypeptide-associated complex (NAC), a dynamic component of the ribosomal exit tunnel, protecting the emerging polypeptides from interaction with other cytoplasmic proteins to ensure appropriate nascent protein targeting (By similarity)
N	A6ZWL1	CC	EGD1 may act as a transcription factor that exert a negative effect on the expression of several genes that are transcribed by RNA polymerase II (By similarity)
N	A6ZWL1	CC	The NAC complex also promotes mitochondrial protein import by enhancing productive ribosome interactions with the outer mitochondrial membrane and blocks the inappropriate interaction of ribosomes translating non-secretory nascent polypeptides with translocation sites in the membrane of the endoplasmic reticulum (By similarity)
N	A6ZWL1	DE	BTF3 homolog EGD1;
N	A6ZWL1	DE	GAL4 DNA-binding enhancer protein 1;
N	A6ZWL1	DE	NAC-beta-1;
N	A6ZWL1	DE	Nascent polypeptide-associated complex subunit beta-1;
N	A6ZWL1	DR	protein transport
N	A6ZXD2	CC	Methyltransferase that catalyzes the formation of N(7)- methylguanine at position 46 (m7G46) in tRNA, a modification required to maintain stability of tRNAs; its absence resulting in tRNA decay
N	A6ZXD2	CC	Both the D-stem and T-stem structures of tRNAs are required for efficient methyltransferase activity (By similarity).
N	A6ZXD2	DE	Transfer RNA methyltransferase 8;
N	A6ZXD2	DE	tRNA (guanine-N(7)-)-methyltransferase;
N	A6ZXD2	DE	tRNA(m7G46)-methyltransferase;
N	A6ZXD2	DR	tRNA (guanine-N7-)-methyltransferase activity
N	A6ZXT5	DE	Pheromone-regulated protein PRM7;
N	A7BJC5	CC	Involved in the isomerization of 5-deoxy-glucuronate (5DG) to 5-dehydro-2-deoxy-D-gluconate (DKG or 2-deoxy-5-keto-D- gluconate) (By similarity).
N	A7BJC5	DE	5-deoxy-glucuronate isomerase;
N	A7BJC5	DE	5DG isomerase;
N	A7BJC5	DR	intramolecular oxidoreductase activity, interconverting aldoses and ketoses
N	A7E8H4	CC	Involved in cytoplasm to vacuole transport (Cvt) vesicles and autophagosomes formation
N	A7E8H4	CC	May mediate the delivery of the vesicles and autophagosomes to the vacuole via the microtubule cytoskeleton (By similarity).
N	A7E8H4	DE	Autophagy-related protein 8;
N	A7E8H4	DE	Autophagy-related ubiquitin-like modifier atg8;
N	A7E8H4	DR	protein transport
N	A7EML8	CC	Required for pre-ribosomal RNA processing
N	A7EML8	CC	Involved in the maturation of the 35S-pre-rRNA and to its cleavage to mature 18S rRNA (By similarity).
N	A7EML8	DE	ATP-dependent rRNA helicase rrp3;
N	A7EML8	DR	ATP-dependent helicase activity
N	A7EML8	DR	rRNA processing
N	A7FD13	CC	Catalyzes the formation of 5-methyl-uridine at position 54 (M-5-U54) in all tRNAs (By similarity).
N	A7FD13	DE	tRNA (uracil-5-)-methyltransferase;
N	A7FD13	DE	tRNA(M-5-U54)-methyltransferase;
N	A7FD13	DR	tRNA (uracil-5-)-methyltransferase activity
N	A7FFN2	DE	Urea amidohydrolase subunit alpha;
N	A7FFN2	DE	Urease subunit alpha;
N	A7FFN2	DR	nickel ion binding
N	A7FFN2	DR	urea metabolic process
N	A7FFN2	DR	urease activity
N	A7FJS2	DE	Methylglyoxal synthase;
N	A7FJS2	DR	methylglyoxal biosynthetic process
N	A7FJS2	DR	methylglyoxal synthase activity
N	A7FNP1	CC	With S4 and S5 plays an important role in translational accuracy (By similarity).
N	A7FNP1	DE	30S ribosomal protein S12;
N	A7FNP1	DR	structural constituent of ribosome
N	A7FNW5	CC	Catalyzes, although with low efficiency, the sulfur transfer reaction from thiosulfate to cyanide (By similarity).
N	A7FNW5	DE	Thiosulfate sulfurtransferase glpE;
N	A7FNW5	DR	glycerol metabolic process
N	A7FNW5	DR	thiosulfate sulfurtransferase activity
N	A7FR34	CC	Endonuclease that specifically degrades the RNA of RNA- DNA hybrids (By similarity).

N	A7FR34	DE	Ribonuclease H;
N	A7FR34	DR	metal ion binding
N	A7FR34	DR	nucleic acid binding
N	A7FR34	DR	ribonuclease H activity
N	A7FRB3	CC	Catalyzes the formation of N(7)-methylguanine at position 46 (m7G46) in tRNA (By similarity).
N	A7FRB3	DE	tRNA (guanine-N(7)-)-methyltransferase;
N	A7FRB3	DE	tRNA(m7G46)-methyltransferase;
N	A7FRB3	DR	tRNA (guanine-N7-)-methyltransferase activity
N	A7FXK9	DE	30S ribosomal protein S21;
N	A7FXK9	DR	structural constituent of ribosome
N	A7FZA5	DE	Lysine--tRNA ligase;
N	A7FZA5	DE	Lysyl-tRNA synthetase;
N	A7FZA5	DR	lysine-tRNA ligase activity
N	A7FZA5	DR	lysyl-tRNA aminoacylation
N	A7FZA5	DR	metal ion binding
N	A7FZA5	DR	nucleic acid binding
N	A7GA53	DE	Adenine aminase;
N	A7GA53	DE	Adenine deaminase;
N	A7GA53	DR	adenine catabolic process
N	A7GA53	DR	adenine deaminase activity
N	A7GJV3	CC	Specifically dimethylates two adjacent adenosines (A1518 and A1519) in the loop of a conserved hairpin near the 3'-end of 16S rRNA in the 30S particle
N	A7GJV3	CC	May play a critical role in biogenesis of 30S subunits (By similarity).
N	A7GJV3	DE	16S rRNA (adenine(1518)-N(6)/adenine(1519)-N(6))-dimethyltransferase;
N	A7GJV3	DE	16S rRNA dimethyladenosine transferase;
N	A7GJV3	DE	16S rRNA dimethylase;
N	A7GJV3	DE	Ribosomal RNA small subunit methyltransferase A;
N	A7GJV3	DE	S-adenosylmethionine-6-N', N'-adenosyl(rRNA) dimethyltransferase;
N	A7GJV3	DR	rRNA (adenine-N6,N6-)-dimethyltransferase activity
N	A7GK01	DE	Cysteine--tRNA ligase;
N	A7GK01	DE	Cysteinyl-tRNA synthetase;
N	A7GK01	DR	cysteine-tRNA ligase activity
N	A7GK01	DR	cysteinyl-tRNA aminoacylation
N	A7GK01	DR	metal ion binding
N	A7GMV2	DE	Phosphoribosyl-ATP pyrophosphatase;
N	A7GMV2	DR	histidine biosynthetic process
N	A7GMV2	DR	phosphoribosyl-ATP diphosphatase activity
N	A7GN41	CC	GTPase that plays an essential role in the late steps of ribosome biogenesis (By similarity).
N	A7GN41	DE	GTP-binding protein EngA;
N	A7GN41	DR	ribosome biogenesis
N	A7GRI3	CC	Catalyzes the reversible formation of acyl-phosphate (acyl-PO(4)) from acyl-[acyl-carrier-protein] (acyl-ACP)
N	A7GRI3	CC	This enzyme utilizes acyl-ACP as fatty acyl donor, but not acyl-CoA (By similarity).
N	A7GRI3	DE	Acyl-ACP phosphotransacylase;
N	A7GRI3	DE	Acyl-[acyl-carrier-protein]--phosphate acyltransferase;
N	A7GRI3	DE	Phosphate acyltransferase;
N	A7GRI3	DE	Phosphate-acyl-ACP acyltransferase;
N	A7GRI3	DR	fatty acid biosynthetic process
N	A7GRI3	DR	phospholipid biosynthetic process
N	A7GRI3	DR	transferase activity, transferring acyl groups other than amino-acyl groups
N	A7GYC8	DE	Acetate kinase;
N	A7GYC8	DE	Acetokinase;
N	A7GYC8	DR	acetate kinase activity
N	A7GYC8	DR	organic acid metabolic process
N	A7H4D8	CC	Converts 2C-methyl-D-erythritol 2,4-cyclodiphosphate (ME-2,4cPP) into 1-hydroxy-2-methyl-2-(E)-butenyl 4-diphosphate (By similarity).
N	A7H4D8	DE	1-hydroxy-2-methyl-2-(E)-butenyl 4-diphosphate synthase;
N	A7H4D8	DE	4-hydroxy-3-methylbut-2-en-1-yl diphosphate synthase;
N	A7H4D8	DR	4 iron, 4 sulfur cluster binding
N	A7H4D8	DR	4-hydroxy-3-methylbut-2-en-1-yl diphosphate synthase activity
N	A7H4D8	DR	iron ion binding
N	A7H4D8	DR	oxidation-reduction process
N	A7H4D8	DR	terpenoid biosynthetic process

N	A7H543	CC	Catalyzes the reversible conversion of 3- phosphohydroxypyruvate to phosphoserine and of 3-hydroxy-2-oxo-4- phosphonooxybutanoate to phosphohydroxythreonine (By similarity).
N	A7H543	DE	Phosphohydroxythreonine aminotransferase;
N	A7H543	DE	Phosphoserine aminotransferase;
N	A7H543	DR	L-serine biosynthetic process
N	A7H543	DR	O-phospho-L-serine:2-oxoglutarate aminotransferase activity
N	A7H543	DR	pyridoxal phosphate binding
N	A7H543	DR	pyridoxine biosynthetic process
N	A7HA27	DE	Maf-like protein Anae109_1366;
N	A7HBM1	CC	One of the primary rRNA binding proteins
N	A7HBM1	CC	It has been suggested to have peptidyltransferase activity; this is somewhat controversial
N	A7HBM1	CC	Makes several contacts with the 16S rRNA in the 70S ribosome (By similarity).
N	A7HBM1	CC	Required for association of the 30S and 50S subunits to form the 70S ribosome, for tRNA binding and peptide bond formation
N	A7HBM1	DE	50S ribosomal protein L2;
N	A7HBM1	DR	structural constituent of ribosome
N	A7HBM1	DR	transferase activity
N	A7HBM2	CC	Protein S19 forms a complex with S13 that binds strongly to the 16S ribosomal RNA (By similarity).
N	A7HBM2	DE	30S ribosomal protein S19;
N	A7HBM2	DR	structural constituent of ribosome
N	A7HM45	CC	Binds 23S rRNA and is also seen to make contacts with the A and possibly P site tRNAs (By similarity).
N	A7HM45	DE	50S ribosomal protein L16;
N	A7HM45	DR	structural constituent of ribosome
N	A7HMA1	CC	Catalyzes the hydrolysis of pseudouridine 5'-phosphate (PsiMP) to ribose 5-phosphate and uracil (By similarity).
N	A7HMA1	DE	Pseudouridine-5'-phosphate glycosidase;
N	A7HMA1	DE	PsiMP glycosidase;
N	A7HMA1	DR	hydrolase activity, acting on glycosyl bonds
N	A7HMA1	DR	metal ion binding
N	A7HPQ2	CC	Modifies the free amino group of the aminoacyl moiety of methionyl-tRNA(fMet)
N	A7HPQ2	CC	The formyl group appears to play a dual role in the initiator identity of N-formylmethionyl-tRNA by: (I) promoting its recognition by IF2 and (II) impairing its binding to EFTu-GTP (By similarity).
N	A7HPQ2	DE	Methionyl-tRNA formyltransferase;
N	A7HPQ2	DR	methionyl-tRNA formyltransferase activity
N	A7HPQ2	DR	methyltransferase activity
N	A7HVU8	CC	Cell wall formation (By similarity).
N	A7HVU8	DE	UDP-N-acetylmuramate--L-alanine ligase;
N	A7HVU8	DE	UDP-N-acetylmuramoyl-L-alanine synthetase;
N	A7HVU8	DR	UDP-N-acetylmuramate-L-alanine ligase activity
N	A7HVU8	DR	cell division
N	A7HVU8	DR	cellular cell wall organization
N	A7HVU8	DR	peptidoglycan biosynthetic process
N	A7HVU8	DR	regulation of cell shape
N	A7HWQ5	CC	DNA-dependent RNA polymerase catalyzes the transcription of DNA into RNA using the four ribonucleoside triphosphates as substrates (By similarity).
N	A7HWQ5	DE	DNA-directed RNA polymerase subunit beta';
N	A7HWQ5	DE	RNA polymerase subunit beta';
N	A7HWQ5	DE	RNAP subunit beta';
N	A7HWQ5	DE	Transcriptase subunit beta';
N	A7HWQ5	DR	DNA-directed RNA polymerase activity
N	A7HX46	CC	Transfers the 4'-phosphopantetheine moiety from coenzyme A to a Ser of acyl-carrier-protein (By similarity).
N	A7HX46	DE	4'-phosphopantetheinyl transferase AcpS;
N	A7HX46	DE	Holo-ACP synthase;
N	A7HX46	DE	Holo-[acyl-carrier-protein] synthase;
N	A7HX46	DR	fatty acid biosynthetic process
N	A7HX46	DR	holo-[acyl-carrier-protein] synthase activity
N	A7HX46	DR	macromolecule biosynthetic process
N	A7HX46	DR	magnesium ion binding
N	A7HZM1	CC	This protein binds to the 23S rRNA, and is important in its secondary structure

N	A7HZM1	CC	It is located near the subunit interface in the base of the L7/L12 stalk, and near the tRNA binding site of the peptidyltransferase center (By similarity).
N	A7HZM1	DE	50S ribosomal protein L6;
N	A7HZM1	DR	structural constituent of ribosome
N	A7I1W6	DE	1-(5-phosphoribosyl)-5-[(5-phosphoribosylamino)methylideneamino] imidazole-4-carboxamide isomerase;
N	A7I1W6	DE	Phosphoribosylformimino-5-aminoimidazole carboxamide ribotide isomerase;
N	A7I1W6	DR	1-(5-phosphoribosyl)-5-[(5-phosphoribosylamino)methylideneamino]imidazole-4-carboxamide isomerase activity
N	A7I1W6	DR	histidine biosynthetic process
N	A7I291	DE	3-dehydroquinate synthase;
N	A7I291	DR	3-dehydroquinate synthase activity
N	A7I291	DR	aromatic amino acid family biosynthetic process
N	A7IGN8	CC	IF-3 binds to the 30S ribosomal subunit and shifts the equilibrum between 70S ribosomes and their 50S and 30S subunits in favor of the free subunits, thus enhancing the availability of 30S subunits on which protein synthesis initiation begins (By similarity)
N	A7IGN8	DE	Translation initiation factor IF-3;
N	A7IGN8	DR	translation initiation factor activity
N	A7IIK6	CC	This is one of the proteins that binds to the 5S RNA in the ribosome where it forms part of the central protuberance (By similarity).
N	A7IIK6	DE	50S ribosomal protein L25;
N	A7IIK6	DE	General stress protein CTC;
N	A7IIK6	DR	5S rRNA binding
N	A7IIK6	DR	structural constituent of ribosome
N	A7M9A4	DE	50S ribosomal protein L2, plastid;
N	A7M9A4	DR	structural constituent of ribosome
N	A7M9A4	DR	transferase activity
N	A7MB45	CC	Activates acetate so that it can be used for lipid synthesis or for energy generation (By similarity).
N	A7MB45	DE	Acyl-CoA synthetase short-chain family member 3, mitochondrial;
N	A7MB45	DR	acetate-CoA ligase activity
N	A7MBG3	CC	Potent and specific inhibitor of CaM-kinase II (CAMK2) (By similarity).
N	A7MBG3	DE	Calcium/calmodulin-dependent protein kinase II inhibitor 1;
N	A7MBG3	DR	protein kinase inhibitor activity
N	A7ME52	CC	Catalyzes the pyrimidine ring opening between N-3 and C- 4 by an unusual flavin hydroperoxide-catalyzed mechanism to yield ureidoacrylate peracid
N	A7ME52	CC	It cleaves pyrimidine rings directly by adding oxygen atoms, making a toxic ureidoacrylate peracid product which can be spontaneously reduced to ureidoacrylate (By similarity)
N	A7ME52	DE	Pyrimidine monooxygenase RutA;
N	A7ME52	DR	oxidation-reduction process
N	A7ME52	DR	oxidoreductase activity, acting on paired donors, with incorporation or reduction of molecular oxygen, reduced flavin or flavoprotein as one donor, and incorporation of one atom of oxygen
N	A7MEU5	CC	Involved in chromosome condensation, segregation and cell cycle progression
N	A7MEU5	CC	It has a calcium binding activity (By similarity).
N	A7MEU5	CC	May participate in facilitating chromosome segregation by condensation DNA from both sides of a centrally located replisome during cell division
N	A7MEU5	CC	Not required for mini-F plasmid partitioning
N	A7MEU5	CC	Overexpression results in anucleate cells
N	A7MEU5	CC	Probably acts via its interaction with mukB and mukE
N	A7MEU5	DE	Chromosome partition protein mukF;
N	A7MEU5	DR	DNA replication
N	A7MEU5	DR	calcium ion binding
N	A7MEU5	DR	cell division
N	A7MEU5	DR	chromosome condensation
N	A7MEU5	DR	chromosome segregation
N	A7MGV3	CC	Specifically methylates position 2 of adenine 2503 in 23S rRNA (By similarity).
N	A7MGV3	DE	23S rRNA m2A2503 methyltransferase;
N	A7MGV3	DE	Ribosomal RNA large subunit methyltransferase N;
N	A7MGV3	DR	4 iron, 4 sulfur cluster binding
N	A7MGV3	DR	RNA methyltransferase activity
N	A7MGV3	DR	metal ion binding
N	A7MGV3	DR	rRNA processing
N	A7MH52	CC	Component of the acetyl coenzyme A carboxylase (ACC) complex

N	A7MH52	CC	Biotin carboxylase (BC) catalyzes the carboxylation of biotin on its carrier protein (BCCP) and then the CO(2) group is transferred by the transcarboxylase to acetyl-CoA to form malonyl- CoA (By similarity).
N	A7MH52	DE	ACCase subunit beta;
N	A7MH52	DE	Acetyl-CoA carboxylase carboxyltransferase subunit beta;
N	A7MH52	DE	Acetyl-coenzyme A carboxylase carboxyl transferase subunit beta;
N	A7MH52	DR	acetyl-CoA carboxylase activity
N	A7MH52	DR	fatty acid biosynthetic process
N	A7MH52	DR	metal ion binding
N	A7MIB1	DE	Protein ApaG;
N	A7MK12	CC	Catalyzes 2 different reactions between oxygene and the acireductone 1,2-dihydroxy-3-keto-5-methylthiopentene (DHK-MTPene) depending upon the metal bound in the active site
N	A7MK12	CC	Fe-containing acireductone dioxygenase (Fe-ARD) produces formate and 2-keto-4- methylthiobutyrate (KMTB), the alpha-ketoacid precursor of methionine in the methionine recycle pathway
N	A7MK12	CC	Ni-containing acireductone dioxygenase (Ni-ARD) produces methylthiopropionate, carbon monoxide and formate, and does not lie on the methionine recycle pathway (By similarity).
N	A7MK12	DE	1,2-dihydroxy-3-keto-5-methylthiopentene dioxygenase;
N	A7MK12	DE	Acireductone dioxygenase (Fe(2+)-requiring);
N	A7MK12	DE	Acireductone dioxygenase (Ni(2+)-requiring);
N	A7MK12	DE	Acireductone dioxygenase;
N	A7MK12	DE	DHK-MTPene dioxygenase;
N	A7MK12	DR	L-methionine salvage from methylthioadenosine
N	A7MK12	DR	acireductone dioxygenase (Ni2+-requiring) activity
N	A7MK12	DR	acireductone dioxygenase [iron(II)-requiring] activity
N	A7MK12	DR	metal ion binding
N	A7MK12	DR	oxidation-reduction process
N	A7MP89	DE	UPF0235 protein ESA_00387;
N	A7MVW6	CC	Catalyzes the synthesis of alpha-ribazole-5'-phosphate from nicotinate mononucleotide (NAMN) and 5,6- dimethylbenzimidazole (DMB).
N	A7MVW6	DE	N(1)-alpha-phosphoribosyltransferase;
N	A7MVW6	DE	NN:DBI PRT;
N	A7MVW6	DE	Nicotinate-nucleotide--dimethylbenzimidazole phosphoribosyltransferase;
N	A7MVW6	DR	cobalamin biosynthetic process
N	A7MVW6	DR	nicotinate-nucleotide-dimethylbenzimidazole phosphoribosyltransferase activity
N	A7MVW6	DR	nucleoside biosynthetic process
N	A7MX54	CC	Prevents misfolding and promotes the refolding and proper assembly of unfolded polypeptides generated under stress conditions (By similarity).
N	A7MX54	DE	60 kDa chaperonin;
N	A7MX54	DE	GroEL protein;
N	A7MX54	DE	Protein Cpn60;
N	A7MX54	DR	protein refolding
N	A7N0W0	DE	DAP epimerase;
N	A7N0W0	DE	Diaminopimelate epimerase;
N	A7N0W0	DR	diaminopimelate epimerase activity
N	A7N0W0	DR	lysine biosynthetic process via diaminopimelate
N	A7N141	CC	Catalyzes the synthesis of the hydroxymethylpyrimidine phosphate (HMP-P) moiety of thiamine from aminoimidazole ribotide (AIR) in a radical S-adenosyl-L-methionine (SAM)-dependent reaction (By similarity).
N	A7N141	DE	HMP-P synthase;
N	A7N141	DE	HMP-phosphate synthase;
N	A7N141	DE	HMPP synthase;
N	A7N141	DE	Hydroxymethylpyrimidine phosphate synthase;
N	A7N141	DE	Phosphomethylpyrimidine synthase;
N	A7N141	DE	Thiamine biosynthesis protein thiC;
N	A7N141	DR	4 iron, 4 sulfur cluster binding
N	A7N141	DR	lyase activity
N	A7N141	DR	metal ion binding
N	A7N141	DR	thiamine biosynthetic process
N	A7NBV5	CC	The ruvA-ruvB complex in the presence of ATP renatures cruciform structure in supercoiled DNA with palindromic sequence, indicating that it may promote strand exchange reactions in homologous recombination

N	A7NBV5	CC	RuvA stimulates, in the presence of DNA, the weak ATPase activity of ruvB (By similarity).
N	A7NBV5	CC	RuvAB is an helicase that mediates the Holliday junction migration by localized denaturation and reannealing
N	A7NBV5	DE	Holliday junction ATP-dependent DNA helicase ruvA;
N	A7NBV5	DR	DNA recombination
N	A7NBV5	DR	four-way junction helicase activity
N	A7ND08	CC	Catalyzes the ATP-dependent amination of UTP to CTP with either L-glutamine or ammonia as the source of nitrogen (By similarity).
N	A7ND08	DE	CTP synthase;
N	A7ND08	DE	CTP synthetase;
N	A7ND08	DE	UTP--ammonia ligase;
N	A7ND08	DR	CTP synthase activity
N	A7ND08	DR	glutamine metabolic process
N	A7ND08	DR	pyrimidine nucleotide biosynthetic process
N	A7NFC5	CC	Necessary for efficient RNA polymerase transcription elongation past template-encoded arresting sites
N	A7NFC5	CC	Cleavage of the nascent transcript by cleavage factors such as greA or greB allows the resumption of elongation from the new 3'terminus
N	A7NFC5	CC	GreA releases sequences of 2 to 3 nucleotides (By similarity).
N	A7NFC5	CC	The arresting sites in DNA have the property of trapping a certain fraction of elongating RNA polymerases that pass through, resulting in locked ternary complexes
N	A7NFC5	DE	Transcript cleavage factor greA;
N	A7NFC5	DE	Transcription elongation factor greA;
N	A7NFC5	DR	transcription elongation regulator activity
N	A7NKV1	DE	Glutamate-1-semialdehyde 2,1-aminomutase;
N	A7NKV1	DE	Glutamate-1-semialdehyde aminotransferase;
N	A7NKV1	DR	chlorophyll biosynthetic process
N	A7NKV1	DR	glutamate-1-semialdehyde 2,1-aminomutase activity
N	A7NKV1	DR	pyridoxal phosphate binding
N	A7NKV1	DR	transaminase activity
N	A7NPD6	CC	NDH-1 shuttles electrons from NADH, via FMN and iron- sulfur (Fe-S) centers, to quinones in the respiratory chain
N	A7NPD6	CC	Couples the redox reaction to proton translocation (for every two electrons transferred, four hydrogen ions are translocated across the cytoplasmic membrane), and thus conserves the redox energy in a proton gradient (By similarity)
N	A7NPD6	CC	The immediate electron acceptor for the enzyme in this species is believed to be ubiquinone
N	A7NPD6	DE	NADH dehydrogenase I subunit N 2;
N	A7NPD6	DE	NADH-quinone oxidoreductase subunit N 2;
N	A7NPD6	DE	NDH-1 subunit N 2;
N	A7NPD6	DR	ATP synthesis coupled electron transport
N	A7NPD6	DR	NADH dehydrogenase (ubiquinone) activity
N	A7NPD6	DR	quinone binding
N	A7S6Y0	CC	Involved in protein trafficking (By similarity).
N	A7S6Y0	DE	Protein SYS1 homolog;
N	A7S6Y0	DR	protein transport
N	A7TJT3	CC	Regulator of type 1 phosphatases which maintains protein phosphatase activity under strict control (By similarity).
N	A7TJT3	DE	Type 1 phosphatases regulator YPI2;
N	A7TR79	CC	Catalyzes the cleavage of L-kynurenine (L-Kyn) and L-3- hydroxykynurenine (L-3OHKyn) into anthranilic acid (AA) and 3- hydroxyanthranilic acid (3-OHAA), respectively (By similarity).
N	A7TR79	DE	Biosynthesis of nicotinic acid protein 5;
N	A7TR79	DE	Kynureninase;
N	A7TR79	DE	L-kynurenine hydrolase;
N	A7TR79	DR	NAD biosynthetic process
N	A7TR79	DR	kynureninase activity
N	A7TR79	DR	pyridoxal phosphate binding
N	A7TR79	DR	tryptophan catabolic process
N	A7TZG3	CC	May act by engaging a cell surface molecule on immature T-cells in the embryonic thymus (By similarity).
N	A7TZG3	DE	Selection and upkeep of intraepithelial T-cells protein 9;
N	A7WXZ6	CC	Converts N-acetylmannosamine-6-phosphate (ManNAc-6-P) to N-acetylglucosamine-6-phosphate (GlcNAc-6-P) (Potential).

N	A7WXZ6	DE	ManNAc-6-P epimerase;
N	A7WXZ6	DE	Putative N-acetylmannosamine-6-phosphate 2-epimerase;
N	A7WXZ6	DR	N-acetylmannosamine metabolic process
N	A7WXZ6	DR	N-acylglucosamine-6-phosphate 2-epimerase activity
N	A7WYX4	CC	Catalyzes the GTP-dependent ribosomal translocation step during translation elongation
N	A7WYX4	CC	Catalyzes the coordinated movement of the two tRNA molecules, the mRNA and conformational changes in the ribosome (By similarity).
N	A7WYX4	CC	During this step, the ribosome changes from the pre-translocational (PRE) to the post- translocational (POST) state as the newly formed A-site-bound peptidyl-tRNA and P-site-bound deacylated tRNA move to the P and E sites, respectively
N	A7WYX4	DE	Elongation factor G;
N	A7WYX4	DR	GTPase activity
N	A7WYX4	DR	translation elongation factor activity
N	A7X2W2	CC	Catalyzes the attachment of glycine to tRNA(Gly) (By similarity).
N	A7X2W2	DE	Glycine--tRNA ligase;
N	A7X2W2	DE	Glycyl-tRNA synthetase;
N	A7X2W2	DR	glycine-tRNA ligase activity
N	A7X2W2	DR	glycyl-tRNA aminoacylation
N	A7X2W2	DR	protein dimerization activity
N	A7X364	CC	This protein binds to 23S rRNA in the presence of protein L20 (By similarity).
N	A7X364	DE	50S ribosomal protein L21;
N	A7X364	DR	structural constituent of ribosome
N	A7X3T8	CC	Catalyzes the decarboxylation of four acetate groups of uroporphyrinogen-III to yield coproporphyrinogen-III (By similarity).
N	A7X3T8	DE	Uroporphyrinogen decarboxylase;
N	A7X3T8	DR	porphyrin biosynthetic process
N	A7X3T8	DR	uroporphyrinogen decarboxylase activity
N	A7XYQ1	CC	Implicated in development of the cochlea (By similarity).
N	A7XYQ1	DE	Jackson circler protein 1;
N	A7XYQ1	DE	Sine oculis-binding protein homolog;
N	A7XYQ1	DR	metal ion binding
N	A7YW81	DE	Transmembrane protein 234;
N	A7YY62	CC	Component of the FTS/Hook/FHIP complex (FHF complex)
N	A7YY62	CC	The FHF complex may function to promote vesicle trafficking and/or fusion via the homotypic vesicular protein sorting complex (the HOPS complex) (By similarity).
N	A7YY62	DE	FTS and Hook-interacting protein;
N	A7YY62	DE	Protein FAM160A2;
N	A7YY62	DR	early endosome to late endosome transport
N	A7YY62	DR	endosome organization
N	A7YY62	DR	endosome to lysosome transport
N	A7YY62	DR	lysosome organization
N	A7YY62	DR	protein transport
N	A7Z259	CC	Probable prenyltransferase that may catalyze the transfer of a prenyl moiety to sn-glycerol-1-phosphate (G1P) (By similarity).
N	A7Z259	DE	Putative glycerol-1-phosphate prenyltransferase;
N	A7Z259	DR	metal ion binding
N	A7Z259	DR	phospholipid biosynthetic process
N	A7Z259	DR	transferase activity, transferring alkyl or aryl (other than methyl) groups
N	A7Z8R4	CC	Transporter involved in the efflux of sodium, potassium, lithium and rubidium (By similarity).
N	A7Z8R4	DE	Sodium, potassium, lithium and rubidium/H(+) antiporter;
N	A7Z8R4	DR	intracellular protein transport
N	A7Z8R4	DR	potassium ion transport
N	A7Z8R4	DR	sodium ion transport
N	A7Z8R4	DR	solute:hydrogen antiporter activity
N	A7Z8R5	CC	Component of the sulfite reductase complex that catalyzes the 6-electron reduction of sulfite to sulfide
N	A7Z8R5	CC	This is one of several activities required for the biosynthesis of L- cysteine from sulfate (By similarity).
N	A7Z8R5	DE	Sulfite reductase [NADPH] hemoprotein beta-component;
N	A7Z8R5	DR	4 iron, 4 sulfur cluster binding
N	A7Z8R5	DR	cysteine biosynthetic process
N	A7Z8R5	DR	oxidation-reduction process
N	A7Z8R5	DR	sulfite reductase (NADPH) activity

N	A7ZCN6	CC	Seems to be the binding site for several of the factors involved in protein synthesis and appears to be essential for accurate translation (By similarity).
N	A7ZCN6	DE	50S ribosomal protein L7/L12;
N	A7ZCN6	DR	structural constituent of ribosome
N	A7ZD62	CC	Key component of the proton channel; it plays a direct role in the translocation of protons across the membrane (By similarity).
N	A7ZD62	DE	ATP synthase F0 sector subunit a;
N	A7ZD62	DE	ATP synthase subunit a;
N	A7ZD62	DE	F-ATPase subunit 6;
N	A7ZD62	DR	ATP synthesis coupled proton transport
N	A7ZD62	DR	hydrogen ion transmembrane transporter activity
N	A7ZEH3	DE	Aspartate--tRNA ligase;
N	A7ZEH3	DE	Aspartyl-tRNA synthetase;
N	A7ZEH3	DR	aspartate-tRNA ligase activity
N	A7ZEH3	DR	aspartyl-tRNA aminoacylation
N	A7ZEH3	DR	nucleic acid binding
N	A7ZI51	DE	Betaine aldehyde dehydrogenase;
N	A7ZI51	DR	betaine biosynthetic process
N	A7ZI51	DR	betaine-aldehyde dehydrogenase activity
N	A7ZI51	DR	oxidation-reduction process
N	A7ZIN6	CC	Displays esterase activity towards short chain fatty esters (acyl chain length of up to 8 carbons)
N	A7ZIN6	CC	Able to hydrolyze triacetylglycerol (triacetin) and tributyrylglycerol (tributyrin), but not trioleylglycerol (triolein) or cholesterol oleate
N	A7ZIN6	CC	Inhibits melA galactosidase activity (By similarity).
N	A7ZIN6	CC	Negatively regulates malT activity by antagonizing maltotriose binding
N	A7ZIN6	DE	Acetyl esterase;
N	A7ZIN6	DR	carboxylesterase activity
N	A7ZIN6	DR	protein homodimerization activity
N	A7ZSH6	CC	Modifies the free amino group of the aminoacyl moiety of methionyl-tRNA(fMet)
N	A7ZSH6	CC	The formyl group appears to play a dual role in the initiator identity of N-formylmethionyl-tRNA by: (I) promoting its recognition by IF2 and (II) impairing its binding to EFTu-GTP (By similarity).
N	A7ZSH6	DE	Methionyl-tRNA formyltransferase;
N	A7ZSH6	DR	methionyl-tRNA formyltransferase activity
N	A7ZSH6	DR	methyltransferase activity
N	A7ZUA1	CC	Necessary for formate dehydrogenase activity (By similarity).
N	A7ZUA1	DE	Protein FdhE;
N	A7ZVS4	CC	Catalyzes a reversible aldol reaction between acetaldehyde and D-glyceraldehyde 3-phosphate to generate 2-deoxy- D-ribose 5-phosphate (By similarity)
N	A7ZVS4	DE	2-deoxy-D-ribose 5-phosphate aldolase;
N	A7ZVS4	DE	Deoxyriboaldolase;
N	A7ZVS4	DE	Deoxyribose-phosphate aldolase;
N	A7ZVS4	DE	Phosphodeoxyriboaldolase;
N	A7ZVS4	DR	deoxyribonucleotide catabolic process
N	A7ZVS4	DR	deoxyribose-phosphate aldolase activity
N	A7ZW53	DE	UPF0289 protein yacF;
N	A7ZWB6	CC	Associates with the EF-Tu.GDP complex and induces the exchange of GDP to GTP
N	A7ZWB6	CC	It remains bound to the aminoacyl-tRNA.EF- Tu.GTP complex up to the GTP hydrolysis stage on the ribosome.
N	A7ZWB6	DE	Elongation factor Ts;
N	A7ZWB6	DR	translation elongation factor activity
N	A7ZXR8	DE	Leucine--tRNA ligase;
N	A7ZXR8	DE	Leucyl-tRNA synthetase;
N	A7ZXR8	DR	leucine-tRNA ligase activity
N	A7ZXR8	DR	leucyl-tRNA aminoacylation
N	A7ZYM0	CC	Activates KDO (a required 8-carbon sugar) for incorporation into bacterial lipopolysaccharide in Gram-negative bacteria (By similarity).
N	A7ZYM0	DE	3-deoxy-manno-octulosonate cytidylyltransferase;
N	A7ZYM0	DE	CMP-2-keto-3-deoxyoctulosonic acid synthase;
N	A7ZYM0	DE	CMP-KDO synthase;
N	A7ZYM0	DR	3-deoxy-manno-octulosonate cytidylyltransferase activity
N	A7ZYM0	DR	lipopolysaccharide biosynthetic process
N	A7ZZE5	CC	Plays a critical role in the incorporation of lipoproteins in the outer membrane after they are released by the lolA protein (By similarity).

N	A7ZZE5	DE	Outer-membrane lipoprotein lolB;
N	A7ZZE5	DR	protein transport
N	A8A1F4	DE	Protein dsrB;
N	A8A1J6	CC	Involved in the regulation of ptsG expression by binding and inactivating mlc (By similarity).
N	A8A1J6	DE	Mlc titration factor A;
N	A8A1J6	DE	Protein mtfA;
N	A8A2L0	CC	Catalyzes the formation of an hydroxyacyl-CoA by addition of water on enoyl-CoA
N	A8A2L0	CC	Also exhibits 3-hydroxyacyl-CoA epimerase and 3-hydroxyacyl-CoA dehydrogenase activities (By similarity).
N	A8A2L0	DE	3-hydroxyacyl-CoA dehydrogenase;
N	A8A2L0	DE	Enoyl-CoA hydratase/3-hydroxybutyryl-CoA epimerase;
N	A8A2L0	DE	Fatty acid oxidation complex subunit alpha;
N	A8A2L0	DR	3-hydroxyacyl-CoA dehydrogenase activity
N	A8A2L0	DR	3-hydroxybutyryl-CoA epimerase activity
N	A8A2L0	DR	enoyl-CoA hydratase activity
N	A8A2L0	DR	fatty acid beta-oxidation
N	A8A3B5	CC	An accessory protein needed during the final step in the assembly of 30S ribosomal subunit, possibly for assembly of the head region
N	A8A3B5	CC	Essential for efficient processing of 16S rRNA
N	A8A3B5	CC	It has affinity for free ribosomal 30S subunits but not for 70S ribosomes (By similarity)
N	A8A3B5	CC	May be needed both before and after rbfA during the maturation of 16S rRNA
N	A8A3B5	CC	Probably interacts with S19
N	A8A3B5	DE	Ribosome maturation factor rimM;
N	A8A3B5	DR	rRNA processing
N	A8A3B5	DR	ribosome binding
N	A8A7N9	CC	Prevents misfolding and promotes the refolding and proper assembly of unfolded polypeptides generated under stress conditions (By similarity).
N	A8A7N9	DE	60 kDa chaperonin;
N	A8A7N9	DE	GroEL protein;
N	A8A7N9	DE	Protein Cpn60;
N	A8A7N9	DR	protein refolding
N	A8A7Q8	CC	May play a role in 30S ribosomal subunit biogenesis
N	A8A7Q8	CC	Unusual circulary permuted GTPase that catalyzes rapid hydrolysis of GTP with a slow catalytic turnover (By similarity).
N	A8A7Q8	DE	Putative ribosome biogenesis GTPase RsgA;
N	A8A7Q8	DR	GTPase activity
N	A8A7Q8	DR	metal ion binding
N	A8A803	DE	Aspartate carbamoyltransferase;
N	A8A803	DE	Aspartate transcarbamylase;
N	A8A803	DR	'de novo' pyrimidine base biosynthetic process
N	A8A803	DR	amino acid binding
N	A8A803	DR	aspartate carbamoyltransferase activity
N	A8A803	DR	cellular amino acid metabolic process
N	A8A803	DR	pyrimidine nucleotide biosynthetic process
N	A8A9B8	CC	Selectively cleaves double-stranded DNA at the second phosphodiester bond 3' to a deoxyinosine leaving behind the intact lesion on the nicked DNA
N	A8A9B8	CC	Acts in DNA repair (By similarity).
N	A8A9B8	DE	Deoxyinosine 3'endonuclease;
N	A8A9B8	DE	Deoxyribonuclease V;
N	A8A9B8	DE	Endonuclease V;
N	A8A9B8	DR	deoxyribonuclease V activity
N	A8ACP7	CC	NAD-binding protein involved in the addition of a carboxymethylaminomethyl (cmnm) group at the wobble position (U34) of certain tRNAs, forming tRNA-cmnm(5)s(2)U34 (By similarity).
N	A8ACP7	DE	Glucose-inhibited division protein A;
N	A8ACP7	DE	tRNA uridine 5-carboxymethylaminomethyl modification enzyme MnmG;
N	A8ACP7	DR	flavin adenine dinucleotide binding
N	A8ACP7	DR	tRNA wobble uridine modification
N	A8ACZ6	CC	Splits dipeptides with a prolyl residue in the C- terminal position (By similarity).
N	A8ACZ6	DE	Imidodipeptidase;
N	A8ACZ6	DE	Proline dipeptidase;
N	A8ACZ6	DE	X-Pro dipeptidase;
N	A8ACZ6	DE	Xaa-Pro dipeptidase;
N	A8ACZ6	DR	cellular process

N	A8ACZ6	DR	dipeptidase activity
N	A8ACZ6	DR	metal ion binding
N	A8ACZ6	DR	metalloexopeptidase activity
N	A8ACZ6	DR	phosphoric triester hydrolase activity
N	A8AHW5	CC	Regulator of peptidoglycan synthesis that is essential for the function of penicillin-binding protein 1B (PBP1b) (By similarity).
N	A8AHW5	DE	PBP activator LpoB;
N	A8AHW5	DE	Penicillin-binding protein activator LpoB;
N	A8AHW5	DR	peptidoglycan biosynthetic process
N	A8AHW5	DR	regulation of cell shape
N	A8AUJ7	CC	Produces ATP from ADP in the presence of a proton gradient across the membrane
N	A8AUJ7	CC	The V-type alpha chain is a catalytic subunit (By similarity).
N	A8AUJ7	DE	V-ATPase subunit A;
N	A8AUJ7	DE	V-type ATP synthase alpha chain;
N	A8AUJ7	DR	ATP biosynthetic process
N	A8AUJ7	DR	ATP hydrolysis coupled proton transport
N	A8AUJ7	DR	hydrogen ion transporting ATP synthase activity, rotational mechanism
N	A8AUJ7	DR	proton-transporting ATPase activity, rotational mechanism
N	A8E1C4	CC	Deneddylase that deregulates the host cell cycle S phase to create a favorable environment allowing efficient viral genome replication
N	A8E1C4	CC	Additionally, acts as a deubiquitinase and cleaves both 'Lys-48' and 'Lys-63'-linked ubiquitin chains (By similarity).
N	A8E1C4	CC	Inhibition of cullins leads to the stabilization of cullin-RING ligase substrates such as host CDKN1A/p21, CDKN1B/p27kip and CDC25A, preventing S phase progression
N	A8E1C4	CC	Interacts with and deneddylates host cullins including CUL1 and CUL4A, thereby reducing their E3 ubiquitin ligase activity
N	A8E1C4	DE	Deneddylase M48;
N	A8E1C4	DR	cysteine-type peptidase activity
N	A8E1C4	DR	interspecies interaction between organisms
N	A8EQZ2	CC	Catalyzes the conversion of glucosamine-6-phosphate to glucosamine-1-phosphate (By similarity).
N	A8EQZ2	DE	Phosphoglucosamine mutase;
N	A8EQZ2	DR	carbohydrate metabolic process
N	A8EQZ2	DR	magnesium ion binding
N	A8EQZ2	DR	phosphoglucosamine mutase activity
N	A8ERI7	CC	Provides the (R)-glutamate required for cell wall biosynthesis (By similarity).
N	A8ERI7	DE	Glutamate racemase;
N	A8ERI7	DR	cellular cell wall organization
N	A8ERI7	DR	glutamate racemase activity
N	A8ERI7	DR	peptidoglycan biosynthetic process
N	A8ERI7	DR	regulation of cell shape
N	A8ERT8	CC	Catalyzes the NADPH-dependent reduction of glutamyl- tRNA(Glu) to glutamate 1-semialdehyde (GSA) (By similarity).
N	A8ERT8	DE	Glutamyl-tRNA reductase;
N	A8ERT8	DR	glutamyl-tRNA reductase activity
N	A8ERT8	DR	oxidation-reduction process
N	A8ERT8	DR	porphyrin biosynthetic process
N	A8ERT8	DR	shikimate 5-dehydrogenase activity
N	A8EV73	CC	F(1)F(0) ATP synthase produces ATP from ADP in the presence of a proton or sodium gradient
N	A8EV73	CC	During catalysis, ATP synthesis in the catalytic domain of F(1) is coupled via a rotary mechanism of the central stalk subunits to proton translocation (By similarity).
N	A8EV73	CC	F-type ATPases consist of two structural domains, F(1) containing the extramembraneous catalytic core and F(0) containing the membrane proton channel, linked together by a central stalk and a peripheral stalk
N	A8EV73	DE	ATP synthase F(1) sector subunit delta;
N	A8EV73	DE	ATP synthase subunit delta;
N	A8EV73	DE	F-ATPase subunit delta;
N	A8EV73	DE	F-type ATPase subunit delta;
N	A8EV73	DR	ATP synthesis coupled proton transport
N	A8EV73	DR	hydrogen ion transporting ATP synthase activity, rotational mechanism
N	A8EXC3	CC	NAD-binding protein involved in the addition of a carboxymethylaminomethyl (cmnm) group at the wobble position (U34) of certain tRNAs, forming tRNA-cmnm(5)s(2)U34 (By similarity).
N	A8EXC3	DE	Glucose-inhibited division protein A;

N	A8EXC3	DE	tRNA uridine 5-carboxymethylaminomethyl modification enzyme MnmG;
N	A8EXC3	DR	flavin adenine dinucleotide binding
N	A8EXC3	DR	tRNA wobble uridine modification
N	A8EXD7	CC	One of the proteins required for the normal export of preproteins out of the cell cytoplasm
N	A8EXD7	CC	It also specifically binds to its receptor secA (By similarity).
N	A8EXD7	CC	It is a molecular chaperone that binds to a subset of precursor proteins, maintaining them in a translocation-competent state
N	A8EXD7	DE	Protein-export protein secB;
N	A8EXD7	DR	protein tetramerization
N	A8EXD7	DR	protein transport
N	A8EXD7	DR	transmembrane transport
N	A8EXD7	DR	unfolded protein binding
N	A8EZN6	CC	Bidirectionally degrades single-stranded DNA into large acid-insoluble oligonucleotides, which are then degraded further into small acid-soluble oligonucleotides (By similarity).
N	A8EZN6	DE	Exodeoxyribonuclease 7 large subunit;
N	A8EZN6	DE	Exodeoxyribonuclease VII large subunit;
N	A8EZN6	DE	Exonuclease VII large subunit;
N	A8EZN6	DR	DNA catabolic process
N	A8EZN6	DR	exodeoxyribonuclease VII activity
N	A8EZN6	DR	nucleic acid binding
N	A8F0A3	DE	50S ribosomal protein L33;
N	A8F0A3	DR	structural constituent of ribosome
N	A8F2H0	CC	Involved in base excision repair of DNA damaged by oxidation or by mutagenic agents
N	A8F2H0	CC	Acts as DNA glycosylase that recognizes and removes damaged bases
N	A8F2H0	CC	Cleaves the DNA backbone by beta-delta elimination to generate a single-strand break at the site of the removed base with both 3'- and 5'-phosphates (By similarity).
N	A8F2H0	CC	Has AP (apurinic/apyrimidinic) lyase activity and introduces nicks in the DNA strand
N	A8F2H0	CC	Has a preference for oxidized purines, such as 7,8-dihydro-8-oxoguanine (8-oxoG)
N	A8F2H0	DE	AP lyase mutM;
N	A8F2H0	DE	DNA-(apurinic or apyrimidinic site) lyase mutM;
N	A8F2H0	DE	Fapy-DNA glycosylase;
N	A8F2H0	DE	Formamidopyrimidine-DNA glycosylase;
N	A8F2H0	DR	base-excision repair
N	A8F2H0	DR	damaged DNA binding
N	A8F2H0	DR	nucleotide-excision repair
N	A8F2H0	DR	oxidized purine base lesion DNA N-glycosylase activity
N	A8F2H0	DR	zinc ion binding
N	A8FH15	DE	DAP epimerase;
N	A8FH15	DE	Diaminopimelate epimerase;
N	A8FH15	DR	diaminopimelate epimerase activity
N	A8FH15	DR	lysine biosynthetic process via diaminopimelate
N	A8FJT1	CC	Specifically methylates the pseudouridine at position 1915 (m3Psi1915) in 23S rRNA (By similarity).
N	A8FJT1	DE	23S rRNA m3Psi1915 methyltransferase;
N	A8FJT1	DE	Ribosomal RNA large subunit methyltransferase H;
N	A8FJT1	DE	rRNA (pseudouridine-N3-)-methyltransferase rlmH;
N	A8FJT1	DR	methyltransferase activity
N	A8FJT1	DR	rRNA processing
N	A8FK34	CC	Binds directly to 23S ribosomal RNA and is necessary for the in vitro assembly process of the 50S ribosomal subunit
N	A8FK34	CC	It is not involved in the protein synthesizing functions of that subunit (By similarity).
N	A8FK34	DE	50S ribosomal protein L20;
N	A8FK34	DR	structural constituent of ribosome
N	A8FKD8	CC	The alpha subunit is responsible for the aldol cleavage of indoleglycerol phosphate to indole and glyceraldehyde 3- phosphate.
N	A8FKD8	DE	Tryptophan synthase alpha chain;
N	A8FKD8	DR	tryptophan synthase activity
N	A8FKR7	CC	Catalyzes the GTP-dependent ribosomal translocation step during translation elongation
N	A8FKR7	CC	Catalyzes the coordinated movement of the two tRNA molecules, the mRNA and conformational changes in the ribosome (By similarity).
N	A8FKR7	CC	During this step, the ribosome changes from the pre-translocational (PRE) to the post- translocational (POST) state as the newly formed A-site-bound peptidyl-tRNA and P-site-bound deacylated tRNA move to the P and E sites, respectively
N	A8FKR7	DE	Elongation factor G;

N	A8FKR7	DR	GTPase activity
N	A8FKR7	DR	translation elongation factor activity
N	A8FQY0	CC	Binds to Cpn60 in the presence of Mg-ATP and suppresses the ATPase activity of the latter (By similarity).
N	A8FQY0	DE	10 kDa chaperonin;
N	A8FQY0	DE	GroES protein;
N	A8FQY0	DE	Protein Cpn10;
N	A8FQY0	DR	protein folding
N	A8FSH2	DE	Glucose-6-phosphate isomerase;
N	A8FSH2	DE	Phosphoglucose isomerase;
N	A8FSH2	DE	Phosphohexose isomerase;
N	A8FSH2	DR	gluconeogenesis
N	A8FSH2	DR	glucose-6-phosphate isomerase activity
N	A8FTI0	CC	ATP-dependent specificity component of the Clp protease
N	A8FTI0	CC	Can perform chaperone functions in the absence of ClpP (By similarity).
N	A8FTI0	CC	It directs the protease to specific substrates
N	A8FTI0	DE	ATP-dependent Clp protease ATP-binding subunit ClpX;
N	A8FTI0	DR	ATPase activity
N	A8FTI0	DR	protein dimerization activity
N	A8FTI0	DR	protein folding
N	A8FTI0	DR	unfolded protein binding
N	A8FTI0	DR	zinc ion binding
N	A8FUW6	DE	Aspartate--tRNA ligase;
N	A8FUW6	DE	Aspartyl-tRNA synthetase;
N	A8FUW6	DR	aspartate-tRNA ligase activity
N	A8FUW6	DR	aspartyl-tRNA aminoacylation
N	A8FUW6	DR	nucleic acid binding
N	A8FW54	CC	Necessary for the introduction of cis unsaturation into fatty acids
N	A8FW54	CC	Catalyzed the dehydration of (3R)-3-hydroxydecanoyl- ACP to E-(2)-decenoyl-ACP and then its isomerization to Z-(3)- decenoyl-ACP (By similarity).
N	A8FW54	DE	3-hydroxydecanoyl-[acyl-carrier-protein] dehydratase;
N	A8FW54	DE	Beta-hydroxydecanoyl thioester dehydrase;
N	A8FW54	DR	3-hydroxydecanoyl-[acyl-carrier-protein] dehydratase activity
N	A8FW54	DR	fatty acid biosynthetic process
N	A8G2S2	DE	Sugar fermentation stimulation protein homolog;
N	A8G4K7	DE	Urea amidohydrolase subunit gamma;
N	A8G4K7	DE	Urease subunit gamma;
N	A8G4K7	DR	nickel ion binding
N	A8G4K7	DR	urea metabolic process
N	A8G4K7	DR	urease activity
N	A8G9L4	CC	Catalyzes the attachment of isoleucine to tRNA(Ile)
N	A8G9L4	CC	As IleRS can inadvertently accommodate and process structurally similar amino acids such as valine, to avoid such errors it has two additional distinct tRNA(Ile)-dependent editing activities
N	A8G9L4	CC	One activity is designated as 'pretransfer' editing and involves the hydrolysis of activated Val-AMP
N	A8G9L4	CC	The other activity is designated 'posttransfer' editing and involves deacylation of mischarged Val-tRNA(Ile) (By similarity).
N	A8G9L4	DE	Isoleucine--tRNA ligase;
N	A8G9L4	DE	Isoleucyl-tRNA synthetase;
N	A8G9L4	DR	isoleucine-tRNA ligase activity
N	A8G9L4	DR	isoleucyl-tRNA aminoacylation
N	A8G9L4	DR	metal ion binding
N	A8GAC2	CC	NQR complex catalyzes the reduction of ubiquinone-1 to ubiquinol by two successive reactions, coupled with the transport of Na(+) ions from the cytoplasm to the periplasm
N	A8GAC2	CC	NqrA to nqrE are probably involved in the second step, the conversion of ubisemiquinone to ubiquinol (By similarity).
N	A8GAC2	DE	NQR complex subunit D;
N	A8GAC2	DE	NQR-1 subunit D;
N	A8GAC2	DE	Na(+)-NQR subunit D;
N	A8GAC2	DE	Na(+)-translocating NADH-quinone reductase subunit D;
N	A8GAC2	DE	Na(+)-translocating NQR subunit D;
N	A8GAC2	DR	oxidation-reduction process
N	A8GAC2	DR	oxidoreductase activity, acting on NADH or NADPH, quinone or similar compound as acceptor

N	A8GAC2	DR	sodium ion transport
N	A8GDZ4	CC	Proton-dependent permease that transports di- and tripeptides (By similarity).
N	A8GDZ4	DE	Dipeptide and tripeptide permease A;
N	A8GDZ4	DR	oligopeptide transport
N	A8GDZ4	DR	peptide transporter activity
N	A8GDZ4	DR	protein transport
N	A8GHY0	CC	Co-chaperone involved in the maturation of iron-sulfur cluster-containing proteins
N	A8GHY0	CC	Seems to help targeting proteins to be folded toward hscA (By similarity).
N	A8GHY0	DE	Co-chaperone protein hscB;
N	A8GHY0	DR	chaperone binding
N	A8GHY0	DR	heat shock protein binding
N	A8GHY0	DR	protein folding
N	A8GKG9	CC	Channel that opens in response to stretch forces in the membrane lipid bilayer
N	A8GKG9	CC	May participate in the regulation of osmotic pressure changes within the cell (By similarity).
N	A8GKG9	DE	Large-conductance mechanosensitive channel;
N	A8GKG9	DR	ion channel activity
N	A8GPE9	CC	One of the primary rRNA binding proteins, this protein initially binds near the 5'-end of the 23S rRNA
N	A8GPE9	CC	It is important during the early stages of 50S assembly
N	A8GPE9	CC	It makes multiple contacts with different domains of the 23S rRNA in the assembled 50S subunit and ribosome (By similarity).
N	A8GPE9	DE	50S ribosomal protein L4;
N	A8GPE9	DR	structural constituent of ribosome
N	A8GRJ1	CC	Involved in the tonB-independent uptake of proteins (By similarity).
N	A8GRJ1	DE	Protein tolB;
N	A8GRJ1	DR	protein import
N	A8GT51	DE	50S ribosomal protein L30;
N	A8GT51	DR	structural constituent of ribosome
N	A8H2P7	DE	UPF0133 protein Spea_1509;
N	A8H966	CC	Methyltransferase required for the conversion of dimethylmenaquinone (DMKH2) to menaquinone (MKH2) and the conversion of 2-polyprenyl-6-methoxy-1,4-benzoquinol (DDMQH2) to 2-polyprenyl-3-methyl-6-methoxy-1,4-benzoquinol (DMQH2) (By similarity).
N	A8H966	DE	Ubiquinone/menaquinone biosynthesis methyltransferase ubiE;
N	A8H966	DR	menaquinone biosynthetic process
N	A8H966	DR	methyltransferase activity
N	A8H966	DR	ubiquinone biosynthetic process
N	A8HS18	CC	F(1)F(0) ATP synthase produces ATP from ADP in the presence of a proton or sodium gradient
N	A8HS18	CC	During catalysis, ATP synthesis in the catalytic domain of F(1) is coupled via a rotary mechanism of the central stalk subunits to proton translocation (By similarity)
N	A8HS18	CC	F-type ATPases consist of two structural domains, F(1) containing the extramembraneous catalytic core and F(0) containing the membrane proton channel, linked together by a central stalk and a peripheral stalk
N	A8HS18	DE	ATP synthase F(1) sector subunit delta;
N	A8HS18	DE	ATP synthase subunit delta;
N	A8HS18	DE	F-ATPase subunit delta;
N	A8HS18	DE	F-type ATPase subunit delta;
N	A8HS18	DR	ATP synthesis coupled proton transport
N	A8HS18	DR	hydrogen ion transporting ATP synthase activity, rotational mechanism
N	A8I489	CC	Involved in saturated fatty acids biosynthesis (By similarity).
N	A8I489	DE	(3R)-hydroxymyristoyl ACP dehydrase;
N	A8I489	DE	(3R)-hydroxymyristoyl-[acyl-carrier-protein] dehydratase;
N	A8I489	DR	fatty acid biosynthetic process
N	A8I489	DR	hydro-lyase activity
N	A8I489	DR	lipid A biosynthetic process
N	A8I4T0	CC	Required for maturation of urease via the functional incorporation of the urease nickel metallocenter (By similarity).
N	A8I4T0	DE	Urease accessory protein ureF;
N	A8I4T0	DR	nickel ion binding
N	A8I4T0	DR	nitrogen compound metabolic process
N	A8IGA3	CC	Involved in mRNA degradation
N	A8IGA3	CC	Hydrolyzes single-stranded polyribonucleotides processively in the 3'- to 5'-direction (By similarity).

N	A8IGA3	DE	Polynucleotide phosphorylase;
N	A8IGA3	DE	Polyribonucleotide nucleotidyltransferase;
N	A8IGA3	DR	3'-5'-exoribonuclease activity
N	A8IGA3	DR	RNA processing
N	A8IGA3	DR	mRNA catabolic process
N	A8IGA3	DR	polyribonucleotide nucleotidyltransferase activity
N	A8KZA9	CC	Specifically methylates the N4 position of cytidine in position 1402 (C1402) of 16S rRNA (By similarity).
N	A8KZA9	DE	16S rRNA m(4)C1402 methyltransferase;
N	A8KZA9	DE	Ribosomal RNA small subunit methyltransferase H;
N	A8KZA9	DE	rRNA (cytosine-N(4)-)-methyltransferase RsmH;
N	A8KZA9	DR	methyltransferase activity
N	A8KZA9	DR	rRNA processing
N	A8LDK0	CC	May play a role in the intracellular transport of hydrophobic ligands (Potential).
N	A8LDK0	DE	UPF0678 fatty acid-binding protein-like protein Franean1_6164;
N	A8LKE7	CC	Involved in mRNA degradation
N	A8LKE7	CC	Hydrolyzes single-stranded polyribonucleotides processively in the 3'- to 5'-direction (By similarity).
N	A8LKE7	DE	Polynucleotide phosphorylase;
N	A8LKE7	DE	Polyribonucleotide nucleotidyltransferase;
N	A8LKE7	DR	3'-5'-exoribonuclease activity
N	A8LKE7	DR	RNA processing
N	A8LKE7	DR	mRNA catabolic process
N	A8LKE7	DR	polyribonucleotide nucleotidyltransferase activity
N	A8LLE0	CC	An essential GTPase that binds both GDP and GTP, with rapid nucleotide exchange
N	A8LLE0	CC	Plays a role in 16S rRNA processing and 30S ribosomal subunit biogenesis and possibly also in cell cycle regulation and energy metabolism (By similarity).
N	A8LLE0	DR	ribosome biogenesis
N	A8LRS7	CC	Catalyzes the transfer of a ribosyl phosphate group from 5-phosphoribose 1-diphosphate to orotate, leading to the formation of orotidine monophosphate (OMP) (By similarity).
N	A8LRS7	DE	Orotate phosphoribosyltransferase;
N	A8LRS7	DR	nucleoside metabolic process
N	A8LRS7	DR	orotate phosphoribosyltransferase activity
N	A8LRS7	DR	pyrimidine nucleotide biosynthetic process
N	A8LXK0	CC	Specifically methylates the N7 position of guanosine in position 518 of 16S rRNA (By similarity).
N	A8LXK0	DE	16S rRNA 7-methylguanosine methyltransferase;
N	A8LXK0	DE	16S rRNA m7G methyltransferase;
N	A8LXK0	DE	Ribosomal RNA small subunit methyltransferase G;
N	A8LXK0	DR	rRNA methyltransferase activity
N	A8LY44	DE	50S ribosomal protein L35;
N	A8LY44	DR	structural constituent of ribosome
N	A8M609	CC	NDH-1 shuttles electrons from NADH, via FMN and iron- sulfur (Fe-S) centers, to quinones in the respiratory chain
N	A8M609	CC	Couples the redox reaction to proton translocation (for every two electrons transferred, four hydrogen ions are translocated across the cytoplasmic membrane), and thus conserves the redox energy in a proton gradient (By similarity)
N	A8M609	CC	The immediate electron acceptor for the enzyme in this species is believed to be a menaquinone
N	A8M609	DE	NADH dehydrogenase I subunit N;
N	A8M609	DE	NADH-quinone oxidoreductase subunit N;
N	A8M609	DE	NDH-1 subunit N;
N	A8M609	DR	ATP synthesis coupled electron transport
N	A8M609	DR	NADH dehydrogenase (ubiquinone) activity
N	A8M609	DR	quinone binding
N	A8MEH2	DE	Histidinol-phosphate aminotransferase;
N	A8MEH2	DE	Imidazole acetol-phosphate transaminase;
N	A8MEH2	DR	1-aminocyclopropane-1-carboxylate synthase activity
N	A8MEH2	DR	histidine biosynthetic process
N	A8MEH2	DR	histidinol-phosphate transaminase activity
N	A8MEH2	DR	pyridoxal phosphate binding
N	A8MI62	DE	UPF0735 ACT domain-containing protein Clos_1956;
N	A8MI62	DR	L-phenylalanine biosynthetic process
N	A8MLE2	CC	One of the early assembly proteins it binds 23S rRNA
N	A8MLE2	CC	Forms the main docking site for trigger factor binding to the ribosome (By similarity)

N	A8MLE2	CC	One of the proteins that surrounds the polypeptide exit tunnel on the outside of the ribosome
N	A8MLE2	DE	50S ribosomal protein L23;
N	A8MLE2	DR	nucleotide binding
N	A8MLE2	DR	structural constituent of ribosome
N	A8MT70	DE	Zinc finger B-box domain-containing protein 1;
N	A8MT70	DR	zinc ion binding
N	A8MVG2	DE	Putative selection and upkeep of intraepithelial T-cells protein 1 homolog;
N	A8MWL7	DE	Transmembrane protein 14D;
N	A8MYR8	CC	May play a role as an effector of the ADP-ribosylation factor-like protein 2, ARL2 (By similarity).
N	A8MYR8	DE	ADP-ribosylation factor-like protein 2-binding protein-like protein;
N	A8MYR8	DE	ARF-like 2-binding protein-like protein;
N	A8MYR8	DE	ARL2BP-like protein;
N	A8NZY7	CC	Catalyzes the synthesis of dihydrouridine, a modified base found in the D-loop of most tRNAs
N	A8NZY7	CC	Specifically modifies U47 in cytoplasmic tRNAs (By similarity).
N	A8NZY7	DE	tRNA-dihydrouridine synthase 3;
N	A8NZY7	DR	flavin adenine dinucleotide binding
N	A8NZY7	DR	nucleic acid binding
N	A8NZY7	DR	oxidation-reduction process
N	A8NZY7	DR	tRNA dihydrouridine synthase activity
N	A8NZY7	DR	tRNA processing
N	A8NZY7	DR	zinc ion binding
N	A8WJR8	CC	Required for oocyte-to-zygote transition in which it phosphorylates oocyte proteins, including mei-1, oma-1, oma-2, mex-5, and mex-6, modifying their activity and/or stability following meiosis
N	A8WJR8	CC	Functions in both spindle positioning and in the posterior localization of cytoplasmic determinants, including pie-1, pos-1, and pgl-1, in early embryos (By similarity)
N	A8WJR8	DE	Dual specificity Yak1-related kinase mbk-2;
N	A8WJR8	DE	Dual specificity tyrosine-phosphorylation-regulated kinase mbk-2;
N	A8WJR8	DE	Minibrain Kinase 2;
N	A8WJR8	DR	multicellular organismal development
N	A8WJR8	DR	protein phosphorylation
N	A8WJR8	DR	protein serine/threonine kinase activity
N	A8WJR8	DR	protein tyrosine kinase activity
N	A8WJR8	DR	response to stimulus
N	A8WJR8	DR	sensory perception of smell
N	A8WLV5	CC	Binds to the 40S ribosome and promotes the binding of methionyl-tRNAi and mRNA
N	A8WLV5	CC	This subunit binds to the 18S rRNA (By similarity).
N	A8WLV5	DE	Probable eukaryotic translation initiation factor 3 subunit G;
N	A8WLV5	DR	nucleotide binding
N	A8WLV5	DR	translation initiation factor activity
N	A8WLV5	DR	zinc ion binding
N	A8XFG2	CC	Core subunit of the mitochondrial membrane respiratory chain NADH dehydrogenase (Complex I) that is believed to belong to the minimal assembly required for catalysis
N	A8XFG2	CC	Complex I functions in the transfer of electrons from NADH to the respiratory chain
N	A8XFG2	CC	The immediate electron acceptor for the enzyme is believed to be ubiquinone (By similarity).
N	A8XFG2	DE	Probable NADH dehydrogenase [ubiquinone] iron-sulfur protein 7, mitochondrial;
N	A8XFG2	DR	4 iron, 4 sulfur cluster binding
N	A8XFG2	DR	NADH dehydrogenase (ubiquinone) activity
N	A8XFG2	DR	electron transport chain
N	A8XFG2	DR	metal ion binding
N	A8XFG2	DR	quinone binding
N	A8Y9E3	CC	NDH shuttles electrons from NAD(P)H:plastoquinone, via FMN and iron-sulfur (Fe-S) centers, to quinones in the photosynthetic chain and possibly in a chloroplast respiratory chain
N	A8Y9E3	CC	Couples the redox reaction to proton translocation, and thus conserves the redox energy in a proton gradient (By similarity).
N	A8Y9E3	CC	The immediate electron acceptor for the enzyme in this species is believed to be plastoquinone
N	A8Y9E3	DE	NAD(P)H dehydrogenase subunit H;
N	A8Y9E3	DE	NAD(P)H-quinone oxidoreductase subunit H, chloroplastic;
N	A8Y9E3	DE	NADH-plastoquinone oxidoreductase 49 kDa subunit;

N	A8Y9E3	DE	NADH-plastoquinone oxidoreductase subunit H;
N	A8Y9E3	DR	oxidation-reduction process
N	A8Y9E3	DR	oxidoreductase activity, acting on NADH or NADPH
N	A8Y9E3	DR	quinone binding
N	A8YTF9	CC	Phosphorylation of dTMP to form dTDP in both de novo and salvage pathways of dTTP synthesis (By similarity).
N	A8YTF9	DE	Thymidylate kinase;
N	A8YTF9	DE	dTMP kinase;
N	A8YTF9	DR	dTDP biosynthetic process
N	A8YTF9	DR	thymidylate kinase activity
N	A8YY76	CC	Key component of the proton channel; it plays a direct role in the translocation of protons across the membrane (By similarity).
N	A8YY76	DE	ATP synthase F0 sector subunit a;
N	A8YY76	DE	ATP synthase subunit a;
N	A8YY76	DE	F-ATPase subunit 6;
N	A8YY76	DR	ATP synthesis coupled proton transport
N	A8YY76	DR	hydrogen ion transmembrane transporter activity
N	A8Z0X4	CC	May play a role in DNA repair
N	A8Z0X4	CC	It may act with recF and recO (By similarity).
N	A8Z0X4	CC	It seems to be involved in an recBC-independent recombinational process of DNA repair
N	A8Z0X4	DE	Recombination protein recR;
N	A8Z0X4	DR	DNA recombination
N	A8Z0X4	DR	metal ion binding
N	A8Z3V1	CC	Responsible for synthesis of pseudouridine from uracil- 55 in the psi GC loop of transfer RNAs (By similarity).
N	A8Z3V1	DE	Psi55 synthase;
N	A8Z3V1	DE	tRNA pseudouridine 55 synthase;
N	A8Z3V1	DE	tRNA pseudouridine synthase B;
N	A8Z3V1	DE	tRNA pseudouridylate synthase;
N	A8Z3V1	DE	tRNA-uridine isomerase;
N	A8Z3V1	DR	pseudouridine synthase activity
N	A8Z3V1	DR	pseudouridine synthesis
N	A8Z3V1	DR	tRNA processing
N	A8Z4N4	DE	Serine protease splF;
N	A8Z4N4	DR	serine-type endopeptidase activity
N	A8Z522	CC	Metallothiol transferase which confers resistance to fosfomycin by catalyzing the addition of a thiol cofactor to fosfomycin
N	A8Z522	CC	L-cysteine is probably the physiological thiol donor (By similarity).
N	A8Z522	DE	Fosfomycin resistance protein;
N	A8Z522	DE	Metallothiol transferase fosB;
N	A8Z522	DR	metal ion binding
N	A8Z522	DR	response to antibiotic
N	A8Z522	DR	transferase activity, transferring alkyl or aryl (other than methyl) groups
N	A8ZUS9	CC	Catalyzes the conversion of dethiobiotin (DTB) to biotin by the insertion of a sulfur atom into dethiobiotin via a radical- based mechanism (By similarity).
N	A8ZUS9	DE	Biotin synthase;
N	A8ZUS9	DR	2 iron, 2 sulfur cluster binding
N	A8ZUS9	DR	4 iron, 4 sulfur cluster binding
N	A8ZUS9	DR	biotin biosynthetic process
N	A8ZUS9	DR	biotin synthase activity
N	A8ZUS9	DR	metal ion binding
N	A8ZXZ4	CC	This is one of the proteins that binds to the 5S RNA in the ribosome where it forms part of the central protuberance (By similarity).
N	A8ZXZ4	DE	50S ribosomal protein L25;
N	A8ZXZ4	DE	General stress protein CTC;
N	A8ZXZ4	DR	5S rRNA binding
N	A8ZXZ4	DR	structural constituent of ribosome
N	A9A565	CC	Catalyzes the attachment of alanine to tRNA(Ala) in a two-step reaction: alanine is first activated by ATP to form Ala- AMP and then transferred to the acceptor end of tRNA(Ala)
N	A9A565	CC	Also edits incorrectly charged Ser-tRNA(Ala) and Gly-tRNA(Ala) via its editing domain (By similarity).
N	A9A565	DE	Alanine--tRNA ligase;
N	A9A565	DE	Alanyl-tRNA synthetase;
N	A9A565	DR	alanine-tRNA ligase activity

N	A9A565	DR	alanyl-tRNA aminoacylation
N	A9A565	DR	metal ion binding
N	A9A6R4	CC	Catalyzes the reversible phosphorylation of UMP to UDP (By similarity).
N	A9A6R4	DE	UMP kinase;
N	A9A6R4	DE	Uridine monophosphate kinase;
N	A9A6R4	DE	Uridylate kinase;
N	A9A6R4	DR	UMP kinase activity
N	A9A6R4	DR	cellular amino acid biosynthetic process
N	A9A6R4	DR	pyrimidine nucleotide biosynthetic process
N	A9A788	CC	Component of the proteasome core, a large protease complex with broad specificity involved in protein degradation (By similarity).
N	A9A788	DE	20S proteasome beta subunit;
N	A9A788	DE	Proteasome core protein PsmB;
N	A9A788	DE	Proteasome subunit beta;
N	A9A788	DR	proteolysis involved in cellular protein catabolic process
N	A9A788	DR	threonine-type endopeptidase activity
N	A9ADK3	CC	Binds to 23S rRNA
N	A9ADK3	CC	Forms part of two intersubunit bridges in the 70S ribosome (By similarity).
N	A9ADK3	DE	50S ribosomal protein L14;
N	A9ADK3	DR	structural constituent of ribosome
N	A9AEU0	DE	Glutamate-1-semialdehyde 2,1-aminomutase;
N	A9AEU0	DE	Glutamate-1-semialdehyde aminotransferase;
N	A9AEU0	DR	glutamate-1-semialdehyde 2,1-aminomutase activity
N	A9AEU0	DR	porphyrin biosynthetic process
N	A9AEU0	DR	pyridoxal phosphate binding
N	A9AEU0	DR	transaminase activity
N	A9AF24	CC	Riboflavin synthase is a bifunctional enzyme complex catalyzing the formation of riboflavin from 5-amino-6-(1'-D)- ribityl-amino-2,4(1H,3H)-pyrimidinedione and L-3,4-dihydrohy-2- butanone-4-phosphate via 6,7-dimethyl-8-lumazine
N	A9AF24	CC	The beta subunit catalyzes the condensation of 5-amino-6-(1'-D)-ribityl-amino-2,4(1H,3H)-pyrimidinedione with L-3,4-dihydrohy-2-butanone-4- phosphate yielding 6,7-dimethyl-8-lumazine (By similarity).
N	A9AF24	DE	6,7-dimethyl-8-ribityllumazine synthase;
N	A9AF24	DE	DMRL synthase;
N	A9AF24	DE	Lumazine synthase;
N	A9AF24	DE	Riboflavin synthase beta chain;
N	A9AF24	DR	riboflavin biosynthetic process
N	A9AF24	DR	riboflavin synthase activity
N	A9AFC7	DE	Aspartate--tRNA ligase;
N	A9AFC7	DE	Aspartyl-tRNA synthetase;
N	A9AFC7	DR	aspartate-tRNA ligase activity
N	A9AFC7	DR	aspartyl-tRNA aminoacylation
N	A9AFC7	DR	nucleic acid binding
N	A9AGE2	CC	Synthesis of 3-octaprenyl-4-hydroxybenzoate (By similarity).
N	A9AGE2	DE	4-HB polyprenyltransferase;
N	A9AGE2	DE	4-hydroxybenzoate octaprenyltransferase;
N	A9AGE2	DR	prenyltransferase activity
N	A9AGE2	DR	ubiquinone biosynthetic process
N	A9AYC1	CC	Catalyzes the reversible formation of acyl-phosphate (acyl-PO(4)) from acyl-[acyl-carrier-protein] (acyl-ACP)
N	A9AYC1	CC	This enzyme utilizes acyl-ACP as fatty acyl donor, but not acyl-CoA (By similarity)
N	A9AYC1	DE	Acyl-ACP phosphotransacylase;
N	A9AYC1	DE	Acyl-[acyl-carrier-protein]--phosphate acyltransferase;
N	A9AYC1	DE	Phosphate acyltransferase;
N	A9AYC1	DE	Phosphate-acyl-ACP acyltransferase;
N	A9AYC1	DR	fatty acid biosynthetic process
N	A9AYC1	DR	phospholipid biosynthetic process
N	A9AYC1	DR	transferase activity, transferring acyl groups other than amino-acyl groups
N	A9B4Z5	CC	NDH-1 shuttles electrons from NADH, via FMN and iron- sulfur (Fe-S) centers, to quinones in the respiratory chain
N	A9B4Z5	CC	Couples the redox reaction to proton translocation (for every two electrons transferred, four hydrogen ions are translocated across the cytoplasmic membrane), and thus conserves the redox energy in a proton gradient (By similarity)
N	A9B4Z5	CC	The immediate electron acceptor for the enzyme in this species is believed to be ubiquinone

N	A9B4Z5	DE	NADH dehydrogenase I subunit B 1;
N	A9B4Z5	DE	NADH-quinone oxidoreductase subunit B 1;
N	A9B4Z5	DE	NDH-1 subunit B 1;
N	A9B4Z5	DR	4 iron, 4 sulfur cluster binding
N	A9B4Z5	DR	NADH dehydrogenase (ubiquinone) activity
N	A9B4Z5	DR	metal ion binding
N	A9B4Z5	DR	oxidation-reduction process
N	A9B4Z5	DR	quinone binding
N	A9B7I9	CC	The natural substrate for this enzyme may be peptidyl- tRNAs which drop off the ribosome during protein synthesis (By similarity).
N	A9B7I9	DE	Peptidyl-tRNA hydrolase;
N	A9B7I9	DR	aminoacyl-tRNA hydrolase activity
N	A9BNJ3	CC	Involved in DNA repair and recF pathway recombination (By similarity).
N	A9BNJ3	DE	DNA repair protein recO;
N	A9BNJ3	DE	Recombination protein O;
N	A9BNJ3	DR	ARF GTPase activator activity
N	A9BNJ3	DR	DNA recombination
N	A9BNJ3	DR	regulation of ARF GTPase activity
N	A9BNJ3	DR	zinc ion binding
N	A9BVK1	CC	Catalyzes the S-adenosylmethionine monomethyl esterification of trans-aconitate (By similarity).
N	A9BVK1	DE	Trans-aconitate 2-methyltransferase;
N	A9BVK1	DR	trans-aconitate 2-methyltransferase activity
N	A9EX55	CC	NDH-1 shuttles electrons from NADH, via FMN and iron- sulfur (Fe-S) centers, to quinones in the respiratory chain
N	A9EX55	CC	Couples the redox reaction to proton translocation (for every two electrons transferred, four hydrogen ions are translocated across the cytoplasmic membrane), and thus conserves the redox energy in a proton gradient (By similarity)
N	A9EX55	CC	The immediate electron acceptor for the enzyme in this species is believed to be ubiquinone
N	A9EX55	DE	NADH dehydrogenase I subunit N 1;
N	A9EX55	DE	NADH-quinone oxidoreductase subunit N 1;
N	A9EX55	DE	NDH-1 subunit N 1;
N	A9EX55	DR	ATP synthesis coupled electron transport
N	A9EX55	DR	NADH dehydrogenase (ubiquinone) activity
N	A9EX55	DR	quinone binding
N	A9FGG6	CC	One of the primary rRNA binding proteins, it binds directly to 16S rRNA central domain where it helps coordinate assembly of the platform of the 30S subunit (By similarity)
N	A9FGG6	DE	30S ribosomal protein S8;
N	A9FGG6	DR	structural constituent of ribosome
N	A9GQU6	DE	Acetylglutamate kinase;
N	A9GQU6	DE	N-acetyl-L-glutamate 5-phosphotransferase;
N	A9GQU6	DR	acetylglutamate kinase activity
N	A9GQU6	DR	arginine biosynthetic process
N	A9GQU6	DR	glutamate 5-kinase activity
N	A9GQU6	DR	proline biosynthetic process
N	A9H311	DE	Histidinol-phosphate aminotransferase;
N	A9H311	DE	Imidazole acetol-phosphate transaminase;
N	A9H311	DR	histidine biosynthetic process
N	A9H311	DR	histidinol-phosphate transaminase activity
N	A9H311	DR	pyridoxal phosphate binding
N	A9HKR8	DE	Acetylglutamate kinase;
N	A9HKR8	DE	N-acetyl-L-glutamate 5-phosphotransferase;
N	A9HKR8	DR	acetylglutamate kinase activity
N	A9HKR8	DR	arginine biosynthetic process
N	A9HKR8	DR	glutamate 5-kinase activity
N	A9HKR8	DR	proline biosynthetic process
N	A9HVA1	CC	Plays an important role in the initiation and regulation of chromosomal replication
N	A9HVA1	CC	Binds to the origin of replication; it binds specifically double-stranded DNA at a 9 bp consensus (dnaA box): 5'-TTATC[CA]A[CA]A-3'
N	A9HVA1	CC	DnaA binds to ATP and to acidic phospholipids (By similarity).
N	A9HVA1	DE	Chromosomal replication initiator protein DnaA;
N	A9HVA1	DR	DNA replication origin binding
N	A9HVA1	DR	DNA-dependent DNA replication initiation
N	A9HVA1	DR	nucleoside-triphosphatase activity

N	A9HVA1	DR	regulation of DNA replication
N	A9ISC1	CC	Involved in protein export
N	A9ISC1	CC	Acts as a chaperone by maintaining the newly synthesized protein in an open conformation (By similarity).
N	A9ISC1	DE	Trigger factor;
N	A9ISC1	DR	cell division
N	A9ISC1	DR	peptidyl-prolyl cis-trans isomerase activity
N	A9ISC1	DR	protein folding
N	A9ISC1	DR	protein transport
N	A9IUA2	CC	Required for the thiolation of cytidine in position 32 of tRNA, to form 2-thiocytidine (s(2)C32) (By similarity).
N	A9IUA2	DE	tRNA 2-thiocytidine biosynthesis protein TtcA;
N	A9IUA2	DR	tRNA processing
N	A9IVC5	CC	Interconversion of serine and glycine (By similarity).
N	A9IVC5	DE	Serine hydroxymethyltransferase;
N	A9IVC5	DE	Serine methylase;
N	A9IVC5	DR	L-serine metabolic process
N	A9IVC5	DR	glycine hydroxymethyltransferase activity
N	A9IVC5	DR	glycine metabolic process
N	A9IVC5	DR	one-carbon metabolic process
N	A9IVC5	DR	pyridoxal phosphate binding
N	A9IWA2	CC	Cell wall formation (By similarity).
N	A9IWA2	DE	D-Ala-D-Ala ligase;
N	A9IWA2	DE	D-alanine--D-alanine ligase;
N	A9IWA2	DE	D-alanylalanine synthetase;
N	A9IWA2	DR	D-alanine-D-alanine ligase activity
N	A9IWA2	DR	cellular cell wall organization
N	A9IWA2	DR	metal ion binding
N	A9IWA2	DR	peptidoglycan biosynthetic process
N	A9IWA2	DR	regulation of cell shape
N	A9JSQ8	DE	Protein FAM195B;
N	A9KBK4	CC	NDH-1 shuttles electrons from NADH, via FMN and iron- sulfur (Fe-S) centers, to quinones in the respiratory chain
N	A9KBK4	CC	Couples the redox reaction to proton translocation (for every two electrons transferred, four hydrogen ions are translocated across the cytoplasmic membrane), and thus conserves the redox energy in a proton gradient (By similarity)
N	A9KBK4	CC	The immediate electron acceptor for the enzyme in this species is believed to be ubiquinone
N	A9KBK4	DE	NADH dehydrogenase I subunit A;
N	A9KBK4	DE	NADH-quinone oxidoreductase subunit A;
N	A9KBK4	DE	NDH-1 subunit A;
N	A9KBK4	DR	NADH dehydrogenase (ubiquinone) activity
N	A9KBK4	DR	oxidation-reduction process
N	A9KBK4	DR	quinone binding
N	A9KGN7	CC	Catalyzes the synthesis of the hydroxymethylpyrimidine phosphate (HMP-P) moiety of thiamine from aminoimidazole ribotide (AIR) in a radical S-adenosyl-L-methionine (SAM)-dependent reaction (By similarity).
N	A9KGN7	DE	HMP-P synthase;
N	A9KGN7	DE	HMP-phosphate synthase;
N	A9KGN7	DE	HMPP synthase;
N	A9KGN7	DE	Hydroxymethylpyrimidine phosphate synthase;
N	A9KGN7	DE	Phosphomethylpyrimidine synthase;
N	A9KGN7	DE	Thiamine biosynthesis protein thiC;
N	A9KGN7	DR	4 iron, 4 sulfur cluster binding
N	A9KGN7	DR	lyase activity
N	A9KGN7	DR	metal ion binding
N	A9KGN7	DR	thiamine biosynthetic process
N	A9KJ36	CC	Catalyzes the attachment of glycine to tRNA(Gly) (By similarity).
N	A9KJ36	DE	Glycine--tRNA ligase;
N	A9KJ36	DE	Glycyl-tRNA synthetase;
N	A9KJ36	DR	glycine-tRNA ligase activity
N	A9KJ36	DR	glycyl-tRNA aminoacylation
N	A9KJ36	DR	protein dimerization activity
N	A9KJS0	DE	Urea amidohydrolase subunit beta;
N	A9KJS0	DE	Urease subunit beta;

N	A9KJS0	DR	nickel ion binding
N	A9KJS0	DR	nitrogen compound metabolic process
N	A9KJS0	DR	urease activity
N	A9KNJ3	DE	Aspartate carbamoyltransferase;
N	A9KNJ3	DE	Aspartate transcarbamylase;
N	A9KNJ3	DR	'de novo' pyrimidine base biosynthetic process
N	A9KNJ3	DR	amino acid binding
N	A9KNJ3	DR	aspartate carbamoyltransferase activity
N	A9KNJ3	DR	cellular amino acid metabolic process
N	A9KNJ3	DR	pyrimidine nucleotide biosynthetic process
N	A9KP86	DE	Glutamate-1-semialdehyde 2,1-aminomutase 1;
N	A9KP86	DE	Glutamate-1-semialdehyde aminotransferase 1;
N	A9KP86	DR	glutamate-1-semialdehyde 2,1-aminomutase activity
N	A9KP86	DR	porphyrin biosynthetic process
N	A9KP86	DR	pyridoxal phosphate binding
N	A9KP86	DR	transaminase activity
N	A9KVD6	DE	Putative hydrolase Sbal195_1503;
N	A9KVD6	DR	DNA replication
N	A9KVD6	DR	DNA-directed DNA polymerase activity
N	A9KVD6	DR	hydrolase activity
N	A9KVD6	DR	metal ion binding
N	A9L0P6	CC	Specifically methylates the cytosine at position 1962 (m5C1962) of 23S rRNA (By similarity).
N	A9L0P6	DE	23S rRNA m5C1962 methyltransferase;
N	A9L0P6	DE	Ribosomal RNA large subunit methyltransferase I;
N	A9L0P6	DE	rRNA (cytosine-C(5)-)-methyltransferase RlmI;
N	A9L0P6	DR	rRNA methyltransferase activity
N	A9L375	DE	D-fructose-1,6-bisphosphate 1-phosphohydrolase class 1;
N	A9L375	DE	FBPase class 1;
N	A9L375	DE	Fructose-1,6-bisphosphatase class 1;
N	A9L375	DR	carbohydrate metabolic process
N	A9L375	DR	fructose 1,6-bisphosphate 1-phosphatase activity
N	A9L375	DR	metal ion binding
N	A9L3W0	CC	Catalyzes the transfer of a dimethylallyl group onto the adenine at position 37 in tRNAs that read codons beginning with uridine, leading to the formation of N6-(dimethylallyl)adenosine (i(6)A) (By similarity).
N	A9L3W0	DE	DMAPP:tRNA dimethylallyltransferase;
N	A9L3W0	DE	Dimethylallyl diphosphate:tRNA dimethylallyltransferase;
N	A9L3W0	DE	IPP transferase;
N	A9L3W0	DE	Isopentenyl-diphosphate:tRNA isopentenyltransferase;
N	A9L3W0	DE	tRNA dimethylallyltransferase;
N	A9L3W0	DR	tRNA dimethylallyltransferase activity
N	A9L3W0	DR	tRNA processing
N	A9LYH4	DE	30S ribosomal protein S2, chloroplastic;
N	A9LYH4	DR	structural constituent of ribosome
N	A9M1D3	CC	Required for maturation of 30S ribosomal subunits (By similarity).
N	A9M1D3	DE	Ribosome maturation factor rimP;
N	A9M1D3	DR	ribosome biogenesis
N	A9M3T2	CC	Oxidative deamination of D-amino acids (By similarity).
N	A9M3T2	DE	D-amino acid dehydrogenase small subunit;
N	A9M3T2	DR	D-amino-acid dehydrogenase activity
N	A9M3T2	DR	oxidation-reduction process
N	A9M3X2	CC	With S4 and S5 plays an important role in translational accuracy (By similarity).
N	A9M3X2	DE	30S ribosomal protein S12;
N	A9M3X2	DR	structural constituent of ribosome
N	A9M7K1	CC	The ruvA-ruvB complex in the presence of ATP renatures cruciform structure in supercoiled DNA with palindromic sequence, indicating that it may promote strand exchange reactions in homologous recombination
N	A9M7K1	CC	RuvA stimulates, in the presence of DNA, the weak ATPase activity of ruvB (By similarity).
N	A9M7K1	CC	RuvAB is an helicase that mediates the Holliday junction migration by localized denaturation and reannealing
N	A9M7K1	DE	Holliday junction ATP-dependent DNA helicase ruvA;
N	A9M7K1	DR	DNA recombination
N	A9M7K1	DR	four-way junction helicase activity

N	A9M9S1	DE	Pantothenate kinase;
N	A9M9S1	DE	Pantothenic acid kinase;
N	A9M9S1	DR	coenzyme A biosynthetic process
N	A9M9S1	DR	pantothenate kinase activity
N	A9MAJ9	CC	Catalyzes the attachment of proline to tRNA(Pro) in a two-step reaction: proline is first activated by ATP to form Pro- AMP and then transferred to the acceptor end of tRNA(Pro) (By similarity).
N	A9MAJ9	DE	Proline--tRNA ligase;
N	A9MAJ9	DE	Prolyl-tRNA synthetase;
N	A9MAJ9	DR	proline-tRNA ligase activity
N	A9MAJ9	DR	prolyl-tRNA aminoacylation
N	A9MF17	CC	Component of the sulfite reductase complex that catalyzes the 6-electron reduction of sulfite to sulfide
N	A9MF17	CC	This is one of several activities required for the biosynthesis of L- cysteine from sulfate (By similarity).
N	A9MF17	DE	Sulfite reductase [NADPH] hemoprotein beta-component;
N	A9MF17	DR	4 iron, 4 sulfur cluster binding
N	A9MF17	DR	cysteine biosynthetic process
N	A9MF17	DR	oxidation-reduction process
N	A9MF17	DR	sulfite reductase (NADPH) activity
N	A9MH00	DE	Dihydroorotase;
N	A9MH00	DR	dihydroorotase activity
N	A9MH00	DR	metal ion binding
N	A9MH00	DR	pyrimidine base biosynthetic process
N	A9MH00	DR	pyrimidine nucleotide biosynthetic process
N	A9MHJ7	CC	Co-chaperone involved in the maturation of iron-sulfur cluster-containing proteins
N	A9MHJ7	CC	Seems to help targeting proteins to be folded toward hscA (By similarity).
N	A9MHJ7	DE	Co-chaperone protein hscB;
N	A9MHJ7	DR	chaperone binding
N	A9MHJ7	DR	heat shock protein binding
N	A9MHJ7	DR	protein folding
N	A9MHV9	CC	Plays a central role in chromosome condensation, segregation and cell cycle progression
N	A9MHV9	CC	Functions as a homodimer, which is essential for chromosome partition
N	A9MHV9	CC	Involved in negative DNA supercoiling in vivo, and by this means organize and compact chromosomes
N	A9MHV9	CC	May achieve or facilitate chromosome segregation by condensation DNA from both sides of a centrally located replisome during cell division (By similarity).
N	A9MHV9	DE	Chromosome partition protein mukB;
N	A9MHV9	DE	Structural maintenance of chromosome-related protein;
N	A9MHV9	DR	cell division
N	A9MHV9	DR	chromosome condensation
N	A9MHV9	DR	chromosome segregation
N	A9MLC3	DE	L-lactate dehydrogenase [cytochrome];
N	A9MLC3	DR	L-lactate dehydrogenase (cytochrome) activity
N	A9MLC3	DR	lactate metabolic process
N	A9MLC3	DR	oxidation-reduction process
N	A9MM62	DE	Universal stress protein B;
N	A9MN71	CC	One of the primary rRNA binding proteins, it binds directly to 16S rRNA where it nucleates assembly of the body of the 30S subunit (By similarity).
N	A9MN71	DE	30S ribosomal protein S4;
N	A9MN71	DR	structural constituent of ribosome
N	A9MNY0	CC	This protein is one of the early assembly proteins of the 50S ribosomal subunit, although it is not seen to bind rRNA by itself
N	A9MNY0	CC	It is important during the early stages of 50S assembly (By similarity).
N	A9MNY0	DE	50S ribosomal protein L13;
N	A9MNY0	DR	structural constituent of ribosome
N	A9MQA5	DE	UPF0289 protein yacF;
N	A9MQH4	CC	Catalyzes the exchange of L-carnitine for gamma- butyrobetaine and related betaines (By similarity).
N	A9MQH4	DE	L-carnitine/gamma-butyrobetaine antiporter;
N	A9MQH4	DR	antiporter activity
N	A9MTI7	CC	Catalyzes the decarboxylative condensation of pimeloyl- CoA and L-alanine to produce 8-amino-7-oxononanoate (AON), coenzyme A, and carbon dioxide (By similarity).
N	A9MTI7	DE	7-KAP synthase;
N	A9MTI7	DE	7-keto-8-amino-pelargonic acid synthase;

N	A9MTI7	DE	8-amino-7-ketopelargonate synthase;
N	A9MTI7	DE	8-amino-7-oxononanoate synthase;
N	A9MTI7	DE	KAPA synthase;
N	A9MTI7	DE	L-alanine--pimeloyl-CoA ligase;
N	A9MTI7	DR	8-amino-7-oxononanoate synthase activity
N	A9MTI7	DR	biotin biosynthetic process
N	A9MTI7	DR	pyridoxal phosphate binding
N	A9MTI7	DR	transferase activity, transferring nitrogenous groups
N	A9MW53	DE	Ureidoglycolate hydrolase;
N	A9MW53	DR	allantoin catabolic process
N	A9MW53	DR	purine base metabolic process
N	A9MW53	DR	ureidoglycolate hydrolase activity
N	A9MXI2	CC	Acts as a chaperone (By similarity).
N	A9MXI2	DE	Chaperone protein DnaK;
N	A9MXI2	DE	Heat shock 70 kDa protein;
N	A9MXI2	DE	Heat shock protein 70;
N	A9MXI2	DR	protein folding
N	A9MXI2	DR	response to stress
N	A9MXI2	DR	unfolded protein binding
N	A9N1X3	CC	Regulates the transcription of several operons and genes involved in the biogenesis of Fe-S clusters and Fe-S-containing proteins (By similarity).
N	A9N1X3	DE	HTH-type transcriptional regulator iscR;
N	A9N1X3	DR	2 iron, 2 sulfur cluster binding
N	A9N1X3	DR	double-stranded DNA binding
N	A9N1X3	DR	metal ion binding
N	A9N1X3	DR	regulation of transcription, DNA-dependent
N	A9N1X3	DR	sequence-specific DNA binding transcription factor activity
N	A9N275	CC	Catalyzes the hydrolysis of N(2)-succinylarginine into N(2)-succinylornithine, ammonia and CO(2) (By similarity).
N	A9N275	DE	N-succinylarginine dihydrolase;
N	A9N275	DR	N-succinylarginine dihydrolase activity
N	A9N275	DR	arginine metabolic process
N	A9N4P2	DE	UPF0301 protein yqgE;
N	A9N4Y6	CC	RNA chaperone that binds small regulatory RNA (sRNAs) and mRNAs to facilitate mRNA translational regulation in response to envelope stress, environmental stress and changes in metabolite concentrations
N	A9N4Y6	CC	Also binds with high specificity to tRNAs (By similarity).
N	A9N4Y6	DE	Host factor-I protein;
N	A9N4Y6	DE	Protein hfq;
N	A9N4Y6	DR	regulation of transcription, DNA-dependent
N	A9N4Y6	DR	response to stress
N	A9N6Z8	CC	Catalytic subunit of the tagatose-1,6-bisphosphate aldolase GatYZ, which catalyzes the reversible aldol condensation of dihydroxyacetone phosphate (DHAP or glycerone-phosphate) with glyceraldehyde 3-phosphate (G3P) to produce tagatose 1,6- bisphosphate (TBP)
N	A9N6Z8	CC	Is involved in the catabolism of galactitol (By similarity).
N	A9N6Z8	CC	Requires GatZ subunit for full activity and stability
N	A9N6Z8	DE	D-tagatose-1,6-bisphosphate aldolase subunit GatY;
N	A9N6Z8	DE	D-tagatose-bisphosphate aldolase class II;
N	A9N6Z8	DE	TagBP aldolase;
N	A9N6Z8	DE	Tagatose-bisphosphate aldolase;
N	A9N6Z8	DR	fructose-bisphosphate aldolase activity
N	A9N6Z8	DR	galactitol metabolic process
N	A9N6Z8	DR	lactose catabolic process via tagatose-6-phosphate
N	A9N6Z8	DR	tagatose-bisphosphate aldolase activity
N	A9N6Z8	DR	zinc ion binding
N	A9N7T9	CC	Involved in chromosome condensation, segregation and cell cycle progression
N	A9N7T9	CC	It has a calcium binding activity (By similarity).
N	A9N7T9	CC	May participate in facilitating chromosome segregation by condensation DNA from both sides of a centrally located replisome during cell division
N	A9N7T9	CC	Not required for mini-F plasmid partitioning
N	A9N7T9	CC	Overexpression results in anucleate cells
N	A9N7T9	CC	Probably acts via its interaction with mukB and mukE
N	A9N7T9	DE	Chromosome partition protein mukF;
N	A9N7T9	DR	DNA replication

N	A9N7T9	DR	calcium ion binding
N	A9N7T9	DR	cell division
N	A9N7T9	DR	chromosome condensation
N	A9N7T9	DR	chromosome segregation
N	A9N7U7	CC	Transfers the gamma-phosphate of ATP to the 4'-position of a tetraacyldisaccharide 1-phosphate intermediate (termed DS-1- P) to form tetraacyldisaccharide 1,4'-bis-phosphate (lipid IVA) (By similarity).
N	A9N7U7	DE	Lipid A 4'-kinase;
N	A9N7U7	DE	Tetraacyldisaccharide 4'-kinase;
N	A9N7U7	DR	lipid A biosynthetic process
N	A9N7U7	DR	tetraacyldisaccharide 4'-kinase activity
N	A9N8H8	CC	Catalyzes the phosphorylation of NAD to NADP
N	A9N8H8	CC	Utilizes ATP and other nucleoside triphosphates as well as inorganic polyphosphate as a source of phosphorus (By similarity).
N	A9N8H8	DE	Poly(P)/ATP NAD kinase;
N	A9N8H8	DE	Probable inorganic polyphosphate/ATP-NAD kinase;
N	A9N8H8	DR	NAD+ kinase activity
N	A9NCE2	DE	UPF0133 protein COXBURSA331_A0772;
N	A9NEF8	CC	Located at the top of the head of the 30S subunit, it contacts several helices of the 16S rRNA
N	A9NEF8	CC	Contacts the tRNAs in the A and P- sites (By similarity).
N	A9NEF8	CC	In the 70S ribosome it contacts the 23S rRNA (bridge B1a) and protein L5 of the 50S subunit (bridge B1b), connecting the 2 subunits; these bridges are implicated in subunit movement
N	A9NEF8	DE	30S ribosomal protein S13;
N	A9NEF8	DR	structural constituent of ribosome
N	A9QQ26	DE	Venom allergen 5;
N	A9QYS2	DE	L-rhamnose isomerase;
N	A9QYS2	DR	L-rhamnose isomerase activity
N	A9QYS2	DR	manganese ion binding
N	A9QYS2	DR	rhamnose metabolic process
N	A9R0U5	DE	30S ribosomal protein S16;
N	A9R0U5	DR	structural constituent of ribosome
N	A9R1J4	CC	Catalyzes the irreversible NADPH-dependent deamination of GMP to IMP
N	A9R1J4	CC	It functions in the conversion of nucleobase, nucleoside and nucleotide derivatives of G to A nucleotides, and in maintaining the intracellular balance of A and G nucleotides (By similarity).
N	A9R1J4	DE	GMP reductase;
N	A9R1J4	DE	Guanosine 5'-monophosphate oxidoreductase;
N	A9R1J4	DE	Guanosine monophosphate reductase;
N	A9R1J4	DR	GMP reductase activity
N	A9R1J4	DR	metal ion binding
N	A9R1J4	DR	nucleotide metabolic process
N	A9R1J4	DR	oxidation-reduction process
N	A9R3Y7	CC	Acts as a radical domain for damaged PFL and possibly other radical proteins (By similarity).
N	A9R3Y7	DE	Autonomous glycyl radical cofactor;
N	A9R3Y7	DR	catalytic activity
N	A9R3Z3	CC	Specifically methylates the adenine in position 37 of tRNA(Val) (By similarity).
N	A9R3Z3	DE	tRNA (adenine-N(6)-)-methyltransferase;
N	A9R3Z3	DE	tRNA m6A37 methyltransferase;
N	A9R3Z3	DR	nucleic acid binding
N	A9R3Z3	DR	tRNA (adenine-N6-)-methyltransferase activity
N	A9R6N5	CC	Conversion of NADPH, generated by peripheral catabolic pathways, to NADH, which can enter the respiratory chain for energy generation (By similarity).
N	A9R6N5	DE	NAD(P)(+) transhydrogenase [B-specific];
N	A9R6N5	DE	Soluble pyridine nucleotide transhydrogenase;
N	A9R6N5	DR	NAD(P)+ transhydrogenase (B-specific) activity
N	A9R6N5	DR	cell redox homeostasis
N	A9R6N5	DR	flavin adenine dinucleotide binding
N	A9R6N5	DR	oxidation-reduction process
N	A9R7S4	CC	Catalyzes the reversible isomerization-deamination of glucosamine 6-phosphate (GlcN6P) to form fructose 6-phosphate (Fru6P) and ammonium ion (By similarity)
N	A9R7S4	DE	GlcN6P deaminase;
N	A9R7S4	DE	Glucosamine-6-phosphate deaminase;

N	A9R7S4	DE	Glucosamine-6-phosphate isomerase;
N	A9R7S4	DR	N-acetylglucosamine metabolic process
N	A9R7S4	DR	glucosamine-6-phosphate deaminase activity
N	A9R7S4	DR	hydrolase activity
N	A9SB31	DE	UPF0497 membrane protein 11;
N	A9ULE9	CC	May function as a co-chaperone.
N	A9ULE9	DE	DnaJ homolog subfamily C member 22;
N	A9ULE9	DR	heat shock protein binding
N	A9ULE9	DR	protein folding
N	A9ULE9	DR	unfolded protein binding
N	A9VGD4	CC	Bidirectionally degrades single-stranded DNA into large acid-insoluble oligonucleotides, which are then degraded further into small acid-soluble oligonucleotides (By similarity).
N	A9VGD4	DE	Exodeoxyribonuclease 7 large subunit;
N	A9VGD4	DE	Exodeoxyribonuclease VII large subunit;
N	A9VGD4	DE	Exonuclease VII large subunit;
N	A9VGD4	DR	DNA catabolic process
N	A9VGD4	DR	exodeoxyribonuclease VII activity
N	A9VGD4	DR	nucleic acid binding
N	A9VGI1	DE	UPF0342 protein BcerKBAB4_0767;
N	A9VJM6	DE	Small, acid-soluble spore protein I;
N	A9VJM6	DR	sporulation resulting in formation of a cellular spore
N	A9VM77	CC	Catalyzes the thiamine diphosphate-dependent decarboxylation of 2-oxoglutarate and the subsequent addition of the resulting succinic semialdehyde-thiamine pyrophosphate anion to isochorismate to yield 2-succinyl-5-enolpyruvyl-6-hydroxy-3- cyclohexene-1-carboxylate (SEPHCHC) (By similarity).
N	A9VM77	DE	2-succinyl-5-enolpyruvyl-6-hydroxy-3-cyclohexene-1-carboxylate synthase;
N	A9VM77	DE	Menaquinone biosynthesis protein menD;
N	A9VM77	DE	SEPHCHC synthase;
N	A9VM77	DR	2-succinyl-5-enolpyruvyl-6-hydroxy-3-cyclohexene-1-carboxylic-acid synthase activity
N	A9VM77	DR	menaquinone biosynthetic process
N	A9VM77	DR	metal ion binding
N	A9VM77	DR	thiamine pyrophosphate binding
N	A9VN96	CC	Participates in a DNA-damage check-point that is active prior to asymmetric division when DNA is damaged
N	A9VN96	CC	DisA forms globular foci that rapidly scan along the chromosomes during sporulation, searching for lesions
N	A9VN96	CC	This triggers a cellular response that culminates in a temporary block in sporulation initiation (By similarity).
N	A9VN96	CC	When a lesion is present, disA pauses at the lesion site
N	A9VN96	DE	DNA integrity scanning protein disA;
N	A9VPA1	DE	50S ribosomal protein L36;
N	A9VPA1	DR	structural constituent of ribosome
N	A9VSD6	CC	Catalyzes the ATP-dependent amination of UTP to CTP with either L-glutamine or ammonia as the source of nitrogen (By similarity).
N	A9VSD6	DE	CTP synthase;
N	A9VSD6	DE	CTP synthetase;
N	A9VSD6	DE	UTP--ammonia ligase;
N	A9VSD6	DR	CTP synthase activity
N	A9VSD6	DR	glutamine metabolic process
N	A9VSD6	DR	pyrimidine nucleotide biosynthetic process
N	A9VZN6	DE	Protein ApaG;
N	A9W3L4	DE	50S ribosomal protein L33;
N	A9W3L4	DR	structural constituent of ribosome
N	A9W606	DE	30S ribosomal protein S9;
N	A9W606	DR	structural constituent of ribosome
N	A9W6R8	CC	Participates actively in the response to hyperosmotic and heat shock by preventing the aggregation of stress-denatured proteins and by disaggregating proteins, also in an autonomous, dnaK-independent fashion
N	A9W6R8	CC	Also involved, together with dnaK and grpE, in the DNA replication of plasmids through activation of initiation proteins (By similarity).
N	A9W6R8	CC	GrpE releases ADP from dnaK; ATP binding to dnaK triggers the release of the substrate protein, thus completing the reaction cycle
N	A9W6R8	CC	Several rounds of ATP-dependent interactions between dnaJ, dnaK and grpE are required for fully efficient folding

N	A9W6R8	CC	Unfolded proteins bind initially to dnaJ; upon interaction with the dnaJ-bound protein, dnaK hydrolyzes its bound ATP, resulting in the formation of a stable complex
N	A9W6R8	DE	Chaperone protein dnaJ;
N	A9W6R8	DR	DNA replication
N	A9W6R8	DR	heat shock protein binding
N	A9W6R8	DR	metal ion binding
N	A9W6R8	DR	protein folding
N	A9W6R8	DR	response to heat
N	A9W6R8	DR	unfolded protein binding
N	A9W8D2	CC	Catalyzes the methylthiolation of an aspartic acid residue of ribosomal protein S12 (By similarity).
N	A9W8D2	DE	Ribosomal protein S12 methylthiotransferase RimO;
N	A9W8D2	DE	Ribosome maturation factor RimO;
N	A9W8D2	DE	S12 MTTase;
N	A9W8D2	DE	S12 methylthiotransferase;
N	A9W8D2	DR	4 iron, 4 sulfur cluster binding
N	A9W8D2	DR	RNA modification
N	A9W8D2	DR	metal ion binding
N	A9W8D2	DR	peptidyl-L-beta-methylthioaspartic acid biosynthetic process from peptidyl-aspartic acid
N	A9W8D2	DR	transferase activity
N	A9WQA3	DE	1-(5-phosphoribosyl)-5-[(5-phosphoribosylamino)methylideneamino] imidazole-4-carboxamide isomerase;
N	A9WQA3	DE	Phosphoribosylformimino-5-aminoimidazole carboxamide ribotide isomerase;
N	A9WQA3	DR	1-(5-phosphoribosyl)-5-[(5-phosphoribosylamino)methylideneamino]imidazole-4-carboxamide isomerase activity
N	A9WQA3	DR	histidine biosynthetic process
N	A9WQA3	DR	phosphoribosylanthranilate isomerase activity
N	A9WQA3	DR	tryptophan biosynthetic process
N	A9WSB2	DE	Aspartate carbamoyltransferase;
N	A9WSB2	DE	Aspartate transcarbamylase;
N	A9WSB2	DR	'de novo' pyrimidine base biosynthetic process
N	A9WSB2	DR	amino acid binding
N	A9WSB2	DR	aspartate carbamoyltransferase activity
N	A9WSB2	DR	cellular amino acid metabolic process
N	A9WSB2	DR	pyrimidine nucleotide biosynthetic process
N	A9WYH0	DE	DTB synthetase;
N	A9WYH0	DE	Dethiobiotin synthase;
N	A9WYH0	DE	Dethiobiotin synthetase;
N	A9WYH0	DR	biotin biosynthetic process
N	A9WYH0	DR	dethiobiotin synthase activity
N	A9WYH0	DR	magnesium ion binding
N	B0B7M3	CC	Produces ATP from ADP in the presence of a proton gradient across the membrane
N	B0B7M3	CC	The V-type beta chain is a regulatory subunit (By similarity).
N	B0B7M3	DE	V-ATPase subunit B;
N	B0B7M3	DE	V-type ATP synthase beta chain;
N	B0B7M3	DR	ATP biosynthetic process
N	B0B7M3	DR	hydrogen ion transporting ATP synthase activity, rotational mechanism
N	B0B7M3	DR	proton-transporting ATPase activity, rotational mechanism
N	B0B874	CC	Major role in the synthesis of nucleoside triphosphates other than ATP
N	B0B874	CC	The ATP gamma phosphate is transferred to the NDP beta phosphate via a ping-pong mechanism, using a phosphorylated active-site intermediate (By similarity).
N	B0B874	DE	NDP kinase;
N	B0B874	DE	Nucleoside diphosphate kinase;
N	B0B874	DE	Nucleoside-2-P kinase;
N	B0B874	DR	CTP biosynthetic process
N	B0B874	DR	GTP biosynthetic process
N	B0B874	DR	UTP biosynthetic process
N	B0B874	DR	metal ion binding
N	B0B874	DR	nucleoside diphosphate kinase activity
N	B0B898	CC	Protein S19 forms a complex with S13 that binds strongly to the 16S ribosomal RNA (By similarity).
N	B0B898	DE	30S ribosomal protein S19;
N	B0B898	DR	structural constituent of ribosome
N	B0BAQ9	CC	Binds to the 23S rRNA (By similarity).
N	B0BAQ9	DE	50S ribosomal protein L9;

N	B0BAQ9	DR	structural constituent of ribosome
N	B0BNS7	CC	Could accelerate the degradation of some genes transcripts potentially through selective RNA binding (By similarity).
N	B0BNS7	DE	Carbon storage regulator homolog;
N	B0BNS7	DR	mRNA catabolic process
N	B0BNS7	DR	regulation of carbohydrate metabolic process
N	B0BRH8	CC	Cell wall formation (By similarity).
N	B0BRH8	DE	UDP-N-acetylmuramate--L-alanine ligase;
N	B0BRH8	DE	UDP-N-acetylmuramoyl-L-alanine synthetase;
N	B0BRH8	DR	UDP-N-acetylmuramate-L-alanine ligase activity
N	B0BRH8	DR	cell division
N	B0BRH8	DR	cellular cell wall organization
N	B0BRH8	DR	peptidoglycan biosynthetic process
N	B0BRH8	DR	regulation of cell shape
N	B0BS44	CC	Catalyzes the isomerization between 2-isopropylmalate and 3-isopropylmalate, via the formation of 2-isopropylmaleate (By similarity).
N	B0BS44	DE	3-isopropylmalate dehydratase large subunit;
N	B0BS44	DE	Alpha-IPM isomerase;
N	B0BS44	DE	Isopropylmalate isomerase;
N	B0BS44	DR	3-isopropylmalate dehydratase activity
N	B0BS44	DR	4 iron, 4 sulfur cluster binding
N	B0BS44	DR	leucine biosynthetic process
N	B0BS44	DR	metal ion binding
N	B0BSV5	CC	One of the primary rRNA binding proteins, it binds directly to 16S rRNA where it nucleates assembly of the body of the 30S subunit (By similarity).
N	B0BSV5	DE	30S ribosomal protein S4;
N	B0BSV5	DR	structural constituent of ribosome
N	B0BX63	CC	An accessory protein needed during the final step in the assembly of 30S ribosomal subunit, possibly for assembly of the head region
N	B0BX63	CC	Essential for efficient processing of 16S rRNA
N	B0BX63	CC	It has affinity for free ribosomal 30S subunits but not for 70S ribosomes (By similarity).
N	B0BX63	CC	May be needed both before and after rbfA during the maturation of 16S rRNA
N	B0BX63	CC	Probably interacts with S19
N	B0BX63	DE	Ribosome maturation factor rimM;
N	B0BX63	DR	rRNA processing
N	B0BX63	DR	ribosome binding
N	B0C3M8	CC	May be involved in photosynthetic membrane biogenesis (By similarity).
N	B0C3M8	DE	Protein thf1;
N	B0C3M8	DR	thylakoid membrane organization
N	B0C7G7	DE	D-fructose-1,6-bisphosphate 1-phosphohydrolase class 1 2;
N	B0C7G7	DE	FBPase class 1 2;
N	B0C7G7	DE	Fructose-1,6-bisphosphatase class 1 2;
N	B0C7G7	DR	fructose 1,6-bisphosphate 1-phosphatase activity
N	B0C7G7	DR	metal ion binding
N	B0C7G7	DR	reductive pentose-phosphate cycle
N	B0CBC7	CC	Catalyzes the ATP-dependent amination of UTP to CTP with either L-glutamine or ammonia as the source of nitrogen (By similarity).
N	B0CBC7	DE	CTP synthase;
N	B0CBC7	DE	CTP synthetase;
N	B0CBC7	DE	UTP--ammonia ligase;
N	B0CBC7	DR	CTP synthase activity
N	B0CBC7	DR	glutamine metabolic process
N	B0CBC7	DR	pyrimidine nucleotide biosynthetic process
N	B0CE03	CC	Carrier of the growing fatty acid chain in fatty acid biosynthesis (By similarity).
N	B0CE03	DE	Acyl carrier protein;
N	B0CE03	DR	acyl carrier activity
N	B0CE03	DR	cofactor binding
N	B0CE03	DR	fatty acid biosynthetic process
N	B0CEZ5	DE	Argininosuccinate lyase;
N	B0CEZ5	DE	Arginosuccinase;
N	B0CEZ5	DR	arginine biosynthetic process via ornithine
N	B0CEZ5	DR	argininosuccinate lyase activity
N	B0CG09	CC	Involved in light-induced Na(+)-dependent proton extrusion
N	B0CG09	CC	Also seems to be involved in CO(2) transport (By similarity).
N	B0CG09	DE	Proton extrusion protein PcxA;

N	B0CG09	DR	proton transport
N	B0CJD1	CC	Catalyzes the specific phosphorylation of the 3-hydroxyl group of shikimic acid using ATP as a cosubstrate (By similarity).
N	B0CJD1	DE	Shikimate kinase;
N	B0CJD1	DR	aromatic amino acid family biosynthetic process
N	B0CJD1	DR	metal ion binding
N	B0CJD1	DR	shikimate kinase activity
N	B0JSE3	CC	With S4 and S5 plays an important role in translational accuracy (By similarity).
N	B0JSE3	DE	30S ribosomal protein S12;
N	B0JSE3	DR	structural constituent of ribosome
N	B0JUA2	DE	UDP-3-O-[3-hydroxymyristoyl] glucosamine N-acyltransferase;
N	B0JUA2	DR	acyltransferase activity
N	B0JUA2	DR	lipid A biosynthetic process
N	B0JWQ2	DE	5-enolpyruvylshikimate-3-phosphate phospholyase;
N	B0JWQ2	DE	Chorismate synthase;
N	B0JWQ2	DR	aromatic amino acid family biosynthetic process
N	B0JWQ2	DR	chorismate synthase activity
N	B0K0Y0	DE	Acetohydroxy-acid isomeroreductase;
N	B0K0Y0	DE	Alpha-keto-beta-hydroxylacil reductoisomerase;
N	B0K0Y0	DE	Ketol-acid reductoisomerase;
N	B0K0Y0	DR	branched chain family amino acid biosynthetic process
N	B0K0Y0	DR	coenzyme binding
N	B0K0Y0	DR	ketol-acid reductoisomerase activity
N	B0K0Y0	DR	oxidation-reduction process
N	B0K365	CC	Catalyzes the reversible reaction in which hydroxymethyl group from 5,10-methylenetetrahydrofolate is tranferred onto alpha-ketoisovalerate to form ketopantoate (By similarity).
N	B0K365	DE	3-methyl-2-oxobutanoate hydroxymethyltransferase;
N	B0K365	DE	Ketopantoate hydroxymethyltransferase;
N	B0K365	DR	3-methyl-2-oxobutanoate hydroxymethyltransferase activity
N	B0K365	DR	metal ion binding
N	B0K365	DR	methyltransferase activity
N	B0K365	DR	pantothenate biosynthetic process
N	B0K414	CC	An essential GTPase which binds GTP, GDP and possibly (p)ppGpp with moderate affinity, with high nucleotide exchange rates and a fairly low GTP hydrolysis rate (By similarity)
N	B0K414	CC	It may play a role in control of the cell cycle, stress response, ribosome biogenesis and in those bacteria that undergo differentiation, in morphogenesis control (Potential)
N	B0K414	DE	GTP-binding protein obg;
N	B0K414	DR	GTPase activity
N	B0K414	DR	magnesium ion binding
N	B0KCI9	CC	Binds directly to 23S rRNA
N	B0KCI9	CC	The L1 stalk is quite mobile in the ribosome, and is involved in E site tRNA release (By similarity).
N	B0KCI9	DE	50S ribosomal protein L1;
N	B0KCI9	DR	RNA processing
N	B0KCI9	DR	regulation of translation
N	B0KCI9	DR	structural constituent of ribosome
N	B0KLP4	DE	UPF0502 protein PputGB1_3531;
N	B0KN58	CC	Catalyzes the rearrangement of 1-deoxy-D-xylulose 5- phosphate (DXP) to produce the thiazole phosphate moiety of thiamine
N	B0KN58	CC	In vitro, sulfur can be provided by H(2)S (By similarity).
N	B0KN58	CC	Sulfur is provided by the thiocarboxylate moiety of the carrier protein ThiS
N	B0KN58	DE	Thiazole synthase;
N	B0KN58	DR	lyase activity
N	B0KN58	DR	thiamine biosynthetic process
N	B0KNF5	CC	Catalyzes the ferrous insertion into protoporphyrin IX (By similarity).
N	B0KNF5	DE	Ferrochelatase;
N	B0KNF5	DE	Heme synthase;
N	B0KNF5	DE	Protoheme ferro-lyase;
N	B0KNF5	DR	ferrochelatase activity
N	B0KNF5	DR	heme biosynthetic process
N	B0KNF5	DR	metal ion binding

N	B0KR91	CC	Catalyzes the reversible phosphatidyl group transfer from one phosphatidylglycerol molecule to another to form cardiolipin (CL) (diphosphatidylglycerol) and glycerol (By similarity).
N	B0KR91	DE	CL synthase;
N	B0KR91	DE	Cardiolipin synthase;
N	B0KR91	DR	phospholipid biosynthetic process
N	B0KR91	DR	phosphotransferase activity, for other substituted phosphate groups
N	B0KZ79	DE	Conotoxin Ec15a;
N	B0KZ79	DR	ion channel inhibitor activity
N	B0RNH1	CC	Provides the cells with the ability to utilize trehalose at high osmolarity by splitting it into glucose molecules that can subsequently be taken up by the phosphotransferase-mediated uptake system (By similarity).
N	B0RNH1	DE	Alpha,alpha-trehalase;
N	B0RNH1	DE	Alpha,alpha-trehalose glucohydrolase;
N	B0RNH1	DE	Periplasmic trehalase;
N	B0RNH1	DR	alpha,alpha-trehalase activity
N	B0RNH1	DR	trehalose metabolic process
N	B0RPG5	CC	Reduction of activated sulfate into sulfite (By similarity).
N	B0RPG5	DE	3'-phosphoadenylylsulfate reductase;
N	B0RPG5	DE	PAPS reductase, thioredoxin dependent;
N	B0RPG5	DE	PAPS sulfotransferase;
N	B0RPG5	DE	PAdoPS reductase;
N	B0RPG5	DE	Phosphoadenosine phosphosulfate reductase;
N	B0RPG5	DR	cysteine biosynthetic process
N	B0RPG5	DR	phosphoadenylyl-sulfate reductase (thioredoxin) activity
N	B0RPG5	DR	sulfate assimilation, phosphoadenylyl sulfate reduction by phosphoadenylyl-sulfate reductase (thioredoxin)
N	B0RTD8	CC	Together with moaA, is involved in the conversion of a guanosine derivative (5'-GTP) into molybdopterin precursor Z (By similarity).
N	B0RTD8	DE	Molybdenum cofactor biosynthesis protein C;
N	B0RTD8	DR	Mo-molybdopterin cofactor biosynthetic process
N	B0S0R2	DE	AIR synthase;
N	B0S0R2	DE	Phosphoribosyl-aminoimidazole synthetase;
N	B0S0R2	DE	Phosphoribosylformylglycinamidine cyclo-ligase;
N	B0S0R2	DR	'de novo' IMP biosynthetic process
N	B0S0R2	DR	phosphoribosylformylglycinamidine cyclo-ligase activity
N	B0S104	CC	Catalyzes the attachment of alanine to tRNA(Ala) in a two-step reaction: alanine is first activated by ATP to form Ala- AMP and then transferred to the acceptor end of tRNA(Ala)
N	B0S104	CC	Also edits incorrectly charged Ser-tRNA(Ala) and Gly-tRNA(Ala) via its editing domain (By similarity).
N	B0S104	DE	Alanine--tRNA ligase;
N	B0S104	DE	Alanyl-tRNA synthetase;
N	B0S104	DR	alanine-tRNA ligase activity
N	B0S104	DR	alanyl-tRNA aminoacylation
N	B0S104	DR	metal ion binding
N	B0S1C3	CC	Catalyzes the transfer of a dimethylallyl group onto the adenine at position 37 in tRNAs that read codons beginning with uridine, leading to the formation of N6-(dimethylallyl)adenosine (i(6)A) (By similarity).
N	B0S1C3	DE	DMAPP:tRNA dimethylallyltransferase;
N	B0S1C3	DE	Dimethylallyl diphosphate:tRNA dimethylallyltransferase;
N	B0S1C3	DE	IPP transferase;
N	B0S1C3	DE	Isopentenyl-diphosphate:tRNA isopentenyltransferase;
N	B0S1C3	DE	tRNA dimethylallyltransferase;
N	B0S1C3	DR	tRNA dimethylallyltransferase activity
N	B0S1C3	DR	tRNA processing
N	B0S1D8	CC	This enzyme is involved in nucleotide metabolism: it produces dUMP, the immediate precursor of thymidine nucleotides and it decreases the intracellular concentration of dUTP so that uracil cannot be incorporated into DNA (By similarity).
N	B0S1D8	DE	Deoxyuridine 5'-triphosphate nucleotidohydrolase;
N	B0S1D8	DE	dUTP pyrophosphatase;
N	B0S1D8	DR	dUTP diphosphatase activity
N	B0S1D8	DR	dUTP metabolic process
N	B0S1D8	DR	metal ion binding
N	B0SB35	DE	Aspartate--tRNA ligase;

N	B0SB35	DE	Aspartyl-tRNA synthetase;
N	B0SB35	DR	aspartate-tRNA ligase activity
N	B0SB35	DR	aspartyl-tRNA aminoacylation
N	B0SB35	DR	nucleic acid binding
N	B0SHL8	CC	Catalyzes the isomerization of sedoheptulose 7-phosphate in D-glycero-D-manno-heptose 7-phosphate (By similarity).
N	B0SHL8	DE	Phosphoheptose isomerase;
N	B0SHL8	DE	Sedoheptulose 7-phosphate isomerase;
N	B0SHL8	DR	D-sedoheptulose 7-phosphate isomerase activity
N	B0SHL8	DR	carbohydrate metabolic process
N	B0SHL8	DR	metal ion binding
N	B0SHL8	DR	sugar binding
N	B0SRX6	DE	GTP cyclohydrolase 1;
N	B0SRX6	DE	GTP cyclohydrolase I;
N	B0SRX6	DR	GTP cyclohydrolase I activity
N	B0SRX6	DR	metal ion binding
N	B0SRX6	DR	one-carbon metabolic process
N	B0SRX6	DR	tetrahydrofolate biosynthetic process
N	B0SSH1	CC	Binds the lower part of the 30S subunit head
N	B0SSH1	CC	Binds mRNA in the 70S ribosome, positioning it for translation (By similarity).
N	B0SSH1	DE	30S ribosomal protein S3;
N	B0SSH1	DR	structural constituent of ribosome
N	B0SUP8	CC	DNA-dependent RNA polymerase catalyzes the transcription of DNA into RNA using the four ribonucleoside triphosphates as substrates (By similarity).
N	B0SUP8	DE	DNA-directed RNA polymerase subunit beta';
N	B0SUP8	DE	RNA polymerase subunit beta';
N	B0SUP8	DE	RNAP subunit beta';
N	B0SUP8	DE	Transcriptase subunit beta';
N	B0SUP8	DR	DNA-directed RNA polymerase activity
N	B0SZS9	CC	Catalyzes the decarboxylative condensation of pimeloyl- CoA and L-alanine to produce 8-amino-7-oxononanoate (AON), coenzyme A and/or converts 2-amino-3-ketobutyrate to glycine and acetyl-CoA (By similarity).
N	B0SZS9	DE	7-KAP synthase;
N	B0SZS9	DE	7-keto-8-amino-pelargonic acid synthase;
N	B0SZS9	DE	8-amino-7-ketopelargonate synthase;
N	B0SZS9	DE	AONS/AKB ligase;
N	B0SZS9	DE	Alpha-oxoamine synthase;
N	B0SZS9	DE	KAPA synthase;
N	B0SZS9	DE	L-alanine--pimeloyl-CoA ligase;
N	B0SZS9	DE	Putative 8-amino-7-oxononanoate synthase/2-amino-3-ketobutyrate coenzyme A ligase;
N	B0SZS9	DR	8-amino-7-oxononanoate synthase activity
N	B0SZS9	DR	biotin biosynthetic process
N	B0SZS9	DR	glycine C-acetyltransferase activity
N	B0SZS9	DR	pyridoxal phosphate binding
N	B0SZS9	DR	transferase activity, transferring nitrogenous groups
N	B0T1T3	DE	UPF0133 protein Caul_4574;
N	B0TEK0	CC	Regulates arginine biosynthesis genes (By similarity).
N	B0TEK0	DE	Arginine repressor;
N	B0TEK0	DR	arginine biosynthetic process
N	B0TEK0	DR	regulation of transcription, DNA-dependent
N	B0TEK0	DR	sequence-specific DNA binding transcription factor activity
N	B0TI63	CC	Catalyzes the conversion of uracil and 5-phospho-alpha- D-ribose 1-diphosphate (PRPP) to UMP and diphosphate (By similarity).
N	B0TI63	DE	UMP pyrophosphorylase;
N	B0TI63	DE	Uracil phosphoribosyltransferase;
N	B0TI63	DR	nucleoside metabolic process
N	B0TI63	DR	uracil phosphoribosyltransferase activity
N	B0TI63	DR	uracil salvage
N	B0TQH2	DE	UPF0161 protein Shal_4312;
N	B0TR47	CC	Catalyzes the reversible adenylation of nicotinate mononucleotide (NaMN) to nicotinic acid adenine dinucleotide (NaAD) (By similarity).
N	B0TR47	DE	Deamido-NAD(+) diphosphorylase;
N	B0TR47	DE	Deamido-NAD(+) pyrophosphorylase;
N	B0TR47	DE	NaMN adenylyltransferase;
N	B0TR47	DE	Nicotinate mononucleotide adenylyltransferase;

N	B0TR47	DE	Probable nicotinate-nucleotide adenylyltransferase;
N	B0TR47	DR	NAD biosynthetic process
N	B0TR47	DR	nicotinate-nucleotide adenylyltransferase activity
N	B0TYH9	CC	Ligates lysine onto the cytidine present at position 34 of the AUA codon-specific tRNA(Ile) that contains the anticodon CAU, in an ATP-dependent manner
N	B0TYH9	CC	Cytidine is converted to lysidine, thus changing the amino acid specificity of the tRNA from methionine to isoleucine (By similarity).
N	B0TYH9	DE	tRNA(Ile)-2-lysyl-cytidine synthase;
N	B0TYH9	DE	tRNA(Ile)-lysidine synthase;
N	B0TYH9	DE	tRNA(Ile)-lysidine synthetase;
N	B0TYH9	DR	ligase activity, forming carbon-nitrogen bonds
N	B0TYH9	DR	tRNA processing
N	B0TYI2	CC	Transfers the N-acyl diglyceride group on what will become the N-terminal cysteine of membrane lipoproteins (By similarity).
N	B0TYI2	DE	Prolipoprotein diacylglyceryl transferase;
N	B0TYI2	DR	lipoprotein biosynthetic process
N	B0TYI2	DR	protein lipoylation
N	B0TYI2	DR	transferase activity, transferring glycosyl groups
N	B0U083	CC	Specifically methylates position 2 of adenine 2503 in 23S rRNA (By similarity).
N	B0U083	DE	23S rRNA m2A2503 methyltransferase;
N	B0U083	DE	Ribosomal RNA large subunit methyltransferase N;
N	B0U083	DR	4 iron, 4 sulfur cluster binding
N	B0U083	DR	RNA methyltransferase activity
N	B0U083	DR	metal ion binding
N	B0U083	DR	rRNA processing
N	B0U1E8	CC	Catalyzes the transfer of a dimethylallyl group onto the adenine at position 37 in tRNAs that read codons beginning with uridine, leading to the formation of N6-(dimethylallyl)adenosine (i(6)A) (By similarity).
N	B0U1E8	DE	DMAPP:tRNA dimethylallyltransferase;
N	B0U1E8	DE	Dimethylallyl diphosphate:tRNA dimethylallyltransferase;
N	B0U1E8	DE	IPP transferase;
N	B0U1E8	DE	Isopentenyl-diphosphate:tRNA isopentenyltransferase;
N	B0U1E8	DE	tRNA dimethylallyltransferase;
N	B0U1E8	DR	tRNA dimethylallyltransferase activity
N	B0U1E8	DR	tRNA processing
N	B0U1Z0	CC	Responsible for the transport of dicarboxylates such as succinate, fumarate, and malate from the periplasm across the membrane (By similarity).
N	B0U1Z0	DE	C4-dicarboxylate transport protein;
N	B0U1Z0	DR	carbohydrate transport
N	B0U1Z0	DR	sodium:dicarboxylate symporter activity
N	B0U3Q8	CC	This protein specifically catalyzes the removal of signal peptides from prolipoproteins (By similarity).
N	B0U3Q8	DE	Lipoprotein signal peptidase;
N	B0U3Q8	DE	Prolipoprotein signal peptidase;
N	B0U3Q8	DE	Signal peptidase II;
N	B0U3Q8	DR	aspartic-type endopeptidase activity
N	B0U6Q7	CC	Catalyzes the 2-thiolation of uridine at the wobble position (U34) of tRNA, leading to the formation of s(2)U34 (By similarity).
N	B0U6Q7	DE	tRNA-specific 2-thiouridylase mnmA;
N	B0U6Q7	DR	tRNA processing
N	B0U6Q7	DR	transferase activity
N	B0UE10	CC	May be involved in the transport of PQQ or its precursor to the periplasm (By similarity).
N	B0UE10	DE	Coenzyme PQQ synthesis protein B;
N	B0UE10	DE	Pyrroloquinoline quinone biosynthesis protein B;
N	B0UE10	DR	pyrroloquinoline quinone biosynthetic process
N	B0UFU2	DE	Flavoprotein wrbA;
N	B0UFU2	DR	oxidoreductase activity
N	B0UJ71	CC	May conjugate Arg from its aminoacyl-tRNA to the N- termini of proteins containing an N-terminal aspartate or glutamate (Potential).
N	B0UJ71	DE	Arginyltransferase;
N	B0UJ71	DE	Putative arginyl-tRNA--protein transferase;
N	B0UJ71	DE	R-transferase;
N	B0UJ71	DR	acyltransferase activity
N	B0UJ71	DR	arginyltransferase activity

N	B0UJ71	DR	protein arginylation
N	B0URM8	CC	Hydrolyzes diadenosine 5',5'''-P1,P4-tetraphosphate to yield ADP (By similarity)
N	B0URM8	DE	Ap4A hydrolase;
N	B0URM8	DE	Bis(5'-nucleosyl)-tetraphosphatase, symmetrical;
N	B0URM8	DE	Diadenosine 5',5'''-P1,P4-tetraphosphate pyrophosphohydrolase;
N	B0URM8	DE	Diadenosine tetraphosphatase;
N	B0URM8	DR	bis(5'-nucleosyl)-tetraphosphatase (symmetrical) activity
N	B0URV9	CC	Phosphorylation of dTMP to form dTDP in both de novo and salvage pathways of dTTP synthesis (By similarity).
N	B0URV9	DE	Thymidylate kinase;
N	B0URV9	DE	dTMP kinase;
N	B0URV9	DR	dTDP biosynthetic process
N	B0URV9	DR	thymidylate kinase activity
N	B0USS1	CC	Digests double-stranded RNA
N	B0USS1	CC	Involved in the processing of ribosomal RNA precursors and of some mRNAs (By similarity).
N	B0USS1	DE	Ribonuclease 3;
N	B0USS1	DE	Ribonuclease III;
N	B0USS1	DR	RNA processing
N	B0USS1	DR	double-stranded RNA binding
N	B0USS1	DR	rRNA catabolic process
N	B0USS1	DR	ribonuclease III activity
N	B0UT85	CC	Catalyzes the last two steps in the biosynthesis of 5- methylaminomethyl-2-thiouridine (mnm(5)s(2)U) at the wobble position (U34) in tRNA
N	B0UT85	CC	Catalyzes the FAD-dependent demodification of cmnm(5)s(2)U34 to nm(5)s(2)U34, followed by the transfer of a methyl group from S-adenosyl-L-methionine to nm(5)s(2)U34, to form mnm(5)s(2)U34 (By similarity).
N	B0UT85	DE	FAD-dependent cmnm(5)s(2)U34 oxidoreductase;
N	B0UT85	DE	tRNA (mnm(5)s(2)U34)-methyltransferase;
N	B0UT85	DE	tRNA 5-methylaminomethyl-2-thiouridine biosynthesis bifunctional protein mnmC;
N	B0UT85	DE	tRNA mnm(5)s(2)U biosynthesis bifunctional protein;
N	B0UT85	DR	oxidation-reduction process
N	B0UT85	DR	oxidoreductase activity, acting on the CH-NH group of donors
N	B0UT85	DR	tRNA (5-methylaminomethyl-2-thiouridylate)-methyltransferase activity
N	B0UUM5	CC	Specifically methylates the guanosine in position 2445 (m2G2445) of 23S rRNA (By similarity).
N	B0UUM5	DE	23S rRNA m2G2445 methyltransferase;
N	B0UUM5	DE	Ribosomal RNA large subunit methyltransferase L;
N	B0UUM5	DE	rRNA (guanine-N(2)-)-methyltransferase rlmL;
N	B0UUM5	DR	rRNA (guanine-N2-)-methyltransferase activity
N	B0UV22	DE	Pantothenate kinase;
N	B0UV22	DE	Pantothenic acid kinase;
N	B0UV22	DR	coenzyme A biosynthetic process
N	B0UV22	DR	pantothenate kinase activity
N	B0UWR3	CC	Participates actively in the response to hyperosmotic and heat shock by preventing the aggregation of stress-denatured proteins and by disaggregating proteins, also in an autonomous, dnaK-independent fashion
N	B0UWR3	CC	Also involved, together with dnaK and grpE, in the DNA replication of plasmids through activation of initiation proteins (By similarity).
N	B0UWR3	CC	GrpE releases ADP from dnaK; ATP binding to dnaK triggers the release of the substrate protein, thus completing the reaction cycle
N	B0UWR3	CC	Several rounds of ATP-dependent interactions between dnaJ, dnaK and grpE are required for fully efficient folding
N	B0UWR3	CC	Unfolded proteins bind initially to dnaJ; upon interaction with the dnaJ-bound protein, dnaK hydrolyzes its bound ATP, resulting in the formation of a stable complex
N	B0UWR3	DE	Chaperone protein dnaJ;
N	B0UWR3	DR	DNA replication
N	B0UWR3	DR	heat shock protein binding
N	B0UWR3	DR	metal ion binding
N	B0UWR3	DR	protein folding
N	B0UWR3	DR	response to heat
N	B0UWR3	DR	unfolded protein binding
N	B0UZC8	DE	von Willebrand factor C domain-containing protein 2-like;

N	B0V5Q4	CC	This protein is one of the two subunits of integration host factor, a specific DNA-binding protein that functions in genetic recombination as well as in transcriptional and translational control (By similarity).
N	B0V5Q4	DE	Integration host factor subunit alpha;
N	B0V5Q4	DR	DNA recombination
N	B0V5Q4	DR	regulation of transcription, DNA-dependent
N	B0V5Q4	DR	regulation of translation
N	B0V6V5	CC	Binds to 23S rRNA
N	B0V6V5	CC	Forms part of two intersubunit bridges in the 70S ribosome (By similarity).
N	B0V6V5	DE	50S ribosomal protein L14;
N	B0V6V5	DR	structural constituent of ribosome
N	B0V732	CC	Catalyzes a trans-dehydration via an enolate intermediate (By similarity).
N	B0V732	DE	3-dehydroquinase;
N	B0V732	DE	3-dehydroquinate dehydratase;
N	B0V732	DE	Type II DHQase;
N	B0V732	DR	3-dehydroquinate dehydratase activity
N	B0V732	DR	aromatic amino acid family biosynthetic process
N	B0V744	CC	Cell wall formation (By similarity).
N	B0V744	DE	UDP-N-acetylenolpyruvoylglucosamine reductase;
N	B0V744	DE	UDP-N-acetylmuramate dehydrogenase;
N	B0V744	DR	UDP-N-acetylmuramate dehydrogenase activity
N	B0V744	DR	cell division
N	B0V744	DR	cellular cell wall organization
N	B0V744	DR	flavin adenine dinucleotide binding
N	B0V744	DR	oxidation-reduction process
N	B0V744	DR	peptidoglycan biosynthetic process
N	B0V744	DR	regulation of cell shape
N	B0V9T8	DE	Allantoate amidinohydrolase;
N	B0V9T8	DE	Probable allantoicase;
N	B0V9T8	DR	allantoicase activity
N	B0V9T8	DR	purine base metabolic process
N	B0VE54	DE	UPF0301 protein ABAYE3454;
N	B0VTM2	CC	Required for accurate and efficient protein synthesis under certain stress conditions
N	B0VTM2	CC	Back- translocation proceeds from a post-translocation (POST) complex to a pre-translocation (PRE) complex, thus giving elongation factor G a second chance to translocate the tRNAs correctly
N	B0VTM2	CC	Binds to ribosomes in a GTP-dependent manner (By similarity).
N	B0VTM2	CC	May act as a fidelity factor of the translation reaction, by catalyzing a one-codon backward translocation of tRNAs on improperly translocated ribosomes
N	B0VTM2	DE	Elongation factor 4;
N	B0VTM2	DE	Ribosomal back-translocase LepA;
N	B0VTM2	DR	GTPase activity
N	B0VZR4	CC	Required for endonucleolytic cleavage during polyadenylation-dependent pre-mRNA 3'-end formation (By similarity).
N	B0VZR4	DE	Protein CLP1 homolog;
N	B0VZR4	DR	mRNA processing
N	B0W6N3	CC	Component of the eukaryotic translation initiation factor 3 (eIF-3) complex, which is involved in protein synthesis and, together with other initiation factors, stimulates binding of mRNA and methionyl-tRNAi to the 40S ribosome (By similarity)
N	B0W6N3	DE	Eukaryotic translation initiation factor 3 subunit 10;
N	B0W6N3	DE	Eukaryotic translation initiation factor 3 subunit A;
N	B0W6N3	DR	translation initiation factor activity
N	B0WS18	DE	Probable cGMP 3',5'-cyclic phosphodiesterase subunit delta;
N	B0WS18	DR	3',5'-cyclic-nucleotide phosphodiesterase activity
N	B0WS18	DR	visual perception
N	B0XQ15	CC	Essential component of the cytosolic iron-sulfur (Fe/S) protein assembly machinery
N	B0XQ15	CC	Required for the maturation of extramitochondrial Fe/S proteins (By similarity).
N	B0XQ15	DE	Probable cytosolic iron-sulfur protein assembly protein 1;
N	B0Y081	CC	Involved in cell death (apoptosis) (By similarity)
N	B0Y081	CC	Required for the apoptotic-like loss of membrane phospholipid asymmetry at stationary phase and facilitates growth under conditions of endoplasmic reticulum stress
N	B0Y081	DE	Metacaspase-1B;
N	B0Y081	DR	cysteine-type endopeptidase activity
N	B0Y7W2	CC	Glucosidase involved in the degradation of cellulosic biomass
N	B0Y7W2	CC	Active on lichenan (By similarity).

N	B0Y7W2	DE	Exo-1,3-beta-glucanase D;
N	B0Y7W2	DE	Probable glucan 1,3-beta-glucosidase D;
N	B0Y7W2	DR	cation binding
N	B0Y7W2	DR	cellular cell wall organization
N	B0Y7W2	DR	glucan exo-1,3-beta-glucosidase activity
N	B0Y7W2	DR	polysaccharide catabolic process
N	B0Z4P1	CC	Required for PSII activity (By similarity).
N	B0Z4P1	DE	PSII 5 kDa protein;
N	B0Z4P1	DE	Photosystem II reaction center protein L;
N	B0Z4P1	DR	photosynthesis
N	B0Z4P6	CC	May help in the organization of the psaE and psaF subunits (By similarity).
N	B0Z4P6	DE	Photosystem I reaction center subunit IX;
N	B0Z4P6	DR	photosynthesis
N	B0Z4U0	CC	This is one of the two reaction center proteins of photosystem II (By similarity).
N	B0Z4U0	DE	32 kDa thylakoid membrane protein;
N	B0Z4U0	DE	Photosystem II protein D1;
N	B0Z4U0	DE	Photosystem Q(B) protein;
N	B0Z4U0	DR	electron transporter, transferring electrons within the cyclic electron transport pathway of photosynthesis activity
N	B0Z4U0	DR	metal ion binding
N	B0Z4U0	DR	photosynthetic electron transport in photosystem II
N	B0Z4U0	DR	response to herbicide
N	B0Z547	CC	Key component of the proton channel; it plays a direct role in the translocation of protons across the membrane (By similarity).
N	B0Z547	DE	ATP synthase F0 sector subunit a;
N	B0Z547	DE	ATP synthase subunit a, chloroplastic;
N	B0Z547	DE	F-ATPase subunit IV;
N	B0Z547	DR	ATP synthesis coupled proton transport
N	B0Z547	DR	hydrogen ion transmembrane transporter activity
N	B0Z549	CC	F(1)F(0) ATP synthase produces ATP from ADP in the presence of a proton or sodium gradient
N	B0Z549	CC	During catalysis, ATP synthesis in the catalytic domain of F(1) is coupled via a rotary mechanism of the central stalk subunits to proton translocation (By similarity).
N	B0Z549	CC	F-type ATPases consist of two structural domains, F(1) containing the extramembraneous catalytic core and F(0) containing the membrane proton channel, linked together by a central stalk and a peripheral stalk
N	B0Z549	DE	ATP synthase F(0) sector subunit b;
N	B0Z549	DE	ATP synthase subunit b, chloroplastic;
N	B0Z549	DE	ATPase subunit I;
N	B0Z549	DR	ATP synthesis coupled proton transport
N	B0Z549	DR	hydrogen ion transmembrane transporter activity
N	B1A933	CC	Binds 16S rRNA, required for the assembly of 30S particles (By similarity).
N	B1A933	DE	30S ribosomal protein S14, chloroplastic;
N	B1A933	DR	structural constituent of ribosome
N	B1GYY9	CC	Regulates the transcription of the pyrimidine nucleotide (pyr) operon in response to exogenous pyrimidines (By similarity).
N	B1GYY9	DE	Bifunctional protein pyrR;
N	B1GYY9	DE	Pyrimidine operon regulatory protein;
N	B1GYY9	DE	Uracil phosphoribosyltransferase;
N	B1GYY9	DR	nucleoside metabolic process
N	B1GYY9	DR	uracil phosphoribosyltransferase activity
N	B1GZ70	DE	D-mannonate hydrolase;
N	B1GZ70	DE	Mannonate dehydratase;
N	B1GZ70	DR	glucuronate catabolic process
N	B1GZ70	DR	mannonate dehydratase activity
N	B1H056	CC	Responsible for the release of ribosomes from messenger RNA at the termination of protein biosynthesis
N	B1H056	CC	May increase the efficiency of translation by recycling ribosomes from one round of translation to another (By similarity).
N	B1H056	DE	Ribosome-recycling factor;
N	B1H056	DE	Ribosome-releasing factor;
N	B1HTK8	CC	Catalyzes a reversible aldol reaction between acetaldehyde and D-glyceraldehyde 3-phosphate to generate 2-deoxy- D-ribose 5-phosphate (By similarity).
N	B1HTK8	DE	2-deoxy-D-ribose 5-phosphate aldolase;
N	B1HTK8	DE	Deoxyriboaldolase;

N	B1HTK8	DE	Deoxyribose-phosphate aldolase;
N	B1HTK8	DE	Phosphodeoxyriboaldolase;
N	B1HTK8	DR	deoxyribonucleotide catabolic process
N	B1HTK8	DR	deoxyribose-phosphate aldolase activity
N	B1I1J5	CC	Binds 23S rRNA and is also seen to make contacts with the A and possibly P site tRNAs (By similarity).
N	B1I1J5	DE	50S ribosomal protein L16;
N	B1I1J5	DR	structural constituent of ribosome
N	B1I383	DE	Dihydrodipicolinate synthase;
N	B1I383	DR	diaminopimelate biosynthetic process
N	B1I383	DR	dihydrodipicolinate synthase activity
N	B1I3J0	CC	Bidirectionally degrades single-stranded DNA into large acid-insoluble oligonucleotides, which are then degraded further into small acid-soluble oligonucleotides (By similarity).
N	B1I3J0	DE	Exodeoxyribonuclease 7 large subunit;
N	B1I3J0	DE	Exodeoxyribonuclease VII large subunit;
N	B1I3J0	DE	Exonuclease VII large subunit;
N	B1I3J0	DR	DNA catabolic process
N	B1I3J0	DR	exodeoxyribonuclease VII activity
N	B1I3J0	DR	nucleic acid binding
N	B1I3W2	CC	Catalyzes the ATP-dependent conversion of 7-carboxy-7- deazaguanine (CDG) to 7-cyano-7-deazaguanine (preQ(0)) (By similarity).
N	B1I3W2	DE	7-cyano-7-carbaguanine synthase;
N	B1I3W2	DE	7-cyano-7-deazaguanine synthase;
N	B1I3W2	DE	PreQ(0) synthase;
N	B1I3W2	DE	Queuosine biosynthesis protein queC;
N	B1I3W2	DR	ligase activity
N	B1I3W2	DR	metal ion binding
N	B1I3W2	DR	queuosine biosynthetic process
N	B1I544	CC	Involved in the synthesis of meso-diaminopimelate (m-DAP or DL-DAP), required for both lysine and peptidoglycan biosynthesis
N	B1I544	CC	Catalyzes the direct conversion of tetrahydrodipicolinate to LL-diaminopimelate, a reaction that requires three enzymes in E.coli (By similarity).
N	B1I544	DE	DAP-aminotransferase;
N	B1I544	DE	LL-DAP-aminotransferase;
N	B1I544	DE	LL-diaminopimelate aminotransferase;
N	B1I544	DR	L,L-diaminopimelate aminotransferase activity
N	B1I544	DR	biosynthetic process
N	B1I544	DR	pyridoxal phosphate binding
N	B1I6Q5	DE	Arginine--tRNA ligase;
N	B1I6Q5	DE	Arginyl-tRNA synthetase;
N	B1I6Q5	DR	arginine-tRNA ligase activity
N	B1I6Q5	DR	arginyl-tRNA aminoacylation
N	B1I843	CC	Required for the formation of a threonylcarbamoyl group on adenosine at position 37 (t(6)A37) in tRNAs that read codons beginning with adenine (By similarity).
N	B1I843	DE	Probable tRNA threonylcarbamoyladenosine biosynthesis protein Gcp;
N	B1I843	DE	t(6)A37 threonylcarbamoyladenosine biosynthesis protein;
N	B1I843	DR	metal ion binding
N	B1I843	DR	metalloendopeptidase activity
N	B1ICH5	DE	5-enolpyruvylshikimate-3-phosphate phospholyase;
N	B1ICH5	DE	Chorismate synthase;
N	B1ICH5	DR	aromatic amino acid family biosynthetic process
N	B1ICH5	DR	chorismate synthase activity
N	B1IE34	CC	Produces ATP from ADP in the presence of a proton gradient across the membrane
N	B1IE34	CC	The catalytic sites are hosted primarily by the beta subunits (By similarity).
N	B1IE34	DE	ATP synthase F1 sector subunit beta;
N	B1IE34	DE	ATP synthase subunit beta;
N	B1IE34	DE	F-ATPase subunit beta;
N	B1IE34	DR	ATP hydrolysis coupled proton transport
N	B1IE34	DR	ATP synthesis coupled proton transport
N	B1IE34	DR	hydrogen ion transporting ATP synthase activity, rotational mechanism
N	B1IE34	DR	hydrogen-exporting ATPase activity, phosphorylative mechanism
N	B1IE34	DR	proton-transporting ATPase activity, rotational mechanism
N	B1IPI6	CC	Transforms N(2)-succinylglutamate into succinate and glutamate (By similarity).
N	B1IPI6	DE	Succinylglutamate desuccinylase;
N	B1IPI6	DR	arginine catabolic process to glutamate

N	B1IPI6	DR	hydrolase activity, acting on ester bonds
N	B1IPI6	DR	succinylglutamate desuccinylase activity
N	B1IPI6	DR	zinc ion binding
N	B1IST8	DE	UPF0283 membrane protein ycjF;
N	B1IT09	CC	Catalyzes the isomerization of L-xylulose-5-phosphate to L-ribulose-5-phosphate
N	B1IT09	CC	Is involved in the anaerobic L-ascorbate utilization (By similarity).
N	B1IT09	DE	L-ascorbate utilization protein E;
N	B1IT09	DE	L-ribulose-5-phosphate 3-epimerase ulaE;
N	B1IT09	DE	L-xylulose-5-phosphate 3-epimerase;
N	B1IT09	DR	L-ribulose-5-phosphate 3-epimerase activity
N	B1IT09	DR	carbohydrate metabolic process
N	B1IT09	DR	intramolecular oxidoreductase activity, interconverting aldoses and ketoses
N	B1IT54	DE	UPF0235 protein yggU;
N	B1IUQ4	CC	Condenses 4-methyl-5-(beta-hydroxyethyl)thiazole monophosphate (THZ-P) and 2-methyl-4-amino-5-hydroxymethyl pyrimidine pyrophosphate (HMP-PP) to form thiamine monophosphate (TMP) (By similarity).
N	B1IUQ4	DE	TMP pyrophosphorylase;
N	B1IUQ4	DE	Thiamine-phosphate pyrophosphorylase;
N	B1IUQ4	DE	Thiamine-phosphate synthase;
N	B1IUQ4	DR	metal ion binding
N	B1IUQ4	DR	thiamine biosynthetic process
N	B1IUQ4	DR	thiamine-phosphate diphosphorylase activity
N	B1IVP8	CC	Acts as a radical domain for damaged PFL and possibly other radical proteins (By similarity).
N	B1IVP8	DE	Autonomous glycyl radical cofactor;
N	B1IVP8	DR	catalytic activity
N	B1IWE3	CC	Major role in the synthesis of nucleoside triphosphates other than ATP
N	B1IWE3	CC	The ATP gamma phosphate is transferred to the NDP beta phosphate via a ping-pong mechanism, using a phosphorylated active-site intermediate (By similarity).
N	B1IWE3	DE	NDP kinase;
N	B1IWE3	DE	Nucleoside diphosphate kinase;
N	B1IWE3	DE	Nucleoside-2-P kinase;
N	B1IWE3	DR	CTP biosynthetic process
N	B1IWE3	DR	GTP biosynthetic process
N	B1IWE3	DR	UTP biosynthetic process
N	B1IWE3	DR	metal ion binding
N	B1IWE3	DR	nucleoside diphosphate kinase activity
N	B1IWN0	CC	Catalyzes the attachment of serine to tRNA(Ser)
N	B1IWN0	CC	Is also able to aminoacylate tRNA(Sec) with serine, to form the misacylated tRNA L-seryl-tRNA(Sec), which will be further converted into selenocysteinyl-tRNA(Sec) (By similarity).
N	B1IWN0	DE	Serine--tRNA ligase;
N	B1IWN0	DE	Seryl-tRNA synthetase;
N	B1IWN0	DE	Seryl-tRNA(Ser/Sec) synthetase;
N	B1IWN0	DR	serine-tRNA ligase activity
N	B1IWN0	DR	seryl-tRNA aminoacylation
N	B1IY62	DE	Malate dehydrogenase [quinone];
N	B1IY62	DE	Probable malate:quinone oxidoreductase;
N	B1IY62	DR	malate dehydrogenase (menaquinone) activity
N	B1IY62	DR	malate dehydrogenase (quinone) activity
N	B1IY62	DR	tricarboxylic acid cycle
N	B1J1C4	CC	Catalyzes the oxidation of either pyridoxine 5'-phosphate (PNP) or pyridoxamine 5'-phosphate (PMP) into pyridoxal 5'-phosphate (PLP) (By similarity).
N	B1J1C4	DE	PNP/PMP oxidase;
N	B1J1C4	DE	Pyridoxal 5'-phosphate synthase;
N	B1J1C4	DE	Pyridoxine/pyridoxamine 5'-phosphate oxidase;
N	B1J1C4	DR	oxidation-reduction process
N	B1J1C4	DR	pyridoxamine-phosphate oxidase activity
N	B1J1C4	DR	pyridoxine biosynthetic process
N	B1J432	CC	Modifies the free amino group of the aminoacyl moiety of methionyl-tRNA(fMet)
N	B1J432	CC	The formyl group appears to play a dual role in the initiator identity of N-formylmethionyl-tRNA by: (I) promoting its recognition by IF2 and (II) impairing its binding to EFTu-GTP (By similarity).
N	B1J432	DE	Methionyl-tRNA formyltransferase;
N	B1J432	DR	methionyl-tRNA formyltransferase activity

N	B1J432	DR	methyltransferase activity
N	B1J522	CC	Catalyzes the attachment of glutamate to tRNA(Glu) in a two-step reaction: glutamate is first activated by ATP to form Glu-AMP and then transferred to the acceptor end of tRNA(Glu) (By similarity).
N	B1J522	DE	Glutamate--tRNA ligase;
N	B1J522	DE	Glutamyl-tRNA synthetase;
N	B1J522	DR	glutamate-tRNA ligase activity
N	B1J522	DR	glutamyl-tRNA aminoacylation
N	B1JIK2	CC	Catalyzes the phosphorylation of methylthioribose into methylthioribose-1-phosphate (By similarity).
N	B1JIK2	DE	MTR kinase;
N	B1JIK2	DE	Methylthioribose kinase;
N	B1JIK2	DR	S-methyl-5-thioribose kinase activity
N	B1JIK2	DR	methionine biosynthetic process
N	B1JIW7	CC	Binds the lower part of the 30S subunit head
N	B1JIW7	CC	Binds mRNA in the 70S ribosome, positioning it for translation (By similarity).
N	B1JIW7	DE	30S ribosomal protein S3;
N	B1JIW7	DR	structural constituent of ribosome
N	B1JL77	CC	Cell wall formation (By similarity).
N	B1JL77	DE	Monofunctional TGase;
N	B1JL77	DE	Monofunctional biosynthetic peptidoglycan transglycosylase;
N	B1JL77	DR	cellular cell wall organization
N	B1JL77	DR	peptidoglycan biosynthetic process
N	B1JL77	DR	regulation of cell shape
N	B1JL77	DR	transferase activity, transferring pentosyl groups
N	B1JPH6	DE	Lysine--tRNA ligase;
N	B1JPH6	DE	Lysyl-tRNA synthetase;
N	B1JPH6	DR	lysine-tRNA ligase activity
N	B1JPH6	DR	lysyl-tRNA aminoacylation
N	B1JPH6	DR	metal ion binding
N	B1JPH6	DR	nucleic acid binding
N	B1JQC1	CC	Plays a role in lysophospholipid acylation
N	B1JQC1	CC	Its physiological function is to regenerate phosphatidylethanolamine from 2-acyl-glycero-3-phosphoethanolamine (2-acyl-GPE) formed by transacylation reactions or degradation by phospholipase A1 (By similarity).
N	B1JQC1	CC	Transfers fatty acids to the 1-position via an enzyme-bound acyl-ACP intermediate in the presence of ATP and magnesium
N	B1JQC1	DE	2-acyl-GPE acyltransferase;
N	B1JQC1	DE	2-acylglycerophosphoethanolamine acyltransferase;
N	B1JQC1	DE	Acyl-ACP synthetase;
N	B1JQC1	DE	Acyl-[acyl-carrier-protein] synthetase;
N	B1JQC1	DE	Acyl-[acyl-carrier-protein]--phospholipid O-acyltransferase;
N	B1JQC1	DE	Bifunctional protein aas;
N	B1JQC1	DE	Long-chain-fatty-acid--[acyl-carrier-protein] ligase;
N	B1JQC1	DR	acyl-[acyl-carrier-protein]-phospholipid O-acyltransferase activity
N	B1JQC1	DR	long-chain fatty acid [acyl-carrier-protein] ligase activity
N	B1JQW9	CC	Reversibly transfers an adenylyl group from ATP to 4'- phosphopantetheine, yielding dephospho-CoA (dPCoA) and pyrophosphate (By similarity).
N	B1JQW9	DE	Dephospho-CoA pyrophosphorylase;
N	B1JQW9	DE	Pantetheine-phosphate adenylyltransferase;
N	B1JQW9	DE	Phosphopantetheine adenylyltransferase;
N	B1JQW9	DR	coenzyme A biosynthetic process
N	B1JQW9	DR	pantetheine-phosphate adenylyltransferase activity
N	B1JRB2	CC	Acts as a radical domain for damaged PFL and possibly other radical proteins (By similarity).
N	B1JRB2	DE	Autonomous glycyl radical cofactor;
N	B1JRB2	DR	catalytic activity
N	B1JSR4	CC	Together with moaA, is involved in the conversion of a guanosine derivative (5'-GTP) into molybdopterin precursor Z (By similarity).
N	B1JSR4	DE	Molybdenum cofactor biosynthesis protein C;
N	B1JSR4	DR	Mo-molybdopterin cofactor biosynthetic process
N	B1JU98	CC	Catalyzes the condensation of ATP and 5-phosphoribose 1- diphosphate to form N'-(5'-phosphoribosyl)-ATP (PR-ATP)
N	B1JU98	CC	Has a crucial role in the pathway because the rate of histidine biosynthesis seems to be controlled primarily by regulation of hisG enzymatic activity (By similarity).

N	B1JU98	DE	ATP phosphoribosyltransferase;
N	B1JU98	DE	ATP-PRTase;
N	B1JU98	DR	ATP phosphoribosyltransferase activity
N	B1JU98	DR	histidine biosynthetic process
N	B1JUK8	DE	Cysteine--tRNA ligase;
N	B1JUK8	DE	Cysteinyl-tRNA synthetase;
N	B1JUK8	DR	cysteine-tRNA ligase activity
N	B1JUK8	DR	cysteinyl-tRNA aminoacylation
N	B1JUK8	DR	metal ion binding
N	B1JZZ7	CC	Catalyzes the addition and repair of the essential 3'- terminal CCA sequence in tRNAs without using a nucleic acid template
N	B1JZZ7	CC	Adds these three nucleotides in the order of C, C, and A to the tRNA nucleotide-73, using CTP and ATP as substrates and producing inorganic pyrophosphate
N	B1JZZ7	CC	Also shows phosphatase, 2'-nucleotidase and 2',3'-cyclic phosphodiesterase activities
N	B1JZZ7	CC	These phosphohydrolase activities are probably involved in the repair of the tRNA 3'-CCA terminus degraded by intracellular RNases (By similarity).
N	B1JZZ7	DE	2',3'-cyclic phosphodiesterase;
N	B1JZZ7	DE	2'-nucleotidase;
N	B1JZZ7	DE	CCA tRNA nucleotidyltransferase;
N	B1JZZ7	DE	CCA-adding enzyme;
N	B1JZZ7	DE	Multifunctional CCA protein;
N	B1JZZ7	DE	Phosphatase;
N	B1JZZ7	DE	tRNA CCA-pyrophosphorylase;
N	B1JZZ7	DE	tRNA adenylyl-/cytidylyl-transferase;
N	B1JZZ7	DE	tRNA nucleotidyltransferase;
N	B1JZZ7	DR	hydrolase activity
N	B1JZZ7	DR	metal ion binding
N	B1JZZ7	DR	tRNA 3'-terminal CCA addition
N	B1JZZ7	DR	tRNA adenylyltransferase activity
N	B1KCW3	CC	Modifies the free amino group of the aminoacyl moiety of methionyl-tRNA(fMet)
N	B1KCW3	CC	The formyl group appears to play a dual role in the initiator identity of N-formylmethionyl-tRNA by: (I) promoting its recognition by IF2 and (II) impairing its binding to EFTu-GTP (By similarity).
N	B1KCW3	DE	Methionyl-tRNA formyltransferase;
N	B1KCW3	DR	methionyl-tRNA formyltransferase activity
N	B1KCW3	DR	methyltransferase activity
N	B1KHT1	DE	Thiopurine S-methyltransferase;
N	B1KHT1	DE	Thiopurine methyltransferase;
N	B1KHT1	DR	thiopurine S-methyltransferase activity
N	B1KL38	CC	Involved in base excision repair of DNA damaged by oxidation or by mutagenic agents
N	B1KL38	CC	Acts as DNA glycosylase that recognizes and removes damaged bases
N	B1KL38	CC	Cleaves the DNA backbone by beta-delta elimination to generate a single-strand break at the site of the removed base with both 3'- and 5'-phosphates (By similarity).
N	B1KL38	CC	Has AP (apurinic/apyrimidinic) lyase activity and introduces nicks in the DNA strand
N	B1KL38	CC	Has a preference for oxidized purines, such as 7,8-dihydro-8-oxoguanine (8-oxoG)
N	B1KL38	DE	AP lyase mutM;
N	B1KL38	DE	DNA-(apurinic or apyrimidinic site) lyase mutM;
N	B1KL38	DE	Fapy-DNA glycosylase;
N	B1KL38	DE	Formamidopyrimidine-DNA glycosylase;
N	B1KL38	DR	base-excision repair
N	B1KL38	DR	damaged DNA binding
N	B1KL38	DR	nucleotide-excision repair
N	B1KL38	DR	oxidized purine base lesion DNA N-glycosylase activity
N	B1KL38	DR	zinc ion binding
N	B1KM58	CC	Converts heme B (protoheme IX) to heme O by substitution of the vinyl group on carbon 2 of heme B porphyrin ring with a hydroxyethyl farnesyl side group (By similarity).
N	B1KM58	DE	Heme B farnesyltransferase 1;
N	B1KM58	DE	Heme O synthase 1;
N	B1KM58	DE	Protoheme IX farnesyltransferase 1;
N	B1KM58	DR	heme O biosynthetic process
N	B1KM58	DR	protoheme IX farnesyltransferase activity
N	B1KNA0	DE	AICAR transformylase;
N	B1KNA0	DE	Bifunctional purine biosynthesis protein purH;
N	B1KNA0	DE	IMP cyclohydrolase;
N	B1KNA0	DE	IMP synthase;

N	B1KNA0	DE	Inosinicase;
N	B1KNA0	DE	Phosphoribosylaminoimidazolecarboxamide formyltransferase;
N	B1KNA0	DR	IMP biosynthetic process
N	B1KNA0	DR	IMP cyclohydrolase activity
N	B1KNA0	DR	phosphoribosylaminoimidazolecarboxamide formyltransferase activity
N	B1KTF0	CC	Cell wall formation (By similarity).
N	B1KTF0	DE	UDP-N-acetylmuramate--L-alanine ligase;
N	B1KTF0	DE	UDP-N-acetylmuramoyl-L-alanine synthetase;
N	B1KTF0	DR	UDP-N-acetylmuramate-L-alanine ligase activity
N	B1KTF0	DR	cell division
N	B1KTF0	DR	cellular cell wall organization
N	B1KTF0	DR	peptidoglycan biosynthetic process
N	B1KTF0	DR	regulation of cell shape
N	B1KY93	DE	Adenosine aminohydrolase;
N	B1KY93	DE	Adenosine deaminase;
N	B1KY93	DR	adenosine deaminase activity
N	B1KY93	DR	metal ion binding
N	B1KY93	DR	purine ribonucleoside monophosphate biosynthetic process
N	B1KZR4	DE	50S ribosomal protein L27;
N	B1KZR4	DR	structural constituent of ribosome
N	B1L9U3	DE	6-phosphofructokinase;
N	B1L9U3	DE	Phosphofructokinase;
N	B1L9U3	DE	Phosphohexokinase;
N	B1L9U3	DR	6-phosphofructokinase activity
N	B1L9U3	DR	fructose 6-phosphate metabolic process
N	B1L9U3	DR	metal ion binding
N	B1LBH9	DE	Aspartate--tRNA ligase;
N	B1LBH9	DE	Aspartyl-tRNA synthetase;
N	B1LBH9	DR	aspartate-tRNA ligase activity
N	B1LBH9	DR	aspartyl-tRNA aminoacylation
N	B1LBH9	DR	nucleic acid binding
N	B1LHB9	CC	This protein binds to the 23S rRNA, and is important in its secondary structure
N	B1LHB9	CC	It is located near the subunit interface in the base of the L7/L12 stalk, and near the tRNA binding site of the peptidyltransferase center (By similarity).
N	B1LHB9	DE	50S ribosomal protein L6;
N	B1LHB9	DR	structural constituent of ribosome
N	B1LHM1	CC	Thiolesterase that catalyzes the hydrolysis of S-D- lactoyl-glutathione to form glutathione and D-lactic acid (By similarity).
N	B1LHM1	DE	Glyoxalase II;
N	B1LHM1	DE	Hydroxyacylglutathione hydrolase;
N	B1LHM1	DR	hydroxyacylglutathione hydrolase activity
N	B1LHM1	DR	zinc ion binding
N	B1LIY1	CC	Catalyzes the NADPH-dependent reduction of glyoxylate and hydroxypyruvate into glycolate and glycerate, respectively (By similarity).
N	B1LIY1	DE	2-ketoacid reductase;
N	B1LIY1	DE	Glyoxylate/hydroxypyruvate reductase A;
N	B1LIY1	DR	cofactor binding
N	B1LIY1	DR	glyoxylate reductase (NADP) activity
N	B1LIY1	DR	hydroxypyruvate reductase activity
N	B1LIY1	DR	oxidation-reduction process
N	B1LM67	CC	Catalyzes the decarboxylative condensation of pimeloyl- CoA and L-alanine to produce 8-amino-7-oxononanoate (AON), coenzyme A, and carbon dioxide (By similarity).
N	B1LM67	DE	7-KAP synthase;
N	B1LM67	DE	7-keto-8-amino-pelargonic acid synthase;
N	B1LM67	DE	8-amino-7-ketopelargonate synthase;
N	B1LM67	DE	8-amino-7-oxononanoate synthase;
N	B1LM67	DE	KAPA synthase;
N	B1LM67	DE	L-alanine--pimeloyl-CoA ligase;
N	B1LM67	DR	8-amino-7-oxononanoate synthase activity
N	B1LM67	DR	biotin biosynthetic process
N	B1LM67	DR	pyridoxal phosphate binding
N	B1LM67	DR	transferase activity, transferring nitrogenous groups
N	B1LMD0	CC	Catalyzes the methylthiolation of an aspartic acid residue of ribosomal protein S12 (By similarity).
N	B1LMD0	DE	Ribosomal protein S12 methylthiotransferase RimO;

N	B1LMD0	DE	Ribosome maturation factor RimO;
N	B1LMD0	DE	S12 MTTase;
N	B1LMD0	DE	S12 methylthiotransferase;
N	B1LMD0	DR	4 iron, 4 sulfur cluster binding
N	B1LMD0	DR	RNA modification
N	B1LMD0	DR	metal ion binding
N	B1LMD0	DR	peptidyl-L-beta-methylthioaspartic acid biosynthetic process from peptidyl-aspartic acid
N	B1LMD0	DR	transferase activity
N	B1LNH2	CC	Specifically methylates position 2 of adenine 2503 in 23S rRNA (By similarity).
N	B1LNH2	DE	23S rRNA m2A2503 methyltransferase;
N	B1LNH2	DE	Ribosomal RNA large subunit methyltransferase N;
N	B1LNH2	DR	4 iron, 4 sulfur cluster binding
N	B1LNH2	DR	RNA methyltransferase activity
N	B1LNH2	DR	metal ion binding
N	B1LNH2	DR	rRNA processing
N	B1LPK8	CC	Represses a number of genes involved in the response to DNA damage (SOS response), including recA and lexA
N	B1LPK8	CC	Binds to the 16 bp palindromic sequence 5'-CTGTATATATATACAG-3'
N	B1LPK8	CC	In the presence of single-stranded DNA, recA interacts with lexA causing an autocatalytic cleavage which disrupts the DNA-binding part of lexA, leading to derepression of the SOS regulon and eventually DNA repair (By similarity).
N	B1LPK8	DE	LexA repressor;
N	B1LPK8	DR	DNA replication
N	B1LPK8	DR	serine-type endopeptidase activity
N	B1LPS5	CC	Catalyzes the dehydration of D-galactonate to 2-keto-3- deoxy-D-galactonate (By similarity).
N	B1LPS5	DE	D-galactonate dehydratase 1;
N	B1LPS5	DR	D-galactonate catabolic process
N	B1LPS5	DR	cellular amino acid catabolic process
N	B1LPS5	DR	galactonate dehydratase activity
N	B1LPS5	DR	metal ion binding
N	B1LQG4	CC	Prevents misfolding and promotes the refolding and proper assembly of unfolded polypeptides generated under stress conditions (By similarity).
N	B1LQG4	DE	60 kDa chaperonin;
N	B1LQG4	DE	GroEL protein;
N	B1LQG4	DE	Protein Cpn60;
N	B1LQG4	DR	protein refolding
N	B1LWQ5	CC	Catalyzes the reversible transfer of the terminal phosphate group between ATP and AMP
N	B1LWQ5	CC	This small ubiquitous enzyme involved in the energy metabolism and nucleotide synthesis, is essential for maintenance and cell growth (By similarity).
N	B1LWQ5	DE	ATP-AMP transphosphorylase;
N	B1LWQ5	DE	Adenylate kinase;
N	B1LWQ5	DR	adenylate kinase activity
N	B1LWQ5	DR	nucleotide biosynthetic process
N	B1MY11	DE	Leucine--tRNA ligase;
N	B1MY11	DE	Leucyl-tRNA synthetase;
N	B1MY11	DR	leucine-tRNA ligase activity
N	B1MY11	DR	leucyl-tRNA aminoacylation
N	B1N065	CC	One of the primary rRNA binding proteins, it binds directly to 16S rRNA where it helps nucleate assembly of the platform of the 30S subunit by binding and bridging several RNA helices of the 16S rRNA (By similarity).
N	B1N065	DE	30S ribosomal protein S15;
N	B1N065	DR	structural constituent of ribosome
N	B1NKT2	CC	Intermediate capsid protein that self assembles to form an icosahedral capsid with a T=13 symmetry, which consists of 230 trimers of VP6, with channels at each of its five-fold vertices
N	B1NKT2	CC	Nacent transcripts are transcribed within the structural confines of this double-layered particle (DLP) and are extruded through the channels at the five-fold axes
N	B1NKT2	CC	The innermost VP2 capsid and the intermediate VP6 capsid remain intact following cell entry to protect the dsRNA from degradation and to prevent unfavorable antiviral responses in the host cell during all the replication cycle of the virus
N	B1NKT2	CC	This capsid constitutes the middle concentric layer of the viral mature particle
N	B1NKT2	CC	VP6 is required for the transcription activity of the DLP (By similarity).
N	B1NKT2	DE	Intermediate capsid protein VP6;

N	B1NKT2	DR	host cell surface receptor binding
N	B1NKT2	DR	metal ion binding
N	B1NKT2	DR	structural molecule activity
N	B1NKT2	DR	viral envelope fusion with host membrane
N	B1VAE8	CC	One of the primary rRNA binding proteins, it binds directly near the 3'-end of the 23S rRNA, where it nucleates assembly of the 50S subunit (By similarity).
N	B1VAE8	DE	50S ribosomal protein L3;
N	B1VAE8	DR	structural constituent of ribosome
N	B1VEZ9	CC	Catalyzes the conversion of glucosamine-6-phosphate to glucosamine-1-phosphate (By similarity).
N	B1VEZ9	DE	Phosphoglucosamine mutase;
N	B1VEZ9	DR	carbohydrate metabolic process
N	B1VEZ9	DR	magnesium ion binding
N	B1VEZ9	DR	phosphoglucosamine mutase activity
N	B1VIS5	DE	UPF0133 protein cu1912;
N	B1VKJ1	DE	30S ribosomal protein S15, chloroplastic;
N	B1VKJ1	DR	structural constituent of ribosome
N	B1VUW6	CC	May play a key role in the regulation of the intracellular concentration of adenosylhomocysteine (By similarity).
N	B1VUW6	DE	Adenosylhomocysteinase;
N	B1VUW6	DE	S-adenosyl-L-homocysteine hydrolase;
N	B1VUW6	DR	adenosylhomocysteinase activity
N	B1VUW6	DR	one-carbon metabolic process
N	B1WSL8	CC	Catalyzes the condensation of ATP and 5-phosphoribose 1- diphosphate to form N'-(5'-phosphoribosyl)-ATP (PR-ATP)
N	B1WSL8	CC	Has a crucial role in the pathway because the rate of histidine biosynthesis seems to be controlled primarily by regulation of hisG enzymatic activity (By similarity).
N	B1WSL8	DE	ATP phosphoribosyltransferase;
N	B1WSL8	DE	ATP-PRTase;
N	B1WSL8	DR	ATP phosphoribosyltransferase activity
N	B1WSL8	DR	histidine biosynthetic process
N	B1WWG9	CC	Binds to Cpn60 in the presence of Mg-ATP and suppresses the ATPase activity of the latter (By similarity).
N	B1WWG9	DE	10 kDa chaperonin;
N	B1WWG9	DE	GroES protein;
N	B1WWG9	DE	Protein Cpn10;
N	B1WWG9	DR	protein folding
N	B1WWJ3	DE	50S ribosomal protein L34;
N	B1WWJ3	DR	structural constituent of ribosome
N	B1WWL8	DE	50S ribosomal protein L27;
N	B1WWL8	DR	structural constituent of ribosome
N	B1WYG5	CC	Catalyzes the isomerization of sedoheptulose 7-phosphate in D-glycero-D-manno-heptose 7-phosphate (By similarity).
N	B1WYG5	DE	Phosphoheptose isomerase;
N	B1WYG5	DE	Sedoheptulose 7-phosphate isomerase;
N	B1WYG5	DR	D-sedoheptulose 7-phosphate isomerase activity
N	B1WYG5	DR	lipopolysaccharide core region biosynthetic process
N	B1WYG5	DR	metal ion binding
N	B1WYG5	DR	sugar binding
N	B1WZP7	CC	NDH-1 shuttles electrons from an unknown electron donor, via FMN and iron-sulfur (Fe-S) centers, to quinones in the respiratory and/or the photosynthetic chain
N	B1WZP7	CC	Couples the redox reaction to proton translocation, and thus conserves the redox energy in a proton gradient
N	B1WZP7	CC	Cyanobacterial NDH-1 also plays a role in inorganic carbon- concentration (By similarity).
N	B1WZP7	CC	The immediate electron acceptor for the enzyme in this species is believed to be plastoquinone
N	B1WZP7	DE	NAD(P)H dehydrogenase I subunit O;
N	B1WZP7	DE	NAD(P)H-quinone oxidoreductase subunit O;
N	B1WZP7	DE	NDH-1 subunit O;
N	B1WZP7	DR	oxidation-reduction process
N	B1WZP7	DR	oxidoreductase activity, acting on NADH or NADPH, quinone or similar compound as acceptor
N	B1WZP7	DR	quinone binding

N	B1WZT6	CC	DNA-dependent RNA polymerase catalyzes the transcription of DNA into RNA using the four ribonucleoside triphosphates as substrates (By similarity).
N	B1WZT6	DE	DNA-directed RNA polymerase subunit beta';
N	B1WZT6	DE	RNA polymerase subunit beta';
N	B1WZT6	DE	RNAP subunit beta';
N	B1WZT6	DE	Transcriptase subunit beta';
N	B1WZT6	DR	DNA-directed RNA polymerase activity
N	B1X6G6	CC	This protein binds specifically to 23S rRNA; its binding is stimulated by other ribosomal proteins, e.g., L4, L17, and L20
N	B1X6G6	CC	It is important during the early stages of 50S assembly
N	B1X6G6	CC	It makes multiple contacts with different domains of the 23S rRNA in the assembled 50S subunit and ribosome (By similarity).
N	B1X6G6	DE	50S ribosomal protein L22;
N	B1X6G6	DR	structural constituent of ribosome
N	B1X6K0	CC	Transport system that facilitates potassium-efflux, possibly by potassium-proton antiport (By similarity).
N	B1X6K0	DE	Glutathione-regulated potassium-efflux system protein kefB;
N	B1X6K0	DE	K(+)/H(+) antiporter;
N	B1X6K0	DE	NEM-activatable K(+)/H(+) antiporter;
N	B1X6K0	DR	catalytic activity
N	B1X6K0	DR	glutathione-regulated potassium exporter activity
N	B1X6K0	DR	solute:hydrogen antiporter activity
N	B1X7B3	CC	Together with moaA, is involved in the conversion of a guanosine derivative (5'-GTP) into molybdopterin precursor Z (By similarity).
N	B1X7B3	DE	Molybdenum cofactor biosynthesis protein C;
N	B1X7B3	DR	Mo-molybdopterin cofactor biosynthetic process
N	B1XBG0	CC	Overproduction of CaiE stimulates the activity of CaiB and CaiD (By similarity).
N	B1XBG0	DE	Carnitine operon protein CaiE;
N	B1XBG0	DR	transferase activity
N	B1XDV3	CC	Binds to the 23S rRNA (By similarity).
N	B1XDV3	DE	50S ribosomal protein L9;
N	B1XDV3	DR	structural constituent of ribosome
N	B1XFA4	CC	Catalyzes the formation of S-adenosylmethionine from methionine and ATP
N	B1XFA4	CC	The overall synthetic reaction is composed of two sequential steps, AdoMet formation and the subsequent tripolyphosphate hydrolysis which occurs prior to release of AdoMet from the enzyme (By similarity).
N	B1XFA4	DE	AdoMet synthase;
N	B1XFA4	DE	Methionine adenosyltransferase;
N	B1XFA4	DE	S-adenosylmethionine synthase;
N	B1XFA4	DR	metal ion binding
N	B1XFA4	DR	methionine adenosyltransferase activity
N	B1XFA4	DR	one-carbon metabolic process
N	B1XGU5	CC	Component of the tagatose-1,6-bisphosphate aldolase kbaYZ that is required for full activity and stability of the Y subunit
N	B1XGU5	CC	Could have a chaperone-like function for the proper and stable folding of kbaY
N	B1XGU5	CC	When expressed alone, kbaZ does not show any aldolase activity (By similarity).
N	B1XGU5	DE	D-tagatose-1,6-bisphosphate aldolase subunit kbaZ;
N	B1XGU5	DR	catalytic activity
N	B1XGU5	DR	galactitol metabolic process
N	B1XHC9	CC	The ruvA-ruvB complex in the presence of ATP renatures cruciform structure in supercoiled DNA with palindromic sequence, indicating that it may promote strand exchange reactions in homologous recombination
N	B1XHC9	CC	RuvA stimulates, in the presence of DNA, the weak ATPase activity of ruvB (By similarity).
N	B1XHC9	CC	RuvAB is an helicase that mediates the Holliday junction migration by localized denaturation and reannealing
N	B1XHC9	DE	Holliday junction ATP-dependent DNA helicase ruvA;
N	B1XHC9	DR	DNA recombination
N	B1XHC9	DR	four-way junction helicase activity
N	B1XHD8	CC	Catalyzes the conversion of 5-methoxyuridine (mo5U) to uridine-5-oxyacetic acid (cmo5U) at position 34 in tRNA
N	B1XHD8	CC	May also participate in the methylation of uridine-5-oxyacetic acid (cmo5U) to uridine-5-oxyacetic acid methyl ester (mcmo5U) (By similarity).
N	B1XHD8	DE	tRNA (cmo5U34)-methyltransferase;
N	B1XHD8	DR	tRNA (uracil) methyltransferase activity

N	B1XHD8	DR	tRNA wobble uridine modification
N	B1XJ13	CC	Responsible for the release of ribosomes from messenger RNA at the termination of protein biosynthesis
N	B1XJ13	CC	May increase the efficiency of translation by recycling ribosomes from one round of translation to another (By similarity).
N	B1XJ13	DE	Ribosome-recycling factor;
N	B1XJ13	DE	Ribosome-releasing factor;
N	B1XQ70	CC	Thiolesterase that catalyzes the hydrolysis of S-D- lactoyl-glutathione to form glutathione and D-lactic acid (By similarity).
N	B1XQ70	DE	Glyoxalase II;
N	B1XQ70	DE	Hydroxyacylglutathione hydrolase;
N	B1XQ70	DR	hydroxyacylglutathione hydrolase activity
N	B1XQ70	DR	zinc ion binding
N	B1XT83	CC	Could be a nuclease that resolves Holliday junction intermediates in genetic recombination (By similarity).
N	B1XT83	DE	Putative Holliday junction resolvase;
N	B1XT83	DR	DNA recombination
N	B1XT83	DR	nuclease activity
N	B1XT83	DR	nucleic acid binding
N	B1XU85	CC	Plays an important role in the de novo pathway of purine nucleotide biosynthesis
N	B1XU85	CC	Catalyzes the first committed step in the biosynthesis of AMP from IMP (By similarity)
N	B1XU85	DE	Adenylosuccinate synthetase;
N	B1XU85	DE	IMP--aspartate ligase;
N	B1XU85	DR	adenylosuccinate synthase activity
N	B1XU85	DR	magnesium ion binding
N	B1XU85	DR	purine nucleotide biosynthetic process
N	B1XUJ8	CC	NDH-1 shuttles electrons from NADH, via FMN and iron- sulfur (Fe-S) centers, to quinones in the respiratory chain
N	B1XUJ8	CC	Couples the redox reaction to proton translocation (for every two electrons transferred, four hydrogen ions are translocated across the cytoplasmic membrane), and thus conserves the redox energy in a proton gradient (By similarity)
N	B1XUJ8	CC	The immediate electron acceptor for the enzyme in this species is believed to be ubiquinone
N	B1XUJ8	DE	NADH dehydrogenase I subunit A;
N	B1XUJ8	DE	NADH-quinone oxidoreductase subunit A;
N	B1XUJ8	DE	NDH-1 subunit A;
N	B1XUJ8	DR	NADH dehydrogenase (ubiquinone) activity
N	B1XUJ8	DR	oxidation-reduction process
N	B1XUJ8	DR	quinone binding
N	B1XUJ9	CC	NDH-1 shuttles electrons from NADH, via FMN and iron- sulfur (Fe-S) centers, to quinones in the respiratory chain
N	B1XUJ9	CC	Couples the redox reaction to proton translocation (for every two electrons transferred, four hydrogen ions are translocated across the cytoplasmic membrane), and thus conserves the redox energy in a proton gradient (By similarity)
N	B1XUJ9	DE	NADH dehydrogenase I subunit B;
N	B1XUJ9	DE	NADH-quinone oxidoreductase subunit B;
N	B1XUJ9	DE	NDH-1 subunit B;
N	B1XUJ9	DR	4 iron, 4 sulfur cluster binding
N	B1XUJ9	DR	NADH dehydrogenase (ubiquinone) activity
N	B1XUJ9	DR	metal ion binding
N	B1XUJ9	DR	oxidation-reduction process
N	B1XUJ9	DR	quinone binding
N	B1XW28	CC	This enzyme is involved in nucleotide metabolism: it produces dUMP, the immediate precursor of thymidine nucleotides and it decreases the intracellular concentration of dUTP so that uracil cannot be incorporated into DNA (By similarity)
N	B1XW28	DE	Deoxyuridine 5'-triphosphate nucleotidohydrolase;
N	B1XW28	DE	dUTP pyrophosphatase;
N	B1XW28	DR	dUTP diphosphatase activity
N	B1XW28	DR	dUTP metabolic process
N	B1XW28	DR	metal ion binding
N	B1XX97	DE	Phosphoribosyl-AMP cyclohydrolase;
N	B1XX97	DR	histidine biosynthetic process
N	B1XX97	DR	phosphoribosyl-AMP cyclohydrolase activity
N	B1Y0H7	CC	Specifically methylates guanosine-37 in various tRNAs (By similarity).
N	B1Y0H7	DE	M1G-methyltransferase;

N	B1Y0H7	DE	tRNA (guanine-N(1)-)-methyltransferase;
N	B1Y0H7	DE	tRNA [GM37] methyltransferase;
N	B1Y0H7	DR	tRNA (guanine-N1-)-methyltransferase activity
N	B1YD57	DE	Shikimate dehydrogenase;
N	B1YD57	DR	aromatic amino acid family biosynthetic process
N	B1YD57	DR	oxidation-reduction process
N	B1YD57	DR	shikimate 5-dehydrogenase activity
N	B1YIY0	CC	Catalyzes the dehydration of methylthioribulose-1- phosphate (MTRu-1-P) into 2,3-diketo-5-methylthiopentyl-1- phosphate (DK-MTP-1-P) (By similarity).
N	B1YIY0	DE	MTRu-1-P dehydratase;
N	B1YIY0	DE	Methylthioribulose-1-phosphate dehydratase;
N	B1YIY0	DR	L-methionine salvage
N	B1YIY0	DR	metal ion binding
N	B1YIY0	DR	methylthioribulose 1-phosphate dehydratase activity
N	B1YJ30	CC	Catalyzes the reversible isomerization-deamination of glucosamine 6-phosphate (GlcN6P) to form fructose 6-phosphate (Fru6P) and ammonium ion (By similarity).
N	B1YJ30	DE	GlcN6P deaminase;
N	B1YJ30	DE	Glucosamine-6-phosphate deaminase;
N	B1YJ30	DE	Glucosamine-6-phosphate isomerase;
N	B1YJ30	DR	N-acetylglucosamine metabolic process
N	B1YJ30	DR	glucosamine-6-phosphate deaminase activity
N	B1YJ30	DR	hydrolase activity
N	B1YKE7	DE	UPF0316 protein Exig_2248;
N	B1YLQ2	CC	Bidirectionally degrades single-stranded DNA into large acid-insoluble oligonucleotides, which are then degraded further into small acid-soluble oligonucleotides (By similarity).
N	B1YLQ2	DE	Exodeoxyribonuclease 7 large subunit;
N	B1YLQ2	DE	Exodeoxyribonuclease VII large subunit;
N	B1YLQ2	DE	Exonuclease VII large subunit;
N	B1YLQ2	DR	DNA catabolic process
N	B1YLQ2	DR	exodeoxyribonuclease VII activity
N	B1YLQ2	DR	nucleic acid binding
N	B1YRR0	CC	Necessary for normal cell division and for the maintenance of normal septation (By similarity).
N	B1YRR0	DE	Probable GTP-binding protein EngB;
N	B1YRR0	DR	barrier septum formation
N	B1YRV7	DE	Imidazoleglycerol-phosphate dehydratase;
N	B1YRV7	DR	histidine biosynthetic process
N	B1YRV7	DR	imidazoleglycerol-phosphate dehydratase activity
N	B1Z778	CC	This protein binds to the 23S rRNA, and is important in its secondary structure
N	B1Z778	CC	It is located near the subunit interface in the base of the L7/L12 stalk, and near the tRNA binding site of the peptidyltransferase center (By similarity).
N	B1Z778	DE	50S ribosomal protein L6;
N	B1Z778	DR	structural constituent of ribosome
N	B1ZFJ0	CC	Catalyzes the NADPH dependent reduction of L-gamma- glutamyl 5-phosphate into L-glutamate 5-semialdehyde and phosphate
N	B1ZFJ0	CC	The product spontaneously undergoes cyclization to form 1-pyrroline-5-carboxylate (By similarity).
N	B1ZFJ0	DE	GSA dehydrogenase;
N	B1ZFJ0	DE	Gamma-glutamyl phosphate reductase;
N	B1ZFJ0	DE	Glutamate-5-semialdehyde dehydrogenase;
N	B1ZFJ0	DE	Glutamyl-gamma-semialdehyde dehydrogenase;
N	B1ZFJ0	DR	glutamate-5-semialdehyde dehydrogenase activity
N	B1ZFJ0	DR	oxidation-reduction process
N	B1ZFJ0	DR	proline biosynthetic process
N	B1ZJM9	CC	Negatively regulates transcription of bacterial ribonucleotide reductase nrd genes and operons by binding to NrdR- boxes (By similarity).
N	B1ZJM9	DE	Transcriptional repressor NrdR;
N	B1ZJM9	DR	transcription repressor activity
N	B1ZJM9	DR	zinc ion binding
N	B2A474	CC	RNaseP catalyzes the removal of the 5'-leader sequence from pre-tRNA to produce the mature 5'-terminus
N	B2A474	CC	It can also cleave other RNA substrates such as 4.5S RNA
N	B2A474	CC	The protein component plays an auxiliary but essential role in vivo by binding to the 5'-leader sequence and broadening the substrate specificity of the ribozyme (By similarity).
N	B2A474	DE	RNase P protein;

N	B2A474	DE	RNaseP protein;
N	B2A474	DE	Ribonuclease P protein component;
N	B2A474	DR	ribonuclease P activity
N	B2A474	DR	tRNA processing
N	B2FQJ3	CC	One of the primary rRNA binding proteins, it binds specifically to the 5'-end of 16S ribosomal RNA (By similarity).
N	B2FQJ3	DE	30S ribosomal protein S17;
N	B2FQJ3	DR	structural constituent of ribosome
N	B2FQK6	CC	Located on the platform of the 30S subunit, it bridges several disparate RNA helices of the 16S rRNA
N	B2FQK6	CC	Forms part of the Shine-Dalgarno cleft in the 70S ribosome (By similarity).
N	B2FQK6	DE	30S ribosomal protein S11;
N	B2FQK6	DR	structural constituent of ribosome
N	B2FUM0	CC	Together with moaC, is involved in the conversion of a guanosine derivative (5'-GTP) into molybdopterin precursor Z (By similarity).
N	B2FUM0	DE	Molybdenum cofactor biosynthesis protein A;
N	B2FUM0	DR	4 iron, 4 sulfur cluster binding
N	B2FUM0	DR	Mo-molybdopterin cofactor biosynthetic process
N	B2FUM0	DR	catalytic activity
N	B2FUM0	DR	metal ion binding
N	B2G6U2	CC	Associates with the EF-Tu.GDP complex and induces the exchange of GDP to GTP
N	B2G6U2	CC	It remains bound to the aminoacyl-tRNA.EF- Tu.GTP complex up to the GTP hydrolysis stage on the ribosome (By similarity).
N	B2G6U2	DE	Elongation factor Ts;
N	B2G6U2	DR	translation elongation factor activity
N	B2GAH9	CC	Binds to Cpn60 in the presence of Mg-ATP and suppresses the ATPase activity of the latter (By similarity).
N	B2GAH9	DE	10 kDa chaperonin;
N	B2GAH9	DE	GroES protein;
N	B2GAH9	DE	Protein Cpn10;
N	B2GAH9	DR	protein folding
N	B2GAI0	CC	Prevents misfolding and promotes the refolding and proper assembly of unfolded polypeptides generated under stress conditions (By similarity).
N	B2GAI0	DE	60 kDa chaperonin;
N	B2GAI0	DE	GroEL protein;
N	B2GAI0	DE	Protein Cpn60;
N	B2GAI0	DR	protein refolding
N	B2GB29	CC	Endonuclease that specifically degrades the RNA of RNA- DNA hybrids (By similarity).
N	B2GB29	DE	RNase HIII;
N	B2GB29	DE	Ribonuclease HIII;
N	B2GB29	DR	RNA metabolic process
N	B2GB29	DR	metal ion binding
N	B2GB29	DR	ribonuclease H activity
N	B2GBB8	CC	One of the primary rRNA binding proteins, it binds directly to 16S rRNA where it helps nucleate assembly of the platform of the 30S subunit by binding and bridging several RNA helices of the 16S rRNA (By similarity).
N	B2GBB8	DE	30S ribosomal protein S15;
N	B2GBB8	DR	structural constituent of ribosome
N	B2GBD0	CC	An essential GTPase which binds GTP, GDP and possibly (p)ppGpp with moderate affinity, with high nucleotide exchange rates and a fairly low GTP hydrolysis rate (By similarity)
N	B2GBD0	CC	It may play a role in control of the cell cycle, stress response, ribosome biogenesis and in those bacteria that undergo differentiation, in morphogenesis control (Potential).
N	B2GBD0	DE	GTP-binding protein obg;
N	B2GBD0	DR	GTPase activity
N	B2GBD0	DR	magnesium ion binding
N	B2GHT7	CC	Catalyzes the condensation of the acetyl group of acetyl-CoA with 3-methyl-2-oxobutanoate (2-oxoisovalerate) to form 3-carboxy-3-hydroxy-4-methylpentanoate (2-isopropylmalate) (By similarity).
N	B2GHT7	DE	2-isopropylmalate synthase;
N	B2GHT7	DE	Alpha-IPM synthase;
N	B2GHT7	DE	Alpha-isopropylmalate synthase;
N	B2GHT7	DR	2-isopropylmalate synthase activity
N	B2GHT7	DR	leucine biosynthetic process

N	B2GIN9	CC	Catalyzes the covalent attachment of the prokaryotic ubiquitin-like protein modifier Pup to the proteasomal substrate proteins, thereby targeting them for proteasomal degradation
N	B2GIN9	CC	The ligation reaction involves the side-chain carboxylate of the C-terminal glutamate of Pup and the side-chain amino group of a substrate lysine (By similarity)
N	B2GIN9	CC	This tagging system is termed pupylation
N	B2GIN9	DE	Proteasome accessory factor A;
N	B2GIN9	DE	Pup--protein ligase;
N	B2GIN9	DE	Pup-conjugating enzyme;
N	B2GIN9	DR	acid-amino acid ligase activity
N	B2GIN9	DR	modification-dependent protein catabolic process
N	B2GIN9	DR	proteasomal protein catabolic process
N	B2GM13	CC	Catalyzes the reversible conversion of 2- phosphoglycerate into phosphoenolpyruvate
N	B2GM13	CC	It is essential for the degradation of carbohydrates via glycolysis (By similarity).
N	B2GM13	DE	2-phospho-D-glycerate hydro-lyase;
N	B2GM13	DE	2-phosphoglycerate dehydratase;
N	B2GM13	DR	magnesium ion binding
N	B2GM13	DR	phosphopyruvate hydratase activity
N	B2HCU4	CC	Exhibits S-adenosyl-L-methionine-dependent methyltransferase activity (By similarity)
N	B2HCU4	DE	Putative S-adenosyl-L-methionine-dependent methyltransferase MMAR_1058;
N	B2HCU4	DR	methyltransferase activity
N	B2HDJ9	CC	Catalyzes the phosphorylation of the position 2 hydroxy group of 4-diphosphocytidyl-2C-methyl-D-erythritol (By similarity).
N	B2HDJ9	DE	4-(cytidine-5'-diphospho)-2-C-methyl-D-erythritol kinase;
N	B2HDJ9	DE	4-diphosphocytidyl-2-C-methyl-D-erythritol kinase;
N	B2HDJ9	DR	4-(cytidine 5'-diphospho)-2-C-methyl-D-erythritol kinase activity
N	B2HDJ9	DR	terpenoid biosynthetic process
N	B2HGR0	CC	Cell division protein that is part of the divisome complex and is recruited early to the Z-ring
N	B2HGR0	CC	Its function overlaps with ftsA (By similarity).
N	B2HGR0	CC	Probably stimulates Z-ring formation, perhaps through the cross-linking of ftsZ protofilaments
N	B2HGR0	DE	Cell division protein sepF;
N	B2HGR0	DR	barrier septum formation
N	B2HMC9	CC	Binds directly to 16S ribosomal RNA (By similarity).
N	B2HMC9	DE	30S ribosomal protein S20;
N	B2HMC9	DR	structural constituent of ribosome
N	B2HTV8	CC	Oxygenase that introduces the hydroxyl group at carbon five of 2-nonaprenyl-3-methyl-6-methoxy-1,4-benzoquinol resulting in the formation of 2-nonaprenyl-3-methyl-5-hydroxy-6-methoxy-1,4- benzoquinol (By similarity).
N	B2HTV8	DE	2-nonaprenyl-3-methyl-6-methoxy-1,4-benzoquinol hydroxylase;
N	B2HTV8	DE	5-demethoxyubiquinone hydroxylase;
N	B2HTV8	DE	DMQ hydroxylase;
N	B2HTV8	DR	monooxygenase activity
N	B2HTV8	DR	oxidation-reduction process
N	B2HTV8	DR	protein metabolic process
N	B2HTV8	DR	transition metal ion binding
N	B2HTV8	DR	ubiquinone biosynthetic process
N	B2HX50	CC	This protein is involved in the repair of mismatches in DNA
N	B2HX50	CC	It is possible that it carries out the mismatch recognition step
N	B2HX50	CC	This protein has a weak ATPase activity (By similarity).
N	B2HX50	DE	DNA mismatch repair protein mutS;
N	B2HX50	DR	mismatch repair
N	B2HX50	DR	mismatched DNA binding
N	B2HX89	CC	Catalyzes the oxidation of erythronate-4-phosphate to 3- hydroxy-2-oxo-4-phosphonooxybutanoate (By similarity).
N	B2HX89	DE	Erythronate-4-phosphate dehydrogenase;
N	B2HX89	DR	4-phosphoerythronate dehydrogenase activity
N	B2HX89	DR	oxidation-reduction process
N	B2HX89	DR	pyridoxine biosynthetic process
N	B2HZB6	DE	Phosphoglycerate kinase;
N	B2HZB6	DR	phosphoglycerate kinase activity
N	B2I3A5	CC	Transfers the N-acyl diglyceride group on what will become the N-terminal cysteine of membrane lipoproteins (By similarity).
N	B2I3A5	DE	Prolipoprotein diacylglyceryl transferase;
N	B2I3A5	DR	lipoprotein biosynthetic process

N	B2I3A5	DR	protein lipoylation
N	B2I3A5	DR	transferase activity, transferring glycosyl groups
N	B2I658	CC	Channel that opens in response to stretch forces in the membrane lipid bilayer
N	B2I658	CC	May participate in the regulation of osmotic pressure changes within the cell (By similarity).
N	B2I658	DE	Large-conductance mechanosensitive channel;
N	B2I658	DR	ion channel activity
N	B2I6G9	CC	Cleaves GlcNAc linked beta-1,4 to MurNAc tripeptides (By similarity).
N	B2I6G9	DE	Beta-N-acetylhexosaminidase;
N	B2I6G9	DE	Beta-hexosaminidase;
N	B2I6G9	DE	N-acetyl-beta-glucosaminidase;
N	B2I6G9	DR	beta-N-acetylhexosaminidase activity
N	B2I6G9	DR	cell division
N	B2I6G9	DR	cellular cell wall organization
N	B2I6G9	DR	peptidoglycan biosynthetic process
N	B2I6G9	DR	regulation of cell shape
N	B2IG63	CC	The natural substrate for this enzyme may be peptidyl- tRNAs which drop off the ribosome during protein synthesis (By similarity).
N	B2IG63	DE	Peptidyl-tRNA hydrolase;
N	B2IG63	DR	aminoacyl-tRNA hydrolase activity
N	B2INJ3	CC	Involved in the biosynthesis of D-alanyl-lipoteichoic acid (LTA)
N	B2INJ3	CC	Activated D-alanyl-Dcp donates its D-alanyl substituent to membrane-associated LTA (By similarity).
N	B2INJ3	DE	D-alanine--poly(phosphoribitol) ligase subunit 2;
N	B2INJ3	DE	D-alanyl carrier protein;
N	B2INJ3	DR	D-alanine-poly(phosphoribitol) ligase activity
N	B2INJ3	DR	acyl carrier activity
N	B2INJ3	DR	cellular cell wall organization
N	B2INJ3	DR	cofactor binding
N	B2INJ3	DR	regulation of cell shape
N	B2INJ3	DR	teichoic acid biosynthetic process
N	B2IR58	CC	Binds as a heterodimer with protein S6 to the central domain of the 16S rRNA, where it helps stabilize the platform of the 30S subunit (By similarity).
N	B2IR58	DE	30S ribosomal protein S18;
N	B2IR58	DR	structural constituent of ribosome
N	B2IWE2	CC	Required for the first step of histidine biosynthesis
N	B2IWE2	CC	May allow the feedback regulation of ATP phosphoribosyltransferase activity by histidine (By similarity).
N	B2IWE2	DE	ATP phosphoribosyltransferase regulatory subunit;
N	B2IWE2	DR	histidine biosynthetic process
N	B2IWE2	DR	histidine-tRNA ligase activity
N	B2IWE2	DR	histidyl-tRNA aminoacylation
N	B2IX54	DE	AICAR transformylase;
N	B2IX54	DE	Bifunctional purine biosynthesis protein purH;
N	B2IX54	DE	IMP cyclohydrolase;
N	B2IX54	DE	IMP synthase;
N	B2IX54	DE	Inosinicase;
N	B2IX54	DE	Phosphoribosylaminoimidazolecarboxamide formyltransferase;
N	B2IX54	DR	IMP biosynthetic process
N	B2IX54	DR	IMP cyclohydrolase activity
N	B2IX54	DR	phosphoribosylaminoimidazolecarboxamide formyltransferase activity
N	B2J5E4	CC	Catalyzes the NADPH-dependent reduction of glutamyl- tRNA(Glu) to glutamate 1-semialdehyde (GSA) (By similarity).
N	B2J5E4	DE	Glutamyl-tRNA reductase;
N	B2J5E4	DR	chlorophyll biosynthetic process
N	B2J5E4	DR	glutamyl-tRNA reductase activity
N	B2J5E4	DR	oxidation-reduction process
N	B2J5E4	DR	shikimate 5-dehydrogenase activity
N	B2J6T8	CC	Binds together with S18 to 16S ribosomal RNA (By similarity).
N	B2J6T8	DE	30S ribosomal protein S6;
N	B2J6T8	DR	structural constituent of ribosome
N	B2JF36	CC	May play a role in 30S ribosomal subunit biogenesis
N	B2JF36	CC	Unusual circulary permuted GTPase that catalyzes rapid hydrolysis of GTP with a slow catalytic turnover (By similarity).
N	B2JF36	DE	Putative ribosome biogenesis GTPase RsgA;

N	B2JF36	DR	GTPase activity
N	B2JF36	DR	metal ion binding
N	B2JI57	CC	One of the primary rRNA binding proteins, it binds specifically to the 5'-end of 16S ribosomal RNA (By similarity).
N	B2JI57	DE	30S ribosomal protein S17;
N	B2JI57	DR	structural constituent of ribosome
N	B2JNF0	DE	PKHD-type hydroxylase Bphy_5374;
N	B2JNF0	DR	L-ascorbic acid binding
N	B2JNF0	DR	iron ion binding
N	B2JNF0	DR	oxidation-reduction process
N	B2JNF0	DR	oxidoreductase activity, acting on paired donors, with incorporation or reduction of molecular oxygen
N	B2JNF0	DR	oxidoreductase activity, acting on single donors with incorporation of molecular oxygen, incorporation of two atoms of oxygen
N	B2JZM8	DE	Histidinol-phosphate aminotransferase;
N	B2JZM8	DE	Imidazole acetol-phosphate transaminase;
N	B2JZM8	DR	histidine biosynthetic process
N	B2JZM8	DR	histidinol-phosphate transaminase activity
N	B2JZM8	DR	pyridoxal phosphate binding
N	B2K0Q4	CC	The glycine cleavage system catalyzes the degradation of glycine
N	B2K0Q4	CC	The H protein shuttles the methylamine group of glycine from the P protein to the T protein (By similarity).
N	B2K0Q4	DE	Glycine cleavage system H protein;
N	B2K0Q4	DR	glycine decarboxylation via glycine cleavage system
N	B2K111	CC	Seems to be the binding site for several of the factors involved in protein synthesis and appears to be essential for accurate translation (By similarity).
N	B2K111	DE	50S ribosomal protein L7/L12;
N	B2K111	DR	structural constituent of ribosome
N	B2K1Z5	DE	Phosphatidylserine decarboxylase alpha chain;
N	B2K1Z5	DE	Phosphatidylserine decarboxylase beta chain;
N	B2K1Z5	DE	Phosphatidylserine decarboxylase proenzyme;
N	B2K1Z5	DR	phosphatidylserine decarboxylase activity
N	B2K1Z5	DR	phospholipid biosynthetic process
N	B2K3F6	CC	Involved in the degradation of phospho-AI-2, thereby terminating induction of the lsr operon and closing the AI-2 signaling cycle
N	B2K3F6	CC	Catalyzes the cleavage of phosphorylated 4,5- dihydroxy-2,3-pentanedione (P-DPD) to 2-phosphoglycolic acid (PG) and another three-carbon compound (By similarity).
N	B2K3F6	DE	AI-2-degrading protein lsrG;
N	B2K3F6	DE	Autoinducer 2-degrading protein lsrG;
N	B2K3F6	DR	antibiotic biosynthetic process
N	B2K3F6	DR	oxidoreductase activity
N	B2K5L5	CC	Catalyzes the conversion of UDP-4-keto-arabinose (UDP- Ara4O) to UDP-4-amino-4-deoxy-L-arabinose (UDP-L-Ara4N)
N	B2K5L5	CC	The modified arabinose is attached to lipid A and is required for resistance to polymyxin and cationic antimicrobial peptides (By similarity).
N	B2K5L5	DE	UDP-(beta-L-threo-pentapyranosyl-4"-ulose diphosphate) aminotransferase;
N	B2K5L5	DE	UDP-4-amino-4-deoxy-L-arabinose aminotransferase;
N	B2K5L5	DE	UDP-4-amino-4-deoxy-L-arabinose--oxoglutarate aminotransferase;
N	B2K5L5	DE	UDP-Ara4O aminotransferase;
N	B2K5L5	DR	lipid A biosynthetic process
N	B2K5L5	DR	pyridoxal phosphate binding
N	B2K5L5	DR	response to antibiotic
N	B2K5L5	DR	transaminase activity
N	B2K6Q3	CC	Catalyzes the transfer of a phosphate group to glutamate to form glutamate 5-phosphate which rapidly cyclizes to 5- oxoproline (By similarity).
N	B2K6Q3	DE	Gamma-glutamyl kinase;
N	B2K6Q3	DE	Glutamate 5-kinase;
N	B2K6Q3	DR	glutamate 5-kinase activity
N	B2K6Q3	DR	proline biosynthetic process
N	B2K6T9	CC	Bidirectionally degrades single-stranded DNA into large acid-insoluble oligonucleotides, which are then degraded further into small acid-soluble oligonucleotides (By similarity).
N	B2K6T9	DE	Exodeoxyribonuclease 7 small subunit;
N	B2K6T9	DE	Exodeoxyribonuclease VII small subunit;
N	B2K6T9	DE	Exonuclease VII small subunit;
N	B2K6T9	DR	DNA catabolic process

N	B2K6T9	DR	exodeoxyribonuclease VII activity
N	B2K6V6	CC	Involved in protein export
N	B2K6V6	CC	Acts as a chaperone by maintaining the newly synthesized protein in an open conformation (By similarity).
N	B2K6V6	DE	Trigger factor;
N	B2K6V6	DR	cell division
N	B2K6V6	DR	peptidyl-prolyl cis-trans isomerase activity
N	B2K6V6	DR	protein folding
N	B2K6V6	DR	protein transport
N	B2K711	CC	Catalyzes the 2-thiolation of uridine at the wobble position (U34) of tRNA(Lys), tRNA(Glu) and tRNA(Gln), leading to the formation of s(2)U34, the first step of tRNA-mnm(5)s(2)U34 synthesis
N	B2K711	CC	Binds ATP and its substrate tRNAs (By similarity).
N	B2K711	CC	Sulfur is provided by iscS, via a sulfur-relay system
N	B2K711	DE	tRNA-specific 2-thiouridylase mnmA;
N	B2K711	DR	tRNA processing
N	B2K711	DR	transferase activity
N	B2K7Z2	CC	Catalyzes a proton abstraction reaction that results in 2,5-elimination of pyruvate from 2-succinyl-5-enolpyruvyl-6- hydroxy-3-cyclohexene-1-carboxylate (SEPHCHC) and the formation of 2-succinyl-6-hydroxy-2,4-cyclohexadiene-1-carboxylate (SHCHC)
N	B2K7Z2	CC	Is also able to catalyze the hydrolysis of the thioester bond in palmitoyl-CoA in vitro (By similarity).
N	B2K7Z2	DE	2-succinyl-6-hydroxy-2,4-cyclohexadiene-1-carboxylate synthase;
N	B2K7Z2	DE	SHCHC synthase;
N	B2K7Z2	DR	2-succinyl-6-hydroxy-2,4-cyclohexadiene-1-carboxylate synthase activity
N	B2K7Z2	DR	menaquinone biosynthetic process
N	B2KB85	CC	Binds directly to 23S ribosomal RNA and is necessary for the in vitro assembly process of the 50S ribosomal subunit
N	B2KB85	CC	It is not involved in the protein synthesizing functions of that subunit (By similarity)
N	B2KB85	DE	50S ribosomal protein L20;
N	B2KB85	DR	structural constituent of ribosome
N	B2LMK2	CC	Component of the acetyl coenzyme A carboxylase (ACC) complex
N	B2LMK2	CC	Biotin carboxylase (BC) catalyzes the carboxylation of biotin on its carrier protein (BCCP) and then the CO(2) group is transferred by the transcarboxylase to acetyl-CoA to form malonyl- CoA (By similarity).
N	B2LMK2	DE	ACCase subunit beta;
N	B2LMK2	DE	Acetyl-CoA carboxylase carboxyltransferase subunit beta;
N	B2LMK2	DE	Acetyl-coenzyme A carboxylase carboxyl transferase subunit beta, chloroplastic;
N	B2LMK2	DR	acetyl-CoA carboxylase activity
N	B2LMK2	DR	fatty acid biosynthetic process
N	B2LMK2	DR	metal ion binding
N	B2RH62	DE	50S ribosomal protein L32;
N	B2RH62	DR	structural constituent of ribosome
N	B2RIK3	CC	Transfers the gamma-phosphate of ATP to the 4'-position of a tetraacyldisaccharide 1-phosphate intermediate (termed DS-1- P) to form tetraacyldisaccharide 1,4'-bis-phosphate (lipid IVA) (By similarity).
N	B2RIK3	DE	Lipid A 4'-kinase;
N	B2RIK3	DE	Tetraacyldisaccharide 4'-kinase;
N	B2RIK3	DR	lipid A biosynthetic process
N	B2RIK3	DR	tetraacyldisaccharide 4'-kinase activity
N	B2RIU1	CC	Endonuclease that specifically degrades the RNA of RNA- DNA hybrids (By similarity).
N	B2RIU1	DE	Ribonuclease HII;
N	B2RIU1	DR	metal ion binding
N	B2RIU1	DR	ribonuclease H activity
N	B2RLM4	CC	Activates KDO (a required 8-carbon sugar) for incorporation into bacterial lipopolysaccharide in Gram-negative bacteria (By similarity).
N	B2RLM4	DE	3-deoxy-manno-octulosonate cytidylyltransferase;
N	B2RLM4	DE	CMP-2-keto-3-deoxyoctulosonic acid synthase;
N	B2RLM4	DE	CMP-KDO synthase;
N	B2RLM4	DR	3-deoxy-manno-octulosonate cytidylyltransferase activity
N	B2RLM4	DR	lipopolysaccharide biosynthetic process
N	B2RN74	CC	Odorant receptor (Potential).
N	B2RN74	DE	Olfactory receptor 11H12;
N	B2RN74	DR	olfactory receptor activity

N	B2RN74	DR	sensory perception of smell
N	B2S1E6	CC	Associates with free 30S ribosomal subunits (but not with 30S subunits that are part of 70S ribosomes or polysomes)
N	B2S1E6	CC	Essential for efficient processing of 16S rRNA
N	B2S1E6	CC	May interact with the 5'-terminal helix region of 16S rRNA (By similarity).
N	B2S1E6	DE	Ribosome-binding factor A;
N	B2S1E6	DR	rRNA processing
N	B2S2Y0	DE	Protein MraZ;
N	B2S673	CC	Binds the lower part of the 30S subunit head
N	B2S673	CC	Binds mRNA in the 70S ribosome, positioning it for translation (By similarity).
N	B2S673	DE	30S ribosomal protein S3;
N	B2S673	DR	structural constituent of ribosome
N	B2SBF0	CC	Catalyzes the conversion of dethiobiotin (DTB) to biotin by the insertion of a sulfur atom into dethiobiotin via a radical- based mechanism (By similarity).
N	B2SBF0	DE	Biotin synthase;
N	B2SBF0	DR	2 iron, 2 sulfur cluster binding
N	B2SBF0	DR	4 iron, 4 sulfur cluster binding
N	B2SBF0	DR	biotin biosynthetic process
N	B2SBF0	DR	biotin synthase activity
N	B2SBF0	DR	metal ion binding
N	B2SCP3	CC	Catalyzes the ferrous insertion into protoporphyrin IX (By similarity).
N	B2SCP3	DE	Ferrochelatase;
N	B2SCP3	DE	Heme synthase;
N	B2SCP3	DE	Protoheme ferro-lyase;
N	B2SCP3	DR	ferrochelatase activity
N	B2SCP3	DR	heme biosynthetic process
N	B2SCP3	DR	metal ion binding
N	B2SFX1	CC	Involved in saturated fatty acids biosynthesis (By similarity).
N	B2SFX1	DE	(3R)-hydroxymyristoyl ACP dehydrase;
N	B2SFX1	DE	(3R)-hydroxymyristoyl-[acyl-carrier-protein] dehydratase;
N	B2SFX1	DR	fatty acid biosynthetic process
N	B2SFX1	DR	hydro-lyase activity
N	B2SFX1	DR	lipid A biosynthetic process
N	B2SKA9	CC	One of the proteins required for the normal export of preproteins out of the cell cytoplasm
N	B2SKA9	CC	It also specifically binds to its receptor secA (By similarity).
N	B2SKA9	CC	It is a molecular chaperone that binds to a subset of precursor proteins, maintaining them in a translocation-competent state
N	B2SKA9	DE	Protein-export protein secB;
N	B2SKA9	DR	protein tetramerization
N	B2SKA9	DR	protein transport
N	B2SKA9	DR	transmembrane transport
N	B2SKA9	DR	unfolded protein binding
N	B2SQT3	CC	One of the primary rRNA binding proteins, it binds directly to 16S rRNA where it nucleates assembly of the body of the 30S subunit (By similarity).
N	B2SQT3	DE	30S ribosomal protein S4;
N	B2SQT3	DR	structural constituent of ribosome
N	B2SQZ9	CC	Associates with the EF-Tu.GDP complex and induces the exchange of GDP to GTP
N	B2SQZ9	CC	It remains bound to the aminoacyl-tRNA.EF- Tu.GTP complex up to the GTP hydrolysis stage on the ribosome (By similarity).
N	B2SQZ9	DE	Elongation factor Ts;
N	B2SQZ9	DR	translation elongation factor activity
N	B2T755	CC	One of the primary rRNA binding proteins, it binds directly to 16S rRNA where it nucleates assembly of the head domain of the 30S subunit
N	B2T755	CC	Is located at the subunit interface close to the decoding center, probably blocks exit of the E-site tRNA (By similarity).
N	B2T755	DE	30S ribosomal protein S7;
N	B2T755	DR	structural constituent of ribosome
N	B2TCK0	CC	Repressor involved in choline regulation of the bet genes (By similarity).
N	B2TCK0	DE	HTH-type transcriptional regulator BetI;
N	B2TCK0	DR	regulation of transcription, DNA-dependent
N	B2TCK0	DR	sequence-specific DNA binding transcription factor activity
N	B2TCK0	DR	specific transcriptional repressor activity
N	B2TIC3	CC	This protein is involved in the repair of mismatches in DNA
N	B2TIC3	CC	It is possible that it carries out the mismatch recognition step

N	B2TIC3	CC	This protein has a weak ATPase activity (By similarity).
N	B2TIC3	DE	DNA mismatch repair protein mutS;
N	B2TIC3	DR	mismatch repair
N	B2TIC3	DR	mismatched DNA binding
N	B2TJ30	CC	An accessory protein needed during the final step in the assembly of 30S ribosomal subunit, possibly for assembly of the head region
N	B2TJ30	CC	Essential for efficient processing of 16S rRNA
N	B2TJ30	CC	It has affinity for free ribosomal 30S subunits but not for 70S ribosomes (By similarity).
N	B2TJ30	CC	May be needed both before and after rbfA during the maturation of 16S rRNA
N	B2TJ30	CC	Probably interacts with S19
N	B2TJ30	DE	Ribosome maturation factor rimM;
N	B2TJ30	DR	rRNA processing
N	B2TJ30	DR	ribosome binding
N	B2TTK3	DE	UPF0502 protein yceH;
N	B2TU77	DE	Leucine--tRNA ligase;
N	B2TU77	DE	Leucyl-tRNA synthetase;
N	B2TU77	DR	leucine-tRNA ligase activity
N	B2TU77	DR	leucyl-tRNA aminoacylation
N	B2U3C0	DE	NH(3)-dependent NAD(+) synthetase;
N	B2U3C0	DR	NAD biosynthetic process
N	B2U3C0	DR	NAD+ synthase (glutamine-hydrolyzing) activity
N	B2U3C0	DR	NAD+ synthase activity
N	B2U3M8	CC	Catalyzes the conversion of 3'-phosphate to a 2',3'- cyclic phosphodiester at the end of RNA
N	B2U3M8	CC	The biological role of this enzyme is unknown but it is likely to function in some aspects of cellular RNA processing (By similarity).
N	B2U3M8	CC	The mechanism of action of the enzyme occurs in 3 steps: (A) adenylation of the enzyme by ATP; (B) transfer of adenylate to an RNA-N3'P to produce RNA-N3'PP5'A; (C) and attack of the adjacent 2'-hydroxyl on the 3'- phosphorus in the diester linkage to produce the cyclic end product
N	B2U3M8	DE	RNA 3'-terminal phosphate cyclase;
N	B2U3M8	DE	RNA cyclase;
N	B2U3M8	DE	RNA-3'-phosphate cyclase;
N	B2U3M8	DR	RNA processing
N	B2U3M8	DR	RNA-3'-phosphate cyclase activity
N	B2UAK3	CC	Transfers the N-acyl diglyceride group on what will become the N-terminal cysteine of membrane lipoproteins (By similarity).
N	B2UAK3	DE	Prolipoprotein diacylglyceryl transferase;
N	B2UAK3	DR	lipoprotein biosynthetic process
N	B2UAK3	DR	protein lipoylation
N	B2UAK3	DR	transferase activity, transferring glycosyl groups
N	B2UB00	CC	DNA ligase that catalyzes the formation of phosphodiester linkages between 5'-phosphoryl and 3'-hydroxyl groups in double-stranded DNA using NAD as a coenzyme and as the energy source for the reaction
N	B2UB00	CC	It is essential for DNA replication and repair of damaged DNA (By similarity).
N	B2UB00	DE	Polydeoxyribonucleotide synthase [NAD+];
N	B2UB00	DR	DNA ligase (NAD+) activity
N	B2UB00	DR	DNA replication
N	B2UB00	DR	metal ion binding
N	B2UTS1	CC	Component of the acetyl coenzyme A carboxylase (ACC) complex
N	B2UTS1	CC	First, biotin carboxylase catalyzes the carboxylation of biotin on its carrier protein (BCCP) and then the CO(2) group is transferred by the carboxyltransferase to acetyl-CoA to form malonyl-CoA (By similarity).
N	B2UTS1	DE	ACCase subunit alpha;
N	B2UTS1	DE	Acetyl-CoA carboxylase carboxyltransferase subunit alpha;
N	B2UTS1	DE	Acetyl-coenzyme A carboxylase carboxyl transferase subunit alpha;
N	B2UTS1	DR	acetyl-CoA carboxylase activity
N	B2UTS1	DR	fatty acid biosynthetic process
N	B2UUI8	DE	Deoxycytidine triphosphate deaminase;
N	B2UUI8	DE	dCTP deaminase;
N	B2UUI8	DR	dCTP deaminase activity
N	B2UUI8	DR	dUTP biosynthetic process
N	B2UXE0	CC	Joins Ado-cobinamide-GDP and alpha-ribazole to generate adenosylcobalamin (Ado-cobalamin) (By similarity).
N	B2UXE0	DE	Cobalamin synthase;

N	B2UXE0	DR	cobalamin 5'-phosphate synthase activity
N	B2UXE0	DR	cobalamin biosynthetic process
N	B2UY33	CC	Cell wall formation (By similarity).
N	B2UY33	DE	D-Ala-D-Ala ligase;
N	B2UY33	DE	D-alanine--D-alanine ligase;
N	B2UY33	DE	D-alanylalanine synthetase;
N	B2UY33	DR	D-alanine-D-alanine ligase activity
N	B2UY33	DR	cellular cell wall organization
N	B2UY33	DR	metal ion binding
N	B2UY33	DR	peptidoglycan biosynthetic process
N	B2UY33	DR	regulation of cell shape
N	B2V0J5	CC	Phosphotransfer between the C1 and C5 carbon atoms of pentose (By similarity).
N	B2V0J5	DE	Phosphodeoxyribomutase;
N	B2V0J5	DE	Phosphopentomutase;
N	B2V0J5	DR	cellular metabolic compound salvage
N	B2V0J5	DR	magnesium ion binding
N	B2V0J5	DR	nucleotide metabolic process
N	B2V0J5	DR	phosphopentomutase activity
N	B2V1U8	CC	Specifically methylates the N7 position of a guanosine in 16S rRNA (By similarity).
N	B2V1U8	DE	16S rRNA 7-methylguanosine methyltransferase;
N	B2V1U8	DE	16S rRNA m7G methyltransferase;
N	B2V1U8	DE	Ribosomal RNA small subunit methyltransferase G;
N	B2V1U8	DR	rRNA methyltransferase activity
N	B2V4F7	CC	Responsible for the release of ribosomes from messenger RNA at the termination of protein biosynthesis
N	B2V4F7	CC	May increase the efficiency of translation by recycling ribosomes from one round of translation to another (By similarity).
N	B2V4F7	DE	Ribosome-recycling factor;
N	B2V4F7	DE	Ribosome-releasing factor;
N	B2V5E8	CC	Modulates recA activity (By similarity).
N	B2V5E8	DE	Regulatory protein recX;
N	B2V5E8	DR	regulation of DNA repair
N	B2V7J9	CC	One of the primary rRNA binding proteins, it binds directly to 16S rRNA central domain where it helps coordinate assembly of the platform of the 30S subunit (By similarity).
N	B2V7J9	DE	30S ribosomal protein S8;
N	B2V7J9	DR	structural constituent of ribosome
N	B2VDY7	CC	H(+)-stimulated, highly selective, manganese uptake system (By similarity).
N	B2VDY7	DE	Probable manganese transport protein mntH;
N	B2VDY7	DR	symporter activity
N	B2VE12	CC	Catalyzes the NADP-dependent rearrangement and reduction of 1-deoxy-D-xylulose-5-phosphate (DXP) to 2-C-methyl-D-erythritol 4-phosphate (MEP) (By similarity).
N	B2VE12	DE	1-deoxy-D-xylulose 5-phosphate reductoisomerase;
N	B2VE12	DE	1-deoxyxylulose-5-phosphate reductoisomerase;
N	B2VE12	DE	2-C-methyl-D-erythritol 4-phosphate synthase;
N	B2VE12	DE	DXP reductoisomerase;
N	B2VE12	DR	1-deoxy-D-xylulose-5-phosphate reductoisomerase activity
N	B2VE12	DR	isoprenoid biosynthetic process
N	B2VE12	DR	metal ion binding
N	B2VE12	DR	oxidation-reduction process
N	B2VFE0	CC	Catalyzes the final step of fatty acid oxidation in which acetyl-CoA is released and the CoA ester of a fatty acid two carbons shorter is formed (By similarity).
N	B2VFE0	DE	3-ketoacyl-CoA thiolase;
N	B2VFE0	DE	Acetyl-CoA acyltransferase;
N	B2VFE0	DE	Beta-ketothiolase;
N	B2VFE0	DE	Fatty acid oxidation complex subunit beta;
N	B2VFE0	DR	acetyl-CoA C-acyltransferase activity
N	B2VFE0	DR	fatty acid metabolic process
N	B2VFE0	DR	lipid catabolic process
N	B2VIS3	DE	UPF0345 protein ETA_25560;
N	B2VVB9	CC	Component of the ERMES/MDM complex, which serves as a molecular tether to connect the endoplasmic reticulum and mitochondria
N	B2VVB9	CC	Components of this complex are involved in the control of mitochondrial shape and protein biogenesis and may function in phospholipid exchange
N	B2VVB9	CC	Essential for establishing and maintaining the structure of mitochondria and maintenance of mtDNA nucleoids (By similarity).

N	B2VVB9	CC	The mdm10-mdm12-mmm1 subcomplex further acts in the TOM40-specific pathway after the action of the mdm12-mmm1 complex
N	B2VVB9	CC	The mdm12-mmm1 subcomplex functions in the major beta-barrel assembly pathway that is responsible for biogenesis of all outer membrane beta-barrel proteins, and acts in a late step after the SAM complex
N	B2VVB9	DE	Maintenance of mitochondrial morphology protein 1;
N	B2W791	CC	Cleaves beta-linked terminal galactosyl residues from gangliosides, glycoproteins, and glycosaminoglycans (By similarity).
N	B2W791	DE	Probable beta-galactosidase B;
N	B2W791	DR	beta-galactosidase activity
N	B2W791	DR	cation binding
N	B2W791	DR	polysaccharide catabolic process
N	B2XWJ4	CC	NDH shuttles electrons from NAD(P)H:plastoquinone, via FMN and iron-sulfur (Fe-S) centers, to quinones in the photosynthetic chain and possibly in a chloroplast respiratory chain
N	B2XWJ4	CC	Couples the redox reaction to proton translocation, and thus conserves the redox energy in a proton gradient (By similarity).
N	B2XWJ4	CC	The immediate electron acceptor for the enzyme in this species is believed to be plastoquinone
N	B2XWJ4	DE	NAD(P)H dehydrogenase subunit 6;
N	B2XWJ4	DE	NAD(P)H-quinone oxidoreductase subunit 6, chloroplastic;
N	B2XWJ4	DE	NADH-plastoquinone oxidoreductase subunit 6;
N	B2XWJ4	DR	NADH dehydrogenase (ubiquinone) activity
N	B2XWJ4	DR	oxidation-reduction process
N	B2XWJ4	DR	quinone binding
N	B3CN07	CC	Nuclease that resolves Holliday junction intermediates in genetic recombination
N	B3CN07	CC	Cleaves the cruciform structure in supercoiled DNA by nicking to strands with the same polarity at sites symmetrically opposed at the junction in the homologous arms and leaves a 5'-terminal phosphate and a 3'-terminal hydroxyl group (By similarity).
N	B3CN07	DE	Crossover junction endodeoxyribonuclease ruvC;
N	B3CN07	DE	Holliday junction nuclease ruvC;
N	B3CN07	DE	Holliday junction resolvase ruvC;
N	B3CN07	DR	DNA recombination
N	B3CN07	DR	crossover junction endodeoxyribonuclease activity
N	B3CN07	DR	metal ion binding
N	B3CN07	DR	nucleic acid binding
N	B3CVD8	CC	Specifically methylates the N4 position of cytidine in position 1402 (C1402) of 16S rRNA (By similarity).
N	B3CVD8	DE	16S rRNA m(4)C1402 methyltransferase;
N	B3CVD8	DE	Ribosomal RNA small subunit methyltransferase H;
N	B3CVD8	DE	rRNA (cytosine-N(4)-)-methyltransferase RsmH;
N	B3CVD8	DR	methyltransferase activity
N	B3CVD8	DR	rRNA processing
N	B3DPH5	CC	Catalyzes the conversion of uracil and 5-phospho-alpha- D-ribose 1-diphosphate (PRPP) to UMP and diphosphate (By similarity).
N	B3DPH5	DE	UMP pyrophosphorylase;
N	B3DPH5	DE	Uracil phosphoribosyltransferase;
N	B3DPH5	DR	nucleoside metabolic process
N	B3DPH5	DR	phosphotransferase activity, alcohol group as acceptor
N	B3DPH5	DR	uracil phosphoribosyltransferase activity
N	B3DPH5	DR	uracil salvage
N	B3DPY3	CC	Binds to Cpn60 in the presence of Mg-ATP and suppresses the ATPase activity of the latter (By similarity).
N	B3DPY3	DE	10 kDa chaperonin;
N	B3DPY3	DE	GroES protein;
N	B3DPY3	DE	Protein Cpn10;
N	B3DPY3	DR	protein folding
N	B3DQG7	CC	Catalyzes the attachment of serine to tRNA(Ser)
N	B3DQG7	CC	Is also able to aminoacylate tRNA(Sec) with serine, to form the misacylated tRNA L-seryl-tRNA(Sec), which will be further converted into selenocysteinyl-tRNA(Sec) (By similarity).
N	B3DQG7	DE	Serine--tRNA ligase;
N	B3DQG7	DE	Seryl-tRNA synthetase;
N	B3DQG7	DE	Seryl-tRNA(Ser/Sec) synthetase;
N	B3DQG7	DR	serine-tRNA ligase activity

N	B3DQG7	DR	seryl-tRNA aminoacylation
N	B3DU87	CC	Catalyzes the attachment of glutamate to tRNA(Glu) in a two-step reaction: glutamate is first activated by ATP to form Glu-AMP and then transferred to the acceptor end of tRNA(Glu) (By similarity).
N	B3DU87	DE	Glutamate--tRNA ligase;
N	B3DU87	DE	Glutamyl-tRNA synthetase;
N	B3DU87	DR	glutamate-tRNA ligase activity
N	B3DU87	DR	glutamyl-tRNA aminoacylation
N	B3E045	DE	UPF0082 protein Minf_0651;
N	B3E849	CC	Binds to the 23S rRNA (By similarity).
N	B3E849	DE	50S ribosomal protein L15;
N	B3E849	DR	structural constituent of ribosome
N	B3EAE4	CC	Required for maturation of 30S ribosomal subunits (By similarity).
N	B3EAE4	DE	Ribosome maturation factor rimP;
N	B3EAE4	DR	ribosome biogenesis
N	B3ECI3	CC	Is required not only for elongation of protein synthesis but also for the initiation of all mRNA translation through initiator tRNA(fMet) aminoacylation (By similarity).
N	B3ECI3	DE	Methionine--tRNA ligase;
N	B3ECI3	DE	Methionyl-tRNA synthetase;
N	B3ECI3	DR	metal ion binding
N	B3ECI3	DR	methionine-tRNA ligase activity
N	B3ECI3	DR	methionyl-tRNA aminoacylation
N	B3EG23	CC	Transfers the gamma-phosphate of ATP to the 4'-position of a tetraacyldisaccharide 1-phosphate intermediate (termed DS-1- P) to form tetraacyldisaccharide 1,4'-bis-phosphate (lipid IVA) (By similarity).
N	B3EG23	DE	Lipid A 4'-kinase;
N	B3EG23	DE	Tetraacyldisaccharide 4'-kinase;
N	B3EG23	DR	lipid A biosynthetic process
N	B3EG23	DR	tetraacyldisaccharide 4'-kinase activity
N	B3EHF8	CC	Required for the formation of a threonylcarbamoyl group on adenosine at position 37 (t(6)A37) in tRNAs that read codons beginning with adenine (By similarity).
N	B3EHF8	DE	Probable tRNA threonylcarbamoyladenosine biosynthesis protein Gcp;
N	B3EHF8	DE	t(6)A37 threonylcarbamoyladenosine biosynthesis protein;
N	B3EHF8	DR	metal ion binding
N	B3EHF8	DR	metalloendopeptidase activity
N	B3ELU5	CC	Catalyzes the dephosphorylation of undecaprenyl diphosphate (UPP)
N	B3ELU5	CC	Confers resistance to bacitracin (By similarity).
N	B3ELU5	DE	Bacitracin resistance protein;
N	B3ELU5	DE	Undecaprenyl pyrophosphate phosphatase;
N	B3ELU5	DE	Undecaprenyl-diphosphatase;
N	B3ELU5	DR	cellular cell wall organization
N	B3ELU5	DR	dephosphorylation
N	B3ELU5	DR	peptidoglycan biosynthetic process
N	B3ELU5	DR	regulation of cell shape
N	B3ELU5	DR	response to antibiotic
N	B3ELU5	DR	undecaprenyl-diphosphatase activity
N	B3EQD1	DE	Phosphoglycerate kinase;
N	B3EQD1	DR	phosphoglycerate kinase activity
N	B3GXD4	DE	3-phosphoshikimate 1-carboxyvinyltransferase;
N	B3GXD4	DE	5-enolpyruvylshikimate-3-phosphate synthase;
N	B3GXD4	DE	EPSP synthase;
N	B3GXD4	DR	3-phosphoshikimate 1-carboxyvinyltransferase activity
N	B3GXD4	DR	aromatic amino acid family biosynthetic process
N	B3GY39	DE	UPF0082 protein APP7_1210;
N	B3GZK8	CC	Cell wall formation
N	B3GZK8	CC	Catalyzes the transfer of a GlcNAc subunit on undecaprenyl-pyrophosphoryl-MurNAc-pentapeptide (lipid intermediate I) to form undecaprenyl-pyrophosphoryl-MurNAc- (pentapeptide)GlcNAc (lipid intermediate II) (By similarity).
N	B3GZK8	DE	UDP-N-acetylglucosamine--N-acetylmuramyl-(pentapeptide) pyrophosphoryl-undecaprenol N-acetylglucosamine transferase;
N	B3GZK8	DE	Undecaprenyl-PP-MurNAc-pentapeptide-UDPGlcNAc GlcNAc transferase;
N	B3GZK8	DR	UDP-N-acetyl-D-glucosamine:N-acetylmuramoyl-L-alanyl-D-glutamyl-meso-2,6-diaminopimelyl-D-alanyl-D-alanine-diphosphoundecaprenol 4-beta-N-acetylglucosaminlytransferase activity
N	B3GZK8	DR	UDP-N-acetylgalactosamine biosynthetic process

N	B3GZK8	DR	carbohydrate binding
N	B3GZK8	DR	cell division
N	B3GZK8	DR	cellular cell wall organization
N	B3GZK8	DR	lipid glycosylation
N	B3GZK8	DR	peptidoglycan biosynthetic process
N	B3GZK8	DR	regulation of cell shape
N	B3GZK8	DR	undecaprenyldiphospho-muramoylpentapeptide beta-N-acetylglucosaminyltransferase activity
N	B3H268	CC	Regulates arginine biosynthesis genes (By similarity).
N	B3H268	DE	Arginine repressor;
N	B3H268	DR	arginine biosynthetic process
N	B3H268	DR	regulation of transcription, DNA-dependent
N	B3H268	DR	sequence-specific DNA binding transcription factor activity
N	B3H2L4	DE	Urea amidohydrolase subunit beta;
N	B3H2L4	DE	Urease subunit beta;
N	B3H2L4	DR	nickel ion binding
N	B3H2L4	DR	nitrogen compound metabolic process
N	B3H2L4	DR	urease activity
N	B3LS11	CC	Is probably involved in a pathway contributing to genomic integrity.
N	B3LS11	DE	Increased recombination centers protein 22;
N	B3LUP1	CC	May be involved in the modulation of rDNA transcription (By similarity).
N	B3LUP1	DE	Regulator of rDNA transcription protein 5;
N	B3LUP1	DR	nucleotide binding
N	B3MMA5	CC	Involved in mitotic DNA repair and meiotic recombination
N	B3MMA5	CC	Essential for interhomolog gene conversion (GC), but may have a less important role in intersister GC than spn-A/Rad51
N	B3MMA5	CC	Functions in the recombinational DNA repair pathway
N	B3MMA5	CC	In the presence of DNA, spn-A/Rad51 enhances the ATPase activity of okr/Rad54 (By similarity).
N	B3MMA5	DE	DNA repair and recombination protein RAD54-like;
N	B3MMA5	DE	Protein okra;
N	B3MMA5	DR	DNA synthesis involved in double-strand break repair via homologous recombination
N	B3MMA5	DR	cell division
N	B3MMA5	DR	chromatin remodeling
N	B3MMA5	DR	helicase activity
N	B3MMA5	DR	meiotic DNA repair synthesis
N	B3MMA5	DR	response to ionizing radiation
N	B3PC22	CC	Catalyzes the ATP-dependent conversion of 7-carboxy-7- deazaguanine (CDG) to 7-cyano-7-deazaguanine (preQ(0)) (By similarity).
N	B3PC22	DE	7-cyano-7-carbaguanine synthase;
N	B3PC22	DE	7-cyano-7-deazaguanine synthase;
N	B3PC22	DE	PreQ(0) synthase;
N	B3PC22	DE	Queuosine biosynthesis protein queC;
N	B3PC22	DR	arginine biosynthetic process
N	B3PC22	DR	argininosuccinate synthase activity
N	B3PC22	DR	metal ion binding
N	B3PC22	DR	queuosine biosynthetic process
N	B3PFK3	DE	Homoserine O-acetyltransferase;
N	B3PFK3	DE	Homoserine O-trans-acetylase;
N	B3PFK3	DE	Homoserine transacetylase;
N	B3PFK3	DR	homoserine O-acetyltransferase activity
N	B3PFK3	DR	methionine biosynthetic process
N	B3PHF4	DE	5-enolpyruvylshikimate-3-phosphate phospholyase;
N	B3PHF4	DE	Chorismate synthase;
N	B3PHF4	DR	aromatic amino acid family biosynthetic process
N	B3PHF4	DR	chorismate synthase activity
N	B3PWT5	CC	One of the primary rRNA binding proteins, it binds directly to 16S rRNA central domain where it helps coordinate assembly of the platform of the 30S subunit (By similarity).
N	B3PWT5	DE	30S ribosomal protein S8;
N	B3PWT5	DR	structural constituent of ribosome
N	B3QS41	CC	Catalyzes the reversible transfer of the terminal phosphate group between ATP and AMP
N	B3QS41	CC	This small ubiquitous enzyme involved in the energy metabolism and nucleotide synthesis, is essential for maintenance and cell growth (By similarity).
N	B3QS41	DE	ATP-AMP transphosphorylase;

N	B3QS41	DE	Adenylate kinase;
N	B3QS41	DR	adenylate kinase activity
N	B3QS41	DR	nucleotide biosynthetic process
N	B3QTQ3	CC	Catalyzes the pyruvoyl-dependent decarboxylation of aspartate to produce beta-alanine (By similarity).
N	B3QTQ3	DE	Aspartate 1-decarboxylase alpha chain;
N	B3QTQ3	DE	Aspartate 1-decarboxylase beta chain;
N	B3QTQ3	DE	Aspartate 1-decarboxylase;
N	B3QTQ3	DE	Aspartate alpha-decarboxylase;
N	B3QTQ3	DR	alanine biosynthetic process
N	B3QTQ3	DR	aspartate 1-decarboxylase activity
N	B3QTQ3	DR	pantothenate biosynthetic process
N	B3QY23	CC	Involved in the binding of tRNA to the ribosomes (By similarity).
N	B3QY23	DE	30S ribosomal protein S10;
N	B3QY23	DR	structural constituent of ribosome
N	B3QYE2	DE	50S ribosomal protein L30;
N	B3QYE2	DR	structural constituent of ribosome
N	B3QYR1	CC	Catalyzes the attachment of glutamate to tRNA(Glu) in a two-step reaction: glutamate is first activated by ATP to form Glu-AMP and then transferred to the acceptor end of tRNA(Glu) (By similarity).
N	B3QYR1	DE	Glutamate--tRNA ligase;
N	B3QYR1	DE	Glutamyl-tRNA synthetase;
N	B3QYR1	DR	glutamate-tRNA ligase activity
N	B3QYR1	DR	glutamyl-tRNA aminoacylation
N	B3QZH3	CC	One of the primary rRNA binding proteins, it binds directly to 16S rRNA where it nucleates assembly of the head domain of the 30S subunit
N	B3QZH3	CC	Is located at the subunit interface close to the decoding center, probably blocks exit of the E-site tRNA (By similarity).
N	B3QZH3	DE	30S ribosomal protein S7;
N	B3QZH3	DR	structural constituent of ribosome
N	B3R0K3	CC	Binds together with S18 to 16S ribosomal RNA (By similarity).
N	B3R0K3	DE	30S ribosomal protein S6;
N	B3R0K3	DR	structural constituent of ribosome
N	B3R7L6	CC	Produces ATP from ADP in the presence of a proton gradient across the membrane
N	B3R7L6	CC	The gamma chain is believed to be important in regulating ATPase activity and the flow of protons through the CF(0) complex (By similarity).
N	B3R7L6	DE	ATP synthase F1 sector gamma subunit;
N	B3R7L6	DE	ATP synthase gamma chain;
N	B3R7L6	DE	F-ATPase gamma subunit;
N	B3R7L6	DR	ATP synthesis coupled proton transport
N	B3R7L6	DR	hydrogen ion transporting ATP synthase activity, rotational mechanism
N	B3R7L6	DR	proton-transporting ATPase activity, rotational mechanism
N	B3RLE6	CC	Catalyzes the interconversion of methylthioribose-1- phosphate (MTR-1-P) into methylthioribulose-1-phosphate (MTRu-1-P) (By similarity).
N	B3RLE6	DE	MTR-1-P isomerase;
N	B3RLE6	DE	Methylthioribose-1-phosphate isomerase;
N	B3RLE6	DE	S-methyl-5-thioribose-1-phosphate isomerase;
N	B3RLE6	DE	Translation initiation factor eIF-2B subunit alpha/beta/delta-like protein;
N	B3RLE6	DR	S-methyl-5-thioribose-1-phosphate isomerase activity
N	B3RLE6	DR	methionine biosynthetic process
N	B4E5X0	CC	An essential GTPase which binds GTP, GDP and possibly (p)ppGpp with moderate affinity, with high nucleotide exchange rates and a fairly low GTP hydrolysis rate (By similarity)
N	B4E5X0	CC	It may play a role in control of the cell cycle, stress response, ribosome biogenesis and in those bacteria that undergo differentiation, in morphogenesis control (Potential).
N	B4E5X0	DE	GTP-binding protein obg;
N	B4E5X0	DR	GTPase activity
N	B4E5X0	DR	magnesium ion binding
N	B4E8N5	CC	May conjugate Arg from its aminoacyl-tRNA to the N- termini of proteins containing an N-terminal aspartate or glutamate (Potential).
N	B4E8N5	DE	Arginyltransferase;
N	B4E8N5	DE	Putative arginyl-tRNA--protein transferase;
N	B4E8N5	DE	R-transferase;
N	B4E8N5	DR	acyltransferase activity
N	B4E8N5	DR	arginyltransferase activity

N	B4E8N5	DR	protein arginylation
N	B4EAW5	CC	GTPase that plays an essential role in the late steps of ribosome biogenesis (By similarity).
N	B4EAW5	DE	GTP-binding protein EngA;
N	B4EAW5	DR	nucleoside-triphosphatase activity
N	B4EAW5	DR	ribosome biogenesis
N	B4EET3	CC	Catalyzes the specific phosphorylation of 1,6-anhydro-N- acetylmuramic acid (anhMurNAc) with the simultaneous cleavage of the 1,6-anhydro ring, generating MurNAc-6-P
N	B4EET3	CC	Is required for the utilization of anhMurNAc either imported from the medium or derived from its own cell wall murein, and thus plays a role in cell wall recycling (By similarity).
N	B4EET3	DE	AnhMurNAc kinase;
N	B4EET3	DE	Anhydro-N-acetylmuramic acid kinase;
N	B4EET3	DR	amino sugar metabolic process
N	B4EET3	DR	kinase activity
N	B4EET3	DR	peptidoglycan turnover
N	B4EET3	DR	phosphotransferase activity, alcohol group as acceptor
N	B4EK77	CC	Catalyzes the conversion of acetaldehyde to acetyl-CoA, using NAD(+) and coenzyme A
N	B4EK77	CC	Is the final enzyme in the meta- cleavage pathway for the degradation of aromatic compounds (By similarity).
N	B4EK77	DE	Acetaldehyde dehydrogenase 1;
N	B4EK77	DE	Acetaldehyde dehydrogenase [acetylating] 1;
N	B4EK77	DR	acetaldehyde dehydrogenase (acetylating) activity
N	B4EK77	DR	aromatic compound catabolic process
N	B4EK77	DR	cellular amino acid metabolic process
N	B4EK77	DR	oxidation-reduction process
N	B4EUV9	DE	Gamma-glutamylcysteine synthetase;
N	B4EUV9	DE	Glutamate--cysteine ligase;
N	B4EUV9	DR	glutamate-cysteine ligase activity
N	B4EUV9	DR	glutathione biosynthetic process
N	B4EV71	DE	Glutamine--tRNA ligase;
N	B4EV71	DE	Glutaminyl-tRNA synthetase;
N	B4EV71	DR	glutamine-tRNA ligase activity
N	B4EV71	DR	glutaminyl-tRNA aminoacylation
N	B4EXE3	CC	Binds to Cpn60 in the presence of Mg-ATP and suppresses the ATPase activity of the latter (By similarity).
N	B4EXE3	DE	10 kDa chaperonin;
N	B4EXE3	DE	GroES protein;
N	B4EXE3	DE	Protein Cpn10;
N	B4EXE3	DR	protein folding
N	B4HSS0	CC	May be involved in N-glycosylation through its association with N-oligosaccharyl transferase (By similarity).
N	B4HSS0	DE	Dolichyl-diphosphooligosaccharide--protein glycosyltransferase subunit 4 homolog;
N	B4HSS0	DR	dolichyl-diphosphooligosaccharide-protein glycotransferase activity
N	B4HSS0	DR	protein N-linked glycosylation
N	B4I5W3	CC	Plays a central role in 2-thiolation of mcm(5)S(2)U at tRNA wobble positions of tRNA(Lys), tRNA(Glu) and tRNA(Gln)
N	B4I5W3	CC	May act by forming a heterodimer with NCS6/CTU1 that ligates sulfur from thiocarboxylated URM1 onto the uridine of tRNAs at wobble position (By similarity).
N	B4I5W3	DE	Cytoplasmic tRNA 2-thiolation protein 2;
N	B4I5W3	DR	tRNA thio-modification
N	B4I5W3	DR	tRNA wobble uridine modification
N	B4I9N7	CC	Catalyzes the formation of N(7)-methylguanine at position 46 (m7G46) in tRNA (By similarity).
N	B4I9N7	DE	tRNA (guanine-N(7)-)-methyltransferase;
N	B4I9N7	DE	tRNA(m7G46)-methyltransferase;
N	B4I9N7	DR	tRNA (guanine-N7-)-methyltransferase activity
N	B4IMF6	CC	Molecular scaffold for [Fe-S] cluster assembly of mitochondrial iron-sulfur proteins (By similarity).
N	B4IMF6	DE	NFU1 iron-sulfur cluster scaffold homolog, mitochondrial;
N	B4IMF6	DR	ATP-dependent helicase activity
N	B4IMF6	DR	iron ion binding
N	B4IMF6	DR	iron-sulfur cluster assembly
N	B4IMF6	DR	iron-sulfur cluster binding

N	B4IMF6	DR	nucleic acid binding
N	B4IMI7	DE	Integrator complex subunit 3 homolog;
N	B4IMI7	DE	SOSS complex subunit A homolog;
N	B4J4G8	CC	Component of the eukaryotic translation initiation factor 3 (eIF-3) complex, which is involved in protein synthesis and, together with other initiation factors, stimulates binding of mRNA and methionyl-tRNAi to the 40S ribosome
N	B4J4G8	CC	This subunit can bind 18S rRNA (By similarity).
N	B4J4G8	DE	Eukaryotic translation initiation factor 3 RNA-binding subunit 1;
N	B4J4G8	DE	Eukaryotic translation initiation factor 3 subunit 4-1;
N	B4J4G8	DE	Eukaryotic translation initiation factor 3 subunit G-1;
N	B4J4G8	DE	eIF-3 RNA-binding subunit 1;
N	B4J4G8	DR	nucleotide binding
N	B4J4G8	DR	translation initiation factor activity
N	B4J4G8	DR	zinc ion binding
N	B4JBN5	CC	Adenylyltransferase that mediates the addition of adenosine 5'-monophosphate (AMP) to specific residues of target proteins (By similarity).
N	B4JBN5	DE	Adenosine monophosphate-protein transferase FICD homolog;
N	B4JBN5	DR	protein adenylylation
N	B4JBN5	DR	protein adenylyltransferase activity
N	B4KG03	DE	Probable cGMP 3',5'-cyclic phosphodiesterase subunit delta;
N	B4KG03	DR	3',5'-cyclic-nucleotide phosphodiesterase activity
N	B4KG03	DR	visual perception
N	B4KRQ4	CC	Regulator of deubiquitinating complexes
N	B4KRQ4	CC	Activates deubiquitination by increasing the catalytic turnover without increasing the affinity of deubiquitinating enzymes for the substrate (By similarity).
N	B4KRQ4	DE	WD repeat-containing protein 48 homolog;
N	B4L629	CC	Catalyzes the oxidative cleavage of the L-tryptophan (L- Trp) pyrrole ring (By similarity).
N	B4L629	DE	Protein vermilion;
N	B4L629	DE	Tryptamin 2,3-dioxygenase;
N	B4L629	DE	Tryptophan 2,3-dioxygenase;
N	B4L629	DE	Tryptophan oxygenase;
N	B4L629	DE	Tryptophan pyrrolase;
N	B4L629	DE	Tryptophanase;
N	B4L629	DR	oxidation-reduction process
N	B4L629	DR	tryptophan 2,3-dioxygenase activity
N	B4L629	DR	tryptophan catabolic process to kynurenine
N	B4LIH0	CC	A calcium channel that regulates synaptic endocytosis and hence couples exo- with endocytosis
N	B4LIH0	CC	Required in the nervous system and necessary in photoreceptor cells (By similarity).
N	B4LIH0	DE	Calcium channel flower;
N	B4LIH0	DR	calcium channel activity
N	B4LIH0	DR	photoreceptor cell differentiation
N	B4LIH0	DR	synaptic vesicle endocytosis
N	B4LNQ8	CC	Component of the eukaryotic translation initiation factor 3 (eIF-3) complex, which is involved in protein synthesis and, together with other initiation factors, stimulates binding of mRNA and methionyl-tRNAi to the 40S ribosome (By similarity).
N	B4LNQ8	DE	Eukaryotic translation initiation factor 3 subunit K;
N	B4LNQ8	DR	regulation of translational initiation
N	B4LNQ8	DR	ribosome binding
N	B4LNQ8	DR	translation initiation factor activity
N	B4N208	CC	Catalyzes the covalent attachment of ubiquitin to other proteins
N	B4N208	CC	Acts as an essential factor of the anaphase promoting complex/cyclosome (APC/C), a cell cycle-regulated ubiquitin ligase that controls progression through mitosis.
N	B4N208	CC	Acts by specifically elongating polyubiquitin chains initiated by the E2 enzyme vih/UbcH10 on APC/C substrates, enhancing the degradation of APC/C substrates by the proteasome and promoting mitotic exit (By similarity).
N	B4N208	DE	Ubiquitin carrier protein S;
N	B4N208	DE	Ubiquitin-conjugating enzyme E2 S;
N	B4N208	DE	Ubiquitin-protein ligase S;
N	B4N208	DR	anaphase-promoting complex-dependent proteasomal ubiquitin-dependent protein catabolic process
N	B4N208	DR	cell division
N	B4N208	DR	exit from mitosis
N	B4N208	DR	post-translational protein modification

N	B4N208	DR	ubiquitin-protein ligase activity
N	B4N4D9	CC	Component of the cytosolic iron-sulfur (Fe/S) protein assembly machinery
N	B4N4D9	CC	May bind and transfer a labile 4Fe-4S cluster to target apoproteins (By similarity)
N	B4N4D9	CC	Required for maturation of extramitochondrial Fe/S proteins
N	B4N4D9	DE	Cytosolic Fe-S cluster assembly factor NUBP2 homolog;
N	B4N4D9	DR	4 iron, 4 sulfur cluster binding
N	B4N4D9	DR	metal ion binding
N	B4P925	CC	Essential arginine methyltransferase that can both catalyze the formation of omega-N monomethylarginine (MMA) and symmetrical dimethylarginine (sDMA)
N	B4P925	CC	Specifically mediates the symmetrical dimethylation of arginine residues in the small nuclear ribonucleoproteins SmD1 and SmD3 (By similarity).
N	B4P925	DE	Protein arginine N-methyltransferase 7;
N	B4P925	DR	protein-arginine omega-N symmetric methyltransferase activity
N	B4PNE2	CC	Catalyzes the interconversion of methylthioribose-1- phosphate (MTR-1-P) into methylthioribulose-1-phosphate (MTRu-1-P) (By similarity).
N	B4PNE2	DE	MTR-1-P isomerase;
N	B4PNE2	DE	Methylthioribose-1-phosphate isomerase;
N	B4PNE2	DE	S-methyl-5-thioribose-1-phosphate isomerase;
N	B4PNE2	DE	Translation initiation factor eIF-2B subunit alpha/beta/delta-like protein;
N	B4PNE2	DR	S-methyl-5-thioribose-1-phosphate isomerase activity
N	B4PNE2	DR	methionine biosynthetic process
N	B4QEJ9	DE	Protein crossbronx-like;
N	B4QEJ9	DR	acid-amino acid ligase activity
N	B4QEJ9	DR	post-translational protein modification
N	B4REI3	CC	Transfers the gamma-phosphate of ATP to the 4'-position of a tetraacyldisaccharide 1-phosphate intermediate (termed DS-1- P) to form tetraacyldisaccharide 1,4'-bis-phosphate (lipid IVA) (By similarity).
N	B4REI3	DE	Lipid A 4'-kinase;
N	B4REI3	DE	Tetraacyldisaccharide 4'-kinase;
N	B4REI3	DR	lipid A biosynthetic process
N	B4REI3	DR	tetraacyldisaccharide 4'-kinase activity
N	B4RH54	CC	Necessary for efficient RNA polymerase transcription elongation past template-encoded arresting sites
N	B4RH54	CC	Cleavage of the nascent transcript by cleavage factors such as greA or greB allows the resumption of elongation from the new 3'terminus
N	B4RH54	CC	GreA releases sequences of 2 to 3 nucleotides (By similarity).
N	B4RH54	CC	The arresting sites in DNA have the property of trapping a certain fraction of elongating RNA polymerases that pass through, resulting in locked ternary complexes
N	B4RH54	DE	Transcript cleavage factor greA;
N	B4RH54	DE	Transcription elongation factor greA;
N	B4RH54	DR	transcription elongation regulator activity
N	B4RIY7	CC	Catalyzes the interconversion of 2-phosphoglycerate and 3-phosphoglycerate (By similarity).
N	B4RIY7	DE	2,3-bisphosphoglycerate-dependent phosphoglycerate mutase;
N	B4RIY7	DE	BPG-dependent PGAM;
N	B4RIY7	DE	Phosphoglyceromutase;
N	B4RIY7	DR	phosphoglycerate mutase activity
N	B4RJZ8	DE	DTB synthetase;
N	B4RJZ8	DE	Dethiobiotin synthase;
N	B4RJZ8	DE	Dethiobiotin synthetase;
N	B4RJZ8	DR	biotin biosynthetic process
N	B4RJZ8	DR	dethiobiotin synthase activity
N	B4RJZ8	DR	magnesium ion binding
N	B4RLX9	DE	3-dehydroquinase;
N	B4RLX9	DE	3-dehydroquinate dehydratase;
N	B4RLX9	DE	Type I DHQase;
N	B4RLX9	DR	3-dehydroquinate dehydratase activity
N	B4RLX9	DR	aromatic amino acid family biosynthetic process
N	B4S3Q6	DE	Glutamate-1-semialdehyde 2,1-aminomutase;
N	B4S3Q6	DE	Glutamate-1-semialdehyde aminotransferase;
N	B4S3Q6	DR	chlorophyll biosynthetic process
N	B4S3Q6	DR	glutamate-1-semialdehyde 2,1-aminomutase activity
N	B4S3Q6	DR	pyridoxal phosphate binding
N	B4S3Q6	DR	transaminase activity
N	B4S517	CC	Involved in the transcription termination process (By similarity).

N	B4S517	DE	N utilization substance protein B homolog;
N	B4S517	DE	Protein nusB;
N	B4S517	DR	regulation of transcription, DNA-dependent
N	B4S517	DR	transcription termination factor activity
N	B4SFQ8	CC	Catalyzes the reversible oxidation of malate to oxaloacetate (By similarity).
N	B4SFQ8	DE	Malate dehydrogenase;
N	B4SFQ8	DR	L-malate dehydrogenase activity
N	B4SFQ8	DR	cellular carbohydrate metabolic process
N	B4SFQ8	DR	malate metabolic process
N	B4SFQ8	DR	tricarboxylic acid cycle
N	B4SNP6	CC	Specifically methylates the N7 position of guanosine in position 527 of 16S rRNA (By similarity).
N	B4SNP6	DE	16S rRNA 7-methylguanosine methyltransferase;
N	B4SNP6	DE	16S rRNA m7G methyltransferase;
N	B4SNP6	DE	Ribosomal RNA small subunit methyltransferase G;
N	B4SNP6	DR	rRNA methyltransferase activity
N	B4SNX3	CC	Subunit of malonate decarboxylase, it is an acyl carrier protein to which acetyl and malonyl thioester residues are bound via a 2'-(5''-phosphoribosyl)-3'-dephospho-CoA prosthetic group and turn over during the catalytic mechanism (By similarity).
N	B4SNX3	DE	Malonate decarboxylase acyl carrier protein;
N	B4SNX3	DE	Malonate decarboxylase subunit delta;
N	B4SQZ7	CC	Bidirectionally degrades single-stranded DNA into large acid-insoluble oligonucleotides, which are then degraded further into small acid-soluble oligonucleotides (By similarity).
N	B4SQZ7	DE	Exodeoxyribonuclease 7 small subunit;
N	B4SQZ7	DE	Exodeoxyribonuclease VII small subunit;
N	B4SQZ7	DE	Exonuclease VII small subunit;
N	B4SQZ7	DR	DNA catabolic process
N	B4SQZ7	DR	exodeoxyribonuclease VII activity
N	B4SRL3	CC	Required for accurate and efficient protein synthesis under certain stress conditions
N	B4SRL3	CC	Back- translocation proceeds from a post-translocation (POST) complex to a pre-translocation (PRE) complex, thus giving elongation factor G a second chance to translocate the tRNAs correctly
N	B4SRL3	CC	Binds to ribosomes in a GTP-dependent manner (By similarity).
N	B4SRL3	CC	May act as a fidelity factor of the translation reaction, by catalyzing a one-codon backward translocation of tRNAs on improperly translocated ribosomes
N	B4SRL3	DE	Elongation factor 4;
N	B4SRL3	DE	Ribosomal back-translocase LepA;
N	B4SRL3	DR	GTPase activity
N	B4SRU3	CC	Catalyzes the dephosphorylation of undecaprenyl diphosphate (UPP)
N	B4SRU3	CC	Confers resistance to bacitracin (By similarity).
N	B4SRU3	DE	Bacitracin resistance protein;
N	B4SRU3	DE	Undecaprenyl pyrophosphate phosphatase;
N	B4SRU3	DE	Undecaprenyl-diphosphatase;
N	B4SRU3	DR	cellular cell wall organization
N	B4SRU3	DR	dephosphorylation
N	B4SRU3	DR	peptidoglycan biosynthetic process
N	B4SRU3	DR	regulation of cell shape
N	B4SRU3	DR	response to antibiotic
N	B4SRU3	DR	undecaprenyl-diphosphatase activity
N	B4ST36	DE	UPF0082 protein Smal_3128;
N	B4SVG0	DE	Arginine--tRNA ligase;
N	B4SVG0	DE	Arginyl-tRNA synthetase;
N	B4SVG0	DR	arginine-tRNA ligase activity
N	B4SVG0	DR	arginyl-tRNA aminoacylation
N	B4SYK7	DE	Leucine--tRNA ligase;
N	B4SYK7	DE	Leucyl-tRNA synthetase;
N	B4SYK7	DR	leucine-tRNA ligase activity
N	B4SYK7	DR	leucyl-tRNA aminoacylation
N	B4SYY7	CC	NDH-1 shuttles electrons from NADH, via FMN and iron- sulfur (Fe-S) centers, to quinones in the respiratory chain
N	B4SYY7	CC	Couples the redox reaction to proton translocation (for every two electrons transferred, four hydrogen ions are translocated across the cytoplasmic membrane), and thus conserves the redox energy in a proton gradient (By similarity).
N	B4SYY7	CC	The immediate electron acceptor for the enzyme in this species is believed to be ubiquinone

N	B4SYY7	DE	NADH dehydrogenase I subunit N;
N	B4SYY7	DE	NADH-quinone oxidoreductase subunit N;
N	B4SYY7	DE	NDH-1 subunit N;
N	B4SYY7	DR	ATP synthesis coupled electron transport
N	B4SYY7	DR	NADH dehydrogenase (ubiquinone) activity
N	B4SYY7	DR	quinone binding
N	B4SZJ7	CC	Catalyzes the conversion of dethiobiotin (DTB) to biotin by the insertion of a sulfur atom into dethiobiotin via a radical- based mechanism (By similarity).
N	B4SZJ7	DE	Biotin synthase;
N	B4SZJ7	DR	2 iron, 2 sulfur cluster binding
N	B4SZJ7	DR	4 iron, 4 sulfur cluster binding
N	B4SZJ7	DR	biotin biosynthetic process
N	B4SZJ7	DR	biotin synthase activity
N	B4SZJ7	DR	metal ion binding
N	B4T2T9	CC	Catalyzes the reversible retro-aldol cleavage of 4- hydroxy-2-ketoheptane-1,7-dioate (HKHD) to pyruvate and succinic semialdehyde (By similarity).
N	B4T2T9	DE	2,4-dihydroxyhept-2-ene-1,7-dioic acid aldolase;
N	B4T2T9	DE	4-hydroxy-2-ketoheptane-1,7-dioate aldolase;
N	B4T2T9	DE	4-hydroxy-2-oxo-heptane-1,7-dioate aldolase;
N	B4T2T9	DE	HHED aldolase;
N	B4T2T9	DE	HKHD aldolase;
N	B4T2T9	DR	2,4-dihydroxyhept-2-ene-1,7-dioate aldolase activity
N	B4T2T9	DR	aldehyde-lyase activity
N	B4T2T9	DR	metal ion binding
N	B4T2T9	DR	phenylacetate catabolic process
N	B4T755	CC	This protein is one of the early assembly proteins of the 50S ribosomal subunit, although it is not seen to bind rRNA by itself
N	B4T755	CC	It is important during the early stages of 50S assembly (By similarity).
N	B4T755	DE	50S ribosomal protein L13;
N	B4T755	DR	structural constituent of ribosome
N	B4T8R5	CC	Bidirectionally degrades single-stranded DNA into large acid-insoluble oligonucleotides, which are then degraded further into small acid-soluble oligonucleotides (By similarity).
N	B4T8R5	DE	Exodeoxyribonuclease 7 small subunit;
N	B4T8R5	DE	Exodeoxyribonuclease VII small subunit;
N	B4T8R5	DE	Exonuclease VII small subunit;
N	B4T8R5	DR	DNA catabolic process
N	B4T8R5	DR	exodeoxyribonuclease VII activity
N	B4T9Y6	CC	Phosphorolytic exoribonuclease that removes nucleotide residues following the -CCA terminus of tRNA and adds nucleotides to the ends of RNA molecules by using nucleoside diphosphates as substrates (By similarity).
N	B4T9Y6	DE	Ribonuclease PH;
N	B4T9Y6	DE	tRNA nucleotidyltransferase;
N	B4T9Y6	DR	3'-5'-exoribonuclease activity
N	B4T9Y6	DR	tRNA nucleotidyltransferase activity
N	B4T9Y6	DR	tRNA processing
N	B4T9Y6	DR	tRNA-specific ribonuclease activity
N	B4TBN7	CC	Formation of pseudouridine at positions 38, 39 and 40 in the anticodon stem and loop of transfer RNAs (By similarity).
N	B4TBN7	DE	tRNA pseudouridine synthase A;
N	B4TBN7	DE	tRNA pseudouridylate synthase I;
N	B4TBN7	DE	tRNA-uridine isomerase I;
N	B4TBN7	DR	pseudouridine synthase activity
N	B4TBN7	DR	pseudouridine synthesis
N	B4TBN7	DR	tRNA processing
N	B4TGH1	DE	Threonine--tRNA ligase;
N	B4TGH1	DE	Threonyl-tRNA synthetase;
N	B4TGH1	DR	metal ion binding
N	B4TGH1	DR	threonine-tRNA ligase activity
N	B4TGH1	DR	threonyl-tRNA aminoacylation
N	B4THE9	CC	Involved in the export of arginine
N	B4THE9	CC	Important to control the intracellular level of arginine and the correct balance between arginine and lysine (By similarity).
N	B4THE9	DE	Arginine exporter protein ArgO;
N	B4THE9	DR	amino acid transport

N	B4TJR9	CC	This protein is one of the early assembly proteins of the 50S ribosomal subunit, although it is not seen to bind rRNA by itself
N	B4TJR9	CC	It is important during the early stages of 50S assembly (By similarity).
N	B4TJR9	DE	50S ribosomal protein L13;
N	B4TJR9	DR	structural constituent of ribosome
N	B4TKN2	CC	Transport system that facilitates potassium-efflux, possibly by potassium-proton antiport (By similarity).
N	B4TKN2	DE	Glutathione-regulated potassium-efflux system protein kefB;
N	B4TKN2	DE	K(+)/H(+) antiporter;
N	B4TKN2	DE	NEM-activatable K(+)/H(+) antiporter;
N	B4TKN2	DR	catalytic activity
N	B4TKN2	DR	glutathione-regulated potassium exporter activity
N	B4TKN2	DR	solute:hydrogen antiporter activity
N	B4TQJ4	CC	Seems to be the binding site for several of the factors involved in protein synthesis and appears to be essential for accurate translation (By similarity).
N	B4TQJ4	DE	50S ribosomal protein L7/L12;
N	B4TQJ4	DR	structural constituent of ribosome
N	B4TSS5	DE	Dihydroorotase;
N	B4TSS5	DR	dihydroorotase activity
N	B4TSS5	DR	metal ion binding
N	B4TSS5	DR	pyrimidine base biosynthetic process
N	B4TSS5	DR	pyrimidine nucleotide biosynthetic process
N	B4TUT6	CC	Participates in cysteine desulfuration mediated by sufS
N	B4TUT6	CC	Cysteine desulfuration mobilizes sulfur from L-cysteine to yield L-alanine and constitutes an essential step in sulfur metabolism for biosynthesis of a variety of sulfur-containing biomolecules
N	B4TUT6	CC	Functions as a sulfur acceptor for sufS, by mediating the direct transfer of the sulfur atom from the S-sulfanylcysteine of sufS, an intermediate product of cysteine desulfuration process (By similarity).
N	B4TUT6	DE	Cysteine desulfuration protein sufE;
N	B4TX11	CC	Involved in mRNA degradation
N	B4TX11	CC	Hydrolyzes single-stranded polyribonucleotides processively in the 3' to 5' direction (By similarity).
N	B4TX11	DE	Exoribonuclease 2;
N	B4TX11	DE	Exoribonuclease II;
N	B4TX11	DE	Ribonuclease II;
N	B4TX11	DR	RNA catabolic process
N	B4TX11	DR	exoribonuclease II activity
N	B4TXB2	CC	Specifically methylates the cytosine at position 967 (m5C967) of 16S rRNA (By similarity).
N	B4TXB2	DE	16S rRNA m5C967 methyltransferase;
N	B4TXB2	DE	Ribosomal RNA small subunit methyltransferase B;
N	B4TXB2	DE	rRNA (cytosine-C(5)-)-methyltransferase rsmB;
N	B4TXB2	DR	rRNA methyltransferase activity
N	B4TXB2	DR	regulation of transcription, DNA-dependent
N	B4U2Z3	CC	Site-specific tyrosine recombinase, which acts by catalyzing the cutting and rejoining of the recombining DNA molecules
N	B4U2Z3	CC	Essential to convert dimers of the bacterial chromosome into monomers to permit their segregation at cell division (By similarity).
N	B4U2Z3	DE	Tyrosine recombinase xerS;
N	B4U2Z3	DR	DNA integration
N	B4U2Z3	DR	DNA recombination
N	B4U2Z3	DR	cell division
N	B4U2Z3	DR	chromosome segregation
N	B4U8K1	CC	Catalyzes the attachment of isoleucine to tRNA(Ile)
N	B4U8K1	CC	As IleRS can inadvertently accommodate and process structurally similar amino acids such as valine, to avoid such errors it has two additional distinct tRNA(Ile)-dependent editing activities
N	B4U8K1	CC	One activity is designated as 'pretransfer' editing and involves the hydrolysis of activated Val-AMP
N	B4U8K1	CC	The other activity is designated 'posttransfer' editing and involves deacylation of mischarged Val-tRNA(Ile) (By similarity).
N	B4U8K1	DE	Isoleucine--tRNA ligase;
N	B4U8K1	DE	Isoleucyl-tRNA synthetase;
N	B4U8K1	DR	isoleucine-tRNA ligase activity

N	B4U8K1	DR	isoleucyl-tRNA aminoacylation
N	B4U8K1	DR	metal ion binding
N	B4U9R1	CC	Catalyzes the transfer of an acyl group from acyl- phosphate (acyl-PO(4)) to glycerol-3-phosphate (G3P) to form lysophosphatidic acid (LPA)
N	B4U9R1	CC	This enzyme utilizes acyl-phosphate as fatty acyl donor, but not acyl-CoA or acyl-ACP (By similarity).
N	B4U9R1	DE	Acyl-PO4 G3P acyltransferase;
N	B4U9R1	DE	Acyl-phosphate--glycerol-3-phosphate acyltransferase;
N	B4U9R1	DE	G3P acyltransferase;
N	B4U9R1	DE	Glycerol-3-phosphate acyltransferase;
N	B4U9R1	DE	LPA synthase;
N	B4U9R1	DE	Lysophosphatidic acid synthase;
N	B4U9R1	DR	phospholipid biosynthetic process
N	B4U9R1	DR	transferase activity, transferring acyl groups other than amino-acyl groups
N	B5BA39	CC	Binds directly to 23S ribosomal RNA and is necessary for the in vitro assembly process of the 50S ribosomal subunit
N	B5BA39	CC	It is not involved in the protein synthesizing functions of that subunit (By similarity).
N	B5BA39	DE	50S ribosomal protein L20;
N	B5BA39	DR	structural constituent of ribosome
N	B5BBD2	DE	Protein yceI;
N	B5BBH3	CC	Interacts with CbpA and inhibits both the DnaJ-like co- chaperone activity and the DNA binding activity of CbpA
N	B5BBH3	CC	Does not inhibit the co-chaperone activity of DnaJ (By similarity).
N	B5BBH3	CC	Together with CbpA, modulates the activity of the DnaK chaperone system
N	B5BBH3	DE	Chaperone modulatory protein CbpM;
N	B5BCW8	CC	ATP-dependent carboxylate-amine ligase (By similarity).
N	B5BCW8	DE	Carboxylate-amine ligase YbdK;
N	B5BCW8	DR	glutamate-cysteine ligase activity
N	B5BCW8	DR	glutathione biosynthetic process
N	B5BDA7	CC	Catalyzes the ATP-dependent transfer of a sulfur to tRNA to produce 4-thiouridine in position 8 of tRNAs, which functions as a near-UV photosensor
N	B5BDA7	CC	Also catalyzes the transfer of sulfur to the sulfur carrier protein ThiS, forming ThiS-thiocarboxylate
N	B5BDA7	CC	The sulfur is donated as persulfide by iscS (By similarity).
N	B5BDA7	CC	This is a step in the synthesis of thiazole, in the thiamine biosynthesis pathway
N	B5BDA7	DE	Sulfur carrier protein ThiS sulfurtransferase;
N	B5BDA7	DE	Thiamine biosynthesis protein thiI;
N	B5BDA7	DE	tRNA 4-thiouridine synthase;
N	B5BDA7	DE	tRNA sulfurtransferase;
N	B5BDA7	DR	thiamine biosynthetic process
N	B5BDA7	DR	transferase activity
N	B5BG11	CC	Catalyzes the phosphorylation of D-glycero-D-manno- heptose 7-phosphate at the C-1 position to form D,D-heptose-1,7- bisphosphate (By similarity).
N	B5BG11	DE	Bifunctional protein hldE;
N	B5BG11	DE	D-beta-D-heptose 1-phosphate adenosyltransferase;
N	B5BG11	DE	D-beta-D-heptose 7-phosphate kinase;
N	B5BG11	DE	D-beta-D-heptose 7-phosphotransferase;
N	B5BG11	DR	biosynthetic process
N	B5BG11	DR	carbohydrate metabolic process
N	B5BG11	DR	kinase activity
N	B5BG11	DR	nucleotidyltransferase activity
N	B5BG11	DR	phosphotransferase activity, alcohol group as acceptor
N	B5BGW2	CC	One of the primary rRNA binding proteins, it binds directly to 16S rRNA where it nucleates assembly of the body of the 30S subunit (By similarity).
N	B5BGW2	DE	30S ribosomal protein S4;
N	B5BGW2	DR	structural constituent of ribosome
N	B5BHZ1	DE	L-threonine 3-dehydrogenase;
N	B5BHZ1	DR	L-threonine 3-dehydrogenase activity
N	B5BHZ1	DR	oxidation-reduction process
N	B5BHZ1	DR	threonine catabolic process
N	B5BHZ1	DR	zinc ion binding
N	B5BKH1	DE	Phosphatidylserine decarboxylase alpha chain;
N	B5BKH1	DE	Phosphatidylserine decarboxylase beta chain;
N	B5BKH1	DE	Phosphatidylserine decarboxylase proenzyme;
N	B5BKH1	DR	phosphatidylserine decarboxylase activity

N	B5BKH1	DR	phospholipid biosynthetic process
N	B5E4P1	CC	Catalyzes the reversible conversion of 2- phosphoglycerate into phosphoenolpyruvate
N	B5E4P1	CC	It is essential for the degradation of carbohydrates via glycolysis (By similarity).
N	B5E4P1	DE	2-phospho-D-glycerate hydro-lyase;
N	B5E4P1	DE	2-phosphoglycerate dehydratase;
N	B5E4P1	DR	magnesium ion binding
N	B5E4P1	DR	phosphopyruvate hydratase activity
N	B5E721	CC	Cell wall formation (By similarity).
N	B5E721	DE	D-Ala-D-Ala ligase;
N	B5E721	DE	D-alanine--D-alanine ligase;
N	B5E721	DE	D-alanylalanine synthetase;
N	B5E721	DR	D-alanine-D-alanine ligase activity
N	B5E721	DR	cellular cell wall organization
N	B5E721	DR	metal ion binding
N	B5E721	DR	peptidoglycan biosynthetic process
N	B5E721	DR	regulation of cell shape
N	B5EB50	DE	Dihydroorotase;
N	B5EB50	DR	dihydroorotase activity
N	B5EB50	DR	metal ion binding
N	B5EB50	DR	pyrimidine nucleotide biosynthetic process
N	B5EBQ4	CC	Cell wall formation (By similarity).
N	B5EBQ4	DE	D-Ala-D-Ala ligase;
N	B5EBQ4	DE	D-alanine--D-alanine ligase;
N	B5EBQ4	DE	D-alanylalanine synthetase;
N	B5EBQ4	DR	D-alanine-D-alanine ligase activity
N	B5EBQ4	DR	cellular cell wall organization
N	B5EBQ4	DR	metal ion binding
N	B5EBQ4	DR	peptidoglycan biosynthetic process
N	B5EBQ4	DR	regulation of cell shape
N	B5EE14	DE	Phosphoribosyl-AMP cyclohydrolase;
N	B5EE14	DR	histidine biosynthetic process
N	B5EE14	DR	phosphoribosyl-AMP cyclohydrolase activity
N	B5EHX2	CC	This is one of the proteins that binds to the 5S RNA in the ribosome where it forms part of the central protuberance (By similarity).
N	B5EHX2	DE	50S ribosomal protein L25;
N	B5EHX2	DE	General stress protein CTC;
N	B5EHX2	DR	5S rRNA binding
N	B5EHX2	DR	structural constituent of ribosome
N	B5EN53	DE	Phenylalanine--tRNA ligase alpha chain;
N	B5EN53	DE	Phenylalanyl-tRNA synthetase alpha chain;
N	B5EN53	DR	metal ion binding
N	B5EN53	DR	phenylalanine-tRNA ligase activity
N	B5EN53	DR	phenylalanyl-tRNA aminoacylation
N	B5EXE7	CC	Phosphorolytic exoribonuclease that removes nucleotide residues following the -CCA terminus of tRNA and adds nucleotides to the ends of RNA molecules by using nucleoside diphosphates as substrates (By similarity).
N	B5EXE7	DE	Ribonuclease PH;
N	B5EXE7	DE	tRNA nucleotidyltransferase;
N	B5EXE7	DR	3'-5'-exoribonuclease activity
N	B5EXE7	DR	tRNA nucleotidyltransferase activity
N	B5EXE7	DR	tRNA processing
N	B5EXE7	DR	tRNA-specific ribonuclease activity
N	B5EXZ5	CC	Is required not only for elongation of protein synthesis but also for the initiation of all mRNA translation through initiator tRNA(fMet) aminoacylation (By similarity).
N	B5EXZ5	DE	Methionine--tRNA ligase;
N	B5EXZ5	DE	Methionyl-tRNA synthetase;
N	B5EXZ5	DR	metal ion binding
N	B5EXZ5	DR	methionine-tRNA ligase activity
N	B5EXZ5	DR	methionyl-tRNA aminoacylation
N	B5EZ89	DE	Leucine--tRNA ligase;
N	B5EZ89	DE	Leucyl-tRNA synthetase;
N	B5EZ89	DR	leucine-tRNA ligase activity
N	B5EZ89	DR	leucyl-tRNA aminoacylation
N	B5EZR7	DE	UPF0115 protein yfcN;
N	B5F0U9	DE	Argininosuccinate lyase;

N	B5F0U9	DE	Arginosuccinase;
N	B5F0U9	DR	arginine biosynthetic process via ornithine
N	B5F0U9	DR	argininosuccinate lyase activity
N	B5F3S9	DE	UPF0745 protein YcgL;
N	B5F744	DE	Dihydrodipicolinate reductase;
N	B5F744	DR	diaminopimelate biosynthetic process
N	B5F744	DR	dihydrodipicolinate reductase activity
N	B5F744	DR	oxidation-reduction process
N	B5F941	DE	Multidrug resistance protein mdtH;
N	B5F941	DR	transmembrane transport
N	B5FA83	CC	Involved in mRNA degradation
N	B5FA83	CC	Hydrolyzes single-stranded polyribonucleotides processively in the 3'- to 5'-direction (By similarity).
N	B5FA83	DE	Polynucleotide phosphorylase;
N	B5FA83	DE	Polyribonucleotide nucleotidyltransferase;
N	B5FA83	DR	3'-5'-exoribonuclease activity
N	B5FA83	DR	RNA processing
N	B5FA83	DR	mRNA catabolic process
N	B5FA83	DR	polyribonucleotide nucleotidyltransferase activity
N	B5FE06	CC	Specifically methylates the cytosine at position 1407 (m5C1407) of 16S rRNA (By similarity).
N	B5FE06	DE	16S rRNA m5C1407 methyltransferase;
N	B5FE06	DE	Ribosomal RNA small subunit methyltransferase F;
N	B5FE06	DE	rRNA (cytosine-C(5)-)-methyltransferase rsmF;
N	B5FE06	DR	rRNA methyltransferase activity
N	B5FFD2	CC	Hydrolyzes D-tyrosyl-tRNA(Tyr) into D-tyrosine and free tRNA(Tyr)
N	B5FFD2	CC	Could be a defense mechanism against a harmful effect of D-tyrosine (By [illegible]
N	B5FFD2	DE	D-tyrosyl-tRNA(Tyr) deacylase;
N	B5FFD2	DR	D-amino acid catabolic process
N	B5FFD2	DR	hydrolase activity, acting on ester bonds
N	B5FG08	CC	Involved in the binding of tRNA to the ribosomes (By similarity).
N	B5FG08	DE	30S ribosomal protein S10;
N	B5FG08	DR	structural constituent of ribosome
N	B5FKZ6	DE	UPF0502 protein yceH;
N	B5FL08	DE	Protein yceI;
N	B5FNB9	CC	Catalyzes the reversible isomerization-deamination of glucosamine 6-phosphate (GlcN6P) to form fructose 6-phosphate (Fru6P) and ammonium ion (By [illegible]
N	B5FNB9	DE	GlcN6P deaminase;
N	B5FNB9	DE	Glucosamine-6-phosphate deaminase;
N	B5FNB9	DE	Glucosamine-6-phosphate isomerase;
N	B5FNB9	DR	N-acetylglucosamine metabolic process
N	B5FNB9	DR	glucosamine-6-phosphate deaminase activity
N	B5FNB9	DR	hydrolase activity
N	B5FP61	CC	Catalyzes the conversion of dethiobiotin (DTB) to biotin by the insertion of a sulfur atom into dethiobiotin via a radical- based mechanism (By similarity).
N	B5FP61	DE	Biotin synthase;
N	B5FP61	DR	2 iron, 2 sulfur cluster binding
N	B5FP61	DR	4 iron, 4 sulfur cluster binding
N	B5FP61	DR	biotin biosynthetic process
N	B5FP61	DR	biotin synthase activity
N	B5FP61	DR	metal ion binding
N	B5FPS9	DE	Triose-phosphate isomerase;
N	B5FPS9	DE	Triosephosphate isomerase;
N	B5FPS9	DR	gluconeogenesis
N	B5FPS9	DR	pentose-phosphate shunt
N	B5FPS9	DR	triose-phosphate isomerase activity
N	B5FSX7	CC	Affects glycogen biosynthesis, gluconeogenesis, cell size and surface properties
N	B5FSX7	CC	Acts to inhibit interaction between the LetD protein and the A subunit of DNA [illegible]
N	B5FSX7	CC	Also required for motility and flagellum biosynthesis through the post-transcriptional activation of flhDC expression
N	B5FSX7	CC	Regulates glycogen synthesis under both aerobic and anaerobic conditions
N	B5FSX7	CC	Seems to accelerate the degradation of glg gene transcripts, potentially through selective RNA binding
N	B5FSX7	CC	This involves binding to and stabilization of the flhDC message by CsrA (By [illegible]
N	B5FSX7	DE	Carbon storage regulator;

N	B5FSX7	DR	mRNA catabolic process
N	B5FSX7	DR	regulation of carbohydrate metabolic process
N	B5QV77	CC	Modulates recA activity (By similarity).
N	B5QV77	DE	Regulatory protein recX;
N	B5QV77	DR	regulation of DNA repair
N	B5QXR2	CC	Provides the (R)-glutamate required for cell wall biosynthesis (By similarity).
N	B5QXR2	DE	Glutamate racemase;
N	B5QXR2	DR	cellular cell wall organization
N	B5QXR2	DR	glutamate racemase activity
N	B5QXR2	DR	peptidoglycan biosynthetic process
N	B5QXR2	DR	regulation of cell shape
N	B5QY87	CC	Could be a mediator in iron transactions between iron acquisition and iron-requiring processes, such as synthesis and/or repair of Fe-S clusters in biosynthetic enzymes (By similarity).
N	B5QY87	DE	Probable Fe(2+)-trafficking protein;
N	B5QY87	DR	iron ion binding
N	B5QZW4	CC	Catalyzes the conversion of glucosamine-6-phosphate to glucosamine-1-phosphate (By similarity).
N	B5QZW4	DE	Phosphoglucosamine mutase;
N	B5QZW4	DR	carbohydrate metabolic process
N	B5QZW4	DR	magnesium ion binding
N	B5QZW4	DR	phosphoglucosamine mutase activity
N	B5R273	CC	Catalyzes the deformylation of 4-deoxy-4-formamido-L- arabinose-phosphoundecaprenol to 4-amino-4-deoxy-L-arabinose- phosphoundecaprenol
N	B5R273	CC	The modified arabinose is attached to lipid A and is required for resistance to polymyxin and cationic antimicrobial peptides (By similarity).
N	B5R273	DE	Probable 4-deoxy-4-formamido-L-arabinose-phosphoundecaprenol deformylase ArnD;
N	B5R273	DR	hydrolase activity, acting on carbon-nitrogen (but not peptide) bonds, in linear amides
N	B5R273	DR	lipid A biosynthetic process
N	B5R273	DR	response to antibiotic
N	B5R2B3	DE	UPF0270 protein yheU;
N	B5R5G1	DE	50S ribosomal protein L28;
N	B5R5G1	DR	structural constituent of ribosome
N	B5RAT3	CC	Participates in cysteine desulfuration mediated by sufS
N	B5RAT3	CC	Cysteine desulfuration mobilizes sulfur from L-cysteine to yield L-alanine and constitutes an essential step in sulfur metabolism for biosynthesis of a variety of sulfur-containing biomolecules
N	B5RAT3	CC	Functions as a sulfur acceptor for sufS, by mediating the direct transfer of the sulfur atom from the S-sulfanylcysteine of sufS, an intermediate product of cysteine desulfuration process (By similarity).
N	B5RAT3	DE	Cysteine desulfuration protein sufE;
N	B5RB01	CC	Catalyzes the transamination of N(2)-succinylornithine and alpha-ketoglutarate into N(2)-succinylglutamate semialdehyde and glutamate
N	B5RB01	CC	Can also act as an acetylornithine aminotransferase (By similarity).
N	B5RB01	DE	Succinylornithine aminotransferase;
N	B5RB01	DE	Succinylornithine transaminase;
N	B5RB01	DR	arginine metabolic process
N	B5RB01	DR	pyridoxal phosphate binding
N	B5RB01	DR	succinylornithine transaminase activity
N	B5RCY8	CC	GTPase that plays an essential role in the late steps of ribosome biogenesis (By similarity).
N	B5RCY8	DE	GTP-binding protein EngA;
N	B5RCY8	DR	ribosome biogenesis
N	B5RGC1	DE	Protein ApaG;
N	B5RGN1	CC	Catalyzes the NADPH-dependent reduction of glyoxylate and hydroxypyruvate into glycolate and glycerate, respectively (By similarity).
N	B5RGN1	DE	Glyoxylate/hydroxypyruvate reductase B;
N	B5RGN1	DR	glyoxylate reductase (NADP) activity
N	B5RGN1	DR	hydroxypyruvate reductase activity
N	B5RGN1	DR	oxidation-reduction process
N	B5RH19	CC	Protein S19 forms a complex with S13 that binds strongly to the 16S ribosomal RNA (By similarity).
N	B5RH19	DE	30S ribosomal protein S19;
N	B5RH19	DR	structural constituent of ribosome
N	B5RHG5	CC	Involved in saturated fatty acids biosynthesis (By similarity).

N	B5RHG5	DE	(3R)-hydroxymyristoyl ACP dehydrase;
N	B5RHG5	DE	(3R)-hydroxymyristoyl-[acyl-carrier-protein] dehydratase;
N	B5RHG5	DR	fatty acid biosynthetic process
N	B5RHG5	DR	hydro-lyase activity
N	B5RHG5	DR	lipid A biosynthetic process
N	B5RKW3	CC	Modifies the free amino group of the aminoacyl moiety of methionyl-tRNA(fMet)
N	B5RKW3	CC	The formyl group appears to play a dual role in the initiator identity of N-formylmethionyl-tRNA by: (I) promoting its recognition by IF2 and (II) impairing its binding to EFTu-GTP (By similarity).
N	B5RKW3	DE	Methionyl-tRNA formyltransferase;
N	B5RKW3	DR	methionyl-tRNA formyltransferase activity
N	B5RKW3	DR	methyltransferase activity
N	B5RPI6	CC	Protein S19 forms a complex with S13 that binds strongly to the 16S ribosomal RNA (By similarity).
N	B5RPI6	DE	30S ribosomal protein S19;
N	B5RPI6	DR	structural constituent of ribosome
N	B5RQP3	CC	Modifies the free amino group of the aminoacyl moiety of methionyl-tRNA(fMet)
N	B5RQP3	CC	The formyl group appears to play a dual role in the initiator identity of N-formylmethionyl-tRNA by: (I) promoting its recognition by IF2 and (II) impairing its binding to EFTu-GTP (By similarity).
N	B5RQP3	DE	Methionyl-tRNA formyltransferase;
N	B5RQP3	DR	methionyl-tRNA formyltransferase activity
N	B5RQP3	DR	methyltransferase activity
N	B5XKU4	DE	5-enolpyruvylshikimate-3-phosphate phospholyase;
N	B5XKU4	DE	Chorismate synthase;
N	B5XKU4	DR	aromatic amino acid family biosynthetic process
N	B5XKU4	DR	chorismate synthase activity
N	B5XNM4	CC	Catalyzes the synthesis of GMP from XMP (By similarity).
N	B5XNM4	DE	GMP synthase [glutamine-hydrolyzing];
N	B5XNM4	DE	GMP synthetase;
N	B5XNM4	DE	Glutamine amidotransferase;
N	B5XNM4	DR	GMP biosynthetic process
N	B5XNM4	DR	GMP synthase (glutamine-hydrolyzing) activity
N	B5XNM4	DR	glutamine metabolic process
N	B5XS09	CC	Catalyzes the decarboxylation of orotidine 5'- monophosphate (OMP) to uridine 5'-monophosphate (UMP) (By similarity).
N	B5XS09	DE	OMP decarboxylase;
N	B5XS09	DE	Orotidine 5'-phosphate decarboxylase;
N	B5XS09	DR	'de novo' UMP biosynthetic process
N	B5XS09	DR	'de novo' pyrimidine base biosynthetic process
N	B5XS09	DR	orotidine-5'-phosphate decarboxylase activity
N	B5XYH9	CC	Required, probably indirectly, for the hydroxylation of 2-octaprenylphenol to 2-octaprenyl-6-hydroxy-phenol, the fourth step in ubiquinone biosynthesis (By similarity).
N	B5XYH9	DE	Probable ubiquinone biosynthesis protein UbiB;
N	B5XYH9	DR	ubiquinone biosynthetic process
N	B5Y1W2	CC	Catalyzes the condensation of the acetyl group of acetyl-CoA with 3-methyl-2-oxobutanoate (2-oxoisovalerate) to form 3-carboxy-3-hydroxy-4-methylpentanoate (2-isopropylmalate) (By similarity).
N	B5Y1W2	DE	2-isopropylmalate synthase;
N	B5Y1W2	DE	Alpha-IPM synthase;
N	B5Y1W2	DE	Alpha-isopropylmalate synthase;
N	B5Y1W2	DR	2-isopropylmalate synthase activity
N	B5Y1W2	DR	leucine biosynthetic process
N	B5Y6G9	CC	Catalyzes the hydrolysis of pseudouridine 5'-phosphate (PsiMP) to ribose 5-phosphate and uracil (By similarity).
N	B5Y6G9	DE	Pseudouridine-5'-phosphate glycosidase;
N	B5Y6G9	DE	PsiMP glycosidase;
N	B5Y6G9	DR	hydrolase activity, acting on glycosyl bonds
N	B5Y6G9	DR	metal ion binding
N	B5YDT8	CC	With S4 and S5 plays an important role in translational accuracy (By similarity).
N	B5YDT8	DE	30S ribosomal protein S12;
N	B5YDT8	DR	structural constituent of ribosome
N	B5YER5	DE	Phenylalanine--tRNA ligase alpha chain;
N	B5YER5	DE	Phenylalanyl-tRNA synthetase alpha chain;
N	B5YER5	DR	metal ion binding

N	B5YER5	DR	phenylalanine-tRNA ligase activity
N	B5YER5	DR	phenylalanyl-tRNA aminoacylation
N	B5YEW7	CC	Binds as a heterodimer with protein S6 to the central domain of the 16S rRNA, where it helps stabilize the platform of the 30S subunit (By similarity).
N	B5YEW7	DE	30S ribosomal protein S18;
N	B5YEW7	DR	structural constituent of ribosome
N	B5YHT9	CC	Associates with free 30S ribosomal subunits (but not with 30S subunits that are part of 70S ribosomes or polysomes)
N	B5YHT9	CC	Essential for efficient processing of 16S rRNA
N	B5YHT9	CC	May interact with the 5'-terminal helix region of 16S rRNA (By similarity).
N	B5YHT9	DE	Ribosome-binding factor A;
N	B5YHT9	DR	rRNA processing
N	B5YI20	CC	F(1)F(0) ATP synthase produces ATP from ADP in the presence of a proton or sodium gradient
N	B5YI20	CC	During catalysis, ATP synthesis in the catalytic domain of F(1) is coupled via a rotary mechanism of the central stalk subunits to proton translocation (By similarity)
N	B5YI20	CC	F-type ATPases consist of two structural domains, F(1) containing the extramembraneous catalytic core and F(0) containing the membrane proton channel, linked together by a central stalk and a peripheral stalk
N	B5YI20	DE	ATP synthase F(0) sector subunit b;
N	B5YI20	DE	ATP synthase subunit b;
N	B5YI20	DE	ATPase subunit I;
N	B5YI20	DE	F-ATPase subunit b;
N	B5YI20	DE	F-type ATPase subunit b;
N	B5YI20	DR	ATP synthesis coupled proton transport
N	B5YI20	DR	hydrogen ion transmembrane transporter activity
N	B5YKK9	CC	Plays an important role in the de novo pathway of purine nucleotide biosynthesis
N	B5YKK9	CC	Catalyzes the first committed step in the biosynthesis of AMP from IMP (By similarity)
N	B5YKK9	DE	Adenylosuccinate synthetase;
N	B5YKK9	DE	IMP--aspartate ligase;
N	B5YKK9	DR	adenylosuccinate synthase activity
N	B5YKK9	DR	magnesium ion binding
N	B5YKK9	DR	purine nucleotide biosynthetic process
N	B5YKM8	DE	Protease HtpX homolog;
N	B5YKM8	DR	metal ion binding
N	B5YKM8	DR	metalloendopeptidase activity
N	B5YT52	CC	Activates KDO (a required 8-carbon sugar) for incorporation into bacterial lipopolysaccharide in Gram-negative bacteria (By similarity).
N	B5YT52	DE	3-deoxy-manno-octulosonate cytidylyltransferase;
N	B5YT52	DE	CMP-2-keto-3-deoxyoctulosonic acid synthase;
N	B5YT52	DE	CMP-KDO synthase;
N	B5YT52	DR	3-deoxy-manno-octulosonate cytidylyltransferase activity
N	B5YT52	DR	lipopolysaccharide biosynthetic process
N	B5YX01	CC	General inhibitor of pancreatic serine proteases: inhibits chymotrypsin, trypsin, elastases, factor X, kallikrein as well as a variety of other proteases (By similarity)
N	B5YX01	DR	serine-type endopeptidase inhibitor activity
N	B5YXA8	CC	RNaseP catalyzes the removal of the 5'-leader sequence from pre-tRNA to produce the mature 5'-terminus
N	B5YXA8	CC	It can also cleave other RNA substrates such as 4.5S RNA
N	B5YXA8	CC	The protein component plays an auxiliary but essential role in vivo by binding to the 5'-leader sequence and broadening the substrate specificity of the ribozyme (By similarity)
N	B5YXA8	DE	RNase P protein;
N	B5YXA8	DE	RNaseP protein;
N	B5YXA8	DE	Ribonuclease P protein component;
N	B5YXA8	DR	ribonuclease P activity
N	B5YXA8	DR	tRNA processing
N	B5YY53	DE	Protein CyaY;
N	B5Z008	CC	Catalyzes the hydrolysis of N-succinyl-L,L- diaminopimelic acid (SDAP), forming succinate and LL-2,6- diaminoheptanedioate (DAP), an intermediate involved in the bacterial biosynthesis of lysine and meso-diaminopimelic acid, an essential component of bacterial cell walls (By similarity).
N	B5Z008	DE	N-succinyl-LL-2,6-diaminoheptanedioate amidohydrolase;
N	B5Z008	DE	SDAP desuccinylase;
N	B5Z008	DE	Succinyl-diaminopimelate desuccinylase;
N	B5Z008	DR	diaminopimelate biosynthetic process

N	B5Z008	DR	metal ion binding
N	B5Z008	DR	metallopeptidase activity
N	B5Z008	DR	succinyl-diaminopimelate desuccinylase activity
N	B5Z0W0	CC	Catalyzes the oxidation of 1-pyrroline, which is spontaneously formed from 4-aminobutanal, leading to 4- aminobutanoate (GABA) (By similarity).
N	B5Z0W0	DE	1-pyrroline dehydrogenase;
N	B5Z0W0	DE	4-aminobutanal dehydrogenase;
N	B5Z0W0	DE	Gamma-aminobutyraldehyde dehydrogenase;
N	B5Z0W0	DR	aminobutyraldehyde dehydrogenase activity
N	B5Z0W0	DR	oxidation-reduction process
N	B5Z0W0	DR	putrescine catabolic process
N	B5Z2U0	CC	Catalyzes the specific phosphorylation of the 3-hydroxyl group of shikimic acid using ATP as a cosubstrate (By similarity).
N	B5Z2U0	DE	Shikimate kinase 2;
N	B5Z2U0	DR	aromatic amino acid family biosynthetic process
N	B5Z2U0	DR	metal ion binding
N	B5Z2U0	DR	shikimate kinase activity
N	B5Z3X9	CC	Catalyzes a salvage reaction resulting in the formation of AMP, that is energically less costly than de novo synthesis (By similarity).
N	B5Z3X9	DE	Adenine phosphoribosyltransferase;
N	B5Z3X9	DR	adenine phosphoribosyltransferase activity
N	B5Z3X9	DR	adenine salvage
N	B5Z3X9	DR	purine ribonucleoside salvage
N	B5Z3Y2	CC	May play a role in DNA repair
N	B5Z3Y2	CC	It may act with recF and recO (By similarity).
N	B5Z3Y2	CC	It seems to be involved in an recBC-independent recombinational process of DNA repair
N	B5Z3Y2	DE	Recombination protein recR;
N	B5Z3Y2	DR	DNA recombination
N	B5Z3Y2	DR	metal ion binding
N	B5Z4C4	CC	Involved in the anomeric conversion of L-fucose (By similarity).
N	B5Z4C4	DE	Fucose 1-epimerase;
N	B5Z4C4	DE	L-fucose mutarotase;
N	B5Z4C4	DE	Type-2 mutarotase;
N	B5Z4C4	DR	carbohydrate transport
N	B5Z4C4	DR	fucose metabolic process
N	B5Z4C4	DR	racemase and epimerase activity, acting on carbohydrates and derivatives
N	B5Z8K3	CC	This protein promotes the GTP-dependent binding of aminoacyl-tRNA to the A-site of ribosomes during protein biosynthesis (By similarity).
N	B5Z8K3	DE	Elongation factor Tu;
N	B5Z8K3	DR	GTPase activity
N	B5Z8K3	DR	translation elongation factor activity
N	B5Z8L0	CC	Involved in mRNA degradation
N	B5Z8L0	CC	Hydrolyzes single-stranded polyribonucleotides processively in the 3'- to 5'-direction (By similarity).
N	B5Z8L0	DE	Polynucleotide phosphorylase;
N	B5Z8L0	DE	Polyribonucleotide nucleotidyltransferase;
N	B5Z8L0	DR	3'-5'-exoribonuclease activity
N	B5Z8L0	DR	RNA processing
N	B5Z8L0	DR	mRNA catabolic process
N	B5Z8L0	DR	polyribonucleotide nucleotidyltransferase activity
N	B5Z8U9	CC	Binds to the 23S rRNA (By similarity).
N	B5Z8U9	DE	50S ribosomal protein L15;
N	B5Z8U9	DR	structural constituent of ribosome
N	B5ZAS4	CC	May play a role in DNA repair
N	B5ZAS4	CC	It may act with recF and recO (By similarity).
N	B5ZAS4	CC	It seems to be involved in an recBC-independent recombinational process of DNA repair
N	B5ZAS4	DE	Recombination protein recR;
N	B5ZAS4	DR	DNA recombination
N	B5ZAS4	DR	metal ion binding
N	B5ZBT6	CC	The ruvA-ruvB complex in the presence of ATP renatures cruciform structure in supercoiled DNA with palindromic sequence, indicating that it may promote strand exchange reactions in homologous recombination
N	B5ZBT6	CC	RuvA stimulates, in the presence of DNA, the weak ATPase activity of ruvB (By similarity).

N	B5ZBT6	CC	RuvAB is an helicase that mediates the Holliday junction migration by localized denaturation and reannealing
N	B5ZBT6	DE	Holliday junction ATP-dependent DNA helicase ruvA;
N	B5ZBT6	DR	DNA recombination
N	B5ZBT6	DR	four-way junction helicase activity
N	B5ZPY6	DE	Anthranilate phosphoribosyltransferase;
N	B5ZPY6	DR	anthranilate phosphoribosyltransferase activity
N	B5ZPY6	DR	tryptophan biosynthetic process
N	B5ZXQ5	CC	Required for accurate and efficient protein synthesis under certain stress conditions
N	B5ZXQ5	CC	Back- translocation proceeds from a post-translocation (POST) complex to a pre-translocation (PRE) complex, thus giving elongation factor G a second chance to translocate the tRNAs correctly
N	B5ZXQ5	CC	Binds to ribosomes in a GTP-dependent manner (By similarity).
N	B5ZXQ5	CC	May act as a fidelity factor of the translation reaction, by catalyzing a one-codon backward translocation of tRNAs on improperly translocated ribosomes
N	B5ZXQ5	DE	Elongation factor 4;
N	B5ZXQ5	DE	Ribosomal back-translocase LepA;
N	B5ZXQ5	DR	GTPase activity
N	B5ZYL3	CC	NDH-1 shuttles electrons from NADH, via FMN and iron- sulfur (Fe-S) centers, to quinones in the respiratory chain
N	B5ZYL3	CC	Couples the redox reaction to proton translocation (for every two electrons transferred, four hydrogen ions are translocated across the cytoplasmic membrane), and thus conserves the redox energy in a proton gradient (By similarity)
N	B5ZYL3	CC	The immediate electron acceptor for the enzyme in this species is believed to be ubiquinone
N	B5ZYL3	DE	NADH dehydrogenase I subunit B;
N	B5ZYL3	DE	NADH-quinone oxidoreductase subunit B;
N	B5ZYL3	DE	NDH-1 subunit B;
N	B5ZYL3	DR	4 iron, 4 sulfur cluster binding
N	B5ZYL3	DR	NADH dehydrogenase (ubiquinone) activity
N	B5ZYL3	DR	metal ion binding
N	B5ZYL3	DR	oxidation-reduction process
N	B5ZYL3	DR	quinone binding
N	B6DCK1	DE	Toxin-like structure LSTX-A12;
N	B6DCK1	DE	U1-lycotoxin-Ls1b;
N	B6EJA3	CC	The beta subunit is responsible for the synthesis of L- tryptophan from indole and L-serine (By similarity).
N	B6EJA3	DE	Tryptophan synthase beta chain;
N	B6EJA3	DR	pyridoxal phosphate binding
N	B6EJA3	DR	tryptophan synthase activity
N	B6EKX9	DE	UPF0246 protein VSAL_I2547;
N	B6EQ13	CC	Catalyzes the oxidation of either pyridoxine 5'-phosphate (PNP) or pyridoxamine 5'-phosphate (PMP) into pyridoxal 5'-phosphate (PLP) (By similarity)
N	B6EQ13	DE	PNP/PMP oxidase;
N	B6EQ13	DE	Pyridoxal 5'-phosphate synthase;
N	B6EQ13	DE	Pyridoxine/pyridoxamine 5'-phosphate oxidase;
N	B6EQ13	DR	oxidation-reduction process
N	B6EQ13	DR	pyridoxamine-phosphate oxidase activity
N	B6EQ13	DR	pyridoxine biosynthetic process
N	B6H460	CC	Mitochondrial GTPase that catalyzes the GTP-dependent ribosomal translocation step during translation elongation
N	B6H460	CC	Catalyzes the coordinated movement of the two tRNA molecules, the mRNA and conformational changes in the ribosome (By similarity).
N	B6H460	CC	During this step, the ribosome changes from the pre-translocational (PRE) to the post-translocational (POST) state as the newly formed A- site-bound peptidyl-tRNA and P-site-bound deacylated tRNA move to the P and E sites, respectively
N	B6H460	DE	Elongation factor G 1, mitochondrial;
N	B6H460	DE	Elongation factor G, mitochondrial;
N	B6H460	DE	Elongation factor G1;
N	B6H460	DR	GTPase activity
N	B6H460	DR	translation elongation factor activity
N	B6HZJ3	CC	May be involved in recombination (By similarity).
N	B6HZJ3	DE	Recombination-associated protein rdgC;
N	B6HZJ3	DR	DNA recombination

N	B6I1M4	CC	Required for the timely initiation of chromosomal replication via direct interactions with the dnaA initiator protein (By similarity).
N	B6I1M4	DE	DnaA initiator-associating protein diaA;
N	B6I1M4	DR	DNA replication
N	B6I1M4	DR	carbohydrate metabolic process
N	B6I1M4	DR	sugar binding
N	B6I3Y4	CC	Responsible for the low-affinity transport of potassium into the cell, with the probable concomitant uptake of protons (symport system) (By similarity).
N	B6I3Y4	DE	Kup system potassium uptake protein;
N	B6I3Y4	DE	Low affinity potassium transport system protein kup;
N	B6I3Y4	DR	potassium ion transmembrane transporter activity
N	B6I3Y4	DR	symporter activity
N	B6I4P5	DE	L-rhamnose isomerase;
N	B6I4P5	DR	L-rhamnose isomerase activity
N	B6I4P5	DR	manganese ion binding
N	B6I4P5	DR	rhamnose metabolic process
N	B6I503	CC	Represses the expression of the murPQ operon involved in the uptake and degradation of N-acetylmuramic acid (MurNAc)
N	B6I503	CC	Binds to two adjacent inverted repeats within the operator region
N	B6I503	CC	MurNAc 6-phosphate, the substrate of murQ, is the specific inducer that weakens binding of murR to the operator (By similarity).
N	B6I503	DE	HTH-type transcriptional regulator murR;
N	B6I503	DE	MurPQ operon repressor;
N	B6I503	DR	carbohydrate metabolic process
N	B6I503	DR	regulation of transcription, DNA-dependent
N	B6I503	DR	sequence-specific DNA binding transcription factor activity
N	B6I503	DR	sugar binding
N	B6I665	CC	Probably involved in ribonucleotide reductase function (By similarity).
N	B6I665	DE	Protein nrdI;
N	B6I7K8	CC	Catalyzes the thiamine diphosphate-dependent decarboxylation of 2-oxoglutarate and the subsequent addition of the resulting succinic semialdehyde-thiamine pyrophosphate anion to isochorismate to yield 2-succinyl-5-enolpyruvyl-6-hydroxy-3- cyclohexene-1-carboxylate (SEPHCHC) (By similarity).
N	B6I7K8	DE	2-succinyl-5-enolpyruvyl-6-hydroxy-3-cyclohexene-1-carboxylate synthase;
N	B6I7K8	DE	Menaquinone biosynthesis protein menD;
N	B6I7K8	DE	SEPHCHC synthase;
N	B6I7K8	DR	2-succinyl-5-enolpyruvyl-6-hydroxy-3-cyclohexene-1-carboxylic-acid synthase activity
N	B6I7K8	DR	menaquinone biosynthetic process
N	B6I7K8	DR	metal ion binding
N	B6I7K8	DR	thiamine pyrophosphate binding
N	B6I8L0	CC	Endonuclease IV plays a role in DNA repair
N	B6I8L0	CC	It cleaves phosphodiester bonds at apurinic or apyrimidinic sites (AP sites) to produce new 5'-ends that are base-free deoxyribose 5-phosphate residues
N	B6I8L0	CC	It preferentially attacks modified AP sites created by bleomycin and neocarzinostatin (By similarity).
N	B6I8L0	DE	Endodeoxyribonuclease IV;
N	B6I8L0	DE	Endonuclease IV;
N	B6I8L0	DE	Probable endonuclease 4;
N	B6I8L0	DR	deoxyribonuclease IV (phage-T4-induced) activity
N	B6I8L0	DR	zinc ion binding
N	B6I919	DE	Dihydroorotate dehydrogenase;
N	B6I919	DE	Dihydroorotate oxidase;
N	B6I919	DR	'de novo' pyrimidine base biosynthetic process
N	B6I919	DR	UMP biosynthetic process
N	B6I919	DR	dihydroorotate oxidase activity
N	B6I919	DR	oxidation-reduction process
N	B6IP33	DE	UPF0178 protein RC1_2062;
N	B6J853	CC	Catalyzes the methylthiolation of N6- (dimethylallyl)adenosine (i(6)A), leading to the formation of 2- methylthio-N6-(dimethylallyl)adenosine (ms(2)i(6)A) at position 37 in tRNAs that read codons beginning with uridine (By similarity).
N	B6J853	DE	(Dimethylallyl)adenosine tRNA methylthiotransferase miaB;
N	B6J853	DE	tRNA-i(6)A37 methylthiotransferase;
N	B6J853	DR	4 iron, 4 sulfur cluster binding
N	B6J853	DR	metal ion binding
N	B6J853	DR	tRNA modification

N	B6J853	DR	transferase activity
N	B6JCI8	DE	Dihydrodipicolinate reductase;
N	B6JCI8	DR	diaminopimelate biosynthetic process
N	B6JCI8	DR	dihydrodipicolinate reductase activity
N	B6JCI8	DR	oxidation-reduction process
N	B6JIP4	CC	This is one of the proteins that binds to the 5S RNA in the ribosome where it forms part of the central protuberance (By similarity).
N	B6JIP4	DE	50S ribosomal protein L25;
N	B6JIP4	DE	General stress protein CTC;
N	B6JIP4	DR	5S rRNA binding
N	B6JIP4	DR	structural constituent of ribosome
N	B6JKP5	DE	Arginine--tRNA ligase;
N	B6JKP5	DE	Arginyl-tRNA synthetase;
N	B6JKP5	DR	arginine-tRNA ligase activity
N	B6JKP5	DR	arginyl-tRNA aminoacylation
N	B6JN39	DE	50S ribosomal protein L10;
N	B6JN39	DR	ribosome biogenesis
N	B6JN39	DR	structural constituent of ribosome
N	B6JNU2	CC	Binds specifically to the ssrA RNA (tmRNA) and is required for stable association of ssrA with ribosomes (By similarity).
N	B6JNU2	DE	SsrA-binding protein;
N	B6K463	CC	Component of the ERMES/MDM complex, which serves as a molecular tether to connect the endoplasmic reticulum and mitochondria
N	B6K463	CC	Can associate with the SAM(core) complex as well as the mdm12-mmm1 complex, both involved in late steps of the major beta-barrel assembly pathway, that is responsible for biogenesis of all outer membrane beta-barrel proteins
N	B6K463	CC	Components of this complex are involved in the control of mitochondrial shape and protein biogenesis and may function in phospholipid exchange
N	B6K463	CC	Functions in the tom40-specific route of the assembly of outer membrane beta-barrel proteins, including the association of tom40 with the receptor tom22 and small TOM proteins
N	B6K463	CC	May act as a switch that shuttles between both complexes and channels precursor proteins into the tom40-specific pathway
N	B6K463	CC	Plays a role in mitochondrial morphology and in the inheritance of mitochondria (By similarity).
N	B6K463	CC	mdm10 is involved in the late assembly steps of the general translocase of the mitochondrial outer membrane (TOM complex)
N	B6K463	DE	Mitochondrial distribution and morphology protein 10;
N	B6K463	DE	Mitochondrial inheritance component mdm10;
N	B6YXJ9	CC	Catalyzes the production of spermidine from putrescine and decarboxylated S-adenosylmethionine (dcSAM), which serves as an aminopropyl donor (By similarity).
N	B6YXJ9	DE	Probable spermidine synthase;
N	B6YXJ9	DE	Putrescine aminopropyltransferase;
N	B6YXJ9	DR	spermidine biosynthetic process
N	B6YXJ9	DR	spermidine synthase activity
N	B7GV08	CC	Participates actively in the response to hyperosmotic and heat shock by preventing the aggregation of stress-denatured proteins and by disaggregating proteins, also in an autonomous, dnaK-independent fashion
N	B7GV08	CC	Also involved, together with dnaK and grpE, in the DNA replication of plasmids through activation of initiation proteins (By similarity).
N	B7GV08	CC	GrpE releases ADP from dnaK; ATP binding to dnaK triggers the release of the substrate protein, thus completing the reaction cycle
N	B7GV08	CC	Several rounds of ATP-dependent interactions between dnaJ, dnaK and grpE are required for fully efficient folding
N	B7GV08	CC	Unfolded proteins bind initially to dnaJ; upon interaction with the dnaJ-bound protein, dnaK hydrolyzes its bound ATP, resulting in the formation of a stable complex.
N	B7GV08	DE	Chaperone protein dnaJ;
N	B7GV08	DR	DNA replication
N	B7GV08	DR	heat shock protein binding
N	B7GV08	DR	metal ion binding
N	B7GV08	DR	protein folding
N	B7GV08	DR	response to heat
N	B7GV08	DR	unfolded protein binding
N	B7GVV0	CC	Catalyzes the hydrolysis of N(2)-succinylarginine into N(2)-succinylornithine, ammonia and CO(2) (By similarity).

N	B7GVV0	DE	N-succinylarginine dihydrolase;
N	B7GVV0	DR	N-succinylarginine dihydrolase activity
N	B7GVV0	DR	arginine metabolic process
N	B7GWA1	DE	Cysteine--tRNA ligase;
N	B7GWA1	DE	Cysteinyl-tRNA synthetase;
N	B7GWA1	DR	cysteine-tRNA ligase activity
N	B7GWA1	DR	cysteinyl-tRNA aminoacylation
N	B7GWA1	DR	metal ion binding
N	B7H746	CC	Cell wall formation (By similarity).
N	B7H746	DE	UDP-N-acetylmuramate--L-alanine ligase;
N	B7H746	DE	UDP-N-acetylmuramoyl-L-alanine synthetase;
N	B7H746	DR	UDP-N-acetylmuramate-L-alanine ligase activity
N	B7H746	DR	cell division
N	B7H746	DR	cellular cell wall organization
N	B7H746	DR	peptidoglycan biosynthetic process
N	B7H746	DR	regulation of cell shape
N	B7HDT6	CC	One of the essential components for the initiation of protein synthesis
N	B7HDT6	CC	Also involved in the hydrolysis of GTP during the formation of the 70S ribosomal complex (By similarity).
N	B7HDT6	CC	Protects formylmethionyl-tRNA from spontaneous hydrolysis and promotes its binding to the 30S ribosomal subunits
N	B7HDT6	DE	Translation initiation factor IF-2;
N	B7HDT6	DR	GTPase activity
N	B7HDT6	DR	translation initiation factor activity
N	B7IIS9	CC	Exchanges the guanine residue with 7-aminomethyl-7- deazaguanine in tRNAs with GU(N) anticodons (tRNA-Asp, -Asn, -His and -Tyr)
N	B7IIS9	CC	After this exchange, a cyclopentendiol moiety is attached to the 7-aminomethyl group of 7-deazaguanine, resulting in the hypermodified nucleoside queuosine (Q) (7-(((4,5-cis- dihydroxy-2-cyclopenten-1-yl)amino)methyl)-7- deazaguanosine) (By similarity)
N	B7IIS9	DE	Guanine insertion enzyme;
N	B7IIS9	DE	Queuine tRNA-ribosyltransferase;
N	B7IIS9	DE	tRNA-guanine transglycosylase;
N	B7IIS9	DR	metal ion binding
N	B7IIS9	DR	queuine tRNA-ribosyltransferase activity
N	B7IIS9	DR	queuosine biosynthetic process
N	B7IL84	CC	The heterodimer acts as both an ATP-dependent DNA helicase and an ATP-dependent, dual-direction single-stranded exonuclease
N	B7IL84	CC	Recognizes the chi site generating a DNA molecule suitable for the initiation of homologous recombination
N	B7IL84	CC	The AddA nuclease domain is required for chi fragment generation; this subunit has the helicase and 3' -> 5' nuclease activities (By similarity).
N	B7IL84	DE	ATP-dependent helicase/nuclease AddA;
N	B7IL84	DE	ATP-dependent helicase/nuclease subunit A;
N	B7IL84	DR	ATP-dependent DNA helicase activity
N	B7IL84	DR	double-strand break repair
N	B7IL84	DR	exonuclease activity
N	B7ILE8	CC	Asymmetrically hydrolyzes Ap4p to yield AMP and ATP (By similarity).
N	B7ILE8	DE	Ap4A hydrolase;
N	B7ILE8	DE	Bis(5'-nucleosyl)-tetraphosphatase prpE [asymmetrical];
N	B7ILE8	DE	Diadenosine 5',5'''-P1,P4-tetraphosphate asymmetrical hydrolase;
N	B7ILE8	DE	Diadenosine tetraphosphatase;
N	B7ILE8	DR	bis(5'-nucleosyl)-tetraphosphatase (asymmetrical) activity
N	B7IS38	DE	UPF0133 protein BCG9842_B5293;
N	B7ISJ0	DE	Imidazolone-5-propionate hydrolase;
N	B7ISJ0	DE	Imidazolonepropionase;
N	B7ISJ0	DR	histidine catabolic process to glutamate and formamide
N	B7ISJ0	DR	imidazolonepropionase activity
N	B7ISJ0	DR	metal ion binding
N	B7IT28	CC	One of the primary rRNA binding proteins, it binds specifically to the 5'-end of 16S ribosomal RNA (By similarity).
N	B7IT28	DE	30S ribosomal protein S17;
N	B7IT28	DR	structural constituent of ribosome
N	B7J0P3	CC	This protein is involved in the repair of mismatches in DNA
N	B7J0P3	CC	It is possible that it carries out the mismatch recognition step
N	B7J0P3	CC	This protein has a weak ATPase activity (By similarity).

N	B7J0P3	DE	DNA mismatch repair protein mutS;
N	B7J0P3	DR	mismatch repair
N	B7J0P3	DR	mismatched DNA binding
N	B7JGN4	CC	F(1)F(0) ATP synthase produces ATP from ADP in the presence of a proton or sodium gradient
N	B7JGN4	CC	During catalysis, ATP synthesis in the catalytic domain of F(1) is coupled via a rotary mechanism of the central stalk subunits to proton translocation (By similarity)
N	B7JGN4	CC	F-type ATPases consist of two structural domains, F(1) containing the extramembraneous catalytic core and F(0) containing the membrane proton channel, linked together by a central stalk and a peripheral stalk
N	B7JGN4	DE	ATP synthase F(0) sector subunit b;
N	B7JGN4	DE	ATP synthase subunit b;
N	B7JGN4	DE	ATPase subunit I;
N	B7JGN4	DE	F-ATPase subunit b;
N	B7JGN4	DE	F-type ATPase subunit b;
N	B7JGN4	DR	ATP synthesis coupled proton transport
N	B7JGN4	DR	hydrogen ion transmembrane transporter activity
N	B7JGY9	CC	GTPase that plays an essential role in the late steps of ribosome biogenesis (By similarity).
N	B7JGY9	DE	GTP-binding protein EngA;
N	B7JGY9	DR	ribosome biogenesis
N	B7JK06	CC	Specifically methylates the N4 position of cytidine in position 1402 (C1402) of 16S rRNA (By similarity).
N	B7JK06	DE	16S rRNA m(4)C1402 methyltransferase;
N	B7JK06	DE	Ribosomal RNA small subunit methyltransferase H;
N	B7JK06	DE	rRNA (cytosine-N(4)-)-methyltransferase RsmH;
N	B7JK06	DR	methyltransferase activity
N	B7JK06	DR	rRNA processing
N	B7JK94	CC	Converts 4-diphosphocytidyl-2-C-methyl-D-erythritol 2- phosphate into 2-C-methyl-D-erythritol 2,4-cyclodiphosphate (MECDP) and CMP (By similarity).
N	B7JK94	DE	2-C-methyl-D-erythritol 2,4-cyclodiphosphate synthase;
N	B7JK94	DE	MECDP-synthase;
N	B7JK94	DR	2-C-methyl-D-erythritol 2,4-cyclodiphosphate synthase activity
N	B7JK94	DR	metal ion binding
N	B7JK94	DR	terpenoid biosynthetic process
N	B7JNF2	DE	Small, acid-soluble spore protein K;
N	B7JNF2	DR	asexual sporulation
N	B7JNF2	DR	sporulation resulting in formation of a cellular spore
N	B7JUZ9	CC	Binds to the 23S rRNA (By similarity).
N	B7JUZ9	DE	50S ribosomal protein L9;
N	B7JUZ9	DR	structural constituent of ribosome
N	B7JX56	DE	Anthranilate phosphoribosyltransferase;
N	B7JX56	DR	anthranilate phosphoribosyltransferase activity
N	B7JX56	DR	tryptophan biosynthetic process
N	B7K1J7	CC	Component of the cytochrome b6-f complex, which mediates electron transfer between photosystem II (PSII) and photosystem I (PSI), cyclic electron flow around PSI, and state transitions (By similarity).
N	B7K1J7	DE	Cytochrome b6-f complex subunit 7;
N	B7K1J7	DE	Cytochrome b6-f complex subunit VII;
N	B7K1J7	DE	Cytochrome b6-f complex subunit petM;
N	B7K1J7	DR	electron transport chain
N	B7K1J7	DR	photosynthesis
N	B7K422	DE	UPF0133 protein PCC8801_2554;
N	B7K8U6	DE	Argininosuccinate lyase;
N	B7K8U6	DE	Arginosuccinase;
N	B7K8U6	DR	arginine biosynthetic process via ornithine
N	B7K8U6	DR	argininosuccinate lyase activity
N	B7KCX9	CC	Plays an important role in the de novo pathway of purine nucleotide biosynthesis
N	B7KCX9	CC	Catalyzes the first committed step in the biosynthesis of AMP from IMP (By similarity)
N	B7KCX9	DE	Adenylosuccinate synthetase;
N	B7KCX9	DE	IMP--aspartate ligase;
N	B7KCX9	DR	adenylosuccinate synthase activity
N	B7KCX9	DR	magnesium ion binding
N	B7KCX9	DR	purine nucleotide biosynthetic process

N	B7KJX8	CC	Catalyzes the condensation of the acetyl group of acetyl-CoA with 3-methyl-2-oxobutanoate (2-oxoisovalerate) to form 3-carboxy-3-hydroxy-4-methylpentanoate (2-isopropylmalate) (By similarity).
N	B7KJX8	DE	2-isopropylmalate synthase;
N	B7KJX8	DE	Alpha-IPM synthase;
N	B7KJX8	DE	Alpha-isopropylmalate synthase;
N	B7KJX8	DR	2-isopropylmalate synthase activity
N	B7KJX8	DR	leucine biosynthetic process
N	B7L0S0	CC	One of the primary rRNA binding proteins, it binds specifically to the 5'-end of 16S ribosomal RNA (By similarity).
N	B7L0S0	DE	30S ribosomal protein S17;
N	B7L0S0	DR	structural constituent of ribosome
N	B7L4C3	CC	Involved in mRNA degradation
N	B7L4C3	CC	Hydrolyzes single-stranded polyribonucleotides processively in the 3' to 5' direction (By similarity).
N	B7L4C3	DE	Exoribonuclease 2;
N	B7L4C3	DE	Exoribonuclease II;
N	B7L4C3	DE	Ribonuclease II;
N	B7L4C3	DR	RNA catabolic process
N	B7L4C3	DR	exoribonuclease II activity
N	B7LGX2	CC	Peptide chain release factor 1 directs the termination of translation in response to the peptide chain termination codons UAG and UAA (By similarity).
N	B7LGX2	DE	Peptide chain release factor 1;
N	B7LGX2	DR	translation release factor activity, codon specific
N	B7LKC4	DE	Histidine--tRNA ligase;
N	B7LKC4	DE	Histidyl-tRNA synthetase;
N	B7LKC4	DR	histidine-tRNA ligase activity
N	B7LKC4	DR	histidyl-tRNA aminoacylation
N	B7LLY4	CC	Binds as a heterodimer with protein S6 to the central domain of the 16S rRNA, where it helps stabilize the platform of the 30S subunit (By similarity).
N	B7LLY4	DE	30S ribosomal protein S18;
N	B7LLY4	DR	structural constituent of ribosome
N	B7LM86	CC	Conversion of glycerol 3-phosphate to dihydroxyacetone
N	B7LM86	CC	Uses fumarate or nitrate as electron acceptor (By similarity).
N	B7LM86	DE	Anaerobic G-3-P dehydrogenase subunit B;
N	B7LM86	DE	Anaerobic G3Pdhase B;
N	B7LM86	DE	Anaerobic glycerol-3-phosphate dehydrogenase subunit B;
N	B7LM86	DR	electron carrier activity
N	B7LM86	DR	oxidation-reduction process
N	B7LM86	DR	sn-glycerol-3-phosphate:ubiquinone-8 oxidoreductase activity
N	B7LMG5	CC	Bidirectionally degrades single-stranded DNA into large acid-insoluble oligonucleotides, which are then degraded further into small acid-soluble oligonucleotides (By similarity).
N	B7LMG5	DE	Exodeoxyribonuclease 7 small subunit;
N	B7LMG5	DE	Exodeoxyribonuclease VII small subunit;
N	B7LMG5	DE	Exonuclease VII small subunit;
N	B7LMG5	DR	DNA catabolic process
N	B7LMG5	DR	exodeoxyribonuclease VII activity
N	B7LRL7	DE	Protein AaeX;
N	B7LSC6	CC	Rhomboid-type serine protease that catalyzes intramembrane proteolysis (By similarity).
N	B7LSC6	DE	Intramembrane serine protease;
N	B7LSC6	DE	Rhomboid protease glpG;
N	B7LSC6	DR	serine-type endopeptidase activity
N	B7LWC1	CC	dGTPase preferentially hydrolyzes dGTP over the other canonical NTPs (By similarity).
N	B7LWC1	DE	Deoxyguanosinetriphosphate triphosphohydrolase;
N	B7LWC1	DE	dGTP triphosphohydrolase;
N	B7LWC1	DR	GTP metabolic process
N	B7LWC1	DR	dGTPase activity
N	B7LWC1	DR	magnesium ion binding
N	B7LX67	CC	Negative regulator of phage lambda lysogenization
N	B7LX67	CC	Acts probably by holding CII on the membrane surface, away from the target promoters, but close to the ftsH protease (By similarity).
N	B7LX67	CC	Contributes to the degradation of the phage regulatory protein CII
N	B7LX67	DE	High frequency lysogenization protein hflD;
N	B7LX89	CC	Cell division inhibitor that blocks the formation of polar Z ring septums
N	B7LX89	CC	Prevents ftsZ polymerization (By similarity).

N	B7LX89	CC	Rapidly oscillates between the poles of the cell to destabilize ftsZ filaments that have formed before they mature into polar Z rings
N	B7LX89	DE	Probable septum site-determining protein minC;
N	B7LX89	DR	barrier septum formation
N	B7LX89	DR	cell morphogenesis
N	B7LX89	DR	regulation of cell cycle
N	B7LX89	DR	regulation of cell division
N	B7LXM2	CC	Catalyzes the 2'-O-methylation at nucleotide C2498 in 23S rRNA (By similarity).
N	B7LXM2	DE	23S rRNA 2'-O-ribose methyltransferase rlmM;
N	B7LXM2	DE	Ribosomal RNA large subunit methyltransferase M;
N	B7LXM2	DR	methyltransferase activity
N	B7LXM2	DR	nucleic acid binding
N	B7LXM2	DR	rRNA processing
N	B7LY03	DE	UPF0263 protein yciU;
N	B7M0M0	CC	Multidrug efflux pump that functions probably as a Na(+)/drug antiporter (By similarity).
N	B7M0M0	DE	Multidrug resistance protein mdtK;
N	B7M0M0	DE	Multidrug-efflux transporter;
N	B7M0M0	DR	antiporter activity
N	B7M0M0	DR	drug transmembrane transporter activity
N	B7M0M0	DR	sodium ion transport
N	B7M1F2	DE	NH(3)-dependent NAD(+) synthetase;
N	B7M1F2	DR	NAD biosynthetic process
N	B7M1F2	DR	NAD+ synthase (glutamine-hydrolyzing) activity
N	B7M1F2	DR	NAD+ synthase activity
N	B7M586	CC	Catalyzes the last two sequential reactions in the de novo biosynthetic pathway for UDP-GlcNAc
N	B7M586	CC	Responsible for the acetylation of Glc-N-1-P to give GlcNAc-1-P and for the uridyl transfer from UTP to GlcNAc-1-P which produces UDP-GlcNAc (By similarity)
N	B7M586	DE	Bifunctional protein glmU;
N	B7M586	DE	Glucosamine-1-phosphate N-acetyltransferase;
N	B7M586	DE	N-acetylglucosamine-1-phosphate uridyltransferase;
N	B7M586	DE	UDP-N-acetylglucosamine pyrophosphorylase;
N	B7M586	DR	UDP-N-acetylglucosamine diphosphorylase activity
N	B7M586	DR	cell morphogenesis
N	B7M586	DR	cellular cell wall organization
N	B7M586	DR	glucosamine-1-phosphate N-acetyltransferase activity
N	B7M586	DR	lipopolysaccharide biosynthetic process
N	B7M586	DR	magnesium ion binding
N	B7M586	DR	peptidoglycan biosynthetic process
N	B7M586	DR	regulation of cell shape
N	B7M7L1	CC	Catalyzes the synthesis of GMP from XMP (By similarity).
N	B7M7L1	DE	GMP synthase [glutamine-hydrolyzing];
N	B7M7L1	DE	GMP synthetase;
N	B7M7L1	DE	Glutamine amidotransferase;
N	B7M7L1	DR	GMP biosynthetic process
N	B7M7L1	DR	GMP synthase (glutamine-hydrolyzing) activity
N	B7M7L1	DR	glutamine metabolic process
N	B7M826	DE	Uncharacterized MFS-type transporter ycaD;
N	B7M826	DR	transmembrane transport
N	B7M826	DR	transporter activity
N	B7MFR2	CC	Catalyzes the reversible adenylation of nicotinate mononucleotide (NaMN) to nicotinic acid adenine dinucleotide (NaAD) (By similarity).
N	B7MFR2	DE	Deamido-NAD(+) diphosphorylase;
N	B7MFR2	DE	Deamido-NAD(+) pyrophosphorylase;
N	B7MFR2	DE	NaMN adenylyltransferase;
N	B7MFR2	DE	Nicotinate mononucleotide adenylyltransferase;
N	B7MFR2	DE	Probable nicotinate-nucleotide adenylyltransferase;
N	B7MFR2	DR	NAD biosynthetic process
N	B7MFR2	DR	nicotinate-nucleotide adenylyltransferase activity
N	B7MHM6	DE	UPF0434 protein ycaR;
N	B7MIQ4	CC	Required for accurate and efficient protein synthesis under certain stress conditions
N	B7MIQ4	CC	Back- translocation proceeds from a post-translocation (POST) complex to a pre-translocation (PRE) complex, thus giving elongation factor G a second chance to translocate the tRNAs correctly
N	B7MIQ4	CC	Binds to ribosomes in a GTP-dependent manner (By similarity).

N	B7MIQ4	CC	May act as a fidelity factor of the translation reaction, by catalyzing a one-codon backward translocation of tRNAs on improperly translocated ribosomes
N	B7MIQ4	DE	Elongation factor 4;
N	B7MIQ4	DE	Ribosomal back-translocase LepA;
N	B7MIQ4	DR	GTPase activity
N	B7MJB5	CC	Cleaves the N-terminal amino acid of tripeptides (By similarity).
N	B7MJB5	DE	Aminotripeptidase;
N	B7MJB5	DE	Peptidase T;
N	B7MJB5	DE	Tripeptidase;
N	B7MJB5	DE	Tripeptide aminopeptidase;
N	B7MJB5	DR	metallopeptidase activity
N	B7MJB5	DR	peptide metabolic process
N	B7MJB5	DR	tripeptide aminopeptidase activity
N	B7MJB5	DR	zinc ion binding
N	B7MQY8	CC	Necessary for formate dehydrogenase activity (By similarity).
N	B7MQY8	DE	Protein FdhD;
N	B7MQY8	DR	formate dehydrogenase (NAD+) activity
N	B7MZ67	CC	Specific inhibitor of chromosomal initiation of replication in vitro
N	B7MZ67	CC	Binds the three 13-mers in the origin (oriC) to block initiation of replication (By similarity).
N	B7MZ67	DE	Chromosome initiation inhibitor;
N	B7MZ67	DE	OriC replication inhibitor;
N	B7MZ67	DR	regulation of transcription, DNA-dependent
N	B7MZ67	DR	sequence-specific DNA binding transcription factor activity
N	B7MZK6	DE	UPF0149 protein ygfB;
N	B7N1C2	CC	One of the primary rRNA binding proteins, it binds directly to 16S rRNA where it nucleates assembly of the head domain of the 30S subunit
N	B7N1C2	CC	Is located at the subunit interface close to the decoding center, probably blocks exit of the E-site tRNA (By similarity).
N	B7N1C2	DE	30S ribosomal protein S7;
N	B7N1C2	DR	structural constituent of ribosome
N	B7N3C1	CC	Component of the SOS system and an inhibitor of cell division
N	B7N3C1	CC	Accumulation of sulA causes rapid cessation of cell division and the appearance of long, non-septate filaments
N	B7N3C1	CC	In the presence of GTP, binds a polymerization-competent form of ftsZ in a 1:1 ratio, thus inhibiting ftsZ polymerization and therefore preventing it from participating in the assembly of the Z ring
N	B7N3C1	CC	This mechanism prevents the premature segregation of damaged DNA to daughter cells during cell division (By similarity).
N	B7N3C1	DE	Cell division inhibitor sulA;
N	B7N3C1	DR	barrier septum formation
N	B7N3C1	DR	negative regulation of cell division
N	B7N3F4	CC	Interacts with CbpA and inhibits both the DnaJ-like co- chaperone activity and the DNA binding activity of CbpA
N	B7N3F4	CC	Does not inhibit the co-chaperone activity of DnaJ (By similarity).
N	B7N3F4	CC	Together with CbpA, modulates the activity of the DnaK chaperone system
N	B7N3F4	DE	Chaperone modulatory protein CbpM;
N	B7N7R3	CC	Catalyzes the reversible transfer of the CoA moiety from gamma-butyrobetainyl-CoA to L-carnitine to generate L-carnitinyl- CoA and gamma-butyrobetaine
N	B7N7R3	CC	Is also able to catalyze the reversible transfer of the CoA moiety from gamma-butyrobetainyl- CoA or L-carnitinyl-CoA to crotonobetaine to generate crotonobetainyl-CoA (By similarity).
N	B7N7R3	DE	Crotonobetainyl-CoA:carnitine CoA-transferase;
N	B7N7R3	DR	transferase activity
N	B7NA78	CC	The UvrABC repair system catalyzes the recognition and processing of DNA lesions
N	B7NA78	CC	A damage recognition complex composed of 2 uvrA and 2 uvrB subunits scans DNA for abnormalities
N	B7NA78	CC	DNA wrap is dependent on ATP binding by uvrB and probably causes local melting of the DNA helix, facilitating insertion of uvrB beta-hairpin between the DNA strands
N	B7NA78	CC	If a lesion is found the uvrA subunits dissociate and the uvrB-DNA preincision complex is formed
N	B7NA78	CC	If no lesion is found, the DNA wraps around the other uvrB subunit that will check the other stand for damage (By similarity).
N	B7NA78	CC	Then uvrB probes one DNA strand for the presence of a lesion
N	B7NA78	CC	This complex is subsequently bound by uvrC and the second uvrB is released

N	B7NA78	CC	Upon binding of the uvrA(2)B(2) complex to a putative damaged site, the DNA wraps around one uvrB monomer
N	B7NA78	DE	Excinuclease ABC subunit B;
N	B7NA78	DE	Protein uvrB;
N	B7NA78	DE	UvrABC system protein B;
N	B7NA78	DR	excinuclease ABC activity
N	B7NA78	DR	helicase activity
N	B7NA78	DR	nucleotide-excision repair
N	B7NA95	DE	UPF0194 membrane protein ybhG;
N	B7NA95	DR	protein secretion
N	B7NA95	DR	protein transporter activity
N	B7NAJ3	DE	Putative transport protein YbjL;
N	B7NAJ3	DR	cation transmembrane transporter activity
N	B7NAJ3	DR	potassium ion transport
N	B7NAQ7	DE	3-phosphoshikimate 1-carboxyvinyltransferase;
N	B7NAQ7	DE	5-enolpyruvylshikimate-3-phosphate synthase;
N	B7NAQ7	DE	EPSP synthase;
N	B7NAQ7	DR	3-phosphoshikimate 1-carboxyvinyltransferase activity
N	B7NAQ7	DR	aromatic amino acid family biosynthetic process
N	B7NAT4	CC	Catalyzes the oxidative demethylation of N-methyl-L- tryptophan (By similarity)
N	B7NAT4	DE	N-methyl-L-tryptophan oxidase;
N	B7NAT4	DR	oxidation-reduction process
N	B7NAT4	DR	oxidoreductase activity
N	B7NB92	CC	Catalyzes the oxidation of either pyridoxine 5'-phosphate (PNP) or pyridoxamine 5'-phosphate (PMP) into pyridoxal 5'-phosphate (PLP) (By similarity)
N	B7NB92	DE	PNP/PMP oxidase;
N	B7NB92	DE	Pyridoxal 5'-phosphate synthase;
N	B7NB92	DE	Pyridoxine/pyridoxamine 5'-phosphate oxidase;
N	B7NB92	DR	oxidation-reduction process
N	B7NB92	DR	pyridoxamine-phosphate oxidase activity
N	B7NB92	DR	pyridoxine biosynthetic process
N	B7NDD8	DE	UPF0306 protein yhbP;
N	B7NDV2	CC	Part of a sulfur-relay system required for 2-thiolation of 5-methylaminomethyl-2-thiouridine (mnm(5)s(2)U) at tRNA wobble positions (By similarity).
N	B7NDV2	DE	Protein tusC;
N	B7NDV2	DE	tRNA 2-thiouridine synthesizing protein C;
N	B7NDV2	DR	tRNA processing
N	B7NHG8	CC	Catalyzes the conversion of L-arabinose to L-ribulose (By similarity).
N	B7NHG8	DE	L-arabinose isomerase;
N	B7NHG8	DR	L-arabinose isomerase activity
N	B7NHG8	DR	L-fucose isomerase activity
N	B7NHG8	DR	arabinose catabolic process
N	B7NHG8	DR	fucose metabolic process
N	B7NHG8	DR	metal ion binding
N	B7NI79	DE	UPF0231 protein yacL;
N	B7NJS2	CC	Catalyzes the transfer of an acyl group from acyl-ACP to glycerol-3-phosphate (G3P) to form lysophosphatidic acid (LPA)
N	B7NJS2	CC	This enzyme can also utilize acyl-CoA as fatty acyl donor, but not acyl-PO(4) (By similarity).
N	B7NJS2	DE	G3P acyltransferase;
N	B7NJS2	DE	Glycerol-3-phosphate acyltransferase;
N	B7NJS2	DE	LPA synthase;
N	B7NJS2	DE	Lysophosphatidic acid synthase;
N	B7NJS2	DR	glycerol-3-phosphate O-acyltransferase activity
N	B7NJS2	DR	phospholipid biosynthetic process
N	B7NK49	CC	Repressor involved in choline regulation of the bet genes (By similarity).
N	B7NK49	DE	HTH-type transcriptional regulator BetI;
N	B7NK49	DR	regulation of transcription, DNA-dependent
N	B7NK49	DR	sequence-specific DNA binding transcription factor activity
N	B7NK49	DR	specific transcriptional repressor activity
N	B7NK71	DE	50S ribosomal protein L36;
N	B7NK71	DR	structural constituent of ribosome
N	B7NLL0	CC	Channel that opens in response to stretch forces in the membrane lipid bilayer
N	B7NLL0	CC	May participate in the regulation of osmotic pressure changes within the cell (By similarity).

N	B7NLL0	DE	Large-conductance mechanosensitive channel;
N	B7NLL0	DR	ion channel activity
N	B7NPE0	CC	Reversibly transfers an adenylyl group from ATP to 4'- phosphopantetheine, yielding dephospho-CoA (dPCoA) and pyrophosphate (By similarity).
N	B7NPE0	DE	Dephospho-CoA pyrophosphorylase;
N	B7NPE0	DE	Pantetheine-phosphate adenylyltransferase;
N	B7NPE0	DE	Phosphopantetheine adenylyltransferase;
N	B7NPE0	DR	coenzyme A biosynthetic process
N	B7NPE0	DR	pantetheine-phosphate adenylyltransferase activity
N	B7NQ04	CC	Catalyzes the transfer of a ribosyl phosphate group from 5-phosphoribose 1-diphosphate to orotate, leading to the formation of orotidine monophosphate (OMP) (By similarity).
N	B7NQ04	DE	Orotate phosphoribosyltransferase;
N	B7NQ04	DR	nucleoside metabolic process
N	B7NQ04	DR	orotate phosphoribosyltransferase activity
N	B7NQ04	DR	pyrimidine nucleotide biosynthetic process
N	B7NU33	CC	Provides the (R)-glutamate required for cell wall biosynthesis (By similarity).
N	B7NU33	DE	Glutamate racemase;
N	B7NU33	DR	cellular cell wall organization
N	B7NU33	DR	glutamate racemase activity
N	B7NU33	DR	peptidoglycan biosynthetic process
N	B7NU33	DR	regulation of cell shape
N	B7PCF6	CC	Has bacteriostatic activity against Gram-positive bacteria, but not against Gram-negative bacteria
N	B7PCF6	CC	Binds and sequesters copper and iron ions
N	B7PCF6	CC	Copper-chelating is crucial for antimicrobial activity against M.luteus (By similarity).
N	B7PCF6	CC	Has fungistatic activity against some but not all fungi
N	B7PCF6	DE	Antimicrobial peptide microplusin;
N	B7PCF6	DR	copper chaperone activity
N	B7PCF6	DR	defense response to Gram-positive bacterium
N	B7PCF6	DR	defense response to fungus
N	B7PCF6	DR	innate immune response
N	B7PCF6	DR	iron ion binding
N	B7PCF6	DR	killing of cells of other organism
N	B7PCF6	DR	metal chelating activity
N	B7UFS6	CC	Catalyzes a proton abstraction reaction that results in 2,5-elimination of pyruvate from 2-succinyl-5-enolpyruvyl-6- hydroxy-3-cyclohexene-1-carboxylate (SEPHCHC) and the formation of 2-succinyl-6-hydroxy-2,4-cyclohexadiene-1-carboxylate (SHCHC)
N	B7UFS6	CC	Is also able to catalyze the hydrolysis of the thioester bond in palmitoyl-CoA in vitro (By similarity).
N	B7UFS6	DE	2-succinyl-6-hydroxy-2,4-cyclohexadiene-1-carboxylate synthase;
N	B7UFS6	DE	SHCHC synthase;
N	B7UFS6	DR	2-succinyl-6-hydroxy-2,4-cyclohexadiene-1-carboxylate synthase activity
N	B7UFS6	DR	menaquinone biosynthetic process
N	B7UGB2	CC	DNA ligase that catalyzes the formation of phosphodiester linkages between 5'-phosphoryl and 3'-hydroxyl groups in double-stranded DNA using NAD as a coenzyme and as the energy source for the reaction
N	B7UGB2	CC	It is essential for DNA replication and repair of damaged DNA (By similarity).
N	B7UGB2	DE	Polydeoxyribonucleotide synthase [NAD+];
N	B7UGB2	DR	DNA ligase (NAD+) activity
N	B7UGB2	DR	DNA replication
N	B7UGB2	DR	metal ion binding
N	B7UHE9	CC	This protein is involved in the repair of mismatches in DNA
N	B7UHE9	CC	It is possible that it carries out the mismatch recognition step
N	B7UHE9	CC	This protein has a weak ATPase activity (By similarity).
N	B7UHE9	DE	DNA mismatch repair protein mutS;
N	B7UHE9	DR	mismatch repair
N	B7UHE9	DR	mismatched DNA binding
N	B7UHV3	CC	The glycine cleavage system catalyzes the degradation of glycine (By similarity).
N	B7UHV3	DE	Aminomethyltransferase;
N	B7UHV3	DE	Glycine cleavage system T protein;
N	B7UHV3	DR	aminomethyltransferase activity
N	B7UHV3	DR	glycine catabolic process
N	B7UHV3	DR	transaminase activity
N	B7UK30	CC	One of the primary rRNA binding proteins, it binds directly to 16S rRNA central domain where it helps coordinate assembly of the platform of the 30S subunit (By similarity).

N	B7UK30	DE	30S ribosomal protein S8;
N	B7UK30	DR	structural constituent of ribosome
N	B7UKQ6	CC	Transfers 2-(5''-triphosphoribosyl)-3'- dephosphocoenzyme-A on a serine residue to the apo-acyl carrier protein (gamma chain) of the citrate lyase to yield holo-acyl carrier protein (By similarity).
N	B7UKQ6	DE	Apo-ACP nucleodityltransferase;
N	B7UKQ6	DE	Holo-ACP synthase;
N	B7UKQ6	DE	Holo-citrate lyase synthase;
N	B7UKQ6	DE	Probable apo-citrate lyase phosphoribosyl-dephospho-CoA transferase;
N	B7UKQ6	DR	holo-citrate lyase synthase activity
N	B7UKQ6	DR	prosthetic group biosynthetic process
N	B7UNU5	CC	Catalyzes the formation of 5-methyl-uridine at position 54 (M-5-U54) in all tRNAs (By similarity).
N	B7UNU5	DE	tRNA (uracil-5-)-methyltransferase;
N	B7UNU5	DE	tRNA(M-5-U54)-methyltransferase;
N	B7UNU5	DR	tRNA (uracil-5-)-methyltransferase activity
N	B7USA1	CC	IF-3 binds to the 30S ribosomal subunit and shifts the equilibrum between 70S ribosomes and their 50S and 30S subunits in favor of the free subunits, thus enhancing the availability of 30S subunits on which protein synthesis initiation begins (By similarity).
N	B7USA1	DE	Translation initiation factor IF-3;
N	B7USA1	DR	translation initiation factor activity
N	B7UWH3	CC	Catalyzes amidations at positions B, D, E, and G on adenosylcobyrinic A,C-diamide
N	B7UWH3	CC	NH(2) groups are provided by glutamine, and one molecule of ATP is hydrogenolyzed for each amidation (By similarity).
N	B7UWH3	DE	Cobyric acid synthase;
N	B7UWH3	DR	catalytic activity
N	B7UWH3	DR	cobalamin biosynthetic process
N	B7UWH3	DR	glutamine metabolic process
N	B7V1Y3	DE	NH(3)-dependent NAD(+) synthetase;
N	B7V1Y3	DR	NAD biosynthetic process
N	B7V1Y3	DR	NAD+ synthase (glutamine-hydrolyzing) activity
N	B7V1Y3	DR	NAD+ synthase activity
N	B7V309	CC	Binds directly to 23S ribosomal RNA and is necessary for the in vitro assembly process of the 50S ribosomal subunit
N	B7V309	CC	It is not involved in the protein synthesizing functions of that subunit (By similarity)
N	B7V309	DE	50S ribosomal protein L20;
N	B7V309	DR	structural constituent of ribosome
N	B7V3H2	CC	Hydrolyzes D-tyrosyl-tRNA(Tyr) into D-tyrosine and free tRNA(Tyr)
N	B7V3H2	CC	Could be a defense mechanism against a harmful effect of D-tyrosine (By similarity)
N	B7V3H2	DE	D-tyrosyl-tRNA(Tyr) deacylase;
N	B7V3H2	DR	D-amino acid catabolic process
N	B7V3H2	DR	hydrolase activity, acting on ester bonds
N	B7V7A1	CC	Specifically methylates the N7 position of guanosine in position 527 of 16S rRNA (By similarity).
N	B7V7A1	DE	16S rRNA 7-methylguanosine methyltransferase;
N	B7V7A1	DE	16S rRNA m7G methyltransferase;
N	B7V7A1	DE	Ribosomal RNA small subunit methyltransferase G;
N	B7V7A1	DR	rRNA methyltransferase activity
N	B7VA96	DE	Homogentisate 1,2-dioxygenase;
N	B7VA96	DE	Homogentisate oxygenase;
N	B7VA96	DE	Homogentisic acid oxidase;
N	B7VA96	DE	Homogentisicase;
N	B7VA96	DR	L-phenylalanine catabolic process
N	B7VA96	DR	homogentisate 1,2-dioxygenase activity
N	B7VA96	DR	metal ion binding
N	B7VA96	DR	oxidation-reduction process
N	B7VA96	DR	tyrosine catabolic process
N	B7VHI0	CC	Promotes RNA polymerase assembly
N	B7VHI0	CC	Latches the N- and C- terminal regions of the beta' subunit thereby facilitating its interaction with the beta and alpha subunits (By similarity).
N	B7VHI0	DE	DNA-directed RNA polymerase subunit omega;
N	B7VHI0	DE	RNA polymerase omega subunit;
N	B7VHI0	DE	RNAP omega subunit;
N	B7VHI0	DE	Transcriptase subunit omega;
N	B7VHI0	DR	DNA-directed RNA polymerase activity

N	B7VMJ2	CC	Involved in the tonB-independent uptake of proteins (By similarity).
N	B7VMJ2	DE	Protein tolB;
N	B7VMJ2	DR	protein import
N	B8CMW5	CC	Catalyzes the removal of elemental sulfur from cysteine to produce alanine (By similarity).
N	B8CMW5	DE	Cysteine desulfurase;
N	B8CMW5	DR	cysteine desulfurase activity
N	B8CMW5	DR	cysteine metabolic process
N	B8CMW5	DR	pyridoxal phosphate binding
N	B8CP85	CC	Participates in the translocation of lipoproteins from the inner membrane to the outer membrane
N	B8CP85	CC	Only forms a complex with a lipoprotein if the residue after the N-terminal Cys is not an aspartate (The Asp acts as a targeting signal to indicate that the lipoprotein should stay in the inner membrane) (By similarity).
N	B8CP85	DE	Outer-membrane lipoprotein carrier protein;
N	B8CP85	DR	protein transport
N	B8CV40	DE	UPF0341 protein swp_4895;
N	B8CWY9	CC	GTPase that plays an essential role in the late steps of ribosome biogenesis (By similarity).
N	B8CWY9	DE	GTP-binding protein EngA;
N	B8CWY9	DR	ribosome biogenesis
N	B8D6W1	DE	Acetylglutamate kinase;
N	B8D6W1	DE	N-acetyl-L-glutamate 5-phosphotransferase;
N	B8D6W1	DR	acetylglutamate kinase activity
N	B8D6W1	DR	arginine biosynthetic process
N	B8D732	DE	50S ribosomal protein L35;
N	B8D732	DR	structural constituent of ribosome
N	B8D845	CC	Protein S19 forms a complex with S13 that binds strongly to the 16S ribosomal RNA (By similarity).
N	B8D845	DE	30S ribosomal protein S19;
N	B8D845	DR	structural constituent of ribosome
N	B8D8L5	DE	30S ribosomal protein S21;
N	B8D8L5	DR	structural constituent of ribosome
N	B8D955	DE	High frequency lysogenization protein hflD homolog;
N	B8D9G5	CC	Catalyzes the conversion of glucosamine-6-phosphate to glucosamine-1-phosphate (By similarity).
N	B8D9G5	DE	Phosphoglucosamine mutase;
N	B8D9G5	DR	carbohydrate metabolic process
N	B8D9G5	DR	magnesium ion binding
N	B8D9G5	DR	phosphoglucosamine mutase activity
N	B8DDM6	DE	Formate--tetrahydrofolate ligase;
N	B8DDM6	DE	Formyltetrahydrofolate synthetase;
N	B8DDM6	DR	folic acid-containing compound biosynthetic process
N	B8DDM6	DR	formate-tetrahydrofolate ligase activity
N	B8DDM6	DR	one-carbon metabolic process
N	B8DE15	CC	Necessary for efficient RNA polymerase transcription elongation past template-encoded arresting sites
N	B8DE15	CC	Cleavage of the nascent transcript by cleavage factors such as greA or greB allows the resumption of elongation from the new 3'terminus
N	B8DE15	CC	GreA releases sequences of 2 to 3 nucleotides (By similarity).
N	B8DE15	CC	The arresting sites in DNA have the property of trapping a certain fraction of elongating RNA polymerases that pass through, resulting in locked ternary complexes
N	B8DE15	DE	Transcript cleavage factor greA;
N	B8DE15	DE	Transcription elongation factor greA;
N	B8DE15	DR	transcription elongation regulator activity
N	B8DM91	CC	Catalyzes the condensation of the acetyl group of acetyl-CoA with 3-methyl-2-oxobutanoate (2-oxoisovalerate) to form 3-carboxy-3-hydroxy-4-methylpentanoate (2-isopropylmalate) (By similarity).
N	B8DM91	DE	2-isopropylmalate synthase;
N	B8DM91	DE	Alpha-IPM synthase;
N	B8DM91	DE	Alpha-isopropylmalate synthase;
N	B8DM91	DR	2-isopropylmalate synthase activity
N	B8DM91	DR	leucine biosynthetic process
N	B8DNA4	DE	50S ribosomal protein L29;
N	B8DNA4	DR	structural constituent of ribosome

N	B8DNQ9	DE	Acetohydroxy-acid isomeroreductase;
N	B8DNQ9	DE	Alpha-keto-beta-hydroxylacil reductoisomerase;
N	B8DNQ9	DE	Ketol-acid reductoisomerase;
N	B8DNQ9	DR	branched chain family amino acid biosynthetic process
N	B8DNQ9	DR	coenzyme binding
N	B8DNQ9	DR	ketol-acid reductoisomerase activity
N	B8DNQ9	DR	oxidation-reduction process
N	B8DTZ2	CC	Prevents misfolding and promotes the refolding and proper assembly of unfolded polypeptides generated under stress conditions (By similarity).
N	B8DTZ2	DE	60 kDa chaperonin;
N	B8DTZ2	DE	GroEL protein;
N	B8DTZ2	DE	Protein Cpn60;
N	B8DTZ2	DR	protein refolding
N	B8DUE9	CC	Nuclease that resolves Holliday junction intermediates in genetic recombination
N	B8DUE9	CC	Cleaves the cruciform structure in supercoiled DNA by nicking to strands with the same polarity at sites symmetrically opposed at the junction in the homologous arms and leaves a 5'-terminal phosphate and a 3'-terminal hydroxyl group (By similarity).
N	B8DUE9	DE	Crossover junction endodeoxyribonuclease ruvC;
N	B8DUE9	DE	Holliday junction nuclease ruvC;
N	B8DUE9	DE	Holliday junction resolvase ruvC;
N	B8DUE9	DR	DNA recombination
N	B8DUE9	DR	crossover junction endodeoxyribonuclease activity
N	B8DUE9	DR	metal ion binding
N	B8DUE9	DR	nucleic acid binding
N	B8DW26	CC	Binds 16S rRNA, required for the assembly of 30S particles and may also be responsible for determining the conformation of the 16S rRNA at the A site (By similarity).
N	B8DW26	DE	30S ribosomal protein S14 type Z;
N	B8DW26	DR	metal ion binding
N	B8DW26	DR	structural constituent of ribosome
N	B8E975	DE	Protein proQ homolog;
N	B8EEA7	DE	5-enolpyruvylshikimate-3-phosphate phospholyase;
N	B8EEA7	DE	Chorismate synthase;
N	B8EEA7	DR	aromatic amino acid family biosynthetic process
N	B8EEA7	DR	chorismate synthase activity
N	B8EPH9	CC	The glycine cleavage system catalyzes the degradation of glycine
N	B8EPH9	CC	The P protein binds the alpha-amino group of glycine through its pyridoxal phosphate cofactor; CO(2) is released and the remaining methylamine moiety is then transferred to the lipoamide cofactor of the H protein (By similarity).
N	B8EPH9	DE	Glycine cleavage system P-protein subunit 1;
N	B8EPH9	DE	Glycine decarboxylase subunit 1;
N	B8EPH9	DE	Probable glycine dehydrogenase [decarboxylating] subunit 1;
N	B8EPH9	DR	glycine dehydrogenase (decarboxylating) activity
N	B8EPH9	DR	oxidation-reduction process
N	B8EPH9	DR	pyridoxal phosphate binding
N	B8EQN3	DE	50S ribosomal protein L28;
N	B8EQN3	DR	structural constituent of ribosome
N	B8ESL2	DE	Argininosuccinate synthase;
N	B8ESL2	DE	Citrulline--aspartate ligase;
N	B8ESL2	DR	arginine biosynthetic process
N	B8ESL2	DR	argininosuccinate synthase activity
N	B8F854	CC	This enzyme is involved in nucleotide metabolism: it produces dUMP, the immediate precursor of thymidine nucleotides and it decreases the intracellular concentration of dUTP so that uracil cannot be incorporated into DNA (By similarity).
N	B8F854	DE	Deoxyuridine 5'-triphosphate nucleotidohydrolase;
N	B8F854	DE	dUTP pyrophosphatase;
N	B8F854	DR	dUTP diphosphatase activity
N	B8F854	DR	dUTP metabolic process
N	B8F854	DR	metal ion binding
N	B8FGA3	CC	Involved in the modulation of the specificity of the ClpAP-mediated ATP-dependent protein degradation (By similarity).
N	B8FGA3	DE	ATP-dependent Clp protease adapter protein ClpS;
N	B8FGA3	DR	protein catabolic process
N	B8G806	CC	Catalyzes the synthesis of alpha-ribazole-5'-phosphate from nicotinate mononucleotide (NAMN) and 5,6- dimethylbenzimidazole (DMB) (By similarity).
N	B8G806	DE	N(1)-alpha-phosphoribosyltransferase;

N	B8G806	DE	NN:DBI PRT;
N	B8G806	DE	Nicotinate-nucleotide--dimethylbenzimidazole phosphoribosyltransferase;
N	B8G806	DR	cobalamin biosynthetic process
N	B8G806	DR	nicotinate-nucleotide-dimethylbenzimidazole phosphoribosyltransferase activity
N	B8G806	DR	nucleoside biosynthetic process
N	B8GJ30	DE	Anthranilate phosphoribosyltransferase;
N	B8GJ30	DR	anthranilate phosphoribosyltransferase activity
N	B8GJ30	DR	tryptophan biosynthetic process
N	B8GP02	CC	One of the essential components for the initiation of protein synthesis
N	B8GP02	CC	Also involved in the hydrolysis of GTP during the formation of the 70S ribosomal complex (By similarity).
N	B8GP02	CC	Protects formylmethionyl-tRNA from spontaneous hydrolysis and promotes its binding to the 30S ribosomal subunits
N	B8GP02	DE	Translation initiation factor IF-2;
N	B8GP02	DR	GTPase activity
N	B8GP02	DR	translation initiation factor activity
N	B8GSV2	DE	Argininosuccinate lyase;
N	B8GSV2	DE	Arginosuccinase;
N	B8GSV2	DR	arginine biosynthetic process via ornithine
N	B8GSV2	DR	argininosuccinate lyase activity
N	B8GX35	DE	Protein CrcB homolog;
N	B8HD44	CC	Catalyzes the transfer of acetyl from acetyl-CoA to desacetylmycothiol (Cys-GlcN-Ins) to form mycothiol (By similarity).
N	B8HD44	DE	MSH acetyltransferase;
N	B8HD44	DE	Mycothiol acetyltransferase;
N	B8HD44	DE	Mycothiol synthase;
N	B8HD44	DR	N-acetyltransferase activity
N	B8HEU5	CC	Catalyzes the reversible conversion of 2- phosphoglycerate into phosphoenolpyruvate
N	B8HEU5	CC	It is essential for the degradation of carbohydrates via glycolysis (By similarity).
N	B8HEU5	DE	2-phospho-D-glycerate hydro-lyase;
N	B8HEU5	DE	2-phosphoglycerate dehydratase;
N	B8HEU5	DR	magnesium ion binding
N	B8HEU5	DR	phosphopyruvate hydratase activity
N	B8HU41	CC	Catalyzes the phosphorylation of the position 2 hydroxy group of 4-diphosphocytidyl-2C-methyl-D-erythritol (By similarity).
N	B8HU41	DE	4-(cytidine-5'-diphospho)-2-C-methyl-D-erythritol kinase;
N	B8HU41	DE	4-diphosphocytidyl-2-C-methyl-D-erythritol kinase;
N	B8HU41	DR	4-(cytidine 5'-diphospho)-2-C-methyl-D-erythritol kinase activity
N	B8HU41	DR	terpenoid biosynthetic process
N	B8HXK4	DE	30S ribosomal protein S2;
N	B8HXK4	DR	structural constituent of ribosome
N	B8HXS5	CC	Involved in DNA repair and recF pathway recombination (By similarity).
N	B8HXS5	DE	DNA repair protein recO;
N	B8HXS5	DE	Recombination protein O;
N	B8HXS5	DR	ARF GTPase activator activity
N	B8HXS5	DR	DNA recombination
N	B8HXS5	DR	regulation of ARF GTPase activity
N	B8HXS5	DR	zinc ion binding
N	B8I004	CC	Catalyzes the dephosphorylation of undecaprenyl diphosphate (UPP)
N	B8I004	CC	Confers resistance to bacitracin (By similarity).
N	B8I004	DE	Bacitracin resistance protein;
N	B8I004	DE	Undecaprenyl pyrophosphate phosphatase;
N	B8I004	DE	Undecaprenyl-diphosphatase;
N	B8I004	DR	cellular cell wall organization
N	B8I004	DR	dephosphorylation
N	B8I004	DR	peptidoglycan biosynthetic process
N	B8I004	DR	regulation of cell shape
N	B8I004	DR	response to antibiotic
N	B8I004	DR	undecaprenyl-diphosphatase activity
N	B8I364	CC	Involved in the hydrolysis of glutamine to glutamate and ammonia
N	B8I364	CC	Channels an ammonia molecule to pdxS (By similarity).
N	B8I364	DE	Glutamine amidotransferase glutaminase subunit pdxT;
N	B8I364	DE	Glutamine amidotransferase subunit pdxT;
N	B8I364	DR	glutamine metabolic process
N	B8I364	DR	transferase activity

N	B8IEM4	CC	Specifically methylates the pseudouridine at position 1915 (m3Psi1915) in 23S rRNA (By similarity).
N	B8IEM4	DE	23S rRNA m3Psi1915 methyltransferase;
N	B8IEM4	DE	Ribosomal RNA large subunit methyltransferase H;
N	B8IEM4	DE	rRNA (pseudouridine-N3-)-methyltransferase rlmH;
N	B8IEM4	DR	methyltransferase activity
N	B8IEM4	DR	rRNA processing
N	B8IU50	CC	Molecular chaperone
N	B8IU50	CC	Has ATPase activity (By similarity).
N	B8IU50	DE	Chaperone protein htpG;
N	B8IU50	DE	Heat shock protein htpG;
N	B8IU50	DE	High temperature protein G;
N	B8IU50	DR	protein folding
N	B8IU50	DR	response to stress
N	B8IU50	DR	unfolded protein binding
N	B8IZD9	CC	Catalyzes the NADPH-dependent reduction of glutamyl- tRNA(Glu) to glutamate 1-semialdehyde (GSA) (By similarity).
N	B8IZD9	DE	Glutamyl-tRNA reductase;
N	B8IZD9	DR	glutamyl-tRNA reductase activity
N	B8IZD9	DR	oxidation-reduction process
N	B8IZD9	DR	porphyrin biosynthetic process
N	B8IZD9	DR	shikimate 5-dehydrogenase activity
N	B8J0S2	DE	Cysteine--tRNA ligase;
N	B8J0S2	DE	Cysteinyl-tRNA synthetase;
N	B8J0S2	DR	cysteine-tRNA ligase activity
N	B8J0S2	DR	cysteinyl-tRNA aminoacylation
N	B8J0S2	DR	metal ion binding
N	B8J1A0	CC	This protein promotes the GTP-dependent binding of aminoacyl-tRNA to the A-site of ribosomes during protein biosynthesis (By similarity).
N	B8J1A0	DE	Elongation factor Tu;
N	B8J1A0	DR	GTPase activity
N	B8J1A0	DR	translation elongation factor activity
N	B8J436	CC	F(1)F(0) ATP synthase produces ATP from ADP in the presence of a proton or sodium gradient
N	B8J436	CC	During catalysis, ATP synthesis in the catalytic domain of F(1) is coupled via a rotary mechanism of the central stalk subunits to proton translocation (By similarity).
N	B8J436	CC	F-type ATPases consist of two structural domains, F(1) containing the extramembraneous catalytic core and F(0) containing the membrane proton channel, linked together by a central stalk and a peripheral stalk
N	B8J436	DE	ATP synthase F(1) sector subunit delta;
N	B8J436	DE	ATP synthase subunit delta;
N	B8J436	DE	F-ATPase subunit delta;
N	B8J436	DE	F-type ATPase subunit delta;
N	B8J436	DR	ATP synthesis coupled proton transport
N	B8J436	DR	hydrogen ion transporting ATP synthase activity, rotational mechanism
N	B8J863	CC	One of the primary rRNA binding proteins
N	B8J863	CC	It has been suggested to have peptidyltransferase activity; this is somewhat controversial
N	B8J863	CC	Makes several contacts with the 16S rRNA in the 70S ribosome (By similarity).
N	B8J863	CC	Required for association of the 30S and 50S subunits to form the 70S ribosome, for tRNA binding and peptide bond formation
N	B8J863	DE	50S ribosomal protein L2;
N	B8J863	DR	structural constituent of ribosome
N	B8J863	DR	transferase activity
N	B8J9L8	CC	Catalyzes the decarboxylation of orotidine 5'- monophosphate (OMP) to uridine 5'-monophosphate (UMP) (By similarity).
N	B8J9L8	DE	OMP decarboxylase;
N	B8J9L8	DE	Orotidine 5'-phosphate decarboxylase;
N	B8J9L8	DR	'de novo' UMP biosynthetic process
N	B8J9L8	DR	'de novo' pyrimidine base biosynthetic process
N	B8J9L8	DR	orotidine-5'-phosphate decarboxylase activity
N	B8JDE1	CC	Involved in DNA repair and recF pathway recombination (By similarity).
N	B8JDE1	DE	DNA repair protein recO;
N	B8JDE1	DE	Recombination protein O;
N	B8JDE1	DR	ARF GTPase activator activity
N	B8JDE1	DR	DNA recombination

N	B8JDE1	DR	regulation of ARF GTPase activity
N	B8JDE1	DR	zinc ion binding
N	B8JFZ2	CC	This enzyme is involved in nucleotide metabolism: it produces dUMP, the immediate precursor of thymidine nucleotides and it decreases the intracellular concentration of dUTP so that uracil cannot be incorporated into DNA (By similarity)
N	B8JFZ2	DE	Deoxyuridine 5'-triphosphate nucleotidohydrolase;
N	B8JFZ2	DE	dUTP pyrophosphatase;
N	B8JFZ2	DR	dUTP diphosphatase activity
N	B8JFZ2	DR	dUTP metabolic process
N	B8JFZ2	DR	metal ion binding
N	B8PZP3	CC	Encapsidates the genome in a ratio of 1 N per 6 ribonucleotides, protecting it from nucleases
N	B8PZP3	CC	During replication, encapsidation by N is coupled to RNA synthesis and all replicative products are resistant to nucleases
N	B8PZP3	CC	N is released in the blood following lysis of measles infected cells, it interacts then with human FCGR2B on immune cells, inducing apoptosis and blocking inflammatory immune response
N	B8PZP3	CC	Ntail binds to a protein on human thymic epithelial cells, termed Nucleoprotein Receptor (NR), inducing growth arrest (By similarity).
N	B8PZP3	CC	The encapsidated genomic RNA is termed the NC and serves as template for transcription and replication
N	B8PZP3	CC	The nucleocapsid (NC) has an helical structure with either 12.35 or 11.64 N per turn, approximately 20nm in diameter, with a hollow central cavity approximately 5nm in diameter
N	B8PZP3	DE	Nucleocapsid protein;
N	B8PZP3	DE	Nucleoprotein;
N	B8PZP3	DR	interspecies interaction between organisms
N	B8PZP3	DR	structural molecule activity
N	B8ZK31	CC	Produces ATP from ADP in the presence of a proton gradient across the membrane
N	B8ZK31	CC	The V-type alpha chain is a catalytic subunit (By similarity).
N	B8ZK31	DE	V-ATPase subunit A;
N	B8ZK31	DE	V-type ATP synthase alpha chain;
N	B8ZK31	DR	ATP biosynthetic process
N	B8ZK31	DR	ATP hydrolysis coupled proton transport
N	B8ZK31	DR	hydrogen ion transporting ATP synthase activity, rotational mechanism
N	B8ZK31	DR	proton-transporting ATPase activity, rotational mechanism
N	B8ZNP1	DE	Pantothenate kinase;
N	B8ZNP1	DE	Pantothenic acid kinase;
N	B8ZNP1	DR	coenzyme A biosynthetic process
N	B8ZNP1	DR	pantothenate kinase activity
N	B8ZQ64	DE	Phosphotagatokinase;
N	B8ZQ64	DE	Tagatose-6-phosphate kinase;
N	B8ZQ64	DR	lactose catabolic process via tagatose-6-phosphate
N	B8ZQ64	DR	tagatose-6-phosphate kinase activity
N	B8ZRK9	DE	Argininosuccinate lyase;
N	B8ZRK9	DE	Arginosuccinase;
N	B8ZRK9	DR	arginine biosynthetic process via ornithine
N	B8ZRK9	DR	argininosuccinate lyase activity
N	B8ZUC1	CC	Catalyzes the conversion of glucosamine-6-phosphate to glucosamine-1-phosphate (By similarity).
N	B8ZUC1	DE	Phosphoglucosamine mutase;
N	B8ZUC1	DR	carbohydrate metabolic process
N	B8ZUC1	DR	magnesium ion binding
N	B8ZUC1	DR	phosphoglucosamine mutase activity
N	B8ZUG3	CC	Involved in the production of pyridoxal phosphate, probably by incorporating ammonia into the pyridine ring (By similarity).
N	B8ZUG3	DE	Pyridoxal biosynthesis lyase pdxS;
N	B8ZUG3	DR	lyase activity
N	B8ZUG3	DR	pyridoxal phosphate biosynthetic process
N	B8ZUM0	CC	Promotes RNA polymerase assembly
N	B8ZUM0	CC	Latches the N- and C- terminal regions of the beta' subunit thereby facilitating its interaction with the beta and alpha subunits (By similarity).
N	B8ZUM0	DE	DNA-directed RNA polymerase subunit omega;
N	B8ZUM0	DE	RNA polymerase omega subunit;
N	B8ZUM0	DE	RNAP omega subunit;

N	B8ZUM0	DE	Transcriptase subunit omega;
N	B8ZUM0	DR	DNA-directed RNA polymerase activity
N	B9A8D7	CC	Slow-binding inhibitor of serine proteases
N	B9A8D7	CC	Active against perkinsin
N	B9A8D7	CC	Active against subtilisin A with a dissociation constant of 0.18 nM
N	B9A8D7	CC	Not active against thermolysin, papain or pepsin.
N	B9A8D7	CC	The inhibitor rapidly binds to the protease forming a weak enzyme- inhibitor complex, and this is followed by a slow isomerization forming a tight-binding enzyme-inhibitor complex
N	B9A8D7	DE	Serine protease inhibitor Cvsi-2;
N	B9A8D7	DR	serine-type endopeptidase inhibitor activity
N	B9DIP1	CC	IGPS catalyzes the conversion of PRFAR and glutamine to IGP, AICAR and glutamate
N	B9DIP1	CC	The hisF subunit catalyzes the cyclization activity that produces IGP and AICAR from PRFAR using the ammonia provided by the hisH subunit (By similarity).
N	B9DIP1	DE	IGP synthase cyclase subunit;
N	B9DIP1	DE	IGP synthase subunit hisF;
N	B9DIP1	DE	IGPS subunit hisF;
N	B9DIP1	DE	ImGP synthase subunit hisF;
N	B9DIP1	DE	Imidazole glycerol phosphate synthase subunit hisF;
N	B9DIP1	DR	histidine biosynthetic process
N	B9DIP1	DR	imidazoleglycerol-phosphate synthase activity
N	B9DIP1	DR	lyase activity
N	B9DMK9	CC	Modulates transcription in response to changes in cellular NADH/NAD(+) redox state (By similarity).
N	B9DMK9	DE	Redox-sensing transcriptional repressor rex;
N	B9DMK9	DR	catalytic activity
N	B9DMK9	DR	response to redox state
N	B9DMK9	DR	transcription repressor activity
N	B9DNC5	DE	50S ribosomal protein L35;
N	B9DNC5	DR	structural constituent of ribosome
N	B9DVD9	CC	Component of the acetyl coenzyme A carboxylase (ACC) complex
N	B9DVD9	CC	First, biotin carboxylase catalyzes the carboxylation of biotin on its carrier protein (BCCP) and then the CO(2) group is transferred by the carboxyltransferase to acetyl-CoA to form malonyl-CoA (By similarity).
N	B9DVD9	DE	ACCase subunit alpha;
N	B9DVD9	DE	Acetyl-CoA carboxylase carboxyltransferase subunit alpha;
N	B9DVD9	DE	Acetyl-coenzyme A carboxylase carboxyl transferase subunit alpha;
N	B9DVD9	DR	acetyl-CoA carboxylase activity
N	B9DVD9	DR	fatty acid biosynthetic process
N	B9DVU8	CC	Converts N-acetylmannosamine-6-phosphate (ManNAc-6-P) to N-acetylglucosamine-6-phosphate (GlcNAc-6-P) (Potential).
N	B9DVU8	DE	ManNAc-6-P epimerase;
N	B9DVU8	DE	Putative N-acetylmannosamine-6-phosphate 2-epimerase;
N	B9DVU8	DR	N-acetylmannosamine metabolic process
N	B9DVU8	DR	N-acylglucosamine-6-phosphate 2-epimerase activity
N	B9DW63	DE	UPF0297 protein SUB1776;
N	B9E104	CC	Catalyzes the acyloin condensation reaction between C atoms 2 and 3 of pyruvate and glyceraldehyde 3-phosphate to yield 1-deoxy-D-xylulose-5-phosphate (DXP) (By similarity).
N	B9E104	DE	1-deoxy-D-xylulose-5-phosphate synthase;
N	B9E104	DE	1-deoxyxylulose-5-phosphate synthase;
N	B9E104	DE	DXP synthase;
N	B9E104	DR	1-deoxy-D-xylulose-5-phosphate synthase activity
N	B9E104	DR	terpenoid biosynthetic process
N	B9E104	DR	thiamine biosynthetic process
N	B9E104	DR	thiamine pyrophosphate binding
N	B9E7A6	CC	Component of the acetyl coenzyme A carboxylase (ACC) complex
N	B9E7A6	CC	Biotin carboxylase (BC) catalyzes the carboxylation of biotin on its carrier protein (BCCP) and then the CO(2) group is transferred by the transcarboxylase to acetyl-CoA to form malonyl- CoA (By similarity).
N	B9E7A6	DE	ACCase subunit beta;
N	B9E7A6	DE	Acetyl-CoA carboxylase carboxyltransferase subunit beta;
N	B9E7A6	DE	Acetyl-coenzyme A carboxylase carboxyl transferase subunit beta;
N	B9E7A6	DR	acetyl-CoA carboxylase activity
N	B9E7A6	DR	fatty acid biosynthetic process
N	B9E7A6	DR	metal ion binding

N	B9E8G2	DE	Thymidine kinase;
N	B9E8G2	DR	DNA replication
N	B9E8G2	DR	metal ion binding
N	B9E8G2	DR	thymidine kinase activity
N	B9EBI5	CC	This protein is involved in the repair of mismatches in DNA
N	B9EBI5	CC	It is required for dam-dependent methyl-directed DNA mismatch repair
N	B9EBI5	CC	May act as a "molecular matchmaker", a protein that promotes the formation of a stable complex between two or more DNA-binding proteins in an ATP-dependent manner without itself being part of a final effector complex (By similarity)
N	B9EBI5	DE	DNA mismatch repair protein mutL;
N	B9EBI5	DR	mismatch repair
N	B9EBI5	DR	mismatched DNA binding
N	B9ENE7	CC	Component of a splicing-dependent multiprotein exon junction complex (EJC) deposited at splice junction on mRNAs
N	B9ENE7	CC	Core components of the EJC, that remains bound to spliced mRNAs throughout all stages of mRNA metabolism, functions to mark the position of the exon-exon junction in the mature mRNA and thereby influences downstream processes of gene expression including mRNA splicing, nuclear mRNA export, subcellular mRNA localization, translation efficiency and nonsense-mediated mRNA decay (NMD) (By similarity)
N	B9ENE7	CC	The EJC is a dynamic structure consisting of a few core proteins and several more peripheral nuclear and cytoplasmic associated factors that join the complex only transiently either during EJC assembly or during subsequent mRNA metabolism
N	B9ENE7	DE	Protein mago nashi homolog;
N	B9ENE7	DR	mRNA processing
N	B9ENE7	DR	mRNA transport
N	B9ENE7	DR	nuclear-transcribed mRNA catabolic process, nonsense-mediated decay
N	B9ENE7	DR	regulation of translation
N	B9ENM6	CC	Ubiquitin-like modifier protein which binds to a number of as yet unidentified target proteins (By similarity).
N	B9ENM6	DE	Ubiquitin-fold modifier 1;
N	B9ENM6	DR	protein ufmylation
N	B9IUK2	DE	UPF0154 protein BCQ_3484;
N	B9IUK2	DR	calcium ion binding
N	B9IWT7	CC	Binds to sigma F and blocks its ability to form an RNA polymerase holoenzyme (E-sigma F)
N	B9IWT7	CC	Phosphorylates SpoIIAA on a serine residue
N	B9IWT7	CC	This phosphorylation may enable SpoIIAA to act as an anti-anti-sigma factor that counteracts SpoIIAB and thus releases sigma F from inhibition (By similarity).
N	B9IWT7	DE	Anti-sigma F factor;
N	B9IWT7	DE	Stage II sporulation protein AB;
N	B9IWT7	DR	negative regulation of transcription, DNA-dependent
N	B9IWT7	DR	protein phosphorylation
N	B9IWT7	DR	protein serine/threonine kinase activity
N	B9IWT7	DR	sigma factor antagonist activity
N	B9IWT7	DR	sporulation resulting in formation of a cellular spore
N	B9IZV2	DE	UPF0346 protein BCQ_2236;
N	B9J159	CC	One of the primary rRNA binding proteins, it binds directly to 16S rRNA where it nucleates assembly of the body of the 30S subunit (By similarity).
N	B9J159	DE	30S ribosomal protein S4;
N	B9J159	DR	structural constituent of ribosome
N	B9J7U0	CC	Negative regulator of class I heat shock genes (grpE- dnaK-dnaJ and groELS operons)
N	B9J7U0	CC	Prevents heat-shock induction of these operons (By similarity).
N	B9J7U0	DE	Heat-inducible transcription repressor hrcA;
N	B9J7U0	DR	regulation of transcription, DNA-dependent
N	B9J7U0	DR	response to stress
N	B9JUH8	CC	Catalyzes the transfer of a phosphate group to glutamate to form glutamate 5-phosphate which rapidly cyclizes to 5- oxoproline (By similarity).
N	B9JUH8	DE	Gamma-glutamyl kinase;
N	B9JUH8	DE	Glutamate 5-kinase;
N	B9JUH8	DR	glutamate 5-kinase activity
N	B9JUH8	DR	proline biosynthetic process
N	B9JWX0	CC	Transfers and isomerizes the ribose moiety from AdoMet to the 7-aminomethyl group of 7-deazaguanine (preQ1-tRNA) to give epoxyqueuosine (oQ-tRNA) (By similarity)
N	B9JWX0	DE	Queuosine biosynthesis protein queA;
N	B9JWX0	DE	S-adenosylmethionine:tRNA ribosyltransferase-isomerase;

N	B9JWX0	DR	isomerase activity
N	B9JWX0	DR	queuosine biosynthetic process
N	B9JWX0	DR	transferase activity
N	B9KBC2	CC	Modifies the free amino group of the aminoacyl moiety of methionyl-tRNA(fMet)
N	B9KBC2	CC	The formyl group appears to play a dual role in the initiator identity of N-formylmethionyl-tRNA by: (I) promoting its recognition by IF2 and (II) impairing its binding to EFTu-GTP (By similarity).
N	B9KBC2	DE	Methionyl-tRNA formyltransferase;
N	B9KBC2	DR	methionyl-tRNA formyltransferase activity
N	B9KBC2	DR	methyltransferase activity
N	B9KJ69	CC	One of the primary rRNA binding proteins, this protein initially binds near the 5'-end of the 23S rRNA
N	B9KJ69	CC	It is important during the early stages of 50S assembly
N	B9KJ69	CC	It makes multiple contacts with different domains of the 23S rRNA in the assembled 50S subunit and ribosome (By similarity).
N	B9KJ69	DE	50S ribosomal protein L4;
N	B9KJ69	DR	structural constituent of ribosome
N	B9KKV1	CC	Phosphorylation of dTMP to form dTDP in both de novo and salvage pathways of dTTP synthesis (By similarity).
N	B9KKV1	DE	Thymidylate kinase;
N	B9KKV1	DE	dTMP kinase;
N	B9KKV1	DR	dTDP biosynthetic process
N	B9KKV1	DR	thymidylate kinase activity
N	B9KSB0	CC	Involved in DNA repair and recF pathway recombination (By similarity).
N	B9KSB0	DE	DNA repair protein recO;
N	B9KSB0	DE	Recombination protein O;
N	B9KSB0	DR	ARF GTPase activator activity
N	B9KSB0	DR	DNA recombination
N	B9KSB0	DR	regulation of ARF GTPase activity
N	B9KSB0	DR	zinc ion binding
N	B9KW01	CC	This protein binds to 23S rRNA in the presence of protein L20 (By similarity).
N	B9KW01	DE	50S ribosomal protein L21;
N	B9KW01	DR	structural constituent of ribosome
N	B9LCT8	CC	The ruvA-ruvB complex in the presence of ATP renatures cruciform structure in supercoiled DNA with palindromic sequence, indicating that it may promote strand exchange reactions in homologous recombination
N	B9LCT8	CC	RuvA stimulates, in the presence of DNA, the weak ATPase activity of ruvB (By similarity).
N	B9LCT8	CC	RuvAB is an helicase that mediates the Holliday junction migration by localized denaturation and reannealing
N	B9LCT8	DE	Holliday junction ATP-dependent DNA helicase ruvA;
N	B9LCT8	DR	DNA recombination
N	B9LCT8	DR	four-way junction helicase activity
N	B9LHE7	CC	Guanylyltransferase that catalyzes the activation of 2- phospho-L-lactate (LP) as (2S)-lactyl-2-diphospho-5'-guanosine (LPPG), via the condensation of LP with GTP
N	B9LHE7	CC	Is involved in the biosynthesis of coenzyme F420, a hydride carrier cofactor (By similarity).
N	B9LHE7	DE	2-phospho-L-lactate guanylyltransferase;
N	B9LHE7	DE	LP guanylyltransferase;
N	B9LHE7	DR	nucleotidyltransferase activity
N	B9LI79	CC	Specifically methylates guanosine-37 in various tRNAs (By similarity).
N	B9LI79	DE	M1G-methyltransferase;
N	B9LI79	DE	tRNA (guanine-N(1)-)-methyltransferase;
N	B9LI79	DE	tRNA [GM37] methyltransferase;
N	B9LI79	DR	tRNA (guanine-N1-)-methyltransferase activity
N	B9LK05	DE	Protein CrcB homolog;
N	B9LQ97	DE	N-(5'-phosphoribosyl)anthranilate isomerase;
N	B9LQ97	DR	phosphoribosylanthranilate isomerase activity
N	B9LQ97	DR	tryptophan biosynthetic process
N	B9LR22	CC	Catalyzes the radical-mediated insertion of two sulfur atoms into the C-6 and C-8 positions of the octanoyl moiety bound to the lipoyl domains of lipoate-dependent enzymes, thereby converting the octanoylated domains into lipoylated derivatives (By similarity).
N	B9LR22	DE	Lipoate synthase;
N	B9LR22	DE	Lipoic acid synthase;

N	B9LR22	DE	Probable lipoyl synthase;
N	B9LR22	DE	Sulfur insertion protein lipA;
N	B9LR22	DR	4 iron, 4 sulfur cluster binding
N	B9LR22	DR	lipoate biosynthetic process
N	B9LR22	DR	lipoate synthase activity
N	B9LR22	DR	metal ion binding
N	B9LUM9	CC	The UvrABC repair system catalyzes the recognition and processing of DNA lesions
N	B9LUM9	CC	The N-terminal half is responsible for the 3' incision and the C-terminal half is responsible for the 5' incision (By similarity).
N	B9LUM9	CC	UvrC both incises the 5' and 3' sides of the lesion
N	B9LUM9	DE	Excinuclease ABC subunit C;
N	B9LUM9	DE	Protein uvrC;
N	B9LUM9	DE	UvrABC system protein C;
N	B9LUM9	DR	excinuclease ABC activity
N	B9LUM9	DR	nucleotide-excision repair
N	B9LZW2	CC	Converts 4-diphosphocytidyl-2-C-methyl-D-erythritol 2- phosphate into 2-C-methyl-D-erythritol 2,4-cyclodiphosphate (MECDP) and CMP (By similarity).
N	B9LZW2	DE	2-C-methyl-D-erythritol 2,4-cyclodiphosphate synthase;
N	B9LZW2	DE	MECDP-synthase;
N	B9LZW2	DR	2-C-methyl-D-erythritol 2,4-cyclodiphosphate synthase activity
N	B9LZW2	DR	metal ion binding
N	B9LZW2	DR	terpenoid biosynthetic process
N	B9M9W4	CC	Specifically methylates the N7 position of guanosine in position 527 of 16S rRNA (By similarity).
N	B9M9W4	DE	16S rRNA 7-methylguanosine methyltransferase;
N	B9M9W4	DE	16S rRNA m7G methyltransferase;
N	B9M9W4	DE	Ribosomal RNA small subunit methyltransferase G;
N	B9M9W4	DR	rRNA methyltransferase activity
N	B9MB77	CC	Protein S19 forms a complex with S13 that binds strongly to the 16S ribosomal RNA (By similarity).
N	B9MB77	DE	30S ribosomal protein S19;
N	B9MB77	DR	structural constituent of ribosome
N	B9ME75	CC	Catalyzes the irreversible NADPH-dependent deamination of GMP to IMP
N	B9ME75	CC	It functions in the conversion of nucleobase, nucleoside and nucleotide derivatives of G to A nucleotides, and in maintaining the intracellular balance of A and G nucleotides (By similarity).
N	B9ME75	DE	GMP reductase;
N	B9ME75	DE	Guanosine 5'-monophosphate oxidoreductase;
N	B9ME75	DE	Guanosine monophosphate reductase;
N	B9ME75	DR	GMP reductase activity
N	B9ME75	DR	oxidation-reduction process
N	B9ME75	DR	purine nucleotide metabolic process
N	B9MGL7	DE	30S ribosomal protein S2;
N	B9MGL7	DR	structural constituent of ribosome
N	B9MJP2	CC	Could be a mediator in iron transactions between iron acquisition and iron-requiring processes, such as synthesis and/or repair of Fe-S clusters in biosynthetic enzymes (By similarity).
N	B9MJP2	DE	Probable Fe(2+)-trafficking protein;
N	B9MJP2	DR	iron ion binding
N	B9ML31	CC	Catalyzes a salvage reaction resulting in the formation of AMP, that is energically less costly than de novo synthesis (By similarity).
N	B9ML31	DE	Adenine phosphoribosyltransferase;
N	B9ML31	DR	adenine phosphoribosyltransferase activity
N	B9ML31	DR	adenine salvage
N	B9ML31	DR	purine ribonucleoside salvage
N	B9MPB0	DE	UPF0145 protein Athe_0545;
N	C0H429	DE	Uncharacterized protein yoyB;
N	C0JAX1	CC	Catalyzes the hydrolysis of sphingomyelin
N	C0JAX1	CC	Induces complement-dependent hemolysis, dermonecrosis, blood vessel permeability and platelet aggregation (By similarity).
N	C0JAX1	DE	Sphingomyelin phosphodiesterase D LdSicTox-alphaIB1avi;
N	C0JAX1	DE	Sphingomyelinase D;
N	C0JAX1	DR	hemolysis in other organism
N	C0JAX1	DR	lipid metabolic process
N	C0JAX1	DR	metal ion binding

N	C0JAX1	DR	sphingomyelin phosphodiesterase D activity
N	C0LGP4	DE	Probable LRR receptor-like serine/threonine-protein kinase At3g47570;
N	C0LGP4	DR	protein phosphorylation
N	C0LGP4	DR	protein serine/threonine kinase activity
N	C0LGP4	DR	receptor activity
N	C0LGR9	DE	Probable LRR receptor-like serine/threonine-protein kinase At4g31250;
N	C0LGR9	DR	protein phosphorylation
N	C0LGR9	DR	protein serine/threonine kinase activity
N	C0LGR9	DR	receptor activity
N	C0MAR3	CC	Could be a nuclease that resolves Holliday junction intermediates in genetic recombination (By similarity).
N	C0MAR3	DE	Putative Holliday junction resolvase;
N	C0MAR3	DR	DNA recombination
N	C0MAR3	DR	nuclease activity
N	C0MAR3	DR	nucleic acid binding
N	C0ME28	DE	50S ribosomal protein L36;
N	C0ME28	DR	structural constituent of ribosome
N	C0MFF9	CC	Catalyzes the folate-dependent formation of 5-methyl- uridine at position 54 (M-5-U54) in all tRNAs (By similarity).
N	C0MFF9	DE	Folate-dependent tRNA (uracil-5-)-methyltransferase;
N	C0MFF9	DE	Folate-dependent tRNA(M-5-U54)-methyltransferase;
N	C0MFF9	DE	Methylenetetrahydrofolate--tRNA-(uracil-5-)-methyltransferase TrmFO;
N	C0MFF9	DR	flavin adenine dinucleotide binding
N	C0MFF9	DR	methylenetetrahydrofolate-tRNA-(uracil-5-)-methyltransferase (FADH2-oxidizing) activity
N	C0MFF9	DR	tRNA processing
N	C0MFQ9	CC	Catalyzes the ATP-dependent amination of UTP to CTP with either L-glutamine or ammonia as the source of nitrogen (By similarity).
N	C0MFQ9	DE	CTP synthase;
N	C0MFQ9	DE	CTP synthetase;
N	C0MFQ9	DE	UTP--ammonia ligase;
N	C0MFQ9	DR	CTP synthase activity
N	C0MFQ9	DR	glutamine metabolic process
N	C0MFQ9	DR	pyrimidine nucleotide biosynthetic process
N	C0PWL9	CC	Affects glycogen biosynthesis, gluconeogenesis, cell size and surface properties
N	C0PWL9	CC	Acts to inhibit interaction between the LetD protein and the A subunit of DNA gyrase
N	C0PWL9	CC	Also required for motility and flagellum biosynthesis through the post-transcriptional activation of flhDC expression
N	C0PWL9	CC	Regulates glycogen synthesis under both aerobic and anaerobic conditions
N	C0PWL9	CC	Seems to accelerate the degradation of glg gene transcripts, potentially through selective RNA binding
N	C0PWL9	CC	This involves binding to and stabilization of the flhDC message by CsrA (By similarity).
N	C0PWL9	DE	Carbon storage regulator;
N	C0PWL9	DR	mRNA catabolic process
N	C0PWL9	DR	regulation of carbohydrate metabolic process
N	C0PY26	CC	The glycine cleavage system catalyzes the degradation of glycine
N	C0PY26	CC	The P protein binds the alpha-amino group of glycine through its pyridoxal phosphate cofactor; CO(2) is released and the remaining methylamine moiety is then transferred to the lipoamide cofactor of the H protein (By similarity).
N	C0PY26	DE	Glycine cleavage system P-protein;
N	C0PY26	DE	Glycine decarboxylase;
N	C0PY26	DE	Glycine dehydrogenase [decarboxylating];
N	C0PY26	DR	glycine dehydrogenase (decarboxylating) activity
N	C0PY26	DR	glycine metabolic process
N	C0PY26	DR	oxidation-reduction process
N	C0PY26	DR	pyridoxal phosphate binding
N	C0PZV0	CC	Modifies the free amino group of the aminoacyl moiety of methionyl-tRNA(fMet)
N	C0PZV0	CC	The formyl group appears to play a dual role in the initiator identity of N-formylmethionyl-tRNA by: (I) promoting its recognition by IF2 and (II) impairing its binding to EFTu-GTP (By similarity).
N	C0PZV0	DE	Methionyl-tRNA formyltransferase;
N	C0PZV0	DR	methionyl-tRNA formyltransferase activity
N	C0PZV0	DR	methyltransferase activity
N	C0Q041	CC	NDH-1 shuttles electrons from NADH, via FMN and iron- sulfur (Fe-S) centers, to quinones in the respiratory chain

N	C0Q041	CC	Couples the redox reaction to proton translocation (for every two electrons transferred, four hydrogen ions are translocated across the cytoplasmic membrane), and thus conserves the redox energy in a proton gradient (By similarity)
N	C0Q041	CC	The immediate electron acceptor for the enzyme in this species is believed to be ubiquinone
N	C0Q041	DE	NADH dehydrogenase I subunit C/D;
N	C0Q041	DE	NADH-quinone oxidoreductase subunit C/D;
N	C0Q041	DE	NDH-1 subunit C/D;
N	C0Q041	DR	NADH dehydrogenase (ubiquinone) activity
N	C0Q041	DR	oxidation-reduction process
N	C0Q041	DR	quinone binding
N	C0Q0D3	CC	Transport system that facilitates potassium-efflux, possibly by potassium-proton antiport (By similarity).
N	C0Q0D3	DE	Glutathione-regulated potassium-efflux system protein kefB;
N	C0Q0D3	DE	K(+)/H(+) antiporter;
N	C0Q0D3	DE	NEM-activatable K(+)/H(+) antiporter;
N	C0Q0D3	DR	catalytic activity
N	C0Q0D3	DR	glutathione-regulated potassium exporter activity
N	C0Q0D3	DR	solute:hydrogen antiporter activity
N	C0Q1G0	DE	Cytidine monophosphokinase;
N	C0Q1G0	DE	Uridine kinase;
N	C0Q1G0	DE	Uridine monophosphokinase;
N	C0Q1G0	DR	phosphotransferase activity, alcohol group as acceptor
N	C0Q1G0	DR	uridine kinase activity
N	C0Q319	CC	Prevents the cell division inhibition by proteins minC and minD at internal division sites while permitting inhibition at polar sites
N	C0Q319	CC	This ensures cell division at the proper site by restricting the formation of a division septum at the midpoint of the long axis of the cell (By similarity).
N	C0Q319	DE	Cell division topological specificity factor;
N	C0Q319	DR	cell division
N	C0Q319	DR	regulation of barrier septum formation
N	C0Q337	CC	Provides the cells with the ability to utilize trehalose at high osmolarity by splitting it into glucose molecules that can subsequently be taken up by the phosphotransferase-mediated uptake system (By similarity).
N	C0Q337	DE	Alpha,alpha-trehalase;
N	C0Q337	DE	Alpha,alpha-trehalose glucohydrolase;
N	C0Q337	DE	Periplasmic trehalase;
N	C0Q337	DR	alpha,alpha-trehalase activity
N	C0Q337	DR	trehalose metabolic process
N	C0Q514	CC	Represses a number of genes involved in the response to DNA damage (SOS response), including recA and lexA
N	C0Q514	CC	Binds to the 16 bp palindromic sequence 5'-CTGTATATATATACAG-3'
N	C0Q514	CC	In the presence of single-stranded DNA, recA interacts with lexA causing an autocatalytic cleavage which disrupts the DNA-binding part of lexA, leading to derepression of the SOS regulon and eventually DNA repair (By similarity).
N	C0Q514	DE	LexA repressor;
N	C0Q514	DR	DNA replication
N	C0Q514	DR	serine-type endopeptidase activity
N	C0Q5S0	CC	Catalyzes the irreversible cleavage of the glycosidic bond in both 5'-methylthioadenosine (MTA) and S- adenosylhomocysteine (SAH/AdoHcy) to adenine and the corresponding thioribose, 5'-methylthioribose and S-ribosylhomocysteine, respectively (By similarity).
N	C0Q5S0	DE	5'-methylthioadenosine nucleosidase;
N	C0Q5S0	DE	5'-methylthioadenosine/S-adenosylhomocysteine nucleosidase;
N	C0Q5S0	DE	AdoHcy nucleosidase;
N	C0Q5S0	DE	MTA nucleosidase;
N	C0Q5S0	DE	MTA/SAH nucleosidase;
N	C0Q5S0	DE	S-adenosylhomocysteine nucleosidase;
N	C0Q5S0	DE	SAH nucleosidase;
N	C0Q5S0	DE	SRH nucleosidase;
N	C0Q5S0	DR	L-methionine salvage from methylthioadenosine
N	C0Q5S0	DR	adenosylhomocysteine nucleosidase activity
N	C0Q5S0	DR	methylthioadenosine nucleosidase activity
N	C0Q5S0	DR	nucleoside catabolic process
N	C0Q6N0	CC	Thiolesterase that catalyzes the hydrolysis of S-D- lactoyl-glutathione to form glutathione and D-lactic acid (By similarity).

N	C0Q6N0	DE	Glyoxalase II;
N	C0Q6N0	DE	Hydroxyacylglutathione hydrolase;
N	C0Q6N0	DR	hydroxyacylglutathione hydrolase activity
N	C0Q6N0	DR	zinc ion binding
N	C0QQM0	CC	Catalyzes the GTP-dependent ribosomal translocation step during translation elongation
N	C0QQM0	CC	Catalyzes the coordinated movement of the two tRNA molecules, the mRNA and conformational changes in the ribosome (By similarity).
N	C0QQM0	CC	During this step, the ribosome changes from the pre-translocational (PRE) to the post- translocational (POST) state as the newly formed A-site-bound peptidyl-tRNA and P-site-bound deacylated tRNA move to the P and E sites, respectively
N	C0QQM0	DE	Elongation factor G;
N	C0QQM0	DR	GTPase activity
N	C0QQM0	DR	translation elongation factor activity
N	C0QVZ6	CC	One of the primary rRNA binding proteins, it binds directly near the 3'-end of the 23S rRNA, where it nucleates assembly of the 50S subunit (By similarity).
N	C0QVZ6	DE	50S ribosomal protein L3;
N	C0QVZ6	DR	structural constituent of ribosome
N	C0R0R3	DE	50S ribosomal protein L28;
N	C0R0R3	DR	structural constituent of ribosome
N	C0R5B9	CC	Allows the formation of correctly charged Asn-tRNA(Asn) or Gln-tRNA(Gln) through the transamidation of misacylated Asp- tRNA(Asn) or Glu-tRNA(Gln) in organisms which lack either or both of asparaginyl-tRNA or glutaminyl-tRNA synthetases
N	C0R5B9	CC	The reaction takes place in the presence of glutamine and ATP through an activated phospho-Asp-tRNA(Asn) or phospho-Glu-tRNA(Gln) (By similarity).
N	C0R5B9	DE	Asp/Glu-ADT subunit B;
N	C0R5B9	DE	Aspartyl/glutamyl-tRNA(Asn/Gln) amidotransferase subunit B;
N	C0R5B9	DR	carbon-nitrogen ligase activity, with glutamine as amido-N-donor
N	C0REI6	CC	Key enzyme in heme biosynthesis
N	C0REI6	CC	Catalyzes the oxidative decarboxylation of propionic acid side chains of rings A and B of coproporphyrinogen III (By similarity).
N	C0REI6	DE	Coprogen oxidase;
N	C0REI6	DE	Coproporphyrinogen-III oxidase, aerobic;
N	C0REI6	DE	Coproporphyrinogenase;
N	C0REI6	DR	coproporphyrinogen oxidase activity
N	C0REI6	DR	oxidation-reduction process
N	C0REI6	DR	porphyrin biosynthetic process
N	C0REI6	DR	protein homodimerization activity
N	C0RJI9	CC	This is 1 of the proteins that binds and probably mediates the attachment of the 5S RNA into the large ribosomal subunit, where it forms part of the central protuberance
N	C0RJI9	CC	Contacts the P site tRNA; the 5S rRNA and some of its associated proteins might help stabilize positioning of ribosome-bound tRNAs (By similarity).
N	C0RJI9	CC	In the 70S ribosome it contacts protein S13 of the 30S subunit (bridge B1b), connecting the 2 subunits; this bridge is implicated in subunit movement
N	C0RJI9	DE	50S ribosomal protein L5;
N	C0RJI9	DR	structural constituent of ribosome
N	C0SPB8	DE	Putative HTH-type transcriptional regulator yvaV;
N	C0Z6S0	DE	Argininosuccinate synthase;
N	C0Z6S0	DE	Citrulline--aspartate ligase;
N	C0Z6S0	DR	arginine biosynthetic process
N	C0Z6S0	DR	argininosuccinate synthase activity
N	C0ZBY1	CC	Involved in peptide bond synthesis
N	C0ZBY1	CC	Probably functions indirectly by altering the affinity of the ribosome for aminoacyl-tRNA, thus increasing their reactivity as acceptors for peptidyl transferase (By similarity).
N	C0ZBY1	CC	Stimulates efficient translation and peptide-bond synthesis on native or reconstituted 70S ribosomes in vitro
N	C0ZBY1	DE	Elongation factor P;
N	C0ZBY1	DR	peptide biosynthetic process
N	C0ZBY1	DR	translation elongation factor activity
N	C0ZP44	DE	FGAM synthase II;
N	C0ZP44	DE	Phosphoribosylformylglycinamidine synthase 2;
N	C0ZP44	DE	Phosphoribosylformylglycinamidine synthase II;
N	C0ZP44	DR	'de novo' IMP biosynthetic process
N	C0ZP44	DR	phosphoribosylformylglycinamidine synthase activity
N	C0ZPH6	DE	UPF0060 membrane protein RER_49640;
N	C0ZW55	DE	50S ribosomal protein L17;

N	C0ZW55	DR	structural constituent of ribosome
N	C0ZXK6	CC	Allows the formation of correctly charged Asn-tRNA(Asn) or Gln-tRNA(Gln) through the transamidation of misacylated Asp- tRNA(Asn) or Glu-tRNA(Gln) in organisms which lack either or both of asparaginyl-tRNA or glutaminyl-tRNA synthetases
N	C0ZXK6	CC	The reaction takes place in the presence of glutamine and ATP through an activated phospho-Asp-tRNA(Asn) or phospho-Glu-tRNA(Gln) (By similarity).
N	C0ZXK6	DE	Asp/Glu-ADT subunit C;
N	C0ZXK6	DE	Aspartyl/glutamyl-tRNA(Asn/Gln) amidotransferase subunit C;
N	C0ZXK6	DR	ligase activity
N	C0ZXK6	DR	regulation of translational fidelity
N	C1A1G4	CC	Negative regulator of class I heat shock genes (grpE- dnaK-dnaJ and groELS operons)
N	C1A1G4	CC	Prevents heat-shock induction of these operons (By similarity).
N	C1A1G4	DE	Heat-inducible transcription repressor hrcA;
N	C1A1G4	DR	regulation of transcription, DNA-dependent
N	C1A1G4	DR	response to stress
N	C1A1M0	CC	Major role in the synthesis of nucleoside triphosphates other than ATP
N	C1A1M0	CC	The ATP gamma phosphate is transferred to the NDP beta phosphate via a ping-pong mechanism, using a phosphorylated active-site intermediate (By similarity).
N	C1A1M0	DE	NDP kinase;
N	C1A1M0	DE	Nucleoside diphosphate kinase;
N	C1A1M0	DE	Nucleoside-2-P kinase;
N	C1A1M0	DR	CTP biosynthetic process
N	C1A1M0	DR	GTP biosynthetic process
N	C1A1M0	DR	UTP biosynthetic process
N	C1A1M0	DR	metal ion binding
N	C1A1M0	DR	nucleoside diphosphate kinase activity
N	C1A3Y1	CC	Involved in mRNA degradation
N	C1A3Y1	CC	Hydrolyzes single-stranded polyribonucleotides processively in the 3'- to 5'-direction (By similarity).
N	C1A3Y1	DE	Polynucleotide phosphorylase;
N	C1A3Y1	DE	Polyribonucleotide nucleotidyltransferase;
N	C1A3Y1	DR	3'-5'-exoribonuclease activity
N	C1A3Y1	DR	RNA processing
N	C1A3Y1	DR	mRNA catabolic process
N	C1A3Y1	DR	polyribonucleotide nucleotidyltransferase activity
N	C1AES0	CC	Links a guanosine 5'-phosphate to molydopterin (MPT) forming molybdopterin guanine dinucleotide (MGD) (By similarity).
N	C1AES0	DE	Probable molybdopterin-guanine dinucleotide biosynthesis protein A;
N	C1AES0	DR	Mo-molybdopterin cofactor biosynthetic process
N	C1AES0	DR	catalytic activity
N	C1AGC7	CC	Furnishes a means for formation of correctly charged Gln-tRNA(Gln) through the transamidation of misacylated Glu- tRNA(Gln) in organisms which lack glutaminyl-tRNA synthetase
N	C1AGC7	CC	The reaction takes place in the presence of glutamine and ATP through an activated gamma-phospho-Glu-tRNA(Gln) (By similarity).
N	C1AGC7	DE	Glu-ADT subunit A;
N	C1AGC7	DE	Glutamyl-tRNA(Gln) amidotransferase subunit A;
N	C1AGC7	DR	carbon-nitrogen ligase activity, with glutamine as amido-N-donor
N	C1AGW8	DE	NADH pyrophosphatase;
N	C1AGW8	DR	NAD+ diphosphatase activity
N	C1AGW8	DR	metal ion binding
N	C1APB4	DE	Malate synthase G;
N	C1APB4	DR	glyoxylate cycle
N	C1APB4	DR	malate synthase activity
N	C1APB4	DR	tricarboxylic acid cycle
N	C1AQY1	DE	UPF0256 protein JTY_2426;
N	C1AQY1	DR	N-acetyltransferase activity
N	C1C5H2	CC	Furnishes a means for formation of correctly charged Gln-tRNA(Gln) through the transamidation of misacylated Glu- tRNA(Gln) in organisms which lack glutaminyl-tRNA synthetase
N	C1C5H2	CC	The reaction takes place in the presence of glutamine and ATP through an activated gamma-phospho-Glu-tRNA(Gln) (By similarity).
N	C1C5H2	DE	Glu-ADT subunit A;
N	C1C5H2	DE	Glutamyl-tRNA(Gln) amidotransferase subunit A;
N	C1C5H2	DR	carbon-nitrogen ligase activity, with glutamine as amido-N-donor

N	C1CF92	CC	Produces ATP from ADP in the presence of a proton gradient across the membrane (By similarity).
N	C1CF92	DE	ATP synthase F1 sector epsilon subunit;
N	C1CF92	DE	ATP synthase epsilon chain;
N	C1CF92	DE	F-ATPase epsilon subunit;
N	C1CF92	DR	ATP synthesis coupled proton transport
N	C1CF92	DR	hydrogen ion transporting ATP synthase activity, rotational mechanism
N	C1CF92	DR	proton-transporting ATPase activity, rotational mechanism
N	C1CH59	DE	Dihydroxy-acid dehydratase;
N	C1CH59	DR	4 iron, 4 sulfur cluster binding
N	C1CH59	DR	branched chain family amino acid biosynthetic process
N	C1CH59	DR	dihydroxy-acid dehydratase activity
N	C1CH59	DR	metal ion binding
N	C1CHL4	DE	30S ribosomal protein S2;
N	C1CHL4	DR	structural constituent of ribosome
N	C1CK08	DE	Agmatine iminohydrolase;
N	C1CK08	DE	Putative agmatine deiminase;
N	C1CK08	DR	agmatine deiminase activity
N	C1CK08	DR	putrescine biosynthetic process
N	C1CLS6	CC	Catalyzes a salvage reaction resulting in the formation of AMP, that is energically less costly than de novo synthesis (By similarity).
N	C1CLS6	DE	Adenine phosphoribosyltransferase;
N	C1CLS6	DR	adenine phosphoribosyltransferase activity
N	C1CLS6	DR	adenine salvage
N	C1CLS6	DR	purine ribonucleoside salvage
N	C1CNH8	CC	Binds to the 23S rRNA (By similarity).
N	C1CNH8	DE	50S ribosomal protein L9;
N	C1CNH8	DR	structural constituent of ribosome
N	C1CP95	CC	Binds 23S rRNA and is also seen to make contacts with the A and possibly P site tRNAs (By similarity).
N	C1CP95	DE	50S ribosomal protein L16;
N	C1CP95	DR	structural constituent of ribosome
N	C1CQU5	CC	Transfers the N-acyl diglyceride group on what will become the N-terminal cysteine of membrane lipoproteins (By similarity).
N	C1CQU5	DE	Prolipoprotein diacylglyceryl transferase;
N	C1CQU5	DR	lipoprotein biosynthetic process
N	C1CQU5	DR	protein lipoylation
N	C1CQU5	DR	transferase activity, transferring glycosyl groups
N	C1CQY1	DE	5-enolpyruvylshikimate-3-phosphate phospholyase;
N	C1CQY1	DE	Chorismate synthase;
N	C1CQY1	DR	aromatic amino acid family biosynthetic process
N	C1CQY1	DR	chorismate synthase activity
N	C1CRS4	CC	Binds specifically to the ssrA RNA (tmRNA) and is required for stable association of ssrA with ribosomes (By similarity).
N	C1CRS4	DE	SsrA-binding protein;
N	C1CSZ4	CC	Modifies the free amino group of the aminoacyl moiety of methionyl-tRNA(fMet)
N	C1CSZ4	CC	The formyl group appears to play a dual role in the initiator identity of N-formylmethionyl-tRNA by: (I) promoting its recognition by IF2 and (II) impairing its binding to EFTu-GTP (By similarity).
N	C1CSZ4	DE	Methionyl-tRNA formyltransferase;
N	C1CSZ4	DR	methionyl-tRNA formyltransferase activity
N	C1CSZ4	DR	methyltransferase activity
N	C1CV29	DE	Phosphoglycerate kinase;
N	C1CV29	DR	phosphoglycerate kinase activity
N	C1D5M0	CC	First step of the lipid cycle reactions in the biosynthesis of the cell wall peptidoglycan (By similarity).
N	C1D5M0	DE	Phospho-N-acetylmuramoyl-pentapeptide-transferase;
N	C1D5M0	DE	UDP-MurNAc-pentapeptide phosphotransferase;
N	C1D5M0	DR	UDP-N-acetylmuramoyl-L-alanyl-D-glutamyl-meso-2,6-diaminopimelyl-D-alanyl-D-alanine:undecaprenyl-phosphate transferase activity
N	C1D5M0	DR	cell division
N	C1D5M0	DR	cellular cell wall organization
N	C1D5M0	DR	peptidoglycan biosynthetic process
N	C1D5M0	DR	phospho-N-acetylmuramoyl-pentapeptide-transferase activity
N	C1D5M0	DR	regulation of cell shape

N	C1D5M8	CC	Allows the formation of correctly charged Asn-tRNA(Asn) or Gln-tRNA(Gln) through the transamidation of misacylated Asp- tRNA(Asn) or Glu-tRNA(Gln) in organisms which lack either or both of asparaginyl-tRNA or glutaminyl-tRNA synthetases
N	C1D5M8	CC	The reaction takes place in the presence of glutamine and ATP through an activated phospho-Asp-tRNA(Asn) or phospho-Glu-tRNA(Gln) (By similarity).
N	C1D5M8	DE	Asp/Glu-ADT subunit B;
N	C1D5M8	DE	Aspartyl/glutamyl-tRNA(Asn/Gln) amidotransferase subunit B;
N	C1D5M8	DR	carbon-nitrogen ligase activity, with glutamine as amido-N-donor
N	C1D6J9	CC	Catalyzes the attachment of valine to tRNA(Val)
N	C1D6J9	CC	As ValRS can inadvertently accommodate and process structurally similar amino acids such as threonine, to avoid such errors, it has a "posttransfer" editing activity that hydrolyzes mischarged Thr-tRNA(Val) in a tRNA-dependent manner (By similarity).
N	C1D6J9	DE	Valine--tRNA ligase;
N	C1D6J9	DE	Valyl-tRNA synthetase;
N	C1D6J9	DR	valine-tRNA ligase activity
N	C1D6J9	DR	valyl-tRNA aminoacylation
N	C1DIX6	CC	Catalyzes the addition and repair of the essential 3'- terminal CCA sequence in tRNAs without using a nucleic acid template
N	C1DIX6	CC	Adds these three nucleotides in the order of C, C, and A to the tRNA nucleotide-73, using CTP and ATP as substrates and producing inorganic pyrophosphate
N	C1DIX6	CC	Also shows phosphatase, 2'-nucleotidase and 2',3'-cyclic phosphodiesterase activities
N	C1DIX6	CC	These phosphohydrolase activities are probably involved in the repair of the tRNA 3'-CCA terminus degraded by intracellular RNases (By similarity).
N	C1DIX6	DE	2',3'-cyclic phosphodiesterase;
N	C1DIX6	DE	2'-nucleotidase;
N	C1DIX6	DE	CCA tRNA nucleotidyltransferase;
N	C1DIX6	DE	CCA-adding enzyme;
N	C1DIX6	DE	Multifunctional CCA protein;
N	C1DIX6	DE	Phosphatase;
N	C1DIX6	DE	tRNA CCA-pyrophosphorylase;
N	C1DIX6	DE	tRNA adenylyl-/cytidylyl-transferase;
N	C1DIX6	DE	tRNA nucleotidyltransferase;
N	C1DIX6	DR	hydrolase activity
N	C1DIX6	DR	metal ion binding
N	C1DIX6	DR	tRNA 3'-terminal CCA addition
N	C1DIX6	DR	tRNA adenylyltransferase activity
N	C1DKN4	DE	50S ribosomal protein L36;
N	C1DKN4	DR	structural constituent of ribosome
N	C1DSX5	CC	Catalyzes the reversible transfer of the terminal phosphate group between ATP and AMP
N	C1DSX5	CC	This small ubiquitous enzyme involved in the energy metabolism and nucleotide synthesis, is essential for maintenance and cell growth (By similarity).
N	C1DSX5	DE	ATP-AMP transphosphorylase;
N	C1DSX5	DE	Adenylate kinase;
N	C1DSX5	DR	adenylate kinase activity
N	C1DSX5	DR	nucleotide biosynthetic process
N	C1EPX5	DE	UPF0223 protein BCA_4066;
N	C1F934	CC	The glycine cleavage system catalyzes the degradation of glycine
N	C1F934	CC	The H protein shuttles the methylamine group of glycine from the P protein to the T protein (By similarity).
N	C1F934	DE	Glycine cleavage system H protein;
N	C1F934	DR	glycine decarboxylation via glycine cleavage system
N	C1KZ32	DE	4-methyl-5-beta-hydroxyethylthiazole kinase;
N	C1KZ32	DE	Hydroxyethylthiazole kinase;
N	C1KZ32	DE	Thz kinase;
N	C1KZ32	DR	hydroxyethylthiazole kinase activity
N	C1KZ32	DR	metal ion binding
N	C1KZ32	DR	thiamine biosynthetic process
N	C1L0D5	CC	Converts N-acetylmannosamine-6-phosphate (ManNAc-6-P) to N-acetylglucosamine-6-phosphate (GlcNAc-6-P) (Potential).
N	C1L0D5	DE	ManNAc-6-P epimerase;
N	C1L0D5	DE	Putative N-acetylmannosamine-6-phosphate 2-epimerase;
N	C1L0D5	DR	N-acetylmannosamine metabolic process
N	C1L0D5	DR	N-acylglucosamine-6-phosphate 2-epimerase activity
N	C1P607	CC	May be a toxic protein; overexpression causes cessation of growth.

N	C1P607	DE	Small toxic protein ibsA;
N	C3K1L3	DE	Histidine--tRNA ligase;
N	C3K1L3	DE	Histidyl-tRNA synthetase;
N	C3K1L3	DR	histidine-tRNA ligase activity
N	C3K1L3	DR	histidyl-tRNA aminoacylation
N	C3KVN0	CC	Catalyzes the reversible transfer of the terminal phosphate group between ATP and AMP
N	C3KVN0	CC	This small ubiquitous enzyme involved in the energy metabolism and nucleotide synthesis, is essential for maintenance and cell growth (By similarity).
N	C3KVN0	DE	ATP-AMP transphosphorylase;
N	C3KVN0	DE	Adenylate kinase;
N	C3KVN0	DR	adenylate kinase activity
N	C3KVN0	DR	metal ion binding
N	C3KVN0	DR	nucleotide biosynthetic process
N	C3KVQ9	CC	DNA-dependent RNA polymerase catalyzes the transcription of DNA into RNA using the four ribonucleoside triphosphates as substrates (By similarity).
N	C3KVQ9	DE	DNA-directed RNA polymerase subunit beta;
N	C3KVQ9	DE	RNA polymerase subunit beta;
N	C3KVQ9	DE	RNAP subunit beta;
N	C3KVQ9	DE	Transcriptase subunit beta;
N	C3KVQ9	DR	DNA-directed RNA polymerase activity
N	C3KVQ9	DR	ribonucleoside binding
N	C3KVX7	DE	Phosphoribosyl-AMP cyclohydrolase;
N	C3KVX7	DR	histidine biosynthetic process
N	C3KVX7	DR	phosphoribosyl-AMP cyclohydrolase activity
N	C3L0J9	DE	50S ribosomal protein L28;
N	C3L0J9	DR	structural constituent of ribosome
N	C3L529	DE	Phosphoribosylaminoimidazole-succinocarboxamide synthase;
N	C3L529	DE	SAICAR synthetase;
N	C3L529	DR	phosphoribosylaminoimidazolesuccinocarboxamide synthase activity
N	C3L529	DR	purine nucleotide biosynthetic process
N	C3LJA1	CC	Binds to the 23S rRNA (By similarity).
N	C3LJA1	DE	50S ribosomal protein L15;
N	C3LJA1	DR	structural constituent of ribosome
N	C3LQ26	CC	Catalyzes the NADP-dependent rearrangement and reduction of 1-deoxy-D-xylulose-5-phosphate (DXP) to 2-C-methyl-D-erythritol 4-phosphate (MEP) (By similarity).
N	C3LQ26	DE	1-deoxy-D-xylulose 5-phosphate reductoisomerase;
N	C3LQ26	DE	1-deoxyxylulose-5-phosphate reductoisomerase;
N	C3LQ26	DE	2-C-methyl-D-erythritol 4-phosphate synthase;
N	C3LQ26	DE	DXP reductoisomerase;
N	C3LQ26	DR	1-deoxy-D-xylulose-5-phosphate reductoisomerase activity
N	C3LQ26	DR	isoprenoid biosynthetic process
N	C3LQ26	DR	metal ion binding
N	C3LQ26	DR	oxidation-reduction process
N	C3M9Z9	DE	UPF0434 protein NGR_c31900;
N	C3MAL6	CC	Converts 2C-methyl-D-erythritol 2,4-cyclodiphosphate (ME-2,4cPP) into 1-hydroxy-2-methyl-2-(E)-butenyl 4-diphosphate (By similarity).
N	C3MAL6	DE	1-hydroxy-2-methyl-2-(E)-butenyl 4-diphosphate synthase;
N	C3MAL6	DE	4-hydroxy-3-methylbut-2-en-1-yl diphosphate synthase;
N	C3MAL6	DR	4 iron, 4 sulfur cluster binding
N	C3MAL6	DR	4-hydroxy-3-methylbut-2-en-1-yl diphosphate synthase activity
N	C3MAL6	DR	iron ion binding
N	C3MAL6	DR	oxidation-reduction process
N	C3MAL6	DR	terpenoid biosynthetic process
N	C3MBW4	DE	UPF0758 protein NGR_c13970;
N	C3MF41	DE	Acetylglutamate kinase;
N	C3MF41	DE	N-acetyl-L-glutamate 5-phosphotransferase;
N	C3MF41	DR	acetylglutamate kinase activity
N	C3MF41	DR	arginine biosynthetic process
N	C3MF41	DR	glutamate 5-kinase activity
N	C3MF41	DR	proline biosynthetic process
N	C3MPZ8	DE	UPF0147 protein LS215_1454;
N	C3MRT0	CC	Catalyzes the synthesis of GMP from XMP (By similarity).
N	C3MRT0	DE	GMP synthase [glutamine-hydrolyzing] subunit B;
N	C3MRT0	DE	GMP synthetase;

N	C3MRT0	DR	GMP biosynthetic process
N	C3MRT0	DR	GMP synthase (glutamine-hydrolyzing) activity
N	C3N0Q3	DE	UPF0282 protein M1627_2198;
N	C3N0Q3	DR	hydrolase activity
N	C3NGV0	CC	Produces ATP from ADP in the presence of a proton gradient across the membrane (By similarity).
N	C3NGV0	DE	V-ATPase subunit E;
N	C3NGV0	DE	V-type proton ATPase subunit E;
N	C3NGV0	DR	ATP biosynthetic process
N	C3NGV0	DR	ATP hydrolysis coupled proton transport
N	C3NGV0	DR	proton-transporting ATPase activity, rotational mechanism
N	C3NHZ7	DE	UPF0200 protein YN1551_1675;
N	C3NHZ7	DR	nucleobase, nucleoside, nucleotide and nucleic acid metabolic process
N	C3NHZ7	DR	nucleobase, nucleoside, nucleotide kinase activity
N	C3NHZ7	DR	nucleoside-triphosphatase activity
N	C3NJ84	CC	Hydrolyzes D-tyrosyl-tRNA(Tyr) into D-tyrosine and free tRNA(Tyr) (By similarity).
N	C3NJ84	DE	D-tyrosyl-tRNA(Tyr) deacylase;
N	C3NJ84	DR	hydrolase activity, acting on ester bonds
N	C3NJ84	DR	metal ion binding
N	C3NL04	CC	Removes 5-oxoproline from various penultimate amino acid residues except L-proline (By similarity).
N	C3NL04	DE	5-oxoprolyl-peptidase;
N	C3NL04	DE	Pyroglutamyl-peptidase I;
N	C3NL04	DE	Pyrrolidone-carboxylate peptidase;
N	C3NL04	DR	cysteine-type peptidase activity
N	C3NMG3	DE	Protease HtpX homolog;
N	C3NMG3	DR	metal ion binding
N	C3NMG3	DR	metalloendopeptidase activity
N	C3P002	DE	UPF0178 protein BAA_3113;
N	C3P210	CC	Catalyzes the reversible conversion of 3- phosphohydroxypyruvate to phosphoserine and of 3-hydroxy-2-oxo-4- phosphonooxybutanoate to phosphohydroxythreonine (By similarity).
N	C3P210	DE	Phosphohydroxythreonine aminotransferase;
N	C3P210	DE	Phosphoserine aminotransferase;
N	C3P210	DR	L-serine biosynthetic process
N	C3P210	DR	O-phospho-L-serine:2-oxoglutarate aminotransferase activity
N	C3P210	DR	pyridoxal phosphate binding
N	C3P2J7	CC	Inhibits the expression or activity of extracellular murein hydrolases by interacting, possibly with LrgB, with the holin-like protein CidA
N	C3P2J7	CC	May be involved in programmed cell death (PCD), possibly triggering PCD in response to antibiotics and environmental stresses (By similarity).
N	C3P2J7	CC	The LrgAB and CidA proteins may affect the proton motive force of the membrane
N	C3P2J7	DE	Antiholin-like protein LrgA;
N	C3P655	CC	Is responsible for channeling the electrons from the oxidation of dihydroorotate from the FMN redox center in the pyrD subunit to the ultimate electron acceptor NAD(+) (By similarity).
N	C3P655	DE	Dihydroorotate dehydrogenase electron transfer subunit;
N	C3P655	DR	2 iron, 2 sulfur cluster binding
N	C3P655	DR	electron carrier activity
N	C3P655	DR	electron transport chain
N	C3P655	DR	flavin adenine dinucleotide binding
N	C3P655	DR	metal ion binding
N	C3P655	DR	oxidoreductase activity
N	C3P655	DR	pyrimidine nucleotide biosynthetic process
N	C3P9H5	CC	Phosphorylation of dTMP to form dTDP in both de novo and salvage pathways of dTTP synthesis (By similarity).
N	C3P9H5	DE	Thymidylate kinase;
N	C3P9H5	DE	dTMP kinase;
N	C3P9H5	DR	dTDP biosynthetic process
N	C3P9H5	DR	thymidylate kinase activity
N	C3PAG3	DE	50S ribosomal protein L35;
N	C3PAG3	DR	structural constituent of ribosome
N	C3PDH0	DE	DAP epimerase;
N	C3PDH0	DE	Diaminopimelate epimerase;
N	C3PDH0	DR	diaminopimelate epimerase activity

N	C3PDH0	DR	lysine biosynthetic process via diaminopimelate
N	C3PGA3	CC	Catalyzes the covalent attachment of the prokaryotic ubiquitin-like protein modifier Pup to the proteasomal substrate proteins, thereby targeting them for proteasomal degradation
N	C3PGA3	CC	The ligation reaction involves the side-chain carboxylate of the C-terminal glutamate of Pup and the side-chain amino group of a substrate lysine (By similarity)
N	C3PGA3	CC	This tagging system is termed pupylation
N	C3PGA3	DE	Proteasome accessory factor A;
N	C3PGA3	DE	Pup--protein ligase;
N	C3PGA3	DE	Pup-conjugating enzyme;
N	C3PGA3	DR	acid-amino acid ligase activity
N	C3PGA3	DR	modification-dependent protein catabolic process
N	C3PGA3	DR	proteasomal protein catabolic process
N	C3PKR1	CC	One of the primary rRNA binding proteins, it binds specifically to the 5'-end of 16S ribosomal RNA (By similarity).
N	C3PKR1	DE	30S ribosomal protein S17;
N	C3PKR1	DR	structural constituent of ribosome
N	C3PM69	CC	Required for the formation of a threonylcarbamoyl group on adenosine at position 37 (t(6)A37) in tRNAs that read codons beginning with adenine (By similarity)
N	C3PM69	DE	Probable tRNA threonylcarbamoyladenosine biosynthesis protein Gcp;
N	C3PM69	DE	t(6)A37 threonylcarbamoyladenosine biosynthesis protein;
N	C3PM69	DR	metal ion binding
N	C3PM69	DR	metalloendopeptidase activity
N	C4K4F8	CC	This protein promotes the GTP-dependent binding of aminoacyl-tRNA to the A-site of ribosomes during protein biosynthesis (By similarity).
N	C4K4F8	DE	Elongation factor Tu;
N	C4K4F8	DR	GTPase activity
N	C4K4F8	DR	translation elongation factor activity
N	C4K6N8	CC	The UvrABC repair system catalyzes the recognition and processing of DNA lesions
N	C4K6N8	CC	A damage recognition complex composed of 2 uvrA and 2 uvrB subunits scans DNA for abnormalities
N	C4K6N8	CC	DNA wrap is dependent on ATP binding by uvrB and probably causes local melting of the DNA helix, facilitating insertion of uvrB beta-hairpin between the DNA strands
N	C4K6N8	CC	If a lesion is found the uvrA subunits dissociate and the uvrB-DNA preincision complex is formed
N	C4K6N8	CC	If no lesion is found, the DNA wraps around the other uvrB subunit that will check the other stand for damage (By similarity).
N	C4K6N8	CC	Then uvrB probes one DNA strand for the presence of a lesion
N	C4K6N8	CC	This complex is subsequently bound by uvrC and the second uvrB is released
N	C4K6N8	CC	Upon binding of the uvrA(2)B(2) complex to a putative damaged site, the DNA wraps around one uvrB monomer
N	C4K6N8	DE	Excinuclease ABC subunit B;
N	C4K6N8	DE	Protein uvrB;
N	C4K6N8	DE	UvrABC system protein B;
N	C4K6N8	DR	excinuclease ABC activity
N	C4K6N8	DR	helicase activity
N	C4K6N8	DR	nucleotide-excision repair
N	C4KIY2	CC	Catalyzes the formation of S-adenosylmethionine from methionine and ATP (By similarity).
N	C4KIY2	DE	AdoMet synthase;
N	C4KIY2	DE	Methionine adenosyltransferase;
N	C4KIY2	DE	S-adenosylmethionine synthase;
N	C4KIY2	DR	methionine adenosyltransferase activity
N	C4KIY2	DR	one-carbon metabolic process
N	C4KZL3	CC	This protein is one of the early assembly proteins of the 50S ribosomal subunit, although it is not seen to bind rRNA by itself
N	C4KZL3	CC	It is important during the early stages of 50S assembly (By similarity).
N	C4KZL3	DE	50S ribosomal protein L13;
N	C4KZL3	DR	structural constituent of ribosome
N	C4L0Z7	DE	UPF0234 protein EAT1b_2037;
N	C4L379	CC	Participates in chromosomal partition during cell division
N	C4L379	CC	May act via the formation of a condensin-like complex containing smc and scpB that pull DNA away from mid-cell into both cell halves (By similarity).
N	C4L379	DE	Segregation and condensation protein A;
N	C4L379	DR	cell division
N	C4L379	DR	chromosome segregation

N	C4LAG9	CC	Regulates arginine biosynthesis genes (By similarity).
N	C4LAG9	DE	Arginine repressor;
N	C4LAG9	DR	arginine biosynthetic process
N	C4LAG9	DR	regulation of transcription, DNA-dependent
N	C4LAG9	DR	sequence-specific DNA binding transcription factor activity
N	C4LDE6	DE	GTP cyclohydrolase 1;
N	C4LDE6	DE	GTP cyclohydrolase I;
N	C4LDE6	DR	GTP cyclohydrolase I activity
N	C4LDE6	DR	metal ion binding
N	C4LDE6	DR	one-carbon metabolic process
N	C4LDE6	DR	tetrahydrofolate biosynthetic process
N	C4LDK0	CC	Catalyzes the dehydration of L-rhamnonate to 2-keto-3- deoxy-L-rhamnonate (KDR) (By similarity).
N	C4LDK0	DE	L-rhamnonate dehydratase;
N	C4LDK0	DR	L-rhamnonate dehydratase activity
N	C4LDK0	DR	cellular amino acid catabolic process
N	C4LDK0	DR	metal ion binding
N	C4LJF0	CC	Catalyzes the isomerization between 2-isopropylmalate and 3-isopropylmalate, via the formation of 2-isopropylmaleate (By similarity).
N	C4LJF0	DE	3-isopropylmalate dehydratase small subunit;
N	C4LJF0	DE	Alpha-IPM isomerase;
N	C4LJF0	DE	Isopropylmalate isomerase;
N	C4LJF0	DR	3-isopropylmalate dehydratase activity
N	C4LJF0	DR	leucine biosynthetic process
N	C4XIK1	DE	UPF0082 protein DMR_30850;
N	C4XJU6	CC	Catalyzes the isomerization of sedoheptulose 7-phosphate in D-glycero-D-manno-heptose 7-phosphate (By similarity).
N	C4XJU6	DE	Phosphoheptose isomerase;
N	C4XJU6	DE	Sedoheptulose 7-phosphate isomerase;
N	C4XJU6	DR	D-sedoheptulose 7-phosphate isomerase activity
N	C4XJU6	DR	carbohydrate metabolic process
N	C4XJU6	DR	metal ion binding
N	C4XJU6	DR	sugar binding
N	C4YKB8	CC	Involved in the control of energetic metabolism and significantly contribute to cell fitness, especially under respiratory growth conditions (By similarity).
N	C4YKB8	DE	Respiratory growth induced protein 1;
N	C4ZEV1	CC	May play a role in 30S ribosomal subunit biogenesis
N	C4ZEV1	CC	Unusual circulary permuted GTPase that catalyzes rapid hydrolysis of GTP with a slow catalytic turnover (By similarity).
N	C4ZEV1	DE	Putative ribosome biogenesis GTPase RsgA;
N	C4ZEV1	DR	GTPase activity
N	C4ZEV1	DR	metal ion binding
N	C4ZQ17	CC	Participates in the translocation of lipoproteins from the inner membrane to the outer membrane
N	C4ZQ17	CC	Only forms a complex with a lipoprotein if the residue after the N-terminal Cys is not an aspartate (The Asp acts as a targeting signal to indicate that the lipoprotein should stay in the inner membrane) (By similarity).
N	C4ZQ17	DE	Outer-membrane lipoprotein carrier protein;
N	C4ZQ17	DR	protein transport
N	C4ZQD8	CC	May reduce aminoacrylate peracid to aminoacrylate
N	C4ZQD8	CC	Required to remove a toxic intermediate produce by the pyrimidine nitrogen degradation (By similarity).
N	C4ZQD8	DE	Putative aminoacrylate peracid reductase RutC;
N	C4ZQD8	DR	oxidation-reduction process
N	C4ZQD8	DR	oxidoreductase activity
N	C4ZQW9	CC	Catalyzes the phosphorylation of D-glycero-D-manno- heptose 7-phosphate at the C-1 position to form D,D-heptose-1,7- bisphosphate (By similarity).
N	C4ZQW9	DE	Bifunctional protein hldE;
N	C4ZQW9	DE	D-beta-D-heptose 1-phosphate adenosyltransferase;
N	C4ZQW9	DE	D-beta-D-heptose 7-phosphate kinase;
N	C4ZQW9	DE	D-beta-D-heptose 7-phosphotransferase;
N	C4ZQW9	DR	biosynthetic process
N	C4ZQW9	DR	carbohydrate metabolic process
N	C4ZQW9	DR	kinase activity
N	C4ZQW9	DR	nucleotidyltransferase activity

N	C4ZQW9	DR	phosphotransferase activity, alcohol group as acceptor
N	C4ZT76	DE	UPF0244 protein yjjX;
N	C4ZX91	CC	Cytoskeletal protein that is involved in cell-shape control through regulation of the length of the long axis (By similarity).
N	C4ZX91	DE	Cytoskeleton protein rodZ;
N	C4ZX91	DR	regulation of cell shape
N	C4ZX91	DR	sequence-specific DNA binding
N	C4ZYC7	CC	Multidrug efflux pump that functions probably as a Na(+)/drug antiporter (By similarity).
N	C4ZYC7	DE	Multidrug resistance protein mdtK;
N	C4ZYC7	DE	Multidrug-efflux transporter;
N	C4ZYC7	DR	antiporter activity
N	C4ZYC7	DR	drug transmembrane transporter activity
N	C4ZYC7	DR	sodium ion transport
N	C4ZZ60	CC	Catalyzes the synthesis of Und-PP-GlcNAc-ManNAcA-Fuc4NAc (Lipid III), the third lipid-linked intermediate involved in ECA synthesis (By similarity).
N	C4ZZ60	DE	4-alpha-L-fucosyltransferase;
N	C4ZZ60	DE	Fuc4NAc transferase;
N	C4ZZ60	DE	TDP-Fuc4NAc:lipid II Fuc4NAc transferase;
N	C4ZZ60	DR	enterobacterial common antigen biosynthetic process
N	C4ZZ60	DR	fucosyltransferase activity
N	C4ZZI8	CC	Not exactly known; may be a structural element that influences the osmotic activation of proP at a post-translational level (By similarity).
N	C4ZZI8	DE	ProP effector;
N	C4ZZP9	CC	Responsible for synthesis of pseudouridine from uracil- 13 in transfer RNAs (By similarity).
N	C4ZZP9	DE	tRNA pseudouridine synthase D;
N	C4ZZP9	DE	tRNA pseudouridylate synthase D;
N	C4ZZP9	DE	tRNA-uridine isomerase D;
N	C4ZZP9	DR	pseudouridine synthase activity
N	C4ZZP9	DR	pseudouridine synthesis
N	C4ZZP9	DR	tRNA processing
N	C4ZZU8	CC	Interacts with the secY protein in vivo
N	C4ZZU8	CC	May bind preferentially to an uncomplexed state of secY, thus functioning either as a chelating agent for excess secY in the cell or as a regulatory factor that negatively controls the translocase function (By similarity).
N	C4ZZU8	DE	Protein syd;
N	C4ZZW1	CC	Catalyzes the 2'-O-methylation at nucleotide C2498 in 23S rRNA (By similarity).
N	C4ZZW1	DE	23S rRNA 2'-O-ribose methyltransferase rlmM;
N	C4ZZW1	DE	Ribosomal RNA large subunit methyltransferase M;
N	C4ZZW1	DR	methyltransferase activity
N	C4ZZW1	DR	nucleic acid binding
N	C4ZZW1	DR	rRNA processing
N	C5A033	CC	Necessary for normal cell division and for the maintenance of normal septation (By similarity).
N	C5A033	DE	Probable GTP-binding protein EngB;
N	C5A033	DR	barrier septum formation
N	C5A134	DE	Glycerol-3-phosphate acyltransferase;
N	C5A134	DR	glycerol-3-phosphate O-acyltransferase activity
N	C5A134	DR	phospholipid biosynthetic process
N	C5A337	CC	Produces ATP from ADP in the presence of a proton gradient across the membrane.
N	C5A337	CC	The archaeal beta chain is a regulatory subunit (By similarity).
N	C5A337	DE	V-ATPase subunit B;
N	C5A337	DE	V-type ATP synthase beta chain;
N	C5A337	DR	ATP hydrolysis coupled proton transport
N	C5A337	DR	ATP synthesis coupled proton transport
N	C5A337	DR	hydrogen ion transporting ATP synthase activity, rotational mechanism
N	C5A337	DR	proton-transporting ATPase activity, rotational mechanism
N	C5A7H4	DE	50S ribosomal protein L39e;
N	C5A7H4	DR	structural constituent of ribosome
N	C5B7L8	CC	Participates actively in the response to hyperosmotic and heat shock by preventing the aggregation of stress-denatured proteins and by disaggregating proteins, also in an autonomous, dnaK-independent fashion
N	C5B7L8	CC	Also involved, together with dnaK and grpE, in the DNA replication of plasmids through activation of initiation proteins (By similarity).

N	C5B7L8	CC	GrpE releases ADP from dnaK; ATP binding to dnaK triggers the release of the substrate protein, thus completing the reaction cycle
N	C5B7L8	CC	Several rounds of ATP-dependent interactions between dnaJ, dnaK and grpE are required for fully efficient folding
N	C5B7L8	CC	Unfolded proteins bind initially to dnaJ; upon interaction with the dnaJ-bound protein, dnaK hydrolyzes its bound ATP, resulting in the formation of a stable complex
N	C5B7L8	DE	Chaperone protein dnaJ;
N	C5B7L8	DR	DNA replication
N	C5B7L8	DR	heat shock protein binding
N	C5B7L8	DR	metal ion binding
N	C5B7L8	DR	protein folding
N	C5B7L8	DR	response to heat
N	C5B7L8	DR	unfolded protein binding
N	C5B995	DE	Glycine--tRNA ligase beta subunit;
N	C5B995	DE	Glycyl-tRNA synthetase beta subunit;
N	C5B995	DR	arginine-tRNA ligase activity
N	C5B995	DR	arginyl-tRNA aminoacylation
N	C5B995	DR	glycine-tRNA ligase activity
N	C5B995	DR	glycyl-tRNA aminoacylation
N	C5BHE2	DE	50S ribosomal protein L10;
N	C5BHE2	DR	ribosome biogenesis
N	C5BHE2	DR	structural constituent of ribosome
N	C5BPP4	DE	Aspartate--tRNA ligase;
N	C5BPP4	DE	Aspartyl-tRNA synthetase;
N	C5BPP4	DR	aspartate-tRNA ligase activity
N	C5BPP4	DR	aspartyl-tRNA aminoacylation
N	C5BPP4	DR	nucleic acid binding
N	C5BQ00	CC	Catalyzes the conversion of glucosamine-6-phosphate to glucosamine-1-phosphate (By similarity).
N	C5BQ00	DE	Phosphoglucosamine mutase;
N	C5BQ00	DR	carbohydrate metabolic process
N	C5BQ00	DR	magnesium ion binding
N	C5BQ00	DR	phosphoglucosamine mutase activity
N	C5BQX3	CC	This protein specifically catalyzes the removal of signal peptides from prolipoproteins (By similarity).
N	C5BQX3	DE	Lipoprotein signal peptidase;
N	C5BQX3	DE	Prolipoprotein signal peptidase;
N	C5BQX3	DE	Signal peptidase II;
N	C5BQX3	DR	aspartic-type endopeptidase activity
N	C5C9Q7	CC	Responsible for the release of ribosomes from messenger RNA at the termination of protein biosynthesis
N	C5C9Q7	CC	May increase the efficiency of translation by recycling ribosomes from one round of translation to another (By similarity).
N	C5C9Q7	DE	Ribosome-recycling factor;
N	C5C9Q7	DE	Ribosome-releasing factor;
N	C5CBV4	CC	Required for correct localization of precursor proteins bearing signal peptides with the twin arginine conserved motif S/T-R-R-X-F-L-K
N	C5CBV4	CC	This sec-independent pathway is termed TAT for twin-arginine translocation system
N	C5CBV4	CC	This system mainly transports proteins with bound cofactors that require folding prior to export (By similarity).
N	C5CBV4	DE	Sec-independent protein translocase protein tatA/E homolog;
N	C5CBV4	DR	protein secretion
N	C5CBV4	DR	protein transporter activity
N	C5CBV4	DR	transmembrane transport
N	C5CF28	CC	This protein is involved in the repair of mismatches in DNA
N	C5CF28	CC	It is required for dam-dependent methyl-directed DNA mismatch repair
N	C5CF28	CC	May act as a "molecular matchmaker", a protein that promotes the formation of a stable complex between two or more DNA-binding proteins in an ATP-dependent manner without itself being part of a final effector complex (By similarity).
N	C5CF28	DE	DNA mismatch repair protein mutL;
N	C5CF28	DR	mismatch repair
N	C5CF28	DR	mismatched DNA binding
N	C5CSH5	CC	Catalyzes the isomerization between 2-isopropylmalate and 3-isopropylmalate, via the formation of 2-isopropylmaleate (By similarity).
N	C5CSH5	DE	3-isopropylmalate dehydratase large subunit;

N	C5CSH5	DE	Alpha-IPM isomerase;
N	C5CSH5	DE	Isopropylmalate isomerase;
N	C5CSH5	DR	3-isopropylmalate dehydratase activity
N	C5CSH5	DR	4 iron, 4 sulfur cluster binding
N	C5CSH5	DR	leucine biosynthetic process
N	C5CSH5	DR	metal ion binding
N	C5D3R7	CC	One of the primary rRNA binding proteins, it binds directly near the 3'-end of the 23S rRNA, where it nucleates assembly of the 50S subunit (By similarity).
N	C5D3R7	DE	50S ribosomal protein L3;
N	C5D3R7	DR	structural constituent of ribosome
N	C5D3Y4	CC	Catalyzes the dephosphorylation of undecaprenyl diphosphate (UPP)
N	C5D3Y4	CC	Confers resistance to bacitracin (By similarity).
N	C5D3Y4	DE	Bacitracin resistance protein;
N	C5D3Y4	DE	Undecaprenyl pyrophosphate phosphatase;
N	C5D3Y4	DE	Undecaprenyl-diphosphatase;
N	C5D3Y4	DR	cellular cell wall organization
N	C5D3Y4	DR	dephosphorylation
N	C5D3Y4	DR	peptidoglycan biosynthetic process
N	C5D3Y4	DR	regulation of cell shape
N	C5D3Y4	DR	response to antibiotic
N	C5D3Y4	DR	undecaprenyl-diphosphatase activity
N	C5D428	CC	Zinc phosphodiesterase, which displays some tRNA 3'-processing endonuclease activity
N	C5D428	CC	Probably involved in tRNA maturation, by removing a 3'-trailer from precursor tRNA (By similarity).
N	C5D428	DE	Ribonuclease Z;
N	C5D428	DE	tRNA 3 endonuclease;
N	C5D428	DR	endoribonuclease activity, producing 5'-phosphomonoesters
N	C5D428	DR	metal ion binding
N	C5D428	DR	tRNA 3'-trailer cleavage
N	C5D8M2	CC	Cell wall formation
N	C5D8M2	CC	Catalyzes the addition of glutamate to the nucleotide precursor UDP-N-acetylmuramoyl-L-alanine (UMA) (By similarity).
N	C5D8M2	DE	D-glutamic acid-adding enzyme;
N	C5D8M2	DE	UDP-N-acetylmuramoyl-L-alanyl-D-glutamate synthetase;
N	C5D8M2	DE	UDP-N-acetylmuramoylalanine--D-glutamate ligase;
N	C5D8M2	DR	UDP-N-acetylmuramoylalanine-D-glutamate ligase activity
N	C5D8M2	DR	cell division
N	C5D8M2	DR	cellular cell wall organization
N	C5D8M2	DR	peptidoglycan biosynthetic process
N	C5D8M2	DR	regulation of cell shape
N	C5FRS7	CC	Component of the 90S pre-ribosome involved in the maturation of rRNAs
N	C5FRS7	CC	Required for early cleavages of the pre-RNAs in the 40S ribosomal subunit maturation pathway (By similarity).
N	C5FRS7	DE	Ribosomal RNA-processing protein 36;
N	C5FRS7	DE	rRNA biogenesis protein RRP36;
N	C5FRS7	DR	rRNA processing
N	C5Y494	DE	UPF0497 membrane protein Sb05g021340;
N	C6A237	DE	Phenylalanine--tRNA ligase beta chain;
N	C6A237	DE	Phenylalanyl-tRNA synthetase beta chain;
N	C6A237	DR	magnesium ion binding
N	C6A237	DR	phenylalanine-tRNA ligase activity
N	C6A237	DR	phenylalanyl-tRNA aminoacylation
N	C6BXD5	DE	UPF0234 protein Desal_2385;
N	C6BYQ6	CC	Binds the 23S rRNA (By similarity).
N	C6BYQ6	DE	50S ribosomal protein L31;
N	C6BYQ6	DR	metal ion binding
N	C6BYQ6	DR	structural constituent of ribosome
N	C6C0I6	DE	5-enolpyruvylshikimate-3-phosphate phospholyase;
N	C6C0I6	DE	Chorismate synthase;
N	C6C0I6	DR	aromatic amino acid family biosynthetic process
N	C6C0I6	DR	chorismate synthase activity
N	C6D9B8	CC	Functions in complex with FlhC as a master transcriptional regulator that regulates transcription of several flagellar and non-flagellar operons by binding to their promoter region

N	C6D9B8	CC	Activates expression of class 2 flagellar genes, including fliA, which is a flagellum-specific sigma factor that turns on the class 3 genes
N	C6D9B8	CC	Also regulates genes whose products function in a variety of physiological pathways (By similarity).
N	C6D9B8	DE	Flagellar transcriptional regulator FlhD;
N	C6D9B8	DR	flagellum assembly
N	C6D9B8	DR	transcription activator activity
N	C6D9J8	CC	Participates actively in the response to hyperosmotic and heat shock by preventing the aggregation of stress-denatured proteins, in association with dnaK and grpE
N	C6D9J8	CC	GrpE releases ADP from dnaK; ATP binding to dnaK triggers the release of the substrate protein, thus completing the reaction cycle
N	C6D9J8	CC	It is the nucleotide exchange factor for dnaK and may function as a thermosensor
N	C6D9J8	CC	Several rounds of ATP- dependent interactions between dnaJ, dnaK and grpE are required for fully efficient folding (By similarity).
N	C6D9J8	CC	Unfolded proteins bind initially to dnaJ; upon interaction with the dnaJ-bound protein, dnaK hydrolyzes its bound ATP, resulting in the formation of a stable complex
N	C6D9J8	DE	HSP-70 cofactor;
N	C6D9J8	DE	Protein grpE;
N	C6D9J8	DR	adenyl-nucleotide exchange factor activity
N	C6D9J8	DR	chaperone binding
N	C6D9J8	DR	protein folding
N	C6D9J8	DR	protein homodimerization activity
N	C6D9J8	DR	response to stress
N	C6DEU3	CC	Cell wall formation
N	C6DEU3	CC	Catalyzes the transfer of a GlcNAc subunit on undecaprenyl-pyrophosphoryl-MurNAc-pentapeptide (lipid intermediate I) to form undecaprenyl-pyrophosphoryl-MurNAc- (pentapeptide)GlcNAc (lipid intermediate II) (By similarity).
N	C6DEU3	DE	UDP-N-acetylglucosamine--N-acetylmuramyl-(pentapeptide) pyrophosphoryl-undecaprenol N-acetylglucosamine transferase;
N	C6DEU3	DE	Undecaprenyl-PP-MurNAc-pentapeptide-UDPGlcNAc GlcNAc transferase;
N	C6DEU3	DR	UDP-N-acetyl-D-glucosamine:N-acetylmuramoyl-L-alanyl-D-glutamyl-meso-2,6-diaminopimelyl-D-alanyl-D-alanine-diphosphoundecaprenol 4-beta-N-acetylglucosaminlytransferase activity
N	C6DEU3	DR	UDP-N-acetylgalactosamine biosynthetic process
N	C6DEU3	DR	carbohydrate binding
N	C6DEU3	DR	cell division
N	C6DEU3	DR	cellular cell wall organization
N	C6DEU3	DR	lipid glycosylation
N	C6DEU3	DR	peptidoglycan biosynthetic process
N	C6DEU3	DR	regulation of cell shape
N	C6DEU3	DR	undecaprenyldiphospho-muramoylpentapeptide beta-N-acetylglucosaminyltransferase activity
N	C6DF77	CC	Catalyzes the condensation of ATP and 5-phosphoribose 1- diphosphate to form N'-(5'-phosphoribosyl)-ATP (PR-ATP)
N	C6DF77	CC	Has a crucial role in the pathway because the rate of histidine biosynthesis seems to be controlled primarily by regulation of hisG enzymatic activity (By similarity).
N	C6DF77	DE	ATP phosphoribosyltransferase;
N	C6DF77	DE	ATP-PRTase;
N	C6DF77	DR	ATP phosphoribosyltransferase activity
N	C6DF77	DR	histidine biosynthetic process
N	C6DF77	DR	magnesium ion binding
N	C6DFE1	CC	Involved in copper homeostasis (By similarity).
N	C6DFE1	DE	Copper homeostasis protein CutC;
N	C6DFE1	DR	copper ion binding
N	C6DFE1	DR	copper ion homeostasis
N	C6DFP4	DE	Glucose-6-phosphate isomerase;
N	C6DFP4	DE	Phosphoglucose isomerase;
N	C6DFP4	DE	Phosphohexose isomerase;
N	C6DFP4	DR	gluconeogenesis
N	C6DFP4	DR	glucose-6-phosphate isomerase activity
N	C6DG62	CC	This is 1 of the proteins that binds and probably mediates the attachment of the 5S RNA into the large ribosomal subunit, where it forms part of the central protuberance
N	C6DG62	CC	Contacts the P site tRNA; the 5S rRNA and some of its associated proteins might help stabilize positioning of ribosome-bound tRNAs (By similarity).

N	C6DG62	CC	In the 70S ribosome it contacts protein S13 of the 30S subunit (bridge B1b), connecting the 2 subunits; this bridge is implicated in subunit movement
N	C6DG62	DE	50S ribosomal protein L5;
N	C6DG62	DR	structural constituent of ribosome
N	C6DJF3	CC	Catalyzes the interconversion of beta-pyran and beta- furan forms of D-ribose (By similarity).
N	C6DJF3	DE	D-ribose pyranase;
N	C6DJF3	DR	carbohydrate metabolic process
N	C6DJF3	DR	carbohydrate transport
N	C6DJF3	DR	intramolecular lyase activity
N	C6E4Q4	CC	One of the primary rRNA binding proteins
N	C6E4Q4	CC	It has been suggested to have peptidyltransferase activity; this is somewhat controversial
N	C6E4Q4	CC	Makes several contacts with the 16S rRNA in the 70S ribosome (By similarity).
N	C6E4Q4	CC	Required for association of the 30S and 50S subunits to form the 70S ribosome, for tRNA binding and peptide bond formation
N	C6E4Q4	DE	50S ribosomal protein L2;
N	C6E4Q4	DR	structural constituent of ribosome
N	C6E4Q4	DR	transferase activity
N	C6E506	CC	Binds to the 23S rRNA (By similarity).
N	C6E506	DE	50S ribosomal protein L9;
N	C6E506	DR	structural constituent of ribosome
N	C6E5S6	DE	Shikimate dehydrogenase;
N	C6E5S6	DR	aromatic amino acid family biosynthetic process
N	C6E5S6	DR	oxidation-reduction process
N	C6E5S6	DR	shikimate 5-dehydrogenase activity
N	C7DQX8	DE	Conotoxin Vi11.3;
N	C8CK75	DE	Conotoxin Ml15b;
N	C8CK75	DR	ion channel inhibitor activity
N	D0ZHC6	CC	Efflux pump driven by the proton motive force
N	D0ZHC6	CC	Confers resistance to a broad spectrum of chemically unrelated drugs (By similarity).
N	D0ZHC6	DE	Multidrug transporter MdfA;
N	D0ZHC6	DR	response to antibiotic
N	D0ZHC6	DR	transmembrane transport
N	D0ZHC6	DR	transporter activity
N	D2HNW6	CC	Transcription factor required to direct islet cell differentiation during endocrine pancreas development
N	D2HNW6	CC	Activates transcription by forming a heterodimer with RFX3 and binding to the X-box in the promoter of target genes (By similarity).
N	D2HNW6	CC	Acts downstream of NEUROG3 and regulates the transcription factors involved in beta-cell maturation and function, thereby restricting the expression of the beta-cell differentiation and specification genes, and thus the beta-cell fate choice
N	D2HNW6	CC	Not required for pancreatic PP (polypeptide-producing) cells differentiation
N	D2HNW6	CC	Specifically required for the differentiation of 4 of the 5 islet cell types and for the production of insulin
N	D2HNW6	DE	DNA-binding protein RFX6;
N	D2HNW6	DE	Regulatory factor X 6;
N	D2HNW6	DR	glucose homeostasis
N	D2HNW6	DR	pancreatic A cell differentiation
N	D2HNW6	DR	pancreatic D cell differentiation
N	D2HNW6	DR	pancreatic E cell differentiation
N	D2HNW6	DR	promoter binding
N	D2HNW6	DR	regulation of insulin secretion
N	D2HNW6	DR	regulation of transcription, DNA-dependent
N	D2HNW6	DR	transcription activator activity
N	D2HNW6	DR	type B pancreatic cell differentiation
N	D2HQI1	CC	Embryonic stem (ES) cell-specific transcription factor required to regulate ES cell pluripotency
N	D2HQI1	CC	Acts as an activator of spontaneous telomere sister chromatid exchange (T-SCE) and telomere elongation in undifferentiated ES cells (By similarity).
N	D2HQI1	CC	Binds telomeres and plays a key role in genomic stability in ES cells by regulating telomere elongation
N	D2HQI1	DE	Zinc finger and SCAN domain-containing protein 4;
N	D2HQI1	DR	regulation of transcription, DNA-dependent
N	D2HQI1	DR	sequence-specific DNA binding transcription factor activity
N	D2HQI1	DR	telomere maintenance via telomere lengthening

N	D2HQI1	DR	viral reproduction
N	D2HQI1	DR	zinc ion binding
N	D2HZB0	CC	Key regulator of mitochondrial calcium uptake required for calcium entry into mitochondrion
N	D2HZB0	CC	May act as a calcium sensor via its EF-hand domains, gating the activity of a calcium channel partner (By similarity).
N	D2HZB0	DE	Calcium uptake protein 1, mitochondrial;
N	D2HZB0	DE	Calcium-binding atopy-related autoantigen 1 homolog;
N	D2HZB0	DR	calcium ion binding
N	D2HZB0	DR	calcium ion import
N	D2HZB0	DR	elevation of mitochondrial calcium ion concentration
N	D2Y240	CC	Ion channel inhibitor (By similarity).
N	D2Y240	DE	Hainantoxin-VIII;
N	D2Y240	DE	Peptide F4-27.90;
N	D2Y240	DR	potassium channel inhibitor activity
N	D2Y261	CC	Probable ion channel inhibitor (By similarity).
N	D2Y261	DE	HNTX-XVI.9;
N	D2Y261	DE	Hainantoxin-XVI.9;
N	D2Y261	DE	Peptide F4-19.87;
N	D2Y261	DR	sodium channel inhibitor activity
N	D2Y290	CC	Probable ion channel inhibitor (By similarity).
N	D2Y290	DE	HNTX-XVI-20;
N	D2Y290	DE	Hainantoxin-XVI-20;
N	D2Y290	DR	sodium channel inhibitor activity
N	D2Y2C8	CC	Putative ion channel inhibitor.
N	D2Y2C8	DE	HNTX-XVII.3;
N	D2Y2C8	DE	Hainantoxin-XVII.3;
N	D2Y2C8	DE	Peptide F2-20.97;
N	D2Y2C8	DR	ion channel inhibitor activity
N	D2Y2G1	CC	Inhibits trypsin and blocks voltage-gated potassium channels (By similarity).
N	D2Y2G1	DE	HNTX-XI-10;
N	D2Y2G1	DE	Hainantoxin-XI-10;
N	D2Y2G1	DR	potassium channel inhibitor activity
N	D2Y2G1	DR	serine-type endopeptidase inhibitor activity
N	D3ZAP3	DE	Uncharacterized protein KIAA1383 homolog;
N	D4CZ44	CC	Secreted metalloproteinase probably acting as a virulence factor (By similarity).
N	D4CZ44	DE	Fungalysin MEP4;
N	D4CZ44	DE	Probable extracellular metalloproteinase 4;
N	D4CZ44	DR	metalloendopeptidase activity
N	D4CZ44	DR	zinc ion binding
N	D5CE34	CC	May reduce aminoacrylate peracid to aminoacrylate
N	D5CE34	CC	Required to remove a toxic intermediate produce by the pyrimidine nitrogen degradation (By similarity).
N	D5CE34	DE	Putative aminoacrylate peracid reductase RutC;
N	D5CE34	DR	oxidation-reduction process
N	D5CE34	DR	oxidoreductase activity
N	D5CZH0	CC	May reduce aminoacrylate peracid to aminoacrylate
N	D5CZH0	CC	Required to remove a toxic intermediate produce by the pyrimidine nitrogen degradation (By similarity).
N	D5CZH0	DE	Putative aminoacrylate peracid reductase RutC;
N	D5CZH0	DR	oxidation-reduction process
N	D5CZH0	DR	oxidoreductase activity
N	E0TZF9	CC	One of two assembly initiator proteins, it binds directly to the 5'-end of the 23S rRNA, where it nucleates assembly of the 50S subunit (By similarity).
N	E0TZF9	DE	50S ribosomal protein L24;
N	E0TZF9	DR	structural constituent of ribosome
N	E1C2Z0	CC	Component of the MMS22L-TONSL complex, a complex that stimulates the recombination-dependent repair of stalled or collapsed replication forks (By similarity).
N	E1C2Z0	DE	Methyl methanesulfonate-sensitivity protein 22-like;
N	E1C2Z0	DE	Protein MMS22-like;
N	E1C2Z0	DR	double-strand break repair via homologous recombination
N	E1C2Z0	DR	replication fork processing
N	O00116	DE	Aging-associated gene 5 protein;
N	O00116	DE	Alkyl-DHAP synthase;
N	O00116	DE	Alkyldihydroxyacetonephosphate synthase, peroxisomal;

N	O00116	DE	Alkylglycerone-phosphate synthase;
N	O00116	DR	alkylglycerone-phosphate synthase activity
N	O00116	DR	ether lipid biosynthetic process
N	O00116	DR	flavin adenine dinucleotide binding
N	O00116	DR	oxidoreductase activity
N	O00311	CC	Seems to phosphorylate critical substrates that regulate the G1/S phase transition and/or DNA replication
N	O00311	CC	Can phosphorylates MCM2 and MCM3.
N	O00311	DE	CDC7-related kinase;
N	O00311	DE	Cell division cycle 7-related protein kinase;
N	O00311	DR	DNA replication
N	O00311	DR	G1/S transition of mitotic cell cycle
N	O00311	DR	M/G1 transition of mitotic cell cycle
N	O00311	DR	cell cycle checkpoint
N	O00311	DR	cell division
N	O00311	DR	metal ion binding
N	O00311	DR	positive regulation of cell proliferation
N	O00311	DR	protein binding
N	O00311	DR	protein phosphorylation
N	O00311	DR	protein serine/threonine kinase activity
N	O00311	DR	regulation of S phase
N	O00526	CC	Component of the asymmetric unit membrane (AUM); a highly specialized biomembrane elaborated by terminally differentiated urothelial cells
N	O00526	CC	May play an important role in regulating the assembly of the AUM (By similarity).
N	O00526	DE	Uroplakin II;
N	O00526	DE	Uroplakin-2;
N	O00526	DR	cellular membrane organization
N	O00526	DR	epithelial cell differentiation
N	O00526	DR	multicellular organismal development
N	O00852	DE	Phosphoglycerate kinase;
N	O00852	DR	phosphoglycerate kinase activity
N	O02161	CC	Essential component of the PAM complex, a complex required for the translocation of transit peptide-containing proteins from the inner membrane into the mitochondrial matrix in an ATP-dependent manner
N	O02161	CC	Recruits mitochondrial HSP70 to drive protein translocation into the matrix using ATP as an energy source (By similarity).
N	O02161	DE	Probable mitochondrial import inner membrane translocase subunit tim-44;
N	O02161	DR	P-P-bond-hydrolysis-driven protein transmembrane transporter activity
N	O02161	DR	embryo development ending in birth or egg hatching
N	O02161	DR	intracellular protein transport
N	O02161	DR	nematode larval development
N	O02161	DR	positive regulation of growth rate
N	O02161	DR	protein binding
N	O02161	DR	receptor-mediated endocytosis
N	O02316	CC	Orphan nuclear receptor.
N	O02316	DE	Nuclear hormone receptor family member nhr-77;
N	O02316	DR	regulation of transcription, DNA-dependent
N	O02316	DR	sequence-specific DNA binding
N	O02316	DR	sequence-specific DNA binding transcription factor activity
N	O02316	DR	steroid hormone receptor activity
N	O02316	DR	zinc ion binding
N	O03064	CC	Produces ATP from ADP in the presence of a proton gradient across the membrane
N	O03064	CC	The catalytic sites are hosted primarily by the beta subunits (By similarity).
N	O03064	DE	ATP synthase F1 sector subunit beta;
N	O03064	DE	ATP synthase subunit beta, chloroplastic;
N	O03064	DE	F-ATPase subunit beta;
N	O03064	DR	ATP hydrolysis coupled proton transport
N	O03064	DR	ATP synthesis coupled proton transport
N	O03064	DR	hydrogen ion transporting ATP synthase activity, rotational mechanism
N	O03064	DR	hydrogen-exporting ATPase activity, phosphorylative mechanism
N	O04019	CC	The 26S protease is involved in the ATP-dependent degradation of ubiquitinated proteins
N	O04019	CC	The regulatory (or ATPase) complex confers ATP dependency and substrate specificity to the 26S complex.
N	O04019	DE	26S protease regulatory subunit 6A homolog B;
N	O04019	DE	26S proteasome AAA-ATPase subunit RPT5b;

N	O04019	DE	Proteasome 26S subunit 6A homolog B;
N	O04019	DE	Regulatory particle triple-A ATPase subunit 5b;
N	O04019	DE	TBP-1 homolog B;
N	O04019	DE	Tat-binding protein 1 homolog B;
N	O04019	DR	embryo sac development
N	O04019	DR	glucose mediated signaling pathway
N	O04019	DR	nucleoside-triphosphatase activity
N	O04019	DR	pollen development
N	O04019	DR	proteasomal protein catabolic process
N	O04019	DR	protein binding
N	O04486	CC	Intracellular vesicle trafficking and protein transport (By similarity).
N	O04486	DE	Ras-related protein RABA2a;
N	O04486	DE	Ras-related protein Rab11C;
N	O04486	DR	protein transport
N	O04486	DR	small GTPase mediated signal transduction
N	O05086	DE	Uncharacterized transposase-like protein HI_1721;
N	O05086	DR	DNA integration
N	O05086	DR	DNA recombination
N	O05086	DR	transposition
N	O05236	DE	Uncharacterized HTH-type transcriptional regulator yugG;
N	O05236	DR	regulation of transcription, DNA-dependent
N	O05236	DR	sequence-specific DNA binding
N	O05236	DR	sequence-specific DNA binding transcription factor activity
N	O05975	DE	Uncharacterized protein RP785;
N	O06051	CC	One of the primary rRNA binding proteins, it binds specifically to the 5'-end of 16S ribosomal RNA (By similarity).
N	O06051	DE	30S ribosomal protein S17;
N	O06051	DR	structural constituent of ribosome
N	O06914	DE	Fumarate reductase iron-sulfur subunit;
N	O06914	DR	2 iron, 2 sulfur cluster binding
N	O06914	DR	3 iron, 4 sulfur cluster binding
N	O06914	DR	4 iron, 4 sulfur cluster binding
N	O06914	DR	electron carrier activity
N	O06914	DR	electron transport chain
N	O06914	DR	metal ion binding
N	O06914	DR	succinate dehydrogenase activity
N	O06914	DR	tricarboxylic acid cycle
N	O06974	DE	UPF0052 protein yvcK;
N	O07005	DE	UPF0311 protein yveG;
N	O07589	DE	UPF0053 protein yhdT;
N	O07589	DR	catalytic activity
N	O07589	DR	flavin adenine dinucleotide binding
N	O07589	DR	oxidation-reduction process
N	O07617	CC	Phosphatase with broad substrate specificity
N	O07617	CC	Does not have phosphoglycerate mutase activtiy.
N	O07617	DE	Uncharacterized phosphatase phoE;
N	O07617	DR	hydrolase activity
N	O08314	CC	Exchanges the guanine residue with 7-aminomethyl-7- deazaguanine in tRNAs with GU(N) anticodons (tRNA-Asp, -Asn, -His and -Tyr)
N	O08314	CC	After this exchange, a cyclopentendiol moiety is attached to the 7-aminomethyl group of 7-deazaguanine, resulting in the hypermodified nucleoside queuosine (Q) (7-(((4,5-cis- dihydroxy-2-cyclopenten-1-yl)amino)methyl)-7- deazaguanosine) (By similarity)
N	O08314	DE	Guanine insertion enzyme;
N	O08314	DE	Queuine tRNA-ribosyltransferase;
N	O08314	DE	tRNA-guanine transglycosylase;
N	O08314	DR	metal ion binding
N	O08314	DR	queuine tRNA-ribosyltransferase activity
N	O08314	DR	queuosine biosynthetic process
N	O08665	CC	Plays a role in growth cones guidance
N	O08665	CC	May function to pattern sensory projections by selectively repelling axons that normally terminate dorsally.
N	O08665	DE	Semaphorin III;
N	O08665	DE	Semaphorin-3A;
N	O08665	DE	Semaphorin-D;
N	O08665	DR	axon guidance

N	O08665	DR	axonal fasciculation
N	O08665	DR	chemorepellent activity
N	O08665	DR	dichotomous subdivision of terminal units involved in salivary gland branching
N	O08665	DR	negative regulation of axon extension involved in axon guidance
N	O08665	DR	negative regulation of epithelial cell migration
N	O08665	DR	receptor activity
N	O08665	DR	regulation of heart rate
N	O08811	CC	ATP-dependent 5'-3' DNA helicase, component of the core- TFIIH basal transcription factor
N	O08811	CC	As part of the mitotic spindle-associated MMXD complex it plays a role in chromosome segregation
N	O08811	CC	Involved in nucleotide excision repair (NER) of DNA by opening DNA around the damage, and in RNA transcription by RNA polymerase II by anchoring the CDK-activating kinase (CAK) complex, composed of CDK7, cyclin H and MAT1, to the core-TFIIH complex
N	O08811	CC	Involved in the regulation of vitamin-D receptor activity
N	O08811	CC	Might have a role in aging process and could play a causative role in the generation of skin cancers (By similarity).
N	O08811	DE	DNA excision repair protein ERCC-2;
N	O08811	DE	DNA repair protein complementing XP-D cells;
N	O08811	DE	TFIIH basal transcription factor complex helicase XPD subunit;
N	O08811	DE	Xeroderma pigmentosum group D-complementing protein;
N	O08811	DR	ATP-dependent DNA helicase activity
N	O08811	DR	UV protection
N	O08811	DR	bone mineralization
N	O08811	DR	cell proliferation
N	O08811	DR	central nervous system myelin formation
N	O08811	DR	chromosome segregation
N	O08811	DR	embryonic cleavage
N	O08811	DR	erythrocyte maturation
N	O08811	DR	extracellular matrix organization
N	O08811	DR	hair cell differentiation
N	O08811	DR	hair follicle maturation
N	O08811	DR	hemopoietic stem cell differentiation
N	O08811	DR	in utero embryonic development
N	O08811	DR	induction of apoptosis
N	O08811	DR	iron-sulfur cluster binding
N	O08811	DR	metal ion binding
N	O08811	DR	multicellular organism growth
N	O08811	DR	negative regulation of apoptosis
N	O08811	DR	positive regulation of DNA binding
N	O08811	DR	post-embryonic development
N	O08811	DR	response to oxidative stress
N	O08811	DR	skin development
N	O08811	DR	spinal cord development
N	O08811	DR	transcription from RNA polymerase II promoter
N	O08811	DR	transcription-coupled nucleotide-excision repair
N	O14050	DE	Uncharacterized mscS family protein C2C4.17c;
N	O14050	DR	cellular response to stress
N	O14050	DR	transmembrane transport
N	O14132	CC	Protein kinase which is essential for spore formation.
N	O14132	DE	Sporulation protein kinase pit1;
N	O14132	DR	ascospore formation
N	O14132	DR	protein phosphorylation
N	O14132	DR	protein serine/threonine kinase activity
N	O14132	DR	signal transduction
N	O14325	DE	Uncharacterized AAA domain-containing protein C16E9.10c;
N	O14325	DR	nucleoside-triphosphatase activity
N	O14342	DE	Uncharacterized protein C2F12.12c;
N	O14442	CC	Catalyzes C14-demethylation of lanosterol which is critical for ergosterol biosynthesis
N	O14442	CC	It transforms lanosterol into 4,4'-dimethyl cholesta-8,14,24-triene-3-beta-ol (By similarity).
N	O14442	DE	Cytochrome P450 51;
N	O14442	DE	Cytochrome P450-14DM;
N	O14442	DE	Cytochrome P450-LIA1;
N	O14442	DE	Eburicol 14-alpha-demethylase;

N	O14442	DE	Sterol 14-alpha demethylase;
N	O14442	DR	electron carrier activity
N	O14442	DR	oxidation-reduction process
N	O14442	DR	sterol 14-demethylase activity
N	O14442	DR	sterol biosynthetic process
N	O14604	CC	Plays an important role in the organization of the cytoskeleton
N	O14604	CC	Binds to and sequesters actin monomers (G actin) and therefore inhibits actin polymerization (By similarity).
N	O14604	DE	Thymosin beta-4, Y-chromosomal;
N	O14604	DR	actin binding
N	O14604	DR	actin cytoskeleton organization
N	O14604	DR	sequestering of actin monomers
N	O15172	DE	L-3-phosphoserine-phosphatase homolog;
N	O15172	DE	Putative phosphoserine phosphatase-like protein;
N	O15172	DR	cell proliferation
N	O15172	DR	phosphoserine phosphatase activity
N	O15226	CC	Interacts with a specific negative regulatory element (NRE) 5'-AATTCCTCTGA-3' to mediate transcriptional repression of certain NK-kappa-B responsive genes
N	O15226	CC	Also involved in the regulation of IL-8 transcription.
N	O15226	CC	Involved in the constitutive silencing of the interferon beta promoter, independently of the virus-induced signals, and in the inhibition of the basal and cytokine-induced iNOS promoter activity
N	O15226	DE	NF-kappa-B-repressing factor;
N	O15226	DE	NFkB-repressing factor;
N	O15226	DE	Protein ITBA4;
N	O15226	DE	Transcription factor NRF;
N	O15226	DR	double-stranded RNA binding
N	O15226	DR	specific transcriptional repressor activity
N	O15360	CC	DNA repair protein that may operate in a postreplication repair or a cell cycle checkpoint function
N	O15360	CC	May be involved in interstrand DNA cross-link repair and in the maintenance of normal chromosome stability.
N	O15360	DE	Fanconi anemia group A protein;
N	O15360	DE	Protein FACA;
N	O15360	DR	protein binding
N	O15360	DR	protein complex assembly
N	O16053	DE	Protein CDV3 homolog;
N	O16053	DE	Protein anon-2C9;
N	O16785	CC	Involved in the regulation of cell adhesion and cytoskeleton organization
N	O16785	CC	Required to recruit unc-89 and myofilaments to newly forming attachments, and also to reposition the attachments so that they form the highly ordered array of dense body and M-line attachments that are characteristic of mature muscle cells.
N	O16785	DE	Actopaxin homolog;
N	O16785	DE	Paralyzed arrest at two-fold protein 6;
N	O16785	DE	Parvin-like protein;
N	O16785	DR	actin binding
N	O16785	DR	cell adhesion
N	O16785	DR	determination of adult lifespan
N	O16785	DR	embryo development ending in birth or egg hatching
N	O16785	DR	nematode larval development
N	O16785	DR	striated muscle contraction involved in embryonic body morphogenesis
N	O16785	DR	striated muscle myosin thick filament assembly
N	O18920	CC	Binds and activates TIE2 receptor by inducing its tyrosine phosphorylation
N	O18920	CC	Appears to play a crucial role in mediating reciprocal interactions between the endothelium and surrounding matrix and mesenchyme
N	O18920	CC	Implicated in endothelial developmental processes later and distinct from that of VEGF
N	O18920	CC	It may play an important role in the heart early development (By similarity).
N	O18920	CC	Mediates blood vessel maturation/stability
N	O18920	DE	Angiopoietin-1;
N	O18920	DR	cell differentiation
N	O19909	CC	Light-harvesting photosynthetic tetrapyrrole chromophore-protein from the phycobiliprotein complex.
N	O19909	DE	C-phycocyanin beta chain;
N	O19909	DR	electron transport chain
N	O19909	DR	mannitol transport

N	O19909	DR	photosynthesis
N	O19909	DR	protein-chromophore linkage
N	O19989	CC	RuBisCO catalyzes two reactions: the carboxylation of D- ribulose 1,5-bisphosphate, the primary event in carbon dioxide fixation, as well as the oxidative fragmentation of the pentose substrate in the photorespiration process
N	O19989	CC	Both reactions occur simultaneously and in competition at the same active site (By similarity).
N	O19989	DE	Ribulose bisphosphate carboxylase large chain;
N	O19989	DE	RuBisCO large subunit;
N	O19989	DR	magnesium ion binding
N	O19989	DR	monooxygenase activity
N	O19989	DR	oxidation-reduction process
N	O19989	DR	photorespiration
N	O19989	DR	reductive pentose-phosphate cycle
N	O19989	DR	ribulose-bisphosphate carboxylase activity
N	O20159	DE	Uncharacterized 79.7 kDa protein in psaB-ycf62 intergenic region;
N	O21543	CC	Core subunit of the mitochondrial membrane respiratory chain NADH dehydrogenase (Complex I) that is believed to belong to the minimal assembly required for catalysis
N	O21543	CC	Complex I functions in the transfer of electrons from NADH to the respiratory chain
N	O21543	CC	The immediate electron acceptor for the enzyme is believed to be ubiquinone (By similarity).
N	O21543	DE	NADH dehydrogenase subunit 3;
N	O21543	DE	NADH-ubiquinone oxidoreductase chain 3;
N	O21543	DR	NADH dehydrogenase (ubiquinone) activity
N	O21543	DR	electron transport chain
N	O22165	CC	Involved in the biogenesis of the 60S ribosomal subunit
N	O22165	CC	Ensures the docking of NOG1 to pre-60S particles (By similarity).
N	O22165	DE	Probable ribosome biogenesis protein RLP24;
N	O22165	DR	ribosome biogenesis
N	O22165	DR	structural constituent of ribosome
N	O23115	CC	Probable transcription factor acting as a positive regulator in the ethylene response pathway
N	O23115	CC	Could bind the primary ethylene response element present in the ETHYLENE-RESPONSE-FACTOR1 promoter.
N	O23115	DE	ETHYLENE INSENSITIVE 3-like 2 protein;
N	O23115	DR	ethylene mediated signaling pathway
N	O23115	DR	sequence-specific DNA binding transcription factor activity
N	O23115	DR	transcription regulator activity
N	O23420	CC	May only reduce GSH-thiol disulfides, but not protein disulfides (Potential).
N	O23420	DE	Monothiol glutaredoxin-S5;
N	O23420	DE	Protein ROXY 12;
N	O23420	DR	2 iron, 2 sulfur cluster binding
N	O23420	DR	cell redox homeostasis
N	O23420	DR	electron carrier activity
N	O23420	DR	metal ion binding
N	O23420	DR	protein disulfide oxidoreductase activity
N	O23651	DE	Terpenoid synthase 1;
N	O23651	DR	lyase activity
N	O23651	DR	magnesium ion binding
N	O24325	CC	Asparagine-specific endopeptidase
N	O24325	CC	Probably involved in the degradation of phaseolin during and after germination.
N	O24325	DE	Legumain-like proteinase;
N	O24325	DE	Vacuolar-processing enzyme;
N	O24325	DR	cysteine-type endopeptidase activity
N	O24388	CC	Inhibitor of cysteine proteases
N	O24388	CC	May protect the plant by inhibiting proteases of invading organisms.
N	O24388	DE	Cysteine protease inhibitor 3;
N	O24388	DR	cysteine-type endopeptidase inhibitor activity
N	O25775	CC	Involved in rRNA and/or ribosome maturation and assembly
N	O25775	CC	May have metal-dependent hydrolase activity (By similarity).
N	O25775	CC	Required for normal 5' and 3' processing of 16S, 23S and 5S rRNAs
N	O25775	DE	Probable rRNA maturation factor;
N	O25775	DR	metal ion binding
N	O25775	DR	metalloendopeptidase activity
N	O25775	DR	rRNA processing

N	O25853	CC	NDH-1 shuttles electrons from NADH, via FMN and iron- sulfur (Fe-S) centers, to quinones in the respiratory chain
N	O25853	CC	Couples the redox reaction to proton translocation (for every two electrons transferred, four hydrogen ions are translocated across the cytoplasmic membrane), and thus conserves the redox energy in a proton gradient (By similarity)
N	O25853	CC	The immediate electron acceptor for the enzyme in this species is believed to be ubiquinone
N	O25853	DE	NADH dehydrogenase I subunit D;
N	O25853	DE	NADH-quinone oxidoreductase subunit D;
N	O25853	DE	NDH-1 subunit D;
N	O25853	DR	NADH dehydrogenase (quinone) activity
N	O25853	DR	oxidation-reduction process
N	O25853	DR	quinone binding
N	O27001	CC	Transcriptional activator of the fmdECB operon (By similarity).
N	O27001	DE	DNA-binding protein tfx;
N	O27001	DR	regulation of transcription, DNA-dependent
N	O27001	DR	sequence-specific DNA binding transcription factor activity
N	O27001	DR	sigma factor activity
N	O27001	DR	transcription initiation, DNA-dependent
N	O27198	DE	UPF0348 protein MTH_1126;
N	O27274	CC	Catalyzes the decarboxylation of sulfopyruvic acid to sulfoacetaldehyde (By similarity)
N	O27274	DE	Sulfopyruvate decarboxylase subunit alpha;
N	O27274	DR	coenzyme M biosynthetic process
N	O27274	DR	sulfopyruvate decarboxylase activity
N	O27274	DR	thiamine pyrophosphate binding
N	O27664	CC	General factor that plays a role in the activation of archaeal genes transcribed by RNA polymerase
N	O27664	CC	Binds specifically to the TATA box promoter element which lies close to the position of transcription initiation (By similarity).
N	O27664	DE	Box A-binding protein;
N	O27664	DE	TATA sequence-binding protein;
N	O27664	DE	TATA-box factor;
N	O27664	DE	TATA-box-binding protein;
N	O27664	DR	RNA polymerase II transcription factor activity
N	O27664	DR	regulation of transcription, DNA-dependent
N	O27664	DR	transcription initiation from RNA polymerase II promoter
N	O27957	DE	Shikimate dehydrogenase;
N	O27957	DR	aromatic amino acid family biosynthetic process
N	O27957	DR	oxidation-reduction process
N	O27957	DR	shikimate 5-dehydrogenase activity
N	O28187	DE	Uncharacterized protein AF_2093;
N	O28512	DE	Uncharacterized protein AF_1762;
N	O28557	DE	Uncharacterized protein AF_1717;
N	O28578	CC	Catalyzes the NADPH-dependent reduction of ketopantoate into pantoic acid (By similarity).
N	O28578	DE	KPA reductase;
N	O28578	DE	Ketopantoate reductase;
N	O28578	DE	Putative 2-dehydropantoate 2-reductase;
N	O28578	DR	2-dehydropantoate 2-reductase activity
N	O28578	DR	oxidation-reduction process
N	O28578	DR	pantothenate biosynthetic process
N	O28965	DE	Triose-phosphate isomerase;
N	O28965	DE	Triosephosphate isomerase;
N	O28965	DR	gluconeogenesis
N	O28965	DR	pentose-phosphate shunt
N	O28965	DR	triose-phosphate isomerase activity
N	O29525	DE	Probable thiamine-monophosphate kinase;
N	O29525	DE	Thiamine-phosphate kinase;
N	O29525	DR	thiamine biosynthetic process
N	O29525	DR	thiamine-phosphate kinase activity
N	O29697	DE	UPF0033 protein AF_0554;
N	O29697	DR	sulfurtransferase activity
N	O29697	DR	tRNA processing
N	O29760	CC	Component of the proteasome core, a large protease complex with broad specificity involved in protein degradation (By similarity).

N	O29760	DE	20S proteasome alpha subunit;
N	O29760	DE	Proteasome core protein PsmA;
N	O29760	DE	Proteasome subunit alpha;
N	O29760	DR	threonine-type endopeptidase activity
N	O29760	DR	ubiquitin-dependent protein catabolic process
N	O30257	DE	Uncharacterized HTH-type transcriptional regulator AF_2414;
N	O30257	DR	sequence-specific DNA binding
N	O31019	DE	Carbamate kinase;
N	O31019	DR	arginine metabolic process
N	O31019	DR	carbamate kinase activity
N	O31019	DR	cellular amino acid biosynthetic process
N	O31046	CC	Catalyzes the condensation of the acetyl group of acetyl-CoA with 3-methyl-2-oxobutanoate (2-oxoisovalerate) to form 3-carboxy-3-hydroxy-4-methylpentanoate (2-isopropylmalate).
N	O31046	DE	2-isopropylmalate synthase;
N	O31046	DE	Alpha-IPM synthase;
N	O31046	DE	Alpha-isopropylmalate synthase;
N	O31046	DR	2-isopropylmalate synthase activity
N	O31046	DR	leucine biosynthetic process
N	O31667	CC	Dephosphorylates 2-hydroxy-3-keto-5-methylthiopentenyl- 1-phosphate (HK-MTPenyl-1-P) yielding 1,2-dihydroxy-3-keto-5- methylthiopentene (DHK-MTPene)
N	O31667	DE	2-hydroxy-3-keto-5-methylthiopentenyl-1-phosphate phosphatase;
N	O31667	DE	HK-MTPenyl-1-P phosphatase;
N	O31667	DR	L-methionine salvage
N	O31667	DR	phosphatase activity
N	O31806	DE	Uncharacterized protein YndB;
N	O31806	DR	response to stress
N	O31889	DE	SPBc2 prophage-derived uncharacterized protein yorZ;
N	O31919	DE	SPBc2 prophage-derived uncharacterized protein yoqT;
N	O32178	CC	Involved in the degradation of long-chain fatty acids.
N	O32178	DE	Probable 3-hydroxyacyl-CoA dehydrogenase;
N	O32178	DR	3-hydroxyacyl-CoA dehydrogenase activity
N	O32178	DR	coenzyme binding
N	O32178	DR	fatty acid metabolic process
N	O32178	DR	lipid catabolic process
N	O32178	DR	oxidation-reduction process
N	O32271	CC	Catalyzes the conversion of UDP-glucose into UDP- glucuronate, one of the precursors of teichuronic acid.
N	O32271	DE	Teichuronic acid biosynthesis protein tuaD;
N	O32271	DE	UDP-Glc dehydrogenase;
N	O32271	DE	UDP-glucose 6-dehydrogenase tuaD;
N	O32271	DR	UDP-glucose 6-dehydrogenase activity
N	O32271	DR	cellular cell wall organization
N	O32271	DR	oxidation-reduction process
N	O32271	DR	response to stress
N	O32507	CC	Catalyzes the NADP(+) dependent oxidation of succinate semialdehyde to succinate (By similarity).
N	O32507	DE	Succinate-semialdehyde dehydrogenase [NADP+];
N	O32507	DR	oxidation-reduction process
N	O32507	DR	oxidoreductase activity
N	O32799	DE	Formate acetyltransferase;
N	O32799	DE	Pyruvate formate-lyase;
N	O32799	DR	formate C-acetyltransferase activity
N	O32799	DR	glucose metabolic process
N	O33104	CC	Allows the formation of correctly charged Asn-tRNA(Asn) or Gln-tRNA(Gln) through the transamidation of misacylated Asp- tRNA(Asn) or Glu-tRNA(Gln) in organisms which lack either or both of asparaginyl-tRNA or glutaminyl-tRNA synthetases
N	O33104	CC	The reaction takes place in the presence of glutamine and ATP through an activated phospho-Asp-tRNA(Asn) or phospho-Glu-tRNA(Gln) (By similarity).
N	O33104	DE	Glu-ADT subunit C;
N	O33104	DE	Glutamyl-tRNA(Gln) amidotransferase subunit C;
N	O33104	DR	ligase activity
N	O33104	DR	regulation of translational fidelity
N	O33778	DE	Phosphoribosyl-AMP cyclohydrolase;
N	O33778	DR	histidine biosynthetic process

N	O33778	DR	phosphoribosyl-AMP cyclohydrolase activity
N	O34101	DE	50S ribosomal protein L32;
N	O34101	DR	structural constituent of ribosome
N	O34163	CC	Carrier of the growing fatty acid chain in fatty acid biosynthesis (By similarity)
N	O34163	CC	Has hemolytic activity forming pores approximatively 1 nm in diameter into erythrocytes
N	O34163	CC	Is able to induce murine colonic lesions and to disrupt the integrity of epithelial cell monolayers.
N	O34163	DE	Acyl carrier protein;
N	O34163	DE	Beta-hemolysin;
N	O34163	DR	acyl carrier activity
N	O34163	DR	cofactor binding
N	O34163	DR	fatty acid biosynthetic process
N	O34163	DR	hemolysis in other organism
N	O34163	DR	phosphopantetheine binding
N	O34346	DE	D-mannonate hydrolase;
N	O34346	DE	Mannonate dehydratase;
N	O34346	DR	glucuronate catabolic process
N	O34346	DR	mannonate dehydratase activity
N	O34767	CC	Converts oxalate to formate and CO(2).
N	O34767	DE	Oxalate decarboxylase oxdD;
N	O34767	DR	metal ion binding
N	O34767	DR	nutrient reservoir activity
N	O34767	DR	oxalate decarboxylase activity
N	O34860	CC	One of 4 functionally non-identical rsbR paralogs, it functions in the environmental signaling branch of the general stress response.
N	O34860	DE	RsbT co-antagonist protein rsbRB;
N	O34860	DE	Stressosome protein rsbRB;
N	O34912	DE	Histidine biosynthesis bifunctional protein HisIE;
N	O34912	DE	Phosphoribosyl-AMP cyclohydrolase;
N	O34912	DE	Phosphoribosyl-ATP pyrophosphatase;
N	O34912	DR	histidine biosynthetic process
N	O34912	DR	phosphoribosyl-AMP cyclohydrolase activity
N	O34912	DR	phosphoribosyl-ATP diphosphatase activity
N	O34970	DE	Probable HTH-type transcriptional regulator yttP;
N	O34970	DE	Stress response protein yttP;
N	O34970	DR	regulation of transcription, DNA-dependent
N	O34970	DR	response to stress
N	O34970	DR	sequence-specific DNA binding transcription factor activity
N	O34970	DR	specific transcriptional repressor activity
N	O35017	DE	Uncharacterized zinc-type alcohol dehydrogenase-like protein YogA;
N	O35017	DR	oxidation-reduction process
N	O35017	DR	oxidoreductase activity
N	O35017	DR	zinc ion binding
N	O35095	CC	Probably involved in signal transduction, in the nervous system, via increasing cell surface localization of GRM5 and positively regulating its signaling
N	O35095	CC	Acts as a negative regulator of Ca(2+)- calmodulin-dependent protein kinase 2 (CaMK2) phosphorylation
N	O35095	CC	May also be involved in neurite outgrowth.
N	O35095	CC	May be involved in bone metabolism
N	O35095	CC	May play a role in modulating melanin-concentrating hormone-mediated functions via its interaction with MCHR1 that interferes with G protein-coupled signal transduction
N	O35095	CC	Required for the spatial learning process
N	O35095	DE	Neurite outgrowth-related protein from the rat brain;
N	O35095	DE	Neurochondrin;
N	O35095	DR	neuron projection development
N	O35095	DR	protein binding
N	O35095	DR	regulation of neuronal synaptic plasticity
N	O35394	CC	General Rab protein regulator required for vesicle formation from the Golgi complex
N	O35394	CC	In addition it inhibits the removal of Rab GTPases from the membrane by GDI1.
N	O35394	CC	May control vesicle docking and fusion by mediating the action of Rab GTPases to the SNARE complexes
N	O35394	DE	PRA1 family protein 1;
N	O35394	DE	Prenylated Rab acceptor protein 1;
N	O35394	DR	proline-rich region binding
N	O35394	DR	protein C-terminus binding

N	O35394	DR	protein binding, bridging
N	O35394	DR	vesicle-mediated transport
N	O35492	CC	Phosphorylates serine- and arginine-rich (SR) proteins of the spliceosomal [illegible]
N	O35492	CC	May be a constituent of a network of regulatory mechanisms that enable SR proteins to control RNA splicing
N	O35492	CC	Phosphorylates serines, threonines and tyrosines.
N	O35492	DE	CDC-like kinase 3;
N	O35492	DE	Dual specificity protein kinase CLK3;
N	O35492	DR	peptidyl-tyrosine phosphorylation
N	O35492	DR	protein autophosphorylation
N	O35492	DR	protein serine/threonine kinase activity
N	O35492	DR	protein tyrosine kinase activity
N	O35547	CC	Activation of long-chain fatty acids for both synthesis of cellular lipids, and degradation via beta-oxidation
N	O35547	CC	Preferentially uses arachidonate and eicosapentaenoate as substrates.
N	O35547	DE	Long-chain acyl-CoA synthetase 4;
N	O35547	DE	Long-chain-fatty-acid--CoA ligase 4;
N	O35547	DR	dendritic spine development
N	O35547	DR	fatty acid metabolic process
N	O35547	DR	fatty acid transport
N	O35547	DR	long-chain fatty acid-CoA ligase activity
N	O35547	DR	response to interleukin-15
N	O35547	DR	response to nutrient
N	O35547	DR	triglyceride metabolic process
N	O35569	CC	Direct ligand for ERBB3 and ERBB4 tyrosine kinase receptors
N	O35569	CC	Concomitantly recruits ERBB1 and ERBB2 coreceptors, resulting in ligand-stimulated tyrosine phosphorylation and activation of the ERBB receptors
N	O35569	CC	May also promote the heterodimerization with the EGF receptor.
N	O35569	DE	Neural- and thymus-derived activator for ERBB kinases;
N	O35569	DE	Neuregulin-2;
N	O35569	DE	Pro-neuregulin-2, membrane-bound isoform;
N	O35569	DR	ErbB-3 class receptor binding
N	O35569	DR	embryo development
N	O35569	DR	epidermal growth factor receptor activity
N	O35569	DR	epidermal growth factor receptor binding
N	O35569	DR	growth factor activity
N	O35936	DE	15-lipoxygenase 2;
N	O35936	DE	8S-lipoxygenase;
N	O35936	DE	Arachidonate 15-lipoxygenase B;
N	O35936	DE	Arachidonate 15-lipoxygenase type II;
N	O35936	DR	arachidonate 15-lipoxygenase activity
N	O35936	DR	iron ion binding
N	O35936	DR	leukotriene biosynthetic process
N	O35936	DR	lipoxygenase activity
N	O35936	DR	negative regulation of cell cycle
N	O35936	DR	negative regulation of cell proliferation
N	O35936	DR	negative regulation of growth
N	O35936	DR	oxidation-reduction process
N	O42188	CC	Lacks PA2 enzymatic activity (By similarity).
N	O42188	DE	Phospholipase A2 homolog;
N	O42188	DR	calcium ion binding
N	O42188	DR	lipid catabolic process
N	O42188	DR	phospholipase A2 activity
N	O42188	DR	phospholipid metabolic process
N	O42242	CC	Facilitates protein transport into the nucleus
N	O42242	CC	Acts at a relatively late stage of nuclear protein import, subsequent to the initial docking of nuclear import ligand at the nuclear envelope
N	O42242	CC	Could be part of a multicomponent system of cytosolic factors that assemble at the pore complex during nuclear import (By similarity).
N	O42242	CC	Interacts with the nucleoporin p62 and with Ran
N	O42242	DE	Nuclear transport factor 2;
N	O42242	DR	protein transport
N	O42626	CC	Controls entry of the cell into the asexual developmental program
N	O42626	CC	Required to repress entry into the conidiation program.
N	O42626	DE	Non-repressible conidiation protein 2;

N	O42626	DE	Serine/threonine-protein kinase nrc-2;
N	O42626	DR	conidium formation
N	O42626	DR	protein phosphorylation
N	O42626	DR	protein serine/threonine kinase activity
N	O42626	DR	sporulation resulting in formation of a cellular spore
N	O42844	CC	Has a role in the regulation of G2/M transition during mitosis.
N	O42844	DE	CAM kinase II;
N	O42844	DE	Calcium/calmodulin-dependent protein kinase type II;
N	O42844	DR	DNA replication checkpoint
N	O42844	DR	calcium ion binding
N	O42844	DR	calmodulin binding
N	O42844	DR	calmodulin-dependent protein kinase activity
N	O42844	DR	cellular response to oxidative stress
N	O42844	DR	negative regulation of G2/M transition of mitotic cell cycle
N	O42844	DR	positive regulation of establishment of bipolar cell polarity resulting in cell shape
N	O42844	DR	protein phosphorylation
N	O42844	DR	stress-activated protein kinase signaling cascade
N	O42943	DE	Uncharacterized ABC transporter ATP-binding protein C16H5.08c;
N	O42943	DR	ATPase activity
N	O42963	CC	Functions as a component of the nuclear pore complex (NPC)
N	O42963	CC	Active directional transport is assured by both, a Phe-Gly (FG) repeat affinity gradient for these transport factors across the NPC and a transport cofactor concentration gradient across the nuclear envelope.
N	O42963	CC	NPC components, collectively referred to as nucleoporins (NUPs), can play the role of both NPC structural components and of docking or interaction partners for transiently associated nuclear transport factors
N	O42963	DE	Nuclear pore protein nup44;
N	O42963	DE	Nucleoporin nup44;
N	O42963	DR	nucleocytoplasmic transport
N	O42963	DR	regulation of mitotic cell cycle
N	O43156	CC	Promotes assembly, stabilizes and maintains the activity of mTORC1 and mTORC2 complexes, which regulate cell growth and survival in response to nutrient and hormonal signals.
N	O43156	DE	Protein SMG10;
N	O43156	DE	TEL2-interacting protein 1 homolog;
N	O43765	CC	Co-chaperone that binds directly to HSC70 and HSP70 and regulates their ATPase activity.
N	O43765	DE	Small glutamine-rich tetratricopeptide repeat-containing protein alpha;
N	O43765	DE	Vpu-binding protein;
N	O43765	DR	interspecies interaction between organisms
N	O43765	DR	protein binding
N	O45687	DE	Alcohol dehydrogenase 2;
N	O45687	DE	Sorbitol dehydrogenase family protein 2;
N	O45687	DR	alcohol dehydrogenase (NAD) activity
N	O45687	DR	determination of adult lifespan
N	O45687	DR	oxidation-reduction process
N	O45687	DR	zinc ion binding
N	O46378	CC	Affects the rate of fibrils formation
N	O46378	CC	May have a primary role in collagen fibrillogenesis (By similarity).
N	O46378	DE	Collagen-binding 59 kDa protein;
N	O46378	DE	Fibromodulin;
N	O46378	DE	KSPG fibromodulin;
N	O46378	DE	Keratan sulfate proteoglycan fibromodulin;
N	O46567	CC	Receptor for glucocorticoids (GC)
N	O46567	CC	Affects inflammatory responses, cellular proliferation and differentiation in [illegible]
N	O46567	CC	Could act as a coactivator for STAT5-dependent transcription upon growth hormone (GH) stimulation and could reveal an essential role of hepatic GR in the control of body growth
N	O46567	CC	Has a dual mode of action: as a transcription factor that binds to glucocorticoid response elements (GRE) and as modulator of other transcription factors
N	O46567	CC	Involved in chromatin remodeling (By similarity)
N	O46567	CC	Involved in nuclear translocation.
N	O46567	CC	Plays a significant role in transactivation
N	O46567	DE	Glucocorticoid receptor;
N	O46567	DE	Nuclear receptor subfamily 3 group C member 1;

N	O46567	DR	chromatin modification
N	O46567	DR	glucocorticoid receptor activity
N	O46567	DR	regulation of transcription, DNA-dependent
N	O46567	DR	sequence-specific DNA binding
N	O46567	DR	sequence-specific DNA binding transcription factor activity
N	O46567	DR	steroid binding
N	O46567	DR	zinc ion binding
N	O46639	CC	Receptor for thyrotropin-releasing hormone
N	O46639	CC	This receptor is mediated by G proteins which activate a phosphatidylinositol-calcium second messenger system.
N	O46639	DE	Thyroliberin receptor;
N	O46639	DE	Thyrotropin-releasing hormone receptor;
N	O46639	DR	thyrotropin-releasing hormone receptor activity
N	O48549	CC	May play an important role in controlling cell growth and proliferation through the selective translation of particular classes of mRNA (By similarity).
N	O48549	DE	40S ribosomal protein S6-1;
N	O48549	DR	structural constituent of ribosome
N	O50422	CC	May directly or indirectly regulate the accessibility of the key branch point intermediate, monoacyl phosphatidylinositol tetramannoside (AcPIM4), to the elongating alpha-1,6 mannosyltransferases which could regulate the lipoarabinomannans (LAMs) biosynthesis (By similarity).
N	O50422	DE	Probable monoacyl phosphatidylinositol tetramannoside-binding protein LpqW;
N	O50422	DR	glycolipid biosynthetic process
N	O50422	DR	phospholipid biosynthetic process
N	O50422	DR	transporter activity
N	O51082	CC	Excises uracil residues from the DNA which can arise as a result of misincorporation of dUMP residues by DNA polymerase or due to deamination of cytosine (By similarity).
N	O51082	DE	Uracil-DNA glycosylase;
N	O51082	DR	base-excision repair
N	O51082	DR	uracil DNA N-glycosylase activity
N	O51314	CC	This protein is one of the early assembly proteins of the 50S ribosomal subunit, although it is not seen to bind rRNA by itself
N	O51314	CC	It is important during the early stages of 50S assembly (By similarity).
N	O51314	DE	50S ribosomal protein L13;
N	O51314	DR	structural constituent of ribosome
N	O51440	CC	One of the primary rRNA binding proteins, it binds specifically to the 5'-end of 16S ribosomal RNA (By similarity).
N	O51440	DE	30S ribosomal protein S17;
N	O51440	DR	structural constituent of ribosome
N	O51875	CC	F(1)F(0) ATP synthase produces ATP from ADP in the presence of a proton or sodium gradient
N	O51875	CC	During catalysis, ATP synthesis in the catalytic domain of F(1) is coupled via a rotary mechanism of the central stalk subunits to proton translocation (By similarity).
N	O51875	CC	F-type ATPases consist of two structural domains, F(1) containing the extramembraneous catalytic core and F(0) containing the membrane proton channel, linked together by a central stalk and a peripheral stalk
N	O51875	DE	ATP synthase F(1) sector subunit delta;
N	O51875	DE	ATP synthase subunit delta;
N	O51875	DE	F-ATPase subunit delta;
N	O51875	DE	F-type ATPase subunit delta;
N	O51875	DR	ATP synthesis coupled proton transport
N	O51875	DR	hydrogen ion transporting ATP synthase activity, rotational mechanism
N	O52348	CC	This is one of the proteins that binds and probably mediates the attachment of the 5S RNA into the large ribosomal subunit, where it forms part of the central protuberance (By similarity).
N	O52348	DE	50S ribosomal protein L18;
N	O52348	DR	structural constituent of ribosome
N	O54715	CC	Vacuolar ATPase is responsible for acidifying a variety of intracellular compartments in eukaryotic cells (By similarity).
N	O54715	DE	C7-1 protein;
N	O54715	DE	V-ATPase Ac45 subunit;
N	O54715	DE	V-ATPase S1 accessory protein;
N	O54715	DE	V-ATPase subunit S1;
N	O54715	DE	V-type proton ATPase subunit S1;
N	O54715	DE	Vacuolar proton pump subunit S1;

N	O54715	DR	ATP hydrolysis coupled proton transport
N	O54715	DR	hydrogen ion transporting ATP synthase activity, rotational mechanism
N	O54715	DR	proton-transporting ATPase activity, rotational mechanism
N	O55101	DE	Synaptogyrin-2;
N	O55709	DE	Putative Bro-N domain-containing protein 069L;
N	O58358	CC	Reversibly transfers an adenylyl group from ATP to 4'- phosphopantetheine, yielding dephospho-CoA (dPCoA) and pyrophosphate (By similarity).
N	O58358	DE	Dephospho-CoA pyrophosphorylase;
N	O58358	DE	Pantetheine-phosphate adenylyltransferase;
N	O58358	DE	Phosphopantetheine adenylyltransferase;
N	O58358	DR	coenzyme A biosynthetic process
N	O58358	DR	pantetheine-phosphate adenylyltransferase activity
N	O59182	DE	UPF0189 protein PH1513;
N	O59921	DE	Alpha,alpha-trehalose-phosphate synthase [UDP-forming];
N	O59921	DE	Trehalose-6-phosphate synthase;
N	O59921	DE	UDP-glucose-glucosephosphate glucosyltransferase;
N	O59921	DR	alpha,alpha-trehalose-phosphate synthase (UDP-forming) activity
N	O59921	DR	trehalose biosynthetic process
N	O59922	DE	PKA regulatory subunit;
N	O59922	DE	cAMP-dependent protein kinase regulatory subunit;
N	O59922	DR	cAMP-dependent protein kinase regulator activity
N	O59922	DR	regulation of protein phosphorylation
N	O60128	DE	40S ribosomal protein S3;
N	O60128	DR	structural constituent of ribosome
N	O60921	CC	Component of the 9-1-1 cell-cycle checkpoint response complex that plays a major role in DNA repair
N	O60921	CC	Acts then as a sliding clamp platform on DNA for several proteins involved in long-patch base excision repair (LP-BER)
N	O60921	CC	The 9-1-1 complex is recruited to DNA lesion upon damage by the RAD17-replication factor C (RFC) clamp loader complex
N	O60921	CC	The 9-1-1 complex stimulates DNA polymerase beta (POLB) activity by increasing its affinity for the 3'-OH end of the primer-template and stabilizes POLB to those sites where LP-BER proceeds; endonuclease FEN1 cleavage activity on substrates with double, nick, or gap flaps of distinct sequences and lengths; and DNA ligase I (LIG1) on long-patch base excision repair substrates.
N	O60921	DE	Checkpoint protein HUS1;
N	O60921	DR	DNA damage checkpoint
N	O60921	DR	DNA replication
N	O60921	DR	protein binding
N	O62643	CC	May play a role in cell-cell or cell-ligand interactions during synaptogenesis and other events in the brain.
N	O62643	DE	Thy-1 antigen;
N	O62643	DE	Thy-1 membrane glycoprotein;
N	O62643	DR	GPI anchor binding
N	O62643	DR	Rho GTPase activator activity
N	O62643	DR	T cell receptor signaling pathway
N	O62643	DR	cell-cell adhesion
N	O62643	DR	cytoskeleton organization
N	O62643	DR	focal adhesion assembly
N	O62643	DR	negative regulation of T cell receptor signaling pathway
N	O62643	DR	negative regulation of axonogenesis
N	O62643	DR	negative regulation of cell migration
N	O62643	DR	negative regulation of protein kinase activity
N	O62643	DR	positive regulation of GTPase activity
N	O62643	DR	positive regulation of T cell activation
N	O62643	DR	positive regulation of release of sequestered calcium ion into cytosol
N	O62643	DR	retinal cone cell development
N	O65438	DE	Cytochrome P450 71A27;
N	O65438	DR	electron carrier activity
N	O65438	DR	monooxygenase activity
N	O65438	DR	oxidation-reduction process
N	O65665	CC	Probably acts as a transcriptional activator
N	O65665	CC	Binds to the GCC-box pathogenesis-related promoter element
N	O65665	CC	May be involved in the regulation of gene expression by stress factors and by components of stress signal transduction pathways (By similarity).

N	O65665	DE	Ethylene-responsive transcription factor ERF060;
N	O65665	DR	ethylene mediated signaling pathway
N	O65665	DR	regulation of transcription, DNA-dependent
N	O65665	DR	sequence-specific DNA binding transcription factor activity
N	O65806	DE	Protein mago nashi homolog;
N	O66403	CC	May function as an integrase.
N	O66403	DE	Probable integrase/recombinase aq_aa09;
N	O66403	DR	DNA integration
N	O66403	DR	DNA recombination
N	O66984	DE	Uncharacterized protein aq_796;
N	O67064	DE	Uncharacterized protein aq_919;
N	O67133	CC	Probably plays a role in an hydrogenase nickel cofactor insertion step (By similarity).
N	O67133	DE	Probable hydrogenase nickel incorporation protein hypA;
N	O67133	DR	electron carrier activity
N	O67133	DR	nickel ion binding
N	O67133	DR	protein modification process
N	O67438	DE	Uncharacterized protein aq_1453;
N	O67631	CC	Catalyzes the addition of meso-diaminopimelic acid to the nucleotide precursor UDP-N-acetylmuramoyl-L-alanyl-D-glutamate (UMAG) in the biosynthesis of bacterial cell-wall peptidoglycan (By similarity).
N	O67631	DE	Meso-A2pm-adding enzyme;
N	O67631	DE	Meso-diaminopimelate-adding enzyme;
N	O67631	DE	UDP-MurNAc-L-Ala-D-Glu:meso-diaminopimelate ligase;
N	O67631	DE	UDP-MurNAc-tripeptide synthetase;
N	O67631	DE	UDP-N-acetylmuramoyl-L-alanyl-D-glutamate--2,6-diaminopimelate ligase;
N	O67631	DE	UDP-N-acetylmuramyl-tripeptide synthetase;
N	O67631	DR	UDP-N-acetylmuramoylalanyl-D-glutamate-2,6-diaminopimelate ligase activity
N	O67631	DR	cell division
N	O67631	DR	cellular cell wall organization
N	O67631	DR	folic acid-containing compound biosynthetic process
N	O67631	DR	peptidoglycan biosynthetic process
N	O67631	DR	regulation of cell shape
N	O67631	DR	tetrahydrofolylpolyglutamate synthase activity
N	O67691	DE	FGAM synthase II;
N	O67691	DE	Phosphoribosylformylglycinamidine synthase 2;
N	O67691	DE	Phosphoribosylformylglycinamidine synthase II;
N	O67691	DR	'de novo' IMP biosynthetic process
N	O67691	DR	phosphoribosylformylglycinamidine synthase activity
N	O68562	CC	May have a structural role in maintaining the cell envelope integrity.
N	O68562	DE	Outer membrane lipoprotein omlA;
N	O69302	CC	This protein is essential for replication of the chromosome
N	O69302	CC	It is also involved in DNA recombination and repair (By similarity).
N	O69302	DE	Helix-destabilizing protein;
N	O69302	DE	Single-stranded DNA-binding protein;
N	O69302	DR	DNA replication
N	O69302	DR	single-stranded DNA binding
N	O70445	CC	Probable E3 ubiquitin-protein ligase
N	O70445	CC	Acts by mediating ubiquitin E3 ligase activity that is required for its tumor suppressor function
N	O70445	CC	Also forms a heterodimer with CSTF1/CSTF-50 to modulate mRNA processing and RNAP II stability by inhibiting pre-mRNA 3' cleavage (By similarity).
N	O70445	CC	Plays a central role in the control of the cell cycle in response to DNA damage
N	O70445	CC	The BRCA1-BARD1 heterodimer specifically mediates the formation of 'Lys-6'-linked polyubiquitin chains and coordinates a diverse range of cellular pathways such as DNA damage repair, ubiquitination and transcriptional regulation to maintain genomic stability
N	O70445	DE	BRCA1-associated RING domain protein 1;
N	O70445	DR	negative regulation of apoptosis
N	O70445	DR	negative regulation of protein export from nucleus
N	O70445	DR	positive regulation of apoptosis
N	O70445	DR	protein K6-linked ubiquitination
N	O70445	DR	protein heterodimerization activity
N	O70445	DR	protein homodimerization activity
N	O70445	DR	regulation of phosphorylation
N	O70445	DR	zinc ion binding

N	O70596	CC	Inward rectifier potassium channels are characterized by a greater tendency to allow potassium to flow into the cell rather than out of it
N	O70596	CC	KCNJ14 gives rise to low-conductance channels with a low affinity to the channel blockers Barium and Cesium.
N	O70596	CC	The inward rectification is mainly due to the blockage of outward current by internal magnesium
N	O70596	CC	Their voltage dependence is regulated by the concentration of extracellular potassium; as external potassium is raised, the voltage range of the channel opening shifts to more positive voltages
N	O70596	DE	ATP-sensitive inward rectifier potassium channel 14;
N	O70596	DE	Inward rectifier K(+) channel Kir2.4;
N	O70596	DE	Potassium channel, inwardly rectifying subfamily J member 14;
N	O70596	DR	inward rectifier potassium channel activity
N	O70900	CC	Escorts unspliced or incompletely spliced viral pre- mRNAs (late transcripts) out of the nucleus of infected cells
N	O70900	CC	Beside KPNB1, also seems to interact with TNPO1/Transportin-1, RANBP5/IPO5 and IPO7/RANBP7 for nuclear import
N	O70900	CC	Conversion from Ran-GTP to Ran-GDP mediates dissociation of the Rev/RRE/XPO1/RAN complex, so that Rev can return to the nucleus for a subsequent round of export
N	O70900	CC	In the nucleus, the conversion from Ran-GDP to Ran-GTP dissociates Rev from KPNB1 and allows Rev's binding to the RRE in viral pre-mRNAs
N	O70900	CC	KPNB1 binds to the GDP bound form of RAN (Ran-GDP) and targets Rev to the nucleus
N	O70900	CC	Rev can then form a complex with XPO1/CRM1 and Ran-GTP, leading to nuclear export of the complex
N	O70900	CC	Rev itself is translated from a fully spliced mRNA that readily exits the nucleus
N	O70900	CC	Rev multimerization on the RRE via cooperative assembly exposes its nuclear export signal (NES) to the surface
N	O70900	CC	Rev's nuclear localization signal (NLS) binds directly to KPNB1/Importin beta-1 without previous binding to KPNA1/Importin alpha-1
N	O70900	CC	The nucleoporin-like HRB/RIP is an essential cofactor that probably indirectly interacts with Rev to release HIV RNAs from the perinuclear region to the cytoplasm (By similarity).
N	O70900	CC	These pre-mRNAs carry a recognition sequence called Rev responsive element (RRE) located in the env gene, that is not present in fully spliced viral mRNAs (early transcripts)
N	O70900	CC	This function is essential since most viral proteins are translated from unspliced or partially spliced pre-mRNAs which cannot exit the nucleus by the pathway used by fully processed cellular mRNAs
N	O70900	DE	Anti-repression transactivator;
N	O70900	DE	Protein Rev;
N	O70900	DE	Regulator of expression of viral proteins;
N	O70900	DR	interspecies interaction between organisms
N	O70900	DR	mRNA transport
N	O70900	DR	regulation of transcription, DNA-dependent
N	O70900	DR	sequence-specific DNA binding transcription factor activity
N	O74524	CC	Converts proline to delta-1-pyrroline-5-carboxylate (By similarity).
N	O74524	DE	Probable proline dehydrogenase, mitochondrial;
N	O74524	DE	Probable proline oxidase;
N	O74524	DR	glutamate biosynthetic process
N	O74524	DR	oxidation-reduction process
N	O74524	DR	proline catabolic process
N	O74524	DR	proline dehydrogenase activity
N	O74957	CC	Required for G1 arrest and mating in response to nitrogen starvation
N	O74957	CC	A member of the RNA-induced transcriptional silencing (RITS) complex which is involved in the biosynthesis of dsRNA from primer siRNAs provided by the RNA-directed RNA polymerase (RDRC) complex
N	O74957	CC	A member of the argonaute siRNA chaperone (ARC) complex which is required for histone H3K9 methylation, heterochromatin assembly and siRNA generation
N	O74957	CC	Ago1 regulation of cytokinesis and cell cycle checkpoints occurs downstream of dcr1
N	O74957	CC	Has a role in the RNA interference (RNAi) pathway which is important for heterochromatin formation, accurate chromosome segregation, centromere cohesion and telomere function during mitosis and meiosis

N	O74957	CC	Has ribonuclease H-like cleavage (slicing) activity towards target messages complementary to siRNA and can direct site-specific cleavage of RNA substrates via siRNA
N	O74957	CC	Promotes histone H3K9 methylation necessary for centromere function
N	O74957	CC	Required for recruitment of swi6 and cohesin to an ectopic dg repeat
N	O74957	CC	Required for silencing at the centromeres and for initiation of transcriptionally silent heterochromatin at the mating type locus
N	O74957	CC	Required, indirectly, for regulated hyperphosphorylation of cdc2
N	O74957	CC	Slicing activity is required for both post-transcriptional and transcriptional gene silencing as well as for histone H3 'Lys-10' methylation spreading, conversion of double-stranded siRNA to single-stranded siRNA and siRNA-dependent association of ago1 with chromatin
N	O74957	CC	The ARC complex contains mostly double-stranded siRNA.
N	O74957	DE	Cell cycle control protein ago1;
N	O74957	DE	Eukaryotic translation initiation factor 2C 2-like protein ago1;
N	O74957	DE	PAZ Piwi domain protein ago1;
N	O74957	DE	Protein argonaute;
N	O74957	DE	Protein slicer;
N	O74957	DE	RNA interference pathway protein ago1;
N	O74957	DR	chromatin silencing at centromere
N	O74957	DR	chromatin silencing by small RNA
N	O74957	DR	conversion of ds siRNA to ss siRNA involved in RNA interference
N	O74957	DR	mitotic sister chromatid segregation
N	O74957	DR	negative regulation of gene-specific transcription from RNA polymerase II promoter
N	O74957	DR	nucleic acid binding
N	O74957	DR	protein binding
N	O74957	DR	regulation of chromatin silencing at centromere
N	O74957	DR	regulation of histone H3-K9 methylation
N	O74957	DR	regulation of nitrogen utilization
N	O75348	CC	Catalytic subunit of the peripheral V1 complex of vacuolar ATPase (V-ATPase)
N	O75348	CC	V-ATPase is responsible for acidifying a variety of intracellular compartments in eukaryotic cells.
N	O75348	DE	V-ATPase 13 kDa subunit 1;
N	O75348	DE	V-ATPase subunit G 1;
N	O75348	DE	V-type proton ATPase subunit G 1;
N	O75348	DE	Vacuolar proton pump subunit G 1;
N	O75348	DE	Vacuolar proton pump subunit M16;
N	O75348	DR	ATPase binding
N	O75348	DR	cellular iron ion homeostasis
N	O75348	DR	insulin receptor signaling pathway
N	O75348	DR	transferrin transport
N	O75414	CC	Major role in the synthesis of nucleoside triphosphates other than ATP
N	O75414	CC	Inhibitor of p53-induced apoptosis.
N	O75414	CC	The ATP gamma phosphate is transferred to the NDP beta phosphate via a ping-pong mechanism, using a phosphorylated active-site intermediate
N	O75414	DE	IPIA-alpha;
N	O75414	DE	Inhibitor of p53-induced apoptosis-alpha;
N	O75414	DE	NDP kinase 6;
N	O75414	DE	Nucleoside diphosphate kinase 6;
N	O75414	DR	CTP biosynthetic process
N	O75414	DR	GTP biosynthetic process
N	O75414	DR	UTP biosynthetic process
N	O75414	DR	anti-apoptosis
N	O75414	DR	metal ion binding
N	O75414	DR	negative regulation of cell growth
N	O75414	DR	negative regulation of mitosis
N	O75414	DR	nucleoside diphosphate kinase activity
N	O77462	CC	Conjugation of reduced glutathione to a wide number of exogenous and endogenous hydrophobic electrophiles (By similarity).
N	O77462	DE	AgGst1-alpha;
N	O77462	DE	GST class-theta;
N	O77462	DE	Glutathione S-transferase 1, isoform A;
N	O77462	DR	glutathione metabolic process
N	O77462	DR	glutathione transferase activity

N	O78761	CC	Component of the ubiquinol-cytochrome c reductase complex (complex III or cytochrome b-c1 complex), which is a respiratory chain that generates an electrochemical potential coupled to ATP synthesis (By similarity).
N	O78761	DE	Complex III subunit 3;
N	O78761	DE	Complex III subunit III;
N	O78761	DE	Cytochrome b-c1 complex subunit 3;
N	O78761	DE	Cytochrome b;
N	O78761	DE	Ubiquinol-cytochrome-c reductase complex cytochrome b subunit;
N	O78761	DR	electron carrier activity
N	O78761	DR	metal ion binding
N	O78761	DR	oxidoreductase activity
N	O78761	DR	respiratory electron transport chain
N	O78788	CC	Component of the ubiquinol-cytochrome c reductase complex (complex III or cytochrome b-c1 complex), which is a respiratory chain that generates an electrochemical potential coupled to ATP synthesis (By similarity).
N	O78788	DE	Complex III subunit 3;
N	O78788	DE	Complex III subunit III;
N	O78788	DE	Cytochrome b-c1 complex subunit 3;
N	O78788	DE	Cytochrome b;
N	O78788	DE	Ubiquinol-cytochrome-c reductase complex cytochrome b subunit;
N	O78788	DR	electron carrier activity
N	O78788	DR	metal ion binding
N	O78788	DR	oxidoreductase activity
N	O78788	DR	respiratory electron transport chain
N	O80323	CC	Self-incompatibility (SI) is the inherited ability of a flowering plant to prevent self-fertilization by discriminating between self and non-self pollen during pollination
N	O80323	CC	In many species, self-incompatibility is controlled by the single, multiallelic locus S
N	O80323	DE	Ribonuclease S-3;
N	O80323	DR	ribonuclease T2 activity
N	O80568	CC	Kinase that can phosphorylate various inositol polyphosphate such as Ins(3,4,5,6)P4 or Ins(1,3,4)P3
N	O80568	CC	Also phosphorylates Ins(1,3,4)P3 on O-5 and O-6 to form Ins(1,3,4,6)P4, an essential molecule in the hexakisphosphate (InsP6) pathway (By similarity).
N	O80568	CC	Phosphorylates Ins(3,4,5,6)P4 at position 1 to form Ins(1,3,4,5,6)P5
N	O80568	CC	This reaction is thought to have regulatory importance, since Ins(3,4,5,6)P4 is an inhibitor of plasma membrane Ca(2+)-activated Cl(-) channels, while Ins(1,3,4,5,6)P5 is not
N	O80568	DE	Inositol 1,3,4-trisphosphate 5/6-kinase 4;
N	O80568	DE	Inositol-tetrakisphosphate 1-kinase 4;
N	O80568	DE	Inositol-triphosphate 5/6-kinase 4;
N	O80568	DE	Ins(1,3,4)P(3) 5/6-kinase 4;
N	O80568	DR	inositol tetrakisphosphate 1-kinase activity
N	O80568	DR	inositol trisphosphate metabolic process
N	O80568	DR	inositol-1,3,4-trisphosphate 5/6-kinase activity
N	O80568	DR	magnesium ion binding
N	O81191	CC	Catalyzes the formation of 1,8-cineole from geranyl diphosphate
N	O81191	CC	The enzyme also produces significant amounts of (+)- and (-)-alpha-pinene, (+)- and (-)-beta-pinene, myrcene and (+)- sabinene.
N	O81191	DE	1,8-cineole synthase, chloroplastic;
N	O81191	DR	lyase activity
N	O81191	DR	magnesium ion binding
N	O81782	DE	B3 domain-containing protein REM6;
N	O81782	DE	Protein REPRODUCTIVE MERISTEM 6;
N	O81782	DR	regulation of transcription, DNA-dependent
N	O83005	CC	The reaction center is a membrane-bound complex that mediates the initial photochemical event in the electron transfer process of photosynthesis.
N	O83005	DE	Photosynthetic reaction center L subunit;
N	O83005	DE	Reaction center protein L chain;
N	O83005	DR	bacteriochlorophyll binding
N	O83005	DR	electron transporter, transferring electrons within the cyclic electron transport pathway of photosynthesis activity
N	O83005	DR	metal ion binding
N	O83005	DR	photosynthetic electron transport in photosystem II
N	O83005	DR	protein-chromophore linkage
N	O83049	CC	The recF protein is involved in DNA metabolism; it is required for DNA replication and normal SOS inducibility

N	O83049	CC	It also seems to bind ATP (By similarity).
N	O83049	CC	RecF binds preferentially to single-stranded, linear DNA
N	O83049	DE	DNA replication and repair protein recF;
N	O83049	DR	DNA replication
N	O83049	DR	single-stranded DNA binding
N	O83273	DE	Uncharacterized protein TP_0245;
N	O83500	DE	Uncharacterized protein TP_0487;
N	O83682	DE	Uncharacterized protein TP_0676;
N	O83767	DE	Uncharacterized protein TP_0788;
N	O84294	CC	This enzyme is involved in nucleotide metabolism: it produces dUMP, the immediate precursor of thymidine nucleotides and it decreases the intracellular concentration of dUTP so that uracil cannot be incorporated into DNA (By similarity)
N	O84294	DE	Deoxyuridine 5'-triphosphate nucleotidohydrolase;
N	O84294	DE	dUTP pyrophosphatase;
N	O84294	DR	dUTP diphosphatase activity
N	O84294	DR	dUTP metabolic process
N	O84294	DR	metal ion binding
N	O86775	DE	50S ribosomal protein L17;
N	O86775	DR	structural constituent of ribosome
N	O86793	CC	Required for the formation of a threonylcarbamoyl group on adenosine at position 37 (t(6)A37) in tRNAs that read codons beginning with adenine (By similarity)
N	O86793	DE	Probable tRNA threonylcarbamoyladenosine biosynthesis protein Gcp;
N	O86793	DE	t(6)A37 threonylcarbamoyladenosine biosynthesis protein;
N	O86793	DR	metal ion binding
N	O86793	DR	metalloendopeptidase activity
N	O87545	CC	DNA gyrase negatively supercoils closed circular double- stranded DNA in an ATP-dependent manner and also catalyzes the interconversion of other topological isomers of double-stranded DNA rings, including catenanes and knotted rings
N	O87545	DE	DNA gyrase subunit B;
N	O87545	DR	DNA topoisomerase (ATP-hydrolyzing) activity
N	O87545	DR	DNA topological change
N	O87786	CC	May help in the organization of the psaL subunit.
N	O87786	DE	Photosystem I reaction center subunit VIII;
N	O87786	DR	photosynthesis
N	O88428	CC	Bifunctional enzyme with both ATP sulfurylase and APS kinase activity, which mediates two steps in the sulfate activation pathway
N	O88428	CC	In mammals, PAPS is the sole source of sulfate; APS appears to be only an intermediate in the sulfate- activation pathway
N	O88428	CC	May have a important role in skeletogenesis during postnatal growth.
N	O88428	CC	The first step is the transfer of a sulfate group to ATP to yield adenosine 5'-phosphosulfate (APS), and the second step is the transfer of a phosphate group from ATP to APS yielding 3'-phosphoadenylylsulfate (PAPS: activated sulfate donor used by sulfotransferase)
N	O88428	DE	3'-phosphoadenosine-5'-phosphosulfate synthase;
N	O88428	DE	APS kinase;
N	O88428	DE	ATP-sulfurylase;
N	O88428	DE	Adenosine-5'-phosphosulfate 3'-phosphotransferase;
N	O88428	DE	Adenylyl-sulfate kinase;
N	O88428	DE	Adenylylsulfate 3'-phosphotransferase;
N	O88428	DE	Bifunctional 3'-phosphoadenosine 5'-phosphosulfate synthase 2;
N	O88428	DE	PAPS synthase 2;
N	O88428	DE	Sulfate adenylate transferase;
N	O88428	DE	Sulfate adenylyltransferase;
N	O88428	DE	Sulfurylase kinase 2;
N	O88428	DR	adenylylsulfate kinase activity
N	O88428	DR	blood coagulation
N	O88428	DR	bone development
N	O88428	DR	sulfate adenylyltransferase (ATP) activity
N	O88428	DR	sulfate assimilation
N	O88487	CC	Acts as one of several non-catalytic accessory components of the cytoplasmic dynein 1 complex that are thought to be involved in linking dynein to cargos and to adapter proteins that regulate dynein function
N	O88487	CC	Cytoplasmic dynein 1 acts as a motor for the intracellular retrograde motility of vesicles and organelles along microtubules

N	O88487	CC	Involved in membrane-transport, such as Golgi apparatus, late endosomes and lysosomes (By similarity).
N	O88487	CC	The intermediate chains mediate the binding of dynein to dynactin via its 150 kDa component (p150- glued) DCNT1
N	O88487	DE	Cytoplasmic dynein 1 intermediate chain 2;
N	O88487	DE	Cytoplasmic dynein intermediate chain 2;
N	O88487	DE	Dynein intermediate chain 2, cytosolic;
N	O88487	DR	motor activity
N	O88545	CC	Component of the COP9 signalosome complex (CSN), a complex involved in various cellular and developmental processes
N	O88545	CC	CSN-dependent phosphorylation of TP53 and JUN promotes and protects degradation by the Ubl system, respectively
N	O88545	CC	Has some glucocorticoid receptor- responsive activity (By similarity).
N	O88545	CC	The CSN complex is an essential regulator of the ubiquitin (Ubl) conjugation pathway by mediating the deneddylation of the cullin subunits of SCF-type E3 ligase complexes, leading to decrease the Ubl ligase activity of SCF-type complexes such as SCF, CSA or DDB2
N	O88545	CC	The complex is also involved in phosphorylation of p53/TP53, c-jun/JUN, IkappaBalpha/NFKBIA, ITPK1 and IRF8, possibly via its association with CK2 and PKD kinases
N	O88545	DE	COP9 signalosome complex subunit 6;
N	O88545	DE	JAB1-containing signalosome subunit 6;
N	O88545	DE	Signalosome subunit 6;
N	O88545	DR	protein binding
N	O88741	CC	May function in a signal transduction pathway responsible for ganglioside-induced neurite differentiation
N	O88741	CC	May also have a role in protecting myelin membranes against free radical-mediated damage.
N	O88741	DE	Ganglioside-induced differentiation-associated protein 1;
N	O88741	DR	response to retinoic acid
N	O89039	CC	Receptor for CXCL12/SDF1 (By similarity).
N	O89039	DE	C-X-C chemokine receptor type 7;
N	O89039	DE	Chemokine orphan receptor 1;
N	O89039	DE	G-protein coupled receptor RDC1 homolog;
N	O89039	DR	G-protein coupled receptor activity
N	O89042	CC	Plays an essential role in the initiation of DNA replication
N	O89042	CC	During the S phase of the cell cycle, the DNA polymerase alpha complex (composed of a catalytic subunit POLA1/p180, a regulatory subunit POLA2/p70 and two primase subunits PRIM1/p49 and PRIM2/p58) is recruited to DNA at the replicative forks via direct interactions with MCM10 and WDHD1
N	O89042	CC	The primase subunit of the polymerase alpha complex initiates DNA synthesis by oligomerising short RNA primers on both leading and lagging strands
N	O89042	CC	The reason this transfer occurs is because the polymerase alpha has limited processivity and lacks intrinsic 3' exonuclease activity for proofreading error, and therefore is not well suited for replicating long complexes (By similarity).
N	O89042	CC	These primers are initially extended by the polymerase alpha catalytic subunit and subsequently transferred to polymerase delta and polymerase epsilon for processive synthesis on the lagging and leading strand, respectively
N	O89042	DE	DNA polymerase alpha catalytic subunit p180;
N	O89042	DE	DNA polymerase alpha catalytic subunit;
N	O89042	DR	DNA-dependent DNA replication initiation
N	O89042	DR	DNA-directed DNA polymerase activity
N	O89042	DR	S phase of mitotic cell cycle
N	O89042	DR	cell proliferation
N	O89042	DR	chromatin binding
N	O89042	DR	double-strand break repair via nonhomologous end joining
N	O89042	DR	double-stranded DNA binding
N	O89042	DR	lagging strand elongation
N	O89042	DR	leading strand elongation
N	O89042	DR	metal ion binding
N	O89042	DR	nucleoside binding
N	O89042	DR	purine nucleotide binding
N	O89042	DR	pyrimidine nucleotide binding
N	O93512	CC	Receptor for persephin

N	O93512	CC	Mediates the GDNF-induced autophosphorylation and activation of the RET receptor (By similarity).
N	O93512	DE	GDNF family receptor alpha-4;
N	O93512	DE	GDNF receptor alpha-4;
N	O93512	DE	GDNFR-alpha-4;
N	O93512	DE	GFR-alpha-4;
N	O93512	DR	receptor activity
N	O93533	CC	One gap junction consists of a cluster of closely packed pairs of transmembrane channels, the connexons, through which materials of low MW diffuse from one cell to a neighboring cell (By similarity).
N	O93533	DE	Connexin-31;
N	O93533	DE	Gap junction beta-6 protein;
N	O93533	DR	cell communication
N	O93533	DR	sensory perception of sound
N	O93655	CC	Proteases F1, F2 and F3 degrade oligopeptides produced by Tricorn (themselves probably produced by the proteasome), yielding free amino acids.
N	O93655	DE	Tricorn protease-interacting factor F3;
N	O93655	DR	aminopeptidase activity
N	O93655	DR	metallopeptidase activity
N	O93655	DR	zinc ion binding
N	O94221	CC	Hydrolyzes a variety of simple alpha-D-galactoside as well as more complex molecules such as oligosaccharides and polysaccharides (By similarity).
N	O94221	DE	Melibiase B;
N	O94221	DE	Probable alpha-galactosidase B;
N	O94221	DR	alpha-galactosidase activity
N	O94221	DR	carbohydrate metabolic process
N	O94221	DR	cation binding
N	O94242	DE	Aspartate--tRNA ligase;
N	O94242	DE	Aspartyl-tRNA synthetase, mitochondrial;
N	O94242	DR	aspartate-tRNA ligase activity
N	O94242	DR	aspartyl-tRNA aminoacylation
N	O94242	DR	mitochondrial translation
N	O94488	CC	Required for cytoplasmic astral microtubule growth during mitosis
N	O94488	CC	Involved in localizing components of the gamma- tubulin complex to the non-spindle pole body (non-SPB) microtubule organizing centers (MTOCs)
N	O94488	CC	Required for new microtubule nucleation from non-SPB sites during interphase.
N	O94488	DE	Microtubule organizer protein 1;
N	O94488	DE	Morphology defective protein 20;
N	O94488	DR	calmodulin binding
N	O94488	DR	establishment of nucleus localization
N	O94488	DR	gamma-tubulin binding
N	O94488	DR	gamma-tubulin complex localization
N	O94488	DR	interphase microtubule nucleation by interphase microtubule organizing center
N	O94488	DR	spindle astral microtubule organization
N	O94504	CC	Able to function as a protein disulfide reductase
N	O94504	CC	Involved in the response to oxidative stress.
N	O94504	DE	Thioredoxin-2, mitochondrial;
N	O94504	DR	arginine biosynthetic process via ornithine
N	O94504	DR	cell redox homeostasis
N	O94504	DR	cellular response to reactive oxygen species
N	O94504	DR	electron carrier activity
N	O94504	DR	electron transport chain
N	O94504	DR	glycerol ether metabolic process
N	O94504	DR	protein binding
N	O94504	DR	protein disulfide oxidoreductase activity
N	O94597	DE	Putative uncharacterized membrane protein C622.07;
N	O94609	CC	Activates ubiquitin by first adenylating its C-terminal glycine residue with ATP, and thereafter linking this residue to the side chain of a cysteine residue in E1, yielding an ubiquitin- E1 thioester and free AMP.
N	O94609	DE	Poly(A)+ RNA transport protein 3;
N	O94609	DE	Ubiquitin-activating enzyme E1 1;
N	O94609	DR	ligase activity
N	O94609	DR	mRNA export from nucleus
N	O94609	DR	protein modification process
N	O94609	DR	small protein activating enzyme activity

N	O94731	CC	Component of the TRAPP I and TRAPP II complexes
N	O94731	CC	Has a role late in meiosis following DNA replication (By similarity).
N	O94731	CC	TRAPP I plays a key role in the late stages of endoplasmic reticulum to Golgi traffic
N	O94731	CC	TRAPP II seems to play a role in intra-Golgi transport
N	O94731	DE	TRAPP subunit trs85-1;
N	O94731	DE	Transport protein particle subunit trs85-1;
N	O94731	DR	intracellular protein transport
N	O94731	DR	vesicle-mediated transport
N	O95479	CC	Oxidizes glucose-6-phosphate and glucose, as well as other hexose-6-phosphates
N	O95479	DE	6-phosphogluconolactonase;
N	O95479	DE	GDH/6PGL endoplasmic bifunctional protein;
N	O95479	DE	Glucose 1-dehydrogenase;
N	O95479	DE	Hexose-6-phosphate dehydrogenase;
N	O95479	DR	6-phosphogluconolactonase activity
N	O95479	DR	glucose 1-dehydrogenase [NAD(P)] activity
N	O95479	DR	glucose-6-phosphate dehydrogenase activity
N	O95989	CC	Cleaves a beta-phosphate from the diphosphate groups in PP-InsP5 (diphosphoinositol pentakisphosphate) and [PP]2-InsP4 (bisdiphosphoinositol tetrakisphosphate), suggesting that it may play a role in signal transduction
N	O95989	CC	Acts as a negative regulator of the ERK1/2 pathway
N	O95989	CC	Also able to catalyze the hydrolysis of dinucleoside oligophosphates, with Ap6A and Ap5A being the preferred substrates
N	O95989	CC	Also able to hydrolyze 5- phosphoribose 1-diphosphate.
N	O95989	CC	InsP6 (inositol hexakisphophate) is not a substrate
N	O95989	CC	The major reaction products are ADP and p4a from Ap6A and ADP and ATP from Ap5A
N	O95989	DE	Diadenosine 5',5'''-P1,P6-hexaphosphate hydrolase 1;
N	O95989	DE	Diphosphoinositol polyphosphate phosphohydrolase 1;
N	O95989	DE	Nucleoside diphosphate-linked moiety X motif 3;
N	O95989	DE	Nudix motif 3;
N	O95989	DR	cell-cell signaling
N	O95989	DR	diadenosine polyphosphate catabolic process
N	O95989	DR	diphosphoinositol polyphosphate catabolic process
N	O95989	DR	diphosphoinositol-polyphosphate diphosphatase activity
N	O95989	DR	magnesium ion binding
N	O98691	CC	NDH shuttles electrons from NAD(P)H:plastoquinone, via FMN and iron-sulfur (Fe-S) centers, to quinones in the photosynthetic chain and possibly in a chloroplast respiratory chain
N	O98691	CC	Couples the redox reaction to proton translocation, and thus conserves the redox energy in a proton gradient (By similarity).
N	O98691	CC	The immediate electron acceptor for the enzyme in this species is believed to be plastoquinone
N	O98691	DE	NAD(P)H dehydrogenase subunit H;
N	O98691	DE	NAD(P)H-quinone oxidoreductase subunit H, chloroplastic;
N	O98691	DE	NADH-plastoquinone oxidoreductase 49 kDa subunit;
N	O98691	DE	NADH-plastoquinone oxidoreductase subunit H;
N	O98691	DR	oxidation-reduction process
N	O98691	DR	oxidoreductase activity, acting on NADH or NADPH
N	O98691	DR	quinone binding
N	O98948	CC	RuBisCO catalyzes two reactions: the carboxylation of D- ribulose 1,5-bisphosphate, the primary event in carbon dioxide fixation, as well as the oxidative fragmentation of the pentose substrate
N	O98948	CC	Both reactions occur simultaneously and in competition at the same active site (By similarity).
N	O98948	DE	Ribulose bisphosphate carboxylase small chain;
N	O98948	DE	RuBisCO small subunit;
N	O98948	DR	monooxygenase activity
N	O98948	DR	oxidation-reduction process
N	O98948	DR	photorespiration
N	O98948	DR	reductive pentose-phosphate cycle
N	O98948	DR	ribulose-bisphosphate carboxylase activity
N	P00197	CC	Ferredoxins are iron-sulfur proteins that transfer electrons in a wide variety of metabolic reactions.
N	P00197	DR	4 iron, 4 sulfur cluster binding
N	P00197	DR	electron carrier activity

N	P00197	DR	electron transport chain
N	P00197	DR	metal ion binding
N	P00208	CC	Ferredoxins are iron-sulfur proteins that transfer electrons in a wide variety of metabolic reactions.
N	P00208	DR	4 iron, 4 sulfur cluster binding
N	P00208	DR	electron carrier activity
N	P00208	DR	electron transport chain
N	P00208	DR	metal ion binding
N	P00209	CC	Ferredoxins are iron-sulfur proteins that transfer electrons in a wide variety of metabolic reactions.
N	P00209	DE	Ferredoxin II;
N	P00209	DE	Ferredoxin-2;
N	P00209	DR	3 iron, 4 sulfur cluster binding
N	P00209	DR	electron carrier activity
N	P00209	DR	electron transport chain
N	P00209	DR	iron ion binding
N	P00347	CC	This transmembrane glycoprotein is involved in the control of cholesterol biosynthesis
N	P00347	CC	It is the rate-limiting enzyme of sterol biosynthesis.
N	P00347	DE	3-hydroxy-3-methylglutaryl-coenzyme A reductase;
N	P00347	DE	HMG-CoA reductase;
N	P00347	DR	cholesterol biosynthetic process
N	P00347	DR	coenzyme A metabolic process
N	P00347	DR	hydroxymethylglutaryl-CoA reductase (NADPH) activity
N	P00347	DR	isoprenoid biosynthetic process
N	P00347	DR	oxidation-reduction process
N	P00389	CC	This enzyme is required for electron transfer from NADP to cytochrome P450 in microsomes
N	P00389	CC	It can also provide electron transfer to heme oxygenase and cytochrome B5.
N	P00389	DE	NADPH--cytochrome P450 reductase;
N	P00389	DR	NADPH-hemoprotein reductase activity
N	P00389	DR	iron ion binding
N	P00389	DR	oxidation-reduction process
N	P00602	CC	PA2 catalyzes the calcium-dependent hydrolysis of the 2- acyl groups in 3-sn-phosphoglycerides.
N	P00602	DE	Phosphatidylcholine 2-acylhydrolase;
N	P00602	DE	Phospholipase A2 isozyme CM-I;
N	P00602	DR	calcium ion binding
N	P00602	DR	lipid catabolic process
N	P00602	DR	phospholipase A2 activity
N	P00602	DR	phospholipid metabolic process
N	P00835	CC	Produces ATP from ADP in the presence of a proton gradient across the membrane
N	P00835	DE	ATP synthase F1 sector epsilon subunit;
N	P00835	DE	ATP synthase epsilon chain, chloroplastic;
N	P00835	DE	F-ATPase epsilon subunit;
N	P00835	DR	ATP synthesis coupled proton transport
N	P00835	DR	hydrogen ion transporting ATP synthase activity, rotational mechanism
N	P00835	DR	proton-transporting ATPase activity, rotational mechanism
N	P00902	DE	Anthranilate synthase component 2;
N	P00902	DE	Anthranilate synthase component II;
N	P00902	DE	Glutamine amido-transferase;
N	P00902	DR	anthranilate synthase activity
N	P00902	DR	glutamine metabolic process
N	P00902	DR	tryptophan biosynthetic process
N	P01134	CC	TGF alpha is a mitogenic polypeptide that is able to bind to the EGF receptor and to act synergistically with TGF beta to promote anchorage-independent cell proliferation in soft agar.
N	P01134	DE	EGF-like TGF;
N	P01134	DE	Protransforming growth factor alpha;
N	P01134	DE	TGF type 1;
N	P01134	DE	TGF-alpha;
N	P01134	DE	Transforming growth factor alpha;
N	P01134	DR	MAP kinase kinase activity
N	P01134	DR	activation of MAPK activity
N	P01134	DR	anti-apoptosis
N	P01134	DR	cytosolic calcium ion homeostasis

N	P01134	DR	epidermal growth factor receptor binding
N	P01134	DR	epidermal growth factor receptor signaling pathway
N	P01134	DR	growth factor activity
N	P01134	DR	negative regulation of neuron apoptosis
N	P01134	DR	neuroprotection
N	P01134	DR	positive regulation of cell division
N	P01134	DR	positive regulation of epidermal growth factor receptor activity
N	P01134	DR	positive regulation of epithelial cell proliferation
N	P01134	DR	positive regulation of mitosis
N	P01134	DR	positive regulation of protein phosphorylation
N	P01134	DR	response to drug
N	P01134	DR	wound healing
N	P01161	CC	Hormone playing a key role in cardiovascular homeostasis through regulation of natriuresis, diuresis, and vasodilatation
N	P01161	CC	Binds the clearance receptor NPR3 (By similarity).
N	P01161	CC	Specifically binds and stimulates the cGMP production of the NPR1 receptor
N	P01161	DE	Atrial natriuretic factor;
N	P01161	DE	Atrial natriuretic peptide;
N	P01161	DE	Atriopeptin I;
N	P01161	DE	Atriopeptin II;
N	P01161	DE	Atriopeptin III;
N	P01161	DE	Atriopeptin-1;
N	P01161	DE	Atriopeptin-2;
N	P01161	DE	Atriopeptin-3;
N	P01161	DE	Auriculin-A;
N	P01161	DE	Auriculin-B;
N	P01161	DE	Prepronatriodilatin;
N	P01161	DR	cGMP biosynthetic process
N	P01161	DR	hormone activity
N	P01161	DR	multicellular organismal water homeostasis
N	P01161	DR	negative regulation of cell growth
N	P01161	DR	peptide hormone receptor binding
N	P01161	DR	receptor guanylyl cyclase signaling pathway
N	P01161	DR	regulation of blood pressure
N	P01161	DR	regulation of blood vessel size
N	P01161	DR	regulation of vasodilation
N	P01161	DR	response to insulin stimulus
N	P01182	CC	Neurophysin 2 specifically binds the midbrain peptide hormone vasopressin.
N	P01182	DE	Neurophysin 2;
N	P01182	DR	neurohypophyseal hormone activity
N	P01388	CC	Produces peripheral paralysis by blocking neuromuscular transmission at the postsynaptic site
N	P01388	CC	Binds to muscular and neuronal (only alpha-7 alpha-8 alpha-9) nicotinic acetylcholine receptors (By similarity).
N	P01388	DE	Long neurotoxin 2;
N	P01388	DE	Neurotoxin B;
N	P01388	DR	acetylcholine receptor inhibitor activity
N	P01388	DR	synaptic transmission
N	P01480	CC	Alpha toxins bind voltage-independently at site-3 of sodium channels and inhibits the inactivation of the activated channels, thereby blocking neuronal transmission
N	P01480	DE	Alpha-mammal toxin Aah3;
N	P01480	DE	Neurotoxin 3;
N	P01480	DE	Neurotoxin III;
N	P01480	DR	defense response
N	P01480	DR	sodium channel inhibitor activity
N	P01757	DE	Ig heavy chain V region J558;
N	P01757	DR	antigen binding
N	P02431	DE	50S ribosomal protein L30;
N	P02431	DR	structural constituent of ribosome
N	P02440	CC	The keratin products of mammalian epidermal derivatives such as wool and hair consist of microfibrils embedded in a rigid matrix of other proteins
N	P02440	CC	The matrix proteins include the high- sulfur and high-tyrosine keratins, having molecular weights of 6- 20 kDa, whereas the microfibrils contain the larger, low-sulfur keratins (40-56 kDa).
N	P02440	DE	Keratin, high-sulfur matrix protein, B2C;

N	P02515	DE	Heat shock protein 22;
N	P02515	DR	determination of adult lifespan
N	P02515	DR	response to heat
N	P02515	DR	response to oxidative stress
N	P02774	CC	Multifunctional protein found in plasma, ascitic fluid, cerebrospinal fluid, and urine and on the surface of many cell types
N	P02774	CC	DBP associates with membrane-bound immunoglobulin on the surface of B-lymphocytes and with IgG Fc receptor on the membranes of T-lymphocytes.
N	P02774	CC	In plasma, it carries the vitamin D sterols and prevents polymerization of actin by binding its monomers
N	P02774	DE	Gc-globulin;
N	P02774	DE	Group-specific component;
N	P02774	DE	Vitamin D-binding protein;
N	P02774	DR	actin binding
N	P02774	DR	hormone biosynthetic process
N	P02774	DR	vitamin D binding
N	P02774	DR	vitamin D metabolic process
N	P02774	DR	vitamin transporter activity
N	P02856	CC	Seed storage protein.
N	P02856	DE	Vicilin, 14 kDa component;
N	P02856	DR	nutrient reservoir activity
N	P03167	CC	Multifunctional protein that may modulate protein degradation pathways, apoptosis, transcription, signal transduction, cell cycle progress, and genetic stability by directly or indirectly interacting with hosts factors
N	P03167	CC	Does not seem to be essential for HBV infection
N	P03167	CC	Effect on apoptosis is controversial depending on the cell types in which the studies have been conducted (By similarity).
N	P03167	CC	May be directly involved in development of cirrhosis and liver cancer (hepatocellular carcinoma)
N	P03167	CC	Most of cytosolic activities involve modulation of cytosolic calcium
N	P03167	DR	interspecies interaction between organisms
N	P03167	DR	viral genome replication
N	P03473	CC	Catalyzes the removal of terminal sialic acid residues from viral and cellular glycoconjugates
N	P03473	CC	Additionally helps virus spread through the circulation by further removing sialic acids from the cell surface
N	P03473	CC	Cleaves off the terminal sialic acids on the glycosylated HA during virus budding to facilitate virus release
N	P03473	CC	Described as a receptor-destroying enzyme because it cleaves a terminal sialic acid from the cellular receptors
N	P03473	CC	Likely to plays a role in the budding process through its association with lipid rafts during intracellular transport
N	P03473	CC	May additionally display a raft-association independent effect on budding
N	P03473	CC	May facilitate viral invasion of the upper airways by cleaving the sialic acid moities on the mucin of the airway epithelial cells
N	P03473	CC	Otherwise, infection would be limited to one round of replication
N	P03473	CC	Plays a role in the determination of host range restriction on replication and virulence
N	P03473	CC	Sialidase activity in late endosome/lysosome traffic seems to enhance virus replication
N	P03473	CC	These cleavages prevent self-aggregation and ensure the efficient spread of the progeny virus from cell to cell
N	P03473	DE	Neuraminidase;
N	P03473	DR	carbohydrate metabolic process
N	P03473	DR	exo-alpha-sialidase activity
N	P03473	DR	metal ion binding
N	P03517	CC	Glycoprotein G1 and glycoprotein G2 interact with each other and are present at the surface of the virion
N	P03517	CC	They are able to attach the virion to a cell receptor and to promote fusion of membranes after endocytosis of the virion (By similarity).
N	P03517	DE	Envelope glycoprotein;
N	P03517	DE	Glycoprotein G1;
N	P03517	DE	Glycoprotein G2;
N	P03517	DE	M polyprotein;
N	P03517	DE	Non-structural protein NSm;
N	P03517	DR	interspecies interaction between organisms
N	P03554	CC	Encodes for at least two polypeptides: protease (PR) and reverse transcriptase (RT)

N	P03554	CC	After budding and infection, the RC-DNA migrates in the nucleus, and is converted into a plasmid-like covalently closed circular DNA (cccDNA) (By similarity)
N	P03554	CC	Neo- synthesized pregenomic RNA (pgRNA) are encapsidated, and reverse-transcribed inside the nucleocapsid
N	P03554	CC	Partial (+)DNA is synthesized from the (-)DNA template and generates the relaxed circular DNA (RC-DNA) genome
N	P03554	CC	Reverse transcriptase is multifunctional enzyme that converts the viral RNA genome into dsDNA in viral cytoplasmic capsids
N	P03554	CC	The protease processes the polyprotein in cis
N	P03554	CC	This enzyme displays a DNA polymerase activity that can copy either DNA or RNA templates, and a ribonuclease H (RNase H) activity that cleaves the RNA strand of RNA-DNA heteroduplexes in a partially processive 3'- to 5'-endonucleasic mode
N	P03554	DE	Aspartic protease;
N	P03554	DE	Endonuclease;
N	P03554	DE	Enzymatic polyprotein;
N	P03554	DE	Reverse transcriptase;
N	P03554	DR	RNA-dependent DNA replication
N	P03554	DR	RNA-directed DNA polymerase activity
N	P03554	DR	aspartic-type endopeptidase activity
N	P03554	DR	endonuclease activity
N	P03657	CC	Isoform G1P plays an essential role in phage assembly
N	P03657	CC	It is required to increase the number of adhesion zones between the inner and outer membranes of the host cell
N	P03657	CC	May be involved with G4P in creating zone through which the phage assembles and budds.
N	P03657	CC	The budding of neo-synthesized phages occurs at these adhesion sites
N	P03657	DE	Gene 1 protein;
N	P03657	DR	viral assembly, maturation, egress, and release
N	P03712	CC	Stabilizes the head shell following the rearrangement of the gpE subunits of the head shell lattice that accompanies expansion of the head
N	P03712	CC	There are approximately 420 copies of protein D per mature phage.
N	P03712	DE	Head decoration protein;
N	P03712	DE	Major capsid protein D;
N	P03738	CC	Gene L protein is a minor tail protein, located at the distal end
N	P03738	CC	It is involved in the assembly of the initiator complex for tail polymerization.
N	P03738	DE	Minor tail protein L;
N	P03998	CC	Coagulogen is a gel-forming protein of hemolymph; it hinders the spread of invaders by immobilizing them.
N	P03998	DE	Coagulin chain A;
N	P03998	DE	Coagulin chain B;
N	P03998	DE	Peptide C;
N	P03998	DR	hemolymph coagulation
N	P04309	DE	Uncharacterized 7.4 kDa protein;
N	P04354	CC	Buffers cytosolic calcium
N	P04354	CC	May stimulate a membrane Ca(2+)-ATPase and a 3',5'-cyclic nucleotide phosphodiesterase.
N	P04354	DE	Calbindin D28;
N	P04354	DE	Vitamin D-dependent calcium-binding protein, avian-type;
N	P04354	DR	calcium ion binding
N	P04354	DR	calcium ion transport
N	P04354	DR	cellular calcium ion homeostasis
N	P04354	DR	sensory perception of pain
N	P04354	DR	vitamin D binding
N	P04451	DE	60S ribosomal protein L23;
N	P04451	DR	structural constituent of ribosome
N	P04586	CC	Integrase performs the integration of the newly synthesized dsDNA copy of the viral genome into the host chromosome
N	P04586	CC	The integrated DNA is called provirus.
N	P04586	DE	Gag-Pol polyprotein;
N	P04586	DE	Integrase;
N	P04586	DE	Pr160Gag-Pol;
N	P04586	DR	RNA-dependent DNA replication
N	P04586	DR	endonuclease activity
N	P04586	DR	entry into host cell
N	P04586	DR	integrase activity

N	P04586	DR	provirus integration
N	P04586	DR	uncoating of virus
N	P04614	CC	Nuclear transcriptional activator of viral gene expression, that is essential for viral transcription from the LTR promoter and replication
N	P04614	CC	Acts as a sequence-specific molecular adapter, directing components of the cellular transcription machinery to the viral RNA to promote processive transcription elongation by the RNA polymerase II (RNA pol II) complex, thereby increasing the level of full-length transcripts
N	P04614	CC	Besides its effect on RNA Pol II processivity, Tat induces chromatin remodeling of proviral genes by recruiting the histone acetyltransferases (HATs) CREBBP, EP300 and PCAF to the chromatin
N	P04614	CC	In the absence of Tat, the RNA Pol II generates short or non-processive transcripts that terminate at approximately 60 bp from the initiation site
N	P04614	CC	In the cytoplasm, Tat is thought to act as a translational activator of HIV-1 mRNAs (By similarity).
N	P04614	CC	In this purpose, it activates EIF2AK2/PKR which, in turns, may phosphorylate and target to degradation the inhibitor IkappaB- alpha which normally retains NF-kappa-B in the cytoplasm of unstimulated cells
N	P04614	CC	Interacts with the cellular capping enzyme RNGTT to mediate co-transcriptional capping of viral mRNAs
N	P04614	CC	Other factors such as HTATSF1/Tat-SF1, SUPT5H/SPT5, and HTATIP2 are also important for Tat's function
N	P04614	CC	Tat associates with the CCNT1/cyclin-T1 component of the P-TEFb complex (CDK9 and CCNT1), which promotes RNA chain elongation
N	P04614	CC	Tat can reactivate a latently infected cell by penetrating in it and transactivating its LTR promoter
N	P04614	CC	Tat protein exerts as well a positive feedback on the translation of its cognate mRNA
N	P04614	CC	The CDK9 component of P-TEFb and other Tat-activated kinases hyperphosphorylate the C-terminus of RNA Pol II that becomes stabilized and much more processive
N	P04614	CC	This also contributes to the increase in proviral transcription rate, especially when the provirus integrates in transcriptionally silent region of the host genome
N	P04614	CC	This binding increases Tat's affinity for a hairpin structure at the 5'-end of all nascent viral mRNAs referred to as the transactivation responsive RNA element (TAR RNA) and allows Tat/P- TEFb complex to bind cooperatively to TAR RNA
N	P04614	CC	Through its interaction with TBP, Tat may be involved in transcription initiation as well
N	P04614	CC	To ensure maximal activation of the LTR, Tat mediates nuclear translocation of NF-kappa-B
N	P04614	DE	Protein Tat;
N	P04614	DE	Transactivating regulatory protein;
N	P04614	DR	evasion by virus of host immune response
N	P04614	DR	metal ion binding
N	P04614	DR	positive regulation of viral transcription
N	P04614	DR	regulation of transcription, DNA-dependent
N	P04614	DR	sequence-specific DNA binding transcription factor activity
N	P04668	CC	Low-potential electron donor to a number of redox enzymes
N	P04668	CC	NifF is the electron donor to nitrogenase.
N	P04668	DR	electron carrier activity
N	P04668	DR	electron transport chain
N	P04668	DR	iron ion binding
N	P04668	DR	nitrogen fixation
N	P04668	DR	oxidoreductase activity
N	P04801	DE	Threonine--tRNA ligase;
N	P04801	DE	Threonyl-tRNA synthetase, cytoplasmic;
N	P04801	DR	protein binding
N	P04801	DR	threonine-tRNA ligase activity
N	P04801	DR	threonyl-tRNA aminoacylation
N	P05004	CC	Produced by macrophages, IFN-alpha have antiviral activities.
N	P05004	DE	Interferon alpha-2;
N	P05100	CC	Hydrolysis of the deoxyribose N-glycosidic bond to excise 3-methyladenine from the damaged DNA polymer formed by alkylation lesions.
N	P05100	DE	3-methyladenine-DNA glycosylase I, constitutive;
N	P05100	DE	DNA-3-methyladenine glycosidase I;
N	P05100	DE	DNA-3-methyladenine glycosylase 1;
N	P05100	DE	DNA-3-methyladenine glycosylase I;
N	P05100	DR	DNA-3-methyladenine glycosylase I activity

N	P05100	DR	base-excision repair
N	P05100	DR	metal ion binding
N	P05100	DR	protein binding
N	P05123	CC	Reversibly catalyzes the transfer of phosphate between ATP and various phosphogens (e.g
N	P05123	CC	Creatine kinase isoenzymes play a central role in energy transduction in tissues with large, fluctuating energy demands, such as skeletal muscle, heart, brain and spermatozoa
N	P05123	CC	creatine phosphate)
N	P05123	DE	Creatine kinase M chain;
N	P05123	DE	Creatine kinase M-type;
N	P05123	DR	creatine kinase activity
N	P05349	CC	RuBisCO catalyzes two reactions: the carboxylation of D- ribulose 1,5-bisphosphate, the primary event in carbon dioxide fixation, as well as the oxidative fragmentation of the pentose substrate
N	P05349	CC	Both reactions occur simultaneously and in competition at the same active site (By similarity).
N	P05349	DE	Ribulose bisphosphate carboxylase small chain 3B, chloroplastic;
N	P05349	DE	RuBisCO small subunit 3B;
N	P05349	DR	monooxygenase activity
N	P05349	DR	oxidation-reduction process
N	P05349	DR	photorespiration
N	P05349	DR	reductive pentose-phosphate cycle
N	P05349	DR	ribulose-bisphosphate carboxylase activity
N	P05575	DR	serine-type endopeptidase inhibitor activity
N	P05588	DR	serine-type endopeptidase inhibitor activity
N	P05653	CC	DNA gyrase negatively supercoils closed circular double- stranded DNA in an ATP-dependent manner and also catalyzes the interconversion of other topological isomers of double-stranded DNA rings, including catenanes and knotted rings
N	P05653	DE	DNA gyrase subunit A;
N	P05653	DR	DNA topoisomerase (ATP-hydrolyzing) activity
N	P05653	DR	DNA topological change
N	P05653	DR	regulation of transcription, DNA-dependent
N	P05653	DR	sequence-specific DNA binding transcription factor activity
N	P05754	DE	40S ribosomal protein S8;
N	P05754	DR	maturation of SSU-rRNA from tricistronic rRNA transcript (SSU-rRNA, 5.8S rRNA, LSU-rRNA)
N	P05754	DR	structural constituent of ribosome
N	P05763	DE	30S ribosomal protein S9P;
N	P05763	DR	structural constituent of ribosome
N	P06392	DE	50S ribosomal protein L33, chloroplastic;
N	P06392	DR	structural constituent of ribosome
N	P06410	CC	NDH shuttles electrons from NAD(P)H:plastoquinone, via FMN and iron-sulfur (Fe-S) centers, to quinones in the photosynthetic chain and possibly in a chloroplast respiratory chain
N	P06410	CC	Couples the redox reaction to proton translocation, and thus conserves the redox energy in a proton gradient (By similarity).
N	P06410	CC	The immediate electron acceptor for the enzyme in this species is believed to be plastoquinone
N	P06410	DE	NAD(P)H dehydrogenase subunit K;
N	P06410	DE	NAD(P)H-quinone oxidoreductase subunit K, chloroplastic;
N	P06410	DE	NADH-plastoquinone oxidoreductase subunit K;
N	P06410	DR	4 iron, 4 sulfur cluster binding
N	P06410	DR	NADH dehydrogenase (ubiquinone) activity
N	P06410	DR	metal ion binding
N	P06410	DR	oxidation-reduction process
N	P06410	DR	quinone binding
N	P06495	DE	Calcium-binding protein;
N	P06495	DE	Calerythrin;
N	P06495	DR	calcium ion binding
N	P06511	CC	PsaA and psaB bind P700, the primary electron donor of photosystem I (PSI), as well as the electron acceptors A0, A1 and FX
N	P06511	CC	Oxidized P700 is reduced on the lumenal side of the thylakoid membrane by plastocyanin
N	P06511	CC	PSI is a plastocyanin-ferredoxin oxidoreductase, converting photonic excitation into a charge separation, which transfers an electron from the donor P700 chlorophyll pair to the spectroscopically characterized acceptors A0, A1, FX, FA and FB in turn

N	P06511	DE	Photosystem I P700 chlorophyll a apoprotein A1;
N	P06511	DR	4 iron, 4 sulfur cluster binding
N	P06511	DR	chlorophyll binding
N	P06511	DR	electron transport chain
N	P06511	DR	metal ion binding
N	P06511	DR	photosynthesis
N	P06511	DR	protein-chromophore linkage
N	P06650	DE	Immunity protein;
N	P06738	CC	Phosphorylase is an important allosteric enzyme in carbohydrate metabolism
N	P06738	CC	Enzymes from different sources differ in their regulatory mechanisms and in their natural substrates
N	P06738	CC	However, all known phosphorylases share catalytic and structural properties.
N	P06738	DE	Glycogen phosphorylase;
N	P06738	DR	glycogen catabolic process
N	P06738	DR	glycogen phosphorylase activity
N	P06738	DR	protein binding
N	P06738	DR	pyridoxal phosphate binding
N	P06793	CC	Minor protein of the capsid that localizes along the inner surface of the virion, within the central cavities beneath the L1 pentamers
N	P06793	CC	During virion assembly, encapsidates the genome by direct interaction with the viral DNA (By similarity).
N	P06793	CC	Later on, promotes late gene expression by interacting with the viral E2 protein and by inhibiting its transcriptional activation functions
N	P06793	CC	Mediates the viral genome import into the nucleus through binding to host importins
N	P06793	CC	Once the virion enters the host cell, escorts the genomic DNA into the nucleus, in particular by promoting virion endosomal escape
N	P06793	CC	Once within the nucleus, L2 localizes viral genomes to PML bodies in order to activate early gene expression for establishment of infection
N	P06793	CC	Plays a role in capsid stabilization through interaction with the major capsid protein L1
N	P06793	DE	Minor capsid protein L2;
N	P06793	DR	structural molecule activity
N	P06935	CC	Capsid protein C self-assembles to form an icosahedral capsid about 30 nm in diameter
N	P06935	CC	Envelope protein E binding to host cell surface receptor is followed by virus internalization through clathrin-mediated endocytosis
N	P06935	CC	Non-structural protein 2A may be involved viral RNA replication and capsid assembly (Potential).
N	P06935	CC	Peptide 2k functions as a signal peptide for NS4B and is required for the interferon antagonism activity of the latter (By similarity).
N	P06935	CC	RNA-directed RNA polymerase NS5 replicates the viral (+) and (-) genome, and performs the capping of genomes in the cytoplasm
N	P06935	CC	Serine protease NS3 displays three enzymatic activities: serine protease, NTPase and RNA helicase
N	P06935	CC	After cleavage of prM, envelope protein E dissociate from small envelope protein M and homodimerizes (By similarity).
N	P06935	CC	Besides its role in genome replication, also prevents the establishment of cellular antiviral state by blocking the interferon-alpha/beta (IFN-alpha/beta) signaling pathway
N	P06935	CC	Envelope protein E is subsequently involved in membrane fusion between virion and host late endosomes
N	P06935	CC	Inhibits host JAK1 and TYK2 phosphorylation, thereby preventing activation of JAK-STAT signaling pathway (By similarity).
N	P06935	CC	NS3 RNA helicase binds RNA and unwinds dsRNA in the 3' to 5' direction (By similarity).
N	P06935	CC	NS3 serine protease, in association with NS2B, performs its autocleavage and cleaves the polyprotein at dibasic sites in the cytoplasm: C-prM, NS2A-NS2B, NS2B-NS3, NS3-NS4A, NS4A-2K and NS4B-NS5
N	P06935	CC	NS5 methylates viral RNA cap at guanine N-7 and ribose 2'-O positions
N	P06935	CC	Synthesized as an homodimer with prM which acts as a chaperone for envelope protein E
N	P06935	CC	The capsid encapsulates the genomic RNA (By similarity).
N	P06935	DE	Capsid protein C;
N	P06935	DE	Core protein;
N	P06935	DE	Envelope protein E;
N	P06935	DE	Flavivirin protease NS2B regulatory subunit;
N	P06935	DE	Flavivirin protease NS3 catalytic subunit;
N	P06935	DE	Genome polyprotein;

N	P06935	DE	Matrix protein;
N	P06935	DE	Non-structural protein 1;
N	P06935	DE	Non-structural protein 2A;
N	P06935	DE	Non-structural protein 2B;
N	P06935	DE	Non-structural protein 3;
N	P06935	DE	Non-structural protein 4A;
N	P06935	DE	Non-structural protein 4B;
N	P06935	DE	Peptide 2k;
N	P06935	DE	Peptide pr;
N	P06935	DE	RNA-directed RNA polymerase NS5;
N	P06935	DE	Serine protease NS3;
N	P06935	DE	Serine protease subunit NS2B;
N	P06935	DE	Small envelope protein M;
N	P06935	DR	ATP-dependent helicase activity
N	P06935	DR	RNA helicase activity
N	P06935	DR	RNA-directed RNA polymerase activity
N	P06935	DR	double-stranded RNA binding
N	P06935	DR	evasion by virus of host immune response
N	P06935	DR	mRNA (guanine-N7-)-methyltransferase activity
N	P06935	DR	mRNA (nucleoside-2'-O-)-methyltransferase activity
N	P06935	DR	metal ion binding
N	P06935	DR	protein binding
N	P06935	DR	serine-type endopeptidase activity
N	P06935	DR	serine-type exopeptidase activity
N	P06935	DR	structural molecule activity
N	P06935	DR	viral genome replication
N	P07167	DE	Limited host range virA protein;
N	P07167	DR	peptidyl-histidine phosphorylation
N	P07167	DR	regulation of transcription, DNA-dependent
N	P07167	DR	two-component response regulator activity
N	P07167	DR	two-component sensor activity
N	P07180	CC	RuBisCO catalyzes two reactions: the carboxylation of D- ribulose 1,5-bisphosphate, the primary event in carbon dioxide fixation, as well as the oxidative fragmentation of the pentose substrate
N	P07180	CC	Both reactions occur simultaneously and in competition at the same active site (By similarity).
N	P07180	DE	Ribulose bisphosphate carboxylase small chain 3A/3C, chloroplastic;
N	P07180	DE	RuBisCO small subunit 3A/3C;
N	P07180	DR	monooxygenase activity
N	P07180	DR	oxidation-reduction process
N	P07180	DR	photorespiration
N	P07180	DR	reductive pentose-phosphate cycle
N	P07180	DR	ribulose-bisphosphate carboxylase activity
N	P07489	CC	Thyroid hormone-binding protein
N	P07489	CC	Probably transports thyroxine from the bloodstream to the brain (By similarity).
N	P07489	DE	Transthyretin;
N	P07489	DR	hormone activity
N	P07635	DE	Beta-fructofuranosidase 7;
N	P07635	DE	DE Flags: Precursor; Fragments;
N	P07635	DE	Invertase 7;
N	P07635	DR	carbohydrate metabolic process
N	P07635	DR	sucrose alpha-glucosidase activity
N	P07795	CC	Core component of nucleosome
N	P07795	CC	DNA accessibility is regulated via a complex set of post-translational modifications of histones, also called histone code, and nucleosome remodeling.
N	P07795	CC	Histones thereby play a central role in transcription regulation, DNA repair, DNA replication and chromosomal stability
N	P07795	CC	Nucleosomes wrap and compact DNA into chromatin, limiting DNA accessibility to the cellular machineries which require DNA as a template
N	P07795	DE	Late histone H2B.2.2;
N	P07795	DR	nucleosome assembly
N	P07855	CC	Fatty acid synthetase catalyzes the formation of long- chain fatty acids from acetyl-CoA, malonyl-CoA and NADPH
N	P07855	CC	This multifunctional protein has 7 catalytic activities and an acyl carrier protein.
N	P07855	DE	Fatty acid synthase;

N	P07855	DR	acyl carrier activity
N	P07855	DR	cofactor binding
N	P07855	DR	fatty acid biosynthetic process
N	P07855	DR	fatty acid synthase activity
N	P07855	DR	phosphopantetheine binding
N	P07859	CC	Regulatory protein of the TOL plasmid xyl operons
N	P07859	CC	XylS activates the xylXYZLTEGFJQKIH operon required for the degradation of toluene, m-xylene and p-xylene.
N	P07859	DE	XylDLEGF operon transcriptional activator;
N	P07859	DR	aromatic compound catabolic process
N	P07859	DR	regulation of transcription, DNA-dependent
N	P07859	DR	sequence-specific DNA binding
N	P07859	DR	sequence-specific DNA binding transcription factor activity
N	P07920	DE	1-aminocyclopropane-1-carboxylate oxidase 2;
N	P07920	DE	ACC oxidase 2;
N	P07920	DE	Ethylene-forming enzyme;
N	P07920	DE	Protein GTOMA;
N	P07920	DR	1-aminocyclopropane-1-carboxylate oxidase activity
N	P07920	DR	L-ascorbic acid binding
N	P07920	DR	ethylene biosynthetic process
N	P07920	DR	metal ion binding
N	P07920	DR	oxidation-reduction process
N	P08045	CC	Binds to poly-G sequences in RNA
N	P08045	CC	May function in post- translational regulation processes.
N	P08045	DE	Xenopus fingers protein;
N	P08045	DE	Zinc finger protein Xfin;
N	P08045	DR	regulation of transcription, DNA-dependent
N	P08045	DR	zinc ion binding
N	P08202	CC	Catalyzes the conversion of L-arabinose to L-ribulose.
N	P08202	DE	L-arabinose isomerase;
N	P08202	DR	L-arabinose catabolic process to xylulose 5-phosphate
N	P08202	DR	L-arabinose isomerase activity
N	P08202	DR	L-fucose isomerase activity
N	P08202	DR	fucose metabolic process
N	P08202	DR	metal ion binding
N	P08372	CC	Not yet known.
N	P08372	DE	Prepilin peptidase-dependent protein C;
N	P08502	CC	Component of the ubiquinol-cytochrome c reductase complex (complex III or cytochrome b-c1 complex), which is a respiratory chain that generates an electrochemical potential coupled to ATP synthesis (By similarity).
N	P08502	DE	Cytochrome b;
N	P08502	DR	electron carrier activity
N	P08502	DR	metal ion binding
N	P08502	DR	oxidoreductase activity
N	P08502	DR	respiratory electron transport chain
N	P08535	CC	Involved in oxygen transport from the lung to the various peripheral tissues.
N	P08535	DE	Beta-globin;
N	P08535	DE	Hemoglobin beta chain;
N	P08535	DE	Hemoglobin subunit beta;
N	P08535	DR	oxygen binding
N	P08535	DR	oxygen transporter activity
N	P08691	CC	Involved in arsenical resistance
N	P08691	CC	Thought to form the channel of an arsenite pump.
N	P08691	DE	Arsenical pump membrane protein;
N	P08691	DR	arsenite transmembrane transporter activity
N	P08691	DR	response to arsenic-containing substance
N	P08861	CC	Efficient protease with alanine specificity but only little elastolytic activity.
N	P08861	DE	Chymotrypsin-like elastase family member 3B;
N	P08861	DE	Elastase IIIB;
N	P08861	DE	Elastase-3B;
N	P08861	DR	cholesterol metabolic process
N	P08861	DR	serine-type endopeptidase activity
N	P08988	CC	Resistance to antibiotics containing the 2-deoxy- streptamine ring including gentamicin, kanamycin, tobramycin, neomycin and apramycin.
N	P08988	DE	Aminocyclitol 3-N-acetyltransferase type IV;

N	P08988	DE	Aminoglycoside N(3')-acetyltransferase IV;
N	P08988	DR	aminoglycoside 3-N-acetyltransferase activity
N	P08988	DR	response to antibiotic
N	P09015	DE	Homeobox protein en-2a;
N	P09015	DE	Homeobox protein engrailed-2a;
N	P09015	DR	anterior/posterior pattern formation
N	P09015	DR	cell fate specification
N	P09015	DR	midbrain development
N	P09015	DR	regulation of transcription, DNA-dependent
N	P09015	DR	sequence-specific DNA binding
N	P09015	DR	sequence-specific DNA binding transcription factor activity
N	P09015	DR	transcription regulator activity
N	P09301	DE	Gene 53 protein;
N	P09384	CC	Involved in the transmission of sensory signals from the chemoreceptors to the flagellar motors
N	P09384	CC	CheA is autophosphorylated; it can transfer its phosphate group to either CheB or CheY
N	P09384	DE	Chemotaxis protein CheA;
N	P09384	DR	peptidyl-histidine phosphorylation
N	P09384	DR	two-component sensor activity
N	P09738	CC	Destroys radicals which are normally produced within the cells and which are toxic to biological systems.
N	P09738	DE	Superoxide dismutase [Mn/Fe];
N	P09738	DR	metal ion binding
N	P09738	DR	oxidation-reduction process
N	P09738	DR	superoxide dismutase activity
N	P09738	DR	superoxide metabolic process
N	P09779	DE	Protein virB6;
N	P09779	DR	protein secretion by the type IV secretion system
N	P09854	CC	Replicates viral genomic DNA
N	P09854	CC	Additionally, the polymerase contains an intrinsic ribonuclease H (RNase H) activity that specifically degrades RNA/DNA heteroduplexes or duplex DNA substrates in the 5' to 3' direction
N	P09854	CC	The replication complex is composed of six viral proteins: the DNA polymerase, processivity factor, primase, primase-associated factor, helicase, and ssDNA-binding protein
N	P09854	CC	Therefore, it can catalyze the excision of the RNA primers that initiate the synthesis of Okazaki fragments at a replication fork during viral DNA replication (By similarity)
N	P09854	DE	DNA polymerase catalytic subunit;
N	P09854	DR	DNA replication
N	P09854	DR	DNA-directed DNA polymerase activity
N	P09854	DR	nucleotide binding
N	P09854	DR	ribonuclease H activity
N	P0A016	CC	Key enzyme in folate metabolism
N	P0A016	CC	Catalyzes an essential reaction for de novo glycine and purine synthesis, and for DNA precursor synthesis (By similarity).
N	P0A016	DE	Dihydrofolate reductase;
N	P0A016	DR	dihydrofolate reductase activity
N	P0A016	DR	glycine biosynthetic process
N	P0A016	DR	nucleotide biosynthetic process
N	P0A016	DR	one-carbon metabolic process
N	P0A016	DR	oxidation-reduction process
N	P0A0B9	DE	Phosphotagatokinase;
N	P0A0B9	DE	Tagatose-6-phosphate kinase;
N	P0A0B9	DR	lactose catabolic process via tagatose-6-phosphate
N	P0A0B9	DR	tagatose-6-phosphate kinase activity
N	P0A0C0	CC	Resistance to gentamicin, tobramycin, and kanamycin
N	P0A0C0	CC	Tobramycin and kanamycin resistance is due to the ACC activity, specified by N-terminal region, and the gentamicin resistance is due to the APH activity encoded by the C-terminal region of the protein (By similarity).
N	P0A0C0	DE	2''-aminoglycoside phosphotransferase;
N	P0A0C0	DE	6'-aminoglycoside N-acetyltransferase;
N	P0A0C0	DE	Bifunctional AAC/APH;
N	P0A0C0	DR	N-acetyltransferase activity
N	P0A0C0	DR	kinase activity
N	P0A0C0	DR	response to antibiotic

N	P0A0T5	CC	Important in the cellular metabolism or defense processes particular to this pathogen (By similarity).
N	P0A0T5	DE	Glutathione peroxidase homolog;
N	P0A0T5	DR	glutathione peroxidase activity
N	P0A0T5	DR	oxidation-reduction process
N	P0A0T5	DR	response to oxidative stress
N	P0A1V2	DE	Insertion element IS629 uncharacterized 12 kDa protein S4062;
N	P0A1V2	DR	transposase activity
N	P0A1V2	DR	transposition, DNA-mediated
N	P0A2B7	CC	Involved in DNA recombination (By similarity).
N	P0A2B7	DE	DNA recombination protein rmuC;
N	P0A2B7	DR	DNA recombination
N	P0A2I3	CC	DNA gyrase negatively supercoils closed circular double- stranded DNA in an ATP-dependent manner and also catalyzes the interconversion of other topological isomers of double-stranded DNA rings, including catenanes and knotted rings
N	P0A2I3	DE	DNA gyrase subunit B;
N	P0A2I3	DR	DNA topoisomerase (ATP-hydrolyzing) activity
N	P0A2I3	DR	DNA topological change
N	P0A2I3	DR	response to antibiotic
N	P0A3B3	CC	Not known; probably interacts with the ribosomes in a GTP dependent manner (By similarity).
N	P0A3B3	DE	GTP-binding protein TypA/BipA;
N	P0A3B3	DE	Tyrosine phosphorylated protein A;
N	P0A3B3	DR	GTPase activity
N	P0A3K0	DE	Uncharacterized low temperature-induced protein all0457;
N	P0A3K8	CC	IF-3 binds to the 30S ribosomal subunit and shifts the equilibrum between 70S ribosomes and their 50S and 30S subunits in favor of the free subunits, thus enhancing the availability of 30S subunits on which protein synthesis initiation begins
N	P0A3K8	DE	Translation initiation factor IF-3;
N	P0A3K8	DR	translation initiation factor activity
N	P0A3U0	CC	Multifunctional protein that promotes group II intron splicing and mobility by acting both on RNA and DNA
N	P0A3U0	CC	After DNA cleavage, retrohoming occurs by a target DNA-primed reverse transcription of the intron RNA that had reverse spliced into the sense strand of the recipient DNA
N	P0A3U0	CC	After splicing, the protein remains bound to the excised intron lariat RNA, forming ribonucleoprotein particles, and cleaving the antisense strand of the recipient DNA in the 3' exon
N	P0A3U0	CC	It also contributes to the recognition of the DNA target site and acts as a repressor of its own translation.
N	P0A3U0	CC	It has three activities: reverse transcriptase (RT) for intron duplication, maturase to promote splicing, and DNA endonuclease for site- specific cleavage of recipient alleles
N	P0A3U0	CC	The intron-encoded protein promotes splicing by facilitating the formation of the catalytically active structure of the intron RNA
N	P0A3U0	DE	DNA endonuclease;
N	P0A3U0	DE	Group II intron-encoded protein ltrA;
N	P0A3U0	DE	RNA maturase;
N	P0A3U0	DE	Reverse-transcriptase;
N	P0A3U0	DR	RNA-dependent DNA replication
N	P0A3U0	DR	RNA-directed DNA polymerase activity
N	P0A3U0	DR	endonuclease activity
N	P0A3U0	DR	intron homing
N	P0A3U0	DR	mRNA processing
N	P0A3V7	CC	VirB proteins are suggested to act at the bacterial surface and there play an important role in directing T-DNA transfer to plant cells.
N	P0A3V7	DE	Protein virB1;
N	P0A3W8	CC	Involved in DNA transformation; mediates the nuclear uptake of single-stranded DNA copies of the transferred DNA (T- DNA) element
N	P0A3W8	CC	Binds single-stranded but not double-stranded DNA regardless of nucleotide sequence composition.
N	P0A3W8	DE	Single-strand DNA-binding protein;
N	P0A3W8	DR	DNA mediated transformation
N	P0A3W8	DR	protein binding
N	P0A3X6	CC	Through the carboxylation of phosphoenolpyruvate (PEP) it forms oxaloacetate, a four-carbon dicarboxylic acid source for the tricarboxylic acid cycle.
N	P0A3X6	DE	Phosphoenolpyruvate carboxylase;

N	P0A3X6	DR	carbon fixation
N	P0A3X6	DR	phosphoenolpyruvate carboxylase activity
N	P0A3X6	DR	tricarboxylic acid cycle
N	P0A4A6	CC	With S4 and S5 plays an important role in translational accuracy (By similarity).
N	P0A4A6	DE	30S ribosomal protein S12;
N	P0A4A6	DR	response to antibiotic
N	P0A4A6	DR	structural constituent of ribosome
N	P0A4L9	CC	DNA gyrase negatively supercoils closed circular double- stranded DNA in an ATP-dependent manner and also catalyzes the interconversion of other topological isomers of double-stranded DNA rings, including catenanes and knotted rings
N	P0A4L9	DE	DNA gyrase subunit B;
N	P0A4L9	DR	DNA topoisomerase (ATP-hydrolyzing) activity
N	P0A4L9	DR	DNA topological change
N	P0A4L9	DR	response to antibiotic
N	P0A4V5	CC	Proteins of the antigen 85 complex are responsible for the high affinity of mycobacteria to fibronectin
N	P0A4V5	CC	Possesses a mycolyltransferase activity required for the biogenesis of trehalose dimycolate (cord factor), a dominant structure necessary for maintaining cell wall integrity (By similarity).
N	P0A4V5	DE	Antigen 85 complex C;
N	P0A4V5	DE	Antigen 85-C;
N	P0A4V5	DE	Fibronectin-binding protein C;
N	P0A4V5	DE	Mycolyl transferase 85C;
N	P0A4V5	DR	acyltransferase activity
N	P0A5E5	DE	Uncharacterized protein Rv1291c/MT1330;
N	P0A5V5	DE	50S ribosomal protein L17;
N	P0A5V5	DR	structural constituent of ribosome
N	P0A5W0	DE	50S ribosomal protein L33 1;
N	P0A5W0	DR	structural constituent of ribosome
N	P0A5Z4	CC	Member of the two-component regulatory system mtrA/mtrB.
N	P0A5Z4	DE	DNA-binding response regulator mtrA;
N	P0A5Z4	DR	growth of symbiont in host
N	P0A5Z4	DR	protein phosphorylation
N	P0A5Z4	DR	regulation of transcription, DNA-dependent
N	P0A5Z4	DR	two-component response regulator activity
N	P0A6N8	DE	Elongation factor P-like protein;
N	P0A6N8	DR	peptide biosynthetic process
N	P0A6N8	DR	translation elongation factor activity
N	P0A6P3	CC	Associates with the EF-Tu.GDP complex and induces the exchange of GDP to GTP
N	P0A6P3	CC	It remains bound to the aminoacyl-tRNA.EF- Tu.GTP complex up to the GTP hydrolysis stage on the ribosome.
N	P0A6P3	DE	Elongation factor Ts;
N	P0A6P3	DR	translation elongation factor activity
N	P0A7D2	CC	The natural substrate for this enzyme may be peptidyl- tRNAs which drop off the ribosome during protein synthesis (By similarity).
N	P0A7D2	DE	Peptidyl-tRNA hydrolase;
N	P0A7D2	DR	aminoacyl-tRNA hydrolase activity
N	P0A7J1	CC	Catalyzes the conversion of D-ribulose 5-phosphate to formate and 3,4-dihydroxy-2-butanone 4-phosphate (By similarity).
N	P0A7J1	DE	3,4-dihydroxy-2-butanone 4-phosphate synthase;
N	P0A7J1	DE	DHBP synthase;
N	P0A7J1	DR	3,4-dihydroxy-2-butanone-4-phosphate synthase activity
N	P0A7J1	DR	metal ion binding
N	P0A7J1	DR	riboflavin biosynthetic process
N	P0A837	DE	Succinyl-CoA ligase [ADP-forming] subunit beta;
N	P0A837	DE	Succinyl-CoA synthetase subunit beta;
N	P0A837	DR	metal ion binding
N	P0A837	DR	succinate-CoA ligase (ADP-forming) activity
N	P0A837	DR	tricarboxylic acid cycle
N	P0A8B1	CC	Required for maturation of 30S ribosomal subunits (By similarity).
N	P0A8B1	DE	Ribosome maturation factor rimP;
N	P0A8B1	DR	ribosome biogenesis
N	P0A8C4	DE	UPF0149 protein ygfB;
N	P0A8C6	DE	UPF0149 protein ygfB;

N	P0A8I9	CC	Specifically methylates the pseudouridine at position 1915 (m3Psi1915) in 23S rRNA (By similarity).
N	P0A8I9	DE	23S rRNA m3Psi1915 methyltransferase;
N	P0A8I9	DE	Ribosomal RNA large subunit methyltransferase H;
N	P0A8I9	DE	rRNA (pseudouridine-N3-)-methyltransferase rlmH;
N	P0A8I9	DR	methyltransferase activity
N	P0A8I9	DR	rRNA methylation
N	P0A8Y9	CC	Displays esterase activity toward palmitoyl-CoA and pNP- butyrate (By similarity).
N	P0A8Y9	DE	Esterase YbdB;
N	P0A8Y9	DR	hydrolase activity
N	P0A914	CC	Thought to play a role in bacterial envelope integrity
N	P0A914	CC	Very strongly associated with the peptidoglycan (By similarity).
N	P0A914	DE	Peptidoglycan-associated lipoprotein;
N	P0A9H1	CC	Excises ethenocytosine and uracil, which can arise by alkylation or deamination of cytosine, respectively, from the corresponding mispairs with guanine in ds-DNA
N	P0A9H1	CC	It is capable of hydrolyzing the carbon-nitrogen bond between the sugar-phosphate backbone of the DNA and the mispaired base
N	P0A9H1	CC	Required for DNA damage lesion repair in stationary-phase cells.
N	P0A9H1	CC	The complementary strand guanine functions in substrate recognition
N	P0A9H1	DE	Double-strand-specific uracil glycosylase;
N	P0A9H1	DE	G/U mismatch-specific DNA glycosylase;
N	P0A9H1	DE	Mismatch-specific uracil DNA-glycosylase;
N	P0A9H1	DR	base-excision repair, AP site formation
N	P0A9H1	DR	pyrimidine-specific mismatch base pair DNA N-glycosylase activity
N	P0A9Y2	CC	Binds to and stimulates the transcription of the CCAAT- containing, cold-shock-inducible promoters of the H-NS and GyrA proteins
N	P0A9Y2	CC	Binds also to the inverted repeat 5'-ATTGG-3' (By similarity).
N	P0A9Y2	DE	7.4 kDa cold shock protein;
N	P0A9Y2	DE	Cold shock protein CspA;
N	P0A9Y2	DR	regulation of transcription, DNA-dependent
N	P0A9Y2	DR	response to stress
N	P0AB41	CC	May be involved in the regulation of biofilm formation (By similarity).
N	P0AB41	DE	Multiple stress resistance protein BhsA;
N	P0AB69	CC	The transhydrogenation between NADH and NADP is coupled to respiration and ATP hydrolysis and functions as a proton pump across the membrane (By similarity).
N	P0AB69	DE	NAD(P) transhydrogenase subunit beta;
N	P0AB69	DE	Nicotinamide nucleotide transhydrogenase subunit beta;
N	P0AB69	DE	Pyridine nucleotide transhydrogenase subunit beta;
N	P0AB69	DR	NAD(P)+ transhydrogenase (AB-specific) activity
N	P0AB69	DR	oxidation-reduction process
N	P0ABG3	DE	CDP-DAG synthase;
N	P0ABG3	DE	CDP-DG synthase;
N	P0ABG3	DE	CDP-diacylglycerol synthase;
N	P0ABG3	DE	CDP-diglyceride pyrophosphorylase;
N	P0ABG3	DE	CDP-diglyceride synthase;
N	P0ABG3	DE	CTP:phosphatidate cytidylyltransferase;
N	P0ABG3	DE	Phosphatidate cytidylyltransferase;
N	P0ABG3	DR	phosphatidate cytidylyltransferase activity
N	P0ABG3	DR	phospholipid biosynthetic process
N	P0ABT2	CC	During stationary phase, binds the chromosome non- specifically, forming a highly ordered and stable dps-DNA co- crystal within which chromosomal DNA is condensed and protected from diverse damages
N	P0ABT2	CC	Also shows a weak catalase activity.
N	P0ABT2	CC	Dps also protects the cell from UV and gamma irradiation, iron and copper toxicity, thermal stress and acid and base shocks
N	P0ABT2	CC	It protects DNA from oxidative damage by sequestering intracellular Fe(2+) ion and storing it in the form of Fe(3+) oxyhydroxide mineral, which can be released after reduction
N	P0ABT2	CC	One hydrogen peroxide oxidizes two Fe(2+) ions, which prevents hydroxyl radical production by the Fenton reaction
N	P0ABT2	DE	DNA protection during starvation protein;
N	P0ABT2	DR	cellular iron ion homeostasis
N	P0ABT2	DR	chromosome condensation
N	P0ABT2	DR	ferric iron binding
N	P0ABT2	DR	oxidation-reduction process

N	P0ABT2	DR	oxidoreductase activity, oxidizing metal ions
N	P0ABT2	DR	protein binding
N	P0ABT2	DR	response to stress
N	P0AC21	CC	Catalyzes the epimerization of carbon 2' of the side chain of dihydroneopterin triphosphate (H2NTP) to form dihydromonapterin triphosphate (H2MTP) (By similarity)
N	P0AC21	DE	D-erythro-7,8-dihydroneopterin triphosphate epimerase;
N	P0AC21	DE	Dihydroneopterin triphosphate 2'-epimerase;
N	P0AC21	DR	dihydroneopterin aldolase activity
N	P0AC21	DR	folic acid-containing compound metabolic process
N	P0AC21	DR	isomerase activity
N	P0AC30	CC	Not known
N	P0AC30	CC	Is coded in an operon essential for cell division.
N	P0AC30	DE	Cell division protein ftsX;
N	P0AD19	DE	Inner membrane protein yohK;
N	P0ADL5	DE	Uncharacterized protein yicN;
N	P0ADT2	DE	UPF0441 protein ygiB;
N	P0ADU2	CC	Can oxidize menadiol to menadione.
N	P0ADU2	DE	Probable quinol monooxygenase ygiN;
N	P0ADU2	DR	antibiotic biosynthetic process
N	P0ADU2	DR	oxidation-reduction process
N	P0ADU2	DR	oxidoreductase activity
N	P0ADU2	DR	protein binding
N	P0AE22	CC	Dephosphorylates several organic phosphomonoesters and catalyzes the transfer of low-energy phosphate groups from phosphomonoesters to hydroxyl groups of various organic compounds
N	P0AE22	CC	Might function as a broad-spectrum dephosphorylating enzyme able to scavenge both 3'- and 5'-nucleotides and also additional organic phosphomonoesters.
N	P0AE22	CC	Preferentially acts on aryl phosphoesters
N	P0AE22	DE	Class B acid phosphatase;
N	P0AE22	DR	acid phosphatase activity
N	P0AE22	DR	metal ion binding
N	P0AE22	DR	protein binding
N	P0AEA5	CC	Converts heme B (protoheme IX) to heme O by substitution of the vinyl group on carbon 2 of heme B porphyrin ring with a hydroxyethyl farnesyl side group.
N	P0AEA5	DE	Heme B farnesyltransferase;
N	P0AEA5	DE	Heme O synthase;
N	P0AEA5	DE	Protoheme IX farnesyltransferase;
N	P0AEA5	DR	heme O biosynthetic process
N	P0AEA5	DR	protoheme IX farnesyltransferase activity
N	P0AEW6	DE	Inosine-guanosine kinase;
N	P0AEW6	DR	hydrolase activity, acting on glycosyl bonds
N	P0AEW6	DR	inosine kinase activity
N	P0AF89	DE	Uncharacterized protein YjfY;
N	P0AFE3	CC	NDH-1 shuttles electrons from NADH, via FMN and iron- sulfur (Fe-S) centers, to quinones in the respiratory chain
N	P0AFE3	CC	Couples the redox reaction to proton translocation (for every two electrons transferred, four hydrogen ions are translocated across the cytoplasmic membrane), and thus conserves the redox energy in a proton gradient (By similarity)
N	P0AFE3	CC	The immediate electron acceptor for the enzyme in this species is believed to be ubiquinone
N	P0AFE3	DE	NADH dehydrogenase I subunit J;
N	P0AFE3	DE	NADH-quinone oxidoreductase subunit J;
N	P0AFE3	DE	NDH-1 subunit J;
N	P0AFE3	DR	NADH dehydrogenase (ubiquinone) activity
N	P0AFE3	DR	oxidation-reduction process
N	P0AFE3	DR	quinone binding
N	P0AFG1	CC	Influences transcription termination and antitermination
N	P0AFG1	CC	Acts as a component of the transcription complex, and interacts with the termination factor rho and RNA polymerase (By similarity).
N	P0AFG1	DE	Transcription antitermination protein nusG;
N	P0AFG1	DR	positive regulation of transcription elongation from RNA polymerase II promoter
N	P0AFG1	DR	transcription antitermination
N	P0AFG1	DR	transcription elongation regulator activity
N	P0AFG1	DR	transcription termination, DNA-dependent

N	P0AFK1	CC	May facilitate the secretion of the peptide antibiotic microcin B17 (MccB17) by completing its maturation
N	P0AFK1	CC	Suppresses the inhibitory activity of the carbon storage regulator (CsrA) (By similarity)
N	P0AFK1	DE	Protein pmbA;
N	P0AG21	CC	In eubacteria ppGpp (guanosine 3'-diphosphate 5-' diphosphate) is a mediator of the stringent response that coordinates a variety of cellular activities in response to changes in nutritional abundance
N	P0AG21	CC	This enzyme catalyzes the formation of pppGpp which is then hydrolyzed to form ppGpp (By similarity).
N	P0AG21	DE	(p)ppGpp synthase;
N	P0AG21	DE	ATP:GTP 3'-pyrophosphotransferase;
N	P0AG21	DE	GTP pyrophosphokinase;
N	P0AG21	DE	ppGpp synthase I;
N	P0AG21	DR	GTP diphosphokinase activity
N	P0AG21	DR	amino acid binding
N	P0AG21	DR	guanosine tetraphosphate metabolic process
N	P0AG21	DR	kinase activity
N	P0AGF8	CC	Acts on both serine and threonine, and properly considered as a hydroxy amino acid deaminase (By similarity).
N	P0AGF8	DE	Threonine deaminase;
N	P0AGF8	DE	Threonine dehydratase catabolic;
N	P0AGF8	DR	L-threonine ammonia-lyase activity
N	P0AGF8	DR	cellular amino acid metabolic process
N	P0AGF8	DR	pyridoxal phosphate binding
N	P0AGN0	CC	Specific, proton motive force-dependent high-affinity transporter for xanthine (By similarity).
N	P0AGN0	DE	Xanthine permease XanP;
N	P0AGN0	DR	transmembrane transport
N	P0AGN0	DR	transporter activity
N	P0C063	CC	This protein is a multifunctional enzyme, able to activate and polymerize the amino acids Pro, Val, Orn and Leu
N	P0C063	CC	Activation sites for these AA consist of individual domains.
N	P0C063	DE	ATP-dependent leucine adenylase;
N	P0C063	DE	ATP-dependent ornithine adenylase;
N	P0C063	DE	ATP-dependent proline adenylase;
N	P0C063	DE	ATP-dependent valine adenylase;
N	P0C063	DE	Gramicidin S synthase 2;
N	P0C063	DE	Gramicidin S synthase II;
N	P0C063	DE	Leucine activase;
N	P0C063	DE	Ornithine activase;
N	P0C063	DE	Proline activase;
N	P0C063	DE	Valine activase;
N	P0C063	DR	acyl carrier activity
N	P0C063	DR	antibiotic biosynthetic process
N	P0C063	DR	cofactor binding
N	P0C063	DR	hydrolase activity, acting on ester bonds
N	P0C063	DR	ligase activity
N	P0C063	DR	phosphopantetheine binding
N	P0C0A9	CC	Overexpression causes the formation of large vacuoles that seemed to be derived from the endoplasmic reticulum.
N	P0C0A9	DE	Small VCP/p97-interacting protein;
N	P0C0D3	CC	This protein binds specifically to 23S rRNA; its binding is stimulated by other ribosomal proteins, e.g., L4, L17, and L20
N	P0C0D3	CC	It is important during the early stages of 50S assembly
N	P0C0D3	CC	It makes multiple contacts with different domains of the 23S rRNA in the assembled 50S subunit and ribosome (By similarity).
N	P0C0D3	DE	50S ribosomal protein L22;
N	P0C0D3	DR	structural constituent of ribosome
N	P0C0M3	CC	May catalyze the synthesis of indole-3-acetic acid (IAA)-amino acid conjugates, providing a mechanism for the plant to cope with the presence of excess auxin (By similarity).
N	P0C0M3	DE	Auxin-responsive GH3-like protein 11;
N	P0C0M3	DE	Probable indole-3-acetic acid-amido synthetase GH3.11;
N	P0C0M3	DR	ligase activity
N	P0C0M3	DR	response to auxin stimulus

N	P0C0M3	DR	response to light stimulus
N	P0C0P9	CC	Cysteine protease able to cleave elastin, insulin, myoglobin, fibronectin, fibrinogen, HMW-kininogen, alpha-1- protease inhibitor and alpha-1-antitrypsin
N	P0C0P9	CC	Along with other extracellular proteases may contribute to the colonization and infection of human tissues.
N	P0C0P9	DE	Extracellular cysteine protease;
N	P0C0P9	DR	cysteine-type endopeptidase activity
N	P0C0Y3	CC	Binds to actin and affects the structure of the cytoskeleton
N	P0C0Y3	CC	At high concentrations, profilin prevents the polymerization of actin, whereas it enhances it at low concentrations
N	P0C0Y3	CC	By binding to PIP2, it inhibits the formation of IP3 and DG (By similarity).
N	P0C0Y3	DE	Fra a 4;
N	P0C0Y3	DR	actin binding
N	P0C0Y3	DR	actin cytoskeleton organization
N	P0C174	CC	Not toxic to mouse.
N	P0C174	DE	Peptide TsPep1;
N	P0C193	CC	Prevents misfolding and promotes the refolding and proper assembly of unfolded polypeptides generated under stress conditions (By similarity).
N	P0C193	DE	60 kDa chaperonin;
N	P0C193	DE	GroEL protein;
N	P0C193	DE	Protein Cpn60;
N	P0C193	DR	protein refolding
N	P0C1A5	CC	Involved in maceration and soft-rotting of plant tissue
N	P0C1A5	CC	Pectate lyases have been implicated as pathogenicity factors which induce maceration or rotting of plant tissue
N	P0C1A5	CC	PelE is sufficient to induce these effects under laboratory conditions.
N	P0C1A5	DE	Pectate lyase E;
N	P0C1A5	DR	metal ion binding
N	P0C1A5	DR	pectate lyase activity
N	P0C1U8	CC	Preferentially cleaves peptide bonds on the carboxyl- terminal side of aspartate and glutamate
N	P0C1U8	CC	Along with other extracellular proteases it is involved in colonization and infection of human tissues
N	P0C1U8	CC	It is the most important protease for degradation of fibronectin-binding protein (FnBP) and surface protein A, which are involved in adherence to host cells
N	P0C1U8	CC	May also protect bacteria against host defense mechanism by cleaving the immunoglobulin classes IgG, IgA and IgM
N	P0C1U8	CC	May be involved in the stability of secreted lipases.
N	P0C1U8	CC	Required for proteolytic maturation of thiol protease sspB and inactivation of sspC, an inhibitor of sspB
N	P0C1U8	DE	Endoproteinase Glu-C;
N	P0C1U8	DE	Glutamyl endopeptidase;
N	P0C1U8	DE	Staphylococcal serine proteinase;
N	P0C1U8	DE	V8 protease;
N	P0C1U8	DE	V8 proteinase;
N	P0C1U8	DR	serine-type endopeptidase activity
N	P0C1Y0	DE	Beta-galactosidase;
N	P0C1Y0	DR	beta-galactosidase activity
N	P0C1Y0	DR	carbohydrate binding
N	P0C1Y0	DR	carbohydrate metabolic process
N	P0C1Y0	DR	cation binding
N	P0C2D5	CC	Catalyzes an oxidative deamination of predominantly hydrophobic and aromatic L-amino acids
N	P0C2D5	CC	Has an ability to induce hemolysis and apoptosis.
N	P0C2D5	CC	Has an ability to induce hemorrhage
N	P0C2D5	CC	Has an antibacterial activity (By similarity)
N	P0C2D5	CC	Inhibits platelet aggregation
N	P0C2D5	DE	Apoxin I-like protein;
N	P0C2D5	DE	L-amino-acid oxidase;
N	P0C2D5	DR	L-amino-acid oxidase activity
N	P0C2D5	DR	blood coagulation
N	P0C2D5	DR	defense response to bacterium
N	P0C2D5	DR	hemolysis in other organism
N	P0C2D5	DR	oxidation-reduction process
N	P0C2P9	DE	Truncated 4.9 kDa accessory protein;

N	P0C2P9	DE	Truncated non-structural protein of 4.9 kDa;
N	P0C2P9	DE	Truncated ns4.9;
N	P0C308	DE	Uncharacterized protein ycf72;
N	P0C498	CC	DNA-dependent RNA polymerase catalyzes the transcription of DNA into RNA using the four ribonucleoside triphosphates as substrates.
N	P0C498	DE	DNA-directed RNA polymerase subunit alpha;
N	P0C498	DE	Plastid-encoded RNA polymerase subunit alpha;
N	P0C498	DE	RNA polymerase subunit alpha;
N	P0C498	DR	DNA-directed RNA polymerase activity
N	P0C498	DR	protein dimerization activity
N	P0C5Y9	CC	Atypical histone H2A which can replace conventional H2A in some nucleosomes
N	P0C5Y9	CC	DNA accessibility is regulated via a complex set of post-translational modifications of histones, also called histone code, and nucleosome remodeling
N	P0C5Y9	CC	Histones thereby play a central role in transcription regulation, DNA repair, DNA replication and chromosomal stability
N	P0C5Y9	CC	Nucleosomes containing this histone are less rigid and organize only 118 base pair of DNA instead of 147 in classical nucleosomes
N	P0C5Y9	CC	Nucleosomes wrap and compact DNA into chromatin, limiting DNA accessibility to the cellular machineries which require DNA as a template
N	P0C5Y9	CC	They are associated with transcriptionally active chromatin and excluded form Barr bodies.
N	P0C5Y9	DE	H2A Barr body-deficient;
N	P0C5Y9	DE	Histone H2A-Bbd type 1;
N	P0C5Y9	DR	nucleosome assembly
N	P0C601	CC	The 2-oxoglutarate dehydrogenase complex catalyzes the overall conversion of 2-oxoglutarate to succinyl-CoA and CO(2)
N	P0C601	CC	It contains multiple copies of three enzymatic components: 2- oxoglutarate dehydrogenase (E1), dihydrolipoamide succinyltransferase (E2) and lipoamide dehydrogenase (E3) (By similarity).
N	P0C601	DE	2-oxoglutarate dehydrogenase E1 component;
N	P0C601	DE	Alpha-ketoglutarate dehydrogenase;
N	P0C601	DR	oxidation-reduction process
N	P0C601	DR	oxoglutarate dehydrogenase (succinyl-transferring) activity
N	P0C601	DR	thiamine pyrophosphate binding
N	P0C6F2	CC	Capsid protein p24 forms the conical core that encapsulates the genomic RNA-nucleocapsid complex in the virion
N	P0C6F2	CC	Gag-Pol polyprotein and Gag polyprotein may regulate their own translation, by the binding genomic RNA in the 5'-UTR
N	P0C6F2	CC	Integrase catalyzes viral DNA integration into the host chromosome, by performing a series of DNA cutting and joining reactions
N	P0C6F2	CC	The aspartyl protease mediates proteolytic cleavages of Gag and Gag-Pol polyproteins during or shortly after the release of the virion from the plasma membrane
N	P0C6F2	CC	A Y-shaped, gapped, recombination intermediate results, with the 5'-ends of the viral DNA strands and the 3' ends of target DNA strands remaining unjoined, flanking a gap of 5 bp
N	P0C6F2	CC	Also cleaves Nef and Vif, probably concomitantly with viral structural proteins on maturation of virus particles (By similarity).
N	P0C6F2	CC	Alternatively, Integrase may catalyze the excision of viral DNA just after strand transfer, this is termed disintegration (By similarity).
N	P0C6F2	CC	At low concentration, Gag-Pol and Gag would promote translation, whereas at high concentration, the polyproteins encapsidate genomic RNA and then shutt off translation (By similarity).
N	P0C6F2	CC	Cleavages take place as an ordered, step-wise cascade to yield mature proteins
N	P0C6F2	CC	Displays maximal activity during the budding process just prior to particle release from the cell
N	P0C6F2	CC	In the second step, the PIC enters cell nucleus
N	P0C6F2	CC	In the third step, termed strand transfer, the integrase protein joins the previously processed 3' ends to the 5' ends of strands of target cellular DNA at the site of integration
N	P0C6F2	CC	Interaction with human PPIA/CYPA protects the virus from restriction by human TRIM5-alpha and from an unknown antiviral activity in human cells
N	P0C6F2	CC	Most core are conical, with only 7% tubular
N	P0C6F2	CC	Since this process occurs at both cuts flanking the HIV genome, a 5 bp duplication of host DNA is produced at the ends of HIV-1 integration
N	P0C6F2	CC	The 5'-ends are produced by integrase-catalyzed staggered cuts, 5 bp apart

N	P0C6F2	CC	The core is constituted by capsid protein hexamer subunits
N	P0C6F2	CC	The core is dissassembled soon after virion entry
N	P0C6F2	CC	The first step in the integration process is 3' processing
N	P0C6F2	CC	The integrase protein removes 2 nucleotides from each 3' end of the viral DNA, leaving recessed CA OH's at the 3' ends
N	P0C6F2	CC	The last step is viral DNA integration into host chromosome
N	P0C6F2	CC	This ability to enter the nucleus is specific of lentiviruses, other retroviruses cannot and rely on cell division to access cell chromosomes
N	P0C6F2	CC	This capsid restriction by TRIM5 is one of the factors which restricts HIV-1 to the human species (By similarity).
N	P0C6F2	CC	This complex is called the pre- integration complex (PIC)
N	P0C6F2	CC	This enzyme activity takes place after virion entry into a cell and reverse transcription of the RNA genome in dsDNA
N	P0C6F2	CC	This involves host DNA repair synthesis in which the 5 bp gaps between the unjoined strands are filled in and then ligated
N	P0C6F2	CC	This process is called maturation
N	P0C6F2	CC	This process is mediated through integrase and Vpr proteins, and allows the virus to infect a non dividing cell
N	P0C6F2	CC	This step requires a complex comprising the viral genome, matrix protein, Vpr and integrase
N	P0C6F2	DE	Capsid protein p24;
N	P0C6F2	DE	Exoribonuclease H;
N	P0C6F2	DE	Gag-Pol polyprotein;
N	P0C6F2	DE	Integrase;
N	P0C6F2	DE	Matrix protein p17;
N	P0C6F2	DE	Nucleocapsid protein p7;
N	P0C6F2	DE	Pr160Gag-Pol;
N	P0C6F2	DE	Retropepsin;
N	P0C6F2	DE	Reverse transcriptase/ribonuclease H;
N	P0C6F2	DE	Spacer peptide p2;
N	P0C6F2	DE	Transframe peptide;
N	P0C6F2	DR	DNA integration
N	P0C6F2	DR	DNA recombination
N	P0C6F2	DR	DNA-directed DNA polymerase activity
N	P0C6F2	DR	RNA-dependent DNA replication
N	P0C6F2	DR	RNA-directed DNA polymerase activity
N	P0C6F2	DR	aspartic-type endopeptidase activity
N	P0C6F2	DR	exoribonuclease H activity
N	P0C6F2	DR	integrase activity
N	P0C6F2	DR	ribonuclease H activity
N	P0C6F2	DR	structural molecule activity
N	P0C6F2	DR	viral procapsid maturation
N	P0C6F2	DR	zinc ion binding
N	P0C6G1	CC	Pathogenicity determinant (By similarity)
N	P0C6G1	CC	May act as a suppressor of RNA-mediated gene silencing, also known as post-transcriptional gene silencing (PTGS), a mechanism of plant viral defense that limits the accumulation of viral RNAs.
N	P0C6G1	DE	Protein AC4;
N	P0C6G1	DE	Protein AL4;
N	P0C6R7	CC	Inhibits ADP-induced human platelet aggregation.
N	P0C6R7	DE	Disintegrin pyramidin-A;
N	P0C6R7	DR	blood coagulation
N	P0C6R7	DR	cell adhesion
N	P0C723	CC	Enhances the ability of BRLF1 to induce lytic infection by cooperating with it to transcriptionally activate the BZLF1 promoter (By similarity).
N	P0C723	DE	Transcriptional activator BRRF1;
N	P0C7K6	CC	May serve as a metalloproteinase inhibitor during glandular storage
N	P0C7K6	CC	Their inhibition may be instantly disengaged, by dilution or physiochemical change, when venom is injected into tissue of the victim (By similarity).
N	P0C7K6	DE	Poly-His-poly-Gly peptide 2;
N	P0C7K6	DR	metalloendopeptidase inhibitor activity
N	P0C7Q1	DE	Coiled-coil domain-containing protein 153;
N	P0C7R5	CC	Catalyzes the reversible oxidation of malate to oxaloacetate (By similarity).
N	P0C7R5	DE	Malate dehydrogenase;
N	P0C7R5	DR	L-malate dehydrogenase activity

N	P0C7R5	DR	cellular carbohydrate metabolic process
N	P0C7R5	DR	malate metabolic process
N	P0C7R5	DR	tricarboxylic acid cycle
N	P0C7X9	CC	Peptide which can recruit, activate and subsequently lyse human neutrophils, thus eliminating the main cellular defense against infection (By similarity).
N	P0C7X9	DE	Phenol-soluble modulin alpha 1 peptide;
N	P0C7Y5	CC	Peptide which can recruit, activate and subsequently lyse human neutrophils, thus eliminating the main cellular defense against infection (By similarity).
N	P0C7Y5	DE	Phenol-soluble modulin alpha 1 peptide;
N	P0C7Y8	CC	Peptide which can recruit, activate and subsequently lyse human neutrophils, thus eliminating the main cellular defense against infection (By similarity).
N	P0C7Y8	DE	Phenol-soluble modulin alpha 1 peptide;
N	P0C867	DE	Putative uncharacterized protein LOC100130201;
N	P0C9E4	DE	Viral histone-like protein A104R;
N	P0C9F1	CC	Plays a role in virus cell tropism, and may be required for efficient virus replication in macrophages (By similarity).
N	P0C9F1	DE	Protein MGF 100-1R;
N	P0C9L1	CC	Plays a role in virus cell tropism, and may be required for efficient virus replication in macrophages (By similarity).
N	P0C9L1	DE	Protein MGF 300-2R;
N	P0C9N0	CC	Plays a role in virus cell tropism, and may be required for efficient virus replication in macrophages (By similarity).
N	P0C9N0	DE	Protein MGF 360-4L;
N	P0C9Q8	CC	Plays a role in virus cell tropism, and may be required for efficient virus replication in macrophages (By similarity).
N	P0C9Q8	DE	Protein MGF 360-15R;
N	P0CA35	DE	Uncharacterized protein B125R;
N	P0CAE3	DE	Transmembrane protein I329L;
N	P0CAL5	DE	Transmembrane protein EP84R;
N	P0CAT2	CC	Mitochondrial carrier required during erythropoiesis
N	P0CAT2	CC	May act by importing glycine into mitochondria or by exchanging glycine for ALA across the mitochondrial inner membrane (By similarity).
N	P0CAT2	CC	Probably involved in the biosynthesis of heme, possibly by facilitating 5-aminolevulinate (ALA) production
N	P0CAT2	DE	Solute carrier family 25 member 38-B;
N	P0CAT2	DR	erythrocyte differentiation
N	P0CAT2	DR	transmembrane transport
N	P0CB88	CC	Accessory subunit of the mitochondrial membrane respiratory chain NADH dehydrogenase (Complex I), that is believed not to be involved in catalysis
N	P0CB88	CC	Complex I functions in the transfer of electrons from NADH to the respiratory chain
N	P0CB88	CC	The immediate electron acceptor for the enzyme is believed to be ubiquinone (By similarity).
N	P0CB88	DE	Complex I-15 kDa;
N	P0CB88	DE	NADH dehydrogenase [ubiquinone] iron-sulfur protein 5;
N	P0CB88	DE	NADH-ubiquinone oxidoreductase 15 kDa subunit;
N	P0CB88	DR	electron transport chain
N	P0CB88	DR	mitochondrial respiratory chain complex I assembly
N	P0CB94	CC	Accessory subunit of the mitochondrial membrane respiratory chain NADH dehydrogenase (Complex I), that is believed not to be involved in catalysis
N	P0CB94	CC	Complex I functions in the transfer of electrons from NADH to the respiratory chain
N	P0CB94	CC	The immediate electron acceptor for the enzyme is believed to be ubiquinone (By similarity).
N	P0CB94	DE	Complex I-B17;
N	P0CB94	DE	NADH dehydrogenase [ubiquinone] 1 beta subcomplex subunit 6;
N	P0CB94	DE	NADH-ubiquinone oxidoreductase B17 subunit;
N	P0CB94	DR	electron transport chain
N	P0CC20	CC	NDH shuttles electrons from NAD(P)H:plastoquinone, via FMN and iron-sulfur (Fe-S) centers, to quinones in the photosynthetic chain and possibly in a chloroplast respiratory chain
N	P0CC20	CC	Couples the redox reaction to proton translocation, and thus conserves the redox energy in a proton gradient (By similarity).
N	P0CC20	CC	The immediate electron acceptor for the enzyme in this species is believed to be plastoquinone
N	P0CC20	DE	NAD(P)H dehydrogenase, subunit 2 A;
N	P0CC20	DE	NAD(P)H-quinone oxidoreductase subunit 2 A, chloroplastic;

N	P0CC20	DE	NADH-plastoquinone oxidoreductase subunit 2 A;
N	P0CC20	DR	ATP synthesis coupled electron transport
N	P0CC20	DR	NADH dehydrogenase (ubiquinone) activity
N	P0CC20	DR	quinone binding
N	P0CE31	CC	Alpha-conotoxins act on postsynaptic membranes, they bind to the nicotinic acetylcholine receptors (nAChR) and thus inhibit them
N	P0CE31	CC	This toxin specifically blocks mammalian neuronal nAChR of the alpha-7/CHRNA7, alpha-3-beta-2/CHRNA3-CHRNB2 and alpha-4-beta-2/CHRNA4-CHRNB2 subtypes (By similarity).
N	P0CE31	DE	Alpha-conotoxin-like Ml20.2;
N	P0CE31	DR	acetylcholine receptor inhibitor activity
N	P0CE31	DR	synaptic transmission
N	P0CF16	DE	UPF0507 protein YML003W;
N	P0CG40	CC	Transcription factor which plays a key role in limb development
N	P0CG40	CC	Positively regulates FGF8 expression in the apical ectodermal ridge (AER) and contributes to limb outgrowth in embryos (By similarity).
N	P0CG40	DE	Transcription factor Sp9;
N	P0CG40	DR	zinc ion binding
N	P0CG64	CC	Ubiquitin exists either covalently attached to another protein, or free (unanchored)
N	P0CG64	CC	Linear polymer chains formed via attachment by the initiator Met lead to cell signaling
N	P0CG64	CC	Polyubiquitin chains, when attached to a target protein, have different functions depending on the Lys residue of the ubiquitin that is linked: Lys-6-linked may be involved in DNA repair; Lys-11-linked is involved in ERAD (endoplasmic reticulum-associated degradation) and in cell-cycle regulation; Lys-29-linked is involved in lysosomal degradation; Lys-33-linked is involved in kinase modification; Lys-48-linked is involved in protein degradation via the proteasome; Lys-63-linked is involved in endocytosis, DNA-damage responses as well as in signaling processes leading to activation of the transcription factor NF-kappa-B
N	P0CG64	CC	Ubiquitin is usually conjugated to Lys residues of target proteins, however, in rare cases, conjugation to Cys or Ser residues has been observed
N	P0CG64	CC	When covalently bound, it is conjugated to target proteins via an isopeptide bond either as a monomer (monoubiquitin), a polymer linked via different Lys residues of the ubiquitin (polyubiquitin chains) or a linear polymer linked via the initiator Met of the ubiquitin (linear polyubiquitin chains)
N	P0CG64	CC	When polyubiquitin is free (unanchored-polyubiquitin), it also has distinct roles, such as in activation of protein kinases, and in signaling (By similarity).
N	P0CG64	DE	Polyubiquitin-C;
N	P0CG64	DE	Ubiquitin;
N	P0CH25	CC	Involved in oxygen transport from the lung to the various peripheral tissues (By similarity).
N	P0CH25	DE	Alpha-1-globin;
N	P0CH25	DE	Hemoglobin alpha-1 chain;
N	P0CH25	DE	Hemoglobin subunit alpha-1;
N	P0CH25	DR	oxygen binding
N	P0CH25	DR	oxygen transporter activity
N	P0CI23	CC	Inhibits the neuronal noradrenaline transporter (NET) (By similarity).
N	P0CI23	DE	Chi-conotoxin-like Ar1311;
N	P0CI41	CC	Omega-conotoxin that blocks voltage-gated calcium channels
N	P0CI41	CC	Does not modify the voltage dependency of activation nor of inactivation.
N	P0CI41	CC	Reversibly blocks calcium current in the dorsal-root ganglion neurons of the rat (high voltage-activated channels but not low voltage-activated channels)
N	P0CI41	DE	Conotoxin CalTx;
N	P0CI41	DE	Omega-conotoxin Cl15a;
N	P0CI41	DE	Omega-conotoxin Cl16a;
N	P0CI41	DR	calcium channel inhibitor activity
N	P0CL23	DE	Putative UPF0377 protein YIL175W;
N	P10219	CC	Participates in the assembly of the infectious particles by decorating the outer surface of the capsid shell and thus forming a layer between the capsid and the tegument
N	P10219	CC	Complexes composed of the capsid protein VP5 and VP26 assemble together in the host cytoplasm and are translocated to the nucleus, where they accumulate and participate in capsid assembly.
N	P10219	DE	Capsid protein VP26;
N	P10294	CC	Inhibits trypsin.
N	P10294	DE	Trypsin inhibitor 1;
N	P10294	DE	Trypsin inhibitor I;

N	P10294	DR	serine-type endopeptidase inhibitor activity
N	P10334	CC	This protein is transferred from male to female during mating and may affect egglaying and behavior after mating.
N	P10334	DE	Accessory gland-specific peptide 26Ab;
N	P10334	DE	Male accessory gland secretory protein 355B;
N	P10334	DR	mating behavior
N	P10432	DE	Putative uncharacterized immunity region protein 9;
N	P10493	CC	Sulfated glycoprotein widely distributed in basement membranes and tightly associated with laminin
N	P10493	CC	Also binds to collagen IV and perlecan
N	P10493	CC	It probably has a role in cell- extracellular matrix interactions.
N	P10493	DR	bioluminescence
N	P10493	DR	calcium ion binding
N	P10493	DR	cell-matrix adhesion
N	P10493	DR	protein binding
N	P10493	DR	protein-chromophore linkage
N	P10630	CC	ATP-dependent RNA helicase which is a subunit of the eIF4F complex involved in cap recognition and is required for mRNA binding to ribosome
N	P10630	CC	In the current model of translation initiation, eIF4A unwinds RNA secondary structures in the 5'-UTR of mRNAs which is necessary to allow efficient binding of the small ribosomal subunit, and subsequent scanning for the initiator codon.
N	P10630	DE	ATP-dependent RNA helicase eIF4A-2;
N	P10630	DE	Eukaryotic initiation factor 4A-II;
N	P10630	DR	ATP-dependent helicase activity
N	P10630	DR	response to protein stimulus
N	P10630	DR	translation initiation factor activity
N	P10937	CC	Converts noradrenaline to adrenaline.
N	P10937	DE	Noradrenaline N-methyltransferase;
N	P10937	DE	Phenylethanolamine N-methyltransferase;
N	P10937	DR	S-adenosylhomocysteine metabolic process
N	P10937	DR	S-adenosylmethionine metabolic process
N	P10937	DR	epinephrine biosynthetic process
N	P10937	DR	norepinephrine metabolic process
N	P10937	DR	phenylethanolamine N-methyltransferase activity
N	P11154	CC	Pyruvate carboxylase catalyzes a 2-step reaction, involving the ATP-dependent carboxylation of the covalently attached biotin in the first step and the transfer of the carboxyl group to pyruvate in the second.
N	P11154	DE	Pyruvate carboxylase 1;
N	P11154	DE	Pyruvic carboxylase 1;
N	P11154	DR	biotin carboxylase activity
N	P11154	DR	gluconeogenesis
N	P11154	DR	metal ion binding
N	P11154	DR	protein binding
N	P11154	DR	pyruvate carboxylase activity
N	P11610	CC	Antigen-presenting protein that binds self and non-self glycolipids and presents them to T-cell receptors on natural killer T-cells.
N	P11610	DE	Antigen-presenting glycoprotein CD1d2;
N	P11610	DE	CD1d.2;
N	P11610	DR	antigen processing and presentation
N	P11610	DR	innate immune response
N	P11610	DR	positive regulation of NK T cell activation
N	P11610	DR	positive regulation of T cell mediated cytotoxicity
N	P11610	DR	positive regulation of interleukin-2 biosynthetic process
N	P11610	DR	positive regulation of interleukin-4 biosynthetic process
N	P11680	CC	A positive regulator of the alternate pathway of complement
N	P11680	CC	It binds to and stabilizes the C3- and C5-convertase enzyme complexes.
N	P11680	DE	Complement factor P;
N	P11680	DR	complement activation, alternative pathway
N	P11708	DE	Cytosolic malate dehydrogenase;
N	P11708	DE	Malate dehydrogenase, cytoplasmic;
N	P11708	DR	L-malate dehydrogenase activity
N	P11708	DR	cellular carbohydrate metabolic process
N	P11708	DR	malate metabolic process
N	P11708	DR	tricarboxylic acid cycle
N	P11894	CC	Binds to the 23S rRNA (By similarity).

N	P11894	DE	50S ribosomal protein L9, chloroplastic;
N	P11894	DR	structural constituent of ribosome
N	P12004	CC	This protein is an auxiliary protein of DNA polymerase delta and is involved in the control of eukaryotic DNA replication by increasing the polymerase's processibility during elongation of the leading strand
N	P12004	CC	Has to be loaded onto DNA in order to be able to stimulate APEX2.
N	P12004	CC	Induces a robust stimulatory effect on the 3'- 5' exonuclease and 3'-phosphodiesterase, but not apurinic- apyrimidinic (AP) endonuclease, APEX2 activities
N	P12004	DE	Proliferating cell nuclear antigen;
N	P12004	DR	DNA polymerase processivity factor activity
N	P12004	DR	DNA strand elongation involved in DNA replication
N	P12004	DR	MutLalpha complex binding
N	P12004	DR	S phase of mitotic cell cycle
N	P12004	DR	cell proliferation
N	P12004	DR	dinucleotide insertion or deletion binding
N	P12004	DR	mismatch repair
N	P12004	DR	nucleotide-excision repair, DNA gap filling
N	P12004	DR	phosphatidylinositol-mediated signaling
N	P12004	DR	positive regulation of deoxyribonuclease activity
N	P12004	DR	purine-specific mismatch base pair DNA N-glycosylase activity
N	P12004	DR	regulation of DNA replication
N	P12004	DR	regulation of transcription involved in G1/S phase of mitotic cell cycle
N	P12004	DR	telomere maintenance via recombination
N	P12004	DR	telomere maintenance via semi-conservative replication
N	P12004	DR	transcription-coupled nucleotide-excision repair
N	P12004	DR	translesion synthesis
N	P12045	CC	Catalyzes the ATP-dependent conversion of 5- aminoimidazole ribonucleotide (AIR) and HCO(3)(-) to N5- carboxyaminoimidazole ribonucleotide (N5-CAIR) (By similarity)
N	P12045	DE	5-(carboxyamino)imidazole ribonucleotide synthetase;
N	P12045	DE	N5-CAIR synthase;
N	P12045	DE	N5-carboxyaminoimidazole ribonucleotide synthase;
N	P12045	DR	'de novo' IMP biosynthetic process
N	P12045	DR	5-(carboxyamino)imidazole ribonucleotide synthase activity
N	P12045	DR	pentose-phosphate shunt
N	P12045	DR	phosphogluconate dehydrogenase (decarboxylating) activity
N	P12045	DR	phosphoribosylaminoimidazole carboxylase activity
N	P12557	CC	Attaches the virus to sialic acid-containing cell receptors and thereby initiating infection
N	P12557	CC	Binding of HN protein to the receptor induces a conformational change that allows the F protein to trigger virion/cell membranes fusion (By similarity).
N	P12557	DE	Hemagglutinin-neuraminidase;
N	P12557	DR	exo-alpha-sialidase activity
N	P12557	DR	host cell surface receptor binding
N	P12557	DR	interspecies interaction between organisms
N	P12557	DR	viral infectious cycle
N	P12583	CC	Binds to sialic acid-containing receptors on the cell surface, bringing about the attachment of the virus particle to the cell
N	P12583	CC	Class I viral fusion protein
N	P12583	CC	Low pH in endosomes induces an irreversible conformational change in HA2, releasing the fusion hydrophobic peptide
N	P12583	CC	Plays a major role in the determination of host range restriction and virulence
N	P12583	CC	Responsible for penetration of the virus into the cell cytoplasm by mediating the fusion of the membrane of the endocytosed virus particle with the endosomal membrane
N	P12583	CC	Several trimers are required to form a competent fusion pore.
N	P12583	CC	This attachment induces virion internalization of about two third of the virus particles through clathrin-dependent endocytosis and about one third through a clathrin- and caveolin- independent pathway
N	P12583	DE	Hemagglutinin HA1 chain;
N	P12583	DE	Hemagglutinin HA2 chain;
N	P12583	DE	Hemagglutinin;
N	P12583	DR	host cell surface receptor binding
N	P12583	DR	viral envelope fusion with host membrane
N	P12601	CC	Binds and inhibits the ubiquitin-like protein G1P2/ISG15, which is an early antiviral protein
N	P12601	CC	Also binds poly(A) and U6 snRNA

N	P12601	CC	Inhibits IFN-beta promoter activation; this inhibition is not dsRNA-binding dependent Prevents EIF2AK2/PKR activation, either by binding double strand RNA or by interacting directly with EIF2AK2/PKR
N	P12601	CC	Inhibits IRF-3 nuclear translocation and activation
N	P12601	CC	Suppresses the RNA silencing-based antiviral response in Drosophila cells (By similarity).
N	P12601	DE	Non-structural protein 1;
N	P12601	DR	evasion by virus of host immune response
N	P12879	CC	One of the primary rRNA binding proteins, it binds directly to 16S rRNA central domain where it helps coordinate assembly of the platform of the 30S subunit (By similarity)
N	P12879	DE	30S ribosomal protein S8;
N	P12879	DR	structural constituent of ribosome
N	P13105	CC	Binds to actin filaments in muscle and non-muscle cells
N	P13105	CC	In non-muscle cells is implicated in stabilizing cytoskeleton actin filaments.
N	P13105	CC	Plays a central role, in association with the troponin complex, in the calcium dependent regulation of vertebrate striated muscle contraction
N	P13105	CC	Smooth muscle contraction is regulated by interaction with caldesmon
N	P13105	DE	Alpha-tropomyosin;
N	P13105	DE	Tropomyosin alpha-1 chain;
N	P13105	DE	Tropomyosin-1;
N	P13105	DR	actin binding
N	P13179	CC	Displays RNA-directed RNA polymerase, mRNA guanylyl transferase, mRNA (guanine-N(7)-)-methyltransferase and poly(A) synthetase activities
N	P13179	CC	Functions either as transcriptase or as replicase
N	P13179	CC	In this mode, the polymerase replicates the whole viral genome without recognizing the transcriptional signals (By similarity).
N	P13179	CC	The replicase mode is dependent on intracellular N protein concentration
N	P13179	CC	The template is composed of the viral RNA tightly encapsidated by the nucleoprotein (N)
N	P13179	CC	The transcriptase synthesizes subsequently five subgenomic RNAs, assuring their capping and polyadenylation by a stuttering mechanism
N	P13179	CC	The viral mRNA guanylyl transferase displays a different biochemical reaction than the cellular enzyme
N	P13179	DE	Large structural protein;
N	P13179	DE	RNA-directed RNA polymerase L;
N	P13179	DE	RNA-directed RNA polymerase;
N	P13179	DE	Transcriptase;
N	P13179	DE	mRNA (guanine-N(7)-)-methyltransferase;
N	P13179	DE	mRNA guanylyltransferase;
N	P13179	DR	RNA-directed RNA polymerase activity
N	P13179	DR	mRNA (guanine-N7-)-methyltransferase activity
N	P13393	CC	General transcription factor that functions at the core of the DNA-binding general transcription factor complex TFIID
N	P13393	CC	Binding of TFIID to a promoter (with or without TATA element) is the initial step in preinitiation complex (PIC) formation
N	P13393	CC	TFIID plays a key role in the regulation of gene expression by RNA polymerase II through different activities such as transcription activator interaction, core promoter recognition and selectivity, TFIIA and TFIIB interaction, chromatin modification (histone acetylation by TAF1), facilitation of DNA opening and initiation of transcription
N	P13393	DE	TATA sequence-binding protein;
N	P13393	DE	TATA-binding factor;
N	P13393	DE	TATA-box factor;
N	P13393	DE	TATA-box-binding protein;
N	P13393	DE	Transcription factor D;
N	P13393	DE	Transcription initiation factor TFIID TBP subunit;
N	P13393	DR	DNA bending activity
N	P13393	DR	RNA polymerase I transcription factor activity
N	P13393	DR	RNA polymerase II transcriptional preinitiation complex assembly
N	P13393	DR	RNA polymerase III transcription factor activity
N	P13393	DR	RNA polymerase III transcriptional preinitiation complex assembly
N	P13393	DR	chromatin binding
N	P13393	DR	general RNA polymerase II transcription factor activity
N	P13393	DR	general transcription from RNA polymerase II promoter
N	P13393	DR	promoter binding
N	P13393	DR	protein binding
N	P13393	DR	regulation of transcription, DNA-dependent

N	P13393	DR	transcription from RNA polymerase I promoter
N	P13393	DR	transposon integration
N	P13567	CC	The UvrABC repair system catalyzes the recognition and processing of DNA lesions
N	P13567	CC	A damage recognition complex composed of 2 UvrA and 2 UvrB subunits scans DNA for abnormalities
N	P13567	CC	UvrA is an ATPase and a DNA-binding protein
N	P13567	CC	When the presence of a lesion has been verified by UvrB, the UvrA molecules dissociate (By similarity).
N	P13567	DE	Excinuclease ABC subunit A;
N	P13567	DE	UvrA protein;
N	P13567	DE	UvrABC system protein A;
N	P13567	DR	ATPase activity
N	P13567	DR	excinuclease ABC activity
N	P13567	DR	metal ion binding
N	P13567	DR	nucleotide-excision repair
N	P13674	CC	Catalyzes the post-translational formation of 4- hydroxyproline in -Xaa-Pro-Gly- sequences in collagens and other proteins.
N	P13674	DE	4-PH alpha-1;
N	P13674	DE	Procollagen-proline,2-oxoglutarate-4-dioxygenase subunit alpha-1;
N	P13674	DE	Prolyl 4-hydroxylase subunit alpha-1;
N	P13674	DR	L-ascorbic acid binding
N	P13674	DR	iron ion binding
N	P13674	DR	oxidation-reduction process
N	P13674	DR	oxidoreductase activity, acting on single donors with incorporation of molecular oxygen, incorporation of two atoms of oxygen
N	P13674	DR	procollagen-proline 4-dioxygenase activity
N	P13726	CC	Initiates blood coagulation by forming a complex with circulating factor VII or VIIa
N	P13726	CC	TF plays a role in normal hemostasis by initiating the cell-surface assembly and propagation of the coagulation protease cascade.
N	P13726	CC	The [TF:VIIa] complex activates factors IX or X by specific limited protolysis
N	P13726	DE	CD142;
N	P13726	DE	Coagulation factor III;
N	P13726	DE	Thromboplastin;
N	P13726	DE	Tissue factor;
N	P13726	DR	activation of blood coagulation via clotting cascade
N	P13726	DR	activation of caspase activity
N	P13726	DR	activation of plasma proteins involved in acute inflammatory response
N	P13726	DR	anti-apoptosis
N	P13726	DR	blood coagulation, extrinsic pathway
N	P13726	DR	cell surface binding
N	P13726	DR	phospholipid binding
N	P13726	DR	positive regulation of angiogenesis
N	P13726	DR	positive regulation of endothelial cell proliferation
N	P13726	DR	positive regulation of platelet-derived growth factor receptor signaling pathway
N	P13726	DR	positive regulation of positive chemotaxis
N	P13726	DR	positive regulation of protein kinase B signaling cascade
N	P13726	DR	protease binding
N	P13770	CC	Catalyzes the recombination between the large inverted repetitions of the plasmid
N	P13770	DE	Recombinase Flp protein;
N	P13770	DR	DNA integration
N	P13770	DR	DNA recombination
N	P14106	CC	C1q associates with the proenzymes C1r and C1s to yield C1, the first component of the serum complement system
N	P14106	CC	The collagen-like regions of C1q interact with the Ca(2+)-dependent C1r(2)C1s(2) proenzyme complex, and efficient activation of C1 takes place on interaction of the globular heads of C1q with the Fc regions of IgG or IgM antibody present in immune complexes.
N	P14106	DE	Complement C1q subcomponent subunit B;
N	P14106	DR	complement activation, classical pathway
N	P14106	DR	innate immune response
N	P14106	DR	protein homodimerization activity
N	P14121	CC	This is one of 5 proteins that mediate the attachment of the 5S rRNA onto the large ribosomal subunit, stabilizing the orientation of adjacent RNA domains.
N	P14121	DE	50S ribosomal protein L30P;
N	P14121	DR	structural constituent of ribosome

N	P14132	DE	40S ribosomal protein 1024;
N	P14132	DE	40S ribosomal protein S9;
N	P14132	DE	Vegetative-specific protein V12;
N	P14132	DR	structural constituent of ribosome
N	P14247	CC	DNA-dependent RNA polymerase catalyzes the transcription of DNA into RNA using the four ribonucleoside triphosphates as substrates.
N	P14247	DE	DNA-directed RNA polymerase subunit A";
N	P14247	DR	DNA-directed RNA polymerase activity
N	P14269	CC	The role of the precursor might be to condense the viral prochromatin for encapsidation by virtue of the two basic domains
N	P14269	CC	Subsequent cleavage within the particle after residue 31 would release the cross-link and would prepare the viral chromatin for the relaxed conformation required during subsequent infection and uncoating
N	P14269	CC	The cleavage seems to be necessary for infectivity.
N	P14269	DE	11 kDa core protein;
N	P14269	DE	Late L2 mu core protein;
N	P14329	CC	Ribosomal protein that binds calmodulin.
N	P14329	DE	22 kDa calmodulin-binding protein;
N	P14329	DE	60S ribosomal protein L19;
N	P14329	DE	Vegetative-specific protein V14;
N	P14329	DR	calmodulin binding
N	P14329	DR	structural constituent of ribosome
N	P14400	DE	Electromotor neuron-associated protein 1;
N	P14465	CC	Fibrinogen has a double function: yielding monomers that polymerize into fibrin and acting as a cofactor in platelet aggregation.
N	P14465	DE	Fibrinogen beta chain;
N	P14465	DE	Fibrinopeptide B;
N	P14465	DR	blood coagulation
N	P14548	CC	Component of the ubiquinol-cytochrome c reductase complex (complex III or cytochrome b-c1 complex), which is a respiratory chain that generates an electrochemical potential coupled to ATP synthesis (By similarity).
N	P14548	DE	Complex III subunit 3;
N	P14548	DE	Complex III subunit III;
N	P14548	DE	Cytochrome b-c1 complex subunit 3;
N	P14548	DE	Cytochrome b;
N	P14548	DE	Ubiquinol-cytochrome-c reductase complex cytochrome b subunit;
N	P14548	DR	electron carrier activity
N	P14548	DR	metal ion binding
N	P14548	DR	oxidoreductase activity
N	P14548	DR	respiratory electron transport chain
N	P14553	CC	Capsid proteins VP1, VP2, and VP3 form a closed capsid enclosing the viral positive strand RNA genome
N	P14553	CC	Protein 2C associates with and induces structural rearrangements of intracellular membranes
N	P14553	CC	Protein 3B is covalently linked to the 5'-end of both the positive-strand and negative-strand genomic RNAs
N	P14553	CC	RNA-directed RNA polymerase 3D-POL replicates genomic and antigenomic RNA by recognizing replications specific signals (By similarity).
N	P14553	CC	The 3AB precursor interacts with the 3CD precursor and with RNA structures found at both the 5'- and 3'-termini of the viral genome
N	P14553	CC	VP1-2A precursor is a component of immature procapsids and corresponds to an extended form of the structural protein VP1
N	P14553	CC	All these proteins contain a beta-sheet structure called beta-barrel jelly roll
N	P14553	CC	It acts as a genome-linked replication primer (By similarity).
N	P14553	CC	It displays RNA- binding, nucleotide binding and NTPase activities (By similarity).
N	P14553	CC	It is proteolytically removed from the precursor by a host protease and does not seem to be found in mature particles (By similarity).
N	P14553	CC	Since the 3AB precursor contains the hydrophobic domain 3A, it probably anchors the whole viral replicase complex to intracellular membranes on which viral RNA synthesis occurs (By similarity).
N	P14553	CC	The C-terminal domain of VP1-2A, protein 2A, acts as an assembly signal that allows multimerization of VP1-2A and formation of pentamers of VP1-VP2-VP3 trimers
N	P14553	CC	The capsid interacts with HAVCR1 to provide virion attachment to target cell (By similarity).

N	P14553	CC	Together they form an icosahedral capsid (T=3) composed of 60 copies of each VP1, VP2, and VP3, with a diameter of approximately 300 Angstroms
N	P14553	CC	VP1 is situated at the 12 fivefold axes, whereas VP2 and VP3 are located at the quasi-sixfold axes
N	P14553	DE	Genome polyprotein;
N	P14553	DE	Picornain 3C;
N	P14553	DE	Protease 3C;
N	P14553	DE	Protein 2A;
N	P14553	DE	Protein 2B;
N	P14553	DE	Protein 2BC;
N	P14553	DE	Protein 2C;
N	P14553	DE	Protein 3A;
N	P14553	DE	Protein 3AB;
N	P14553	DE	Protein 3ABC;
N	P14553	DE	Protein 3ABCD;
N	P14553	DE	Protein 3B;
N	P14553	DE	Protein 3CD;
N	P14553	DE	Protein VP0;
N	P14553	DE	Protein VP1-2A;
N	P14553	DE	Protein VP1;
N	P14553	DE	Protein VP2;
N	P14553	DE	Protein VP3;
N	P14553	DE	Protein VP4;
N	P14553	DE	RNA-directed RNA polymerase 3D-POL;
N	P14553	DE	Virion protein 1;
N	P14553	DE	Virion protein 2;
N	P14553	DE	Virion protein 3;
N	P14553	DE	Virion protein 4;
N	P14553	DR	RNA helicase activity
N	P14553	DR	RNA-directed RNA polymerase activity
N	P14553	DR	RNA-protein covalent cross-linking
N	P14553	DR	cysteine-type endopeptidase activity
N	P14553	DR	evasion by virus of host immune response
N	P14553	DR	structural molecule activity
N	P14553	DR	viral genome replication
N	P14592	DE	24 kDa antigen;
N	P14669	CC	Inhibitor of phospholipase A2, also possesses anti- coagulant properties.
N	P14669	DE	35-alpha calcimedin;
N	P14669	DE	Annexin III;
N	P14669	DE	Lipocortin III;
N	P14669	DE	Placental anticoagulant protein III;
N	P14669	DR	calcium ion binding
N	P14669	DR	calcium-dependent phospholipid binding
N	P14669	DR	hippocampus development
N	P14669	DR	organ regeneration
N	P14669	DR	phospholipase A2 inhibitor activity
N	P14669	DR	positive regulation of DNA metabolic process
N	P14669	DR	response to glucocorticoid stimulus
N	P14669	DR	response to growth factor stimulus
N	P14870	CC	Recognizes the double-stranded sequences GGATG and CATCC and cleaves respectively 14 bases after G-1 and 13 bases before C- 1.
N	P14870	DE	Endonuclease FokI;
N	P14870	DE	Type II restriction enzyme FokI;
N	P14870	DE	Type IIS restriction enzyme FokI;
N	P14870	DE	Type-2 restriction enzyme FokI;
N	P14870	DR	DNA restriction-modification system
N	P14870	DR	Type II site-specific deoxyribonuclease activity
N	P14921	CC	Transcription factor.
N	P14921	DE	Protein C-ets-1;
N	P14921	DR	PML body organization
N	P14921	DR	RNA polymerase II transcription factor activity
N	P14921	DR	cell motility
N	P14921	DR	immune response
N	P14921	DR	induction of apoptosis
N	P14921	DR	negative regulation of cell cycle

N	P14921	DR	negative regulation of cell proliferation
N	P14921	DR	positive regulation of cellular component movement
N	P14921	DR	positive regulation of erythrocyte differentiation
N	P14921	DR	positive regulation of gene-specific transcription from RNA polymerase II promoter
N	P14921	DR	protein binding
N	P14921	DR	response to antibiotic
N	P14921	DR	sequence-specific DNA binding
N	P14921	DR	sequence-specific DNA binding transcription factor activity
N	P14921	DR	transcription factor binding
N	P14921	DR	transcription from RNA polymerase II promoter
N	P15063	CC	Seems to act as an inhibitor of cellular proliferation (By similarity).
N	P15063	DE	Protein B-Myc;
N	P15063	DR	regulation of transcription, DNA-dependent
N	P15063	DR	sequence-specific DNA binding transcription factor activity
N	P15067	CC	Hydrolyzes the alpha-1,6-glucosidic linkages in glycogen which has first been partially depolymerized by phosphorylase
N	P15067	CC	Shows only very little activity with native glycogen.
N	P15067	DE	Glycogen debranching enzyme;
N	P15067	DE	Glycogen operon protein GlgX;
N	P15067	DR	cation binding
N	P15067	DR	glycogen catabolic process
N	P15067	DR	glycogen debranching enzyme activity
N	P15067	DR	hydrolase activity, hydrolyzing O-glycosyl compounds
N	P15067	DR	response to DNA damage stimulus
N	P15098	CC	The three proteins TGB1, TGB2 and TGB3 are required for virus movement.
N	P15098	DE	7 kDa protein;
N	P15098	DE	ORF4 protein;
N	P15098	DE	TGB3 protein;
N	P15098	DE	Triple gene block 3 protein;
N	P15098	DR	spread of virus in host, cell to cell
N	P15183	CC	Capsid protein self-assembles to form an icosahedral capsid with a T=3 symmetry, about 32-35 nm in diameter, and consisting of 180 capsid proteins.
N	P15183	DE	Capsid protein;
N	P15183	DE	Coat protein;
N	P15183	DR	structural molecule activity
N	P15406	CC	Required for genome encapsidation
N	P15406	CC	Forms ribonucleoprotein complexes along with TGB1 helicase and viral RNA.
N	P15406	DE	Capsid protein;
N	P15406	DE	Coat protein;
N	P15406	DR	structural molecule activity
N	P15570	CC	Induces local and distal defense responses (incompatible hypersensitive reaction) in plants from the solanaceae and cruciferae families
N	P15570	CC	Elicits leaf necrosis and causes the accumulation of pathogenesis-related proteins
N	P15570	CC	Might interact with the lipidic molecules of the plasma membrane.
N	P15570	DE	Beta-elicitin cryptogein;
N	P15570	DR	defense response
N	P15570	DR	modulation by symbiont of host defense-related programmed cell death
N	P15732	CC	Catalyzes the covalent attachment of ubiquitin to other proteins
N	P15732	CC	Mediates the selective degradation of short-lived and abnormal proteins.
N	P15732	DE	Ubiquitin carrier protein;
N	P15732	DE	Ubiquitin-conjugating enzyme E2-16 kDa;
N	P15732	DE	Ubiquitin-protein ligase;
N	P15732	DR	post-translational protein modification
N	P15732	DR	protein polyubiquitination
N	P15732	DR	response to stress
N	P15732	DR	ubiquitin-protein ligase activity
N	P15777	CC	S1 attaches the virion to the cell membrane by binding to 9-O-acetylated sialic acid containing proteins, initiating the infection (By similarity).
N	P15777	DE	Peplomer protein;
N	P15777	DE	S glycoprotein;
N	P15777	DE	Spike glycoprotein;
N	P15777	DE	Spike protein S1;
N	P15777	DE	Spike protein S2;
N	P15777	DR	cellular membrane fusion
N	P15777	DR	virion attachment, binding of host cell surface receptor

N	P15788	CC	Ferredoxins are iron-sulfur proteins that transfer electrons in a wide variety of metabolic reactions.
N	P15788	DR	2 iron, 2 sulfur cluster binding
N	P15788	DR	electron carrier activity
N	P15788	DR	electron transport chain
N	P15788	DR	metal ion binding
N	P15992	CC	Not known
N	P15992	CC	One of the major polypeptides produced on heat shock.
N	P15992	DE	26 kDa heat shock protein;
N	P15992	DE	Heat shock protein 26;
N	P15992	DR	protein folding
N	P15992	DR	response to stress
N	P15992	DR	unfolded protein binding
N	P16079	CC	This protein found in the seeds of many leguminous and non-leguminous plants is the source of sulfur-containing amino acids in seed meals.
N	P16079	DE	Legumin type B acidic chain;
N	P16079	DE	Legumin type B alpha chain;
N	P16079	DE	Legumin type B basic chain;
N	P16079	DE	Legumin type B beta chain;
N	P16079	DE	Legumin type B;
N	P16079	DR	nutrient reservoir activity
N	P16295	CC	Factor IX is a vitamin K-dependent plasma protein that participates in the intrinsic pathway of blood coagulation by converting factor X to its active form in the presence of Ca(2+) ions, phospholipids, and factor VIIIa.
N	P16295	DE	Christmas factor;
N	P16295	DE	Coagulation factor IX;
N	P16295	DR	blood coagulation
N	P16295	DR	serine-type endopeptidase activity
N	P16517	DE	Early protein GP16.7;
N	P16813	DE	Uncharacterized protein UL39;
N	P17021	CC	May be involved in transcriptional regulation.
N	P17021	DE	Zinc finger protein 17;
N	P17021	DE	Zinc finger protein HPF3;
N	P17021	DE	Zinc finger protein KOX10;
N	P17021	DR	regulation of transcription, DNA-dependent
N	P17021	DR	zinc ion binding
N	P17065	CC	Guanine nucleotide exchange factor for SEC4, catalyzing the dissociation of GDP from SEC4 and also potently promoting binding of GTP
N	P17065	CC	Activation of SEC4 by SEC2 is needed for the directed transport of vesicles to sites of exocytosis
N	P17065	CC	Binds the Rab GTPase YPT32, but does not have exhange activity on YPT32.
N	P17065	DE	GDP-GTP exchange factor SEC2;
N	P17065	DE	Rab guanine nucleotide exchange factor SEC2;
N	P17065	DR	guanyl-nucleotide exchange factor activity
N	P17065	DR	protein transport
N	P17178	CC	Catalyzes the first step in the oxidation of the side chain of sterol intermediates; the 27-hydroxylation of 5-beta- cholestane-3-alpha,7-alpha,12-alpha-triol
N	P17178	CC	Has also a vitamin D3- 25-hydroxylase activity.
N	P17178	DE	5-beta-cholestane-3-alpha,7-alpha,12-alpha-triol 27-hydroxylase;
N	P17178	DE	Cytochrome P-450C27/25;
N	P17178	DE	Cytochrome P450 27;
N	P17178	DE	Sterol 26-hydroxylase, mitochondrial;
N	P17178	DE	Sterol 27-hydroxylase;
N	P17178	DE	Vitamin D(3) 25-hydroxylase;
N	P17178	DR	cholestanetriol 26-monooxygenase activity
N	P17178	DR	cholesterol 26-hydroxylase activity
N	P17178	DR	cholesterol metabolic process
N	P17178	DR	electron carrier activity
N	P17178	DR	oxidation-reduction process
N	P17178	DR	steroid catabolic process
N	P17178	DR	vitamin D3 25-hydroxylase activity
N	P17400	CC	The large envelope protein exists in two topological conformations, one which is termed 'external' or Le-HBsAg and the other 'internal' or Li-HBsAg
N	P17400	CC	In its external conformation the protein attaches the virus to cell receptors and thereby initiating infection

N	P17400	CC	In its internal conformation the protein plays a role in virion morphogenesis and mediates the contact with the nucleocapsid like a matrix protein (By similarity).
N	P17400	CC	The large envelope protein probably also assumes fusion between virion and host membranes
N	P17400	CC	This interaction determines the species specificity and liver tropism
N	P17400	DE	L glycoprotein;
N	P17400	DE	Large S protein;
N	P17400	DE	Large envelope protein;
N	P17400	DE	Large surface protein;
N	P17400	DE	Major surface antigen;
N	P17400	DR	viral reproduction
N	P17553	CC	Ligand for members of the frizzled family of seven transmembrane receptors
N	P17553	CC	Wnt-3 and Wnt-3a play distinct roles in cell-cell signaling during morphogenesis of the developing neural tube.
N	P17553	DE	Proto-oncogene Int-4;
N	P17553	DE	Proto-oncogene Wnt-3;
N	P17553	DR	Spemann organizer formation at the anterior end of the primitive streak
N	P17553	DR	Wnt receptor signaling pathway, calcium modulating pathway
N	P17553	DR	anterior/posterior axis specification
N	P17553	DR	axon guidance
N	P17553	DR	dorsal/ventral axis specification
N	P17553	DR	embryonic forelimb morphogenesis
N	P17553	DR	embryonic hindlimb morphogenesis
N	P17553	DR	head morphogenesis
N	P17553	DR	mesoderm formation
N	P17553	DR	negative regulation of axon extension involved in axon guidance
N	P17553	DR	organ morphogenesis
N	P17553	DR	positive regulation of collateral sprouting in absence of injury
N	P17553	DR	positive regulation of gene expression
N	P17553	DR	protein domain specific binding
N	P17553	DR	signal transducer activity
N	P17603	CC	Flagellin is the subunit protein which polymerizes to form the filaments of archaeal flagella.
N	P17603	DE	24 kDa flagellin;
N	P17603	DR	cellular component movement
N	P17603	DR	structural molecule activity
N	P17672	CC	Implicated in the regulation of ecdysone-triggered gene hierarchies
N	P17672	CC	Probably plays a key role in mediating the regulation of the larval molt by 20-OH-ecdysone.
N	P17672	DE	Ecdysone-induced protein 75B, isoform A;
N	P17672	DE	Nuclear receptor subfamily 1 group D member 3, isoform A;
N	P17672	DR	antimicrobial humoral response
N	P17672	DR	ecdysis, chitin-based cuticle
N	P17672	DR	ecdysone-mediated induction of salivary gland cell autophagic cell death
N	P17672	DR	regulation of ecdysteroid metabolic process
N	P17672	DR	regulation of transcription, DNA-dependent
N	P17672	DR	sequence-specific DNA binding
N	P17672	DR	sequence-specific DNA binding transcription factor activity
N	P17672	DR	steroid hormone receptor activity
N	P17672	DR	thyroid hormone receptor activity
N	P17672	DR	zinc ion binding
N	P17676	CC	Important transcriptional activator in the regulation of genes involved in immune and inflammatory responses
N	P17676	CC	Functions in brown adipose tissue (BAT) differentiation (By similarity).
N	P17676	CC	It probably plays a role in the regulation of acute-phase reaction, inflammation and hemopoiesis
N	P17676	CC	NF-IL6 also binds to regulatory regions of several acute-phase and cytokines genes
N	P17676	CC	Specifically binds to an IL-1 response element in the IL-6 gene
N	P17676	CC	The consensus recognition site is 5'-T[TG]NNGNAA[TG]-3'
N	P17676	DE	C/EBP beta;
N	P17676	DE	CCAAT/enhancer-binding protein beta;
N	P17676	DE	Liver activator protein;
N	P17676	DE	Nuclear factor NF-IL6;
N	P17676	DE	Transcription factor 5;
N	P17676	DR	acute-phase response

N	P17676	DR	immune response
N	P17676	DR	sequence-specific enhancer binding RNA polymerase II transcription factor activity
N	P17677	CC	This protein is associated with nerve growth
N	P17677	CC	It is a major component of the motile "growth cones" that form the tips of elongating axons.
N	P17677	DE	Axonal membrane protein GAP-43;
N	P17677	DE	Growth-associated protein 43;
N	P17677	DE	Neural phosphoprotein B-50;
N	P17677	DE	Neuromodulin;
N	P17677	DR	activation of protein kinase C activity by G-protein coupled receptor protein signaling pathway
N	P17677	DR	calmodulin binding
N	P17677	DR	cell differentiation
N	P17677	DR	nervous system development
N	P17677	DR	regulation of growth
N	P17677	DR	response to wounding
N	P17684	CC	Conantokins inhibit N-methyl-D-aspartate (NMDA) receptors
N	P17684	CC	Induces sleep-like symptoms in young mice and hyperactivity in older mice.
N	P17684	CC	This toxin inhibits both NR2A and NR2B subunits of N- methyl-D-aspartate (NMDA) receptor-mediated calcium influx in central nervous system neurons
N	P17684	DE	Conantokin-T;
N	P17684	DR	acetylcholine receptor inhibitor activity
N	P17684	DR	ion channel inhibitor activity
N	P17684	DR	synaptic transmission
N	P17989	DE	1,3-1,4-beta-D-glucan 4-glucanohydrolase;
N	P17989	DE	Beta-glucanase;
N	P17989	DE	Endo-beta-1,3-1,4 glucanase;
N	P17989	DE	Mixed linkage beta-glucanase;
N	P17989	DR	carbohydrate metabolic process
N	P17989	DR	licheninase activity
N	P18424	CC	Central enzyme in the extracellular metabolism of plasma lipoproteins
N	P18424	CC	Also produced in the brain by primary astrocytes, and esterifies free cholesterol on nascent APOE-containing lipoproteins secreted from glia and influences cerebral spinal fluid (CSF) APOE- and APOA1 levels
N	P18424	CC	Has a preference for plasma 16:0-18:2 or 18:O-18:2 phosphatidylcholines.
N	P18424	CC	Required for remodeling high-density lipoprotein particles into their spherical forms (By similarity)
N	P18424	CC	Synthesized mainly in the liver and secreted into plasma where it converts cholesterol and phosphatidylcholines (lecithins) to cholesteryl esters and lysophosphatidylcholines on the surface of high and low density lipoproteins (HDLs and LDLs)
N	P18424	CC	The cholesterol ester is then transported back to the liver
N	P18424	CC	Together with APOE and the cholesterol transporter ABCA1, plays a key role in the maturation of glial-derived, nascent lipoproteins
N	P18424	DE	Lecithin-cholesterol acyltransferase;
N	P18424	DE	Phosphatidylcholine-sterol acyltransferase;
N	P18424	DE	Phospholipid-cholesterol acyltransferase;
N	P18424	DR	cholesterol metabolic process
N	P18424	DR	phosphatidylcholine-sterol O-acyltransferase activity
N	P18424	DR	phospholipase A2 activity
N	P18424	DR	response to copper ion
N	P18424	DR	response to glucocorticoid stimulus
N	P18434	DE	Gastric H(+)/K(+) ATPase subunit beta;
N	P18434	DE	Potassium-transporting ATPase subunit beta;
N	P18434	DE	Proton pump beta chain;
N	P18434	DR	ATP biosynthetic process
N	P18434	DR	proton transport
N	P18434	DR	sodium:potassium-exchanging ATPase activity
N	P18801	CC	Bypasses host T-cell signaling by inducing a transcriptional program nearly identical to that of anti-CD3 cell activation
N	P18801	CC	Extracellular Nef protein targets CD4(+) T-lymphocytes for apoptosis by interacting with CXCR4 surface receptors (By similarity).
N	P18801	CC	Factor of infectivity and pathogenicity, required for optimal virus replication
N	P18801	CC	Alters numerous pathways of T- lymphocytes function and downregulates immunity surface molecules in order to evade host defense and increase viral infectivity

N	P18801	CC	Alters the functionality of other immunity cells, like dendritic cells, monocytes/macrophages and NK cells
N	P18801	CC	Increasing surface FasL molecules and decreasing surface MHC-I molecules on infected CD4(+) cells send attacking cytotoxic CD8+ T-lymphocytes into apoptosis (By similarity).
N	P18801	CC	Interaction with TCR-zeta chain up-regulates the Fas ligand (FasL)
N	P18801	CC	One of the earliest and most abundantly expressed viral proteins (By similarity).
N	P18801	DE	C-terminal core protein;
N	P18801	DE	Negative factor;
N	P18801	DE	Protein Nef;
N	P18801	DR	SH3 domain binding
N	P18801	DR	evasion by virus of host immune response
N	P18801	DR	regulation of defense response to virus by virus
N	P19067	CC	This molybdenum-iron protein is part of the nitrogenase complex that catalyzes the key enzymatic reactions in nitrogen fixation.
N	P19067	DE	Dinitrogenase;
N	P19067	DE	Nitrogenase component I;
N	P19067	DE	Nitrogenase molybdenum-iron protein beta chain;
N	P19067	DR	iron-sulfur cluster binding
N	P19067	DR	metal ion binding
N	P19067	DR	nitrogen fixation
N	P19067	DR	nitrogenase activity
N	P19067	DR	oxidation-reduction process
N	P19132	CC	Stores iron in a soluble, non-toxic, readily available form
N	P19132	CC	Also plays a role in delivery of iron to cells
N	P19132	CC	Has ferroxidase activity
N	P19132	CC	Important for iron homeostasis
N	P19132	CC	Iron is taken up in the ferrous form and deposited as ferric hydroxides after oxidation
N	P19132	CC	Mediates iron uptake in capsule cells of the developing kidney (By similarity).
N	P19132	DE	Ferritin H subunit;
N	P19132	DE	Ferritin heavy chain;
N	P19132	DR	cellular iron ion homeostasis
N	P19132	DR	ferric iron binding
N	P19132	DR	ferroxidase activity
N	P19132	DR	immune response
N	P19132	DR	iron ion transport
N	P19132	DR	negative regulation of cell proliferation
N	P19132	DR	negative regulation of necrotic cell death
N	P19132	DR	oxidation-reduction process
N	P19132	DR	protein binding
N	P19141	CC	Crystallins are the dominant structural components of the vertebrate eye lens.
N	P19141	DE	Beta-B3 crystallin;
N	P19141	DE	Beta-crystallin B3;
N	P19141	DR	structural constituent of eye lens
N	P19170	CC	Protein S19 forms a complex with S13 that binds strongly to the 16S ribosomal RNA (By similarity).
N	P19170	DE	30S ribosomal protein S19, chloroplastic;
N	P19170	DR	structural constituent of ribosome
N	P19205	CC	This enzyme catalyzes the hydrolysis of the N-terminal peptide bond of an N-acetylated peptide to generate an N- acetylated amino acid and a peptide with a free N-terminus
N	P19205	CC	It preferentially cleaves off Ac-Ala, Ac-Met and Ac-Ser.
N	P19205	DE	Acyl-peptide hydrolase;
N	P19205	DE	Acylamino-acid-releasing enzyme;
N	P19205	DE	Acylaminoacyl-peptidase;
N	P19205	DR	serine-type endopeptidase activity
N	P19435	DE	Uncharacterized 23.9 kDa protein in glnII region;
N	P19506	CC	Counteracts the innate antiviral activity of APOBEC3G
N	P19506	CC	Binds viral RNA and affects the stability of viral nucleoprotein core
N	P19506	CC	Forms a complex with host APOBEC3G thus preventing the entry of this lethally hypermutating enzyme into progeny virions
N	P19506	CC	Functions as an adapter molecule, recruiting APOBEC3G to the ubiquitin-proteasome machinery
N	P19506	CC	May play a role in viral morphology (By similarity).
N	P19506	CC	Targets APOBEC3G for degradation through the assembly with elongin BC complex, CUL5 and RBX1

N	P19506	DE	SOR protein;
N	P19506	DE	Virion infectivity factor;
N	P19506	DR	interspecies interaction between organisms
N	P19506	DR	viral infectious cycle
N	P19507	CC	Nuclear transcriptional activator of viral gene expression, that is essential for viral transcription from the LTR promoter and replication
N	P19507	CC	Acts as a sequence-specific molecular adapter, directing components of the cellular transcription machinery to the viral RNA to promote processive transcription elongation by the RNA polymerase II (RNA pol II) complex, thereby increasing the level of full-length transcripts
N	P19507	CC	In the absence of Tat, the RNA Pol II generates short or non-processive transcripts that terminate at approximately 60 bp from the initiation site
N	P19507	CC	Tat associates with the CCNT1/cyclin-T1 component of the P-TEFb complex (CDK9 and CCNT1), which promotes RNA chain elongation
N	P19507	CC	The CDK9 component of P-TEFb and other Tat-activated kinases hyperphosphorylate the C-terminus of RNA Pol II that becomes stabilized and much more processive (By similarity).
N	P19507	CC	This binding increases Tat's affinity for a hairpin structure at the 5'-end of all nascent viral mRNAs referred to as the transactivation responsive RNA element (TAR RNA) and allows Tat/P- TEFb complex to bind cooperatively to TAR RNA
N	P19507	DE	Protein Tat;
N	P19507	DE	Transactivating regulatory protein;
N	P19507	DR	interspecies interaction between organisms
N	P19507	DR	regulation of transcription, DNA-dependent
N	P19507	DR	sequence-specific DNA binding transcription factor activity
N	P19532	CC	Transcription factor that specifically recognizes and binds E-box sequences (3'-CANNTG-5')
N	P19532	CC	Efficient DNA-binding requires dimerization with itself or with another MiT/TFE family member such as TFEB or MITF
N	P19532	CC	In association with TFEB, activates the expression of CD40L in T-cells, thereby playing a role in T- cell-dependent antibody responses in activated CD4(+) T-cells and thymus-dependent humoral immunity
N	P19532	CC	It also binds very well to a USF/MLTF site.
N	P19532	CC	Specifically recognizes the MUE3 box, a subset of E-boxes, present in the immunoglobulin enhancer
N	P19532	DE	Class E basic helix-loop-helix protein 33;
N	P19532	DE	Transcription factor E3;
N	P19532	DR	humoral immune response
N	P19532	DR	positive regulation of gene-specific transcription from RNA polymerase II promoter
N	P19532	DR	promoter binding
N	P19532	DR	sequence-specific DNA binding transcription factor activity
N	P19532	DR	transcription regulator activity
N	P19555	CC	Involved in the transport of the viral pre-integration (PIC) complex to the nucleus during the early stages of the infection
N	P19555	CC	May also act directly at the nuclear pore complex, by binding nucleoporins phenylalanine-glycine (FG)-repeat regions (By similarity).
N	P19555	CC	May interact with karyopherin alpha/KPNA1 and KPNA2 to increase their affinity for proteins containing basic-type nuclear localization signal, including the viral matrix protein MA, thus facilitating the translocation of the viral genome into the nucleus
N	P19555	CC	This function is crucial for viral infection of non- dividing macrophages
N	P19555	DE	Protein Vpr;
N	P19555	DE	R ORF protein;
N	P19555	DE	Viral protein R;
N	P19555	DR	RNA-dependent DNA replication
N	P19555	DR	entry into host cell
N	P19555	DR	ion channel activity
N	P19555	DR	provirus integration
N	P19555	DR	uncoating of virus
N	P19697	CC	Binds to sialic acid-containing receptors on the cell surface, bringing about the attachment of the virus particle to the cell
N	P19697	CC	Class I viral fusion protein
N	P19697	CC	Low pH in endosomes induces an irreversible conformational change in HA2, releasing the fusion hydrophobic peptide
N	P19697	CC	Plays a major role in the determination of host range restriction and virulence

N	P19697	CC	Responsible for penetration of the virus into the cell cytoplasm by mediating the fusion of the membrane of the endocytosed virus particle with the endosomal membrane
N	P19697	CC	Several trimers are required to form a competent fusion pore.
N	P19697	CC	This attachment induces virion internalization of about two third of the virus particles through clathrin-dependent endocytosis and about one third through a clathrin- and caveolin- independent pathway
N	P19697	DE	Hemagglutinin HA1 chain;
N	P19697	DE	Hemagglutinin HA2 chain;
N	P19697	DE	Hemagglutinin;
N	P19697	DR	host cell surface receptor binding
N	P19697	DR	viral envelope fusion with host membrane
N	P20052	CC	Cyclin partner of the cyclin-dependent kinase (CDK) PHO85
N	P20052	CC	Negatively regulates the expression of phosphate- starvation-responsive genes under phosphate-rich conditions
N	P20052	CC	PHO80-PHO85 also phosphorylates and inactivates the calcineurin-responsive transcription factor CRZ1, linking PHO85 to calcium signaling.
N	P20052	CC	PHO80-PHO85 phosphorylates and inactivates protein kinase RIM15 by retaining it in the cytoplasm, antagonizing RIM15-induced entry into stationary phase
N	P20052	CC	The PHO80-PHO85 cyclin-CDK holoenzyme phosphorylates and inactivates the transcription factor PHO4, by preventing its association with the transcription factor PHO2 and the nuclear import receptor PSE1, and by promoting association with the nuclear export receptor MSN5, excluding PHO4 from the nucleus
N	P20052	DE	Aminoglycoside antibiotic sensitivity protein 3;
N	P20052	DE	PHO85 cyclin PHO80;
N	P20052	DE	Phosphate system cyclin PHO80;
N	P20052	DR	cellular metal ion homeostasis
N	P20052	DR	cyclin-dependent protein kinase regulator activity
N	P20052	DR	negative regulation of calcium-mediated signaling
N	P20052	DR	negative regulation of macroautophagy
N	P20052	DR	negative regulation of phosphate metabolic process
N	P20052	DR	negative regulation of transcription from RNA polymerase II promoter
N	P20052	DR	regulation of cyclin-dependent protein kinase activity
N	P20052	DR	regulation of protein localization
N	P20059	CC	Binds heme and transports it to the liver for breakdown and iron recovery, after which the free hemopexin returns to the circulation.
N	P20059	DR	metal ion binding
N	P20195	CC	Essential for virus function.
N	P20195	DE	Uncharacterized protein A-132;
N	P20432	CC	Conjugation of reduced glutathione to a wide number of exogenous and endogenous hydrophobic electrophiles
N	P20432	CC	Has DDT dehydrochlorinase activity.
N	P20432	DE	DDT-dehydrochlorinase;
N	P20432	DE	GST class-theta;
N	P20432	DE	Glutathione S-transferase 1-1;
N	P20432	DR	DDT-dehydrochlorinase activity
N	P20432	DR	glutathione transferase activity
N	P20432	DR	protein binding
N	P20566	DE	Uncharacterized 8.4 kDa protein;
N	P20692	DE	Prephenate dehydrogenase;
N	P20692	DR	amino acid binding
N	P20692	DR	oxidation-reduction process
N	P20692	DR	prephenate dehydrogenase (NADP+) activity
N	P20692	DR	prephenate dehydrogenase activity
N	P20692	DR	tyrosine biosynthetic process
N	P20734	CC	This enzyme is a serine protease capable of degrading the native triple helix of collagen
N	P20734	DE	Collagenolytic protease 36 kDa C;
N	P20734	DR	collagen catabolic process
N	P20734	DR	serine-type peptidase activity
N	P21342	CC	The alpha subunit may be involved in the regulation of PFP by Fru-2,6-P.
N	P21342	DE	6-phosphofructokinase, pyrophosphate dependent;
N	P21342	DE	Pyrophosphate--fructose 6-phosphate 1-phosphotransferase subunit alpha;
N	P21342	DE	Pyrophosphate-dependent 6-phosphofructose-1-kinase;
N	P21342	DR	6-phosphofructokinase activity
N	P21342	DR	diphosphate-fructose-6-phosphate 1-phosphotransferase activity
N	P21407	DR	aspartic-type endopeptidase activity

N	P21407	DR	dUTP metabolic process
N	P21407	DR	nucleic acid binding
N	P21444	CC	The surface protein (SU) attaches the virus to the host cell by binding to its [illegible]
N	P21444	CC	Fusion occurs at the host cell plasma membrane (By similarity).
N	P21444	CC	This interaction triggers the refolding of the transmembrane protein (TM) and is thought to activate its fusogenic potential by unmasking its fusion peptide
N	P21444	DE	DE Flags: Precursor; Fragment;
N	P21444	DE	Env polyprotein;
N	P21444	DE	Envelope glycoprotein;
N	P21444	DE	Glycoprotein 70;
N	P21444	DE	Surface protein;
N	P21444	DR	interspecies interaction between organisms
N	P21444	DR	structural molecule activity
N	P21444	DR	viral infectious cycle
N	P21768	CC	Involved in oxygen transport from the lung to the various peripheral tissues.
N	P21768	DE	Alpha-1/2-globin;
N	P21768	DE	Hemoglobin alpha-1/2 chain;
N	P21768	DE	Hemoglobin subunit alpha-1/2;
N	P21768	DR	oxygen binding
N	P21768	DR	oxygen transporter activity
N	P21788	CC	May play an important role in mitosis of sea urchin egg
N	P21788	CC	May function as a Ca(2+)-dependent intracellular modulator of microtubule [illegible]
N	P21788	DE	15 kDa calcium-binding protein;
N	P21788	DR	calcium ion binding
N	P22062	CC	Catalyzes the methyl esterification of L-isoaspartyl and D-aspartyl residues in peptides and proteins that result from spontaneous decomposition of normal L-aspartyl and L-asparaginyl residues
N	P22062	CC	Acts on microtubule-associated protein 2, calreticulin, clathrin light chains a and b, Ubiquitin carboxyl- terminal hydrolase isozyme L1, phosphatidylethanolamine-binding protein 1, stathmin, beta-synuclein and alpha-synuclein (By similarity)
N	P22062	CC	It plays a role in the repair and/or degradation of damaged proteins
N	P22062	DE	L-isoaspartyl protein carboxyl methyltransferase;
N	P22062	DE	Protein L-isoaspartyl/D-aspartyl methyltransferase;
N	P22062	DE	Protein-L-isoaspartate(D-aspartate) O-methyltransferase;
N	P22062	DE	Protein-beta-aspartate methyltransferase;
N	P22062	DR	S-adenosylhomocysteine metabolic process
N	P22062	DR	S-adenosylmethionine metabolic process
N	P22062	DR	protein-L-isoaspartate (D-aspartate) O-methyltransferase activity
N	P22092	CC	Binds to sialic acid-containing receptors on the cell surface, bringing about the attachment of the virus particle to the cell
N	P22092	CC	Class I viral fusion protein
N	P22092	CC	Low pH in endosomes induce an irreversible conformational change in HA2, releasing the fusion hydrophobic peptide
N	P22092	CC	Plays a major role in the determination of host range restriction and virulence
N	P22092	CC	Responsible for penetration of the virus into the cell cytoplasm by mediating the fusion of the membrane of the endocytosed virus particle with the endosomal membrane
N	P22092	CC	Several trimers are required to form a competent fusion pore.
N	P22092	DE	Hemagglutinin HA1 chain;
N	P22092	DE	Hemagglutinin HA2 chain;
N	P22092	DE	Hemagglutinin;
N	P22092	DR	host cell surface receptor binding
N	P22092	DR	viral envelope fusion with host membrane
N	P22106	DE	Asparagine synthetase B [glutamine-hydrolyzing];
N	P22106	DR	amino acid binding
N	P22106	DR	asparaginase activity
N	P22106	DR	asparagine biosynthetic process
N	P22106	DR	asparagine synthase (glutamine-hydrolyzing) activity
N	P22106	DR	cellular amino acid catabolic process
N	P22106	DR	glutamine metabolic process
N	P22220	DE	Arginine decarboxylase;
N	P22220	DR	arginine catabolic process
N	P22220	DR	arginine decarboxylase activity
N	P22220	DR	putrescine biosynthetic process
N	P22220	DR	spermidine biosynthetic process

N	P22760	CC	Arylacetamide deacetylation is an important enzyme activity in the metabolic activation of arylamine substrates to ultimate carcinogens
N	P22760	CC	Displays cellular triglyceride lipase activity in liver
N	P22760	CC	Displays major serine hydrolase activity in liver microsomes
N	P22760	CC	Hydrolyzes also flutamide, which is an antiandrogen drug used for the treatment of prostate cancer that occasionaly causes severe hepatotoxicity
N	P22760	CC	Increases intracellular fatty acids derived from hydrolysis of newly formed triglyceride stores.
N	P22760	DE	Arylacetamide deacetylase;
N	P22760	DR	deacetylase activity
N	P22760	DR	positive regulation of triglyceride catabolic process
N	P22760	DR	serine hydrolase activity
N	P22760	DR	triglyceride lipase activity
N	P22792	CC	The 83 kDa subunit binds and stabilizes the catalytic subunit at 37 degrees Celsius and keeps it in circulation
N	P22792	CC	Under some circumstances it may be an allosteric modifier of the catalytic subunit
N	P22792	DE	Carboxypeptidase N 83 kDa chain;
N	P22792	DE	Carboxypeptidase N large subunit;
N	P22792	DE	Carboxypeptidase N polypeptide 2;
N	P22792	DE	Carboxypeptidase N regulatory subunit;
N	P22792	DE	Carboxypeptidase N subunit 2;
N	P22792	DR	enzyme regulator activity
N	P22792	DR	protein stabilization
N	P22917	DE	Uncharacterized 17.8 kDa protein in arn-motA intergenic region;
N	P23268	CC	Odorant receptor (Potential).
N	P23268	DE	Olfactory receptor 1082;
N	P23268	DE	Olfactory receptor-like protein F12;
N	P23275	CC	Odorant receptor (Potential)
N	P23275	CC	May be involved in the molecular processes underlying fasciculation and targeting of olfactory axons.
N	P23275	DE	Odorant receptor OR3;
N	P23275	DE	Olfactory receptor 15;
N	P23275	DE	Olfactory receptor 256-17;
N	P23275	DR	olfactory receptor activity
N	P23275	DR	sensory perception of smell
N	P23529	CC	Mediates the binding, internalization, and catabolism of lipoprotein particles
N	P23529	CC	It can serve as a ligand for the LDL (apo B/E) receptor and for the specific apo-E receptor (chylomicron remnant) of hepatic tissues.
N	P23529	DE	Apolipoprotein E;
N	P23529	DR	heparin binding
N	P23579	CC	DNA-dependent RNA polymerase catalyzes the transcription of DNA into RNA using the four ribonucleoside triphosphates as substrates.
N	P23579	DE	DNA-directed RNA polymerase subunit beta;
N	P23579	DE	Plastid-encoded RNA polymerase subunit beta;
N	P23579	DE	RNA polymerase subunit beta;
N	P23579	DR	DNA-directed RNA polymerase activity
N	P23579	DR	ribonucleoside binding
N	P23941	CC	This methylase recognizes the double-stranded sequence GGATCC, causes specific methylation on C-5 on both strands, and protects the DNA from cleavage by the BamHI endonuclease.
N	P23941	DE	Modification methylase BamHI;
N	P23941	DE	N(4)- cytosine-specific methyltransferase BamHI;
N	P23941	DR	DNA restriction-modification system
N	P23941	DR	N-methyltransferase activity
N	P23941	DR	site-specific DNA-methyltransferase (cytosine-N4-specific) activity
N	P24109	CC	Nuclear transcriptional activator of viral gene expression, that is essential for viral transcription from the LTR promoter and replication
N	P24109	CC	Acts as a sequence-specific molecular adapter, directing components of the cellular transcription machinery to the viral RNA to promote processive transcription elongation by the RNA polymerase II (RNA pol II) complex, thereby increasing the level of full-length transcripts
N	P24109	CC	In the absence of Tat, the RNA Pol II generates short or non-processive transcripts that terminate at approximately 60 bp from the initiation site
N	P24109	CC	Tat associates with the CCNT1/cyclin-T1 component of the P-TEFb complex (CDK9 and CCNT1), which promotes RNA chain elongation

N	P24109	CC	The CDK9 component of P-TEFb and other Tat-activated kinases hyperphosphorylate the C-terminus of RNA Pol II that becomes stabilized and much more processive (By similarity).
N	P24109	CC	This binding increases Tat's affinity for a hairpin structure at the 5'-end of all nascent viral mRNAs referred to as the transactivation responsive RNA element (TAR RNA) and allows Tat/P- TEFb complex to bind cooperatively to TAR RNA
N	P24109	DE	Protein Tat;
N	P24109	DE	Transactivating regulatory protein;
N	P24109	DR	interspecies interaction between organisms
N	P24109	DR	regulation of transcription, DNA-dependent
N	P24109	DR	sequence-specific DNA binding transcription factor activity
N	P24137	CC	Component of the oligopeptide permease, a binding protein-dependent transport system
N	P24137	CC	Necessary for genetic competence but not sporulation
N	P24137	CC	Probably responsible for energy coupling to the transport system.
N	P24137	DE	Oligopeptide transport ATP-binding protein OppF;
N	P24137	DE	Stage 0 sporulation protein KE;
N	P24137	DR	ATPase activity
N	P24137	DR	establishment of competence for transformation
N	P24137	DR	peptide transport
N	P24137	DR	protein transport
N	P24137	DR	sporulation resulting in formation of a cellular spore
N	P24186	CC	Catalyzes the oxidation of 5,10- methylenetetrahydrofolate to 5,10- methenyltetrahydrofolate and then the hydrolysis of 5,10- methenyltetrahydrofolate to 10- formyltetrahydrofolate
N	P24186	CC	This enzyme is specific for NADP.
N	P24186	DE	Bifunctional protein FolD;
N	P24186	DE	Methenyltetrahydrofolate cyclohydrolase;
N	P24186	DE	Methylenetetrahydrofolate dehydrogenase;
N	P24186	DR	folic acid-containing compound biosynthetic process
N	P24186	DR	histidine biosynthetic process
N	P24186	DR	methenyltetrahydrofolate cyclohydrolase activity
N	P24186	DR	methionine biosynthetic process
N	P24186	DR	methylenetetrahydrofolate dehydrogenase (NADP+) activity
N	P24186	DR	one-carbon metabolic process
N	P24186	DR	oxidation-reduction process
N	P24186	DR	purine nucleotide biosynthetic process
N	P24219	CC	Sigma factors are initiation factors that promote the attachment of RNA polymerase to specific initiation sites and are then released
N	P24219	CC	The open complex (sigma-54 and core RNA polymerase) serves as the receptor for receipt of the melting signal from the remotely bound activator protein levR for the expression of the levanase operon.
N	P24219	CC	This sigma factor is responsible for the expression of the levanase operon
N	P24219	DE	RNA polymerase sigma-54 factor;
N	P24219	DR	DNA-directed RNA polymerase activity
N	P24219	DR	regulation of transcription, DNA-dependent
N	P24219	DR	sequence-specific DNA binding transcription factor activity
N	P24219	DR	sigma factor activity
N	P24219	DR	transcription initiation, DNA-dependent
N	P24483	CC	Adrenodoxin transfers electrons from adrenodoxin reductase to the cholesterol side chain cleavage cytochrome P450.
N	P24483	DE	Adrenal ferredoxin;
N	P24483	DE	Adrenodoxin, mitochondrial;
N	P24483	DE	Ferredoxin-1;
N	P24483	DR	2 iron, 2 sulfur cluster binding
N	P24483	DR	NADPH oxidation
N	P24483	DR	adrenal gland development
N	P24483	DR	cellular response to epinephrine stimulus
N	P24483	DR	cellular response to leptin stimulus
N	P24483	DR	cellular response to organic cyclic compound
N	P24483	DR	electron carrier activity
N	P24483	DR	electron transport chain
N	P24483	DR	enzyme binding
N	P24483	DR	iron ion binding
N	P24483	DR	positive regulation of oxidoreductase activity
N	P24483	DR	response to gonadotropin stimulus

N	P24517	CC	May play a role in the repair of endogenous alkylation damage.
N	P24517	DE	DNA repair protein RadA;
N	P24517	DE	DNA repair protein Sms;
N	P24517	DR	ATP-dependent peptidase activity
N	P24517	DR	damaged DNA binding
N	P24517	DR	metal ion binding
N	P24517	DR	serine-type endopeptidase activity
N	P24589	CC	Involved in oxygen transport from the lung to the various peripheral tissues.
N	P24589	DE	Alpha-A-globin;
N	P24589	DE	Hemoglobin alpha-A chain;
N	P24589	DE	Hemoglobin subunit alpha-A;
N	P24589	DR	oxygen binding
N	P24589	DR	oxygen transporter activity
N	P24685	CC	Usually encoded in the trnK tRNA gene intron
N	P24685	CC	Probably assists in splicing its own and other chloroplast group II introns (By similarity)
N	P24685	DE	Intron maturase;
N	P24685	DR	mRNA processing
N	P24685	DR	tRNA processing
N	P24723	CC	This is calcium-independent, phospholipid-dependent, serine- and threonine-specific enzyme.
N	P24723	DE	Protein kinase C eta type;
N	P24723	DR	enzyme binding
N	P24723	DR	intracellular signal transduction
N	P24723	DR	metal ion binding
N	P24723	DR	platelet activation
N	P24723	DR	protein kinase C activity
N	P24723	DR	protein phosphorylation
N	P25050	CC	Acts synergistically with Tip to stimulate NF-kappa-B activity and interleukin-2 gene expression by binding to host TRAF proteins
N	P25050	CC	Activation of NF-kappa-B protects lymphocytes from apoptosis, thereby facilitating viral induced cell transformation.
N	P25050	DE	Collagen-like protein;
N	P25050	DE	Saimiri transformation-associated protein;
N	P25050	DR	interspecies interaction between organisms
N	P25229	CC	Binds actin in a Ca(2+)-independent manner.
N	P25229	DE	Actin-binding protein chain A;
N	P25229	DE	F-actin-capping protein subunit alpha-1;
N	P25229	DR	actin binding
N	P25229	DR	actin cytoskeleton organization
N	P25353	CC	Mediates extracellular nucleotide derived phosphate hydrolysis along with NPP2 and PHO5.
N	P25353	DE	Alkaline phosphodiesterase 1;
N	P25353	DE	Ectonucleotide pyrophosphatase/phosphodiesterase 1;
N	P25353	DE	Nucleotide pyrophosphatase;
N	P25353	DR	cellular response to phosphate starvation
N	P25353	DR	nucleoside triphosphate metabolic process
N	P25353	DR	nucleoside-triphosphatase activity
N	P25353	DR	nucleoside-triphosphate diphosphatase activity
N	P25353	DR	nucleotide diphosphatase activity
N	P25353	DR	phosphodiesterase I activity
N	P25402	CC	Mediates assembly of pili by forming soluble multimeric complexes with pili subunits as an intermediate step in the assembly process
N	P25402	CC	This protein is involved in K99 pili assembly.
N	P25402	DE	Chaperone protein fanE;
N	P25402	DR	cellular cell wall organization
N	P25669	CC	Produces peripheral paralysis by blocking neuromuscular transmission at the postsynaptic site
N	P25669	CC	Binds to muscular and neuronal (only alpha-7 alpha-8 alpha-9) nicotinic acetylcholine receptors (By similarity).
N	P25669	DE	Long neurotoxin 2;
N	P25669	DR	acetylcholine receptor inhibitor activity
N	P25669	DR	synaptic transmission
N	P25788	CC	The proteasome is a multicatalytic proteinase complex which is characterized by its ability to cleave peptides with Arg, Phe, Tyr, Leu, and Glu adjacent to the leaving group at neutral or slightly basic pH

N	P25788	CC	Binds to the C-terminus of CDKN1A and thereby mediates its degradation
N	P25788	CC	Negatively regulates the membrane trafficking of the cell-surface thromboxane A2 receptor (TBXA2R) isoform 2.
N	P25788	CC	The proteasome has an ATP-dependent proteolytic activity
N	P25788	DE	Macropain subunit C8;
N	P25788	DE	Multicatalytic endopeptidase complex subunit C8;
N	P25788	DE	Proteasome component C8;
N	P25788	DE	Proteasome subunit alpha type-3;
N	P25788	DR	DNA damage response, signal transduction by p53 class mediator resulting in cell cycle arrest
N	P25788	DR	G1/S transition of mitotic cell cycle
N	P25788	DR	M/G1 transition of mitotic cell cycle
N	P25788	DR	S phase of mitotic cell cycle
N	P25788	DR	anaphase-promoting complex-dependent proteasomal ubiquitin-dependent protein catabolic process
N	P25788	DR	interspecies interaction between organisms
N	P25788	DR	mRNA metabolic process
N	P25788	DR	negative regulation of ubiquitin-protein ligase activity involved in mitotic cell cycle
N	P25788	DR	positive regulation of ubiquitin-protein ligase activity involved in mitotic cell cycle
N	P25788	DR	protein binding
N	P25788	DR	regulation of apoptosis
N	P25788	DR	regulation of cellular amino acid metabolic process
N	P25788	DR	threonine-type endopeptidase activity
N	P25788	DR	viral reproduction
N	P25811	CC	Exhibits a very high intrinsic GTPase hydrolysis rate
N	P25811	CC	Involved in the addition of a carboxymethylaminomethyl (cmnm) group at the wobble position (U34) of certain tRNAs, forming tRNA- cmnm(5)s(2)U34 (By similarity)
N	P25811	DE	tRNA modification GTPase mnmE;
N	P25811	DR	GTPase activity
N	P25811	DR	metal ion binding
N	P25811	DR	small GTPase mediated signal transduction
N	P25811	DR	tRNA modification
N	P25874	CC	UCP are mitochondrial transporter proteins that create proton leaks across the inner mitochondrial membrane, thus uncoupling oxidative phosphorylation from ATP synthesis
N	P25874	CC	As a result, energy is dissipated in the form of heat.
N	P25874	DE	Mitochondrial brown fat uncoupling protein 1;
N	P25874	DE	Solute carrier family 25 member 7;
N	P25874	DE	Thermogenin;
N	P25874	DR	brown fat cell differentiation
N	P25874	DR	cellular lipid metabolic process
N	P25874	DR	respiratory electron transport chain
N	P25934	DE	Phosphopentokinase;
N	P25934	DE	Phosphoribulokinase, 41 kDa subunit;
N	P25934	DR	phosphoribulokinase activity
N	P25934	DR	reductive pentose-phosphate cycle
N	P25945	CC	Interacts with outer membrane receptor proteins that carry out high-affinity binding and energy dependent uptake into the periplasmic space of specific substrates such as cobalamin, and various iron compounds (such as iron dicitrate, enterochelin, aerobactin, etc.)
N	P25945	CC	In the absence of tonB these receptors bind their substrates but do not carry out active transport
N	P25945	CC	It could act to transduce energy from the cytoplasmic membrane to specific energy-requiring processes in the outer membrane, resulting in the release into the periplasm of ligands bound by these outer membrane proteins.
N	P25945	CC	TonB also interacts with some colicins and is involved in the energy- dependent, irreversible steps of bacteriophages phi 80 and T1 infection
N	P25945	DE	Protein tonB;
N	P25945	DR	bacteriocin transport
N	P25945	DR	iron ion transmembrane transporter activity
N	P25945	DR	protein transporter activity
N	P26182	CC	Actins are highly conserved proteins that are involved in various types of cell motility and are ubiquitously expressed in all eukaryotic cells.
N	P26267	CC	The pyruvate dehydrogenase complex catalyzes the overall conversion of pyruvate to acetyl-CoA and CO(2)

N	P26267	CC	It contains multiple copies of three enzymatic components: pyruvate dehydrogenase (E1), dihydrolipoamide acetyltransferase (E2) and lipoamide dehydrogenase (E3)
N	P26267	DE	Pyruvate dehydrogenase E1 component subunit alpha type I, mitochondrial;
N	P26267	DR	oxidation-reduction process
N	P26267	DR	pyruvate dehydrogenase (acetyl-transferring) activity
N	P26767	CC	Probably activates the RNA polymerase sigma-35 factor at the stage II of sporulation
N	P26767	DE	Putative sporulation sigma factor-processing peptidase;
N	P26767	DR	asexual sporulation
N	P26767	DR	aspartic-type endopeptidase activity
N	P26767	DR	sporulation resulting in formation of a cellular spore
N	P26772	CC	Eukaryotic CPN10 homolog which is essential for mitochondrial protein biogenesis, together with CPN60
N	P26772	CC	Binds to CPN60 in the presence of Mg-ATP and suppresses the ATPase activity of the latter.
N	P26772	DE	10 kDa chaperonin;
N	P26772	DE	10 kDa heat shock protein, mitochondrial;
N	P26772	DE	Chaperonin 10;
N	P26772	DR	chaperone binding
N	P26772	DR	protein folding
N	P26772	DR	response to stress
N	P26837	DE	Uncharacterized protein in sul1 3'region;
N	P26929	DE	Urea amidohydrolase subunit alpha;
N	P26929	DE	Urease subunit alpha;
N	P26929	DR	nickel ion binding
N	P26929	DR	urea metabolic process
N	P26929	DR	urease activity
N	P26989	DE	1,4-alpha-D-glucan glucohydrolase;
N	P26989	DE	Glucan 1,4-alpha-glucosidase;
N	P26989	DE	Glucoamylase GLA1;
N	P26989	DR	glucan 1,4-alpha-glucosidase activity
N	P26989	DR	polysaccharide catabolic process
N	P27054	CC	Defense against chitin containing fungal pathogens.
N	P27054	DE	Endochitinase PR4;
N	P27054	DR	cell wall macromolecule catabolic process
N	P27054	DR	chitin binding
N	P27054	DR	chitin catabolic process
N	P27054	DR	chitinase activity
N	P27054	DR	defense response
N	P27061	DE	Acid phosphatase 1;
N	P27061	DR	acid phosphatase activity
N	P27075	DE	60S ribosomal protein L41;
N	P27075	DE	60S ribosomal protein L44;
N	P27075	DR	response to antibiotic
N	P27075	DR	response to cycloheximide
N	P27075	DR	structural constituent of ribosome
N	P27282	CC	P123 and P123' are short-lived polyproteins, accumulating during early stage of infection
N	P27282	CC	nsP2 has two separate domain with different biological activities
N	P27282	CC	nsP4 is a RNA dependent RNA polymerase
N	P27282	CC	After these early events, P123 and P123' are cleaved sequentially into nsP1, nsP2 and nsP3/nsP3'
N	P27282	CC	By interacting with nsP4, they start viral genome replication into antigenome
N	P27282	CC	It replicates genomic and antigenomic RNA by recognizing replications specific signals
N	P27282	CC	P123 is directly translated from the genome, whereas P123' is a product of the cleavage of P1234
N	P27282	CC	The C-terminal section harbors a protease that specifically cleaves and releases the four mature proteins (By similarity).
N	P27282	CC	The N-terminal section is part of the RNA polymerase complex and has RNA trisphosphatase and RNA helicase activity
N	P27282	CC	They localize the viral replication complex to the cytoplasmic surface of modified endosomes and lysosomes
N	P27282	CC	This 26S mRNA encodes for structural proteins
N	P27282	CC	This sequence of delayed processing would allow correct assembly and membrane association of the RNA polymerase complex (By similarity).
N	P27282	CC	Transcribes also a 26S subgenomic mRNA by initiating RNA synthesis internally on antigenomic RNA

N	P27282	CC	nsP4 is a short-lived protein regulated by several ways: the opal codon readthrough and degradation by ubiquitin pathway (By similarity).
N	P27282	DE	Non-structural polyprotein;
N	P27282	DE	Non-structural protein 1;
N	P27282	DE	Non-structural protein 2;
N	P27282	DE	Non-structural protein 3';
N	P27282	DE	Non-structural protein 3;
N	P27282	DE	Non-structural protein 4;
N	P27282	DE	Polyprotein nsP1234;
N	P27282	DE	Protease nsP2;
N	P27282	DE	RNA-directed RNA polymerase nsP4;
N	P27282	DE	mRNA-capping enzyme nsP1;
N	P27282	DR	RNA-directed RNA polymerase activity
N	P27282	DR	cysteine-type endopeptidase activity
N	P27282	DR	helicase activity
N	P27282	DR	methyltransferase activity
N	P27282	DR	polynucleotide 5'-phosphatase activity
N	P27282	DR	viral genome replication
N	P27321	CC	Specific inhibition of calpain (calcium-dependent cysteine protease)
N	P27321	CC	Plays a key role in postmortem tenderization of meat and have been proposed to be involved in muscle protein degradation in living tissue.
N	P27321	DE	Calpain inhibitor;
N	P27321	DE	Calpastatin;
N	P27321	DR	calcium-dependent cysteine-type endopeptidase inhibitor activity
N	P27599	CC	The heterodimer glycoprotein H-glycoprotein L is required for the fusion of viral and plasma membranes leading to virus entry into the host cell
N	P27599	CC	Membrane fusion is mediated by the fusion machinery composed at least of gB and the heterodimer gH/gL (By similarity).
N	P27599	DE	Envelope glycoprotein H;
N	P27621	CC	Catalyzes the addition of a single glycerol-P residue to the prenolpyrophosphate-linked dissacharide, as a primer for polymerisation by tagF (Potential).
N	P27621	DE	Major teichoic acid biosynthesis protein B;
N	P27621	DE	Putative CDP-glycerol:glycerophosphate glycerophosphotransferase;
N	P27621	DR	CDP-glycerol glycerophosphotransferase activity
N	P27621	DR	cellular cell wall organization
N	P27621	DR	teichoic acid biosynthetic process
N	P27621	DR	transferase activity, transferring glycosyl groups
N	P27654	CC	Seems to have esterase activity
N	P27654	CC	Prefers ester of fatty acids from 4 to 16 carbon atoms.
N	P27654	DE	Temperature shock-inducible protein 1;
N	P27654	DR	fungal-type cell wall organization
N	P27654	DR	hydrolase activity
N	P27654	DR	response to stress
N	P27654	DR	structural constituent of cell wall
N	P27661	CC	Variant histone H2A which replaces conventional H2A in a subset of nucleosomes
N	P27661	CC	DNA accessibility is regulated via a complex set of post-translational modifications of histones, also called histone code, and nucleosome remodeling
N	P27661	CC	Histones thereby play a central role in transcription regulation, DNA repair, DNA replication and chromosomal stability
N	P27661	CC	Nucleosomes wrap and compact DNA into chromatin, limiting DNA accessibility to the cellular machineries which require DNA as a template
N	P27661	CC	Required for checkpoint-mediated arrest of cell cycle progression in response to low doses of ionizing radiation and for efficient repair of DNA double strand breaks (DSBs) specifically when modified by C- terminal phosphorylation.
N	P27661	DE	Histone H2A.x;
N	P27661	DR	damaged DNA binding
N	P27661	DR	double-strand break repair via homologous recombination
N	P27661	DR	nucleosome assembly
N	P27661	DR	spermatogenesis
N	P27678	CC	Shows particularly broad specificity; although bonds involving phenylalanine and leucine are preferred, many others are also cleaved to some extent.
N	P27678	DE	Pepsin I/II;
N	P27678	DR	aspartic-type endopeptidase activity
N	P27761	DE	Amb a 1.3;
N	P27761	DE	Antigen Amb a I;

N	P27761	DE	Pollen allergen Amb a 1.3;
N	P27835	CC	Could be involved in ECA polysaccharide chain elongation.
N	P27835	DE	Putative ECA polymerase;
N	P27835	DR	enterobacterial common antigen biosynthetic process
N	P27882	CC	FAD-dependent sulfhydryl oxidase that catalyzes disulfide bond formation
N	P27882	CC	Forms a redox cycle with MIA40 that involves a disulfide relay system
N	P27882	CC	Important for maintaining the cysteine residues in MIA40 in an oxidized state
N	P27882	CC	Reduced ERV1 is reoxidized by cytochrome c
N	P27882	CC	Required for the import and folding of small cysteine-containing proteins in the mitochondrial intermembrane space (IMS)
N	P27882	CC	Required for the maturation of cytoplasmic, but not of mitochondrial Fe/S proteins
N	P27882	DE	14 kDa regulatory protein;
N	P27882	DE	Essential for respiration and vegetative growth protein 1;
N	P27882	DE	Mitochondrial FAD-linked sulfhydryl oxidase ERV1;
N	P27882	DR	cellular iron ion homeostasis
N	P27882	DR	cellular response to oxidative stress
N	P27882	DR	flavin-linked sulfhydryl oxidase activity
N	P27882	DR	oxidation-reduction process
N	P27882	DR	protein import into mitochondrial intermembrane space
N	P27898	CC	Transcription factor postulated to regulate the biosynthetic pathway of a flavonoid-derived pigment in certain floral tissues.
N	P27898	DE	Myb-related protein P;
N	P28147	CC	General transcription factor that functions at the core of the DNA-binding multiprotein factor TFIID
N	P28147	CC	Binding of TFIID to the TATA box is the initial transcriptional step of the pre-initiation complex (PIC), playing a role in the activation of eukaryotic genes transcribed by RNA polymerase II.
N	P28147	DE	TATA sequence-binding protein 1;
N	P28147	DE	TATA-binding factor 1;
N	P28147	DE	TATA-box factor 1;
N	P28147	DE	TATA-box-binding protein 1;
N	P28147	DE	Transcription initiation factor TFIID TBP-1 subunit;
N	P28147	DR	RNA polymerase II transcription factor activity
N	P28147	DR	protein binding
N	P28147	DR	regulation of transcription, DNA-dependent
N	P28147	DR	transcription initiation from RNA polymerase II promoter
N	P28219	DE	Tyrosine-protein phosphatase 27;
N	P28219	DR	protein dephosphorylation
N	P28219	DR	protein tyrosine phosphatase activity
N	P28229	CC	One gap junction consists of a cluster of closely packed pairs of transmembrane channels, the connexons, through which materials of low MW diffuse from one cell to a neighboring cell.
N	P28229	DE	Connexin-45;
N	P28229	DE	Gap junction alpha-7 protein;
N	P28229	DE	Gap junction gamma-1 protein;
N	P28229	DR	cardiac muscle tissue development
N	P28229	DR	cell development
N	P28229	DR	protein binding
N	P28229	DR	synaptic transmission
N	P28229	DR	vasculogenesis
N	P28229	DR	visual perception
N	P28355	CC	Single-stranded-DNA-specific exonuclease
N	P28355	CC	Required for many types of recombinational events, although the stringency of the requirement for recJ appears to vary with the type of recombinational event monitored and the other recombination gene products which are available.
N	P28355	DE	Single-stranded-DNA-specific exonuclease recJ;
N	P28355	DR	5'-3' exonuclease activity
N	P28355	DR	DNA recombination
N	P28355	DR	manganese ion binding
N	P28355	DR	nucleic acid binding
N	P28386	CC	RuBisCO catalyzes two reactions: the carboxylation of D- ribulose 1,5-bisphosphate, the primary event in carbon dioxide fixation, as well as the oxidative fragmentation of the pentose substrate in the photorespiration process
N	P28386	CC	Both reactions occur simultaneously and in competition at the same active site (By similarity).

N	P28386	DE	Ribulose bisphosphate carboxylase large chain;
N	P28386	DE	RuBisCO large subunit;
N	P28386	DR	magnesium ion binding
N	P28386	DR	monooxygenase activity
N	P28386	DR	oxidation-reduction process
N	P28386	DR	photorespiration
N	P28386	DR	reductive pentose-phosphate cycle
N	P28386	DR	ribulose-bisphosphate carboxylase activity
N	P28419	CC	RuBisCO catalyzes two reactions: the carboxylation of D- ribulose 1,5-bisphosphate, the primary event in carbon dioxide fixation, as well as the oxidative fragmentation of the pentose substrate in the photorespiration process
N	P28419	CC	Both reactions occur simultaneously and in competition at the same active site (By similarity).
N	P28419	DE	Ribulose bisphosphate carboxylase large chain;
N	P28419	DE	RuBisCO large subunit;
N	P28419	DR	magnesium ion binding
N	P28419	DR	monooxygenase activity
N	P28419	DR	oxidation-reduction process
N	P28419	DR	photorespiration
N	P28419	DR	reductive pentose-phosphate cycle
N	P28419	DR	ribulose-bisphosphate carboxylase activity
N	P28504	CC	Hirudin is a potent thrombin-specific protease inhibitor
N	P28504	CC	It forms a stable non-covalent complex with alpha- thrombin, thereby abolishing its ability to cleave fibrinogen.
N	P28504	DR	serine-type endopeptidase inhibitor activity
N	P28609	CC	Participates actively in the response to hyperosmotic and heat shock by preventing the aggregation of stress-denatured proteins, in association with dnaK and grpE
N	P28609	CC	GrpE releases ADP from dnaK; ATP binding to dnaK triggers the release of the substrate protein, thus completing the reaction cycle
N	P28609	CC	It is the nucleotide exchange factor for dnaK and may function as a thermosensor
N	P28609	CC	Several rounds of ATP- dependent interactions between dnaJ, dnaK and grpE are required for fully efficient folding (By similarity).
N	P28609	CC	Unfolded proteins bind initially to dnaJ; upon interaction with the dnaJ-bound protein, dnaK hydrolyzes its bound ATP, resulting in the formation of a stable complex
N	P28609	DE	HSP-70 cofactor;
N	P28609	DE	Protein grpE;
N	P28609	DR	adenyl-nucleotide exchange factor activity
N	P28609	DR	chaperone binding
N	P28609	DR	protein folding
N	P28609	DR	protein homodimerization activity
N	P28609	DR	response to stress
N	P28734	CC	Important for the metabolism of amino acids and Krebs- cycle related organic acids
N	P28734	CC	In plants, it is involved in nitrogen metabolism and in aspects of carbon and energy metabolism.
N	P28734	DE	Aspartate aminotransferase, cytoplasmic;
N	P28734	DE	Transaminase A;
N	P28734	DR	2-oxoglutarate metabolic process
N	P28734	DR	L-aspartate:2-oxoglutarate aminotransferase activity
N	P28734	DR	aspartate metabolic process
N	P28734	DR	biosynthetic process
N	P28734	DR	glutamate metabolic process
N	P28734	DR	pyridoxal phosphate binding
N	P28760	CC	Destroys radicals which are normally produced within the cells and which are toxic to biological systems.
N	P28760	DE	Superoxide dismutase [Mn];
N	P28760	DR	metal ion binding
N	P28760	DR	oxidation-reduction process
N	P28760	DR	superoxide dismutase activity
N	P28760	DR	superoxide metabolic process
N	P28879	CC	Alpha-conotoxins act on postsynaptic membranes, they bind to the nicotinic acetylcholine receptors (nAChR) and thus inhibit them
N	P28879	CC	Has no effect on the release of catecholamines evoked by nicotine.
N	P28879	DE	Alpha-conotoxin S2;
N	P28879	DE	Alpha-conotoxin SII;
N	P28879	DR	acetylcholine receptor inhibitor activity

N	P28879	DR	synaptic transmission
N	P29110	CC	May have a structural role to stabilize the lipid body during desiccation of the seed by preventing coalescence of the oil
N	P29110	CC	May also provide recognition signals for specific lipase anchorage in lipolysis during seedling growth.
N	P29110	CC	Probably interacts with both lipid and phospholipid moieties of lipid bodies
N	P29110	DE	Oleosin Bn-III;
N	P29244	DE	Globin CTT-W;
N	P29244	DR	oxygen binding
N	P29244	DR	oxygen transporter activity
N	P29398	CC	DNA-dependent RNA polymerase catalyzes the transcription of DNA into RNA using the four ribonucleoside triphosphates as substrates.
N	P29398	DE	DNA-directed RNA polymerase subunit beta;
N	P29398	DE	RNA polymerase subunit beta;
N	P29398	DE	RNAP subunit beta;
N	P29398	DE	Transcriptase subunit beta;
N	P29398	DR	DNA-directed RNA polymerase activity
N	P29398	DR	ribonucleoside binding
N	P29422	CC	Decomposes hydrogen peroxide into water and oxygen; serves to protect cells from the toxic effects of hydrogen peroxide.
N	P29422	DR	catalase activity
N	P29422	DR	hydrogen peroxide catabolic process
N	P29422	DR	oxidation-reduction process
N	P29521	CC	This protein promotes the GTP-dependent binding of aminoacyl-tRNA to the A-site of ribosomes during protein biosynthesis.
N	P29521	DE	EF-1-alpha;
N	P29521	DE	Elongation factor 1-alpha;
N	P29521	DR	GTPase activity
N	P29521	DR	translation elongation factor activity
N	P29527	CC	May have a structural role to stabilize the lipid body during desiccation of the seed by preventing coalescence of the oil
N	P29527	CC	May also provide recognition signals for specific lipase anchorage in lipolysis during seedling growth (By similarity).
N	P29527	CC	Probably interacts with both lipid and phospholipid moieties of lipid bodies
N	P29527	DE	Oleosin 18.2 kDa;
N	P29963	CC	Required for the export of heme to the periplasm for the biogenesis of c-type cytochromes (Potential).
N	P29963	DE	Cytochrome c-type biogenesis protein HelD;
N	P29963	DE	Heme exporter protein D;
N	P29963	DR	cytochrome complex assembly
N	P29973	CC	Visual signal transduction is mediated by a G-protein coupled cascade using cGMP as second messenger
N	P29973	CC	This protein can be activated by cyclic GMP which leads to an opening of the cation channel and thereby causing a depolarization of rod photoreceptors.
N	P29973	DE	CNG channel alpha-1;
N	P29973	DE	Cyclic nucleotide-gated cation channel 1;
N	P29973	DE	Cyclic nucleotide-gated channel alpha-1;
N	P29973	DE	Cyclic nucleotide-gated channel, photoreceptor;
N	P29973	DE	Rod photoreceptor cGMP-gated channel subunit alpha;
N	P29973	DE	cGMP-gated cation channel alpha-1;
N	P29973	DR	ion channel activity
N	P29973	DR	response to stimulus
N	P29973	DR	visual perception
N	P30160	DE	Uncharacterized tatC-like protein ycf43;
N	P30557	CC	Receptor for prostaglandin E2 (PGE2); the EP3 receptor may be involved in inhibition of gastric acid secretion, modulation of neurotransmitter release in central and peripheral neurons, inhibition of sodium and water reabsorption in kidney tubulus and contraction in uterine smooth muscle
N	P30557	CC	The activity of this receptor can couple to both the inhibition of adenylate cyclase mediated by G(i) proteins, and to an elevation of intracellular calcium
N	P30557	CC	The various forms can interact with different second messenger systems.
N	P30557	DE	PGE receptor EP3 subtype;
N	P30557	DE	PGE2 receptor EP3 subtype;
N	P30557	DE	Prostaglandin E2 receptor EP3 subtype;
N	P30557	DE	Prostanoid EP3 receptor;

N	P30557	DR	G-protein signaling, coupled to cAMP nucleotide second messenger
N	P30557	DR	activation of phospholipase C activity by G-protein coupled receptor protein signaling pathway coupled to IP3 second messenger
N	P30557	DR	bicarbonate transport
N	P30557	DR	elevation of cytosolic calcium ion concentration
N	P30557	DR	fever generation
N	P30557	DR	positive regulation of fever generation
N	P30557	DR	prostaglandin E receptor activity
N	P30557	DR	protein binding
N	P30713	CC	Catalyzes the inactivation of reactive sulfate esters in carcinogenic arylmethanols
N	P30713	CC	Highest activity towards ethacrynic acid and cumene hydroperoxide.
N	P30713	DE	GST class-theta-2;
N	P30713	DE	Glutathione S-transferase 12;
N	P30713	DE	Glutathione S-transferase Yrs-Yrs;
N	P30713	DE	Glutathione S-transferase theta-2;
N	P30713	DR	glutathione metabolic process
N	P30713	DR	glutathione transferase activity
N	P31005	DE	NAD-dependent methanol dehydrogenase;
N	P31005	DR	metal ion binding
N	P31005	DR	methanol dehydrogenase activity
N	P31005	DR	oxidation-reduction process
N	P31261	CC	Sequence-specific transcription factor which is part of a developmental regulatory system that provides cells with specific positional identities on the anterior-posterior axis
N	P31261	DE	Homeobox protein Hbox-2.8;
N	P31261	DE	Homeobox protein Hox-A2;
N	P31261	DE	NvHbox-2.8;
N	P31261	DR	multicellular organismal development
N	P31261	DR	regulation of transcription, DNA-dependent
N	P31261	DR	sequence-specific DNA binding
N	P31261	DR	sequence-specific DNA binding transcription factor activity
N	P31261	DR	transcription regulator activity
N	P31272	CC	Sequence-specific transcription factor which is part of a developmental regulatory system that provides cells with specific positional identities on the anterior-posterior axis
N	P31272	DE	Homeobox protein Hox-B9;
N	P31272	DR	multicellular organismal development
N	P31272	DR	regulation of transcription, DNA-dependent
N	P31272	DR	sequence-specific DNA binding
N	P31272	DR	sequence-specific DNA binding transcription factor activity
N	P31272	DR	transcription activator activity
N	P31336	CC	May be a component of the oxygen-evolving complex.
N	P31336	DE	Light-regulated unknown 11 kDa protein;
N	P31336	DE	Photosystem II 5 kDa protein, chloroplastic;
N	P31336	DR	photosynthesis
N	P31391	CC	Receptor for somatostatin-14
N	P31391	CC	It is functionally coupled not only to inhibition of adenylate cyclase, but also to activation of both arachidonate release and mitogen-activated protein (MAP) kinase cascade
N	P31391	CC	Mediates antiproliferative action of somatostatin in tumor cells.
N	P31391	CC	The activity of this receptor is mediated by G proteins which inhibits adenylyl cyclase
N	P31391	DE	Somatostatin receptor type 4;
N	P31391	DR	G-protein signaling, coupled to cyclic nucleotide second messenger
N	P31391	DR	negative regulation of cell proliferation
N	P31391	DR	somatostatin receptor activity
N	P31423	CC	Receptor for glutamate
N	P31423	CC	The activity of this receptor is mediated by a G-protein that inhibits adenylate cyclase activity.
N	P31423	DE	Metabotropic glutamate receptor 4;
N	P31423	DR	G-protein coupled receptor activity
N	P31423	DR	gamma-aminobutyric acid secretion
N	P31423	DR	negative regulation of apoptosis
N	P31423	DR	negative regulation of cell proliferation
N	P31423	DR	negative regulation of synaptic transmission
N	P31423	DR	neuron projection development
N	P31423	DR	neuroprotection
N	P31423	DR	synaptic transmission, GABAergic
N	P31423	DR	synaptic transmission, glutamatergic

N	P31433	DE	Uncharacterized protein yicH;
N	P31494	CC	Expression of the urease operon increases the likelihood of bacterial survival by contibuting to acid resistance in vitro and in vivo in BALB/c mice
N	P31494	CC	Y.enterocolitica enters the body via an oral path and must survive the acidic stomach before being able to colonize the intestinal mucosa (PubMed:7558281).
N	P31494	DE	Urea amidohydrolase subunit alpha;
N	P31494	DE	Urease subunit alpha;
N	P31494	DR	nickel ion binding
N	P31494	DR	urea metabolic process
N	P31494	DR	urease activity
N	P31610	CC	Probable component of the transcriptional machinery present in the inner capsid
N	P31610	CC	Displays dsRNA binding activity and may play an important role in the sorting of viral RNA and virion assembly
N	P31610	CC	Together with the RNA-directed RNA polymerase P1 and capping enzyme P5, forms an transcriptional complex positioned near the channels situated at each of the five-fold vertices of the core (By similarity).
N	P31610	DE	60 kDa core protein;
N	P31809	CC	Unknown
N	P31809	CC	In case of murine coronavirus (MHV) infection, serves as receptor for MHV S1 spike glycoprotein.
N	P31809	DE	Biliary glycoprotein 1;
N	P31809	DE	Biliary glycoprotein D;
N	P31809	DE	CD66a;
N	P31809	DE	Carcinoembryonic antigen-related cell adhesion molecule 1;
N	P31809	DE	Murine hepatitis virus receptor;
N	P31809	DR	interspecies interaction between organisms
N	P31809	DR	receptor activity
N	P31956	DE	Penicillin G acylase subunit alpha;
N	P31956	DE	Penicillin G acylase subunit beta;
N	P31956	DE	Penicillin G acylase;
N	P31956	DE	Penicillin G amidase;
N	P31956	DE	Penicillin G amidohydrolase;
N	P31956	DR	antibiotic biosynthetic process
N	P31956	DR	metal ion binding
N	P31956	DR	penicillin amidase activity
N	P31956	DR	response to antibiotic
N	P32162	DE	UPF0381 protein yiiS;
N	P32247	CC	Role in sperm cell division, maturation, or function
N	P32247	CC	This receptor mediates its action by association with G proteins that activate a phosphatidylinositol-calcium second messenger system.
N	P32247	DE	Bombesin receptor subtype-3;
N	P32247	DR	adult feeding behavior
N	P32247	DR	bombesin receptor activity
N	P32247	DR	glucose metabolic process
N	P32247	DR	regulation of blood pressure
N	P32399	DE	Uncharacterized protein yhgE;
N	P32486	CC	Involved in the synthesis of (1->6)- and (1->3)-beta-D- glucan polymers of the yeast cell wall in vivo
N	P32486	CC	It is required for full activity of beta-glucan synthase in vitro
N	P32486	CC	May act as a transglucosidase and contribute to the construction of a protein-bound glucan-structure that acts as an acceptor site for the addition of (1->6)-beta-D-glucan at the cell surface.
N	P32486	CC	May be involved in the maturation and transport of cell wall proteins (CWP) to the cell wall
N	P32486	DE	Beta-glucan synthesis-associated protein KRE6;
N	P32486	DE	Killer toxin-resistance protein 6;
N	P32486	DR	1,6-beta-glucan biosynthetic process
N	P32486	DR	fungal-type cell wall organization
N	P32486	DR	glucosidase activity
N	P32541	CC	The surface protein (SU) attaches the virus to the host cell by binding to its receptor
N	P32541	CC	Fusion occurs at the host cell plasma membrane (By similarity).
N	P32541	CC	This interaction triggers the refolding of the transmembrane protein (TM) and is thought to activate its fusogenic potential by unmasking its fusion peptide
N	P32541	DE	Env polyprotein;
N	P32541	DE	Envelope glycoprotein;

N	P32541	DE	Glycoprotein 45;
N	P32541	DE	Glycoprotein 90;
N	P32541	DE	Surface protein;
N	P32541	DE	Transmembrane protein;
N	P32541	DR	interspecies interaction between organisms
N	P32541	DR	structural molecule activity
N	P32638	DE	Glyceraldehyde-3-phosphate dehydrogenase;
N	P32638	DR	glyceraldehyde-3-phosphate dehydrogenase (NAD+) (phosphorylating) activity
N	P32638	DR	oxidation-reduction process
N	P32720	CC	Part of the binding-protein-dependent transport system AlsBAC for D-allose; probably responsible for the translocation of the substrate across the membrane.
N	P32720	DE	D-allose transport system permease protein AlsC;
N	P32720	DR	carbohydrate transport
N	P32720	DR	transporter activity
N	P32772	DE	Protein UGX2;
N	P32772	DR	protein binding
N	P32847	CC	Band 3 has two functional domains: its integral domain mediates a 1:1 exchange of inorganic anions across the membrane, whereas its cytoplasmic domain provides binding sites for cytoskeletal and other proteins (By similarity).
N	P32847	DE	Anion exchange protein 1;
N	P32847	DE	Anion exchanger 1;
N	P32847	DE	Band 3 anion exchange protein;
N	P32847	DE	Solute carrier family 4 member 1;
N	P32847	DR	inorganic anion exchanger activity
N	P33150	CC	Cadherins are calcium dependent cell adhesion proteins
N	P33150	CC	May act as a negative regulator of neural cell growth.
N	P33150	CC	They preferentially interact with themselves in a homophilic manner in connecting cells; cadherins may thus contribute to the sorting of heterogeneous cell types.
N	P33150	DE	Cadherin-13;
N	P33150	DE	T-cadherin;
N	P33150	DE	Truncated cadherin;
N	P33150	DR	calcium ion binding
N	P33156	CC	Can catalyze the hydrolysis of ATP in the presence of single-stranded DNA, the ATP-dependent uptake of single-stranded DNA by duplex DNA, and the ATP-dependent hybridization of homologous single-stranded DNAs
N	P33156	CC	It interacts with LexA causing its activation and leading to its autocatalytic cleavage.
N	P33156	DE	Protein RecA;
N	P33156	DE	Recombinase A;
N	P33156	DR	DNA recombination
N	P33156	DR	DNA-dependent ATPase activity
N	P33156	DR	single-stranded DNA binding
N	P33197	DE	Isocitrate dehydrogenase [NADP];
N	P33197	DE	NADP(+)-specific ICDH;
N	P33197	DE	Oxalosuccinate decarboxylase;
N	P33197	DR	glyoxylate cycle
N	P33197	DR	isocitrate dehydrogenase (NADP+) activity
N	P33197	DR	magnesium ion binding
N	P33197	DR	tricarboxylic acid cycle
N	P33289	CC	Involved in peroxisome biosynthesis
N	P33289	CC	Essential for growth on oleic acid and methanol as sole carbon source.
N	P33289	CC	Necessary for matrix peroxisomal proteins import
N	P33289	DE	Peroxisomal biogenesis factor 6;
N	P33289	DE	Peroxisome biosynthesis protein PAS5;
N	P33289	DR	nucleoside-triphosphatase activity
N	P33289	DR	peroxisome organization
N	P33655	CC	DNA primase is the polymerase that synthesizes small RNA primers for the Okazaki fragments on both template strands at replication forks during chromosomal DNA synthesis.
N	P33655	DE	DNA primase;
N	P33655	DR	DNA primase activity
N	P33655	DR	DNA replication, synthesis of RNA primer
N	P33655	DR	zinc ion binding
N	P33663	DE	UPF0124 protein PA4543;
N	P33751	CC	Confers resistance to cinnamic acid.
N	P33751	DE	Phenylacrylic acid decarboxylase;

N	P33751	DR	carboxy-lyase activity
N	P33751	DR	cinnamic acid catabolic process
N	P33886	CC	Dosage-dependent regulator of mitosis with serine/ threonine protein kinase activity
N	P33886	CC	It may interact with cdc25, wee1 and win1
N	P33886	CC	May activate sty1.
N	P33886	CC	May play a role in the integration of nutritional sensing with the control over entry into mitosis
N	P33886	DE	Protein kinase sty2;
N	P33886	DE	Protein kinase wis1;
N	P33886	DR	G1 to G0 transition
N	P33886	DR	MAP kinase kinase activity
N	P33886	DR	cell division
N	P33886	DR	cellular response to hydrogen peroxide
N	P33886	DR	positive regulation of establishment of bipolar cell polarity resulting in cell shape
N	P33886	DR	protein binding
N	P33886	DR	protein phosphorylation
N	P33886	DR	protein serine/threonine kinase activity
N	P33886	DR	regulation of mitotic cell cycle
N	P33886	DR	regulation of translation in response to stress
N	P33886	DR	response to arsenic-containing substance
N	P33886	DR	response to osmotic stress
N	P33886	DR	stress-activated MAPK cascade
N	P33898	DE	Putative glyceraldehyde-3-phosphate dehydrogenase C;
N	P33898	DR	glyceraldehyde-3-phosphate dehydrogenase (NAD+) (phosphorylating) activity
N	P33898	DR	oxidation-reduction process
N	P33909	CC	Positive regulation of conjugal transfer of Ti plasmids.
N	P33909	DE	Transcriptional activator protein traR;
N	P33909	DR	quorum sensing
N	P33909	DR	regulation of transcription, DNA-dependent
N	P33909	DR	sequence-specific DNA binding
N	P33909	DR	sequence-specific DNA binding transcription factor activity
N	P33909	DR	two-component response regulator activity
N	P34296	DE	Uncharacterized protein C06E1.1;
N	P34296	DR	protein binding
N	P34306	DE	Uncharacterized protein C06E1.11;
N	P34610	DE	Putative serine protease pcp-1;
N	P34610	DR	serine-type peptidase activity
N	P34698	DE	Uncharacterized protein in CYP107B1 3'region;
N	P34698	DR	transmembrane transport
N	P34812	CC	Light-harvesting photosynthetic bile pigment-protein from the phycobiliprotein complex
N	P34812	CC	Allophycocyanin has a maximum absorption at approximately 650 nanometers.
N	P34812	DE	Allophycocyanin subunit B18;
N	P34812	DE	Allophycocyanin subunit beta-18;
N	P34812	DR	electron transport chain
N	P34812	DR	photosynthesis
N	P34812	DR	protein-chromophore linkage
N	P34896	CC	Interconversion of serine and glycine.
N	P34896	DE	Glycine hydroxymethyltransferase;
N	P34896	DE	Serine hydroxymethyltransferase, cytosolic;
N	P34896	DE	Serine methylase;
N	P34896	DR	L-serine catabolic process
N	P34896	DR	carnitine biosynthetic process
N	P34896	DR	folic acid metabolic process
N	P34896	DR	glycine hydroxymethyltransferase activity
N	P34896	DR	one-carbon metabolic process
N	P34896	DR	protein homodimerization activity
N	P34896	DR	purine base biosynthetic process
N	P34896	DR	pyridoxal phosphate binding
N	P34932	DE	Heat shock 70 kDa protein 4;
N	P34932	DE	Heat shock 70-related protein APG-2;
N	P34932	DR	cellular chaperone-mediated protein complex assembly
N	P34932	DR	protein import into mitochondrial outer membrane
N	P34932	DR	response to unfolded protein
N	P35309	CC	Protamines substitute for histones in the chromatin of sperm during the haploid phase of spermatogenesis

N	P35309	CC	They compact sperm DNA into a highly condensed, stable and inactive complex.
N	P35309	DE	Cysteine-rich protamine;
N	P35309	DE	Sperm protamine P1;
N	P35309	DR	chromosome condensation
N	P35309	DR	multicellular organismal development
N	P35436	CC	NMDA receptor subtype of glutamate-gated ion channels possesses high calcium permeability and voltage-dependent sensitivity to magnesium
N	P35436	CC	Activation requires binding of agonist to both types of subunits.
N	P35436	DE	Glutamate [NMDA] receptor subunit epsilon-1;
N	P35436	DE	N-methyl D-aspartate receptor subtype 2A;
N	P35436	DR	N-methyl-D-aspartate selective glutamate receptor activity
N	P35436	DR	calcium channel activity
N	P35436	DR	directional locomotion
N	P35436	DR	dopamine metabolic process
N	P35436	DR	locomotory behavior
N	P35436	DR	negative regulation of protein catabolic process
N	P35436	DR	positive regulation of apoptosis
N	P35436	DR	protein localization
N	P35436	DR	regulation of excitatory postsynaptic membrane potential
N	P35436	DR	regulation of sensory perception of pain
N	P35436	DR	response to amphetamine
N	P35436	DR	response to drug
N	P35436	DR	response to ethanol
N	P35436	DR	response to wounding
N	P35436	DR	sensory perception of pain
N	P35436	DR	serotonin metabolic process
N	P35436	DR	startle response
N	P35436	DR	visual learning
N	P35436	DR	zinc ion binding
N	P35508	CC	Casein kinases are operationally defined by their preferential utilization of acidic proteins such as caseins as substrates
N	P35508	CC	It can phosphorylate a large number of proteins
N	P35508	CC	Participates in Wnt signaling (By similarity).
N	P35508	DE	Casein kinase I isoform delta;
N	P35508	DR	Wnt receptor signaling pathway
N	P35508	DR	circadian regulation of gene expression
N	P35508	DR	positive regulation of proteasomal ubiquitin-dependent protein catabolic process
N	P35508	DR	protein phosphorylation
N	P35508	DR	protein serine/threonine kinase activity
N	P35508	DR	regulation of circadian rhythm
N	P35510	CC	This is a key enzyme of plant metabolism catalyzing the first reaction in the biosynthesis from L-phenylalanine of a wide variety of natural products based on the phenylpropane skeleton.
N	P35510	DE	Phenylalanine ammonia-lyase 1;
N	P35510	DR	L-phenylalanine catabolic process
N	P35510	DR	biosynthetic process
N	P35510	DR	drought recovery
N	P35510	DR	lignin catabolic process
N	P35510	DR	phenylalanine ammonia-lyase activity
N	P35510	DR	pollen development
N	P35510	DR	response to UV-B
N	P35510	DR	response to karrikin
N	P35510	DR	response to oxidative stress
N	P35510	DR	salicylic acid catabolic process
N	P35567	CC	Plays a key role in the control of the eukaryotic cell cycle
N	P35567	CC	Component of the kinase complex that phosphorylates the repetitive C-terminus of RNA polymerase II (By similarity).
N	P35567	CC	It is required in higher cells for entry into S-phase and mitosis
N	P35567	DE	Cell division control protein 2 homolog 1;
N	P35567	DE	Cell division control protein 2-A;
N	P35567	DE	Cell division protein kinase 1-A;
N	P35567	DE	Cyclin-dependent kinase 1-A;
N	P35567	DE	p34 protein kinase 1;
N	P35567	DR	RNA polymerase II carboxy-terminal domain kinase activity
N	P35567	DR	cell division

N	P35567	DR	cyclin-dependent protein kinase activity
N	P35567	DR	protein binding
N	P35567	DR	protein phosphorylation
N	P35833	CC	Granulocyte/macrophage colony-stimulating factors are cytokines that act in hematopoiesis by controlling the production, differentiation, and function of 2 related white cell populations of the blood, the granulocytes and the monocytes-macrophages
N	P35833	CC	This CSF induces granulocytes.
N	P35833	DE	Granulocyte colony-stimulating factor;
N	P35833	DR	cytokine activity
N	P35833	DR	growth factor activity
N	P35833	DR	immune response
N	P35959	CC	Bypasses host T-cell signaling by inducing a transcriptional program nearly identical to that of anti-CD3 cell activation
N	P35959	CC	Extracellular Nef protein targets CD4(+) T-lymphocytes for apoptosis by interacting with CXCR4 surface receptors (By similarity).
N	P35959	CC	Factor of infectivity and pathogenicity, required for optimal virus replication
N	P35959	CC	Alters numerous pathways of T-lymphocytes function and downregulates immunity surface molecules in order to evade host defense and increase viral infectivity
N	P35959	CC	Alters the functionality of other immunity cells, like dendritic cells, monocytes/macrophages and NK cells
N	P35959	CC	Increasing surface FasL molecules and decreasing surface MHC-I molecules on infected CD4(+) cells send attacking cytotoxic CD8+ T-lymphocytes into apoptosis (By similarity).
N	P35959	CC	Interaction with TCR-zeta chain up-regulates the Fas ligand (FasL)
N	P35959	CC	One of the earliest and most abundantly expressed viral proteins (By similarity).
N	P35959	DE	C-terminal core protein;
N	P35959	DE	Negative factor;
N	P35959	DE	Protein Nef;
N	P35959	DR	SH3 domain binding
N	P35959	DR	evasion by virus of host immune response
N	P35959	DR	regulation of defense response to virus by virus
N	P36026	DE	Deubiquitinating enzyme 11;
N	P36026	DE	Ubiquitin carboxyl-terminal hydrolase 11;
N	P36026	DE	Ubiquitin thiolesterase 11;
N	P36026	DE	Ubiquitin-specific-processing protease 11;
N	P36026	DR	ubiquitin thiolesterase activity
N	P36026	DR	ubiquitin-dependent protein catabolic process
N	P36026	DR	ubiquitin-specific protease activity
N	P36087	DE	Uncharacterized protein YKL070W;
N	P36335	CC	This protein is a cell adhesion molecule involved in neuron-neuron adhesion, neurite fasciculation, outgrowth of neurites, etc.
N	P36335	DE	Neural cell adhesion molecule 1-B;
N	P36335	DR	cell adhesion
N	P36454	CC	One of the primary rRNA binding proteins, it binds directly to 16S rRNA where it nucleates assembly of the body of the 30S subunit (By similarity).
N	P36454	DE	30S ribosomal protein S4, chloroplastic;
N	P36454	DR	structural constituent of ribosome
N	P36787	CC	E2 regulates viral transcription and DNA replication
N	P36787	CC	It binds to the E2RE response element (5'-ACCNNNNNNGGT-3') present in multiple copies in the regulatory region
N	P36787	CC	It can either activate or repress transcription depending on E2RE's position with regards to proximal promoter elements
N	P36787	CC	Repression occurs by sterically hindering the assembly of the transcription initiation complex
N	P36787	CC	The E1-E2 complex binds to the origin of DNA replication.
N	P36787	DE	Regulatory protein E2;
N	P36787	DR	DNA replication
N	P36787	DR	nucleotide binding
N	P36787	DR	regulation of DNA replication
N	P36787	DR	regulation of transcription, DNA-dependent
N	P36787	DR	sequence-specific DNA binding transcription factor activity
N	P36787	DR	viral reproduction
N	P36821	CC	E7 protein has both transforming and trans-activating activities
N	P36821	CC	Disrupts the function of host retinoblastoma protein RB1/pRb, which is a key regulator of the cell cycle

N	P36821	CC	Inactivation of the ability of RB1 to arrest the cell cycle is critical for cellular transformation, uncontrolled cellular growth and proliferation induced by viral infection
N	P36821	CC	Induces the disassembly of the E2F1 transcription factors from RB1, with subsequent transcriptional activation of E2F1-regulated S-phase genes
N	P36821	CC	Interferes with histone deacetylation mediated by HDAC1 and HDAC2, leading to activation of transcription (By similarity).
N	P36821	CC	Stimulation of progression from G1 to S phase allows the virus to efficiently use the cellular DNA replicating machinery to achieve viral genome replication
N	P36821	DR	interspecies interaction between organisms
N	P36821	DR	metal ion binding
N	P36821	DR	regulation of transcription, DNA-dependent
N	P36821	DR	sequence-specific DNA binding transcription factor activity
N	P36961	CC	Kills Lactococci.
N	P36961	DE	Bacteriocin lactococcin-G subunit alpha;
N	P36961	DR	defense response to bacterium
N	P36976	CC	May play an important role in the synaptic function of specific neuronal systems
N	P36976	CC	Associates with proteins involved in vesicle docking and membrane fusion.
N	P36976	DE	Synaptosomal-associated 25 kDa protein;
N	P36976	DE	Synaptosomal-associated protein 25;
N	P37417	CC	Cell wall formation.
N	P37417	DE	UDP-N-acetylenolpyruvoylglucosamine reductase;
N	P37417	DE	UDP-N-acetylmuramate dehydrogenase;
N	P37417	DR	UDP-N-acetylmuramate dehydrogenase activity
N	P37417	DR	cell division
N	P37417	DR	cellular cell wall organization
N	P37417	DR	flavin adenine dinucleotide binding
N	P37417	DR	oxidation-reduction process
N	P37417	DR	peptidoglycan biosynthetic process
N	P37417	DR	regulation of cell shape
N	P37538	DE	Uncharacterized protein yaaQ;
N	P37640	DE	Putative HTH-type transcriptional regulator yhjB;
N	P37640	DR	protein binding
N	P37640	DR	regulation of transcription, DNA-dependent
N	P37640	DR	sequence-specific DNA binding
N	P37640	DR	sequence-specific DNA binding transcription factor activity
N	P37640	DR	two-component response regulator activity
N	P37681	DE	Putative outer membrane protein yiaT;
N	P37685	CC	Catalyzes the NADP-dependent oxidation of diverse aldehydes such as chloroacetaldehyde, acetaldehyde, propionaldehyde, benzaldehyde, mafosfamide, 4- hydroperoxycyclophosphamide
N	P37685	CC	Its preferred substrates are acetaldehyde and chloroacetaldehyde.
N	P37685	DE	Aldehyde dehydrogenase B;
N	P37685	DR	aldehyde dehydrogenase (NAD) activity
N	P37685	DR	oxidation-reduction process
N	P37685	DR	protein binding
N	P37685	DR	response to DNA damage stimulus
N	P37685	DR	response to ethanol
N	P37695	CC	Probably activates the acyl carrier protein (ACP) domain of hetM, by transferring the 4'-phosphopantetheinyl moiety of coenzyme A (CoA) to a serine residue
N	P37695	CC	May be required for maintaining vegetative growth and probably acts via hetN to inhibit differentiation.
N	P37695	DE	4'-phosphopantetheinyl transferase hetI;
N	P37695	DR	holo-[acyl-carrier-protein] synthase activity
N	P37695	DR	macromolecule biosynthetic process
N	P37695	DR	magnesium ion binding
N	P38061	DE	60S ribosomal protein L32;
N	P38061	DR	structural constituent of ribosome
N	P38076	DE	Cysteine synthase;
N	P38076	DE	O-acetylserine (thiol)-lyase;
N	P38076	DE	O-acetylserine sulfhydrylase;
N	P38076	DR	cysteine biosynthetic process from serine
N	P38076	DR	cysteine synthase activity
N	P38076	DR	pyridoxal phosphate binding
N	P38076	DR	transferase activity
N	P38079	DE	Protein YRO2;

N	P38079	DR	ion channel activity
N	P38180	DE	Uncharacterized protein YBL081W;
N	P38180	DR	protein binding
N	P38266	DE	Altered inheritance of mitochondria protein 3;
N	P38266	DR	actin cortical patch assembly
N	P38266	DR	protein binding
N	P38274	CC	Involved in the control of the cell cycle at the G2/M (mitosis) transition
N	P38274	CC	Acts as a negative regulator of the filamentous growth-signaling pathway through inhibition of STE20.
N	P38274	CC	Cooperates with HSL1 to hyperphosphorylate SWE1, thereby targeting SWE1 for polyubiquitination and subsequent degradation
N	P38274	DE	Protein arginine N-methyltransferase HSL7;
N	P38274	DR	G2/M transition of mitotic cell cycle
N	P38274	DR	cell division
N	P38274	DR	cell morphogenesis
N	P38274	DR	histone-arginine N-methyltransferase activity
N	P38274	DR	identical protein binding
N	P38274	DR	positive regulation of mitosis
N	P38274	DR	protein-arginine omega-N monomethyltransferase activity
N	P38274	DR	protein-arginine omega-N symmetric methyltransferase activity
N	P38375	CC	Involved in protein export
N	P38375	CC	Interacts with secA and secE to allow the translocation of proteins across the plasma membrane, by forming part of a channel.
N	P38375	DE	Preprotein translocase subunit secY;
N	P38375	DR	P-P-bond-hydrolysis-driven protein transmembrane transporter activity
N	P38511	CC	This protein binds specifically to 23S rRNA; its binding is stimulated by other ribosomal proteins, e.g., L4, L17, and L20
N	P38511	CC	It is important during the early stages of 50S assembly
N	P38511	CC	It makes multiple contacts with different domains of the 23S rRNA in the assembled 50S subunit and ribosome (By similarity).
N	P38511	DE	50S ribosomal protein L22;
N	P38511	DR	structural constituent of ribosome
N	P38891	CC	Catalyzes the first reaction in the catabolism of the essential branched chain amino acids leucine, isoleucine, and valine
N	P38891	CC	Appears to be involved in the regulation of the transition from G1 to S phase in the cell cycle
N	P38891	CC	High copy suppressor of a temperature-sensitive mutation in the ABC transporter, ATM1.
N	P38891	DE	Branched-chain-amino-acid aminotransferase, mitochondrial;
N	P38891	DE	Protein ECA39;
N	P38891	DE	Protein TWT1;
N	P38891	DR	L-isoleucine transaminase activity
N	P38891	DR	L-leucine transaminase activity
N	P38891	DR	L-valine transaminase activity
N	P38891	DR	branched chain family amino acid biosynthetic process
N	P38891	DR	branched chain family amino acid catabolic process
N	P38891	DR	cellular response to UV
N	P38891	DR	mitotic cell cycle G1/S transition DNA damage checkpoint
N	P38958	CC	Required for the biogenesis of cytochrome c oxidase
N	P38958	CC	Probably for its assembly into an active holoenzyme.
N	P38958	DE	Protein PET100, mitochondrial;
N	P38958	DR	aerobic respiration
N	P38958	DR	mitochondrial respiratory chain complex IV assembly
N	P38958	DR	unfolded protein binding
N	P39149	CC	Catalyzes the conversion of uracil and 5-phospho-alpha- D-ribose 1-diphosphate (PRPP) to UMP and diphosphate (By similarity).
N	P39149	DE	UMP pyrophosphorylase;
N	P39149	DE	Uracil phosphoribosyltransferase;
N	P39149	DR	nucleoside metabolic process
N	P39149	DR	uracil phosphoribosyltransferase activity
N	P39149	DR	uracil salvage
N	P39151	DE	Stage II sporulation protein R;
N	P39151	DR	sporulation resulting in formation of a cellular spore
N	P39434	CC	Murein-degrading enzyme

N	P39434	CC	Catalyzes the cleavage of the glycosidic bonds between N-acetylmuramic acid and N- acetylglucosamine residues in peptidoglycan
N	P39434	CC	May play a role in recycling of muropeptides during cell elongation and/or cell division (By similarity).
N	P39434	DE	Peptidoglycan lytic exotransglycosylase;
N	P39434	DE	Soluble lytic murein transglycosylase;
N	P39434	DR	carbon-oxygen lyase activity, acting on polysaccharides
N	P39434	DR	cellular cell wall organization
N	P39434	DR	hydrolase activity, hydrolyzing O-glycosyl compounds
N	P39434	DR	lytic transglycosylase activity
N	P39434	DR	peptidoglycan metabolic process
N	P39514	CC	This endonuclease is specific to the nrdB gene splice junction and is involved in intron homing (By similarity).
N	P39514	DE	Defective intron-associated endonuclease 3;
N	P39514	DR	endonuclease activity
N	P39514	DR	intron homing
N	P39696	CC	Dispensable for transformability
N	P39696	CC	Not known if it can act as a pyrroline-5-carboxylate reductase.
N	P39696	DE	ComE operon protein 4;
N	P39696	DR	oxidation-reduction process
N	P39696	DR	proline biosynthetic process
N	P39696	DR	pyrroline-5-carboxylate reductase activity
N	P39785	CC	Functions as a terminase.
N	P39785	DE	PBSX phage terminase small subunit;
N	P39785	DR	DNA packaging
N	P39918	DE	Replication-associated recombination protein A;
N	P39918	DR	nucleoside-triphosphatase activity
N	P40034	CC	Histone demethylase that specifically demethylates 'Lys- 36' of histone H3, thereby playing a central role in histone code
N	P40034	CC	Does not demethylate H3 'Lys-4' nor 'Lys-79'.
N	P40034	DE	JmjC domain-containing histone demethylation protein 1;
N	P40034	DE	Jumonji/ARID domain-containing protein 1;
N	P40034	DE	[Histone-H3]-lysine-36 demethylase 1;
N	P40034	DR	histone demethylase activity (H3-K36 specific)
N	P40034	DR	methylated histone residue binding
N	P40034	DR	oxidation-reduction process
N	P40034	DR	oxidoreductase activity, acting on single donors with incorporation of molecular oxygen, incorporation of two atoms of oxygen
N	P40034	DR	zinc ion binding
N	P40042	CC	May be involved in the modulation of rDNA transcription.
N	P40042	DE	Regulator of rDNA transcription protein 13;
N	P40304	CC	The proteasome is a multicatalytic proteinase complex which is characterized by its ability to cleave peptides with Arg, Phe, Tyr, Leu, and Glu adjacent to the leaving group at neutral or slightly basic pH
N	P40304	CC	The proteasome has an ATP-dependent proteolytic activity.
N	P40304	DE	Proteasome 26 kDa subunit;
N	P40304	DE	Proteasome subunit beta type-1;
N	P40304	DR	protein binding
N	P40304	DR	proteolysis involved in cellular protein catabolic process
N	P40304	DR	threonine-type endopeptidase activity
N	P40343	CC	Component of the ESCRT-0 complex which is the sorting receptor for ubiquitinated cargo proteins at the multivesicular body (MVB) and recruits ESCRT-I to the MVB outer membrane
N	P40343	CC	Allows VPS10 to return to the (trans-Golgi network) TGN from the PVC
N	P40343	CC	Controls exit from the prevacuolar compartment (PVC) in both the forward direction to the vacuole and the return to the Golgi
N	P40343	CC	Might also function as an alternate adapter in the COPIb clathrin-like coat.
N	P40343	DE	Golgi retention defective protein 11;
N	P40343	DE	Vacuolar protein sorting-associated protein 27;
N	P40343	DR	late endosome to vacuole transport
N	P40343	DR	phosphatidylinositol-3-phosphate binding
N	P40343	DR	protein retention in Golgi apparatus
N	P40343	DR	protein targeting to vacuole
N	P40343	DR	ubiquitin binding
N	P40343	DR	zinc ion binding

N	P40462	CC	Putative zinc aminopeptidase which may be involved in ribosome biogenesis.
N	P40462	DE	108 kDa translation machinery-associated protein;
N	P40462	DE	Protein TMA108;
N	P40462	DR	aminopeptidase activity
N	P40462	DR	metallopeptidase activity
N	P40462	DR	ribosome biogenesis
N	P40462	DR	zinc ion binding
N	P40536	DE	Putative uncharacterized protein YIL032C;
N	P40653	DE	SRY-related protein MG44;
N	P40692	CC	Heterodimerizes with PMS2 to form MutL alpha, a component of the post-replicative DNA mismatch repair system (MMR)
N	P40692	CC	Also implicated in DNA damage signaling, a process which induces cell cycle arrest and can lead to apoptosis in case of major DNA damages
N	P40692	CC	Assembly of the MutL-MutS- heteroduplex ternary complex in presence of RFC and PCNA is sufficient to activate endonuclease activity of PMS2
N	P40692	CC	DNA methylation would prevent cleavage and therefore assure that only the newly mutated DNA strand is going to be corrected
N	P40692	CC	DNA repair is initiated by MutS alpha (MSH2-MSH6) or MutS beta (MSH2-MSH6) binding to a dsDNA mismatch, then MutL alpha is recruited to the heteroduplex
N	P40692	CC	Heterodimerizes with MLH3 to form MutL gamma which plays a role in meiosis.
N	P40692	CC	It introduces single-strand breaks near the mismatch and thus generates new entry points for the exonuclease EXO1 to degrade the strand containing the mismatch
N	P40692	CC	MutL alpha (MLH1-PMS2) interacts physically with the clamp loader subunits of DNA polymerase III, suggesting that it may play a role to recruit the DNA polymerase III to the site of the MMR
N	P40692	DE	DNA mismatch repair protein Mlh1;
N	P40692	DE	MutL protein homolog 1;
N	P40692	DR	ATPase activity
N	P40692	DR	mismatch repair
N	P40692	DR	protein binding
N	P40692	DR	somatic hypermutation of immunoglobulin genes
N	P41016	CC	Affects cell viability at low temperatures.
N	P41016	DE	Cold shock protein CspB;
N	P41016	DR	regulation of transcription, DNA-dependent
N	P41016	DR	response to stress
N	P41067	CC	Involved in F pilus assembly.
N	P41067	DE	Protein traB;
N	P41182	CC	Transcriptional repressor which is required for germinal center formation and antibody affinity maturation
N	P41182	CC	Probably plays an important role in lymphomagenesis.
N	P41182	DE	B-cell lymphoma 5 protein;
N	P41182	DE	B-cell lymphoma 6 protein;
N	P41182	DE	Protein LAZ-3;
N	P41182	DE	Zinc finger and BTB domain-containing protein 27;
N	P41182	DE	Zinc finger protein 51;
N	P41182	DR	chromatin binding
N	P41182	DR	negative regulation of B cell apoptosis
N	P41182	DR	negative regulation of S phase of mitotic cell cycle
N	P41182	DR	negative regulation of cell growth
N	P41182	DR	negative regulation of transcription from RNA polymerase II promoter
N	P41182	DR	positive regulation of apoptosis
N	P41182	DR	protein binding
N	P41182	DR	protein import into nucleus, translocation
N	P41182	DR	regulation of germinal center formation
N	P41182	DR	response to DNA damage stimulus
N	P41182	DR	sequence-specific DNA binding
N	P41182	DR	sequence-specific DNA binding transcription factor activity
N	P41182	DR	transcription repressor activity
N	P41182	DR	zinc ion binding
N	P41262	DE	Hemoglobin III;
N	P41262	DE	Hemoglobin-3;
N	P41262	DR	oxygen binding
N	P41262	DR	oxygen transporter activity
N	P41345	CC	May play a key role in regulating the relative amounts of cyclic and non-cyclic electron flow to meet the demands of the plant for ATP and reducing power

N	P41345	CC	Is involved in nitrate assimilation.
N	P41345	DE	Ferredoxin--NADP reductase, root isozyme, chloroplastic;
N	P41345	DR	electron transport chain
N	P41345	DR	ferredoxin-NADP+ reductase activity
N	P41345	DR	flavin adenine dinucleotide binding
N	P41345	DR	photosynthesis
N	P41352	CC	Tubulin is the major constituent of microtubules
N	P41352	CC	It binds two moles of GTP, one at an exchangeable site on the beta chain and one at a non-exchangeable site on the alpha-chain.
N	P41352	DE	Beta-tubulin;
N	P41352	DE	Tubulin beta chain;
N	P41352	DR	GTPase activity
N	P41352	DR	microtubule-based movement
N	P41352	DR	protein polymerization
N	P41352	DR	structural molecule activity
N	P41444	DE	Fibroblast growth factor homolog;
N	P41444	DR	growth factor activity
N	P41472	DE	Uncharacterized 11.5 kDa protein in IAP2-VLF1 intergenic region;
N	P41589	DE	Melanotropin alpha;
N	P41589	DR	hormone activity
N	P41767	CC	Mitochondrial peptide chain release factor that directs the termination of translation in response to the peptide chain termination codons UAA and UAG.
N	P41767	DE	Peptide chain release factor 1, mitochondrial;
N	P41767	DR	translation release factor activity, codon specific
N	P41863	DE	CalliFMRFamide-8;
N	P41863	DR	neuropeptide signaling pathway
N	P41935	DE	Homeobox protein ceh-10;
N	P41935	DR	cell fate specification
N	P41935	DR	neuron migration
N	P41935	DR	regulation of cell migration
N	P41935	DR	regulation of transcription, DNA-dependent
N	P41935	DR	sequence-specific DNA binding
N	P41935	DR	sequence-specific DNA binding transcription factor activity
N	P41935	DR	tail morphogenesis
N	P41935	DR	transcription regulator activity
N	P42306	DE	Uncharacterized MFS-type transporter yxiO;
N	P42369	CC	Participates actively in the response to hyperosmotic and heat shock by preventing the aggregation of stress-denatured proteins, in association with dnaK and grpE
N	P42369	CC	GrpE releases ADP from dnaK; ATP binding to dnaK triggers the release of the substrate protein, thus completing the reaction cycle
N	P42369	CC	It is the nucleotide exchange factor for dnaK and may function as a thermosensor
N	P42369	CC	Several rounds of ATP- dependent interactions between dnaJ, dnaK and grpE are required for fully efficient folding (By similarity).
N	P42369	CC	Unfolded proteins bind initially to dnaJ; upon interaction with the dnaJ-bound protein, dnaK hydrolyzes its bound ATP, resulting in the formation of a stable complex
N	P42369	DE	HSP-70 cofactor;
N	P42369	DE	Protein grpE;
N	P42369	DR	adenyl-nucleotide exchange factor activity
N	P42369	DR	chaperone binding
N	P42369	DR	protein folding
N	P42369	DR	protein homodimerization activity
N	P42369	DR	response to stress
N	P42516	CC	Converts p-coumaric acid into p-coumaryl CoA
N	P42516	CC	This is necessary for the activation of the photoactive yellow protein (PYP) chromophore.
N	P42516	DE	4-coumarate--CoA ligase;
N	P42516	DE	4-coumaroyl-CoA synthase;
N	P42516	DR	4-coumarate-CoA ligase activity
N	P42516	DR	phenylpropanoid metabolic process
N	P42675	CC	Hydrolyzes oligopeptides such as neurotensin, bradykinin and dynorphin A (By similarity).
N	P42675	DE	Microsomal endopeptidase;
N	P42675	DE	Mitochondrial oligopeptidase M;
N	P42675	DE	Neurolysin, mitochondrial;
N	P42675	DE	Neurotensin endopeptidase;

N	P42675	DR	metal ion binding
N	P42675	DR	metalloendopeptidase activity
N	P42689	DE	Tyrosine-protein kinase SRK3;
N	P42689	DR	non-membrane spanning protein tyrosine kinase activity
N	P42689	DR	protein phosphorylation
N	P42728	CC	The actions of the proteins tfxB, tfxD and tfxF are implicated in the processing of the inactive trifolitoxin (tfxA) precursor into the active peptide.
N	P42728	DE	Trifolitoxin-processing protein tfxF;
N	P42783	CC	May participate in wall plasticization and/or intussusception of in cell wall turnover
N	P42783	DE	Wall-associated proteinase;
N	P42783	DR	serine-type peptidase activity
N	P42881	CC	Catalyzes the initial step in the synthesis of dolichol- P-P-oligosaccharides.
N	P42881	DE	GlcNAc-1-P transferase;
N	P42881	DE	N-acetylglucosamine-1-phosphate transferase;
N	P42881	DE	UDP-N-acetylglucosamine--dolichyl-phosphate N-acetylglucosaminephosphotransferase;
N	P42881	DR	UDP-N-acetylglucosamine-dolichyl-phosphate N-acetylglucosaminephosphotransferase activity
N	P42881	DR	phospho-N-acetylmuramoyl-pentapeptide-transferase activity
N	P42881	DR	protein N-linked glycosylation
N	P42881	DR	transferase activity, transferring glycosyl groups
N	P43053	CC	DNA gyrase negatively supercoils closed circular double- stranded DNA in an ATP-dependent manner and also catalyzes the interconversion of other topological isomers of double-stranded DNA rings, including catenanes and knotted rings
N	P43053	DE	DNA gyrase subunit B;
N	P43053	DR	DNA topoisomerase (ATP-hydrolyzing) activity
N	P43053	DR	DNA topological change
N	P43151	CC	Guanine nucleotide-binding proteins (G proteins) are involved as modulators or transducers in various transmembrane signaling systems.
N	P43151	DE	Putative guanine nucleotide-binding protein subunit alpha;
N	P43151	DR	G-protein coupled receptor protein signaling pathway
N	P43151	DR	aminoacyl-tRNA ligase activity
N	P43151	DR	signal transducer activity
N	P43151	DR	tRNA aminoacylation for protein translation
N	P43172	DE	FMRFamide-like neuropeptide AF9;
N	P43172	DR	neuropeptide signaling pathway
N	P43462	CC	Probably involved in the positive regulation of the thc operon for the degradation of the thiocarbamate herbicide EPTC.
N	P43462	DE	Probable thc operon regulatory protein;
N	P43462	DR	regulation of transcription, DNA-dependent
N	P43462	DR	sequence-specific DNA binding
N	P43462	DR	sequence-specific DNA binding transcription factor activity
N	P43810	CC	Involved in cell division, probably involved in intracellular septation (By similarity)
N	P43810	DE	Probable intracellular septation protein;
N	P43810	DR	barrier septum formation
N	P43852	DE	AICAR transformylase;
N	P43852	DE	Bifunctional purine biosynthesis protein purH;
N	P43852	DE	IMP cyclohydrolase;
N	P43852	DE	IMP synthase;
N	P43852	DE	Inosinicase;
N	P43852	DE	Phosphoribosylaminoimidazolecarboxamide formyltransferase;
N	P43852	DR	IMP biosynthetic process
N	P43852	DR	IMP cyclohydrolase activity
N	P43852	DR	phosphoribosylaminoimidazolecarboxamide formyltransferase activity
N	P43868	CC	Catalyzes the ferrous insertion into protoporphyrin IX.
N	P43868	DE	Ferrochelatase;
N	P43868	DE	Heme synthase;
N	P43868	DE	Protoheme ferro-lyase;
N	P43868	DR	ferrochelatase activity
N	P43868	DR	heme biosynthetic process
N	P43868	DR	metal ion binding
N	P44213	DE	Putative uncharacterized protein HI_1486 in Mu-like prophage FluMu region;
N	P44373	CC	One of the primary rRNA binding proteins, it binds directly to 16S rRNA where it nucleates assembly of the body of the 30S subunit (By similarity).
N	P44373	DE	30S ribosomal protein S4;
N	P44373	DR	structural constituent of ribosome

N	P44564	CC	Negative regulator of replication initiation
N	P44564	CC	Could block the allosteric transition of dnaA from an inactive form to an active form (By similarity).
N	P44564	CC	Required for sequestration, a process that blocks secondary initiation events
N	P44564	DE	Protein seqA homolog;
N	P44564	DR	negative regulation of DNA-dependent DNA replication initiation
N	P44757	DE	Uncharacterized HTH-type transcriptional regulator HI_0570;
N	P44757	DR	regulation of transcription, DNA-dependent
N	P44757	DR	sequence-specific DNA binding transcription factor activity
N	P44757	DR	specific transcriptional repressor activity
N	P44940	DE	Uncharacterized protein HI_0929;
N	P44940	DR	catalytic activity
N	P45107	CC	Involved in acetate metabolism (By similarity).
N	P45107	DE	Phosphate acetyltransferase;
N	P45107	DE	Phosphotransacetylase;
N	P45107	DR	phosphate acetyltransferase activity
N	P45291	CC	Formation of pseudouridine at positions 38, 39 and 40 in the anticodon stem and loop of transfer RNAs (By similarity).
N	P45291	DE	tRNA pseudouridine synthase A;
N	P45291	DE	tRNA pseudouridylate synthase I;
N	P45291	DE	tRNA-uridine isomerase I;
N	P45291	DR	pseudouridine synthase activity
N	P45291	DR	pseudouridine synthesis
N	P45291	DR	tRNA processing
N	P45359	DE	Acetoacetyl-CoA thiolase;
N	P45359	DE	Acetyl-CoA acetyltransferase;
N	P45359	DR	acetyl-CoA C-acetyltransferase activity
N	P45480	DE	GSH synthetase;
N	P45480	DE	Glutathione synthase;
N	P45480	DE	Glutathione synthetase;
N	P45480	DR	glutathione synthase activity
N	P45480	DR	metal ion binding
N	P45522	CC	Transport system that facilitates potassium-efflux, possibly by potassium-proton antiport
N	P45522	DE	Glutathione-regulated potassium-efflux system protein kefB;
N	P45522	DE	K(+)/H(+) antiporter;
N	P45522	DE	NEM-activable K(+)/H(+) antiporter;
N	P45522	DR	catalytic activity
N	P45522	DR	glutathione-regulated potassium exporter activity
N	P45522	DR	solute:hydrogen antiporter activity
N	P45663	CC	Beta toxins bind voltage-independently at site-4 of sodium channels and shift the voltage of activation toward more negative potentials thereby affecting sodium channel activation and promoting spontaneous and repetitive firing
N	P45663	CC	At high concentrations, it does displace the (beta) mammal-specific toxin Cn2 from rat brain synaptosomes.
N	P45663	CC	This toxin is lethal to crustaceans (freshwater crayfish (Cambarellus montezumae spp.)), it provokes a reversible paralysis to insects (crickets (Achaeta spp.)), but is not toxic to mice
N	P45663	DE	Beta-toxin Cn5;
N	P45663	DR	defense response
N	P45663	DR	sodium channel inhibitor activity
N	P45822	CC	Produces ATP from ADP in the presence of a proton gradient across the membrane
N	P45822	DE	ATP synthase F1 sector epsilon subunit;
N	P45822	DE	ATP synthase epsilon chain;
N	P45822	DE	F-ATPase epsilon subunit;
N	P45822	DR	ATP synthesis coupled proton transport
N	P45822	DR	hydrogen ion transporting ATP synthase activity, rotational mechanism
N	P45822	DR	proton-transporting ATPase activity, rotational mechanism
N	P45845	CC	Responsible for the post-translational oxidative deamination of peptidyl lysine residues in precursors to fibrous collagen and elastin.
N	P45845	DE	Lysyl oxidase;
N	P45845	DE	Protein-lysine 6-oxidase;
N	P45845	DR	copper ion binding
N	P45845	DR	oxidation-reduction process
N	P45845	DR	protein-lysine 6-oxidase activity
N	P45919	DE	Uncharacterized protein yqbC;

N	P46024	DE	80 kDa D15 antigen;
N	P46024	DE	Outer membrane protein D15;
N	P46024	DE	Protective surface antigen D15;
N	P46079	DE	Uncharacterized protein alr2393;
N	P46085	DE	Hemagglutinin component HA-22A;
N	P46085	DE	Hemagglutinin component HA-22B;
N	P46085	DE	Hemagglutinin component HA-23A;
N	P46085	DE	Hemagglutinin component HA-23B;
N	P46085	DE	Hemagglutinin component HA-53;
N	P46085	DE	Hemagglutinin components HA-22/23/53;
N	P46325	DE	Uncharacterized protein yxbA;
N	P46439	CC	Conjugation of reduced glutathione to a wide number of exogenous and endogenous hydrophobic electrophiles.
N	P46439	DE	GST class-mu 5;
N	P46439	DE	Glutathione S-transferase Mu 5;
N	P46439	DR	glutathione transferase activity
N	P46439	DR	xenobiotic metabolic process
N	P46555	CC	Converts D-glucuronic acid residues adjacent to N- sulfate sugar residues to L-iduronic acids (By similarity).
N	P46555	DE	D-glucuronyl C5-epimerase;
N	P46555	DE	Heparin/heparan sulfate:glucuronic acid C5-epimerase;
N	P46555	DR	glycosaminoglycan biosynthetic process
N	P46555	DR	positive regulation of growth rate
N	P46555	DR	positive regulation of multicellular organism growth
N	P46555	DR	racemase and epimerase activity, acting on carbohydrates and derivatives
N	P46910	CC	Activates, in anaerobic conditions, the transcription of the fermentative operons lctEP and alsDS, of the hmp gene encoding a flavohemoglobin-like protein, the nitrite reductase operon nasDE and the heme biosynthesis genes hemN and hemZ
N	P46910	DE	Anaerobic respiration and fermentation modulator;
N	P46910	DE	Probable transcription regulator ArfM;
N	P46924	DE	Probable HTH-type transcriptional regulator leuO;
N	P46924	DR	regulation of transcription, DNA-dependent
N	P46924	DR	sequence-specific DNA binding transcription factor activity
N	P47019	CC	With ARX1, involved in proper assembly of pre-ribosomal particles during the biogenesis of the 60S ribosomal subunit
N	P47019	CC	Accompanies the pre-60S particles to the cytoplasm.
N	P47019	DE	Ribosome biogenesis protein ALB1;
N	P47019	DR	protein binding
N	P47019	DR	ribosomal large subunit biogenesis
N	P47313	DE	Uncharacterized lipoprotein MG067;
N	P47313	DR	RNA helicase activity
N	P47331	CC	Catalyzes the ATP- as well as the pyrophosphate- dependent phosphorylation of a specific serine residue in HPr, a phosphocarrier protein of the phosphoenolpyruvate-dependent sugar phosphotransferase system (PTS)
N	P47331	CC	HprK/P also catalyzes the pyrophosphate-producing, inorganic phosphate-dependent dephosphorylation (phosphorolysis) of seryl-phosphorylated HPr (P-Ser-HPr) (By similarity).
N	P47331	DE	HPr kinase/phosphorylase;
N	P47331	DE	HPr(Ser) kinase/phosphorylase;
N	P47331	DR	carbohydrate metabolic process
N	P47331	DR	metal ion binding
N	P47331	DR	protein serine/threonine kinase activity
N	P47331	DR	regulation of carbohydrate metabolic process
N	P47331	DR	two-component sensor activity
N	P47427	DE	Uncharacterized protein MG181;
N	P47427	DR	cobalamin biosynthetic process
N	P47427	DR	cobalt ion transmembrane transporter activity
N	P47479	DE	Uncharacterized protein MG237;
N	P47497	DE	Uncharacterized protein MG255;
N	P47520	CC	In eubacteria ppGpp (guanosine 3'-diphosphate 5-' diphosphate) is a mediator of the stringent response that coordinates a variety of cellular activities in response to changes in nutritional abundance
N	P47520	CC	It may also be capable of catalyzing the synthesis of ppGpp (By similarity).
N	P47520	CC	This enzyme catalyzes the degradation of ppGpp into GDP
N	P47520	DE	(ppGpp)ase;

N	P47520	DE	Penta-phosphate guanosine-3'-pyrophosphohydrolase;
N	P47520	DE	Probable guanosine-3',5'-bis(diphosphate) 3'-pyrophosphohydrolase;
N	P47520	DR	guanosine tetraphosphate metabolic process
N	P47520	DR	guanosine-3',5'-bis(diphosphate) 3'-diphosphatase activity
N	P47583	CC	DNA-dependent RNA polymerase catalyzes the transcription of DNA into RNA using the four ribonucleoside triphosphates as substrates.
N	P47583	DE	DNA-directed RNA polymerase subunit beta;
N	P47583	DE	RNA polymerase subunit beta;
N	P47583	DE	RNAP subunit beta;
N	P47583	DE	Transcriptase subunit beta;
N	P47583	DR	DNA-directed RNA polymerase activity
N	P47583	DR	ribonucleoside binding
N	P47657	CC	This protein is one of the early assembly proteins of the 50S ribosomal subunit, although it is not seen to bind rRNA by itself
N	P47657	CC	It is important during the early stages of 50S assembly (By similarity).
N	P47657	DE	50S ribosomal protein L13;
N	P47657	DR	structural constituent of ribosome
N	P47662	DE	Uncharacterized protein MG423;
N	P47662	DR	hydrolase activity, acting on ester bonds
N	P47662	DR	metal ion binding
N	P47691	CC	May play a role in stationary phase survival (By similarity).
N	P47691	DE	Alpha-D-glucosyl-1-phosphate uridylyltransferase;
N	P47691	DE	UDP-glucose pyrophosphorylase;
N	P47691	DE	UTP--glucose-1-phosphate uridylyltransferase;
N	P47691	DE	Uridine diphosphoglucose pyrophosphorylase;
N	P47691	DR	UDP-glucose metabolic process
N	P47691	DR	UTP:glucose-1-phosphate uridylyltransferase activity
N	P47691	DR	biosynthetic process
N	P47872	CC	This is a receptor for secretin
N	P47872	CC	The activity of this receptor is mediated by G proteins which activate adenylyl cyclase
N	P47872	DE	Secretin receptor;
N	P47872	DR	secretin receptor activity
N	P47938	CC	Participates in various redox reactions through the reversible oxidation of its active center dithiol to a disulfide and catalyzes dithiol-disulfide exchange reactions
N	P47938	CC	As a reducing substrate of peroxiredoxin 1, thioredoxin 2 is preferred over thioredoxin 1
N	P47938	CC	Required for female meiosis and early embryonic development.
N	P47938	DE	Protein deadhead;
N	P47938	DE	Thioredoxin-1;
N	P47938	DR	cell redox homeostasis
N	P47938	DR	electron carrier activity
N	P47938	DR	electron transport chain
N	P47938	DR	glycerol ether metabolic process
N	P47938	DR	protein binding
N	P47938	DR	protein disulfide oxidoreductase activity
N	P47938	DR	response to DNA damage stimulus
N	P48032	CC	Complexes with metalloproteinases (such as collagenases) and irreversibly inactivates them by binding to their catalytic zinc cofactor
N	P48032	CC	May form part of a tissue-specific acute response to remodeling stimuli.
N	P48032	DE	Metalloproteinase inhibitor 3;
N	P48032	DE	Tissue inhibitor of metalloproteinases 3;
N	P48032	DR	central nervous system development
N	P48032	DR	induction of apoptosis
N	P48032	DR	metal ion binding
N	P48032	DR	metalloendopeptidase inhibitor activity
N	P48032	DR	response to amino acid stimulus
N	P48032	DR	response to estrogen stimulus
N	P48032	DR	response to folic acid
N	P48032	DR	response to mechanical stimulus
N	P48032	DR	response to organic cyclic compound
N	P48032	DR	tissue regeneration
N	P48159	DE	60S ribosomal protein L23;
N	P48159	DR	mitotic spindle elongation
N	P48159	DR	protein binding
N	P48159	DR	structural constituent of ribosome
N	P48324	DE	Uncharacterized 24.3 kDa protein in psbH-rpl11 intergenic region;

N	P48374	CC	Topoisomerase IV is essential for chromosome segregation
N	P48374	CC	It relaxes supercoiled DNA
N	P48374	CC	Performs the decatenation events required during the replication of a circular DNA molecule (By similarity).
N	P48374	DE	DNA topoisomerase 4 subunit A;
N	P48374	DE	Topoisomerase IV subunit A;
N	P48374	DR	DNA topoisomerase (ATP-hydrolyzing) activity
N	P48374	DR	DNA topological change
N	P48374	DR	regulation of transcription, DNA-dependent
N	P48374	DR	sequence-specific DNA binding transcription factor activity
N	P48453	CC	Calcium-dependent, calmodulin-stimulated protein phosphatase
N	P48453	CC	This subunit may have a role in the calmodulin activation of calcineurin.
N	P48453	DE	CAM-PRP catalytic subunit;
N	P48453	DE	Calmodulin-dependent calcineurin A subunit beta isoform;
N	P48453	DE	Serine/threonine-protein phosphatase 2B catalytic subunit beta isoform;
N	P48453	DR	T cell differentiation
N	P48453	DR	T cell homeostasis
N	P48453	DR	calmodulin binding
N	P48453	DR	heart development
N	P48453	DR	metal ion binding
N	P48453	DR	negative regulation of T cell mediated cytotoxicity
N	P48453	DR	protein dephosphorylation
N	P48453	DR	protein serine/threonine phosphatase activity
N	P48453	DR	regulation of gene expression
N	P48453	DR	response to cytokine stimulus
N	P48556	CC	Acts as a regulatory subunit of the 26S proteasome which is involved in the ATP-dependent degradation of ubiquitinated proteins
N	P48556	CC	Necessary for activation of the CDC28 kinase.
N	P48556	DE	26S proteasome non-ATPase regulatory subunit 8;
N	P48556	DE	26S proteasome regulatory subunit RPN12;
N	P48556	DE	26S proteasome regulatory subunit S14;
N	P48556	DR	DNA damage response, signal transduction by p53 class mediator resulting in cell cycle arrest
N	P48556	DR	G1/S transition of mitotic cell cycle
N	P48556	DR	M/G1 transition of mitotic cell cycle
N	P48556	DR	S phase of mitotic cell cycle
N	P48556	DR	anaphase-promoting complex-dependent proteasomal ubiquitin-dependent protein catabolic process
N	P48556	DR	mRNA metabolic process
N	P48556	DR	negative regulation of ubiquitin-protein ligase activity involved in mitotic cell cycle
N	P48556	DR	positive regulation of ubiquitin-protein ligase activity involved in mitotic cell cycle
N	P48556	DR	protein binding
N	P48556	DR	regulation of apoptosis
N	P48556	DR	regulation of cellular amino acid metabolic process
N	P48556	DR	viral reproduction
N	P48573	CC	Catalyzes the isomerization between 2-isopropylmalate and 3-isopropylmalate, via the formation of 2-isopropylmaleate.
N	P48573	DE	3-isopropylmalate dehydratase large subunit;
N	P48573	DE	Alpha-IPM isomerase;
N	P48573	DE	Isopropylmalate isomerase;
N	P48573	DR	3-isopropylmalate dehydratase activity
N	P48573	DR	4 iron, 4 sulfur cluster binding
N	P48573	DR	leucine biosynthetic process
N	P48573	DR	metal ion binding
N	P48763	CC	Involved in pH regulation to eliminate acids generated by active metabolism or to counter adverse environmental conditions
N	P48763	CC	Major proton extruding system driven by the inward sodium ion chemical gradient
N	P48763	CC	Seems to play an important role in colonic sodium absorption.
N	P48763	DE	Na(+)/H(+) exchanger 2;
N	P48763	DE	Sodium/hydrogen exchanger 2;
N	P48763	DE	Solute carrier family 9 member 2;
N	P48763	DR	sodium:hydrogen antiporter activity
N	P48988	CC	Interacts with centromeric heterochromatin in chromosomes and binds to a specific subset of alphoid satellite DNA, called the CENP-B box

N	P48988	CC	May organize arrays of centromere satellite DNA into a higher order structure which then directs centromere formation and kinetochore assembly in mammalian chromosomes (By similarity).
N	P48988	DE	Centromere protein B;
N	P48988	DE	Major centromere autoantigen B;
N	P48988	DR	chromatin binding
N	P49290	CC	Mediates tyrosine nitration of secondary granule proteins in mature resting eosinophils (By similarity).
N	P49290	DE	Eosinophil peroxidase heavy chain;
N	P49290	DE	Eosinophil peroxidase light chain;
N	P49290	DE	Eosinophil peroxidase;
N	P49290	DR	hydrogen peroxide catabolic process
N	P49290	DR	oxidation-reduction process
N	P49290	DR	peroxidase activity
N	P49599	DE	Protein phosphatase 2C 57;
N	P49599	DE	Protein phosphatase 2C PPH1;
N	P49599	DR	metal ion binding
N	P49599	DR	photosynthetic electron transport chain
N	P49599	DR	photosystem stoichiometry adjustment
N	P49599	DR	protein dephosphorylation
N	P49599	DR	protein serine/threonine phosphatase activity
N	P49626	CC	Participates in the regulation of the accumulation of its own mRNA.
N	P49626	DE	60S ribosomal protein L4-B;
N	P49626	DR	structural constituent of ribosome
N	P49653	CC	Binding of this ligand-gated ion channel to ATP mediates synaptic transmission between neurons and from neurons to smooth muscle.
N	P49653	DE	ATP receptor;
N	P49653	DE	P2X purinoceptor 2;
N	P49653	DE	Purinergic receptor;
N	P49653	DR	cadmium ion binding
N	P49653	DR	cobalt ion binding
N	P49653	DR	copper ion binding
N	P49653	DR	extracellular ATP-gated cation channel activity
N	P49653	DR	identical protein binding
N	P49653	DR	mercury ion binding
N	P49653	DR	nickel ion binding
N	P49653	DR	phosphatidylinositol binding
N	P49653	DR	protein heterooligomerization
N	P49653	DR	protein homooligomerization
N	P49653	DR	receptor activity
N	P49653	DR	regulation of action potential in neuron
N	P49653	DR	zinc ion binding
N	P49807	CC	Inhibits signal transduction by increasing the GTPase activity of G protein alpha subunits thereby driving them into their inactive GDP-bound form.
N	P49807	DE	Regulator of G-protein signaling 11;
N	P49807	DR	GTPase activator activity
N	P49807	DR	negative regulation of signal transduction
N	P49807	DR	regulation of G-protein coupled receptor protein signaling pathway
N	P49807	DR	signal transducer activity
N	P49851	DE	Uncharacterized acyl-CoA thioester hydrolase YkhA;
N	P49851	DR	hydrolase activity
N	P49875	CC	Granulocyte/macrophage colony-stimulating factors are cytokines that act in hematopoiesis by controlling the production, differentiation, and function of 2 related white cell populations of the blood, the granulocytes and the monocytes-macrophages (By similarity).
N	P49875	DE	Hematopoietic growth factor;
N	P49875	DE	Interleukin-3;
N	P49875	DE	Mast cell growth factor;
N	P49875	DE	Multipotential colony-stimulating factor;
N	P49875	DE	P-cell-stimulating factor;
N	P49875	DR	cytokine activity
N	P49875	DR	growth factor activity
N	P49875	DR	immune response
N	P49875	DR	interleukin-3 receptor binding
N	P49875	DR	positive regulation of cell proliferation

N	P49875	DR	positive regulation of survival gene product expression
N	P50167	DE	D-arabinitol 2-dehydrogenase [ribulose-forming];
N	P50167	DR	D-arabinitol 2-dehydrogenase activity
N	P50167	DR	carbohydrate metabolic process
N	P50167	DR	oxidation-reduction process
N	P50212	CC	Binds to one of the cis-acting domain of the insulin gene enhancer
N	P50212	CC	May be involved in subtype specialization of primary motoneurons.
N	P50212	DE	Insulin gene enhancer protein ISL-2B;
N	P50212	DR	multicellular organismal development
N	P50212	DR	regulation of transcription, DNA-dependent
N	P50212	DR	sequence-specific DNA binding
N	P50212	DR	sequence-specific DNA binding transcription factor activity
N	P50212	DR	transcription regulator activity
N	P50212	DR	zinc ion binding
N	P50433	CC	Interconversion of serine and glycine.
N	P50433	DE	Glycine hydroxymethyltransferase;
N	P50433	DE	Serine hydroxymethyltransferase, mitochondrial;
N	P50433	DE	Serine methylase;
N	P50433	DR	L-serine metabolic process
N	P50433	DR	glycine hydroxymethyltransferase activity
N	P50433	DR	glycine metabolic process
N	P50433	DR	one-carbon metabolic process
N	P50433	DR	pyridoxal phosphate binding
N	P50481	CC	Binds to and activates the promoter of the alpha- glycoprotein gene, and synergistically enhances transcription from the prolactin promoter in cooperation with Pit-1
N	P50481	DE	Homeobox protein LIM-3;
N	P50481	DE	Homeobox protein P-LIM;
N	P50481	DE	LIM homeobox protein 3;
N	P50481	DE	LIM/homeobox protein Lhx3;
N	P50481	DR	dorsal/ventral pattern formation
N	P50481	DR	medial motor column neuron differentiation
N	P50481	DR	motor axon guidance
N	P50481	DR	negative regulation of apoptosis
N	P50481	DR	pituitary gland development
N	P50481	DR	positive regulation of transcription from RNA polymerase II promoter
N	P50481	DR	protein binding
N	P50481	DR	protein binding transcription factor activity
N	P50481	DR	sequence-specific DNA binding
N	P50481	DR	sequence-specific DNA binding transcription factor activity
N	P50481	DR	transcription regulator activity
N	P50481	DR	zinc ion binding
N	P50558	CC	This is 1 of the proteins that binds and probably mediates the attachment of the 5S RNA into the large ribosomal subunit, where it forms part of the central protuberance
N	P50558	CC	In the 70S ribosome it contacts protein S13 of the 30S subunit (bridge B1b), connecting the 2 subunits; this bridge is implicated in subunit movement
N	P50558	CC	May contact the P site tRNA; the 5S rRNA and some of its associated proteins might help stabilize positioning of ribosome-bound tRNAs (By similarity).
N	P50558	DE	50S ribosomal protein L5P;
N	P50558	DR	structural constituent of ribosome
N	P50583	CC	Asymmetrically hydrolyzes Ap4A to yield AMP and ATP
N	P50583	CC	Plays a major role in maintaining homeostasis.
N	P50583	DE	Ap4A hydrolase;
N	P50583	DE	Bis(5'-nucleosyl)-tetraphosphatase [asymmetrical];
N	P50583	DE	Diadenosine 5',5'''-P1,P4-tetraphosphate asymmetrical hydrolase;
N	P50583	DE	Diadenosine tetraphosphatase;
N	P50583	DE	Nucleoside diphosphate-linked moiety X motif 2;
N	P50583	DE	Nudix motif 2;
N	P50583	DR	bis(5'-nucleosyl)-tetraphosphatase (asymmetrical) activity
N	P50583	DR	bis(5'-nucleosyl)-tetraphosphatase (symmetrical) activity
N	P50583	DR	induction of apoptosis
N	P50583	DR	nucleobase, nucleoside, nucleotide and nucleic acid metabolic process
N	P50779	CC	E7 protein has both transforming and trans-activating activities
N	P50779	CC	Disrupts the function of host retinoblastoma protein RB1/pRb, which is a key regulator of the cell cycle

N	P50779	CC	Inactivation of the ability of RB1 to arrest the cell cycle is critical for cellular transformation, uncontrolled cellular growth and proliferation induced by viral infection
N	P50779	CC	Induces the disassembly of the E2F1 transcription factors from RB1, with subsequent transcriptional activation of E2F1-regulated S-phase genes
N	P50779	CC	Interferes with histone deacetylation mediated by HDAC1 and HDAC2, leading to activation of transcription (By similarity).
N	P50779	CC	Stimulation of progression from G1 to S phase allows the virus to efficiently use the cellular DNA replicating machinery to achieve viral genome replication
N	P50779	DR	interspecies interaction between organisms
N	P50779	DR	metal ion binding
N	P50779	DR	regulation of transcription, DNA-dependent
N	P50779	DR	sequence-specific DNA binding transcription factor activity
N	P51083	CC	The primary product of this enzyme is 4,2',4',6'- tetrahydroxychalcone (also termed naringenin-chalcone or chalcone) which can under specific conditions spontaneously isomerize into naringenin.
N	P51083	DE	Chalcone synthase 1;
N	P51083	DE	Naringenin-chalcone synthase 1;
N	P51083	DR	acyltransferase activity
N	P51083	DR	flavonoid biosynthetic process
N	P51083	DR	naringenin-chalcone synthase activity
N	P51191	DE	Uncharacterized protein ycf37;
N	P51251	CC	DNA-dependent RNA polymerase catalyzes the transcription of DNA into RNA using the four ribonucleoside triphosphates as substrates.
N	P51251	DE	DNA-directed RNA polymerase subunit beta';
N	P51251	DE	Plastid-encoded RNA polymerase subunit beta';
N	P51251	DE	RNA polymerase subunit beta';
N	P51251	DR	DNA-directed RNA polymerase activity
N	P51325	DE	Photosystem II 10 kDa phosphoprotein;
N	P51325	DE	Photosystem II reaction center protein H;
N	P51325	DR	phosphate binding
N	P51325	DR	photosynthesis
N	P51325	DR	protein stabilization
N	P51367	CC	Light-harvesting photosynthetic bile pigment-protein from the phycobiliprotein complex (By similarity).
N	P51367	DE	R-phycoerythrin beta chain;
N	P51367	DR	electron transport chain
N	P51367	DR	photosynthesis
N	P51367	DR	protein-chromophore linkage
N	P51413	DE	60S ribosomal protein L17-2;
N	P51413	DR	structural constituent of ribosome
N	P51489	CC	Photoreceptor required for image-forming vision at low light intensity
N	P51489	CC	Light-induced isomerization of 11-cis to all-trans retinal triggers a conformational change leading to G-protein activation and release of all-trans retinal (By similarity)
N	P51489	CC	Required for photoreceptor cell viability after birth
N	P51489	DR	G-protein coupled receptor activity
N	P51489	DR	metal ion binding
N	P51489	DR	photoreceptor activity
N	P51489	DR	protein-chromophore linkage
N	P51489	DR	rhodopsin mediated phototransduction
N	P51512	CC	Endopeptidase that degrades various components of the extracellular matrix, such as collagen type III and fibronectin
N	P51512	CC	Activates progelatinase A
N	P51512	CC	However, upon interaction with CSPG4, it may be involved in degradation and invasion of type I collagen by melanoma cells.
N	P51512	CC	Involved in the matrix remodeling of blood vessels
N	P51512	CC	Isoform short cleaves fibronectin and also collagen type III, but at lower rate
N	P51512	CC	It has no effect on type I, II, IV and V collagen
N	P51512	DE	Matrix metalloproteinase-16;
N	P51512	DE	Membrane-type matrix metalloproteinase 3;
N	P51512	DE	Membrane-type-3 matrix metalloproteinase;
N	P51512	DR	calcium ion binding
N	P51512	DR	collagen catabolic process
N	P51512	DR	enzyme activator activity
N	P51512	DR	metalloendopeptidase activity
N	P51512	DR	zinc ion binding

N	P52242	DR	serine-type endopeptidase inhibitor activity
N	P52328	CC	Sigma factors are initiation factors that promote the attachment of RNA polymerase to specific initiation sites and are then released
N	P52328	CC	This is the primary sigma factor of this bacterium (By similarity).
N	P52328	DE	RNA polymerase sigma factor rpoD;
N	P52328	DR	regulation of transcription, DNA-dependent
N	P52328	DR	sequence-specific DNA binding transcription factor activity
N	P52328	DR	sigma factor activity
N	P52328	DR	transcription initiation, DNA-dependent
N	P52328	DR	transcription regulator activity
N	P52347	CC	Major protein of the icosahedral capsid.
N	P52347	DE	Major capsid protein;
N	P52347	DR	structural molecule activity
N	P52518	DE	Uncharacterized protein U55B;
N	P52665	CC	Regulator of the budABC operon for 2,3-butanediol synthesis (By similarity).
N	P52665	DE	Bud operon transcriptional regulator;
N	P52665	DE	HTH-type transcriptional regulator BudR;
N	P52665	DR	regulation of transcription, DNA-dependent
N	P52665	DR	sequence-specific DNA binding transcription factor activity
N	P53070	CC	Involved in the 5-carboxymethylaminomethyl modification (mnm(5)s(2)U34) of the wobble uridine base in mitochondrial tRNAs.
N	P53070	DE	Mitochondrial translation optimization protein 1;
N	P53070	DR	flavin adenine dinucleotide binding
N	P53070	DR	identical protein binding
N	P53070	DR	mitochondrial tRNA wobble uridine modification
N	P53093	CC	May be involved in proper membrane localization of Rab GTPases.
N	P53093	DE	Protein YIP4;
N	P53093	DE	YPT-interacting protein 4;
N	P53093	DR	Rab GTPase binding
N	P53093	DR	identical protein binding
N	P53210	DE	Uncharacterized protein YGR017W;
N	P53238	CC	Required for polar bud growth and cell wall abscission.
N	P53238	DE	Penta-EF hand domain-containing protein 1;
N	P53238	DR	axial cellular bud site selection
N	P53238	DR	bipolar cellular bud site selection
N	P53238	DR	calcium ion binding
N	P53238	DR	identical protein binding
N	P53238	DR	zinc ion binding
N	P53269	DE	Putative uncharacterized protein YGR115C;
N	P53277	CC	Involved in pre-mRNA splicing and cell cycle control
N	P53277	CC	As a component of the NTC complex (or PRP19-associated complex), associates to the spliceosome to mediate conformational rearrangement or to stabilize the structure of the spliceosome after U4 snRNA dissociation, which leads to spliceosome maturation
N	P53277	CC	The cell cycle arrest of SYF2 defective cells may be due to the inefficient splicing of TUB1.
N	P53277	DE	PRP19 complex protein 31;
N	P53277	DE	Pre-mRNA-splicing factor SYF2;
N	P53277	DE	Synthetic lethal with CDC40 protein 2;
N	P53277	DR	nuclear mRNA splicing, via spliceosome
N	P53277	DR	protein binding
N	P53625	CC	Involved in the maturation of Asn-linked oligosaccharides
N	P53625	CC	Progressively trim alpha-1,2-linked mannose residues from Man(9)GlcNAc(2) to produce Man(5)GlcNAc(2).
N	P53625	DE	Man(9)-alpha-mannosidase;
N	P53625	DE	Mannosidase-1;
N	P53625	DE	Mannosyl-oligosaccharide alpha-1,2-mannosidase isoform B;
N	P53625	DR	calcium ion binding
N	P53625	DR	determination of adult lifespan
N	P53625	DR	mannosyl-oligosaccharide 1,2-alpha-mannosidase activity
N	P53625	DR	response to anesthetic
N	P53685	CC	NAD-dependent histone deacetylase involved in telomeric silencing
N	P53685	CC	Acts as a sensor of NAD(+) levels and regulator of NAD(+) biosynthesis
N	P53685	CC	Histone deacetylase proteins act via the formation of large multiprotein complexes that are responsible for the deacetylation of lysine residues on the N-terminal part of the core histones (H2A, H2B, H3 and H4)

N	P53685	CC	Histone deacetylation gives a tag for epigenetic repression and plays an important role in transcriptional regulation, cell cycle progression and developmental events
N	P53685	CC	Regulates the gene expression of de novo NAD(+) biosynthesis genes.
N	P53685	CC	Required to repress middle sporulation genes during vegetative growth
N	P53685	CC	Restores silencing at HMR in SIR2 mutants when overexpressed
N	P53685	DE	Homologous to SIR2 protein 1;
N	P53685	DE	NAD-dependent deacetylase HST1;
N	P53685	DR	NAD-dependent histone deacetylase activity
N	P53685	DR	NAD-independent histone deacetylase activity
N	P53685	DR	chromatin silencing
N	P53685	DR	histone deacetylation
N	P53685	DR	negative regulation of mitotic recombination
N	P53685	DR	protein binding
N	P53685	DR	sporulation resulting in formation of a cellular spore
N	P53685	DR	zinc ion binding
N	P53735	DE	Protein ZRG17;
N	P53735	DE	Zinc-regulated gene 17 protein;
N	P53735	DR	protein binding
N	P53735	DR	zinc ion transmembrane transporter activity
N	P53817	CC	Tumor suppressor that may be involved in interferon- dependent cell death
N	P53817	CC	Also has weak lysophospholipase activity (By similarity).
N	P53817	CC	Specifically catalyzes the release of fatty acids from phospholipids in adipose tissue
N	P53817	DE	Group XVI phospholipase A2;
N	P53817	DE	H-rev 107 protein;
N	P53817	DE	HRAS-like suppressor 3;
N	P53817	DR	lipid catabolic process
N	P53817	DR	negative regulation of cell cycle
N	P53817	DR	phospholipase A2 activity
N	P53821	DE	Putative UPF0320 protein YNL337W;
N	P53967	DE	Putative uncharacterized protein YNL028W;
N	P54022	DE	50S ribosomal protein L18e;
N	P54022	DR	structural constituent of ribosome
N	P54273	DE	Xylose isomerase;
N	P54273	DR	D-xylose metabolic process
N	P54273	DR	metal ion binding
N	P54273	DR	pentose-phosphate shunt
N	P54273	DR	xylose isomerase activity
N	P54332	DE	Phage-like element PBSX protein xkdM;
N	P54368	CC	Binds to, and destabilizes, ornithine decarboxylase which is then degraded
N	P54368	CC	Also inhibits cellular uptake of polyamines by inactivating the polyamine uptake transporter
N	P54368	CC	SMAD1/OAZ1/PSMB4 complex mediates the degradation of the CREBBP/EP300 repressor SNIP1.
N	P54368	DE	Ornithine decarboxylase antizyme 1;
N	P54368	DR	ornithine decarboxylase inhibitor activity
N	P54368	DR	polyamine biosynthetic process
N	P54368	DR	regulation of cellular amino acid metabolic process
N	P54377	CC	The glycine cleavage system catalyzes the degradation of glycine
N	P54377	CC	The P protein binds the alpha-amino group of glycine through its pyridoxal phosphate cofactor; CO(2) is released and the remaining methylamine moiety is then transferred to the lipoamide cofactor of the H protein (By similarity).
N	P54377	DE	Glycine cleavage system P-protein subunit 2;
N	P54377	DE	Glycine decarboxylase subunit 2;
N	P54377	DE	Probable glycine dehydrogenase [decarboxylating] subunit 2;
N	P54377	DR	glycine dehydrogenase (decarboxylating) activity
N	P54377	DR	oxidation-reduction process
N	P54377	DR	pyridoxal phosphate binding
N	P54662	CC	Regulating factor for the production of extracellular proteases
N	P54662	CC	The N-terminal region acts as an inhibitor, whereas the C-terminal region carries enhancing activity.
N	P54662	DE	Transcriptional regulatory protein degU;
N	P54662	DR	regulation of transcription, DNA-dependent
N	P54662	DR	sequence-specific DNA binding
N	P54662	DR	sequence-specific DNA binding transcription factor activity
N	P54662	DR	two-component response regulator activity

N	P54713	CC	Accessory subunit of the mitochondrial membrane respiratory chain NADH dehydrogenase (Complex I), that is believed not to be involved in catalysis
N	P54713	CC	Complex I functions in the transfer of electrons from NADH to the respiratory chain
N	P54713	CC	The immediate electron acceptor for the enzyme is believed to be ubiquinone (By similarity).
N	P54713	DE	Complex I-42kD;
N	P54713	DE	NADH dehydrogenase [ubiquinone] 1 alpha subcomplex subunit 10;
N	P54713	DE	NADH-ubiquinone oxidoreductase 42 kDa subunit;
N	P54713	DR	electron transport chain
N	P54956	DE	Uncharacterized protein yxeQ;
N	P54956	DR	2-methylcitrate dehydratase activity
N	P54956	DR	propionate catabolic process
N	P55024	CC	This is a copper-containing oxidase that functions in the formation of pigments such as melanins and other polyphenolic compounds
N	P55024	CC	Catalyzes the rate-limiting conversions of tyrosine to DOPA, DOPA to DOPA-quinone and possibly 5,6-dihydroxyindole to indole-5,6 quinone.
N	P55024	DE	Monophenol monooxygenase;
N	P55024	DR	melanin biosynthetic process
N	P55024	DR	monophenol monooxygenase activity
N	P55024	DR	oxidation-reduction process
N	P55075	CC	Stimulates growth of the cells in an autocrine manner
N	P55075	CC	Mediates hormonal action on the growth of cancer cells.
N	P55075	DE	Androgen-induced growth factor;
N	P55075	DE	Fibroblast growth factor 8;
N	P55075	DE	Heparin-binding growth factor 8;
N	P55075	DR	bone development
N	P55075	DR	dopaminergic neuron differentiation
N	P55075	DR	fibroblast growth factor receptor signaling pathway
N	P55075	DR	gonad development
N	P55075	DR	growth factor activity
N	P55075	DR	insulin receptor signaling pathway
N	P55075	DR	mesonephros development
N	P55075	DR	metanephros development
N	P55075	DR	neuroepithelial cell differentiation
N	P55075	DR	odontogenesis
N	P55075	DR	positive regulation of cell division
N	P55075	DR	positive regulation of cell proliferation
N	P55075	DR	type 1 fibroblast growth factor receptor binding
N	P55075	DR	type 2 fibroblast growth factor receptor binding
N	P55089	CC	Acts in vitro to stimulate the secretion of adrenocorticotropic hormone (ACTH)
N	P55089	CC	Binds with high affinity to CRF Receptor types 1, 2-alpha, and 2-beta.
N	P55089	DR	neuropeptide hormone activity
N	P55121	CC	Involved in fatty acylation of the protoxin (lktA) at two internal lysine residues, thereby converting it to the active toxin
N	P55121	CC	The acyl donor is ACP (By similarity).
N	P55121	DE	Leukotoxin C;
N	P55121	DE	Leukotoxin-activating lysine-acyltransferase lktC serotype A11;
N	P55121	DE	Toxin-activating protein C;
N	P55121	DR	acyltransferase activity
N	P55121	DR	hemolysis in other organism
N	P55121	DR	toxin metabolic process
N	P55289	CC	Cadherins are calcium dependent cell adhesion proteins
N	P55289	CC	They preferentially interact with themselves in a homophilic manner in connecting cells; cadherins may thus contribute to the sorting of heterogeneous cell types
N	P55289	DE	BR-cadherin;
N	P55289	DE	Brain cadherin;
N	P55289	DE	Cadherin-12;
N	P55289	DE	N-cadherin 2;
N	P55289	DE	Neural type cadherin 2;
N	P55289	DR	adherens junction organization
N	P55289	DR	calcium ion binding
N	P55289	DR	cell junction assembly
N	P55289	DR	homophilic cell adhesion
N	P55505	DE	Uncharacterized protein y4jE;
N	P55507	DE	Uncharacterized protein y4jG;

N	P55663	DE	Uncharacterized hydrolase y4tI;
N	P55663	DR	hydrolase activity
N	P55691	CC	Possible binding-protein with either a transport or enzymatic activity.
N	P55691	DE	Uncharacterized protein y4wM;
N	P55691	DR	transporter activity
N	P55699	DE	Uncharacterized protein y4xG;
N	P55812	DE	Ubiquitin-like protein FUBI;
N	P55927	CC	Potent inhibitor of the A-type voltage-gated potassium channels
N	P55927	CC	Most potent inhibitor of Kv1.2/KCNA2 channels
N	P55927	CC	Reversibly block the Shaker B potassium-channels (Kv1.1 sub- family).
N	P55927	DE	DE Flags: Precursor; Fragment;
N	P55927	DE	Pandinotoxin-alpha;
N	P55927	DE	Potassium channel toxin alpha-KTx 7.1;
N	P55927	DE	Potassium channel-blocking toxin 2;
N	P55927	DE	Toxin PiTX-K-alpha;
N	P55927	DR	potassium channel inhibitor activity
N	P55946	CC	The metallothioneins are involved in the cellular sequestration of toxic metal ions and regulation of essential trace elements.
N	P55946	DE	Metallothionein;
N	P55946	DR	metal ion binding
N	P55975	CC	Associates with the EF-Tu.GDP complex and induces the exchange of GDP to GTP
N	P55975	CC	It remains bound to the aminoacyl-tRNA.EF- Tu.GTP complex up to the GTP hydrolysis stage on the ribosome.
N	P55975	DE	Elongation factor Ts;
N	P55975	DR	translation elongation factor activity
N	P55985	CC	Responsible for synthesis of pseudouridine from uracil- 13 in transfer RNAs (By similarity).
N	P55985	DE	tRNA pseudouridine synthase D;
N	P55985	DE	tRNA pseudouridylate synthase D;
N	P55985	DE	tRNA-uridine isomerase D;
N	P55985	DR	pseudouridine synthase activity
N	P55985	DR	pseudouridine synthesis
N	P55985	DR	tRNA processing
N	P56061	CC	Catalyzes the condensation of pantoate with beta-alanine in an ATP-dependent reaction via a pantoyl-adenylate intermediate (By similarity).
N	P56061	DE	Pantoate--beta-alanine ligase;
N	P56061	DE	Pantoate-activating enzyme;
N	P56061	DE	Pantothenate synthetase;
N	P56061	DR	pantoate-beta-alanine ligase activity
N	P56061	DR	pantothenate biosynthetic process
N	P56069	DE	Cystathionine gamma-synthase;
N	P56069	DE	O-succinylhomoserine (thiol)-lyase;
N	P56069	DR	cystathionine gamma-synthase activity
N	P56069	DR	methionine biosynthetic process
N	P56069	DR	pyridoxal phosphate binding
N	P56097	CC	This protein is essential to the cell-division process
N	P56097	CC	Binds to and hydrolyzes GTP
N	P56097	CC	Involved in the synthesis of the septal peptidoglycan (By similarity).
N	P56097	CC	It seems to assemble into a dynamic ring on the inner surface of the cytoplasmic membrane at the place where division will occur, and the formation of the ring is the signal for septation to begin
N	P56097	DE	Cell division protein ftsZ;
N	P56097	DR	GTPase activity
N	P56097	DR	barrier septum formation
N	P56097	DR	protein polymerization
N	P56241	CC	Antibacterial peptide, that adopts an alpha helical conformation which can disrupt bacterial membranes
N	P56241	CC	Each caerin displays a different antimicrobial specificity.
N	P56241	DR	defense response to bacterium
N	P56277	DE	MTCP-1 type A;
N	P56277	DE	Mature T-cell proliferation 1 neighbor protein;
N	P56277	DE	Mature T-cell proliferation-1 type A;
N	P56277	DE	Protein p8 MTCP-1;
N	P56277	DR	cell proliferation

N	P56306	CC	Component of the cytochrome b6-f complex, which mediates electron transfer between photosystem II (PSII) and photosystem I (PSI), cyclic electron flow around PSI, and state transitions
N	P56306	CC	PetL is important for photoautotrophic growth as well as for electron transfer efficiency and stability of the cytochrome b6-f complex (By similarity).
N	P56306	DE	Cytochrome b6-f complex subunit 6;
N	P56306	DE	Cytochrome b6-f complex subunit VI;
N	P56306	DE	Cytochrome b6-f complex subunit petL;
N	P56306	DR	electron carrier activity
N	P56306	DR	electron transport chain
N	P56306	DR	photosynthesis
N	P56325	DE	Photosystem II reaction center protein M;
N	P56325	DR	photosynthesis, light reaction
N	P56348	CC	This protein is a component of the reaction center of photosystem II.
N	P56348	DE	Photosystem II reaction center protein K;
N	P56348	DR	photosynthesis
N	P56425	CC	Exerts a potent antimicrobial activity.
N	P56425	DE	Antibacterial peptide BMAP-34;
N	P56425	DE	Cathelicidin-7;
N	P56425	DR	defense response to bacterium
N	P56589	CC	Involved in peroxisome biosynthesis and integrity
N	P56589	CC	As a docking factor for PEX19, is necessary for the import of peroxisomal membrane proteins in the peroxisomes.
N	P56589	CC	Assembles membrane vesicles before the matrix proteins are translocated
N	P56589	DE	Peroxisomal assembly protein PEX3;
N	P56589	DE	Peroxisomal biogenesis factor 3;
N	P56589	DR	protein binding
N	P56589	DR	protein import into peroxisome membrane
N	P56589	DR	transmembrane transport
N	P56825	CC	Hemocyanins are copper-containing oxygen carriers occurring freely dissolved in the hemolymph of many mollusks and arthropods.
N	P56825	DE	Hemocyanin, units E and F;
N	P56825	DR	metal ion binding
N	P56825	DR	oxidoreductase activity
N	P56825	DR	oxygen transporter activity
N	P56848	CC	Catalyzes the phosphorylation of the position 2 hydroxy group of 4-diphosphocytidyl-2C-methyl-D-erythritol (By similarity).
N	P56848	DE	4-(cytidine-5'-diphospho)-2-C-methyl-D-erythritol kinase;
N	P56848	DE	4-diphosphocytidyl-2-C-methyl-D-erythritol kinase, chloroplastic;
N	P56848	DR	4-(cytidine 5'-diphospho)-2-C-methyl-D-erythritol kinase activity
N	P56848	DR	terpenoid biosynthetic process
N	P57360	CC	Binds tightly to ds-DNA, increases its thermal stability and inhibits transcription (By similarity).
N	P57360	DE	DNA-binding protein H-NS homolog;
N	P57360	DR	regulation of transcription, DNA-dependent
N	P57460	CC	Involved in protein export
N	P57460	CC	Participates in an early event of protein translocation (By similarity).
N	P57460	DE	Protein-export membrane protein secG;
N	P57460	DR	P-P-bond-hydrolysis-driven protein transmembrane transporter activity
N	P57460	DR	protein secretion
N	P57484	CC	Could accelerate the degradation of some genes transcripts potentially through selective RNA binding (By similarity).
N	P57484	DE	Carbon storage regulator homolog;
N	P57484	DR	mRNA catabolic process
N	P57484	DR	regulation of carbohydrate metabolic process
N	P57563	CC	Removes the formyl group from the N-terminal Met of newly synthesized proteins
N	P57563	CC	N-terminal L-methionine is a prerequisite for activity but the enzyme has broad specificity at other positions (By similarity).
N	P57563	CC	Requires at least a dipeptide for an efficient rate of reaction
N	P57563	DE	Peptide deformylase;
N	P57563	DE	Polypeptide deformylase;
N	P57563	DR	iron ion binding
N	P57563	DR	peptide deformylase activity
N	P57591	CC	One of the primary rRNA binding proteins, it binds directly near the 3'-end of the 23S rRNA, where it nucleates assembly of the 50S subunit (By similarity).

N	P57591	DE	50S ribosomal protein L3;
N	P57591	DR	structural constituent of ribosome
N	P57667	CC	3'-to-5' exoribonuclease specific for small oligoribonucleotides.
N	P57667	DE	Oligoribonuclease;
N	P57667	DR	exonuclease activity
N	P57667	DR	nucleic acid binding
N	P57739	CC	Plays a major role in tight junction-specific obliteration of the intercellular space, through calcium- independent cell-adhesion activity (By similarity).
N	P57739	DR	calcium-independent cell-cell adhesion
N	P57739	DR	identical protein binding
N	P57739	DR	structural molecule activity
N	P57828	DE	AICAR transformylase;
N	P57828	DE	Bifunctional purine biosynthesis protein purH;
N	P57828	DE	IMP cyclohydrolase;
N	P57828	DE	IMP synthase;
N	P57828	DE	Inosinicase;
N	P57828	DE	Phosphoribosylaminoimidazolecarboxamide formyltransferase;
N	P57828	DR	IMP biosynthetic process
N	P57828	DR	IMP cyclohydrolase activity
N	P57828	DR	phosphoribosylaminoimidazolecarboxamide formyltransferase activity
N	P57903	CC	Catalyzes a trans-dehydration via an enolate intermediate (By similarity).
N	P57903	DE	3-dehydroquinase;
N	P57903	DE	3-dehydroquinate dehydratase;
N	P57903	DE	Type II DHQase;
N	P57903	DR	3-dehydroquinate dehydratase activity
N	P57903	DR	aromatic amino acid family biosynthetic process
N	P58017	DE	UPF0147 protein SSO6503;
N	P58160	CC	Transfers 2-(5''-triphosphoribosyl)-3'- dephosphocoenzyme-A on a serine residue to the apo-acyl carrier protein (gamma chain) of the citrate lyase to yield holo-acyl carrier protein (By similarity).
N	P58160	DE	Apo-ACP nucleodityltransferase;
N	P58160	DE	Holo-ACP synthase;
N	P58160	DE	Holo-citrate lyase synthase;
N	P58160	DE	Probable apo-citrate lyase phosphoribosyl-dephospho-CoA transferase;
N	P58160	DR	holo-citrate lyase synthase activity
N	P58160	DR	prosthetic group biosynthetic process
N	P58272	CC	No specific function has so far been attributed to this initiation factor; however, it seems to stimulate more or less all the activities of the other two initiation factors, IF-2 and IF-3
N	P58272	DE	Translation initiation factor IF-1, chloroplastic;
N	P58272	DR	translation initiation factor activity
N	P58372	DE	60S ribosomal protein L30;
N	P58372	DR	structural constituent of ribosome
N	P58478	CC	Molecular chaperone
N	P58478	CC	Has ATPase activity (By similarity).
N	P58478	DE	Chaperone protein htpG;
N	P58478	DE	Heat shock protein htpG;
N	P58478	DE	High temperature protein G;
N	P58478	DR	protein folding
N	P58478	DR	response to stress
N	P58478	DR	unfolded protein binding
N	P58583	DE	Photosystem I reaction center subunit PsaK 1;
N	P58583	DE	Photosystem I subunit X 1;
N	P58583	DR	photosynthesis
N	P58693	DE	Asparagine--tRNA ligase;
N	P58693	DE	Asparaginyl-tRNA synthetase;
N	P58693	DR	asparagine-tRNA ligase activity
N	P58693	DR	asparaginyl-tRNA aminoacylation
N	P58693	DR	aspartate-tRNA ligase activity
N	P58693	DR	aspartyl-tRNA aminoacylation
N	P58693	DR	nucleic acid binding
N	P59204	CC	Has a role in maltotetraose utilization.
N	P59204	DE	Maltodextrose utilization protein malA;
N	P59362	CC	Catalyzes the first step in hexosamine metabolism, converting fructose-6P into glucosamine-6P using glutamine as a nitrogen source (By similarity).
N	P59362	DE	D-fructose-6-phosphate amidotransferase;

N	P59362	DE	Glucosamine--fructose-6-phosphate aminotransferase [isomerizing];
N	P59362	DE	Glucosamine-6-phosphate synthase;
N	P59362	DE	Hexosephosphate aminotransferase;
N	P59362	DE	L-glutamine-D-fructose-6-phosphate amidotransferase;
N	P59362	DR	carbohydrate biosynthetic process
N	P59362	DR	glutamine metabolic process
N	P59362	DR	glutamine-fructose-6-phosphate transaminase (isomerizing) activity
N	P59362	DR	sugar binding
N	P59379	CC	Located on the platform of the 30S subunit, it bridges several disparate RNA helices of the 16S rRNA
N	P59379	CC	Forms part of the Shine-Dalgarno cleft in the 70S ribosome (By similarity).
N	P59379	DE	30S ribosomal protein S11;
N	P59379	DR	structural constituent of ribosome
N	P59416	DE	3-phosphoshikimate 1-carboxyvinyltransferase;
N	P59416	DE	5-enolpyruvylshikimate-3-phosphate synthase;
N	P59416	DE	EPSP synthase;
N	P59416	DR	3-phosphoshikimate 1-carboxyvinyltransferase activity
N	P59416	DR	aromatic amino acid family biosynthetic process
N	P59466	DE	Tryptophan--tRNA ligase;
N	P59466	DE	Tryptophanyl-tRNA synthetase;
N	P59466	DR	tryptophan-tRNA ligase activity
N	P59466	DR	tryptophanyl-tRNA aminoacylation
N	P59645	CC	Induces a hyperpolarization-activated chloride current when expressed in Xenopus oocytes
N	P59645	CC	May be a modulator capable of activating endogenous oocyte channels.
N	P59645	DE	FXYD domain-containing ion transport regulator 3;
N	P59645	DR	ion channel activity
N	P59664	DE	Phenylalanine--tRNA ligase beta chain;
N	P59664	DE	Phenylalanyl-tRNA synthetase beta chain;
N	P59664	DR	magnesium ion binding
N	P59664	DR	phenylalanine-tRNA ligase activity
N	P59664	DR	phenylalanyl-tRNA aminoacylation
N	P59664	DR	tRNA processing
N	P59804	DE	ESAT-6-like protein esxL;
N	P59960	DE	Protein dipZ;
N	P59960	DR	antioxidant activity
N	P59960	DR	cell redox homeostasis
N	P59960	DR	cytochrome complex assembly
N	P59960	DR	electron carrier activity
N	P59960	DR	glycerol ether metabolic process
N	P59960	DR	oxidation-reduction process
N	P59960	DR	protein disulfide oxidoreductase activity
N	P59994	CC	Pore-forming (alpha) subunit of voltage-gated rapidly inactivating A-type potassium channels
N	P59994	CC	Channel properties are modulated by interactions with other alpha subunits and with regulatory subunits.
N	P59994	CC	May contribute to I(To) current in heart and I(Sa) current in neurons
N	P59994	DE	Potassium voltage-gated channel subfamily D member 1;
N	P59994	DE	Voltage-gated potassium channel subunit Kv4.1;
N	P59994	DR	voltage-gated potassium channel activity
N	P60143	CC	Required for PSII activity (By similarity).
N	P60143	DE	PSII 5 kDa protein;
N	P60143	DE	Photosystem II reaction center protein L;
N	P60143	DR	photosynthesis
N	P60159	CC	Core subunit of the mitochondrial membrane respiratory chain NADH dehydrogenase (Complex I) that is believed to belong to the minimal assembly required for catalysis
N	P60159	CC	Complex I functions in the transfer of electrons from NADH to the respiratory chain
N	P60159	CC	The immediate electron acceptor for the enzyme is believed to be ubiquinone (By similarity).
N	P60159	DE	NADH dehydrogenase subunit 3;
N	P60159	DE	NADH-ubiquinone oxidoreductase chain 3;
N	P60159	DR	NADH dehydrogenase (ubiquinone) activity
N	P60159	DR	electron transport chain
N	P60164	CC	Binds and inhibits voltage-sensitive potassium channels
N	P60164	CC	Inhibits the vertebrate potassium channels Kv1.1, Kv1.2 and Kv1.3 with low affinity

N	P60164	DE	Parabutoxin-1;
N	P60164	DE	Potassium channel toxin alpha-KTx 11.1;
N	P60164	DR	potassium channel inhibitor activity
N	P60341	CC	Responsible for synthesis of pseudouridine from uracil- 55 in the psi GC loop of transfer RNAs (By similarity).
N	P60341	DE	Psi55 synthase;
N	P60341	DE	tRNA pseudouridine 55 synthase;
N	P60341	DE	tRNA pseudouridine synthase B;
N	P60341	DE	tRNA pseudouridylate synthase;
N	P60341	DE	tRNA-uridine isomerase;
N	P60341	DR	pseudouridine synthase activity
N	P60341	DR	pseudouridine synthesis
N	P60341	DR	tRNA processing
N	P60356	DE	UPF0297 protein lin1538;
N	P60368	CC	In the hair cortex, hair keratin intermediate filaments are embedded in an interfilamentous matrix, consisting of hair keratin-associated proteins (KRTAP), which are essential for the formation of a rigid and resistant hair shaft through their extensive disulfide bond cross-linking with abundant cysteine residues of hair keratins
N	P60368	CC	The matrix proteins include the high- sulfur and high-glycine-tyrosine keratins.
N	P60368	DE	High sulfur keratin-associated protein 10.2;
N	P60368	DE	Keratin-associated protein 10-2;
N	P60368	DE	Keratin-associated protein 10.2;
N	P60368	DE	Keratin-associated protein 18-2;
N	P60368	DE	Keratin-associated protein 18.2;
N	P60383	CC	Modulates transcription in response to changes in cellular NADH/NAD(+) redox state (By similarity).
N	P60383	DE	Redox-sensing transcriptional repressor rex;
N	P60383	DR	catalytic activity
N	P60383	DR	response to redox state
N	P60383	DR	transcription repressor activity
N	P60389	CC	Modulates transcription in response to changes in cellular NADH/NAD(+) redox state (By similarity).
N	P60389	DE	Redox-sensing transcriptional repressor rex;
N	P60389	DR	catalytic activity
N	P60389	DR	response to redox state
N	P60389	DR	transcription repressor activity
N	P60422	CC	One of the primary rRNA binding proteins
N	P60422	CC	It has been suggested to have peptidyltransferase activity; this is highly controversial
N	P60422	CC	Located near the base of the L1 stalk, it is probably also mobile
N	P60422	CC	Required for association of the 30S and 50S subunits to form the 70S ribosome, for tRNA binding and peptide bond formation
N	P60422	DE	50S ribosomal protein L2;
N	P60422	DR	protein binding
N	P60422	DR	structural constituent of ribosome
N	P60422	DR	transferase activity
N	P60422	DR	zinc ion binding
N	P60666	CC	IGPS catalyzes the conversion of PRFAR and glutamine to IGP, AICAR and glutamate
N	P60666	CC	The hisF subunit catalyzes the cyclization activity that produces IGP and AICAR from PRFAR using the ammonia provided by the hisH subunit (By similarity).
N	P60666	DE	IGP synthase cyclase subunit;
N	P60666	DE	IGP synthase subunit hisF;
N	P60666	DE	IGPS subunit hisF;
N	P60666	DE	ImGP synthase subunit hisF;
N	P60666	DE	Imidazole glycerol phosphate synthase subunit hisF;
N	P60666	DR	histidine biosynthetic process
N	P60666	DR	imidazoleglycerol-phosphate synthase activity
N	P60666	DR	lyase activity
N	P60793	CC	Required for accurate and efficient protein synthesis under certain stress conditions
N	P60793	CC	Back- translocation proceeds from a post-translocation (POST) complex to a pre-translocation (PRE) complex, thus giving elongation factor G a second chance to translocate the tRNAs correctly
N	P60793	CC	Binds to ribosomes in a GTP-dependent manner (By similarity).
N	P60793	CC	May act as a fidelity factor of the translation reaction, by catalyzing a one-codon backward translocation of tRNAs on improperly translocated ribosomes
N	P60793	DE	Elongation factor 4;

N	P60793	DE	Ribosomal back-translocase LepA;
N	P60793	DR	GTPase activity
N	P60859	CC	Catalyzes the sequential NAD-dependent oxidations of L- histidinol to L-histidinaldehyde and then to L-histidine (By similarity).
N	P60859	DE	Histidinol dehydrogenase;
N	P60859	DR	histidine biosynthetic process
N	P60859	DR	histidinol dehydrogenase activity
N	P60859	DR	oxidation-reduction process
N	P60859	DR	zinc ion binding
N	P60869	DE	Putative transport protein YbjL;
N	P60869	DR	cation transmembrane transporter activity
N	P60869	DR	potassium ion transport
N	P61017	CC	Protein transport
N	P61017	CC	Probably involved in vesicular traffic (By similarity).
N	P61017	DE	Ras-related protein Rab-4B;
N	P61017	DR	protein transport
N	P61017	DR	small GTPase mediated signal transduction
N	P61039	CC	Component of the cytochrome b6-f complex, which mediates electron transfer between photosystem II (PSII) and photosystem I (PSI), cyclic electron flow around PSI, and state transitions (By similarity).
N	P61039	DE	Cytochrome b6-f complex subunit 8;
N	P61039	DE	Cytochrome b6-f complex subunit VIII;
N	P61039	DE	Cytochrome b6-f complex subunit petN;
N	P61039	DR	cytochrome complex assembly
N	P61039	DR	electron transport chain
N	P61039	DR	electron transporter, transferring electrons within cytochrome b6/f complex of photosystem II activity
N	P61039	DR	photosynthesis
N	P61216	CC	TFIID beta-specific TAFII (By similarity).
N	P61216	DE	Transcription initiation factor TFIID 18 kDa subunit;
N	P61216	DE	Transcription initiation factor TFIID subunit 13;
N	P61216	DR	RNA polymerase II transcription factor activity
N	P61216	DR	transcription from RNA polymerase II promoter
N	P61239	DE	30S ribosomal protein S27ae;
N	P61239	DR	metal ion binding
N	P61239	DR	structural constituent of ribosome
N	P61326	CC	Component of a splicing-dependent multiprotein exon junction complex (EJC) deposited at splice junction on mRNAs
N	P61326	CC	Core components of the EJC, that remains bound to spliced mRNAs throughout all stages of mRNA metabolism, functions to mark the position of the exon-exon junction in the mature mRNA and thereby influences downstream processes of gene expression including mRNA splicing, nuclear mRNA export, subcellular mRNA localization, translation efficiency and nonsense-mediated mRNA decay (NMD)
N	P61326	CC	Remains associated with the mRNA after its export to the cytoplasm and require translation of the mRNA for removal
N	P61326	CC	The EJC is a dynamic structure consisting of a few core proteins and several more peripheral nuclear and cytoplasmic associated factors that join the complex only transiently either during EJC assembly or during subsequent mRNA metabolism
N	P61326	CC	The heterodimer MAGOH-RBM8A interacts with PYM that function to enhance the translation of EJC-bearing spliced mRNAs by recruiting them to the ribosomal 48S preinitiation complex.
N	P61326	DE	Protein mago nashi homolog;
N	P61326	DR	mRNA 3'-end processing
N	P61326	DR	mRNA export from nucleus
N	P61326	DR	nuclear mRNA splicing, via spliceosome
N	P61326	DR	nuclear-transcribed mRNA catabolic process, nonsense-mediated decay
N	P61326	DR	protein binding
N	P61326	DR	regulation of translation
N	P61326	DR	termination of RNA polymerase II transcription
N	P61508	CC	Forms pore that permeabilize the cell membrane
N	P61508	CC	May function both in the prey capture strategy as well as protection from infectious organisms arising from prey ingestion (By similarity).
N	P61508	CC	Potently inhibits the growth of bacteria, yeast and Leishmania
N	P61508	CC	Promotes efflux of calcium from synaptosomes, causes hemolysis, and dissipates voltage gradients across muscle membrane

N	P61508	DE	Lycotoxin II;
N	P61508	DE	Lycotoxin-2;
N	P61508	DE	M-LCTX-Hc2a;
N	P61508	DE	M-lycotoxin-Hc2a;
N	P61508	DR	defense response to bacterium
N	P61508	DR	hemolysis in other organism
N	P61645	CC	Catalyzes the polycondensation of alpha-2,8-linked sialic acid required for the synthesis of polysialic acid (PSA), which is present on the embryonic neural cell adhesion molecule (N-CAM), necessary for plasticity of neural cells (By similarity)
N	P61645	DE	Alpha-2,8-sialyltransferase 8D;
N	P61645	DE	CMP-N-acetylneuraminate-poly-alpha-2,8-sialyltransferase;
N	P61645	DE	Polysialyltransferase-1;
N	P61645	DE	Sialyltransferase 8D;
N	P61645	DE	Sialytransferase St8Sia IV;
N	P61645	DR	protein glycosylation
N	P61662	DE	Sugar fermentation stimulation protein homolog;
N	P61688	CC	No specific function has so far been attributed to this initiation factor; however, it seems to stimulate more or less all the activities of the other two initiation factors, IF-2 and IF-3
N	P61688	DE	Translation initiation factor IF-1;
N	P61688	DR	translation initiation factor activity
N	P62029	CC	The glycine cleavage system catalyzes the degradation of glycine
N	P62029	CC	The P protein binds the alpha-amino group of glycine through its pyridoxal phosphate cofactor; CO(2) is released and the remaining methylamine moiety is then transferred to the lipoamide cofactor of the H protein (By similarity).
N	P62029	DE	Glycine cleavage system P-protein subunit 2;
N	P62029	DE	Glycine decarboxylase subunit 2;
N	P62029	DE	Probable glycine dehydrogenase [decarboxylating] subunit 2;
N	P62029	DR	glycine dehydrogenase (decarboxylating) activity
N	P62029	DR	oxidation-reduction process
N	P62029	DR	pyridoxal phosphate binding
N	P62153	CC	Calmodulin mediates the control of a large number of enzymes and other proteins by Ca(2+)
N	P62153	CC	Among the enzymes to be stimulated by the calmodulin-Ca(2+) complex are a number of protein kinases and phosphatases.
N	P62153	DE	Calmodulin-A;
N	P62153	DR	calcium ion binding
N	P62163	CC	Calmodulin mediates the control of a large number of enzymes and other proteins by Ca(2+)
N	P62163	CC	Among the enzymes to be stimulated by the calmodulin-Ca(2+) complex are a number of protein kinases and phosphatases.
N	P62163	DE	Calmodulin-2;
N	P62163	DR	calcium ion binding
N	P62229	DE	30S ribosomal protein S16;
N	P62229	DR	structural constituent of ribosome
N	P62369	CC	Converts 4-diphosphocytidyl-2C-methyl-D-erythritol 2- phosphate into 2C-methyl-D-erythritol 2,4-cyclodiphosphate and CMP (By similarity).
N	P62369	DE	2-C-methyl-D-erythritol 2,4-cyclodiphosphate synthase;
N	P62369	DE	MECDP-synthase;
N	P62369	DR	2-C-methyl-D-erythritol 2,4-cyclodiphosphate synthase activity
N	P62369	DR	terpenoid biosynthetic process
N	P62742	CC	Gamma chains make up the fetal hemoglobin F, in combination with alpha chains
N	P62742	DE	Gamma-2-globin;
N	P62742	DE	Hemoglobin gamma-2 chain;
N	P62742	DE	Hemoglobin gamma-G chain;
N	P62742	DE	Hemoglobin subunit gamma-2;
N	P62742	DR	oxygen binding
N	P62742	DR	oxygen transporter activity
N	P62847	CC	Required for processing of pre-rRNA and maturation of 40S ribosomal subunits.
N	P62847	DE	40S ribosomal protein S24;
N	P62847	DR	endocrine pancreas development
N	P62847	DR	erythrocyte homeostasis
N	P62847	DR	nucleotide binding
N	P62847	DR	rRNA processing
N	P62847	DR	ribosomal small subunit biogenesis
N	P62847	DR	structural constituent of ribosome

N	P62847	DR	translation initiation factor binding
N	P62847	DR	translational elongation
N	P62847	DR	translational termination
N	P62847	DR	viral transcription
N	P62890	DE	60S ribosomal protein L30;
N	P62890	DR	structural constituent of ribosome
N	P62924	CC	mRNA-binding protein involved in translation elongation
N	P62924	CC	Functions as a regulator of apoptosis (By similarity).
N	P62924	CC	Has an important function at the level of mRNA turnover, probably acting downstream of decapping
N	P62924	CC	Involved in actin dynamics and cell cycle progression, mRNA decay and probably in a pathway involved in stress response and maintenance of cell wall integrity
N	P62924	DE	Eukaryotic translation initiation factor 5A;
N	P62924	DR	peptidyl-lysine modification to hypusine
N	P62924	DR	positive regulation of translational elongation
N	P62924	DR	positive regulation of translational termination
N	P62924	DR	ribosome binding
N	P62924	DR	translation elongation factor activity
N	P62924	DR	translational frameshifting
N	P63028	CC	Involved in calcium binding and microtubule stabilization (By similarity).
N	P63028	DE	21 kDa polypeptide;
N	P63028	DE	Translationally-controlled tumor protein;
N	P63028	DR	anti-apoptosis
N	P63028	DR	stem cell maintenance
N	P63028	DR	transcription factor binding
N	P63076	CC	May be a signaling molecule in the induction and patterning of the embryonic brain
N	P63076	DE	Fibroblast growth factor 17;
N	P63076	DR	growth factor activity
N	P63259	CC	Actins are highly conserved proteins that are involved in various types of cell motility and are ubiquitously expressed in all eukaryotic cells.
N	P63259	DE	Actin, cytoplasmic 2, N-terminally processed;
N	P63259	DE	Actin, cytoplasmic 2;
N	P63259	DE	Gamma-actin;
N	P63259	DR	response to calcium ion
N	P63427	DE	Probable acyl-CoA dehydrogenase fadE25;
N	P63427	DR	acyl-CoA dehydrogenase activity
N	P63427	DR	flavin adenine dinucleotide binding
N	P63427	DR	oxidation-reduction process
N	P63550	DE	Ribulokinase;
N	P63550	DR	L-arabinose catabolic process
N	P63550	DR	ribulokinase activity
N	P63552	DE	Arginine deiminase;
N	P63552	DE	Arginine dihydrolase;
N	P63552	DR	arginine catabolic process
N	P63552	DR	arginine deiminase activity
N	P63602	CC	Catalyzes the specific phosphorylation of the 3-hydroxyl group of shikimic acid using ATP as a cosubstrate (By similarity).
N	P63602	DE	Shikimate kinase 1;
N	P63602	DR	aromatic amino acid family biosynthetic process
N	P63602	DR	metal ion binding
N	P63602	DR	shikimate kinase activity
N	P63613	DE	5-enolpyruvylshikimate-3-phosphate phospholyase;
N	P63613	DE	Chorismate synthase;
N	P63613	DR	aromatic amino acid family biosynthetic process
N	P63613	DR	chorismate synthase activity
N	P63658	CC	F(1)F(0) ATP synthase produces ATP from ADP in the presence of a proton or sodium gradient
N	P63658	CC	During catalysis, ATP synthesis in the catalytic domain of F(1) is coupled via a rotary mechanism of the central stalk subunits to proton translocation (By similarity)
N	P63658	CC	F-type ATPases consist of two structural domains, F(1) containing the extramembraneous catalytic core and F(0) containing the membrane proton channel, linked together by a central stalk and a peripheral stalk
N	P63658	DE	ATP synthase F(1) sector subunit delta;
N	P63658	DE	ATP synthase subunit delta;
N	P63658	DE	F-ATPase subunit delta;

N	P63658	DE	F-type ATPase subunit delta;
N	P63658	DR	ATP synthesis coupled proton transport
N	P63658	DR	hydrogen ion transporting ATP synthase activity, rotational mechanism
N	P63701	DE	Uncharacterized protein NMA0420;
N	P63725	CC	Membrane-anchoring subunit of succinate dehydrogenase (SDH) (By similarity).
N	P63725	DE	Cytochrome b-556;
N	P63725	DE	Succinate dehydrogenase cytochrome b556 subunit;
N	P63725	DR	electron carrier activity
N	P63725	DR	electron transport chain
N	P63725	DR	metal ion binding
N	P63725	DR	succinate dehydrogenase activity
N	P63725	DR	tricarboxylic acid cycle
N	P63843	CC	DNA-binding protein that represses the expression of many genes that are induced as cells make the transition from rapid exponential growth to stationary phase (By similarity).
N	P63843	CC	At low GTP concentration it no longer binds GTP and stop to act as a transcriptional repressor (By similarity).
N	P63843	CC	It is a GTP-binding protein that senses the intracellular GTP concentration as an indicator of nutritional limitations
N	P63843	DE	GTP-sensing transcriptional pleiotropic repressor CodY;
N	P63843	DR	regulation of transcription, DNA-dependent
N	P63843	DR	sequence-specific DNA binding transcription factor activity
N	P63843	DR	transcription repressor activity
N	P63870	DE	Cysteine synthase;
N	P63870	DE	O-acetylserine (thiol)-lyase;
N	P63870	DE	O-acetylserine sulfhydrylase;
N	P63870	DR	cysteine biosynthetic process from serine
N	P63870	DR	cysteine synthase activity
N	P63870	DR	pyridoxal phosphate binding
N	P63870	DR	transferase activity
N	P63963	CC	DNA primase is the polymerase that synthesizes small RNA primers for the Okazaki fragments on both template strands at replication forks during chromosomal DNA synthesis.
N	P63963	DE	DNA primase;
N	P63963	DR	DNA primase activity
N	P63963	DR	DNA replication, synthesis of RNA primer
N	P63963	DR	zinc ion binding
N	P64275	CC	Necessary for efficient RNA polymerase transcription elongation past template-encoded arresting sites
N	P64275	CC	Cleavage of the nascent transcript by cleavage factors such as greA or greB allows the resumption of elongation from the new 3'terminus
N	P64275	CC	GreA releases sequences of 2 to 3 nucleotides (By similarity).
N	P64275	CC	The arresting sites in DNA have the property of trapping a certain fraction of elongating RNA polymerases that pass through, resulting in locked ternary complexes
N	P64275	DE	Transcript cleavage factor greA;
N	P64275	DE	Transcription elongation factor greA;
N	P64275	DR	transcription elongation regulator activity
N	P64328	CC	Catalyzes the NADPH-dependent reduction of glutamyl- tRNA(Glu) to glutamate 1-semialdehyde (GSA) (By similarity).
N	P64328	DE	Glutamyl-tRNA reductase;
N	P64328	DR	glutamyl-tRNA reductase activity
N	P64328	DR	oxidation-reduction process
N	P64328	DR	porphyrin biosynthetic process
N	P64328	DR	shikimate 5-dehydrogenase activity
N	P64728	DE	Uncharacterized protein Mb0520;
N	P64954	DE	Uncharacterized protein Mb2232;
N	P65030	DE	Uncharacterized protein Mb2628;
N	P65098	DE	Isocitrate dehydrogenase [NADP];
N	P65098	DE	NADP(+)-specific ICDH;
N	P65098	DE	Oxalosuccinate decarboxylase;
N	P65098	DR	glyoxylate cycle
N	P65098	DR	isocitrate dehydrogenase (NADP+) activity
N	P65098	DR	isocitrate metabolic process
N	P65098	DR	magnesium ion binding
N	P65098	DR	tricarboxylic acid cycle

N	P65110	CC	No specific function has so far been attributed to this initiation factor; however, it seems to stimulate more or less all the activities of the other two initiation factors, IF-2 and IF-3
N	P65110	DE	Translation initiation factor IF-1;
N	P65110	DR	translation initiation factor activity
N	P65416	DE	Putative 3-methyladenine DNA glycosylase;
N	P65416	DR	alkylbase DNA N-glycosylase activity
N	P65416	DR	base-excision repair
N	P65439	DE	Protein MraZ;
N	P65462	CC	Cell wall formation (By similarity).
N	P65462	DE	UDP-N-acetylenolpyruvoylglucosamine reductase;
N	P65462	DE	UDP-N-acetylmuramate dehydrogenase;
N	P65462	DR	UDP-N-acetylmuramate dehydrogenase activity
N	P65462	DR	cell division
N	P65462	DR	cellular cell wall organization
N	P65462	DR	flavin adenine dinucleotide binding
N	P65462	DR	oxidation-reduction process
N	P65462	DR	peptidoglycan biosynthetic process
N	P65462	DR	regulation of cell shape
N	P65483	CC	Cell wall formation
N	P65483	CC	Catalyzes the transfer of a GlcNAc subunit on undecaprenyl-pyrophosphoryl-MurNAc-pentapeptide (lipid intermediate I) to form undecaprenyl-pyrophosphoryl-MurNAc- (pentapeptide)GlcNAc (lipid intermediate II) (By similarity)
N	P65483	DE	UDP-N-acetylglucosamine--N-acetylmuramyl-(pentapeptide) pyrophosphoryl-undecaprenol N-acetylglucosamine transferase;
N	P65483	DE	Undecaprenyl-PP-MurNAc-pentapeptide-UDPGlcNAc GlcNAc transferase;
N	P65483	DR	UDP-N-acetyl-D-glucosamine:N-acetylmuramoyl-L-alanyl-D-glutamyl-meso-2,6-diaminopimelyl-D-alanyl-D-alanine-diphosphoundecaprenol 4-beta-N-acetylglucosaminlytransferase activity
N	P65483	DR	UDP-N-acetylgalactosamine biosynthetic process
N	P65483	DR	carbohydrate binding
N	P65483	DR	cell division
N	P65483	DR	cellular cell wall organization
N	P65483	DR	lipid glycosylation
N	P65483	DR	peptidoglycan biosynthetic process
N	P65483	DR	regulation of cell shape
N	P65483	DR	undecaprenyldiphospho-muramoylpentapeptide beta-N-acetylglucosaminyltransferase activity
N	P65492	CC	This protein is involved in the repair of mismatches in DNA
N	P65492	CC	It is required for dam-dependent methyl-directed DNA mismatch repair
N	P65492	CC	May act as a "molecular matchmaker", a protein that promotes the formation of a stable complex between two or more DNA-binding proteins in an ATP-dependent manner without itself being part of a final effector complex (By similarity)
N	P65492	DE	DNA mismatch repair protein mutL;
N	P65492	DR	mismatch repair
N	P65492	DR	mismatched DNA binding
N	P65621	DE	Aspartate carbamoyltransferase;
N	P65621	DE	Aspartate transcarbamylase;
N	P65621	DR	'de novo' pyrimidine base biosynthetic process
N	P65621	DR	amino acid binding
N	P65621	DR	aspartate carbamoyltransferase activity
N	P65621	DR	cellular amino acid metabolic process
N	P65621	DR	pyrimidine nucleotide biosynthetic process
N	P65840	CC	Responsible for synthesis of pseudouridine from uracil- 516 in 16S ribosomal RNA (By similarity).
N	P65840	DE	16S pseudouridine 516 synthase;
N	P65840	DE	16S pseudouridylate 516 synthase;
N	P65840	DE	Ribosomal small subunit pseudouridine synthase A;
N	P65840	DE	rRNA pseudouridylate synthase A;
N	P65840	DE	rRNA-uridine isomerase A;
N	P65840	DR	pseudouridine synthase activity
N	P65840	DR	pseudouridine synthesis
N	P65840	DR	rRNA processing
N	P65921	CC	Catalyzes the ATP-dependent amination of UTP to CTP with either L-glutamine or ammonia as the source of nitrogen (By similarity).
N	P65921	DE	CTP synthase;

N	P65921	DE	CTP synthetase;
N	P65921	DE	UTP--ammonia ligase;
N	P65921	DR	CTP synthase activity
N	P65921	DR	glutamine metabolic process
N	P65921	DR	pyrimidine nucleotide biosynthetic process
N	P65948	CC	Transfers and isomerizes the ribose moiety from AdoMet to the 7-aminomethyl group of 7-deazaguanine (preQ1-tRNA) to give epoxyqueuosine (oQ-tRNA) (By similarity).
N	P65948	DE	Queuosine biosynthesis protein queA;
N	P65948	DE	S-adenosylmethionine:tRNA ribosyltransferase-isomerase;
N	P65948	DR	isomerase activity
N	P65948	DR	queuosine biosynthetic process
N	P65948	DR	transferase activity
N	P66094	CC	Binds directly to 23S rRNA
N	P66094	CC	The L1 stalk is quite mobile in the ribosome, and is involved in E site tRNA release (By similarity).
N	P66094	DE	50S ribosomal protein L1;
N	P66094	DR	RNA processing
N	P66094	DR	regulation of translation
N	P66094	DR	structural constituent of ribosome
N	P66099	CC	Binds directly to 23S rRNA
N	P66099	CC	The L1 stalk is quite mobile in the ribosome, and is involved in E site tRNA release (By similarity).
N	P66099	DE	50S ribosomal protein L1;
N	P66099	DR	RNA processing
N	P66099	DR	regulation of translation
N	P66099	DR	structural constituent of ribosome
N	P66258	DE	50S ribosomal protein L34;
N	P66258	DR	structural constituent of ribosome
N	P66333	CC	Involved in the binding of tRNA to the ribosomes (By similarity).
N	P66333	DE	30S ribosomal protein S10;
N	P66333	DR	structural constituent of ribosome
N	P66361	CC	Located on the platform of the 30S subunit, it bridges several disparate RNA helices of the 16S rRNA
N	P66361	CC	Forms part of the Shine-Dalgarno cleft in the 70S ribosome (By similarity).
N	P66361	DE	30S ribosomal protein S11;
N	P66361	DR	structural constituent of ribosome
N	P66407	CC	Binds 16S rRNA, required for the assembly of 30S particles and may also be responsible for determining the conformation of the 16S rRNA at the A site (By similarity).
N	P66407	DE	30S ribosomal protein S14;
N	P66407	DR	structural constituent of ribosome
N	P66520	DE	30S ribosomal protein S21;
N	P66520	DR	structural constituent of ribosome
N	P66749	CC	The ruvA-ruvB complex in the presence of ATP renatures cruciform structure in supercoiled DNA with palindromic sequence, indicating that it may promote strand exchange reactions in homologous recombination
N	P66749	CC	RuvA stimulates, in the presence of DNA, the weak ATPase activity of ruvB (By similarity).
N	P66749	CC	RuvAB is an helicase that mediates the Holliday junction migration by localized denaturation and reannealing
N	P66749	DE	Holliday junction ATP-dependent DNA helicase ruvA;
N	P66749	DR	DNA recombination
N	P66749	DR	four-way junction helicase activity
N	P66764	CC	Catalyzes the formation of S-adenosylmethionine from methionine and ATP
N	P66764	CC	The overall synthetic reaction is composed of two sequential steps, AdoMet formation and the subsequent tripolyphosphate hydrolysis which occurs prior to release of AdoMet from the enzyme (By similarity).
N	P66764	DE	AdoMet synthase;
N	P66764	DE	Methionine adenosyltransferase;
N	P66764	DE	S-adenosylmethionine synthase;
N	P66764	DR	metal ion binding
N	P66764	DR	methionine adenosyltransferase activity
N	P66764	DR	one-carbon metabolic process
N	P66810	CC	Sigma factors are initiation factors that promote the attachment of RNA polymerase to specific initiation sites and are then released (By similarity).
N	P66810	DE	Probable RNA polymerase sigma-C factor;

N	P66810	DR	regulation of transcription, DNA-dependent
N	P66810	DR	sequence-specific DNA binding transcription factor activity
N	P66810	DR	sigma factor activity
N	P66810	DR	transcription initiation, DNA-dependent
N	P66858	CC	Binds specifically to the ssrA RNA (tmRNA) and is required for stable association of ssrA with ribosomes (By similarity).
N	P66858	DE	SsrA-binding protein;
N	P66889	CC	Required for correct localization of precursor proteins bearing signal peptides with the twin arginine conserved motif S/T-R-R-X-F-L-K
N	P66889	CC	This sec-independent pathway is termed TAT for twin-arginine translocation system
N	P66889	CC	This system mainly transports proteins with bound cofactors that require folding prior to export (By similarity).
N	P66889	DE	Sec-independent protein translocase protein tatA/E homolog;
N	P66889	DR	protein secretion
N	P66889	DR	protein transporter activity
N	P66889	DR	transmembrane transport
N	P67093	DE	Universal stress protein G;
N	P67093	DR	response to stress
N	P67172	DE	UPF0079 ATP-binding protein Mb3456c;
N	P67174	DE	UPF0082 protein BR1717;
N	P67216	CC	Required for maturation of 30S ribosomal subunits (By similarity).
N	P67216	DE	Ribosome maturation factor rimP;
N	P67216	DR	ribosome biogenesis
N	P67220	CC	Required for maturation of 30S ribosomal subunits (By similarity).
N	P67220	DE	Ribosome maturation factor rimP;
N	P67220	DR	ribosome biogenesis
N	P67399	CC	Catalyzes the conversion of uracil and 5-phospho-alpha- D-ribose 1-diphosphate (PRPP) to UMP and diphosphate (By similarity).
N	P67399	DE	UMP pyrophosphorylase;
N	P67399	DE	Uracil phosphoribosyltransferase;
N	P67399	DR	nucleoside metabolic process
N	P67399	DR	uracil phosphoribosyltransferase activity
N	P67399	DR	uracil salvage
N	P67591	DE	Tryptophan--tRNA ligase;
N	P67591	DE	Tryptophanyl-tRNA synthetase;
N	P67591	DR	tryptophan-tRNA ligase activity
N	P67591	DR	tryptophanyl-tRNA aminoacylation
N	P67595	DE	Tryptophan--tRNA ligase;
N	P67595	DE	Tryptophanyl-tRNA synthetase;
N	P67595	DR	tryptophan-tRNA ligase activity
N	P67595	DR	tryptophanyl-tRNA aminoacylation
N	P67640	DE	UPF0735 ACT domain-containing protein SAV1643;
N	P67640	DR	L-phenylalanine biosynthetic process
N	P67865	CC	Plays critical roles in virus replication, from virus entry and uncoating to assembly and budding of the virus particle
N	P67865	CC	During virus entry into cell, the M2 ion channel acidifies the internal virion core, inducing M1 dissociation from the RNP
N	P67865	CC	Forms a continuous shell on the inner side of the lipid bilayer in virion, where it binds the RNP
N	P67865	CC	Interaction of viral NEP with M1-RNP is thought to promote nuclear export of the complex, which is targeted to the virion assembly site at the apical plasma membrane in polarized epithelial cells
N	P67865	CC	Interactions with NA and HA may bring M1, a non-raft-associated protein, into lipid rafts
N	P67865	CC	M1 binding to ribonucleocapsids (RNPs) in nucleus seems to inhibit viral transcription
N	P67865	CC	M1-free RNPs are transported to the nucleus, where viral transcription and replication can take place (By similarity).
N	P67865	DE	Matrix protein 1;
N	P67865	DR	evasion by virus of host immune response
N	P67865	DR	structural molecule activity
N	P68193	CC	Although this protein associates with the 30S subunit of the ribosome it is not considered to be a bona fide ribosomal protein (By similarity).
N	P68193	DE	30S ribosomal protein S22;
N	P68193	DE	Stationary-phase-induced ribosome-associated protein;
N	P68193	DR	structural constituent of ribosome

N	P68248	CC	Pancreatic hormone is synthesized in pancreatic islets of Langerhans and acts as a regulator of pancreatic and gastrointestinal functions.
N	P68248	DE	Pancreatic hormone;
N	P68248	DE	Pancreatic polypeptide;
N	P68248	DR	hormone activity
N	P68302	CC	Metallothioneins have a high content of cysteine residues that bind various heavy metals; these proteins are transcriptionally regulated by both heavy metals and glucocorticoids
N	P68302	DE	Metallothionein-2;
N	P68302	DE	Metallothionein-II;
N	P68302	DR	metal ion binding
N	P68384	DR	serine-type endopeptidase inhibitor activity
N	P68531	CC	Subunits I, II and III form the functional core of the enzyme complex.
N	P68531	DE	Cytochrome c oxidase polypeptide III;
N	P68531	DE	Cytochrome c oxidase subunit 3;
N	P68531	DR	cytochrome-c oxidase activity
N	P68531	DR	mitochondrial electron transport, cytochrome c to oxygen
N	P69148	CC	Myoactive; cardioexcitatory substance
N	P69148	CC	Pharmacological activities include augmentation, induction, and regularization of cardiac contraction.
N	P69148	DR	neuropeptide signaling pathway
N	P69239	CC	Core subunit of the mitochondrial membrane respiratory chain NADH dehydrogenase (Complex I) that is believed to belong to the minimal assembly required for catalysis
N	P69239	CC	Complex I functions in the transfer of electrons from NADH to the respiratory chain
N	P69239	CC	The immediate electron acceptor for the enzyme is believed to be ubiquinone (By similarity).
N	P69239	DE	NADH dehydrogenase subunit 4L;
N	P69239	DE	NADH-ubiquinone oxidoreductase chain 4L;
N	P69239	DR	ATP synthesis coupled electron transport
N	P69239	DR	NADH dehydrogenase (ubiquinone) activity
N	P69289	CC	Encapsidates the negative strand viral RNA, protecting it from nucleases
N	P69289	CC	Dissociation of M1 from RNP unmask nucleoprotein's nuclear localization signals, targeting the RNP to the nucleus (By similarity).
N	P69289	CC	It is possible that the nucleoprotein binds directly exportin-1 (XPO1) and plays an active role in RNP nuclear export
N	P69289	CC	Later in the infection, nucleus export of RNP are mediated through viral proteins NEP interacting with M1 which binds nucleoproteins
N	P69289	CC	M1 interaction with RNP seems to hide nucleoprotein's nuclear localization signals
N	P69289	CC	NP comprises at least 2 nuclear localization signals and is responsible of the active RNP import into the nucleus through the cellular importin alpha/beta pathway
N	P69289	CC	Soon after a virion infects a new cell, M1 dissociates from the RNP under acidification of the virion driven by M2 protein
N	P69289	CC	The RNP needs to be localized in the nucleus to start an infectious cycle, but is too large to diffuse through the nuclear pore complex
N	P69289	CC	The encapsidated genomic RNA is termed the ribonucleoprotein (RNP) and serves as template for transcription and replication
N	P69289	DE	Nucleocapsid protein;
N	P69289	DE	Nucleoprotein;
N	P69289	DR	interspecies interaction between organisms
N	P69289	DR	structural molecule activity
N	P69361	CC	The VP7 protein is one of the five proteins (with VP1, VP3, VP4, and VP6) which form the inner capsid of the virus.
N	P69361	DE	Core protein VP7;
N	P69361	DR	host cell surface receptor binding
N	P69361	DR	structural molecule activity
N	P69361	DR	viral envelope fusion with host membrane
N	P69406	CC	Positive regulator of capsular polysaccharide synthesis
N	P69406	CC	RcsA and rcsB form a complex to promote transcription of the genes for capsule synthesis (By similarity).
N	P69406	DE	Colanic acid capsular biosynthesis activation protein A;
N	P69406	DR	regulation of transcription, DNA-dependent
N	P69406	DR	sequence-specific DNA binding
N	P69406	DR	sequence-specific DNA binding transcription factor activity
N	P69406	DR	two-component response regulator activity
N	P69423	CC	Required for correct localization of precursor proteins bearing signal peptides with the twin arginine conserved motif S/T-R-R-X-F-L-K

N	P69423	CC	This sec-independent pathway is termed TAT for twin-arginine translocation system
N	P69423	CC	This system mainly transports proteins with bound cofactors that require folding prior to export (By similarity).
N	P69423	DE	Sec-independent protein translocase protein tatC;
N	P69423	DR	intracellular protein transmembrane transport
N	P69423	DR	proton motive force dependent protein transmembrane transporter activity
N	P69475	DE	Coat protein;
N	P69475	DR	structural molecule activity
N	P69505	CC	Di-iron-containing protein involved in the repair of iron-sulfur clusters damaged by oxidative and nitrosative stress conditions (By similarity).
N	P69505	DE	Iron-sulfur cluster repair protein YtfE;
N	P69505	DR	metal ion binding
N	P69505	DR	response to stress
N	P69625	DE	Uncharacterized 9.7 kDa protein in cox-rep intergenic region;
N	P69686	CC	One of the two reaction center proteins of photosystem II (PSII), D2 is needed for assembly of a stable PSII complex (By similarity).
N	P69686	DE	PSII D2 protein;
N	P69686	DE	Photosystem II D2 protein;
N	P69686	DE	Photosystem Q(A) protein;
N	P69686	DR	electron transporter, transferring electrons within the cyclic electron transport pathway of photosynthesis activity
N	P69686	DR	metal ion binding
N	P69686	DR	photosynthetic electron transport in photosystem II
N	P69733	CC	Escorts unspliced or incompletely spliced viral pre- mRNAs (late transcripts) out of the nucleus of infected cells
N	P69733	CC	These pre-mRNAs carry a recognition sequence called Rev responsive element (RRE) located in the env gene, that is not present in fully spliced viral mRNAs (early transcripts)
N	P69733	CC	This function is essential since most viral proteins are translated from unspliced or partially spliced pre-mRNAs which cannot exit the nucleus by the pathway used by fully processed cellular mRNAs (By similarity).
N	P69733	DE	Protein Rev;
N	P69733	DE	Regulator of expression of viral proteins;
N	P69733	DR	mRNA transport
N	P69733	DR	regulation of transcription, DNA-dependent
N	P69733	DR	sequence-specific DNA binding transcription factor activity
N	P69733	DR	specific transcriptional repressor activity
N	P69749	CC	Delta-conotoxins bind to site 6 of voltage-gated sodium channels and inhibit the inactivation process (By similarity).
N	P69749	DE	Delta-BVIA;
N	P69749	DE	Delta-conotoxin-like BVIA;
N	P69749	DR	sodium channel inhibitor activity
N	P69798	CC	The phosphoenolpyruvate-dependent sugar phosphotransferase system (sugar PTS), a major carbohydrate active -transport system, catalyzes the phosphorylation of incoming sugar substrates concomitantly with their translocation across the cell membrane
N	P69798	CC	This system is involved in mannose transport (By similarity).
N	P69798	DE	Mannose-specific phosphotransferase enzyme IIA component;
N	P69798	DE	Mannose-specific phosphotransferase enzyme IIB component;
N	P69798	DE	PTS system mannose-specific EIIA component;
N	P69798	DE	PTS system mannose-specific EIIAB component;
N	P69798	DE	PTS system mannose-specific EIIB component;
N	P69798	DR	kinase activity
N	P69798	DR	phosphoenolpyruvate-dependent sugar phosphotransferase system
N	P69798	DR	protein-N(PI)-phosphohistidine-sugar phosphotransferase activity
N	P69887	DE	Triose-phosphate isomerase;
N	P69887	DE	Triosephosphate isomerase;
N	P69887	DR	gluconeogenesis
N	P69887	DR	pentose-phosphate shunt
N	P69887	DR	triose-phosphate isomerase activity
N	P69891	CC	Gamma chains make up the fetal hemoglobin F, in combination with alpha chains
N	P69891	DE	Gamma-1-globin;
N	P69891	DE	Hb F Agamma;
N	P69891	DE	Hemoglobin gamma-1 chain;
N	P69891	DE	Hemoglobin gamma-A chain;
N	P69891	DE	Hemoglobin subunit gamma-1;
N	P69891	DR	blood coagulation

N	P69891	DR	oxygen binding
N	P69891	DR	oxygen transporter activity
N	P69891	DR	protein binding
N	P70182	CC	Participates in the biosynthesis of phosphatidylinositol-4,5-bisphosphate
N	P70182	CC	Acts as an activator of TUT1 adenylyltransferase activity in nuclear speckles, thereby regulating mRNA polyadenylation of a select set of mRNAs
N	P70182	CC	Contributes to the activation of PLD2.
N	P70182	CC	Mediates RAC1-dependent reorganization of actin filaments
N	P70182	DE	68 kDa type I phosphatidylinositol-4-phosphate 5-kinase;
N	P70182	DE	PI4P5KIbeta;
N	P70182	DE	PIP5K1-alpha;
N	P70182	DE	PIP5KIalpha;
N	P70182	DE	Phosphatidylinositol-4-phosphate 5-kinase type I alpha;
N	P70182	DE	Phosphatidylinositol-4-phosphate 5-kinase type I beta;
N	P70182	DE	Phosphatidylinositol-4-phosphate 5-kinase type-1 alpha;
N	P70182	DE	PtdIns(4)P-5-kinase 1 alpha;
N	P70182	DR	1-phosphatidylinositol-4-phosphate 5-kinase activity
N	P70182	DR	fibroblast migration
N	P70423	CC	Mediates the uptake of the cationic amino acids arginine, lysine and ornithine in a sodium-independent manner.
N	P70423	DE	Cationic amino acid transporter 3;
N	P70423	DE	Cationic amino acid transporter y+;
N	P70423	DE	Solute carrier family 7 member 3;
N	P70423	DR	L-lysine transmembrane transporter activity
N	P70423	DR	arginine transmembrane transporter activity
N	P70423	DR	cationic amino acid transmembrane transporter activity
N	P70668	CC	Endogenous activator of intestinal guanylate cyclase
N	P70668	CC	It stimulates this enzyme through the same receptor binding region as the heat-stable enterotoxins
N	P70668	CC	May be a potent physiological regulator of intestinal fluid and electrolyte [illegible]
N	P70668	CC	May be an autocrine/paracrine regulator of intestinal salt and water transport.
N	P70668	DE	Guanylate cyclase activator 2B;
N	P70668	DE	Uroguanylin;
N	P70668	DR	enzyme activator activity
N	P70668	DR	ion homeostasis
N	P70668	DR	regulation of cyclic nucleotide biosynthetic process
N	P70668	DR	water homeostasis
N	P70901	CC	The vlp and vsp proteins are antigenically distinct proteins, only one vlp or vsp gene is transcriptionally active at any one time
N	P70901	CC	Switching between these genes is a mechanism of host immune reponse evasion.
N	P70901	DE	Variable large protein 12;
N	P71381	DE	Anthranilate synthase component II;
N	P71381	DE	Glutamine amido-transferase;
N	P71381	DR	anthranilate synthase activity
N	P71381	DR	glutamine metabolic process
N	P71381	DR	tryptophan biosynthetic process
N	P71724	DE	Phosphoglyceromutase;
N	P71724	DE	Uncharacterized phosphoglycerate mutase family protein Rv2419c;
N	P71724	DR	isomerase activity
N	P71955	DE	Uncharacterized protein Rv2658c/MT2734.1;
N	P72255	DE	UPF0758 protein;
N	P72830	DE	6-phosphofructokinase 1;
N	P72830	DE	Phosphofructokinase 1;
N	P72830	DE	Phosphohexokinase 1;
N	P72830	DR	6-phosphofructokinase activity
N	P72830	DR	fructose 6-phosphate metabolic process
N	P72830	DR	metal ion binding
N	P72848	CC	Key enzyme in heme biosynthesis
N	P72848	CC	Catalyzes the oxidative decarboxylation of propionic acid side chains of rings A and B of coproporphyrinogen III (By similarity).
N	P72848	DE	Coprogen oxidase;
N	P72848	DE	Coproporphyrinogen-III oxidase, aerobic;
N	P72848	DE	Coproporphyrinogenase;
N	P72848	DR	coproporphyrinogen oxidase activity
N	P72848	DR	oxidation-reduction process

N	P72848	DR	porphyrin biosynthetic process
N	P72848	DR	protein homodimerization activity
N	P73056	DE	Uncharacterized monothiol glutaredoxin ycf64-like;
N	P73056	DR	2 iron, 2 sulfur cluster binding
N	P73056	DR	cell redox homeostasis
N	P73056	DR	electron carrier activity
N	P73056	DR	metal ion binding
N	P73056	DR	protein disulfide oxidoreductase activity
N	P73161	CC	Catalyzes the formation of N(7)-methylguanine at position 46 (m7G46) in tRNA (By similarity).
N	P73161	DE	tRNA (guanine-N(7)-)-methyltransferase;
N	P73161	DE	tRNA(m7G46)-methyltransferase;
N	P73161	DR	tRNA (guanine-N7-)-methyltransferase activity
N	P73320	CC	One of the primary rRNA binding proteins, it binds directly near the 3'-end of the 23S rRNA, where it nucleates assembly of the 50S subunit (By similarity).
N	P73320	DE	50S ribosomal protein L3;
N	P73320	DR	structural constituent of ribosome
N	P73859	DE	KaiB-like protein 1;
N	P73859	DR	rhythmic process
N	P74267	DE	50S ribosomal protein L27;
N	P74267	DR	structural constituent of ribosome
N	P74794	DE	UPF0150 protein ssl0738;
N	P74857	DE	Probable secretion system apparatus ATP synthase SsaN;
N	P74857	DR	ATP biosynthetic process
N	P74857	DR	protein secretion by the type III secretion system
N	P74857	DR	proton-transporting ATPase activity, rotational mechanism
N	P74921	CC	Acts on leucine, isoleucine and valine (By similarity).
N	P74921	DE	Probable branched-chain-amino-acid aminotransferase;
N	P74921	DR	L-isoleucine transaminase activity
N	P74921	DR	L-leucine transaminase activity
N	P74921	DR	L-valine transaminase activity
N	P74921	DR	branched chain family amino acid biosynthetic process
N	P75084	DE	Uncharacterized protein MG027 homolog;
N	P75084	DR	regulation of transcription, DNA-dependent
N	P75150	DE	Uncharacterized lipoprotein MG439 homolog 1;
N	P75452	DE	Uncharacterized protein MPN_110;
N	P75452	DR	hydrolase activity
N	P75452	DR	identical protein binding
N	P75684	DE	Uncharacterized protein yagP;
N	P75933	CC	Involved in the assembly process of the P-ring formation
N	P75933	CC	It may associate with flgF on the rod constituting a structure essential for the P-ring assembly or may act as a modulator protein for the P-ring assembly.
N	P75933	DE	Flagella basal body P-ring formation protein flgA;
N	P75933	DR	ciliary or flagellar motility
N	P75933	DR	flagellum organization
N	P78406	CC	Binds mRNA
N	P78406	CC	May function in nucleocytoplasmic transport and in directly or indirectly attaching cytoplasmic mRNPs to the cytoskeleton.
N	P78406	DE	Rae1 protein homolog;
N	P78406	DE	mRNA export factor;
N	P78406	DE	mRNA-associated protein mrnp 41;
N	P78406	DR	carbohydrate metabolic process
N	P78406	DR	glucose transport
N	P78406	DR	mRNA export from nucleus
N	P78406	DR	microtubule binding
N	P78406	DR	regulation of glucose transport
N	P78406	DR	transmembrane transport
N	P78406	DR	viral reproduction
N	P78556	CC	Chemotactic factor that attracts lymphocytes and, slightly, neutrophils, but not monocytes
N	P78556	CC	C-terminal processed forms have been shown to be equally chemotactically active for leukocytes
N	P78556	CC	Inhibits proliferation of myeloid progenitors in colony formation assays
N	P78556	CC	May be involved in formation and function of the mucosal lymphoid tissues by attracting lymphocytes and dendritic cells towards epithelial cells
N	P78556	CC	Possesses antibacterial activity E.coli ATCC 25922 and S.aureus ATCC 29213.

N	P78556	DE	Beta-chemokine exodus-1;
N	P78556	DE	C-C motif chemokine 20;
N	P78556	DE	CC chemokine LARC;
N	P78556	DE	CCL20(1-64);
N	P78556	DE	CCL20(1-67);
N	P78556	DE	CCL20(2-70);
N	P78556	DE	Liver and activation-regulated chemokine;
N	P78556	DE	MIP-3-alpha;
N	P78556	DE	Macrophage inflammatory protein 3 alpha;
N	P78556	DE	Small-inducible cytokine A20;
N	P78556	DR	cell-cell signaling
N	P78556	DR	chemokine activity
N	P78556	DR	defense response to bacterium
N	P78556	DR	immune response
N	P78556	DR	inflammatory response
N	P78556	DR	signal transduction
N	P78763	DE	Uncharacterized mitochondrial carrier C83.13;
N	P78763	DR	mitochondrial transport
N	P78937	CC	Catalyzes the first intracellular reaction of sulfate assimilation, forming adenosine-5'-phosphosulfate (APS) from inorganic sulfate and ATP
N	P78937	CC	Plays an important role in sulfate activation as a component of the biosynthesis pathway of sulfur- containing amino acids (By similarity).
N	P78937	DE	ATP-sulfurylase;
N	P78937	DE	Sulfate adenylate transferase;
N	P78937	DE	Sulfate adenylyltransferase;
N	P78937	DR	cysteine biosynthetic process
N	P78937	DR	methionine biosynthetic process
N	P78937	DR	sulfate adenylyltransferase (ATP) activity
N	P78937	DR	sulfate assimilation
N	P79071	DE	60S ribosomal protein L6;
N	P79071	DR	structural constituent of ribosome
N	P79175	CC	Receptor for the chemotactic and inflammatory peptide anaphylatoxin C5a
N	P79175	CC	This receptor stimulates chemotaxis, granule enzyme release and superoxide anion production.
N	P79175	DE	C5a anaphylatoxin chemotactic receptor;
N	P79175	DR	C5a anaphylatoxin receptor activity
N	P79339	CC	Participates in at least several B-cell activation processes as well as of other cell types
N	P79339	CC	It also regulates the expression of the low affinity Fc receptor for IgE (CD23) on both lymphocytes and monocytes (By similarity).
N	P79339	CC	It enhances both secretion and cell surface expression of IgE and IgG1
N	P79339	CC	It induces the expression of class II MHC molecules on resting B-cells
N	P79339	CC	It is a costimulator of DNA-synthesis
N	P79339	DE	B-cell stimulatory factor 1;
N	P79339	DE	Interleukin-4;
N	P79339	DE	Lymphocyte stimulatory factor 1;
N	P79339	DR	B cell activation
N	P79339	DR	cytokine activity
N	P79339	DR	growth factor activity
N	P79339	DR	immune response
N	P79339	DR	interleukin-4 receptor binding
N	P79339	DR	negative regulation of apoptosis
N	P79339	DR	negative regulation of osteoclast differentiation
N	P79339	DR	positive regulation of B cell proliferation
N	P79339	DR	positive regulation of MHC class II biosynthetic process
N	P79339	DR	positive regulation of T cell proliferation
N	P79339	DR	positive regulation of isotype switching to IgE isotypes
N	P79339	DR	positive regulation of isotype switching to IgG isotypes
N	P79339	DR	positive regulation of transcription from RNA polymerase II promoter
N	P79339	DR	regulation of phosphorylation
N	P79809	CC	Visual pigments such as rhodopsin and porphyropsin are light-absorbing molecules that mediate vision
N	P79809	CC	Porphyropsin consists of opsin covalently linked to 11-cis 3,4- didehydroretinal.
N	P79809	CC	Rhodopsin consists of an apoprotein, opsin, covalently linked to 11-cis-retinal
N	P79809	CC	This receptor is coupled to the activation of phospholipase C
N	P79809	DR	G-protein coupled receptor activity

N	P79809	DR	photoreceptor activity
N	P79809	DR	phototransduction
N	P79809	DR	protein-chromophore linkage
N	P79809	DR	visual perception
N	P79944	CC	Functions as a transcriptional activator playing a crucial role during development
N	P79944	CC	Activates wnt8, t/bra, chrd and mix-A/mix.1 expression.
N	P79944	CC	Functions in gastrulation, regulating mesoderm differentiation
N	P79944	DE	Eomesodermin;
N	P79944	DR	cell differentiation
N	P79944	DR	endoderm formation
N	P79944	DR	mesoderm formation
N	P79944	DR	regulation of transcription, DNA-dependent
N	P79944	DR	sequence-specific DNA binding transcription factor activity
N	P79944	DR	transcription activator activity
N	P80288	CC	Electron donor for nitrous-oxide reductase.
N	P80288	DE	Cytochrome c-550;
N	P80288	DE	Cytochrome c550;
N	P80288	DR	electron carrier activity
N	P80288	DR	electron transport chain
N	P80528	CC	Thiol protease.
N	P80528	DE	Cathepsin L-like cysteine proteinase;
N	P80528	DE	Newly excysted juvenile protein 4;
N	P80528	DR	cysteine-type peptidase activity
N	P80682	CC	Component of the cuticle of the larva of Tenebrio molitor.
N	P80682	DE	Larval cuticle protein A2B;
N	P80682	DR	structural constituent of cuticle
N	P80717	DE	1,4-beta-D-xylan xylanohydrolase B;
N	P80717	DE	Endo-1,4-beta-xylanase B;
N	P80717	DE	Xylanase B;
N	P80717	DR	endo-1,4-beta-xylanase activity
N	P80717	DR	xylan catabolic process
N	P81104	CC	PPIases accelerate the folding of proteins during protein synthesis.
N	P81104	DE	70 kDa peptidyl-prolyl isomerase;
N	P81104	DE	Cyclophilin;
N	P81104	DE	Peptidyl-prolyl cis-trans isomerase;
N	P81104	DR	calmodulin binding
N	P81104	DR	peptidyl-prolyl cis-trans isomerase activity
N	P81104	DR	protein folding
N	P81162	CC	Serine protease inhibitor.
N	P81162	DE	Protease inhibitor carrapatin;
N	P81162	DR	serine-type endopeptidase inhibitor activity
N	P81240	CC	Excitatory insect beta-toxins induce a spastic paralysis
N	P81240	CC	It is more similar to the excitatory toxins in its mode of action and the depressant toxins in its primary structure.
N	P81240	CC	It operates by inducing a fast contraction paralysis without depressant activity
N	P81240	CC	They bind voltage-independently at site-4 of sodium channels and shift the voltage of activation toward more negative potentials thereby affecting sodium channel activation and promoting spontaneous and repetitive firing (By similarity)
N	P81240	CC	This toxin is active only on insects
N	P81240	DE	Insect toxin LqhIT5;
N	P81240	DR	defense response
N	P81240	DR	sodium channel inhibitor activity
N	P81528	CC	Hydrolyzes the cell walls of mycobacteria
N	P81528	CC	May play an important role in cell wall growth and cell separation.
N	P81528	DE	38 kDa autolysin;
N	P81528	DE	Beta-glycosidase;
N	P81528	DE	Peptidoglycan hydrolase;
N	P81528	DR	barrier septum formation
N	P81528	DR	cellular cell wall organization
N	P81528	DR	defense response to bacterium
N	P81528	DR	hydrolase activity, acting on glycosyl bonds
N	P81921	CC	Complexes with Or83b to form odorant-sensing units, providing sensitive and prolonged odorant signaling and calcium permeability
N	P81921	CC	They are necessary and sufficient to promote functional reconstitution of odor-evoked signaling in sensory neurons that normally respond only to carbon dioxide

N	P81921	DE	Odorant receptor 47a;
N	P81921	DR	odorant binding
N	P81921	DR	olfactory receptor activity
N	P81921	DR	sensory perception of smell
N	P81988	DE	Inorganic pyrophosphatase;
N	P81988	DE	Pyrophosphate phospho-hydrolase;
N	P81988	DR	inorganic diphosphatase activity
N	P82006	CC	Structural component of the virion.
N	P82006	DE	14.5 kDa structural polyprotein;
N	P82166	CC	Component of the cuticle of migratory locust which contains more than 100 different structural proteins.
N	P82166	DE	Cuticle protein 19.8;
N	P82166	DR	structural constituent of cuticle
N	P82291	DE	Soluble cytochrome b558;
N	P82291	DR	electron transport chain
N	P82610	CC	Catalyzes the transfer of a methyl group from 5- methyltetrahydrofolate to homocysteine resulting in methionine formation.
N	P82610	DE	5-methyltetrahydropteroyltriglutamate--homocysteine methyltransferase;
N	P82610	DE	Cobalamin-independent methionine synthase;
N	P82610	DE	Methionine synthase, vitamin-B12 independent isozyme;
N	P82610	DR	5-methyltetrahydropteroyltriglutamate-homocysteine S-methyltransferase activity
N	P82610	DR	induction by symbiont of host defense response
N	P82610	DR	methionine biosynthetic process
N	P82610	DR	response to heat
N	P82610	DR	zinc ion binding
N	P82837	CC	Antibacterial activity against Gram-positive bacterium S.aureus and Gram-negative bacterium E.coli.
N	P82837	DE	Brevinin-1Be;
N	P82837	DR	defense response to bacterium
N	P82914	DE	28S ribosomal protein S15, mitochondrial;
N	P82914	DR	structural constituent of ribosome
N	P83065	CC	This is one of the proteins that binds and probably mediates the attachment of the 5S RNA into the large ribosomal subunit, where it forms part of the central protuberance
N	P83065	CC	Contacts the P site tRNA; the 5S rRNA and some of its associated proteins might help stabilize positioning of ribosome-bound tRNAs (By similarity).
N	P83065	CC	In the 70S ribosome it contacts protein S13 of the 30S subunit (bridge B1b), connecting the two subunits; this bridge is implicated in subunit movement
N	P83065	DE	50S ribosomal protein L5;
N	P83143	CC	Possesses antifungal activity against P.infestans but not F.graminearum.
N	P83143	DE	Antifungal protein 2 large subunit;
N	P83143	DR	defense response to bacterium
N	P83143	DR	defense response to fungus
N	P83143	DR	killing of cells of other organism
N	P83143	DR	xenobiotic metabolic process
N	P83427	CC	Has antibacterial activity, preferentially against Gram- negative bacteria.
N	P83427	DR	defense response to bacterium
N	P83427	DR	innate immune response
N	P83575	DE	NADP phosphatase 1;
N	P83575	DE	NADP phosphatase I;
N	P83575	DR	hydrolase activity
N	P83627	CC	Inhibits secondary vitellogenesis in females
N	P83627	CC	Has no hyperglycemic or molt-inhibiting activity.
N	P83627	DE	Vitellogenesis-inhibiting hormone;
N	P83627	DR	neuropeptide hormone activity
N	P83627	DR	neuropeptide signaling pathway
N	P83709	CC	Acts as a chaperone (By similarity).
N	P83709	DE	Chaperone protein DnaK;
N	P83709	DE	Heat shock 70 kDa protein;
N	P83709	DE	Heat shock protein 70;
N	P83709	DR	response to stress
N	P83749	CC	Functional homolog of SRP receptor
N	P83749	CC	Probably involved in the reception and insertion of a subset of proteins at the cytoplasmic membrane (By similarity).
N	P83749	DE	Cell division protein ftsY;
N	P83749	DR	SRP-dependent cotranslational protein targeting to membrane

N	P83749	DR	cell division
N	P83749	DR	nucleoside-triphosphatase activity
N	P83749	DR	protein binding
N	P83900	CC	Guanine nucleotide exchange factor (GEF) for RAP1A, RAP2A and MRAS/M-Ras-GTP
N	P83900	CC	Its association with MRAS inhibits Rap1 activation (By similarity).
N	P83900	DE	M-Ras-regulated Rap GEF;
N	P83900	DE	Rap guanine nucleotide exchange factor 5;
N	P83900	DR	guanyl-nucleotide exchange factor activity
N	P83900	DR	regulation of small GTPase mediated signal transduction
N	P83900	DR	small GTPase mediated signal transduction
N	P83922	CC	Mediates visceral muscle contractile activity (myotropic activity).
N	P83922	DE	Periviscerokinin-1;
N	P83922	DR	neuropeptide signaling pathway
N	P84181	CC	Probable role in the gustatory response (By similarity).
N	P84181	DE	Putative gustatory receptor 22d;
N	P84181	DR	G-protein coupled receptor activity
N	P84181	DR	sensory perception of taste
N	P84182	CC	Displays antimicrobial activity against E.coli, S.aureus and P.aeruginosa.
N	P84182	DE	Antimicrobial peptide OEP3121;
N	P84182	DR	defense response to bacterium
N	P84205	CC	Involved in oxygen transport from gills to the various peripheral tissues.
N	P84205	DE	Hemoglobin anodic alpha chain;
N	P84205	DE	Hemoglobin anodic subunit alpha;
N	P84205	DR	oxygen binding
N	P84205	DR	oxygen transporter activity
N	P84439	CC	Mediates visceral muscle contractile activity (myotropic activity).
N	P84439	DE	Pea-PVK-2-like peptide;
N	P84439	DE	Periviscerokinin-2.1;
N	P84439	DE	Periviscerokinin-3;
N	P84439	DE	SheLa-PVK-3;
N	P84439	DR	neuropeptide signaling pathway
N	P84452	CC	Inhibits trypsin.
N	P84452	DE	Trypsin inhibitor 5;
N	P84452	DE	Trypsin inhibitor V;
N	P84452	DR	serine-type endopeptidase inhibitor activity
N	P84621	CC	Has oxidoreductase activity
N	P84621	CC	The Hdl and Mvh subunits may together mediate electron transfer from hydrogen to an unidentified electron acceptor on the cytoplasmic side of the membrane.
N	P84621	DE	Heterodisulfide reductase subunit C-like protein;
N	P84621	DR	4 iron, 4 sulfur cluster binding
N	P84621	DR	electron carrier activity
N	P84621	DR	metal ion binding
N	P84621	DR	oxidation-reduction process
N	P84621	DR	oxidoreductase activity
N	P84630	CC	Reversibly blocks Shaker B potassium channels, with a dissociation constant of 200 nM
N	P84630	DE	Potassium channel toxin alpha-KTx 13.3;
N	P84630	DR	potassium channel inhibitor activity
N	P84671	CC	Myoactive (By similarity).
N	P84671	DE	DerVe-Capa-PK;
N	P84671	DE	Derve-PK-5;
N	P84671	DE	FXPRL-amide;
N	P84671	DE	Pyrokinin-5;
N	P84671	DR	neuropeptide hormone activity
N	P84671	DR	neuropeptide signaling pathway
N	P84797	CC	Has antifungal activity against M.arachidicola with an IC(50) of 125 uM, but not against F.oxysporum
N	P84797	CC	Has antibacterial activity against the Gram- negative bacterium P.fluorescens
N	P84797	CC	Has mitogenic activity towards murine splenocytes
N	P84797	CC	Inhibits human immunodeficiency virus-1 (HIV-1) reverse transcriptase with an IC(50) of 60 uM
N	P84797	CC	Lacks antiproliferative activity towards HepG2 hepatoma cells
N	P84797	CC	No antibacterial activity against the Gram-positive bacteria B.subtilis, B.cereus, B.megaterium, M.phei and S.aureus, or against the Gram-negative bacteria E.aerogenes, E.coli, P.aeruginosa, P.fluorescens and P.vulgaris.

N	P84797	DR	defense response to Gram-negative bacterium
N	P84797	DR	defense response to fungus
N	P84797	DR	killing of cells of other organism
N	P84797	DR	positive regulation of cell division
N	P84797	DR	positive regulation of mitosis
N	P84894	CC	Produces in vitro relaxation of rat arterial smooth muscle and constriction of intestinal smooth muscle (By similarity).
N	P84894	DE	[Val1,Thr6]-bradykinin;
N	P84894	DR	defense response
N	P84976	DE	Glycine-rich RNA-binding protein 2;
N	P84976	DR	nucleotide binding
N	P85354	CC	May play a role in plant defense
N	P85354	CC	Probably has no oxalate oxidase activity even if the active site is conserved (By similarity).
N	P85354	DE	Germin-like protein 4;
N	P85354	DR	metal ion binding
N	P85502	CC	Structural protein.
N	P85502	DE	ORF23 protein;
N	P85502	DE	Structural protein 2;
N	P85548	CC	Myoactive (By similarity).
N	P85548	DE	BlaDu-Capa-PKb;
N	P85548	DE	FXPRL-amide;
N	P85548	DE	Pyrokinin-5b;
N	P85548	DR	neuropeptide hormone activity
N	P85548	DR	neuropeptide signaling pathway
N	P85573	CC	Mediates visceral muscle contractile activity (myotropic activity).
N	P85573	DE	CryKy-PVK-3;
N	P85573	DE	Periviscerokinin-3;
N	P85573	DR	neuropeptide signaling pathway
N	P85840	DE	Venom allergen 5;
N	P85929	CC	Major role in the synthesis of nucleoside triphosphates other than ATP
N	P85929	CC	The ATP gamma phosphate is transferred to the NDP beta phosphate via a ping-pong mechanism, using a phosphorylated active-site intermediate.
N	P85929	DE	NDP kinase I;
N	P85929	DE	Nucleoside diphosphate kinase 1;
N	P85929	DE	Nucleoside diphosphate kinase I;
N	P85929	DR	metal ion binding
N	P85929	DR	nucleoside diphosphate kinase activity
N	P86017	CC	Antimicrobial peptide (By similarity).
N	P86017	DE	Esculentin-2Ra;
N	P86017	DR	defense response to bacterium
N	P86018	CC	Shows antibacterial activity against representative Gram-negative and Gram-positive bacterial species, and hemolytic activity (By similarity).
N	P86018	DE	Esculentin-1R;
N	P86018	DR	defense response to bacterium
N	P86018	DR	hemolysis in other organism
N	P86049	DE	Probable RNA-binding protein 46;
N	P86049	DE	RNA-binding motif protein 46;
N	P86049	DR	nucleotide binding
N	P86060	DE	Unknown protein 2;
N	P86072	DE	1,4-alpha-D-glucan glucanohydrolase;
N	P86072	DE	Alpha-amylase 1;
N	P86072	DR	alpha-amylase activity
N	P86072	DR	carbohydrate metabolic process
N	P86072	DR	metal ion binding
N	P86135	CC	Inhibits the formation of NO by neuronal nitric oxide synthase with an IC(50) of 81 ug/ml.
N	P86135	DE	Signiferin-3.1;
N	P86135	DR	defense response
N	P86135	DR	negative regulation of nitric-oxide synthase activity
N	P86168	CC	Has antifungal activity against S.sclerotiorum (MIC=32.4 ug/ml after 288 hours) and B.cinerea (MIC=10.8 ug/ml after 288 hours), and weak antifungal activity against F.solani, A.fumigatus and G.candidum
N	P86168	CC	Inhibits the production of interleukin-2 by activated CD4+ T-cells with an IC(50) of 167.3 nM

N	P86168	CC	Lacks antifungal activity against P.ultimum, T.viride and R.solani
N	P86168	CC	Lacks cytotoxic activity against human peripheral blood mononuclear cells.
N	P86168	DE	Colutellin-A;
N	P86168	DR	defense response to fungus
N	P86168	DR	killing of cells of other organism
N	P86168	DR	negative regulation of interleukin-2 production
N	P86303	DE	Tachykinin-like peptide-VI;
N	P86390	CC	Involved in oxygen transport from the lung to the various peripheral tissues.
N	P86390	DE	Beta-2-globin;
N	P86390	DE	Hemoglobin beta-2 chain;
N	P86390	DE	Hemoglobin beta-II chain;
N	P86390	DE	Hemoglobin subunit beta-2;
N	P86390	DR	oxygen binding
N	P86390	DR	oxygen transporter activity
N	P86391	CC	Involved in oxygen transport from the lung to the various peripheral tissues.
N	P86391	DE	Beta-2-globin;
N	P86391	DE	Hemoglobin beta-2 chain;
N	P86391	DE	Hemoglobin beta-II chain;
N	P86391	DE	Hemoglobin subunit beta-2;
N	P86391	DR	oxygen binding
N	P86391	DR	oxygen transporter activity
N	P86577	DE	Tachykinin-related peptide 3;
N	P86577	DR	neuropeptide signaling pathway
N	P86648	CC	Mediates visceral muscle contractile activity (myotropic activity) (By similarity).
N	P86648	DE	Periviscerokinin-1;
N	P86648	DR	neuropeptide signaling pathway
N	P86778	CC	In muscle, parvalbumin is thought to be involved in relaxation after contraction
N	P86778	CC	It binds two calcium ions (By similarity).
N	P86778	DE	Parvalbumin beta 1;
N	P86778	DR	calcium ion binding
N	P87023	CC	Involved in the synthesis of (1->6)- and (1->3)-beta-D- glucan polymers of the yeast cell wall in vivo
N	P87023	CC	It is required for full activity of beta-glucan synthase in vitro
N	P87023	CC	It may be a beta- glucan synthase, part of a multiprotein glucan synthase or a modulator
N	P87023	DE	Beta-glucan synthesis-associated protein KRE6;
N	P87023	DR	cellular cell wall organization
N	P87220	CC	Subunit of the peripheral V1 complex of vacuolar ATPase
N	P87220	CC	V-ATPase is responsible for acidifying a variety of intracellular compartments in eukaryotic cells, thus providing most of the energy required for transport processes in the vacuolar system (By similarity).
N	P87220	DE	V-ATPase subunit D;
N	P87220	DE	V-type proton ATPase subunit D;
N	P87220	DE	Vacuolar proton pump subunit D;
N	P87220	DR	ATP hydrolysis coupled proton transport
N	P87220	DR	proton-transporting ATPase activity, rotational mechanism
N	P87228	DE	Putative D-3-phosphoglycerate dehydrogenase;
N	P87228	DR	L-serine biosynthetic process
N	P87228	DR	oxidation-reduction process
N	P87228	DR	phosphoglycerate dehydrogenase activity
N	P87238	DE	CUE domain-containing protein 4, mitochondrial;
N	P87347	DE	Oocyte maturation factor mos;
N	P87347	DE	Serine/threonine-protein kinase mos;
N	P87347	DR	protein phosphorylation
N	P87347	DR	protein serine/threonine kinase activity
N	P87745	CC	The large envelope protein exists in two topological conformations, one which is termed 'external' or Le-HBsAg and the other 'internal' or Li-HBsAg
N	P87745	CC	In its external conformation the protein attaches the virus to cell receptors and thereby initiating infection
N	P87745	CC	In its internal conformation the protein plays a role in virion morphogenesis and mediates the contact with the nucleocapsid like a matrix protein (By similarity).
N	P87745	CC	The large envelope protein probably also assumes fusion between virion and host membranes
N	P87745	CC	This interaction determines the species specificity and liver tropism
N	P87745	DE	L glycoprotein;
N	P87745	DE	Large S protein;

N	P87745	DE	Large envelope protein;
N	P87745	DE	Large surface protein;
N	P87745	DE	Major surface antigen;
N	P87745	DR	viral reproduction
N	P92657	CC	Component of the ubiquinol-cytochrome c reductase complex (complex III or cytochrome b-c1 complex), which is a respiratory chain that generates an electrochemical potential coupled to ATP synthesis (By similarity).
N	P92657	DE	Complex III subunit 3;
N	P92657	DE	Complex III subunit III;
N	P92657	DE	Cytochrome b-c1 complex subunit 3;
N	P92657	DE	Cytochrome b;
N	P92657	DE	Ubiquinol-cytochrome-c reductase complex cytochrome b subunit;
N	P92657	DR	electron carrier activity
N	P92657	DR	metal ion binding
N	P92657	DR	oxidoreductase activity
N	P92657	DR	respiratory electron transport chain
N	P92994	CC	Controls carbon flux to pigments essential for pollination or UV protection, to numerous pytoalexins synthesized by plants when challenged by pathogens, and to lignins
N	P92994	DE	Cinnamic acid 4-hydroxylase;
N	P92994	DE	Cytochrome P450 73;
N	P92994	DE	Cytochrome P450C4H;
N	P92994	DE	Trans-cinnamate 4-monooxygenase;
N	P92994	DR	electron carrier activity
N	P92994	DR	lignin metabolic process
N	P92994	DR	oxidation-reduction process
N	P92994	DR	pollen development
N	P92994	DR	response to karrikin
N	P92994	DR	trans-cinnamate 4-monooxygenase activity
N	P93015	CC	Trans-acting factor that binds specifically to the consensus nucleotide sequence 5'-TNCGTACAA-3' of AP1 promoter
N	P93015	CC	Binds specifically to the 5'-GTAC-3' core sequence
N	P93015	CC	Promotes both vegetative phase change and flowering
N	P93015	CC	Regulates phase-specific patterns of leaf epidermal differentiation and flowering time, but does not seem to affect leaf shape.
N	P93015	DE	Squamosa promoter-binding-like protein 3;
N	P93015	DR	cell differentiation
N	P93015	DR	flower development
N	P93015	DR	inflorescence development
N	P93015	DR	metal ion binding
N	P93015	DR	positive regulation of flower development
N	P93015	DR	regulation of vegetative phase change
N	P93015	DR	sequence-specific DNA binding transcription factor activity
N	P94026	CC	Methylates 'Lys-14' of the large subunit of RuBisCO.
N	P94026	DE	Ribulose-1,5 bisphosphate carboxylase/oxygenase large subunit N-methyltransferase, chloroplastic;
N	P94026	DE	RuBisCO LSMT;
N	P94026	DE	RuBisCO methyltransferase;
N	P94026	DE	[Ribulose-bisphosphate carboxylase]-lysine N-methyltransferase;
N	P94026	DR	[ribulose-bisphosphate carboxylase]-lysine N-methyltransferase activity
N	P94462	CC	Removes the formyl group from the N-terminal Met of newly synthesized proteins
N	P94462	CC	N-terminal L-methionine is a prerequisite for activity but the enzyme has broad specificity at other positions (By similarity).
N	P94462	CC	Requires at least a dipeptide for an efficient rate of reaction
N	P94462	DE	Peptide deformylase 1;
N	P94462	DE	Polypeptide deformylase 1;
N	P94462	DR	iron ion binding
N	P94462	DR	peptide deformylase activity
N	P94666	CC	Can catalyze the hydrolysis of ATP in the presence of single-stranded DNA, the ATP-dependent uptake of single-stranded DNA by duplex DNA, and the ATP-dependent hybridization of homologous single-stranded DNAs
N	P94666	CC	It interacts with LexA causing its activation and leading to its autocatalytic cleavage
N	P94666	DE	Protein RecA;
N	P94666	DE	Recombinase A;
N	P94666	DR	DNA recombination
N	P94666	DR	DNA-dependent ATPase activity

N	P94666	DR	single-stranded DNA binding
N	P94907	CC	Catalyzes the condensation of the acetyl group of acetyl-CoA with 3-methyl-2-oxobutanoate (2-oxoisovalerate) to form 3-carboxy-3-hydroxy-4-methylpentanoate (2-isopropylmalate) (By similarity).
N	P94907	DE	2-isopropylmalate synthase;
N	P94907	DE	Alpha-IPM synthase;
N	P94907	DE	Alpha-isopropylmalate synthase;
N	P94907	DR	2-isopropylmalate synthase activity
N	P94907	DR	leucine biosynthetic process
N	P95315	DE	Uncharacterized PPE family protein PPE36;
N	P95669	CC	Binds and compact DNA (95 to 150 base pairs) to form nucleosome-like structures that contain positive DNA supercoils
N	P95669	CC	Increases the resistance of DNA to thermal denaturation (By similarity).
N	P95669	DE	Archaeal histone HAN1 subunit A;
N	P95669	DR	sequence-specific DNA binding
N	P96174	DE	Aspartate carbamoyltransferase catalytic chain;
N	P96174	DE	Aspartate transcarbamylase;
N	P96174	DR	'de novo' pyrimidine base biosynthetic process
N	P96174	DR	amino acid binding
N	P96174	DR	aspartate carbamoyltransferase activity
N	P96174	DR	cellular amino acid metabolic process
N	P96174	DR	pyrimidine nucleotide biosynthetic process
N	P96581	DE	Uncharacterized protein ydaH;
N	P96632	DE	Uncharacterized protein ydcO;
N	P96686	CC	Member of the two-component regulatory system ydfH/ydfI
N	P96686	CC	Regulates the transcription of ydfJ by binding to its promoter region.
N	P96686	DE	Transcriptional regulatory protein ydfI;
N	P96686	DR	regulation of transcription, DNA-dependent
N	P96686	DR	sequence-specific DNA binding
N	P96686	DR	sequence-specific DNA binding transcription factor activity
N	P96686	DR	two-component response regulator activity
N	P97028	DE	Uncharacterized protein ygaN;
N	P97280	CC	May act as a carrier of hyaluronan in serum or as a binding protein between hyaluronan and other matrix protein, including those on cell surfaces in tissues to regulate the localization, synthesis and degradation of hyaluronan which are essential to cells undergoing biological processes (By similarity).
N	P97280	DE	ITI heavy chain H3;
N	P97280	DE	Inter-alpha-inhibitor heavy chain 3;
N	P97280	DE	Inter-alpha-trypsin inhibitor heavy chain H3;
N	P97280	DR	hyaluronan metabolic process
N	P97280	DR	serine-type endopeptidase inhibitor activity
N	P97402	CC	Branching enzyme that converts linear into branched poly-N-acetyllactosaminoglycans
N	P97402	CC	Introduces the blood group I antigen during embryonic development
N	P97402	CC	It is closely associated with the development and maturation of erythroid cells.
N	P97402	DE	I-branching enzyme;
N	P97402	DE	Large I antigen-forming beta-1,6-N-acetylglucosaminyltransferase;
N	P97402	DE	N-acetylglucosaminyltransferase;
N	P97402	DE	N-acetyllactosaminide beta-1,6-N-acetylglucosaminyl-transferase;
N	P97402	DR	N-acetyllactosaminide beta-1,6-N-acetylglucosaminyltransferase activity
N	P97541	DE	Heat shock 20 kDa-like protein p20;
N	P97541	DE	Heat shock protein beta-6;
N	P97541	DR	protein homodimerization activity
N	P97541	DR	regulation of muscle contraction
N	P97541	DR	response to heat
N	P97541	DR	structural constituent of eye lens
N	P98044	CC	Cytochrome c oxidase is the component of the respiratory chain that catalyzes the reduction of oxygen to water
N	P98044	CC	Subunit 2 transfers the electrons from cytochrome c via its binuclear copper A center to the bimetallic center of the catalytic subunit 1.
N	P98044	CC	Subunits 1- 3 form the functional core of the enzyme complex
N	P98044	DE	Cytochrome c oxidase polypeptide II;
N	P98044	DE	Cytochrome c oxidase subunit 2;
N	P98044	DR	copper ion binding
N	P98044	DR	cytochrome-c oxidase activity
N	P98044	DR	electron carrier activity

N	P98044	DR	respiratory electron transport chain
N	P99159	CC	Catalyzes the condensation reaction of fatty acid synthesis by the addition to an acyl acceptor of two carbons from malonyl-ACP
N	P99159	CC	Catalyzes the first condensation reaction which initiates fatty acid synthesis and may therefore play a role in governing the total rate of fatty acid production
N	P99159	CC	Has some substrate preference for butyryl- and isobutyryl-CoA
N	P99159	CC	Its substrate specificity determines the biosynthesis of branched- chain of fatty acids
N	P99159	CC	Possesses both acetoacetyl-ACP synthase and acetyl transacylase activities
N	P99159	DE	3-oxoacyl-[acyl-carrier-protein] synthase 3;
N	P99159	DE	3-oxoacyl-[acyl-carrier-protein] synthase III;
N	P99159	DE	Beta-ketoacyl-ACP synthase III;
N	P99159	DR	3-oxoacyl-[acyl-carrier-protein] synthase activity
N	P99159	DR	beta-ketoacyl-acyl-carrier-protein synthase III activity
N	P99159	DR	fatty acid biosynthetic process
N	Q00103	DE	Uncharacterized protein ORF72;
N	Q00103	DR	zinc ion binding
N	Q00234	CC	This is a copper-containing oxidase that functions in the formation of pigments such as melanins and other polyphenolic compounds.
N	Q00234	DE	Monophenol monooxygenase;
N	Q00234	DR	melanin biosynthetic process
N	Q00234	DR	metal ion binding
N	Q00234	DR	monophenol monooxygenase activity
N	Q00234	DR	oxidation-reduction process
N	Q00651	CC	Integrins alpha-4/beta-1 (VLA-4 or LPAM-2) and alpha- 4/beta-7 (LPAM-1) are receptors for fibronectin
N	Q00651	CC	Integrin alpha- 4/beta-7 is also a receptor for MADCAM1
N	Q00651	CC	Integrin alpha-4/beta-1 recognizes the sequence Q-I-D-S in VCAM1
N	Q00651	CC	Integrin alpha-4/beta-7 is also a receptor for MADCAM1
N	Q00651	CC	It may also participate in cytolytic T-cell interactions with target cells
N	Q00651	CC	It recognizes the sequence L-D-T in MADCAM1
N	Q00651	CC	Mice homozygous for a null mutation of the alpha-4 subunit gene die by day E11-E14 from detachment and rupture of the epicardium and coronary arteries.
N	Q00651	CC	On activated endothelial cells integrin VLA-4 triggers homotypic aggregation for most VLA- 4-positive leukocyte cell lines
N	Q00651	CC	They are also receptors for VCAM1
N	Q00651	CC	They recognize one or more domains within the alternatively spliced CS-1 and CS-5 regions of fibronectin
N	Q00651	DE	CD49 antigen-like family member D;
N	Q00651	DE	CD49d;
N	Q00651	DE	Integrin alpha-4;
N	Q00651	DE	Integrin alpha-IV;
N	Q00651	DE	LPAM subunit alpha;
N	Q00651	DE	Lymphocyte Peyer patch adhesion molecules subunit alpha;
N	Q00651	DE	VLA-4 subunit alpha;
N	Q00651	DR	blood vessel remodeling
N	Q00651	DR	cell migration
N	Q00651	DR	cell-matrix adhesion
N	Q00651	DR	chorio-allantoic fusion
N	Q00651	DR	face development
N	Q00651	DR	fibronectin binding
N	Q00651	DR	heterophilic cell-cell adhesion
N	Q00651	DR	integrin-mediated signaling pathway
N	Q00651	DR	leukocyte cell-cell adhesion
N	Q00651	DR	mesenchyme development
N	Q00651	DR	receptor activity
N	Q00651	DR	visceral serous pericardium development
N	Q00747	DE	Protein LE25;
N	Q00747	DR	embryo development
N	Q00970	CC	Performs an essential function in the repair of oxidatively damaged mtDNA that is required for the maintenance of the mitochondrial genome
N	Q00970	CC	Binds to DNA (By similarity).
N	Q00970	DE	Mitochondrial genome maintenance protein MGM101;
N	Q00970	DR	mitochondrial genome maintenance
N	Q00996	DE	Surface glycoprotein CD59 homolog;

N	Q01020	CC	Component of the molecular motor that translocates genomic DNA in empty capsid during DNA packaging
N	Q01020	CC	Heterodimerizes with small terminase protein to be docked on capsid portal protein
N	Q01020	CC	May have or induce an endonuclease activity to cleave the genome concatemer after encapsidation (By similarity).
N	Q01020	CC	The latter forms a ring in which genomic DNA in translocated into the capsid
N	Q01020	DE	DNA-packaging protein 29;
N	Q01020	DE	Terminase large subunit;
N	Q01020	DE	Tripartite terminase subunit UL15 homolog;
N	Q01020	DR	DNA packaging
N	Q01229	DE	Protein B11;
N	Q01327	DE	Ornithine carbamoyltransferase;
N	Q01327	DR	amino acid binding
N	Q01327	DR	arginine biosynthetic process
N	Q01327	DR	ornithine carbamoyltransferase activity
N	Q01449	DE	Myosin light chain 2a;
N	Q01449	DE	Myosin regulatory light chain 2, atrial isoform;
N	Q01449	DE	Myosin regulatory light chain 7;
N	Q01449	DR	ATPase activity, coupled
N	Q01449	DR	actin filament-based movement
N	Q01449	DR	calcium ion binding
N	Q01449	DR	microfilament motor activity
N	Q01449	DR	smooth muscle contraction
N	Q01457	CC	Provides resistance to bacteriophage by abortive infection.
N	Q01457	DE	Abortive phage resistance protein AbiC;
N	Q01550	DR	structural molecule activity
N	Q01769	CC	Essential component of the RNA polymerase transcription and replication complex
N	Q01769	CC	Binds the viral ribonucleocapsid and positions the L polymerase on the template
N	Q01769	CC	May acts as a chaperone for newly synthesized free N protein, so-called N(0)
N	Q01769	CC	Plays a role in virion assembly (By similarity).
N	Q01769	DE	Phosphoprotein;
N	Q01769	DR	RNA-directed RNA polymerase activity
N	Q01819	CC	Cell adhesion protein involved in target recognition during neuromuscular development
N	Q01819	CC	Mediates homophilic cellular adhesion.
N	Q01819	DR	axonal fasciculation
N	Q01819	DR	homophilic cell adhesion
N	Q01819	DR	synaptic target attraction
N	Q01980	CC	Transcriptional activator of anaerobic gene expression
N	Q01980	CC	Also required for the anaerobic degradation of benzoate.
N	Q01980	CC	For aromatic acid degradation
N	Q01980	DE	Anaerobic aromatic degradation regulator;
N	Q01980	DE	Transcriptional activatory protein AadR;
N	Q01980	DR	regulation of transcription, DNA-dependent
N	Q01980	DR	sequence-specific DNA binding transcription factor activity
N	Q01SK1	DE	UPF0182 protein Acid_6445;
N	Q01UN9	CC	NDH-1 shuttles electrons from NADH, via FMN and iron- sulfur (Fe-S) centers, to quinones in the respiratory chain
N	Q01UN9	CC	Couples the redox reaction to proton translocation (for every two electrons transferred, four hydrogen ions are translocated across the cytoplasmic membrane), and thus conserves the redox energy in a proton gradient (By similarity)
N	Q01UN9	CC	The immediate electron acceptor for the enzyme in this species is believed to be ubiquinone
N	Q01UN9	DE	NADH dehydrogenase I subunit D 2;
N	Q01UN9	DE	NADH-quinone oxidoreductase subunit D 2;
N	Q01UN9	DE	NDH-1 subunit D 2;
N	Q01UN9	DR	NADH dehydrogenase (quinone) activity
N	Q01UN9	DR	oxidation-reduction process
N	Q01UN9	DR	quinone binding
N	Q01YD0	CC	Catalyzes the interconversion of 2-phosphoglycerate and 3-phosphoglycerate (By similarity).
N	Q01YD0	DE	2,3-bisphosphoglycerate-dependent phosphoglycerate mutase;
N	Q01YD0	DE	BPG-dependent PGAM;
N	Q01YD0	DE	Phosphoglyceromutase;
N	Q01YD0	DR	phosphoglycerate mutase activity

N	Q020J1	CC	Responsible for the transport of dicarboxylates such as succinate, fumarate, and malate from the periplasm across the membrane (By similarity).
N	Q020J1	DE	C4-dicarboxylate transport protein;
N	Q020J1	DR	carbohydrate transport
N	Q020J1	DR	sodium:dicarboxylate symporter activity
N	Q02223	CC	Receptor for TNFSF13B/BLyS/BAFF and TNFSF13/APRIL
N	Q02223	CC	Activates NF-kappa-B and JNK.
N	Q02223	CC	Promotes B-cell survival and plays a role in the regulation of humoral immunity
N	Q02223	DE	B-cell maturation protein;
N	Q02223	DE	CD269;
N	Q02223	DE	Tumor necrosis factor receptor superfamily member 17;
N	Q02223	DR	cell proliferation
N	Q02223	DR	multicellular organismal development
N	Q02283	CC	Probable transcription activator involved in leaf development
N	Q02283	CC	Binds to the DNA sequence 5'-CAAT[AT]ATTG-3'.
N	Q02283	DE	HD-ZIP protein 5;
N	Q02283	DE	HD-ZIP protein ATHB-1;
N	Q02283	DE	Homeobox-leucine zipper protein HAT5;
N	Q02283	DE	Homeodomain transcription factor ATHB-1;
N	Q02283	DE	Homeodomain-leucine zipper protein HAT5;
N	Q02283	DR	leaf morphogenesis
N	Q02283	DR	protein homodimerization activity
N	Q02283	DR	regulation of transcription, DNA-dependent
N	Q02283	DR	response to blue light
N	Q02283	DR	response to salt stress
N	Q02283	DR	sequence-specific DNA binding
N	Q02283	DR	sequence-specific DNA binding transcription factor activity
N	Q02283	DR	transcription activator activity
N	Q02586	DE	Polyketide synthase CurC;
N	Q02586	DR	antibiotic biosynthetic process
N	Q02655	CC	Component of the ubiquinol-cytochrome c reductase complex (complex III or cytochrome b-c1 complex), which is a respiratory chain that generates an electrochemical potential coupled to ATP synthesis (By similarity).
N	Q02655	DE	Complex III subunit 3;
N	Q02655	DE	Complex III subunit III;
N	Q02655	DE	Cytochrome b-c1 complex subunit 3;
N	Q02655	DE	Cytochrome b;
N	Q02655	DE	Ubiquinol-cytochrome-c reductase complex cytochrome b subunit;
N	Q02655	DR	electron carrier activity
N	Q02655	DR	metal ion binding
N	Q02655	DR	oxidoreductase activity
N	Q02655	DR	respiratory electron transport chain
N	Q02831	DE	Uncharacterized protein YPL077C;
N	Q02831	DR	protein binding
N	Q02843	CC	Matrix protein p17 targets Gag and Gag-Pol polyproteins to the plasma membrane via a multipartite membrane binding signal, that includes its [illegible]
N	Q02843	CC	Nucleocapsid protein p7 encapsulates and protects viral dimeric unspliced (genomic) RNA
N	Q02843	CC	Also mediates nuclear localization of the preintegration complex
N	Q02843	CC	Binds these RNAs through its zinc fingers (By similarity).
N	Q02843	CC	Implicated in the release from host cell mediated by Vpu (By similarity).
N	Q02843	DE	Capsid protein p24;
N	Q02843	DE	Gag polyprotein;
N	Q02843	DE	Matrix protein p17;
N	Q02843	DE	Nucleocapsid protein p7;
N	Q02843	DE	Spacer peptide p1;
N	Q02843	DE	Spacer peptide p2;
N	Q02843	DR	interspecies interaction between organisms
N	Q02843	DR	structural molecule activity
N	Q02843	DR	viral reproduction
N	Q02843	DR	zinc ion binding
N	Q02879	CC	General transcription factor that functions at the core of the DNA-binding multiprotein factor TFIID

N	Q02879	CC	Binding of TFIID to the TATA box is the initial transcriptional step of the pre-initiation complex (PIC), playing a role in the activation of eukaryotic genes transcribed by RNA polymerase II.
N	Q02879	DE	TATA sequence-binding protein 2;
N	Q02879	DE	TATA-binding factor 2;
N	Q02879	DE	TATA-box factor 2;
N	Q02879	DE	TATA-box-binding protein 2;
N	Q02879	DE	Transcription initiation factor TFIID TBP-2 subunit;
N	Q02879	DR	RNA polymerase II transcription factor activity
N	Q02879	DR	regulation of transcription, DNA-dependent
N	Q02879	DR	transcription initiation from RNA polymerase II promoter
N	Q02DL4	DE	Homoserine kinase;
N	Q02DL4	DR	homoserine kinase activity
N	Q02DL4	DR	threonine biosynthetic process
N	Q02FV2	DE	Sugar fermentation stimulation protein homolog;
N	Q02HR9	CC	Required for accurate and efficient protein synthesis under certain stress conditions
N	Q02HR9	CC	Back- translocation proceeds from a post-translocation (POST) complex to a pre-translocation (PRE) complex, thus giving elongation factor G a second chance to translocate the tRNAs correctly
N	Q02HR9	CC	Binds to ribosomes in a GTP-dependent manner (By similarity).
N	Q02HR9	CC	May act as a fidelity factor of the translation reaction, by catalyzing a one-codon backward translocation of tRNAs on improperly translocated ribosomes
N	Q02HR9	DE	Elongation factor 4;
N	Q02HR9	DE	Ribosomal back-translocase LepA;
N	Q02HR9	DR	GTPase activity
N	Q02JE0	DE	UPF0260 protein PA14_47410;
N	Q02R72	DE	50S ribosomal protein L36 2;
N	Q02R72	DR	structural constituent of ribosome
N	Q02TR5	CC	Catalyzes the decarboxylative condensation of pimeloyl- CoA and L-alanine to produce 8-amino-7-oxononanoate (AON), coenzyme A, and carbon dioxide (By similarity).
N	Q02TR5	DE	7-KAP synthase;
N	Q02TR5	DE	7-keto-8-amino-pelargonic acid synthase;
N	Q02TR5	DE	8-amino-7-ketopelargonate synthase;
N	Q02TR5	DE	8-amino-7-oxononanoate synthase;
N	Q02TR5	DE	KAPA synthase;
N	Q02TR5	DE	L-alanine--pimeloyl-CoA ligase;
N	Q02TR5	DR	8-amino-7-oxononanoate synthase activity
N	Q02TR5	DR	biotin biosynthetic process
N	Q02TR5	DR	pyridoxal phosphate binding
N	Q02TR5	DR	transferase activity, transferring nitrogenous groups
N	Q02ZY9	CC	Specifically methylates the N4 position of cytidine in position 1402 (C1402) of 16S rRNA (By similarity).
N	Q02ZY9	DE	16S rRNA m(4)C1402 methyltransferase;
N	Q02ZY9	DE	Ribosomal RNA small subunit methyltransferase H;
N	Q02ZY9	DE	rRNA (cytosine-N(4)-)-methyltransferase RsmH;
N	Q02ZY9	DR	methyltransferase activity
N	Q02ZY9	DR	rRNA processing
N	Q030Z9	CC	Participates in both the initiation and recycling phases of transcription
N	Q030Z9	CC	In the presence of the delta subunit, RNAP displays an increased specificity of transcription, a decreased affinity for nucleic acids, and an increased efficiency of RNA synthesis because of enhanced recycling (By similarity).
N	Q030Z9	DE	Probable DNA-directed RNA polymerase subunit delta;
N	Q030Z9	DE	RNAP delta factor;
N	Q030Z9	DR	DNA-directed RNA polymerase activity
N	Q03133	DE	6-deoxyerythronolide B synthase III;
N	Q03133	DE	Erythronolide synthase, modules 5 and 6;
N	Q03133	DR	acyl carrier activity
N	Q03133	DR	acyltransferase activity
N	Q03133	DR	antibiotic biosynthetic process
N	Q03133	DR	cofactor binding
N	Q03133	DR	erythronolide synthase activity
N	Q03133	DR	hydrolase activity, acting on ester bonds
N	Q03133	DR	oxidoreductase activity
N	Q03133	DR	phosphopantetheine binding
N	Q03191	CC	Involved in the maintenance and repair of the intestinal mucosa

N	Q03191	CC	Promotes the mobility of epithelial cells in healing processes (motogen) (By similarity).
N	Q03191	DE	Intestinal trefoil factor;
N	Q03191	DE	Polypeptide P1.B;
N	Q03191	DE	Trefoil factor 3;
N	Q03191	DR	response to peptide hormone stimulus
N	Q03191	DR	wound healing
N	Q03196	DE	Cysteine proteinase inhibitor;
N	Q03196	DR	cysteine-type endopeptidase inhibitor activity
N	Q03196	DR	response to stress
N	Q03522	CC	Cell wall formation
N	Q03522	CC	Catalyzes the addition of glutamate to the nucleotide precursor UDP-N-acetylmuramoyl-L-alanine (UMA) (By similarity).
N	Q03522	DE	D-glutamic acid-adding enzyme;
N	Q03522	DE	UDP-N-acetylmuramoyl-L-alanyl-D-glutamate synthetase;
N	Q03522	DE	UDP-N-acetylmuramoylalanine--D-glutamate ligase;
N	Q03522	DR	UDP-N-acetylmuramoylalanine-D-glutamate ligase activity
N	Q03522	DR	cell division
N	Q03522	DR	cellular cell wall organization
N	Q03522	DR	peptidoglycan biosynthetic process
N	Q03522	DR	regulation of cell shape
N	Q03E93	CC	Catalyzes the hydrolysis of both 2',3'-cyclic AMP and 2',3'-cyclic GMP into 3'-AMP and 3'-GMP, respectively, at the 3'- terminal of RNA (By similarity).
N	Q03E93	DE	2',3'-cyclic-nucleotide 2'-phosphodiesterase 2;
N	Q03E93	DR	2',3'-cyclic-nucleotide 2'-phosphodiesterase activity
N	Q03EB0	CC	DNA-dependent RNA polymerase catalyzes the transcription of DNA into RNA using the four ribonucleoside triphosphates as substrates.
N	Q03EB0	DE	DNA-directed RNA polymerase subunit beta';
N	Q03EB0	DE	RNA polymerase subunit beta';
N	Q03EB0	DE	RNAP subunit beta';
N	Q03EB0	DE	Transcriptase subunit beta';
N	Q03EB0	DR	DNA-directed RNA polymerase activity
N	Q03F22	CC	One of the primary rRNA binding proteins, it binds directly to 16S rRNA where it helps nucleate assembly of the platform of the 30S subunit by binding and bridging several RNA helices of the 16S rRNA (By similarity).
N	Q03F22	DE	30S ribosomal protein S15;
N	Q03F22	DR	structural constituent of ribosome
N	Q03F28	CC	Necessary for normal cell division and for the maintenance of normal septation (By similarity).
N	Q03F28	DE	Probable GTP-binding protein EngB;
N	Q03F28	DR	barrier septum formation
N	Q03FW3	CC	Specifically methylates guanosine-37 in various tRNAs (By similarity).
N	Q03FW3	DE	M1G-methyltransferase;
N	Q03FW3	DE	tRNA (guanine-N(1)-)-methyltransferase;
N	Q03FW3	DE	tRNA [GM37] methyltransferase;
N	Q03FW3	DR	tRNA (guanine-N1-)-methyltransferase activity
N	Q03GC2	CC	Involved in base excision repair of DNA damaged by oxidation or by mutagenic agents.
N	Q03GC2	CC	Acts as DNA glycosylase that recognizes and removes damaged bases
N	Q03GC2	CC	Cleaves the DNA backbone by beta-delta elimination to generate a single-strand break at the site of the removed base with both 3'- and 5'-phosphates (By similarity).
N	Q03GC2	CC	Has AP (apurinic/apyrimidinic) lyase activity and introduces nicks in the DNA strand.
N	Q03GC2	CC	Has a preference for oxidized purines, such as 7,8-dihydro-8-oxoguanine (8-oxoG).
N	Q03GC2	DE	AP lyase mutM;
N	Q03GC2	DE	DNA-(apurinic or apyrimidinic site) lyase mutM;
N	Q03GC2	DE	Fapy-DNA glycosylase;
N	Q03GC2	DE	Formamidopyrimidine-DNA glycosylase;
N	Q03GC2	DR	base-excision repair
N	Q03GC2	DR	damaged DNA binding
N	Q03GC2	DR	nucleotide-excision repair
N	Q03GC2	DR	oxidized purine base lesion DNA N-glycosylase activity
N	Q03GC2	DR	zinc ion binding
N	Q03H06	CC	Binds to Cpn60 in the presence of Mg-ATP and suppresses the ATPase activity of the latter.
N	Q03H06	DE	10 kDa chaperonin;
N	Q03H06	DE	GroES protein;
N	Q03H06	DE	Protein Cpn10;

N	Q03H06	DR	protein folding
N	Q03I91	CC	Binds to the 23S rRNA (By similarity).
N	Q03I91	DE	50S ribosomal protein L9;
N	Q03I91	DR	structural constituent of ribosome
N	Q03J80	CC	Allows the formation of correctly charged Asn-tRNA(Asn) or Gln-tRNA(Gln) through the transamidation of misacylated Asp- tRNA(Asn) or Glu-tRNA(Gln) in organisms which lack either or both of asparaginyl-tRNA or glutaminyl-tRNA synthetases
N	Q03J80	CC	The reaction takes place in the presence of glutamine and ATP through an activated phospho-Asp-tRNA(Asn) or phospho-Glu-tRNA(Gln) (By similarity).
N	Q03J80	DE	Asp/Glu-ADT subunit C;
N	Q03J80	DE	Aspartyl/glutamyl-tRNA(Asn/Gln) amidotransferase subunit C;
N	Q03J80	DR	ligase activity
N	Q03J80	DR	regulation of translational fidelity
N	Q03PV4	CC	Catalyzes the GTP-dependent ribosomal translocation step during translation elongation
N	Q03PV4	CC	Catalyzes the coordinated movement of the two tRNA molecules, the mRNA and conformational changes in the ribosome (By similarity).
N	Q03PV4	CC	During this step, the ribosome changes from the pre-translocational (PRE) to the post- translocational (POST) state as the newly formed A-site-bound peptidyl-tRNA and P-site-bound deacylated tRNA move to the P and E sites, respectively
N	Q03PV4	DE	Elongation factor G;
N	Q03PV4	DR	GTPase activity
N	Q03PV4	DR	translation elongation factor activity
N	Q03S45	DE	Formate--tetrahydrofolate ligase;
N	Q03S45	DE	Formyltetrahydrofolate synthetase;
N	Q03S45	DR	folic acid-containing compound biosynthetic process
N	Q03S45	DR	formate-tetrahydrofolate ligase activity
N	Q03S45	DR	one-carbon metabolic process
N	Q03SR1	CC	Modulates transcription in response to changes in cellular NADH/NAD(+) redox state (By similarity).
N	Q03SR1	DE	Redox-sensing transcriptional repressor rex;
N	Q03SR1	DR	catalytic activity
N	Q03SR1	DR	response to redox state
N	Q03SR1	DR	transcription repressor activity
N	Q043R9	CC	Endonuclease that specifically degrades the RNA of RNA- DNA hybrids (By similarity).
N	Q043R9	DE	Ribonuclease HII;
N	Q043R9	DR	metal ion binding
N	Q043R9	DR	ribonuclease H activity
N	Q04418	CC	Forms an interface between the RNA polymerase II enzyme and chaperone/scaffolding proteins, suggesting that it is required to connect RNA polymerase II to regulators of protein complex formation (By similarity).
N	Q04418	DE	RNA polymerase II-associated protein RBA50;
N	Q04418	DE	RNA polymerase II-associated protein of 50 kDa;
N	Q04418	DR	protein binding
N	Q04418	DR	transcription from RNA polymerase II promoter
N	Q04451	CC	Catalyzes the attachment of glycine to tRNA(Gly)
N	Q04451	CC	Is also able produce diadenosine tetraphosphate (Ap4A), a universal pleiotropic signaling molecule needed for cell regulation pathways, by direct condensation of 2 ATPs (By similarity).
N	Q04451	DE	AP-4-A synthetase;
N	Q04451	DE	Diadenosine tetraphosphate synthetase;
N	Q04451	DE	Glycine--tRNA ligase;
N	Q04451	DE	Glycyl-tRNA synthetase;
N	Q04451	DR	diadenosine tetraphosphate biosynthetic process
N	Q04451	DR	glycine-tRNA ligase activity
N	Q04451	DR	glycyl-tRNA aminoacylation
N	Q04451	DR	protein dimerization activity
N	Q046C2	CC	One of the primary rRNA binding proteins
N	Q046C2	CC	It has been suggested to have peptidyltransferase activity; this is somewhat controversial
N	Q046C2	CC	Makes several contacts with the 16S rRNA in the 70S ribosome (By similarity).
N	Q046C2	CC	Required for association of the 30S and 50S subunits to form the 70S ribosome, for tRNA binding and peptide bond formation
N	Q046C2	DE	50S ribosomal protein L2;
N	Q046C2	DR	structural constituent of ribosome
N	Q046C2	DR	transferase activity

N	Q04749	CC	Component of TORC2, which regulates cell cycle-dependent polarization of the actin-cytoskeleton and cell wall integrity
N	Q04749	CC	TORC2 controls polarity of the actin cytoskeleton, which is required for orienting the secretory pathway toward discrete growth sites, via the RHO1/PKC1/MAPK cell integrity pathway.
N	Q04749	DE	Adheres voraciously to TOR2 protein 2;
N	Q04749	DE	TORC2 subunit AVO2;
N	Q04749	DE	Target of rapamycin complex 2 subunit AVO2;
N	Q04749	DR	TOR signaling cascade
N	Q04749	DR	establishment or maintenance of actin cytoskeleton polarity
N	Q04749	DR	fungal-type cell wall organization
N	Q04749	DR	protein binding
N	Q04749	DR	regulation of cell growth
N	Q04785	CC	Gp-Ib, a surface membrane protein of platelets, participates in the formation of platelet plugs by binding to von Willebrand factor, which is already bound to the subendothelium
N	Q04785	DE	Antigen CD42b-beta;
N	Q04785	DE	CD42c;
N	Q04785	DE	GP-Ib beta;
N	Q04785	DE	Platelet glycoprotein Ib beta chain;
N	Q04785	DR	blood coagulation
N	Q04785	DR	cell adhesion
N	Q04851	DE	Outer surface protein A;
N	Q04C21	CC	DNA-dependent RNA polymerase catalyzes the transcription of DNA into RNA using the four ribonucleoside triphosphates as substrates.
N	Q04C21	DE	DNA-directed RNA polymerase subunit beta';
N	Q04C21	DE	RNA polymerase subunit beta';
N	Q04C21	DE	RNAP subunit beta';
N	Q04C21	DE	Transcriptase subunit beta';
N	Q04C21	DR	DNA-directed RNA polymerase activity
N	Q04EG2	DE	UPF0173 metal-dependent hydrolase OEOE_1287;
N	Q04EG2	DR	hydrolase activity
N	Q04FP6	CC	An accessory protein needed during the final step in the assembly of 30S ribosomal subunit, possibly for assembly of the head region
N	Q04FP6	CC	Essential for efficient processing of 16S rRNA
N	Q04FP6	CC	It has affinity for free ribosomal 30S subunits but not for 70S ribosomes (By similarity)
N	Q04FP6	CC	May be needed both before and after rbfA during the maturation of 16S rRNA
N	Q04FP6	CC	Probably interacts with S19
N	Q04FP6	DE	Ribosome maturation factor rimM;
N	Q04FP6	DR	rRNA processing
N	Q04FP6	DR	ribosome binding
N	Q04GA9	CC	May be required for sporulation (By similarity).
N	Q04GA9	DE	Putative sporulation transcription regulator whiA;
N	Q04GM3	CC	Catalyzes the attachment of serine to tRNA(Ser)
N	Q04GM3	CC	Is also able to aminoacylate tRNA(Sec) with serine, to form the misacylated tRNA L-seryl-tRNA(Sec), which will be further converted into selenocysteinyl-tRNA(Sec) (By similarity).
N	Q04GM3	DE	Serine--tRNA ligase;
N	Q04GM3	DE	Seryl-tRNA synthetase;
N	Q04GM3	DE	Seryl-tRNA(Ser/Sec) synthetase;
N	Q04GM3	DR	serine-tRNA ligase activity
N	Q04GM3	DR	seryl-tRNA aminoacylation
N	Q04IN8	CC	Sulfhydryl-activated toxin that causes cytolysis by forming pores in cholesterol containing host membranes
N	Q04IN8	CC	After binding to target membranes, the protein undergoes a major conformation change, leading to its insertion in the host membrane and formation of an oligomeric pore complex
N	Q04IN8	CC	Can be reversibly inactivated by oxidation (By similarity).
N	Q04IN8	CC	Cholesterol may be required for binding to host membranes, membrane insertion and pore formation
N	Q04IN8	DE	Pneumolysin;
N	Q04IN8	DE	Thiol-activated cytolysin;
N	Q04IN8	DR	cholesterol binding
N	Q04IN8	DR	hemolysis in other organism
N	Q04M96	DE	UPF0398 protein SPD_0338;
N	Q04ZG7	DE	Threonine--tRNA ligase;
N	Q04ZG7	DE	Threonyl-tRNA synthetase;

N	Q04ZG7	DR	metal ion binding
N	Q04ZG7	DR	threonine-tRNA ligase activity
N	Q04ZG7	DR	threonyl-tRNA aminoacylation
N	Q04ZS1	CC	GTPase that plays an essential role in the late steps of ribosome biogenesis (By similarity).
N	Q04ZS1	DE	GTP-binding protein EngA;
N	Q04ZS1	DR	ribosome biogenesis
N	Q05059	CC	Envelope glycoprotein that binds to the potential host cell entry receptors TNFRSF14/HVEM, PVRL1 and 3-O-sulfated heparan sulfate
N	Q05059	CC	May trigger fusion with host membrane, by recruiting the fusion machinery composed of gB and gH/gL (By similarity).
N	Q05059	DE	Envelope glycoprotein D;
N	Q05059	DR	interspecies interaction between organisms
N	Q05059	DR	metal ion binding
N	Q05203	CC	Flagellin is the subunit protein which polymerizes to form the filaments of bacterial flagella.
N	Q05203	DR	ciliary or flagellar motility
N	Q05203	DR	structural molecule activity
N	Q05283	DE	Gene 69 protein;
N	Q05291	DE	Gene 76 protein;
N	Q05397	CC	Non-receptor protein-tyrosine kinase implicated in signaling pathways involved in cell motility, proliferation and apoptosis
N	Q05397	CC	Activated by tyrosine-phosphorylation in response to either integrin clustering induced by cell adhesion or antibody cross-linking, or via G-protein coupled receptor (GPCR) occupancy by ligands such as bombesin or lysophosphatidic acid, or via LDL receptor occupancy
N	Q05397	CC	Microtubule-induced dephosphorylation at Tyr- 397 is crucial for the induction of focal adhesion disassembly
N	Q05397	CC	Plays a potential role in oncogenic transformations resulting in increased kinase activity
N	Q05397	DE	Focal adhesion kinase 1;
N	Q05397	DE	Protein-tyrosine kinase 2;
N	Q05397	DR	SH2 domain binding
N	Q05397	DR	axon guidance
N	Q05397	DR	blood coagulation
N	Q05397	DR	cellular component disassembly involved in apoptosis
N	Q05397	DR	integrin-mediated signaling pathway
N	Q05397	DR	non-membrane spanning protein tyrosine kinase activity
N	Q05397	DR	protein phosphorylation
N	Q05397	DR	signal complex assembly
N	Q05397	DR	signal transducer activity
N	Q053M7	CC	Catalyzes the attachment of proline to tRNA(Pro) in a two-step reaction: proline is first activated by ATP to form Pro- AMP and then transferred to the acceptor end of tRNA(Pro)
N	Q053M7	CC	As ProRS can inadvertently accommodate and process non-cognate amino acids such as alanine and cysteine, to avoid such errors it has two additional distinct editing activities against alanine
N	Q053M7	CC	One activity is designated as 'pretransfer' editing and involves the tRNA(Pro)-independent hydrolysis of activated Ala-AMP
N	Q053M7	CC	The misacylated Cys- tRNA(Pro) is not edited by ProRS (By similarity).
N	Q053M7	CC	The other activity is designated 'posttransfer' editing and involves deacylation of mischarged Ala-tRNA(Pro)
N	Q053M7	DE	Proline--tRNA ligase;
N	Q053M7	DE	Prolyl-tRNA synthetase;
N	Q053M7	DR	proline-tRNA ligase activity
N	Q053M7	DR	prolyl-tRNA aminoacylation
N	Q057A7	CC	One of the primary rRNA binding proteins
N	Q057A7	CC	It has been suggested to have peptidyltransferase activity; this is somewhat controversial
N	Q057A7	CC	Makes several contacts with the 16S rRNA in the 70S ribosome (By similarity).
N	Q057A7	CC	Required for association of the 30S and 50S subunits to form the 70S ribosome, for tRNA binding and peptide bond formation
N	Q057A7	DE	50S ribosomal protein L2;
N	Q057A7	DR	structural constituent of ribosome
N	Q057A7	DR	transferase activity

N	Q05800	CC	RuBisCO catalyzes two reactions: the carboxylation of D- ribulose 1,5-bisphosphate, the primary event in carbon dioxide fixation, as well as the oxidative fragmentation of the pentose substrate in the photorespiration process
N	Q05800	CC	Both reactions occur simultaneously and in competition at the same active site (By similarity).
N	Q05800	DE	Ribulose bisphosphate carboxylase large chain;
N	Q05800	DE	RuBisCO large subunit;
N	Q05800	DR	magnesium ion binding
N	Q05800	DR	monooxygenase activity
N	Q05800	DR	oxidation-reduction process
N	Q05800	DR	photorespiration
N	Q05800	DR	reductive pentose-phosphate cycle
N	Q05800	DR	ribulose-bisphosphate carboxylase activity
N	Q05858	CC	Is important for morphogenesis of limb and kidney and may be involved in determining dorsoventral neural tube polarity and motor neuron induction
N	Q05858	CC	It may also have a function in differentiated cells or be involved in maintaining specific differentiated states.
N	Q05858	DE	Limb deformity protein;
N	Q05858	DR	actin binding
N	Q05858	DR	actin cytoskeleton organization
N	Q05979	CC	Catalyzes the cleavage of L-kynurenine (L-Kyn) and L-3- hydroxykynurenine (L-3OHKyn) into anthranilic acid (AA) and 3- hydroxyanthranilic acid (3-OHAA), respectively.
N	Q05979	DE	Biosynthesis of nicotinic acid protein 5;
N	Q05979	DE	Kynureninase;
N	Q05979	DE	L-kynurenine hydrolase;
N	Q05979	DR	de novo NAD biosynthetic process from tryptophan
N	Q05979	DR	kynureninase activity
N	Q05979	DR	protein binding
N	Q05979	DR	pyridoxal phosphate binding
N	Q05979	DR	tryptophan catabolic process
N	Q05985	CC	RuBisCO catalyzes two reactions: the carboxylation of D- ribulose 1,5-bisphosphate, the primary event in carbon dioxide fixation, as well as the oxidative fragmentation of the pentose substrate in the photorespiration process
N	Q05985	CC	Both reactions occur simultaneously and in competition at the same active site (By similarity).
N	Q05985	DE	Ribulose bisphosphate carboxylase large chain;
N	Q05985	DE	RuBisCO large subunit;
N	Q05985	DR	magnesium ion binding
N	Q05985	DR	monooxygenase activity
N	Q05985	DR	oxidation-reduction process
N	Q05985	DR	photorespiration
N	Q05985	DR	reductive pentose-phosphate cycle
N	Q05985	DR	ribulose-bisphosphate carboxylase activity
N	Q06177	DE	Translation machinery-associated protein 10;
N	Q06749	CC	Depolymerizes alginate by cleaving the beta-1,4 glycosidic bond
N	Q06749	CC	May enhance the production of alginate by controlling the length of the polymer chain during export.
N	Q06749	DE	Alginate lyase;
N	Q06749	DE	Poly(beta-D-mannuronate) lyase;
N	Q06749	DE	Poly(mana) alginate lyase;
N	Q06749	DR	alginic acid catabolic process
N	Q06749	DR	poly(beta-D-mannuronate) lyase activity
N	Q06GM7	DE	30S ribosomal protein S11, chloroplastic;
N	Q06GM7	DR	structural constituent of ribosome
N	Q06GR9	CC	DNA-dependent RNA polymerase catalyzes the transcription of DNA into RNA using the four ribonucleoside triphosphates as substrates.
N	Q06GR9	DE	DNA-directed RNA polymerase subunit beta';
N	Q06GR9	DE	Plastid-encoded RNA polymerase subunit beta';
N	Q06GR9	DE	RNA polymerase subunit beta';
N	Q06GR9	DR	DNA-directed RNA polymerase activity
N	Q06GT7	CC	One of the primary rRNA binding proteins, it binds directly to 16S rRNA where it nucleates assembly of the head domain of the 30S subunit (By similarity).
N	Q06GT7	DE	30S ribosomal protein S7, chloroplastic;
N	Q06GT7	DR	structural constituent of ribosome

N	Q06R94	DE	50S ribosomal protein L16, chloroplastic;
N	Q06R94	DR	structural constituent of ribosome
N	Q06RD2	CC	PsaA and psaB bind P700, the primary electron donor of photosystem I (PSI), as well as the electron acceptors A0, A1 and FX
N	Q06RD2	CC	Oxidized P700 is reduced on the lumenal side of the thylakoid membrane by plastocyanin
N	Q06RD2	CC	PSI is a plastocyanin-ferredoxin oxidoreductase, converting photonic excitation into a charge separation, which transfers an electron from the donor P700 chlorophyll pair to the spectroscopically characterized acceptors A0, A1, FX, FA and FB in turn
N	Q06RD2	DE	Photosystem I P700 chlorophyll a apoprotein A2;
N	Q06RD2	DR	4 iron, 4 sulfur cluster binding
N	Q06RD2	DR	chlorophyll binding
N	Q06RD2	DR	electron transport chain
N	Q06RD2	DR	metal ion binding
N	Q06RD2	DR	photosynthesis
N	Q06RD2	DR	protein-chromophore linkage
N	Q06RD6	CC	One of the two reaction center proteins of photosystem II (PSII), D2 is needed for assembly of a stable PSII complex (By similarity).
N	Q06RD6	DE	PSII D2 protein;
N	Q06RD6	DE	Photosystem II D2 protein;
N	Q06RD6	DE	Photosystem Q(A) protein;
N	Q06RD6	DR	electron transporter, transferring electrons within the cyclic electron transport pathway of photosynthesis activity
N	Q06RD6	DR	metal ion binding
N	Q06RD6	DR	photosynthetic electron transport in photosystem II
N	Q07251	DE	L-lactate dehydrogenase;
N	Q07251	DR	L-lactate dehydrogenase activity
N	Q07251	DR	oxidation-reduction process
N	Q07351	DE	Zinc finger protein STP4;
N	Q07351	DR	sequence-specific DNA binding
N	Q07351	DR	zinc ion binding
N	Q07409	CC	Contactins mediate cell surface interactions during nervous system development
N	Q07409	CC	Has some neurite outgrowth-promoting activity (By similarity).
N	Q07409	DE	Brain-derived immunoglobulin superfamily protein 1;
N	Q07409	DE	Contactin-3;
N	Q07409	DE	Plasmacytoma-associated neuronal glycoprotein;
N	Q07409	DR	cell adhesion
N	Q07590	CC	Not yet known, shows ATPase activity.
N	Q07590	DE	Protein SAV;
N	Q07590	DR	nucleoside-triphosphatase activity
N	Q07788	DE	Protein COS7;
N	Q07788	DR	protein binding
N	Q07788	DR	receptor activity
N	Q07DX6	CC	Plays a central role during spermatogenesis by repressing transposable elements and prevent their mobilization, which is essential for the germline integrity
N	Q07DX6	CC	Acts via the piRNA metabolic process, which mediates the repression of transposable elements during meiosis by forming complexes composed of piRNAs and Piwi proteins and govern the methylation and subsequent repression of transposons
N	Q07DX6	CC	Its association with pi-bodies suggests a participation in the primary piRNAs metabolic process
N	Q07DX6	CC	May act by mediating protein-protein interactions during germ cell maturation (By similarity).
N	Q07DX6	CC	Required prior to the pachytene stage to facilitate the production of multiple types of piRNAs, including those associated with repeats involved in regulation of retrotransposons
N	Q07DX6	DE	Ankyrin repeat, SAM and basic leucine zipper domain-containing protein 1;
N	Q07DX6	DE	Germ cell-specific ankyrin, SAM and basic leucine zipper domain-containing protein;
N	Q07DX6	DR	DNA methylation involved in gamete generation
N	Q07DX6	DR	cell differentiation
N	Q07DX6	DR	gene silencing by RNA
N	Q07DX6	DR	multicellular organismal development
N	Q07DX6	DR	piRNA metabolic process
N	Q07DX6	DR	spermatogenesis
N	Q07TB7	CC	Prevents misfolding and promotes the refolding and proper assembly of unfolded polypeptides generated under stress conditions (By similarity).
N	Q07TB7	DE	60 kDa chaperonin 1;

N	Q07TB7	DE	GroEL protein 1;
N	Q07TB7	DE	Protein Cpn60 1;
N	Q07TB7	DR	protein refolding
N	Q07Z00	CC	Transfers the 4'-phosphopantetheine moiety from coenzyme A to a Ser of acyl-carrier-protein (By similarity).
N	Q07Z00	DE	4'-phosphopantetheinyl transferase AcpS;
N	Q07Z00	DE	Holo-ACP synthase;
N	Q07Z00	DE	Holo-[acyl-carrier-protein] synthase;
N	Q07Z00	DR	fatty acid biosynthetic process
N	Q07Z00	DR	holo-[acyl-carrier-protein] synthase activity
N	Q07Z00	DR	macromolecule biosynthetic process
N	Q07Z00	DR	magnesium ion binding
N	Q08094	CC	Thin filament-associated protein that is implicated in the regulation and modulation of smooth muscle contraction
N	Q08094	CC	It is capable of binding to actin, calmodulin, troponin C and tropomyosin
N	Q08094	CC	The interaction of calponin with actin inhibits the actomyosin Mg-ATPase activity
N	Q08094	DE	Calponin H2, smooth muscle;
N	Q08094	DE	Neutral calponin;
N	Q08094	DR	actin binding
N	Q08094	DR	actomyosin structure organization
N	Q08094	DR	calmodulin binding
N	Q08143	CC	Transcription factor that is required for post- fertilization events
N	Q08143	CC	It is required for the developmental events that occur in the female organ after fertilization.
N	Q08143	DE	Sporulation minus regulator 2;
N	Q08143	DR	single fertilization
N	Q08455	CC	Plays an essential role for the viral pathogenicity.
N	Q08455	DE	Non-virion protein;
N	Q08477	CC	Cytochromes P450 are a group of heme-thiolate monooxygenases
N	Q08477	CC	This enzyme requires molecular oxygen and NADPH for the omega-hydroxylation of LTB4, a potent chemoattractant for polymorphonuclear leukocytes
N	Q08477	DE	Cytochrome P450 4F3;
N	Q08477	DE	Cytochrome P450-LTB-omega;
N	Q08477	DE	Leukotriene-B(4) 20-monooxygenase 2;
N	Q08477	DE	Leukotriene-B(4) omega-hydroxylase 2;
N	Q08477	DR	electron carrier activity
N	Q08477	DR	leukotriene metabolic process
N	Q08477	DR	leukotriene-B4 20-monooxygenase activity
N	Q08477	DR	oxidation-reduction process
N	Q08477	DR	oxygen binding
N	Q08477	DR	xenobiotic metabolic process
N	Q08585	CC	The light-harvesting complex (LHC) functions as a light receptor, it captures and delivers excitation energy to photosystems with which it is closely associated
N	Q08585	CC	Energy is transferred from the carotenoid and chlorophyll C (or B) to chlorophyll A and the photosynthetic reaction centers where it is used to synthesize ATP and reducing power.
N	Q08585	DE	Fucoxanthin-chlorophyll a-c binding protein B, chloroplastic;
N	Q08585	DR	chlorophyll binding
N	Q08585	DR	photosynthesis
N	Q08585	DR	protein-chromophore linkage
N	Q086G9	CC	One of the primary rRNA binding proteins, it binds directly to 16S rRNA where it helps nucleate assembly of the platform of the 30S subunit by binding and bridging several RNA helices of the 16S rRNA (By similarity).
N	Q086G9	DE	30S ribosomal protein S15;
N	Q086G9	DR	structural constituent of ribosome
N	Q089N8	CC	This is one of the proteins that binds and probably mediates the attachment of the 5S RNA into the large ribosomal subunit, where it forms part of the central protuberance (By similarity).
N	Q089N8	DE	50S ribosomal protein L18;
N	Q089N8	DR	structural constituent of ribosome
N	Q08CA1	DE	UPF0580 protein C15orf58 homolog;
N	Q08DM1	CC	Actin-bundling protein
N	Q08DM1	CC	May function in mitogen- activated protein kinase pathway (By similarity).
N	Q08DM1	DE	Erythrocyte membrane protein band 4.9;
N	Q08DM1	DR	actin binding

N	Q08DM1	DR	actin filament capping
N	Q08DM1	DR	cytoskeleton organization
N	Q08DT6	DE	39S ribosomal protein L47, mitochondrial;
N	Q08DT6	DR	structural constituent of ribosome
N	Q08HI0	CC	Core subunit of the mitochondrial membrane respiratory chain NADH dehydrogenase (Complex I) that is believed to belong to the minimal assembly required for catalysis
N	Q08HI0	CC	Complex I functions in the transfer of electrons from NADH to the respiratory chain
N	Q08HI0	CC	The immediate electron acceptor for the enzyme is believed to be ubiquinone (By similarity).
N	Q08HI0	DE	NADH dehydrogenase subunit 4L;
N	Q08HI0	DE	NADH-ubiquinone oxidoreductase chain 4L;
N	Q08HI0	DR	ATP synthesis coupled electron transport
N	Q08HI0	DR	NADH dehydrogenase (ubiquinone) activity
N	Q09113	CC	Spike-forming protein that mediates virion attachment to the host epithelial cell receptors and plays a major role in cell penetration, determination of host range restriction and virulence
N	Q09113	CC	VP8* forms the head of the spikes
N	Q09113	CC	According to the considered strain, VP4 seems to essentially target sialic acid and/or the integrin heterodimer ITGA2/ITGB1 (By similarity).
N	Q09113	CC	In sialic acid-dependent strains, VP8* binds to host cell sialic acid, most probably a ganglioside, providing the initial contact (By similarity).
N	Q09113	CC	It is the viral hemagglutinin and an important target of neutralizing antibodies
N	Q09113	CC	Rotavirus entry into the host cell probably involves multiple sequential contacts between the outer capsid proteins VP4 and VP7, and the cell receptors
N	Q09113	DE	Hemagglutinin;
N	Q09113	DE	Outer capsid protein VP4;
N	Q09113	DE	Outer capsid protein VP5*;
N	Q09113	DE	Outer capsid protein VP8*;
N	Q09113	DR	interspecies interaction between organisms
N	Q09113	DR	viral infectious cycle
N	Q09123	DE	Fructokinase;
N	Q09123	DR	cellular carbohydrate catabolic process
N	Q09123	DR	fructokinase activity
N	Q09123	DR	metal ion binding
N	Q09125	CC	RuBisCO catalyzes two reactions: the carboxylation of D- ribulose 1,5-bisphosphate, the primary event in carbon dioxide fixation, as well as the oxidative fragmentation of the pentose substrate
N	Q09125	CC	Both reactions occur simultaneously and in competition at the same active site (By similarity).
N	Q09125	DE	Ribulose bisphosphate carboxylase small chain;
N	Q09125	DE	RuBisCO small subunit;
N	Q09125	DR	monooxygenase activity
N	Q09125	DR	oxidation-reduction process
N	Q09125	DR	photorespiration
N	Q09125	DR	reductive pentose-phosphate cycle
N	Q09125	DR	ribulose-bisphosphate carboxylase activity
N	Q09248	CC	Modulates cytoplasmic dynein binding to an organelle, and plays a role in prometaphase chromosome alignment and spindle organization during mitosis
N	Q09248	CC	May play a role in synapse formation during brain development (By similarity).
N	Q09248	DE	Probable dynactin subunit 2;
N	Q09248	DR	centrosomal and pronuclear rotation
N	Q09248	DR	chromosome segregation
N	Q09248	DR	embryo development ending in birth or egg hatching
N	Q09248	DR	establishment of mitotic spindle orientation
N	Q09248	DR	identical protein binding
N	Q09248	DR	motor activity
N	Q09248	DR	pronuclear migration
N	Q09343	DE	Uncharacterized protein W02B3.7;
N	Q09354	DE	Uncharacterized protein T15H9.4;
N	Q09508	CC	Flavoprotein (FP) subunit of succinate dehydrogenase (SDH) that is involved in complex II of the mitochondrial electron transport chain and is responsible for transferring electrons from succinate to ubiquinone (coenzyme Q) (By similarity).
N	Q09508	DE	Flavoprotein subunit of complex II;
N	Q09508	DE	Succinate dehydrogenase [ubiquinone] flavoprotein subunit, mitochondrial;
N	Q09508	DR	body morphogenesis

N	Q09508	DR	electron carrier activity
N	Q09508	DR	electron transport chain
N	Q09508	DR	embryo development ending in birth or egg hatching
N	Q09508	DR	flavin adenine dinucleotide binding
N	Q09508	DR	positive regulation of growth rate
N	Q09508	DR	succinate dehydrogenase (ubiquinone) activity
N	Q09508	DR	tricarboxylic acid cycle
N	Q09531	CC	GTPase-activating protein for the ADP ribosylation factor family (Potential).
N	Q09531	DE	Uncharacterized protein F07F6.4;
N	Q09531	DR	ARF GTPase activator activity
N	Q09531	DR	regulation of ARF GTPase activity
N	Q09531	DR	zinc ion binding
N	Q09651	DE	Coiled-coil domain-containing protein 130 homolog;
N	Q09651	DR	embryo development ending in birth or egg hatching
N	Q09651	DR	protein binding
N	Q09675	DE	Uncharacterized protein C5H10.02c;
N	Q09675	DR	cellular response to oxidative stress
N	Q09675	DR	peptidase activity
N	Q09898	CC	Part of a signaling pathway
N	Q09898	CC	Required for initiation of medial ring constriction and septation.
N	Q09898	DE	Serine/threonine-protein kinase sid2;
N	Q09898	DR	maintenance of endoplasmic reticulum location involved in endoplasmic reticulum polarization at cell division site
N	Q09898	DR	protein binding
N	Q09898	DR	protein phosphorylation
N	Q09898	DR	protein serine/threonine kinase activity
N	Q09898	DR	septation initiation signaling cascade
N	Q09G39	CC	Produces ATP from ADP in the presence of a proton gradient across the membrane
N	Q09G39	CC	The catalytic sites are hosted primarily by the beta subunits (By similarity).
N	Q09G39	DE	ATP synthase F1 sector subunit beta;
N	Q09G39	DE	ATP synthase subunit beta, chloroplastic;
N	Q09G39	DE	F-ATPase subunit beta;
N	Q09G39	DR	ATP hydrolysis coupled proton transport
N	Q09G39	DR	ATP synthesis coupled proton transport
N	Q09G39	DR	hydrogen ion transporting ATP synthase activity, rotational mechanism
N	Q09G39	DR	hydrogen-exporting ATPase activity, phosphorylative mechanism
N	Q09G39	DR	proton-transporting ATPase activity, rotational mechanism
N	Q09MC9	CC	NDH shuttles electrons from NAD(P)H:plastoquinone, via FMN and iron-sulfur (Fe-S) centers, to quinones in the photosynthetic chain and possibly in a chloroplast respiratory chain
N	Q09MC9	CC	Couples the redox reaction to proton translocation, and thus conserves the redox energy in a proton gradient (By similarity).
N	Q09MC9	CC	The immediate electron acceptor for the enzyme in this species is believed to be plastoquinone
N	Q09MC9	DE	NAD(P)H dehydrogenase subunit 5;
N	Q09MC9	DE	NAD(P)H-quinone oxidoreductase subunit 5, chloroplastic;
N	Q09MC9	DE	NADH-plastoquinone oxidoreductase subunit 5;
N	Q09MC9	DR	ATP synthesis coupled electron transport
N	Q09MC9	DR	NADH dehydrogenase (ubiquinone) activity
N	Q09MC9	DR	quinone binding
N	Q0A5S1	DE	Dihydrodipicolinate synthase;
N	Q0A5S1	DR	diaminopimelate biosynthetic process
N	Q0A5S1	DR	dihydrodipicolinate synthase activity
N	Q0ABG4	CC	One of two assembly initiator proteins, it binds directly to the 5'-end of the 23S rRNA, where it nucleates assembly of the 50S subunit (By similarity).
N	Q0ABG4	DE	50S ribosomal protein L24;
N	Q0ABG4	DR	structural constituent of ribosome
N	Q0ADD7	CC	Specifically methylates the N7 position of guanosine in position 527 of 16S rRNA (By similarity).
N	Q0ADD7	DE	16S rRNA 7-methylguanosine methyltransferase;
N	Q0ADD7	DE	16S rRNA m7G methyltransferase;
N	Q0ADD7	DE	Ribosomal RNA small subunit methyltransferase G;
N	Q0ADD7	DR	rRNA methyltransferase activity
N	Q0AGQ8	CC	Specifically dimethylates two adjacent adenosines (A1518 and A1519) in the loop of a conserved hairpin near the 3'-end of 16S rRNA in the 30S particle

N	Q0AGQ8	CC	May play a critical role in biogenesis of 30S subunits (By similarity).
N	Q0AGQ8	DE	16S rRNA (adenine(1518)-N(6)/adenine(1519)-N(6))-dimethyltransferase;
N	Q0AGQ8	DE	16S rRNA dimethyladenosine transferase;
N	Q0AGQ8	DE	16S rRNA dimethylase;
N	Q0AGQ8	DE	Ribosomal RNA small subunit methyltransferase A;
N	Q0AGQ8	DE	S-adenosylmethionine-6-N', N'-adenosyl(rRNA) dimethyltransferase;
N	Q0AGQ8	DR	rRNA (adenine-N6,N6-)-dimethyltransferase activity
N	Q0AHX8	CC	This enzyme is involved in nucleotide metabolism: it produces dUMP, the immediate precursor of thymidine nucleotides and it decreases the intracellular concentration of dUTP so that uracil cannot be incorporated into DNA (By similarity).
N	Q0AHX8	DE	Deoxyuridine 5'-triphosphate nucleotidohydrolase;
N	Q0AHX8	DE	dUTP pyrophosphatase;
N	Q0AHX8	DR	dUTP diphosphatase activity
N	Q0AHX8	DR	dUTP metabolic process
N	Q0AHX8	DR	metal ion binding
N	Q0AIA2	CC	One of the proteins required for the normal export of preproteins out of the cell cytoplasm
N	Q0AIA2	CC	It also specifically binds to its receptor secA (By similarity).
N	Q0AIA2	CC	It is a molecular chaperone that binds to a subset of precursor proteins, maintaining them in a translocation-competent state
N	Q0AIA2	DE	Protein-export protein secB;
N	Q0AIA2	DR	protein tetramerization
N	Q0AIA2	DR	protein transport
N	Q0AIA2	DR	transmembrane transport
N	Q0AIA2	DR	unfolded protein binding
N	Q0AJ91	CC	Required for the formation of a threonylcarbamoyl group on adenosine at position 37 (t(6)A37) in tRNAs that read codons beginning with adenine (By similarity).
N	Q0AJ91	DE	Probable tRNA threonylcarbamoyladenosine biosynthesis protein Gcp;
N	Q0AJ91	DE	t(6)A37 threonylcarbamoyladenosine biosynthesis protein;
N	Q0AJ91	DR	metal ion binding
N	Q0AJ91	DR	metalloendopeptidase activity
N	Q0AWZ8	CC	Involved in the modulation of the chemotaxis system; catalyzes the demethylation of specific methylglutamate residues introduced into the chemoreceptors (methyl-accepting chemotaxis proteins) by CheR (By similarity).
N	Q0AWZ8	DE	Chemotaxis response regulator protein-glutamate methylesterase 3;
N	Q0AWZ8	DR	protein-glutamate methylesterase activity
N	Q0AWZ8	DR	regulation of transcription, DNA-dependent
N	Q0AWZ8	DR	two-component response regulator activity
N	Q0AZ84	CC	Catalyzes the synthesis of the hydroxymethylpyrimidine phosphate (HMP-P) moiety of thiamine from aminoimidazole ribotide (AIR) in a radical S-adenosyl-L-methionine (SAM)-dependent reaction (By similarity).
N	Q0AZ84	DE	HMP-P synthase;
N	Q0AZ84	DE	HMP-phosphate synthase;
N	Q0AZ84	DE	HMPP synthase;
N	Q0AZ84	DE	Hydroxymethylpyrimidine phosphate synthase;
N	Q0AZ84	DE	Phosphomethylpyrimidine synthase;
N	Q0AZ84	DE	Thiamine biosynthesis protein thiC;
N	Q0AZ84	DR	4 iron, 4 sulfur cluster binding
N	Q0AZ84	DR	lyase activity
N	Q0AZ84	DR	metal ion binding
N	Q0AZ84	DR	thiamine biosynthetic process
N	Q0BC36	CC	Key enzyme in the regulation of glycerol uptake and metabolism.
N	Q0BC36	DE	ATP:glycerol 3-phosphotransferase;
N	Q0BC36	DE	Glycerokinase;
N	Q0BC36	DE	Glycerol kinase;
N	Q0BC36	DR	glycerol kinase activity
N	Q0BC36	DR	glycerol-3-phosphate metabolic process
N	Q0BED6	CC	Catalyzes the formation of 4-diphosphocytidyl-2-C- methyl-D-erythritol from CTP and 2-C-methyl-D-erythritol 4- phosphate (MEP) (By similarity).
N	Q0BED6	DE	2-C-methyl-D-erythritol 4-phosphate cytidylyltransferase;
N	Q0BED6	DE	4-diphosphocytidyl-2C-methyl-D-erythritol synthase;
N	Q0BED6	DE	MEP cytidylyltransferase;
N	Q0BED6	DR	2-C-methyl-D-erythritol 4-phosphate cytidylyltransferase activity
N	Q0BED6	DR	isoprenoid biosynthetic process
N	Q0BEG9	CC	Involved in cell division; probably involved in intracellular septation (By similarity).

N	Q0BEG9	DE	Probable intracellular septation protein;
N	Q0BEG9	DR	barrier septum formation
N	Q0BH18	CC	Catalyzes the reversible formation of acyl-phosphate (acyl-PO(4)) from acyl-[acyl-carrier-protein] (acyl-ACP)
N	Q0BH18	CC	This enzyme utilizes acyl-ACP as fatty acyl donor, but not acyl-CoA (By similarity)
N	Q0BH18	DE	Acyl-ACP phosphotransacylase;
N	Q0BH18	DE	Acyl-[acyl-carrier-protein]--phosphate acyltransferase;
N	Q0BH18	DE	Phosphate acyltransferase;
N	Q0BH18	DE	Phosphate-acyl-ACP acyltransferase;
N	Q0BH18	DR	fatty acid biosynthetic process
N	Q0BH18	DR	phospholipid biosynthetic process
N	Q0BH18	DR	transferase activity, transferring acyl groups other than amino-acyl groups
N	Q0BLS0	DE	UPF0176 protein FTH_1091;
N	Q0BMM0	CC	Provides the sole de novo source of dTMP for DNA biosynthesis (By similarity).
N	Q0BMM0	DE	Thymidylate synthase;
N	Q0BMM0	DR	dTMP biosynthetic process
N	Q0BMM0	DR	thymidylate synthase activity
N	Q0BNQ2	DE	50S ribosomal protein L17;
N	Q0BNQ2	DR	structural constituent of ribosome
N	Q0BNT0	CC	One of the primary rRNA binding proteins, it binds directly to 16S rRNA where it nucleates assembly of the head domain of the 30S subunit
N	Q0BNT0	CC	Is located at the subunit interface close to the decoding center, probably blocks exit of the E-site tRNA (By similarity).
N	Q0BNT0	DE	30S ribosomal protein S7;
N	Q0BNT0	DR	structural constituent of ribosome
N	Q0BRE9	CC	An essential GTPase which binds GTP, GDP and possibly (p)ppGpp with moderate affinity, with high nucleotide exchange rates and a fairly low GTP hydrolysis rate (By similarity)
N	Q0BRE9	CC	It may play a role in control of the cell cycle, stress response, ribosome biogenesis and in those bacteria that undergo differentiation, in morphogenesis control (Potential)
N	Q0BRE9	DE	GTP-binding protein obg;
N	Q0BRE9	DR	GTPase activity
N	Q0BRE9	DR	magnesium ion binding
N	Q0BTF8	CC	May conjugate Arg from its aminoacyl-tRNA to the N- termini of proteins containing an N-terminal aspartate or glutamate (Potential).
N	Q0BTF8	DE	Arginyltransferase;
N	Q0BTF8	DE	Putative arginyl-tRNA--protein transferase;
N	Q0BTF8	DE	R-transferase;
N	Q0BTF8	DR	acyltransferase activity
N	Q0BTF8	DR	arginyltransferase activity
N	Q0BTF8	DR	protein arginylation
N	Q0C9R3	CC	Component of the EKC/KEOPS complex which promotes both telomere uncapping and telomere elongation (By similarity)
N	Q0C9R3	CC	The complex is required for efficient recruitment of transcriptional coactivators (By similarity).
N	Q0C9R3	DE	Protein cgi121;
N	Q0CVW0	CC	Modulates the mitochondrial matrix zinc pool (By similarity).
N	Q0CVW0	DE	Mitochondrial zinc maintenance protein 1, mitochondrial;
N	Q0CY48	CC	ATP-binding RNA helicase involved in the biogenesis of 60S ribosomal subunits and is required for the normal formation of 25S and 5.8S rRNAs (By similarity)
N	Q0CY48	DE	ATP-dependent RNA helicase dbp9;
N	Q0CY48	DR	ATP-dependent helicase activity
N	Q0CY48	DR	rRNA processing
N	Q0G9X7	CC	Produces ATP from ADP in the presence of a proton gradient across the membrane
N	Q0G9X7	CC	The alpha chain is a regulatory subunit (By similarity).
N	Q0G9X7	DE	ATP synthase F1 sector subunit alpha;
N	Q0G9X7	DE	ATP synthase subunit alpha, chloroplastic;
N	Q0G9X7	DE	F-ATPase subunit alpha;
N	Q0G9X7	DR	ATP hydrolysis coupled proton transport
N	Q0G9X7	DR	ATP synthesis coupled proton transport
N	Q0G9X7	DR	hydrogen ion transporting ATP synthase activity, rotational mechanism
N	Q0G9X7	DR	proton-transporting ATPase activity, rotational mechanism
N	Q0G9X9	CC	This protein is a component of the reaction center of photosystem II.
N	Q0G9X9	DE	Photosystem II reaction center protein K;
N	Q0G9X9	DR	photosynthesis

N	Q0H8Z0	DE	Ribosomal protein S3, mitochondrial;
N	Q0H8Z0	DR	structural constituent of ribosome
N	Q0HG53	DE	Glutamate-1-semialdehyde 2,1-aminomutase;
N	Q0HG53	DE	Glutamate-1-semialdehyde aminotransferase;
N	Q0HG53	DR	glutamate-1-semialdehyde 2,1-aminomutase activity
N	Q0HG53	DR	porphyrin biosynthetic process
N	Q0HG53	DR	pyridoxal phosphate binding
N	Q0HG53	DR	transaminase activity
N	Q0HJ42	CC	Activates KDO (a required 8-carbon sugar) for incorporation into bacterial lipopolysaccharide in Gram-negative bacteria (By similarity).
N	Q0HJ42	DE	3-deoxy-manno-octulosonate cytidylyltransferase;
N	Q0HJ42	DE	CMP-2-keto-3-deoxyoctulosonic acid synthase;
N	Q0HJ42	DE	CMP-KDO synthase;
N	Q0HJ42	DR	3-deoxy-manno-octulosonate cytidylyltransferase activity
N	Q0HJ42	DR	lipopolysaccharide biosynthetic process
N	Q0HPG0	CC	Produces ATP from ADP in the presence of a proton gradient across the membrane
N	Q0HPG0	CC	The gamma chain is believed to be important in regulating ATPase activity and the flow of protons through the CF(0) complex (By similarity).
N	Q0HPG0	DE	ATP synthase F1 sector gamma subunit;
N	Q0HPG0	DE	ATP synthase gamma chain;
N	Q0HPG0	DE	F-ATPase gamma subunit;
N	Q0HPG0	DR	ATP synthesis coupled proton transport
N	Q0HPG0	DR	hydrogen ion transporting ATP synthase activity, rotational mechanism
N	Q0HPG0	DR	proton-transporting ATPase activity, rotational mechanism
N	Q0HQI7	CC	Tetrapolymerization of the monopyrrole PBG into the hydroxymethylbilane pre-uroporphyrinogen in several discrete steps.
N	Q0HQI7	DE	Hydroxymethylbilane synthase;
N	Q0HQI7	DE	Porphobilinogen deaminase;
N	Q0HQI7	DE	Pre-uroporphyrinogen synthase;
N	Q0HQI7	DR	hydroxymethylbilane synthase activity
N	Q0HQI7	DR	peptidyl-pyrromethane cofactor linkage
N	Q0HQI7	DR	porphyrin biosynthetic process
N	Q0HS05	DE	Protein ApaG;
N	Q0HVA7	CC	Required for the thiolation of cytidine in position 32 of tRNA, to form 2-thiocytidine (s(2)C32) (By similarity).
N	Q0HVA7	DE	tRNA 2-thiocytidine biosynthesis protein TtcA;
N	Q0HVA7	DR	tRNA processing
N	Q0HXU4	DE	Leucine--tRNA ligase;
N	Q0HXU4	DE	Leucyl-tRNA synthetase;
N	Q0HXU4	DR	leucine-tRNA ligase activity
N	Q0HXU4	DR	leucyl-tRNA aminoacylation
N	Q0HYV7	DE	PKHD-type hydroxylase Shewmr7_0698;
N	Q0HYV7	DR	L-ascorbic acid binding
N	Q0HYV7	DR	iron ion binding
N	Q0HYV7	DR	oxidation-reduction process
N	Q0HYV7	DR	oxidoreductase activity, acting on paired donors, with incorporation or reduction of molecular oxygen
N	Q0HYV7	DR	oxidoreductase activity, acting on single donors with incorporation of molecular oxygen, incorporation of two atoms of oxygen
N	Q0I1Z1	CC	Catalyzes the reversible conversion of 2- phosphoglycerate into phosphoenolpyruvate
N	Q0I1Z1	CC	It is essential for the degradation of carbohydrates via glycolysis (By similarity).
N	Q0I1Z1	DE	2-phospho-D-glycerate hydro-lyase;
N	Q0I1Z1	DE	2-phosphoglycerate dehydratase;
N	Q0I1Z1	DR	magnesium ion binding
N	Q0I1Z1	DR	phosphopyruvate hydratase activity
N	Q0I5V1	DE	D-serine deaminase;
N	Q0I5V1	DE	Probable D-serine dehydratase;
N	Q0I5V1	DR	D-amino acid metabolic process
N	Q0I5V1	DR	D-serine ammonia-lyase activity
N	Q0I5V1	DR	pyridoxal phosphate binding
N	Q0I891	CC	Converts heme B (protoheme IX) to heme O by substitution of the vinyl group on carbon 2 of heme B porphyrin ring with a hydroxyethyl farnesyl side group (By similarity).
N	Q0I891	DE	Heme B farnesyltransferase;
N	Q0I891	DE	Heme O synthase;
N	Q0I891	DE	Protoheme IX farnesyltransferase;

N	Q0I891	DR	heme O biosynthetic process
N	Q0I891	DR	protoheme IX farnesyltransferase activity
N	Q0ICR3	CC	Seems to play a role in the dimerization of PSII (By similarity).
N	Q0ICR3	DE	Photosystem II reaction center protein T;
N	Q0ICR3	DR	photosynthesis
N	Q0ID56	CC	With S4 and S5 plays an important role in translational accuracy (By similarity).
N	Q0ID56	DE	30S ribosomal protein S12;
N	Q0ID56	DR	structural constituent of ribosome
N	Q0IIH8	CC	May have pro-apoptotic function (By similarity).
N	Q0IIH8	DE	Protein CARP;
N	Q0IIH8	DE	Protein FAM188A;
N	Q0IIH8	DR	calcium ion binding
N	Q0INY7	CC	RuBisCO catalyzes two reactions: the carboxylation of D- ribulose 1,5-bisphosphate, the primary event in carbon dioxide fixation, as well as the oxidative fragmentation of the pentose substrate
N	Q0INY7	CC	Both reactions occur simultaneously and in competition at the same active site (By similarity).
N	Q0INY7	DE	Ribulose bisphosphate carboxylase small chain C;
N	Q0INY7	DE	Ribulose bisphosphate carboxylase small chain, chloroplastic;
N	Q0INY7	DE	RuBisCO small subunit C;
N	Q0INY7	DE	RuBisCO small subunit;
N	Q0INY7	DR	monooxygenase activity
N	Q0INY7	DR	oxidation-reduction process
N	Q0INY7	DR	photorespiration
N	Q0INY7	DR	reductive pentose-phosphate cycle
N	Q0INY7	DR	ribulose-bisphosphate carboxylase activity
N	Q0ITW7	DE	Alcohol dehydrogenase 2;
N	Q0ITW7	DR	alcohol dehydrogenase (NAD) activity
N	Q0ITW7	DR	oxidation-reduction process
N	Q0ITW7	DR	zinc ion binding
N	Q0J0N4	DE	Beta-glucosidase 30;
N	Q0J0N4	DR	beta-glucosidase activity
N	Q0J0N4	DR	carbohydrate metabolic process
N	Q0J0N4	DR	cation binding
N	Q0J361	CC	Mediates both low-affinity uptake and efflux of sugar across the plasma membrane (By similarity).
N	Q0J361	DE	Bidirectional sugar transporter SWEET7a;
N	Q0J361	DR	sugar transmembrane transporter activity
N	Q0KA17	CC	Associates with the EF-Tu.GDP complex and induces the exchange of GDP to GTP
N	Q0KA17	CC	It remains bound to the aminoacyl-tRNA.EF- Tu.GTP complex up to the GTP hydrolysis stage on the ribosome.
N	Q0KA17	DE	Elongation factor Ts;
N	Q0KA17	DR	translation elongation factor activity
N	Q0MQH3	CC	Accessory subunit of the mitochondrial membrane respiratory chain NADH dehydrogenase (Complex I), that is believed not to be involved in catalysis
N	Q0MQH3	CC	Complex I functions in the transfer of electrons from NADH to the respiratory chain
N	Q0MQH3	CC	The immediate electron acceptor for the enzyme is believed to be ubiquinone (By similarity).
N	Q0MQH3	DE	Complex I-15 kDa;
N	Q0MQH3	DE	NADH dehydrogenase [ubiquinone] iron-sulfur protein 5;
N	Q0MQH3	DE	NADH-ubiquinone oxidoreductase 15 kDa subunit;
N	Q0MQH3	DR	electron transport chain
N	Q0MQH3	DR	mitochondrial respiratory chain complex I assembly
N	Q0Q472	CC	Component of the viral envelope that plays a central role in virus morphogenesis and assembly via its interactions with other viral proteins (By similarity).
N	Q0Q472	DE	E1 glycoprotein;
N	Q0Q472	DE	Matrix glycoprotein;
N	Q0Q472	DE	Membrane glycoprotein;
N	Q0Q472	DE	Membrane protein;
N	Q0Q472	DR	evasion by virus of host immune response
N	Q0Q472	DR	viral infectious cycle
N	Q0RBC4	CC	Acts as a chaperone (By similarity).
N	Q0RBC4	DE	Chaperone protein DnaK;
N	Q0RBC4	DE	Heat shock 70 kDa protein;
N	Q0RBC4	DE	Heat shock protein 70;

N	Q0RBC4	DR	protein folding
N	Q0RBC4	DR	response to stress
N	Q0RBC4	DR	unfolded protein binding
N	Q0RF89	CC	Modifies the free amino group of the aminoacyl moiety of methionyl-tRNA(fMet)
N	Q0RF89	CC	The formyl group appears to play a dual role in the initiator identity of N-formylmethionyl-tRNA by: (I) promoting its recognition by IF2 and (II) impairing its binding to EFTu-GTP (By similarity).
N	Q0RF89	DE	Methionyl-tRNA formyltransferase;
N	Q0RF89	DR	methionyl-tRNA formyltransferase activity
N	Q0RF89	DR	methyltransferase activity
N	Q0RJJ1	CC	Catalyzes the dephosphorylation of undecaprenyl diphosphate (UPP)
N	Q0RJJ1	CC	Confers resistance to bacitracin (By similarity).
N	Q0RJJ1	DE	Bacitracin resistance protein 2;
N	Q0RJJ1	DE	Undecaprenyl pyrophosphate phosphatase 2;
N	Q0RJJ1	DE	Undecaprenyl-diphosphatase 2;
N	Q0RJJ1	DR	cellular cell wall organization
N	Q0RJJ1	DR	dephosphorylation
N	Q0RJJ1	DR	peptidoglycan biosynthetic process
N	Q0RJJ1	DR	regulation of cell shape
N	Q0RJJ1	DR	response to antibiotic
N	Q0RJJ1	DR	undecaprenyl-diphosphatase activity
N	Q0S275	CC	Catalyzes the NADP-dependent rearrangement and reduction of 1-deoxy-D-xylulose-5-phosphate (DXP) to 2-C-methyl-D-erythritol 4-phosphate (MEP) (By similarity)
N	Q0S275	DE	1-deoxy-D-xylulose 5-phosphate reductoisomerase;
N	Q0S275	DE	1-deoxyxylulose-5-phosphate reductoisomerase;
N	Q0S275	DE	2-C-methyl-D-erythritol 4-phosphate synthase;
N	Q0S275	DE	DXP reductoisomerase;
N	Q0S275	DR	1-deoxy-D-xylulose-5-phosphate reductoisomerase activity
N	Q0S275	DR	isoprenoid biosynthetic process
N	Q0S275	DR	metal ion binding
N	Q0S275	DR	oxidation-reduction process
N	Q0S2D6	DE	Acylphosphatase;
N	Q0S2D6	DE	Acylphosphate phosphohydrolase;
N	Q0S2D6	DR	acylphosphatase activity
N	Q0S3E6	DE	50S ribosomal protein L17;
N	Q0S3E6	DR	structural constituent of ribosome
N	Q0SI81	DE	CMP kinase;
N	Q0SI81	DE	Cytidine monophosphate kinase;
N	Q0SI81	DE	Cytidylate kinase;
N	Q0SI81	DR	cytidylate kinase activity
N	Q0SQE8	CC	Protein S19 forms a complex with S13 that binds strongly to the 16S ribosomal RNA (By similarity).
N	Q0SQE8	DE	30S ribosomal protein S19;
N	Q0SQE8	DR	structural constituent of ribosome
N	Q0SRD5	CC	Initiates the rapid degradation of small, acid-soluble proteins during spore germination (By similarity).
N	Q0SRD5	DE	GPR endopeptidase;
N	Q0SRD5	DE	Germination protease;
N	Q0SRD5	DE	Germination proteinase;
N	Q0SRD5	DE	Spore protease;
N	Q0SRD5	DR	peptidase activity
N	Q0SRD5	DR	spore germination
N	Q0SS42	CC	Catalyzes the attachment of alanine to tRNA(Ala) in a two-step reaction: alanine is first activated by ATP to form Ala- AMP and then transferred to the acceptor end of tRNA(Ala)
N	Q0SS42	CC	Also edits incorrectly charged Ser-tRNA(Ala) and Gly-tRNA(Ala) via its editing domain (By similarity).
N	Q0SS42	DE	Alanine--tRNA ligase;
N	Q0SS42	DE	Alanyl-tRNA synthetase;
N	Q0SS42	DR	alanine-tRNA ligase activity
N	Q0SS42	DR	alanyl-tRNA aminoacylation
N	Q0SS42	DR	metal ion binding
N	Q0SXB9	DE	Phosphatidylserine decarboxylase alpha chain;
N	Q0SXB9	DE	Phosphatidylserine decarboxylase beta chain;
N	Q0SXB9	DE	Phosphatidylserine decarboxylase proenzyme;

N	Q0SXB9	DR	phosphatidylserine decarboxylase activity
N	Q0SXB9	DR	phospholipid biosynthetic process
N	Q0SXC1	DE	Uncharacterized protein YjeA;
N	Q0SXC1	DR	lysine-tRNA ligase activity
N	Q0SXC1	DR	lysyl-tRNA aminoacylation
N	Q0T0S8	CC	Catalyzes the formation of N(7)-methylguanine at position 46 (m7G46) in tRNA (By similarity).
N	Q0T0S8	DE	tRNA (guanine-N(7)-)-methyltransferase;
N	Q0T0S8	DE	tRNA(m7G46)-methyltransferase;
N	Q0T0S8	DR	tRNA (guanine-N7-)-methyltransferase activity
N	Q0T0U3	CC	Could be a nuclease that resolves Holliday junction intermediates in genetic recombination.
N	Q0T0U3	DE	Putative Holliday junction resolvase;
N	Q0T0U3	DR	DNA recombination
N	Q0T0U3	DR	nuclease activity
N	Q0T0U3	DR	nucleic acid binding
N	Q0T3U0	DE	Aspartate--tRNA ligase;
N	Q0T3U0	DE	Aspartyl-tRNA synthetase;
N	Q0T3U0	DR	aspartate-tRNA ligase activity
N	Q0T3U0	DR	aspartyl-tRNA aminoacylation
N	Q0T3U0	DR	nucleic acid binding
N	Q0T4Y5	DE	Peptide methionine sulfoxide reductase MsrB;
N	Q0T4Y5	DE	Peptide-methionine (R)-S-oxide reductase;
N	Q0T4Y5	DR	metal ion binding
N	Q0T4Y5	DR	oxidation-reduction process
N	Q0T4Y5	DR	peptide-methionine (R)-S-oxide reductase activity
N	Q0T4Y5	DR	peptide-methionine-(S)-S-oxide reductase activity
N	Q0T7D7	CC	Catalyzes the hydrolysis of 4-amino-2-methyl-5- hydroxymethylpyrimidine pyrophosphate (HMP-PP) to 4-amino-2- methyl-5-hydroxymethylpyrimidine phosphate (HMP-P) (By similarity).
N	Q0T7D7	DE	HMP-PP phosphatase;
N	Q0T7D7	DR	hydrolase activity
N	Q0TCW1	DE	UPF0102 protein yraN;
N	Q0TCW1	DR	nuclease activity
N	Q0TEI3	CC	Catalyzes the attachment of alanine to tRNA(Ala) in a two-step reaction: alanine is first activated by ATP to form Ala- AMP and then transferred to the acceptor end of tRNA(Ala)
N	Q0TEI3	CC	Also edits incorrectly charged Ser-tRNA(Ala) and Gly-tRNA(Ala) via its editing domain (By similarity).
N	Q0TEI3	DE	Alanine--tRNA ligase;
N	Q0TEI3	DE	Alanyl-tRNA synthetase;
N	Q0TEI3	DR	alanine-tRNA ligase activity
N	Q0TEI3	DR	alanyl-tRNA aminoacylation
N	Q0TEI3	DR	metal ion binding
N	Q0TF48	CC	Phosphorylates B6 vitamers; functions in a salvage pathway
N	Q0TF48	CC	Uses pyridoxal, pyridoxine, and pyridoxamine as substrates (By similarity).
N	Q0TF48	DE	PN/PL/PM kinase;
N	Q0TF48	DE	Pyridoxal kinase;
N	Q0TF48	DE	Pyridoxamine kinase;
N	Q0TF48	DE	Pyridoxine kinase;
N	Q0TF48	DE	Vitamin B6 kinase;
N	Q0TF48	DR	metal ion binding
N	Q0TF48	DR	pyridoxal kinase activity
N	Q0TF48	DR	pyridoxine biosynthetic process
N	Q0TIF9	CC	Peptide chain release factor 1 directs the termination of translation in response to the peptide chain termination codons UAG and UAA (By similarity).
N	Q0TIF9	DE	Peptide chain release factor 1;
N	Q0TIF9	DR	translation release factor activity, codon specific
N	Q0TIY4	CC	Catalyzes the reversible formation of acyl-phosphate (acyl-PO(4)) from acyl-[acyl-carrier-protein] (acyl-ACP)
N	Q0TIY4	CC	This enzyme utilizes acyl-ACP as fatty acyl donor, but not acyl-CoA (By similarity).
N	Q0TIY4	DE	Acyl-ACP phosphotransacylase;
N	Q0TIY4	DE	Acyl-[acyl-carrier-protein]--phosphate acyltransferase;
N	Q0TIY4	DE	Phosphate acyltransferase;
N	Q0TIY4	DE	Phosphate-acyl-ACP acyltransferase;

N	Q0TIY4	DR	fatty acid biosynthetic process
N	Q0TIY4	DR	phospholipid biosynthetic process
N	Q0TIY4	DR	transferase activity, transferring acyl groups other than amino-acyl groups
N	Q0TK32	CC	Together with lptD, is involved in the assembly of LPS in the outer leaflet of the outer membrane (By similarity).
N	Q0TK32	DE	LPS-assembly lipoprotein lptE;
N	Q0TK32	DR	Gram-negative-bacterium-type cell outer membrane assembly
N	Q0TL85	CC	Poorly processive, error-prone DNA polymerase involved in untargeted mutagenesis
N	Q0TL85	CC	Copies undamaged DNA at stalled replication forks, which arise in vivo from mismatched or misaligned primer ends
N	Q0TL85	CC	Exhibits no 3'-5' exonuclease (proofreading) activity
N	Q0TL85	CC	May be involved in translesional synthesis, in conjunction with the beta clamp from polIII (By similarity).
N	Q0TL85	CC	These misaligned primers can be extended by polIV
N	Q0TL85	DE	DNA polymerase IV;
N	Q0TL85	DR	DNA replication
N	Q0TL85	DR	DNA-directed DNA polymerase activity
N	Q0TL85	DR	damaged DNA binding
N	Q0TL85	DR	metal ion binding
N	Q0TLC3	CC	Endonuclease that specifically degrades the RNA of RNA- DNA hybrids (By similarity).
N	Q0TLC3	DE	Ribonuclease H;
N	Q0TLC3	DR	metal ion binding
N	Q0TLC3	DR	nucleic acid binding
N	Q0TLC3	DR	ribonuclease H activity
N	Q0TP38	CC	Modulates recA activity (By similarity).
N	Q0TP38	DE	Regulatory protein recX;
N	Q0TP38	DR	regulation of DNA repair
N	Q0TPT1	CC	Catalyzes the hydrolysis of both 2',3'-cyclic AMP and 2',3'-cyclic GMP into 3'-AMP and 3'-GMP, respectively, at the 3'- terminal of RNA (By similarity).
N	Q0TPT1	DE	2',3'-cyclic-nucleotide 2'-phosphodiesterase;
N	Q0TPT1	DR	2',3'-cyclic-nucleotide 2'-phosphodiesterase activity
N	Q0TQZ6	CC	Binds specifically to the ssrA RNA (tmRNA) and is required for stable association of ssrA with ribosomes (By similarity).
N	Q0TQZ6	DE	SsrA-binding protein;
N	Q0TR61	CC	May catalyze the methylation of C-1 in cobalt-precorrin- 5 and the subsequent extrusion of acetic acid from the resulting intermediate to form cobalt-precorrin-6A
N	Q0TR61	DE	Putative cobalt-precorrin-6A synthase [deacetylating];
N	Q0TR61	DR	cobalamin biosynthetic process
N	Q0TR61	DR	transferase activity
N	Q0TT99	DE	3-phosphoshikimate 1-carboxyvinyltransferase;
N	Q0TT99	DE	5-enolpyruvylshikimate-3-phosphate synthase;
N	Q0TT99	DE	EPSP synthase;
N	Q0TT99	DR	3-phosphoshikimate 1-carboxyvinyltransferase activity
N	Q0TT99	DR	aromatic amino acid family biosynthetic process
N	Q0TUD0	CC	The UvrABC repair system catalyzes the recognition and processing of DNA lesions
N	Q0TUD0	CC	A damage recognition complex composed of 2 uvrA and 2 uvrB subunits scans DNA for abnormalities
N	Q0TUD0	CC	DNA wrap is dependent on ATP binding by uvrB and probably causes local melting of the DNA helix, facilitating insertion of uvrB beta-hairpin between the DNA strands
N	Q0TUD0	CC	If a lesion is found the uvrA subunits dissociate and the uvrB-DNA preincision complex is formed
N	Q0TUD0	CC	If no lesion is found, the DNA wraps around the other uvrB subunit that will check the other stand for damage (By similarity).
N	Q0TUD0	CC	Then uvrB probes one DNA strand for the presence of a lesion
N	Q0TUD0	CC	This complex is subsequently bound by uvrC and the second uvrB is released
N	Q0TUD0	CC	Upon binding of the uvrA(2)B(2) complex to a putative damaged site, the DNA wraps around one uvrB monomer
N	Q0TUD0	DE	Excinuclease ABC subunit B;
N	Q0TUD0	DE	Protein uvrB;
N	Q0TUD0	DE	UvrABC system protein B;
N	Q0TUD0	DR	excinuclease ABC activity
N	Q0TUD0	DR	helicase activity
N	Q0TUD0	DR	nucleotide-excision repair

N	Q0UUN6	CC	Catalyzes the dehydration of methylthioribulose-1- phosphate (MTRu-1-P) into 2,3-diketo-5-methylthiopentyl-1- phosphate (DK-MTP-1-P) (By similarity).
N	Q0UUN6	DE	MTRu-1-P dehydratase;
N	Q0UUN6	DE	Methylthioribulose-1-phosphate dehydratase;
N	Q0UUN6	DR	L-methionine salvage
N	Q0UUN6	DR	metal ion binding
N	Q0UUN6	DR	methylthioribulose 1-phosphate dehydratase activity
N	Q0V8N6	CC	The ubiquitous expression and the conservation of the sequence in distant animal species suggest that the gene codes for a protein with housekeeping functions (By similarity)
N	Q0V8N6	DE	Solute carrier family 10 member 3;
N	Q0V8N6	DR	bile acid:sodium symporter activity
N	Q0V8N6	DR	organic anion transport
N	Q0V9P1	CC	Inactivates histamine by N-methylation
N	Q0V9P1	CC	Plays an important role in degrading histamine and in regulating the airway response to histamine (By similarity).
N	Q0V9P1	DE	Histamine N-methyltransferase;
N	Q0V9P1	DR	histamine N-methyltransferase activity
N	Q0VC58	CC	Monovalent cation channel required for maintenance of rapid intracellular calcium release
N	Q0VC58	CC	May act as a potassium counter-ion channel that functions in synchronization with calcium release from intracellular stores (By similarity).
N	Q0VC58	DE	Transmembrane protein 38B;
N	Q0VC58	DE	Trimeric intracellular cation channel type B;
N	Q0VC58	DR	potassium channel activity
N	Q0VCL3	CC	E2-like enzyme involved in autophagy and mitochondrial homeostasis
N	Q0VCL3	CC	ATG7 (E1-like enzyme) facilitates this reaction by forming an E1- E2 complex with ATG3 (By similarity).
N	Q0VCL3	CC	Also acts as an autocatalytic E2-like enzyme, catalyzing the conjugation of ATG12 to itself, ATG12 conjugation to ATG3 playing a role in mitochondrial homeostasis but not in autophagy
N	Q0VCL3	CC	Catalyzes the conjugation of ATG8-like proteins (GABARAP, GABARAPL1, GABARAPL2 or MAP1LC3A) to phosphatidylethanolamine (PE)
N	Q0VCL3	CC	PE-conjugation to ATG8-like proteins is essential for autophagy
N	Q0VCL3	CC	Preferred substrate is MAP1LC3A
N	Q0VCL3	DE	Autophagy-related protein 3;
N	Q0VCL3	DE	Ubiquitin-like-conjugating enzyme ATG3;
N	Q0VCL3	DR	Atg12 ligase activity
N	Q0VCL3	DR	Atg8 ligase activity
N	Q0VCL3	DR	autophagic vacuole assembly
N	Q0VCL3	DR	mitochondrial fragmentation involved in apoptosis
N	Q0VCL3	DR	protein transport
N	Q0VL48	CC	Necessary for normal cell division and for the maintenance of normal septation (By similarity).
N	Q0VL48	DE	Probable GTP-binding protein EngB;
N	Q0VL48	DR	barrier septum formation
N	Q0VP77	DE	AIR synthase;
N	Q0VP77	DE	Phosphoribosyl-aminoimidazole synthetase;
N	Q0VP77	DE	Phosphoribosylformylglycinamidine cyclo-ligase;
N	Q0VP77	DR	'de novo' IMP biosynthetic process
N	Q0VP77	DR	phosphoribosylformylglycinamidine cyclo-ligase activity
N	Q0VQG9	DE	UPF0341 protein ABO_1131;
N	Q0VTN6	DE	UPF0391 membrane protein ABO_0024;
N	Q0WJT1	CC	Nitric oxide-sensitive repressor of genes involved in protecting the cell against nitrosative stress
N	Q0WJT1	CC	May require iron for activity (By similarity).
N	Q0WJT1	DE	HTH-type transcriptional repressor nsrR;
N	Q0WJT1	DR	2 iron, 2 sulfur cluster binding
N	Q0WJT1	DR	metal ion binding
N	Q0WRJ7	CC	PPIases accelerate the folding of proteins
N	Q0WRJ7	CC	Involved in the accumulation of the PSII complex.
N	Q0WRJ7	CC	It catalyzes the cis-trans isomerization of proline imidic peptide bonds in oligopeptides
N	Q0WRJ7	DE	FKBP-type peptidyl-prolyl cis-trans isomerase 6, chloroplastic;
N	Q0WRJ7	DR	oxidoreductase activity
N	Q0WRJ7	DR	peptidyl-prolyl cis-trans isomerase activity
N	Q0WRJ7	DR	photosystem II assembly

N	Q0WRJ7	DR	protein binding
N	Q0WRJ7	DR	protein folding
N	Q0WVN5	CC	Thought to be a Golgi-localized beta-glycan synthase that polymerize the backbones of noncellulosic polysaccharides (hemicelluloses) of plant cell wall.
N	Q0WVN5	DE	Cellulose synthase-like protein G3;
N	Q0WVN5	DR	cellular cell wall organization
N	Q0WVN5	DR	cellulose biosynthetic process
N	Q0WVN5	DR	cellulose synthase (UDP-forming) activity
N	Q10222	CC	Component of the cleavage and polyadenylation factor (CPF) complex, which plays a key role in polyadenylation-dependent pre-mRNA 3'-end formation and cooperates with cleavage factors including the CFIA complex and NAB4/CFIB.
N	Q10222	DE	mRNA cleavage and polyadenylation specificity factor complex subunit pta1;
N	Q10222	DR	mRNA processing
N	Q10926	DE	Uncharacterized protein B0302.2;
N	Q10QL5	CC	May play a role in responses to biotic and abiotic stresses (By similarity).
N	Q10QL5	DE	BTH-induced protein phosphatase 2C 1;
N	Q10QL5	DE	Probable protein phosphatase 2C BIPP2C1;
N	Q10QL5	DR	metal ion binding
N	Q10QL5	DR	phosphoprotein phosphatase activity
N	Q10UY0	CC	The glycine cleavage system catalyzes the degradation of glycine
N	Q10UY0	CC	The H protein shuttles the methylamine group of glycine from the P protein to the T protein (By similarity).
N	Q10UY0	DE	Glycine cleavage system H protein;
N	Q10UY0	DR	glycine decarboxylation via glycine cleavage system
N	Q10Y25	CC	Component of the kaiABC clock protein complex, which constitutes the main circadian regulator in cyanobacteria
N	Q10Y25	CC	In the complex, it decreases the phosphorylation status of kaiC
N	Q10Y25	CC	It has no effect on kaiC by itself, but instead needs the presence of both kaiA and kaiC, suggesting that it acts by antagonizing the interaction between kaiA and kaiC (By similarity).
N	Q10Y25	CC	The kaiABC complex may act as a promoter-non-specific transcription regulator that represses transcription, possibly by acting on the state of chromosome compaction
N	Q10Y25	DE	Circadian clock protein kaiB;
N	Q10Y25	DR	circadian rhythm
N	Q10YG4	CC	This protein is involved in the repair of mismatches in DNA
N	Q10YG4	CC	It is possible that it carries out the mismatch recognition step
N	Q10YG4	CC	This protein has a weak ATPase activity (By similarity).
N	Q10YG4	DE	DNA mismatch repair protein mutS;
N	Q10YG4	DR	mismatch repair
N	Q10YG4	DR	mismatched DNA binding
N	Q10YS9	CC	This b-type cytochrome is tightly associated with the reaction center of photosystem II and possibly is part of the water-oxidation complex (By similarity).
N	Q10YS9	DE	Cytochrome b559 subunit beta;
N	Q10YS9	DE	PSII reaction center subunit VI;
N	Q10YS9	DR	photosynthetic electron transport chain
N	Q11097	DE	Uncharacterized protein C02B8.7;
N	Q11113	DE	Uncharacterized protein C03B1.6;
N	Q116J4	DE	UPF0367 protein Tery_1229;
N	Q118J2	CC	Zinc phosphodiesterase, which displays some tRNA 3'-processing endonuclease activity
N	Q118J2	CC	Probably involved in tRNA maturation, by removing a 3'-trailer from precursor tRNA (By similarity).
N	Q118J2	DE	Ribonuclease Z;
N	Q118J2	DE	tRNA 3 endonuclease;
N	Q118J2	DR	endoribonuclease activity, producing 5'-phosphomonoesters
N	Q118J2	DR	metal ion binding
N	Q118J2	DR	tRNA 3'-trailer cleavage
N	Q11CB5	CC	Catalyzes the interconversion of 2-phosphoglycerate and 3-phosphoglycerate (By similarity).
N	Q11CB5	DE	2,3-bisphosphoglycerate-dependent phosphoglycerate mutase;
N	Q11CB5	DE	BPG-dependent PGAM;
N	Q11CB5	DE	Phosphoglyceromutase;
N	Q11CB5	DR	phosphoglycerate mutase activity
N	Q11CL2	CC	Protease subunit of a proteasome-like degradation complex believed to be a general protein degrading machinery (By similarity).
N	Q11CL2	DE	ATP-dependent protease subunit HslV;

N	Q11CL2	DR	metal ion binding
N	Q11CL2	DR	proteolysis involved in cellular protein catabolic process
N	Q11CL2	DR	threonine-type endopeptidase activity
N	Q11HC6	CC	Required for correct localization of precursor proteins bearing signal peptides with the twin arginine conserved motif S/T-R-R-X-F-L-K
N	Q11HC6	CC	This sec-independent pathway is termed TAT for twin-arginine translocation system
N	Q11HC6	CC	This system mainly transports proteins with bound cofactors that require folding prior to export (By similarity).
N	Q11HC6	DE	Sec-independent protein translocase protein tatB homolog;
N	Q11HC6	DR	protein secretion
N	Q11HC6	DR	protein transport by the Tat complex
N	Q11HC6	DR	protein transporter activity
N	Q11IE7	DE	Putative 3-methyladenine DNA glycosylase;
N	Q11IE7	DR	alkylbase DNA N-glycosylase activity
N	Q11IE7	DR	base-excision repair
N	Q11QA3	DE	50S ribosomal protein L10;
N	Q11QA3	DR	ribosome biogenesis
N	Q11QA3	DR	structural constituent of ribosome
N	Q11QA6	CC	DNA-dependent RNA polymerase catalyzes the transcription of DNA into RNA using the four ribonucleoside triphosphates as substrates.
N	Q11QA6	DE	DNA-directed RNA polymerase subunit beta';
N	Q11QA6	DE	RNA polymerase subunit beta';
N	Q11QA6	DE	RNAP subunit beta';
N	Q11QA6	DE	Transcriptase subunit beta';
N	Q11QA6	DR	DNA-directed RNA polymerase activity
N	Q11QC7	CC	This protein binds to the 23S rRNA, and is important in its secondary structure
N	Q11QC7	CC	It is located near the subunit interface in the base of the L7/L12 stalk, and near the tRNA binding site of the peptidyltransferase center (By similarity).
N	Q11QC7	DE	50S ribosomal protein L6;
N	Q11QC7	DR	structural constituent of ribosome
N	Q11R71	CC	Activates KDO (a required 8-carbon sugar) for incorporation into bacterial lipopolysaccharide in Gram-negative bacteria (By similarity).
N	Q11R71	DE	3-deoxy-manno-octulosonate cytidylyltransferase;
N	Q11R71	DE	CMP-2-keto-3-deoxyoctulosonic acid synthase;
N	Q11R71	DE	CMP-KDO synthase;
N	Q11R71	DR	3-deoxy-manno-octulosonate cytidylyltransferase activity
N	Q11R71	DR	lipopolysaccharide biosynthetic process
N	Q12348	CC	Component of the COP9 signalosome (CSN) complex that acts as an regulator of the ubiquitin (Ubl) conjugation pathway by mediating the deneddylation of the cullin subunit of SCF-type E3 ubiquitin-protein ligase complexes
N	Q12348	CC	The CSN complex is involved in the regulation of the mating pheromone response
N	Q12348	DE	COP9 signalosome complex subunit 10;
N	Q12348	DR	adaptation of signaling pathway by response to pheromone involved in conjugation with cellular fusion
N	Q12348	DR	cullin deneddylation
N	Q12348	DR	protein binding
N	Q12425	CC	Involved in the assembly of mitochondrial and cytoplasmic iron-sulfur proteins
N	Q12425	CC	Probably involved in the binding of an intermediate of Fe/S cluster assembly.
N	Q12425	DE	Iron-sulfur assembly protein 2;
N	Q12425	DR	biotin biosynthetic process
N	Q12425	DR	iron-sulfur cluster assembly
N	Q12425	DR	iron-sulfur cluster binding
N	Q12425	DR	protein maturation
N	Q12425	DR	structural molecule activity
N	Q12493	CC	Component of the central kinetochore, which mediates the attachment of the centromere to the mitotic spindle by forming essential interactions between the microtubule-associated outer kinetochore proteins and the centromere-associated inner kinetochore proteins
N	Q12493	CC	Required for establishing bipolar spindle- microtubule attachments and proper chromosome segregation.
N	Q12493	DE	Central kinetochore subunit NKP1;
N	Q12493	DE	Non-essential kinetochore protein 1;
N	Q12493	DR	cell division
N	Q12493	DR	protein binding

N	Q12530	CC	Functions as part of ribonuclease MRP (RNase MRP), which is involved in rRNA processing in mitochondria.
N	Q12530	DE	RNA-processing protein RMP1;
N	Q12530	DE	RNase MRP 23.6 kDa subunit;
N	Q12530	DE	Ribonuclease MRP protein subunit RMP1;
N	Q12530	DR	mRNA cleavage
N	Q12530	DR	maturation of 5.8S rRNA from tricistronic rRNA transcript (SSU-rRNA, 5.8S rRNA, LSU-rRNA)
N	Q12545	CC	Catalyzes the oxidation of 3-carboxy-2-hydroxy-4- methylpentanoate (3-isopropylmalate) to 3-carboxy-4-methyl-2- oxopentanoate
N	Q12545	CC	The product decarboxylates to 4-methyl-2 oxopentanoate.
N	Q12545	DE	3-isopropylmalate dehydrogenase;
N	Q12545	DE	Beta-IPM dehydrogenase;
N	Q12545	DR	3-isopropylmalate dehydrogenase activity
N	Q12545	DR	leucine biosynthetic process
N	Q12545	DR	magnesium ion binding
N	Q12545	DR	oxidation-reduction process
N	Q12578	DE	Imidazoleglycerol-phosphate dehydratase;
N	Q12578	DR	histidine biosynthetic process
N	Q12578	DR	imidazoleglycerol-phosphate dehydratase activity
N	Q12609	CC	May be involved in the hydroxylation of averantin to form 5'-hydroxyaverantin, oxidation of averufin to 1- hydroxyversicolorone and its oxidation to versiconal hemiacetal acetate
N	Q12609	DE	Cytochrome P450 60A2;
N	Q12609	DE	Probable sterigmatocystin biosynthesis P450 monooxygenase stcF;
N	Q12609	DR	electron carrier activity
N	Q12609	DR	monooxygenase activity
N	Q12609	DR	oxidation-reduction process
N	Q126J2	CC	Carrier of the growing fatty acid chain in fatty acid biosynthesis (By similarity).
N	Q126J2	DE	Acyl carrier protein;
N	Q126J2	DR	acyl carrier activity
N	Q126J2	DR	cofactor binding
N	Q126J2	DR	fatty acid biosynthetic process
N	Q126J2	DR	phosphopantetheine binding
N	Q12765	CC	Regulates exocytosis in mast cells
N	Q12765	CC	Increases both the extent of secretion and the sensitivity of mast cells to stimulation with calcium (By similarity).
N	Q12765	DR	dipeptidase activity
N	Q12887	CC	Converts protoheme IX and farnesyl diphosphate to heme O (By similarity).
N	Q12887	DE	Heme O synthase;
N	Q12887	DE	Protoheme IX farnesyltransferase, mitochondrial;
N	Q12887	DR	heme O biosynthetic process
N	Q12887	DR	heme a biosynthetic process
N	Q12887	DR	mitochondrial electron transport, cytochrome c to oxygen
N	Q12887	DR	protoheme IX farnesyltransferase activity
N	Q12887	DR	respiratory chain complex IV assembly
N	Q12950	DE	Forkhead box protein D4;
N	Q12950	DE	Forkhead-related protein FKHL9;
N	Q12950	DE	Forkhead-related transcription factor 5;
N	Q12950	DE	Myeloid factor-alpha;
N	Q12950	DR	DNA bending activity
N	Q12950	DR	axon extension involved in axon guidance
N	Q12950	DR	cartilage development
N	Q12950	DR	dichotomous subdivision of terminal units involved in ureteric bud branching
N	Q12950	DR	double-stranded DNA binding
N	Q12950	DR	embryo development
N	Q12950	DR	enteric nervous system development
N	Q12950	DR	iridophore differentiation
N	Q12950	DR	lateral line nerve glial cell development
N	Q12950	DR	melanocyte differentiation
N	Q12950	DR	negative regulation of gene-specific transcription from RNA polymerase II promoter
N	Q12950	DR	neural crest cell migration
N	Q12950	DR	pattern specification process
N	Q12950	DR	peripheral nervous system development
N	Q12950	DR	positive regulation of BMP signaling pathway
N	Q12950	DR	positive regulation of kidney development

N	Q12950	DR	positive regulation of transcription from RNA polymerase II promoter
N	Q12950	DR	promoter binding
N	Q12950	DR	regulation of sequence-specific DNA binding transcription factor activity
N	Q12950	DR	sequence-specific enhancer binding RNA polymerase II transcription factor activity
N	Q12950	DR	specific RNA polymerase II transcription factor activity
N	Q12950	DR	specific transcriptional repressor activity
N	Q12950	DR	sympathetic nervous system development
N	Q12950	DR	transcription activator activity
N	Q12950	DR	transcription factor binding
N	Q12GX1	CC	One of the primary rRNA binding proteins, it binds directly near the 3'-end of the 23S rRNA, where it nucleates assembly of the 50S subunit (By similarity).
N	Q12GX1	DE	50S ribosomal protein L3;
N	Q12GX1	DR	structural constituent of ribosome
N	Q12PA3	CC	The UvrABC repair system catalyzes the recognition and processing of DNA lesions
N	Q12PA3	CC	The N-terminal half is responsible for the 3' incision and the C-terminal half is responsible for the 5' incision (By similarity).
N	Q12PA3	CC	UvrC both incises the 5' and 3' sides of the lesion
N	Q12PA3	DE	Excinuclease ABC subunit C;
N	Q12PA3	DE	Protein uvrC;
N	Q12PA3	DE	UvrABC system protein C;
N	Q12PA3	DR	excinuclease ABC activity
N	Q12PA3	DR	nucleotide-excision repair
N	Q12QK6	CC	NQR complex catalyzes the reduction of ubiquinone-1 to ubiquinol by two successive reactions, coupled with the transport of Na(+) ions from the cytoplasm to the periplasm
N	Q12QK6	CC	NqrA to nqrE are probably involved in the second step, the conversion of ubisemiquinone to ubiquinol (By similarity).
N	Q12QK6	DE	NQR complex subunit A;
N	Q12QK6	DE	NQR-1 subunit A;
N	Q12QK6	DE	Na(+)-NQR subunit A;
N	Q12QK6	DE	Na(+)-translocating NADH-quinone reductase subunit A;
N	Q12QK6	DE	Na(+)-translocating NQR subunit A;
N	Q12QK6	DR	oxidation-reduction process
N	Q12QK6	DR	oxidoreductase activity, acting on NADH or NADPH, quinone or similar compound as acceptor
N	Q12QK6	DR	sodium ion transport
N	Q12R24	CC	Involved in rRNA and/or ribosome maturation and assembly
N	Q12R24	CC	May have metal-dependent hydrolase activity (By similarity).
N	Q12R24	CC	Required for normal 5' and 3' processing of 16S, 23S and 5S rRNAs
N	Q12R24	DE	Probable rRNA maturation factor;
N	Q12R24	DR	metal ion binding
N	Q12R24	DR	metalloendopeptidase activity
N	Q12R24	DR	rRNA processing
N	Q12S01	CC	Synthesis of 3-octaprenyl-4-hydroxybenzoate (By similarity).
N	Q12S01	DE	4-HB polyprenyltransferase;
N	Q12S01	DE	4-hydroxybenzoate octaprenyltransferase;
N	Q12S01	DR	prenyltransferase activity
N	Q12S01	DR	ubiquinone biosynthetic process
N	Q12SW9	CC	Binds directly to 23S rRNA
N	Q12SW9	CC	The L1 stalk is quite mobile in the ribosome, and is involved in E site tRNA release (By similarity).
N	Q12SW9	DE	50S ribosomal protein L1;
N	Q12SW9	DR	RNA processing
N	Q12SW9	DR	regulation of translation
N	Q12SW9	DR	structural constituent of ribosome
N	Q12VF1	CC	Specifically catalyzes the NAD or NADP-dependent dehydrogenation of L-aspartate to iminoaspartate (By similarity).
N	Q12VF1	DE	Probable L-aspartate dehydrogenase;
N	Q12VF1	DR	NAD biosynthetic process
N	Q12VF1	DR	NADP catabolic process
N	Q12VF1	DR	aspartate dehydrogenase activity
N	Q12VF1	DR	oxidation-reduction process
N	Q12ZG1	DE	Dihydrodipicolinate reductase;
N	Q12ZG1	DR	diaminopimelate biosynthetic process
N	Q12ZG1	DR	dihydrodipicolinate reductase activity
N	Q12ZG1	DR	oxidation-reduction process

N	Q12ZT1	CC	With S4 and S12 plays an important role in translational accuracy (By similarity)
N	Q12ZT1	DE	30S ribosomal protein S5P;
N	Q12ZT1	DR	structural constituent of ribosome
N	Q13045	CC	May play a role as coactivator in transcriptional activation by hormone-activated nuclear receptors (NR) and acts in cooperation with NCOA2 and CARM1
N	Q13045	CC	Involved in early embryonic development (By similarity)
N	Q13045	CC	Involved in estrogen hormone signaling
N	Q13045	CC	May play a role in regulation of cytoskeletal rearrangements involved in cytokinesis and cell migration.
N	Q13045	DE	Protein flightless-1 homolog;
N	Q13045	DR	actin binding
N	Q13045	DR	multicellular organismal development
N	Q13045	DR	muscle contraction
N	Q13445	CC	Potential role in protein trafficking.
N	Q13445	DE	Interleukin-1 receptor-like 1 ligand;
N	Q13445	DE	Putative T1/ST2 receptor-binding protein;
N	Q13445	DE	Transmembrane emp24 domain-containing protein 1;
N	Q13445	DR	cell-cell signaling
N	Q13445	DR	receptor binding
N	Q13445	DR	signal transduction
N	Q13465	DE	MDS1 and EVI1 complex locus protein MDS1;
N	Q13465	DE	Myelodysplasia syndrome 1 protein;
N	Q13465	DE	Myelodysplasia syndrome-associated protein 1;
N	Q13465	DR	sequence-specific DNA binding transcription factor activity
N	Q13541	CC	Regulates eIF4E activity by preventing its assembly into the eIF4F complex
N	Q13541	CC	Mediates the regulation of protein translation by hormones, growth factors and other stimuli that signal through the MAP kinase and mTORC1 pathways.
N	Q13541	DE	Eukaryotic translation initiation factor 4E-binding protein 1;
N	Q13541	DE	Phosphorylated heat- and acid-stable protein regulated by insulin 1;
N	Q13541	DE	eIF4E-binding protein 1;
N	Q13541	DR	G1/S transition of mitotic cell cycle
N	Q13541	DR	TOR signaling cascade
N	Q13541	DR	insulin receptor signaling pathway
N	Q13541	DR	positive regulation of mitotic cell cycle
N	Q13547	CC	Responsible for the deacetylation of lysine residues on the N-terminal part of the core histones (H2A, H2B, H3 and H4)
N	Q13547	CC	Component of the BRG1-RB1-HDAC1 complex, which negatively regulates the CREST- mediated transcription in resting neurons
N	Q13547	CC	Deacetylates 'Lys-310' in RELA and thereby inhibits the transcriptional activity of NF-kappa-B.
N	Q13547	CC	Deacetylates SP proteins, SP1 and SP3, and regulates their function
N	Q13547	CC	Deacetylates TSHZ3 and regulates its transcriptional repressor activity
N	Q13547	CC	Histone deacetylases act via the formation of large multiprotein complexes
N	Q13547	CC	Histone deacetylation gives a tag for epigenetic repression and plays an important role in transcriptional regulation, cell cycle progression and developmental events
N	Q13547	CC	Upon calcium stimulation, HDAC1 is released from the complex and CREBBP is recruited, which facilitates transcriptional activation
N	Q13547	DE	Histone deacetylase 1;
N	Q13547	DR	NAD-dependent histone deacetylase activity (H3-K14 specific)
N	Q13547	DR	NAD-dependent histone deacetylase activity (H3-K9 specific)
N	Q13547	DR	NAD-dependent histone deacetylase activity (H4-K16 specific)
N	Q13547	DR	anti-apoptosis
N	Q13547	DR	blood coagulation
N	Q13547	DR	chromatin remodeling
N	Q13547	DR	embryonic digit morphogenesis
N	Q13547	DR	epidermal cell differentiation
N	Q13547	DR	eyelid development in camera-type eye
N	Q13547	DR	fungiform papilla formation
N	Q13547	DR	hair follicle placode formation
N	Q13547	DR	histone H3 deacetylation
N	Q13547	DR	histone H4 deacetylation
N	Q13547	DR	histone deacetylase activity (H3-K16 specific)
N	Q13547	DR	histone deacetylase binding
N	Q13547	DR	identical protein binding
N	Q13547	DR	negative regulation by host of viral transcription

N	Q13547	DR	negative regulation of cell cycle
N	Q13547	DR	negative regulation of gene-specific transcription from RNA polymerase II promoter
N	Q13547	DR	nerve growth factor receptor signaling pathway
N	Q13547	DR	odontogenesis of dentine-containing tooth
N	Q13547	DR	positive regulation of cell proliferation
N	Q13547	DR	positive regulation of gene-specific transcription from RNA polymerase II promoter
N	Q13547	DR	positive regulation of receptor biosynthetic process
N	Q13547	DR	sequence-specific DNA binding transcription factor activity
N	Q13547	DR	specific transcriptional repressor activity
N	Q13547	DR	transcription activator activity
N	Q13547	DR	transcription factor binding
N	Q136H4	CC	Catalyzes the attachment of serine to tRNA(Ser)
N	Q136H4	CC	Is also able to aminoacylate tRNA(Sec) with serine, to form the misacylated tRNA L-seryl-tRNA(Sec), which will be further converted into selenocysteinyl-tRNA(Sec) (By similarity).
N	Q136H4	DE	Serine--tRNA ligase;
N	Q136H4	DE	Seryl-tRNA synthetase;
N	Q136H4	DE	Seryl-tRNA(Ser/Sec) synthetase;
N	Q136H4	DR	serine-tRNA ligase activity
N	Q136H4	DR	seryl-tRNA aminoacylation
N	Q136W1	CC	Transfers the 4'-phosphopantetheine moiety from coenzyme A to a Ser of acyl-carrier-protein (By similarity).
N	Q136W1	DE	4'-phosphopantetheinyl transferase AcpS;
N	Q136W1	DE	Holo-ACP synthase;
N	Q136W1	DE	Holo-[acyl-carrier-protein] synthase;
N	Q136W1	DR	fatty acid biosynthetic process
N	Q136W1	DR	holo-[acyl-carrier-protein] synthase activity
N	Q136W1	DR	macromolecule biosynthetic process
N	Q136W1	DR	magnesium ion binding
N	Q13829	CC	Substrate-specific adapter of a BCR (BTB-CUL3-RBX1) E3 ubiquitin-protein ligase complex involved in regulation of cytoskeleton structure
N	Q13829	CC	Its interaction with RHOB may regulate apoptosis
N	Q13829	CC	May enhance the PCNA-dependent DNA polymerase delta activity.
N	Q13829	CC	The BCR(BACURD2) E3 ubiquitin ligase complex mediates the ubiquitination of RHOA, leading to its degradation by the proteasome, thereby regulating the actin cytoskeleton and cell migration
N	Q13829	DE	BTB/POZ domain-containing adapter for CUL3-mediated RhoA degradation protein 2;
N	Q13829	DE	BTB/POZ domain-containing protein TNFAIP1;
N	Q13829	DE	Protein B12;
N	Q13829	DE	Tumor necrosis factor, alpha-induced protein 1, endothelial;
N	Q13829	DR	DNA replication
N	Q13829	DR	GTP-Rho binding
N	Q13829	DR	cell migration
N	Q13829	DR	embryo development
N	Q13829	DR	immune response
N	Q13829	DR	negative regulation of Rho protein signal transduction
N	Q13829	DR	proteasomal ubiquitin-dependent protein catabolic process
N	Q13829	DR	protein ubiquitination
N	Q13829	DR	stress fiber assembly
N	Q13829	DR	voltage-gated potassium channel activity
N	Q13885	CC	Tubulin is the major constituent of microtubules
N	Q13885	CC	It binds two moles of GTP, one at an exchangeable site on the beta chain and one at a non-exchangeable site on the alpha-chain (By similarity).
N	Q13885	DE	Tubulin beta-2A chain;
N	Q13885	DR	'de novo' posttranslational protein folding
N	Q13885	DR	GTPase activity
N	Q13885	DR	microtubule-based movement
N	Q13885	DR	protein binding
N	Q13885	DR	protein polymerization
N	Q13885	DR	structural molecule activity
N	Q13EL3	CC	Catalyzes the formation of N(7)-methylguanine at position 46 (m7G46) in tRNA (By similarity).
N	Q13EL3	DE	tRNA (guanine-N(7)-)-methyltransferase;
N	Q13EL3	DE	tRNA(m7G46)-methyltransferase;
N	Q13EL3	DR	tRNA (guanine-N7-)-methyltransferase activity

N	Q13TI6	CC	This is one of the proteins that binds and probably mediates the attachment of the 5S RNA into the large ribosomal subunit, where it forms part of the central protuberance (By similarity).
N	Q13TI6	DE	50S ribosomal protein L18;
N	Q13TI6	DR	structural constituent of ribosome
N	Q13TY3	DE	Protein MraZ;
N	Q13W50	CC	Catalyzes the conversion of glucosamine-6-phosphate to glucosamine-1-phosphate (By similarity).
N	Q13W50	DE	Phosphoglucosamine mutase;
N	Q13W50	DR	carbohydrate metabolic process
N	Q13W50	DR	magnesium ion binding
N	Q13W50	DR	phosphoglucosamine mutase activity
N	Q14119	CC	Possible transcription factor
N	Q14119	CC	Specifically binds to the CT/GC-rich region of the interleukin-3 promoter and mediates tax transactivation of IL-3.
N	Q14119	DE	Putative transcription factor DB1;
N	Q14119	DE	Vascular endothelial zinc finger 1;
N	Q14119	DE	Zinc finger protein 161;
N	Q14119	DR	cellular defense response
N	Q14119	DR	regulation of transcription from RNA polymerase II promoter
N	Q14119	DR	specific RNA polymerase II transcription factor activity
N	Q14119	DR	zinc ion binding
N	Q142T1	CC	Removes the pyruvyl group from chorismate, with concomitant aromatization of the ring, to provide 4- hydroxybenzoate (4HB) for the ubiquinone pathway (By similarity).
N	Q142T1	DE	Probable chorismate--pyruvate lyase;
N	Q142T1	DR	chorismate lyase activity
N	Q142T1	DR	ubiquinone biosynthetic process
N	Q14439	CC	Orphan receptor.
N	Q14439	DE	Probable G-protein coupled receptor 176;
N	Q14439	DR	G-protein coupled receptor activity
N	Q14439	DR	synaptic transmission
N	Q14865	CC	DNA-binding protein that binds to the 5'-AATA[CT]-3' core sequence
N	Q14865	CC	May be involved in lipid stores.
N	Q14865	CC	Overexpression leads to induction of smooth muscle marker genes, suggesting that it may act as a regulator of smooth muscle cell differentiation and proliferation
N	Q14865	CC	Probably acts as a transcription regulator
N	Q14865	CC	Represses the cytomegalovirus enhancer
N	Q14865	DE	ARID domain-containing protein 5B;
N	Q14865	DE	AT-rich interactive domain-containing protein 5B;
N	Q14865	DE	MRF1-like protein;
N	Q14865	DE	Modulator recognition factor 2;
N	Q14865	DR	negative regulation of transcription, DNA-dependent
N	Q14865	DR	transcription repressor activity
N	Q148G4	CC	Putative deoxyribonuclease (By similarity).
N	Q148G4	DE	Putative deoxyribonuclease TATDN1;
N	Q148G4	DR	endodeoxyribonuclease activity, producing 5'-phosphomonoesters
N	Q14CN4	CC	Has a role in hair formation
N	Q14CN4	CC	Specific component of keratin intermediate filaments in the inner root sheath (IRS) of the hair follicle (Probable).
N	Q14CN4	DE	Cytokeratin-72;
N	Q14CN4	DE	Keratin, type II cytoskeletal 72;
N	Q14CN4	DE	Type II inner root sheath-specific keratin-K6irs2;
N	Q14CN4	DE	Type-II keratin Kb35;
N	Q14CN4	DR	structural molecule activity
N	Q14D33	DE	Uncharacterized protein C2orf85;
N	Q14FG6	CC	DNA-dependent RNA polymerase catalyzes the transcription of DNA into RNA using the four ribonucleoside triphosphates as substrates.
N	Q14FG6	DE	DNA-directed RNA polymerase subunit beta';
N	Q14FG6	DE	Plastid-encoded RNA polymerase subunit beta';
N	Q14FG6	DE	RNA polymerase subunit beta';
N	Q14FG6	DR	DNA-directed RNA polymerase activity
N	Q14FH0	CC	F(1)F(0) ATP synthase produces ATP from ADP in the presence of a proton or sodium gradient
N	Q14FH0	CC	During catalysis, ATP synthesis in the catalytic domain of F(1) is coupled via a rotary mechanism of the central stalk subunits to proton translocation (By similarity).

N	Q14FH0	CC	F-type ATPases consist of two structural domains, F(1) containing the extramembraneous catalytic core and F(0) containing the membrane proton channel, linked together by a central stalk and a peripheral stalk
N	Q14FH0	DE	ATP synthase F(0) sector subunit c;
N	Q14FH0	DE	ATP synthase subunit c, chloroplastic;
N	Q14FH0	DE	ATPase subunit III;
N	Q14FH0	DE	F-ATPase subunit c;
N	Q14FH0	DE	F-type ATPase subunit c;
N	Q14FH0	DE	Lipid-binding protein;
N	Q14FH0	DR	ATP hydrolysis coupled proton transport
N	Q14FH0	DR	ATP synthesis coupled proton transport
N	Q14FH0	DR	hydrogen ion transmembrane transporter activity
N	Q14FH0	DR	lipid binding
N	Q14GK9	CC	Catalyzes the pyruvoyl-dependent decarboxylation of aspartate to produce beta-alanine (By similarity).
N	Q14GK9	DE	Aspartate 1-decarboxylase alpha chain;
N	Q14GK9	DE	Aspartate 1-decarboxylase beta chain;
N	Q14GK9	DE	Aspartate 1-decarboxylase;
N	Q14GK9	DE	Aspartate alpha-decarboxylase;
N	Q14GK9	DR	alanine biosynthetic process
N	Q14GK9	DR	aspartate 1-decarboxylase activity
N	Q14GK9	DR	pantothenate biosynthetic process
N	Q14GR4	CC	Catalyzes the interconversion of 2-phosphoglycerate and 3-phosphoglycerate (By similarity).
N	Q14GR4	DE	2,3-bisphosphoglycerate-independent phosphoglycerate mutase;
N	Q14GR4	DE	BPG-independent PGAM;
N	Q14GR4	DE	Phosphoglyceromutase;
N	Q14GR4	DR	manganese ion binding
N	Q14GR4	DR	phosphoglycerate mutase activity
N	Q14GX0	CC	Participates actively in the response to hyperosmotic and heat shock by preventing the aggregation of stress-denatured proteins and by disaggregating proteins, also in an autonomous, dnaK-independent fashion
N	Q14GX0	CC	Also involved, together with dnaK and grpE, in the DNA replication of plasmids through activation of initiation proteins (By similarity).
N	Q14GX0	CC	GrpE releases ADP from dnaK; ATP binding to dnaK triggers the release of the substrate protein, thus completing the reaction cycle
N	Q14GX0	CC	Several rounds of ATP-dependent interactions between dnaJ, dnaK and grpE are required for fully efficient folding
N	Q14GX0	CC	Unfolded proteins bind initially to dnaJ; upon interaction with the dnaJ-bound protein, dnaK hydrolyzes its bound ATP, resulting in the formation of a stable complex
N	Q14GX0	DE	Chaperone protein dnaJ;
N	Q14GX0	DR	DNA replication
N	Q14GX0	DR	heat shock protein binding
N	Q14GX0	DR	metal ion binding
N	Q14GX0	DR	protein folding
N	Q14GX0	DR	response to heat
N	Q14GX0	DR	unfolded protein binding
N	Q14J06	CC	First step of the lipid cycle reactions in the biosynthesis of the cell wall peptidoglycan (By similarity).
N	Q14J06	DE	Phospho-N-acetylmuramoyl-pentapeptide-transferase;
N	Q14J06	DE	UDP-MurNAc-pentapeptide phosphotransferase;
N	Q14J06	DR	UDP-N-acetylmuramoyl-L-alanyl-D-glutamyl-meso-2,6-diaminopimelyl-D-alanyl-D-alanine:undecaprenyl-phosphate transferase activity
N	Q14J06	DR	cell division
N	Q14J06	DR	cellular cell wall organization
N	Q14J06	DR	peptidoglycan biosynthetic process
N	Q14J06	DR	phospho-N-acetylmuramoyl-pentapeptide-transferase activity
N	Q14J06	DR	regulation of cell shape
N	Q15262	CC	Regulation of processes involving cell contact and adhesion such as growth control, tumor invasion, and metastasis
N	Q15262	CC	Beta-catenin may be a substrate for the catalytic activity of PTP- kappa.
N	Q15262	CC	Forms complexes with beta-catenin and gamma-catenin/plakoglobin
N	Q15262	DE	Protein-tyrosine phosphatase kappa;
N	Q15262	DE	R-PTP-kappa;
N	Q15262	DE	Receptor-type tyrosine-protein phosphatase kappa;

N	Q15262	DR	beta-catenin binding
N	Q15262	DR	cell migration
N	Q15262	DR	cellular response to UV
N	Q15262	DR	cellular response to reactive oxygen species
N	Q15262	DR	focal adhesion assembly
N	Q15262	DR	gamma-catenin binding
N	Q15262	DR	negative regulation of cell cycle
N	Q15262	DR	negative regulation of cell migration
N	Q15262	DR	negative regulation of keratinocyte proliferation
N	Q15262	DR	protein dephosphorylation
N	Q15262	DR	protein kinase binding
N	Q15262	DR	protein localization at cell surface
N	Q15262	DR	transforming growth factor beta receptor signaling pathway
N	Q15262	DR	transmembrane receptor protein tyrosine phosphatase activity
N	Q15434	DE	RNA-binding motif, single-stranded-interacting protein 2;
N	Q15434	DE	Suppressor of CDC2 with RNA-binding motif 3;
N	Q15434	DR	RNA processing
N	Q15434	DR	nucleotide binding
N	Q15569	CC	Dual specificity protein kinase activity catalyzing autophosphorylation and phosphorylation of exogenous substrates on both serine/threonine and tyrosine residues
N	Q15569	CC	Probably plays a central role at and after the meiotic phase of spermatogenesis (By similarity).
N	Q15569	DE	Dual specificity testis-specific protein kinase 1;
N	Q15569	DE	Testicular protein kinase 1;
N	Q15569	DR	cell junction assembly
N	Q15569	DR	metal ion binding
N	Q15569	DR	protein binding
N	Q15569	DR	protein phosphorylation
N	Q15569	DR	protein serine/threonine kinase activity
N	Q15569	DR	protein tyrosine kinase activity
N	Q15569	DR	spermatogenesis
N	Q15NA2	DE	UPF0391 membrane protein Patl_4137;
N	Q15P51	CC	Provides the sole de novo source of dTMP for DNA biosynthesis (By similarity).
N	Q15P51	DE	Thymidylate synthase;
N	Q15P51	DR	dTMP biosynthetic process
N	Q15P51	DR	thymidylate synthase activity
N	Q15TR4	CC	Catalyzes the methylthiolation of N6- (dimethylallyl)adenosine (i(6)A), leading to the formation of 2- methylthio-N6-(dimethylallyl)adenosine (ms(2)i(6)A) at position 37 in tRNAs that read codons beginning with uridine (By similarity).
N	Q15TR4	DE	(Dimethylallyl)adenosine tRNA methylthiotransferase miaB;
N	Q15TR4	DE	tRNA-i(6)A37 methylthiotransferase;
N	Q15TR4	DR	4 iron, 4 sulfur cluster binding
N	Q15TR4	DR	metal ion binding
N	Q15TR4	DR	tRNA modification
N	Q15TR4	DR	transferase activity
N	Q15Z11	DE	Protein CyaY;
N	Q164Y5	CC	Part of the ABC transporter complex UgpABCE involved in sn-glycerol-3-phosphate import
N	Q164Y5	CC	Responsible for energy coupling to the transport system (By similarity).
N	Q164Y5	DE	sn-glycerol-3-phosphate import ATP-binding protein UgpC;
N	Q164Y5	DR	glycerol-3-phosphate-transporting ATPase activity
N	Q165U5	CC	Catalyzes the attachment of alanine to tRNA(Ala) in a two-step reaction: alanine is first activated by ATP to form Ala- AMP and then transferred to the acceptor end of tRNA(Ala)
N	Q165U5	CC	Also edits incorrectly charged Ser-tRNA(Ala) and Gly-tRNA(Ala) via its editing domain (By similarity).
N	Q165U5	DE	Alanine--tRNA ligase;
N	Q165U5	DE	Alanyl-tRNA synthetase;
N	Q165U5	DR	alanine-tRNA ligase activity
N	Q165U5	DR	alanyl-tRNA aminoacylation
N	Q165U5	DR	metal ion binding
N	Q16982	DE	Neurocalcin;
N	Q16982	DR	calcium ion binding
N	Q16AD2	CC	This is 1 of the proteins that binds and probably mediates the attachment of the 5S RNA into the large ribosomal subunit, where it forms part of the central protuberance

N	Q16AD2	CC	Contacts the P site tRNA; the 5S rRNA and some of its associated proteins might help stabilize positioning of ribosome-bound tRNAs (By similarity).
N	Q16AD2	CC	In the 70S ribosome it contacts protein S13 of the 30S subunit (bridge B1b), connecting the 2 subunits; this bridge is implicated in subunit movement
N	Q16AD2	DE	50S ribosomal protein L5;
N	Q16AD2	DR	structural constituent of ribosome
N	Q16CZ1	DE	Maf-like protein RD1_0437;
N	Q17027	CC	Responsible for the anti Gram-positive activity of immune hemolymph (By similarity).
N	Q17027	DR	defense response to bacterium
N	Q17027	DR	innate immune response
N	Q17QM9	CC	Suppresses cannabinoid receptor CNR1-mediated tonic inhibition of voltage-gated calcium channels (By similarity).
N	Q17QM9	DE	CB1 cannabinoid receptor-interacting protein 1;
N	Q17WI2	CC	Nuclease that resolves Holliday junction intermediates in genetic recombination
N	Q17WI2	CC	Cleaves the cruciform structure in supercoiled DNA by nicking to strands with the same polarity at sites symmetrically opposed at the junction in the homologous arms and leaves a 5'-terminal phosphate and a 3'-terminal hydroxyl group (By similarity).
N	Q17WI2	DE	Crossover junction endodeoxyribonuclease ruvC;
N	Q17WI2	DE	Holliday junction nuclease ruvC;
N	Q17WI2	DE	Holliday junction resolvase ruvC;
N	Q17WI2	DR	DNA recombination
N	Q17WI2	DR	crossover junction endodeoxyribonuclease activity
N	Q17WI2	DR	metal ion binding
N	Q17WI2	DR	nucleic acid binding
N	Q181G8	CC	Catalyzes the phosphorylation of the position 2 hydroxy group of 4-diphosphocytidyl-2C-methyl-D-erythritol (By similarity).
N	Q181G8	DE	4-(cytidine-5'-diphospho)-2-C-methyl-D-erythritol kinase;
N	Q181G8	DE	4-diphosphocytidyl-2-C-methyl-D-erythritol kinase;
N	Q181G8	DR	4-(cytidine 5'-diphospho)-2-C-methyl-D-erythritol kinase activity
N	Q181G8	DR	homoserine kinase activity
N	Q181G8	DR	terpenoid biosynthetic process
N	Q181G8	DR	threonine metabolic process
N	Q182C0	CC	Involved in DNA repair and recF pathway recombination (By similarity).
N	Q182C0	DE	DNA repair protein recO;
N	Q182C0	DE	Recombination protein O;
N	Q182C0	DR	ARF GTPase activator activity
N	Q182C0	DR	DNA recombination
N	Q182C0	DR	regulation of ARF GTPase activity
N	Q182C0	DR	zinc ion binding
N	Q187P6	CC	Component of the acetyl coenzyme A carboxylase (ACC) complex
N	Q187P6	CC	Biotin carboxylase (BC) catalyzes the carboxylation of biotin on its carrier protein (BCCP) and then the CO(2) group is transferred by the transcarboxylase to acetyl-CoA to form malonyl- CoA (By similarity).
N	Q187P6	DE	ACCase subunit beta;
N	Q187P6	DE	Acetyl-CoA carboxylase carboxyltransferase subunit beta;
N	Q187P6	DE	Acetyl-coenzyme A carboxylase carboxyl transferase subunit beta;
N	Q187P6	DR	acetyl-CoA carboxylase activity
N	Q187P6	DR	fatty acid biosynthetic process
N	Q187P6	DR	metal ion binding
N	Q18CH5	CC	This protein binds to the 23S rRNA, and is important in its secondary structure
N	Q18CH5	CC	It is located near the subunit interface in the base of the L7/L12 stalk, and near the tRNA binding site of the peptidyltransferase center (By similarity).
N	Q18CH5	DE	50S ribosomal protein L6;
N	Q18CH5	DR	structural constituent of ribosome
N	Q18PF5	CC	Functions as an ammonia transporter
N	Q18PF5	CC	May play a role in the elimination of ammonia in the gill.
N	Q18PF5	DE	Ammonium transporter Rh type C 1;
N	Q18PF5	DE	Rh family type C glycoprotein 1;
N	Q18PF5	DE	Rh type C glycoprotein 1;
N	Q18PF5	DE	Rhesus blood group family type C glycoprotein 1;
N	Q18PF5	DR	ammonium transmembrane transporter activity
N	Q1AWG4	DE	Threonine--tRNA ligase;
N	Q1AWG4	DE	Threonyl-tRNA synthetase;
N	Q1AWG4	DR	metal ion binding
N	Q1AWG4	DR	threonine-tRNA ligase activity

N	Q1AWG4	DR	threonyl-tRNA aminoacylation
N	Q1AYN2	DE	Protein CrcB homolog 1;
N	Q1BD76	CC	Exhibits S-adenosyl-L-methionine-dependent methyltransferase activity (By similarity)
N	Q1BD76	DE	Putative S-adenosyl-L-methionine-dependent methyltransferase Mmcs_1044;
N	Q1BD76	DR	methyltransferase activity
N	Q1BNL1	CC	No specific function has so far been attributed to this initiation factor; however, it seems to stimulate more or less all the activities of the other two initiation factors, IF-2 and IF-3
N	Q1BNL1	DE	Translation initiation factor IF-1 2;
N	Q1BNL1	DR	translation initiation factor activity
N	Q1BWA2	CC	May catalyze the methylation of C-1 in cobalt-precorrin- 5 and the subsequent extrusion of acetic acid from the resulting intermediate to form cobalt-precorrin-6A
N	Q1BWA2	DE	Putative cobalt-precorrin-6A synthase [deacetylating];
N	Q1BWA2	DR	cobalamin biosynthetic process
N	Q1BWA2	DR	transferase activity
N	Q1BWM2	CC	Functions in the N-end rule pathway of protein degradation where it conjugates Leu, Phe and, less efficiently, Met from aminoacyl-tRNAs to the N-termini of proteins containing an N-terminal arginine or lysine (By similarity).
N	Q1BWM2	DE	L/F-transferase;
N	Q1BWM2	DE	Leucyl/phenylalanyl-tRNA--protein transferase;
N	Q1BWM2	DE	Leucyltransferase;
N	Q1BWM2	DE	Phenyalanyltransferase;
N	Q1BWM2	DR	acyltransferase activity
N	Q1BWM2	DR	leucyltransferase activity
N	Q1BWM2	DR	protein catabolic process
N	Q1BY04	DE	30S ribosomal protein S16;
N	Q1BY04	DR	structural constituent of ribosome
N	Q1BY28	DE	3-phosphoshikimate 1-carboxyvinyltransferase;
N	Q1BY28	DE	5-enolpyruvylshikimate-3-phosphate synthase;
N	Q1BY28	DE	EPSP synthase;
N	Q1BY28	DR	3-phosphoshikimate 1-carboxyvinyltransferase activity
N	Q1BY28	DR	aromatic amino acid family biosynthetic process
N	Q1C475	CC	Nucleotidase with a broad substrate specificity as it can dephosphorylate various ribo- and deoxyribonucleoside 5'- monophosphates and ribonucleoside 3'-monophosphates with highest affinity to 3'-AMP
N	Q1C475	CC	Also hydrolyzes polyphosphate (exopolyphosphatase activity) with the preference for short-chain- length substrates (P20-25)
N	Q1C475	CC	Might be involved in the regulation of dNTP and NTP pools, and in the turnover of 3'-mononucleotides produced by numerous intracellular RNases (T1, T2, and F) during the degradation of various RNAs (By similarity).
N	Q1C475	DE	5'/3'-nucleotidase;
N	Q1C475	DE	Exopolyphosphatase;
N	Q1C475	DE	Multifunctional protein surE;
N	Q1C475	DE	Nucleoside monophosphate phosphohydrolase;
N	Q1C475	DR	3'-nucleotidase activity
N	Q1C475	DR	5'-nucleotidase activity
N	Q1C475	DR	exopolyphosphatase activity
N	Q1C475	DR	metal ion binding
N	Q1C475	DR	nucleotide binding
N	Q1C479	CC	Required for the cell division process (By similarity).
N	Q1C479	DE	Cell division protein ftsB homolog;
N	Q1C479	DR	cell division
N	Q1C5J6	CC	Catalyzes the synthesis of GMP from XMP (By similarity).
N	Q1C5J6	DE	GMP synthase [glutamine-hydrolyzing];
N	Q1C5J6	DE	GMP synthetase;
N	Q1C5J6	DE	Glutamine amidotransferase;
N	Q1C5J6	DR	GMP biosynthetic process
N	Q1C5J6	DR	GMP synthase (glutamine-hydrolyzing) activity
N	Q1C5J6	DR	glutamine metabolic process
N	Q1C7J3	CC	Catalyzes the conversion of L-arabinose to L-ribulose (By similarity).
N	Q1C7J3	DE	L-arabinose isomerase;
N	Q1C7J3	DR	L-arabinose isomerase activity
N	Q1C7J3	DR	L-fucose isomerase activity
N	Q1C7J3	DR	arabinose catabolic process
N	Q1C7J3	DR	fucose metabolic process
N	Q1C7J3	DR	metal ion binding

N	Q1C8A7	CC	Transforms N(2)-succinylglutamate into succinate and glutamate.
N	Q1C8A7	DE	Succinylglutamate desuccinylase;
N	Q1C8A7	DR	arginine catabolic process to glutamate
N	Q1C8A7	DR	hydrolase activity, acting on ester bonds
N	Q1C8A7	DR	succinylglutamate desuccinylase activity
N	Q1C8A7	DR	zinc ion binding
N	Q1C9V1	CC	Part of the ABC transporter complex MglABC involved in galactose/methyl galactoside import
N	Q1C9V1	CC	Responsible for energy coupling to the transport system (By similarity).
N	Q1C9V1	DE	Galactose/methyl galactoside import ATP-binding protein MglA;
N	Q1C9V1	DR	monosaccharide-transporting ATPase activity
N	Q1CEC9	CC	Involved in resistance toward heavy metals (By similarity).
N	Q1CEC9	DE	Divalent-cation tolerance protein CutA;
N	Q1CEC9	DR	metal ion binding
N	Q1CEC9	DR	response to metal ion
N	Q1CFE4	CC	Modifies, by uridylylation or deuridylylation the PII (glnB) regulatory protein (By similarity).
N	Q1CFE4	DE	PII uridylyl-transferase;
N	Q1CFE4	DE	Uridylyl-removing enzyme;
N	Q1CFE4	DE	[Protein-PII] uridylyltransferase;
N	Q1CFE4	DR	[protein-PII] uridylyltransferase activity
N	Q1CFE4	DR	amino acid binding
N	Q1CFE4	DR	nitrogen compound metabolic process
N	Q1CGL5	DE	UPF0319 protein YPN_2537;
N	Q1CHR3	CC	NDH-1 shuttles electrons from NADH, via FMN and iron- sulfur (Fe-S) centers, to quinones in the respiratory chain
N	Q1CHR3	CC	Couples the redox reaction to proton translocation (for every two electrons transferred, four hydrogen ions are translocated across the cytoplasmic membrane), and thus conserves the redox energy in a proton gradient (By similarity)
N	Q1CHR3	CC	The immediate electron acceptor for the enzyme in this species is believed to be ubiquinone
N	Q1CHR3	DE	NADH dehydrogenase I subunit N;
N	Q1CHR3	DE	NADH-quinone oxidoreductase subunit N;
N	Q1CHR3	DE	NDH-1 subunit N;
N	Q1CHR3	DR	ATP synthesis coupled electron transport
N	Q1CHR3	DR	NADH dehydrogenase (ubiquinone) activity
N	Q1CHR3	DR	quinone binding
N	Q1CN19	CC	Could be involved in the degradation of phospho-AI-2 (By similarity).
N	Q1CN19	DE	Uncharacterized aldolase lsrF;
N	Q1CN19	DR	lyase activity
N	Q1CN78	CC	DNA-dependent RNA polymerase catalyzes the transcription of DNA into RNA using the four ribonucleoside triphosphates as substrates.
N	Q1CN78	DE	DNA-directed RNA polymerase subunit beta;
N	Q1CN78	DE	RNA polymerase subunit beta;
N	Q1CN78	DE	RNAP subunit beta;
N	Q1CN78	DE	Transcriptase subunit beta;
N	Q1CN78	DR	DNA-directed RNA polymerase activity
N	Q1CN78	DR	ribonucleoside binding
N	Q1CZH6	DE	Anthranilate phosphoribosyltransferase;
N	Q1CZH6	DR	anthranilate phosphoribosyltransferase activity
N	Q1CZH6	DR	tryptophan biosynthetic process
N	Q1D375	CC	Involved in rRNA and/or ribosome maturation and assembly
N	Q1D375	CC	May have metal-dependent hydrolase activity (By similarity).
N	Q1D375	CC	Required for normal 5' and 3' processing of 16S, 23S and 5S rRNAs
N	Q1D375	DE	Probable rRNA maturation factor;
N	Q1D375	DR	metal ion binding
N	Q1D375	DR	metalloendopeptidase activity
N	Q1D375	DR	rRNA processing
N	Q1DI07	CC	ATP-binding RNA helicase involved in transcription elongation and required for the export of mRNA out of the nucleus
N	Q1DI07	CC	May be involved in rDNA and telomeric silencing, and maintenance of genome integrity (By similarity).
N	Q1DI07	CC	SUB2 plays also a role in pre-mRNA splicing and spliceosome assembly
N	Q1DI07	DE	ATP-dependent RNA helicase SUB2;
N	Q1DI07	DR	ATP-dependent helicase activity

N	Q1DI07	DR	mRNA processing
N	Q1DI07	DR	mRNA transport
N	Q1DTG2	CC	Variant histone H2A which can replace H2A in some nucleosomes
N	Q1DTG2	CC	DNA accessibility is regulated via a complex set of post-translational modifications of histones, also called histone code, and nucleosome remodeling
N	Q1DTG2	CC	Histones thereby play a central role in transcription regulation, DNA repair, DNA replication and chromosomal stability
N	Q1DTG2	CC	Involved in chromosome stability (By similarity).
N	Q1DTG2	CC	Near telomeres, it may counteract gene silencing caused by the spread of heterochromatin proteins
N	Q1DTG2	CC	Nucleosomes wrap and compact DNA into chromatin, limiting DNA accessibility to the cellular machineries which require DNA as a template
N	Q1DTG2	CC	Required for the RNA polymerase II and SPT15/TBP recruitment to the target genes
N	Q1DTG2	CC	This variant is enriched at promoters, it may keep them in a repressed state until the appropriate activation signal is received
N	Q1DTG2	DE	Histone H2A.Z;
N	Q1DTG2	DR	nucleosome assembly
N	Q1G948	DE	UPF0297 protein Ldb1606;
N	Q1G9L4	CC	Specifically methylates guanosine-37 in various tRNAs (By similarity).
N	Q1G9L4	DE	M1G-methyltransferase;
N	Q1G9L4	DE	tRNA (guanine-N(1)-)-methyltransferase;
N	Q1G9L4	DE	tRNA [GM37] methyltransferase;
N	Q1G9L4	DR	tRNA (guanine-N1-)-methyltransferase activity
N	Q1G9L7	CC	This protein is located at the 30S-50S ribosomal subunit interface and may play a role in the structure and function of the aminoacyl-tRNA binding site (By similarity).
N	Q1G9L7	DE	50S ribosomal protein L19;
N	Q1G9L7	DR	structural constituent of ribosome
N	Q1GAQ4	CC	Binds directly to 16S ribosomal RNA (By similarity).
N	Q1GAQ4	DE	30S ribosomal protein S20;
N	Q1GAQ4	DR	structural constituent of ribosome
N	Q1GBK5	CC	Binds 16S rRNA, required for the assembly of 30S particles and may also be responsible for determining the conformation of the 16S rRNA at the A site (By similarity).
N	Q1GBK5	DE	30S ribosomal protein S14 type Z;
N	Q1GBK5	DR	metal ion binding
N	Q1GBK5	DR	structural constituent of ribosome
N	Q1GHL5	DE	FGAM synthase II;
N	Q1GHL5	DE	Phosphoribosylformylglycinamidine synthase 2;
N	Q1GHL5	DE	Phosphoribosylformylglycinamidine synthase II;
N	Q1GHL5	DR	'de novo' IMP biosynthetic process
N	Q1GHL5	DR	phosphoribosylformylglycinamidine synthase activity
N	Q1GIV5	CC	Required for accurate and efficient protein synthesis under certain stress conditions
N	Q1GIV5	CC	Back- translocation proceeds from a post-translocation (POST) complex to a pre-translocation (PRE) complex, thus giving elongation factor G a second chance to translocate the tRNAs correctly
N	Q1GIV5	CC	Binds to ribosomes in a GTP-dependent manner (By similarity).
N	Q1GIV5	CC	May act as a fidelity factor of the translation reaction, by catalyzing a one-codon backward translocation of tRNAs on improperly translocated ribosomes
N	Q1GIV5	DE	Elongation factor 4;
N	Q1GIV5	DE	Ribosomal back-translocase LepA;
N	Q1GIV5	DR	GTPase activity
N	Q1GPA3	CC	Protein S19 forms a complex with S13 that binds strongly to the 16S ribosomal RNA (By similarity).
N	Q1GPA3	DE	30S ribosomal protein S19;
N	Q1GPA3	DR	structural constituent of ribosome
N	Q1GPB4	CC	This protein binds to the 23S rRNA, and is important in its secondary structure
N	Q1GPB4	CC	It is located near the subunit interface in the base of the L7/L12 stalk, and near the tRNA binding site of the peptidyltransferase center (By similarity).
N	Q1GPB4	DE	50S ribosomal protein L6;
N	Q1GPB4	DR	structural constituent of ribosome
N	Q1GRR0	CC	Involved in saturated fatty acids biosynthesis.
N	Q1GRR0	DE	(3R)-hydroxymyristoyl ACP dehydrase;
N	Q1GRR0	DE	(3R)-hydroxymyristoyl-[acyl-carrier-protein] dehydratase;
N	Q1GRR0	DR	fatty acid biosynthetic process
N	Q1GRR0	DR	hydro-lyase activity
N	Q1GRR0	DR	lipid A biosynthetic process

N	Q1GSP2	CC	This is one of the proteins that binds to the 5S RNA in the ribosome where it forms part of the central protuberance (By similarity).
N	Q1GSP2	DE	50S ribosomal protein L25;
N	Q1GSP2	DE	General stress protein CTC;
N	Q1GSP2	DR	5S rRNA binding
N	Q1GSP2	DR	structural constituent of ribosome
N	Q1H042	CC	Involved in cell division; probably involved in intracellular septation (By similarity)
N	Q1H042	DE	Probable intracellular septation protein;
N	Q1H042	DR	barrier septum formation
N	Q1H1K6	DE	UPF0059 membrane protein Mfla_1363;
N	Q1H1T1	DE	50S ribosomal protein L36 2;
N	Q1H1T1	DR	structural constituent of ribosome
N	Q1H4X6	DE	Protein smg homolog;
N	Q1HK80	CC	Core subunit of the mitochondrial membrane respiratory chain NADH dehydrogenase (Complex I) that is believed to belong to the minimal assembly required for catalysis
N	Q1HK80	CC	Complex I functions in the transfer of electrons from NADH to the respiratory chain
N	Q1HK80	CC	The immediate electron acceptor for the enzyme is believed to be ubiquinone (By similarity).
N	Q1HK80	DE	NADH dehydrogenase subunit 5;
N	Q1HK80	DE	NADH-ubiquinone oxidoreductase chain 5;
N	Q1HK80	DR	ATP synthesis coupled electron transport
N	Q1HK80	DR	NADH dehydrogenase (ubiquinone) activity
N	Q1I2I2	CC	F(1)F(0) ATP synthase produces ATP from ADP in the presence of a proton or sodium gradient
N	Q1I2I2	CC	During catalysis, ATP synthesis in the catalytic domain of F(1) is coupled via a rotary mechanism of the central stalk subunits to proton translocation (By similarity)
N	Q1I2I2	CC	F-type ATPases consist of two structural domains, F(1) containing the extramembraneous catalytic core and F(0) containing the membrane proton channel, linked together by a central stalk and a peripheral stalk
N	Q1I2I2	DE	ATP synthase F(0) sector subunit c;
N	Q1I2I2	DE	ATP synthase subunit c;
N	Q1I2I2	DE	F-ATPase subunit c;
N	Q1I2I2	DE	F-type ATPase subunit c;
N	Q1I2I2	DE	Lipid-binding protein;
N	Q1I2I2	DR	ATP hydrolysis coupled proton transport
N	Q1I2I2	DR	ATP synthesis coupled proton transport
N	Q1I2I2	DR	hydrogen ion transmembrane transporter activity
N	Q1I2I2	DR	lipid binding
N	Q1I2I4	CC	F(1)F(0) ATP synthase produces ATP from ADP in the presence of a proton or sodium gradient
N	Q1I2I4	CC	During catalysis, ATP synthesis in the catalytic domain of F(1) is coupled via a rotary mechanism of the central stalk subunits to proton translocation (By similarity)
N	Q1I2I4	CC	F-type ATPases consist of two structural domains, F(1) containing the extramembraneous catalytic core and F(0) containing the membrane proton channel, linked together by a central stalk and a peripheral stalk
N	Q1I2I4	DE	ATP synthase F(1) sector subunit delta;
N	Q1I2I4	DE	ATP synthase subunit delta;
N	Q1I2I4	DE	F-ATPase subunit delta;
N	Q1I2I4	DE	F-type ATPase subunit delta;
N	Q1I2I4	DR	ATP synthesis coupled proton transport
N	Q1I2I4	DR	hydrogen ion transporting ATP synthase activity, rotational mechanism
N	Q1I7P1	CC	One of the components of the high-affinity ATP-driven potassium transport (or KDP) system, which catalyzes the hydrolysis of ATP coupled with the exchange of hydrogen and potassium ions
N	Q1I7P1	CC	The C subunit may be involved in assembly of the KDP complex (By similarity).
N	Q1I7P1	DE	ATP phosphohydrolase [potassium-transporting] C chain;
N	Q1I7P1	DE	Potassium-binding and translocating subunit C;
N	Q1I7P1	DE	Potassium-translocating ATPase C chain;
N	Q1I7P1	DE	Potassium-transporting ATPase C chain;
N	Q1I7P1	DR	potassium-transporting ATPase activity
N	Q1I7Z8	CC	NDH-1 shuttles electrons from NADH, via FMN and iron- sulfur (Fe-S) centers, to quinones in the respiratory chain
N	Q1I7Z8	CC	Couples the redox reaction to proton translocation (for every two electrons transferred, four hydrogen ions are translocated across the cytoplasmic membrane), and thus conserves the redox energy in a proton gradient (By similarity)

N	Q1I7Z8	CC	The immediate electron acceptor for the enzyme in this species is believed to be ubiquinone
N	Q1I7Z8	DE	NADH dehydrogenase I subunit C/D;
N	Q1I7Z8	DE	NADH-quinone oxidoreductase subunit C/D;
N	Q1I7Z8	DE	NDH-1 subunit C/D;
N	Q1I7Z8	DR	NADH dehydrogenase (ubiquinone) activity
N	Q1I7Z8	DR	oxidation-reduction process
N	Q1I7Z8	DR	quinone binding
N	Q1IC11	DE	Phenylalanine--tRNA ligase alpha chain;
N	Q1IC11	DE	Phenylalanyl-tRNA synthetase alpha chain;
N	Q1IC11	DR	metal ion binding
N	Q1IC11	DR	phenylalanine-tRNA ligase activity
N	Q1IC11	DR	phenylalanyl-tRNA aminoacylation
N	Q1IDA4	CC	Catalyzes the interconversion of methylthioribose-1- phosphate (MTR-1-P) into methylthioribulose-1-phosphate (MTRu-1-P) (By similarity).
N	Q1IDA4	DE	MTR-1-P isomerase;
N	Q1IDA4	DE	Methylthioribose-1-phosphate isomerase;
N	Q1IDA4	DE	S-methyl-5-thioribose-1-phosphate isomerase;
N	Q1IDA4	DR	S-methyl-5-thioribose-1-phosphate isomerase activity
N	Q1IDA4	DR	methionine biosynthetic process
N	Q1IHW2	CC	The natural substrate for this enzyme may be peptidyl- tRNAs which drop off the ribosome during protein synthesis (By similarity).
N	Q1IHW2	DE	Peptidyl-tRNA hydrolase;
N	Q1IHW2	DR	aminoacyl-tRNA hydrolase activity
N	Q1IL91	CC	NDH-1 shuttles electrons from NADH, via FMN and iron- sulfur (Fe-S) centers, to quinones in the respiratory chain
N	Q1IL91	CC	Couples the redox reaction to proton translocation (for every two electrons transferred, four hydrogen ions are translocated across the cytoplasmic membrane), and thus conserves the redox energy in a proton gradient (By similarity)
N	Q1IL91	CC	The immediate electron acceptor for the enzyme in this species is believed to be ubiquinone
N	Q1IL91	DE	NADH dehydrogenase I subunit B 2;
N	Q1IL91	DE	NADH-quinone oxidoreductase subunit B 2;
N	Q1IL91	DE	NDH-1 subunit B 2;
N	Q1IL91	DR	4 iron, 4 sulfur cluster binding
N	Q1IL91	DR	NADH dehydrogenase (ubiquinone) activity
N	Q1IL91	DR	metal ion binding
N	Q1IL91	DR	oxidation-reduction process
N	Q1IL91	DR	quinone binding
N	Q1J7K5	DE	Asparagine--tRNA ligase;
N	Q1J7K5	DE	Asparaginyl-tRNA synthetase;
N	Q1J7K5	DR	asparagine-tRNA ligase activity
N	Q1J7K5	DR	asparaginyl-tRNA aminoacylation
N	Q1J7K5	DR	aspartate-tRNA ligase activity
N	Q1J7K5	DR	aspartyl-tRNA aminoacylation
N	Q1J7K5	DR	nucleic acid binding
N	Q1J7Q5	CC	Transfers the N-acyl diglyceride group on what will become the N-terminal cysteine of membrane lipoproteins (By similarity).
N	Q1J7Q5	DE	Prolipoprotein diacylglyceryl transferase;
N	Q1J7Q5	DR	lipoprotein biosynthetic process
N	Q1J7Q5	DR	protein lipoylation
N	Q1J7Q5	DR	transferase activity, transferring glycosyl groups
N	Q1JAI0	CC	Endonuclease that resolves Holliday junction intermediates in genetic recombination
N	Q1JAI0	CC	Cleaves mobile four-strand junctions by introducing symmetrical nicks in paired strands
N	Q1JAI0	CC	Promotes annealing of linear ssDNA with homologous dsDNA
N	Q1JAI0	CC	Required for DNA repair, homologous recombination and chromosome segregation (By similarity).
N	Q1JAI0	DE	Holliday junction resolvase recU;
N	Q1JAI0	DE	Recombination protein U homolog;
N	Q1JAI0	DR	DNA recombination
N	Q1JAI0	DR	endonuclease activity
N	Q1JAI0	DR	metal ion binding
N	Q1JAR2	DE	Arginine deiminase;
N	Q1JAR2	DE	Arginine dihydrolase;
N	Q1JAR2	DR	arginine catabolic process

N	Q1JAR2	DR	arginine deiminase activity
N	Q1JFE2	CC	Component of the acetyl coenzyme A carboxylase (ACC) complex
N	Q1JFE2	CC	Biotin carboxylase (BC) catalyzes the carboxylation of biotin on its carrier protein (BCCP) and then the CO(2) group is transferred by the transcarboxylase to acetyl-CoA to form malonyl- CoA (By similarity).
N	Q1JFE2	DE	ACCase subunit beta;
N	Q1JFE2	DE	Acetyl-CoA carboxylase carboxyltransferase subunit beta;
N	Q1JFE2	DE	Acetyl-coenzyme A carboxylase carboxyl transferase subunit beta;
N	Q1JFE2	DR	acetyl-CoA carboxylase activity
N	Q1JFE2	DR	fatty acid biosynthetic process
N	Q1JFE2	DR	metal ion binding
N	Q1JH90	CC	Catalyzes the transfer of a dimethylallyl group onto the adenine at position 37 in tRNAs that read codons beginning with uridine, leading to the formation of N6-(dimethylallyl)adenosine (i(6)A) (By similarity).
N	Q1JH90	DE	DMAPP:tRNA dimethylallyltransferase;
N	Q1JH90	DE	Dimethylallyl diphosphate:tRNA dimethylallyltransferase;
N	Q1JH90	DE	IPP transferase;
N	Q1JH90	DE	Isopentenyl-diphosphate:tRNA isopentenyltransferase;
N	Q1JH90	DE	tRNA dimethylallyltransferase;
N	Q1JH90	DR	tRNA dimethylallyltransferase activity
N	Q1JH90	DR	tRNA processing
N	Q1JNZ9	DE	50S ribosomal protein L30;
N	Q1JNZ9	DR	structural constituent of ribosome
N	Q1JP18	CC	Involved in the binding of tRNA to the ribosomes (By similarity).
N	Q1JP18	DE	30S ribosomal protein S10;
N	Q1JP18	DR	structural constituent of ribosome
N	Q1KVT9	CC	Binds 16S rRNA, required for the assembly of 30S particles (By similarity).
N	Q1KVT9	DE	30S ribosomal protein S14, chloroplastic;
N	Q1KVT9	DR	structural constituent of ribosome
N	Q1KXQ4	CC	Apoprotein for the two 4Fe-4S centers FA and FB of photosystem I (PSI); essential for photochemical activity
N	Q1KXQ4	CC	FB is the terminal electron acceptor of PSI, donating electrons to ferredoxin
N	Q1KXQ4	CC	PSI is a plastocyanin-ferredoxin oxidoreductase, converting photonic excitation into a charge separation, which transfers an electron from the donor P700 chlorophyll pair to the spectroscopically characterized acceptors A0, A1, FX, FA and FB in turn
N	Q1KXQ4	CC	Required for binding of psaD and psaE to PSI
N	Q1KXQ4	CC	The C-terminus interacts with psaA/B/D and helps assemble the protein into the PSI complex
N	Q1KXQ4	DE	9 kDa polypeptide;
N	Q1KXQ4	DE	Photosystem I iron-sulfur center;
N	Q1KXQ4	DE	Photosystem I subunit VII;
N	Q1KXQ4	DR	4 iron, 4 sulfur cluster binding
N	Q1KXQ4	DR	electron carrier activity
N	Q1KXQ4	DR	metal ion binding
N	Q1KXQ4	DR	photosynthetic electron transport in photosystem I
N	Q1L8D2	DE	Glycoprotein endo-alpha-1,2-mannosidase-like protein;
N	Q1L8D2	DR	hydrolase activity
N	Q1L8F9	DE	UPF0536 protein C12orf66 homolog;
N	Q1LKM2	CC	Carrier of the growing fatty acid chain in fatty acid biosynthesis (By similarity).
N	Q1LKM2	DE	Acyl carrier protein;
N	Q1LKM2	DR	acyl carrier activity
N	Q1LKM2	DR	cofactor binding
N	Q1LKM2	DR	fatty acid biosynthetic process
N	Q1LKM2	DR	phosphopantetheine binding
N	Q1LP27	DE	Dihydroorotate dehydrogenase;
N	Q1LP27	DE	Dihydroorotate oxidase;
N	Q1LP27	DR	'de novo' pyrimidine base biosynthetic process
N	Q1LP27	DR	UMP biosynthetic process
N	Q1LP27	DR	dihydroorotate oxidase activity
N	Q1LP27	DR	oxidation-reduction process
N	Q1LPG5	CC	This protein is involved in the repair of mismatches in DNA
N	Q1LPG5	CC	It is possible that it carries out the mismatch recognition step
N	Q1LPG5	CC	This protein has a weak ATPase activity (By similarity).
N	Q1LPG5	DE	DNA mismatch repair protein mutS;
N	Q1LPG5	DR	mismatch repair

N	Q1LPG5	DR	mismatched DNA binding
N	Q1LQ15	DE	D-fructose-1,6-bisphosphate 1-phosphohydrolase class 1 1;
N	Q1LQ15	DE	FBPase class 1 1;
N	Q1LQ15	DE	Fructose-1,6-bisphosphatase class 1 1;
N	Q1LQ15	DR	carbohydrate metabolic process
N	Q1LQ15	DR	fructose 1,6-bisphosphate 1-phosphatase activity
N	Q1LQ15	DR	metal ion binding
N	Q1LSJ9	CC	An essential GTPase which binds GTP, GDP and possibly (p)ppGpp with moderate affinity, with high nucleotide exchange rates and a fairly low GTP hydrolysis rate (By similarity)
N	Q1LSJ9	CC	It may play a role in control of the cell cycle, stress response, ribosome biogenesis and in those bacteria that undergo differentiation, in morphogenesis control (Potential)
N	Q1LSJ9	DE	GTP-binding protein obg;
N	Q1LSJ9	DR	GTPase activity
N	Q1LSJ9	DR	magnesium ion binding
N	Q1LT98	CC	NDH-1 shuttles electrons from NADH, via FMN and iron- sulfur (Fe-S) centers, to quinones in the respiratory chain
N	Q1LT98	CC	Couples the redox reaction to proton translocation (for every two electrons transferred, four hydrogen ions are translocated across the cytoplasmic membrane), and thus conserves the redox energy in a proton gradient (By similarity)
N	Q1LT98	CC	The immediate electron acceptor for the enzyme in this species is believed to be ubiquinone
N	Q1LT98	DE	NADH dehydrogenase I subunit K;
N	Q1LT98	DE	NADH-quinone oxidoreductase subunit K;
N	Q1LT98	DE	NDH-1 subunit K;
N	Q1LT98	DR	ATP synthesis coupled electron transport
N	Q1LT98	DR	NADH dehydrogenase (quinone) activity
N	Q1LT98	DR	quinone binding
N	Q1LTI6	CC	Transfers the 4'-phosphopantetheine moiety from coenzyme A to a Ser of acyl-carrier-protein (By similarity).
N	Q1LTI6	DE	4'-phosphopantetheinyl transferase AcpS;
N	Q1LTI6	DE	Holo-ACP synthase;
N	Q1LTI6	DE	Holo-[acyl-carrier-protein] synthase;
N	Q1LTI6	DR	fatty acid biosynthetic process
N	Q1LTI6	DR	holo-[acyl-carrier-protein] synthase activity
N	Q1LTI6	DR	macromolecule biosynthetic process
N	Q1LTI6	DR	magnesium ion binding
N	Q1LTK8	DE	UPF0434 protein BCI_0256;
N	Q1LTL2	CC	Catalyzes the reversible conversion of 3- phosphohydroxypyruvate to phosphoserine and of 3-hydroxy-2-oxo-4- phosphonooxybutanoate to phosphohydroxythreonine (By similarity).
N	Q1LTL2	DE	Phosphohydroxythreonine aminotransferase;
N	Q1LTL2	DE	Phosphoserine aminotransferase;
N	Q1LTL2	DR	L-serine biosynthetic process
N	Q1LTL2	DR	O-phospho-L-serine:2-oxoglutarate aminotransferase activity
N	Q1LTL2	DR	pyridoxal phosphate binding
N	Q1LTL2	DR	pyridoxine biosynthetic process
N	Q1LUD1	DE	Transmembrane protein 168-A;
N	Q1LWG3	CC	Component of the large subunit of the mitochondrial ribosome (Potential).
N	Q1LWG3	DE	39S ribosomal protein L36, mitochondrial;
N	Q1LWG3	DR	structural constituent of ribosome
N	Q1MAK5	DE	30S ribosomal protein S16;
N	Q1MAK5	DR	structural constituent of ribosome
N	Q1MCN6	CC	Part of the ABC transporter complex UgpABCE involved in sn-glycerol-3-phosphate import
N	Q1MCN6	CC	Responsible for energy coupling to the transport system (By similarity).
N	Q1MCN6	DE	sn-glycerol-3-phosphate import ATP-binding protein UgpC 1;
N	Q1MCN6	DR	glycerol-3-phosphate-transporting ATPase activity
N	Q1MH54	DE	30S ribosomal protein S2;
N	Q1MH54	DR	structural constituent of ribosome
N	Q1MPZ8	CC	Catalyzes the oxidation of 5,10- methylenetetrahydrofolate to 5,10-methenyltetrahydrofolate and then the hydrolysis of 5,10-methenyltetrahydrofolate to 10- formyltetrahydrofolate (By similarity).
N	Q1MPZ8	DE	Bifunctional protein FolD;
N	Q1MPZ8	DE	Methenyltetrahydrofolate cyclohydrolase;

N	Q1MPZ8	DE	Methylenetetrahydrofolate dehydrogenase;
N	Q1MPZ8	DR	folic acid-containing compound biosynthetic process
N	Q1MPZ8	DR	histidine biosynthetic process
N	Q1MPZ8	DR	methenyltetrahydrofolate cyclohydrolase activity
N	Q1MPZ8	DR	methionine biosynthetic process
N	Q1MPZ8	DR	methylenetetrahydrofolate dehydrogenase (NADP+) activity
N	Q1MPZ8	DR	one-carbon metabolic process
N	Q1MPZ8	DR	oxidation-reduction process
N	Q1MPZ8	DR	purine nucleotide biosynthetic process
N	Q1MTQ7	CC	Probable plus-end-directed motor enzyme involved in cytoskeleton movements (By similarity).
N	Q1MTQ7	DE	Kinesin-like motor protein C15D4.01c;
N	Q1MTQ7	DR	ATPase activity
N	Q1MTQ7	DR	microtubule motor activity
N	Q1MTQ7	DR	microtubule sliding
N	Q1MTQ7	DR	mitotic anaphase B
N	Q1MTQ7	DR	protein binding
N	Q1MTQ7	DR	spindle elongation
N	Q1PEF6	CC	Involved in ubiquitination and subsequent proteasomal degradation of target proteins
N	Q1PEF6	CC	In the SCF complex, it serves as an adapter that links the F-box protein to CUL1 (By similarity).
N	Q1PEF6	CC	The functional specificity of this complex depends of the type of F- box protein
N	Q1PEF6	CC	Together with CUL1, RBX1 and a F- box protein, it forms a SCF E3 ubiquitin ligase complex
N	Q1PEF6	DE	SKP1-like protein 6;
N	Q1PEF6	DR	ubiquitin-dependent protein catabolic process
N	Q1PFI4	DE	F-box protein At1g60400;
N	Q1QL34	CC	Allows the formation of correctly charged Asn-tRNA(Asn) or Gln-tRNA(Gln) through the transamidation of misacylated Asp- tRNA(Asn) or Glu-tRNA(Gln) in organisms which lack either or both of asparaginyl-tRNA or glutaminyl-tRNA synthetases
N	Q1QL34	CC	The reaction takes place in the presence of glutamine and ATP through an activated phospho-Asp-tRNA(Asn) or phospho-Glu-tRNA(Gln) (By similarity).
N	Q1QL34	DE	Asp/Glu-ADT subunit B;
N	Q1QL34	DE	Aspartyl/glutamyl-tRNA(Asn/Gln) amidotransferase subunit B;
N	Q1QL34	DR	carbon-nitrogen ligase activity, with glutamine as amido-N-donor
N	Q1QN82	DE	Phosphatidylserine decarboxylase alpha chain;
N	Q1QN82	DE	Phosphatidylserine decarboxylase beta chain;
N	Q1QN82	DE	Phosphatidylserine decarboxylase proenzyme;
N	Q1QN82	DR	cofactor binding
N	Q1QN82	DR	phosphatidylethanolamine biosynthetic process
N	Q1QN82	DR	phosphatidylserine decarboxylase activity
N	Q1QR47	CC	Part of the ABC transporter complex CcmAB involved in the biogenesis of c-type cytochromes; once thought to export heme, this seems not to be the case, but its exact role is uncertain
N	Q1QR47	CC	Responsible for energy coupling to the transport system (By similarity).
N	Q1QR47	DE	Cytochrome c biogenesis ATP-binding export protein CcmA;
N	Q1QR47	DE	Heme exporter protein A;
N	Q1QR47	DR	cytochrome complex assembly
N	Q1QR47	DR	heme-transporting ATPase activity
N	Q1QSZ3	CC	One of the primary rRNA binding proteins, it binds directly to 16S rRNA where it helps nucleate assembly of the platform of the 30S subunit by binding and bridging several RNA helices of the 16S rRNA (By similarity).
N	Q1QSZ3	DE	30S ribosomal protein S15;
N	Q1QSZ3	DR	structural constituent of ribosome
N	Q1QX69	CC	Involved in lipid A export and possibly also in glycerophospholipid export and for biogenesis of the outer membrane
N	Q1QX69	CC	Transmembrane domains (TMD) form a pore in the inner membrane and the ATP-binding domain (NBD) is responsible for energy generation (By similarity).
N	Q1QX69	DE	Lipid A export ATP-binding/permease protein MsbA;
N	Q1QX69	DR	ATPase activity, coupled to transmembrane movement of substances
N	Q1QX69	DR	lipid transport
N	Q1R0G5	CC	Binds to 23S rRNA
N	Q1R0G5	CC	Forms part of two intersubunit bridges in the 70S ribosome (By similarity).
N	Q1R0G5	DE	50S ribosomal protein L14;
N	Q1R0G5	DR	structural constituent of ribosome

N	Q1R420	CC	Necessary for formate dehydrogenase activity (By similarity).
N	Q1R420	DE	Protein FdhD;
N	Q1R420	DR	formate dehydrogenase (NAD+) activity
N	Q1R7C6	CC	The glycine cleavage system catalyzes the degradation of glycine (By similarity).
N	Q1R7C6	DE	Aminomethyltransferase;
N	Q1R7C6	DE	Glycine cleavage system T protein;
N	Q1R7C6	DR	aminomethyltransferase activity
N	Q1R7C6	DR	glycine catabolic process
N	Q1R7C6	DR	transaminase activity
N	Q1R824	CC	Probably involved in ribonucleotide reductase function (By similarity).
N	Q1R824	DE	Protein nrdI;
N	Q1R8A9	CC	Catalyzes the phosphorylation of NAD to NADP
N	Q1R8A9	CC	Utilizes ATP and other nucleoside triphosphates as well as inorganic polyphosphate as a source of phosphorus (By similarity).
N	Q1R8A9	DE	Poly(P)/ATP NAD kinase;
N	Q1R8A9	DE	Probable inorganic polyphosphate/ATP-NAD kinase;
N	Q1R8A9	DR	NAD+ kinase activity
N	Q1R984	CC	Involved in the removal of murein from the sacculus
N	Q1R984	CC	May also facilitate integration of nascent murein strands into the sacculus by cleaving the peptide bonds between neighboring strands in mature murein (By similarity).
N	Q1R984	DE	D-alanyl-D-alanine-endopeptidase;
N	Q1R984	DE	DD-endopeptidase;
N	Q1R984	DE	Penicillin-insensitive murein endopeptidase;
N	Q1R984	DR	metal ion binding
N	Q1R984	DR	metallopeptidase activity
N	Q1R984	DR	serine-type endopeptidase activity
N	Q1RBN8	DE	UPF0056 inner membrane protein marC;
N	Q1REK9	CC	Involved in base excision repair of DNA damaged by oxidation or by mutagenic agents
N	Q1REK9	CC	Acts as DNA glycosylase that recognizes and removes damaged bases
N	Q1REK9	CC	Cleaves the DNA backbone by beta-delta elimination to generate a single-strand break at the site of the removed base with both 3'- and 5'- phosphates (By similarity).
N	Q1REK9	CC	Has AP (apurinic/apyrimidinic) lyase activity and introduces nicks in the DNA strand
N	Q1REK9	CC	Has a preference for oxidized pyrimidines, such as thymine glycol, 5,6-dihydrouracil and 5,6-dihydrothymine
N	Q1REK9	DE	DNA glycosylase/AP lyase Nei;
N	Q1REK9	DE	DNA-(apurinic or apyrimidinic site) lyase Nei;
N	Q1REK9	DE	Endonuclease 8;
N	Q1REK9	DE	Endonuclease VIII;
N	Q1REK9	DR	DNA-(apurinic or apyrimidinic site) lyase activity
N	Q1REK9	DR	base-excision repair
N	Q1REK9	DR	damaged DNA binding
N	Q1REK9	DR	hydrolase activity, hydrolyzing N-glycosyl compounds
N	Q1REK9	DR	nucleotide-excision repair
N	Q1REK9	DR	zinc ion binding
N	Q1RG44	CC	Probable regulatory factor involved in maltose metabolism (By similarity).
N	Q1RG44	DE	Sugar fermentation stimulation protein A;
N	Q1RHG2	DE	UPF0416 protein RBE_1121;
N	Q1RJD8	DE	Thioredoxin reductase;
N	Q1RJD8	DR	flavin adenine dinucleotide binding
N	Q1RJD8	DR	oxidation-reduction process
N	Q1RJD8	DR	removal of superoxide radicals
N	Q1RJD8	DR	thioredoxin-disulfide reductase activity
N	Q1RMH4	CC	General Rab protein regulator required for vesicle formation from the Golgi complex
N	Q1RMH4	CC	In addition it inhibits the removal of Rab GTPases from the membrane by GDI1 (By similarity).
N	Q1RMH4	CC	May control vesicle docking and fusion by mediating the action of Rab GTPases to the SNARE complexes
N	Q1RMH4	DE	PRA1 family protein 1;
N	Q1RMH4	DE	Prenylated Rab acceptor protein 1;
N	Q1WRR6	CC	Catalyzes the NADPH dependent reduction of L-gamma- glutamyl 5-phosphate into L-glutamate 5-semialdehyde and phosphate
N	Q1WRR6	CC	The product spontaneously undergoes cyclization to form 1-pyrroline-5-carboxylate
N	Q1WRR6	DE	GSA dehydrogenase;
N	Q1WRR6	DE	Gamma-glutamyl phosphate reductase;
N	Q1WRR6	DE	Glutamate-5-semialdehyde dehydrogenase;

N	Q1WRR6	DE	Glutamyl-gamma-semialdehyde dehydrogenase;
N	Q1WRR6	DR	glutamate-5-semialdehyde dehydrogenase activity
N	Q1WRR6	DR	oxidation-reduction process
N	Q1WRR6	DR	proline biosynthetic process
N	Q1X711	CC	Promotes the survival of neuronal populations that are all located either in the central nervous system or directly connected to it (By similarity).
N	Q1X711	DE	Brain-derived neurotrophic factor;
N	Q1X711	DE	DE Flags: Precursor; Fragment;
N	Q1X711	DR	growth factor activity
N	Q1XDE8	DE	50S ribosomal protein L12, chloroplastic;
N	Q1XDE8	DR	structural constituent of ribosome
N	Q1XDN4	CC	Binds directly to 16S ribosomal RNA (By similarity).
N	Q1XDN4	DE	30S ribosomal protein S20, chloroplastic;
N	Q1XDN4	DR	structural constituent of ribosome
N	Q1XFL1	DE	ZAR1-like protein;
N	Q1XFL1	DR	bile acid:sodium symporter activity
N	Q1ZXE6	CC	Component of the anaphase promoting complex/cyclosome (APC/C), a cell cycle-regulated E3 ubiquitin-protein ligase complex that controls progression through mitosis and the G1 phase of the cell cycle (By similarity).
N	Q1ZXE6	DE	Anaphase-promoting complex subunit 6;
N	Q1ZXE6	DE	Cell division cycle protein 16 homolog;
N	Q1ZXE6	DR	cell division
N	Q1ZXI3	DE	Putative uncharacterized protein DDB_G0278881;
N	Q20459	CC	Probable metalloprotease (By similarity).
N	Q20459	DE	Nematode astacin 25;
N	Q20459	DE	Zinc metalloproteinase nas-25;
N	Q20459	DR	metalloendopeptidase activity
N	Q20459	DR	zinc ion binding
N	Q20F03	DE	30S ribosomal protein S11, chloroplastic;
N	Q20F03	DR	structural constituent of ribosome
N	Q20FQ1	CC	Component of the ubiquinol-cytochrome c reductase complex (complex III or cytochrome b-c1 complex), which is a respiratory chain that generates an electrochemical potential coupled to ATP synthesis (By similarity).
N	Q20FQ1	DE	Complex III subunit 3;
N	Q20FQ1	DE	Complex III subunit III;
N	Q20FQ1	DE	Cytochrome b-c1 complex subunit 3;
N	Q20FQ1	DE	Cytochrome b;
N	Q20FQ1	DE	Ubiquinol-cytochrome-c reductase complex cytochrome b subunit;
N	Q20FQ1	DR	electron carrier activity
N	Q20FQ1	DR	metal ion binding
N	Q20FQ1	DR	oxidoreductase activity
N	Q20FQ1	DR	respiratory electron transport chain
N	Q20NV4	CC	Implicated in endonuclease cleavage of capped RNA primers
N	Q20NV4	CC	Dispensable for viral transcription, but not replication (By similarity).
N	Q20NV4	CC	Displays an elongation factor activity in viral RNA synthesis
N	Q20NV4	DE	Polymerase acidic protein;
N	Q20NV4	DE	RNA-directed RNA polymerase subunit P2;
N	Q20NV4	DR	RNA-directed RNA polymerase activity
N	Q210F5	DE	Urea amidohydrolase subunit gamma;
N	Q210F5	DE	Urease subunit gamma;
N	Q210F5	DR	nickel ion binding
N	Q210F5	DR	urea metabolic process
N	Q210F5	DR	urease activity
N	Q213W6	CC	Catalyzes the folate-dependent formation of 5-methyl- uridine at position 54 (M-5-U54) in all tRNAs (By similarity).
N	Q213W6	DE	Folate-dependent tRNA (uracil-5-)-methyltransferase;
N	Q213W6	DE	Folate-dependent tRNA(M-5-U54)-methyltransferase;
N	Q213W6	DE	Methylenetetrahydrofolate--tRNA-(uracil-5-)-methyltransferase TrmFO;
N	Q213W6	DR	flavin adenine dinucleotide binding
N	Q213W6	DR	methylenetetrahydrofolate-tRNA-(uracil-5-)-methyltransferase (FADH2-oxidizing) activity
N	Q213W6	DR	tRNA processing
N	Q21407	DE	Putative glycerophosphocholine phosphodiesterase GPCPD1 homolog K10B3.6;
N	Q21407	DR	carbohydrate binding
N	Q21407	DR	glycerol metabolic process

N	Q21407	DR	glycerophosphodiester phosphodiesterase activity
N	Q21407	DR	lipid metabolic process
N	Q214R5	DE	Putative 3-methyladenine DNA glycosylase;
N	Q214R5	DR	alkylbase DNA N-glycosylase activity
N	Q214R5	DR	base-excision repair
N	Q215B8	DE	Anthranilate phosphoribosyltransferase;
N	Q215B8	DR	anthranilate phosphoribosyltransferase activity
N	Q215B8	DR	tryptophan biosynthetic process
N	Q215E3	CC	Catalyzes the NADP-dependent rearrangement and reduction of 1-deoxy-D-xylulose-5-phosphate (DXP) to 2-C-methyl-D-erythritol 4-phosphate (MEP) (By similarity)
N	Q215E3	DE	1-deoxy-D-xylulose 5-phosphate reductoisomerase;
N	Q215E3	DE	1-deoxyxylulose-5-phosphate reductoisomerase;
N	Q215E3	DE	2-C-methyl-D-erythritol 4-phosphate synthase;
N	Q215E3	DE	DXP reductoisomerase;
N	Q215E3	DR	1-deoxy-D-xylulose-5-phosphate reductoisomerase activity
N	Q215E3	DR	isoprenoid biosynthetic process
N	Q215E3	DR	metal ion binding
N	Q215E3	DR	oxidation-reduction process
N	Q215E6	CC	Responsible for the release of ribosomes from messenger RNA at the termination of protein biosynthesis
N	Q215E6	CC	May increase the efficiency of translation by recycling ribosomes from one round of translation to another (By similarity).
N	Q215E6	DE	Ribosome-recycling factor;
N	Q215E6	DE	Ribosome-releasing factor;
N	Q21890	DE	Uncharacterized protein R102.4;
N	Q21890	DR	cellular amino acid metabolic process
N	Q21890	DR	lyase activity
N	Q21890	DR	pyridoxal phosphate binding
N	Q219R0	CC	Uses Mg-ATP and reduced ferredoxin to reduce ring D of protochlorophyllide (Pchlide) to form chlorophyllide a (Chlide) (By similarity)
N	Q219R0	CC	This reaction is light-independent.
N	Q219R0	DE	DPOR subunit B;
N	Q219R0	DE	LI-POR subunit B;
N	Q219R0	DE	Light-independent protochlorophyllide reductase subunit B;
N	Q219R0	DR	bacteriochlorophyll biosynthetic process
N	Q219R0	DR	oxidation-reduction process
N	Q219R0	DR	oxidoreductase activity, acting on iron-sulfur proteins as donors
N	Q219R0	DR	photosynthesis, dark reaction
N	Q21DY7	CC	Synthesis of 3-octaprenyl-4-hydroxybenzoate (By similarity).
N	Q21DY7	DE	4-HB polyprenyltransferase;
N	Q21DY7	DE	4-hydroxybenzoate octaprenyltransferase;
N	Q21DY7	DR	prenyltransferase activity
N	Q21DY7	DR	ubiquinone biosynthetic process
N	Q21FH2	CC	Involved in rRNA and/or ribosome maturation and assembly
N	Q21FH2	CC	May have metal-dependent hydrolase activity (By similarity).
N	Q21FH2	CC	Required for normal 5' and 3' processing of 16S, 23S and 5S rRNAs
N	Q21FH2	DE	Probable rRNA maturation factor;
N	Q21FH2	DR	metal ion binding
N	Q21FH2	DR	metalloendopeptidase activity
N	Q21FH2	DR	rRNA processing
N	Q21H92	CC	May play a role in 30S ribosomal subunit biogenesis
N	Q21H92	CC	Unusual circulary permuted GTPase that catalyzes rapid hydrolysis of GTP with a slow catalytic turnover (By similarity).
N	Q21H92	DE	Putative ribosome biogenesis GTPase RsgA;
N	Q21H92	DR	GTPase activity
N	Q21H92	DR	metal ion binding
N	Q21LV8	CC	Binds to the 23S rRNA (By similarity).
N	Q21LV8	DE	50S ribosomal protein L9;
N	Q21LV8	DR	structural constituent of ribosome
N	Q21M35	CC	Located on the platform of the 30S subunit, it bridges several disparate RNA helices of the 16S rRNA
N	Q21M35	CC	Forms part of the Shine-Dalgarno cleft in the 70S ribosome (By similarity).
N	Q21M35	DE	30S ribosomal protein S11;
N	Q21M35	DR	structural constituent of ribosome
N	Q21MA4	DE	Aspartate--tRNA ligase;

N	Q21MA4	DE	Aspartyl-tRNA synthetase;
N	Q21MA4	DR	aspartate-tRNA ligase activity
N	Q21MA4	DR	aspartyl-tRNA aminoacylation
N	Q21MA4	DR	nucleic acid binding
N	Q21MZ8	DE	Phosphoribosylaminoimidazole-succinocarboxamide synthase;
N	Q21MZ8	DE	SAICAR synthetase;
N	Q21MZ8	DR	phosphoribosylaminoimidazolesuccinocarboxamide synthase activity
N	Q21MZ8	DR	purine nucleotide biosynthetic process
N	Q21U91	CC	IGPS catalyzes the conversion of PRFAR and glutamine to IGP, AICAR and glutamate
N	Q21U91	CC	The hisF subunit catalyzes the cyclization activity that produces IGP and AICAR from PRFAR using the ammonia provided by the hisH subunit (By similarity).
N	Q21U91	DE	IGP synthase cyclase subunit;
N	Q21U91	DE	IGP synthase subunit hisF;
N	Q21U91	DE	IGPS subunit hisF;
N	Q21U91	DE	ImGP synthase subunit hisF;
N	Q21U91	DE	Imidazole glycerol phosphate synthase subunit hisF;
N	Q21U91	DR	histidine biosynthetic process
N	Q21U91	DR	imidazoleglycerol-phosphate synthase activity
N	Q21U91	DR	lyase activity
N	Q21X08	CC	Participates actively in the response to hyperosmotic and heat shock by preventing the aggregation of stress-denatured proteins, in association with dnaK and grpE
N	Q21X08	CC	GrpE releases ADP from dnaK; ATP binding to dnaK triggers the release of the substrate protein, thus completing the reaction cycle
N	Q21X08	CC	It is the nucleotide exchange factor for dnaK and may function as a thermosensor
N	Q21X08	CC	Several rounds of ATP- dependent interactions between dnaJ, dnaK and grpE are required for fully efficient folding (By similarity).
N	Q21X08	CC	Unfolded proteins bind initially to dnaJ; upon interaction with the dnaJ-bound protein, dnaK hydrolyzes its bound ATP, resulting in the formation of a stable complex
N	Q21X08	DE	HSP-70 cofactor;
N	Q21X08	DE	Protein grpE;
N	Q21X08	DR	adenyl-nucleotide exchange factor activity
N	Q21X08	DR	chaperone binding
N	Q21X08	DR	protein folding
N	Q21X08	DR	protein homodimerization activity
N	Q21X08	DR	response to stress
N	Q22000	DE	Probable 3',5'-cyclic phosphodiesterase pde-4;
N	Q22000	DR	3',5'-cyclic-nucleotide phosphodiesterase activity
N	Q22000	DR	metal ion binding
N	Q22000	DR	negative regulation of cAMP-mediated signaling
N	Q22000	DR	negative regulation of locomotion
N	Q22000	DR	signal transduction
N	Q23469	DE	UPF0392 protein ZK381.2;
N	Q23946	CC	Sequence-specific transcription factor which is part of a developmental regulatory system that provides cells with specific positional identities on the anterior-posterior axis
N	Q23946	CC	Binds the consensus region 5'-TTAAT[GT][GA]-3'
N	Q23946	CC	It activates the synthesis of the decapentaplegic (DPP) growth factor (By similarity)
N	Q23946	CC	This homeotic protein controls development of the cells in the posterior thoracic and first abdominal segments
N	Q23946	DE	Homeotic protein ultrabithorax;
N	Q23946	DR	multicellular organismal development
N	Q23946	DR	regulation of transcription, DNA-dependent
N	Q23946	DR	sequence-specific DNA binding transcription factor activity
N	Q23949	CC	Member of the regulatory pathway controlling female somatic sexual differentiation, regulated by Sxl
N	Q23949	CC	Activates dsx female-specific splicing by promoting the formation of a splicing enhancer complex which consists of tra, tra2 and sr proteins.
N	Q23949	DE	Female-specific protein transformer;
N	Q23949	DR	cell differentiation
N	Q23949	DR	female sex differentiation
N	Q23949	DR	mRNA processing
N	Q24118	DE	Protein linotte;
N	Q24118	DE	Protein pigeon;
N	Q24337	CC	Acts as an enhancer of the rudimentary gene
N	Q24337	CC	Has a role in pyrimidine biosynthesis and the cell cycle.
N	Q24337	DE	Protein enhancer of rudimentary;

N	Q24337	DR	protein binding
N	Q24337	DR	pyrimidine nucleotide biosynthetic process
N	Q24472	CC	Functions in cell cycle regulation
N	Q24472	CC	Component of the DREAM complex, a multiprotein complex that can both act as a transcription activator or repressor depending on the context
N	Q24472	CC	During development, the complex represses transcription of developmentally controlled E2F target genes.
N	Q24472	CC	In follicle cells, the complex plays a central role in the site- specific DNA replication at the chorion loci
N	Q24472	DE	Retinoblastoma family protein;
N	Q24472	DR	DNA endoreduplication
N	Q24472	DR	G1 phase of mitotic cell cycle
N	Q24472	DR	eggshell chorion gene amplification
N	Q24472	DR	endomitotic cell cycle
N	Q24472	DR	negative regulation of DNA replication
N	Q24472	DR	negative regulation of S phase of mitotic cell cycle
N	Q24472	DR	negative regulation of apoptosis
N	Q24472	DR	negative regulation of cell proliferation
N	Q24472	DR	negative regulation of transcription from RNA polymerase II promoter
N	Q24472	DR	promoter binding
N	Q24472	DR	regulation of DNA binding
N	Q24472	DR	transcription factor binding
N	Q24509	CC	Mediates endoplasmic reticulum to Golgi transport (By similarity).
N	Q24509	DE	Sed5 protein;
N	Q24509	DR	SNAP receptor activity
N	Q24509	DR	intracellular protein transport
N	Q24509	DR	neurotransmitter secretion
N	Q24509	DR	phagocytosis, engulfment
N	Q24509	DR	protein homodimerization activity
N	Q24817	DE	DE Flags: Precursor; Fragment;
N	Q24817	DE	Rho-related protein racD;
N	Q24817	DR	small GTPase mediated signal transduction
N	Q24JP1	DE	Protein alcS;
N	Q24PG6	DE	Sugar fermentation stimulation protein homolog;
N	Q24QI7	CC	Required for the first step of histidine biosynthesis
N	Q24QI7	CC	May allow the feedback regulation of ATP phosphoribosyltransferase activity by histidine (By similarity).
N	Q24QI7	DE	ATP phosphoribosyltransferase regulatory subunit;
N	Q24QI7	DR	histidine biosynthetic process
N	Q24QI7	DR	histidine-tRNA ligase activity
N	Q24QI7	DR	histidyl-tRNA aminoacylation
N	Q24UY9	CC	Involved in the transcription termination process (By similarity).
N	Q24UY9	DE	N utilization substance protein B homolog;
N	Q24UY9	DE	Protein nusB;
N	Q24UY9	DR	regulation of transcription, DNA-dependent
N	Q24UY9	DR	transcription termination factor activity
N	Q250M9	CC	One of the primary rRNA binding proteins
N	Q250M9	CC	It has been suggested to have peptidyltransferase activity; this is somewhat controversial
N	Q250M9	CC	Makes several contacts with the 16S rRNA in the 70S ribosome (By similarity).
N	Q250M9	CC	Required for association of the 30S and 50S subunits to form the 70S ribosome, for tRNA binding and peptide bond formation
N	Q250M9	DE	50S ribosomal protein L2;
N	Q250M9	DR	structural constituent of ribosome
N	Q250M9	DR	transferase activity
N	Q27562	CC	The proteasome is a multicatalytic proteinase complex which is characterized by its ability to cleave peptides with Arg, Phe, Tyr, Leu, and Glu adjacent to the leaving group at neutral or slightly basic pH
N	Q27562	CC	The proteasome has an ATP-dependent proteolytic activity.
N	Q27562	DE	Proteasome subunit C2;
N	Q27562	DE	Proteasome subunit alpha type-1;
N	Q27562	DR	threonine-type endopeptidase activity
N	Q27562	DR	ubiquitin-dependent protein catabolic process
N	Q27U48	CC	Tubulin is the major constituent of microtubules
N	Q27U48	CC	It binds two moles of GTP, one at an exchangeable site on the beta chain and one at a non-exchangeable site on the alpha-chain (By similarity).

N	Q27U48	DE	Beta-1-tubulin;
N	Q27U48	DE	Tubulin beta-1 chain;
N	Q27U48	DR	GTPase activity
N	Q27U48	DR	microtubule-based movement
N	Q27U48	DR	protein polymerization
N	Q27U48	DR	structural molecule activity
N	Q28066	CC	Controls the classical pathway of complement activation
N	Q28066	CC	It also accelerates the degradation of the C4bC2a complex (C3 convertase) by dissociating the complement fragment C2a
N	Q28066	CC	It also interacts with serum amyloid P component.
N	Q28066	CC	It binds as a cofactor to C3b/C4b inactivator (C3bINA), which then hydrolyzes the complement fragment C4b
N	Q28066	DE	C4b-binding protein beta chain;
N	Q28066	DR	complement activation, classical pathway
N	Q28066	DR	innate immune response
N	Q28224	CC	May mediate the control of various cellular processes by insulin
N	Q28224	CC	Activates phosphatidylinositol 3-kinase when bound to the regulatory p85 subunit (By similarity).
N	Q28224	CC	When phosphorylated by the insulin receptor binds specifically to various cellular proteins containing SH2 domains such as phosphatidylinositol 3-kinase p85 subunit or GRB2
N	Q28224	DE	Insulin receptor substrate 1;
N	Q28224	DR	SH2 domain binding
N	Q28224	DR	insulin receptor binding
N	Q28224	DR	insulin receptor signaling pathway
N	Q28224	DR	insulin-like growth factor receptor binding
N	Q28224	DR	insulin-like growth factor receptor signaling pathway
N	Q28224	DR	phosphatidylinositol 3-kinase binding
N	Q28224	DR	signal transducer activity
N	Q28566	CC	Participates in the regulation of gene transcription
N	Q28566	CC	Binds DNA in a non-specific manner, yet also specifically recognizes the core sequence 5'-CAC[GA]TG-3'
N	Q28566	CC	Seems to activate the transcription of growth-related genes (By similarity).
N	Q28566	DE	Myc proto-oncogene protein;
N	Q28566	DE	Proto-oncogene c-Myc;
N	Q28566	DE	Transcription factor p64;
N	Q28566	DR	DNA fragmentation involved in apoptotic nuclear change
N	Q28566	DR	activation of caspase activity
N	Q28566	DR	activation of pro-apoptotic gene products
N	Q28566	DR	induction of apoptosis by intracellular signals
N	Q28566	DR	negative regulation of survival gene product expression
N	Q28566	DR	positive regulation of cell proliferation
N	Q28566	DR	regulation of transcription, DNA-dependent
N	Q28566	DR	release of cytochrome c from mitochondria
N	Q28566	DR	response to radiation
N	Q28566	DR	sequence-specific DNA binding transcription factor activity
N	Q28566	DR	transcription activator activity
N	Q28806	CC	ICAM proteins are ligands for the leukocyte adhesion protein LFA-1 (integrin alpha-L/beta-2)
N	Q28806	CC	During leukocyte trans- endothelial migration, ICAM1 engagement promotes the assembly of endothelial apical cups through ARHGEF26/SGEF and RHOG activation (By similarity).
N	Q28806	DE	Intercellular adhesion molecule 1;
N	Q28896	CC	May play a role in carrying and orienting carbohydrate, as well as having a more specific role.
N	Q28896	DE	CAMPATH-1 antigen;
N	Q28896	DE	Epididymal secretory protein E5;
N	Q28FQ5	CC	DNA repair enzyme that can remove a variety of covalent adducts from DNA through hydrolysis of a 5'-phosphodiester bond, giving rise to DNA with a free 5' phosphate
N	Q28FQ5	CC	Catalyzes the hydrolysis of dead-end complexes between DNA and the topoisomerase 2 (top2) active site tyrosine residue
N	Q28FQ5	CC	Hydrolyzes 5'- phosphoglycolates on protruding 5' ends on DNA double-strand breaks (DSBs) due to DNA damage by radiation and free radicals (By similarity).
N	Q28FQ5	DE	5'-Tyr-DNA phosphodiesterase;
N	Q28FQ5	DE	5'-tyrosyl-DNA phosphodiesterase;
N	Q28FQ5	DE	TRAF and TNF receptor-associated protein homolog;

N	Q28FQ5	DE	Tyr-DNA phosphodiesterase 2;
N	Q28FQ5	DE	Tyrosyl-DNA phosphodiesterase 2;
N	Q28FQ5	DR	5'-tyrosyl-DNA phosphodiesterase activity
N	Q28FQ5	DR	double-strand break repair
N	Q28FQ5	DR	metal ion binding
N	Q28FQ5	DR	nuclease activity
N	Q28FQ5	DR	receptor activity
N	Q28H04	CC	Ubiquitin-like protein that can be covalently attached to proteins as a monomer or as a lysine-linked polymer
N	Q28H04	CC	Covalent attachment via an isopeptide bond to its substrates requires prior activation by the E1 complex sae1-sae2 and linkage to the E2 enzyme ube2i, and can be promoted by an E3 ligase such as pias1-4
N	Q28H04	CC	Polymeric sumo2 chains are also susceptible to polyubiquitination which functions as a signal for proteasomal degradation of modified proteins (By similarity)
N	Q28H04	CC	This post-translational modification on lysine residues of proteins plays a crucial role in a number of cellular processes such as nuclear transport, DNA replication and repair, mitosis and signal transduction
N	Q28H04	DE	Small ubiquitin-related modifier 2;
N	Q28HC9	DE	Protein FAM103A1;
N	Q28IL7	DE	Oligosaccharyltransferase complex subunit ostc;
N	Q28N58	CC	Catalyzes amidations at positions B, D, E, and G on adenosylcobyrinic A,C-diamide
N	Q28N58	CC	NH(2) groups are provided by glutamine, and one molecule of ATP is hydrogenolyzed for each amidation (By similarity).
N	Q28N58	DE	Cobyric acid synthase;
N	Q28N58	DR	catalytic activity
N	Q28N58	DR	cobalamin biosynthetic process
N	Q28N58	DR	glutamine metabolic process
N	Q28U74	DE	Succinyl-CoA ligase [ADP-forming] subunit beta;
N	Q28U74	DE	Succinyl-CoA synthetase subunit beta;
N	Q28U74	DR	metal ion binding
N	Q28U74	DR	succinate-CoA ligase (ADP-forming) activity
N	Q28U74	DR	tricarboxylic acid cycle
N	Q28UJ8	CC	Catalyzes the methylthiolation of N6- (dimethylallyl)adenosine (i(6)A), leading to the formation of 2- methylthio-N6-(dimethylallyl)adenosine (ms(2)i(6)A) at position 37 in tRNAs that read codons beginning with uridine (By similarity).
N	Q28UJ8	DE	(Dimethylallyl)adenosine tRNA methylthiotransferase miaB;
N	Q28UJ8	DE	tRNA-i(6)A37 methylthiotransferase;
N	Q28UJ8	DR	4 iron, 4 sulfur cluster binding
N	Q28UJ8	DR	metal ion binding
N	Q28UJ8	DR	tRNA modification
N	Q28UJ8	DR	transferase activity
N	Q28UW8	CC	Catalyzes the GTP-dependent ribosomal translocation step during translation elongation
N	Q28UW8	CC	Catalyzes the coordinated movement of the two tRNA molecules, the mRNA and conformational changes in the ribosome (By similarity).
N	Q28UW8	CC	During this step, the ribosome changes from the pre-translocational (PRE) to the post- translocational (POST) state as the newly formed A-site-bound peptidyl-tRNA and P-site-bound deacylated tRNA move to the P and E sites, respectively
N	Q28UW8	DE	Elongation factor G;
N	Q28UW8	DR	GTPase activity
N	Q28UW8	DR	translation elongation factor activity
N	Q28XT3	CC	Catalyzes the cofactor-independent reversible oxidation of gamma-hydroxybutyrate (GHB) to succinic semialdehyde (SSA) coupled to reduction of 2-ketoglutarate (2-KG) to D-2- hydroxyglutarate (D-2-HG)
N	Q28XT3	CC	L-3-hydroxybutyrate (L-3-OHB) is also a substrate for HOT when using 2-KG as hydrogen acceptor, resulting in the formation of D-2-HG (By similarity).
N	Q28XT3	DE	Probable hydroxyacid-oxoacid transhydrogenase, mitochondrial;
N	Q28XT3	DR	hydroxyacid-oxoacid transhydrogenase activity
N	Q28XT3	DR	metal ion binding
N	Q28XT3	DR	molecular hydrogen transport
N	Q28XT3	DR	oxidation-reduction process
N	Q29865	CC	Involved in the presentation of foreign antigens to the immune system.
N	Q29865	DE	HLA class I histocompatibility antigen, Cw-18 alpha chain;
N	Q29865	DE	MHC class I antigen Cw*18;
N	Q29865	DR	antigen processing and presentation of peptide antigen via MHC class I
N	Q29865	DR	immune response

N	Q29865	DR	interspecies interaction between organisms
N	Q299E6	CC	A humoral factor that may play a role in stress tolerance (By similarity).
N	Q299E6	DE	Protein Turandot X;
N	Q299E6	DR	innate immune response
N	Q299E6	DR	response to bacterium
N	Q299E6	DR	response to heat
N	Q299E6	DR	response to oxidative stress
N	Q29JX6	CC	Mediates response to the active Hedgehog (Hh) protein signal in embryos, functioning upstream or at the level of patched (ptc) (By similarity).
N	Q29JX6	DE	Interference hedgehog;
N	Q29JX6	DR	heparin binding
N	Q29JX6	DR	protein homodimerization activity
N	Q29JX6	DR	smoothened signaling pathway
N	Q29L58	CC	Required for polyglutamylation of axonemal tubulin in sensory cilia (By similarity).
N	Q29L58	CC	Plays a role in anterograde intraflagellar transport (IFT), the process by which cilia precursors are transported from the base of the cilium to the site of their incorporation at the tip (By similarity).
N	Q29L58	DE	TPR repeat protein 30 homolog;
N	Q29L58	DE	Tetratricopeptide repeat protein 30 homolog;
N	Q29L58	DR	intraflagellar transport
N	Q29L58	DR	protein polyglutamylation
N	Q29L58	DR	transporter activity
N	Q2A167	CC	Plays an important role in the de novo pathway of purine nucleotide biosynthesis
N	Q2A167	CC	Catalyzes the first committed step in the biosynthesis of AMP from IMP (By similarity).
N	Q2A167	DE	Adenylosuccinate synthetase;
N	Q2A167	DE	IMP--aspartate ligase;
N	Q2A167	DR	adenylosuccinate synthase activity
N	Q2A167	DR	magnesium ion binding
N	Q2A167	DR	purine nucleotide biosynthetic process
N	Q2A1N3	DE	30S ribosomal protein S16;
N	Q2A1N3	DR	structural constituent of ribosome
N	Q2ES50	CC	Serine protease inhibitor (By similarity).
N	Q2ES50	DE	Kunitz protease inhibitor 1;
N	Q2ES50	DE	Kunitz protease inhibitor I;
N	Q2ES50	DR	serine-type endopeptidase inhibitor activity
N	Q2FET8	DE	Galactose-6-phosphate isomerase subunit lacB;
N	Q2FET8	DR	galactose-6-phosphate isomerase activity
N	Q2FET8	DR	lactose catabolic process
N	Q2FF22	CC	Produces ATP from ADP in the presence of a proton gradient across the membrane.
N	Q2FF22	CC	The alpha chain is a regulatory subunit (By similarity).
N	Q2FF22	DE	ATP synthase F1 sector subunit alpha;
N	Q2FF22	DE	ATP synthase subunit alpha;
N	Q2FF22	DE	F-ATPase subunit alpha;
N	Q2FF22	DR	ATP hydrolysis coupled proton transport
N	Q2FF22	DR	ATP synthesis coupled proton transport
N	Q2FF22	DR	hydrogen ion transporting ATP synthase activity, rotational mechanism
N	Q2FF22	DR	proton-transporting ATPase activity, rotational mechanism
N	Q2FF35	CC	Condenses 4-methyl-5-(beta-hydroxyethyl)thiazole monophosphate (THZ-P) and 2-methyl-4-amino-5-hydroxymethyl pyrimidine pyrophosphate (HMP-PP) to form thiamine monophosphate (TMP) (By similarity).
N	Q2FF35	DE	TMP pyrophosphorylase;
N	Q2FF35	DE	Thiamine-phosphate pyrophosphorylase;
N	Q2FF35	DE	Thiamine-phosphate synthase;
N	Q2FF35	DR	metal ion binding
N	Q2FF35	DR	thiamine biosynthetic process
N	Q2FF35	DR	thiamine-phosphate diphosphorylase activity
N	Q2FF62	DE	Protein sprT-like;
N	Q2FF62	DR	metal ion binding
N	Q2FFT2	DE	Serine protease splD;
N	Q2FFT2	DR	serine-type endopeptidase activity
N	Q2FH42	DE	Dihydrodipicolinate reductase;
N	Q2FH42	DR	diaminopimelate biosynthetic process
N	Q2FH42	DR	dihydrodipicolinate reductase activity
N	Q2FH42	DR	oxidation-reduction process

N	Q2FH63	CC	The alpha subunit is responsible for the aldol cleavage of indoleglycerol phosphate to indole and glyceraldehyde 3- phosphate.
N	Q2FH63	DE	Tryptophan synthase alpha chain;
N	Q2FH63	DR	tryptophan synthase activity
N	Q2FHQ9	DE	Protein MraZ;
N	Q2FKN8	CC	Member of the two-component regulatory system walK/walR that regulates genes involved in autolysis, biofilm formation and cell wall metabolism (By similarity).
N	Q2FKN8	DE	Transcriptional regulatory protein walR;
N	Q2FKN8	DR	regulation of transcription, DNA-dependent
N	Q2FKN8	DR	two-component response regulator activity
N	Q2FPK3	DE	Threonine--tRNA ligase;
N	Q2FPK3	DE	Threonyl-tRNA synthetase;
N	Q2FPK3	DR	threonine-tRNA ligase activity
N	Q2FPK3	DR	threonyl-tRNA aminoacylation
N	Q2FPK3	DR	zinc ion binding
N	Q2FQU2	CC	Involved in the modulation of the chemotaxis system; catalyzes the demethylation of specific methylglutamate residues introduced into the chemoreceptors (methyl-accepting chemotaxis proteins) by CheR (By similarity).
N	Q2FQU2	DE	Chemotaxis response regulator protein-glutamate methylesterase 2;
N	Q2FQU2	DR	protein-glutamate methylesterase activity
N	Q2FQU2	DR	regulation of transcription, DNA-dependent
N	Q2FQU2	DR	two-component response regulator activity
N	Q2FTS5	DE	Putative thymidine phosphorylase;
N	Q2FTS5	DR	pyrimidine base metabolic process
N	Q2FTS5	DR	pyrimidine nucleoside metabolic process
N	Q2FTS5	DR	thymidine phosphorylase activity
N	Q2FVN1	CC	Probably required for nitrate uptake under anoxic conditions
N	Q2FVN1	CC	Also possibly involved in excretion of nitrite produced by the dissimilatory reduction of nitrate (By similarity).
N	Q2FVN1	DE	Probable nitrate transporter narT;
N	Q2FVN1	DR	nitrate assimilation
N	Q2FVN1	DR	transmembrane transport
N	Q2FW10	CC	Protein S19 forms a complex with S13 that binds strongly to the 16S ribosomal RNA (By similarity).
N	Q2FW10	DE	30S ribosomal protein S19;
N	Q2FW10	DR	structural constituent of ribosome
N	Q2FX12	CC	Dephosphorylates the phosphotyrosine-containing proteins (By similarity).
N	Q2FX12	DE	Low molecular weight protein-tyrosine-phosphatase ptpA;
N	Q2FX12	DE	Phosphotyrosine phosphatase A;
N	Q2FX12	DR	protein dephosphorylation
N	Q2FX12	DR	protein tyrosine phosphatase activity
N	Q2FYK3	CC	Required for hemolysin production
N	Q2FYK3	CC	Contributes to virulence in both silkworm-infection model and mice.
N	Q2FYK3	DE	Conserved virulence factor C;
N	Q2FYK3	DR	iron ion binding
N	Q2FZ29	CC	Protease subunit of a proteasome-like degradation complex believed to be a general protein degrading machinery (By similarity).
N	Q2FZ29	DE	ATP-dependent protease subunit HslV;
N	Q2FZ29	DR	metal ion binding
N	Q2FZ29	DR	proteolysis involved in cellular protein catabolic process
N	Q2FZ29	DR	response to stress
N	Q2FZ29	DR	threonine-type endopeptidase activity
N	Q2FZD3	DE	MutS2 protein;
N	Q2FZD3	DR	damaged DNA binding
N	Q2FZD3	DR	maintenance of fidelity involved in DNA-dependent DNA replication
N	Q2FZD3	DR	mismatch repair
N	Q2FZD3	DR	mismatched DNA binding
N	Q2G0Q0	CC	Involved in the hydrolysis of glutamine to glutamate and ammonia
N	Q2G0Q0	CC	Channels an ammonia molecule to pdxS (By similarity).
N	Q2G0Q0	DE	Glutamine amidotransferase glutaminase subunit pdxT;
N	Q2G0Q0	DE	Glutamine amidotransferase subunit pdxT;
N	Q2G0Q0	DR	glutamine metabolic process
N	Q2G0Q0	DR	transferase activity

N	Q2G7S5	CC	Catalyzes the formation of the alpha-1,6-glucosidic linkages in glycogen by scission of a 1,4-alpha-linked oligosaccharide from growing alpha-1,4-glucan chains and the subsequent attachment of the oligosaccharide to the alpha-1,6 position (By similarity)
N	Q2G7S5	DE	1,4-alpha-D-glucan:1,4-alpha-D-glucan 6-glucosyl-transferase;
N	Q2G7S5	DE	1,4-alpha-glucan-branching enzyme;
N	Q2G7S5	DE	Glycogen-branching enzyme;
N	Q2G7S5	DR	1,4-alpha-glucan branching enzyme activity
N	Q2G7S5	DR	cation binding
N	Q2G7S5	DR	glycogen biosynthetic process
N	Q2G7S5	DR	hydrolase activity, hydrolyzing O-glycosyl compounds
N	Q2GE40	CC	Catalyzes the attachment of proline to tRNA(Pro) in a two-step reaction: proline is first activated by ATP to form Pro- AMP and then transferred to the acceptor end of tRNA(Pro) (By similarity).
N	Q2GE40	DE	Proline--tRNA ligase;
N	Q2GE40	DE	Prolyl-tRNA synthetase;
N	Q2GE40	DR	proline-tRNA ligase activity
N	Q2GE40	DR	prolyl-tRNA aminoacylation
N	Q2GEF4	DE	Phosphatidylserine decarboxylase alpha chain;
N	Q2GEF4	DE	Phosphatidylserine decarboxylase beta chain;
N	Q2GEF4	DE	Phosphatidylserine decarboxylase proenzyme;
N	Q2GEF4	DR	cofactor binding
N	Q2GEF4	DR	phosphatidylethanolamine biosynthetic process
N	Q2GEF4	DR	phosphatidylserine decarboxylase activity
N	Q2GFV4	CC	This protein is involved in the repair of mismatches in DNA
N	Q2GFV4	CC	It is required for dam-dependent methyl-directed DNA mismatch repair
N	Q2GFV4	CC	May act as a "molecular matchmaker", a protein that promotes the formation of a stable complex between two or more DNA-binding proteins in an ATP-dependent manner without itself being part of a final effector complex (By similarity)
N	Q2GFV4	DE	DNA mismatch repair protein mutL;
N	Q2GFV4	DR	mismatch repair
N	Q2GFV4	DR	mismatched DNA binding
N	Q2GJF3	DE	50S ribosomal protein L33;
N	Q2GJF3	DR	structural constituent of ribosome
N	Q2GLH3	CC	Interconversion of serine and glycine.
N	Q2GLH3	DE	Serine hydroxymethyltransferase;
N	Q2GLH3	DE	Serine methylase;
N	Q2GLH3	DR	L-serine metabolic process
N	Q2GLH3	DR	glycine hydroxymethyltransferase activity
N	Q2GLH3	DR	glycine metabolic process
N	Q2GLH3	DR	one-carbon metabolic process
N	Q2GLH3	DR	pyridoxal phosphate binding
N	Q2HDP2	CC	Catalyzes the synthesis of dihydrouridine, a modified base found in the D-loop of most tRNAs
N	Q2HDP2	CC	Specifically modifies U47 in cytoplasmic tRNAs (By similarity).
N	Q2HDP2	DE	tRNA-dihydrouridine synthase 3;
N	Q2HDP2	DR	flavin adenine dinucleotide binding
N	Q2HDP2	DR	metal ion binding
N	Q2HDP2	DR	oxidation-reduction process
N	Q2HDP2	DR	tRNA dihydrouridine synthase activity
N	Q2HDP2	DR	tRNA processing
N	Q2HE94	CC	Component of the eukaryotic translation initiation factor 3 (eIF-3) complex, which is involved in protein synthesis and, together with other initiation factors, stimulates binding of mRNA and methionyl-tRNAi to the 40S ribosome (By similarity)
N	Q2HE94	DE	Eukaryotic translation initiation factor 3 subunit M;
N	Q2HE94	DR	translation initiation factor activity
N	Q2HJH3	CC	Exhibits ester hydrolase activity on the substrate p- nitrophenyl acetate (By similarity)
N	Q2HJH3	DE	Ester hydrolase C11orf54 homolog;
N	Q2HJH3	DR	hydrolase activity, acting on ester bonds
N	Q2HJH3	DR	zinc ion binding
N	Q2IHV2	CC	Catalyzes the oxidation of either pyridoxine 5'-phosphate (PNP) or pyridoxamine 5'-phosphate (PMP) into pyridoxal 5'-phosphate (PLP) (By similarity)
N	Q2IHV2	DE	PNP/PMP oxidase;
N	Q2IHV2	DE	Pyridoxal 5'-phosphate synthase;
N	Q2IHV2	DE	Pyridoxine/pyridoxamine 5'-phosphate oxidase;
N	Q2IHV2	DR	oxidation-reduction process

N	Q2IHV2	DR	pyridoxamine-phosphate oxidase activity
N	Q2IHV2	DR	pyridoxine biosynthetic process
N	Q2IL06	CC	NDH-1 shuttles electrons from NADH, via FMN and iron- sulfur (Fe-S) centers, to quinones in the respiratory chain
N	Q2IL06	CC	Couples the redox reaction to proton translocation (for every two electrons transferred, four hydrogen ions are translocated across the cytoplasmic membrane), and thus conserves the redox energy in a proton gradient (By similarity)
N	Q2IL06	CC	The immediate electron acceptor for the enzyme in this species is believed to be ubiquinone
N	Q2IL06	DE	NADH dehydrogenase I subunit D 1;
N	Q2IL06	DE	NADH-quinone oxidoreductase subunit D 1;
N	Q2IL06	DE	NDH-1 subunit D 1;
N	Q2IL06	DR	NADH dehydrogenase (quinone) activity
N	Q2IL06	DR	oxidation-reduction process
N	Q2IL06	DR	quinone binding
N	Q2INW2	DE	1-(5-phosphoribosyl)-5-[(5-phosphoribosylamino)methylideneamino] imidazole-4-carboxamide isomerase;
N	Q2INW2	DE	Phosphoribosylformimino-5-aminoimidazole carboxamide ribotide isomerase;
N	Q2INW2	DR	1-(5-phosphoribosyl)-5-[(5-phosphoribosylamino)methylideneamino]imidazole-4-carboxamide isomerase activity
N	Q2INW2	DR	histidine biosynthetic process
N	Q2ISF7	CC	This is one of the proteins that binds to the 5S RNA in the ribosome where it forms part of the central protuberance (By similarity).
N	Q2ISF7	DE	50S ribosomal protein L25;
N	Q2ISF7	DE	General stress protein CTC;
N	Q2ISF7	DR	5S rRNA binding
N	Q2ISF7	DR	structural constituent of ribosome
N	Q2IUB0	DE	UPF0260 protein RPB_3505;
N	Q2IUG5	CC	Heme chaperone required for the biogenesis of c-type cytochromes
N	Q2IUG5	CC	Transiently binds heme delivered by CcmC and transfers the heme to apo-cytochromes in a process facilitated by CcmF and CcmH (By similarity).
N	Q2IUG5	DE	Cytochrome c maturation protein E;
N	Q2IUG5	DE	Cytochrome c-type biogenesis protein CcmE;
N	Q2IUG5	DE	Heme chaperone CcmE;
N	Q2IUG5	DR	cytochrome complex assembly
N	Q2IUG5	DR	metal ion binding
N	Q2IUG5	DR	protein-heme linkage
N	Q2J2D8	CC	Catalyzes the dephosphorylation of undecaprenyl diphosphate (UPP)
N	Q2J2D8	CC	Confers resistance to bacitracin (By similarity).
N	Q2J2D8	DE	Bacitracin resistance protein;
N	Q2J2D8	DE	Undecaprenyl pyrophosphate phosphatase;
N	Q2J2D8	DE	Undecaprenyl-diphosphatase;
N	Q2J2D8	DR	cellular cell wall organization
N	Q2J2D8	DR	dephosphorylation
N	Q2J2D8	DR	peptidoglycan biosynthetic process
N	Q2J2D8	DR	regulation of cell shape
N	Q2J2D8	DR	response to antibiotic
N	Q2J2D8	DR	undecaprenyl-diphosphatase activity
N	Q2J734	CC	One of the primary rRNA binding proteins, it binds directly to 16S rRNA where it helps nucleate assembly of the platform of the 30S subunit by binding and bridging several RNA helices of the 16S rRNA (By similarity).
N	Q2J734	DE	30S ribosomal protein S15;
N	Q2J734	DR	structural constituent of ribosome
N	Q2JFG6	CC	Binds to 23S rRNA
N	Q2JFG6	CC	Forms part of two intersubunit bridges in the 70S ribosome (By similarity).
N	Q2JFG6	DE	50S ribosomal protein L14;
N	Q2JFG6	DR	structural constituent of ribosome
N	Q2JIL0	CC	Binds to the 23S rRNA (By similarity).
N	Q2JIL0	DE	50S ribosomal protein L15;
N	Q2JIL0	DR	structural constituent of ribosome
N	Q2JJ68	CC	NDH-1 shuttles electrons from an unknown electron donor, via FMN and iron-sulfur (Fe-S) centers, to quinones in the respiratory and/or the photosynthetic chain
N	Q2JJ68	CC	Couples the redox reaction to proton translocation, and thus conserves the redox energy in a proton gradient

N	Q2JJ68	CC	Cyanobacterial NDH-1 also plays a role in inorganic carbon- concentration (By similarity).
N	Q2JJ68	CC	The immediate electron acceptor for the enzyme in this species is believed to be plastoquinone
N	Q2JJ68	DE	NAD(P)H dehydrogenase I subunit L;
N	Q2JJ68	DE	NAD(P)H-quinone oxidoreductase subunit L;
N	Q2JJ68	DE	NDH-1 subunit L;
N	Q2JJ68	DR	oxidation-reduction process
N	Q2JJ68	DR	oxidoreductase activity, acting on NADH or NADPH, quinone or similar compound as acceptor
N	Q2JJ68	DR	quinone binding
N	Q2JJR4	CC	Cell wall formation
N	Q2JJR4	CC	Catalyzes the transfer of a GlcNAc subunit on undecaprenyl-pyrophosphoryl-MurNAc-pentapeptide (lipid intermediate I) to form undecaprenyl-pyrophosphoryl-MurNAc- (pentapeptide)GlcNAc (lipid intermediate II) (By similarity).
N	Q2JJR4	DE	UDP-N-acetylglucosamine--N-acetylmuramyl-(pentapeptide) pyrophosphoryl-undecaprenol N-acetylglucosamine transferase;
N	Q2JJR4	DE	Undecaprenyl-PP-MurNAc-pentapeptide-UDPGlcNAc GlcNAc transferase;
N	Q2JJR4	DR	UDP-N-acetyl-D-glucosamine:N-acetylmuramoyl-L-alanyl-D-glutamyl-meso-2,6-diaminopimelyl-D-alanyl-D-alanine-diphosphoundecaprenol 4-beta-N-acetylglucosaminlytransferase activity
N	Q2JJR4	DR	UDP-N-acetylgalactosamine biosynthetic process
N	Q2JJR4	DR	carbohydrate binding
N	Q2JJR4	DR	cell division
N	Q2JJR4	DR	cellular cell wall organization
N	Q2JJR4	DR	lipid glycosylation
N	Q2JJR4	DR	peptidoglycan biosynthetic process
N	Q2JJR4	DR	regulation of cell shape
N	Q2JJR4	DR	undecaprenyldiphospho-muramoylpentapeptide beta-N-acetylglucosaminyltransferase activity
N	Q2JMZ3	CC	Functions in the N-end rule pathway of protein degradation where it conjugates Leu, Phe and, less efficiently, Met from aminoacyl-tRNAs to the N-termini of proteins containing an N-terminal arginine or lysine (By similarity).
N	Q2JMZ3	DE	L/F-transferase;
N	Q2JMZ3	DE	Leucyl/phenylalanyl-tRNA--protein transferase;
N	Q2JMZ3	DE	Leucyltransferase;
N	Q2JMZ3	DE	Phenyalanyltransferase;
N	Q2JMZ3	DR	acyltransferase activity
N	Q2JMZ3	DR	leucyltransferase activity
N	Q2JMZ3	DR	protein catabolic process
N	Q2JNH4	DE	Maf-like protein CYB_0709;
N	Q2JQ65	DE	Anthranilate phosphoribosyltransferase;
N	Q2JQ65	DR	anthranilate phosphoribosyltransferase activity
N	Q2JQ65	DR	tryptophan biosynthetic process
N	Q2JR49	CC	Binds directly to 23S ribosomal RNA and is necessary for the in vitro assembly process of the 50S ribosomal subunit
N	Q2JR49	CC	It is not involved in the protein synthesizing functions of that subunit (By similarity).
N	Q2JR49	DE	50S ribosomal protein L20;
N	Q2JR49	DR	structural constituent of ribosome
N	Q2JTW6	CC	Catalyzes the decarboxylation of orotidine 5'- monophosphate (OMP) to uridine 5'-monophosphate (UMP) (By similarity).
N	Q2JTW6	DE	OMP decarboxylase;
N	Q2JTW6	DE	Orotidine 5'-phosphate decarboxylase;
N	Q2JTW6	DR	'de novo' UMP biosynthetic process
N	Q2JTW6	DR	'de novo' pyrimidine base biosynthetic process
N	Q2JTW6	DR	orotidine-5'-phosphate decarboxylase activity
N	Q2JV84	CC	One of the primary rRNA binding proteins
N	Q2JV84	CC	It has been suggested to have peptidyltransferase activity; this is somewhat controversial
N	Q2JV84	CC	Makes several contacts with the 16S rRNA in the 70S ribosome (By similarity).
N	Q2JV84	CC	Required for association of the 30S and 50S subunits to form the 70S ribosome, for tRNA binding and peptide bond formation
N	Q2JV84	DE	50S ribosomal protein L2;
N	Q2JV84	DR	structural constituent of ribosome
N	Q2JV84	DR	transferase activity
N	Q2K2Y1	CC	This protein binds to 23S rRNA in the presence of protein L20 (By similarity).

N	Q2K2Y1	DE	50S ribosomal protein L21;
N	Q2K2Y1	DR	structural constituent of ribosome
N	Q2K302	CC	Involved in peptide bond synthesis
N	Q2K302	CC	Probably functions indirectly by altering the affinity of the ribosome for aminoacyl-tRNA, thus increasing their reactivity as acceptors for peptidyl transferase (By similarity)
N	Q2K302	CC	Stimulates efficient translation and peptide-bond synthesis on native or reconstituted 70S ribosomes in vitro
N	Q2K302	DE	Elongation factor P;
N	Q2K302	DR	peptide biosynthetic process
N	Q2K302	DR	translation elongation factor activity
N	Q2K433	DE	Xylose isomerase;
N	Q2K433	DR	D-xylose metabolic process
N	Q2K433	DR	metal ion binding
N	Q2K433	DR	pentose-phosphate shunt
N	Q2K433	DR	xylose isomerase activity
N	Q2K4E6	DE	TPR repeat-containing protein RHE_CH03534.1;
N	Q2K7G6	CC	Catalyzes the synthesis of alpha-ribazole-5'-phosphate from nicotinate mononucleotide (NAMN) and 5,6- dimethylbenzimidazole (DMB).
N	Q2K7G6	DE	N(1)-alpha-phosphoribosyltransferase;
N	Q2K7G6	DE	NN:DBI PRT;
N	Q2K7G6	DE	Nicotinate-nucleotide--dimethylbenzimidazole phosphoribosyltransferase;
N	Q2K7G6	DR	cobalamin biosynthetic process
N	Q2K7G6	DR	nicotinate-nucleotide-dimethylbenzimidazole phosphoribosyltransferase activity
N	Q2K7G6	DR	nucleoside biosynthetic process
N	Q2K9W4	DE	30S ribosomal protein S9;
N	Q2K9W4	DR	structural constituent of ribosome
N	Q2KA54	CC	The UvrABC repair system catalyzes the recognition and processing of DNA lesions
N	Q2KA54	CC	The N-terminal half is responsible for the 3' incision and the C-terminal half is responsible for the 5' incision (By similarity).
N	Q2KA54	CC	UvrC both incises the 5' and 3' sides of the lesion
N	Q2KA54	DE	Excinuclease ABC subunit C;
N	Q2KA54	DE	Protein uvrC;
N	Q2KA54	DE	UvrABC system protein C;
N	Q2KA54	DR	excinuclease ABC activity
N	Q2KA54	DR	nucleotide-excision repair
N	Q2KCH7	CC	Putative member of a two-component regulatory system.
N	Q2KCH7	DE	Probable chemotaxis protein CheY;
N	Q2KCH7	DR	regulation of transcription, DNA-dependent
N	Q2KCH7	DR	two-component response regulator activity
N	Q2KW23	CC	No specific function has so far been attributed to this initiation factor; however, it seems to stimulate more or less all the activities of the other two initiation factors, IF-2 and IF-3
N	Q2KW23	DE	Translation initiation factor IF-1 2;
N	Q2KW23	DR	translation initiation factor activity
N	Q2KYQ6	CC	Could be a mediator in iron transactions between iron acquisition and iron-requiring processes, such as synthesis and/or repair of Fe-S clusters in biosynthetic enzymes (By similarity).
N	Q2KYQ6	DE	Probable Fe(2+)-trafficking protein;
N	Q2KYQ6	DR	iron ion binding
N	Q2L0A9	CC	One of the proteins required for the normal export of preproteins out of the cell cytoplasm
N	Q2L0A9	CC	It also specifically binds to its receptor secA (By similarity).
N	Q2L0A9	CC	It is a molecular chaperone that binds to a subset of precursor proteins, maintaining them in a translocation-competent state
N	Q2L0A9	DE	Protein-export protein secB;
N	Q2L0A9	DR	protein tetramerization
N	Q2L0A9	DR	protein transport
N	Q2L0A9	DR	transmembrane transport
N	Q2L0A9	DR	unfolded protein binding
N	Q2L937	DE	30S ribosomal protein S11, chloroplastic;
N	Q2L937	DR	structural constituent of ribosome
N	Q2L957	CC	NDH shuttles electrons from NAD(P)H:plastoquinone, via FMN and iron-sulfur (Fe-S) centers, to quinones in the photosynthetic chain and possibly in a chloroplast respiratory chain
N	Q2L957	CC	Couples the redox reaction to proton translocation, and thus conserves the redox energy in a proton gradient (By similarity).

N	Q2L957	CC	The immediate electron acceptor for the enzyme in this species is believed to be plastoquinone
N	Q2L957	DE	NAD(P)H dehydrogenase subunit 4L;
N	Q2L957	DE	NAD(P)H-quinone oxidoreductase subunit 4L, chloroplastic;
N	Q2L957	DE	NADH-plastoquinone oxidoreductase subunit 4L;
N	Q2L957	DR	ATP synthesis coupled electron transport
N	Q2L957	DR	oxidoreductase activity, acting on NADH or NADPH
N	Q2L957	DR	quinone binding
N	Q2LR03	CC	Necessary for efficient RNA polymerase transcription elongation past template-encoded arresting sites
N	Q2LR03	CC	Cleavage of the nascent transcript by cleavage factors such as greA or greB allows the resumption of elongation from the new 3'terminus
N	Q2LR03	CC	GreA releases sequences of 2 to 3 nucleotides (By similarity).
N	Q2LR03	CC	The arresting sites in DNA have the property of trapping a certain fraction of elongating RNA polymerases that pass through, resulting in locked ternary complexes
N	Q2LR03	DE	Transcript cleavage factor greA;
N	Q2LR03	DE	Transcription elongation factor greA;
N	Q2LR03	DR	transcription elongation regulator activity
N	Q2LUS9	CC	Catalyzes the formation of 4-diphosphocytidyl-2-C- methyl-D-erythritol from CTP and 2-C-methyl-D-erythritol 4- phosphate (MEP) (By similarity).
N	Q2LUS9	DE	2-C-methyl-D-erythritol 4-phosphate cytidylyltransferase;
N	Q2LUS9	DE	4-diphosphocytidyl-2C-methyl-D-erythritol synthase;
N	Q2LUS9	DE	MEP cytidylyltransferase;
N	Q2LUS9	DR	2-C-methyl-D-erythritol 4-phosphate cytidylyltransferase activity
N	Q2LUS9	DR	isoprenoid biosynthetic process
N	Q2LVI9	DE	Glycine--tRNA ligase beta subunit;
N	Q2LVI9	DE	Glycyl-tRNA synthetase beta subunit;
N	Q2LVI9	DR	arginine-tRNA ligase activity
N	Q2LVI9	DR	arginyl-tRNA aminoacylation
N	Q2LVI9	DR	glycine-tRNA ligase activity
N	Q2LVI9	DR	glycyl-tRNA aminoacylation
N	Q2LVL0	CC	Involved in lipid A export and possibly also in glycerophospholipid export and for biogenesis of the outer membrane
N	Q2LVL0	CC	Transmembrane domains (TMD) form a pore in the inner membrane and the ATP-binding domain (NBD) is responsible for energy generation (By similarity).
N	Q2LVL0	DE	Lipid A export ATP-binding/permease protein MsbA;
N	Q2LVL0	DR	ATPase activity, coupled to transmembrane movement of substances
N	Q2LVL0	DR	lipid transport
N	Q2LWC5	CC	Increases the formation of ribosomal termination complexes and stimulates activities of RF-1 and RF-2
N	Q2LWC5	CC	It binds guanine nucleotides and has strong preference for UGA stop codons
N	Q2LWC5	CC	It may interact directly with the ribosome
N	Q2LWC5	CC	The stimulation of RF- 1 and RF-2 is significantly reduced by GTP and GDP, but not by GMP (By similarity).
N	Q2LWC5	DE	Peptide chain release factor 3;
N	Q2LWC5	DR	GTPase activity
N	Q2LWC5	DR	translation release factor activity, codon specific
N	Q2M389	CC	Component of the WASH complex, a complex present at the surface of endosomes that recruits and activates the Arp2/3 complex to induce actin polymerization
N	Q2M389	CC	The WASH complex plays a key role in the fission of tubules that serve as transport intermediates during endosome sorting (By similarity).
N	Q2M389	DE	WASH complex subunit 7;
N	Q2M389	DR	endosome transport
N	Q2MI88	CC	May be involved in proton extrusion
N	Q2MI88	CC	Indirectly promotes efficient inorganic carbon uptake into chloroplasts (By similarity)
N	Q2MI88	DE	Chloroplast envelope membrane protein;
N	Q2MI88	DR	proton transport
N	Q2MI91	CC	Component of the acetyl coenzyme A carboxylase (ACC) complex
N	Q2MI91	CC	Biotin carboxylase (BC) catalyzes the carboxylation of biotin on its carrier protein (BCCP) and then the CO(2) group is transferred by the transcarboxylase to acetyl-CoA to form malonyl- CoA (By similarity)
N	Q2MI91	CC	Is up-regulated upon chromoplast differentiation, presumably for fatty acid biosynthesis
N	Q2MI91	DE	ACCase subunit beta;
N	Q2MI91	DE	Acetyl-CoA carboxylase carboxyltransferase subunit beta;
N	Q2MI91	DE	Acetyl-coenzyme A carboxylase carboxyl transferase subunit beta, chloroplastic;

N	Q2MI91	DR	acetyl-CoA carboxylase activity
N	Q2MI91	DR	fatty acid biosynthetic process
N	Q2MI91	DR	metal ion binding
N	Q2MIK8	CC	With S4 and S5 plays an important role in translational accuracy
N	Q2MIK8	CC	Located at the interface of the 30S and 50S subunits (By similarity).
N	Q2MIK8	DE	30S ribosomal protein S12, chloroplastic;
N	Q2MIK8	DR	structural constituent of ribosome
N	Q2N5Q5	CC	DNA-dependent RNA polymerase catalyzes the transcription of DNA into RNA using the four ribonucleoside triphosphates as substrates (By similarity).
N	Q2N5Q5	DE	DNA-directed RNA polymerase subunit beta';
N	Q2N5Q5	DE	RNA polymerase subunit beta';
N	Q2N5Q5	DE	RNAP subunit beta';
N	Q2N5Q5	DE	Transcriptase subunit beta';
N	Q2N5Q5	DR	DNA-directed RNA polymerase activity
N	Q2N659	CC	Catalyzes the condensation of pantoate with beta-alanine in an ATP-dependent reaction via a pantoyl-adenylate intermediate (By similarity).
N	Q2N659	DE	Pantoate--beta-alanine ligase;
N	Q2N659	DE	Pantoate-activating enzyme;
N	Q2N659	DE	Pantothenate synthetase;
N	Q2N659	DR	pantoate-beta-alanine ligase activity
N	Q2N659	DR	pantothenate biosynthetic process
N	Q2N9B2	CC	One of the primary rRNA binding proteins, this protein initially binds near the 5'-end of the 23S rRNA
N	Q2N9B2	CC	It is important during the early stages of 50S assembly
N	Q2N9B2	CC	It makes multiple contacts with different domains of the 23S rRNA in the assembled 50S subunit and ribosome (By similarity).
N	Q2N9B2	DE	50S ribosomal protein L4;
N	Q2N9B2	DR	structural constituent of ribosome
N	Q2NF86	CC	Produces ATP from ADP in the presence of a proton gradient across the membrane
N	Q2NF86	DE	V-ATPase subunit F;
N	Q2NF86	DE	V-type ATP synthase subunit F;
N	Q2NF86	DR	ATP biosynthetic process
N	Q2NF86	DR	ATP hydrolysis coupled proton transport
N	Q2NF86	DR	hydrogen ion transporting ATP synthase activity, rotational mechanism
N	Q2NF86	DR	proton-transporting ATPase activity, rotational mechanism
N	Q2NHU7	DE	UPF0219 protein Msp_0117;
N	Q2NHU7	DR	hydroxymethylglutaryl-CoA synthase activity
N	Q2NHU7	DR	isoprenoid biosynthetic process
N	Q2NHU7	DR	transferase activity, transferring acyl groups other than amino-acyl groups
N	Q2NIA4	CC	Required for the formation of a threonylcarbamoyl group on adenosine at position 37 (t(6)A37) in tRNAs that read codons beginning with adenine (By similarity).
N	Q2NIA4	DE	Probable bifunctional tRNA threonylcarbamoyladenosine biosynthesis protein;
N	Q2NIA4	DE	Probable serine/threonine-protein kinase BUD32 homolog;
N	Q2NIA4	DE	Probable tRNA threonylcarbamoyladenosine biosynthesis protein KAE1 homolog;
N	Q2NIA4	DE	t(6)A37 threonylcarbamoyladenosine biosynthesis protein KAE1 homolog;
N	Q2NIA4	DR	metalloendopeptidase activity
N	Q2NIA4	DR	protein phosphorylation
N	Q2NIA4	DR	protein tyrosine kinase activity
N	Q2NIA4	DR	zinc ion binding
N	Q2NIN4	CC	Ligates lysine onto the cytidine present at position 34 of the AUA codon-specific tRNA(Ile) that contains the anticodon CAU, in an ATP-dependent manner
N	Q2NIN4	CC	Cytidine is converted to lysidine, thus changing the amino acid specificity of the tRNA from methionine to isoleucine (By similarity).
N	Q2NIN4	DE	tRNA(Ile)-2-lysyl-cytidine synthase;
N	Q2NIN4	DE	tRNA(Ile)-lysidine synthase;
N	Q2NIN4	DE	tRNA(Ile)-lysidine synthetase;
N	Q2NIN4	DR	ligase activity, forming carbon-nitrogen bonds
N	Q2NIN4	DR	tRNA processing
N	Q2NTA8	CC	Provides the D-alanine required for cell wall biosynthesis (By similarity).
N	Q2NTA8	DE	Alanine racemase;
N	Q2NTA8	DR	alanine metabolic process
N	Q2NTA8	DR	alanine racemase activity
N	Q2NTA8	DR	cellular cell wall organization
N	Q2NTA8	DR	peptidoglycan biosynthetic process
N	Q2NTA8	DR	regulation of cell shape

N	Q2NW08	CC	Increases the formation of ribosomal termination complexes and stimulates activities of RF-1 and RF-2
N	Q2NW08	CC	It binds guanine nucleotides and has strong preference for UGA stop codons
N	Q2NW08	CC	It may interact directly with the ribosome
N	Q2NW08	CC	The stimulation of RF- 1 and RF-2 is significantly reduced by GTP and GDP, but not by GMP (By similarity).
N	Q2NW08	DE	Peptide chain release factor 3;
N	Q2NW08	DR	GTPase activity
N	Q2NW08	DR	translation release factor activity, codon specific
N	Q2NY86	DE	UPF0391 membrane protein XOO3986;
N	Q2P5W1	DE	Aspartate carbamoyltransferase;
N	Q2P5W1	DE	Aspartate transcarbamylase;
N	Q2P5W1	DR	'de novo' pyrimidine base biosynthetic process
N	Q2P5W1	DR	amino acid binding
N	Q2P5W1	DR	aspartate carbamoyltransferase activity
N	Q2P5W1	DR	cellular amino acid metabolic process
N	Q2P5W1	DR	pyrimidine nucleotide biosynthetic process
N	Q2P6P9	DE	Phosphoriboisomerase A;
N	Q2P6P9	DE	Ribose-5-phosphate isomerase A;
N	Q2P6P9	DR	pentose-phosphate shunt, non-oxidative branch
N	Q2P6P9	DR	ribose-5-phosphate isomerase activity
N	Q2P7Q7	CC	F(1)F(0) ATP synthase produces ATP from ADP in the presence of a proton or sodium gradient
N	Q2P7Q7	CC	During catalysis, ATP synthesis in the catalytic domain of F(1) is coupled via a rotary mechanism of the central stalk subunits to proton translocation (By similarity)
N	Q2P7Q7	CC	F-type ATPases consist of two structural domains, F(1) containing the extramembraneous catalytic core and F(0) containing the membrane proton channel, linked together by a central stalk and a peripheral stalk
N	Q2P7Q7	DE	ATP synthase F(1) sector subunit delta;
N	Q2P7Q7	DE	ATP synthase subunit delta;
N	Q2P7Q7	DE	F-ATPase subunit delta;
N	Q2P7Q7	DE	F-type ATPase subunit delta;
N	Q2P7Q7	DR	ATP synthesis coupled proton transport
N	Q2P7Q7	DR	hydrogen ion transporting ATP synthase activity, rotational mechanism
N	Q2PMN2	DE	NAD(P)H dehydrogenase, chain 4;
N	Q2PMN2	DE	NAD(P)H-quinone oxidoreductase chain 4, chloroplastic;
N	Q2PMN2	DE	NADH-plastoquinone oxidoreductase chain 4;
N	Q2PMN2	DR	ATP synthesis coupled electron transport
N	Q2PMN2	DR	NADH dehydrogenase (ubiquinone) activity
N	Q2PMN2	DR	quinone binding
N	Q2QD64	CC	Cleaves peptides in various proteins in a process that requires ATP hydrolysis
N	Q2QD64	CC	Has a chymotrypsin-like activity
N	Q2QD64	CC	Plays a major role in the degradation of misfolded proteins (By similarity).
N	Q2QD64	DE	ATP-dependent Clp protease proteolytic subunit;
N	Q2QD64	DE	Endopeptidase Clp;
N	Q2QD64	DR	serine-type endopeptidase activity
N	Q2QLG6	CC	Involved in the costimulatory signal essential for T- cell receptor (TCR)-mediated T-cell activation
N	Q2QLG6	CC	Forms a stable heterooligomeric complex with CAV2 that targets to lipid rafts and drives caveolae formation
N	Q2QLG6	CC	Interacts directly with G- protein alpha subunits and can functionally regulate their activity
N	Q2QLG6	CC	Its binding to DPP4 induces T-cell proliferation and NF-kappa-B activation in a T-cell receptor/CD3-dependent manner
N	Q2QLG6	CC	May act as a scaffolding protein within caveolar membranes
N	Q2QLG6	CC	Recruits CTNNB1 to caveolar membranes and may regulate CTNNB1-mediated signaling through the Wnt pathway (By similarity).
N	Q2QLG6	DR	T cell costimulation
N	Q2R483	DE	2,3-diketo-5-methylthio-1-phosphopentane phosphatase;
N	Q2R483	DE	Enolase-phosphatase E1;
N	Q2R483	DE	MTRu-1-P dehydratase;
N	Q2R483	DE	Methylthioribulose-1-phosphate dehydratase;
N	Q2R483	DE	Probable bifunctional methylthioribulose-1-phosphate dehydratase/enolase-phosphatase E1;
N	Q2R483	DR	L-methionine salvage from methylthioadenosine

N	Q2R483	DR	acireductone synthase activity
N	Q2R483	DR	metal ion binding
N	Q2R483	DR	methylthioribulose 1-phosphate dehydratase activity
N	Q2R483	DR	phosphoglycolate phosphatase activity
N	Q2RHM6	CC	Catalyzes the radical-mediated insertion of two sulfur atoms into the C-6 and C-8 positions of the octanoyl moiety bound to the lipoyl domains of lipoate-dependent enzymes, thereby converting the octanoylated domains into lipoylated derivatives (By similarity).
N	Q2RHM6	DE	Lipoate synthase;
N	Q2RHM6	DE	Lipoic acid synthase;
N	Q2RHM6	DE	Lipoyl synthase;
N	Q2RHM6	DE	Sulfur insertion protein lipA;
N	Q2RHM6	DR	4 iron, 4 sulfur cluster binding
N	Q2RHM6	DR	lipoate biosynthetic process
N	Q2RHM6	DR	lipoate synthase activity
N	Q2RHM6	DR	metal ion binding
N	Q2RHR4	CC	Catalyzes the synthesis of the hydroxymethylpyrimidine phosphate (HMP-P) moiety of thiamine from aminoimidazole ribotide (AIR) in a radical S-adenosyl-L-methionine (SAM)-dependent reaction (By similarity).
N	Q2RHR4	DE	HMP-P synthase 3;
N	Q2RHR4	DE	HMP-phosphate synthase 3;
N	Q2RHR4	DE	HMPP synthase 3;
N	Q2RHR4	DE	Hydroxymethylpyrimidine phosphate synthase 3;
N	Q2RHR4	DE	Phosphomethylpyrimidine synthase 3;
N	Q2RHR4	DE	Thiamine biosynthesis protein thiC 3;
N	Q2RHR4	DR	4 iron, 4 sulfur cluster binding
N	Q2RHR4	DR	lyase activity
N	Q2RHR4	DR	metal ion binding
N	Q2RHR4	DR	thiamine biosynthetic process
N	Q2RP10	DE	Acetylglutamate kinase;
N	Q2RP10	DE	N-acetyl-L-glutamate 5-phosphotransferase;
N	Q2RP10	DR	acetylglutamate kinase activity
N	Q2RP10	DR	arginine biosynthetic process
N	Q2RP10	DR	glutamate 5-kinase activity
N	Q2RP10	DR	proline biosynthetic process
N	Q2RQV5	CC	With S4 and S5 plays an important role in translational accuracy (By similarity).
N	Q2RQV5	DE	30S ribosomal protein S12;
N	Q2RQV5	DR	structural constituent of ribosome
N	Q2RQW2	CC	One of the early assembly proteins it binds 23S rRNA
N	Q2RQW2	CC	Forms the main docking site for trigger factor binding to the ribosome (By similarity).
N	Q2RQW2	CC	One of the proteins that surrounds the polypeptide exit tunnel on the outside of the ribosome
N	Q2RQW2	DE	50S ribosomal protein L23;
N	Q2RQW2	DR	nucleotide binding
N	Q2RQW2	DR	structural constituent of ribosome
N	Q2RS50	CC	Synthesizes alpha-1,4-glucan chains using ADP-glucose.
N	Q2RS50	DE	Glycogen synthase;
N	Q2RS50	DE	Starch [bacterial glycogen] synthase;
N	Q2RS50	DR	glycogen biosynthetic process
N	Q2RS50	DR	starch synthase activity
N	Q2RS75	CC	The UvrABC repair system catalyzes the recognition and processing of DNA lesions.
N	Q2RS75	CC	The N-terminal half is responsible for the 3' incision and the C-terminal half is responsible for the 5' incision (By similarity).
N	Q2RS75	CC	UvrC both incises the 5' and 3' sides of the lesion
N	Q2RS75	DE	Excinuclease ABC subunit C;
N	Q2RS75	DE	Protein uvrC;
N	Q2RS75	DE	UvrABC system protein C;
N	Q2RS75	DR	excinuclease ABC activity
N	Q2RS75	DR	nucleotide-excision repair
N	Q2RTE2	CC	Catalyzes the dephosphorylation of undecaprenyl diphosphate (UPP)
N	Q2RTE2	CC	Confers resistance to bacitracin (By similarity).
N	Q2RTE2	DE	Bacitracin resistance protein 2;
N	Q2RTE2	DE	Undecaprenyl pyrophosphate phosphatase 2;
N	Q2RTE2	DE	Undecaprenyl-diphosphatase 2;
N	Q2RTE2	DR	cellular cell wall organization

N	Q2RTE2	DR	dephosphorylation
N	Q2RTE2	DR	peptidoglycan biosynthetic process
N	Q2RTE2	DR	regulation of cell shape
N	Q2RTE2	DR	response to antibiotic
N	Q2RTE2	DR	undecaprenyl-diphosphatase activity
N	Q2RWE3	DE	Histidine--tRNA ligase;
N	Q2RWE3	DE	Histidyl-tRNA synthetase;
N	Q2RWE3	DR	histidine-tRNA ligase activity
N	Q2RWE3	DR	histidyl-tRNA aminoacylation
N	Q2S1Q6	CC	DNA-dependent RNA polymerase catalyzes the transcription of DNA into RNA using the four ribonucleoside triphosphates as substrates.
N	Q2S1Q6	DE	DNA-directed RNA polymerase subunit beta';
N	Q2S1Q6	DE	RNA polymerase subunit beta';
N	Q2S1Q6	DE	RNAP subunit beta';
N	Q2S1Q6	DE	Transcriptase subunit beta';
N	Q2S1Q6	DR	DNA-directed RNA polymerase activity
N	Q2S919	CC	Binds 23S rRNA and is also seen to make contacts with the A and possibly P site tRNAs (By similarity).
N	Q2S919	DE	50S ribosomal protein L16;
N	Q2S919	DR	structural constituent of ribosome
N	Q2SJB9	DE	Protein CrcB homolog;
N	Q2SMB5	DE	Imidazoleglycerol-phosphate dehydratase;
N	Q2SMB5	DR	histidine biosynthetic process
N	Q2SMB5	DR	imidazoleglycerol-phosphate dehydratase activity
N	Q2SML5	CC	Required for maturation of 30S ribosomal subunits (By similarity).
N	Q2SML5	DE	Ribosome maturation factor rimP;
N	Q2SML5	DR	ribosome biogenesis
N	Q2ST53	CC	This protein binds directly to 23S ribosomal RNA (By similarity).
N	Q2ST53	DE	50S ribosomal protein L11;
N	Q2ST53	DR	structural constituent of ribosome
N	Q2SU20	CC	DNA-dependent RNA polymerase catalyzes the transcription of DNA into RNA using the four ribonucleoside triphosphates as substrates.
N	Q2SU20	DE	DNA-directed RNA polymerase subunit beta';
N	Q2SU20	DE	RNA polymerase subunit beta';
N	Q2SU20	DE	RNAP subunit beta';
N	Q2SU20	DE	Transcriptase subunit beta';
N	Q2SU20	DR	DNA-directed RNA polymerase activity
N	Q2SWZ7	CC	Associates with the EF-Tu.GDP complex and induces the exchange of GDP to GTP
N	Q2SWZ7	CC	It remains bound to the aminoacyl-tRNA.EF- Tu.GTP complex up to the GTP hydrolysis stage on the ribosome.
N	Q2SWZ7	DE	Elongation factor Ts;
N	Q2SWZ7	DR	translation elongation factor activity
N	Q2SYJ5	CC	Prevents misfolding and promotes the refolding and proper assembly of unfolded polypeptides generated under stress conditions (By similarity).
N	Q2SYJ5	DE	60 kDa chaperonin 1;
N	Q2SYJ5	DE	GroEL protein 1;
N	Q2SYJ5	DE	Protein Cpn60 1;
N	Q2SYJ5	DR	protein refolding
N	Q2SYS4	CC	Interconversion of serine and glycine.
N	Q2SYS4	DE	Serine hydroxymethyltransferase 1;
N	Q2SYS4	DE	Serine methylase 1;
N	Q2SYS4	DR	L-serine metabolic process
N	Q2SYS4	DR	glycine hydroxymethyltransferase activity
N	Q2SYS4	DR	glycine metabolic process
N	Q2SYS4	DR	one-carbon metabolic process
N	Q2SYS4	DR	pyridoxal phosphate binding
N	Q2T096	CC	Catalyzes the pyruvoyl-dependent decarboxylation of aspartate to produce beta-alanine (By similarity).
N	Q2T096	DE	Aspartate 1-decarboxylase alpha chain;
N	Q2T096	DE	Aspartate 1-decarboxylase beta chain;
N	Q2T096	DE	Aspartate 1-decarboxylase;
N	Q2T096	DE	Aspartate alpha-decarboxylase;
N	Q2T096	DR	alanine biosynthetic process
N	Q2T096	DR	aspartate 1-decarboxylase activity
N	Q2T096	DR	pantothenate biosynthetic process

N	Q2T8T6	CC	Part of the ABC transporter complex RbsABCD involved in ribose import
N	Q2T8T6	CC	Responsible for energy coupling to the transport system (By similarity).
N	Q2T8T6	DE	Ribose import ATP-binding protein RbsA 2;
N	Q2T8T6	DR	monosaccharide-transporting ATPase activity
N	Q2TA14	CC	Cleaves C-terminal amino acids linked to proline in peptides such as angiotensin II, III and des-Arg9-bradykinin
N	Q2TA14	CC	This cleavage occurs at acidic pH, but enzymatic activity is retained with some substrates at neutral pH (By similarity).
N	Q2TA14	DE	Lysosomal Pro-X carboxypeptidase;
N	Q2TA14	DE	Proline carboxypeptidase;
N	Q2TA14	DE	Prolylcarboxypeptidase;
N	Q2TA14	DR	carboxypeptidase activity
N	Q2TA14	DR	serine-type peptidase activity
N	Q2U561	CC	Plays a role in maintenance of chromatin structure during RNA polymerase II transcription elongation thereby repressing transcription initiation from cryptic promoters
N	Q2U561	CC	Mediates the reassembly of nucleosomes onto the promoters of at least a selected set of genes during repression; the nucleosome reassembly is essential for transcriptional repression (By similarity).
N	Q2U561	DE	Chromatin elongation factor spt6;
N	Q2U561	DE	Transcription elongation factor spt6;
N	Q2U561	DR	regulation of transcription from RNA polymerase II promoter
N	Q2U561	DR	transcription elongation regulator activity
N	Q2U9E7	CC	Component of the Mediator complex, a coactivator involved in the regulated transcription of nearly all RNA polymerase II-dependent genes
N	Q2U9E7	CC	Mediator functions as a bridge to convey information from gene-specific regulatory proteins to the basal RNA polymerase II transcription machinery
N	Q2U9E7	CC	Mediator is recruited to promoters by direct interactions with regulatory proteins and serves as a scaffold for the assembly of a functional preinitiation complex with RNA polymerase II and the general transcription factors (By similarity).
N	Q2U9E7	DE	Mediator complex subunit 6;
N	Q2U9E7	DE	Mediator of RNA polymerase II transcription subunit 6;
N	Q2U9E7	DR	RNA polymerase II transcription mediator activity
N	Q2U9E7	DR	regulation of transcription from RNA polymerase II promoter
N	Q2UKT0	CC	ATPase required for the post-translational delivery of tail-anchored (TA) proteins to the endoplasmic reticulum
N	Q2UKT0	CC	ATP binding drives the homodimer towards the closed dimer state, facilitating recognition of newly synthesized TA membrane proteins
N	Q2UKT0	CC	ATP hydolysis is required for insertion
N	Q2UKT0	CC	Recognizes and selectively binds the transmembrane domain of TA proteins in the cytosol
N	Q2UKT0	CC	Subsequently, the homodimer reverts towards the open dimer state, lowering its affinity for the membrane-bound receptor, and returning it to the cytosol to initiate a new round of targeting (By similarity).
N	Q2UKT0	CC	This complex then targets to the endoplasmic reticulum by membrane-bound receptors, where the tail- anchored protein is released for insertion
N	Q2UKT0	CC	This process is regulated by ATP binding and hydrolysis
N	Q2UKT0	DE	ATPase get3;
N	Q2UKT0	DE	Arsenical pump-driving ATPase;
N	Q2UKT0	DE	Arsenite-stimulated ATPase;
N	Q2UKT0	DE	Golgi to ER traffic protein 3;
N	Q2UKT0	DE	Guided entry of tail-anchored proteins 3;
N	Q2UKT0	DR	cellular metal ion homeostasis
N	Q2UKT0	DR	hydrolase activity
N	Q2UKT0	DR	metal ion binding
N	Q2UMX6	CC	Involved in degradation of plant cell walls
N	Q2UMX6	CC	Hydrolyzes of the feruloyl-arabinose ester bond in arabinoxylans as well as the feruloyl-galactose and feruloyl-arabinose ester bonds in pectin (By similarity).
N	Q2UMX6	DE	Ferulic acid esterase B-2;
N	Q2UMX6	DE	Probable feruloyl esterase B-2;
N	Q2UMX6	DR	feruloyl esterase activity
N	Q2UMX6	DR	xylan catabolic process
N	Q2UTN6	CC	Histone methyltransferase that methylates histone H3 to form H3K36me
N	Q2UTN6	CC	Involved in transcription elongation as well as in transcription repression (By similarity).
N	Q2UTN6	DE	Histone-lysine N-methyltransferase, H3 lysine-36 specific;
N	Q2UTN6	DE	SET domain-containing protein 2;

N	Q2UTN6	DR	histone-lysine N-methyltransferase activity
N	Q2UTN6	DR	regulation of transcription, DNA-dependent
N	Q2V460	DE	Putative defensin-like protein 39;
N	Q2V460	DR	defense response to fungus
N	Q2V460	DR	killing of cells of other organism
N	Q2VEH9	CC	Controls the interaction of photosystem II (PSII) cores with the light-harvesting antenna (By similarity).
N	Q2VEH9	DE	Photosystem II reaction center protein Z;
N	Q2VEH9	DR	photosynthesis
N	Q2VEH9	DR	photosystem II stabilization
N	Q2VPM4	CC	Transcriptional corepressor which does not bind directly to DNA and may regulate transcription through recruitment of histone deacetylases to gene promoters
N	Q2VPM4	CC	May be required for segmental gene expression during hindbrain development (By similarity).
N	Q2VPM4	CC	Regulates cell adhesion, migration and proliferation
N	Q2VPM4	DE	Zinc finger protein 703-B;
N	Q2VPM4	DR	adherens junction assembly
N	Q2VPM4	DR	negative regulation of homotypic cell-cell adhesion
N	Q2VPM4	DR	nucleic acid binding
N	Q2VPM4	DR	positive regulation of cell migration
N	Q2VPM4	DR	regulation of canonical Wnt receptor signaling pathway
N	Q2VPM4	DR	regulation of cell cycle
N	Q2VPM4	DR	regulation of transforming growth factor beta receptor signaling pathway
N	Q2VPM4	DR	specific transcriptional repressor activity
N	Q2VPM4	DR	zinc ion binding
N	Q2VZN3	CC	Produces ATP from ADP in the presence of a proton gradient across the membrane
N	Q2VZN3	DE	ATP synthase F1 sector epsilon subunit;
N	Q2VZN3	DE	ATP synthase epsilon chain;
N	Q2VZN3	DE	F-ATPase epsilon subunit;
N	Q2VZN3	DR	ATP synthesis coupled proton transport
N	Q2VZN3	DR	hydrogen ion transporting ATP synthase activity, rotational mechanism
N	Q2VZN3	DR	proton-transporting ATPase activity, rotational mechanism
N	Q2W000	CC	Catalyzes the ATP-dependent conversion of 7-carboxy-7- deazaguanine (CDG) to 7-cyano-7-deazaguanine (preQ(0)) (By similarity).
N	Q2W000	DE	7-cyano-7-carbaguanine synthase;
N	Q2W000	DE	7-cyano-7-deazaguanine synthase;
N	Q2W000	DE	PreQ(0) synthase;
N	Q2W000	DE	Queuosine biosynthesis protein queC;
N	Q2W000	DR	ligase activity
N	Q2W000	DR	metal ion binding
N	Q2W000	DR	queuosine biosynthetic process
N	Q2XXP2	CC	Blocks ryanodine receptors, and potassium channels (By similarity).
N	Q2XXP2	DE	CRISP-VAR10;
N	Q2XXP2	DE	Cysteine-rich secretory protein VAR10;
N	Q2XXP2	DE	DE Flags: Precursor; Fragment;
N	Q2XXP2	DR	calcium channel inhibitor activity
N	Q2XXP2	DR	potassium channel inhibitor activity
N	Q2Y8G6	CC	Key component of the proton channel; it plays a direct role in the translocation of protons across the membrane (By similarity).
N	Q2Y8G6	DE	ATP synthase F0 sector subunit a;
N	Q2Y8G6	DE	ATP synthase subunit a;
N	Q2Y8G6	DE	F-ATPase subunit 6;
N	Q2Y8G6	DR	ATP synthesis coupled proton transport
N	Q2Y8G6	DR	hydrogen ion transmembrane transporter activity
N	Q2Y8V8	DE	UPF0061 protein Nmul_A1510;
N	Q2YAA4	CC	NDH-1 shuttles electrons from NADH, via FMN and iron- sulfur (Fe-S) centers, to quinones in the respiratory chain
N	Q2YAA4	CC	Couples the redox reaction to proton translocation (for every two electrons transferred, four hydrogen ions are translocated across the cytoplasmic membrane), and thus conserves the redox energy in a proton gradient (By similarity)
N	Q2YAA4	CC	The immediate electron acceptor for the enzyme in this species is believed to be ubiquinone
N	Q2YAA4	DE	NADH dehydrogenase I subunit B 1;
N	Q2YAA4	DE	NADH-quinone oxidoreductase subunit B 1;
N	Q2YAA4	DE	NDH-1 subunit B 1;

N	Q2YAA4	DR	4 iron, 4 sulfur cluster binding
N	Q2YAA4	DR	NADH dehydrogenase (ubiquinone) activity
N	Q2YAA4	DR	metal ion binding
N	Q2YAA4	DR	oxidation-reduction process
N	Q2YAA4	DR	quinone binding
N	Q2YD57	CC	Negatively regulates transcription of bacterial ribonucleotide reductase nrd genes and operons by binding to NrdR- boxes (By similarity).
N	Q2YD57	DE	Transcriptional repressor NrdR;
N	Q2YD57	DR	transcription repressor activity
N	Q2YD57	DR	zinc ion binding
N	Q2YDF6	DE	28S ribosomal protein S28, mitochondrial;
N	Q2YDF6	DE	28S ribosomal protein S35, mitochondrial;
N	Q2YDJ4	CC	Hydrolyzes phosphoramidate and acyl-adenylate substrates.
N	Q2YDJ4	DE	Histidine triad nucleotide-binding protein 3;
N	Q2YDJ4	DR	hydrolase activity
N	Q2YHT5	DE	CD200 cell surface glycoprotein receptor 1-B;
N	Q2YHT5	DE	Cell surface glycoprotein CD200 receptor 1-B;
N	Q2YHT5	DE	Cell surface glycoprotein OX2 receptor 1-B;
N	Q2YHT5	DR	receptor activity
N	Q2YJQ3	CC	Modifies the free amino group of the aminoacyl moiety of methionyl-tRNA(fMet)
N	Q2YJQ3	CC	The formyl group appears to play a dual role in the initiator identity of N-formylmethionyl-tRNA by: (I) promoting its recognition by IF2 and (II) impairing its binding to EFTu-GTP (By similarity).
N	Q2YJQ3	DE	Methionyl-tRNA formyltransferase;
N	Q2YJQ3	DR	methionyl-tRNA formyltransferase activity
N	Q2YJQ3	DR	methyltransferase activity
N	Q2YKK2	CC	Catalyzes the last two sequential reactions in the de novo biosynthetic pathway for UDP-GlcNAc
N	Q2YKK2	CC	Responsible for the acetylation of Glc-N-1-P to give GlcNAc-1-P and for the uridyl transfer from UTP to GlcNAc-1-P which produces UDP-GlcNAc (By similarity)
N	Q2YKK2	DE	Bifunctional protein glmU;
N	Q2YKK2	DE	Glucosamine-1-phosphate N-acetyltransferase;
N	Q2YKK2	DE	N-acetylglucosamine-1-phosphate uridyltransferase;
N	Q2YKK2	DE	UDP-N-acetylglucosamine pyrophosphorylase;
N	Q2YKK2	DR	UDP-N-acetylglucosamine diphosphorylase activity
N	Q2YKK2	DR	cell morphogenesis
N	Q2YKK2	DR	cellular cell wall organization
N	Q2YKK2	DR	glucosamine-1-phosphate N-acetyltransferase activity
N	Q2YKK2	DR	lipopolysaccharide biosynthetic process
N	Q2YKK2	DR	magnesium ion binding
N	Q2YKK2	DR	peptidoglycan biosynthetic process
N	Q2YKK2	DR	regulation of cell shape
N	Q2YKL4	CC	Allows the formation of correctly charged Asn-tRNA(Asn) or Gln-tRNA(Gln) through the transamidation of misacylated Asp- tRNA(Asn) or Glu-tRNA(Gln) in organisms which lack either or both of asparaginyl-tRNA or glutaminyl-tRNA synthetases
N	Q2YKL4	CC	The reaction takes place in the presence of glutamine and ATP through an activated phospho-Asp-tRNA(Asn) or phospho-Glu-tRNA(Gln) (By similarity).
N	Q2YKL4	DE	Asp/Glu-ADT subunit C;
N	Q2YKL4	DE	Aspartyl/glutamyl-tRNA(Asn/Gln) amidotransferase subunit C;
N	Q2YKL4	DR	ligase activity
N	Q2YKL4	DR	regulation of translational fidelity
N	Q2YLH0	DE	AICAR transformylase;
N	Q2YLH0	DE	Bifunctional purine biosynthesis protein purH;
N	Q2YLH0	DE	IMP cyclohydrolase;
N	Q2YLH0	DE	IMP synthase;
N	Q2YLH0	DE	Inosinicase;
N	Q2YLH0	DE	Phosphoribosylaminoimidazolecarboxamide formyltransferase;
N	Q2YLH0	DR	IMP biosynthetic process
N	Q2YLH0	DR	IMP cyclohydrolase activity
N	Q2YLH0	DR	phosphoribosylaminoimidazolecarboxamide formyltransferase activity
N	Q2YLM3	CC	Catalyzes the transfer of a phosphate group to glutamate to form glutamate 5-phosphate which rapidly cyclizes to 5- oxoproline.
N	Q2YLM3	DE	Gamma-glutamyl kinase;
N	Q2YLM3	DE	Glutamate 5-kinase;
N	Q2YLM3	DR	glutamate 5-kinase activity

N	Q2YLM3	DR	proline biosynthetic process
N	Q2YLS2	CC	The 2-oxoglutarate dehydrogenase complex catalyzes the overall conversion of 2-oxoglutarate to succinyl-CoA and CO(2)
N	Q2YLS2	CC	It contains multiple copies of three enzymatic components: 2- oxoglutarate dehydrogenase (E1), dihydrolipoamide succinyltransferase (E2) and lipoamide dehydrogenase (E3) (By similarity).
N	Q2YLS2	DE	2-oxoglutarate dehydrogenase E1 component;
N	Q2YLS2	DE	Alpha-ketoglutarate dehydrogenase;
N	Q2YLS2	DR	oxidation-reduction process
N	Q2YLS2	DR	oxoglutarate dehydrogenase (succinyl-transferring) activity
N	Q2YLS2	DR	thiamine pyrophosphate binding
N	Q2YNJ9	DE	Arginine--tRNA ligase;
N	Q2YNJ9	DE	Arginyl-tRNA synthetase;
N	Q2YNJ9	DR	arginine-tRNA ligase activity
N	Q2YNJ9	DR	arginyl-tRNA aminoacylation
N	Q2YQY9	DE	1-(5-phosphoribosyl)-5-[(5-phosphoribosylamino)methylideneamino] imidazole-4-carboxamide isomerase;
N	Q2YQY9	DE	Phosphoribosylformimino-5-aminoimidazole carboxamide ribotide isomerase;
N	Q2YQY9	DR	1-(5-phosphoribosyl)-5-[(5-phosphoribosylamino)methylideneamino]imidazole-4-carboxamide isomerase activity
N	Q2YQY9	DR	histidine biosynthetic process
N	Q2YR96	CC	Catalyzes the ATP-dependent conversion of 7-carboxy-7- deazaguanine (CDG) to 7-cyano-7-deazaguanine (preQ(0)) (By similarity).
N	Q2YR96	DE	7-cyano-7-carbaguanine synthase;
N	Q2YR96	DE	7-cyano-7-deazaguanine synthase;
N	Q2YR96	DE	PreQ(0) synthase;
N	Q2YR96	DE	Queuosine biosynthesis protein queC;
N	Q2YR96	DR	ligase activity
N	Q2YR96	DR	metal ion binding
N	Q2YR96	DR	queuosine biosynthetic process
N	Q2YSD7	DE	Putative ATP:guanido phosphotransferase SAB0474;
N	Q2YSD7	DR	kinase activity
N	Q2YTA9	CC	IF-3 binds to the 30S ribosomal subunit and shifts the equilibrum between 70S ribosomes and their 50S and 30S subunits in favor of the free subunits, thus enhancing the availability of 30S subunits on which protein synthesis initiation begins
N	Q2YTA9	DE	Translation initiation factor IF-3;
N	Q2YTA9	DR	translation initiation factor activity
N	Q2YTB5	CC	ATP-dependent specificity component of the Clp protease
N	Q2YTB5	CC	Can perform chaperone functions in the absence of ClpP (By similarity).
N	Q2YTB5	CC	It directs the protease to specific substrates
N	Q2YTB5	DE	ATP-dependent Clp protease ATP-binding subunit ClpX;
N	Q2YTB5	DR	ATPase activity
N	Q2YTB5	DR	protein dimerization activity
N	Q2YTB5	DR	protein folding
N	Q2YTB5	DR	unfolded protein binding
N	Q2YTB5	DR	zinc ion binding
N	Q2YU22	DE	Glutamate-1-semialdehyde 2,1-aminomutase 2;
N	Q2YU22	DE	Glutamate-1-semialdehyde aminotransferase 2;
N	Q2YU22	DR	glutamate-1-semialdehyde 2,1-aminomutase activity
N	Q2YU22	DR	porphyrin biosynthetic process
N	Q2YU22	DR	pyridoxal phosphate binding
N	Q2YU22	DR	transaminase activity
N	Q2YUH3	DE	Probable DEAD-box ATP-dependent RNA helicase SAB1965c;
N	Q2YUH3	DR	ATP-dependent helicase activity
N	Q2YW62	CC	Promotes bacterial attachment to multiple substrates, such as fibronectin (Fn), fibrinogen (Fg), elastin peptides and tropoelastin
N	Q2YW62	CC	Promotes adherence to and aggregation of activated platelets (By similarity).
N	Q2YW62	CC	This confers to S.aureus the ability to invade endothelial cells
N	Q2YW62	DE	Fibronectin-binding protein A;
N	Q2YW62	DR	cell adhesion
N	Q2YXG5	DE	Carbamoyl-phosphate synthase large chain;
N	Q2YXG5	DE	Carbamoyl-phosphate synthetase ammonia chain;
N	Q2YXG5	DR	arginine biosynthetic process
N	Q2YXG5	DR	carbamoyl-phosphate synthase (glutamine-hydrolyzing) activity
N	Q2YXG5	DR	metal ion binding

N	Q2YXG5	DR	pyrimidine nucleotide biosynthetic process
N	Q2YY45	CC	Multidrug efflux pump that acts independently of norA and is one of the factors that confers resistance against diverse quinolones and chemical compounds (By similarity).
N	Q2YY45	DE	Quinolone resistance protein norB;
N	Q2YY45	DR	response to antibiotic
N	Q2YY45	DR	transmembrane transport
N	Q30QX0	CC	Allows the formation of correctly charged Asn-tRNA(Asn) or Gln-tRNA(Gln) through the transamidation of misacylated Asp- tRNA(Asn) or Glu-tRNA(Gln) in organisms which lack either or both of asparaginyl-tRNA or glutaminyl-tRNA synthetases
N	Q30QX0	CC	The reaction takes place in the presence of glutamine and ATP through an activated phospho-Asp-tRNA(Asn) or phospho-Glu-tRNA(Gln) (By similarity).
N	Q30QX0	DE	Asp/Glu-ADT subunit B;
N	Q30QX0	DE	Aspartyl/glutamyl-tRNA(Asn/Gln) amidotransferase subunit B;
N	Q30QX0	DR	carbon-nitrogen ligase activity, with glutamine as amido-N-donor
N	Q30T61	DE	Acetohydroxy-acid isomeroreductase;
N	Q30T61	DE	Alpha-keto-beta-hydroxylacil reductoisomerase;
N	Q30T61	DE	Ketol-acid reductoisomerase;
N	Q30T61	DR	branched chain family amino acid biosynthetic process
N	Q30T61	DR	coenzyme binding
N	Q30T61	DR	ketol-acid reductoisomerase activity
N	Q30T61	DR	oxidation-reduction process
N	Q30WM4	DE	Branched-chain carboxylic acid kinase;
N	Q30WM4	DE	Probable butyrate kinase;
N	Q30WM4	DR	butyrate kinase activity
N	Q30YX6	CC	The ruvA-ruvB complex in the presence of ATP renatures cruciform structure in supercoiled DNA with palindromic sequence, indicating that it may promote strand exchange reactions in homologous recombination
N	Q30YX6	CC	RuvA stimulates, in the presence of DNA, the weak ATPase activity of ruvB (By similarity).
N	Q30YX6	CC	RuvAB is an helicase that mediates the Holliday junction migration by localized denaturation and reannealing
N	Q30YX6	DE	Holliday junction ATP-dependent DNA helicase ruvA;
N	Q30YX6	DR	DNA recombination
N	Q30YX6	DR	four-way junction helicase activity
N	Q30ZT5	CC	ATPase subunit of a proteasome-like degradation complex; this subunit has chaperone activity
N	Q30ZT5	CC	HslU recognizes the N-terminal part of its protein substrates and unfolds these before they are guided to HslV for hydrolysis (By similarity).
N	Q30ZT5	CC	The binding of ATP and its subsequent hydrolysis by HslU are essential for unfolding of protein substrates subsequently hydrolyzed by HslV
N	Q30ZT5	DE	ATP-dependent protease ATPase subunit HslU;
N	Q30ZT5	DE	Unfoldase HslU;
N	Q30ZT5	DR	ATPase activity
N	Q30ZT5	DR	peptidase activity, acting on L-amino acid peptides
N	Q30ZT5	DR	response to stress
N	Q314N8	DE	UPF0597 protein Dde_0807;
N	Q318J4	CC	Binds to 23S rRNA
N	Q318J4	CC	Forms part of two intersubunit bridges in the 70S ribosome (By similarity).
N	Q318J4	DE	50S ribosomal protein L14;
N	Q318J4	DR	structural constituent of ribosome
N	Q318Q5	CC	Binds directly to 16S ribosomal RNA (By similarity).
N	Q318Q5	DE	30S ribosomal protein S20;
N	Q318Q5	DR	structural constituent of ribosome
N	Q319M2	CC	Catalyzes the phosphorylation of NAD to NADP
N	Q319M2	CC	Utilizes ATP and other nucleoside triphosphates as well as inorganic polyphosphate as a source of phosphorus (By similarity).
N	Q319M2	DE	Poly(P)/ATP NAD kinase 2;
N	Q319M2	DE	Probable inorganic polyphosphate/ATP-NAD kinase 2;
N	Q319M2	DR	NAD+ kinase activity
N	Q319X7	CC	Catalyzes amidations at positions B, D, E, and G on adenosylcobyrinic A,C-diamide
N	Q319X7	CC	NH(2) groups are provided by glutamine, and one molecule of ATP is hydrogenolyzed for each amidation (By similarity).
N	Q319X7	DE	Cobyric acid synthase;
N	Q319X7	DR	catalytic activity
N	Q319X7	DR	cobalamin biosynthetic process

N	Q319X7	DR	glutamine metabolic process
N	Q31BZ1	CC	Uses Mg-ATP and reduced ferredoxin to reduce ring D of protochlorophyllide (Pchlide) to form chlorophyllide a (Chlide) (By similarity)
N	Q31BZ1	CC	This reaction is light-independent.
N	Q31BZ1	DE	DPOR subunit L;
N	Q31BZ1	DE	LI-POR subunit L;
N	Q31BZ1	DE	Light-independent protochlorophyllide reductase iron-sulfur ATP-binding protein;
N	Q31BZ1	DR	4 iron, 4 sulfur cluster binding
N	Q31BZ1	DR	chlorophyll biosynthetic process
N	Q31BZ1	DR	metal ion binding
N	Q31BZ1	DR	oxidation-reduction process
N	Q31BZ1	DR	oxidoreductase activity, acting on iron-sulfur proteins as donors
N	Q31BZ1	DR	photosynthesis, dark reaction
N	Q31C13	CC	Responsible for the release of ribosomes from messenger RNA at the termination of protein biosynthesis
N	Q31C13	CC	May increase the efficiency of translation by recycling ribosomes from one round of translation to another (By similarity).
N	Q31C13	DE	Ribosome-recycling factor;
N	Q31C13	DE	Ribosome-releasing factor;
N	Q31HI0	CC	Catalyzes the oxidation of 3-carboxy-2-hydroxy-4- methylpentanoate (3-isopropylmalate) to 3-carboxy-4-methyl-2- oxopentanoate
N	Q31HI0	CC	The product decarboxylates to 4-methyl-2 oxopentanoate (By similarity).
N	Q31HI0	DE	3-isopropylmalate dehydrogenase;
N	Q31HI0	DE	Beta-IPM dehydrogenase;
N	Q31HI0	DR	3-isopropylmalate dehydrogenase activity
N	Q31HI0	DR	leucine biosynthetic process
N	Q31HI0	DR	magnesium ion binding
N	Q31HI0	DR	oxidation-reduction process
N	Q31IW7	CC	This protein binds to the 23S rRNA, and is important in its secondary structure
N	Q31IW7	CC	It is located near the subunit interface in the base of the L7/L12 stalk, and near the tRNA binding site of the peptidyltransferase center (By similarity).
N	Q31IW7	DE	50S ribosomal protein L6;
N	Q31IW7	DR	structural constituent of ribosome
N	Q31TI1	DE	Aspartate carbamoyltransferase;
N	Q31TI1	DE	Aspartate transcarbamylase;
N	Q31TI1	DR	'de novo' pyrimidine base biosynthetic process
N	Q31TI1	DR	amino acid binding
N	Q31TI1	DR	aspartate carbamoyltransferase activity
N	Q31TI1	DR	cellular amino acid metabolic process
N	Q31TI1	DR	pyrimidine nucleotide biosynthetic process
N	Q31WA8	CC	Efflux pump whose substrates are p-hydroxybenzoic acid (pHBA), 6-hydroxy-2-naphthoic and 2-hydroxycinnamate
N	Q31WA8	CC	Could function as a metabolic relief valve, allowing to eliminate certain compounds when they accumulate to high levels in the cell (By similarity).
N	Q31WA8	DE	p-hydroxybenzoic acid efflux pump subunit AaeB;
N	Q31WA8	DE	pHBA efflux pump protein B;
N	Q31XA6	CC	Nucleotidase with a broad substrate specificity as it can dephosphorylate various ribo- and deoxyribonucleoside 5'- monophosphates and ribonucleoside 3'-monophosphates with highest affinity to 3'-AMP
N	Q31XA6	CC	Also hydrolyzes polyphosphate (exopolyphosphatase activity) with the preference for short-chain- length substrates (P20-25)
N	Q31XA6	CC	Might be involved in the regulation of dNTP and NTP pools, and in the turnover of 3'-mononucleotides produced by numerous intracellular RNases (T1, T2, and F) during the degradation of various RNAs (By similarity).
N	Q31XA6	DE	5'/3'-nucleotidase;
N	Q31XA6	DE	Exopolyphosphatase;
N	Q31XA6	DE	Multifunctional protein surE;
N	Q31XA6	DE	Nucleoside monophosphate phosphohydrolase;
N	Q31XA6	DR	3'-nucleotidase activity
N	Q31XA6	DR	5'-nucleotidase activity
N	Q31XA6	DR	exopolyphosphatase activity
N	Q31XA6	DR	metal ion binding
N	Q31XA6	DR	nucleotide binding

N	Q31Y68	CC	DNA ligase that catalyzes the formation of phosphodiester linkages between 5'-phosphoryl and 3'-hydroxyl groups in double-stranded DNA using NAD as a coenzyme and as the energy source for the reaction
N	Q31Y68	CC	It is essential for DNA replication and repair of damaged DNA (By similarity).
N	Q31Y68	DE	Polydeoxyribonucleotide synthase [NAD+];
N	Q31Y68	DR	DNA ligase (NAD+) activity
N	Q31Y68	DR	DNA replication
N	Q31Y68	DR	metal ion binding
N	Q321N6	CC	Transforms N(2)-succinylglutamate into succinate and glutamate.
N	Q321N6	DE	Succinylglutamate desuccinylase;
N	Q321N6	DR	arginine catabolic process to glutamate
N	Q321N6	DR	hydrolase activity, acting on ester bonds
N	Q321N6	DR	succinylglutamate desuccinylase activity
N	Q321N6	DR	zinc ion binding
N	Q325G3	CC	ATP-dependent specificity component of the Clp protease
N	Q325G3	CC	Can perform chaperone functions in the absence of ClpP (By similarity).
N	Q325G3	CC	It directs the protease to specific substrates
N	Q325G3	DE	ATP-dependent Clp protease ATP-binding subunit ClpX;
N	Q325G3	DR	ATPase activity
N	Q325G3	DR	protein dimerization activity
N	Q325G3	DR	protein folding
N	Q325G3	DR	unfolded protein binding
N	Q325G3	DR	zinc ion binding
N	Q328K0	CC	Catalyzes the isomerization of L-ribulose 5-phosphate to D-xylulose 5-phosphate
N	Q328K0	CC	Is involved in the anaerobic L-ascorbate utilization (By similarity).
N	Q328K0	DE	L-ascorbate utilization protein F;
N	Q328K0	DE	L-ribulose-5-phosphate 4-epimerase UlaF;
N	Q328K0	DE	Phosphoribulose isomerase;
N	Q328K0	DR	L-ribulose-phosphate 4-epimerase activity
N	Q328K0	DR	metal ion binding
N	Q329T3	DE	Asparagine synthetase A;
N	Q329T3	DE	Aspartate--ammonia ligase;
N	Q329T3	DR	aminoacyl-tRNA ligase activity
N	Q329T3	DR	asparagine biosynthetic process
N	Q329T3	DR	aspartate-ammonia ligase activity
N	Q329T3	DR	tRNA aminoacylation for protein translation
N	Q32B33	CC	One of the early assembly proteins it binds 23S rRNA
N	Q32B33	CC	Forms the main docking site for trigger factor binding to the ribosome (By similarity)
N	Q32B33	CC	One of the proteins that surrounds the polypeptide exit tunnel on the outside of the ribosome
N	Q32B33	DE	50S ribosomal protein L23;
N	Q32B33	DR	nucleotide binding
N	Q32B33	DR	structural constituent of ribosome
N	Q32BX2	CC	Activator of cell division through the inhibition of FtsZ GTPase activity, therefore promoting FtsZ assembly into bundles of protofilaments necessary for the formation of the division Z ring
N	Q32BX2	CC	It is recruited early at mid-cell but it is not essential for cell division (By similarity)
N	Q32BX2	DE	Cell division protein ZapA;
N	Q32BX2	DE	Z ring-associated protein ZapA;
N	Q32BX2	DR	barrier septum formation
N	Q32CG5	CC	Reduction of activated sulfate into sulfite.
N	Q32CG5	DE	3'-phosphoadenylylsulfate reductase;
N	Q32CG5	DE	PAPS reductase, thioredoxin dependent;
N	Q32CG5	DE	PAPS sulfotransferase;
N	Q32CG5	DE	PAdoPS reductase;
N	Q32CG5	DE	Phosphoadenosine phosphosulfate reductase;
N	Q32CG5	DR	cysteine biosynthetic process
N	Q32CG5	DR	phosphoadenylyl-sulfate reductase (thioredoxin) activity
N	Q32CG5	DR	sulfate assimilation, phosphoadenylyl sulfate reduction by phosphoadenylyl-sulfate reductase (thioredoxin)
N	Q32FI6	DE	Phenylalanine--tRNA ligase beta chain;
N	Q32FI6	DE	Phenylalanyl-tRNA synthetase beta chain;
N	Q32FI6	DR	magnesium ion binding
N	Q32FI6	DR	phenylalanine-tRNA ligase activity
N	Q32FI6	DR	phenylalanyl-tRNA aminoacylation

N	Q32FI6	DR	tRNA processing
N	Q32GS9	CC	The beta subunit is responsible for the synthesis of L- tryptophan from indole and L-serine.
N	Q32GS9	DE	Tryptophan synthase beta chain;
N	Q32GS9	DR	pyridoxal phosphate binding
N	Q32GS9	DR	tryptophan synthase activity
N	Q32J26	CC	Catalyzes the transfer of a phosphate group to glutamate to form glutamate 5-phosphate which rapidly cyclizes to 5- oxoproline.
N	Q32J26	DE	Gamma-glutamyl kinase;
N	Q32J26	DE	Glutamate 5-kinase;
N	Q32J26	DR	glutamate 5-kinase activity
N	Q32J26	DR	proline biosynthetic process
N	Q32K67	CC	Hydrolyzes both purine and pyrimidine ribonucleosides with a broad-substrate specificity (By similarity).
N	Q32K67	DE	Non-specific ribonucleoside hydrolase rihC;
N	Q32K67	DE	Purine/pyrimidine ribonucleoside hydrolase;
N	Q32K67	DR	hydrolase activity, hydrolyzing N-glycosyl compounds
N	Q32KN8	CC	Tubulin is the major constituent of microtubules
N	Q32KN8	CC	It binds two moles of GTP, one at an exchangeable site on the beta chain and one at a non-exchangeable site on the alpha-chain (By similarity).
N	Q32KN8	DE	Alpha-tubulin 3;
N	Q32KN8	DE	Tubulin alpha-3 chain;
N	Q32KN8	DR	GTPase activity
N	Q32KN8	DR	microtubule-based movement
N	Q32KN8	DR	protein polymerization
N	Q32KN8	DR	structural molecule activity
N	Q32LA4	DE	Uracil phosphoribosyltransferase homolog;
N	Q32LA4	DR	nucleoside metabolic process
N	Q32LB9	DE	Uncharacterized protein C4orf14 homolog;
N	Q32LZ8	CC	Lipid hydrolase (By similarity).
N	Q32LZ8	DE	Patatin-like phospholipase domain-containing protein 5;
N	Q32LZ8	DR	hydrolase activity
N	Q32LZ8	DR	lipid catabolic process
N	Q32NI8	CC	Condensing enzyme that catalyzes the synthesis of very long chain fatty acids (By similarity).
N	Q32NI8	DE	3-keto acyl-CoA synthase elovl5;
N	Q32NI8	DE	Elongation of very long chain fatty acids protein 5;
N	Q32NI8	DR	fatty acid elongase activity
N	Q32NI8	DR	very long-chain fatty acid biosynthetic process
N	Q32NX4	DE	Na(+)/K(+)-transporting ATPase subunit beta-1-interacting protein 4;
N	Q32NX4	DE	Sodium/potassium-transporting ATPase subunit beta-1-interacting protein 4;
N	Q32PG7	DE	Transmembrane protein 175;
N	Q32RJ3	CC	Component of the cytochrome b6-f complex, which mediates electron transfer between photosystem II (PSII) and photosystem I (PSI), cyclic electron flow around PSI, and state transitions (By similarity).
N	Q32RJ3	DE	Apocytochrome f;
N	Q32RJ3	DR	electron carrier activity
N	Q32RJ3	DR	electron transport chain
N	Q32RJ3	DR	photosynthesis
N	Q32RK6	CC	Uses Mg-ATP and reduced ferredoxin to reduce ring D of protochlorophyllide (Pchlide) to form chlorophyllide a (Chlide) (By similarity)
N	Q32RK6	CC	This reaction is light-independent.
N	Q32RK6	DE	DPOR subunit N;
N	Q32RK6	DE	LI-POR subunit N;
N	Q32RK6	DE	Light-independent protochlorophyllide reductase subunit N;
N	Q32RK6	DR	chlorophyll biosynthetic process
N	Q32RK6	DR	oxidation-reduction process
N	Q32RK6	DR	oxidoreductase activity, acting on iron-sulfur proteins as donors
N	Q32RK6	DR	photosynthesis, dark reaction
N	Q32RM0	CC	Apoprotein for the two 4Fe-4S centers FA and FB of photosystem I (PSI); essential for photochemical activity
N	Q32RM0	CC	FB is the terminal electron acceptor of PSI, donating electrons to ferredoxin
N	Q32RM0	CC	PSI is a plastocyanin-ferredoxin oxidoreductase, converting photonic excitation into a charge separation, which transfers an electron from the donor P700 chlorophyll pair to the spectroscopically characterized acceptors A0, A1, FX, FA and FB in turn

N	Q32RM0	CC	Required for binding of psaD and psaE to PSI
N	Q32RM0	CC	The C-terminus interacts with psaA/B/D and helps assemble the protein into the PSI complex
N	Q32RM0	DE	9 kDa polypeptide;
N	Q32RM0	DE	Photosystem I iron-sulfur center;
N	Q32RM0	DE	Photosystem I subunit VII;
N	Q32RM0	DR	4 iron, 4 sulfur cluster binding
N	Q32RM0	DR	electron carrier activity
N	Q32RM0	DR	metal ion binding
N	Q32RM0	DR	photosynthetic electron transport in photosystem I
N	Q32RT0	CC	One of the two reaction center proteins of photosystem II (PSII), D2 is needed for assembly of a stable PSII complex (By similarity).
N	Q32RT0	DE	PSII D2 protein;
N	Q32RT0	DE	Photosystem II D2 protein;
N	Q32RT0	DE	Photosystem Q(A) protein;
N	Q32RT0	DR	electron transporter, transferring electrons within the cyclic electron transport pathway of photosynthesis activity
N	Q32RT0	DR	metal ion binding
N	Q32RT0	DR	photosynthetic electron transport in photosystem II
N	Q32RY5	CC	Seems to be required for the assembly of the photosystem I complex (By similarity).
N	Q32RY5	DE	Photosystem I assembly protein ycf3;
N	Q32RY5	DR	photosynthesis
N	Q32S05	CC	NDH shuttles electrons from NAD(P)H:plastoquinone, via FMN and iron-sulfur (Fe-S) centers, to quinones in the photosynthetic chain and possibly in a chloroplast respiratory chain
N	Q32S05	CC	Couples the redox reaction to proton translocation, and thus conserves the redox energy in a proton gradient (By similarity).
N	Q32S05	CC	The immediate electron acceptor for the enzyme in this species is believed to be plastoquinone
N	Q32S05	DE	NAD(P)H dehydrogenase subunit 4L;
N	Q32S05	DE	NAD(P)H-quinone oxidoreductase subunit 4L, chloroplastic;
N	Q32S05	DE	NADH-plastoquinone oxidoreductase subunit 4L;
N	Q32S05	DR	ATP synthesis coupled electron transport
N	Q32S05	DR	oxidoreductase activity, acting on NADH or NADPH
N	Q32S05	DR	quinone binding
N	Q332R6	CC	Binds to 23S rRNA (By similarity).
N	Q332R6	DE	50S ribosomal protein L23, chloroplastic;
N	Q332R6	DR	nucleotide binding
N	Q332R6	DR	structural constituent of ribosome
N	Q33528	CC	RuBisCO catalyzes two reactions: the carboxylation of D- ribulose 1,5-bisphosphate, the primary event in carbon dioxide fixation, as well as the oxidative fragmentation of the pentose substrate in the photorespiration process
N	Q33528	CC	Both reactions occur simultaneously and in competition at the same active site (By similarity).
N	Q33528	DE	Ribulose bisphosphate carboxylase large chain;
N	Q33528	DE	RuBisCO large subunit;
N	Q33528	DR	magnesium ion binding
N	Q33528	DR	monooxygenase activity
N	Q33528	DR	oxidation-reduction process
N	Q33528	DR	photorespiration
N	Q33528	DR	reductive pentose-phosphate cycle
N	Q33528	DR	ribulose-bisphosphate carboxylase activity
N	Q33820	CC	Cytochrome c oxidase is the component of the respiratory chain that catalyzes the reduction of oxygen to water
N	Q33820	CC	CO I is the catalytic subunit of the enzyme
N	Q33820	CC	Electrons originating in cytochrome c are transferred via the copper A center of subunit 2 and heme A of subunit 1 to the bimetallic center formed by heme A3 and copper B
N	Q33820	CC	Subunits 1- 3 form the functional core of the enzyme complex
N	Q33820	DE	Cytochrome c oxidase polypeptide I;
N	Q33820	DE	Cytochrome c oxidase subunit 1;
N	Q33820	DR	aerobic respiration
N	Q33820	DR	cytochrome-c oxidase activity
N	Q33820	DR	electron carrier activity
N	Q33820	DR	electron transport chain
N	Q33BZ0	DE	50S ribosomal protein L2, chloroplastic;

N	Q33BZ0	DR	structural constituent of ribosome
N	Q33BZ0	DR	transferase activity
N	Q34941	CC	Cytochrome c oxidase is the component of the respiratory chain that catalyzes the reduction of oxygen to water
N	Q34941	CC	CO I is the catalytic subunit of the enzyme
N	Q34941	CC	Electrons originating in cytochrome c are transferred via the copper A center of subunit 2 and heme A of subunit 1 to the bimetallic center formed by heme A3 and copper B
N	Q34941	CC	Subunits 1- 3 form the functional core of the enzyme complex
N	Q34941	DE	Cytochrome c oxidase polypeptide I;
N	Q34941	DE	Cytochrome c oxidase subunit 1;
N	Q34941	DR	aerobic respiration
N	Q34941	DR	cytochrome-c oxidase activity
N	Q34941	DR	electron carrier activity
N	Q34941	DR	electron transport chain
N	Q36610	CC	RuBisCO catalyzes two reactions: the carboxylation of D- ribulose 1,5-bisphosphate, the primary event in carbon dioxide fixation, as well as the oxidative fragmentation of the pentose substrate in the photorespiration process
N	Q36610	CC	Both reactions occur simultaneously and in competition at the same active site (By similarity).
N	Q36610	DE	Ribulose bisphosphate carboxylase large chain;
N	Q36610	DE	RuBisCO large subunit;
N	Q36610	DR	magnesium ion binding
N	Q36610	DR	monooxygenase activity
N	Q36610	DR	oxidation-reduction process
N	Q36610	DR	photorespiration
N	Q36610	DR	reductive pentose-phosphate cycle
N	Q36610	DR	ribulose-bisphosphate carboxylase activity
N	Q36852	CC	Mitochondrial membrane ATP synthase (F(1)F(0) ATP synthase or Complex V) produces ATP from ADP in the presence of a proton gradient across the membrane which is generated by electron transport complexes of the respiratory chain
N	Q36852	CC	A homomeric c-ring of probably 10 subunits is part of the complex rotary element
N	Q36852	CC	During catalysis, ATP synthesis in the catalytic domain of F(1) is coupled via a rotary mechanism of the central stalk subunits to proton translocation
N	Q36852	CC	F-type ATPases consist of two structural domains, F(1) - containing the extramembraneous catalytic core and F(0) - containing the membrane proton channel, linked together by a central stalk and a peripheral stalk
N	Q36852	CC	Part of the complex F(0) domain
N	Q36852	DE	ATP synthase subunit 9, mitochondrial;
N	Q36852	DE	Lipid-binding protein;
N	Q36852	DR	ATP hydrolysis coupled proton transport
N	Q36852	DR	ATP synthesis coupled proton transport
N	Q36852	DR	hydrogen ion transmembrane transporter activity
N	Q36852	DR	lipid binding
N	Q36907	DE	Uncharacterized protein ycf73;
N	Q37717	CC	Core subunit of the mitochondrial membrane respiratory chain NADH dehydrogenase (Complex I) that is believed to belong to the minimal assembly required for catalysis
N	Q37717	CC	Complex I functions in the transfer of electrons from NADH to the respiratory chain
N	Q37717	CC	The immediate electron acceptor for the enzyme is believed to be ubiquinone (By similarity).
N	Q37717	DE	NADH dehydrogenase subunit 1;
N	Q37717	DE	NADH-ubiquinone oxidoreductase chain 1;
N	Q37717	DR	NADH dehydrogenase (ubiquinone) activity
N	Q37717	DR	electron transport chain
N	Q37787	CC	Core subunit of the mitochondrial membrane respiratory chain NADH dehydrogenase (Complex I) that is believed to belong to the minimal assembly required for catalysis
N	Q37787	CC	Complex I functions in the transfer of electrons from NADH to the respiratory chain
N	Q37787	CC	The immediate electron acceptor for the enzyme is believed to be ubiquinone (By similarity).
N	Q37787	DE	Complex I-27kD;
N	Q37787	DE	NADH dehydrogenase subunit 9;
N	Q37787	DE	NADH-ubiquinone oxidoreductase 27 kDa subunit;
N	Q37787	DR	NADH dehydrogenase (ubiquinone) activity
N	Q37787	DR	electron transport chain
N	Q38680	CC	Non-catalytic subunit of the peripheral V1 complex of vacuolar ATPase

N	Q38680	CC	V-ATPase is responsible for acidifying a variety of intracellular compartments in eukaryotic cells.
N	Q38680	DE	V-ATPase subunit B 2;
N	Q38680	DE	V-type proton ATPase subunit B 2;
N	Q38680	DE	Vacuolar proton pump subunit B 2;
N	Q38680	DR	ATP hydrolysis coupled proton transport
N	Q38680	DR	hydrogen ion transporting ATP synthase activity, rotational mechanism
N	Q38680	DR	proton-transporting ATPase activity, rotational mechanism
N	Q38869	CC	May play a role in signal transduction pathways that involve calcium as a second messenger
N	Q38869	CC	Functions as regulator of the calcium-mediated abscisic acid (ABA) signaling pathway
N	Q38869	CC	Phosphorylates ABA-responsive transcription factors ABF1 and ABF4 in vitro
N	Q38869	CC	Phosphorylates the nuclear zinc finger Di19 in vitro.
N	Q38869	DE	Calcium-dependent protein kinase 4;
N	Q38869	DE	Calmodulin-domain protein kinase CDPK isoform 4;
N	Q38869	DR	calcium ion binding
N	Q38869	DR	positive regulation of abscisic acid mediated signaling pathway
N	Q38869	DR	protein binding
N	Q38869	DR	protein phosphorylation
N	Q38869	DR	protein serine/threonine kinase activity
N	Q38UV3	CC	Formation of pseudouridine at positions 38, 39 and 40 in the anticodon stem and loop of transfer RNAs (By similarity).
N	Q38UV3	DE	tRNA pseudouridine synthase A;
N	Q38UV3	DE	tRNA pseudouridylate synthase I;
N	Q38UV3	DE	tRNA-uridine isomerase I;
N	Q38UV3	DR	pseudouridine synthase activity
N	Q38UV3	DR	pseudouridine synthesis
N	Q38UV3	DR	tRNA processing
N	Q38ZE2	DE	Pantothenate kinase;
N	Q38ZE2	DE	Pantothenic acid kinase;
N	Q38ZE2	DR	coenzyme A biosynthetic process
N	Q38ZE2	DR	pantothenate kinase activity
N	Q39027	DE	MAP kinase 7;
N	Q39027	DE	Mitogen-activated protein kinase 7;
N	Q39027	DR	MAP kinase activity
N	Q39027	DR	protein binding
N	Q39027	DR	protein phosphorylation
N	Q39027	DR	response to hydrogen peroxide
N	Q39445	CC	Tubulin is the major constituent of microtubules
N	Q39445	CC	It binds two moles of GTP, one at an exchangeable site on the beta chain and one at a non-exchangeable site on the alpha-chain.
N	Q39445	DE	Beta-tubulin;
N	Q39445	DE	Tubulin beta chain;
N	Q39445	DR	GTPase activity
N	Q39445	DR	microtubule-based movement
N	Q39445	DR	protein polymerization
N	Q39445	DR	structural molecule activity
N	Q39769	DE	Glyceraldehyde-3-phosphate dehydrogenase, cytosolic;
N	Q39769	DR	glyceraldehyde-3-phosphate dehydrogenase (NAD+) (phosphorylating) activity
N	Q39769	DR	oxidation-reduction process
N	Q39BG3	DE	UPF0502 protein Bcep18194_B0081;
N	Q39EE7	CC	NDH-1 shuttles electrons from NADH, via FMN and iron- sulfur (Fe-S) centers, to quinones in the respiratory chain
N	Q39EE7	CC	Couples the redox reaction to proton translocation (for every two electrons transferred, four hydrogen ions are translocated across the cytoplasmic membrane), and thus conserves the redox energy in a proton gradient (By similarity)
N	Q39EE7	CC	The immediate electron acceptor for the enzyme in this species is believed to be ubiquinone
N	Q39EE7	DE	NADH dehydrogenase I subunit C;
N	Q39EE7	DE	NADH-quinone oxidoreductase subunit C;
N	Q39EE7	DE	NDH-1 subunit C;
N	Q39EE7	DR	NADH dehydrogenase (ubiquinone) activity
N	Q39EE7	DR	oxidation-reduction process
N	Q39EE7	DR	quinone binding

N	Q39GT3	CC	Represses a number of genes involved in the response to DNA damage (SOS response), including recA and lexA
N	Q39GT3	CC	In the presence of single-stranded DNA, recA interacts with lexA causing an autocatalytic cleavage which disrupts the DNA-binding part of lexA, leading to derepression of the SOS regulon and eventually DNA repair (By similarity).
N	Q39GT3	DE	LexA repressor;
N	Q39GT3	DR	DNA replication
N	Q39GT3	DR	serine-type endopeptidase activity
N	Q39JR1	CC	Part of the ABC transporter complex AraFGH involved in arabinose import
N	Q39JR1	CC	Responsible for energy coupling to the transport system (By similarity).
N	Q39JR1	DE	Arabinose import ATP-binding protein AraG 1;
N	Q39JR1	DR	L-arabinose transport
N	Q39JR1	DR	L-arabinose-importing ATPase activity
N	Q39KC4	DE	3-dehydroquinate synthase;
N	Q39KC4	DR	3-dehydroquinate synthase activity
N	Q39KC4	DR	aromatic amino acid family biosynthetic process
N	Q39KF0	CC	With S4 and S12 plays an important role in translational accuracy (By similarity).
N	Q39KF0	DE	30S ribosomal protein S5;
N	Q39KF0	DR	structural constituent of ribosome
N	Q39UF9	CC	Catalyzes the conversion of glucosamine-6-phosphate to glucosamine-1-phosphate (By similarity).
N	Q39UF9	DE	Phosphoglucosamine mutase;
N	Q39UF9	DR	carbohydrate metabolic process
N	Q39UF9	DR	magnesium ion binding
N	Q39UF9	DR	phosphoglucosamine mutase activity
N	Q39VA3	CC	Responsible for synthesis of pseudouridine from uracil- 55 in the psi GC loop of transfer RNAs (By similarity).
N	Q39VA3	DE	Psi55 synthase;
N	Q39VA3	DE	tRNA pseudouridine 55 synthase;
N	Q39VA3	DE	tRNA pseudouridine synthase B;
N	Q39VA3	DE	tRNA pseudouridylate synthase;
N	Q39VA3	DE	tRNA-uridine isomerase;
N	Q39VA3	DR	pseudouridine synthase activity
N	Q39VA3	DR	pseudouridine synthesis
N	Q39VA3	DR	tRNA processing
N	Q39VY5	CC	Catalyzes the decarboxylation of orotidine 5'- monophosphate (OMP) to uridine 5'-monophosphate (UMP) (By similarity).
N	Q39VY5	DE	OMP decarboxylase;
N	Q39VY5	DE	Orotidine 5'-phosphate decarboxylase;
N	Q39VY5	DR	'de novo' UMP biosynthetic process
N	Q39VY5	DR	'de novo' pyrimidine base biosynthetic process
N	Q39VY5	DR	orotidine-5'-phosphate decarboxylase activity
N	Q39W87	CC	Associates with the EF-Tu.GDP complex and induces the exchange of GDP to GTP.
N	Q39W87	CC	It remains bound to the aminoacyl-tRNA.EF- Tu.GTP complex up to the GTP hydrolysis stage on the ribosome.
N	Q39W87	DE	Elongation factor Ts;
N	Q39W87	DR	translation elongation factor activity
N	Q39ZB0	CC	NDH-1 shuttles electrons from NADH, via FMN and iron- sulfur (Fe-S) centers, to quinones in the respiratory chain
N	Q39ZB0	CC	Couples the redox reaction to proton translocation (for every two electrons transferred, four hydrogen ions are translocated across the cytoplasmic membrane), and thus conserves the redox energy in a proton gradient (By similarity).
N	Q39ZB0	CC	The immediate electron acceptor for the enzyme in this species is believed to be ubiquinone
N	Q39ZB0	DE	NADH dehydrogenase I subunit K 1;
N	Q39ZB0	DE	NADH-quinone oxidoreductase subunit K 1;
N	Q39ZB0	DE	NDH-1 subunit K 1;
N	Q39ZB0	DR	ATP synthesis coupled electron transport
N	Q39ZB0	DR	NADH dehydrogenase (quinone) activity
N	Q39ZB0	DR	quinone binding
N	Q3A4N9	DE	Phenylalanine--tRNA ligase alpha chain;
N	Q3A4N9	DE	Phenylalanyl-tRNA synthetase alpha chain;
N	Q3A4N9	DR	metal ion binding
N	Q3A4N9	DR	phenylalanine-tRNA ligase activity
N	Q3A4N9	DR	phenylalanyl-tRNA aminoacylation

N	Q3A6R6	CC	Catalyzes the attachment of proline to tRNA(Pro) in a two-step reaction: proline is first activated by ATP to form Pro- AMP and then transferred to the acceptor end of tRNA(Pro)
N	Q3A6R6	CC	As ProRS can inadvertently accommodate and process non-cognate amino acids such as alanine and cysteine, to avoid such errors it has two additional distinct editing activities against alanine
N	Q3A6R6	CC	One activity is designated as 'pretransfer' editing and involves the tRNA(Pro)-independent hydrolysis of activated Ala-AMP
N	Q3A6R6	CC	The misacylated Cys- tRNA(Pro) is not edited by ProRS (By similarity).
N	Q3A6R6	CC	The other activity is designated 'posttransfer' editing and involves deacylation of mischarged Ala-tRNA(Pro)
N	Q3A6R6	DE	Proline--tRNA ligase;
N	Q3A6R6	DE	Prolyl-tRNA synthetase;
N	Q3A6R6	DR	proline-tRNA ligase activity
N	Q3A6R6	DR	prolyl-tRNA aminoacylation
N	Q3ABS6	DE	Threonine--tRNA ligase;
N	Q3ABS6	DE	Threonyl-tRNA synthetase;
N	Q3ABS6	DR	metal ion binding
N	Q3ABS6	DR	threonine-tRNA ligase activity
N	Q3ABS6	DR	threonyl-tRNA aminoacylation
N	Q3AC47	DE	Acetate kinase;
N	Q3AC47	DE	Acetokinase;
N	Q3AC47	DR	acetate kinase activity
N	Q3AC47	DR	organic acid metabolic process
N	Q3AFM4	CC	Catalyzes the phosphorylation of the position 2 hydroxy group of 4-diphosphocytidyl-2C-methyl-D-erythritol (By similarity).
N	Q3AFM4	DE	4-(cytidine-5'-diphospho)-2-C-methyl-D-erythritol kinase;
N	Q3AFM4	DE	4-diphosphocytidyl-2-C-methyl-D-erythritol kinase;
N	Q3AFM4	DR	4-(cytidine 5'-diphospho)-2-C-methyl-D-erythritol kinase activity
N	Q3AFM4	DR	terpenoid biosynthetic process
N	Q3AHR1	DE	UPF0082 protein Syncc9605_2132;
N	Q3AIH3	DE	UDP-3-O-[3-hydroxymyristoyl] glucosamine N-acyltransferase;
N	Q3AIH3	DR	acyltransferase activity
N	Q3AIH3	DR	lipid A biosynthetic process
N	Q3ALS4	DE	Acetylglutamate kinase;
N	Q3ALS4	DE	N-acetyl-L-glutamate 5-phosphotransferase;
N	Q3ALS4	DR	acetylglutamate kinase activity
N	Q3ALS4	DR	arginine biosynthetic process
N	Q3ALS4	DR	glutamate 5-kinase activity
N	Q3ALS4	DR	proline biosynthetic process
N	Q3AN03	CC	Interconversion of serine and glycine.
N	Q3AN03	DE	Serine hydroxymethyltransferase;
N	Q3AN03	DE	Serine methylase;
N	Q3AN03	DR	L-serine metabolic process
N	Q3AN03	DR	glycine hydroxymethyltransferase activity
N	Q3AN03	DR	glycine metabolic process
N	Q3AN03	DR	one-carbon metabolic process
N	Q3AN03	DR	pyridoxal phosphate binding
N	Q3AS97	DE	50S ribosomal protein L27;
N	Q3AS97	DR	structural constituent of ribosome
N	Q3AT62	DE	1-(5-phosphoribosyl)-5-[(5-phosphoribosylamino)methylideneamino] imidazole-4-carboxamide isomerase;
N	Q3AT62	DE	Phosphoribosylformimino-5-aminoimidazole carboxamide ribotide isomerase;
N	Q3AT62	DR	1-(5-phosphoribosyl)-5-[(5-phosphoribosylamino)methylideneamino]imidazole-4-carboxamide isomerase activity
N	Q3AT62	DR	histidine biosynthetic process
N	Q3AZM2	CC	F(1)F(0) ATP synthase produces ATP from ADP in the presence of a proton or sodium gradient
N	Q3AZM2	CC	During catalysis, ATP synthesis in the catalytic domain of F(1) is coupled via a rotary mechanism of the central stalk subunits to proton translocation (By similarity).
N	Q3AZM2	CC	F-type ATPases consist of two structural domains, F(1) containing the extramembraneous catalytic core and F(0) containing the membrane proton channel, linked together by a central stalk and a peripheral stalk
N	Q3AZM2	DE	ATP synthase F(1) sector subunit delta;
N	Q3AZM2	DE	ATP synthase subunit delta;

N	Q3AZM2	DE	F-ATPase subunit delta;
N	Q3AZM2	DE	F-type ATPase subunit delta;
N	Q3AZM2	DR	ATP synthesis coupled proton transport
N	Q3AZM2	DR	hydrogen ion transporting ATP synthase activity, rotational mechanism
N	Q3B1H3	CC	This protein binds directly to 23S ribosomal RNA (By similarity).
N	Q3B1H3	DE	50S ribosomal protein L11;
N	Q3B1H3	DR	structural constituent of ribosome
N	Q3B213	CC	Catalyzes the 1,3-allylic rearrangement of the homoallylic substrate isopentenyl (IPP) to its allylic isomer, dimethylallyl diphosphate (DMAPP) (By similarity).
N	Q3B213	DE	IPP isomerase;
N	Q3B213	DE	Isopentenyl pyrophosphate isomerase;
N	Q3B213	DE	Isopentenyl-diphosphate delta-isomerase;
N	Q3B213	DR	isopentenyl-diphosphate delta-isomerase activity
N	Q3B213	DR	isoprenoid biosynthetic process
N	Q3B213	DR	oxidoreductase activity
N	Q3B2T5	CC	Participates actively in the response to hyperosmotic and heat shock by preventing the aggregation of stress-denatured proteins and by disaggregating proteins, also in an autonomous, dnaK-independent fashion
N	Q3B2T5	CC	Also involved, together with dnaK and grpE, in the DNA replication of plasmids through activation of initiation proteins (By similarity).
N	Q3B2T5	CC	GrpE releases ADP from dnaK; ATP binding to dnaK triggers the release of the substrate protein, thus completing the reaction cycle
N	Q3B2T5	CC	Several rounds of ATP-dependent interactions between dnaJ, dnaK and grpE are required for fully efficient folding
N	Q3B2T5	CC	Unfolded proteins bind initially to dnaJ; upon interaction with the dnaJ-bound protein, dnaK hydrolyzes its bound ATP, resulting in the formation of a stable complex
N	Q3B2T5	DE	Chaperone protein dnaJ;
N	Q3B2T5	DR	DNA replication
N	Q3B2T5	DR	heat shock protein binding
N	Q3B2T5	DR	metal ion binding
N	Q3B2T5	DR	protein folding
N	Q3B2T5	DR	response to heat
N	Q3B2T5	DR	unfolded protein binding
N	Q3B4B5	CC	Presumably involved in the processing and regular turnover of intracellular proteins
N	Q3B4B5	CC	Catalyzes the removal of unsubstituted N-terminal amino acids from various peptides (By similarity).
N	Q3B4B5	DE	Leucine aminopeptidase;
N	Q3B4B5	DE	Leucyl aminopeptidase;
N	Q3B4B5	DE	Probable cytosol aminopeptidase;
N	Q3B4B5	DR	aminopeptidase activity
N	Q3B4B5	DR	manganese ion binding
N	Q3B4B5	DR	metalloexopeptidase activity
N	Q3B5J2	CC	Catalyzes the interconversion of 2-phosphoglycerate and 3-phosphoglycerate (By similarity).
N	Q3B5J2	DE	2,3-bisphosphoglycerate-dependent phosphoglycerate mutase;
N	Q3B5J2	DE	BPG-dependent PGAM;
N	Q3B5J2	DE	Phosphoglyceromutase;
N	Q3B5J2	DR	phosphoglycerate mutase activity
N	Q3BAR4	CC	This is one of the two reaction center proteins of photosystem II (By similarity).
N	Q3BAR4	DE	32 kDa thylakoid membrane protein;
N	Q3BAR4	DE	Photosystem II protein D1;
N	Q3BAR4	DE	Photosystem Q(B) protein;
N	Q3BAR4	DR	electron transporter, transferring electrons within the cyclic electron transport pathway of photosynthesis activity
N	Q3BAR4	DR	metal ion binding
N	Q3BAR4	DR	photosynthetic electron transport in photosystem II
N	Q3BAR4	DR	response to herbicide
N	Q3BRK2	DE	Phosphatidylserine decarboxylase alpha chain;
N	Q3BRK2	DE	Phosphatidylserine decarboxylase beta chain;
N	Q3BRK2	DE	Phosphatidylserine decarboxylase proenzyme;
N	Q3BRK2	DR	phosphatidylserine decarboxylase activity
N	Q3BRK2	DR	phospholipid biosynthetic process
N	Q3BRL8	CC	Component of the acetyl coenzyme A carboxylase (ACC) complex

N	Q3BRL8	CC	Biotin carboxylase (BC) catalyzes the carboxylation of biotin on its carrier protein (BCCP) and then the CO(2) group is transferred by the transcarboxylase to acetyl-CoA to form malonyl- CoA (By similarity).
N	Q3BRL8	DE	ACCase subunit beta;
N	Q3BRL8	DE	Acetyl-CoA carboxylase carboxyltransferase subunit beta;
N	Q3BRL8	DE	Acetyl-coenzyme A carboxylase carboxyl transferase subunit beta;
N	Q3BRL8	DR	acetyl-CoA carboxylase activity
N	Q3BRL8	DR	fatty acid biosynthetic process
N	Q3BRL8	DR	metal ion binding
N	Q3BUF6	DE	Histidinol-phosphate aminotransferase;
N	Q3BUF6	DE	Imidazole acetol-phosphate transaminase;
N	Q3BUF6	DR	histidine biosynthetic process
N	Q3BUF6	DR	histidinol-phosphate transaminase activity
N	Q3BUF6	DR	pyridoxal phosphate binding
N	Q3BXC4	CC	Forms oxaloacetate, a four-carbon dicarboxylic acid source for the tricarboxylic acid cycle.
N	Q3BXC4	DE	Phosphoenolpyruvate carboxylase;
N	Q3BXC4	DR	carbon fixation
N	Q3BXC4	DR	phosphoenolpyruvate carboxylase activity
N	Q3BXC4	DR	tricarboxylic acid cycle
N	Q3BYD2	DE	Phosphoribosylaminoimidazole-succinocarboxamide synthase;
N	Q3BYD2	DE	SAICAR synthetase;
N	Q3BYD2	DR	phosphoribosylaminoimidazolesuccinocarboxamide synthase activity
N	Q3BYD2	DR	purine nucleotide biosynthetic process
N	Q3E740	DE	Uncharacterized protein YGL258W-A;
N	Q3E740	DR	aspartic-type endopeptidase activity
N	Q3EDD7	DE	Probable protein cornichon homolog 2;
N	Q3EDD7	DR	intracellular signal transduction
N	Q3HM41	CC	Involved in transcription initiation and cap-stealing mechanism, in which cellular capped pre-mRNA are used to generate primers for viral transcription
N	Q3HM41	CC	Binds the cap of the target pre- RNA which is subsequently cleaved by PB1
N	Q3HM41	CC	May play a role in genome replication (By similarity).
N	Q3HM41	DE	Polymerase basic protein 2;
N	Q3HM41	DE	RNA-directed RNA polymerase subunit P3;
N	Q3HM41	DR	RNA-directed RNA polymerase activity
N	Q3IFT7	CC	Catalyzes the NAD-dependent reduction of succinylglutamate semialdehyde into succinylglutamate (By similarity).
N	Q3IFT7	DE	N-succinylglutamate 5-semialdehyde dehydrogenase 1;
N	Q3IFT7	DE	Succinylglutamic semialdehyde dehydrogenase 1;
N	Q3IFT7	DR	arginine catabolic process
N	Q3IFT7	DR	oxidation-reduction process
N	Q3IFT7	DR	succinylglutamate-semialdehyde dehydrogenase activity
N	Q3IHN9	CC	NQR complex catalyzes the reduction of ubiquinone-1 to ubiquinol by two successive reactions, coupled with the transport of Na(+) ions from the cytoplasm to the periplasm
N	Q3IHN9	CC	NqrA to nqrE are probably involved in the second step, the conversion of ubisemiquinone to ubiquinol (By similarity).
N	Q3IHN9	DE	NQR complex subunit E;
N	Q3IHN9	DE	NQR-1 subunit E;
N	Q3IHN9	DE	Na(+)-NQR subunit E;
N	Q3IHN9	DE	Na(+)-translocating NADH-quinone reductase subunit E;
N	Q3IHN9	DE	Na(+)-translocating NQR subunit E;
N	Q3IHN9	DR	oxidoreductase activity, acting on NADH or NADPH, quinone or similar compound as acceptor
N	Q3IHN9	DR	respiratory electron transport chain
N	Q3IHN9	DR	sodium ion transport
N	Q3II81	CC	Binds as a heterodimer with protein S6 to the central domain of the 16S rRNA, where it helps stabilize the platform of the 30S subunit (By similarity).
N	Q3II81	DE	30S ribosomal protein S18;
N	Q3II81	DR	structural constituent of ribosome
N	Q3IJS5	CC	Catalyzes the isomerization between 2-isopropylmalate and 3-isopropylmalate, via the formation of 2-isopropylmaleate.
N	Q3IJS5	DE	3-isopropylmalate dehydratase small subunit;
N	Q3IJS5	DE	Alpha-IPM isomerase;
N	Q3IJS5	DE	Isopropylmalate isomerase;
N	Q3IJS5	DR	3-isopropylmalate dehydratase activity

N	Q3IJS5	DR	leucine biosynthetic process
N	Q3IKE8	CC	Catalyzes the ATP-dependent conversion of 7-carboxy-7- deazaguanine (CDG) to 7-cyano-7-deazaguanine (preQ(0)) (By similarity).
N	Q3IKE8	DE	7-cyano-7-carbaguanine synthase;
N	Q3IKE8	DE	7-cyano-7-deazaguanine synthase;
N	Q3IKE8	DE	PreQ(0) synthase;
N	Q3IKE8	DE	Queuosine biosynthesis protein queC;
N	Q3IKE8	DR	ligase activity
N	Q3IKE8	DR	metal ion binding
N	Q3IKE8	DR	queuosine biosynthetic process
N	Q3IKQ1	CC	Catalyzes the reversible transfer of the terminal phosphate group between ATP and AMP
N	Q3IKQ1	CC	This small ubiquitous enzyme involved in the energy metabolism and nucleotide synthesis, is essential for maintenance and cell growth (By similarity).
N	Q3IKQ1	DE	ATP-AMP transphosphorylase;
N	Q3IKQ1	DE	Adenylate kinase;
N	Q3IKQ1	DR	adenylate kinase activity
N	Q3IKQ1	DR	nucleotide biosynthetic process
N	Q3IMV8	DE	CMP kinase;
N	Q3IMV8	DE	Cytidine monophosphate kinase;
N	Q3IMV8	DE	Cytidylate kinase;
N	Q3IMV8	DR	cytidylate kinase activity
N	Q3IMV8	DR	shikimate kinase activity
N	Q3IZG8	CC	Binds specifically to the ssrA RNA (tmRNA) and is required for stable association of ssrA with ribosomes (By similarity).
N	Q3IZG8	DE	SsrA-binding protein;
N	Q3J2B9	CC	Specifically dimethylates two adjacent adenosines (A1518 and A1519) in the loop of a conserved hairpin near the 3'-end of 16S rRNA in the 30S particle
N	Q3J2B9	CC	May play a critical role in biogenesis of 30S subunits (By similarity).
N	Q3J2B9	DE	16S rRNA (adenine(1518)-N(6)/adenine(1519)-N(6))-dimethyltransferase;
N	Q3J2B9	DE	16S rRNA dimethyladenosine transferase;
N	Q3J2B9	DE	16S rRNA dimethylase;
N	Q3J2B9	DE	Ribosomal RNA small subunit methyltransferase A;
N	Q3J2B9	DE	S-adenosylmethionine-6-N', N'-adenosyl(rRNA) dimethyltransferase;
N	Q3J2B9	DR	rRNA (adenine-N6,N6-)-dimethyltransferase activity
N	Q3J5R9	CC	One of the primary rRNA binding proteins
N	Q3J5R9	CC	It has been suggested to have peptidyltransferase activity; this is somewhat controversial
N	Q3J5R9	CC	Makes several contacts with the 16S rRNA in the 70S ribosome (By similarity).
N	Q3J5R9	CC	Required for association of the 30S and 50S subunits to form the 70S ribosome, for tRNA binding and peptide bond formation
N	Q3J5R9	DE	50S ribosomal protein L2;
N	Q3J5R9	DR	structural constituent of ribosome
N	Q3J5R9	DR	transferase activity
N	Q3J787	CC	Cell wall formation
N	Q3J787	CC	Catalyzes the addition of glutamate to the nucleotide precursor UDP-N-acetylmuramoyl-L-alanine (UMA) (By similarity).
N	Q3J787	DE	D-glutamic acid-adding enzyme;
N	Q3J787	DE	UDP-N-acetylmuramoyl-L-alanyl-D-glutamate synthetase;
N	Q3J787	DE	UDP-N-acetylmuramoylalanine--D-glutamate ligase;
N	Q3J787	DR	UDP-N-acetylmuramoylalanine-D-glutamate ligase activity
N	Q3J787	DR	cell division
N	Q3J787	DR	cellular cell wall organization
N	Q3J787	DR	peptidoglycan biosynthetic process
N	Q3J787	DR	regulation of cell shape
N	Q3J7D1	CC	Catalyzes the conversion of 5-methoxyuridine (mo5U) to uridine-5-oxyacetic acid (cmo5U) at position 34 in tRNA
N	Q3J7D1	CC	May also participate in the methylation of uridine-5-oxyacetic acid (cmo5U) to uridine-5-oxyacetic acid methyl ester (mcmo5U) (By similarity).
N	Q3J7D1	DE	tRNA (cmo5U34)-methyltransferase;
N	Q3J7D1	DR	tRNA (uracil) methyltransferase activity
N	Q3J7D1	DR	tRNA wobble uridine modification
N	Q3J7H0	CC	Catalyzes the condensation of ATP and 5-phosphoribose 1- diphosphate to form N'-(5'-phosphoribosyl)-ATP (PR-ATP)
N	Q3J7H0	CC	Has a crucial role in the pathway because the rate of histidine biosynthesis seems to be controlled primarily by regulation of hisG enzymatic activity (By similarity).

N	Q3J7H0	DE	ATP phosphoribosyltransferase;
N	Q3J7H0	DE	ATP-PRTase;
N	Q3J7H0	DR	ATP phosphoribosyltransferase activity
N	Q3J7H0	DR	histidine biosynthetic process
N	Q3J7H2	DE	Histidinol-phosphate aminotransferase 2;
N	Q3J7H2	DE	Imidazole acetol-phosphate transaminase 2;
N	Q3J7H2	DR	histidine biosynthetic process
N	Q3J7H2	DR	histidinol-phosphate transaminase activity
N	Q3J7H2	DR	pyridoxal phosphate binding
N	Q3J7W3	CC	Catalyzes the transfer of endogenously produced octanoic acid from octanoyl-acyl-carrier-protein onto the lipoyl domains of lipoate-dependent enzymes
N	Q3J7W3	CC	Lipoyl-ACP can also act as a substrate although octanoyl-ACP is likely to be the physiological substrate (By similarity).
N	Q3J7W3	DE	Lipoate-protein ligase B;
N	Q3J7W3	DE	Lipoyl/octanoyl transferase;
N	Q3J7W3	DE	Octanoyl-[acyl-carrier-protein]-protein N-octanoyltransferase;
N	Q3J7W3	DE	Octanoyltransferase;
N	Q3J7W3	DR	lipoate biosynthetic process
N	Q3J7W3	DR	lipoyl(octanoyl) transferase activity
N	Q3J7W3	DR	octanoyltransferase activity
N	Q3J7W3	DR	protein modification process
N	Q3J869	DE	Dihydrodipicolinate synthase;
N	Q3J869	DR	diaminopimelate biosynthetic process
N	Q3J869	DR	dihydrodipicolinate synthase activity
N	Q3JCZ3	CC	Catalyzes the methyl esterification of L-isoaspartyl residues in peptides and proteins that result from spontaneous decomposition of normal L-aspartyl and L-asparaginyl residues
N	Q3JCZ3	CC	It plays a role in the repair and/or degradation of damaged proteins (By similarity).
N	Q3JCZ3	DE	L-isoaspartyl protein carboxyl methyltransferase 1;
N	Q3JCZ3	DE	Protein L-isoaspartyl methyltransferase 1;
N	Q3JCZ3	DE	Protein-L-isoaspartate O-methyltransferase 1;
N	Q3JCZ3	DE	Protein-beta-aspartate methyltransferase 1;
N	Q3JCZ3	DR	protein-L-isoaspartate (D-aspartate) O-methyltransferase activity
N	Q3JST3	DE	Phosphoriboisomerase A;
N	Q3JST3	DE	Ribose-5-phosphate isomerase A;
N	Q3JST3	DR	pentose-phosphate shunt, non-oxidative branch
N	Q3JST3	DR	ribose-5-phosphate isomerase activity
N	Q3JUJ4	CC	Catalyzes two reactions: the first one is the production of beta-formyl glycinamide ribonucleotide (GAR) from formate, ATP and beta GAR; the second, a side reaction, is the production of acetyl phosphate and ADP from acetate and ATP (By similarity).
N	Q3JUJ4	DE	5'-phosphoribosylglycinamide transformylase 2;
N	Q3JUJ4	DE	Formate-dependent GAR transformylase;
N	Q3JUJ4	DE	GAR transformylase 2;
N	Q3JUJ4	DE	Phosphoribosylglycinamide formyltransferase 2;
N	Q3JUJ4	DR	hydroxymethyl-, formyl- and related transferase activity
N	Q3JUJ4	DR	ligase activity
N	Q3JUJ4	DR	magnesium ion binding
N	Q3JUJ4	DR	purine ribonucleotide biosynthetic process
N	Q3JUY1	CC	Is required not only for elongation of protein synthesis but also for the initiation of all mRNA translation through initiator tRNA(fMet) aminoacylation (By similarity).
N	Q3JUY1	DE	Methionine--tRNA ligase;
N	Q3JUY1	DE	Methionyl-tRNA synthetase;
N	Q3JUY1	DR	metal ion binding
N	Q3JUY1	DR	methionine-tRNA ligase activity
N	Q3JUY1	DR	methionyl-tRNA aminoacylation
N	Q3JXS3	CC	ATP-dependent carboxylate-amine ligase (By similarity).
N	Q3JXS3	DE	Carboxylate-amine ligase BURPS1710b_0214;
N	Q3JXS3	DR	glutamate-cysteine ligase activity
N	Q3JXS3	DR	glutathione biosynthetic process
N	Q3K0Y6	CC	Part of the ABC transporter complex PotABCD involved in spermidine/putrescine import
N	Q3K0Y6	CC	Responsible for energy coupling to the transport system (By similarity).
N	Q3K0Y6	DE	Spermidine/putrescine import ATP-binding protein PotA;
N	Q3K0Y6	DR	polyamine-transporting ATPase activity
N	Q3K1B8	CC	Catalyzes the folate-dependent formation of 5-methyl- uridine at position 54 (M-5-U54) in all tRNAs (By similarity).

N	Q3K1B8	DE	Folate-dependent tRNA (uracil-5-)-methyltransferase;
N	Q3K1B8	DE	Folate-dependent tRNA(M-5-U54)-methyltransferase;
N	Q3K1B8	DE	Methylenetetrahydrofolate--tRNA-(uracil-5-)-methyltransferase TrmFO;
N	Q3K1B8	DR	flavin adenine dinucleotide binding
N	Q3K1B8	DR	methylenetetrahydrofolate-tRNA-(uracil-5-)-methyltransferase (FADH2-oxidizing) activity
N	Q3K1B8	DR	tRNA processing
N	Q3K1L5	CC	Cell wall formation
N	Q3K1L5	CC	Adds enolpyruvyl to UDP-N- acetylglucosamine (By similarity).
N	Q3K1L5	DE	Enoylpyruvate transferase 1;
N	Q3K1L5	DE	UDP-N-acetylglucosamine 1-carboxyvinyltransferase 1;
N	Q3K1L5	DE	UDP-N-acetylglucosamine enolpyruvyl transferase 1;
N	Q3K1L5	DR	UDP-N-acetylgalactosamine biosynthetic process
N	Q3K1L5	DR	UDP-N-acetylglucosamine 1-carboxyvinyltransferase activity
N	Q3K1L5	DR	cell division
N	Q3K1L5	DR	cellular cell wall organization
N	Q3K1L5	DR	peptidoglycan biosynthetic process
N	Q3K1L5	DR	regulation of cell shape
N	Q3K4M8	DE	UPF0758 protein Pfl01_5539;
N	Q3K5T0	CC	Hydrolyzes diadenosine 5',5'''-P1,P4-tetraphosphate to yield ADP (By similarity)
N	Q3K5T0	DE	Ap4A hydrolase;
N	Q3K5T0	DE	Bis(5'-nucleosyl)-tetraphosphatase, symmetrical;
N	Q3K5T0	DE	Diadenosine 5',5'''-P1,P4-tetraphosphate pyrophosphohydrolase;
N	Q3K5T0	DE	Diadenosine tetraphosphatase;
N	Q3K5T0	DR	bis(5'-nucleosyl)-tetraphosphatase (symmetrical) activity
N	Q3K6W5	CC	Catalyzes the phosphorylation of the position 2 hydroxy group of 4-diphosphocytidyl-2C-methyl-D-erythritol (By similarity).
N	Q3K6W5	DE	4-(cytidine-5'-diphospho)-2-C-methyl-D-erythritol kinase;
N	Q3K6W5	DE	4-diphosphocytidyl-2-C-methyl-D-erythritol kinase;
N	Q3K6W5	DR	4-(cytidine 5'-diphospho)-2-C-methyl-D-erythritol kinase activity
N	Q3K6W5	DR	terpenoid biosynthetic process
N	Q3KF35	CC	Catalyzes the attachment of glutamate to tRNA(Glu) in a two-step reaction: glutamate is first activated by ATP to form Glu-AMP and then transferred to the acceptor end of tRNA(Glu) (By similarity).
N	Q3KF35	DE	Glutamate--tRNA ligase;
N	Q3KF35	DE	Glutamyl-tRNA synthetase;
N	Q3KF35	DR	glutamate-tRNA ligase activity
N	Q3KF35	DR	glutamyl-tRNA aminoacylation
N	Q3KHY6	DE	ATP-sulfurylase small subunit;
N	Q3KHY6	DE	Sulfate adenylate transferase;
N	Q3KHY6	DE	Sulfate adenylyltransferase subunit 2;
N	Q3KHY6	DR	sulfate adenylyltransferase (ATP) activity
N	Q3KHY6	DR	sulfate reduction
N	Q3KIX5	CC	Binds to the 23S rRNA (By similarity).
N	Q3KIX5	DE	50S ribosomal protein L9;
N	Q3KIX5	DR	structural constituent of ribosome
N	Q3LHN0	CC	In the hair cortex, hair keratin intermediate filaments are embedded in an interfilamentous matrix, consisting of hair keratin-associated proteins (KRTAP), which are essential for the formation of a rigid and resistant hair shaft through their extensive disulfide bond cross-linking with abundant cysteine residues of hair keratins
N	Q3LHN0	CC	The matrix proteins include the high- sulfur and high-glycine-tyrosine keratins (By similarity).
N	Q3LHN0	DE	Keratin-associated protein 25-1;
N	Q3MA96	CC	This protein specifically catalyzes the removal of signal peptides from prolipoproteins (By similarity).
N	Q3MA96	DE	Lipoprotein signal peptidase;
N	Q3MA96	DE	Prolipoprotein signal peptidase;
N	Q3MA96	DE	Signal peptidase II;
N	Q3MA96	DR	aspartic-type endopeptidase activity
N	Q3MBQ2	CC	Phosphorylation of dTMP to form dTDP in both de novo and salvage pathways of dTTP synthesis (By similarity).
N	Q3MBQ2	DE	Thymidylate kinase;
N	Q3MBQ2	DE	dTMP kinase;
N	Q3MBQ2	DR	dTDP biosynthetic process
N	Q3MBQ2	DR	thymidylate kinase activity

N	Q3MGE7	CC	DNA ligase that catalyzes the formation of phosphodiester linkages between 5'-phosphoryl and 3'-hydroxyl groups in double-stranded DNA using NAD as a coenzyme and as the energy source for the reaction
N	Q3MGE7	CC	It is essential for DNA replication and repair of damaged DNA (By similarity).
N	Q3MGE7	DE	Polydeoxyribonucleotide synthase [NAD+];
N	Q3MGE7	DR	DNA ligase (NAD+) activity
N	Q3MGE7	DR	DNA replication
N	Q3MGE7	DR	metal ion binding
N	Q3MHG7	CC	Catalyzes the oxidation of uric acid to 5- hydroxyisourate, which is further processed to form (S)-allantoin (By similarity).
N	Q3MHG7	DE	Urate oxidase;
N	Q3MHG7	DR	oxidation-reduction process
N	Q3MHG7	DR	purine base metabolic process
N	Q3MHG7	DR	urate oxidase activity
N	Q3MIF2	CC	Putative Polycomb group (PcG) protein
N	Q3MIF2	CC	Binds histone H3 peptides that are monomethylated or dimethylated on 'Lys-4', 'Lys-9' or 'Lys-27' (By similarity).
N	Q3MIF2	CC	Binds to monomethylated and dimethylated 'Lys-20' on histone H4
N	Q3MIF2	CC	Its association with a chromatin-remodeling complex suggests that it may contribute to prevent expression of genes that trigger the cell into mitosis
N	Q3MIF2	CC	PcG proteins maintain the transcriptionally repressive state of genes, probably via a modification of chromatin, rendering it heritably changed in its expressibility
N	Q3MIF2	DE	L(3)mbt-like protein 2;
N	Q3MIF2	DE	Lethal(3)malignant brain tumor-like protein 2;
N	Q3MIF2	DR	chromatin modification
N	Q3MIF2	DR	methylated histone residue binding
N	Q3MIF2	DR	zinc ion binding
N	Q3SJP4	CC	NDH-1 shuttles electrons from NADH, via FMN and iron- sulfur (Fe-S) centers, to quinones in the respiratory chain
N	Q3SJP4	CC	Couples the redox reaction to proton translocation (for every two electrons transferred, four hydrogen ions are translocated across the cytoplasmic membrane), and thus conserves the redox energy in a proton gradient (By similarity)
N	Q3SJP4	CC	The immediate electron acceptor for the enzyme in this species is believed to be ubiquinone
N	Q3SJP4	DE	NADH dehydrogenase I subunit N;
N	Q3SJP4	DE	NADH-quinone oxidoreductase subunit N;
N	Q3SJP4	DE	NDH-1 subunit N;
N	Q3SJP4	DR	ATP synthesis coupled electron transport
N	Q3SJP4	DR	NADH dehydrogenase (ubiquinone) activity
N	Q3SJP4	DR	quinone binding
N	Q3SK65	DE	Glucose-6-phosphate isomerase 1;
N	Q3SK65	DE	Phosphoglucose isomerase 1;
N	Q3SK65	DE	Phosphohexose isomerase 1;
N	Q3SK65	DR	gluconeogenesis
N	Q3SK65	DR	glucose-6-phosphate isomerase activity
N	Q3SKN6	CC	Responsible for the release of ribosomes from messenger RNA at the termination of protein biosynthesis
N	Q3SKN6	CC	May increase the efficiency of translation by recycling ribosomes from one round of translation to another (By similarity).
N	Q3SKN6	DE	Ribosome-recycling factor;
N	Q3SKN6	DE	Ribosome-releasing factor;
N	Q3SLP9	CC	One of the primary rRNA binding proteins, it binds directly near the 3'-end of the 23S rRNA, where it nucleates assembly of the 50S subunit (By similarity).
N	Q3SLP9	DE	50S ribosomal protein L3;
N	Q3SLP9	DR	structural constituent of ribosome
N	Q3SQN5	DE	UPF0178 protein Nwi_2152;
N	Q3SSR2	CC	Catalyzes the oxidation of the C8 methyl side group on heme O porphyrin ring into a formyl group (By similarity).
N	Q3SSR2	DE	Cytochrome aa3-controlling protein;
N	Q3SSR2	DE	Heme A synthase;
N	Q3SSR2	DR	heme biosynthetic process
N	Q3SSR2	DR	oxidation-reduction process
N	Q3SSR2	DR	oxidoreductase activity
N	Q3SSR2	DR	protein complex assembly

N	Q3SWX8	CC	Core histone-binding subunit that may target chromatin remodeling factors, histone acetyltransferases and histone deacetylases to their histone substrates in a manner that is regulated by nucleosomal DNA
N	Q3SWX8	CC	Component of several complexes which regulate chromatin metabolism
N	Q3SWX8	CC	These include the type B histone acetyltransferase (HAT) complex, which is required for chromatin assembly following DNA replication; the core histone deacetylase (HDAC) complex, which promotes histone deacetylation and consequent transcriptional repression; the nucleosome remodeling and histone deacetylase complex (the NuRD complex), which promotes transcriptional repression by histone deacetylation and nucleosome remodeling; and the PRC2/EED-EZH2 complex, which promotes repression of homeotic genes during development; and the NURF (nucleosome remodeling factor) complex (By similarity).
N	Q3SWX8	DE	Histone-binding protein RBBP7;
N	Q3SWX8	DE	Nucleosome-remodeling factor subunit RBAP46;
N	Q3SWX8	DE	Retinoblastoma-binding protein 7;
N	Q3SWX8	DR	DNA replication
N	Q3SWX8	DR	cellular heat acclimation
N	Q3SWX8	DR	chromatin modification
N	Q3SWX8	DR	negative regulation of cell growth
N	Q3T003	DE	60S ribosomal protein L18a;
N	Q3T003	DR	structural constituent of ribosome
N	Q3T0X6	DE	40S ribosomal protein S16;
N	Q3T0X6	DR	structural constituent of ribosome
N	Q3T0Z2	CC	Ileal protein which stimulates gastric acid and pepsinogen secretion
N	Q3T0Z2	CC	Seems to be able to bind to bile salts and bilirubins (By similarity).
N	Q3T0Z2	DE	Fatty acid-binding protein 6;
N	Q3T0Z2	DE	Gastrotropin;
N	Q3T0Z2	DE	Ileal lipid-binding protein;
N	Q3T0Z2	DR	lipid binding
N	Q3T0Z2	DR	transporter activity
N	Q3TFQ1	DE	CLL deletion region gene 6 protein homolog;
N	Q3TFQ1	DE	Chronic lymphocytic leukemia deletion region gene 6 protein homolog;
N	Q3TPR7	CC	Possible tumor suppressor which may play a role in cell growth (By similarity).
N	Q3TPR7	DE	Transmembrane protein 184C;
N	Q3TPR7	DE	Transmembrane protein 34;
N	Q3U4B4	CC	Plays a highly specific role in the last step of keratinocyte differentiation
N	Q3U4B4	CC	May have an essential function in lipid metabolism of the most differentiated epidermal layers (By similarity).
N	Q3U4B4	DE	Lipase member N;
N	Q3U4B4	DE	Lipase-like abhydrolase domain-containing protein 4;
N	Q3U4B4	DR	hydrolase activity
N	Q3U4B4	DR	lipid catabolic process
N	Q3U4G3	DE	Uncharacterized protein C3orf21 homolog;
N	Q3U4G3	DR	transferase activity, transferring glycosyl groups
N	Q3V2K1	DE	Uncharacterized protein CXorf65 homolog;
N	Q3V4S8	DE	Uncharacterized protein ORF82;
N	Q3V7G2	CC	Cell wall formation (By similarity).
N	Q3V7G2	DE	Monofunctional TGase;
N	Q3V7G2	DE	Monofunctional biosynthetic peptidoglycan transglycosylase;
N	Q3V7G2	DR	cellular cell wall organization
N	Q3V7G2	DR	peptidoglycan biosynthetic process
N	Q3V7G2	DR	regulation of cell shape
N	Q3V7G2	DR	transferase activity, transferring pentosyl groups
N	Q3YQX7	CC	Digests double-stranded RNA
N	Q3YQX7	CC	Involved in the processing of ribosomal RNA precursors and of some mRNAs (By similarity).
N	Q3YQX7	DE	Ribonuclease 3;
N	Q3YQX7	DE	Ribonuclease III;
N	Q3YQX7	DR	RNA processing
N	Q3YQX7	DR	double-stranded RNA binding
N	Q3YQX7	DR	rRNA catabolic process
N	Q3YQX7	DR	ribonuclease III activity
N	Q3YU12	CC	Catalyzes a reversible aldol reaction between acetaldehyde and D-glyceraldehyde 3-phosphate to generate 2-deoxy- D-ribose 5-phosphate (By similarity).
N	Q3YU12	DE	2-deoxy-D-ribose 5-phosphate aldolase;

N	Q3YU12	DE	Deoxyriboaldolase;
N	Q3YU12	DE	Deoxyribose-phosphate aldolase;
N	Q3YU12	DE	Phosphodeoxyriboaldolase;
N	Q3YU12	DR	deoxyribonucleotide catabolic process
N	Q3YU12	DR	deoxyribose-phosphate aldolase activity
N	Q3YUE4	CC	Binds to the 23S rRNA (By similarity).
N	Q3YUE4	DE	50S ribosomal protein L9;
N	Q3YUE4	DR	structural constituent of ribosome
N	Q3YV72	DE	Rhamnulokinase;
N	Q3YV72	DE	Rhamnulose kinase;
N	Q3YV72	DR	rhamnose catabolic process
N	Q3YV72	DR	rhamnulokinase activity
N	Q3YWA3	CC	Catalyzes, although with low efficiency, the sulfur transfer reaction from thiosulfate to cyanide (By similarity).
N	Q3YWA3	DE	Thiosulfate sulfurtransferase glpE;
N	Q3YWA3	DR	glycerol metabolic process
N	Q3YWA3	DR	thiosulfate sulfurtransferase activity
N	Q3Z0G5	CC	Catalyzes the sequential NAD-dependent oxidations of L- histidinol to L-histidinaldehyde and then to L-histidine (By similarity).
N	Q3Z0G5	DE	Histidinol dehydrogenase;
N	Q3Z0G5	DR	histidine biosynthetic process
N	Q3Z0G5	DR	histidinol dehydrogenase activity
N	Q3Z0G5	DR	oxidation-reduction process
N	Q3Z0G5	DR	zinc ion binding
N	Q3Z146	CC	Involved in the breakdown of putrescine via hydrolysis of the gamma-glutamyl linkage of gamma-glutamyl-gamma- aminobutyrate (By similarity).
N	Q3Z146	DE	Gamma-Glu-GABA hydrolase;
N	Q3Z146	DE	Gamma-glutamyl-gamma-aminobutyrate hydrolase;
N	Q3Z146	DR	gamma-glutamyl-gamma-aminobutyrate hydrolase activity
N	Q3Z146	DR	glutamine metabolic process
N	Q3Z3L1	DE	CMP kinase;
N	Q3Z3L1	DE	Cytidine monophosphate kinase;
N	Q3Z3L1	DE	Cytidylate kinase;
N	Q3Z3L1	DR	cytidylate kinase activity
N	Q3Z4T0	CC	Catalyzes a salvage reaction resulting in the formation of AMP, that is energically less costly than de novo synthesis (By similarity).
N	Q3Z4T0	DE	Adenine phosphoribosyltransferase;
N	Q3Z4T0	DR	adenine phosphoribosyltransferase activity
N	Q3Z4T0	DR	adenine salvage
N	Q3Z4T0	DR	purine ribonucleoside salvage
N	Q3Z5H3	CC	Component of the acetyl coenzyme A carboxylase (ACC) complex
N	Q3Z5H3	CC	First, biotin carboxylase catalyzes the carboxylation of biotin on its carrier protein (BCCP) and then the CO(2) group is transferred by the carboxyltransferase to acetyl-CoA to form malonyl-CoA (By similarity).
N	Q3Z5H3	DE	ACCase subunit alpha;
N	Q3Z5H3	DE	Acetyl-CoA carboxylase carboxyltransferase subunit alpha;
N	Q3Z5H3	DE	Acetyl-coenzyme A carboxylase carboxyl transferase subunit alpha;
N	Q3Z5H3	DR	acetyl-CoA carboxylase activity
N	Q3Z5H3	DR	fatty acid biosynthetic process
N	Q3Z7Z8	CC	NDH-1 shuttles electrons from NADH, via FMN and iron- sulfur (Fe-S) centers, to quinones in the respiratory chain
N	Q3Z7Z8	CC	Couples the redox reaction to proton translocation (for every two electrons transferred, four hydrogen ions are translocated across the cytoplasmic membrane), and thus conserves the redox energy in a proton gradient (By similarity)
N	Q3Z7Z8	CC	The immediate electron acceptor for the enzyme in this species is believed to be ubiquinone
N	Q3Z7Z8	DE	NADH dehydrogenase I subunit D;
N	Q3Z7Z8	DE	NADH-quinone oxidoreductase subunit D;
N	Q3Z7Z8	DE	NDH-1 subunit D;
N	Q3Z7Z8	DR	NADH dehydrogenase (quinone) activity
N	Q3Z7Z8	DR	oxidation-reduction process
N	Q3Z7Z8	DR	quinone binding
N	Q3Z8X7	CC	Involved in DNA repair and recF pathway recombination (By similarity).
N	Q3Z8X7	DE	DNA repair protein recO;
N	Q3Z8X7	DE	Recombination protein O;

N	Q3Z8X7	DR	ARF GTPase activator activity
N	Q3Z8X7	DR	DNA recombination
N	Q3Z8X7	DR	regulation of ARF GTPase activity
N	Q3Z8X7	DR	zinc ion binding
N	Q3Z9G6	CC	Is required not only for elongation of protein synthesis but also for the initiation of all mRNA translation through initiator tRNA(fMet) aminoacylation (By similarity)
N	Q3Z9G6	DE	Methionine--tRNA ligase;
N	Q3Z9G6	DE	Methionyl-tRNA synthetase;
N	Q3Z9G6	DR	metal ion binding
N	Q3Z9G6	DR	methionine-tRNA ligase activity
N	Q3Z9G6	DR	methionyl-tRNA aminoacylation
N	Q3Z9L4	CC	This protein is located at the 30S-50S ribosomal subunit interface and may play a role in the structure and function of the aminoacyl-tRNA binding site (By similarity)
N	Q3Z9L4	DE	50S ribosomal protein L19;
N	Q3Z9L4	DR	structural constituent of ribosome
N	Q3ZBK5	CC	Acts as a negative regulator of innate and adaptive immunity by maintaining immune homeostasis
N	Q3ZBK5	CC	Inhibits JUN/AP1 and NF-kappa-B activation
N	Q3ZBK5	CC	Negative regulator of Toll-like receptor and T-cell receptor function
N	Q3ZBK5	CC	Prevents hyperresponsiveness of the immune system and maintains immune homeostasis
N	Q3ZBK5	CC	Promotes Fas-induced apoptosis (By similarity).
N	Q3ZBK5	DE	TNF alpha-induced protein 8-like protein 2;
N	Q3ZBK5	DE	TNFAIP8-like protein 2;
N	Q3ZBK5	DE	Tumor necrosis factor alpha-induced protein 8-like protein 2;
N	Q3ZBK5	DR	innate immune response
N	Q3ZCB6	CC	Probable regulator of exocrine pancreas development (By similarity).
N	Q3ZCB6	DE	Exocrine differentiation and proliferation factor;
N	Q3ZCB6	DE	Pancreatic progenitor cell differentiation and proliferation factor;
N	Q3ZCB6	DR	cell differentiation
N	Q3ZCB6	DR	multicellular organismal development
N	Q3ZCT8	DE	Kelch domain-containing protein 6;
N	Q3ZCT8	DE	Kelch repeat and BTB domain-containing protein 12;
N	Q3ZJ19	CC	This protein is a component of the reaction center of photosystem II.
N	Q3ZJ19	DE	Photosystem II reaction center protein J;
N	Q3ZJ19	DR	photosynthesis
N	Q3ZXE0	CC	Specifically methylates the N7 position of a guanosine in 16S rRNA (By similarity)
N	Q3ZXE0	DE	16S rRNA 7-methylguanosine methyltransferase;
N	Q3ZXE0	DE	16S rRNA m7G methyltransferase;
N	Q3ZXE0	DE	Ribosomal RNA small subunit methyltransferase G;
N	Q3ZXE0	DR	rRNA methyltransferase activity
N	Q3ZXU3	CC	One of the essential components for the initiation of protein synthesis
N	Q3ZXU3	CC	Also involved in the hydrolysis of GTP during the formation of the 70S ribosomal complex (By similarity).
N	Q3ZXU3	CC	Protects formylmethionyl-tRNA from spontaneous hydrolysis and promotes its binding to the 30S ribosomal subunits
N	Q3ZXU3	DE	Translation initiation factor IF-2;
N	Q3ZXU3	DR	GTPase activity
N	Q3ZXU3	DR	translation initiation factor activity
N	Q40392	CC	Disease resistance protein
N	Q40392	CC	Resistance proteins guard the plant against pathogens that contain an appropriate avirulence protein via a direct or indirect interaction with this avirulence protein
N	Q40392	CC	That triggers a defense system including the hypersensitive response, which restricts the pathogen growth.
N	Q40392	DE	TMV resistance protein N;
N	Q40392	DR	plant-type hypersensitive response
N	Q40392	DR	transmembrane receptor activity
N	Q42328	DE	Defensin-like protein 195;
N	Q42328	DE	Trypsin inhibitor ATTI-1;
N	Q42328	DR	defense response to fungus
N	Q42328	DR	ion channel inhibitor activity
N	Q42328	DR	killing of cells of other organism
N	Q42569	DE	Cytochrome P450 90A1;
N	Q42569	DR	brassinosteroid biosynthetic process
N	Q42569	DR	brassinosteroid homeostasis
N	Q42569	DR	electron carrier activity

N	Q42569	DR	monooxygenase activity
N	Q42569	DR	oxidation-reduction process
N	Q42569	DR	pollen exine formation
N	Q42569	DR	positive regulation of flower development
N	Q42569	DR	response to UV-B
N	Q42569	DR	tapetal cell differentiation
N	Q42602	DE	Cytochrome P450 89A2;
N	Q42602	DR	electron carrier activity
N	Q42602	DR	monooxygenase activity
N	Q42602	DR	oxidation-reduction process
N	Q42870	CC	Converts lysophosphatidic acid (LPA) into phosphatidic acid by incorporating acyl moiety at the 2 position
N	Q42870	CC	This enzyme uses erucoyl-CoA as an acyl donor.
N	Q42870	DE	1-AGP acyltransferase;
N	Q42870	DE	1-acyl-sn-glycerol-3-phosphate acyltransferase;
N	Q42870	DE	Lysophosphatidic acid acyltransferase;
N	Q42870	DR	1-acylglycerol-3-phosphate O-acyltransferase activity
N	Q42870	DR	phospholipid biosynthetic process
N	Q42908	CC	Catalyzes the interconversion of 2-phosphoglycerate and 3-phosphoglycerate (By similarity).
N	Q42908	DE	2,3-bisphosphoglycerate-independent phosphoglycerate mutase;
N	Q42908	DE	BPG-independent PGAM;
N	Q42908	DE	Phosphoglyceromutase;
N	Q42908	DR	manganese ion binding
N	Q42908	DR	phosphoglycerate mutase activity
N	Q42908	DR	response to stress
N	Q43495	DE	Protein 108;
N	Q43922	CC	Converts dehydroshikimate to protocatechuate.
N	Q43922	DE	3-DHS dehydratase;
N	Q43922	DE	3-dehydroshikimate dehydratase;
N	Q43922	DR	lyase activity
N	Q43922	DR	quinate metabolic process
N	Q44297	CC	In the forward direction acts also on secondary amine dicarboxylates such as N-(1-carboxyethyl)methionine and N-(1- carboxyethyl)phenylalanine
N	Q44297	CC	In the reverse direction, the enzyme also acts on neutral amino acids as an amino donor
N	Q44297	CC	They include L-amino acids such as 2-aminopentanoic acid, 2-aminobutyric acid, 2-aminohexanoic acid, 3-chloroalanine, O-acetylserine, methionine, isoleucine, valine, phenylalanine, leucine and alanine.
N	Q44297	DE	N-(1-D-carboxyethyl)-L-norvaline dehydrogenase;
N	Q44297	DE	Opine dehydrogenase;
N	Q44297	DR	glycerol-3-phosphate catabolic process
N	Q44297	DR	opine dehydrogenase activity
N	Q44297	DR	oxidation-reduction process
N	Q44297	DR	oxidoreductase activity, acting on the CH-OH group of donors, NAD or NADP as acceptor
N	Q44586	CC	Component of the NCC cation-efflux system that confers resistance to nickel, cobalt and cadmium
N	Q44586	CC	May form a membrane tunnel, which allows ion transport across the membrane.
N	Q44586	DE	Nickel-cobalt-cadmium resistance protein NccA;
N	Q44586	DR	cation transmembrane transporter activity
N	Q44586	DR	response to cadmium ion
N	Q45618	CC	Involved in the transposition of the insertion sequence (By similarity).
N	Q45618	DE	Putative transposase for insertion sequence element IS5376;
N	Q45618	DR	DNA integration
N	Q45618	DR	DNA recombination
N	Q45618	DR	recombinase activity
N	Q45618	DR	transposition
N	Q45747	CC	Promotes colloidosmotic lysis by binding to the midgut epithelial cells of insects.
N	Q45747	DE	131 kDa crystal protein;
N	Q45747	DE	Crystaline entomocidal protoxin;
N	Q45747	DE	Insecticidal delta-endotoxin CryID(b);
N	Q45747	DE	Pesticidal crystal protein cry1Db;
N	Q45747	DR	defense response
N	Q45747	DR	receptor binding
N	Q45747	DR	sporulation resulting in formation of a cellular spore

N	Q45983	CC	Required for biosynthesis of LAH modification in the post-translational modification of Campylobacter coli flagellin.
N	Q45983	DE	Post-translational flagellin modification protein A;
N	Q45983	DR	oxidation-reduction process
N	Q45983	DR	oxidoreductase activity
N	Q45UF8	CC	Spike-forming protein that mediates virion attachment to the host epithelial cell receptors and plays a major role in cell penetration, determination of host range restriction and virulence
N	Q45UF8	CC	VP8* forms the head of the spikes (By similarity).
N	Q45UF8	CC	Rotavirus entry into the host cell probably involves multiple sequential contacts between the outer capsid proteins VP4 and VP7, and the cell receptors (By similarity)
N	Q45UF8	DE	Hemagglutinin;
N	Q45UF8	DE	Outer capsid protein VP4;
N	Q45UF8	DE	Outer capsid protein VP5*;
N	Q45UF8	DE	Outer capsid protein VP8*;
N	Q45UF8	DR	interspecies interaction between organisms
N	Q45UF8	DR	viral infectious cycle
N	Q46036	CC	Involved in the storage or transport of lipids necessary for membrane maintenance under stressful conditions
N	Q46036	CC	Displays a binding preference for lysophospholipids (By similarity).
N	Q46036	DE	Outer membrane lipoprotein blc;
N	Q46036	DR	lipid binding
N	Q46036	DR	transporter activity
N	Q46403	DE	Late transcription unit A protein;
N	Q464Z5	CC	Involved in the binding of tRNA to the ribosomes (By similarity).
N	Q464Z5	DE	30S ribosomal protein S10P;
N	Q464Z5	DR	structural constituent of ribosome
N	Q465V4	DE	30S ribosomal protein S8e;
N	Q465V4	DR	structural constituent of ribosome
N	Q465Y5	CC	Participates actively in the response to hyperosmotic and heat shock by preventing the aggregation of stress-denatured proteins, in association with dnaK and grpE
N	Q465Y5	CC	GrpE releases ADP from dnaK; ATP binding to dnaK triggers the release of the substrate protein, thus completing the reaction cycle
N	Q465Y5	CC	It is the nucleotide exchange factor for dnaK and may function as a thermosensor
N	Q465Y5	CC	Several rounds of ATP- dependent interactions between dnaJ, dnaK and grpE are required for fully efficient folding (By similarity).
N	Q465Y5	CC	Unfolded proteins bind initially to dnaJ; upon interaction with the dnaJ-bound protein, dnaK hydrolyzes its bound ATP, resulting in the formation of a stable complex
N	Q465Y5	DE	HSP-70 cofactor;
N	Q465Y5	DE	Protein grpE;
N	Q465Y5	DR	adenyl-nucleotide exchange factor activity
N	Q465Y5	DR	chaperone binding
N	Q465Y5	DR	protein folding
N	Q465Y5	DR	protein homodimerization activity
N	Q465Y5	DR	response to stress
N	Q46E46	DE	Histidinol-phosphate aminotransferase;
N	Q46E46	DE	Imidazole acetol-phosphate transaminase;
N	Q46E46	DR	histidine biosynthetic process
N	Q46E46	DR	histidinol-phosphate transaminase activity
N	Q46E46	DR	pyridoxal phosphate binding
N	Q46FV4	CC	Catalyzes the reversible reduction of methylene-H(4)MPT to methyl-H(4)MPT (By similarity).
N	Q46FV4	DE	5,10-methylenetetrahydromethanopterin reductase;
N	Q46FV4	DE	Coenzyme F420-dependent N(5),N(10)-methylenetetrahydromethanopterin reductase;
N	Q46FV4	DE	Methylene-H(4)MPT reductase;
N	Q46FV4	DR	coenzyme F420-dependent N5,N10-methenyltetrahydromethanopterin reductase activity
N	Q46FV4	DR	methanogenesis
N	Q46FV4	DR	one-carbon metabolic process
N	Q46GB9	CC	Catalyzes the reversible transfer of the terminal phosphate group between ATP and AMP
N	Q46GB9	CC	This small ubiquitous enzyme involved in the energy metabolism and nucleotide synthesis, is essential for maintenance and cell growth (By similarity).
N	Q46GB9	DE	ATP-AMP transphosphorylase;
N	Q46GB9	DE	Adenylate kinase;
N	Q46GB9	DR	adenylate kinase activity

N	Q46GB9	DR	nucleotide biosynthetic process
N	Q46L08	CC	The UvrABC repair system catalyzes the recognition and processing of DNA lesions
N	Q46L08	CC	The N-terminal half is responsible for the 3' incision and the C-terminal half is responsible for the 5' incision (By similarity).
N	Q46L08	CC	UvrC both incises the 5' and 3' sides of the lesion
N	Q46L08	DE	Excinuclease ABC subunit C;
N	Q46L08	DE	Protein uvrC;
N	Q46L08	DE	UvrABC system protein C;
N	Q46L08	DR	excinuclease ABC activity
N	Q46L08	DR	nucleotide-excision repair
N	Q46L27	CC	Part of the ABC transporter complex PstSACB involved in phosphate import
N	Q46L27	CC	Responsible for energy coupling to the transport system (By similarity).
N	Q46L27	DE	ABC phosphate transporter;
N	Q46L27	DE	Phosphate import ATP-binding protein PstB;
N	Q46L27	DE	Phosphate-transporting ATPase;
N	Q46L27	DR	inorganic phosphate transmembrane transporter activity
N	Q46L27	DR	phosphate transmembrane-transporting ATPase activity
N	Q470E8	CC	Involved in saturated fatty acids biosynthesis.
N	Q470E8	DE	(3R)-hydroxymyristoyl ACP dehydrase;
N	Q470E8	DE	(3R)-hydroxymyristoyl-[acyl-carrier-protein] dehydratase;
N	Q470E8	DR	fatty acid biosynthetic process
N	Q470E8	DR	hydro-lyase activity
N	Q470E8	DR	lipid A biosynthetic process
N	Q47CN0	CC	This protein is one of the two subunits of integration host factor, a specific DNA-binding protein that functions in genetic recombination as well as in transcriptional and translational control (By similarity).
N	Q47CN0	DE	IHF-alpha 2;
N	Q47CN0	DE	Integration host factor subunit alpha 2;
N	Q47CN0	DR	DNA recombination
N	Q47CN0	DR	regulation of transcription, DNA-dependent
N	Q47CN0	DR	regulation of translation
N	Q47GC1	CC	Catalyzes the cleavage of the C5-C6 bond of 2-hydroxy-6- oxononadienedioate and 2-hydroxy-6-oxononatrienedioate, a dienol ring fission product of the bacterial meta-cleavage pathway for degradation of phenylpropionic acid (By similarity).
N	Q47GC1	DE	2-hydroxy-6-ketonona-2,4-diene-1,9-dioic acid 5,6-hydrolase 2;
N	Q47GC1	DE	2-hydroxy-6-oxonona-2,4,7-triene-1,9-dioic acid 5,6-hydrolase 2;
N	Q47GC1	DE	2-hydroxy-6-oxonona-2,4-diene-1,9-dioic acid 5,6-hydrolase 2;
N	Q47GC1	DE	2-hydroxy-6-oxononadienedioate/2-hydroxy-6-oxononatrienedioate hydrolase 2;
N	Q47GC1	DR	aromatic compound catabolic process
N	Q47GC1	DR	hydrolase activity, acting on acid carbon-carbon bonds, in ketonic substances
N	Q47K71	CC	Plays an important role in the initiation and regulation of chromosomal replication
N	Q47K71	CC	Binds to the origin of replication; it binds specifically double-stranded DNA at a 9 bp consensus (dnaA box): 5'-TTATC[CA]A[CA]A-3'
N	Q47K71	CC	DnaA binds to ATP and to acidic phospholipids (By similarity).
N	Q47K71	DE	Chromosomal replication initiator protein DnaA;
N	Q47K71	DR	DNA replication origin binding
N	Q47K71	DR	DNA-dependent DNA replication initiation
N	Q47K71	DR	nucleoside-triphosphatase activity
N	Q47K71	DR	regulation of DNA replication
N	Q47KV5	CC	Catalyzes the condensation of pantoate with beta-alanine in an ATP-dependent reaction via a pantoyl-adenylate intermediate (By similarity).
N	Q47KV5	DE	Pantoate--beta-alanine ligase;
N	Q47KV5	DE	Pantoate-activating enzyme;
N	Q47KV5	DE	Pantothenate synthetase;
N	Q47KV5	DR	pantoate-beta-alanine ligase activity
N	Q47KV5	DR	pantothenate biosynthetic process
N	Q47N84	DE	Argininosuccinate synthase;
N	Q47N84	DE	Citrulline--aspartate ligase;
N	Q47N84	DR	arginine biosynthetic process
N	Q47N84	DR	argininosuccinate synthase activity
N	Q47VS4	CC	Catalyzes the phosphorylation of the 3'-hydroxyl group of dephosphocoenzyme A to form coenzyme A (By similarity).
N	Q47VS4	DE	Dephospho-CoA kinase;
N	Q47VS4	DE	Dephosphocoenzyme A kinase;
N	Q47VS4	DR	coenzyme A biosynthetic process

N	Q47VS4	DR	dephospho-CoA kinase activity
N	Q47W35	CC	Required for the formation of a threonylcarbamoyl group on adenosine at position 37 (t(6)A37) in tRNAs that read codons beginning with adenine (By similarity)
N	Q47W35	DE	Probable tRNA threonylcarbamoyladenosine biosynthesis protein Gcp;
N	Q47W35	DE	t(6)A37 threonylcarbamoyladenosine biosynthesis protein;
N	Q47W35	DR	metal ion binding
N	Q47W35	DR	metalloendopeptidase activity
N	Q47WD4	DE	UPF0061 protein CPS_4238;
N	Q48456	CC	May function as a transporter.
N	Q48456	DE	Uncharacterized 49.5 kDa protein in cps region;
N	Q488Z2	DE	50S ribosomal protein L36;
N	Q488Z2	DR	structural constituent of ribosome
N	Q48AB7	CC	Converts heme B (protoheme IX) to heme O by substitution of the vinyl group on carbon 2 of heme B porphyrin ring with a hydroxyethyl farnesyl side group (By similarity)
N	Q48AB7	DE	Heme B farnesyltransferase;
N	Q48AB7	DE	Heme O synthase;
N	Q48AB7	DE	Protoheme IX farnesyltransferase;
N	Q48AB7	DR	heme O biosynthetic process
N	Q48AB7	DR	protoheme IX farnesyltransferase activity
N	Q48HL2	CC	Part of the ABC transporter complex PhnCDE involved in phosphonates import
N	Q48HL2	CC	Responsible for energy coupling to the transport system (By similarity).
N	Q48HL2	DE	Phosphonates import ATP-binding protein PhnC 2;
N	Q48HL2	DR	phosphonate transmembrane-transporting ATPase activity
N	Q48KP5	CC	Catalyzes the decarboxylation of orotidine 5'- monophosphate (OMP) to uridine 5'-monophosphate (UMP) (By similarity).
N	Q48KP5	DE	OMP decarboxylase;
N	Q48KP5	DE	Orotidine 5'-phosphate decarboxylase;
N	Q48KP5	DR	'de novo' UMP biosynthetic process
N	Q48KP5	DR	'de novo' pyrimidine base biosynthetic process
N	Q48KP5	DR	orotidine-5'-phosphate decarboxylase activity
N	Q48SU8	CC	Cell wall formation
N	Q48SU8	CC	Adds enolpyruvyl to UDP-N- acetylglucosamine (By similarity).
N	Q48SU8	DE	Enoylpyruvate transferase 2;
N	Q48SU8	DE	UDP-N-acetylglucosamine 1-carboxyvinyltransferase 2;
N	Q48SU8	DE	UDP-N-acetylglucosamine enolpyruvyl transferase 2;
N	Q48SU8	DR	UDP-N-acetylgalactosamine biosynthetic process
N	Q48SU8	DR	UDP-N-acetylglucosamine 1-carboxyvinyltransferase activity
N	Q48SU8	DR	cell division
N	Q48SU8	DR	cellular cell wall organization
N	Q48SU8	DR	peptidoglycan biosynthetic process
N	Q48SU8	DR	regulation of cell shape
N	Q48TZ6	DE	UPF0176 protein M28_Spy0697;
N	Q49184	CC	DHPS catalyzes the formation of the immediate precursor of folic acid
N	Q49184	CC	Implicated in resistance to sulfonamide.
N	Q49184	CC	The type II enzyme is stable whereas type I DHPS loses its activity rapidly (By similarity)
N	Q49184	DE	Dihydropteroate pyrophosphorylase type I;
N	Q49184	DE	Dihydropteroate synthase type I;
N	Q49184	DE	Dihydropteroate synthase type-1;
N	Q49184	DR	dihydropteroate synthase activity
N	Q49184	DR	folic acid biosynthetic process
N	Q49184	DR	metal ion binding
N	Q49184	DR	response to antibiotic
N	Q491Y3	DE	Triose-phosphate isomerase;
N	Q491Y3	DE	Triosephosphate isomerase;
N	Q491Y3	DR	gluconeogenesis
N	Q491Y3	DR	pentose-phosphate shunt
N	Q491Y3	DR	triose-phosphate isomerase activity
N	Q492A5	DE	3-dehydroquinate synthase;
N	Q492A5	DR	3-dehydroquinate synthase activity
N	Q492A5	DR	aromatic amino acid family biosynthetic process
N	Q49329	DE	Uncharacterized protein MG269.1;
N	Q493D9	CC	Accelerates the degradation of transcripts by removing pyrophosphate from the 5'-end of triphosphorylated RNA, leading to a more labile monophosphorylated state that can stimulate subsequent ribonuclease cleavage (By similarity).

N	Q493D9	DE	(Di)nucleoside polyphosphate hydrolase;
N	Q493D9	DE	RNA pyrophosphohydrolase;
N	Q493D9	DR	hydrolase activity
N	Q493I5	CC	One of the primary rRNA binding proteins, it binds directly to 16S rRNA where it nucleates assembly of the body of the 30S subunit (By similarity).
N	Q493I5	DE	30S ribosomal protein S4;
N	Q493I5	DR	structural constituent of ribosome
N	Q493K9	CC	Involved in the binding of tRNA to the ribosomes (By similarity).
N	Q493K9	DE	30S ribosomal protein S10;
N	Q493K9	DR	structural constituent of ribosome
N	Q494E6	CC	Riboflavin synthase is a bifunctional enzyme complex catalyzing the formation of riboflavin from 5-amino-6-(1'-D)- ribityl-amino-2,4(1H,3H)-pyrimidinedione and L-3,4-dihydrohy-2- butanone-4-phosphate via 6,7-dimethyl-8-lumazine
N	Q494E6	CC	The beta subunit catalyzes the condensation of 5-amino-6-(1'-D)-ribityl-amino-2,4(1H,3H)-pyrimidinedione with L-3,4-dihydrohy-2-butanone-4- phosphate yielding 6,7-dimethyl-8-lumazine (By similarity).
N	Q494E6	DE	6,7-dimethyl-8-ribityllumazine synthase;
N	Q494E6	DE	DMRL synthase;
N	Q494E6	DE	Lumazine synthase;
N	Q494E6	DE	Riboflavin synthase beta chain;
N	Q494E6	DR	riboflavin biosynthetic process
N	Q494E6	DR	riboflavin synthase activity
N	Q49536	CC	Responsible for the antigenic diversity for host adaptation.
N	Q49536	DE	Variant surface antigen D;
N	Q49536	DE	VlpD prolipoprotein;
N	Q498L2	CC	May act as a general inhibitor of transcription (By similarity).
N	Q498L2	DE	SAFB-like transcription modulator;
N	Q498L2	DR	nucleotide binding
N	Q49AH0	CC	Trophic factor for dopamine neurons
N	Q49AH0	CC	Prevents the 6- hydroxydopamine (6-OHDA)-induced degeneration of dopaminergic neurons
N	Q49AH0	CC	When administered after 6-OHDA-lesioning, restores the dopaminergic function and prevents the degeneration of dopaminergic neurons in substantia nigra (By similarity)
N	Q49AH0	DE	ARMET-like protein 1;
N	Q49AH0	DE	Cerebral dopamine neurotrophic factor;
N	Q49AH0	DE	Conserved dopamine neurotrophic factor;
N	Q49AH0	DR	growth factor activity
N	Q49YJ6	CC	Riboflavin synthase is a bifunctional enzyme complex catalyzing the formation of riboflavin from 5-amino-6-(1'-D)- ribityl-amino-2,4(1H,3H)-pyrimidinedione and L-3,4-dihydrohy-2- butanone-4-phosphate via 6,7-dimethyl-8-lumazine
N	Q49YJ6	CC	The beta subunit catalyzes the condensation of 5-amino-6-(1'-D)-ribityl-amino-2,4(1H,3H)-pyrimidinedione with L-3,4-dihydrohy-2-butanone-4- phosphate yielding 6,7-dimethyl-8-lumazine (By similarity).
N	Q49YJ6	DE	6,7-dimethyl-8-ribityllumazine synthase;
N	Q49YJ6	DE	DMRL synthase;
N	Q49YJ6	DE	Lumazine synthase;
N	Q49YJ6	DE	Riboflavin synthase beta chain;
N	Q49YJ6	DR	riboflavin biosynthetic process
N	Q49YJ6	DR	riboflavin synthase activity
N	Q49ZE2	CC	DNA-dependent RNA polymerase catalyzes the transcription of DNA into RNA using the four ribonucleoside triphosphates as substrates.
N	Q49ZE2	DE	DNA-directed RNA polymerase subunit alpha;
N	Q49ZE2	DE	RNA polymerase subunit alpha;
N	Q49ZE2	DE	RNAP subunit alpha;
N	Q49ZE2	DE	Transcriptase subunit alpha;
N	Q49ZE2	DR	DNA-directed RNA polymerase activity
N	Q49ZE2	DR	protein dimerization activity
N	Q49ZG7	CC	One of the primary rRNA binding proteins, this protein initially binds near the 5'-end of the 23S rRNA
N	Q49ZG7	CC	It is important during the early stages of 50S assembly
N	Q49ZG7	CC	It makes multiple contacts with different domains of the 23S rRNA in the assembled 50S subunit and ribosome (By similarity).
N	Q49ZG7	DE	50S ribosomal protein L4;
N	Q49ZG7	DR	structural constituent of ribosome
N	Q49ZR0	DE	Lysostaphin resistance protein A;

N	Q49ZU9	DE	Fd-NADP+ reductase;
N	Q49ZU9	DE	Ferredoxin--NADP reductase;
N	Q49ZU9	DR	ferredoxin-NADP+ reductase activity
N	Q49ZU9	DR	flavin adenine dinucleotide binding
N	Q49ZU9	DR	oxidation-reduction process
N	Q4A180	CC	Plays an important role in the initiation and regulation of chromosomal replication
N	Q4A180	CC	Binds to the origin of replication; it binds specifically double-stranded DNA at a 9 bp consensus (dnaA box): 5'-TTATC[CA]A[CA]A-3'
N	Q4A180	CC	DnaA binds to ATP and to acidic phospholipids (By similarity).
N	Q4A180	DE	Chromosomal replication initiator protein DnaA;
N	Q4A180	DR	DNA replication origin binding
N	Q4A180	DR	DNA-dependent DNA replication initiation
N	Q4A180	DR	nucleoside-triphosphatase activity
N	Q4A180	DR	regulation of DNA replication
N	Q4A8K7	CC	Involved in rRNA and/or ribosome maturation and assembly
N	Q4A8K7	CC	May have metal-dependent hydrolase activity (By similarity).
N	Q4A8K7	CC	Required for normal 5' and 3' processing of 16S, 23S and 5S rRNAs
N	Q4A8K7	DE	Probable rRNA maturation factor;
N	Q4A8K7	DR	metal ion binding
N	Q4A8K7	DR	metalloendopeptidase activity
N	Q4A8K7	DR	rRNA processing
N	Q4FNA5	CC	Part of the Sec protein translocase complex
N	Q4FNA5	CC	Has a central role in coupling the hydrolysis of ATP to the transfer of proteins into and across the cell membrane, serving both as a receptor for the preprotein-secB complex and as an ATP-driven molecular motor driving the stepwise translocation of polypeptide chains across the membrane (By similarity).
N	Q4FNA5	CC	Interacts with the secYEG preprotein conducting channel
N	Q4FNA5	DE	Protein translocase subunit secA;
N	Q4FNA5	DR	protein import
N	Q4FNA5	DR	protein targeting
N	Q4FNA5	DR	transmembrane transport
N	Q4FRX3	DE	NAD-dependent malic enzyme;
N	Q4FRX3	DR	malate dehydrogenase (oxaloacetate-decarboxylating) activity
N	Q4FRX3	DR	malate metabolic process
N	Q4FRX3	DR	metal ion binding
N	Q4FRX3	DR	oxidation-reduction process
N	Q4FZF3	DE	DEAD box protein 49;
N	Q4FZF3	DE	Probable ATP-dependent RNA helicase DDX49;
N	Q4FZF3	DR	ATP-dependent helicase activity
N	Q4FZU3	DE	Coiled-coil domain-containing protein 55;
N	Q4G0G2	DE	Putative uncharacterized protein C3orf47;
N	Q4G3D9	DE	30S ribosomal protein S18, chloroplastic;
N	Q4G3D9	DR	structural constituent of ribosome
N	Q4I7S1	CC	Component of the SWR1 complex which mediates the ATP- dependent exchange of histone H2A for the H2A variant HZT1 leading to transcriptional regulation of selected genes by chromatin remodeling
N	Q4I7S1	CC	Component of the NuA4 histone acetyltransferase complex which is involved in transcriptional activation of selected genes principally by acetylation of nucleosomal histones H4 and H2A
N	Q4I7S1	CC	The NuA4 complex is also involved in DNA repair
N	Q4I7S1	CC	Yaf9 may also be required for viability in conditions in which the structural integrity of the spindle is compromised (By similarity).
N	Q4I7S1	DE	Protein AF-9 homolog;
N	Q4I7S1	DR	chromatin modification
N	Q4I7S1	DR	regulation of transcription, DNA-dependent
N	Q4I830	CC	ATP-binding RNA helicase involved in ribosome assembly (By similarity).
N	Q4I830	DE	ATP-dependent RNA helicase DRS1;
N	Q4I830	DR	ATP-dependent helicase activity
N	Q4I830	DR	ribosome biogenesis
N	Q4J8I5	CC	IGPS catalyzes the conversion of PRFAR and glutamine to IGP, AICAR and glutamate
N	Q4J8I5	CC	The hisH subunit provides the glutamine amidotransferase activity that produces the ammonia necessary to hisF for the synthesis of IGP and AICAR (By similarity)
N	Q4J8I5	DE	IGP synthase glutamine amidotransferase subunit;
N	Q4J8I5	DE	IGP synthase subunit hisH;
N	Q4J8I5	DE	IGPS subunit hisH;

N	Q4J8I5	DE	ImGP synthase subunit hisH;
N	Q4J8I5	DE	Imidazole glycerol phosphate synthase subunit hisH;
N	Q4J8I5	DR	glutamine metabolic process
N	Q4J8I5	DR	histidine biosynthetic process
N	Q4J8I5	DR	transferase activity, transferring pentosyl groups
N	Q4JAG1	CC	Required for the formation of a threonylcarbamoyl group on adenosine at position 37 (t(6)A37) in tRNAs that read codons beginning with adenine (By similarity).
N	Q4JAG1	DE	Probable tRNA threonylcarbamoyladenosine biosynthesis protein KAE1 homolog;
N	Q4JAG1	DE	t(6)A37 threonylcarbamoyladenosine biosynthesis protein KAE1 homolog;
N	Q4JAG1	DR	metal ion binding
N	Q4JAG1	DR	metalloendopeptidase activity
N	Q4JC76	DE	3-dehydroquinate synthase;
N	Q4JC76	DR	3-dehydroquinate synthase activity
N	Q4JC76	DR	aromatic amino acid family biosynthetic process
N	Q4JIM4	CC	Probable catalytic subunit of the gamma-secretase complex, an endoprotease complex that catalyzes the intramembrane cleavage of integral membrane proteins such as Notch receptors and APP (beta-amyloid precursor protein)
N	Q4JIM4	CC	May also play a role in hematopoiesis (By similarity).
N	Q4JIM4	CC	May play a role in intracellular signaling and gene expression or in linking chromatin to the nuclear membrane
N	Q4JIM4	CC	Requires the other members of the gamma-secretase complex to have a protease activity
N	Q4JIM4	CC	Stimulates cell-cell adhesion though its association with the E-cadherin/catenin complex
N	Q4JIM4	CC	Under conditions of apoptosis or calcium influx, cleaves E-cadherin promoting the disassembly of the E-cadherin/catenin complex and increasing the pool of cytoplasmic beta-catenin, thus negatively regulating Wnt signaling
N	Q4JIM4	DE	Presenilin-1 CTF subunit;
N	Q4JIM4	DE	Presenilin-1 NTF subunit;
N	Q4JIM4	DE	Presenilin-1;
N	Q4JIM4	DR	Notch receptor processing
N	Q4JIM4	DR	Notch signaling pathway
N	Q4JIM4	DR	amyloid precursor protein catabolic process
N	Q4JIM4	DR	anti-apoptosis
N	Q4JIM4	DR	beta-amyloid metabolic process
N	Q4JIM4	DR	cadherin binding
N	Q4JIM4	DR	endopeptidase activity
N	Q4JIM4	DR	membrane protein ectodomain proteolysis
N	Q4JIM4	DR	smooth endoplasmic reticulum calcium ion homeostasis
N	Q4JQW5	CC	Transcriptional activator of immediate-early (IE) gene products (alpha genes)
N	Q4JQW5	CC	Acts as a key activator of lytic infection by initiating the lytic program through the assembly of the transcriptional regulatory VP16-induced complex composed of VP16 and two cellular factors, HCFC1 and POU2F 1
N	Q4JQW5	CC	VP16-induced complex represents a regulatory switch: when it is on, it promotes IE-gene expression and thus lytic infection, and when it is off, it limits IE-gene transcription favoring latent infection (By similarity).
N	Q4JQW5	DE	Alpha trans-inducing protein;
N	Q4JQW5	DE	ORF10 protein;
N	Q4JQW5	DE	Tegument protein 10;
N	Q4JQW5	DE	Tegument protein VP16 homolog;
N	Q4JQW5	DR	regulation of transcription, DNA-dependent
N	Q4JTF1	CC	Binds to Cpn60 in the presence of Mg-ATP and suppresses the ATPase activity of the latter.
N	Q4JTF1	DE	10 kDa chaperonin;
N	Q4JTF1	DE	GroES protein;
N	Q4JTF1	DE	Protein Cpn10;
N	Q4JTF1	DR	protein folding
N	Q4JUI9	CC	Peptide chain release factor 1 directs the termination of translation in response to the peptide chain termination codons UAG and UAA (By similarity).
N	Q4JUI9	DE	Peptide chain release factor 1;
N	Q4JUI9	DR	translation release factor activity, codon specific
N	Q4JUL8	CC	DNA ligase that catalyzes the formation of phosphodiester linkages between 5'-phosphoryl and 3'-hydroxyl groups in double-stranded DNA using NAD as a coenzyme and as the energy source for the reaction
N	Q4JUL8	CC	It is essential for DNA replication and repair of damaged DNA (By similarity).
N	Q4JUL8	DE	Polydeoxyribonucleotide synthase [NAD+];
N	Q4JUL8	DR	DNA ligase (NAD+) activity

N	Q4JUL8	DR	DNA replication
N	Q4JUL8	DR	metal ion binding
N	Q4JVU1	CC	Part of the Sec protein translocase complex
N	Q4JVU1	CC	Has a central role in coupling the hydrolysis of ATP to the transfer of proteins into and across the cell membrane, serving as an ATP-driven molecular motor driving the stepwise translocation of polypeptide chains across the membrane (By similarity)
N	Q4JVU1	CC	Interacts with the secYEG preprotein conducting channel
N	Q4JVU1	DE	Protein translocase subunit secA 2;
N	Q4JVU1	DR	protein import
N	Q4JVU1	DR	protein targeting
N	Q4JVU1	DR	transmembrane transport
N	Q4JX93	DE	UPF0271 protein jk0407;
N	Q4JX93	DR	carbohydrate metabolic process
N	Q4JX93	DR	catalytic activity
N	Q4K898	CC	Catalyzes the formation of 5-methyl-uridine at position 1939 (M-5-U1939) in 23S rRNA (By similarity).
N	Q4K898	DE	23S rRNA (uracil-5-)-methyltransferase RumA;
N	Q4K898	DE	23S rRNA(M-5-U1939)-methyltransferase;
N	Q4K898	DR	4 iron, 4 sulfur cluster binding
N	Q4K898	DR	RNA methyltransferase activity
N	Q4K898	DR	metal ion binding
N	Q4K898	DR	rRNA processing
N	Q4KHT3	CC	Required for accurate and efficient protein synthesis under certain stress conditions
N	Q4KHT3	CC	Back- translocation proceeds from a post-translocation (POST) complex to a pre-translocation (PRE) complex, thus giving elongation factor G a second chance to translocate the tRNAs correctly
N	Q4KHT3	CC	Binds to ribosomes in a GTP-dependent manner (By similarity).
N	Q4KHT3	CC	May act as a fidelity factor of the translation reaction, by catalyzing a one-codon backward translocation of tRNAs on improperly translocated ribosomes
N	Q4KHT3	DE	Elongation factor 4;
N	Q4KHT3	DE	Ribosomal back-translocase LepA;
N	Q4KHT3	DR	GTPase activity
N	Q4KWH8	CC	The production of the second messenger molecules diacylglycerol (DAG) and inositol 1,4,5-trisphosphate (IP3) is mediated by calcium-activated phosphatidylinositol-specific phospholipase C enzymes.
N	Q4KWH8	DE	1-phosphatidylinositol-4,5-bisphosphate phosphodiesterase eta-1;
N	Q4KWH8	DE	Phosphoinositide phospholipase C-eta-1;
N	Q4KWH8	DE	Phospholipase C-eta-1;
N	Q4KWH8	DE	Phospholipase C-like protein 3;
N	Q4KWH8	DR	calcium ion binding
N	Q4KWH8	DR	calcium-dependent phospholipase C activity
N	Q4KWH8	DR	lipid catabolic process
N	Q4KWH8	DR	phosphatidylinositol phospholipase C activity
N	Q4KWH8	DR	phosphatidylinositol-mediated signaling
N	Q4KWH8	DR	signal transducer activity
N	Q4L3G8	CC	Acts as a processive, ATP-dependent zinc metallopeptidase for both cytoplasmic and membrane proteins
N	Q4L3G8	CC	Plays a role in the quality control of integral membrane proteins (By similarity).
N	Q4L3G8	DE	ATP-dependent zinc metalloprotease FtsH;
N	Q4L3G8	DR	metalloendopeptidase activity
N	Q4L3G8	DR	nucleoside-triphosphatase activity
N	Q4L3G8	DR	protein catabolic process
N	Q4L3G8	DR	zinc ion binding
N	Q4L6T4	CC	Required for accurate and efficient protein synthesis under certain stress conditions
N	Q4L6T4	CC	Back- translocation proceeds from a post-translocation (POST) complex to a pre-translocation (PRE) complex, thus giving elongation factor G a second chance to translocate the tRNAs correctly
N	Q4L6T4	CC	Binds to ribosomes in a GTP-dependent manner (By similarity).
N	Q4L6T4	CC	May act as a fidelity factor of the translation reaction, by catalyzing a one-codon backward translocation of tRNAs on improperly translocated ribosomes
N	Q4L6T4	DE	Elongation factor 4;
N	Q4L6T4	DE	Ribosomal back-translocase LepA;
N	Q4L6T4	DR	GTPase activity
N	Q4L6W8	CC	Catalyzes the 2-thiolation of uridine at the wobble position (U34) of tRNA, leading to the formation of s(2)U34 (By similarity).

N	Q4L6W8	DE	tRNA-specific 2-thiouridylase mnmA;
N	Q4L6W8	DR	tRNA processing
N	Q4L6W8	DR	transferase activity
N	Q4L7I0	CC	Involved in the biosynthesis of cell wall peptidoglycan
N	Q4L7I0	CC	Responsible for the elongation of the glycan strands using lipid- linked disaccharide-pentapeptide as the substrate (By similarity).
N	Q4L7I0	DE	Monofunctional glycosyltransferase;
N	Q4L7I0	DE	Peptidoglycan TGase;
N	Q4L7I0	DR	cellular cell wall organization
N	Q4L7I0	DR	peptidoglycan biosynthetic process
N	Q4L7I0	DR	regulation of cell shape
N	Q4L7I0	DR	transferase activity, transferring glycosyl groups
N	Q4L7K3	DE	UPF0421 protein SH1063;
N	Q4N3P0	DE	40S ribosomal protein S8;
N	Q4N3P0	DR	structural constituent of ribosome
N	Q4P5F5	CC	Essential component of the cytosolic iron-sulfur (Fe/S) protein assembly machinery
N	Q4P5F5	CC	Required for the maturation of extramitochondrial Fe/S proteins (By similarity).
N	Q4P5F5	DE	Probable cytosolic iron-sulfur protein assembly protein 1;
N	Q4PDF6	CC	Catalytic subunit of the SLX1-SLX4 structure-specific endonuclease that resolves DNA secondary structures generated during DNA repair and recombination
N	Q4PDF6	CC	Has endonuclease activity towards branched DNA substrates, introducing single-strand cuts in duplex DNA close to junctions with ss-DNA (By similarity).
N	Q4PDF6	DE	Structure-specific endonuclease subunit SLX1;
N	Q4PDF6	DR	DNA recombination
N	Q4PDF6	DR	endonuclease activity
N	Q4QM29	DE	50S ribosomal protein L27;
N	Q4QM29	DR	structural constituent of ribosome
N	Q4QMA9	CC	This is 1 of the proteins that binds and probably mediates the attachment of the 5S RNA into the large ribosomal subunit, where it forms part of the central protuberance
N	Q4QMA9	CC	Contacts the P site tRNA; the 5S rRNA and some of its associated proteins might help stabilize positioning of ribosome-bound tRNAs (By similarity).
N	Q4QMA9	CC	In the 70S ribosome it contacts protein S13 of the 30S subunit (bridge B1b), connecting the 2 subunits; this bridge is implicated in subunit movement
N	Q4QMA9	DE	50S ribosomal protein L5;
N	Q4QMA9	DR	structural constituent of ribosome
N	Q4QMZ9	CC	Specifically dimethylates two adjacent adenosines (A1518 and A1519) in the loop of a conserved hairpin near the 3'-end of 16S rRNA in the 30S particle
N	Q4QMZ9	CC	May play a critical role in biogenesis of 30S subunits (By similarity).
N	Q4QMZ9	DE	16S rRNA (adenine(1518)-N(6)/adenine(1519)-N(6))-dimethyltransferase;
N	Q4QMZ9	DE	16S rRNA dimethyladenosine transferase;
N	Q4QMZ9	DE	16S rRNA dimethylase;
N	Q4QMZ9	DE	Ribosomal RNA small subunit methyltransferase A;
N	Q4QMZ9	DE	S-adenosylmethionine-6-N', N'-adenosyl(rRNA) dimethyltransferase;
N	Q4QMZ9	DR	rRNA (adenine-N6,N6-)-dimethyltransferase activity
N	Q4QP20	CC	NQR complex catalyzes the reduction of ubiquinone-1 to ubiquinol by two successive reactions, coupled with the transport of Na(+) ions from the cytoplasm to the periplasm
N	Q4QP20	CC	NqrA to nqrE are probably involved in the second step, the conversion of ubisemiquinone to ubiquinol (By similarity).
N	Q4QP20	DE	NQR complex subunit E;
N	Q4QP20	DE	NQR-1 subunit E;
N	Q4QP20	DE	Na(+)-NQR subunit E;
N	Q4QP20	DE	Na(+)-translocating NADH-quinone reductase subunit E;
N	Q4QP20	DE	Na(+)-translocating NQR subunit E;
N	Q4QP20	DR	oxidoreductase activity, acting on NADH or NADPH, quinone or similar compound as acceptor
N	Q4QP20	DR	respiratory electron transport chain
N	Q4QP20	DR	sodium ion transport
N	Q4QR85	CC	Non-catalytic component of the 20S PRMT5-containing methyltransferase complex, which modifies specific arginines to dimethylarginines in several spliceosomal Sm proteins
N	Q4QR85	CC	Might play a role in transcription regulation
N	Q4QR85	CC	The 20S PRMT5-containing methyltransferase complex also methylates the Piwi proteins (PIWIL1, PIWIL2 and PIWIL4), methylation of Piwi proteins being required for the interaction with Tudor domain- containing proteins and subsequent localization to the meiotic nuage (By similarity).

N	Q4QR85	CC	This modification targets Sm proteins to the survival of motor neurons (SMN) complex for assembly into small nuclear ribonucleoprotein core particles
N	Q4QR85	DE	Methylosome protein 50;
N	Q4QR85	DE	WD repeat-containing protein 77;
N	Q4R6B2	CC	Probably involved in sperm cell hyperactivation via its association with [illegible]
N	Q4R6B2	CC	Sperm cell hyperactivation is needed for sperm motility which is essential late in the preparation of sperm for fertilization (By similarity).
N	Q4R6B2	DE	Cation channel sperm-associated protein subunit gamma;
N	Q4R6B2	DR	cell differentiation
N	Q4R6B2	DR	multicellular organismal development
N	Q4R6B2	DR	spermatogenesis
N	Q4R6Y6	CC	Part of the SNAPc complex required for the transcription of both RNA polymerase II and III small-nuclear RNA genes
N	Q4R6Y6	CC	Binds to the proximal sequence element (PSE), a non-TATA-box basal promoter element common to these 2 types of genes
N	Q4R6Y6	CC	Recruits TBP and BRF2 to the U6 snRNA TATA box (By similarity).
N	Q4R6Y6	DE	SNAPc subunit 3;
N	Q4R6Y6	DE	Small nuclear RNA-activating complex polypeptide 3;
N	Q4R6Y6	DE	snRNA-activating protein complex subunit 3;
N	Q4R998	DE	MAGE-B18 antigen;
N	Q4R998	DE	Melanoma-associated antigen B18;
N	Q4R9D1	CC	Accepts ubiquitin from the E1 complex and catalyzes its covalent attachment to other proteins
N	Q4R9D1	CC	Acts as an essential factor of the anaphase promoting complex/cyclosome (APC/C), a cell cycle-regulated ubiquitin ligase that controls progression through mitosis
N	Q4R9D1	CC	Acts by initiating 'Lys-11'-linked polyubiquitin chains on APC/C substrates, leading to the degradation of APC/C substrates by the proteasome and promoting mitotic exit (By similarity).
N	Q4R9D1	CC	In vitro catalyzes 'Lys- 11'- and 'Lys-48'-linked polyubiquitination
N	Q4R9D1	DE	Ubiquitin carrier protein C;
N	Q4R9D1	DE	Ubiquitin-conjugating enzyme E2 C;
N	Q4R9D1	DE	Ubiquitin-protein ligase C;
N	Q4R9D1	DR	anaphase-promoting complex-dependent proteasomal ubiquitin-dependent protein catabolic process
N	Q4R9D1	DR	cell division
N	Q4R9D1	DR	exit from mitosis
N	Q4R9D1	DR	free ubiquitin chain polymerization
N	Q4R9D1	DR	post-translational protein modification
N	Q4R9D1	DR	protein K11-linked ubiquitination
N	Q4R9D1	DR	protein K48-linked ubiquitination
N	Q4R9D1	DR	ubiquitin-protein ligase activity
N	Q4TWH8	CC	Non-structural polyprotein 1AB contains the viral protease participating in the cleavage of the polyprotein into functional products
N	Q4TWH8	CC	It contains also the activities necessary for replication of genomic RNA, as well as transcription of subgenomic mRNA (By similarity).
N	Q4TWH8	DE	Non-structural polyprotein 1AB;
N	Q4TWH8	DE	Protein p19;
N	Q4TWH8	DE	Protein p20;
N	Q4TWH8	DE	RNA-directed RNA polymerase p57;
N	Q4TWH8	DE	Serine protease p27;
N	Q4TWH8	DE	Transmembrane protein 1A;
N	Q4TWH8	DR	RNA-directed RNA polymerase activity
N	Q4TWH8	DR	cysteine-type peptidase activity
N	Q4TWH8	DR	serine-type endopeptidase activity
N	Q4TWH8	DR	serine-type exopeptidase activity
N	Q4TWH8	DR	viral genome replication
N	Q4UJM4	CC	Component of the ubiquinol-cytochrome c reductase complex (complex III or cytochrome b-c1 complex), which is a respiratory chain that generates an electrochemical potential coupled to ATP synthesis (By similarity).
N	Q4UJM4	DE	Cytochrome b;
N	Q4UJM4	DR	electron carrier activity
N	Q4UJM4	DR	metal ion binding
N	Q4UJM4	DR	oxidoreductase activity
N	Q4UJM4	DR	respiratory electron transport chain

N	Q4ULR1	CC	Binds specifically to the ssrA RNA (tmRNA) and is required for stable association of ssrA with ribosomes (By similarity).
N	Q4ULR1	DE	SsrA-binding protein;
N	Q4UMZ7	CC	Part of the ABC transporter complex LolCDE involved in the translocation of mature outer membrane-directed lipoproteins, from the inner membrane to the periplasmic chaperone, LolA
N	Q4UMZ7	CC	Responsible for the formation of the LolA-lipoprotein complex in an ATP-dependent manner (By similarity).
N	Q4UMZ7	DE	Lipoprotein-releasing system ATP-binding protein LolD;
N	Q4UMZ7	DR	ATPase activity
N	Q4UMZ7	DR	lipoprotein transporter activity
N	Q4UN32	DE	SURF1-like protein;
N	Q4UPQ3	CC	Catalyzes the transfer of a ribosyl phosphate group from 5-phosphoribose 1-diphosphate to orotate, leading to the formation of orotidine monophosphate (OMP) (By similarity).
N	Q4UPQ3	DE	Orotate phosphoribosyltransferase;
N	Q4UPQ3	DR	nucleoside metabolic process
N	Q4UPQ3	DR	orotate phosphoribosyltransferase activity
N	Q4UPQ3	DR	pyrimidine nucleotide biosynthetic process
N	Q4UPU5	CC	Required for the formation of a threonylcarbamoyl group on adenosine at position 37 (t(6)A37) in tRNAs that read codons beginning with adenine (By similarity).
N	Q4UPU5	DE	Probable tRNA threonylcarbamoyladenosine biosynthesis protein Gcp;
N	Q4UPU5	DE	t(6)A37 threonylcarbamoyladenosine biosynthesis protein;
N	Q4UPU5	DR	metal ion binding
N	Q4UPU5	DR	metalloendopeptidase activity
N	Q4UQT7	CC	Negatively regulates transcription of bacterial ribonucleotide reductase nrd genes and operons by binding to NrdR- boxes (By similarity).
N	Q4UQT7	DE	Transcriptional repressor NrdR;
N	Q4UQT7	DR	transcription repressor activity
N	Q4UQT7	DR	zinc ion binding
N	Q4UTC7	CC	Is probably involved in the electron transfer between its heme and the molybdenum cofactor of the reductase catalytic subunit yedY (By similarity).
N	Q4UTC7	DE	Flavocytochrome yedZ;
N	Q4UTC7	DE	Sulfoxide reductase heme-binding subunit yedZ;
N	Q4UTC7	DR	electron carrier activity
N	Q4UTC7	DR	electron transport chain
N	Q4UTC7	DR	flavin adenine dinucleotide binding
N	Q4UTC7	DR	iron ion binding
N	Q4UTC7	DR	oxidoreductase activity
N	Q4UWY7	DE	Aspartate carbamoyltransferase;
N	Q4UWY7	DE	Aspartate transcarbamylase;
N	Q4UWY7	DR	'de novo' pyrimidine base biosynthetic process
N	Q4UWY7	DR	amino acid binding
N	Q4UWY7	DR	aspartate carbamoyltransferase activity
N	Q4UWY7	DR	cellular amino acid metabolic process
N	Q4UWY7	DR	pyrimidine nucleotide biosynthetic process
N	Q4UY06	DE	ATP-sulfurylase small subunit;
N	Q4UY06	DE	Sulfate adenylate transferase;
N	Q4UY06	DE	Sulfate adenylyltransferase subunit 2;
N	Q4UY06	DR	sulfate adenylyltransferase (ATP) activity
N	Q4UY06	DR	sulfate reduction
N	Q4V7C1	CC	Required for anchoring microtubules to the centrosomes (By similarity).
N	Q4V7C1	DE	FGFR1 oncogene partner;
N	Q4V7C1	DR	microtubule anchoring
N	Q4VYA5	CC	Catalyzes the hydrolysis of 5-hydroxyisourate (HIU) to 2-oxo-4-hydroxy-4-carboxy-5-ureidoimidazoline (OHCU).
N	Q4VYA5	DE	5-hydroxyisourate hydrolase;
N	Q4VYA5	DE	HIU hydrolase;
N	Q4VYA5	DE	Transthyretin-like protein;
N	Q4VYA5	DE	Transthyretin-related protein;
N	Q4VYA5	DR	hydroxyisourate hydrolase activity
N	Q4VYA5	DR	purine base metabolic process
N	Q4WDD0	CC	Involved in pre-mRNA splicing
N	Q4WDD0	CC	May do so by stabilizing the catalytic center or the position of the RNA substrate (By similarity).

N	Q4WDD0	CC	May function at or prior to the first catalytic step of splicing at the catalytic center of the spliceosome
N	Q4WDD0	DE	Pre-mRNA-splicing factor cwc21;
N	Q4WDD0	DR	mRNA processing
N	Q4WDE0	CC	Component of the ERMES/MDM complex, which serves as a molecular tether to connect the endoplasmic reticulum and mitochondria
N	Q4WDE0	CC	Components of this complex are involved in the control of mitochondrial shape and protein biogenesis and may function in phospholipid exchange
N	Q4WDE0	CC	Essential for establishing and maintaining the structure of mitochondria and maintenance of mtDNA nucleoids (By similarity).
N	Q4WDE0	CC	The mdm10-mdm12-mmm1 subcomplex further acts in the TOM40-specific pathway after the action of the mdm12-mmm1 complex
N	Q4WDE0	CC	The mdm12-mmm1 subcomplex functions in the major beta-barrel assembly pathway that is responsible for biogenesis of all outer membrane beta-barrel proteins, and acts in a late step after the SAM complex
N	Q4WDE0	DE	Maintenance of mitochondrial morphology protein 1;
N	Q4WEU2	CC	DNA-dependent RNA polymerase catalyzes the transcription of DNA into RNA using the four ribonucleoside triphosphates as substrates
N	Q4WEU2	CC	Specific core component of RNA polymerase III which synthesizes small RNAs, such as 5S rRNA and tRNAs (By similarity).
N	Q4WEU2	DE	DNA-directed RNA polymerase III subunit rpc3;
N	Q4WEU2	DE	RNA polymerase III subunit C3;
N	Q4WEU2	DR	DNA-directed RNA polymerase activity
N	Q4WKH9	CC	DNA helicase which participates in several chromatin remodeling complexes, including the SWR1 and the INO80 complexes
N	Q4WKH9	CC	Also involved in pre-rRNA processing (By similarity).
N	Q4WKH9	CC	The INO80 complex remodels chromatin by shifting nucleosomes and is involved in DNA repair
N	Q4WKH9	CC	The SWR1 complex mediates the ATP-dependent exchange of histone H2A for the H2A variant HZT1 leading to transcriptional regulation of selected genes by chromatin remodeling
N	Q4WKH9	DE	RuvB-like helicase 2;
N	Q4WKH9	DR	DNA helicase activity
N	Q4WKH9	DR	chromatin modification
N	Q4WKH9	DR	rRNA processing
N	Q4WT68	CC	May be involved in mitochondrial DNA stability (By similarity).
N	Q4WT68	DE	Mitochondrial outer membrane protein iml2;
N	Q4WXX5	CC	Histone chaperone that facilitates histone deposition and histone exchange and removal during nucleosome assembly and disassembly (By similarity).
N	Q4WXX5	DE	Anti-silencing function protein 1;
N	Q4WXX5	DE	Histone chaperone asf1;
N	Q4WXX5	DR	chromatin assembly or disassembly
N	Q4WXX5	DR	chromatin modification
N	Q4X0I8	CC	Has a role in mitochondrial fission
N	Q4X0I8	CC	Has a role in outer membrane fission but not matrix separation (By similarity).
N	Q4X0I8	DE	Mitochondria fission 1 protein;
N	Q4X2S8	CC	Plays an important role in the salvage pathway for purine nucleotide biosynthesis
N	Q4X2S8	CC	Catalyzes the first committed step in the biosynthesis of AMP from IMP (By similarity).
N	Q4X2S8	DE	Adenylosuccinate synthetase;
N	Q4X2S8	DE	IMP--aspartate ligase;
N	Q4X2S8	DR	adenylosuccinate synthase activity
N	Q4X2S8	DR	magnesium ion binding
N	Q4X2S8	DR	purine nucleotide biosynthetic process
N	Q4ZQ90	DE	3,4-dihydroxy-5-hexaprenylbenzoate methyltransferase;
N	Q4ZQ90	DE	3-demethylubiquinone-9 3-methyltransferase;
N	Q4ZQ90	DE	DHHB methyltransferase;
N	Q4ZQ90	DR	2-polyprenyl-6-methoxy-1,4-benzoquinone methyltransferase activity
N	Q4ZQ90	DR	3-demethylubiquinone-9 3-O-methyltransferase activity
N	Q4ZQ90	DR	ubiquinone biosynthetic process
N	Q4ZRI4	CC	NDH-1 shuttles electrons from NADH, via FMN and iron- sulfur (Fe-S) centers, to quinones in the respiratory chain
N	Q4ZRI4	CC	Couples the redox reaction to proton translocation (for every two electrons transferred, four hydrogen ions are translocated across the cytoplasmic membrane), and thus conserves the redox energy in a proton gradient (By similarity).

N	Q4ZRI4	CC	The immediate electron acceptor for the enzyme in this species is believed to be ubiquinone
N	Q4ZRI4	DE	NADH dehydrogenase I subunit K;
N	Q4ZRI4	DE	NADH-quinone oxidoreductase subunit K;
N	Q4ZRI4	DE	NDH-1 subunit K;
N	Q4ZRI4	DR	ATP synthesis coupled electron transport
N	Q4ZRI4	DR	NADH dehydrogenase (quinone) activity
N	Q4ZRI4	DR	quinone binding
N	Q4ZSF3	CC	Part of the ABC transporter complex XylFGH involved in xylose import
N	Q4ZSF3	CC	Responsible for energy coupling to the transport system (By similarity).
N	Q4ZSF3	DE	Xylose import ATP-binding protein XylG;
N	Q4ZSF3	DR	D-xylose-importing ATPase activity
N	Q4ZSF5	DE	Xylose isomerase;
N	Q4ZSF5	DR	D-xylose metabolic process
N	Q4ZSF5	DR	metal ion binding
N	Q4ZSF5	DR	pentose-phosphate shunt
N	Q4ZSF5	DR	xylose isomerase activity
N	Q4ZVL1	CC	Endonuclease that specifically degrades the RNA of RNA- DNA hybrids (By similarity).
N	Q4ZVL1	DE	Ribonuclease H;
N	Q4ZVL1	DR	metal ion binding
N	Q4ZVL1	DR	nucleic acid binding
N	Q4ZVL1	DR	ribonuclease H activity
N	Q4ZWL3	CC	Nuclease that resolves Holliday junction intermediates in genetic recombination
N	Q4ZWL3	CC	Cleaves the cruciform structure in supercoiled DNA by nicking to strands with the same polarity at sites symmetrically opposed at the junction in the homologous arms and leaves a 5'-terminal phosphate and a 3'-terminal hydroxyl group (By similarity)
N	Q4ZWL3	DE	Crossover junction endodeoxyribonuclease ruvC;
N	Q4ZWL3	DE	Holliday junction nuclease ruvC;
N	Q4ZWL3	DE	Holliday junction resolvase ruvC;
N	Q4ZWL3	DR	DNA recombination
N	Q4ZWL3	DR	crossover junction endodeoxyribonuclease activity
N	Q4ZWL3	DR	metal ion binding
N	Q4ZWL3	DR	nucleic acid binding
N	Q4ZWV3	CC	Forms oxaloacetate, a four-carbon dicarboxylic acid source for the tricarboxylic acid cycle.
N	Q4ZWV3	DE	Phosphoenolpyruvate carboxylase;
N	Q4ZWV3	DR	carbon fixation
N	Q4ZWV3	DR	phosphoenolpyruvate carboxylase activity
N	Q4ZWV3	DR	tricarboxylic acid cycle
N	Q4ZX15	CC	Catalyzes the decarboxylation of oxaloacetate into pyruvate
N	Q4ZX15	CC	Seems to play a role in maintaining cellular concentrations of bicarbonate and pyruvate (By similarity).
N	Q4ZX15	DE	Oxaloacetate decarboxylase;
N	Q4ZX15	DR	metal ion binding
N	Q4ZX15	DR	oxaloacetate decarboxylase activity
N	Q4ZZU5	DE	Amino-acid acetyltransferase;
N	Q4ZZU5	DE	N-acetylglutamate synthase;
N	Q4ZZU5	DR	acetyl-CoA:L-glutamate N-acetyltransferase activity
N	Q4ZZU5	DR	arginine biosynthetic process
N	Q50192	DE	Leucine--tRNA ligase;
N	Q50192	DE	Leucyl-tRNA synthetase;
N	Q50192	DR	leucine-tRNA ligase activity
N	Q50192	DR	leucyl-tRNA aminoacylation
N	Q503I8	CC	Specifically deglycosylates the denatured form of N- linked glycoproteins in the cytoplasm and assists their proteasome-mediated degradation
N	Q503I8	CC	Can recognize misfolded proteins in the endoplasmic reticulun that are exported in the cytosol to be destroyed and deglycosylate them, while it has no activity toward native proteins
N	Q503I8	CC	Cleaves the beta-aspartyl- glucosamine (GlcNAc) of the glycan and the amide side chain of Asn, converting Asn to Asp
N	Q503I8	CC	Deglycosylation is prerequisite for subsequent proteasome-mediated degradation of some, but not all, misfolded glycoproteins (By similarity).
N	Q503I8	CC	Prefers proteins containing high- mannose over those bearing complex type oligosaccharides

N	Q503I8	DE	N-glycanase 1;
N	Q503I8	DE	Peptide-N(4)-(N-acetyl-beta-glucosaminyl)asparagine amidase;
N	Q503I8	DE	Peptide:N-glycanase;
N	Q503I8	DR	glycoprotein catabolic process
N	Q503I8	DR	metal ion binding
N	Q503I8	DR	peptide-N4-(N-acetyl-beta-glucosaminyl)asparagine amidase activity
N	Q503R4	CC	Probable substrate-specific adapter of an E3 ubiquitin- protein ligase complex which mediates the ubiquitination and subsequent proteasomal degradation of target proteins (By similarity).
N	Q503R4	DE	Kelch-like protein 36;
N	Q503U1	DE	LYR motif-containing protein 5A;
N	Q50400	CC	Probable electron acceptor for methylamine dehydrogenase.
N	Q50400	DR	copper ion binding
N	Q50400	DR	electron carrier activity
N	Q50400	DR	electron transport chain
N	Q50KA9	CC	Major role in the synthesis of nucleoside triphosphates other than ATP
N	Q50KA9	CC	Involved in cell proliferation, differentiation and development, signal transduction, G protein- coupled receptor endocytosis, and gene expression
N	Q50KA9	CC	Possesses nucleoside-diphosphate kinase, serine/threonine-specific protein kinase, geranyl and farnesyl pyrophosphate kinase, histidine protein kinase and 3'-5' exonuclease activities
N	Q50KA9	CC	Required for neural development including neural patterning and cell fate determination (By similarity).
N	Q50KA9	CC	The ATP gamma phosphate is transferred to the NDP beta phosphate via a ping-pong mechanism, using a phosphorylated active-site intermediate
N	Q50KA9	DE	NDP kinase A;
N	Q50KA9	DE	Nucleoside diphosphate kinase A;
N	Q50KA9	DR	CTP biosynthetic process
N	Q50KA9	DR	GTP biosynthetic process
N	Q50KA9	DR	UTP biosynthetic process
N	Q50KA9	DR	cell differentiation
N	Q50KA9	DR	nervous system development
N	Q50KA9	DR	nucleoside diphosphate kinase activity
N	Q51473	CC	This protein is one of the two subunits of integration host factor, a specific DNA-binding protein that functions in genetic recombination as well as in transcriptional and translational control (By similarity).
N	Q51473	DE	Integration host factor subunit beta;
N	Q51473	DR	DNA recombination
N	Q51473	DR	regulation of transcription, DNA-dependent
N	Q51473	DR	regulation of translation
N	Q52086	CC	Catalyzes the hydrolytic dehalogenation of small (R)-2- haloalkanoic acids to yield the corresponding (S)-2- hydroxyalkanoic acids
N	Q52086	CC	Acts on acids of short chain lengths, C(2) to C(4), with inversion of configuration [illegible]
N	Q52086	DE	(R)-2-haloacid dehalogenase;
N	Q52086	DE	D-2-haloacid dehalogenase;
N	Q52086	DR	(R)-2-haloacid dehalogenase activity
N	Q52R90	CC	Indispensable for the control of thyroid structure and metabolism (By similarity).
N	Q52R90	DE	Thyroid-stimulating hormone subunit beta;
N	Q52R90	DE	Thyrotropin beta chain;
N	Q52R90	DE	Thyrotropin subunit beta;
N	Q52R90	DR	hormone activity
N	Q53120	CC	This photoactive protein is a photoreceptor with kinetics similar to that of [illegible]
N	Q53120	DE	Photoactive yellow protein;
N	Q53120	DR	photoreceptor activity
N	Q53120	DR	phototransduction
N	Q53120	DR	protein-chromophore linkage
N	Q53120	DR	regulation of transcription, DNA-dependent
N	Q53B56	CC	This three-finger toxin produces peripheral paralysis by blocking neuromuscular transmission at the postsynaptic site
N	Q53B56	CC	Binds to muscular and neuronal (only alpha-7 alpha-8 alpha-9) nicotinic acetylcholine receptors (By similarity).
N	Q53B56	DE	Long neurotoxin OH-57;
N	Q53B56	DR	acetylcholine receptor inhibitor activity
N	Q53B56	DR	synaptic transmission
N	Q53LU4	CC	Lignin degradation and detoxification of lignin-derived products (By similarity).

N	Q53LU4	DE	Benzenediol:oxygen oxidoreductase 18;
N	Q53LU4	DE	Diphenol oxidase 18;
N	Q53LU4	DE	Urishiol oxidase 18;
N	Q53LU4	DR	copper ion binding
N	Q53LU4	DR	laccase activity
N	Q53LU4	DR	lignin catabolic process
N	Q53LU4	DR	oxidation-reduction process
N	Q53RY4	DE	Keratinocyte-associated protein 3;
N	Q54450	CC	Can bind free heme and also acquire it from hemoglobin
N	Q54450	CC	Conveys heme from hemoglobin to the HasR receptor which releases it into the bacterium
N	Q54450	CC	HasR alone can take up heme but the synergy between HasA and HasR increases heme uptake 100-fold.
N	Q54450	DE	Heme acquisition system protein A;
N	Q54450	DE	Hemophore HasA;
N	Q54450	DR	metal ion binding
N	Q54CB2	CC	Probably mediates the nuclear export of actin and profilin-actin complexes (By similarity).
N	Q54CB2	DR	protein transport
N	Q54CH1	DE	Arrestin domain-containing protein A;
N	Q54CH1	DR	signal transduction
N	Q54CH1	DR	zinc ion binding
N	Q54DR7	DE	Putative uncharacterized protein DDB_G0292176;
N	Q54DX7	CC	Acyltransferase which mediates the conversion of 1-acyl- sn-glycero-3-phosphocholine (LPC) into phosphatidylcholine (PC)
N	Q54DX7	CC	Involved in the remodeling of the acyl groups of cardiolipin in the mitochondrial inner membrane, which affects the assembly and stability of respiratory chain complex IV and its supercomplex forms (By similarity).
N	Q54DX7	CC	Required for normal phospholipid content of mitochondrial membranes
N	Q54DX7	DE	1-acylglycerophosphocholine O-acyltransferase;
N	Q54DX7	DE	Putative lysophosphatidylcholine acyltransferase;
N	Q54DX7	DE	Tafazzin homolog;
N	Q54DX7	DR	1-acylglycerophosphocholine O-acyltransferase activity
N	Q54IC9	CC	May be involved in the degradation process of specific misfolded endoplasmic reticulum (ER) luminal proteins (By similarity).
N	Q54IC9	DE	Probable derlin-1 homolog;
N	Q54IC9	DR	response to unfolded protein
N	Q54J77	DE	Frizzled and smoothened-like protein F;
N	Q54JB3	CC	Converts protoheme IX and farnesyl diphosphate to heme O (By similarity).
N	Q54JB3	DE	Heme O synthase;
N	Q54JB3	DE	Probable protoheme IX farnesyltransferase, mitochondrial;
N	Q54JB3	DR	heme biosynthetic process
N	Q54JB3	DR	protoheme IX farnesyltransferase activity
N	Q54NK8	DE	ATP-binding domain 1 family member C homolog;
N	Q54NK8	DE	GPN-loop GTPase 3 homolog;
N	Q54NZ4	CC	Part of the AP-4 complex, an adaptor-related complex which might be implicated in vesicular transport between intracellular compartments appearing upon cell differentiation such as prespore vesicles (PSVs) (By similarity).
N	Q54NZ4	DE	AP-4 adapter complex subunit sigma;
N	Q54NZ4	DE	AP-4 complex subunit sigma;
N	Q54NZ4	DE	Adapter-related protein complex 4 subunit sigma;
N	Q54NZ4	DE	Sigma subunit of AP-4;
N	Q54NZ4	DE	Sigma4-adaptin;
N	Q54NZ4	DR	protein transporter activity
N	Q54Q02	DE	Putative uncharacterized protein DDB_G0284297;
N	Q54Q40	CC	May be a zinc metalloprotease that specifically cleaves ubiquitin chains (By similarity)
N	Q54Q40	DE	Developmental gene 1039 protein;
N	Q54Q40	DE	Probable ubiquitin thiolesterase DG1039;
N	Q54Q40	DR	metal ion binding
N	Q54Q40	DR	metallopeptidase activity
N	Q54Q92	DE	LMBR1 domain-containing protein 2 homolog A;
N	Q54QS0	CC	E3 UFM1-protein ligase that mediates ufmylation of target proteins (By similarity)
N	Q54QS0	DE	E3 UFM1-protein ligase 1 homolog;
N	Q54QS0	DR	ligase activity
N	Q54QU3	DE	Uncharacterized protein DDB_G0283639;

N	Q54SG3	DE	Uncharacterized transmembrane protein DDB_G0282483;
N	Q54SR6	DE	Putative uncharacterized protein DDB_G0282269;
N	Q54TF8	CC	ATP-binding RNA helicase required for normal differentiation and development.
N	Q54TF8	DE	ATP-dependent RNA helicase ddx19;
N	Q54TF8	DE	ATP-dependent RNA helicase helC;
N	Q54TF8	DE	DEAD box protein 19;
N	Q54TF8	DR	ATP-dependent helicase activity
N	Q54TF8	DR	multicellular organismal development
N	Q54VG1	CC	Guanine nucleotide-binding proteins (G proteins) are involved as modulators or transducers in various transmembrane signaling systems.
N	Q54VG1	DE	G alpha-12;
N	Q54VG1	DE	Guanine nucleotide-binding protein alpha-12 subunit;
N	Q54VG1	DR	G-protein coupled receptor protein signaling pathway
N	Q54VG1	DR	signal transducer activity
N	Q54W16	DE	UPF0406 protein;
N	Q54X53	DE	60S ribosomal protein L21;
N	Q54X53	DR	response to bacterium
N	Q54X53	DR	structural constituent of ribosome
N	Q54XM0	DE	Uncharacterized CDP-alcohol phosphatidyltransferase class-I family protein 2;
N	Q54XM0	DR	phospholipid biosynthetic process
N	Q54XM0	DR	phosphotransferase activity, for other substituted phosphate groups
N	Q54YB7	DE	Myb-like protein F;
N	Q54ZS8	CC	Involved in the 3'-end formation of mRNA precursors (pre-mRNA) by the addition of a poly(A) tail of 200-250 nt to the upstream cleavage product (By similarity).
N	Q54ZS8	DE	Poly(A)-binding protein 2;
N	Q54ZS8	DE	Poly(A)-binding protein II;
N	Q54ZS8	DE	Polyadenylate-binding protein 2;
N	Q54ZS8	DR	mRNA polyadenylation
N	Q54ZS8	DR	nucleotide binding
N	Q54ZS8	DR	poly(A) RNA binding
N	Q55332	CC	Stabilizes the structure of photosystem II oxygen- evolving complex (OEC), the ion environment of oxygen evolution and protects the OEC against heat-induced inactivation
N	Q55332	CC	May modulate the Cl(-) requirement for oxygen evolution.
N	Q55332	DE	PS II complex 12 kDa extrinsic protein;
N	Q55332	DE	Photosystem II 12 kDa extrinsic protein;
N	Q55332	DR	electron transport chain
N	Q55332	DR	photosynthesis
N	Q55332	DR	photosystem II stabilization
N	Q559T8	DE	Probable serine/threonine-protein kinase DDB_G0272282;
N	Q559T8	DR	cell communication
N	Q559T8	DR	phosphatidylinositol binding
N	Q559T8	DR	protein phosphorylation
N	Q559T8	DR	protein serine/threonine kinase activity
N	Q559W3	DE	Uncharacterized protein DDB_G0272420;
N	Q559W3	DR	nucleic acid binding
N	Q559W3	DR	zinc ion binding
N	Q55BZ5	DE	Acid ceramidase-like protein A;
N	Q55BZ5	DE	Protein dcd1A;
N	Q55C57	DE	Glycogen synthase kinase-like kinase A;
N	Q55C57	DE	Probable serine/threonine-protein kinase glkA;
N	Q55C57	DR	protein phosphorylation
N	Q55C57	DR	protein serine/threonine kinase activity
N	Q55C57	DR	protein tyrosine kinase activity
N	Q55C57	DR	tau-protein kinase activity
N	Q55E94	CC	Mitochondrial GTPase that catalyzes the GTP-dependent ribosomal translocation step during translation elongation
N	Q55E94	CC	Catalyzes the coordinated movement of the two tRNA molecules, the mRNA and conformational changes in the ribosome (By similarity).
N	Q55E94	CC	During this step, the ribosome changes from the pre-translocational (PRE) to the post-translocational (POST) state as the newly formed A- site-bound peptidyl-tRNA and P-site-bound deacylated tRNA move to the P and E sites, respectively
N	Q55E94	DE	Elongation factor G 1, mitochondrial;
N	Q55E94	DE	Elongation factor G, mitochondrial;
N	Q55E94	DE	Elongation factor G1;
N	Q55E94	DR	GTPase activity

N	Q55E94	DR	translation elongation factor activity
N	Q55EF8	DE	Transmembrane protein DDB_G0269096;
N	Q55FF3	CC	Conjugation of reduced glutathione to a wide number of exogenous and endogenous hydrophobic electrophiles (By similarity).
N	Q55FF3	DE	GST class-alpha 1;
N	Q55FF3	DE	Putative glutathione S-transferase alpha-1;
N	Q55FF3	DR	glutathione transferase activity
N	Q56214	CC	The ruvA-ruvB complex in the presence of ATP renatures cruciform structure in supercoiled DNA with palindromic sequence, indicating that it may promote strand exchange reactions in homologous recombination
N	Q56214	CC	It can promote Holliday junction migration alone.
N	Q56214	CC	RuvAB is an helicase that mediates the Holliday junction migration by localized denaturation and reannealing
N	Q56214	CC	RuvB is a Mg(2+)-dependent, DNA-dependent ATPase with an equal preference for supercoiled and linear duplex DNA
N	Q56214	DE	Holliday junction ATP-dependent DNA helicase ruvB;
N	Q56214	DR	DNA recombination
N	Q56214	DR	four-way junction helicase activity
N	Q56316	DE	Pyruvate oxidoreductase delta chain;
N	Q56316	DE	Pyruvate synthase subunit porD;
N	Q56316	DE	Pyruvic-ferredoxin oxidoreductase subunit delta;
N	Q56316	DR	4 iron, 4 sulfur cluster binding
N	Q56316	DR	electron carrier activity
N	Q56316	DR	electron transport chain
N	Q56316	DR	metal ion binding
N	Q56316	DR	oxidoreductase activity, acting on the aldehyde or oxo group of donors, iron-sulfur protein as acceptor
N	Q56425	CC	Required for maturation of 30S ribosomal subunits (By similarity).
N	Q56425	DE	Ribosome maturation factor rimP;
N	Q56425	DR	ribosome biogenesis
N	Q56694	CC	Catalyzes the oxidation of long-chain aliphatic aldehydes to acids
N	Q56694	CC	May be implicated in controlling luminescence as it catalyzes the oxidation of the fatty aldehyde substrate for the light-emitting reaction.
N	Q56694	DE	NADP-dependent fatty aldehyde dehydrogenase;
N	Q56694	DR	aldehyde dehydrogenase (NADP+) activity
N	Q56694	DR	oxidation-reduction process
N	Q56715	CC	This protein binds to the 23S rRNA, and is important in its secondary structure
N	Q56715	CC	It is located near the subunit interface in the base of the L7/L12 stalk, and near the tRNA binding site of the peptidyltransferase center (By similarity).
N	Q56715	DE	50S ribosomal protein L6;
N	Q57624	CC	Allows the formation of correctly charged Asn-tRNA(Asn) or Gln-tRNA(Gln) through the transamidation of misacylated Asp- tRNA(Asn) or Glu-tRNA(Gln) in organisms which lack either or both of asparaginyl-tRNA or glutaminyl-tRNA synthetases
N	Q57624	CC	The reaction takes place in the presence of glutamine and ATP through an activated phospho-Asp-tRNA(Asn) or phospho-Glu-tRNA(Gln) (By similarity).
N	Q57624	DE	Asp/Glu-ADT subunit B;
N	Q57624	DE	Aspartyl/glutamyl-tRNA(Asn/Gln) amidotransferase subunit B;
N	Q57624	DR	carbon-nitrogen ligase activity, with glutamine as amido-N-donor
N	Q57673	CC	Produces ATP from ADP in the presence of a proton gradient across the membrane
N	Q57673	DE	V-ATPase subunit E;
N	Q57673	DE	V-type ATP synthase subunit E;
N	Q57673	DR	ATP biosynthetic process
N	Q57673	DR	ATP hydrolysis coupled proton transport
N	Q57673	DR	proton-transporting ATPase activity, rotational mechanism
N	Q57727	CC	Prenyltransferase that catalyzes the transfer of the geranylgeranyl moiety of geranylgeranyl diphosphate (GGPP) to the C2 hydroxyl of (S)-3-O-geranylgeranylglyceryl phosphate (GGGP)
N	Q57727	CC	This reaction is the second ether-bond-formation step in the biosynthesis of archaeal membrane lipids (By similarity).
N	Q57727	DE	(S)-2,3-di-O-geranylgeranylglyceryl phosphate synthase;
N	Q57727	DE	DGGGP synthase;
N	Q57727	DE	Digeranylgeranylglyceryl phosphate synthase;
N	Q57727	DE	Geranylgeranylglycerol-phosphate geranylgeranyltransferase;
N	Q57727	DR	geranylgeranylglycerol-phosphate geranylgeranyltransferase activity
N	Q57727	DR	magnesium ion binding

N	Q57727	DR	phospholipid biosynthetic process
N	Q57727	DR	prenyltransferase activity
N	Q57842	DE	Probable phosphomannomutase;
N	Q57842	DR	carbohydrate metabolic process
N	Q57842	DR	magnesium ion binding
N	Q57842	DR	phosphomannomutase activity
N	Q57866	DE	Uncharacterized ATP-binding protein MJ0423;
N	Q57889	DE	Uncharacterized protein MJ0447;
N	Q57950	DE	Uncharacterized ZPR1-like protein MJ0530;
N	Q57950	DR	zinc ion binding
N	Q57C89	CC	DNA ligase that catalyzes the formation of phosphodiester linkages between 5'-phosphoryl and 3'-hydroxyl groups in double-stranded DNA using NAD as a coenzyme and as the energy source for the reaction
N	Q57C89	CC	It is essential for DNA replication and repair of damaged DNA (By similarity).
N	Q57C89	DE	Polydeoxyribonucleotide synthase [NAD+];
N	Q57C89	DR	DNA ligase (NAD+) activity
N	Q57C89	DR	DNA replication
N	Q57C89	DR	metal ion binding
N	Q57CZ9	DE	Anthranilate phosphoribosyltransferase;
N	Q57CZ9	DR	anthranilate phosphoribosyltransferase activity
N	Q57CZ9	DR	tryptophan biosynthetic process
N	Q57D97	DE	UPF0283 membrane protein BruAb1_1038;
N	Q57DG3	CC	Together with moaC, is involved in the conversion of a guanosine derivative (5'-GTP) into molybdopterin precursor Z (By similarity).
N	Q57DG3	DE	Molybdenum cofactor biosynthesis protein A;
N	Q57DG3	DR	4 iron, 4 sulfur cluster binding
N	Q57DG3	DR	Mo-molybdopterin cofactor biosynthetic process
N	Q57DG3	DR	catalytic activity
N	Q57DG3	DR	metal ion binding
N	Q57DZ4	CC	May conjugate Arg from its aminoacyl-tRNA to the N- termini of proteins containing an N-terminal aspartate or glutamate (Potential).
N	Q57DZ4	DE	Arginyltransferase;
N	Q57DZ4	DE	Putative arginyl-tRNA--protein transferase;
N	Q57DZ4	DE	R-transferase;
N	Q57DZ4	DR	acyltransferase activity
N	Q57DZ4	DR	arginyltransferase activity
N	Q57DZ4	DR	protein arginylation
N	Q57F30	CC	Catalyzes the condensation of pantoate with beta-alanine in an ATP-dependent reaction via a pantoyl-adenylate intermediate (By similarity).
N	Q57F30	DE	Pantoate--beta-alanine ligase;
N	Q57F30	DE	Pantoate-activating enzyme;
N	Q57F30	DE	Pantothenate synthetase;
N	Q57F30	DR	pantoate-beta-alanine ligase activity
N	Q57F30	DR	pantothenate biosynthetic process
N	Q57HF3	DE	6-phosphofructokinase;
N	Q57HF3	DE	Phosphofructokinase;
N	Q57HF3	DE	Phosphohexokinase;
N	Q57HF3	DR	6-phosphofructokinase activity
N	Q57HF3	DR	fructose 6-phosphate metabolic process
N	Q57HF3	DR	metal ion binding
N	Q57IG5	DE	Glycine--tRNA ligase alpha subunit;
N	Q57IG5	DE	Glycyl-tRNA synthetase alpha subunit;
N	Q57IG5	DR	glycine-tRNA ligase activity
N	Q57IG5	DR	glycyl-tRNA aminoacylation
N	Q57L18	CC	Binds specifically to the ssrA RNA (tmRNA) and is required for stable association of ssrA with ribosomes (By similarity).
N	Q57L18	DE	SsrA-binding protein;
N	Q58004	DE	UPF0718 protein MJ0584;
N	Q58059	DE	Uncharacterized protein MJ0642;
N	Q58059	DR	high-affinity iron ion transport
N	Q58059	DR	iron ion transmembrane transporter activity
N	Q58112	DE	Uncharacterized protein MJ0701;
N	Q58112	DR	DNA primase activity
N	Q58112	DR	DNA replication, synthesis of RNA primer
N	Q58217	DE	Uncharacterized protein MJ0807;

N	Q58217	DR	lyase activity
N	Q58299	DE	Uncharacterized protein MJ0889;
N	Q58342	CC	Binds and compact DNA (95 to 150 base pairs) to form nucleosome-like structures that contain positive DNA supercoils
N	Q58342	CC	Increases the resistance of DNA to thermal denaturation (By similarity).
N	Q58342	DE	Probable archaeal histone 2;
N	Q58342	DR	sequence-specific DNA binding
N	Q58606	CC	This methylase recognizes the double-stranded sequence CCGG, causes specific methylation on C-? on both strands, and protects the DNA from cleavage by the MjaVI endonuclease (Potential).
N	Q58606	DE	Modification methylase MjaVI;
N	Q58606	DE	N-4 cytosine-specific methyltransferase MjaVI;
N	Q58606	DR	DNA restriction-modification system
N	Q58606	DR	N-methyltransferase activity
N	Q58606	DR	site-specific DNA-methyltransferase (cytosine-N4-specific) activity
N	Q58610	DE	Uncharacterized protein MJ1213;
N	Q58933	CC	Specifically phosphorylates seryl-tRNA(Sec) to O- phosphoseryl-tRNA(Sec), an activated intermediate for selenocysteine biosynthesis.
N	Q58933	DE	L-seryl-tRNA(Sec) kinase;
N	Q58933	DE	O-phosphoseryl-tRNA(Sec) kinase;
N	Q58933	DR	kinase activity
N	Q58933	DR	phosphotransferase activity, alcohol group as acceptor
N	Q589R5	DE	Triose-phosphate isomerase;
N	Q589R5	DE	Triosephosphate isomerase;
N	Q589R5	DR	fatty acid biosynthetic process
N	Q589R5	DR	gluconeogenesis
N	Q589R5	DR	pentose-phosphate shunt
N	Q589R5	DR	triose-phosphate isomerase activity
N	Q58FF8	CC	Molecular chaperone
N	Q58FF8	CC	Has ATPase activity (By similarity).
N	Q58FF8	DE	Heat shock protein 90-beta b;
N	Q58FF8	DE	Heat shock protein 90Bb;
N	Q58FF8	DE	Putative heat shock protein HSP 90-beta 2;
N	Q58FF8	DR	protein folding
N	Q58FF8	DR	response to stress
N	Q58FF8	DR	unfolded protein binding
N	Q59024	CC	Possesses two activities: a DNA synthesis (polymerase) and an exonucleolytic activity that degrades single stranded DNA in the 3'- to 5'-direction
N	Q59024	CC	Has a template-primer preference which is characteristic of a replicative DNA polymerase.
N	Q59024	DE	DNA polymerase II large subunit;
N	Q59024	DR	DNA catabolic process
N	Q59024	DR	DNA replication
N	Q59024	DR	DNA-directed DNA polymerase activity
N	Q59024	DR	exonuclease activity
N	Q591M6	CC	Core subunit of the mitochondrial membrane respiratory chain NADH dehydrogenase (Complex I) that is believed to belong to the minimal assembly required for catalysis
N	Q591M6	CC	Complex I functions in the transfer of electrons from NADH to the respiratory chain
N	Q591M6	CC	The immediate electron acceptor for the enzyme is believed to be ubiquinone (By similarity).
N	Q591M6	DE	NADH dehydrogenase subunit 4L;
N	Q591M6	DE	NADH-ubiquinone oxidoreductase chain 4L;
N	Q591M6	DR	ATP synthesis coupled electron transport
N	Q591M6	DR	NADH dehydrogenase (ubiquinone) activity
N	Q59284	DE	SDAP desuccinylase;
N	Q59284	DE	Succinyl-diaminopimelate desuccinylase;
N	Q59284	DR	diaminopimelate biosynthetic process
N	Q59284	DR	metal ion binding
N	Q59284	DR	metallopeptidase activity
N	Q59284	DR	succinyl-diaminopimelate desuccinylase activity
N	Q59334	CC	Catalyzes an early step in the biosynthesis of tetrapyrroles
N	Q59334	CC	Binds two molecules of 5-aminolevulinate per subunit, each at a distinct site, and catalyzes their condensation to form porphobilinogen.
N	Q59334	DE	Delta-aminolevulinic acid dehydratase;
N	Q59334	DE	Porphobilinogen synthase;

N	Q59334	DR	heme biosynthetic process
N	Q59334	DR	metal ion binding
N	Q59334	DR	porphobilinogen synthase activity
N	Q59478	CC	Degrades alginates that contain guluronic acid.
N	Q59478	DE	Alginate lyase;
N	Q59478	DE	Poly(beta-D-mannuronate) lyase;
N	Q59478	DE	Poly(mana) alginate lyase;
N	Q59478	DR	poly(beta-D-mannuronate) lyase activity
N	Q59KI4	CC	DNA helicase component of the INO80 complex which remodels chromatin by shifting nucleosomes and is involved in DNA repair (By similarity).
N	Q59KI4	DE	Putative DNA helicase INO80;
N	Q59KI4	DR	chromatin modification
N	Q59KI4	DR	helicase activity
N	Q59ZB1	DE	Adenosine aminohydrolase;
N	Q59ZB1	DE	Adenosine deaminase;
N	Q59ZB1	DR	adenosine deaminase activity
N	Q59ZB1	DR	metal ion binding
N	Q59ZB1	DR	purine ribonucleoside monophosphate biosynthetic process
N	Q5B0C0	CC	Involved in osmoadaptation.
N	Q5B0C0	DE	Heat shock 70 kDa protein;
N	Q5B0C0	DR	protein folding
N	Q5B0C0	DR	response to stress
N	Q5B0C0	DR	unfolded protein binding
N	Q5B3X3	CC	Component of the coenzyme Q biosynthetic pathway
N	Q5B3X3	CC	May play a role in organizing a multi-subunit COQ enzyme complex required for coenzyme Q biosynthesis
N	Q5B3X3	CC	Required for steady-state levels of other COQ polypeptides (By similarity).
N	Q5B3X3	DE	Coenzyme Q biosynthesis protein 4;
N	Q5B3X3	DE	Ubiquinone biosynthesis protein coq4, mitochondrial;
N	Q5B3X3	DR	ubiquinone biosynthetic process
N	Q5B538	CC	Required for the post-translational delivery of tail- anchored (TA) proteins to the endoplasmic reticulum
N	Q5B538	CC	Acts as a membrane receptor for soluble get3, which recognizes and selectively binds the transmembrane domain of TA proteins in the cytosol (By similarity).
N	Q5B538	DE	Guided entry of tail-anchored proteins 1;
N	Q5B538	DE	Protein get1;
N	Q5B8X0	CC	Catalyzes the formation of N(1)-methylguanine at position 9 (m1G9) in cytoplasmic tRNAs (By similarity).
N	Q5B8X0	DE	tRNA (guanine-N(1)-)-methyltransferase;
N	Q5B8X0	DE	tRNA methyltransferase 10;
N	Q5B8X0	DR	tRNA (guanine-N1-)-methyltransferase activity
N	Q5BCU8	CC	Serine/threonine protein kinase probably involved in the cytoplasm to vacuole transport (Cvt) and in autophagy, where it may be required for the formation of autophagosomes (By similarity).
N	Q5BCU8	DE	Autophagy-related protein 1;
N	Q5BCU8	DE	Serine/threonine-protein kinase atg1;
N	Q5BCU8	DR	protein phosphorylation
N	Q5BCU8	DR	protein serine/threonine kinase activity
N	Q5BCU8	DR	protein transport
N	Q5BKF8	CC	Transcriptional regulator which participates in regulating the pre-mRNA splicing process (By similarity).
N	Q5BS23	DE	Uncharacterized protein SJCHGC06711;
N	Q5DTT8	DE	Paraneoplastic antigen-like protein 5;
N	Q5E2C7	CC	Catalyzes the transfer of a dimethylallyl group onto the adenine at position 37 in tRNAs that read codons beginning with uridine, leading to the formation of N6-(dimethylallyl)adenosine (i(6)A) (By similarity).
N	Q5E2C7	DE	DMAPP:tRNA dimethylallyltransferase;
N	Q5E2C7	DE	Dimethylallyl diphosphate:tRNA dimethylallyltransferase;
N	Q5E2C7	DE	IPP transferase;
N	Q5E2C7	DE	Isopentenyl-diphosphate:tRNA isopentenyltransferase;
N	Q5E2C7	DE	tRNA dimethylallyltransferase;
N	Q5E2C7	DR	tRNA dimethylallyltransferase activity
N	Q5E2C7	DR	tRNA processing
N	Q5E491	DE	UPF0260 protein VF_1660;

N	Q5E4P6	CC	Specifically methylates the cytosine at position 1962 (m5C1962) of 23S rRNA (By similarity).
N	Q5E4P6	DE	23S rRNA m5C1962 methyltransferase;
N	Q5E4P6	DE	Ribosomal RNA large subunit methyltransferase I;
N	Q5E4P6	DE	rRNA (cytosine-C(5)-)-methyltransferase RlmI;
N	Q5E4P6	DR	rRNA methyltransferase activity
N	Q5E839	CC	Reduction of activated sulfate into sulfite.
N	Q5E839	DE	3'-phosphoadenylylsulfate reductase;
N	Q5E839	DE	PAPS reductase, thioredoxin dependent;
N	Q5E839	DE	PAPS sulfotransferase;
N	Q5E839	DE	PAdoPS reductase;
N	Q5E839	DE	Phosphoadenosine phosphosulfate reductase;
N	Q5E839	DR	cysteine biosynthetic process
N	Q5E839	DR	phosphoadenylyl-sulfate reductase (thioredoxin) activity
N	Q5E839	DR	sulfate assimilation, phosphoadenylyl sulfate reduction by phosphoadenylyl-sulfate reductase (thioredoxin)
N	Q5E887	DE	D-fructose-1,6-bisphosphate 1-phosphohydrolase class 1;
N	Q5E887	DE	FBPase class 1;
N	Q5E887	DE	Fructose-1,6-bisphosphatase class 1;
N	Q5E887	DR	carbohydrate metabolic process
N	Q5E887	DR	fructose 1,6-bisphosphate 1-phosphatase activity
N	Q5E887	DR	metal ion binding
N	Q5E9C8	CC	May be involved in the binding and intracellular trafficking of cobalamin (vitamin B12) (By similarity).
N	Q5E9C8	DE	Methylmalonic aciduria and homocystinuria type C protein homolog;
N	Q5E9C8	DR	cobalamin binding
N	Q5E9Z9	CC	Seems to be involved in cell adhesion through trans- homophilic and -heterophilic interactions, the latter including specifically interactions with PVRL2/nectin-1 (By similarity).
N	Q5E9Z9	DE	Poliovirus receptor-related protein 4;
N	Q5E9Z9	DR	cell adhesion
N	Q5EFR1	CC	Suppresses the host immune response through NF-kappa-B inactivation
N	Q5EFR1	CC	Possesses ankyrin repeat domains required for NF- kappa-B binding but lacks the regulatory regions required for dissociation from NF-kappa-B and degradation
N	Q5EFR1	CC	Therefore, prevents host NF-kappa-B release and subsequent activation (By similarity)
N	Q5EFR1	DE	I-Kappa-B like protein I1;
N	Q5EFR1	DR	interspecies interaction between organisms
N	Q5F5C2	CC	Condenses 4-methyl-5-(beta-hydroxyethyl)thiazole monophosphate (THZ-P) and 2-methyl-4-amino-5-hydroxymethyl pyrimidine pyrophosphate (HMP-PP) to form thiamine monophosphate (TMP) (By similarity).
N	Q5F5C2	DE	TMP pyrophosphorylase;
N	Q5F5C2	DE	Thiamine-phosphate pyrophosphorylase;
N	Q5F5C2	DE	Thiamine-phosphate synthase;
N	Q5F5C2	DR	metal ion binding
N	Q5F5C2	DR	thiamine biosynthetic process
N	Q5F5C2	DR	thiamine-phosphate diphosphorylase activity
N	Q5F7V9	CC	Nucleotidase that shows phosphatase activity on nucleoside 5'-monophosphates (By similarity).
N	Q5F7V9	DE	5'-nucleotidase surE;
N	Q5F7V9	DE	Nucleoside 5'-monophosphate phosphohydrolase;
N	Q5F7V9	DR	5'-nucleotidase activity
N	Q5F7V9	DR	metal ion binding
N	Q5F7V9	DR	nucleotide binding
N	Q5FAC7	CC	Cell wall formation
N	Q5FAC7	CC	Essential for cell wall synthesis.
N	Q5FAC7	CC	Synthesis of cross-linked peptidoglycan from the lipid intermediates
N	Q5FAC7	CC	The enzyme has a penicillin-insensitive transglycosylase N-terminal domain (formation of linear glycan strands) and a penicillin-sensitive transpeptidase C-terminal domain (cross-linking of the peptide subunits) (By similarity)
N	Q5FAC7	DE	DD-transpeptidase;
N	Q5FAC7	DE	Penicillin-binding protein 1A;
N	Q5FAC7	DE	Penicillin-insensitive transglycosylase;
N	Q5FAC7	DE	Penicillin-sensitive transpeptidase;
N	Q5FAC7	DE	Peptidoglycan TGase;
N	Q5FAC7	DR	cellular cell wall organization

N	Q5FAC7	DR	penicillin binding
N	Q5FAC7	DR	peptidase activity
N	Q5FAC7	DR	peptidoglycan biosynthetic process
N	Q5FAC7	DR	regulation of cell shape
N	Q5FAC7	DR	response to antibiotic
N	Q5FAC7	DR	transferase activity, transferring pentosyl groups
N	Q5FB95	DE	ERGIC53-like protein;
N	Q5FB95	DE	LMAN1-like protein;
N	Q5FB95	DE	Lectin mannose-binding 1-like;
N	Q5FB95	DE	Protein ERGIC-53-like;
N	Q5FB95	DE	Sublingual acinar membrane protein;
N	Q5FB95	DR	sugar binding
N	Q5FG20	CC	DNA ligase that catalyzes the formation of phosphodiester linkages between 5'-phosphoryl and 3'-hydroxyl groups in double-stranded DNA using NAD as a coenzyme and as the energy source for the reaction
N	Q5FG20	CC	It is essential for DNA replication and repair of damaged DNA (By similarity).
N	Q5FG20	DE	Polydeoxyribonucleotide synthase [NAD+];
N	Q5FG20	DR	DNA ligase (NAD+) activity
N	Q5FG20	DR	DNA replication
N	Q5FG20	DR	metal ion binding
N	Q5FGQ8	CC	Participates actively in the response to hyperosmotic and heat shock by preventing the aggregation of stress-denatured proteins and by disaggregating proteins, also in an autonomous, dnaK-independent fashion
N	Q5FGQ8	CC	Also involved, together with dnaK and grpE, in the DNA replication of plasmids through activation of initiation proteins (By similarity).
N	Q5FGQ8	CC	GrpE releases ADP from dnaK; ATP binding to dnaK triggers the release of the substrate protein, thus completing the reaction cycle
N	Q5FGQ8	CC	Several rounds of ATP-dependent interactions between dnaJ, dnaK and grpE are required for fully efficient folding
N	Q5FGQ8	CC	Unfolded proteins bind initially to dnaJ; upon interaction with the dnaJ-bound protein, dnaK hydrolyzes its bound ATP, resulting in the formation of a stable complex
N	Q5FGQ8	DE	Chaperone protein dnaJ;
N	Q5FGQ8	DR	DNA replication
N	Q5FGQ8	DR	heat shock protein binding
N	Q5FGQ8	DR	metal ion binding
N	Q5FGQ8	DR	protein folding
N	Q5FGQ8	DR	response to heat
N	Q5FGQ8	DR	unfolded protein binding
N	Q5FKD1	DE	Protein CrcB homolog 2;
N	Q5FNS2	CC	Catalyzes the complicated ring closure reaction between the two acyclic compounds 1-deoxy-D-xylulose-5-phosphate (DXP) and 3-amino-2-oxopropyl phosphate (1-amino-acetone-3-phosphate or AAP) to form pyridoxine 5'-phosphate (PNP) and inorganic phosphate (By similarity).
N	Q5FNS2	DE	PNP synthase;
N	Q5FNS2	DE	Pyridoxine 5'-phosphate synthase;
N	Q5FNS2	DR	pyridoxine 5'-phosphate synthase activity
N	Q5FNS2	DR	pyridoxine biosynthetic process
N	Q5FNY6	CC	Produces ATP from ADP in the presence of a proton gradient across the membrane
N	Q5FNY6	CC	The alpha chain is a regulatory subunit (By similarity).
N	Q5FNY6	DE	ATP synthase F1 sector subunit alpha 2;
N	Q5FNY6	DE	ATP synthase subunit alpha 2;
N	Q5FNY6	DE	F-ATPase subunit alpha 2;
N	Q5FNY6	DR	ATP hydrolysis coupled proton transport
N	Q5FNY6	DR	ATP synthesis coupled proton transport
N	Q5FNY6	DR	hydrogen ion transporting ATP synthase activity, rotational mechanism
N	Q5FNY6	DR	proton-transporting ATPase activity, rotational mechanism
N	Q5FUJ7	CC	Cell wall formation
N	Q5FUJ7	CC	Catalyzes the addition of glutamate to the nucleotide precursor UDP-N-acetylmuramoyl-L-alanine (UMA) (By similarity).
N	Q5FUJ7	DE	D-glutamic acid-adding enzyme;
N	Q5FUJ7	DE	UDP-N-acetylmuramoyl-L-alanyl-D-glutamate synthetase;
N	Q5FUJ7	DE	UDP-N-acetylmuramoylalanine--D-glutamate ligase;
N	Q5FUJ7	DR	UDP-N-acetylmuramoylalanine-D-glutamate ligase activity
N	Q5FUJ7	DR	cell division
N	Q5FUJ7	DR	cellular cell wall organization

N	Q5FUJ7	DR	peptidoglycan biosynthetic process
N	Q5FUJ7	DR	regulation of cell shape
N	Q5FWT5	DE	Glutaminyl-tRNA synthase-like protein 1;
N	Q5FWT5	DE	Glutamyl-tRNA(Gln) amidotransferase subunit A homolog;
N	Q5FWT5	DR	carbon-nitrogen ligase activity, with glutamine as amido-N-donor
N	Q5GC92	CC	Maximin-S1 has no antimicrobial activity
N	Q5GC92	CC	Maximin-S3 has an activity against mycoplasma but has no activity against common Gram-positive and Gram-negative bacteria nor fungi
N	Q5GC92	CC	Maximin-S5 has an activity against mycoplasma but has no activity against common Gram-positive and Gram-negative bacteria nor fungi
N	Q5GC92	CC	Has no hemolytic activity (By similarity).
N	Q5GC92	CC	Has no hemolytic activity.
N	Q5GC92	DE	Maximin-S1;
N	Q5GC92	DE	Maximin-S2;
N	Q5GC92	DE	Maximin-S3;
N	Q5GC92	DE	Maximin-S4;
N	Q5GC92	DE	Maximin-S5;
N	Q5GC92	DE	Maximins-S type B/C;
N	Q5GC92	DR	defense response to bacterium
N	Q5GWT3	CC	One of the primary rRNA binding proteins, it binds directly near the 3'-end of the 23S rRNA, where it nucleates assembly of the 50S subunit (By similarity).
N	Q5GWT3	DE	50S ribosomal protein L3;
N	Q5GWT3	DR	structural constituent of ribosome
N	Q5H2R3	CC	Catalyzes the attachment of glutamate to tRNA(Glu) in a two-step reaction: glutamate is first activated by ATP to form Glu-AMP and then transferred to the acceptor end of tRNA(Glu) (By similarity).
N	Q5H2R3	DE	Glutamate--tRNA ligase;
N	Q5H2R3	DE	Glutamyl-tRNA synthetase;
N	Q5H2R3	DR	glutamate-tRNA ligase activity
N	Q5H2R3	DR	glutamyl-tRNA aminoacylation
N	Q5H4J1	DE	Maf-like protein XOO0876;
N	Q5H6Q3	CC	Catalyzes the formation of the alpha-1,6-glucosidic linkages in glycogen by scission of a 1,4-alpha-linked oligosaccharide from growing alpha-1,4-glucan chains and the subsequent attachment of the oligosaccharide to the alpha-1,6 position (By similarity).
N	Q5H6Q3	DE	1,4-alpha-D-glucan:1,4-alpha-D-glucan 6-glucosyl-transferase 2;
N	Q5H6Q3	DE	1,4-alpha-glucan-branching enzyme 2;
N	Q5H6Q3	DE	Glycogen-branching enzyme 2;
N	Q5H6Q3	DR	1,4-alpha-glucan branching enzyme activity
N	Q5H6Q3	DR	cation binding
N	Q5H6Q3	DR	glycogen biosynthetic process
N	Q5H6Q3	DR	hydrolase activity, hydrolyzing O-glycosyl compounds
N	Q5H9J7	DE	Brain-expressed X-linked protein 5;
N	Q5H9J7	DE	NGFRAP1-like protein 1;
N	Q5H9J7	DE	Nerve growth factor receptor-associated protein 2;
N	Q5H9J7	DE	Protein BEX5;
N	Q5HAN4	CC	NAD-binding protein involved in the addition of a carboxymethylaminomethyl (cmnm) group at the wobble position (U34) of certain tRNAs, forming tRNA-cmnm(5)s(2)U34 (By similarity).
N	Q5HAN4	DE	Glucose-inhibited division protein A;
N	Q5HAN4	DE	tRNA uridine 5-carboxymethylaminomethyl modification enzyme MnmG;
N	Q5HAN4	DR	flavin adenine dinucleotide binding
N	Q5HAN4	DR	tRNA wobble uridine modification
N	Q5HBC6	CC	Specifically dimethylates two adjacent adenosines (A1518 and A1519) in the loop of a conserved hairpin near the 3'-end of 16S rRNA in the 30S particle
N	Q5HBC6	CC	May play a critical role in biogenesis of 30S subunits (By similarity).
N	Q5HBC6	DE	16S rRNA (adenine(1518)-N(6)/adenine(1519)-N(6))-dimethyltransferase;
N	Q5HBC6	DE	16S rRNA dimethyladenosine transferase;
N	Q5HBC6	DE	16S rRNA dimethylase;
N	Q5HBC6	DE	Ribosomal RNA small subunit methyltransferase A;
N	Q5HBC6	DE	S-adenosylmethionine-6-N', N'-adenosyl(rRNA) dimethyltransferase;
N	Q5HBC6	DR	rRNA (adenine-N6,N6-)-dimethyltransferase activity
N	Q5HCL3	CC	Probably part of an ABC transporter complex
N	Q5HCL3	CC	Responsible for energy coupling to the transport system (By similarity).
N	Q5HCL3	DE	Putative ABC transporter ATP-binding protein SACOL2708;
N	Q5HCL3	DR	ATPase activity

N	Q5HCL3	DR	hydrolase activity, acting on acid anhydrides, catalyzing transmembrane movement of substances
N	Q5HDD1	CC	Transforms pimelate into pimeloyl-CoA (By similarity).
N	Q5HDD1	DE	6-carboxyhexanoate--CoA ligase;
N	Q5HDD1	DE	Pimeloyl-CoA synthase;
N	Q5HDD1	DR	6-carboxyhexanoate-CoA ligase activity
N	Q5HDD1	DR	biotin biosynthetic process
N	Q5HF34	CC	Cell wall formation (By similarity).
N	Q5HF34	DE	UDP-N-acetylmuramate--L-alanine ligase;
N	Q5HF34	DE	UDP-N-acetylmuramoyl-L-alanine synthetase;
N	Q5HF34	DR	UDP-N-acetylmuramate-L-alanine ligase activity
N	Q5HF34	DR	cell division
N	Q5HF34	DR	cellular cell wall organization
N	Q5HF34	DR	peptidoglycan biosynthetic process
N	Q5HF34	DR	regulation of cell shape
N	Q5HFM1	CC	Catalyzes the specific phosphorylation of the 3-hydroxyl group of shikimic acid using ATP as a cosubstrate (By similarity).
N	Q5HFM1	DE	Shikimate kinase;
N	Q5HFM1	DR	aromatic amino acid family biosynthetic process
N	Q5HFM1	DR	metal ion binding
N	Q5HFM1	DR	shikimate kinase activity
N	Q5HKN9	DE	Imidazoleglycerol-phosphate dehydratase;
N	Q5HKN9	DR	histidine biosynthetic process
N	Q5HKN9	DR	imidazoleglycerol-phosphate dehydratase activity
N	Q5HLW3	DE	Urea amidohydrolase subunit gamma;
N	Q5HLW3	DE	Urease subunit gamma;
N	Q5HLW3	DR	nickel ion binding
N	Q5HLW3	DR	urea metabolic process
N	Q5HLW3	DR	urease activity
N	Q5HMG7	CC	Required for the formation of a threonylcarbamoyl group on adenosine at position 37 (t(6)A37) in tRNAs that read codons beginning with adenine (By similarity)
N	Q5HMG7	CC	Critical mediator involved in the modification of cell wall peptidoglycan synthesis and/or cell division
N	Q5HMG7	CC	Essential for cell viability (By similarity).
N	Q5HMG7	DE	Probable tRNA threonylcarbamoyladenosine biosynthesis protein Gcp;
N	Q5HMG7	DE	t(6)A37 threonylcarbamoyladenosine biosynthesis protein;
N	Q5HMG7	DR	metal ion binding
N	Q5HMG7	DR	metalloendopeptidase activity
N	Q5HNR0	CC	The ruvA-ruvB complex in the presence of ATP renatures cruciform structure in supercoiled DNA with palindromic sequence, indicating that it may promote strand exchange reactions in homologous recombination
N	Q5HNR0	CC	RuvAB is an helicase that mediates the Holliday junction migration by localized denaturation and reannealing (By similarity).
N	Q5HNR0	DE	Holliday junction ATP-dependent DNA helicase ruvB;
N	Q5HNR0	DR	DNA recombination
N	Q5HNR0	DR	four-way junction helicase activity
N	Q5HNW1	CC	Binds directly to 16S ribosomal RNA (By similarity).
N	Q5HNW1	DE	30S ribosomal protein S20;
N	Q5HNW1	DR	structural constituent of ribosome
N	Q5HPK8	CC	Decomposes hydrogen peroxide into water and oxygen; serves to protect cells from the toxic effects of hydrogen peroxide (By similarity).
N	Q5HPK8	DR	catalase activity
N	Q5HPK8	DR	hydrogen peroxide catabolic process
N	Q5HPK8	DR	oxidation-reduction process
N	Q5HPL2	CC	Catalyzes the ATP-dependent phosphorylation of L- homoserine to L-homoserine phosphate (By similarity).
N	Q5HPL2	DE	Homoserine kinase;
N	Q5HPL2	DR	homoserine kinase activity
N	Q5HPL2	DR	threonine biosynthetic process
N	Q5HQ78	CC	Removes the formyl group from the N-terminal Met of newly synthesized proteins
N	Q5HQ78	CC	N-terminal L-methionine is a prerequisite for activity but the enzyme has broad specificity at other positions (By similarity).
N	Q5HQ78	CC	Requires at least a dipeptide for an efficient rate of reaction
N	Q5HQ78	DE	Peptide deformylase;
N	Q5HQ78	DE	Polypeptide deformylase;

N	Q5HQ78	DR	iron ion binding
N	Q5HQ78	DR	peptide deformylase activity
N	Q5HQG8	DE	UPF0413 protein SERP0581;
N	Q5HZL1	DE	ERI1 exoribonuclease 2;
N	Q5HZL1	DE	Exonuclease domain-containing protein 1;
N	Q5HZL1	DR	exonuclease activity
N	Q5HZL1	DR	nucleic acid binding
N	Q5HZL1	DR	zinc ion binding
N	Q5I6M2	CC	Mitogen-activated protein kinase involved in a signal transduction pathway that is activated by changes in the osmolarity of the extracellular environment
N	Q5I6M2	CC	Controls osmotic regulation of transcription of target genes (By similarity).
N	Q5I6M2	DE	MAP kinase HOG1;
N	Q5I6M2	DE	MAP kinase STK1;
N	Q5I6M2	DE	Mitogen-activated protein kinase HOG1;
N	Q5I6M2	DR	MAP kinase activity
N	Q5I6M2	DR	protein phosphorylation
N	Q5J3Q5	CC	Probable transcription factor (By similarity).
N	Q5J3Q5	DE	Forkhead box protein E1;
N	Q5J3Q5	DR	regulation of transcription, DNA-dependent
N	Q5J3Q5	DR	sequence-specific DNA binding
N	Q5J3Q5	DR	sequence-specific DNA binding transcription factor activity
N	Q5JH81	CC	Relaxes both positive and negative superturns and exhibits a strong decatenase [illegible]
N	Q5JH81	CC	The B subunit binds ATP (By similarity).
N	Q5JH81	DE	Type 2 DNA topoisomerase 6 subunit B;
N	Q5JH81	DE	Type II DNA topoisomerase VI subunit B;
N	Q5JH81	DR	DNA topoisomerase (ATP-hydrolyzing) activity
N	Q5JH81	DR	DNA topological change
N	Q5JJG3	CC	One of the primary rRNA binding proteins, it binds directly to 16S rRNA central domain where it helps coordinate assembly of the platform of the 30S subunit [illegible]
N	Q5JJG3	DE	30S ribosomal protein S8P;
N	Q5JJG3	DR	structural constituent of ribosome
N	Q5JPB2	DE	Zinc finger protein 831;
N	Q5JPB2	DR	nucleic acid binding
N	Q5JPB2	DR	zinc ion binding
N	Q5K654	CC	Involved in pre-mRNA splicing and cell cycle progression
N	Q5K654	CC	Required for the spliceosome assembly and initiation of the DNA replication (By similarity).
N	Q5K654	DE	Pre-mRNA-splicing factor CLF1;
N	Q5K654	DR	mRNA processing
N	Q5KB85	CC	Component of the cytosolic Fe/S protein assembly machinery
N	Q5KB85	CC	May play a role in the transfer of pre-assembled Fe/S clusters to target apoproteins (By similarity).
N	Q5KB85	CC	Required for maturation of extramitochondrial Fe/S proteins
N	Q5KB85	DE	Cytosolic Fe-S cluster assembly factor NAR1;
N	Q5KB85	DE	Nuclear architecture-related protein 1;
N	Q5KB85	DR	4 iron, 4 sulfur cluster binding
N	Q5KB85	DR	iron-sulfur cluster assembly
N	Q5KB85	DR	metal ion binding
N	Q5KFI6	CC	Involved in the import of GDP-mannose from the cytoplasm into the Golgi [illegible]
N	Q5KFI6	CC	Involved in capsule synthesis.
N	Q5KFI6	DE	GDP-mannose transporter 1;
N	Q5KFI6	DR	nucleotide-sugar transmembrane transporter activity
N	Q5KN36	CC	ATP-binding RNA helicase involved in translation initiation
N	Q5KN36	CC	Remodels RNA in response to ADP and ATP concentrations by facilitating disruption, but also formation of RNA duplexes (By similarity).
N	Q5KN36	DE	ATP-dependent RNA helicase ded1;
N	Q5KN36	DR	ATP-dependent helicase activity
N	Q5KN36	DR	translation initiation factor activity
N	Q5KVM7	CC	Catalyzes the radical-mediated insertion of two sulfur atoms into the C-6 and C-8 positions of the octanoyl moiety bound to the lipoyl domains of lipoate-dependent enzymes, thereby converting the octanoylated domains into lipoylated derivatives (By similarity).
N	Q5KVM7	DE	Lipoate synthase;
N	Q5KVM7	DE	Lipoic acid synthase;
N	Q5KVM7	DE	Lipoyl synthase;

N	Q5KVM7	DE	Sulfur insertion protein lipA;
N	Q5KVM7	DR	4 iron, 4 sulfur cluster binding
N	Q5KVM7	DR	lipoate biosynthetic process
N	Q5KVM7	DR	lipoate synthase activity
N	Q5KVM7	DR	metal ion binding
N	Q5KWR3	CC	Transfers and isomerizes the ribose moiety from AdoMet to the 7-aminomethyl group of 7-deazaguanine (preQ1-tRNA) to give epoxyqueuosine (oQ-tRNA) (By similarity).
N	Q5KWR3	DE	Queuosine biosynthesis protein queA;
N	Q5KWR3	DE	S-adenosylmethionine:tRNA ribosyltransferase-isomerase;
N	Q5KWR3	DR	isomerase activity
N	Q5KWR3	DR	queuosine biosynthetic process
N	Q5KWR3	DR	transferase activity
N	Q5L407	CC	Seems to be the binding site for several of the factors involved in protein synthesis and appears to be essential for accurate translation (By similarity).
N	Q5L407	DE	50S ribosomal protein L7/L12;
N	Q5L407	DR	structural constituent of ribosome
N	Q5L4W6	CC	ATP-dependent specificity component of the Clp protease
N	Q5L4W6	CC	Can perform chaperone functions in the absence of ClpP (By similarity).
N	Q5L4W6	CC	It directs the protease to specific substrates
N	Q5L4W6	DE	ATP-dependent Clp protease ATP-binding subunit ClpX;
N	Q5L4W6	DR	ATPase activity
N	Q5L4W6	DR	protein dimerization activity
N	Q5L4W6	DR	protein folding
N	Q5L4W6	DR	unfolded protein binding
N	Q5L4W6	DR	zinc ion binding
N	Q5L588	CC	Catalyzes the conversion of glucosamine-6-phosphate to glucosamine-1-phosphate (By similarity).
N	Q5L588	DE	Phosphoglucosamine mutase;
N	Q5L588	DR	carbohydrate metabolic process
N	Q5L588	DR	magnesium ion binding
N	Q5L588	DR	phosphoglucosamine mutase activity
N	Q5L627	CC	One of the essential components for the initiation of protein synthesis
N	Q5L627	CC	Also involved in the hydrolysis of GTP during the formation of the 70S ribosomal complex (By similarity).
N	Q5L627	CC	Protects formylmethionyl-tRNA from spontaneous hydrolysis and promotes its binding to the 30S ribosomal subunits
N	Q5L627	DE	Translation initiation factor IF-2;
N	Q5L627	DR	GTPase activity
N	Q5L627	DR	translation initiation factor activity
N	Q5LNH0	CC	F(1)F(0) ATP synthase produces ATP from ADP in the presence of a proton or sodium gradient
N	Q5LNH0	CC	During catalysis, ATP synthesis in the catalytic domain of F(1) is coupled via a rotary mechanism of the central stalk subunits to proton translocation (By similarity).
N	Q5LNH0	CC	F-type ATPases consist of two structural domains, F(1) containing the extramembraneous catalytic core and F(0) containing the membrane proton channel, linked together by a central stalk and a peripheral stalk
N	Q5LNH0	DE	ATP synthase F(0) sector subunit c;
N	Q5LNH0	DE	ATP synthase subunit c;
N	Q5LNH0	DE	F-ATPase subunit c;
N	Q5LNH0	DE	F-type ATPase subunit c;
N	Q5LNH0	DE	Lipid-binding protein;
N	Q5LNH0	DR	ATP hydrolysis coupled proton transport
N	Q5LNH0	DR	ATP synthesis coupled proton transport
N	Q5LNH0	DR	hydrogen ion transmembrane transporter activity
N	Q5LNH0	DR	lipid binding
N	Q5LW59	CC	One of the primary rRNA binding proteins
N	Q5LW59	CC	It has been suggested to have peptidyltransferase activity; this is somewhat controversial
N	Q5LW59	CC	Makes several contacts with the 16S rRNA in the 70S ribosome (By similarity).
N	Q5LW59	CC	Required for association of the 30S and 50S subunits to form the 70S ribosome, for tRNA binding and peptide bond formation
N	Q5LW59	DE	50S ribosomal protein L2;
N	Q5LW59	DR	structural constituent of ribosome
N	Q5LW59	DR	transferase activity
N	Q5LXR5	CC	Protein S19 forms a complex with S13 that binds strongly to the 16S ribosomal RNA (By similarity).

N	Q5LXR5	DE	30S ribosomal protein S19;
N	Q5LXR5	DR	structural constituent of ribosome
N	Q5M0X5	CC	This protein specifically catalyzes the removal of signal peptides from prolipoproteins (By similarity).
N	Q5M0X5	DE	Lipoprotein signal peptidase;
N	Q5M0X5	DE	Prolipoprotein signal peptidase;
N	Q5M0X5	DE	Signal peptidase II;
N	Q5M0X5	DR	aspartic-type endopeptidase activity
N	Q5M117	CC	Catalyzes the ATP-dependent phosphorylation of L- homoserine to L-homoserine phosphate (By similarity).
N	Q5M117	DE	Homoserine kinase;
N	Q5M117	DR	homoserine kinase activity
N	Q5M117	DR	threonine biosynthetic process
N	Q5M376	CC	Cell wall formation
N	Q5M376	CC	Adds enolpyruvyl to UDP-N- acetylglucosamine (By similarity).
N	Q5M376	DE	Enoylpyruvate transferase 2;
N	Q5M376	DE	UDP-N-acetylglucosamine 1-carboxyvinyltransferase 2;
N	Q5M376	DE	UDP-N-acetylglucosamine enolpyruvyl transferase 2;
N	Q5M376	DR	UDP-N-acetylgalactosamine biosynthetic process
N	Q5M376	DR	UDP-N-acetylglucosamine 1-carboxyvinyltransferase activity
N	Q5M376	DR	cell division
N	Q5M376	DR	cellular cell wall organization
N	Q5M376	DR	peptidoglycan biosynthetic process
N	Q5M376	DR	regulation of cell shape
N	Q5M782	CC	Mediates the import of RPA complex into the nucleus, via its interaction with importin beta (By similarity).
N	Q5M782	DE	RPA-interacting protein B;
N	Q5M782	DR	metal ion binding
N	Q5M9G5	DE	Coiled-coil domain-containing protein 117;
N	Q5MBK3	CC	Pectinolytic enzymes consist of four classes of enzymes: pectine lyase, polygalacturonase, pectin methylesterase and rhamnogalacturonase
N	Q5MBK3	CC	Among pectinolytic enzymes, pectine lyase is the most important in depolymerization of pectin, since it cleaves internal glycosidic bonds of highly methylated pectins (By similarity).
N	Q5MBK3	DE	Probable pectin lyase C;
N	Q5MBK3	DR	cellular cell wall organization
N	Q5MBK3	DR	pectin catabolic process
N	Q5MBK3	DR	pectin lyase activity
N	Q5MZB9	DE	Photosystem II 13 kDa protein;
N	Q5MZB9	DE	Photosystem II reaction center Psb28 protein;
N	Q5MZB9	DE	Photosystem II reaction center W protein;
N	Q5MZB9	DR	photosynthesis
N	Q5N1P1	CC	Synthesizes alpha-1,4-glucan chains using ADP-glucose.
N	Q5N1P1	DE	Glycogen synthase;
N	Q5N1P1	DE	Starch [bacterial glycogen] synthase;
N	Q5N1P1	DR	glycogen biosynthetic process
N	Q5N1P1	DR	starch synthase activity
N	Q5N1P5	CC	Involved in protein export
N	Q5N1P5	CC	Acts as a chaperone by maintaining the newly synthesized protein in an open conformation (By similarity).
N	Q5N1P5	DE	Trigger factor;
N	Q5N1P5	DR	cell division
N	Q5N1P5	DR	peptidyl-prolyl cis-trans isomerase activity
N	Q5N1P5	DR	protein folding
N	Q5N1P5	DR	protein transport
N	Q5N3H1	CC	The UvrABC repair system catalyzes the recognition and processing of DNA lesions
N	Q5N3H1	CC	A damage recognition complex composed of 2 uvrA and 2 uvrB subunits scans DNA for abnormalities
N	Q5N3H1	CC	DNA wrap is dependent on ATP binding by uvrB and probably causes local melting of the DNA helix, facilitating insertion of uvrB beta-hairpin between the DNA strands
N	Q5N3H1	CC	If a lesion is found the uvrA subunits dissociate and the uvrB-DNA preincision complex is formed
N	Q5N3H1	CC	If no lesion is found, the DNA wraps around the other uvrB subunit that will check the other stand for damage (By similarity).
N	Q5N3H1	CC	Then uvrB probes one DNA strand for the presence of a lesion

N	Q5N3H1	CC	This complex is subsequently bound by uvrC and the second uvrB is released
N	Q5N3H1	CC	Upon binding of the uvrA(2)B(2) complex to a putative damaged site, the DNA wraps around one uvrB monomer
N	Q5N3H1	DE	Excinuclease ABC subunit B;
N	Q5N3H1	DE	Protein uvrB;
N	Q5N3H1	DE	UvrABC system protein B;
N	Q5N3H1	DR	excinuclease ABC activity
N	Q5N3H1	DR	helicase activity
N	Q5N3H1	DR	nucleotide-excision repair
N	Q5N643	DE	Arginine--tRNA ligase;
N	Q5N643	DE	Arginyl-tRNA synthetase;
N	Q5N643	DR	arginine-tRNA ligase activity
N	Q5N643	DR	arginyl-tRNA aminoacylation
N	Q5NA06	CC	The coatomer is a cytosolic protein complex that binds to dilysine motifs and reversibly associates with Golgi non- clathrin-coated vesicles, which further mediate biosynthetic protein transport from the ER, via the Golgi up to the trans Golgi network
N	Q5NA06	CC	Coatomer complex is required for budding from Golgi membranes, and is essential for the retrograde Golgi-to-ER transport of dilysine-tagged proteins (By similarity)
N	Q5NA06	DE	Coatomer subunit zeta-3;
N	Q5NA06	DE	Zeta-3-coat protein;
N	Q5NA06	DR	protein transport
N	Q5NA06	DR	vesicle-mediated transport
N	Q5NAV3	DE	Zinc finger CCCH domain-containing protein 5;
N	Q5NAV3	DE	Zinc finger CCCH domain-containing protein ZFN-like 3;
N	Q5NAV3	DR	zinc ion binding
N	Q5NFT3	CC	Catalyzes the ferrous insertion into protoporphyrin IX.
N	Q5NFT3	DE	Ferrochelatase;
N	Q5NFT3	DE	Heme synthase;
N	Q5NFT3	DE	Protoheme ferro-lyase;
N	Q5NFT3	DR	ferrochelatase activity
N	Q5NFT3	DR	heme biosynthetic process
N	Q5NFT3	DR	metal ion binding
N	Q5NHX1	CC	One of the primary rRNA binding proteins, it binds directly to 16S rRNA where it nucleates assembly of the head domain of the 30S subunit
N	Q5NHX1	CC	Is located at the subunit interface close to the decoding center, probably blocks exit of the E-site tRNA (By similarity).
N	Q5NHX1	DE	30S ribosomal protein S7;
N	Q5NHX1	DR	structural constituent of ribosome
N	Q5NID1	CC	DNA-dependent RNA polymerase catalyzes the transcription of DNA into RNA using the four ribonucleoside triphosphates as substrates.
N	Q5NID1	DE	DNA-directed RNA polymerase subunit beta';
N	Q5NID1	DE	RNA polymerase subunit beta';
N	Q5NID1	DE	RNAP subunit beta';
N	Q5NID1	DE	Transcriptase subunit beta';
N	Q5NID1	DR	DNA-directed RNA polymerase activity
N	Q5NQN1	DE	UPF0301 protein ZMO0349;
N	Q5NR81	CC	Catalyzes the formation of N(7)-methylguanine at position 46 (m7G46) in tRNA (By similarity).
N	Q5NR81	DE	tRNA (guanine-N(7)-)-methyltransferase;
N	Q5NR81	DE	tRNA(m7G46)-methyltransferase;
N	Q5NR81	DR	tRNA (guanine-N7-)-methyltransferase activity
N	Q5P4P5	CC	Exhibits a very high intrinsic GTPase hydrolysis rate
N	Q5P4P5	CC	Involved in the addition of a carboxymethylaminomethyl (cmnm) group at the wobble position (U34) of certain tRNAs, forming tRNA- cmnm(5)s(2)U34 (By similarity)
N	Q5P4P5	DE	tRNA modification GTPase mnmE;
N	Q5P4P5	DR	GTPase activity
N	Q5P4P5	DR	metal ion binding
N	Q5P4P5	DR	tRNA modification
N	Q5P7Z5	DE	50S ribosomal protein L27;
N	Q5P7Z5	DR	structural constituent of ribosome
N	Q5P9A5	CC	This is one of the proteins that binds to the 5S RNA in the ribosome where it forms part of the central protuberance (By similarity).
N	Q5P9A5	DE	50S ribosomal protein L25;
N	Q5P9A5	DE	General stress protein CTC;
N	Q5P9A5	DR	5S rRNA binding

N	Q5P9A5	DR	structural constituent of ribosome
N	Q5PA96	DE	50S ribosomal protein L32;
N	Q5PA96	DR	structural constituent of ribosome
N	Q5PBR8	CC	One of the proteins required for the normal export of preproteins out of the cell cytoplasm
N	Q5PBR8	CC	It also specifically binds to its receptor secA (By similarity).
N	Q5PBR8	CC	It is a molecular chaperone that binds to a subset of precursor proteins, maintaining them in a translocation-competent state
N	Q5PBR8	DE	Protein-export protein secB;
N	Q5PBR8	DR	protein tetramerization
N	Q5PBR8	DR	protein transport
N	Q5PBR8	DR	transmembrane transport
N	Q5PBR8	DR	unfolded protein binding
N	Q5PCF3	CC	Catalyzes the hydrolysis of the pyrophosphate bond of UDP-2,3-diacylglucosamine to yield 2,3-diacylglucosamine 1- phosphate (lipid X) and UMP (By similarity).
N	Q5PCF3	DE	UDP-2,3-diacylglucosamine hydrolase;
N	Q5PCF3	DR	lipid A biosynthetic process
N	Q5PCF3	DR	pyrophosphatase activity
N	Q5PDA1	CC	Catalyzes the decarboxylation of S-adenosylmethionine to S-adenosylmethioninamine (dcAdoMet), the propylamine donor required for the synthesis of the polyamines spermine and spermidine from the diamine putrescine (By similarity).
N	Q5PDA1	DE	S-adenosylmethionine decarboxylase alpha chain;
N	Q5PDA1	DE	S-adenosylmethionine decarboxylase beta chain;
N	Q5PDA1	DE	S-adenosylmethionine decarboxylase proenzyme;
N	Q5PDA1	DR	adenosylmethionine decarboxylase activity
N	Q5PDA1	DR	spermidine biosynthetic process
N	Q5PEN3	CC	Accelerates the degradation of transcripts by removing pyrophosphate from the 5'-end of triphosphorylated RNA, leading to a more labile monophosphorylated state that can stimulate subsequent ribonuclease cleavage (By similarity).
N	Q5PEN3	DE	(Di)nucleoside polyphosphate hydrolase;
N	Q5PEN3	DE	RNA pyrophosphohydrolase;
N	Q5PEN3	DR	hydrolase activity
N	Q5PFV0	CC	Inhibits RpoS proteolysis by regulating RssB activity, thereby increasing the stability of the sigma stress factor RpoS especially during phosphate and magnesium starvation, but also in stationary phase and during nitrogen starvation
N	Q5PFV0	CC	Its effect on RpoS stability is due to its interaction with RssB, which probably blocks the interaction of RssB with RpoS, and the consequent delivery of the RssB-RpoS complex to the ClpXP protein degradation pathway (By similarity).
N	Q5PFV0	DE	Anti-adapter protein iraP;
N	Q5PFV0	DR	response to stress
N	Q5PLF2	CC	Catalyzes the phosphorylation of N-acetylmannosamine (ManNAc) to ManNAc-6-P (By similarity).
N	Q5PLF2	DE	ManNAc kinase;
N	Q5PLF2	DE	N-acetyl-D-mannosamine kinase;
N	Q5PLF2	DE	N-acetylmannosamine kinase;
N	Q5PLF2	DR	N-acylmannosamine kinase activity
N	Q5PLF2	DR	metal ion binding
N	Q5PNG3	CC	Catalyzes the formation of 2'O-methylated cytidine (Cm32) or 2'O-methylated uridine (Um32) at position 32 in tRNA (By similarity).
N	Q5PNG3	DE	tRNA (cytidine/uridine-2'-O-)-methyltransferase TrmJ;
N	Q5PNG3	DE	tRNA Cm32/Um32 methyltransferase;
N	Q5PNG3	DR	RNA methyltransferase activity
N	Q5PNG3	DR	tRNA processing
N	Q5QQ52	CC	Catalyzes the first step in biosynthesis of glycosaminoglycan
N	Q5QQ52	CC	Initial enzyme in the biosynthesis of chondroitin sulfate and dermatan sulfate proteoglycans in fibroblasts and chondrocytes (By similarity).
N	Q5QQ52	CC	Transfers D-xylose from UDP-D-xylose to specific serine residues of the core protein
N	Q5QQ52	DE	Peptide O-xylosyltransferase;
N	Q5QQ52	DE	Squashed vulva protein 6;
N	Q5QQ52	DE	Xylosyltransferase sqv-6;
N	Q5QQ52	DR	acetylglucosaminyltransferase activity
N	Q5QQ52	DR	protein xylosyltransferase activity
N	Q5QTZ0	CC	Responsible for synthesis of pseudouridine from uracil- 55 in the psi GC loop of transfer RNAs (By similarity).
N	Q5QTZ0	DE	Psi55 synthase;

N	Q5QTZ0	DE	tRNA pseudouridine 55 synthase;
N	Q5QTZ0	DE	tRNA pseudouridine synthase B;
N	Q5QTZ0	DE	tRNA pseudouridylate synthase;
N	Q5QTZ0	DE	tRNA-uridine isomerase;
N	Q5QTZ0	DR	pseudouridine synthase activity
N	Q5QTZ0	DR	pseudouridine synthesis
N	Q5QTZ0	DR	tRNA processing
N	Q5QVT8	CC	Involved in peptide bond synthesis
N	Q5QVT8	CC	Probably functions indirectly by altering the affinity of the ribosome for aminoacyl-tRNA, thus increasing their reactivity as acceptors for peptidyl transferase (By similarity)
N	Q5QVT8	CC	Stimulates efficient translation and peptide-bond synthesis on native or reconstituted 70S ribosomes in vitro
N	Q5QVT8	DE	Elongation factor P;
N	Q5QVT8	DR	peptide biosynthetic process
N	Q5QVT8	DR	translation elongation factor activity
N	Q5QVT9	CC	Methylates ribosomal protein L11 (By similarity).
N	Q5QVT9	DE	Ribosomal protein L11 methyltransferase;
N	Q5QVT9	DR	protein methyltransferase activity
N	Q5QW47	CC	Binds specifically to the ssrA RNA (tmRNA) and is required for stable association of ssrA with ribosomes (By similarity).
N	Q5QW47	DE	SsrA-binding protein;
N	Q5R461	DE	Oligodendrocytic myelin paranodal and inner loop protein;
N	Q5R461	DE	Transmembrane protein 10;
N	Q5R4G5	CC	Involved in membrane protein trafficking at the base of the ciliary organelle
N	Q5R4G5	CC	May be required for proper retinal function and organization (By similarity).
N	Q5R4G5	CC	May regulate cilia assembly and disassembly and subsequent ciliary signaling events such as the Wnt signaling cascade
N	Q5R4G5	CC	Mediates recruitment onto plasma membrane of the BBSome complex which would constitute a coat complex required for sorting of specific membrane proteins to the primary cilia
N	Q5R4G5	DE	ADP-ribosylation factor-like protein 6;
N	Q5R4G5	DR	cell projection organization
N	Q5R4G5	DR	metal ion binding
N	Q5R4G5	DR	small GTPase mediated signal transduction
N	Q5R4K3	CC	Serine/threonine kinase required for the mitogen or stress-induced phosphorylation of the transcription factors CREB (cAMP response element-binding protein) and ATF1 (activating transcription factor-1)
N	Q5R4K3	CC	Directly represses transcription via phosphorylation of 'Ser-1' of histone H2A
N	Q5R4K3	CC	Essential role in the control of RELA transcriptional activity in response to TNF
N	Q5R4K3	CC	May also phosphorylate 'Ser-28' of histone H3
N	Q5R4K3	CC	Mediates the mitogen- and stress-induced phosphorylation of high mobility group protein 14 (HMG-14) (By similarity).
N	Q5R4K3	CC	Phosphorylates 'Ser-10' of histone H3 in response to mitogenics, stress stimuli and epidemal growth-factor (EGF), which results in the transcriptional activation of several immediate early genes, including proto-oncogenes c-fos/FOS and c-jun/JUN
N	Q5R4K3	DE	Ribosomal protein S6 kinase alpha-5;
N	Q5R4K3	DE	S6K-alpha-5;
N	Q5R4K3	DR	magnesium ion binding
N	Q5R4K3	DR	protein serine/threonine kinase activity
N	Q5R537	CC	Thrombin, which cleaves bonds after Arg and Lys, converts fibrinogen to fibrin and activates factors V, VII, VIII, XIII, and, in complex with thrombomodulin, protein C
N	Q5R537	CC	Functions in blood homeostasis, inflammation and wound healing (By similarity).
N	Q5R537	DE	Activation peptide fragment 1;
N	Q5R537	DE	Activation peptide fragment 2;
N	Q5R537	DE	Coagulation factor II;
N	Q5R537	DE	Prothrombin;
N	Q5R537	DE	Thrombin heavy chain;
N	Q5R537	DE	Thrombin light chain;
N	Q5R537	DR	acute-phase response
N	Q5R537	DR	calcium ion binding
N	Q5R5J8	CC	The ZDHHC9-GOLGA7 complex is a palmitoyltransferase specific for HRAS and NRAS (By similarity).
N	Q5R5J8	DE	Palmitoyltransferase ZDHHC9;
N	Q5R5J8	DE	Zinc finger DHHC domain-containing protein 9;
N	Q5R5J8	DR	acyltransferase activity

N	Q5R5J8	DR	zinc ion binding
N	Q5R637	CC	Molecular chaperone; assists the folding of proteins upon ATP hydrolysis
N	Q5R637	CC	As part of the BBS/CCT complex may play a role in the assembly of BBSome, a complex involved in ciliogenesis regulating transports vesicles to the cilia
N	Q5R637	CC	Known to play a role, in vitro, in the folding of actin and tubulin (By similarity).
N	Q5R637	DE	T-complex protein 1 subunit delta;
N	Q5R637	DE	TCP-1-delta;
N	Q5R637	DR	protein folding
N	Q5R637	DR	unfolded protein binding
N	Q5R6B5	CC	ALDHs play a major role in the detoxification of alcohol-derived acetaldehyde
N	Q5R6B5	CC	They are involved in the metabolism of corticosteroids, biogenic amines, neurotransmitters, and lipid peroxidation (By similarity).
N	Q5R6B5	DE	Aldehyde dehydrogenase X, mitochondrial;
N	Q5R6B5	DE	Aldehyde dehydrogenase family 1 member B1;
N	Q5R6B5	DR	aldehyde dehydrogenase (NAD) activity
N	Q5R6B5	DR	oxidation-reduction process
N	Q5R947	CC	Component of the 60-80S U3 small nucleolar ribonucleoprotein (U3 snoRNP)
N	Q5R947	CC	Required for the early cleavages during pre-18S ribosomal RNA processing (By similarity).
N	Q5R947	DE	U3 small nucleolar ribonucleoprotein protein IMP4;
N	Q5R947	DE	U3 snoRNP protein IMP4;
N	Q5R947	DR	aminoacyl-tRNA ligase activity
N	Q5R947	DR	rRNA processing
N	Q5R9H0	CC	Transfers the glycosyl residue from UDP-Glc to the non- reducing end of alpha-1,4-glucan (By similarity).
N	Q5R9H0	DE	Glycogen [starch] synthase, muscle;
N	Q5R9H0	DR	glycogen (starch) synthase activity
N	Q5R9H0	DR	glycogen biosynthetic process
N	Q5R9M7	CC	Modulates endosomal trafficking
N	Q5R9M7	CC	Acts as a major regulator of membrane delivery during cytokinesis (By similarity)
N	Q5R9M7	DE	Ras-related protein Rab-11A;
N	Q5R9M7	DR	GTPase activity
N	Q5R9M7	DR	protein transport
N	Q5R9M7	DR	small GTPase mediated signal transduction
N	Q5RAP3	DE	Tetraspanin-4;
N	Q5RAT9	CC	Cell surface proteoglycan that bears heparan sulfate (By similarity).
N	Q5RAT9	DR	cytoskeletal protein binding
N	Q5RBC3	CC	Glycosyltransferase required for the biosynthesis of heparan-sulfate
N	Q5RBC3	CC	The EXT1/EXT2 complex possesses substantially higher glycosyltransferase activity than EXT1 or EXT2 alone (By similarity).
N	Q5RBC3	DE	Exostosin-1;
N	Q5RBC3	DE	Glucuronosyl-N-acetylglucosaminyl-proteoglycan/N-acetylglucosaminyl-proteoglycan 4-alpha-N-acetylglucosaminyltransferase;
N	Q5RBC3	DE	Multiple exostoses protein 1 homolog;
N	Q5RBC3	DR	N-acetylglucosaminyl-proteoglycan 4-beta-glucuronosyltransferase activity
N	Q5RBC3	DR	glucuronosyl-N-acetylglucosaminyl-proteoglycan 4-alpha-N-acetylglucosaminyltransferase activity
N	Q5RBM8	DE	RNA-binding motif protein 12;
N	Q5RBM8	DE	RNA-binding protein 12;
N	Q5RBM8	DR	nucleotide binding
N	Q5RBU7	CC	Cleaves C-terminal amino acids linked to proline in peptides such as angiotensin II, III and des-Arg9-bradykinin
N	Q5RBU7	CC	This cleavage occurs at acidic pH, but enzymatic activity is retained with some substrates at neutral pH (By similarity).
N	Q5RBU7	DE	Lysosomal Pro-X carboxypeptidase;
N	Q5RBU7	DE	Proline carboxypeptidase;
N	Q5RBU7	DE	Prolylcarboxypeptidase;
N	Q5RBU7	DR	carboxypeptidase activity
N	Q5RBU7	DR	serine-type peptidase activity
N	Q5RBU8	CC	Involved with pre-mRNA processing
N	Q5RBU8	CC	Forms complexes (ribonucleosomes) with at least 20 other different hnRNP and heterogeneous nuclear RNA in the nucleous (By similarity).
N	Q5RBU8	DE	Heterogeneous nuclear ribonucleoproteins A2/B1;
N	Q5RBU8	DE	hnRNP A2/B1;
N	Q5RBU8	DR	mRNA processing

N	Q5RBU8	DR	nucleotide binding
N	Q5RBZ8	DE	Transmembrane protein 41B;
N	Q5RCG1	DE	Transmembrane protein 192;
N	Q5RD60	CC	Functions as a coactivator for aryl hydrocarbon and nuclear receptors (NR)
N	Q5RD60	CC	Coactivator function for nuclear receptors and LEF1/CTNNB1 involves differential utilization of two different activation regions (By similarity).
N	Q5RD60	CC	During ER-activation acts synergistically in combination with other NCOA2-binding proteins, such as EP300, CREBBP and CARM1
N	Q5RD60	CC	Functions as a secondary coactivator in LEF1- mediated transcriptional activation via its interaction with CTNNB1
N	Q5RD60	CC	Involved in the transcriptional activation of target genes in the Wnt/CTNNB1 pathway
N	Q5RD60	CC	Recruited to promoters through its contact with the N-terminal basic helix-loop-helix-Per-Arnt-Sim (PAS) domain of transcription factors or coactivators, such as NCOA2
N	Q5RD60	DE	Calcium-binding and coiled-coil domain-containing protein 1;
N	Q5RD60	DR	Wnt receptor signaling pathway
N	Q5RDC9	CC	Antiporter transporting nucleotide sugars such as UDP-N- acetylglucosamine (UDP-GlcNAc), UDP-glucose (UDP-Glc) and GDP- mannose (GDP-Man) pooled in the cytosol into the lumen of the Golgi in exchange for the corresponding nucleosides monophosphates (UMP for UDP-sugars and GMP for GDP-sugars)
N	Q5RDC9	CC	May take part in heparan sulfate synthesis by supplying UDP-Glc-NAc, the donor substrate, and thus be involved in growth factor signaling (By similarity).
N	Q5RDC9	DE	Solute carrier family 35 member D2;
N	Q5RDC9	DE	UDP-N-acetylglucosamine/UDP-glucose/GDP-mannose transporter;
N	Q5RDC9	DR	carbohydrate transport
N	Q5RDD3	CC	Accepts electrons from ETF and reduces ubiquinone (By similarity).
N	Q5RDD3	DE	ETF dehydrogenase;
N	Q5RDD3	DE	ETF-ubiquinone oxidoreductase;
N	Q5RDD3	DE	Electron transfer flavoprotein-ubiquinone oxidoreductase, mitochondrial;
N	Q5RDD3	DE	Electron-transferring-flavoprotein dehydrogenase;
N	Q5RDD3	DR	4 iron, 4 sulfur cluster binding
N	Q5RDD3	DR	electron transport chain
N	Q5RDD3	DR	electron-transferring-flavoprotein dehydrogenase activity
N	Q5RDD3	DR	metal ion binding
N	Q5REI9	DE	Tumor protein p53-inducible protein 11;
N	Q5REI9	DE	p53-induced gene 11 protein;
N	Q5REY3	CC	Involved in redox regulation of the cell
N	Q5REY3	CC	Protects radical-sensitive enzymes from oxidative damage by a radical-generating system (By similarity).
N	Q5REY3	DE	Peroxiredoxin-3;
N	Q5REY3	DE	Thioredoxin-dependent peroxide reductase, mitochondrial;
N	Q5REY3	DR	cell redox homeostasis
N	Q5REY3	DR	oxidation-reduction process
N	Q5REY3	DR	peroxidase activity
N	Q5REY3	DR	peroxiredoxin activity
N	Q5REY6	CC	Regulates a signal transduction pathway linking plasma membrane receptors to the assembly of focal adhesions and actin stress fibers
N	Q5REY6	CC	Activated by ARHGEF2, which promotes the exchange of GDP for GTP
N	Q5REY6	CC	Essential for the SPATA13-mediated regulation of cell migration and adhesion assembly and disassembly (By similarity).
N	Q5REY6	CC	May be an activator of PLCE1
N	Q5REY6	DE	Transforming protein RhoA;
N	Q5REY6	DR	small GTPase mediated signal transduction
N	Q5RGJ8	CC	Catalyzes the formation of mannose 6-phosphate (M6P) markers on high mannose type oligosaccharides in the Golgi apparatus
N	Q5RGJ8	CC	M6P residues are required to bind to the M6P receptors (MPR), which mediate the vesicular transport of lysosomal enzymes to the endosomal/prelysosomal compartment (By similarity).
N	Q5RGJ8	DE	GlcNAc-1-phosphotransferase subunits alpha/beta;
N	Q5RGJ8	DE	N-acetylglucosamine-1-phosphotransferase subunit alpha;
N	Q5RGJ8	DE	N-acetylglucosamine-1-phosphotransferase subunit beta;
N	Q5RGJ8	DE	N-acetylglucosamine-1-phosphotransferase subunits alpha/beta;
N	Q5RGJ8	DE	Stealth protein gnptab;
N	Q5RGJ8	DE	UDP-N-acetylglucosamine-1-phosphotransferase subunits alpha/beta;
N	Q5RGJ8	DR	UDP-N-acetylglucosamine-lysosomal-enzyme N-acetylglucosaminephosphotransferase activity

N	Q5RGJ8	DR	chondrocyte development
N	Q5RGJ8	DR	embryonic cranial skeleton morphogenesis
N	Q5RGJ8	DR	metal ion binding
N	Q5RGJ8	DR	transcription factor binding
N	Q5RKI9	CC	Responsible for the release of ribosomes from messenger RNA at the termination of protein biosynthesis
N	Q5RKI9	CC	May increase the efficiency of translation by recycling ribosomes from one round of translation to another (By similarity).
N	Q5RKI9	DE	Ribosome-recycling factor, mitochondrial;
N	Q5RKI9	DE	Ribosome-releasing factor, mitochondrial;
N	Q5RKI9	DR	regulation of transcription, DNA-dependent
N	Q5RKI9	DR	sequence-specific DNA binding transcription factor activity
N	Q5SHG2	CC	Component of the acetyl coenzyme A carboxylase (ACC) complex
N	Q5SHG2	CC	Biotin carboxylase (BC) catalyzes the carboxylation of biotin on its carrier protein (BCCP) and then the CO(2) group is transferred by the transcarboxylase to acetyl-CoA to form malonyl- CoA (By similarity).
N	Q5SHG2	DE	ACCase subunit beta;
N	Q5SHG2	DE	Acetyl-CoA carboxylase carboxyltransferase subunit beta;
N	Q5SHG2	DE	Acetyl-coenzyme A carboxylase carboxyl transferase subunit beta;
N	Q5SHG2	DR	acetyl-CoA carboxylase activity
N	Q5SHG2	DR	fatty acid biosynthetic process
N	Q5SHG2	DR	metal ion binding
N	Q5SLS1	CC	Plays an important role in the de novo pathway of purine nucleotide biosynthesis
N	Q5SLS1	CC	Catalyzes the first committed step in the biosynthesis of AMP from IMP (By similarity).
N	Q5SLS1	DE	Adenylosuccinate synthetase;
N	Q5SLS1	DE	IMP--aspartate ligase;
N	Q5SLS1	DR	adenylosuccinate synthase activity
N	Q5SLS1	DR	magnesium ion binding
N	Q5SLS1	DR	purine nucleotide biosynthetic process
N	Q5SV42	CC	Regulates the activity of the neutrophil proteases (By similarity).
N	Q5SV42	DE	Leukocyte elastase inhibitor C;
N	Q5SV42	DE	Serine protease inhibitor EIC;
N	Q5SV42	DR	serine-type endopeptidase inhibitor activity
N	Q5SYB0	CC	Stabilizes membrane-bound GPSM1, and thereby promotes its interaction with GNAI1
N	Q5SYB0	DE	FERM and PDZ domain-containing protein 1;
N	Q5SYB0	DE	FERM domain-containing protein 2;
N	Q5T230	CC	Acts as a transcriptional coactivator of ATF2.
N	Q5T230	DE	Undifferentiated embryonic cell transcription factor 1;
N	Q5T230	DR	RNA polymerase II transcription factor activity
N	Q5T230	DR	male gonad development
N	Q5T230	DR	positive regulation of transcription from RNA polymerase II promoter
N	Q5T230	DR	protein binding
N	Q5T230	DR	transcription coactivator activity
N	Q5T7P6	DE	Transmembrane protein 78;
N	Q5T9G4	DE	Uncharacterized protein C6orf81;
N	Q5TBC7	DE	Bcl-2-like protein 15;
N	Q5U2V9	DE	Transmembrane protein 39A;
N	Q5U7I5	CC	Thyroid hormone-binding protein
N	Q5U7I5	CC	Probably transports thyroxine from the bloodstream to the brain (By similarity).
N	Q5U7I5	DE	Transthyretin;
N	Q5U7I5	DR	hormone activity
N	Q5UP83	DE	Putative KilA-N domain-containing protein L4;
N	Q5UPE0	DE	Uncharacterized protein L65;
N	Q5UPX7	CC	DNA-dependent RNA polymerase catalyzes the transcription of DNA into RNA using the four ribonucleoside triphosphates as substrates (By similarity).
N	Q5UPX7	DE	DNA-directed RNA polymerase subunit 5;
N	Q5UPX7	DR	DNA-directed RNA polymerase activity
N	Q5UPZ5	DE	Uncharacterized protein L309;
N	Q5UQK8	DE	Uncharacterized protein L412;
N	Q5UQK8	DR	nucleic acid binding
N	Q5UQK8	DR	zinc ion binding
N	Q5UQU8	DE	Uncharacterized protein L357;
N	Q5UXN1	CC	Catalyzes the radical-mediated transfer of the hydroxybenzyl group from 4-hydroxyphenylpyruvate (HPP) to 5-amino- 6-ribitylamino-2,4(1H,3H)-pyrimidinedione to form 7,8-didemethyl- 8-hydroxy-5-deazariboflavin (FO) (By similarity).

N	Q5UXN1	DE	7,8-didemethyl-8-hydroxy-5-deazariboflavin synthase subunit 2;
N	Q5UXN1	DE	FO synthase subunit 2;
N	Q5UXN1	DR	4 iron, 4 sulfur cluster binding
N	Q5UXN1	DR	metal ion binding
N	Q5UXN1	DR	transferase activity, transferring alkyl or aryl (other than methyl) groups
N	Q5VU97	CC	May regulate voltage-dependent calcium channels (By similarity).
N	Q5VU97	DE	Cache domain-containing protein 1;
N	Q5VU97	DE	VWFA and cache domain-containing protein 1;
N	Q5VU97	DR	calcium ion transport
N	Q5WFK7	CC	Modifies the free amino group of the aminoacyl moiety of methionyl-tRNA(fMet)
N	Q5WFK7	CC	The formyl group appears to play a dual role in the initiator identity of N-formylmethionyl-tRNA by: (I) promoting its recognition by IF2 and (II) impairing its binding to EFTu-GTP (By similarity).
N	Q5WFK7	DE	Methionyl-tRNA formyltransferase;
N	Q5WFK7	DR	methionyl-tRNA formyltransferase activity
N	Q5WFK7	DR	methyltransferase activity
N	Q5WFX1	CC	Can catalyze the hydrolysis of ATP in the presence of single-stranded DNA, the ATP-dependent uptake of single-stranded DNA by duplex DNA, and the ATP-dependent hybridization of homologous single-stranded DNAs
N	Q5WFX1	CC	It interacts with LexA causing its activation and leading to its autocatalytic cleavage (By similarity).
N	Q5WFX1	DE	Protein RecA;
N	Q5WFX1	DE	Recombinase A;
N	Q5WFX1	DR	DNA recombination
N	Q5WFX1	DR	DNA-dependent ATPase activity
N	Q5WFX1	DR	single-stranded DNA binding
N	Q5WGA5	CC	Catalyzes the pyruvoyl-dependent decarboxylation of aspartate to produce beta-alanine (By similarity).
N	Q5WGA5	DE	Aspartate 1-decarboxylase alpha chain;
N	Q5WGA5	DE	Aspartate 1-decarboxylase beta chain;
N	Q5WGA5	DE	Aspartate 1-decarboxylase;
N	Q5WGA5	DE	Aspartate alpha-decarboxylase;
N	Q5WGA5	DR	alanine biosynthetic process
N	Q5WGA5	DR	aspartate 1-decarboxylase activity
N	Q5WGA5	DR	pantothenate biosynthetic process
N	Q5WQ00	CC	This protein is essential for plasmid replication; it is involved in copy control functions (By similarity).
N	Q5WQ00	DE	Probable replication-associated protein repA1;
N	Q5WQ00	DR	DNA replication
N	Q5WQ00	DR	plasmid maintenance
N	Q5WXF0	CC	Part of the ABC transporter complex PotABCD involved in spermidine/putrescine import
N	Q5WXF0	CC	Responsible for energy coupling to the transport system (By similarity).
N	Q5WXF0	DE	Spermidine/putrescine import ATP-binding protein PotA;
N	Q5WXF0	DR	polyamine-transporting ATPase activity
N	Q5X3K0	CC	Catalyzes the 1,3-allylic rearrangement of the homoallylic substrate isopentenyl (IPP) to its allylic isomer, dimethylallyl diphosphate (DMAPP) (By similarity).
N	Q5X3K0	DE	IPP isomerase;
N	Q5X3K0	DE	Isopentenyl pyrophosphate isomerase;
N	Q5X3K0	DE	Isopentenyl-diphosphate delta-isomerase;
N	Q5X3K0	DR	isopentenyl-diphosphate delta-isomerase activity
N	Q5X3K0	DR	isoprenoid biosynthetic process
N	Q5X3K0	DR	oxidoreductase activity
N	Q5XA26	CC	Required for the formation of a threonylcarbamoyl group on adenosine at position 37 (t(6)A37) in tRNAs that read codons beginning with adenine (By similarity).
N	Q5XA26	DE	Probable tRNA threonylcarbamoyladenosine biosynthesis protein Gcp;
N	Q5XA26	DE	t(6)A37 threonylcarbamoyladenosine biosynthesis protein;
N	Q5XA26	DR	metal ion binding
N	Q5XA26	DR	metalloendopeptidase activity
N	Q5XAP1	CC	Essential for recycling GMP and indirectly, cGMP (By similarity).
N	Q5XAP1	DE	Guanylate kinase;
N	Q5XAP1	DR	guanylate kinase activity
N	Q5XAP1	DR	purine nucleotide metabolic process
N	Q5XCA6	DE	2-amino-4-hydroxy-6-hydroxymethyldihydropteridine pyrophosphokinase;
N	Q5XCA6	DE	6-hydroxymethyl-7,8-dihydropterin pyrophosphokinase;

N	Q5XCA6	DE	7,8-dihydro-6-hydroxymethylpterin-pyrophosphokinase;
N	Q5XCA6	DR	2-amino-4-hydroxy-6-hydroxymethyldihydropteridine diphosphokinase activity
N	Q5XCA6	DR	folic acid biosynthetic process
N	Q5XCA6	DR	kinase activity
N	Q5XFN2	CC	Desmin are class-III intermediate filaments found in muscle cells
N	Q5XFN2	CC	In adult striated muscle they form a fibrous network connecting myofibrils to each other and to the plasma membrane from the periphery of the Z-line structures
N	Q5XFN2	DR	structural molecule activity
N	Q5XGY9	CC	Required for proper homologous chromosome pairing and efficient cross-over and intragenic recombination during meiosis
N	Q5XGY9	CC	Stimulates both dmc1- and rad51-mediated homologous strand assimilation, which is required for the resolution of meiotic double-strand breaks (By similarity)
N	Q5XGY9	DE	Meiotic nuclear division protein 1 homolog;
N	Q5XGY9	DR	DNA recombination
N	Q5XH36	CC	Possible role in neuronal acetylcholine receptor transport (By similarity).
N	Q5XH36	DE	Cysteine-rich with EGF-like domain protein 2-A;
N	Q5XH36	DR	calcium ion binding
N	Q5XIF4	CC	Ubiquitin-like protein which can be covalently attached to target lysines either as a monomer or as a lysine-linked polymer
N	Q5XIF4	CC	Covalent attachment to its substrates requires prior activation by the E1 complex SAE1-SAE2 and linkage to the E2 enzyme UBE2I, and can be promoted by an E3 ligase such as PIAS1-4, RANBP2 or CBX4 (By similarity).
N	Q5XIF4	CC	Does not seem to be involved in protein degradation and may function as an antagonist of ubiquitin in the degradation process
N	Q5XIF4	CC	Plays a role in a number of cellular processes such as nuclear transport, DNA replication and repair, mitosis and signal transduction
N	Q5XIF4	DE	Small ubiquitin-related modifier 3;
N	Q5XIP1	CC	Required for normal chromosome segregation during cell division and genomic stability (By similarity)
N	Q5XIP1	CC	May function in recognizing stalled ribosomes and triggering endonucleolytic cleavage of the mRNA, a mechanism to release non-functional ribosomes and degrade damaged mRNAs
N	Q5XIP1	CC	May have ribonuclease activity (Potential).
N	Q5XIP1	DE	Protein pelota homolog;
N	Q5XIP1	DR	cell division
N	Q5XIP1	DR	endonuclease activity
N	Q5XIP1	DR	metal ion binding
N	Q5Y4X0	CC	Insect active toxin causing rapid but reversible paralysis in crickets
N	Q5Y4X0	CC	Does not show effect on mammalian voltage-gated calcium channels (By similarity)
N	Q5Y4X0	CC	No activity shown in mammals
N	Q5Y4X0	DE	U2-AGTX-Ao1p;
N	Q5Y4X0	DE	U2-agatoxin-Ao1p;
N	Q5YJU0	CC	Usually encoded in the trnK tRNA gene intron
N	Q5YJU0	CC	Probably assists in splicing its own and other chloroplast group II introns (By similarity)
N	Q5YJU0	DE	Intron maturase;
N	Q5YJU0	DR	mRNA processing
N	Q5YJU0	DR	tRNA processing
N	Q5YNA5	CC	Catalyzes the formation of N(7)-methylguanine at position 46 (m7G46) in tRNA (By similarity).
N	Q5YNA5	DE	tRNA (guanine-N(7)-)-methyltransferase;
N	Q5YNA5	DE	tRNA(m7G46)-methyltransferase;
N	Q5YNA5	DR	tRNA (guanine-N7-)-methyltransferase activity
N	Q5YS38	CC	Specifically methylates guanosine-37 in various tRNAs (By similarity).
N	Q5YS38	DE	M1G-methyltransferase;
N	Q5YS38	DE	tRNA (guanine-N(1)-)-methyltransferase;
N	Q5YS38	DE	tRNA [GM37] methyltransferase;
N	Q5YS38	DR	tRNA (guanine-N1-)-methyltransferase activity
N	Q5ZID0	CC	Redox sensor protein
N	Q5ZID0	CC	Undergoes restructuring and subcellular redistribution in response to changes in intracellular NADPH/NADP(+) levels (By similarity).
N	Q5ZID0	DE	NmrA-like family domain-containing protein 1;
N	Q5ZID0	DR	catalytic activity
N	Q5ZJL4	CC	Polynucleotide kinase that can phosphorylate the 5'- hydroxyl groups of double-stranded RNA (dsRNA), single-stranded RNA (ssRNA), double stranded DNA (dsDNA) and double-stranded DNA:RNA hybrids

N	Q5ZJL4	CC	Also phosphorylates the 5'-terminus of exogenously introduced short interfering RNAs (siRNAs), which is a necessary prerequisite for their incorporation into the RNA- induced silencing complex (RISC)
N	Q5ZJL4	CC	Appears to have roles in both tRNA splicing and mRNA 3'-end formation
N	Q5ZJL4	CC	Component of the pre-mRNA cleavage complex II (CF-II), which seems to be required for mRNA 3'-end formation
N	Q5ZJL4	CC	Component of the tRNA splicing endonuclease complex
N	Q5ZJL4	CC	However endogenous siRNAs and microRNAs (miRNAs) that are produced by the cleavage of dsRNA precursors by DICER1 already contain a 5'-phosphate group, so this protein may be dispensible for normal RNA-mediated gene silencing (By similarity)
N	Q5ZJL4	CC	Phosphorylates the 5'-terminus of the tRNA 3'-exon during tRNA splicing; this phosphorylation event is a prerequisite for the subsequent ligation of the two exon halves and the production of a mature tRNA
N	Q5ZJL4	CC	dsRNA is phosphorylated more efficiently than dsDNA, and the RNA component of a DNA:RNA hybrid is phosphorylated more efficiently than the DNA component
N	Q5ZJL4	DE	Polynucleotide kinase Clp1;
N	Q5ZJL4	DE	Polyribonucleotide 5'-hydroxyl-kinase Clp1;
N	Q5ZJL4	DE	Pre-mRNA cleavage complex II protein Clp1;
N	Q5ZJL4	DR	ATP-dependent polydeoxyribonucleotide 5'-hydroxyl-kinase activity
N	Q5ZJL4	DR	ATP-dependent polyribonucleotide 5'-hydroxyl-kinase activity
N	Q5ZJL4	DR	mRNA processing
N	Q5ZJL4	DR	siRNA loading onto RISC involved in RNA interference
N	Q5ZJL4	DR	tRNA splicing, via endonucleolytic cleavage and ligation
N	Q5ZJL4	DR	targeting of mRNA for destruction involved in RNA interference
N	Q5ZL55	CC	Probable core component of the endosomal sorting required for transport complex III (ESCRT-III) which is involved in multivesicular bodies (MVBs) formation and sorting of endosomal cargo proteins into MVBs
N	Q5ZL55	CC	In the ESCRT-III complex, it probably serves as an acceptor for the ESCRT-II complex on endosomal membranes (By similarity).
N	Q5ZL55	CC	MVBs contain intraluminal vesicles (ILVs) that are generated by invagination and scission from the limiting membrane of the endosome and mostly are delivered to lysosomes enabling degradation of membrane proteins, such as stimulated growth factor receptors, lysosomal enzymes and lipids
N	Q5ZL55	DE	Charged multivesicular body protein 6;
N	Q5ZL55	DE	Chromatin-modifying protein 6;
N	Q5ZL55	DR	protein transport
N	Q5ZLC7	CC	May be involved in microtubule polymerization, and spindle function by stabilizing microtubules and anchoring them at centrosomes (By similarity).
N	Q5ZLC7	DE	Microtubule-associated protein RP/EB family member 1;
N	Q5ZLC7	DR	cell division
N	Q5ZLC7	DR	microtubule plus-end binding
N	Q5ZLC7	DR	negative regulation of microtubule polymerization
N	Q5ZLC7	DR	protein localization to microtubule
N	Q5ZM25	CC	Hydrolyzes ATP, and can also hydrolyze GTP with lower efficiency
N	Q5ZM25	CC	Has lower affinity for GTP (By similarity).
N	Q5ZM25	DE	Obg-like ATPase 1;
N	Q5ZM25	DR	hydrolase activity
N	Q5ZM73	CC	Mitochondrial GTPase involved in mitochondrial trafficking
N	Q5ZM73	CC	Probably involved in control of anterograde transport of mitochondria and their subcellular distribution (By similarity).
N	Q5ZM73	DE	Mitochondrial Rho GTPase 1;
N	Q5ZM73	DE	Ras homolog gene family member T1;
N	Q5ZM73	DR	calcium ion binding
N	Q5ZM73	DR	cellular homeostasis
N	Q5ZM73	DR	hydrolase activity
N	Q5ZM73	DR	mitochondrion transport along microtubule
N	Q5ZM73	DR	small GTPase mediated signal transduction
N	Q5ZWN7	CC	Produces ATP from ADP in the presence of a proton gradient across the membrane
N	Q5ZWN7	CC	The alpha chain is a regulatory subunit (By similarity).
N	Q5ZWN7	DE	ATP synthase F1 sector subunit alpha 1;
N	Q5ZWN7	DE	ATP synthase subunit alpha 1;
N	Q5ZWN7	DE	F-ATPase subunit alpha 1;
N	Q5ZWN7	DR	ATP hydrolysis coupled proton transport
N	Q5ZWN7	DR	ATP synthesis coupled proton transport
N	Q5ZWN7	DR	hydrogen ion transporting ATP synthase activity, rotational mechanism

N	Q5ZWN7	DR	proton-transporting ATPase activity, rotational mechanism
N	Q5ZX60	CC	Increases the formation of ribosomal termination complexes and stimulates activities of RF-1 and RF-2
N	Q5ZX60	CC	It binds guanine nucleotides and has strong preference for UGA stop codons
N	Q5ZX60	CC	It may interact directly with the ribosome
N	Q5ZX60	CC	The stimulation of RF- 1 and RF-2 is significantly reduced by GTP and GDP, but not by GMP (By similarity).
N	Q5ZX60	DE	Peptide chain release factor 3;
N	Q5ZX60	DR	GTPase activity
N	Q5ZX60	DR	translation release factor activity, codon specific
N	Q5ZYQ1	CC	Seems to be the binding site for several of the factors involved in protein synthesis and appears to be essential for accurate translation (By similarity).
N	Q5ZYQ1	DE	50S ribosomal protein L7/L12;
N	Q5ZYQ1	DR	structural constituent of ribosome
N	Q600A8	DE	Glucose-6-phosphate isomerase;
N	Q600A8	DE	Phosphoglucose isomerase;
N	Q600A8	DE	Phosphohexose isomerase;
N	Q600A8	DR	gluconeogenesis
N	Q600A8	DR	glucose-6-phosphate isomerase activity
N	Q601J0	CC	Located on the platform of the 30S subunit, it bridges several disparate RNA helices of the 16S rRNA
N	Q601J0	CC	Forms part of the Shine-Dalgarno cleft in the 70S ribosome (By similarity).
N	Q601J0	DE	30S ribosomal protein S11;
N	Q601J0	DR	structural constituent of ribosome
N	Q60276	DE	Uncharacterized protein MJECL14;
N	Q605D1	CC	Binds to the 23S rRNA (By similarity).
N	Q605D1	DE	50S ribosomal protein L15;
N	Q605D1	DR	structural constituent of ribosome
N	Q606K9	DE	Maf-like protein MCA2007;
N	Q60841	CC	Extracellular matrix serine protease that plays a role in layering of neurons in the cerebral cortex and cerebellum
N	Q60841	CC	Affects migration of sympathetic preganglionic neurons in the spinal cord, where it seems to act as a barrier to neuronal migration
N	Q60841	CC	Binding to the extracellular domains of lipoprotein receptors VLDLR and ApoER2 induces tyrosine phosphorylation of Dab1 and modulation of Tau phosphorylation
N	Q60841	CC	Enzymatic activity is important for the modulation of cell adhesion
N	Q60841	CC	Regulates microtubule function in neurons and neuronal migration
N	Q60841	DE	Reeler protein;
N	Q60841	DR	axon guidance
N	Q60841	DR	cell adhesion
N	Q60841	DR	cerebral cortex tangential migration
N	Q60841	DR	dendrite development
N	Q60841	DR	glial cell differentiation
N	Q60841	DR	metal ion binding
N	Q60841	DR	neuron migration
N	Q60841	DR	peptidyl-tyrosine phosphorylation
N	Q60841	DR	positive regulation of protein kinase activity
N	Q60841	DR	positive regulation of small GTPase mediated signal transduction
N	Q60841	DR	protein serine/threonine/tyrosine kinase activity
N	Q60841	DR	response to pain
N	Q60841	DR	serine-type peptidase activity
N	Q60841	DR	spinal cord patterning
N	Q61062	CC	May play a role in the signal transduction pathway mediated by multiple Wnt genes
N	Q61062	DE	DSH homolog 3;
N	Q61062	DE	Dishevelled-3;
N	Q61062	DE	Segment polarity protein dishevelled homolog DVL-3;
N	Q61062	DR	cochlea morphogenesis
N	Q61062	DR	intracellular signal transduction
N	Q61062	DR	outflow tract septum morphogenesis
N	Q61062	DR	planar cell polarity pathway involved in neural tube closure
N	Q61062	DR	signal transducer activity
N	Q61194	CC	Phosphorylates PtdIns, PtdIns4P and PtdIns(4,5)P2
N	Q61194	CC	May be a downstream effector in insulin signaling cascade.
N	Q61194	CC	May be involved in mitosis and UV-induced damage response (By similarity)
N	Q61194	CC	May play a role in clathrin-coated endocytic vesicle formation and EGF signaling cascade

N	Q61194	DE	PI3K-C2-alpha;
N	Q61194	DE	Phosphatidylinositol-4-phosphate 3-kinase C2 domain-containing subunit alpha;
N	Q61194	DE	Phosphoinositide 3-kinase-C2-alpha;
N	Q61194	DE	PtdIns-3-kinase C2 subunit alpha;
N	Q61194	DR	1-phosphatidylinositol-3-kinase activity
N	Q61194	DR	cell communication
N	Q61194	DR	phosphatidylinositol binding
N	Q61194	DR	phosphatidylinositol-4-phosphate 3-kinase activity
N	Q61194	DR	phosphatidylinositol-mediated signaling
N	Q61458	CC	Regulates CDK7, the catalytic subunit of the CDK- activating kinase (CAK) enzymatic complex
N	Q61458	CC	CAK activates the cyclin-associated kinases CDK1, CDK2, CDK4 and CDK6 by threonine phosphorylation
N	Q61458	CC	CAK complexed to the core-TFIIH basal transcription factor activates RNA polymerase II by serine phosphorylation of the repetitive C-terminus domain (CTD) of its large subunit (POLR2A), allowing its escape from the promoter and elongation of the transcripts
N	Q61458	CC	Involved in cell cycle control and in RNA transcription by RNA polymerase II
N	Q61458	CC	Its expression and activity are constant throughout the cell cycle.
N	Q61458	DR	transcription from RNA polymerase II promoter
N	Q61R02	CC	Probable ATP-binding RNA helicase.
N	Q61R02	DE	DEAD box protein 55;
N	Q61R02	DE	Probable ATP-dependent RNA helicase DDX55 homolog;
N	Q61R02	DR	ATP-dependent helicase activity
N	Q626H5	CC	Involved as a receptor for mab-20/sema-2a in the formation or stabilization of cell-cell contacts at several stages of epithelial morphogenesis
N	Q626H5	CC	During male tail morphogenesis, involved in precursor cell sorting and in the formation of distinct sensory rays (By similarity).
N	Q626H5	CC	In early embryonic development, required for proper ventral closure of the [illegible]
N	Q626H5	DR	cell differentiation
N	Q626H5	DR	multicellular organismal development
N	Q626H5	DR	receptor activity
N	Q62909	CC	Promotes ATP-dependent removal of tubulin dimers from microtubules
N	Q62909	CC	Regulates the turnover of microtubules at the kinetochore and functions in chromosome segregation during mitosis (By similarity).
N	Q62909	DE	Kinesin-like protein KIF2C;
N	Q62909	DE	Kinesin-related protein 2;
N	Q62909	DE	Mitotic centromere-associated kinesin;
N	Q62909	DR	cell division
N	Q62909	DR	chromosome segregation
N	Q62909	DR	microtubule motor activity
N	Q62909	DR	microtubule plus-end binding
N	Q62909	DR	microtubule-based movement
N	Q62J08	CC	Catalyzes the ATP-dependent amination of UTP to CTP with either L-glutamine or ammonia as the source of nitrogen (By similarity).
N	Q62J08	DE	CTP synthase;
N	Q62J08	DE	CTP synthetase;
N	Q62J08	DE	UTP--ammonia ligase;
N	Q62J08	DR	CTP synthase activity
N	Q62J08	DR	glutamine metabolic process
N	Q62J08	DR	pyrimidine nucleotide biosynthetic process
N	Q62LW6	CC	Multidrug efflux pump (By similarity).
N	Q62LW6	DE	Multidrug-efflux transporter;
N	Q62LW6	DE	Probable multidrug resistance protein norM;
N	Q62LW6	DR	antiporter activity
N	Q62LW6	DR	drug transmembrane transporter activity
N	Q62LW6	DR	ion transport
N	Q62MR4	CC	Furnishes a means for formation of correctly charged Gln-tRNA(Gln) through the transamidation of misacylated Glu- tRNA(Gln) in organisms which lack glutaminyl-tRNA synthetase
N	Q62MR4	CC	The reaction takes place in the presence of glutamine and ATP through an activated gamma-phospho-Glu-tRNA(Gln) (By similarity).
N	Q62MR4	DE	Glu-ADT subunit A;
N	Q62MR4	DE	Glutamyl-tRNA(Gln) amidotransferase subunit A;
N	Q62MR4	DR	carbon-nitrogen ligase activity, with glutamine as amido-N-donor

N	Q635P2	CC	Dephosphorylates 2-hydroxy-3-keto-5-methylthiopentenyl- 1-phosphate (HK-MTPenyl-1-P) yielding 1,2-dihydroxy-3-keto-5- methylthiopentene (DHK-MTPene) (By similarity)
N	Q635P2	DE	2-hydroxy-3-keto-5-methylthiopentenyl-1-phosphate phosphatase;
N	Q635P2	DE	HK-MTPenyl-1-P phosphatase;
N	Q635P2	DR	L-methionine salvage
N	Q635P2	DR	phosphatase activity
N	Q635S6	CC	Catalyzes the transfer of a methyl group from 5- methyltetrahydrofolate to homocysteine resulting in methionine formation (By similarity).
N	Q635S6	DE	5-methyltetrahydropteroyltriglutamate--homocysteine methyltransferase;
N	Q635S6	DE	Cobalamin-independent methionine synthase;
N	Q635S6	DE	Methionine synthase, vitamin-B12 independent isozyme;
N	Q635S6	DR	5-methyltetrahydropteroyltriglutamate-homocysteine S-methyltransferase activity
N	Q635S6	DR	methionine biosynthetic process
N	Q635S6	DR	zinc ion binding
N	Q63802	CC	May act as a negative regulator of entry into mitosis (G2 to M transition) by protecting the nucleus from cytoplasmically activated cyclin B1-complexed CDK1 before the onset of mitosis
N	Q63802	CC	A correlated decrease in protein level occurs at M/G1 phase, probably due to its degradation
N	Q63802	CC	Its activity increases during S and G2 phases and decreases at M phase when it is hyperphosphorylated
N	Q63802	CC	Phosphorylation of cyclin B1-CDK1 occurs exclusively on 'Tyr-15' and phosphorylation of monomeric CDK1 does not occur (By similarity).
N	Q63802	CC	Specifically phosphorylates and inactivates cyclin B1-complexed CDK1 reaching a maximum during G2 phase and a minimum as cells enter M phase
N	Q63802	DE	Wee1-like protein kinase;
N	Q63802	DE	Wee1A kinase;
N	Q63802	DR	cell division
N	Q63802	DR	magnesium ion binding
N	Q63802	DR	non-membrane spanning protein tyrosine kinase activity
N	Q63802	DR	protein phosphorylation
N	Q63802	DR	protein serine/threonine kinase activity
N	Q63CR4	DE	UPF0291 protein BCE33L1708;
N	Q63GI2	CC	Catalyzes the attachment of proline to tRNA(Pro) in a two-step reaction: proline is first activated by ATP to form Pro- AMP and then transferred to the acceptor end of tRNA(Pro) (By similarity).
N	Q63GI2	DE	Proline--tRNA ligase 2;
N	Q63GI2	DE	Prolyl-tRNA synthetase 2;
N	Q63GI2	DR	proline-tRNA ligase activity
N	Q63GI2	DR	prolyl-tRNA aminoacylation
N	Q63S99	CC	Transfers the 4'-phosphopantetheine moiety from coenzyme A to a Ser of acyl-carrier-protein (By similarity).
N	Q63S99	DE	4'-phosphopantetheinyl transferase AcpS;
N	Q63S99	DE	Holo-ACP synthase;
N	Q63S99	DE	Holo-[acyl-carrier-protein] synthase;
N	Q63S99	DR	fatty acid biosynthetic process
N	Q63S99	DR	holo-[acyl-carrier-protein] synthase activity
N	Q63S99	DR	macromolecule biosynthetic process
N	Q63S99	DR	magnesium ion binding
N	Q63SP8	CC	Catalyzes the ATP-dependent amination of UTP to CTP with either L-glutamine or ammonia as the source of nitrogen (By similarity).
N	Q63SP8	DE	CTP synthase;
N	Q63SP8	DE	CTP synthetase;
N	Q63SP8	DE	UTP--ammonia ligase;
N	Q63SP8	DR	CTP synthase activity
N	Q63SP8	DR	glutamine metabolic process
N	Q63SP8	DR	pyrimidine nucleotide biosynthetic process
N	Q63SX4	CC	Catalyzes the attachment of glutamate to tRNA(Glu) in a two-step reaction: glutamate is first activated by ATP to form Glu-AMP and then transferred to the acceptor end of tRNA(Glu) (By similarity).
N	Q63SX4	DE	Glutamate--tRNA ligase;
N	Q63SX4	DE	Glutamyl-tRNA synthetase;
N	Q63SX4	DR	glutamate-tRNA ligase activity
N	Q63SX4	DR	glutamyl-tRNA aminoacylation
N	Q63V40	CC	ATP-dependent specificity component of the Clp protease

N	Q63V40	CC	Can perform chaperone functions in the absence of ClpP (By similarity).
N	Q63V40	CC	It directs the protease to specific substrates
N	Q63V40	DE	ATP-dependent Clp protease ATP-binding subunit ClpX;
N	Q63V40	DR	ATPase activity
N	Q63V40	DR	protein dimerization activity
N	Q63V40	DR	protein folding
N	Q63V40	DR	unfolded protein binding
N	Q63V40	DR	zinc ion binding
N	Q64261	CC	Probably involved in the control of the cell cycle
N	Q64261	CC	Interacts with D-type G1 cyclins (By similarity).
N	Q64261	DE	Cell division protein kinase 6;
N	Q64261	DE	Cyclin-dependent kinase 6;
N	Q64261	DE	Serine/threonine-protein kinase PLSTIRE;
N	Q64261	DR	cell division
N	Q64261	DR	cyclin-dependent protein kinase activity
N	Q64261	DR	protein phosphorylation
N	Q64408	CC	Has steroid 11-beta-hydroxylase activity
N	Q64408	CC	In addition to this activity, the 18 or 19-hydroxylation of steroids and the aromatization of androstendione to estrone have also been ascribed to cytochrome P450 XIB
N	Q64408	DE	Aldosterone synthase;
N	Q64408	DE	Cytochrome P450 11B1, mitochondrial;
N	Q64408	DE	Cytochrome P450C11;
N	Q64408	DE	Steroid 11-beta-hydroxylase;
N	Q64408	DR	electron carrier activity
N	Q64408	DR	oxidation-reduction process
N	Q64408	DR	steroid 11-beta-monooxygenase activity
N	Q64408	DR	steroid biosynthetic process
N	Q64437	CC	Could function in retinol oxidation for the synthesis of retinoic acid, a hormone important for cellular differentiation.
N	Q64437	DE	Alcohol dehydrogenase 7;
N	Q64437	DE	Alcohol dehydrogenase class 4 mu/sigma chain;
N	Q64437	DE	Alcohol dehydrogenase class IV mu/sigma chain;
N	Q64437	DE	Gastric alcohol dehydrogenase;
N	Q64437	DE	Retinol dehydrogenase;
N	Q64437	DR	ethanol catabolic process
N	Q64437	DR	retinoic acid metabolic process
N	Q64437	DR	retinol dehydrogenase activity
N	Q64437	DR	retinol metabolic process
N	Q64475	CC	Core component of nucleosome
N	Q64475	CC	DNA accessibility is regulated via a complex set of post-translational modifications of histones, also called histone code, and nucleosome remodeling.
N	Q64475	CC	Histones thereby play a central role in transcription regulation, DNA repair, DNA replication and chromosomal stability
N	Q64475	CC	Nucleosomes wrap and compact DNA into chromatin, limiting DNA accessibility to the cellular machineries which require DNA as a template
N	Q64475	DE	Histone H2B type 1-B;
N	Q64475	DR	nucleosome assembly
N	Q64525	CC	Core component of nucleosome
N	Q64525	CC	DNA accessibility is regulated via a complex set of post-translational modifications of histones, also called histone code, and nucleosome remodeling.
N	Q64525	CC	Histones thereby play a central role in transcription regulation, DNA repair, DNA replication and chromosomal stability
N	Q64525	CC	Nucleosomes wrap and compact DNA into chromatin, limiting DNA accessibility to the cellular machineries which require DNA as a template
N	Q64525	DE	Histone H2B type 2-B;
N	Q64525	DR	nucleosome assembly
N	Q645T6	CC	Receptor that may play a role in the perception of bitterness and is gustducin-linked
N	Q645T6	CC	May play a role in sensing the chemical composition of the gastrointestinal content
N	Q645T6	CC	The activity of this receptor may stimulate alpha gustducin, mediate PLC-beta-2 activation and lead to the gating of TRPM5 (By similarity).
N	Q645T6	DE	Taste receptor type 2 member 50;
N	Q645T6	DR	G-protein coupled receptor activity
N	Q645T6	DR	sensory perception of taste
N	Q645Y8	CC	Gustducin-coupled receptor implicated in the perception of bitter compounds in the oral cavity and the gastrointestinal tract

N	Q645Y8	CC	In airway epithelial cells, binding of denatonium increases the intracellular calcium ion concentration and stimulates ciliary beat frequency (By similarity).
N	Q645Y8	CC	Signals through PLCB2 and the calcium-regulated cation channel TRPM5 (By similarity)
N	Q645Y8	DE	Taste receptor type 2 member 4;
N	Q645Y8	DR	G-protein coupled receptor activity
N	Q645Y8	DR	sensory perception of taste
N	Q64634	CC	UDPGT is of major importance in the conjugation and subsequent elimination of potentially toxic xenobiotics and endogenous compounds.
N	Q64634	DE	UDP-glucuronosyltransferase 1-8;
N	Q64634	DR	4-chlorobiphenyl metabolic process
N	Q64634	DR	bilirubin conjugation
N	Q64634	DR	biphenyl catabolic process
N	Q64634	DR	cellular response to ethanol
N	Q64634	DR	cellular response to glucocorticoid stimulus
N	Q64634	DR	cellular response to organic cyclic compound
N	Q64634	DR	coumarin catabolic process
N	Q64634	DR	enzyme binding
N	Q64634	DR	estrogen catabolic process
N	Q64634	DR	glucuronosyltransferase activity
N	Q64634	DR	liver development
N	Q64634	DR	organ regeneration
N	Q64634	DR	protein glucuronidation
N	Q64634	DR	response to drug
N	Q64634	DR	response to lipopolysaccharide
N	Q64634	DR	response to nutrient
N	Q64634	DR	response to peptide hormone stimulus
N	Q64634	DR	response to starvation
N	Q64770	CC	Early protein essential for viral replication
N	Q64770	CC	Activates transcription by blocking host retinoblastoma protein (RB) and inhibiting the SUMO pathway
N	Q64770	CC	Blocks the SUMO pathway by targeting the E1 enzyme (SAE1/UBA2 heterodimer) to the ubiquitin-proteasome machinery
N	Q64770	CC	Inhibition of host RB leads to the activation of E2F1-dependent transcription and, in particiuliar, of E2F1-regulated S-phase genes
N	Q64770	CC	Inhibits HDAC1 sumoylation, thereby interfering with histone deacetylation mediated by HDAC1, leading to activation of transcription
N	Q64770	CC	Mediates SAE1 degradation possibly through the formation of complexes with either CUL2-elongin BC complex-RBX1 or CUL5-elongin BC complex-RBX1
N	Q64770	CC	Mediates induction of heat-shock response
N	Q64770	CC	Seems to have an antiapoptotic function.
N	Q64770	CC	Stimulation of progression from G1 to S phase allows the virus to efficiently use the cellular DNA replicating machinery to achieve viral genome replication
N	Q64770	CC	Strong and global transcriptional activator of both viral and cellular genes
N	Q64770	CC	The degradation of UBA2 is probably a consequent effect of SAE1 disappearance
N	Q64770	DE	Gallus-anti morte protein;
N	Q64770	DE	Protein GAM-1;
N	Q64770	DR	interspecies interaction between organisms
N	Q64LH1	CC	Binds to actin and affects the structure of the cytoskeleton
N	Q64LH1	CC	At high concentrations, profilin prevents the polymerization of actin, whereas it enhances it at low concentrations
N	Q64LH1	CC	By binding to PIP2, it inhibits the formation of IP3 and DG (By similarity).
N	Q64LH1	DE	Amb a 8;
N	Q64LH1	DE	Pollen allergen Amb a 8;
N	Q64LH1	DR	actin binding
N	Q64LH1	DR	actin cytoskeleton organization
N	Q64NF1	DE	Insertion sequence IS21-like putative ATP-binding protein;
N	Q64NF1	DR	regulation of transcription, DNA-dependent
N	Q64NF1	DR	transcription factor binding
N	Q64PM4	DE	Asparagine synthetase A;
N	Q64PM4	DE	Aspartate--ammonia ligase;
N	Q64PM4	DR	aminoacyl-tRNA ligase activity
N	Q64PM4	DR	asparagine biosynthetic process
N	Q64PM4	DR	aspartate-ammonia ligase activity
N	Q64PM4	DR	tRNA aminoacylation for protein translation
N	Q64TK0	DE	CinA-like protein;

N	Q64TK0	DR	Mo-molybdopterin cofactor biosynthetic process
N	Q64VI6	CC	Participates actively in the response to hyperosmotic and heat shock by preventing the aggregation of stress-denatured proteins, in association with dnaK and grpE
N	Q64VI6	CC	GrpE releases ADP from dnaK; ATP binding to dnaK triggers the release of the substrate protein, thus completing the reaction cycle
N	Q64VI6	CC	It is the nucleotide exchange factor for dnaK and may function as a thermosensor
N	Q64VI6	CC	Several rounds of ATP- dependent interactions between dnaJ, dnaK and grpE are required for fully efficient folding (By similarity).
N	Q64VI6	CC	Unfolded proteins bind initially to dnaJ; upon interaction with the dnaJ-bound protein, dnaK hydrolyzes its bound ATP, resulting in the formation of a stable complex
N	Q64VI6	DE	HSP-70 cofactor;
N	Q64VI6	DE	Protein grpE;
N	Q64VI6	DR	adenyl-nucleotide exchange factor activity
N	Q64VI6	DR	chaperone binding
N	Q64VI6	DR	protein folding
N	Q64VI6	DR	protein homodimerization activity
N	Q64VI6	DR	response to stress
N	Q64WC2	CC	Catalyzes the oxidation of either pyridoxine 5'-phosphate (PNP) or pyridoxamine 5'-phosphate (PMP) into pyridoxal 5'-phosphate (PLP) (By similarity)
N	Q64WC2	DE	PNP/PMP oxidase;
N	Q64WC2	DE	Pyridoxal 5'-phosphate synthase;
N	Q64WC2	DE	Pyridoxine/pyridoxamine 5'-phosphate oxidase;
N	Q64WC2	DR	oxidation-reduction process
N	Q64WC2	DR	pyridoxamine-phosphate oxidase activity
N	Q64WC2	DR	pyridoxine biosynthetic process
N	Q650H8	CC	Hydrolyzes D-tyrosyl-tRNA(Tyr) into D-tyrosine and free tRNA(Tyr)
N	Q650H8	CC	Could be a defense mechanism against a harmful effect of D-tyrosine (By similarity)
N	Q650H8	DE	D-tyrosyl-tRNA(Tyr) deacylase;
N	Q650H8	DR	D-amino acid catabolic process
N	Q650H8	DR	hydrolase activity, acting on ester bonds
N	Q65139	DE	Uncharacterized protein A118R;
N	Q65192	DE	NifS-like protein;
N	Q65192	DR	catalytic activity
N	Q65192	DR	pyridoxal phosphate binding
N	Q65729	CC	Nuclear inclusion protein B is a RNA-dependent RNA polymerase that plays an essential role in the virus replication (By similarity).
N	Q65729	DE	49 kDa proteinase;
N	Q65729	DE	49 kDa-Pro;
N	Q65729	DE	Capsid protein;
N	Q65729	DE	Coat protein;
N	Q65729	DE	Genome polyprotein;
N	Q65729	DE	Nuclear inclusion protein A;
N	Q65729	DE	Nuclear inclusion protein B;
N	Q65729	DE	RNA-directed RNA polymerase;
N	Q65729	DR	RNA-directed RNA polymerase activity
N	Q65729	DR	cysteine-type peptidase activity
N	Q65729	DR	nucleotide binding
N	Q65729	DR	viral genome replication
N	Q65QT7	CC	Involved in the transport of maltose and maltodextrins (By similarity).
N	Q65QT7	DE	Maltose-inducible porin;
N	Q65QT7	DR	porin activity
N	Q65QT7	DR	sugar:hydrogen symporter activity
N	Q65R93	DE	UPF0352 protein MS1910;
N	Q65RC3	CC	Catalyzes the conversion of uracil and 5-phospho-alpha- D-ribose 1-diphosphate (PRPP) to UMP and diphosphate (By similarity).
N	Q65RC3	DE	UMP pyrophosphorylase;
N	Q65RC3	DE	Uracil phosphoribosyltransferase;
N	Q65RC3	DR	nucleoside metabolic process
N	Q65RC3	DR	uracil phosphoribosyltransferase activity
N	Q65RC3	DR	uracil salvage
N	Q65S59	CC	An essential GTPase which binds GTP, GDP and possibly (p)ppGpp with moderate affinity, with high nucleotide exchange rates and a fairly low GTP hydrolysis rate (By similarity)
N	Q65S59	CC	It may play a role in control of the cell cycle, stress response, ribosome biogenesis and in those bacteria that undergo differentiation, in morphogenesis control (Potential)

N	Q65S59	DE	GTP-binding protein obg;
N	Q65S59	DR	GTPase activity
N	Q65S59	DR	magnesium ion binding
N	Q65SS8	CC	Negatively regulates transcription of bacterial ribonucleotide reductase nrd genes and operons by binding to NrdR- boxes (By similarity).
N	Q65SS8	DE	Transcriptional repressor NrdR;
N	Q65SS8	DR	transcription repressor activity
N	Q65SS8	DR	zinc ion binding
N	Q65UK6	CC	Specifically methylates the guanosine in position 2445 (m2G2445) of 23S rRNA (By similarity).
N	Q65UK6	DE	23S rRNA m2G2445 methyltransferase;
N	Q65UK6	DE	Ribosomal RNA large subunit methyltransferase L;
N	Q65UK6	DE	rRNA (guanine-N(2)-)-methyltransferase rlmL;
N	Q65UK6	DR	rRNA (guanine-N2-)-methyltransferase activity
N	Q65UP1	CC	The ruvA-ruvB complex in the presence of ATP renatures cruciform structure in supercoiled DNA with palindromic sequence, indicating that it may promote strand exchange reactions in homologous recombination
N	Q65UP1	CC	RuvA stimulates, in the presence of DNA, the weak ATPase activity of ruvB (By similarity).
N	Q65UP1	CC	RuvAB is an helicase that mediates the Holliday junction migration by localized denaturation and reannealing
N	Q65UP1	DE	Holliday junction ATP-dependent DNA helicase ruvA;
N	Q65UP1	DR	DNA recombination
N	Q65UP1	DR	four-way junction helicase activity
N	Q660T5	CC	Is required not only for elongation of protein synthesis but also for the initiation of all mRNA translation through initiator tRNA(fMet) aminoacylation (By similarity).
N	Q660T5	DE	Methionine--tRNA ligase;
N	Q660T5	DE	Methionyl-tRNA synthetase;
N	Q660T5	DR	metal ion binding
N	Q660T5	DR	methionine-tRNA ligase activity
N	Q660T5	DR	methionyl-tRNA aminoacylation
N	Q66117	CC	RNA-dependent RNA polymerase which replicates the viral genome composed of 3 RNA segments, RNA1, RNA2 and RNA3 (Potential).
N	Q66117	DE	RNA-directed RNA polymerase 2a;
N	Q66117	DE	protein 2a;
N	Q66117	DR	RNA-directed RNA polymerase activity
N	Q66117	DR	nucleotide binding
N	Q66117	DR	viral genome replication
N	Q661F0	CC	This protein specifically catalyzes the removal of signal peptides from prolipoproteins (By similarity).
N	Q661F0	DE	Lipoprotein signal peptidase;
N	Q661F0	DE	Prolipoprotein signal peptidase;
N	Q661F0	DE	Signal peptidase II;
N	Q661F0	DR	aspartic-type endopeptidase activity
N	Q661P9	CC	Catalyzes the attachment of tyrosine to tRNA(Tyr) in a two-step reaction: tyrosine is first activated by ATP to form Tyr- AMP and then transferred to the acceptor end of tRNA(Tyr) (By similarity).
N	Q661P9	DE	Tyrosine--tRNA ligase;
N	Q661P9	DE	Tyrosyl-tRNA synthetase;
N	Q661P9	DR	tyrosine-tRNA ligase activity
N	Q661P9	DR	tyrosyl-tRNA aminoacylation
N	Q662P0	CC	Associates with the EF-Tu.GDP complex and induces the exchange of GDP to GTP.
N	Q662P0	CC	It remains bound to the aminoacyl-tRNA.EF- Tu.GTP complex up to the GTP hydrolysis stage on the ribosome.
N	Q662P0	DE	Elongation factor Ts;
N	Q662P0	DR	translation elongation factor activity
N	Q66647	CC	This protein may be an helicase and is required for replication of viral DNA (By similarity).
N	Q66647	DE	Probable ATP-dependent helicase 44;
N	Q66647	DR	DNA replication
N	Q66647	DR	helicase activity
N	Q66647	DR	viral genome replication
N	Q66AS1	CC	Prevents the cell division inhibition by proteins minC and minD at internal division sites while permitting inhibition at polar sites

N	Q66AS1	CC	This ensures cell division at the proper site by restricting the formation of a division septum at the midpoint of the long axis of the cell (By similarity).
N	Q66AS1	DE	Cell division topological specificity factor;
N	Q66AS1	DR	cell division
N	Q66AS1	DR	regulation of barrier septum formation
N	Q66H24	DE	Uncharacterized protein C9orf40 homolog;
N	Q66HC7	CC	Required for MAP kinase p38 (MAPK11, MAPK12, MAPK13 and/or MAPK14) activation during gastrulation
N	Q66HC7	CC	Required for down- regulation of E-cadherin during gastrulation by regulating E-cadherin protein level downstream from NCK-interacting kinase (NIK) and independently of the regulation of transcription by Fgf signaling and Snail
N	Q66HC7	CC	Required for starvation-induced ATG9A trafficking during autophagy (By similarity)
N	Q66HC7	DE	Protein FAM48A;
N	Q66HE2	CC	Proton-linked monocarboxylate transporter
N	Q66HE2	CC	Catalyzes the rapid transport across the plasma membrane of many monocarboxylates (By similarity).
N	Q66HE2	DE	Monocarboxylate transporter 13;
N	Q66HE2	DE	Solute carrier family 16 member 13;
N	Q66HE2	DR	symporter activity
N	Q66I12	DE	Coiled-coil domain-containing protein 47;
N	Q66J36	CC	May act as a transcriptional regulator of a number of proteins of the circadian clock (By similarity).
N	Q66J36	DE	Nuclear factor interleukin-3-regulated protein;
N	Q66J36	DR	circadian rhythm
N	Q66J36	DR	protein dimerization activity
N	Q66J36	DR	regulation of transcription, DNA-dependent
N	Q66J36	DR	sequence-specific DNA binding
N	Q66J36	DR	sequence-specific DNA binding transcription factor activity
N	Q66J36	DR	transcription regulator activity
N	Q67HX4	DE	Protein psbN;
N	Q67HX4	DR	photosynthesis
N	Q67N73	CC	DNA polymerase involved in damage-induced mutagenesis and translesion synthesis (TLS)
N	Q67N73	CC	It is not the major replicative DNA polymerase (By similarity).
N	Q67N73	DE	Error-prone DNA polymerase;
N	Q67N73	DR	3'-5' exonuclease activity
N	Q67N73	DR	DNA replication
N	Q67N73	DR	DNA-directed DNA polymerase activity
N	Q67Q99	CC	Nuclease that resolves Holliday junction intermediates in genetic recombination
N	Q67Q99	CC	Cleaves the cruciform structure in supercoiled DNA by nicking to strands with the same polarity at sites symmetrically opposed at the junction in the homologous arms and leaves a 5'-terminal phosphate and a 3'-terminal hydroxyl group (By similarity)
N	Q67Q99	DE	Crossover junction endodeoxyribonuclease ruvC;
N	Q67Q99	DE	Holliday junction nuclease ruvC;
N	Q67Q99	DE	Holliday junction resolvase ruvC;
N	Q67Q99	DR	DNA recombination
N	Q67Q99	DR	crossover junction endodeoxyribonuclease activity
N	Q67Q99	DR	metal ion binding
N	Q67Q99	DR	nucleic acid binding
N	Q67QZ4	CC	Cleaves peptides in various proteins in a process that requires ATP hydrolysis
N	Q67QZ4	CC	Has a chymotrypsin-like activity
N	Q67QZ4	CC	Plays a major role in the degradation of misfolded proteins (By similarity).
N	Q67QZ4	DE	ATP-dependent Clp protease proteolytic subunit 2;
N	Q67QZ4	DE	Endopeptidase Clp 2;
N	Q67QZ4	DR	serine-type endopeptidase activity
N	Q67SJ9	CC	ATP-dependent specificity component of the Clp protease
N	Q67SJ9	CC	Can perform chaperone functions in the absence of ClpP (By similarity).
N	Q67SJ9	CC	It directs the protease to specific substrates
N	Q67SJ9	DE	ATP-dependent Clp protease ATP-binding subunit ClpX;
N	Q67SJ9	DR	ATPase activity
N	Q67SJ9	DR	protein dimerization activity
N	Q67SJ9	DR	protein folding
N	Q67SJ9	DR	unfolded protein binding
N	Q67SJ9	DR	zinc ion binding

N	Q67XZ3	CC	6-fructan exohydrolase that can use phlein, levan, neokestose, levanbiose, 6-kestose, and 1-kestose as substrates.
N	Q67XZ3	DE	6-fructan exohydrolase;
N	Q67XZ3	DE	Beta-fructofuranosidase 5;
N	Q67XZ3	DE	Beta-fructofuranosidase, insoluble isoenzyme CWINV3;
N	Q67XZ3	DE	Cell wall beta-fructosidase 3;
N	Q67XZ3	DE	Cell wall invertase 3;
N	Q67XZ3	DE	Sucrose hydrolase 3;
N	Q67XZ3	DR	carbohydrate metabolic process
N	Q67XZ3	DR	fructan beta-fructosidase activity
N	Q67YC0	CC	Catalyzes the specific cleavage of pyrophosphate.
N	Q67YC0	DE	AtPPsPase1;
N	Q67YC0	DE	Inorganic pyrophosphatase 1;
N	Q67YC0	DE	PPi phosphatase 1;
N	Q67YC0	DE	Protein PHOSPHATE STARVATION-INDUCED GENE 2;
N	Q67YC0	DE	Pyrophosphate-specific phosphatase 1;
N	Q67YC0	DR	cellular response to phosphate starvation
N	Q67YC0	DR	inorganic diphosphatase activity
N	Q67YC0	DR	metal ion binding
N	Q67YC0	DR	phosphatase activity
N	Q67YC0	DR	protein tetramerization
N	Q68WD3	CC	This is one of the proteins that binds to the 5S RNA in the ribosome where it forms part of the central protuberance (By similarity).
N	Q68WD3	DE	50S ribosomal protein L25;
N	Q68WD3	DE	General stress protein CTC;
N	Q68WD3	DR	5S rRNA binding
N	Q68WD3	DR	structural constituent of ribosome
N	Q68WK6	CC	The pyruvate dehydrogenase complex catalyzes the overall conversion of pyruvate to acetyl-CoA and CO(2)
N	Q68WK6	CC	It contains multiple copies of three enzymatic components: pyruvate dehydrogenase (E1), dihydrolipoamide acetyltransferase (E2) and lipoamide dehydrogenase (E3) (By similarity).
N	Q68WK6	DE	Dihydrolipoamide acetyltransferase component of pyruvate dehydrogenase complex;
N	Q68WK6	DE	Dihydrolipoyllysine-residue acetyltransferase component of pyruvate dehydrogenase complex;
N	Q68WK6	DR	dihydrolipoyllysine-residue acetyltransferase activity
N	Q68WK6	DR	pyruvate metabolic process
N	Q68XJ4	CC	Necessary for efficient export of extracytoplasmic proteins
N	Q68XJ4	CC	Binds to the signal sequence when it emerges from the ribosomes (By similarity).
N	Q68XJ4	DE	Fifty-four homolog;
N	Q68XJ4	DE	Signal recognition particle protein;
N	Q68XJ4	DR	7S RNA binding
N	Q68XJ4	DR	SRP-dependent cotranslational protein targeting to membrane
N	Q68XJ4	DR	nucleoside-triphosphatase activity
N	Q68XX7	DE	Phosphate acetyltransferase;
N	Q68XX7	DE	Phosphotransacetylase;
N	Q68XX7	DR	phosphate acetyltransferase activity
N	Q69ZB0	CC	Required for the organization of the mitotic spindle
N	Q69ZB0	CC	Maintains the structural integrity of centrosomes during mitosis (By similarity).
N	Q69ZB0	DE	Leucine-rich repeat and coiled-coil domain-containing protein 1;
N	Q69ZB0	DR	cell division
N	Q69ZI1	CC	Acts as a scaffold protein, contributes to Rac-induced signal transduction such as JNKs (MAPK8 and MAPK9) activation and induces apoptosis
N	Q69ZI1	CC	In fibroblasts, induces apoptosis.
N	Q69ZI1	CC	Within a signaling complex, it probably recruits protein kinases such as MAP3K10 or MAP3K11 which are in turn activated leading to the sequential activation of MAP2K4, MAP2K7 and JNKs (MAPK8 and MAPK9) (By similarity).
N	Q69ZI1	DE	Plenty of SH3s;
N	Q69ZI1	DE	Protein POSH;
N	Q69ZI1	DE	Putative E3 ubiquitin-protein ligase SH3RF1;
N	Q69ZI1	DE	SH3 domain-containing RING finger protein 1;
N	Q69ZI1	DE	SH3 multiple domains protein 2;
N	Q69ZI1	DR	MAP-kinase scaffold activity
N	Q69ZI1	DR	ligase activity
N	Q69ZI1	DR	regulation of JNK cascade

N	Q69ZI1	DR	zinc ion binding
N	Q69ZN7	CC	Calcium/phospholipid-binding protein that plays a role in the plasmalemma repair mechanism of endothelial cells that permits rapid resealing of membranes disrupted by mechanical stress
N	Q69ZN7	CC	Implicated in VEGF signal transduction by regulating the levels of the receptor KDR
N	Q69ZN7	CC	Involved in endocytic recycling
N	Q69ZN7	DE	Fer-1-like protein 3;
N	Q69ZN7	DR	cellular response to heat
N	Q69ZN7	DR	phospholipid binding
N	Q69ZN7	DR	plasma membrane repair
N	Q69ZN7	DR	protein binding
N	Q69ZN7	DR	regulation of vascular endothelial growth factor receptor signaling pathway
N	Q6A902	CC	Can catalyze the hydrolysis of ATP in the presence of single-stranded DNA, the ATP-dependent uptake of single-stranded DNA by duplex DNA, and the ATP-dependent hybridization of homologous single-stranded DNAs
N	Q6A902	CC	It interacts with LexA causing its activation and leading to its autocatalytic cleavage (By similarity).
N	Q6A902	DE	Protein RecA;
N	Q6A902	DE	Recombinase A;
N	Q6A902	DR	DNA recombination
N	Q6A902	DR	DNA-dependent ATPase activity
N	Q6A902	DR	single-stranded DNA binding
N	Q6A9C3	DE	L-lactate dehydrogenase;
N	Q6A9C3	DR	L-lactate dehydrogenase activity
N	Q6A9C3	DR	oxidation-reduction process
N	Q6AB49	CC	Ligates lysine onto the cytidine present at position 34 of the AUA codon-specific tRNA(Ile) that contains the anticodon CAU, in an ATP-dependent manner
N	Q6AB49	CC	Cytidine is converted to lysidine, thus changing the amino acid specificity of the tRNA from methionine to isoleucine (By similarity).
N	Q6AB49	DE	tRNA(Ile)-2-lysyl-cytidine synthase;
N	Q6AB49	DE	tRNA(Ile)-lysidine synthase;
N	Q6AB49	DE	tRNA(Ile)-lysidine synthetase;
N	Q6AB49	DR	ligase activity, forming carbon-nitrogen bonds
N	Q6AB49	DR	tRNA processing
N	Q6ADB9	DE	Homoserine O-acetyltransferase;
N	Q6ADB9	DE	Homoserine O-trans-acetylase;
N	Q6ADB9	DE	Homoserine transacetylase;
N	Q6ADB9	DR	homoserine O-acetyltransferase activity
N	Q6ADB9	DR	methionine biosynthetic process
N	Q6ADQ8	CC	The natural substrate for this enzyme may be peptidyl- tRNAs which drop off the ribosome during protein synthesis (By similarity).
N	Q6ADQ8	DE	Peptidyl-tRNA hydrolase;
N	Q6ADQ8	DR	aminoacyl-tRNA hydrolase activity
N	Q6AEE4	CC	Catalyzes the 2-thiolation of uridine at the wobble position (U34) of tRNA, leading to the formation of s(2)U34 (By similarity).
N	Q6AEE4	DE	tRNA-specific 2-thiouridylase mnmA;
N	Q6AEE4	DR	tRNA processing
N	Q6AEE4	DR	transferase activity
N	Q6AGI2	CC	Part of the Sec protein translocase complex
N	Q6AGI2	CC	Has a central role in coupling the hydrolysis of ATP to the transfer of proteins into and across the cell membrane, serving as an ATP-driven molecular motor driving the stepwise translocation of polypeptide chains across the membrane (By similarity).
N	Q6AGI2	CC	Interacts with the secYEG preprotein conducting channel
N	Q6AGI2	DE	Protein translocase subunit secA;
N	Q6AGI2	DR	protein import
N	Q6AGI2	DR	protein targeting
N	Q6AGI2	DR	transmembrane transport
N	Q6AHC2	CC	Catalyzes the thiamine diphosphate-dependent decarboxylation of 2-oxoglutarate and the subsequent addition of the resulting succinic semialdehyde-thiamine pyrophosphate anion to isochorismate to yield 2-succinyl-5-enolpyruvyl-6-hydroxy-3- cyclohexene-1-carboxylate (SEPHCHC) (By similarity).
N	Q6AHC2	DE	2-succinyl-5-enolpyruvyl-6-hydroxy-3-cyclohexene-1-carboxylate synthase;
N	Q6AHC2	DE	Menaquinone biosynthesis protein menD;
N	Q6AHC2	DE	SEPHCHC synthase;
N	Q6AHC2	DR	2-succinyl-5-enolpyruvyl-6-hydroxy-3-cyclohexene-1-carboxylic-acid synthase activity

N	Q6AHC2	DR	menaquinone biosynthetic process
N	Q6AHC2	DR	metal ion binding
N	Q6AHC2	DR	thiamine pyrophosphate binding
N	Q6APL9	DE	Uncharacterized transporter DP0976;
N	Q6APL9	DR	cation transmembrane transporter activity
N	Q6APL9	DR	potassium ion transport
N	Q6APZ3	CC	Transfers the N-acyl diglyceride group on what will become the N-terminal cysteine of membrane lipoproteins (By similarity).
N	Q6APZ3	DE	Prolipoprotein diacylglyceryl transferase;
N	Q6APZ3	DR	lipoprotein biosynthetic process
N	Q6APZ3	DR	protein lipoylation
N	Q6APZ3	DR	transferase activity, transferring glycosyl groups
N	Q6AT25	DE	Zinc finger CCCH domain-containing protein 34;
N	Q6AT25	DR	zinc ion binding
N	Q6AXU3	CC	3'-> 5'-exoribonuclease involved in ribosome biogenesis in the processing of the 12S pre-rRNA
N	Q6AXU3	CC	Displays a strong specificity for a 3'-end containing a free hydroxyl group (By similarity).
N	Q6AXU3	DE	Interferon-stimulated 20 kDa exonuclease-like 2;
N	Q6AXU3	DR	exonuclease activity
N	Q6AXU3	DR	nucleic acid binding
N	Q6AXU3	DR	ribosome biogenesis
N	Q6AYG7	CC	Plays a role in the inducible expression of cytokine genes in T-cells, especially by increasing NFAT-driven IL-4 production (By similarity).
N	Q6AYG7	DE	NFATC2-interacting protein;
N	Q6AYG7	DE	Nuclear factor of activated T-cells, cytoplasmic 2-interacting protein;
N	Q6AYH2	DE	Coiled-coil domain-containing protein 95;
N	Q6AYH2	DE	INO80 complex subunit E;
N	Q6AYH6	DE	UPF0510 protein INM02;
N	Q6B0Y1	DE	Putative uncharacterized protein YML009W-B;
N	Q6B2U8	DE	Uncharacterized protein YAL064C-A;
N	Q6BQR8	CC	DNA polymerase II participates in chromosomal DNA replication.
N	Q6BQR8	DE	DNA polymerase II subunit 2;
N	Q6BQR8	DE	DNA polymerase epsilon subunit B;
N	Q6BQR8	DR	DNA replication
N	Q6BQR8	DR	DNA-directed DNA polymerase activity
N	Q6BRA6	CC	Involved in cytoplasm to vacuole transport (Cvt) and pexophagy (By similarity).
N	Q6BRA6	DE	Autophagy-related protein 11;
N	Q6BRA6	DR	protein transport
N	Q6BUT3	CC	May act as a negative regulator of salt tolerance (By similarity).
N	Q6BUT3	DE	Stress response protein NST1;
N	Q6BUT3	DR	response to stress
N	Q6BX45	CC	PPIases accelerate the folding of proteins
N	Q6BX45	CC	It catalyzes the cis-trans isomerization of proline imidic peptide bonds in oligopeptides (By similarity).
N	Q6BX45	DE	FK506-binding protein 1;
N	Q6BX45	DE	Peptidyl-prolyl cis-trans isomerase;
N	Q6BX45	DE	Rapamycin-binding protein;
N	Q6BX45	DR	peptidyl-prolyl cis-trans isomerase activity
N	Q6BX45	DR	protein folding
N	Q6BXC7	CC	Bifunctional enzyme that catalyzes the enolization of 2,3-diketo-5-methylthiopentyl-1-phosphate (DK-MTP-1-P) into the intermediate 2-hydroxy-3-keto-5-methylthiopentenyl-1-phosphate (HK-MTPenyl-1-P), which is then dephosphorylated to form the acireductone 1,2-dihydroxy-3-keto-5-methylthiopentene (DHK-MTPene) (By similarity).
N	Q6BXC7	DE	2,3-diketo-5-methylthio-1-phosphopentane phosphatase;
N	Q6BXC7	DE	Enolase-phosphatase E1;
N	Q6BXC7	DR	L-methionine salvage from methylthioadenosine
N	Q6BXC7	DR	acireductone synthase activity
N	Q6BXC7	DR	metal ion binding
N	Q6C6Y4	CC	F-actin-capping proteins bind in a Ca(2+)-independent manner to the fast growing ends of actin filaments (barbed end) thereby blocking the exchange of subunits at these ends.
N	Q6C6Y4	CC	Unlike other capping proteins (such as gelsolin and severin), these proteins do not sever actin filaments (By similarity).
N	Q6C6Y4	DE	F-actin-capping protein subunit alpha;
N	Q6C6Y4	DR	actin binding
N	Q6C6Y4	DR	actin cytoskeleton organization

N	Q6C6Y4	DR	actin filament capping
N	Q6CCU3	CC	Involved in cell wall synthesis where it is required for glycosylation
N	Q6CCU3	CC	Involved in cell cycle progression through cell- size checkpoint (By similarity).
N	Q6CCU3	DE	GDP-mannose pyrophosphorylase;
N	Q6CCU3	DE	GTP-mannose-1-phosphate guanylyltransferase;
N	Q6CCU3	DE	Mannose-1-phosphate guanyltransferase;
N	Q6CCU3	DR	biosynthetic process
N	Q6CCU3	DR	mannose-1-phosphate guanylyltransferase activity
N	Q6CCU8	CC	Required for multiple vacuole delivery pathways including the cytoplasm to vacuole transport (Cvt), autophagy, pexophagy and endocytosis (By similarity).
N	Q6CCU8	DE	Vacuolar fusion protein MON1;
N	Q6CCU8	DR	protein transport
N	Q6CFD2	CC	Catalyzes the first intracellular reaction of sulfate assimilation, forming adenosine-5'-phosphosulfate (APS) from inorganic sulfate and ATP
N	Q6CFD2	CC	Plays an important role in sulfate activation as a component of the biosynthesis pathway of sulfur- containing amino acids (By similarity).
N	Q6CFD2	DE	ATP-sulfurylase;
N	Q6CFD2	DE	Sulfate adenylate transferase;
N	Q6CFD2	DE	Sulfate adenylyltransferase;
N	Q6CFD2	DR	cysteine biosynthetic process
N	Q6CFD2	DR	kinase activity
N	Q6CFD2	DR	methionine biosynthetic process
N	Q6CFD2	DR	sulfate adenylyltransferase (ATP) activity
N	Q6CFD2	DR	sulfate assimilation
N	Q6CFR3	CC	Component of the cytosolic Fe/S protein assembly machinery
N	Q6CFR3	CC	May play a role in the transfer of pre-assembled Fe/S clusters to target apoproteins (By similarity).
N	Q6CFR3	CC	Required for maturation of extramitochondrial Fe/S proteins
N	Q6CFR3	DE	Cytosolic Fe-S cluster assembly factor NAR1;
N	Q6CFR3	DE	Nuclear architecture-related protein 1;
N	Q6CFR3	DR	4 iron, 4 sulfur cluster binding
N	Q6CFR3	DR	iron-sulfur cluster assembly
N	Q6CFR3	DR	metal ion binding
N	Q6CHP5	DE	Guanine nucleotide-binding protein subunit gamma;
N	Q6CHP5	DR	G-protein coupled receptor protein signaling pathway
N	Q6CHP5	DR	signal transducer activity
N	Q6CKY3	CC	Required for the proteolytic cleavage of the transcription factor RIM101 in response to alkaline ambient pH (By similarity).
N	Q6CKY3	DE	Calpain-like protease palB/RIM13;
N	Q6CKY3	DE	Cysteine protease RIM13;
N	Q6CKY3	DR	calcium-dependent cysteine-type endopeptidase activity
N	Q6CND0	CC	Involved in deacetylation of histones, chromatin assembly and chromosome [illegible]
N	Q6CND0	CC	Component of the NuA4 histone acetyltransferase complex which is involved in transcriptional activation of selected genes principally by acetylation of nucleosomal histone H4 and H2A
N	Q6CND0	CC	May act as a transcriptional oscillator, directing histone deacetylases to specific chromosomal domains
N	Q6CND0	CC	The NuA4 complex is also involved in DNA repair (By similarity).
N	Q6CND0	DE	Chromatin modification-related protein EAF3;
N	Q6CND0	DR	chromatin assembly or disassembly
N	Q6CND0	DR	chromatin binding
N	Q6CND0	DR	chromatin modification
N	Q6CQL7	DE	1-(5-phosphoribosyl)-5-[(5-phosphoribosylamino)methylideneamino] imidazole-4-carboxamide isomerase;
N	Q6CQL7	DE	5-proFAR isomerase;
N	Q6CQL7	DE	Phosphoribosylformimino-5-aminoimidazole carboxamide ribotide isomerase;
N	Q6CQL7	DR	1-(5-phosphoribosyl)-5-[(5-phosphoribosylamino)methylideneamino]imidazole-4-carboxamide isomerase activity
N	Q6CQL7	DR	histidine biosynthetic process
N	Q6CSP9	CC	GTPase that associates with pre-60S ribosomal subunits in the nucleolus and is required for their nuclear export and maturation (By similarity).
N	Q6CSP9	DE	Nucleolar GTP-binding protein 2;
N	Q6CSP9	DR	ribosome biogenesis
N	Q6CU61	CC	Involved in the partitioning of the mitochondrial organelle and mitochondrial DNA (mtDNA) inheritance (By similarity).

N	Q6CU61	DE	Protein DML1;
N	Q6CU61	DR	protein polymerization
N	Q6CVX2	DE	Altered inheritance of mitochondria protein 39, mitochondrial;
N	Q6CW75	CC	Adds a GMP to the 5'-end of tRNA(His) after transcription and RNase P cleavage (By similarity).
N	Q6CW75	DE	tRNA(His) guanylyltransferase;
N	Q6CW75	DE	tRNA-histidine guanylyltransferase;
N	Q6CW75	DR	tRNA guanylyltransferase activity
N	Q6CW75	DR	tRNA modification
N	Q6CWW7	CC	Constituent of COPII-coated endoplasmic reticulum- derived transport vesicles
N	Q6CWW7	CC	Facilitates retrograde transport from the Golgi to the endoplasmic reticulum (By similarity).
N	Q6CWW7	CC	Required for efficient transport of a subset of secretory proteins to the Golgi
N	Q6CWW7	DE	Endoplasmic reticulum vesicle protein 25;
N	Q6CWW7	DR	protein transport
N	Q6CWW7	DR	vesicle-mediated transport
N	Q6CXB9	CC	Component of the EKC/KEOPS complex which promotes both telomere uncapping and telomere elongation (By similarity)
N	Q6CXB9	CC	Important for bud site selection (By similarity).
N	Q6CXB9	CC	The complex is required for efficient recruitment of transcriptional coactivators
N	Q6CXB9	DE	Serine/threonine-protein kinase BUD32;
N	Q6CXB9	DR	lipopolysaccharide biosynthetic process
N	Q6CXB9	DR	protein phosphorylation
N	Q6CXB9	DR	protein serine/threonine kinase activity
N	Q6CXB9	DR	protein tyrosine kinase activity
N	Q6CZW6	CC	This protein promotes the GTP-dependent binding of aminoacyl-tRNA to the A-site of ribosomes during protein biosynthesis (By similarity).
N	Q6CZW6	DE	Elongation factor Tu;
N	Q6CZW6	DR	GTPase activity
N	Q6CZW6	DR	translation elongation factor activity
N	Q6D035	DE	Phosphatidylserine decarboxylase alpha chain;
N	Q6D035	DE	Phosphatidylserine decarboxylase beta chain;
N	Q6D035	DE	Phosphatidylserine decarboxylase proenzyme;
N	Q6D035	DR	phosphatidylserine decarboxylase activity
N	Q6D035	DR	phospholipid biosynthetic process
N	Q6D0E0	CC	Specifically dimethylates two adjacent adenosines (A1518 and A1519) in the loop of a conserved hairpin near the 3'-end of 16S rRNA in the 30S particle
N	Q6D0E0	CC	May play a critical role in biogenesis of 30S subunits (By similarity).
N	Q6D0E0	DE	16S rRNA (adenine(1518)-N(6)/adenine(1519)-N(6))-dimethyltransferase;
N	Q6D0E0	DE	16S rRNA dimethyladenosine transferase;
N	Q6D0E0	DE	16S rRNA dimethylase;
N	Q6D0E0	DE	Ribosomal RNA small subunit methyltransferase A;
N	Q6D0E0	DE	S-adenosylmethionine-6-N', N'-adenosyl(rRNA) dimethyltransferase;
N	Q6D0E0	DR	rRNA (adenine-N6,N6-)-dimethyltransferase activity
N	Q6D0G7	CC	Catalyzes the oxidation of 3-carboxy-2-hydroxy-4- methylpentanoate (3-isopropylmalate) to 3-carboxy-4-methyl-2- oxopentanoate
N	Q6D0G7	CC	The product decarboxylates to 4-methyl-2 oxopentanoate.
N	Q6D0G7	DE	3-isopropylmalate dehydrogenase;
N	Q6D0G7	DE	Beta-IPM dehydrogenase;
N	Q6D0G7	DR	3-isopropylmalate dehydrogenase activity
N	Q6D0G7	DR	leucine biosynthetic process
N	Q6D0G7	DR	magnesium ion binding
N	Q6D0G7	DR	oxidation-reduction process
N	Q6D1A3	CC	Reduction of activated sulfate into sulfite.
N	Q6D1A3	DE	3'-phosphoadenylylsulfate reductase;
N	Q6D1A3	DE	PAPS reductase, thioredoxin dependent;
N	Q6D1A3	DE	PAPS sulfotransferase;
N	Q6D1A3	DE	PAdoPS reductase;
N	Q6D1A3	DE	Phosphoadenosine phosphosulfate reductase;
N	Q6D1A3	DR	cysteine biosynthetic process
N	Q6D1A3	DR	phosphoadenylyl-sulfate reductase (thioredoxin) activity
N	Q6D1A3	DR	sulfate assimilation, phosphoadenylyl sulfate reduction by phosphoadenylyl-sulfate reductase (thioredoxin)
N	Q6D808	DE	50S ribosomal protein L36 2;
N	Q6D808	DR	structural constituent of ribosome

N	Q6D8C9	CC	Endonuclease that specifically degrades the RNA of RNA- DNA hybrids (By similarity).
N	Q6D8C9	DE	Ribonuclease HII;
N	Q6D8C9	DR	metal ion binding
N	Q6D8C9	DR	ribonuclease H activity
N	Q6D9C5	DE	50S ribosomal protein L27;
N	Q6D9C5	DR	structural constituent of ribosome
N	Q6DCX2	CC	Required for RNA-mediated gene silencing (RNAi) by the RNA-induced silencing complex (RISC)
N	Q6DCX2	CC	Binding of RISC to a partially complementary mRNA results in silencing through inhibition of translation, and this is independent of endonuclease activity
N	Q6DCX2	CC	Binding of RISC to a perfectly complementary mRNA generally results in silencing due to endonucleolytic cleavage of the mRNA specifically by eif2c2/ago2
N	Q6DCX2	CC	The 'minimal RISC' appears to include eif2c2/ago2 bound to a short guide RNA such as a microRNA (miRNA) or short interfering RNA (siRNA)
N	Q6DCX2	CC	The inhibition of translational initiation leads to the accumulation of the affected mRNA in cytoplasmic processing bodies (P-bodies), where mRNA degradation may subsequently occur (By similarity).
N	Q6DCX2	CC	The precise mechanism of gene silencing depends on the degree of complementarity between the miRNA or siRNA and its target
N	Q6DCX2	CC	These guide RNAs direct RISC to complementary mRNAs that are targets for RISC- mediated gene silencing
N	Q6DCX2	DE	Argonaute2;
N	Q6DCX2	DE	Eukaryotic translation initiation factor 2C 2;
N	Q6DCX2	DE	Protein argonaute-2;
N	Q6DCX2	DE	Protein slicer;
N	Q6DCX2	DR	RNA 7-methylguanosine cap binding
N	Q6DCX2	DR	endoribonuclease activity, cleaving siRNA-paired mRNA
N	Q6DCX2	DR	mRNA cleavage involved in gene silencing by miRNA
N	Q6DCX2	DR	metal ion binding
N	Q6DCX2	DR	negative regulation of translation involved in gene silencing by miRNA
N	Q6DCX2	DR	negative regulation of translational initiation
N	Q6DCX2	DR	pre-microRNA processing
N	Q6DCX2	DR	siRNA binding
N	Q6DD06	CC	Protects drg1 from proteolytic degradation (By similarity).
N	Q6DD06	DE	DRG family-regulatory protein 1;
N	Q6DD06	DE	Zinc finger CCCH domain-containing protein 15;
N	Q6DD06	DR	nucleic acid binding
N	Q6DD06	DR	zinc ion binding
N	Q6DER2	DE	60S ribosomal protein L36;
N	Q6DER2	DR	structural constituent of ribosome
N	Q6DIP9	DE	WASH complex subunit strumpellin;
N	Q6DPE5	CC	Encapsidates the negative strand viral RNA, protecting it from nucleases
N	Q6DPE5	CC	Dissociation of M1 from RNP unmask nucleoprotein's nuclear localization signals, targeting the RNP to the nucleus (By similarity).
N	Q6DPE5	CC	It is possible that the nucleoprotein binds directly exportin-1 (XPO1) and plays an active role in RNP nuclear export
N	Q6DPE5	CC	Later in the infection, nucleus export of RNP are mediated through viral proteins NEP interacting with M1 which binds nucleoproteins
N	Q6DPE5	CC	M1 interaction with RNP seems to hide nucleoprotein's nuclear localization signals
N	Q6DPE5	CC	NP comprises at least 2 nuclear localization signals and is responsible of the active RNP import into the nucleus through the cellular importin alpha/beta pathway
N	Q6DPE5	CC	Soon after a virion infects a new cell, M1 dissociates from the RNP under acidification of the virion driven by M2 protein
N	Q6DPE5	CC	The RNP needs to be localized in the nucleus to start an infectious cycle, but is too large to diffuse through the nuclear pore complex
N	Q6DPE5	CC	The encapsidated genomic RNA is termed the ribonucleoprotein (RNP) and serves as template for transcription and replication
N	Q6DPE5	DE	Nucleoprotein;
N	Q6DPE5	DR	interspecies interaction between organisms
N	Q6DPE5	DR	structural molecule activity
N	Q6DPT9	CC	Forms a proton-selective ion channel that is necessary for the efficient release of the viral genome during virus entry
N	Q6DPT9	CC	Acidification of the endosome triggers M2 ion channel activity
N	Q6DPT9	CC	After attaching to the cell surface, the virion enters the cell by endocytosis

N	Q6DPT9	CC	Also plays a role in viral proteins secretory pathway
N	Q6DPT9	CC	Elevates the intravesicular pH of normally acidic compartments, such as trans-Golgi network, preventing newly formed hemagglutinin from premature switching to the fusion-active conformation (By similarity).
N	Q6DPT9	CC	The influx of protons into virion interior is believed to disrupt interactions between the viral ribonucleoprotein (RNP), matrix protein 1 (M1), and lipid bilayers, thereby freeing the viral genome from interaction with viral proteins and enabling RNA segments to migrate to the host cell nucleus, where influenza virus RNA transcription and replication occur
N	Q6DPT9	DE	Matrix protein 2;
N	Q6DPT9	DE	Proton channel protein M2;
N	Q6DPT9	DR	hydrogen ion transmembrane transporter activity
N	Q6DPT9	DR	interspecies interaction between organisms
N	Q6DPT9	DR	ion channel activity
N	Q6DT45	CC	Hydrolyzes semenogelin-1 thus leading to the liquefaction of the seminal coagulum (By similarity).
N	Q6DT45	DE	Kallikrein-3;
N	Q6DT45	DE	Prostate-specific antigen;
N	Q6DT45	DE	Semenogelase;
N	Q6DT45	DR	serine-type endopeptidase activity
N	Q6EIJ3	CC	Usually encoded in the trnK tRNA gene intron
N	Q6EIJ3	CC	Probably assists in splicing its own and other chloroplast group II introns (By similarity)
N	Q6EIJ3	DE	Intron maturase;
N	Q6EIJ3	DR	mRNA processing
N	Q6EIJ3	DR	tRNA processing
N	Q6ENP4	CC	NDH shuttles electrons from NAD(P)H:plastoquinone, via FMN and iron-sulfur (Fe-S) centers, to quinones in the photosynthetic chain and possibly in a chloroplast respiratory chain
N	Q6ENP4	CC	Couples the redox reaction to proton translocation, and thus conserves the redox energy in a proton gradient (By similarity).
N	Q6ENP4	CC	The immediate electron acceptor for the enzyme in this species is believed to be plastoquinone
N	Q6ENP4	DE	NAD(P)H dehydrogenase subunit 6;
N	Q6ENP4	DE	NAD(P)H-quinone oxidoreductase subunit 6, chloroplastic;
N	Q6ENP4	DE	NADH-plastoquinone oxidoreductase subunit 6;
N	Q6ENP4	DR	NADH dehydrogenase (ubiquinone) activity
N	Q6ENP4	DR	oxidation-reduction process
N	Q6ENP4	DR	quinone binding
N	Q6EV77	CC	Catalyzes the addition of fucose in alpha 1-6 linkage to the first GlcNAc residue, next to the peptide chains in N-glycans (By similarity).
N	Q6EV77	DE	Alpha-(1,6)-fucosyltransferase;
N	Q6EV77	DE	Fucosyltransferase 8;
N	Q6EV77	DE	GDP-L-Fuc:N-acetyl-beta-D-glucosaminide alpha1,6-fucosyltransferase;
N	Q6EV77	DE	GDP-fucose--glycoprotein fucosyltransferase;
N	Q6EV77	DE	Glycoprotein 6-alpha-L-fucosyltransferase;
N	Q6EV77	DE	alpha1-6FucT;
N	Q6EV77	DR	SH3 domain binding
N	Q6EV77	DR	glycoprotein 6-alpha-L-fucosyltransferase activity
N	Q6EV77	DR	protein glycosylation in Golgi
N	Q6EW16	CC	One of the primary rRNA binding proteins, it binds directly to 16S rRNA central domain where it helps coordinate assembly of the platform of the 30S subunit (By similarity)
N	Q6EW16	DE	30S ribosomal protein S8, chloroplastic;
N	Q6EW16	DR	structural constituent of ribosome
N	Q6EWG8	CC	The movement protein is assembled into tubules that allow the transport of virions from cell to cell (By similarity).
N	Q6EWG8	DE	Capsid protein VP20;
N	Q6EWG8	DE	Capsid protein VP24;
N	Q6EWG8	DE	Capsid protein VP25;
N	Q6EWG8	DE	Movement protein;
N	Q6EWG8	DE	RNA2 polyprotein;
N	Q6EWG8	DR	spread of virus in host, cell to cell
N	Q6EYJ5	CC	Seems to play a role in the dimerization of PSII (By similarity).
N	Q6EYJ5	DE	Photosystem II reaction center protein T;
N	Q6EYJ5	DR	photosynthesis
N	Q6F147	CC	Negative regulator of class I heat shock genes (grpE- dnaK-dnaJ and groELS operons)

N	Q6F147	CC	Prevents heat-shock induction of these operons (By similarity).
N	Q6F147	DE	Heat-inducible transcription repressor hrcA;
N	Q6F147	DR	regulation of transcription, DNA-dependent
N	Q6F147	DR	response to stress
N	Q6F705	CC	Cell wall formation (By similarity).
N	Q6F705	DE	D-Ala-D-Ala ligase;
N	Q6F705	DE	D-alanine--D-alanine ligase;
N	Q6F705	DE	D-alanylalanine synthetase;
N	Q6F705	DR	D-alanine-D-alanine ligase activity
N	Q6F705	DR	cellular cell wall organization
N	Q6F705	DR	metal ion binding
N	Q6F705	DR	peptidoglycan biosynthetic process
N	Q6F705	DR	regulation of cell shape
N	Q6FCI6	DE	Phosphoriboisomerase A;
N	Q6FCI6	DE	Ribose-5-phosphate isomerase A;
N	Q6FCI6	DR	pentose-phosphate shunt, non-oxidative branch
N	Q6FCI6	DR	ribose-5-phosphate isomerase activity
N	Q6FFH9	CC	Required for the formation of a threonylcarbamoyl group on adenosine at position 37 (t(6)A37) in tRNAs that read codons beginning with adenine (By similarity).
N	Q6FFH9	DE	t(6)A37 threonylcarbamoyladenosine biosynthesis protein RimN;
N	Q6FFH9	DE	tRNA threonylcarbamoyladenosine biosynthesis protein RimN;
N	Q6FFH9	DR	tRNA processing
N	Q6FUJ7	CC	Involved in pre-mRNA splicing and cell cycle progression (By similarity).
N	Q6FUJ7	DE	Pre-mRNA-splicing factor SYF1;
N	Q6FUJ7	DR	mRNA processing
N	Q6FX63	CC	Required for proper assembly of pre-ribosomal particles during the biogenesis of the 60S ribosomal subunit (By similarity).
N	Q6FX63	DE	2'-O-ribose RNA methyltransferase;
N	Q6FX63	DE	AdoMet-dependent rRNA methyltransferase SPB1;
N	Q6FX63	DE	S-adenosyl-L-methionine-dependent methyltransferase;
N	Q6FX63	DR	methyltransferase activity
N	Q6FX63	DR	nucleic acid binding
N	Q6FX63	DR	rRNA methylation
N	Q6FZ21	DE	UPF0102 protein BQ09720;
N	Q6FZ21	DR	nuclease activity
N	Q6FZD8	CC	This is one of the proteins that binds and probably mediates the attachment of the 5S RNA into the large ribosomal subunit, where it forms part of the central protuberance (By similarity).
N	Q6FZD8	DE	50S ribosomal protein L18;
N	Q6FZD8	DR	structural constituent of ribosome
N	Q6FZK7	CC	Allows the formation of correctly charged Asn-tRNA(Asn) or Gln-tRNA(Gln) through the transamidation of misacylated Asp- tRNA(Asn) or Glu-tRNA(Gln) in organisms which lack either or both of asparaginyl-tRNA or glutaminyl-tRNA synthetases
N	Q6FZK7	CC	The reaction takes place in the presence of glutamine and ATP through an activated phospho-Asp-tRNA(Asn) or phospho-Glu-tRNA(Gln) (By similarity).
N	Q6FZK7	DE	Asp/Glu-ADT subunit B;
N	Q6FZK7	DE	Aspartyl/glutamyl-tRNA(Asn/Gln) amidotransferase subunit B;
N	Q6FZK7	DR	carbon-nitrogen ligase activity, with glutamine as amido-N-donor
N	Q6FZU8	CC	Phosphorylation of dTMP to form dTDP in both de novo and salvage pathways of dTTP synthesis (By similarity).
N	Q6FZU8	DE	Thymidylate kinase;
N	Q6FZU8	DE	dTMP kinase;
N	Q6FZU8	DR	dTDP biosynthetic process
N	Q6FZU8	DR	thymidylate kinase activity
N	Q6G0F8	CC	This is one of the proteins that binds to the 5S RNA in the ribosome where it forms part of the central protuberance (By similarity).
N	Q6G0F8	DE	50S ribosomal protein L25;
N	Q6G0F8	DE	General stress protein CTC;
N	Q6G0F8	DR	5S rRNA binding
N	Q6G0F8	DR	structural constituent of ribosome
N	Q6G5R1	CC	The ruvA-ruvB complex in the presence of ATP renatures cruciform structure in supercoiled DNA with palindromic sequence, indicating that it may promote strand exchange reactions in homologous recombination
N	Q6G5R1	CC	RuvAB is an helicase that mediates the Holliday junction migration by localized denaturation and reannealing (By similarity).

N	Q6G5R1	DE	Holliday junction ATP-dependent DNA helicase ruvB;
N	Q6G5R1	DR	DNA recombination
N	Q6G5R1	DR	four-way junction helicase activity
N	Q6G5Z1	CC	Probably part of an ABC transporter complex
N	Q6G5Z1	CC	Responsible for energy coupling to the transport system (By similarity).
N	Q6G5Z1	DE	Putative ABC transporter ATP-binding protein SAS2569;
N	Q6G5Z1	DR	ATPase activity
N	Q6G5Z1	DR	hydrolase activity, acting on acid anhydrides, catalyzing transmembrane movement of substances
N	Q6G625	CC	Part of the Sec protein translocase complex
N	Q6G625	CC	Has a central role in coupling the hydrolysis of ATP to the transfer of proteins into and across the cell membrane, serving as an ATP-driven molecular motor driving the stepwise translocation of polypeptide chains across the membrane (By similarity)
N	Q6G625	CC	Interacts with the secYEG preprotein conducting channel
N	Q6G625	DE	Protein translocase subunit secA 2;
N	Q6G625	DR	P-P-bond-hydrolysis-driven protein transmembrane transporter activity
N	Q6G625	DR	protein import
N	Q6G625	DR	protein targeting
N	Q6G773	CC	One of the early assembly proteins it binds 23S rRNA
N	Q6G773	CC	Forms the main docking site for trigger factor binding to the ribosome (By similarity)
N	Q6G773	CC	One of the proteins that surrounds the polypeptide exit tunnel on the outside of the ribosome
N	Q6G773	DE	50S ribosomal protein L23;
N	Q6G773	DR	nucleotide binding
N	Q6G773	DR	structural constituent of ribosome
N	Q6G7J2	CC	Peptide chain release factor 1 directs the termination of translation in response to the peptide chain termination codons UAG and UAA (By similarity).
N	Q6G7J2	DE	Peptide chain release factor 1;
N	Q6G7J2	DR	translation release factor activity, codon specific
N	Q6G8P0	CC	Negatively regulates transcription of bacterial ribonucleotide reductase nrd genes and operons by binding to NrdR- boxes (By similarity).
N	Q6G8P0	DE	Transcriptional repressor NrdR;
N	Q6G8P0	DR	transcription repressor activity
N	Q6G8P0	DR	zinc ion binding
N	Q6GB63	CC	May be required for sporulation (By similarity).
N	Q6GB63	DE	Putative sporulation transcription regulator whiA;
N	Q6GBK3	DE	Mrp complex subunit E2;
N	Q6GBK3	DE	Putative NADH-ubiquinone oxidoreductase subunit mnhE2;
N	Q6GBK3	DE	Putative antiporter subunit mnhE2;
N	Q6GBK3	DR	antiporter activity
N	Q6GBK3	DR	cation transmembrane transporter activity
N	Q6GBZ0	CC	Could be involved in septation (By similarity).
N	Q6GBZ0	DE	Putative septation protein spoVG;
N	Q6GBZ0	DR	barrier septum formation
N	Q6GBZ0	DR	sporulation resulting in formation of a cellular spore
N	Q6GDK4	CC	Catalyzes the reversible reaction in which hydroxymethyl group from 5,10-methylenetetrahydrofolate is tranferred onto alpha-ketoisovalerate to form ketopantoate (By similarity).
N	Q6GDK4	DE	3-methyl-2-oxobutanoate hydroxymethyltransferase;
N	Q6GDK4	DE	Ketopantoate hydroxymethyltransferase;
N	Q6GDK4	DR	3-methyl-2-oxobutanoate hydroxymethyltransferase activity
N	Q6GDK4	DR	metal ion binding
N	Q6GDK4	DR	methyltransferase activity
N	Q6GDK4	DR	pantothenate biosynthetic process
N	Q6GEN5	DE	Galactose-6-phosphate isomerase subunit lacB;
N	Q6GEN5	DR	galactose-6-phosphate isomerase activity
N	Q6GEN5	DR	lactose catabolic process
N	Q6GEW8	CC	F(1)F(0) ATP synthase produces ATP from ADP in the presence of a proton or sodium gradient
N	Q6GEW8	CC	During catalysis, ATP synthesis in the catalytic domain of F(1) is coupled via a rotary mechanism of the central stalk subunits to proton translocation (By similarity)
N	Q6GEW8	CC	F-type ATPases consist of two structural domains, F(1) containing the extramembraneous catalytic core and F(0) containing the membrane proton channel, linked together by a central stalk and a peripheral stalk
N	Q6GEW8	DE	ATP synthase F(0) sector subunit b;

N	Q6GEW8	DE	ATP synthase subunit b;
N	Q6GEW8	DE	ATPase subunit I;
N	Q6GEW8	DE	F-ATPase subunit b;
N	Q6GEW8	DE	F-type ATPase subunit b;
N	Q6GEW8	DR	ATP synthesis coupled proton transport
N	Q6GEW8	DR	hydrogen ion transmembrane transporter activity
N	Q6GFH7	DE	UPF0435 protein SAR1970;
N	Q6GFX9	CC	Catalyzes the attachment of tyrosine to tRNA(Tyr) in a two-step reaction: tyrosine is first activated by ATP to form Tyr- AMP and then transferred to the acceptor end of tRNA(Tyr) (By similarity).
N	Q6GFX9	DE	Tyrosine--tRNA ligase;
N	Q6GFX9	DE	Tyrosyl-tRNA synthetase;
N	Q6GFX9	DR	tyrosine-tRNA ligase activity
N	Q6GFX9	DR	tyrosyl-tRNA aminoacylation
N	Q6GG60	CC	An essential GTPase which binds GTP, GDP and possibly (p)ppGpp with moderate affinity, with high nucleotide exchange rates and a fairly low GTP hydrolysis rate (By similarity)
N	Q6GG60	CC	It may play a role in control of the cell cycle, stress response, ribosome biogenesis and in those bacteria that undergo differentiation, in morphogenesis control (Potential)
N	Q6GG60	DE	GTP-binding protein obg;
N	Q6GG60	DR	GTPase activity
N	Q6GG60	DR	magnesium ion binding
N	Q6GHJ0	DE	Succinyl-CoA ligase [ADP-forming] subunit beta;
N	Q6GHJ0	DE	Succinyl-CoA synthetase subunit beta;
N	Q6GHJ0	DR	metal ion binding
N	Q6GHJ0	DR	succinate-CoA ligase (ADP-forming) activity
N	Q6GHJ0	DR	tricarboxylic acid cycle
N	Q6GHN0	CC	Catalyzes the transfer of a ribosyl phosphate group from 5-phosphoribose 1-diphosphate to orotate, leading to the formation of orotidine monophosphate (OMP) (By similarity)
N	Q6GHN0	DE	Orotate phosphoribosyltransferase;
N	Q6GHN0	DR	nucleoside metabolic process
N	Q6GHN0	DR	orotate phosphoribosyltransferase activity
N	Q6GHN0	DR	pyrimidine nucleotide biosynthetic process
N	Q6GI10	DE	Glycinamide ribonucleotide synthetase;
N	Q6GI10	DE	Phosphoribosylamine--glycine ligase;
N	Q6GI10	DE	Phosphoribosylglycinamide synthetase;
N	Q6GI10	DR	metal ion binding
N	Q6GI10	DR	phosphoribosylamine-glycine ligase activity
N	Q6GI10	DR	purine base biosynthetic process
N	Q6GI10	DR	purine nucleotide biosynthetic process
N	Q6GJI5	CC	Involved in initiation control of chromosome replication (By similarity).
N	Q6GJI5	DE	Initiation-control protein yabA;
N	Q6GJI5	DR	DNA replication
N	Q6GK90	DE	Formate acetyltransferase;
N	Q6GK90	DE	Pyruvate formate-lyase;
N	Q6GK90	DR	formate C-acetyltransferase activity
N	Q6GK90	DR	glucose metabolic process
N	Q6GKC4	DE	Acetylglutamate kinase;
N	Q6GKC4	DE	N-acetyl-L-glutamate 5-phosphotransferase;
N	Q6GKC4	DR	acetylglutamate kinase activity
N	Q6GKC4	DR	arginine biosynthetic process
N	Q6GKH9	CC	Catalyzes the irreversible reduction of 2,3-butanediol to (S)-acetoin in the presence of NADH (By similarity).
N	Q6GKH9	DE	Acetoin(diacetyl) reductase;
N	Q6GKH9	DE	Diacetyl reductase [(S)-acetoin forming];
N	Q6GKH9	DE	Meso-2,3-butanediol dehydrogenase;
N	Q6GKH9	DR	acetoin catabolic process
N	Q6GKH9	DR	diacetyl reductase ((S)-acetoin forming) activity
N	Q6GKH9	DR	oxidation-reduction process
N	Q6GKS1	CC	Specifically methylates the pseudouridine at position 1915 (m3Psi1915) in 23S rRNA (By similarity).
N	Q6GKS1	DE	23S rRNA m3Psi1915 methyltransferase;
N	Q6GKS1	DE	Ribosomal RNA large subunit methyltransferase H;
N	Q6GKS1	DE	rRNA (pseudouridine-N3-)-methyltransferase rlmH;
N	Q6GKS1	DR	methyltransferase activity

N	Q6GKS1	DR	rRNA methylation
N	Q6GL44	CC	May play a role as regulator of the innate immune system
N	Q6GL44	CC	Has deubiquitinating activity that is directed towards 'Lys-63'-linked polyubiquitin chains (By similarity).
N	Q6GL44	DE	Deubiquitinating enzyme A;
N	Q6GL44	DE	OTU domain-containing protein 5;
N	Q6GL44	DR	cysteine-type peptidase activity
N	Q6GMF8	CC	Not expected to have protease activity (By similarity).
N	Q6GMF8	DE	Rhomboid family member 1;
N	Q6GMF8	DR	serine-type endopeptidase activity
N	Q6GMG8	CC	Probable role in regulating transcription of specific genes (By similarity).
N	Q6GMG8	DE	LPS-induced TNF-alpha factor homolog;
N	Q6GMG8	DE	Lipopolysaccharide-induced tumor necrosis factor-alpha factor homolog;
N	Q6GPP1	CC	May be involved in telomere length regulation Promotes assembly, stabilizes and maintains the activity of TORC complexes, which regulate cell growth and survival in response to nutrient and hormonal signals (By similarity).
N	Q6GPP1	DE	Telomere length regulation protein TEL2 homolog;
N	Q6GUR1	CC	Cytochromes P450 are a group of heme-thiolate monooxygenases
N	Q6GUR1	CC	In liver microsomes, this enzyme is involved in an NADPH-dependent electron transport pathway
N	Q6GUR1	CC	It oxidizes a variety of structurally unrelated compounds, including steroids, fatty acids, and xenobiotics.
N	Q6GUR1	DE	Cytochrome P450 1A1;
N	Q6GUR1	DR	aromatase activity
N	Q6GUR1	DR	electron carrier activity
N	Q6GUR1	DR	oxidation-reduction process
N	Q6GV10	CC	Catalyzes the formation of S-adenosylmethionine from methionine and ATP
N	Q6GV10	CC	The overall synthetic reaction is composed of two sequential steps, AdoMet formation and the subsequent tripolyphosphate hydrolysis which occurs prior to release of AdoMet from the enzyme (By similarity).
N	Q6GV10	DE	AdoMet synthase;
N	Q6GV10	DE	Methionine adenosyltransferase;
N	Q6GV10	DE	S-adenosylmethionine synthase;
N	Q6GV10	DR	metal ion binding
N	Q6GV10	DR	methionine adenosyltransferase activity
N	Q6GV10	DR	one-carbon metabolic process
N	Q6HAW8	DE	UPF0340 protein BT9727_4999;
N	Q6HBF3	CC	Catalyzes the reversible conversion of 2- phosphoglycerate into phosphoenolpyruvate
N	Q6HBF3	CC	It is essential for the degradation of carbohydrates via glycolysis (By similarity).
N	Q6HBF3	DE	2-phospho-D-glycerate hydro-lyase;
N	Q6HBF3	DE	2-phosphoglycerate dehydratase;
N	Q6HBF3	DR	magnesium ion binding
N	Q6HBF3	DR	phosphopyruvate hydratase activity
N	Q6HER5	CC	Cell division protein that is part of the divisome complex and is recruited early to the Z-ring
N	Q6HER5	CC	Its function overlaps with ftsA (By similarity).
N	Q6HER5	CC	Probably stimulates Z-ring formation, perhaps through the cross-linking of ftsZ protofilaments
N	Q6HER5	DE	Cell division protein sepF;
N	Q6HER5	DR	barrier septum formation
N	Q6IF99	CC	Odorant receptor (Potential).
N	Q6IF99	DE	Olfactory receptor 10K2;
N	Q6IF99	DE	Olfactory receptor OR1-4;
N	Q6IF99	DR	olfactory receptor activity
N	Q6IF99	DR	sensory perception of smell
N	Q6IG03	CC	Has a role in hair formation
N	Q6IG03	CC	Specific component of keratin intermediate filaments in the inner root sheath (IRS) of the hair follicle (By similarity).
N	Q6IG03	DE	Cytokeratin-73;
N	Q6IG03	DE	Keratin, type II cytoskeletal 73;
N	Q6IG03	DE	Type II inner root sheath-specific keratin-K6irs3;
N	Q6IG03	DE	Type-II keratin Kb36;
N	Q6IG03	DR	structural molecule activity
N	Q6IPR1	DE	LYR motif-containing protein 5;
N	Q6ITB0	CC	Serine protease inhibitor (Potential).

N	Q6ITB0	DE	Carinatin-1;
N	Q6ITB0	DR	serine-type endopeptidase inhibitor activity
N	Q6K0P9	CC	Major mediator of the tumor suppressor activity of IFN in breast cancer cells
N	Q6K0P9	CC	Promotes ubiquitination and subsequent degradation of HDAC1, which in turn enhances maspin expression, and impairs invasive activity of cancer cells.
N	Q6K0P9	CC	Promotes ubiquitination and subsequent degradation of MDM2, which leads to p53/TP53 stabilization
N	Q6K0P9	DE	Interferon-inducible protein X;
N	Q6K0P9	DE	Pyrin and HIN domain-containing protein 1;
N	Q6K6N7	DE	Probable protein phosphatase 2C 14;
N	Q6K6N7	DR	metal ion binding
N	Q6K6N7	DR	phosphoprotein phosphatase activity
N	Q6K953	CC	Has a glutathione-disulfide oxidoreductase activity in the presence of NADPH and glutathione reductase
N	Q6K953	CC	Reduces low molecular weight disulfides and proteins (By similarity).
N	Q6K953	DE	Glutaredoxin-C2 homolog 2;
N	Q6K953	DE	Glutaredoxin-C4, chloroplastic;
N	Q6K953	DR	cell redox homeostasis
N	Q6K953	DR	electron carrier activity
N	Q6K953	DR	electron transport chain
N	Q6K953	DR	protein disulfide oxidoreductase activity
N	Q6KHH5	CC	One of the primary rRNA binding proteins, it binds directly to 16S rRNA where it helps nucleate assembly of the platform of the 30S subunit by binding and bridging several RNA helices of the 16S rRNA (By similarity).
N	Q6KHH5	DE	30S ribosomal protein S15;
N	Q6KHH5	DR	structural constituent of ribosome
N	Q6KI81	CC	Produces ATP from ADP in the presence of a proton gradient across the membrane
N	Q6KI81	CC	The gamma chain is believed to be important in regulating ATPase activity and the flow of protons through the CF(0) complex.
N	Q6KI81	DE	ATP synthase F1 sector gamma subunit;
N	Q6KI81	DE	ATP synthase gamma chain;
N	Q6KI81	DE	F-ATPase gamma subunit;
N	Q6KI81	DR	ATP synthesis coupled proton transport
N	Q6KI81	DR	hydrogen ion transporting ATP synthase activity, rotational mechanism
N	Q6KI81	DR	proton-transporting ATPase activity, rotational mechanism
N	Q6L0L2	DE	UPF0215 protein PTO0905;
N	Q6L0R4	CC	With S4 and S5 plays an important role in translational accuracy
N	Q6L0R4	CC	Located at the interface of the 30S and 50S subunits (By similarity).
N	Q6L0R4	DE	30S ribosomal protein S12P;
N	Q6L0R4	DR	structural constituent of ribosome
N	Q6L708	CC	Plays a major role in tight junction-specific obliteration of the intercellular space, through calcium- independent cell-adhesion activity (By similarity).
N	Q6L708	DR	calcium-independent cell-cell adhesion
N	Q6L708	DR	identical protein binding
N	Q6L708	DR	structural molecule activity
N	Q6LFS0	CC	Catalyzes the oxidation of either pyridoxine 5'-phosphate (PNP) or pyridoxamine 5'-phosphate (PMP) into pyridoxal 5'-phosphate (PLP) (By similarity).
N	Q6LFS0	DE	PNP/PMP oxidase;
N	Q6LFS0	DE	Pyridoxal 5'-phosphate synthase;
N	Q6LFS0	DE	Pyridoxine/pyridoxamine 5'-phosphate oxidase;
N	Q6LFS0	DR	oxidation-reduction process
N	Q6LFS0	DR	pyridoxamine-phosphate oxidase activity
N	Q6LFS0	DR	pyridoxine biosynthetic process
N	Q6LLT9	CC	Conversion of NADPH, generated by peripheral catabolic pathways, to NADH, which can enter the respiratory chain for energy generation (By similarity).
N	Q6LLT9	DE	NAD(P)(+) transhydrogenase [B-specific];
N	Q6LLT9	DE	Soluble pyridine nucleotide transhydrogenase;
N	Q6LLT9	DR	NAD(P)+ transhydrogenase (B-specific) activity
N	Q6LLT9	DR	cell redox homeostasis
N	Q6LLT9	DR	flavin adenine dinucleotide binding
N	Q6LLT9	DR	oxidation-reduction process
N	Q6LMV7	DE	30S ribosomal protein S16;
N	Q6LMV7	DR	structural constituent of ribosome
N	Q6LN41	CC	Ligates lysine onto the cytidine present at position 34 of the AUA codon-specific tRNA(Ile) that contains the anticodon CAU, in an ATP-dependent manner

N	Q6LN41	CC	Cytidine is converted to lysidine, thus changing the amino acid specificity of the tRNA from methionine to isoleucine (By similarity).
N	Q6LN41	DE	tRNA(Ile)-2-lysyl-cytidine synthase;
N	Q6LN41	DE	tRNA(Ile)-lysidine synthase;
N	Q6LN41	DE	tRNA(Ile)-lysidine synthetase;
N	Q6LN41	DR	ligase activity, forming carbon-nitrogen bonds
N	Q6LN41	DR	tRNA processing
N	Q6LQ38	DE	Acylphosphatase;
N	Q6LQ38	DE	Acylphosphate phosphohydrolase;
N	Q6LQ38	DR	acylphosphatase activity
N	Q6LU16	CC	Negatively regulates transcription of bacterial ribonucleotide reductase nrd genes and operons by binding to NrdR- boxes (By similarity).
N	Q6LU16	DE	Transcriptional repressor NrdR;
N	Q6LU16	DR	transcription repressor activity
N	Q6LU16	DR	zinc ion binding
N	Q6LWJ8	CC	Sliding clamp subunit
N	Q6LWJ8	CC	Responsible for tethering the catalytic subunit of DNA polymerase to DNA during high-speed replication (By similarity).
N	Q6LWJ8	DE	DNA polymerase sliding clamp;
N	Q6LWJ8	DE	Proliferating cell nuclear antigen homolog;
N	Q6LWJ8	DR	DNA polymerase processivity factor activity
N	Q6LWJ8	DR	DNA replication
N	Q6LWJ8	DR	regulation of DNA replication
N	Q6LX61	CC	Catalyzes the deamination of 5-methylthioadenosine and S-adenosyl-L-homocysteine into 5-methylthioinosine and S-inosyl-L- homocysteine, respectively.
N	Q6LX61	CC	Is also able to deaminate adenosine (By similarity).
N	Q6LX61	DE	5-methylthioadenosine/S-adenosylhomocysteine deaminase;
N	Q6LX61	DE	MTA/SAH deaminase;
N	Q6LX61	DR	S-adenosylhomocysteine deaminase activity
N	Q6LX61	DR	metal ion binding
N	Q6LXW0	DE	UPF0280 protein MMP1236;
N	Q6LXW0	DR	thiamine biosynthetic process
N	Q6M116	CC	Catalyzes the methyl esterification of L-isoaspartyl residues in peptides and proteins that result from spontaneous decomposition of normal L-aspartyl and L-asparaginyl residues.
N	Q6M116	CC	It plays a role in the repair and/or degradation of damaged proteins (By similarity).
N	Q6M116	DE	L-isoaspartyl protein carboxyl methyltransferase;
N	Q6M116	DE	Protein L-isoaspartyl methyltransferase;
N	Q6M116	DE	Protein-L-isoaspartate O-methyltransferase;
N	Q6M116	DE	Protein-beta-aspartate methyltransferase;
N	Q6M116	DR	protein-L-isoaspartate (D-aspartate) O-methyltransferase activity
N	Q6MB12	CC	Catalyzes the decarboxylation of four acetate groups of uroporphyrinogen-III to yield coproporphyrinogen-III (By similarity).
N	Q6MB12	DE	Uroporphyrinogen decarboxylase;
N	Q6MB12	DR	porphyrin biosynthetic process
N	Q6MB12	DR	uroporphyrinogen decarboxylase activity
N	Q6MVL6	DE	Putative uncharacterized protein B8J22.070;
N	Q6N4Q4	CC	This protein promotes the GTP-dependent binding of aminoacyl-tRNA to the A-site of ribosomes during protein biosynthesis (By similarity).
N	Q6N4Q4	DE	Elongation factor Tu;
N	Q6N4Q4	DR	GTPase activity
N	Q6N4Q4	DR	translation elongation factor activity
N	Q6N693	CC	Interconversion of serine and glycine.
N	Q6N693	DE	Serine hydroxymethyltransferase 1;
N	Q6N693	DE	Serine methylase 1;
N	Q6N693	DR	L-serine metabolic process
N	Q6N693	DR	glycine hydroxymethyltransferase activity
N	Q6N693	DR	glycine metabolic process
N	Q6N693	DR	one-carbon metabolic process
N	Q6N693	DR	pyridoxal phosphate binding
N	Q6NBF3	CC	Catalyzes the ferrous insertion into protoporphyrin IX.
N	Q6NBF3	DE	Ferrochelatase;
N	Q6NBF3	DE	Heme synthase;
N	Q6NBF3	DE	Protoheme ferro-lyase;
N	Q6NBF3	DR	ferrochelatase activity
N	Q6NBF3	DR	heme biosynthetic process

N	Q6NBF3	DR	metal ion binding
N	Q6NDM1	CC	Involved in base excision repair of DNA damaged by oxidation or by mutagenic agents
N	Q6NDM1	CC	Acts as DNA glycosylase that recognizes and removes damaged bases
N	Q6NDM1	CC	Cleaves the DNA backbone by beta-delta elimination to generate a single-strand break at the site of the removed base with both 3'- and 5'-phosphates (By similarity).
N	Q6NDM1	CC	Has AP (apurinic/apyrimidinic) lyase activity and introduces nicks in the DNA strand
N	Q6NDM1	CC	Has a preference for oxidized purines, such as 7,8-dihydro-8-oxoguanine (8-oxoG)
N	Q6NDM1	DE	AP lyase mutM;
N	Q6NDM1	DE	DNA-(apurinic or apyrimidinic site) lyase mutM;
N	Q6NDM1	DE	Fapy-DNA glycosylase;
N	Q6NDM1	DE	Formamidopyrimidine-DNA glycosylase;
N	Q6NDM1	DR	base-excision repair
N	Q6NDM1	DR	damaged DNA binding
N	Q6NDM1	DR	nucleotide-excision repair
N	Q6NDM1	DR	oxidized purine base lesion DNA N-glycosylase activity
N	Q6NDM1	DR	zinc ion binding
N	Q6NDM2	CC	Methyltransferase required for the conversion of dimethylmenaquinone (DMKH2) to menaquinone (MKH2) and the conversion of 2-polyprenyl-6-methoxy-1,4-benzoquinol (DDMQH2) to 2-polyprenyl-3-methyl-6-methoxy-1,4-benzoquinol (DMQH2) (By similarity).
N	Q6NDM2	DE	Ubiquinone/menaquinone biosynthesis methyltransferase ubiE;
N	Q6NDM2	DR	menaquinone biosynthetic process
N	Q6NDM2	DR	methyltransferase activity
N	Q6NDM2	DR	ubiquinone biosynthetic process
N	Q6NGI5	CC	Specifically methylates guanosine-37 in various tRNAs (By similarity).
N	Q6NGI5	DE	M1G-methyltransferase;
N	Q6NGI5	DE	tRNA (guanine-N(1)-)-methyltransferase;
N	Q6NGI5	DE	tRNA [GM37] methyltransferase;
N	Q6NGI5	DR	tRNA (guanine-N1-)-methyltransferase activity
N	Q6NGR0	CC	Catalyzes the methylthiolation of N6- (dimethylallyl)adenosine (i(6)A), leading to the formation of 2- methylthio-N6-(dimethylallyl)adenosine (ms(2)i(6)A) at position 37 in tRNAs that read codons beginning with uridine (By similarity).
N	Q6NGR0	DE	(Dimethylallyl)adenosine tRNA methylthiotransferase miaB;
N	Q6NGR0	DE	tRNA-i(6)A37 methylthiotransferase;
N	Q6NGR0	DR	4 iron, 4 sulfur cluster binding
N	Q6NGR0	DR	metal ion binding
N	Q6NGR0	DR	tRNA modification
N	Q6NGR0	DR	transferase activity
N	Q6NHG5	CC	Regulates arginine biosynthesis genes (By similarity).
N	Q6NHG5	DE	Arginine repressor;
N	Q6NHG5	DR	arginine biosynthetic process
N	Q6NHG5	DR	regulation of transcription, DNA-dependent
N	Q6NHG5	DR	sequence-specific DNA binding transcription factor activity
N	Q6NPT8	CC	Component of SCF(ASK-cullin-F-box) E3 ubiquitin ligase complexes, which may mediate the ubiquitination and subsequent proteasomal degradation of target proteins (By similarity).
N	Q6NPT8	DE	F-box protein PP2-B1;
N	Q6NPT8	DE	Protein PHLOEM PROTEIN 2-LIKE B1;
N	Q6NPT8	DE	SKP1-interacting partner 21;
N	Q6NUD8	CC	Probable peripherally associated component of the endosomal sorting required for transport complex III (ESCRT-III) which is involved in multivesicular bodies (MVBs) formation and sorting of endosomal cargo proteins into MVBs
N	Q6NUD8	CC	MVBs contain intraluminal vesicles (ILVs) that are generated by invagination and scission from the limiting membrane of the endosome and mostly are delivered to lysosomes enabling degradation of membrane proteins, such as stimulated growth factor receptors, lysosomal enzymes and lipids (By similarity).
N	Q6NUD8	DE	Charged multivesicular body protein 1a;
N	Q6NUD8	DE	Chromatin-modifying protein 1a;
N	Q6NUD8	DR	protein transport
N	Q6NV72	DE	WD repeat-containing protein C2orf44 homolog;
N	Q6NXR2	DE	STAG3-like protein 1;
N	Q6NXR2	DE	STAG3-like protein 2;
N	Q6NXR2	DE	STAG3-like protein 3;
N	Q6NXR2	DE	Stromal antigen 3-like protein 1;
N	Q6NXR2	DE	Stromal antigen 3-like protein 2;

N	Q6NXR2	DE	Stromal antigen 3-like protein 3;
N	Q6NYZ4	CC	Hydrolyzes the ester bond at the sn-1 position of glycerophospholipids and produces 2-acyl lysophospholipids (By similarity).
N	Q6NYZ4	DE	Phospholipase A1 member A;
N	Q6NYZ4	DR	hydrolase activity
N	Q6NYZ4	DR	lipid catabolic process
N	Q6P0H6	CC	Component of the COP9 signalosome complex (CSN), a complex involved in various cellular and developmental processes
N	Q6P0H6	CC	The CSN complex is an essential regulator of the ubiquitin (Ubl) conjugation pathway by mediating the deneddylation of the cullin subunits of E3 ligase complexes, leading to modify the Ubl ligase activity (By similarity).
N	Q6P0H6	DE	COP9 signalosome complex subunit 4;
N	Q6P0H6	DE	Signalosome subunit 4;
N	Q6P1V3	CC	Probable substrate-recognition component of a SCF-like ECS (Elongin-Cullin-SOCS-box protein) E3 ubiquitin-protein ligase complex which mediates the ubiquitination and subsequent proteasomal degradation of target proteins (By similarity).
N	Q6P1V3	DE	WD repeat and SOCS box-containing protein 1;
N	Q6P1V3	DR	intracellular signal transduction
N	Q6P3V2	CC	May be involved in transcriptional regulation.
N	Q6P3V2	DE	Zinc finger protein 585A;
N	Q6P3V2	DR	nucleic acid binding
N	Q6P3V2	DR	regulation of transcription, DNA-dependent
N	Q6P3V2	DR	zinc ion binding
N	Q6P7L9	CC	Component of the eukaryotic translation initiation factor 3 (eIF-3) complex, which is involved in protein synthesis and, together with other initiation factors, stimulates binding of mRNA and methionyl-tRNAi to the 40S ribosome (By similarity).
N	Q6P7L9	DE	Eukaryotic translation initiation factor 3 subunit 6;
N	Q6P7L9	DE	Eukaryotic translation initiation factor 3 subunit E;
N	Q6P7L9	DR	translation initiation factor activity
N	Q6P9B6	DE	TLD domain-containing protein KIAA1609;
N	Q6P9B6	DR	protein binding
N	Q6P9V9	CC	Tubulin is the major constituent of microtubules
N	Q6P9V9	CC	It binds two moles of GTP, one at an exchangeable site on the beta chain and one at a non-exchangeable site on the alpha-chain.
N	Q6P9V9	DE	Alpha-tubulin 2;
N	Q6P9V9	DE	Tubulin alpha-1B chain;
N	Q6P9V9	DE	Tubulin alpha-2 chain;
N	Q6P9V9	DR	GTPase activity
N	Q6P9V9	DR	microtubule-based movement
N	Q6P9V9	DR	protein polymerization
N	Q6P9V9	DR	structural molecule activity
N	Q6PA35	CC	May be required for the assembly pathway of mitochondrial outer membrane proteins (By similarity).
N	Q6PA35	DE	Sorting and assembly machinery component 50 homolog B;
N	Q6PA79	CC	Phosphorylates uridine and cytidine to uridine monophosphate and cytidine monophosphate
N	Q6PA79	CC	Can use ATP or GTP as a phosphate donor (By similarity).
N	Q6PA79	CC	Does not phosphorylate deoxyribonucleosides or purine ribonucleosides
N	Q6PA79	DE	Cytidine monophosphokinase 1-A;
N	Q6PA79	DE	Uridine monophosphokinase 1-A;
N	Q6PA79	DE	Uridine-cytidine kinase 1-A;
N	Q6PA79	DR	phosphotransferase activity, alcohol group as acceptor
N	Q6PA79	DR	uridine kinase activity
N	Q6PAM0	CC	AMPK is responsible for the regulation of fatty acid synthesis by phosphorylation of acetyl-CoA carboxylase
N	Q6PAM0	CC	Also regulates cholesterol synthesis via phosphorylation and inactivation of hydroxymethylglutaryl-CoA reductase and hormone- sensitive lipase
N	Q6PAM0	CC	It may also serve as an adapter molecule for the catalytic alpha-subunit (By [illegible]
N	Q6PAM0	CC	This is a regulatory subunit, may be a positive regulator of AMPK activity
N	Q6PAM0	DE	5'-AMP-activated protein kinase subunit beta-2;
N	Q6PAM0	DE	AMPK subunit beta-2;
N	Q6PAM0	DR	AMP-activated protein kinase activity
N	Q6PAM0	DR	fatty acid biosynthetic process
N	Q6PAM0	DR	response to stress
N	Q6PB06	DE	Aminoglycoside phosphotransferase domain-containing protein 1;

N	Q6PB06	DR	kinase activity
N	Q6PBJ2	DE	UPF0468 protein C16orf80 homolog;
N	Q6PEW1	CC	Transcriptional coactivator in the bone morphogenetic protein (BMP)-signaling pathway
N	Q6PEW1	CC	It contributes to the BMP-induced enhancement of cholinergic-neuron-specific gene expression (By similarity).
N	Q6PEW1	CC	It positively modulates BMP signaling by interacting with SMAD1 and associating with CBP in the transcription complex
N	Q6PEW1	DE	Smad-interacting zinc finger protein 1;
N	Q6PEW1	DE	Zinc finger CCHC domain-containing protein 12;
N	Q6PEW1	DR	nucleic acid binding
N	Q6PEW1	DR	zinc ion binding
N	Q6PFH3	CC	May be involved in ubiquitination and degradation through a DBB1-CUL4 E3 protein-ubiquitin ligase (By similarity).
N	Q6PFH3	DE	DDB1- and CUL4-associated factor 15;
N	Q6PIU2	CC	Hydrolyzes 2-acetyl monoalkylglycerol ether, the penultimate precursor of the pathway for de novo synthesis of platelet-activating factor
N	Q6PIU2	CC	Also involved in organ detoxification by hydrolyzing exogenous organophosphorus compounds
N	Q6PIU2	CC	May be responsible for cholesterol ester hydrolysis in macrophages, thereby contributing to the development of atherosclerosis
N	Q6PIU2	CC	May contribute to cancer pathogenesis by promoting tumor cell migration.
N	Q6PIU2	DE	Arylacetamide deacetylase-like 1;
N	Q6PIU2	DE	Neutral cholesterol ester hydrolase 1;
N	Q6PIU2	DR	carboxylesterase activity
N	Q6PIU2	DR	lipid catabolic process
N	Q6PJG2	DE	Uncharacterized protein C14orf43;
N	Q6PTD4	CC	Alpha-conotoxins act on postsynaptic membranes, they bind to the nicotinic acetylcholine receptors (nAChR) and thus inhibit them (By similarity).
N	Q6PTD4	DE	Alpha-conotoxin-like Lp1.2;
N	Q6PTD4	DR	acetylcholine receptor inhibitor activity
N	Q6PTD4	DR	synaptic transmission
N	Q6Q2J0	CC	Catalyzes the oxidative deamination of biogenic and xenobiotic amines and has important functions in the metabolism of neuroactive and vasoactive amines in the central nervous system and peripheral tissues
N	Q6Q2J0	CC	MAOA preferentially oxidizes biogenic amines such as 5-hydroxytryptamine (5-HT), norepinephrine and epinephrine (By similarity).
N	Q6Q2J0	DE	Amine oxidase [flavin-containing] A;
N	Q6Q2J0	DE	Monoamine oxidase type A;
N	Q6Q2J0	DR	neurotransmitter catabolic process
N	Q6Q2J0	DR	oxidation-reduction process
N	Q6Q2J0	DR	oxidoreductase activity
N	Q6QLQ5	CC	Binds bacterial lipopolysaccharide (LPS)
N	Q6QLQ5	CC	Has hemolytic activity (in vitro)
N	Q6QLQ5	CC	Has potent antimicrobial activity against Gram-positive and Gram-negative bacteria (in vitro)
N	Q6QLQ5	CC	May play a role in the innate immune response.
N	Q6QLQ5	DE	Cathelicidin-1;
N	Q6QLQ5	DE	Fowlicidin-1;
N	Q6QLQ5	DR	defense response to bacterium
N	Q6QLQ5	DR	innate immune response
N	Q6RY07	CC	Degrades chitin and chitotriose
N	Q6RY07	CC	Contributes to the response to IL-13 and inflammation in response to IL-13
N	Q6RY07	CC	Its function in the inflammatory response and in protecting cells against apoptosis is inhibited by allosamidin, suggesting that the function of this protein depends on carbohydrate binding (By similarity).
N	Q6RY07	CC	May participate in the defense against nematodes, fungi and other pathogens
N	Q6RY07	CC	Plays a role in T helper cell type 2 (Th2) immune response
N	Q6RY07	CC	Protects lung epithelial cells against apoptosis and promotes phosphorylation of AKT1
N	Q6RY07	CC	Stimulates chemokine production by pulmonary epithelial cells
N	Q6RY07	DE	Acidic mammalian chitinase;
N	Q6RY07	DR	cation binding
N	Q6RY07	DR	chitin binding
N	Q6RY07	DR	chitin catabolic process
N	Q6RY07	DR	chitinase activity
N	Q6RY07	DR	positive regulation of chemokine secretion

N	Q6RY07	DR	production of molecular mediator involved in inflammatory response
N	Q6S001	CC	Microtubule-associated force-producing protein that plays a role in organelle transport
N	Q6S001	CC	Its motor activity is directed toward the microtubule's plus end (By similarity).
N	Q6S001	DE	Kinesin family member 11;
N	Q6S001	DE	Kinesin-related protein 11;
N	Q6S001	DR	microtubule motor activity
N	Q6S001	DR	microtubule-based movement
N	Q6TND0	CC	Usually encoded in the trnK tRNA gene intron
N	Q6TND0	CC	Probably assists in splicing its own and other chloroplast group II introns (By similarity)
N	Q6TND0	DE	Intron maturase;
N	Q6TND0	DR	mRNA processing
N	Q6TND0	DR	tRNA processing
N	Q6UVK1	CC	Proteoglycan playing a role in cell proliferation and migration which stimulates endothelial cells motility during microvascular morphogenesis
N	Q6UVK1	CC	Binds through its extracellular N- terminus growth factors, extracellular matrix proteases modulating their activity
N	Q6UVK1	CC	Cell surface receptor for collagen alpha 2(VI) which may confer cells ability to migrate on that substrate
N	Q6UVK1	CC	Functions also as a signal transducing protein by binding through its cytoplasmic C-terminus scaffolding and signaling proteins
N	Q6UVK1	CC	May activate FAK and ERK1/ERK2 signaling cascades.
N	Q6UVK1	CC	May also inhibit neurite outgrowth and growth cone collapse during axon regeneration
N	Q6UVK1	CC	May modulate the plasminogen system by enhancing plasminogen activation and inhibiting angiostatin
N	Q6UVK1	CC	May promote retraction fiber formation and cell polarization through Rho GTPase activation
N	Q6UVK1	CC	May regulate MPP16-dependent degradation and invasion of type I collagen participating in melanoma cells invasion properties
N	Q6UVK1	CC	May stimulate alpha-4, beta-1 integrin- mediated adhesion and spreading by recruiting and activating a signaling cascade through CDC42, ACK1 and BCAR1
N	Q6UVK1	DE	Chondroitin sulfate proteoglycan 4;
N	Q6UVK1	DE	Chondroitin sulfate proteoglycan NG2;
N	Q6UVK1	DE	Melanoma chondroitin sulfate proteoglycan;
N	Q6UVK1	DE	Melanoma-associated chondroitin sulfate proteoglycan;
N	Q6UVK1	DR	cell differentiation
N	Q6UVK1	DR	intracellular signal transduction
N	Q6UVK1	DR	positive regulation of peptidyl-tyrosine phosphorylation
N	Q6UVK1	DR	protein kinase binding
N	Q6UVK1	DR	signal transducer activity
N	Q6UVK1	DR	tissue remodeling
N	Q6UXK2	CC	Required for axon extension during neural development (By similarity).
N	Q6UXK2	DE	Immunoglobulin superfamily containing leucine-rich repeat protein 2;
N	Q6UXK2	DE	Leucine-rich repeat domain and immunoglobulin domain-containing axon extension protein;
N	Q6UXK2	DR	positive regulation of axon extension
N	Q6W8Q2	CC	Antioxidant protein that seems to contribute to the inhibition of germination during stress (By similarity).
N	Q6W8Q2	DE	1-Cys peroxiredoxin PER1;
N	Q6W8Q2	DE	Rehydrin homolog;
N	Q6W8Q2	DE	Thioredoxin peroxidase;
N	Q6W8Q2	DR	cell redox homeostasis
N	Q6W8Q2	DR	oxidation-reduction process
N	Q6W8Q2	DR	peroxidase activity
N	Q6W8Q2	DR	peroxiredoxin activity
N	Q6X7S9	CC	Interacts with EP300 and acts as a repressor of MYOD- dependent transcription and muscle differentiation
N	Q6X7S9	CC	Acts as a repressor of TGFB/SMAD transcriptional responses
N	Q6X7S9	CC	Inhibits EP300 histone acetyltransferase activity
N	Q6X7S9	CC	May act as a repressor of the TGFB/SMAD3-dependent signaling by selectively blocking formation of TGFB-induced SMAD3-SMAD4 complex (By similarity).
N	Q6X7S9	DE	CREBBP/EP300 inhibitor 2;
N	Q6X7S9	DE	EID-1-like inhibitor of differentiation 2;
N	Q6X7S9	DE	EP300-interacting inhibitor of differentiation 2;
N	Q6X7S9	DR	SMAD protein complex assembly
N	Q6X7S9	DR	cell differentiation

N	Q6X7S9	DR	muscle organ development
N	Q6X7S9	DR	negative regulation of transforming growth factor beta receptor signaling pathway
N	Q6X7S9	DR	regulation of cell proliferation
N	Q6X7S9	DR	transforming growth factor beta receptor complex assembly
N	Q6XTD8	CC	Inhibits post-transcriptional processing of cellular pre-mRNA, by binding and inhibiting two cellular proteins that are required for the 3'-end processing of cellular pre-mRNAs: the 30 kDa cleavage and polyadenylation specificity factor (CPSF4) and the poly(A)-binding protein 2 (PABPN1)
N	Q6XTD8	CC	Cellular protein synthesis is thereby shut off very early after virus infection
N	Q6XTD8	CC	This results in the accumulation of unprocessed 3' end pre-mRNAs which can't be exported from the nucleus
N	Q6XTD8	CC	Viral protein synthesis is not affected by the inhibition of the cellular 3' end processing machinery because the poly(A) tails of viral mRNAs are produced by the viral polymerase through a stuttering mechanism (By similarity).
N	Q6XTD8	DE	Non-structural protein 1;
N	Q6XTD8	DR	evasion by virus of host immune response
N	Q6XTK0	CC	Inhibits post-transcriptional processing of cellular pre-mRNA, by binding and inhibiting two cellular proteins that are required for the 3'-end processing of cellular pre-mRNAs: the 30 kDa cleavage and polyadenylation specificity factor (CPSF4) and the poly(A)-binding protein 2 (PABPN1)
N	Q6XTK0	CC	Cellular protein synthesis is thereby shut off very early after virus infection
N	Q6XTK0	CC	This results in the accumulation of unprocessed 3' end pre-mRNAs which can't be exported from the nucleus
N	Q6XTK0	CC	Viral protein synthesis is not affected by the inhibition of the cellular 3' end processing machinery because the poly(A) tails of viral mRNAs are produced by the viral polymerase through a stuttering mechanism (By similarity).
N	Q6XTK0	DE	Non-structural protein 1;
N	Q6XTK0	DR	evasion by virus of host immune response
N	Q6YK37	CC	Catalyzes the depolymerization of methylglucuronoxylan (MeGAXn)
N	Q6YK37	CC	It cleaves the beta-1,4-xylosidic bond penultimate to that linking carbon one of the xylose residue substituted with alpha-1,2-linked 4-O-methyl-D-glucuronate (MeGA) (By similarity).
N	Q6YK37	DE	Endoxylanase xynC;
N	Q6YK37	DE	Glucuronoxylan xylanohydrolase;
N	Q6YK37	DE	Glucuronoxylanase xynC;
N	Q6YK37	DR	cation binding
N	Q6YK37	DR	glucosylceramidase activity
N	Q6YK37	DR	glucuronoarabinoxylan endo-1,4-beta-xylanase activity
N	Q6YK37	DR	lysosome organization
N	Q6YK37	DR	sphingolipid metabolic process
N	Q6YK37	DR	xylan catabolic process
N	Q6YPX7	DE	Phenylalanine--tRNA ligase beta chain;
N	Q6YPX7	DE	Phenylalanyl-tRNA synthetase beta chain;
N	Q6YPX7	DR	magnesium ion binding
N	Q6YPX7	DR	phenylalanine-tRNA ligase activity
N	Q6YPX7	DR	phenylalanyl-tRNA aminoacylation
N	Q6YPX7	DR	tRNA processing
N	Q6YXP2	CC	Seems to be required for the assembly of the photosystem I complex (By similarity)
N	Q6YXP2	DE	Photosystem I assembly protein ycf3;
N	Q6YXP2	DR	photosynthesis
N	Q6Z3L4	CC	Transcription factor which may be involved in developmental processes (By similarity)
N	Q6Z3L4	DE	Protein WOX11/12;
N	Q6Z3L4	DE	WUSCHEL-related homeobox 10;
N	Q6Z3L4	DR	multicellular organismal development
N	Q6Z3L4	DR	regulation of transcription, DNA-dependent
N	Q6Z3L4	DR	sequence-specific DNA binding
N	Q6Z3L4	DR	sequence-specific DNA binding transcription factor activity
N	Q6ZMM2	DE	ADAMTS-like protein 5;
N	Q6ZMM2	DE	Thrombospondin type-1 domain-containing protein 6;
N	Q6ZMM2	DR	metalloendopeptidase activity
N	Q6ZMM2	DR	zinc ion binding
N	Q6ZVW7	DE	IL-17 receptor E-like;
N	Q6ZVW7	DE	IL-17RE-like;
N	Q6ZVW7	DE	Putative interleukin-17 receptor E-like;
N	Q6ZW76	DE	Ankyrin repeat and SAM domain-containing protein 3;

N	Q71DC3	DE	Neuropeptide-like 3;
N	Q71DC3	DE	SHA-peptide;
N	Q71DC3	DE	VVI-amide peptide;
N	Q71DC3	DR	neuropeptide signaling pathway
N	Q71RU5	CC	Core subunit of the mitochondrial membrane respiratory chain NADH dehydrogenase (Complex I) that is believed to belong to the minimal assembly required for catalysis
N	Q71RU5	CC	Complex I functions in the transfer of electrons from NADH to the respiratory chain
N	Q71RU5	CC	The immediate electron acceptor for the enzyme is believed to be ubiquinone (By similarity).
N	Q71RU5	DE	NADH dehydrogenase subunit 4L;
N	Q71RU5	DE	NADH-ubiquinone oxidoreductase chain 4L;
N	Q71RU5	DR	ATP synthesis coupled electron transport
N	Q71RU5	DR	NADH dehydrogenase (ubiquinone) activity
N	Q71WG1	CC	This protein binds to the 23S rRNA, and is important in its secondary structure
N	Q71WG1	CC	It is located near the subunit interface in the base of the L7/L12 stalk, and near the tRNA binding site of the peptidyltransferase center (By similarity).
N	Q71WG1	DE	50S ribosomal protein L6;
N	Q71WG1	DR	structural constituent of ribosome
N	Q71XV0	CC	Channel that opens in response to stretch forces in the membrane lipid bilayer
N	Q71XV0	CC	May participate in the regulation of osmotic pressure changes within the cell (By similarity).
N	Q71XV0	DE	Large-conductance mechanosensitive channel;
N	Q71XV0	DR	ion channel activity
N	Q71Y27	CC	Catalyzes a reversible aldol reaction between acetaldehyde and D-glyceraldehyde 3-phosphate to generate 2-deoxy- D-ribose 5-phosphate (By similarity).
N	Q71Y27	DE	2-deoxy-D-ribose 5-phosphate aldolase;
N	Q71Y27	DE	Deoxyriboaldolase;
N	Q71Y27	DE	Deoxyribose-phosphate aldolase;
N	Q71Y27	DE	Phosphodeoxyriboaldolase;
N	Q71Y27	DR	deoxyribonucleotide catabolic process
N	Q71Y27	DR	deoxyribose-phosphate aldolase activity
N	Q71Z52	CC	Catalyzes the formation of N(7)-methylguanine at position 46 (m7G46) in tRNA (By similarity).
N	Q71Z52	DE	tRNA (guanine-N(7)-)-methyltransferase;
N	Q71Z52	DE	tRNA(m7G46)-methyltransferase;
N	Q71Z52	DR	tRNA (guanine-N7-)-methyltransferase activity
N	Q71ZH3	DE	Cytidine monophosphokinase;
N	Q71ZH3	DE	Uridine kinase;
N	Q71ZH3	DE	Uridine monophosphokinase;
N	Q71ZH3	DR	phosphotransferase activity, alcohol group as acceptor
N	Q71ZH3	DR	uridine kinase activity
N	Q721J0	DE	Phosphoriboisomerase A;
N	Q721J0	DE	Ribose-5-phosphate isomerase A;
N	Q721J0	DR	pentose-phosphate shunt, non-oxidative branch
N	Q721J0	DR	ribose-5-phosphate isomerase activity
N	Q728U8	CC	Cell wall formation
N	Q728U8	CC	Catalyzes the transfer of a GlcNAc subunit on undecaprenyl-pyrophosphoryl-MurNAc-pentapeptide (lipid intermediate I) to form undecaprenyl-pyrophosphoryl-MurNAc- (pentapeptide)GlcNAc (lipid intermediate II) (By similarity).
N	Q728U8	DE	UDP-N-acetylglucosamine--N-acetylmuramyl-(pentapeptide) pyrophosphoryl-undecaprenol N-acetylglucosamine transferase;
N	Q728U8	DE	Undecaprenyl-PP-MurNAc-pentapeptide-UDPGlcNAc GlcNAc transferase;
N	Q728U8	DR	UDP-N-acetyl-D-glucosamine:N-acetylmuramoyl-L-alanyl-D-glutamyl-meso-2,6-diaminopimelyl-D-alanyl-D-alanine-diphosphoundecaprenol 4-beta-N-acetylglucosaminlytransferase activity
N	Q728U8	DR	UDP-N-acetylgalactosamine biosynthetic process
N	Q728U8	DR	carbohydrate binding
N	Q728U8	DR	cell division
N	Q728U8	DR	cellular cell wall organization
N	Q728U8	DR	lipid glycosylation
N	Q728U8	DR	peptidoglycan biosynthetic process
N	Q728U8	DR	regulation of cell shape
N	Q728U8	DR	undecaprenyldiphospho-muramoylpentapeptide beta-N-acetylglucosaminyltransferase activity

N	Q72BL2	CC	Catalyzes the ATP-dependent amination of UTP to CTP with either L-glutamine or ammonia as the source of nitrogen (By similarity).
N	Q72BL2	DE	CTP synthase;
N	Q72BL2	DE	CTP synthetase;
N	Q72BL2	DE	UTP--ammonia ligase;
N	Q72BL2	DR	CTP synthase activity
N	Q72BL2	DR	glutamine metabolic process
N	Q72BL2	DR	pyrimidine nucleotide biosynthetic process
N	Q72C17	CC	ATPase subunit of a proteasome-like degradation complex; this subunit has chaperone activity
N	Q72C17	CC	HslU recognizes the N-terminal part of its protein substrates and unfolds these before they are guided to HslV for hydrolysis (By similarity).
N	Q72C17	CC	The binding of ATP and its subsequent hydrolysis by HslU are essential for unfolding of protein substrates subsequently hydrolyzed by HslV
N	Q72C17	DE	ATP-dependent protease ATPase subunit HslU;
N	Q72C17	DE	Unfoldase HslU;
N	Q72C17	DR	ATPase activity
N	Q72C17	DR	peptidase activity, acting on L-amino acid peptides
N	Q72CF5	CC	One of the primary rRNA binding proteins, it binds directly to 16S rRNA where it nucleates assembly of the body of the 30S subunit (By similarity).
N	Q72CF5	DE	30S ribosomal protein S4;
N	Q72CF5	DR	structural constituent of ribosome
N	Q72CG2	DE	50S ribosomal protein L30;
N	Q72CG2	DR	structural constituent of ribosome
N	Q72I05	CC	One of the primary rRNA binding proteins, this protein initially binds near the 5'-end of the 23S rRNA
N	Q72I05	CC	It is important during the early stages of 50S assembly
N	Q72I05	CC	It makes multiple contacts with different domains of the 23S rRNA in the assembled 50S subunit and ribosome (By similarity).
N	Q72I05	DE	50S ribosomal protein L4;
N	Q72I05	DR	structural constituent of ribosome
N	Q72JR1	CC	Catalyzes the interconversion of methylthioribose-1- phosphate (MTR-1-P) into methylthioribulose-1-phosphate (MTRu-1-P) (By similarity).
N	Q72JR1	DE	MTR-1-P isomerase;
N	Q72JR1	DE	Methylthioribose-1-phosphate isomerase;
N	Q72JR1	DE	S-methyl-5-thioribose-1-phosphate isomerase;
N	Q72JR1	DR	S-methyl-5-thioribose-1-phosphate isomerase activity
N	Q72JR1	DR	methionine biosynthetic process
N	Q72NP0	CC	Catalyzes the phosphorylation of pantothenate (Pan), the first step in CoA biosynthesis (By similarity).
N	Q72NP0	DE	Pantothenic acid kinase;
N	Q72NP0	DE	Type III pantothenate kinase;
N	Q72NP0	DR	coenzyme A biosynthetic process
N	Q72NP0	DR	metal ion binding
N	Q72NP0	DR	pantothenate kinase activity
N	Q72NP0	DR	transcription activator activity
N	Q72PR5	DE	FGAM synthase II;
N	Q72PR5	DE	Phosphoribosylformylglycinamidine synthase 2;
N	Q72PR5	DE	Phosphoribosylformylglycinamidine synthase II;
N	Q72PR5	DR	'de novo' IMP biosynthetic process
N	Q72PR5	DR	phosphoribosylformylglycinamidine synthase activity
N	Q72R81	CC	Catalyzes the addition of meso-diaminopimelic acid to the nucleotide precursor UDP-N-acetylmuramoyl-L-alanyl-D-glutamate (UMAG) in the biosynthesis of bacterial cell-wall peptidoglycan (By similarity).
N	Q72R81	DE	Meso-A2pm-adding enzyme;
N	Q72R81	DE	Meso-diaminopimelate-adding enzyme;
N	Q72R81	DE	UDP-MurNAc-L-Ala-D-Glu:meso-diaminopimelate ligase;
N	Q72R81	DE	UDP-MurNAc-tripeptide synthetase;
N	Q72R81	DE	UDP-N-acetylmuramoyl-L-alanyl-D-glutamate--2,6-diaminopimelate ligase;
N	Q72R81	DE	UDP-N-acetylmuramyl-tripeptide synthetase;
N	Q72R81	DR	UDP-N-acetylmuramoylalanyl-D-glutamate-2,6-diaminopimelate ligase activity
N	Q72R81	DR	cell division
N	Q72R81	DR	cellular cell wall organization
N	Q72R81	DR	peptidoglycan biosynthetic process
N	Q72R81	DR	regulation of cell shape

N	Q72RH7	CC	Catalyzes the oxidation of 3-carboxy-2-hydroxy-4- methylpentanoate (3-isopropylmalate) to 3-carboxy-4-methyl-2- oxopentanoate
N	Q72RH7	CC	The product decarboxylates to 4-methyl-2 oxopentanoate.
N	Q72RH7	DE	3-isopropylmalate dehydrogenase;
N	Q72RH7	DE	Beta-IPM dehydrogenase;
N	Q72RH7	DR	3-isopropylmalate dehydrogenase activity
N	Q72RH7	DR	leucine biosynthetic process
N	Q72RH7	DR	magnesium ion binding
N	Q72RH7	DR	oxidation-reduction process
N	Q72ZC1	DE	UPF0173 metal-dependent hydrolase BCE_4747;
N	Q72ZC1	DR	hydrolase activity
N	Q730M0	CC	Participates actively in the response to hyperosmotic and heat shock by preventing the aggregation of stress-denatured proteins, in association with dnaK and grpE
N	Q730M0	CC	GrpE releases ADP from dnaK; ATP binding to dnaK triggers the release of the substrate protein, thus completing the reaction cycle
N	Q730M0	CC	It is the nucleotide exchange factor for dnaK and may function as a thermosensor
N	Q730M0	CC	Several rounds of ATP- dependent interactions between dnaJ, dnaK and grpE are required for fully efficient folding (By similarity).
N	Q730M0	CC	Unfolded proteins bind initially to dnaJ; upon interaction with the dnaJ-bound protein, dnaK hydrolyzes its bound ATP, resulting in the formation of a stable complex
N	Q730M0	DE	HSP-70 cofactor;
N	Q730M0	DE	Protein grpE;
N	Q730M0	DR	adenyl-nucleotide exchange factor activity
N	Q730M0	DR	chaperone binding
N	Q730M0	DR	protein folding
N	Q730M0	DR	protein homodimerization activity
N	Q730M0	DR	response to stress
N	Q734S7	DE	Putative regulator of ribonuclease activity;
N	Q734S7	DR	regulation of RNA metabolic process
N	Q734S7	DR	ribonuclease inhibitor activity
N	Q73AY2	CC	Methyltransferase required for the conversion of dimethylmenaquinone (DMKH2) to menaquinone (MKH2) (By similarity).
N	Q73AY2	DE	Menaquinone biosynthesis methyltransferase ubiE;
N	Q73AY2	DR	menaquinone biosynthetic process
N	Q73AY2	DR	methyltransferase activity
N	Q73FJ4	CC	Involved in the hydrolysis of glutamine to glutamate and ammonia
N	Q73FJ4	CC	Channels an ammonia molecule to pdxS (By similarity).
N	Q73FJ4	DE	Glutamine amidotransferase glutaminase subunit pdxT;
N	Q73FJ4	DE	Glutamine amidotransferase subunit pdxT;
N	Q73FJ4	DR	glutamine metabolic process
N	Q73FJ4	DR	transferase activity
N	Q73HS5	CC	Catalyzes the ATP-dependent amination of UTP to CTP with either L-glutamine or ammonia as the source of nitrogen (By similarity).
N	Q73HS5	DE	CTP synthase;
N	Q73HS5	DE	CTP synthetase;
N	Q73HS5	DE	UTP--ammonia ligase;
N	Q73HS5	DR	CTP synthase activity
N	Q73HS5	DR	glutamine metabolic process
N	Q73HS5	DR	pyrimidine nucleotide biosynthetic process
N	Q73J84	CC	Catalyzes the ATP-dependent amination of UTP to CTP with either L-glutamine or ammonia as the source of nitrogen (By similarity).
N	Q73J84	DE	CTP synthase;
N	Q73J84	DE	CTP synthetase;
N	Q73J84	DE	UTP--ammonia ligase;
N	Q73J84	DR	CTP synthase activity
N	Q73J84	DR	glutamine metabolic process
N	Q73J84	DR	pyrimidine nucleotide biosynthetic process
N	Q73JG0	CC	This protein specifically catalyzes the removal of signal peptides from prolipoproteins (By similarity).
N	Q73JG0	DE	Lipoprotein signal peptidase;
N	Q73JG0	DE	Prolipoprotein signal peptidase;
N	Q73JG0	DE	Signal peptidase II;
N	Q73JG0	DR	aspartic-type endopeptidase activity
N	Q73JJ8	CC	DNA-dependent RNA polymerase catalyzes the transcription of DNA into RNA using the four ribonucleoside triphosphates as substrates.

N	Q73JJ8	DE	DNA-directed RNA polymerase subunit beta';
N	Q73JJ8	DE	RNA polymerase subunit beta';
N	Q73JJ8	DE	RNAP subunit beta';
N	Q73JJ8	DE	Transcriptase subunit beta';
N	Q73JJ8	DR	DNA-directed RNA polymerase activity
N	Q73MH0	CC	DNA ligase that catalyzes the formation of phosphodiester linkages between 5'-phosphoryl and 3'-hydroxyl groups in double-stranded DNA using NAD as a coenzyme and as the energy source for the reaction
N	Q73MH0	CC	It is essential for DNA replication and repair of damaged DNA (By similarity).
N	Q73MH0	DE	Polydeoxyribonucleotide synthase [NAD+];
N	Q73MH0	DR	DNA ligase (NAD+) activity
N	Q73MH0	DR	DNA replication
N	Q73MH0	DR	metal ion binding
N	Q73SG5	DE	UPF0336 protein MAP_4109;
N	Q73SG5	DR	oxidoreductase activity
N	Q73UI3	CC	Exhibits S-adenosyl-L-methionine-dependent methyltransferase activity (By similarity).
N	Q73UI3	DE	Putative S-adenosyl-L-methionine-dependent methyltransferase MAP_3385;
N	Q73UI3	DR	methyltransferase activity
N	Q73XR5	CC	Catalyzes the reversible adenylation of nicotinate mononucleotide (NaMN) to nicotinic acid adenine dinucleotide (NaAD) (By similarity).
N	Q73XR5	DE	Deamido-NAD(+) diphosphorylase;
N	Q73XR5	DE	Deamido-NAD(+) pyrophosphorylase;
N	Q73XR5	DE	NaMN adenylyltransferase;
N	Q73XR5	DE	Nicotinate mononucleotide adenylyltransferase;
N	Q73XR5	DE	Probable nicotinate-nucleotide adenylyltransferase;
N	Q73XR5	DR	NAD biosynthetic process
N	Q73XR5	DR	nicotinate-nucleotide adenylyltransferase activity
N	Q73ZR2	CC	The glycine cleavage system catalyzes the degradation of glycine
N	Q73ZR2	CC	The H protein shuttles the methylamine group of glycine from the P protein to the T protein (By similarity).
N	Q73ZR2	DE	Glycine cleavage system H protein;
N	Q73ZR2	DR	glycine decarboxylation via glycine cleavage system
N	Q741F1	CC	Catalyzes the conversion of D-ribulose 5-phosphate to formate and 3,4-dihydroxy-2-butanone 4-phosphate (By similarity).
N	Q741F1	DE	3,4-dihydroxy-2-butanone 4-phosphate synthase;
N	Q741F1	DE	DHBP synthase;
N	Q741F1	DE	GTP cyclohydrolase II;
N	Q741F1	DE	GTP cyclohydrolase-2;
N	Q741F1	DE	Riboflavin biosynthesis protein ribBA;
N	Q741F1	DR	3,4-dihydroxy-2-butanone-4-phosphate synthase activity
N	Q741F1	DR	GTP cyclohydrolase II activity
N	Q741F1	DR	metal ion binding
N	Q741F1	DR	riboflavin biosynthetic process
N	Q748Y5	CC	Seems to be the binding site for several of the factors involved in protein synthesis and appears to be essential for accurate translation (By similarity).
N	Q748Y5	DE	50S ribosomal protein L7/L12;
N	Q748Y5	DR	structural constituent of ribosome
N	Q74JJ0	DE	UPF0324 membrane protein LJ_1117;
N	Q74MR6	CC	This protein binds to the 23S rRNA, and is important in its secondary structure
N	Q74MR6	CC	It is located near the subunit interface in the base of the L7/L12 stalk, and near the tRNA binding site of the peptidyltransferase center (By similarity).
N	Q74MR6	DE	50S ribosomal protein L6P;
N	Q74MR6	DR	structural constituent of ribosome
N	Q751Z4	CC	May be involved in decane metabolism and autophagy
N	Q751Z4	CC	Involved in the biosynthesis of sterol glucoside (By similarity).
N	Q751Z4	DE	Autophagy-related protein 26;
N	Q751Z4	DE	Sterol 3-beta-glucosyltransferase;
N	Q751Z4	DR	lipid glycosylation
N	Q751Z4	DR	protein transport
N	Q751Z4	DR	sterol 3-beta-glucosyltransferase activity
N	Q751Z4	DR	sterol biosynthetic process
N	Q753I0	CC	The GINS complex plays an essential role in the initiation of DNA replication
N	Q753I0	CC	Has a role in chromosome segregation (By similarity).
N	Q753I0	DE	DNA replication complex GINS protein SLD5;
N	Q753I0	DR	DNA replication

N	Q753I0	DR	chromosome segregation
N	Q755C8	CC	Formation of pseudouridine at positions 27 and 28 in the anticodon stem and loop of transfer RNAs; at positions 34 and 36 of intron-containing precursor tRNA(Ile) and at position 35 in the intron-containing tRNA(Tyr) (By similarity).
N	Q755C8	DE	tRNA pseudouridine synthase 1;
N	Q755C8	DE	tRNA pseudouridylate synthase 1;
N	Q755C8	DE	tRNA-uridine isomerase 1;
N	Q755C8	DR	metal ion binding
N	Q755C8	DR	pseudouridine synthase activity
N	Q755C8	DR	pseudouridine synthesis
N	Q755C8	DR	tRNA processing
N	Q755G5	CC	DNA helicase which participates in several chromatin remodeling complexes, including the SWR1 and the INO80 complexes
N	Q755G5	CC	Also involved in pre-rRNA processing (By similarity).
N	Q755G5	CC	The INO80 complex remodels chromatin by shifting nucleosomes and is involved in DNA repair
N	Q755G5	CC	The SWR1 complex mediates the ATP-dependent exchange of histone H2A for the H2A variant HZT1 leading to transcriptional regulation of selected genes by chromatin remodeling
N	Q755G5	DE	RuvB-like helicase 2;
N	Q755G5	DR	DNA helicase activity
N	Q755G5	DR	chromatin modification
N	Q755G5	DR	rRNA processing
N	Q756C4	DE	Protein EFR3;
N	Q75AX7	CC	Component of the SRB8-11 complex
N	Q75AX7	CC	It may inhibit the association of the Mediator complex with RNA polymerase II to form the holoenzyme complex
N	Q75AX7	CC	The SRB8-11 complex is a regulatory module of the Mediator complex which is itself involved in regulation of basal and activated RNA polymerase II-dependent transcription
N	Q75AX7	CC	The SRB8-11 complex may be involved in the transcriptional repression of a subset of genes regulated by Mediator
N	Q75AX7	CC	The SRB8-11 complex phosphorylates the C-terminal domain (CTD) of the largest subunit of RNA polymerase II (By similarity).
N	Q75AX7	DE	RNA polymerase II holoenzyme cyclin-like subunit;
N	Q75BH9	CC	GDP-GTP exchange factor component of the mitotic exit network (MEN)
N	Q75BH9	CC	Fine-tunes the timing of the mitotic exit and couples this event with cytokinesis
N	Q75BH9	CC	May also be involved in proprotein-processing in the secretory pathway (By similarity).
N	Q75BH9	DE	Guanine nucleotide exchange factor LTE1;
N	Q75BH9	DR	cell division
N	Q75BH9	DR	guanyl-nucleotide exchange factor activity
N	Q75BH9	DR	regulation of small GTPase mediated signal transduction
N	Q75BH9	DR	small GTPase mediated signal transduction
N	Q75DY0	CC	Tetrapolymerization of the monopyrrole PBG into the hydroxymethylbilane pre-uroporphyrinogen in several discrete steps (By similarity).
N	Q75DY0	DE	Hydroxymethylbilane synthase;
N	Q75DY0	DE	Porphobilinogen deaminase;
N	Q75DY0	DE	Pre-uroporphyrinogen synthase;
N	Q75DY0	DR	heme biosynthetic process
N	Q75DY0	DR	hydroxymethylbilane synthase activity
N	Q75DY0	DR	peptidyl-pyrromethane cofactor linkage
N	Q75FW3	DE	3-dehydroquinate synthase;
N	Q75FW3	DR	3-dehydroquinate synthase activity
N	Q75FW3	DR	aromatic amino acid family biosynthetic process
N	Q75ZQ2	CC	Required for maturation of urease via the functional incorporation of the urease nickel metallocenter (By similarity).
N	Q75ZQ2	DE	Urease accessory protein ureD;
N	Q75ZQ2	DR	nickel ion binding
N	Q75ZQ2	DR	nitrogen compound metabolic process
N	Q788J2	DE	Polyprotein p42;
N	Q788J2	DE	Protein CM2;
N	Q788J2	DE	Protein M1';
N	Q7A0D7	CC	Toxic component of a toxin-antitoxin (TA) module
N	Q7A0D7	CC	Cleavages occur preferentially in a U-rich region with a consensus sequence of 5'-[ACG]UU[ACG]-3' in single- stranded RNA (By similarity).

N	Q7A0D7	CC	Ribosome-independent, sequence-specific endoribonuclease that cleaves mRNA, thus inhibiting protein synthesis and inducing bacterial stasis
N	Q7A0D7	DE	Endoribonuclease MazF;
N	Q7A0D7	DE	mRNA interferase MazF;
N	Q7A0D7	DR	endonuclease activity
N	Q7A150	CC	Involved in heme (porphyrin) scavenging
N	Q7A150	CC	Binds Fe(2+) and Fe(3+) heme but the largest fraction is Fe(2+) heme
N	Q7A150	CC	Functions as a high-affinity heme binding protein and probably has a role in relaying heme-iron from cell wall-anchored isd proteins receptors to the putative probable isdF (By similarity).
N	Q7A150	DE	High-affinity heme uptake system protein isdE;
N	Q7A150	DE	Iron-regulated surface determinant protein E;
N	Q7A150	DE	Staphylococcal iron-regulated protein F;
N	Q7A150	DR	high-affinity iron ion transport
N	Q7A150	DR	iron ion transmembrane transporter activity
N	Q7A150	DR	metal ion binding
N	Q7A3I5	DE	D-fructose-1,6-bisphosphate 1-phosphohydrolase class 3;
N	Q7A3I5	DE	FBPase class 3;
N	Q7A3I5	DE	Fructose-1,6-bisphosphatase class 3;
N	Q7A3I5	DR	fructose 1,6-bisphosphate 1-phosphatase activity
N	Q7A3I5	DR	gluconeogenesis
N	Q7A551	DE	Putative universal stress protein SA1532;
N	Q7A551	DR	response to stress
N	Q7A6M9	DE	Organic hydroperoxide resistance protein-like;
N	Q7A6M9	DR	response to stress
N	Q7BWI3	CC	May be involved in signal transduction
N	Q7BWI3	CC	Participates in the kaiABC clock protein complex, which constitutes the main circadian regulator in cyanobacteria, via its interaction with kaiC
N	Q7BWI3	CC	Required for robustness of the circadian rhythm of gene expression and is involved in clock outputs (By similarity).
N	Q7BWI3	DE	Adaptive-response sensory-kinase sasA;
N	Q7BWI3	DR	peptidyl-histidine phosphorylation
N	Q7BWI3	DR	rhythmic process
N	Q7BWI3	DR	two-component sensor activity
N	Q7CQK1	CC	Catalyzes the excretion of spermidine (By similarity).
N	Q7CQK1	DE	Spermidine export protein mdtJ;
N	Q7CR64	DE	UPF0325 protein yaeH;
N	Q7EZW6	CC	Thought to be a Golgi-localized beta-glycan synthase that polymerize the backbones of noncellulosic polysaccharides (hemicelluloses) of plant cell wall.
N	Q7EZW6	DE	Cellulose synthase-like protein D3;
N	Q7EZW6	DR	cellular cell wall organization
N	Q7EZW6	DR	cellulose biosynthetic process
N	Q7EZW6	DR	cellulose synthase (UDP-forming) activity
N	Q7FAT5	DE	Cyclin-P2-1;
N	Q7FAT5	DR	cell division
N	Q7G9E7	DE	Putative F-box protein At1g52480;
N	Q7GZA1	DE	Protein psbN;
N	Q7GZA1	DR	photosynthesis
N	Q7IS11	CC	Usually encoded in the trnK tRNA gene intron
N	Q7IS11	CC	Probably assists in splicing its own and other chloroplast group II introns (By similarity).
N	Q7IS11	DE	Intron maturase;
N	Q7IS11	DR	mRNA processing
N	Q7IS11	DR	tRNA processing
N	Q7J189	CC	Seems to play a role in the dimerization of PSII (By similarity).
N	Q7J189	DE	Photosystem II reaction center protein T;
N	Q7J189	DR	photosynthesis
N	Q7J1C3	CC	Required for PSII activity (By similarity).
N	Q7J1C3	DE	PSII 5 kDa protein;
N	Q7J1C3	DE	Photosystem II reaction center protein L;
N	Q7J1C3	DR	photosynthesis
N	Q7K2Y9	CC	Mediates the side-chain deamidation of N-terminal glutamine residues to glutamate, an important step in N-end rule pathway of protein degradation
N	Q7K2Y9	CC	Conversion of the resulting N- terminal glutamine to glutamate renders the protein susceptible to arginylation, polyubiquitination and degradation as specified by the N-end rule

N	Q7K2Y9	CC	Does not act on substrates with internal or C- terminal glutamine and does not act on non-glutamine residues in any position (By similarity).
N	Q7K2Y9	DE	N-terminal Gln amidase;
N	Q7K2Y9	DE	Nt(Q)-amidase;
N	Q7K2Y9	DE	Protein N-terminal glutamine amidohydrolase;
N	Q7K2Y9	DE	Protein NH2-terminal glutamine deamidase;
N	Q7K2Y9	DE	Protein tungus;
N	Q7K2Y9	DR	hydrolase activity, acting on carbon-nitrogen (but not peptide) bonds, in linear amides
N	Q7K2Y9	DR	olfactory learning
N	Q7K2Y9	DR	protein binding
N	Q7LZI6	CC	This platelet antagonist specifically and reversibly binds to a site on platelet GPIb close to or identical with the site for vWF binding
N	Q7LZI6	CC	It inhibits the binding of vWF to platelets.
N	Q7LZI6	DE	Tokaracetin subunit beta;
N	Q7LZI6	DR	sugar binding
N	Q7M459	CC	Water-borne pheromone that attract the marine mollusk Aplysia into breeding aggregations and coordinate male and female reproductive behavior within the aggregation (By similarity).
N	Q7M459	DR	mating pheromone activity
N	Q7M459	DR	sexual reproduction
N	Q7M4F3	CC	Component of the abdominal endocuticle.
N	Q7M4F3	DE	Endocuticle structural glycoprotein SgAbd-2;
N	Q7M4F3	DR	structural constituent of cuticle
N	Q7M8E0	CC	Binds the lower part of the 30S subunit head
N	Q7M8E0	CC	Binds mRNA in the 70S ribosome, positioning it for translation (By similarity).
N	Q7M8E0	DE	30S ribosomal protein S3;
N	Q7M8E0	DR	structural constituent of ribosome
N	Q7M936	CC	Catalyzes the conversion of D-ribulose 5-phosphate to formate and 3,4-dihydroxy-2-butanone 4-phosphate (By similarity).
N	Q7M936	DE	3,4-dihydroxy-2-butanone 4-phosphate synthase;
N	Q7M936	DE	DHBP synthase;
N	Q7M936	DR	3,4-dihydroxy-2-butanone-4-phosphate synthase activity
N	Q7M936	DR	GTP cyclohydrolase II activity
N	Q7M936	DR	metal ion binding
N	Q7M936	DR	riboflavin biosynthetic process
N	Q7MF12	CC	Can catalyze the oxidation of choline to betaine aldehyde and betaine aldehyde to glycine betaine (By similarity).
N	Q7MF12	DE	Choline dehydrogenase;
N	Q7MF12	DR	choline dehydrogenase activity
N	Q7MF12	DR	flavin adenine dinucleotide binding
N	Q7MF12	DR	glycine betaine biosynthetic process from choline
N	Q7MF12	DR	oxidation-reduction process
N	Q7MGH6	CC	F(1)F(0) ATP synthase produces ATP from ADP in the presence of a proton or sodium gradient
N	Q7MGH6	CC	During catalysis, ATP synthesis in the catalytic domain of F(1) is coupled via a rotary mechanism of the central stalk subunits to proton translocation (By similarity).
N	Q7MGH6	CC	F-type ATPases consist of two structural domains, F(1) containing the extramembraneous catalytic core and F(0) containing the membrane proton channel, linked together by a central stalk and a peripheral stalk
N	Q7MGH6	DE	ATP synthase F(0) sector subunit b;
N	Q7MGH6	DE	ATP synthase subunit b;
N	Q7MGH6	DE	ATPase subunit I;
N	Q7MGH6	DE	F-ATPase subunit b;
N	Q7MGH6	DE	F-type ATPase subunit b;
N	Q7MGH6	DR	ATP synthesis coupled proton transport
N	Q7MGH6	DR	hydrogen ion transmembrane transporter activity
N	Q7MHP1	CC	Catalyzes the complicated ring closure reaction between the two acyclic compounds 1-deoxy-D-xylulose-5-phosphate (DXP) and 3-amino-2-oxopropyl phosphate (1-amino-acetone-3-phosphate or AAP) to form pyridoxine 5'-phosphate (PNP) and inorganic phosphate (By similarity).
N	Q7MHP1	DE	PNP synthase;
N	Q7MHP1	DE	Pyridoxine 5'-phosphate synthase;
N	Q7MHP1	DR	pyridoxine 5'-phosphate synthase activity
N	Q7MHP1	DR	pyridoxine biosynthetic process
N	Q7MNP1	CC	Sequence-specific endonuclease that cleaves unmethylated GATC sequences

N	Q7MNP1	CC	It is involved in DNA mismatch repair (By similarity).
N	Q7MNP1	DE	DNA mismatch repair protein mutH;
N	Q7MNP1	DE	Methyl-directed mismatch repair protein;
N	Q7MNP1	DR	DNA modification
N	Q7MNP1	DR	endonuclease activity
N	Q7MPY4	CC	Involved in iron-sulfur cluster biogenesis
N	Q7MPY4	CC	Binds a 4Fe- 4S cluster, can transfer this cluster to apoproteins, and thereby intervenes in the maturation of Fe/S proteins
N	Q7MPY4	CC	Could also act as a scaffold/chaperone for damaged Fe/S proteins (By similarity).
N	Q7MPY4	DE	Fe/S biogenesis protein nfuA;
N	Q7MPY4	DR	4 iron, 4 sulfur cluster binding
N	Q7MPY4	DR	iron ion binding
N	Q7MPY4	DR	iron-sulfur cluster assembly
N	Q7MPY4	DR	protein maturation
N	Q7MU43	CC	Binds directly to 16S ribosomal RNA (By similarity).
N	Q7MU43	DE	30S ribosomal protein S20;
N	Q7MU43	DR	structural constituent of ribosome
N	Q7MVD7	CC	Catalyzes the attachment of valine to tRNA(Val)
N	Q7MVD7	CC	As ValRS can inadvertently accommodate and process structurally similar amino acids such as threonine, to avoid such errors, it has a "posttransfer" editing activity that hydrolyzes mischarged Thr-tRNA(Val) in a tRNA-dependent manner (By similarity)
N	Q7MVD7	DE	Valine--tRNA ligase;
N	Q7MVD7	DE	Valyl-tRNA synthetase;
N	Q7MVD7	DR	valine-tRNA ligase activity
N	Q7MVD7	DR	valyl-tRNA aminoacylation
N	Q7MXM0	DE	Aspartate--tRNA ligase;
N	Q7MXM0	DE	Aspartyl-tRNA synthetase;
N	Q7MXM0	DR	aspartate-tRNA ligase activity
N	Q7MXM0	DR	aspartyl-tRNA aminoacylation
N	Q7MXM0	DR	nucleic acid binding
N	Q7MXZ1	CC	Plays an important role in the initiation and regulation of chromosomal replication
N	Q7MXZ1	CC	Binds to the origin of replication; it binds specifically double-stranded DNA at a 9 bp consensus (dnaA box): 5'-TTATC[CA]A[CA]A-3'
N	Q7MXZ1	CC	DnaA binds to ATP and to acidic phospholipids (By similarity).
N	Q7MXZ1	DE	Chromosomal replication initiator protein DnaA;
N	Q7MXZ1	DR	DNA replication origin binding
N	Q7MXZ1	DR	DNA-dependent DNA replication initiation
N	Q7MXZ1	DR	nucleoside-triphosphatase activity
N	Q7MXZ1	DR	regulation of DNA replication
N	Q7MYY7	CC	One of the essential components for the initiation of protein synthesis
N	Q7MYY7	CC	Also involved in the hydrolysis of GTP during the formation of the 70S ribosomal complex (By similarity).
N	Q7MYY7	CC	Protects formylmethionyl-tRNA from spontaneous hydrolysis and promotes its binding to the 30S ribosomal subunits
N	Q7MYY7	DE	Translation initiation factor IF-2;
N	Q7MYY7	DR	GTPase activity
N	Q7MYY7	DR	translation initiation factor activity
N	Q7N2H0	CC	Catalyzes the hydrolysis of N(2)-succinylarginine into N(2)-succinylornithine, ammonia and CO(2) (By similarity).
N	Q7N2H0	DE	N-succinylarginine dihydrolase;
N	Q7N2H0	DR	N-succinylarginine dihydrolase activity
N	Q7N2H0	DR	arginine metabolic process
N	Q7N2U4	CC	Catalyzes the synthesis of alpha-ribazole-5'-phosphate from nicotinate mononucleotide (NAMN) and 5,6- dimethylbenzimidazole (DMB).
N	Q7N2U4	DE	N(1)-alpha-phosphoribosyltransferase;
N	Q7N2U4	DE	NN:DBI PRT;
N	Q7N2U4	DE	Nicotinate-nucleotide--dimethylbenzimidazole phosphoribosyltransferase;
N	Q7N2U4	DR	cobalamin biosynthetic process
N	Q7N2U4	DR	nicotinate-nucleotide-dimethylbenzimidazole phosphoribosyltransferase activity
N	Q7N2U4	DR	nucleoside biosynthetic process
N	Q7N8N7	DE	UDP-3-O-[3-hydroxymyristoyl] glucosamine N-acyltransferase;
N	Q7N8N7	DR	acyltransferase activity
N	Q7N8N7	DR	lipid A biosynthetic process

N	Q7N8N8	CC	Molecular chaperone that interacts specifically with outer membrane proteins, thus maintaining the solubility of early folding intermediates during passage through the periplasm (By similarity).
N	Q7N8N8	DE	Chaperone protein skp;
N	Q7N8N8	DR	unfolded protein binding
N	Q7NB34	DE	50S ribosomal protein L33 1;
N	Q7NB34	DR	structural constituent of ribosome
N	Q7NBP5	CC	Catalyzes the attachment of proline to tRNA(Pro) in a two-step reaction: proline is first activated by ATP to form Pro- AMP and then transferred to the acceptor end of tRNA(Pro) (By similarity).
N	Q7NBP5	DE	Proline--tRNA ligase;
N	Q7NBP5	DE	Prolyl-tRNA synthetase;
N	Q7NBP5	DR	proline-tRNA ligase activity
N	Q7NBP5	DR	prolyl-tRNA aminoacylation
N	Q7NDU3	DE	UPF0284 protein glr4139;
N	Q7NDU3	DR	cobalamin biosynthetic process
N	Q7NDU3	DR	nicotinate-nucleotide-dimethylbenzimidazole phosphoribosyltransferase activity
N	Q7NEW2	CC	Cleaves peptides in various proteins in a process that requires ATP hydrolysis
N	Q7NEW2	CC	Has a chymotrypsin-like activity
N	Q7NEW2	CC	Plays a major role in the degradation of misfolded proteins (By similarity).
N	Q7NEW2	DE	ATP-dependent Clp protease proteolytic subunit 1;
N	Q7NEW2	DE	Endopeptidase Clp 1;
N	Q7NEW2	DR	serine-type endopeptidase activity
N	Q7NFA4	CC	Converts 2C-methyl-D-erythritol 2,4-cyclodiphosphate (ME-2,4cPP) into 1-hydroxy-2-methyl-2-(E)-butenyl 4-diphosphate (By similarity).
N	Q7NFA4	DE	1-hydroxy-2-methyl-2-(E)-butenyl 4-diphosphate synthase;
N	Q7NFA4	DE	4-hydroxy-3-methylbut-2-en-1-yl diphosphate synthase;
N	Q7NFA4	DR	4 iron, 4 sulfur cluster binding
N	Q7NFA4	DR	4-hydroxy-3-methylbut-2-en-1-yl diphosphate synthase activity
N	Q7NFA4	DR	iron ion binding
N	Q7NFA4	DR	oxidation-reduction process
N	Q7NFA4	DR	terpenoid biosynthetic process
N	Q7NFM6	DE	UPF0133 protein glr3498;
N	Q7NGV3	CC	Binds directly to 23S ribosomal RNA and is necessary for the in vitro assembly process of the 50S ribosomal subunit
N	Q7NGV3	CC	It is not involved in the protein synthesizing functions of that subunit (By similarity).
N	Q7NGV3	DE	50S ribosomal protein L20;
N	Q7NGV3	DR	structural constituent of ribosome
N	Q7NNG8	CC	Catalyzes the last two sequential reactions in the de novo biosynthetic pathway for UDP-GlcNAc
N	Q7NNG8	CC	Responsible for the acetylation of Glc-N-1-P to give GlcNAc-1-P and for the uridyl transfer from UTP to GlcNAc-1-P which produces UDP-GlcNAc (By similarity).
N	Q7NNG8	DE	Bifunctional protein glmU;
N	Q7NNG8	DE	Glucosamine-1-phosphate N-acetyltransferase;
N	Q7NNG8	DE	N-acetylglucosamine-1-phosphate uridyltransferase;
N	Q7NNG8	DE	UDP-N-acetylglucosamine pyrophosphorylase;
N	Q7NNG8	DR	UDP-N-acetylglucosamine diphosphorylase activity
N	Q7NNG8	DR	cell morphogenesis
N	Q7NNG8	DR	cellular cell wall organization
N	Q7NNG8	DR	glucosamine-1-phosphate N-acetyltransferase activity
N	Q7NNG8	DR	lipopolysaccharide biosynthetic process
N	Q7NNG8	DR	magnesium ion binding
N	Q7NNG8	DR	peptidoglycan biosynthetic process
N	Q7NNG8	DR	regulation of cell shape
N	Q7NZD3	CC	Methyltransferase required for the conversion of dimethylmenaquinone (DMKH2) to menaquinone (MKH2) and the conversion of 2-polyprenyl-6-methoxy-1,4-benzoquinol (DDMQH2) to 2-polyprenyl-3-methyl-6-methoxy-1,4-benzoquinol (DMQH2) (By similarity).
N	Q7NZD3	DE	Ubiquinone/menaquinone biosynthesis methyltransferase ubiE;
N	Q7NZD3	DR	menaquinone biosynthetic process
N	Q7NZD3	DR	methyltransferase activity
N	Q7NZD3	DR	ubiquinone biosynthetic process
N	Q7PRA4	CC	Required for polyglutamylation of axonemal tubulin in sensory cilia (By similarity).

N	Q7PRA4	CC	Plays a role in anterograde intraflagellar transport (IFT), the process by which cilia precursors are transported from the base of the cilium to the site of their incorporation at the tip (By similarity).
N	Q7PRA4	DE	TPR repeat protein 30 homolog;
N	Q7PRA4	DE	Tetratricopeptide repeat protein 30 homolog;
N	Q7PRA4	DR	intraflagellar transport
N	Q7PRA4	DR	protein polyglutamylation
N	Q7PRA4	DR	transporter activity
N	Q7SAC2	CC	Probable mitochondrial mRNA stabilization factor (By similarity).
N	Q7SAC2	DE	ATPase synthesis protein 25, mitochondrial;
N	Q7SAK5	CC	Involved in pre-mRNA splicing and cell cycle progression (By similarity).
N	Q7SAK5	DE	Pre-mRNA-splicing factor syf-1;
N	Q7SAK5	DR	mRNA processing
N	Q7T3N6	DE	60S ribosomal protein L15;
N	Q7T3N6	DR	structural constituent of ribosome
N	Q7TMQ7	DE	WD repeat-containing protein 91;
N	Q7TNV9	CC	Has antibacterial activity (By similarity).
N	Q7TNV9	DE	Beta-defensin 14;
N	Q7TNV9	DE	Defensin, beta 14;
N	Q7TNV9	DR	defense response to bacterium
N	Q7TUZ8	CC	Binds as a heterodimer with protein S6 to the central domain of the 16S rRNA, where it helps stabilize the platform of the 30S subunit (By similarity).
N	Q7TUZ8	DE	30S ribosomal protein S18;
N	Q7TUZ8	DR	structural constituent of ribosome
N	Q7TZ12	CC	Protein modifier that is covalently attached to lysine residues of substrate proteins, thereby targeting them for proteasomal degradation
N	Q7TZ12	CC	The tagging system is termed pupylation (By similarity).
N	Q7TZ12	DE	Bacterial ubiquitin-like modifier;
N	Q7TZ12	DE	Prokaryotic ubiquitin-like protein Pup;
N	Q7U2U1	CC	Converts the D-glycero-alpha-D-manno-heptose 1,7- bisphosphate intermediate into D-glycero-alpha-D-manno-heptose 1- phosphate by removing the phosphate group at the C-7 position (By similarity).
N	Q7U2U1	DE	D,D-heptose 1,7-bisphosphate phosphatase;
N	Q7U2U1	DE	D-glycero-D-manno-heptose 1,7-bisphosphate phosphatase;
N	Q7U2U1	DR	carbohydrate metabolic process
N	Q7U2U1	DR	phosphatase activity
N	Q7U437	CC	Phosphorylation of dTMP to form dTDP in both de novo and salvage pathways of dTTP synthesis (By similarity).
N	Q7U437	DE	Thymidylate kinase;
N	Q7U437	DE	dTMP kinase;
N	Q7U437	DR	dTDP biosynthetic process
N	Q7U437	DR	thymidylate kinase activity
N	Q7U4D9	CC	Catalyzes the radical-mediated insertion of two sulfur atoms into the C-6 and C-8 positions of the octanoyl moiety bound to the lipoyl domains of lipoate-dependent enzymes, thereby converting the octanoylated domains into lipoylated derivatives (By similarity).
N	Q7U4D9	DE	Lipoate synthase 1;
N	Q7U4D9	DE	Lipoic acid synthase 1;
N	Q7U4D9	DE	Lipoyl synthase 1;
N	Q7U4D9	DE	Sulfur insertion protein lipA1;
N	Q7U4D9	DR	4 iron, 4 sulfur cluster binding
N	Q7U4D9	DR	lipoate biosynthetic process
N	Q7U4D9	DR	lipoate synthase activity
N	Q7U4D9	DR	metal ion binding
N	Q7U4J9	CC	One of the primary rRNA binding proteins, this protein initially binds near the 5'-end of the 23S rRNA
N	Q7U4J9	CC	It is important during the early stages of 50S assembly
N	Q7U4J9	CC	It makes multiple contacts with different domains of the 23S rRNA in the assembled 50S subunit and ribosome (By similarity).
N	Q7U4J9	DE	50S ribosomal protein L4;
N	Q7U4J9	DR	structural constituent of ribosome
N	Q7U4P7	CC	Catalyzes the two-electron reduction of biliverdin IX- alpha at the C15 methine bridge (By similarity).
N	Q7U4P7	DE	15,16-dihydrobiliverdin:ferredoxin oxidoreductase;
N	Q7U4P7	DR	15,16-dihydrobiliverdin:ferredoxin oxidoreductase activity

N	Q7U4P7	DR	cobalt ion binding
N	Q7U4P7	DR	oxidation-reduction process
N	Q7U4P7	DR	phytochromobilin biosynthetic process
N	Q7U8W4	CC	Produces ATP from ADP in the presence of a proton gradient across the membrane
N	Q7U8W4	CC	The gamma chain is believed to be important in regulating ATPase activity and the flow of protons through the CF(0) complex.
N	Q7U8W4	DE	ATP synthase F1 sector gamma subunit;
N	Q7U8W4	DE	ATP synthase gamma chain;
N	Q7U8W4	DE	F-ATPase gamma subunit;
N	Q7U8W4	DR	ATP synthesis coupled proton transport
N	Q7U8W4	DR	hydrogen ion transporting ATP synthase activity, rotational mechanism
N	Q7U8W4	DR	proton-transporting ATPase activity, rotational mechanism
N	Q7UDG0	DE	Putative protein hokF;
N	Q7UJK0	CC	Has an important function as a repair enzyme for proteins that have been inactivated by oxidation
N	Q7UJK0	CC	Catalyzes the reversible oxidation-reduction of methionine sulfoxide in proteins to methionine (By similarity).
N	Q7UJK0	DE	Peptide Met(O) reductase 1;
N	Q7UJK0	DE	Peptide methionine sulfoxide reductase MsrA 1;
N	Q7UJK0	DE	Peptide-methionine (S)-S-oxide reductase 1;
N	Q7UJK0	DE	Protein-methionine-S-oxide reductase 1;
N	Q7UJK0	DR	oxidation-reduction process
N	Q7UJK0	DR	peptide-methionine-(S)-S-oxide reductase activity
N	Q7UJK0	DR	protein metabolic process
N	Q7UQJ2	DE	UPF0365 protein RB6291;
N	Q7UR95	CC	Involved in mRNA degradation
N	Q7UR95	CC	Hydrolyzes single-stranded polyribonucleotides processively in the 3'- to 5'-direction (By similarity).
N	Q7UR95	DE	Polynucleotide phosphorylase;
N	Q7UR95	DE	Polyribonucleotide nucleotidyltransferase;
N	Q7UR95	DR	3'-5'-exoribonuclease activity
N	Q7UR95	DR	RNA processing
N	Q7UR95	DR	mRNA catabolic process
N	Q7UR95	DR	polyribonucleotide nucleotidyltransferase activity
N	Q7V1V0	DE	30S ribosomal protein S2;
N	Q7V1V0	DR	structural constituent of ribosome
N	Q7V2D0	CC	RuBisCO catalyzes two reactions: the carboxylation of D- ribulose 1,5-bisphosphate, the primary event in carbon dioxide fixation, as well as the oxidative fragmentation of the pentose substrate in the photorespiration process
N	Q7V2D0	CC	Both reactions occur simultaneously and in competition at the same active site (By similarity).
N	Q7V2D0	DE	Ribulose bisphosphate carboxylase large chain;
N	Q7V2D0	DE	RuBisCO large subunit;
N	Q7V2D0	DR	magnesium ion binding
N	Q7V2D0	DR	monooxygenase activity
N	Q7V2D0	DR	oxidation-reduction process
N	Q7V2D0	DR	photorespiration
N	Q7V2D0	DR	reductive pentose-phosphate cycle
N	Q7V2D0	DR	ribulose-bisphosphate carboxylase activity
N	Q7V3M9	CC	Catalyzes the biosynthesis of agmatine from arginine (By similarity).
N	Q7V3M9	DE	Biosynthetic arginine decarboxylase;
N	Q7V3M9	DR	arginine catabolic process
N	Q7V3M9	DR	arginine decarboxylase activity
N	Q7V3M9	DR	metal ion binding
N	Q7V3M9	DR	spermidine biosynthetic process
N	Q7V3T5	CC	Acts as a chaperone (By similarity).
N	Q7V3T5	DE	Chaperone protein dnaK2;
N	Q7V3T5	DE	Heat shock 70 kDa protein 2;
N	Q7V3T5	DE	Heat shock protein 70-2;
N	Q7V3T5	DR	protein folding
N	Q7V3T5	DR	response to stress
N	Q7V3T5	DR	unfolded protein binding
N	Q7V531	CC	This protein binds to the 23S rRNA, and is important in its secondary structure
N	Q7V531	CC	It is located near the subunit interface in the base of the L7/L12 stalk, and near the tRNA binding site of the peptidyltransferase center (By similarity).

N	Q7V531	DE	50S ribosomal protein L6;
N	Q7V531	DR	structural constituent of ribosome
N	Q7V7L7	CC	The UvrABC repair system catalyzes the recognition and processing of DNA lesions
N	Q7V7L7	CC	The N-terminal half is responsible for the 3' incision and the C-terminal half is responsible for the 5' incision (By similarity).
N	Q7V7L7	CC	UvrC both incises the 5' and 3' sides of the lesion
N	Q7V7L7	DE	Excinuclease ABC subunit C;
N	Q7V7L7	DE	Protein uvrC;
N	Q7V7L7	DE	UvrABC system protein C;
N	Q7V7L7	DR	excinuclease ABC activity
N	Q7V7L7	DR	nucleotide-excision repair
N	Q7V926	CC	May play a key role in the regulation of the intracellular concentration of adenosylhomocysteine (By similarity).
N	Q7V926	DE	Adenosylhomocysteinase;
N	Q7V926	DE	S-adenosyl-L-homocysteine hydrolase;
N	Q7V926	DR	adenosylhomocysteinase activity
N	Q7V926	DR	one-carbon metabolic process
N	Q7VEU0	CC	Catalyzes the 1,3-allylic rearrangement of the homoallylic substrate isopentenyl (IPP) to its highly electrophilic allylic isomer, dimethylallyl diphosphate (DMAPP) (By similarity).
N	Q7VEU0	DE	IPP isomerase;
N	Q7VEU0	DE	IPP:DMAPP isomerase;
N	Q7VEU0	DE	Isopentenyl pyrophosphate isomerase;
N	Q7VEU0	DE	Isopentenyl-diphosphate Delta-isomerase;
N	Q7VEU0	DR	hydrolase activity
N	Q7VEU0	DR	isopentenyl-diphosphate delta-isomerase activity
N	Q7VEU0	DR	isoprenoid biosynthetic process
N	Q7VEU0	DR	metal ion binding
N	Q7VFL1	CC	Interconversion of serine and glycine.
N	Q7VFL1	DE	Serine hydroxymethyltransferase;
N	Q7VFL1	DE	Serine methylase;
N	Q7VFL1	DR	L-serine metabolic process
N	Q7VFL1	DR	glycine hydroxymethyltransferase activity
N	Q7VFL1	DR	glycine metabolic process
N	Q7VFL1	DR	one-carbon metabolic process
N	Q7VFL1	DR	pyridoxal phosphate binding
N	Q7VH68	DE	UDP-3-O-[3-hydroxymyristoyl] glucosamine N-acyltransferase;
N	Q7VH68	DR	acyltransferase activity
N	Q7VH68	DR	lipid A biosynthetic process
N	Q7VIA7	CC	Furnishes a means for formation of correctly charged Gln-tRNA(Gln) through the transamidation of misacylated Glu- tRNA(Gln) in organisms which lack glutaminyl-tRNA synthetase
N	Q7VIA7	CC	The reaction takes place in the presence of glutamine and ATP through an activated gamma-phospho-Glu-tRNA(Gln) (By similarity).
N	Q7VIA7	DE	Glu-ADT subunit A;
N	Q7VIA7	DE	Glutamyl-tRNA(Gln) amidotransferase subunit A;
N	Q7VIA7	DR	carbon-nitrogen ligase activity, with glutamine as amido-N-donor
N	Q7VIS3	DE	Glycine--tRNA ligase alpha subunit;
N	Q7VIS3	DE	Glycyl-tRNA synthetase alpha subunit;
N	Q7VIS3	DR	glycine-tRNA ligase activity
N	Q7VIS3	DR	glycyl-tRNA aminoacylation
N	Q7VJ81	CC	Seems to be the binding site for several of the factors involved in protein synthesis and appears to be essential for accurate translation (By similarity).
N	Q7VJ81	DE	50S ribosomal protein L7/L12;
N	Q7VJ81	DR	structural constituent of ribosome
N	Q7VMB7	CC	Participates actively in the response to hyperosmotic and heat shock by preventing the aggregation of stress-denatured proteins, in association with dnaK and grpE
N	Q7VMB7	CC	GrpE releases ADP from dnaK; ATP binding to dnaK triggers the release of the substrate protein, thus completing the reaction cycle
N	Q7VMB7	CC	It is the nucleotide exchange factor for dnaK and may function as a thermosensor
N	Q7VMB7	CC	Several rounds of ATP- dependent interactions between dnaJ, dnaK and grpE are required for fully efficient folding (By similarity).
N	Q7VMB7	CC	Unfolded proteins bind initially to dnaJ; upon interaction with the dnaJ-bound protein, dnaK hydrolyzes its bound ATP, resulting in the formation of a stable complex
N	Q7VMB7	DE	HSP-70 cofactor;
N	Q7VMB7	DE	Protein grpE;

N	Q7VMB7	DR	adenyl-nucleotide exchange factor activity
N	Q7VMB7	DR	chaperone binding
N	Q7VMB7	DR	protein folding
N	Q7VMB7	DR	protein homodimerization activity
N	Q7VMB7	DR	response to stress
N	Q7VMZ4	CC	Required to facilitate the formation of correct disulfide bonds in some periplasmic proteins and for the assembly of the periplasmic c-type cytochromes
N	Q7VMZ4	CC	Acts by transferring electrons from cytoplasmic thioredoxin to the periplasm
N	Q7VMZ4	CC	This transfer involves a cascade of disulfide bond formation and reduction steps (By similarity).
N	Q7VMZ4	DE	Disulfide reductase;
N	Q7VMZ4	DE	Protein-disulfide reductase;
N	Q7VMZ4	DE	Thiol:disulfide interchange protein DsbD;
N	Q7VMZ4	DR	cell redox homeostasis
N	Q7VMZ4	DR	cytochrome complex assembly
N	Q7VMZ4	DR	electron carrier activity
N	Q7VMZ4	DR	electron transport chain
N	Q7VMZ4	DR	glycerol ether metabolic process
N	Q7VMZ4	DR	protein disulfide oxidoreductase activity
N	Q7VMZ4	DR	protein-disulfide reductase activity
N	Q7VPN5	CC	Methylates ribosomal protein L11 (By similarity).
N	Q7VPN5	DE	Ribosomal protein L11 methyltransferase;
N	Q7VPN5	DR	protein methyltransferase activity
N	Q7VQD8	CC	Binds to 23S rRNA
N	Q7VQD8	CC	Forms part of two intersubunit bridges in the 70S ribosome (By similarity).
N	Q7VQD8	DE	50S ribosomal protein L14;
N	Q7VQD8	DR	structural constituent of ribosome
N	Q7VQN2	CC	This protein binds to 23S rRNA in the presence of protein L20 (By similarity).
N	Q7VQN2	DE	50S ribosomal protein L21;
N	Q7VQN2	DR	structural constituent of ribosome
N	Q7VQR0	DE	30S ribosomal protein S21;
N	Q7VQR0	DR	structural constituent of ribosome
N	Q7VRE2	CC	Catalyzes the NADP-dependent rearrangement and reduction of 1-deoxy-D-xylulose-5-phosphate (DXP) to 2-C-methyl-D-erythritol 4-phosphate (MEP) (By similarity).
N	Q7VRE2	DE	1-deoxy-D-xylulose 5-phosphate reductoisomerase;
N	Q7VRE2	DE	1-deoxyxylulose-5-phosphate reductoisomerase;
N	Q7VRE2	DE	2-C-methyl-D-erythritol 4-phosphate synthase;
N	Q7VRE2	DE	DXP reductoisomerase;
N	Q7VRE2	DR	1-deoxy-D-xylulose-5-phosphate reductoisomerase activity
N	Q7VRE2	DR	isoprenoid biosynthetic process
N	Q7VRE2	DR	metal ion binding
N	Q7VRE2	DR	oxidation-reduction process
N	Q7VRV2	CC	NDH-1 shuttles electrons from NADH, via FMN and iron- sulfur (Fe-S) centers, to quinones in the respiratory chain
N	Q7VRV2	CC	Couples the redox reaction to proton translocation (for every two electrons transferred, four hydrogen ions are translocated across the cytoplasmic membrane), and thus conserves the redox energy in a proton gradient (By similarity).
N	Q7VRV2	CC	The immediate electron acceptor for the enzyme in this species is believed to be ubiquinone
N	Q7VRV2	DE	NADH dehydrogenase I subunit A;
N	Q7VRV2	DE	NADH-quinone oxidoreductase subunit A;
N	Q7VRV2	DE	NDH-1 subunit A;
N	Q7VRV2	DR	NADH dehydrogenase (ubiquinone) activity
N	Q7VRV2	DR	oxidation-reduction process
N	Q7VRV2	DR	quinone binding
N	Q7VRY3	CC	Catalyzes the NADPH-dependent reduction of glutamyl- tRNA(Glu) to glutamate 1-semialdehyde (GSA) (By similarity).
N	Q7VRY3	DE	Glutamyl-tRNA reductase;
N	Q7VRY3	DR	glutamyl-tRNA reductase activity
N	Q7VRY3	DR	oxidation-reduction process
N	Q7VRY3	DR	porphyrin biosynthetic process
N	Q7VRY3	DR	shikimate 5-dehydrogenase activity
N	Q7VUS7	CC	Catalyzes a trans-dehydration via an enolate intermediate (By similarity).
N	Q7VUS7	DE	3-dehydroquinase;
N	Q7VUS7	DE	3-dehydroquinate dehydratase;

N	Q7VUS7	DE	Type II DHQase;
N	Q7VUS7	DR	3-dehydroquinate dehydratase activity
N	Q7VUS7	DR	aromatic amino acid family biosynthetic process
N	Q7VXN4	CC	Required for the formation of a threonylcarbamoyl group on adenosine at position 37 (t(6)A37) in tRNAs that read codons beginning with adenine (By similarity).
N	Q7VXN4	DE	Probable tRNA threonylcarbamoyladenosine biosynthesis protein Gcp;
N	Q7VXN4	DE	t(6)A37 threonylcarbamoyladenosine biosynthesis protein;
N	Q7VXN4	DR	metal ion binding
N	Q7VXN4	DR	metalloendopeptidase activity
N	Q7W179	CC	The natural substrate for this enzyme may be peptidyl- tRNAs which drop off the ribosome during protein synthesis (By similarity).
N	Q7W179	DE	Peptidyl-tRNA hydrolase;
N	Q7W179	DR	aminoacyl-tRNA hydrolase activity
N	Q7W2B6	DE	3-dehydroquinate synthase;
N	Q7W2B6	DR	3-dehydroquinate synthase activity
N	Q7W2B6	DR	aromatic amino acid family biosynthetic process
N	Q7W2B7	CC	Catalyzes the specific phosphorylation of the 3-hydroxyl group of shikimic acid using ATP as a cosubstrate (By similarity).
N	Q7W2B7	DE	Shikimate kinase;
N	Q7W2B7	DR	aromatic amino acid family biosynthetic process
N	Q7W2B7	DR	metal ion binding
N	Q7W2B7	DR	shikimate kinase activity
N	Q7W2D9	CC	This is one of the proteins that binds and probably mediates the attachment of the 5S RNA into the large ribosomal subunit, where it forms part of the central protuberance (By similarity).
N	Q7W2D9	DE	50S ribosomal protein L18;
N	Q7W2D9	DR	structural constituent of ribosome
N	Q7W2F2	CC	Protein S19 forms a complex with S13 that binds strongly to the 16S ribosomal RNA (By similarity).
N	Q7W2F2	DE	30S ribosomal protein S19;
N	Q7W2F2	DR	structural constituent of ribosome
N	Q7W9J5	CC	Cleaves GlcNAc linked beta-1,4 to MurNAc tripeptides (By similarity).
N	Q7W9J5	DE	Beta-N-acetylhexosaminidase;
N	Q7W9J5	DE	Beta-hexosaminidase;
N	Q7W9J5	DE	N-acetyl-beta-glucosaminidase;
N	Q7W9J5	DR	beta-N-acetylhexosaminidase activity
N	Q7W9J5	DR	cell division
N	Q7W9J5	DR	cellular cell wall organization
N	Q7W9J5	DR	peptidoglycan biosynthetic process
N	Q7W9J5	DR	regulation of cell shape
N	Q7WBA1	CC	Necessary for formate dehydrogenase activity (By similarity).
N	Q7WBA1	DE	Protein FdhD homolog;
N	Q7WBA1	DR	formate dehydrogenase (NAD+) activity
N	Q7WD94	CC	Catalyzes the reversible oxidation of malate to oxaloacetate (By similarity).
N	Q7WD94	DE	Malate dehydrogenase;
N	Q7WD94	DR	L-malate dehydrogenase activity
N	Q7WD94	DR	cellular carbohydrate metabolic process
N	Q7WD94	DR	malate metabolic process
N	Q7WD94	DR	tricarboxylic acid cycle
N	Q7WGH4	CC	Cell wall formation (By similarity).
N	Q7WGH4	DE	UDP-N-acetylenolpyruvoylglucosamine reductase;
N	Q7WGH4	DE	UDP-N-acetylmuramate dehydrogenase;
N	Q7WGH4	DR	UDP-N-acetylmuramate dehydrogenase activity
N	Q7WGH4	DR	cell division
N	Q7WGH4	DR	cellular cell wall organization
N	Q7WGH4	DR	flavin adenine dinucleotide binding
N	Q7WGH4	DR	oxidation-reduction process
N	Q7WGH4	DR	peptidoglycan biosynthetic process
N	Q7WGH4	DR	regulation of cell shape
N	Q7WGH9	CC	Negative regulator of class I heat shock genes (grpE- dnaK-dnaJ and groELS operons).
N	Q7WGH9	CC	Prevents heat-shock induction of these operons (By similarity).
N	Q7WGH9	DE	Heat-inducible transcription repressor hrcA;
N	Q7WGH9	DR	regulation of transcription, DNA-dependent
N	Q7WGH9	DR	response to stress
N	Q7WGX3	CC	3'-to-5' exoribonuclease specific for small oligoribonucleotides (By similarity).

N	Q7WGX3	DE	Oligoribonuclease;
N	Q7WGX3	DR	exonuclease activity
N	Q7WGX3	DR	nucleic acid binding
N	Q7WUM8	CC	Is involved in NO detoxification in an aerobic process, termed nitric oxide dioxygenase (NOD) reaction that utilizes O(2) and NAD(P)H to convert NO to nitrate, which protects the bacterium from various noxious nitrogen compounds
N	Q7WUM8	CC	Therefore, plays a central role in the inducible response to nitrosative stress (By similarity).
N	Q7WUM8	DE	Flavohemoglobin;
N	Q7WUM8	DE	Flavohemoprotein;
N	Q7WUM8	DE	Hemoglobin-like protein;
N	Q7WUM8	DE	NO oxygenase;
N	Q7WUM8	DE	Nitric oxide dioxygenase;
N	Q7WUM8	DR	nitric oxide dioxygenase activity
N	Q7WUM8	DR	oxidation-reduction process
N	Q7WUM8	DR	oxygen binding
N	Q7WUM8	DR	oxygen transporter activity
N	Q7WUM8	DR	response to toxin
N	Q7WXC1	CC	Part of the ABC transporter complex CcmAB involved in the biogenesis of c-type cytochromes; once thought to export heme, this seems not to be the case, but its exact role is uncertain
N	Q7WXC1	CC	Responsible for energy coupling to the transport system (By similarity).
N	Q7WXC1	DE	Cytochrome c biogenesis ATP-binding export protein CcmA;
N	Q7WXC1	DE	Heme exporter protein A;
N	Q7WXC1	DR	cytochrome complex assembly
N	Q7WXC1	DR	heme-transporting ATPase activity
N	Q7X9H9	CC	Probable transcription factor (By similarity).
N	Q7X9H9	DE	Agamous-like MADS-box protein AGL93;
N	Q7X9H9	DR	protein binding
N	Q7X9H9	DR	regulation of transcription, DNA-dependent
N	Q7X9H9	DR	sequence-specific DNA binding
N	Q7X9H9	DR	sequence-specific DNA binding transcription factor activity
N	Q7XLY8	CC	Possesses E3 ubiquitin-protein ligase in vitro.
N	Q7XLY8	DE	E3 ubiquitin-protein ligase Os04g0590900;
N	Q7XLY8	DE	RING-H2 finger protein Os04g0590900;
N	Q7XLY8	DR	ubiquitin-protein ligase activity
N	Q7XLY8	DR	zinc ion binding
N	Q7Y201	CC	Acts in the modification of cell walls via demethylesterification of cell wall pectin (By similarity).
N	Q7Y201	DE	Pectin methylesterase 13;
N	Q7Y201	DE	Pectin methylesterase inhibitor 13;
N	Q7Y201	DE	Pectinesterase 13;
N	Q7Y201	DE	Pectinesterase inhibitor 13;
N	Q7Y201	DE	Probable pectinesterase/pectinesterase inhibitor 13;
N	Q7Y201	DR	aspartyl esterase activity
N	Q7Y201	DR	cell wall modification
N	Q7Y201	DR	enzyme inhibitor activity
N	Q7Y201	DR	pectinesterase activity
N	Q7YJV7	CC	Component of the cytochrome b6-f complex, which mediates electron transfer between photosystem II (PSII) and photosystem I (PSI), cyclic electron flow around PSI, and state transitions
N	Q7YJV7	CC	PetL is important for photoautotrophic growth as well as for electron transfer efficiency and stability of the cytochrome b6-f complex (By similarity).
N	Q7YJV7	DE	Cytochrome b6-f complex subunit 6;
N	Q7YJV7	DE	Cytochrome b6-f complex subunit VI;
N	Q7YJV7	DE	Cytochrome b6-f complex subunit petL;
N	Q7YJV7	DR	electron carrier activity
N	Q7YJV7	DR	electron transport chain
N	Q7YJV7	DR	photosynthesis
N	Q7YJW0	CC	Component of the cytochrome b6-f complex, which mediates electron transfer between photosystem II (PSII) and photosystem I (PSI), cyclic electron flow around PSI, and state transitions (By similarity).
N	Q7YJW0	DE	Apocytochrome f;
N	Q7YJW0	DR	electron carrier activity
N	Q7YJW0	DR	electron transport chain

N	Q7YJW0	DR	photosynthesis
N	Q7YJY8	CC	This is one of the two reaction center proteins of photosystem II (By similarity).
N	Q7YJY8	DE	32 kDa thylakoid membrane protein;
N	Q7YJY8	DE	Photosystem II protein D1;
N	Q7YJY8	DE	Photosystem Q(B) protein;
N	Q7YJY8	DR	electron transporter, transferring electrons within the cyclic electron transport pathway of photosynthesis activity
N	Q7YJY8	DR	metal ion binding
N	Q7YJY8	DR	photosynthetic electron transport in photosystem II
N	Q7YJY8	DR	response to herbicide
N	Q7YRH1	DE	Ribonuclease-like protein 9;
N	Q7YRH1	DR	nucleic acid binding
N	Q7YRH1	DR	pancreatic ribonuclease activity
N	Q7YZT6	CC	Catalyzes the production of trehalose from glucose-6- phosphate and UDP-glucose in a 2 step process.
N	Q7YZT6	DE	Alpha,alpha-trehalose-phosphate synthase [UDP-forming] 1;
N	Q7YZT6	DE	Trehalose-6-phosphate synthase 1;
N	Q7YZT6	DE	UDP-glucose-glucosephosphate glucosyltransferase 1;
N	Q7YZT6	DR	alpha,alpha-trehalose-phosphate synthase (UDP-forming) activity
N	Q7YZT6	DR	determination of adult lifespan
N	Q7YZT6	DR	trehalose biosynthetic process
N	Q7Z6G3	DE	EF-hand calcium-binding protein 2;
N	Q7Z6G3	DE	N-terminal EF-hand calcium-binding protein 2;
N	Q7Z6G3	DE	Neuronal calcium-binding protein 2;
N	Q7Z6G3	DE	Synaptotagmin-interacting protein 2;
N	Q7Z6G3	DR	antibiotic biosynthetic process
N	Q7Z6G3	DR	calcium ion binding
N	Q7Z6G3	DR	oxidoreductase activity
N	Q7Z6G3	DR	protein binding
N	Q7ZV50	DE	Magnesium transporter protein 1;
N	Q7ZV50	DR	cell redox homeostasis
N	Q7ZV50	DR	magnesium ion transport
N	Q7ZWG6	CC	Has been shown to act both as an intestinal proton- coupled high-affinity folate transporter and as an intestinal heme transporter which mediates heme uptake from the gut lumen into duodenal epithelial cells
N	Q7ZWG6	CC	Shows a higher affinity for folate than heme (By similarity).
N	Q7ZWG6	DE	Heme carrier protein 1;
N	Q7ZWG6	DE	Proton-coupled folate transporter;
N	Q7ZWG6	DE	Solute carrier family 46 member 1;
N	Q7ZWG6	DR	folic acid binding
N	Q7ZWG6	DR	transmembrane transport
N	Q7ZWU7	CC	May play a role as calcium-regulated intrinsic membrane protein (By similarity).
N	Q7ZWU7	DE	Extended synaptotagmin-2-B;
N	Q7ZXV5	CC	Protein-lysine N-methyltransferase that methylates both histones and non-histone proteins
N	Q7ZXV5	CC	Has also methyltransferase activity toward non-histone proteins such as p53/TP53 and RB1 (By similarity).
N	Q7ZXV5	CC	Specifically methylates histone H3 'Lys-4' (H3K4me) and dimethylates histone H3 'Lys-36' (H3K36me2)
N	Q7ZXV5	DE	Histone methyltransferase SMYD2-A;
N	Q7ZXV5	DE	N-lysine methyltransferase SMYD2-A;
N	Q7ZXV5	DE	SET and MYND domain-containing protein 2A;
N	Q7ZXV5	DR	RNA polymerase II core binding
N	Q7ZXV5	DR	histone methyltransferase activity (H3-K36 specific)
N	Q7ZXV5	DR	negative regulation of cell proliferation
N	Q7ZXV5	DR	negative regulation of gene-specific transcription from RNA polymerase II promoter
N	Q7ZXV5	DR	peptidyl-lysine dimethylation
N	Q7ZXV5	DR	peptidyl-lysine monomethylation
N	Q7ZXV5	DR	regulation of DNA damage response, signal transduction by p53 class mediator
N	Q7ZXV5	DR	zinc ion binding
N	Q7ZY11	CC	Functions in the early steps of protein synthesis of a small number of specific mRNAs
N	Q7ZY11	CC	Acts by directing the binding of methionyl-tRNAi to 40S ribosomal subunits
N	Q7ZY11	CC	In contrast to the eIF- 2 complex, it binds methionyl-tRNAi to 40 S subunits in a codon- dependent manner, whereas the eIF-2 complex binds methionyl-tRNAi to 40 S subunits in a GTP-dependent manner

N	Q7ZY11	CC	May act by impiging the expression of specific proteins (By similarity).
N	Q7ZY11	DE	Eukaryotic translation initiation factor 2A;
N	Q7ZY11	DR	regulation of translation
N	Q7ZY11	DR	translation initiation factor activity
N	Q803A7	CC	Converts O-phosphoseryl-tRNA(Sec) to selenocysteinyl- tRNA(Sec) required for selenoprotein biosynthesis (By similarity).
N	Q803A7	DE	O-phosphoseryl-tRNA(Sec) selenium transferase;
N	Q803A7	DE	Sec synthase;
N	Q803A7	DE	Selenocysteine synthase;
N	Q803A7	DE	Selenocysteinyl-tRNA(Sec) synthase;
N	Q803A7	DE	Sep-tRNA:Sec-tRNA synthase;
N	Q803A7	DE	Soluble liver antigen/liver pancreas antigen-like;
N	Q803A7	DE	UGA suppressor tRNA-associated protein homolog;
N	Q803A7	DR	pyridoxal phosphate binding
N	Q803A7	DR	transferase activity, transferring selenium-containing groups
N	Q803Q4	DE	DEP domain-containing protein 1A;
N	Q803Q4	DR	GTPase activator activity
N	Q803Q4	DR	intracellular signal transduction
N	Q80WG5	CC	Involved in B-cell development
N	Q80WG5	CC	Required for the pro-B cell to pre-B cell transition.
N	Q80WG5	DE	Leucine-rich repeat-containing protein 8A;
N	Q80WG5	DR	pre-B cell differentiation
N	Q80XN0	DE	3-hydroxybutyrate dehydrogenase;
N	Q80XN0	DE	D-beta-hydroxybutyrate dehydrogenase, mitochondrial;
N	Q80XN0	DR	3-hydroxybutyrate dehydrogenase activity
N	Q80XN0	DR	oxidation-reduction process
N	Q80XU8	CC	Promotes neurite outgrowth in hippocampal neurons
N	Q80XU8	CC	May play a role in redistributing DLG4 to the cell periphery.
N	Q80XU8	DE	Leucine-rich repeat and fibronectin type-III domain-containing protein 4;
N	Q80YS6	CC	Can cross-link actin filaments into both network and bundle structures
N	Q80YS6	CC	May function as an adapter molecule that links other proteins, such as SRC and PKC to the actin cytoskeleton (By similarity).
N	Q80YS6	CC	May modulate changes in actin filament integrity and induce lamellipodia formation
N	Q80YS6	DE	110 kDa actin filament-associated protein;
N	Q80YS6	DE	Actin filament-associated protein 1;
N	Q80YS6	DR	SH3 domain binding
N	Q80YS6	DR	actin binding
N	Q814F6	CC	Exhibits a very high intrinsic GTPase hydrolysis rate
N	Q814F6	CC	Involved in the addition of a carboxymethylaminomethyl (cmnm) group at the wobble position (U34) of certain tRNAs, forming tRNA- cmnm(5)s(2)U34 (By similarity).
N	Q814F6	DE	tRNA modification GTPase mnmE;
N	Q814F6	DR	GTPase activity
N	Q814F6	DR	metal ion binding
N	Q814F6	DR	tRNA modification
N	Q814Q1	CC	Catalyzes the production of spermidine from putrescine and decarboxylated S-adenosylmethionine (dcSAM), which serves as an aminopropyl donor (By similarity).
N	Q814Q1	DE	Putrescine aminopropyltransferase 1;
N	Q814Q1	DE	Spermidine synthase 1;
N	Q814Q1	DR	spermidine biosynthetic process
N	Q814Q1	DR	spermidine synthase activity
N	Q81835	CC	Promotes both transcription and replication of genomic RNA
N	Q81835	CC	Following virus entry into host cell, provides nuclear import of HDV RNPs thanks to its nuclear localization signal
N	Q81835	CC	May interact with host RNA polymerase II thereby changing its template requirement from DNA to RNA
N	Q81835	CC	RNA pol II complex would then acts as a RNA-directed RNA polymerase, and transcribe and replicate HDV genome (By similarity).
N	Q81835	DE	Small delta antigen;
N	Q81835	DR	interspecies interaction between organisms
N	Q818F0	CC	Participates actively in the response to hyperosmotic and heat shock by preventing the aggregation of stress-denatured proteins and by disaggregating proteins, also in an autonomous, dnaK-independent fashion
N	Q818F0	CC	Also involved, together with dnaK and grpE, in the DNA replication of plasmids through activation of initiation proteins (By similarity).

N	Q818F0	CC	GrpE releases ADP from dnaK; ATP binding to dnaK triggers the release of the substrate protein, thus completing the reaction cycle
N	Q818F0	CC	Several rounds of ATP-dependent interactions between dnaJ, dnaK and grpE are required for fully efficient folding
N	Q818F0	CC	Unfolded proteins bind initially to dnaJ; upon interaction with the dnaJ-bound protein, dnaK hydrolyzes its bound ATP, resulting in the formation of a stable complex
N	Q818F0	DE	Chaperone protein dnaJ;
N	Q818F0	DR	DNA replication
N	Q818F0	DR	heat shock protein binding
N	Q818F0	DR	metal ion binding
N	Q818F0	DR	protein folding
N	Q818F0	DR	response to heat
N	Q818F0	DR	unfolded protein binding
N	Q818W3	DE	Ornithine carbamoyltransferase;
N	Q818W3	DR	amino acid binding
N	Q818W3	DR	arginine biosynthetic process
N	Q818W3	DR	ornithine carbamoyltransferase activity
N	Q819E7	CC	Dephosphorylates 2-hydroxy-3-keto-5-methylthiopentenyl- 1-phosphate (HK-MTPenyl-1-P) yielding 1,2-dihydroxy-3-keto-5- methylthiopentene (DHK-MTPene) (By similarity)
N	Q819E7	DE	2-hydroxy-3-keto-5-methylthiopentenyl-1-phosphate phosphatase;
N	Q819E7	DE	HK-MTPenyl-1-P phosphatase;
N	Q819E7	DR	L-methionine salvage
N	Q819E7	DR	phosphatase activity
N	Q819V8	CC	Digests double-stranded RNA
N	Q819V8	CC	Involved in the processing of ribosomal RNA precursors and of some mRNAs (By similarity).
N	Q819V8	DE	Ribonuclease 3;
N	Q819V8	DE	Ribonuclease III;
N	Q819V8	DR	RNA processing
N	Q819V8	DR	double-stranded RNA binding
N	Q819V8	DR	rRNA catabolic process
N	Q819V8	DR	ribonuclease III activity
N	Q81G41	CC	Involved in the biosynthesis of D-alanyl-lipoteichoic acid (LTA)
N	Q81G41	CC	Activated D-alanyl-Dcp donates its D-alanyl substituent to membrane-associated LTA (By similarity).
N	Q81G41	DE	D-alanine--poly(phosphoribitol) ligase subunit 2;
N	Q81G41	DE	D-alanyl carrier protein;
N	Q81G41	DR	D-alanine-poly(phosphoribitol) ligase activity
N	Q81G41	DR	acyl carrier activity
N	Q81G41	DR	cellular cell wall organization
N	Q81G41	DR	cofactor binding
N	Q81G41	DR	teichoic acid biosynthetic process
N	Q81JY5	CC	Catalyzes the conversion of uracil and 5-phospho-alpha- D-ribose 1-diphosphate (PRPP) to UMP and diphosphate (By similarity).
N	Q81JY5	DE	UMP pyrophosphorylase;
N	Q81JY5	DE	Uracil phosphoribosyltransferase;
N	Q81JY5	DR	nucleoside metabolic process
N	Q81JY5	DR	uracil phosphoribosyltransferase activity
N	Q81JY5	DR	uracil salvage
N	Q81LC2	CC	Necessary for normal cell division and for the maintenance of normal septation (By similarity).
N	Q81LC2	DE	Probable GTP-binding protein EngB;
N	Q81LC2	DR	barrier septum formation
N	Q81LT9	CC	Involved in DNA repair and recF pathway recombination (By similarity).
N	Q81LT9	DE	DNA repair protein recO;
N	Q81LT9	DE	Recombination protein O;
N	Q81LT9	DR	ARF GTPase activator activity
N	Q81LT9	DR	DNA recombination
N	Q81LT9	DR	regulation of ARF GTPase activity
N	Q81LT9	DR	zinc ion binding
N	Q81PP9	CC	Catalyzes the hydrolysis of N-formyl-L-kynurenine to L- kynurenine (By similarity)
N	Q81PP9	DE	Kynurenine formamidase;
N	Q81PP9	DE	N-formylkynurenine formamidase;
N	Q81PP9	DR	anthranilate metabolic process
N	Q81PP9	DR	arylformamidase activity

N	Q81PP9	DR	formamidase activity
N	Q81PP9	DR	tryptophan catabolic process to kynurenine
N	Q81V80	CC	Catalyzes the decarboxylative condensation of pimeloyl- CoA and L-alanine to produce 8-amino-7-oxononanoate (AON), coenzyme A and/or converts 2-amino-3-ketobutyrate to glycine and acetyl-CoA (By similarity).
N	Q81V80	DE	7-KAP synthase 1;
N	Q81V80	DE	7-keto-8-amino-pelargonic acid synthase 1;
N	Q81V80	DE	8-amino-7-ketopelargonate synthase 1;
N	Q81V80	DE	AONS/AKB ligase 1;
N	Q81V80	DE	Alpha-oxoamine synthase 1;
N	Q81V80	DE	KAPA synthase 1;
N	Q81V80	DE	L-alanine--pimeloyl-CoA ligase 1;
N	Q81V80	DE	Putative 8-amino-7-oxononanoate synthase/2-amino-3-ketobutyrate coenzyme A ligase 1;
N	Q81V80	DR	8-amino-7-oxononanoate synthase activity
N	Q81V80	DR	biosynthetic process
N	Q81V80	DR	glycine C-acetyltransferase activity
N	Q81V80	DR	pyridoxal phosphate binding
N	Q81V80	DR	transferase activity, transferring nitrogenous groups
N	Q81VT4	CC	One of the primary rRNA binding proteins, it binds directly to 16S rRNA where it nucleates assembly of the head domain of the 30S subunit
N	Q81VT4	CC	Is located at the subunit interface close to the decoding center, probably blocks exit of the E-site tRNA (By similarity).
N	Q81VT4	DE	30S ribosomal protein S7;
N	Q81VT4	DR	structural constituent of ribosome
N	Q81WH2	CC	Modifies the free amino group of the aminoacyl moiety of methionyl-tRNA(fMet)
N	Q81WH2	CC	The formyl group appears to play a dual role in the initiator identity of N-formylmethionyl-tRNA by: (I) promoting its recognition by IF2 and (II) impairing its binding to EFTu-GTP (By similarity).
N	Q81WH2	DE	Methionyl-tRNA formyltransferase;
N	Q81WH2	DR	methionyl-tRNA formyltransferase activity
N	Q81WH2	DR	methyltransferase activity
N	Q81XB6	DE	Protein psiE homolog;
N	Q81XB6	DR	cellular response to phosphate starvation
N	Q81YW4	CC	Modulates recA activity (By similarity).
N	Q81YW4	DE	Regulatory protein recX;
N	Q81YW4	DR	regulation of DNA repair
N	Q81ZF8	DE	1-pyrroline-5-carboxylate dehydrogenase;
N	Q81ZF8	DE	P5C dehydrogenase;
N	Q81ZF8	DR	1-pyrroline-5-carboxylate dehydrogenase activity
N	Q81ZF8	DR	oxidation-reduction process
N	Q81ZF8	DR	proline biosynthetic process
N	Q820S3	CC	The ruvA-ruvB complex in the presence of ATP renatures cruciform structure in supercoiled DNA with palindromic sequence, indicating that it may promote strand exchange reactions in homologous recombination
N	Q820S3	CC	RuvA stimulates, in the presence of DNA, the weak ATPase activity of ruvB (By similarity).
N	Q820S3	CC	RuvAB is an helicase that mediates the Holliday junction migration by localized denaturation and reannealing
N	Q820S3	DE	Holliday junction ATP-dependent DNA helicase ruvA;
N	Q820S3	DR	DNA recombination
N	Q820S3	DR	four-way junction helicase activity
N	Q824N5	CC	With S4 and S12 plays an important role in translational accuracy (By similarity).
N	Q824N5	DE	30S ribosomal protein S5;
N	Q824N5	DR	structural constituent of ribosome
N	Q827G1	CC	Key enzyme in the regulation of glycerol uptake and metabolism.
N	Q827G1	DE	ATP:glycerol 3-phosphotransferase 2;
N	Q827G1	DE	Glycerokinase 2;
N	Q827G1	DE	Glycerol kinase 2;
N	Q827G1	DR	glycerol kinase activity
N	Q827G1	DR	glycerol-3-phosphate metabolic process
N	Q82AP6	CC	Catalyzes the transfer of endogenously produced octanoic acid from octanoyl-acyl-carrier-protein onto the lipoyl domains of lipoate-dependent enzymes
N	Q82AP6	CC	Lipoyl-ACP can also act as a substrate although octanoyl-ACP is likely to be the physiological substrate (By similarity).
N	Q82AP6	DE	Lipoate-protein ligase B;

N	Q82AP6	DE	Lipoyl/octanoyl transferase;
N	Q82AP6	DE	Octanoyl-[acyl-carrier-protein]-protein N-octanoyltransferase;
N	Q82AP6	DE	Octanoyltransferase;
N	Q82AP6	DR	lipoate biosynthetic process
N	Q82AP6	DR	lipoyl(octanoyl) transferase activity
N	Q82AP6	DR	octanoyltransferase activity
N	Q82AP6	DR	protein modification process
N	Q82AY2	DE	Uncharacterized lipoprotein SAV_5923;
N	Q82FW3	DE	FGAM synthase I;
N	Q82FW3	DE	Phosphoribosylformylglycinamidine synthase 1;
N	Q82FW3	DE	Phosphoribosylformylglycinamidine synthase I;
N	Q82FW3	DR	'de novo' IMP biosynthetic process
N	Q82FW3	DR	cobalamin biosynthetic process
N	Q82FW3	DR	glutamine metabolic process
N	Q82FW3	DR	phosphoribosylformylglycinamidine synthase activity
N	Q82M40	CC	Catalyzes the transfer of the CoA moiety from formyl-CoA to oxalate (By similarity).
N	Q82M40	DE	Formyl-CoA transferase;
N	Q82M40	DE	Formyl-coenzyme A transferase;
N	Q82M40	DR	formyl-CoA transferase activity
N	Q82ML4	CC	Catalyzes the acyloin condensation reaction between C atoms 2 and 3 of pyruvate and glyceraldehyde 3-phosphate to yield 1-deoxy-D-xylulose-5-phosphate (DXP) (By similarity).
N	Q82ML4	DE	1-deoxy-D-xylulose-5-phosphate synthase 1;
N	Q82ML4	DE	1-deoxyxylulose-5-phosphate synthase 1;
N	Q82ML4	DE	DXP synthase 1;
N	Q82ML4	DR	1-deoxy-D-xylulose-5-phosphate synthase activity
N	Q82ML4	DR	oxidoreductase activity, acting on the aldehyde or oxo group of donors, disulfide as acceptor
N	Q82ML4	DR	terpenoid biosynthetic process
N	Q82ML4	DR	thiamine biosynthetic process
N	Q82NZ4	CC	Cleaves peptides in various proteins in a process that requires ATP hydrolysis
N	Q82NZ4	CC	Has a chymotrypsin-like activity
N	Q82NZ4	CC	Plays a major role in the degradation of misfolded proteins (By similarity).
N	Q82NZ4	DE	ATP-dependent Clp protease proteolytic subunit 1;
N	Q82NZ4	DE	Endopeptidase Clp 1;
N	Q82NZ4	DR	serine-type endopeptidase activity
N	Q82VV9	CC	Nucleotidase that shows phosphatase activity on nucleoside 5'-monophosphates (By similarity).
N	Q82VV9	DE	5'-nucleotidase surE;
N	Q82VV9	DE	Nucleoside 5'-monophosphate phosphohydrolase;
N	Q82VV9	DR	5'-nucleotidase activity
N	Q82VV9	DR	metal ion binding
N	Q82VV9	DR	nucleotide binding
N	Q831A5	CC	Produces ATP from ADP in the presence of a proton gradient across the membrane
N	Q831A5	CC	The catalytic sites are hosted primarily by the beta subunits (By similarity).
N	Q831A5	DE	ATP synthase F1 sector subunit beta;
N	Q831A5	DE	ATP synthase subunit beta;
N	Q831A5	DE	F-ATPase subunit beta;
N	Q831A5	DR	ATP hydrolysis coupled proton transport
N	Q831A5	DR	ATP synthesis coupled proton transport
N	Q831A5	DR	hydrogen ion transporting ATP synthase activity, rotational mechanism
N	Q831A5	DR	hydrogen-exporting ATPase activity, phosphorylative mechanism
N	Q831A5	DR	proton-transporting ATPase activity, rotational mechanism
N	Q831F5	DE	Thymidine kinase;
N	Q831F5	DR	DNA replication
N	Q831F5	DR	metal ion binding
N	Q831F5	DR	thymidine kinase activity
N	Q83262	CC	RNA-dependent RNA polymerase which replicates the viral genome composed of 3 RNA segments, RNA1, RNA2 and RNA3 (Potential).
N	Q83262	DE	RNA-directed RNA polymerase 2a;
N	Q83262	DE	protein 2a;
N	Q83262	DR	RNA-directed RNA polymerase activity
N	Q83262	DR	nucleotide binding
N	Q83262	DR	viral genome replication
N	Q834G2	CC	Zinc phosphodiesterase, which displays some tRNA 3'-processing endonuclease activity

N	Q834G2	CC	Probably involved in tRNA maturation, by removing a 3'-trailer from precursor tRNA (By similarity).
N	Q834G2	DE	Ribonuclease Z;
N	Q834G2	DE	tRNA 3 endonuclease;
N	Q834G2	DR	endoribonuclease activity, producing 5'-phosphomonoesters
N	Q834G2	DR	metal ion binding
N	Q834G2	DR	tRNA 3'-trailer cleavage
N	Q835X3	DE	UPF0756 membrane protein EF_1246;
N	Q839E3	CC	Catalyzes the reversible transfer of the terminal phosphate group between ATP and AMP
N	Q839E3	CC	This small ubiquitous enzyme involved in the energy metabolism and nucleotide synthesis, is essential for maintenance and cell growth (By similarity).
N	Q839E3	DE	ATP-AMP transphosphorylase;
N	Q839E3	DE	Adenylate kinase;
N	Q839E3	DR	adenylate kinase activity
N	Q83BE2	CC	The ruvA-ruvB complex in the presence of ATP renatures cruciform structure in supercoiled DNA with palindromic sequence, indicating that it may promote strand exchange reactions in homologous recombination
N	Q83BE2	CC	RuvA stimulates, in the presence of DNA, the weak ATPase activity of ruvB (By similarity).
N	Q83BE2	CC	RuvAB is an helicase that mediates the Holliday junction migration by localized denaturation and reannealing
N	Q83BE2	DE	Holliday junction ATP-dependent DNA helicase ruvA;
N	Q83BE2	DR	DNA recombination
N	Q83BE2	DR	four-way junction helicase activity
N	Q83BM9	CC	Furnishes a means for formation of correctly charged Gln-tRNA(Gln) through the transamidation of misacylated Glu- tRNA(Gln) in organisms which lack glutaminyl-tRNA synthetase
N	Q83BM9	CC	The reaction takes place in the presence of glutamine and ATP through an activated gamma-phospho-Glu-tRNA(Gln) (By similarity).
N	Q83BM9	DE	Glu-ADT subunit A;
N	Q83BM9	DE	Glutamyl-tRNA(Gln) amidotransferase subunit A;
N	Q83BM9	DR	carbon-nitrogen ligase activity, with glutamine as amido-N-donor
N	Q83G46	CC	Catalyzes the acyloin condensation reaction between C atoms 2 and 3 of pyruvate and glyceraldehyde 3-phosphate to yield 1-deoxy-D-xylulose-5-phosphate (DXP) (By similarity).
N	Q83G46	DE	1-deoxy-D-xylulose-5-phosphate synthase;
N	Q83G46	DE	1-deoxyxylulose-5-phosphate synthase;
N	Q83G46	DE	DXP synthase;
N	Q83G46	DR	1-deoxy-D-xylulose-5-phosphate synthase activity
N	Q83G46	DR	terpenoid biosynthetic process
N	Q83G46	DR	thiamine biosynthetic process
N	Q83HK9	CC	Endonuclease that specifically degrades the RNA of RNA- DNA hybrids (By similarity).
N	Q83HK9	DE	Ribonuclease H;
N	Q83HK9	DR	metal ion binding
N	Q83HK9	DR	nucleic acid binding
N	Q83HK9	DR	ribonuclease H activity
N	Q83HW9	CC	3'-to-5' exoribonuclease specific for small oligoribonucleotides (By similarity).
N	Q83HW9	DE	Oligoribonuclease;
N	Q83HW9	DR	exonuclease activity
N	Q83HW9	DR	nucleic acid binding
N	Q83JD1	CC	Required for the formation of a threonylcarbamoyl group on adenosine at position 37 (t(6)A37) in tRNAs that read codons beginning with adenine (By similarity).
N	Q83JD1	DE	t(6)A37 threonylcarbamoyladenosine biosynthesis protein RimN;
N	Q83JD1	DE	tRNA threonylcarbamoyladenosine biosynthesis protein RimN;
N	Q83JD1	DR	tRNA processing
N	Q83LP3	CC	Catalyzes the reversible conversion of 3- phosphohydroxypyruvate to phosphoserine and of 3-hydroxy-2-oxo-4- phosphonooxybutanoate to phosphohydroxythreonine (By similarity).
N	Q83LP3	DE	Phosphohydroxythreonine aminotransferase;
N	Q83LP3	DE	Phosphoserine aminotransferase;
N	Q83LP3	DR	L-serine biosynthetic process
N	Q83LP3	DR	O-phospho-L-serine:2-oxoglutarate aminotransferase activity
N	Q83LP3	DR	pyridoxal phosphate binding

N	Q83LP3	DR	pyridoxine biosynthetic process
N	Q83MK8	DE	D-serine deaminase;
N	Q83MK8	DE	D-serine dehydratase;
N	Q83MK8	DR	D-amino acid metabolic process
N	Q83MK8	DR	D-serine ammonia-lyase activity
N	Q83MK8	DR	pyridoxal phosphate binding
N	Q83S42	CC	The UvrABC repair system catalyzes the recognition and processing of DNA lesions
N	Q83S42	CC	A damage recognition complex composed of 2 uvrA and 2 uvrB subunits scans DNA for abnormalities
N	Q83S42	CC	DNA wrap is dependent on ATP binding by uvrB and probably causes local melting of the DNA helix, facilitating insertion of uvrB beta-hairpin between the DNA strands
N	Q83S42	CC	If a lesion is found the uvrA subunits dissociate and the uvrB-DNA preincision complex is formed
N	Q83S42	CC	If no lesion is found, the DNA wraps around the other uvrB subunit that will check the other stand for damage (By similarity).
N	Q83S42	CC	Then uvrB probes one DNA strand for the presence of a lesion
N	Q83S42	CC	This complex is subsequently bound by uvrC and the second uvrB is released
N	Q83S42	CC	Upon binding of the uvrA(2)B(2) complex to a putative damaged site, the DNA wraps around one uvrB monomer
N	Q83S42	DE	Excinuclease ABC subunit B;
N	Q83S42	DE	Protein uvrB;
N	Q83S42	DE	UvrABC system protein B;
N	Q83S42	DR	excinuclease ABC activity
N	Q83S42	DR	helicase activity
N	Q83S42	DR	nucleotide-excision repair
N	Q83SQ8	CC	Overproduction of CaiE stimulates the activity of CaiB and CaiD (By similarity).
N	Q83SQ8	DE	Carnitine operon protein CaiE;
N	Q83SQ8	DR	transferase activity
N	Q84BL4	CC	Binds directly to 23S ribosomal RNA and is necessary for the in vitro assembly process of the 50S ribosomal subunit
N	Q84BL4	CC	It is not involved in the protein synthesizing functions of that subunit (By similarity)
N	Q84BL4	DE	50S ribosomal protein L20;
N	Q84BL4	DR	structural constituent of ribosome
N	Q84G06	CC	Hydrolyzes phosphonopyruvate
N	Q84G06	CC	Not active towards phosphoenolpyruvate, glycerophosphate, phospho-L-serine or phosphoglycolic acid.
N	Q84G06	DE	Phosphonopyruvate hydrolase;
N	Q84G06	DR	metal ion binding
N	Q84G06	DR	phosphonopyruvate hydrolase activity
N	Q84JG2	CC	Component of a multiprotein complex equivalent of the SWI/SNF complex, an ATP-dependent chromatin-remodeling complex, which is required for the positive and negative regulation of gene expression of a large number of genes
N	Q84JG2	CC	It changes chromatin structure by altering DNA-histone contacts within a nucleosome, leading eventually to a change in nucleosome position, thus facilitating or repressing binding of gene-specific transcription factors
N	Q84JG2	CC	May be a positive regulator of ABA signaling.
N	Q84JG2	CC	May play an essential role in the transition from the vegetative to the reproductive phase of development
N	Q84JG2	DE	SWI/SNF complex subunit SWI3B;
N	Q84JG2	DE	Transcription regulatory protein SWI3B;
N	Q84JG2	DR	chromatin modification
N	Q84JG2	DR	identical protein binding
N	Q84JG2	DR	multicellular organismal development
N	Q84N48	CC	Required for the splicing of group IIB introns in chloroplasts
N	Q84N48	CC	Forms splicing particles with CRS2
N	Q84N48	CC	Interacts with RNA and confers intron specificity of the splicing particles.
N	Q84N48	DE	CRS2-associated factor 2, chloroplastic;
N	Q84N48	DE	Chloroplastic group IIA intron splicing facilitator CRS2-associated factor 2;
N	Q84N48	DR	mRNA processing
N	Q84QC0	CC	Mediates resistance to sphinganine-analog mycotoxins (SAMs) by restoring the sphingolipid biosynthesis
N	Q84QC0	CC	Could salvage the transport of GPI-anchored proteins from the endoplasmic reticulum to the Golgi apparatus in ceramides-depleted cells after SAM exposure (By similarity)
N	Q84QC0	DE	ASC1-like protein 3;
N	Q84QC0	DE	Alternaria stem canker resistance-like protein 3;

N	Q84QC0	DR	lipid biosynthetic process
N	Q84WV9	CC	Converts glutamine and N-terminal glutamyl residues in peptides to 5-oxoproline and 5-oxoproline residues
N	Q84WV9	CC	Not involved in the major pathway for 5-oxoproline production.
N	Q84WV9	DE	Glutaminyl cyclase;
N	Q84WV9	DE	Glutaminyl-peptide cyclotransferase;
N	Q84WV9	DR	acyltransferase activity
N	Q84WV9	DR	glutaminyl-peptide cyclotransferase activity
N	Q84WV9	DR	peptidyl-pyroglutamic acid biosynthetic process, using glutaminyl-peptide cyclotransferase
N	Q84ZX5	DE	Art v 1;
N	Q84ZX5	DE	Defensin-like protein 1;
N	Q84ZX5	DE	Major pollen allergen Art v 1;
N	Q84ZX5	DR	defense response
N	Q851L5	CC	Hydrolyzes certain amino acid conjugates of the plant growth regulator indole-3-acetic acid (IAA) (By similarity).
N	Q851L5	DE	IAA-amino acid hydrolase ILR1-like 3;
N	Q851L5	DR	hydrolase activity
N	Q85FX0	CC	Component of the cytochrome b6-f complex, which mediates electron transfer between photosystem II (PSII) and photosystem I (PSI), cyclic electron flow around PSI, and state transitions (By similarity).
N	Q85FX0	DE	Apocytochrome f;
N	Q85FX0	DR	electron carrier activity
N	Q85FX0	DR	electron transport chain
N	Q85FX0	DR	photosynthesis
N	Q85FX8	CC	Component of the cytochrome b6-f complex, which mediates electron transfer between photosystem II (PSII) and photosystem I (PSI), cyclic electron flow around PSI, and state transitions (By similarity).
N	Q85FX8	DE	Cytochrome b6-f complex subunit 7;
N	Q85FX8	DE	Cytochrome b6-f complex subunit VII;
N	Q85FX8	DE	Cytochrome b6-f complex subunit petM;
N	Q85FX8	DR	electron transport chain
N	Q85FX8	DR	photosynthesis
N	Q85PQ7	CC	Core subunit of the mitochondrial membrane respiratory chain NADH dehydrogenase (Complex I) that is believed to belong to the minimal assembly required for catalysis
N	Q85PQ7	CC	Complex I functions in the transfer of electrons from NADH to the respiratory chain
N	Q85PQ7	CC	The immediate electron acceptor for the enzyme is believed to be ubiquinone (By similarity).
N	Q85PQ7	DE	NADH dehydrogenase subunit 2;
N	Q85PQ7	DE	NADH-ubiquinone oxidoreductase chain 2;
N	Q85PQ7	DR	NADH dehydrogenase (ubiquinone) activity
N	Q85PQ7	DR	mitochondrial electron transport, NADH to ubiquinone
N	Q85RV3	CC	Component of the ubiquinol-cytochrome c reductase complex (complex III or cytochrome b-c1 complex), which is a respiratory chain that generates an electrochemical potential coupled to ATP synthesis (By similarity).
N	Q85RV3	DE	Complex III subunit 3;
N	Q85RV3	DE	Complex III subunit III;
N	Q85RV3	DE	Cytochrome b-c1 complex subunit 3;
N	Q85RV3	DE	Cytochrome b;
N	Q85RV3	DE	Ubiquinol-cytochrome-c reductase complex cytochrome b subunit;
N	Q85RV3	DR	electron carrier activity
N	Q85RV3	DR	metal ion binding
N	Q85RV3	DR	oxidoreductase activity
N	Q85RV3	DR	respiratory electron transport chain
N	Q86A14	DE	Probable splicing factor 3A subunit 1;
N	Q86A14	DR	nuclear mRNA splicing, via spliceosome
N	Q86CR6	CC	Ubiquitin-like protein involved in autophagy vesicles formation
N	Q86CR6	CC	Conjugated to atg5 through an ubiquitin-like conjugating system involving also atg7 as an E1-like activating enzyme is essential for its function (By similarity).
N	Q86CR6	CC	Required for atg8 association to the vesicle membranes
N	Q86CR6	DE	APG12-like;
N	Q86CR6	DE	Autophagy-related protein 12;
N	Q86CR6	DE	Ubiquitin-like protein atg12;
N	Q86CR6	DR	autophagic vacuole assembly
N	Q86CR6	DR	protein transport

N	Q86HS8	CC	Component of the Mediator complex, a coactivator involved in the regulated transcription of nearly all RNA polymerase II-dependent genes
N	Q86HS8	CC	Mediator functions as a bridge to convey information from gene-specific regulatory proteins to the basal RNA polymerase II transcription machinery
N	Q86HS8	CC	Mediator is recruited to promoters by direct interactions with regulatory proteins and serves as a scaffold for the assembly of a functional preinitiation complex with RNA polymerase II and the general transcription factors (By similarity).
N	Q86HS8	DE	Mediator complex subunit 30;
N	Q86HS8	DE	Putative mediator of RNA polymerase II transcription subunit 30;
N	Q86JM7	CC	P2X receptors are ATP-gated ion channels that play a role in intracellular calcium signaling
N	Q86JM7	CC	Exclusively selective for ATP over other nucleotides
N	Q86JM7	CC	Found to be permeable to chloride ions
N	Q86JM7	CC	Inhibited by copper and sodium ions.
N	Q86JM7	CC	Insensitive to P2 receptor antagonists PPADS, suramin and 2',3'-O-(2,4,6-trinitrophenyl)-ATP but inhibited by nanomolar concentrations of copper and sodium ion
N	Q86JM7	CC	Inward currents evoked by intracellular ATP and ATP analogs
N	Q86JM7	CC	It has been reported that p2xA is not essential for osmoregulation (PubMed:19833731), however this information is in contradiction with another source (PubMed:17625565) which indicates that p2xA is required for osmoregulation
N	Q86JM7	CC	More permeable to ammonium than either sodium or potassium ions and less permeable to choline
N	Q86JM7	CC	Not required for the purinergic response to extracellular nucleotides
N	Q86JM7	DE	P2X receptor A;
N	Q86JM7	DR	cellular water homeostasis
N	Q86JM7	DR	hypotonic response
N	Q86JM7	DR	ligand-gated ion channel activity
N	Q86JM7	DR	receptor activity
N	Q86JM7	DR	regulation of calcium-mediated signaling
N	Q86L05	DE	60S ribosomal protein L10a;
N	Q86L05	DR	RNA processing
N	Q86L05	DR	structural constituent of ribosome
N	Q86TS7	DE	UPF0730 protein CS0DE013YM09;
N	Q86V97	DE	Kelch repeat and BTB domain-containing protein 6;
N	Q86V97	DR	protein binding
N	Q86XA9	DE	HEAT repeat-containing protein 5A;
N	Q86YN6	CC	Plays a role of stimulator of transcription factors and nuclear receptors activities
N	Q86YN6	CC	Activates transcritional activity of estrogen receptor alpha, nuclear respiratory factor 1 (NRF1) and glucocorticoid receptor in the presence of glucocorticoids
N	Q86YN6	CC	May be involved in fat oxidation and non-oxidative glucose metabolism and in the regulation of energy expenditure.
N	Q86YN6	CC	May play a role in constitutive non-adrenergic-mediated mitochondrial biogenesis as suggested by increased basal oxygen consumption and mitochondrial number when overexpressed
N	Q86YN6	DE	PGC-1-beta;
N	Q86YN6	DE	PGC-1-related estrogen receptor alpha coactivator;
N	Q86YN6	DE	PPAR-gamma coactivator 1-beta;
N	Q86YN6	DE	PPARGC-1-beta;
N	Q86YN6	DE	Peroxisome proliferator-activated receptor gamma coactivator 1-beta;
N	Q86YN6	DR	AF-2 domain binding
N	Q86YN6	DR	RNA polymerase II transcription mediator activity
N	Q86YN6	DR	estrogen receptor binding
N	Q86YN6	DR	estrogen receptor signaling pathway
N	Q86YN6	DR	ligand-dependent nuclear receptor transcription coactivator activity
N	Q86YN6	DR	nucleotide binding
N	Q86YN6	DR	positive regulation of transcription from RNA polymerase II promoter
N	Q86YN6	DR	receptor activator activity
N	Q874J6	CC	Histone H3-like variant which exclusively replaces conventional H3 in the nucleosome core of centromeric chromatin at the inner plate of the kinetochore
N	Q874J6	CC	May serve as an epigenetic mark that propagates centromere identity through replication and cell division (By similarity).
N	Q874J6	CC	Required for recruitment and assembly of kinetochore proteins, mitotic progression and chromosome segregation
N	Q874J6	DE	CENP-A homolog;
N	Q874J6	DE	Histone H3-like centromeric protein CSE4;

N	Q874J6	DR	nucleosome assembly
N	Q87D63	DE	GTP cyclohydrolase 1;
N	Q87D63	DE	GTP cyclohydrolase I;
N	Q87D63	DR	GTP cyclohydrolase I activity
N	Q87D63	DR	metal ion binding
N	Q87D63	DR	one-carbon metabolic process
N	Q87D63	DR	tetrahydrofolate biosynthetic process
N	Q87D75	DE	Putative fis-like DNA-binding protein;
N	Q87D75	DR	regulation of transcription, DNA-dependent
N	Q87D75	DR	sequence-specific DNA binding transcription factor activity
N	Q87DY8	CC	Catalyzes the ATP-dependent amination of UTP to CTP with either L-glutamine or ammonia as the source of nitrogen (By similarity).
N	Q87DY8	DE	CTP synthase;
N	Q87DY8	DE	CTP synthetase;
N	Q87DY8	DE	UTP--ammonia ligase;
N	Q87DY8	DR	CTP synthase activity
N	Q87DY8	DR	glutamine metabolic process
N	Q87DY8	DR	pyrimidine nucleotide biosynthetic process
N	Q87E50	CC	ATP-dependent specificity component of the Clp protease
N	Q87E50	CC	Can perform chaperone functions in the absence of ClpP (By similarity).
N	Q87E50	CC	It directs the protease to specific substrates
N	Q87E50	DE	ATP-dependent Clp protease ATP-binding subunit ClpX;
N	Q87E50	DR	ATPase activity
N	Q87E50	DR	protein dimerization activity
N	Q87E50	DR	protein folding
N	Q87E50	DR	unfolded protein binding
N	Q87E50	DR	zinc ion binding
N	Q87E51	CC	Cleaves peptides in various proteins in a process that requires ATP hydrolysis
N	Q87E51	CC	Has a chymotrypsin-like activity
N	Q87E51	CC	Plays a major role in the degradation of misfolded proteins (By similarity).
N	Q87E51	DE	ATP-dependent Clp protease proteolytic subunit;
N	Q87E51	DE	Endopeptidase Clp;
N	Q87E51	DR	serine-type endopeptidase activity
N	Q87FD6	CC	Specifically catalyzes the cleavage of the D-lactyl ether substituent of MurNAc 6-phosphate, producing GlcNAc 6- phosphate and D-lactate
N	Q87FD6	CC	Together with AnmK, is also required for the utilization of anhydro-N-acetylmuramic acid (anhMurNAc) either imported from the medium or derived from its own cell wall murein, and thus plays a role in cell wall recycling (By similarity).
N	Q87FD6	DE	MurNAc-6-P etherase 2;
N	Q87FD6	DE	N-acetylmuramic acid 6-phosphate etherase 2;
N	Q87FD6	DE	N-acetylmuramic acid 6-phosphate hydrolase 2;
N	Q87FD6	DE	N-acetylmuramic acid 6-phosphate lyase 2;
N	Q87FD6	DR	amino sugar catabolic process
N	Q87FD6	DR	hydro-lyase activity
N	Q87FD6	DR	sugar binding
N	Q87KM9	CC	Synthesis of 3-octaprenyl-4-hydroxybenzoate (By similarity).
N	Q87KM9	DE	4-HB polyprenyltransferase;
N	Q87KM9	DE	4-hydroxybenzoate octaprenyltransferase;
N	Q87KM9	DR	prenyltransferase activity
N	Q87KM9	DR	ubiquinone biosynthetic process
N	Q87LT0	CC	Specifically methylates guanosine-37 in various tRNAs (By similarity).
N	Q87LT0	DE	M1G-methyltransferase;
N	Q87LT0	DE	tRNA (guanine-N(1)-)-methyltransferase;
N	Q87LT0	DE	tRNA [GM37] methyltransferase;
N	Q87LT0	DR	tRNA (guanine-N1-)-methyltransferase activity
N	Q87M23	CC	The enzymes which catalyze the reversible phosphorolysis of pyrimidine nucleosides are involved in the degradation of these compounds and in their utilization as carbon and energy sources, or in the rescue of pyrimidine bases for nucleotide synthesis (By similarity).
N	Q87M23	DE	Thymidine phosphorylase;
N	Q87M23	DR	pyrimidine base metabolic process
N	Q87M23	DR	pyrimidine nucleoside metabolic process
N	Q87M23	DR	pyrimidine-nucleoside phosphorylase activity
N	Q87M23	DR	thymidine phosphorylase activity

N	Q87MD6	CC	Modifies, by uridylylation or deuridylylation the PII (glnB) regulatory protein (By similarity).
N	Q87MD6	DE	PII uridylyl-transferase;
N	Q87MD6	DE	Uridylyl-removing enzyme;
N	Q87MD6	DE	[Protein-PII] uridylyltransferase;
N	Q87MD6	DR	[protein-PII] uridylyltransferase activity
N	Q87MD6	DR	amino acid binding
N	Q87MD6	DR	nitrogen compound metabolic process
N	Q87QM0	DE	High frequency lysogenization protein hflD homolog;
N	Q87R80	CC	Cleaves peptides in various proteins in a process that requires ATP hydrolysis
N	Q87R80	CC	Has a chymotrypsin-like activity
N	Q87R80	CC	Plays a major role in the degradation of misfolded proteins (By similarity).
N	Q87R80	DE	ATP-dependent Clp protease proteolytic subunit;
N	Q87R80	DE	Endopeptidase Clp;
N	Q87R80	DR	serine-type endopeptidase activity
N	Q87TB0	CC	Promotes RNA polymerase assembly
N	Q87TB0	CC	Latches the N- and C- terminal regions of the beta' subunit thereby facilitating its interaction with the beta and alpha subunits (By similarity).
N	Q87TB0	DE	DNA-directed RNA polymerase subunit omega;
N	Q87TB0	DE	RNA polymerase omega subunit;
N	Q87TB0	DE	RNAP omega subunit;
N	Q87TB0	DE	Transcriptase subunit omega;
N	Q87TB0	DR	DNA-directed RNA polymerase activity
N	Q87V59	CC	Transfers 2'-(5-triphosphoribosyl)-3'-dephosphocoenzyme- A to the apo-[acyl-carrier-protein] of the malonate decarboxylase to yield holo-[acyl-carrier-protein] (By similarity).
N	Q87V59	DE	Holo-ACP synthase;
N	Q87V59	DE	Malonate decarboxylase holo-[acyl-carrier-protein] synthase;
N	Q87V59	DE	Phosphoribosyl-dephospho-CoA transferase;
N	Q87V59	DR	nucleotidyltransferase activity
N	Q87YG5	CC	Catalyzes the reversible formation of acyl-phosphate (acyl-PO(4)) from acyl-[acyl-carrier-protein] (acyl-ACP)
N	Q87YG5	CC	This enzyme utilizes acyl-ACP as fatty acyl donor, but not acyl-CoA (By similarity).
N	Q87YG5	DE	Acyl-ACP phosphotransacylase;
N	Q87YG5	DE	Acyl-[acyl-carrier-protein]--phosphate acyltransferase;
N	Q87YG5	DE	Phosphate acyltransferase;
N	Q87YG5	DE	Phosphate-acyl-ACP acyltransferase;
N	Q87YG5	DR	fatty acid biosynthetic process
N	Q87YG5	DR	phospholipid biosynthetic process
N	Q87YG5	DR	transferase activity, transferring acyl groups other than amino-acyl groups
N	Q88421	DE	Uncharacterized protein ORF12;
N	Q885T7	CC	Catalyzes the interconversion of methylthioribose-1- phosphate (MTR-1-P) into methylthioribulose-1-phosphate (MTRu-1-P) (By similarity).
N	Q885T7	DE	MTR-1-P isomerase;
N	Q885T7	DE	Methylthioribose-1-phosphate isomerase;
N	Q885T7	DE	S-methyl-5-thioribose-1-phosphate isomerase;
N	Q885T7	DR	S-methyl-5-thioribose-1-phosphate isomerase activity
N	Q885T7	DR	methionine biosynthetic process
N	Q889F0	CC	Catalyzes the transfer of a phosphate group to glutamate to form glutamate 5-phosphate which rapidly cyclizes to 5- oxoproline.
N	Q889F0	DE	Gamma-glutamyl kinase;
N	Q889F0	DE	Glutamate 5-kinase;
N	Q889F0	DR	glutamate 5-kinase activity
N	Q889F0	DR	proline biosynthetic process
N	Q88BD7	CC	Catalyzes the transfer of a ribosyl phosphate group from 5-phosphoribose 1-diphosphate to orotate, leading to the formation of orotidine monophosphate (OMP) (By similarity).
N	Q88BD7	DE	Orotate phosphoribosyltransferase;
N	Q88BD7	DR	nucleoside metabolic process
N	Q88BD7	DR	orotate phosphoribosyltransferase activity
N	Q88BD7	DR	pyrimidine nucleotide biosynthetic process
N	Q88EA1	CC	Bifunctional enzyme which can phosphorylate or dephosphorylate isocitrate dehydrogenase (IDH) on a specific serine residue
N	Q88EA1	CC	This is a regulatory mechanism which enables bacteria to bypass the Krebs cycle via the glyoxylate shunt in response to the source of carbon

N	Q88EA1	CC	When bacteria are grown on glucose, IDH is fully active and unphosphorylated, but when grown on acetate or ethanol, the activity of IDH declines drastically concomitant with its phosphorylation (By similarity).
N	Q88EA1	DE	IDH kinase/phosphatase;
N	Q88EA1	DE	Isocitrate dehydrogenase kinase/phosphatase;
N	Q88EA1	DR	[isocitrate dehydrogenase (NADP+)] kinase activity
N	Q88EA1	DR	glucose metabolic process
N	Q88EA1	DR	glyoxylate cycle
N	Q88EA1	DR	phosphoprotein phosphatase activity
N	Q88EA1	DR	tricarboxylic acid cycle
N	Q88EI4	CC	Catalyzes the NAD-dependent reduction of succinylglutamate semialdehyde into succinylglutamate (By similarity).
N	Q88EI4	DE	N-succinylglutamate 5-semialdehyde dehydrogenase;
N	Q88EI4	DE	Succinylglutamic semialdehyde dehydrogenase;
N	Q88EI4	DR	arginine catabolic process
N	Q88EI4	DR	oxidation-reduction process
N	Q88EI4	DR	succinylglutamate-semialdehyde dehydrogenase activity
N	Q88PK5	CC	Co-chaperone involved in the maturation of iron-sulfur cluster-containing proteins
N	Q88PK5	CC	Seems to help targeting proteins to be folded toward hscA (By similarity).
N	Q88PK5	DE	Co-chaperone protein hscB homolog;
N	Q88PK5	DR	chaperone binding
N	Q88PK5	DR	heat shock protein binding
N	Q88PK5	DR	protein folding
N	Q88QH1	CC	Catalyzes the conversion of GTP to 2,5-diamino-6- ribosylamino-4(3H)-pyrimidinone 5'-phosphate (DARP), formate and pyrophosphate (By similarity).
N	Q88QH1	DE	GTP cyclohydrolase II;
N	Q88QH1	DE	GTP cyclohydrolase-2;
N	Q88QH1	DR	GTP cyclohydrolase II activity
N	Q88QH1	DR	metal ion binding
N	Q88QH1	DR	riboflavin biosynthetic process
N	Q88R93	CC	Part of the ABC transporter complex SsuABC involved in aliphatic sulfonates import
N	Q88R93	CC	Responsible for energy coupling to the transport system (By similarity).
N	Q88R93	DE	Aliphatic sulfonates import ATP-binding protein SsuB;
N	Q88R93	DR	ATPase activity
N	Q88R93	DR	transporter activity
N	Q88S50	CC	Involved in the anomeric conversion of L-rhamnose (By similarity).
N	Q88S50	DE	L-rhamnose mutarotase;
N	Q88S50	DE	Rhamnose 1-epimerase;
N	Q88S50	DE	Type-3 mutarotase;
N	Q88S50	DR	racemase and epimerase activity, acting on carbohydrates and derivatives
N	Q88S50	DR	rhamnose metabolic process
N	Q88VC8	CC	Reversibly transfers an adenylyl group from ATP to 4'- phosphopantetheine, yielding dephospho-CoA (dPCoA) and pyrophosphate (By similarity).
N	Q88VC8	DE	Dephospho-CoA pyrophosphorylase;
N	Q88VC8	DE	Pantetheine-phosphate adenylyltransferase;
N	Q88VC8	DE	Phosphopantetheine adenylyltransferase;
N	Q88VC8	DR	coenzyme A biosynthetic process
N	Q88VC8	DR	pantetheine-phosphate adenylyltransferase activity
N	Q88WL3	CC	Modifies the free amino group of the aminoacyl moiety of methionyl-tRNA(fMet)
N	Q88WL3	CC	The formyl group appears to play a dual role in the initiator identity of N-formylmethionyl-tRNA by: (I) promoting its recognition by IF2 and (II) impairing its binding to EFTu-GTP (By similarity).
N	Q88WL3	DE	Methionyl-tRNA formyltransferase;
N	Q88WL3	DR	methionyl-tRNA formyltransferase activity
N	Q88WL3	DR	methyltransferase activity
N	Q892S8	DE	Deoxyguanosinetriphosphate triphosphohydrolase-like protein;
N	Q892S8	DR	GTP metabolic process
N	Q892S8	DR	dGTPase activity
N	Q892S8	DR	magnesium ion binding
N	Q892Z2	DE	Uncharacterized RNA methyltransferase CTC_01941;
N	Q892Z2	DR	4 iron, 4 sulfur cluster binding
N	Q892Z2	DR	RNA methyltransferase activity
N	Q892Z2	DR	RNA processing
N	Q892Z2	DR	metal ion binding
N	Q89420	CC	E2 regulates viral transcription and DNA replication

N	Q89420	CC	It binds to the E2RE response element (5'-ACCNNNNNNGGT-3') present in multiple copies in the regulatory region
N	Q89420	CC	It can either activate or repress transcription depending on E2RE's position with regards to proximal promoter elements
N	Q89420	CC	Repression occurs by sterically hindering the assembly of the transcription initiation complex
N	Q89420	CC	The E1-E2 complex binds to the origin of DNA replication.
N	Q89420	DE	Regulatory protein E2;
N	Q89420	DR	DNA replication
N	Q89420	DR	nucleotide binding
N	Q89420	DR	regulation of DNA replication
N	Q89420	DR	regulation of transcription, DNA-dependent
N	Q89420	DR	sequence-specific DNA binding transcription factor activity
N	Q89420	DR	viral reproduction
N	Q899S0	CC	Specifically methylates the N7 position of a guanosine in 16S rRNA (By similarity)
N	Q899S0	DE	16S rRNA 7-methylguanosine methyltransferase;
N	Q899S0	DE	16S rRNA m7G methyltransferase;
N	Q899S0	DE	Ribosomal RNA small subunit methyltransferase G;
N	Q899S0	DR	rRNA methyltransferase activity
N	Q89A71	CC	One of the primary rRNA binding proteins
N	Q89A71	CC	It has been suggested to have peptidyltransferase activity; this is somewhat controversial
N	Q89A71	CC	Makes several contacts with the 16S rRNA in the 70S ribosome (By similarity).
N	Q89A71	CC	Required for association of the 30S and 50S subunits to form the 70S ribosome, for tRNA binding and peptide bond formation
N	Q89A71	DE	50S ribosomal protein L2;
N	Q89A71	DR	structural constituent of ribosome
N	Q89A71	DR	transferase activity
N	Q89AD1	CC	5'-3' exonuclease acting preferentially on double- stranded DNA (By similarity).
N	Q89AD1	DE	5'-3' exonuclease;
N	Q89AD1	DR	5'-3' exonuclease activity
N	Q89AE1	DE	Protein tldD homolog;
N	Q89AI3	CC	ATPase required for the correct placement of the division site
N	Q89AI3	CC	Cell division inhibitors minC and minD act in concert to form an inhibitor capable of blocking formation of the polar Z ring septums
N	Q89AI3	CC	Rapidly oscillates between the poles of the cell to destabilize ftsZ filaments that have formed before they mature into polar Z rings (By similarity).
N	Q89AI3	DE	Cell division inhibitor minD;
N	Q89AI3	DE	Septum site-determining protein minD;
N	Q89AI3	DR	ATPase activity
N	Q89AI3	DR	barrier septum site selection
N	Q89AX6	DE	Histidine biosynthesis bifunctional protein HisIE;
N	Q89AX6	DE	Phosphoribosyl-AMP cyclohydrolase;
N	Q89AX6	DE	Phosphoribosyl-ATP pyrophosphatase;
N	Q89AX6	DR	histidine biosynthetic process
N	Q89AX6	DR	phosphoribosyl-AMP cyclohydrolase activity
N	Q89AX6	DR	phosphoribosyl-ATP diphosphatase activity
N	Q89B22	CC	Histone-like DNA-binding protein which is capable of wrapping DNA to stabilize it, and thus to prevent its denaturation under extreme environmental conditions (By similarity)
N	Q89B22	DE	DNA-binding protein HU;
N	Q89B22	DR	chromosome condensation
N	Q89B30	CC	This protein is required for chromosomal replication
N	Q89B30	CC	It forms, in concert with dnaB protein and other prepriming proteins dnaT, N, N', N'' a prepriming protein complex on the specific site of the template DNA recognized by protein N' (By similarity).
N	Q89B30	DE	DNA replication protein dnaC;
N	Q89B30	DR	DNA replication, synthesis of RNA primer
N	Q89B30	DR	nucleoside-triphosphatase activity
N	Q89DI7	CC	Riboflavin synthase is a bifunctional enzyme complex catalyzing the formation of riboflavin from 5-amino-6-(1'-D)- ribityl-amino-2,4(1H,3H)-pyrimidinedione and L-3,4-dihydrohy-2- butanone-4-phosphate via 6,7-dimethyl-8-lumazine
N	Q89DI7	CC	The beta subunit catalyzes the condensation of 5-amino-6-(1'-D)-ribityl-amino-2,4(1H,3H)-pyrimidinedione with L-3,4-dihydrohy-2-butanone-4- phosphate yielding 6,7-dimethyl-8-lumazine (By similarity).
N	Q89DI7	DE	6,7-dimethyl-8-ribityllumazine synthase 2;
N	Q89DI7	DE	DMRL synthase 2;

N	Q89DI7	DE	Lumazine synthase 2;
N	Q89DI7	DE	Riboflavin synthase 2 beta chain;
N	Q89DI7	DR	riboflavin biosynthetic process
N	Q89DI7	DR	riboflavin synthase activity
N	Q89KE5	DE	30S ribosomal protein S9;
N	Q89KE5	DR	structural constituent of ribosome
N	Q89KU5	CC	Catalyzes the ATP-dependent amination of UTP to CTP with either L-glutamine or ammonia as the source of nitrogen (By similarity).
N	Q89KU5	DE	CTP synthase;
N	Q89KU5	DE	CTP synthetase;
N	Q89KU5	DE	UTP--ammonia ligase;
N	Q89KU5	DR	CTP synthase activity
N	Q89KU5	DR	glutamine metabolic process
N	Q89KU5	DR	pyrimidine nucleotide biosynthetic process
N	Q8AWA7	DE	Keratin, type 1 cytoskeletal 11;
N	Q8AWA7	DE	Type I keratin 11;
N	Q8AWA7	DR	structural molecule activity
N	Q8B9L3	CC	Involved in late/very late gene activation (By similarity).
N	Q8B9L3	DE	Late expression factor 11;
N	Q8B9L3	DR	viral infectious cycle
N	Q8BDG3	CC	Minor protein of the capsid that localizes along the inner surface of the virion, within the central cavities beneath the L1 pentamers
N	Q8BDG3	CC	During virion assembly, encapsidates the genome by direct interaction with the viral DNA (By similarity).
N	Q8BDG3	CC	Later on, promotes late gene expression by interacting with the viral E2 protein and by inhibiting its transcriptional activation functions
N	Q8BDG3	CC	Mediates the viral genome import into the nucleus through binding to host importins
N	Q8BDG3	CC	Once the virion enters the host cell, escorts the genomic DNA into the nucleus, in particular by promoting virion endosomal escape
N	Q8BDG3	CC	Once within the nucleus, L2 localizes viral genomes to PML bodies in order to activate early gene expression for establishment of infection
N	Q8BDG3	CC	Plays a role in capsid stabilization through interaction with the major capsid protein L1
N	Q8BDG3	DE	Minor capsid protein L2;
N	Q8BDG3	DR	structural molecule activity
N	Q8BFR1	DE	Zinc finger CCCH-type antiviral protein 1-like;
N	Q8BFR1	DR	nucleic acid binding
N	Q8BFR1	DR	zinc ion binding
N	Q8BGY4	DE	Kelch-like protein 26;
N	Q8BH58	CC	May be a allosteric regulator of serine/threonine- protein phosphatase 2A (PP2A)
N	Q8BH58	CC	Inhibits catalytic activity of the PP2A(D) core complex in vitro
N	Q8BH58	CC	May play a role in the regulation of ATM/ATR signaling pathway controlling DNA replication and repair (By similarity).
N	Q8BH58	CC	The PP2A(C):TIPRL complex does not show phosphatase activity
N	Q8BH58	DE	TIP41-like protein;
N	Q8BHS3	CC	Involved in pre-mRNA splicing (Probable)
N	Q8BHS3	CC	May translocate the cytosolic calcium-binding protein PDCD6 in the nucleus (By similarity).
N	Q8BHS3	DE	Pre-mRNA-splicing factor RBM22;
N	Q8BHS3	DE	RNA-binding motif protein 22;
N	Q8BHS3	DR	mRNA processing
N	Q8BHS3	DR	nucleotide binding
N	Q8BHS3	DR	zinc ion binding
N	Q8BHX3	CC	Component of the chromosomal passenger complex (CPC), a complex that acts as a key regulator of mitosis
N	Q8BHX3	CC	In the complex, it may be required to direct the CPC to centromeric DNA
N	Q8BHX3	CC	Major effector of the TTK kinase in the control of attachment-error-correction and chromosome alignment (By similarity).
N	Q8BHX3	CC	The CPC complex has essential functions at the centromere in ensuring correct chromosome alignment and segregation and is required for chromatin-induced microtubule stabilization and spindle assembly
N	Q8BHX3	DE	Cell division cycle-associated protein 8;
N	Q8BHX3	DR	cell division
N	Q8BIF0	CC	May function as a homophilic adhesion molecule
N	Q8BIF0	CC	Does not seem to be involved in docking of leukocytes to the vessel wall or in lymphocyte diapedesis

N	Q8BIF0	CC	Functions in leukocyte-endothelial cell interactions during leukocyte extravasation, and in particular, at the diapedesis step
N	Q8BIF0	CC	In PubMed:17344467 showed heterophilic aggregation.
N	Q8BIF0	DE	CD99 antigen-like protein 2;
N	Q8BIF0	DE	MIC2-like protein 1;
N	Q8BIF0	DR	cell adhesion
N	Q8BRM6	DE	UPF0623 protein;
N	Q8BRM6	DR	protein binding
N	Q8BRM6	DR	spermatogenesis
N	Q8BW49	DE	TPR repeat protein 12;
N	Q8BW49	DE	Tetratricopeptide repeat protein 12;
N	Q8BXQ2	CC	Component of the GPI transamidase complex
N	Q8BXQ2	CC	Essential for transfer of GPI to proteins, particularly for formation of carbonyl intermediates (By similarity).
N	Q8BXQ2	DE	GPI transamidase component PIG-T;
N	Q8BXQ2	DE	Neuronal development-associated protein 7;
N	Q8BXQ2	DE	Phosphatidylinositol-glycan biosynthesis class T protein;
N	Q8BXQ2	DR	attachment of GPI anchor to protein
N	Q8BXQ2	DR	neuron apoptosis
N	Q8BXQ2	DR	neuron differentiation
N	Q8BXZ1	CC	Probable disulfide isomerase, which participates in the folding of proteins containing disulfide bonds
N	Q8BXZ1	CC	May act as a dithiol oxidase (By similarity).
N	Q8BXZ1	DE	Protein disulfide-isomerase TMX3;
N	Q8BXZ1	DE	Thioredoxin domain-containing protein 10;
N	Q8BXZ1	DE	Thioredoxin-related transmembrane protein 3;
N	Q8BXZ1	DR	cell redox homeostasis
N	Q8BXZ1	DR	electron carrier activity
N	Q8BXZ1	DR	glycerol ether metabolic process
N	Q8BXZ1	DR	protein disulfide isomerase activity
N	Q8BXZ1	DR	protein disulfide oxidoreductase activity
N	Q8C138	DE	Uncharacterized protein C6orf105 homolog;
N	Q8C3J5	CC	Involved in cytoskeletal rearrangements required for lymphocyte migration in response of chemokines
N	Q8C3J5	CC	Activates RAC1 and RAC2 small GTPases, probably by functioning as a guanine nucleotide exchange factor (GEF), which exchanges bound GDP for free GTP
N	Q8C3J5	CC	May participate in IL2 transcriptional activation via the activation of RAC2.
N	Q8C3J5	DE	Dedicator of cytokinesis protein 2;
N	Q8C3J5	DE	Protein Hch;
N	Q8C3J5	DR	GTPase binding
N	Q8C3J5	DR	Rac GTPase activator activity
N	Q8C3J5	DR	Rac guanyl-nucleotide exchange factor activity
N	Q8C3J5	DR	T cell receptor binding
N	Q8C3J5	DR	alpha-beta T cell proliferation
N	Q8C3J5	DR	cytoskeleton organization
N	Q8C3J5	DR	establishment of T cell polarity
N	Q8C3J5	DR	immunological synapse formation
N	Q8C3J5	DR	membrane raft polarization
N	Q8C3J5	DR	negative thymic T cell selection
N	Q8C3J5	DR	positive regulation of Rac protein signal transduction
N	Q8C3J5	DR	positive thymic T cell selection
N	Q8C9A2	DE	Putative endonuclease FLJ39025 homolog;
N	Q8C9A2	DR	endonuclease activity
N	Q8CBY3	DE	Leukocyte receptor cluster member 8 homolog;
N	Q8CEG8	DE	Deubiquitinating enzyme 27;
N	Q8CEG8	DE	Ubiquitin carboxyl-terminal hydrolase 27;
N	Q8CEG8	DE	Ubiquitin thiolesterase 27;
N	Q8CEG8	DE	Ubiquitin-specific-processing protease 27;
N	Q8CEG8	DE	X-linked ubiquitin carboxyl-terminal hydrolase 27;
N	Q8CEG8	DR	cysteine-type peptidase activity
N	Q8CEG8	DR	ubiquitin thiolesterase activity
N	Q8CEG8	DR	ubiquitin-dependent protein catabolic process
N	Q8CFU8	DE	Diabetes and obesity-regulated protein;
N	Q8CFU8	DE	Tumor protein p53-inducible nuclear protein 2;

N	Q8CG48	CC	Central component of the condensin complex, a complex required for conversion of interphase chromatin into mitotic-like condense chromosomes
N	Q8CG48	CC	The condensin complex probably introduces positive supercoils into relaxed DNA in the presence of type I topoisomerases and converts nicked DNA into positive knotted forms in the presence of type II topoisomerases (By similarity).
N	Q8CG48	DE	Chromosome-associated protein E;
N	Q8CG48	DE	FGF-inducible protein 16;
N	Q8CG48	DE	SMC protein 2;
N	Q8CG48	DE	Structural maintenance of chromosomes protein 2;
N	Q8CG48	DE	XCAP-E homolog;
N	Q8CG48	DR	cell division
N	Q8CJ44	CC	High-affinity sodium/citrate cotransporter that mediates citrate entry into cells
N	Q8CJ44	CC	May facilitate the utilization of circulating citrate for the generation of metabolic energy and for the synthesis of fatty acids and cholesterol.
N	Q8CJ44	CC	The transport process is electrogenic; it is the trivalent form of citrate rather than the divalent form that is recognized as a substrate
N	Q8CJ44	DE	Na(+)/citrate cotransporter;
N	Q8CJ44	DE	Sodium-coupled citrate transporter;
N	Q8CJ44	DE	Sodium-dependent citrate transporter;
N	Q8CJ44	DE	Solute carrier family 13 member 5;
N	Q8CJ44	DR	citrate transmembrane transporter activity
N	Q8CJ44	DR	sodium:dicarboxylate symporter activity
N	Q8CJ44	DR	succinate transmembrane transporter activity
N	Q8CNJ9	CC	Involved in saturated fatty acids biosynthesis.
N	Q8CNJ9	DE	(3R)-hydroxymyristoyl ACP dehydrase;
N	Q8CNJ9	DE	(3R)-hydroxymyristoyl-[acyl-carrier-protein] dehydratase;
N	Q8CNJ9	DR	fatty acid biosynthetic process
N	Q8CNJ9	DR	hydro-lyase activity
N	Q8CNJ9	DR	lipid A biosynthetic process
N	Q8CR71	CC	Catalyzes the reversible transfer of the terminal phosphate of ATP to form a long-chain polyphosphate (polyP).
N	Q8CR71	DE	ATP-polyphosphate phosphotransferase;
N	Q8CR71	DE	Polyphosphate kinase;
N	Q8CR71	DE	Polyphosphoric acid kinase;
N	Q8CR71	DR	polyphosphate biosynthetic process
N	Q8CR71	DR	polyphosphate kinase activity
N	Q8CRG3	CC	One of the primary rRNA binding proteins
N	Q8CRG3	CC	It has been suggested to have peptidyltransferase activity; this is somewhat controversial
N	Q8CRG3	CC	Makes several contacts with the 16S rRNA in the 70S ribosome (By similarity).
N	Q8CRG3	CC	Required for association of the 30S and 50S subunits to form the 70S ribosome, for tRNA binding and peptide bond formation
N	Q8CRG3	DE	50S ribosomal protein L2;
N	Q8CRG3	DR	structural constituent of ribosome
N	Q8CRG3	DR	transferase activity
N	Q8CS62	DE	UPF0173 metal-dependent hydrolase SE_1382;
N	Q8CS62	DR	hydrolase activity
N	Q8CSQ2	CC	Catalyzes the ATP-dependent phosphorylation of L- homoserine to L-homoserine phosphate (By similarity).
N	Q8CSQ2	DE	Homoserine kinase;
N	Q8CSQ2	DR	homoserine kinase activity
N	Q8CSQ2	DR	threonine biosynthetic process
N	Q8CTU2	DE	Serine acetyltransferase;
N	Q8CTU2	DR	cysteine biosynthetic process from serine
N	Q8CTU2	DR	serine O-acetyltransferase activity
N	Q8CWN3	DE	50S ribosomal protein L32;
N	Q8CWN3	DR	structural constituent of ribosome
N	Q8CX20	CC	Formation of pseudouridine at positions 38, 39 and 40 in the anticodon stem and loop of transfer RNAs (By similarity).
N	Q8CX20	DE	tRNA pseudouridine synthase A;
N	Q8CX20	DE	tRNA pseudouridylate synthase I;
N	Q8CX20	DE	tRNA-uridine isomerase I;
N	Q8CX20	DR	pseudouridine synthase activity
N	Q8CX20	DR	pseudouridine synthesis
N	Q8CX20	DR	tRNA processing
N	Q8CXB4	CC	The UvrABC repair system catalyzes the recognition and processing of DNA lesions

N	Q8CXB4	CC	The N-terminal half is responsible for the 3' incision and the C-terminal half is responsible for the 5' incision (By similarity).
N	Q8CXB4	CC	UvrC both incises the 5' and 3' sides of the lesion
N	Q8CXB4	DE	Excinuclease ABC subunit C;
N	Q8CXB4	DE	Protein uvrC;
N	Q8CXB4	DE	UvrABC system protein C;
N	Q8CXB4	DR	excinuclease ABC activity
N	Q8CXB4	DR	nucleotide-excision repair
N	Q8CXQ7	CC	Prevents misfolding and promotes the refolding and proper assembly of unfolded polypeptides generated under stress conditions (By similarity).
N	Q8CXQ7	DE	60 kDa chaperonin;
N	Q8CXQ7	DE	GroEL protein;
N	Q8CXQ7	DE	Protein Cpn60;
N	Q8CXQ7	DR	protein refolding
N	Q8CY46	CC	Formation of pseudouridine at positions 38, 39 and 40 in the anticodon stem and loop of transfer RNAs (By similarity).
N	Q8CY46	DE	tRNA pseudouridine synthase A;
N	Q8CY46	DE	tRNA pseudouridylate synthase I;
N	Q8CY46	DE	tRNA-uridine isomerase I;
N	Q8CY46	DR	pseudouridine synthase activity
N	Q8CY46	DR	pseudouridine synthesis
N	Q8CY46	DR	tRNA processing
N	Q8D201	CC	One of two assembly initiator proteins, it binds directly to the 5'-end of the 23S rRNA, where it nucleates assembly of the 50S subunit (By similarity).
N	Q8D201	DE	50S ribosomal protein L24;
N	Q8D201	DR	structural constituent of ribosome
N	Q8D3A0	CC	Part of the ABC transporter complex LolCDE involved in the translocation of lipoproteins, in an ATP-dependent manner (By similarity).
N	Q8D3A0	DE	Lipoprotein-releasing system ATP-binding protein LolD;
N	Q8D3A0	DR	ATPase activity
N	Q8D3A0	DR	lipoprotein transporter activity
N	Q8D921	CC	Catalyzes the synthesis of alpha-ribazole-5'-phosphate from nicotinate mononucleotide (NAMN) and 5,6- dimethylbenzimidazole (DMB).
N	Q8D921	DE	N(1)-alpha-phosphoribosyltransferase;
N	Q8D921	DE	NN:DBI PRT;
N	Q8D921	DE	Nicotinate-nucleotide--dimethylbenzimidazole phosphoribosyltransferase;
N	Q8D921	DR	cobalamin biosynthetic process
N	Q8D921	DR	nicotinate-nucleotide-dimethylbenzimidazole phosphoribosyltransferase activity
N	Q8D921	DR	nucleoside biosynthetic process
N	Q8DB42	DE	5-enolpyruvylshikimate-3-phosphate phospholyase;
N	Q8DB42	DE	Chorismate synthase;
N	Q8DB42	DR	aromatic amino acid family biosynthetic process
N	Q8DB42	DR	chorismate synthase activity
N	Q8DBW4	CC	Catalyzes the conversion of glucosamine-6-phosphate to glucosamine-1-phosphate (By similarity).
N	Q8DBW4	DE	Phosphoglucosamine mutase;
N	Q8DBW4	DR	carbohydrate metabolic process
N	Q8DBW4	DR	magnesium ion binding
N	Q8DBW4	DR	phosphoglucosamine mutase activity
N	Q8DC50	CC	Modulates recA activity (By similarity).
N	Q8DC50	DE	Regulatory protein recX;
N	Q8DC50	DR	regulation of DNA repair
N	Q8DCK7	DE	Glucose-6-phosphate isomerase;
N	Q8DCK7	DE	Phosphoglucose isomerase;
N	Q8DCK7	DE	Phosphohexose isomerase;
N	Q8DCK7	DR	gluconeogenesis
N	Q8DCK7	DR	glucose-6-phosphate isomerase activity
N	Q8DCV6	CC	3'-to-5' exoribonuclease specific for small oligoribonucleotides (By similarity).
N	Q8DCV6	DE	Oligoribonuclease;
N	Q8DCV6	DR	exonuclease activity
N	Q8DCV6	DR	nucleic acid binding
N	Q8DDK1	CC	Could be part of a sulfur-relay system (By similarity).
N	Q8DDK1	DE	Sulfurtransferase tusA homolog;
N	Q8DDK1	DR	sulfurtransferase activity
N	Q8DDK1	DR	tRNA processing

N	Q8DG25	CC	Involved in protein export
N	Q8DG25	CC	Acts as a chaperone by maintaining the newly synthesized protein in an open conformation (By similarity).
N	Q8DG25	DE	Trigger factor;
N	Q8DG25	DR	cell division
N	Q8DG25	DR	peptidyl-prolyl cis-trans isomerase activity
N	Q8DG25	DR	protein folding
N	Q8DG25	DR	protein transport
N	Q8DMA5	DE	1-(5-phosphoribosyl)-5-[(5-phosphoribosylamino)methylideneamino] imidazole-4-carboxamide isomerase;
N	Q8DMA5	DE	Phosphoribosylformimino-5-aminoimidazole carboxamide ribotide isomerase;
N	Q8DMA5	DR	1-(5-phosphoribosyl)-5-[(5-phosphoribosylamino)methylideneamino]imidazole-4-carboxamide isomerase activity
N	Q8DMA5	DR	histidine biosynthetic process
N	Q8DNL1	CC	Converts the preformed base xanthine, a product of nucleic acid breakdown, to xanthosine 5'-monophosphate (XMP), so it can be reused for RNA or DNA synthesis (By similarity).
N	Q8DNL1	DE	Xanthine phosphoribosyltransferase;
N	Q8DNL1	DR	purine ribonucleoside salvage
N	Q8DNL1	DR	xanthine metabolic process
N	Q8DNL1	DR	xanthine phosphoribosyltransferase activity
N	Q8DS31	CC	Binds to the 23S rRNA (By similarity).
N	Q8DS31	DE	50S ribosomal protein L15;
N	Q8DS31	DR	structural constituent of ribosome
N	Q8DSY0	CC	Binds directly to 23S rRNA
N	Q8DSY0	CC	The L1 stalk is quite mobile in the ribosome, and is involved in E site tRNA release (By similarity).
N	Q8DSY0	DE	50S ribosomal protein L1;
N	Q8DSY0	DR	RNA processing
N	Q8DSY0	DR	regulation of translation
N	Q8DSY0	DR	structural constituent of ribosome
N	Q8DUK7	CC	Catalyzes the ATP-dependent conversion of 7-carboxy-7- deazaguanine (CDG) to 7-cyano-7-deazaguanine (preQ(0)) (By similarity).
N	Q8DUK7	DE	7-cyano-7-carbaguanine synthase;
N	Q8DUK7	DE	7-cyano-7-deazaguanine synthase;
N	Q8DUK7	DE	PreQ(0) synthase;
N	Q8DUK7	DE	Queuosine biosynthesis protein queC;
N	Q8DUK7	DR	ligase activity
N	Q8DUK7	DR	metal ion binding
N	Q8DUK7	DR	queuosine biosynthetic process
N	Q8DWW6	CC	The ruvA-ruvB complex in the presence of ATP renatures cruciform structure in supercoiled DNA with palindromic sequence, indicating that it may promote strand exchange reactions in homologous recombination
N	Q8DWW6	CC	RuvA stimulates, in the presence of DNA, the weak ATPase activity of ruvB (By similarity).
N	Q8DWW6	CC	RuvAB is an helicase that mediates the Holliday junction migration by localized denaturation and reannealing
N	Q8DWW6	DE	Holliday junction ATP-dependent DNA helicase ruvA;
N	Q8DWW6	DR	DNA recombination
N	Q8DWW6	DR	four-way junction helicase activity
N	Q8DY25	CC	Furnishes a means for formation of correctly charged Gln-tRNA(Gln) through the transamidation of misacylated Glu- tRNA(Gln) in organisms which lack glutaminyl-tRNA synthetase
N	Q8DY25	CC	The reaction takes place in the presence of glutamine and ATP through an activated gamma-phospho-Glu-tRNA(Gln) (By similarity).
N	Q8DY25	DE	Glu-ADT subunit A;
N	Q8DY25	DE	Glutamyl-tRNA(Gln) amidotransferase subunit A;
N	Q8DY25	DR	carbon-nitrogen ligase activity, with glutamine as amido-N-donor
N	Q8DZY3	DE	L-lactate dehydrogenase;
N	Q8DZY3	DR	L-lactate dehydrogenase activity
N	Q8DZY3	DR	oxidation-reduction process
N	Q8E3W3	DE	UPF0161 protein gbs1641;
N	Q8E720	CC	Involved in saturated fatty acids biosynthesis.
N	Q8E720	DE	(3R)-hydroxymyristoyl ACP dehydrase;
N	Q8E720	DE	(3R)-hydroxymyristoyl-[acyl-carrier-protein] dehydratase;

N	Q8E720	DR	fatty acid biosynthetic process
N	Q8E720	DR	hydro-lyase activity
N	Q8E720	DR	lipid A biosynthetic process
N	Q8E942	CC	Together with moaA, is involved in the conversion of a guanosine derivative (5'-GTP) into molybdopterin precursor Z (By similarity).
N	Q8E942	DE	Molybdenum cofactor biosynthesis protein C;
N	Q8E942	DR	Mo-molybdopterin cofactor biosynthetic process
N	Q8E9P6	CC	Cell wall formation
N	Q8E9P6	CC	Catalyzes the addition of glutamate to the nucleotide precursor UDP-N-acetylmuramoyl-L-alanine (UMA) (By similarity).
N	Q8E9P6	DE	D-glutamic acid-adding enzyme;
N	Q8E9P6	DE	UDP-N-acetylmuramoyl-L-alanyl-D-glutamate synthetase;
N	Q8E9P6	DE	UDP-N-acetylmuramoylalanine--D-glutamate ligase;
N	Q8E9P6	DR	UDP-N-acetylmuramoylalanine-D-glutamate ligase activity
N	Q8E9P6	DR	cell division
N	Q8E9P6	DR	cellular cell wall organization
N	Q8E9P6	DR	peptidoglycan biosynthetic process
N	Q8E9P6	DR	regulation of cell shape
N	Q8EBH1	DE	Glucose-6-phosphate isomerase;
N	Q8EBH1	DE	Phosphoglucose isomerase;
N	Q8EBH1	DE	Phosphohexose isomerase;
N	Q8EBH1	DR	gluconeogenesis
N	Q8EBH1	DR	glucose-6-phosphate isomerase activity
N	Q8EEF3	CC	The ruvA-ruvB complex in the presence of ATP renatures cruciform structure in supercoiled DNA with palindromic sequence, indicating that it may promote strand exchange reactions in homologous recombination
N	Q8EEF3	CC	RuvAB is an helicase that mediates the Holliday junction migration by localized denaturation and reannealing (By similarity).
N	Q8EEF3	DE	Holliday junction ATP-dependent DNA helicase ruvB;
N	Q8EEF3	DR	DNA recombination
N	Q8EEF3	DR	four-way junction helicase activity
N	Q8EFB1	CC	Catalyzes the sequential NAD-dependent oxidations of L- histidinol to L-histidinaldehyde and then to L-histidine (By similarity).
N	Q8EFB1	DE	Histidinol dehydrogenase;
N	Q8EFB1	DR	histidine biosynthetic process
N	Q8EFB1	DR	histidinol dehydrogenase activity
N	Q8EFB1	DR	oxidation-reduction process
N	Q8EFB1	DR	zinc ion binding
N	Q8EFB2	DE	Histidinol-phosphate aminotransferase;
N	Q8EFB2	DE	Imidazole acetol-phosphate transaminase;
N	Q8EFB2	DR	histidine biosynthetic process
N	Q8EFB2	DR	histidinol-phosphate transaminase activity
N	Q8EFB2	DR	pyridoxal phosphate binding
N	Q8EKX1	CC	Binds to the 23S rRNA (By similarity).
N	Q8EKX1	DE	50S ribosomal protein L9;
N	Q8EKX1	DR	structural constituent of ribosome
N	Q8EL25	DE	UPF0324 membrane protein OB3406;
N	Q8EQS7	CC	DNA motor protein, which is both required to move DNA out of the region of the septum during cell division and for the septum formation
N	Q8EQS7	CC	Tracks DNA in an ATP-dependent manner by generating positive supercoils in front of it and negative supercoils behind it (By similarity).
N	Q8EQS7	DE	DNA translocase ftsK;
N	Q8EQS7	DR	cell division
N	Q8EQS7	DR	chromosome segregation
N	Q8EQS7	DR	nucleoside-triphosphatase activity
N	Q8ETX2	CC	One of two assembly initiator proteins, it binds directly to the 5'-end of the 23S rRNA, where it nucleates assembly of the 50S subunit (By similarity).
N	Q8ETX2	DE	50S ribosomal protein L24;
N	Q8ETX2	DR	structural constituent of ribosome
N	Q8EWT2	CC	Endonuclease IV plays a role in DNA repair
N	Q8EWT2	CC	It cleaves phosphodiester bonds at apurinic or apyrimidinic sites (AP sites) to produce new 5'-ends that are base-free deoxyribose 5-phosphate residues
N	Q8EWT2	CC	It preferentially attacks modified AP sites created by bleomycin and neocarzinostatin (By similarity).
N	Q8EWT2	DE	Endodeoxyribonuclease IV;

N	Q8EWT2	DE	Endonuclease IV;
N	Q8EWT2	DE	Probable endonuclease 4;
N	Q8EWT2	DR	deoxyribonuclease IV (phage-T4-induced) activity
N	Q8EWT2	DR	zinc ion binding
N	Q8F0T5	DE	UPF0234 protein LA_3406;
N	Q8FBS5	CC	Functions as an ATPase
N	Q8FBS5	CC	May play a role in metal insertion (metal-chelatase) or as a chaperone (By similarity)
N	Q8FBS5	DE	ATPase ravA;
N	Q8FBS5	DE	Regulatory ATPase variant A;
N	Q8FBS5	DR	ATPase activity
N	Q8FBS5	DR	hydrolase activity, acting on acid anhydrides, catalyzing transmembrane movement of substances
N	Q8FE69	DE	UPF0267 protein yqfB;
N	Q8FGL1	DE	Flagellar hook-basal body complex protein FliE;
N	Q8FGL1	DR	ciliary or flagellar motility
N	Q8FGL1	DR	motor activity
N	Q8FGL1	DR	structural molecule activity
N	Q8FLA3	CC	Required for anaerobic carnitine reduction
N	Q8FLA3	CC	May bring reductant to CaiA (By similarity).
N	Q8FLA3	DE	Protein fixA;
N	Q8FLA3	DR	electron carrier activity
N	Q8FLA3	DR	electron transport chain
N	Q8FNT1	DE	Protein MraZ;
N	Q8FS75	CC	Binds 23S rRNA and is also seen to make contacts with the A and possibly P site tRNAs (By similarity).
N	Q8FS75	DE	50S ribosomal protein L16;
N	Q8FS75	DR	structural constituent of ribosome
N	Q8FSD6	CC	Catalyzes the decarboxylation of four acetate groups of uroporphyrinogen-III to yield coproporphyrinogen-III (By similarity).
N	Q8FSD6	DE	Uroporphyrinogen decarboxylase;
N	Q8FSD6	DR	porphyrin biosynthetic process
N	Q8FSD6	DR	uroporphyrinogen decarboxylase activity
N	Q8FSJ0	CC	Catalyzes a reversible aldol reaction between acetaldehyde and D-glyceraldehyde 3-phosphate to generate 2-deoxy- D-ribose 5-phosphate (By similarity)
N	Q8FSJ0	DE	2-deoxy-D-ribose 5-phosphate aldolase;
N	Q8FSJ0	DE	Deoxyriboaldolase;
N	Q8FSJ0	DE	Deoxyribose-phosphate aldolase;
N	Q8FSJ0	DE	Phosphodeoxyriboaldolase;
N	Q8FSJ0	DR	deoxyribonucleotide catabolic process
N	Q8FSJ0	DR	deoxyribose-phosphate aldolase activity
N	Q8FV25	DE	2,3,4,5-tetrahydropyridine-2,6-dicarboxylate N-succinyltransferase;
N	Q8FV25	DE	THDP succinyltransferase;
N	Q8FV25	DE	THP succinyltransferase;
N	Q8FV25	DE	Tetrahydrodipicolinate N-succinyltransferase;
N	Q8FV25	DE	Tetrahydropicolinate succinylase;
N	Q8FV25	DR	2,3,4,5-tetrahydropyridine-2,6-dicarboxylate N-succinyltransferase activity
N	Q8FV25	DR	diaminopimelate biosynthetic process
N	Q8FYW3	CC	Catalyzes the conversion of N5-carboxyaminoimidazole ribonucleotide (N5-CAIR) to 4-carboxy-5-aminoimidazole ribonucleotide (CAIR) (By similarity).
N	Q8FYW3	DE	5-(carboxyamino)imidazole ribonucleotide mutase;
N	Q8FYW3	DE	N5-CAIR mutase;
N	Q8FYW3	DE	N5-carboxyaminoimidazole ribonucleotide mutase;
N	Q8FYW3	DR	'de novo' IMP biosynthetic process
N	Q8FYW3	DR	5-(carboxyamino)imidazole ribonucleotide mutase activity
N	Q8FYW3	DR	phosphoribosylaminoimidazole carboxylase activity
N	Q8FZP5	CC	Cell wall formation (By similarity).
N	Q8FZP5	DE	D-Ala-D-Ala ligase B;
N	Q8FZP5	DE	D-alanine--D-alanine ligase B;
N	Q8FZP5	DE	D-alanylalanine synthetase B;
N	Q8FZP5	DR	D-alanine-D-alanine ligase activity
N	Q8FZP5	DR	cellular cell wall organization
N	Q8FZP5	DR	metal ion binding
N	Q8FZP5	DR	peptidoglycan biosynthetic process
N	Q8FZP5	DR	regulation of cell shape
N	Q8G075	CC	Catalyzes the GTP-dependent ribosomal translocation step during translation elongation

N	Q8G075	CC	Catalyzes the coordinated movement of the two tRNA molecules, the mRNA and conformational changes in the ribosome (By similarity).
N	Q8G075	CC	During this step, the ribosome changes from the pre-translocational (PRE) to the post- translocational (POST) state as the newly formed A-site-bound peptidyl-tRNA and P-site-bound deacylated tRNA move to the P and E sites, respectively
N	Q8G075	DE	Elongation factor G;
N	Q8G075	DR	GTPase activity
N	Q8G075	DR	translation elongation factor activity
N	Q8G308	CC	This protein specifically catalyzes the removal of signal peptides from prolipoproteins (By similarity).
N	Q8G308	DE	Lipoprotein signal peptidase;
N	Q8G308	DE	Prolipoprotein signal peptidase;
N	Q8G308	DE	Signal peptidase II;
N	Q8G308	DR	aspartic-type endopeptidase activity
N	Q8G312	CC	Modifies, by uridylylation or deuridylylation the PII (glnB) regulatory protein (By similarity).
N	Q8G312	DE	PII uridylyl-transferase;
N	Q8G312	DE	Uridylyl-removing enzyme;
N	Q8G312	DE	[Protein-PII] uridylyltransferase;
N	Q8G312	DR	[protein-PII] uridylyltransferase activity
N	Q8G312	DR	amino acid binding
N	Q8G312	DR	nitrogen compound metabolic process
N	Q8G4G1	DE	Probable serine/threonine-protein kinase pknA2;
N	Q8G4G1	DR	penicillin binding
N	Q8G4G1	DR	protein phosphorylation
N	Q8G4G1	DR	protein serine/threonine kinase activity
N	Q8G816	DE	Carbamoyl-phosphate synthase small chain;
N	Q8G816	DE	Carbamoyl-phosphate synthetase glutamine chain;
N	Q8G816	DR	arginine biosynthetic process
N	Q8G816	DR	carbamoyl-phosphate synthase (glutamine-hydrolyzing) activity
N	Q8G816	DR	glutamine metabolic process
N	Q8G816	DR	pyrimidine nucleotide biosynthetic process
N	Q8GC81	CC	Specifically catalyzes the cleavage of the D-lactyl ether substituent of MurNAc 6-phosphate, producing GlcNAc 6- phosphate and D-lactate
N	Q8GC81	CC	Together with AnmK, is also required for the utilization of anhydro-N-acetylmuramic acid (anhMurNAc) either imported from the medium or derived from its own cell wall murein, and thus plays a role in cell wall recycling (By similarity).
N	Q8GC81	DE	MurNAc-6-P etherase;
N	Q8GC81	DE	N-acetylmuramic acid 6-phosphate etherase;
N	Q8GC81	DE	N-acetylmuramic acid 6-phosphate hydrolase;
N	Q8GC81	DE	N-acetylmuramic acid 6-phosphate lyase;
N	Q8GC81	DR	amino sugar catabolic process
N	Q8GC81	DR	hydro-lyase activity
N	Q8GC81	DR	sugar binding
N	Q8GPG1	CC	May transfer electrons to the iron-sulfur centers of ddhB.
N	Q8GPG1	DE	DMS DH heme subunit;
N	Q8GPG1	DE	DMS DH subunit gamma;
N	Q8GPG1	DE	Dimethylsulfide dehydrogenase subunit gamma;
N	Q8GPG1	DE	Dimethylsulfide heme subunit;
N	Q8GPG1	DR	electron transport chain
N	Q8GPI4	CC	Antibacterial peptide active against a broad range of lactic acid bacteria, L.monocytogenes and many epidemiologically unrelated strains of S.aureus involved in bovine mastitis.
N	Q8GPI4	DE	Bacteriocin aureocin A53;
N	Q8GPI4	DR	defense response to bacterium
N	Q8GY63	CC	Converts adenosine 3'-phosphate 5'-phosphosulfate (PAPS) to adenosine 5'-phosphosulfate (APS) and 3'(2')-phosphoadenosine 5'- phosphate (PAP) to AMP (By similarity)
N	Q8GY63	CC	Is also able to hydrolyze inositol 1,4-bisphosphate (By similarity).
N	Q8GY63	DE	3'(2'),5'-bisphosphate nucleotidase 3;
N	Q8GY63	DE	3'(2'),5'-bisphosphonucleoside 3'(2')-phosphohydrolase 3;
N	Q8GY63	DE	Inositol polyphosphate 1-phosphatase 3;
N	Q8GY63	DE	Inositol-1,4-bisphosphate 1-phosphatase 3;
N	Q8GY63	DE	Probable SAL3 phosphatase;
N	Q8GY63	DR	3'(2'),5'-bisphosphate nucleotidase activity

N	Q8GY63	DR	inositol-1,4-bisphosphate 1-phosphatase activity
N	Q8GY63	DR	metal ion binding
N	Q8GY63	DR	sulfur compound metabolic process
N	Q8H852	CC	The coatomer is a cytosolic protein complex that binds to dilysine motifs and reversibly associates with Golgi non- clathrin-coated vesicles, which further mediate biosynthetic protein transport from the ER, via the Golgi up to the trans Golgi network
N	Q8H852	CC	Coatomer complex is required for budding from Golgi membranes, and is essential for the retrograde Golgi-to-ER transport of dilysine-tagged proteins (By similarity)
N	Q8H852	DE	Coatomer subunit gamma-1;
N	Q8H852	DE	Gamma-1-COP;
N	Q8H852	DE	Gamma-1-coat protein;
N	Q8H852	DR	intracellular protein transport
N	Q8H852	DR	structural molecule activity
N	Q8H852	DR	vesicle-mediated transport
N	Q8HQQ3	CC	Usually encoded in the trnK tRNA gene intron
N	Q8HQQ3	CC	Probably assists in splicing its own and other chloroplast group II introns (By similarity)
N	Q8HQQ3	DE	Intron maturase;
N	Q8HQQ3	DR	mRNA processing
N	Q8HQQ3	DR	tRNA processing
N	Q8HVA1	CC	Usually encoded in the trnK tRNA gene intron
N	Q8HVA1	CC	Probably assists in splicing its own and other chloroplast group II introns (By similarity)
N	Q8HVA1	DE	Intron maturase;
N	Q8HVA1	DR	mRNA processing
N	Q8HVA1	DR	tRNA processing
N	Q8HVL8	CC	NDH shuttles electrons from NAD(P)H:plastoquinone, via FMN and iron-sulfur (Fe-S) centers, to quinones in the photosynthetic chain and possibly in a chloroplast respiratory chain
N	Q8HVL8	CC	Couples the redox reaction to proton translocation, and thus conserves the redox energy in a proton gradient (By similarity).
N	Q8HVL8	CC	The immediate electron acceptor for the enzyme in this species is believed to be plastoquinone
N	Q8HVL8	DE	NAD(P)H dehydrogenase subunit I;
N	Q8HVL8	DE	NAD(P)H-quinone oxidoreductase subunit I, chloroplastic;
N	Q8HVL8	DE	NADH-plastoquinone oxidoreductase subunit I;
N	Q8HVL8	DE	NDH subunit I;
N	Q8HVL8	DR	4 iron, 4 sulfur cluster binding
N	Q8HVL8	DR	NADH dehydrogenase (ubiquinone) activity
N	Q8HVL8	DR	electron carrier activity
N	Q8HVL8	DR	metal ion binding
N	Q8HVL8	DR	oxidation-reduction process
N	Q8HVL8	DR	quinone binding
N	Q8HVR8	CC	NDH shuttles electrons from NAD(P)H:plastoquinone, via FMN and iron-sulfur (Fe-S) centers, to quinones in the photosynthetic chain and possibly in a chloroplast respiratory chain
N	Q8HVR8	CC	Couples the redox reaction to proton translocation, and thus conserves the redox energy in a proton gradient (By similarity).
N	Q8HVR8	CC	The immediate electron acceptor for the enzyme in this species is believed to be plastoquinone
N	Q8HVR8	DE	NAD(P)H dehydrogenase subunit I;
N	Q8HVR8	DE	NAD(P)H-quinone oxidoreductase subunit I, chloroplastic;
N	Q8HVR8	DE	NADH-plastoquinone oxidoreductase subunit I;
N	Q8HVR8	DE	NDH subunit I;
N	Q8HVR8	DR	4 iron, 4 sulfur cluster binding
N	Q8HVR8	DR	NADH dehydrogenase (ubiquinone) activity
N	Q8HVR8	DR	electron carrier activity
N	Q8HVR8	DR	metal ion binding
N	Q8HVR8	DR	oxidation-reduction process
N	Q8HVR8	DR	quinone binding
N	Q8HVY4	CC	DNA-dependent RNA polymerase catalyzes the transcription of DNA into RNA using the four ribonucleoside triphosphates as substrates.
N	Q8HVY4	DE	DNA-directed RNA polymerase subunit beta';
N	Q8HVY4	DE	Plastid-encoded RNA polymerase subunit beta';
N	Q8HVY4	DE	RNA polymerase subunit beta';
N	Q8HVY4	DR	DNA-directed RNA polymerase activity

N	Q8HXW6	CC	Removes thioester-linked fatty acyl groups such as palmitate from modified cysteine residues in proteins or peptides during lysosomal degradation
N	Q8HXW6	CC	Prefers acyl chain lengths of 14 to 18 carbons (By similarity).
N	Q8HXW6	DE	Palmitoyl-protein hydrolase 1;
N	Q8HXW6	DE	Palmitoyl-protein thioesterase 1;
N	Q8HXW6	DR	DNA fragmentation involved in apoptotic nuclear change
N	Q8HXW6	DR	cofactor metabolic process
N	Q8HXW6	DR	cofactor transport
N	Q8HXW6	DR	lipid catabolic process
N	Q8HXW6	DR	lysosomal lumen acidification
N	Q8HXW6	DR	membrane raft organization
N	Q8HXW6	DR	negative regulation of cell growth
N	Q8HXW6	DR	negative regulation of neuron apoptosis
N	Q8HXW6	DR	nervous system development
N	Q8HXW6	DR	palmitoyl-(protein) hydrolase activity
N	Q8HXW6	DR	palmitoyl-CoA hydrolase activity
N	Q8HXW6	DR	positive regulation of pinocytosis
N	Q8HXW6	DR	positive regulation of receptor-mediated endocytosis
N	Q8HXW6	DR	protein depalmitoylation
N	Q8HXW6	DR	protein transport
N	Q8IG39	CC	Putative nucleoside deaminase
N	Q8IG39	CC	May catalyze the hydrolytic deamination of adenosine or some similar substrate and play a role in purine metabolism (By similarity).
N	Q8IG39	DE	Adenosine deaminase-like protein;
N	Q8IG39	DR	deaminase activity
N	Q8IG39	DR	hydrolase activity
N	Q8IG39	DR	metal ion binding
N	Q8IG39	DR	purine ribonucleoside monophosphate biosynthetic process
N	Q8ISP0	CC	Located at the top of the head of the 40S subunit, it contacts several helices of the 18S rRNA (By similarity).
N	Q8ISP0	DE	40S ribosomal protein S18;
N	Q8ISP0	DR	structural constituent of ribosome
N	Q8ITC7	CC	Acts as a receptor for the neuropeptides CAP-1 and CAP- 2, but not CAP-3
N	Q8ITC7	CC	Probably a component of signal transduction pathway that leads to Malpighian tubule fluid secretion in response to these ligands.
N	Q8ITC7	DE	Cap2b receptor;
N	Q8ITC7	DE	Neuropeptides capa receptor;
N	Q8ITC7	DR	body fluid secretion
N	Q8ITC7	DR	neuromedin U receptor activity
N	Q8IVF1	DE	Protein FAM22A;
N	Q8IWX7	CC	May play a role in sarcomere formation during muscle cell development (By similarity)
N	Q8IWX7	CC	May act as co-chaperone for HSP90 (Potential)
N	Q8IWX7	CC	May be necessary for proper folding of myosin (Potential).
N	Q8IWX7	DE	Protein unc-45 homolog B;
N	Q8IWX7	DR	cell differentiation
N	Q8IWX7	DR	muscle organ development
N	Q8IYM0	DE	Protein FAM186B;
N	Q8J282	CC	Involved in autophagy, in the development of aerial hyphae, in pigmentation and in the differentiation of female reproductive organs
N	Q8J282	CC	May be also involved in cytoplasm to vacuole transport (Cvt) vesicles formation (By similarity)
N	Q8J282	CC	May mediate the delivery of the vesicles and autophagosomes to the vacuole via the microtubule cytoskeleton (By similarity).
N	Q8J282	DE	Autophagy-related protein 8;
N	Q8J282	DE	Autophagy-related ubiquitin-like modifier ATG8;
N	Q8J282	DE	Induced during the incompatibility reaction protein 7;
N	Q8J282	DR	protein transport
N	Q8JTH0	CC	Attaches the virus to host cellular receptor, inducing endocytosis of the virion
N	Q8JTH0	CC	In the endosome, the acidic pH induces conformational changes in the glycoprotein trimer, which trigger fusion between virus and cell membrane
N	Q8JTH0	CC	There is convincing in vitro evidence that the muscular form of the nicotinic acetylcholine receptor (nAChR), the neuronal cell adhesion molecule (NCAM), and the p75 neurotrophin receptor (p75NTR) bind glycoprotein G and thereby facilitate rabies virus entry into cells (By similarity).
N	Q8JTH0	DE	Glycoprotein G;

N	Q8JZS9	DE	39S ribosomal protein L48, mitochondrial;
N	Q8JZS9	DR	structural constituent of ribosome
N	Q8K3Z9	CC	Essential component of the nuclear pore complex (NPC)
N	Q8K3Z9	CC	The repeat-containing domain may be involved in anchoring components of the pore complex to the pore membrane
N	Q8K3Z9	CC	When overexpressed in cells induces the formation of cytoplasmic annulate lamellae (AL) (By similarity).
N	Q8K3Z9	DE	Nuclear envelope pore membrane protein POM 121;
N	Q8K3Z9	DE	Nucleoporin Nup121;
N	Q8K3Z9	DE	Pore membrane protein of 121 kDa;
N	Q8K3Z9	DR	mRNA transport
N	Q8K3Z9	DR	protein transport
N	Q8K3Z9	DR	transmembrane transport
N	Q8K593	CC	Catalyzes the conversion of phosphatidic acid (PA) to diacylglycerol (DG)
N	Q8K593	CC	In addition it hydrolyzes lysophosphatidic acid (LPA), ceramide-1-phosphate (C-1-P) and sphingosine-1- phosphate (S-1-P) (By similarity).
N	Q8K593	DE	Lipid phosphate phosphohydrolase 2;
N	Q8K593	DE	Phosphatidate phosphohydrolase type 2c;
N	Q8K593	DE	Phosphatidic acid phosphatase 2c;
N	Q8K593	DR	phosphatidate phosphatase activity
N	Q8K6Z3	CC	Transfers and isomerizes the ribose moiety from AdoMet to the 7-aminomethyl group of 7-deazaguanine (preQ1-tRNA) to give epoxyqueuosine (oQ-tRNA) (By similarity)
N	Q8K6Z3	DE	Queuosine biosynthesis protein queA;
N	Q8K6Z3	DE	S-adenosylmethionine:tRNA ribosyltransferase-isomerase;
N	Q8K6Z3	DR	isomerase activity
N	Q8K6Z3	DR	queuosine biosynthetic process
N	Q8K6Z3	DR	transferase activity
N	Q8K7C0	CC	Interferes with activator-stimulated transcription by interaction with the RNA polymerase alpha-CTD
N	Q8K7C0	CC	May function to globally reduce transcription of genes involved in growth- and development-promoting processes and to increase transcription of genes involved in thiol homeostasis, during periods of extreme stress (By similarity).
N	Q8K7C0	DE	Regulatory protein spx;
N	Q8K873	CC	Forms oxaloacetate, a four-carbon dicarboxylic acid source for the tricarboxylic acid cycle.
N	Q8K873	DE	Phosphoenolpyruvate carboxylase;
N	Q8K873	DR	carbon fixation
N	Q8K873	DR	phosphoenolpyruvate carboxylase activity
N	Q8K873	DR	tricarboxylic acid cycle
N	Q8K8E4	CC	Has an important function as a repair enzyme for proteins that have been inactivated by oxidation
N	Q8K8E4	CC	Catalyzes the reversible oxidation-reduction of methionine sulfoxide in proteins to methionine (By similarity).
N	Q8K8E4	DE	Peptide Met(O) reductase;
N	Q8K8E4	DE	Peptide methionine sulfoxide reductase MsrA;
N	Q8K8E4	DE	Peptide-methionine (S)-S-oxide reductase;
N	Q8K8E4	DE	Protein-methionine-S-oxide reductase;
N	Q8K8E4	DR	oxidation-reduction process
N	Q8K8E4	DR	peptide-methionine-(S)-S-oxide reductase activity
N	Q8K8E4	DR	protein metabolic process
N	Q8K9Y5	CC	NDH-1 shuttles electrons from NADH, via FMN and iron- sulfur (Fe-S) centers, to quinones in the respiratory chain
N	Q8K9Y5	CC	Couples the redox reaction to proton translocation (for every two electrons transferred, four hydrogen ions are translocated across the cytoplasmic membrane), and thus conserves the redox energy in a proton gradient (By similarity)
N	Q8K9Y5	CC	The immediate electron acceptor for the enzyme in this species is believed to be ubiquinone
N	Q8K9Y5	DE	NADH dehydrogenase I subunit C/D;
N	Q8K9Y5	DE	NADH-quinone oxidoreductase subunit C/D;
N	Q8K9Y5	DE	NDH-1 subunit C/D;
N	Q8K9Y5	DR	NADH dehydrogenase (ubiquinone) activity
N	Q8K9Y5	DR	oxidation-reduction process
N	Q8K9Y5	DR	quinone binding
N	Q8KED5	CC	Has antioxidant activity
N	Q8KED5	CC	Could remove peroxides or H(2)O(2) (By similarity).

N	Q8KED5	DE	Probable thiol peroxidase;
N	Q8KED5	DR	cell redox homeostasis
N	Q8KED5	DR	oxidation-reduction process
N	Q8KED5	DR	thioredoxin peroxidase activity
N	Q8KEW8	CC	Binds specifically to the ssrA RNA (tmRNA) and is required for stable association of ssrA with ribosomes (By similarity).
N	Q8KEW8	DE	SsrA-binding protein;
N	Q8KF55	DE	1-(5-phosphoribosyl)-5-[(5-phosphoribosylamino)methylideneamino] imidazole-4-carboxamide isomerase;
N	Q8KF55	DE	Phosphoribosylformimino-5-aminoimidazole carboxamide ribotide isomerase;
N	Q8KF55	DR	1-(5-phosphoribosyl)-5-[(5-phosphoribosylamino)methylideneamino]imidazole-4-carboxamide isomerase activity
N	Q8KF55	DR	histidine biosynthetic process
N	Q8KG17	DE	50S ribosomal protein L10;
N	Q8KG17	DR	ribosome biogenesis
N	Q8KG17	DR	structural constituent of ribosome
N	Q8KG38	CC	Catalyzes the first step in hexosamine metabolism, converting fructose-6P into glucosamine-6P using glutamine as a nitrogen source (By similarity).
N	Q8KG38	DE	D-fructose-6-phosphate amidotransferase;
N	Q8KG38	DE	Glucosamine--fructose-6-phosphate aminotransferase [isomerizing];
N	Q8KG38	DE	Glucosamine-6-phosphate synthase;
N	Q8KG38	DE	Hexosephosphate aminotransferase;
N	Q8KG38	DE	L-glutamine-D-fructose-6-phosphate amidotransferase;
N	Q8KG38	DR	carbohydrate biosynthetic process
N	Q8KG38	DR	glutamine metabolic process
N	Q8KG38	DR	glutamine-fructose-6-phosphate transaminase (isomerizing) activity
N	Q8KG38	DR	sugar binding
N	Q8L3R2	CC	Potential calcium sensor (By similarity).
N	Q8L3R2	DE	Calmodulin-like protein 41;
N	Q8L3R2	DE	Probable calcium-binding protein CML41;
N	Q8L3R2	DR	calcium ion binding
N	Q8L910	CC	May constitute a glutathione peroxidase-like protective system against oxidative stresses (By similarity).
N	Q8L910	DE	Probable glutathione peroxidase 4;
N	Q8L910	DR	glutathione peroxidase activity
N	Q8L910	DR	oxidation-reduction process
N	Q8L910	DR	response to oxidative stress
N	Q8LBC0	DE	Cyclin-T1-3;
N	Q8LBC0	DR	cell division
N	Q8LBC0	DR	protein binding
N	Q8LW53	CC	Uses Mg-ATP and reduced ferredoxin to reduce ring D of protochlorophyllide (Pchlide) to form chlorophyllide a (Chlide) (By similarity)
N	Q8LW53	CC	This reaction is light-independent.
N	Q8LW53	DE	DPOR subunit N;
N	Q8LW53	DE	LI-POR subunit N;
N	Q8LW53	DE	Light-independent protochlorophyllide reductase subunit N;
N	Q8LW53	DR	chlorophyll biosynthetic process
N	Q8LW53	DR	oxidation-reduction process
N	Q8LW53	DR	oxidoreductase activity, acting on iron-sulfur proteins as donors
N	Q8LW53	DR	photosynthesis, dark reaction
N	Q8MCL8	CC	Usually encoded in the trnK tRNA gene intron
N	Q8MCL8	CC	Probably assists in splicing its own and other chloroplast group II introns (By similarity).
N	Q8MCL8	DE	Intron maturase;
N	Q8MCL8	DR	mRNA processing
N	Q8MCL8	DR	tRNA processing
N	Q8MIB3	CC	Xenobiotic transporter that may play an important role in the exclusion of xenobiotics from the brain
N	Q8MIB3	CC	May be involved in brain-to-blood efflux (By similarity).
N	Q8MIB3	DE	ATP-binding cassette sub-family G member 2;
N	Q8MIB3	DE	Brain multidrug resistance protein;
N	Q8MIB3	DE	CD338;
N	Q8MIB3	DR	ATPase activity
N	Q8MIQ5	CC	May stabilize HDL (high density lipoprotein) structure by its association with lipids, and affect the HDL metabolism.
N	Q8MIQ5	DE	Apolipoprotein A-II;

N	Q8MIQ5	DE	Apolipoprotein A2;
N	Q8MIQ5	DR	positive regulation of interleukin-8 biosynthetic process
N	Q8MIQ5	DR	protein heterodimerization activity
N	Q8MJZ2	CC	May act as receptor for class I MHC antigens (By similarity).
N	Q8MJZ2	DE	Leukocyte immunoglobulin-like receptor E;
N	Q8MJZ2	DE	Leukocyte immunoglobulin-like receptor subfamily A member 6;
N	Q8MJZ2	DR	receptor activity
N	Q8N394	DE	Transmembrane and TPR repeat-containing protein 2;
N	Q8N801	DE	Uncharacterized protein C2orf61;
N	Q8NBX0	DE	Probable saccharopine dehydrogenase;
N	Q8NBX0	DR	oxidation-reduction process
N	Q8NBX0	DR	saccharopine dehydrogenase (NAD+, L-glutamate-forming) activity
N	Q8NCW5	DE	Apolipoprotein A-I-binding protein;
N	Q8NCW5	DE	YjeF N-terminal domain-containing protein 1;
N	Q8NCW5	DR	protein binding
N	Q8NCX0	DE	Coiled-coil domain-containing protein 150;
N	Q8NE01	CC	Probable metal transporter (By similarity).
N	Q8NE01	DE	Ancient conserved domain-containing protein 3;
N	Q8NE01	DE	Metal transporter CNNM3;
N	Q8NE01	DR	ion transport
N	Q8NE01	DR	protein binding
N	Q8NGG1	CC	Odorant receptor (Potential).
N	Q8NGG1	DE	Olfactory receptor 8J2;
N	Q8NGG1	DR	olfactory receptor activity
N	Q8NGG1	DR	sensory perception of smell
N	Q8NGU4	CC	Odorant receptor (Potential).
N	Q8NGU4	DE	Putative olfactory receptor 2I1;
N	Q8NGU4	DE	Putative olfactory receptor 2I2;
N	Q8NGU4	DE	Putative olfactory receptor 2I3;
N	Q8NGU4	DE	Putative olfactory receptor 2I4;
N	Q8NGU4	DR	olfactory receptor activity
N	Q8NGU4	DR	sensory perception of smell
N	Q8NHP1	CC	Can reduce the dialdehyde protein-binding form of aflatoxin B1 (AFB1) to the non-binding AFB1 dialcohol
N	Q8NHP1	CC	May be involved in protection of liver against the toxic and carcinogenic effects of AFB1, a potent hepatocarcinogen (By similarity).
N	Q8NHP1	DE	AFB1 aldehyde reductase 3;
N	Q8NHP1	DE	Aflatoxin B1 aldehyde reductase member 4;
N	Q8NHP1	DE	Aldoketoreductase 7-like;
N	Q8NHP1	DR	oxidation-reduction process
N	Q8NHP1	DR	oxidoreductase activity
N	Q8NHR7	DE	Uncharacterized protein C15orf43;
N	Q8NI37	DE	Protein phosphatase PTC7 homolog;
N	Q8NI37	DE	T-cell activation protein phosphatase 2C-like;
N	Q8NI37	DE	T-cell activation protein phosphatase 2C;
N	Q8NI37	DR	metal ion binding
N	Q8NI37	DR	phosphoprotein phosphatase activity
N	Q8NQH1	CC	Condenses 4-methyl-5-(beta-hydroxyethyl)thiazole monophosphate (THZ-P) and 4-amino-5-hydroxymethyl pyrimidine pyrophosphate (HMP-PP) to form thiamine monophosphate (TMP) (By similarity).
N	Q8NQH1	DE	HMP kinase;
N	Q8NQH1	DE	HMP-P kinase;
N	Q8NQH1	DE	HMP-phosphate kinase;
N	Q8NQH1	DE	HMPP kinase;
N	Q8NQH1	DE	Hydroxymethylpyrimidine kinase;
N	Q8NQH1	DE	Hydroxymethylpyrimidine phosphate kinase;
N	Q8NQH1	DE	Hydroxymethylpyrimidine/phosphomethylpyrimidine kinase;
N	Q8NQH1	DE	Multifunctional protein thiED;
N	Q8NQH1	DE	TMP pyrophosphorylase;
N	Q8NQH1	DE	TMP-PPase;
N	Q8NQH1	DE	Thiamine-phosphate pyrophosphorylase;
N	Q8NQH1	DE	Thiamine-phosphate synthase;
N	Q8NQH1	DR	hydroxymethylpyrimidine kinase activity
N	Q8NQH1	DR	metal ion binding
N	Q8NQH1	DR	phosphomethylpyrimidine kinase activity

N	Q8NQH1	DR	thiamine biosynthetic process
N	Q8NQH1	DR	thiamine-phosphate diphosphorylase activity
N	Q8NSM4	CC	Modulates the activities of several enzymes which are inactive in their acetylated form (By similarity).
N	Q8NSM4	DE	NAD-dependent deacetylase;
N	Q8NSM4	DE	Regulatory protein SIR2 homolog;
N	Q8NSM4	DR	chromatin silencing
N	Q8NSM4	DR	hydrolase activity, acting on carbon-nitrogen (but not peptide) bonds, in linear amides
N	Q8NSM4	DR	protein deacetylation
N	Q8NSM4	DR	zinc ion binding
N	Q8NW03	DE	Protein CrcB homolog 2;
N	Q8NZ29	DE	Putative competence-damage inducible protein;
N	Q8NZ29	DR	Mo-molybdopterin cofactor biosynthetic process
N	Q8NZH6	CC	Removes N-terminal dipeptides sequentially from polypeptides having unsubstituted N-termini provided that the penultimate residue is proline.
N	Q8NZH6	DE	X-Pro dipeptidyl-peptidase;
N	Q8NZH6	DE	X-prolyl-dipeptidyl aminopeptidase;
N	Q8NZH6	DE	Xaa-Pro dipeptidyl-peptidase;
N	Q8NZH6	DR	aminopeptidase activity
N	Q8NZH6	DR	dipeptidyl-peptidase activity
N	Q8NZH6	DR	serine-type peptidase activity
N	Q8P3N0	CC	Specifically methylates the N7 position of guanosine in position 527 of 16S rRNA (By similarity).
N	Q8P3N0	DE	16S rRNA 7-methylguanosine methyltransferase;
N	Q8P3N0	DE	16S rRNA m7G methyltransferase;
N	Q8P3N0	DE	Ribosomal RNA small subunit methyltransferase G;
N	Q8P3N0	DR	rRNA methyltransferase activity
N	Q8P5L1	CC	Catalyzes the oxidation of 3-carboxy-2-hydroxy-4- methylpentanoate (3-isopropylmalate) to 3-carboxy-4-methyl-2- oxopentanoate
N	Q8P5L1	CC	The product decarboxylates to 4-methyl-2 oxopentanoate.
N	Q8P5L1	DE	3-isopropylmalate dehydrogenase;
N	Q8P5L1	DE	Beta-IPM dehydrogenase;
N	Q8P5L1	DR	3-isopropylmalate dehydrogenase activity
N	Q8P5L1	DR	leucine biosynthetic process
N	Q8P5L1	DR	magnesium ion binding
N	Q8P5L1	DR	oxidation-reduction process
N	Q8PAW4	CC	Involved in saturated fatty acids biosynthesis.
N	Q8PAW4	DE	(3R)-hydroxymyristoyl ACP dehydrase;
N	Q8PAW4	DE	(3R)-hydroxymyristoyl-[acyl-carrier-protein] dehydratase;
N	Q8PAW4	DR	fatty acid biosynthetic process
N	Q8PAW4	DR	hydro-lyase activity
N	Q8PAW4	DR	lipid A biosynthetic process
N	Q8PH04	CC	Catalyzes the isomerization between 2-isopropylmalate and 3-isopropylmalate, via the formation of 2-isopropylmaleate.
N	Q8PH04	DE	3-isopropylmalate dehydratase small subunit;
N	Q8PH04	DE	Alpha-IPM isomerase;
N	Q8PH04	DE	Isopropylmalate isomerase;
N	Q8PH04	DR	3-isopropylmalate dehydratase activity
N	Q8PH04	DR	leucine biosynthetic process
N	Q8PHP7	DE	UPF0213 protein XAC3202;
N	Q8PHP7	DR	nuclease activity
N	Q8PIX4	DE	Maf-like protein XAC2771;
N	Q8PJ05	DE	Deoxycytidine triphosphate deaminase;
N	Q8PJ05	DE	dCTP deaminase;
N	Q8PJ05	DR	dCTP deaminase activity
N	Q8PJ05	DR	dUTP biosynthetic process
N	Q8PLY5	DE	3-phosphoshikimate 1-carboxyvinyltransferase;
N	Q8PLY5	DE	5-enolpyruvylshikimate-3-phosphate synthase;
N	Q8PLY5	DE	EPSP synthase;
N	Q8PLY5	DR	3-phosphoshikimate 1-carboxyvinyltransferase activity
N	Q8PLY5	DR	aromatic amino acid family biosynthetic process
N	Q8PM39	CC	Catalyzes the phosphorylation of NAD to NADP
N	Q8PM39	CC	Utilizes ATP and other nucleoside triphosphates as well as inorganic polyphosphate as a source of phosphorus (By similarity).
N	Q8PM39	DE	Poly(P)/ATP NAD kinase;

N	Q8PM39	DE	Probable inorganic polyphosphate/ATP-NAD kinase;
N	Q8PM39	DR	NAD+ kinase activity
N	Q8PMY1	CC	Specifically methylates guanosine-37 in various tRNAs (By similarity).
N	Q8PMY1	DE	M1G-methyltransferase;
N	Q8PMY1	DE	tRNA (guanine-N(1)-)-methyltransferase;
N	Q8PMY1	DE	tRNA [GM37] methyltransferase;
N	Q8PMY1	DR	tRNA (guanine-N1-)-methyltransferase activity
N	Q8PP46	CC	Provides the sole de novo source of dTMP for DNA biosynthesis (By similarity).
N	Q8PP46	DE	Thymidylate synthase;
N	Q8PP46	DR	dTMP biosynthetic process
N	Q8PP46	DR	thymidylate synthase activity
N	Q8PPA3	CC	Involved in the biosynthesis of lipid A, a phosphorylated glycolipid that anchors the lipopolysaccharide to the outer membrane of the cell (By similarity).
N	Q8PPA3	DE	UDP-3-O-[3-hydroxymyristoyl] N-acetylglucosamine deacetylase;
N	Q8PPA3	DE	UDP-3-O-acyl-GlcNAc deacetylase;
N	Q8PPA3	DR	UDP-3-O-[3-hydroxymyristoyl] N-acetylglucosamine deacetylase activity
N	Q8PPA3	DR	lipid A biosynthetic process
N	Q8PQE8	CC	NAD-binding protein involved in the addition of a carboxymethylaminomethyl (cmnm) group at the wobble position (U34) of certain tRNAs, forming tRNA-cmnm(5)s(2)U34 (By similarity).
N	Q8PQE8	DE	Glucose-inhibited division protein A;
N	Q8PQE8	DE	tRNA uridine 5-carboxymethylaminomethyl modification enzyme MnmG;
N	Q8PQE8	DR	flavin adenine dinucleotide binding
N	Q8PQE8	DR	tRNA wobble uridine modification
N	Q8PUQ8	CC	DNA-dependent RNA polymerase catalyzes the transcription of DNA into RNA using the four ribonucleoside triphosphates as substrates.
N	Q8PUQ8	DE	DNA-directed RNA polymerase subunit H;
N	Q8PUQ8	DR	DNA-directed RNA polymerase activity
N	Q8PV17	CC	Located on the platform of the 30S subunit (By similarity).
N	Q8PV17	DE	30S ribosomal protein S11P;
N	Q8PV17	DR	structural constituent of ribosome
N	Q8PX17	DE	Histidinol-phosphate aminotransferase;
N	Q8PX17	DE	Imidazole acetol-phosphate transaminase;
N	Q8PX17	DR	histidine biosynthetic process
N	Q8PX17	DR	histidinol-phosphate transaminase activity
N	Q8PX17	DR	pyridoxal phosphate binding
N	Q8PX50	CC	Involved in protein export
N	Q8PX50	CC	The function of the beta subunit is unknown, but it may be involved in stabilization of the trimeric complex (By similarity).
N	Q8PX50	DE	Preprotein translocase subunit secG;
N	Q8PX50	DE	Protein transport protein Sec61 subunit beta homolog;
N	Q8PX50	DR	protein transport
N	Q8PX50	DR	transmembrane transport
N	Q8PYZ0	DE	UPF0284 protein MM_0708;
N	Q8PYZ0	DR	cobalamin biosynthetic process
N	Q8PYZ0	DR	nicotinate-nucleotide-dimethylbenzimidazole phosphoribosyltransferase activity
N	Q8Q024	CC	Catalyzes the attachment of valine to tRNA(Val)
N	Q8Q024	CC	As ValRS can inadvertently accommodate and process structurally similar amino acids such as threonine, to avoid such errors, it has a "posttransfer" editing activity that hydrolyzes mischarged Thr-tRNA(Val) in a tRNA-dependent manner (By similarity).
N	Q8Q024	DE	Valine--tRNA ligase;
N	Q8Q024	DE	Valyl-tRNA synthetase;
N	Q8Q024	DR	valine-tRNA ligase activity
N	Q8Q024	DR	valyl-tRNA aminoacylation
N	Q8R0J7	CC	Component of the ESCRT-I complex, a regulator of vesicular trafficking process
N	Q8R0J7	CC	May be involved in cell growth and differentiation (By similarity).
N	Q8R0J7	CC	Required for the sorting of endocytic ubiquitinated cargos into multivesicular bodies
N	Q8R0J7	DE	ESCRT-I complex subunit VPS37B;
N	Q8R0J7	DE	Vacuolar protein sorting-associated protein 37B;
N	Q8R0J7	DR	protein transport
N	Q8R197	CC	In fatty acid biosynthesis chain termination and release of the free fatty acid product is achieved by hydrolysis of the thio ester by a thioesterase I, a component of the fatty acid synthetase complex

N	Q8R197	CC	However, in the mammary glands of non-ruminant mammals, and in the uropygial gland of certain waterfowl there exists a second thioesterase which releases medium-chain length fatty acids (C8 to C2) (By similarity).
N	Q8R197	CC	The chain length of the released fatty acid is usually C16
N	Q8R197	DE	Oleoyl-ACP hydrolase;
N	Q8R197	DE	S-acyl fatty acid synthase thioesterase, medium chain;
N	Q8R197	DE	Thioesterase II;
N	Q8R197	DE	Thioesterase domain-containing protein 1;
N	Q8R197	DR	fatty acid biosynthetic process
N	Q8R197	DR	myristoyl-[acyl-carrier-protein] hydrolase activity
N	Q8R197	DR	oleoyl-[acyl-carrier-protein] hydrolase activity
N	Q8R197	DR	palmitoyl-[acyl-carrier-protein] hydrolase activity
N	Q8R1C3	CC	Increases the transcriptional activity of NFKB1 by facilitating its nuclear translocation, DNA-binding and associated apoptotic response, when overexpressed (By similarity).
N	Q8R1C3	DE	EGFR-coamplified and overexpressed protein;
N	Q8R1C3	DE	Vesicular, overexpressed in cancer, prosurvival protein 1;
N	Q8R431	CC	Converts monoacylglycerides to free fatty acids and glycerol
N	Q8R431	CC	Hydrolyzes the endocannabinoid 2-arachidonoylglycerol, and thereby contributes to the regulation of endocannabinoid signaling, nociperception and perception of pain
N	Q8R431	CC	Regulates the levels of fatty acids that serve as signaling molecules and promote cancer cell migration, invasion and tumor growth (By similarity).
N	Q8R431	DE	Monoacylglycerol lipase;
N	Q8R431	DE	Monoglyceride lipase;
N	Q8R431	DR	acylglycerol catabolic process
N	Q8R431	DR	acylglycerol lipase activity
N	Q8R431	DR	arachidonic acid metabolic process
N	Q8R431	DR	fatty acid biosynthetic process
N	Q8R431	DR	lipid binding
N	Q8R431	DR	regulation of inflammatory response
N	Q8R431	DR	regulation of sensory perception of pain
N	Q8R431	DR	regulation of signal transduction
N	Q8R6I7	DE	UPF0236 protein TTE0033/TTE0744/TTE0838/TTE0852/TTE1082/TTE1247/TTE1519/TTE1678/TTE1739/TTE1823/TTE2212;
N	Q8R749	CC	Cell wall formation (By similarity).
N	Q8R749	DE	UDP-N-acetylmuramate--L-alanine ligase;
N	Q8R749	DE	UDP-N-acetylmuramoyl-L-alanine synthetase;
N	Q8R749	DR	UDP-N-acetylmuramate-L-alanine ligase activity
N	Q8R749	DR	cell division
N	Q8R749	DR	cellular cell wall organization
N	Q8R749	DR	peptidoglycan biosynthetic process
N	Q8R749	DR	regulation of cell shape
N	Q8R933	DE	Uncharacterized RNA methyltransferase TTE1797;
N	Q8R933	DR	4 iron, 4 sulfur cluster binding
N	Q8R933	DR	RNA methyltransferase activity
N	Q8R933	DR	RNA processing
N	Q8R933	DR	metal ion binding
N	Q8R9R6	DE	Dihydroorotase;
N	Q8R9R6	DR	dihydroorotase activity
N	Q8R9R6	DR	metal ion binding
N	Q8R9R6	DR	pyrimidine nucleotide biosynthetic process
N	Q8RDQ4	CC	Cell wall formation (By similarity).
N	Q8RDQ4	DE	D-Ala-D-Ala ligase;
N	Q8RDQ4	DE	D-alanine--D-alanine ligase;
N	Q8RDQ4	DE	D-alanylalanine synthetase;
N	Q8RDQ4	DR	D-alanine-D-alanine ligase activity
N	Q8RDQ4	DR	cellular cell wall organization
N	Q8RDQ4	DR	metal ion binding
N	Q8RDQ4	DR	peptidoglycan biosynthetic process
N	Q8RDQ4	DR	regulation of cell shape
N	Q8RY83	DE	Probable purine permease 13;
N	Q8RY83	DR	purine base transmembrane transporter activity
N	Q8S8M5	CC	Involved in regulation of actin and microtubule organization
N	Q8S8M5	CC	Part of a WAVE complex that activates the Arp2/3 complex (By similarity).
N	Q8S8M5	DE	Abl interactor-like protein 1;

N	Q8S8M5	DE	Protein ABIL1;
N	Q8S8M5	DR	protein binding
N	Q8SR53	CC	Molecular chaperone; assists the folding of proteins upon ATP hydrolysis (By similarity).
N	Q8SR53	DE	T-complex protein 1 subunit eta;
N	Q8SR53	DR	protein folding
N	Q8SR53	DR	unfolded protein binding
N	Q8SRC3	CC	Signal-recognition-particle assembly has a crucial role in targeting secretory proteins to the rough endoplasmic reticulum membrane
N	Q8SRC3	CC	It must be involved intimately in the translocation of a wide variety of protein substrates (By similarity).
N	Q8SRC3	DE	Signal recognition particle SEC65 subunit;
N	Q8SRC3	DR	7S RNA binding
N	Q8SRC3	DR	SRP-dependent cotranslational protein targeting to membrane
N	Q8SSG3	CC	Core component of nucleosome
N	Q8SSG3	CC	DNA accessibility is regulated via a complex set of post-translational modifications of histones, also called histone code, and nucleosome remodeling.
N	Q8SSG3	CC	Histones thereby play a central role in transcription regulation, DNA repair, DNA replication and chromosomal stability
N	Q8SSG3	CC	Nucleosomes wrap and compact DNA into chromatin, limiting DNA accessibility to the cellular machineries which require DNA as a template
N	Q8SSG3	DE	Histone H2A;
N	Q8SSG3	DR	sequence-specific DNA binding
N	Q8SW18	DE	Homeobox protein HD-10;
N	Q8SW18	DR	regulation of transcription, DNA-dependent
N	Q8SW18	DR	sequence-specific DNA binding
N	Q8SW18	DR	sequence-specific DNA binding transcription factor activity
N	Q8SW79	DE	UPF0328 protein ECU03_0080;
N	Q8SWA6	DE	Homeobox protein HD-11;
N	Q8SWA6	DR	regulation of transcription, DNA-dependent
N	Q8SWA6	DR	sequence-specific DNA binding
N	Q8SWA6	DR	sequence-specific DNA binding transcription factor activity
N	Q8SWA6	DR	transcription regulator activity
N	Q8T2Q0	DE	Putative ZDHHC-type palmitoyltransferase 6;
N	Q8T2Q0	DE	Zinc finger DHHC domain-containing protein 6;
N	Q8T2Q0	DR	acyltransferase activity
N	Q8T2Q0	DR	zinc ion binding
N	Q8T498	CC	Probable GTPase-activating protein, which may act as a negative regulator for some member of the Ras family
N	Q8T498	CC	They decrease the signaling activity of Ras by stimulating its intinsic GTPase activity, thereby lowering the levels of GTP-bound, active Ras (By similarity).
N	Q8T498	DE	Probable Ras GTPase-activating protein;
N	Q8T498	DR	GTPase activator activity
N	Q8T498	DR	inter-male aggressive behavior
N	Q8T498	DR	negative regulation of Ras protein signal transduction
N	Q8T498	DR	protein binding
N	Q8T498	DR	signal transduction
N	Q8TAE8	CC	Acts as a negative regulator of G1 to S cell cycle phase progression by inhibiting cyclin-dependent kinases
N	Q8TAE8	CC	Acts as a repressor of the orphan nuclear receptor NR4A1 by inhibiting AB domain-mediated transcriptional activity
N	Q8TAE8	CC	Inhibitory effects are additive with GADD45 proteins but occurs also in the absence of GADD45 proteins
N	Q8TAE8	CC	May be involved in the hormone-mediated regulation of NR4A1 transcriptional activity
N	Q8TAE8	DE	CKII beta-associating protein;
N	Q8TAE8	DE	CR6-interacting factor 1;
N	Q8TAE8	DE	Growth arrest and DNA damage-inducible proteins-interacting protein 1;
N	Q8TAE8	DE	Papillomavirus L2-interacting nuclear protein 1;
N	Q8TAE8	DE	p53-responsive gene 6 protein;
N	Q8TAE8	DR	interspecies interaction between organisms
N	Q8TAE8	DR	protein binding
N	Q8TF32	CC	May be involved in transcriptional regulation.
N	Q8TF32	DE	Zinc finger protein 431;
N	Q8TF32	DR	regulation of transcription, DNA-dependent
N	Q8TF32	DR	zinc ion binding
N	Q8TFG8	DE	Putative uncharacterized membrane protein PB15E9.02c;

N	Q8THJ9	DE	Acetylglutamate kinase;
N	Q8THJ9	DE	N-acetyl-L-glutamate 5-phosphotransferase;
N	Q8THJ9	DR	acetylglutamate kinase activity
N	Q8THJ9	DR	arginine biosynthetic process
N	Q8TIY5	CC	Structure-specific nuclease with 5'-flap endonuclease and 5'-3' exonuclease activities involved in DNA replication and repair
N	Q8TIY5	CC	Acts as a genome stabilization factor that prevents flaps from equilibrating into structures that lead to duplications and deletions
N	Q8TIY5	CC	Also involved in the base excision repair (BER) pathway
N	Q8TIY5	CC	Also possesses 5'-3' exonuclease activity on nicked or gapped double-stranded DNA (By similarity).
N	Q8TIY5	CC	Binds the unpaired 3'-DNA end and kinks the DNA to facilitate 5' cleavage specificity
N	Q8TIY5	CC	Cleaves one nucleotide into the double- stranded DNA from the junction in flap DNA, leaving a nick for ligation
N	Q8TIY5	CC	During DNA replication, cleaves the 5'-overhanging flap structure that is generated by displacement synthesis when DNA polymerase encounters the 5'-end of a downstream Okazaki fragment
N	Q8TIY5	DE	Flap endonuclease 1;
N	Q8TIY5	DE	Flap structure-specific endonuclease 1;
N	Q8TIY5	DR	DNA replication
N	Q8TIY5	DR	endonuclease activity
N	Q8TIY5	DR	exonuclease activity
N	Q8TIY5	DR	metal ion binding
N	Q8TLP3	CC	The beta subunit is responsible for the synthesis of L- tryptophan from indole and L-serine.
N	Q8TLP3	DE	Tryptophan synthase beta chain 1;
N	Q8TLP3	DR	pyridoxal phosphate binding
N	Q8TLP3	DR	tryptophan synthase activity
N	Q8TPW4	CC	Catalyzes the synthesis of the hydroxymethylpyrimidine phosphate (HMP-P) moiety of thiamine from aminoimidazole ribotide (AIR) in a radical S-adenosyl-L-methionine (SAM)-dependent reaction (By similarity).
N	Q8TPW4	DE	HMP-P synthase 1;
N	Q8TPW4	DE	HMP-phosphate synthase 1;
N	Q8TPW4	DE	HMPP synthase 1;
N	Q8TPW4	DE	Hydroxymethylpyrimidine phosphate synthase 1;
N	Q8TPW4	DE	Phosphomethylpyrimidine synthase 1;
N	Q8TPW4	DE	Thiamine biosynthesis protein thiC 1;
N	Q8TPW4	DR	4 iron, 4 sulfur cluster binding
N	Q8TPW4	DR	lyase activity
N	Q8TPW4	DR	metal ion binding
N	Q8TPW4	DR	thiamine biosynthetic process
N	Q8TRK8	DE	P-Rib-PP synthase;
N	Q8TRK8	DE	PRPP synthase;
N	Q8TRK8	DE	Phosphoribosyl pyrophosphate synthase;
N	Q8TRK8	DE	Ribose-phosphate pyrophosphokinase;
N	Q8TRK8	DR	kinase activity
N	Q8TRK8	DR	magnesium ion binding
N	Q8TRK8	DR	nucleoside metabolic process
N	Q8TRK8	DR	nucleotide biosynthetic process
N	Q8TRK8	DR	ribose phosphate diphosphokinase activity
N	Q8TRU7	CC	One of the primary rRNA binding proteins, it binds directly near the 3'-end of the 23S rRNA, where it nucleates assembly of the 50S subunit (By similarity).
N	Q8TRU7	DE	50S ribosomal protein L3P;
N	Q8TRU7	DR	structural constituent of ribosome
N	Q8TS38	CC	Catalyzes the NAD-dependent oxidative cleavage of spermidine and the subsequent transfer of the butylamine moiety of spermidine to the epsilon-amino group of a specific lysine residue of the eIF-5A precursor protein to form the intermediate deoxyhypusine residue (By similarity).
N	Q8TS38	DE	Probable deoxyhypusine synthase 1;
N	Q8TS38	DR	deoxyhypusine synthase activity
N	Q8TS38	DR	peptidyl-lysine modification to hypusine
N	Q8TS38	DR	spermidine catabolic process to deoxyhypusine, using deoxyhypusine synthase
N	Q8TS62	DE	Histidine--tRNA ligase;
N	Q8TS62	DE	Histidyl-tRNA synthetase;
N	Q8TS62	DR	histidine-tRNA ligase activity

N	Q8TS62	DR	histidyl-tRNA aminoacylation
N	Q8TS66	CC	Joins Ado-cobinamide-GDP and alpha-ribazole to generate adenosylcobalamin (Ado-cobalamin) (By similarity).
N	Q8TS66	DE	Cobalamin synthase;
N	Q8TS66	DR	cobalamin 5'-phosphate synthase activity
N	Q8TS66	DR	cobalamin biosynthetic process
N	Q8TWY3	CC	Catalyzes the radical-mediated transfer of the hydroxybenzyl group from 4-hydroxyphenylpyruvate (HPP) to 5-amino- 6-ribitylamino-2,4(1H,3H)-pyrimidinedione to form 7,8-didemethyl- 8-hydroxy-5-deazariboflavin (FO) (By similarity).
N	Q8TWY3	DE	7,8-didemethyl-8-hydroxy-5-deazariboflavin synthase subunit 1;
N	Q8TWY3	DE	FO synthase subunit 1;
N	Q8TWY3	DR	4 iron, 4 sulfur cluster binding
N	Q8TWY3	DR	coenzyme biosynthetic process
N	Q8TWY3	DR	metal ion binding
N	Q8TWY3	DR	transferase activity, transferring alkyl or aryl (other than methyl) groups
N	Q8TXF4	CC	Part of a complex that catalyzes the reversible cleavage of acetyl-CoA, allowing autotrophic growth from CO(2) (By similarity).
N	Q8TXF4	DE	ACDS complex acyltransferase;
N	Q8TXF4	DE	ACDS complex subunit beta;
N	Q8TXF4	DE	Acetyl-CoA decarbonylase/synthase complex subunit beta;
N	Q8TXF4	DR	acetyl-CoA metabolic process
N	Q8TXF4	DR	acyltransferase activity
N	Q8TXF4	DR	carbon-monoxide dehydrogenase (acceptor) activity
N	Q8TXF4	DR	iron-sulfur cluster binding
N	Q8TXF4	DR	metal ion binding
N	Q8TY87	DE	30S ribosomal protein S27e;
N	Q8TY87	DR	metal ion binding
N	Q8TY87	DR	structural constituent of ribosome
N	Q8TZD9	DE	30S ribosomal protein S15P/S13e;
N	Q8TZD9	DR	structural constituent of ribosome
N	Q8U147	CC	Catalyzes the NAD(P)H-dependent reduction of dihydroxyacetonephosphate (DHAP or glycerone phosphate) to glycerol-1-phosphate (G1P)
N	Q8U147	CC	The G1P thus generated is used as the glycerophosphate backbone of phospholipids in the cellular membranes of Archaea (By similarity).
N	Q8U147	DE	Enantiomeric glycerophosphate synthase;
N	Q8U147	DE	G1P dehydrogenase;
N	Q8U147	DE	Glycerol-1-phosphate dehydrogenase [NAD(P)+];
N	Q8U147	DE	sn-glycerol-1-phosphate dehydrogenase;
N	Q8U147	DR	glycerol-1-phosphate dehydrogenase [NAD(P)+] activity
N	Q8U147	DR	metal ion binding
N	Q8U147	DR	oxidation-reduction process
N	Q8U147	DR	phospholipid biosynthetic process
N	Q8U259	DE	Probable L-threonine 3-dehydrogenase;
N	Q8U259	DR	L-threonine 3-dehydrogenase activity
N	Q8U259	DR	oxidation-reduction process
N	Q8U259	DR	threonine catabolic process
N	Q8U259	DR	zinc ion binding
N	Q8U2L3	DE	50S ribosomal protein L34e;
N	Q8U2L3	DR	structural constituent of ribosome
N	Q8U9K5	CC	The ruvA-ruvB complex in the presence of ATP renatures cruciform structure in supercoiled DNA with palindromic sequence, indicating that it may promote strand exchange reactions in homologous recombination
N	Q8U9K5	CC	RuvA stimulates, in the presence of DNA, the weak ATPase activity of ruvB (By similarity).
N	Q8U9K5	CC	RuvAB is an helicase that mediates the Holliday junction migration by localized denaturation and reannealing
N	Q8U9K5	DE	Holliday junction ATP-dependent DNA helicase ruvA;
N	Q8U9K5	DR	DNA recombination
N	Q8U9K5	DR	four-way junction helicase activity
N	Q8UJ92	DE	Pantothenate kinase;
N	Q8UJ92	DE	Pantothenic acid kinase;
N	Q8UJ92	DR	coenzyme A biosynthetic process
N	Q8UJ92	DR	pantothenate kinase activity
N	Q8UN02	CC	Seems to be important for efficient replication in vivo.
N	Q8UN02	DE	Glycosylated Gag polyprotein;

N	Q8UN02	DE	Nextended-MA-p12;
N	Q8UN02	DR	nucleic acid binding
N	Q8UN02	DR	structural molecule activity
N	Q8UN02	DR	virion assembly
N	Q8UN02	DR	zinc ion binding
N	Q8UUH9	CC	PA2 catalyzes the calcium-dependent hydrolysis of the 2- acyl groups in 3-sn-phosphoglycerides
N	Q8UUH9	CC	Acts presynaptically (By similarity).
N	Q8UUH9	CC	Inhibits neuromuscular transmission by blocking acetylcholine release from the nerve termini
N	Q8UUH9	DE	Phosphatidylcholine 2-acylhydrolase;
N	Q8UUH9	DE	Phospholipase A2 PC9;
N	Q8UUH9	DR	calcium ion binding
N	Q8UUH9	DR	lipid catabolic process
N	Q8UUH9	DR	phospholipase A2 activity
N	Q8UUH9	DR	phospholipid metabolic process
N	Q8UUI2	CC	PA2 catalyzes the calcium-dependent hydrolysis of the 2- acyl groups in 3-sn-phosphoglycerides (By similarity).
N	Q8UUI2	DE	Phosphatidylcholine 2-acylhydrolase;
N	Q8UUI2	DE	Phospholipase A2 PC16;
N	Q8UUI2	DR	calcium ion binding
N	Q8UUI2	DR	lipid catabolic process
N	Q8UUI2	DR	phospholipase A2 activity
N	Q8UUI2	DR	phospholipid metabolic process
N	Q8UW74	CC	Transcriptional repressor
N	Q8UW74	CC	Demarcates the prospective midbrain-hindbrain boundary (MHB) region in the neuroectoderm in early gastrulae embryos by repressing transcription of a number of target genes.
N	Q8UW74	CC	Represses transcription from both N box- and E box-containing promoters (By similarity).
N	Q8UW74	DE	HES-related protein 1-A';
N	Q8UW74	DE	HES-related protein 1-A;
N	Q8UW74	DE	Hairy and enhancer of split 7.1-A;
N	Q8UW74	DE	Transcription factor HES-7.1-A;
N	Q8UW74	DR	midbrain-hindbrain boundary development
N	Q8UW74	DR	negative regulation of transcription from RNA polymerase II promoter
N	Q8UW74	DR	transcription repressor activity
N	Q8V9S9	CC	Plays an essential role in the assembly of the icosahedral capsid of the virus (By similarity).
N	Q8V9S9	DE	Protein B602L;
N	Q8VCF5	DE	Oculospanin;
N	Q8VCF5	DE	Tetraspanin-10;
N	Q8VDQ9	DE	Protein KRI1 homolog;
N	Q8VHK9	CC	Plays a role in degradation and deadenylation of mRNAs containing in their 3'-UTR the consensus ARE sequence element
N	Q8VHK9	CC	May function in sex development and spermatogenesis (By similarity).
N	Q8VHK9	DE	DEAH box protein 36;
N	Q8VHK9	DE	MLE-like protein 1;
N	Q8VHK9	DE	Probable ATP-dependent RNA helicase DHX36;
N	Q8VHK9	DE	RNA helicase associated with AU-rich element ARE;
N	Q8VHK9	DR	ATP-dependent helicase activity
N	Q8VHK9	DR	nucleic acid binding
N	Q8VS92	DE	Gal-1-P uridylyltransferase;
N	Q8VS92	DE	Galactose-1-phosphate uridylyltransferase;
N	Q8VS92	DE	UDP-glucose--hexose-1-phosphate uridylyltransferase;
N	Q8VS92	DR	UDP-glucose:hexose-1-phosphate uridylyltransferase activity
N	Q8VS92	DR	galactose metabolic process
N	Q8VXV7	CC	Probable histone demethylase that reduces the levels of histone H3 'Lys-4' methylation in chromatin of the floral repressor FLOWERING LOCUS C (FLC) and the sporophytically silenced floral repressor FWA
N	Q8VXV7	CC	Required for cytosine methylation of FWA.
N	Q8VXV7	CC	Seems to act in partial redundancy with FLOWERING LOCUS D (FLD) to repress FLC expression
N	Q8VXV7	DE	Flavin-containing amine oxidase domain-containing protein 1;
N	Q8VXV7	DE	Lysine-specific histone demethylase 1 homolog 1;
N	Q8VXV7	DE	Protein LSD1-LIKE 1;

N	Q8VXV7	DR	histone H3-K4 methylation
N	Q8VXV7	DR	histone deacetylation
N	Q8VXV7	DR	oxidation-reduction process
N	Q8VXV7	DR	oxidoreductase activity
N	Q8VXV7	DR	root development
N	Q8VZ87	CC	The light-harvesting complex (LHC) functions as a light receptor, it captures and delivers excitation energy to photosystems with which it is closely associated.
N	Q8VZ87	DE	Chlorophyll a-b binding protein 3, chloroplastic;
N	Q8VZ87	DE	Chlorophyll a-b protein 180;
N	Q8VZ87	DE	LHCII type I CAB-3;
N	Q8VZ87	DR	chlorophyll binding
N	Q8VZ87	DR	metal ion binding
N	Q8VZ87	DR	photosynthesis, light harvesting
N	Q8VZ87	DR	protein-chromophore linkage
N	Q8W9N6	CC	Core subunit of the mitochondrial membrane respiratory chain NADH dehydrogenase (Complex I) that is believed to belong to the minimal assembly required for catalysis
N	Q8W9N6	CC	Complex I functions in the transfer of electrons from NADH to the respiratory chain
N	Q8W9N6	CC	The immediate electron acceptor for the enzyme is believed to be ubiquinone (By similarity).
N	Q8W9N6	DE	NADH dehydrogenase subunit 1;
N	Q8W9N6	DE	NADH-ubiquinone oxidoreductase chain 1;
N	Q8W9N6	DR	NADH dehydrogenase (ubiquinone) activity
N	Q8W9N6	DR	electron transport chain
N	Q8WJN0	CC	Usually encoded in the trnK tRNA gene intron
N	Q8WJN0	CC	Probably assists in splicing its own and other chloroplast group II introns (By similarity)
N	Q8WJN0	DE	Intron maturase;
N	Q8WJN0	DR	mRNA processing
N	Q8WJN0	DR	tRNA processing
N	Q8WJR3	CC	Usually encoded in the trnK tRNA gene intron
N	Q8WJR3	CC	Probably assists in splicing its own and other chloroplast group II introns (By similarity)
N	Q8WJR3	DE	Intron maturase;
N	Q8WJR3	DR	mRNA processing
N	Q8WJR3	DR	tRNA processing
N	Q8WMN5	CC	Constitutes one of the E3 ubiquitin-protein ligases that mediate monoubiquitination of 'Lys-119' of histone H2A, thereby playing a central role in histone code and gene regulation
N	Q8WMN5	CC	Compared to RNF2/RING2, it does not have the main E3 ubiquitin ligase activity on histone H2A, and it may rather act as a modulator of RNF2/RING2 activity (By similarity).
N	Q8WMN5	CC	Essential component of the Polycomb group (PcG) multiprotein PRC1 complex, a complex required to maintain the transcriptionally repressive state of many genes, including Hox genes, throughout development
N	Q8WMN5	CC	H2A 'Lys-119' ubiquitination gives a specific tag for epigenetic transcriptional repression and participates in X chromosome inactivation of female mammals
N	Q8WMN5	CC	PcG PRC1 complex act via chromatin remodeling and modification of histones, rendering chromatin heritably changed in its expressibility
N	Q8WMN5	DE	E3 ubiquitin-protein ligase RING1;
N	Q8WMN5	DE	Polycomb complex protein RING1;
N	Q8WMN5	DE	RING finger protein 1;
N	Q8WMN5	DR	zinc ion binding
N	Q8WNZ6	CC	Protamines substitute for histones in the chromatin of sperm during the haploid phase of spermatogenesis
N	Q8WNZ6	CC	They compact sperm DNA into a highly condensed, stable and inactive complex (By similarity).
N	Q8WNZ6	DE	Sperm protamine P1;
N	Q8WNZ6	DR	cell differentiation
N	Q8WNZ6	DR	chromosome condensation
N	Q8WNZ6	DR	multicellular organismal development
N	Q8WNZ6	DR	spermatogenesis
N	Q8WUJ3	CC	May be involved in hearing.
N	Q8WUJ3	DE	Protein KIAA1199;
N	Q8WVI0	DE	UPF0640 protein C3orf78;
N	Q8WWV6	CC	Functions as a receptor for the Fc fragment of IgA and IgM
N	Q8WWV6	CC	Binds IgA and IgM with high affinity and mediates their endocytosis
N	Q8WWV6	CC	May function in the immune response to microbes mediated by IgA and IgM.

N	Q8WWV6	DE	CD351;
N	Q8WWV6	DE	Fc alpha/mu receptor;
N	Q8WWV6	DE	High affinity immunoglobulin alpha and immunoglobulin mu Fc receptor;
N	Q8WWV6	DR	receptor activity
N	Q8WXC6	DE	Myeloma-overexpressed gene 2 protein;
N	Q8WXG9	CC	Receptor that may have an important role in the development of the central nervous system.
N	Q8WXG9	DE	G-protein coupled receptor 98;
N	Q8WXG9	DE	Monogenic audiogenic seizure susceptibility protein 1 homolog;
N	Q8WXG9	DE	Usher syndrome type-2C protein;
N	Q8WXG9	DE	Very large G-protein coupled receptor 1;
N	Q8WXG9	DR	G-protein coupled receptor activity
N	Q8WXG9	DR	calcium ion binding
N	Q8WXG9	DR	cell communication
N	Q8WXG9	DR	cell-cell adhesion
N	Q8WXG9	DR	maintenance of organ identity
N	Q8WXG9	DR	neuropeptide signaling pathway
N	Q8WXG9	DR	photoreceptor cell maintenance
N	Q8X485	CC	Involved in the degradation and recycling of damaged RNA
N	Q8X485	CC	It is itself a target for degradation by the ATP-dependent protease La (By similarity)
N	Q8X485	DE	Endoribonuclease symE;
N	Q8X485	DR	RNA metabolic process
N	Q8X485	DR	endoribonuclease activity
N	Q8X6T2	CC	Interacts with the secY protein in vivo
N	Q8X6T2	CC	May bind preferentially to an uncomplexed state of secY, thus functioning either as a chelating agent for excess secY in the cell or as a regulatory factor that negatively controls the translocase function (By similarity).
N	Q8X6T2	DE	Protein syd;
N	Q8X711	CC	Cell wall formation (By similarity).
N	Q8X711	DE	UDP-N-acetylenolpyruvoylglucosamine reductase;
N	Q8X711	DE	UDP-N-acetylmuramate dehydrogenase;
N	Q8X711	DR	UDP-N-acetylmuramate dehydrogenase activity
N	Q8X711	DR	cell division
N	Q8X711	DR	cellular cell wall organization
N	Q8X711	DR	flavin adenine dinucleotide binding
N	Q8X711	DR	oxidation-reduction process
N	Q8X711	DR	peptidoglycan biosynthetic process
N	Q8X711	DR	regulation of cell shape
N	Q8X8H6	CC	Phosphorylation of dTMP to form dTDP in both de novo and salvage pathways of dTTP synthesis (By similarity).
N	Q8X8H6	DE	Thymidylate kinase;
N	Q8X8H6	DE	dTMP kinase;
N	Q8X8H6	DR	dTDP biosynthetic process
N	Q8X8H6	DR	thymidylate kinase activity
N	Q8XA01	CC	Catalyzes the isomerization between 2-isopropylmalate and 3-isopropylmalate, via the formation of 2-isopropylmaleate.
N	Q8XA01	DE	3-isopropylmalate dehydratase small subunit;
N	Q8XA01	DE	Alpha-IPM isomerase;
N	Q8XA01	DE	Isopropylmalate isomerase;
N	Q8XA01	DR	3-isopropylmalate dehydratase activity
N	Q8XA01	DR	leucine biosynthetic process
N	Q8XAY9	CC	Part of the ABC transporter complex lsrABCD involved in autoinducer 2 (AI-2) import
N	Q8XAY9	CC	Binds AI-2 and delivers it to the lsrC and lsrD permeases (By similarity).
N	Q8XAY9	DE	AI-2-binding protein lsrB;
N	Q8XAY9	DE	Autoinducer 2-binding protein lsrB;
N	Q8XBY1	CC	Part of a cation efflux system that mediates resistance to copper and silver (By similarity).
N	Q8XBY1	DE	Cation efflux system protein CusA;
N	Q8XBY1	DR	cation transmembrane transporter activity
N	Q8XBY1	DR	copper ion transport
N	Q8XJP2	CC	Might take part in the signal recognition particle (SRP) pathway
N	Q8XJP2	CC	May be a regulatory protein.
N	Q8XJP2	CC	This is inferred from the conservation of its genetic proximity to ftsY/ffh
N	Q8XJP2	DE	UPF0122 protein CPE1714;
N	Q8XJP2	DR	regulation of transcription, DNA-dependent

N	Q8XJP2	DR	sequence-specific DNA binding transcription factor activity
N	Q8XJP2	DR	sigma factor activity
N	Q8XJP2	DR	transcription initiation, DNA-dependent
N	Q8XJZ6	DE	Peptide methionine sulfoxide reductase MsrB;
N	Q8XJZ6	DE	Peptide-methionine (R)-S-oxide reductase;
N	Q8XJZ6	DR	oxidation-reduction process
N	Q8XJZ6	DR	peptide-methionine (R)-S-oxide reductase activity
N	Q8XJZ6	DR	peptide-methionine-(S)-S-oxide reductase activity
N	Q8XKF6	DE	UPF0213 protein CPE1444;
N	Q8XKF6	DR	nuclease activity
N	Q8XKT3	DE	DegV domain-containing protein CPE1310;
N	Q8XKU7	CC	Binds specifically to the ssrA RNA (tmRNA) and is required for stable association of ssrA with ribosomes (By similarity).
N	Q8XKU7	DE	SsrA-binding protein;
N	Q8XLG8	CC	Involved in saturated fatty acids biosynthesis.
N	Q8XLG8	DE	(3R)-hydroxymyristoyl ACP dehydrase;
N	Q8XLG8	DE	(3R)-hydroxymyristoyl-[acyl-carrier-protein] dehydratase;
N	Q8XLG8	DR	fatty acid biosynthetic process
N	Q8XLG8	DR	hydro-lyase activity
N	Q8XLG8	DR	lipid A biosynthetic process
N	Q8XPD7	DE	DegV domain-containing protein CPE0026;
N	Q8XT76	CC	Catalyzes the formation of the alpha-1,6-glucosidic linkages in glycogen by scission of a 1,4-alpha-linked oligosaccharide from growing alpha-1,4-glucan chains and the subsequent attachment of the oligosaccharide to the alpha-1,6 position (By similarity).
N	Q8XT76	DE	1,4-alpha-D-glucan:1,4-alpha-D-glucan 6-glucosyl-transferase;
N	Q8XT76	DE	1,4-alpha-glucan-branching enzyme;
N	Q8XT76	DE	Glycogen-branching enzyme;
N	Q8XT76	DR	1,4-alpha-glucan branching enzyme activity
N	Q8XT76	DR	cation binding
N	Q8XT76	DR	glycogen biosynthetic process
N	Q8XT76	DR	hydrolase activity, hydrolyzing O-glycosyl compounds
N	Q8XV20	DE	50S ribosomal protein L29;
N	Q8XV20	DR	structural constituent of ribosome
N	Q8XV22	CC	Binds to 23S rRNA
N	Q8XV22	CC	Forms part of two intersubunit bridges in the 70S ribosome (By similarity).
N	Q8XV22	DE	50S ribosomal protein L14;
N	Q8XV22	DR	structural constituent of ribosome
N	Q8XW52	DE	AIR synthase;
N	Q8XW52	DE	Phosphoribosyl-aminoimidazole synthetase;
N	Q8XW52	DE	Phosphoribosylformylglycinamidine cyclo-ligase;
N	Q8XW52	DR	'de novo' IMP biosynthetic process
N	Q8XW52	DR	phosphoribosylformylglycinamidine cyclo-ligase activity
N	Q8XZR1	CC	Participates in the degradation of poly-3- hydroxybutyrate (PHB)
N	Q8XZR1	CC	It works downstream of poly(3- hydroxybutyrate) depolymerase, hydrolyzing D(-)-3-hydroxybutyrate oligomers of various length (3HB-oligomers) into 3HB-monomers (By similarity).
N	Q8XZR1	DE	3HB-oligomer hydrolase;
N	Q8XZR1	DE	D-(-)-3-hydroxybutyrate oligomer hydrolase;
N	Q8XZR1	DR	butyrate metabolic process
N	Q8XZR1	DR	hydroxybutyrate-dimer hydrolase activity
N	Q8XZV4	CC	Responsible for synthesis of pseudouridine from uracil- 55 in the psi GC loop of transfer RNAs (By similarity).
N	Q8XZV4	DE	Psi55 synthase;
N	Q8XZV4	DE	tRNA pseudouridine 55 synthase;
N	Q8XZV4	DE	tRNA pseudouridine synthase B;
N	Q8XZV4	DE	tRNA pseudouridylate synthase;
N	Q8XZV4	DE	tRNA-uridine isomerase;
N	Q8XZV4	DR	pseudouridine synthase activity
N	Q8XZV4	DR	pseudouridine synthesis
N	Q8XZV4	DR	tRNA processing
N	Q8Y449	CC	Catalyzes the reversible transfer of the terminal phosphate group between ATP and AMP
N	Q8Y449	CC	This small ubiquitous enzyme involved in the energy metabolism and nucleotide synthesis, is essential for maintenance and cell growth (By similarity).
N	Q8Y449	DE	ATP-AMP transphosphorylase;

N	Q8Y449	DE	Adenylate kinase;
N	Q8Y449	DR	adenylate kinase activity
N	Q8Y495	CC	Catalyzes the ATP-dependent amination of UTP to CTP with either L-glutamine or ammonia as the source of nitrogen (By similarity).
N	Q8Y495	DE	CTP synthase;
N	Q8Y495	DE	CTP synthetase;
N	Q8Y495	DE	UTP--ammonia ligase;
N	Q8Y495	DR	CTP synthase activity
N	Q8Y495	DR	glutamine metabolic process
N	Q8Y495	DR	pyrimidine nucleotide biosynthetic process
N	Q8Y611	CC	Endonuclease that resolves Holliday junction intermediates in genetic recombination
N	Q8Y611	CC	Cleaves mobile four-strand junctions by introducing symmetrical nicks in paired strands
N	Q8Y611	CC	Promotes annealing of linear ssDNA with homologous dsDNA
N	Q8Y611	CC	Required for DNA repair, homologous recombination and chromosome segregation (By similarity).
N	Q8Y611	DE	Holliday junction resolvase recU;
N	Q8Y611	DE	Recombination protein U homolog;
N	Q8Y611	DR	DNA recombination
N	Q8Y611	DR	endonuclease activity
N	Q8Y611	DR	metal ion binding
N	Q8Y6U0	CC	Catalyzes the ATP-dependent transfer of a sulfur to tRNA to produce 4-thiouridine in position 8 of tRNAs, which functions as a near-UV photosensor
N	Q8Y6U0	CC	Also catalyzes the transfer of sulfur to the sulfur carrier protein ThiS, forming ThiS-thiocarboxylate
N	Q8Y6U0	CC	The sulfur is donated as persulfide by iscS (By similarity).
N	Q8Y6U0	CC	This is a step in the synthesis of thiazole, in the thiamine biosynthesis pathway
N	Q8Y6U0	DE	Probable tRNA sulfurtransferase;
N	Q8Y6U0	DE	Sulfur carrier protein ThiS sulfurtransferase;
N	Q8Y6U0	DE	Thiamine biosynthesis protein thiI;
N	Q8Y6U0	DE	tRNA 4-thiouridine synthase;
N	Q8Y6U0	DR	thiamine biosynthetic process
N	Q8Y6U0	DR	transferase activity
N	Q8Y7J7	CC	DNA-binding protein that represses the expression of many genes that are induced as cells make the transition from rapid exponential growth to stationary phase (By similarity).
N	Q8Y7J7	CC	At low GTP concentration it no longer binds GTP and stop to act as a transcriptional repressor (By similarity).
N	Q8Y7J7	CC	It is a GTP-binding protein that senses the intracellular GTP concentration as an indicator of nutritional limitations
N	Q8Y7J7	DE	GTP-sensing transcriptional pleiotropic repressor CodY;
N	Q8Y7J7	DR	regulation of transcription, DNA-dependent
N	Q8Y7J7	DR	sequence-specific DNA binding transcription factor activity
N	Q8Y7J7	DR	transcription repressor activity
N	Q8YCY1	DE	Ornithine cyclodeaminase;
N	Q8YCY1	DR	arginine metabolic process
N	Q8YCY1	DR	ornithine cyclodeaminase activity
N	Q8YDR6	CC	Probably part of an ABC transporter complex.
N	Q8YDR6	DE	Putative binding protein BMEII0109;
N	Q8YDR6	DR	sulfur compound metabolic process
N	Q8YGN1	DE	FGAM synthase II;
N	Q8YGN1	DE	Phosphoribosylformylglycinamidine synthase 2;
N	Q8YGN1	DE	Phosphoribosylformylglycinamidine synthase II;
N	Q8YGN1	DR	'de novo' IMP biosynthetic process
N	Q8YGN1	DR	phosphoribosylformylglycinamidine synthase activity
N	Q8YHH3	CC	Generates undecaprenyl pyrophosphate (UPP) from isopentenyl pyrophosphate (IPP)
N	Q8YHH3	CC	UPP is the precursor of glycosyl carrier lipid in the biosynthesis of bacterial cell wall polysaccharide components such as peptidoglycan and lipopolysaccharide (By similarity).
N	Q8YHH3	DE	Di-trans,poly-cis-decaprenylcistransferase;
N	Q8YHH3	DE	UPP synthase;
N	Q8YHH3	DE	Undecaprenyl diphosphate synthase;
N	Q8YHH3	DE	Undecaprenyl pyrophosphate synthase;
N	Q8YHH3	DR	cell division
N	Q8YHH3	DR	cellular cell wall organization
N	Q8YHH3	DR	di-trans,poly-cis-decaprenylcistransferase activity
N	Q8YHH3	DR	peptidoglycan biosynthetic process
N	Q8YHH3	DR	regulation of cell shape

N	Q8YIT2	CC	Part of the ABC transporter complex MacAB involved in macrolide export
N	Q8YIT2	CC	Transmembrane domains (TMD) form a pore in the inner membrane and the ATP-binding domain (NBD) is responsible for energy generation (By similarity).
N	Q8YIT2	DE	Macrolide export ATP-binding/permease protein MacB;
N	Q8YIT2	DR	ATPase activity
N	Q8YIT2	DR	hydrolase activity, acting on acid anhydrides, catalyzing transmembrane movement of substances
N	Q8YIT2	DR	response to antibiotic
N	Q8YJ42	CC	Transaldolase is important for the balance of metabolites in the pentose-phosphate pathway (By similarity).
N	Q8YJ42	DE	Probable transaldolase;
N	Q8YJ42	DR	pentose-phosphate shunt
N	Q8YJ42	DR	sedoheptulose-7-phosphate:D-glyceraldehyde-3-phosphate glyceronetransferase activity
N	Q8YLZ0	CC	Zinc phosphodiesterase, which displays some tRNA 3'-processing endonuclease activity
N	Q8YLZ0	CC	Probably involved in tRNA maturation, by removing a 3'-trailer from precursor tRNA (By similarity).
N	Q8YLZ0	DE	Ribonuclease Z;
N	Q8YLZ0	DE	tRNA 3 endonuclease;
N	Q8YLZ0	DR	endoribonuclease activity, producing 5'-phosphomonoesters
N	Q8YLZ0	DR	metal ion binding
N	Q8YLZ0	DR	tRNA 3'-trailer cleavage
N	Q8YM38	CC	Involved in the synthesis of meso-diaminopimelate (m-DAP or DL-DAP), required for both lysine and peptidoglycan biosynthesis
N	Q8YM38	CC	Catalyzes the direct conversion of tetrahydrodipicolinate to LL-diaminopimelate, a reaction that requires three enzymes in E.coli (By similarity).
N	Q8YM38	DE	DAP-aminotransferase 1;
N	Q8YM38	DE	LL-DAP-aminotransferase 1;
N	Q8YM38	DE	LL-diaminopimelate aminotransferase 1;
N	Q8YM38	DR	L,L-diaminopimelate aminotransferase activity
N	Q8YM38	DR	biosynthetic process
N	Q8YM38	DR	pyridoxal phosphate binding
N	Q8YP43	CC	Cleaves peptides in various proteins in a process that requires ATP hydrolysis
N	Q8YP43	CC	Has a chymotrypsin-like activity
N	Q8YP43	CC	Plays a major role in the degradation of misfolded proteins (By similarity).
N	Q8YP43	DE	Endopeptidase Clp 3;
N	Q8YP43	DE	Probable ATP-dependent Clp protease proteolytic subunit 3;
N	Q8YP43	DR	serine-type endopeptidase activity
N	Q8YPK0	DE	50S ribosomal protein L36;
N	Q8YPK0	DR	structural constituent of ribosome
N	Q8YU89	CC	Catalyzes the hydrolysis of 1,4-dihydroxy-2-naphthoyl- CoA (DHNA-CoA) to 1,4-dihydroxy-2-naphthoate (DHNA), a reaction involved in phylloquinone (vitamin K1) biosynthesis (By similarity).
N	Q8YU89	DE	1,4-dihydroxy-2-naphthoyl-CoA hydrolase;
N	Q8YU89	DE	DHNA-CoA hydrolase;
N	Q8YU89	DE	DHNA-CoA thioesterase;
N	Q8YU89	DR	thiolester hydrolase activity
N	Q8YYI9	CC	Transfers the fatty acyl group on membrane lipoproteins (By similarity).
N	Q8YYI9	DE	ALP N-acyltransferase;
N	Q8YYI9	DE	Apolipoprotein N-acyltransferase;
N	Q8YYI9	DR	N-acyltransferase activity
N	Q8YYI9	DR	hydrolase activity, acting on carbon-nitrogen (but not peptide) bonds
N	Q8YYI9	DR	lipoprotein biosynthetic process
N	Q8YYI9	DR	nitrogen compound metabolic process
N	Q8YZ99	CC	Thiolesterase that catalyzes the hydrolysis of S-D- lactoyl-glutathione to form glutathione and D-lactic acid (By similarity).
N	Q8YZ99	DE	Glyoxalase II;
N	Q8YZ99	DE	Hydroxyacylglutathione hydrolase;
N	Q8YZ99	DR	hydroxyacylglutathione hydrolase activity
N	Q8YZ99	DR	zinc ion binding
N	Q8YZI3	CC	Cell wall formation
N	Q8YZI3	CC	Catalyzes the transfer of a GlcNAc subunit on undecaprenyl-pyrophosphoryl-MurNAc-pentapeptide (lipid intermediate I) to form undecaprenyl-pyrophosphoryl-MurNAc- (pentapeptide)GlcNAc (lipid intermediate II) (By similarity).
N	Q8YZI3	DE	UDP-N-acetylglucosamine--N-acetylmuramyl-(pentapeptide) pyrophosphoryl-undecaprenol N-acetylglucosamine transferase;

N	Q8YZI3	DE	Undecaprenyl-PP-MurNAc-pentapeptide-UDPGlcNAc GlcNAc transferase;
N	Q8YZI3	DR	UDP-N-acetyl-D-glucosamine:N-acetylmuramoyl-L-alanyl-D-glutamyl-meso-2,6-diaminopimelyl-D-alanyl-D-alanine-diphosphoundecaprenol 4-beta-N-acetylglucosaminlytransferase activity
N	Q8YZI3	DR	UDP-N-acetylgalactosamine biosynthetic process
N	Q8YZI3	DR	carbohydrate binding
N	Q8YZI3	DR	cell division
N	Q8YZI3	DR	cellular cell wall organization
N	Q8YZI3	DR	lipid glycosylation
N	Q8YZI3	DR	peptidoglycan biosynthetic process
N	Q8YZI3	DR	regulation of cell shape
N	Q8YZI3	DR	undecaprenyldiphospho-muramoylpentapeptide beta-N-acetylglucosaminyltransferase activity
N	Q8Z075	CC	NDH-1 shuttles electrons from NAD(P)H, via FMN and iron- sulfur (Fe-S) centers, to quinones in the respiratory chain
N	Q8Z075	CC	Couples the redox reaction to proton translocation (for every two electrons transferred, four hydrogen ions are translocated across the cytoplasmic membrane), and thus conserves the redox energy in a proton gradient (By similarity)
N	Q8Z075	CC	The immediate electron acceptor for the enzyme in this species is believed to be plastoquinone
N	Q8Z075	DE	NAD(P)H dehydrogenase I, chain 6;
N	Q8Z075	DE	NAD(P)H-quinone oxidoreductase chain 6;
N	Q8Z075	DE	NDH-1, chain 6;
N	Q8Z075	DR	NADH dehydrogenase (ubiquinone) activity
N	Q8Z075	DR	oxidation-reduction process
N	Q8Z075	DR	quinone binding
N	Q8Z0U8	CC	Increases the formation of ribosomal termination complexes and stimulates activities of RF-1 and RF-2
N	Q8Z0U8	CC	It binds guanine nucleotides and has strong preference for UGA stop codons
N	Q8Z0U8	CC	It may interact directly with the ribosome
N	Q8Z0U8	CC	The stimulation of RF- 1 and RF-2 is significantly reduced by GTP and GDP, but not by GMP (By similarity).
N	Q8Z0U8	DE	Peptide chain release factor 3;
N	Q8Z0U8	DR	GTPase activity
N	Q8Z0U8	DR	translation release factor activity, codon specific
N	Q8Z2F0	CC	Catalyzes the interconversion of 2-phosphoglycerate and 3-phosphoglycerate (By similarity).
N	Q8Z2F0	DE	2,3-bisphosphoglycerate-independent phosphoglycerate mutase;
N	Q8Z2F0	DE	BPG-independent PGAM;
N	Q8Z2F0	DE	Phosphoglyceromutase;
N	Q8Z2F0	DR	manganese ion binding
N	Q8Z2F0	DR	phosphoglycerate mutase activity
N	Q8Z2Q2	CC	Catalyzes the first step in hexosamine metabolism, converting fructose-6P into glucosamine-6P using glutamine as a nitrogen source (By similarity).
N	Q8Z2Q2	DE	D-fructose-6-phosphate amidotransferase;
N	Q8Z2Q2	DE	Glucosamine--fructose-6-phosphate aminotransferase [isomerizing];
N	Q8Z2Q2	DE	Glucosamine-6-phosphate synthase;
N	Q8Z2Q2	DE	Hexosephosphate aminotransferase;
N	Q8Z2Q2	DE	L-glutamine-D-fructose-6-phosphate amidotransferase;
N	Q8Z2Q2	DR	carbohydrate biosynthetic process
N	Q8Z2Q2	DR	glutamine metabolic process
N	Q8Z2Q2	DR	glutamine-fructose-6-phosphate transaminase (isomerizing) activity
N	Q8Z2Q2	DR	sugar binding
N	Q8Z2W6	DE	CDP-diacylglycerol phosphatidylhydrolase;
N	Q8Z2W6	DE	CDP-diacylglycerol pyrophosphatase;
N	Q8Z2W6	DE	CDP-diglyceride hydrolase;
N	Q8Z2W6	DR	CDP-diacylglycerol diphosphatase activity
N	Q8Z2W6	DR	phospholipid biosynthetic process
N	Q8Z4W4	CC	DNA ligase that catalyzes the formation of phosphodiester linkages between 5'-phosphoryl and 3'-hydroxyl groups in double-stranded DNA using NAD as a coenzyme and as the energy source for the reaction
N	Q8Z4W4	CC	It is essential for DNA replication and repair of damaged DNA (By similarity).
N	Q8Z4W4	DE	Polydeoxyribonucleotide synthase [NAD+];
N	Q8Z4W4	DR	DNA ligase (NAD+) activity
N	Q8Z4W4	DR	DNA replication

N	Q8Z4W4	DR	metal ion binding
N	Q8Z538	CC	Catalyzes the transfer of the L-Ara4N moiety of the glycolipid undecaprenyl phosphate-alpha-L-Ara4N to lipid A
N	Q8Z538	CC	The modified arabinose is attached to lipid A and is required for resistance to polymyxin and cationic antimicrobial peptides.
N	Q8Z538	DE	4-amino-4-deoxy-L-arabinose lipid A transferase;
N	Q8Z538	DE	Lipid IV(A) 4-amino-4-deoxy-L-arabinosyltransferase;
N	Q8Z538	DE	Melittin resistance protein pqaB;
N	Q8Z538	DE	Polymyxin resistance protein pmrK;
N	Q8Z538	DE	Undecaprenyl phosphate-alpha-4-amino-4-deoxy-L-arabinose arabinosyl transferase;
N	Q8Z538	DE	Undecaprenyl phosphate-alpha-L-Ara4N transferase;
N	Q8Z538	DR	lipid A biosynthetic process
N	Q8Z538	DR	mannosyltransferase activity
N	Q8Z538	DR	protein O-linked glycosylation
N	Q8Z864	CC	Part of the ABC transporter complex GsiABCD involved in glutathione import
N	Q8Z864	CC	Responsible for energy coupling to the transport system (By similarity).
N	Q8Z864	DE	Glutathione import ATP-binding protein GsiA;
N	Q8Z864	DR	ATPase activity
N	Q8Z864	DR	peptide transport
N	Q8Z8D2	CC	Involved in base excision repair of DNA damaged by oxidation or by mutagenic agents
N	Q8Z8D2	CC	Acts as DNA glycosylase that recognizes and removes damaged bases
N	Q8Z8D2	CC	Cleaves the DNA backbone by beta-delta elimination to generate a single-strand break at the site of the removed base with both 3'- and 5'- phosphates (By similarity)
N	Q8Z8D2	CC	Has AP (apurinic/apyrimidinic) lyase activity and introduces nicks in the DNA strand
N	Q8Z8D2	CC	Has a preference for oxidized pyrimidines, such as thymine glycol, 5,6-dihydrouracil and 5,6-dihydrothymine
N	Q8Z8D2	DE	DNA glycosylase/AP lyase Nei;
N	Q8Z8D2	DE	DNA-(apurinic or apyrimidinic site) lyase Nei;
N	Q8Z8D2	DE	Endonuclease 8;
N	Q8Z8D2	DE	Endonuclease VIII;
N	Q8Z8D2	DR	DNA-(apurinic or apyrimidinic site) lyase activity
N	Q8Z8D2	DR	base-excision repair
N	Q8Z8D2	DR	damaged DNA binding
N	Q8Z8D2	DR	hydrolase activity, hydrolyzing N-glycosyl compounds
N	Q8Z8D2	DR	nucleotide-excision repair
N	Q8Z8D2	DR	zinc ion binding
N	Q8Z9C1	DE	Sugar fermentation stimulation protein A;
N	Q8Z9E2	CC	Catalyzes the production of spermidine from putrescine and decarboxylated S-adenosylmethionine (dcSAM), which serves as an aminopropyl donor (By similarity)
N	Q8Z9E2	DE	Putrescine aminopropyltransferase;
N	Q8Z9E2	DE	Spermidine synthase;
N	Q8Z9E2	DR	spermidine biosynthetic process
N	Q8Z9E2	DR	spermidine synthase activity
N	Q8Z9G9	CC	Cell wall formation
N	Q8Z9G9	CC	Catalyzes the transfer of a GlcNAc subunit on undecaprenyl-pyrophosphoryl-MurNAc-pentapeptide (lipid intermediate I) to form undecaprenyl-pyrophosphoryl-MurNAc- (pentapeptide)GlcNAc (lipid intermediate II) (By similarity)
N	Q8Z9G9	DE	UDP-N-acetylglucosamine--N-acetylmuramyl-(pentapeptide) pyrophosphoryl-undecaprenol N-acetylglucosamine transferase;
N	Q8Z9G9	DE	Undecaprenyl-PP-MurNAc-pentapeptide-UDPGlcNAc GlcNAc transferase;
N	Q8Z9G9	DR	UDP-N-acetyl-D-glucosamine:N-acetylmuramoyl-L-alanyl-D-glutamyl-meso-2,6-diaminopimelyl-D-alanyl-D-alanine-diphosphoundecaprenol 4-beta-N-acetylglucosaminlytransferase activity
N	Q8Z9G9	DR	UDP-N-acetylgalactosamine biosynthetic process
N	Q8Z9G9	DR	carbohydrate binding
N	Q8Z9G9	DR	cell division
N	Q8Z9G9	DR	cellular cell wall organization
N	Q8Z9G9	DR	lipid glycosylation
N	Q8Z9G9	DR	peptidoglycan biosynthetic process
N	Q8Z9G9	DR	regulation of cell shape
N	Q8Z9G9	DR	undecaprenyldiphospho-muramoylpentapeptide beta-N-acetylglucosaminyltransferase activity
N	Q8ZAH6	CC	Conversion of glycerol 3-phosphate to dihydroxyacetone
N	Q8ZAH6	CC	Uses fumarate or nitrate as electron acceptor (By similarity).
N	Q8ZAH6	DE	Anaerobic G-3-P dehydrogenase subunit B;

N	Q8ZAH6	DE	Anaerobic G3Pdhase B;
N	Q8ZAH6	DE	Anaerobic glycerol-3-phosphate dehydrogenase subunit B;
N	Q8ZAH6	DR	electron carrier activity
N	Q8ZAH6	DR	oxidation-reduction process
N	Q8ZAH6	DR	sn-glycerol-3-phosphate:ubiquinone-8 oxidoreductase activity
N	Q8ZDJ9	DE	UPF0304 protein YPO2563/y1624/YP_2374;
N	Q8ZG80	CC	Necessary for the introduction of cis unsaturation into fatty acids
N	Q8ZG80	CC	Catalyzed the dehydration of (3R)-3-hydroxydecanoyl- ACP to E-(2)-decenoyl-ACP and then its isomerization to Z-(3)- decenoyl-ACP (By similarity).
N	Q8ZG80	DE	3-hydroxydecanoyl-[acyl-carrier-protein] dehydratase;
N	Q8ZG80	DE	Beta-hydroxydecanoyl thioester dehydrase;
N	Q8ZG80	DR	3-hydroxydecanoyl-[acyl-carrier-protein] dehydratase activity
N	Q8ZG80	DR	fatty acid biosynthetic process
N	Q8ZHG8	CC	Catalyzes the biosynthesis of agmatine from arginine (By similarity).
N	Q8ZHG8	DE	Biosynthetic arginine decarboxylase;
N	Q8ZHG8	DR	arginine catabolic process
N	Q8ZHG8	DR	arginine decarboxylase activity
N	Q8ZHG8	DR	metal ion binding
N	Q8ZHG8	DR	putrescine biosynthetic process
N	Q8ZHG8	DR	spermidine biosynthetic process
N	Q8ZIQ2	DE	Purine nucleoside phosphorylase deoD-type;
N	Q8ZIQ2	DR	nucleoside metabolic process
N	Q8ZIQ2	DR	purine-nucleoside phosphorylase activity
N	Q8ZJH2	CC	Necessary for efficient RNA polymerase transcription elongation past template-encoded arresting sites
N	Q8ZJH2	CC	Cleavage of the nascent transcript by cleavage factors such as greA or greB allows the resumption of elongation from the new 3'terminus
N	Q8ZJH2	CC	GreB releases sequences of up to 9 nucleotides in length (By similarity).
N	Q8ZJH2	CC	The arresting sites in DNA have the property of trapping a certain fraction of elongating RNA polymerases that pass through, resulting in locked ternary complexes
N	Q8ZJH2	DE	Transcript cleavage factor greB;
N	Q8ZJH2	DE	Transcription elongation factor greB;
N	Q8ZJH2	DR	transcription elongation regulator activity
N	Q8ZL42	CC	Catalyzes the formation of phosphodiester linkages between 5'-phosphoryl and 3'-hydroxyl groups in double-stranded DNA using NAD as a coenzyme and as the energy source for the reaction (By similarity).
N	Q8ZL42	DE	DNA ligase B;
N	Q8ZL42	DE	Polydeoxyribonucleotide synthase [NAD+] B;
N	Q8ZL42	DR	DNA ligase (NAD+) activity
N	Q8ZL42	DR	DNA replication
N	Q8ZLJ2	CC	Necessary for efficient RNA polymerase transcription elongation past template-encoded arresting sites
N	Q8ZLJ2	CC	Cleavage of the nascent transcript by cleavage factors such as greA or greB allows the resumption of elongation from the new 3'terminus
N	Q8ZLJ2	CC	GreB releases sequences of up to 9 nucleotides in length (By similarity).
N	Q8ZLJ2	CC	The arresting sites in DNA have the property of trapping a certain fraction of elongating RNA polymerases that pass through, resulting in locked ternary complexes
N	Q8ZLJ2	DE	Transcript cleavage factor greB;
N	Q8ZLJ2	DE	Transcription elongation factor greB;
N	Q8ZLJ2	DR	transcription elongation regulator activity
N	Q8ZM46	DE	UPF0235 protein yggU;
N	Q8ZNM4	CC	Catalyzes the synthesis of dihydrouridine, a modified base found in the D-loop of most tRNAs (By similarity).
N	Q8ZNM4	DE	tRNA-dihydrouridine synthase C;
N	Q8ZNM4	DR	flavin adenine dinucleotide binding
N	Q8ZNM4	DR	oxidation-reduction process
N	Q8ZNM4	DR	tRNA dihydrouridine synthase activity
N	Q8ZNM4	DR	tRNA processing
N	Q8ZTB7	CC	Signal-recognition-particle assembly, binds directly to 7S RNA and mediates binding of the 54 kDa subunit of the SRP (By similarity).
N	Q8ZTB7	DE	Signal recognition particle 19 kDa protein;
N	Q8ZTB7	DR	7S RNA binding
N	Q8ZTB7	DR	SRP-dependent cotranslational protein targeting to membrane
N	Q8ZTY6	CC	Involved in ribosome biogenesis; more specifically in 18S rRNA pseudouridylation and in cleavage of pre-rRNA (By similarity).

N	Q8ZTY6	DE	Ribosome biogenesis protein Nop10;
N	Q8ZTY6	DR	rRNA processing
N	Q8ZVM1	CC	Component of the proteasome core, a large protease complex with broad specificity involved in protein degradation (By similarity).
N	Q8ZVM1	DE	20S proteasome alpha subunit;
N	Q8ZVM1	DE	Proteasome core protein PsmA;
N	Q8ZVM1	DE	Proteasome subunit alpha;
N	Q8ZVM1	DR	threonine-type endopeptidase activity
N	Q8ZVM1	DR	ubiquitin-dependent protein catabolic process
N	Q8ZYK3	CC	Part of the RFC clamp loader complex which loads the PCNA sliding clamp onto DNA (By similarity).
N	Q8ZYK3	DE	Clamp loader large subunit;
N	Q8ZYK3	DE	RFC large subunit;
N	Q8ZYK3	DE	Replication factor C large subunit;
N	Q8ZYK3	DR	DNA replication
N	Q8ZYK3	DR	nucleoside-triphosphatase activity
N	Q90252	CC	Binds to mesotocin and may play a role in the regulation of water and salt transport
N	Q90252	DE	Mesotocin receptor;
N	Q90252	DR	oxytocin receptor activity
N	Q90252	DR	vasopressin receptor activity
N	Q90XG0	DE	Triose-phosphate isomerase B;
N	Q90XG0	DE	Triosephosphate isomerase B;
N	Q90XG0	DR	fatty acid biosynthetic process
N	Q90XG0	DR	gluconeogenesis
N	Q90XG0	DR	pentose-phosphate shunt
N	Q90XG0	DR	triose-phosphate isomerase activity
N	Q90YB1	CC	Efficiently joins single-strand breaks in a double- stranded polydeoxynucleotide in an ATP-dependent reaction
N	Q90YB1	CC	Involved in DNA nonhomologous end joining (NHEJ) required for double-strand break repair and V(D)J recombination (By similarity).
N	Q90YB1	DE	DNA ligase 4;
N	Q90YB1	DE	DNA ligase IV;
N	Q90YB1	DE	Polydeoxyribonucleotide synthase [ATP] 4;
N	Q90YB1	DR	DNA ligase (ATP) activity
N	Q90YB1	DR	DNA replication
N	Q90YB1	DR	cell division
N	Q90YB1	DR	metal ion binding
N	Q90YC2	CC	May play a role in gastrulation-related cell movement.
N	Q90YC2	DE	Matrix metalloproteinase-21;
N	Q90YC2	DR	calcium ion binding
N	Q90YC2	DR	metalloendopeptidase activity
N	Q90YC2	DR	zinc ion binding
N	Q90YR7	DE	40S ribosomal protein S7;
N	Q90YR7	DR	structural constituent of ribosome
N	Q91090	CC	The absence of paraoxonase activity in turkey and chicken blood and in turkey liver indicates that PON2, if expressed, does not hydrolyze paraoxon.
N	Q91090	DE	A-esterase 2;
N	Q91090	DE	Aromatic esterase 2;
N	Q91090	DE	Serum aryldialkylphosphatase 2;
N	Q91090	DE	Serum paraoxonase/arylesterase 2;
N	Q91090	DR	aryldialkylphosphatase activity
N	Q91090	DR	arylesterase activity
N	Q91090	DR	metal ion binding
N	Q91364	DE	Prolactin II;
N	Q91364	DE	Prolactin-2;
N	Q91364	DR	hormone activity
N	Q91642	CC	Hydrolyzes dipeptides containing N-terminal aspartate residues.
N	Q91642	DE	Alpha-aspartyl dipeptidase;
N	Q91642	DE	Asp-specific dipeptidase;
N	Q91642	DE	Dipeptidase E;
N	Q91642	DR	dipeptidase activity
N	Q91642	DR	serine-type peptidase activity
N	Q91690	CC	May act as a transcriptional activator
N	Q91690	CC	Implicated in the transduction of Shh signal (By similarity).
N	Q91690	DE	Zinc finger protein GLI1;

N	Q91690	DR	cell differentiation
N	Q91690	DR	multicellular organismal development
N	Q91690	DR	transcription activator activity
N	Q91690	DR	zinc ion binding
N	Q918W3	CC	RNA replication
N	Q918W3	CC	The central part of this protein possibly functions as an ATP-binding helicase (Probable)
N	Q918W3	DE	RNA replication protein;
N	Q918W3	DE	RNA-directed RNA polymerase;
N	Q918W3	DR	RNA processing
N	Q918W3	DR	RNA-directed RNA polymerase activity
N	Q918W3	DR	helicase activity
N	Q918W3	DR	mRNA methyltransferase activity
N	Q918W3	DR	metal ion binding
N	Q918W3	DR	oxidation-reduction process
N	Q918W3	DR	oxidoreductase activity, acting on single donors with incorporation of molecular oxygen, incorporation of two atoms of oxygen
N	Q918W3	DR	viral genome replication
N	Q91FF5	CC	Probable endonuclease (Potential).
N	Q91FF5	DE	Probable RAD2-like endonuclease 369L;
N	Q91FF5	DR	endonuclease activity
N	Q91FF5	DR	metal ion binding
N	Q91G63	DE	Uncharacterized protein 034R;
N	Q91VX5	CC	May be signaling molecules that resemble neuropeptides
N	Q91VX5	CC	Ligand for alpha-neurexins.
N	Q91VX5	DE	Neurexophilin-3;
N	Q91VX5	DR	receptor binding
N	Q91Z84	CC	Associates with IL12B to form the IL-23 interleukin, an heterodimeric cytokine which functions in innate and adaptive immunity
N	Q91Z84	CC	IL-23 binds to an heterodimeric receptor complex composed of IL12RB1 and IL23R, activates the Jak- Stat signaling cascade, stimulates memory rather than naive T- cells and promotes production of proinflammatory cytokines
N	Q91Z84	CC	IL-23 induces autoimmune inflammation and thus may be responsible for autoimmune inflammatory diseases and may be important for tumorigenesis (By similarity)
N	Q91Z84	CC	IL-23 may constitute with IL-17 an acute response to infection in peripheral tissues
N	Q91Z84	DE	IL-23 subunit alpha;
N	Q91Z84	DE	Interleukin-23 subunit alpha;
N	Q91Z84	DE	Interleukin-23 subunit p19;
N	Q91Z84	DR	cytokine activity
N	Q91Z84	DR	inflammatory response
N	Q91Z84	DR	innate immune response
N	Q91Z84	DR	response to virus
N	Q91Z84	DR	tissue remodeling
N	Q91ZB7	CC	Orphan receptor
N	Q91ZB7	CC	May regulate nociceptor function and/or development, including the sensation or modulation of pain.
N	Q91ZB7	DE	Evolutionary breakpoint transcript 3 protein;
N	Q91ZB7	DE	Mas-related G-protein coupled receptor member E;
N	Q91ZB7	DR	G-protein coupled receptor activity
N	Q92193	CC	Actins are highly conserved proteins that are involved in various types of cell motility and are ubiquitously expressed in all eukaryotic cells.
N	Q921A3	CC	Ubiquitin-like protein modifier which can be covalently attached to target protein and subsequently leads to their degradation by the 26S proteasome, in a NUB1L-dependent manner
N	Q921A3	CC	May be involved in dendritic cell (DC) maturation, the process by which immature dendritic cells differentiate into fully competent antigen-presenting cells that initiate T cell responses
N	Q921A3	CC	May be involved in the formation of aggresomes when proteasome is saturated or impaired
N	Q921A3	CC	Mediates apoptosis in a caspase-dependent manner, especially in renal epithelium and tubular cells during renal diseases (By similarity).
N	Q921A3	CC	Mediates mitotic non-disjunction and chromosome instability, in long-term in vitro culture and cancers, by abbreviating mitotic phase and impairing the kinetochore localization of MAD2L1 during the prometaphase stage of the cell cycle
N	Q921A3	CC	Probably functions as a survival factor
N	Q921A3	CC	Promotes the expression of the proteasome subunit beta type-9 (PSMB9/LMP2)

N	Q921A3	CC	Regulates TNF- alpha-induced and LPS-mediated activation of the central mediator of innate immunity NF-kappa-B by promoting TNF-alpha-mediated proteasomal degradation of ubiquitinated-I-kappa-B-alpha
N	Q921A3	CC	Required for TNF-alpha-induced p65 nuclear translocation in renal tubular epithelial cells (RTECs)
N	Q921A3	DE	Diubiquitin;
N	Q921A3	DE	Ubiquitin D;
N	Q921A3	DE	Ubiquitin-like protein FAT10;
N	Q921A3	DR	aggresome assembly
N	Q921A3	DR	myeloid dendritic cell differentiation
N	Q921A3	DR	negative regulation of mitotic prometaphase
N	Q921A3	DR	positive regulation of I-kappaB kinase/NF-kappaB cascade
N	Q921A3	DR	positive regulation of apoptosis
N	Q921A3	DR	proteasome binding
N	Q921A3	DR	protein ubiquitination
N	Q921A3	DR	response to organic nitrogen
N	Q921A3	DR	ubiquitin-dependent protein catabolic process
N	Q922E4	DE	CTP:phosphoethanolamine cytidylyltransferase;
N	Q922E4	DE	Ethanolamine-phosphate cytidylyltransferase;
N	Q922E4	DE	Phosphorylethanolamine transferase;
N	Q922E4	DR	ethanolamine-phosphate cytidylyltransferase activity
N	Q922E4	DR	phospholipid biosynthetic process
N	Q923Y6	CC	Orphan receptor
N	Q923Y6	CC	Although some trace amines have clearly defined roles as neurotransmitters in invertebrates, the extent to which they function as true neurotransmitters in vertebrates has remained speculative
N	Q923Y6	CC	Could be a receptor for trace amines
N	Q923Y6	CC	Trace amines are biogenic amines present in very low levels in mammalian [illegible]
N	Q923Y6	CC	Trace amines are likely to be involved in a variety of physiological functions that have yet to be fully understood.
N	Q923Y6	DE	Trace amine receptor 3;
N	Q923Y6	DE	Trace amine receptor 9;
N	Q923Y6	DE	Trace amine-associated receptor 9;
N	Q923Y6	DR	G-protein coupled receptor activity
N	Q924T8	CC	Receptor for cysteinyl leukotrienes mediating constriction of the microvascular smooth muscle during an inflammatory response
N	Q924T8	CC	This response is mediated via a G-protein that activates a phosphatidylinositol-calcium second messenger system (By similarity).
N	Q924T8	DE	Cysteinyl leukotriene receptor 1;
N	Q924T8	DR	inflammatory response
N	Q924T8	DR	leukotriene receptor activity
N	Q924T8	DR	positive regulation of vasoconstriction
N	Q924W9	CC	May be involved in gene regulation within neural lineage cells potentially by direct DNA binding or by binding to other transcription factors.
N	Q924W9	DE	Cysteine-rich protein TTG-1;
N	Q924W9	DE	LIM domain only protein 1;
N	Q924W9	DE	Rhombotin-1;
N	Q924W9	DE	T-cell translocation protein 1;
N	Q924W9	DR	zinc ion binding
N	Q92664	CC	Interacts with the internal control region (ICR) of approximately 50 bases within the 5S RNA genes, is required for correct transcription of these genes by RNA polymerase III
N	Q92664	CC	Also binds the transcribed 5S RNA's
N	Q92664	CC	May initiate transcription of the 5S ribosomal RNA gene and maintain the stability of transcription of other genes.
N	Q92664	DE	Transcription factor IIIA;
N	Q92664	DR	RNA polymerase III transcription factor activity
N	Q92664	DR	protein binding
N	Q92664	DR	rRNA transcription
N	Q92664	DR	transcription from RNA polymerase III promoter
N	Q92664	DR	zinc ion binding
N	Q92C05	CC	The glycine cleavage system catalyzes the degradation of glycine
N	Q92C05	CC	The P protein binds the alpha-amino group of glycine through its pyridoxal phosphate cofactor; CO(2) is released and the remaining methylamine moiety is then transferred to the lipoamide cofactor of the H protein (By similarity).
N	Q92C05	DE	Glycine cleavage system P-protein subunit 1;

N	Q92C05	DE	Glycine decarboxylase subunit 1;
N	Q92C05	DE	Probable glycine dehydrogenase [decarboxylating] subunit 1;
N	Q92C05	DR	glycine dehydrogenase (decarboxylating) activity
N	Q92C05	DR	oxidation-reduction process
N	Q92C05	DR	pyridoxal phosphate binding
N	Q92C68	CC	Catalyzes the transfer of an acyl group from acyl- phosphate (acyl-PO(4)) to glycerol-3-phosphate (G3P) to form lysophosphatidic acid (LPA)
N	Q92C68	CC	This enzyme utilizes acyl-phosphate as fatty acyl donor, but not acyl-CoA or acyl-ACP (By similarity).
N	Q92C68	DE	Acyl-PO4 G3P acyltransferase;
N	Q92C68	DE	Acyl-phosphate--glycerol-3-phosphate acyltransferase;
N	Q92C68	DE	G3P acyltransferase;
N	Q92C68	DE	Glycerol-3-phosphate acyltransferase;
N	Q92C68	DE	LPA synthase;
N	Q92C68	DE	Lysophosphatidic acid synthase;
N	Q92C68	DR	phospholipid biosynthetic process
N	Q92C68	DR	transferase activity, transferring acyl groups other than amino-acyl groups
N	Q92D49	CC	Involved in the biosynthesis of D-alanyl-lipoteichoic acid (LTA)
N	Q92D49	CC	Activated D-alanyl-Dcp donates its D-alanyl substituent to membrane-associated LTA (By similarity).
N	Q92D49	DE	D-alanine--poly(phosphoribitol) ligase subunit 2;
N	Q92D49	DE	D-alanyl carrier protein;
N	Q92D49	DR	D-alanine-poly(phosphoribitol) ligase activity
N	Q92D49	DR	acyl carrier activity
N	Q92D49	DR	cellular cell wall organization
N	Q92D49	DR	cofactor binding
N	Q92D49	DR	teichoic acid biosynthetic process
N	Q92F56	CC	Ligates lysine onto the cytidine present at position 34 of the AUA codon-specific tRNA(Ile) that contains the anticodon CAU, in an ATP-dependent manner
N	Q92F56	CC	Cytidine is converted to lysidine, thus changing the amino acid specificity of the tRNA from methionine to isoleucine (By similarity).
N	Q92F56	DE	Bifunctional protein tilS/hprT;
N	Q92F56	DE	HGPRTase;
N	Q92F56	DE	Hypoxanthine-guanine phosphoribosyltransferase;
N	Q92F56	DE	tRNA(Ile)-2-lysyl-cytidine synthase;
N	Q92F56	DE	tRNA(Ile)-lysidine synthase;
N	Q92F56	DE	tRNA(Ile)-lysidine synthetase;
N	Q92F56	DR	guanine phosphoribosyltransferase activity
N	Q92F56	DR	hypoxanthine phosphoribosyltransferase activity
N	Q92F56	DR	ligase activity, forming carbon-nitrogen bonds
N	Q92F56	DR	metal ion binding
N	Q92F56	DR	purine ribonucleoside salvage
N	Q92F56	DR	tRNA processing
N	Q92G41	CC	Ferredoxins are iron-sulfur proteins that transfer electrons in a wide variety of metabolic reactions (By similarity).
N	Q92G41	DR	3 iron, 4 sulfur cluster binding
N	Q92G41	DR	4 iron, 4 sulfur cluster binding
N	Q92G41	DR	electron carrier activity
N	Q92G41	DR	electron transport chain
N	Q92G41	DR	metal ion binding
N	Q92G41	DR	oxidoreductase activity
N	Q92HI5	DE	Uncharacterized protein RC0786;
N	Q92JF2	DE	Uncharacterized protein RC0115;
N	Q92JL6	DE	Uncharacterized protein RC0051;
N	Q92NM4	CC	Cell wall formation (By similarity).
N	Q92NM4	DE	D-Ala-D-Ala ligase;
N	Q92NM4	DE	D-alanine--D-alanine ligase;
N	Q92NM4	DE	D-alanylalanine synthetase;
N	Q92NM4	DR	D-alanine-D-alanine ligase activity
N	Q92NM4	DR	cellular cell wall organization
N	Q92NM4	DR	metal ion binding
N	Q92NM4	DR	peptidoglycan biosynthetic process
N	Q92NM4	DR	regulation of cell shape

N	Q92QK7	CC	Furnishes a means for formation of correctly charged Gln-tRNA(Gln) through the transamidation of misacylated Glu- tRNA(Gln) in organisms which lack glutaminyl-tRNA synthetase
N	Q92QK7	CC	The reaction takes place in the presence of glutamine and ATP through an activated gamma-phospho-Glu-tRNA(Gln) (By similarity).
N	Q92QK7	DE	Glu-ADT subunit A;
N	Q92QK7	DE	Glutamyl-tRNA(Gln) amidotransferase subunit A;
N	Q92QK7	DR	carbon-nitrogen ligase activity, with glutamine as amido-N-donor
N	Q931D7	CC	Necessary for formate dehydrogenase activity (By similarity).
N	Q931D7	DE	Protein FdhE homolog;
N	Q935S7	CC	Plays a central role in chromosome condensation, segregation and cell cycle progression
N	Q935S7	CC	Functions as a homodimer, which is essential for chromosome partition
N	Q935S7	CC	Involved in negative DNA supercoiling in vivo, and by this means organize and compact chromosomes
N	Q935S7	CC	May achieve or facilitate chromosome segregation by condensation DNA from both sides of a centrally located replisome during cell division (By similarity).
N	Q935S7	DE	Chromosome partition protein mukB;
N	Q935S7	DE	Structural maintenance of chromosome-related protein;
N	Q935S7	DR	cell division
N	Q935S7	DR	chromosome condensation
N	Q935S7	DR	chromosome segregation
N	Q93WU7	CC	Transcription factor
N	Q93WU7	CC	Interacts specifically with the W box (5'-(T)TGAC[CT]-3'), a frequently occurring elicitor- responsive cis-acting element (By similarity).
N	Q93WU7	DE	Probable WRKY transcription factor 58;
N	Q93WU7	DE	WRKY DNA-binding protein 58;
N	Q93WU7	DR	regulation of defense response
N	Q93WU7	DR	sequence-specific DNA binding
N	Q93WU7	DR	sequence-specific DNA binding transcription factor activity
N	Q941Z0	CC	The enzyme apparently serves as a quinone reductase in connection with conjugation reactions of hydroquinones involved in detoxification pathways (By similarity)
N	Q941Z0	DE	Probable NADPH:quinone oxidoreductase 1;
N	Q941Z0	DR	NAD(P)H dehydrogenase (quinone) activity
N	Q941Z0	DR	oxidation-reduction process
N	Q944W2	CC	Responsible for the transport of sucrose into the cell, with the concomitant uptake of protons (symport system)
N	Q944W2	CC	May also transport other glucosides (By similarity).
N	Q944W2	DE	Sucrose permease 3;
N	Q944W2	DE	Sucrose transport protein SUT3;
N	Q944W2	DE	Sucrose transporter 3;
N	Q944W2	DE	Sucrose-proton symporter 3;
N	Q944W2	DR	sucrose transmembrane transporter activity
N	Q944W2	DR	symporter activity
N	Q94587	CC	Involved in calcium binding and microtubule stabilization (By similarity).
N	Q94587	DE	Translationally-controlled tumor protein homolog;
N	Q94AZ4	CC	Potential calcium sensor (By similarity).
N	Q94AZ4	DE	Calmodulin-like protein 13;
N	Q94AZ4	DE	Probable calcium-binding protein CML13;
N	Q94AZ4	DR	calcium ion binding
N	Q94B55	CC	No E3 ubiquitin-protein ligase activity observed when associated with the E2 enzyme UBC8 in vitro.
N	Q94B55	DE	Ankyrin repeat domain and RING finger-containing protein XBAT31;
N	Q94B55	DE	Protein XB3 homolog 1;
N	Q94B55	DE	Putative E3 ubiquitin-protein ligase XBAT31;
N	Q94B55	DR	ligase activity
N	Q94B55	DR	protein binding
N	Q94B55	DR	zinc ion binding
N	Q94IR2	CC	Cleaves a variety of carotenoids at the 9-10 and 9'-10' double bonds
N	Q94IR2	CC	Probably not involved in abscisic acid biosynthesis.
N	Q94IR2	DE	Carotenoid 9,10(9',10')-cleavage dioxygenase 1;
N	Q94IR2	DE	Neoxanthin cleavage enzyme NC2;
N	Q94IR2	DR	metal ion binding
N	Q94IR2	DR	oxidation-reduction process
N	Q94IR2	DR	oxidoreductase activity, acting on single donors with incorporation of molecular oxygen, incorporation of two atoms of oxygen

N	Q94JL3	DE	Basic helix-loop-helix protein 112;
N	Q94JL3	DE	Transcription factor EN 64;
N	Q94JL3	DE	Transcription factor bHLH112;
N	Q94JL3	DE	bHLH transcription factor bHLH112;
N	Q94JL3	DR	transcription regulator activity
N	Q95136	CC	This is one of the five types (D1 to D5) of receptors for dopamine
N	Q95136	CC	The activity of this receptor is mediated by G proteins which activate adenylyl cyclase
N	Q95136	DE	D(1A) dopamine receptor;
N	Q95136	DE	Dopamine D1 receptor;
N	Q95136	DR	dopamine receptor activity
N	Q95196	CC	Participates in the formation of a gel matrix (sperm coagulum) entrapping the accessory gland secretions and ejaculated spermatozoa.
N	Q95196	DE	Semenogelin II;
N	Q95196	DE	Semenogelin-2;
N	Q95196	DR	sexual reproduction
N	Q95196	DR	structural molecule activity
N	Q95GN9	CC	No specific function has so far been attributed to this initiation factor; however, it seems to stimulate more or less all the activities of the other two initiation factors, IF-2 and IF-3
N	Q95GN9	DE	Translation initiation factor IF-1, chloroplastic;
N	Q95GN9	DR	translation initiation factor activity
N	Q95LM0	CC	Probable redox-inactive chaperone involved in spermatogenesis (By similarity).
N	Q95LM0	DE	Protein disulfide-isomerase-like protein of the testis;
N	Q95LM0	DR	cell differentiation
N	Q95LM0	DR	cell redox homeostasis
N	Q95LM0	DR	isomerase activity
N	Q95LM0	DR	multicellular organismal development
N	Q95LM0	DR	spermatogenesis
N	Q95M53	CC	Cooperates with LY96 to mediate the innate immune response to bacterial lipoproteins and other microbial cell wall components
N	Q95M53	CC	Acts via MYD88 and TRAF6, leading to NF-kappa-B activation, cytokine secretion and the inflammatory response
N	Q95M53	CC	Cooperates with TLR1 to mediate the innate immune response to bacterial lipoproteins or lipopeptides
N	Q95M53	CC	May also promote apoptosis in response to lipoproteins (By similarity).
N	Q95M53	DE	CD282;
N	Q95M53	DE	Toll-like receptor 2;
N	Q95M53	DR	inflammatory response
N	Q95M53	DR	innate immune response
N	Q95M53	DR	transmembrane receptor activity
N	Q95SS8	DE	Transmembrane protein 70 homolog, mitochondrial;
N	Q95SS8	DR	mitochondrial proton-transporting ATP synthase complex assembly
N	Q962X7	CC	Necessary for protein translocation in the endoplasmic reticulum (By similarity).
N	Q962X7	DE	Protein transport protein Sec61 subunit gamma;
N	Q962X7	DR	P-P-bond-hydrolysis-driven protein transmembrane transporter activity
N	Q962X7	DR	protein targeting to ER
N	Q962X9	DE	Protein BUD31 homolog;
N	Q962X9	DE	Protein G10 homolog;
N	Q96680	DE	Maturation protein;
N	Q96680	DE	Protein IVA2;
N	Q96680	DR	viral transcription
N	Q966L8	CC	Transcription factor essential for the identity of ASE chemosensory neurons
N	Q966L8	CC	Acts by activating expression of genes that are specific for ASE chemosensory neurons, such as seven- transmembrane receptors or guanylate cyclase
N	Q966L8	CC	Also required to control the left/right asymmetry in ASE chemosensory neurons by regulating expression of genes such as cog-1 and ceh-36.
N	Q966L8	DE	Abnormal chemotaxis protein 1;
N	Q966L8	DE	Transcription factor che-1;
N	Q966L8	DR	zinc ion binding
N	Q96CK0	CC	Transcriptional repressor
N	Q96CK0	CC	May repress NR5A1, PPARG, NR1H3, NR4A2, ESR1 and NR3C1 transcriptional activity.
N	Q96CK0	DE	67 kDa zinc finger protein;
N	Q96CK0	DE	Zinc finger protein 653;
N	Q96CK0	DE	Zinc finger protein Zip67;
N	Q96CK0	DR	zinc ion binding

N	Q96DE9	DE	Protein CXorf40B;
N	Q96I45	DE	Transmembrane protein 141;
N	Q96N68	DE	Putative uncharacterized protein C18orf15;
N	Q96SK3	CC	May be involved in transcriptional regulation.
N	Q96SK3	DE	Zinc finger protein 607;
N	Q96SK3	DR	regulation of transcription, DNA-dependent
N	Q96SK3	DR	zinc ion binding
N	Q96WN6	CC	Positive regulation of the major cellulase and xylanase genes (cbh1, cbh2, egl2 and xyn2)
N	Q96WN6	CC	Binds to the 5'-GGCTAATAA-3' site present in the cbh1 promoter.
N	Q96WN6	DE	Transcription factor ACEII;
N	Q96WN6	DR	positive regulation of transcription, DNA-dependent
N	Q96WN6	DR	sequence-specific DNA binding transcription factor activity
N	Q96WN6	DR	transcription activator activity
N	Q96WN6	DR	zinc ion binding
N	Q971E0	DE	Protein ST1413;
N	Q971E0	DR	Mo-molybdopterin cofactor biosynthetic process
N	Q971S9	CC	Binds to the signal sequence of presecretory protein when they emerge from the ribosomes (By similarity).
N	Q971S9	DE	Signal recognition 54 kDa protein;
N	Q971S9	DR	7S RNA binding
N	Q971S9	DR	SRP-dependent cotranslational protein targeting to membrane
N	Q971S9	DR	nucleoside-triphosphatase activity
N	Q971Z4	DE	Acylphosphatase;
N	Q971Z4	DE	Acylphosphate phosphohydrolase;
N	Q971Z4	DR	acylphosphatase activity
N	Q976K0	CC	Involved in both the arginine and lysine biosynthetic pathways (By similarity).
N	Q976K0	DE	Acetylornithine/acetyl-lysine aminotransferase;
N	Q976K0	DR	N2-acetyl-L-ornithine:2-oxoglutarate 5-aminotransferase activity
N	Q976K0	DR	arginine biosynthetic process
N	Q976K0	DR	lysine biosynthetic process
N	Q976K0	DR	pyridoxal phosphate binding
N	Q97A09	CC	Endonuclease IV plays a role in DNA repair
N	Q97A09	CC	It cleaves phosphodiester bonds at apurinic or apyrimidinic sites (AP sites) to produce new 5'-ends that are base-free deoxyribose 5-phosphate residues
N	Q97A09	CC	It preferentially attacks modified AP sites created by bleomycin and neocarzinostatin (By similarity).
N	Q97A09	DE	Endodeoxyribonuclease IV;
N	Q97A09	DE	Endonuclease IV;
N	Q97A09	DE	Probable endonuclease 4;
N	Q97A09	DR	deoxyribonuclease IV (phage-T4-induced) activity
N	Q97A09	DR	zinc ion binding
N	Q97KI3	CC	Catalyzes the condensation of ATP and 5-phosphoribose 1- diphosphate to form N'-(5'-phosphoribosyl)-ATP (PR-ATP)
N	Q97KI3	CC	Has a crucial role in the pathway because the rate of histidine biosynthesis seems to be controlled primarily by regulation of hisG enzymatic activity (By similarity)
N	Q97KI3	DE	ATP phosphoribosyltransferase;
N	Q97KI3	DE	ATP-PRTase;
N	Q97KI3	DR	ATP phosphoribosyltransferase activity
N	Q97KI3	DR	histidine biosynthetic process
N	Q97KL8	CC	Catalyzes a trans-dehydration via an enolate intermediate (By similarity).
N	Q97KL8	DE	3-dehydroquinase;
N	Q97KL8	DE	3-dehydroquinate dehydratase;
N	Q97KL8	DE	Type II DHQase;
N	Q97KL8	DR	3-dehydroquinate dehydratase activity
N	Q97KL8	DR	aromatic amino acid family biosynthetic process
N	Q97KZ2	DE	UPF0272 protein CA_C0774;
N	Q97LP1	CC	May be required for sporulation (By similarity).
N	Q97LP1	DE	Putative sporulation transcription regulator whiA;
N	Q97LP1	DR	endonuclease activity
N	Q97R22	DE	Thymidine kinase;
N	Q97R22	DR	DNA replication
N	Q97R22	DR	metal ion binding
N	Q97R22	DR	thymidine kinase activity

N	Q97RS5	CC	Condenses 4-methyl-5-(beta-hydroxyethyl)thiazole monophosphate (THZ-P) and 2-methyl-4-amino-5-hydroxymethyl pyrimidine pyrophosphate (HMP-PP) to form thiamine monophosphate (TMP) (By similarity).
N	Q97RS5	DE	TMP pyrophosphorylase 1;
N	Q97RS5	DE	TMP-PPase 1;
N	Q97RS5	DE	Thiamine-phosphate pyrophosphorylase 1;
N	Q97RS5	DE	Thiamine-phosphate synthase 1;
N	Q97RS5	DR	metal ion binding
N	Q97RS5	DR	thiamine biosynthetic process
N	Q97RS5	DR	thiamine-phosphate diphosphorylase activity
N	Q97SU1	CC	Catalyzes the reversible transfer of the terminal phosphate group between ATP and AMP
N	Q97SU1	CC	This small ubiquitous enzyme involved in the energy metabolism and nucleotide synthesis, is essential for maintenance and cell growth (By similarity).
N	Q97SU1	DE	ATP-AMP transphosphorylase;
N	Q97SU1	DE	Adenylate kinase;
N	Q97SU1	DR	adenylate kinase activity
N	Q97TC5	CC	Ligates lysine onto the cytidine present at position 34 of the AUA codon-specific tRNA(Ile) that contains the anticodon CAU, in an ATP-dependent manner
N	Q97TC5	CC	Cytidine is converted to lysidine, thus changing the amino acid specificity of the tRNA from methionine to isoleucine (By similarity).
N	Q97TC5	DE	tRNA(Ile)-2-lysyl-cytidine synthase;
N	Q97TC5	DE	tRNA(Ile)-lysidine synthase;
N	Q97TC5	DE	tRNA(Ile)-lysidine synthetase;
N	Q97TC5	DR	ligase activity, forming carbon-nitrogen bonds
N	Q97TC5	DR	tRNA processing
N	Q97WG9	CC	Involved in DNA double-strand break repair (DSBR)
N	Q97WG9	CC	The rad50/mre11 complex possesses single-strand endonuclease activity and ATP-dependent double-strand-specific 3'-5' exonuclease activity (By similarity).
N	Q97WG9	DE	DNA double-strand break repair protein mre11;
N	Q97WG9	DR	endonuclease activity
N	Q97WG9	DR	exonuclease activity
N	Q97ZM1	DE	Ornithine carbamoyltransferase;
N	Q97ZM1	DR	amino acid binding
N	Q97ZM1	DR	arginine biosynthetic process
N	Q97ZM1	DR	ornithine carbamoyltransferase activity
N	Q98300	CC	Essential for virus replication (By similarity).
N	Q98300	DE	Protein MC134;
N	Q98300	DE	Viral replication protein A28-like;
N	Q98300	DR	viral reproduction
N	Q986Q8	CC	Specifically catalyzes the cleavage of the D-lactyl ether substituent of MurNAc 6-phosphate, producing GlcNAc 6- phosphate and D-lactate
N	Q986Q8	CC	Together with AnmK, is also required for the utilization of anhydro-N-acetylmuramic acid (anhMurNAc) either imported from the medium or derived from its own cell wall murein, and thus plays a role in cell wall recycling (By similarity).
N	Q986Q8	DE	MurNAc-6-P etherase;
N	Q986Q8	DE	N-acetylmuramic acid 6-phosphate etherase;
N	Q986Q8	DE	N-acetylmuramic acid 6-phosphate hydrolase;
N	Q986Q8	DE	N-acetylmuramic acid 6-phosphate lyase;
N	Q986Q8	DR	amino sugar catabolic process
N	Q986Q8	DR	hydro-lyase activity
N	Q986Q8	DR	sugar binding
N	Q98965	CC	Shows cytolytic activity (By similarity).
N	Q98965	DE	Cardiotoxin-6;
N	Q98FE3	DE	50S ribosomal protein L36;
N	Q98FE3	DR	structural constituent of ribosome
N	Q98G94	CC	Probable protein methyltransferase
N	Q98G94	CC	May methylate a Gln residue in target proteins (By similarity).
N	Q98G94	DE	Protein methyltransferase hemK homolog;
N	Q98G94	DR	nucleic acid binding
N	Q98G94	DR	protein methyltransferase activity
N	Q98KA8	CC	Catalyzes the addition of meso-diaminopimelic acid to the nucleotide precursor UDP-N-acetylmuramoyl-L-alanyl-D-glutamate (UMAG) in the biosynthesis of bacterial cell-wall peptidoglycan (By similarity).
N	Q98KA8	DE	Meso-A2pm-adding enzyme;

N	Q98KA8	DE	Meso-diaminopimelate-adding enzyme;
N	Q98KA8	DE	UDP-MurNAc-L-Ala-D-Glu:meso-diaminopimelate ligase;
N	Q98KA8	DE	UDP-MurNAc-tripeptide synthetase;
N	Q98KA8	DE	UDP-N-acetylmuramoyl-L-alanyl-D-glutamate--2,6-diaminopimelate ligase;
N	Q98KA8	DE	UDP-N-acetylmuramyl-tripeptide synthetase;
N	Q98KA8	DR	UDP-N-acetylmuramoylalanyl-D-glutamate-2,6-diaminopimelate ligase activity
N	Q98KA8	DR	cell division
N	Q98KA8	DR	cellular cell wall organization
N	Q98KA8	DR	peptidoglycan biosynthetic process
N	Q98KA8	DR	regulation of cell shape
N	Q98N46	CC	One of two assembly initiator proteins, it binds directly to the 5'-end of the 23S rRNA, where it nucleates assembly of the 50S subunit (By similarity).
N	Q98N46	DE	50S ribosomal protein L24;
N	Q98N46	DR	structural constituent of ribosome
N	Q98PA0	DE	UPF0337 protein msl9551;
N	Q98PZ5	CC	Binds 16S rRNA, required for the assembly of 30S particles and may also be responsible for determining the conformation of the 16S rRNA at the A site (By similarity).
N	Q98PZ5	DE	30S ribosomal protein S14 type Z;
N	Q98PZ5	DR	metal ion binding
N	Q98PZ5	DR	structural constituent of ribosome
N	Q98Q13	CC	Could be a nuclease that resolves Holliday junction intermediates in genetic recombination.
N	Q98Q13	DE	Putative Holliday junction resolvase;
N	Q98Q13	DR	DNA recombination
N	Q98Q13	DR	nuclease activity
N	Q98Q13	DR	nucleic acid binding
N	Q98QQ2	DE	UPF0348 protein MYPU_3090;
N	Q98R70	CC	Binds the 23S rRNA (By similarity).
N	Q98R70	DE	50S ribosomal protein L31;
N	Q98R70	DR	structural constituent of ribosome
N	Q98SV0	CC	Might be responsible for some of the extracellular antioxidant defense properties of selenium.
N	Q98SV0	DE	Selenoprotein Pb;
N	Q98SV0	DR	response to oxidative stress
N	Q98SV0	DR	selenium binding
N	Q99795	CC	May play a role in cell-cell recognition and signaling.
N	Q99795	DE	Cell surface A33 antigen;
N	Q99795	DE	Glycoprotein A33;
N	Q99795	DR	receptor activity
N	Q99JT2	CC	Mediator of cell growth
N	Q99JT2	CC	Modulates apoptosis (By similarity).
N	Q99JT2	DE	Mammalian STE20-like protein kinase 4;
N	Q99JT2	DE	STE20-like kinase MST4;
N	Q99JT2	DE	Serine/threonine-protein kinase MST4;
N	Q99JT2	DR	magnesium ion binding
N	Q99JT2	DR	protein phosphorylation
N	Q99JT2	DR	protein serine/threonine kinase activity
N	Q99JT2	DR	regulation of apoptosis
N	Q99KJ8	CC	Modulates cytoplasmic dynein binding to an organelle, and plays a role in prometaphase chromosome alignment and spindle organization during mitosis
N	Q99KJ8	CC	Involved in anchoring microtubules to centrosomes
N	Q99KJ8	CC	May play a role in synapse formation during brain development.
N	Q99KJ8	DE	50 kDa dynein-associated polypeptide;
N	Q99KJ8	DE	Dynactin complex 50 kDa subunit;
N	Q99KJ8	DE	Dynactin subunit 2;
N	Q99KJ8	DE	Growth cone membrane protein 23-48K;
N	Q99KJ8	DE	p50 dynamitin;
N	Q99KJ8	DR	motor activity
N	Q99KJ8	DR	protein binding
N	Q99M64	CC	Together with PI4K2B and the type III PI4Ks (PIK4CA and PIK4CB) it contributes to the overall PI4-kinase activity of the cell
N	Q99M64	CC	Contributes to the production of InsP3 in stimulated cells.
N	Q99M64	CC	The phosphorylation of phosphatidylinositol (PI) to PI4P is the first committed step in the generation of phosphatidylinositol 4,5-bisphosphate (PIP2), a precursor of the second messenger inositol 1,4,5-trisphosphate (InsP3)

N	Q99M64	DE	55 kDa type II phosphatidylinositol 4-kinase;
N	Q99M64	DE	Phosphatidylinositol 4-kinase type 2-alpha;
N	Q99M64	DE	Phosphatidylinositol 4-kinase type II-alpha;
N	Q99M64	DR	1-phosphatidylinositol 4-kinase activity
N	Q99M64	DR	basophil degranulation
N	Q99M64	DR	protein complex binding
N	Q99MD9	CC	Required for DNA replication, normal cell cycle progression and cell proliferation
N	Q99MD9	CC	Forms a cytoplasmic complex with HSP90 and linker H1 histones and stimulates HSP90 ATPase activity
N	Q99MD9	CC	NASP and H1 histone are subsequently released from the complex and translocate to the nucleus where the histone is released for binding to DNA.
N	Q99MD9	DE	Nuclear autoantigenic sperm protein;
N	Q99MD9	DR	DNA replication
N	Q99MD9	DR	Hsp90 protein binding
N	Q99MD9	DR	blastocyst development
N	Q99MD9	DR	cell proliferation
N	Q99MD9	DR	histone binding
N	Q99MD9	DR	histone exchange
N	Q99MD9	DR	protein transport
N	Q99PP9	CC	May play a role in the regulation of keratinocyte differentiation.
N	Q99PP9	DE	Estrogen-responsive B box protein;
N	Q99PP9	DE	Tripartite motif-containing protein 16;
N	Q99PP9	DR	zinc ion binding
N	Q99Q02	CC	Involved in sarA attenuation
N	Q99Q02	CC	Affects resistance to oxacillin and teicoplanin, as well as the synthesis of virulence factors (By similarity).
N	Q99Q02	DE	Regulatory protein msrR;
N	Q99QY9	CC	Part of the Sec protein translocase complex
N	Q99QY9	CC	Has a central role in coupling the hydrolysis of ATP to the transfer of proteins into and across the cell membrane, serving as an ATP-driven molecular motor driving the stepwise translocation of polypeptide chains across the membrane (By similarity)
N	Q99QY9	CC	Interacts with the secYEG preprotein conducting channel
N	Q99QY9	DE	Protein translocase subunit secA 2;
N	Q99QY9	DR	P-P-bond-hydrolysis-driven protein transmembrane transporter activity
N	Q99QY9	DR	protein import
N	Q99QY9	DR	protein targeting
N	Q99UA3	CC	Part of a transport system that may be involved in oligopeptide uptake
N	Q99UA3	CC	Important for S.aureus growth and survival during the infection process (By similarity)
N	Q99UA3	CC	Probably responsible for energy coupling to the transport system
N	Q99UA3	DE	Putative oligopeptide transport ATP-binding protein oppF2;
N	Q99UA3	DR	ATPase activity
N	Q99UA3	DR	peptide transport
N	Q99UA3	DR	protein transport
N	Q99WG0	DE	Uncharacterized lipoprotein SAV0442;
N	Q99WG6	DE	Uncharacterized lipoprotein SAV0436;
N	Q99YL4	CC	Divisome component that associates with the complex late in its assembly, after the Z-ring is formed, and is dependent on divIC and PBP2B for its recruitment to the divisome
N	Q99YL4	CC	Also contributes to the recruitment of PBP1 to the division complex
N	Q99YL4	CC	Its main role could be the removal of PBP1 from the cell pole after pole maturation is completed
N	Q99YL4	CC	Not essential for septum formation (By similarity).
N	Q99YL4	CC	Together with ezrA, is a key component of the system that regulates PBP1 localization during cell cycle progression
N	Q99YL4	DE	Cell cycle protein gpsB;
N	Q99YL4	DE	Guiding PBP1-shuttling protein;
N	Q99YL4	DR	cell division
N	Q99YL4	DR	regulation of cell shape
N	Q99ZB6	CC	Has a role in maltotetraose utilization (By similarity).
N	Q99ZB6	DE	Maltodextrose utilization protein malA;
N	Q9A088	CC	Necessary for normal cell division and for the maintenance of normal septation (By similarity).
N	Q9A088	DE	Probable GTP-binding protein EngB;
N	Q9A088	DR	barrier septum formation
N	Q9A229	CC	IGPS catalyzes the conversion of PRFAR and glutamine to IGP, AICAR and glutamate

N	Q9A229	CC	The hisF subunit catalyzes the cyclization activity that produces IGP and AICAR from PRFAR using the ammonia provided by the hisH subunit (By similarity).
N	Q9A229	DE	IGP synthase cyclase subunit;
N	Q9A229	DE	IGP synthase subunit hisF;
N	Q9A229	DE	IGPS subunit hisF;
N	Q9A229	DE	ImGP synthase subunit hisF;
N	Q9A229	DE	Imidazole glycerol phosphate synthase subunit hisF;
N	Q9A229	DR	histidine biosynthetic process
N	Q9A229	DR	imidazoleglycerol-phosphate synthase activity
N	Q9A229	DR	lyase activity
N	Q9A3K2	CC	With S4 and S5 plays an important role in translational accuracy (By similarity).
N	Q9A3K2	DE	30S ribosomal protein S12;
N	Q9A3K2	DR	structural constituent of ribosome
N	Q9ABF8	CC	May play a role in DNA repair
N	Q9ABF8	CC	It may act with recF and recO (By similarity).
N	Q9ABF8	CC	It seems to be involved in an recBC-independent recombinational process of DNA repair
N	Q9ABF8	DE	Recombination protein recR;
N	Q9ABF8	DR	DNA recombination
N	Q9ABF8	DR	metal ion binding
N	Q9ABW5	CC	Catalyzes the decarboxylation of orotidine 5'- monophosphate (OMP) to uridine 5'-monophosphate (UMP) (By similarity).
N	Q9ABW5	DE	OMP decarboxylase;
N	Q9ABW5	DE	Orotidine 5'-phosphate decarboxylase;
N	Q9ABW5	DR	'de novo' UMP biosynthetic process
N	Q9ABW5	DR	'de novo' pyrimidine base biosynthetic process
N	Q9ABW5	DR	orotidine-5'-phosphate decarboxylase activity
N	Q9ABZ9	CC	Required for the formation of a threonylcarbamoyl group on adenosine at position 37 (t(6)A37) in tRNAs that read codons beginning with adenine (By similarity).
N	Q9ABZ9	DE	Probable tRNA threonylcarbamoyladenosine biosynthesis protein Gcp;
N	Q9ABZ9	DE	t(6)A37 threonylcarbamoyladenosine biosynthesis protein;
N	Q9ABZ9	DR	metal ion binding
N	Q9ABZ9	DR	metalloendopeptidase activity
N	Q9AC57	DE	Shikimate dehydrogenase;
N	Q9AC57	DR	aromatic amino acid family biosynthetic process
N	Q9AC57	DR	oxidation-reduction process
N	Q9AC57	DR	shikimate 5-dehydrogenase activity
N	Q9B9E4	CC	Component of the ubiquinol-cytochrome c reductase complex (complex III or cytochrome b-c1 complex), which is a respiratory chain that generates an electrochemical potential coupled to ATP synthesis (By similarity).
N	Q9B9E4	DE	Complex III subunit 3;
N	Q9B9E4	DE	Complex III subunit III;
N	Q9B9E4	DE	Cytochrome b-c1 complex subunit 3;
N	Q9B9E4	DE	Cytochrome b;
N	Q9B9E4	DE	Ubiquinol-cytochrome-c reductase complex cytochrome b subunit;
N	Q9B9E4	DR	electron carrier activity
N	Q9B9E4	DR	metal ion binding
N	Q9B9E4	DR	oxidoreductase activity
N	Q9B9E4	DR	respiratory electron transport chain
N	Q9BAC0	CC	Probably a ribosomal protein or a ribosome-associated protein (By similarity).
N	Q9BAC0	DE	Probable 30S ribosomal protein 3, chloroplastic;
N	Q9BAC0	DR	structural constituent of ribosome
N	Q9BP45	DE	Conotoxin PnMLKM-D0211;
N	Q9BP45	DE	DE Flags: Precursor; Fragment;
N	Q9BP72	DE	Conotoxin ArMSGL-0124;
N	Q9BP72	DR	ion channel inhibitor activity
N	Q9BPU6	CC	May have a function in neuronal differentiation and/or axon growth.
N	Q9BPU6	DE	CRMP3-associated molecule;
N	Q9BPU6	DE	Collapsin response mediator protein 5;
N	Q9BPU6	DE	Dihydropyrimidinase-related protein 5;
N	Q9BPU6	DE	UNC33-like phosphoprotein 6;
N	Q9BPU6	DR	axon guidance
N	Q9BPU6	DR	hydrolase activity, acting on carbon-nitrogen (but not peptide) bonds
N	Q9BPU6	DR	signal transduction
N	Q9BQ13	DE	BTB/POZ domain-containing protein KCTD14;
N	Q9BQ13	DR	voltage-gated potassium channel activity

N	Q9BS26	CC	Mediates thiol-dependent retention in the early secretory pathway, forming mixed disulfides with substrate proteins through its conserved CRFS motif
N	Q9BS26	CC	Inhibits the calcium channel activity of ITPR1
N	Q9BS26	CC	May have a role in the control of oxidative protein folding in the endoplasmic reticulum
N	Q9BS26	CC	Required to retain ERO1L and ERO1LB in the endoplasmic reticulum.
N	Q9BS26	DE	ER protein 44;
N	Q9BS26	DE	Endoplasmic reticulum resident protein 44;
N	Q9BS26	DE	Thioredoxin domain-containing protein 4;
N	Q9BS26	DR	cell redox homeostasis
N	Q9BS26	DR	glycoprotein metabolic process
N	Q9BS26	DR	protein binding
N	Q9BS26	DR	protein disulfide isomerase activity
N	Q9BS26	DR	protein folding
N	Q9BS26	DR	response to unfolded protein
N	Q9BSJ6	DE	Protein FAM64A;
N	Q9BSJ6	DR	protein binding
N	Q9BV68	DE	RING finger protein 126;
N	Q9BV68	DR	protein binding
N	Q9BV68	DR	zinc ion binding
N	Q9BVC4	CC	Subunit of both mTORC1 and mTORC2, which regulates cell growth and survival in response to nutrient and hormonal signals
N	Q9BVC4	CC	Activated mTORC1 up-regulates protein synthesis by phosphorylating key regulators of mRNA translation and ribosome synthesis
N	Q9BVC4	CC	Amino-acid-signaling to mTORC1 requires its relocalization to the lysosomes mediated by the Ragulator complex and the Rag GTPases
N	Q9BVC4	CC	Growth factor-stimulated mTORC1 activation involves a AKT1- mediated phosphorylation of TSC1-TSC2, which leads to the activation of the RHEB GTPase that potently activates the protein kinase activity of mTORC1
N	Q9BVC4	CC	In nutrient- poor conditions, stabilizes the MTOR-RPTOR interaction and favors RPTOR-mediated inhibition of MTOR activity
N	Q9BVC4	CC	Within mTORC1, LST8 interacts directly with MTOR and enhances its kinase activity
N	Q9BVC4	CC	mTORC1 is activated in response to growth factors or amino-acids
N	Q9BVC4	CC	mTORC1 phosphorylates EIF4EBP1 and releases it from inhibiting the elongation initiation factor 4E (eiF4E)
N	Q9BVC4	CC	mTORC1 phosphorylates and activates S6K1 at 'Thr-389', which then promotes protein synthesis by phosphorylating PDCD4 and targeting it for degradation
N	Q9BVC4	CC	mTORC2 also modulates the phosphorylation of PRKCA on 'Ser-657'.
N	Q9BVC4	CC	mTORC2 is also activated by growth factors, but seems to be nutrient-insensitive
N	Q9BVC4	CC	mTORC2 plays a critical role in AKT1 'Ser-473' phosphorylation, which may facilitate the phosphorylation of the activation loop of AKT1 on 'Thr-308' by PDK1 which is a prerequisite for full activation
N	Q9BVC4	CC	mTORC2 promotes the serum- induced formation of stress-fibers or F-actin
N	Q9BVC4	CC	mTORC2 regulates the phosphorylation of SGK1 at 'Ser-422'
N	Q9BVC4	CC	mTORC2 seems to function upstream of Rho GTPases to regulate the actin cytoskeleton, probably by activating one or more Rho-type guanine nucleotide exchange factors
N	Q9BVC4	DE	G protein beta subunit-like;
N	Q9BVC4	DE	Mammalian lethal with SEC13 protein 8;
N	Q9BVC4	DE	Protein GbetaL;
N	Q9BVC4	DE	TORC subunit LST8;
N	Q9BVC4	DE	Target of rapamycin complex subunit LST8;
N	Q9BVC4	DR	T cell costimulation
N	Q9BVC4	DR	insulin receptor signaling pathway
N	Q9BVC4	DR	nerve growth factor receptor signaling pathway
N	Q9BVC4	DR	phosphatidylinositol-mediated signaling
N	Q9BVC4	DR	positive regulation of TOR signaling cascade
N	Q9BVC4	DR	protein binding
N	Q9BVC4	DR	regulation of actin cytoskeleton organization
N	Q9BXU3	DE	Testis-expressed sequence 13A protein;
N	Q9BXU3	DR	zinc ion binding
N	Q9BY49	CC	Participates in chain elongation of fatty acids
N	Q9BY49	CC	Has no 2,4-dienoyl-CoA reductase activity.
N	Q9BY49	DE	2,4-dienoyl-CoA reductase-related protein;
N	Q9BY49	DE	Peroxisomal trans-2-enoyl-CoA reductase;
N	Q9BY49	DR	fatty acid biosynthetic process

N	Q9BY49	DR	oxidation-reduction process
N	Q9BY49	DR	regulation of apoptosis
N	Q9BY49	DR	trans-2-enoyl-CoA reductase (NADPH) activity
N	Q9C168	CC	Occurs in almost all aerobically respiring organisms and serves to protect cells from the toxic effects of hydrogen peroxide (By similarity).
N	Q9C168	DR	catalase activity
N	Q9C168	DR	conidium formation
N	Q9C168	DR	hydrogen peroxide catabolic process
N	Q9C168	DR	oxidation-reduction process
N	Q9C245	CC	Acts as a sulfur carrier required for 2-thiolation of mcm(5)S(2)U at tRNA wobble positions
N	Q9C245	CC	Indirectly involved in oxidative stress response and regulation of budding and haploid invasive growth (By similarity).
N	Q9C245	CC	May also act as an ubiquitin-like protein that is covalently conjugated to other proteins such as AHP1; the relevance of such function is however unclear in vivo
N	Q9C245	CC	Prior mcm(5) tRNA modification by the elongator complex is required for 2-thiolation
N	Q9C245	CC	Serves as sulfur donor in tRNA 2-thiolation reaction by being thiocarboxylated (-COSH) at its C-terminus by UBA4
N	Q9C245	CC	The sulfur is then transferred to tRNA to form 2-thiolation of mcm(5)S(2)U
N	Q9C245	DE	Ubiquitin-related modifier 1;
N	Q9C245	DR	tRNA thio-modification
N	Q9C5K8	CC	Repressor of jasmonate responses negatively regulated by the proteasome in an SCF(COI1) E3 ubiquitin-protein ligase complex-dependent manner (By similarity).
N	Q9C5K8	DE	Jasmonate ZIM domain-containing protein 12;
N	Q9C5K8	DE	Protein TIFY 3B;
N	Q9C5K8	DR	defense response
N	Q9C5K8	DR	protein binding
N	Q9C889	CC	May be involved in both secretory and endocytic intracellular trafficking in the endosomal/prevacuolar compartments (By similarity).
N	Q9C889	DE	PRA1 family protein F2;
N	Q9C889	DR	protein binding
N	Q9C889	DR	vesicle-mediated transport
N	Q9CBJ5	DE	50S ribosomal protein L33 2;
N	Q9CBJ5	DR	structural constituent of ribosome
N	Q9CC12	DE	Acetylornithine aminotransferase;
N	Q9CC12	DR	N2-acetyl-L-ornithine:2-oxoglutarate 5-aminotransferase activity
N	Q9CC12	DR	arginine biosynthetic process
N	Q9CC12	DR	pyridoxal phosphate binding
N	Q9CCS0	CC	Involved in peptide bond synthesis
N	Q9CCS0	CC	Probably functions indirectly by altering the affinity of the ribosome for aminoacyl-tRNA, thus increasing their reactivity as acceptors for peptidyl transferase (By similarity).
N	Q9CCS0	CC	Stimulates efficient translation and peptide-bond synthesis on native or reconstituted 70S ribosomes in vitro
N	Q9CCS0	DE	Elongation factor P;
N	Q9CCS0	DR	peptide biosynthetic process
N	Q9CCS0	DR	translation elongation factor activity
N	Q9CDW1	CC	Involved in the binding of tRNA to the ribosomes (By similarity).
N	Q9CDW1	DE	30S ribosomal protein S10;
N	Q9CDW1	DR	structural constituent of ribosome
N	Q9CE00	DE	Putative glutamine amidotransferase-like protein yvdE;
N	Q9CE00	DR	glutamine metabolic process
N	Q9CE00	DR	hydrolase activity
N	Q9CE00	DR	transferase activity
N	Q9CFB6	CC	An accessory protein needed during the final step in the assembly of 30S ribosomal subunit, possibly for assembly of the head region
N	Q9CFB6	CC	Essential for efficient processing of 16S rRNA
N	Q9CFB6	CC	It has affinity for free ribosomal 30S subunits but not for 70S ribosomes (By similarity).
N	Q9CFB6	CC	May be needed both before and after rbfA during the maturation of 16S rRNA
N	Q9CFB6	CC	Probably interacts with S19
N	Q9CFB6	DE	Ribosome maturation factor rimM;
N	Q9CFB6	DR	rRNA processing
N	Q9CFB6	DR	ribosome binding
N	Q9CFF3	CC	Part of a stress-induced multi-chaperone system, it is involved in the recovery of the cell from heat-induced damage, in cooperation with DnaK, DnaJ and GrpE
N	Q9CFF3	CC	Acts before DnaK, in the processing of protein aggregates

N	Q9CFF3	CC	Protein binding stimulates the ATPase activity; ATP hydrolysis unfolds the denatured protein aggregates, which probably helps expose new hydrophobic binding sites on the surface of ClpB-bound aggregates, contributing to the solubilization and refolding of denatured protein aggregates by DnaK (By similarity)
N	Q9CFF3	DE	Chaperone protein ClpB;
N	Q9CFF3	DR	nucleoside-triphosphatase activity
N	Q9CFF3	DR	protein processing
N	Q9CFF3	DR	response to heat
N	Q9CI63	DE	Prophage ps2 probable integrase;
N	Q9CI63	DR	DNA integration
N	Q9CI63	DR	DNA recombination
N	Q9CJS5	CC	Carrier of the growing fatty acid chain in fatty acid biosynthesis (By similarity).
N	Q9CJS5	DE	Acyl carrier protein;
N	Q9CJS5	DR	acyl carrier activity
N	Q9CJS5	DR	cofactor binding
N	Q9CJS5	DR	fatty acid biosynthetic process
N	Q9CJS5	DR	phosphopantetheine binding
N	Q9CK89	DE	50S ribosomal protein L10;
N	Q9CK89	DR	ribosome biogenesis
N	Q9CK89	DR	structural constituent of ribosome
N	Q9CKD7	CC	Putative quercetin 2,3-dioxygenase (By similarity).
N	Q9CKD7	DE	Pirin-like protein PM1685;
N	Q9CKD7	DE	Putative quercetin 2,3-dioxygenase PM1685;
N	Q9CKD7	DE	Putative quercetinase;
N	Q9CKD7	DR	metal ion binding
N	Q9CKD7	DR	oxidation-reduction process
N	Q9CKD7	DR	quercetin 2,3-dioxygenase activity
N	Q9CKN5	CC	The phosphoenolpyruvate-dependent sugar phosphotransferase system (sugar PTS), a major carbohydrate active -transport system, catalyzes the phosphorylation of incoming sugar substrates concomitantly with their translocation across the cell membrane
N	Q9CKN5	CC	Is also able to take up anhydro-N-acetylmuramic acid (anhMurNAc), but cannot phosphorylate the carbon 6, probably because of the 1,6-anhydro ring (By similarity)
N	Q9CKN5	CC	This system is involved in N-acetylmuramic acid (MurNAc) transport, yielding cytoplasmic MurNAc-6-P
N	Q9CKN5	DE	EIIBC-MurNAc;
N	Q9CKN5	DE	N-acetylmuramic acid permease IIC component;
N	Q9CKN5	DE	N-acetylmuramic acid-specific phosphotransferase enzyme IIB component;
N	Q9CKN5	DE	PTS system N-acetylmuramic acid-specific EIIB component;
N	Q9CKN5	DE	PTS system N-acetylmuramic acid-specific EIIBC component;
N	Q9CKN5	DE	PTS system N-acetylmuramic acid-specific EIIC component;
N	Q9CKN5	DR	kinase activity
N	Q9CKN5	DR	phosphoenolpyruvate-dependent sugar phosphotransferase system
N	Q9CKN5	DR	protein-N(PI)-phosphohistidine-sugar phosphotransferase activity
N	Q9CKN5	DR	sugar:hydrogen symporter activity
N	Q9CME6	CC	Catalyzes the phosphorylation of D-glycero-D-manno- heptose 7-phosphate at the C-1 position to form D,D-heptose-1,7- bisphosphate (By similarity).
N	Q9CME6	DE	Bifunctional protein hldE;
N	Q9CME6	DE	D-beta-D-heptose 1-phosphate adenosyltransferase;
N	Q9CME6	DE	D-beta-D-heptose 7-phosphate kinase;
N	Q9CME6	DE	D-beta-D-heptose 7-phosphotransferase;
N	Q9CME6	DR	kinase activity
N	Q9CME6	DR	lipopolysaccharide biosynthetic process
N	Q9CME6	DR	nucleotidyltransferase activity
N	Q9CME6	DR	phosphotransferase activity, alcohol group as acceptor
N	Q9CN18	CC	This protein is one of the two subunits of integration host factor, a specific DNA-binding protein that functions in genetic recombination as well as in transcriptional and translational control (By similarity).
N	Q9CN18	DE	Integration host factor subunit alpha;
N	Q9CN18	DR	DNA recombination
N	Q9CN18	DR	regulation of transcription, DNA-dependent
N	Q9CN18	DR	regulation of translation
N	Q9CQJ0	DE	Thioesterase superfamily member 5;
N	Q9CQJ0	DR	hydrolase activity
N	Q9CQU3	CC	Involved in the retrieval of endoplasmic reticulum membrane proteins from the early Golgi compartment (By similarity).

N	Q9CQU3	DE	Protein RER1;
N	Q9CR30	CC	Cleaves 'Lys-63'-linked poly-ubiquitin chains, and with lesser efficiency 'Lys-48'-linked poly-ubiquitin chains (in vitro)
N	Q9CR30	CC	May act as a deubiquitinating enzyme (By similarity).
N	Q9CR30	DE	Josephin domain-containing protein 2;
N	Q9CWF2	CC	Tubulin is the major constituent of microtubules
N	Q9CWF2	CC	It binds two moles of GTP, one at an exchangeable site on the beta chain and one at a non-exchangeable site on the alpha-chain (By similarity)
N	Q9CWF2	CC	TUBB2B is implicated in neuronal migration (By similarity).
N	Q9CWF2	DE	Tubulin beta-2B chain;
N	Q9CWF2	DR	GTPase activity
N	Q9CWF2	DR	microtubule-based movement
N	Q9CWF2	DR	protein polymerization
N	Q9CWF2	DR	structural molecule activity
N	Q9CWH0	CC	May play a role in maintaining cell pluripotentiality May play a role in embryo preimplantation development.
N	Q9CWH0	DE	Developmental pluripotency-associated protein 2;
N	Q9CWH0	DE	Pluripotent embryonic stem cell-related gene 1 protein;
N	Q9CWH0	DR	nucleic acid binding
N	Q9CWV1	CC	May have a role in the control of cell proliferation
N	Q9CWV1	CC	Appears to be involved in the activation of the prereplicative complex (pre-RC) during G(1) phase by recruiting CDC6 to the origin recognition complex (ORC)
N	Q9CWV1	CC	Binds chromatin throughout the cell cycle (By similarity).
N	Q9CWV1	DE	DNA replication licensing factor MCM8;
N	Q9CWV1	DE	Minichromosome maintenance 8;
N	Q9CWV1	DR	DNA replication
N	Q9CWV1	DR	nucleoside-triphosphatase activity
N	Q9CY25	CC	Part of the MIS12 complex which is required for normal chromosome alignment and segregation and for kinetochore formation during mitosis (By similarity).
N	Q9CY25	DE	Protein MIS12 homolog;
N	Q9CY25	DR	cell division
N	Q9CY25	DR	chromosome segregation
N	Q9CY25	DR	kinetochore assembly
N	Q9D1G3	CC	Negatively regulates N-terminal palmitoylation of SHH by HHAT/SKN.
N	Q9D1G3	DE	Glycerol uptake/transporter homolog;
N	Q9D1G3	DE	Hedgehog acyltransferase-like protein;
N	Q9D1G3	DE	Protein-cysteine N-palmitoyltransferase HHAT-like protein;
N	Q9D1G3	DR	negative regulation of N-terminal protein palmitoylation
N	Q9D1G3	DR	protein binding
N	Q9D2Q3	DE	Uncharacterized protein C10orf88 homolog;
N	Q9D2Q3	DR	identical protein binding
N	Q9D6Z1	CC	Required for 60S ribosomal subunit biogenesis (By similarity).
N	Q9D6Z1	DE	Nucleolar protein 56;
N	Q9D6Z1	DE	Nucleolar protein 5A;
N	Q9D6Z1	DR	ribosome biogenesis
N	Q9D7R7	CC	Hydrolyzes a variety of proteins.
N	Q9D7R7	DE	Pepsinogen C;
N	Q9D7R7	DR	aspartic-type endopeptidase activity
N	Q9D8X1	CC	May play a role in copper homeostasis
N	Q9D8X1	CC	Can bind one Cu(1+) per subunit (By similarity).
N	Q9D8X1	DE	Copper homeostasis protein cutC homolog;
N	Q9D8X1	DR	copper ion binding
N	Q9D8X1	DR	copper ion homeostasis
N	Q9D9F8	DE	Mirror-image polydactyly gene 1 protein homolog;
N	Q9D9Z5	CC	May be involved in ubiquitination and subsequent proteasomal degradation of target proteins
N	Q9D9Z5	CC	Component of the DDD- E2 complexes which may provide a platform for interaction with CUL4A and WD repeat proteins (By similarity).
N	Q9D9Z5	DE	DET1- and DDB1-associated protein 1;
N	Q9DA80	DE	Radial spoke head protein 3 homolog B;
N	Q9DA80	DE	Radial spoke head-like protein 2B;
N	Q9DCH2	CC	Component of ribonuclease P, a protein complex that generates mature tRNA molecules by cleaving their 5'-ends (By similarity).
N	Q9DCH2	DE	RNaseP protein p20;
N	Q9DCH2	DE	Ribonuclease P protein subunit p20;

N	Q9DCH2	DR	nucleic acid binding
N	Q9DCH2	DR	ribonuclease P activity
N	Q9DCH2	DR	tRNA processing
N	Q9EA41	CC	RNA-dependent RNA polymerase which is responsible for replication and transcription of virus segments
N	Q9EA41	CC	Binds the promoter sequence of the encapsidated viral RNA
N	Q9EA41	CC	Cleaves cellular pre-mRNA to generate primers for viral transcription (By similarity)
N	Q9EA41	CC	Displays an endonuclease activity involved in cap-stealing
N	Q9EA41	DE	Polymerase basic protein 1;
N	Q9EA41	DE	RNA-directed RNA polymerase catalytic subunit;
N	Q9EA41	DE	RNA-directed RNA polymerase subunit P1;
N	Q9EA41	DR	RNA-directed RNA polymerase activity
N	Q9EA41	DR	nucleotide binding
N	Q9EA41	DR	viral genome replication
N	Q9EP93	CC	Putative pheromone receptor implicated in the regulation of social and reproductive behavior.
N	Q9EP93	DE	Pheromone receptor VN5;
N	Q9EP93	DE	Vomeronasal receptor 5;
N	Q9EP93	DE	Vomeronasal type-1 receptor 53;
N	Q9EP93	DE	Vomeronasal type-1 receptor B3;
N	Q9EP93	DR	pheromone receptor activity
N	Q9EP93	DR	response to pheromone
N	Q9EP93	DR	sensory perception of chemical stimulus
N	Q9ESI9	CC	Factor that induces terminal differentiation of late- developing B-cells to immunoglobulin secreting cells.
N	Q9ESI9	DE	Eosinophil differentiation factor;
N	Q9ESI9	DE	Interleukin-5;
N	Q9ESI9	DE	T-cell replacing factor;
N	Q9ESI9	DR	cytokine activity
N	Q9ESI9	DR	growth factor activity
N	Q9ESI9	DR	immune response
N	Q9ESI9	DR	interleukin-5 receptor binding
N	Q9ESK4	CC	Seems to be involved in p53/TP53 activation and p53/TP53-dependent apoptotic pathways, probably by enhancing acetylation of p53/TP53
N	Q9ESK4	CC	Component of a mSin3A-like corepressor complex, which is probably involved in deacetylation of nucleosomal histones
N	Q9ESK4	CC	ING2 activity seems to be modulated by binding to phosphoinositides (PtdInsPs) (By similarity).
N	Q9ESK4	DE	Inhibitor of growth 1-like protein;
N	Q9ESK4	DE	Inhibitor of growth protein 2;
N	Q9ESK4	DR	chromatin modification
N	Q9ESK4	DR	regulation of growth
N	Q9ESK4	DR	transcription activator activity
N	Q9ESK4	DR	zinc ion binding
N	Q9ESL8	CC	Induces hepatocellular proliferation
N	Q9ESL8	CC	Has no biological effect on the heart (By similarity).
N	Q9ESL8	DE	Fibroblast growth factor 16;
N	Q9ESL8	DR	growth factor activity
N	Q9EV32	CC	Pasteurella leukotoxins are exotoxins that attack host leukocytes and especially polymorphonuclear cells, by causing cell rupture
N	Q9EV32	CC	It has also weak hemolytic activity.
N	Q9EV32	CC	The leukotoxin binds to the host LFA-1 integrin and induces a signaling cascade leading to many biological effects, including tyrosine phosphorylation of the CD18 tail, elevation of the intracellular Ca(2+) and lysis of the host cell (By similarity)
N	Q9EV32	CC	This leukotoxin is a major contributor to the pathogenesis of lung injury in ovine pneumonic pasteurellosis
N	Q9EV32	DR	calcium ion binding
N	Q9EV32	DR	hemolysis in other organism
N	Q9EXD4	DE	Uncharacterized protein MG269.1 homolog;
N	Q9EYV5	CC	Positively regulates the expression of about fifteen genes involved in acid resistance such as gadA, gadB and gadC
N	Q9EYV5	CC	Depending on the conditions (growth phase and medium), can repress gadW (By similarity)
N	Q9EYV5	CC	Negatively regulates perA expression in acidic conditions and positively regulates it in alkaline conditions.

N	Q9EYV5	DE	HTH-type transcriptional regulator gadX;
N	Q9EYV5	DR	regulation of transcription, DNA-dependent
N	Q9EYV5	DR	sequence-specific DNA binding
N	Q9EYV5	DR	sequence-specific DNA binding transcription factor activity
N	Q9F672	CC	Can catalyze the hydrolysis of ATP in the presence of single-stranded DNA, the ATP-dependent uptake of single-stranded DNA by duplex DNA, and the ATP-dependent hybridization of homologous single-stranded DNAs
N	Q9F672	CC	It interacts with LexA causing its activation and leading to its autocatalytic cleavage (By similarity).
N	Q9F672	DE	Protein RecA;
N	Q9F672	DE	Recombinase A;
N	Q9F672	DR	DNA recombination
N	Q9F672	DR	DNA-dependent ATPase activity
N	Q9F672	DR	single-stranded DNA binding
N	Q9FF66	CC	Accepts the ubiquitin from the E1 complex and catalyzes its covalent attachment to other proteins.
N	Q9FF66	DE	Ubiquitin carrier protein 22;
N	Q9FF66	DE	Ubiquitin-conjugating enzyme E2 22;
N	Q9FF66	DR	post-translational protein modification
N	Q9FF66	DR	ubiquitin-dependent protein catabolic process
N	Q9FF66	DR	ubiquitin-protein ligase activity
N	Q9FFN9	CC	Might act as an E3 ubiquitin-protein ligase, or as part of E3 complex, which accepts ubiquitin from specific E2 ubiquitin- conjugating enzymes and then transfers it to substrates (By similarity).
N	Q9FFN9	DE	ARIADNE-like protein ARI13;
N	Q9FFN9	DE	Probable E3 ubiquitin-protein ligase ARI13;
N	Q9FFN9	DE	Protein ariadne homolog 13;
N	Q9FFN9	DR	ligase activity
N	Q9FFN9	DR	zinc ion binding
N	Q9FGD6	CC	Transcription factor that binds specifically to a 5'- AA[AG]G-3' consensus core sequence (By similarity).
N	Q9FGD6	DE	Dof zinc finger protein DOF5.8;
N	Q9FGD6	DR	zinc ion binding
N	Q9FGE7	CC	Sequence-specific RNA-binding protein that regulates translation and mRNA stability by binding the 3'-UTR of target mRNAs (By similarity).
N	Q9FGE7	DE	Pumilio homolog 26;
N	Q9FGE7	DR	regulation of translation
N	Q9FKI0	CC	Cross-links actin filaments (F-actin)
N	Q9FKI0	CC	May regulate actin cytoarchitecture, cell cycle, cell division, cell elongation and cytoplasmic tractus (By similarity).
N	Q9FKI0	CC	Stabilizes and prevents F-actin depolymerization mediated by profilin
N	Q9FKI0	DE	Fimbrin-like protein 2;
N	Q9FKI0	DR	actin cytoskeleton organization
N	Q9FKI0	DR	actin filament binding
N	Q9FKI0	DR	pollen germination
N	Q9FKI0	DR	pollen tube growth
N	Q9FLT3	CC	May play a role in plant defense
N	Q9FLT3	CC	Probably has no oxalate oxidase activity even if the active site is conserved.
N	Q9FLT3	DE	Germin-like protein subfamily 3 member 4;
N	Q9FLT3	DR	manganese ion binding
N	Q9FLT3	DR	nutrient reservoir activity
N	Q9FQY8	CC	Catalyzes the conversion of caffeic acid to ferulic acid and of 5-hydroxyferulic acid to sinapic acid
N	Q9FQY8	CC	The resulting products may subsequently be converted to the corresponding alcohols that are incorporated into lignins.
N	Q9FQY8	DE	Caffeic acid 3-O-methyltransferase;
N	Q9FQY8	DE	S-adenosysl-L-methionine:caffeic acid 3-O-methyltransferase;
N	Q9FQY8	DR	caffeate O-methyltransferase activity
N	Q9FQY8	DR	lignin biosynthetic process
N	Q9FQY8	DR	protein dimerization activity
N	Q9FXI7	CC	Core component of nucleosome
N	Q9FXI7	CC	DNA accessibility is regulated via a complex set of post-translational modifications of histones, also called histone code, and nucleosome remodeling (By similarity)
N	Q9FXI7	CC	Histones thereby play a central role in transcription regulation, DNA repair, DNA replication and chromosomal stability

N	Q9FXI7	CC	Nucleosomes wrap and compact DNA into chromatin, limiting DNA accessibility to the cellular machineries which require DNA as a template
N	Q9FXI7	DE	Histone H3-like 2;
N	Q9FXI7	DE	Male gamete-specific histone H3;
N	Q9FXI7	DR	double fertilization forming a zygote and endosperm
N	Q9FXI7	DR	nucleosome assembly
N	Q9FXI7	DR	pollen sperm cell differentiation
N	Q9FY99	CC	Catalyzes the rate-limiting step of the oxidative pentose-phosphate pathway, which represents a route for the dissimilation of carbohydrates besides glycolysis
N	Q9FY99	CC	The main function of this enzyme is to provide reducing power (NADPH) and pentose phosphates for fatty acid and nucleic acid synthesis which are involved in membrane synthesis and cell division.
N	Q9FY99	DE	Glucose-6-phosphate 1-dehydrogenase 2, chloroplastic;
N	Q9FY99	DR	glucose-6-phosphate dehydrogenase activity
N	Q9FY99	DR	pentose-phosphate shunt, oxidative branch
N	Q9GPS1	DE	LYR motif-containing protein 7;
N	Q9GZU0	DE	HBV X-transactivated gene 12 protein;
N	Q9GZU0	DE	HBV XAg-transactivated protein 12;
N	Q9GZU0	DE	Uncharacterized protein C6orf62;
N	Q9GZZ1	CC	Probable catalytic component of the NAA11-NAA15 complex which displays alpha (N-terminal) acetyltransferase activity.
N	Q9GZZ1	DE	N-acetyltransferase 13;
N	Q9GZZ1	DE	N-acetyltransferase 5;
N	Q9GZZ1	DE	N-acetyltransferase san homolog;
N	Q9GZZ1	DE	N-alpha-acetyltransferase 50, NatE catalytic subunit;
N	Q9GZZ1	DR	N-acetyltransferase activity
N	Q9GZZ1	DR	N-terminal protein amino acid acetylation
N	Q9GZZ1	DR	protein binding
N	Q9H7E2	CC	Scaffolding protein that specifically recognizes and binds dimethylarginine-containing proteins
N	Q9H7E2	CC	In cytoplasm, may play a role in the assembly and/or disassembly of mRNA stress granules and in the regulation of translation of target mRNAs by binding Arg/Gly-rich motifs (GAR) in dimethylarginine-containing proteins.
N	Q9H7E2	CC	In nucleus, acts as a coactivator: recognizes and binds asymmetric dimethylation on the core histone tails associated with transcriptional activation (H3R17me2a and H4R3me2a) and recruits proteins at these arginine- methylated loci
N	Q9H7E2	DE	Tudor domain-containing protein 3;
N	Q9H7E2	DR	chromatin binding
N	Q9H7E2	DR	chromatin modification
N	Q9H7E2	DR	methylated histone residue binding
N	Q9H7E2	DR	nucleic acid binding
N	Q9H7E2	DR	transcription coactivator activity
N	Q9H7J1	CC	Acts as a glycogen-targeting subunit for PP1
N	Q9H7J1	CC	PP1 is involved in glycogen metabolism and contributes to the activation of glycogen synthase leading to an increase in glycogen synthesis.
N	Q9H7J1	DE	Protein phosphatase 1 regulatory subunit 3E;
N	Q9H7J1	DR	glycogen metabolic process
N	Q9H814	CC	A phosphoprotein adapter involved in the XPO1-mediated U snRNA export from the nucleus
N	Q9H814	CC	Binds also to telomerase RNA.
N	Q9H814	CC	Binds strongly to m7G-capped U1 and U5 small nuclear RNAs (snRNAs) in a sequence-unspecific manner and phosphorylation- independent manner (By similarity)
N	Q9H814	CC	Binds strongly to m7G-capped U3, U8 and U13 precursor snoRNAs and weakly to trimethylated (TMG)-capped U3, U8 and U13 snoRNAs
N	Q9H814	CC	Bridge components required for U snRNA export, the cap binding complex (CBC)-bound snRNA on the one hand and the GTPase Ran in its active GTP-bound form together with the export receptor XPO1 on the other
N	Q9H814	CC	Involved in the U3 snoRNA transport from nucleoplasm to Cajal bodies
N	Q9H814	CC	It is recycled back to the nucleus via the importin alpha/beta heterodimeric import receptor
N	Q9H814	CC	Its compartmentalized phosphorylation cycle may also contribute to the directionality of export
N	Q9H814	CC	Its phosphorylation in the nucleus is required for U snRNA export complex assembly and export, while its dephosphorylation in the cytoplasm causes export complex disassembly
N	Q9H814	CC	Plays also a role in the biogenesis of U3 small nucleolar RNA (snoRNA)

N	Q9H814	CC	The directionality of nuclear export is thought to be conferred by an asymmetric distribution of the GTP- and GDP-bound forms of Ran between the cytoplasm and nucleus
N	Q9H814	DE	Phosphorylated adapter RNA export protein;
N	Q9H814	DE	RNA U small nuclear RNA export adapter protein;
N	Q9H814	DR	ncRNA metabolic process
N	Q9H814	DR	protein transport
N	Q9H814	DR	snRNA export from nucleus
N	Q9H814	DR	spliceosomal snRNP assembly
N	Q9H8H3	CC	Probable methyltransferase (By similarity).
N	Q9H8H3	DE	Methyltransferase-like protein 7A;
N	Q9H8H3	DE	Protein AAM-B;
N	Q9H8H3	DR	methyltransferase activity
N	Q9H8Y8	CC	May be involved in assembly and membrane stacking of the Golgi cisternae, and in the process by which Golgi stacks reform after mitotic breakdown
N	Q9H8Y8	CC	May regulate the intracellular transport and presentation of a defined set of transmembrane proteins, such as transmembrane TGFA.
N	Q9H8Y8	DE	Golgi phosphoprotein 6;
N	Q9H8Y8	DE	Golgi reassembly-stacking protein 2;
N	Q9H8Y8	DE	Golgi reassembly-stacking protein of 55 kDa;
N	Q9H9Y6	CC	DNA-dependent RNA polymerase catalyzes the transcription of DNA into RNA using the four ribonucleoside triphosphates as substrates
N	Q9H9Y6	CC	Pol I is composed of mobile elements and RPA2 is part of the core element with the central large cleft and probably a clamp element that moves to open and close the cleft (By similarity).
N	Q9H9Y6	CC	Proposed to contribute to the polymerase catalytic activity and forms the polymerase active center together with the largest subunit
N	Q9H9Y6	CC	Second largest core component of RNA polymerase I which synthesizes ribosomal RNA precursors
N	Q9H9Y6	DE	DNA-directed RNA polymerase I 135 kDa polypeptide;
N	Q9H9Y6	DE	DNA-directed RNA polymerase I subunit RPA2;
N	Q9H9Y6	DE	RNA polymerase I subunit 2;
N	Q9H9Y6	DR	DNA-directed RNA polymerase activity
N	Q9H9Y6	DR	metal ion binding
N	Q9H9Y6	DR	protein binding
N	Q9H9Y6	DR	ribonucleoside binding
N	Q9H9Y6	DR	termination of RNA polymerase I transcription
N	Q9H9Y6	DR	transcription elongation from RNA polymerase I promoter
N	Q9H9Y6	DR	transcription initiation from RNA polymerase I promoter
N	Q9HBV2	CC	May be involved in sperm-egg fusion.
N	Q9HBV2	DE	Sperm acrosomal membrane-associated protein 32;
N	Q9HBV2	DE	Sperm acrosome membrane-associated protein 1;
N	Q9HC24	DE	Protein S1R;
N	Q9HC24	DE	Transmembrane BAX inhibitor motif-containing protein 4;
N	Q9HC24	DR	protein binding
N	Q9HCM1	DE	Uncharacterized protein C12orf35;
N	Q9HDU6	CC	Catalyzes the NADPH-dependent reduction of ketopantoate into pantoic acid (By similarity).
N	Q9HDU6	DE	KPA reductase;
N	Q9HDU6	DE	Ketopantoate reductase;
N	Q9HDU6	DE	Probable 2-dehydropantoate 2-reductase;
N	Q9HDU6	DR	2-dehydropantoate 2-reductase activity
N	Q9HDU6	DR	oxidation-reduction process
N	Q9HDU6	DR	pantothenate biosynthetic process
N	Q9HDU6	DR	pyrimidine nucleotide biosynthetic process
N	Q9HDX6	DE	Uncharacterized protein PB1A10.14;
N	Q9HE05	CC	E4 ubiquitin chain-elongation enzyme specifically involved in polyubiquitin chain assembly
N	Q9HE05	CC	Binds to cdc48 and elongates mono- and diubiquitinated ERAD substrates presented by the ufd1-npl4-cdc48 (UNC) AAA ATPase complex to a chain length of 4 to 6 ubiquitin moieties
N	Q9HE05	CC	Delivers these polyubiquitinated substrates to downstream ERAD components, which target them to the proteasome
N	Q9HE05	CC	Enhances ubiquitination at 'Lys-48', but not at 'Lys-29' of the Ub moiety (By similarity)
N	Q9HE05	DE	UB fusion protein 2;
N	Q9HE05	DE	Ubiquitin conjugation factor E4;

N	Q9HE05	DE	Ubiquitin fusion degradation protein 2;
N	Q9HE05	DR	ubiquitin-dependent protein catabolic process
N	Q9HE05	DR	ubiquitin-ubiquitin ligase activity
N	Q9HH82	DE	50S ribosomal protein L32e;
N	Q9HH82	DR	structural constituent of ribosome
N	Q9HIY5	CC	Endonuclease that removes tRNA introns
N	Q9HIY5	CC	Cleaves pre-tRNA at the 5'- and 3'-splice sites to release the intron
N	Q9HIY5	CC	Recognizes a pseudosymmetric substrate in which 2 bulged loops of 3 bases are separated by a stem of 4 bp (By similarity).
N	Q9HIY5	CC	The products are an intron and two tRNA half-molecules bearing 2',3' cyclic phosphate and 5'-OH termini
N	Q9HIY5	DE	tRNA-intron endonuclease;
N	Q9HIY5	DE	tRNA-splicing endonuclease;
N	Q9HIY5	DR	nucleic acid binding
N	Q9HIY5	DR	tRNA splicing, via endonucleolytic cleavage and ligation
N	Q9HIY5	DR	tRNA-intron endonuclease activity
N	Q9HLR8	CC	Involved in DNA double-strand break repair (DSBR)
N	Q9HLR8	CC	Rad50 provides an ATP-dependent control of mre11 by unwinding and/or repositioning DNA ends into the mre11 active site (By similarity).
N	Q9HLR8	CC	The rad50/mre11 complex possesses single-strand endonuclease activity and ATP-dependent double-strand-specific 3'-5' exonuclease activity
N	Q9HLR8	DE	DNA double-strand break repair rad50 ATPase;
N	Q9HLR8	DR	nuclease activity
N	Q9HLR8	DR	zinc ion binding
N	Q9HPG3	DE	Anthranilate phosphoribosyltransferase;
N	Q9HPG3	DR	anthranilate phosphoribosyltransferase activity
N	Q9HPG3	DR	tryptophan biosynthetic process
N	Q9HQ95	DE	UPF0212 protein VNG_1264C;
N	Q9HRI2	DE	Uncharacterized aldolase VNG_0683C;
N	Q9HRI2	DR	lyase activity
N	Q9HS70	CC	Hydrolyzes D-tyrosyl-tRNA(Tyr) into D-tyrosine and free tRNA(Tyr) (By similarity).
N	Q9HS70	DE	D-tyrosyl-tRNA(Tyr) deacylase;
N	Q9HS70	DR	hydrolase activity, acting on ester bonds
N	Q9HS70	DR	metal ion binding
N	Q9HSC0	CC	The beta subunit is responsible for the synthesis of L- tryptophan from indole and L-serine.
N	Q9HSC0	DE	Tryptophan synthase beta chain;
N	Q9HSC0	DR	pyridoxal phosphate binding
N	Q9HSC0	DR	tryptophan synthase activity
N	Q9HV61	DE	UPF0337 protein PA4738;
N	Q9I190	CC	Part of the ABC transporter complex MacAB involved in macrolide export
N	Q9I190	CC	Transmembrane domains (TMD) form a pore in the inner membrane and the ATP-binding domain (NBD) is responsible for energy generation (By similarity).
N	Q9I190	DE	Macrolide export ATP-binding/permease protein MacB;
N	Q9I190	DR	ATPase activity
N	Q9I190	DR	hydrolase activity, acting on acid anhydrides, catalyzing transmembrane movement of substances
N	Q9I190	DR	response to antibiotic
N	Q9I2W4	CC	Catalyzes both methylations at C-2 and C-7 of uroporphyrinogen III leading to precorrin-1 and precorrin-2; their oxidative esterification gives respectively factor I octamethyl ester and sirohydrochlorin (By similarity).
N	Q9I2W4	DE	Urogen III methylase;
N	Q9I2W4	DE	Uroporphyrinogen III methylase;
N	Q9I2W4	DE	Uroporphyrinogen-III C-methyltransferase;
N	Q9I2W4	DR	cobalamin biosynthetic process
N	Q9I2W4	DR	oxidation-reduction process
N	Q9I2W4	DR	uroporphyrin-III C-methyltransferase activity
N	Q9I3H9	CC	May play a role in DNA repair
N	Q9I3H9	CC	It may act with recF and recO (By similarity).
N	Q9I3H9	CC	It seems to be involved in an recBC-independent recombinational process of DNA repair
N	Q9I3H9	DE	Recombination protein recR;
N	Q9I3H9	DR	DNA recombination
N	Q9I3H9	DR	metal ion binding
N	Q9I869	CC	Kinesin family member that is involved in spindle formation and the movements of chromosomes during mitosis and meiosis

N	Q9I869	CC	Binds to microtubules and to DNA.
N	Q9I869	DE	Chromokinesin kid-A;
N	Q9I869	DE	Kinesin-like protein KIF22-A;
N	Q9I869	DR	microtubule motor activity
N	Q9I869	DR	microtubule-based movement
N	Q9I8D8	CC	Cytokine that binds to TNFRSF5
N	Q9I8D8	CC	Involved in immunoglobulin class switching (By similarity).
N	Q9I8D8	CC	Mediates B-cell proliferation in the absence of co-stimulus as well as IgE production in the presence of IL-4
N	Q9I8D8	DE	CD40 ligand, membrane form;
N	Q9I8D8	DE	CD40 ligand, soluble form;
N	Q9I8D8	DE	CD40 ligand;
N	Q9I8D8	DE	Tumor necrosis factor ligand superfamily member 5;
N	Q9I8D8	DR	B cell proliferation
N	Q9I8D8	DR	CD40 receptor binding
N	Q9I8D8	DR	cytokine activity
N	Q9I8D8	DR	inflammatory response
N	Q9I8D8	DR	platelet activation
N	Q9I8D8	DR	tumor necrosis factor receptor binding
N	Q9JHA8	DE	Protein G7c;
N	Q9JII2	DE	PRL-like protein L;
N	Q9JII2	DE	Placental prolactin-like protein L;
N	Q9JII2	DE	Prolactin-5A1;
N	Q9JII2	DR	hormone activity
N	Q9JJJ7	CC	Modulates the processing of Wnt proteins
N	Q9JJJ7	CC	Probable protein-cysteine N-palmitoyltransferase that palmitoylates Wnt family members
N	Q9JJJ7	DE	Probable protein-cysteine N-palmitoyltransferase porcupine;
N	Q9JJJ7	DR	Wnt receptor signaling pathway
N	Q9JJJ7	DR	acyltransferase activity
N	Q9JJJ7	DR	glycoprotein metabolic process
N	Q9JJJ7	DR	protein binding
N	Q9JLB5	CC	Ionotropic receptor with a probable role in the modulation of auditory stimuli
N	Q9JLB5	CC	Agonist binding may induce an extensive change in conformation that affects all subunits and leads to opening of an ion-conducting channel across the plasma membrane
N	Q9JLB5	CC	In the ear, this leads to a reduction in basilar membrane motion, altering the activity of auditory nerve fibers and reducing the range of dynamic hearing
N	Q9JLB5	CC	The channel is permeable to a range of divalent cations including calcium, the influx of which may activate a potassium current which hyperpolarizes the cell membrane
N	Q9JLB5	CC	This may protect against acoustic trauma.
N	Q9JLB5	DE	NACHR alpha-10;
N	Q9JLB5	DE	Neuronal acetylcholine receptor subunit alpha-10;
N	Q9JLB5	DE	Nicotinic acetylcholine receptor subunit alpha-10;
N	Q9JLB5	DR	calcium channel activity
N	Q9JLB5	DR	receptor activity
N	Q9JLB5	DR	synaptic transmission, cholinergic
N	Q9JLC4	DE	VPS10 domain-containing receptor SorCS1;
N	Q9JLR9	DE	HIG1 domain family member 1A;
N	Q9JLR9	DE	Hypoxia-inducible gene 1 protein;
N	Q9JLR9	DR	response to stress
N	Q9JU12	DE	UPF0301 protein NMA1550;
N	Q9JUE9	CC	Ligates lysine onto the cytidine present at position 34 of the AUA codon-specific tRNA(Ile) that contains the anticodon CAU, in an ATP-dependent manner
N	Q9JUE9	CC	Cytidine is converted to lysidine, thus changing the amino acid specificity of the tRNA from methionine to isoleucine (By similarity).
N	Q9JUE9	DE	tRNA(Ile)-2-lysyl-cytidine synthase;
N	Q9JUE9	DE	tRNA(Ile)-lysidine synthase;
N	Q9JUE9	DE	tRNA(Ile)-lysidine synthetase;
N	Q9JUE9	DR	ligase activity, forming carbon-nitrogen bonds
N	Q9JUE9	DR	tRNA processing
N	Q9JVT3	CC	Cleaves GlcNAc linked beta-1,4 to MurNAc tripeptides (By similarity).
N	Q9JVT3	DE	Beta-N-acetylhexosaminidase;
N	Q9JVT3	DE	Beta-hexosaminidase;
N	Q9JVT3	DE	N-acetyl-beta-glucosaminidase;
N	Q9JVT3	DR	beta-N-acetylhexosaminidase activity
N	Q9JVT3	DR	cell division

N	Q9JVT3	DR	cellular cell wall organization
N	Q9JVT3	DR	peptidoglycan biosynthetic process
N	Q9JVT3	DR	regulation of cell shape
N	Q9JWS8	DE	Phosphoglycerate kinase;
N	Q9JWS8	DR	phosphoglycerate kinase activity
N	Q9JXF7	CC	Condenses 4-methyl-5-(beta-hydroxyethyl)thiazole monophosphate (THZ-P) and 2-methyl-4-amino-5-hydroxymethyl pyrimidine pyrophosphate (HMP-PP) to form thiamine monophosphate (TMP) (By similarity).
N	Q9JXF7	DE	TMP pyrophosphorylase;
N	Q9JXF7	DE	Thiamine-phosphate pyrophosphorylase;
N	Q9JXF7	DE	Thiamine-phosphate synthase;
N	Q9JXF7	DR	metal ion binding
N	Q9JXF7	DR	thiamine biosynthetic process
N	Q9JXF7	DR	thiamine-phosphate diphosphorylase activity
N	Q9JXQ5	DE	Glycine--tRNA ligase beta subunit;
N	Q9JXQ5	DE	Glycyl-tRNA synthetase beta subunit;
N	Q9JXQ5	DR	arginine-tRNA ligase activity
N	Q9JXQ5	DR	arginyl-tRNA aminoacylation
N	Q9JXQ5	DR	glycine-tRNA ligase activity
N	Q9JXQ5	DR	glycyl-tRNA aminoacylation
N	Q9JXS6	CC	RNaseP catalyzes the removal of the 5'-leader sequence from pre-tRNA to produce the mature 5'-terminus
N	Q9JXS6	CC	It can also cleave other RNA substrates such as 4.5S RNA
N	Q9JXS6	CC	The protein component plays an auxiliary but essential role in vivo by binding to the 5'-leader sequence and broadening the substrate specificity of the ribozyme (By similarity)
N	Q9JXS6	DE	RNase P protein;
N	Q9JXS6	DE	RNaseP protein;
N	Q9JXS6	DE	Ribonuclease P protein component;
N	Q9JXS6	DR	ribonuclease P activity
N	Q9JXS6	DR	tRNA processing
N	Q9JYV1	CC	Nuclease that resolves Holliday junction intermediates in genetic recombination
N	Q9JYV1	CC	Cleaves the cruciform structure in supercoiled DNA by nicking to strands with the same polarity at sites symmetrically opposed at the junction in the homologous arms and leaves a 5'-terminal phosphate and a 3'-terminal hydroxyl group (By similarity)
N	Q9JYV1	DE	Crossover junction endodeoxyribonuclease ruvC;
N	Q9JYV1	DE	Holliday junction nuclease ruvC;
N	Q9JYV1	DE	Holliday junction resolvase ruvC;
N	Q9JYV1	DR	DNA recombination
N	Q9JYV1	DR	crossover junction endodeoxyribonuclease activity
N	Q9JYV1	DR	metal ion binding
N	Q9JYV1	DR	nucleic acid binding
N	Q9K0C7	CC	An essential GTPase that binds both GDP and GTP, with rapid nucleotide exchange
N	Q9K0C7	CC	Plays a role in 16S rRNA processing and 30S ribosomal subunit biogenesis and possibly also in cell cycle regulation and energy metabolism (By similarity).
N	Q9K0C7	DR	ribosome biogenesis
N	Q9K6H2	CC	F(1)F(0) ATP synthase produces ATP from ADP in the presence of a proton or sodium gradient
N	Q9K6H2	CC	During catalysis, ATP synthesis in the catalytic domain of F(1) is coupled via a rotary mechanism of the central stalk subunits to proton translocation (By similarity)
N	Q9K6H2	CC	F-type ATPases consist of two structural domains, F(1) containing the extramembraneous catalytic core and F(0) containing the membrane proton channel, linked together by a central stalk and a peripheral stalk
N	Q9K6H2	DE	ATP synthase F(1) sector subunit delta;
N	Q9K6H2	DE	ATP synthase subunit delta;
N	Q9K6H2	DE	F-ATPase subunit delta;
N	Q9K6H2	DE	F-type ATPase subunit delta;
N	Q9K6H2	DR	ATP synthesis coupled proton transport
N	Q9K6H2	DR	hydrogen ion transporting ATP synthase activity, rotational mechanism
N	Q9K846	DE	UPF0756 membrane protein BH3161;
N	Q9K8E9	CC	Catalyzes the oxidation of 3-carboxy-2-hydroxy-4- methylpentanoate (3-isopropylmalate) to 3-carboxy-4-methyl-2- oxopentanoate
N	Q9K8E9	CC	The product decarboxylates to 4-methyl-2 oxopentanoate.
N	Q9K8E9	DE	3-isopropylmalate dehydrogenase;
N	Q9K8E9	DE	Beta-IPM dehydrogenase;
N	Q9K8E9	DR	3-isopropylmalate dehydrogenase activity

N	Q9K8E9	DR	leucine biosynthetic process
N	Q9K8E9	DR	magnesium ion binding
N	Q9K8E9	DR	oxidation-reduction process
N	Q9K974	CC	May be involved in recombinational repair of damaged DNA (By similarity).
N	Q9K974	DE	DNA repair protein recN;
N	Q9K974	DE	Recombination protein N;
N	Q9K974	DR	DNA recombination
N	Q9KDP2	CC	Required for the insertion of integral membrane proteins into the membrane
N	Q9KDP2	CC	May also be involved in protein secretion processes (By similarity).
N	Q9KDP2	DE	Membrane protein oxaA 2;
N	Q9KDP2	DR	protein insertion into membrane
N	Q9KET5	CC	Catalyzes the condensation reaction of fatty acid synthesis by the addition to an acyl acceptor of two carbons from malonyl-ACP
N	Q9KET5	CC	Catalyzes the first condensation reaction which initiates fatty acid synthesis and may therefore play a role in governing the total rate of fatty acid production
N	Q9KET5	CC	Its substrate specificity determines the biosynthesis of branched- chain and/or straight-chain of fatty acids (By similarity).
N	Q9KET5	CC	Possesses both acetoacetyl-ACP synthase and acetyl transacylase activities
N	Q9KET5	DE	3-oxoacyl-[acyl-carrier-protein] synthase 3 protein 2;
N	Q9KET5	DE	3-oxoacyl-[acyl-carrier-protein] synthase III protein 2;
N	Q9KET5	DE	Beta-ketoacyl-ACP synthase III 2;
N	Q9KET5	DR	3-oxoacyl-[acyl-carrier-protein] synthase activity
N	Q9KET5	DR	beta-ketoacyl-acyl-carrier-protein synthase III activity
N	Q9KET5	DR	fatty acid biosynthetic process
N	Q9KGG0	CC	Participates in a DNA-damage check-point that is active prior to asymmetric division when DNA is damaged
N	Q9KGG0	CC	DisA forms globular foci that rapidly scan along the chromosomes during sporulation, searching for lesions
N	Q9KGG0	CC	This triggers a cellular response that culminates in a temporary block in sporulation initiation (By similarity).
N	Q9KGG0	CC	When a lesion is present, disA pauses at the lesion site
N	Q9KGG0	DE	DNA integrity scanning protein disA;
N	Q9KI14	CC	Binds to type I collagen via alpha-2(I) or alpha-1(I) chains, although its affinity for the alpha-1(I) chain is significantly higher
N	Q9KI14	CC	Involved in bacterial adherence to transcutaneous drivelines from explanted ventricular assist devices.
N	Q9KI14	DE	Serine-aspartate repeat-containing protein F;
N	Q9KI14	DR	cell adhesion
N	Q9KMA6	CC	Toxic component of a toxin-antitoxin (TA) module
N	Q9KMA6	CC	Inhibits translation by cleavage of mRNA.
N	Q9KMA6	DE	Toxin higB-2;
N	Q9KNA8	CC	Involved in repair of UV radiation-induced DNA damage
N	Q9KNA8	CC	Catalyzes the light-dependent monomerization (300-600 nm) of cyclobutyl pyrimidine dimers (in cis-syn configuration), which are formed between adjacent bases on the same DNA strand upon exposure to ultraviolet radiation.
N	Q9KNA8	DE	DNA photolyase;
N	Q9KNA8	DE	Deoxyribodipyrimidine photo-lyase;
N	Q9KNA8	DE	Photoreactivating enzyme;
N	Q9KNA8	DR	deoxyribodipyrimidine photo-lyase activity
N	Q9KNA8	DR	protein-chromophore linkage
N	Q9KRA6	CC	May conjugate Arg from its aminoacyl-tRNA to the N- termini of proteins containing an N-terminal aspartate or glutamate (Potential).
N	Q9KRA6	DE	Arginyltransferase;
N	Q9KRA6	DE	Putative arginyl-tRNA--protein transferase;
N	Q9KRA6	DE	R-transferase;
N	Q9KRA6	DR	acyltransferase activity
N	Q9KRA6	DR	arginyltransferase activity
N	Q9KRA6	DR	protein arginylation
N	Q9KTX8	CC	Probable chaperone
N	Q9KTX8	CC	Has a low intrinsic ATPase activity which is markedly stimulated by hscB (By similarity).
N	Q9KTX8	DE	Chaperone protein hscA homolog;
N	Q9KTX8	DR	ATPase activity
N	Q9KTX8	DR	iron-sulfur cluster assembly
N	Q9KTX8	DR	protein folding
N	Q9KTX8	DR	unfolded protein binding

N	Q9KVD1	CC	Catalyzes two steps in the biosynthesis of coenzyme A
N	Q9KVD1	CC	In the first step cysteine is conjugated to 4'-phosphopantothenate to form 4-phosphopantothenoylcysteine, in the latter compound is decarboxylated to form 4'-phosphopantotheine (By similarity).
N	Q9KVD1	DE	Coenzyme A biosynthesis bifunctional protein CoaBC;
N	Q9KVD1	DE	DNA/pantothenate metabolism flavoprotein;
N	Q9KVD1	DE	PPC synthetase;
N	Q9KVD1	DE	Phosphopantothenate--cysteine ligase;
N	Q9KVD1	DE	Phosphopantothenoylcysteine decarboxylase;
N	Q9KVD1	DE	Phosphopantothenoylcysteine synthase;
N	Q9KVD1	DR	coenzyme A biosynthetic process
N	Q9KVD1	DR	metal ion binding
N	Q9KVD1	DR	pantothenate catabolic process
N	Q9KVD1	DR	phosphopantothenate--cysteine ligase activity
N	Q9KVD1	DR	phosphopantothenoylcysteine decarboxylase activity
N	Q9KWZ4	CC	Catalyzes the formation of N(7)-methylguanine at position 46 (m7G46) in tRNA (By similarity).
N	Q9KWZ4	DE	tRNA (guanine-N(7)-)-methyltransferase;
N	Q9KWZ4	DE	tRNA(m7G46)-methyltransferase;
N	Q9KWZ4	DR	tRNA (guanine-N7-)-methyltransferase activity
N	Q9L0Q7	CC	Converts 4-diphosphocytidyl-2-C-methyl-D-erythritol 2- phosphate into 2-C-methyl-D-erythritol 2,4-cyclodiphosphate (MECDP) and CMP (By similarity).
N	Q9L0Q7	DE	2-C-methyl-D-erythritol 2,4-cyclodiphosphate synthase;
N	Q9L0Q7	DE	MECDP-synthase;
N	Q9L0Q7	DR	2-C-methyl-D-erythritol 2,4-cyclodiphosphate synthase activity
N	Q9L0Q7	DR	metal ion binding
N	Q9L0Q7	DR	terpenoid biosynthetic process
N	Q9L6M9	DE	Dienelactone hydrolase;
N	Q9L6M9	DE	Putative carboxymethylenebutenolidase;
N	Q9L6M9	DR	carboxymethylenebutenolidase activity
N	Q9L8R8	CC	Hydrolyzes acyl homoserine lactones with varying lengths of acyl chains, with a slight preference for substrates without 3- oxo substitution at the C3 position
N	Q9L8R8	CC	Has only residual activity towards non-acyl lactones, and no activity towards non-cyclic esters.
N	Q9L8R8	DE	AHL-lactonase;
N	Q9L8R8	DE	Acyl-homoserine lactonase;
N	Q9L8R8	DE	N-acyl homoserine lactonase;
N	Q9L8R8	DR	hydrolase activity
N	Q9L8R8	DR	metal ion binding
N	Q9LCK1	CC	DNA gyrase negatively supercoils closed circular double- stranded DNA in an ATP-dependent manner and also catalyzes the interconversion of other topological isomers of double-stranded DNA rings, including catenanes and knotted rings
N	Q9LCK1	DE	DNA gyrase subunit B;
N	Q9LCK1	DR	DNA topoisomerase (ATP-hydrolyzing) activity
N	Q9LCK1	DR	DNA topological change
N	Q9LDE3	DE	F-box/kelch-repeat protein At1g23390;
N	Q9LDY5	CC	PPIases accelerate the folding of proteins
N	Q9LDY5	CC	It catalyzes the cis-trans isomerization of proline imidic peptide bonds in oligopeptides (By similarity).
N	Q9LDY5	DE	Probable FKBP-type peptidyl-prolyl cis-trans isomerase 5, chloroplastic;
N	Q9LDY5	DR	peptidyl-prolyl cis-trans isomerase activity
N	Q9LDY5	DR	protein folding
N	Q9LEQ3	CC	Putative cyclic nucleotide-gated ion channel.
N	Q9LEQ3	DE	Cyclic nucleotide- and calmodulin-regulated ion channel 18;
N	Q9LEQ3	DE	Putative cyclic nucleotide-gated ion channel 18;
N	Q9LEQ3	DR	calmodulin binding
N	Q9LEQ3	DR	cellular calcium ion homeostasis
N	Q9LEQ3	DR	ion channel activity
N	Q9LEQ3	DR	pollen tube growth
N	Q9LFT5	CC	Controls stomatal patterning (By similarity).
N	Q9LFT5	DE	EPF-like protein 1;
N	Q9LFT5	DE	EPIDERMAL PATTERNING FACTOR-like protein 1;
N	Q9LFT5	DR	guard cell differentiation
N	Q9LHJ9	DE	Probable protein phosphatase 2C 38;
N	Q9LHJ9	DR	metal ion binding

N	Q9LHJ9	DR	protein dephosphorylation
N	Q9LHJ9	DR	protein serine/threonine phosphatase activity
N	Q9LIR2	DE	Putative F-box/FBD/LRR-repeat protein At3g23955;
N	Q9LM71	CC	PPIases accelerate the folding of proteins
N	Q9LM71	CC	It catalyzes the cis-trans isomerization of proline imidic peptide bonds in oligopeptides (By similarity).
N	Q9LM71	DE	Probable FKBP-type peptidyl-prolyl cis-trans isomerase 1, chloroplastic;
N	Q9LM71	DR	peptidyl-prolyl cis-trans isomerase activity
N	Q9LM71	DR	protein folding
N	Q9LMA1	CC	Required for the establishment of systemic acquired resistance (SAR)
N	Q9LMA1	CC	Confers a salicylic acid-dependent (SA) resistance to virulent pathogens such as P.syringae pv tomato and H.parasitica.
N	Q9LMA1	CC	Not involved in local defense mechanisms
N	Q9LMA1	DE	Probable flavin-containing monooxygenase 1;
N	Q9LMA1	DR	defense response signaling pathway, resistance gene-dependent
N	Q9LMA1	DR	defense response signaling pathway, resistance gene-independent
N	Q9LMA1	DR	defense response to bacterium
N	Q9LMA1	DR	defense response to fungus
N	Q9LMA1	DR	flavin adenine dinucleotide binding
N	Q9LMA1	DR	flavin-containing monooxygenase activity
N	Q9LMA1	DR	oxidation-reduction process
N	Q9LMA1	DR	plant-type hypersensitive response
N	Q9LMA1	DR	systemic acquired resistance
N	Q9LQN6	CC	Involved in leaf development regulation.
N	Q9LQN6	DE	Probable protein phosphatase 2C 4;
N	Q9LQN6	DE	Protein POLTERGEIST-LIKE 5;
N	Q9LQN6	DE	Protein phosphatase 2C PLL5;
N	Q9LQN6	DR	leaf development
N	Q9LQN6	DR	metal ion binding
N	Q9LQN6	DR	phosphoprotein phosphatase activity
N	Q9LQV2	CC	RNA-dependent direct polymerase involved in antiviral silencing
N	Q9LQV2	CC	Involved in cucumber mosaic virus (CMV) silencing
N	Q9LQV2	CC	Not involved in the production of siRNAs derived from a single- stranded 336-nucleotide satellite RNA of CMV.
N	Q9LQV2	CC	Required for the biogenesis of viral secondary siRNAs, process that follows the production of primary siRNAs derived from viral RNA replication
N	Q9LQV2	CC	Required for the production of some small RNAs (mainly 21 and some 22 nucleotides) derived from the crucifer-infecting tobamovirus (TMV-cg)
N	Q9LQV2	CC	Required for turnip mosaic virus (TuMV) silencing and accumulation of viral siRNAs
N	Q9LQV2	CC	Specifically targets the positive-strand of the 3 RNA genomes of CMV and preferentially amplifies the 5'-terminal siRNAs of each viral genomic RNA
N	Q9LQV2	DE	RNA-dependent RNA polymerase 1;
N	Q9LQV2	DE	RNA-directed RNA polymerase 1;
N	Q9LQV2	DR	RNA-directed RNA polymerase activity
N	Q9LQV2	DR	defense response
N	Q9LQV2	DR	gene silencing by RNA
N	Q9LQV2	DR	nucleotide binding
N	Q9LQV2	DR	positive regulation of posttranscriptional gene silencing
N	Q9LQV2	DR	response to salicylic acid stimulus
N	Q9LQV2	DR	response to virus
N	Q9LUL5	DE	Pentatricopeptide repeat-containing protein At3g14330;
N	Q9LUP9	DE	Putative F-box protein At3g17480;
N	Q9LUQ9	CC	Component of SCF(ASK-cullin-F-box) E3 ubiquitin ligase complexes, which may mediate the ubiquitination and subsequent proteasomal degradation of target proteins (By similarity).
N	Q9LUQ9	DE	F-box/kelch-repeat protein At3g16740;
N	Q9LVX0	CC	Might act as an E3 ubiquitin-protein ligase, or as part of E3 complex, which accepts ubiquitin from specific E2 ubiquitin- conjugating enzymes and then transfers it to substrates (By similarity).
N	Q9LVX0	DE	ARIADNE-like protein ARI3;
N	Q9LVX0	DE	Probable E3 ubiquitin-protein ligase ARI3;
N	Q9LVX0	DE	Protein ariadne homolog 3;
N	Q9LVX0	DR	ligase activity
N	Q9LVX0	DR	zinc ion binding
N	Q9LWV3	CC	Transcriptional activator that binds specifically to the DNA sequence 5'-[AG]CCCAC-3'

N	Q9LWV3	CC	Binding to the C-repeat/DRE element mediates high salinity- and dehydration-inducible transcription (By similarity).
N	Q9LWV3	DE	Dehydration-responsive element-binding protein 1C;
N	Q9LWV3	DE	Protein C-repeat-binding factor 1;
N	Q9LWV3	DE	Protein DREB1C;
N	Q9LWV3	DR	regulation of transcription, DNA-dependent
N	Q9LWV3	DR	response to stress
N	Q9LWV3	DR	sequence-specific DNA binding transcription factor activity
N	Q9LXI4	DE	Purple acid phosphatase 21;
N	Q9LXI4	DR	acid phosphatase activity
N	Q9LXI4	DR	metal ion binding
N	Q9M0G4	DE	Probable tyrosine decarboxylase 2;
N	Q9M0G4	DR	aromatic-L-amino-acid decarboxylase activity
N	Q9M0G4	DR	cellular amino acid metabolic process
N	Q9M0G4	DR	pyridoxal phosphate binding
N	Q9M0G4	DR	response to stress
N	Q9M0G4	DR	tyrosine decarboxylase activity
N	Q9M0U8	DE	Putative F-box/LRR-repeat protein 21;
N	Q9M1Y3	CC	Component of SCF(ASK-cullin-F-box) E3 ubiquitin ligase complexes, which may mediate the ubiquitination and subsequent proteasomal degradation of target proteins (By similarity).
N	Q9M1Y3	DE	F-box/ankyrin repeat protein SKIP35;
N	Q9M1Y3	DE	SKP1-interacting partner 35;
N	Q9M2Q4	DE	UPF0202 protein At3g57940;
N	Q9M3K7	DE	30S ribosomal protein S18, chloroplastic;
N	Q9M3K7	DR	structural constituent of ribosome
N	Q9M9U0	DE	Putative glycosyltransferase 4;
N	Q9M9U0	DR	transferase activity, transferring hexosyl groups
N	Q9M9W3	DE	Serine/threonine-protein phosphatase PP1 isozyme 9;
N	Q9M9W3	DR	metal ion binding
N	Q9M9W3	DR	phosphoprotein phosphatase activity
N	Q9MTJ3	CC	DNA-dependent RNA polymerase catalyzes the transcription of DNA into RNA using the four ribonucleoside triphosphates as substrates.
N	Q9MTJ3	DE	DNA-directed RNA polymerase subunit alpha;
N	Q9MTJ3	DE	Plastid-encoded RNA polymerase subunit alpha;
N	Q9MTJ3	DE	RNA polymerase subunit alpha;
N	Q9MTJ3	DR	DNA-directed RNA polymerase activity
N	Q9MTJ3	DR	protein dimerization activity
N	Q9MUU7	CC	No specific function has so far been attributed to this initiation factor; however, it seems to stimulate more or less all the activities of the other two initiation factors, IF-2 and IF-3
N	Q9MUU7	DE	Translation initiation factor IF-1, chloroplastic;
N	Q9MUU7	DR	translation initiation factor activity
N	Q9MYL0	CC	Receptor for obesity factor (leptin)
N	Q9MYL0	CC	Can also mediate the ERK/FOS signaling pathway (By similarity).
N	Q9MYL0	CC	Involved in the regulation of fat metabolism and, in a hematopoietic pathway, required for normal lymphopoiesis
N	Q9MYL0	CC	May play a role in reproduction
N	Q9MYL0	CC	On ligand binding, mediates signaling through JAK2/STAT3 (By similarity)
N	Q9MYL0	DE	CD295;
N	Q9MYL0	DE	Leptin receptor;
N	Q9MYL0	DE	OB receptor;
N	Q9MYL0	DR	cytokine receptor activity
N	Q9MZ06	CC	Acts as a carrier protein that release fibroblast- binding factors (FGFs) from the extracellular matrix (EM) storage and thus enhance the mitogenic activity of FGFs
N	Q9MZ06	CC	Enhances FGF2 signaling during tissue repair, angiogenesis and in tumor growth (By similarity).
N	Q9MZ06	DE	FGF-binding protein 1;
N	Q9MZ06	DE	Fibroblast growth factor-binding protein 1;
N	Q9MZ06	DR	growth factor binding
N	Q9NNW7	CC	Maintains thioredoxin in a reduced state
N	Q9NNW7	CC	Implicated in the defenses against oxidative stress
N	Q9NNW7	CC	May play a role in redox- regulated cell signaling.
N	Q9NNW7	DE	Selenoprotein Z;
N	Q9NNW7	DE	Thioredoxin reductase 2, mitochondrial;
N	Q9NNW7	DE	Thioredoxin reductase TR3;

N	Q9NNW7	DR	cell redox homeostasis
N	Q9NNW7	DR	flavin adenine dinucleotide binding
N	Q9NNW7	DR	oxidation-reduction process
N	Q9NNW7	DR	protein binding
N	Q9NNW7	DR	response to oxygen radical
N	Q9NNW7	DR	thioredoxin-disulfide reductase activity
N	Q9NP81	CC	Catalyzes the attachment of serine to tRNA(Ser)
N	Q9NP81	CC	Is also able to aminoacylate tRNA(Sec) with serine, to form the misacylated tRNA L-seryl-tRNA(Sec), which will be further converted into selenocysteinyl-tRNA(Sec) (By similarity).
N	Q9NP81	DE	Serine--tRNA ligase;
N	Q9NP81	DE	Seryl-tRNA synthetase, mitochondrial;
N	Q9NP81	DE	Seryl-tRNA(Ser/Sec) synthetase;
N	Q9NP81	DR	protein binding
N	Q9NP81	DR	serine-tRNA ligase activity
N	Q9NP81	DR	seryl-tRNA aminoacylation
N	Q9NQ75	CC	Possible docking protein which may play a role for tyrosine-kinase-based signaling related to cell adhesion
N	Q9NQ75	CC	Regulates FAK activity, focal adhesion integrity, and cell spreading.
N	Q9NQ75	DE	Cas scaffolding protein family member 4;
N	Q9NQ75	DE	HEF-like protein;
N	Q9NQ75	DE	HEF1-EFS-p130Cas-like protein;
N	Q9NQ75	DR	cell adhesion
N	Q9NQ75	DR	two-component sensor activity
N	Q9NQW1	CC	As a component of the coat protein complex II (COPII), may function in vesicle budding and cargo export from the endoplasmic reticulum.
N	Q9NQW1	DE	Protein transport protein Sec31B;
N	Q9NQW1	DE	SEC31-like protein 2;
N	Q9NQW1	DE	SEC31-related protein B;
N	Q9NQW1	DR	protein transport
N	Q9NQW1	DR	vesicle-mediated transport
N	Q9NS39	CC	Does not act on glutamate receptor or on serotonin receptor subtype 2C
N	Q9NS39	CC	Capable of binding to dsRNA but also to ssRNA.
N	Q9NS39	DE	Double-stranded RNA-specific editase B2;
N	Q9NS39	DE	RNA-dependent adenosine deaminase 3;
N	Q9NS39	DE	RNA-editing deaminase 2;
N	Q9NS39	DE	RNA-editing enzyme 2;
N	Q9NS39	DE	dsRNA adenosine deaminase B2;
N	Q9NS39	DR	adenosine deaminase activity
N	Q9NS39	DR	double-stranded RNA binding
N	Q9NS39	DR	mRNA processing
N	Q9NS39	DR	metal ion binding
N	Q9NS39	DR	single-stranded RNA binding
N	Q9NS93	DE	Seven span transmembrane protein;
N	Q9NS93	DE	Transmembrane 7 superfamily member 3;
N	Q9NVR2	CC	Component of the Integrator complex, a complex involved in the small nuclear RNAs (snRNA) U1 and U2 transcription and in their 3'-box-dependent processing
N	Q9NVR2	CC	The Integrator complex is associated with the C-terminal domain (CTD) of RNA polymerase II largest subunit (POLR2A) and is recruited to the U1 and U2 snRNAs genes.
N	Q9NVR2	DE	Integrator complex subunit 10;
N	Q9NVR2	DR	protein binding
N	Q9NVR2	DR	snRNA processing
N	Q9NXE4	CC	Catalyzes the hydrolysis of membrane sphingomyelin to form phosphorylcholine and ceramide.
N	Q9NXE4	DE	Neutral sphingomyelinase 3;
N	Q9NXE4	DE	Neutral sphingomyelinase III;
N	Q9NXE4	DE	Sphingomyelin phosphodiesterase 4;
N	Q9NXE4	DR	metal ion binding
N	Q9NXE4	DR	protein binding
N	Q9NXE4	DR	sphingomyelin catabolic process
N	Q9NXE4	DR	sphingomyelin phosphodiesterase D activity
N	Q9NXE4	DR	sphingomyelin phosphodiesterase activity
N	Q9NYZ4	CC	Putative adhesion molecule that mediates sialic-acid dependent binding to cells
N	Q9NYZ4	CC	Also binds to alpha-2,6-linked sialic acid

N	Q9NYZ4	CC	Preferentially binds to alpha-2,3- linked sialic acid
N	Q9NYZ4	CC	The sialic acid recognition site may be masked by cis interactions with sialic acids on the same cell surface.
N	Q9NYZ4	DE	CD329;
N	Q9NYZ4	DE	Sialic acid-binding Ig-like lectin 8;
N	Q9NYZ4	DE	Sialoadhesin family member 2;
N	Q9NYZ4	DR	cell adhesion
N	Q9NYZ4	DR	sugar binding
N	Q9NYZ4	DR	transmembrane receptor activity
N	Q9NZ32	DE	Actin-related protein 10;
N	Q9NZ32	DE	Actin-related protein 11;
N	Q9NZN3	CC	Plays a role in endocytic transport.
N	Q9NZN3	DE	EH domain-containing protein 3;
N	Q9NZN3	DE	PAST homolog 3;
N	Q9NZN3	DR	GTPase activity
N	Q9NZN3	DR	blood coagulation
N	Q9NZN3	DR	calcium ion binding
N	Q9NZN3	DR	endocytic recycling
N	Q9NZN3	DR	nucleic acid binding
N	Q9NZN3	DR	protein binding
N	Q9NZN3	DR	protein homooligomerization
N	Q9P203	DE	BTB/POZ domain-containing protein 7;
N	Q9P281	DE	BAH and coiled-coil domain-containing protein 1;
N	Q9P281	DE	BAH domain-containing protein 2;
N	Q9P281	DE	Bromo adjacent homology domain-containing protein 2;
N	Q9P2F5	DE	Storkhead-box protein 2;
N	Q9P2F5	DR	embryo development
N	Q9P2F5	DR	maternal placenta development
N	Q9P2K5	CC	Transcriptional repressor of the myelin basic protein gene (MBP)
N	Q9P2K5	CC	Binds to the proximal MB1 element 5'-TTGTCC-3' of the MBP promoter
N	Q9P2K5	CC	Its binding to MB1 and function are inhibited by PURA (By similarity).
N	Q9P2K5	DE	Myelin expression factor 2;
N	Q9P2K5	DR	nucleotide binding
N	Q9P373	CC	Probable cysteine protease necessary for autophagy and cytoplasm to vacuole transport (Cvt) (By similarity).
N	Q9P373	DE	Autophagy-related protein 4;
N	Q9P373	DE	Probable cysteine protease atg4;
N	Q9P373	DR	cellular response to nitrogen starvation
N	Q9P373	DR	cysteine-type peptidase activity
N	Q9P373	DR	protein transport
N	Q9P6R1	CC	Involved in cytoskeletal organization and cellular growth
N	Q9P6R1	CC	May exert its effects on the cytoskeleton directly, or indirectly via proline-binding proteins such as profilin or proteins possessing SH3 domains
N	Q9P6R1	CC	Plays a role in actin patch assembly by enhancing the ability of myo1 to stimulate actin polymerization by the Arp2/3 complex.
N	Q9P6R1	DR	actin binding
N	Q9P6R1	DR	actin cortical patch assembly
N	Q9P6R1	DR	actin nucleation
N	Q9P6R1	DR	positive regulation of actin nucleation
N	Q9P6S4	DE	Meiotic expression up-regulated protein 25;
N	Q9P7D1	DE	Uncharacterized protein P4H10.18c;
N	Q9P869	CC	This protein causes expression of glucose-repressible genes upon glucose deprivation.
N	Q9P869	CC	It interacts and has functional relationship to the protein-kinase SNF1 (By similarity).
N	Q9P869	DE	Nuclear protein SNF4;
N	Q9P869	DR	carbohydrate metabolic process
N	Q9P869	DR	catalytic activity
N	Q9PAE6	CC	Cell wall formation (By similarity).
N	Q9PAE6	DE	UDP-N-acetylenolpyruvoylglucosamine reductase;
N	Q9PAE6	DE	UDP-N-acetylmuramate dehydrogenase;
N	Q9PAE6	DR	UDP-N-acetylmuramate dehydrogenase activity
N	Q9PAE6	DR	cell division
N	Q9PAE6	DR	cellular cell wall organization
N	Q9PAE6	DR	flavin adenine dinucleotide binding
N	Q9PAE6	DR	oxidation-reduction process
N	Q9PAE6	DR	peptidoglycan biosynthetic process

N	Q9PAE6	DR	regulation of cell shape
N	Q9PB76	CC	Key enzyme in the regulation of glycerol uptake and metabolism.
N	Q9PB76	DE	ATP:glycerol 3-phosphotransferase;
N	Q9PB76	DE	Glycerokinase;
N	Q9PB76	DE	Glycerol kinase;
N	Q9PB76	DR	glycerol kinase activity
N	Q9PB76	DR	glycerol-3-phosphate metabolic process
N	Q9PC79	CC	The ruvA-ruvB complex in the presence of ATP renatures cruciform structure in supercoiled DNA with palindromic sequence, indicating that it may promote strand exchange reactions in homologous recombination
N	Q9PC79	CC	RuvAB is an helicase that mediates the Holliday junction migration by localized denaturation and reannealing (By similarity).
N	Q9PC79	DE	Holliday junction ATP-dependent DNA helicase ruvB;
N	Q9PC79	DR	DNA recombination
N	Q9PC79	DR	four-way junction helicase activity
N	Q9PDK4	CC	The beta subunit is responsible for the synthesis of L- tryptophan from indole and L-serine.
N	Q9PDK4	DE	Tryptophan synthase beta chain;
N	Q9PDK4	DR	pyridoxal phosphate binding
N	Q9PDK4	DR	tryptophan synthase activity
N	Q9PE42	CC	Involved in protein export
N	Q9PE42	CC	Acts as a chaperone by maintaining the newly synthesized protein in an open conformation (By similarity).
N	Q9PE42	DE	Trigger factor;
N	Q9PE42	DR	cell division
N	Q9PE42	DR	peptidyl-prolyl cis-trans isomerase activity
N	Q9PE42	DR	protein folding
N	Q9PE42	DR	protein transport
N	Q9PGR9	CC	Catalyzes the reversible reaction in which hydroxymethyl group from 5,10-methylenetetrahydrofolate is tranferred onto alpha-ketoisovalerate to form ketopantoate (By similarity).
N	Q9PGR9	DE	3-methyl-2-oxobutanoate hydroxymethyltransferase;
N	Q9PGR9	DE	Ketopantoate hydroxymethyltransferase;
N	Q9PGR9	DR	3-methyl-2-oxobutanoate hydroxymethyltransferase activity
N	Q9PGR9	DR	metal ion binding
N	Q9PGR9	DR	methyltransferase activity
N	Q9PGR9	DR	pantothenate biosynthetic process
N	Q9PIX9	DE	UPF0102 protein Cj0148c;
N	Q9PIX9	DR	nuclease activity
N	Q9PK27	CC	Catalyzes the specific phosphorylation of the 3-hydroxyl group of shikimic acid using ATP as a cosubstrate (By similarity).
N	Q9PK27	DE	Shikimate kinase;
N	Q9PK27	DR	aromatic amino acid family biosynthetic process
N	Q9PK27	DR	metal ion binding
N	Q9PK27	DR	shikimate kinase activity
N	Q9PKY3	CC	Converts 2C-methyl-D-erythritol 2,4-cyclodiphosphate (ME-2,4cPP) into 1-hydroxy-2-methyl-2-(E)-butenyl 4-diphosphate (By similarity).
N	Q9PKY3	DE	1-hydroxy-2-methyl-2-(E)-butenyl 4-diphosphate synthase;
N	Q9PKY3	DE	4-hydroxy-3-methylbut-2-en-1-yl diphosphate synthase;
N	Q9PKY3	DR	4 iron, 4 sulfur cluster binding
N	Q9PKY3	DR	4-hydroxy-3-methylbut-2-en-1-yl diphosphate synthase activity
N	Q9PKY3	DR	iron ion binding
N	Q9PKY3	DR	oxidation-reduction process
N	Q9PKY3	DR	terpenoid biosynthetic process
N	Q9PL45	DE	Polymorphic membrane protein G;
N	Q9PL45	DE	Probable outer membrane protein pmpG;
N	Q9PLC0	CC	Catalyzes the phosphorylation of the position 2 hydroxy group of 4-diphosphocytidyl-2C-methyl-D-erythritol (By similarity).
N	Q9PLC0	DE	4-(cytidine-5'-diphospho)-2-C-methyl-D-erythritol kinase;
N	Q9PLC0	DE	4-diphosphocytidyl-2-C-methyl-D-erythritol kinase;
N	Q9PLC0	DR	4-(cytidine 5'-diphospho)-2-C-methyl-D-erythritol kinase activity
N	Q9PLC0	DR	terpenoid biosynthetic process
N	Q9PLH1	CC	Involved in peptide bond synthesis
N	Q9PLH1	CC	Probably functions indirectly by altering the affinity of the ribosome for aminoacyl-tRNA, thus increasing their reactivity as acceptors for peptidyl transferase (By similarity).

N	Q9PLH1	CC	Stimulates efficient translation and peptide-bond synthesis on native or reconstituted 70S ribosomes in vitro
N	Q9PLH1	DE	Elongation factor P 2;
N	Q9PLH1	DR	peptide biosynthetic process
N	Q9PLH1	DR	translation elongation factor activity
N	Q9PMP8	CC	Transfers the 4'-phosphopantetheine moiety from coenzyme A to a Ser of acyl-carrier-protein (By similarity).
N	Q9PMP8	DE	4'-phosphopantetheinyl transferase AcpS;
N	Q9PMP8	DE	Holo-ACP synthase;
N	Q9PMP8	DE	Holo-[acyl-carrier-protein] synthase;
N	Q9PMP8	DR	fatty acid biosynthetic process
N	Q9PMP8	DR	holo-[acyl-carrier-protein] synthase activity
N	Q9PMP8	DR	macromolecule biosynthetic process
N	Q9PMP8	DR	magnesium ion binding
N	Q9PNL3	CC	Condenses 4-methyl-5-(beta-hydroxyethyl)thiazole monophosphate (THZ-P) and 2-methyl-4-amino-5-hydroxymethyl pyrimidine pyrophosphate (HMP-PP) to form thiamine monophosphate (TMP) (By similarity).
N	Q9PNL3	DE	TMP pyrophosphorylase;
N	Q9PNL3	DE	Thiamine-phosphate pyrophosphorylase;
N	Q9PNL3	DE	Thiamine-phosphate synthase;
N	Q9PNL3	DR	metal ion binding
N	Q9PNL3	DR	thiamine biosynthetic process
N	Q9PNL3	DR	thiamine-phosphate diphosphorylase activity
N	Q9PP06	CC	Catalyzes a salvage reaction resulting in the formation of AMP, that is energically less costly than de novo synthesis.
N	Q9PP06	DE	Adenine phosphoribosyltransferase;
N	Q9PP06	DR	adenine phosphoribosyltransferase activity
N	Q9PP06	DR	adenine salvage
N	Q9PP06	DR	purine ribonucleoside salvage
N	Q9PPT8	CC	Binds as a heterodimer with protein S6 to the central domain of the 16S rRNA, where it helps stabilize the platform of the 30S subunit (By similarity).
N	Q9PPT8	DE	30S ribosomal protein S18;
N	Q9PPT8	DR	structural constituent of ribosome
N	Q9PQQ5	CC	This protein binds specifically to 23S rRNA; its binding is stimulated by other ribosomal proteins, e.g., L4, L17, and L20
N	Q9PQQ5	CC	It is important during the early stages of 50S assembly
N	Q9PQQ5	CC	It makes multiple contacts with different domains of the 23S rRNA in the assembled 50S subunit and ribosome (By similarity).
N	Q9PQQ5	DE	50S ribosomal protein L22;
N	Q9PQQ5	DR	structural constituent of ribosome
N	Q9PR42	CC	The UvrABC repair system catalyzes the recognition and processing of DNA lesions
N	Q9PR42	CC	A damage recognition complex composed of 2 UvrA and 2 UvrB subunits scans DNA for abnormalities
N	Q9PR42	CC	UvrA is an ATPase and a DNA-binding protein
N	Q9PR42	CC	When the presence of a lesion has been verified by UvrB, the UvrA molecules dissociate (By similarity).
N	Q9PR42	DE	Excinuclease ABC subunit A;
N	Q9PR42	DE	UvrA protein;
N	Q9PR42	DE	UvrABC system protein A;
N	Q9PR42	DR	ATPase activity
N	Q9PR42	DR	excinuclease ABC activity
N	Q9PR42	DR	metal ion binding
N	Q9PR42	DR	nucleotide-excision repair
N	Q9PRA6	CC	NAD-binding protein involved in the addition of a carboxymethylaminomethyl (cmnm) group at the wobble position (U34) of certain tRNAs, forming tRNA-cmnm(5)s(2)U34 (By similarity).
N	Q9PRA6	DE	Glucose-inhibited division protein A;
N	Q9PRA6	DE	tRNA uridine 5-carboxymethylaminomethyl modification enzyme MnmG;
N	Q9PRA6	DR	flavin adenine dinucleotide binding
N	Q9PRA6	DR	tRNA wobble uridine modification
N	Q9PUI7	CC	Involved in cannabinoid-induced CNS effects
N	Q9PUI7	CC	Acts by inhibiting adenylate cyclase (By similarity).
N	Q9PUI7	DE	Cannabinoid receptor 1;
N	Q9PUI7	DR	cannabinoid receptor activity
N	Q9QSK4	DE	Putative helicase 022L;

N	Q9QSK4	DR	helicase activity
N	Q9QW30	CC	Functions as a receptor for membrane-bound ligands Jagged1, Jagged2 and Delta1 to regulate cell-fate determination
N	Q9QW30	CC	Affects the implementation of differentiation, proliferation and apoptotic programs
N	Q9QW30	CC	May play an essential role in postimplantation development, probably in some aspect of cell specification and/or differentiation (By similarity).
N	Q9QW30	CC	Upon ligand activation through the released notch intracellular domain (NICD) it forms a transcriptional activator complex with RBPJ/RBPSUH and activates genes of the enhancer of split locus
N	Q9QW30	DE	Neurogenic locus notch homolog protein 2;
N	Q9QW30	DE	Notch 2 extracellular truncation;
N	Q9QW30	DE	Notch 2 intracellular domain;
N	Q9QW30	DR	Notch signaling pathway
N	Q9QW30	DR	calcium ion binding
N	Q9QW30	DR	cell cycle arrest
N	Q9QW30	DR	cell fate determination
N	Q9QW30	DR	negative regulation of cell proliferation
N	Q9QW30	DR	organ morphogenesis
N	Q9QW30	DR	positive regulation of Ras protein signal transduction
N	Q9QW30	DR	receptor activity
N	Q9QW30	DR	regulation of developmental process
N	Q9QW30	DR	tissue regeneration
N	Q9QWM1	CC	Binds to promoters containing the sequence element 5'-AACGACCGACCTTGAG-3'
N	Q9QWM1	CC	May play a role in embryonic development (By similarity).
N	Q9QWM1	CC	Plays a role in the regulation of gene expression in liver and pancreas
N	Q9QWM1	DE	FTZ-F1 beta;
N	Q9QWM1	DE	Liver receptor homolog 1;
N	Q9QWM1	DE	Nuclear receptor subfamily 5 group A member 2;
N	Q9QWM1	DR	lipid binding
N	Q9QWM1	DR	steroid hormone receptor activity
N	Q9QWM1	DR	transcription activator activity
N	Q9QWM1	DR	zinc ion binding
N	Q9R0M6	CC	Involved in the transport of proteins between the endosomes and the trans-Golgi network (By similarity).
N	Q9R0M6	DE	Ras-related protein Rab-9A;
N	Q9R0M6	DR	GTPase activity
N	Q9R0M6	DR	protein transport
N	Q9R0M6	DR	small GTPase mediated signal transduction
N	Q9R1T7	CC	Enhances all basic T-cell responses to a foreign antigen, namely proliferation, secretion of lymphokines, up- regulation of molecules that mediate cell-cell interaction, and effective help for antibody secretion by B-cells
N	Q9R1T7	CC	Does not up- regulate the production of interleukin-2, but superinduces the synthesis of interleukin-10
N	Q9R1T7	CC	Essential both for efficient interaction between T and B-cells and for normal antibody responses to T-cell dependent antigens
N	Q9R1T7	CC	Plays a critical role in CD40-mediated class switching of immunoglobin isotypes (By similarity).
N	Q9R1T7	CC	Prevents the apoptosis of pre- activated T-cells
N	Q9R1T7	DE	Activation-inducible lymphocyte immunomediatory molecule;
N	Q9R1T7	DE	CD278;
N	Q9R1T7	DE	Inducible T-cell costimulator;
N	Q9R1T7	DR	T cell costimulation
N	Q9R1T7	DR	T cell tolerance induction
N	Q9R1T7	DR	cell-cell adhesion
N	Q9R233	CC	Involved in the association of MHC class I with transporter associated with antigen processing (TAP) and in the assembly of MHC class I with peptide (peptide loading)
N	Q9R233	DE	TAP-associated protein;
N	Q9R233	DE	TAP-binding protein;
N	Q9R233	DR	antigen processing and presentation of endogenous peptide antigen via MHC class I
N	Q9R233	DR	antigen processing and presentation of exogenous peptide antigen via MHC class I, TAP-dependent
N	Q9R233	DR	defense response
N	Q9R233	DR	peptide antigen stabilization
N	Q9RDV3	CC	Specifically methylates guanosine-37 in various tRNAs (By similarity).
N	Q9RDV3	DE	M1G-methyltransferase;

N	Q9RDV3	DE	tRNA (guanine-N(1)-)-methyltransferase;
N	Q9RDV3	DE	tRNA [GM37] methyltransferase;
N	Q9RDV3	DR	tRNA (guanine-N1-)-methyltransferase activity
N	Q9RFF5	DE	Urea amidohydrolase subunit gamma;
N	Q9RFF5	DE	Urease subunit gamma;
N	Q9RFF5	DR	nickel ion binding
N	Q9RFF5	DR	urea metabolic process
N	Q9RFF5	DR	urease activity
N	Q9RHW9	DE	FGAM synthase II;
N	Q9RHW9	DE	Phosphoribosylformylglycinamidine synthase 2;
N	Q9RHW9	DE	Phosphoribosylformylglycinamidine synthase II;
N	Q9RHW9	DR	'de novo' IMP biosynthetic process
N	Q9RHW9	DR	phosphoribosylformylglycinamidine synthase activity
N	Q9RJM5	DE	NH(3)-dependent NAD(+) synthetase;
N	Q9RJM5	DR	NAD biosynthetic process
N	Q9RJM5	DR	NAD+ synthase (glutamine-hydrolyzing) activity
N	Q9RJM5	DR	NAD+ synthase activity
N	Q9RL99	CC	Catalyzes the formation of S-adenosylmethionine from methionine and ATP
N	Q9RL99	CC	The overall synthetic reaction is composed of two sequential steps, AdoMet formation and the subsequent tripolyphosphate hydrolysis which occurs prior to release of AdoMet from the enzyme (By similarity).
N	Q9RL99	DE	AdoMet synthase;
N	Q9RL99	DE	Methionine adenosyltransferase;
N	Q9RL99	DE	S-adenosylmethionine synthase;
N	Q9RL99	DR	metal ion binding
N	Q9RL99	DR	methionine adenosyltransferase activity
N	Q9RL99	DR	one-carbon metabolic process
N	Q9RNL5	CC	Required for the insertion of integral membrane proteins into the membrane
N	Q9RNL5	CC	Involved in integration of membrane proteins that insert dependently and independently of the Sec translocase complex (By similarity).
N	Q9RNL5	CC	Probably plays an essential role in the integration of proteins of the respiratory chain complexes
N	Q9RNL5	DE	Inner membrane protein oxaA;
N	Q9RNL5	DR	protein insertion into membrane
N	Q9RQF8	CC	Catalyzes a salvage reaction resulting in the formation of AMP, that is energically less costly than de novo synthesis.
N	Q9RQF8	DE	Adenine phosphoribosyltransferase;
N	Q9RQF8	DR	adenine phosphoribosyltransferase activity
N	Q9RQF8	DR	adenine salvage
N	Q9RQF8	DR	purine ribonucleoside salvage
N	Q9RR89	CC	Catalyzes the phosphorylation of the position 2 hydroxy group of 4-diphosphocytidyl-2C-methyl-D-erythritol (By similarity).
N	Q9RR89	DE	4-(cytidine-5'-diphospho)-2-C-methyl-D-erythritol kinase;
N	Q9RR89	DE	4-diphosphocytidyl-2-C-methyl-D-erythritol kinase;
N	Q9RR89	DR	4-(cytidine 5'-diphospho)-2-C-methyl-D-erythritol kinase activity
N	Q9RR89	DR	terpenoid biosynthetic process
N	Q9RR92	DE	Acetate kinase;
N	Q9RR92	DE	Acetokinase;
N	Q9RR92	DR	acetate kinase activity
N	Q9RR92	DR	organic acid metabolic process
N	Q9RSL3	CC	It is located near the subunit interface in the base of the L7/L12 stalk, and near the tRNA binding site of the peptidyltransferase center (By similarity)
N	Q9RSL3	CC	This protein binds to the 23S rRNA, and is important in its secondary structure.
N	Q9RSL3	DE	50S ribosomal protein L6;
N	Q9RSL3	DR	structural constituent of ribosome
N	Q9RT62	DE	DegV domain-containing protein DR_1903;
N	Q9RVW0	CC	DNA-dependent RNA polymerase catalyzes the transcription of DNA into RNA using the four ribonucleoside triphosphates as substrates.
N	Q9RVW0	DE	DNA-directed RNA polymerase subunit beta';
N	Q9RVW0	DE	RNA polymerase subunit beta';
N	Q9RVW0	DE	RNAP subunit beta';
N	Q9RVW0	DE	Transcriptase subunit beta';
N	Q9RVW0	DR	DNA-directed RNA polymerase activity
N	Q9RYG9	DE	Aldehyde dehydrogenase;
N	Q9RYG9	DR	aldehyde dehydrogenase (NAD) activity

N	Q9RYG9	DR	oxidation-reduction process
N	Q9S0S3	CC	Exhibits lactonase activity
N	Q9S0S3	CC	Acts in cells with perturbed membrane integrity and is possibly related to the membrane homeostasis
N	Q9S0S3	CC	Contributes to bacitracin resistance (By similarity).
N	Q9S0S3	DE	Lactonase drp35;
N	Q9S0S3	DR	hydrolase activity
N	Q9S0S3	DR	metal ion binding
N	Q9S0S3	DR	response to antibiotic
N	Q9S597	CC	Promotes colloidosmotic lysis by binding to the midgut epithelial cells of insects.
N	Q9S597	DE	94 kDa crystal protein;
N	Q9S597	DE	Crystaline entomocidal protoxin;
N	Q9S597	DE	Insecticidal delta-endotoxin CryXXVIIA(a);
N	Q9S597	DE	Pesticidal crystal protein cry27Aa;
N	Q9S597	DR	defense response
N	Q9S597	DR	receptor binding
N	Q9S597	DR	sporulation resulting in formation of a cellular spore
N	Q9SIA1	DE	MATE efflux family protein 8;
N	Q9SIA1	DE	Protein DETOXIFICATION 1-like 4;
N	Q9SIA1	DE	Protein DTX5;
N	Q9SIA1	DR	antiporter activity
N	Q9SIA1	DR	drug transmembrane transporter activity
N	Q9SJF0	CC	Phosphatase involved in elongation process, probably by acting as a regulator of brassinolide signaling.
N	Q9SJF0	DE	BSU1-like protein 2;
N	Q9SJF0	DE	Serine/threonine-protein phosphatase BSL2;
N	Q9SJF0	DR	iron ion binding
N	Q9SJF0	DR	manganese ion binding
N	Q9SJF0	DR	phosphoprotein phosphatase activity
N	Q9SPL5	CC	Antimicrobial peptides 2b, 2c and 2d have antibacterial and antifungal activity against a range of species (By similarity).
N	Q9SPL5	DE	Antimicrobial peptide 2a;
N	Q9SPL5	DE	Antimicrobial peptide 2b;
N	Q9SPL5	DE	Antimicrobial peptide 2c-1;
N	Q9SPL5	DE	Antimicrobial peptide 2c-2;
N	Q9SPL5	DE	Antimicrobial peptide 2c-3;
N	Q9SPL5	DE	Antimicrobial peptide 2d;
N	Q9SPL5	DE	MiAMP2c-1;
N	Q9SPL5	DE	MiAMP2c-2;
N	Q9SPL5	DE	MiAMP2c-3;
N	Q9SPL5	DE	Vicilin-like antimicrobial peptides 2-1;
N	Q9SPL5	DR	defense response to bacterium
N	Q9SPL5	DR	defense response to fungus
N	Q9SPL5	DR	killing of cells of other organism
N	Q9SPL5	DR	nutrient reservoir activity
N	Q9SSD1	CC	May participate in the initiation of asymmetric divisions that form stomata by sensing positional cues during epidermal development.
N	Q9SSD1	DE	Protein TOO MANY MOUTHS;
N	Q9SSD1	DE	Receptor-like protein 17;
N	Q9SSD1	DR	asymmetric cell division
N	Q9SSD1	DR	protein binding
N	Q9SSD1	DR	response to abscisic acid stimulus
N	Q9STL2	DE	Cytochrome P450 71A21;
N	Q9STL2	DR	electron carrier activity
N	Q9STL2	DR	monooxygenase activity
N	Q9STL2	DR	oxidation-reduction process
N	Q9SUE7	CC	Histone methyltransferase (By similarity).
N	Q9SUE7	DE	Histone-lysine N-methyltransferase ATX4;
N	Q9SUE7	DE	Protein SET DOMAIN GROUP 16;
N	Q9SUE7	DE	TRX-homolog protein 4;
N	Q9SUE7	DE	Trithorax 4;
N	Q9SUE7	DE	Trithorax-homolog protein 4;
N	Q9SUE7	DR	histone-lysine N-methyltransferase activity
N	Q9SUE7	DR	zinc ion binding

N	Q9SUR9	CC	Functions in R gene-mediated resistance, but participates in a lower extent than SGT1B to RPP5-mediated resistance
N	Q9SUR9	CC	Not required for RPM1, RPS2, RPS4 and RPS5-mediated resistance
N	Q9SUR9	CC	Probably required for SCF-mediated ubiquitination, by coupling HSP90 to SCF complex for ubiquitination of HSP90 client proteins.
N	Q9SUR9	DE	Protein SGT1 homolog A;
N	Q9SUR9	DE	Suppressor of G2 allele of SKP1 homolog A;
N	Q9SUR9	DR	cellular response to auxin stimulus
N	Q9SUR9	DR	innate immune response
N	Q9SUR9	DR	protein binding
N	Q9SUR9	DR	regulation of defense response to fungus, incompatible interaction
N	Q9SUT0	DE	Probable cysteine proteinase At4g11310;
N	Q9SUT0	DR	cysteine-type endopeptidase activity
N	Q9T9J5	CC	Component of the ubiquinol-cytochrome c reductase complex (complex III or cytochrome b-c1 complex), which is a respiratory chain that generates an electrochemical potential coupled to ATP synthesis (By similarity).
N	Q9T9J5	DE	Complex III subunit 3;
N	Q9T9J5	DE	Complex III subunit III;
N	Q9T9J5	DE	Cytochrome b-c1 complex subunit 3;
N	Q9T9J5	DE	Cytochrome b;
N	Q9T9J5	DE	Ubiquinol-cytochrome-c reductase complex cytochrome b subunit;
N	Q9T9J5	DR	electron carrier activity
N	Q9T9J5	DR	metal ion binding
N	Q9T9J5	DR	oxidoreductase activity
N	Q9T9J5	DR	respiratory electron transport chain
N	Q9TDK9	CC	Component of the ubiquinol-cytochrome c reductase complex (complex III or cytochrome b-c1 complex), which is a respiratory chain that generates an electrochemical potential coupled to ATP synthesis (By similarity).
N	Q9TDK9	DE	Complex III subunit 3;
N	Q9TDK9	DE	Complex III subunit III;
N	Q9TDK9	DE	Cytochrome b-c1 complex subunit 3;
N	Q9TDK9	DE	Cytochrome b;
N	Q9TDK9	DE	Ubiquinol-cytochrome-c reductase complex cytochrome b subunit;
N	Q9TDK9	DR	electron carrier activity
N	Q9TDK9	DR	metal ion binding
N	Q9TDK9	DR	oxidoreductase activity
N	Q9TDK9	DR	respiratory electron transport chain
N	Q9TF37	CC	Component of the ubiquinol-cytochrome c reductase complex (complex III or cytochrome b-c1 complex), which is a respiratory chain that generates an electrochemical potential coupled to ATP synthesis (By similarity).
N	Q9TF37	DE	Complex III subunit 3;
N	Q9TF37	DE	Complex III subunit III;
N	Q9TF37	DE	Cytochrome b-c1 complex subunit 3;
N	Q9TF37	DE	Cytochrome b;
N	Q9TF37	DE	Ubiquinol-cytochrome-c reductase complex cytochrome b subunit;
N	Q9TF37	DR	electron carrier activity
N	Q9TF37	DR	metal ion binding
N	Q9TF37	DR	oxidoreductase activity
N	Q9TF37	DR	respiratory electron transport chain
N	Q9TL01	CC	Component of the cytochrome b6-f complex, which mediates electron transfer between photosystem II (PSII) and photosystem I (PSI), cyclic electron flow around PSI, and state transitions (By similarity).
N	Q9TL01	DE	Cytochrome b6-f complex subunit 8;
N	Q9TL01	DE	Cytochrome b6-f complex subunit VIII;
N	Q9TL01	DE	Cytochrome b6-f complex subunit petN;
N	Q9TL01	DR	cytochrome complex assembly
N	Q9TL01	DR	electron transport chain
N	Q9TL01	DR	electron transporter, transferring electrons within cytochrome b6/f complex of photosystem II activity
N	Q9TL01	DR	photosynthesis
N	Q9TLQ8	CC	IGPS catalyzes the conversion of PRFAR and glutamine to IGP, AICAR and glutamate
N	Q9TLQ8	CC	The hisH subunit provides the glutamine amidotransferase activity that produces the ammonia necessary to hisF for the synthesis of IGP and AICAR (By similarity).
N	Q9TLQ8	DE	IGP synthase glutamine amidotransferase subunit;
N	Q9TLQ8	DE	IGP synthase subunit hisH;

N	Q9TLQ8	DE	IGPS subunit hisH;
N	Q9TLQ8	DE	ImGP synthase subunit hisH;
N	Q9TLQ8	DE	Imidazole glycerol phosphate synthase subunit hisH;
N	Q9TLQ8	DR	glutamine metabolic process
N	Q9TLQ8	DR	histidine biosynthetic process
N	Q9TLQ8	DR	transferase activity, transferring pentosyl groups
N	Q9TLT6	CC	Protein S19 forms a complex with S13 that binds strongly to the 16S ribosomal RNA (By similarity).
N	Q9TLT6	DE	30S ribosomal protein S19, chloroplastic;
N	Q9TLT6	DR	structural constituent of ribosome
N	Q9TLT8	DE	30S ribosomal protein S3, chloroplastic;
N	Q9TLT8	DR	structural constituent of ribosome
N	Q9TM07	CC	Possible role in chlorophyll and/or carotenoid binding.
N	Q9TM07	DE	Uncharacterized protein ycf17;
N	Q9TM07	DR	chlorophyll binding
N	Q9TM07	DR	protein-chromophore linkage
N	Q9TUI4	CC	Pore-forming (alpha) subunit of voltage-gated inwardly rectifying potassium channel
N	Q9TUI4	CC	Channel properties are modulated by cAMP and subunit assembly
N	Q9TUI4	CC	Mediates the rapidly activating component of the delayed rectifying potassium current in heart (IKr) (By similarity).
N	Q9TUI4	DE	Eag-related protein 1;
N	Q9TUI4	DE	Ether-a-go-go-related gene potassium channel 1;
N	Q9TUI4	DE	Ether-a-go-go-related protein 1;
N	Q9TUI4	DE	Potassium voltage-gated channel subfamily H member 2;
N	Q9TUI4	DE	Voltage-gated potassium channel subunit Kv11.1;
N	Q9TUI4	DR	voltage-gated potassium channel activity
N	Q9TVD0	CC	Important role in determination of the surface properties of the casein micelles (By similarity).
N	Q9TVD0	DE	Beta-casein;
N	Q9TVD0	DR	transporter activity
N	Q9TVU5	CC	Protein transport
N	Q9TVU5	CC	Probably involved in vesicular traffic from ER to Golgi (By similarity).
N	Q9TVU5	DE	Ras-related protein Rab-1;
N	Q9TVU5	DE	Small GTP-binding protein rab1;
N	Q9TVU5	DR	protein transport
N	Q9TVU5	DR	small GTPase mediated signal transduction
N	Q9TXD8	CC	Peptide isomerase that inverts the chirality at the Ser- 81 of omega-Aga IVB
N	Q9TXD8	CC	Acts cofactor-independently.
N	Q9TXD8	DE	Venom peptide isomerase heavy chain;
N	Q9TXD8	DR	isomerase activity
N	Q9TXD8	DR	serine-type endopeptidase activity
N	Q9U5Z8	CC	Acts as a regulatory subunit of the 26 proteasome which is involved in the ATP-dependent degradation of ubiquitinated proteins (By similarity).
N	Q9U5Z8	DE	26S proteasome regulatory subunit RPN3;
N	Q9U5Z8	DE	26S proteasome subunit S3;
N	Q9U5Z8	DE	Diphenol oxidase A2 component;
N	Q9U5Z8	DE	Probable 26S proteasome non-ATPase regulatory subunit 3;
N	Q9U5Z8	DR	enzyme regulator activity
N	Q9U5Z8	DR	regulation of protein catabolic process
N	Q9U8Q4	CC	Catalyzes the ADP ribosylation of double-stranded DNA by targeting the N2 amino group of dG residues
N	Q9U8Q4	CC	Induces apoptosis in a range of human cell lines.
N	Q9U8Q4	DE	NAD--DNA ADP-ribosyltransferase;
N	Q9U8Q4	DR	DNA ADP-ribosylation
N	Q9U8Q4	DR	NAD DNA ADP-ribosyltransferase activity
N	Q9U8Q4	DR	induction of apoptosis
N	Q9UBM7	CC	Production of cholesterol by reduction of C7-C8 double bond of 7-dehydrocholesterol (7-DHC).
N	Q9UBM7	DE	7-DHC reductase;
N	Q9UBM7	DE	7-dehydrocholesterol reductase;
N	Q9UBM7	DE	Putative sterol reductase SR-2;
N	Q9UBM7	DE	Sterol Delta(7)-reductase;
N	Q9UBM7	DR	7-dehydrocholesterol reductase activity
N	Q9UBM7	DR	cholesterol biosynthetic process
N	Q9UBM7	DR	oxidation-reduction process

N	Q9UBM7	DR	protein binding
N	Q9UBQ5	CC	Component of the eukaryotic translation initiation factor 3 (eIF-3) complex, which is required for several steps in the initiation of protein synthesis
N	Q9UBQ5	CC	The eIF-3 complex associates with the 40S ribosome and facilitates the recruitment of eIF-1, eIF-1A, eIF-2:GTP:methionyl-tRNAi and eIF-5 to form the 43S preinitiation complex (43S PIC)
N	Q9UBQ5	CC	The eIF-3 complex is also required for disassembly and recycling of posttermination ribosomal complexes and subsequently prevents premature joining of the 40S and 60S ribosomal subunits prior to initiation.
N	Q9UBQ5	CC	The eIF-3 complex stimulates mRNA recruitment to the 43S PIC and scanning of the mRNA for AUG recognition
N	Q9UBQ5	DE	Eukaryotic translation initiation factor 3 subunit 12;
N	Q9UBQ5	DE	Eukaryotic translation initiation factor 3 subunit K;
N	Q9UBQ5	DE	Muscle-specific gene M9 protein;
N	Q9UBQ5	DR	protein binding
N	Q9UBQ5	DR	regulation of translational initiation
N	Q9UBQ5	DR	ribosome binding
N	Q9UBQ5	DR	translation initiation factor activity
N	Q9UGU5	CC	Negatively regulates Wnt/beta-catenin signaling during development (By similarity)
N	Q9UGU5	DE	HMG box-containing protein 4;
N	Q9UGU5	DE	HMG domain-containing protein 4;
N	Q9UGU5	DE	High mobility group protein 2-like 1;
N	Q9UGU5	DE	Protein HMGBCG;
N	Q9UGU5	DR	Wnt receptor signaling pathway
N	Q9UGU5	DR	endosome to lysosome transport
N	Q9UGU5	DR	negative regulation of Wnt receptor signaling pathway
N	Q9UHI8	CC	Cleaves aggrecan, a cartilage proteoglycan, and may be involved in its turnover (By similarity)
N	Q9UHI8	CC	Active metalloprotease, which may be associated with various inflammatory processes as well as development of cancer cachexia
N	Q9UHI8	CC	Has angiogenic inhibitor activity
N	Q9UHI8	CC	May play a critical role in follicular rupture.
N	Q9UHI8	DE	A disintegrin and metalloproteinase with thrombospondin motifs 1;
N	Q9UHI8	DR	heparin binding
N	Q9UHI8	DR	integrin-mediated signaling pathway
N	Q9UHI8	DR	negative regulation of cell proliferation
N	Q9UHI8	DR	zinc ion binding
N	Q9UHX3	CC	Receptor probably involved in cell attachment.
N	Q9UHX3	DE	CD312;
N	Q9UHX3	DE	EGF-like module receptor 2;
N	Q9UHX3	DE	EGF-like module-containing mucin-like hormone receptor-like 2;
N	Q9UHX3	DR	G-protein coupled receptor activity
N	Q9UHX3	DR	calcium ion binding
N	Q9UHX3	DR	cell adhesion
N	Q9UHX3	DR	neuropeptide signaling pathway
N	Q9UKC9	CC	Probably recognizes and binds to some phosphorylated proteins and promotes their ubiquitination and degradation
N	Q9UKC9	CC	Binds to hepatitis C virus non-structural protein 5A (NS5A) in a reaction crucial for hepatitis C virus RNA replication.
N	Q9UKC9	DE	F-box and leucine-rich repeat protein 2;
N	Q9UKC9	DE	F-box protein FBL2/FBL3;
N	Q9UKC9	DE	F-box/LRR-repeat protein 2;
N	Q9UKC9	DR	interspecies interaction between organisms
N	Q9UKC9	DR	ubiquitin-protein ligase activity
N	Q9ULC3	DE	Ras-related protein Rab-23;
N	Q9ULC3	DR	protein transport
N	Q9ULC3	DR	small GTPase mediated signal transduction
N	Q9UPR6	DE	Zinc finger RNA-binding protein 2;
N	Q9UPR6	DR	nucleic acid binding
N	Q9UPR6	DR	zinc ion binding
N	Q9UQ88	CC	Appears to play multiple roles in cell cycle progression, cytokinesis and apoptosis
N	Q9UQ88	CC	The p110 isoforms have been suggested to be involved in pre-mRNA splicing, potentially by phosphorylating the splicing protein SFRS7
N	Q9UQ88	CC	The p58 isoform may act as a negative regulator of normal cell cycle progression.
N	Q9UQ88	DE	Cell division cycle 2-like protein kinase 2;

N	Q9UQ88	DE	Cell division protein kinase 11A;
N	Q9UQ88	DE	Cyclin-dependent kinase 11A;
N	Q9UQ88	DE	Galactosyltransferase-associated protein kinase p58/GTA;
N	Q9UQ88	DE	PITSLRE serine/threonine-protein kinase CDC2L2;
N	Q9UQ88	DR	cyclin-dependent protein kinase activity
N	Q9UQ88	DR	protein binding
N	Q9UQ88	DR	protein phosphorylation
N	Q9UQ88	DR	regulation of cell growth
N	Q9UQ88	DR	regulation of mRNA processing
N	Q9UQ88	DR	regulation of transcription, DNA-dependent
N	Q9US09	CC	Involved in vacuolar transport and vacuole pH homeostasis
N	Q9US09	CC	Also required for cytokinesis.
N	Q9US09	DE	Protein btn1;
N	Q9US09	DR	actin cortical patch localization
N	Q9US09	DR	amino acid transport
N	Q9US09	DR	cellular protein localization
N	Q9US09	DR	cellular response to heat
N	Q9US09	DR	late endosome to vacuole transport
N	Q9US09	DR	membrane raft distribution
N	Q9US09	DR	regulation of cell cycle cytokinesis
N	Q9US09	DR	vacuolar acidification
N	Q9US09	DR	vacuole organization
N	Q9UT27	CC	Involved in cell wall biogenesis
N	Q9UT27	CC	Has a role in the addition of Gal-beta1,3 moieties to galactomannans and their subsequent pyruvylation.
N	Q9UT27	DE	PvGal synthesis protein 1;
N	Q9UT27	DE	Pyruvyl transferase 1;
N	Q9UT27	DE	Pyruvylated Gal-beta-1,3-epitope synthesis protein 1;
N	Q9UT27	DR	4,6-pyruvylated galactose residue biosynthetic process
N	Q9UT27	DR	cellular cell wall organization
N	Q9UT27	DR	fungal-type cell wall biogenesis
N	Q9UT27	DR	pyruvyltransferase activity
N	Q9UTB5	CC	May be involved in the regulation of phospholipid biosynthesis and interorganelle trafficking of phosphatidylserine (By similarity).
N	Q9UTB5	DE	Phosphatidylserine decarboxylase 1 alpha chain;
N	Q9UTB5	DE	Phosphatidylserine decarboxylase 1 beta chain;
N	Q9UTB5	DE	Phosphatidylserine decarboxylase proenzyme 1, mitochondrial;
N	Q9UTB5	DR	phosphatidylethanolamine biosynthetic process
N	Q9UTB5	DR	phosphatidylserine decarboxylase activity
N	Q9UTB5	DR	regulation of cell cycle cytokinesis
N	Q9UTB5	DR	regulation of cell shape
N	Q9UU82	DE	Probable sterol O-acyltransferase 2;
N	Q9UU82	DE	Sterol-ester synthase 2;
N	Q9UU82	DR	cholesterol O-acyltransferase activity
N	Q9UUD8	DE	Uncharacterized peptidase C18A7.01;
N	Q9UUD8	DR	cellular process
N	Q9UUD8	DR	metal ion binding
N	Q9UUD8	DR	metalloexopeptidase activity
N	Q9UUF3	DE	Probable tyrosine-protein phosphatase C17A3.03c;
N	Q9UUF3	DR	protein tyrosine phosphatase activity
N	Q9UUF3	DR	signal transduction
N	Q9UWW8	CC	Produces ATP from ADP in the presence of a proton gradient across the membrane
N	Q9UWW8	CC	The archaeal beta chain is a regulatory subunit.
N	Q9UWW8	DE	V-ATPase subunit B;
N	Q9UWW8	DE	V-type ATP synthase beta chain;
N	Q9UWW8	DR	ATP hydrolysis coupled proton transport
N	Q9UWW8	DR	ATP synthesis coupled proton transport
N	Q9UWW8	DR	hydrogen ion transporting ATP synthase activity, rotational mechanism
N	Q9UWW8	DR	proton-transporting ATPase activity, rotational mechanism
N	Q9UWX9	DE	Acetohydroxy-acid isomeroreductase 1;
N	Q9UWX9	DE	Alpha-keto-beta-hydroxylacil reductoisomerase 1;
N	Q9UWX9	DE	Ketol-acid reductoisomerase 1;
N	Q9UWX9	DR	branched chain family amino acid biosynthetic process
N	Q9UWX9	DR	coenzyme binding
N	Q9UWX9	DR	ketol-acid reductoisomerase activity

N	Q9UWX9	DR	oxidation-reduction process
N	Q9V1F8	DE	Intracellular protease 1;
N	Q9V1F8	DE	Intracellular protease I;
N	Q9V1F8	DR	hydrolase activity, acting on glycosyl bonds
N	Q9V1F8	DR	peptidase activity
N	Q9V3J8	CC	Essential for viability.
N	Q9V3J8	DE	Protein will die slowly;
N	Q9V629	CC	Involved in the rasiRNA pathway and most likely acts downstream of siRNA biogenesis.
N	Q9V629	DE	Protein cutoff;
N	Q9V629	DR	dorsal/ventral pattern formation
N	Q9V629	DR	germarium-derived female germ-line cyst formation
N	Q9V629	DR	karyosome formation
N	Q9V629	DR	ovarian fusome organization
N	Q9V629	DR	production of siRNA involved in RNA interference
N	Q9V629	DR	protein binding
N	Q9V6H2	CC	Complexes with Or83b to form odorant-sensing units, providing sensitive and prolonged odorant signaling and calcium permeability
N	Q9V6H2	CC	They are necessary and sufficient to promote functional reconstitution of odor-evoked signaling in sensory neurons that normally respond only to carbon dioxide (By similarity)
N	Q9V6H2	DE	Odorant receptor 49b;
N	Q9V6H2	DR	odorant binding
N	Q9V6H2	DR	olfactory receptor activity
N	Q9V6H2	DR	sensory perception of smell
N	Q9V8K2	CC	Component of the exocyst complex involved in the docking of exocytic vesicles with fusion sites on the plasma membrane.
N	Q9V8K2	DE	Exocyst complex component 3;
N	Q9V8K2	DE	Exocyst complex component Sec6;
N	Q9V8K2	DR	border follicle cell migration
N	Q9V8K2	DR	instar larval development
N	Q9V8K2	DR	photoreceptor cell maintenance
N	Q9V8K2	DR	photoreceptor cell morphogenesis
N	Q9V8K2	DR	plasma membrane organization
N	Q9V8K2	DR	positive regulation of exocytosis
N	Q9V8K2	DR	protein binding
N	Q9V8K2	DR	protein transport
N	Q9V8K2	DR	rhabdomere membrane biogenesis
N	Q9V8K2	DR	synaptic vesicle docking involved in exocytosis
N	Q9V8K2	DR	synaptic vesicle targeting
N	Q9VD51	CC	Probable RNA-dependent helicase
N	Q9VD51	CC	Functions in cell growth and proliferation
N	Q9VD51	CC	May have a role in ribosome biogenesis and, consequently, in protein biosynthesis
N	Q9VD51	DE	Probable ATP-dependent RNA helicase pitchoune;
N	Q9VD51	DR	ATP-dependent helicase activity
N	Q9VEJ2	CC	Involved in nucleolar processing of pre-18S ribosomal RNA
N	Q9VEJ2	CC	Has a role in the nuclear export of 40S pre-ribosomal subunit to the cytoplasm (By similarity).
N	Q9VEJ2	DE	Nucleolar complex protein 14 homolog;
N	Q9VEJ2	DE	Nucleolar protein 14 homolog;
N	Q9VEJ2	DR	protein binding
N	Q9VEJ2	DR	rRNA processing
N	Q9VEJ2	DR	ribosomal small subunit biogenesis
N	Q9VEJ2	DR	snoRNA binding
N	Q9VEP1	CC	Probable methyltransferase (By similarity).
N	Q9VEP1	DE	Putative ribosomal RNA methyltransferase CG5220;
N	Q9VEP1	DE	rRNA (uridine-2'-O-)-methyltransferase;
N	Q9VEP1	DR	methyltransferase activity
N	Q9VEP1	DR	nucleic acid binding
N	Q9VEP1	DR	protein binding
N	Q9VEP1	DR	rRNA methylation
N	Q9VH19	CC	Essential for the coordination of mitotic progression, and also plays a role in cell migration.
N	Q9VH19	DE	Invadolysin;
N	Q9VH19	DE	Leishmanolysin-like peptidase;
N	Q9VH19	DR	brain development

N	Q9VH19	DR	cell adhesion
N	Q9VH19	DR	cell division
N	Q9VH19	DR	centrosome duplication
N	Q9VH19	DR	chromatin remodeling
N	Q9VH19	DR	germ cell migration
N	Q9VH19	DR	gonad development
N	Q9VH19	DR	imaginal disc development
N	Q9VH19	DR	metalloendopeptidase activity
N	Q9VH19	DR	mitotic centrosome separation
N	Q9VH19	DR	mitotic chromosome condensation
N	Q9VH19	DR	mitotic spindle organization
N	Q9VH19	DR	positive regulation of mitotic metaphase/anaphase transition
N	Q9VH19	DR	zinc ion binding
N	Q9VIM5	CC	Functions as actin-binding component of the Arp2/3 complex which is involved in regulation of actin polymerization and together with an activating nucleation-promoting factor (NPF) mediates the formation of branched actin networks
N	Q9VIM5	CC	Seems to contact the mother actin filament (By similarity).
N	Q9VIM5	DE	Arp2/3 complex 34 kDa subunit;
N	Q9VIM5	DE	Probable actin-related protein 2/3 complex subunit 2;
N	Q9VIM5	DR	actin binding
N	Q9VIM5	DR	cell projection assembly
N	Q9VIM5	DR	cortical actin cytoskeleton organization
N	Q9VIM5	DR	regulation of actin filament polymerization
N	Q9VIM5	DR	regulation of cell shape
N	Q9VMV6	DE	SelT-like protein;
N	Q9VMV6	DR	cell redox homeostasis
N	Q9VMV6	DR	selenium binding
N	Q9VP22	CC	Cyclin-dependent kinase which displays CTD kinase activity: hyperphosphorylates the C-terminal heptapeptide repeat domain (CTD) of the largest RNA polymerase II subunit, thereby acting as a key regulator of transcription elongation
N	Q9VP22	DE	Cell division protein kinase 12;
N	Q9VP22	DE	Cyclin-dependent kinase 12;
N	Q9VP22	DR	RNA polymerase II carboxy-terminal domain kinase activity
N	Q9VP22	DR	cyclin binding
N	Q9VP22	DR	cyclin-dependent protein kinase activity
N	Q9VP22	DR	phagocytosis, engulfment
N	Q9VP22	DR	phosphorylation of RNA polymerase II C-terminal domain
N	Q9W0Y6	CC	Receptor-activated non-selective cation channel involved in detection of pain sensation due to high temperature
N	Q9W0Y6	CC	Involved in heat nociception by being activated by noxious temperature of 38 degrees Celsius.
N	Q9W0Y6	DE	Transient receptor potential cation channel protein painless;
N	Q9W0Y6	DR	behavioral response to pain
N	Q9W0Y6	DR	detection of mechanical stimulus involved in sensory perception of pain
N	Q9W0Y6	DR	detection of temperature stimulus involved in sensory perception of pain
N	Q9W0Y6	DR	feeding behavior
N	Q9W0Y6	DR	ion channel activity
N	Q9W0Y6	DR	negative gravitaxis
N	Q9W0Y6	DR	regulation of female receptivity
N	Q9W0Y6	DR	response to heat
N	Q9W3C0	CC	Involved in nucleolar processing of pre-18S ribosomal RNA (By similarity).
N	Q9W3C0	DE	Probable U3 small nucleolar RNA-associated protein 11;
N	Q9W3C0	DE	U3 snoRNA-associated protein 11;
N	Q9W3C0	DR	rRNA processing
N	Q9W7J6	CC	Lethal neurotoxin, binds and inhibits neuronal nicotinic acetylcholine receptors (nAChR) (By similarity).
N	Q9W7J6	DE	Alpha-neurotoxin 7;
N	Q9W7J6	DE	Short neurotoxin 7;
N	Q9W7J6	DR	acetylcholine receptor inhibitor activity
N	Q9W7J6	DR	synaptic transmission
N	Q9WUQ3	CC	Promotes formation and maturation of oligodendrocytes, especially within the brain
N	Q9WUQ3	CC	Cooperates with OLIG2 to establish the pMN domain of the embryonic neural tube (By similarity).
N	Q9WUQ3	DE	Olg-1 bHLH protein;
N	Q9WUQ3	DE	Oligodendrocyte transcription factor 1;

N	Q9WUQ3	DR	transcription regulator activity
N	Q9WYZ1	CC	Protease subunit of a proteasome-like degradation complex believed to be a general protein degrading machinery.
N	Q9WYZ1	DE	ATP-dependent protease subunit HslV;
N	Q9WYZ1	DR	metal ion binding
N	Q9WYZ1	DR	proteolysis involved in cellular protein catabolic process
N	Q9WYZ1	DR	threonine-type endopeptidase activity
N	Q9WZ25	CC	Catalyzes the isomerization between 2-isopropylmalate and 3-isopropylmalate, via the formation of 2-isopropylmaleate.
N	Q9WZ25	DE	3-isopropylmalate dehydratase small subunit 2;
N	Q9WZ25	DE	Alpha-IPM isomerase 2;
N	Q9WZ25	DE	Isopropylmalate isomerase 2;
N	Q9WZ25	DR	3-isopropylmalate dehydratase activity
N	Q9WZ25	DR	leucine biosynthetic process
N	Q9WZN3	CC	One of the essential components for the initiation of protein synthesis
N	Q9WZN3	CC	Also involved in the hydrolysis of GTP during the formation of the 70S ribosomal complex (By similarity).
N	Q9WZN3	CC	Protects formylmethionyl-tRNA from spontaneous hydrolysis and promotes its binding to the 30S ribosomal subunits
N	Q9WZN3	DE	Translation initiation factor IF-2;
N	Q9WZN3	DR	GTPase activity
N	Q9WZN3	DR	translation initiation factor activity
N	Q9X1K5	DE	DAP decarboxylase;
N	Q9X1K5	DE	Diaminopimelate decarboxylase;
N	Q9X1K5	DR	diaminopimelate decarboxylase activity
N	Q9X1K5	DR	lysine biosynthetic process via diaminopimelate
N	Q9X521	CC	Binds directly to 23S rRNA
N	Q9X521	CC	The L1 stalk is quite mobile in the ribosome, and is involved in E site tRNA release (By similarity).
N	Q9X521	DE	50S ribosomal protein L1;
N	Q9X521	DR	RNA processing
N	Q9X521	DR	regulation of translation
N	Q9X521	DR	structural constituent of ribosome
N	Q9XDB4	CC	Catalyzes the attachment of isoleucine to tRNA(Ile)
N	Q9XDB4	CC	As IleRS can inadvertently accommodate and process structurally similar amino acids such as valine, to avoid such errors it has two additional distinct tRNA(Ile)-dependent editing activities
N	Q9XDB4	CC	One activity is designated as 'pretransfer' editing and involves the hydrolysis of activated Val-AMP
N	Q9XDB4	CC	The other activity is designated 'posttransfer' editing and involves deacylation of mischarged Val-tRNA(Ile) (By similarity).
N	Q9XDB4	DE	Isoleucine--tRNA ligase;
N	Q9XDB4	DE	Isoleucyl-tRNA synthetase;
N	Q9XDB4	DR	isoleucine-tRNA ligase activity
N	Q9XDB4	DR	isoleucyl-tRNA aminoacylation
N	Q9XDB4	DR	metal ion binding
N	Q9XG56	CC	Core component of nucleosome
N	Q9XG56	CC	DNA accessibility is regulated via a complex set of post-translational modifications of histones, also called histone code, and nucleosome remodeling.
N	Q9XG56	CC	Histones thereby play a central role in transcription regulation, DNA repair, DNA replication and chromosomal stability
N	Q9XG56	CC	Nucleosomes wrap and compact DNA into chromatin, limiting DNA accessibility to the cellular machineries which require DNA as a template
N	Q9XG56	DE	Histone H2A.1;
N	Q9XG56	DR	nucleosome assembly
N	Q9XH37	CC	Probable transcription activator that binds to the DNA sequence 5'-CAAT[AT]ATTG-3'
N	Q9XH37	CC	May be involved in the regulation of gibberellin signaling.
N	Q9XH37	DE	HD-ZIP protein HOX4;
N	Q9XH37	DE	Homeobox-leucine zipper protein HOX4;
N	Q9XH37	DE	Homeodomain transcription factor HOX4;
N	Q9XH37	DR	regulation of transcription, DNA-dependent
N	Q9XH37	DR	sequence-specific DNA binding
N	Q9XH37	DR	sequence-specific DNA binding transcription factor activity
N	Q9XH37	DR	transcription regulator activity

N	Q9XP34	CC	Component of the ubiquinol-cytochrome c reductase complex (complex III or cytochrome b-c1 complex), which is a respiratory chain that generates an electrochemical potential coupled to ATP synthesis (By similarity).
N	Q9XP34	DE	Complex III subunit 3;
N	Q9XP34	DE	Complex III subunit III;
N	Q9XP34	DE	Cytochrome b-c1 complex subunit 3;
N	Q9XP34	DE	Cytochrome b;
N	Q9XP34	DE	Ubiquinol-cytochrome-c reductase complex cytochrome b subunit;
N	Q9XP34	DR	electron carrier activity
N	Q9XP34	DR	metal ion binding
N	Q9XP34	DR	oxidoreductase activity
N	Q9XP34	DR	respiratory electron transport chain
N	Q9XT49	CC	Serotonin transporter whose primary function in the central nervous system involves the regulation of serotonergic signaling via transport of serotonin molecules from the synaptic cleft back into the pre-synaptic terminal for re-utilization
N	Q9XT49	CC	Plays a key role in mediating regulation of the availability of serotonin to other receptors of serotonergic systems
N	Q9XT49	CC	Terminates the action of serotonin and recycles it in a sodium-dependent manner (By similarity).
N	Q9XT49	DE	5HT transporter;
N	Q9XT49	DE	Sodium-dependent serotonin transporter;
N	Q9XT49	DE	Solute carrier family 6 member 4;
N	Q9XT49	DR	actin filament binding
N	Q9XT49	DR	protein oligomerization
N	Q9XT49	DR	serotonin transmembrane transporter activity
N	Q9XT49	DR	serotonin uptake
N	Q9XT49	DR	serotonin:sodium symporter activity
N	Q9XXC8	CC	Catalyzes the reduction of trans-2-enoyl-CoA to acyl- CoA
N	Q9XXC8	CC	May have a role in the mitochondrial synthesis of fatty acids (By similarity).
N	Q9XXC8	DE	Probable trans-2-enoyl-CoA reductase 2, mitochondrial;
N	Q9XXC8	DR	fatty acid biosynthetic process
N	Q9XXC8	DR	oxidation-reduction process
N	Q9XXC8	DR	protein binding
N	Q9XXC8	DR	trans-2-enoyl-CoA reductase (NADPH) activity
N	Q9XXC8	DR	zinc ion binding
N	Q9Y1U3	CC	Depressant insect beta-toxins cause a transient contraction paralysis followed by a slow flaccid paralysis
N	Q9Y1U3	CC	They bind voltage-independently at site-4 of sodium channels and shift the voltage of activation toward more negative potentials thereby affecting sodium channel activation and promoting spontaneous and repetitive firing (By similarity).
N	Q9Y1U3	DE	Toxin BmKITc;
N	Q9Y1U3	DR	defense response
N	Q9Y1U3	DR	sodium channel inhibitor activity
N	Q9Y2T7	CC	Major constituent of messenger ribonucleoprotein particles (mRNPs)
N	Q9Y2T7	CC	Binds to Y-box consensus promoter element
N	Q9Y2T7	CC	Binds to full length mRNA with high affinity in a sequence-independent manner
N	Q9Y2T7	CC	Binds to short RNA sequences containing the consensus site 5'-UCCAUCA-3' with low affinity and limited sequence specificity
N	Q9Y2T7	CC	Involved in the regulation of the stability and/or translation of germ cell mRNAs
N	Q9Y2T7	CC	Its binding with maternal mRNAs is necessary for its cytoplasmic retention
N	Q9Y2T7	CC	May mark specific mRNAs (those transcribed from Y-box promoters) in the nucleus for cytoplasmic storage, thereby linking transcription and mRNA storage/translational delay (By similarity).
N	Q9Y2T7	DE	DNA-binding protein C;
N	Q9Y2T7	DE	Germ cell-specific Y-box-binding protein;
N	Q9Y2T7	DE	MSY2 homolog;
N	Q9Y2T7	DE	Y-box-binding protein 2;
N	Q9Y2T7	DR	regulation of transcription, DNA-dependent
N	Q9Y2T7	DR	transcription from RNA polymerase II promoter
N	Q9Y2T7	DR	translational attenuation
N	Q9Y3Q8	CC	Transcriptional repressor.
N	Q9Y3Q8	DE	TSC22 domain family protein 4;
N	Q9Y3Q8	DE	TSC22-related-inducible leucine zipper protein 2;
N	Q9Y3Q8	DE	Tsc-22-like protein THG-1;
N	Q9Y3Q8	DR	protein binding

N	Q9Y3Q8	DR	regulation of transcription, DNA-dependent
N	Q9Y3Q8	DR	sequence-specific DNA binding transcription factor activity
N	Q9Y3Q8	DR	transcription repressor activity
N	Q9Y4U1	CC	May be involved in the binding and intracellular trafficking of cobalamin (vitamin B12)
N	Q9Y4U1	DE	Methylmalonic aciduria and homocystinuria type C protein;
N	Q9Y4U1	DR	cobalamin binding
N	Q9Y5H6	CC	Potential calcium-dependent cell-adhesion protein
N	Q9Y5H6	CC	May be involved in the establishment and maintenance of specific neuronal connections in the brain.
N	Q9Y5H6	DE	PCDH-alpha-8;
N	Q9Y5H6	DE	Protocadherin alpha-8;
N	Q9Y5H6	DR	calcium ion binding
N	Q9Y5H6	DR	homophilic cell adhesion
N	Q9Y5H6	DR	nervous system development
N	Q9Y5H6	DR	protein binding
N	Q9Y5M8	CC	Component of the SRP (signal recognition particle) receptor
N	Q9Y5M8	CC	Ensures, in conjunction with the signal recognition particle, the correct targeting of the nascent secretory proteins to the endoplasmic reticulum membrane system
N	Q9Y5M8	CC	Has GTPase activity
N	Q9Y5M8	CC	May mediate the membrane association of SRPR (By similarity).
N	Q9Y5M8	DE	Protein APMCF1;
N	Q9Y5M8	DE	Signal recognition particle receptor subunit beta;
N	Q9Y5M8	DR	protein binding
N	Q9Y5M8	DR	receptor activity
N	Q9Y5U9	CC	May be involved in protein transport between endoplasmic reticulum and Golgi apparatus (By similarity).
N	Q9Y5U9	DE	Immediate early response 3-interacting protein 1;
N	Q9Y6Z5	DE	Protein HGC6.4;
N	Q9Y6Z5	DE	Putative uncharacterized protein C6orf124;
N	Q9Y897	CC	Catalyzes the oxidation of 3-carboxy-2-hydroxy-4- methylpentanoate (3-isopropylmalate) to 3-carboxy-4-methyl-2- oxopentanoate
N	Q9Y897	CC	The product decarboxylates to 4-methyl-2 oxopentanoate.
N	Q9Y897	DE	3-isopropylmalate dehydrogenase;
N	Q9Y897	DE	Beta-IPM dehydrogenase;
N	Q9Y897	DR	3-isopropylmalate dehydrogenase activity
N	Q9Y897	DR	leucine biosynthetic process
N	Q9Y897	DR	magnesium ion binding
N	Q9Y897	DR	oxidation-reduction process
N	Q9YAD5	CC	Transcription factor that plays a role in the activation of archaeal genes transcribed by RNA polymerase
N	Q9YAD5	CC	Facilitates transcription initiation by enhancing TATA-box recognition by TATA-box-binding protein (tbp), and transcription factor B (tfb) and RNA polymerase recruitment
N	Q9YAD5	CC	It dynamically alters the nucleic acid-binding properties of RNA polymerases by stabilizing the initiation complex and destabilizing elongation complexes
N	Q9YAD5	CC	Not absolutely required for transcription in vitro, but particularly important in cases where tbp or tfb function is not optimal
N	Q9YAD5	CC	Seems to translocate with the RNA polymerase following initiation and acts by binding to the non template strand of the transcription bubble in elongation complexes (By similarity).
N	Q9YAD5	DE	TFIIE subunit alpha homolog;
N	Q9YAD5	DE	Transcription factor E;
N	Q9YAD5	DE	Transcription initiation factor TFIIE;
N	Q9YAD5	DR	RNA polymerase II transcription factor activity
N	Q9YAD5	DR	regulation of transcription, DNA-dependent
N	Q9YAD5	DR	transcription initiation from RNA polymerase II promoter
N	Q9YAD5	DR	zinc ion binding
N	Q9YAS4	CC	Converts HMG-CoA to mevalonate (By similarity).
N	Q9YAS4	DE	3-hydroxy-3-methylglutaryl-coenzyme A reductase;
N	Q9YAS4	DE	HMG-CoA reductase;
N	Q9YAS4	DR	coenzyme A metabolic process
N	Q9YAS4	DR	hydroxymethylglutaryl-CoA reductase (NADPH) activity
N	Q9YAS4	DR	isoprenoid biosynthetic process
N	Q9YAS4	DR	oxidation-reduction process
N	Q9YBD6	CC	The natural substrate for this enzyme may be peptidyl- tRNAs which drop off the ribosome during protein synthesis (By similarity).

N	Q9YBD6	DE	Peptidyl-tRNA hydrolase;
N	Q9YBD6	DR	aminoacyl-tRNA hydrolase activity
N	Q9YF40	CC	Catalyzes the reversible phosphorylation of UMP to UDP (By similarity).
N	Q9YF40	DE	UMP kinase;
N	Q9YF40	DE	Uridine monophosphate kinase;
N	Q9YF40	DE	Uridylate kinase;
N	Q9YF40	DR	UMP kinase activity
N	Q9YF40	DR	cellular amino acid biosynthetic process
N	Q9YF40	DR	pyrimidine nucleotide biosynthetic process
N	Q9YFI6	DE	Dihydroorotate dehydrogenase;
N	Q9YFI6	DE	Dihydroorotate oxidase;
N	Q9YFI6	DR	'de novo' pyrimidine base biosynthetic process
N	Q9YFI6	DR	UMP biosynthetic process
N	Q9YFI6	DR	dihydroorotate oxidase activity
N	Q9YFI6	DR	oxidation-reduction process
N	Q9YGI2	CC	Binds to the nicotinic acetylcholine receptor (By similarity).
N	Q9YGI2	DE	Probable weak neurotoxin NNAM1;
N	Q9YGI2	DR	acetylcholine receptor inhibitor activity
N	Q9YGI2	DR	synaptic transmission
N	Q9YH13	CC	Sequence-specific transcription factor which is part of a developmental regulatory system that provides cells with specific positional identities on the anterior-posterior axis (By similarity).
N	Q9YH13	DE	Homeobox protein Hox-C8;
N	Q9YH13	DR	multicellular organismal development
N	Q9YH13	DR	regulation of transcription, DNA-dependent
N	Q9YH13	DR	sequence-specific DNA binding
N	Q9YH13	DR	sequence-specific DNA binding transcription factor activity
N	Q9YH13	DR	transcription regulator activity
N	Q9Z0L0	DE	5T4 oncofetal trophoblast glycoprotein;
N	Q9Z0L0	DE	5T4 oncotrophoblast glycoprotein;
N	Q9Z0L0	DE	Trophoblast glycoprotein;
N	Q9Z0N7	CC	Probably important in cardiac repolarization
N	Q9Z0N7	CC	Associates with KCNE1 (MinK) to form the I(Ks) cardiac potassium current
N	Q9Z0N7	CC	Elicits a rapidly activating, potassium-selective outward current.
N	Q9Z0N7	DE	IKs producing slow voltage-gated potassium channel subunit alpha KvLQT1;
N	Q9Z0N7	DE	Potassium voltage-gated channel subfamily KQT member 1;
N	Q9Z0N7	DE	Voltage-gated potassium channel subunit Kv7.1;
N	Q9Z0N7	DR	negative regulation of insulin secretion
N	Q9Z0N7	DR	outward rectifier potassium channel activity
N	Q9Z0N7	DR	response to chemical stimulus
N	Q9Z1Z1	CC	Phosphorylates the alpha subunit of eukaryotic translation-initiation factor 2 (EIF2), leading to its inactivation and thus to a rapid reduction of translational initiation and repression of global protein synthesis
N	Q9Z1Z1	CC	Serves as a critical effector of unfolded protein response (UPR)-induced G1 growth arrest due to the loss of cyclin D1 (By similarity).
N	Q9Z1Z1	DE	Eukaryotic translation initiation factor 2-alpha kinase 3;
N	Q9Z1Z1	DE	PRKR-like endoplasmic reticulum kinase;
N	Q9Z1Z1	DE	Pancreatic eIF2-alpha kinase;
N	Q9Z1Z1	DR	ER overload response
N	Q9Z1Z1	DR	endocrine pancreas development
N	Q9Z1Z1	DR	endoplasmic reticulum unfolded protein response
N	Q9Z1Z1	DR	eukaryotic translation initiation factor 2alpha kinase activity
N	Q9Z1Z1	DR	negative regulation of translational initiation in response to stress
N	Q9Z1Z1	DR	protein autophosphorylation
N	Q9Z1Z1	DR	protein homooligomerization
N	Q9Z265	CC	Regulates cell cycle checkpoints and apoptosis in response to DNA damage, particularly to DNA double-strand breaks
N	Q9Z265	CC	Inhibits CDC25C phosphatase by phosphorylation, preventing the entry into mitosis
N	Q9Z265	CC	May also play a role in meiosis
N	Q9Z265	CC	Regulates the TP53 tumor suppressor through phosphorylation at 'Thr-20' and 'Ser-23'
N	Q9Z265	DE	Serine/threonine-protein kinase Chk2;
N	Q9Z265	DR	metal ion binding
N	Q9Z265	DR	protein serine/threonine kinase activity
N	Q9Z265	DR	response to gamma radiation
N	Q9Z265	DR	signal transduction in response to DNA damage

N	Q9Z280	CC	Implicated as a critical step in numerous cellular pathways, including signal transduction, membrane trafficking, and the regulation of mitosis
N	Q9Z280	CC	May be involved in the regulation of perinuclear intravesicular membrane traffic.
N	Q9Z280	DE	Choline phosphatase 1;
N	Q9Z280	DE	Phosphatidylcholine-hydrolyzing phospholipase D1;
N	Q9Z280	DE	Phospholipase D1;
N	Q9Z280	DR	NAPE-specific phospholipase D activity
N	Q9Z280	DR	cell communication
N	Q9Z280	DR	defense response to Gram-positive bacterium
N	Q9Z280	DR	lipid catabolic process
N	Q9Z280	DR	phosphatidylinositol binding
N	Q9Z280	DR	phospholipase D activity
N	Q9Z7K9	DE	30S ribosomal protein S2;
N	Q9Z7K9	DR	structural constituent of ribosome
N	Q9Z8Z9	DE	Pentose-5-phosphate 3-epimerase;
N	Q9Z8Z9	DE	Ribulose-phosphate 3-epimerase;
N	Q9Z8Z9	DR	carbohydrate metabolic process
N	Q9Z8Z9	DR	ribulose-phosphate 3-epimerase activity
N	Q9ZDB1	DE	Leucine--tRNA ligase;
N	Q9ZDB1	DE	Leucyl-tRNA synthetase;
N	Q9ZDB1	DR	leucine-tRNA ligase activity
N	Q9ZDB1	DR	leucyl-tRNA aminoacylation
N	Q9ZDC9	DE	Putative carboxypeptidase RP402;
N	Q9ZDC9	DR	carboxypeptidase activity
N	Q9ZDC9	DR	serine-type peptidase activity
N	Q9ZE17	DE	Aspartate--tRNA ligase;
N	Q9ZE17	DE	Aspartyl-tRNA synthetase;
N	Q9ZE17	DR	aspartate-tRNA ligase activity
N	Q9ZE17	DR	aspartyl-tRNA aminoacylation
N	Q9ZE17	DR	nucleic acid binding
N	Q9ZHA4	DE	UPF0124 protein in ftsZ 3'region;
N	Q9ZJX1	CC	Responsible for the release of ribosomes from messenger RNA at the termination of protein biosynthesis
N	Q9ZJX1	CC	May increase the efficiency of translation by recycling ribosomes from one round of translation to another (By similarity).
N	Q9ZJX1	DE	Ribosome-recycling factor;
N	Q9ZJX1	DE	Ribosome-releasing factor;
N	Q9ZK47	DE	Carbon starvation protein A homolog;
N	Q9ZK47	DR	cellular response to starvation
N	Q9ZK59	CC	Cell wall formation
N	Q9ZK59	CC	Catalyzes the transfer of a GlcNAc subunit on undecaprenyl-pyrophosphoryl-MurNAc-pentapeptide (lipid intermediate I) to form undecaprenyl-pyrophosphoryl-MurNAc- (pentapeptide)GlcNAc (lipid intermediate II) (By similarity)
N	Q9ZK59	DE	UDP-N-acetylglucosamine--N-acetylmuramyl-(pentapeptide) pyrophosphoryl-undecaprenol N-acetylglucosamine transferase;
N	Q9ZK59	DE	Undecaprenyl-PP-MurNAc-pentapeptide-UDPGlcNAc GlcNAc transferase;
N	Q9ZK59	DR	UDP-N-acetyl-D-glucosamine:N-acetylmuramoyl-L-alanyl-D-glutamyl-meso-2,6-diaminopimelyl-D-alanyl-D-alanine-diphosphoundecaprenol 4-beta-N-acetylglucosaminlytransferase activity
N	Q9ZK59	DR	UDP-N-acetylgalactosamine biosynthetic process
N	Q9ZK59	DR	carbohydrate binding
N	Q9ZK59	DR	cell division
N	Q9ZK59	DR	cellular cell wall organization
N	Q9ZK59	DR	lipid glycosylation
N	Q9ZK59	DR	peptidoglycan biosynthetic process
N	Q9ZK59	DR	regulation of cell shape
N	Q9ZK59	DR	undecaprenyldiphospho-muramoylpentapeptide beta-N-acetylglucosaminyltransferase activity
N	Q9ZL42	CC	Catalyzes the conversion of GTP to 2,5-diamino-6- ribosylamino-4(3H)-pyrimidinone 5'-phosphate (DARP), formate and pyrophosphate (By similarity).
N	Q9ZL42	DE	GTP cyclohydrolase II;
N	Q9ZL42	DE	GTP cyclohydrolase-2;
N	Q9ZL42	DR	GTP cyclohydrolase II activity
N	Q9ZL42	DR	metal ion binding
N	Q9ZL42	DR	riboflavin biosynthetic process

N	Q9ZML9	CC	NAD-binding protein involved in the addition of a carboxymethylaminomethyl (cmnm) group at the wobble position (U34) of certain tRNAs, forming tRNA-cmnm(5)s(2)U34 (By similarity).
N	Q9ZML9	DE	Glucose-inhibited division protein A;
N	Q9ZML9	DE	tRNA uridine 5-carboxymethylaminomethyl modification enzyme MnmG;
N	Q9ZML9	DR	flavin adenine dinucleotide binding
N	Q9ZML9	DR	tRNA wobble uridine modification
N	Q9ZMP8	DE	Lysine--tRNA ligase;
N	Q9ZMP8	DE	Lysyl-tRNA synthetase;
N	Q9ZMP8	DR	lysine-tRNA ligase activity
N	Q9ZMP8	DR	lysyl-tRNA aminoacylation
N	Q9ZMP8	DR	metal ion binding
N	Q9ZMP8	DR	nucleic acid binding
N	Q9ZPE9	DE	5'-adenylylsulfate reductase-like 6;
N	Q9ZPE9	DE	APR-like 6;
N	Q9ZPE9	DE	Adenosine 5'-phosphosulfate reductase-like 6;
N	Q9ZPE9	DR	cell redox homeostasis
N	Q9ZVL3	CC	Stimulates the transcription of various genes by recognizing and binding to a CCAAT motif in promoters (By similarity).
N	Q9ZVL3	DE	Nuclear transcription factor Y subunit C-3;
N	Q9ZVL3	DR	protein binding
N	Q9ZVL3	DR	sequence-specific DNA binding
N	A0ALD9	DE	2-phospho-D-glycerate hydro-lyase;
N	A0ALD9	DE	2-phosphoglycerate dehydratase;
N	A0ALD9	CC	Catalyzes the reversible conversion of 2- phosphoglycerate into phosphoenolpyruvate
N	A0ALD9	CC	It is essential for the degradation of carbohydrates via glycolysis (By similarity).
N	A0ALD9	DR	magnesium ion binding
N	A0ALD9	DR	phosphopyruvate hydratase activity
N	A1L3G9	DE	Transmembrane protein 194A;
N	A1RXF3	DE	Translation initiation factor 6;
N	A1RXF3	CC	Binds to the 50S ribosomal subunit and prevents its association with the 30S ribosomal subunit to form the 70S initiation complex (By similarity).
N	A1RXF3	DR	ribosome binding
N	A1RXF3	DR	translation initiation factor activity
N	A1RXF3	DR	mature ribosome assembly
N	A2RG22	DE	Probable rRNA maturation factor;
N	A2RG22	CC	Involved in rRNA and/or ribosome maturation and assembly
N	A2RG22	CC	Required for normal 5' and 3' processing of 16S, 23S and 5S rRNAs
N	A2RG22	CC	May have metal-dependent hydrolase activity (By similarity).
N	A2RG22	DR	metal ion binding
N	A2RG22	DR	metalloendopeptidase activity
N	A2RG22	DR	rRNA processing
N	A2RJT9	DE	Dihydroorotate dehydrogenase A;
N	A2RJT9	DE	DHOdehase A;
N	A2RJT9	DE	Dihydroorotate oxidase A;
N	A2RJT9	DR	dihydroorotate oxidase activity
N	A2RJT9	DR	'de novo' pyrimidine base biosynthetic process
N	A2RJT9	DR	oxidation-reduction process
N	A2RJT9	DR	UMP biosynthetic process
N	A3D2L9	DE	Ribonuclease HII;
N	A3D2L9	CC	Endonuclease that specifically degrades the RNA of RNA- DNA hybrids (By similarity).
N	A3D2L9	DR	metal ion binding
N	A3D2L9	DR	ribonuclease H activity
N	A3PB16	DE	NAD(P)H-quinone oxidoreductase subunit 3;
N	A3PB16	DE	NAD(P)H dehydrogenase subunit 3;
N	A3PB16	DE	NADH-plastoquinone oxidoreductase subunit 3;
N	A3PB16	DE	NDH-1 subunit 3;
N	A3PB16	CC	NDH-1 shuttles electrons from an unknown electron donor, via FMN and iron-sulfur (Fe-S) centers, to quinones in the respiratory and/or the photosynthetic chain
N	A3PB16	CC	The immediate electron acceptor for the enzyme in this species is believed to be plastoquinone
N	A3PB16	CC	Couples the redox reaction to proton translocation, and thus conserves the redox energy in a proton gradient

N	A3PB16	CC	Cyanobacterial NDH-1 also plays a role in inorganic carbon- concentration (By similarity).
N	A3PB16	DR	NADH dehydrogenase (ubiquinone) activity
N	A3PB16	DR	quinone binding
N	A3PB16	DR	oxidation-reduction process
N	A4G709	DE	50S ribosomal protein L9;
N	A4G709	CC	Binds to the 23S rRNA (By similarity).
N	A4G709	DR	structural constituent of ribosome
N	A4IJL3	DE	30S ribosomal protein S11;
N	A4IJL3	CC	Located on the platform of the 30S subunit, it bridges several disparate RNA helices of the 16S rRNA
N	A4IJL3	CC	Forms part of the Shine-Dalgarno cleft in the 70S ribosome (By similarity).
N	A4IJL3	DR	structural constituent of ribosome
N	A4SQX0	DE	Probable 4-deoxy-4-formamido-L-arabinose-phosphoundecaprenol deformylase ArnD;
N	A4SQX0	CC	Catalyzes the deformylation of 4-deoxy-4-formamido-L- arabinose-phosphoundecaprenol to 4-amino-4-deoxy-L-arabinose- phosphoundecaprenol
N	A4SQX0	CC	The modified arabinose is attached to lipid A and is required for resistance to polymyxin and cationic antimicrobial peptides (By similarity).
N	A4SQX0	DR	hydrolase activity, acting on carbon-nitrogen (but not peptide) bonds, in linear amides
N	A4SQX0	DR	lipid A biosynthetic process
N	A4SQX0	DR	response to antibiotic
N	A5E0U9	DE	ATP-dependent RNA helicase DBP8;
N	A5E0U9	CC	ATP-binding RNA helicase involved in 40S ribosomal subunit biogenesis and is required for the normal formation of 18S rRNAs through pre-rRNA processing at A0, A1 and A2 sites
N	A5E0U9	CC	Required for vegetative growth (By similarity).
N	A5E0U9	DR	ATP-dependent helicase activity
N	A5E0U9	DR	rRNA processing
N	A6LPS9	DE	50S ribosomal protein L30;
N	A6LPS9	DR	structural constituent of ribosome
N	A6VCJ8	DE	30S ribosomal protein S15;
N	A6VCJ8	CC	One of the primary rRNA binding proteins, it binds directly to 16S rRNA where it helps nucleate assembly of the platform of the 30S subunit by binding and bridging several RNA helices of the 16S rRNA (By similarity).
N	A6VCJ8	DR	structural constituent of ribosome
N	A7FHL4	DE	Transcriptional regulator slyA;
N	A7FHL4	CC	Transcription regulator that can both activate or repress expression (By similarity).
N	A7FHL4	DR	sequence-specific DNA binding transcription factor activity
N	A7FHL4	DR	regulation of transcription, DNA-dependent
N	A7ZZM6	DE	Exoribonuclease 2;
N	A7ZZM6	DE	Exoribonuclease II;
N	A7ZZM6	DE	Ribonuclease II;
N	A7ZZM6	CC	Involved in mRNA degradation
N	A7ZZM6	CC	Hydrolyzes single-stranded polyribonucleotides processively in the 3' to 5' direction (By similarity).
N	A7ZZM6	DR	exoribonuclease II activity
N	A7ZZM6	DR	RNA catabolic process
N	A8M519	DE	50S ribosomal protein L14;
N	A8M519	CC	Binds to 23S rRNA
N	A8M519	CC	Forms part of two intersubunit bridges in the 70S ribosome (By similarity).
N	A8M519	DR	structural constituent of ribosome
N	A8Y7P6	DE	Trypsin inhibitor B6;
N	A8Y7P6	DE	Trypsin inhibitor 6;
N	A8Y7P6	CC	Inhibits trypsin (By similarity).
N	A8Y7P6	DR	serine-type endopeptidase inhibitor activity
N	A9N141	DE	Cysteine desulfuration protein sufE;
N	A9N141	CC	Participates in cysteine desulfuration mediated by sufS
N	A9N141	CC	Cysteine desulfuration mobilizes sulfur from L-cysteine to yield L-alanine and constitutes an essential step in sulfur metabolism for biosynthesis of a variety of sulfur-containing biomolecules
N	A9N141	CC	Functions as a sulfur acceptor for sufS, by mediating the direct transfer of the sulfur atom from the S-sulfanylcysteine of sufS, an intermediate product of cysteine desulfuration process (By similarity).
N	A9R2Y6	DE	Na(+)-translocating NADH-quinone reductase subunit A;
N	A9R2Y6	DE	Na(+)-NQR subunit A;

N	A9R2Y6	DE	Na(+)-translocating NQR subunit A;
N	A9R2Y6	DE	NQR complex subunit A;
N	A9R2Y6	DE	NQR-1 subunit A;
N	A9R2Y6	CC	NQR complex catalyzes the reduction of ubiquinone-1 to ubiquinol by two successive reactions, coupled with the transport of Na(+) ions from the cytoplasm to the periplasm
N	A9R2Y6	CC	NqrA to nqrE are probably involved in the second step, the conversion of ubisemiquinone to ubiquinol (By similarity).
N	A9R2Y6	DR	oxidoreductase activity, acting on NADH or NADPH, quinone or similar compound as acceptor
N	A9R2Y6	DR	oxidation-reduction process
N	A9R2Y6	DR	sodium ion transport
N	A9R3T6	DE	Acid shock protein;
N	A9R3T6	CC	Required for growth and/or survival at acidic conditions (By similarity).
N	A9R3T6	DR	response to acidity
N	A9R5M0	DE	Glycogen synthase;
N	A9R5M0	DE	Starch [bacterial glycogen] synthase;
N	A9R5M0	CC	Synthesizes alpha-1,4-glucan chains using ADP-glucose (By similarity).
N	A9R5M0	DR	starch synthase activity
N	A9R5M0	DR	glycogen biosynthetic process
N	A9VP76	DE	30S ribosomal protein S10;
N	A9VP76	CC	Involved in the binding of tRNA to the ribosomes (By similarity).
N	A9VP76	DR	structural constituent of ribosome
N	B0SNK3	DE	Glutamate-1-semialdehyde 2,1-aminomutase;
N	B0SNK3	DE	Glutamate-1-semialdehyde aminotransferase;
N	B0SNK3	DR	glutamate-1-semialdehyde 2,1-aminomutase activity
N	B0SNK3	DR	pyridoxal phosphate binding
N	B0SNK3	DR	transaminase activity
N	B0SNK3	DR	porphyrin biosynthetic process
N	B2KC93	DE	Ribosome maturation factor rimP;
N	B2KC93	CC	Required for maturation of 30S ribosomal subunits (By similarity).
N	B2KC93	DR	ribosome biogenesis
N	B2SFE5	DE	50S ribosomal protein L19;
N	B2SFE5	CC	This protein is located at the 30S-50S ribosomal subunit interface and may play a role in the structure and function of the aminoacyl-tRNA binding site (By similarity).
N	B2SFE5	DR	structural constituent of ribosome
N	B3GYU5	DE	50S ribosomal protein L1;
N	B3GYU5	CC	Binds directly to 23S rRNA
N	B3GYU5	CC	The L1 stalk is quite mobile in the ribosome, and is involved in E site tRNA release (By similarity).
N	B3GYU5	DR	structural constituent of ribosome
N	B3GYU5	DR	regulation of translation
N	B3GYU5	DR	RNA processing
N	B4KBH3	DE	Molybdopterin synthase catalytic subunit;
N	B4KBH3	DE	Molybdenum cofactor synthesis protein 2 large subunit;
N	B4KBH3	DE	Molybdenum cofactor synthesis protein 2B;
N	B4KBH3	CC	Catalytic subunit of the molybdopterin synthase complex, a complex that catalyzes the conversion of precursor Z into molybdopterin
N	B4KBH3	CC	Acts by mediating the incorporation of 2 sulfur atoms from thiocarboxylated MOCS2A into precursor Z to generate a dithiolene group (By similarity).
N	B4KBH3	DR	Mo-molybdopterin synthase activity
N	B4KBH3	DR	transferase activity
N	B4KBH3	DR	Mo-molybdopterin cofactor biosynthetic process
N	B4M357	DE	Ubiquitin-like modifier-activating enzyme 5;
N	B4M357	DE	Ubiquitin-activating enzyme 5;
N	B4M357	CC	E1-like enzyme which activates UFM1 (By similarity).
N	B4M357	DR	cofactor binding
N	B4M357	DR	metal ion binding
N	B4M357	DR	oxidoreductase activity, acting on the CH-OH group of donors, NAD or NADP as acceptor
N	B4N369	DE	Mitotic-spindle organizing protein 1;
N	B4N369	DE	Mitotic-spindle organizing protein associated with a ring of gamma-tubulin 1;
N	B4N369	CC	Required for gamma-tubulin complex recruitment to the centrosome (By similarity).
N	B5XIE3	DE	Probable tRNA threonylcarbamoyladenosine biosynthesis protein Gcp;
N	B5XIE3	DE	t(6)A37 threonylcarbamoyladenosine biosynthesis protein;

N	B5XIE3	CC	Required for the formation of a threonylcarbamoyl group on adenosine at position 37 (t(6)A37) in tRNAs that read codons beginning with adenine (By similarity)
N	B5XIE3	DR	metal ion binding
N	B5XIE3	DR	metalloendopeptidase activity
N	B5ZBS9	DE	Urease subunit alpha;
N	B5ZBS9	DE	Urea amidohydrolase subunit alpha;
N	B5ZBS9	DR	nickel ion binding
N	B5ZBS9	DR	urease activity
N	B5ZBS9	DR	urea metabolic process
N	B5ZS20	DE	Ribonuclease HII;
N	B5ZS20	CC	Endonuclease that specifically degrades the RNA of RNA- DNA hybrids (By similarity).
N	B5ZS20	DR	metal ion binding
N	B5ZS20	DR	ribonuclease H activity
N	B7ULM0	DE	Protein tolB;
N	B7ULM0	CC	Involved in the tonB-independent uptake of proteins (By similarity).
N	B7ULM0	DR	protein import
N	B8DHZ4	DE	Ribonuclease PH;
N	B8DHZ4	DE	tRNA nucleotidyltransferase;
N	B8DHZ4	CC	Phosphorolytic exoribonuclease that removes nucleotide residues following the -CCA terminus of tRNA and adds nucleotides to the ends of RNA molecules by using nucleoside diphosphates as substrates (By similarity).
N	B8DHZ4	DR	3'-5'-exoribonuclease activity
N	B8DHZ4	DR	tRNA nucleotidyltransferase activity
N	B8DHZ4	DR	tRNA-specific ribonuclease activity
N	B8DHZ4	DR	tRNA processing
N	B8GSD2	DE	Beta-hexosaminidase;
N	B8GSD2	DE	Beta-N-acetylhexosaminidase;
N	B8GSD2	DE	N-acetyl-beta-glucosaminidase;
N	B8GSD2	CC	Cleaves GlcNAc linked beta-1,4 to MurNAc tripeptides (By similarity).
N	B8GSD2	DR	beta-N-acetylhexosaminidase activity
N	B8GSD2	DR	cell division
N	B8GSD2	DR	cellular cell wall organization
N	B8GSD2	DR	peptidoglycan biosynthetic process
N	B8GSD2	DR	regulation of cell shape
N	C1DK16	DE	Thymidylate synthase;
N	C1DK16	CC	Provides the sole de novo source of dTMP for DNA biosynthesis (By similarity).
N	C1DK16	DR	thymidylate synthase activity
N	C1DK16	DR	dTMP biosynthetic process
N	C3K225	DE	Ketol-acid reductoisomerase;
N	C3K225	DE	Acetohydroxy-acid isomeroreductase;
N	C3K225	DE	Alpha-keto-beta-hydroxylacil reductoisomerase;
N	C3K225	DR	coenzyme binding
N	C3K225	DR	ketol-acid reductoisomerase activity
N	C3K225	DR	branched chain family amino acid biosynthetic process
N	C3K225	DR	oxidation-reduction process
N	C4KIJ3	DE	Prefoldin subunit alpha;
N	C4KIJ3	DE	GimC subunit alpha;
N	C4KIJ3	CC	Molecular chaperone capable of stabilizing a range of proteins
N	C4KIJ3	CC	Seems to fulfill an ATP-independent, HSP70-like function in archaeal de novo protein folding (By similarity).
N	C4KIJ3	DR	unfolded protein binding
N	C4KIJ3	DR	protein folding
N	O26103	DE	4-hydroxythreonine-4-phosphate dehydrogenase;
N	O26103	DE	4-(phosphohydroxy)-L-threonine dehydrogenase;
N	O26103	CC	Catalyzes the NAD(P)-dependent oxidation of 4- (phosphohydroxy)-L-threonine (HTP) into 2-amino-3-oxo-4- (phosphohydroxy)butyric acid which spontaneously decarboxylates to form 3-amino-2-oxopropyl phosphate (AHAP) (By similarity)
N	O26103	DR	4-hydroxythreonine-4-phosphate dehydrogenase activity
N	O26103	DR	metal ion binding
N	O26103	DR	oxidation-reduction process
N	O26103	DR	pyridoxine biosynthetic process
N	O26368	DE	30S ribosomal protein S27ae;
N	O26368	DR	metal ion binding
N	O26368	DR	structural constituent of ribosome

N	O95232	DE	Luc7-like protein 3;
N	O95232	DE	Cisplatin resistance-associated-overexpressed protein;
N	O95232	DE	Okadaic acid-inducible phosphoprotein OA48-18;
N	O95232	DE	cAMP regulatory element-associated protein 1;
N	O95232	DE	CRE-associated protein 1;
N	O95232	CC	Binds cAMP regulatory element DNA sequence
N	O95232	CC	May play a role in RNA splicing.
N	O95232	DR	protein binding
N	O95232	DR	mRNA processing
N	O95232	DR	response to stress
N	P02899	DE	Uncharacterized kinetoplast minicircle 51 polypeptide;
N	P05560	DR	serine-type endopeptidase inhibitor activity
N	Q0IH73	DE	Short-chain dehydrogenase/reductase family 42E member 1;
N	Q0IH73	DR	3-beta-hydroxy-delta5-steroid dehydrogenase activity
N	Q0IH73	DR	oxidation-reduction process
N	Q0IH73	DR	steroid biosynthetic process
N	P0A1U5	DE	Uncharacterized protein yjaG;
N	P12615	DE	12S seed storage globulin 1;
N	P12615	DE	12S seed storage globulin 1 acidic chain;
N	P12615	DE	12S seed storage globulin 1 basic chain;
N	P12615	CC	This is a seed storage protein.
N	P12615	DR	nutrient reservoir activity
N	P14091	DE	Cathepsin E;
N	P14091	DE	Cathepsin E form I;
N	P14091	DE	Cathepsin E form II;
N	P14091	CC	May have a role in immune function
N	P14091	CC	Probably involved in the processing of antigenic peptides during MHC class II-mediated antigen presentation
N	P14091	CC	May play a role in activation-induced lymphocyte depletion in the thymus, and in neuronal degeneration and glial cell activation in the brain.
N	P14091	DR	aspartic-type endopeptidase activity
N	P14091	DR	antigen processing and presentation of exogenous peptide antigen via MHC class II
N	P14318	DE	Muscle-specific protein 20;
N	P14318	DR	protein binding
N	P14318	DR	cell adhesion
N	P14318	DR	myoblast fusion
N	P14318	DR	regulation of cell shape
N	P18295	DE	Non-structural protein 1;
N	P18295	CC	Inhibits post-transcriptional processing of cellular pre-mRNA, by binding and inhibiting two cellular proteins that are required for the 3'-end processing of cellular pre-mRNAs: the 30 kDa cleavage and polyadenylation specificity factor (CPSF4) and the poly(A)-binding protein 2 (PABPN1)
N	P18295	CC	This results in the accumulation of unprocessed 3' end pre-mRNAs which can't be exported from the nucleus
N	P18295	CC	Cellular protein synthesis is thereby shut off very early after virus infection
N	P18295	CC	Viral protein synthesis is not affected by the inhibition of the cellular 3' end processing machinery because the poly(A) tails of viral mRNAs are produced by the viral polymerase through a stuttering mechanism (By similarity).
N	P18295	DR	evasion by virus of host immune response
N	P36831	CC	E7 protein has both transforming and trans-activating activities
N	P36831	CC	Disrupts the function of host retinoblastoma protein RB1/pRb, which is a key regulator of the cell cycle
N	P36831	CC	Induces the disassembly of the E2F1 transcription factors from RB1, with subsequent transcriptional activation of E2F1-regulated S-phase genes
N	P36831	CC	Inactivation of the ability of RB1 to arrest the cell cycle is critical for cellular transformation, uncontrolled cellular growth and proliferation induced by viral infection
N	P36831	CC	Stimulation of progression from G1 to S phase allows the virus to efficiently use the cellular DNA replicating machinery to achieve viral genome replication
N	P36831	CC	Interferes with histone deacetylation mediated by HDAC1 and HDAC2, leading to activation of transcription (By similarity).
N	P36831	DR	metal ion binding
N	P36831	DR	sequence-specific DNA binding transcription factor activity
N	P36831	DR	evasion by virus of host immune response
N	P36831	DR	regulation of transcription, DNA-dependent
N	P38583	DE	Uncharacterized 9.1 kDa protein in BM1 immunity protein 3'region;

N	P38764	DE	26S proteasome regulatory subunit RPN1;
N	P38764	DE	HMG-CoA reductase degradation protein 2;
N	P38764	DE	Proteasome non-ATPase subunit 1;
N	P38764	CC	Acts as a regulatory subunit of the 26S proteasome which is involved in the ATP-dependent degradation of ubiquitinated proteins.
N	P38764	DR	enzyme regulator activity
N	P38764	DR	protein binding, bridging
N	P38764	DR	regulation of protein catabolic process
N	P38764	DR	ubiquitin-dependent protein catabolic process
N	P42252	DE	Sec-independent protein translocase protein tatCd;
N	P42252	CC	Required for correct localization of precursor proteins bearing signal peptides with the twin arginine conserved motif S/T-R-R-X-F-L-K
N	P42252	CC	Required for phoD secretion
N	P42252	CC	TatCd promotes membrane localization of tatAd via domain specific interactions
N	P42252	CC	TatCd is required for stabile production of tatAd as well as for its maintenance.
N	P42252	DR	protein transport
N	P42252	DR	transmembrane transport
N	P51833	DE	Ribonuclease 3;
N	P51833	DE	Ribonuclease III;
N	P51833	CC	Digests double-stranded RNA
N	P51833	CC	Involved in the processing of ribosomal RNA precursors and of some mRNAs (By similarity).
N	P51833	DR	double-stranded RNA binding
N	P51833	DR	ribonuclease III activity
N	P51833	DR	RNA processing
N	P51833	DR	rRNA catabolic process
N	P56379	DE	6.8 kDa mitochondrial proteolipid;
N	P58404	CC	Binds calmodulin in a calcium dependent manner
N	P58404	CC	May function as scaffolding or signaling protein.
N	P58404	DR	calmodulin binding
N	Q00239	DE	Nitrogenase molybdenum-iron protein alpha chain;
N	Q00239	DE	Dinitrogenase;
N	Q00239	DE	Nitrogenase component I;
N	Q00239	CC	This molybdenum-iron protein is part of the nitrogenase complex that catalyzes the key enzymatic reactions in nitrogen fixation.
N	Q00239	DR	iron-sulfur cluster binding
N	Q00239	DR	metal ion binding
N	Q00239	DR	nitrogenase activity
N	Q00239	DR	nitrogen fixation
N	Q00239	DR	oxidation-reduction process
N	Q04ZD2	DE	Anthranilate phosphoribosyltransferase;
N	Q04ZD2	DR	anthranilate phosphoribosyltransferase activity
N	Q04ZD2	DR	tryptophan biosynthetic process
N	Q06GR4	DE	Photosystem II CP43 chlorophyll apoprotein;
N	Q06GR4	DE	PSII 43 kDa protein;
N	Q06GR4	DE	Photosystem II 44 kDa reaction center protein;
N	Q06GR4	DE	Protein CP-43;
N	Q06GR4	CC	One of the components of the core antenna complex of photosystem II
N	Q06GR4	CC	It binds chlorophyll and helps catalyze the primary light-induced photochemical processes of photosystem II (By similarity).
N	Q06GR4	DR	chlorophyll binding
N	Q06GR4	DR	electron transporter, transferring electrons within the cyclic electron transport pathway of photosynthesis activity
N	Q06GR4	DR	photosynthetic electron transport in photosystem II
N	Q06GR4	DR	protein-chromophore linkage
N	Q09596	DE	Probable glutathione S-transferase 5;
N	Q09596	DE	GST class-sigma;
N	Q09596	CC	Conjugation of reduced glutathione to a wide number of exogenous and endogenous hydrophobic electrophiles (By similarity).
N	Q09596	DR	glutathione transferase activity
N	Q09596	DR	determination of adult lifespan
N	Q0BZX9	DE	Nucleoside diphosphate kinase;
N	Q0BZX9	DE	NDP kinase;
N	Q0BZX9	DE	Nucleoside-2-P kinase;
N	Q0BZX9	CC	Major role in the synthesis of nucleoside triphosphates other than ATP

N	Q0BZX9	CC	The ATP gamma phosphate is transferred to the NDP beta phosphate via a ping-pong mechanism, using a phosphorylated active-site intermediate.
N	Q0BZX9	DR	metal ion binding
N	Q0BZX9	DR	nucleoside diphosphate kinase activity
N	Q0BZX9	DR	CTP biosynthetic process
N	Q0BZX9	DR	GTP biosynthetic process
N	Q0BZX9	DR	UTP biosynthetic process
N	Q14BV6	DE	UPF0249 protein ydjC homolog;
N	Q14BV6	DR	hydrolase activity, acting on carbon-nitrogen (but not peptide) bonds
N	Q14BV6	DR	carbohydrate metabolic process
N	Q1CWZ9	DE	Chemotaxis response regulator protein-glutamate methylesterase 5;
N	Q1CWZ9	CC	Involved in the modulation of the chemotaxis system; catalyzes the demethylation of specific methylglutamate residues introduced into the chemoreceptors (methyl-accepting chemotaxis proteins) by CheR (By similarity).
N	Q1CWZ9	DR	protein-glutamate methylesterase activity
N	Q1CWZ9	DR	two-component response regulator activity
N	Q1CWZ9	DR	regulation of transcription, DNA-dependent
N	Q1RGA8	DE	Phospho-N-acetylmuramoyl-pentapeptide-transferase;
N	Q1RGA8	DE	UDP-MurNAc-pentapeptide phosphotransferase;
N	Q1RGA8	CC	First step of the lipid cycle reactions in the biosynthesis of the cell wall peptidoglycan (By similarity).
N	Q1RGA8	DR	phospho-N-acetylmuramoyl-pentapeptide-transferase activity
N	Q1RGA8	DR	UDP-N-acetylmuramoyl-L-alanyl-D-glutamyl-meso-2,6-diaminopimelyl-D-alanyl-D-alanine:undecaprenyl-phosphate transferase activity
N	Q1RGA8	DR	cell division
N	Q1RGA8	DR	cellular cell wall organization
N	Q1RGA8	DR	peptidoglycan biosynthetic process
N	Q1RGA8	DR	regulation of cell shape
N	Q27793	DE	Bifunctional dihydrofolate reductase-thymidylate synthase;
N	Q27793	DE	Dihydrofolate reductase;
N	Q27793	DE	Thymidylate synthase;
N	Q27793	CC	Bifunctional enzyme
N	Q27793	CC	Involved in de novo dTMP biosynthesis
N	Q27793	CC	Key enzyme in folate metabolism
N	Q27793	CC	Catalyzes an essential reaction for de novo glycine and purine synthesis, DNA precursor synthesis, and for the conversion of dUMP to dTMP.
N	Q27793	DR	dihydrofolate reductase activity
N	Q27793	DR	thymidylate synthase activity
N	Q27793	DR	dTMP biosynthetic process
N	Q27793	DR	glycine biosynthetic process
N	Q27793	DR	oxidation-reduction process
N	Q29L39	DE	Protein purity of essence;
N	Q29L39	CC	Has a role in growth of the perineurial glial layer of the larval peripheral nerve
N	Q29L39	CC	May have a role in male fertility and eye development or function
N	Q29L39	CC	May bind calmodulin (By similarity).
N	Q29L39	DR	calmodulin binding
N	Q29L39	DR	ubiquitin-protein ligase activity
N	Q29L39	DR	zinc ion binding
N	Q29L39	DR	nervous system development
N	Q29L39	DR	response to stimulus
N	Q29L39	DR	sperm individualization
N	Q29L39	DR	visual perception
N	Q2L8Y5	DE	30S ribosomal protein S12, chloroplastic;
N	Q2L8Y5	CC	With S4 and S5 plays an important role in translational accuracy
N	Q2L8Y5	CC	Located at the interface of the 30S and 50S subunits (By similarity).
N	Q2L8Y5	DR	structural constituent of ribosome
N	Q2NQI4	DE	33 kDa chaperonin;
N	Q2NQI4	DE	Heat shock protein 33 homolog;
N	Q2NQI4	CC	Redox regulated molecular chaperone
N	Q2NQI4	CC	Protects both thermally unfolding and oxidatively damaged proteins from irreversible aggregation
N	Q2NQI4	CC	Plays an important role in the bacterial defense system toward oxidative stress (By similarity).
N	Q2NQI4	DR	unfolded protein binding
N	Q2NQI4	DR	protein folding

N	Q2P9K6	DE	Fructose-1,6-bisphosphatase class 1;
N	Q2P9K6	DE	FBPase class 1;
N	Q2P9K6	DE	D-fructose-1,6-bisphosphate 1-phosphohydrolase class 1;
N	Q2P9K6	DR	fructose 1,6-bisphosphate 1-phosphatase activity
N	Q2P9K6	DR	metal ion binding
N	Q2P9K6	DR	carbohydrate metabolic process
N	Q30X45	DE	GTP cyclohydrolase folE2;
N	Q30X45	CC	Converts GTP to 7,8-dihydroneopterin triphosphate (By similarity).
N	Q30X45	DR	GTP cyclohydrolase I activity
N	Q30X45	DR	oxidoreductase activity
N	Q30X45	DR	oxidation-reduction process
N	Q30X45	DR	purine base metabolic process
N	Q46CG5	DE	Glutamyl-tRNA synthetase;
N	Q46CG5	DE	Glutamate--tRNA ligase;
N	Q46CG5	CC	Catalyzes the attachment of glutamate to tRNA(Glu) in a two-step reaction: glutamate is first activated by ATP to form Glu-AMP and then transferred to the acceptor end of tRNA(Glu) (By similarity).
N	Q46CG5	DR	glutamate-tRNA ligase activity
N	Q46CG5	DR	glutamyl-tRNA aminoacylation
N	Q48E75	DE	Ribosome maturation factor rimP;
N	Q48E75	CC	Required for maturation of 30S ribosomal subunits (By similarity).
N	Q48E75	DR	ribosome biogenesis
N	Q4R8X1	DE	Endoplasmic reticulum-Golgi intermediate compartment protein 3;
N	Q4R8X1	CC	Possible role in transport between endoplasmic reticulum and Golgi (By similarity).
N	Q4R8X1	DR	vesicle-mediated transport
N	Q4WTT7	DE	GPI mannosyltransferase 4;
N	Q4WTT7	DE	GPI mannosyltransferase IV;
N	Q4WTT7	CC	Alpha-1,2-mannosyltransferase involved in glycosylphosphatidylinositol-anchor biosynthesis
N	Q4WTT7	CC	Transfers a fourth mannose to trimannosyl-GPIs during GPI precursor assembly
N	Q4WTT7	CC	The presence of a fourth mannose in GPI is essential in fungi (By similarity).
N	Q4WTT7	DR	transferase activity, transferring glycosyl groups
N	Q4WTT7	DR	GPI anchor biosynthetic process
N	Q9V2C6	DE	2-phosphoglycerate kinase;
N	Q9V2C6	DR	kinase activity
N	Q9V2C6	DR	phosphotransferase activity, carboxyl group as acceptor
N	Q9A634	DE	2,3-bisphosphoglycerate-dependent phosphoglycerate mutase;
N	Q9A634	DE	BPG-dependent PGAM;
N	Q9A634	DE	Phosphoglyceromutase;
N	Q9A634	CC	Catalyzes the interconversion of 2-phosphoglycerate and 3-phosphoglycerate (By similarity).
N	Q9A634	DR	phosphoglycerate mutase activity
N	Q98BJ6	DE	Apolipoprotein N-acyltransferase;
N	Q98BJ6	DE	ALP N-acyltransferase;
N	Q98BJ6	CC	Transfers the fatty acyl group on membrane lipoproteins (By similarity).
N	Q98BJ6	DR	hydrolase activity, acting on carbon-nitrogen (but not peptide) bonds
N	Q98BJ6	DR	N-acyltransferase activity
N	Q98BJ6	DR	lipoprotein biosynthetic process
N	Q98BJ6	DR	nitrogen compound metabolic process
N	Q8Z4N0	DE	Cysteine desulfurase;
N	Q8Z4N0	CC	Catalyzes the removal of elemental sulfur and selenium atoms from cysteine and selenocysteine to produce alanine
N	Q8Z4N0	CC	Functions as a sulfur delivery protein for NAD, biotin and Fe-S cluster synthesis
N	Q8Z4N0	CC	Transfers sulfur on 'Cys-456' of ThiI in a transpersulfidation reaction
N	Q8Z4N0	CC	Functions also as a selenium delivery protein in the pathway for the biosynthesis of selenophosphate (By similarity).
N	Q8Z4N0	DR	cysteine desulfurase activity
N	Q8Z4N0	DR	pyridoxal phosphate binding
N	Q8Z4N0	DR	cysteine metabolic process
N	Q8XXY2	DE	Tryptophan synthase alpha chain;
N	Q8XXY2	CC	The alpha subunit is responsible for the aldol cleavage of indoleglycerol phosphate to indole and glyceraldehyde 3- phosphate.
N	Q8XXY2	DR	tryptophan synthase activity
N	Q8TE23	DE	Taste receptor type 1 member 2;
N	Q8TE23	DE	G-protein coupled receptor 71;

N	Q8TE23	DE	Sweet taste receptor T1R2;
N	Q8TE23	CC	Putative taste receptor
N	Q8TE23	CC	TAS1R2/TAS1R3 recognizes diverse natural and synthetic sweeteners.
N	Q8TE23	DR	protein heterodimerization activity
N	Q8TE23	DR	taste receptor activity
N	Q8TE23	DR	detection of chemical stimulus involved in sensory perception of sweet taste
N	Q8PC64	DE	4-diphosphocytidyl-2-C-methyl-D-erythritol kinase;
N	Q8PC64	DE	4-(cytidine-5'-diphospho)-2-C-methyl-D-erythritol kinase;
N	Q8PC64	CC	Catalyzes the phosphorylation of the position 2 hydroxy group of 4-diphosphocytidyl-2C-methyl-D-erythritol (By similarity).
N	Q8PC64	DR	4-(cytidine 5'-diphospho)-2-C-methyl-D-erythritol kinase activity
N	Q8PC64	DR	terpenoid biosynthetic process
N	Q8G1B4	DE	NADH-quinone oxidoreductase subunit D;
N	Q8G1B4	DE	NADH dehydrogenase I subunit D;
N	Q8G1B4	DE	NDH-1 subunit D;
N	Q8G1B4	CC	NDH-1 shuttles electrons from NADH, via FMN and iron- sulfur (Fe-S) centers, to quinones in the respiratory chain
N	Q8G1B4	CC	The immediate electron acceptor for the enzyme in this species is believed to be ubiquinone
N	Q8G1B4	CC	Couples the redox reaction to proton translocation (for every two electrons transferred, four hydrogen ions are translocated across the cytoplasmic membrane), and thus conserves the redox energy in a proton gradient (By similarity)
N	Q8G1B4	DR	NADH dehydrogenase (quinone) activity
N	Q8G1B4	DR	quinone binding
N	Q8G1B4	DR	oxidation-reduction process
N	Q8DGJ7	DE	Bifunctional protein pyrR;
N	Q8DGJ7	DE	Pyrimidine operon regulatory protein;
N	Q8DGJ7	DE	Uracil phosphoribosyltransferase;
N	Q8DGJ7	CC	Regulates the transcription of the pyrimidine nucleotide (pyr) operon in response to exogenous pyrimidines (By similarity).
N	Q8DGJ7	DR	uracil phosphoribosyltransferase activity
N	Q8DGJ7	DR	nucleoside metabolic process
N	Q88M09	DE	Methylthioribose-1-phosphate isomerase;
N	Q88M09	DE	MTR-1-P isomerase;
N	Q88M09	DE	S-methyl-5-thioribose-1-phosphate isomerase;
N	Q88M09	CC	Catalyzes the interconversion of methylthioribose-1- phosphate (MTR-1-P) into methylthioribulose-1-phosphate (MTRu-1-P) (By similarity).
N	Q88M09	DR	S-methyl-5-thioribose-1-phosphate isomerase activity
N	Q88M09	DR	methionine biosynthetic process
N	Q7VIA8	DE	Chorismate synthase;
N	Q7VIA8	DE	5-enolpyruvylshikimate-3-phosphate phospholyase;
N	Q7VIA8	DR	chorismate synthase activity
N	Q7VIA8	DR	aromatic amino acid family biosynthetic process
N	Q7UDT8	DE	4-hydroxy-3-methylbut-2-enyl diphosphate reductase;
N	Q7UDT8	CC	Converts 1-hydroxy-2-methyl-2-(E)-butenyl 4-diphosphate into isopentenyl diphosphate (IPP) and dimethylallyl diphosphate (DMAPP) (By similarity).
N	Q7UDT8	DR	3 iron, 4 sulfur cluster binding
N	Q7UDT8	DR	4-hydroxy-3-methylbut-2-en-1-yl diphosphate reductase activity
N	Q7UDT8	DR	metal ion binding
N	Q7UDT8	DR	isopentenyl diphosphate biosynthetic process, mevalonate-independent pathway
N	Q7UDT8	DR	oxidation-reduction process
N	Q7SHE8	DE	Chromosome segregation in meiosis protein 3;
N	Q7SHE8	CC	Forms a fork protection complex (FPC) with tof-1 and which is required for chromosome segregation during meiosis and DNA damage repair
N	Q7SHE8	CC	FPC coordinates leading and lagging strand synthesis and moves with the replication fork
N	Q7SHE8	CC	FPC stabilizes replication forks in a configuration that is recognized by replication checkpoint sensors (By similarity).
N	Q7SHE8	DR	replication fork protection
N	Q75BG6	DE	J domain-containing protein 1;
N	Q75BG6	CC	Probable chaperone (By similarity).
N	Q75BG6	DR	heat shock protein binding
N	Q75BG6	DR	unfolded protein binding
N	Q75BG6	DR	protein folding
N	Q6LZH4	DE	Ketol-acid reductoisomerase;
N	Q6LZH4	DE	Acetohydroxy-acid isomeroreductase;

N	Q6LZH4	DE	Alpha-keto-beta-hydroxylacil reductoisomerase;
N	Q6LZH4	DR	coenzyme binding
N	Q6LZH4	DR	ketol-acid reductoisomerase activity
N	Q6LZH4	DR	branched chain family amino acid biosynthetic process
N	Q6LZH4	DR	oxidation-reduction process
N	Q6CZ80	DE	Putative agmatine deiminase;
N	Q6CZ80	DE	Agmatine iminohydrolase;
N	Q6CZ80	DR	agmatine deiminase activity
N	Q6CZ80	DR	putrescine biosynthetic process
N	Q6DF67	DE	Isochorismatase domain-containing protein 2, mitochondrial;
N	Q6DF67	DR	catalytic activity
N	Q66HG0	DE	E3 ubiquitin-protein ligase RNF13;
N	Q66HG0	DE	RING finger protein 13;
N	Q66HG0	CC	E3 ubiquitin-protein ligase that may play a role in controlling cell proliferation (By similarity).
N	Q66HG0	DR	ubiquitin-protein ligase activity
N	Q66HG0	DR	zinc ion binding
N	Q66HG0	DR	protein autoubiquitination
N	Q5WDT6	DE	Homoserine kinase;
N	Q5WDT6	CC	Catalyzes the ATP-dependent phosphorylation of L- homoserine to L-homoserine phosphate (By similarity).
N	Q5WDT6	DR	homoserine kinase activity
N	Q5WDT6	DR	threonine biosynthetic process
N	Q5KWB0	DE	Acetyl-coenzyme A carboxylase carboxyl transferase subunit alpha;
N	Q5KWB0	DE	ACCase subunit alpha;
N	Q5KWB0	DE	Acetyl-CoA carboxylase carboxyltransferase subunit alpha;
N	Q5KWB0	CC	Component of the acetyl coenzyme A carboxylase (ACC) complex
N	Q5KWB0	CC	First, biotin carboxylase catalyzes the carboxylation of biotin on its carrier protein (BCCP) and then the CO(2) group is transferred by the carboxyltransferase to acetyl-CoA to form malonyl-CoA (By similarity).
N	Q5KWB0	DR	acetyl-CoA carboxylase activity
N	Q5KWB0	DR	fatty acid biosynthetic process
N	Q5F384	DE	Protein YIPF3;
N	Q5F384	DE	YIP1 family member 3;
N	Q5F384	CC	May play a role in hematopoiesis (By similarity).
N	Q5F384	DR	cell differentiation
N	Q4ZYY8	DE	Oligoribonuclease;
N	Q4ZYY8	CC	3'-to-5' exoribonuclease specific for small oligoribonucleotides (By similarity).
N	Q4ZYY8	DR	exonuclease activity
N	Q4ZYY8	DR	nucleic acid binding
N	Q06817	DE	Glycyl-tRNA synthetase 2;
N	Q06817	DE	Diadenosine tetraphosphate synthetase;
N	Q06817	DE	AP-4-A synthetase;
N	Q06817	DE	Glycine--tRNA ligase 2;
N	Q06817	CC	Catalyzes the attachment of glycine to tRNA(Gly)
N	Q06817	CC	Is also able produce diadenosine tetraphosphate (Ap4A), a universal pleiotropic signaling molecule needed for cell regulation pathways, by direct condensation of 2 ATPs (By similarity).
N	Q06817	DR	glycine-tRNA ligase activity
N	Q06817	DR	protein dimerization activity
N	Q06817	DR	diadenosine tetraphosphate biosynthetic process
N	Q06817	DR	glycyl-tRNA aminoacylation
N	B0CEQ4	DE	Glycyl-tRNA synthetase alpha subunit;
N	B0CEQ4	DE	Glycine--tRNA ligase alpha subunit;
N	B0CEQ4	DR	glycine-tRNA ligase activity
N	B0CEQ4	DR	glycyl-tRNA aminoacylation
N	A1TUT1	DE	Glycyl-tRNA synthetase alpha subunit;
N	A1TUT1	DE	Glycine--tRNA ligase alpha subunit;
N	A1TUT1	DR	glycine-tRNA ligase activity
N	A1TUT1	DR	glycyl-tRNA aminoacylation
N	B9MCM3	DE	Glycyl-tRNA synthetase alpha subunit;
N	B9MCM3	DE	Glycine--tRNA ligase alpha subunit;
N	B9MCM3	DR	glycine-tRNA ligase activity
N	B9MCM3	DR	glycyl-tRNA aminoacylation
N	A1W3F2	DE	Glycyl-tRNA synthetase alpha subunit;

N	A1W3F2	DE	Glycine--tRNA ligase alpha subunit;
N	A1W3F2	DR	glycine-tRNA ligase activity
N	A1W3F2	DR	glycyl-tRNA aminoacylation
N	A3N398	DE	Glycyl-tRNA synthetase alpha subunit;
N	A3N398	DE	Glycine--tRNA ligase alpha subunit;
N	A3N398	DR	glycine-tRNA ligase activity
N	A3N398	DR	glycyl-tRNA aminoacylation
N	B3GZ52	DE	Glycyl-tRNA synthetase alpha subunit;
N	B3GZ52	DE	Glycine--tRNA ligase alpha subunit;
N	B3GZ52	DR	glycine-tRNA ligase activity
N	B3GZ52	DR	glycyl-tRNA aminoacylation
N	B0BSX2	DE	Glycyl-tRNA synthetase alpha subunit;
N	B0BSX2	DE	Glycine--tRNA ligase alpha subunit;
N	B0BSX2	DR	glycine-tRNA ligase activity
N	B0BSX2	DR	glycyl-tRNA aminoacylation
N	A6VLE0	DE	Glycyl-tRNA synthetase alpha subunit;
N	A6VLE0	DE	Glycine--tRNA ligase alpha subunit;
N	A6VLE0	DR	glycine-tRNA ligase activity
N	A6VLE0	DR	glycyl-tRNA aminoacylation
N	A0KEK0	DE	Glycyl-tRNA synthetase alpha subunit;
N	A0KEK0	DE	Glycine--tRNA ligase alpha subunit;
N	A0KEK0	DR	glycine-tRNA ligase activity
N	A0KEK0	DR	glycyl-tRNA aminoacylation
N	A4STG2	DE	Glycyl-tRNA synthetase alpha subunit;
N	A4STG2	DE	Glycine--tRNA ligase alpha subunit;
N	A4STG2	DR	glycine-tRNA ligase activity
N	A4STG2	DR	glycyl-tRNA aminoacylation
N	Q8UHP1	DE	Glycyl-tRNA synthetase alpha subunit;
N	Q8UHP1	DE	Glycine--tRNA ligase alpha subunit;
N	Q8UHP1	DR	glycine-tRNA ligase activity
N	Q8UHP1	DR	glycyl-tRNA aminoacylation
N	B6EGT2	DE	Glycyl-tRNA synthetase alpha subunit;
N	B6EGT2	DE	Glycine--tRNA ligase alpha subunit;
N	B6EGT2	DR	glycine-tRNA ligase activity
N	B6EGT2	DR	glycyl-tRNA aminoacylation
N	B4S278	DE	Glycyl-tRNA synthetase alpha subunit;
N	B4S278	DE	Glycine--tRNA ligase alpha subunit;
N	B4S278	DR	glycine-tRNA ligase activity
N	B4S278	DR	glycyl-tRNA aminoacylation
N	B8JDD9	DE	Glycyl-tRNA synthetase alpha subunit;
N	B8JDD9	DE	Glycine--tRNA ligase alpha subunit;
N	B8JDD9	DR	glycine-tRNA ligase activity
N	B8JDD9	DR	glycyl-tRNA aminoacylation
N	Q2IQJ6	DE	Glycyl-tRNA synthetase alpha subunit;
N	Q2IQJ6	DE	Glycine--tRNA ligase alpha subunit;
N	Q2IQJ6	DR	glycine-tRNA ligase activity
N	Q2IQJ6	DR	glycyl-tRNA aminoacylation
N	B4UG88	DE	Glycyl-tRNA synthetase alpha subunit;
N	B4UG88	DE	Glycine--tRNA ligase alpha subunit;
N	B4UG88	DR	glycine-tRNA ligase activity
N	B4UG88	DR	glycyl-tRNA aminoacylation
N	Q3M502	DE	Glycyl-tRNA synthetase alpha subunit;
N	Q3M502	DE	Glycine--tRNA ligase alpha subunit;
N	Q3M502	DR	glycine-tRNA ligase activity
N	Q3M502	DR	glycyl-tRNA aminoacylation
N	O67081	DE	Glycyl-tRNA synthetase alpha subunit;
N	O67081	DE	Glycine--tRNA ligase alpha subunit;
N	O67081	DR	glycine-tRNA ligase activity
N	O67081	DR	glycyl-tRNA aminoacylation
N	A8EW78	DE	Glycyl-tRNA synthetase alpha subunit;
N	A8EW78	DE	Glycine--tRNA ligase alpha subunit;
N	A8EW78	DR	glycine-tRNA ligase activity
N	A8EW78	DR	glycyl-tRNA aminoacylation
N	A7Z6U1	DE	Glycyl-tRNA synthetase alpha subunit;
N	A7Z6U1	DE	Glycine--tRNA ligase alpha subunit;

N	A7Z6U1	DR	glycine-tRNA ligase activity
N	A7Z6U1	DR	glycyl-tRNA aminoacylation
N	Q9KD49	DE	Glycyl-tRNA synthetase alpha subunit;
N	Q9KD49	DE	Glycine--tRNA ligase alpha subunit;
N	Q9KD49	DR	glycine-tRNA ligase activity
N	Q9KD49	DR	glycyl-tRNA aminoacylation
N	Q65H75	DE	Glycyl-tRNA synthetase alpha subunit;
N	Q65H75	DE	Glycine--tRNA ligase alpha subunit;
N	Q65H75	DR	glycine-tRNA ligase activity
N	Q65H75	DR	glycyl-tRNA aminoacylation
N	A8FFB1	DE	Glycyl-tRNA synthetase alpha subunit;
N	A8FFB1	DE	Glycine--tRNA ligase alpha subunit;
N	A8FFB1	DR	glycine-tRNA ligase activity
N	A8FFB1	DR	glycyl-tRNA aminoacylation
N	Q5WHD6	DE	Glycyl-tRNA synthetase alpha subunit;
N	Q5WHD6	DE	Glycine--tRNA ligase alpha subunit;
N	Q5WHD6	DR	glycine-tRNA ligase activity
N	Q5WHD6	DR	glycyl-tRNA aminoacylation
N	P54380	DE	Glycyl-tRNA synthetase alpha subunit;
N	P54380	DE	Glycine--tRNA ligase alpha subunit;
N	P54380	DR	glycine-tRNA ligase activity
N	P54380	DR	glycyl-tRNA aminoacylation
