# Supplementary material for: METSP: A Maximum-Entropy Classifier Based Text Mining Tool for Transporter-Substrate Identification with Semistructured Text
Source: Biomed Res Int. 2015 Oct 1;2015:254838. doi: 10.1155/2015/254838 (PMC4606149; doi:10.1155/2015/254838)
Supplement: Supplementary file 1 — In Additional file 1, the 6955 TSPs were manually collected from UniProt, TransportDB, and TCDB. In Additional file 2, the training set includes 41,332 instances in the format of “label + accession number + field flag + a sentence.” In Additional file 3, the 3942 human TSPs were extracted by METSP. [file 254838.f1.zip › 254838.f1/254838-3.pdf]

Additional file 3 – The 3942 human TSPs were extracted by METSP.

UniProt_AC	CompoundID_KEGG
A0AV02	C00238
A0AV02	C00698
A0AV02	C01373
A0PJK1	C00293
A0PJK1	C01330
A1A5B4	C00076
A1A5B4	C00698
A5D6W6	C01356
A5PKW4	C00215
A5PKW4	C00242
A5X5Y0	C01373
A6NFH5	C01356
A6NH11	C05005
A6NH57	C00035
A6NH57	C00044
A6NIM6	C00098
A6NJY1	C00282
A6NK97	C00059
A6NK97	C00468
A6NM10	C00001
A6NNN8	C00045
A6NNN8	C01330
A6NP61	C01330
A6NP61	C01558
A8K7I4	C00076
A8K7I4	C00698
A8MPY1	C00334
A8MPY1	C00698
A8MTJ3	C00012
A8MTJ3	C00076
A8MTJ3	C00215
A8MTJ3	C00293
A8MTJ3	C00403
A8MTJ3	C00575
A8MTJ3	C00942
A8MTJ3	C01330
A8MTJ3	C01373
A8MTJ3	C01501
A8MTJ3	C11477
A8MTJ3	C15906
A8MTJ3	C16048
A8MYU2	C00076
A8MYU2	C00238
B2C4J0	C00174
B2C4J0	C00326
B7U540	C00238

O00141	C00238
O00141	C00698
O00141	C01330
O00159	C00293
O00159	C00723
O00168	C00698
O00180	C00238
O00217	C00004
O00217	C00399
O00217	C05359
O00244	C00050
O00244	C00070
O00291	C11033
O00291	C12269
O00299	C00698
O00305	C00076
O00337	C00212
O00337	C00396
O00337	C00697
O00337	C00801
O00337	C01330
O00337	C03169
O00337	C07207
O00337	C07210
O00341	C00025
O00341	C00045
O00341	C00302
O00341	C00376
O00341	C00698
O00341	C01330
O00341	C02028
O00391	C00145
O00400	C00024
O00468	C01330
O00476	C00009
O00476	C00293
O00476	C00366
O00476	C01330
O00483	C00004
O00483	C00399
O00483	C05359
O00522	C00007
O00555	C00076
O00555	C00403
O00562	C01194
O00591	C00334
O00591	C00698
O00624	C00009

O00624	C01330
O00631	C00076
O12163	C01356
O12163	C01373
O14520	C00001
O14520	C00116
O14559	C00293
O14559	C00723
O14561	C00004
O14561	C00399
O14561	C05359
O14618	C00070
O14649	C00174
O14649	C00238
O14735	C00055
O14735	C01194
O14745	C00051
O14745	C00282
O14745	C00698
O14745	C01330
O14764	C00334
O14764	C00698
O14791	C00698
O14791	C01356
O14827	C00035
O14827	C00044
O14827	C00076
O14827	C00215
O14827	C00242
O14842	C00076
O14842	C00293
O14842	C00723
O14863	C00038
O14910	C00334
O14925	C00012
O14958	C00076
O14975	C00162
O14980	C16439
O14981	C14439
O14983	C00076
O14983	C00305
O15068	C00215
O15068	C00242
O15078	C00376
O15118	C00187
O15118	C00370
O15130	C16112
O15162	C00076

O15162	C00865
O15211	C00215
O15211	C00242
O15239	C00004
O15239	C00399
O15239	C05359
O15243	C18181
O15244	C00114
O15244	C00179
O15244	C00547
O15244	C00780
O15244	C00791
O15244	C01373
O15244	C03758
O15244	C06818
O15244	C06821
O15244	C06911
O15244	C06952
O15244	C06994
O15244	C07151
O15244	C07233
O15244	C07512
O15244	C11273
O15244	C11310
O15244	C13258
O15244	C13736
O15245	C00114
O15245	C00315
O15245	C00455
O15245	C00750
O15245	C01373
O15245	C01416
O15245	C01479
O15245	C06526
O15245	C06952
O15245	C07073
O15245	C07368
O15245	C07401
O15245	C12269
O15245	C13815
O15247	C00698
O15315	C00039
O15315	C00271
O15374	C00022
O15374	C00033
O15374	C00060
O15374	C00164
O15374	C00174

O15374	C01432
O15374	C01450
O15374	C16434
O15374	C16436
O15374	C16439
O15375	C00022
O15375	C00033
O15375	C00060
O15375	C00164
O15375	C01432
O15375	C01450
O15375	C16434
O15375	C16436
O15375	C16439
O15399	C00076
O15399	C00302
O15399	C00305
O15399	C12269
O15403	C00022
O15403	C00033
O15403	C00060
O15403	C00164
O15403	C00174
O15403	C01432
O15403	C01450
O15403	C16434
O15403	C16436
O15403	C16439
O15427	C00022
O15427	C00033
O15427	C00060
O15427	C00164
O15427	C01432
O15427	C01450
O15427	C16434
O15427	C16436
O15427	C16439
O15431	C00070
O15432	C00070
O15438	C01558
O15439	C00698
O15440	C00215
O15457	C00039
O15496	C00187
O15496	C06254
O15504	C00293
O15528	C00076
O15547	C01373

O15554	C00076
O15554	C00238
O15554	C06922
O43181	C00004
O43181	C00399
O43181	C05359
O43196	C00039
O43246	C00045
O43246	C01602
O43246	C16440
O43306	C00001
O43315	C00001
O43315	C00060
O43315	C00116
O43315	C00396
O43315	C00706
O43315	C15587
O43448	C00238
O43462	C00370
O43502	C00039
O43502	C00271
O43511	C00059
O43511	C00698
O43511	C00708
O43511	C01330
O43520	C00865
O43520	C01558
O43525	C00238
O43526	C00238
O43526	C07485
O43526	C14206
O43542	C00039
O43543	C00039
O43592	C00066
O43615	C00012
O43615	C13028
O43633	C11106
O43674	C00004
O43674	C00399
O43674	C05359
O43676	C00004
O43676	C00399
O43676	C05359
O43677	C00004
O43677	C00399
O43677	C05359
O43678	C00004
O43678	C00399

O43678	C05359
O43681	C06697
O43687	C00076
O43687	C00575
O43707	C00282
O43707	C01330
O43739	C00242
O43761	C03758
O43768	C00723
O43772	C00162
O43772	C00487
O43808	C00147
O43808	C00215
O43819	C00070
O43819	C00524
O43826	C00293
O43861	C00865
O43861	C01373
O43868	C00237
O43868	C00299
O43868	C00801
O43868	C01330
O43868	C15586
O43868	C15587
O43889	C00076
O43920	C00004
O43920	C00399
O43920	C05359
O60229	C00035
O60229	C00044
O60266	C00001
O60285	C00293
O60312	C00865
O60343	C00293
O60343	C00723
O60344	C00012
O60359	C00076
O60359	C00302
O60359	C11033
O60391	C00076
O60391	C00302
O60391	C00305
O60391	C12269
O60423	C00865
O60493	C01330
O60494	C00023
O60494	C01708
O60494	C01834

O60503	C00001
O60656	C15584
O60664	C00159
O60669	C00022
O60669	C00033
O60669	C00060
O60669	C00164
O60669	C01432
O60669	C01450
O60669	C16434
O60669	C16436
O60669	C16439
O60706	C00238
O60721	C00076
O60721	C00205
O60721	C00238
O60721	C00376
O60721	C01330
O60733	C00219
O60733	C00533
O60733	C00840
O60733	C00865
O60741	C00215
O60741	C00238
O60741	C01330
O60779	C00378
O60779	C00504
O60830	C00012
O60831	C00025
O60840	C00076
O60894	C00012
O60894	C06865
O60895	C00012
O60895	C06865
O60896	C00012
O60896	C06865
O60928	C00238
O60931	C00491
O60931	C01420
O60939	C01330
O60939	C13603
O70891	C01356
O70891	C01373
O70901	C01356
O70901	C01373
O75027	C00032
O75110	C00865
O75145	C00082

O75185	C00076
O75185	C00305
O75190	C00237
O75251	C00004
O75251	C00399
O75251	C05359
O75306	C00004
O75306	C00399
O75306	C05359
O75311	C00037
O75311	C00698
O75334	C00082
O75351	C00187
O75351	C00238
O75367	C00301
O75367	C08353
O75380	C00004
O75380	C00399
O75380	C05359
O75387	C00045
O75387	C01330
O75387	C02057
O75417	C00271
O75438	C00004
O75438	C00399
O75438	C05359
O75469	C06708
O75489	C00004
O75489	C00399
O75489	C05359
O75604	C00496
O75643	C00046
O75694	C00293
O75746	C00025
O75746	C00049
O75746	C00076
O75746	C00302
O75746	C16433
O75751	C01373
O75762	C01373
O75771	C00039
O75787	C00080
O75880	C00070
O75899	C00076
O75899	C00137
O75899	C00238
O75899	C00334
O75908	C00187

O75915	C00025
O75915	C00245
O75915	C00302
O75947	C00282
O75947	C05359
O75952	C00076
O75962	C00035
O75962	C00044
O75964	C00282
O75964	C05359
O76024	C00076
O76054	C02477
O76061	C00009
O76061	C00076
O76082	C00487
O76082	C01330
O76082	C01373
O76082	C07512
O76090	C00076
O76090	C00698
O91085	C01356
O91085	C01373
O94759	C00076
O94759	C01330
O94759	C01373
O94761	C00039
O94762	C00039
O94778	C00001
O94778	C01319
O94823	C00865
O94827	C00215
O94827	C00242
O94875	C00001
O94875	C01330
O94956	C00403
O94956	C00584
O94956	C00696
O94956	C01330
O94956	C02166
O94956	C04741
O94956	C05122
O94956	C05963
O95045	C00214
O95045	C00299
O95045	C00526
O95045	C03169
O95045	C16633
O95069	C00238

O95073	C00039
O95139	C00004
O95139	C00399
O95139	C05359
O95149	C00046
O95166	C00334
O95167	C00004
O95167	C00399
O95167	C05359
O95168	C00004
O95168	C00399
O95168	C05359
O95169	C00004
O95169	C00399
O95169	C05359
O95178	C00004
O95178	C00399
O95178	C05359
O95180	C00076
O95180	C00291
O95180	C07222
O95182	C00004
O95182	C00399
O95182	C05359
O95214	C18181
O95236	C01356
O95255	C00051
O95255	C02441
O95259	C00238
O95259	C13240
O95264	C00780
O95264	C01373
O95267	C00035
O95267	C00044
O95267	C00076
O95267	C00165
O95267	C00215
O95267	C00242
O95279	C00174
O95279	C00238
O95298	C00004
O95298	C00399
O95298	C05359
O95299	C00004
O95299	C00399
O95299	C05359
O95342	C01330
O95342	C01558

O95396	C00087
O95397	C05005
O95398	C00215
O95398	C00242
O95398	C00575
O95436	C00009
O95436	C01330
O95445	C00187
O95445	C01356
O95477	C00187
O95477	C00575
O95477	C00865
O95528	C00031
O95528	C00282
O95528	C00293
O95528	C11477
O95622	C00001
O95750	C00293
O95833	C00698
O95907	C00022
O95907	C00033
O95907	C00060
O95907	C00164
O95907	C01432
O95907	C01450
O95907	C16434
O95907	C16436
O95907	C16439
O95954	C00504
O95970	C00238
O96000	C00004
O96000	C00399
O96000	C05359
P00395	C00007
P00395	C00524
P00395	C05359
P00403	C00007
P00403	C00524
P00403	C05359
P00414	C00007
P00414	C00524
P00414	C05359
P00450	C00023
P00450	C00070
P00505	C00162
P00734	C00076
P00740	C01065
P00750	C00471

P00797	C00873
P00797	C01330
P00797	C02246
P00813	C00212
P00813	C15587
P01130	C00187
P01130	C01834
P01133	C00076
P01133	C00305
P01185	C00001
P01185	C01330
P01241	C00293
P01258	C00009
P01258	C00076
P01258	C06865
P01270	C00293
P01275	C00293
P01275	C00723
P01303	C00076
P01308	C00045
P01308	C00293
P01584	C00293
P01588	C01330
P01588	C18200
P01903	C00012
P01906	C00012
P01911	C00012
P01912	C00012
P02008	C00007
P02042	C00007
P02100	C00007
P02144	C00007
P02647	C00157
P02647	C00187
P02647	C00865
P02649	C00187
P02649	C00865
P02652	C00187
P02652	C00865
P02654	C00187
P02654	C00865
P02655	C00187
P02655	C00865
P02656	C00187
P02656	C00865
P02689	C01356
P02708	C01373
P02708	C01996

P02730	C00288
P02730	C00698
P02730	C01708
P02749	C00422
P02768	C00001
P02768	C00038
P02768	C00076
P02768	C00238
P02768	C00486
P02768	C01330
P02768	C01558
P02775	C00293
P02787	C00023
P02787	C00032
P02787	C00288
P02787	C14819
P02788	C00023
P02788	C00288
P02788	C14819
P02790	C00023
P02790	C00032
P02792	C00023
P02794	C00023
P03170	C00012
P03180	C00012
P03886	C00004
P03886	C00399
P03886	C05359
P03891	C00004
P03891	C00399
P03891	C05359
P03897	C00004
P03897	C00399
P03897	C05359
P03901	C00004
P03901	C00399
P03901	C05359
P03905	C00004
P03905	C00399
P03905	C05359
P03915	C00004
P03915	C00399
P03915	C05359
P03923	C00004
P03923	C00399
P03923	C05359
P03928	C00282
P03928	C05359

P04054	C00076
P04054	C00293
P04083	C00076
P04114	C00187
P04156	C00023
P04180	C00187
P04196	C00032
P04196	C00326
P04196	C00374
P04196	C00520
P04196	C00925
P04196	C02148
P04229	C00012
P04271	C00076
P04271	C00238
P04278	C00377
P04798	C00005
P04798	C05359
P04839	C00080
P04899	C00076
P04920	C00288
P05019	C00039
P05019	C00182
P05019	C00293
P05019	C00723
P05023	C00238
P05023	C01330
P05026	C00238
P05026	C01330
P05090	C01356
P05106	C01356
P05141	C00147
P05141	C00215
P05177	C00005
P05177	C05359
P05305	C00533
P05496	C00282
P05496	C05359
P05538	C00012
P05543	C08215
P05771	C00007
P05771	C01834
P05814	C00076
P05814	C00367
P05919	C01356
P05919	C01373
P05921	C01356
P05921	C01373

P05923	C01356
P05923	C01373
P05924	C01356
P05924	C01373
P05925	C01356
P05925	C01373
P05937	C00076
P05937	C04212
P05947	C01356
P05947	C01373
P05948	C01356
P05948	C01373
P05949	C01356
P05949	C01373
P05953	C00524
P06213	C00293
P06213	C00723
P06241	C00076
P06307	C00012
P06307	C00174
P06340	C00012
P06576	C00282
P06576	C05359
P06727	C00187
P06727	C00865
P06756	C01356
P06881	C00076
P06927	C01356
P06927	C02690
P06927	C04911
P07108	C00040
P07108	C02843
P07148	C01356
P07355	C00076
P07510	C01996
P07602	C01356
P07738	C00007
P07738	C01708
P07738	C03339
P07919	C00390
P07919	C00524
P07919	C05359
P07996	C00638
P08069	C00723
P08133	C00076
P08172	C00020
P08172	C00238
P08172	C01996

P08173	C00020
P08173	C00238
P08173	C01996
P08183	C06708
P08195	C00045
P08195	C00050
P08195	C00062
P08195	C00076
P08195	C00078
P08195	C00082
P08195	C00097
P08195	C00123
P08195	C00303
P08195	C00355
P08195	C01330
P08195	C02057
P08195	C02465
P08195	C08215
P08195	C16439
P08195	C18672
P08235	C00001
P08235	C00238
P08519	C01356
P08582	C00023
P08684	C00005
P08684	C05359
P08754	C00044
P08754	C00238
P08803	C01356
P08803	C01373
P08804	C01356
P08804	C01373
P08805	C01356
P08805	C01373
P08806	C01356
P08806	C01373
P08807	C01356
P08807	C01373
P08808	C01356
P08808	C01373
P08912	C00020
P08912	C00238
P08912	C01996
P08913	C00076
P08913	C00238
P09105	C00007
P09131	C01330
P09131	C01558

P09298	C00326
P09327	C00681
P09327	C04637
P09455	C00473
P09486	C00076
P0C0P6	C00076
P0C6Z6	C00012
P0C737	C00012
P0C738	C00012
P0C739	C00012
P0C7X4	C00023
P0C851	C04637
P0CAP9	C00012
P10082	C00012
P10632	C00005
P10632	C05359
P10644	C00001
P10909	C00187
P10909	C00524
P11166	C00031
P11166	C00293
P11166	C05422
P11168	C00031
P11168	C00293
P11168	C01330
P11169	C00031
P11169	C00293
P11169	C05422
P11215	C13603
P11229	C00020
P11229	C00238
P11229	C01996
P11230	C01996
P11388	C00039
P11473	C00076
P11597	C00187
P11597	C00865
P11712	C00005
P11712	C05359
P11717	C00009
P11717	C00159
P12107	C00211
P12235	C00147
P12235	C00215
P12236	C00147
P12236	C00215
P12270	C00293
P12515	C01356

P12515	C01373
P12516	C01356
P12516	C01373
P12517	C01356
P12517	C01373
P12518	C01356
P12518	C01373
P12955	C00211
P13569	C00288
P13569	C00575
P13569	C00698
P13584	C00005
P13584	C05359
P13637	C00238
P13637	C01330
P13686	C00174
P13686	C00898
P13727	C00388
P13760	C00012
P13761	C00012
P13762	C00012
P13765	C00012
P13861	C00001
P13866	C00293
P13866	C01330
P13942	C00211
P14207	C00504
P14334	C00012
P14345	C00012
P14415	C00238
P14415	C01330
P14415	C13603
P14416	C00076
P14416	C00238
P14416	C01330
P14416	C03758
P14618	C00293
P14672	C00031
P14672	C00293
P14672	C00723
P14867	C00334
P14867	C00698
P14920	C00740
P14927	C00390
P14927	C00524
P14927	C05359
P15090	C01356
P15328	C00504

P15382	C00238
P16112	C00866
P16152	C00051
P16260	C00010
P16389	C00238
P16444	C05951
P16444	C05952
P16499	C00076
P16615	C00076
P16615	C00305
P16671	C00162
P16671	C00187
P16671	C00638
P16671	C01834
P17081	C00293
P17516	C00069
P17516	C01558
P17516	C01792
P17568	C00004
P17568	C00399
P17568	C05359
P17612	C00001
P17658	C00238
P17787	C00076
P17787	C01373
P17787	C01996
P17844	C00046
P18428	C00338
P18428	C01356
P18505	C00334
P18505	C00698
P18507	C00334
P18507	C00698
P18806	C01356
P18806	C01373
P18859	C00282
P18859	C05359
P19224	C15584
P19367	C00293
P19404	C00004
P19404	C00399
P19404	C05359
P19440	C00051
P19440	C00736
P19544	C00039
P19544	C00046
P19554	C01356
P19554	C01373

P19634	C00080
P19634	C00282
P19634	C01330
P19634	C06821
P19793	C00777
P20020	C00076
P20020	C00305
P20036	C00012
P20039	C00012
P20061	C00175
P20062	C00175
P20062	C05776
P20309	C00020
P20309	C00238
P20309	C01996
P20382	C16030
P20585	C00039
P20591	C00076
P20645	C00009
P20645	C00159
P20648	C00080
P20648	C00238
P20648	C00282
P20813	C00005
P20813	C05359
P20815	C00005
P20815	C05359
P20823	C00293
P20853	C00005
P20853	C05359
P20882	C01356
P20882	C01373
P21399	C00023
P21439	C00157
P21439	C06708
P21554	C00076
P21673	C06702
P21728	C00238
P21754	C00034
P21754	C00076
P21757	C00187
P21817	C00076
P21817	C08705
P21917	C00076
P21917	C00238
P21917	C00282
P21917	C01330
P22001	C00238

P22059	C00187
P22059	C01356
P22307	C01356
P22413	C00013
P22413	C00215
P22413	C00293
P22459	C00238
P22460	C00238
P22466	C00723
P22466	C18181
P22612	C00001
P22626	C00046
P22680	C00187
P22694	C00001
P22732	C00293
P22732	C01496
P22748	C00288
P22748	C01330
P23297	C00076
P23297	C00238
P23327	C00076
P23415	C00037
P23415	C00698
P23416	C00037
P23416	C00698
P23588	C00046
P23634	C00076
P23634	C00305
P23975	C00547
P23975	C00706
P23975	C01330
P24046	C00334
P24046	C00698
P24462	C00005
P24462	C05359
P24539	C05359
P24723	C00076
P24723	C00716
P24723	C00865
P25100	C00076
P25101	C00293
P25705	C05359
P25963	C00187
P26678	C00076
P27169	C00187
P27352	C00175
P27986	C00293
P28288	C00215

P28288	C00638
P28289	C00839
P28331	C00004
P28331	C00399
P28331	C05359
P28472	C00334
P28472	C00698
P28476	C00334
P28476	C00698
P29373	C00777
P29475	C00076
P29972	C00001
P29972	C00011
P29972	C00014
P29972	C00116
P29972	C00238
P29972	C00533
P29972	C00942
P29972	C01373
P29973	C00215
P29973	C00942
P29973	C01373
P30044	C00027
P30044	C00342
P30049	C05359
P30301	C00001
P30518	C00001
P30531	C00334
P30531	C00698
P30531	C01330
P30532	C01373
P30532	C01996
P30536	C00032
P30536	C00187
P30626	C00076
P30825	C00045
P30825	C01602
P30825	C16440
P30872	C16022
P30874	C00076
P30926	C01373
P30926	C01996
P30968	C18183
P30989	C01836
P31321	C00001
P31323	C00001
P31327	C00014
P31327	C00086

P31415	C00076
P31639	C00293
P31639	C01330
P31641	C00045
P31641	C00099
P31641	C00245
P31641	C00698
P31641	C01330
P31644	C00334
P31644	C00698
P31645	C00780
P31645	C01330
P31749	C00293
P31749	C00638
P31749	C00723
P31751	C00293
P31751	C00638
P32119	C00342
P32121	C00723
P32189	C00116
P32297	C01373
P32297	C01996
P32322	C00003
P32322	C00006
P32418	C00076
P32418	C01330
P33056	C00039
P33260	C00005
P33260	C05359
P33527	C00051
P33527	C00951
P33527	C01937
P33527	C02166
P33527	C03033
P33806	C00039
P33827	C00046
P33849	C00039
P33897	C00215
P34903	C00334
P34903	C00698
P35222	C00076
P35367	C00388
P35367	C02012
P35408	C01780
P35498	C01330
P35499	C01330
P35520	C00283
P35520	C00716

P35520	C00736
P35523	C00698
P35557	C00293
P35557	C00723
P35568	C00293
P35575	C00293
P35610	C00187
P35613	C00060
P35613	C00376
P35613	C01432
P35658	C00293
P35670	C00070
P35790	C01356
P35913	C00076
P35966	C01356
P35966	C01373
P36021	C00060
P36021	C00174
P36021	C02465
P36021	C07639
P36021	C08215
P36404	C00076
P36404	C01996
P36542	C05359
P36544	C00076
P36544	C00698
P36544	C01373
P36544	C01996
P37088	C01330
P37088	C06821
P37198	C00293
P37231	C00638
P37231	C01834
P37840	C00547
P37840	C00780
P37840	C03758
P38571	C00287
P38571	C00356
P38571	C01834
P38919	C00046
P39086	C00302
P39086	C01373
P39905	C03758
P40126	C17936
P40126	C17937
P40145	C00001
P40222	C00076
P40261	C06708

P40879	C00059
P40879	C00288
P40879	C00698
P41143	C00076
P41143	C00238
P41145	C00076
P41145	C00238
P41180	C00076
P41181	C00001
P41181	C00116
P41181	C00840
P41222	C00376
P41222	C00486
P41222	C00500
P41222	C00777
P41222	C08215
P41231	C00075
P41231	C13742
P41439	C00504
P41440	C00080
P41440	C00504
P41440	C01937
P41595	C00076
P41743	C00293
P42261	C00302
P42261	C01373
P42262	C00302
P42262	C01373
P42263	C00302
P42263	C01373
P42658	C00238
P43003	C00025
P43003	C00045
P43003	C00302
P43003	C00402
P43003	C01330
P43003	C02028
P43003	C16433
P43004	C00025
P43004	C00045
P43004	C00302
P43004	C00402
P43004	C01330
P43004	C02028
P43004	C16433
P43005	C00025
P43005	C00045
P43005	C00302

P43005	C00402
P43005	C01330
P43005	C02028
P43005	C16433
P43007	C00041
P43007	C00045
P43007	C00065
P43007	C00148
P43007	C00188
P43007	C00491
P43007	C00698
P43007	C00716
P43007	C00736
P43007	C01015
P43007	C01330
P43007	C01401
P43007	C02028
P43155	C00024
P43246	C00039
P43487	C00044
P43652	C01834
P43652	C02477
P43681	C00076
P43681	C01373
P43681	C01996
P45844	C00187
P45844	C00326
P45844	C00370
P45844	C00865
P45844	C01356
P46059	C00012
P46059	C00080
P46059	C00098
P46059	C00282
P46063	C00039
P46063	C00305
P46063	C00434
P46098	C00780
P46098	C01373
P46721	C00403
P46721	C01330
P46721	C01558
P46721	C11363
P46934	C01330
P47224	C00215
P47224	C00242
P47710	C00009
P47710	C00076

P47869	C00334
P47869	C00698
P47870	C00334
P47870	C00698
P47871	C00293
P47871	C00723
P47871	C01501
P48029	C00300
P48029	C00698
P48029	C01330
P48047	C05359
P48048	C00238
P48048	C13881
P48050	C00238
P48051	C00238
P48051	C00293
P48051	C00723
P48058	C00302
P48058	C01373
P48061	C00076
P48065	C00334
P48065	C00698
P48065	C00719
P48065	C01330
P48066	C00334
P48066	C00698
P48066	C01330
P48067	C00037
P48067	C00698
P48067	C01330
P48167	C00037
P48167	C00698
P48169	C00334
P48169	C00698
P48201	C00282
P48201	C05359
P48454	C00391
P48544	C00238
P48547	C00238
P48549	C00238
P48553	C01330
P48651	C00716
P48664	C00025
P48664	C00045
P48664	C00049
P48664	C00302
P48664	C00402
P48664	C01330

P48664	C02028
P48664	C16433
P48723	C00012
P48751	C00288
P48751	C00698
P48764	C00080
P48764	C00282
P48764	C01330
P48995	C00076
P48995	C01373
P49146	C00076
P49279	C00023
P49279	C00034
P49279	C00050
P49279	C00062
P49279	C00088
P49279	C00282
P49279	C01413
P49279	C02148
P49281	C00023
P49281	C00034
P49281	C00038
P49281	C00050
P49281	C00070
P49281	C00175
P49281	C00282
P49281	C00291
P49281	C00572
P49281	C01413
P49281	C02148
P49281	C06267
P49281	C06696
P49321	C01429
P49447	C05359
P49448	C00302
P49675	C00187
P49763	C00374
P49768	C00076
P49790	C00293
P49792	C00293
P49810	C00076
P49821	C00004
P49821	C00399
P49821	C05359
P49840	C00293
P49908	C01529
P49914	C00045
P49914	C00214

P49914	C00504
P49914	C06265
P50120	C00473
P50238	C00038
P50443	C00059
P50613	C00039
P50993	C00238
P50993	C01330
P50995	C00076
P51161	C01558
P51164	C00080
P51164	C00238
P51164	C00282
P51164	C01330
P51168	C01330
P51168	C06821
P51170	C01330
P51170	C06821
P51172	C01330
P51172	C06821
P51531	C00039
P51532	C00039
P51575	C00076
P51575	C01373
P51659	C00370
P51693	C00211
P51693	C00374
P51787	C00238
P51787	C00575
P51787	C00698
P51787	C07485
P51787	C14206
P51788	C00698
P51790	C00080
P51790	C00698
P51793	C00080
P51793	C00698
P51795	C00080
P51795	C00698
P51798	C00080
P51798	C00698
P51800	C00698
P51801	C00698
P51810	C00076
P51810	C00355
P51811	C00045
P51811	C01330
P51828	C00001

P51970	C00004
P51970	C00399
P51970	C05359
P52306	C00035
P52306	C00044
P52569	C00045
P52569	C01602
P52569	C16440
P52701	C00039
P52735	C00215
P52735	C00242
P52789	C00293
P52790	C00293
P52948	C00293
P52952	C00076
P52952	C01330
P53007	C00149
P53007	C00158
P53675	C00293
P53794	C00137
P53794	C01330
P53985	C00022
P53985	C00033
P53985	C00060
P53985	C00164
P53985	C01432
P53985	C01450
P53985	C16434
P53985	C16436
P53985	C16439
P54105	C00215
P54105	C00698
P54132	C00039
P54132	C00305
P54132	C00434
P54219	C00706
P54284	C00076
P54289	C00076
P54368	C06702
P54707	C00080
P54707	C00238
P54707	C00282
P54709	C00238
P54709	C01330
P54710	C00238
P54710	C01330
P54750	C00575
P54750	C00942

P55011	C00014
P55011	C00238
P55011	C00698
P55011	C01330
P55011	C06859
P55017	C00698
P55017	C01330
P55036	C00496
P55042	C00076
P55055	C00187
P55055	C01356
P55056	C01356
P55058	C00865
P55058	C01356
P55064	C00001
P55064	C00011
P55072	C00305
P55073	C08215
P55087	C00001
P55157	C00287
P55157	C00422
P55157	C00865
P55157	C01356
P55259	C00326
P55263	C00147
P55263	C00212
P55263	C00801
P56134	C05359
P56181	C00004
P56181	C00399
P56181	C05359
P56211	C00238
P56211	C00293
P56373	C00076
P56373	C01373
P56381	C05359
P56385	C00282
P56385	C05359
P56539	C00238
P56539	C01330
P56556	C00004
P56556	C00399
P56556	C05359
P56559	C00187
P56696	C00238
P56696	C07485
P56696	C14206
P57057	C00093

P57103	C00076
P57103	C01330
P57727	C01330
P57740	C00293
P57789	C00238
P57796	C00076
P58743	C00059
P59095	C01356
P59535	C00076
P59535	C01373
P59537	C00076
P59537	C01373
P59632	C00238
P60528	C00012
P61073	C00076
P61073	C00496
P61073	C00575
P61586	C00035
P61586	C00044
P61916	C00187
P61916	C00865
P61916	C05005
P62158	C00076
P62158	C08705
P62166	C00076
P62826	C00044
P62826	C00046
P62942	C00076
P62942	C08705
P62955	C00076
P62955	C00302
P62955	C11033
P63092	C00001
P63096	C00035
P63172	C00778
P63252	C00238
P68106	C00076
P68106	C08705
P68871	C00007
P68871	C00533
P69699	C01356
P69699	C01373
P69700	C01356
P69700	C01373
P69891	C00007
P69892	C00007
P69905	C00007
P78329	C00005

P78329	C05359
P78334	C00334
P78334	C00698
P78348	C00076
P78348	C00174
P78348	C01330
P78348	C01373
P78348	C06821
P78352	C00174
P78352	C00238
P78352	C12269
P78363	C00376
P78363	C00473
P78363	C02110
P78381	C00052
P78381	C00215
P78381	C00282
P78381	C11477
P78382	C00055
P78382	C00128
P78382	C00174
P78382	C00282
P78382	C11477
P78383	C00052
P78383	C11477
P78406	C00293
P78411	C00238
P78508	C00238
P78562	C00009
P80075	C00076
P80188	C00023
P81172	C00023
P82251	C00045
P82251	C00326
P82251	C00491
P82251	C01330
P82251	C01420
P82980	C00473
P89433	C00326
P98155	C01356
P98194	C00034
P98194	C00076
P98194	C00305
P98196	C00865
P98198	C00865
Q00169	C00157
Q00169	C01194
Q00325	C00009

Q00325	C00080
Q00341	C01356
Q00839	C00039
Q008S8	C00215
Q008S8	C00242
Q00975	C00076
Q00975	C00403
Q01118	C01330
Q01362	C00076
Q01484	C13603
Q01650	C00045
Q01650	C00050
Q01650	C00062
Q01650	C00078
Q01650	C00082
Q01650	C00097
Q01650	C00355
Q01650	C01330
Q01650	C02057
Q01650	C02465
Q01650	C08215
Q01650	C16439
Q01650	C18672
Q01668	C00076
Q01668	C00403
Q01814	C00076
Q01814	C00305
Q01959	C00706
Q01959	C01330
Q01959	C03758
Q02094	C00011
Q02094	C00014
Q02094	C01371
Q02156	C00076
Q02156	C00716
Q02156	C00865
Q02246	C00238
Q02641	C00076
Q02880	C00039
Q02978	C00026
Q02978	C00149
Q02978	C00311
Q02978	C00697
Q02978	C01432
Q03135	C00076
Q03181	C00162
Q03181	C00293
Q03468	C00039

Q03518	C00012
Q03518	C00098
Q03518	C00326
Q03519	C00012
Q03519	C00098
Q03519	C00326
Q03721	C00238
Q04446	C00182
Q04609	C00504
Q04656	C00070
Q04671	C00082
Q04671	C00158
Q04671	C05606
Q04671	C06697
Q04759	C00076
Q04759	C00716
Q04759	C00865
Q04828	C01558
Q04844	C01373
Q04844	C01996
Q05513	C00076
Q05513	C00716
Q05513	C00865
Q05586	C00076
Q05586	C00302
Q05586	C00305
Q05586	C12269
Q05655	C00076
Q05655	C00716
Q05655	C00865
Q05901	C01373
Q05901	C01996
Q05932	C00504
Q05940	C00388
Q05940	C00547
Q05940	C00706
Q05940	C00780
Q05940	C03758
Q06055	C00282
Q06055	C05359
Q06432	C00076
Q06495	C00009
Q06495	C01330
Q06609	C00039
Q06609	C00271
Q06830	C00342
Q06830	C07292
Q07001	C01373

Q07001	C01996
Q07666	C00046
Q07699	C01330
Q07812	C00524
Q07837	C00045
Q07837	C00491
Q07837	C01330
Q07837	C01420
Q07869	C00162
Q07889	C00035
Q07889	C00044
Q07890	C00035
Q07890	C00044
Q07954	C00187
Q07954	C01834
Q08289	C00076
Q08289	C13603
Q08357	C00009
Q08357	C00174
Q08357	C01330
Q08357	C01356
Q08462	C00001
Q08828	C00001
Q08945	C00039
Q08945	C00434
Q08945	C06911
Q08AI6	C00045
Q08AI6	C01330
Q09327	C00326
Q09328	C00326
Q09428	C00238
Q09428	C00723
Q09470	C00238
Q0D2K0	C00034
Q0D2K0	C00175
Q0D2K0	C00305
Q0D2K0	C13881
Q0D2K0	C13884
Q0GE19	C01330
Q0GE19	C01558
Q0VD83	C01356
Q10588	C00076
Q10588	C13050
Q12767	C01373
Q12769	C00046
Q12769	C00293
Q12769	C00895
Q12770	C00370

Q12791	C00076
Q12791	C00238
Q12802	C00044
Q12802	C00215
Q12802	C00242
Q12809	C00238
Q12809	C13240
Q12864	C00012
Q12879	C00076
Q12879	C00302
Q12879	C00305
Q12879	C12269
Q12904	C00293
Q12904	C01501
Q12907	C00159
Q12908	C00403
Q12908	C01330
Q12908	C01558
Q12908	C05122
Q12959	C00238
Q12967	C00215
Q12967	C00242
Q12980	C00697
Q12983	C00076
Q13002	C00302
Q13002	C01373
Q13126	C00147
Q13126	C01733
Q13126	C06702
Q13127	C00076
Q13127	C00238
Q13133	C00187
Q13133	C01356
Q13136	C00082
Q13144	C00035
Q13183	C00042
Q13183	C00158
Q13183	C01330
Q13183	C02028
Q13201	C00752
Q13224	C00076
Q13224	C00302
Q13224	C00305
Q13224	C12269
Q13286	C00062
Q13286	C00076
Q13303	C00238
Q13303	C13603

Q13322	C00293
Q13336	C00086
Q13352	C08215
Q13433	C00038
Q13459	C00076
Q13488	C00282
Q13501	C00238
Q13507	C00076
Q13507	C01373
Q13520	C00001
Q13520	C00174
Q13522	C00182
Q13563	C00076
Q13563	C00698
Q13563	C01330
Q13563	C01373
Q13586	C00076
Q13621	C00238
Q13621	C00698
Q13621	C01330
Q13621	C06859
Q13671	C00035
Q13671	C00044
Q13671	C00215
Q13671	C00242
Q13698	C00076
Q13698	C00403
Q13733	C00238
Q13733	C01330
Q13790	C00187
Q13790	C00287
Q13790	C01356
Q13796	C01330
Q13829	C00238
Q13932	C00076
Q13932	C00391
Q13932	C00403
Q13972	C00035
Q13972	C00044
Q13972	C00215
Q14003	C00238
Q14008	C00046
Q14028	C00205
Q14028	C00215
Q14028	C00575
Q14028	C00942
Q14028	C01373
Q14032	C00040

Q14032	C01356
Q14032	C01558
Q14061	C00070
Q14123	C00575
Q14123	C00942
Q14183	C00076
Q14184	C00076
Q14184	C00293
Q14184	C00723
Q14289	C00076
Q14289	C00238
Q14397	C00293
Q14409	C00116
Q14410	C00116
Q14435	C00009
Q14493	C01429
Q14500	C00238
Q14524	C01330
Q14524	C11692
Q14542	C00262
Q14542	C00396
Q14542	C00801
Q14542	C15587
Q14562	C00046
Q14565	C00039
Q14571	C00076
Q14571	C00575
Q14571	C01245
Q14573	C00076
Q14573	C01245
Q14643	C00076
Q14643	C00137
Q14643	C01245
Q14644	C00076
Q14644	C00137
Q14654	C00238
Q14681	C00238
Q14721	C00238
Q14722	C00238
Q14728	C06570
Q14802	C00698
Q14849	C00187
Q14894	C08215
Q14916	C00009
Q14916	C01330
Q14940	C00080
Q14940	C00282
Q14940	C01330

Q14957	C00076
Q14957	C00302
Q14957	C00305
Q14957	C12269
Q14973	C00174
Q14973	C00403
Q14973	C01330
Q14973	C01558
Q14973	C05122
Q14C86	C00723
Q14CN2	C00076
Q14CN2	C00698
Q15012	C00801
Q15043	C00023
Q15043	C00038
Q15049	C01373
Q15077	C00015
Q15077	C00075
Q15080	C00005
Q15080	C00007
Q15109	C00012
Q15121	C00293
Q15121	C00723
Q15276	C00215
Q15311	C00051
Q15311	C01661
Q15388	C00012
Q15391	C00075
Q15413	C00076
Q15413	C08705
Q15438	C00242
Q15545	C00750
Q15642	C00723
Q15651	C00037
Q15651	C00293
Q15669	C01321
Q15700	C00238
Q15700	C12269
Q15722	C00075
Q15722	C00076
Q15726	C00076
Q15751	C00035
Q15751	C00044
Q15758	C00045
Q15758	C01330
Q15758	C02028
Q15788	C08215
Q15822	C01373

Q15822	C01996
Q15825	C01373
Q15825	C01996
Q15842	C00238
Q15843	C00496
Q15848	C00187
Q15848	C00293
Q15849	C00086
Q15849	C00840
Q15858	C01330
Q15858	C11692
Q15878	C00076
Q15878	C00291
Q15878	C00403
Q16206	C00003
Q16206	C05359
Q16222	C00140
Q16222	C01074
Q16280	C00215
Q16280	C00575
Q16280	C01373
Q16281	C00076
Q16281	C00215
Q16281	C00575
Q16281	C00942
Q16281	C01373
Q16281	C06958
Q16322	C00215
Q16322	C00238
Q16322	C00575
Q16322	C01373
Q16348	C00012
Q16348	C00080
Q16348	C00098
Q16348	C00282
Q16445	C00334
Q16445	C00698
Q16515	C00174
Q16515	C01330
Q16515	C01373
Q16515	C06821
Q16518	C00376
Q16558	C00076
Q16558	C00238
Q16558	C13837
Q16572	C01996
Q16627	C00076
Q16627	C12405

Q16635	C05359
Q16661	C00001
Q16678	C00005
Q16678	C05359
Q16718	C00004
Q16718	C00399
Q16718	C05359
Q16720	C00076
Q16720	C00305
Q16795	C00004
Q16795	C00399
Q16795	C01330
Q16795	C05359
Q16878	C00736
Q17RG1	C00238
Q1EHB4	C00022
Q1EHB4	C00060
Q1EHB4	C00163
Q1EHB4	C00246
Q1EHB4	C00253
Q1EHB4	C01330
Q1EHB4	C01432
Q29974	C00012
Q2I0M4	C00076
Q2I0M4	C00238
Q2M3G0	C01661
Q2M3M2	C00159
Q2M3M2	C00293
Q2M3M2	C01330
Q2M3M2	C10906
Q2NKJ3	C00039
Q2NKJ3	C00271
Q2Y0W8	C00174
Q2Y0W8	C00288
Q2Y0W8	C01330
Q2Y0W8	C12603
Q2Y0W8	C15473
Q30134	C00012
Q30167	C00012
Q30201	C00023
Q32M45	C00076
Q32M45	C00698
Q3KNW5	C00377
Q3KNW5	C01330
Q3KNW5	C01558
Q3KNW5	C02592
Q3L8U1	C00039
Q3SYG4	C00035

Q3SYG4	C00044
Q3ZCQ8	C00012
Q401N2	C00038
Q495M3	C00037
Q495M3	C00045
Q495M3	C16435
Q495N2	C00045
Q4G0N8	C00080
Q4G0N8	C00282
Q4G0N8	C01330
Q4G0X4	C00238
Q4KMQ2	C00076
Q4KMQ2	C00698
Q4KMZ8	C00238
Q4KMZ8	C01330
Q4U2R8	C00059
Q4U2R8	C00076
Q4U2R8	C00174
Q4U2R8	C00242
Q4U2R8	C00366
Q4U2R8	C00736
Q4U2R8	C00954
Q4U2R8	C01330
Q4U2R8	C01586
Q4U2R8	C01926
Q4U2R8	C02028
Q4U2R8	C02181
Q4U2R8	C05151
Q4U2R8	C05551
Q4U2R8	C05658
Q4U2R8	C06810
Q4U2R8	C06909
Q4U2R8	C06952
Q4U2R8	C07017
Q4U2R8	C07072
Q4U2R8	C07372
Q4U2R8	C11277
Q4U2R8	C11309
Q4U2R8	C12600
Q4U2R8	C13008
Q4VNC0	C01373
Q4VNC1	C01373
Q4ZJI4	C00282
Q504Y0	C00038
Q504Y0	C00050
Q53GD3	C00114
Q53S99	C00504
Q53TN4	C14819

Q5EBM0	C00396
Q5I7T1	C00238
Q5JRA6	C00211
Q5JS13	C00215
Q5JS13	C00242
Q5JSL3	C00035
Q5JSL3	C00044
Q5JSL3	C00215
Q5JSL3	C00242
Q5JUK3	C00076
Q5JUK3	C00237
Q5JUK3	C00238
Q5JUK3	C00698
Q5JUK3	C01330
Q5JVL4	C00076
Q5JXX5	C00037
Q5JXX5	C00698
Q5K4L6	C00162
Q5K4L6	C00638
Q5NUL3	C00293
Q5PT55	C01330
Q5PT55	C01558
Q5SRD1	C00012
Q5SRE5	C00293
Q5SW96	C00187
Q5SW96	C01834
Q5T2N8	C11106
Q5T2W1	C00009
Q5T2W1	C00187
Q5T2W1	C00487
Q5T2W1	C00698
Q5T2W1	C01330
Q5T9A4	C11106
Q5TA50	C05005
Q5TAH2	C00080
Q5TAH2	C00282
Q5TAH2	C01330
Q5TF39	C00293
Q5TF39	C01330
Q5TH69	C00215
Q5TH69	C00242
Q5VTU8	C05359
Q5VU97	C00076
Q5VV41	C00035
Q5VV41	C00044
Q5VXU1	C00238
Q5VXU1	C01330
Q5VYX0	C00706

Q5VYX0	C13512
Q5W0U4	C00076
Q5XXA6	C00076
Q5XXA6	C00698
Q63HQ0	C18340
Q658P3	C00023
Q68DU8	C00238
Q693B1	C00238
Q695T7	C00045
Q695T7	C00698
Q695T7	C01330
Q695T7	C16439
Q6AI14	C00080
Q6AI14	C00238
Q6AI14	C00282
Q6AI14	C01330
Q6AI14	C11591
Q6AI14	C15473
Q6B0K9	C00007
Q6DN90	C00044
Q6DN90	C00215
Q6DN90	C00242
Q6FHJ7	C00009
Q6FHJ7	C01330
Q6FI04	C00018
Q6GPH4	C00524
Q6IAA8	C00187
Q6IWH7	C00076
Q6IWH7	C00698
Q6J4K2	C00076
Q6J4K2	C00238
Q6J4K2	C01330
Q6J4K2	C01373
Q6KCM7	C00009
Q6KCM7	C00076
Q6KCM7	C00147
Q6MZM0	C00070
Q6NUK1	C00009
Q6NUK1	C00076
Q6NUK1	C00147
Q6NVV3	C00034
Q6NVV3	C00070
Q6NVV3	C00175
Q6NVV3	C00305
Q6NVV3	C13881
Q6NVV3	C13884
Q6NVV3	C14818
Q6NXT4	C00038

Q6P1K1	C00032
Q6P1M0	C00162
Q6P1M0	C00638
Q6P1N0	C00076
Q6P4A7	C00023
Q6P4A7	C01373
Q6P4Q7	C00050
Q6P5W5	C00038
Q6P9H4	C01330
Q6P9H4	C01780
Q6PCB7	C00162
Q6PCB7	C00638
Q6PCB7	C00723
Q6PI25	C00302
Q6PI25	C11033
Q6PI47	C00238
Q6PIL6	C00076
Q6PIL6	C00238
Q6PIS1	C00072
Q6PIS1	C01330
Q6PIU1	C00238
Q6PL18	C11106
Q6PML9	C00038
Q6PML9	C01373
Q6PXP3	C00293
Q6PXP3	C00586
Q6PXP3	C01394
Q6PXP3	C01496
Q6PXP3	C01582
Q6PXP3	C11477
Q6Q788	C01356
Q6RUJ8	C00076
Q6U841	C00288
Q6U841	C00698
Q6U841	C01330
Q6UVM3	C00076
Q6UVM3	C00238
Q6UVM3	C00698
Q6UVM3	C01330
Q6UVM3	C07518
Q6UWJ1	C00080
Q6UWJ1	C00282
Q6UWJ1	C01330
Q6UXH1	C01996
Q6XR72	C00038
Q6XR72	C01373
Q6YBV0	C00041
Q6YBV0	C00045

Q6YBV0	C00078
Q6YBV0	C01330
Q6YBV0	C01401
Q6YBV0	C16435
Q6ZMD2	C01356
Q6ZMH5	C00038
Q6ZMH5	C00050
Q6ZMJ2	C00023
Q6ZMT4	C01429
Q6ZNA5	C14818
Q6ZNA5	C14819
Q6ZNW5	C00394
Q6ZQN7	C00575
Q6ZQN7	C01330
Q6ZQN7	C01443
Q6ZQN7	C01937
Q6ZQN7	C06956
Q6ZQN7	C14206
Q6ZRH7	C01373
Q6ZRS2	C01429
Q6ZSM3	C00060
Q6ZSZ5	C00215
Q6ZSZ5	C00242
Q6ZWB6	C00238
Q70625	C01356
Q70625	C01373
Q70HW3	C00019
Q70J99	C00076
Q70Z35	C01194
Q70Z44	C01373
Q719H9	C00238
Q71RS6	C00076
Q71RS6	C00238
Q71RS6	C01330
Q71RS6	C01373
Q75007	C01356
Q75007	C01373
Q75T13	C00137
Q75V66	C00076
Q75V66	C00698
Q76EJ3	C00015
Q76EJ3	C00029
Q76EJ3	C00035
Q76EJ3	C00043
Q76EJ3	C00096
Q76EJ3	C00144
Q76EJ3	C00159
Q76EJ3	C00215

Q77376	C01356
Q77376	C01373
Q77NP2	C00326
Q79669	C01356
Q79669	C01373
Q7L0Q8	C00044
Q7L0Q8	C00215
Q7L0Q8	C00242
Q7L273	C00238
Q7LBE3	C00059
Q7LBE3	C00209
Q7LBE3	C00288
Q7LBE3	C00320
Q7LBE3	C00698
Q7LBE3	C11591
Q7LBE5	C00293
Q7LBE5	C00723
Q7LBR1	C11106
Q7LDG7	C00035
Q7LDG7	C00044
Q7LDG7	C00076
Q7LDG7	C00215
Q7LDG7	C00242
Q7RTM1	C00076
Q7RTP0	C00034
Q7RTP0	C00175
Q7RTP0	C00305
Q7RTP0	C13881
Q7RTP0	C13884
Q7RTP0	C14818
Q7RTS1	C00076
Q7RTT9	C00212
Q7RTT9	C00214
Q7RTT9	C00294
Q7RTT9	C00299
Q7RTT9	C00387
Q7RTT9	C00475
Q7RTT9	C00547
Q7RTT9	C00698
Q7RTT9	C00780
Q7RTT9	C00801
Q7RTT9	C01330
Q7RTT9	C01373
Q7RTT9	C03758
Q7RTT9	C11310
Q7RTX7	C00076
Q7RTX7	C01373
Q7RTX9	C00060

Q7RTY0	C00060
Q7RTY1	C00060
Q7Z2D5	C00865
Q7Z2E3	C00020
Q7Z2H8	C00037
Q7Z2H8	C00045
Q7Z2H8	C01401
Q7Z2H8	C16435
Q7Z2W7	C01373
Q7Z3B4	C00293
Q7Z3S7	C00076
Q7Z418	C00076
Q7Z418	C00238
Q7Z418	C07518
Q7Z475	C00045
Q7Z4N2	C00076
Q7Z4N2	C00376
Q7Z4N2	C01373
Q7Z4Y8	C00282
Q7Z4Y8	C05359
Q7Z5Y7	C00238
Q861H8	C00012
Q86SH2	C01330
Q86SH2	C01558
Q86TD4	C00076
Q86U42	C00895
Q86U42	C01445
Q86UD5	C00080
Q86UD5	C00282
Q86UD5	C01330
Q86UG4	C00403
Q86UK0	C01356
Q86UT5	C00001
Q86UW1	C01558
Q86UW2	C01558
Q86V24	C00162
Q86V24	C00293
Q86V24	C18189
Q86VB7	C00076
Q86VB7	C00082
Q86VB7	C00137
Q86VB7	C00536
Q86VL8	C00059
Q86VL8	C00080
Q86VL8	C00282
Q86VL8	C00468
Q86VL8	C00791
Q86VL8	C01373

Q86VL8	C06810
Q86VL8	C06952
Q86VL8	C07019
Q86VL8	C07151
Q86VL8	C07401
Q86VL8	C07512
Q86VL8	C11158
Q86VP3	C00524
Q86VW0	C00076
Q86VW1	C00487
Q86VW1	C01330
Q86VW1	C01373
Q86VW1	C01661
Q86VW1	C07512
Q86VW2	C00215
Q86VW2	C00242
Q86W47	C00076
Q86W47	C00238
Q86WA9	C00059
Q86WA9	C01330
Q86WA9	C11591
Q86WV5	C00039
Q86WV5	C00271
Q86XP0	C00219
Q86XP0	C01595
Q86XQ3	C00076
Q86XQ3	C01373
Q86Y82	C00187
Q86YB8	C00145
Q86YL7	C00001
Q86YL7	C00045
Q86YL7	C00504
Q86YR7	C00215
Q86YR7	C00242
Q86YT5	C00158
Q86YT5	C00187
Q86YT5	C01330
Q89843	C01356
Q89843	C01373
Q8AYW1	C00174
Q8AYW1	C00326
Q8HNQ4	C00282
Q8HNQ4	C05359
Q8IU99	C00076
Q8IUH4	C00305
Q8IUH5	C00305
Q8IUH5	C01834
Q8IUN9	C00076

Q8IUN9	C00716
Q8IUN9	C01074
Q8IUN9	C01582
Q8IV61	C00076
Q8IV61	C00215
Q8IV61	C00242
Q8IV77	C00215
Q8IV77	C00575
Q8IV77	C01373
Q8IVB4	C00080
Q8IVB4	C00282
Q8IVB4	C01330
Q8IVH4	C00194
Q8IVJ1	C00305
Q8IVV8	C00238
Q8IVV8	C01330
Q8IVW8	C01356
Q8IVW8	C06124
Q8IWA5	C00114
Q8IWL2	C01356
Q8IWT1	C01330
Q8IWU4	C00038
Q8IWU4	C00723
Q8IWW8	C00282
Q8IWZ6	C00035
Q8IWZ6	C00044
Q8IXF9	C00001
Q8IY34	C00012
Q8IY34	C00098
Q8IY34	C00282
Q8IY34	C00768
Q8IYB8	C00046
Q8IZD6	C00179
Q8IZF0	C00076
Q8IZF0	C00238
Q8IZF0	C01330
Q8IZF0	C01373
Q8IZM9	C00045
Q8IZM9	C01330
Q8IZS8	C00076
Q8IZT8	C00925
Q8IZY2	C00187
Q8IZY2	C00865
Q8MH63	C00045
Q8N130	C00009
Q8N130	C01330
Q8N139	C01356
Q8N142	C00020

Q8N142	C00130
Q8N142	C00215
Q8N142	C15587
Q8N1C3	C00334
Q8N1C3	C00698
Q8N1F7	C00293
Q8N1I0	C00035
Q8N1I0	C00044
Q8N1I0	C00215
Q8N1I0	C00242
Q8N1M1	C00076
Q8N1M1	C00698
Q8N1S5	C00038
Q8N1S5	C00050
Q8N1W1	C00215
Q8N1W1	C00242
Q8N2C7	C01330
Q8N2C7	C01373
Q8N2C7	C01836
Q8N2C7	C16094
Q8N335	C00003
Q8N335	C00116
Q8N335	C01330
Q8N335	C06696
Q8N370	C00045
Q8N370	C00698
Q8N370	C01330
Q8N3I7	C00035
Q8N3I7	C00044
Q8N3R9	C00334
Q8N4E7	C00023
Q8N4M1	C00114
Q8N4T8	C00472
Q8N4V1	C00305
Q8N4Y2	C00076
Q8N5I3	C00238
Q8N5J4	C00023
Q8N5Z5	C00238
Q8N608	C00238
Q8N668	C00070
Q8N695	C00022
Q8N695	C00033
Q8N695	C00060
Q8N695	C00163
Q8N695	C00164
Q8N695	C00174
Q8N695	C00180
Q8N695	C00186

Q8N695	C00246
Q8N695	C00256
Q8N695	C00698
Q8N695	C00708
Q8N695	C00803
Q8N695	C00805
Q8N695	C01330
Q8N695	C01432
Q8N695	C01450
Q8N695	C14042
Q8N697	C00012
Q8N697	C00098
Q8N697	C00282
Q8N697	C00768
Q8N6M3	C01356
Q8N6T7	C00293
Q8N7X0	C00007
Q8N8D7	C00238
Q8N8D7	C01330
Q8N8Q9	C00305
Q8N8R3	C00487
Q8N8Y2	C00282
Q8N9B8	C00215
Q8N9B8	C00242
Q8N9F0	C00174
Q8N9F0	C03758
Q8N9F0	C07164
Q8NB49	C00865
Q8NBI2	C14818
Q8NBI2	C14819
Q8NBQ7	C00001
Q8NBS3	C00238
Q8NBS3	C00282
Q8NBS3	C00288
Q8NBS3	C01330
Q8NBS3	C11477
Q8NBS3	C13563
Q8NBU5	C11106
Q8NBW4	C00045
Q8NBW4	C01330
Q8NC69	C00238
Q8NCC5	C00934
Q8NCK7	C00060
Q8NCM2	C00238
Q8NCM2	C13240
Q8NCS7	C00114
Q8NCX3	C06702
Q8NDB2	C00076

Q8NDX2	C00009
Q8NDX2	C00025
Q8NDX2	C00302
Q8NDX2	C01330
Q8NE01	C00050
Q8NEC5	C00076
Q8NEC5	C01373
Q8NER1	C00076
Q8NER1	C01373
Q8NET8	C00076
Q8NET8	C01373
Q8NEW0	C00038
Q8NEW0	C01373
Q8NFF2	C00076
Q8NFF2	C00238
Q8NFF2	C01330
Q8NFH1	C01356
Q8NFH3	C00293
Q8NFH4	C00293
Q8NFH5	C00293
Q8NFJ9	C00035
Q8NFJ9	C00044
Q8NFM4	C00001
Q8NFU0	C00076
Q8NFU0	C00698
Q8NFU1	C00076
Q8NFU1	C00698
Q8NFZ0	C00039
Q8NG04	C00288
Q8NG04	C00698
Q8NHE4	C00080
Q8NHE4	C00282
Q8NHX9	C00076
Q8NHX9	C13051
Q8NI99	C00723
Q8TAA5	C00012
Q8TAA5	C13028
Q8TAD4	C00038
Q8TAD4	C00175
Q8TAD4	C00723
Q8TAE7	C00238
Q8TAM2	C00035
Q8TAM2	C00044
Q8TB24	C00035
Q8TB24	C00044
Q8TB24	C00215
Q8TB24	C00242
Q8TB61	C00053

Q8TB61	C00212
Q8TBB6	C00045
Q8TBC3	C00238
Q8TBE1	C00302
Q8TBE1	C11033
Q8TBN0	C00215
Q8TBN0	C00242
Q8TC29	C00076
Q8TC92	C00003
Q8TC92	C05359
Q8TCC7	C00468
Q8TCC7	C05551
Q8TCC7	C06810
Q8TCC7	C06952
Q8TCC7	C06999
Q8TCU3	C00025
Q8TCU3	C00045
Q8TCU3	C00049
Q8TCU3	C00174
Q8TCU3	C00302
Q8TCU3	C01330
Q8TCU3	C16433
Q8TCU5	C00076
Q8TCU5	C00302
Q8TCU5	C00305
Q8TCU5	C12269
Q8TCU6	C01194
Q8TD20	C00031
Q8TD20	C00293
Q8TD22	C00023
Q8TD22	C00158
Q8TD22	C01373
Q8TD43	C00076
Q8TD43	C00238
Q8TD43	C01373
Q8TD43	C13971
Q8TDB8	C00293
Q8TDD1	C00046
Q8TDF6	C00035
Q8TDF6	C00044
Q8TDF6	C00165
Q8TDF6	C00215
Q8TDF6	C01373
Q8TDG4	C00039
Q8TDN1	C00238
Q8TDN2	C00238
Q8TDS7	C00076
Q8TDS7	C00099

Q8TE04	C00010
Q8TE54	C00059
Q8TE54	C00174
Q8TE54	C00209
Q8TE54	C00288
Q8TE54	C00698
Q8TE54	C01330
Q8TE54	C11591
Q8TE68	C00242
Q8TED4	C00934
Q8TEM1	C00293
Q8TEU7	C00215
Q8TEU7	C00242
Q8TF62	C00865
Q8TF71	C00045
Q8TF71	C00060
Q8TF71	C00539
Q8TF71	C01021
Q8TF71	C01330
Q8WTV0	C00187
Q8WTV0	C00287
Q8WTV0	C00338
Q8WTW4	C00697
Q8WUG5	C00023
Q8WUG5	C01373
Q8WUM0	C00046
Q8WUM0	C00293
Q8WUM0	C00895
Q8WUM9	C00009
Q8WUM9	C00174
Q8WUM9	C01330
Q8WUM9	C01356
Q8WUX1	C00037
Q8WUX1	C00045
Q8WUX1	C00303
Q8WUX1	C00716
Q8WUX1	C00768
Q8WUX1	C01330
Q8WUX1	C01401
Q8WUX1	C16438
Q8WUX2	C01373
Q8WV28	C10923
Q8WVF5	C00238
Q8WWA0	C00293
Q8WWA0	C00723
Q8WWG9	C00012
Q8WWG9	C00238
Q8WWI5	C00114

Q8WWM9	C00007
Q8WWN9	C00007
Q8WWN9	C00242
Q8WWT9	C01330
Q8WWT9	C02028
Q8WWT9	C06265
Q8WWX8	C00031
Q8WWX8	C00137
Q8WWX8	C00293
Q8WWX8	C01330
Q8WWX8	C01394
Q8WWX8	C01719
Q8WWZ4	C01356
Q8WWZ7	C00187
Q8WXA8	C01373
Q8WXG6	C00035
Q8WXG6	C00044
Q8WXH2	C00076
Q8WXH2	C08705
Q8WXS4	C00076
Q8WXS5	C00076
Q8WXS5	C00302
Q8WXS5	C11033
Q8WY07	C00045
Q8WY07	C00062
Q8WY07	C01330
Q8WY07	C01602
Q8WY07	C16440
Q8WYA0	C00487
Q8WYP3	C00035
Q8WYP3	C00044
Q8WYP3	C00215
Q8WYP3	C00242
Q8WZ19	C00238
Q8WZ55	C00238
Q8WZ55	C00698
Q8WZA2	C00215
Q8WZA2	C00242
Q8WZA2	C00575
Q90037	C00174
Q90037	C00326
Q92482	C00001
Q92482	C00116
Q92496	C01356
Q92504	C00038
Q92536	C00045
Q92536	C00062
Q92536	C00237

Q92536	C00303
Q92536	C00376
Q92536	C01330
Q92536	C01602
Q92536	C16439
Q92538	C00215
Q92538	C00242
Q92581	C00076
Q92581	C00080
Q92581	C00238
Q92581	C00282
Q92581	C01330
Q92614	C00039
Q92621	C00293
Q92643	C00145
Q92673	C00187
Q92673	C01356
Q92673	C01834
Q92681	C00293
Q92736	C00076
Q92736	C08705
Q92752	C01330
Q92806	C00238
Q92823	C01330
Q92841	C00046
Q92887	C06911
Q92888	C00215
Q92888	C00242
Q92911	C00708
Q92911	C01330
Q92911	C08215
Q92952	C00076
Q92952	C00238
Q92953	C00238
Q92959	C00584
Q92959	C00639
Q92959	C00696
Q92959	C01330
Q92959	C04741
Q92974	C00035
Q92974	C00044
Q92974	C00215
Q92974	C00242
Q93034	C00076
Q93050	C00282
Q93084	C00076
Q93084	C00305
Q93086	C00076

Q93086	C01373
Q95HM8	C00012
Q969I6	C00045
Q969I6	C01330
Q969R2	C01356
Q969S0	C00043
Q969S0	C00190
Q969S0	C11477
Q969T4	C01330
Q96A29	C00035
Q96A46	C00023
Q96A54	C00162
Q96A54	C00293
Q96A54	C18189
Q96A58	C00035
Q96A58	C00044
Q96AA3	C00930
Q96AA3	C01356
Q96BD0	C00403
Q96BD0	C00468
Q96BD0	C01330
Q96BD0	C01829
Q96BD0	C02465
Q96BD0	C05122
Q96BD0	C07639
Q96BD0	C08215
Q96BI1	C00282
Q96BI1	C01373
Q96BI1	C06527
Q96BI1	C07625
Q96BR1	C00238
Q96BW9	C00012
Q96C36	C00004
Q96C36	C00006
Q96CF2	C11106
Q96CX2	C00238
Q96D31	C00076
Q96D96	C00007
Q96D96	C00282
Q96DA6	C00012
Q96DA6	C13028
Q96DR4	C01356
Q96DR7	C00035
Q96DR7	C00044
Q96DW6	C00037
Q96DZ5	C00293
Q96EB6	C00003
Q96EB6	C00004

Q96EE3	C00293
Q96EP9	C01330
Q96EP9	C01558
Q96F81	C00012
Q96F81	C00187
Q96F85	C00076
Q96FC9	C00039
Q96FC9	C00131
Q96FC9	C00271
Q96FL8	C00059
Q96FL8	C00282
Q96FL8	C00468
Q96FL8	C00791
Q96FL8	C00875
Q96FL8	C06810
Q96FL8	C06952
Q96FL8	C07019
Q96FL8	C07151
Q96FL8	C07401
Q96FL8	C07512
Q96FL8	C08099
Q96FL8	C11158
Q96FL8	C13258
Q96FL8	C14701
Q96FT7	C00174
Q96FT7	C01330
Q96FT7	C01373
Q96FT7	C06821
Q96FZ7	C11106
Q96G79	C00282
Q96G79	C05227
Q96G79	C11477
Q96GZ6	C01373
Q96H72	C00038
Q96H72	C00050
Q96HA1	C00293
Q96HE7	C00145
Q96J66	C00051
Q96J66	C00377
Q96J66	C00951
Q96J66	C01227
Q96J66	C01921
Q96J66	C01937
Q96J66	C02166
Q96J66	C02538
Q96J66	C05122
Q96J92	C00238
Q96JA3	C05005

Q96JJ6	C00076
Q96JJ6	C08705
Q96JW4	C00305
Q96KC2	C00035
Q96KC2	C00044
Q96KK3	C00238
Q96L42	C00238
Q96L42	C13240
Q96LB2	C00388
Q96LB2	C07625
Q96LR9	C01356
Q96MG2	C00076
Q96MP8	C00238
Q96N16	C00334
Q96N87	C00045
Q96N87	C00698
Q96N87	C01330
Q96NB2	C00023
Q96NB2	C01373
Q96NL6	C01330
Q96NT5	C00023
Q96NT5	C00032
Q96NT5	C00504
Q96NY7	C00001
Q96NY7	C00698
Q96P56	C00076
Q96P56	C01373
Q96PD7	C01356
Q96PH1	C00007
Q96PH1	C00076
Q96PH1	C00282
Q96PH1	C00752
Q96PR1	C00238
Q96PS8	C00001
Q96PS8	C00086
Q96PS8	C00116
Q96PU5	C01330
Q96Q91	C00288
Q96Q91	C01330
Q96Q91	C12603
Q96QD8	C00045
Q96QD8	C01330
Q96QE2	C00080
Q96QE2	C00137
Q96QE2	C00282
Q96QE3	C11106
Q96QF0	C00215
Q96QF0	C00242

Q96QI5	C00925
Q96QT4	C00076
Q96QT4	C00305
Q96QT4	C00572
Q96QT4	C00716
Q96QT4	C01373
Q96QZ7	C00174
Q96R05	C00473
Q96RD7	C00076
Q96RK4	C00035
Q96RK4	C00044
Q96RN1	C00059
Q96RN1	C00209
Q96RN1	C00698
Q96RN1	C11591
Q96RP8	C00238
Q96RU7	C00293
Q96S06	C01834
Q96S37	C00366
Q96S66	C00698
Q96SB3	C00302
Q96SB3	C11033
Q96SB3	C12269
Q96SF2	C00076
Q96SF2	C00238
Q96SI1	C00238
Q96SN7	C00076
Q96SQ9	C06708
Q96SU4	C01356
Q96T49	C00752
Q96T54	C00174
Q96T54	C00238
Q96T55	C00238
Q96T83	C00080
Q96T83	C00238
Q96T83	C00282
Q96T83	C01330
Q96T83	C01373
Q96TA2	C00038
Q99250	C01330
Q99418	C00242
Q99429	C00302
Q99429	C00698
Q99437	C00282
Q99497	C00076
Q99523	C00293
Q99567	C00293
Q99571	C00076

Q99571	C01330
Q99571	C01373
Q99572	C00076
Q99572	C01330
Q99572	C01373
Q99578	C00035
Q99578	C00044
Q99584	C00070
Q99595	C00012
Q99624	C00041
Q99624	C00045
Q99624	C00064
Q99624	C00135
Q99624	C00152
Q99624	C00303
Q99624	C01330
Q99653	C00238
Q99700	C00046
Q99712	C00238
Q99720	C00238
Q99720	C01356
Q99726	C00038
Q99758	C00187
Q99766	C00282
Q99808	C00212
Q99808	C00801
Q99808	C13961
Q99884	C01330
Q99884	C16435
Q99928	C00334
Q99928	C00698
Q9B1D4	C00390
Q9B1D4	C00524
Q9B1D4	C05359
Q9BD17	C00012
Q9BD37	C00012
Q9BPW4	C01356
Q9BPX6	C00076
Q9BQ13	C00238
Q9BQ31	C00238
Q9BQE4	C00293
Q9BQE5	C01356
Q9BQS7	C00023
Q9BQS7	C00070
Q9BQS7	C14819
Q9BQT8	C00036
Q9BQT8	C00158
Q9BQT8	C00322

Q9BQT8	C00489
Q9BQT8	C02656
Q9BQT8	C06104
Q9BQT8	C16588
Q9BR39	C00076
Q9BR39	C08705
Q9BRI3	C00038
Q9BRI3	C01373
Q9BRQ5	C00076
Q9BRV3	C11477
Q9BRY0	C00038
Q9BS26	C00076
Q9BS91	C00215
Q9BS91	C00282
Q9BS91	C05227
Q9BS91	C11477
Q9BSA4	C00076
Q9BSA4	C00698
Q9BSW2	C00076
Q9BT67	C00023
Q9BT67	C02148
Q9BU23	C01834
Q9BU40	C00376
Q9BUM1	C00293
Q9BUR5	C00187
Q9BUR5	C01356
Q9BUX1	C01373
Q9BV35	C00009
Q9BV35	C00076
Q9BV35	C00147
Q9BVG9	C00716
Q9BVL2	C00293
Q9BVV7	C00012
Q9BW27	C00293
Q9BWE0	C00039
Q9BWG6	C01330
Q9BWM7	C00023
Q9BWM7	C01373
Q9BWW8	C01356
Q9BWW9	C01356
Q9BX79	C00473
Q9BX84	C00076
Q9BX84	C00305
Q9BX84	C00716
Q9BX84	C01373
Q9BXB4	C01356
Q9BXB5	C01356
Q9BXC9	C00035

Q9BXC9	C00044
Q9BXI2	C00077
Q9BXI2	C01602
Q9BXP2	C00238
Q9BXP2	C00698
Q9BXP2	C01373
Q9BXS9	C00059
Q9BXT2	C00076
Q9BXU8	C00023
Q9BXW6	C01356
Q9BY07	C00288
Q9BY07	C01330
Q9BY07	C12603
Q9BY32	C00081
Q9BY43	C11106
Q9BYP7	C01330
Q9BYP7	C17061
Q9BYT9	C00076
Q9BYT9	C00698
Q9BYW1	C00293
Q9BYW1	C11477
Q9BZ29	C00035
Q9BZ29	C00044
Q9BZ29	C00215
Q9BZ29	C00242
Q9BZ71	C01194
Q9BZC7	C00187
Q9BZD2	C00147
Q9BZD2	C00212
Q9BZD2	C00262
Q9BZD2	C00299
Q9BZD2	C00801
Q9BZD2	C07210
Q9BZD2	C08431
Q9BZD2	C12806
Q9BZF1	C01356
Q9BZF2	C01356
Q9BZF3	C01356
Q9BZV2	C00378
Q9BZV2	C00504
Q9BZW2	C00059
Q9BZW2	C01330
Q9BZY2	C00076
Q9C0H2	C00076
Q9C0H2	C00698
Q9C0K1	C00038
Q9GIP4	C00045
Q9GIX6	C00012

Q9GJ56	C00012
Q9GZN6	C00698
Q9GZN6	C01330
Q9GZQ4	C00076
Q9GZQ4	C00698
Q9GZU1	C00023
Q9GZU1	C00076
Q9GZU1	C01373
Q9GZV3	C00114
Q9GZV3	C01330
Q9GZV9	C00009
Q9GZZ6	C00076
Q9GZZ6	C00238
Q9H015	C00487
Q9H015	C01330
Q9H015	C01373
Q9H015	C05570
Q9H015	C07512
Q9H078	C00238
Q9H0C2	C00147
Q9H0C2	C00215
Q9H0H5	C00059
Q9H0U3	C00305
Q9H0X2	C00076
Q9H0X2	C01373
Q9H0X9	C00187
Q9H172	C00187
Q9H172	C01356
Q9H173	C00215
Q9H1C4	C00215
Q9H1D0	C00076
Q9H1D0	C01373
Q9H1H9	C00159
Q9H1H9	C18340
Q9H1K4	C00080
Q9H1K4	C00302
Q9H1N7	C00053
Q9H1N7	C00212
Q9H1P3	C01356
Q9H1V8	C00037
Q9H1V8	C00045
Q9H1V8	C00698
Q9H1V8	C01330
Q9H1V8	C01401
Q9H1V8	C16435
Q9H1V8	C16439
Q9H221	C00187
Q9H221	C00370

Q9H222	C00187
Q9H222	C00370
Q9H251	C00076
Q9H252	C00238
Q9H252	C13240
Q9H2B4	C00059
Q9H2C2	C00370
Q9H2D1	C00504
Q9H2H9	C00045
Q9H2H9	C00303
Q9H2H9	C01330
Q9H2J7	C00045
Q9H2J7	C00698
Q9H2J7	C01330
Q9H2J7	C16435
Q9H2J7	C16439
Q9H2S1	C00076
Q9H2S1	C00238
Q9H2V7	C01356
Q9H2X3	C00012
Q9H2X9	C00238
Q9H2X9	C00698
Q9H2X9	C01330
Q9H2Y9	C00403
Q9H310	C00014
Q9H310	C01371
Q9H313	C00023
Q9H313	C00698
Q9H3F6	C00238
Q9H3M0	C00238
Q9H427	C00174
Q9H427	C00238
Q9H444	C11106
Q9H598	C00037
Q9H598	C00045
Q9H598	C00282
Q9H598	C00334
Q9H668	C00039
Q9H668	C00271
Q9H6E5	C00075
Q9H6E5	C00895
Q9H6F2	C00076
Q9H6F2	C00238
Q9H6F2	C01373
Q9H6S3	C00242
Q9H6Z4	C00215
Q9H6Z4	C00242
Q9H7F0	C01373

Q9H814	C00044
Q9H814	C00046
Q9H814	C00562
Q9H8M5	C00034
Q9H8M5	C00050
Q9H8M5	C00070
Q9H8M5	C00175
Q9H8M5	C00305
Q9H8M5	C01373
Q9H8M5	C02148
Q9H8M5	C13881
Q9H8M5	C13884
Q9H8M5	C14818
Q9H8V3	C00215
Q9H8V3	C00242
Q9H936	C00025
Q9H936	C00080
Q9H936	C00302
Q9H999	C00010
Q9H9B4	C00023
Q9H9B4	C01373
Q9HAB3	C00255
Q9HAB3	C01330
Q9HAP6	C00174
Q9HAP6	C00334
Q9HAS3	C00214
Q9HAS3	C00294
Q9HAS3	C00396
Q9HAS3	C00801
Q9HAS3	C01330
Q9HAS3	C07210
Q9HAS3	C07234
Q9HAS3	C07650
Q9HAS3	C15587
Q9HAV7	C00012
Q9HAV7	C13028
Q9HB14	C00238
Q9HB14	C07515
Q9HB15	C00238
Q9HB15	C07515
Q9HB89	C00076
Q9HB89	C00698
Q9HBA0	C00076
Q9HBA0	C00391
Q9HBA0	C01373
Q9HBG4	C00174
Q9HBG4	C00282
Q9HBL8	C00005

Q9HBL8	C00006
Q9HBR0	C00045
Q9HBR0	C01330
Q9HBY8	C00238
Q9HBY8	C01330
Q9HC10	C00076
Q9HC21	C12652
Q9HC58	C00076
Q9HC58	C00238
Q9HC58	C01330
Q9HC96	C00293
Q9HCE9	C00076
Q9HCE9	C00698
Q9HCG7	C00550
Q9HCG7	C05005
Q9HCJ1	C00009
Q9HCJ1	C00013
Q9HCK8	C00039
Q9HCP6	C00116
Q9HCX4	C00076
Q9HCX4	C01373
Q9HD20	C01373
Q9HD23	C00305
Q9HD26	C00174
Q9HD26	C00698
Q9HD42	C11106
Q9HD89	C00736
Q9HDC5	C00076
Q9HDC5	C08705
Q9IDV3	C01356
Q9IDV3	C01373
Q9MY54	C00012
Q9MY56	C00012
Q9NNX6	C00012
Q9NP58	C00032
Q9NP58	C01413
Q9NP59	C00023
Q9NP78	C00012
Q9NP78	C00098
Q9NP86	C00076
Q9NP91	C00037
Q9NP91	C00045
Q9NP91	C00076
Q9NP91	C00148
Q9NP91	C00698
Q9NP91	C01330
Q9NP91	C16435
Q9NP94	C00038

Q9NPA1	C00076
Q9NPA1	C00238
Q9NPC2	C00174
Q9NPC2	C00238
Q9NPD5	C00403
Q9NPD5	C00951
Q9NPD5	C01330
Q9NPD5	C01558
Q9NPD5	C01937
Q9NPD5	C02166
Q9NPD5	C02465
Q9NPD5	C04555
Q9NPD5	C05122
Q9NPD5	C11363
Q9NPG2	C00007
Q9NPH5	C00007
Q9NPH5	C00238
Q9NPI9	C00238
Q9NPQ8	C00035
Q9NPQ8	C00044
Q9NPY2	C00315
Q9NPY2	C06702
Q9NQ11	C01373
Q9NQ40	C00255
Q9NQ40	C01330
Q9NQ90	C00076
Q9NQ90	C00698
Q9NQ92	C01429
Q9NQA5	C00076
Q9NQA5	C01373
Q9NQR9	C00293
Q9NQS5	C00162
Q9NQW8	C00076
Q9NQW8	C00215
Q9NQW8	C00575
Q9NQW8	C00942
Q9NQW8	C01373
Q9NQW8	C06958
Q9NR50	C00035
Q9NR82	C00238
Q9NR82	C07473
Q9NRA2	C00174
Q9NRA2	C00282
Q9NRA2	C01330
Q9NRD0	C00242
Q9NRG9	C00293
Q9NRJ3	C00076
Q9NRK6	C00032

Q9NRK6	C00098
Q9NRM0	C00282
Q9NRM0	C00293
Q9NRM0	C00366
Q9NRM0	C01496
Q9NRM0	C11477
Q9NRQ2	C00076
Q9NRQ2	C00865
Q9NRU3	C00050
Q9NRX5	C00065
Q9NRY6	C00076
Q9NRY6	C00865
Q9NRY7	C00076
Q9NRY7	C00865
Q9NS18	C00145
Q9NS40	C00238
Q9NS40	C13240
Q9NS69	C00012
Q9NS82	C00045
Q9NS82	C00065
Q9NS82	C01330
Q9NSA0	C00059
Q9NSA0	C00468
Q9NSA0	C01330
Q9NSA0	C04555
Q9NSA1	C00293
Q9NSA2	C00238
Q9NSD5	C00334
Q9NSD5	C00698
Q9NSD5	C01330
Q9NSY2	C01356
Q9NTG1	C00076
Q9NTI2	C00865
Q9NTM9	C00070
Q9NTN3	C00015
Q9NTN3	C00174
Q9NTN3	C01074
Q9NUL3	C00046
Q9NUL7	C00046
Q9NUL7	C00305
Q9NUM3	C00038
Q9NUM3	C00050
Q9NUP9	C00334
Q9NUV9	C00035
Q9NUV9	C00044
Q9NUV9	C00305
Q9NVC3	C00045
Q9NVC3	C01330

Q9NVE7	C00010
Q9NVI7	C11106
Q9NVN3	C00035
Q9NVN3	C00044
Q9NVN3	C00215
Q9NVN3	C00242
Q9NVN3	C00575
Q9NVP2	C01429
Q9NVT9	C00050
Q9NVV0	C00076
Q9NVV0	C00238
Q9NVV0	C01373
Q9NW15	C00076
Q9NW15	C00698
Q9NW64	C00076
Q9NWB1	C00046
Q9NWF4	C00255
Q9NWF4	C01330
Q9NX18	C00042
Q9NX18	C00399
Q9NX18	C05359
Q9NXV2	C00238
Q9NY12	C01373
Q9NY26	C00038
Q9NY37	C01330
Q9NY37	C01373
Q9NY37	C06821
Q9NY46	C01330
Q9NY47	C00076
Q9NY64	C00031
Q9NY64	C00293
Q9NY64	C00723
Q9NY64	C01496
Q9NY64	C11477
Q9NY72	C01330
Q9NY91	C00293
Q9NY91	C01330
Q9NYB5	C00403
Q9NYB5	C00468
Q9NYB5	C00951
Q9NYB5	C01330
Q9NYB5	C02465
Q9NYB5	C07639
Q9NYB5	C08215
Q9NYB5	C11363
Q9NYG8	C00219
Q9NYG8	C00238
Q9NYV7	C00076

Q9NYV7	C01373
Q9NYW0	C00076
Q9NYW0	C01373
Q9NYW1	C00076
Q9NYW1	C01373
Q9NYW3	C00076
Q9NYW3	C01373
Q9NYW5	C00076
Q9NYW5	C01373
Q9NYW6	C00076
Q9NYW6	C01373
Q9NYZ2	C00023
Q9NYZ2	C00032
Q9NZ53	C00572
Q9NZ53	C05359
Q9NZA1	C00698
Q9NZC9	C00039
Q9NZD2	C05005
Q9NZI1	C00076
Q9NZI1	C00238
Q9NZI2	C00076
Q9NZI2	C00238
Q9NZJ9	C00046
Q9NZL6	C00215
Q9NZL6	C00242
Q9NZM6	C01373
Q9NZN1	C00076
Q9NZQ8	C00076
Q9NZQ8	C00238
Q9NZQ8	C01330
Q9NZQ8	C01373
Q9NZQ8	C13971
Q9NZU7	C00076
Q9NZU7	C00391
Q9NZV8	C00238
Q9NZZ3	C11106
Q9P0J7	C00238
Q9P0L9	C00076
Q9P0L9	C01373
Q9P0X4	C00076
Q9P0X4	C00291
Q9P0X4	C07222
Q9P1Z3	C00215
Q9P1Z3	C00238
Q9P1Z3	C01330
Q9P241	C00865
Q9P241	C01373
Q9P246	C00076

Q9P2D8	C01330
Q9P2D8	C01373
Q9P2D8	C01836
Q9P2D8	C16094
Q9P2E2	C12269
Q9P2K9	C01356
Q9P2U7	C00009
Q9P2U7	C00025
Q9P2U7	C00302
Q9P2U7	C01330
Q9P2U8	C00009
Q9P2U8	C00025
Q9P2U8	C00302
Q9P2U8	C01330
Q9QSQ8	C01356
Q9QSQ8	C01373
Q9TQE0	C00012
Q9UBD6	C00014
Q9UBD6	C00706
Q9UBD6	C01371
Q9UBG0	C00211
Q9UBL9	C00076
Q9UBL9	C01373
Q9UBN1	C00076
Q9UBN1	C00302
Q9UBN1	C11033
Q9UBN4	C00076
Q9UBN4	C01373
Q9UBN4	C13881
Q9UBP9	C00187
Q9UBP9	C01356
Q9UBP9	C02690
Q9UBS5	C00076
Q9UBS5	C00137
Q9UBS5	C00238
Q9UBS5	C00334
Q9UBU3	C18181
Q9UBX3	C00009
Q9UBX3	C00042
Q9UBX3	C00059
Q9UBX3	C00094
Q9UBX3	C00149
Q9UBX3	C00320
Q9UBX3	C00383
Q9UBX3	C02028
Q9UBY0	C00080
Q9UBY0	C00282
Q9UBY0	C01330

Q9UBY8	C01356
Q9UCZ3	C00238
Q9UDW1	C00390
Q9UDW1	C00524
Q9UDW1	C05359
Q9UDX3	C00751
Q9UDX4	C00751
Q9UES8	C00777
Q9UF02	C00076
Q9UF02	C00302
Q9UF02	C11033
Q9UF72	C06911
Q9UG63	C00023
Q9UGH3	C00072
Q9UGH3	C00282
Q9UGH3	C00701
Q9UGH3	C01330
Q9UGI6	C00076
Q9UGI6	C00238
Q9UGJ0	C00293
Q9UGK8	C00215
Q9UGK8	C00242
Q9UGM1	C00076
Q9UGM1	C00238
Q9UGQ2	C00076
Q9UGQ3	C00031
Q9UGQ3	C00293
Q9UHC3	C00174
Q9UHC3	C01330
Q9UHC3	C01373
Q9UHC3	C06821
Q9UHC6	C00238
Q9UHC9	C00187
Q9UHC9	C01356
Q9UHI5	C00041
Q9UHI5	C00045
Q9UHI5	C00050
Q9UHI5	C00062
Q9UHI5	C00078
Q9UHI5	C00079
Q9UHI5	C00082
Q9UHI5	C00097
Q9UHI5	C00303
Q9UHI5	C00716
Q9UHI5	C00736
Q9UHI5	C01330
Q9UHI5	C01373
Q9UHI5	C01401

Q9UHI5	C02057
Q9UHI5	C16439
Q9UHI5	C18672
Q9UHI7	C00072
Q9UHI7	C00701
Q9UHI7	C01330
Q9UHI7	C05422
Q9UHV5	C00215
Q9UHV5	C00242
Q9UHW9	C00238
Q9UHW9	C00698
Q9UHW9	C01330
Q9UI32	C00303
Q9UI33	C01330
Q9UI33	C11692
Q9UI40	C00076
Q9UI40	C00205
Q9UI40	C00238
Q9UI40	C00376
Q9UI40	C01330
Q9UIA0	C00242
Q9UID3	C01356
Q9UIG8	C00059
Q9UIG8	C00082
Q9UIG8	C00403
Q9UIG8	C00468
Q9UIG8	C00840
Q9UIG8	C01330
Q9UIG8	C04741
Q9UIG8	C05122
Q9UIG8	C06956
Q9UIG8	C18095
Q9UIX4	C00238
Q9UIZ6	C00012
Q9UJ41	C00035
Q9UJ41	C00044
Q9UJ41	C00215
Q9UJ71	C00076
Q9UJ71	C00159
Q9UJ90	C00238
Q9UJ96	C00238
Q9UJS0	C00025
Q9UJS0	C00049
Q9UJS0	C00076
Q9UJS0	C00302
Q9UJS0	C16433
Q9UJU2	C00039
Q9UK17	C00238

Q9UK73	C00293
Q9UKA9	C00046
Q9UKF7	C01194
Q9UKF7	C01356
Q9UKG1	C00293
Q9UKG4	C00059
Q9UKG4	C01330
Q9UKL6	C00157
Q9UKU0	C00162
Q9UKU0	C01356
Q9UKW4	C00215
Q9UKW4	C00242
Q9UKX7	C00293
Q9UL51	C00215
Q9UL51	C00238
Q9UL51	C01330
Q9UL62	C00076
Q9UL62	C01373
Q9ULA0	C00012
Q9ULD8	C00238
Q9ULD8	C13240
Q9ULF5	C00038
Q9ULF5	C00050
Q9ULG1	C00039
Q9ULI0	C11106
Q9ULQ1	C00076
Q9ULQ1	C13051
Q9ULS6	C00238
Q9ULU8	C00076
Q9ULZ1	C13662
Q9UM01	C00045
Q9UM01	C00062
Q9UM01	C00237
Q9UM01	C00303
Q9UM01	C01330
Q9UM01	C16439
Q9UM21	C00293
Q9UMX9	C05606
Q9UMZ3	C01194
Q9UN42	C00238
Q9UN42	C01330
Q9UN57	C00076
Q9UN57	C00238
Q9UN76	C00045
Q9UN76	C00698
Q9UN76	C01330
Q9UN76	C16435
Q9UN88	C00334

Q9UN88	C00698
Q9UNQ0	C00032
Q9UNQ0	C01661
Q9UNQ0	C01907
Q9UNQ0	C06708
Q9UNQ0	C11190
Q9UNQ0	C11195
Q9UNX9	C00238
Q9UNX9	C13881
Q9UNY4	C00039
Q9UP52	C00023
Q9UP79	C00009
Q9UP95	C00238
Q9UP95	C00698
Q9UP95	C01330
Q9UPI3	C00032
Q9UPI3	C00076
Q9UPR5	C00076
Q9UPR5	C01330
Q9UPY5	C00045
Q9UPY5	C00076
Q9UPY5	C00302
Q9UPY5	C01330
Q9UPY5	C01420
Q9UQ05	C00238
Q9UQ05	C13240
Q9UQ53	C00420
Q9UQ90	C00038
Q9UQC9	C00076
Q9UQC9	C00698
Q9UQD0	C01330
Q9UQN3	C11106
Q9Y210	C00076
Q9Y210	C01373
Q9Y217	C00076
Q9Y217	C00238
Q9Y217	C01194
Q9Y226	C01373
Q9Y227	C00015
Q9Y227	C00035
Q9Y227	C00044
Q9Y227	C00063
Q9Y227	C00075
Q9Y227	C00112
Q9Y227	C00459
Q9Y230	C00039
Q9Y230	C00271
Q9Y230	C05426

Q9Y233	C00575
Q9Y233	C00942
Q9Y253	C00396
Q9Y257	C00238
Q9Y261	C00293
Q9Y265	C00039
Q9Y265	C00271
Q9Y265	C05426
Q9Y267	C01373
Q9Y277	C00147
Q9Y289	C00120
Q9Y289	C00725
Q9Y289	C00864
Q9Y289	C01330
Q9Y294	C01429
Q9Y2C5	C00009
Q9Y2C5	C01330
Q9Y2D2	C00013
Q9Y2D2	C00015
Q9Y2D2	C00043
Q9Y2D2	C00282
Q9Y2D2	C00299
Q9Y2D2	C11477
Q9Y2E8	C00080
Q9Y2E8	C00282
Q9Y2E8	C01330
Q9Y2G3	C00865
Q9Y2P4	C00162
Q9Y2P4	C00638
Q9Y2P5	C00162
Q9Y2P5	C01558
Q9Y2Q0	C00865
Q9Y2Q0	C01373
Q9Y2T6	C00076
Q9Y2U2	C00238
Q9Y2W3	C00293
Q9Y2W3	C11477
Q9Y2W7	C00039
Q9Y2W7	C00076
Q9Y2W7	C00238
Q9Y2W7	C00305
Q9Y2Y8	C00388
Q9Y2Y8	C02166
Q9Y342	C00238
Q9Y342	C01356
Q9Y345	C00037
Q9Y345	C00698
Q9Y345	C01330

Q9Y375	C00004
Q9Y375	C00399
Q9Y375	C05359
Q9Y3D7	C00012
Q9Y3D7	C13028
Q9Y3E7	C11106
Q9Y3Q4	C00215
Q9Y3Q4	C00238
Q9Y3Q4	C01330
Q9Y487	C00080
Q9Y487	C00282
Q9Y4G8	C00215
Q9Y4G8	C00242
Q9Y4H2	C00293
Q9Y4H2	C00638
Q9Y597	C00238
Q9Y5I7	C00305
Q9Y5N2	C00391
Q9Y5P4	C00195
Q9Y5S1	C00076
Q9Y5S1	C01373
Q9Y5S8	C00080
Q9Y5S8	C00215
Q9Y5S8	C00747
Q9Y5S8	C05359
Q9Y5T3	C00076
Q9Y5T3	C07222
Q9Y5X9	C00187
Q9Y5Y0	C00023
Q9Y5Y0	C00032
Q9Y5Y0	C00486
Q9Y5Y9	C01330
Q9Y5Y9	C11692
Q9Y5Z9	C00828
Q9Y619	C00077
Q9Y619	C01602
Q9Y662	C00925
Q9Y663	C00925
Q9Y666	C00238
Q9Y666	C00698
Q9Y666	C01330
Q9Y691	C00076
Q9Y691	C00238
Q9Y691	C13603
Q9Y694	C00059
Q9Y694	C00072
Q9Y694	C00468
Q9Y694	C00489

Q9Y694	C00584
Q9Y694	C00805
Q9Y694	C01330
Q9Y694	C04555
Q9Y694	C06570
Q9Y694	C06816
Q9Y694	C06859
Q9Y694	C07394
Q9Y694	C07649
Q9Y696	C00698
Q9Y698	C00076
Q9Y698	C00302
Q9Y698	C11033
Q9Y6D5	C00215
Q9Y6D5	C00242
Q9Y6D6	C00215
Q9Y6D6	C00242
Q9Y6F6	C00076
Q9Y6F6	C00306
Q9Y6H6	C00012
Q9Y6H6	C00238
Q9Y6H6	C00575
Q9Y6H6	C00698
Q9Y6J6	C00012
Q9Y6J6	C00238
Q9Y6K0	C00114
Q9Y6K0	C00865
Q9Y6L6	C00059
Q9Y6L6	C00403
Q9Y6L6	C00468
Q9Y6L6	C00584
Q9Y6L6	C00951
Q9Y6L6	C01330
Q9Y6L6	C01558
Q9Y6L6	C01844
Q9Y6L6	C01937
Q9Y6L6	C02465
Q9Y6L6	C04555
Q9Y6L6	C05122
Q9Y6L6	C05952
Q9Y6L6	C05963
Q9Y6L6	C08215
Q9Y6M5	C00038
Q9Y6M5	C00076
Q9Y6M5	C01413
Q9Y6M7	C00288
Q9Y6M7	C01330
Q9Y6M7	C12603

Q9Y6M9	C00004
Q9Y6M9	C00399
Q9Y6M9	C05359
Q9Y6N3	C00076
Q9Y6N3	C00698
Q9Y6R1	C00288
Q9Y6R1	C01330
Q9Y6R1	C12603
